# Decoding the Cellular Response to Methylthioadenosine Uncovers Novel Targets for Chronic Liver Disease

**DOI:** 10.3390/cells15191720

**Published:** 2026-09-22

**Authors:** Irene Blázquez-García, Laura Guerrero, Mary Dayne S. Tai, Américo Cerqueira, Ana Cuervo, Alberto Paradela, Fernando J. Corrales

**Affiliations:** 1 Functional Proteomics Laboratory, National Center for Biotechnology (CNB-CSIC), Darwin 3, 28049 Madrid, Spain; irene.blazquez@cnb.csic.es (I.B.-G.); laura.guerrero@ugent.be (L.G.); americo.cerqueira@csic.es (A.C.); alberto.paradela@cnb.csic.es (A.P.); 2 Leukemia Therapy Resistance Lab, Department of Biomolecular Medicine, Faculty of Medicine and Health Sciences, Ghent University, 9000 Ghent, Belgium; 3 Department of Macromolecular Structure, National Center for Biotechnology (CNB-CSIC), Darwin 3, 28049 Madrid, Spain; md.tai@cnb.csic.es (M.D.S.T.); acuervo@cnb.csic.es (A.C.)

**Keywords:** proteomics, methylthioadenosine, cholestasis, liver

## Abstract

Progressive familial intrahepatic cholestasis type 3 (PFIC3), caused by MDR3 deficiency (MDR2 in mice), is a lethal pediatric liver disease characterized by early-onset persistent cholestasis, progressive fibrosis, cirrhosis, and eventual liver failure. MDR3 is a phosphatidylcholine transporter located in the canalicular membrane of hepatocytes, and its deficiency leads to the accumulation of free bile acids, which damage epithelial cells due to their detergent-like effect. Therapeutic options remain limited, highlighting the need to identify molecular drivers of disease progression and novel pharmacological strategies. To investigate the molecular alterations associated with this deficiency, our laboratory performed functional proteomic analyses in patient samples and murine hepatic organoids, revealing consistent reprogramming of one-carbon metabolism (OCM). Based on these findings, we explored the therapeutic potential and molecular mechanisms of 5′-methylthioadenosine (MTA), a metabolite involved in this pathway. Using hepatocellular carcinoma cells (PLCs), we combined quantitative shotgun proteomics with the proteome integral solubility alteration (PISA) assay to characterize functional responses and identify direct protein targets of MTA. To reinforce the physiological relevance of our findings, we extended the PISA analysis to liver protein extracts from the Mdr2^−/−^ mouse model of chronic cholestatic injury, enabling validation of candidate target interactions observed in cellular extracts. Proteomic profiling revealed that MTA induces significant alterations in cell division processes, epigenetic regulatory networks, and energy metabolism, thereby promoting an adaptive phenotype characterized by metabolic plasticity, microenvironmental reorganization, and immunomodulation. These findings provide a robust molecular framework for understanding the hepatic response to MTA and shed new light on the mechanisms that may underlie its beneficial effects. In addition, they provide a valuable platform for further investigation of the therapeutic potential of this compound and its prospective translation into clinical practice.

## 1. Introduction

Liver diseases are becoming increasingly prevalent worldwide, accounting for more than 2 million deaths annually and therefore imposing a substantial burden on society and healthcare systems [1]. Some risk factors for liver diseases are well known, such as alcohol consumption, infection with hepatitis A, B, and C viruses, obesity, diabetes, the intake of hepatotoxins such as aflatoxin, and genetic determinants, among others [2,3,4,5,6,7]. In particular, the disruption of bile flow from the hepatocyte to the small intestine or cholestasis leads to the accumulation of biliary components that induce cellular damage in the liver parenchyma and the biliary tree. The pathological presentation involves impaired hepatobiliary function, fibrosis, cirrhosis, and hepatocellular carcinoma (HCC) or cholangiocarcinoma in the most severe cases. Among different etiologies, genetic cholestasis syndromes represent a significant clinical challenge due to their early onset, rapid progression, and lack of treatments [8,9].

Progressive familial intrahepatic cholestasis (PFIC) is a severe, rare disease occurring in one in 10,000–20,000 births [10], presenting different subtypes according to the affected gene. The impaired activity of the encoded proteins involved in the transport of bile acids or phospholipids is ultimately the triggering factor of PFIC. PFIC3 results from mutations in the ABCB4 gene that encodes the MDR3 protein (MDR2 was the former identification of the mouse ortholog). MDR3 translocates phosphatidylcholine (PC) from the hepatocytes into bile through a mechanism dependent on ATP hydrolysis [11,12]. PC is an essential component of bile that maintains solubilized cholesterol and bile salts, preventing potential damage to cholangiocytes. Therapeutic options are lacking and currently involve palliative treatment with ursodeoxycholic acid or obeticholic acid, but liver transplantation remains the gold-standard intervention for the most severe cases [10,13].

To deepen our understanding of PFIC and identify new potential clinical treatments, the molecular underpinnings of the disease have been extensively characterized [14,15]. Comprehensive analysis of liver proteome dynamics revealed profound alterations in critical cellular pathways associated with cholestasis and uncovered candidate protein drivers with potential pathogenic and therapeutic relevance, opening new opportunities for precision-based clinical strategies [16,17]. Among the alterations, reshaping of one-carbon metabolism (OCM) was raised as a cornerstone for disease progression. OCM is a masterpiece of hepatocyte biology that connects intermediate metabolism and epigenetic regulation through the synthesis of S-adenosylmethionine (SAMe) [18,19]. Impairment of OCM leads to hepatocyte dedifferentiation and proliferation and contributes to the progression of chronic liver damage [20,21]. Interestingly, it was demonstrated that the administration of SAMe prevented liver damage in experimental models [22,23,24] and improved the survival of cirrhotic patients [25]. More recently, it was proposed that part of the beneficial effect of SAMe could be attributed to another OCM metabolite: methylthioadenosine (MTA) [26].

MTA is produced during the synthesis of polyamines from decarboxylated SAMe and initiates the purine and methionine salvage pathways through the action of MTA phosphorylase (MTAP). Several studies have demonstrated that MTA can ameliorate disease progression by targeting inflammatory pathways, highlighting its therapeutic potential across multiple disorders. MTA reverses brain autoimmune disease and reduces liver inflammation and fibrosis by suppressing T-cell activation in vitro and in vivo through the inhibition of NFκB activation. Moreover, it suppresses the production of proinflammatory cytokines and genes (IFNγ, TNFα, and iNOS) and increases the production of anti-inflammatory cytokines (IL10) in a cAMP-independent manner [27,28]. Oral administration of MTA in Mdr2^−/−^-deficient mice, a PFIC3 mouse model, significantly reduced hepatomegaly and liver injury by mechanisms independent of its interaction with adenosine receptors, whose expression is negligible in liver cells [29].

In order to provide further mechanistic support to the putative beneficial effect of MTA in chronic liver diseases, we have performed a systematic analysis of the reshaping of the liver proteome in response to MTA. We describe here the interaction of MTA with specific protein targets and the subsequent regulation of critical cellular pathways that suggest the role of this compound in the preservation of quiescent and differentiated phenotypes of hepatocytes.

## 2. Materials and Methods

This study combined complementary mass spectrometry-based proteomic approaches to investigate both the cellular response to MTA and its direct effects on protein stability. Differential proteomic analysis was performed to characterize proteome remodeling following MTA treatment in PLC cells. In parallel, protein integral solubility alteration (PISA) assays were carried out in intact PLC cells and in native protein extracts prepared from PLC cells and mouse liver tissue to identify MTA-induced changes in protein thermal stability. All samples were subsequently processed by quantitative LC-MS/MS.

### 2.1. Cell Culture

PLC cells were from the ATCC (ATCC-CRL-8024) and were cultured at 37 °C in high-glucose DMEM supplemented with 10% FBS in a humidified atmosphere containing 5% CO_2_. Cells were grown until 80% confluency prior to different treatments or collection.

### 2.2. Mouse Liver Samples

Liver tissue was collected from 12-month-old wild-type (WT) and Mdr2^−/−^ mice. Animals were grown at the CNB-CSIC animal facility, and all experimental procedures were approved by the institutional ethics committee (PROEX 251.6/23).

### 2.3. Sample Preparation

Three different sample types were generated for the proteomic analyses performed in this study. Whole-cell lysates from MTA-treated PLC cells were used to characterize the cellular proteomic response to treatment (n = 3). In parallel, intact PLC cells and native protein extracts prepared from PLC cells or mouse liver tissue were used for protein integral solubility alteration (PISA) assays (n = 4) to evaluate MTA-induced changes in protein thermal stability.

PLC cells were treated with MTA at final concentrations of 10 μM or 50 μM for 24 or 48 h. PBS-treated cells served as controls. Following treatment, cells were washed with PBS, harvested, and lysed using SDS/TEAB (2.5% SDS [Cytiva, Marlborough, MA, USA], 50 mM triethylammonium bicarbonate [Thermo Fisher Scientific, Waltham, MA, USA]). Protein concentration was determined using a Pierce 660 nm Protein Assay, following the indications of the manufacturer (Thermo Fisher Scientific, Waltham, MA, USA), supplemented with the ionic detergent compatibility reagent to ensure compatibility with SDS-containing samples. A total of 50 μg of protein per sample was used for downstream proteomic analysis.

For PISA analysis, PLC cells were incubated with 200 μM MTA or PBS for 15 or 30 min before being subjected to the PISA assay. For protein extracts, washed PLC cell pellets and 5mg mouse liver tissue were lysed under mild non-denaturing conditions using PBS to preserve native protein conformations and protein–protein interactions. Samples were homogenized using three freeze/thaw cycles in liquid nitrogen, followed by clarification by centrifugation at 21,000× *g* for 10 min. Protein concentration was determined using a Pierce 660nm Protein Assay, following the indications of the manufacturer (Thermo Fisher Scientific), and protein concentrations were normalized across all samples prior to incubation with MTA for 15 or 30 min at room temperature, followed by the PISA assay.

### 2.4. Western Blot Analysis

PLC cells were treated with MTA as described in the sample preparation section. Following treatment, cells were washed with PBS, harvested, and lysed using SDS/TEAB buffer (2.5% SDS, 50 mM triethylammonium bicarbonate). Protein concentration was determined using the Pierce 660 nm Protein Assay, following the manufacturer’s instructions (Thermo Fisher Scientific, Waltham, MA, USA). Then, 10 µg of the protein samples were resolved by 10% acrylamide/bis(acrylamide) (29:1) SDS–PAGE alongside a Precision Plus Protein Dual Color molecular weight marker (Bio-Rad, Hercules, CA, USA), and transferred using 15 mM Tris, 192 mM glycine, and 20% *v*/*v* methanol onto nitrocellulose membranes (0.45 μm, 1620115, Bio-Rad) for immunoblot analysis with antibodies (Table 1). The resulting bands were quantified using Fiji/ImageJ software (version 1.54p). Whole Western blot membranes are shown in Supplementary Figure S3.

### 2.5. Protein Integral Solubility Alteration (PISA) Assay

PISA experiments were performed in both intact PLC cells and native protein extracts, following the workflow described by Zhang et al. [30]. Briefly, samples were incubated with 200 μM MTA or PBS for controls under the experimental conditions described above and divided into four equal aliquots per condition. Each aliquot was exposed to different temperatures (53, 55, 58, and 61 °C) for 5 min, and then cooled at room temperature for 6 min. After thermal challenge, the aliquots from each condition were pooled into a unique sample. To separate the soluble protein fraction from heat-induced aggregates, lysates were ultracentrifuged at 100,000× *g* for 30 min at 12 °C. The resulting supernatant, corresponding to the soluble proteome, was collected and lyophilized. Lyophilized samples were subsequently resuspended in denaturing lysis buffer containing 2.5% SDS/TEAB for downstream proteomic processing.

### 2.6. Proteomic Sample Preparation

Whole-cell lysates generated for differential proteomic analysis were processed using S-Trap micro spin columns (Protifi, Fairport, NY, USA) according to the manufacturer’s instructions. Briefly, proteins were reduced, alkylated, and loaded into the S-Trap matrix, washed, and subjected to on-column overnight digestion at 37 °C with trypsin (1:15 enzyme-to-substrate ratio). The resulting peptides were eluted, vacuum-dried, and desalted using C18 StageTips (Empore Octadecyl C18 extraction disks, SUPELCO, Bellefonte, PA, USA) before liquid chromatography–tandem mass spectrometry (LC-MS/MS) analysis.

Samples generated from the PISA workflow were processed using an automated magnetic-bead-based digestion protocol implemented on an Opentrons OT-2 liquid handling platform [31]. Briefly, proteins were reduced, alkylated, and captured onto magnetic beads prior to enzymatic digestion with trypsin/Lys-C. Following digestion, peptides were recovered, dried, and reconstituted for LC-MS/MS analysis.

### 2.7. LC-MS/MS Analysis

Peptides from both workflows were reconstituted in 2% acetonitrile (ACN) and 0.1% formic acid (FA) prior to LC-MS/MS analysis. For differential proteomic analysis, 500 ng of peptides per sample were loaded onto a 0.3 mm × 5 mm trap column, then switched online to a 50 cm EASY-Spray HPLC column (Thermo Fisher Scientific). Separation was performed in an UltiMate 3000 RSLCnano system (Thermo Fisher Scientific). Peptides were eluted over a 120-min gradient. Mobile phase A was 0.1% formic acid in water, whereas mobile phase B was 0.1% formic acid in 80:20 acetonitrile/water. All mobile phases were LC-MS-grade. The flow rate was maintained at 250 nL/min throughout the separation. The Orbitrap Exploris 240 mass spectrometer (Thermo Fisher Scientific) operated in Data-Independent Acquisition (DIA) mode. Full MS1 survey scans were acquired at a resolution of 60,000 across a mass range of 350–1200 *m/z*. For MS2 acquisition, a scheme of custom variable isolation windows (including a 2 *m/z* overlap) was utilized to optimize precursor sampling. Fragmentation was performed via higher-energy collisional dissociation (HCD) using a normalized collision energy (NCE) of 30, and MS2 fragment spectra were acquired at a resolution of 30,000.

For the PISA assay, 200 ng of peptide mixtures resulting from the tryptic digestion of the protein extracts were analyzed using a Vanquish Neo UHPLC system (Thermo Fisher Scientific) coupled to an Orbitrap Eclipse Tribrid mass spectrometer (Thermo Fisher Scientific). Peptides were separated on a 50 cm µPAC Neo C18 analytical column (180 µm bed width, 100–300 Å pore size; Thermo Fisher Scientific) using a 140-min gradient. Mobile phase A consisted of 0.1% formic acid in water, whereas mobile phase B consisted of 0.1% formic acid in 80:20 acetonitrile/water. The flow rate was maintained at 250 nL/min throughout the separation. The Orbitrap Eclipse Tribrid mass spectrometer (Thermo Fisher Scientific) was operated in Data-Independent Acquisition (DIA) mode, and both MS1 and MS2 spectra were acquired in the Orbitrap. Full MS1 survey scans were acquired at a resolution of 120,000 across a mass range of 350–1250 *m/z*. For MS2 acquisition, a scheme of custom variable isolation windows (including a 2 *m/z* overlap) was utilized to optimize precursor sampling. Fragmentation was performed via higher-energy collisional dissociation (HCD) using a normalized collision energy (NCE) of 30, and MS2 fragment spectra were acquired in the Orbitrap at a resolution of 30,000 across a scan range of 125–1500 *m/z*.

### 2.8. Proteomic Data Processing and Statistical Analysis

Raw MS data files were processed using Spectronaut software (version 19, Biognosys, Schlieren, Switzerland). Data generated from PLC cells were searched against the Homo sapiens UniProt reference proteome database (downloaded on 12 June 2024), while mouse liver samples were searched against the Mus musculus UniProt reference proteome database (downloaded on 31 October 2024). The search parameters included carbamidomethylation of cysteines as a fixed modification, and oxidation of methionines and protein N-terminal acetylation as variable modifications. False discovery rate (FDR) thresholds for protein, peptide, and peptide spectrum match (PSM) identifications were set at 1% (FDR < 0.01). Data were normalized using the default automatic normalization settings, and missing values were handled using the Global Imputation algorithm incorporated in Spectronaut. To determine the statistical significance of differentially abundant or differentially stabilized proteins, an unpaired two-tailed Student’s *t*-test was performed. Across all experiments, proteins were considered significantly altered if they presented a q-value ≤ 0.05 (equivalent to a 5% false discovery rate). Furthermore, for the differential proteomic analysis characterizing the cellular response to MTA at 24 and 48 h, an additional threshold was applied: differentially expressed proteins were strictly defined as those meeting both the q-value ≤ 0.05 criterion and an absolute log_2_ fold change (|log_2_FC| ≥ 0.26). The mass spectrometry proteomics data have been deposited in the ProteomeXchange Consortium via the jPOST partner repository, with the dataset identifier PXD082684 (jPOST ID: JPST004847).

### 2.9. Bioinformatics and Functional Enrichment Analysis

Downstream bioinformatic analyses and data visualization were performed using R software (version 4.4.2). Principal component analysis (PCA) was conducted to evaluate the variance and overall reproducibility among biological replicates. Volcano plots were generated in R to visualize the magnitude (log_2_ fold change) and statistical significance (−log_10_ q-value) of the proteomic and stability changes. Functional enrichment analysis was performed using the Metascape platform (http://metascape.org), employing a multiple gene list comparison approach to identify shared and distinct enriched Gene Ontology (GO) terms and pathways across experimental conditions. Finally, bar charts representing the log_2_ fold change (log_2_FC) of specific candidate proteins were generated using GraphPad Prism (version 10.1.0).

### 2.10. Differential Scanning Fluorimetry

Human PAH was recombinantly expressed and purified from *E. coli* K12 TB1 cells (New England BioLabs, Ipswich, MA, USA) using a protocol previously described by Flydal et al. [32]. Thermal denaturation curves of PAH were obtained at different MTA concentrations using differential scanning fluorimetry (DSF). Samples with 1.92 µM (subunit) PAH in the presence of 0, 25, 50, or 100 µM MTA were prepared in 20 mM Na-Hepes pH7.0, 200 mM NaCl buffer with 7x SYPRO Orange dye (Merck, Darmstadt, Germany) prior to loading into a 384-well plate (Applied Biosystems). The plate was heated stepwise from 20 °C to 100 °C at a heating rate of 1.8 °C/min using QuantStudio5 (Applied Biosystems, (Applied Biosystems, Foster City, CA, USA). The thermal denaturation of PAH was monitored by detecting the increase in SYPRO Orange fluorescence (λex = 465 nm, λem = 610 nm) upon its binding to exposed hydrophobic patches. Data analysis was performed using DSF Harmonizer [MDST2, https://github.com/Pipa19/DSF-Harmonizer, (accessed on 14 July 2026)] and GraphPad Prism 10.1.1 to obtain the melting temperatures of the regulatory (*T*_m1_) and the catalytic and oligomerization (*T*_m2_) domains.

## 3. Results

Methylthioadenosine (MTA) is a naturally occurring metabolite synthesized by the consecutive activity of spermidine and spermine synthases during polyamine synthesis. In addition to its essential role as a precursor of purines and methionine through the purine and methionine salvage pathways, several studies suggest a beneficial effect of this molecule in different pathological conditions, including cancer, neurodegenerative diseases, and liver diseases [22,23,27,28,29]. To systematically investigate the mechanisms driving MTA’s effects and to identify the key proteins involved, we have analyzed the proteome changes in liver cells induced by exposure to this compound using complementary methods.

### 3.1. Proteome Rewiring Induced by Cellular Exposure to MTA

PLC cells were incubated with increasing concentrations of MTA for 24 and 48 h, and the proteomes obtained following treatment were subjected to quantitative proteomic analysis. A total of 7224 proteins were identified across all experimental conditions (Supplementary Table S1). Among these, 373 and 429 and 797 and 932 proteins were found to be differentially expressed following treatment with 10 μM and 50 μM MTA (at 24 and 48 h), following treatment with 10 μM and 50 μM MTA, respectively, which are within the therapeutic window commonly accepted for preclinical studies of drug candidates (Figure 1A,D).

Principal component analysis (PCA) performed based on the top 25% most variable proteins demonstrated a clear separation of the experimental groups. Notably, principal component 1 (PC1) primarily reflected the temporal response to MTA exposure, whereas principal component 2 (PC2) was mainly associated with MTA concentration, indicating both time- and dose-dependent effects on the cellular proteome (Figure 1B). Despite the strong discriminatory power of the differentially expressed proteins (DEPs), only a limited overlap was observed among the DEP sets identified under the different experimental conditions. Roughly 50% of the regulated proteins were unique to each condition, whereas only 18 proteins were consistently modulated across all MTA concentrations and exposure times (Figure 1C). Nevertheless, functional enrichment analysis revealed a remarkable convergence at the pathway level, with all conditions showing regulation of key cellular processes, including cell proliferation, epigenetic regulation, RNA processing and translation, proteostasis, energy and nucleotide metabolism, and extracellular matrix remodeling (Figure 1E). Consistent with the proteomic changes induced by MTA, these functional alterations also exhibited clear dose- and time-dependent patterns, indicating that MTA elicits a progressive and coordinated cellular response.

These effects are mediated by several proteins that trigger the response to MTA and delineate the final phenotype (Figure 2 and Figure S2). MTA can induce epigenetic remodeling, resulting in the early production of amphiregulin (AREG) at low MTA concentrations, leading to upregulation at 50 μM and paracrine activation of EGFR, stimulating downstream signaling (Figure 2A). Although the FC of EGFR is below the accepted threshold in this study, it has been considered based on the statistical significance and the biological relevance of its modulation. MTA induces a coordinated reprogramming of hepatocyte function by simultaneously modulating metabolic pathways, intracellular signaling, and inflammatory responses. At 10 μM, MTA suppresses key glycolytic and gluconeogenic enzymes (PFKM and PCK1) while selectively upregulating regulatory enzymes, such as PFKFB2, at both 10 and 50 μM, indicating metabolic rewiring (Figure 2B). At the signaling level, at 24 h, MTA inhibits kinase-driven pathways, including MAPK and TLR signaling (MAP2K5, MAPKAPK3/5, and IRAK1), while activating phosphatase-mediated negative regulators (PPP2R5B, PPP3CB, and PTPRF) (Figure 2C). These results are further supported by the downregulation of PTEN (Supplementary Figure S1). Additionally, MTA modulates lipid signaling via SPHK1 and promotes mitochondrial and stress adaptation pathways (FASTKD3 and STK11IP). Overall, these coordinated effects shift hepatocytes toward a low-inflammatory, metabolically restrained, and homeostatic state. Furthermore, the early regulation of TNFRSF10A, TNFAIP2, IL2RG, IL1RL2, and IL10RB reveals that MTA participates in the reprogramming of the hepatic immune response, acting as a modulator that integrates inflammatory, cytokine, and apoptotic signals to preserve cellular homeostasis (Figure 2D).

The coordinated upregulation of EGFR, IGFBP1, and IGFBP3 suggests that MTA promotes a hepatoprotective adaptive program characterized by enhanced cell survival signaling, metabolic reprogramming, and lipid homeostasis. Rather than inducing a purely proliferative response, MTA appears to establish a balanced state that integrates stress adaptation, growth control, and protection against lipotoxic and inflammatory damage, thereby contributing to the maintenance of hepatic cellular homeostasis. These observations parallel the alterations that indicate effects on cellular architecture. The coordinated upregulation of syndecans (also assessed by Western blot) and keratin family members, together with the remodeling of tubulin isoforms and the downregulation at 24 h of WASF1, suggests that MTA induces a profound reorganization of the hepatocyte cytoskeleton and cell–matrix interactions (Figure 2E and Figure S1). These changes are consistent with the establishment of a protective and structurally stabilized cellular state characterized by enhanced adhesion, reduced cytoskeletal dynamics, and increased resistance to stress. Thus, beyond its immunometabolic effects, MTA appears to act as a regulator of hepatocellular architecture and tissue homeostasis.

### 3.2. Early Events Triggered by MTA Exposure in Liver Cells

Next, we wanted to investigate the early events triggered by MTA in PLC cells that lead to the above-described expression changes. To this end, we performed a PISA assay [33] to look for proteins that change their thermal stability upon incubation with the compound, with the readout being the amount of soluble protein remaining after the thermal challenge. Ideally, the change in stability would be associated with the binding of MTA to the protein, hence enabling us to identify direct MTA targets. However, in the context of functional cells, there may be other alternative explanations behind an eventual increase or decrease in protein stability, including post-translational modifications or changes in their protein–protein interaction profiles [34]. The experiment was performed using a high MTA concentration (200 μM) to favor interactions with its protein targets, incubating for 15 and 30 min. Overall, 1781 protein groups were identified and quantified (Supplementary Table S2), resulting in 167 changes in protein stability (94 decreases and 73 increases) after 15 min and 150 changes (118 decreases and 32 increases) after 30 min (Figure 3A,B). Functional enrichment analysis revealed that the differentially expressed proteins are mainly involved in nucleotide metabolism, energy production, redox balance, RNA metabolism and protein translation, and protein homeostasis (Figure 3C).

The analysis of protein stability changes in hepatic cells following exposure to MTA for 15 and 30 min points to a dynamic and coordinated modulation of regulatory, metabolic, and structural processes. The effect evolves from an initial phase dominated by signaling and RNA regulators toward a deeper reprogramming of the cellular proteome. MTA exposure induced an increase in the stability of proteins involved in transcriptional and post-transcriptional regulation, notably GTF2F1, YBX1, ILF2, and EWSR1, together with the regulator YAP1, which could be one of the major early signaling nodes (Figure 4A). These proteins suggest that MTA can induce a rapid activation of mechanisms controlling gene expression. Concomitantly, the stability of proteins involved in the redox response, such as TXN (at 30 min) and ROMO1 (Figure S2B), increases, suggesting an early alteration of oxidative balance. Stabilization of mitochondrial proteins, including PDHX and TIMM13, is also detected, pointing to an initial adaptation of energy metabolism. In contrast, even at this early stage, a decrease in the stability of proteins involved in anabolic metabolism is observed, including HMGCS1 and MVD (lipid/cholesterol synthesis) and GART and PFAS (nucleotide synthesis), suggesting a rapid inhibition of biosynthetic pathways. Likewise, the stability of proteins associated with proteostasis and intracellular trafficking, such as UBE2V2, WDR48, and RABGGTB, as well as the autophagy regulator ATG16L1, is reduced. An early increase in the stability of proteins involved in ribosome biogenesis and translational control is observed, including NOP2, GTPBP4, RSL1D1, and YTHDF3, together with RNA-processing regulators such as STAU1 and TIA1. This pattern points to a reprogramming of post-transcriptional control, with possible activation of selective translation mechanisms. In parallel, the stability of proteins associated with nuclear regulation and chromatin dynamics, such as HCFC1 and SSRP1, as well as the signaling receptor EGFR, increases, indicating the consolidation of regulatory programs initiated during the early phase (Figure 4D). At the metabolic level, increased stability of key mitochondrial proteins is detected, including MT-ATP6, UQCRC1, COX6C, and ATP5 (transiently), as well as the enzyme GLS, suggesting a shift toward a metabolism that is more dependent on mitochondrial function. In contrast, a generalized change in the stability of proteins involved in redox homeostasis and central metabolism is observed, notably TALDO1 and IDH1, together with detoxification enzymes such as GSTP1, PRDX6, and GLO1 (Figure 4B). This pattern indicates a reduction in cellular reducing capacity. Moreover, a significant decrease is detected in key proteostasis proteins (mainly at 15 min), including chaperones such as HSP90AA1, HSP90AB1, and HSPA1A/B, and components of the ubiquitin-proteasome system, such as PSMD3, PSMD14, and UBE2D2/UBE2D3, suggesting a reduction in global protein turnover (Figure 4C). Finally, a marked reduction is observed in proteins associated with the cytoskeleton and cell adhesion, including VIM, FLNA, FSCN1, and PFN1, together with a pronounced decrease in CD44, which represents one of the most significant changes. In parallel, the stability of proteins involved in cell proliferation and nucleotide synthesis, such as PCNA (at 15 min), CTPS1, GART, and PFAS, is reduced (Figure 4D,E and Figure S2).

Taken together, these results indicate that MTA induces changes in the stability of specific proteins, which might drive a progressive response in liver cells. The mechanisms involve the early activation of gene expression regulators such as YAP1 and YBX1, followed by a reprogramming of RNA control and translation, mediated by proteins such as YTHDF3 and NOP2. These modifications define a state characterized by reduced proliferative capacity, metabolic reprogramming, and diminished structural plasticity, in good agreement with the regulatory effects induced by MTA in PLC cells after 24 and 48 h.

### 3.3. Identification of the Protein Targets of MTA in Liver Cell Extracts

We next sought to identify the direct protein targets of MTA while avoiding confounding effects arising from PTM-induced changes in thermal stability or MTA-driven alterations of the interactome in intact cells. To this end, protein extracts from PLC cells and from WT and Mdr2^−/−^ livers were incubated with MTA, and the soluble protein fraction remaining after a thermal challenge was analyzed. Protein extracts were incubated with 200 mM MTA for 15 or 30 min prior to heating. After centrifugation, 4530, 1729, and 1827 protein groups were identified in the soluble fractions from PLC cells, the WT liver, and the Mdr2^−/−^ liver, respectively. The lower number of proteins identified in liver extracts compared to cells likely reflects a lower efficiency of the extraction protocol used for tissue samples. In PLC cells, 547 and 512 differentially stable proteins were identified after 15 and 30 min of incubation with MTA, respectively. In the WT liver, 123 and 34 differential proteins were detected, whereas 19 and 28 differential proteins were identified in Mdr2^−/−^ livers at the corresponding time points (Figure 5; Supplementary Tables S3, S4, and S5, respectively).

Overall, most candidate target proteins were functionally associated with adenine nucleotide binding, cytoskeleton and microfilament organization, methyltransferase activity, calcium homeostasis, and protease activity and stability. These functional categories suggest early molecular events that may underlie the late cellular effects of MTA observed in PLC cells. Despite these shared functional features, overlap among candidate targets across experimental conditions was limited, suggesting predominantly low-affinity interactions that may contribute to variability across biological matrices.

Notably, seven proteins were consistently stabilized upon MTA incubation across all conditions, suggesting that they act as direct MTA targets (Figure 5C). Four of these candidates were expected, given their direct biochemical relationship with MTA. Spermine synthase (SMS) and spermidine synthase (SRM) catalyze polyamine biosynthesis, producing MTA as a byproduct, which in turn acts as a potent feedback inhibitor of the former and, to a lesser extent, the latter [35,36]. Methylthioadenosine phosphorylase (MTAP) catalyzes the hydrolysis of MTA into methylthioribose-1-phosphate, while adenosylhomocysteinase (AHCY) converts S-adenosylhomocysteine into L-homocysteine and adenosine, a reaction also inhibited by MTA [37].

In contrast, phenylalanine hydroxylase (PAH) was consistently stabilized by MTA, representing an unexpected finding, as no direct functional link between MTA and PAH has been previously reported, we next investigated whether MTA directly stabilizes recombinant human PAH. Using differential scanning fluorimetry (DSF), we monitored the thermal unfolding of PAH in the absence and presence of increasing MTA concentrations. In agreement with previous studies [38], PAH exhibited a biphasic unfolding profile (Figure 6A), corresponding to the sequential denaturation of the regulatory domain (RD; *T*_m1_ = 46.83 ± 0.19 °C) and the combined catalytic and oligomerization domains (CD+OD; *T*_m2_ = 59.70 ± 0.56 °C). MTA increased *T*_m2_ in a concentration-dependent manner by up to approximately 3 °C (Figure 6A,B), while having no significant effect on *T*_m1_. These findings indicate that MTA interacts directly with PAH and primarily stabilizes the CD+OD region of the protein.

In summary, incubation of protein extracts from PLC cells and liver samples from WT and Mdr2^−/−^ mice enabled the identification of established MTA binders, as well as additional protein targets whose modulation may contribute to both early molecular events and downstream cellular effects. Importantly, we also identified PAH as an unexpected MTA target, highlighting a potential new therapeutic application that warrants further investigation.

## 4. Discussion

PFIC3 is a severe chronic liver disease characterized by a strong inflammatory component [39]. In this context, essential hepatocyte processes are significantly regulated, leading to a reconfiguration of liver homeostasis [16]. Among these alterations, metabolic rewiring emerges as a key mechanism driving disease progression, particularly affecting one-carbon metabolism (OCM). OCM constitutes a critical link between intermediate metabolism and epigenetic regulation [18] and contributes to maintaining the quiescent, differentiated phenotype of hepatocytes [40,41,42,43]. The hepatoprotective properties of several OCM-related metabolites have been investigated, including S-adenosylmethionine (SAMe) [24,25] and methylthioadenosine (MTA) [26]. To further elucidate the molecular mechanisms underlying the proposed pharmacological effects of MTA in the liver, we performed a systematic analysis of the cellular response to MTA, focusing on proteome dynamics.

The PLC cellular proteome undergoes a marked dose- and time-dependent remodeling following MTA exposure, with significant effects at 10 mM, supporting its pharmacological relevance. By integrating thermal proteome profiling with longitudinal proteomic analyses, our approach enabled the identification of both direct MTA-interacting proteins and the early molecular events that precede the extensive proteome rewiring observed after 24 and 48 h of treatment. Changes in protein thermal stability likely reflect direct ligand engagement or alterations in protein PTM profiles or complex assembly, thereby providing mechanistic insights into the primary molecular interactions driving downstream cellular responses.

A prominent finding was the stabilization of multiple methyltransferases, including PRMT5, PRMT9, HNMT, TPMT, INMT, and PNPT1, together with the methylosome component WDR77, following the incubation of protein extracts derived from PLC cells, as well as WT and Mdr2^−/−^ mouse livers, with MTA. These observations are consistent with previous biochemical and structural studies identifying MTA as a competitive inhibitor or regulatory ligand of several SAM-dependent methyltransferases, including PRMT5 and WDR77 [44]. Given the central role of these enzymes in the transfer of methyl groups to protein, RNA, and DNA substrates, their stabilization by MTA is expected to induce widespread alterations in cellular methylation networks. Consistent with this hypothesis, previous quantitative methyl-proteomics studies demonstrated that MTA exposure profoundly modifies protein methylation patterns [45], potentially affecting chromatin organization, epigenetic regulation, RNA processing, and signal transduction pathways.

In addition to methyltransferases, MTA induced the stabilization of several ATP- and adenosine nucleotide-binding proteins, including IFI35, MAPK family members, CDK2, IRAK4, BLVRB, and ADK. The effect of MTA on proteins suggests that its biological activity may involve the modulation of kinase-dependent signaling cascades, nucleotide metabolism, and inflammatory responses. In particular, the engagement of proteins such as IRAK4 and MAPKs provides a plausible mechanistic link between MTA exposure and the anti-inflammatory and cytoprotective effects reported in different experimental systems [27,28]. Moreover, modulation of ADK activity could influence intracellular adenosine homeostasis, further amplifying downstream effects on energy metabolism and signaling [46].

The stabilization of numerous RNA-binding proteins, including multiple ribosomal subunits and translation-associated factors, points to an early and coordinated regulation of RNA metabolism. This observation is particularly noteworthy given that several RNA-binding proteins contain established or putative arginine methylation sites that regulate RNA–protein interactions, ribonucleoprotein complex assembly, and translational efficiency [47]. The convergence of thermal stabilization signatures on proteins involved in ribosome function, RNA processing, and protein synthesis is consistent with previous reports indicating that MTA rapidly impacts translational programs and post-transcriptional regulatory networks [45,48]. These findings support the existence of an early adaptive response aimed at reshaping cellular gene expression before overt proteome remodeling becomes detectable.

MTA might also regulate proteostasis, as suggested by the stabilization of the molecular chaperone PFDN6 and several components of the ubiquitin-conjugation machinery, including UBE2O, UBE2N, UBE2L3, and ISG15. The engagement of these proteins suggests an early impact of MTA on protein quality control pathways, encompassing both protein folding and selective protein degradation. Since ubiquitination orchestrates the turnover of damaged proteins and regulates multiple signaling pathways [49], MTA-dependent stabilization of E2 ubiquitin-conjugating enzymes could contribute to the extensive proteomic adaptations observed at longer MTA incubation times.

Finally, MTA stabilized multiple proteins involved in the maintenance of epithelial architecture, cellular polarity, and cytoskeletal organization, including keratins, DSP, PKP1, and WASF2. These interactions suggest that MTA could rapidly influence structural and mechanical features of the cell, potentially affecting cell–cell adhesion, cytoskeletal dynamics, and tissue organization. Such remodeling may constitute an early adaptive mechanism that enhances cellular resilience under stress conditions and precedes the activation of survival and repair programs [50]. Collectively, these data support a model in which MTA acts as a pleiotropic metabolic regulator that simultaneously targets methylation-dependent processes, RNA metabolism, proteostasis networks, signaling pathways, and cytoskeletal organization.

Exposure of PLC cells to methylthioadenosine (MTA) induces the coordinated stabilization of a set of proteins involved in cytokine signaling, inflammatory responses, and the control of apoptosis, including TNFRSF10A, TNFAIP2, IL2RG, IL1RL2, and IL10RB. The combined analysis of these factors suggests that MTA does not act on proteins of a single molecular axis but rather reorganizes a complex functional network that integrates pro- and anti-inflammatory signals with mechanisms of cell survival [51]. TNFAIP2, a TNFα-inducible gene, acts as a marker of early inflammatory activation [52], whereas IL1RL2 (the IL-36 receptor) amplifies pro-inflammatory signals through the NF-κB and MAPK pathways [53]. The alteration of these components in the presence of MTA suggests that this molecule regulates the intensity of the hepatic inflammatory response, potentially limiting the excessive activation of pro-inflammatory pathways. Complementarily, the stabilization of IL2RG and IL10RB suggests a reorganization of cytokine-dependent signaling through the JAK/STAT pathway. IL2RG, the common γ-chain shared by multiple interleukin receptors, is associated with proliferative and pro-inflammatory signaling [54], whereas IL10RB participates in IL-10 signaling, a key cytokine involved in the resolution of inflammation and liver injury repair [55]. The simultaneous stabilization and potential modulation of both subunits suggest that MTA contributes to establishing a dynamic balance between immune activation and anti-inflammatory mechanisms, thereby promoting a homeostatic state in hepatocytes. On the other hand, the stabilization of TNFRSF10A (DR4) introduces an additional component related to the control of apoptosis. This receptor, activated by TRAIL, plays a fundamental role in the elimination of damaged or stressed cells and has been proposed to have positive effects in cholestatic liver disease [56]. Taken together, these results support the hypothesis that MTA might act as a key immunometabolic regulator in hepatocytes, capable of coordinating three fundamental processes: (i) the activation of NF-κB-mediated inflammatory responses, (ii) the balance of cytokine signaling through the JAK/STAT pathway, and (iii) the control of apoptosis through TNF-family receptors.

The proteome remodeling in PLC cells after 24–48 h suggests that exposure to MTA induces a coordinated cellular reprogramming program encompassing metabolism, signaling, structural organization, and immunomodulation. Within this context, the identification of amphiregulin (AREG) as a differentially expressed protein emerges as a key element for the mechanistic interpretation of this response. AREG, a ligand of the epidermal growth factor receptor (EGFR), has been widely implicated in liver regeneration, cell survival [57], and adaptation to cholestatic liver injury [58]. From a mechanistic perspective, we propose that MTA initially acts as a metabolic signal that alters the balance of the methionine cycle and the availability of methyl-group donors. This shift would affect epigenetic regulation, as suggested by the observed modulation of histone methyltransferases and demethylases, as well as chromatin remodeling components (e.g., INO80 and BRG1). This reconfigured epigenetic state would favor the expression of early-response genes, including AREG. Once induced, AREG could act in an autocrine or paracrine manner by activating EGFR in hepatocytes. Once activated by the ligand, EGFR is internalized and targeted for lysosomal degradation to turn off growth signals. The increase in amphiregulin can also be associated with the early and transient upregulation of TGFβ-1 [59]. Activation of EGFR and TGFβ-1 can act in a coordinated manner, mediating a wound-healing-like process. These regulatory mechanisms are likely mediated by an increase in stonin proteins, which serve as key adaptors in the intracellular trafficking machinery, facilitating the clathrin-mediated internalization of membrane receptors [60]. The induction of AREG may not only activate canonical EGFR signaling but could also cooperate with semaphorin-dependent pathways through shared regulation of Rho-family GTPases, cytoskeletal remodeling, and extracellular matrix dynamics. Such crosstalk may contribute to the phenotypic plasticity and adaptive remodeling observed in MTA-treated PLC cells. Activation of this pathway is consistent with multiple modules detected in the proteome, including MAPK signaling components (BRAF and MAPKAPK) and regulators of small GTPases (RhoGAPs and chimaerins). This network suggests that the AREG/EGFR axis functions as a central amplifier that translates the initial metabolic signal into a multifaceted cellular response. Paralleling the findings from the PISA assay, one of the most prominent effects of this axis is metabolic reprogramming. The presence of glucose transporters (GLUT2/3/6) and key regulatory enzymes of glycolysis (PFKFB1/2) and gluconeogenesis (PEPCK), together with multiple mitochondrial proteins, indicates a dynamic adjustment of energy metabolism. In parallel, the regulation of lipid-metabolizing enzymes (ceramide synthase and sphingosine kinase) suggests a role for bioactive lipids in integrating survival and stress-response signals. Furthermore, the strong representation of membrane proteoglycans (syndecans and glypicans), together with extracellular matrix components (fibronectin, thrombospondin, and nidogen), indicates that activation of the AREG–EGFR axis is also associated with remodeling of the cellular microenvironment. In parallel, modulation of immune-related pathways is observed, including TGF-β1, interleukin receptors, and chemokines such as CXCL16. This is consistent with the reported role of AREG in promoting tissue-repair states characterized by controlled inflammation [61], suggesting that MTA may induce a more tolerogenic hepatocyte phenotype.

Despite the results discussed above, we acknowledge several limitations of this study. The use of cellular models provides a feasible approach to disentangle the molecular mechanisms underlying the key proteins driving biological effects. However, cell lines differ from primary cells in several respects and lack the physiological context of the whole organism. In addition, although proteomic analyses offer a detailed snapshot of the proteome changes that shape the cellular response to MTA, encompassing both initiating events and compensatory adaptations, distinguishing between these different layers of molecular regulation and understanding how they converge to produce the final phenotype remains challenging. It must also be noted that changes in the stability of proteins induced by MTA might result from direct binding, alteration of the interaction pattern with other biomolecules, or changes in PTM profiles. While the change in stability can be considered as a readout of the regulation of the protein in all cases, further support would be needed to assess direct binding for PAH, AHCY, MTAP, WDR77, TPMT, SRM, and SMS. Therefore, further functional validation in primary cells and ultimately in whole-organism models is required to confirm the biological relevance of these findings.

## 5. Conclusions

Taken together, we propose a model in which MTA induces a hepatocellular “rewiring” state through the activation of AREG as a central regulatory node. This axis links metabolic cues with signaling, epigenetic, and structural remodeling programs, thereby promoting an adaptive phenotype characterized by metabolic plasticity, microenvironmental reorganization, and immunomodulation. These findings provide a strong molecular framework for understanding the hepatic response to MTA and offer valuable insights into the mechanisms underlying its beneficial effects. Moreover, they establish a promising basis for further exploring the therapeutic potential of this compound and its possible applications in patient care.

## Figures and Tables

**Figure 1:**
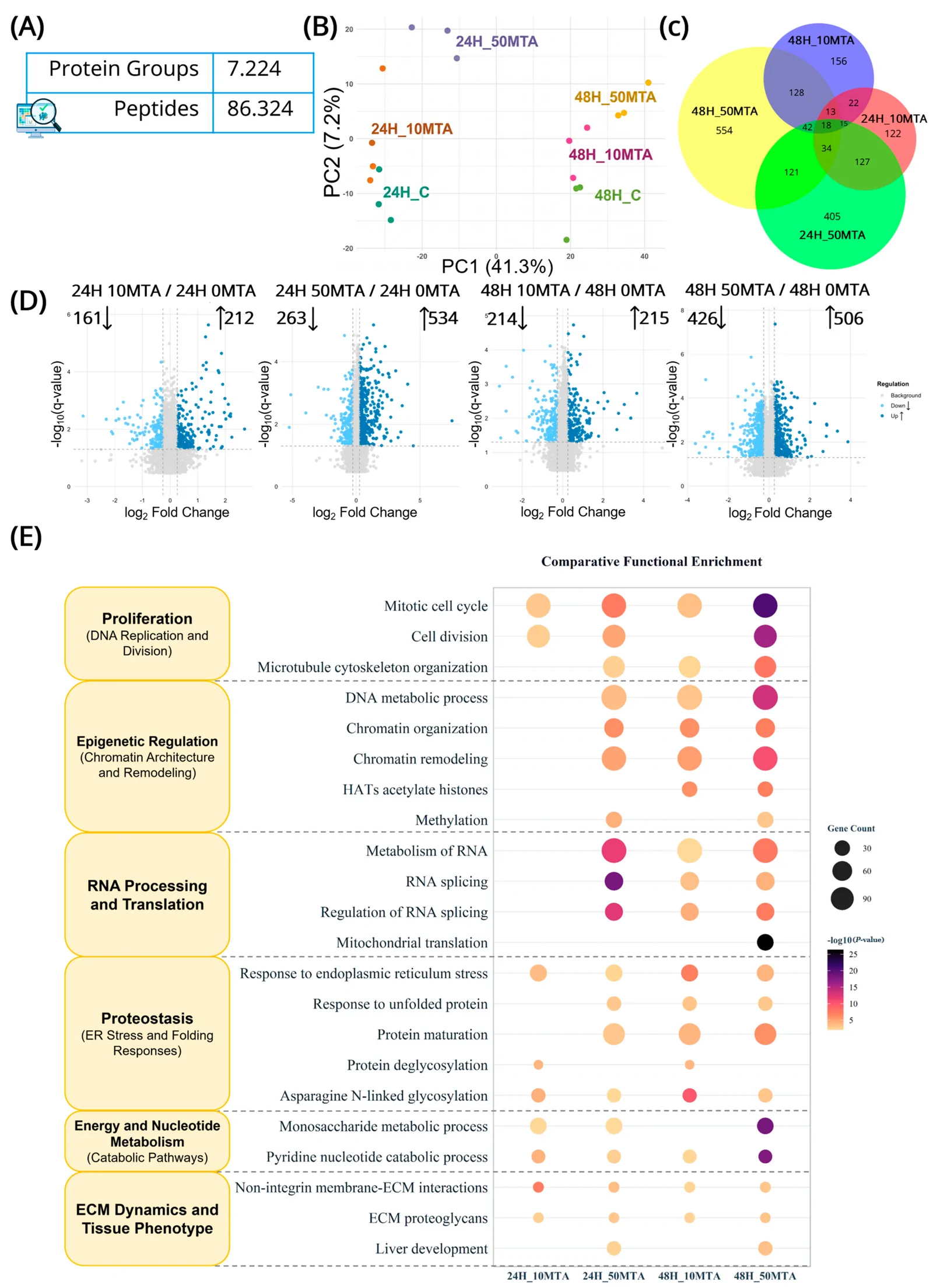
Dose- and time-dependent proteome rewiring induced by exposure to MTA in PLC cells. (**A**) Summary of the total number of identified and quantified protein groups and peptides across all experimental conditions. (**B**) Principal component analysis (PCA) showing the separation of samples according to treatment and time point. (**C**) Venn diagram showing the overlap of differentially expressed proteins across the studied comparisons. (**D**) Volcano plots illustrating the statistical significance (−log_10_(q-value)) versus magnitude of change (log_2_FC) across the four experimental comparisons. Significantly regulated proteins (thresholds: *q* < 0.05 and |log_2_FC| > 0.26) are highlighted as indicated in the embedded legends. (**E**) Comparative functional enrichment analysis of the significantly altered proteins across the four comparisons. Specific enriched pathway terms were collapsed into broader biological categories to highlight the major functional shifts. Statistical significance in (**D**) was determined using an unpaired two-tailed Student’s *t*-test.

**Figure 2:**
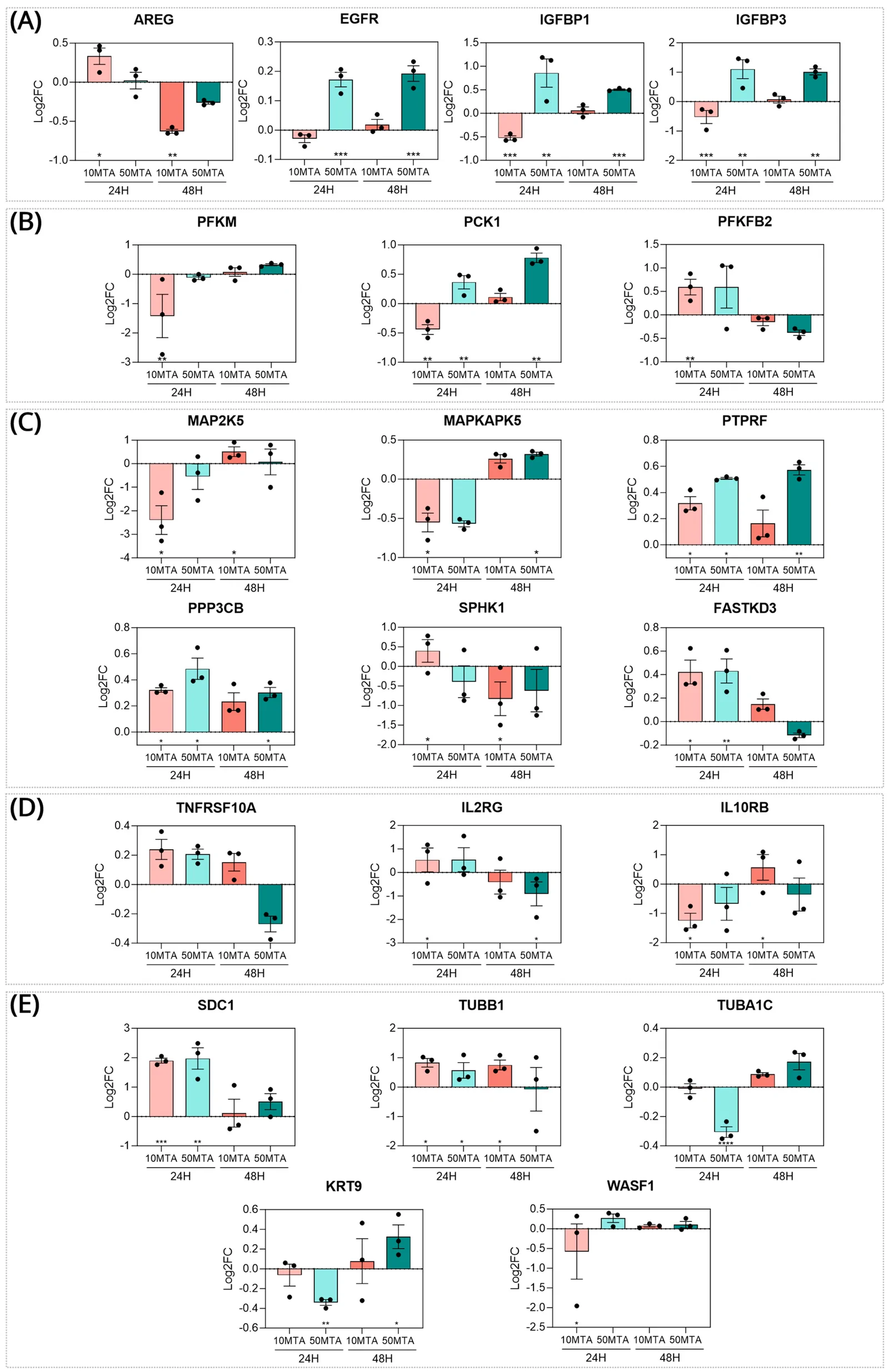
Differential expression of key functional protein clusters across 10 µM and 50 µM MTA treatments at 24 and 48 h. Bar plots represent the log_2_ fold change (log_2_FC) of selected differentially expressed proteins, calculated as the log_2_ ratio of protein abundance in each indicated treatment condition relative to its respective control (MTA-treated/control). Proteins are grouped by biological function to illustrate pathway-specific responses: (**A**) growth factors and receptors, (**B**) metabolism, (**C**) intracellular signaling, (**D**) cytokine signaling and inflammatory response, and (**E**) extracellular matrix and cytoskeleton. Error bars indicate SEM across 3 biological replicates. Statistical significance was determined by Student’s *t*-test (* q < 0.05, ** q < 0.01, *** q < 0.001, and **** q < 0.0001).

**Figure 3:**
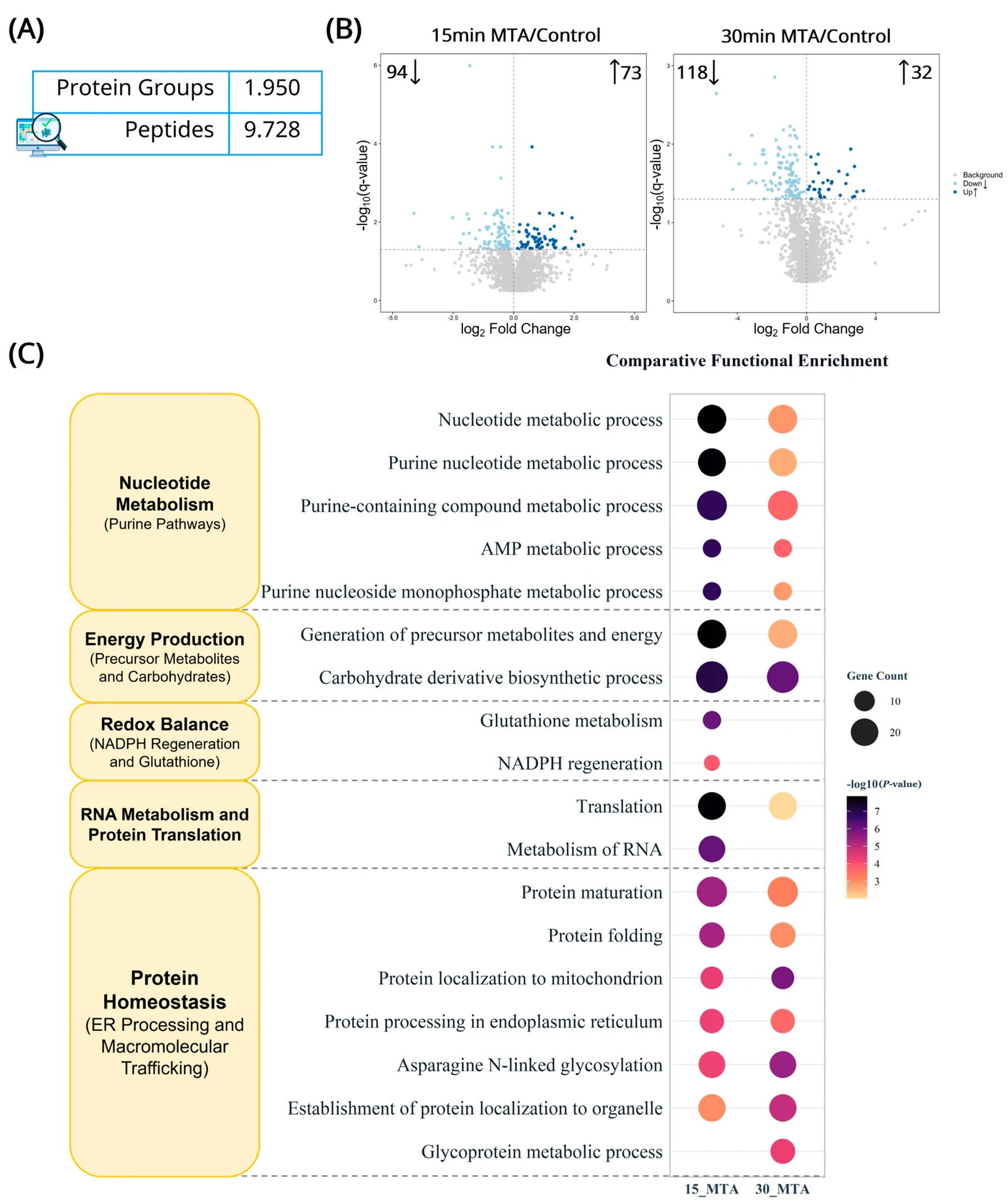
Early proteome stability events triggered by MTA exposure in intact PLC cells. (**A**) Summary of the total number of identified and quantified protein groups and peptides across the experimental conditions. (**B**) Volcano plots illustrating the statistical significance (−log10(q-value)) versus the magnitude of stability change (log_2_FC) between MTA-treated and control cells. Proteins exhibiting statistically significant shifts in stability (threshold: q ≤ 0.05) are highlighted as indicated in the embedded legends. (**C**) Comparative functional enrichment analysis of proteins showing significant stability changes. Specific enriched pathways and Gene Ontology (GO) terms were collapsed into broader biological categories to highlight the major functional networks engaged during these early cellular events. Statistical significance in (**B**) was determined using an unpaired two-tailed Student’s *t*-test.

**Figure 4:**
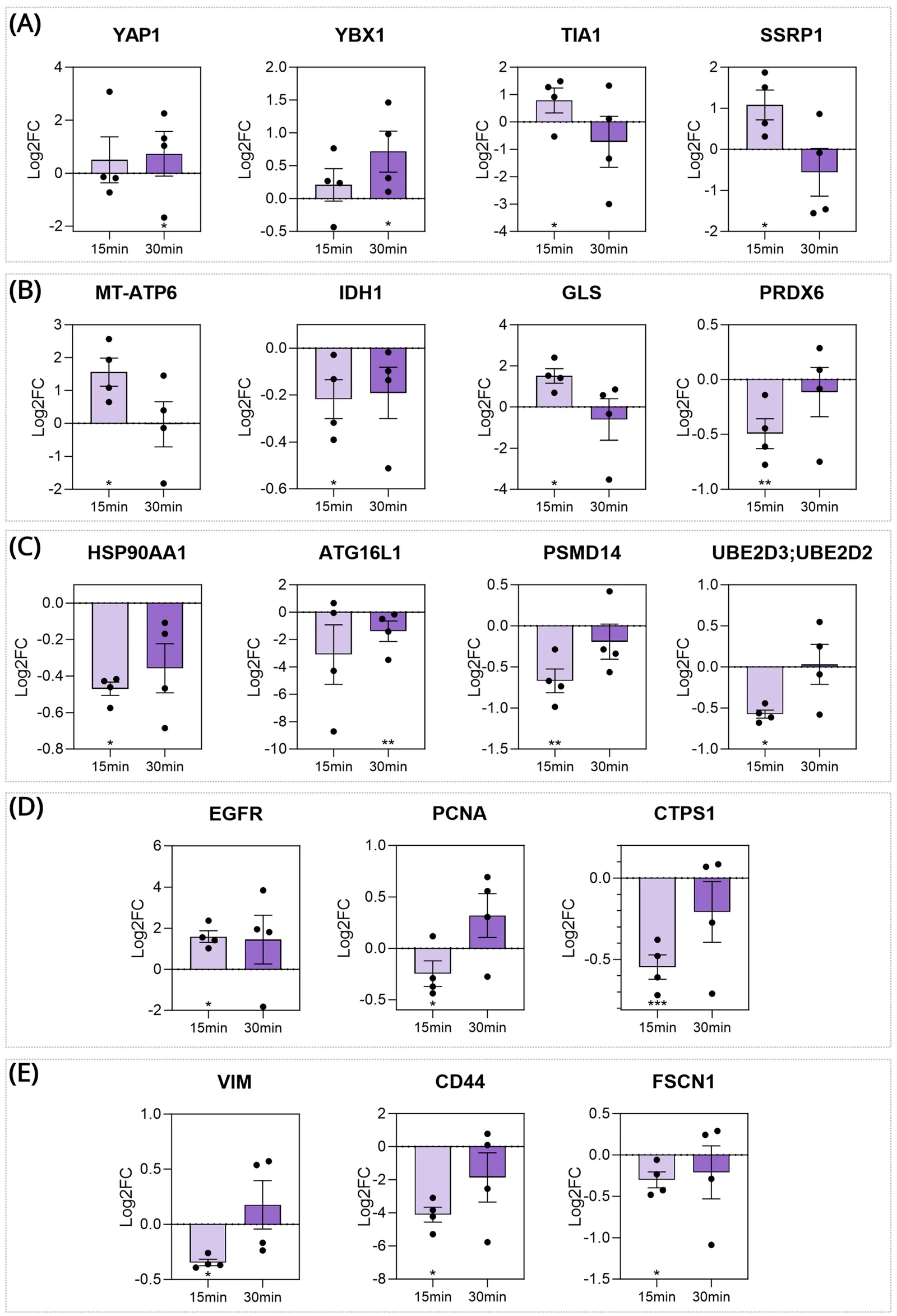
Functional clustering of proteins exhibiting different stability following short-term treatment in PLC cells with 200 µM MTA. Bar charts display the log_2_ fold change (log_2_FC) representing the relative stability of a representative subset of proteins that showed statistically significant differences at 15 and 30 min. The log_2_FC is defined as the log_2_ ratio of soluble abundance in each treated sample compared to its corresponding control. Proteins are clustered into five major functional domains: (**A**) gene regulation and RNA processing; (**B**) metabolism, redox, and mitochondrial function; (**C**) proteostasis and degradation; (**D**) cell signaling and proliferation; and (**E**) cytoskeleton and cell adhesion. Error bars represent the SEM of 4 biological replicates. Statistical significance of stability changes was determined by a Student’s *t*-test (* q < 0.05, ** q < 0.01, and *** q < 0.001).

**Figure 5:**
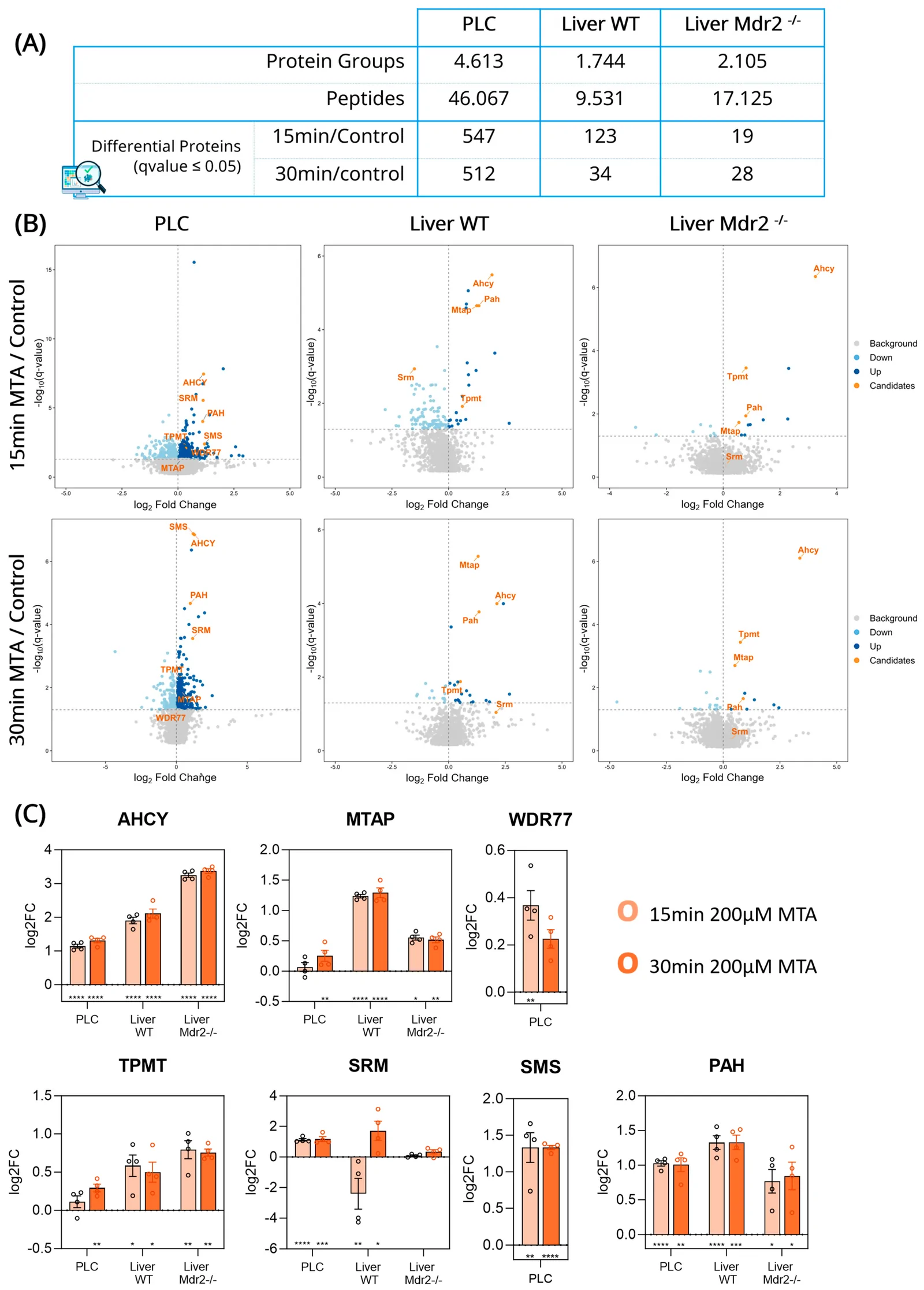
Identification of direct protein targets of MTA in liver cell and tissue extracts. (**A**) Summary of the total number of identified and quantified protein groups and peptides across the different biological systems analyzed (PLC cell extracts, wild-type (WT) mouse liver tissue, and Mdr2^−/−^ mouse liver tissue). The number of proteins exhibiting statistically significant stability changes (q-value ≤ 0.05) in each comparative analysis is also indicated. (**B**) Volcano plots illustrating the statistical significance (−log_10_(q-value)) versus the magnitude of stability shift (log_2_FC) induced by MTA treatment in the extracts. Candidate direct protein targets of MTA are highlighted as indicated in the embedded legends. (**C**) Bar charts representing the relative soluble abundance (log_2_FC) of the selected candidate target proteins following the PISA assay, indicative of their altered stability across all experimental conditions and biological models. Error bars represent the SEM of 4 independent replicates. Statistical significance for the candidate targets in (**B**,**C**) was determined using an unpaired two-tailed Student’s *t*-test (* q < 0.05, ** q < 0.01, and *** q < 0.001, **** q < 0.0001).

**Figure 6:**
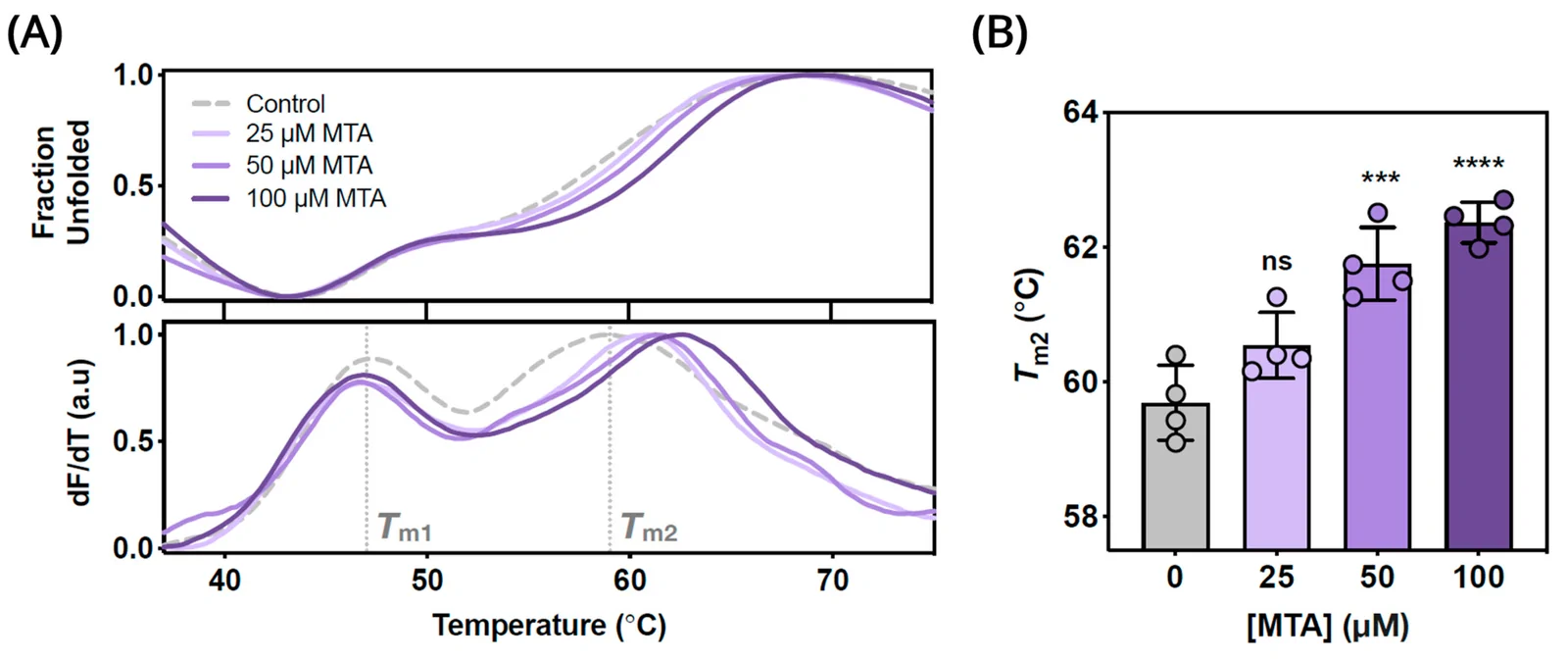
Differential scanning fluorimetry (DSF)-monitored thermal unfolding of PAH in the presence of varying MTA concentrations. (**A**) Representative thermograms for PAH (1.92 µM subunit) alone (gray stippled line) or in the presence of 25, 50, or 100 μM MTA (light to dark violet lines). PAH displays a biphasic transition of unfolding (*T*_m1_ and *T*_m2_; gray dotted lines), corresponding to the sequential denaturation of the regulatory domains (*T*_m1_) and the catalytic and oligomerization domains (CD+OD; *T*_m2_). MTA increases the *T*_m2_ of PAH in a dose-dependent manner, indicating the binding and stabilizing effect of MTA on PAH-CD+OD. (**B**) Concentration-dependent stabilization of PAH-CD+OD by MTA. The thermal unfolding of PAH-CD+OD at varying MTA concentrations (0–100 µM) was monitored by following the second melting temperature transition (*T*_m2_) of PAH. Data shown represent the mean ± SD for four replicate samples. The *T*_m2_ of PAH at varying MTA concentrations was compared to that recorded in the absence of MTA using one-way ANOVA and Tukey’s post hoc HSD test (*** *p* < 0.003; **** *p* < 0.0001).

**Table 1:** Antibodies used for Western blot analysis.

Antibodies	Dilution	Host	Ref	Company
Anti-CD138 (SDC1)	1/125	Rabbit	36-2900	Invitrogen (Allentown, PA, USA)
Anti-PTEN	1/1000	Mouse	14642	Cell Signaling (Danvers, MA, USA)
Anti-GAPDH	1/2000	Mouse	ab8245	Abcam (Cambridge, UK)
Goat Anti-Rabbit IgG (HRP)	1/2000	Goat	P0448	DAKO (Glostrup, Denmark)
Goat Anti-Mouse IgG (HRP)	1/2000	Goat	P0447	DAKO

## Data Availability

The mass spectrometry proteomics data have been deposited in the ProteomeXchange Consortium via the jPOST partner repository, with the dataset identifier PXD082684 (jPOST ID: JPST004847).

## Supplementary Materials

The following supporting information can be downloaded at: https://www.mdpi.com/article/10.3390/cells15191720/s1, Figure S1. Altered stability and validation of key functional protein clusters following MTA treatment in PLC cells. (A–C) Bar charts represent the relative soluble abundance (log_2_FC) of selected proteins after the PISA assay, indicative of their altered stability. Proteins are grouped by biological function to illustrate pathway-specific responses: (A) Intracellular Signaling, (B) Cytokine Signaling and Inflammatory Response, and (C) Extracellular Matrix and Cytoskeleton. (D) Quantification of protein expression by Western blot. Band intensities were quantified by densitometry and normalized to GAPDH (for SDC1) or total protein levels using Ponceau S staining (for PTEN). Normalized values were then expressed relative to the respective experimental control and plotted as log_2_ fold change (log_2_FC). The horizontal dashed line at y = 0 indicates the experimental control baseline level. All data are presented as mean ± SEM from three independent replicates per condition (n = 3). Statistical significance was assessed using an unpaired two-tailed Student’s *t*-test (* p/q ≤ 0.05, ** q ≤ 0.01, *** q ≤ 0.001). Figure S2. Functional clustering of proteins exhibiting differential stability following short-term treatment in PLC cells with 200µM MTA. Bar charts display the log2 fold change (log2FC) representing the relative stability of a representative subset of proteins that showed statistically significant differences between 15min/Control and 30min/Control. Proteins are clustered into five major functional domains: (A) Gene Regulation and RNA Processing; (B) Metabolism, Redox and Mitochondrial Function; (C) Proteostasis and Degradation; (D) Cell Signaling and Proliferation; (E) Cytoskeleton and Cell Adhesion. Error bars represent SEM of 4 biological replicates. Statistical significance of stability changes was determined by by Student’s *t*-test (* q ≤ 0.05, ** q ≤ 0.01, *** 1 ≤ 0.001). Figure S3. Uncropped Western blot membranes. Full-length immunoblots corresponding to the representative cropped panels shown in Figure S1. Membranes display the expression levels of (A) SDC1 and (B) PTEN and the loading controls (GAPDH or Ponceau S) in PLC cells following MTA treatment. As shown in the lanes, each experimental condition includes three independent biological replicates (n = 3). Molecular weight markers (kDa) are indicated on the left side of each membrane. Table S1. Quantitative and statistical data for the proteomic analysis of PLC cells treated with 10µM and 50µM MTA for 24 and 48H. The table provides detailed quantitative and statistical metrics for all identified protein groups across the analyzed comparisons. Columns denote the specific comparison (group1/group2), protein group IDs, average log2 fold change (AVG Log2 FC), unadjusted pvalue, adjusted pvalue (qvalue), corresponding gene names, protein descriptions and names, number of unique total peptides, and linear fold change (FC). Rows highlighted in orange indicate statistically significant differentially expressed proteins (DEPs), defined by a qvalue ≤ 0.05 and an absolute fold change threshold of |log2FC| ≥ 0.26. Table S2. Quantitative and statistical data for the intact-cell PISA assay of PLC cells following short-term treatment with 200 µM MTA. The table provides detailed quantitative and statistical metrics for all identified protein groups across the analyzed comparisons. Columns denote the specific comparison (group1/group2), protein group IDs, average log2 fold change (AVG Log2 FC), unadjusted pvalue, adjusted pvalue (qvalue), corresponding gene names, protein descriptions and names, number of unique total peptides, and linear fold change (FC). Rows highlighted in orange indicate statistically significant differentially expressed proteins (DEPs), defined by a qvalue ≤ 0.05. Table S3. Quantitative and statistical data for the PISA assay in PLC cell extracts following short-term treatment with 200 µM MTA. The table provides detailed quantitative and statistical metrics for all identified protein groups across the analyzed comparisons. Columns denote the specific comparison (group1/group2), protein group IDs, average log2 fold change (AVG Log2 FC), unadjusted pvalue, adjusted pvalue (qvalue), corresponding gene names, protein descriptions and names, number of unique total peptides, and linear fold change (FC). Rows highlighted in orange indicate statistically significant differentially expressed proteins (DEPs), defined by a qvalue ≤ 0.05. Table S4. Quantitative and statistical data for the PISA assay in wild-type (WT) mouse liver tissue extracts following short-term treatment with 200 µM MTA. The table provides detailed quantitative and statistical metrics for all identified protein groups across the analyzed comparisons. Columns denote the specific comparison (group1/group2), protein group IDs, average log2 fold change (AVG Log2 FC), unadjusted pvalue, adjusted pvalue (qvalue), corresponding gene names, protein descriptions and names, number of unique total peptides, and linear fold change (FC). Rows highlighted in orange indicate statistically significant differentially expressed proteins (DEPs), defined by a qvalue ≤ 0.05. Table S5. Quantitative and statistical data for the PISA assay in Mdr2-/- mouse liver tissue extracts following short-term treatment with 200 µM MTA. The table provides detailed quantitative and statistical metrics for all identified protein groups across the analyzed comparisons. Columns denote the specific comparison (group1/group2), protein group IDs, average log2 fold change (AVG Log2 FC), unadjusted pvalue, adjusted pvalue (qvalue), corresponding gene names, protein descriptions and names, number of unique total peptides, and linear fold change (FC). Rows highlighted in orange indicate statistically significant differentially expressed proteins (DEPs), defined by a qvalue ≤ 0.05.
