# Supplementary material for: Decoding the Cellular Response to Methylthioadenosine Uncovers Novel Targets for Chronic Liver Disease
Source: Cells. 2026 Sep 22;15(19):1720. doi: 10.3390/cells15191720 (PMC13664110; doi:10.3390/cells15191720)
Supplement: Supplementary file 1 [file cells-15-01720-s001.zip › SupplementaryFigures R1 01.pdf]

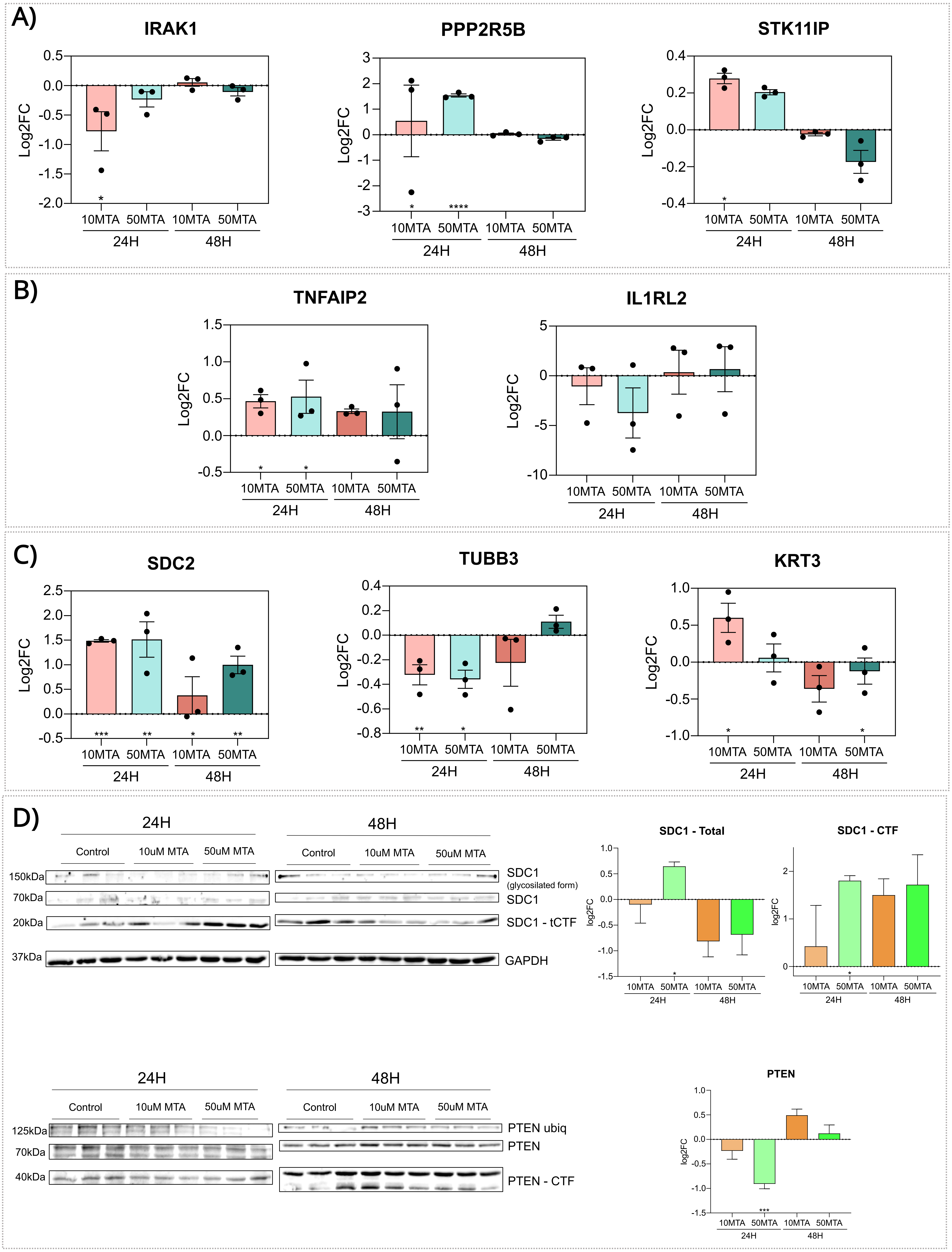
A)
IRAK1
PPP2R5B
STK11IP
B)
TNFAIP2
IL1RL2
C)
SDC2
TUBB3
KRT3
Log2FC
10MTA 50MTA 10MTA 50MTA
24H
48H
D)
24H
48H
Control
10uM MTA
50uM MTA
150kDa
70kDa
20kDa
37kDa
SDC1
(glycosilated form)
SDC1
SDC1 - tCTF
GAPDH
SDC1 - Total
SDC1 - CTF
log2FC
125kDa
70kDa
40kDa
PTEN ubiq
PTEN
PTEN - CTF
PTEN

Figure S1. Altered stability and validation of key functional protein clusters following MTA treatment in PLC cells. (A-C) Bar charts represent the relative soluble abundance (log_2FC) of selected proteins after the PISA assay, indicative of their altered stability. Proteins are grouped by biological function to illustrate pathway-specific responses: (A) Intracellular Signaling, (B) Cytokine Signaling and Inflammatory Response, and (C) Extracellular Matrix and Cytoskeleton. (D) Quantification of protein expression by Western blot. Band intensities were quantified by densitometry and normalized to GAPDH (for SDC1) or total protein levels using Ponceau S staining (for PTEN). Normalized values were then expressed relative to the respective experimental control and plotted as log_2 fold change (log_2FC). The horizontal dashed line at y = 0 indicates the experimental control baseline level. All data are presented as mean ± SEM from three independent replicates per condition (n = 3). Statistical significance was assessed using an unpaired two-tailed Student's t-test (* $p/q \leq 0.05$, ** $q \leq 0.01$, *** $q \leq 0.001$).

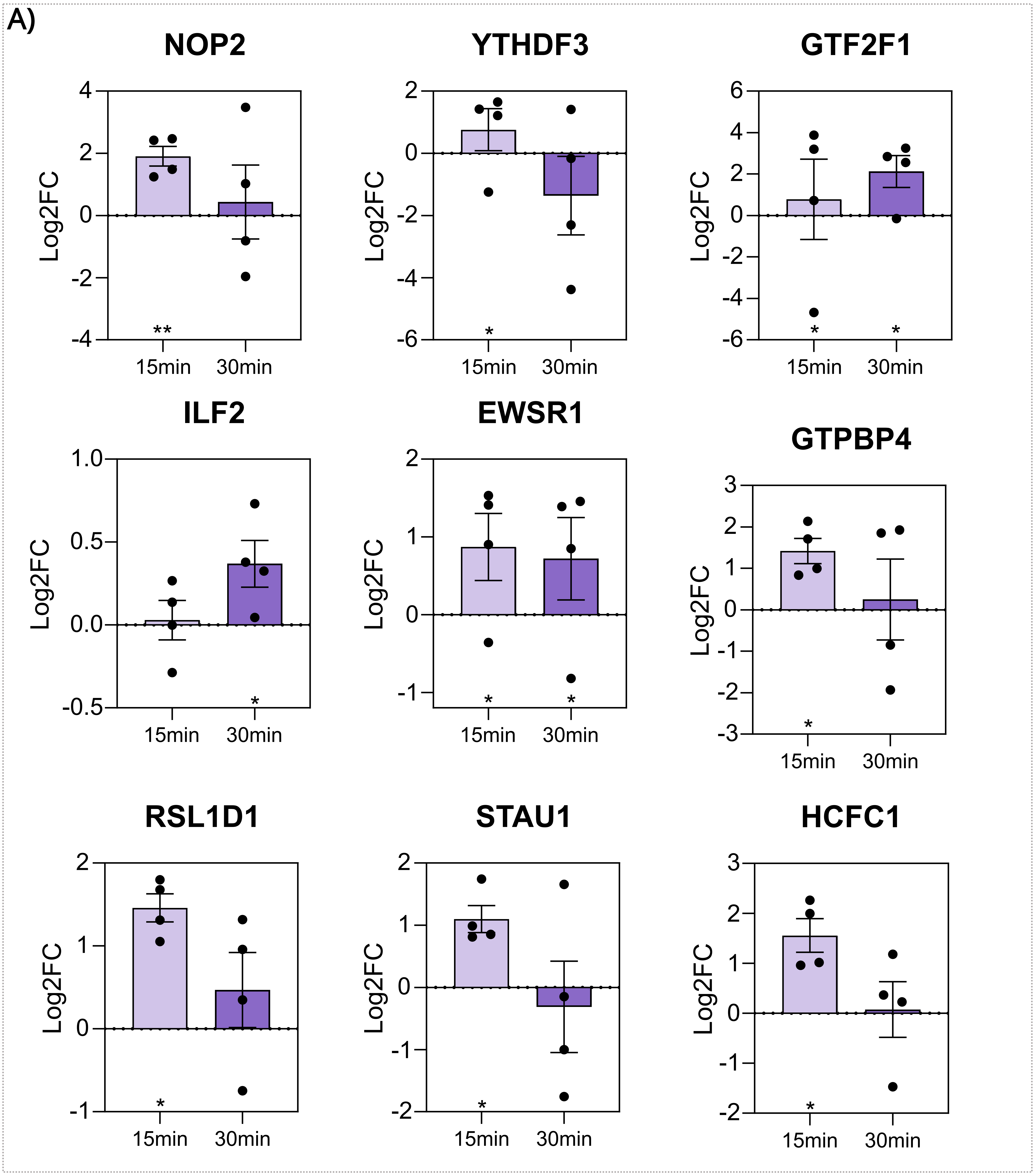
A)
NOP2
YTHDF3
GTF2F1
ILF2
EWSR1
GTPBP4
RSL1D1
STAU1
HCFC1
Log2FC
15min
30min
**
*

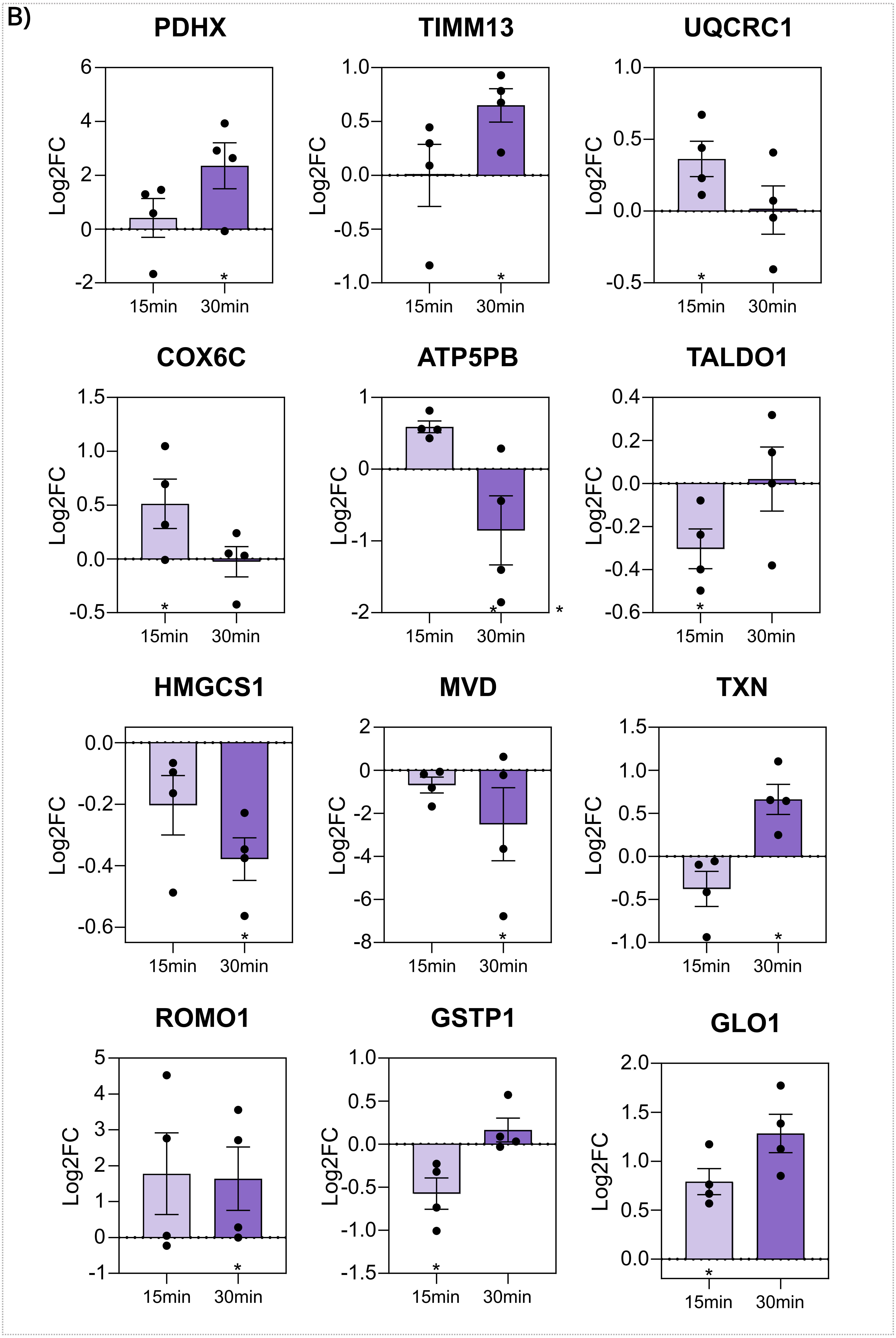
B)
PDHX
TIMM13
UQCRC1
COX6C
ATP5PB
TALDO1
HMGCS1
MVD
TXN
ROMO1
GSTP1
GLO1
Log2FC
15min
30min
*

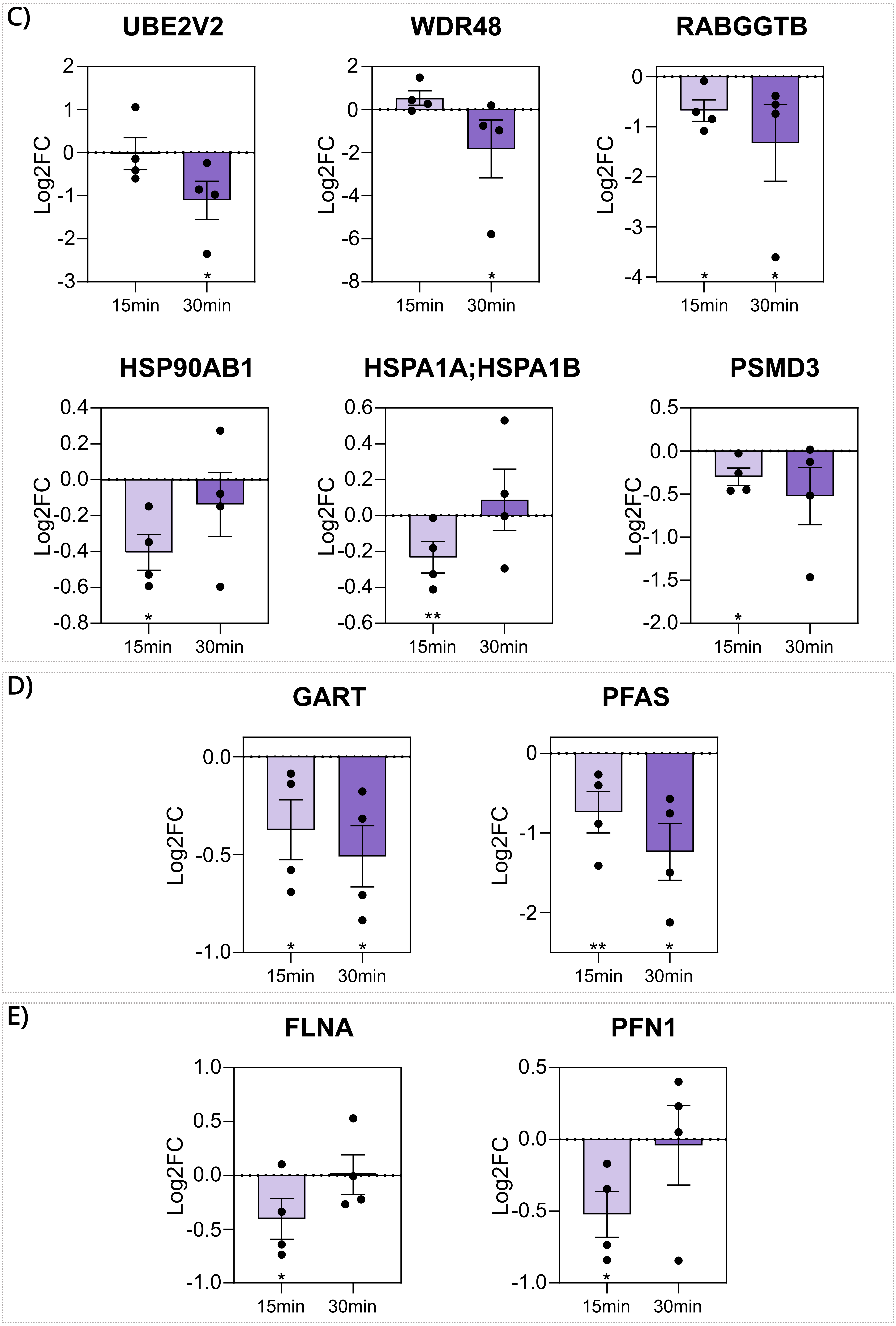

C)
UBE2V2
WDR48
RABGGTB
HSP90AB1
HSPA1A;HSPA1B
PSMD3
D)
GART
PFAS
E)
FLNA
PFN1
Log2FC
15min
30min
*
**

Figure S2. Functional clustering of proteins exhibiting differential stability following short-term treatment in PLC cells with 200µM MTA. Bar charts display the log2 fold change (log2FC) representing the relative stability of a representative subset of proteins that showed statistically significant differences between 15min/Control and 30min/Control. Proteins are clustered into five major functional domains: **(A)** Gene Regulation and RNA Processing; **(B)** Metabolism, Redox and Mitochondrial Function; **(C)** Proteostasis and Degradation; **(D)** Cell Signaling and Proliferation; **(E)** Cytoskeleton and Cell Adhesion. Error bars represent SEM of 4 biological replicates. Statistical significance of stability changes was determined by by Student's t-test (*q ≤ 0.05, **q ≤ 0.01, ***1 ≤ 0.001).

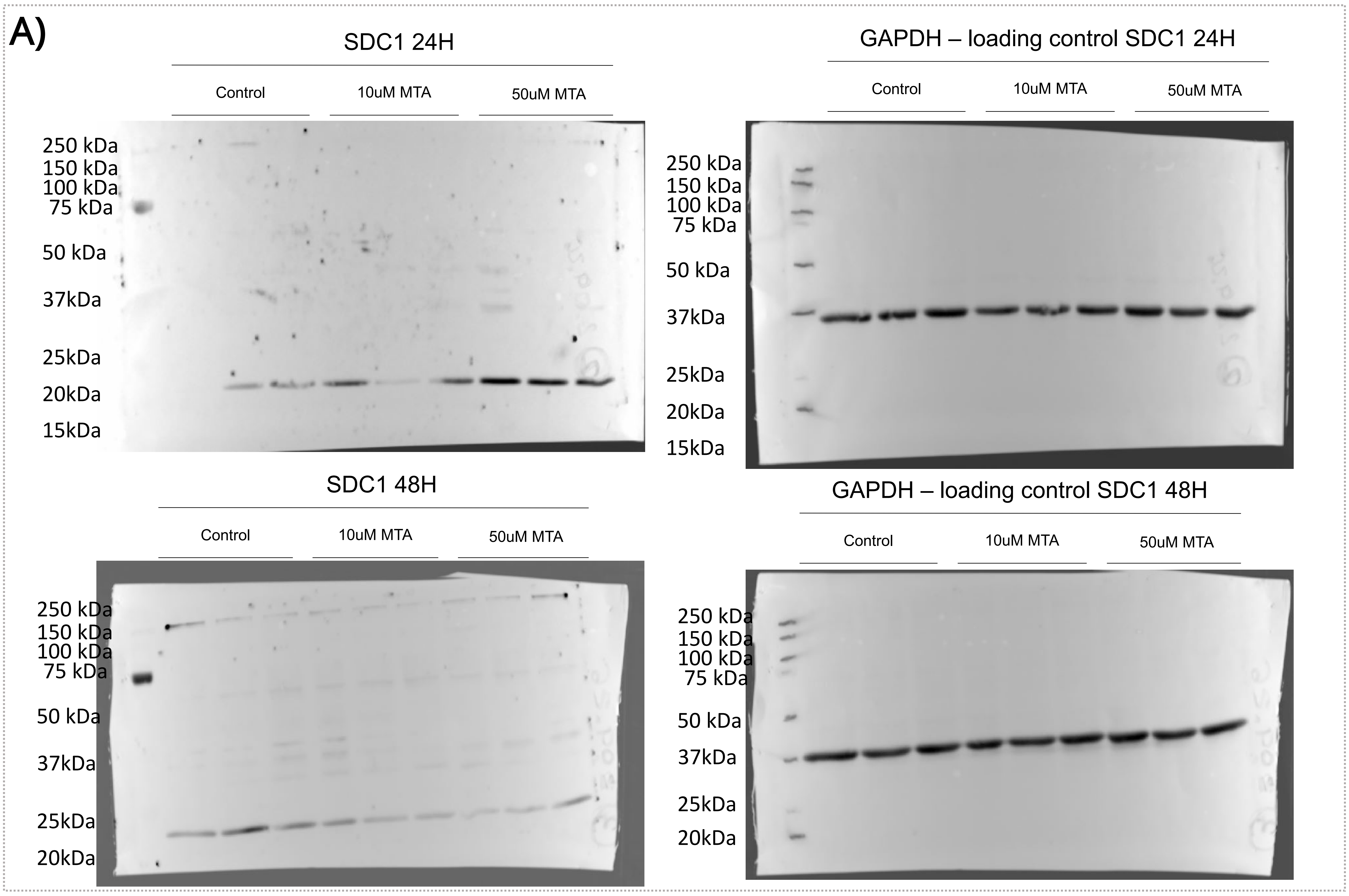
A)
SDC1 24H
Control
10uM MTA
50uM MTA
250 kDa
150 kDa
100 kDa
75 kDa
50 kDa
37kDa
25kDa
20kDa
15kDa
GAPDH – loading control SDC1 24H
Control
10uM MTA
50uM MTA
250 kDa
150 kDa
100 kDa
75 kDa
50 kDa
37kDa
25kDa
20kDa
15kDa
SDC1 48H
Control
10uM MTA
50uM MTA
250 kDa
150 kDa
100 kDa
75 kDa
50 kDa
37kDa
25kDa
20kDa
GAPDH – loading control SDC1 48H
Control
10uM MTA
50uM MTA
250 kDa
150 kDa
100 kDa
75 kDa
50 kDa
37kDa
25kDa
20kDa

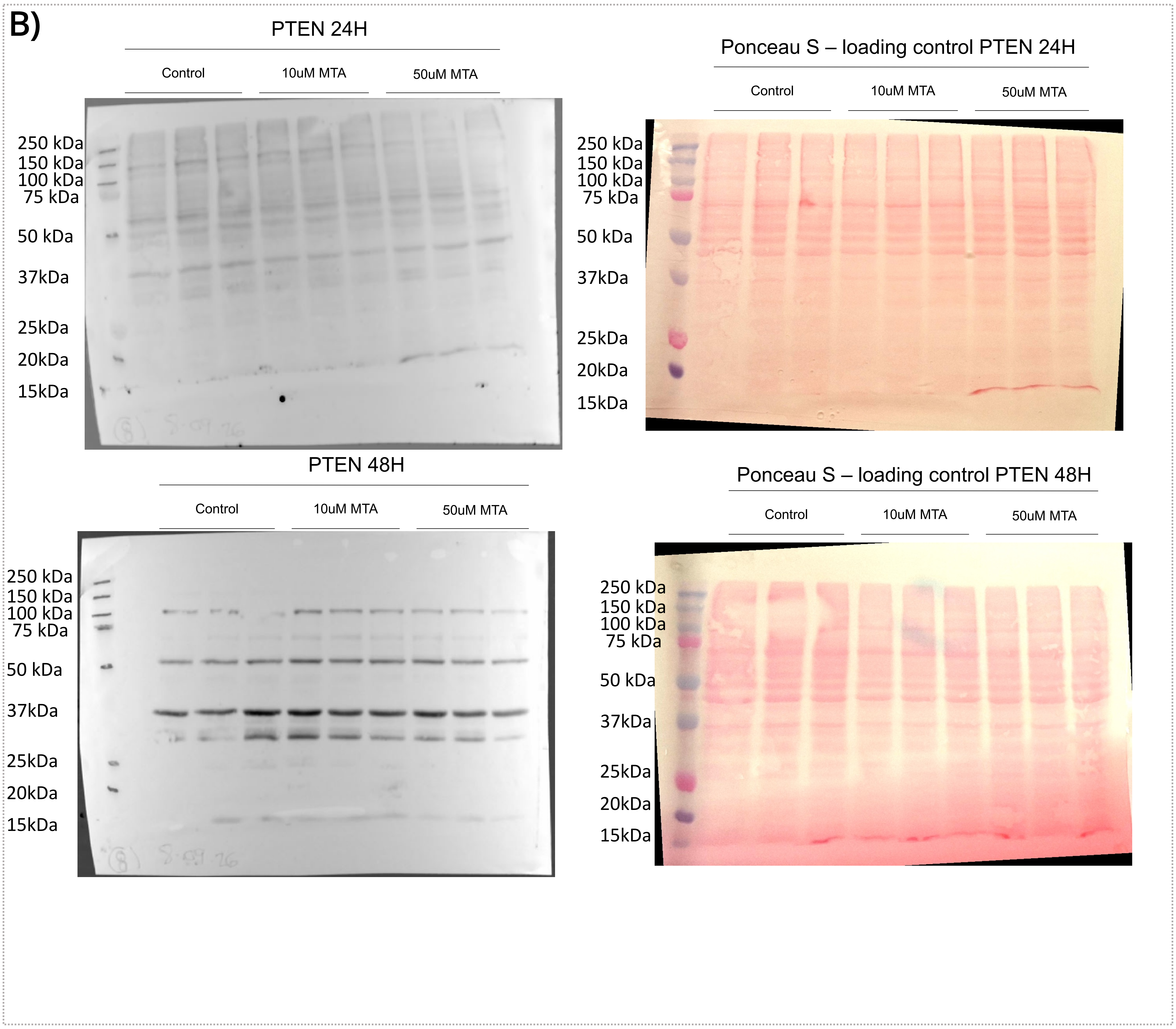
B)
PTEN 24H
Control
10uM MTA
50uM MTA
250 kDa
150 kDa
100 kDa
75 kDa
50 kDa
37kDa
25kDa
20kDa
15kDa
Ponceau S – loading control PTEN 24H
Control
10uM MTA
50uM MTA
250 kDa
150 kDa
100 kDa
75 kDa
50 kDa
37kDa
25kDa
20kDa
15kDa
PTEN 48H
Control
10uM MTA
50uM MTA
250 kDa
150 kDa
100 kDa
75 kDa
50 kDa
37kDa
25kDa
20kDa
15kDa
Ponceau S – loading control PTEN 48H
Control
10uM MTA
50uM MTA
250 kDa
150 kDa
100 kDa
75 kDa
50 kDa
37kDa
25kDa
20kDa
15kDa

Figure S3. Uncropped Western blot membranes. Full-length immunoblots corresponding to the representative cropped panels shown in Figure S1. Membranes display the expression levels of (A) SDC1 and (B) PTEN and the loading controls (GAPDH or Ponceau S) in PLC cells following MTA treatment. As shown in the lanes, each experimental condition includes three independent biological replicates (n = 3). Molecular weight markers (kDa) are indicated on the left side of each membrane.
