# Supplementary material for: Decoding the Cellular Response to Methylthioadenosine Uncovers Novel Targets for Chronic Liver Disease
Source: Cells. 2026 Sep 22;15(19):1720. doi: 10.3390/cells15191720 (PMC13664110; doi:10.3390/cells15191720)
Supplement: Supplementary file 1 [file cells-15-01720-s001.zip › Table S1_CellularResponsetoMTA_PLC.xlsx.pdf]

Compariso	ProteinGro	AVG Log2 F	Pvalue	Qvalue	Genes	ProteinDes	ProteinNar	# Unic	FC
48H 50MT/	P01042	0.298441	1.15E-12	4.25E-08	KNG1	Kininogen-:	KNG1_HUN	1	1.229815
48H 50MT/	P02769	-0.87822	3.58E-10	1.36E-06	ALB	Serum albu	ALBU_BOVI	56	0.54404
24H 10MT/	Q6YHK3	1.385853	2.51E-09	2.32E-06	CD109	CD109 anti	CD109_HU	36	2.613264
24H 10MT/	Q7Z5H4	1.283289	2.05E-08	6.06E-06	VN1R5	Vomeronas	VN1R5_HU	1	2.433932
24H 50MT/	Q9BRK4	1.359526	2.51E-08	6.60E-06	LZTS2	Leucine zip	LZTS2_HUN	6	2.566009
24H 10MT/	Q6PL24	-0.02837	6.43E-08	1.04E-05	TMED8	Protein TM	TMED8_HU	1	0.980526
24H 50MT/	Q9NXZ2	-0.17779	1.20E-07	1.39E-05	DDX43	Probable A'	DDX43_HU	29	0.884056
24H 50MT/	P49842	-0.91447	1.40E-07	1.44E-05	STK19	Inactive ser	STK19_HUN	3	0.530539
48H 50MT/	P62341	-3.10652	1.45E-07	1.45E-05	SELENOT	Thioredoxi	SELT_HUM/	2	0.116103
24H 50MT/	Q9H078	0.112104	1.58E-07	1.48E-05	CLPB	Mitochond	CLPB_HUM	23	1.080804
48H 50MT/	Q5ZPR3	0.099906	2.13E-07	1.71E-05	CD276	CD276 anti	CD276_HU	7	1.071704
24H 50MT/	P04439	0.843198	2.15E-07	1.72E-05	HLA-A	HLA class I I	HLAA_HUN	2	1.794022
48H 50MT/	Q9NZ45	0.38946	2.36E-07	1.80E-05	CISD1	CDGSH iror	CISD1_HUN	5	1.309903
48H 10MT/	P43652	0.539575	2.50E-07	1.86E-05	AFM	Afamin	AFAM_HUN	1	1.453544
48H 50MT/	P13674	0.591776	2.58E-07	1.88E-05	P4HA1	Prolyl 4-hy	P4HA1_HU	23	1.507101
48H 50MT/	P15924	0.159221	2.79E-07	1.95E-05	DSP	Desmoplak	DESP_HUM	135	1.116684
48H 50MT/	P50454	0.095605	2.82E-07	1.95E-05	SERPINH1	Serpin H1	SERPH_HUI	19	1.068513
48H 50MT/	Q9P0U1	-0.76094	3.90E-07	2.27E-05	TOMM7	Mitochond	TOM7_HUN	2	0.59011
24H 10MT/	P29323	1.858917	3.93E-07	2.28E-05	EPHB2	Ephrin type	EPHB2_HUI	1	3.627354
48H 50MT/	P28331	0.159606	4.08E-07	2.30E-05	NDUFS1	NADH-ubiq	NDUS1_HU	35	1.116982
48H 50MT/	Q9BX66	0.413728	4.16E-07	2.32E-05	SORBS1	Sorbin and	SRBS1_HUN	31	1.332123
48H 50MT/	Q9NW13	-0.25252	4.22E-07	2.33E-05	RBM28	RNA-bindir	RBM28_HU	24	0.839429
48H 50MT/	Q1KMD3	0.210131	4.73E-07	2.44E-05	HNRNPUL2	Heterogene	HNRL2_HU	27	1.156793
24H 50MT/	Q460N5	1.049088	4.91E-07	2.51E-05	PARP14	Protein mo	PAR14_HUI	1	2.069222
48H 50MT/	Q16698	0.26259	5.05E-07	2.54E-05	DECR1	2,4-dienoyl	DECR_HUN	15	1.199631
24H 50MT/	Q12860	0.56907	5.87E-07	2.79E-05	CNTN1	Contactin-:	CNTN1_HU	37	1.483567
24H 50MT/	Q9UNE7	-0.22377	6.00E-07	2.81E-05	STUB1	E3 ubiquiti	CHIP_HUM	16	0.856328
24H 50MT/	Q02880	0.168854	6.28E-07	2.87E-05	TOP2B	DNA topois	TOP2B_HUI	42	1.124165
24H 50MT/	P04181	0.196619	6.34E-07	2.87E-05	OAT	Ornithine a	OAT_HUMA	21	1.14601
24H 50MT/	Q15435	-0.20154	7.07E-07	3.03E-05	PPP1R7	Protein phc	PP1R7_HUI	18	0.869622
24H 50MT/	P29323	2.259985	8.03E-07	3.23E-05	EPHB2	Ephrin type	EPHB2_HUI	1	4.789866
48H 50MT/	P02753	0.675908	8.28E-07	3.27E-05	RBP4	Retinol-bin	RET4_HUM	7	1.597602
24H 50MT/	Q9Y2L5	0.139106	8.47E-07	3.32E-05	TRAPPC8	Trafficking	TPPC8_HUI	12	1.101223
48H 10MT/	P19827	0.28435	8.77E-07	3.38E-05	ITIH1	Inter-alpha	ITIH1_HUN	1	1.217861
48H 50MT/	O14618	0.263187	1.06E-06	3.72E-05	CCS	Copper cha	CCS_HUMA	8	1.200127
24H 50MT/	Q86UP2	0.080501	1.07E-06	3.74E-05	KTN1	Kinectin	KTN1_HUN	68	1.057385
24H 50MT/	Q15173	1.538248	1.11E-06	3.83E-05	PPP2R5B	Serine/thre	2A5B_HUN	2	2.904415
24H 50MT/	Q9UJV9	0.199486	1.16E-06	3.90E-05	DDX41	Probable A'	DDX41_HU	29	1.148289
48H 50MT/	P78310	0.141112	1.17E-06	3.90E-05	CXADR	Coxsackievi	CXAR_HUM	19	1.102755
48H 50MT/	P36776	0.272408	1.18E-06	3.93E-05	LONP1	Lon protea:	LONM_HUN	45	1.207822
24H 50MT/	P02675	0.44362	1.18E-06	3.93E-05	FGB	Fibrinogen	FIBB_HUM/	22	1.360013
48H 10MT/	Q9NZP5	1.048264	1.26E-06	4.03E-05	OR5AC2	Olfactory re	O5AC2_HU	1	2.06804
24H 50MT/	P84098	0.134235	1.29E-06	4.11E-05	RPL19	Large ribos	RL19_HUM	6	1.09751
24H 50MT/	O00217	0.122881	1.35E-06	4.21E-05	NDUFS8	NADH dehy	NDUS8_HU	6	1.088907
48H 50MT/	P06737	0.399124	1.34E-06	4.21E-05	PYGL	Glycogen p	PYGL_HUM	37	1.318707
48H 50MT/	P06396	0.347028	1.38E-06	4.27E-05	GSN	Gelsolin	GELS_HUM	18	1.271938
24H 50MT/	Q14151	0.245213	1.41E-06	4.32E-05	SAFB2	Scaffold att	SAFB2_HUN	26	1.185268
24H 10MT/	Q92597	-0.2368	1.44E-06	4.36E-05	NDRG1	Protein ND	NDRG1_HU	12	0.848627
48H 50MT/	P19827	0.466059	1.49E-06	4.41E-05	ITIH1	Inter-alpha	ITIH1_HUN	1	1.381331
48H 50MT/	Q6P0N0	-1.13135	1.49E-06	4.42E-05	MIS18BP1	Mis18-binc	M18BP_HU	4	0.456488
48H 50MT/	P02774	-0.62589	1.61E-06	4.61E-05	GC	Vitamin D-I	VTDB_HUN	5	0.648018

24H 10MT/	Q9Y5V3	-0.32699	1.61E-06	4.61E-05	MAGED1	Melanoma-	MAGD1_HU	10	0.797199
48H 50MT/	P11169	0.40503	1.61E-06	4.61E-05	SLC2A3	Solute carri	GTR3_HUM	7	1.324117
24H 50MT/	Q9UJ41	-0.13439	1.62E-06	4.62E-05	RABGEF1	Rab5 GDP/	RABX5_HU	11	0.911057
48H 50MT/	Q02952	0.345014	1.63E-06	4.63E-05	AKAP12	A-kinase an	AKA12_HU	79	1.270164
24H 10MT/	P15882	1.309642	1.72E-06	4.78E-05	CHN1	N-chimaeri	CHIN_HUM	2	2.4788
24H 50MT/	Q9NZP5	1.539774	1.75E-06	4.84E-05	OR5AC2	Olfactory re	O5AC2_HU	1	2.907489
48H 50MT/	Q9NXW2	-0.40545	1.86E-06	5.00E-05	DNAJB12	DnaJ homo	DJB12_HU	10	0.754999
24H 50MT/	Q9UH99	0.086999	2.15E-06	5.37E-05	SUN2	SUN domai	SUN2_HUM	19	1.062159
48H 50MT/	Q53EP0	0.143586	2.20E-06	5.41E-05	FNDC3B	Fibronectir	FND3B_HU	33	1.104647
24H 50MT/	Q9H8H3	0.225447	2.25E-06	5.46E-05	TMT1A	Thiol S-met	TMT1A_HU	7	1.169139
48H 50MT/	O60885	0.167175	2.38E-06	5.65E-05	BRD4	Bromodom	BRD4_HUM	23	1.122858
48H 50MT/	O94808	1.09942	2.52E-06	5.85E-05	GFPT2	Glutamine-	GFPT2_HU	2	2.142686
24H 10MT/	Q14118	1.196333	2.63E-06	5.96E-05	DAG1	Dystroglyca	DAG1_HUM	12	2.291564
24H 50MT/	P12268	0.135178	3.02E-06	6.44E-05	IMPDH2	Inosine-5'-r	IMDH2_HU	29	1.098228
48H 50MT/	P04259	0.689008	3.04E-06	6.47E-05	KRT6B	Keratin, typ	K2C6B_HU	2	1.612175
24H 50MT/	Q16698	0.102824	3.19E-06	6.65E-05	DECR1	2,4-dienoyl	DECR_HUM	15	1.073873
48H 50MT/	O00468	0.253965	3.38E-06	6.85E-05	AGRN	Agrin	AGRIN_HU	42	1.19248
48H 50MT/	Q96QD8	-0.80161	3.60E-06	7.10E-05	SLC38A2	Sodium-co	S38A2_HU	5	0.573709
24H 50MT/	P78509	0.337051	3.73E-06	7.20E-05	RELN	Reelin	RELN_HUM	48	1.263172
48H 50MT/	Q9Y2R9	-0.34913	3.78E-06	7.27E-05	MRPS7	Small ribos	RT07_HUM	12	0.785059
48H 10MT/	P08779	-0.61513	4.09E-06	7.62E-05	KRT16	Keratin, typ	K1C16_HU	23	0.65287
48H 50MT/	Q16658	0.392429	4.15E-06	7.67E-05	FSCN1	Fascin	FSCN1_HU	19	1.312601
24H 50MT/	Q9NYF8	0.369184	4.15E-06	7.68E-05	BCLAF1	Bcl-2-assoc	BCLF1_HU	20	1.291622
24H 10MT/	P48200	-0.12469	4.24E-06	7.76E-05	IREB2	Iron-respor	IREB2_HU	27	0.917203
24H 50MT/	Q99714	0.223374	4.24E-06	7.76E-05	HSD17B10	3-hydroxya	HCD2_HUM	18	1.167461
24H 50MT/	Q9BUQ8	0.248372	4.48E-06	7.96E-05	DDX23	Probable A	DDX23_HU	32	1.187866
48H 10MT/	Q5ZPR3	-0.02724	4.50E-06	7.99E-05	CD276	CD276 anti	CD276_HU	7	0.981298
24H 10MT/	Q9Y6N7	0.144905	4.58E-06	8.08E-05	ROBO1	Roundabou	ROBO1_HU	39	1.105658
24H 10MT/	P98160	1.306717	4.80E-06	8.34E-05	HSPG2	Basement m	PGBM_HU	19	2.47378
48H 50MT/	P43360	-1.52227	4.99E-06	8.47E-05	MAGEA6	Melanoma-	MAGA6_HU	2	0.348137
48H 10MT/	P98194	-0.0018	4.98E-06	8.47E-05	ATP2C1	Calcium-tra	AT2C1_HU	16	0.998752
48H 50MT/	P19367	0.479377	5.05E-06	8.52E-05	HK1	Hexokinase	HXK1_HUM	35	1.394141
48H 50MT/	O43708	0.219172	5.06E-06	8.53E-05	GSTZ1	Maleylacet	MAAI_HUM	5	1.164066
24H 50MT/	P35659	0.191505	5.08E-06	8.55E-05	DEK	Protein DE	DEK_HUMA	16	1.141955
24H 50MT/	Q8NH16	1.176754	5.13E-06	8.60E-05	OR2L2	Olfactory re	OR2L2_HU	1	2.260675
24H 50MT/	O00330	0.154241	5.20E-06	8.68E-05	PDHX	Pyruvate de	ODPX_HUM	18	1.112836
24H 50MT/	Q96I99	0.051279	5.31E-06	8.77E-05	SUCLG2	Succinate--	SUCB2_HU	19	1.036183
48H 50MT/	Q8NH16	1.240474	5.28E-06	8.77E-05	OR2L2	Olfactory re	OR2L2_HU	1	2.362762
24H 50MT/	O14662	0.140538	5.33E-06	8.78E-05	STX16	Syntaxin-1	STX16_HU	8	1.102316
48H 10MT/	O00560	0.116368	5.33E-06	8.79E-05	SDCBP	Syntenin-1	SDCB1_HU	8	1.084002
48H 50MT/	Q8NC96	-0.17694	5.40E-06	8.86E-05	NECAP1	Adaptin ear	NECP1_HU	9	0.884579
24H 50MT/	Q6ZXV5	0.074208	5.44E-06	8.88E-05	TMTC3	Protein O-n	TMTC3_HU	17	1.052783
48H 50MT/	O94822	-0.17744	5.69E-06	9.09E-05	LTN1	E3 ubiquiti	LTN1_HUM	40	0.884269
48H 50MT/	P05165	0.288856	5.73E-06	9.13E-05	PCCA	Propionyl-(	PCCA_HUM	24	1.221671
24H 10MT/	Q9NZP5	1.739809	5.74E-06	9.14E-05	OR5AC2	Olfactory re	O5AC2_HU	1	3.33991
48H 50MT/	Q9NQR4	0.205118	5.93E-06	9.29E-05	NIT2	Omega-ami	NIT2_HUM	16	1.15278
48H 50MT/	Q9NZP5	1.389634	6.38E-06	9.68E-05	OR5AC2	Olfactory re	O5AC2_HU	1	2.620122
48H 50MT/	P50416	0.332636	6.61E-06	9.90E-05	CPT1A	Carnitine O	CPT1A_HU	28	1.259312
24H 50MT/	P30519	0.165097	6.64E-06	9.92E-05	HMOX2	Heme oxyg	HMOX2_HU	15	1.121241
48H 50MT/	Q9NYK5	-0.23678	6.75E-06	0.0001	MRPL39	Large ribos	RM39_HUM	17	0.848639
24H 50MT/	Q15438	1.914587	6.79E-06	0.000101	CYTH1	Cytohesin-	CYH1_HUM	3	3.770059
24H 50MT/	P56537	-0.18499	6.86E-06	0.000101	EIF6	Eukaryotic	IF6_HUMA	10	0.879657

48H 10MTA	Q71DI3	-1.93541	7.01E-06	0.000102	H3C15	Histone H3	H32_HUMA	1	0.261447
48H 50MTA	P09972	0.790684	7.05E-06	0.000103	ALDOC	Fructose-bi	ALDOC_HU	8	1.729894
48H 50MTA	P00450	0.64959	7.07E-06	0.000103	CP	Ceruloplasr	CERU_HUM	16	1.568722
24H 50MTA	P19525	0.060169	7.50E-06	0.000105	EIF2AK2	Interferon-i	E2AK2_HUI	18	1.042588
24H 10MTA	Q6ZSC3	1.768558	7.67E-06	0.000107	RBM43	RNA-bindir	RBM43_HU	1	3.407132
48H 50MTA	P22307	0.256858	7.68E-06	0.000107	SCP2	Sterol carri	SCP2_HUM	24	1.194874
24H 50MTA	Q14692	0.095842	7.75E-06	0.000107	BMS1	Ribosome b	BMS1_HUM	33	1.068689
24H 50MTA	P12755	0.300484	7.94E-06	0.000109	SKI	Ski oncoger	SKI_HUMAI	2	1.231558
48H 10MTA	Q13404	0.122829	8.07E-06	0.00011	UBE2V1	Ubiquitin-c	UB2V1_HU	9	1.088868
48H 10MTA	Q9NP61	0.104338	8.55E-06	0.000114	ARFGAP3	ADP-ribosy	ARFG3_HUI	13	1.075001
24H 10MTA	Q04828	-0.20781	8.84E-06	0.000116	AKR1C1	Aldo-keto r	AK1C1_HUI	21	0.86585
48H 50MTA	O75694	-0.19482	8.91E-06	0.000116	NUP155	Nuclear por	NU155_HU	46	0.87368
48H 50MTA	Q8TAQ2	0.127454	9.28E-06	0.000119	SMARCC2	SWI/SNF cc	SMRC2_HU	20	1.092364
48H 10MTA	P20226	0.271869	9.33E-06	0.000119	TBP	TATA-box-b	TBP_HUMA	5	1.207371
48H 50MTA	Q14137	-0.22996	9.49E-06	0.00012	BOP1	Ribosome b	BOP1_HUM	26	0.85266
48H 50MTA	Q14011	0.177514	9.64E-06	0.000121	CIRBP	Cold-induc	CIRBP_HUM	4	1.130933
48H 50MTA	Q09666	0.269797	1.00E-05	0.000124	AHNAK	Neuroblast	AHNK_HUM	240	1.205638
48H 50MTA	Q15120	0.282345	1.01E-05	0.000124	PDK3	[Pyruvate d	PDK3_HUM	13	1.21617
48H 50MTA	Q8N5I9	-1.16515	1.02E-05	0.000125	NOPCHAP1	NOP protei	NOPC1_HU	2	0.445917
24H 50MTA	O14745	0.169888	1.02E-05	0.000125	NHERF1	Na(+)/H(+) e	NHRF1_HU	23	1.124971
24H 50MTA	Q13011	0.127161	1.06E-05	0.000128	ECH1	Delta(3,5)-I	ECH1_HUM	16	1.092142
48H 50MTA	P35580	0.08353	1.08E-05	0.00013	MYH10	Myosin-10	MYH10_HU	90	1.059608
48H 50MTA	P31327	0.349863	1.08E-05	0.00013	CPS1	Carbamoyl-	CPSM_HUM	84	1.27444
48H 10MTA	O95260	0.063364	1.09E-05	0.00013	ATE1	Arginyl-tRN	ATE1_HUM	12	1.044899
24H 50MTA	P12235	0.158101	1.10E-05	0.000131	SLC25A4	ADP/ATP tr	ADT1_HUM	8	1.115818
48H 50MTA	O95490	0.255762	1.11E-05	0.000131	ADGRL2	Adhesion G	AGRL2_HUI	28	1.193966
24H 50MTA	Q9NSE4	0.071971	1.14E-05	0.000133	IARS2	Isoleucine--	SYIM_HUM	36	1.051152
48H 50MTA	Q9NP61	0.345251	1.14E-05	0.000133	ARFGAP3	ADP-ribosy	ARFG3_HUI	13	1.270372
24H 50MTA	P35520	-0.19661	1.15E-05	0.000134	CBS	Cystathioni	CBS_HUMA	22	0.872596
48H 50MTA	O95478	-0.36517	1.15E-05	0.000134	NSA2	Ribosome b	NSA2_HUM	8	0.776378
24H 50MTA	P78347	0.168006	1.16E-05	0.000135	GTF2I	General tra	GTF2I_HUM	45	1.123504
48H 10MTA	P14210	0.864009	1.16E-05	0.000135	HGF	Hepatocyte	HGF_HUMA	1	1.820089
24H 50MTA	Q92878	0.142627	1.17E-05	0.000135	RAD50	DNA repair	RAD50_HU	48	1.103913
24H 50MTA	P16435	0.217554	1.19E-05	0.000136	POR	NADPH--cyt	NCPR_HUM	39	1.16276
24H 50MTA	Q7Z5H4	1.383689	1.19E-05	0.000136	VN1R5	Vomeronas	VN1R5_HU	1	2.609348
48H 50MTA	P05091	0.259123	1.19E-05	0.000136	ALDH2	Aldehyde d	ALDH2_HU	26	1.196751
48H 50MTA	Q3ZCQ3	0.112903	1.20E-05	0.000137	FAM174B	Membrane	F174B_HUI	1	1.081402
48H 50MTA	O00471	0.1172	1.22E-05	0.000139	EXOC5	Exocyst cor	EXOC5_HUI	24	1.084628
48H 10MTA	Q9Y4Z0	0.394779	1.24E-05	0.00014	LSM4	U6 snRNA-a	LSM4_HUM	5	1.314741
24H 50MTA	O75494	0.224045	1.25E-05	0.000141	SRSF10	Serine/argi	SRS10_HUM	6	1.168004
24H 50MTA	Q96RQ1	0.333847	1.25E-05	0.000141	ERGIC2	Endoplasm	ERGI2_HUM	6	1.260369
24H 50MTA	Q15424	0.150463	1.27E-05	0.000142	SAFB	Scaffold att	SAFB1_HUI	14	1.109926
24H 50MTA	Q8TEX9	-0.18792	1.33E-05	0.000147	IPO4	Importin-4	IPO4_HUM	47	0.877871
24H 10MTA	Q8WXX5	0.146939	1.35E-05	0.000148	DNAJC9	DnaJ homo	DNJC9_HUI	15	1.107218
48H 50MTA	P15151	0.404799	1.36E-05	0.000148	PVR	Poliovirus r	PVR_HUMA	6	1.323904
24H 50MTA	P14678	0.285595	1.37E-05	0.000149	SNRPB	Small nucle	RSMB_HUM	12	1.218913
24H 50MTA	Q9Y255	-0.19516	1.38E-05	0.000149	PRELID1	PRELI doma	PRLD1_HUI	3	0.873477
48H 50MTA	Q96AC1	0.236174	1.38E-05	0.00015	FERMT2	Fermitin fa	FERM2_HU	27	1.177865
48H 50MTA	Q9Y314	-0.23883	1.38E-05	0.00015	NOSIP	Nitric oxide	NOSIP_HUM	12	0.847433
24H 50MTA	Q15637	0.21419	1.39E-05	0.00015	SF1	Splicing fac	SF01_HUM	17	1.160053
24H 50MTA	P02671	0.364565	1.40E-05	0.00015	FGA	Fibrinogen	FIBA_HUMA	25	1.287493
24H 50MTA	Q13451	0.199654	1.41E-05	0.000151	FKBP5	Peptidyl-pr	FKBP5_HUI	23	1.148423

48H 50MT/	Q8WUF5	0.186514	1.43E-05	0.000152	PPP1R13L	RelA-associ	IASPP_HUN	20	1.13801
48H 50MT/	Q14444	-0.24048	1.44E-05	0.000153	CAPRIN1	Caprin-1	CAPR1_HUI	13	0.846461
48H 10MT/	Q9Y5K5	0.031098	1.47E-05	0.000155	UCHL5	Ubiquitin c	UCHL5_HU	16	1.021789
48H 50MT/	Q96M27	0.312101	1.47E-05	0.000155	PRRC1	Protein PRI	PRRC1_HUI	10	1.241515
48H 50MT/	Q6DKI1	-0.45515	1.51E-05	0.000157	RPL7L1	Ribosomal	RL7L_HUM	12	0.729433
24H 50MT/	Q96QZ7	0.145622	1.52E-05	0.000157	MAGI1	Membrane-	MAGI1_HU	19	1.106207
48H 50MT/	Q9Y5J1	-0.27776	1.53E-05	0.000159	UTP18	U3 small nu	UTP18_HUI	19	0.824872
24H 50MT/	P02679	0.428793	1.55E-05	0.00016	FGG	Fibrinogen	FIBG_HUM/	20	1.346107
48H 50MT/	Q9Y490	0.114908	1.58E-05	0.000161	TLN1	Talin-1	TLN1_HUM	110	1.082906
48H 50MT/	P55317	-0.36924	1.59E-05	0.000162	FOXA1	Hepatocyte	FOXA1_HUI	2	0.774188
48H 50MT/	P08574	0.157483	1.59E-05	0.000162	CYC1	Cytochrom	CY1_HUMA	8	1.11534
24H 50MT/	O94808	1.194236	1.59E-05	0.000162	GFPT2	Glutamine-	GFPT2_HUI	2	2.288236
24H 50MT/	Q92544	0.173394	1.61E-05	0.000163	TM9SF4	Transmemb	TM9S4_HU	17	1.127708
48H 50MT/	Q9P2E9	0.075808	1.62E-05	0.000164	RRBP1	Ribosome-I	RRBP1_HUI	45	1.053951
24H 50MT/	Q13057	-0.17866	1.62E-05	0.000164	COASY	Bifunctiona	COASY_HUI	15	0.883524
24H 10MT/	Q9BU61	0.050328	1.65E-05	0.000166	NDUFAF3	NADH dehy	NDUF3_HU	6	1.0355
48H 50MT/	Q9UBR2	0.165098	1.67E-05	0.000167	CTSZ	Cathepsin Z	CATZ_HUM	8	1.121242
24H 50MT/	P18084	0.263005	1.67E-05	0.000167	ITGB5	Integrin be	ITB5_HUM/	24	1.199976
24H 10MT/	Q8NH16	1.134334	1.68E-05	0.000168	OR2L2	Olfactory re	OR2L2_HUI	1	2.195172
24H 10MT/	Q02880	0.07311	1.69E-05	0.000168	TOP2B	DNA topois	TOP2B_HUI	42	1.051982
24H 50MT/	P78527	0.108069	1.71E-05	0.000169	PRKDC	DNA-depen	PRKDC_HU	210	1.077785
24H 50MT/	Q9Y6N7	0.107555	1.71E-05	0.000169	ROBO1	Roundabou	ROBO1_HU	39	1.077401
24H 50MT/	Q14498	0.375102	1.71E-05	0.000169	RBM39	RNA-bindir	RBM39_HU	18	1.296932
24H 50MT/	O43490	0.340324	1.72E-05	0.000169	PROM1	Prominin-1	PROM1_HU	21	1.266041
24H 50MT/	Q14118	1.07745	1.74E-05	0.000171	DAG1	Dystroglyca	DAG1_HUN	12	2.110303
24H 50MT/	O00338	0.577971	1.76E-05	0.000171	SULT1C2	Sulfotransf	ST1C2_HUI	1	1.492749
24H 50MT/	O75964	0.807624	1.77E-05	0.000172	ATP5MG	ATP syntha	ATP5L_HUI	6	1.750327
48H 50MT/	Q9UBP0	0.077463	1.78E-05	0.000172	SPAST	Spastin	SPAST_HUN	15	1.055161
48H 10MT/	Q9BXP5	0.116488	1.80E-05	0.000174	SRRT	Serrate RN/	SRRT_HUM	34	1.084092
48H 50MT/	Q8TED0	-0.21067	1.80E-05	0.000174	UTP15	U3 small nu	UTP15_HUI	25	0.864136
24H 50MT/	Q9Y625	0.865267	1.82E-05	0.000175	GPC6	Glypican-6	GPC6_HUN	3	1.821676
24H 50MT/	O00567	0.057736	1.82E-05	0.000175	NOP56	Nucleolar p	NOP56_HU	27	1.040831
48H 50MT/	Q9Y3U8	-0.20036	1.83E-05	0.000176	RPL36	Large ribos	RL36_HUM	5	0.870332
24H 50MT/	Q9NSI2	-0.15614	1.86E-05	0.000178	SLX9	Ribosome b	SLX9_HUM	7	0.897426
48H 50MT/	Q9BQQ3	2.240285	1.86E-05	0.000178	GORASP1	Golgi reass	GORS1_HU	1	4.724905
48H 10MT/	Q96A57	0.428735	1.87E-05	0.000179	TMEM230	Transmemb	TM230_HU	3	1.346053
24H 50MT/	P11310	0.199033	1.88E-05	0.000179	ACADM	Medium-ch	ACADM_HU	19	1.147929
48H 50MT/	P42771	-0.57903	1.89E-05	0.00018	CDKN2A	Cyclin-depe	CDN2A_HU	2	0.669412
48H 50MT/	P83111	0.08434	1.91E-05	0.000181	LACTB	Serine beta	LACTB_HUN	23	1.060203
24H 50MT/	P63092;Q5	0.065417	1.92E-05	0.000181	GNAS	Guanine nu	GNAS2_HU	14	1.046387
48H 50MT/	O95759	0.149147	1.95E-05	0.000183	TBC1D8	TBC1 doma	TBCD8_HUI	24	1.108913
24H 50MT/	P55084	0.120347	1.96E-05	0.000184	HADHB	Trifunction	ECHB_HUM	30	1.086996
24H 50MT/	Q9H2F9	0.911544	1.96E-05	0.000184	CCDC68	Coiled-coil	CCD68_HU	1	1.881057
48H 50MT/	Q15436	0.14784	1.98E-05	0.000185	SEC23A	Protein trai	SC23A_HUI	30	1.107909
24H 50MT/	Q5T2D3	-0.30768	1.99E-05	0.000185	OTUD3	OTU domai	OTUD3_HU	1	0.807941
48H 50MT/	P21912	0.197484	1.99E-05	0.000185	SDHB	Succinate d	SDHB_HUN	10	1.146697
24H 50MT/	P15882	1.710417	2.00E-05	0.000185	CHN1	N-chimaeri	CHIN_HUM	2	3.272555
48H 50MT/	P51570	0.18166	2.00E-05	0.000186	GALK1	Galactokina	GALK1_HUI	14	1.134188
48H 50MT/	P24941	-0.24319	2.01E-05	0.000186	CDK2	Cyclin-depe	CDK2_HUN	17	0.844876
24H 50MT/	P53355	0.789052	2.05E-05	0.000189	DAPK1	Death-asso	DAPK1_HU	8	1.727939
48H 50MT/	Q9BXP5	0.070647	2.15E-05	0.000194	SRRT	Serrate RN/	SRRT_HUM	34	1.050188
48H 50MT/	Q9UMX0	0.185644	2.15E-05	0.000194	UBQLN1	Ubiquilin-1	UBQL1_HU	15	1.137324

24H 50MTA	Q7Z4R8	3.097147	2.19E-05	0.000196	C6orf120	UPF0669 p	CF120_HUI	1	8.557249
24H 50MTA	P61313	0.332642	2.22E-05	0.000197	RPL15	Large ribos	RL15_HUM	10	1.259317
48H 10MTA	Q12765	0.08155	2.28E-05	0.000201	SCRN1	Secernin-1	SCRN1_HUI	20	1.058154
48H 10MTA	O75449	0.296264	2.29E-05	0.000201	KATNA1	Katanin p6	KTNA1_HUI	9	1.22796
48H 50MTA	Q8WUM4	0.097163	2.32E-05	0.000203	PDCD6IP	Programme	PDC6I_HUI	48	1.069668
48H 50MTA	P60880	0.290728	2.33E-05	0.000204	SNAP25	Synaptosor	SNP25_HUI	9	1.223257
24H 50MTA	O00746	0.171742	2.36E-05	0.000205	NME4	Nucleoside	NDKM_HUI	11	1.126418
24H 50MTA	P14625	0.15956	2.36E-05	0.000205	HSP90B1	Endoplasm	ENPL_HUM	52	1.116947
48H 50MTA	Q96GQ7	-0.28205	2.36E-05	0.000205	DDX27	Probable A	DDX27_HU	31	0.82242
48H 50MTA	Q9NR30	-0.33259	2.37E-05	0.000205	DDX21	Nucleolar R	DDX21_HU	43	0.794107
24H 50MTA	Q10567	-0.11029	2.42E-05	0.000207	AP1B1	AP-1 compl	AP1B1_HUI	27	0.926401
24H 50MTA	P46776	0.142086	2.44E-05	0.000208	RPL27A	Large ribos	RL27A_HUI	7	1.103499
48H 50MTA	Q12860	0.461982	2.44E-05	0.000209	CNTN1	Contactin-1	CNTN1_HU	37	1.377433
24H 50MTA	P61604	0.287547	2.47E-05	0.00021	HSPE1	10 kDa heat	CH10_HUM	13	1.220563
24H 50MTA	P42166	0.160232	2.47E-05	0.00021	TMPO	Lamina-ass	LAP2A_HUI	35	1.117467
48H 50MTA	Q6PL18	-0.50165	2.49E-05	0.00021	ATAD2	ATPase fam	ATAD2_HUI	28	0.7063
48H 50MTA	Q8WTV0	0.565156	2.48E-05	0.00021	SCARB1	Scavenger r	SCRB1_HUI	8	1.479547
48H 50MTA	Q96IV0	0.462474	2.49E-05	0.000211	NGLY1	Peptide-N(	NGLY1_HUI	11	1.377902
48H 50MTA	P60174	0.327078	2.49E-05	0.000211	TPI1	Triosephos	TPIS_HUMA	18	1.25447
24H 50MTA	P99999	0.194928	2.51E-05	0.000212	CYCS	Cytochrom	CYC_HUMA	8	1.144667
48H 50MTA	Q96KP1	0.071497	2.52E-05	0.000212	EXOC2	Exocyst cor	EXOC2_HUI	19	1.050807
24H 10MTA	P28838	-0.08503	2.54E-05	0.000213	LAP3	Cytosol am	AMPL_HUM	29	0.942765
24H 50MTA	Q53EZ4	0.052746	2.56E-05	0.000214	CEP55	Centrosom	CEP55_HUI	13	1.037238
24H 50MTA	Q00839	0.165955	2.57E-05	0.000214	HNRNPU	Heterogene	HNRPU_HU	38	1.121908
48H 50MTA	P04818	-0.57392	2.60E-05	0.000216	TYMS	Thymidylat	TYSY_HUMA	12	0.671789
48H 50MTA	O43148	0.135245	2.61E-05	0.000216	RNMT	mRNA cap g	MCES_HUM	13	1.098279
48H 50MTA	Q9NPF0	-0.43909	2.63E-05	0.000217	CD320	CD320 anti	CD320_HU	5	0.737602
48H 10MTA	Q04941	0.069741	2.64E-05	0.000218	PLP2	Proteolipid	PLP2_HUM	1	1.049528
48H 50MTA	Q69YQ0	-0.37777	2.65E-05	0.000218	SPECC1L	Cytospin-A	CYTSA_HUM	23	0.769625
48H 10MTA	O75907	-0.86559	2.65E-05	0.000218	DGAT1	Diacylglyce	DGAT1_HU	5	0.54882
24H 50MTA	P58511	1.77548	2.69E-05	0.00022	SMIM11	Small integ	SIM11_HUI	1	3.423518
48H 50MTA	P52292	-0.38329	2.72E-05	0.000222	KPNA2	Importin su	IMA1_HUM	24	0.76669
48H 50MTA	P06493	-0.28848	2.75E-05	0.000223	CDK1	Cyclin-depe	CDK1_HUM	17	0.818762
48H 50MTA	Q99848	-0.26008	2.76E-05	0.000224	EBNA1BP2	Probable rR	EBP2_HUM	13	0.835042
48H 50MTA	Q0IIM8	0.431915	2.77E-05	0.000224	TBC1D8B	TBC1 doma	TBC8B_HUI	11	1.349023
24H 50MTA	Q99828	-0.54894	2.77E-05	0.000224	CIB1	Calcium an	CIB1_HUMA	4	0.683523
48H 10MTA	P00747	0.690077	2.78E-05	0.000224	PLG	Plasminoge	PLMN_HUM	3	1.61337
24H 50MTA	P02786	0.274226	2.82E-05	0.000226	TFRC	Transferrin	TFR1_HUM	36	1.209346
48H 10MTA	Q16630	0.105981	2.87E-05	0.000229	CPSF6	Cleavage an	CPSF6_HUI	11	1.076226
24H 10MTA	Q92785	0.142868	2.89E-05	0.00023	DPF2	Zinc finger	REQU_HUM	14	1.104098
24H 50MTA	Q4G0N4	0.205329	2.91E-05	0.00023	NADK2	NAD kinase	NAKD2_HU	18	1.152949
24H 50MTA	Q8N9T8	0.114946	2.92E-05	0.000231	KRI1	Protein KRI	KRI1_HUMA	12	1.082935
48H 50MTA	Q9Y4C8	-0.40377	2.92E-05	0.000231	RBM19	Probable R	RBM19_HU	15	0.75588
24H 50MTA	P04843	0.129416	2.94E-05	0.000232	RPN1	Dolichyl-di	RPN1_HUM	32	1.093851
48H 50MTA	Q9NXN4	-0.27467	2.95E-05	0.000232	GDAP2	Ganglioside	GDAP2_HU	6	0.826641
48H 50MTA	P08833	0.503703	2.98E-05	0.000234	IGFBP1	Insulin-like	IBP1_HUMA	7	1.417848
24H 50MTA	Q96EP5	0.152096	3.00E-05	0.000235	DAZAP1	DAZ-associ	DAZP1_HUI	12	1.111183
24H 10MTA	P36871	-0.18342	3.05E-05	0.000237	PGM1	Phosphoglu	PGM1_HUM	28	0.880615
48H 50MTA	Q86U90	-0.36525	3.05E-05	0.000237	YRDC	Threonylca	YRDC_HUM	12	0.776333
24H 50MTA	Q86Y46	2.509698	3.07E-05	0.000238	KRT73	Keratin, typ	K2C73_HUI	1	5.695009
48H 50MTA	Q70UQ0	0.111057	3.08E-05	0.000238	IKBIP	Inhibitor of	IKIP_HUMA	14	1.080019
24H 50MTA	P31327	0.229568	3.18E-05	0.000244	CPS1	Carbamoyl-	CPSM_HUM	84	1.172484

24H 50MT/	Q9BQE3	-0.30483	3.19E-05	0.000244	TUBA1C	Tubulin alp	TBA1C_HUI	4	0.809536
24H 50MT/	Q9ULD2	0.155695	3.22E-05	0.000246	MTUS1	Microtubul	MTUS1_HU	32	1.113958
48H 50MT/	O14965	-0.50839	3.24E-05	0.000246	AURKA	Aurora kina	AURKA_HU	15	0.703005
48H 10MT/	Q10471	0.089465	3.26E-05	0.000248	GALNT2	Polypeptid	GALT2_HUI	26	1.063975
48H 50MT/	P51398	-0.38793	3.27E-05	0.000248	DAP3	Small ribos	RT29_HUM	18	0.764224
24H 50MT/	Q8N126	2.543975	3.29E-05	0.000249	CADM3	Cell adhesi	CADM3_HU	1	5.831938
24H 50MT/	Q8WWV3	0.234817	3.31E-05	0.00025	RTN4IP1	Reticulon-4	RT4I1_HUN	13	1.176757
24H 10MT/	P02751	0.793813	3.35E-05	0.000252	FN1	Fibronectir	FINC_HUM/	77	1.73365
24H 50MT/	P46100	0.237183	3.38E-05	0.000253	ATRX	Transcripti	ATRX_HUM	44	1.178689
48H 50MT/	Q02252	0.243628	3.47E-05	0.000258	ALDH6A1	Methylmal	MMSA_HUI	19	1.183966
48H 50MT/	Q9NRX2	-0.31626	3.47E-05	0.000258	MRPL17	Large ribos	RM17_HUN	10	0.803152
24H 50MT/	P08754	0.134124	3.51E-05	0.000259	GNAI3	Guanine nu	GNAI3_HUI	9	1.097427
48H 50MT/	Q9BVP2	-0.19382	3.52E-05	0.00026	GNL3	Guanine nu	GNL3_HUN	24	0.874287
24H 50MT/	Q14789	0.076444	3.56E-05	0.000261	GOLGB1	Golgin subf	GOGB1_HU	92	1.054416
24H 50MT/	P04818	-0.4343	3.57E-05	0.000261	TYMS	Thymidylat	TYSY_HUM/	12	0.740051
24H 10MT/	P11047	0.094666	3.59E-05	0.000262	LAMC1	Laminin sul	LAMC1_HU	40	1.067818
24H 50MT/	Q96HJ9	2.079968	3.59E-05	0.000262	FMC1	Protein FM	FMC1_HUN	1	4.227978
48H 50MT/	P63167	0.350049	3.61E-05	0.000263	DYNLL1	Dynein ligh	DYL1_HUM	1	1.274604
48H 50MT/	P05546	1.065605	3.64E-05	0.000264	SERPIND1	Heparin co	HEP2_HUM	2	2.093047
48H 50MT/	P02751	0.497558	3.65E-05	0.000264	FN1	Fibronectir	FINC_HUM/	77	1.411822
48H 50MT/	Q9BQG0	-0.13263	3.67E-05	0.000265	MYBBP1A	Myb-bindir	MBB1A_HU	48	0.912168
48H 50MT/	P20340	0.174999	3.70E-05	0.000266	RAB6A	Ras-related	RAB6A_HUI	11	1.128964
24H 50MT/	P49916	0.208369	3.73E-05	0.000268	LIG3	DNA ligase :	DNLI3_HUN	20	1.155381
48H 10MT/	Q12888	0.025564	3.74E-05	0.000269	TP53BP1	TP53-bindi	TP53B_HUI	51	1.017877
24H 50MT/	Q9HAB8	-0.06962	3.75E-05	0.000269	PPCS	Phosphopa	PPCS_HUM	15	0.952888
48H 50MT/	Q13576	0.109348	3.77E-05	0.00027	IQGAP2	Ras GTPase-	IQGA2_HUI	66	1.07874
48H 10MT/	Q8NH16	0.730105	3.81E-05	0.000271	OR2L2	Olfactory re	OR2L2_HUI	1	1.65876
48H 50MT/	Q13445	0.193669	3.81E-05	0.000271	TMED1	Transmemb	TMED1_HU	5	1.143669
24H 10MT/	Q9BR76	-0.17972	3.82E-05	0.000272	CORO1B	Coronin-1E	COR1B_HU	17	0.882874
48H 10MT/	P05156	-0.56417	3.85E-05	0.000273	CFI	Compleme	CFAI_HUM/	2	0.676346
24H 10MT/	P18827	1.903807	3.89E-05	0.000275	SDC1	Syndecan-1	SDC1_HUN	3	3.741992
48H 10MT/	Q8IZQ5	-0.42443	3.89E-05	0.000275	SELENOH	Selenoprot	SELH_HUM	2	0.745132
24H 50MT/	P20700	0.149767	3.90E-05	0.000275	LMNB1	Lamin-B1	LMNB1_HU	31	1.10939
48H 50MT/	O14772	0.207626	3.91E-05	0.000276	FPGT	Fucose-1-pl	FPGT_HUM	27	1.154786
48H 50MT/	Q99541	1.112076	4.00E-05	0.000279	PLIN2	Perilipin-2	PLIN2_HUN	13	2.161564
48H 50MT/	Q14126	0.184827	4.03E-05	0.00028	DSG2	Desmoglein	DSG2_HUM	25	1.136681
48H 50MT/	Q9BYD2	-0.26594	4.04E-05	0.000281	MRPL9	Large ribos	RM09_HUN	7	0.831655
48H 50MT/	P35558	0.78511	4.04E-05	0.000281	PCK1	Phosphoen	PCKGC_HU	18	1.723224
48H 50MT/	P05156	-0.55691	4.05E-05	0.000281	CFI	Compleme	CFAI_HUM/	2	0.679756
48H 50MT/	P50135	0.036925	4.06E-05	0.000281	HNMT	Histamine I	HNMT_HUI	10	1.025925
48H 50MT/	P49755	0.089498	4.07E-05	0.000282	TMED10	Transmemb	TMEDA_HU	7	1.064
48H 50MT/	Q9Y678	0.125286	4.11E-05	0.000284	COPG1	Coatomer s	COPG1_HU	42	1.090724
24H 50MT/	Q01780	0.105651	4.12E-05	0.000284	EXOSC10	Exosome cc	EXOSX_HUI	38	1.07598
24H 50MT/	P41247	-0.8587	4.13E-05	0.000285	PNPLA4	Patatin-like	PLPL4_HUN	2	0.55145
24H 50MT/	Q8IX12	0.167831	4.18E-05	0.000287	CCAR1	Cell divisior	CCAR1_HUI	30	1.123368
48H 50MT/	P07602	0.222468	4.18E-05	0.000287	PSAP	Prosaposin	SAP_HUMA	17	1.166728
48H 10MT/	P02774	0.418327	4.20E-05	0.000288	GC	Vitamin D-I	VTDB_HUM	5	1.336377
24H 50MT/	P11413	-0.17772	4.21E-05	0.000288	G6PD	Glucose-6-r	G6PD_HUN	34	0.8841
24H 50MT/	P01889	0.239724	4.22E-05	0.000289	HLA-B	HLA class I I	HLAB_HUM	8	1.180766
48H 50MT/	Q14807	-0.4806	4.23E-05	0.000289	KIF22	Kinesin-like	KIF22_HUN	25	0.716678
48H 50MT/	P16219	0.398102	4.24E-05	0.000289	ACADS	Short-chair	ACADS_HUI	9	1.317773
48H 50MT/	P02788	-0.19776	4.25E-05	0.00029	LTF	Lactotransf	TRFL_HUM/	2	0.871906

48H 50MT/	Q96K17	0.207584	4.25E-05	0.00029	BTF3L4	Transcripti	BT3L4_HUI	10	1.154752
24H 50MT/	Q8NBS9	0.092428	4.26E-05	0.00029	TXNDC5	Thioredoxi	TXND5_HU	21	1.066163
24H 50MT/	P01111	0.015517	4.30E-05	0.000292	NRAS	GTPase NRa	RASN_HUM	8	1.010814
24H 50MT/	Q96HP4	0.193576	4.34E-05	0.000293	OXNAD1	Oxidoreduc	OXND1_HU	4	1.143595
48H 50MT/	Q6P1L8	-0.39023	4.44E-05	0.000297	MRPL14	Large ribos	RM14_HUM	5	0.76301
24H 50MT/	P78536	0.187944	4.46E-05	0.000298	ADAM17	Disintegrin	ADA17_HU	8	1.139139
48H 50MT/	P78509	0.277556	4.51E-05	0.0003	RELN	Reelin	RELN_HUM	48	1.212139
24H 10MT/	Q9P2M7	0.050587	4.52E-05	0.0003	CGN	Cingulin	CING_HUM	44	1.035686
24H 50MT/	Q9H857	0.17698	4.54E-05	0.000301	NT5DC2	5'-nucleoti	NT5D2_HU	25	1.130515
48H 50MT/	P0C0S8;P2	-0.29842	4.57E-05	0.000302	H2AC11;H2	Histone H2	H2A1_HUM	8	0.813144
48H 50MT/	Q9ULW0	-0.45378	4.57E-05	0.000302	TPX2	Targeting p	TPX2_HUM	31	0.730127
48H 10MT/	Q8IVS8	-2.78831	4.59E-05	0.000303	GLYCTK	Glycerate k	GLCTK_HUI	2	0.144756
48H 10MT/	Q96IY1	0.168219	4.61E-05	0.000304	NSL1	Kinetochor	NSL1_HUM	5	1.123671
48H 50MT/	Q13601	-0.39015	4.63E-05	0.000304	KRR1	KRR1 small	KRR1_HUM	13	0.76305
48H 50MT/	Q9NXH9	-0.30431	4.62E-05	0.000304	TRMT1	tRNA (guan	TRM1_HUM	14	0.809828
24H 50MT/	O60488	0.229125	4.70E-05	0.000307	ACSL4	Long-chain	ACSL4_HUI	33	1.172124
48H 10MT/	Q02952	0.143174	4.74E-05	0.000309	AKAP12	A-kinase an	AKA12_HUI	79	1.104332
24H 50MT/	Q9UEU0	0.052318	4.76E-05	0.00031	VTI1B	Vesicle trar	VTI1B_HUM	8	1.036929
48H 50MT/	P47756	0.157294	4.77E-05	0.00031	CAPZB	F-actin-cap	CAPZB_HUI	19	1.115193
48H 10MT/	Q92917	0.011608	4.80E-05	0.000311	GPKOW	G-patch do	GPKOW_HU	19	1.008078
48H 50MT/	A8CG34	-0.28999	4.82E-05	0.000312	POM121C	Nuclear env	P121C_HUI	13	0.817909
24H 50MT/	Q9UKY7	-0.14551	4.84E-05	0.000313	CDV3	Protein CD'	CDV3_HUM	13	0.904062
24H 50MT/	P98174	2.59312	4.86E-05	0.000314	FGD1	FYVE, RhoG	FGD1_HUM	2	6.034023
48H 50MT/	Q14978	-0.29784	4.89E-05	0.000315	NOLC1	Nucleolar a	NOLC1_HU	10	0.81347
48H 50MT/	Q16222	0.073534	4.89E-05	0.000315	UAP1	UDP-N-acet	UAP1_HUM	9	1.052291
48H 10MT/	Q9Y4P8	0.154946	4.89E-05	0.000315	WIPI2	WD repeat	WIPI2_HUM	8	1.11338
24H 50MT/	Q6P1X5	0.289275	4.90E-05	0.000315	TAF2	Transcripti	TAF2_HUM	11	1.222026
48H 50MT/	Q9BYD1	-0.33564	4.91E-05	0.000315	MRPL13	Large ribos	RM13_HUM	12	0.792435
48H 10MT/	Q6ZSJ8	-0.43639	4.94E-05	0.000317	C1orf122	Uncharacte	CA122_HUI	4	0.738982
48H 50MT/	P36871	0.313278	4.97E-05	0.000318	PGM1	Phosphoglu	PGM1_HUM	28	1.242528
48H 10MT/	O15379	-0.12019	4.98E-05	0.000318	HDAC3	Histone dea	HDAC3_HU	7	0.920067
24H 50MT/	P27144	0.20036	5.03E-05	0.00032	AK4	Adenylate k	KAD4_HUM	14	1.148985
48H 50MT/	Q93034	0.113795	5.06E-05	0.000321	CUL5	Cullin-5	CUL5_HUM	35	1.082071
48H 50MT/	O75691	-0.28029	5.08E-05	0.000322	UTP20	Small subu	UTP20_HUI	83	0.823424
48H 50MT/	Q9Y230	0.107951	5.11E-05	0.000323	RUVBL2	RuvB-like 2	RUVB2_HU	28	1.077696
24H 50MT/	P17302	-0.43962	5.12E-05	0.000323	GJA1	Gap junctic	CXA1_HUM	8	0.737326
48H 50MT/	Q9Y305	0.083571	5.12E-05	0.000324	ACOT9	Acyl-coenzy	ACOT9_HUI	20	1.059638
24H 50MT/	O94953	0.877549	5.13E-05	0.000324	KDM4B	Lysine-spec	KDM4B_HU	8	1.837252
48H 50MT/	P01889	0.557444	5.15E-05	0.000325	HLA-B	HLA class I I	HLAB_HUM	8	1.47166
24H 50MT/	Q10471	0.133596	5.15E-05	0.000325	GALNT2	Polypeptid	GALT2_HUI	26	1.097025
24H 50MT/	P61916	0.149499	5.19E-05	0.000326	NPC2	NPC intrace	NPC2_HUM	7	1.109184
24H 50MT/	Q9Y5A7	0.154224	5.19E-05	0.000326	NUB1	NEDD8 ulti	NUB1_HUM	8	1.112823
48H 10MT/	Q02809	0.149441	5.22E-05	0.000328	PLOD1	Procollager	PLOD1_HU	27	1.109139
48H 10MT/	Q9NUQ6	0.059479	5.26E-05	0.000329	SPATS2L	SPATS2-like	SPS2L_HUM	18	1.04209
48H 50MT/	Q9NVI1	-0.25953	5.30E-05	0.00033	FANCI	Fanconi an	FANCI_HUM	53	0.835358
24H 50MT/	Q9BW91	0.103936	5.34E-05	0.000332	NUDT9	ADP-ribose	NUDT9_HU	10	1.074701
48H 50MT/	Q9P0S3	-0.44339	5.42E-05	0.000335	ORMDL1	ORM1-like	ORML1_HU	4	0.735403
24H 50MT/	P23677	0.163772	5.42E-05	0.000336	ITPKA	Inositol-tri	IP3KA_HUM	10	1.120212
48H 50MT/	Q9BZE1	-0.28267	5.43E-05	0.000336	MRPL37	Large ribos	RM37_HUM	19	0.822066
24H 50MT/	Q70UQ0	0.114552	5.45E-05	0.000336	IKBIP	Inhibitor of	IKIP_HUMA	14	1.082638
48H 50MT/	Q15459	0.075	5.45E-05	0.000336	SF3A1	Splicing fac	SF3A1_HUM	34	1.053361
24H 10MT/	Q9NPR2	0.939089	5.46E-05	0.000337	SEMA4B	Semaphori	SEM4B_HU	7	1.917317

24H 50MT/	Q99798	0.085125	5.48E-05	0.000337	ACO2	Aconitate h	ACON_HUM	37	1.060779
48H 50MT/	Q9Y394	0.21187	5.51E-05	0.000338	DHRS7	Dehydroge	DHRS7_HU	14	1.158188
24H 50MT/	P49411	0.097218	5.52E-05	0.000339	TUFM	Elongation	EFTU_HUM	31	1.069709
24H 50MT/	Q13740	0.161666	5.53E-05	0.000339	ALCAM	CD166 anti	CD166_HU	17	1.118578
24H 50MT/	Q9H4G0	0.489832	5.56E-05	0.00034	EPB41L1	Band 4.1-lil	E41L1_HUM	4	1.404281
48H 10MT/	Q96MV1	-0.50648	5.57E-05	0.000341	TLCD4	TLC domair	TLCD4_HUI	4	0.703939
24H 50MT/	Q9UI14	0.621448	5.64E-05	0.000344	RABAC1	Prenylated	PRAF1_HUI	4	1.538419
48H 10MT/	P0C0S8;P2	-0.30109	5.66E-05	0.000344	H2AC11;H2	Histone H2	H2A1_HUM	8	0.81164
48H 10MT/	P50453	0.115916	5.69E-05	0.000345	SERPINB9	Serpin B9	SPB9_HUM	16	1.083663
24H 50MT/	Q8IZV5	0.263988	5.72E-05	0.000346	RDH10	Retinol deh	RDH10_HU	9	1.200794
48H 50MT/	Q03701	-0.19571	5.78E-05	0.000348	CEBPZ	CCAAT/enh	CEBPZ_HUM	36	0.873144
48H 50MT/	Q12834	-0.65653	5.82E-05	0.00035	CDC20	Cell divisio	CDC20_HU	11	0.634402
24H 50MT/	P31350	-0.44723	5.88E-05	0.000352	RRM2	Ribonuclec	RIR2_HUMA	19	0.733451
48H 50MT/	P78527	0.05562	5.92E-05	0.000353	PRKDC	DNA-depen	PRKDC_HU	210	1.039305
24H 50MT/	P43304	0.084457	5.97E-05	0.000355	GPD2	Glycerol-3-	GPDM_HUI	36	1.060289
48H 50MT/	P00533	0.192981	6.01E-05	0.000356	EGFR	Epidermal g	EGFR_HUM	40	1.143123
48H 50MT/	Q8IYU2	-1.17797	6.06E-05	0.000359	HACE1	E3 ubiquiti	HACE1_HUI	2	0.441972
24H 50MT/	Q8N4Q1	0.618332	6.13E-05	0.000361	CHCHD4	Mitochond	MIA40_HUI	4	1.535099
24H 50MT/	P55347	0.235645	6.15E-05	0.000362	PKNOX1	Homeobox	PKNX1_HU	1	1.177433
24H 50MT/	Q96DE5	-0.76895	6.17E-05	0.000362	ANAPC16	Anaphase-p	APC16_HUI	4	0.586844
48H 50MT/	P35249	-0.2167	6.21E-05	0.000364	RFC4	Replicatior	RFC4_HUM	18	0.86053
48H 50MT/	Q13332	0.843969	6.28E-05	0.000366	PTPRS	Receptor-ty	PTPRS_HUM	3	1.794982
24H 50MT/	Q9Y383	0.187145	6.35E-05	0.000368	LUC7L2	Putative RN	LC7L2_HUM	8	1.138509
24H 50MT/	Q15459	0.076098	6.42E-05	0.000371	SF3A1	Splicing fac	SF3A1_HUM	34	1.054163
48H 50MT/	Q96HR9	0.326258	6.44E-05	0.000372	REEP6	Receptor ex	REEP6_HUM	6	1.253758
24H 50MT/	P41252	0.037455	6.48E-05	0.000373	IARS1	Isoleucine-	SYIC_HUMA	60	1.026302
48H 50MT/	Q02809	0.399485	6.48E-05	0.000373	PLOD1	Procollager	PLOD1_HU	27	1.319037
48H 50MT/	P17302	-0.56288	6.48E-05	0.000373	GJA1	Gap junctic	CXA1_HUM	8	0.67695
24H 50MT/	Q9Y6D6	0.123102	6.49E-05	0.000373	ARFGEF1	Brefeldin A	BIG1_HUM	27	1.089074
48H 10MT/	O43633	0.243178	6.56E-05	0.000375	CHMP2A	Charged mu	CHM2A_HU	7	1.183597
24H 10MT/	P01009	-0.1036	6.56E-05	0.000375	SERPINA1	Alpha-1-ant	A1AT_HUM	31	0.930708
24H 50MT/	P13010	0.089354	6.57E-05	0.000376	XRCC5	X-ray repair	XRCC5_HUI	43	1.063893
48H 10MT/	Q9GZM5	-0.03164	6.59E-05	0.000376	YIPF3	Protein YIP	YIPF3_HUM	8	0.978305
48H 50MT/	O95602	-0.21993	6.67E-05	0.00038	POLR1A	DNA-direct	RPA1_HUM	50	0.858605
48H 50MT/	Q13813	0.083542	6.68E-05	0.00038	SPTAN1	Spectrin alp	SPTN1_HUI	146	1.059616
24H 50MT/	Q15428	0.286154	6.70E-05	0.00038	SF3A2	Splicing fac	SF3A2_HUM	9	1.219385
48H 50MT/	Q7Z7N9	0.273359	6.70E-05	0.00038	TMEM179E	Transmemb	T179B_HUI	2	1.208619
48H 50MT/	Q8N0Y2	-0.49437	6.81E-05	0.000385	ZNF444	Zinc finger	ZN444_HUI	3	0.709873
48H 50MT/	Q5T0N5	0.164752	6.89E-05	0.000387	FNBP1L	Formin-bin	FBP1L_HUM	17	1.120974
24H 10MT/	Q9H3K6	-0.34688	6.89E-05	0.000387	BOLA2	BolA-like pr	BOLA2_HUI	5	0.786284
24H 50MT/	Q9Y3B4	0.325743	6.92E-05	0.000388	SF3B6	Splicing fac	SF3B6_HUM	6	1.25331
24H 10MT/	P19022	0.228557	6.95E-05	0.000389	CDH2	Cadherin-2	CADH2_HU	15	1.171663
48H 50MT/	Q14690	-0.24449	6.97E-05	0.00039	PDCD11	Protein RRI	RRP5_HUM	86	0.844116
48H 50MT/	Q9NX62	0.224702	7.01E-05	0.000392	BPNT2	Golgi-reside	IMPA3_HUI	12	1.168536
48H 10MT/	Q9BYV8	0.279178	7.03E-05	0.000392	CEP41	Centrosom	CEP41_HUI	8	1.213504
24H 50MT/	Q7Z4H8	0.129709	7.11E-05	0.000395	POGLUT3	Protein O-g	PLGT3_HUI	21	1.094073
24H 50MT/	P45880	0.121054	7.21E-05	0.000399	VDAC2	Voltage-dep	VDAC2_HU	14	1.087529
48H 50MT/	P17858	0.350473	7.24E-05	0.0004	PFKL	ATP-depend	PFKAL_HUM	30	1.274978
48H 50MT/	O75844	-0.19755	7.27E-05	0.000401	ZMPSTE24	CAAX preny	FACE1_HUM	15	0.872028
48H 50MT/	P02787	0.509116	7.27E-05	0.000401	TF	Serotransfe	TRFE_HUMA	40	1.423178
48H 50MT/	P42345	0.067972	7.28E-05	0.000401	MTOR	Serine/thre	MTOR_HUM	62	1.048242
48H 50MT/	P30520	-0.17334	7.31E-05	0.000402	ADSS2	Adenylosuc	PURA2_HU	17	0.886789

48H 50MT/	Q9BVI4	-0.20319	7.36E-05	0.000404	NOC4L	Nucleolar c	NOC4L_HU	23	0.868626
24H 10MT/	Q2VYF4	0.815252	7.36E-05	0.000404	LETM2	LETM1 dom	LETM2_HU	1	1.759605
24H 50MT/	Q9UHJ6	0.248665	7.40E-05	0.000405	SHPK	Sedoheptul	SHPK_HUM	11	1.188107
48H 50MT/	Q9BTT0	0.287589	7.42E-05	0.000406	ANP32E	Acidic leuci	AN32E_HU	8	1.220599
48H 50MT/	Q15906	-0.12394	7.48E-05	0.000408	VPS72	Vacuolar pr	VPS72_HU	2	0.917681
24H 50MT/	Q9HCC0	0.163002	7.50E-05	0.000409	MCCC2	Methylcrot	MCCB_HUM	24	1.119614
24H 50MT/	P45973	0.134693	7.55E-05	0.00041	CBX5	Chromobo:	CBX5_HUM	9	1.097859
48H 50MT/	Q9NQW6	-0.34835	7.56E-05	0.000411	ANLN	Anillin	ANLN_HUM	37	0.785482
48H 10MT/	Q09666	0.165776	7.62E-05	0.000413	AHNAK	Neuroblast	AHNK_HUM	240	1.121769
24H 10MT/	P00366	0.570877	7.65E-05	0.000414	GLUD1	Glutamate	DHE3_BOV	1	1.485426
48H 50MT/	Q99856	0.124546	7.66E-05	0.000415	ARID3A	AT-rich inte	ARI3A_HUM	17	1.090165
24H 50MT/	O60216	0.171813	7.69E-05	0.000415	RAD21	Double-stra	RAD21_HU	22	1.126473
48H 50MT/	Q6UVJ0	-0.34737	7.71E-05	0.000416	SASS6	Spindle ass	SAS6_HUM	12	0.786016
48H 10MT/	Q8ND24	0.092819	7.71E-05	0.000416	RNF214	RING finger	RN214_HU	20	1.066452
24H 50MT/	P05114	0.6052	7.71E-05	0.000416	HMGN1	Non-histon	HMGN1_HU	3	1.52119
24H 10MT/	P08754	0.112789	7.72E-05	0.000416	GNAI3	Guanine nu	GNAI3_HU	9	1.081317
24H 50MT/	Q9UPP1	1.50367	7.76E-05	0.000418	PHF8	Histone lysi	PHF8_HUM	4	2.835631
24H 50MT/	Q9H329	0.224442	7.77E-05	0.000418	EPB41L4B	Band 4.1-lil	E41LB_HUM	3	1.168325
24H 50MT/	Q92667	0.185703	7.82E-05	0.000419	AKAP1	A-kinase an	AKAP1_HU	21	1.137371
48H 10MT/	Q8IZN3	-0.19505	7.90E-05	0.000423	ZDHHC14	Palmitoyltr	ZDH14_HU	3	0.873544
24H 50MT/	O00429	-0.09302	7.92E-05	0.000423	DNM1L	Dynamin-1	DNM1L_HU	44	0.937556
48H 50MT/	P42704	-0.23901	7.93E-05	0.000423	LRPPRC	Leucine-ric	LPPRC_HU	94	0.847324
48H 50MT/	O75170	0.259957	7.94E-05	0.000424	PPP6R2	Serine/thre	PP6R2_HU	30	1.197443
48H 50MT/	P61158	0.081889	7.97E-05	0.000425	ACTR3	Actin-relate	ARP3_HUM	18	1.058403
24H 50MT/	Q13418	-0.16955	8.03E-05	0.000427	ILK	Integrin-lin	ILK_HUMA	17	0.889117
48H 50MT/	P62256	0.137732	8.11E-05	0.00043	UBE2H	Ubiquitin-c	UBE2H_HU	3	1.100174
48H 50MT/	Q86VP1	-0.41374	8.12E-05	0.00043	TAX1BP1	Tax1-bindi	TAXB1_HU	11	0.750672
48H 50MT/	P60866	-0.06252	8.16E-05	0.000431	RPS20	Small ribos	RS20_HUM	5	0.957591
48H 50MT/	Q14254	0.151741	8.16E-05	0.000431	FLOT2	Flotillin-2	FLOT2_HUM	22	1.110909
48H 50MT/	P15121	0.121619	8.17E-05	0.000431	AKR1B1	Aldo-keto r	ALDR_HUM	19	1.087955
48H 10MT/	Q8NHU6	0.233249	8.22E-05	0.000433	TDRD7	Tudor dom	TDRD7_HU	7	1.175479
24H 50MT/	Q9Y2W1	0.123762	8.23E-05	0.000433	THRAP3	Thyroid hoi	TR150_HU	22	1.089573
24H 50MT/	A0A0B4J2D	0.147036	8.28E-05	0.000435	GATD3B;G/	Putative glu	GAL3B_HU	8	1.107292
24H 50MT/	O95159	0.141454	8.29E-05	0.000435	ZFPL1	Zinc finger	ZFPL1_HUM	10	1.103017
24H 50MT/	Q02543	0.520884	8.37E-05	0.000439	RPL18A	Large ribos	RL18A_HU	8	1.434834
48H 50MT/	P07148	0.615778	8.38E-05	0.000439	FABP1	Fatty acid-b	FABPL_HUM	7	1.532384
48H 50MT/	O60341	-0.00948	8.39E-05	0.000439	KDM1A	Lysine-spec	KDM1A_HU	23	0.993451
48H 10MT/	Q96ER3	0.310405	8.39E-05	0.000439	SAAL1	Protein SA/	SAAL1_HUM	6	1.240056
24H 10MT/	P25325	-0.1826	8.41E-05	0.000439	MPST	3-mercapto	THTM_HUM	15	0.881111
48H 50MT/	P52758	0.174204	8.47E-05	0.000441	RIDA	2-iminobut	RIDA_HUM	5	1.128342
48H 50MT/	P13797	0.167168	8.49E-05	0.000442	PLS3	Plastin-3	PLST_HUM/	38	1.122852
48H 50MT/	P04217	0.789397	8.51E-05	0.000443	A1BG	Alpha-1B-gl	A1BG_HUM	2	1.728352
48H 10MT/	Q9UBL3	0.024501	8.55E-05	0.000444	ASH2L	Set1/Ash2 l	ASH2L_HU	13	1.017128
48H 50MT/	O00560	0.086879	8.58E-05	0.000445	SDCBP	Syntenin-1	SDCB1_HU	8	1.06207
24H 10MT/	P00439	-0.30315	8.63E-05	0.000446	PAH	Phenylalan	PH4H_HUM	30	0.810479
48H 10MT/	Q9UPN3	0.029149	8.63E-05	0.000446	MACF1	Microtubul	MACF1_HU	190	1.02041
48H 50MT/	Q7Z460	0.150624	8.63E-05	0.000446	CLASP1	CLIP-associ	CLAP1_HU	40	1.110049
24H 50MT/	Q5JRA6	0.25629	8.82E-05	0.000453	MIA3	Transport a	TGO1_HUM	43	1.194403
48H 50MT/	Q9UGP8	0.100334	8.88E-05	0.000455	SEC63	Translocati	SEC63_HUM	34	1.072022
24H 50MT/	P14314	0.078934	8.88E-05	0.000455	PRKCSH	Glucosidas	GLU2B_HU	24	1.056237
24H 10MT/	P08833	-0.52315	8.90E-05	0.000455	IGFBP1	Insulin-like	IBP1_HUM/	7	0.69585
48H 50MT/	Q9NP72	0.018653	8.93E-05	0.000456	RAB18	Ras-related	RAB18_HU	12	1.013013

24H 50MTA	Q01970	0.004844	8.96E-05	0.000458	PLCB3	1-phosphat	PLCB3_HUI	25	1.003363
48H 50MTA	Q9BRJ7	-0.17154	8.96E-05	0.000458	NUDT16L1	Tudor-inter	TIRR_HUMA	11	0.887894
24H 10MTA	P12277	-0.20824	8.98E-05	0.000458	CKB	Creatine kir	KCRB_HUM	20	0.865592
24H 50MTA	Q66GS9	1.752179	8.98E-05	0.000458	CEP135	Centrosom	CP135_HUI	1	3.36867
24H 10MTA	Q8WU79	-0.08291	8.99E-05	0.000459	SMAP2	Stromal me	SMAP2_HU	13	0.944152
24H 50MTA	P11498	0.200661	8.99E-05	0.000459	PC	Pyruvate ca	PYC_HUMA	45	1.149225
48H 50MTA	Q5JTH9	-0.1564	9.03E-05	0.00046	RRP12	RRP12-like	RRP12_HUI	45	0.897259
24H 10MTA	P33992	0.070202	9.05E-05	0.00046	MCM5	DNA replica	MCM5_HUI	41	1.049864
48H 50MTA	Q9P016	0.250952	9.05E-05	0.00046	THYN1	Thymocyte	THYN1_HUI	10	1.189992
48H 50MTA	Q9P2J5	0.114208	9.06E-05	0.00046	LARS1	Leucine--tR	SYLC_HUMA	55	1.082381
24H 50MTA	P33991	0.037801	9.07E-05	0.000461	MCM4	DNA replica	MCM4_HUI	45	1.026548
24H 50MTA	P19367	0.252905	9.10E-05	0.000462	HK1	Hexokinase	HXK1_HUM	35	1.191604
24H 50MTA	Q8IYU8	0.111063	9.10E-05	0.000462	MICU2	Calcium up	MICU2_HU	13	1.080024
24H 10MTA	Q12860	0.404634	9.24E-05	0.000467	CNTN1	Contactin-1	CNTN1_HU	37	1.323753
48H 50MTA	Q96DH6	0.191939	9.26E-05	0.000468	MSI2	RNA-bindir	MSI2H_HUI	6	1.142298
48H 10MTA	P61247	0.060041	9.29E-05	0.000468	RPS3A	Small ribos	RS3A_HUM	18	1.042496
48H 50MTA	Q86W92	0.224852	9.29E-05	0.000468	PPFIBP1	Liprin-beta	LIPB1_HUM	28	1.168658
24H 50MTA	Q9H8Y8	0.154163	9.33E-05	0.000469	GORASP2	Golgi reasse	GORS2_HU	15	1.112776
24H 10MTA	P55347	0.250623	9.36E-05	0.00047	PKNOX1	Homeobox	PKNX1_HU	1	1.18972
48H 50MTA	Q9HA64	0.174644	9.36E-05	0.00047	FN3KRP	Ketosamine	KT3K_HUM	9	1.128686
24H 50MTA	P62995	0.330976	9.39E-05	0.000471	TRA2B	Transforme	TRA2B_HUI	6	1.257864
24H 50MTA	Q9UBB4	-0.16768	9.40E-05	0.000472	ATXN10	Ataxin-10	ATX10_HUI	22	0.890275
48H 50MTA	O75521	0.084483	9.41E-05	0.000472	ECI2	Enoyl-CoA c	ECI2_HUMA	18	1.060307
48H 50MTA	P33993	-0.14989	9.44E-05	0.000473	MCM7	DNA replica	MCM7_HUI	38	0.901316
48H 10MTA	Q9NZN8	0.318932	9.46E-05	0.000474	CNOT2	CCR4-NOT t	CNOT2_HU	11	1.247406
24H 10MTA	O75592	-0.25521	9.48E-05	0.000474	MYCBP2	E3 ubiquiti	MYCB2_HU	11	0.837864
24H 50MTA	Q9NPJ3	0.186442	9.52E-05	0.000475	ACOT13	Acyl-coenzy	ACO13_HU	7	1.137954
48H 50MTA	Q01082	0.090302	9.58E-05	0.000477	SPTBN1	Spectrin be	SPTB2_HUI	127	1.064593
24H 50MTA	Q9H7B2	0.221293	9.58E-05	0.000477	RPF2	Ribosome p	RPF2_HUM	17	1.165778
48H 50MTA	P50440	0.364311	9.63E-05	0.000479	GATM	Glycine am	GATM_HUM	19	1.287267
48H 50MTA	Q9Y4Z0	0.383171	9.69E-05	0.000481	LSM4	U6 snRNA-a	LSM4_HUM	5	1.304206
24H 50MTA	P21741	1.05746	9.70E-05	0.000481	MDK	Midkine	MK_HUMAI	2	2.081265
48H 10MTA	P10619	-0.29629	9.71E-05	0.000481	CTSA	Lysosomal p	PPGB_HUM	13	0.814347
24H 10MTA	O75954	0.017969	9.73E-05	0.000482	TSPAN9	Tetraspanir	TSN9_HUM	2	1.012533
24H 50MTA	Q06210	-0.13685	9.75E-05	0.000482	GFPT1	Glutamine-	GFPT1_HUI	39	0.909503
24H 50MTA	Q3YEC7	0.293602	9.78E-05	0.000483	RABL6	Rab-like pro	RABL6_HUI	18	1.225697
24H 50MTA	Q96LD4	0.411781	9.81E-05	0.000484	TRIM47	E3 ubiquiti	TRI47_HUM	7	1.330328
48H 50MTA	Q6ZN17	0.091101	9.83E-05	0.000485	LIN28B	Protein lin-	LN28B_HUI	9	1.065182
24H 50MTA	O60264	0.085436	9.89E-05	0.000487	SMARCA5	SWI/SNF-re	SMCA5_HU	48	1.061009
48H 50MTA	Q92896	0.207694	9.90E-05	0.000487	GLG1	Golgi appar	GSLG1_HUI	40	1.154841
24H 50MTA	Q8N983	-0.17955	9.91E-05	0.000487	MRPL43	Large ribos	RM43_HUM	5	0.88298
24H 50MTA	Q9P2M7	0.152971	9.92E-05	0.000487	CGN	Cingulin	CING_HUM	44	1.111857
24H 50MTA	P02787	0.922025	9.95E-05	0.000488	TF	Serotransfe	TRFE_HUMA	40	1.894774
24H 50MTA	O75533	0.150913	9.96E-05	0.000489	SF3B1	Splicing fac	SF3B1_HUM	66	1.110272
48H 10MTA	Q00587	0.224121	0.0001	0.000492	CDC42EP1	Cdc42 effec	BORG5_HU	6	1.168065
24H 50MTA	P11177	0.17732	0.000101	0.000493	PDHB	Pyruvate de	ODPB_HUM	17	1.130781
24H 50MTA	Q9NPR2	1.567436	0.000102	0.000495	SEMA4B	Semaphori	SEM4B_HU	7	2.963775
48H 10MTA	Q6NUK1	0.06294	0.000102	0.000495	SLC25A24	Mitochond	SCMC1_HU	20	1.044592
24H 50MTA	O75400	0.215765	0.000103	0.000499	PRPF40A	Pre-mRNA-p	PR40A_HUI	18	1.161319
48H 50MTA	P17844	-0.21954	0.000103	0.0005	DDX5	Probable AT	DDX5_HUM	33	0.858842
24H 50MTA	P83111	0.160319	0.000103	0.0005	LACTB	Serine beta	LACTB_HUI	23	1.117534
48H 50MTA	Q9Y5N6	-0.71606	0.000104	0.0005	ORC6	Origin reco	ORC6_HUM	4	0.608758

48H 10MTA	Q15436	0.080622	0.000104	0.000501	SEC23A	Protein tra	SC23A_HUI	30	1.057474
24H 10MTA	P34741	1.485384	0.000105	0.000505	SDC2	Syndecan-2	SDC2_HUM	2	2.799918
48H 50MTA	O43633	0.299433	0.000105	0.000506	CHMP2A	Charged mu	CHM2A_HU	7	1.23066
24H 50MTA	Q9H4A3	0.125961	0.000106	0.000508	WNK1	Serine/thre	WNK1_HUI	36	1.091234
48H 50MTA	P12830	0.538313	0.000106	0.000508	CDH1	Cadherin-1	CADH1_HU	7	1.452273
24H 50MTA	O95445	0.378261	0.000106	0.000508	APOM	Apolipopro	APOM_HUN	4	1.299774
48H 50MTA	P49023	0.209954	0.000107	0.000511	PXN	Paxillin	PAXI_HUMA	18	1.156651
24H 50MTA	Q14678	0.219086	0.000108	0.000514	KANK1	KN motif ar	KANK1_HU	23	1.163996
24H 50MTA	Q8NF37	0.097965	0.000108	0.000514	LPCAT1	Lysophospl	PCAT1_HUI	14	1.070263
24H 50MTA	P24752	0.189132	0.000108	0.000514	ACAT1	Acetyl-CoA	THIL_HUMA	20	1.140077
24H 50MTA	Q9NU22	0.167246	0.000108	0.000514	MDN1	Midasin	MDN1_HUI	115	1.122913
48H 50MTA	Q9BV36	-0.57879	0.000109	0.000516	MLPH	Melanophil	MELPH_HU	16	0.669523
48H 50MTA	P82933	-0.24442	0.000109	0.000516	MRPS9	Small ribos	RT09_HUM	18	0.844155
48H 50MTA	P57737	0.055736	0.000109	0.000516	CORO7	Coronin-7	CORO7_HU	23	1.039389
48H 50MTA	Q8WU79	0.234664	0.000109	0.000516	SMAP2	Stromal me	SMAP2_HU	13	1.176632
48H 50MTA	Q9NRZ9	-0.45284	0.000109	0.000516	HELLS	Lymphoid-s	HELLS_HUN	30	0.730601
24H 50MTA	Q5ZPR3	0.352065	0.000109	0.000518	CD276	CD276 anti	CD276_HU	7	1.276386
48H 10MTA	Q9BYN8	0.040846	0.000109	0.000519	MRPS26	Small ribos	RT26_HUM	9	1.028717
48H 50MTA	P31930	0.094094	0.000109	0.000519	UQCRC1	Cytochrom	QCR1_HUM	17	1.067395
48H 50MTA	O95684	-0.15615	0.00011	0.000521	CEP43	Centrosom	CEP43_HUI	15	0.897416
48H 50MTA	O00541	-0.18078	0.000111	0.000523	PES1	Pescadillo h	PESC_HUM	21	0.882226
48H 10MTA	P63167	0.389723	0.000111	0.000524	DYNLL1	Dynein ligh	DYL1_HUM	1	1.310142
24H 10MTA	P12268	0.069644	0.000112	0.000525	IMPDH2	Inosine-5'-r	IMDH2_HU	29	1.049457
24H 50MTA	O15460	0.568088	0.000112	0.000525	P4HA2	Prolyl 4-hy	P4HA2_HU	16	1.482557
24H 50MTA	O94925	0.085854	0.000112	0.000525	GLS	Glutaminas	GLSK_HUM	21	1.061316
24H 50MTA	P63162	4.035272	0.000112	0.000526	SNRPN	Small nucle	RSMN_HUN	1	16.396
48H 50MTA	P22314	0.123361	0.000112	0.000526	UBA1	Ubiquitin-l	UBA1_HUM	46	1.089269
48H 50MTA	Q9Y4F1	0.171639	0.000113	0.000529	FARP1	FERM, ARH	FARP1_HUI	22	1.126337
24H 50MTA	O60341	0.117463	0.000114	0.000531	KDM1A	Lysine-spec	KDM1A_HU	23	1.084826
24H 50MTA	P49207	0.160909	0.000114	0.000533	RPL34	Large ribos	RL34_HUM	2	1.117991
48H 10MTA	Q92769	0.149167	0.000114	0.000534	HDAC2	Histone dea	HDAC2_HU	8	1.108929
48H 50MTA	P09012	0.238903	0.000115	0.000535	SNRPA	U1 small nu	SNRPA_HUI	10	1.180095
48H 10MTA	Q15031	0.056444	0.000115	0.000536	LARS2	Leucine--tR	SYLM_HUM	25	1.0399
48H 50MTA	P50995	0.110179	0.000115	0.000536	ANXA11	Annexin A1	ANX11_HU	19	1.079362
24H 50MTA	P11169	0.263969	0.000116	0.000538	SLC2A3	Solute carri	GTR3_HUM	7	1.200778
48H 50MTA	O75643	-0.19983	0.000116	0.000539	SNRNP200	U5 small nu	U520_HUM	116	0.870656
48H 10MTA	Q92841	0.026786	0.000116	0.000539	DDX17	Probable A	DDX17_HU	27	1.01874
48H 10MTA	Q9BST9	0.093211	0.000117	0.00054	RTKN	Rhotekin	RTKN_HUM	11	1.066742
48H 50MTA	P31350	-0.40148	0.000118	0.000543	RRM2	Ribonuclec	RIR2_HUMA	19	0.757081
48H 50MTA	Q9NY93	-0.35785	0.000118	0.000544	DDX56	Probable A	DDX56_HU	22	0.780329
24H 50MTA	Q6KC79	0.138007	0.000119	0.000545	NIPBL	Nipped-B-li	NIPBL_HUN	47	1.100384
24H 50MTA	Q9Y230	0.05128	0.000119	0.000545	RUVBL2	RuvB-like 2	RUVB2_HU	28	1.036184
24H 50MTA	Q92597	0.98208	0.000119	0.000545	NDRG1	Protein ND	NDRG1_HU	12	1.975311
48H 50MTA	Q14669	-0.22383	0.000119	0.000546	TRIP12	E3 ubiquiti	TRIPC_HUN	55	0.856291
24H 50MTA	Q5VWN6	0.434769	0.00012	0.000547	TASOR2	Protein TAS	TASO2_HUI	3	1.351694
48H 10MTA	Q9Y241	-0.47879	0.00012	0.000549	HIGD1A	HIG1 doma	HIG1A_HUI	3	0.717581
24H 50MTA	Q9H0D6	0.071423	0.000122	0.000554	XRN2	5'-3' exorib	XRN2_HUM	38	1.050752
24H 50MTA	Q96H79	0.23038	0.000122	0.000555	ZC3HAV1L	Zinc finger	ZCCHL_HUI	13	1.173144
48H 10MTA	Q7Z7N9	0.225188	0.000123	0.000557	TMEM179B	Transmemb	T179B_HUI	2	1.168929
24H 50MTA	Q9BR76	-0.15324	0.000123	0.000558	CORO1B	Coronin-1B	COR1B_HU	17	0.899228
48H 50MTA	Q53GA4	-0.96007	0.000123	0.000559	PHLDA2	Pleckstrin h	PHLA2_HUI	1	0.514032
24H 50MTA	Q96S66	0.047741	0.000123	0.000559	CLCC1	Chloride ch	CLCC1_HUI	20	1.033645

48H 50MT/	P30626	0.283816	0.000123	0.000559	SRI	Sorcin	SORCN_HU	8	1.217411
24H 50MT/	Q68E01	0.22123	0.000124	0.000559	INTS3	Integrator (	INT3_HUM/	30	1.165727
48H 50MT/	Q9H8H0	-0.23021	0.000124	0.00056	NOL11	Nucleolar p	NOL11_HU	24	0.85251
48H 10MT/	Q9UMX0	0.174469	0.000125	0.000565	UBQLN1	Ubiquilin-1	UBQL1_HU	15	1.128549
48H 50MT/	P00813	0.185616	0.000126	0.000566	ADA	Adenosine (	ADA_HUM/	14	1.137303
48H 50MT/	Q9H6R4	-0.29205	0.000126	0.000566	NOL6	Nucleolar p	NOL6_HUM	26	0.816743
24H 10MT/	Q02952	0.050661	0.000126	0.000567	AKAP12	A-kinase an	AKA12_HU	79	1.03574
24H 50MT/	P51572	0.143912	0.000126	0.000568	BCAP31	B-cell recep	BAP31_HU	11	1.104897
48H 10MT/	Q01813	0.13915	0.000126	0.000568	PFKP	ATP-depen(	PFKAP_HU	27	1.101256
48H 10MT/	P78509	0.088643	0.000128	0.000571	RELN	Reelin	RELN_HUM	48	1.06337
24H 50MT/	Q02218	0.009512	0.000128	0.000571	OGDH	2-oxogluta	ODO1_HUM	41	1.006615
24H 50MT/	Q92797	0.137575	0.000128	0.000571	SYMPK	Symplekin	SYMPK_HU	40	1.100054
24H 50MT/	Q96KC8	0.14349	0.000128	0.000574	DNAJC1	DnaJ homo	DNJC1_HU	10	1.104574
48H 10MT/	Q9BUB5	-0.1324	0.000129	0.000574	MKNK1	MAP kinase	MKNK1_HU	3	0.912313
48H 50MT/	Q96RR1	-0.58305	0.000129	0.000576	TWNK	Twinkle mt	PEO1_HUM	13	0.667551
24H 50MT/	Q9Y277	0.072945	0.000129	0.000576	VDAC3	Voltage-de	VDAC3_HU	13	1.051861
48H 50MT/	Q16851	0.150235	0.000129	0.000577	UGP2	UTP--gluco	UGPA_HUM	25	1.10975
48H 50MT/	O15344	0.205667	0.00013	0.000578	MID1	E3 ubiquiti	TRI18_HUM	20	1.15322
48H 50MT/	P53350	-0.55869	0.00013	0.000578	PLK1	Serine/thre	PLK1_HUM	21	0.678917
24H 50MT/	Q9Y617	-0.01835	0.00013	0.00058	PSAT1	Phosphoser	SERC_HUM	20	0.987358
24H 10MT/	P17936	-0.49212	0.000131	0.000582	IGFBP3	Insulin-like	IBP3_HUM/	5	0.710979
48H 50MT/	Q9UNY4	-0.44888	0.000131	0.000582	TTF2	Transcripti	TTF2_HUM/	32	0.732609
24H 50MT/	Q9UMN6	0.307903	0.000131	0.000582	KMT2B	Histone-lys	KMT2B_HU	12	1.237907
48H 50MT/	Q9UKD2	-0.19808	0.000131	0.000582	MRTO4	mRNA turn	MRT4_HUM	15	0.871709
48H 50MT/	Q9BRJ2	-0.30428	0.000132	0.000586	MRPL45	Large ribos	RM45_HUM	14	0.809845
24H 50MT/	P00533	0.172286	0.000132	0.000586	EGFR	Epidermal g	EGFR_HUM	40	1.126842
24H 50MT/	P51531	0.238056	0.000133	0.000587	SMARCA2	Probable gl	SMCA2_HU	12	1.179403
24H 50MT/	P25205	0.004799	0.000133	0.000587	MCM3	DNA replica	MCM3_HU	37	1.003332
48H 10MT/	Q86W92	0.104688	0.000133	0.000587	PPFIBP1	Liprin-beta	LIPB1_HUM	28	1.075262
24H 50MT/	Q86WQ0	-0.27797	0.000134	0.000589	NR2C2AP	Nuclear rec	NR2CA_HU	5	0.824749
48H 50MT/	Q3YEC7	0.269775	0.000134	0.000591	RABL6	Rab-like pr(	RABL6_HU	18	1.20562
24H 50MT/	P08648	0.191283	0.000134	0.000591	ITGA5	Integrin alp	ITA5_HUM/	19	1.141778
24H 50MT/	Q9P016	-0.00565	0.000134	0.000591	THYN1	Thymocyte	THYN1_HU	10	0.996093
24H 50MT/	Q9Y3Y2	0.016891	0.000135	0.000593	CHTOP	Chromatin	CHTOP_HU	6	1.011777
48H 50MT/	P29317	-0.75823	0.000135	0.000594	EPHA2	Ephrin type	EPHA2_HU	17	0.591221
48H 50MT/	Q9NZJ9	-0.21558	0.000135	0.000594	NUDT4	Diphospho	NUDT4_HU	6	0.8612
48H 50MT/	P15882	0.73526	0.000136	0.000596	CHN1	N-chimaeri	CHIN_HUM	2	1.664697
48H 50MT/	Q8WXE9	0.69463	0.000136	0.000597	STON2	Stonin-2	STON2_HU	1	1.618469
48H 10MT/	P42166	0.072436	0.000136	0.000597	TMPO	Lamina-ass	LAP2A_HU	35	1.051491
24H 50MT/	Q07065	0.118366	0.000137	0.0006	CKAP4	Cytoskeleto	CKAP4_HU	29	1.085505
48H 50MT/	Q86SQ4	-0.55395	0.000138	0.0006	ADGRG6	Adhesion G	AGRG6_HU	8	0.681153
48H 50MT/	P82650	-0.17401	0.000138	0.000601	MRPS22	Small ribos	RT22_HUM	15	0.886376
24H 10MT/	P07919	-0.48657	0.000138	0.000602	UQCRH	Cytochrom	QCR6_HUM	3	0.713721
48H 50MT/	P02675	0.18472	0.000139	0.000604	FGB	Fibrinogen	FIBB_HUM/	22	1.136596
48H 10MT/	Q9HA64	0.200807	0.00014	0.000609	FN3KRP	Ketosamine	KT3K_HUM	9	1.149341
48H 50MT/	Q08623	0.360381	0.000142	0.000611	PUDP	Pseudourid	HDHD1_HU	11	1.283765
24H 50MT/	P07686	0.076262	0.000142	0.000611	HEXB	Beta-hexos	HEXB_HUM	22	1.054283
48H 50MT/	P55157	0.319034	0.000142	0.000612	MTTP	Microsoma	MTP_HUM/	36	1.247495
48H 50MT/	Q9Y5Z4	0.203377	0.000142	0.000612	HEBP2	Heme-bind	HEBP2_HU	9	1.15139
48H 10MT/	Q9NV06	0.042079	0.000143	0.000615	DCAF13	DDB1- and	DCA13_HU	14	1.029596
48H 50MT/	P14314	0.116996	0.000144	0.000616	PRKCSH	Glucosidas	GLU2B_HU	24	1.084474
48H 50MT/	O75976	0.241843	0.000145	0.00062	CPD	Carboxype	CBPD_HUM	44	1.182503

24H 50MT/	Q8N5M1	0.157147	0.000146	0.000625	ATPAF2	ATP syntha:	ATPF2_HUI	8	1.11508
24H 50MT/	P54886	0.248383	0.000148	0.000629	ALDH18A1	Delta-1-pyr	P5CS_HUM	42	1.187875
24H 50MT/	Q9P0J0	0.095135	0.000148	0.000629	NDUFA13	NADH dehy	NDUAD_HU	9	1.068165
48H 50MT/	Q99988	-0.8907	0.000148	0.000629	GDF15	Growth/dif	GDF15_HU	7	0.539352
48H 50MT/	Q15047	-0.19002	0.000148	0.000629	SETDB1	Histone-lys	SETB1_HUI	13	0.876592
48H 50MT/	Q9BXS6	-0.37481	0.000148	0.00063	NUSAP1	Nucleolar a	NUSAP_HU	11	0.771209
24H 50MT/	Q3SXM5	0.127638	0.000148	0.00063	HSDL1	Inactive hy	HSDL1_HU	7	1.092504
48H 50MT/	Q15417	0.078981	0.000149	0.000632	CNN3	Calponin-3	CNN3_HUN	15	1.056272
24H 50MT/	P09651	0.081605	0.000149	0.000632	HNRNPA1	Heterogene	ROA1_HUN	22	1.058194
48H 10MT/	Q15835	-2.47841	0.000149	0.000633	GRK1	Rhodopsin	GRK1_HUN	1	0.179443
24H 50MT/	P08397	-0.19092	0.000149	0.000633	HMBS	Porphobili	HEM3_HUN	17	0.876047
24H 50MT/	Q9HD23	0.265492	0.00015	0.000633	MRS2	Magnesium	MRS2_HUN	15	1.202046
24H 50MT/	Q13217	0.19768	0.00015	0.000635	DNAJC3	DnaJ homo	DNJC3_HU	21	1.146853
24H 50MT/	Q53GQ0	0.209515	0.000152	0.000641	HSD17B12	Very-long-c	DHB12_HU	14	1.1563
24H 50MT/	P07237	0.110471	0.000152	0.000642	P4HB	Protein dis	PDIA1_HUI	35	1.079581
24H 10MT/	Q7Z4H8	0.13421	0.000153	0.000645	POGLUT3	Protein O-g	PLGT3_HUI	21	1.097492
24H 10MT/	P63092;Q5	0.027418	0.000154	0.000645	GNAS	Guanine nu	GNAS2_HU	14	1.019186
48H 50MT/	Q9H8V3	-0.4853	0.000154	0.000645	ECT2	Protein ECT	ECT2_HUM	28	0.714349
24H 50MT/	Q92621	0.030797	0.000154	0.000647	NUP205	Nuclear po	NU205_HU	72	1.021576
24H 10MT/	Q9BXW9	0.159174	0.000154	0.000647	FANCD2	Fanconi an	FACD2_HUI	34	1.116648
24H 10MT/	Q15011	-0.45575	0.000155	0.000648	HERPUD1	Homocyste	HERP1_HUI	5	0.72913
24H 10MT/	Q5VSG8	0.286454	0.000155	0.000649	MANEAL	Glycoprote	MANEL_HU	1	1.219639
48H 50MT/	O60763	0.141105	0.000155	0.000649	USO1	General ves	USO1_HUN	42	1.102749
48H 50MT/	P11182	0.110062	0.000155	0.00065	DBT	Lipoamide	ODB2_HUN	22	1.079275
48H 50MT/	Q9P287	0.120829	0.000156	0.000651	BCCIP	BRCA2 and	BCCIP_HUN	14	1.087359
24H 50MT/	P61978	0.082078	0.000156	0.000652	HNRNPK	Heterogene	HNRPK_HU	27	1.058541
24H 50MT/	Q96BD5	0.139907	0.000156	0.000653	PHF21A	PHD finger	PF21A_HUI	5	1.101834
48H 50MT/	Q96BI1	0.380191	0.000157	0.000655	SLC22A18	Solute carri	S22AI_HUN	4	1.301514
24H 50MT/	Q9BXW7	0.242754	0.000157	0.000656	HDHD5	Haloacid de	HDHD5_HU	16	1.183249
24H 50MT/	Q5QP82	-2.97317	0.000158	0.000656	DCAF10	DDB1- and	DCA10_HU	1	0.127346
24H 10MT/	Q9UBB4	-0.17499	0.000158	0.000656	ATXN10	Ataxin-10	ATX10_HUI	22	0.885774
48H 10MT/	Q92575	0.239071	0.000158	0.000657	UBXN4	UBX domai	UBXN4_HU	9	1.180232
24H 50MT/	Q96IV0	0.103191	0.000158	0.000658	NGLY1	Peptide-N(	NGLY1_HUI	11	1.074146
24H 50MT/	P46940	-0.08325	0.000158	0.000658	IQGAP1	Ras GTPase	IQGA1_HUI	83	0.943929
48H 50MT/	P15104	-0.30039	0.000158	0.000658	GLUL	Glutamine	GLNA_HUM	7	0.812031
48H 50MT/	Q99798	0.083185	0.000159	0.000659	ACO2	Aconitate h	ACON_HUM	37	1.059354
48H 50MT/	Q9BV44	-0.01518	0.00016	0.000661	THUMPD3	tRNA (guan	THUM3_HU	14	0.989537
48H 50MT/	Q96DM3	-0.02549	0.00016	0.000663	RMC1	Regulator c	RMC1_HUN	11	0.982489
24H 10MT/	Q04446	-0.15209	0.000161	0.000664	GBE1	1,4-alpha-g	GLGB_HUM	27	0.899948
48H 50MT/	P52569	0.375552	0.000163	0.00067	SLC7A2	Cationic an	CTR2_HUM	8	1.297336
24H 50MT/	Q9H9Y6	0.067517	0.000163	0.000671	POLR1B	DNA-direct	RPA2_HUM	31	1.047912
24H 10MT/	Q01650	0.415488	0.000164	0.000672	SLC7A5	Large neutr	LAT1_HUM	8	1.33375
48H 50MT/	Q9NYU2	0.163838	0.000165	0.000675	UGGT1	UDP-glucos	UGGG1_HU	67	1.120263
48H 50MT/	Q9Y6N7	0.161473	0.000166	0.000678	ROBO1	Roundabou	ROBO1_HU	39	1.118428
48H 50MT/	P55011	-0.34649	0.000166	0.000679	SLC12A2	Solute carri	S12A2_HUI	31	0.786493
24H 50MT/	Q9NV35	0.443589	0.000166	0.000679	NUDT15	Nucleotide	NUD15_HU	7	1.359983
24H 50MT/	Q9H0V1	0.428769	0.000167	0.000683	TMEM168	Transmemb	TM168_HU	3	1.346084
24H 50MT/	Q6ZW76	1.591539	0.000168	0.000684	ANKS3	Ankyrin rep	ANKS3_HUI	1	3.013707
24H 10MT/	Q149N8	0.082704	0.00017	0.00069	SHPRH	E3 ubiquiti	SHPRH_HU	12	1.059001
48H 50MT/	O43175	0.12839	0.00017	0.00069	PHGDH	D-3-phosph	SERA_HUM	24	1.093073
24H 50MT/	P68431	0.692693	0.000171	0.000691	H3C1	Histone H3	H31_HUMA	6	1.616297
24H 50MT/	Q6NW29	1.236664	0.000171	0.000691	RWDD4	RWD doma	RWDD4_HU	3	2.356529

48H 10MT/	Q15154	0.112288	0.000171	0.000692	PCM1	Pericentrio	PCM1_HUM	73	1.080941
48H 50MT/	P07099	0.096173	0.000171	0.000692	EPHX1	Epoxide hy	HYEP_HUM	19	1.068934
24H 50MT/	Q629K1	0.095336	0.000171	0.000693	TRIQK	Triple QxxK	TRIQK_HUM	1	1.068314
24H 50MT/	P04075	0.13139	0.000171	0.000693	ALDOA	Fructose-bi	ALDOA_HU	29	1.095349
48H 50MT/	Q01581	-0.35613	0.000171	0.000693	HMGCS1	Hydroxyme	HMCS1_HU	23	0.781257
24H 50MT/	Q9HD45	0.183148	0.000171	0.000693	TM9SF3	Transmemb	TM9S3_HU	13	1.135359
48H 10MT/	Q15417	0.077641	0.000173	0.000696	CNN3	Calponin-3	CNN3_HUM	15	1.055291
24H 50MT/	P55157	0.249649	0.000173	0.000696	MTTP	Microsoma	MTP_HUM/	36	1.188918
48H 50MT/	P21399	0.1836	0.000173	0.000696	ACO1	Cytoplasmi	ACOHC_HU	41	1.135715
48H 50MT/	O00232	0.114184	0.000174	0.0007	PSMD12	26S protea	PSD12_HU	26	1.082363
24H 50MT/	P35558	0.373083	0.000175	0.000702	PCK1	Phosphoen	PCKGC_HU	18	1.295118
48H 50MT/	Q9NVJ2	0.178636	0.000175	0.000702	ARL8B	ADP-ribosy	ARL8B_HU	6	1.131813
24H 50MT/	Q6P2Q9	0.086248	0.000176	0.000705	PRPF8	Pre-mRNA-|	PRP8_HUM	107	1.061606
24H 10MT/	P46379	-0.05105	0.000177	0.000707	BAG6	Large prolir	BAG6_HUM	36	0.965231
24H 50MT/	Q9Y6Y8	0.045594	0.000177	0.000707	SEC23IP	SEC23-inter	S23IP_HUM	27	1.032108
24H 50MT/	Q99623	0.103132	0.000178	0.00071	PHB2	Prohibitin-	PHB2_HUM	19	1.074102
48H 50MT/	P51452	-0.13976	0.000179	0.000712	DUSP3	Dual specifi	DUS3_HUM	8	0.907671
48H 50MT/	P37173	-0.62092	0.000179	0.000713	TGFBR2	TGF-beta re	TGFR2_HU	12	0.650258
24H 50MT/	Q13427	0.279996	0.00018	0.000715	PPIG	Peptidyl-pr	PPIG_HUM	8	1.214192
48H 10MT/	Q92878	0.02765	0.00018	0.000715	RAD50	DNA repair	RAD50_HU	48	1.01935
48H 50MT/	Q13838	0.14459	0.000181	0.000717	DDX39B	Spliceosom	DX39B_HU	22	1.105416
48H 50MT/	Q10471	0.190867	0.000181	0.000718	GALNT2	Polypeptid	GALT2_HU	26	1.14145
24H 50MT/	P50440	0.228997	0.000181	0.000718	GATM	Glycine am	GATM_HUM	19	1.17202
24H 50MT/	Q9UKV3	0.278159	0.000182	0.000721	ACIN1	Apoptotic	ACINU_HU	29	1.212646
48H 50MT/	Q9NUL7	-0.49018	0.000183	0.000722	DDX28	Probable A	DDX28_HU	11	0.711937
48H 50MT/	P16118	0.614955	0.000183	0.000723	PFKFB1	6-phospho	F261_HUM	6	1.53151
24H 50MT/	P61513	0.301251	0.000184	0.000724	RPL37A	Large ribos	RL37A_HU	3	1.232213
24H 50MT/	Q9NRV9	-0.08001	0.000184	0.000724	HEBP1	Heme-bind	HEBP1_HU	8	0.946051
24H 50MT/	P51858	0.116524	0.000185	0.000727	HDGF	Hepatoma-	HDGF_HUM	22	1.08412
48H 50MT/	O95786	0.16682	0.000186	0.000732	RIGI	Antiviral in	RIGI_HUM/	16	1.122581
24H 50MT/	Q9HC52	0.6004	0.000188	0.000737	CBX8	Chromobo	CBX8_HUM	9	1.516137
24H 50MT/	Q9UBK8	-0.22608	0.00019	0.000741	MTRR	Methionine	MTRR_HUM	10	0.854956
24H 50MT/	P61026	0.163651	0.00019	0.000742	RAB10	Ras-related	RAB10_HU	11	1.120118
24H 50MT/	Q7LGA3	0.117828	0.00019	0.000743	HS2ST1	Heparan su	HS2ST_HUM	13	1.0851
48H 50MT/	P07355	0.120299	0.000191	0.000745	ANXA2	Annexin A2	ANXA2_HU	28	1.08696
48H 50MT/	Q12888	0.071458	0.000192	0.000747	TP53BP1	TP53-bindi	TP53B_HU	51	1.050778
48H 50MT/	P02771	-0.55983	0.000192	0.000747	AFP	Alpha-feto	FETA_HUM/	3	0.678383
48H 50MT/	P14923	0.166484	0.000193	0.000749	JUP	Junction pl	PLAK_HUM	30	1.12232
48H 50MT/	P0C0L4	0.830823	0.000193	0.00075	C4A	Complemei	CO4A_HUM	10	1.7787
48H 50MT/	Q16630	0.104843	0.000194	0.000753	CPSF6	Cleavage ar	CPSF6_HU	11	1.075377
24H 50MT/	P00338	0.089411	0.000194	0.000753	LDHA	L-lactate de	LDHA_HUM	27	1.063936
24H 50MT/	Q15149	0.026263	0.000195	0.000754	PLEC	Plectin	PLEC_HUM	199	1.018371
24H 50MT/	P49406	0.027242	0.000195	0.000755	MRPL19	Large ribos	RM19_HUM	12	1.019062
24H 50MT/	Q92841	-0.14394	0.000195	0.000756	DDX17	Probable A	DDX17_HU	27	0.905042
48H 50MT/	Q8N3Z3	-0.46262	0.000196	0.000756	GTPBP8	GTP-bindin	GTPB8_HU	3	0.725667
24H 50MT/	Q13061	-0.70498	0.000196	0.000757	TRDN	Triadin	TRDN_HUM	1	0.613453
24H 50MT/	O60513	-2.52657	0.000197	0.000761	B4GALT4	Beta-1,4-ga	B4GT4_HU	2	0.17355
24H 10MT/	P01889	0.123528	0.000198	0.000762	HLA-B	HLA class I	HLAB_HUM	8	1.089396
48H 50MT/	P08195	-0.20832	0.000199	0.000766	SLC3A2	Amino acid	4F2_HUMA	32	0.865542
24H 50MT/	P47914	0.69429	0.0002	0.000766	RPL29	Large ribos	RL29_HUM	2	1.618088
24H 10MT/	P49773	-0.361	0.0002	0.000767	HINT1	Adenosine	HINT1_HU	9	0.778623
24H 50MT/	Q9UHB6	0.140744	0.0002	0.000768	LIMA1	LIM domai	LIMA1_HU	22	1.102473

24H 50MT/	Q15776;Q4	-2.18933	0.000202	0.000771	ZKSCAN8;ZI	Zinc finger	ZKSC8_HUI	1	0.219254
24H 50MT/	A0AVI4	-0.98914	0.000202	0.000773	TMEM129	E3 ubiquiti	TM129_HU	1	0.503779
48H 50MT/	Q9BYC9	-0.44216	0.000204	0.000777	MRPL20	Large ribos	RM20_HUI	4	0.736032
48H 50MT/	Q9H477	0.611477	0.000204	0.000777	RBKS	Ribokinase	RBSK_HUM	6	1.527823
24H 50MT/	P07947	0.124053	0.000204	0.000777	YES1	Tyrosine-pr	YES_HUMA	18	1.089792
48H 50MT/	Q06787	0.127253	0.000205	0.000779	FMR1	Fragile X m	FMR1_HUI	11	1.092212
24H 50MT/	Q9NPB8	-0.27232	0.000205	0.000781	GPCPD1	Glycerophc	GPCP1_HU	6	0.827986
24H 50MT/	Q9H2U2	0.039581	0.000206	0.000781	PPA2	Inorganic p	IPYR2_HUI	14	1.027815
48H 50MT/	P50570	0.161226	0.000206	0.000782	DNM2	Dynamin-2	DYN2_HUI	43	1.118237
48H 10MT/	Q15388	-0.39242	0.000206	0.000782	TOMM20	Mitochond	TOM20_HU	3	0.761852
48H 50MT/	Q53EL6	0.557338	0.000206	0.000783	PDCD4	Programme	PDCD4_HU	12	1.471551
48H 50MT/	Q8NBJ5	0.111068	0.000207	0.000783	COLGALT1	Procollager	GT251_HU	26	1.080027
24H 50MT/	P60842	-0.15318	0.000207	0.000785	EIF4A1	Eukaryotic	IF4A1_HUI	30	0.899267
48H 50MT/	Q9H6F5	-0.42036	0.000208	0.000786	CCDC86	Coiled-coil	CCD86_HU	17	0.747236
48H 50MT/	Q8IWB7	0.200167	0.000208	0.000786	WDFY1	WD repeat	WDFY1_HU	15	1.148831
48H 50MT/	Q9HB40	0.226857	0.000209	0.000788	SCPEP1	Retinoid-in	RISC_HUM/	11	1.170282
48H 10MT/	Q99661	0.116997	0.000212	0.000796	KIF2C	Kinesin-like	KIF2C_HUI	22	1.084475
24H 10MT/	Q8NCR0	-0.57791	0.000213	0.000798	B3GALNT2	UDP-GalNA	B3GL2_HU	2	0.669931
24H 50MT/	P50570	0.077689	0.000213	0.000799	DNM2	Dynamin-2	DYN2_HUI	43	1.055326
48H 10MT/	P07902	-0.2138	0.000215	0.000804	GALT	Galactose-1	GALT_HUM	8	0.862263
48H 10MT/	P42765	0.170586	0.000215	0.000804	ACAA2	3-ketoacyl-	THIM_HUI	20	1.125516
24H 50MT/	Q9H5Z1	0.095914	0.000215	0.000805	DHX35	Probable A	DHX35_HU	15	1.068743
48H 50MT/	Q9NTJ3	-0.15258	0.000215	0.000805	SMC4	Structural r	SMC4_HUI	56	0.89964
48H 10MT/	Q14839	0.099856	0.000216	0.000805	CHD4	Chromodo	CHD4_HUI	68	1.071666
24H 50MT/	Q86UU1	-0.22178	0.000216	0.000806	PHLDB1	Pleckstrin I	PHLB1_HU	12	0.857507
24H 50MT/	Q15154	0.115883	0.000217	0.000808	PCM1	Pericentrio	PCM1_HUI	73	1.083638
24H 50MT/	Q96PU5	0.18081	0.000218	0.000811	NEDD4L	E3 ubiquiti	NED4L_HU	27	1.13352
24H 50MT/	Q9H3K6	-0.34999	0.000218	0.000811	BOLA2	BolA-like pr	BOLA2_HU	5	0.78459
24H 10MT/	P42330	-0.29575	0.000219	0.000813	AKR1C3	Aldo-keto r	AK1C3_HU	11	0.81465
48H 10MT/	Q15404	0.060812	0.000219	0.000813	RSU1	Ras suppres	RSU1_HUI	11	1.043052
48H 50MT/	O00411	-0.12245	0.000219	0.000813	POLRMT	DNA-direct	RPOM_HUI	32	0.918624
48H 50MT/	P12694	0.227589	0.000219	0.000813	BCKDHA	2-oxoisoval	ODBA_HUI	13	1.170877
48H 50MT/	O00584	0.651483	0.000219	0.000813	RNASET2	Ribonuclea	RNT2_HUI	11	1.570782
24H 50MT/	Q02809	0.15982	0.000219	0.000813	PLOD1	Procollager	PLOD1_HU	27	1.117148
24H 50MT/	P53007	0.319478	0.000219	0.000814	SLC25A1	Tricarboxyl	TXTP_HUM	10	1.247879
24H 50MT/	Q2VYF4	0.970757	0.00022	0.000815	LETM2	LETM1 dom	LETM2_HU	1	1.959869
24H 50MT/	Q9P013	0.603514	0.00022	0.000815	CWC15	Spliceosom	CWC15_HU	9	1.519413
24H 50MT/	O60294	0.076215	0.00022	0.000817	LCMT2	tRNA wybu	TYW4_HUI	9	1.054249
24H 50MT/	O95232	0.284765	0.000221	0.000819	LUC7L3	Luc7-like pi	LC7L3_HUI	10	1.218212
48H 50MT/	P55061	-0.53758	0.000222	0.00082	TMBIM6	Bax inhibit	BI1_HUMA	3	0.688924
48H 50MT/	Q16877	1.126717	0.000223	0.000823	PFKFB4	6-phospho	F264_HUM	9	2.183612
48H 50MT/	P35221	0.187103	0.000223	0.000823	CTNNA1	Catenin alp	CTNA1_HU	44	1.138475
48H 10MT/	P84243	-1.49291	0.000223	0.000823	H3-3A	Histone H3	H33_HUM/	1	0.355296
24H 50MT/	P20933	1.840814	0.000224	0.000825	AGA	N(4)-(beta-I	ASPG_HUM	1	3.582121
48H 50MT/	Q92917	0.000841	0.000224	0.000826	GPKOW	G-patch do	GPKOW_HU	19	1.000583
48H 10MT/	P60880	0.144543	0.000226	0.00083	SNAP25	Synaptosor	SNP25_HU	9	1.105381
24H 50MT/	Q5VSG8	-0.0735	0.000226	0.00083	MANEAL	Glycoprote	MANEL_HU	1	0.950329
24H 50MT/	Q9NQ94	0.135372	0.000226	0.00083	A1CF	APOBEC1 c	A1CF_HUM	33	1.098376
48H 50MT/	Q7L3T8	0.215975	0.000227	0.000831	PARS2	Probable pi	SYPM_HUI	11	1.161488
48H 50MT/	Q8WZA9	-0.22672	0.000227	0.000832	IRGQ	Immunity-r	IRGQ_HUM	17	0.854576
24H 50MT/	P49914	0.049959	0.000227	0.000833	MTHFS	5-formyltet	MTHFS_HU	8	1.035235
48H 50MT/	P63272	-0.20708	0.000229	0.000838	SUPT4H1	Transcripti	SPT4H_HUI	5	0.866287

24H 50MTA	P05388	0.147729	0.000229	0.000838	RPLP0	Large ribos	RLA0_HUM	13	1.107824
48H 50MTA	Q6P996	0.141445	0.000231	0.000842	PDXDC1	Pyridoxal-d	PDXD1_HU	33	1.10301
24H 50MTA	Q12906	0.08042	0.000231	0.000843	ILF3	Interleukin	ILF3_HUMA	38	1.057326
24H 50MTA	Q12959	0.151142	0.000231	0.000844	DLG1	Disks large	DLG1_HUM	22	1.110448
24H 50MTA	Q16540	0.175919	0.000232	0.000846	MRPL23	Large ribos	RM23_HUM	9	1.129684
24H 50MTA	Q9HC35	0.165381	0.000232	0.000846	EML4	Echinoderm	EMAL4_HU	37	1.121462
24H 50MTA	O75976	0.168016	0.000234	0.000851	CPD	Carboxypep	CBPD_HUM	44	1.123513
24H 50MTA	O00479	0.774508	0.000234	0.000851	HMGN4	High mobil	HMGN4_HU	1	1.710606
24H 50MTA	Q14353	0.320489	0.000235	0.000852	GAMT	Guanidinoa	GAMT_HUM	10	1.248754
48H 50MTA	P02649	0.218399	0.000235	0.000853	APOE	Apolipopro	APOE_HUM	17	1.163442
24H 10MTA	P04217	1.984646	0.000237	0.000855	A1BG	Alpha-1B-gl	A1BG_HUM	2	3.957655
48H 10MTA	Q9P2J5	0.047573	0.000237	0.000856	LARS1	Leucine--tR	SYLC_HUMA	55	1.033525
48H 50MTA	P26038	0.187176	0.000237	0.000857	MSN	Moesin	MOES_HUM	27	1.138533
48H 50MTA	Q9H4L7	-0.07789	0.000238	0.000857	SMARCAD1	SWI/SNF-re	SMRCD_HU	19	0.947444
48H 50MTA	Q99829	0.15568	0.000238	0.000858	CPNE1	Copine-1	CPNE1_HU	19	1.113946
24H 50MTA	Q8NEB9	0.123028	0.000239	0.00086	PIK3C3	Phosphatid	PK3C3_HU	20	1.089018
24H 50MTA	Q9HD20	0.06618	0.00024	0.000865	ATP13A1	Endoplasm	AT131_HU	31	1.046941
48H 50MTA	P50453	0.413352	0.00024	0.000865	SERPINB9	Serpin B9	SPB9_HUM	16	1.331776
48H 10MTA	O95671	0.108888	0.000241	0.000867	ASMTL	Probable bi	ASML_HUM	20	1.078396
24H 50MTA	P13674	0.45213	0.000242	0.000869	P4HA1	Prolyl 4-hy	P4HA1_HU	23	1.368059
48H 50MTA	P43155	0.229407	0.000243	0.00087	CRAT	Carnitine O	CACP_HUM	16	1.172353
24H 50MTA	P22061	0.06782	0.000243	0.00087	PCMT1	Protein-L-is	PIMT_HUM	15	1.048132
48H 10MTA	P08708	0.230298	0.000244	0.000872	RPS17	Small ribos	RS17_HUM	9	1.173077
48H 50MTA	O60675	-0.24734	0.000244	0.000873	MAFK	Transcripti	MAFK_HUM	4	0.842449
48H 50MTA	P01008	0.209185	0.000244	0.000873	SERPINC1	Antithromb	ANT3_HUM	6	1.156035
24H 50MTA	Q13247	0.095487	0.000246	0.000879	SRSF6	Serine/argi	SRSF6_HUM	9	1.068426
24H 50MTA	Q9P035	0.160903	0.000247	0.000881	HACD3	Very-long-c	HACD3_HU	11	1.117987
48H 50MTA	Q9H9J2	-0.2357	0.000248	0.000883	MRPL44	Large ribos	RM44_HUM	14	0.849275
48H 10MTA	Q2TAA2	0.165139	0.000248	0.000883	IAH1	Isoamyl ace	IAH1_HUM	8	1.121274
24H 10MTA	O75494	0.152267	0.000248	0.000884	SRSF10	Serine/argi	SRS10_HUM	6	1.111314
24H 10MTA	Q13561	-0.14405	0.000248	0.000884	DCTN2	Dynactin su	DCTN2_HU	21	0.904973
48H 50MTA	Q9NQ94	0.108594	0.000249	0.000885	A1CF	APOBEC1 co	A1CF_HUM	33	1.078177
48H 50MTA	O43324	-0.24282	0.00025	0.000887	EEF1E1	Eukaryotic	MCA3_HUM	10	0.845092
48H 50MTA	O95239	-0.28819	0.00025	0.000888	KIF4A	Chromosor	KIF4A_HUM	34	0.818931
48H 50MTA	Q13162	0.055651	0.00025	0.000888	PRDX4	Peroxiredo	PRDX4_HU	13	1.039328
48H 10MTA	Q9UKX7	0.039926	0.000251	0.000888	NUP50	Nuclear po	NUP50_HU	18	1.028061
48H 50MTA	Q9GZT3	-0.20983	0.000252	0.000891	SLIRP	SRA stem-lo	SLIRP_HUM	9	0.864639
48H 50MTA	P55265	0.065527	0.000253	0.000893	ADAR	Double-stra	DSRAD_HU	33	1.046467
48H 50MTA	P02042	-0.08639	0.000253	0.000894	HBD	Hemoglobi	HBD_HUMA	3	0.941876
24H 10MTA	P00338	-0.1403	0.000254	0.000895	LDHA	L-lactate de	LDHA_HUM	27	0.907327
48H 50MTA	P48735	0.485518	0.000254	0.000897	IDH2	Isocitrate d	IDHP_HUM	28	1.400088
24H 50MTA	P80404	0.248832	0.000255	0.000897	ABAT	4-aminobu	GABT_HUM	11	1.188245
48H 50MTA	P68366	0.162392	0.000255	0.000899	TUBA4A	Tubulin alp	TBA4A_HU	4	1.119141
24H 10MTA	P49914	0.07645	0.000255	0.0009	MTHFS	5-formyltet	MTHFS_HU	8	1.05442
48H 50MTA	Q96EL2	-0.64962	0.000256	0.0009	MRPS24	Small ribos	RT24_HUM	6	0.637449
24H 50MTA	P11597	2.114077	0.000256	0.000902	CETP	Cholesteryl	CETP_HUM	1	4.329129
48H 10MTA	P28331	0.035574	0.000256	0.000902	NDUFS1	NADH-ubiq	NDUS1_HU	35	1.024964
48H 50MTA	Q96RQ1	0.149272	0.000256	0.000902	ERGIC2	Endoplasm	ERGI2_HUM	6	1.10901
48H 50MTA	Q5T3I0	-0.59613	0.000256	0.000903	GPATCH4	G patch do	GPTC4_HU	11	0.661524
24H 50MTA	P00558	0.137583	0.000257	0.000904	PGK1	Phosphogly	PGK1_HUM	37	1.10006
24H 50MTA	Q9H6E4	0.104836	0.000259	0.000908	CCDC134	Coiled-coil	CC134_HU	7	1.075372
24H 10MTA	O94808	1.122393	0.000261	0.000913	GFPT2	Glutamine-	GFPT2_HU	2	2.177078

48H 50MT/	O95865	0.132479	0.000261	0.000914	DDAH2	Putative hy	DDAH2_HU	14	1.096176
24H 50MT/	P09525	0.136735	0.000261	0.000914	ANXA4	Annexin A4	ANXA4_HU	24	1.099414
48H 10MT/	P82673	0.086499	0.000262	0.000915	MRPS35	Small ribos	RT35_HUM	11	1.061791
48H 50MT/	P09525	0.517221	0.000262	0.000915	ANXA4	Annexin A4	ANXA4_HU	24	1.431195
48H 50MT/	Q9NRR3	0.36383	0.000263	0.000917	CDC42SE2	CDC42 sma	C42S2_HUI	1	1.286837
24H 50MT/	Q9NRX5	0.764499	0.000263	0.000918	SERINC1	Serine inco	SERC1_HUN	4	1.69878
48H 10MT/	O75665	-0.15243	0.000264	0.00092	OFD1	Centriole a	OFD1_HUN	11	0.899735
48H 50MT/	P21291	-0.16645	0.000264	0.00092	CSRP1	Cysteine an	CSRP1_HUI	8	0.891034
48H 10MT/	Q7L014	-0.01311	0.000265	0.000922	DDX46	Probable A	DDX46_HU	42	0.990954
48H 50MT/	P11142	-0.28156	0.000266	0.000925	HSPA8	Heat shock	HSP7C_HUI	27	0.822703
48H 50MT/	P41134	-0.919	0.000267	0.000926	ID1	DNA-bindir	ID1_HUMA	4	0.528875
24H 50MT/	Q8N442	-0.20434	0.000267	0.000926	GUF1	Translation	GUF1_HUN	10	0.867938
48H 50MT/	P14635	-0.40647	0.000268	0.000928	CCNB1	G2/mitotic	CCNB1_HU	10	0.75447
48H 50MT/	Q9HBU6	-0.51503	0.00027	0.000934	ETNK1	Ethanolami	EKI1_HUM/	8	0.69978
48H 10MT/	Q13416	0.028339	0.00027	0.000934	ORC2	Origin reco	ORC2_HUN	13	1.019837
24H 50MT/	Q13685	-0.18873	0.00027	0.000934	AAMP	Angio-asso	AAMP_HUN	12	0.877376
24H 50MT/	P60880	0.488419	0.00027	0.000934	SNAP25	Synaptosor	SNP25_HUI	9	1.402906
24H 50MT/	Q96KP4	-0.17582	0.000271	0.000938	CNDP2	Cytosolic n	CNDP2_HU	26	0.885264
24H 50MT/	P11166	0.460806	0.000272	0.000938	SLC2A1	Solute carri	GTR1_HUN	8	1.376311
24H 50MT/	O75947	0.150669	0.000272	0.000939	ATP5PD	ATP syntha	ATP5H_HUI	12	1.110084
48H 50MT/	O75368	0.243983	0.000272	0.00094	SH3BGRL	Adapter SH	SH3L1_HUI	4	1.184258
24H 50MT/	P05023	0.059097	0.000272	0.00094	ATP1A1	Sodium/po	AT1A1_HUI	41	1.041814
48H 50MT/	P12109	0.95179	0.000273	0.000941	COL6A1	Collagen al	CO6A1_HU	3	1.934271
24H 50MT/	P41229	0.443686	0.000273	0.000943	KDM5C	Lysine-spec	KDM5C_HU	15	1.360075
24H 50MT/	Q86TI2	0.117671	0.000275	0.000946	DPP9	Dipeptidyl	DPP9_HUN	32	1.084982
24H 50MT/	P36776	0.201639	0.000275	0.000946	LONP1	Lon protea	LONM_HUN	45	1.150004
48H 50MT/	Q15004	-0.75232	0.000276	0.00095	PCLAF	PCNA-asso	PAF15_HUI	8	0.593649
48H 10MT/	Q9H4L7	0.149008	0.000276	0.00095	SMARCAD1	SWI/SNF-re	SMRCD_HU	19	1.108807
24H 50MT/	P63010	0.035614	0.000278	0.000953	AP2B1	AP-2 compl	AP2B1_HUI	45	1.024993
24H 50MT/	P48735	0.240717	0.000279	0.000956	IDH2	Isocitrate d	IDHP_HUM	28	1.18158
24H 50MT/	Q9NVP1	0.194539	0.00028	0.000959	DDX18	ATP-depen	DDX18_HU	29	1.144359
48H 50MT/	O96008	-0.17586	0.00028	0.000959	TOMM40	Mitochond	TOM40_HU	11	0.885243
48H 50MT/	Q14677	0.064467	0.000281	0.00096	CLINT1	Clathrin int	EPN4_HUM	19	1.045698
24H 50MT/	Q6SZW1	-0.11879	0.000281	0.00096	SARM1	NAD(+) hyd	SARM1_HU	28	0.920959
48H 50MT/	O95563	0.203965	0.000282	0.000961	MPC2	Mitochond	MPC2_HUN	7	1.15186
48H 10MT/	Q96J02	0.039813	0.000282	0.000961	ITCH	E3 ubiquiti	ITCH_HUM/	28	1.02798
48H 50MT/	Q13501	-0.50549	0.000283	0.000964	SQSTM1	Sequestoso	SQSTM_HU	18	0.704423
48H 50MT/	P60983	0.247877	0.000285	0.00097	GMFB	Glia matura	GMFB_HUN	8	1.187458
48H 50MT/	P63092;Q5	0.134456	0.000285	0.00097	GNAS	Guanine nu	GNAS2_HU	14	1.097679
24H 50MT/	P21291	-0.38799	0.000286	0.000973	CSRP1	Cysteine an	CSRP1_HUI	8	0.764194
48H 50MT/	Q9UHI6	-0.26706	0.000286	0.000973	DDX20	Probable A	DDX20_HU	21	0.831009
24H 50MT/	Q8WTV0	0.30483	0.000287	0.000975	SCARB1	Scavenger r	SCRB1_HUI	8	1.235273
48H 50MT/	Q96DV4	-0.26449	0.000289	0.000978	MRPL38	Large ribos	RM38_HUN	14	0.832496
48H 50MT/	Q15738	-0.15511	0.000289	0.000978	NSDHL	Sterol-4-alp	NSDHL_HU	15	0.898066
48H 50MT/	Q9BZE4	-0.25916	0.000289	0.000978	GTPBP4	GTP-bindin	GTPB4_HUI	33	0.835573
24H 50MT/	O00469	0.942403	0.00029	0.00098	PLOD2	Procollager	PLOD2_HU	17	1.921726
48H 50MT/	O96013	0.202814	0.00029	0.000981	PAK4	Serine/thre	PAK4_HUN	13	1.150941
48H 10MT/	Q01469	0.11373	0.000291	0.000983	FABP5	Fatty acid-k	FABP5_HUI	10	1.082022
48H 50MT/	Q9Y2H6	0.027414	0.000291	0.000983	FNDC3A	Fibronectir	FND3A_HU	27	1.019183
48H 10MT/	P27694	0.130201	0.000292	0.000985	RPA1	Replicatior	RFA1_HUM	30	1.094446
48H 50MT/	Q9HC38	0.188057	0.000292	0.000985	GLOD4	Glyoxalase	GLOD4_HU	16	1.139229
24H 50MT/	P35222	0.073237	0.000292	0.000985	CTNNB1	Catenin bet	CTNB1_HUI	35	1.052075

24H 50MT/	Q16527	0.173124	0.000293	0.000986	CSRP2	Cysteine an	CSRP2_HUI	8	1.127497
24H 50MT/	Q9H0A0	0.135152	0.000293	0.000986	NAT10	RNA cytidir	NAT10_HUI	48	1.098209
48H 10MT/	Q9Y248	0.222635	0.000292	0.000986	GINS2	DNA replica	PSF2_HUM	3	1.166863
48H 50MT/	Q66K14	0.324747	0.000293	0.000987	TBC1D9B	TBC1 doma	TBC9B_HUI	26	1.252445
48H 50MT/	Q13616	0.045022	0.000293	0.000987	CUL1	Cullin-1	CUL1_HUM	36	1.031699
24H 50MT/	O43390	0.137153	0.000294	0.000988	HNRNPR	Heterogene	HNRPR_HU	23	1.099733
24H 50MT/	Q05048	0.210522	0.000294	0.000989	CSTF1	Cleavage sti	CSTF1_HUM	14	1.157107
48H 10MT/	Q8IU81	0.119037	0.000295	0.000992	IRF2BP1	Interferon r	I2BP1_HUM	18	1.086009
24H 50MT/	Q5QJE6	-0.06286	0.000296	0.000992	DNTTIP2	Deoxynucle	TDIF2_HUM	20	0.957367
48H 10MT/	O94905	0.086198	0.000297	0.000994	ERLIN2	Erlin-2	ERLN2_HUI	19	1.061569
48H 10MT/	P80217	0.127715	0.000297	0.000995	IFI35	Interferon-i	IN35_HUM	6	1.092562
24H 10MT/	O60858	-0.9764	0.000298	0.000997	TRIM13	E3 ubiquiti	TRI13_HUM	4	0.508246
24H 50MT/	P12277	0.191593	0.000299	0.001	CKB	Creatine kir	KCRB_HUM	20	1.142024
48H 10MT/	O00124	0.27907	0.000301	0.001004	UBXN8	UBX domai	UBXN8_HU	4	1.213413
24H 10MT/	Q92530	-0.26561	0.000301	0.001004	PSMF1	Proteasom	PSMF1_HU	8	0.831848
48H 50MT/	P54136	0.024926	0.000301	0.001004	RARS1	Arginine--tl	SYRC_HUM	47	1.017427
24H 50MT/	P07307	0.39319	0.000302	0.001006	ASGR2	Asialoglyco	ASGR2_HUI	10	1.313294
48H 10MT/	P63220	0.282108	0.000302	0.001007	RPS21	Small ribos	RS21_HUM	6	1.215971
48H 50MT/	Q9NNW5	-0.23772	0.000303	0.001008	WDR6	tRNA (34-2'	WDR6_HUI	26	0.848086
24H 50MT/	P28482	0.025905	0.000303	0.001008	MAPK1	Mitogen-ac	MK01_HUM	25	1.018118
48H 50MT/	Q9HBH0	0.03851	0.000303	0.001009	RHOF	Rho-relatec	RHOF_HUM	10	1.027052
24H 50MT/	P01024	0.125753	0.000303	0.001009	C3	Compleme	CO3_HUM/	58	1.091077
48H 10MT/	P52756	-0.02348	0.000304	0.001011	RBM5	RNA-bindir	RBM5_HUM	14	0.983855
24H 10MT/	Q9P283	0.479107	0.000305	0.001012	SEMA5B	Semaphori	SEM5B_HU	1	1.393881
24H 50MT/	Q13043	-0.2901	0.000306	0.001016	STK4	Serine/thre	STK4_HUM	22	0.817847
48H 50MT/	Q9P0M9	-0.57964	0.000306	0.001016	MRPL27	Large ribos	RM27_HUM	5	0.66913
48H 50MT/	Q92618	-0.27552	0.000307	0.001017	ZNF516	Zinc finger	ZN516_HUI	8	0.826154
24H 50MT/	P62873	0.108215	0.000307	0.001018	GNB1	Guanine nu	GBB1_HUM	14	1.077893
48H 50MT/	P53621	0.156347	0.000308	0.001018	COPA	Coatomer s	COPA_HUM	68	1.114462
48H 50MT/	Q9ULC5	0.190375	0.000308	0.001019	ACSL5	Long-chain	ACSL5_HUM	20	1.14106
24H 50MT/	Q8N8A6	0.09396	0.000308	0.001019	DDX51	ATP-depen	DDX51_HU	20	1.067296
24H 50MT/	Q8NBX0	0.161381	0.000308	0.00102	SCCPDH	Saccharopi	SCPDL_HUI	12	1.118357
24H 50MT/	Q92616	-0.10145	0.000309	0.00102	GCN1	Stalled ribc	GCN1_HUM	146	0.932094
24H 50MT/	Q5SVS4	1.305012	0.000309	0.001021	SLC25A30	Kidney mit	KMCP1_HU	1	2.470858
24H 50MT/	P30876	0.067538	0.00031	0.001022	POLR2B	DNA-direct	RPB2_HUM	41	1.047927
48H 10MT/	Q9NVJ2	0.078558	0.00031	0.001022	ARL8B	ADP-ribosy	ARL8B_HUI	6	1.055962
48H 50MT/	Q53RE8	-0.36069	0.00031	0.001023	ANKRD39	Ankyrin rep	ANR39_HU	3	0.778794
24H 10MT/	Q5W111	0.189608	0.000312	0.001028	SPRYD7	SPRY doma	SPRY7_HUM	5	1.140454
24H 50MT/	O95759	0.144062	0.000312	0.001028	TBC1D8	TBC1 doma	TBCD8_HUI	24	1.105012
48H 50MT/	Q9GZT4	0.412814	0.000315	0.001035	SRR	Serine race	SRR_HUMA	4	1.33128
48H 10MT/	P09972	0.115161	0.000316	0.001035	ALDOC	Fructose-bi	ALDOC_HU	8	1.083096
48H 50MT/	O75616	-0.41432	0.000316	0.001036	ERAL1	GTPase Era,	ERAL1_HUM	11	0.750373
48H 10MT/	O43324	-0.21845	0.000319	0.001043	EEF1E1	Eukaryotic	MCA3_HUM	10	0.859487
24H 10MT/	Q96T58	0.097757	0.000319	0.001044	SPEN	Msx2-intera	MINT_HUM	26	1.070109
24H 50MT/	Q9UM21	0.156753	0.000321	0.001048	MGAT4A	Alpha-1,3-r	MGT4A_HU	2	1.114776
24H 50MT/	Q9UHQ4	0.363166	0.000321	0.001048	BCAP29	B-cell recep	BAP29_HUI	5	1.286246
48H 50MT/	P49903	0.201305	0.000321	0.001048	SEPHS1	Selenide, w	SPS1_HUM	11	1.149738
48H 50MT/	O60832	-0.15661	0.000321	0.001049	DKC1	H/ACA ribo	DKC1_HUM	27	0.897128
24H 50MT/	P62081	0.12696	0.000323	0.001053	RPS7	Small ribos	RS7_HUMA	10	1.09199
24H 50MT/	Q9HB40	0.199046	0.000323	0.001053	SCPEP1	Retinoid-in	RISC_HUM/	11	1.147939
48H 50MT/	P28827	0.255337	0.000323	0.001054	PTPRM	Receptor-ty	PTPRM_HU	11	1.193615
48H 50MT/	Q00341	0.114383	0.000324	0.001055	HDLBP	Vigilin	VIGLN_HUM	61	1.082512

24H	50MT/	P11216	-0.12782	0.000326	0.001058	PYGB	Glycogen p	PYGB_HUM	30	0.915215
48H	50MT/	P0DP23;PC	0.437584	0.000327	0.001061	CALM1;CAL	Calmodulir	CALM1_HU	8	1.354334
24H	50MT/	Q96MW1	-0.1902	0.000327	0.001061	CCDC43	Coiled-coil	CCD43_HU	7	0.876481
48H	10MT/	Q9BRK5	0.226398	0.000327	0.001062	SDF4	45 kDa calc	CAB45_HU	13	1.16991
48H	10MT/	Q9NQR4	0.098376	0.000328	0.001064	NIT2	Omega-ami	NIT2_HUM/	16	1.070567
24H	50MT/	Q8IUE6	0.478431	0.000328	0.001064	H2AC21	Histone H2	H2A2B_HU	3	1.393227
48H	50MT/	Q9P2K5	1.043816	0.00033	0.001067	MYEF2	Myelin expı	MYEF2_HU	1	2.061674
24H	50MT/	O14772	0.22229	0.000331	0.001069	FPGT	Fucose-1-pl	FPGT_HUM	27	1.166584
24H	50MT/	Q15363	0.546486	0.000331	0.00107	TMED2	Transmemk	TMED2_HU	5	1.460524
24H	50MT/	Q9GZU7	0.108977	0.000332	0.001072	CTDSP1	Carboxy-tei	CTDS1_HU	3	1.078463
48H	50MT/	Q9Y3D9	-0.33378	0.000333	0.001075	MRPS23	Small ribos	RT23_HUM	11	0.793456
48H	10MT/	Q9BQ39	0.08061	0.000333	0.001075	DDX50	ATP-depen(	DDX50_HU	25	1.057465
24H	50MT/	P21399	0.144022	0.000333	0.001075	ACO1	Cytoplasmi	ACOHC_HU	41	1.104981
24H	50MT/	P07225	0.528248	0.000333	0.001076	PROS1	Vitamin K-c	PROS_HUM	9	1.442177
24H	50MT/	P02753	0.214502	0.000334	0.001076	RBP4	Retinol-bin	RET4_HUM	7	1.160303
48H	10MT/	P35580	0.005057	0.000336	0.001083	MYH10	Myosin-10	MYH10_HU	90	1.003512
48H	50MT/	Q16527	0.331962	0.000337	0.001085	CSRP2	Cysteine an	CSRP2_HU	8	1.258724
24H	10MT/	Q9NRX1	0.109851	0.000338	0.001087	PNO1	RNA-bindir	PNO1_HUM	7	1.079117
24H	50MT/	Q14697	0.162643	0.000339	0.001089	GANAB	Neutral alp	GANAB_HU	50	1.119336
48H	50MT/	O95671	0.382693	0.000339	0.001089	ASMTL	Probable bi	ASML_HUM	20	1.303773
48H	10MT/	P00374	0.128583	0.00034	0.001091	DHFR	Dihydrofol	DYR_HUMA	8	1.093219
24H	50MT/	A6NJ78	0.232695	0.000341	0.001092	METTL15	12S rRNA N	MET15_HU	13	1.175028
24H	50MT/	P61019	0.119948	0.000341	0.001092	RAB2A	Ras-related	RAB2A_HU	14	1.086695
48H	50MT/	P55060	-0.16698	0.000343	0.001096	CSE1L	Exportin-2	XPO2_HUM	52	0.890704
24H	50MT/	Q5TEU4	0.325681	0.000345	0.0011	NDUFAF5	Arginine-hy	NDUF5_HU	3	1.253256
48H	50MT/	O95159	0.07742	0.000345	0.001101	ZFPL1	Zinc finger	ZFPL1_HUM	10	1.055129
24H	50MT/	Q9P2E9	0.114965	0.000346	0.001105	RRBP1	Ribosome-l	RRBP1_HU	45	1.082949
24H	50MT/	P08833	0.91505	0.000346	0.001105	IGFBP1	Insulin-like	IBP1_HUMA	7	1.885635
24H	10MT/	P35520	-0.16813	0.00035	0.001112	CBS	Cystathioni	CBS_HUMA	22	0.889994
48H	50MT/	Q99442	-0.15692	0.00035	0.001113	SEC62	Translocati	SEC62_HUM	9	0.896941
24H	50MT/	Q96FV2	0.337511	0.000351	0.001114	SCRN2	Secernin-2	SCRN2_HU	9	1.263575
24H	10MT/	Q9NPJ3	0.062709	0.000351	0.001114	ACOT13	Acyl-coenzy	ACO13_HU	7	1.044425
48H	50MT/	P46019	0.164928	0.000353	0.00112	PHKA2	Phosphoryl	KPB2_HUM	17	1.121111
48H	10MT/	Q9UK41	0.211845	0.000353	0.00112	VPS28	Vacuolar pı	VPS28_HU	11	1.158168
24H	50MT/	Q9P2D0	0.590213	0.000353	0.00112	IBTK	Inhibitor of	IBTK_HUM/	8	1.505469
48H	50MT/	P41567	-0.3469	0.000354	0.001121	EIF1	Eukaryotic	EIF1_HUM/	10	0.786274
48H	10MT/	Q9UMR2	0.046917	0.000354	0.001122	DDX19B	ATP-depen(	DD19B_HU	2	1.033055
24H	50MT/	O14832	0.844638	0.000355	0.001123	PHYH	Phytanoyl-	PAHX_HUM	9	1.795815
48H	50MT/	Q06323	0.216644	0.000355	0.001124	PSME1	Proteasom	PSME1_HU	19	1.162028
48H	50MT/	Q9NW81	-0.17182	0.000357	0.001127	DMAC2	Distal mem	DMAC2_HU	3	0.887724
24H	10MT/	Q92667	0.076938	0.000358	0.001128	AKAP1	A-kinase an	AKAP1_HU	21	1.054777
48H	10MT/	P52272	0.036569	0.000358	0.001129	HNRNPM	Heterogene	HNRPM_HU	41	1.025672
24H	50MT/	Q53GA4	-0.48819	0.000359	0.001131	PHLDA2	Pleckstrin	PHLA2_HU	1	0.71292
48H	50MT/	Q8NEM2	-0.57195	0.00036	0.001132	SHCBP1	SHC SH2 dc	SHCBP_HU	20	0.672706
24H	10MT/	P11168	0.578872	0.000363	0.001139	SLC2A2	Solute carri	GTR2_HUM	7	1.493681
24H	50MT/	P51793;P5	2.414587	0.000365	0.001144	CLCN4;CLC	H(+)/Cl(-) ex	CLCN4_HU	2	5.331667
24H	50MT/	Q86TX2	0.120364	0.000365	0.001144	ACOT1	Acyl-coenzy	ACOT1_HU	19	1.087009
48H	10MT/	P60983	0.246939	0.000365	0.001145	GMFB	Glia matura	GMFB_HUM	8	1.186686
24H	50MT/	P82912	-0.1941	0.000366	0.001146	MRPS11	Small ribos	RT11_HUM	5	0.874118
48H	50MT/	Q15166	0.142128	0.000366	0.001146	PON3	Serum para	PON3_HUM	10	1.103532
24H	10MT/	Q96GF1	-1.69297	0.000368	0.001149	RNF185	E3 ubiquiti	RN185_HU	2	0.309289
48H	50MT/	Q13451	0.093878	0.000368	0.00115	FKBP5	Peptidyl-pr	FKBP5_HU	23	1.067235

24H 50MT/	B9A064	-5.1187	0.000368	0.00115	IGLL5	Immunoglo	IGLL5_HUM	1	0.028782
48H 50MT/	Q9NPJ3	0.258231	0.000369	0.001152	ACOT13	Acyl-coenzy	ACO13_HU	7	1.196011
24H 10MT/	Q8WXE9	1.470226	0.00037	0.001154	STON2	Stonin-2	STON2_HUI	1	2.770653
48H 50MT/	Q9BVM2	0.274169	0.000372	0.001157	DPCD	Protein DP(	DPCD_HUM	7	1.209298
48H 10MT/	Q16777;Q6	-3.12465	0.000373	0.001159	H2AC20;H2	Histone H2	H2A2C_HU	1	0.114654
48H 50MT/	Q7Z4W1	0.155329	0.000373	0.00116	DCXR	L-xylulose r	DCXR_HUM	10	1.113676
48H 50MT/	Q04656	0.160365	0.000374	0.001162	ATP7A	Copper-trai	ATP7A_HUI	22	1.11757
48H 10MT/	Q9P287	0.082662	0.000375	0.001164	BCCIP	BRCA2 and	BCCIP_HUM	14	1.05897
48H 50MT/	O94905	0.106045	0.000375	0.001164	ERLIN2	Erlin-2	ERLN2_HUI	19	1.076274
48H 50MT/	P31689	-0.28683	0.000378	0.00117	DNAJA1	DnaJ homo	DNJA1_HUI	18	0.819702
48H 50MT/	P46939	0.100381	0.00038	0.001173	UTRN	Utrophin	UTRN_HUM	137	1.072056
48H 50MT/	Q9UPN3	0.005542	0.00038	0.001173	MACF1	Microtubul	MACF1_HU	190	1.003849
48H 50MT/	Q03154	0.401634	0.00038	0.001173	ACY1	Aminoacyla	ACY1_HUM	17	1.321003
24H 50MT/	Q9NZ01	0.154724	0.000381	0.001174	TECR	Very-long-c	TECR_HUM	12	1.113209
24H 50MT/	P49590	0.293296	0.000381	0.001175	HARS2	Histidine--t	SYHM_HUM	15	1.225436
48H 10MT/	O96019	0.137424	0.000383	0.001179	ACTL6A	Actin-like p	ACL6A_HUI	15	1.099939
48H 50MT/	Q96FX7	-0.40375	0.000384	0.001182	TRMT61A	tRNA (aden	TRM61_HU	7	0.755891
48H 50MT/	Q6IBS0	0.267436	0.000386	0.001185	TWF2	Twinfilin-2	TWF2_HUM	17	1.203667
48H 50MT/	Q09161	0.06002	0.000386	0.001186	NCBP1	Nuclear cap	NCBP1_HU	28	1.04248
24H 50MT/	P01009	0.504994	0.000386	0.001186	SERPINA1	Alpha-1-ant	A1AT_HUM	31	1.419117
24H 50MT/	A6NHR9	0.071598	0.000387	0.001187	SMCHD1	Structural r	SMHD1_HU	74	1.05088
24H 10MT/	Q96B54	0.059509	0.000387	0.001187	ZNF428	Zinc finger	ZN428_HUI	4	1.042111
24H 10MT/	Q8TCE6	-0.24939	0.000387	0.001188	DENND10	DENN doma	DEN10_HU	1	0.841252
48H 50MT/	Q7Z4H8	0.084425	0.000387	0.001189	POGLUT3	Protein O-g	PLGT3_HUI	21	1.060265
24H 50MT/	P26373	0.139365	0.000389	0.001191	RPL13	Large ribos	RL13_HUM	7	1.10142
48H 50MT/	O76054	0.175457	0.000389	0.001192	SEC14L2	SEC14-like	S14L2_HUI	10	1.129322
48H 10MT/	P16455	-0.02697	0.000392	0.001199	MGMT	Methylated	MGMT_HUI	5	0.981476
48H 50MT/	P49419	0.168343	0.000393	0.001202	ALDH7A1	Alpha-amin	AL7A1_HUI	24	1.123767
24H 50MT/	P02760	0.151703	0.000394	0.001202	AMBP	Protein AM	AMBP_HUM	11	1.11088
48H 10MT/	O60675	-0.21653	0.000395	0.001204	MAFK	Transcripti	MAFK_HUM	4	0.860631
24H 50MT/	O00291	0.13799	0.000395	0.001205	HIP1	Huntingtin	HIP1_HUM	22	1.100371
24H 50MT/	Q5T8D3	0.1211	0.000396	0.001207	ACBD5	Acyl-CoA-bi	ACBD5_HU	13	1.087564
48H 50MT/	O43390	0.038146	0.000396	0.001207	HNRNPR	Heterogene	HNRPR_HU	23	1.026793
48H 50MT/	P82979	0.078366	0.000397	0.001208	SARNP	SAP domair	SARNP_HUI	8	1.055821
48H 50MT/	P07306	0.290494	0.000397	0.001209	ASGR1	Asialoglyco	ASGR1_HUI	14	1.223059
24H 50MT/	P51149	0.0538	0.000398	0.001211	RAB7A	Ras-related	RAB7A_HU	17	1.037995
24H 10MT/	Q15170	0.194123	0.000398	0.001211	TCEAL1	Transcripti	TCAL1_HUI	1	1.144029
24H 50MT/	Q13177	-0.08517	0.000398	0.001212	PAK2	Serine/thre	PAK2_HUM	21	0.942677
24H 50MT/	Q6UXV4	0.14571	0.000399	0.001212	APOOL	MICOS com	MIC27_HUI	12	1.106275
24H 50MT/	P82979	0.123925	0.000399	0.001213	SARNP	SAP domair	SARNP_HUI	8	1.089696
24H 50MT/	P53602	-0.14859	0.0004	0.001214	MVD	Diphospho	MVD1_HUM	16	0.902131
48H 10MT/	P49247	0.068956	0.0004	0.001215	RPIA	Ribose-5-pl	RPIA_HUM.	5	1.048957
24H 50MT/	Q9Y467	0.782959	0.0004	0.001215	SALL2	Sal-like pro	SALL2_HUM	4	1.720656
48H 50MT/	Q9BYT8	0.091654	0.000401	0.001216	NLN	Neurolysin,	NEUL_HUM	19	1.065591
24H 50MT/	Q9UHY1	-0.14935	0.000403	0.00122	NRBP1	Nuclear rec	NRBP_HUM	11	0.901658
48H 50MT/	P40939	0.10102	0.000403	0.001222	HADHA	Trifunction	ECHA_HUM	37	1.072531
24H 50MT/	O43172	0.131934	0.000404	0.001222	PRPF4	U4/U6 sma	PRP4_HUM	19	1.095762
48H 50MT/	P40123	0.200527	0.000404	0.001222	CAP2	Adenylyl cy	CAP2_HUM	15	1.149118
48H 50MT/	Q15014	-0.30381	0.000404	0.001222	MORF4L2	Mortality fa	MO4L2_HU	8	0.810109
48H 10MT/	Q9UHB9	0.025879	0.000404	0.001223	SRP68	Signal reco	SRP68_HUI	31	1.0181
48H 50MT/	P09110	0.144479	0.000405	0.001224	ACAA1	3-ketoacyl-	THIK_HUM.	18	1.105332
24H 10MT/	P52735	-0.07494	0.000406	0.001225	VAV2	Guanine nu	VAV2_HUM	25	0.949379

24H 50MT/	Q86XX4	0.301156	0.000406	0.001226	FRAS1	Extracellula	FRAS1_HUI	31	1.232132
48H 50MT/	Q9GZN7	2.023069	0.000406	0.001226	ROGDI	Protein rog	ROGDI_HUI	2	4.064474
48H 10MT/	Q96K17	0.229729	0.000407	0.001228	BTF3L4	Transcripti	BT3L4_HUI	10	1.172614
24H 50MT/	Q9HBH5	0.15682	0.000407	0.001228	RDH14	Retinol deh	RDH14_HU	12	1.114827
48H 10MT/	Q04828	-0.2487	0.000407	0.001228	AKR1C1	Aldo-keto r	AK1C1_HUI	21	0.841655
48H 50MT/	Q10570	0.068013	0.000408	0.00123	CPSF1	Cleavage ar	CPSF1_HUI	43	1.048272
48H 10MT/	P46781	0.046011	0.000409	0.001233	RPS9	Small ribos	RS9_HUMA	14	1.032407
48H 50MT/	Q86XX4	0.155936	0.000411	0.001236	FRAS1	Extracellula	FRAS1_HUI	31	1.114144
24H 50MT/	P22413	0.200001	0.000411	0.001237	ENPP1	Ectonucleo	ENPP1_HUI	13	1.148699
48H 50MT/	Q9GZR7	-0.20748	0.000413	0.001241	DDX24	ATP-depen	DDX24_HU	23	0.866046
48H 50MT/	Q9H3K2	0.221158	0.000414	0.001243	GHITM	Growth ho	GHITM_HU	8	1.165669
24H 50MT/	P08185	1.921918	0.000416	0.001247	SERPINA6	Corticoster	CBG_HUM/	1	3.789266
24H 50MT/	P49903	0.134812	0.000418	0.001251	SEPHS1	Selenide, w	SPS1_HUM	11	1.09795
24H 10MT/	P53396	-0.0693	0.000419	0.001254	ACLY	ATP-citrate	ACLY_HUM	56	0.953098
48H 10MT/	Q7Z6B7	-0.00961	0.000419	0.001255	SRGAP1	SLIT-ROBO	SRGP1_HUI	13	0.993358
48H 50MT/	Q92575	0.245666	0.00042	0.001256	UBXN4	UBX domai	UBXN4_HU	9	1.18564
48H 50MT/	Q92520	0.12489	0.000421	0.001258	FAM3C	Protein FAI	FAM3C_HU	8	1.090424
24H 50MT/	Q86X29	0.296833	0.000421	0.001258	LSR	Lipolysis-st	LSR_HUMA	10	1.228445
24H 50MT/	Q9BRX8	0.273258	0.000422	0.001259	PRXL2A	Peroxiredo	PXL2A_HUI	9	1.208534
24H 10MT/	Q6KC79	0.052435	0.000422	0.001259	NIPBL	Nipped-B-li	NIPBL_HUI	47	1.037014
48H 10MT/	Q6GQQ9	0.042264	0.000422	0.00126	OTUD7B	OTU domai	OTU7B_HU	10	1.029728
24H 50MT/	P20962	0.39943	0.000422	0.00126	PTMS	Parathymo	PTMS_HUI	1	1.318987
24H 50MT/	P29966	0.24178	0.000423	0.001261	MARCKS	Myristoylat	MARCS_HU	13	1.18245
48H 50MT/	P81605	0.347683	0.000423	0.001261	DCD	Dermcidin	DCD_HUM/	2	1.272515
24H 50MT/	Q9Y2X9	0.136016	0.000425	0.001265	ZNF281	Zinc finger	ZN281_HUI	19	1.098866
48H 50MT/	Q16540	-0.26774	0.000425	0.001266	MRPL23	Large ribos	RM23_HUI	9	0.830622
24H 50MT/	Q9BV36	-0.54448	0.000425	0.001266	MLPH	Melanophil	MELPH_HU	16	0.685637
48H 50MT/	Q9BRK5	0.316839	0.000427	0.00127	SDF4	45 kDa calc	CAB45_HUI	13	1.245598
24H 50MT/	O43491	0.088434	0.000428	0.001272	EPB41L2	Band 4.1-lil	E41L2_HUI	49	1.063215
24H 50MT/	P31942	0.137159	0.000429	0.001275	HNRNPH3	Heterogene	HNRH3_HU	12	1.099737
48H 10MT/	Q9BTC8	0.148705	0.000429	0.001275	MTA3	Metastasis-	MTA3_HUI	14	1.108574
24H 50MT/	Q9GZZ1	-0.15872	0.00043	0.001275	NAA50	N-alpha-ac	NAA50_HU	13	0.89582
24H 50MT/	Q96AE4	0.130217	0.00043	0.001275	FUBP1	Far upstrea	FUBP1_HUI	28	1.094458
48H 50MT/	Q9NRW1	-0.01347	0.000431	0.001276	RAB6B	Ras-related	RAB6B_HUI	2	0.990706
48H 50MT/	Q15758	-0.25786	0.000432	0.001281	SLC1A5	Neutral am	AAAT_HUM	11	0.83633
24H 50MT/	Q9HCN4	0.102979	0.000434	0.001284	GPN1	GPN-loop G	GPN1_HUI	4	1.073989
24H 50MT/	Q9UHX1	0.056645	0.000435	0.001288	PUF60	Poly(U)-bin	PUF60_HUI	24	1.040044
48H 10MT/	P47756	0.060345	0.000436	0.001288	CAPZB	F-actin-cap	CAPZB_HUI	19	1.042715
24H 50MT/	Q9HCD5	0.16411	0.000436	0.001288	NCOA5	Nuclear rec	NCOA5_HU	19	1.120474
24H 10MT/	Q15459	0.062194	0.000436	0.001289	SF3A1	Splicing fac	SF3A1_HUI	34	1.044052
48H 50MT/	Q969X6	-0.32404	0.000437	0.001291	UTP4	U3 small nu	UTP4_HUI	22	0.798827
48H 50MT/	Q9BQE3	0.175447	0.000437	0.001291	TUBA1C	Tubulin alp	TBA1C_HUI	4	1.129315
24H 50MT/	Q03154	0.310748	0.000437	0.001291	ACY1	Aminoacyla	ACY1_HUM	17	1.240351
48H 50MT/	P06865	0.047175	0.000438	0.001293	HEXA	Beta-hexos	HEXA_HUM	18	1.033239
48H 50MT/	P49327	-0.18689	0.000438	0.001293	FASN	Fatty acid s	FAS_HUMA	123	0.878496
48H 50MT/	Q96J02	-0.13925	0.000438	0.001293	ITCH	E3 ubiquiti	ITCH_HUM/	28	0.907994
24H 50MT/	P49792	0.040188	0.000439	0.001293	RANBP2	E3 SUMO-p	RBP2_HUM	96	1.028248
24H 50MT/	Q9BSH5	0.20653	0.000441	0.001299	HDHD3	Haloacid de	HDHD3_HU	10	1.15391
48H 10MT/	Q96AC1	0.118381	0.000442	0.001301	FERMT2	Fermitin fa	FERM2_HU	27	1.085516
48H 10MT/	P78316	-0.01609	0.000442	0.001301	NOP14	Nucleolar p	NOP14_HU	22	0.988906
48H 50MT/	Q9UGY1	-0.68363	0.000443	0.001301	NOL12	Nucleolar p	NOL12_HU	4	0.622597
48H 10MT/	Q9Y2K1	-1.61475	0.000443	0.001302	ZBTB1	Zinc finger	ZBTB1_HUI	2	0.326522

24H 10MT/	Q7Z6Z7	-0.03753	0.000444	0.001302	HUWE1	E3 ubiquiti	HUWE1_HU	111	0.974319
24H 10MT/	Q96I99	0.061764	0.000444	0.001303	SUCLG2	Succinate--	SUCB2_HUI	19	1.043741
48H 10MT/	Q01518	-0.12621	0.000445	0.001305	CAP1	Adenylyl cy	CAP1_HUM	30	0.916232
48H 50MT/	Q9BVJ6	-0.22146	0.000447	0.001309	UTP14A	U3 small nu	UT14A_HUI	18	0.857698
48H 50MT/	Q9Y5M8	-0.14477	0.000447	0.001309	SRPRB	Signal reco	SRPRB_HUI	15	0.904523
48H 50MT/	Q13464	0.10388	0.000447	0.001309	ROCK1	Rho-associa	ROCK1_HU	42	1.07466
48H 50MT/	Q9H583	-0.26139	0.000447	0.001309	HEATR1	HEAT repea	HEAT1_HUI	76	0.834282
24H 10MT/	Q5SVS4	-0.23332	0.000448	0.001311	SLC25A30	Kidney mit	KMCP1_HU	1	0.850676
24H 50MT/	Q9H1X3	2.443682	0.000449	0.001312	DNAJC25	DnaJ homo	DJC25_HUI	3	5.440283
48H 10MT/	Q96RR1	-0.20821	0.000449	0.001313	TWNK	Twinkle mt	PEO1_HUM	13	0.865608
24H 50MT/	Q96M86	-0.19067	0.000454	0.001323	DNHD1	Dynein hea	DNHD1_HU	2	0.876197
24H 50MT/	P78540	0.197523	0.000455	0.001324	ARG2	Arginase-2,	ARGI2_HUI	9	1.146728
24H 10MT/	Q8NBT2	-0.50312	0.000456	0.001327	SPC24	Kinetochor	SPC24_HUI	4	0.705578
24H 50MT/	Q9UNY4	-0.36457	0.000457	0.001329	TTF2	Transcripti	TTF2_HUM/	32	0.776698
24H 50MT/	Q15170	0.436538	0.000457	0.001329	TCEAL1	Transcripti	TCAL1_HUI	1	1.353352
48H 50MT/	Q5VWQ0	-0.00998	0.000458	0.001331	RSBN1	Lysine-spec	RSBN1_HUI	13	0.993104
24H 50MT/	O96013	0.15058	0.000459	0.001335	PAK4	Serine/thre	PAK4_HUM	13	1.110016
48H 50MT/	Q96M86	-0.07535	0.00046	0.001335	DNHD1	Dynein hea	DNHD1_HU	2	0.949114
24H 50MT/	Q5W111	0.151228	0.00046	0.001335	SPRYD7	SPRY doma	SPRY7_HUI	5	1.110514
24H 50MT/	O00422	0.33322	0.000461	0.001337	SAP18	Histone dea	SAP18_HUI	7	1.259822
24H 50MT/	Q9H081	0.606413	0.000461	0.001337	MIS12	Protein MIS	MIS12_HUI	1	1.522469
24H 10MT/	P49589	-0.08965	0.000463	0.001339	CARS1	Cysteine--tI	SYCC_HUM	34	0.939749
48H 50MT/	Q7Z6V5	0.249369	0.000463	0.00134	ADAT2	tRNA-speci	ADAT2_HUI	4	1.188687
48H 10MT/	Q96T88	0.075563	0.000466	0.001345	UHRF1	E3 ubiquiti	UHRF1_HU	31	1.053772
24H 50MT/	P68371	-0.16292	0.000466	0.001345	TUBB4B	Tubulin bet	TBB4B_HUI	30	0.893216
24H 10MT/	P40692	0.143033	0.000467	0.001348	MLH1	DNA misma	MLH1_HUM	31	1.104224
48H 10MT/	O15382	0.073285	0.000468	0.001349	BCAT2	Branched-c	BCAT2_HUI	17	1.05211
48H 50MT/	Q9Y570	0.194333	0.000468	0.00135	PPME1	Protein ph	PPME1_HU	18	1.144195
48H 50MT/	Q12765	0.144849	0.000473	0.001359	SCRN1	Secernin-1	SCRN1_HUI	20	1.105615
24H 50MT/	Q2M389	0.29767	0.000473	0.00136	WASHC4	WASH com	WASC4_HU	28	1.229158
24H 50MT/	Q9UK22	0.08974	0.000474	0.00136	FBXO2	F-box only	FBX2_HUM	11	1.064178
48H 50MT/	Q9NVS2	-0.29803	0.000474	0.001361	MRPS18A	Large ribos	RT18A_HUI	7	0.813361
48H 50MT/	Q13112	-0.26532	0.000475	0.001363	CHAF1B	Chromatin	CAF1B_HUI	14	0.832012
24H 50MT/	Q9Y512	0.137915	0.000475	0.001363	SAMM50	Sorting and	SAM50_HU	23	1.100313
24H 50MT/	Q5JRX3	0.108232	0.000476	0.001363	PITRM1	Presequen	PREP_HUM	33	1.077906
24H 50MT/	P67812	0.057424	0.000476	0.001364	SEC11A	Signal pept	SC11A_HUI	6	1.040606
48H 10MT/	Q96QC0	0.019064	0.000477	0.001366	PPP1R10	Serine/thre	PP1RA_HUI	22	1.013302
24H 50MT/	Q9BRK5	0.203689	0.000478	0.001367	SDF4	45 kDa calc	CAB45_HUI	13	1.151639
24H 50MT/	Q9BYC9	-0.23382	0.000478	0.001367	MRPL20	Large ribos	RM20_HUM	4	0.850379
48H 10MT/	P61077	0.316608	0.000479	0.001369	UBE2D3	Ubiquitin-c	UB2D3_HU	4	1.245399
24H 10MT/	Q96HJ9	2.108309	0.00048	0.001372	FMC1	Protein FM	FMC1_HUM	1	4.311857
24H 50MT/	Q8NC96	-0.22993	0.000481	0.001374	NECAP1	Adaptin ear	NECP1_HUI	9	0.852678
48H 50MT/	P16278	0.141415	0.000482	0.001374	GLB1	Beta-galact	BGAL_HUM	14	1.102986
24H 50MT/	Q6YHK3	1.265032	0.000482	0.001375	CD109	CD109 anti	CD109_HU	36	2.403326
24H 50MT/	O60885	0.192428	0.000483	0.001378	BRD4	Bromodom	BRD4_HUM	23	1.142685
48H 50MT/	Q6RW13	0.24212	0.000486	0.001383	AGTRAP	Type-1 angi	ATRAP_HUI	4	1.182729
24H 50MT/	P07814	0.036308	0.000486	0.001383	EPRS1	Bifunctiona	SYEP_HUM/	85	1.025486
24H 50MT/	Q9NRZ7	0.153397	0.000487	0.001385	AGPAT3	1-acyl-sn-gl	PLCC_HUM	9	1.112185
24H 50MT/	Q9HAZ1	0.772639	0.000488	0.001387	CLK4	Dual specifi	CLK4_HUM	3	1.708392
48H 10MT/	Q9NZM1	0.026175	0.000489	0.001389	MYOF	Myoferlin	MYOF_HUN	87	1.018309
24H 50MT/	Q9H3N1	0.045512	0.000491	0.001392	TMX1	Thioredoxi	TMX1_HUM	14	1.032049
48H 50MT/	Q04446	0.341684	0.000491	0.001394	GBE1	1,4-alpha-g	GLGB_HUM	27	1.267235

24H 50MTA	Q9BSE5	0.206082	0.000492	0.001395	AGMAT	Guanidino	GDAH_HUM	14	1.153551
48H 10MTA	A6NNW6	0.636628	0.000493	0.001398	ENO4	Enolase 4	ENO4_HUM	1	1.554691
48H 10MTA	Q14527	0.086148	0.000493	0.001398	HLTF	Helicase-lik	HLTF_HUM	28	1.061532
48H 10MTA	O75528	0.527905	0.000494	0.001399	TADA3	Transcripti	TADA3_HUI	5	1.441834
48H 50MTA	Q5VWN6	0.255591	0.000498	0.001408	TASOR2	Protein TAS	TASO2_HUI	3	1.193825
24H 50MTA	O14639	-0.11463	0.000499	0.00141	ABLIM1	Actin-bindi	ABLM1_HU	21	0.923616
24H 50MTA	Q15287	0.362529	0.0005	0.001411	RNPS1	RNA-bindir	RNPS1_HUI	6	1.285677
48H 50MTA	P53618	0.131419	0.0005	0.001412	COPB1	Coatomer s	COPB_HUM	46	1.09537
48H 50MTA	Q14527	0.157647	0.000501	0.001413	HLTF	Helicase-lik	HLTF_HUM	28	1.115466
48H 50MTA	P52789	0.477511	0.000501	0.001413	HK2	Hexokinase	HXK2_HUM	31	1.39234
48H 50MTA	P28161	0.252227	0.000502	0.001415	GSTM2	Glutathion	GSTM2_HU	16	1.191045
24H 10MTA	Q8IY50	0.93874	0.000505	0.001419	SLC35F3	Solute carri	S35F3_HUI	1	1.916854
48H 50MTA	O75794	-0.27589	0.000505	0.00142	CDC123	Translation	CD123_HU	12	0.825942
24H 10MTA	Q96BS2	-0.17774	0.000505	0.00142	TESC	Calcineurin	CHP3_HUM	8	0.884086
24H 10MTA	P11388	0.127207	0.000506	0.001422	TOP2A	DNA topois	TOP2A_HUI	73	1.092177
48H 50MTA	Q9H1K1	0.120095	0.000507	0.001424	ISCU	Iron-sulfur	ISCU_HUM.	6	1.086806
48H 50MTA	Q4G0N4	0.06157	0.00051	0.001429	NADK2	NAD kinase	NAKD2_HU	18	1.043601
24H 50MTA	Q5T160	0.141567	0.00051	0.001429	RARS2	Probable ar	SYRM_HUM	21	1.103103
48H 50MTA	P98179	-0.35487	0.000511	0.001431	RBM3	RNA-bindir	RBM3_HUM	5	0.781939
24H 50MTA	Q9Y2B0	0.127	0.000512	0.001434	CNPY2	Protein car	CNPY2_HUI	11	1.09202
24H 50MTA	P25685	-0.13028	0.000513	0.001435	DNAJB1	DnaJ homo	DNJB1_HUI	17	0.913656
48H 50MTA	O95487	0.071542	0.000513	0.001435	SEC24B	Protein trai	SC24B_HUI	28	1.050839
48H 50MTA	Q9H6D7	-0.2628	0.000514	0.001438	HAUS4	HAUS augm	HAUS4_HU	10	0.833466
48H 50MTA	P51659	0.069511	0.000514	0.001438	HSD17B4	Peroxisoma	DHB4_HUM	30	1.049361
48H 50MTA	Q66K89	0.45288	0.000515	0.001439	E4F1	Transcripti	E4F1_HUM	1	1.36877
24H 50MTA	Q15233	0.050175	0.000515	0.00144	NONO	Non-POU d	NONO_HUM	22	1.035391
48H 10MTA	Q9UBK7;Q	-0.07477	0.000515	0.00144	RABL2A;RA	Rab-like pr	RBL2A_HUI	3	0.949491
24H 10MTA	Q86VD7	-0.22074	0.000516	0.001441	SLC25A42	Mitochond	S2542_HUI	4	0.858126
24H 50MTA	Q9H0A8	-0.21272	0.000517	0.001442	COMMD4	COMM don	COMD4_HU	7	0.862906
48H 50MTA	Q9NV70	0.070003	0.000519	0.001447	EXOC1	Exocyst cor	EXOC1_HUI	19	1.049719
24H 50MTA	P29992	0.015607	0.00052	0.001449	GNA11	Guanine nu	GNA11_HU	14	1.010877
48H 50MTA	Q15833	0.163826	0.00052	0.001449	STXBP2	Syntaxin-bi	STXB2_HUM	25	1.120254
48H 50MTA	Q8WYA6	0.200388	0.00052	0.001449	CTNNBL1	Beta-cateni	CTBL1_HUM	21	1.149007
48H 50MTA	P21796	0.080764	0.000522	0.001451	VDAC1	Non-selecti	VDAC1_HU	19	1.057578
48H 10MTA	P50995	0.064675	0.000522	0.001451	ANXA11	Annexin A1	ANX11_HU	19	1.04585
48H 50MTA	P41252	0.0479	0.000524	0.001454	IARS1	Isoleucine-	SYIC_HUM/	60	1.033759
24H 50MTA	P52789	0.490963	0.000524	0.001455	HK2	Hexokinase	HXK2_HUM	31	1.405383
24H 10MTA	P62258	-0.21901	0.000524	0.001455	YWHAE	14-3-3 prot	1433E_HUI	21	0.859156
48H 10MTA	P18583	0.04071	0.000525	0.001456	SON	Protein SOI	SON_HUM/	39	1.02862
48H 50MTA	Q96AH0	-1.05049	0.000525	0.001456	NABP1	SOSS comp	SOSB2_HUI	2	0.482805
24H 50MTA	Q8IY81	0.064213	0.000526	0.001458	FTSJ3	pre-rRNA 2'	SPB1_HUM	27	1.045515
48H 50MTA	P61978	0.100229	0.000526	0.001459	HNRNPK	Heterogene	HNRPK_HU	27	1.071943
48H 50MTA	Q99081	-1.80078	0.00053	0.001465	TCF12	Transcripti	HTF4_HUM	3	0.287019
48H 50MTA	Q07954	0.778788	0.00053	0.001465	LRP1	Prolow-der	LRP1_HUM	40	1.715689
24H 10MTA	Q9BT23	0.133231	0.00053	0.001466	LIMD2	LIM domair	LIMD2_HUI	1	1.096747
24H 50MTA	P50748	-0.20038	0.000533	0.001471	KNTC1	Kinetochor	KNTC1_HUI	36	0.870324
24H 50MTA	Q9Y2X3	0.069569	0.000533	0.001471	NOP58	Nucleolar p	NOP58_HU	24	1.049403
24H 50MTA	Q9NVJ2	0.271348	0.000534	0.001472	ARL8B	ADP-ribosy	ARL8B_HUI	6	1.206935
24H 50MTA	Q9BU89	-0.31225	0.000534	0.001474	DOHH	Deoxyhypu	DOHH_HUM	10	0.805382
48H 50MTA	Q96A26	0.330947	0.000536	0.001477	FAM162A	Protein FAI	F162A_HUI	6	1.257839
48H 50MTA	Q9UNF1	0.132736	0.000539	0.001483	MAGED2	Melanoma-	MAGD2_HU	21	1.096371
48H 50MTA	Q9UL26	-0.00185	0.000539	0.001484	RAB22A	Ras-related	RB22A_HUI	8	0.998718

24H 50MT/	O00264	0.313774	0.00054	0.001485	PGRMC1	Membrane	PGRC1_HU	9	1.242955
48H 50MT/	Q96GA7	0.516201	0.00054	0.001485	SDSL	Serine dehy	SDSL_HUM	12	1.430184
48H 50MT/	Q93100	0.043358	0.000542	0.001488	PHKB	Phosphoryl	KPBB_HUM	12	1.03051
24H 10MT/	Q99611	-0.22994	0.000542	0.001488	SEPHS2	Selenide, w	SPS2_HUM	17	0.852672
48H 50MT/	Q9HCU5	-0.1288	0.000543	0.00149	PREB	Guanine nu	SEC12_HUM	14	0.914593
48H 50MT/	P00367	0.06614	0.000543	0.001491	GLUD1	Glutamate	DHE3_HUM	29	1.046912
24H 50MT/	Q9BUN8	0.142422	0.000544	0.001491	DERL1	Derlin-1	DERL1_HUM	7	1.103757
48H 10MT/	P02545	0.038389	0.000544	0.001492	LMNA	Prelamin-A	LMNA_HUM	40	1.026967
48H 50MT/	Q9UBU6	0.066077	0.000544	0.001492	FAM8A1	Protein FAM	FA8A1_HUM	10	1.046867
48H 50MT/	P48444	0.12067	0.000546	0.001496	ARCN1	Coatomer s	COPD_HUM	32	1.087239
48H 50MT/	Q16181	0.129352	0.000546	0.001496	SEPTIN7	Septin-7	SEPT7_HUM	19	1.093802
24H 50MT/	Q13435	0.162393	0.000546	0.001497	SF3B2	Splicing fac	SF3B2_HUM	39	1.119142
48H 10MT/	P33947	-1.2056	0.000547	0.001498	KDELR2	ER lumen p	ERD22_HUM	6	0.433588
48H 10MT/	Q9HBM1	0.164778	0.000547	0.001498	SPC25	Kinetochor	SPC25_HUM	8	1.120994
48H 50MT/	Q12907	0.149852	0.000549	0.001501	LMAN2	Vesicular ir	LMAN2_HU	16	1.109455
48H 50MT/	Q9BTE3	-0.2531	0.000549	0.001502	MCMBP	Mini-chron	MCMBP_HU	26	0.839091
24H 50MT/	Q9NX55	-1.19096	0.00055	0.001503	HYPK	Huntingtin	HYPK_HUM	2	0.438011
24H 10MT/	Q9BX95	-0.31345	0.00055	0.001504	SGPP1	Sphingosin	SGPP1_HUM	1	0.804715
48H 10MT/	Q14691	0.025965	0.000551	0.001505	GINS1	DNA replica	PSF1_HUM	7	1.018161
48H 50MT/	P61165	2.8222	0.000551	0.001505	TMEM258	Transmemb	TM258_HU	1	7.072399
24H 50MT/	Q969H8	0.190842	0.000551	0.001505	MYDGF	Myeloid-de	MYDGF_HU	7	1.14143
48H 10MT/	Q96IW7	0.317731	0.000552	0.001506	SEC22A	Vesicle-traf	SC22A_HUM	4	1.246369
48H 50MT/	O75874	0.152684	0.000552	0.001507	IDH1	Isocitrate d	IDHC_HUM	23	1.111636
48H 50MT/	Q9H8H2	-0.32072	0.000553	0.001507	DDX31	ATP-depend	DDX31_HU	18	0.800668
48H 50MT/	Q9BUT1	0.240669	0.000553	0.001508	BDH2	Dehydroge	DHRS6_HU	5	1.181541
24H 10MT/	Q3SXM5	0.082966	0.000554	0.001509	HSDL1	Inactive hy	HSDL1_HUM	7	1.059193
48H 50MT/	Q9UKV3	0.078809	0.000555	0.001511	ACIN1	Apoptotic	ACINU_HUM	29	1.056146
48H 50MT/	Q9Y2L1	0.077438	0.000555	0.001511	DIS3	Exosome co	RRP44_HU	38	1.055142
24H 10MT/	P11169	0.285219	0.00056	0.001521	SLC2A3	Solute carri	GTR3_HUM	7	1.218596
24H 10MT/	Q11206	-1.03445	0.000561	0.001521	ST3GAL4	CMP-N-acet	SIA4C_HUM	3	0.488202
48H 50MT/	Q9BVA0	0.259602	0.000561	0.001521	KATNB1	Katanin p8	KTNB1_HUM	10	1.197148
48H 50MT/	O94925	-0.17774	0.000561	0.001521	GLS	Glutaminas	GLSK_HUM	21	0.884088
48H 10MT/	Q8IZ21	0.047823	0.000561	0.001522	PHACTR4	Phosphatas	PHAR4_HU	10	1.033704
24H 50MT/	Q9UNK0	0.266281	0.000562	0.001523	STX8	Syntaxin-8	STX8_HUM	5	1.202704
48H 50MT/	P30536	0.463405	0.000563	0.001525	TSPO	Translocato	TSPO_HUM	2	1.378793
48H 50MT/	O95905	-0.2858	0.000563	0.001525	ECD	Protein ecd	ECD_HUMA	18	0.820284
24H 50MT/	Q16891	0.063916	0.000563	0.001525	IMMT	MICOS com	MIC60_HUM	35	1.045299
48H 50MT/	Q9BRT2	-0.10192	0.000564	0.001526	UQCC2	Ubiquinol-	UQCC2_HU	6	0.931794
48H 50MT/	Q6PIU2	-0.22492	0.000565	0.001527	NCEH1	Neutral cho	NCEH1_HU	18	0.855644
48H 50MT/	P13667	0.12984	0.000565	0.001529	PDIA4	Protein dis	PDIA4_HUM	41	1.094173
48H 50MT/	Q9Y2S7	-0.26109	0.000566	0.00153	POLDIP2	Polymerase	PDIP2_HUM	16	0.83446
24H 50MT/	O43837	0.176395	0.000568	0.001534	IDH3B	Isocitrate d	IDH3B_HUM	17	1.130057
48H 50MT/	Q96ND0	0.424066	0.000571	0.00154	FAM210A	Protein FAM	F210A_HUM	3	1.341703
48H 10MT/	Q9BVA0	0.248779	0.000572	0.001542	KATNB1	Katanin p8	KTNB1_HUM	10	1.188201
48H 10MT/	Q13616	0.011041	0.000572	0.001542	CUL1	Cullin-1	CUL1_HUM	36	1.007682
24H 50MT/	P00450	0.851973	0.000573	0.001543	CP	Ceruloplasr	CERU_HUM	16	1.804967
48H 10MT/	Q1KMD3	0.026417	0.000574	0.001545	HNRNPUL2	Heterogene	HNRL2_HU	27	1.018479
24H 50MT/	Q13595	0.42573	0.000575	0.001546	TRA2A	Transforme	TRA2A_HUM	8	1.343252
48H 50MT/	Q6PK04	-0.72209	0.000576	0.001548	CCDC137	Coiled-coil	CC137_HUM	6	0.606218
48H 50MT/	O43837	0.039971	0.000576	0.001549	IDH3B	Isocitrate d	IDH3B_HUM	17	1.028093
48H 50MT/	O94901	0.088763	0.000576	0.001549	SUN1	SUN domai	SUN1_HUM	30	1.063458
48H 50MT/	Q9P0M6	0.391017	0.000577	0.00155	MACROH2/	Core histor	H2AW_HUM	8	1.311318

24H 50MT/	Q96HE7	0.22286	0.000577	0.001551	ERO1A	ERO1-like p	ERO1A_HU	24	1.167045
24H 50MT/	Q8IY17	0.314104	0.000579	0.001554	PNPLA6	Patatin-like	PLPL6_HUI	22	1.24324
24H 10MT/	O43581	0.359259	0.000579	0.001555	SYT7	Synaptotag	SYT7_HUM/	6	1.282767
48H 50MT/	Q5TH69	0.357199	0.000581	0.001558	ARFGEF3	Brefeldin A	BIG3_HUM	24	1.280937
24H 50MT/	Q01628	0.497516	0.000581	0.001559	IFITM3	Interferon-i	IFM3_HUM	3	1.411781
24H 50MT/	Q9NUJ1	0.167698	0.000582	0.001561	ABHD10	Palmitoyl-p	ABHDA_HU	11	1.123265
48H 10MT/	Q9UI26	0.062276	0.000583	0.001562	IPO11	Importin-1	IPO11_HUI	27	1.044112
24H 50MT/	Q96SQ9	0.210102	0.000583	0.001564	CYP2S1	Cytochrom	CP2S1_HUI	10	1.15677
48H 50MT/	Q9H5K3	-0.22555	0.000585	0.001568	POMK	Protein O-n	SG196_HUI	5	0.855266
24H 10MT/	P51812	0.014234	0.000586	0.001569	RPS6KA3	Ribosomal	KS6A3_HUI	34	1.009915
48H 50MT/	P47985	0.102347	0.000586	0.001569	UQCRFS1	Cytochrom	UCRI_HUM	11	1.073518
24H 50MT/	Q9HC21	-0.91034	0.000586	0.001569	SLC25A19	Mitochond	TPC_HUMA	2	0.53206
24H 50MT/	Q6RW13	0.260901	0.000586	0.001569	AGTRAP	Type-1 angi	ATRAP_HUI	4	1.198226
48H 50MT/	A0AVT1	0.110631	0.000587	0.00157	UBA6	Ubiquitin-l	UBA6_HUM	43	1.0797
24H 50MT/	P05141	0.133355	0.000588	0.001572	SLC25A5	ADP/ATP tr	ADT2_HUM	20	1.096841
48H 10MT/	P07355	0.09423	0.000589	0.001573	ANXA2	Annexin A2	ANXA2_HU	28	1.067495
48H 50MT/	Q9BY77	-0.08191	0.000589	0.001574	POLDIP3	Polymerase	PDIP3_HUI	17	0.944803
48H 50MT/	P10155	0.182847	0.00059	0.001575	RO60	RNA-bindir	RO60_HUM	20	1.135122
48H 50MT/	P35606	0.130852	0.00059	0.001575	COPB2	Coatomer s	COPB2_HU	43	1.09494
48H 50MT/	Q96MV1	-0.52727	0.00059	0.001575	TLCD4	TLC domair	TLCD4_HUI	4	0.693868
24H 10MT/	P34949	-0.1072	0.000591	0.001577	MPI	Mannose-6	MPI_HUMA	15	0.928387
48H 50MT/	O95900	-0.3433	0.000592	0.001579	TRUB2	Pseudourid	TRUB2_HUI	6	0.788234
48H 10MT/	O75533	0.023809	0.000592	0.00158	SF3B1	Splicing fac	SF3B1_HUI	66	1.01664
24H 50MT/	P05386	0.188076	0.000595	0.001584	RPLP1	Large ribos	RLA1_HUM	4	1.139244
48H 10MT/	P78406	0.161453	0.000595	0.001585	RAE1	mRNA expc	RAE1L_HUI	11	1.118413
48H 50MT/	P11166	0.44224	0.000595	0.001586	SLC2A1	Solute carri	GTR1_HUM	8	1.358712
24H 10MT/	P78527	0.054306	0.000596	0.001586	PRKDC	DNA-depen	PRKDC_HU	210	1.038359
24H 50MT/	P13804	0.087026	0.000598	0.001589	ETFA	Electron tra	ETFA_HUM/	16	1.062178
48H 50MT/	Q7Z7H5	0.23455	0.000599	0.001591	TMED4	Transmemb	TMED4_HU	8	1.17654
48H 50MT/	O43615	-0.15456	0.000599	0.001592	TIMM44	Mitochond	TIM44_HUI	22	0.898405
48H 50MT/	Q9NPA0	-0.1643	0.000601	0.001595	EMC7	Endoplasm	EMC7_HUM	7	0.892364
24H 50MT/	P50454	0.142352	0.000602	0.001597	SERPINH1	Serpin H1	SERPH_HUI	19	1.103703
24H 50MT/	Q8N3C0	0.08465	0.000605	0.0016	ASCC3	Activating s	ASCC3_HUI	63	1.06043
48H 50MT/	O15379	-0.21941	0.000606	0.001602	HDAC3	Histone dea	HDAC3_HU	7	0.858916
48H 50MT/	P23284	0.072156	0.000606	0.001602	PPIB	Peptidyl-pr	PPIB_HUM/	13	1.051287
48H 10MT/	Q92918	-0.09037	0.000607	0.001604	MAP4K1	Mitogen-ac	M4K1_HUM	1	0.939283
48H 50MT/	Q96G61	-0.23805	0.000609	0.001607	NUDT11	Diphospho	NUD11_HU	1	0.847893
48H 50MT/	Q3ZCQ8	-0.17224	0.000609	0.001608	TIMM50	Mitochond	TIM50_HUI	11	0.887465
48H 50MT/	Q14674	-0.50938	0.00061	0.00161	ESPL1	Separin	ESPL1_HUI	15	0.702523
48H 50MT/	Q86X76	0.15832	0.000611	0.00161	NIT1	Deaminate	NIT1_HUM/	11	1.115987
24H 10MT/	Q9Y4F1	0.026939	0.000611	0.001612	FARP1	FERM, ARH	FARP1_HUI	22	1.018848
24H 50MT/	Q9BQG0	0.044769	0.000613	0.001616	MYBBP1A	Myb-bindir	MBB1A_HU	48	1.031518
48H 50MT/	P08134	0.079521	0.000614	0.001617	RHOC	Rho-relatec	RHOC_HUM	10	1.056667
48H 50MT/	P46087	-0.22903	0.000615	0.001619	NOP2	28S rRNA (c	NOP2_HUM	25	0.853206
48H 10MT/	Q96FN4	0.185794	0.000615	0.001619	CPNE2	Copine-2	CPNE2_HUI	8	1.137443
24H 50MT/	Q9BQ39	0.264961	0.000615	0.001619	DDX50	ATP-depen	DDX50_HU	25	1.201604
24H 50MT/	P49750	0.026195	0.000616	0.001622	YLPM1	YLP motif-c	YLPM1_HU	34	1.018323
48H 10MT/	Q13561	0.025275	0.000617	0.001622	DCTN2	Dynactin su	DCTN2_HU	21	1.017674
48H 50MT/	Q86UP2	0.040597	0.000617	0.001622	KTN1	Kinectin	KTN1_HUM	68	1.028539
24H 10MT/	Q92769	0.090387	0.000617	0.001622	HDAC2	Histone dea	HDAC2_HU	8	1.064656
24H 50MT/	P13667	0.120651	0.000618	0.001623	PDIA4	Protein disi	PDIA4_HUI	41	1.087225
48H 10MT/	O76080	-0.39487	0.000618	0.001623	ZFAND5	AN1-type zi	ZFAN5_HUI	3	0.760559

48H 50MTA	P20810	0.234239	0.000619	0.001625	CAST	Calpastatin	ICAL_HUMA	22	1.176286
24H 50MTA	P45954	0.137845	0.000621	0.001629	ACADSB	Short/bran	ACDSB_HUI	17	1.10026
24H 50MTA	O95490	0.238305	0.000623	0.001632	ADGRL2	Adhesion G	AGRL2_HUI	28	1.179606
48H 50MTA	Q9Y3B7	-0.27566	0.000624	0.001634	MRPL11	Large ribos	RM11_HUM	10	0.826073
24H 50MTA	Q9H5V9	0.276417	0.000625	0.001637	STEEP1	STING ER ex	STEEP_HUM	4	1.211183
24H 50MTA	Q9H2V7	0.054351	0.000625	0.001637	SPNS1	Protein spi	SPNS1_HUI	8	1.038392
24H 10MTA	Q15084	-0.12894	0.000627	0.001639	PDIA6	Protein dis	PDIA6_HUN	18	0.914502
24H 50MTA	Q15771	0.456026	0.000627	0.00164	RAB30	Ras-related	RAB30_HUI	1	1.371758
24H 50MTA	P33908	0.500369	0.000629	0.001642	MAN1A1	Mannosyl-c	MA1A1_HU	16	1.414575
48H 50MTA	Q7Z5H4	0.908402	0.00063	0.001645	VN1R5	Vomeronas	VN1R5_HU	1	1.876965
48H 10MTA	P15514	-0.62671	0.00063	0.001646	AREG	Amphiregu	AREG_HUM	3	0.647653
24H 50MTA	P21796	0.124302	0.000632	0.001649	VDAC1	Non-selecti	VDAC1_HU	19	1.08998
48H 10MTA	O95759	-0.00808	0.000634	0.001652	TBC1D8	TBC1 doma	TBCD8_HUI	24	0.994418
24H 50MTA	Q6DKJ4	-0.1962	0.000634	0.001653	NXN	Nucleoredo	NXN_HUMA	20	0.872845
48H 50MTA	Q9BQ39	0.083737	0.000637	0.001658	DDX50	ATP-depend	DDX50_HU	25	1.05976
24H 50MTA	Q14566	0.074316	0.000639	0.001661	MCM6	DNA replica	MCM6_HUI	43	1.052862
48H 50MTA	Q96GD4	-0.58878	0.000639	0.001662	AURKB	Aurora kina	AURKB_HU	11	0.664903
48H 50MTA	Q96HV5	-0.32264	0.000639	0.001663	TMEM41A	Transmemb	TM41A_HU	3	0.799606
48H 50MTA	O43592	0.154387	0.00064	0.001663	XPOT	Exportin-T	XPOT_HUM	29	1.112949
24H 50MTA	Q8N183	0.204768	0.00064	0.001663	NDUFAF2	NADH dehy	NDUF2_HU	10	1.152501
24H 50MTA	P35527	-0.33958	0.00064	0.001663	KRT9	Keratin, typ	K1C9_HUM	31	0.79027
24H 10MTA	O95239	0.121561	0.00064	0.001663	KIF4A	Chromosor	KIF4A_HUN	34	1.087911
48H 10MTA	Q99959	0.043816	0.000642	0.001667	PKP2	Plakophilin	PKP2_HUM	27	1.030837
48H 50MTA	P01024	0.622267	0.000643	0.001668	C3	Complemer	CO3_HUMA	58	1.539292
48H 50MTA	A1L0T0	0.128525	0.000644	0.001669	ILVBL	2-hydroxya	HACL2_HUI	22	1.093175
48H 50MTA	Q05682	0.180924	0.000644	0.001669	CALD1	Caldesmon	CALD1_HUI	19	1.13361
48H 50MTA	Q9GZM5	0.047404	0.000644	0.001669	YIPF3	Protein YIP	YIPF3_HUM	8	1.033404
48H 50MTA	P41214	-0.12281	0.000645	0.001671	EIF2D	Eukaryotic	EIF2D_HUN	10	0.918398
48H 50MTA	Q8NEU8	0.108556	0.000646	0.001672	APPL2	DCC-intera	DP13B_HU	15	1.078149
48H 50MTA	P63162	2.833106	0.000646	0.001673	SNRPN	Small nucle	RSMN_HUN	1	7.126069
48H 50MTA	O95926	-0.75065	0.000648	0.001676	SYF2	Pre-mRNA-s	SYF2_HUMA	4	0.594337
48H 50MTA	Q13617	0.041682	0.000648	0.001676	CUL2	Cullin-2	CUL2_HUM	24	1.029313
24H 50MTA	P02649	0.236818	0.000648	0.001676	APOE	Apolipopro	APOE_HUM	17	1.17839
48H 10MTA	Q9NW82	0.054997	0.000649	0.001679	WDR70	WD repeat-	WDR70_HU	18	1.038857
24H 50MTA	Q15691	-0.1083	0.00065	0.00168	MAPRE1	Microtubul	MARE1_HU	18	0.927681
48H 50MTA	O14524	-0.8324	0.00065	0.00168	NEMP1	Nuclear env	NEMP1_HU	5	0.561594
24H 10MTA	Q9BX66	-0.14203	0.000652	0.001682	SORBS1	Sorbin and	SRBS1_HUI	31	0.90624
48H 10MTA	P42771	-0.33635	0.000652	0.001683	CDKN2A	Cyclin-depe	CDN2A_HU	2	0.792042
24H 50MTA	B1AK53	2.7761	0.000652	0.001683	ESPN	Espin	ESPN_HUM	1	6.849983
48H 50MTA	Q14696	0.163688	0.000653	0.001684	MESD	LRP chaper	MESD_HUM	12	1.120147
24H 50MTA	Q96T37	0.135224	0.000656	0.001691	RBM15	RNA-bindir	RBM15_HU	22	1.098264
48H 50MTA	Q9UBM7	-0.17782	0.000657	0.001692	DHCR7	7-dehydroc	DHCR7_HU	7	0.884036
48H 50MTA	P12268	0.138888	0.00066	0.001698	IMPDH2	Inosine-5'-r	IMDH2_HU	29	1.101056
48H 10MTA	Q53GA4	-0.28204	0.000663	0.001704	PHLDA2	Pleckstrin h	PHLA2_HUI	1	0.822428
48H 50MTA	P49588	0.152641	0.000664	0.001706	AARS1	Alanine--tR	SYAC_HUM	44	1.111603
48H 50MTA	Q96FC7	0.217363	0.000665	0.001707	PHYHIPL	Phytanoyl-(	PHIPL_HUN	9	1.162607
24H 50MTA	Q8TB61	0.259817	0.000666	0.00171	SLC35B2	Adenosine 3	S35B2_HUI	11	1.197327
48H 50MTA	O95861	0.163407	0.000671	0.001719	BPNT1	3'(2'),5'-bis	BPNT1_HUI	19	1.119929
24H 50MTA	Q8NEW0	0.098902	0.000672	0.00172	SLC30A7	Zinc transp	ZNT7_HUM	8	1.070958
48H 50MTA	P54886	0.260633	0.000675	0.001725	ALDH18A1	Delta-1-pyr	P5CS_HUM	42	1.198004
48H 50MTA	Q15404	0.047173	0.000679	0.001731	RSU1	Ras suppres	RSU1_HUM	11	1.033239
48H 50MTA	O15400	0.072388	0.00068	0.001734	STX7	Syntaxin-7	STX7_HUM	10	1.051456

24H 10MT/	Q460N5	1.020924	0.000681	0.001734	PARP14	Protein mo	PAR14_HUI	1	2.029219
24H 50MT/	P17931	-0.20693	0.000684	0.001741	LGALS3	Galectin-3	LEG3_HUM	6	0.86638
48H 50MT/	Q8WZA0	0.145693	0.000684	0.001741	LZIC	Protein LZI	LZIC_HUMA	9	1.106262
24H 50MT/	P46019	0.195495	0.000685	0.001742	PHKA2	Phosphoryl	KPB2_HUM	17	1.145117
48H 50MT/	Q02880	0.124549	0.000685	0.001742	TOP2B	DNA topois	TOP2B_HUI	42	1.090167
24H 50MT/	P11387	0.185081	0.000685	0.001743	TOP1	DNA topois	TOP1_HUM	30	1.13688
24H 50MT/	Q13423	0.172692	0.000685	0.001743	NNT	NAD(P) trar	NNTM_HUI	37	1.127159
48H 50MT/	O60271	0.12411	0.000686	0.001744	SPAG9	C-Jun-amin	JIP4_HUMA	45	1.089835
48H 10MT/	P09012	0.19486	0.000687	0.001745	SNRPA	U1 small nu	SNRPA_HUI	10	1.144613
48H 50MT/	O75175	0.123155	0.000687	0.001746	CNOT3	CCR4-NOT t	CNOT3_HU	8	1.089114
48H 50MT/	Q14566	-0.14605	0.000687	0.001746	MCM6	DNA replica	MCM6_HUI	43	0.903724
48H 50MT/	Q9NQZ2	-0.31631	0.000689	0.001748	UTP3	Something	SAS10_HUI	11	0.803119
48H 50MT/	Q4KMQ1	-0.47798	0.00069	0.00175	TPRN	Taperin	TPRN_HUM	11	0.717981
24H 50MT/	Q6ZSJ8	-0.592	0.00069	0.001751	C1orf122	Uncharacte	CA122_HUI	4	0.663423
48H 50MT/	P14868	0.16963	0.00069	0.001751	DARS1	Aspartate--t	SYDC_HUM	36	1.12477
48H 10MT/	Q6DKJ4	0.107993	0.00069	0.001751	NXN	Nucleoredo	NXN_HUMA	20	1.077728
48H 50MT/	Q9NVS9	0.134426	0.00069	0.001751	PNPO	Pyridoxine-	PNPO_HUM	13	1.097656
48H 50MT/	P08779	0.139491	0.000691	0.001751	KRT16	Keratin, typ	K1C16_HUI	23	1.101516
24H 50MT/	Q9BPU6	1.780806	0.000691	0.001751	DPYSL5	Dihydropyr	DPYL5_HUI	1	3.436181
48H 50MT/	Q9BPW8	0.305886	0.000692	0.001754	NIPSNAP1	Protein Nip	NIPS1_HUM	11	1.236177
48H 10MT/	Q8NFH5	0.153654	0.000694	0.001756	NUP35	Nucleopori	NUP35_HU	11	1.112383
48H 50MT/	Q5TA50	-1.14152	0.000697	0.001762	CPTP	Ceramide-1	CPTP_HUM	1	0.453281
48H 50MT/	Q9H300	0.182151	0.000698	0.001764	PARL	Presenilin-a	PARL_HUM	6	1.134574
24H 50MT/	Q9Y6A9	0.098841	0.000698	0.001764	SPCS1	Signal pept	SPCS1_HUI	2	1.070913
48H 50MT/	Q9GZR2	-0.18618	0.000699	0.001766	REXO4	RNA exonu	REXO4_HUI	13	0.878931
24H 50MT/	P32119	0.108563	0.0007	0.001767	PRDX2	Peroxiredo	PRDX2_HU	13	1.078154
48H 50MT/	Q9UM54	0.119462	0.000703	0.001772	MYO6	Unconvent	MYO6_HUM	56	1.086329
24H 50MT/	P18206	-0.17846	0.000704	0.001774	VCL	Vinculin	VINC_HUM	52	0.883647
24H 50MT/	Q8TDD1	0.1664	0.000704	0.001774	DDX54	ATP-depen	DDX54_HU	37	1.122254
48H 50MT/	Q9BQ69	0.298403	0.000706	0.001777	MACROD1	ADP-ribose	MACD1_HU	4	1.229782
48H 10MT/	Q7Z460	0.060354	0.000706	0.001778	CLASP1	CLIP-associ	CLAP1_HUI	40	1.042722
48H 50MT/	Q9UJV9	0.015581	0.000709	0.001784	DDX41	Probable A	DDX41_HU	29	1.010858
48H 50MT/	Q92536	-0.54735	0.000711	0.001787	SLC7A6	Y+L amino a	YLAT2_HUM	3	0.684276
48H 50MT/	P35790	-0.53052	0.000713	0.00179	CHKA	Choline kin	CHKA_HUM	11	0.692305
24H 50MT/	O95747	0.059269	0.000713	0.00179	OXSR1	Serine/thre	OXSR1_HUI	16	1.041938
48H 10MT/	O94808	0.700354	0.000714	0.001792	GFPT2	Glutamine-	GFPT2_HUI	2	1.624903
24H 50MT/	P55265	0.101029	0.000715	0.001793	ADAR	Double-stra	DSRAD_HU	33	1.072538
24H 50MT/	Q8N2K0	0.196669	0.000715	0.001793	ABHD12	Lysophospl	ABD12_HU	13	1.146049
48H 10MT/	Q9GZN7	2.049768	0.000715	0.001793	ROGDI	Protein rog	ROGDI_HUI	2	4.140394
48H 10MT/	O15355	0.049399	0.000715	0.001793	PPM1G	Protein pho	PPM1G_HU	15	1.034834
24H 50MT/	Q96SY0	0.145303	0.000717	0.001797	INTS14	Integrator c	INT14_HUM	10	1.105963
24H 50MT/	Q0ZGT2	-0.0397	0.000718	0.001797	NEXN	Nexilin	NEXN_HUM	9	0.97286
48H 10MT/	P54277	-0.06738	0.000718	0.001799	PMS1	PMS1 prote	PMS1_HUM	16	0.954373
24H 50MT/	O94826	0.021851	0.00072	0.001803	TOMM70	Mitochond	TOM70_HU	26	1.015261
48H 10MT/	P62316	0.046286	0.000721	0.001804	SNRPD2	Small nucle	SMD2_HUM	8	1.032603
24H 50MT/	Q9P283	0.39739	0.000721	0.001804	SEMA5B	Semaphori	SEM5B_HU	1	1.317123
24H 50MT/	O95563	0.451689	0.000725	0.001809	MPC2	Mitochond	MPC2_HUM	7	1.36764
48H 50MT/	O00161	0.251168	0.000726	0.001812	SNAP23	Synaptosor	SNP23_HUI	10	1.19017
48H 50MT/	Q9P021	-0.83726	0.000728	0.001814	CRIPT	Cysteine-ric	CRIPT_HUM	2	0.559704
48H 50MT/	Q9NUQ8	-0.18127	0.000728	0.001814	ABCF3	ATP-bindin	ABCF3_HUI	34	0.881924
24H 50MT/	Q9Y2G5	0.07256	0.000728	0.001814	POFUT2	GDP-fucose	OFUT2_HUI	15	1.051581
48H 50MT/	Q15345	-0.19032	0.000728	0.001815	LRRC41	Leucine-ric	LRC41_HUI	1	0.876411

48H	50MT/	Q86VQ1	-1.29396	0.00073	0.001818	GLCCI1	Glucocorti	GLCI1_HUM	2	0.40783
48H	50MT/	Q9UJU6	0.177204	0.00073	0.001819	DBNL	Drebrin-lik	DBNL_HUM	13	1.130691
24H	50MT/	Q9BTC8	0.115867	0.000732	0.001822	MTA3	Metastasis-	MTA3_HUM	14	1.083626
48H	10MT/	Q66K74	0.072748	0.000733	0.001822	MAP1S	Microtubul	MAP1S_HU	20	1.051718
24H	50MT/	Q9BPZ7	0.395224	0.000734	0.001824	MAPKAP1	Target of ra	SIN1_HUM/	5	1.315147
24H	10MT/	Q06210	-0.10945	0.000734	0.001825	GFPT1	Glutamine-	GFPT1_HUI	39	0.92694
48H	50MT/	Q96S66	-0.18368	0.000734	0.001825	CLCC1	Chloride ch	CLCC1_HUI	20	0.880452
48H	10MT/	Q5T6F2	0.076778	0.000735	0.001826	UBAP2	Ubiquitin-a	UBAP2_HU	11	1.05466
24H	10MT/	P01023	-0.21775	0.000736	0.001827	A2M	Alpha-2-ma	A2MG_HUI	57	0.859908
48H	50MT/	O43924	0.232318	0.000736	0.001828	PDE6D	Retinal rod	PDE6D_HU	4	1.174721
48H	50MT/	Q9BUF5	0.086652	0.000737	0.00183	TUBB6	Tubulin bet	TBB6_HUM	16	1.061903
24H	50MT/	Q86Y56	-0.11784	0.000739	0.001833	DNAAF5	Dynein axo	DAAF5_HUI	35	0.921566
48H	10MT/	Q9NRV9	0.063966	0.00074	0.001835	HEBP1	Heme-bind	HEBP1_HUI	8	1.045335
24H	50MT/	P46087	0.069919	0.00074	0.001835	NOP2	28S rRNA (c	NOP2_HUM	25	1.049658
24H	10MT/	Q9NQ29	0.109098	0.000742	0.001839	LUC7L	Putative RN	LUC7L_HUI	7	1.078554
48H	10MT/	O60264	0.043441	0.000745	0.001844	SMARCA5	SWI/SNF-re	SMCA5_HU	48	1.030569
24H	50MT/	O43447	0.177361	0.000746	0.001847	PPIH	Peptidyl-pr	PPIH_HUM	8	1.130814
48H	50MT/	Q7Z6Z7	0.098816	0.000747	0.001848	HUWE1	E3 ubiquiti	HUWE1_HU	111	1.070895
24H	50MT/	Q92769	0.080115	0.000747	0.001849	HDAC2	Histone dea	HDAC2_HU	8	1.057102
24H	10MT/	P04114	-0.24007	0.000749	0.001853	APOB	Apolipoprc	APOB_HUM	89	0.846707
24H	50MT/	P25686	-0.34056	0.000751	0.001855	DNAJB2	DnaJ homo	DNJB2_HUI	6	0.789734
24H	50MT/	P14635	-0.33185	0.000753	0.001858	CCNB1	G2/mitotic	CCNB1_HU	10	0.794517
48H	10MT/	Q16658	0.085299	0.000755	0.001861	FSCN1	Fascin	FSCN1_HUI	19	1.060908
48H	50MT/	Q01813	0.381301	0.000755	0.001861	PFKP	ATP-depen	PFKAP_HUI	27	1.302515
24H	50MT/	Q9H8P0	0.041779	0.000756	0.001863	SRD5A3	Polyprenol	PORED_HU	2	1.029383
48H	50MT/	P02545	0.046495	0.000756	0.001863	LMNA	Prelamin-A	LMNA_HUM	40	1.032753
24H	10MT/	P35268	0.199341	0.000757	0.001863	RPL22	Large ribos	RL22_HUM	4	1.148173
48H	50MT/	O43772	0.184757	0.000757	0.001864	SLC25A20	Mitochond	MCAT_HUM	15	1.136625
48H	10MT/	P51114	0.046762	0.000758	0.001866	FXR1	RNA-bindir	FXR1_HUM	21	1.032944
48H	50MT/	Q08257	0.149988	0.000761	0.00187	CRYZ	Quinone ox	QOR_HUM/	17	1.10956
48H	10MT/	Q8IXM6	-0.15327	0.000763	0.001875	NRM	Nurim	NRM_HUM	5	0.899209
48H	10MT/	Q6PF05	3.6677	0.000764	0.001877	TTC23L	Tetratricop	TT23L_HUM	1	12.70831
24H	50MT/	Q8WW33	-0.02973	0.000765	0.001879	GTSF1	Gametocyt	GTSF1_HUI	5	0.979601
24H	50MT/	Q9NYU2	0.122693	0.000768	0.001882	UGGT1	UDP-glucos	UGGG1_HU	67	1.088765
48H	50MT/	Q14376	0.262664	0.000768	0.001882	GALE	UDP-glucos	GALE_HUM	13	1.199692
24H	10MT/	Q9NX62	0.103093	0.000772	0.001887	BPNT2	Golgi-resid	IMPA3_HUI	12	1.074074
48H	50MT/	P14859	0.091682	0.000772	0.001888	POU2F1	POU domai	PO2F1_HUI	9	1.065611
48H	50MT/	Q9NZJ0	-0.98565	0.000773	0.001889	DTL	Denticleles	DTL_HUMA	4	0.504999
24H	50MT/	O95716	0.225039	0.000773	0.00189	RAB3D	Ras-related	RAB3D_HU	8	1.168809
24H	50MT/	P00387	0.120367	0.000774	0.00189	CYB5R3	NADH-cyto	NB5R3_HU	19	1.087011
24H	50MT/	Q99541	0.476517	0.000774	0.001891	PLIN2	Perilipin-2	PLIN2_HUM	13	1.391381
48H	10MT/	P30536	0.373963	0.000775	0.001892	TSPO	Translocatc	TSPO_HUM	2	1.295908
48H	10MT/	Q53H96	0.091515	0.000776	0.001896	PYCR3	Pyrroline-5	P5CR3_HUI	9	1.065488
48H	50MT/	O96019	0.07694	0.000778	0.001899	ACTL6A	Actin-like p	ACL6A_HUI	15	1.054779
48H	50MT/	P55084	0.058758	0.000779	0.0019	HADHB	Trifunction	ECHB_HUM	30	1.041569
24H	50MT/	Q8IY50	0.88093	0.000779	0.0019	SLC35F3	Solute carri	S35F3_HUI	1	1.841563
24H	50MT/	Q01658	0.319779	0.00078	0.001902	DR1	Protein Dr1	NC2B_HUM	6	1.248139
48H	50MT/	Q9BVA1	0.040006	0.00078	0.001902	TUBB2B	Tubulin bet	TBB2B_HUI	1	1.028118
24H	50MT/	P26640	-0.13524	0.000781	0.001904	VARS1	Valine--tRN	SYVC_HUM	60	0.910516
24H	50MT/	Q12929	-0.19899	0.000784	0.001908	EPS8	Epidermal g	EPS8_HUM	32	0.871161
24H	50MT/	Q6NUQ1	0.365086	0.000786	0.001912	RINT1	RAD50-inte	RINT1_HUM	9	1.287958
48H	50MT/	Q9UHG3	0.117186	0.000788	0.001916	PCYOX1	Prenylcyste	PCYOX_HUI	16	1.084617

24H 50MTA	P05455	0.148417	0.000788	0.001917	SSB	Lupus La pr	LA_HUMAN	29	1.108353
48H 50MTA	Q7L4I2	0.228363	0.00079	0.00192	RSRC2	Arginine/se	RSRC2_HUI	8	1.171505
24H 50MTA	Q15738	0.052978	0.000791	0.001921	NSDHL	Sterol-4-alp	NSDHL_HU	15	1.037404
48H 50MTA	Q8TEX9	-0.23655	0.000791	0.001921	IPO4	Importin-4	IPO4_HUM	47	0.848774
24H 50MTA	P61018	0.83874	0.000792	0.001922	RAB4B	Ras-related	RAB4B_HUI	3	1.788487
24H 50MTA	Q6ZSC3	1.280976	0.000792	0.001923	RBM43	RNA-bindir	RBM43_HU	1	2.430033
24H 50MTA	Q3KQZ1	0.594291	0.000794	0.001925	SLC25A35	Solute carri	S2535_HUI	5	1.50973
48H 50MTA	Q9Y3Y2	0.171099	0.000794	0.001926	CHTOP	Chromatin	CHTOP_HU	6	1.125916
48H 50MTA	P04632	0.155666	0.000796	0.001927	CAPNS1	Calpain sma	CPNS1_HUI	11	1.113936
24H 50MTA	O95210	0.402508	0.000797	0.001929	STBD1	Starch-binc	STBD1_HUI	5	1.321804
48H 10MTA	Q0IIM8	0.012771	0.0008	0.001934	TBC1D8B	TBC1 doma	TBC8B_HUI	11	1.008892
24H 50MTA	Q86WA6	0.221615	0.000801	0.001935	BPHL	Valacyclovi	BPHL_HUM	5	1.166038
48H 50MTA	Q96EK6	0.174371	0.000802	0.001938	GNPNAT1	Glucosamir	GNA1_HUM	11	1.128472
24H 50MTA	Q08AE8	-0.25307	0.000802	0.001938	SPIRE1	Protein spi	SPIR1_HUM	5	0.839109
24H 50MTA	P36542	0.068937	0.000803	0.001939	ATP5F1C	ATP syntha	ATPG_HUM	10	1.048944
48H 50MTA	Q15435	0.082002	0.000804	0.00194	PPP1R7	Protein pho	PP1R7_HUI	18	1.058486
24H 50MTA	Q8N7H5	0.076979	0.000804	0.001941	PAF1	RNA polym	PAF1_HUM	21	1.054807
24H 10MTA	Q92466	0.411299	0.000807	0.001946	DDB2	DNA damag	DDB2_HUM	5	1.329883
48H 10MTA	Q9BYI3	0.4295	0.000808	0.001947	HYCC1	Hyccin	HYCCI_HUM	2	1.346767
24H 50MTA	Q8N1F7	0.05349	0.000808	0.001947	NUP93	Nuclear po	NUP93_HU	39	1.037773
24H 50MTA	Q8IUH4	-1.02111	0.000808	0.001947	ZDHHC13	Palmitoyltr	ZDH13_HU	2	0.492737
24H 50MTA	Q5RKV6	0.077932	0.000809	0.001949	EXOSC6	Exosome cc	EXOS6_HUI	8	1.055504
24H 10MTA	P01111	0.039441	0.000809	0.001949	NRAS	GTPase NRa	RASN_HUM	8	1.027715
24H 10MTA	O75976	-0.13056	0.000811	0.001952	CPD	Carboxypep	CBPD_HUM	44	0.913477
48H 10MTA	Q96M96	0.011039	0.000812	0.001954	FGD4	FYVE, RhoG	FGD4_HUM	21	1.007681
48H 10MTA	P39060	0.065863	0.000813	0.001955	COL18A1	Collagen alp	COIA1_HUM	14	1.046711
24H 50MTA	P23246	0.178078	0.000816	0.001959	SFPQ	Splicing fac	SFPQ_HUM	25	1.131376
24H 10MTA	P28074	0.041256	0.000816	0.00196	PSMB5	Proteasome	PSB5_HUM	15	1.029009
24H 10MTA	O15355	0.065936	0.000818	0.001963	PPM1G	Protein pho	PPM1G_HU	15	1.046764
48H 10MTA	Q9NYZ2	0.626392	0.000818	0.001963	SLC25A37	Mitoferrin-	MFRN1_HU	1	1.5437
24H 50MTA	P26368	0.216857	0.000821	0.001968	U2AF2	Splicing fac	U2AF2_HUI	13	1.162199
48H 50MTA	Q5TKA1	-0.28735	0.000821	0.001968	LIN9	Protein lin-	LIN9_HUMA	2	0.819404
24H 10MTA	Q16539	-0.05813	0.000822	0.001969	MAPK14	Mitogen-ac	MK14_HUM	13	0.96051
24H 10MTA	O00468	0.402028	0.000824	0.001972	AGRN	Agrin	AGRIN_HUI	42	1.321364
24H 50MTA	P31146	0.61068	0.000825	0.001974	CORO1A	Coronin-1A	COR1A_HU	3	1.526979
48H 10MTA	P04632	0.112002	0.000826	0.001977	CAPNS1	Calpain sma	CPNS1_HUI	11	1.080727
48H 50MTA	Q9NP64	-0.17579	0.000827	0.001978	ZCCHC17	Zinc finger	ZCC17_HUI	3	0.885285
24H 50MTA	Q8TEL6	-0.43918	0.000828	0.001979	TRPC4AP	Short trans	TP4AP_HUI	7	0.737554
48H 10MTA	P51884	0.73262	0.000828	0.00198	LUM	Lumican	LUM_HUMA	4	1.661653
48H 50MTA	Q6JQN1	0.22534	0.000829	0.001981	ACAD10	Acyl-CoA de	ACD10_HU	25	1.169052
24H 50MTA	Q9BRS2	0.415065	0.00083	0.001982	RIOK1	Serine/thre	RIOK1_HUM	11	1.333358
48H 50MTA	Q9HD23	0.168164	0.00083	0.001982	MRS2	Magnesium	MRS2_HUM	15	1.123628
48H 50MTA	Q96C23	0.056855	0.00083	0.001983	GALM	Galactose n	GALM_HUM	8	1.040196
24H 50MTA	Q8N0X7	0.586615	0.000831	0.001983	SPART	Spartin	SPART_HUM	3	1.501719
48H 10MTA	Q9BV44	-0.00987	0.000834	0.001989	THUMPD3	tRNA (guan	THUM3_HU	14	0.993184
48H 50MTA	Q15020	0.094778	0.000834	0.001989	SART3	Squamous	SART3_HUM	38	1.067901
48H 10MTA	Q8IXB1	0.011279	0.000835	0.00199	DNAJC10	DnaJ homo	DJC10_HUI	21	1.007849
24H 10MTA	Q9C0A1	-1.90743	0.000837	0.001994	ZFHX2	Zinc finger	ZFHX2_HUI	1	0.266567
48H 50MTA	P09327	0.30973	0.000839	0.001999	VIL1	Villin-1	VILI_HUMA	23	1.239476
24H 50MTA	P16278	0.133547	0.000841	0.002001	GLB1	Beta-galact	BGAL_HUM	14	1.096988
24H 10MTA	O60218	0.107447	0.000843	0.002004	AKR1B10	Aldo-keto r	AK1BA_HUI	1	1.07732
48H 50MTA	Q9NVQ4	-0.51976	0.000848	0.002011	FAIM	Fas apoptot	FAIM1_HUI	4	0.697488

24H 10MTA	Q6FI81	-0.14214	0.000848	0.002011	CIAPIN1	Anamorsin	CPIN1_HUM	20	0.906177
24H 50MTA	Q96SZ6	0.043011	0.000848	0.002012	CDK5RAP1	Mitochond	CK5P1_HU	13	1.030262
24H 10MTA	Q96P47	-0.09911	0.000849	0.002013	AGAP3	Arf-GAP wit	AGAP3_HU	5	0.933612
24H 50MTA	P16615	0.061403	0.00085	0.002014	ATP2A2	Sarcoplasm	AT2A2_HU	45	1.04348
24H 10MTA	P28749	0.302765	0.000851	0.002016	RBL1	Retinoblast	RBL1_HUM	12	1.233506
24H 50MTA	Q92572	-0.16154	0.000852	0.002018	AP3S1	AP-3 compl	AP3S1_HU	7	0.894068
24H 50MTA	P42785	0.120447	0.000853	0.002018	PRCP	Lysosomal	PCP_HUMA	12	1.087071
24H 50MTA	Q14137	0.040479	0.000855	0.002023	BOP1	Ribosome b	BOP1_HUM	26	1.028455
48H 50MTA	P49591	0.134582	0.000858	0.002027	SARS1	Serine--tRN	SYSC_HUMA	34	1.097775
24H 50MTA	P07954	0.140549	0.000859	0.002029	FH	Fumarate h	FUMH_HUM	20	1.102324
24H 50MTA	Q9GZL7	0.068264	0.000865	0.002039	WDR12	Ribosome b	WDR12_HU	18	1.048454
48H 50MTA	Q14764	0.329811	0.000866	0.00204	MVP	Major vault	MVP_HUMA	44	1.256849
48H 10MTA	Q9Y5Z4	0.149834	0.000867	0.002041	HEBP2	Heme-bind	HEBP2_HU	9	1.109442
48H 10MTA	Q99986	0.062493	0.000868	0.002044	VRK1	Serine/thre	VRK1_HUM	13	1.044269
48H 50MTA	Q9NWU1	0.123811	0.00087	0.002047	OXSM	3-oxoacyl-[	OXSM_HUM	14	1.08961
24H 50MTA	Q5SY16	0.022008	0.000871	0.002048	NOL9	Polynucleo	NOL9_HUM	21	1.015372
48H 50MTA	P78540	-0.22795	0.000871	0.002048	ARG2	Arginase-2,	ARGI2_HUM	9	0.853847
24H 50MTA	Q9BS26	0.085208	0.000872	0.002049	ERP44	Endoplasm	ERP44_HU	17	1.060841
24H 50MTA	O14966	-0.03465	0.000873	0.00205	RAB29	Ras-related	RAB7L_HU	4	0.976266
48H 50MTA	Q9H9T3	0.035114	0.000873	0.00205	ELP3	Elongator c	ELP3_HUM	18	1.024638
48H 10MTA	Q5EBL4	0.109781	0.000874	0.002052	RILPL1	RILP-like pr	RIPL1_HUM	4	1.079065
24H 50MTA	O14910	0.122265	0.000874	0.002052	LIN7A	Protein lin-	LIN7A_HUM	9	1.088442
24H 50MTA	O43505	0.188187	0.000877	0.002057	B4GAT1	Beta-1,4-gl	B4GA1_HU	13	1.139331
24H 50MTA	Q53GS9	0.085425	0.000877	0.002057	USP39	Ubiquitin c	UBP39_HU	15	1.061
24H 50MTA	Q10570	0.197122	0.000879	0.002059	CPSF1	Cleavage ar	CPSF1_HUM	43	1.146409
24H 50MTA	Q6P1Q0	-0.26943	0.000879	0.00206	LETMD1	LETM1 dom	LTMD1_HU	5	0.829649
24H 50MTA	P49720	0.104313	0.00088	0.002063	PSMB3	Proteasom	PSB3_HUM	12	1.074982
48H 50MTA	Q9BY76	-1.39901	0.000881	0.002063	ANGPTL4	Angiopoiet	ANGL4_HU	1	0.379188
48H 50MTA	Q92552	-0.28333	0.000881	0.002064	MRPS27	Small ribos	RT27_HUM	19	0.821691
48H 50MTA	Q01469	0.21136	0.000882	0.002065	FABP5	Fatty acid-b	FABP5_HU	10	1.157779
24H 50MTA	Q92900	-0.12541	0.000884	0.002069	UPF1	Regulator c	RENT1_HU	51	0.916745
48H 50MTA	P43490	0.057897	0.000885	0.002071	NAMPT	Nicotinami	NAMPT_HU	25	1.040947
48H 50MTA	Q15067	0.076144	0.000886	0.002071	ACOX1	Peroxisoma	ACOX1_HU	26	1.054197
48H 50MTA	Q9NRL2	-0.2906	0.000887	0.002073	BAZ1A	Bromodom	BAZ1A_HU	24	0.81756
24H 50MTA	P49427	-0.12698	0.000888	0.002074	CDC34	Ubiquitin-c	UB2R1_HU	5	0.915749
48H 50MTA	Q9ULU4	0.185705	0.000889	0.002075	ZMYND8	MYND-type	ZMYD8_HU	23	1.137373
48H 50MTA	P04040	0.059082	0.00089	0.002077	CAT	Catalase	CATA_HUM	26	1.041803
24H 10MTA	P15848	0.102722	0.00089	0.002077	ARSB	Arylsulfatas	ARSB_HUM	9	1.073798
24H 50MTA	Q8N7X0	2.043742	0.000891	0.002078	ADGB	Androglobi	ADGB_HUM	1	4.123136
48H 50MTA	Q15118	0.497628	0.000891	0.002079	PDK1	[Pyruvate d	PDK1_HUM	8	1.41189
48H 10MTA	P21796	0.03308	0.000893	0.002083	VDAC1	Non-selecti	VDAC1_HU	19	1.023194
24H 50MTA	Q8N567	0.141468	0.000894	0.002084	ZCCHC9	Zinc finger	ZCHC9_HU	3	1.103027
24H 50MTA	O75475	0.302366	0.000895	0.002084	PSIP1	PC4 and SF	PSIP1_HUM	14	1.233165
48H 50MTA	Q9UMR2	0.026843	0.000895	0.002085	DDX19B	ATP-depend	DD19B_HU	2	1.01878
24H 10MTA	P49720	0.073306	0.000895	0.002085	PSMB3	Proteasom	PSB3_HUM	12	1.052125
48H 10MTA	Q6P996	0.077821	0.000896	0.002087	PDXDC1	Pyridoxal-d	PDXD1_HU	33	1.055422
24H 10MTA	Q8N8A6	0.008392	0.000901	0.002094	DDX51	ATP-depend	DDX51_HU	20	1.005834
48H 10MTA	Q9BPX6	-0.06862	0.000901	0.002094	MICU1	Calcium up	MICU1_HU	8	0.953548
48H 50MTA	Q9Y399	-0.34003	0.000901	0.002094	MRPS2	Small ribos	RT02_HUM	10	0.790025
24H 50MTA	P38571	0.189939	0.000905	0.002101	LIPA	Lysosomal a	LICH_HUMA	5	1.140716
48H 10MTA	Q9UK39	0.228294	0.000906	0.002102	NOCT	Nocturnin	NOCT_HUM	1	1.171449
48H 50MTA	Q5T2R2	-0.53798	0.000907	0.002104	PDSS1	All trans-pc	DPS1_HUM	3	0.688734

24H 50MT/	Q8WVZ9	0.166051	0.000909	0.002106	KBTBD7	Kelch repea	KBTB7_HUI	1	1.121983
24H 50MT/	O60218	0.013462	0.000909	0.002107	AKR1B10	Aldo-keto r	AK1BA_HUI	1	1.009375
48H 10MT/	Q99856	0.072589	0.00091	0.002109	ARID3A	AT-rich inte	ARI3A_HUN	17	1.051602
48H 10MT/	P78527	-0.00116	0.000911	0.002111	PRKDC	DNA-depen	PRKDC_HU	210	0.999193
24H 50MT/	Q9NRX1	0.223457	0.000913	0.002113	PNO1	RNA-bindir	PNO1_HUN	7	1.167528
48H 50MT/	P27348	-0.03966	0.000916	0.002117	YWHAQ	14-3-3 prot	1433T_HUI	13	0.972886
24H 50MT/	Q8IY67	0.016773	0.000916	0.002117	RAVER1	Ribonuclec	RAVR1_HUI	17	1.011694
24H 10MT/	O95602	0.037562	0.00092	0.002125	POLR1A	DNA-direct	RPA1_HUN	50	1.026378
24H 50MT/	O94905	-0.00015	0.00092	0.002126	ERLIN2	Erlin-2	ERLN2_HUI	19	0.999895
48H 50MT/	Q2TAA2	0.298065	0.000922	0.002128	IAH1	Isoamyl ace	IAH1_HUM	8	1.229494
48H 50MT/	P00492	0.085362	0.000922	0.002128	HPRT1	Hypoxanth	HPRT_HUM	13	1.060954
48H 10MT/	Q9Y4F1	0.030145	0.000922	0.002128	FARP1	FERM, ARH	FARP1_HUI	22	1.021115
48H 50MT/	O43813	0.257807	0.000923	0.002128	LANCL1	Glutathion	LANC1_HUI	15	1.19566
24H 50MT/	Q9BRA0	0.124818	0.000924	0.002131	NAA38	N-alpha-ace	NAA38_HU	4	1.09037
24H 50MT/	P48651	0.225075	0.000924	0.002132	PTDSS1	Phosphatic	PTSS1_HUN	7	1.168838
48H 50MT/	P49406	-0.34199	0.000925	0.002133	MRPL19	Large ribos	RM19_HUN	12	0.788955
24H 50MT/	P49821	0.082547	0.000925	0.002133	NDUFV1	NADH dehy	NDUV1_HU	20	1.058886
24H 10MT/	Q0VGL1	0.051763	0.000925	0.002133	LAMTOR4	Ragulator c	LTOR4_HUI	3	1.036531
24H 50MT/	P48723	0.152327	0.000927	0.002136	HSPA13	Heat shock	HSP13_HUI	14	1.111361
24H 50MT/	Q9P2T1	0.147906	0.000928	0.002137	GMPR2	GMP reduc	GMPR2_HU	15	1.10796
24H 10MT/	O43447	0.075393	0.000929	0.002138	PPIH	Peptidyl-pr	PPIH_HUM	8	1.053648
48H 50MT/	O75896	-0.35281	0.00093	0.002138	TUSC2	Tumor sup	TUSC2_HUI	4	0.783059
48H 50MT/	Q9BYD3	-0.32198	0.000931	0.002141	MRPL4	Large ribos	RM04_HUN	12	0.799969
48H 50MT/	P46109	0.276023	0.000932	0.002143	CRKL	Crk-like prc	CRKL_HUM	14	1.210852
48H 50MT/	O94888	0.113063	0.000933	0.002144	UBXN7	UBX domai	UBXN7_HU	15	1.081522
48H 50MT/	Q92979	-0.14434	0.000933	0.002144	EMG1	Ribosomal	NEP1_HUM	15	0.904793
48H 10MT/	O43772	0.00015	0.000934	0.002146	SLC25A20	Mitochond	MCAT_HUN	15	1.000104
48H 50MT/	Q8N766	-0.12283	0.000935	0.002147	EMC1	ER membra	EMC1_HUN	37	0.918387
48H 50MT/	P49321	0.127394	0.000941	0.002157	NASP	Nuclear aut	NASP_HUM	35	1.092319
48H 50MT/	O75832	0.210805	0.000944	0.002161	PSMD10	26S protea	PSD10_HUI	9	1.157334
48H 10MT/	Q8WU79	0.118163	0.000944	0.002161	SMAP2	Stromal me	SMAP2_HU	13	1.085352
48H 50MT/	Q6L8Q7	-0.10849	0.000944	0.002161	PDE12	2',5'-phosp	PDE12_HUI	23	0.927556
24H 50MT/	Q9ULR0	0.172735	0.000944	0.002161	ISY1	Pre-mRNA-s	ISY1_HUMA	6	1.127193
24H 50MT/	Q9Y624	0.171593	0.000945	0.002164	F11R	Junctional	JAM1_HUN	10	1.126302
24H 10MT/	P09960	-0.07005	0.000946	0.002164	LTA4H	Leukotrien	LKHA4_HUI	33	0.952605
24H 10MT/	Q9BRJ6	0.098476	0.000949	0.00217	C7orf50	Uncharacte	CG050_HU	9	1.070642
48H 10MT/	Q96DM3	-0.04399	0.000951	0.002173	RMC1	Regulator c	RMC1_HUN	11	0.969971
48H 50MT/	Q32P28	0.046697	0.000951	0.002173	P3H1	Prolyl 3-hy	P3H1_HUM	19	1.032897
48H 10MT/	Q9P0L0	0.125654	0.000952	0.002175	VAPA	Vesicle-ass	VAPA_HUM	9	1.091002
24H 50MT/	Q6EMK4	0.781898	0.000953	0.002175	VASN	Vasorin	VASN_HUM	4	1.719391
48H 50MT/	P22413	0.173765	0.000956	0.00218	ENPP1	Ectonucleo	ENPP1_HUI	13	1.127998
48H 50MT/	P46060	-0.18648	0.000956	0.00218	RANGAP1	Ran GTPase	RAGP1_HU	33	0.87875
48H 10MT/	P28340	0.030132	0.00096	0.002185	POLD1	DNA polym	DPOD1_HU	46	1.021105
24H 10MT/	P27694	0.09807	0.00096	0.002186	RPA1	Replicatior	RFA1_HUM	30	1.070341
48H 50MT/	P34949	0.361391	0.000963	0.002189	MPI	Mannose-6	MPI_HUMA	15	1.284664
48H 50MT/	Q06481	0.284177	0.000967	0.002195	APLP2	Amyloid be	APLP2_HUI	16	1.217715
24H 10MT/	O75054	0.471827	0.00097	0.002201	IGSF3	Immunoglc	IGSF3_HUN	12	1.386865
24H 50MT/	P49585	0.043933	0.00097	0.002201	PCYT1A	Choline-ph	PCY1A_HUI	14	1.03092
48H 50MT/	P53634	0.088723	0.000973	0.002206	CTSC	Dipeptidyl	CATC_HUM	11	1.063429
24H 10MT/	Q9BY77	0.064918	0.000974	0.002208	POLDIP3	Polymerase	PDIP3_HUN	17	1.046025
48H 50MT/	Q5SRE5	-0.22729	0.000974	0.002208	NUP188	Nucleopori	NU188_HU	51	0.854236
48H 50MT/	Q8NFH3	-0.17534	0.000975	0.002208	NUP43	Nucleopori	NUP43_HU	11	0.885556

48H 50MT/	P09001	-0.32846	0.000975	0.002209	MRPL3	Large ribos	RM03_HUM	11	0.796386
48H 10MT/	P02771	-0.34437	0.000977	0.002211	AFP	Alpha-fetop	FETA_HUM/	3	0.787652
48H 50MT/	Q8WUD6	-0.09373	0.000977	0.002212	CHPT1	Cholinephc	CHPT1_HUI	1	0.937099
48H 10MT/	Q5JRA6	0.064811	0.000978	0.002213	MIA3	Transport a	TGO1_HUM	43	1.045948
24H 50MT/	Q93063	0.195505	0.00098	0.002216	EXT2	Exostosin-2	EXT2_HUM	10	1.145125
48H 10MT/	Q9UGP8	0.04864	0.00098	0.002216	SEC63	Translocati	SEC63_HUI	34	1.034289
24H 50MT/	P05091	0.141868	0.000982	0.002219	ALDH2	Aldehyde d	ALDH2_HU	26	1.103333
24H 50MT/	Q6P4E1	0.256811	0.000983	0.00222	GOLM2	Protein GO	GOLM2_HU	9	1.194834
48H 50MT/	Q9UK41	0.213819	0.000983	0.002221	VPS28	Vacuolar pı	VPS28_HUI	11	1.159754
24H 50MT/	Q9HB21	0.149522	0.000984	0.002223	PLEKHA1	Pleckstrin I	PKHA1_HU	10	1.109202
24H 10MT/	O43491	0.018648	0.000984	0.002223	EPB41L2	Band 4.1-lil	E41L2_HUI	49	1.01301
48H 10MT/	P21127	0.010728	0.000988	0.002229	CDK11B	Cyclin-depe	CD11B_HU	21	1.007464
24H 50MT/	Q96E39	0.298063	0.000988	0.002229	RBMXL1	RNA bindin	RMXL1_HU	3	1.229492
24H 10MT/	Q9P016	0.042155	0.000989	0.00223	THYN1	Thymocyte	THYN1_HUI	10	1.02965
24H 10MT/	O00159	0.124646	0.00099	0.002232	MYO1C	Unconvent	MYO1C_HU	43	1.09024
48H 50MT/	Q92878	0.134724	0.00099	0.002232	RAD50	DNA repair	RAD50_HU	48	1.097883
48H 50MT/	P60604	0.040446	0.000991	0.002233	UBE2G2	Ubiquitin-c	UB2G2_HU	4	1.028432
48H 50MT/	P13647	0.239224	0.000991	0.002233	KRT5	Keratin, typ	K2C5_HUM	14	1.180357
24H 50MT/	Q8IY37	0.074761	0.000993	0.002235	DHX37	Probable A	DHX37_HU	32	1.053186
24H 50MT/	P51610	0.065199	0.000995	0.002237	HCFC1	Host cell fa	HCFC1_HUI	40	1.046229
48H 50MT/	Q9Y620	-0.67455	0.000996	0.00224	RAD54B	DNA repair	RA54B_HUI	12	0.626526
48H 10MT/	P37802	0.077801	0.000998	0.002242	TAGLN2	Transgelin-	TAGL2_HUI	15	1.055408
48H 50MT/	Q9Y6V7	-0.28549	0.001002	0.002249	DDX49	Probable A	DDX49_HU	19	0.82046
24H 50MT/	P00366	0.355101	0.001002	0.002249	GLUD1	Glutamate	DHE3_BOV	1	1.279075
48H 10MT/	Q8WXG6	-0.33731	0.001002	0.00225	MADD	MAP kinase	MADD_HUI	7	0.791517
24H 50MT/	Q5VT25	-0.29099	0.001003	0.002251	CDC42BPA	Serine/thre	MRCKA_HU	7	0.817342
24H 50MT/	Q9Y2G8	0.041048	0.001004	0.002254	DNAJC16	DnaJ homo	DJC16_HUI	14	1.028861
48H 50MT/	P40222	-0.16449	0.001008	0.002258	TXLNA	Alpha-taxili	TXLNA_HUI	25	0.892245
48H 10MT/	Q10567	0.075846	0.001008	0.002258	AP1B1	AP-1 compl	AP1B1_HUI	27	1.053979
48H 50MT/	P40763	0.138199	0.001009	0.002259	STAT3	Signal trans	STAT3_HUN	37	1.10053
48H 10MT/	Q7Z5H4	0.566305	0.001011	0.002263	VN1R5	Vomeronas	VN1R5_HU	1	1.480727
24H 50MT/	Q8IWS0	0.131373	0.001013	0.002267	PHF6	PHD finger	PHF6_HUM	9	1.095335
24H 50MT/	P07305	1.402245	0.001014	0.002267	H1-0	Histone H1	H10_HUM/	4	2.643125
24H 50MT/	Q12797	0.218176	0.001016	0.002271	ASPH	Aspartyl/as	ASPH_HUM	23	1.163262
48H 50MT/	Q96HE7	0.402648	0.001019	0.002275	ERO1A	ERO1-like p	ERO1A_HUI	24	1.321932
24H 50MT/	P02662	-0.48329	0.00102	0.002277	CSN1S1	Alpha-S1-ca	CASA1_BOV	4	0.715344
48H 10MT/	Q96CP7	-0.91175	0.001021	0.002278	TLCD1	TLC domair	TLCD1_HUI	4	0.53154
24H 50MT/	P11168	0.543073	0.001024	0.002284	SLC2A2	Solute carri	GTR2_HUM	7	1.457073
48H 50MT/	Q7L2E3	-0.20823	0.001025	0.002285	DHX30	ATP-depenc	DHX30_HU	64	0.865597
48H 50MT/	Q9Y6H1	0.239786	0.001025	0.002286	CHCHD2	Coiled-coil-	CHCH2_HU	4	1.180817
48H 50MT/	P09884	-0.22847	0.001026	0.002286	POLA1	DNA polym	DPOLA_HU	35	0.853539
48H 10MT/	Q96HW7	0.066783	0.001026	0.002286	INTS4	Integrator (	INT4_HUM/	19	1.047379
48H 10MT/	Q9BSR8	0.252096	0.001026	0.002287	YIPF4	Protein YIP	YIPF4_HUM	3	1.190936
24H 10MT/	Q9NR46	-0.16849	0.001027	0.002288	SH3GLB2	Endophilin	SHLB2_HUI	11	0.889771
24H 10MT/	Q3LXA3	-0.16104	0.001029	0.002291	TKFC	Triokinase/	TKFC_HUM	18	0.894382
24H 10MT/	P61970	-0.2138	0.00103	0.002292	NUTF2	Nuclear tra	NTF2_HUM	6	0.862263
24H 50MT/	P10155	0.128501	0.001031	0.002294	RO60	RNA-bindir	RO60_HUM	20	1.093157
48H 10MT/	Q6UW68	0.169252	0.001031	0.002294	TMEM205	Transmemk	TM205_HU	5	1.124475
24H 50MT/	Q9BXP2	-0.05955	0.001032	0.002294	SLC12A9	Solute carri	S12A9_HUI	7	0.959561
48H 50MT/	Q5T0D9	-0.66397	0.001032	0.002295	TPRG1L	Tumor prot	TPRGL_HUI	4	0.631138
24H 50MT/	O75843	-0.37558	0.001032	0.002295	AP1G2	AP-1 compl	AP1G2_HU	4	0.770798
24H 50MT/	A0A0K0K1/	0.070116	0.001033	0.002297	TRBV6-5	T cell recep	TVB65_HUI	1	1.049801

48H 50MT/	P23470	0.095579	0.001033	0.002297	PTPRG	Receptor-ty	PTPRG_HUI	14	1.068494
24H 50MT/	Q86UN3	1.742895	0.001033	0.002297	RTN4RL2	Reticulon-4	R4RL2_HUI	2	3.347061
48H 50MT/	O94874	0.039821	0.001035	0.002299	UFL1	E3 UFM1-pı	UFL1_HUM	37	1.027986
48H 50MT/	O00186	0.108391	0.001036	0.002302	STXBP3	Syntaxin-bi	STXB3_HUI	19	1.078026
24H 50MT/	O95400	0.041283	0.001038	0.002305	CD2BP2	CD2 antige	CD2B2_HU	12	1.029028
24H 10MT/	Q03013	-0.30053	0.001041	0.00231	GSTM4	Glutathion	GSTM4_HU	9	0.811956
48H 50MT/	Q9BQ52	-0.17559	0.001043	0.002314	ELAC2	Zinc phospl	RNZ2_HUM	31	0.885408
48H 50MT/	P61353	-0.143	0.001045	0.002317	RPL27	Large ribos	RL27_HUM	7	0.905635
48H 10MT/	P60604	0.1649	0.001047	0.00232	UBE2G2	Ubiquitin-c	UB2G2_HU	4	1.121089
24H 50MT/	P49756	0.058953	0.001048	0.002321	RBM25	RNA-bindir	RBM25_HU	27	1.041709
48H 10MT/	P05141	-0.15012	0.001049	0.002322	SLC25A5	ADP/ATP tr	ADT2_HUM	20	0.901176
48H 50MT/	O00488	-0.2769	0.001051	0.002324	ZNF593	Zinc finger	ZN593_HUI	4	0.825362
24H 50MT/	Q15118	0.514623	0.001053	0.002328	PDK1	[Pyruvate d	PDK1_HUM	8	1.42862
24H 50MT/	Q9UHG3	0.235968	0.001056	0.002333	PCYOX1	Prenylcyste	PCYOX_HUI	16	1.177697
48H 50MT/	Q96T88	-0.37879	0.001056	0.002333	UHRF1	E3 ubiquiti	UHRF1_HU	31	0.769084
48H 50MT/	Q9UK39	0.532132	0.001056	0.002334	NOCT	Nocturnin	NOCT_HUM	1	1.446065
48H 50MT/	P29350	0.224598	0.001057	0.002334	PTPN6	Tyrosine-pr	PTN6_HUM	14	1.168451
24H 10MT/	Q8WZA9	-0.15987	0.001058	0.002335	IRGQ	Immunity-r	IRGQ_HUM	17	0.895109
24H 50MT/	Q14642	0.632379	0.001058	0.002335	INPP5A	Inositol pol	I5P1_HUM	6	1.550119
48H 10MT/	Q6PD74	0.132237	0.00106	0.002338	AAGAB	Alpha- and	AAGAB_HU	6	1.095992
48H 50MT/	Q8WVK7	-0.34921	0.00106	0.002338	SKA2	Spindle and	SKA2_HUM	1	0.785016
24H 50MT/	O14907	-0.1178	0.001061	0.002339	TAX1BP3	Tax1-bindir	TX1B3_HUI	4	0.921595
48H 10MT/	P20810	0.207992	0.001063	0.002341	CAST	Calpastatin	ICAL_HUM	22	1.155079
48H 50MT/	P55201	-1.76769	0.001066	0.002346	BRPF1	Peregrin	BRPF1_HUI	4	0.293678
24H 10MT/	P07148	-0.07248	0.001067	0.002347	FABP1	Fatty acid-l	FABPL_HUI	7	0.951
48H 10MT/	Q13043	0.074872	0.001067	0.002347	STK4	Serine/thre	STK4_HUM	22	1.053267
48H 10MT/	P27105	0.15418	0.001072	0.002355	STOM	Stomatin	STOM_HUM	7	1.112789
24H 50MT/	Q9P2R7	0.15779	0.001073	0.002356	SUCLA2	Succinate--	SUCB1_HUI	20	1.115577
24H 50MT/	Q8WZA9	-0.21361	0.001073	0.002356	IRGQ	Immunity-r	IRGQ_HUM	17	0.86238
48H 50MT/	P42695	-0.31613	0.001075	0.002359	NCAPD3	Condensin-	CNDD3_HU	22	0.803222
24H 50MT/	Q9NPH2	-0.08883	0.001075	0.00236	ISYNA1	Inositol-3-p	INO1_HUM	14	0.940285
24H 10MT/	Q96PZ7	0.06922	0.001078	0.002365	CSMD1	CUB and su	CSMD1_HU	1	1.049149
24H 10MT/	Q01780	0.041571	0.00108	0.002368	EXOSC10	Exosome cc	EXOSX_HUI	38	1.029234
24H 50MT/	O15254	0.11156	0.001082	0.00237	ACOX3	Peroxisoma	ACOX3_HU	15	1.080396
24H 10MT/	Q53EL6	-0.13983	0.001083	0.002371	PDCD4	Programme	PDCD4_HU	12	0.907625
48H 50MT/	P30047	0.396602	0.001084	0.002372	GCHFR	GTP cycloh	GFRP_HUM	5	1.316404
48H 10MT/	P33240	0.100896	0.001089	0.002381	CSTF2	Cleavage sti	CSTF2_HUI	17	1.072439
24H 50MT/	Q6DKI1	0.046994	0.00109	0.002382	RPL7L1	Ribosomal	RL7L_HUM	12	1.03311
48H 50MT/	O14495	-0.57256	0.001091	0.002385	PLPP3	Phospholip	PLPP3_HUI	5	0.672425
48H 50MT/	Q99808	-0.15518	0.001094	0.002391	SLC29A1	Equilibrati	S29A1_HUI	7	0.898023
48H 50MT/	Q96AE7	-0.50015	0.001095	0.002392	TTC17	Tetratricop	TTC17_HUI	12	0.707033
48H 50MT/	P06733	0.169492	0.001096	0.002393	ENO1	Alpha-enol	ENOA_HUM	28	1.124662
48H 50MT/	Q9NSK0	0.20274	0.001103	0.002406	KLC4	Kinesin ligh	KLC4_HUM	16	1.150882
48H 10MT/	Q9Y3I0	0.032826	0.001104	0.002406	RTCB	RNA-splicir	RTCB_HUM	24	1.023014
48H 50MT/	Q8NEB9	0.093384	0.001105	0.002409	PIK3C3	Phosphatid	PK3C3_HUI	20	1.066869
24H 50MT/	O75962	-0.23731	0.001107	0.002412	TRIO	Triple funct	TRIO_HUM	29	0.848324
24H 50MT/	O75146	-0.07188	0.001109	0.002415	HIP1R	Huntingtin	HIP1R_HUI	19	0.951395
24H 50MT/	P48634	0.023457	0.001109	0.002415	PRRC2A	Protein PRI	PRC2A_HUI	44	1.016392
24H 10MT/	P04899	0.08122	0.001109	0.002416	GNAI2	Guanine nu	GNAI2_HUI	13	1.057912
24H 50MT/	P01019	0.235771	0.001112	0.002421	AGT	Angiotensir	ANGT_HUM	12	1.177536
48H 10MT/	P62277	0.113593	0.001113	0.002422	RPS13	Small ribos	RS13_HUM	10	1.081919
48H 10MT/	P46459	0.019243	0.001113	0.002423	NSF	Vesicle-fusi	NSF_HUMA	43	1.013428

24H 50MT/	Q14980	0.123476	0.001119	0.002431	NUMA1	Nuclear mi	NUMA1_HU	76	1.089357
24H 50MT/	Q15286	0.160707	0.00112	0.002433	RAB35	Ras-related	RAB35_HU	9	1.117834
24H 10MT/	Q9Y625	0.727125	0.001121	0.002434	GPC6	Glypican-6	GPC6_HUM	3	1.655337
48H 10MT/	Q8WUM0	-0.00454	0.001122	0.002436	NUP133	Nuclear po	NU133_HU	46	0.99686
24H 50MT/	P84085	0.125589	0.001123	0.002437	ARF5	ADP-ribosy	ARF5_HUM	6	1.090953
24H 50MT/	Q9UBT2	0.080296	0.001123	0.002438	UBA2	SUMO-activ	SAE2_HUM	30	1.057235
48H 10MT/	Q8WUM4	0.039936	0.001124	0.002439	PDCD6IP	Programme	PDC6I_HUI	48	1.028068
48H 50MT/	Q86Y39	-0.18132	0.001124	0.002439	NDUFA11	NADH dehy	NDUAB_HU	7	0.881893
24H 50MT/	Q6IBS0	0.186592	0.001125	0.00244	TWF2	Twinfilin-2	TWF2_HUM	17	1.138072
24H 50MT/	P0CG12	1.699159	0.001126	0.00244	DERPC	Decreased e	DERPC_HU	12	3.247117
48H 50MT/	Q9P1F3	-0.37804	0.001129	0.002446	ABRACL	Costars fam	ABRAL_HUI	2	0.769482
24H 10MT/	Q9Y5X3	-0.12263	0.001131	0.002449	SNX5	Sorting nex	SNX5_HUM	14	0.918512
24H 10MT/	P46013	0.150038	0.001133	0.002453	MKI67	Proliferatic	KI67_HUM/	119	1.109599
24H 50MT/	O75153	-0.18589	0.001136	0.002457	CLUH	Clustered m	CLU_HUMA	45	0.879106
24H 50MT/	P25787	0.038759	0.001135	0.002457	PSMA2	Proteasom	PSA2_HUM	14	1.02723
48H 50MT/	Q8IUF8	-0.21988	0.001136	0.002457	RIOX2	Ribosomal	RIOX2_HUI	12	0.858635
24H 50MT/	Q7L014	0.333596	0.001138	0.002459	DDX46	Probable AT	DDX46_HU	42	1.260151
48H 10MT/	Q8IWV8	0.12327	0.001138	0.00246	UBR2	E3 ubiquiti	UBR2_HUM	24	1.089201
24H 10MT/	P11216	-0.11233	0.00114	0.002463	PYGB	Glycogen p	PYGB_HUM	30	0.925095
48H 10MT/	Q6P161	0.167534	0.001144	0.002468	MRPL54	Large ribos	RM54_HUI	4	1.123137
48H 50MT/	P60033	0.201888	0.001145	0.002469	CD81	CD81 antig	CD81_HUM	2	1.150202
24H 50MT/	Q8N3U4	0.022562	0.001145	0.00247	STAG2	Cohesin sul	STAG2_HUI	25	1.015762
48H 50MT/	Q96BS2	0.580473	0.001146	0.002471	TESC	Calcineurin	CHP3_HUM	8	1.49534
48H 50MT/	Q9BSR8	0.223449	0.001147	0.002472	YIPF4	Protein YIP	YIPF4_HUM	3	1.167521
24H 50MT/	Q13424	1.132414	0.001148	0.002474	SNTA1	Alpha-1-syr	SNTA1_HUI	1	2.192253
24H 50MT/	P61088	-0.21418	0.001149	0.002474	UBE2N	Ubiquitin-c	UBE2N_HU	9	0.862033
24H 50MT/	Q969V3	0.057902	0.001148	0.002474	NCLN	BOS compl	NCLN_HUM	22	1.040951
48H 50MT/	P08709	0.359439	0.001149	0.002475	F7	Coagulatio	FA7_HUMA	4	1.282927
24H 50MT/	P00918	-0.12367	0.00115	0.002475	CA2	Carbonic ar	CAH2_HUM	16	0.917847
24H 50MT/	Q8N5K1	0.073589	0.00115	0.002475	CISD2	CDGSH iror	CISD2_HUI	7	1.052332
24H 50MT/	Q9P2I0	0.075958	0.001152	0.002479	CPSF2	Cleavage ar	CPSF2_HUI	31	1.05406
48H 10MT/	P46782	0.166126	0.001154	0.002482	RPS5	Small ribos	RS5_HUMA	10	1.122042
48H 50MT/	Q15287	0.082996	0.001154	0.002482	RNPS1	RNA-bindir	RNPS1_HUI	6	1.059216
48H 50MT/	P25786	0.134838	0.001154	0.002482	PSMA1	Proteasom	PSA1_HUM	15	1.09797
24H 50MT/	P52597	0.101762	0.001154	0.002482	HNRNPF	Heterogene	HNRPF_HU	18	1.073083
24H 50MT/	O43813	0.148142	0.001155	0.002483	LANCL1	Glutathion	LANC1_HUI	15	1.108141
24H 50MT/	P10114	0.504035	0.001155	0.002483	RAP2A	Ras-related	RAP2A_HUI	2	1.418174
24H 50MT/	Q12931	0.100912	0.001156	0.002483	TRAP1	Heat shock	TRAP1_HUI	34	1.072451
48H 10MT/	Q9Y570	0.101122	0.001156	0.002484	PPME1	Protein ph	PPME1_HU	18	1.072607
24H 10MT/	O60566	0.062295	0.001167	0.002502	BUB1B	Mitotic che	BUB1B_HU	25	1.044125
48H 50MT/	P22626	0.140371	0.001169	0.002505	HNRNPA2B	Heterogene	ROA2_HUM	24	1.102189
24H 10MT/	O00422	0.239153	0.00117	0.002506	SAP18	Histone dea	SAP18_HUI	7	1.1803
24H 50MT/	P14618	0.128558	0.00117	0.002506	PKM	Pyruvate ki	KPYM_HUM	43	1.0932
24H 10MT/	Q15029	0.068408	0.001172	0.002508	EFTUD2	116 kDa U5	U5S1_HUM	51	1.048559
24H 50MT/	Q9Y320	-0.16751	0.001172	0.002509	TMX2	Thioredoxi	TMX2_HUM	13	0.890376
24H 50MT/	Q96PK6	0.13558	0.001173	0.00251	RBM14	RNA-bindir	RBM14_HU	25	1.098534
48H 50MT/	Q5GLZ8	0.108824	0.001174	0.002512	HERC4	Probable E3	HERC4_HUI	27	1.078349
48H 50MT/	P00734	0.747642	0.001174	0.002512	F2	Prothromb	THRB_HUM	26	1.679047
48H 50MT/	Q9C004	-0.56287	0.001175	0.002512	SPRY4	Protein spr	SPY4_HUM	4	0.676952
48H 10MT/	O43765	0.073824	0.001177	0.002516	SGTA	Small gluta	SGTA_HUM	11	1.052502
24H 50MT/	P29401	0.087337	0.001178	0.002518	TKT	Transketola	TKT_HUMA	29	1.062407
48H 50MT/	Q14687	-0.39831	0.001179	0.002518	GSE1	Genetic sup	GSE1_HUM	5	0.758747

24H 50MT/	Q9H2G4	0.020502	0.001182	0.002524	TSPYL2	Testis-speci	TSYL2_HUN	5	1.014313
48H 50MT/	P08648	0.261785	0.001183	0.002525	ITGA5	Integrin alp	ITA5_HUM/	19	1.198961
24H 50MT/	P21912	0.160313	0.001183	0.002525	SDHB	Succinate d	SDHB_HUN	10	1.11753
48H 50MT/	Q99661	-0.2244	0.001183	0.002525	KIF2C	Kinesin-like	KIF2C_HUN	22	0.855951
24H 50MT/	Q6JQN1	0.322911	0.001184	0.002526	ACAD10	Acyl-CoA de	ACD10_HU	25	1.250852
24H 10MT/	P41247	1.271331	0.001184	0.002527	PNPLA4	Patatin-like	PLPL4_HUN	2	2.413842
48H 10MT/	Q13085	-0.02438	0.001186	0.002529	ACACA	Acetyl-CoA	ACACA_HUI	82	0.983243
48H 50MT/	Q9UNI6	-0.20626	0.001186	0.002529	DUSP12	Dual specifi	DUS12_HU	10	0.86678
48H 50MT/	Q9H981	-0.3359	0.001187	0.002531	ACTR8	Actin-relate	ARP8_HUN	13	0.79229
24H 10MT/	P06744	-0.17285	0.001188	0.002532	GPI	Glucose-6-p	G6PI_HUM	23	0.887091
24H 50MT/	P28331	0.132493	0.001189	0.002533	NDUFS1	NADH-ubiq	NDUS1_HU	35	1.096186
48H 10MT/	Q8N163	0.053973	0.001189	0.002533	CCAR2	Cell cycle a	CCAR2_HUI	34	1.03812
48H 50MT/	Q9Y289	-0.49826	0.001191	0.002536	SLC5A6	Sodium-de	SC5A6_HUI	4	0.707958
24H 50MT/	Q9Y287	0.352498	0.001192	0.002537	ITM2B	Integral me	ITM2B_HUI	6	1.276769
24H 50MT/	Q13501	-0.23077	0.001198	0.002545	SQSTM1	Sequestoso	SQSTM_HU	18	0.852183
24H 50MT/	Q13439	0.118299	0.0012	0.002549	GOLGA4	Golgin subf	GOGA4_HU	41	1.085454
24H 50MT/	Q9UL25	0.070945	0.001202	0.00255	RAB21	Ras-related	RAB21_HUI	11	1.050405
24H 50MT/	P61011	0.072789	0.001203	0.002552	SRP54	Signal reco	SRP54_HUI	22	1.051748
48H 50MT/	P35573	0.084712	0.001203	0.002552	AGL	Glycogen d	GDE_HUM/	77	1.060476
24H 50MT/	Q6NVY1	0.113636	0.001203	0.002552	HIBCH	3-hydroxyi:	HIBCH_HUI	15	1.081951
48H 50MT/	Q12797	0.215366	0.001205	0.002555	ASPH	Aspartyl/as	ASPH_HUM	23	1.160999
24H 50MT/	Q15418	0.184162	0.001206	0.002556	RPS6KA1	Ribosomal	KS6A1_HUI	15	1.136157
24H 10MT/	Q9P253	-0.0489	0.001207	0.002558	VPS18	Vacuolar pr	VPS18_HUI	27	0.966671
24H 50MT/	O95197	0.303861	0.001209	0.00256	RTN3	Reticulon-3	RTN3_HUM	7	1.234444
48H 50MT/	Q9C0C4	0.535767	0.001209	0.00256	SEMA4C	Semaphori	SEM4C_HU	5	1.449713
48H 50MT/	Q96BR5	-0.15654	0.001209	0.002561	COA7	Cytochrom	COA7_HUM	5	0.897172
24H 50MT/	Q9H3H1	-0.02116	0.001212	0.002565	TRIT1	tRNA dimet	MOD5_HUI	17	0.98544
24H 50MT/	Q14839	0.171696	0.001216	0.002571	CHD4	Chromodoi	CHD4_HUN	68	1.126382
48H 50MT/	Q92830	-0.59974	0.001216	0.002571	KAT2A	Histone ace	KAT2A_HUI	11	0.659873
48H 50MT/	O60749	0.046206	0.001219	0.002577	SNX2	Sorting nex	SNX2_HUN	22	1.032546
24H 50MT/	A1L0T0	0.169348	0.001221	0.002579	ILVBL	2-hydroxya	HACL2_HUI	22	1.12455
24H 50MT/	Q8NEJ9	0.154094	0.001221	0.002579	NGDN	Neuroguidi	NGDN_HUN	11	1.112723
24H 10MT/	P10301	0.252542	0.001221	0.002579	RRAS	Ras-related	RRAS_HUM	7	1.191304
24H 50MT/	Q5HYK7	3.604251	0.001222	0.002579	SH3D19	SH3 domai	SH319_HUI	1	12.16151
48H 10MT/	Q9H098	0.032243	0.001223	0.002581	FAM107B	Protein FAI	F107B_HUI	7	1.022601
24H 50MT/	Q9NP72	-0.1406	0.001225	0.002585	RAB18	Ras-related	RAB18_HUI	12	0.907141
48H 50MT/	Q99536	0.141512	0.001228	0.002588	VAT1	Synaptic ve	VAT1_HUM	16	1.103061
24H 50MT/	P46779	0.183113	0.00123	0.002591	RPL28	Large ribos	RL28_HUM	5	1.135331
48H 50MT/	Q53SF7	0.060486	0.001235	0.0026	COBLL1	Cordon-ble	COBL1_HUI	15	1.042817
24H 50MT/	Q06136	-0.05676	0.001237	0.002603	KDSR	3-ketodihy	KDSR_HUM	5	0.961423
24H 50MT/	O75648	0.267004	0.001242	0.00261	TRMU	Mitochond	MTU1_HUN	13	1.203307
48H 10MT/	Q9NXB9	-0.48243	0.001242	0.00261	ELOVL2	Very long cl	ELOV2_HUI	4	0.715772
48H 50MT/	O75208	0.317599	0.001245	0.002614	COQ9	Ubiquinon	COQ9_HUN	7	1.246255
48H 50MT/	Q6NUK1	0.165407	0.001245	0.002615	SLC25A24	Mitochond	SCMC1_HU	20	1.121482
24H 50MT/	P39060	0.282061	0.001247	0.002617	COL18A1	Collagen al	COIA1_HUN	14	1.215931
48H 10MT/	P18615	0.050228	0.001247	0.002617	NELFE	Negative el	NELFE_HUN	15	1.035428
24H 10MT/	P60228	0.051208	0.001248	0.002619	EIF3E	Eukaryotic	EIF3E_HUN	25	1.036132
48H 50MT/	Q9H4A4	0.152644	0.00125	0.002621	RNPEP	Aminopept	AMPB_HUN	29	1.111605
24H 50MT/	O60830	0.068378	0.001257	0.002633	TIMM17B	Mitochond	TI17B_HUN	4	1.048537
24H 50MT/	P02647	0.373718	0.001257	0.002633	APOA1	Apolipopro	APOA1_HU	9	1.295688
24H 10MT/	Q00839	0.078556	0.001258	0.002634	HNRNPU	Heterogene	HNRPU_HU	38	1.055961
24H 50MT/	Q14671	-0.00496	0.001258	0.002634	PUM1	Pumilio ho	PUM1_HUN	25	0.996567

24H 50MTA	P20340	0.187664	0.001259	0.002635	RAB6A	Ras-related	RAB6A_HUI	11	1.138918
24H 50MTA	Q9NXH9	-0.21593	0.00126	0.002636	TRMT1	tRNA (guan	TRM1_HUN	14	0.860988
24H 50MTA	P49321	0.045329	0.00126	0.002637	NASP	Nuclear aut	NASP_HUM	35	1.031919
48H 10MTA	Q9UK76	0.134299	0.00126	0.002637	JPT1	Jupiter mic	JUPI1_HUN	7	1.09756
24H 50MTA	Q96CD2	-0.12634	0.001265	0.002644	PPCDC	Phosphopa	COAC_HUN	2	0.91615
48H 10MTA	P48436	0.890786	0.001266	0.002645	SOX9	Transcripti	SOX9_HUM	1	1.854186
48H 10MTA	Q05048	0.077865	0.001266	0.002645	CSTF1	Cleavage sti	CSTF1_HUN	14	1.055455
48H 10MTA	P27695	0.053245	0.001267	0.002647	APEX1	DNA repair	APEX1_HUI	16	1.037596
24H 10MTA	P01137	1.549275	0.001268	0.002648	TGFB1	Transformi	TGFB1_HUI	2	2.9267
24H 10MTA	O14786	1.963254	0.00127	0.00265	NRP1	Neuropilin-	NRP1_HUN	11	3.899405
24H 50MTA	Q7KZ85	0.04393	0.001272	0.002654	SUPT6H	Transcripti	SPT6H_HUI	55	1.030918
24H 50MTA	O43143	0.072997	0.001274	0.002657	DHX15	ATP-depend	DHX15_HU	36	1.0519
24H 10MTA	P60174	-0.17279	0.001281	0.002668	TPI1	Triosephos	TPIS_HUMA	18	0.887126
48H 10MTA	Q16181	0.102306	0.001282	0.002669	SEPTIN7	Septin-7	SEPT7_HUN	19	1.073488
48H 50MTA	Q8NBJ7	0.151059	0.001286	0.002676	SUMF2	Inactive C-a	SUMF2_HU	10	1.110384
24H 50MTA	P0DP23;P0	0.602439	0.001287	0.002676	CALM1;CAL	Calmodulir	CALM1_HU	8	1.518281
24H 50MTA	P62753	0.029737	0.001287	0.002677	RPS6	Small ribos	RS6_HUMA	10	1.020826
24H 50MTA	Q6UX07	0.481317	0.001287	0.002677	DHRS13	Dehydroge	DHR13_HU	9	1.396018
48H 10MTA	Q9NVV0	-0.57646	0.001291	0.002683	TMEM38B	Trimeric int	TM38B_HU	3	0.670609
48H 50MTA	P18850	-0.81699	0.001294	0.002686	ATF6	Cyclic AMP	ATF6A_HUN	2	0.567624
48H 10MTA	Q96HR8	0.032167	0.001295	0.002688	NAF1	H/ACA ribo	NAF1_HUM	4	1.022547
24H 50MTA	O95989	0.083788	0.001295	0.002689	NUDT3	Diphospho	NUDT3_HU	5	1.059797
48H 50MTA	Q9HC21	-0.90162	0.001296	0.002689	SLC25A19	Mitochond	TPC_HUMA	2	0.535286
24H 50MTA	Q9BXI6	0.419228	0.001296	0.002689	TBC1D10A	TBC1 doma	TB10A_HUI	12	1.337211
24H 50MTA	Q5SXM8	0.158259	0.001297	0.00269	DNLZ	DNL-type zi	DNLZ_HUM	1	1.11594
24H 10MTA	Q8N567	0.016126	0.001298	0.00269	ZCCHC9	Zinc finger	ZCHC9_HUI	3	1.011241
24H 50MTA	O75446	0.655514	0.001299	0.002692	SAP30	Histone dea	SAP30_HUI	3	1.575177
24H 10MTA	P04632	-0.1353	0.001301	0.002696	CAPNS1	Calpain sma	CPNS1_HUI	11	0.910478
48H 10MTA	P0DMV1;P0	0.216746	0.001302	0.002697	CT45A8;CT4	Cancer/test	CT458_HUI	9	1.162109
24H 50MTA	O95551	0.197909	0.001304	0.0027	TDP2	Tyrosyl-DNA	TYDP2_HUI	11	1.147034
48H 50MTA	P51114	0.018238	0.001305	0.002701	FXR1	RNA-bindir	FXR1_HUM	21	1.012722
24H 50MTA	P21333	-0.17639	0.001306	0.002701	FLNA	Filamin-A	FLNA_HUM	104	0.884916
48H 50MTA	Q13438	0.021457	0.001307	0.002704	OS9	Protein OS-	OS9_HUMA	20	1.014984
24H 50MTA	Q8TDN6	0.134261	0.001312	0.00271	BRIX1	Ribosome b	BRX1_HUN	20	1.097531
24H 10MTA	Q8WUA2	0.162566	0.001312	0.002711	PPIL4	Peptidyl-pr	PPIL4_HUN	20	1.119276
48H 50MTA	Q8NC54	0.342837	0.001313	0.002712	KCT2	Keratinocy	KCT2_HUM	3	1.268248
24H 50MTA	Q9Y5Z9	-0.01288	0.001313	0.002712	UBIAD1	UbiA preny	UBIA1_HUN	5	0.991115
48H 50MTA	Q9NWT1	-0.2053	0.001314	0.002713	PAK1IP1	p21-activat	PK1IP_HUN	16	0.867361
48H 50MTA	P27694	0.116633	0.001316	0.002716	RPA1	Replicatior	RFA1_HUM	30	1.084202
48H 10MTA	Q66K14	0.050279	0.001317	0.002717	TBC1D9B	TBC1 doma	TBC9B_HUI	26	1.035465
48H 10MTA	Q92896	0.08909	0.001317	0.002717	GLG1	Golgi appar	GSLG1_HUI	40	1.063699
48H 50MTA	Q9UJK0	-0.4216	0.001322	0.002723	TSR3	18S rRNA ar	TSR3_HUM	6	0.746595
24H 50MTA	O43251	0.521366	0.001328	0.002731	RBFOX2	RNA bindin	RFOX2_HUI	7	1.435314
24H 50MTA	O60716	0.04717	0.001329	0.002732	CTNND1	Catenin del	CTND1_HU	40	1.033236
48H 50MTA	P09417	0.067915	0.001332	0.002738	QDPR	Dihydropte	DHPR_HUN	10	1.048201
48H 10MTA	Q9UQ35	0.092034	0.001333	0.002739	SRRM2	Serine/argi	SRRM2_HU	45	1.065872
24H 50MTA	P51687	0.226214	0.001333	0.002739	SUOX	Sulfite oxid	SUOX_HUN	11	1.169761
24H 10MTA	Q9UNE7	-0.04678	0.001335	0.002741	STUB1	E3 ubiquiti	CHIP_HUM	16	0.968092
48H 10MTA	Q9UJA5	-0.02788	0.001336	0.002744	TRMT6	tRNA (aden	TRM6_HUN	19	0.980858
24H 50MTA	Q9NRC8	1.731755	0.001339	0.002747	SIRT7	NAD-depen	SIR7_HUMA	2	3.321315
24H 50MTA	Q9BU61	0.088252	0.001339	0.002747	NDUFAF3	NADH dehy	NDUF3_HU	6	1.063081
24H 50MTA	Q8N4T8	0.231167	0.00134	0.002748	CBR4	3-oxoacyl-[	CBR4_HUM	5	1.173784

48H 10MT/	Q53FT3	0.10959	0.00134	0.002748	HIKESHI	Protein Hik	HIKES_HUN	5	1.078922
48H 50MT/	A1X283	0.129619	0.00134	0.002748	SH3PXD2B	SH3 and PX	SPD2B_HU	13	1.094005
24H 50MT/	Q15291	0.18666	0.001342	0.002752	RBBP5	Retinoblast	RBBP5_HU	15	1.138125
48H 10MT/	P09884	0.070732	0.001343	0.002754	POLA1	DNA polym	DPOLA_HU	35	1.050249
48H 10MT/	Q13164	-1.31571	0.001345	0.002756	MAPK7	Mitogen-ac	MK07_HUN	1	0.401729
48H 50MT/	Q9BPX6	-0.0315	0.001347	0.002758	MICU1	Calcium up	MICU1_HU	8	0.978403
48H 10MT/	Q01082	0.004037	0.001348	0.002759	SPTBN1	Spectrin be	SPTB2_HUN	127	1.002802
24H 50MT/	Q9NQX6	0.566768	0.001351	0.002763	ZNF331	Zinc finger	ZN331_HU	4	1.481201
48H 50MT/	Q16555	0.053044	0.001352	0.002765	DPYSL2	Dihydropyr	DPYL2_HU	17	1.037452
48H 50MT/	Q6NUK4	-0.54026	0.001354	0.002769	REEP3	Receptor e	REEP3_HUN	3	0.687648
48H 50MT/	Q6I9Y2	0.191084	0.001355	0.00277	THOC7	THO compl	THOC7_HU	8	1.141621
48H 10MT/	Q7Z4W1	0.094348	0.001357	0.002772	DCXR	L-xylulose r	DCXR_HUN	10	1.067582
48H 50MT/	Q13148	0.10147	0.001358	0.002773	TARDBP	TAR DNA-bi	TADBP_HU	14	1.072866
48H 50MT/	Q9Y5T4	-0.51501	0.00136	0.002776	DNAJC15	DnaJ homo	DJC15_HU	3	0.699788
24H 50MT/	Q9BV38	0.101318	0.001363	0.00278	WDR18	WD repeat-	WDR18_HU	13	1.072753
24H 50MT/	Q99959	0.166184	0.001363	0.00278	PKP2	Plakophilir	PKP2_HUN	27	1.122086
48H 50MT/	Q9H254	0.926706	0.001364	0.002781	SPTBN4	Spectrin be	SPTN4_HU	2	1.900931
24H 50MT/	Q9P287	0.047866	0.001364	0.002781	BCCIP	BRCA2 and	BCCIP_HUN	14	1.033735
48H 50MT/	P51948	-0.18734	0.001366	0.002783	MNAT1	CDK-activa	MAT1_HUN	11	0.878223
24H 10MT/	P20336	0.244167	0.001369	0.002789	RAB3A	Ras-related	RAB3A_HU	1	1.184409
24H 50MT/	Q8WXE1	1.763577	0.00137	0.002789	ATRIP	ATR-interac	ATRIP_HUN	1	3.395389
24H 50MT/	Q13561	-0.11987	0.001372	0.002793	DCTN2	Dynactin su	DCTN2_HU	21	0.920273
48H 10MT/	Q15370	0.215584	0.001374	0.002796	ELOB	Elongin-B	ELOB_HUM	10	1.161174
48H 50MT/	Q8IWE2	0.287614	0.001374	0.002796	FAM114A1	Protein NO	NXP20_HU	14	1.22062
48H 50MT/	P16455	0.083317	0.001375	0.002796	MGMT	Methylatec	MGMT_HU	5	1.059451
24H 50MT/	O00571	-0.11841	0.001375	0.002797	DDX3X	ATP-depenc	DDX3X_HU	44	0.921203
24H 50MT/	Q7Z2K8	0.26183	0.001376	0.002797	GPRIN1	G protein-r	GRIN1_HU	10	1.198999
24H 50MT/	P17706	0.202865	0.001375	0.002797	PTPN2	Tyrosine-pr	PTN2_HUN	9	1.150982
24H 50MT/	Q9Y5K6	-0.10087	0.001375	0.002797	CD2AP	CD2-associ	CD2AP_HU	27	0.932472
48H 10MT/	P49903	0.154162	0.00138	0.002802	SEPHS1	Selenide, w	SPS1_HUM	11	1.112775
24H 50MT/	Q9H6R3	0.167608	0.001383	0.002806	ACSS3	Acyl-CoA sy	ACSS3_HUN	17	1.123195
24H 50MT/	P38646	0.123994	0.001383	0.002807	HSPA9	Stress-70 pi	GRP75_HU	32	1.089748
48H 50MT/	P55196	0.123495	0.001384	0.002808	AFDN	Afadin	AFAD_HUN	58	1.08937
24H 50MT/	P20020	0.094878	0.001384	0.002808	ATP2B1	Plasma mer	AT2B1_HU	37	1.067975
24H 50MT/	O43826	0.190342	0.00139	0.002815	SLC37A4	Glucose-6-j	G6PT1_HU	3	1.141034
24H 50MT/	Q9H999	-0.0342	0.001391	0.002818	PANK3	Pantothena	PANK3_HU	10	0.976576
48H 10MT/	Q8TB72	0.226707	0.001391	0.002818	PUM2	Pumilio ho	PUM2_HUN	10	1.170161
24H 50MT/	Q96BM9	0.185431	0.001392	0.002818	ARL8A	ADP-ribosy	ARL8A_HU	10	1.137157
48H 10MT/	Q99942	0.619868	0.001392	0.002819	RNF5	E3 ubiquiti	RNF5_HUM	1	1.536735
24H 50MT/	Q5T653	-0.02134	0.001393	0.002819	MRPL2	Large ribos	RM02_HUN	7	0.985321
24H 50MT/	P06756	0.050003	0.001393	0.00282	ITGAV	Integrin alp	ITAV_HUM/	37	1.035267
48H 50MT/	P50613	-0.48109	0.001393	0.00282	CDK7	Cyclin-depe	CDK7_HUN	14	0.716436
24H 50MT/	Q5GLZ8	0.098108	0.001394	0.00282	HERC4	Probable E	HERC4_HU	27	1.070369
48H 10MT/	Q52LJ0	0.083612	0.001396	0.002824	FAM98B	Protein FAI	FA98B_HU	12	1.059667
48H 50MT/	P29966	0.167085	0.001397	0.002825	MARCKS	Myristoylat	MARCS_HU	13	1.122787
48H 10MT/	Q9NVP2	-0.00445	0.001397	0.002825	ASF1B	Histone cha	ASF1B_HUN	4	0.99692
24H 10MT/	Q96LD4	0.32861	0.001397	0.002825	TRIM47	E3 ubiquiti	TRI47_HUN	7	1.255803
24H 10MT/	P40205	-0.2123	0.001397	0.002825	MYCNOS	N-cym prot	NCYM_HUN	1	0.863159
24H 50MT/	P21675	0.222238	0.0014	0.002829	TAF1	Transcripti	TAF1_HUM	11	1.166542
24H 50MT/	P14866	0.102062	0.0014	0.002829	HNRNPL	Heterogene	HNRPL_HU	23	1.073307
48H 10MT/	P35269	0.095189	0.0014	0.002829	GTF2F1	General tra	T2FA_HUM	15	1.068205
48H 50MT/	Q96EC8	0.353952	0.001402	0.002832	YIPF6	Protein YIP	YIPF6_HUN	4	1.278057

48H 50MT/	Q9BXW9	-0.34353	0.001403	0.002834	FANCD2	Fanconi an	FACD2_HU	34	0.78811
48H 10MT/	Q96M86	-0.21622	0.001404	0.002835	DNHD1	Dynein hea	DNHD1_HU	2	0.860818
24H 10MT/	Q8TCA0	-0.05818	0.001405	0.002836	LRRC20	Leucine-ric	LRC20_HU	10	0.960478
24H 50MT/	Q9BX68	0.117793	0.001406	0.002839	HINT2	Adenosine	HINT2_HU	4	1.085074
24H 10MT/	Q9NQT4	0.225339	0.001408	0.00284	EXOSC5	Exosome cc	EXOS5_HU	7	1.169052
24H 50MT/	O95772	0.126915	0.001408	0.002841	STARD3NL	STARD3 N-t	STR3N_HU	3	1.091956
48H 50MT/	Q9H4I9	1.043101	0.001408	0.002841	SMDT1	Essential M	EMRE_HUM	2	2.060651
24H 50MT/	Q53EL6	0.714038	0.001408	0.002841	PDCD4	Programme	PDCD4_HU	12	1.640389
24H 50MT/	Q96T76	-0.1665	0.001409	0.002842	MMS19	MMS19 nu	MMS19_HU	37	0.891
24H 10MT/	O95747	0.028541	0.001409	0.002843	OXSR1	Serine/thre	OXSR1_HU	16	1.01998
24H 50MT/	Q15819	0.001516	0.00141	0.002844	UBE2V2	Ubiquitin-c	UB2V2_HU	5	1.001051
48H 10MT/	P04259	-0.54988	0.001411	0.002846	KRT6B	Keratin, typ	K2C6B_HU	2	0.683078
24H 50MT/	Q8N6N3	-0.07595	0.001412	0.002848	C1orf52	UPF0690 p	CA052_HU	7	0.948719
48H 50MT/	P36957	0.019778	0.001413	0.002849	DLST	Dihydrolip	ODO2_HUM	11	1.013803
24H 10MT/	Q8TBQ9	0.204291	0.001416	0.002853	TMEM167A	Protein kisl	KISHA_HUM	3	1.15212
24H 50MT/	P98194	0.01825	0.001416	0.002853	ATP2C1	Calcium-tra	AT2C1_HU	16	1.01273
48H 10MT/	P54136	0.02769	0.001419	0.002857	RARS1	Arginine--t	SYRC_HUM	47	1.019378
24H 50MT/	P78310	0.064565	0.001421	0.002859	CXADR	Coxsackievi	CXAR_HUM	19	1.045769
48H 50MT/	O75083	0.131685	0.001421	0.00286	WDR1	WD repeat-	WDR1_HU	28	1.095573
24H 50MT/	Q5TC12	0.093932	0.001421	0.00286	ATPAF1	ATP synthas	ATPF1_HUM	9	1.067275
48H 50MT/	Q8TEQ6	-0.14412	0.001421	0.00286	GEMIN5	Gem-associ	GEMI5_HU	49	0.904931
24H 50MT/	P33992	0.066336	0.001425	0.002865	MCM5	DNA replica	MCM5_HU	41	1.047054
24H 10MT/	P50454	0.191594	0.001426	0.002867	SERPINH1	Serpin H1	SERPH_HU	19	1.142025
48H 50MT/	P05198	0.035007	0.001429	0.00287	EIF2S1	Eukaryotic	IF2A_HUM/	25	1.024562
48H 50MT/	Q8WWV3	0.110866	0.001429	0.002872	RTN4IP1	Reticulon-4	RT4I1_HUM	13	1.079876
48H 50MT/	P30044	0.146662	0.00143	0.002873	PRDX5	Peroxiredo	PRDX5_HU	11	1.107005
24H 50MT/	Q8WXF0	-0.59363	0.001432	0.002875	SRSF12	Serine/argi	SRS12_HUM	2	0.662671
24H 50MT/	Q6ZTW0	0.337653	0.001432	0.002876	TPGS1	Tubulin pol	TPGS1_HU	6	1.263699
24H 10MT/	O95819	0.20058	0.001432	0.002876	MAP4K4	Mitogen-ac	M4K4_HUM	31	1.149161
48H 50MT/	P12532;P1	0.804142	0.001433	0.002877	CKMT1A;Ck	Creatine kir	KCRU_HUM	1	1.746107
24H 10MT/	Q8NEJ9	0.094653	0.001433	0.002877	NGDN	Neuroguidi	NGDN_HUM	11	1.067808
24H 50MT/	P55010	-0.2085	0.001435	0.002878	EIF5	Eukaryotic	IF5_HUMAI	17	0.865434
48H 50MT/	Q9BW61	-1.18956	0.001435	0.002878	DDA1	DET1- and D	DDA1_HUM	2	0.438436
48H 50MT/	Q9GZT9	0.242245	0.001435	0.002878	EGLN1	Egl nine ho	EGLN1_HU	11	1.182832
24H 50MT/	Q9H8V3	-0.30899	0.001435	0.002879	ECT2	Protein ECT	ECT2_HUM	28	0.807206
24H 50MT/	Q8NHZ8	-0.69426	0.00144	0.002885	CDC26	Anaphase-p	CDC26_HU	1	0.618026
48H 50MT/	O95373	-0.10743	0.001443	0.002889	IPO7	Importin-7	IPO7_HUM	43	0.928243
24H 10MT/	Q01581	-0.12468	0.001443	0.002889	HMGCS1	Hydroxyme	HMCS1_HU	23	0.917207
48H 50MT/	Q96EY7	-0.15452	0.001443	0.002889	PTCD3	Small ribos	PTCD3_HU	26	0.898433
24H 50MT/	P50416	0.103736	0.001444	0.00289	CPT1A	Carnitine O	CPT1A_HU	28	1.074552
48H 50MT/	P24752	0.166507	0.001445	0.002892	ACAT1	Acetyl-CoA	THIL_HUM/	20	1.122338
24H 50MT/	Q08378	0.013257	0.001447	0.002893	GOLGA3	Golgin subf	GOGA3_HU	46	1.009231
48H 50MT/	Q5UIP0	-0.27013	0.001447	0.002894	RIF1	Telomere-a	RIF1_HUM/	48	0.829243
48H 50MT/	Q10567	0.071706	0.001448	0.002894	AP1B1	AP-1 compl	AP1B1_HU	27	1.050959
24H 10MT/	Q9NSV4	0.097945	0.00145	0.002897	DIAPH3	Protein dia	DIAP3_HUM	15	1.070248
24H 50MT/	Q15172	0.026198	0.001454	0.002903	PPP2R5A	Serine/thre	2A5A_HUM	14	1.018325
24H 50MT/	Q9H1E5	0.326681	0.001455	0.002905	TMX4	Thioredoxi	TMX4_HUM	5	1.254125
48H 50MT/	P0DPD7	0.124176	0.001455	0.002905	EEF1AKMT4	EEF1A lysin	EFMT4_HU	10	1.089885
48H 50MT/	Q7KZF4	0.050551	0.001455	0.002905	SND1	Staphyloco	SND1_HUM	50	1.03566
24H 50MT/	A6NDG6	-0.01598	0.001458	0.002909	PGP	Glycerol-3-	PGP_HUM/	14	0.988984
48H 50MT/	O00571	-0.13871	0.001459	0.00291	DDX3X	ATP-depend	DDX3X_HU	44	0.908334
24H 10MT/	Q13057	-0.06746	0.00146	0.002911	COASY	Bifunctiona	COASY_HU	15	0.954317

24H 50MT/	P51991	0.029763	0.001465	0.002919	HNRNPA3	Heterogene	ROA3_HUM	22	1.020845
48H 50MT/	P62258	0.211832	0.001467	0.002921	YWHAE	14-3-3 prot	1433E_HUI	21	1.158158
48H 10MT/	Q92782	0.898167	0.001467	0.002922	DPF1	Zinc finger	DPF1_HUM	1	1.863696
48H 10MT/	Q5T5Y3	0.316057	0.00147	0.002927	CAMSAP1	Calmodulir	CAMP1_HU	4	1.244923
48H 50MT/	P0DMV8;P(	-0.16672	0.001471	0.002927	HSPA1A;HS	Heat shock	HS71A_HUI	25	0.890865
24H 10MT/	Q8N126	2.190202	0.001473	0.002932	CADM3	Cell adhesi	CADM3_HU	1	4.563694
24H 10MT/	O60341	0.108783	0.001476	0.002935	KDM1A	Lysine-spec	KDM1A_HU	23	1.078318
48H 10MT/	P02753	0.162446	0.001477	0.002937	RBP4	Retinol-bin	RET4_HUM	7	1.119183
24H 50MT/	P09211	-0.24249	0.001479	0.00294	GSTP1	Glutathion	GSTP1_HUI	16	0.845287
48H 10MT/	P78310	0.066609	0.001481	0.002942	CXADR	Coxsackievi	CXAR_HUM	19	1.047252
24H 50MT/	Q9Y2V7	0.145384	0.001481	0.002942	COG6	Conserved	COG6_HUM	20	1.106025
24H 10MT/	P00918	-0.25818	0.001483	0.002945	CA2	Carbonic ar	CAH2_HUM	16	0.836144
24H 50MT/	Q1KMD3	0.343078	0.001484	0.002945	HNRNPUL2	Heterogene	HNRL2_HU	27	1.26846
48H 10MT/	Q9UKV3	0.012789	0.001486	0.002949	ACIN1	Apoptotic	ACINU_HUI	29	1.008904
48H 50MT/	Q96IU4	0.186544	0.001486	0.002949	ABHD14B	Putative pr	ABHEB_HUI	6	1.138035
48H 10MT/	Q9BW61	-0.96936	0.001488	0.002952	DDA1	DET1- and I	DDA1_HUM	2	0.510731
48H 10MT/	P23919	-0.01145	0.001489	0.002954	DTYMK	Thymidylat	KTHY_HUM	19	0.992092
48H 10MT/	Q9NP79	0.14336	0.001492	0.002958	VTA1	Vacuolar pı	VTA1_HUM	11	1.104474
24H 50MT/	O75396	0.214293	0.001493	0.002959	SEC22B	Vesicle-traf	SC22B_HUI	11	1.160135
48H 50MT/	Q969V3	-0.18345	0.001493	0.002959	NCLN	BOS compl	NCLN_HUM	22	0.880598
24H 50MT/	Q92979	0.107374	0.001494	0.00296	EMG1	Ribosomal	NEP1_HUM	15	1.077265
48H 50MT/	A4D1E9	-0.46174	0.001495	0.002961	GTPBP10	GTP-bindin	GTPBA_HUI	12	0.72611
48H 10MT/	Q15021	0.025236	0.001495	0.002962	NCAPD2	Condensin	CND1_HUM	53	1.017646
48H 50MT/	O43379	-0.24831	0.001497	0.002966	WDR62	WD repeat-	WDR62_HU	9	0.841883
24H 10MT/	P20933	1.415176	0.001498	0.002966	AGA	N(4)-(beta-I	ASPG_HUM	1	2.666923
48H 10MT/	P10643	-0.83299	0.001498	0.002966	C7	Compleme	CO7_HUM/	2	0.561366
24H 50MT/	Q9H9B4	0.154976	0.001498	0.002966	SFXN1	Sideroflexir	SFXN1_HUI	12	1.113403
48H 50MT/	P98174	0.026824	0.0015	0.002969	FGD1	FYVE, RhoG	FGD1_HUM	2	1.018767
24H 10MT/	Q9UHN6	0.163574	0.001503	0.002974	CEMIP2	Inactive cel	CEIP2_HUM	40	1.120059
48H 50MT/	P63010	0.036333	0.001505	0.002976	AP2B1	AP-2 compl	AP2B1_HUI	45	1.025504
24H 10MT/	Q15233	0.075044	0.001509	0.002981	NONO	Non-POU d	NONO_HUI	22	1.053393
24H 50MT/	Q5TH69	0.050765	0.00151	0.002982	ARFGEF3	Brefeldin A	BIG3_HUM	24	1.035814
48H 50MT/	P05455	0.123722	0.001516	0.00299	SSB	Lupus La pr	LA_HUMAN	29	1.089542
24H 10MT/	Q6NUQ1	0.330659	0.001519	0.002995	RINT1	RAD50-inte	RINT1_HUM	9	1.257588
48H 50MT/	Q2TAL8	0.077558	0.00152	0.002997	QRICH1	Transcripti	QRIC1_HUI	13	1.05523
24H 50MT/	Q6XR72	1.424442	0.001521	0.002998	SLC30A10	Calcium/m	ZNT10_HUI	1	2.684107
24H 50MT/	Q86Y39	0.073326	0.001524	0.003002	NDUFA11	NADH dehy	NDUAB_HU	7	1.052139
48H 50MT/	Q8WX92	0.075771	0.001526	0.003004	NELFB	Negative el	NELFB_HUI	13	1.053924
24H 10MT/	P09525	-0.0975	0.001528	0.003007	ANXA4	Annexin A4	ANXA4_HU	24	0.934651
48H 10MT/	Q16698	0.092146	0.001529	0.003009	DECR1	2,4-dienoyl	DECR_HUM	15	1.065955
48H 50MT/	Q9HCE1	0.124172	0.00153	0.003011	MOV10	Helicase M(	MOV10_HU	44	1.089882
24H 50MT/	Q9UDR5	0.324936	0.001532	0.003013	AASS	Alpha-amir	AASS_HUM	13	1.252609
48H 50MT/	Q8TEV9	-0.17271	0.001534	0.003015	SMCR8	Guanine nu	SMCR8_HU	1	0.887174
48H 10MT/	O14618	0.05828	0.001535	0.003017	CCS	Copper cha	CCS_HUMA	8	1.041223
24H 10MT/	P23229	0.112686	0.001538	0.003021	ITGA6	Integrin alp	ITA6_HUM/	24	1.081239
48H 50MT/	P23786	0.067647	0.001538	0.003022	CPT2	Carnitine O	CPT2_HUM	21	1.048006
48H 50MT/	Q9Y2Z4	-0.25209	0.001542	0.003027	YARS2	Tyrosine--tI	SYYM_HUM	18	0.839677
48H 50MT/	Q8WXF1	0.096741	0.001543	0.003029	PSPC1	Paraspeckl	PSPC1_HUI	14	1.069355
48H 10MT/	P49321	0.096591	0.001544	0.003029	NASP	Nuclear aut	NASP_HUM	35	1.069244
48H 50MT/	P04746;P0	-1.03826	0.001546	0.003033	AMY2A;AM	Pancreatic	AMYP_HUM	1	0.486913
24H 10MT/	P78347	0.035032	0.001547	0.003034	GTF2I	General tra	GTF2I_HUM	45	1.024579
48H 50MT/	Q12788	-0.16125	0.001549	0.003037	TBL3	Transducin	TBL3_HUM	35	0.89425

24H	50MT/	P62993	-0.14998	0.001549	0.003037	GRB2	Growth fac	GRB2_HUN	13	0.901265
48H	50MT/	Q96I24	0.006511	0.00155	0.003038	FUBP3	Far upstrea	FUBP3_HUI	24	1.004523
24H	50MT/	P04792	0.097968	0.001554	0.003043	HSPB1	Heat shock	HSPB1_HUI	16	1.070265
48H	10MT/	Q96EC8	0.280625	0.001555	0.003044	YIPF6	Protein YIP	YIPF6_HUN	4	1.214721
48H	50MT/	P33527	0.041697	0.001556	0.003045	ABCC1	Multidrug r	MRP1_HUN	23	1.029324
24H	50MT/	Q9NPA8	-0.65402	0.001559	0.00305	ENY2	Transcripti	ENY2_HUM	1	0.635508
48H	50MT/	Q9NR19	0.202603	0.001562	0.003054	ACSS2	Acetyl-coer	ACSA_HUM	12	1.150773
48H	10MT/	O14772	0.070051	0.001562	0.003054	FPGT	Fucose-1-pl	FPGT_HUM	27	1.049754
48H	50MT/	Q9Y6M1	0.108891	0.001562	0.003054	IGF2BP2	Insulin-like	IF2B2_HUN	20	1.078399
48H	10MT/	Q15345	-0.50948	0.001563	0.003054	LRRC41	Leucine-ric	LRC41_HUI	1	0.702474
24H	10MT/	Q96ES7	0.170041	0.001563	0.003055	SGF29	SAGA-assoc	SGF29_HUI	4	1.125091
24H	50MT/	Q9H0U3	0.100136	0.001569	0.003064	MAGT1	Magnesium	MAGT1_HU	7	1.071874
24H	50MT/	P31153	-0.15909	0.001569	0.003064	MAT2A	S-adenosylr	METK2_HU	16	0.895589
24H	10MT/	Q9UGQ3	0.619342	0.00157	0.003065	SLC2A6	Solute carri	GTR6_HUM	1	1.536174
48H	50MT/	O94979	0.096507	0.00157	0.003065	SEC31A	Protein trai	SC31A_HUI	36	1.069182
24H	50MT/	O95758	-0.13914	0.001576	0.003073	PTBP3	Polypyrimi	PTBP3_HUI	13	0.908061
24H	50MT/	Q96F25	0.467925	0.001576	0.003073	ALG14	UDP-N-acet	ALG14_HUI	3	1.383118
48H	50MT/	Q6P444	-0.30885	0.001577	0.003075	MTFR2	Mitochond	MTFR2_HU	6	0.807286
48H	10MT/	Q6DKI1	-0.19561	0.001577	0.003076	RPL7L1	Ribosomal	RL7L_HUM	12	0.873203
24H	50MT/	O43688	0.261523	0.001579	0.003077	PLPP2	Phospholip	PLPP2_HUI	6	1.198744
48H	10MT/	Q15056	0.153574	0.001579	0.003078	EIF4H	Eukaryotic	IF4H_HUM/	9	1.112321
48H	10MT/	Q8IXI2	0.108511	0.001579	0.003078	RHOT1	Mitochond	MIRO1_HU	8	1.078115
24H	50MT/	O95994	-0.08806	0.001586	0.003086	AGR2	Anterior gr	AGR2_HUN	5	0.940789
48H	10MT/	Q96GA7	0.205935	0.001587	0.003087	SDSL	Serine dehy	SDSL_HUM	12	1.153434
24H	50MT/	P62310	-0.36291	0.001587	0.003088	LSM3	U6 snRNA-a	LSM3_HUN	3	0.777596
48H	50MT/	Q15018	0.077589	0.00159	0.003091	ABRAXAS2	BRISC comp	ABRX2_HUI	11	1.055253
48H	50MT/	Q9HDC9	0.109481	0.00159	0.003091	APMAP	Adipocyte p	APMAP_HU	21	1.07884
48H	10MT/	Q9H254	1.227537	0.00159	0.003091	SPTBN4	Spectrin be	SPTN4_HUI	2	2.341668
48H	10MT/	Q9BYE7	0.351021	0.001591	0.003093	PCGF6	Polycomb g	PCGF6_HUI	3	1.275463
48H	10MT/	Q14657	-0.09691	0.001592	0.003093	LAGE3	EKC/KEOPS	LAGE3_HUI	2	0.935036
24H	50MT/	Q06330	0.148992	0.001592	0.003093	RBPJ	Recombinii	SUH_HUM/	5	1.108795
24H	50MT/	P26599	0.104773	0.001596	0.003098	PTBP1	Polypyrimi	PTBP1_HUI	18	1.075325
48H	50MT/	Q6YHK3	0.536122	0.001597	0.0031	CD109	CD109 anti	CD109_HU	36	1.45007
48H	50MT/	Q86UE4	0.033016	0.001599	0.003102	MTDH	Protein LYF	LYRIC_HUN	22	1.023149
48H	50MT/	Q8WVM8	0.104843	0.001601	0.003104	SCFD1	Sec1 family	SCFD1_HUI	25	1.075378
24H	50MT/	P18827	2.059176	0.001601	0.003105	SDC1	Syndecan-1	SDC1_HUM	3	4.167481
48H	50MT/	P04003	-0.06558	0.001602	0.003106	C4BPA	C4b-bindin	C4BPA_HUI	1	0.955563
24H	50MT/	Q5HYK3	0.391239	0.001604	0.003108	COQ5	2-methoxy-	COQ5_HUN	12	1.311519
48H	50MT/	Q8N983	-0.35129	0.001604	0.003108	MRPL43	Large ribos	RM43_HUN	5	0.783883
24H	50MT/	Q8IY95	-0.0249	0.001607	0.003111	TMEM192	Transmemb	TM192_HU	3	0.982891
48H	50MT/	P08758	0.054032	0.001608	0.003112	ANXA5	Annexin A5	ANXA5_HU	23	1.038162
48H	10MT/	Q8WXE9	0.587615	0.001608	0.003112	STON2	Stonin-2	STON2_HUI	1	1.50276
48H	10MT/	O60762	-0.11878	0.001608	0.003112	DPM1	Dolichol-pl	DPM1_HUN	13	0.920968
48H	10MT/	P11142	-0.14303	0.001611	0.003117	HSPA8	Heat shock	HSP7C_HUI	27	0.905617
48H	10MT/	P48668	-0.26185	0.001612	0.003118	KRT6C	Keratin, typ	K2C6C_HUI	32	0.834017
24H	50MT/	O43395	0.227326	0.001613	0.003119	PRPF3	U4/U6 sma	PRPF3_HUI	23	1.170663
48H	50MT/	Q6SJ93	-0.52602	0.001614	0.00312	FAM111B	Serine prot	F111B_HUI	8	0.69447
48H	10MT/	Q8N999	-0.35054	0.001614	0.00312	RLIG1	RNA ligase 1	RLIG1_HUN	6	0.784289
48H	10MT/	Q96J94	-0.34578	0.001615	0.003122	PIWIL1	Piwi-like pr	PIWL1_HUI	1	0.786881
48H	50MT/	Q86U42	0.144274	0.001617	0.003125	PABPN1	Polyadenyl	PABP2_HUI	8	1.105174
24H	50MT/	P01023	0.195119	0.001625	0.003135	A2M	Alpha-2-ma	A2MG_HUN	57	1.144818
48H	50MT/	Q5T8D3	0.181214	0.001625	0.003136	ACBD5	Acyl-CoA-bi	ACBD5_HU	13	1.133838

24H	50MT/	Q9H0V9	0.226056	0.001625	0.003136	LMAN2L	VIP36-like p	LMA2L_HU	12	1.169633
48H	50MT/	Q6PF05	3.353737	0.00163	0.003143	TTC23L	Tetratricop	TT23L_HUM	1	10.22293
48H	50MT/	P00918	-0.18221	0.001631	0.003143	CA2	Carbonic ar	CAH2_HUM	16	0.881354
48H	10MT/	Q13573	0.066763	0.001632	0.003144	SNW1	SNW doma	SNW1_HUM	24	1.047364
48H	50MT/	Q8TAE8	-0.22114	0.001632	0.003145	GADD45GI	Large ribos	G45IP_HUM	7	0.857886
24H	50MT/	Q3KQV9	0.162691	0.001636	0.003149	UAP1L1	UDP-N-acet	UAP1L_HU	10	1.119373
48H	50MT/	Q15526	0.177903	0.001637	0.00315	SURF1	Surfeit locu	SURF1_HU	7	1.131238
24H	50MT/	P14868	0.13434	0.001637	0.00315	DARS1	Aspartate--t	SYDC_HUM	36	1.09759
48H	50MT/	Q01780	-0.14029	0.001638	0.003151	EXOSC10	Exosome cc	EXOSX_HU	38	0.907339
48H	50MT/	Q14432	0.218623	0.001638	0.003151	PDE3A	cGMP-inhil	PDE3A_HU	10	1.163622
24H	50MT/	Q9NPJ6	0.134529	0.001639	0.003151	MED4	Mediator o	MED4_HUM	9	1.097734
48H	50MT/	Q9UBU9	-0.13417	0.001639	0.003151	NXF1	Nuclear RN	NXF1_HUM	25	0.911195
24H	50MT/	Q9UBS0	0.091655	0.001641	0.003154	RPS6KB2	Ribosomal	KS6B2_HU	4	1.065592
48H	50MT/	O00762	-0.495	0.001642	0.003156	UBE2C	Ubiquitin-c	UBE2C_HU	8	0.70956
24H	50MT/	Q96FV9	0.205455	0.001642	0.003156	THOC1	THO compl	THOC1_HU	24	1.15305
24H	10MT/	Q13185	0.103433	0.001645	0.003161	CBX3	Chromobo:	CBX3_HUM	8	1.074327
48H	10MT/	Q9H223	-0.01372	0.001646	0.003162	EHD4	EH domain-	EHD4_HUM	21	0.990537
24H	50MT/	Q6UW68	0.29481	0.001647	0.003164	TMEM205	Transmemb	TM205_HU	5	1.226724
24H	50MT/	O60934	0.177168	0.001648	0.003165	NBN	Nibrin	NBN_HUM/	22	1.130662
48H	50MT/	Q9BYN0	-0.5603	0.001651	0.00317	SRXN1	Sulfiredoxi	SRXN1_HU	5	0.67816
48H	50MT/	O43688	-0.37282	0.001652	0.00317	PLPP2	Phospholip	PLPP2_HU	6	0.772273
48H	10MT/	Q9H3S7	0.023967	0.001652	0.00317	PTPN23	Tyrosine-pr	PTN23_HU	32	1.016751
24H	50MT/	Q6UXH1	0.14752	0.001653	0.003173	CRELD2	Protein dis	CREL2_HUM	5	1.107664
24H	50MT/	O00161	0.089669	0.001657	0.003177	SNAP23	Synaptosor	SNP23_HU	10	1.064126
24H	50MT/	Q92820	0.170061	0.001658	0.003178	GGH	Gamma-glu	GGH_HUM/	11	1.125106
24H	50MT/	Q13509	-0.35539	0.001658	0.003178	TUBB3	Tubulin bet	TBB3_HUM	4	0.78166
48H	10MT/	Q06609	0.137966	0.001658	0.003178	RAD51	DNA repair	RAD51_HU	10	1.100352
48H	50MT/	Q14240	0.315755	0.001665	0.003188	EIF4A2	Eukaryotic	IF4A2_HUM	12	1.244663
48H	50MT/	Q7Z4H3	0.076682	0.001666	0.003189	HDDC2	5'-deoxynu	HDDC2_HU	11	1.05459
48H	10MT/	Q86VP1	-0.05053	0.001667	0.003189	TAX1BP1	Tax1-bindir	TAXB1_HU	11	0.965584
48H	50MT/	Q86TP1	0.00294	0.001668	0.003192	PRUNE1	Exopolyphc	PRUN1_HU	12	1.00204
24H	10MT/	Q15014	0.125811	0.001675	0.003202	MORF4L2	Mortality fa	MO4L2_HU	8	1.091121
24H	50MT/	Q8TE02	-0.3138	0.001676	0.003203	ELP5	Elongator c	ELP5_HUM	5	0.804519
24H	10MT/	P07305	-0.35654	0.001681	0.003209	H1-0	Histone H1	H10_HUM/	4	0.781034
48H	50MT/	P60059	0.386681	0.001683	0.003213	SEC61G	Protein trai	SC61G_HU	2	1.307382
24H	50MT/	O75477	0.127998	0.001684	0.003213	ERLIN1	Erlin-1	ERLN1_HU	10	1.092776
48H	10MT/	P31942	0.028169	0.001685	0.003215	HNRNPH3	Heterogene	HNRH3_HU	12	1.019717
24H	50MT/	Q15029	0.064881	0.001687	0.003219	EFTUD2	116 kDa U5	U5S1_HUM	51	1.045999
48H	10MT/	P46977	-0.17246	0.001688	0.003219	STT3A	Dolichyl-di	STT3A_HUM	16	0.887326
48H	50MT/	Q8IXH7	0.083466	0.00169	0.003222	NELFCD	Negative ele	NELFD_HU	14	1.059561
48H	10MT/	O00442	0.073626	0.001693	0.003226	RTCA	RNA 3'-tern	RTCA_HUM	15	1.052358
24H	50MT/	Q9ULX3	0.283551	0.001694	0.003226	NOB1	RNA-bindir	NOB1_HUM	9	1.217187
48H	10MT/	P00395	-0.55274	0.001695	0.003227	MT-CO1	Cytochrom	COX1_HUM	4	0.681724
48H	50MT/	O00566	-0.12983	0.001696	0.003227	MPHOSPH1	U3 small nu	MPP10_HU	16	0.913937
48H	10MT/	Q8N8N7	0.050215	0.001696	0.003227	PTGR2	Prostaglanc	PTGR2_HU	9	1.035419
48H	10MT/	Q92616	0.025342	0.001696	0.003227	GCN1	Stalled ribc	GCN1_HUM	146	1.017721
24H	10MT/	O60925	-1.17537	0.001697	0.003229	PFDN1	Prefoldin su	PFD1_HUM	2	0.442769
48H	50MT/	Q9Y4W2	-0.16366	0.001698	0.00323	LAS1L	Ribosomal	LAS1L_HUM	29	0.892756
48H	50MT/	P07814	0.086984	0.001701	0.003233	EPRS1	Bifunctiona	SYEP_HUM/	85	1.062147
24H	50MT/	Q9HCK8	0.070142	0.001707	0.003241	CHD8	Chromodor	CHD8_HUM	28	1.04982
48H	10MT/	P61165	2.227272	0.001707	0.003241	TMEM258	Transmemb	TM258_HU	1	4.682477
48H	10MT/	P35237	0.043312	0.001708	0.003242	SERPINB6	Serpin B6	SPB6_HUM	18	1.030477

48H 50MT/	Q9NTJ5	-0.13107	0.001712	0.003248	SACM1L	Phosphatid	SAC1_HUM	28	0.913155
48H 10MT/	Q8N7H5	0.030354	0.001713	0.003248	PAF1	RNA polym	PAF1_HUM	21	1.021262
24H 10MT/	O75414	0.068373	0.001714	0.00325	NME6	Nucleoside	NDK6_HUM	4	1.048533
48H 10MT/	Q15738	-0.144	0.001714	0.00325	NSDHL	Sterol-4-alp	NSDHL_HU	15	0.905007
48H 50MT/	Q9H3H1	0.002941	0.001714	0.00325	TRIT1	tRNA dimet	MOD5_HUI	17	1.002041
24H 50MT/	Q9BRR6	0.098308	0.001715	0.003251	ADPGK	ADP-depen	ADPGK_HU	11	1.070517
48H 50MT/	Q9P015	-0.31223	0.001716	0.003252	MRPL15	Large ribos	RM15_HUM	17	0.805397
48H 50MT/	O75027	0.024815	0.001718	0.003253	ABCB7	Iron-sulfur	ABCB7_HUI	17	1.017349
24H 10MT/	P02753	0.105993	0.001719	0.003254	RBP4	Retinol-bin	RET4_HUM	7	1.076235
48H 50MT/	Q9P2X3	0.283175	0.001719	0.003254	IMPACT	Protein IMI	IMPCT_HUI	6	1.21687
24H 10MT/	P06396	0.192973	0.001723	0.003261	GSN	Gelsolin	GELS_HUM	18	1.143117
24H 50MT/	O14733	-0.21257	0.001728	0.003269	MAP2K7	Dual specifi	MP2K7_HU	11	0.863001
24H 50MT/	P22626	0.161673	0.001729	0.00327	HNRNPA2B	Heterogene	ROA2_HUM	24	1.118584
24H 10MT/	Q99584	-0.22511	0.001729	0.00327	S100A13	Protein S1(	S10AD_HUI	5	0.855529
24H 50MT/	Q96FW1	0.077809	0.00173	0.00327	OTUB1	Ubiquitin t	OTUB1_HU	14	1.055414
24H 50MT/	Q96AY3	0.060646	0.00173	0.00327	FKBP10	Peptidyl-pr	FKB10_HUI	27	1.042932
48H 10MT/	Q9NX62	0.044724	0.001731	0.003272	BPNT2	Golgi-resid	IMPA3_HUI	12	1.031486
24H 10MT/	Q9UBT2	0.05543	0.001732	0.003273	UBA2	SUMO-activ	SAE2_HUM	30	1.039169
24H 50MT/	Q9Y343	0.059294	0.001735	0.003277	SNX24	Sorting nex	SNX24_HUI	2	1.041956
48H 10MT/	P14543	0.117464	0.001736	0.003277	NID1	Nidogen-1	NID1_HUM	17	1.084826
24H 50MT/	P53990	-0.12756	0.001738	0.003281	IST1	IST1 homol	IST1_HUM/	11	0.915381
24H 50MT/	Q9Y221	0.224438	0.001739	0.003282	NIP7	60S riboson	NIP7_HUM	9	1.168322
24H 10MT/	Q8TEQ6	0.091148	0.00174	0.003284	GEMIN5	Gem-associ	GEMI5_HUI	49	1.065217
24H 50MT/	Q9UNI6	-0.10319	0.001741	0.003285	DUSP12	Dual specifi	DUS12_HU	10	0.930972
48H 50MT/	Q9H0R1	0.534551	0.001743	0.003288	AP5M1	AP-5 compl	AP5M1_HU	2	1.448492
48H 10MT/	Q7L590	-1.22586	0.001746	0.003292	MCM10	Protein MC	MCM10_HU	1	0.427543
48H 50MT/	Q9H7C9	0.238916	0.001749	0.003295	AAMDC	Mth938 do	AAMDC_HU	7	1.180105
48H 50MT/	Q9ULX9	-0.48398	0.001751	0.003299	MAFF	Transcripti	MAFF_HUM	4	0.715004
24H 50MT/	Q5TBA9	0.890801	0.001752	0.003299	FRY	Protein furi	FRY_HUMA	4	1.854206
24H 50MT/	P09543	0.119179	0.001754	0.003302	CNP	2',3'-cyclic-	CN37_HUM	20	1.086117
24H 10MT/	Q96MF7	0.223634	0.001754	0.003302	NSMCE2	E3 SUMO-p	NSE2_HUM	4	1.167671
48H 50MT/	P00387	0.145845	0.001755	0.003303	CYB5R3	NADH-cyto	NB5R3_HU	19	1.106379
24H 50MT/	P40926	0.081379	0.001756	0.003305	MDH2	Malate deh	MDHM_HU	15	1.058029
48H 50MT/	P63220	0.136313	0.001757	0.003306	RPS21	Small ribos	RS21_HUM	6	1.099093
24H 10MT/	O60658	-0.02754	0.001759	0.003308	PDE8A	High affinit	PDE8A_HUI	14	0.981094
48H 50MT/	Q9Y6Y8	0.103424	0.001762	0.003313	SEC23IP	SEC23-inte	S23IP_HUM	27	1.07432
24H 50MT/	Q9BUJ2	0.118076	0.001764	0.003316	HNRNPUL1	Heterogene	HNRL1_HU	25	1.085287
24H 10MT/	Q01433	-0.12131	0.001765	0.003316	AMPD2	AMP deami	AMPD2_HU	28	0.919349
48H 50MT/	Q9HB71	-0.11353	0.001765	0.003316	CACYBP	Calcyclin-b	CYBP_HUM	18	0.924324
24H 50MT/	P24278	0.290718	0.001766	0.003317	ZBTB25	Zinc finger	ZBT25_HUI	1	1.223249
24H 50MT/	P12956	0.07179	0.001768	0.00332	XRCC6	X-ray repair	XRCC6_HUI	43	1.05102
48H 50MT/	Q8N5V2	0.127715	0.00177	0.003323	NGEF	Ephexin-1	NGEF_HUM	12	1.092562
24H 10MT/	Q9UHX1	0.043546	0.00177	0.003323	PUF60	Poly(U)-bin	PUF60_HUI	24	1.030644
48H 10MT/	Q8N2Z9	-0.23904	0.001771	0.003324	CENPS	Centromer	CENPS_HUI	2	0.847307
48H 50MT/	Q9NRH1	-0.55373	0.001771	0.003325	YAE1	Protein YAI	YAE1_HUM	2	0.681257
24H 50MT/	P27824	0.036405	0.001772	0.003325	CANX	Calnexin	CALX_HUM	36	1.025555
24H 50MT/	P23786	0.095776	0.001775	0.003329	CPT2	Carnitine O	CPT2_HUM	21	1.06864
48H 10MT/	Q13099	0.363469	0.00178	0.003336	IFT88	Intraflagell	IFT88_HUM	2	1.286516
24H 10MT/	Q8N5M1	0.106148	0.001786	0.003344	ATPAF2	ATP syntha	ATPF2_HUI	8	1.076351
48H 50MT/	O43657	-0.36162	0.001791	0.003352	TSPAN6	Tetraspanir	TSN6_HUM	5	0.778291
48H 50MT/	Q96HS1	-0.16155	0.001792	0.003352	PGAM5	Serine/thre	PGAM5_HU	14	0.894062
48H 10MT/	Q5QJE6	-0.03809	0.001792	0.003352	DNTTIP2	Deoxynucle	TDIF2_HUM	20	0.973943

24H 50MT/	Q86YH6	0.170657	0.001792	0.003353	PDSS2	All trans-pc	DLP1_HUM	16	1.125571
24H 50MT/	Q93096	0.207018	0.001795	0.003356	PTP4A1	Protein tyr	TP4A1_HUI	5	1.1543
24H 50MT/	Q9Y3D0	-0.55749	0.001796	0.003358	CIAO2B	Cytosolic ir	CIA2B_HUM	4	0.679485
24H 50MT/	P12236	0.241358	0.001802	0.003367	SLC25A6	ADP/ATP tr	ADT3_HUM	4	1.182105
48H 50MT/	O60784	0.24823	0.001803	0.003368	TOM1	Target of M	TOM1_HUM	13	1.187749
24H 50MT/	Q9Y276	0.00872	0.001804	0.003368	BCS1L	Mitochond	BCS1_HUM	19	1.006063
24H 50MT/	P30084	0.10871	0.001805	0.003369	ECHS1	Enoyl-CoA I	ECHM_HUM	18	1.078264
24H 50MT/	Q15036	-0.16543	0.001806	0.00337	SNX17	Sorting nex	SNX17_HUI	17	0.891662
48H 10MT/	O94842	0.045701	0.001807	0.003372	TOX4	TOX high m	TOX4_HUM	10	1.032185
48H 10MT/	Q96A26	0.062665	0.001807	0.003372	FAM162A	Protein FAI	F162A_HUI	6	1.044393
24H 50MT/	P54252	-0.46111	0.001808	0.003372	ATXN3	Ataxin-3	ATX3_HUM	5	0.726426
24H 50MT/	O60422	0.189237	0.001812	0.003377	ONECUT3	One cut do	ONEC3_HU	3	1.140161
48H 50MT/	P57088	-0.186	0.001812	0.003377	TMEM33	Transmemb	TMM33_HU	11	0.879037
24H 50MT/	Q9H5H4	0.018638	0.001813	0.003378	ZNF768	Zinc finger	ZN768_HUI	8	1.013003
24H 50MT/	P0DPD7	0.228059	0.001815	0.003381	EEF1AKMT4	EEF1A lysin	EFMT4_HUI	10	1.171258
48H 10MT/	P0DI83	0.032304	0.001816	0.003383	RAB34	Ras-related	NARR_HUM	3	1.022644
24H 10MT/	P63098	0.044502	0.001817	0.003384	PPP3R1	Calcineurir	CANB1_HU	5	1.031327
48H 50MT/	Q8N3C0	0.045719	0.00182	0.003388	ASCC3	Activating s	ASCC3_HUI	63	1.032198
24H 50MT/	O14657	-0.28212	0.001821	0.003389	TOR1B	Torsin-1B	TOR1B_HUI	8	0.82238
48H 10MT/	Q96SK2	0.066077	0.001821	0.003389	TMEM209	Transmemb	TM209_HU	9	1.046866
24H 50MT/	Q00325	0.026849	0.001822	0.00339	SLC25A3	Solute carri	S25A3_HUI	15	1.018785
24H 10MT/	Q9NYB0	-0.03156	0.001822	0.00339	TERF2IP	Telomeric r	TE2IP_HUM	10	0.978361
24H 10MT/	P17858	0.06507	0.001822	0.00339	PFKL	ATP-depenc	PFKAL_HUI	30	1.046135
48H 50MT/	P57740	-0.13566	0.001823	0.00339	NUP107	Nuclear poi	NU107_HU	41	0.910251
24H 10MT/	P05114	0.170902	0.001823	0.003391	HMGN1	Non-histon	HMGN1_HU	3	1.125762
24H 50MT/	Q9BXF6	0.391745	0.001824	0.003391	RAB11FIP5	Rab11 fami	RFIP5_HUM	12	1.311979
24H 50MT/	Q9BV57	0.295051	0.001824	0.003393	ADI1	Acireducto	MTND_HUI	10	1.226928
24H 50MT/	P20336	0.198115	0.001827	0.003397	RAB3A	Ras-related	RAB3A_HUI	1	1.147198
48H 50MT/	Q9P283	0.233437	0.001829	0.003398	SEMA5B	Semaphori	SEM5B_HU	1	1.175632
24H 50MT/	Q9UMS0	0.350683	0.001832	0.003403	NFU1	NFU1 iron-:	NFU1_HUM	6	1.275164
48H 50MT/	Q6YP21	0.092508	0.001834	0.003405	KYAT3	Kynurenine	KAT3_HUM	14	1.066222
24H 50MT/	Q9NUP9	-0.07571	0.001834	0.003405	LIN7C	Protein lin-	LIN7C_HUM	5	0.948876
48H 50MT/	Q9Y3B9	-0.24004	0.001834	0.003405	RRP15	RRP15-like	RRP15_HUI	9	0.846724
24H 50MT/	O94992	0.117221	0.001836	0.003406	HEXIM1	Protein HE)	HEXI1_HUM	8	1.084644
24H 50MT/	Q7Z7H5	0.053507	0.001836	0.003407	TMED4	Transmemb	TMED4_HU	8	1.037784
48H 50MT/	Q96ME1	0.044265	0.001837	0.003409	FBXL18	F-box/LRR-I	FXL18_HUI	20	1.031158
48H 50MT/	Q9H8Y8	0.207884	0.001841	0.003414	GORASP2	Golgi reass	GORS2_HU	15	1.154993
24H 50MT/	P63279	0.093625	0.001842	0.003415	UBE2I	SUMO-conj	UBC9_HUM	12	1.067048
24H 50MT/	Q93099	-0.13193	0.001843	0.003416	HGD	Homogenti	HGD_HUM/	17	0.912611
48H 50MT/	E9PAV3;Q1	-0.19112	0.001848	0.003423	NACA	Nascent po	NACAM_HU	5	0.875927
24H 50MT/	P46734	-0.1952	0.001851	0.003426	MAP2K3	Dual specifi	MP2K3_HU	13	0.873451
24H 50MT/	Q99536	-0.00292	0.001853	0.003429	VAT1	Synaptic ve	VAT1_HUM	16	0.997979
48H 50MT/	Q9Y4B6	-0.25829	0.001853	0.003429	DCAF1	DDB1- and	DCAF1_HUI	27	0.836078
48H 10MT/	Q9NWW5	0.279942	0.001853	0.00343	CLN6	Ceroid-lipo	CLN6_HUM	1	1.214146
24H 10MT/	P54619	0.046026	0.001854	0.00343	PRKAG1	5'-AMP-acti	AAKG1_HU	11	1.032417
48H 50MT/	Q9BY42	-0.21844	0.001855	0.003431	RTF2	Replicatior	RTF2_HUM	8	0.859497
48H 50MT/	Q9C0C2	0.303796	0.001856	0.003433	TNKS1BP1	182 kDa ta	TB182_HUI	35	1.234388
24H 10MT/	Q7Z4R8	2.144567	0.001858	0.003435	C6orf120	UPF0669 p	CF120_HUI	1	4.421596
24H 50MT/	P21397	0.221515	0.001859	0.003436	MAOA	Amine oxid	AOFA_HUM	10	1.165958
24H 50MT/	Q09161	0.074167	0.00186	0.003437	NCBP1	Nuclear ca	NCBP1_HU	28	1.052753
24H 10MT/	Q9NRA8	-0.19751	0.00186	0.003438	EIF4ENIF1	Eukaryotic	4ET_HUMA	15	0.872054
48H 50MT/	O75400	0.08919	0.001862	0.003441	PRPF40A	Pre-mRNA-	PR40A_HUI	18	1.063773

24H	50MT/	P18583	0.266395	0.001863	0.003441	SON	Protein SOI	SON_HUM/	39	1.202798
24H	50MT/	Q8IYB8	0.01589	0.001869	0.00345	SUPV3L1	ATP-depen	SUV3_HUN	25	1.011075
48H	10MT/	Q13813	0.024828	0.00187	0.003452	SPTAN1	Spectrin al	SPTN1_HUI	146	1.017359
48H	50MT/	Q53FA7	0.100505	0.001873	0.003455	TP53I3	Quinone ox	QORX_HUN	13	1.072149
48H	50MT/	P07307	0.192243	0.001875	0.003458	ASGR2	Asialoglyco	ASGR2_HUI	10	1.142538
48H	50MT/	Q5T6V5	0.070984	0.001875	0.003458	QNG1	Queuosine	QNG1_HUN	15	1.050433
24H	10MT/	P61088	-0.1827	0.001876	0.003459	UBE2N	Ubiquitin-c	UBE2N_HU	9	0.881051
48H	50MT/	Q16777;Q(	-2.94818	0.001879	0.003463	H2AC20;H2	Histone H2	H2A2C_HU	1	0.129571
24H	50MT/	Q9BQ95	0.106148	0.001881	0.003465	ECSIT	Evolutiona	ECSIT_HUN	11	1.076351
24H	10MT/	Q9Y3E7	0.099605	0.001881	0.003465	CHMP3	Charged mu	CHMP3_HU	5	1.07148
48H	10MT/	Q9UBB9	0.040894	0.001882	0.003466	TFIP11	Tuftelin-int	TFP11_HUN	27	1.028751
24H	10MT/	P62888	0.074303	0.001886	0.003471	RPL30	Large ribos	RL30_HUM	5	1.052853
48H	10MT/	Q53SF7	0.038239	0.001887	0.003472	COBLL1	Cordon-ble	COBL1_HUI	15	1.02686
48H	50MT/	Q9H8H3	0.21767	0.001887	0.003472	TMT1A	Thiol S-met	TMT1A_HU	7	1.162854
24H	10MT/	P62316	0.086556	0.001888	0.003473	SNRPD2	Small nucle	SMD2_HUN	8	1.061832
24H	50MT/	Q96JG8	0.75182	0.00189	0.003476	MAGED4	Melanoma-	MAGD4_HU	4	1.683915
48H	50MT/	O00743	0.127792	0.001891	0.003477	PPP6C	Serine/thre	PPP6_HUN	14	1.09262
48H	50MT/	Q92879	0.071699	0.001892	0.003478	CELF1	CUGBP Elav	CELF1_HUN	10	1.050954
48H	50MT/	P00403	-0.02891	0.001896	0.003483	MT-CO2	Cytochrom	COX2_HUN	7	0.980161
24H	50MT/	O60508	0.029104	0.001897	0.003484	CDC40	Pre-mRNA-|	PRP17_HUI	24	1.020378
48H	10MT/	Q96AG3	1.74773	0.001897	0.003484	SLC25A46	Mitochond	S2546_HUI	2	3.358297
48H	10MT/	Q8WU90	0.117637	0.001898	0.003486	ZC3H15	Zinc finger	ZC3HF_HUI	15	1.084956
24H	10MT/	Q5ZPR3	0.288493	0.0019	0.003487	CD276	CD276 anti	CD276_HU	7	1.221364
48H	10MT/	P12236	-0.25318	0.0019	0.003487	SLC25A6	ADP/ATP tr	ADT3_HUN	4	0.839044
24H	50MT/	Q9P1U1	0.359891	0.001901	0.003489	ACTR3B	Actin-relate	ARP3B_HUI	7	1.283329
24H	10MT/	Q4UJ75;Q5	0.461014	0.001902	0.003489	ANKRD20A	Putative an	A20A4_HUI	1	1.376509
24H	50MT/	Q8NI27	0.201867	0.001905	0.003494	THOC2	THO compl	THOC2_HU	41	1.150186
48H	50MT/	P00558	0.534253	0.001907	0.003495	PGK1	Phosphogly	PGK1_HUN	37	1.448192
24H	50MT/	Q9NZW5	0.069717	0.001908	0.003497	PALS2	Protein PAI	PALS2_HUI	24	1.049511
24H	50MT/	Q5TEC6	0.055038	0.00191	0.003499	H3-7	Histone H3	H37_HUM/	1	1.038886
24H	10MT/	Q14123	-3.14972	0.001916	0.003506	PDE1C	Dual specifi	PDE1C_HUI	1	0.112678
24H	10MT/	Q9P003	-0.36606	0.001916	0.003506	CNIH4	Protein cor	CNIH4_HUI	1	0.775899
48H	50MT/	Q99426	0.24556	0.001916	0.003507	TBCB	Tubulin-fol	TBCB_HUM	13	1.185553
48H	10MT/	P60510	0.141673	0.001925	0.003519	PPP4C	Serine/thre	PP4C_HUN	13	1.103183
24H	50MT/	Q99459	0.061405	0.001927	0.003523	CDC5L	Cell divisio	CDC5L_HUI	33	1.043482
48H	50MT/	Q9NXE8	-0.2844	0.001929	0.003524	CWC25	Pre-mRNA-:	CWC25_HU	7	0.821084
24H	50MT/	Q86UE4	0.089237	0.001931	0.003527	MTDH	Protein LYF	LYRIC_HUN	22	1.063808
48H	50MT/	P10253	0.349825	0.001934	0.00353	GAA	Lysosomal	LYAG_HUM	15	1.274406
48H	10MT/	P23284	0.040293	0.001934	0.00353	PPIB	Peptidyl-pr	PPIB_HUM/	13	1.028322
24H	10MT/	O00232	0.064476	0.001938	0.003534	PSMD12	26S protea:	PSD12_HUI	26	1.045705
24H	50MT/	Q7L8L6	0.059984	0.001938	0.003534	FASTKD5	FAST kinase	FAKD5_HUI	24	1.042454
24H	50MT/	Q9NZM1	-0.15936	0.001938	0.003534	MYOF	Myoferlin	MYOF_HUN	87	0.895422
24H	50MT/	Q02790	0.067404	0.00194	0.003536	FKBP4	Peptidyl-pr	FKBP4_HUI	34	1.047829
48H	10MT/	Q15012	0.198077	0.001941	0.003537	LAPTM4A	Lysosomal-	LAP4A_HUI	1	1.147168
48H	50MT/	Q9H4A3	0.280486	0.001943	0.003539	WNK1	Serine/thre	WNK1_HUI	36	1.214604
48H	50MT/	Q8TDZ2	0.146255	0.001943	0.003539	MICAL1	[F-actin]-m	MICA1_HUI	22	1.106693
48H	50MT/	Q9Y2R4	-0.29479	0.001944	0.00354	DDX52	Probable A	DDX52_HU	18	0.815193
48H	10MT/	P67870	0.03628	0.001945	0.003542	CSNK2B	Casein kina	CSK2B_HUI	10	1.025466
24H	10MT/	P04798	0.062029	0.001946	0.003543	CYP1A1	Cytochrom	CP1A1_HUI	10	1.043933
24H	50MT/	Q8IYS2	0.187354	0.001947	0.003544	KIAA2013	Uncharacte	K2013_HUI	13	1.138674
48H	10MT/	P30626	0.128856	0.001948	0.003545	SRI	Sorcin	SORCN_HU	8	1.093427
48H	50MT/	Q86WR0	0.066198	0.001948	0.003545	CCDC25	Coiled-coil	CCD25_HU	6	1.046954

48H 10MTA	O43592	0.096207	0.00195	0.003547	XPOT	Exportin-T	XPOT_HUM	29	1.068959
48H 10MTA	Q96NL8	0.466741	0.001952	0.003549	CFAP418	Cilia- and fl	CF418_HUI	1	1.381984
24H 50MTA	Q9BSD7	0.136679	0.001952	0.003549	NTPCR	Cancer-rela	NTPCR_HUI	10	1.099371
48H 50MTA	P11021	0.060114	0.001954	0.003552	HSPA5	Endoplasm	BIP_HUMA	35	1.042548
48H 50MTA	Q9HAW4	-0.82157	0.001956	0.003555	CLSPN	Claspin	CLSPN_HUI	6	0.565826
48H 50MTA	Q9UNE7	-0.17832	0.001956	0.003555	STUB1	E3 ubiquiti	CHIP_HUM	16	0.88373
48H 50MTA	Q8TED9	-0.26386	0.001957	0.003555	AFAP1L1	Actin filam	AF1L1_HUI	1	0.832859
48H 50MTA	P35222	0.277153	0.001957	0.003555	CTNNB1	Catenin bet	CTNB1_HUI	35	1.211801
48H 50MTA	Q16790	1.491521	0.001958	0.003556	CA9	Carbonic ar	CAH9_HUM	3	2.811853
48H 50MTA	O14562	0.233907	0.00196	0.003559	UBFD1	Ubiquitin d	UBFD1_HU	7	1.176015
24H 10MTA	Q06546	0.094528	0.001961	0.00356	GABPA	GA-binding	GABPA_HU	9	1.067716
48H 10MTA	Q969X6	-0.19479	0.001967	0.003569	UTP4	U3 small nu	UTP4_HUM	22	0.8737
48H 50MTA	Q5T1M5	0.065911	0.001969	0.003571	FKBP15	FK506-binc	FKB15_HUI	25	1.046746
24H 50MTA	P30086	0.222323	0.001971	0.003574	PEBP1	Phosphatid	PEBP1_HUI	13	1.16661
24H 50MTA	P49841	-0.08669	0.001974	0.003578	GSK3B	Glycogen sy	GSK3B_HUI	13	0.941679
24H 10MTA	Q14145	0.11377	0.001975	0.003579	KEAP1	Kelch-like E	KEAP1_HUI	20	1.082052
48H 50MTA	P22234	0.117241	0.001976	0.003579	PAICS	Bifunctiona	PUR6_HUM	23	1.084659
48H 10MTA	Q86UY8	-0.27959	0.001976	0.003579	NT5DC3	5'-nucleoti	NT5D3_HU	6	0.823822
48H 50MTA	P49643	-0.27712	0.001977	0.003581	PRIM2	DNA primas	PRI2_HUMA	20	0.825238
24H 50MTA	Q96GN5	1.660638	0.001979	0.003583	CDCA7L	Cell divisio	CDA7L_HUI	2	3.161564
24H 10MTA	P35221	0.058847	0.001981	0.003586	CTNNA1	Catenin alp	CTNA1_HUI	44	1.041633
48H 50MTA	P46013	-0.22699	0.001985	0.00359	MKI67	Proliferatic	KI67_HUMA	119	0.854414
24H 50MTA	P52306	-0.13686	0.001985	0.003591	RAP1GDS1	Rap1 GTPas	GDS1_HUM	14	0.909495
24H 10MTA	P36543	-0.13106	0.001986	0.003592	ATP6V1E1	V-type prot	VATE1_HUI	12	0.913159
24H 50MTA	P39656	0.172791	0.001991	0.003599	DDOST	Dolichyl-di	OST48_HUI	16	1.127237
24H 50MTA	P31040	0.20936	0.001995	0.003605	SDHA	Succinate d	SDHA_HUM	24	1.156175
24H 50MTA	Q9UPQ8	0.217688	0.002001	0.003613	DOLK	Dolichol ki	DOLK_HUM	4	1.162868
48H 50MTA	O43447	0.163484	0.002006	0.00362	PPIH	Peptidyl-pr	PPIH_HUM	8	1.119989
24H 50MTA	Q9BTU6	0.026531	0.002007	0.00362	PI4K2A	Phosphatid	P4K2A_HUI	22	1.01856
24H 50MTA	Q13951	-0.2217	0.002007	0.003621	CBFB	Core-bindir	PEBB_HUM	7	0.857557
24H 50MTA	Q07021	0.105185	0.002008	0.003622	C1QBP	Compleme	C1QBP_HU	9	1.075633
48H 50MTA	Q6XD76	-3.04838	0.00201	0.003624	ASCL4	Achaete-scu	ASCL4_HUI	1	0.120877
48H 10MTA	P11169	0.073634	0.002011	0.003625	SLC2A3	Solute carri	GTR3_HUM	7	1.052364
24H 50MTA	Q9NWL6	0.405782	0.002011	0.003626	ASNSD1	Asparagine	ASND1_HU	4	1.324807
48H 10MTA	Q9UHX1	0.084808	0.002017	0.003635	PUF60	Poly(U)-bin	PUF60_HUI	24	1.060547
24H 10MTA	Q13217	0.083084	0.002018	0.003635	DNAJC3	DnaJ homo	DNJC3_HUI	21	1.05928
24H 10MTA	P10643	2.18759	0.002018	0.003635	C7	Compleme	CO7_HUMA	2	4.55544
48H 50MTA	P54577	0.023048	0.002023	0.00364	YARS1	Tyrosine--tl	SYYC_HUMA	36	1.016104
48H 50MTA	P61289	-0.15534	0.002028	0.003646	PSME3	Proteasom	PSME3_HU	17	0.897921
48H 10MTA	Q06330	0.014308	0.002031	0.003649	RBPJ	Recombinii	SUH_HUMA	5	1.009967
48H 10MTA	P57740	0.003599	0.002032	0.003651	NUP107	Nuclear po	NU107_HU	41	1.002498
48H 50MTA	Q52LJ0	0.133961	0.002034	0.003653	FAM98B	Protein FAI	FA98B_HUI	12	1.097302
48H 50MTA	O00178	0.315117	0.002036	0.003655	GTPBP1	GTP-bindin	GTPB1_HUI	17	1.244112
24H 50MTA	O95881	0.301926	0.002038	0.003658	TXNDC12	Thioredoxi	TXD12_HUI	7	1.232789
48H 50MTA	Q8TB61	0.166356	0.002039	0.00366	SLC35B2	Adenosine :	S35B2_HUI	11	1.122221
24H 50MTA	Q4KWH8	0.462384	0.002042	0.003662	PLCH1	1-phosphat	PLCH1_HUI	3	1.377817
24H 50MTA	Q16537	-0.13475	0.002042	0.003663	PPP2R5E	Serine/thre	2A5E_HUM	14	0.91083
24H 50MTA	Q16181	0.04399	0.002048	0.00367	SEPTIN7	Septin-7	SEPT7_HUI	19	1.030962
48H 10MTA	P60059	0.512613	0.002048	0.00367	SEC61G	Protein trai	SC61G_HUI	2	1.426631
24H 10MTA	P00403	-0.10354	0.002052	0.003674	MT-CO2	Cytochrom	COX2_HUM	7	0.930745
48H 50MTA	Q14839	0.060478	0.002053	0.003676	CHD4	Chromodoi	CHD4_HUM	68	1.042811
48H 50MTA	P62195	0.014361	0.002054	0.003676	PSMC5	26S protea:	PRS8_HUM	28	1.010004

48H 10MT/	Q92820	-0.11676	0.002068	0.003696	GGH	Gamma-glu	GGH_HUM/	11	0.922255
24H 10MT/	P05455	0.0905	0.002069	0.003699	SSB	Lupus La pr	LA_HUMAN	29	1.064739
24H 50MT/	P53621	0.013458	0.002075	0.003706	COPA	Coatomer s	COPA_HUM	68	1.009372
48H 50MT/	P18085	0.102772	0.00208	0.003713	ARF4	ADP-ribosy	ARF4_HUM	7	1.073835
24H 10MT/	Q96PU5	0.144822	0.002084	0.003718	NEDD4L	E3 ubiquiti	NED4L_HUI	27	1.105594
48H 50MT/	Q92797	0.099057	0.002085	0.00372	SYMPK	Symplekin	SYMPK_HU	40	1.071073
24H 50MT/	O43157	0.211237	0.002086	0.003722	PLXNB1	Plexin-B1	PLXB1_HUI	21	1.157681
24H 50MT/	Q9Y6A1	0.201621	0.002087	0.003723	POMT1	Protein O-n	POMT1_HU	4	1.14999
24H 10MT/	Q07021	0.100996	0.002088	0.003724	C1QBP	Complemer	C1QBP_HU	9	1.072514
24H 10MT/	Q96KC8	0.053216	0.002092	0.00373	DNAJC1	DnaJ homo	DNJC1_HUI	10	1.037575
24H 50MT/	Q16718	0.140904	0.002095	0.003733	NDUFA5	NADH dehy	NDUA5_HU	6	1.102596
24H 10MT/	Q9Y3C8	-0.3024	0.002104	0.003746	UFC1	Ubiquitin-f	UFC1_HUM	7	0.810905
48H 50MT/	Q8N9N7	-0.10725	0.002105	0.003747	LRRC57	Leucine-ric	LRC57_HUI	7	0.928354
48H 50MT/	Q13895	-0.17855	0.002105	0.003747	BYSL	Bystin	BYST_HUM/	20	0.883592
48H 50MT/	Q5VSL9	-0.00613	0.00211	0.003754	STRIP1	Striatin-int	STRP1_HUI	14	0.995758
24H 10MT/	Q93063	0.072482	0.002115	0.003759	EXT2	Exostosin-2	EXT2_HUM	10	1.051524
48H 50MT/	Q8TDN6	-0.25623	0.002117	0.003761	BRIX1	Ribosome b	BRX1_HUM	20	0.837275
48H 50MT/	Q08AD1	-0.79291	0.00212	0.003765	CAMSAP2	Calmodulir	CAMP2_HU	9	0.57718
48H 50MT/	Q9H9C1	0.206272	0.002121	0.003765	VIPAS39	Spermatog	SPE39_HUI	14	1.153703
48H 50MT/	Q6ZT62	0.370169	0.002122	0.003767	BARGIN	Bargin	BGIN_HUM	3	1.292504
48H 50MT/	Q07817	-0.2281	0.002127	0.003773	BCL2L1	Bcl-2-like p	B2CL1_HUI	5	0.853756
24H 50MT/	P27816	-0.11141	0.002132	0.00378	MAP4	Microtubul	MAP4_HUM	41	0.925681
48H 50MT/	Q86Y37	-0.17604	0.002133	0.00378	CACUL1	CDK2-assoc	CACL1_HUI	4	0.885129
24H 50MT/	O95202	0.071655	0.002135	0.003782	LETM1	Mitochond	LETM1_HUI	34	1.050922
48H 10MT/	Q9Y508	0.195196	0.002135	0.003782	RNF114	E3 ubiquiti	RN114_HU	6	1.14488
48H 10MT/	Q14683	-0.00838	0.002135	0.003783	SMC1A	Structural r	SMC1A_HU	54	0.994211
48H 50MT/	P14543	0.137648	0.002137	0.003785	NID1	Nidogen-1	NID1_HUM	17	1.10011
24H 50MT/	P53801	-0.26099	0.002139	0.003787	PTTG1IP	Pituitary tu	PTTG_HUM	1	0.834516
48H 50MT/	Q9NWV8	0.287634	0.002141	0.003789	BABAM1	BRISC and E	BABA1_HUI	6	1.220637
48H 50MT/	Q14139	0.079509	0.002141	0.003789	UBE4A	Ubiquitin c	UBE4A_HUI	30	1.056658
48H 50MT/	Q14141	0.219988	0.002141	0.003789	SEPTIN6	Septin-6	SEPT6_HUM	8	1.164724
24H 50MT/	Q9Y2W2	0.111582	0.002142	0.003789	WBP11	WW domai	WBP11_HU	21	1.080412
24H 50MT/	Q14689	-0.36834	0.002142	0.00379	DIP2A	Disco-inter	DIP2A_HUM	9	0.774675
48H 50MT/	P62826	-0.1878	0.002143	0.003791	RAN	GTP-bindin	RAN_HUM/	11	0.877943
24H 50MT/	P53597	0.327509	0.002144	0.003791	SUCLG1	Succinate--	SUCA_HUM	9	1.254845
24H 50MT/	P42224	-0.09771	0.002145	0.003793	STAT1	Signal trans	STAT1_HUM	37	0.934516
48H 50MT/	Q86WA8	0.215023	0.002148	0.003796	LONP2	Lon protea:	LONP2_HU	28	1.160722
24H 50MT/	Q9H0E2	-0.20106	0.002153	0.003804	TOLLIP	Toll-interac	TOLIP_HUM	7	0.869911
24H 50MT/	Q9UNL2	-0.11605	0.002153	0.003804	SSR3	Translocon	SSRG_HUM	2	0.92271
48H 50MT/	Q7Z3U7	0.075923	0.002155	0.003806	MON2	Protein MC	MON2_HUI	37	1.054035
24H 50MT/	P35268	0.122314	0.00216	0.003813	RPL22	Large ribos	RL22_HUM	4	1.088479
24H 10MT/	Q96F25	0.159678	0.002168	0.003824	ALG14	UDP-N-acet	ALG14_HUI	3	1.117038
48H 50MT/	Q13426	0.06258	0.002169	0.003824	XRCC4	DNA repair	XRCC4_HUI	9	1.044331
48H 10MT/	Q7Z3T8	0.066412	0.002179	0.003838	ZFYVE16	Zinc finger	ZFY16_HUM	28	1.04711
48H 10MT/	Q9Y6W3	-0.00934	0.002179	0.003838	CAPN7	Calpain-7	CAN7_HUM	18	0.993546
24H 50MT/	P38919	0.11494	0.002182	0.003841	EIF4A3	Eukaryotic	IF4A3_HUM	24	1.08293
48H 50MT/	Q03252	-0.1152	0.002185	0.003845	LMNB2	Lamin-B2	LMNB2_HU	27	0.923252
48H 50MT/	Q14114	-0.49022	0.002188	0.00385	LRP8	Low-densit	LRP8_HUM	5	0.711918
48H 50MT/	P45984	0.211341	0.002189	0.00385	MAPK9	Mitogen-ac	MK09_HUM	9	1.157764
48H 50MT/	Q5H9R7	0.029792	0.002189	0.003851	PPP6R3	Serine/thre	PP6R3_HUI	35	1.020865
24H 50MT/	Q9H0S4	0.069191	0.002192	0.003855	DDX47	Probable A'	DDX47_HU	18	1.049128
48H 50MT/	Q6NT16	0.42959	0.002193	0.003855	SLC18B1	MFS-type tı	S18B1_HUI	2	1.346851

24H 10MTA	Q08209	0.042144	0.002196	0.003859	PPP3CA	Protein pho	PP2BA_HUI	6	1.029643
24H 50MTA	Q14344	0.028061	0.002196	0.003859	GNA13	Guanine nu	GNA13_HU	14	1.019641
24H 50MTA	Q9Y4P3	0.125735	0.002196	0.003859	TBL2	Transducin	TBL2_HUM	21	1.091063
24H 50MTA	Q96PC5	0.118825	0.002199	0.003863	MIA2	Melanoma	MIA2_HUM	27	1.08585
48H 50MTA	P16152	0.120301	0.002204	0.003868	CBR1	Carbonyl re	CBR1_HUM	17	1.086961
48H 10MTA	Q9NQ88	-0.00902	0.002204	0.003868	TIGAR	Fructose-2,	TIGAR_HUM	9	0.993767
48H 50MTA	Q07065	0.065543	0.002205	0.003868	CKAP4	Cytoskeleto	CKAP4_HUI	29	1.046479
48H 50MTA	Q9Y2V7	0.068171	0.002206	0.003869	COG6	Conserved o	COG6_HUM	20	1.048387
48H 50MTA	P28347	-0.34303	0.002206	0.003869	TEAD1	Transcripti	TEAD1_HUI	4	0.788384
24H 10MTA	Q9HAC7	-1.46682	0.002207	0.003871	SUGCT	Succinate--	SUCHY_HUI	1	0.361779
48H 50MTA	P20742	-0.95887	0.002209	0.003874	PZP	Pregnancy z	PZP_HUMA	3	0.514458
24H 10MTA	O00264	-0.16673	0.00221	0.003875	PGRMC1	Membrane-	PGRC1_HU	9	0.890859
24H 10MTA	Q16864	-0.5729	0.00221	0.003875	ATP6V1F	V-type prot	VATF_HUM	3	0.672263
24H 10MTA	Q15418	0.085088	0.002214	0.003879	RPS6KA1	Ribosomal	KS6A1_HUI	15	1.060752
48H 50MTA	Q6NUQ1	0.085823	0.002216	0.003881	RINT1	RAD50-inte	RINT1_HUM	9	1.061293
48H 10MTA	Q14562	0.008683	0.002217	0.003882	DHX8	ATP-depend	DHX8_HUM	35	1.006037
24H 10MTA	P46100	0.102183	0.002223	0.00389	ATRX	Transcripti	ATRX_HUM	44	1.073397
48H 50MTA	Q16762	0.3589	0.002223	0.00389	TST	Thiosulfate	THTR_HUM	15	1.282448
48H 50MTA	Q9HBL7	0.617016	0.002224	0.003891	PLGRKT	Plasminoge	PLRKT_HUM	5	1.5337
24H 50MTA	P31629	0.329917	0.002228	0.003896	HIVEP2	Transcripti	ZEP2_HUM	4	1.256941
48H 50MTA	P11234	0.13152	0.002229	0.003897	RALB	Ras-related	RALB_HUM	10	1.095447
24H 50MTA	Q9UK99	0.197638	0.00223	0.003899	FBXO3	F-box only p	FBX3_HUM	10	1.14682
48H 50MTA	Q9Y4R8	-0.26941	0.002232	0.003902	TELO2	Telomere le	TELO2_HUM	24	0.829657
24H 50MTA	Q99856	0.15112	0.002237	0.003907	ARID3A	AT-rich inte	ARI3A_HUM	17	1.110431
48H 50MTA	Q2VPB7	-0.23705	0.002238	0.003909	AP5B1	AP-5 compl	AP5B1_HUI	11	0.848479
48H 50MTA	Q9NVM6	-0.02582	0.00224	0.003912	DNAJC17	DnaJ homo	DJC17_HUI	5	0.982259
24H 10MTA	P25205	0.022993	0.002245	0.003917	MCM3	DNA replica	MCM3_HUI	37	1.016065
24H 10MTA	P16152	-0.12215	0.00225	0.003923	CBR1	Carbonyl re	CBR1_HUM	17	0.91882
24H 10MTA	Q8IYB1	0.205706	0.002251	0.003925	MB21D2	Nucleotidy	M21D2_HU	10	1.153251
48H 50MTA	Q9UIA9	0.044524	0.002253	0.003927	XPO7	Exportin-7	XPO7_HUM	37	1.031343
24H 50MTA	P09382	-0.15658	0.002254	0.003929	LGALS1	Galectin-1	LEG1_HUM	9	0.897148
48H 50MTA	Q9UNQ2	-0.2953	0.002254	0.003929	DIMT1	Dimethylad	DIM1_HUM	13	0.814905
24H 50MTA	O14786	1.456636	0.002257	0.003933	NRP1	Neuropilin-	NRP1_HUM	11	2.744677
48H 10MTA	Q9Y266	0.1035	0.00226	0.003937	NUDC	Nuclear mi	NUDC_HUM	19	1.074377
48H 50MTA	P10619	-0.22785	0.002262	0.003939	CTSA	Lysosomal p	PPGB_HUM	13	0.853908
48H 10MTA	Q04695	-0.35304	0.002263	0.003939	KRT17	Keratin, typ	K1C17_HUI	15	0.782935
48H 10MTA	P52306	0.005987	0.002263	0.003939	RAP1GDS1	Rap1 GTPas	GDS1_HUM	14	1.004158
24H 50MTA	P08621	0.158679	0.002264	0.00394	SNRNP70	U1 small nu	RU17_HUM	14	1.116265
24H 10MTA	Q9HAV0	0.328422	0.00227	0.003945	GNB4	Guanine nu	GBB4_HUM	6	1.255639
48H 10MTA	Q96RU2	-0.10263	0.002271	0.003946	USP28	Ubiquitin c	UBP28_HU	20	0.931336
48H 50MTA	P35240	0.068386	0.002276	0.003952	NF2	Merlin	MERL_HUM	10	1.048543
48H 50MTA	Q9NPD3	-0.24492	0.002277	0.003953	EXOSC4	Exosome co	EXOS4_HUI	8	0.843861
24H 50MTA	Q86U90	-0.32875	0.002278	0.003954	YRDC	Threonylca	YRDC_HUM	12	0.796224
24H 10MTA	P08237	-1.07222	0.002279	0.003956	PFKM	ATP-depend	PFKAM_HU	1	0.475587
48H 10MTA	O60828	0.018195	0.00228	0.003956	PQBP1	Polyglutam	PQBP1_HU	11	1.012692
48H 50MTA	P48147	0.143036	0.002288	0.003967	PREP	Prolyl endo	PPCE_HUM	35	1.104226
24H 50MTA	Q96N64	1.156257	0.002291	0.003971	PWWP2A	PWWP dom	PWP2A_HU	2	2.228784
24H 50MTA	O95793	-0.12891	0.002294	0.003973	STAU1	Double-stra	STAU1_HUI	28	0.914525
48H 50MTA	P28332	0.236379	0.002295	0.003974	ADH6	Alcohol deh	ADH6_HUM	11	1.178033
48H 50MTA	O43491	0.138254	0.002296	0.003976	EPB41L2	Band 4.1-lik	E41L2_HUM	49	1.100572
24H 50MTA	P07910	0.165858	0.002296	0.003976	HNRNPC	Heterogene	HNRPC_HU	15	1.121833
24H 50MTA	Q15022	0.085672	0.002298	0.003978	SUZ12	Polycomb p	SUZ12_HUI	9	1.061182

48H 50MTA	Q9Y248	-0.06324	0.002299	0.003978	GINS2	DNA replica	PSF2_HUM	3	0.957113
24H 50MTA	Q8WVM8	0.032181	0.002303	0.003983	SCFD1	Sec1 family	SCFD1_HUI	25	1.022557
48H 10MTA	Q9NV70	1.99E-05	0.002304	0.003984	EXOC1	Exocyst cor	EXOC1_HUI	19	1.000014
48H 50MTA	Q8IX12	0.063868	0.002306	0.003986	CCAR1	Cell divisio	CCAR1_HUI	30	1.045264
48H 10MTA	Q9BQQ3	3.050551	0.002308	0.003988	GORASP1	Golgi reasse	GORS1_HU	1	8.285283
24H 50MTA	Q2NL82	0.051238	0.002309	0.003988	TSR1	Pre-rRNA-p	TSR1_HUM	23	1.036153
48H 50MTA	Q14980	-0.12227	0.002309	0.003989	NUMA1	Nuclear mi	NUMA1_HU	76	0.91874
48H 10MTA	P62195	0.011304	0.002312	0.003992	PSMC5	26S protea	PRS8_HUM	28	1.007866
48H 10MTA	P17858	0.057286	0.002314	0.003994	PFKL	ATP-depen	PFKAL_HUI	30	1.040507
24H 50MTA	Q9BXP5	0.06316	0.002314	0.003994	SRRT	Serrate RNA	SRRT_HUM	34	1.044752
24H 50MTA	Q6P2I3	0.083799	0.002314	0.003995	FAHD2B	Oxaloaceta	FAH2B_HUI	1	1.059805
24H 50MTA	P30153	-0.13992	0.002323	0.004006	PPP2R1A	Serine/thre	2AAA_HUM	28	0.907568
24H 10MTA	O95104	0.103651	0.002324	0.004008	SCAF4	SR-related a	SCAF4_HUI	14	1.074489
24H 50MTA	Q5EBL4	0.261899	0.002325	0.00401	RILPL1	RILP-like pr	RIPL1_HUN	4	1.199056
48H 50MTA	P02792	-1.22056	0.002327	0.004011	FTL	Ferritin ligh	FRIL_HUMA	3	0.429116
24H 50MTA	P26651	1.73074	0.002328	0.004012	ZFP36	mRNA deca	TTP_HUMA	2	3.318979
48H 10MTA	E9PRG8	0.147593	0.002329	0.004014	C11orf98	Uncharacte	CK098_HUI	4	1.10772
48H 50MTA	O75127	-0.35878	0.00233	0.004014	PTCD1	Pentatrico	PTCD1_HUI	12	0.779824
24H 10MTA	Q6ZN17	0.081523	0.00233	0.004014	LIN28B	Protein lin-	LN28B_HUI	9	1.058135
24H 10MTA	Q14344	0.067688	0.002335	0.004021	GNA13	Guanine nu	GNA13_HU	14	1.048035
24H 50MTA	Q9Y2S2	-0.00081	0.002335	0.004021	CRYL1	Lambda-cry	CRYL1_HUI	8	0.999441
24H 50MTA	Q9P0B6	0.792765	0.002335	0.004021	CCDC167	Coiled-coil	CC167_HUI	1	1.732392
48H 10MTA	P46939	0.035693	0.002338	0.004024	UTRN	Utrophin	UTRN_HUM	137	1.025049
24H 50MTA	Q96IW7	0.273244	0.00234	0.004027	SEC22A	Vesicle-traf	SC22A_HUI	4	1.208523
24H 50MTA	Q14008	-0.09999	0.00234	0.004027	CKAP5	Cytoskelet	CKAP5_HUI	75	0.933039
24H 50MTA	P04899	0.104353	0.002341	0.004028	GNAI2	Guanine nu	GNAI2_HUI	13	1.075012
48H 50MTA	Q659C4	-0.42173	0.002343	0.004031	LARP1B	La-related p	LAR1B_HUI	5	0.746531
24H 50MTA	Q9H467	-0.1216	0.002345	0.004033	CUEDC2	CUE domai	CUED2_HU	4	0.919169
48H 50MTA	Q9BYV8	0.275805	0.002351	0.00404	CEP41	Centrosom	CEP41_HUI	8	1.21067
48H 50MTA	Q13740	-0.00748	0.002357	0.004048	ALCAM	CD166 anti	CD166_HU	17	0.994829
24H 50MTA	P32929	-0.15761	0.002365	0.004058	CTH	Cystathioni	CGL_HUMA	12	0.896509
24H 50MTA	Q9NZJ7	0.07741	0.002365	0.004058	MTCH1	Mitochond	MTCH1_HU	11	1.055122
48H 50MTA	Q9HD33	-0.29483	0.002366	0.004059	MRPL47	Large ribos	RM47_HUN	8	0.815169
24H 50MTA	Q495W5	-0.20729	0.002367	0.004059	FUT11	Alpha-(1,3)	FUT11_HUI	5	0.866165
48H 50MTA	Q9UDY2	-0.07285	0.002368	0.00406	TJP2	Tight juncti	ZO2_HUMA	35	0.950757
24H 10MTA	Q13151	0.141296	0.00237	0.004063	HNRNPA0	Heterogene	ROA0_HUM	8	1.102895
48H 50MTA	Q00534	-0.27207	0.00237	0.004063	CDK6	Cyclin-depe	CDK6_HUM	12	0.828128
48H 10MTA	Q9BRD0	0.035755	0.002372	0.004064	BUD13	BUD13 hon	BUD13_HU	9	1.025093
48H 50MTA	O75449	0.213043	0.002373	0.004066	KATNA1	Katanin p6	KTNA1_HUI	9	1.159131
24H 50MTA	Q96GS4	0.340696	0.002377	0.004071	BORCS6	BLOC-1-rela	BORC6_HU	11	1.266367
48H 10MTA	P63313	0.15644	0.002377	0.004071	TMSB10	Thymosin b	TYB10_HUN	1	1.114533
48H 10MTA	O00231	-0.00518	0.002379	0.004072	PSMD11	26S protea	PSD11_HUI	29	0.996416
24H 50MTA	P53634	0.156556	0.00238	0.004074	CTSC	Dipeptidyl	CATC_HUM	11	1.114623
48H 50MTA	Q9H892	0.7139	0.002381	0.004075	TTC12	Tetratricop	TTC12_HUN	1	1.640233
48H 10MTA	Q9Y2W2	0.086582	0.002384	0.00408	WBP11	WW domai	WBP11_HU	21	1.061852
48H 50MTA	Q14353	0.384168	0.002384	0.00408	GAMT	Guanidinoa	GAMT_HUN	10	1.305107
48H 50MTA	Q9NVV0	-0.45536	0.002386	0.004082	TMEM38B	Trimeric int	TM38B_HU	3	0.72933
48H 50MTA	Q92621	-0.09071	0.002386	0.004082	NUP205	Nuclear po	NU205_HU	72	0.939059
48H 50MTA	Q15293	0.19937	0.0024	0.004099	RCN1	Reticulocal	RCN1_HUM	15	1.148197
48H 10MTA	O60502	0.057528	0.002404	0.004104	OGA	Protein O-G	OGA_HUMA	30	1.040681
24H 10MTA	Q9NRL2	0.081351	0.002408	0.004109	BAZ1A	Bromodom	BAZ1A_HUI	24	1.058008
48H 50MTA	P53992	0.07663	0.002409	0.004111	SEC24C	Protein tra	SC24C_HUI	35	1.054552

24H 50MT/	O14773	0.271781	0.002411	0.004113	TPP1	Tripeptidyl	TPP1_HUM	11	1.207297
24H 10MT/	Q9Y287	0.296361	0.002413	0.004116	ITM2B	Integral me	ITM2B_HUI	6	1.228043
48H 10MT/	Q92685	-0.2849	0.002414	0.004116	ALG3	Dol-P-Man:	ALG3_HUM	3	0.820801
24H 10MT/	Q969H8	0.13486	0.002414	0.004116	MYDGF	Myeloid-de	MYDGF_HU	7	1.097986
24H 10MT/	Q9NYZ2	0.796112	0.002414	0.004117	SLC25A37	Mitoferrin-	MFRN1_HU	1	1.736415
24H 50MT/	Q93034	0.084684	0.002415	0.004117	CUL5	Cullin-5	CUL5_HUM	35	1.060456
24H 50MT/	Q9NX40	-0.12102	0.002417	0.00412	OCIAD1	OCIA doma	OCAD1_HU	9	0.91954
24H 50MT/	O43861	0.172004	0.002419	0.004121	ATP9B	Probable pl	ATP9B_HUI	4	1.126622
24H 50MT/	P09493	0.362516	0.00242	0.004122	TPM1	Tropomyos	TPM1_HUM	6	1.285666
48H 50MT/	Q9BW19	-0.27301	0.00242	0.004122	KIFC1	Kinesin-like	KIFC1_HUM	21	0.827589
24H 10MT/	Q6NUS6	0.168869	0.002421	0.004123	TCTN3	Tectonic-3	TECT3_HUM	6	1.124177
24H 50MT/	Q96J02	-0.11563	0.002421	0.004123	ITCH	E3 ubiquiti	ITCH_HUM/	28	0.922979
48H 50MT/	O15160	-0.20072	0.002426	0.004129	POLR1C	DNA-direct	RPAC1_HUI	17	0.870114
24H 50MT/	P06213	0.027734	0.002432	0.004137	INSR	Insulin rece	INSR_HUM/	20	1.01941
24H 50MT/	Q9HD15	-0.19302	0.002439	0.004145	SRA1	Steroid rec	SRA1_HUM	9	0.874774
24H 10MT/	Q5R3I4	-0.15663	0.002441	0.004147	TTC38	Tetratricop	TTC38_HUI	16	0.89712
48H 10MT/	O00161	0.139179	0.002444	0.004151	SNAP23	Synaptosor	SNP23_HUI	10	1.101278
24H 50MT/	Q16850	0.141447	0.002444	0.004151	CYP51A1	Lanosterol	CP51A_HUI	23	1.103011
24H 50MT/	P62495	-0.10033	0.002448	0.004156	ETF1	Eukaryotic	ERF1_HUM	20	0.932821
48H 10MT/	Q6ZNC8	0.613741	0.002451	0.00416	MBOAT1	Lysophospl	MBOA1_HU	1	1.530222
48H 10MT/	P49023	0.036515	0.002452	0.004161	PXN	Paxillin	PAXI_HUM/	18	1.025633
24H 10MT/	Q15051	1.176	0.002456	0.004165	IQCB1	IQ calmodu	IQCB1_HUI	2	2.259495
24H 10MT/	Q9NX20	0.097138	0.002459	0.004169	MRPL16	Large ribos	RM16_HUM	9	1.069649
24H 50MT/	O60518	0.041167	0.002462	0.004173	RANBP6	Ran-bindin	RNBP6_HU	9	1.028946
48H 50MT/	O15347	0.208908	0.002464	0.004175	HMGB3	High mobil	HMGB3_HU	7	1.155813
48H 50MT/	Q8WUM0	-0.11518	0.002465	0.004177	NUP133	Nuclear poi	NU133_HU	46	0.923266
48H 50MT/	P40261	0.24612	0.002468	0.004181	NNMT	Nicotinami	NNMT_HUI	11	1.186013
48H 50MT/	P30740	0.115576	0.002471	0.004183	SERPINB1	Leukocyte	ILEU_HUM/	19	1.083408
24H 50MT/	P34741	1.597662	0.002471	0.004183	SDC2	Syndecan-2	SDC2_HUM	2	3.026523
24H 50MT/	Q9Y6X3	0.120423	0.002476	0.004189	MAU2	MAU2 chro	SCC4_HUM	10	1.087054
24H 50MT/	P04844	0.12758	0.00248	0.004194	RPN2	Dolichyl-di	RPN2_HUM	29	1.092459
24H 10MT/	Q8IXQ5	-0.02405	0.00248	0.004194	KLHL7	Kelch-like p	KLHL7_HUI	11	0.983466
48H 10MT/	Q9H8Y8	0.220284	0.002481	0.004194	GORASP2	Golgi reass	GORS2_HU	15	1.164963
24H 50MT/	P30837	0.060615	0.002484	0.004196	ALDH1B1	Aldehyde d	AL1B1_HUI	16	1.04291
48H 50MT/	Q96Q89	-0.4588	0.002484	0.004197	KIF20B	Kinesin-like	KI20B_HUM	22	0.727591
24H 50MT/	P60953	-0.05633	0.002486	0.004198	CDC42	Cell divisio	CDC42_HU	8	0.96171
24H 50MT/	Q6ZRP7	0.24451	0.002487	0.004199	QSOX2	Sulfhydryl	QSOX2_HU	20	1.184691
48H 10MT/	P05546	0.791751	0.002488	0.0042	SERPIND1	Heparin co	HEP2_HUM	2	1.731174
48H 10MT/	Q8NBJ5	0.066502	0.002488	0.0042	COLGALT1	Procollager	GT251_HUI	26	1.047174
48H 50MT/	Q15262	0.212541	0.002493	0.004207	PTPRK	Receptor-ty	PTPRK_HUI	15	1.158727
24H 50MT/	O96008	0.063674	0.002494	0.004207	TOMM40	Mitochond	TOM40_HU	11	1.045124
48H 10MT/	O75976	0.042531	0.002496	0.00421	CPD	Carboxyper	CBPD_HUM	44	1.029919
24H 10MT/	Q9H3R0	-1.64197	0.002496	0.00421	KDM4C	Lysine-spec	KDM4C_HU	2	0.320418
48H 50MT/	Q9NZM5	-0.39977	0.002502	0.004216	NOP53	Ribosome b	NOP53_HU	8	0.757981
24H 50MT/	P51159	0.312162	0.002502	0.004217	RAB27A	Ras-related	RB27A_HUI	5	1.241567
48H 10MT/	P04217	0.432052	0.002503	0.004217	A1BG	Alpha-1B-gl	A1BG_HUM	2	1.349151
48H 10MT/	O75915	0.168209	0.002507	0.004223	ARL6IP5	PRA1 family	PRAF3_HUI	6	1.123663
24H 50MT/	Q9Y6N5	0.082129	0.002513	0.00423	SQOR	Sulfide:quir	SQOR_HUM	30	1.058579
48H 50MT/	Q96NC0	0.255551	0.00252	0.00424	ZMAT2	Zinc finger	ZMAT2_HU	4	1.193792
24H 50MT/	Q8NDT2	0.175054	0.002521	0.004241	RBM15B	Putative RN	RB15B_HUI	8	1.129007
24H 50MT/	A0A024RB(	-0.21675	0.002523	0.004244	NUDT4B	Diphospho	NUD4B_HU	1	0.860499
48H 50MT/	Q9UH99	0.052361	0.002524	0.004245	SUN2	SUN domai	SUN2_HUM	19	1.03696

24H 50MT/	P62847	0.061738	0.002525	0.004246	RPS24	Small ribos	RS24_HUM	5	1.043722
48H 50MT/	Q9H7B2	-0.16395	0.002528	0.004249	RPF2	Ribosome p	RPF2_HUM	17	0.892576
48H 10MT/	P22087	-0.16616	0.002535	0.004259	FBL	rRNA 2'-O-n	FBRL_HUM	15	0.891213
48H 50MT/	Q9Y673	0.020877	0.002538	0.004262	ALG5	Dolichyl-ph	ALG5_HUM	12	1.014576
48H 50MT/	Q7Z5L2	0.125844	0.002539	0.004264	R3HCC1L	Coiled-coil	R3HCL_HUI	2	1.091146
48H 10MT/	Q92618	0.012354	0.002541	0.004267	ZNF516	Zinc finger p	ZN516_HUI	8	1.0086
24H 50MT/	P49419	0.07524	0.002542	0.004267	ALDH7A1	Alpha-amin	AL7A1_HUI	24	1.053536
24H 50MT/	Q86VD7	-0.23247	0.002547	0.004275	SLC25A42	Mitochond	S2542_HUI	4	0.851175
48H 50MT/	Q9Y2Q5	-0.03664	0.002552	0.004282	LAMTOR2	Ragulator c	LTOR2_HUI	6	0.974922
24H 50MT/	Q92945	0.056764	0.002555	0.004284	KHSRP	Far upstrea	FUBP2_HUI	28	1.04013
48H 10MT/	Q96CS2	0.278473	0.002557	0.004287	HAUS1	HAUS augm	HAUS1_HU	8	1.212911
24H 50MT/	P14927	0.083807	0.002557	0.004287	UQCRB	Cytochrom	QCR7_HUM	8	1.059811
48H 50MT/	Q13084	-0.19592	0.002558	0.004287	MRPL28	Large ribos	RM28_HUM	14	0.873013
48H 10MT/	Q99733	0.039206	0.00256	0.004291	NAP1L4	Nucleosom	NP1L4_HUI	14	1.027548
48H 10MT/	O94992	0.096138	0.002563	0.004294	HEXIM1	Protein HE>	HEXI1_HUM	8	1.068908
24H 50MT/	Q9Y265	0.032476	0.002569	0.004302	RUVBL1	RuvB-like 1	RUVB1_HU	25	1.022766
24H 50MT/	Q9NPL8	0.188251	0.00257	0.004303	TIMMDC1	Complex I a	TIDC1_HUM	10	1.139382
24H 10MT/	P08195	0.148445	0.002571	0.004304	SLC3A2	Amino acid	4F2_HUMA	32	1.108374
24H 50MT/	Q14108	0.210927	0.002571	0.004304	SCARB2	Lysosome n	SCRB2_HUI	11	1.157432
48H 50MT/	Q2TAK8	-2.75512	0.002571	0.004304	PWWP3A	PWWP dom	PWP3A_HU	2	0.148124
24H 50MT/	P37802	-0.27555	0.002572	0.004304	TAGLN2	Transgelin-:	TAGL2_HUI	15	0.826134
48H 10MT/	Q86XP3	0.016242	0.002572	0.004305	DDX42	ATP-depend	DDX42_HU	31	1.011321
24H 50MT/	Q8NB15	0.190403	0.002573	0.004305	ZNF511	Zinc finger p	ZN511_HUI	5	1.141082
48H 50MT/	P02533	0.354653	0.002574	0.004306	KRT14	Keratin, typ	K1C14_HUI	33	1.278678
48H 50MT/	Q96EP5	0.151282	0.002574	0.004306	DAZAP1	DAZ-associa	DAZP1_HUI	12	1.110556
24H 50MT/	Q96KP1	-0.06144	0.002577	0.004309	EXOC2	Exocyst cor	EXOC2_HUI	19	0.958307
48H 50MT/	P33908	0.20198	0.002577	0.004309	MAN1A1	Mannosyl-c	MA1A1_HU	16	1.150276
24H 10MT/	O95861	-0.09574	0.002579	0.004311	BPNT1	3'(2'),5'-bisp	BPNT1_HUI	19	0.935795
48H 10MT/	Q96BW9	0.092119	0.002579	0.004311	TAMM41	Phosphatic	TAM41_HU	6	1.065935
24H 10MT/	P21399	-0.126	0.00258	0.004312	ACO1	Cytoplasmi	ACOHC_HU	41	0.91637
48H 10MT/	Q86YN1	-0.27778	0.002581	0.004314	DOLPP1	Dolichyldip	DOPP1_HU	3	0.824857
48H 10MT/	P15924	0.020523	0.002583	0.004316	DSP	Desmoplak	DESP_HUM	135	1.014327
24H 50MT/	P06737	0.088917	0.002584	0.004317	PYGL	Glycogen p	PYGL_HUM	37	1.063571
24H 50MT/	Q99797	0.128828	0.002586	0.004318	MIPEP	Mitochond	MIPEP_HUI	20	1.093405
24H 10MT/	Q9H2A7	0.554009	0.002585	0.004318	CXCL16	C-X-C motif	CXL16_HUI	2	1.468159
48H 10MT/	P35052	0.457026	0.002589	0.004322	GPC1	Glypican-1	GPC1_HUM	2	1.372709
48H 10MT/	Q9H0U4	0.071309	0.00259	0.004324	RAB1B	Ras-related	RAB1B_HUI	9	1.050669
48H 50MT/	Q13243	0.167679	0.002591	0.004325	SRSF5	Serine/argi	SRSF5_HUM	8	1.12325
48H 10MT/	Q5T0N5	0.018674	0.002591	0.004325	FNBP1L	Formin-bin	FBP1L_HUM	17	1.013028
24H 10MT/	Q9BU89	-0.31499	0.002591	0.004325	DOHH	Deoxyhypu	DOHH_HUM	10	0.803854
24H 10MT/	Q9H4I9	0.139613	0.002594	0.004328	SMDT1	Essential M	EMRE_HUM	2	1.10161
24H 10MT/	O00161	0.064108	0.002596	0.004331	SNAP23	Synaptosor	SNP23_HUI	10	1.045438
48H 10MT/	Q9NYY8	-0.12956	0.002598	0.004333	FASTKD2	FAST kinase	FAKD2_HUI	17	0.914108
24H 10MT/	P28161	-0.07879	0.002601	0.004337	GSTM2	Glutathion	GSTM2_HU	16	0.946849
48H 50MT/	Q6RFH5	-0.25056	0.002603	0.00434	WDR74	WD repeat-	WDR74_HU	17	0.840572
24H 50MT/	Q8N6T3	0.050128	0.002603	0.00434	ARFGAP1	ADP-ribosy	ARFG1_HUI	15	1.035357
48H 50MT/	Q8N697	-0.17741	0.002605	0.004343	SLC15A4	Solute carri	S15A4_HUI	2	0.884288
48H 10MT/	P82912	-0.02786	0.002606	0.004343	MRPS11	Small ribos	RT11_HUM	5	0.980875
24H 10MT/	P32119	-0.07215	0.002608	0.004346	PRDX2	Peroxiredo	PRDX2_HU	13	0.951217
48H 50MT/	P31942	0.136349	0.002608	0.004346	HNRNPH3	Heterogene	HNRH3_HU	12	1.09912
48H 10MT/	Q8WYA6	0.119754	0.002608	0.004346	CTNNBL1	Beta-cateni	CTBL1_HUM	21	1.08655
48H 50MT/	P30041	0.056171	0.002609	0.004347	PRDX6	Peroxiredo	PRDX6_HU	19	1.039703

24H 50MTA	Q9UJX4	0.035717	0.00261	0.004347	ANAPC5	Anaphase-p	APC5_HUM	25	1.025066
24H 10MTA	O15258	0.08325	0.002612	0.00435	RER1	Protein REF	RER1_HUM	6	1.059402
24H 50MTA	Q9Y2Z4	0.104327	0.002616	0.004354	YARS2	Tyrosine--tI	SYYM_HUM	18	1.074993
24H 50MTA	P30048	0.039115	0.002616	0.004355	PRDX3	Thioredoxi	PRDX3_HU	11	1.027483
48H 10MTA	P22307	0.05319	0.002621	0.00436	SCP2	Sterol carri	SCP2_HUM	24	1.037556
48H 10MTA	Q9HC38	0.117078	0.002621	0.00436	GLOD4	Glyoxalase	GLOD4_HU	16	1.084536
24H 50MTA	P22307	0.111898	0.002623	0.004362	SCP2	Sterol carri	SCP2_HUM	24	1.080649
48H 50MTA	P16435	0.075322	0.002624	0.004364	POR	NADPH--cyt	NCPR_HUM	39	1.053596
48H 50MTA	Q8TEM1	0.161883	0.002624	0.004364	NUP210	Nuclear por	PO210_HU	45	1.118746
24H 10MTA	P35226	0.10286	0.002625	0.004365	BMI1	Polycomb c	BMI1_HUM	4	1.0739
48H 10MTA	Q5T6V5	0.080696	0.002626	0.004365	QNG1	Queuosine	QNG1_HUM	15	1.057528
24H 50MTA	P00734	0.449953	0.002628	0.004367	F2	Prothromb	THRB_HUM	26	1.365996
24H 50MTA	Q9P2X3	0.189423	0.002629	0.004368	IMPACT	Protein IMI	IMPCT_HUI	6	1.140307
48H 10MTA	Q9BUR4	-0.04377	0.002631	0.004371	WRAP53	Telomerase	TCAB1_HUI	6	0.970117
24H 50MTA	Q92786	0.210893	0.002636	0.004376	PROX1	Prospero h	PROX1_HU	17	1.157404
48H 10MTA	P82979	0.008372	0.002642	0.004384	SARNP	SAP domair	SARNP_HUI	8	1.00582
24H 50MTA	Q9UIC8	-0.11392	0.002644	0.004387	LCMT1	Leucine car	LCMT1_HU	10	0.924076
24H 50MTA	P06865	0.05375	0.002645	0.004388	HEXA	Beta-hexosa	HEXA_HUM	18	1.037959
24H 50MTA	P59190	-0.80399	0.002647	0.00439	RAB15	Ras-related	RAB15_HUI	1	0.572761
48H 50MTA	Q9Y3L5	0.128985	0.00265	0.004393	RAP2C	Ras-related	RAP2C_HUI	4	1.093524
24H 10MTA	Q9NXR5	-1.85641	0.002654	0.004399	ANKRD10	Ankyrin rep	ANR10_HU	2	0.276164
24H 50MTA	P30050	-0.12974	0.002659	0.004405	RPL12	Large ribos	RL12_HUM	6	0.913997
24H 50MTA	Q9NVA4	-3.93852	0.00266	0.004406	TMEM184C	Transmemb	T184C_HUI	1	0.065221
48H 50MTA	O96007	-0.05285	0.002667	0.004414	MOCS2	Molybdopt	MOC2B_HU	4	0.964027
24H 10MTA	Q8WXE1	2.018531	0.002671	0.004418	ATRIP	ATR-interac	ATRIP_HUM	1	4.05171
48H 10MTA	Q9BWD1	0.020859	0.002676	0.004425	ACAT2	Acetyl-CoA	THIC_HUMA	17	1.014563
24H 10MTA	Q5GLZ8	0.100015	0.002677	0.004425	HERC4	Probable E3	HERC4_HUI	27	1.071785
48H 50MTA	Q6ZN54	0.368241	0.002682	0.004432	DEF8	Differential	DEFI8_HUM	2	1.290778
24H 50MTA	Q9Y6I9	0.174298	0.002687	0.004438	TEX264	Testis-expre	TX264_HUI	10	1.128415
48H 10MTA	Q9NRF9	0.123202	0.00269	0.004441	POLE3	DNA polym	DPOE3_HU	4	1.089149
48H 10MTA	Q08623	0.126148	0.00269	0.004442	PUDP	Pseudouric	HDHD1_HU	11	1.091376
48H 50MTA	Q13404	0.12237	0.002692	0.004444	UBE2V1	Ubiquitin-c	UB2V1_HU	9	1.088522
48H 10MTA	P34949	-0.01122	0.002693	0.004445	MPI	Mannose-6	MPI_HUMA	15	0.992253
48H 10MTA	P06576	-0.19785	0.002696	0.004448	ATP5F1B	ATP syntha	ATPB_HUM	28	0.87185
48H 10MTA	P43243	0.027793	0.002698	0.004451	MATR3	Matrin-3	MATR3_HU	31	1.019451
24H 10MTA	Q9BTT0	0.190455	0.002698	0.004451	ANP32E	Acidic leuci	AN32E_HUI	8	1.141124
48H 50MTA	P07686	0.100658	0.002711	0.004468	HEXB	Beta-hexosa	HEXB_HUM	22	1.072262
24H 10MTA	Q9NRV9	-0.06837	0.002711	0.004468	HEBP1	Heme-bind	HEBP1_HUI	8	0.953715
48H 10MTA	Q7L4I2	0.171652	0.002718	0.004477	RSRC2	Arginine/se	RSRC2_HUI	8	1.126348
48H 10MTA	Q9BTT0	0.102567	0.002722	0.004482	ANP32E	Acidic leuci	AN32E_HUI	8	1.073682
24H 10MTA	O95159	0.057424	0.002725	0.004485	ZFPL1	Zinc finger	ZFPL1_HUM	10	1.040606
48H 50MTA	Q9Y448	-0.4693	0.002725	0.004485	KNSTRN	Small kinet	SKAP_HUM	5	0.722314
24H 50MTA	P31937	0.172141	0.002726	0.004486	HIBADH	3-hydroxyis	3HIDH_HUI	12	1.126729
24H 50MTA	P55210	-0.30726	0.002727	0.004487	CASP7	Caspase-7	CASP7_HUI	7	0.808175
48H 50MTA	O76003	0.144301	0.002727	0.004487	GLRX3	Glutaredox	GLRX3_HUI	17	1.105195
24H 50MTA	Q99442	0.142812	0.00273	0.004491	SEC62	Translocati	SEC62_HUM	9	1.104055
24H 50MTA	P57105	0.134998	0.002734	0.004497	SYNJ2BP	Synaptojan	SYJ2B_HUM	4	1.098092
48H 50MTA	O43719	0.018801	0.002736	0.004499	HTATSF1	17S U2 SnR	HTSF1_HUM	20	1.013117
48H 10MTA	Q08J23	0.058845	0.002738	0.004501	NSUN2	RNA cytosir	NSUN2_HU	41	1.041632
24H 50MTA	O75390	0.189992	0.002739	0.004503	CS	Citrate synt	CISY_HUMA	18	1.140757
48H 10MTA	P55268	0.003896	0.002741	0.004505	LAMB2	Laminin sul	LAMB2_HU	28	1.002704
24H 50MTA	Q6QNY0	-0.15739	0.002741	0.004506	BLOC1S3	Biogenesis	BL1S3_HUM	7	0.896646

48H 50MTA	Q6IPR3	-0.09584	0.002743	0.004507	TYW3	tRNA wybu	TYW3_HUM	10	0.935724
24H 10MTA	Q08752	0.101966	0.002746	0.00451	PPID	Peptidyl-pr	PPID_HUM	21	1.073235
48H 50MTA	P12956	0.101424	0.002747	0.004513	XRCC6	X-ray repair	XRCC6_HUI	43	1.072832
24H 50MTA	O60942	0.164079	0.002748	0.004513	RNGTT	mRNA-capp	MCE1_HUM	11	1.120451
48H 10MTA	P37268	0.15518	0.002749	0.004514	FDFT1	Squalene sy	FDFT_HUM	15	1.11356
48H 50MTA	Q9NP92	-0.20081	0.002751	0.004517	MRPS30	Large ribos	RT30_HUM	12	0.870062
24H 10MTA	P52179	-2.31042	0.002754	0.00452	MYOM1	Myomesin-	MYOM1_HU	2	0.201601
48H 50MTA	P26583	0.443897	0.00276	0.004528	HMGB2	High mobil	HMGB2_HU	7	1.360274
48H 50MTA	Q8WVJ2	0.107184	0.002762	0.00453	NUDCD2	NudC doma	NUDC2_HU	6	1.077123
24H 10MTA	Q96AY3	0.073763	0.002765	0.004535	FKBP10	Peptidyl-pr	FKB10_HUI	27	1.052458
48H 50MTA	Q9NYL9	0.070967	0.002772	0.004542	TMOD3	Tropomodu	TMOD3_HU	18	1.050421
24H 10MTA	O95336	-0.19551	0.002774	0.004544	PGLS	6-phospho	6PGL_HUM	12	0.873265
48H 10MTA	Q15369	0.210564	0.002777	0.004548	ELOC	Elongin-C	ELOC_HUM	5	1.157141
48H 10MTA	Q5BKZ1	0.151875	0.002779	0.00455	ZNF326	DBIRD com	ZN326_HUI	19	1.111012
24H 50MTA	P31930	0.076324	0.002784	0.004557	UQCRC1	Cytochrom	QCR1_HUM	17	1.054328
24H 50MTA	Q9UHB4	-0.39019	0.002785	0.004558	NDOR1	NADPH-dep	NDOR1_HU	12	0.76303
48H 10MTA	Q13586	-0.0078	0.002787	0.004561	STIM1	Stromal int	STIM1_HUI	20	0.99461
48H 50MTA	Q9UIG0	-0.25905	0.002787	0.004561	BAZ1B	Tyrosine-pr	BAZ1B_HUI	42	0.835641
24H 50MTA	P17936	1.168291	0.002787	0.004561	IGFBP3	Insulin-like	IBP3_HUMA	5	2.247453
24H 50MTA	Q16625	0.146556	0.002789	0.004563	OCLN	Occludin	OCLN_HUM	8	1.106924
48H 50MTA	P62136	-0.09674	0.002789	0.004563	PPP1CA	Serine/thre	PP1A_HUM	7	0.935146
24H 50MTA	P15311	0.151141	0.002792	0.004566	EZR	Ezrin	EZRI_HUMA	25	1.110447
24H 50MTA	P61254	0.16452	0.002793	0.004568	RPL26	Large ribos	RL26_HUM	8	1.120793
24H 50MTA	Q8NFD5	0.034774	0.002796	0.004572	ARID1B	AT-rich inte	ARI1B_HUM	26	1.024396
48H 50MTA	Q8TEQ8	-0.14	0.002796	0.004572	PIGO	GPI ethano	PIGO_HUM	11	0.907522
24H 50MTA	P08559	0.084646	0.002796	0.004572	PDHA1	Pyruvate de	ODPA_HUM	21	1.060428
24H 10MTA	Q8N1F7	0.044734	0.002801	0.004577	NUP93	Nuclear po	NUP93_HU	39	1.031493
24H 50MTA	P30405	0.116594	0.002802	0.004579	PPIF	Peptidyl-pr	PPIF_HUMA	5	1.084172
48H 50MTA	Q13823	-0.26074	0.002809	0.004586	GNL2	Nucleolar G	NOG2_HUM	21	0.834659
24H 50MTA	P30613	0.141922	0.00281	0.004587	PKLR	Pyruvate ki	KPYR_HUM	4	1.103374
48H 50MTA	P01034	0.908582	0.002814	0.004591	CST3	Cystatin-C	CYTC_HUM	1	1.877199
24H 50MTA	Q8WZ82	-0.2714	0.002822	0.004602	OVCA2	Esterase OV	OVCA2_HU	8	0.828513
48H 10MTA	P50440	0.022642	0.002823	0.004602	GATM	Glycine am	GATM_HUM	19	1.015818
24H 10MTA	Q9HCN4	0.11001	0.002827	0.004607	GPN1	GPN-loop G	GPN1_HUM	4	1.079236
48H 10MTA	Q9Y5B9	0.022825	0.002828	0.004609	SUPT16H	FACT comp	SP16H_HUI	43	1.015947
24H 50MTA	Q9NPQ8	-0.0919	0.002829	0.004611	RIC8A	Synembryn	RIC8A_HUM	17	0.938285
48H 50MTA	Q13206	-0.17689	0.002835	0.004619	DDX10	Probable A	DDX10_HU	30	0.884607
24H 50MTA	Q08211	0.074838	0.002836	0.00462	DHX9	ATP-depend	DHX9_HUM	64	1.053242
48H 50MTA	O43583	0.209309	0.002838	0.004621	DENR	Density-reg	DENR_HUM	9	1.156134
48H 50MTA	P20645	0.253203	0.00284	0.004624	M6PR	Cation-dep	MPRD_HUM	7	1.191851
24H 50MTA	Q9HA77	0.169308	0.002844	0.004629	CARS2	Probable cy	SYCM_HUM	16	1.124519
48H 50MTA	P49247	-0.05446	0.002846	0.004631	RPIA	Ribose-5-ph	RPIA_HUMA	5	0.962955
24H 50MTA	Q15025	0.28835	0.002848	0.004634	TNIP1	TNFAIP3-in	TNIP1_HUM	6	1.221243
48H 50MTA	P51884	0.326742	0.002851	0.004637	LUM	Lumican	LUM_HUMA	4	1.254178
48H 50MTA	O75394	-0.22878	0.002854	0.00464	MRPL33	Large ribos	RM33_HUM	2	0.853358
24H 50MTA	O43660	0.139396	0.002857	0.004645	PLRG1	Pleiotropic	PLRG1_HUI	22	1.101444
48H 50MTA	P49189	0.126534	0.002857	0.004645	ALDH9A1	4-trimethyl	AL9A1_HUI	21	1.091668
24H 50MTA	O15062	-0.22408	0.00286	0.004648	ZBTB5	Zinc finger a	ZBTB5_HUM	1	0.85614
24H 50MTA	P04217	1.697453	0.00286	0.004648	A1BG	Alpha-1B-gl	A1BG_HUM	2	3.24328
48H 50MTA	P62873	0.138595	0.002862	0.00465	GNB1	Guanine nu	GBB1_HUM	14	1.100832
24H 10MTA	Q16527	0.186602	0.002864	0.004652	CSRP2	Cysteine an	CSRP2_HUI	8	1.13808
48H 50MTA	Q96DB5	0.096976	0.002868	0.004657	RMDN1	Regulator c	RMD1_HUM	13	1.06953

48H 50MT/	Q16186	0.262619	0.002872	0.004661	ADRM1	Proteasom	ADRM1_HU	9	1.199654
48H 50MT/	P61326	0.561149	0.002873	0.004662	MAGOH	Protein ma	MGN_HUM	1	1.475444
48H 50MT/	O14745	0.251089	0.002874	0.004663	NHERF1	Na(+)/H(+) e	NHRF1_HU	23	1.190105
48H 50MT/	Q8NDT2	-0.02754	0.002875	0.004665	RBM15B	Putative RN	RB15B_HU	8	0.981093
48H 50MT/	P78406	0.123478	0.002878	0.004668	RAE1	mRNA expo	RAE1L_HUN	11	1.089358
48H 10MT/	P41208	0.196028	0.002879	0.00467	CETN2	Centrin-2	CETN2_HUI	5	1.14554
24H 50MT/	Q9NWV4	-0.2825	0.002881	0.004672	CZIB	CXXC motif	CZIB_HUM/	5	0.822167
24H 50MT/	P62805	0.118898	0.002884	0.004676	H4C1	Histone H4	H4_HUMAN	12	1.085905
24H 50MT/	P63098	-0.10717	0.002886	0.004677	PPP3R1	Calcineurin	CANB1_HU	5	0.928407
24H 10MT/	O00203	-0.04254	0.002886	0.004678	AP3B1	AP-3 compl	AP3B1_HU	37	0.970941
24H 10MT/	P05386	-0.40113	0.002889	0.00468	RPLP1	Large ribos	RLA1_HUM	4	0.757263
48H 10MT/	Q6PK04	-0.11738	0.00289	0.004682	CCDC137	Coiled-coil	CC137_HU	6	0.92186
24H 50MT/	Q96I99	0.136902	0.002891	0.004683	SUCLG2	Succinate--	SUCB2_HU	19	1.099541
48H 50MT/	Q7Z4Q2	-0.09811	0.002894	0.004687	HEATR3	HEAT repea	HEAT3_HUI	20	0.934259
24H 50MT/	P11047	-0.02919	0.002895	0.004688	LAMC1	Laminin sul	LAMC1_HU	40	0.979972
48H 10MT/	P23193	0.095398	0.002901	0.004694	TCEA1	Transcripti	TCEA1_HUN	17	1.06836
24H 50MT/	Q92598	-0.15856	0.002901	0.004694	HSPH1	Heat shock	HS105_HU	44	0.895917
48H 50MT/	P32456	0.097714	0.002905	0.004698	GBP2	Guanylate-l	GBP2_HUM	11	1.070076
48H 50MT/	Q9BX46	-0.17804	0.002905	0.004699	RBM24	RNA-bindir	RBM24_HU	6	0.883901
48H 50MT/	P13639	-0.15073	0.002906	0.004699	EEF2	Elongation	EF2_HUMA	57	0.900796
24H 10MT/	Q96SZ5	0.038556	0.002906	0.004699	ADO	2-aminoeth	AEDO_HUM	5	1.027085
48H 50MT/	Q12849	-0.24167	0.00291	0.004703	GRSF1	G-rich sequ	GRSF1_HUI	23	0.845766
24H 50MT/	P62714	-0.13903	0.002911	0.004704	PPP2CB	Serine/thre	PP2AB_HUI	12	0.908132
48H 10MT/	P30622	0.00714	0.002912	0.004705	CLIP1	CAP-Gly do	CLIP1_HUN	44	1.004962
48H 50MT/	Q6PJG6	-0.27521	0.002916	0.00471	BRAT1	BRCA1-assc	BRAT1_HUI	37	0.826331
24H 50MT/	Q9NTI5	0.095655	0.002917	0.004711	PDS5B	Sister chro	PDS5B_HUI	41	1.068551
24H 10MT/	Q9Y624	-0.09662	0.002921	0.004716	F11R	Junctional	JAM1_HUM	10	0.935222
24H 50MT/	Q9BTT0	0.109067	0.002929	0.004724	ANP32E	Acidic leuci	AN32E_HUI	8	1.07853
24H 50MT/	Q16864	-0.85589	0.002929	0.004725	ATP6V1F	V-type prot	VATF_HUM	3	0.552523
48H 50MT/	Q9BX74	-1.92415	0.002933	0.004729	TM2D1	TM2 domai	TM2D1_HU	1	0.263496
48H 10MT/	Q9NVF7	-0.17237	0.002933	0.004729	FBXO28	F-box only	FBX28_HUI	5	0.887383
48H 50MT/	Q96G03	0.092699	0.002937	0.004733	PGM2	Phosphope	PGM2_HUN	30	1.066363
24H 50MT/	Q96HR3	1.560674	0.002937	0.004733	MED30	Mediator o	MED30_HU	2	2.949915
24H 50MT/	Q08623	0.144454	0.002942	0.004739	PUDP	Pseudourid	HDHD1_HU	11	1.105312
24H 10MT/	P62917	-0.09204	0.002947	0.004745	RPL8	Large ribos	RL8_HUMA	14	0.938194
48H 10MT/	Q92879	0.096912	0.002953	0.00475	CELF1	CUGBP Elav	CELF1_HUN	10	1.069482
48H 50MT/	P10321	0.241144	0.002954	0.004751	HLA-C	HLA class I	HLAC_HUM	3	1.18193
48H 50MT/	Q86XP3	0.037064	0.002956	0.004753	DDX42	ATP-depen	DDX42_HU	31	1.026024
24H 50MT/	Q9BW60	-0.27959	0.002956	0.004753	ELOVL1	Very long c	ELOV1_HUI	4	0.823824
24H 10MT/	Q9UMX5	-0.28095	0.002963	0.004761	NENF	Neudesin	NENF_HUM	7	0.823046
24H 50MT/	Q8NBQ5	0.089406	0.002966	0.004765	HSD17B11	Estradiol 1	DHB11_HU	14	1.063932
48H 50MT/	Q92609	0.16057	0.002966	0.004765	TBC1D5	TBC1 doma	TBCD5_HUI	14	1.117728
48H 50MT/	O43852	0.15988	0.00297	0.00477	CALU	Calumenin	CALU_HUM	21	1.117194
24H 50MT/	O60313	0.178351	0.002971	0.00477	OPA1	Dynamin-li	OPA1_HUM	46	1.13159
24H 50MT/	Q9NPG3	-0.07954	0.002975	0.004775	UBN1	Ubinuclein	UBN1_HUM	3	0.946362
24H 50MT/	O43426	-0.93298	0.00298	0.00478	SYNJ1	Synaptojan	SYNJ1_HUN	6	0.523774
48H 50MT/	Q12792	0.059769	0.002982	0.004781	TWF1	Twinfilin-1	TWF1_HUM	15	1.042299
24H 10MT/	Q01970	0.017344	0.002982	0.004782	PLCB3	1-phosphat	PLCB3_HUI	25	1.012095
24H 50MT/	P62851	-0.23889	0.002983	0.004783	RPS25	Small ribos	RS25_HUM	4	0.847399
24H 50MT/	Q8WUR7	-1.1666	0.002984	0.004783	C15orf40	UPF0235 p	CO040_HU	2	0.445471
24H 50MT/	Q4G176	0.195727	0.002984	0.004783	ACSF3	Malonate--	ACSF3_HUI	18	1.145301
48H 50MT/	Q96RQ3	0.087711	0.002985	0.004784	MCCC1	Methylcrot	MCCA_HUM	19	1.062683

48H 10MTA	P30048	-0.17879	0.002987	0.004787	PRDX3	Thioredoxin	PRDX3_HU	11	0.883444
24H 10MTA	Q9NV35	0.218823	0.002998	0.004801	NUDT15	Nucleotide	NUD15_HU	7	1.163784
48H 50MTA	Q92598	-0.18269	0.002999	0.004802	HSPH1	Heat shock	HS105_HU	44	0.881059
24H 50MTA	Q9BVL4	0.482452	0.003006	0.00481	SELENOO	Protein ade	SELO_HUM	11	1.397116
24H 50MTA	P51659	0.180616	0.003007	0.004811	HSD17B4	Peroxisoma	DHB4_HUM	30	1.133367
24H 10MTA	P08397	-0.09625	0.003007	0.004812	HMBS	Porphobilin	HEM3_HUM	17	0.935461
24H 10MTA	Q9UNL2	-0.19941	0.00301	0.004816	SSR3	Translocon	SSRG_HUM	2	0.870908
24H 50MTA	P35221	0.098305	0.003013	0.004817	CTNNA1	Catenin alp	CTNA1_HU	44	1.070515
48H 10MTA	Q8N573	0.468076	0.003015	0.004821	OXR1	Oxidation r	OXR1_HUM	2	1.383264
48H 10MTA	Q9H5K3	-0.21394	0.003017	0.004822	POMK	Protein O-m	SG196_HU	5	0.862176
48H 50MTA	O76021	-0.20368	0.003024	0.004831	RSL1D1	Ribosomal	RL1D1_HU	22	0.868331
48H 50MTA	O95479	0.695594	0.003025	0.004832	H6PD	GDH/6PGL	G6PE_HUM	25	1.619551
24H 50MTA	Q4G0J3	0.073419	0.003027	0.004834	LARP7	La-related p	LARP7_HU	17	1.052208
48H 50MTA	P35611	0.196616	0.003031	0.004839	ADD1	Alpha-addu	ADDA_HUM	16	1.146008
48H 10MTA	P27816	0.114676	0.003035	0.004842	MAP4	Microtubul	MAP4_HUM	41	1.082732
24H 10MTA	Q9H7L9	-0.04127	0.003038	0.004846	SUDS3	Sin3 histon	SDS3_HUM	8	0.971799
24H 50MTA	Q9NQ29	0.170051	0.003044	0.004852	LUC7L	Putative RN	LUC7L_HU	7	1.125098
48H 10MTA	P52758	-0.04736	0.003047	0.004856	RIDA	2-iminobut	RIDA_HUM	5	0.967706
48H 10MTA	P45984	0.143447	0.003052	0.004861	MAPK9	Mitogen-ac	MK09_HUM	9	1.104541
48H 50MTA	Q9UEY8	0.210343	0.003055	0.004864	ADD3	Gamma-add	ADDG_HUM	16	1.156964
48H 10MTA	P08047	0.402599	0.003056	0.004865	SP1	Transcripti	SP1_HUMA	3	1.321887
24H 50MTA	P00367	0.102191	0.003057	0.004866	GLUD1	Glutamate	DHE3_HUM	29	1.073402
48H 50MTA	O75884	0.205931	0.003058	0.004866	RBBP9	Serine hydr	RBBP9_HU	8	1.15343
24H 50MTA	Q15436	0.057955	0.003058	0.004866	SEC23A	Protein tra	SC23A_HU	30	1.040989
24H 50MTA	Q8IV08	0.308048	0.003061	0.00487	PLD3	5'-3' exonu	PLD3_HUM	6	1.238032
48H 10MTA	Q8TAQ2	0.081514	0.003062	0.004871	SMARCC2	SWI/SNF co	SMRC2_HU	20	1.058128
48H 50MTA	Q92551	0.017873	0.003064	0.004874	IP6K1	Inositol hex	IP6K1_HUM	3	1.012466
48H 50MTA	P51572	0.131898	0.003066	0.004875	BCAP31	B-cell recep	BAP31_HU	11	1.095734
24H 10MTA	O95070	0.200632	0.00307	0.00488	YIF1A	Protein YIF	YIF1A_HUM	4	1.149202
48H 10MTA	P58511	-1.04423	0.003073	0.004884	SMIM11	Small integ	SIM11_HU	1	0.484902
48H 10MTA	P01889	0.16999	0.003076	0.004887	HLA-B	HLA class I	HLAB_HUM	8	1.125051
24H 50MTA	P51151	0.234618	0.003079	0.004891	RAB9A	Ras-related	RAB9A_HU	7	1.176595
24H 50MTA	P62318	0.075359	0.003081	0.004893	SNRPD3	Small nucle	SMD3_HUM	5	1.053623
48H 50MTA	P22694	-0.00328	0.003083	0.004894	PRKACB	cAMP-depe	KAPCB_HU	12	0.997726
24H 10MTA	Q9HCM4	0.088723	0.00309	0.004903	EPB41L5	Band 4.1-lik	E41L5_HUM	12	1.063429
48H 50MTA	O95394	0.104537	0.003091	0.004904	PGM3	Phosphoac	AGM1_HUM	21	1.075149
48H 50MTA	Q8IYI6	0.122969	0.003095	0.004909	EXOC8	Exocyst cor	EXOC8_HU	19	1.088974
48H 10MTA	Q9BVS4	0.001424	0.003095	0.004909	RIOK2	Serine/thre	RIOK2_HUM	11	1.000987
48H 50MTA	Q15370	0.215989	0.0031	0.004915	ELOB	Elongin-B	ELOB_HUM	10	1.1615
24H 10MTA	Q96FF7	-1.96085	0.003099	0.004915	MISP3	Uncharacte	MISP3_HU	1	0.256878
24H 50MTA	Q9Y570	0.042725	0.003099	0.004915	PPME1	Protein pho	PPME1_HU	18	1.030058
24H 50MTA	P33552	-0.18656	0.003104	0.004919	CKS2	Cyclin-depe	CKS2_HUM	2	0.878698
48H 50MTA	O00244	0.244225	0.003108	0.004922	ATOX1	Copper tra	ATOX1_HU	6	1.184456
24H 10MTA	Q9NVJ2	0.130887	0.003108	0.004922	ARL8B	ADP-ribosy	ARL8B_HU	6	1.094967
48H 50MTA	Q9H3S7	0.002005	0.00311	0.004926	PTPN23	Tyrosine-pr	PTN23_HU	32	1.001391
24H 50MTA	P62136	-0.14505	0.003113	0.004928	PPP1CA	Serine/thre	PP1A_HUM	7	0.904346
24H 10MTA	P14550	-0.10566	0.003114	0.004929	AKR1A1	Aldo-keto r	AK1A1_HU	19	0.929379
48H 10MTA	Q5VV42	0.091686	0.003114	0.004929	CDKAL1	Threonylca	CDKAL_HU	15	1.065615
48H 50MTA	P05981	1.666471	0.003115	0.00493	HPN	Serine prot	HEPS_HUM	6	3.174371
48H 10MTA	Q12906	-0.00543	0.003116	0.00493	ILF3	Interleukin	ILF3_HUMA	38	0.996244
24H 10MTA	P08134	0.070756	0.003119	0.004934	RHOC	Rho-related	RHOC_HUM	10	1.050267
24H 50MTA	Q13523	0.493266	0.003119	0.004934	PRP4K	Serine/thre	PRP4K_HU	20	1.407628

48H 10MTA	Q9NYL9	0.063937	0.003123	0.004939	TMOD3	Tropomodu	TMOD3_HU	18	1.045314
48H 10MTA	P20700	0.012048	0.003126	0.004942	LMNB1	Lamin-B1	LMNB1_HU	31	1.008386
24H 50MTA	Q13769	0.153371	0.003128	0.004943	THOC5	THO compl	THOC5_HU	23	1.112165
24H 50MTA	Q96CB9	0.219349	0.00313	0.004947	NSUN4	5-methylcy	NSUN4_HU	5	1.164208
48H 10MTA	O14545	0.179305	0.003132	0.004949	TRAFD1	TRAF-type z	TRAD1_HU	9	1.132338
48H 50MTA	P16070	-1.06253	0.003138	0.004956	CD44	CD44 antig	CD44_HUM	1	0.47879
24H 10MTA	P62310	-0.45878	0.003145	0.004964	LSM3	U6 snRNA-a	LSM3_HUM	3	0.727602
24H 50MTA	Q06787	0.026875	0.003145	0.004964	FMR1	Fragile X m	FMR1_HUM	11	1.018803
48H 10MTA	Q9H892	1.149853	0.003146	0.004964	TTC12	Tetratricop	TTC12_HUM	1	2.218914
48H 50MTA	Q96ST2	0.105694	0.003148	0.004966	IWS1	Protein IWS	IWS1_HUM	13	1.076012
48H 10MTA	Q6RFH5	-0.07227	0.003148	0.004966	WDR74	WD repeat-	WDR74_HU	17	0.951143
48H 50MTA	Q9UNM6	0.077235	0.003148	0.004966	PSMD13	26S protea	PSD13_HU	27	1.054994
24H 50MTA	Q9UKM9	0.129121	0.003151	0.00497	RALY	RNA-bindir	RALY_HUM	11	1.093627
48H 50MTA	P25398	-0.21507	0.003162	0.004982	RPS12	Small ribos	RS12_HUM	9	0.861502
24H 50MTA	O14874	0.012791	0.003162	0.004983	BCKDK	Branched-c	BCKD_HUM	8	1.008905
48H 50MTA	Q8NI36	-0.05325	0.003163	0.004983	WDR36	WD repeat-	WDR36_HU	30	0.963765
48H 50MTA	P52272	0.042727	0.003164	0.004984	HNRNPM	Heterogene	HNRPM_HU	41	1.030059
48H 10MTA	Q5UCC4	0.409678	0.003164	0.004984	EMC10	ER membra	EMC10_HU	3	1.328389
24H 50MTA	O95104	0.059428	0.003167	0.004986	SCAF4	SR-related a	SCAF4_HUM	14	1.042053
24H 10MTA	P35658	0.030463	0.00317	0.00499	NUP214	Nuclear po	NU214_HU	48	1.02134
24H 50MTA	P26440	-0.02257	0.00317	0.00499	IVD	Isovaleryl-C	IVD_HUMA	12	0.984479
24H 10MTA	Q9P2E9	-0.08282	0.003175	0.004995	RRBP1	Ribosome-b	RRBP1_HU	45	0.944207
24H 50MTA	Q99426	0.121654	0.003176	0.004996	TBCB	Tubulin-fol	TBCB_HUM	13	1.087981
48H 10MTA	Q15005	0.116903	0.003179	0.004999	SPCS2	Signal pept	SPCS2_HUM	10	1.084405
24H 50MTA	O00442	-0.08898	0.003183	0.005004	RTCA	RNA 3'-term	RTCA_HUM	15	0.940189
48H 50MTA	O15091	-0.27555	0.003186	0.005008	PRORP	Mitochond	MRPP3_HU	17	0.826133
24H 50MTA	Q8WXA3	-0.26673	0.003188	0.00501	RUFY2	RUN and FY	RUFY2_HU	9	0.8312
48H 50MTA	P07339	0.106334	0.00319	0.005011	CTSD	Cathepsin D	CATD_HUM	16	1.076489
48H 50MTA	Q96A65	0.158007	0.00319	0.005011	EXOC4	Exocyst cor	EXOC4_HU	32	1.115745
48H 50MTA	Q9BSE5	0.102858	0.00319	0.005011	AGMAT	Guanidino	GDAH_HUM	14	1.073899
24H 10MTA	P52732	0.061968	0.003192	0.005013	KIF11	Kinesin-like	KIF11_HUM	30	1.043889
24H 10MTA	Q9NZI7	-0.11209	0.003192	0.005014	UBP1	Upstream-b	UBIP1_HUM	4	0.925246
48H 50MTA	Q3B726	-0.23386	0.003195	0.005016	POLR1F	DNA-direct	RPA43_HU	5	0.850356
48H 50MTA	Q8NF50	1.040793	0.003201	0.005023	DOCK8	Dedicator c	DOCK8_HU	3	2.057359
48H 10MTA	Q9Y5Y2	0.076096	0.003205	0.005028	NUBP2	Cytosolic Fe	NUBP2_HU	11	1.054161
48H 50MTA	Q9H2U2	0.13484	0.003206	0.005028	PPA2	Inorganic p	IPYR2_HUM	14	1.097971
24H 10MTA	Q9BSD7	0.013111	0.003213	0.005036	NTPCR	Cancer-rela	NTPCR_HU	10	1.009129
48H 50MTA	P54578	0.129655	0.003224	0.00505	USP14	Ubiquitin c	UBP14_HU	24	1.094032
48H 10MTA	Q99615	0.033614	0.003226	0.005053	DNAJC7	DnaJ homo	DNJC7_HU	27	1.023573
48H 50MTA	P55039	0.048826	0.003227	0.005054	DRG2	Developme	DRG2_HUM	17	1.034423
24H 10MTA	Q9H0W9	-0.13664	0.003228	0.005055	C11orf54	Ester hydro	CK054_HU	13	0.909633
24H 10MTA	Q9BUK6	-0.14387	0.003232	0.005059	MSTO1	Protein mis	MSTO1_HU	17	0.905087
48H 10MTA	Q86WR0	0.04522	0.003233	0.00506	CCDC25	Coiled-coil	CCD25_HU	6	1.03184
48H 50MTA	Q8N0V3	-0.73421	0.003235	0.005063	RBFA	Putative rib	RBFA_HUM	2	0.601145
24H 10MTA	Q9H5Q4	-0.16707	0.003238	0.005067	TFB2M	Dimethylac	TFB2M_HU	19	0.890652
24H 50MTA	P60891	0.013202	0.003241	0.00507	PRPS1	Ribose-pho	PRPS1_HU	14	1.009193
48H 50MTA	P62879	0.137198	0.003243	0.005073	GNB2	Guanine nu	GBB2_HUM	8	1.099767
24H 10MTA	P00352	-0.13248	0.003246	0.005077	ALDH1A1	Aldehyde d	AL1A1_HU	32	0.912261
24H 50MTA	Q5JTV8	0.047585	0.003248	0.005078	TOR1AIP1	Torsin-1A-i	TOIP1_HUM	13	1.033533
24H 50MTA	P35232	0.084456	0.003251	0.005081	PHB1	Prohibitin	PHB1_HUM	20	1.060288
24H 50MTA	Q9H3P7	0.015481	0.003252	0.005082	ACBD3	Golgi reside	GCP60_HU	18	1.010788
48H 50MTA	P21266	0.193687	0.003252	0.005082	GSTM3	Glutathion	GSTM3_HU	18	1.143683

24H 50MTA	P25208	0.335135	0.003255	0.005084	NFYB	Nuclear tra	NFYB_HUM	2	1.261496
48H 50MTA	Q9BU61	-0.04705	0.003257	0.005088	NDUFAF3	NADH dehy	NDUF3_HU	6	0.967916
48H 50MTA	Q17R98	0.226918	0.003258	0.005089	ZNF827	Zinc finger	ZN827_HU	2	1.170332
48H 10MTA	Q9Y490	0.032603	0.003259	0.005089	TLN1	Talin-1	TLN1_HUM	110	1.022856
48H 10MTA	Q6N069	-0.00061	0.00326	0.00509	NAA16	N-alpha-ace	NAA16_HU	13	0.99958
48H 10MTA	Q8TBA6	0.00921	0.003259	0.00509	GOLGA5	Golgin subf	GOGA5_HU	19	1.006404
24H 50MTA	P38159	0.254136	0.003266	0.005099	RBMX	RNA-bindir	RBMX_HUM	12	1.192621
24H 50MTA	Q15942	-0.44622	0.00327	0.005105	ZYX	Zyxin	ZYX_HUMA	18	0.733961
24H 50MTA	P30533	0.155305	0.003272	0.005105	LRPAP1	Alpha-2-ma	AMRP_HUM	14	1.113657
24H 50MTA	Q96MB7	1.823765	0.003272	0.005106	HARBI1	Putative nu	HARB1_HU	1	3.540039
48H 50MTA	P28074	0.018066	0.003273	0.005107	PSMB5	Proteasom	PSB5_HUM	15	1.012601
48H 50MTA	Q5NDL2	-0.38475	0.003273	0.005107	EOGT	EGF domair	EOGT_HUM	8	0.765912
48H 10MTA	O75486	0.680689	0.003277	0.005111	SUPT3H	Transcripti	SUPT3_HUI	2	1.602906
48H 50MTA	Q92538	0.036562	0.003278	0.005112	GBF1	Golgi-speci	GBF1_HUM	70	1.025667
24H 50MTA	P46939	0.018703	0.003281	0.005115	UTRN	Utrophin	UTRN_HUM	137	1.013048
48H 10MTA	A5PLL7	-0.36347	0.003282	0.005116	PEDS1	Plasmanyle	PDES1_HUI	4	0.777291
24H 50MTA	Q13190	0.052773	0.003282	0.005116	STX5	Syntaxin-5	STX5_HUM	13	1.037256
48H 50MTA	Q86UX2	-0.76369	0.003283	0.005116	ITIH5	Inter-alpha	ITIH5_HUM	1	0.588987
24H 50MTA	O60524	0.121091	0.003287	0.00512	NEMF	Ribosome c	NEMF_HUM	19	1.087557
24H 50MTA	Q9NQT4	0.229282	0.003291	0.005125	EXOSC5	Exosome cc	EXOS5_HUI	7	1.172251
48H 50MTA	Q9H2W6	-0.1867	0.003295	0.00513	MRPL46	Large ribos	RM46_HUM	11	0.878616
48H 10MTA	P83881;Q9	0.128469	0.003298	0.005132	RPL36A;RP	Large ribos	RL36A_HUI	4	1.093133
24H 50MTA	O15228	0.108315	0.003299	0.005132	GNPAT	Dihydroxya	GNPAT_HU	26	1.077968
48H 50MTA	P05161	-0.00609	0.0033	0.005134	ISG15	Ubiquitin-l	ISG15_HUM	5	0.995787
48H 10MTA	Q8N3F8	0.060518	0.003301	0.005134	MICALL1	MICAL-like	MILK1_HUI	4	1.04284
24H 10MTA	Q9Y305	0.077823	0.003303	0.005137	ACOT9	Acyl-coenzy	ACOT9_HUI	20	1.055424
48H 10MTA	P00918	-0.19388	0.003307	0.005141	CA2	Carbonic ar	CAH2_HUM	16	0.874252
48H 10MTA	Q15526	0.130379	0.003309	0.005144	SURF1	Surfeit locu	SURF1_HUI	7	1.094581
24H 50MTA	Q75QN2	0.16899	0.003316	0.005152	INTS8	Integrator c	INT8_HUMA	15	1.124271
48H 50MTA	Q99567	-0.0366	0.003317	0.005154	NUP88	Nuclear poi	NUP88_HU	20	0.974947
48H 10MTA	P24539	-0.12567	0.003319	0.005155	ATP5PB	ATP synthas	AT5F1_HUM	11	0.916581
24H 50MTA	P61923	-0.52847	0.003324	0.005162	COPZ1	Coatomer s	COPZ1_HUI	6	0.693292
24H 50MTA	Q8WUF5	-0.03611	0.003324	0.005162	PPP1R13L	RelA-associ	IASPP_HUM	20	0.975278
48H 10MTA	O00154	0.097826	0.003326	0.005164	ACOT7	Cytosolic ac	BACH_HUM	13	1.07016
24H 50MTA	O60907	0.123731	0.003329	0.005167	TBL1X	F-box-like/V	TBL1X_HUM	13	1.089549
48H 10MTA	Q9NRN7	0.03327	0.00333	0.005168	AASDHPPT	L-aminoadi	ADPPT_HUI	11	1.023329
24H 50MTA	P33993	0.023142	0.003337	0.005177	MCM7	DNA replica	MCM7_HUI	38	1.01617
48H 50MTA	Q05397	0.150343	0.003339	0.005178	PTK2	Focal adhes	FAK1_HUM	20	1.109833
24H 50MTA	O75529	-2.32911	0.00334	0.005179	TAF5L	TAF5-like RI	TAF5L_HUM	1	0.199007
24H 50MTA	Q9NY27	0.112545	0.003344	0.005183	PPP4R2	Serine/thre	PP4R2_HUI	14	1.081134
24H 50MTA	O00308	0.65468	0.003351	0.005191	WWP2	NEDD4-like	WWP2_HU	5	1.574267
24H 50MTA	Q9H900	-0.27506	0.003353	0.005193	ZWILCH	Protein zwi	ZWILC_HUI	13	0.826418
48H 50MTA	Q562E7	0.067878	0.003358	0.005198	WDR81	WD repeat-	WDR81_HU	24	1.048174
48H 10MTA	Q9UI09	0.107688	0.00336	0.005199	NDUFA12	NADH dehy	NDUAC_HU	9	1.0775
24H 50MTA	Q6ZT62	0.236576	0.00336	0.0052	BARGIN	Bargin	BGIN_HUM	3	1.178193
24H 50MTA	Q96ME7	0.211015	0.00336	0.0052	ZNF512	Zinc finger	ZN512_HUI	12	1.157502
48H 50MTA	P19525	0.065451	0.003361	0.0052	EIF2AK2	Interferon-i	E2AK2_HUI	18	1.046412
48H 10MTA	Q99439	0.115619	0.003363	0.005202	CNN2	Calponin-2	CNN2_HUM	14	1.08344
48H 50MTA	Q96AY3	0.191348	0.003368	0.005209	FKBP10	Peptidyl-pr	FKB10_HUI	27	1.14183
48H 50MTA	Q15046	0.014166	0.003375	0.005216	KARS1	Lysine--tRN	SYK_HUMA	29	1.009867
24H 50MTA	O43290	0.159694	0.003376	0.005217	SART1	U4/U6.U5 t	SNUT1_HUI	30	1.117051
48H 10MTA	Q9Y5J1	-0.17749	0.003378	0.00522	UTP18	U3 small nu	UTP18_HUI	19	0.884243

48H 10MT/	Q3YEC7	0.095642	0.003379	0.005221	RABL6	Rab-like pr	RABL6_HU	18	1.068541
24H 50MT/	P06576	0.07658	0.003383	0.005225	ATP5F1B	ATP synthas	ATPB_HUM	28	1.054516
48H 10MT/	Q3ZCM7	-0.50985	0.003385	0.005228	TUBB8	Tubulin bet	TBB8_HUM	3	0.702295
24H 50MT/	Q9H2F3	0.440173	0.003388	0.005232	HSD3B7	3 beta-hydr	3BHS7_HU	9	1.356767
24H 10MT/	P14543	0.260327	0.003393	0.005237	NID1	Nidogen-1	NID1_HUM	17	1.19775
48H 50MT/	P60510	0.128536	0.003392	0.005237	PPP4C	Serine/thre	PP4C_HUM	13	1.093184
48H 50MT/	Q9HCN4	0.157127	0.003393	0.005237	GPN1	GPN-loop G	GPN1_HUM	4	1.115065
48H 10MT/	Q9NWS0	0.052159	0.003394	0.005238	PIH1D1	PIH1 doma	PIHD1_HU	9	1.036815
24H 10MT/	O75648	0.108908	0.003394	0.005238	TRMU	Mitochond	MTU1_HUM	13	1.078412
24H 10MT/	Q75QN2	0.140791	0.003396	0.005241	INTS8	Integrator	INT8_HUM	15	1.102509
48H 10MT/	P55036	0.044612	0.003397	0.005241	PSMD4	26S protea	PSMD4_HU	14	1.031406
48H 50MT/	Q9HD15	0.109151	0.003402	0.005246	SRA1	Steroid rec	SRA1_HUM	9	1.078593
48H 50MT/	Q9NZW5	0.04935	0.003406	0.005251	PALS2	Protein PAI	PALS2_HU	24	1.034798
48H 50MT/	Q8NCW5	0.016947	0.003408	0.005253	NAXE	NAD(P)H-hy	NNRE_HUM	10	1.011816
48H 50MT/	Q9Y6A4	0.090994	0.003408	0.005253	CFAP20	Cilia- and fl	CFA20_HU	11	1.065104
48H 10MT/	Q9Y2U8	0.071795	0.003409	0.005254	LEMD3	Inner nucle	MAN1_HUM	25	1.051024
24H 50MT/	Q8N6L1	0.411368	0.00341	0.005254	KRTCAP2	Keratinocyt	KTAP2_HU	1	1.329946
48H 50MT/	A4D1P6	0.151268	0.00341	0.005255	WDR91	WD repeat-	WDR91_HU	20	1.110545
24H 50MT/	Q12907	0.131104	0.003413	0.005258	LMAN2	Vesicular in	LMAN2_HU	16	1.095131
48H 50MT/	P62834	0.363501	0.003415	0.005261	RAP1A	Ras-related	RAP1A_HU	3	1.286544
48H 50MT/	P56556	-0.17146	0.003423	0.00527	NDUFA6	NADH dehy	NDUA6_HU	6	0.887944
24H 10MT/	Q5SXM8	0.194346	0.003423	0.00527	DNLZ	DNL-type zi	DNLZ_HUM	1	1.144205
24H 50MT/	P41567	-0.31818	0.003429	0.005276	EIF1	Eukaryotic	EIF1_HUM/	10	0.802083
48H 50MT/	P48059	0.101258	0.003432	0.005279	LIMS1	LIM and ser	LIMS1_HUI	12	1.072708
24H 50MT/	Q9H8Y5	0.134333	0.003436	0.005283	ANKZF1	tRNA endor	ANKZ1_HU	12	1.097585
48H 50MT/	Q96D71	-0.01234	0.003436	0.005283	REPS1	RalBP1-ass	REPS1_HUI	11	0.991481
48H 10MT/	P27540	0.099071	0.003438	0.005285	ARNT	Aryl hydroc	ARNT_HUM	6	1.071083
24H 50MT/	P52735	-0.08474	0.003438	0.005285	VAV2	Guanine nu	VAV2_HUM	25	0.942953
24H 50MT/	Q15773	0.426306	0.003438	0.005285	MLF2	Myeloid leu	MLF2_HUM	6	1.343788
24H 10MT/	P29144	-0.09109	0.00344	0.005286	TPP2	Tripeptidyl	TPP2_HUM	50	0.938816
48H 50MT/	Q9H9B4	0.10878	0.003444	0.005291	SFXN1	Sideroflexir	SFXN1_HU	12	1.078316
48H 10MT/	Q9NYZ3	2.04448	0.003449	0.005298	GTSE1	G2 and S pl	GTSE1_HUI	2	4.125245
24H 50MT/	Q9NWB6	0.882275	0.00346	0.005311	ARGLU1	Arginine an	ARGL1_HU	2	1.84328
24H 50MT/	Q9Y666	0.064661	0.003461	0.005312	SLC12A7	Solute carri	S12A7_HU	17	1.045839
48H 50MT/	Q9NS91	-0.27688	0.003462	0.005313	RAD18	E3 ubiquiti	RAD18_HU	15	0.825373
24H 50MT/	Q9BTX1	0.059543	0.003471	0.005324	NDC1	Nucleopori	NDC1_HUM	17	1.042135
48H 50MT/	Q9UEU0	0.114931	0.003474	0.005327	VTI1B	Vesicle trar	VTI1B_HUM	8	1.082923
24H 50MT/	Q5TAX3	0.203515	0.00348	0.005335	TUT4	Terminal ur	TUT4_HUM	9	1.151501
48H 50MT/	Q86TS9	-0.59762	0.003491	0.005348	MRPL52	Large ribos	RM52_HUM	2	0.660842
48H 50MT/	Q9H0W9	0.190093	0.003493	0.00535	C11orf54	Ester hydro	CK054_HU	13	1.140837
48H 50MT/	Q9P0V9	0.166359	0.003493	0.00535	SEPTIN10	Septin-10	SEP10_HUI	15	1.122223
24H 50MT/	Q96E11	0.334499	0.003493	0.00535	MRRF	Ribosome-r	RRFM_HUM	8	1.26094
48H 50MT/	O75915	0.172694	0.003494	0.00535	ARL6IP5	PRA1 family	PRAF3_HUI	6	1.127162
48H 50MT/	Q96ST3	-0.02937	0.003498	0.005356	SIN3A	Paired amp	SIN3A_HUM	31	0.979846
48H 50MT/	Q96CP7	-0.5758	0.003499	0.005357	TLCD1	TLC domair	TLCD1_HUI	4	0.670914
24H 50MT/	P61769	-0.0164	0.0035	0.005357	B2M	Beta-2-mic	B2MG_HUM	3	0.988694
24H 50MT/	P98160	0.898629	0.003508	0.005366	HSPG2	Basement r	PGBM_HUM	19	1.864293
24H 50MT/	Q7Z2T5	0.154078	0.003509	0.005367	TRMT1L	TRMT1-like	TRM1L_HU	27	1.11271
48H 50MT/	Q9H6S1	0.380434	0.003522	0.005381	AZI2	5-azacytidi	AZI2_HUM/	5	1.301734
24H 50MT/	P09972	0.442882	0.003522	0.005381	ALDOC	Fructose-bi	ALDOC_HU	8	1.359317
48H 50MT/	A6NNA2	2.809529	0.003535	0.005396	SRRM3	Serine/argi	SRRM3_HU	1	7.010558
48H 50MT/	P30530	0.676326	0.003542	0.005402	AXL	Tyrosine-pr	UFO_HUM/	8	1.598065

48H	50MT/	P27816	0.159584	0.003542	0.005402	MAP4	Microtubul	MAP4_HUN	41	1.116965
24H	50MT/	Q9BVK6	0.154688	0.003545	0.005406	TMED9	Transmemt	TMED9_HU	10	1.113181
48H	50MT/	Q96BW9	0.034302	0.003546	0.005406	TAMM41	Phosphatic	TAM41_HU	6	1.024061
24H	10MT/	Q96MM7	0.106624	0.003546	0.005406	HS6ST2	Heparan-su	H6ST2_HUI	3	1.076706
48H	50MT/	Q15382	-0.1379	0.003548	0.005409	RHEB	GTP-bindin	RHEB_HUM	7	0.908838
24H	10MT/	Q9NSI2	-0.06822	0.003549	0.00541	SLX9	Ribosome k	SLX9_HUM	7	0.953814
24H	50MT/	P14854	0.226219	0.003552	0.005412	COX6B1	Cytochrom	CX6B1_HUI	4	1.169766
24H	10MT/	Q6P4A8	0.046723	0.003554	0.005415	PLBD1	Phospholip	PLBL1_HUN	10	1.032916
24H	50MT/	Q9NRW4	-0.35332	0.003555	0.005416	DUSP22	Dual specifi	DUS22_HU	2	0.782783
48H	10MT/	O00303	-0.00037	0.003556	0.005417	EIF3F	Eukaryotic	EIF3F_HUM	11	0.999744
48H	10MT/	P63244	0.09495	0.003557	0.005417	RACK1	Small ribos	RACK1_HUI	20	1.068028
48H	10MT/	Q7Z7H5	0.203613	0.00356	0.00542	TMED4	Transmemt	TMED4_HU	8	1.151579
48H	50MT/	Q9Y6I3	0.093461	0.003559	0.00542	EPN1	Epsin-1	EPN1_HUM	10	1.066927
48H	10MT/	Q5VWQ0	-0.07867	0.003561	0.005421	RSBN1	Lysine-spec	RSBN1_HUI	13	0.946932
24H	50MT/	Q8NE01	-0.17376	0.003562	0.005423	CNNM3	Metal trans	CNNM3_HU	19	0.886528
48H	10MT/	Q5TA50	-0.44688	0.003565	0.005426	CPTP	Ceramide-1	CPTP_HUM	1	0.733628
24H	50MT/	Q9NVZ3	-0.25239	0.003569	0.00543	NECAP2	Adaptin ear	NECP2_HUI	7	0.839506
24H	10MT/	Q16836	-0.0992	0.00357	0.00543	HADH	Hydroxyacy	HCDH_HUN	10	0.933548
48H	10MT/	P57076	0.17401	0.003572	0.005432	CFAP298	Cilia- and fl	CF298_HUI	8	1.12819
48H	50MT/	Q9UI14	0.25636	0.003576	0.005436	RABAC1	Prenylated	PRAF1_HUI	4	1.194462
48H	50MT/	Q14573	0.185949	0.003578	0.005438	ITPR3	Inositol 1,4	ITPR3_HUN	23	1.137565
48H	50MT/	P06280	-0.27591	0.003579	0.005439	GLA	Alpha-galac	AGAL_HUM	13	0.82593
48H	50MT/	Q8NHU6	0.191644	0.003581	0.005441	TDRD7	Tudor dom	TDRD7_HU	7	1.142065
24H	50MT/	P54709	0.118806	0.003582	0.005443	ATP1B3	Sodium/po	AT1B3_HUI	12	1.085836
24H	10MT/	O75534	0.051471	0.003584	0.005445	CSDE1	Cold shock	CSDE1_HUI	44	1.036321
24H	50MT/	P60900	-0.07434	0.003593	0.005454	PSMA6	Proteasom	PSA6_HUM	14	0.949776
48H	50MT/	P11717	0.043295	0.003597	0.005458	IGF2R	Cation-inde	MPRI_HUM	85	1.030465
48H	10MT/	Q9H9C1	0.069841	0.003601	0.005462	VIPAS39	Spermatog	SPE39_HUN	14	1.049601
48H	10MT/	Q15050	0.004301	0.003606	0.005468	RRS1	Ribosome k	RRS1_HUM	16	1.002986
24H	10MT/	Q14155	-0.02109	0.003609	0.005472	ARHGEF7	Rho guanin	ARHG7_HU	8	0.985485
48H	50MT/	P23193	0.095259	0.00361	0.005472	TCEA1	Transcripti	TCEA1_HUN	17	1.068257
48H	50MT/	Q14116	0.244894	0.00361	0.005472	IL18	Interleukin	IL18_HUM/	10	1.185006
48H	50MT/	P08069	0.111755	0.00361	0.005472	IGF1R	Insulin-like	IGF1R_HUN	16	1.080542
48H	10MT/	Q15126	0.015562	0.003611	0.005473	PMVK	Phosphom	PMVK_HUN	11	1.010845
24H	10MT/	O60739	-0.61073	0.003611	0.005474	EIF1B	Eukaryotic	EIF1B_HUM	1	0.654864
48H	50MT/	Q8IV50	-3.18321	0.003617	0.005481	LYSMD2	LysM and p	LYSM2_HUI	1	0.110092
48H	50MT/	P62316	0.05972	0.003618	0.005482	SNRPD2	Small nucle	SMD2_HUN	8	1.042264
48H	50MT/	P04075	0.334845	0.003619	0.005484	ALDOA	Fructose-bi	ALDOA_HU	29	1.261242
24H	50MT/	Q96BR5	0.140827	0.003624	0.00549	COA7	Cytochrom	COA7_HUM	5	1.102537
24H	50MT/	Q9HDC9	0.125349	0.003624	0.00549	APMAP	Adipocyte p	APMAP_HU	21	1.090771
48H	10MT/	Q9H0V1	0.198293	0.003627	0.005493	TMEM168	Transmemt	TM168_HU	3	1.14734
24H	50MT/	P49641	0.061765	0.003628	0.005495	MAN2A2	Alpha-mani	MA2A2_HU	10	1.043742
48H	50MT/	Q9NUQ2	0.175518	0.003629	0.005495	AGPAT5	1-acyl-sn-gl	PLCE_HUM	12	1.12937
48H	50MT/	Q9BQB6	0.364383	0.003636	0.0055	VKORC1	Vitamin K e	VKOR1_HU	3	1.287331
24H	50MT/	Q8WWC4	0.368607	0.003637	0.005501	MAIP1	m-AAA prot	MAIP1_HUI	7	1.291105
24H	50MT/	O75643	0.114215	0.00364	0.005504	SNRNP200	U5 small nu	U520_HUM	116	1.082386
48H	50MT/	Q8IY50	0.661504	0.003641	0.005504	SLC35F3	Solute carri	S35F3_HUN	1	1.581731
24H	50MT/	P05452	-1.78561	0.003644	0.005508	CLEC3B	Tetranectir	TETN_HUM	3	0.290053
48H	10MT/	P57737	0.019411	0.00365	0.005515	CORO7	Coronin-7	CORO7_HU	23	1.013545
24H	50MT/	P48200	0.123295	0.003651	0.005516	IREB2	Iron-respor	IREB2_HUN	27	1.08922
48H	50MT/	Q16822	0.287035	0.003654	0.005518	PCK2	Phosphoen	PCKGM_HU	30	1.22013
48H	50MT/	P48200	0.095787	0.003654	0.005518	IREB2	Iron-respor	IREB2_HUN	27	1.068648

48H 10MTA	P46108	0.041786	0.003655	0.005519	CRK	Adapter mc	CRK_HUMA	13	1.029387
24H 50MTA	Q9Y237	-0.21246	0.003657	0.005522	PIN4	Peptidyl-pr	PIN4_HUM	6	0.863063
24H 50MTA	Q86W50	0.288737	0.003662	0.005526	METTL16	RNA N6-ade	MET16_HU	11	1.22157
48H 10MTA	Q00534	0.096696	0.003667	0.005532	CDK6	Cyclin-depe	CDK6_HUM	12	1.069322
48H 10MTA	P26038	0.090042	0.003684	0.005553	MSN	Moesin	MOES_HUM	27	1.064401
48H 10MTA	Q14696	0.054474	0.003685	0.005553	MESD	LRP chaper	MESD_HUM	12	1.038481
24H 10MTA	Q86YS6	-0.20998	0.003685	0.005553	RAB43	Ras-related	RAB43_HU	8	0.864551
48H 10MTA	P55265	0.014702	0.003687	0.005555	ADAR	Double-stra	DSRAD_HU	33	1.010243
24H 50MTA	Q9P0U4	0.054568	0.003688	0.005555	CXXC1	CXXC-type	CXXC1_HU	12	1.038548
48H 50MTA	Q9HCU9	-0.24397	0.003694	0.005561	BRMS1	Breast canc	BRMS1_HU	4	0.844417
48H 10MTA	Q7Z6Z7	0.036216	0.003695	0.005563	HUWE1	E3 ubiquiti	HUWE1_HU	111	1.025421
48H 50MTA	Q9H0V1	0.279328	0.003695	0.005563	TMEM168	Transmemb	TM168_HU	3	1.21363
24H 50MTA	Q8TBQ9	0.095966	0.003696	0.005564	TMEM167A	Protein kish	KISHA_HUM	3	1.06878
48H 50MTA	Q15173	-0.15704	0.0037	0.005568	PPP2R5B	Serine/thre	2A5B_HUM	2	0.896861
48H 10MTA	O14562	0.112311	0.0037	0.005568	UBFD1	Ubiquitin d	UBFD1_HU	7	1.080959
48H 50MTA	Q96IW7	0.520683	0.003703	0.005571	SEC22A	Vesicle-traf	SC22A_HU	4	1.434634
48H 50MTA	Q9BQT8	0.068697	0.003703	0.005571	SLC25A21	Mitochond	ODC_HUMA	7	1.048769
48H 50MTA	P34741	1.019429	0.003707	0.005576	SDC2	Syndecan-2	SDC2_HUM	2	2.027117
48H 50MTA	P06730	0.079965	0.00371	0.005579	EIF4E	Eukaryotic	IF4E_HUMA	9	1.056993
48H 50MTA	Q00613	0.013318	0.003711	0.005581	HSF1	Heat shock	HSF1_HUM	3	1.009274
48H 50MTA	Q92544	0.113476	0.003712	0.005581	TM9SF4	Transmemb	TM9S4_HU	17	1.081832
24H 50MTA	P53999	-0.13335	0.003718	0.005588	SUB1	Activated R	TCP4_HUM	8	0.911709
48H 50MTA	P56270	-0.28862	0.003725	0.005596	MAZ	Myc-associ	MAZ_HUMA	6	0.818683
48H 50MTA	P62495	-0.10017	0.003726	0.005597	ETF1	Eukaryotic	ERF1_HUM	20	0.932921
48H 50MTA	O43242	0.053599	0.003731	0.005603	PSMD3	26S protea	PSMD3_HU	31	1.03785
48H 10MTA	Q9UIA9	0.015733	0.003735	0.005607	XPO7	Exportin-7	XPO7_HUM	37	1.010965
48H 10MTA	P78318	0.069122	0.00374	0.005613	IGBP1	Immunoglo	IGBP1_HUM	15	1.049078
24H 50MTA	Q06481	0.135836	0.003742	0.005615	APLP2	Amyloid be	APLP2_HU	16	1.098729
48H 50MTA	P52788	0.198485	0.003745	0.005618	SMS	Spermine sy	SPSY_HUMA	19	1.147492
48H 50MTA	P15848	-0.23126	0.003747	0.005621	ARSB	Arylsulfatas	ARSB_HUM	9	0.851889
48H 50MTA	P04920	-0.31589	0.003749	0.005623	SLC4A2	Anion exch	B3A2_HUM	21	0.803354
48H 10MTA	Q86X76	0.083814	0.003754	0.005629	NIT1	Deaminate	NIT1_HUMA	11	1.059816
48H 10MTA	Q96B36	0.131205	0.003756	0.005631	AKT1S1	Proline-rich	AKTS1_HUM	4	1.095208
48H 50MTA	Q99549	-0.07492	0.003758	0.005633	MPHOSPH8	M-phase ph	MPP8_HUM	11	0.949396
24H 10MTA	O43795	0.073719	0.003758	0.005633	MYO1B	Unconvent	MYO1B_HU	43	1.052426
48H 10MTA	Q8WV24	0.389874	0.003766	0.005642	PHLDA1	Pleckstrin h	PHLA1_HU	3	1.310279
48H 50MTA	Q8NEY8	-0.32962	0.003767	0.005643	PPHLN1	Periphilin-1	PPHLN_HU	8	0.795747
24H 50MTA	Q5F1R6	-0.26181	0.00377	0.005647	DNAJC21	DnaJ homo	DJC21_HU	11	0.834038
24H 50MTA	P01137	1.522996	0.003771	0.005648	TGFB1	Transformi	TGFB1_HU	2	2.873872
24H 50MTA	Q6NUQ4	0.070862	0.003772	0.00565	TMEM214	Transmemb	TM214_HU	29	1.050344
48H 50MTA	Q14493	-0.52011	0.003775	0.005653	SLBP	Histone RN	SLBP_HUM	4	0.697319
24H 50MTA	Q7L590	-0.1648	0.003776	0.005653	MCM10	Protein MC	MCM10_HU	1	0.892051
48H 10MTA	P14314	0.027803	0.003778	0.005655	PRKCSH	Glucosidas	GLU2B_HU	24	1.019458
48H 50MTA	Q86T03	-0.14502	0.003782	0.005661	PIP4P1	Type 1 phos	PP4P1_HU	5	0.904365
48H 10MTA	P35908	-0.42795	0.003783	0.005661	KRT2	Keratin, typ	K22E_HUM	31	0.743319
24H 10MTA	Q99614	-0.13355	0.003784	0.005662	TTC1	Tetratricop	TTC1_HUM	13	0.911586
24H 50MTA	P19256	0.17921	0.003791	0.005671	CD58	Lymphocyt	LFA3_HUM	2	1.132264
24H 50MTA	Q92925	0.105382	0.003792	0.005672	SMARCD2	SWI/SNF-re	SMRD2_HU	14	1.075779
48H 50MTA	Q4G0X4	-0.07878	0.003793	0.005672	KCTD21	BTB/POZ dc	KCD21_HU	6	0.946855
24H 10MTA	Q16658	0.059091	0.003793	0.005672	FSCN1	Fascin	FSCN1_HU	19	1.041809
48H 50MTA	P00439	-0.18804	0.003794	0.005672	PAH	Phenylalan	PH4H_HUM	30	0.877795
24H 10MTA	Q9NZ63	-0.10297	0.003795	0.005674	C9orf78	Splicing fac	TLS1_HUMA	9	0.931114

24H 50MT/	P15151	0.105881	0.003798	0.005677	PVR	Poliovirus r	PVR_HUMA	6	1.076151
48H 50MT/	Q9NV06	-0.15371	0.003799	0.005678	DCAF13	DDB1- and	DCA13_HU	14	0.898938
48H 10MT/	Q9Y295	0.077082	0.003801	0.00568	DRG1	Developme	DRG1_HUM	23	1.054882
24H 50MT/	P55263	0.114328	0.00381	0.00569	ADK	Adenosine l	ADK_HUMA	23	1.082471
48H 50MT/	Q9NRN7	-0.13027	0.003812	0.005691	AASDHPPT	L-aminoadi	ADPPT_HU	11	0.913658
48H 10MT/	Q8NBZ7	0.033431	0.003813	0.005693	UXS1	UDP-glucur	UXS1_HUM	11	1.023443
48H 50MT/	P17050	0.200865	0.003814	0.005694	NAGA	Alpha-N-ac	NAGAB_HU	10	1.149387
48H 50MT/	O60488	0.109789	0.003816	0.005696	ACSL4	Long-chain	ACSL4_HUM	33	1.079071
24H 50MT/	Q9C0E8	0.280505	0.003817	0.005697	LNPK	Endoplasm	LNP_HUMA	12	1.21462
24H 50MT/	Q96A26	0.128825	0.003819	0.005698	FAM162A	Protein FAM	F162A_HUI	6	1.093403
24H 10MT/	O14647	0.22653	0.003821	0.005701	CHD2	Chromodor	CHD2_HUM	14	1.170017
24H 50MT/	P22102	-0.10142	0.003822	0.005701	GART	Trifunction	PUR2_HUM	42	0.932118
24H 10MT/	Q15118	0.000781	0.003824	0.005703	PDK1	[Pyruvate d	PDK1_HUM	8	1.000541
48H 10MT/	Q02241	0.146812	0.003832	0.00571	KIF23	Kinesin-like	KIF23_HUM	17	1.10712
24H 50MT/	Q9H6U8	0.253472	0.003839	0.005719	ALG9	Alpha-1,2-r	ALG9_HUM	7	1.192073
24H 50MT/	O95182	0.174422	0.003842	0.005723	NDUFA7	NADH dehy	NDUA7_HU	6	1.128512
48H 50MT/	O15037	0.249776	0.003843	0.005723	KHNYN	Protein KH	KHNYN_HU	4	1.189022
48H 50MT/	O00231	0.009898	0.003844	0.005724	PSMD11	26S protea	PSD11_HUI	29	1.006884
24H 50MT/	Q14032	0.525186	0.003845	0.005725	BAAT	Bile acid-Co	BAAT_HUM	3	1.439119
24H 50MT/	O75420	-1.16056	0.00385	0.005731	GIGYF1	GRB10-inte	GGYF1_HUI	2	0.447338
24H 50MT/	Q9NR45	0.023743	0.003851	0.005732	NANS	Sialic acid s	SIAS_HUMA	16	1.016594
24H 50MT/	Q5T6V5	0.031726	0.00386	0.00574	QNG1	Queuosine	QNG1_HUM	15	1.022234
48H 10MT/	P01008	0.045609	0.003863	0.005745	SERPINC1	Antithrombi	ANT3_HUM	6	1.032119
48H 10MT/	P35659	0.026605	0.00387	0.005753	DEK	Protein DEI	DEK_HUMA	16	1.018612
24H 10MT/	P53680	0.095016	0.003872	0.005755	AP2S1	AP-2 compl	AP2S1_HUI	7	1.068077
48H 50MT/	Q02218	0.041073	0.003873	0.005755	OGDH	2-oxoglutar	ODO1_HUM	41	1.028878
24H 50MT/	Q9UK61	0.072183	0.003873	0.005755	TASOR	Protein TAS	TASOR_HUI	40	1.051306
24H 50MT/	Q8WVE0	-0.26679	0.003875	0.005756	EEF1AKMT1	EEF1A lysin	EFMT1_HUI	1	0.831165
48H 50MT/	P28070	0.047144	0.003879	0.005761	PSMB4	Proteasom	PSB4_HUM	10	1.033217
24H 50MT/	Q96MM7	0.074158	0.003884	0.005765	HS6ST2	Heparan-su	H6ST2_HUI	3	1.052747
24H 10MT/	Q8NCM8	0.023637	0.003884	0.005766	DYNC2H1	Cytoplasmi	DYHC2_HU	33	1.016519
48H 50MT/	Q8WTT2	-0.2443	0.003894	0.005776	NOC3L	Nucleolar c	NOC3L_HU	20	0.844223
48H 50MT/	P36405	0.078419	0.003899	0.00578	ARL3	ADP-ribosy	ARL3_HUM	11	1.05586
48H 50MT/	Q96JN8	0.33204	0.003902	0.005784	NEURL4	Neuralized-	NEUL4_HUI	2	1.258792
24H 50MT/	P35249	-0.098	0.003905	0.005786	RFC4	Replication	RFC4_HUM	18	0.934328
48H 50MT/	P78560	0.347455	0.003911	0.005793	CRADD	Death dom	CRADD_HU	6	1.272314
48H 50MT/	Q06033	0.086729	0.003915	0.005798	ITIH3	Inter-alpha	ITIH3_HUM	4	1.061959
48H 50MT/	Q9HAV0	0.176216	0.003916	0.005798	GNB4	Guanine nu	GBB4_HUM	6	1.129916
24H 10MT/	Q9NTX5	-0.08067	0.003919	0.0058	ECHDC1	Ethylmalon	ECHD1_HU	14	0.94562
48H 10MT/	O75940	0.021535	0.003926	0.005807	SMNDC1	Survival of	SPF30_HUM	6	1.015039
24H 50MT/	P53680	0.107457	0.003935	0.005815	AP2S1	AP-2 compl	AP2S1_HUI	7	1.077328
48H 10MT/	Q9UNF1	0.034664	0.003935	0.005815	MAGED2	Melanoma-	MAGD2_HU	21	1.024318
48H 50MT/	P49757	0.177215	0.00394	0.005821	NUMB	Protein nur	NUMB_HUI	11	1.130699
24H 50MT/	P61081	0.244259	0.003945	0.005826	UBE2M	NEDD8-cor	UBC12_HU	9	1.184484
24H 10MT/	Q66K14	-0.07234	0.003946	0.005827	TBC1D9B	TBC1 doma	TBC9B_HUI	26	0.951095
48H 10MT/	Q86VS8	0.039342	0.003946	0.005827	HOOK3	Protein Ho	HOOK3_HU	21	1.027645
48H 50MT/	Q9H0X4	0.442152	0.00395	0.005832	FAM234A	Protein FAM	F234A_HUI	6	1.358629
48H 10MT/	P61326	0.560496	0.00395	0.005832	MAGOH	Protein ma	MGN_HUM	1	1.474776
24H 10MT/	P09543	0.056837	0.003954	0.005836	CNP	2',3'-cyclic-	CN37_HUM	20	1.040182
48H 50MT/	Q12769	-0.10393	0.003957	0.005839	NUP160	Nuclear po	NU160_HU	41	0.930497
24H 10MT/	P33991	0.04399	0.00396	0.005843	MCM4	DNA replica	MCM4_HUI	45	1.030962
48H 50MT/	Q9BXW7	0.186233	0.003961	0.005844	HDHD5	Haloacid de	HDHD5_HU	16	1.137789

24H 50MT/	Q9H019	0.236577	0.003964	0.005847	MTFR1L	Mitochond	MFR1L_HU	4	1.178194
48H 10MT/	Q9HD15	0.057169	0.003971	0.005855	SRA1	Steroid rec	SRA1_HUM	9	1.040422
48H 50MT/	P29279	0.456434	0.003978	0.005863	CCN2	CCN family	CCN2_HUM	5	1.372146
24H 50MT/	Q9NQX3	0.148204	0.003988	0.005874	GPHN	Gephyrin	GEPH_HUM	22	1.108189
48H 10MT/	Q8NB16	0.159099	0.00399	0.005876	MLKL	Mixed linea	MLKL_HUM	11	1.11659
48H 10MT/	Q9NR81	-0.799	0.003991	0.005876	ARHGEF3	Rho guanin	ARHG3_HU	1	0.574746
24H 50MT/	P82914	0.10652	0.003995	0.005879	MRPS15	Small ribos	RT15_HUM	7	1.076628
24H 10MT/	Q13247	0.087513	0.003996	0.00588	SRSF6	Serine/argi	SRSF6_HUM	9	1.062537
24H 10MT/	P57740	0.070328	0.003997	0.00588	NUP107	Nuclear po	NU107_HU	41	1.049956
48H 10MT/	P62979	0.108156	0.003998	0.005881	RPS27A	Ubiquitin-r	RS27A_HUI	8	1.077849
24H 50MT/	Q9NQS7	-0.21418	0.003998	0.005882	INCENP	Inner centr	INCE_HUM/	12	0.862036
24H 50MT/	Q5T8P6	0.035747	0.004005	0.00589	RBM26	RNA-bindir	RBM26_HU	15	1.025087
48H 50MT/	P05543	-0.41419	0.004011	0.005894	SERPINA7	Thyroxine-l	THBG_HUM	3	0.75044
24H 50MT/	Q8NEY8	0.142908	0.004012	0.005895	PPHLN1	Periphilin-:	PPHLN_HU	8	1.104128
24H 50MT/	Q15024	0.09119	0.004015	0.005899	EXOSC7	Exosome cc	EXOS7_HUI	10	1.065248
24H 10MT/	P46778	-0.18214	0.004019	0.005903	RPL21	Large ribos	RL21_HUM	8	0.881395
24H 10MT/	Q9H4H8	-0.00684	0.004019	0.005904	FAM83D	Protein FAI	FA83D_HUI	12	0.995271
24H 10MT/	Q96M27	-0.06736	0.004025	0.005908	PRRC1	Protein PRI	PRRC1_HUI	10	0.954382
48H 10MT/	P52732	0.09089	0.004026	0.00591	KIF11	Kinesin-like	KIF11_HUM	30	1.065027
48H 10MT/	P22314	0.045099	0.004028	0.005911	UBA1	Ubiquitin-l	UBA1_HUM	46	1.031754
48H 10MT/	P01042	0.794028	0.004031	0.005915	KNG1	Kininogen-:	KNG1_HUM	1	1.733909
24H 10MT/	P62995	0.135333	0.004033	0.005917	TRA2B	Transforme	TRA2B_HUI	6	1.098346
24H 10MT/	Q14684	0.011711	0.004034	0.005919	RRP1B	Ribosomal	RRP1B_HUI	30	1.00815
48H 50MT/	Q96I51	-0.23515	0.004036	0.005921	RCC1L	RCC1-like G	RCC1L_HUI	13	0.849598
48H 10MT/	Q8IWS0	0.176191	0.004036	0.005921	PHF6	PHD finger	PHF6_HUM	9	1.129897
48H 50MT/	Q14103	0.109766	0.004037	0.005921	HNRNPD	Heterogene	HNRPD_HU	15	1.079053
24H 50MT/	P45974	0.021253	0.00404	0.005925	USP5	Ubiquitin c	UBP5_HUM	35	1.01484
24H 50MT/	P09012	0.053433	0.004043	0.005927	SNRPA	U1 small nu	SNRPA_HUI	10	1.037731
48H 10MT/	P22234	0.073895	0.004045	0.005929	PAICS	Bifunctiona	PUR6_HUM	23	1.052555
48H 50MT/	P19256	0.11046	0.004047	0.005931	CD58	Lymphocyt	LFA3_HUM	2	1.079573
48H 50MT/	O00124	-0.14661	0.004048	0.005932	UBXN8	UBX domai	UBXN8_HU	4	0.903372
24H 50MT/	Q9BX10	0.450728	0.004056	0.005941	GTPBP2	GTP-bindin	GTPB2_HUI	4	1.36673
24H 50MT/	P13196	0.090098	0.004065	0.005951	ALAS1	5-aminolev	HEM1_HUM	7	1.064442
48H 50MT/	O95456	-0.16998	0.00407	0.005956	PSMG1	Proteasom	PSMG1_HU	13	0.888853
48H 50MT/	P27707	-0.13029	0.004081	0.00597	DCK	Deoxycytid	DCK_HUM/	11	0.913651
24H 50MT/	Q96CW1	0.056139	0.004083	0.005973	AP2M1	AP-2 compl	AP2M1_HU	22	1.03968
48H 10MT/	O95406	1.058449	0.004084	0.005974	CNIH1	Protein cor	CNIH1_HUI	1	2.082692
24H 10MT/	Q8N7X0	-0.35664	0.004085	0.005974	ADGB	Androglobi	ADGB_HUM	1	0.780984
48H 50MT/	P20290	-0.22244	0.004085	0.005975	BTF3	Transcripti	BTF3_HUM	11	0.857117
24H 50MT/	P35658	0.019127	0.004087	0.005977	NUP214	Nuclear po	NU214_HU	48	1.013346
24H 10MT/	Q9Y617	0.023182	0.004095	0.005985	PSAT1	Phosphose	SERC_HUM	20	1.016198
48H 50MT/	P04424	0.132168	0.004098	0.005988	ASL	Argininosu	ARLY_HUM	13	1.095939
24H 10MT/	Q9NPJ6	0.022914	0.004098	0.005988	MED4	Mediator o	MED4_HUM	9	1.01601
24H 10MT/	P52292	0.066651	0.0041	0.00599	KPNA2	Importin su	IMA1_HUM	24	1.047282
24H 10MT/	P07108	-0.42503	0.004105	0.005995	DBI	Acyl-CoA-bi	ACBP_HUM	4	0.744821
48H 10MT/	P16070	-0.99283	0.004108	0.005999	CD44	CD44 antig	CD44_HUM	1	0.50249
24H 50MT/	Q9UPS6	2.707589	0.004112	0.006003	SETD1B	Histone-lys	SET1B_HUM	3	6.53229
48H 10MT/	Q9C0C9	0.00877	0.004113	0.006004	UBE2O	(E3-indeper	UBE2O_HU	30	1.006098
48H 10MT/	O43837	0.022774	0.004114	0.006005	IDH3B	Isocitrate d	IDH3B_HUI	17	1.015911
24H 50MT/	P60510	0.010942	0.004115	0.006006	PPP4C	Serine/thre	PP4C_HUM	13	1.007614
48H 50MT/	Q9BQ70	-0.31082	0.00412	0.006011	TCF25	Ribosome c	TCF25_HUM	16	0.806181
24H 50MT/	P67936	-0.20682	0.004121	0.006012	TPM4	Tropomyos	TPM4_HUM	11	0.866448

24H 50MT/	Q07020	0.161282	0.004123	0.006015	RPL18	Large ribos	RL18_HUM	7	1.11828
24H 50MT/	Q8IUH3	0.025406	0.004126	0.006017	RBM45	RNA-bindir	RBM45_HU	11	1.017766
24H 50MT/	Q9H840	-0.58909	0.00413	0.006021	GEMIN7	Gem-associ	GEMI7_HUI	1	0.664761
48H 50MT/	Q9NQX7	0.258499	0.004143	0.006036	ITM2C	Integral me	ITM2C_HUI	5	1.196234
24H 50MT/	Q2Q1W2	0.039946	0.004146	0.006039	TRIM71	E3 ubiquiti	LIN41_HUN	16	1.028075
48H 50MT/	Q9NPI0	-0.54296	0.00415	0.006044	TMEM138	Transmemb	TM138_HU	1	0.686359
48H 50MT/	P46940	0.074881	0.004153	0.006047	IQGAP1	Ras GTPase-	IQGA1_HUI	83	1.053274
48H 50MT/	O95486	0.025202	0.004155	0.006049	SEC24A	Protein tra	SC24A_HUI	27	1.017622
24H 50MT/	P08697	0.252765	0.004158	0.006052	SERPINF2	Alpha-2-an	A2AP_HUM	14	1.191488
24H 10MT/	Q8NBL1	-0.18048	0.004176	0.006072	POGLUT1	Protein O-g	PGLT1_HUI	12	0.882409
48H 10MT/	Q9P0S3	-0.17422	0.004177	0.006072	ORMDL1	ORM1-like	ORML1_HU	4	0.886244
48H 50MT/	P99999	0.186983	0.004183	0.006079	CYCS	Cytochrom	CYC_HUMA	8	1.138381
48H 50MT/	P07942	0.05463	0.004184	0.00608	LAMB1	Laminin sul	LAMB1_HU	43	1.038593
48H 10MT/	Q9Y5M8	-0.11239	0.004184	0.00608	SRPRB	Signal reco	SRPRB_HUI	15	0.925057
48H 50MT/	P11388	-0.18411	0.004186	0.006083	TOP2A	DNA topois	TOP2A_HUI	73	0.880192
48H 50MT/	Q6P2I3	0.17126	0.004193	0.006089	FAHD2B	Oxaloaceta	FAH2B_HUI	1	1.126041
24H 50MT/	P18077	0.155179	0.004195	0.006091	RPL35A	Large ribos	RL35A_HUI	4	1.11356
48H 50MT/	Q9H4A6	0.093435	0.004195	0.006092	GOLPH3	Golgi phos	GOLP3_HU	10	1.066907
48H 10MT/	P49354	0.107872	0.004196	0.006093	FNTA	Protein farr	FNTA_HUM	14	1.077637
24H 50MT/	Q99943	0.13015	0.004205	0.006101	AGPAT1	1-acyl-sn-gl	PLCA_HUM	4	1.094407
24H 50MT/	Q6UW63	0.100226	0.004209	0.006105	POGLUT2	Protein O-g	PLGT2_HUI	18	1.071942
24H 10MT/	Q9NZM3	-0.11167	0.004233	0.006132	ITSN2	Intersectin	ITSN2_HUN	23	0.925514
48H 50MT/	O94842	-0.00218	0.004238	0.006137	TOX4	TOX high m	TOX4_HUM	10	0.99849
24H 10MT/	Q13405	-0.07811	0.004242	0.006142	MRPL49	Large ribos	RM49_HUN	7	0.947295
24H 50MT/	A6NJ88	1.552605	0.004244	0.006144	SAGE2P	Putative SA	SGE2P_HUI	1	2.933464
48H 50MT/	Q9H900	-0.22676	0.004252	0.006152	ZWILCH	Protein zwi	ZWILC_HUI	13	0.854553
24H 50MT/	Q3KRA6	-0.20964	0.004254	0.006153	C2orf76	UPF0538 p	CB076_HUI	3	0.864756
24H 10MT/	P30153	-0.11229	0.004261	0.00616	PPP2R1A	Serine/thre	2AAA_HUM	28	0.92512
24H 50MT/	P05165	0.361405	0.004262	0.006162	PCCA	Propionyl-(	PCCA_HUM	24	1.284676
48H 50MT/	Q9P209	0.38982	0.004263	0.006163	CEP72	Centrosom	CEP72_HUI	8	1.31023
48H 50MT/	P63165	0.155903	0.004267	0.006167	SUMO1	Small ubiq	SUMO1_HU	3	1.114119
48H 50MT/	Q8IYS2	0.022796	0.004276	0.006177	KIAA2013	Uncharacte	K2013_HUI	13	1.015927
48H 50MT/	Q14653	0.15976	0.004278	0.00618	IRF3	Interferon	IRF3_HUM/	9	1.117101
24H 10MT/	P50225	-1.60606	0.004279	0.006181	SULT1A1	Sulfotransf	ST1A1_HUI	2	0.328494
48H 10MT/	Q9UEU0	0.171929	0.00428	0.006182	VTI1B	Vesicle tran	VTI1B_HUN	8	1.126564
48H 50MT/	Q8TBC4	0.176526	0.004284	0.006185	UBA3	NEDD8-acti	UBA3_HUM	19	1.130159
48H 10MT/	Q15018	0.140474	0.004285	0.006186	ABRAXAS2	BRISC comp	ABRX2_HUI	11	1.102267
48H 50MT/	O75528	0.353951	0.004287	0.006188	TADA3	Transcripti	TADA3_HUI	5	1.278056
48H 10MT/	O60885	0.083987	0.004288	0.006189	BRD4	Bromodom	BRD4_HUM	23	1.059943
48H 10MT/	O94906	0.044381	0.004288	0.006189	PRPF6	Pre-mRNA-	PRP6_HUM	38	1.03124
24H 50MT/	Q96P16	0.140083	0.004289	0.006189	RPRD1A	Regulation	RPR1A_HUI	14	1.101969
24H 50MT/	Q9H8S9	0.530012	0.004288	0.006189	MOB1A	MOB kinase	MOB1A_HU	1	1.443941
24H 10MT/	P17931	-0.12439	0.004289	0.00619	LGALS3	Galectin-3	LEG3_HUM	6	0.917394
48H 10MT/	Q0ZGT2	0.103916	0.00429	0.00619	NEXN	Nexilin	NEXN_HUM	9	1.074687
48H 50MT/	Q86VP6	0.061368	0.0043	0.0062	CAND1	Cullin-asso	CAND1_HU	58	1.043454
48H 10MT/	Q2M1Z3	-1.43166	0.004301	0.0062	ARHGAP31	Rho GTPase	RHG31_HU	2	0.370705
24H 10MT/	P23458	0.123461	0.004303	0.006202	JAK1	Tyrosine-pr	JAK1_HUM	29	1.089345
24H 10MT/	Q9HAS0	0.009244	0.004304	0.006203	C17orf75	Protein Njn	NJMU_HUN	9	1.006428
24H 50MT/	Q15365	-0.00368	0.004307	0.006206	PCBP1	Poly(rC)-bi	PCBP1_HUI	14	0.997454
24H 50MT/	P52434	0.07064	0.004308	0.006206	POLR2H	DNA-direct	RPAB3_HUI	7	1.050182
24H 50MT/	Q9BSK2	0.111864	0.004308	0.006206	SLC25A33	Solute carri	S2533_HUI	8	1.080623
24H 10MT/	Q9UKY4	-0.06064	0.004308	0.006207	POMT2	Protein O-n	POMT2_HU	3	0.958837

24H 50MT/	Q15650	-0.25367	0.004313	0.006211	TRIP4	Activating s	TRIP4_HUN	16	0.83876
48H 10MT/	Q99523	-0.01931	0.004315	0.006212	SORT1	Sortilin	SORT_HUM	15	0.986706
48H 10MT/	Q13444	0.059489	0.004318	0.006216	ADAM15	Disintegrin	ADA15_HU	7	1.042097
24H 50MT/	Q13433	0.205375	0.004318	0.006216	SLC39A6	Zinc transp	S39A6_HUI	1	1.152986
24H 50MT/	Q8NBP0	0.61273	0.004319	0.006217	TTC13	Tetratricop	TTC13_HUI	8	1.52915
24H 50MT/	Q9Y680	0.318565	0.004322	0.006219	FKBP7	Peptidyl-pr	FKBP7_HUI	6	1.24709
24H 50MT/	P56182	0.274682	0.004327	0.006224	RRP1	Ribosomal	RRP1_HUM	13	1.209727
24H 50MT/	P46977	0.141073	0.004333	0.00623	STT3A	Dolichyl-di	STT3A_HUN	16	1.102725
48H 10MT/	Q9UJU6	0.117351	0.004335	0.006232	DBNL	Drebrin-like	DBNL_HUM	13	1.084741
24H 50MT/	P54259	-0.92188	0.004336	0.006233	ATN1	Atrophin-1	ATN1_HUM	1	0.527821
24H 50MT/	Q02252	0.095773	0.004338	0.006235	ALDH6A1	Methylmal	MMSA_HUI	19	1.068638
48H 50MT/	Q8TEP8	-0.06649	0.004346	0.006243	CEP192	Centrosom	CE192_HUI	10	0.954957
24H 50MT/	Q9UM22	0.329476	0.004349	0.006246	EPDR1	Mammaliar	EPDR1_HUI	2	1.256557
48H 10MT/	P49792	0.005502	0.004349	0.006246	RANBP2	E3 SUMO-p	RBP2_HUM	96	1.003821
48H 10MT/	O75643	-0.10661	0.004351	0.006249	SNRNP200	U5 small nu	U520_HUM	116	0.928771
48H 10MT/	P46379	0.03504	0.004351	0.006249	BAG6	Large prolir	BAG6_HUM	36	1.024585
48H 10MT/	Q9H790	-2.79882	0.004352	0.006249	EXO5	Exonucleas	EXO5_HUM	1	0.143705
48H 10MT/	Q2M389	-0.02955	0.004353	0.006251	WASHC4	WASH com	WASC4_HU	28	0.979727
48H 50MT/	Q14847	0.11597	0.004354	0.006251	LASP1	LIM and SH	LASP1_HUN	12	1.083703
48H 50MT/	O15294	-0.12758	0.004359	0.006257	OGT	UDP-N-acet	OGT1_HUM	36	0.915364
24H 10MT/	Q9HA64	-0.07508	0.00436	0.006257	FN3KRP	Ketosamine	KT3K_HUM	9	0.949289
48H 50MT/	Q9BWH2	-0.40106	0.004361	0.00626	FUNDC2	FUN14 don	FUND2_HU	5	0.757301
48H 50MT/	Q15493	0.434127	0.004368	0.006265	RGN	Regucalcin	RGN_HUM/	7	1.351093
48H 10MT/	P42574	0.0858	0.00437	0.006268	CASP3	Caspase-3	CASP3_HUI	7	1.061276
48H 50MT/	Q9NQZ5	-0.37894	0.004371	0.006268	STARD7	StAR-relate	STAR7_HUN	9	0.769004
24H 50MT/	Q9Y5B9	0.1484	0.004372	0.006269	SUPT16H	FACT comp	SP16H_HUI	43	1.108339
48H 10MT/	O00743	0.10634	0.004377	0.006275	PPP6C	Serine/thre	PPP6_HUM	14	1.076494
24H 50MT/	Q14684	0.029094	0.004377	0.006275	RRP1B	Ribosomal	RRP1B_HUI	30	1.020371
24H 50MT/	Q52LW3	-0.12455	0.00438	0.006279	ARHGAP29	Rho GTPase	RHG29_HU	43	0.917287
24H 50MT/	Q9Y2H5	-0.40214	0.00439	0.006288	PLEKHA6	Pleckstrin I	PKHA6_HU	4	0.756735
48H 50MT/	P29144	-0.01689	0.004395	0.006293	TPP2	Tripeptidyl	TPP2_HUM	50	0.988362
48H 10MT/	P05109	-0.88309	0.004397	0.006295	S100A8	Protein S1(	S10A8_HUI	1	0.542206
24H 10MT/	O43903	-0.18727	0.004401	0.006299	GAS2	Growth arr	GAS2_HUM	4	0.878268
24H 50MT/	Q9Y2A7	0.088696	0.004403	0.0063	NCKAP1	Nck-associa	NCKP1_HU	37	1.063409
24H 50MT/	O95487	0.011382	0.004407	0.006304	SEC24B	Protein trai	SC24B_HUI	28	1.007921
48H 50MT/	Q13884	0.09836	0.004408	0.006306	SNTB1	Beta-1-synt	SNTB1_HUI	18	1.070556
48H 10MT/	Q96IZ6	1.18557	0.004409	0.006306	METTL2A	tRNA N(3)-r	MET2A_HU	1	2.274533
48H 50MT/	Q7Z2Z2	0.077384	0.00441	0.006307	EFL1	Elongation	EFL1_HUM/	20	1.055103
48H 50MT/	P52701	-0.11194	0.00441	0.006308	MSH6	DNA misma	MSH6_HUN	51	0.925343
24H 10MT/	O96013	0.038684	0.004413	0.006311	PAK4	Serine/thre	PAK4_HUM	13	1.027176
48H 50MT/	P26358	-0.21106	0.004413	0.006312	DNMT1	DNA (cytosi	DNMT1_HU	51	0.863899
24H 50MT/	Q9UEY8	0.168035	0.004416	0.006313	ADD3	Gamma-ad(	ADDG_HUN	16	1.123527
48H 50MT/	Q5VV42	0.072512	0.00442	0.006317	CDKAL1	Threonylca	CDKAL_HUI	15	1.051546
24H 50MT/	Q9BV81	-0.05557	0.004425	0.006322	EMC6	ER membra	EMC6_HUN	2	0.962214
48H 10MT/	Q9NYP7	0.004768	0.004431	0.006329	ELOVL5	Very long cl	ELOV5_HUI	2	1.00331
48H 50MT/	Q9H977	0.521796	0.004432	0.00633	WDR54	WD repeat-	WDR54_HU	3	1.435741
24H 10MT/	Q6IN85	0.054116	0.004432	0.00633	PPP4R3A	Serine/thre	P4R3A_HUI	17	1.038223
24H 10MT/	O95214	0.081854	0.004443	0.00634	LEPROTL1	Leptin rece	LERL1_HUN	1	1.058377
48H 50MT/	Q9Y287	-0.0884	0.004455	0.006353	ITM2B	Integral me	ITM2B_HUI	6	0.940563
24H 50MT/	Q8N4P2	0.687637	0.00446	0.006359	IFT70B	Intraflagell	IT70B_HUN	2	1.610643
48H 10MT/	O14524	-0.39625	0.004469	0.006369	NEMP1	Nuclear env	NEMP1_HU	5	0.759831
48H 10MT/	Q9BSE5	0.066721	0.004471	0.006372	AGMAT	Guanidino	GDAH_HUN	14	1.047334

24H 50MT/	Q6ZRS2	0.14728	0.004475	0.006375	SRCAP	Helicase SR	SRCAP_HUI	17	1.10748
24H 50MT/	Q86U42	0.094847	0.004475	0.006375	PABPN1	Polyadenyl	PABP2_HUI	8	1.067952
48H 50MT/	Q9UBQ0	-0.29453	0.004476	0.006376	VPS29	Vacuolar pı	VPS29_HUI	10	0.815337
48H 10MT/	Q07820	0.210304	0.004477	0.006378	MCL1	Induced my	MCL1_HUN	3	1.156932
48H 50MT/	Q9UBE0	0.057513	0.004478	0.006379	SAE1	SUMO-activ	SAE1_HUM	23	1.04067
24H 50MT/	Q9HC38	0.091049	0.004479	0.00638	GLOD4	Glyoxalase	GLOD4_HU	16	1.065144
24H 10MT/	A0AV96	-0.15768	0.004486	0.006386	RBM47	RNA-bindir	RBM47_HU	16	0.896465
48H 10MT/	O75844	-0.21277	0.004488	0.006388	ZMPSTE24	CAAX preny	FACE1_HUN	15	0.862881
24H 10MT/	Q9P2D0	0.275029	0.004489	0.00639	IBTK	Inhibitor o	IBTK_HUM/	8	1.210018
48H 10MT/	Q9UHD9	0.153864	0.004493	0.006393	UBQLN2	Ubiquilin-2	UBQL2_HU	10	1.112545
48H 10MT/	O43292	0.040781	0.0045	0.006401	GPAA1	Glycosylph	GPAA1_HU	11	1.028671
24H 10MT/	Q9HAU5	-0.09521	0.0045	0.006402	UPF2	Regulator c	RENT2_HUI	24	0.936133
48H 50MT/	O00425	0.065276	0.004503	0.006405	IGF2BP3	Insulin-like	IF2B3_HUN	23	1.046285
48H 10MT/	P11388	0.10849	0.004506	0.006408	TOP2A	DNA topois	TOP2A_HUI	73	1.0781
48H 50MT/	Q6FI81	0.001566	0.004508	0.00641	CIAPIN1	Anamorsin	CPIN1_HUI	20	1.001086
48H 50MT/	Q3LXA3	0.185009	0.004509	0.00641	TKFC	Triokinase/	TKFC_HUM	18	1.136824
24H 50MT/	Q9H0C8	-0.15853	0.00451	0.006411	ILKAP	Integrin-lin	ILKAP_HUN	13	0.89594
48H 50MT/	Q71RC2	-0.17151	0.004516	0.006419	LARP4	La-related p	LARP4_HUI	20	0.88791
48H 50MT/	P08697	0.250854	0.004521	0.006424	SERPINF2	Alpha-2-ant	A2AP_HUM	14	1.189912
24H 10MT/	P63208	-0.26113	0.004523	0.006426	SKP1	S-phase kin	SKP1_HUM	6	0.834433
48H 10MT/	Q6IQ49	-0.59739	0.004524	0.006427	SDE2	Splicing reg	SDE2_HUM	3	0.66095
48H 50MT/	Q9ULX3	-0.15982	0.004528	0.006431	NOB1	RNA-bindir	NOB1_HUN	9	0.895134
24H 50MT/	Q9Y305	0.016259	0.00453	0.006432	ACOT9	Acyl-coenzy	ACOT9_HUI	20	1.011334
48H 10MT/	P35443	-0.70738	0.004531	0.006433	THBS4	Thrombosp	TSP4_HUM	2	0.612431
48H 10MT/	Q14254	0.053398	0.004535	0.006438	FLOT2	Flotillin-2	FLOT2_HUN	22	1.037706
24H 50MT/	Q6RFH5	0.144439	0.004536	0.006439	WDR74	WD repeat-	WDR74_HU	17	1.105301
24H 50MT/	P29350	0.022027	0.004537	0.00644	PTPN6	Tyrosine-pr	PTN6_HUM	14	1.015385
48H 10MT/	Q58FF7	-1.17076	0.004539	0.006442	HSP90AB3I	Putative he	H90B3_HU	5	0.444187
24H 10MT/	Q13618	0.129671	0.004554	0.006458	CUL3	Cullin-3	CUL3_HUM	35	1.094044
48H 10MT/	P49736	0.070304	0.004557	0.00646	MCM2	DNA replica	MCM2_HUI	36	1.049938
48H 50MT/	Q13506	-0.5498	0.004557	0.00646	NAB1	NGFI-A-bin	NAB1_HUN	5	0.683113
48H 10MT/	P21912	0.089573	0.004558	0.006461	SDHB	Succinate d	SDHB_HUM	10	1.064055
24H 50MT/	Q9H892	-1.95347	0.004558	0.006461	TTC12	Tetratricop	TTC12_HUN	1	0.258194
48H 10MT/	Q17R98	0.282373	0.004559	0.006461	ZNF827	Zinc finger	ZN827_HUI	2	1.216194
48H 50MT/	P17252	0.234801	0.00456	0.006461	PRKCA	Protein kin	KPCA_HUM	15	1.176744
24H 50MT/	P27695	0.049164	0.004561	0.006463	APEX1	DNA repair	APEX1_HUI	16	1.034665
24H 10MT/	P48634	-0.07239	0.004571	0.006474	PRRC2A	Protein PRI	PRC2A_HUI	44	0.951062
24H 10MT/	P06213	0.053111	0.004574	0.006479	INSR	Insulin rece	INSR_HUM/	20	1.0375
24H 50MT/	Q9UNH6	0.185508	0.004576	0.006481	SNX7	Sorting nex	SNX7_HUM	14	1.137218
24H 50MT/	Q13557	-0.01494	0.004586	0.006491	CAMK2D	Calcium/ca	KCC2D_HU	10	0.989697
48H 50MT/	P61619	0.047458	0.004593	0.0065	SEC61A1	Protein trai	S61A1_HUI	7	1.033442
24H 10MT/	Q9H0V1	0.399178	0.004598	0.006504	TMEM168	Transmemb	TM168_HU	3	1.318756
48H 50MT/	Q14680	-0.38709	0.0046	0.006506	MELK	Maternal er	MELK_HUM	13	0.764669
48H 50MT/	Q96FZ7	0.083666	0.004602	0.006508	CHMP6	Charged mı	CHMP6_HU	6	1.059707
48H 10MT/	Q9NP64	-0.218	0.004603	0.006509	ZCCHC17	Zinc finger	ZCC17_HUI	3	0.859755
24H 50MT/	O14530	-0.08315	0.004605	0.006511	TXNDC9	Thioredoxi	TXND9_HU	10	0.943991
48H 50MT/	Q96ER3	0.153111	0.004606	0.006511	SAAL1	Protein SA/	SAAL1_HUN	6	1.111965
48H 50MT/	Q9Y6E2	-0.18428	0.004612	0.006517	BZW2	eIF5-mimic	5MP1_HUN	22	0.880091
48H 50MT/	P84022	0.073295	0.004618	0.006523	SMAD3	Mothers ag	SMAD3_HU	5	1.052117
48H 10MT/	P00748	-0.09157	0.004618	0.006523	F12	Coagulatio	FA12_HUM	5	0.938502
24H 50MT/	Q96HS1	0.035133	0.004628	0.006534	PGAM5	Serine/thre	PGAM5_HU	14	1.024651
24H 50MT/	Q6ZT12	0.055884	0.004631	0.006536	UBR3	E3 ubiquiti	UBR3_HUM	23	1.039496

24H 10MTA	P06132	-0.16059	0.004631	0.006537	UROD	Uroporphy	DCUP_HUM	15	0.894662
24H 50MTA	O95786	0.161269	0.004633	0.006538	RIGI	Antiviral in	RIGI_HUMA	16	1.11827
48H 50MTA	Q8WVM0	-0.44129	0.004633	0.006538	TFB1M	Dimethylac	TFB1M_HU	15	0.736476
48H 50MTA	Q8IYL3	-0.15536	0.004634	0.006539	C1orf174	UPF0688 p	CA174_HU	1	0.897911
24H 10MTA	Q9NUJ3	-0.83694	0.004637	0.006542	TCP11L1	T-complex	T11L1_HUM	3	0.559828
48H 50MTA	O95249	0.097618	0.004647	0.006553	GOSR1	Golgi SNAP	GOSR1_HU	12	1.070005
24H 50MTA	Q06265	0.120319	0.00465	0.006556	EXOSC9	Exosome cc	EXOS9_HU	11	1.086975
48H 50MTA	Q9NZN8	0.293106	0.004651	0.006556	CNOT2	CCR4-NOT t	CNOT2_HU	11	1.225275
24H 10MTA	Q02543	0.237579	0.004652	0.006557	RPL18A	Large ribos	RL18A_HU	8	1.179013
24H 50MTA	O14981	0.040955	0.004654	0.006559	BTAF1	TATA-bindi	BTAF1_HUM	47	1.028795
48H 10MTA	Q9NUL5	-0.51821	0.004656	0.00656	SHFL	Shiftless an	SHFL_HUM	4	0.698239
24H 50MTA	Q9Y2L1	0.039178	0.004656	0.00656	DIS3	Exosome cc	RRP44_HU	38	1.027528
48H 10MTA	Q6NXT4	-0.00989	0.00466	0.006563	SLC30A6	Zinc transp	ZNT6_HUM	4	0.993168
48H 50MTA	P31937	0.158373	0.004662	0.006565	HIBADH	3-hydroxyi	3HIDH_HU	12	1.116028
24H 50MTA	P30101	0.139221	0.004664	0.006567	PDIA3	Protein dis	PDIA3_HUM	40	1.10131
24H 50MTA	P62269	0.14343	0.004664	0.006567	RPS18	Small ribos	RS18_HUM	11	1.104528
48H 50MTA	Q9H875	-2.76808	0.004665	0.006567	PRKRIP1	PRKR-intera	PKRI1_HUM	1	0.1468
48H 50MTA	Q14746	0.016983	0.004669	0.006572	COG2	Conserved	COG2_HUM	25	1.011841
48H 10MTA	O95425	0.049939	0.00467	0.006573	SVIL	Supervillin	SVIL_HUMA	34	1.035221
48H 10MTA	Q9BXR0	0.045392	0.004672	0.006575	QTRT1	Queuine tR	TGT_HUMA	10	1.031963
48H 50MTA	Q6P9B6	0.089633	0.004678	0.00658	MEAK7	MTOR-asso	MEAK7_HU	10	1.064099
24H 10MTA	O95084	0.665107	0.004678	0.00658	PRSS23	Serine prot	PRS23_HU	4	1.585686
48H 50MTA	Q9H0H0	0.048569	0.004679	0.006581	INTS2	Integrator	INT2_HUMA	11	1.034239
48H 10MTA	Q9P016	0.032565	0.00468	0.006582	THYN1	Thymocyte	THYN1_HU	10	1.022829
24H 50MTA	Q9BUR5	0.127403	0.004682	0.006584	APOO	MICOS com	MIC26_HU	5	1.092326
24H 50MTA	Q14204	-0.06455	0.004685	0.006586	DYNC1H1	Cytoplasmi	DYHC1_HU	233	0.956242
24H 50MTA	Q8N573	0.659253	0.004686	0.006587	OXR1	Oxidation r	OXR1_HUM	2	1.579264
24H 10MTA	O00746	0.157776	0.004688	0.006588	NME4	Nucleoside	NDKM_HU	11	1.115566
48H 50MTA	Q14657	-0.01564	0.004688	0.006588	LAGE3	EKC/KEOPS	LAGE3_HU	2	0.989217
24H 10MTA	O60563	-0.42269	0.004689	0.006589	CCNT1	Cyclin-T1	CCNT1_HU	1	0.746031
24H 50MTA	Q6P1Q9	-0.41948	0.00469	0.006589	METTL2B	tRNA N(3)-r	MET2B_HU	6	0.747697
24H 50MTA	Q86W34	0.590769	0.004691	0.00659	AMZ2	Archaemet	AMZ2_HUM	3	1.506049
24H 10MTA	Q96BD5	-0.22343	0.004692	0.006591	PHF21A	PHD finger	PF21A_HU	5	0.856524
24H 50MTA	Q9P0T7	0.279828	0.004694	0.006593	TMEM9	Proton-trar	TMEM9_HU	4	1.21405
24H 50MTA	Q8WVK2	1.639039	0.004698	0.006596	SNRNP27	U4/U6.U5 s	SNR27_HU	2	3.114583
48H 10MTA	Q9Y2S7	-0.14167	0.004699	0.006597	POLDIP2	Polymerase	PDIP2_HUM	16	0.906466
24H 10MTA	P04183	0.115264	0.004705	0.006604	TK1	Thymidine	KITH_HUMA	12	1.083173
48H 50MTA	Q9BUA3	0.243445	0.004707	0.006606	SPINDOC	Spindlin int	SPNDC_HU	3	1.183816
48H 50MTA	Q8TBZ6	-0.58779	0.004714	0.006612	TRMT10A	tRNA meth	TM10A_HU	3	0.665363
24H 50MTA	Q9Y6M9	0.116269	0.004714	0.006612	NDUFB9	NADH dehy	NDUB9_HU	11	1.083928
48H 50MTA	P17931	0.205234	0.004715	0.006613	LGALS3	Galectin-3	LEG3_HUM	6	1.152873
24H 10MTA	Q15058	0.163576	0.004715	0.006613	KIF14	Kinesin-like	KIF14_HUM	15	1.12006
48H 10MTA	P33992	0.069024	0.004718	0.006615	MCM5	DNA replica	MCM5_HU	41	1.049006
48H 50MTA	P49790	-0.1234	0.004718	0.006615	NUP153	Nuclear po	NU153_HU	43	0.918023
24H 10MTA	Q96IZ6	-1.52956	0.004721	0.006619	METTL2A	tRNA N(3)-r	MET2A_HU	1	0.346382
24H 10MTA	Q9Y5X2	0.064337	0.004732	0.006631	SNX8	Sorting nex	SNX8_HUM	18	1.045604
48H 50MTA	P17936	1.017509	0.004734	0.006632	IGFBP3	Insulin-like	IBP3_HUMA	5	2.024421
24H 10MTA	P62191	0.032465	0.004735	0.006633	PSMC1	26S protea	PRS4_HUM	25	1.022758
48H 10MTA	Q9BYC9	-0.18303	0.004738	0.006636	MRPL20	Large ribos	RM20_HUM	4	0.880851
48H 10MTA	O43143	-0.13	0.004744	0.006642	DHX15	ATP-depend	DHX15_HU	36	0.913829
24H 50MTA	Q9UHD8	-0.14407	0.004754	0.006652	SEPTIN9	Septin-9	SEPT9_HUM	28	0.904965
48H 50MTA	Q8IWA0	-0.26073	0.004757	0.006655	WDR75	WD repeat-	WDR75_HU	26	0.834667

48H 50MT/	P53990	0.079534	0.00476	0.006658	IST1	IST1 homol	IST1_HUM/	11	1.056677
48H 50MT/	P15291	0.318754	0.004766	0.006662	B4GALT1	Beta-1,4-ga	B4GT1_HUI	8	1.247253
48H 50MT/	Q8N8S7	0.044102	0.004769	0.006665	ENAH	Protein ena	ENAH_HUM	19	1.031041
24H 50MT/	Q9ULX6	0.135144	0.004773	0.006669	AKAP8L	A-kinase an	AKP8L_HUI	11	1.098203
24H 10MT/	P67812	0.06762	0.004776	0.006671	SEC11A	Signal pept	SC11A_HUI	6	1.047986
24H 50MT/	Q9BV10	0.193765	0.004776	0.006671	ALG12	Dol-P-Man:	ALG12_HUI	4	1.143745
24H 10MT/	Q9NX47	0.178045	0.004776	0.006671	MARCHF5	E3 ubiquiti	MARH5_HU	13	1.13135
24H 50MT/	P55061	-0.963	0.004778	0.006672	TMBIM6	Bax inhibit	BI1_HUMA	3	0.512989
48H 50MT/	P12814	0.061439	0.004779	0.006673	ACTN1	Alpha-actir	ACTN1_HUI	58	1.043506
24H 10MT/	Q9UDW1	0.236292	0.004782	0.006676	UQCR10	Cytochrom	QCR9_HUM	2	1.177961
24H 10MT/	P45973	0.09873	0.004784	0.006677	CBX5	Chromobo:	CBX5_HUM	9	1.070831
48H 50MT/	Q9Y3D3	-0.20098	0.004784	0.006677	MRPS16	Small ribos	RT16_HUM	4	0.869962
24H 50MT/	O95218	-0.21713	0.004787	0.006681	ZRANB2	Zinc finger	ZRAB2_HUI	14	0.860275
24H 10MT/	Q13616	0.03984	0.00479	0.006683	CUL1	Cullin-1	CUL1_HUM	36	1.028
48H 10MT/	Q7L7V1	-0.07237	0.00479	0.006683	DHX32	Putative pr	DHX32_HU	16	0.951072
24H 50MT/	Q9NW64	0.058757	0.004795	0.006688	RBM22	Pre-mRNA-	RBM22_HU	12	1.041568
24H 50MT/	O15145	-0.25356	0.004802	0.006694	ARPC3	Actin-relate	ARPC3_HUI	8	0.838821
24H 10MT/	P53007	0.158069	0.004802	0.006694	SLC25A1	Tricarboxyl	TXTP_HUM	10	1.115793
24H 50MT/	Q9NX38	-0.7873	0.004803	0.006695	ABITRAM	Protein Abi	ABITM_HUI	1	0.579425
48H 10MT/	Q13200	0.024874	0.004805	0.006696	PSMD2	26S protea:	PSMD2_HU	53	1.017391
48H 50MT/	P60953	-0.16373	0.004807	0.006699	CDC42	Cell divisior	CDC42_HU	8	0.892714
48H 10MT/	Q7Z4H8	0.076241	0.004808	0.0067	POGLUT3	Protein O-g	PLGT3_HUI	21	1.054267
24H 50MT/	P22033	0.152721	0.00481	0.006701	MMUT	Methylmal	MUTA_HUM	24	1.111664
24H 50MT/	Q9NZ08	0.0327	0.004825	0.006716	ERAP1	Endoplasm	ERAP1_HUI	27	1.022925
48H 10MT/	O14976	0.093656	0.004831	0.006722	GAK	Cyclin-G-as	GAK_HUM/	26	1.067071
24H 50MT/	Q4KMQ2	0.289328	0.004832	0.006723	ANO6	Anoctamin	ANO6_HUM	16	1.222071
48H 50MT/	Q14147	-0.44757	0.004833	0.006723	DHX34	Probable A	DHX34_HU	12	0.733275
24H 50MT/	P35237	0.021822	0.004834	0.006724	SERPINB6	Serpin B6	SPB6_HUM	18	1.015241
24H 50MT/	Q13907	0.17908	0.004836	0.006726	IDI1	Isopenteny	IDI1_HUM/	10	1.132162
48H 10MT/	Q6YN16	0.029015	0.00484	0.00673	HSDL2	Hydroxyste	HSDL2_HUI	27	1.020315
24H 10MT/	Q7L1Q6	0.096451	0.004848	0.006736	BZW1	eIF5-mimic	5MP2_HUM	19	1.06914
24H 50MT/	O75531	-0.45253	0.004851	0.006738	BANF1	Barrier-to-a	BAF_HUMA	4	0.730762
24H 50MT/	Q6UWZ7	0.39673	0.004854	0.006741	ABRAXAS1	BRCA1-A cc	ABRX1_HUI	3	1.316521
24H 50MT/	P25325	-0.10535	0.004858	0.006745	MPST	3-mercapto	THTM_HUM	15	0.929577
24H 10MT/	O14578	0.119081	0.004865	0.006751	CIT	Citron Rho-	CTRO_HUM	26	1.086043
48H 50MT/	Q2TB90	0.365448	0.004867	0.006753	HKDC1	Hexokinase	HKDC1_HU	18	1.288282
24H 50MT/	Q9H7C9	-0.01273	0.004868	0.006754	AAMDC	Mth938 do	AAMDC_HU	7	0.991213
48H 50MT/	P25685	-0.17756	0.004868	0.006754	DNAJB1	DnaJ homo	DNJB1_HUI	17	0.884197
24H 50MT/	Q15527	-0.26536	0.004873	0.006759	SURF2	Surfeit locu	SURF2_HUI	4	0.83199
48H 50MT/	Q8N5L8	-0.00226	0.004874	0.00676	RPP25L	Ribonuclea	RP25L_HUI	1	0.998432
24H 10MT/	P10114	0.760778	0.004874	0.00676	RAP2A	Ras-related	RAP2A_HUI	2	1.694404
24H 10MT/	Q6DD88	0.158638	0.004876	0.006761	ATL3	Atlastin-3	ATLA3_HUI	26	1.116233
48H 10MT/	O43396	0.117617	0.004878	0.006763	TXNL1	Thioredoxi	TXNL1_HUI	16	1.084941
48H 50MT/	Q03001	0.107184	0.004885	0.00677	DST	Dystonin	DYST_HUM	42	1.077124
24H 50MT/	P15104	-0.06669	0.004888	0.006774	GLUL	Glutamine	GLNA_HUM	7	0.954827
24H 50MT/	Q12905	0.064429	0.00489	0.006776	ILF2	Interleukin	ILF2_HUM/	20	1.045671
24H 50MT/	O60664	-0.10145	0.0049	0.006788	PLIN3	Perilipin-3	PLIN3_HUM	20	0.932098
48H 10MT/	O60232	0.217809	0.004904	0.006792	ZNRD2	Protein ZNI	ZNRD2_HU	5	1.162966
48H 50MT/	Q9H9S5	-0.27873	0.004909	0.006797	FKRP	Ribitol 5-pl	FKRP_HUM	5	0.824315
24H 10MT/	Q9NS69	-0.46685	0.00491	0.006798	TOMM22	Mitochond	TOM22_HU	5	0.72354
48H 50MT/	P78346	-0.11386	0.004917	0.006805	RPP30	Ribonuclea	RPP30_HUI	14	0.924113
24H 50MT/	P54253	-0.6024	0.004932	0.00682	ATXN1	Ataxin-1	ATX1_HUM	2	0.658657

24H 10MTA	Q01082	0.018961	0.004939	0.006827	SPTBN1	Spectrin be	SPTB2_HUI	127	1.01323
48H 50MTA	Q13011	0.151286	0.004939	0.006827	ECH1	Delta(3,5)-I	ECH1_HUM	16	1.110559
24H 10MTA	Q9Y678	-0.07822	0.004944	0.006833	COPG1	Coatomer s	COPG1_HU	42	0.947222
24H 50MTA	P52569	0.211075	0.00495	0.006839	SLC7A2	Cationic an	CTR2_HUM	8	1.15755
48H 10MTA	O94776	0.079665	0.004951	0.006839	MTA2	Metastasis-	MTA2_HUN	24	1.056773
48H 10MTA	P52434	0.132361	0.004956	0.006844	POLR2H	DNA-direct	RPAB3_HUI	7	1.096086
24H 50MTA	O95249	0.179452	0.00496	0.006849	GOSR1	Golgi SNAP	GOSR1_HU	12	1.132454
48H 10MTA	Q96SI9	0.05997	0.004961	0.006849	STRBP	Spermatid	STRBP_HUI	22	1.042444
24H 50MTA	Q9H9E3	0.100857	0.004978	0.006866	COG4	Conserved	COG4_HUN	26	1.07241
48H 50MTA	Q9NTX5	0.101264	0.004981	0.00687	ECHDC1	Ethylmalor	ECHD1_HU	14	1.072713
48H 10MTA	Q9Y2R9	-0.09095	0.004986	0.006874	MRPS7	Small ribos	RT07_HUM	12	0.938902
24H 50MTA	Q86TG7	-0.26484	0.004988	0.006876	PEG10	Retrotrans	PEG10_HUI	6	0.832291
48H 50MTA	O14777	-0.19281	0.004988	0.006876	NDC80	Kinetochor	NDC80_HU	23	0.874899
48H 10MTA	P09417	0.026947	0.005	0.006888	QDPR	Dihydropte	DHPR_HUN	10	1.018854
24H 10MTA	Q9BUQ8	0.089859	0.005003	0.006892	DDX23	Probable A	DDX23_HU	32	1.064266
24H 50MTA	Q14687	0.029464	0.005004	0.006893	GSE1	Genetic sup	GSE1_HUM	5	1.020633
24H 50MTA	O43169	0.247484	0.005015	0.006905	CYB5B	Cytochrom	CYB5B_HUI	6	1.187135
48H 50MTA	Q13232	0.044734	0.005024	0.006916	NME3	Nucleoside	NDK3_HUN	8	1.031493
48H 10MTA	P30154	0.109738	0.005027	0.006919	PPP2R1B	Serine/thre	2AAB_HUM	22	1.079032
48H 10MTA	Q8WW59	-0.15174	0.005027	0.006919	SPRYD4	SPRY doma	SPRY4_HUI	13	0.900166
48H 50MTA	P13861	0.140989	0.005031	0.006922	PRKAR2A	cAMP-depe	KAP2_HUM	21	1.102661
48H 10MTA	P50542	0.143548	0.005034	0.006926	PEX5	Peroxisom	PEX5_HUM	22	1.104618
48H 50MTA	Q08378	0.055016	0.005037	0.006928	GOLGA3	Golgin subf	GOGA3_HU	46	1.03887
24H 50MTA	Q6P1J9	0.08538	0.005038	0.006928	CDC73	Parafibrom	CDC73_HU	27	1.060967
24H 50MTA	Q8IWV7	-0.1505	0.005037	0.006928	UBR1	E3 ubiquiti	UBR1_HUN	28	0.900941
24H 50MTA	Q6PL24	-0.16173	0.005038	0.006929	TMED8	Protein TM	TMED8_HU	1	0.89395
24H 10MTA	Q8N4Q1	-0.0845	0.005041	0.006932	CHCHD4	Mitochond	MIA40_HUI	4	0.943109
24H 10MTA	Q02241	0.253416	0.005042	0.006933	KIF23	Kinesin-like	KIF23_HUN	17	1.192027
24H 10MTA	P13716	-0.15173	0.00505	0.00694	ALAD	Delta-amin	HEM2_HUN	8	0.900169
48H 10MTA	Q96FC7	0.088688	0.005057	0.006948	PHYHIPL	Phytanoyl-	PHIPL_HUN	9	1.063403
48H 10MTA	O75351	0.049172	0.005058	0.006949	VPS4B	Vacuolar pı	VPS4B_HUI	16	1.034671
24H 10MTA	P14868	0.048871	0.005061	0.006951	DARS1	Aspartate--	SYDC_HUM	36	1.034455
48H 10MTA	Q16513	0.096847	0.005066	0.006957	PKN2	Serine/thre	PKN2_HUN	42	1.069434
24H 50MTA	Q4UJ75;Q5	0.306952	0.005068	0.006959	ANKRD20A	Putative an	A20A4_HUI	1	1.237091
24H 10MTA	P11498	-0.09124	0.005069	0.00696	PC	Pyruvate ca	PYC_HUMA	45	0.938718
24H 50MTA	Q10472	0.135175	0.005073	0.006963	GALNT1	Polypeptid	GALT1_HUI	14	1.098226
48H 50MTA	Q8IU81	-0.12809	0.005073	0.006963	IRF2BP1	Interferon	I2BP1_HUN	18	0.915041
48H 50MTA	P14174	0.526556	0.005078	0.006968	MIF	Macrophag	MIF_HUMA	5	1.440486
48H 50MTA	Q76EJ3	-0.43935	0.005079	0.006968	SLC35D2	Nucleotide	S35D2_HUI	3	0.737467
24H 50MTA	P84103	0.26092	0.00508	0.006969	SRSF3	Serine/argi	SRSF3_HUI	6	1.198242
48H 50MTA	Q969J3	0.123346	0.005083	0.006972	BORCS5	BLOC-1-rela	BORC5_HU	5	1.089258
48H 50MTA	Q96G28	0.138608	0.005085	0.006975	CFAP36	Cilia- and fl	CFA36_HUI	10	1.100842
24H 50MTA	Q08170	0.150083	0.005092	0.006982	SRSF4	Serine/argi	SRSF4_HUN	4	1.109633
48H 50MTA	P01130	-0.13108	0.005097	0.006987	LDLR	Low-densit	LDLR_HUM	18	0.913146
24H 10MTA	P58546	-0.54224	0.005113	0.007003	MTPN	Myotrophi	MTPN_HUN	2	0.686703
48H 50MTA	Q92769	0.111582	0.005116	0.007007	HDAC2	Histone dea	HDAC2_HU	8	1.080412
24H 50MTA	Q16539	-0.07902	0.005117	0.007008	MAPK14	Mitogen-ac	MK14_HUN	13	0.946698
48H 50MTA	Q96FV2	0.270233	0.005132	0.007023	SCRN2	Secernin-2	SCRN2_HUI	9	1.206003
24H 50MTA	Q00653	-0.26468	0.005143	0.007034	NFKB2	Nuclear fac	NFKB2_HUI	33	0.832386
48H 50MTA	Q86YN1	-0.14501	0.005144	0.007035	DOLPP1	Dolichyldip	DOPP1_HU	3	0.904374
24H 50MTA	Q68CQ4	0.142047	0.005151	0.007042	UTP25	U3 small nu	UTP25_HUI	20	1.10347
48H 50MTA	Q00839	0.050626	0.005152	0.007043	HNRNPU	Heterogene	HNRPU_HU	38	1.035714

24H 50MTA	P67870	0.19291	0.005153	0.007043	CSNK2B	Casein kina	CSK2B_HUI	10	1.143067
48H 10MTA	Q12893	0.030385	0.005162	0.007051	TMEM115	Transmemb	TM115_HU	6	1.021284
48H 50MTA	P00747	0.212216	0.005162	0.007051	PLG	Plasminoge	PLMN_HUN	3	1.158466
24H 50MTA	Q9UBW7	0.276559	0.005165	0.007054	ZMYM2	Zinc finger	ZMYM2_HU	16	1.211303
24H 50MTA	Q9BYW2	0.318312	0.005167	0.007056	SETD2	Histone-lys	SETD2_HUN	13	1.246871
48H 50MTA	Q9NXB9	-0.35938	0.005167	0.007056	ELOVL2	Very long cl	ELOV2_HUI	4	0.779497
48H 10MTA	Q8TF05	-0.01245	0.005168	0.007056	PPP4R1	Serine/thre	PP4R1_HUI	32	0.991406
24H 10MTA	Q13315	0.036375	0.00517	0.007058	ATM	Serine-prot	ATM_HUMA	48	1.025534
24H 10MTA	Q2M389	0.145121	0.005174	0.007062	WASHC4	WASH com	WASC4_HU	28	1.105824
48H 50MTA	P0DMV1;P(	0.060418	0.005177	0.007065	CT45A8;CT4	Cancer/test	CT458_HUI	9	1.042768
24H 10MTA	Q6P1A2	0.114154	0.005179	0.007066	LPCAT3	Lysophospl	MBOA5_HU	8	1.08234
24H 50MTA	Q9HCM9	-1.6501	0.00518	0.007067	TRIM39	E3 ubiquiti	TRI39_HUN	1	0.318619
24H 50MTA	Q9UL45	-0.40837	0.005187	0.007074	BLOC1S6	Biogenesis	BL1S6_HUN	2	0.753472
48H 50MTA	Q15334	0.371434	0.00519	0.007077	LLGL1	Lethal(2) gi	L2GL1_HUI	8	1.293638
24H 50MTA	Q99956	0.365412	0.00519	0.007077	DUSP9	Dual specifi	DUS9_HUM	12	1.28825
24H 10MTA	P35558	-0.4371	0.005196	0.007083	PCK1	Phosphoen	PCKGC_HU	18	0.738617
24H 50MTA	Q14152	0.056906	0.005199	0.007086	EIF3A	Eukaryotic	EIF3A_HUM	68	1.040232
24H 50MTA	Q8IYS1	0.091361	0.005199	0.007086	PM20D2	Xaa-Arg dip	P20D2_HU	9	1.065375
48H 50MTA	P54132	-0.55709	0.005203	0.007089	BLM	RecQ-like D	BLM_HUMA	11	0.679672
48H 50MTA	P55268	0.259881	0.00521	0.007096	LAMB2	Laminin sul	LAMB2_HU	28	1.19738
24H 10MTA	Q9H0M0	-1.33707	0.005211	0.007097	WWP1	NEDD4-like	WWP1_HU	2	0.395825
24H 10MTA	Q15276	-0.01942	0.005219	0.007106	RABEP1	Rab GTPase	RABE1_HUI	12	0.986629
48H 50MTA	A6NCE7;Q9	-0.07705	0.005221	0.007108	MAP1LC3B	Microtubul	MP3B2_HU	1	0.947993
48H 50MTA	P23468	0.487382	0.005227	0.007114	PTPRD	Receptor-ty	PTPRD_HUI	8	1.401898
48H 10MTA	Q15691	0.083315	0.005234	0.007121	MAPRE1	Microtubul	MARE1_HU	18	1.05945
48H 50MTA	P62917	-0.21412	0.005235	0.007122	RPL8	Large ribos	RL8_HUMA	14	0.862074
24H 50MTA	Q9Y3D7	0.197741	0.005238	0.007125	PAM16	Mitochond	TIM16_HUI	3	1.146901
48H 50MTA	P40925	0.155533	0.005247	0.007133	MDH1	Malate deh	MDHC_HUI	13	1.113833
24H 50MTA	Q7Z2K6	0.119804	0.005248	0.007133	ERMP1	Endoplasm	ERMP1_HU	17	1.086587
48H 50MTA	Q13868	-0.01985	0.005251	0.007136	EXOSC2	Exosome cc	EXOS2_HUI	12	0.986334
48H 10MTA	Q14671	0.110735	0.00525	0.007136	PUM1	Pumilio ho	PUM1_HUN	25	1.079778
24H 50MTA	Q5VTL8	0.720305	0.005251	0.007136	PRPF38B	Pre-mRNA-	PR38B_HUI	11	1.647531
48H 10MTA	P49368	0.086417	0.005253	0.007138	CCT3	T-complex	TCPG_HUM	39	1.06173
24H 10MTA	O43175	-0.08857	0.005258	0.007143	PHGDH	D-3-phosph	SERA_HUM	24	0.940452
48H 50MTA	Q8TCJ2	0.050615	0.005263	0.007148	STT3B	Dolichyl-di	STT3B_HUN	14	1.035706
48H 10MTA	Q96LI5	0.542145	0.005265	0.007149	CNOT6L	CCR4-NOT t	CNO6L_HU	2	1.456135
24H 50MTA	P56270	-0.31016	0.005266	0.00715	MAZ	Myc-associ	MAZ_HUMA	6	0.806554
24H 50MTA	Q99807	0.154024	0.005269	0.007152	COQ7	5-demetho	COQ7_HUN	4	1.112669
48H 10MTA	Q9NS86	-0.01357	0.005269	0.007152	LANCL2	LanC-like p	LANC2_HUI	14	0.990641
48H 50MTA	Q8WVV4	0.403433	0.005269	0.007152	POF1B	Protein POI	POF1B_HUI	6	1.322652
48H 10MTA	Q96G25	1.288795	0.005275	0.007158	MED8	Mediator o	MED8_HUN	2	2.443239
24H 50MTA	Q16795	0.034886	0.005276	0.007158	NDUFA9	NADH dehy	NDUA9_HU	17	1.024476
24H 50MTA	O43822	-0.14664	0.005279	0.007161	CFAP410	Cilia- and fl	CF410_HUI	1	0.903354
24H 10MTA	Q9NZM1	0.084823	0.00528	0.007162	MYOF	Myoferlin	MYOF_HUN	87	1.060558
48H 10MTA	O76027	1.515501	0.005285	0.007166	ANXA9	Annexin A9	ANXA9_HU	3	2.858982
24H 50MTA	Q2PZI1	0.225051	0.005286	0.007167	DPY19L1	Protein C-m	D19L1_HUI	13	1.168819
48H 10MTA	P62834	0.241815	0.005286	0.007168	RAP1A	Ras-related	RAP1A_HUI	3	1.18248
48H 10MTA	Q9NUU7	0.122471	0.005291	0.007172	DDX19A	ATP-depend	DD19A_HU	26	1.088597
48H 50MTA	Q6ZSC3	1.7515	0.005294	0.007174	RBM43	RNA-bindir	RBM43_HU	1	3.367085
24H 50MTA	P23921	0.096645	0.00531	0.007191	RRM1	Ribonuclec	RIR1_HUMA	30	1.069284
48H 10MTA	Q05BQ5	-0.81292	0.005312	0.007193	MBTD1	MBT domai	MBTD1_HU	1	0.569229
24H 50MTA	P42765	0.131135	0.005319	0.007199	ACAA2	3-ketoacyl-	THIM_HUM	20	1.095155

24H 50MT/	Q9BXW6	-0.33202	0.005321	0.0072	OSBPL1A	Oxysterol-b	OSBL1_HUI	7	0.794421
24H 50MT/	P50225	-1.17503	0.005325	0.007203	SULT1A1	Sulfotransf	ST1A1_HUI	2	0.442876
48H 10MT/	Q96AG4	0.011007	0.005329	0.007206	LRRC59	Leucine-ric	LRC59_HUI	13	1.007659
24H 10MT/	P61018	0.66609	0.005331	0.007207	RAB4B	Ras-related	RAB4B_HUI	3	1.586767
24H 50MT/	Q15042	0.005298	0.005343	0.00722	RAB3GAP1	Rab3 GTPas	RB3GP_HU	40	1.003679
48H 50MT/	P00761	0.319147	0.005348	0.007226	NaN	Trypsin (Lal	TRYP_PIG	7	1.247593
24H 10MT/	Q9NV70	0.028469	0.00535	0.007228	EXOC1	Exocyst cor	EXOC1_HUI	19	1.019929
24H 50MT/	Q8TBE9	0.696108	0.005351	0.007229	NANP	N-acylneur	NANP_HUM	4	1.620129
24H 50MT/	O95336	-0.16296	0.005358	0.007235	PGLS	6-phospho	6PGL_HUM	12	0.893189
48H 10MT/	Q92870	-0.17399	0.00536	0.007238	APBB2	Amyloid be	APBB2_HUI	1	0.88639
24H 10MT/	P14735	-0.11011	0.005362	0.007239	IDE	Insulin-deg	IDE_HUMA	47	0.926517
48H 10MT/	A6NNA2	2.766248	0.005364	0.007242	SRRM3	Serine/argi	SRRM3_HU	1	6.803364
48H 50MT/	P33981	-0.27582	0.005364	0.007242	TTK	Dual specifi	TTK_HUMA	19	0.82598
48H 50MT/	Q96SQ9	0.309143	0.005367	0.007243	CYP2S1	Cytochrom	CP2S1_HUI	10	1.238971
24H 10MT/	Q8TF68	0.045766	0.00537	0.007246	ZNF384	Zinc finger	ZN384_HUI	6	1.032231
24H 50MT/	Q12846	0.105247	0.005373	0.00725	STX4	Syntaxin-4	STX4_HUM	11	1.075679
48H 10MT/	Q9H5Z1	0.072403	0.005374	0.00725	DHX35	Probable A	DHX35_HU	15	1.051467
48H 10MT/	Q9NPF0	-0.14483	0.00538	0.007255	CD320	CD320 anti	CD320_HU	5	0.904486
24H 10MT/	Q9Y6V0	1.510528	0.005383	0.007258	PCLO	Protein pic	PCLO_HUM	2	2.849142
24H 50MT/	P11117	0.117633	0.005386	0.007261	ACP2	Lysosomal	PPAL_HUM	11	1.084953
24H 50MT/	Q96QE5	0.202674	0.005389	0.007265	TEFM	Transcripti	TEFM_HUM	8	1.15083
48H 50MT/	P25205	-0.17194	0.00539	0.007266	MCM3	DNA replica	MCM3_HUI	37	0.887648
24H 50MT/	Q9UMX0	0.052082	0.005391	0.007267	UBQLN1	Ubiquilin-1	UBQL1_HU	15	1.03676
48H 10MT/	Q96DE5	0.001967	0.005392	0.007267	ANAPC16	Anaphase-p	APC16_HUI	4	1.001364
24H 50MT/	Q96FN4	0.415662	0.005394	0.007269	CPNE2	Copine-2	CPNE2_HUI	8	1.333911
48H 50MT/	P02786	0.043798	0.005399	0.007274	TFRC	Transferrin	TFR1_HUM	36	1.030824
48H 50MT/	Q32P44	-0.0711	0.005402	0.007277	EML3	Echinoderm	EMAL3_HU	16	0.951912
48H 10MT/	Q9H0X4	0.207802	0.005409	0.007284	FAM234A	Protein FAI	F234A_HUI	6	1.154927
24H 10MT/	P00883	-0.24258	0.005409	0.007284	ALDOA	Fructose-bi	ALDOA_RAI	2	0.845231
48H 10MT/	Q6VY07	-0.04369	0.00541	0.007284	PACS1	Phosphofu	PACS1_HUI	16	0.970171
24H 50MT/	Q03001	-0.0571	0.005411	0.007286	DST	Dystonin	DYST_HUM	42	0.961192
48H 50MT/	Q6SZW1	-0.12865	0.005413	0.007287	SARM1	NAD(+) hyd	SARM1_HU	28	0.91469
48H 10MT/	Q8IYB8	-0.01574	0.005413	0.007287	SUPV3L1	ATP-depend	SUV3_HUM	25	0.989152
48H 50MT/	Q96QD9	0.128378	0.005416	0.00729	FYTTD1	UAP56-inte	UIF_HUMA	7	1.093064
24H 50MT/	Q9NTX5	-0.10504	0.005416	0.00729	ECHDC1	Ethylmalor	ECHD1_HU	14	0.929777
24H 50MT/	A6NHX0	-0.23541	0.005422	0.007296	CASTOR2	Cytosolic a	CAST2_HUI	9	0.849442
24H 10MT/	Q8NEB9	-0.00356	0.005425	0.007299	PIK3C3	Phosphatid	PK3C3_HUI	20	0.997534
24H 50MT/	Q9UHV9	0.20922	0.005426	0.007299	PFDN2	Prefoldin s	PFD2_HUM	7	1.156063
24H 50MT/	Q15019	-0.0838	0.005428	0.007302	SEPTIN2	Septin-2	SEPT2_HUI	16	0.943567
24H 50MT/	Q9Y5J1	0.181735	0.005436	0.007311	UTP18	U3 small nu	UTP18_HUI	19	1.134247
48H 50MT/	P54802	0.092544	0.005438	0.007313	NAGLU	Alpha-N-ac	ANAG_HUM	19	1.066249
24H 50MT/	Q96SB8	0.294053	0.005439	0.007314	SMC6	Structural	SMC6_HUM	9	1.22608
48H 10MT/	Q9H7C9	0.010959	0.00544	0.007315	AAMDC	Mth938 do	AAMDC_HU	7	1.007625
48H 50MT/	Q92576	0.103349	0.005443	0.007318	PHF3	PHD finger	PHF3_HUM	36	1.074264
48H 50MT/	Q12929	0.075298	0.005448	0.007321	EPS8	Epidermal	EPS8_HUM	32	1.053579
24H 10MT/	Q9H329	0.010783	0.005448	0.007321	EPB41L4B	Band 4.1-lil	E41LB_HUI	3	1.007502
24H 50MT/	Q9Y6H1	0.231152	0.005453	0.007326	CHCHD2	Coiled-coil	CHCH2_HU	4	1.173772
24H 50MT/	P04003	-1.14057	0.005459	0.007333	C4BPA	C4b-bindin	C4BPA_HUI	1	0.45358
48H 50MT/	Q2M389	-0.03525	0.005461	0.007334	WASHC4	WASH com	WASC4_HU	28	0.97586
24H 10MT/	P60903	-0.62552	0.005462	0.007335	S100A10	Protein S1C	S10AA_HUI	3	0.648186
48H 50MT/	P04004	0.306347	0.005463	0.007336	VTN	Vitronectir	VTNC_HUM	9	1.236573
48H 10MT/	O95347	0.005365	0.005463	0.007336	SMC2	Structural	SMC2_HUM	48	1.003725

24H	10MTA	Q8WWI1	0.095164	0.005463	0.007336	LMO7	LIM domain	LMO7_HUM	44	1.068187
24H	10MTA	Q9Y6M7	0.158405	0.005475	0.007347	SLC4A7	Sodium bic	S4A7_HUM	15	1.116052
24H	10MTA	P61221	0.076863	0.005481	0.007354	ABCE1	ATP-bindin	ABCE1_HU	28	1.054722
48H	50MTA	Q96CS3	-0.11364	0.005482	0.007355	FAF2	FAS-associa	FAF2_HUM	18	0.924255
48H	10MTA	Q8NCF5	0.032341	0.005483	0.007356	NFATC2IP	NFATC2-int	NF2IP_HUM	5	1.02267
48H	50MTA	Q14181	-0.20785	0.005486	0.007358	POLA2	DNA polym	DPOA2_HU	12	0.865826
24H	50MTA	P09417	0.016483	0.005486	0.007358	QDPR	Dihydropte	DHPR_HUM	10	1.01149
24H	10MTA	P07199	0.126805	0.005488	0.007359	CENPB	Major centr	CENPB_HU	5	1.091873
24H	50MTA	O75909	0.128166	0.0055	0.007373	CCNK	Cyclin-K	CCNK_HUM	9	1.092903
48H	50MTA	P35637	0.054155	0.005501	0.007373	FUS	RNA-bindin	FUS_HUMA	12	1.03825
48H	10MTA	Q9Y5L4	0.16841	0.005504	0.007376	TIMM13	Mitochond	TIM13_HU	7	1.123819
48H	50MTA	Q8NFW8	0.0652	0.005511	0.007383	CMAS	N-acylneura	NEUA_HUM	14	1.04623
48H	10MTA	O15371	0.143973	0.005514	0.007386	EIF3D	Eukaryotic	EIF3D_HUM	24	1.104944
48H	50MTA	Q6EMK4	0.705357	0.005515	0.007386	VASN	Vasorin	VASN_HUM	4	1.630548
24H	50MTA	P31271;P3	1.37487	0.005515	0.007387	HOXA13;H	Homeobox	HXA13_HU	1	2.593446
24H	50MTA	Q9Y2Q9	-0.1903	0.005517	0.007388	MRPS28	Small ribos	RT28_HUM	6	0.876423
48H	50MTA	P39687	0.230851	0.005522	0.007394	ANP32A	Acidic leuci	AN32A_HU	10	1.173527
24H	50MTA	P30414	0.325226	0.005526	0.007397	NKTR	NK-tumor r	NKTR_HUM	8	1.252861
48H	50MTA	P05388	-0.15127	0.005526	0.007398	RPLP0	Large ribos	RLA0_HUM	13	0.900456
24H	50MTA	Q15059	0.332707	0.005529	0.0074	BRD3	Bromodom	BRD3_HUM	8	1.259374
48H	50MTA	Q15061	-0.14161	0.005529	0.0074	WDR43	WD repeat-	WDR43_HU	25	0.906504
48H	50MTA	Q96AB3	0.287042	0.005536	0.007408	ISOC2	Isochorism	ISOC2_HUM	7	1.220136
24H	50MTA	Q9UJX2	0.070732	0.005538	0.00741	CDC23	Cell divisio	CDC23_HU	24	1.05025
48H	50MTA	Q9HCE6	0.194185	0.005544	0.007416	ARHGEF10L	Rho guanin	ARGAL_HU	10	1.144078
48H	50MTA	O95218	0.190735	0.005544	0.007416	ZRANB2	Zinc finger	ZRAB2_HU	14	1.141345
24H	10MTA	Q92791	0.129574	0.005545	0.007416	P3H4	Endoplasm	SC65_HUM	7	1.093971
24H	50MTA	Q5JTD0	-0.05929	0.005546	0.007417	TJAP1	Tight juncti	TJAP1_HUM	9	0.959735
48H	10MTA	Q658Y4	0.009899	0.005546	0.007417	FAM91A1	Protein FAM	F91A1_HU	17	1.006885
48H	50MTA	Q9NWL6	-0.05618	0.005547	0.007418	ASNSD1	Asparagine	ASND1_HU	4	0.961809
24H	10MTA	Q9BX93	-0.11381	0.00555	0.00742	PLA2G12B	Group XIIB	PG12B_HU	2	0.924142
24H	10MTA	Q96KR1	0.015537	0.00556	0.00743	ZFR	Zinc finger	ZFR_HUMA	33	1.010828
48H	50MTA	P39060	0.006977	0.005561	0.007431	COL18A1	Collagen al	COIA1_HUM	14	1.004848
24H	50MTA	Q9Y4W6	0.123993	0.00557	0.007441	AFG3L2	Mitochond	AFG32_HU	34	1.089746
48H	50MTA	Q969X5	0.077772	0.005571	0.007442	ERGIC1	Endoplasm	ERGI1_HUM	9	1.055387
48H	10MTA	Q9Y6X3	0.147233	0.005576	0.007447	MAU2	MAU2 chro	SCC4_HUM	10	1.107444
48H	50MTA	P35237	0.206792	0.00558	0.007449	SERPINB6	Serpin B6	SPB6_HUM	18	1.154119
48H	50MTA	Q9UKK3	0.087158	0.005581	0.00745	PARP4	Protein mo	PARP4_HU	35	1.062275
48H	10MTA	Q9HB58	-1.29823	0.005583	0.007452	SP110	Sp110 nucl	SP110_HU	2	0.406624
48H	50MTA	Q9P0S9	0.123288	0.005584	0.007453	TMEM14C	Transmemb	TM14C_HU	3	1.089214
48H	10MTA	Q99735	-0.36926	0.005586	0.007455	MGST2	Microsoma	MGST2_HU	2	0.774181
24H	50MTA	Q9BSJ8	0.000631	0.005589	0.007457	ESYT1	Extended sy	ESYT1_HUM	45	1.000437
48H	10MTA	Q9ULG6	-0.53596	0.005598	0.007468	CCPG1	Cell cycle p	CCPG1_HU	4	0.689699
48H	10MTA	Q13123	0.025671	0.005599	0.007469	IK	Protein Red	RED_HUMA	19	1.017953
24H	50MTA	P36507	-0.09284	0.005603	0.007473	MAP2K2	Dual specifi	MP2K2_HU	29	0.937675
48H	10MTA	P04004	0.19925	0.005605	0.007474	VTN	Vitronectin	VTNC_HUM	9	1.148101
48H	10MTA	Q8N1G2	0.019229	0.005609	0.007477	CMTR1	Cap-specifi	CMTR1_HU	33	1.013418
48H	50MTA	Q96P63	-0.21833	0.00561	0.007479	SERPINB12	Serpin B12	SPB12_HU	3	0.859559
24H	10MTA	P31939	-0.0868	0.005616	0.007485	ATIC	Bifunctiona	PUR9_HUM	41	0.941612
48H	10MTA	P00761	0.376303	0.005621	0.007489	NaN	Trypsin (Lal	TRYP_PIG	7	1.298011
48H	50MTA	O00217	0.054463	0.005622	0.00749	NDUFS8	NADH dehy	NDUS8_HU	6	1.038472
48H	50MTA	Q9UBL3	0.064698	0.005623	0.00749	ASH2L	Set1/Ash2 I	ASH2L_HU	13	1.045866
48H	10MTA	P13797	0.044982	0.005626	0.007493	PLS3	Plastin-3	PLST_HUMA	38	1.03167

24H 10MT/	P17987	0.013364	0.005626	0.007493	TCP1	T-complex	TCPA_HUM	32	1.009306
48H 10MT/	Q9Y6X4	0.043078	0.005626	0.007493	FAM169A	Soluble lam	F169A_HUI	12	1.030309
24H 50MT/	Q9UJK0	0.502698	0.005625	0.007493	TSR3	18S rRNA ai	TSR3_HUM	6	1.41686
48H 50MT/	Q92597	1.350534	0.005626	0.007493	NDRG1	Protein ND	NDRG1_HU	12	2.550065
24H 50MT/	P43307	0.147142	0.005638	0.007501	SSR1	Translocon	SSRA_HUM	6	1.107373
48H 10MT/	Q99732	0.226263	0.005638	0.007501	LITAF	Lipopolysa	LITAF_HUM	1	1.169801
48H 10MT/	O43815	0.07477	0.005639	0.007502	STRN	Striatin	STRN_HUM	18	1.053193
48H 50MT/	Q9NRX5	0.160418	0.00564	0.007502	SERINC1	Serine inco	SERC1_HUI	4	1.117611
24H 10MT/	Q5SRE5	0.081477	0.005658	0.007521	NUP188	Nucleopori	NU188_HU	51	1.058101
48H 50MT/	Q8N1B4	0.075434	0.005662	0.007524	VPS52	Vacuolar pi	VPS52_HUI	18	1.053678
24H 50MT/	Q96BY9	-0.60419	0.005672	0.007534	SARAF	Store-opera	SARAF_HUI	1	0.657842
48H 50MT/	Q5EBL4	0.291112	0.005673	0.007536	RILPL1	RILP-like pr	RIPL1_HUM	4	1.223583
24H 10MT/	P62273	-0.49643	0.005675	0.007537	RPS29	Small ribos	RS29_HUM	4	0.708859
24H 10MT/	Q9P287	0.026945	0.005676	0.007537	BCCIP	BRCA2 and	BCCIP_HUM	14	1.018852
48H 50MT/	P52926	0.125971	0.005679	0.00754	HMGA2	High mobil	HMGA2_HU	4	1.091242
24H 10MT/	Q9H0V9	0.15661	0.00568	0.007542	LMAN2L	VIP36-like	LMA2L_HU	12	1.114665
48H 10MT/	O00213	0.069555	0.005682	0.007543	APBB1	Amyloid be	APBB1_HUI	3	1.049393
48H 50MT/	O95302	0.106233	0.005683	0.007543	FKBP9	Peptidyl-pr	FKBP9_HUI	14	1.076414
48H 50MT/	P48668	0.17505	0.005692	0.007552	KRT6C	Keratin, typ	K2C6C_HUI	32	1.129003
24H 10MT/	P04406	-0.09174	0.005701	0.007559	GAPDH	Glyceraldel	G3P_HUM/	23	0.938393
24H 50MT/	Q9HAV0	0.472263	0.005709	0.007567	GNB4	Guanine nu	GBB4_HUM	6	1.387284
24H 50MT/	Q9H2G2	-0.09119	0.00571	0.007568	SLK	STE20-like	SLK_HUMA	36	0.938747
48H 10MT/	P09132	0.106279	0.005711	0.007568	SRP19	Signal reco	SRP19_HUI	4	1.076449
24H 50MT/	O14949	0.153848	0.005711	0.007569	UQCRQ	Cytochrom	QCR8_HUM	3	1.112533
24H 50MT/	Q8TF74	-0.1703	0.005714	0.007572	WIPF2	WAS/WASL	WIPF2_HUI	11	0.888661
24H 50MT/	P07919	0.402725	0.005715	0.007573	UQCRH	Cytochrom	QCR6_HUM	3	1.322003
48H 50MT/	Q5T653	-0.17046	0.005716	0.007574	MRPL2	Large ribos	RM02_HUM	7	0.88856
24H 50MT/	Q13586	0.04995	0.005721	0.00758	STIM1	Stromal int	STIM1_HUI	20	1.035229
48H 50MT/	Q92696	-0.15458	0.005723	0.00758	RABGGTA	Geranylgera	PGTA_HUM	21	0.898391
48H 50MT/	O75955	0.096763	0.005724	0.007581	FLOT1	Flotillin-1	FLOT1_HUI	23	1.069371
24H 50MT/	P09874	0.083479	0.005727	0.007584	PARP1	Poly [ADP-r	PARP1_HUI	53	1.05957
48H 10MT/	Q96EY8	0.003911	0.005728	0.007585	MMAB	Corrinoid a	MMAB_HUI	7	1.002715
48H 10MT/	P29144	0.017428	0.00574	0.007598	TPP2	Tripeptidyl	TPP2_HUM	50	1.012154
48H 50MT/	P55735	0.142643	0.005741	0.007599	SEC13	Protein SEC	SEC13_HUI	11	1.103926
24H 10MT/	Q86U44	0.067426	0.005743	0.0076	METTL3	N6-adenosi	MTA70_HU	15	1.047846
48H 50MT/	Q06210	0.078556	0.005754	0.007609	GFPT1	Glutamine-	GFPT1_HUI	39	1.05596
24H 50MT/	Q76M96	0.054024	0.005757	0.007613	CCDC80	Coiled-coil	CCD80_HU	1	1.038157
24H 50MT/	P63172	-0.41187	0.005757	0.007613	DYNLT1	Dynein ligh	DYLT1_HUI	5	0.751647
48H 10MT/	Q9UK61	0.008195	0.005758	0.007613	TASOR	Protein TAS	TASOR_HUI	40	1.005696
24H 50MT/	Q9BUL9	0.229065	0.005766	0.007621	RPP25	Ribonuclea	RPP25_HUI	8	1.172075
24H 50MT/	P06744	0.014759	0.005768	0.007622	GPI	Glucose-6-	G6PI_HUM	23	1.010283
24H 50MT/	Q9NX02	0.048217	0.005768	0.007623	NLRP2	NACHT, LRF	NALP2_HUI	46	1.033986
24H 10MT/	O60942	0.174164	0.005771	0.007626	RNGTT	mRNA-capp	MCE1_HUM	11	1.128311
24H 50MT/	O00584	0.399229	0.005773	0.007627	RNASET2	Ribonuclea	RNT2_HUM	11	1.318803
48H 10MT/	Q9Y3Y2	0.125328	0.005781	0.007636	CHTOP	Chromatin	CHTOP_HU	6	1.090755
24H 50MT/	Q15051	1.183428	0.005791	0.007644	IQCB1	IQ calmodu	IQCB1_HUI	2	2.271158
24H 50MT/	Q15427	0.209696	0.005794	0.007645	SF3B4	Splicing fac	SF3B4_HUI	8	1.156444
48H 10MT/	Q7Z4S6	0.042102	0.005797	0.007649	KIF21A	Kinesin-like	KI21A_HUI	40	1.029613
24H 50MT/	P53618	0.082333	0.005801	0.007652	COPB1	Coatomer s	COPB_HUM	46	1.058729
48H 10MT/	P43358	0.066044	0.005805	0.007656	MAGEA4	Melanoma-	MAGA4_HU	11	1.046842
48H 10MT/	Q9Y3C6	0.120018	0.005811	0.007662	PPIL1	Peptidyl-pr	PPIL1_HUM	7	1.086749
48H 10MT/	Q8TBF4	0.243961	0.005813	0.007664	ZCRB1	Zinc finger	ZCRB1_HUI	4	1.18424

48H	50MT/	Q13111	-0.5715	0.005816	0.007666	CHAF1A	Chromatin	CAF1A_HUI	13	0.672917
24H	50MT/	Q9H7Z7	0.220773	0.005818	0.007667	PTGES2	Prostaglan(	PGES2_HUI	14	1.165358
24H	10MT/	P55263	0.10157	0.005822	0.00767	ADK	Adenosine I	ADK_HUM/	23	1.072941
24H	10MT/	O43924	-0.10542	0.005823	0.007671	PDE6D	Retinal rod	PDE6D_HU	4	0.929531
24H	50MT/	O75063	0.494851	0.005832	0.00768	FAM20B	Glycosamir	XYLK_HUM	7	1.409175
24H	10MT/	P51553	-0.17496	0.005835	0.007682	IDH3G	Isocitrate d	IDH3G_HUI	12	0.885794
48H	10MT/	Q96ME1	0.020359	0.005837	0.007684	FBXL18	F-box/LRR-i	FXL18_HUI	20	1.014212
48H	50MT/	Q6P3X3	-0.1016	0.005839	0.007685	TTC27	Tetratricop	TTC27_HUI	21	0.932002
24H	50MT/	O60739	-0.7808	0.005843	0.007689	EIF1B	Eukaryotic	EIF1B_HUM	1	0.582045
24H	50MT/	O00257	0.133483	0.005844	0.007689	CBX4	E3 SUMO-p	CBX4_HUM	6	1.096939
48H	50MT/	Q9Y4G8	0.066793	0.005845	0.007689	RAPGEF2	Rap guanin	RPGF2_HUI	6	1.047386
24H	50MT/	Q9NS56	0.551015	0.005846	0.00769	TOPORS	E3 ubiquiti	TOPRS_HUI	7	1.465116
24H	10MT/	P18085	0.150158	0.005851	0.007696	ARF4	ADP-ribosy	ARF4_HUM	7	1.109691
48H	10MT/	P68402	-0.17378	0.005851	0.007696	PAFAH1B2	Platelet-act	PA1B2_HUI	8	0.886518
48H	10MT/	Q9NWV8	0.298193	0.005855	0.007698	BABAM1	BRISC and I	BABA1_HUI	6	1.229603
24H	50MT/	O94901	0.112132	0.005855	0.007698	SUN1	SUN domai	SUN1_HUM	30	1.080824
48H	50MT/	Q53S33	0.333165	0.005859	0.007702	BOLA3	BolA-like pı	BOLA3_HUI	3	1.259774
48H	10MT/	P78524	-1.50945	0.005859	0.007702	DENND2B	DENN dom	DEN2B_HU	1	0.351246
24H	50MT/	Q8N3Z3	-0.05693	0.005862	0.007704	GTPBP8	GTP-bindin	GTPB8_HUI	3	0.96131
48H	10MT/	Q15776;Q4	-0.81825	0.005863	0.007705	ZKSCAN8;ZI	Zinc finger	ZKSC8_HUI	1	0.567129
48H	50MT/	Q7L576	0.050682	0.00587	0.007712	CYFIP1	Cytoplasmi	CYFP1_HUI	46	1.035755
24H	50MT/	Q9Y676	0.092823	0.005874	0.007717	MRPS18B	Small ribos	RT18B_HUI	9	1.066455
48H	50MT/	Q04941	0.032048	0.005875	0.007717	PLP2	Proteolipid	PLP2_HUM	1	1.022463
48H	10MT/	Q96NC0	0.220361	0.005877	0.007719	ZMAT2	Zinc finger	ZMAT2_HU	4	1.165025
24H	50MT/	P50148	0.18324	0.00588	0.007722	GNAQ	Guanine nu	GNAQ_HUM	2	1.135431
24H	10MT/	Q9UBS4	0.027815	0.005886	0.007727	DNAJB11	DnaJ homo	DJB11_HUI	15	1.019467
24H	50MT/	Q96IZ5	0.973612	0.005886	0.007727	RBM41	RNA-bindir	RBM41_HU	2	1.963751
48H	50MT/	P00352	0.069372	0.005897	0.007737	ALDH1A1	Aldehyde d	AL1A1_HUI	32	1.04926
24H	10MT/	P04156	0.275591	0.005898	0.007737	PRNP	Major prior	PRIO_HUM	4	1.21049
24H	50MT/	P40205	7.503059	0.005897	0.007737	MYCNOS	N-cym prot	NCYM_HUM	1	181.4035
24H	50MT/	Q14254	0.116944	0.005899	0.007738	FLOT2	Flotillin-2	FLOT2_HUI	22	1.084435
24H	50MT/	Q9NWH9	0.20044	0.005909	0.007749	SLTM	SAFB-like tr	SLTM_HUM	17	1.149049
48H	10MT/	Q15046	0.024834	0.00591	0.00775	KARS1	Lysine--tRN	SYK_HUMA	29	1.017363
48H	50MT/	Q9UBD5	-0.20966	0.005912	0.007751	ORC3	Origin reco	ORC3_HUM	16	0.864738
24H	50MT/	Q6UXN9	0.113937	0.005917	0.007755	WDR82	WD repeat-	WDR82_HU	13	1.082177
24H	50MT/	Q8NG27	0.501397	0.00592	0.007758	PJA1	E3 ubiquiti	PJA1_HUM	1	1.415584
24H	50MT/	P51608	0.327465	0.00592	0.007758	MECP2	Methyl-Cp(	MECP2_HU	12	1.254807
24H	50MT/	Q96FZ2	-0.10472	0.005924	0.007761	HMCES	Abasic site	HMCES_HU	11	0.929986
48H	50MT/	P36955	0.254524	0.005925	0.007762	SERPINF1	Pigment ep	PEDF_HUM	10	1.192942
48H	50MT/	O15440	0.122248	0.005925	0.007762	ABCC5	ATP-bindin	MRP5_HUM	16	1.088429
48H	50MT/	Q969G3	0.079955	0.005929	0.007765	SMARCE1	SWI/SNF-re	SMCE1_HU	12	1.056985
24H	50MT/	A8MXV4	0.275117	0.00593	0.007766	NUDT19	Acyl-coenzy	NUD19_HU	16	1.210092
48H	50MT/	Q9H8M2	-1.41694	0.005931	0.007767	BRD9	Bromodom	BRD9_HUM	1	0.374505
24H	50MT/	P51965	0.134252	0.005932	0.007767	UBE2E1	Ubiquitin-c	UB2E1_HUI	1	1.097524
24H	10MT/	Q9UPW5	0.02015	0.005936	0.007772	AGTPBP1	Cytosolic c	CBPC1_HUI	1	1.014065
48H	10MT/	P17936	0.077343	0.005937	0.007773	IGFBP3	Insulin-like	IBP3_HUM/	5	1.055073
48H	10MT/	Q6P2I3	0.330593	0.005938	0.007775	FAHD2B	Oxaloaceta	FAH2B_HUI	1	1.25753
24H	10MT/	P53814	0.401626	0.00594	0.007776	SMTN	Smoothelir	SMTN_HUM	13	1.320996
48H	10MT/	P14859	0.075383	0.00594	0.007776	POU2F1	POU domai	PO2F1_HUI	9	1.053641
24H	10MT/	Q93099	-0.08208	0.005957	0.007792	HGD	Homogenti	HGD_HUM/	17	0.944696
24H	50MT/	P17707	1.52715	0.005958	0.007793	AMD1	S-adenosylı	DCAM_HUI	4	2.882159
24H	50MT/	Q02318	0.218583	0.005961	0.007797	CYP27A1	Sterol 26-h	CP27A_HUI	9	1.16359

48H 50MT/	Q8WW12	0.055646	0.005968	0.007802	PCNP	PEST protei	PCNP_HUM	6	1.039324
24H 50MT/	P11021	0.102583	0.005974	0.007807	HSPA5	Endoplasm	BIP_HUMA	35	1.073694
48H 10MT/	Q9H1K1	0.124572	0.005973	0.007807	ISCU	Iron-sulfur	ISCU_HUM	6	1.090184
48H 10MT/	Q9HAZ1	-0.24853	0.005981	0.007813	CLK4	Dual specifi	CLK4_HUM	3	0.841755
48H 10MT/	O95786	0.02218	0.005982	0.007814	RIGI	Antiviral in	RIGI_HUM/	16	1.015493
48H 10MT/	P40763	0.083124	0.005989	0.007821	STAT3	Signal trans	STAT3_HUM	37	1.059309
48H 50MT/	Q9NRF9	-0.03101	0.005991	0.007822	POLE3	DNA polym	DPOE3_HU	4	0.978734
24H 10MT/	P48556	0.100082	0.005992	0.007823	PSMD8	26S protea:	PSMD8_HU	13	1.071834
48H 50MT/	P61077	0.201244	0.005994	0.007823	UBE2D3	Ubiquitin-c	UB2D3_HU	4	1.149689
24H 50MT/	Q7Z3C6	0.211253	0.005994	0.007823	ATG9A	Autophagy-	ATG9A_HU	17	1.157693
24H 10MT/	P52948	0.021381	0.005996	0.007823	NUP98	Nuclear po	NUP98_HU	54	1.014931
24H 50MT/	O43852	0.098084	0.006002	0.007829	CALU	Calumenin	CALU_HUM	21	1.070351
48H 50MT/	O43325	-0.93489	0.006004	0.00783	LYRM1	LYR motif-c	LYRM1_HU	1	0.523084
48H 50MT/	Q9ULV4	0.085328	0.006005	0.007832	CORO1C	Coronin-1C	COR1C_HU	19	1.060929
24H 50MT/	Q13868	0.194534	0.006018	0.007844	EXOSC2	Exosome cc	EXOS2_HU	12	1.144355
24H 50MT/	P57737	-0.13396	0.006019	0.007845	CORO7	Coronin-7	CORO7_HU	23	0.911324
24H 50MT/	Q17R98	0.421585	0.006022	0.007848	ZNF827	Zinc finger	ZN827_HU	2	1.339398
24H 10MT/	P30044	-0.11004	0.006025	0.007851	PRDX5	Peroxiredo	PRDX5_HU	11	0.926563
24H 50MT/	P22234	0.019749	0.006035	0.007861	PAICS	Bifunctiona	PUR6_HUM	23	1.013783
24H 50MT/	Q9Y6K5	-0.6402	0.006041	0.007867	OAS3	2'-5'-oligoa	OAS3_HUM	7	0.641624
48H 50MT/	Q99436	0.082399	0.006042	0.007868	PSMB7	Proteasom	PSB7_HUM	12	1.058777
24H 50MT/	P13073	-0.01453	0.006044	0.00787	COX4I1	Cytochrom	COX41_HU	10	0.989976
48H 50MT/	P49915	-0.09314	0.006046	0.007871	GMPS	GMP synth	GUAA_HUM	35	0.93748
48H 50MT/	P08243	0.009669	0.006048	0.007873	ASNS	Asparagine	ASNS_HUM	26	1.006724
48H 50MT/	Q9NR12	0.043172	0.006052	0.007875	PDLIM7	PDZ and LIN	PDLI7_HUM	12	1.030377
48H 50MT/	Q9UDY4	0.128402	0.006054	0.007877	DNAJB4	DnaJ homo	DNJB4_HU	11	1.093083
48H 10MT/	O95716	0.077021	0.006056	0.007879	RAB3D	Ras-related	RAB3D_HU	8	1.054837
24H 10MT/	P10606	-0.34383	0.006069	0.007893	COX5B	Cytochrom	COX5B_HU	6	0.787947
24H 10MT/	Q9NZJ5	0.06679	0.00607	0.007893	EIF2AK3	Eukaryotic	E2AK3_HU	6	1.047384
48H 10MT/	P29320	-0.16998	0.006071	0.007894	EPHA3	Ephrin type	EPHA3_HU	1	0.888855
24H 50MT/	Q9H7N4	0.319422	0.006072	0.007894	SCAF1	Splicing fac	SFR19_HUN	7	1.24783
48H 50MT/	Q9NWK9	-0.54197	0.006072	0.007894	ZNHIT6	Box C/D snc	BCD1_HUM	12	0.686833
48H 50MT/	Q96GD0	0.049112	0.006072	0.007895	PDXP	Chronophi	PLPP_HUM	9	1.034628
48H 10MT/	P17480	0.058964	0.006078	0.0079	UBTF	Nucleolar t	UBF1_HUM	23	1.041718
48H 10MT/	Q93009	0.025407	0.00608	0.007902	USP7	Ubiquitin c	UBP7_HUM	54	1.017767
48H 10MT/	Q15437	0.04546	0.006084	0.007906	SEC23B	Protein trai	SC23B_HU	29	1.032012
48H 50MT/	P42892	0.06186	0.006088	0.00791	ECE1	Endothelin	ECE1_HUM	8	1.043811
48H 50MT/	P26196	-0.13318	0.006091	0.007913	DDX6	Probable A	DDX6_HUM	25	0.911819
48H 50MT/	Q9UDW1	0.087702	0.006091	0.007914	UQCR10	Cytochrom	QCR9_HUM	2	1.062676
24H 50MT/	Q58WW2	0.279885	0.006096	0.007918	DCAF6	DDB1- and	DCAF6_HU	14	1.214098
24H 10MT/	Q9H0G5	1.249565	0.006098	0.00792	NSRP1	Nuclear spe	NSRP1_HU	4	2.377698
48H 50MT/	Q96M96	-0.06301	0.006099	0.007921	FGD4	FYVE, RhoG	FGD4_HUM	21	0.957265
48H 50MT/	P10586	0.573475	0.00611	0.007932	PTPRF	Receptor-ty	PTPRF_HUN	25	1.488104
24H 50MT/	Q7L523	0.149959	0.006112	0.007935	RRAGA	Ras-related	RRAGA_HU	10	1.109538
48H 50MT/	Q9Y6N5	0.046682	0.006116	0.007937	SQOR	Sulfide:qui	SQOR_HUM	30	1.032887
24H 10MT/	Q9NUJ7	-0.43162	0.00612	0.007941	PLCXD1	PI-PLC X do	PLCX1_HU	4	0.741427
24H 50MT/	Q9Y673	0.162642	0.006122	0.007941	ALG5	Dolichyl-ph	ALG5_HUM	12	1.119335
24H 50MT/	P07306	0.074924	0.006135	0.007955	ASGR1	Asialoglyco	ASGR1_HU	14	1.053305
24H 50MT/	Q96T58	0.167054	0.006136	0.007955	SPEN	Msx2-intera	MINT_HUM	26	1.122763
24H 50MT/	Q9UGQ3	0.344808	0.006137	0.007956	SLC2A6	Solute carri	GTR6_HUM	1	1.269982
48H 50MT/	Q9BTX1	-0.10235	0.006137	0.007956	NDC1	Nucleopori	NDC1_HUM	17	0.931513
48H 50MT/	Q9NPD8	-0.45383	0.006139	0.007958	UBE2T	Ubiquitin-c	UBE2T_HU	7	0.730104

24H 10MT/	Q9HC21	-0.68756	0.00614	0.007958	SLC25A19	Mitochond	TPC_HUMA	2	0.620901
24H 10MT/	P05556	0.107668	0.00615	0.007969	ITGB1	Integrin bet	ITB1_HUM/	25	1.077485
24H 50MT/	Q8TBN0	-0.00061	0.006155	0.007975	RAB3IL1	Guanine nu	R3GEF_HUI	6	0.99958
48H 10MT/	Q5VSL9	0.026569	0.006155	0.007975	STRIP1	Striatin-int	STRP1_HUI	14	1.018587
48H 10MT/	Q13547	0.08422	0.006155	0.007975	HDAC1	Histone dea	HDAC1_HU	12	1.060115
24H 50MT/	Q8WXE9	1.617491	0.00616	0.00798	STON2	Stonin-2	STON2_HUI	1	3.068408
24H 50MT/	Q16881	-0.19328	0.006162	0.007981	TXNRD1	Thioredoxi	TRXR1_HUI	28	0.874615
48H 50MT/	O14795	0.140427	0.006165	0.007984	UNC13B	Protein unc	UN13B_HU	13	1.102231
48H 10MT/	P61978	0.014019	0.006167	0.007986	HNRNPK	Heterogene	HNRPK_HU	27	1.009765
24H 10MT/	Q9UBP6	-0.0245	0.006167	0.007986	METTL1	tRNA (guan	TRMB_HUM	10	0.983159
24H 50MT/	Q8IXK0	0.223431	0.006172	0.00799	PHC2	Polyhomec	PHC2_HUM	6	1.167507
48H 50MT/	Q96IY1	0.146423	0.006172	0.00799	NSL1	Kinetochor	NSL1_HUM	5	1.106822
48H 10MT/	Q9C0E2	0.104582	0.006173	0.007992	XPO4	Exportin-4	XPO4_HUM	25	1.075183
24H 50MT/	Q9Y2Z2	0.120299	0.006181	0.008	MTO1	Protein MT	MTO1_HUM	18	1.08696
48H 50MT/	Q14151	0.053222	0.006187	0.008005	SAFB2	Scaffold att	SAFB2_HUI	26	1.037579
24H 50MT/	O75312	-0.15945	0.006192	0.008009	ZPR1	Zinc finger	ZPR1_HUM	17	0.895365
48H 50MT/	Q9UM00	-0.23249	0.006193	0.00801	TMCO1	Calcium loa	TMCO1_HU	3	0.851164
48H 50MT/	Q99470	0.293325	0.006201	0.008015	SDF2	Stromal cel	SDF2_HUM	5	1.225461
48H 50MT/	Q9BYG3	-0.19964	0.006201	0.008015	NIFK	MKI67 FHA	MK67I_HUI	17	0.870768
24H 50MT/	O75381	1.464942	0.006201	0.008015	PEX14	Peroxisoma	PEX14_HUI	2	2.760525
24H 50MT/	Q9UBM7	0.131799	0.006202	0.008016	DHCR7	7-dehydroc	DHCR7_HU	7	1.095659
48H 50MT/	P21127	0.00683	0.006204	0.008018	CDK11B	Cyclin-depe	CD11B_HU	21	1.004745
48H 10MT/	P78344	0.05625	0.006209	0.008021	EIF4G2	Eukaryotic	IF4G2_HUN	46	1.039759
24H 50MT/	Q8TEB7	0.176985	0.006208	0.008021	RNF128	E3 ubiquiti	RN128_HU	1	1.130519
24H 50MT/	Q15006	0.021022	0.006212	0.008025	EMC2	ER membra	EMC2_HUN	10	1.014678
24H 10MT/	Q7Z6B7	0.036388	0.006213	0.008026	SRGAP1	SLIT-ROBO	SRGP1_HUI	13	1.025543
48H 10MT/	Q969G3	0.11173	0.006215	0.008028	SMARCE1	SWI/SNF-re	SMCE1_HU	12	1.080523
24H 50MT/	P17480	0.021052	0.006221	0.008034	UBTF	Nucleolar t	UBF1_HUM	23	1.014699
24H 50MT/	Q08257	0.092454	0.006226	0.008039	CRYZ	Quinone ox	QOR_HUM/	17	1.066182
24H 50MT/	P07099	0.196648	0.006249	0.00806	EPHX1	Epoxide hy	HYEP_HUM	19	1.146032
48H 50MT/	P42785	0.0378	0.006254	0.008064	PRCP	Lysosomal	PCP_HUMA	12	1.026547
48H 50MT/	Q5JPE7	-0.10162	0.006255	0.008064	NOMO2	BOS compl	NOMO2_HU	44	0.931985
48H 50MT/	Q14790	0.209789	0.006266	0.008075	CASP8	Caspase-8	CASP8_HUI	17	1.156519
48H 50MT/	Q9NUQ3	-0.28833	0.006275	0.008082	TXLNG	Gamma-tax	TXLNG_HUI	17	0.818851
24H 50MT/	Q15075	-0.08463	0.006276	0.008083	EEA1	Early endos	EEA1_HUM	63	0.943025
24H 10MT/	Q7Z2K8	0.098752	0.006278	0.008086	GPRIN1	G protein-r	GRIN1_HUI	10	1.070847
24H 10MT/	P49184	1.848092	0.006281	0.008089	DNASE1L1	Deoxyribor	DNSL1_HUI	1	3.600236
24H 50MT/	Q14573	0.041567	0.006285	0.008091	ITPR3	Inositol 1,4	ITPR3_HUN	23	1.029231
48H 50MT/	Q6ZSJ8	-0.70358	0.006285	0.008092	C1orf122	Uncharacte	CA122_HUI	4	0.614045
48H 50MT/	Q01415	0.058943	0.006287	0.008094	GALK2	N-acetylgal	GALK2_HUI	10	1.041703
48H 50MT/	O43251	0.233435	0.006288	0.008094	RBFOX2	RNA bindin	RFOX2_HUI	7	1.175631
48H 10MT/	Q05513	0.115433	0.006288	0.008094	PRKCZ	Protein kin	KPCZ_HUM	5	1.0833
24H 10MT/	Q9H1X3	1.775676	0.006288	0.008094	DNAJC25	DnaJ homo	DJC25_HUI	3	3.423984
48H 50MT/	P43363	0.159052	0.006292	0.008098	MAGEA10	Melanoma-	MAGAA_HU	5	1.116553
24H 50MT/	P51452	-0.17362	0.006295	0.0081	DUSP3	Dual specifi	DUS3_HUM	8	0.886617
24H 50MT/	Q9UL26	0.242301	0.006295	0.0081	RAB22A	Ras-related	RB22A_HUI	8	1.182877
48H 10MT/	Q9BV86	0.172946	0.006296	0.0081	NTMT1	N-terminal	NTM1A_HU	8	1.127358
24H 10MT/	Q9UM22	0.293317	0.006305	0.008108	EPDR1	Mammaliar	EPDR1_HUI	2	1.225454
24H 50MT/	Q9NQZ5	-0.25154	0.00631	0.008113	STARD7	StAR-relate	STAR7_HUN	9	0.840001
24H 10MT/	P82914	0.058025	0.006312	0.008115	MRPS15	Small ribos	RT15_HUM	7	1.041039
48H 50MT/	Q15185	0.141516	0.006312	0.008115	PTGES3	Prostaglanc	TEBP_HUM	6	1.103064
24H 50MT/	Q93008	-0.07782	0.006319	0.00812	USP9X	Ubiquitin c	USP9X_HUI	64	0.94749

48H 10MT/	Q06033	0.651529	0.006319	0.00812	ITIH3	Inter-alpha	ITIH3_HUN	4	1.570832
48H 10MT/	Q16186	0.286736	0.006322	0.008122	ADRM1	Proteasom	ADRM1_HU	9	1.219877
48H 50MT/	Q14974	-0.10445	0.006332	0.008131	KPNB1	Importin su	IMB1_HUM	39	0.93016
24H 50MT/	Q6P1N9	-0.02962	0.006336	0.008136	TATDN1	Deoxyribor	TATD1_HUI	14	0.97968
24H 10MT/	Q16181	0.079783	0.006348	0.008148	SEPTIN7	Septin-7	SEPT7_HUN	19	1.056859
48H 10MT/	P17302	-0.26384	0.006351	0.008151	GJA1	Gap junctic	CXA1_HUM	8	0.83287
48H 10MT/	P35241	0.031516	0.006355	0.008155	RDX	Radixin	RADI_HUM	39	1.022086
48H 50MT/	Q99497	0.062882	0.006358	0.008157	PARK7	Parkinson c	PARK7_HUI	16	1.044551
48H 10MT/	O75764	0.497167	0.00636	0.008159	TCEA3	Transcripti	TCEA3_HUN	3	1.411439
48H 50MT/	Q9UNZ2	0.043234	0.006363	0.008162	NSFL1C	NSFL1 cofa	NSF1C_HUI	17	1.030421
48H 10MT/	Q8IUR0	-0.14547	0.006365	0.008163	TRAPPC5	Trafficking	TPPC5_HUI	5	0.904088
48H 50MT/	Q8NDD1	-1.19495	0.006367	0.008164	C1orf131	Uncharacte	CA131_HUI	2	0.436803
48H 50MT/	Q93062	-0.20626	0.006367	0.008164	RBPMS	RNA-bindir	RBPMS_HU	8	0.866779
24H 50MT/	P49761	0.382316	0.006372	0.008168	CLK3	Dual specifi	CLK3_HUM	5	1.303432
48H 10MT/	P12268	0.082816	0.006372	0.008168	IMPDH2	Inosine-5'-r	IMDH2_HU	29	1.059084
48H 50MT/	Q8TAD8	-0.10792	0.006372	0.008168	SNIP1	Smad nucle	SNIP1_HUN	5	0.927927
24H 10MT/	Q9Y5B9	0.129263	0.006372	0.008168	SUPT16H	FACT comp	SP16H_HUI	43	1.093735
48H 10MT/	Q6ZN54	0.276715	0.006375	0.008171	DEF8	Differential	DEFI8_HUN	2	1.211433
24H 50MT/	Q86UA1	0.287664	0.006386	0.008182	PRPF39	Pre-mRNA-|	PRP39_HUI	13	1.220663
24H 50MT/	Q8IWV8	-0.00867	0.006389	0.008183	UBR2	E3 ubiquiti	UBR2_HUM	24	0.994005
24H 50MT/	P06899;Q1	0.046082	0.00639	0.008185	H2BC11;H2	Histone H2	H2B1J_HUI	2	1.032458
48H 10MT/	Q92538	0.014994	0.006395	0.008189	GBF1	Golgi-speci	GBF1_HUM	70	1.010447
48H 50MT/	O75529	-0.39056	0.006404	0.008199	TAF5L	TAF5-like RI	TAF5L_HUN	1	0.762832
48H 10MT/	Q8TCD5	0.12402	0.006406	0.0082	NT5C	5'(3')-deoxy	NT5C_HUM	9	1.089767
24H 50MT/	Q96HP0	-0.20975	0.006408	0.008202	DOCK6	Dedicator c	DOCK6_HU	4	0.86469
48H 50MT/	P05155	0.454071	0.006414	0.008208	SERPING1	Plasma pro	IC1_HUMA	8	1.3699
48H 10MT/	P06396	0.115776	0.006427	0.008222	GSN	Gelsolin	GELS_HUM	18	1.083558
48H 10MT/	P00387	0.037987	0.00643	0.008224	CYB5R3	NADH-cyto	NB5R3_HU	19	1.02668
24H 50MT/	Q9Y263	-0.08566	0.006434	0.008226	PLAA	Phospholip	PLAP_HUM	26	0.942357
24H 50MT/	O76094	0.137761	0.006442	0.008233	SRP72	Signal reco	SRP72_HUI	28	1.100197
48H 10MT/	P17405	-0.35131	0.006443	0.008234	SMPD1	Sphingomy	ASM_HUM/	5	0.783873
48H 10MT/	Q96QG7	0.128506	0.006445	0.008235	MTMR9	Myotubula	MTMR9_HU	7	1.093161
24H 50MT/	P10588	0.331513	0.006446	0.008236	NR2F6	Nuclear rec	NR2F6_HUI	7	1.258333
48H 10MT/	O75122	0.120888	0.006448	0.008237	CLASP2	CLIP-associ	CLAP2_HUI	34	1.087404
48H 50MT/	Q3B7T1	-0.72541	0.006454	0.008243	EDRF1	Erythroid d	EDRF1_HUI	12	0.604825
24H 50MT/	Q9NY97	0.106478	0.006459	0.008248	B3GNT2	N-acetyllac	B3GN2_HU	1	1.076597
48H 50MT/	Q5SNT2	-0.56522	0.006461	0.008249	TMEM201	Transmemk	TM201_HU	9	0.675851
48H 10MT/	O60291	0.233809	0.006465	0.008252	MGRN1	E3 ubiquiti	MGRN1_HU	7	1.175935
48H 10MT/	O76071	0.168183	0.00647	0.008256	CIAO1	Probable cy	CIAO1_HUN	14	1.123643
24H 50MT/	Q14157	0.078958	0.006477	0.008262	UBAP2L	Ubiquitin-a	UBP2L_HUI	21	1.056255
48H 10MT/	P0DP23;PC	0.346047	0.006479	0.008265	CALM1;CAL	Calmodulir	CALM1_HU	8	1.271073
48H 10MT/	Q8N4T8	-0.1402	0.006482	0.008267	CBR4	3-oxoacyl-[	CBR4_HUM	5	0.907394
48H 10MT/	P09496	0.09442	0.006482	0.008267	CLTA	Clathrin lig	CLCA_HUM	5	1.067636
48H 10MT/	Q13451	0.026604	0.006495	0.008278	FKBP5	Peptidyl-pr	FKBP5_HUI	23	1.018612
48H 10MT/	P18850	-0.5901	0.0065	0.008282	ATF6	Cyclic AMP	ATF6A_HUN	2	0.664298
24H 50MT/	O43809	0.1412	0.006501	0.008283	NUDT21	Cleavage ar	CPSF5_HUN	15	1.102822
24H 10MT/	Q9BW19	0.054876	0.006501	0.008284	KIFC1	Kinesin-like	KIFC1_HUN	21	1.03877
24H 50MT/	Q13352	0.935464	0.006502	0.008284	ITGB3BP	Centromere	CENPR_HUI	1	1.912505
24H 50MT/	Q9Y6I3	0.179023	0.006504	0.008286	EPN1	Epsin-1	EPN1_HUM	10	1.132117
48H 50MT/	O75122	0.045878	0.006516	0.008297	CLASP2	CLIP-associ	CLAP2_HUI	34	1.032311
48H 50MT/	Q04637	0.006089	0.006517	0.008298	EIF4G1	Eukaryotic	IF4G1_HUN	55	1.00423
24H 10MT/	Q13740	0.08573	0.006518	0.008299	ALCAM	CD166 anti	CD166_HU	17	1.061224

48H 10MT/	P31930	0.018202	0.00652	0.0083	UQCRC1	Cytochrom	QCR1_HUM	17	1.012696
48H 50MT/	O43464	0.177845	0.006523	0.008302	HTRA2	Serine prot	HTRA2_HUI	8	1.131193
48H 10MT/	P23381	0.05801	0.006527	0.008306	WARS1	Tryptophar	SYWC_HUM	23	1.041029
48H 10MT/	P49757	0.071121	0.006529	0.008309	NUMB	Protein nui	NUMB_HUI	11	1.050533
24H 50MT/	Q6DD88	0.126872	0.006529	0.008309	ATL3	Atlastin-3	ATLA3_HUM	26	1.091923
48H 50MT/	Q9UHB9	0.03602	0.006534	0.008313	SRP68	Signal reco	SRP68_HUI	31	1.025282
24H 50MT/	P14859	0.1678	0.006541	0.00832	POU2F1	POU domai	PO2F1_HUI	9	1.123344
48H 10MT/	Q06546	-0.01846	0.006557	0.008334	GABPA	GA-binding	GABPA_HU	9	0.987287
24H 50MT/	Q96IZ7	0.474111	0.006557	0.008334	RSRC1	Serine/Argi	RSRC1_HUI	2	1.389062
24H 10MT/	Q86XL3	0.099798	0.00656	0.008337	ANKLE2	Ankyrin rep	ANKL2_HUI	21	1.071623
24H 10MT/	O00571	-0.0535	0.006565	0.008342	DDX3X	ATP-depenc	DDX3X_HU	44	0.963599
48H 50MT/	P82673	-0.15881	0.006566	0.008342	MRPS35	Small ribos	RT35_HUM	11	0.895763
48H 10MT/	Q14139	0.059435	0.006566	0.008342	UBE4A	Ubiquitin c	UBE4A_HUI	30	1.042058
24H 50MT/	P36021	0.180015	0.006569	0.008344	SLC16A2	Monocarbo	MOT8_HUM	2	1.132896
48H 10MT/	O75144	0.259481	0.006572	0.008346	ICOSLG	ICOS ligand	ICOSL_HUM	1	1.197048
48H 50MT/	O14967	0.148416	0.006573	0.008346	CLGN	Calmegin	CLGN_HUM	9	1.108352
24H 10MT/	P63010	0.061581	0.006572	0.008346	AP2B1	AP-2 compl	AP2B1_HUI	45	1.043609
24H 50MT/	P22830	0.10779	0.006574	0.008347	FECH	Ferrochelat	HEMH_HUI	12	1.077577
24H 50MT/	Q6UX53	0.143414	0.00658	0.008352	TMT1B	Thiol S-met	TMT1B_HU	10	1.104516
24H 50MT/	Q14202	0.228291	0.006584	0.008356	ZMYM3	Zinc finger	ZMYM3_HU	34	1.171446
48H 50MT/	Q86U38	-0.35942	0.006592	0.008363	NOP9	Nucleolar p	NOP9_HUM	20	0.779478
48H 50MT/	P07225	0.344991	0.006596	0.008366	PROS1	Vitamin K-c	PROS_HUM	9	1.270143
48H 10MT/	Q9BW85	0.056348	0.006596	0.008366	YJU2	Splicing fac	YJU2_HUM	12	1.03983
48H 50MT/	Q15836	0.203523	0.006598	0.008367	VAMP3	Vesicle-assc	VAMP3_HU	5	1.151507
24H 10MT/	O43426	-0.57166	0.006601	0.008371	SYNJ1	Synaptojan	SYNJ1_HUM	6	0.672841
24H 50MT/	Q6PIU2	-0.22157	0.006604	0.008374	NCEH1	Neutral chc	NCEH1_HU	18	0.857631
24H 10MT/	Q13601	0.040953	0.006606	0.008376	KRR1	KRR1 small	KRR1_HUM	13	1.028793
24H 50MT/	P29597	-0.5638	0.006606	0.008376	TYK2	Non-recept	TYK2_HUM	4	0.676517
48H 50MT/	O75718	0.006827	0.00661	0.008379	CRTAP	Cartilage-as	CRTAP_HUI	13	1.004743
48H 50MT/	Q5EBL8	-0.22006	0.006611	0.00838	PDZD11	PDZ domai	PDZ11_HUI	3	0.858531
48H 50MT/	P21980	0.27767	0.006612	0.008381	TGM2	Protein-glu	TGM2_HUM	18	1.212235
48H 50MT/	Q9NWV4	0.046225	0.006618	0.008387	CZIB	CXXC motif	CZIB_HUM/	5	1.03256
48H 50MT/	Q8NC60	-0.28741	0.006619	0.008387	NOA1	Nitric oxide	NOA1_HUM	14	0.819374
24H 50MT/	P10253	0.096616	0.006622	0.00839	GAA	Lysosomal	LYAG_HUM	15	1.069262
24H 50MT/	Q86T24	-0.24943	0.006624	0.00839	ZBTB33	Transcripti	KAISO_HUM	1	0.841229
24H 10MT/	Q99442	0.082885	0.006625	0.008391	SEC62	Translocati	SEC62_HUM	9	1.059134
48H 50MT/	Q9NRG4	-0.28296	0.006627	0.008392	SMYD2	N-lysine me	SMYD2_HU	5	0.821904
48H 50MT/	P11172	-0.12431	0.006629	0.008393	UMPS	Uridine 5'-r	UMPS_HUM	24	0.917441
48H 50MT/	P50895	-0.01026	0.006633	0.008398	BCAM	Basal cell ac	BCAM_HUM	16	0.99291
24H 50MT/	Q9Y5Y2	-0.05912	0.006642	0.008407	NUBP2	Cytosolic F	NUBP2_HU	11	0.959847
24H 10MT/	Q9UJX6	0.065627	0.006647	0.008411	ANAPC2	Anaphase-p	ANC2_HUM	21	1.046539
24H 50MT/	P31689	-0.18991	0.006647	0.008411	DNAJA1	DnaJ homo	DNJA1_HUI	18	0.876662
24H 50MT/	P78356	1.134044	0.006649	0.008414	PIP4K2B	Phosphatid	PI42B_HUM	2	2.19473
24H 10MT/	P19367	0.021559	0.00665	0.008414	HK1	Hexokinase	HXK1_HUM	35	1.015055
48H 50MT/	O00764	0.082836	0.006657	0.008421	PDXK	Pyridoxal k	PDXK_HUM	16	1.059098
24H 10MT/	Q07065	0.025859	0.006658	0.008423	CKAP4	Cytoskeletc	CKAP4_HUI	29	1.018086
48H 50MT/	Q99519	-0.2505	0.006662	0.008427	NEU1	Sialidase-1	NEUR1_HU	14	0.840602
24H 50MT/	Q56P03	0.952065	0.006667	0.008432	EAPP	E2F-associa	EAPP_HUM	2	1.93464
48H 50MT/	Q96DR4	-0.71087	0.006678	0.008441	STARD4	StAR-relate	STAR4_HUM	2	0.610952
48H 10MT/	Q8IZL8	-0.02994	0.006681	0.008444	PELP1	Proline-, gl	PELP1_HUM	24	0.979461
48H 10MT/	O43252	0.082817	0.006682	0.008445	PAPSS1	Bifunctiona	PAPS1_HUI	21	1.059084
48H 50MT/	Q96AG3	1.772594	0.006684	0.008446	SLC25A46	Mitochond	S2546_HUI	2	3.416677

24H	50MT/	P52292	-0.15842	0.006684	0.008446	KPNA2	Importin su	IMA1_HUM	24	0.896005
24H	10MT/	Q9NPH2	-0.12397	0.006687	0.008448	ISYNA1	Inositol-3-p	INO1_HUM	14	0.917658
48H	10MT/	Q9Y697	0.032369	0.006689	0.008449	NFS1	Cysteine de	NFS1_HUM	20	1.02269
48H	10MT/	P13010	0.040605	0.00669	0.008449	XRCC5	X-ray repair	XRCC5_HU	43	1.028545
48H	50MT/	P53680	0.230993	0.00669	0.008449	AP2S1	AP-2 compl	AP2S1_HU	7	1.173642
24H	50MT/	Q86YP4	0.190503	0.006696	0.008456	GATAD2A	Transcripti	P66A_HUM	22	1.141161
48H	50MT/	Q96EK4	-0.45484	0.0067	0.00846	THAP11	THAP doma	THA11_HU	7	0.72959
24H	50MT/	P21127	0.071002	0.006701	0.00846	CDK11B	Cyclin-depe	CD11B_HU	21	1.050446
24H	50MT/	Q9UBR2	0.117932	0.006702	0.008461	CTSZ	Cathepsin Z	CATZ_HUM	8	1.085178
24H	50MT/	O75695	-0.26146	0.006703	0.008462	RP2	Protein XRI	XRP2_HUM	10	0.834244
48H	10MT/	O14949	0.266917	0.006705	0.008463	UQCRQ	Cytochrom	QCR8_HUM	3	1.203234
24H	10MT/	P24278	0.262766	0.006709	0.008467	ZBTB25	Zinc finger	ZBT25_HUI	1	1.199777
48H	50MT/	Q8N6H7	0.018263	0.006712	0.00847	ARFGAP2	ADP-ribosy	ARFG2_HU	16	1.012739
24H	10MT/	Q9UKG9	-0.18188	0.006715	0.008473	CROT	Peroxisoma	OCTC_HUM	10	0.881556
48H	10MT/	O60610	0.041433	0.006723	0.00848	DIAPH1	Protein dia	DIAP1_HUI	44	1.029136
24H	10MT/	Q9H063	-1.14831	0.006724	0.008481	MAF1	Repressor c	MAF1_HUM	2	0.451152
24H	10MT/	Q96EU7	0.120684	0.006731	0.008487	C1GALT1C1	C1GALT1-sp	C1GLC_HU	9	1.08725
24H	50MT/	Q05D32	0.229507	0.006732	0.008487	CTDSPL2	CTD small p	CTSL2_HUM	9	1.172435
48H	10MT/	P53675	-0.09221	0.006738	0.008493	CLTCL1	Clathrin he	CLH2_HUM	23	0.938084
48H	50MT/	O00442	0.108565	0.006743	0.008497	RTCA	RNA 3'-term	RTCA_HUM	15	1.078156
24H	50MT/	O14497	0.102366	0.006753	0.008506	ARID1A	AT-rich inte	ARI1A_HUM	34	1.073532
24H	50MT/	Q00577	-0.25269	0.006755	0.008508	PURA	Transcripti	PURA_HUM	5	0.839327
48H	10MT/	O43395	-0.01706	0.006762	0.008514	PRPF3	U4/U6 sma	PRPF3_HUI	23	0.988242
24H	50MT/	P43243	0.016687	0.006764	0.008515	MATR3	Matrin-3	MATR3_HU	31	1.011634
24H	10MT/	P05387	-0.48317	0.006768	0.008519	RPLP2	Large ribos	RLA2_HUM	7	0.715405
24H	50MT/	P06730	0.100882	0.006769	0.00852	EIF4E	Eukaryotic	IF4E_HUMA	9	1.072429
48H	50MT/	Q9Y5P6	0.006734	0.006772	0.008522	GMPPB	Mannose-1	GMPPB_HU	13	1.004679
24H	50MT/	Q9NQT5	0.120144	0.006776	0.008525	EXOSC3	Exosome cc	EXOS3_HUI	10	1.086843
48H	10MT/	Q8WVQ1	0.055002	0.006804	0.008553	CANT1	Soluble cal	CANT1_HUI	13	1.038861
24H	50MT/	Q9Y6M1	0.046986	0.006805	0.008553	IGF2BP2	Insulin-like	IF2B2_HUM	20	1.033104
24H	10MT/	P49591	-0.1445	0.006809	0.008557	SARS1	Serine--tRN	SYSC_HUMA	34	0.904692
24H	10MT/	Q6NZ67	-0.1649	0.006813	0.00856	MZT2B	Mitotic-spi	MZT2B_HU	6	0.891991
24H	50MT/	P23528	0.213785	0.006814	0.008561	CFL1	Cofilin-1	COF1_HUM	18	1.159727
24H	50MT/	Q9H845	0.227193	0.006814	0.008561	ACAD9	Complex I a	ACAD9_HU	25	1.170555
48H	50MT/	Q9HB21	0.106335	0.00682	0.008566	PLEKHA1	Pleckstrin h	PKHA1_HU	10	1.07649
48H	10MT/	P55061	-0.36343	0.006824	0.00857	TMBIM6	Bax inhibit	BI1_HUMA	3	0.777313
24H	50MT/	P17858	0.122799	0.00684	0.008585	PFKL	ATP-depen	PFKAL_HUM	30	1.088846
48H	50MT/	Q9UHE5	-0.20701	0.006846	0.00859	NAT8	N-acetyltra	NAT8_HUM	7	0.866329
48H	50MT/	Q5SYC1	0.315114	0.006853	0.008596	CLVS2	Clavesin-2	CLVS2_HUM	4	1.24411
24H	50MT/	Q3B8N5	-0.85298	0.006861	0.008605	PROX2	Prospero h	PROX2_HU	2	0.553639
24H	10MT/	P55010	-0.15984	0.006867	0.008612	EIF5	Eukaryotic	IF5_HUMA	17	0.895123
48H	50MT/	P04049	0.273626	0.006869	0.008613	RAF1	RAF proto-c	RAF1_HUM	16	1.208842
48H	10MT/	O43290	0.017888	0.00687	0.008614	SART1	U4/U6.U5 t	SNUT1_HU	30	1.012476
24H	10MT/	Q9NPQ8	0.095965	0.00687	0.008614	RIC8A	Synembryn	RIC8A_HUM	17	1.06878
48H	50MT/	P52895	0.315508	0.006881	0.008624	AKR1C2	Aldo-keto r	AK1C2_HUI	3	1.24445
24H	50MT/	A4D1P6	0.074816	0.006883	0.008625	WDR91	WD repeat-	WDR91_HU	20	1.053226
24H	10MT/	Q9BU19	0.058708	0.006884	0.008626	ZNF692	Zinc finger	ZN692_HUI	1	1.041532
48H	10MT/	Q9NUP7	-0.29999	0.006891	0.008632	TRMT13	tRNA:m(4)X	TRM13_HU	7	0.812257
48H	10MT/	Q8WZ82	-0.0322	0.006894	0.008635	OVCA2	Esterase OV	OVCA2_HU	8	0.977926
48H	50MT/	O43818	-0.19556	0.006899	0.008637	RRP9	U3 small nu	U3IP2_HUM	13	0.873233
24H	50MT/	Q14CZ7	0.438383	0.006902	0.008639	FASTKD3	FAST kinase	FAKD3_HUI	6	1.355085
24H	10MT/	Q9NVS9	-0.13956	0.006903	0.008641	PNPO	Pyridoxine-	PNPO_HUM	13	0.907797

48H 50MT/	Q05639	0.060242	0.006906	0.008643	EEF1A2	Elongation	EF1A2_HUI	8	1.042641
24H 50MT/	P42704	-0.00293	0.006909	0.008645	LRPPRC	Leucine-ric	LPPRC_HUI	94	0.997969
48H 50MT/	Q01433	0.016355	0.00691	0.008646	AMPD2	AMP deami	AMPD2_HU	28	1.011401
24H 50MT/	P15848	0.148711	0.006913	0.008648	ARSB	Arylsulfata:	ARSB_HUM	9	1.108579
24H 50MT/	Q6IAN0	0.249046	0.006915	0.008652	DHRS7B	Dehydroge	DRS7B_HUI	9	1.188421
24H 10MT/	O95630	0.084042	0.006919	0.008655	STAMBP	STAM-bindi	STABP_HUI	11	1.059984
48H 10MT/	Q86XX4	0.015971	0.00692	0.008656	FRAS1	Extracellul:	FRAS1_HUI	31	1.011132
24H 10MT/	P49336	0.54106	0.006926	0.008662	CDK8	Cyclin-depe	CDK8_HUM	6	1.455041
24H 50MT/	P05166	0.226426	0.006928	0.008663	PCCB	Propionyl-(	PCCB_HUM	19	1.169933
48H 50MT/	O43427	0.087862	0.006928	0.008663	FIBP	Acidic fibrc	FIBP_HUM/	13	1.062794
24H 10MT/	Q9NX55	-0.57907	0.006932	0.008666	HYPK	Huntingtin	HYPK_HUM	2	0.669394
48H 10MT/	Q3ZAQ7	0.868868	0.006937	0.008671	VMA21	Vacuolar A	VMA21_HU	2	1.826229
48H 50MT/	O14936	0.110854	0.006943	0.008675	CASK	Peripheral	CSKP_HUM	18	1.079867
24H 50MT/	Q6NYC8	0.045836	0.006945	0.008677	PPP1R18	Phostensin	PPR18_HUI	8	1.032281
48H 50MT/	A6NNW6	0.886596	0.006961	0.008693	ENO4	Enolase 4	ENO4_HUM	1	1.848808
24H 50MT/	Q9BV86	0.031743	0.006961	0.008693	NTMT1	N-terminal	NTM1A_HU	8	1.022246
48H 10MT/	Q9BQT8	-0.02305	0.006972	0.008702	SLC25A21	Mitochond	ODC_HUM/	7	0.984148
24H 50MT/	Q9BUF5	-0.11787	0.006974	0.008704	TUBB6	Tubulin bet	TBB6_HUM	16	0.921545
48H 50MT/	P50750	-0.00391	0.006976	0.008705	CDK9	Cyclin-depe	CDK9_HUM	10	0.99729
48H 10MT/	Q8N183	0.171854	0.006976	0.008705	NDUFAF2	NADH dehy	NDUF2_HU	10	1.126505
48H 50MT/	P15169	-0.35786	0.006979	0.008707	CPN1	Carboxype	CBPN_HUM	1	0.780321
48H 10MT/	Q7Z569	0.128806	0.00698	0.008707	BRAP	BRCA1-assc	BRAP_HUM	9	1.093388
48H 50MT/	P07858	0.136632	0.006983	0.00871	CTSB	Cathepsin E	CATB_HUM	11	1.099336
48H 10MT/	P11182	-0.00633	0.006985	0.008711	DBT	Lipoamide	ODB2_HUM	22	0.995625
48H 10MT/	Q99829	0.051046	0.006987	0.008714	CPNE1	Copine-1	CPNE1_HUI	19	1.036015
24H 50MT/	Q5TFE4	0.084228	0.006988	0.008714	NT5DC1	5'-nucleoti	NT5D1_HU	18	1.060121
48H 10MT/	Q9UBQ5	0.068411	0.006992	0.008718	EIF3K	Eukaryotic	EIF3K_HUM	11	1.048561
24H 50MT/	O14925	-0.02329	0.006996	0.008722	TIMM23	Mitochond	TIM23_HUI	7	0.983986
48H 10MT/	Q99567	-0.03798	0.006998	0.008724	NUP88	Nuclear po	NUP88_HU	20	0.974018
48H 50MT/	Q9H0V9	0.010102	0.007001	0.008727	LMAN2L	VIP36-like	LMA2L_HU	12	1.007026
48H 10MT/	Q15785	0.051841	0.007005	0.00873	TOMM34	Mitochond	TOM34_HU	16	1.036587
24H 50MT/	Q9BRJ6	0.100495	0.007007	0.008732	C7orf50	Uncharacte	CG050_HU	9	1.072141
48H 10MT/	Q86UL3	0.028631	0.007007	0.008732	GPAT4	Glycerol-3-	GPAT4_HUI	6	1.020044
24H 50MT/	Q9NUU7	-0.05188	0.007011	0.008736	DDX19A	ATP-depen	DD19A_HU	26	0.964676
24H 50MT/	P54819	0.072663	0.007012	0.008737	AK2	Adenylate k	KAD2_HUM	14	1.051656
24H 50MT/	Q6I9Y2	0.156057	0.007015	0.00874	THOC7	THO compl	THOC7_HU	8	1.114238
48H 50MT/	Q96GG9	0.066026	0.007015	0.00874	DCUN1D1	DCN1-like	DCNL1_HU	10	1.046829
24H 50MT/	P18754	0.004817	0.00703	0.008754	RCC1	Regulator c	RCC1_HUM	20	1.003344
24H 10MT/	Q04724	0.036128	0.007031	0.008754	TLE1	Transducin	TLE1_HUM/	7	1.025358
24H 50MT/	Q7L2H7	-0.14479	0.007031	0.008754	EIF3M	Eukaryotic	EIF3M_HUM	19	0.904513
48H 50MT/	Q8WUU5	-0.52634	0.00703	0.008754	GATAD1	GATA zinc fi	GATD1_HU	4	0.694313
24H 10MT/	O75864	-0.34531	0.007036	0.008757	PPP1R37	Protein ph	PPR37_HUI	2	0.787138
24H 10MT/	Q9Y3B4	0.196477	0.007037	0.008757	SF3B6	Splicing fac	SF3B6_HUI	6	1.145897
48H 50MT/	Q9ULT8	-0.11291	0.007038	0.008758	HECTD1	E3 ubiquiti	HECD1_HU	64	0.924724
24H 10MT/	Q9UPX8	0.16049	0.007039	0.008759	SHANK2	SH3 and mu	SHAN2_HU	6	1.117667
24H 10MT/	O95456	0.074348	0.00704	0.008759	PSMG1	Proteasom	PSMG1_HU	13	1.052885
48H 50MT/	Q9NY12	-0.15033	0.007041	0.00876	GAR1	H/ACA ribo	GAR1_HUM	8	0.901046
24H 50MT/	Q9UDX5	0.240141	0.007043	0.008761	MTFP1	Mitochond	MTFP1_HU	8	1.181108
24H 10MT/	Q6P1N9	-0.03442	0.007046	0.008762	TATDN1	Deoxyribor	TATD1_HUI	14	0.976422
48H 50MT/	Q9UKM7	-0.0146	0.007056	0.00877	MAN1B1	Endoplasm	MA1B1_HU	13	0.989933
24H 10MT/	Q14247	-0.07507	0.00706	0.008774	CTTN	Src substra	SRC8_HUM	19	0.949295
24H 50MT/	P62683;P6	-1.0172	0.007064	0.008777	ERVK-21;HI	Endogenou	GAK21_HU	1	0.494073

48H 50MT/	P43652	0.118395	0.007064	0.008777	AFM	Afamin	AFAM_HUN	1	1.085526
24H 50MT/	O00148	0.021112	0.007075	0.008787	DDX39A	ATP-depen	DX39A_HU	12	1.014741
48H 50MT/	Q9BZV1	0.155376	0.007079	0.00879	UBXN6	UBX domai	UBXN6_HU	12	1.113712
48H 50MT/	P48723	-0.10183	0.00708	0.008791	HSPA13	Heat shock	HSP13_HUI	14	0.931853
48H 10MT/	Q15427	-0.26543	0.007081	0.008792	SF3B4	Splicing fac	SF3B4_HUI	8	0.831953
24H 10MT/	Q9BRQ8	0.17878	0.007082	0.008792	AIFM2	Ferroptosis	FSP1_HUM	15	1.131926
48H 50MT/	Q5VW32	-0.01314	0.007094	0.008806	BROX	BRO1 doma	BROX_HUN	7	0.990935
24H 50MT/	P25098	0.008552	0.007095	0.008807	GRK2	Beta-adren	ARBK1_HUI	7	1.005946
48H 50MT/	Q9BQ48	-0.31159	0.007102	0.008813	MRPL34	Large ribos	RM34_HUN	2	0.805753
48H 10MT/	Q92797	0.015371	0.007107	0.008817	SYMPK	Symplekin	SYMPK_HU	40	1.010711
24H 50MT/	Q8TCZ2	1.208724	0.007107	0.008817	CD99L2	CD99 antig	C99L2_HUI	1	2.311332
48H 50MT/	Q9P2D0	0.166764	0.007109	0.00882	IBTK	Inhibitor of	IBTK_HUM/	8	1.122538
48H 50MT/	Q9BUQ8	-0.00175	0.007116	0.008825	DDX23	Probable A'	DDX23_HU	32	0.998789
48H 50MT/	Q92997	0.029636	0.007118	0.008827	DVL3	Segment pc	DVL3_HUM	6	1.020755
48H 50MT/	Q9H0U9	-0.25954	0.007126	0.008836	TSPYL1	Testis-speci	TSYL1_HUN	9	0.835357
24H 10MT/	Q14694	-0.0659	0.007128	0.008837	USP10	Ubiquitin c	UBP10_HU	25	0.955351
48H 10MT/	Q96FX8	-0.88112	0.007129	0.008839	PERP	p53 apopto	PERP_HUM	1	0.542945
48H 50MT/	P35241	0.035493	0.00713	0.00884	RDX	Radixin	RADI_HUM	39	1.024907
48H 10MT/	P19338	0.137422	0.007131	0.00884	NCL	Nucleolin	NUCL_HUN	27	1.099938
48H 50MT/	Q96MX3	-1.22701	0.007131	0.00884	ZNF48	Zinc finger	ZNF48_HUI	1	0.427202
24H 50MT/	P04114	0.169661	0.00714	0.008848	APOB	Apolipoprc	APOB_HUN	89	1.124795
24H 50MT/	Q9BRJ2	-0.03926	0.007142	0.008849	MRPL45	Large ribos	RM45_HUN	14	0.973152
48H 50MT/	P13010	0.105254	0.007147	0.008853	XRCC5	X-ray repair	XRCC5_HUI	43	1.075684
24H 50MT/	O15440	-0.11269	0.00715	0.008855	ABCC5	ATP-bindin	MRP5_HUN	16	0.924861
48H 50MT/	Q96NU7	0.421901	0.007154	0.008858	AMDHD1	Probable in	HUTI_HUM	8	1.339692
48H 50MT/	O75131	0.053872	0.007153	0.008858	CPNE3	Copine-3	CPNE3_HUI	23	1.038047
24H 10MT/	Q8WZ82	-0.18289	0.007163	0.008865	OVCA2	Esterase OV	OVCA2_HU	8	0.880936
48H 10MT/	Q8IWT0	0.04062	0.007166	0.008868	ZBTB8OS	Protein arc	ARCH_HUN	2	1.028556
48H 50MT/	Q9H2G4	0.190369	0.007172	0.008874	TSPYL2	Testis-speci	TSYL2_HUN	5	1.141055
24H 50MT/	P18887	0.309869	0.007176	0.008877	XRCC1	DNA repair	XRCC1_HUI	11	1.239595
48H 50MT/	P82914	-0.12914	0.007177	0.008878	MRPS15	Small ribos	RT15_HUM	7	0.914377
24H 50MT/	Q9BQ04	0.178978	0.007181	0.008881	RBM4B	RNA-bindir	RBM4B_HU	15	1.132082
48H 10MT/	Q9H9B4	0.084611	0.007182	0.008881	SFXN1	Sideroflexir	SFXN1_HUI	12	1.060402
48H 10MT/	Q8IYB3	0.12899	0.007184	0.008884	SRRM1	Serine/argi	SRRM1_HU	7	1.093528
24H 50MT/	Q1MSJ5	0.682558	0.007184	0.008884	CSPP1	Centrosom	CSPP1_HUI	7	1.604983
48H 10MT/	P00439	-0.16259	0.007186	0.008885	PAH	Phenylalan	PH4H_HUN	30	0.893422
24H 10MT/	O75223	0.03299	0.00719	0.008889	GGCT	Gamma-glu	GGCT_HUN	8	1.023131
48H 50MT/	P18124	-0.14533	0.007222	0.008918	RPL7	Large ribos	RL7_HUMA	17	0.904171
48H 50MT/	O75607	-0.20012	0.007223	0.008919	NPM3	Nucleoplas	NPM3_HUN	7	0.870478
24H 10MT/	Q9NRW7	-0.14902	0.007224	0.008919	VPS45	Vacuolar pı	VPS45_HUI	21	0.901862
24H 10MT/	O75570	-0.89304	0.007224	0.008919	MTRF1	Peptide cha	RF1M_HUN	3	0.538478
48H 50MT/	Q92820	0.076804	0.007227	0.008921	GGH	Gamma-glu	GGH_HUM/	11	1.054679
48H 50MT/	O75533	0.022494	0.007231	0.008925	SF3B1	Splicing fac	SF3B1_HUI	66	1.015714
48H 50MT/	Q6P1N9	0.148711	0.007232	0.008925	TATDN1	Deoxyribor	TATD1_HUI	14	1.108578
24H 50MT/	Q9Y639	0.241336	0.007243	0.008935	NPTN	Neuroplast	NPTN_HUM	7	1.182087
48H 10MT/	Q9P0J7	0.148219	0.007245	0.008937	KCMF1	E3 ubiquiti	KCMF1_HU	5	1.108201
48H 10MT/	A0AVT1	0.044116	0.00725	0.008942	UBA6	Ubiquitin-l	UBA6_HUN	43	1.031051
48H 50MT/	Q96KB5	-0.21832	0.00725	0.008942	PBK	Lymphokin	TOPK_HUN	15	0.859569
24H 50MT/	Q9UI12	0.018287	0.007252	0.008943	ATP6V1H	V-type prot	VATH_HUN	20	1.012757
24H 50MT/	P49189	0.052214	0.00726	0.008948	ALDH9A1	4-trimethyl	AL9A1_HUI	21	1.036855
24H 10MT/	Q8TCG1	0.111299	0.00726	0.008948	CIP2A	Protein CIP	CIP2A_HUN	19	1.0802
24H 10MT/	O43818	0.066946	0.007261	0.008949	RRP9	U3 small nu	U3IP2_HUN	13	1.047497

24H 10MTA	Q9BPW8	-0.09111	0.00726	0.008949	NIPSNAP1	Protein Nip	NIPS1_HUM	11	0.938798
24H 50MTA	Q9H074	-0.17594	0.007261	0.008949	PAIP1	Polyadenyl	PAIP1_HUM	14	0.885193
24H 10MTA	O94761	0.37565	0.007266	0.008954	RECQL4	ATP-depend	RECQ4_HU	1	1.297424
48H 10MTA	P20930	-1.05382	0.007266	0.008954	FLG	Filaggrin	FILA_HUMA	1	0.48169
48H 50MTA	P08754	0.081156	0.007275	0.008963	GNAI3	Guanine nu	GNAI3_HUI	9	1.057865
24H 50MTA	Q9UBU6	0.201433	0.007286	0.008973	FAM8A1	Protein FAI	FA8A1_HUI	10	1.14984
48H 10MTA	Q9H2H9	-0.40922	0.007288	0.008975	SLC38A1	Sodium-cou	S38A1_HUI	6	0.753028
48H 50MTA	Q09028	-0.09193	0.007293	0.00898	RBBP4	Histone-bir	RBBP4_HUI	15	0.938268
24H 50MTA	Q13393	0.105766	0.007297	0.008983	PLD1	Phospholip	PLD1_HUM	24	1.076066
48H 10MTA	P60033	0.23624	0.007298	0.008984	CD81	CD81 antig	CD81_HUM	2	1.177919
24H 10MTA	Q5MIZ7	0.033611	0.007298	0.008984	PPP4R3B	Serine/thre	P4R3B_HUI	18	1.023571
24H 10MTA	O95210	0.359133	0.007303	0.008988	STBD1	Starch-bind	STBD1_HUI	5	1.282655
48H 50MTA	Q15369	0.181455	0.007307	0.008992	ELOC	Elongin-C	ELOC_HUM	5	1.134027
24H 50MTA	Q96RQ3	0.153299	0.007307	0.008992	MCCC1	Methylcrot	MCCA_HUM	19	1.112109
48H 50MTA	Q86XI2	-0.35664	0.007308	0.008992	NCAPG2	Condensin-	CNDG2_HU	15	0.78098
24H 50MTA	P63208	-0.2356	0.007309	0.008993	SKP1	S-phase kin	SKP1_HUM	6	0.849332
48H 50MTA	Q8WUH6	0.153723	0.00731	0.008994	TMEM263	Transmemb	TM263_HU	4	1.112437
48H 10MTA	Q9BWU0	0.106819	0.007314	0.008998	SLC4A1AP	Kanadaptin	NADAP_HU	18	1.076851
24H 50MTA	P11279	0.178285	0.007317	0.009	LAMP1	Lysosome-a	LAMP1_HU	6	1.131538
48H 50MTA	P12004	-0.16794	0.007317	0.009	PCNA	Proliferatin	PCNA_HUM	13	0.890113
24H 10MTA	O75165	0.037483	0.007319	0.009001	DNAJC13	DnaJ homo	DJC13_HUI	62	1.026321
48H 10MTA	P81605	0.188741	0.007329	0.009011	DCD	Dermcidin	DCD_HUMA	2	1.139768
24H 50MTA	P48739	-0.1079	0.007329	0.009012	PITPNB	Phosphatid	PIPNB_HUI	21	0.927939
24H 10MTA	O75356	-0.46524	0.007339	0.00902	ENTPD5	Nucleoside	ENTP5_HUI	4	0.724352
48H 10MTA	P35251	0.038614	0.007343	0.009023	RFC1	Replication	RFC1_HUM	26	1.027127
24H 50MTA	Q9NVM9	0.196268	0.007343	0.009023	INTS13	Integrator	INT13_HUI	16	1.145731
24H 50MTA	Q7Z2W4	0.020657	0.007346	0.009025	ZC3HAV1	Zinc finger	ZCCHV_HUI	37	1.014421
24H 50MTA	P41236	-0.05129	0.007347	0.009025	PPP1R2	Protein pho	IPP2_HUMA	9	0.965071
48H 50MTA	Q9NVR5	0.251074	0.007352	0.009031	DNAAF2	Protein kin	KTU_HUMA	5	1.190092
48H 50MTA	O75534	-0.15997	0.007355	0.009033	CSDE1	Cold shock	CSDE1_HUI	44	0.895047
24H 50MTA	Q15269	0.12699	0.007358	0.009036	PWP2	Periodic try	PWP2_HUM	27	1.092013
24H 50MTA	Q14683	0.104449	0.007363	0.00904	SMC1A	Structural	SMC1A_HU	54	1.075083
48H 50MTA	P78504	-0.05103	0.007363	0.00904	JAG1	Protein jag	JAG1_HUM	4	0.965247
48H 50MTA	O15126	0.124316	0.007364	0.009041	SCAMP1	Secretory c	SCAM1_HU	5	1.089991
24H 50MTA	Q8NBJ5	0.119316	0.007365	0.009042	COLGALT1	Procollager	GT251_HUI	26	1.08622
24H 50MTA	P50502	-0.11264	0.007372	0.009048	ST13	Hsc70-inter	F10A1_HUI	11	0.924892
24H 10MTA	Q8WVY7	0.053691	0.007375	0.00905	UBLCP1	Ubiquitin-l	UBCP1_HU	14	1.037917
24H 50MTA	Q96HC4	-0.05062	0.007377	0.009052	PDLIM5	PDZ and LIN	PDLI5_HUM	23	0.965524
48H 10MTA	Q9Y230	0.057253	0.007379	0.009053	RUVBL2	RuvB-like 2	RUVB2_HU	28	1.040483
24H 50MTA	P31150	-0.18734	0.007383	0.009057	GDI1	Rab GDP di	GDIA_HUM	13	0.878225
48H 10MTA	P21291	-0.01056	0.007387	0.00906	CSRP1	Cysteine an	CSRP1_HUI	8	0.992706
48H 50MTA	O95696	-0.92029	0.007391	0.009063	BRD1	Bromodom	BRD1_HUM	2	0.528404
48H 50MTA	Q14520	0.188898	0.007397	0.009069	HABP2	Hyaluronar	HABP2_HU	10	1.139892
24H 50MTA	Q92882	-0.19512	0.007398	0.00907	OSTF1	Osteoclast-	OSTF1_HUI	7	0.8735
24H 50MTA	C9JLW8	-0.09555	0.0074	0.009071	MCRIP1	Mapk-regul	MCRI1_HUI	4	0.935912
24H 50MTA	O14975	0.14404	0.007401	0.009073	SLC27A2	Long-chain	S27A2_HUI	15	1.104995
48H 50MTA	P31146	0.359733	0.007407	0.009078	CORO1A	Coronin-1A	COR1A_HU	3	1.283188
24H 10MTA	Q9BWH2	0.237895	0.007413	0.009082	FUNDC2	FUN14 dom	FUND2_HU	5	1.179271
24H 50MTA	Q86X40	-0.83018	0.007415	0.009084	LRRC28	Leucine-ric	LRC28_HUI	2	0.56246
48H 50MTA	Q9BVW5	-0.65838	0.007417	0.009085	TIPIN	TIMELESS-ir	TIPIN_HUM	3	0.633589
24H 50MTA	P28288	0.059651	0.007419	0.009087	ABCD3	ATP-bindin	ABCD3_HU	33	1.042214
48H 10MTA	Q99873	0.079969	0.007429	0.009096	PRMT1	Protein arg	ANM1_HUM	23	1.056995

48H 10MT/	P09525	0.083303	0.00743	0.009096	ANXA4	Annexin A4	ANXA4_HU	24	1.059441
48H 10MT/	Q5JPH6	0.058549	0.00743	0.009097	EARS2	Nondiscrim	SYEM_HUM	14	1.041418
24H 10MT/	Q8N1G4	-0.09969	0.007431	0.009097	LRRC47	Leucine-ric	LRC47_HU	23	0.933234
48H 10MT/	O43299	-0.09698	0.007437	0.009101	AP5Z1	AP-5 compl	AP5Z1_HU	8	0.934989
48H 10MT/	P48051;P4	-1.64897	0.007441	0.009105	KCNJ6;KCN	G protein-a	KCNJ6_HU	1	0.318867
24H 50MT/	Q9NWV8	0.102983	0.007441	0.009105	BABAM1	BRISC and E	BABA1_HU	6	1.073992
48H 50MT/	P14854	0.165268	0.007444	0.009108	COX6B1	Cytochrom	CX6B1_HU	4	1.121374
24H 50MT/	Q9NY61	0.129471	0.007445	0.009109	AATF	Protein AA	AATF_HUM	17	1.093893
24H 50MT/	O75928	0.585137	0.00745	0.009113	PIAS2	E3 SUMO-p	PIAS2_HUM	4	1.500182
48H 50MT/	P53365	0.000251	0.007458	0.00912	ARFIP2	Arfaptin-2	ARFP2_HU	12	1.000174
24H 10MT/	Q8WVE0	-0.03896	0.007458	0.00912	EEF1AKMT1	EEF1A lysin	EFMT1_HU	1	0.97336
48H 50MT/	Q9NR81	-0.73008	0.007458	0.00912	ARHGEF3	Rho guanin	ARHG3_HU	1	0.60287
24H 50MT/	Q6P9B6	0.103603	0.007462	0.009123	MEAK7	MTOR-asso	MEAK7_HU	10	1.074454
24H 50MT/	Q9NR30	0.038274	0.007462	0.009123	DDX21	Nucleolar R	DDX21_HU	43	1.026884
24H 50MT/	Q96MU7	0.378429	0.007463	0.009124	YTHDC1	YTH domair	YTDC1_HU	7	1.299926
24H 50MT/	Q96E29	0.044974	0.007466	0.009127	MTERF3	Transcripti	MTEF3_HU	9	1.031664
48H 10MT/	Q96HR9	-0.05047	0.00747	0.00913	REEP6	Receptor e:	REEP6_HUI	6	0.965622
24H 50MT/	Q9BUH6	-0.08877	0.007476	0.009137	PAXX	Protein PA	PAXX_HUM	9	0.940325
48H 10MT/	Q96JM3	0.04188	0.007481	0.009141	CHAMP1	Chromosor	CHAP1_HU	17	1.029455
48H 10MT/	O00170	0.045726	0.007495	0.009154	AIP	AH recepto	AIP_HUMA	15	1.032202
48H 50MT/	Q9NUG6	-0.86424	0.007499	0.009157	PDRG1	p53 and DN	PDRG1_HU	2	0.549336
48H 10MT/	P14635	0.046867	0.0075	0.009158	CCNB1	G2/mitotic	CCNB1_HU	10	1.033019
48H 50MT/	Q9H0S4	-0.10391	0.007508	0.009164	DDX47	Probable A	DDX47_HU	18	0.930509
48H 50MT/	Q8IXT5	0.001549	0.007522	0.009176	RBM12B	RNA-bindir	RB12B_HU	22	1.001074
48H 10MT/	Q9H3P7	0.08266	0.007524	0.009178	ACBD3	Golgi reside	GCP60_HU	18	1.058969
48H 50MT/	Q8TDQ7	0.199125	0.00753	0.009184	GNPDA2	Glucosamir	GNPI2_HU	6	1.148002
48H 10MT/	Q9Y2Z4	-0.18932	0.007534	0.009189	YARS2	Tyrosine--t	SYYM_HUM	18	0.877017
48H 50MT/	Q9HBL8	0.436893	0.007536	0.00919	NMRAL1	NmrA-like f	NMRL1_HU	4	1.353686
24H 50MT/	O43741	0.406625	0.007537	0.009191	PRKAB2	5'-AMP-acti	AAKB2_HU	4	1.325581
48H 50MT/	P82970	0.705503	0.00754	0.009194	HMGN5	High mobil	HMGN5_HU	5	1.630714
24H 10MT/	P53602	-0.14492	0.007547	0.0092	MVD	Diphospho	MVD1_HUI	16	0.904429
48H 10MT/	P60953	-0.16334	0.00755	0.009203	CDC42	Cell divisio	CDC42_HU	8	0.892956
48H 50MT/	Q8TD16	-0.25886	0.007559	0.00921	BICD2	Protein bic	BICD2_HUI	16	0.835749
24H 10MT/	O75874	-0.12253	0.007559	0.00921	IDH1	Isocitrate d	IDHC_HUM	23	0.918578
48H 10MT/	P68104	-0.20801	0.007569	0.00922	EEF1A1	Elongation	EF1A1_HUI	30	0.865731
24H 10MT/	O43251	0.169363	0.007578	0.009227	RBFOX2	RNA bindin	RFOX2_HU	7	1.124562
48H 50MT/	Q58WW2	0.10556	0.007583	0.009232	DCAF6	DDB1- and	DCAF6_HU	14	1.075912
48H 50MT/	O95551	0.298946	0.007589	0.009238	TDP2	Tyrosyl-DN	TYDP2_HU	11	1.230245
24H 10MT/	Q96NU7	0.043786	0.00759	0.009238	AMDHD1	Probable in	HUTI_HUM	8	1.030815
48H 10MT/	Q99943	0.170062	0.007593	0.00924	AGPAT1	1-acyl-sn-gl	PLCA_HUM	4	1.125107
24H 50MT/	Q3KQU3	-0.12505	0.007595	0.009242	MAP7D1	MAP7 dom	MA7D1_HU	10	0.916972
48H 10MT/	P15169	-0.52937	0.00761	0.009256	CPN1	Carboxype	CBPN_HUM	1	0.692857
48H 50MT/	P41235	0.079556	0.007615	0.009261	HNF4A	Hepatocyte	HNF4A_HU	11	1.056692
48H 50MT/	Q9NRG9	-0.17957	0.007618	0.009264	AAAS	Aladin	AAAS_HUM	26	0.882967
24H 50MT/	P50897	0.137004	0.007628	0.009273	PPT1	Palmitoyl-p	PPT1_HUM	11	1.099619
24H 50MT/	Q0VGL1	0.026119	0.00763	0.009275	LAMTOR4	Ragulator c	LTOR4_HU	3	1.018269
48H 50MT/	Q9Y2I7	0.125913	0.007633	0.009278	PIKFYVE	1-phosphat	FYV1_HUM	16	1.091198
24H 50MT/	Q15020	0.098622	0.007633	0.009279	SART3	Squamous	SART3_HUI	38	1.07075
24H 10MT/	P36954	-0.37799	0.007635	0.00928	POLR2I	DNA-direct	RPB9_HUM	6	0.769511
24H 50MT/	Q9ULG6	-0.00344	0.00764	0.009285	CCPG1	Cell cycle p	CCPG1_HU	4	0.99762
48H 10MT/	P16219	0.148407	0.007644	0.009287	ACADS	Short-chair	ACADS_HU	9	1.108345
24H 50MT/	Q8N6M0	-0.01733	0.007647	0.009291	OTUD6B	Deubiquiti	OTU6B_HU	8	0.988056

48H 10MTA	P55327	0.011769	0.007651	0.009294	TPD52	Tumor prot	TPD52_HU	9	1.008191
48H 10MTA	Q15003	0.064712	0.007654	0.009296	NCAPH	Condensin	CND2_HUN	21	1.045876
48H 10MTA	Q15942	0.046807	0.007653	0.009296	ZYX	Zyxin	ZYX_HUMA	18	1.032976
48H 50MTA	Q15814	-0.09353	0.007654	0.009296	TBCC	Tubulin-spe	TBCC_HUM	12	0.937228
48H 50MTA	Q9BUJ2	0.16069	0.007655	0.009297	HNRNPUL1	Heterogene	HNRL1_HU	25	1.117821
24H 50MTA	Q99471	-0.71809	0.007666	0.009308	PFDN5	Prefoldin su	PFD5_HUM	4	0.607901
48H 10MTA	Q9NTX5	0.037026	0.007666	0.009308	ECHDC1	Ethylmalor	ECHD1_HU	14	1.025997
48H 50MTA	Q99956	0.224223	0.007668	0.009309	DUSP9	Dual specifi	DUS9_HUM	12	1.168148
48H 10MTA	A8MXV4	-0.18179	0.007669	0.00931	NUDT19	Acyl-coenzy	NUD19_HU	16	0.881607
48H 50MTA	Q9P265	-0.09913	0.00767	0.00931	DIP2B	Disco-inter	DIP2B_HUN	34	0.933598
24H 10MTA	O00442	-0.07945	0.00767	0.00931	RTCA	RNA 3'-tern	RTCA_HUM	15	0.946415
48H 50MTA	P42765	0.267244	0.007674	0.009314	ACAA2	3-ketoacyl-	THIM_HUM	20	1.203507
24H 10MTA	P83111	0.107536	0.007679	0.009318	LACTB	Serine beta	LACTB_HUN	23	1.077386
48H 50MTA	Q15904	-0.22129	0.00769	0.009327	ATP6AP1	V-type prot	VAS1_HUM	12	0.857795
24H 10MTA	Q14498	0.074081	0.007692	0.00933	RBM39	RNA-bindir	RBM39_HU	18	1.05269
48H 10MTA	Q96H79	0.07827	0.007702	0.009339	ZC3HAV1L	Zinc finger	ZCCHL_HUI	13	1.055751
48H 50MTA	P06744	0.415215	0.007704	0.009341	GPI	Glucose-6-p	G6PI_HUM	23	1.333498
48H 50MTA	Q9Y5Y2	-0.01677	0.007705	0.009341	NUBP2	Cytosolic Fe	NUBP2_HU	11	0.988442
48H 50MTA	G2XKQ0	0.042282	0.007706	0.009341	SUMO1P1	Small ubiqu	SUMO5_HU	3	1.029742
24H 50MTA	O75489	0.127763	0.007712	0.009347	NDUFS3	NADH dehy	NDUS3_HU	13	1.092598
48H 10MTA	Q13438	-0.01037	0.007715	0.009349	OS9	Protein OS-	OS9_HUMA	20	0.992838
48H 50MTA	Q9BYI3	0.282972	0.007726	0.00936	HYCC1	Hyccin	HYCCI_HUN	2	1.216698
24H 10MTA	Q9H3F6	0.463208	0.007727	0.00936	KCTD10	BTB/POZ dc	BACD3_HU	2	1.378604
48H 50MTA	A2RU67	-0.12872	0.00774	0.009373	FAM234B	Protein FAN	F234B_HUI	1	0.914641
24H 50MTA	Q8TD30	0.305574	0.007744	0.009376	GPT2	Alanine am	ALAT2_HUN	10	1.23591
24H 50MTA	Q9H501	0.212145	0.007744	0.009376	ESF1	ESF1 homo	ESF1_HUMA	17	1.158409
24H 10MTA	O43314	-0.18797	0.007751	0.009382	PPIP5K2	Inositol hex	VIP2_HUMA	10	0.877842
24H 50MTA	Q9Y6G9	-0.11847	0.007752	0.009382	DYNC1LI1	Cytoplasmi	DC1L1_HUI	27	0.921166
24H 10MTA	Q9NVI1	0.078415	0.007757	0.009387	FANCI	Fanconi an	FANCI_HUN	53	1.055857
48H 50MTA	Q9Y646	0.30431	0.007762	0.009392	CPQ	Carboxypep	CBPQ_HUM	4	1.234828
24H 50MTA	Q53T59	-0.12175	0.007775	0.009404	HS1BP3	HCLS1-binc	H1BP3_HU	17	0.919073
48H 10MTA	Q8IV63	0.026884	0.007781	0.009408	VRK3	Serine/thre	VRK3_HUM	3	1.018809
48H 50MTA	Q9NUJ3	-0.24022	0.007783	0.00941	TCP11L1	T-complex p	T11L1_HUN	3	0.846617
24H 10MTA	Q8WUH6	0.45118	0.007785	0.009412	TMEM263	Transmemb	TM263_HU	4	1.367158
24H 10MTA	O60885	0.102365	0.007792	0.009418	BRD4	Bromodom	BRD4_HUM	23	1.073532
24H 50MTA	Q96EY8	0.291195	0.007798	0.009423	MMAB	Corrinoid a	MMAB_HUI	7	1.223653
24H 50MTA	P56192	0.022894	0.007799	0.009424	MARS1	Methionine	SYMC_HUM	43	1.015996
24H 50MTA	Q9UBU9	0.041211	0.0078	0.009425	NXF1	Nuclear RN	NXF1_HUM	25	1.028977
48H 50MTA	P56181	-0.48821	0.007801	0.009425	NDUFV3	NADH dehy	NDUV3_HU	1	0.712911
48H 10MTA	Q14181	0.0018	0.007807	0.009431	POLA2	DNA polym	DPOA2_HU	12	1.001248
24H 10MTA	Q9UNW1	0.152515	0.007808	0.009432	MINPP1	Multiple in	MINP1_HU	11	1.111505
24H 50MTA	P78417	0.054255	0.007811	0.009434	GSTO1	Glutathion	GSTO1_HUI	19	1.038322
48H 50MTA	O15511	0.248749	0.007814	0.009436	ARPC5	Actin-relate	ARPC5_HUI	5	1.188176
24H 50MTA	P31939	-0.07991	0.007814	0.009436	ATIC	Bifunctiona	PUR9_HUM	41	0.946115
24H 50MTA	Q6DKK2	0.406197	0.007828	0.009448	TTC19	Tetratricop	TTC19_HUN	9	1.325188
24H 10MTA	P11117	0.072341	0.007831	0.00945	ACP2	Lysosomal a	PPAL_HUM	11	1.051421
24H 50MTA	Q7Z3B4	0.002358	0.007832	0.00945	NUP54	Nucleopori	NUP54_HU	19	1.001636
24H 50MTA	Q9UDW1	0.118196	0.00784	0.009457	UQCR10	Cytochrom	QCR9_HUM	2	1.085377
24H 50MTA	P24539	0.192431	0.007844	0.009459	ATP5PB	ATP syntha	AT5F1_HUN	11	1.142687
24H 50MTA	Q9Y646	0.664408	0.007844	0.009459	CPQ	Carboxypep	CBPQ_HUM	4	1.584918
24H 50MTA	P16219	0.158569	0.007855	0.009466	ACADS	Short-chair	ACADS_HUI	9	1.11618
48H 50MTA	P23229	-0.05112	0.007856	0.009467	ITGA6	Integrin alp	ITA6_HUMA	24	0.965187

48H 10MTA	Q9NVZ3	0.013299	0.007863	0.009475	NECAP2	Adaptin ear	NECP2_HU	7	1.009261
48H 50MTA	P17028	-0.31435	0.007865	0.009476	ZNF24	Zinc finger p	ZNF24_HU	7	0.804212
24H 10MTA	Q6PKC3	-0.07842	0.007865	0.009476	TXNDC11	Thioredoxin	TXD11_HU	9	0.947093
48H 10MTA	P53602	0.031478	0.007869	0.009479	MVD	Diphospho	MVD1_HUM	16	1.022058
24H 10MTA	Q93052	-0.1369	0.00787	0.00948	LPP	Lipoma-pre	LPP_HUMA	15	0.909469
48H 50MTA	Q92905	0.034651	0.00788	0.009489	COPS5	COP9 signa	CSN5_HUM	16	1.024309
24H 10MTA	Q16773	-0.49808	0.007883	0.009492	KYAT1	Kynurenine	KAT1_HUM	3	0.708048
24H 10MTA	Q9UPN3	0.018886	0.00789	0.009497	MACF1	Microtubul	MACF1_HU	190	1.013177
48H 50MTA	Q16775	0.097724	0.007902	0.009506	HAGH	Hydroxyacy	GLO2_HUM	8	1.070084
24H 50MTA	Q07954	0.384601	0.007905	0.009509	LRP1	Prolow-der	LRP1_HUM	40	1.305498
24H 50MTA	Q14681	-1.63032	0.00791	0.009514	KCTD2	BTB/POZ do	KCTD2_HU	1	0.323017
48H 50MTA	P0C264	0.049242	0.007911	0.009514	SBK3	Uncharacte	SBK3_HUM	1	1.034721
24H 10MTA	Q99956	-0.24414	0.007914	0.009516	DUSP9	Dual specifi	DUS9_HUM	12	0.844316
48H 10MTA	O95396	0.041334	0.007915	0.009518	MOCS3	Adenylyltra	MOCS3_HU	13	1.029065
48H 50MTA	Q9BXC9	1.083769	0.00792	0.009521	BBS2	Bardet-Bied	BBS2_HUM	1	2.119566
24H 50MTA	P38117	0.117778	0.007923	0.009524	ETFB	Electron tra	ETFB_HUM	17	1.085063
48H 50MTA	Q9Y294	0.030877	0.007924	0.009524	ASF1A	Histone cha	ASF1A_HUI	5	1.021633
24H 50MTA	Q8N5Y8	0.505827	0.007926	0.009525	PARP16	Protein mo	PAR16_HU	3	1.419937
48H 10MTA	Q14151	0.039387	0.007933	0.00953	SAFB2	Scaffold att	SAFB2_HUI	26	1.027677
48H 50MTA	Q92522	0.281472	0.007941	0.009537	H1-10	Histone H1	H1X_HUMA	6	1.215434
24H 50MTA	Q05639	-0.05584	0.007944	0.009539	EEF1A2	Elongation	EF1A2_HUI	8	0.962035
48H 10MTA	P35250	0.028707	0.007945	0.009539	RFC2	Replication	RFC2_HUM	19	1.020098
24H 10MTA	Q9UHY8	-0.18391	0.007945	0.009539	FEZ2	Fasciculatio	FEZ2_HUM	5	0.880311
24H 10MTA	P62906	0.074737	0.007946	0.00954	RPL10A	Large ribos	RL10A_HU	13	1.053169
48H 10MTA	P62256	0.02773	0.007952	0.009545	UBE2H	Ubiquitin-c	UBE2H_HU	3	1.019407
24H 50MTA	Q9Y223	-0.0007	0.007954	0.009546	GNE	Bifunctiona	GLCNE_HU	24	0.999515
24H 10MTA	O43242	0.0183	0.007956	0.009548	PSMD3	26S protea	PSMD3_HU	31	1.012765
48H 10MTA	P56277	-0.22078	0.007959	0.00955	CMC4	Cx9C motif	CMC4_HUM	1	0.858099
24H 50MTA	Q9BRQ6	0.264457	0.007962	0.009551	CHCHD6	MICOS com	MIC25_HU	7	1.201184
24H 50MTA	Q9H8M5	0.219053	0.007967	0.009556	CNNM2	Metal trans	CNNM2_HU	12	1.163969
24H 50MTA	Q86VP6	0.080565	0.00797	0.009558	CAND1	Cullin-asso	CAND1_HU	58	1.057432
48H 50MTA	Q9NYZ2	0.398162	0.007976	0.009562	SLC25A37	Mitoferrin-	MFRN1_HU	1	1.317828
24H 10MTA	O00487	-0.11967	0.007981	0.009566	PSMD14	26S protea	PSDE_HUM	15	0.920401
24H 10MTA	Q9NWV4	-0.17168	0.007982	0.009566	CZIB	CXXC motif	CZIB_HUM	5	0.887806
24H 50MTA	Q9BVN2	-0.25379	0.007988	0.009573	RUSC1	AP-4 compl	RUSC1_HU	3	0.838691
24H 50MTA	P00966	0.058465	0.007996	0.009581	ASS1	Argininosu	ASSY_HUM	27	1.041357
24H 50MTA	Q15629	-0.05947	0.008008	0.009592	TRAM1	Translocati	TRAM1_HU	6	0.959619
24H 10MTA	Q9H7B4	-0.09938	0.008015	0.009598	SMYD3	Histone-lys	SMYD3_HU	6	0.933437
24H 10MTA	Q9HBK9	-0.10611	0.00802	0.009602	AS3MT	Arsenite me	AS3MT_HU	24	0.929089
48H 50MTA	O60337	3.867207	0.00802	0.009603	MARCHF6	E3 ubiquiti	MARH6_HU	1	14.59303
24H 50MTA	Q16186	0.29996	0.008022	0.009604	ADRM1	Proteasom	ADRM1_HU	9	1.231111
24H 50MTA	P32519	0.176761	0.008026	0.009605	ELF1	ETS-related	ELF1_HUM	7	1.130343
24H 10MTA	P22830	0.050665	0.008034	0.009614	FECH	Ferrochelat	HEMH_HUM	12	1.035742
48H 10MTA	P40123	0.083414	0.008045	0.009622	CAP2	Adenylyl cy	CAP2_HUM	15	1.059522
48H 50MTA	Q9NRY4	0.068247	0.008051	0.009629	ARHGAP35	Rho GTPase	RHG35_HU	27	1.048442
48H 50MTA	P09382	0.021935	0.008053	0.00963	LGALS1	Galectin-1	LEG1_HUM	9	1.01532
24H 50MTA	O43581	0.712916	0.008068	0.009643	SYT7	Synaptotag	SYT7_HUM	6	1.639114
24H 50MTA	Q13206	0.147836	0.008074	0.009649	DDX10	Probable A	DDX10_HU	30	1.107907
24H 50MTA	Q96BS2	-0.08084	0.008076	0.00965	TESC	Calcineurin	CHP3_HUM	8	0.945504
24H 10MTA	P19525	0.042656	0.008079	0.009654	EIF2AK2	Interferon-i	E2AK2_HUI	18	1.030008
24H 50MTA	Q13263	0.086836	0.008084	0.009658	TRIM28	Transcripti	TIF1B_HUM	33	1.062039
24H 50MTA	Q7Z333	0.07933	0.008086	0.009661	SETX	Probable h	SETX_HUM	17	1.056527

48H	50MTA	Q9Y5X1	0.146455	0.008094	0.009668	SNX9	Sorting nex	SNX9_HUM	18	1.106846
24H	50MTA	Q16877	1.359647	0.008096	0.00967	PFKFB4	6-phosphof	F264_HUM	9	2.566224
24H	10MTA	Q6SZW1	-0.06425	0.008099	0.009673	SARM1	NAD(+) hyd	SARM1_HU	28	0.95644
48H	50MTA	Q8TBX8	-0.12542	0.0081	0.009673	PIP4K2C	Phosphatid	PI42C_HUM	17	0.916734
24H	10MTA	Q9UPS6	1.016972	0.008105	0.009678	SETD1B	Histone-lys	SET1B_HUM	3	2.023668
48H	50MTA	Q9Y624	0.115661	0.008107	0.00968	F11R	Junctional	JAM1_HUM	10	1.083472
48H	50MTA	O43252	0.124633	0.008108	0.009681	PAPSS1	Bifunctiona	PAPS1_HUI	21	1.09023
48H	10MTA	Q96ST3	-0.04529	0.008127	0.0097	SIN3A	Paired amp	SIN3A_HUM	31	0.969095
48H	10MTA	O15318	0.139544	0.008129	0.009701	POLR3G	DNA-direct	RPC7_HUM	3	1.101557
48H	10MTA	P55957	-0.05947	0.008133	0.009704	BID	BH3-intera	BID_HUMA	4	0.959615
24H	50MTA	P15559	-0.14166	0.008135	0.009705	NQO1	NAD(P)H de	NQO1_HUM	9	0.906473
48H	50MTA	P45877	0.443831	0.008137	0.009706	PPIC	Peptidyl-pr	PPIC_HUMA	2	1.360211
48H	50MTA	P07384	0.154924	0.00814	0.009708	CAPN1	Calpain-1 c	CAN1_HUM	38	1.113363
48H	50MTA	P25054	0.03168	0.008144	0.009711	APC	Adenomato	APC_HUMA	7	1.022202
24H	50MTA	P30154	0.123752	0.008144	0.009711	PPP2R1B	Serine/thre	2AAB_HUM	22	1.089565
24H	50MTA	O14647	0.249	0.008145	0.009712	CHD2	Chromodo	CHD2_HUM	14	1.188383
24H	10MTA	P16118	-0.29541	0.008153	0.009718	PFKFB1	6-phosphof	F261_HUM	6	0.814841
48H	10MTA	Q01970	-0.00129	0.008154	0.009719	PLCB3	1-phosphat	PLCB3_HUI	25	0.999106
48H	50MTA	Q9H2J4	-0.1129	0.008159	0.009722	PDCL3	Phosducin-	PDCL3_HUI	15	0.92473
48H	10MTA	O75347	0.05371	0.008163	0.009726	TBCA	Tubulin-spe	TBCA_HUM	10	1.037931
48H	50MTA	Q9Y2G5	0.166849	0.008164	0.009726	POFUT2	GDP-fucose	OFUT2_HUI	15	1.122604
24H	50MTA	Q86V81	0.069795	0.008172	0.009735	ALYREF	THO compl	THOC4_HU	10	1.049567
24H	50MTA	Q14126	0.149365	0.008173	0.009736	DSG2	Desmoglein	DSG2_HUM	25	1.109081
24H	10MTA	Q5EBL4	0.134573	0.008176	0.009738	RILPL1	RILP-like pr	RIPL1_HUM	4	1.097768
48H	10MTA	Q9ULX9	-0.18114	0.008184	0.009745	MAFF	Transcripti	MAFF_HUM	4	0.882006
48H	50MTA	O00469	0.774385	0.008185	0.009746	PLOD2	Procollager	PLOD2_HU	17	1.710461
24H	50MTA	Q01973	0.213038	0.008186	0.009746	ROR1	Inactive tyr	ROR1_HUM	8	1.159127
24H	50MTA	P36551	0.156754	0.00819	0.009749	CPOX	Oxygen-dep	HEM6_HUM	17	1.114776
48H	50MTA	Q9NQW7	0.157258	0.008191	0.009749	XPNPEP1	Xaa-Pro am	XPP1_HUM	28	1.115166
24H	50MTA	Q9NRL3	-0.0507	0.008195	0.009752	STRN4	Striatin-4	STRN4_HUI	14	0.965467
48H	50MTA	Q92786	0.195222	0.008196	0.009753	PROX1	Prospero h	PROX1_HU	17	1.1449
24H	50MTA	P38435	0.067531	0.008197	0.009753	GGCX	Vitamin K-c	VKGC_HUM	16	1.047921
48H	10MTA	P50416	0.026424	0.008198	0.009753	CPT1A	Carnitine O	CPT1A_HUI	28	1.018484
48H	10MTA	Q15054	-0.01696	0.008199	0.009754	POLD3	DNA polym	DPOD3_HU	11	0.988312
48H	10MTA	Q9UKE5	0.093032	0.008205	0.009759	TNIK	TRAF2 and	TNIK_HUMA	13	1.066609
24H	50MTA	P35680	0.038759	0.008207	0.00976	HNF1B	Hepatocyte	HNF1B_HU	6	1.02723
48H	10MTA	Q9BYG3	-0.02503	0.008207	0.00976	NIFK	MKI67 FHA	MK67I_HUI	17	0.982802
48H	50MTA	O75352	-0.14478	0.008218	0.00977	MPDU1	Mannose-P	MPU1_HUM	4	0.90452
48H	50MTA	Q15008	0.012426	0.008232	0.009782	PSMD6	26S protea	PSMD6_HU	30	1.00865
48H	50MTA	Q8N1F8	-0.17094	0.008234	0.009785	STK11IP	Serine/thre	S11IP_HUM	14	0.888264
48H	10MTA	P49585	-0.00786	0.008239	0.009789	PCYT1A	Choline-ph	PCY1A_HUI	14	0.994565
48H	50MTA	P21397	0.081661	0.00824	0.009789	MAOA	Amine oxid	AOFA_HUM	10	1.058236
48H	50MTA	Q9Y679	-0.06815	0.00824	0.009789	AUP1	Lipid dropl	AUP1_HUM	13	0.95386
48H	10MTA	P29992	-0.07741	0.008245	0.009795	GNA11	Guanine nu	GNA11_HU	14	0.947757
24H	10MTA	Q4KMQ2	0.26378	0.008248	0.009797	ANO6	Anoctamin	ANO6_HUM	16	1.20062
24H	50MTA	O60551	0.423075	0.008265	0.009813	NMT2	Glycylpepti	NMT2_HUM	1	1.340783
24H	10MTA	Q6P1X5	0.323419	0.00827	0.009816	TAF2	Transcripti	TAF2_HUM	11	1.251292
48H	50MTA	Q7Z6J9	-0.01907	0.008275	0.009821	TSEN54	tRNA-splici	SEN54_HUI	7	0.986868
48H	10MTA	Q9Y6D9	0.06606	0.008276	0.009821	MAD1L1	Mitotic spi	MD1L1_HU	22	1.046854
24H	50MTA	O76061	0.776256	0.008277	0.009823	STC2	Stanniocalc	STC2_HUM	3	1.71268
24H	50MTA	Q9UPX8	0.206945	0.008278	0.009824	SHANK2	SH3 and mu	SHAN2_HU	6	1.154242
48H	50MTA	P61224	0.075329	0.00828	0.009825	RAP1B	Ras-related	RAP1B_HUI	10	1.053602

48H 10MTA	Q9H6R3	0.042986	0.008284	0.009828	ACSS3	Acyl-CoA sy	ACSS3_HUI	17	1.030244
24H 50MTA	P37173	-0.14115	0.00829	0.009833	TGFBR2	TGF-beta re	TGFR2_HUI	12	0.906797
48H 10MTA	O60658	0.12394	0.008294	0.009837	PDE8A	High affinit	PDE8A_HUI	14	1.089707
48H 50MTA	Q12931	0.044935	0.008295	0.009838	TRAP1	Heat shock	TRAP1_HUI	34	1.031637
24H 10MTA	Q5HYK7	2.694887	0.008297	0.00984	SH3D19	SH3 domai	SH319_HUI	1	6.475031
24H 50MTA	Q68CQ7	0.309898	0.008306	0.009847	GLT8D1	Glycosyltra	GL8D1_HU	9	1.23962
24H 50MTA	Q9Y3C8	-0.22167	0.008307	0.009848	UFC1	Ubiquitin-f	UFC1_HUM	7	0.857575
24H 50MTA	Q9HD33	0.252511	0.008318	0.009857	MRPL47	Large ribos	RM47_HUM	8	1.191278
48H 50MTA	O43318	0.237539	0.008319	0.009858	MAP3K7	Mitogen-ac	M3K7_HUM	8	1.17898
48H 10MTA	Q9UG56	-0.3671	0.008322	0.009861	PISD	Phosphatid	PISD_HUMA	7	0.775341
48H 10MTA	Q9UNS2	0.063646	0.008323	0.009861	COPS3	COP9 signa	CSN3_HUM	18	1.045104
24H 50MTA	Q92576	0.231816	0.008325	0.009863	PHF3	PHD finger	PHF3_HUM	36	1.174312
48H 50MTA	P12277	0.715518	0.008329	0.009866	CKB	Creatine ki	KCRB_HUM	20	1.642073
24H 50MTA	P35613	0.088164	0.008334	0.009871	BSG	Basigin	BASI_HUMA	9	1.063016
24H 50MTA	Q6ZWJ1	0.118031	0.008336	0.009872	STXBP4	Syntaxin-bi	STXB4_HUI	10	1.085253
48H 10MTA	P21333	0.028596	0.008339	0.009874	FLNA	Filamin-A	FLNA_HUM	104	1.020019
24H 50MTA	Q9UKG1	0.031831	0.008346	0.009881	APPL1	DCC-intera	DP13A_HU	20	1.022308
24H 10MTA	Q1XH10	-0.61184	0.008351	0.009885	SKIDA1	SKI/DACH d	SKDA1_HUI	2	0.65436
48H 50MTA	Q92973	-0.14522	0.008352	0.009885	TNPO1	Transportir	TNPO1_HU	31	0.904244
48H 50MTA	O43808	-0.10372	0.008361	0.009893	SLC25A17	Peroxisoma	PM34_HUM	8	0.930633
48H 10MTA	Q96D71	-0.04651	0.008372	0.009901	REPS1	RalBP1-ass	REPS1_HUI	11	0.968273
24H 50MTA	Q86X83	-0.19218	0.008375	0.009904	COMMD2	COMM dom	COMD2_HU	8	0.875283
24H 10MTA	Q9UN37	-0.08355	0.008377	0.009905	VPS4A	Vacuolar pr	VPS4A_HUI	13	0.943734
48H 50MTA	Q9H0U4	0.081365	0.008381	0.009908	RAB1B	Ras-related	RAB1B_HUI	9	1.058018
48H 10MTA	Q96HA7	0.09323	0.008382	0.009908	TONSL	Tonsoku-lik	TONSL_HUI	11	1.066756
48H 50MTA	Q7L5Y1	0.1704	0.008385	0.009911	ENOSF1	Mitochond	ENOF1_HUI	13	1.125371
24H 50MTA	Q9GZV4	0.013059	0.008385	0.009911	EIF5A2	Eukaryotic	IF5A2_HUM	7	1.009093
24H 50MTA	Q99549	0.128629	0.008389	0.009914	MPHOSPH8	M-phase ph	MPP8_HUM	11	1.093254
24H 10MTA	Q15274	-0.10607	0.008395	0.00992	QPRT	Nicotinate-	NADC_HUM	8	0.929114
24H 50MTA	P62312	0.266768	0.008405	0.00993	LSM6	U6 snRNA-a	LSM6_HUM	6	1.203109
48H 50MTA	Q86VN1	0.109708	0.008413	0.009937	VPS36	Vacuolar pr	VPS36_HUI	17	1.07901
48H 10MTA	O60337	3.370406	0.008417	0.009939	MARCHF6	E3 ubiquiti	MARH6_HU	1	10.34173
24H 10MTA	Q9BWJ5	-0.33087	0.008421	0.009943	SF3B5	Splicing fac	SF3B5_HUI	4	0.795059
24H 50MTA	Q96H20	-0.15488	0.008422	0.009944	SNF8	Vacuolar-sc	SNF8_HUM	7	0.89821
48H 50MTA	Q15776;Q4	-0.69784	0.008427	0.009948	ZKSCAN8;Z	Zinc finger	ZKSC8_HUI	1	0.616494
24H 10MTA	Q14126	0.246993	0.008436	0.009958	DSG2	Desmoglei	DSG2_HUM	25	1.186731
48H 10MTA	P02749	-0.33992	0.008437	0.009958	APOH	Beta-2-glyc	APOH_HUM	1	0.790087
24H 50MTA	Q01581	-0.12428	0.008438	0.009959	HMGCS1	Hydroxyme	HMCS1_HU	23	0.917462
24H 50MTA	O43396	0.097361	0.008444	0.009964	TXNL1	Thioredoxi	TXNL1_HUI	16	1.069814
48H 50MTA	P05067	-0.03066	0.008464	0.009984	APP	Amyloid-be	A4_HUMAN	10	0.978971
24H 50MTA	P40855	-1.0181	0.008465	0.009986	PEX19	Peroxisoma	PEX19_HUI	1	0.493767
24H 50MTA	P17050	0.257013	0.008466	0.009986	NAGA	Alpha-N-ac	NAGAB_HU	10	1.195002
48H 50MTA	P62140	0.098666	0.008471	0.009989	PPP1CB	Serine/thre	PP1B_HUM	9	1.070783
48H 50MTA	Q16134	0.107219	0.008477	0.009993	ETFDH	Electron tra	ETFD_HUM	18	1.07715
48H 10MTA	Q86UB9	-0.43634	0.008482	0.009996	TMEM135	Transmemb	TM135_HU	7	0.739009
48H 50MTA	Q14318	0.148694	0.008485	0.009998	FKBP8	Peptidyl-pr	FKBP8_HUI	10	1.108565
48H 10MTA	P52597	0.031514	0.008487	0.009999	HNRNPF	Heterogene	HNRPF_HU	18	1.022084
24H 50MTA	Q969Z0	0.162513	0.0085	0.01001	TBRG4	FAST kinase	FAKD4_HUI	19	1.119235
24H 10MTA	Q9H5K3	0.267941	0.008503	0.010012	POMK	Protein O-n	SG196_HUI	5	1.204088
48H 50MTA	Q8IUC4	0.200829	0.008504	0.010013	RHPN2	Rhophilin-2	RHPN2_HU	21	1.149358
24H 10MTA	Q92616	-0.05497	0.008507	0.010017	GCN1	Stalled ribo	GCN1_HUM	146	0.962615
48H 10MTA	Q9NZ45	0.178222	0.008508	0.010017	CISD1	CDGSH iron	CISD1_HUM	5	1.131488

48H 10MTA	Q9NRL2	-0.068	0.008509	0.010018	BAZ1A	Bromodom	BAZ1A_HUI	24	0.953962
24H 50MTA	Q53FA7	-0.08941	0.008512	0.010021	TP53I3	Quinone ox	QORX_HUN	13	0.93991
24H 50MTA	P40937	0.05852	0.00852	0.010027	RFC5	Replicatior	RFC5_HUM	22	1.041397
24H 10MTA	Q6Y1H2	0.428353	0.008521	0.010028	HACD2	Very-long-c	HACD2_HU	4	1.345696
48H 10MTA	Q86X10	0.034979	0.008529	0.010035	RALGAPB	Ral GTPase-	RLGPB_HUI	30	1.024542
48H 50MTA	O14730	0.236337	0.008531	0.010036	RIOK3	Serine/thre	RIOK3_HUN	7	1.177998
24H 50MTA	P46783	-0.09982	0.008531	0.010036	RPS10	Small ribos	RS10_HUM	7	0.933151
48H 10MTA	Q9Y6I3	0.006615	0.008534	0.010038	EPN1	Epsin-1	EPN1_HUM	10	1.004596
24H 50MTA	Q99676	0.34887	0.008536	0.01004	ZNF184	Zinc finger	ZN184_HUI	1	1.273563
48H 50MTA	Q8IYA6	-0.69575	0.008543	0.010044	CKAP2L	Cytoskeletc	CKP2L_HUI	5	0.617387
24H 50MTA	Q9Y2Z9	0.252657	0.008543	0.010044	COQ6	Ubiquinon	COQ6_HUN	11	1.191399
24H 10MTA	Q7KZ85	0.017988	0.008545	0.010045	SUPT6H	Transcripti	SPT6H_HUI	55	1.012546
24H 50MTA	O75363	0.094472	0.008549	0.010049	BCAS1	Breast carci	BCAS1_HUI	5	1.067674
48H 50MTA	P55347	0.218516	0.008552	0.010052	PKNOX1	Homeobox	PKNX1_HU	1	1.163536
48H 10MTA	Q00403	0.086932	0.008553	0.010053	GTF2B	Transcripti	TF2B_HUM	17	1.062109
24H 10MTA	Q96RU2	0.121778	0.008555	0.010054	USP28	Ubiquitin c	UBP28_HU	20	1.088075
24H 50MTA	Q9H0W9	0.068022	0.008564	0.010061	C11orf54	Ester hydro	CK054_HUI	13	1.048278
24H 10MTA	Q9HC07	0.100011	0.008568	0.010064	TMEM165	Putative di	TM165_HU	5	1.071782
48H 10MTA	Q9Y673	-0.02336	0.008568	0.010064	ALG5	Dolichyl-pl	ALG5_HUM	12	0.983939
48H 10MTA	Q8WWX9	-1.23664	0.008571	0.010067	SELENOM	Selenoprot	SELM_HUM	1	0.42436
24H 50MTA	O15260	-0.06575	0.008579	0.010073	SURF4	Surfeit locu	SURF4_HUI	6	0.955447
24H 50MTA	Q9NV66	0.445639	0.008585	0.010079	TYW1	S-adenosyl-	TYW1_HUN	9	1.361917
24H 10MTA	Q9HAB8	-0.0812	0.008589	0.010082	PPCS	Phosphopa	PPCS_HUM	15	0.945269
24H 50MTA	Q15437	0.008358	0.008602	0.010092	SEC23B	Protein tra	SC23B_HUI	29	1.00581
48H 50MTA	Q9BRA2	0.160883	0.00861	0.0101	TXNDC17	Thioredoxi	TXD17_HUI	5	1.117971
48H 50MTA	Q12904	0.068874	0.008619	0.010109	AIMP1	Aminoacyl	AIMP1_HUI	18	1.048897
24H 10MTA	P46060	0.10411	0.008625	0.010114	RANGAP1	Ran GTPase	RAGP1_HU	33	1.074831
24H 10MTA	P55786	-0.08115	0.008627	0.010116	NPEPPS	Puromycin	PSA_HUMA	43	0.945304
48H 10MTA	Q8N1A0	0.226613	0.00863	0.010117	KRT222	Keratin-like	KT222_HUI	3	1.170085
24H 50MTA	P62877	0.348003	0.008636	0.010122	RBX1	E3 ubiquiti	RBX1_HUM	2	1.272797
48H 10MTA	Q9NPJ3	0.015314	0.008645	0.010131	ACOT13	Acyl-coenzy	ACO13_HU	7	1.010671
24H 10MTA	Q9NTN9	-0.14063	0.008654	0.010139	SEMA4G	Semaphori	SEM4G_HU	19	0.907125
24H 10MTA	Q9NY35	0.74253	0.008657	0.010141	CLDND1	Claudin do	CLDN1_HU	3	1.673107
24H 50MTA	P48444	0.047217	0.008659	0.010144	ARCN1	Coatomer s	COPD_HUN	32	1.033269
24H 10MTA	Q9UEU0	0.005453	0.008666	0.010149	VTI1B	Vesicle trar	VTI1B_HUN	8	1.003787
48H 10MTA	P23229	-0.054	0.008671	0.010152	ITGA6	Integrin alp	ITA6_HUMA	24	0.963264
48H 50MTA	Q92506	0.049481	0.008671	0.010152	HSD17B8	(3R)-3-hydr	DHB8_HUN	6	1.034892
24H 10MTA	A0MZ66	0.017648	0.008673	0.010155	SHTN1	Shootin-1	SHOT1_HUI	28	1.012308
24H 50MTA	P22695	0.114935	0.008675	0.010157	UQCRC2	Cytochrom	QCR2_HUN	12	1.082926
24H 50MTA	Q13162	0.050108	0.008687	0.010168	PRDX4	Peroxiredo	PRDX4_HU	13	1.035343
48H 10MTA	Q6ZSZ5	-0.03775	0.008687	0.010168	ARHGEF18	Rho guanin	ARHGI_HUI	15	0.974171
24H 10MTA	P61019	0.086291	0.008689	0.010169	RAB2A	Ras-related	RAB2A_HUI	14	1.061637
24H 50MTA	Q9UKN8	0.086764	0.008698	0.010177	GTF3C4	General tra	TF3C4_HUN	25	1.061985
24H 50MTA	Q08426	0.112197	0.008699	0.010178	EHHADH	Peroxisoma	ECHP_HUM	31	1.080873
24H 50MTA	O75880	0.139976	0.008701	0.01018	SCO1	Protein SCC	SCO1_HUM	8	1.101887
48H 10MTA	O14745	0.095634	0.008706	0.010184	NHERF1	Na(+)/H(+) e	NHRF1_HU	23	1.068535
24H 10MTA	Q5RKV6	0.062144	0.008709	0.010187	EXOSC6	Exosome cc	EXOS6_HUI	8	1.044016
48H 50MTA	P62330	0.135048	0.008709	0.010187	ARF6	ADP-ribosy	ARF6_HUM	8	1.098129
24H 50MTA	P54802	0.109468	0.00871	0.010188	NAGLU	Alpha-N-ac	ANAG_HUN	19	1.078831
24H 50MTA	Q9UIF9	0.218134	0.00872	0.010195	BAZ2A	Bromodom	BAZ2A_HUI	26	1.163228
24H 50MTA	Q92539	-0.09664	0.008732	0.010206	LPIN2	Phosphatid	LPIN2_HUN	11	0.935212
48H 10MTA	Q96HY6	0.005115	0.008734	0.010207	DDRGK1	DDRGK dor	DDRGK_HU	8	1.003552

48H	10MT/	O76054	0.000381	0.008737	0.010209	SEC14L2	SEC14-like	S14L2_HUI	10	1.000264
24H	50MT/	Q14680	0.162172	0.008744	0.010216	MELK	Maternal er	MELK_HUN	13	1.118971
48H	50MT/	Q9BZE2	0.040763	0.008747	0.010218	PUS3	tRNA pseud	PUS3_HUN	6	1.028658
24H	10MT/	Q9BYJ9	0.00083	0.008753	0.010224	YTHDF1	YTH domair	YTHD1_HUI	9	1.000575
48H	10MT/	O60942	0.060536	0.008757	0.010226	RNGTT	mRNA-capp	MCE1_HUN	11	1.042853
48H	50MT/	Q5VZ89	-0.23748	0.008763	0.010232	DENND4C	DENN doma	DEN4C_HU	13	0.848228
24H	10MT/	Q13557	0.004726	0.008768	0.010236	CAMK2D	Calcium/ca	KCC2D_HU	10	1.003281
48H	50MT/	Q9HAN9	0.093358	0.00877	0.010237	NMNAT1	Nicotinami	NMNA1_HU	5	1.06685
48H	10MT/	P61769	-0.50648	0.008773	0.010239	B2M	Beta-2-micı	B2MG_HUN	3	0.703936
48H	50MT/	Q96CW6	-0.51679	0.008772	0.010239	SLC7A6OS	Probable R	S7A6O_HUI	3	0.698923
24H	50MT/	Q96S52	0.188056	0.008773	0.010239	PIGS	GPI transan	PIGS_HUM/	16	1.139228
24H	10MT/	Q00535	-0.10626	0.008776	0.010241	CDK5	Cyclin-depe	CDK5_HUN	13	0.928996
48H	50MT/	Q8IZN3	-0.13483	0.008781	0.010247	ZDHHC14	Palmitoyltr	ZDH14_HU	3	0.910778
48H	10MT/	O15372	-0.13711	0.008783	0.010248	EIF3H	Eukaryotic	EIF3H_HUN	21	0.909337
24H	50MT/	Q9NZ45	0.187262	0.008784	0.010248	CISD1	CDGSH iror	CISD1_HUN	5	1.138601
48H	50MT/	P14866	0.059803	0.008788	0.010252	HNRNPL	Heterogene	HNRPL_HU	23	1.042323
48H	50MT/	Q6NTF9	0.308575	0.00879	0.010254	RHBDD2	Rhomboid	RHBD2_HU	1	1.238484
48H	10MT/	P53999	0.154542	0.008791	0.010254	SUB1	Activated R	TCP4_HUM	8	1.113068
48H	10MT/	Q9BV73	0.006901	0.008795	0.010258	CEP250	Centrosom	CP250_HUI	18	1.004795
48H	50MT/	Q9BRR6	-0.11228	0.0088	0.010262	ADPGK	ADP-depen	ADPGK_HU	11	0.925124
24H	50MT/	Q6ZN17	0.000544	0.008804	0.010266	LIN28B	Protein lin-	LN28B_HUI	9	1.000377
24H	50MT/	Q9NP77	-0.0458	0.008815	0.010274	SSU72	RNA polym	SSU72_HUI	5	0.968749
48H	50MT/	P08236	0.090742	0.008815	0.010274	GUSB	Beta-glucur	BGLR_HUM	6	1.064918
24H	50MT/	Q9BYD1	-0.08195	0.008821	0.010279	MRPL13	Large ribos	RM13_HUN	12	0.944777
24H	10MT/	P22314	-0.06846	0.008823	0.010281	UBA1	Ubiquitin-l	UBA1_HUN	46	0.953658
24H	10MT/	P01019	-0.14833	0.008826	0.010284	AGT	Angiotensir	ANGT_HUN	12	0.902297
24H	50MT/	P29590	0.121052	0.008833	0.010291	PML	Protein PM	PML_HUM/	11	1.087528
24H	10MT/	Q6Y7W6	-0.07012	0.008834	0.010292	GIGYF2	GRB10-inte	GGYF2_HUI	31	0.95256
48H	50MT/	Q16576	-0.28488	0.008849	0.010305	RBBP7	Histone-bir	RBBP7_HUI	12	0.820809
48H	50MT/	Q9UDT6	0.01711	0.008849	0.010305	CLIP2	CAP-Gly do	CLIP2_HUN	31	1.011931
24H	50MT/	Q6PI48	0.078086	0.008854	0.01031	DARS2	Aspartate--	SYDM_HUN	31	1.055617
24H	50MT/	Q9BVG8	-0.43514	0.008858	0.010313	KIFC3	Kinesin-like	KIFC3_HUN	16	0.739624
48H	10MT/	P31641	-1.21017	0.008862	0.010317	SLC6A6	Sodium- an	SC6A6_HUI	2	0.432218
24H	50MT/	P10619	0.179414	0.008864	0.010318	CTSA	Lysosomal	PPGB_HUM	13	1.132424
48H	50MT/	Q13951	-0.11652	0.008869	0.010322	CBFB	Core-bindir	PEBB_HUM	7	0.922412
24H	50MT/	P82970	0.263972	0.008873	0.010325	HMGN5	High mobil	HMGN5_HU	5	1.20078
48H	50MT/	P02538	0.397448	0.008878	0.01033	KRT6A	Keratin, typ	K2C6A_HUI	2	1.317176
48H	50MT/	O43172	-0.1207	0.00888	0.010331	PRPF4	U4/U6 sma	PRP4_HUN	19	0.919742
48H	50MT/	Q8N511	-0.41994	0.008899	0.010349	TMEM199	Transmemb	TM199_HU	4	0.747455
48H	10MT/	Q9H7E9	0.046085	0.008899	0.010349	C8orf33	UPF0488 p	CH033_HU	4	1.03246
24H	10MT/	Q13868	0.192178	0.0089	0.01035	EXOSC2	Exosome co	EXOS2_HUI	12	1.142488
48H	10MT/	O75477	0.163321	0.008901	0.010351	ERLIN1	Erlin-1	ERLN1_HUI	10	1.119862
24H	10MT/	Q9UNK0	0.037745	0.008903	0.010352	STX8	Syntaxin-8	STX8_HUM	5	1.026508
48H	10MT/	Q9UNQ0	-0.15725	0.008912	0.01036	ABCG2	Broad subst	ABCG2_HU	9	0.896735
48H	50MT/	Q9NVV5	0.072078	0.008917	0.010363	AIG1	Androgen-i	AIG1_HUM	3	1.051229
48H	10MT/	Q8IY50	0.56819	0.008927	0.010373	SLC35F3	Solute carri	S35F3_HUN	1	1.482662
24H	50MT/	O95453	0.193475	0.008928	0.010374	PARN	Poly(A)-spe	PARN_HUN	22	1.143515
24H	50MT/	Q16543	-0.22664	0.008928	0.010374	CDC37	Hsp90 co-c	CDC37_HU	9	0.854622
24H	50MT/	Q14258	-0.11505	0.008929	0.010374	TRIM25	E3 ubiquiti	TRI25_HUN	30	0.92335
24H	50MT/	Q9Y342	-0.612	0.00893	0.010376	PLLP	Plasmolipir	PLLP_HUM	2	0.65429
24H	50MT/	P17655	-0.03079	0.008945	0.010388	CAPN2	Calpain-2 c	CAN2_HUM	22	0.978882
48H	10MT/	Q8IV61	1.337865	0.008946	0.010388	RASGRP3	Ras guanyl-	GRP3_HUM	2	2.527769

48H 10MT/	P49591	0.065467	0.008959	0.010399	SARS1	Serine--tRN	SYSC_HUM/	34	1.046424
48H 50MT/	Q96H79	0.110943	0.008971	0.010409	ZC3HAV1L	Zinc finger (	ZCCHL_HUI	13	1.079934
48H 10MT/	Q9P2M7	0.038824	0.008971	0.010409	CGN	Cingulin	CING_HUM	44	1.027276
48H 50MT/	Q15398	-0.35985	0.008971	0.010409	DLGAP5	Disks large-	DLGP5_HU	16	0.779247
24H 50MT/	Q9BRF8	-0.02184	0.008973	0.010411	CPPED1	Serine/thre	CPPED_HUI	12	0.984976
24H 50MT/	Q13362	-0.10412	0.008978	0.010416	PPP2R5C	Serine/thre	2A5G_HUM	15	0.930375
48H 50MT/	Q16270	0.594241	0.008981	0.010419	IGFBP7	Insulin-like	IBP7_HUM/	6	1.509678
24H 10MT/	Q7L5Y1	-0.07275	0.008983	0.01042	ENOSF1	Mitochond	ENOF1_HUI	13	0.950825
24H 10MT/	Q9NTJ3	0.092196	0.008992	0.010429	SMC4	Structural r	SMC4_HUM	56	1.065992
48H 10MT/	P07602	0.049067	0.008993	0.01043	PSAP	Prosaposin	SAP_HUMA	17	1.034595
24H 50MT/	Q9Y6A5	-0.19777	0.008998	0.010434	TACC3	Transformi	TACC3_HUI	15	0.871895
24H 10MT/	P21675	0.09809	0.009001	0.010437	TAF1	Transcripti	TAF1_HUM	11	1.070356
48H 50MT/	O95825	0.151596	0.00901	0.010444	CRYZL1	Quinone ox	QORL1_HU	12	1.110798
48H 50MT/	O60313	0.041032	0.009014	0.010447	OPA1	Dynamin-li	OPA1_HUM	46	1.02885
48H 10MT/	Q8IYS1	-0.15832	0.009018	0.010451	PM20D2	Xaa-Arg dip	P20D2_HU	9	0.89607
24H 10MT/	O14979	0.060831	0.009019	0.010451	HNRNPDL	Heterogene	HNRDL_HU	14	1.043067
24H 50MT/	Q92805	0.011581	0.00902	0.010452	GOLGA1	Golgin subf	GOGA1_HU	23	1.00806
24H 50MT/	O95477	1.865415	0.00903	0.010462	ABCA1	Phospholip	ABCA1_HUI	2	3.643729
24H 50MT/	Q9Y6V0	1.194407	0.009035	0.010466	PCLO	Protein pic	PCLO_HUM	2	2.288507
24H 10MT/	Q9Y5Z9	-0.04619	0.009044	0.010473	UBIAD1	UbiA preny	UBIA1_HUI	5	0.968487
48H 50MT/	L0R8F8	-0.55014	0.009057	0.010485	MIEF1	Mitochond	MIDUO_HU	3	0.682955
48H 10MT/	Q9BVC3	-0.02458	0.009059	0.010487	DSCC1	Sister chror	DCC1_HUM	5	0.983103
48H 10MT/	Q16637	0.240115	0.00906	0.010488	SMN1	Survival mc	SMN_HUM/	8	1.181087
24H 50MT/	Q12765	-0.14185	0.009067	0.010494	SCRN1	Secernin-1	SCRN1_HUI	20	0.906359
48H 50MT/	Q16831	-0.07973	0.00907	0.010496	UPP1	Uridine pho	UPP1_HUM	16	0.946232
24H 10MT/	Q6NVY1	0.095786	0.009071	0.010497	HIBCH	3-hydroxyi:	HIBCH_HUI	15	1.068647
48H 50MT/	Q13492	0.092498	0.009076	0.010502	PICALM	Phosphatid	PICAL_HUM	17	1.066215
24H 10MT/	Q9NPF0	-0.09752	0.009078	0.010504	CD320	CD320 anti	CD320_HU	5	0.934636
24H 50MT/	Q96N21	-0.59274	0.009086	0.010508	TEPSIN	AP-4 compl	AP4AT_HUI	1	0.663083
24H 10MT/	Q9Y376	-0.02547	0.009087	0.01051	CAB39	Calcium-bi	CAB39_HUI	13	0.982497
24H 50MT/	Q29RF7	0.097101	0.009091	0.010513	PDS5A	Sister chror	PDS5A_HUI	51	1.069622
48H 10MT/	P62258	0.12252	0.009097	0.010518	YWHAE	14-3-3 prot	1433E_HUI	21	1.088635
48H 10MT/	Q9Y6E2	-0.10613	0.009099	0.01052	BZW2	eIF5-mimic	5MP1_HUM	22	0.92908
48H 50MT/	Q14197	-0.19041	0.009105	0.010524	MRPL58	Large ribos	ICT1_HUM/	6	0.876355
48H 50MT/	P38432	-0.10763	0.009106	0.010526	COIL	Coilin	COIL_HUM/	11	0.92811
48H 50MT/	P51149	-0.10257	0.00912	0.010538	RAB7A	Ras-related	RAB7A_HUI	17	0.931373
24H 10MT/	O15397	0.127992	0.009122	0.01054	IPO8	Importin-8	IPO8_HUM	23	1.092771
24H 50MT/	Q9BZV1	0.007048	0.009125	0.010543	UBXN6	UBX domai	UBXN6_HU	12	1.004897
24H 50MT/	Q9H300	0.02349	0.009129	0.010547	PARL	Presenilin-a	PARL_HUM	6	1.016415
48H 50MT/	Q8NET6	0.621149	0.00913	0.010547	CHST13	Carbohydra	CHSTD_HUI	5	1.5381
24H 50MT/	Q8N8S7	0.012509	0.009134	0.01055	ENAH	Protein ena	ENAH_HUM	19	1.008708
48H 10MT/	Q8N1F7	0.043645	0.009136	0.010552	NUP93	Nuclear po	NUP93_HU	39	1.030715
48H 10MT/	Q7Z417	0.124412	0.009139	0.010554	NUFIP2	FMR1-inter	NUFP2_HU	18	1.090063
48H 50MT/	A3KN83	-0.17634	0.009144	0.010559	SBNO1	Protein stra	SBNO1_HU	24	0.884945
24H 50MT/	P62875	0.224213	0.009148	0.010561	POLR2L	DNA-direct	RPAB5_HUI	3	1.16814
24H 50MT/	Q9BVP2	-0.0882	0.009148	0.010561	GNL3	Guanine nu	GNL3_HUM	24	0.940698
48H 10MT/	Q4VC31	-0.00987	0.009149	0.010561	MIX23	Protein MI)	MIX23_HUI	6	0.993181
48H 50MT/	Q9H444	0.029175	0.009158	0.010569	CHMP4B	Charged mu	CHM4B_HU	8	1.020428
48H 10MT/	Q99816	0.116462	0.009161	0.010571	TSG101	Tumor susc	TS101_HUM	16	1.084073
24H 50MT/	Q9HAD4	0.12041	0.009161	0.010571	WDR41	WD repeat-	WDR41_HU	7	1.087044
48H 50MT/	Q92616	0.002995	0.009162	0.010572	GCN1	Stalled ribo	GCN1_HUM	146	1.002078
24H 10MT/	Q9H300	0.044293	0.009163	0.010573	PARL	Presenilin-a	PARL_HUM	6	1.031178

24H 10MT/	Q96JK2	0.069823	0.009166	0.010575	DCAF5	DDB1- and	DCAF5_HUI	1	1.049588
48H 10MT/	P25786	0.096027	0.009166	0.010575	PSMA1	Proteasom	PSA1_HUM	15	1.068826
24H 50MT/	Q16401	-0.07743	0.009166	0.010575	PSMD5	26S protea	PSMD5_HU	24	0.947745
48H 50MT/	Q8N392	0.379763	0.009173	0.010581	ARHGAP18	Rho GTPase	RHG18_HU	15	1.301128
48H 10MT/	Q8WWV3	0.044416	0.009174	0.010582	RTN4IP1	Reticulon-4	RT4I1_HUM	13	1.031266
48H 10MT/	Q92905	0.035065	0.009179	0.010586	COPS5	COP9 signa	CSN5_HUM	16	1.024603
48H 10MT/	Q9Y2U9	0.157863	0.009184	0.010589	KLHDC2	Kelch doma	KLDC2_HUI	4	1.115633
48H 10MT/	Q969N2	-0.19414	0.009188	0.010592	PIGT	GPI transan	PIGT_HUM/	19	0.874095
48H 10MT/	A7KAX9	0.651817	0.00919	0.010593	ARHGAP32	Rho GTPase	RHG32_HU	3	1.571145
48H 50MT/	O00423	0.050044	0.009191	0.010594	EML1	Echinoderm	EMAL1_HU	11	1.035297
24H 50MT/	Q13315	0.057994	0.009194	0.010596	ATM	Serine-prot	ATM_HUM/	48	1.041017
24H 10MT/	O95490	0.052317	0.009201	0.0106	ADGRL2	Adhesion G	AGRL2_HUI	28	1.036929
48H 10MT/	Q15165	-0.29883	0.009201	0.0106	PON2	Serum para	PON2_HUM	16	0.812913
24H 10MT/	Q86WQ0	-0.09639	0.009201	0.0106	NR2C2AP	Nuclear rec	NR2CA_HU	5	0.935371
48H 50MT/	Q9UHR4	-0.16577	0.009204	0.010602	BAIAP2L1	BAR/IMD d	BI2L1_HUM	19	0.891453
48H 10MT/	Q9Y520	0.026276	0.009205	0.010603	PRRC2C	Protein PRI	PRC2C_HUI	62	1.01838
24H 50MT/	P30520	-0.08117	0.009209	0.010606	ADSS2	Adenylosuc	PURA2_HU	17	0.945288
48H 10MT/	Q9Y4W6	-0.08578	0.009213	0.010609	AFG3L2	Mitochond	AFG32_HUI	34	0.942273
24H 50MT/	Q99873	0.086817	0.009215	0.010611	PRMT1	Protein arg	ANM1_HUM	23	1.062024
24H 10MT/	Q9UHG3	0.098239	0.009216	0.010612	PCYOX1	Prenylcyste	PCYOX_HUI	16	1.070466
48H 50MT/	Q9UKV8	0.09063	0.009218	0.010612	ago-02	Protein arg	AGO2_HUM	14	1.064835
48H 50MT/	O60610	0.063358	0.009222	0.010616	DIAPH1	Protein dia	DIAP1_HUM	44	1.044895
48H 50MT/	P13693	-0.00906	0.009221	0.010616	TPT1	Translation	TCTP_HUM	7	0.99374
24H 50MT/	Q8N5J2	-0.02227	0.009224	0.010617	MINDY1	Ubiquitin c	MINY1_HUI	2	0.98468
24H 10MT/	P25787	0.033261	0.009227	0.010619	PSMA2	Proteasom	PSA2_HUM	14	1.023323
48H 50MT/	Q9H967	-0.41688	0.00923	0.010622	WDR76	WD repeat-	WDR76_HU	10	0.749044
48H 50MT/	Q8N490	0.028133	0.009233	0.010625	PNKD	Probable hy	PNKD_HUM	4	1.019692
48H 10MT/	P19971	-0.02022	0.009238	0.010628	TYMP	Thymidine	TYPH_HUM	4	0.986085
24H 50MT/	Q8NE62	0.025261	0.009245	0.010635	CHDH	Choline del	CHDH_HUM	19	1.017664
24H 50MT/	O00273	-0.27795	0.009248	0.010637	DFFA	DNA fragme	DFFA_HUM	14	0.824763
24H 10MT/	P49327	-0.08254	0.009253	0.01064	FASN	Fatty acid s	FAS_HUMA	123	0.944394
48H 10MT/	Q5T3I0	-0.24498	0.009258	0.010643	GPATCH4	G patch do	GPTC4_HUI	11	0.843825
24H 10MT/	Q8N697	-0.01582	0.009257	0.010643	SLC15A4	Solute carri	S15A4_HUI	2	0.989092
48H 10MT/	Q8IVP5	0.791596	0.009257	0.010643	FUNDC1	FUN14 dom	FUND1_HU	1	1.730989
24H 50MT/	Q9BWS9	0.20476	0.009261	0.010646	CHID1	Chitinase d	CHID1_HUI	9	1.152495
48H 10MT/	P53597	-0.10042	0.009263	0.010647	SUCLG1	Succinate--	SUCA_HUM	9	0.932764
24H 50MT/	P62834	0.252842	0.009267	0.010649	RAP1A	Ras-related	RAP1A_HU	3	1.191552
24H 50MT/	O75940	0.209668	0.009272	0.010653	SMNDC1	Survival of r	SPF30_HUM	6	1.156422
48H 10MT/	Q15185	0.09508	0.009276	0.010656	PTGES3	Prostagland	TEBP_HUM	6	1.068125
24H 50MT/	Q9P0M9	0.2851	0.009284	0.010662	MRPL27	Large ribos	RM27_HUM	5	1.218495
48H 10MT/	O60271	0.068159	0.009284	0.010662	SPAG9	C-Jun-amin	JIP4_HUM/	45	1.048378
48H 50MT/	Q9UPY6	0.518147	0.009285	0.010663	WASF3	Actin-bindi	WASF3_HU	3	1.432115
48H 50MT/	P12235	0.124419	0.009291	0.010668	SLC25A4	ADP/ATP tr	ADT1_HUM	8	1.090069
24H 10MT/	Q96DZ1	-0.05828	0.009295	0.010672	ERLEC1	Endoplasm	ERLEC_HUM	14	0.960409
48H 50MT/	Q96LB3	-0.03068	0.009304	0.01068	IFT74	Intraflagell	IFT74_HUM	9	0.978961
24H 10MT/	Q4KWH8	0.431901	0.009309	0.010684	PLCH1	1-phosphat	PLCH1_HUI	3	1.34901
48H 50MT/	O00330	0.00179	0.00931	0.010685	PDHX	Pyruvate de	ODPX_HUM	18	1.001242
48H 10MT/	Q9BUJ2	0.095607	0.009317	0.01069	HNRNPUL1	Heterogene	HNRL1_HU	25	1.068515
24H 10MT/	P98174	1.399658	0.009323	0.010693	FGD1	FYVE, RhoG	FGD1_HUM	2	2.638391
24H 50MT/	Q9UPU5	-0.04061	0.009324	0.010694	USP24	Ubiquitin c	UBP24_HU	61	0.972246
24H 10MT/	P62993	-0.13769	0.009339	0.010707	GRB2	Growth fac	GRB2_HUM	13	0.908974
24H 50MT/	Q5VV42	0.218742	0.009363	0.010729	CDKAL1	Threonylca	CDKAL_HUI	15	1.163719

48H 50MT/	Q99460	0.058037	0.009363	0.010729	PSMD1	26S protea	PSMD1_HU	42	1.041048
48H 50MT/	Q5T2D3	-0.00295	0.009367	0.010732	OTUD3	OTU domai	OTUD3_HU	1	0.997956
24H 10MT/	Q9UI30	-0.12656	0.009369	0.010734	TRMT112	Multifuncti	TR112_HU	4	0.916014
48H 50MT/	Q14257	0.394895	0.009372	0.010736	RCN2	Reticulocal	RCN2_HUM	13	1.314847
24H 50MT/	Q14696	0.038948	0.009375	0.010739	MESD	LRP chaper	MESD_HUM	12	1.027364
48H 50MT/	Q9NWX5	-0.06241	0.009376	0.010739	ASB6	Ankyrin rep	ASB6_HUM	2	0.957665
48H 10MT/	Q8NBJ7	0.056611	0.009377	0.010739	SUMF2	Inactive C-a	SUMF2_HU	10	1.040019
24H 50MT/	P11234	-0.00184	0.009383	0.010744	RALB	Ras-related	RALB_HUM	10	0.998724
24H 10MT/	Q6XZF7	0.024064	0.00939	0.010749	DNMBP	Dynamin-b	DNMBP_HU	16	1.01682
48H 10MT/	O43242	0.053001	0.009393	0.010752	PSMD3	26S protea	PSMD3_HU	31	1.037421
48H 50MT/	Q9UJ04	-0.05678	0.00941	0.010767	TSPYL4	Testis-speci	TSYL4_HUM	2	0.96141
48H 50MT/	P33991	-0.08595	0.009412	0.010768	MCM4	DNA replica	MCM4_HU	45	0.942166
48H 10MT/	B9A064	0.288047	0.009415	0.01077	IGLL5	Immunoglo	IGLL5_HUM	1	1.220986
48H 50MT/	Q01970	0.04533	0.009426	0.01078	PLCB3	1-phosphat	PLCB3_HU	25	1.031919
48H 10MT/	Q9NQW6	-0.02455	0.009427	0.010781	ANLN	Anillin	ANLN_HUM	37	0.98313
48H 50MT/	Q969Z0	-0.11798	0.009432	0.010785	TBRG4	FAST kinase	FAKD4_HU	19	0.921478
48H 50MT/	Q460N5	0.622016	0.009432	0.010786	PARP14	Protein mo	PAR14_HU	1	1.539024
48H 50MT/	Q8N573	0.677457	0.009438	0.01079	OXR1	Oxidation r	OXR1_HUM	2	1.599318
48H 50MT/	Q15437	0.078508	0.009439	0.010791	SEC23B	Protein tra	SC23B_HU	29	1.055925
48H 10MT/	Q8N3E9	-0.91922	0.009442	0.010794	PLCD3	1-phosphat	PLCD3_HU	2	0.528795
24H 50MT/	Q8IX90	-0.24309	0.009444	0.010795	SKA3	Spindle anc	SKA3_HUM	6	0.844934
24H 10MT/	P57105	0.110034	0.009445	0.010796	SYNJ2BP	Synaptojan	SYJ2B_HUM	4	1.079254
48H 50MT/	O94915	0.004215	0.009448	0.010797	FRYL	Protein fur	FRYL_HUM	26	1.002926
48H 10MT/	P35680	0.005964	0.009467	0.010815	HNF1B	Hepatocyte	HNF1B_HU	6	1.004143
48H 50MT/	P78536	-0.02494	0.009475	0.010822	ADAM17	Disintegrin	ADA17_HU	8	0.982858
24H 10MT/	Q15054	-0.02673	0.009478	0.010825	POLD3	DNA polym	DPOD3_HU	11	0.98164
48H 50MT/	P41250	0.106071	0.009481	0.010827	GARS1	Glycine--tR	GARS_HUM	35	1.076293
48H 50MT/	Q9Y4P3	-0.00621	0.009492	0.010837	TBL2	Transducin	TBL2_HUM	21	0.995701
24H 10MT/	O43760	0.125879	0.009493	0.010838	SYNGR2	Synaptogyr	SNG2_HUM	4	1.091172
24H 10MT/	A0A0K0K1/	-0.11119	0.009496	0.010839	TRBV6-5	T cell recep	TVB65_HU	1	0.925824
24H 50MT/	Q16512	0.208563	0.009498	0.010841	PKN1	Serine/thre	PKN1_HUM	17	1.155536
24H 10MT/	Q9Y2G8	0.054384	0.009499	0.010842	DNAJC16	DnaJ homo	DJC16_HU	14	1.038415
24H 50MT/	Q9BT23	0.2061	0.009506	0.010846	LIMD2	LIM domai	LIMD2_HU	1	1.153566
24H 50MT/	P17174	-0.08287	0.00951	0.01085	GOT1	Aspartate a	AATC_HUM	23	0.944177
24H 10MT/	P53618	0.081046	0.009514	0.010853	COPB1	Coatomer s	COPB_HUM	46	1.057785
48H 10MT/	Q8N8S7	0.133231	0.009515	0.010853	ENAH	Protein ena	ENAH_HUM	19	1.096747
48H 10MT/	Q9ULV4	0.022118	0.009515	0.010853	CORO1C	Coronin-1C	COR1C_HU	19	1.015449
48H 10MT/	P78563	-0.57351	0.009521	0.010856	ADARB1	Double-stra	RED1_HUM	1	0.671982
24H 50MT/	Q12888	0.086062	0.009526	0.01086	TP53BP1	TP53-bindi	TP53B_HU	51	1.061469
48H 50MT/	Q9UIM3	-0.94187	0.009531	0.010864	FKBPL	FK506-binc	FKBPL_HU	3	0.520556
24H 50MT/	Q15643	0.136137	0.009532	0.010865	TRIP11	Thyroid rec	TRIPB_HUM	35	1.098959
48H 10MT/	Q9NXH9	-0.04698	0.00954	0.010871	TRMT1	tRNA (guan	TRM1_HUM	14	0.96796
48H 50MT/	O14579	0.206608	0.009542	0.010872	COPE	Coatomer s	COPE_HUM	13	1.153972
48H 10MT/	Q9NPD8	-0.01338	0.009555	0.010885	UBE2T	Ubiquitin-c	UBE2T_HU	7	0.990771
48H 50MT/	Q86XK2	-0.38529	0.009557	0.010887	FBXO11	F-box only	FBX11_HU	3	0.765624
48H 50MT/	Q96LR5	0.14962	0.009558	0.010887	UBE2E2	Ubiquitin-c	UB2E2_HU	5	1.109278
48H 50MT/	Q9H832	0.149261	0.00956	0.010889	UBE2Z	Ubiquitin-c	UBE2Z_HU	10	1.109001
24H 10MT/	P39748	-0.09727	0.009565	0.010894	FEN1	Flap endon	FEN1_HUM	15	0.934802
48H 10MT/	Q5H9R7	0.028982	0.009567	0.010894	PPP6R3	Serine/thre	PP6R3_HU	35	1.020292
24H 10MT/	Q9HCK4	0.298278	0.009571	0.010897	ROBO2	Roundabou	ROBO2_HU	9	1.229676
24H 50MT/	Q92542	0.094742	0.009573	0.010897	NCSTN	Nicastrin	NICA_HUM	12	1.067875
48H 50MT/	O43598	0.140675	0.009575	0.010899	DNPH1	5-hydroxyn	DNPH1_HU	10	1.102421

24H 50MTA	Q2TAL8	0.200694	0.00958	0.010904	QRICH1	Transcripti	QRIC1_HUI	13	1.149251
24H 50MTA	Q96NU7	0.122629	0.009584	0.010906	AMDHD1	Probable in	HUTI_HUM	8	1.088717
24H 10MTA	P23258	0.039854	0.009589	0.01091	TUBG1	Tubulin gar	TBG1_HUM	24	1.02801
48H 50MTA	Q66PJ3	0.338218	0.009597	0.010917	ARL6IP4	ADP-ribosy	AR6P4_HUI	5	1.264194
24H 50MTA	P28838	-0.0752	0.009606	0.010923	LAP3	Cytosol am	AMPL_HUM	29	0.949213
48H 50MTA	Q7L592	-0.14361	0.009606	0.010923	NDUFAF7	Protein arg	NDUF7_HU	14	0.905249
24H 50MTA	Q8N5I9	-0.51173	0.009608	0.010925	NOPCHAP1	NOP protei	NOPC1_HU	2	0.701381
48H 10MTA	Q5TGL8	-0.99909	0.009609	0.010925	PXDC1	PX domain	PXDC1_HU	2	0.500317
48H 10MTA	Q8IWF2	-0.35507	0.009616	0.010932	FOXRED2	FAD-depen	FXRD2_HUI	4	0.781831
24H 50MTA	P40925	0.05497	0.009619	0.010933	MDH1	Malate deh	MDHC_HUI	13	1.038837
48H 10MTA	Q86T03	-0.06773	0.00962	0.010934	PIP4P1	Type 1 phos	PP4P1_HUI	5	0.954136
48H 10MTA	O60739	0.300607	0.009622	0.010935	EIF1B	Eukaryotic	EIF1B_HUM	1	1.231663
24H 50MTA	Q9BPW8	0.186284	0.009629	0.010942	NIPSNAP1	Protein Nip	NIPS1_HUM	11	1.137829
48H 50MTA	P51148	-0.00086	0.00963	0.010943	RAB5C	Ras-related	RAB5C_HUI	6	0.999407
48H 10MTA	P06730	0.030136	0.009638	0.01095	EIF4E	Eukaryotic	IF4E_HUMA	9	1.021108
48H 50MTA	Q53HL2	-0.37757	0.009646	0.010956	CDCA8	Borealin	BOREA_HUI	4	0.769732
24H 10MTA	O75934	-0.30886	0.009653	0.01096	BCAS2	Pre-mRNA-s	SPF27_HUM	11	0.807277
48H 50MTA	Q9NY97	-1.06876	0.009653	0.01096	B3GNT2	N-acetyllac	B3GN2_HU	1	0.47673
24H 50MTA	P02751	0.844006	0.009653	0.01096	FN1	Fibronectir	FINC_HUMA	77	1.795027
24H 10MTA	P49736	0.056316	0.009655	0.010962	MCM2	DNA replica	MCM2_HUI	36	1.039807
48H 10MTA	P78346	-0.13257	0.009665	0.010972	RPP30	Ribonuclea	RPP30_HUI	14	0.912206
24H 10MTA	Q9BX68	-0.14985	0.009671	0.010976	HINT2	Adenosine	HINT2_HUM	4	0.901345
24H 10MTA	P09496	-0.49347	0.009676	0.010981	CLTA	Clathrin lig	CLCA_HUM	5	0.710317
48H 10MTA	Q13409	0.060715	0.009679	0.010983	DYNC1I2	Cytoplasmi	DC1I2_HUM	19	1.042982
24H 10MTA	Q5VWN6	0.37252	0.009679	0.010983	TASOR2	Protein TAS	TASO2_HUI	3	1.294613
48H 10MTA	Q9NYH9	-0.01637	0.009701	0.011001	UTP6	U3 small nu	UTP6_HUM	22	0.988715
24H 50MTA	Q8TAQ2	0.00113	0.009705	0.011004	SMARCC2	SWI/SNF cc	SMRC2_HU	20	1.000783
24H 50MTA	P05067	0.208462	0.009708	0.011006	APP	Amyloid-be	A4_HUMAN	10	1.155455
24H 50MTA	Q86UU0	-0.19873	0.009709	0.011006	BCL9L	B-cell CLL/l	BCL9L_HUM	12	0.87132
48H 50MTA	Q9Y343	-0.61642	0.009711	0.011007	SNX24	Sorting nex	SNX24_HUI	2	0.652289
48H 10MTA	O43583	0.207323	0.009712	0.011007	DENR	Density-reg	DENR_HUM	9	1.154544
48H 10MTA	Q9NP97	0.056222	0.009721	0.011017	DYNLRB1	Dynein ligh	DLRB1_HUI	5	1.03974
48H 50MTA	Q15428	0.099717	0.009724	0.01102	SF3A2	Splicing fac	SF3A2_HUM	9	1.071563
48H 50MTA	Q92665	-0.17032	0.009725	0.01102	MRPS31	Small ribos	RT31_HUM	15	0.888643
24H 50MTA	O15230	0.038807	0.009733	0.011027	LAMA5	Laminin sul	LAMA5_HU	57	1.027264
48H 10MTA	Q5VWN6	0.309776	0.009739	0.011033	TASOR2	Protein TAS	TASO2_HUI	3	1.239515
48H 50MTA	P08865	-0.15584	0.009741	0.011035	RPSA	Small ribos	RSSA_HUM	18	0.897611
48H 50MTA	Q13685	-0.17257	0.009746	0.011039	AAMP	Angio-asso	AAMP_HUM	12	0.887263
48H 10MTA	P18206	0.028716	0.009747	0.01104	VCL	Vinculin	VINC_HUM	52	1.020104
24H 50MTA	P14384	0.243792	0.009748	0.01104	CPM	Carboxypep	CBPM_HUM	9	1.184101
48H 10MTA	Q8NDI1	0.013401	0.009751	0.011043	EHBP1	EH domain	EHBP1_HUI	20	1.009332
48H 50MTA	P49257	0.060449	0.009752	0.011043	LMAN1	Protein ERG	LMAN1_HU	19	1.04279
48H 10MTA	P09669	0.109343	0.009757	0.011046	COX6C	Cytochrom	COX6C_HU	8	1.078737
48H 50MTA	P28482	0.066211	0.009769	0.011056	MAPK1	Mitogen-ac	MK01_HUM	25	1.046964
24H 10MTA	O43306	0.109674	0.00977	0.011056	ADCY6	Adenylate c	ADCY6_HUI	6	1.078984
48H 50MTA	Q5VT25	-0.65349	0.009771	0.011057	CDC42BPA	Serine/thre	MRCKA_HU	7	0.635741
24H 10MTA	G2XKQ0	-0.06316	0.009774	0.011059	SUMO1P1	Small ubiqu	SUMO5_HU	3	0.957164
48H 10MTA	Q71RG4	0.062823	0.009778	0.011062	TMUB2	Transmemb	TMUB2_HU	4	1.044508
48H 10MTA	Q9BZG8	0.00093	0.009781	0.011063	DPH1	2-(3-amino	DPH1_HUM	10	1.000645
24H 50MTA	O43772	0.122061	0.009782	0.011063	SLC25A20	Mitochond	MCAT_HUM	15	1.088289
48H 10MTA	P42694	-0.06883	0.009805	0.011083	HELZ	Probable h	HELZ_HUM	13	0.953412
24H 10MTA	Q15654	-0.18774	0.009807	0.011084	TRIP6	Thyroid rec	TRIP6_HUM	8	0.877981

48H	10MT/	P19404	0.113198	0.009811	0.011088	NDUFV2	NADH dehy	NDUV2_HU	9	1.081624
48H	10MT/	Q00535	0.003473	0.009815	0.011091	CDK5	Cyclin-depe	CDK5_HUM	13	1.00241
48H	50MT/	Q0VDG4	0.214098	0.009827	0.0111	SCRN3	Secernin-3	SCRN3_HU	9	1.159979
24H	50MT/	Q9H9F9	-0.01951	0.009834	0.011105	ACTR5	Actin-relate	ARP5_HUM	10	0.986566
24H	50MT/	Q99729	0.178755	0.009837	0.011109	HNRNPAB	Heterogene	ROAA_HUM	11	1.131907
48H	50MT/	P61966	0.19322	0.009844	0.011115	AP1S1	AP-1 compl	AP1S1_HU	7	1.143312
48H	50MT/	Q96EY4	-0.30001	0.009848	0.01112	TMA16	Translation	TMA16_HU	8	0.812248
48H	50MT/	P82663	-0.17319	0.009853	0.011124	MRPS25	Small ribos	RT25_HUM	10	0.88688
24H	50MT/	Q14966	0.04896	0.009862	0.01113	ZNF638	Zinc finger	ZN638_HU	46	1.034519
48H	50MT/	Q8NFQ8	-0.1305	0.00987	0.011137	TOR1AIP2	Torsin-1A-i	TOIP2_HUM	15	0.913517
24H	50MT/	O75165	0.067609	0.009872	0.011139	DNAJC13	DnaJ homo	DJC13_HU	62	1.047978
48H	10MT/	P52815	-0.02735	0.00988	0.011146	MRPL12	Large ribos	RM12_HUM	5	0.981223
48H	50MT/	Q5T9A4	-0.26371	0.009881	0.011146	ATAD3B	ATPase fam	ATD3B_HU	15	0.832942
48H	50MT/	Q9NQ55	-0.18295	0.009883	0.011147	PPAN	Suppressor	SSF1_HUM	12	0.880899
48H	50MT/	O76062	0.424262	0.009898	0.011161	TM7SF2	Delta(14)-s	ERG24_HU	4	1.341886
48H	50MT/	Q8WVK2	0.222761	0.009899	0.011161	SNRNP27	U4/U6.U5 s	SNR27_HU	2	1.166965
24H	10MT/	P40222	-0.09499	0.009901	0.011163	TXLNA	Alpha-taxili	TXLNA_HU	25	0.936281
48H	50MT/	O00193	0.379252	0.009905	0.011165	SMAP	Small acidi	SMAP_HUM	3	1.300668
24H	50MT/	A8CG34	0.017765	0.009904	0.011165	POM121C	Nuclear env	P121C_HU	13	1.01239
24H	50MT/	Q9UHL0	1.465219	0.009909	0.011168	DDX25	ATP-depend	DDX25_HU	1	2.761053
24H	50MT/	P38432	0.099994	0.009914	0.011172	COIL	Coilin	COIL_HUM	11	1.071769
24H	50MT/	Q9NX58	0.143483	0.009915	0.011172	LYAR	Cell growth	LYAR_HUM	11	1.104569
24H	50MT/	Q9HCJ6	-0.23776	0.009922	0.011178	VAT1L	Synaptic ve	VAT1L_HU	12	0.84806
24H	10MT/	Q92541	0.032395	0.009927	0.011182	RTF1	RNA polym	RTF1_HUM	18	1.022708
48H	50MT/	Q86UT6	0.167129	0.009928	0.011182	NLRX1	NLR family	NLRX1_HU	10	1.122822
48H	10MT/	P14618	-0.12231	0.00994	0.011192	PKM	Pyruvate ki	KPYM_HUM	43	0.918717
48H	50MT/	Q99941	-0.40098	0.00994	0.011192	ATF6B	Cyclic AMP	ATF6B_HU	6	0.757342
24H	50MT/	Q96AT9	0.09775	0.009945	0.011197	RPE	Ribulose-pl	RPE_HUMA	7	1.070103
24H	50MT/	P52294	0.282057	0.009963	0.011213	KPNA1	Importin su	IMA5_HUM	8	1.215928
48H	10MT/	Q6NTF9	-0.02926	0.009973	0.011222	RHBDD2	Rhomboid	RHBD2_HU	1	0.979921
24H	50MT/	Q03169	0.562521	0.009978	0.011226	TNFAIP2	Tumor necr	TNAP2_HU	2	1.476848
48H	50MT/	Q6NUM9	-0.13165	0.009979	0.011227	RETSAT	All-trans-re	RETST_HUM	15	0.912786
48H	10MT/	Q9UIC8	0.129776	0.009982	0.01123	LCMT1	Leucine car	LCMT1_HU	10	1.094124
48H	10MT/	Q13445	0.15652	0.009983	0.011231	TMED1	Transmemb	TMED1_HU	5	1.114595
24H	10MT/	Q9BYC9	-0.14161	0.009986	0.011232	MRPL20	Large ribos	RM20_HUM	4	0.90651
48H	50MT/	Q15527	-0.23909	0.009993	0.011238	SURF2	Surfeit locu	SURF2_HU	4	0.847281
24H	10MT/	O75694	0.068434	0.009993	0.011238	NUP155	Nuclear po	NU155_HU	46	1.048578
48H	10MT/	Q3B726	-0.0451	0.009994	0.011238	POLR1F	DNA-direct	RPA43_HU	5	0.969224
48H	10MT/	O96000	0.103068	0.009998	0.011242	NDUFB10	NADH dehy	NDUBA_HU	11	1.074055
48H	10MT/	P36776	0.057833	0.01	0.011243	LONP1	Lon protea	LONM_HUM	45	1.040901
48H	10MT/	P08962	-0.26822	0.010037	0.011276	CD63	CD63 antig	CD63_HUM	2	0.830346
48H	10MT/	Q96MM7	-0.16701	0.010041	0.011278	HS6ST2	Heparan-su	H6ST2_HU	3	0.890686
48H	50MT/	O14735	0.041904	0.010042	0.011279	CDIPT	CDP-diacyl	CDIPT_HUM	6	1.029472
48H	10MT/	P55209	0.215428	0.010055	0.011289	NAP1L1	Nucleosom	NP1L1_HU	13	1.161048
48H	50MT/	P35914	0.209866	0.010056	0.01129	HMGCL	Hydroxyme	HMGCL_HU	11	1.15658
24H	50MT/	Q14019	0.230556	0.010058	0.01129	COTL1	Coactosin-l	COTL1_HU	8	1.173287
24H	50MT/	Q03252	-0.00273	0.010067	0.011299	LMNB2	Lamin-B2	LMNB2_HU	27	0.998108
48H	50MT/	O95819	-0.13411	0.010069	0.011301	MAP4K4	Mitogen-ac	M4K4_HUM	31	0.911234
24H	50MT/	Q9Y5P4	0.044858	0.010074	0.011304	CERT1	Ceramide t	CERT_HUM	13	1.031582
48H	10MT/	Q8WW12	0.012338	0.01008	0.011309	PCNP	PEST prote	PCNP_HUM	6	1.008589
24H	50MT/	O43719	0.04751	0.010081	0.01131	HTATSF1	17S U2 SnR	HTSF1_HU	20	1.03348
48H	10MT/	P17980	0.068409	0.010087	0.011314	PSMC3	26S protea	PRS6A_HU	28	1.048559

24H 50MTA	O43598	-0.03814	0.010088	0.011315	DNPH1	5-hydroxyn	DNPH1_HU	10	0.97391
24H 50MTA	Q9UPY3	-0.10152	0.01009	0.011317	DICER1	Endoribonu	DICER_HUM	25	0.932051
48H 50MTA	P38606	0.05164	0.010104	0.01133	ATP6V1A	V-type prot	VATA_HUM	36	1.036443
24H 50MTA	Q5T3I0	0.034256	0.010105	0.01133	GPATCH4	G patch do	GPTC4_HU	11	1.024029
24H 50MTA	A7MBM2	0.84234	0.010113	0.011336	DISP2	Protein dis	DISP2_HUM	2	1.792955
48H 50MTA	Q9UI43	0.163778	0.010116	0.011338	MRM2	rRNA meth	MRM2_HU	1	1.120217
24H 50MTA	O95295	-0.85478	0.010118	0.01134	SNAPIN	SNARE-asso	SNAPN_HU	3	0.552948
48H 50MTA	Q9BZ95	-0.27574	0.010121	0.011342	NSD3	Histone-lys	NSD3_HUM	4	0.826026
24H 10MTA	P61457	-0.09064	0.010131	0.011352	PCBD1	Pterin-4-al	PHS_HUMA	7	0.939107
48H 50MTA	P17275	-0.75287	0.010132	0.011353	JUNB	Transcripti	JUNB_HUM	4	0.593422
24H 50MTA	Q14554	0.176943	0.010135	0.011355	PDIA5	Protein dis	PDIA5_HUM	14	1.130486
48H 50MTA	Q12874	0.014013	0.010139	0.011358	SF3A3	Splicing fac	SF3A3_HUM	26	1.00976
48H 50MTA	Q15043	-0.21919	0.01014	0.011359	SLC39A14	Metal catio	S39AE_HUM	4	0.85905
24H 50MTA	Q9NZU5	-0.12099	0.010142	0.01136	LMCD1	LIM and cys	LMCD1_HU	21	0.919555
24H 50MTA	P62256	0.023437	0.010145	0.011362	UBE2H	Ubiquitin-c	UBE2H_HU	3	1.016378
48H 50MTA	Q14152	-0.10692	0.010144	0.011362	EIF3A	Eukaryotic	EIF3A_HUM	68	0.928567
48H 50MTA	P69905	0.167197	0.01015	0.011366	HBA1	Hemoglobi	HBA_HUMA	5	1.122875
24H 50MTA	O00116	0.030914	0.010153	0.011369	AGPS	Alkyldihydr	ADAS_HUM	32	1.021659
24H 50MTA	P63173	0.048105	0.010156	0.01137	RPL38	Large ribos	RL38_HUM	6	1.033906
24H 50MTA	P48147	0.057335	0.010162	0.011375	PREP	Prolyl endo	PPCE_HUM	35	1.040542
48H 10MTA	O00193	0.36441	0.010163	0.011376	SMAP	Small acidi	SMAP_HUM	3	1.287355
24H 50MTA	P17676	0.112297	0.010176	0.011388	CEBPB	CCAAT/enh	CEBPB_HU	6	1.080948
48H 10MTA	O60870	-0.02872	0.010177	0.011388	KIN	DNA/RNA-b	KIN17_HUM	8	0.980292
24H 50MTA	Q02338	0.423557	0.01018	0.01139	BDH1	D-beta-hyd	BDH_HUMA	5	1.341231
24H 10MTA	P27824	-0.17117	0.010187	0.011398	CANX	Calnexin	CALX_HUM	36	0.888122
24H 50MTA	Q68CZ2	-0.10673	0.010188	0.011398	TNS3	Tensin-3	TENS3_HUM	36	0.92869
24H 50MTA	O43670	0.107381	0.010193	0.011403	ZNF207	BUB3-inter	ZN207_HU	7	1.077271
24H 50MTA	Q69YQ0	-0.25965	0.010199	0.011409	SPECC1L	Cytospin-A	CYTSA_HUM	23	0.835288
48H 10MTA	Q9UQN3	0.158308	0.010204	0.011412	CHMP2B	Charged mu	CHM2B_HU	2	1.115978
24H 50MTA	Q8N766	0.081277	0.010214	0.011421	EMC1	ER membra	EMC1_HUM	37	1.057954
48H 10MTA	Q6Y1H2	-0.39491	0.010216	0.011422	HACD2	Very-long-c	HACD2_HU	4	0.760539
24H 50MTA	P07942	-0.1478	0.010227	0.011432	LAMB1	Laminin sul	LAMB1_HU	43	0.902626
48H 50MTA	P55036	0.068936	0.010228	0.011433	PSMD4	26S protea	PSMD4_HU	14	1.048943
24H 50MTA	Q9H553	0.125111	0.01023	0.011434	ALG2	Alpha-1,3/1	ALG2_HUM	13	1.090592
48H 10MTA	Q9HCD5	0.043631	0.010234	0.011437	NCOA5	Nuclear rec	NCOA5_HU	19	1.030704
48H 10MTA	O95777	-0.07246	0.010235	0.011439	LSM8	U6 snRNA-a	LSM8_HUM	7	0.951014
48H 50MTA	Q9HAT2	0.200667	0.010239	0.011441	SIAE	Sialate O-ac	SIAE_HUMA	5	1.14923
48H 10MTA	P42025	0.064085	0.010245	0.011446	ACTR1B	Beta-centra	ACTY_HUM	15	1.045422
48H 50MTA	Q9Y223	0.077979	0.010252	0.01145	GNE	Bifunctiona	GLCNE_HU	24	1.055538
48H 10MTA	Q8NDX6	0.158325	0.010259	0.011456	ZNF740	Zinc finger	ZN740_HU	1	1.115991
24H 50MTA	P41223	-0.08858	0.010261	0.011456	BUD31	Protein BU	BUD31_HU	8	0.940447
24H 10MTA	Q92989	0.086582	0.010262	0.011456	CLP1	Polyribonu	CLP1_HUM	12	1.061852
48H 10MTA	Q9BT78	0.051918	0.010262	0.011456	COPS4	COP9 signa	CSN4_HUM	25	1.036642
24H 10MTA	Q9BTE7	0.146439	0.010268	0.011462	DCUN1D5	DCN1-like p	DCNL5_HU	9	1.106834
24H 50MTA	P52564	0.235687	0.010272	0.011463	MAP2K6	Dual specifi	MP2K6_HU	2	1.177467
24H 50MTA	Q13363	0.093618	0.010271	0.011463	CTBP1	C-terminal-	CTBP1_HU	14	1.067043
48H 50MTA	P22830	0.069689	0.010288	0.011477	FECH	Ferrochelat	HEMH_HUM	12	1.04949
48H 50MTA	Q86YS7	0.04546	0.01029	0.011478	C2CD5	C2 domain-	C2CD5_HU	13	1.032012
48H 10MTA	Q96EL2	-0.57987	0.010296	0.011485	MRPS24	Small ribos	RT24_HUM	6	0.669026
48H 10MTA	P13489	0.122821	0.010306	0.011492	RNH1	Ribonuclea	RINI_HUMA	23	1.088862
48H 50MTA	Q9Y5Q8	-0.11838	0.01031	0.011495	GTF3C5	General tra	TF3C5_HUM	18	0.92122
24H 50MTA	Q32P28	0.029812	0.010312	0.011496	P3H1	Prolyl 3-hy	P3H1_HUM	19	1.020879

48H 50MTA	Q6P587	-0.0404	0.010312	0.011496	FAHD1	Oxaloaceta	FAHD1_HU	11	0.972386
48H 10MTA	Q9UBS4	-0.09909	0.010317	0.011502	DNAJB11	DnaJ homo	DJB11_HUI	15	0.933623
48H 10MTA	P00740	1.191742	0.010325	0.011508	F9	Coagulatio	FA9_HUMA	1	2.284284
24H 50MTA	Q9UPN3	-0.11866	0.01033	0.011511	MACF1	Microtubul	MACF1_HU	190	0.921043
48H 50MTA	O95260	0.086225	0.010336	0.011515	ATE1	Arginyl-tRN	ATE1_HUM	12	1.061589
48H 50MTA	Q9Y2A7	0.124596	0.010337	0.011515	NCKAP1	Nck-associa	NCKP1_HU	37	1.090203
24H 10MTA	Q53T59	-0.0537	0.010338	0.011517	HS1BP3	HCLS1-binc	H1BP3_HU	17	0.963459
48H 50MTA	Q9NRP0	0.002104	0.010343	0.011521	OSTC	Oligosacch	OSTC_HUM	3	1.001459
48H 10MTA	Q8TDQ7	0.031592	0.010347	0.011524	GNPDA2	Glucosamir	GNPI2_HUI	6	1.02214
48H 50MTA	Q99700	0.052449	0.010347	0.011524	ATXN2	Ataxin-2	ATX2_HUM	25	1.037024
24H 50MTA	P52209	-0.10155	0.010351	0.011528	PGD	6-phospho	6PGD_HUN	25	0.932032
48H 10MTA	Q9NX40	0.004153	0.010353	0.011531	OCIAD1	OCIA doma	OCAD1_HU	9	1.002883
48H 50MTA	O94811	0.330025	0.010354	0.011531	TPPP	Tubulin pol	TPPP_HUM	3	1.257035
48H 50MTA	O43396	0.11618	0.010354	0.011531	TXNL1	Thioredoxi	TXNL1_HUI	16	1.083861
24H 10MTA	Q96M89	0.481142	0.01037	0.011544	CCDC138	Coiled-coil	CC138_HUI	4	1.395848
48H 50MTA	Q96EU6	-0.22857	0.010373	0.011546	RRP36	Ribosomal	RRP36_HUI	8	0.853479
48H 50MTA	Q676U5	-0.1353	0.010385	0.011556	ATG16L1	Autophagy-	A16L1_HUI	13	0.910483
48H 50MTA	Q9NS23	-3.61168	0.010386	0.011556	RASSF1	Ras associat	RASF1_HUI	1	0.081804
24H 50MTA	P07858	0.060793	0.010393	0.011562	CTSB	Cathepsin E	CATB_HUM	11	1.043039
48H 50MTA	Q96GF1	-1.03826	0.010398	0.011566	RNF185	E3 ubiquiti	RN185_HU	2	0.486915
48H 10MTA	Q16342	0.138168	0.01041	0.011578	PDCD2	Programme	PDCD2_HU	7	1.100507
24H 50MTA	Q9UIW2	0.045509	0.010414	0.011581	PLXNA1	Plexin-A1	PLXA1_HUI	8	1.032047
48H 50MTA	Q9BRX8	0.094181	0.010418	0.011583	PRXL2A	Peroxiredo	PXL2A_HUI	9	1.067459
24H 50MTA	Q9H6W3	0.137259	0.010421	0.011585	RIOX1	Ribosomal	RIOX1_HUI	13	1.099813
48H 50MTA	Q969Z3	0.279061	0.010429	0.011594	MTARC2	Mitochond	MARC2_HU	4	1.213405
24H 50MTA	Q11206	0.78604	0.010434	0.011599	ST3GAL4	CMP-N-acet	SIA4C_HUN	3	1.724335
48H 50MTA	P27361	0.041913	0.010444	0.011605	MAPK3	Mitogen-ac	MK03_HUN	15	1.029478
48H 50MTA	Q96IF1	-0.26666	0.010443	0.011605	AJUBA	LIM domair	AJUBA_HUI	16	0.831239
24H 50MTA	O75167	-0.08228	0.010445	0.011605	PHACTR2	Phosphatas	PHAR2_HU	3	0.944562
24H 50MTA	Q9BZJ0	0.126048	0.010447	0.011607	CRNKL1	Crooked ne	CRNL1_HUI	24	1.0913
24H 50MTA	P55072	0.043749	0.010451	0.01161	VCP	Transitiona	TERA_HUM	57	1.030789
48H 10MTA	P49184	-0.72508	0.010453	0.011612	DNASE1L1	Deoxyribor	DNSL1_HUI	1	0.604965
48H 10MTA	Q96BM9	-0.30752	0.010456	0.011613	ARL8A	ADP-ribosy	ARL8A_HUI	10	0.808032
24H 50MTA	Q12789	0.075095	0.010466	0.011622	GTF3C1	General tra	TF3C1_HUI	59	1.053431
24H 50MTA	O15397	0.105495	0.010471	0.011626	IPO8	Importin-8	IPO8_HUM	23	1.075864
48H 10MTA	O15400	0.017214	0.010477	0.011632	STX7	Syntaxin-7	STX7_HUM	10	1.012003
24H 10MTA	P18084	0.097344	0.010479	0.011633	ITGB5	Integrin bet	ITB5_HUMA	24	1.069802
48H 10MTA	Q8N2K0	-0.11949	0.010482	0.011635	ABHD12	Lysophospl	ABD12_HU	13	0.920513
48H 50MTA	P30260	-0.20872	0.010488	0.01164	CDC27	Cell divisio	CDC27_HU	20	0.865304
48H 10MTA	P42704	-0.09924	0.010491	0.011642	LRPPRC	Leucine-ric	LPPRC_HUI	94	0.933526
48H 50MTA	Q9BSC4	-0.11713	0.010496	0.011646	NOL10	Nucleolar p	NOL10_HU	25	0.922023
48H 10MTA	O43795	-0.07207	0.010503	0.011652	MYO1B	Unconvent	MYO1B_HU	43	0.951275
24H 50MTA	Q7L3T8	0.186737	0.010508	0.011656	PARS2	Probable pı	SYPM_HUN	11	1.138186
24H 50MTA	Q6PCB0	0.463303	0.010509	0.011657	VWA1	von Willebı	VWA1_HUN	5	1.378695
48H 50MTA	P49354	0.102181	0.010517	0.011663	FNTA	Protein farı	FNTA_HUM	14	1.073395
48H 10MTA	P50454	-0.06012	0.010524	0.011671	SERPINH1	Serpin H1	SERPH_HUI	19	0.959187
48H 10MTA	Q96BI3	-0.78652	0.010536	0.01168	APH1A	Gamma-sec	APH1A_HU	2	0.57974
24H 50MTA	Q9UBL3	0.062284	0.010538	0.011681	ASH2L	Set1/Ash2 I	ASH2L_HUI	13	1.044117
24H 50MTA	Q8NBJ9	0.241684	0.010542	0.011685	SIDT2	SID1 transn	SIDT2_HUN	4	1.182372
24H 50MTA	Q9Y3A4	-0.09214	0.010545	0.011686	RRP7A	Ribosomal	RRP7A_HUI	11	0.93813
48H 50MTA	P18669	0.230178	0.010546	0.011687	PGAM1	Phosphogly	PGAM1_HU	20	1.17298
48H 50MTA	Q658Y4	0.042544	0.010547	0.011687	FAM91A1	Protein FAI	F91A1_HUI	17	1.029928

24H	50MTA	Q13136	-0.00806	0.01055	0.011689	PPFIA1	Liprin-alph	LIPA1_HUM	28	0.994427
48H	50MTA	Q5QJE6	-0.18128	0.010561	0.011696	DNTTIP2	Deoxynucle	TDIF2_HUM	20	0.88192
48H	50MTA	Q9UPW6	-0.7072	0.010561	0.011696	SATB2	DNA-bindir	SATB2_HUM	5	0.612507
24H	10MTA	Q9UIG0	0.078463	0.010564	0.011698	BAZ1B	Tyrosine-pr	BAZ1B_HUI	42	1.055892
48H	50MTA	P54725	0.146562	0.010574	0.011708	RAD23A	UV excision	RD23A_HU	7	1.106928
24H	50MTA	O15400	0.108275	0.010591	0.011723	STX7	Syntaxin-7	STX7_HUM	10	1.077938
48H	10MTA	P39023	0.061601	0.010595	0.011726	RPL3	Large ribos	RL3_HUMA	21	1.043623
24H	50MTA	Q16134	0.197302	0.010598	0.011727	ETFDH	Electron tra	ETFD_HUM	18	1.146552
48H	50MTA	P53355	0.587501	0.010601	0.011729	DAPK1	Death-asso	DAPK1_HU	8	1.502642
24H	50MTA	Q92989	0.036864	0.010609	0.011735	CLP1	Polyribonu	CLP1_HUM	12	1.025881
24H	50MTA	Q9BTV4	0.005168	0.010614	0.011737	TMEM43	Transmemb	TMM43_HU	20	1.003588
24H	50MTA	Q9BTY2	0.037151	0.010615	0.011738	FUCA2	Plasma alpł	FUCO2_HU	13	1.026086
48H	10MTA	Q9NPG8	-0.44317	0.010625	0.011748	ZDHHC4	Palmitoyltr	ZDHC4_HU	1	0.735518
48H	50MTA	Q96JJ7	-0.11096	0.010627	0.011749	TMX3	Protein dis	TMX3_HUM	12	0.925975
24H	50MTA	Q9H1D9	-0.21091	0.010628	0.01175	POLR3F	DNA-direct	RPC6_HUM	6	0.863994
24H	50MTA	Q13564	0.014695	0.01063	0.011751	NAE1	NEDD8-acti	ULA1_HUM	18	1.010238
48H	50MTA	Q9ULK4	0.0856	0.010636	0.011756	MED23	Mediator o	MED23_HU	28	1.061129
48H	50MTA	Q9UBK7;Q	-0.06748	0.010638	0.011758	RABL2A;RA	Rab-like pro	RBL2A_HUI	3	0.954306
48H	10MTA	P60002	0.292773	0.010642	0.011761	ELOF1	Transcripti	ELOF1_HUM	1	1.224993
48H	50MTA	Q9BT22	-0.11809	0.010651	0.011768	ALG1	Chitobiosyl	ALG1_HUM	10	0.921408
48H	50MTA	P08962	-0.28834	0.010654	0.01177	CD63	CD63 antig	CD63_HUM	2	0.818843
48H	10MTA	P33316	0.094613	0.010653	0.01177	DUT	Deoxyuridi	DUT_HUMA	9	1.067779
24H	50MTA	Q14764	0.077193	0.010656	0.011771	MVP	Major vault	MVP_HUM	44	1.054963
48H	10MTA	P68431	-0.42747	0.010659	0.011773	H3C1	Histone H3	H31_HUMA	6	0.743562
48H	10MTA	Q9Y282	0.074894	0.010682	0.011793	ERGIC3	Endoplasm	ERGI3_HUM	9	1.053283
24H	10MTA	Q14642	0.497453	0.010702	0.01181	INPP5A	Inositol pol	I5P1_HUM	6	1.411719
48H	50MTA	Q96BW5	0.435592	0.010703	0.01181	PTER	Phosphotri	PTER_HUM	12	1.352466
24H	50MTA	P40222	-0.09665	0.010712	0.011818	TXLNA	Alpha-taxili	TXLNA_HUI	25	0.935201
24H	50MTA	Q96NC0	0.132002	0.010712	0.011818	ZMAT2	Zinc finger	ZMAT2_HU	4	1.095814
24H	50MTA	P04080	-0.0972	0.010714	0.011819	CSTB	Cystatin-B	CYTB_HUM	6	0.934843
24H	50MTA	Q92508	0.125264	0.010723	0.011826	PIEZO1	Piezo-type	PIEZ1_HUM	3	1.090707
24H	50MTA	Q9Y679	0.048969	0.010737	0.011837	AUP1	Lipid dropl	AUP1_HUM	13	1.034525
48H	50MTA	O14578	-0.11861	0.010738	0.011838	CIT	Citron Rho-	CTRO_HUM	26	0.921073
48H	10MTA	P0C264	0.332592	0.010739	0.011838	SBK3	Uncharacte	SBK3_HUM	1	1.259274
48H	50MTA	P05141	-0.11604	0.010741	0.01184	SLC25A5	ADP/ATP tr	ADT2_HUM	20	0.922715
24H	50MTA	P56556	0.230526	0.010745	0.011843	NDUFA6	NADH dehy	NDUA6_HU	6	1.173262
48H	50MTA	Q96EY8	-0.05794	0.010746	0.011843	MMAB	Corrinoid a	MMAB_HUI	7	0.960637
24H	50MTA	C4AMC7;Q	-0.17305	0.010749	0.011845	WASH3P;W	Putative W	WASH3_HU	11	0.886965
24H	10MTA	Q9Y3C4	-0.20539	0.010751	0.011847	TPRKB	EKC/KEOPS	TPRKB_HUI	6	0.867305
48H	50MTA	Q9Y3B3	0.077726	0.010752	0.011848	TMED7	Transmemb	TMED7_HU	7	1.055354
24H	50MTA	P07384	0.037153	0.010754	0.011849	CAPN1	Calpain-1 c	CAN1_HUM	38	1.026087
24H	50MTA	P02686	-0.00842	0.010756	0.01185	MBP	Myelin basi	MBP_HUM	2	0.994183
48H	10MTA	P35606	0.04783	0.010757	0.01185	COPB2	Coatomer s	COPB2_HU	43	1.033709
24H	50MTA	Q9UJS0	0.047905	0.010757	0.01185	SLC25A13	Electrogeni	S2513_HUI	29	1.033763
48H	50MTA	Q9Y2Q9	-0.26217	0.010757	0.01185	MRPS28	Small ribos	RT28_HUM	6	0.833831
48H	50MTA	O94973	0.022035	0.010767	0.011859	AP2A2	AP-2 compl	AP2A2_HUI	29	1.015391
48H	10MTA	O43924	0.249023	0.01077	0.011861	PDE6D	Retinal rod	PDE6D_HU	4	1.188402
48H	50MTA	P54652	0.033498	0.010771	0.011861	HSPA2	Heat shock	HSP72_HUI	22	1.02349
24H	50MTA	O00213	0.697446	0.010772	0.011862	APBB1	Amyloid be	APBB1_HUI	3	1.621631
48H	50MTA	Q9Y3C6	0.062167	0.010785	0.011872	PPIL1	Peptidyl-pr	PPIL1_HUM	7	1.044033
24H	50MTA	Q15907	0.078001	0.010786	0.011873	RAB11B	Ras-related	RB11B_HUI	2	1.055554
24H	50MTA	Q01130	0.367259	0.010787	0.011874	SRSF2	Serine/argi	SRSF2_HUM	5	1.289899

48H 50MT/	Q9NRG0	-0.64035	0.010789	0.011875	CHRAC1	Chromatin	CHRC1_HU	3	0.641556
24H 10MT/	P53999	-0.05924	0.010792	0.011878	SUB1	Activated R	TCP4_HUM	8	0.959767
24H 50MT/	Q8WY22	0.023632	0.010795	0.011879	BRI3BP	BRI3-bindi	BRI3B_HUM	3	1.016515
24H 10MT/	P27361	0.016887	0.010797	0.011879	MAPK3	Mitogen-ac	MK03_HUM	15	1.011774
48H 10MT/	Q9Y6P5	-1.25491	0.010796	0.011879	SESN1	Sestrin-1	SESN1_HUM	1	0.41902
24H 10MT/	P04080	-0.15822	0.010799	0.011879	CSTB	Cystatin-B	CYTB_HUM	6	0.896132
48H 50MT/	P14625	0.137809	0.010799	0.011879	HSP90B1	Endoplasm	ENPL_HUM	52	1.100233
48H 10MT/	Q6ZSC3	1.327431	0.010801	0.011881	RBM43	RNA-bindir	RBM43_HU	1	2.509554
48H 50MT/	Q9H0E2	0.17564	0.010803	0.011883	TOLLIP	Toll-interac	TOLIP_HUM	7	1.129465
24H 10MT/	Q9H081	0.103861	0.010805	0.011883	MIS12	Protein MIS	MIS12_HUI	1	1.074645
48H 50MT/	O15382	0.030161	0.010807	0.011884	BCAT2	Branched-c	BCAT2_HUI	17	1.021126
24H 10MT/	Q10567	-0.01807	0.010808	0.011885	AP1B1	AP-1 compl	AP1B1_HUI	27	0.987555
48H 50MT/	Q9BUB5	-0.34019	0.010809	0.011886	MKNK1	MAP kinase	MKNK1_HU	3	0.789937
48H 50MT/	Q15388	-0.17986	0.010816	0.011892	TOMM20	Mitochond	TOM20_HU	3	0.882789
48H 50MT/	Q86WA6	0.220148	0.01082	0.011893	BPHL	Valacyclovi	BPHL_HUM	5	1.164853
48H 10MT/	Q8TED9	-0.20044	0.01082	0.011893	AFAP1L1	Actin filam	AF1L1_HUM	1	0.870287
24H 10MT/	Q9Y5U2	-0.55695	0.010829	0.011899	TSSC4	U5 small nu	TSSC4_HUM	3	0.679736
48H 50MT/	P48163	0.125079	0.010849	0.011915	ME1	NADP-depe	MAOX_HUM	20	1.090567
24H 50MT/	Q14520	0.232149	0.010849	0.011915	HABP2	Hyaluronar	HABP2_HU	10	1.174583
24H 50MT/	Q6P1A2	0.076032	0.010849	0.011915	LPCAT3	Lysophospl	MBOA5_HU	8	1.054115
24H 50MT/	Q9BYN0	0.026075	0.01085	0.011915	SRXN1	Sulfiredoxi	SRXN1_HUI	5	1.018238
24H 10MT/	Q8NE62	-0.18749	0.010854	0.011917	CHDH	Choline del	CHDH_HUM	19	0.878134
48H 10MT/	O96017	0.073644	0.010861	0.011924	CHEK2	Serine/thre	CHK2_HUM	14	1.052372
48H 10MT/	Q9Y6J0	0.629713	0.010867	0.011929	CABIN1	Calcineurin	CABIN_HUM	1	1.547257
24H 50MT/	Q86VM9	0.101117	0.01087	0.011931	ZC3H18	Zinc finger	ZCH18_HUI	12	1.072603
24H 50MT/	O75197	1.879073	0.010872	0.011932	LRP5	Low-densit	LRP5_HUM	2	3.678386
48H 10MT/	Q96MW1	0.180883	0.010873	0.011932	CCDC43	Coiled-coil	CCD43_HU	7	1.133578
48H 50MT/	P02671	-0.07194	0.010876	0.011934	FGA	Fibrinogen	FIBA_HUM/	25	0.951357
48H 50MT/	P54709	-0.15846	0.010883	0.01194	ATP1B3	Sodium/po	AT1B3_HUI	12	0.89598
48H 10MT/	Q9BU23	-0.28642	0.010886	0.011942	LMF2	Lipase matu	LMF2_HUM	8	0.819934
24H 10MT/	P40937	0.081912	0.010888	0.011945	RFC5	Replication	RFC5_HUM	22	1.05842
24H 50MT/	Q9UBQ0	0.301278	0.01089	0.011946	VPS29	Vacuolar pr	VPS29_HUI	10	1.232235
48H 50MT/	Q09472	0.17454	0.010896	0.011949	EP300	Histone ace	EP300_HUI	7	1.128604
48H 50MT/	P05166	0.108923	0.010896	0.01195	PCCB	Propionyl-(	PCCB_HUM	19	1.078423
48H 10MT/	P60174	0.054723	0.010903	0.011955	TPI1	Triosephos	TPIS_HUM/	18	1.03866
48H 50MT/	Q9HBM1	-0.02222	0.010904	0.011955	SPC25	Kinetochor	SPC25_HUI	8	0.984717
48H 50MT/	O75204	-0.18312	0.01091	0.01196	TMEM127	Transmemb	TM127_HU	1	0.880796
24H 50MT/	Q16643	0.067903	0.010911	0.011961	DBN1	Drebrin	DREB_HUM	23	1.048192
48H 50MT/	Q9NTU7	-0.2427	0.010911	0.011961	CBLN4	Cerebellin-	CBLN4_HUI	1	0.845163
48H 10MT/	P52655	0.274897	0.010934	0.011982	GTF2A1	Transcripti	TF2AA_HUM	2	1.209907
24H 50MT/	Q9NVH0	0.143225	0.010934	0.011982	EXD2	Exonucleas	EXD2_HUM	17	1.104371
24H 50MT/	P28370	0.124633	0.010937	0.011985	SMARCA1	Probable gl	SMCA1_HU	24	1.090231
48H 50MT/	Q9UQ35	0.172522	0.010938	0.011985	SRRM2	Serine/argi	SRRM2_HU	45	1.127027
24H 10MT/	Q8NEW0	0.092862	0.010943	0.01199	SLC30A7	Zinc transp	ZNT7_HUM	8	1.066484
24H 50MT/	Q15031	0.152356	0.010956	0.011999	LARS2	Leucine--tR	SYLM_HUM	25	1.111383
48H 10MT/	P27986	0.773834	0.010956	0.011999	PIK3R1	Phosphatid	P85A_HUM	2	1.709808
24H 50MT/	O14979	0.032197	0.01096	0.012002	HNRNPDL	Heterogene	HNRDL_HU	14	1.022568
24H 50MT/	Q9BZE1	0.097109	0.010966	0.012007	MRPL37	Large ribos	RM37_HUM	19	1.069628
48H 50MT/	Q8IXJ6	-1.27361	0.01097	0.012011	SIRT2	NAD-depen	SIR2_HUM/	2	0.413625
24H 10MT/	Q643R3	-0.2628	0.010975	0.012016	LPCAT4	Lysophospl	LPCT4_HUM	4	0.833471
24H 10MT/	P46734	0.014898	0.010977	0.012017	MAP2K3	Dual specifi	MP2K3_HU	13	1.01038
48H 50MT/	Q8TEY7	-0.28107	0.010985	0.012023	USP33	Ubiquitin c	UBP33_HU	11	0.822978

48H 50MTA	P17812	-0.19588	0.010996	0.012032	CTPS1	CTP syntha:	PYRG1_HUI	29	0.873043
24H 10MTA	O95551	-0.09464	0.010997	0.012033	TDP2	Tyrosyl-DN.	TYDP2_HUI	11	0.936505
24H 10MTA	P06576	0.015064	0.010998	0.012033	ATP5F1B	ATP syntha:	ATPB_HUM	28	1.010497
24H 50MTA	Q86VU5	-1.06767	0.011	0.012035	COMTD1	Catechol O-	CMTD1_HU	1	0.477088
24H 50MTA	P18085	0.099387	0.011006	0.01204	ARF4	ADP-ribosy	ARF4_HUM	7	1.071318
48H 50MTA	Q04206	0.09075	0.011007	0.01204	RELA	Transcripti	TF65_HUM	15	1.064924
48H 50MTA	Q8TCD5	0.155811	0.011019	0.01205	NT5C	5'(3')-deoxy	NT5C_HUM	9	1.114048
48H 10MTA	P17252	0.036831	0.011025	0.012055	PRKCA	Protein kin	KPCA_HUM	15	1.025858
24H 50MTA	P62899	0.008306	0.011026	0.012056	RPL31	Large ribos	RL31_HUM	5	1.005774
24H 10MTA	Q9P000	-0.10628	0.011027	0.012056	COMMD9	COMM don	COMD9_HU	8	0.928982
24H 10MTA	Q9Y2A7	0.07514	0.011027	0.012056	NCKAP1	Nck-associa	NCKP1_HU	37	1.053463
24H 50MTA	Q9BVQ7	-0.18313	0.011029	0.012057	AFG2B	ATPase fam	AFG2B_HUI	21	0.880787
48H 50MTA	Q5VTL8	0.270781	0.011032	0.01206	PRPF38B	Pre-mRNA-	PR38B_HUI	11	1.206461
24H 50MTA	Q9UGP4	-0.04421	0.011038	0.012064	LIMD1	LIM domair	LIMD1_HUI	14	0.969823
24H 10MTA	Q8N8R3	-0.90663	0.011048	0.012071	SLC25A29	Mitochond	S2529_HUI	2	0.533429
24H 10MTA	P43034	0.066908	0.011052	0.012073	PAFAH1B1	Platelet-act	LIS1_HUMA	20	1.047469
48H 50MTA	Q9H6R0	0.20576	0.011053	0.012074	DHX33	ATP-depen	DHX33_HU	7	1.153294
24H 10MTA	Q9H706	-1.40072	0.011054	0.012075	GAREM1	GRB2-assoc	GARE1_HUI	2	0.378741
24H 10MTA	P59190	-0.07586	0.011065	0.012086	RAB15	Ras-related	RAB15_HUI	1	0.948773
48H 10MTA	Q86Y56	0.065315	0.011066	0.012086	DNAAF5	Dynein axo	DAAF5_HUI	35	1.046313
24H 50MTA	Q14457	0.021015	0.011068	0.012087	BECN1	Beclin-1	BECN1_HUI	7	1.014673
48H 50MTA	Q9Y315	0.097707	0.01107	0.012087	DERA	Deoxyribos	DEOC_HUM	17	1.070071
24H 10MTA	Q13163	-2.12381	0.011077	0.012095	MAP2K5	Dual specifi	MP2K5_HU	2	0.229441
24H 50MTA	Q9UJQ4	0.432627	0.01108	0.012097	SALL4	Sal-like pro	SALL4_HUM	10	1.349689
48H 10MTA	Q9BPX3	0.072737	0.011084	0.0121	NCAPG	Condensin	CND3_HUM	27	1.05171
48H 10MTA	P50579	0.069519	0.011087	0.012102	METAP2	Methionine	MAP2_HUM	22	1.049367
48H 50MTA	Q96T37	-0.04029	0.011087	0.012102	RBM15	RNA-bindir	RBM15_HU	22	0.972457
24H 50MTA	Q09666	-0.12004	0.011089	0.012103	AHNAK	Neuroblast	AHNK_HUM	240	0.920165
48H 50MTA	Q9Y5S9	0.277839	0.011093	0.012106	RBM8A	RNA-bindir	RBM8A_HU	8	1.212377
48H 50MTA	O75190	-0.11415	0.011096	0.012107	DNAJB6	DnaJ homo	DNJB6_HUI	7	0.923928
24H 10MTA	Q96N64	0.812185	0.011109	0.012118	PWWP2A	PWWP don	PWP2A_HU	2	1.755869
48H 10MTA	Q9Y3E7	0.150749	0.011111	0.012119	CHMP3	Charged mu	CHMP3_HU	5	1.110145
48H 50MTA	P48436	0.535639	0.011118	0.012125	SOX9	Transcripti	SOX9_HUM	1	1.449584
48H 10MTA	Q9P270	0.12414	0.011131	0.012136	SLAIN2	SLAIN moti	SLAI2_HUM	11	1.089858
24H 50MTA	Q16762	0.180962	0.011132	0.012137	TST	Thiosulfate	THTR_HUM	15	1.133639
48H 50MTA	Q9H974	-0.13607	0.011133	0.012138	QTRT2	Queuine tR	QTRT2_HUI	14	0.909996
48H 50MTA	Q9Y3I0	0.005586	0.011134	0.012138	RTCB	RNA-splicir	RTCB_HUM	24	1.003879
48H 10MTA	Q13505	0.052939	0.011137	0.01214	MTX1	Metaxin-1	MTX1_HUM	11	1.037376
48H 10MTA	Q96GN5	-0.13766	0.011138	0.01214	CDCA7L	Cell divisior	CDA7L_HUI	2	0.908995
24H 10MTA	O15144	0.058857	0.011139	0.01214	ARPC2	Actin-relate	ARPC2_HUI	19	1.04164
24H 10MTA	Q6UXN9	0.138475	0.011143	0.012144	WDR82	WD repeat-	WDR82_HU	13	1.100741
48H 50MTA	Q9Y5Z9	-0.43568	0.01115	0.012148	UBIAD1	UbiA preny	UBIA1_HUM	5	0.739345
48H 10MTA	P08758	0.018795	0.011158	0.012154	ANXA5	Annexin A5	ANXA5_HU	23	1.013113
48H 10MTA	Q8WVM8	0.027519	0.01116	0.012156	SCFD1	Sec1 family	SCFD1_HUI	25	1.019258
24H 50MTA	Q96EY1	0.031658	0.011166	0.012161	DNAJA3	DnaJ homo	DNJA3_HUI	15	1.022186
24H 50MTA	Q13112	0.069924	0.011168	0.012162	CHAF1B	Chromatin	CAF1B_HUI	14	1.049661
48H 50MTA	Q8WVI0	-0.27999	0.011172	0.012165	UQCC5	Ubiquinol-c	UQCC5_HU	1	0.823595
48H 50MTA	Q9NR45	0.108626	0.011188	0.012175	NANS	Sialic acid s	SIAS_HUMA	16	1.078201
24H 10MTA	Q8N6L1	0.315673	0.01119	0.012176	KRTCAP2	Keratinocy	KTAP2_HUI	1	1.244592
24H 50MTA	P62854	0.209586	0.011198	0.012183	RPS26	Small ribos	RS26_HUM	5	1.156356
48H 50MTA	Q96EU7	0.035263	0.0112	0.012185	C1GALT1C1	C1GALT1-sp	C1GLC_HUI	9	1.024743
48H 50MTA	Q06330	-0.0909	0.011203	0.012187	RBPJ	Recombinin	SUH_HUMA	5	0.938934

48H 10MT/	Q14318	0.067067	0.011203	0.012187	FKBP8	Peptidyl-pr	FKBP8_HUI	10	1.047585
24H 10MT/	Q8TBC3	0.465604	0.011206	0.012189	SHKBP1	SH3KBP1-b	SHKB1_HUI	5	1.380895
48H 10MT/	O00468	0.088325	0.011206	0.012189	AGRN	Agrin	AGRIN_HUI	42	1.063135
48H 10MT/	Q8N131	0.131483	0.011209	0.01219	TMEM123	Porimin	PORIM_HU	1	1.095419
48H 50MT/	Q9NP79	0.071424	0.011208	0.01219	VTA1	Vacuolar pr	VTA1_HUM	11	1.050753
48H 50MT/	Q8NEZ2	0.297372	0.011227	0.012205	VPS37A	Vacuolar pr	VP37A_HUI	10	1.228904
24H 50MT/	P43007	0.066019	0.011231	0.012209	SLC1A4	Neutral am	SATT_HUM/	5	1.046824
48H 10MT/	Q5BJH2	-0.14985	0.011241	0.012218	TMEM128	Transmemk	TM128_HU	1	0.901346
48H 10MT/	P35221	0.038207	0.011263	0.012234	CTNNA1	Catenin alp	CTNA1_HUI	44	1.026837
48H 50MT/	P50914	-0.14985	0.011269	0.012238	RPL14	Large ribos	RL14_HUM	4	0.901347
24H 50MT/	Q02978	0.159594	0.011269	0.012238	SLC25A11	Mitochond	M2OM_HU	17	1.116973
24H 50MT/	P41235	0.140206	0.011272	0.012241	HNF4A	Hepatocyte	HNF4A_HU	11	1.102063
24H 50MT/	Q13535	0.165244	0.011279	0.012247	ATR	Serine/thre	ATR_HUMA	28	1.121356
48H 50MT/	P83916	0.195633	0.011289	0.012257	CBX1	Chromobo:	CBX1_HUM	5	1.145227
48H 10MT/	P55196	0.016663	0.011292	0.01226	AFDN	Afadin	AFAD_HUM	58	1.011617
24H 50MT/	Q9UJU6	-0.07127	0.011297	0.012263	DBNL	Drebrin-lik	DBNL_HUM	13	0.951803
48H 50MT/	Q9Y512	-0.00785	0.011297	0.012263	SAMM50	Sorting and	SAM50_HU	23	0.994571
24H 10MT/	Q00796	-0.10297	0.011298	0.012264	SORD	Sorbitol de	DHSO_HUM	15	0.931114
48H 50MT/	Q8N7R7	0.12268	0.011302	0.012266	CCNYL1	Cyclin-Y-lik	CCYL1_HUI	5	1.088756
24H 10MT/	Q8WVV9	-0.03588	0.011303	0.012266	HNRNPLL	Heterogene	HNRLL_HUI	16	0.975438
48H 50MT/	Q5HYJ3	0.046671	0.011308	0.012269	FAM76B	Protein FAI	FA76B_HUI	3	1.032879
24H 10MT/	Q96BF6	0.225236	0.011313	0.012272	NACC2	Nucleus acc	NACC2_HU	3	1.168968
24H 50MT/	P0CG38	0.345483	0.011313	0.012272	POTEI	POTE ankyr	POTEI_HUM	3	1.270576
48H 10MT/	O95721	0.158527	0.011317	0.012275	SNAP29	Synaptosor	SNP29_HUI	10	1.116147
24H 10MT/	P00167	-0.22407	0.011316	0.012275	CYB5A	Cytochrom	CYB5_HUM	4	0.856145
48H 50MT/	O14832	0.329902	0.011324	0.012281	PHYH	Phytanoyl-(	PAHX_HUM	9	1.256928
24H 50MT/	P82930	0.13114	0.011341	0.012296	MRPS34	Small ribos	RT34_HUM	12	1.095159
24H 50MT/	O75746	0.048648	0.011345	0.012298	SLC25A12	Electrogeni	S2512_HUI	25	1.034295
48H 10MT/	Q562E7	0.034315	0.01135	0.012303	WDR81	WD repeat-	WDR81_HU	24	1.024071
24H 50MT/	P62917	0.091386	0.011359	0.012311	RPL8	Large ribos	RL8_HUMA	14	1.065393
48H 50MT/	Q13564	0.111019	0.011361	0.012314	NAE1	NEDD8-acti	ULA1_HUM	18	1.079991
24H 10MT/	Q16644	-0.76531	0.011372	0.012322	MAPKAPK3	MAP kinase	MAPK3_HU	3	0.588326
24H 10MT/	Q13325	0.171936	0.011397	0.012345	IFIT5	Interferon-i	IFIT5_HUM	12	1.126569
48H 50MT/	Q9BQE4	-0.4844	0.011399	0.012346	SELENOS	Selenoprot	SELS_HUM/	6	0.714792
48H 50MT/	Q9P2M7	0.070964	0.011401	0.012347	CGN	Cingulin	CING_HUM	44	1.050418
48H 10MT/	Q8IXT5	-0.02985	0.01141	0.012356	RBM12B	RNA-bindir	RB12B_HUI	22	0.979524
48H 10MT/	P00734	0.126745	0.011414	0.012359	F2	Prothromb	THRB_HUM	26	1.091827
48H 50MT/	O95104	0.212555	0.011417	0.012363	SCAF4	SR-related a	SCAF4_HUI	14	1.158738
24H 50MT/	O75718	0.086159	0.01142	0.012365	CRTAP	Cartilage-as	CRTAP_HUI	13	1.06154
48H 50MT/	Q01130	0.496529	0.011426	0.01237	SRSF2	Serine/argi	SRSF2_HUI	5	1.410815
48H 10MT/	Q6PI98	-0.03323	0.011436	0.012378	INO80C	INO80 com	IN80C_HUI	3	0.977228
48H 10MT/	Q9BSJ2	0.013328	0.011445	0.012384	TUBGCP2	Gamma-tul	GCP2_HUM	35	1.009281
24H 10MT/	P35240	0.041722	0.011445	0.012384	NF2	Merlin	MERL_HUM	10	1.029342
48H 10MT/	P48509	-0.23138	0.011446	0.012384	CD151	CD151 anti	CD151_HU	2	0.851817
24H 50MT/	Q92922	0.079084	0.01145	0.012388	SMARCC1	SWI/SNF cc	SMRC1_HU	33	1.056347
48H 50MT/	Q86XZ4	0.091084	0.011453	0.012391	SPATS2	Spermatog	SPAS2_HUI	11	1.065171
24H 10MT/	Q9UNA4	1.36296	0.011453	0.012391	POLI	DNA polym	POLI_HUM/	1	2.572123
48H 10MT/	Q96E11	0.032578	0.011454	0.012391	MRRF	Ribosome-r	RRFM_HUM	8	1.022838
24H 10MT/	P02786	0.057222	0.011456	0.012392	TFRC	Transferrin	TFR1_HUM	36	1.04046
24H 10MT/	P14678	0.088061	0.011483	0.012413	SNRPB	Small nucle	RSMB_HUM	12	1.06294
24H 10MT/	O95997;Q	0.080038	0.011485	0.012415	PTTG1;PTT(	Securin;Sec	PTTG1_HUI	1	1.057046
48H 50MT/	O43826	0.102181	0.011489	0.012418	SLC37A4	Glucose-6-|	G6PT1_HUI	3	1.073395

48H 50MT/	Q9H2H9	-0.44691	0.01149	0.012418	SLC38A1	Sodium-co	S38A1_HU	6	0.733612
48H 10MT/	P24941	0.043199	0.011494	0.012421	CDK2	Cyclin-depe	CDK2_HUN	17	1.030396
24H 10MT/	Q9BPZ7	0.069698	0.011494	0.012421	MAPKAP1	Target of ra	SIN1_HUM/	5	1.049497
48H 10MT/	O95926	-0.58209	0.011501	0.012427	SYF2	Pre-mRNA-	SYF2_HUM/	4	0.667997
24H 10MT/	Q8IX12	0.048534	0.011503	0.012428	CCAR1	Cell divisio	CCAR1_HU	30	1.034214
48H 50MT/	O15143	0.016241	0.011517	0.01244	ARPC1B	Actin-relate	ARC1B_HU	16	1.011321
48H 10MT/	Q5VZE5	0.03076	0.011523	0.012446	NAA35	N-alpha-ac	NAA35_HU	20	1.02155
24H 50MT/	Q6YN16	0.082444	0.011527	0.012448	HSDL2	Hydroxyste	HSDL2_HU	27	1.05881
24H 50MT/	P02545	0.104672	0.011531	0.012451	LMNA	Prelamin-A	LMNA_HUN	40	1.07525
24H 50MT/	P61960	-0.43311	0.011536	0.012456	UFM1	Ubiquitin-f	UFM1_HUN	5	0.740665
48H 10MT/	Q13112	0.024263	0.011537	0.012457	CHAF1B	Chromatin	CAF1B_HU	14	1.01696
24H 50MT/	Q9Y3C4	-0.19215	0.011542	0.012461	TPRKB	EKC/KEOPS	TPRKB_HU	6	0.875303
48H 50MT/	P00338	0.275784	0.011569	0.012482	LDHA	L-lactate de	LDHA_HUN	27	1.210652
48H 10MT/	Q9H944	-0.00884	0.011572	0.012485	MED20	Mediator o	MED20_HU	9	0.993889
24H 10MT/	Q9NYH9	0.124991	0.011579	0.01249	UTP6	U3 small nu	UTP6_HUM	22	1.090501
24H 50MT/	Q9Y5K8	-0.0753	0.011589	0.012496	ATP6V1D	V-type prot	VATD_HUM	7	0.949142
24H 50MT/	Q9BZD4	-0.24494	0.011598	0.012502	NUF2	Kinetochor	NUF2_HUN	12	0.84385
48H 10MT/	Q92804	0.29264	0.011603	0.012504	TAF15	TATA-bindi	RBP56_HU	7	1.22488
48H 10MT/	Q96AB3	0.099733	0.011603	0.012504	ISOC2	Isochorism	ISOC2_HUN	7	1.071575
48H 50MT/	Q9Y6A5	-0.25894	0.011602	0.012504	TACC3	Transformi	TACC3_HU	15	0.8357
48H 10MT/	Q02978	-0.08431	0.011605	0.012505	SLC25A11	Mitochond	M2OM_HU	17	0.943233
48H 50MT/	P35613	0.254458	0.011612	0.012511	BSG	Basigin	BASI_HUM/	9	1.192887
48H 50MT/	P28676	0.366997	0.011613	0.012513	GCA	Grancalcin	GRAN_HUN	7	1.289666
24H 50MT/	O00743	0.082754	0.011614	0.012513	PPP6C	Serine/thre	PPP6_HUM	14	1.059038
24H 10MT/	O15230	0.25512	0.011616	0.012514	LAMA5	Laminin sul	LAMA5_HU	57	1.193435
24H 50MT/	Q53EP0	0.111952	0.011627	0.012524	FNDC3B	Fibronectir	FND3B_HU	33	1.080689
48H 50MT/	Q8WWK9	-0.38304	0.011648	0.012541	CKAP2	Cytoskeleto	CKAP2_HU	9	0.766819
24H 50MT/	A6ND36	-5.24081	0.011657	0.012547	FAM83G	Protein FAI	FA83G_HU	1	0.026446
24H 50MT/	Q9NWZ5	-0.18206	0.011661	0.01255	UCKL1	Uridine-cyt	UCKL1_HU	14	0.881445
48H 10MT/	O95340	0.230119	0.011678	0.012561	PAPSS2	Bifunctiona	PAPS2_HU	9	1.172932
48H 50MT/	Q96GA3	-0.32013	0.01168	0.012563	LTV1	Protein LTV	LTV1_HUM	17	0.800996
48H 10MT/	Q9Y265	0.046012	0.01168	0.012563	RUVBL1	RuvB-like 1	RUVB1_HU	25	1.032407
24H 10MT/	O95989	-0.00554	0.011689	0.012569	NUDT3	Diphospho	NUDT3_HU	5	0.996165
48H 10MT/	Q15262	0.023806	0.01169	0.01257	PTPRK	Receptor-ty	PTPRK_HU	15	1.016638
24H 10MT/	P55795	0.013983	0.011699	0.012578	HNRNPH2	Heterogene	HNRH2_HU	9	1.009739
48H 50MT/	P61204	0.03306	0.011706	0.012584	ARF3	ADP-ribosy	ARF3_HUM	12	1.02318
24H 50MT/	Q6MZT1	1.723914	0.011707	0.012584	RGS7BP	Regulator c	R7BP_HUN	1	3.303313
48H 50MT/	Q58FF8	-1.40117	0.011731	0.012604	HSP90AB2I	Putative he	H90B2_HU	2	0.378621
24H 10MT/	Q9Y672	0.011973	0.011734	0.012607	ALG6	Dolichyl py	ALG6_HUM	7	1.008333
24H 10MT/	P62847	0.122715	0.011746	0.012616	RPS24	Small ribos	RS24_HUM	5	1.088782
24H 10MT/	Q05209	0.079918	0.011746	0.012616	PTPN12	Tyrosine-pr	PTN12_HU	15	1.056958
48H 50MT/	Q04760	0.099958	0.011751	0.01262	GLO1	Lactoylglut	LGUL_HUM	12	1.071742
48H 10MT/	Q9H300	0.08103	0.01176	0.012625	PARL	Presenilin-a	PARL_HUM	6	1.057773
48H 10MT/	P49459	0.012577	0.011761	0.012626	UBE2A	Ubiquitin-c	UBE2A_HU	3	1.008756
24H 50MT/	Q99643	0.991059	0.01179	0.012652	SDHC	Succinate d	C560_HUN	3	1.987643
24H 50MT/	O95831	0.13495	0.011792	0.012654	AIFM1	Apoptosis-i	AIFM1_HU	24	1.098055
24H 10MT/	Q13451	0.075121	0.011804	0.012664	FKBP5	Peptidyl-pr	FKBP5_HU	23	1.05345
48H 10MT/	P06213	-0.01838	0.01181	0.012667	INSR	Insulin rece	INSR_HUM/	20	0.987338
48H 50MT/	P31483	-0.04921	0.011809	0.012667	TIA1	Cytotoxic g	TIA1_HUM/	10	0.966465
24H 50MT/	Q9NUQ3	-0.05994	0.01181	0.012667	TXLNG	Gamma-tax	TXLNG_HU	17	0.959302
48H 10MT/	Q6UB35	0.008441	0.01181	0.012667	MTHFD1L	Monofunct	C1TM_HUN	29	1.005868
24H 10MT/	Q99567	0.048644	0.011817	0.012673	NUP88	Nuclear po	NUP88_HU	20	1.034293

48H 50MT/	Q13363	0.015173	0.011818	0.012674	CTBP1	C-terminal-	CTBP1_HUI	14	1.010573
24H 10MT/	Q3MHD2	0.067845	0.011825	0.012679	LSM12	Protein LSN	LSM12_HU	5	1.04815
24H 10MT/	Q15691	0.05024	0.011835	0.012687	MAPRE1	Microtubul	MARE1_HU	18	1.035438
48H 50MT/	Q9H479	0.234384	0.011838	0.012689	FN3K	Fructosami	FN3K_HUM	8	1.176404
48H 10MT/	O43181	0.109807	0.011844	0.012694	NDUFS4	NADH dehy	NDUS4_HU	5	1.079084
24H 10MT/	P35052	1.133202	0.011845	0.012695	GPC1	Glypican-1	GPC1_HUM	2	2.193451
48H 50MT/	Q14108	0.089451	0.01185	0.012697	SCARB2	Lysosome n	SCRB2_HUI	11	1.063965
48H 10MT/	Q13724	-0.08762	0.01185	0.012697	MOGS	Mannosyl-c	MOGS_HUN	33	0.941075
48H 10MT/	O75340	0.044181	0.011851	0.012698	PDCD6	Programme	PDCD6_HU	9	1.031098
24H 10MT/	O75170	-0.13587	0.011863	0.012708	PPP6R2	Serine/thre	PP6R2_HUI	30	0.910121
24H 50MT/	Q16222	-0.04787	0.011871	0.012713	UAP1	UDP-N-acet	UAP1_HUM	9	0.967363
48H 10MT/	Q66PJ3	0.347224	0.011876	0.012717	ARL6IP4	ADP-ribosy	AR6P4_HUI	5	1.27211
48H 10MT/	P19447	-0.07638	0.011877	0.012718	ERCC3	General tra	ERCC3_HUI	19	0.948436
48H 10MT/	Q96R06	0.111084	0.011878	0.012718	SPAG5	Sperm-asso	SPAG5_HUI	24	1.08004
24H 10MT/	Q9Y5Z4	-0.11201	0.011878	0.012718	HEBP2	Heme-bind	HEBP2_HUI	9	0.9253
24H 10MT/	O43657	0.123369	0.01188	0.012719	TSPAN6	Tetraspanir	TSN6_HUM	5	1.089276
48H 10MT/	Q13310	0.058028	0.011882	0.01272	PABPC4	Polyadenyl	PABP4_HUI	21	1.041042
24H 50MT/	P37268	0.048206	0.011884	0.012721	FDFT1	Squalene sy	FDFT_HUM	15	1.033978
48H 50MT/	A6NH11	0.360556	0.011887	0.012723	GLTPD2	Glycolipid	GLTD2_HUI	6	1.283921
24H 10MT/	Q9NUQ7	-0.06867	0.011889	0.012724	UFSP2	Ufm1-speci	UFSP2_HUI	12	0.953519
48H 10MT/	O43663	0.061811	0.011891	0.012725	PRC1	Protein reg	PRC1_HUM	16	1.043775
48H 50MT/	Q9H3F6	0.394398	0.011897	0.01273	KCTD10	BTB/POZ dc	BACD3_HU	2	1.314394
24H 10MT/	P30405	0.040174	0.011897	0.01273	PPIF	Peptidyl-pr	PPIF_HUM/	5	1.028238
48H 10MT/	Q01085	-0.13505	0.011899	0.012731	TIAL1	Nucleolysir	TIAR_HUM/	9	0.910635
24H 10MT/	P49642	0.140779	0.011904	0.012735	PRIM1	DNA prima:	PRI1_HUM/	18	1.1025
24H 50MT/	Q13576	0.044888	0.011905	0.012736	IQGAP2	Ras GTPase-	IQGA2_HUI	66	1.031603
24H 50MT/	Q9HD34	0.012217	0.01191	0.012739	LYRM4	LYR motif-c	LYRM4_HU	4	1.008504
24H 10MT/	Q8WV07	-0.1149	0.011911	0.012739	LTO1	Protein LTC	LTO1_HUM	3	0.923447
24H 10MT/	Q9H0A0	0.059491	0.011911	0.012739	NAT10	RNA cytidir	NAT10_HUI	48	1.042098
24H 10MT/	P23528	0.176644	0.011915	0.012741	CFL1	Cofilin-1	COF1_HUM	18	1.130252
48H 10MT/	P46087	-0.11255	0.011919	0.012744	NOP2	28S rRNA (c	NOP2_HUN	25	0.924949
24H 10MT/	Q9NW68	-0.50327	0.011921	0.012746	BSDC1	BSD domai	BSDC1_HUI	3	0.705505
24H 10MT/	O00244	-0.15837	0.011924	0.012749	ATOX1	Copper trai	ATOX1_HUI	6	0.896035
24H 10MT/	Q9UPY8	-0.0969	0.011926	0.01275	MAPRE3	Microtubul	MARE3_HU	1	0.935037
48H 50MT/	P52429	1.087165	0.011928	0.012751	DGKE	Diacylglyce	DGKE_HUN	1	2.124561
24H 50MT/	P28799	0.144716	0.011934	0.012755	GRN	Progranulir	GRN_HUM/	9	1.105513
48H 50MT/	Q6DKJ4	0.09378	0.011942	0.01276	NXN	Nucleoredc	NXN_HUM/	20	1.067162
24H 50MT/	Q9H0B6	-0.11433	0.011945	0.012763	KLC2	Kinesin ligh	KLC2_HUM	22	0.923814
24H 50MT/	Q96EU7	0.009776	0.011946	0.012764	C1GALT1C1	C1GALT1-s|	C1GLC_HUI	9	1.006799
24H 10MT/	P78310	0.032352	0.011947	0.012764	CXADR	Coxsackievi	CXAR_HUM	19	1.022678
24H 10MT/	Q8NCC3	0.021759	0.011949	0.012766	PLA2G15	Lysosomal		PAG15_HU	8	1.015197
48H 10MT/	Q9BZV1	0.119595	0.011964	0.012778	UBXN6	UBX domai	UBXN6_HU	12	1.08643
24H 50MT/	Q9UET6	-0.09112	0.011968	0.01278	FTSJ1	tRNA (cytid	TRM7_HUN	6	0.938797
24H 50MT/	Q9BXS4	0.149198	0.011969	0.012781	TMEM59	Transmemk	TMM59_HU	4	1.108953
24H 50MT/	Q9UQ35	0.221984	0.011985	0.012794	SRRM2	Serine/argi	SRRM2_HU	45	1.166337
24H 50MT/	P49356	-0.11637	0.011987	0.012796	FNTB	Protein fari	FNTB_HUM	7	0.922506
24H 50MT/	Q9Y2Z0	-0.04315	0.011993	0.0128	SUGT1	Protein SG	SGT1_HUM	24	0.970534
48H 10MT/	P49761	0.250302	0.011995	0.012802	CLK3	Dual specifi	CLK3_HUM	5	1.189456
48H 50MT/	Q02750	0.131911	0.011999	0.012806	MAP2K1	Dual specifi	MP2K1_HU	10	1.095744
48H 50MT/	P09960	0.162534	0.012005	0.01281	LTA4H	Leukotrien	LKHA4_HUI	33	1.119251
24H 10MT/	P04920	-0.13136	0.012011	0.012812	SLC4A2	Anion exch	B3A2_HUM	21	0.91297
24H 10MT/	Q15149	0.055926	0.012011	0.012812	PLEC	Plectin	PLEC_HUM	199	1.039526

48H 50MT/	Q9Y2W2	0.053481	0.012013	0.012813	WBP11	WW domai	WBP11_HU	21	1.037766
24H 50MT/	P04798	0.14688	0.012016	0.012815	CYP1A1	Cytochrom	CP1A1_HU	10	1.107173
48H 10MT/	Q13488	-0.14909	0.012018	0.012816	TCIRG1	V-type prot	VPP3_HUM	3	0.901821
24H 10MT/	P25705	0.009224	0.012021	0.012818	ATP5F1A	ATP synthas	ATPA_HUM	30	1.006414
48H 10MT/	Q9NUJ1	-0.14271	0.012025	0.012822	ABHD10	Palmitoyl-p	ABHDA_HU	11	0.905818
48H 10MT/	Q9BUF5	0.01076	0.012026	0.012822	TUBB6	Tubulin bet	TBB6_HUM	16	1.007486
24H 50MT/	Q9HAS0	-0.04751	0.012034	0.012829	C17orf75	Protein Njn	NJMU_HUM	9	0.967604
48H 10MT/	P05452	-1.0142	0.012036	0.012829	CLEC3B	Tetranectin	TETN_HUM	3	0.495104
48H 50MT/	Q92759	-0.16552	0.012035	0.012829	GTF2H4	General tra	TF2H4_HU	10	0.891607
24H 50MT/	Q9Y3B8	0.014253	0.012037	0.01283	REXO2	Oligoribonu	ORN_HUM/	10	1.009928
48H 10MT/	P61254	0.088045	0.012044	0.012836	RPL26	Large ribos	RL26_HUM	8	1.062929
48H 50MT/	Q8WYA0	0.003934	0.012069	0.012854	IFT81	Intraflagell	IFT81_HUM	2	1.00273
48H 50MT/	Q5BJF2	-0.31445	0.012073	0.012858	TMEM97	Sigma intra	SGMR2_HU	2	0.804158
48H 50MT/	Q04726	0.221767	0.01208	0.012864	TLE3	Transducin	TLE3_HUM/	11	1.166161
24H 50MT/	Q9Y5M8	0.051692	0.012081	0.012865	SRPRB	Signal reco	SRPRB_HU	15	1.03648
48H 10MT/	Q9H4K7	-0.22462	0.012089	0.012872	MTG2	Mitochond	MTG2_HUM	5	0.855822
24H 10MT/	O75531	-0.31518	0.012095	0.012876	BANF1	Barrier-to-a	BAF_HUMA	4	0.803751
24H 10MT/	Q93062	-0.13829	0.012097	0.012877	RBPMS	RNA-bindir	RBPMS_HU	8	0.908596
48H 10MT/	P14854	0.272821	0.012098	0.012878	COX6B1	Cytochrom	CX6B1_HU	4	1.208168
48H 10MT/	P09110	0.085225	0.012099	0.012878	ACAA1	3-ketoacyl-	THIK_HUM/	18	1.060853
24H 10MT/	Q99808	0.138306	0.012107	0.012885	SLC29A1	Equilibrativ	S29A1_HU	7	1.100612
24H 50MT/	Q9BTL3	0.064835	0.012107	0.012885	RAMAC	RNA guanir	RAMAC_HU	5	1.045965
24H 10MT/	Q8IV08	0.168755	0.012109	0.012887	PLD3	5'-3' exonu	PLD3_HUM	6	1.124088
24H 50MT/	P01893	-0.13631	0.012114	0.01289	HLA-H	Putative HL	HLAH_HUM	1	0.909843
48H 10MT/	O43490	0.057811	0.012119	0.012895	PROM1	Prominin-1	PROM1_HU	21	1.040885
48H 50MT/	Q9UJC3	0.145039	0.01212	0.012895	HOOK1	Protein Ho	HOOK1_HU	22	1.105761
24H 10MT/	O00148	0.020356	0.012129	0.012904	DDX39A	ATP-depend	DX39A_HU	12	1.01421
48H 50MT/	Q6YN16	0.063046	0.012129	0.012904	HSDL2	Hydroxyste	HSDL2_HU	27	1.044669
48H 50MT/	Q05048	0.042116	0.012135	0.012908	CSTF1	Cleavage sti	CSTF1_HUM	14	1.029623
24H 10MT/	P34931	0.192944	0.01214	0.012911	HSPA1L	Heat shock	HS71L_HU	3	1.143094
48H 10MT/	O14933	0.451654	0.012151	0.012921	UBE2L6	Ubiquitin/I	UB2L6_HU	1	1.367608
24H 50MT/	P42677	-0.10285	0.012152	0.012921	RPS27	Small ribos	RS27_HUM	2	0.931192
48H 50MT/	Q96CM8	0.555465	0.012157	0.012924	ACSF2	Medium-ch	ACSF2_HUM	13	1.469643
24H 10MT/	Q9Y230	0.052787	0.012171	0.012934	RUVBL2	RuvB-like 2	RUVB2_HU	28	1.037267
24H 50MT/	P49591	-0.11149	0.012175	0.012938	SARS1	Serine--tRN	SYSC_HUM/	34	0.925631
48H 10MT/	Q9HCN4	0.122879	0.012183	0.012944	GPN1	GPN-loop G	GPN1_HUM	4	1.088906
48H 10MT/	P78347	0.044185	0.012185	0.012946	GTF2I	General tra	GTF2I_HUM	45	1.031101
24H 50MT/	Q9BYB4	-0.12646	0.012186	0.012947	GNB1L	Guanine nu	GNB1L_HU	9	0.916076
48H 50MT/	Q9UHB6	0.071232	0.012188	0.012947	LIMA1	LIM domair	LIMA1_HU	22	1.050614
48H 50MT/	O43402	0.053722	0.012193	0.012952	EMC8	ER membra	EMC8_HUM	5	1.037939
48H 50MT/	O95831	0.001394	0.012197	0.012954	AIFM1	Apoptosis-i	AIFM1_HU	24	1.000967
24H 10MT/	P85298	1.418197	0.012205	0.012961	ARHGAP8	Rho GTPase	RHG08_HU	1	2.672514
24H 50MT/	P25705	0.042065	0.01221	0.012964	ATP5F1A	ATP synthas	ATPA_HUM	30	1.029586
48H 50MT/	Q9UHX1	0.031087	0.012215	0.012968	PUF60	Poly(U)-bin	PUF60_HU	24	1.021782
48H 50MT/	O00165	-0.13515	0.012224	0.012976	HAX1	HCLS1-asso	HAX1_HUM	10	0.910575
48H 50MT/	Q9Y261	0.236409	0.012231	0.012982	FOXA2	Hepatocyte	FOXA2_HU	3	1.178057
48H 50MT/	Q01968	0.105622	0.012239	0.012989	OCRL	Inositol pol	OCRL_HUM	26	1.075958
48H 50MT/	Q53H82	0.180089	0.01224	0.01299	LACTB2	Endoribonu	LACB2_HU	9	1.132954
24H 50MT/	O43325	0.993293	0.012243	0.012992	LYRM1	LYR motif-c	LYRM1_HU	1	1.990724
48H 50MT/	O95347	-0.09426	0.012245	0.012993	SMC2	Structural r	SMC2_HUM	48	0.936751
48H 50MT/	Q9BT09	0.123629	0.012254	0.013001	CNPY3	Protein can	CNPY3_HU	8	1.089472
24H 10MT/	Q9BV73	0.116646	0.012263	0.013009	CEP250	Centrosom	CP250_HU	18	1.084211

48H	10MT/	Q8N653	-0.25298	0.012265	0.013009	LZTR1	Leucine-zip	LZTR1_HUM	11	0.839162
48H	50MT/	Q9NZ63	-0.31528	0.012266	0.01301	C9orf78	Splicing fac	TLS1_HUM/	9	0.803696
24H	10MT/	P50452	0.171614	0.012267	0.013011	SERPINB8	Serpin B8	SPB8_HUM	7	1.126318
24H	10MT/	Q5TAP6	-0.22609	0.012268	0.013011	UTP14C	U3 small nu	UT14C_HU	8	0.854948
24H	50MT/	P07737	-0.08745	0.012269	0.013012	PFN1	Profilin-1	PROF1_HU	11	0.941183
48H	10MT/	Q7LBC6	0.085398	0.012276	0.013018	KDM3B	Lysine-spec	KDM3B_HU	31	1.06098
24H	50MT/	P48047	0.119739	0.012285	0.013026	ATP5PO	ATP syntha	ATPO_HUM	10	1.086538
48H	10MT/	Q8NI27	-0.12827	0.012289	0.013028	THOC2	THO compl	THOC2_HU	41	0.914925
24H	50MT/	Q92484	0.055794	0.012298	0.013035	SMPDL3A	Cyclic GMP	ASM3A_HU	11	1.039431
24H	50MT/	Q9UBV2	0.018773	0.012301	0.013037	SEL1L	Protein sel-	SE1L1_HUM	24	1.013098
24H	50MT/	O43684	0.017022	0.012303	0.013039	BUB3	Mitotic che	BUB3_HUM	12	1.011868
24H	50MT/	O94979	-0.00752	0.012305	0.01304	SEC31A	Protein tra	SC31A_HUI	36	0.9948
24H	50MT/	Q9UL46	0.105289	0.012305	0.01304	PSME2	Proteasom	PSME2_HU	14	1.07571
48H	50MT/	Q9BXI6	0.222711	0.012316	0.013049	TBC1D10A	TBC1 doma	TB10A_HUI	12	1.166924
48H	10MT/	O43491	0.085745	0.012331	0.013063	EPB41L2	Band 4.1-lil	E41L2_HUM	49	1.061236
48H	50MT/	O95425	0.041019	0.012335	0.013065	SVIL	Supervillin	SVIL_HUM/	34	1.02884
48H	50MT/	Q08426	0.216919	0.012335	0.013065	EHHADH	Peroxisoma	ECHP_HUM	31	1.162248
24H	50MT/	P40123	0.042363	0.012337	0.013067	CAP2	Adenylyl cy	CAP2_HUM	15	1.029799
24H	10MT/	P68366	0.01389	0.012346	0.013075	TUBA4A	Tubulin alp	TBA4A_HUI	4	1.009675
24H	10MT/	Q9UPP1	0.591965	0.012347	0.013075	PHF8	Histone lysi	PHF8_HUM	4	1.507298
48H	10MT/	Q6NZY4	0.106236	0.012355	0.013082	ZCCHC8	Zinc finger (	ZCHC8_HUI	19	1.076416
48H	50MT/	Q9Y277	0.045199	0.012356	0.013082	VDAC3	Voltage-de	VDAC3_HU	13	1.031825
24H	10MT/	Q9C086	-1.26933	0.012362	0.013085	INO80B	INO80 com	IN80B_HUI	2	0.414853
24H	10MT/	P61165	-0.99707	0.012364	0.013087	TMEM258	Transmemb	TM258_HU	1	0.501017
24H	10MT/	Q53GQ0	0.099822	0.012386	0.013104	HSD17B12	Very-long-c	DHB12_HU	14	1.071641
48H	50MT/	P51665	0.080775	0.012397	0.013113	PSMD7	26S protea	PSMD7_HU	12	1.057586
48H	10MT/	Q96K37	-0.23864	0.012401	0.013117	SLC35E1	Solute carri	S35E1_HUI	6	0.847545
48H	10MT/	P06132	-0.11598	0.012407	0.013122	UROD	Uroporphy	DCUP_HUM	15	0.922753
48H	50MT/	P08247	0.090012	0.012408	0.013123	SYP	Synaptophy	SYPH_HUM	3	1.064379
24H	10MT/	Q9NQW6	0.10109	0.012419	0.013132	ANLN	Anillin	ANLN_HUM	37	1.072584
48H	10MT/	Q5SW96	-0.19894	0.012432	0.013141	LDLRAP1	Low densit	ARH_HUM/	2	0.871191
48H	50MT/	P62906	-0.14986	0.012433	0.013142	RPL10A	Large ribos	RL10A_HUI	13	0.901338
48H	10MT/	O95258	-0.33158	0.012435	0.013143	SLC25A14	Brain mitoc	UCP5_HUM	3	0.794667
24H	50MT/	Q14240	-0.1696	0.012435	0.013143	EIF4A2	Eukaryotic	IF4A2_HUM	12	0.88909
48H	10MT/	Q8WUF5	0.096033	0.012438	0.013144	PPP1R13L	RelA-associ	IASPP_HUM	20	1.06883
24H	10MT/	Q02978	0.105789	0.012444	0.01315	SLC25A11	Mitochond	M2OM_HU	17	1.076083
48H	50MT/	Q8WVY7	0.107429	0.012446	0.013152	UBLCP1	Ubiquitin-l	UBCP1_HU	14	1.077307
48H	50MT/	Q15438	0.808888	0.012449	0.013154	CYTH1	Cytohesin-1	CYH1_HUM	3	1.751861
24H	50MT/	Q04726	0.298343	0.012456	0.01316	TLE3	Transducin	TLE3_HUM/	11	1.229731
24H	50MT/	P48556	0.058857	0.012463	0.013167	PSMD8	26S protea	PSMD8_HU	13	1.04164
48H	50MT/	Q9NSG2	-0.50747	0.012464	0.013168	FIRRM	FIGNL1-inte	FIRRM_HUI	9	0.703455
48H	50MT/	O60508	-0.02459	0.012468	0.013169	CDC40	Pre-mRNA-|	PRP17_HUI	24	0.9831
48H	50MT/	Q9BU76	-0.12796	0.012468	0.013169	MMTAG2	Multiple m	MMTA2_HU	5	0.915122
24H	50MT/	O43823	0.137162	0.012471	0.013172	AKAP8	A-kinase an	AKAP8_HUI	21	1.09974
48H	50MT/	Q9GZN8	-0.10484	0.012478	0.013178	ADISSP	Adipose-sec	ADSSP_HUI	9	0.92991
48H	50MT/	Q7L211	-0.41513	0.012481	0.013181	ABHD13	Protein ABI	ABHDD_HU	3	0.749951
24H	10MT/	O60934	0.107421	0.012489	0.013186	NBN	Nibrin	NBN_HUM/	22	1.077301
48H	50MT/	Q9Y320	-0.34784	0.012489	0.013186	TMX2	Thioredoxi	TMX2_HUM	13	0.785759
48H	50MT/	P13056	-1.08959	0.012504	0.013198	NR2C1	Nuclear rec	NR2C1_HU	2	0.469893
48H	50MT/	P26641	-0.174	0.012507	0.013198	EEF1G	Elongation	EF1G_HUM	27	0.88638
48H	50MT/	Q96GW9	-0.20102	0.012506	0.013198	MARS2	Methionine	SYMM_HUI	13	0.869935
48H	50MT/	O76027	1.780435	0.012507	0.013199	ANXA9	Annexin A9	ANXA9_HU	3	3.435298

24H 50MT/	Q92609	0.310979	0.012511	0.013201	TBC1D5	TBC1 doma	TBCD5_HU	14	1.240549
48H 10MT/	Q13907	-0.14735	0.012511	0.013201	IDI1	Isopenteny	IDI1_HUM/	10	0.90291
48H 10MT/	Q7L5Y9	0.006712	0.012517	0.013204	MAEA	E3 ubiquiti	MAEA_HUN	11	1.004663
24H 50MT/	P78560	0.348763	0.012532	0.013218	CRADD	Death dom	CRADD_HU	6	1.273469
24H 50MT/	Q8NFF5	-0.17656	0.012537	0.013223	FLAD1	FAD syntha	FAD1_HUN	16	0.884811
48H 50MT/	P22670	-0.41833	0.01255	0.013234	RFX1	MHC class I	RFX1_HUM	9	0.748291
48H 50MT/	Q8WVC0	-0.16677	0.012556	0.013237	LEO1	RNA polym	LEO1_HUM	11	0.890837
48H 10MT/	A2RTX5	-0.31208	0.012558	0.013238	TARS3	Threonine-	SYTC2_HUN	9	0.805478
24H 50MT/	Q13618	0.063303	0.01256	0.013241	CUL3	Cullin-3	CUL3_HUN	35	1.044855
48H 10MT/	Q9Y5S9	0.263748	0.012569	0.013247	RBM8A	RNA-bindir	RBM8A_HU	8	1.200594
24H 50MT/	O15382	0.100972	0.012569	0.013247	BCAT2	Branched-c	BCAT2_HUI	17	1.072496
24H 10MT/	P06756	0.057499	0.01258	0.013255	ITGAV	Integrin alp	ITAV_HUM/	37	1.04066
24H 10MT/	O95684	0.031869	0.012588	0.013262	CEP43	Centrosom	CEP43_HUI	15	1.022336
24H 50MT/	Q8N6R0	-0.21646	0.012588	0.013262	METTL13	eEF1A lysin	EFNMT_HU	17	0.860676
48H 10MT/	Q86UP2	-0.03843	0.012603	0.013273	KTN1	Kinectin	KTN1_HUN	68	0.973712
24H 50MT/	Q14676	0.195965	0.012612	0.013281	MDC1	Mediator o	MDC1_HUI	43	1.14549
48H 10MT/	Q99961	0.069045	0.012613	0.013282	SH3GL1	Endophilin	SH3G1_HU	12	1.049022
48H 50MT/	P36551	0.001648	0.012615	0.013284	CPOX	Oxygen-dep	HEM6_HUN	17	1.001143
24H 50MT/	Q92688	-0.18654	0.012621	0.013289	ANP32B	Acidic leuci	AN32B_HU	4	0.87871
24H 10MT/	O75886	0.124079	0.012623	0.013289	STAM2	Signal trans	STAM2_HU	13	1.089812
48H 50MT/	P30038	0.088422	0.012636	0.013302	ALDH4A1	Delta-1-pyr	AL4A1_HUI	13	1.063207
48H 50MT/	P48637	0.080066	0.012644	0.013308	GSS	Glutathion	GSHB_HUN	26	1.057066
48H 50MT/	O75764	0.403796	0.012652	0.013313	TCEA3	Transcripti	TCEA3_HUI	3	1.322984
24H 50MT/	Q96C24	0.404975	0.012653	0.013314	SYTL4	Synaptotag	SYTL4_HUN	7	1.324066
48H 50MT/	Q86UX6	0.242163	0.012658	0.013317	STK32C	Serine/thre	ST32C_HUI	5	1.182764
48H 10MT/	P82921	0.040187	0.012664	0.013323	MRPS21	Small ribos	RT21_HUM	3	1.028247
24H 10MT/	P62875	0.150481	0.012667	0.013325	POLR2L	DNA-direct	RPAB5_HUI	3	1.10994
24H 10MT/	Q9UNS1	0.1506	0.012686	0.013342	TIMELESS	Protein tim	TIM_HUMA	18	1.110031
24H 10MT/	P82970	-0.17649	0.012687	0.013342	HMGN5	High mobil	HMGN5_HU	5	0.88485
48H 10MT/	Q13263	0.018167	0.012687	0.013342	TRIM28	Transcripti	TIF1B_HUN	33	1.012672
48H 50MT/	Q86X55	0.000487	0.012689	0.013344	CARM1	Histone-arg	CARM1_HU	14	1.000338
48H 10MT/	Q9NSC2	0.063661	0.012693	0.013346	SALL1	Sal-like pro	SALL1_HUN	4	1.045114
48H 10MT/	P15509	-0.15756	0.012694	0.013347	CSF2RA	Granulocyt	CSF2R_HUN	1	0.896543
48H 50MT/	P09601	0.17487	0.012697	0.013349	HMOX1	Heme oxyg	HMOX1_HU	10	1.128862
48H 50MT/	P26368	0.176634	0.012697	0.013349	U2AF2	Splicing fac	U2AF2_HUI	13	1.130244
24H 10MT/	Q8IY67	-0.07324	0.012696	0.013349	RAVER1	Ribonuclec	RAVR1_HUI	17	0.950502
24H 10MT/	P10599	-0.17501	0.0127	0.01335	TXN	Thioredoxi	THIO_HUM	7	0.885763
48H 10MT/	P13674	0.087935	0.0127	0.01335	P4HA1	Prolyl 4-hy	P4HA1_HU	23	1.062847
48H 10MT/	P29597	-0.30275	0.012707	0.013356	TYK2	Non-recept	TYK2_HUM	4	0.810705
24H 10MT/	O60907	0.131694	0.01271	0.013358	TBL1X	F-box-like/\	TBL1X_HUN	13	1.095579
24H 10MT/	Q9H0R1	0.310718	0.012723	0.013366	AP5M1	AP-5 compl	AP5M1_HU	2	1.240325
48H 50MT/	P61020	0.107773	0.01273	0.013373	RAB5B	Ras-related	RAB5B_HUI	4	1.077564
48H 10MT/	Q9NR12	0.053188	0.012735	0.013377	PDLIM7	PDZ and LIN	PDLI7_HUN	12	1.037555
24H 50MT/	P00488	-1.09413	0.012743	0.013383	F13A1	Coagulatio	F13A_HUM	1	0.468417
48H 10MT/	Q02447	-0.18257	0.012748	0.013385	SP3	Transcripti	SP3_HUMA	6	0.881129
48H 50MT/	O95182	-0.01205	0.012765	0.0134	NDUFA7	NADH dehy	NDUA7_HU	6	0.991679
48H 50MT/	O14907	-0.0269	0.012777	0.013408	TAX1BP3	Tax1-bindir	TX1B3_HUI	4	0.981529
24H 50MT/	Q9Y6D9	-0.08211	0.012776	0.013408	MAD1L1	Mitotic spi	MD1L1_HU	22	0.944673
48H 50MT/	Q9BTM9	0.137406	0.012784	0.013414	URM1	Ubiquitin-r	URM1_HUN	1	1.099925
24H 10MT/	Q7L576	0.047396	0.012789	0.013417	CYFIP1	Cytoplasmi	CYFP1_HUN	46	1.033398
48H 50MT/	Q8IXI2	0.293582	0.012792	0.013418	RHOT1	Mitochond	MIRO1_HU	8	1.225679
24H 50MT/	O95365	-0.74304	0.012796	0.013419	ZBTB7A	Zinc finger	ZBT7A_HUN	4	0.59748

24H 50MT/	Q9Y4L1	0.035417	0.012808	0.01343	HYOU1	Hypoxia up	HYOU1_HU	44	1.024853
48H 10MT/	Q9BVT8	-0.13318	0.01282	0.013441	TMUB1	Transmemb	TMUB1_HU	3	0.911816
48H 50MT/	P00568	0.15603	0.012821	0.013441	AK1	Adenylate k	KAD1_HUM	9	1.114217
48H 50MT/	Q9H5Z1	0.146917	0.012829	0.013448	DHX35	Probable A	DHX35_HU	15	1.107201
24H 50MT/	P82094	0.036849	0.01284	0.013455	TMF1	TATA eleme	TMF1_HUM	34	1.025871
24H 50MT/	Q9BQG2	0.445022	0.012858	0.013471	NUDT12	NAD-cappe	NUD12_HU	4	1.361335
48H 50MT/	P13489	0.096552	0.012871	0.013481	RNH1	Ribonuclea	RINI_HUM/	23	1.069215
48H 50MT/	Q5BKT4;Q5	-0.44181	0.012872	0.013483	ALG10;ALG	Dol-P-Glc:(	AG10A_HU	3	0.736213
24H 50MT/	Q9UGI8	-0.12856	0.012874	0.013484	TES	Testin	TES_HUMA	22	0.914745
48H 50MT/	Q8N335	0.117548	0.012878	0.013487	GPD1L	Glycerol-3-	GPD1L_HU	12	1.084889
48H 50MT/	O43490	0.085477	0.012888	0.013494	PROM1	Prominin-1	PROM1_HU	21	1.061039
24H 10MT/	Q96EL3	-0.30516	0.012889	0.013494	MRPL53	Large ribos	RM53_HUN	5	0.809351
48H 10MT/	B7U540	-0.95816	0.01289	0.013494	KCNJ18	Inward rect	KCJ18_HUN	1	0.514712
48H 50MT/	Q9Y262	-0.17499	0.012906	0.013507	EIF3L	Eukaryotic	EIF3L_HUM	32	0.885772
48H 50MT/	Q8WVX9	-0.1585	0.012912	0.013509	FAR1	Fatty acyl-(	FACR1_HUI	19	0.895959
48H 10MT/	Q7Z7F0	0.755572	0.012911	0.013509	KHDC4	KH homolo	KHDC4_HU	2	1.6883
24H 50MT/	Q9HAN9	0.241785	0.012915	0.013512	NMNAT1	Nicotinami	NMNA1_HU	5	1.182455
48H 10MT/	P16615	0.028049	0.012921	0.013517	ATP2A2	Sarcoplasm	AT2A2_HUI	45	1.019632
48H 50MT/	P45954	0.094398	0.012921	0.013517	ACADSB	Short/bran	ACDSB_HUI	17	1.067619
24H 50MT/	Q9BVC3	-0.2157	0.012927	0.01352	DSCC1	Sister chror	DCC1_HUM	5	0.86113
24H 10MT/	Q13485	-0.16868	0.012943	0.013531	SMAD4	Mothers ag	SMAD4_HU	10	0.889658
48H 10MT/	Q7Z3Y8	1.211521	0.012957	0.013542	KRT27	Keratin, typ	K1C27_HUI	2	2.315817
48H 50MT/	O43292	-0.03502	0.01296	0.013543	GPAA1	Glycosylph	GPAA1_HU	11	0.97602
48H 10MT/	Q96P70	0.038639	0.01296	0.013543	IPO9	Importin-9	IPO9_HUM	28	1.027145
24H 10MT/	Q92917	-0.08894	0.012963	0.013546	GPKOW	G-patch do	GPKOW_HU	19	0.940211
24H 10MT/	P98194	0.027884	0.012964	0.013547	ATP2C1	Calcium-tra	AT2C1_HUI	16	1.019515
24H 10MT/	E9PAV3;Q1	0.071138	0.01297	0.013552	NACA	Nascent po	NACAM_HU	5	1.050545
48H 50MT/	Q9Y2R5	-0.27828	0.012972	0.013554	MRPS17	Small ribos	RT17_HUM	4	0.824574
24H 10MT/	Q96FZ2	0.003706	0.012974	0.013555	HMCES	Abasic site	HMCES_HU	11	1.002572
24H 50MT/	Q92599	0.093427	0.012987	0.013567	SEPTIN8	Septin-8	SEPT8_HUN	7	1.066902
24H 50MT/	Q9H4I3	-0.18499	0.012989	0.013568	TRABD	TraB domai	TRABD_HUI	10	0.879655
48H 10MT/	Q92945	0.013133	0.012995	0.013573	KHSRP	Far upstrea	FUBP2_HUI	28	1.009145
48H 50MT/	P07910	0.047043	0.012998	0.013575	HNRNPC	Heterogene	HNRPC_HU	15	1.033145
24H 50MT/	P46013	0.049717	0.013001	0.013578	MKI67	Proliferatic	KI67_HUM/	119	1.035062
24H 50MT/	Q9Y3E7	0.051136	0.013002	0.013579	CHMP3	Charged mu	CHMP3_HU	5	1.03608
24H 50MT/	O43765	0.092909	0.013014	0.013587	SGTA	Small gluta	SGTA_HUM	11	1.066519
48H 50MT/	Q99942	-0.10895	0.013013	0.013587	RNF5	E3 ubiquiti	RNF5_HUM	1	0.927264
24H 50MT/	Q12904	0.047642	0.013015	0.013587	AIMP1	Aminoacyl	AIMP1_HUI	18	1.033574
48H 10MT/	Q14847	0.05451	0.013018	0.013589	LASP1	LIM and SH	LASP1_HUN	12	1.038506
24H 10MT/	P62306	0.111764	0.01302	0.013591	SNRPF	Small nucle	RUXF_HUM	7	1.080549
24H 10MT/	Q96PC3	0.659198	0.013022	0.013592	AP1S3	AP-1 compl	AP1S3_HUI	2	1.579205
24H 50MT/	Q8NET6	0.861521	0.013029	0.013598	CHST13	Carbohydra	CHSTD_HUI	5	1.816953
24H 50MT/	Q9NRG9	0.111727	0.013038	0.013605	AAAS	Aladin	AAAS_HUM	26	1.080521
24H 50MT/	Q07955	0.106785	0.013043	0.013609	SRSF1	Serine/argi	SRSF1_HUN	14	1.076826
48H 10MT/	P33991	0.041792	0.013051	0.013616	MCM4	DNA replica	MCM4_HUI	45	1.029392
48H 10MT/	P01024	-0.01053	0.013056	0.013619	C3	Compleme	CO3_HUM/	58	0.99273
48H 10MT/	Q32P41	-0.00061	0.013057	0.013619	TRMT5	tRNA (guan	TRM5_HUN	14	0.999576
48H 10MT/	Q92536	-0.2998	0.013057	0.01362	SLC7A6	Y+L amino a	YLAT2_HUN	3	0.812367
48H 50MT/	A5PLL7	0.188556	0.013067	0.013629	PEDS1	Plasmanyle	PDES1_HUI	4	1.139623
48H 50MT/	Q13283	-0.17665	0.013079	0.013639	G3BP1	Ras GTPase	G3BP1_HU	23	0.884754
48H 10MT/	Q5TFE4	-0.00071	0.01308	0.01364	NT5DC1	5'-nucleoti	NT5D1_HU	18	0.999506
24H 10MT/	Q99549	0.111597	0.01309	0.013648	MPHOSPH8	M-phase ph	MPP8_HUN	11	1.080423

48H 10MT/	Q9BY41	0.173	0.013091	0.013648	HDAC8	Histone dea	HDAC8_HU	5	1.1274
48H 10MT/	O00461	-0.1803	0.013092	0.013649	GOLIM4	Golgi integ	GOLI4_HUN	12	0.882522
24H 50MT/	Q13867	0.094564	0.013113	0.013665	BLMH	Bleomycin	BLMH_HUN	14	1.067742
24H 50MT/	Q9NRH1	-0.21087	0.013123	0.013673	YAE1	Protein YAE	YAE1_HUM	2	0.864013
24H 50MT/	P15260	0.58211	0.013124	0.013673	IFNGR1	Interferon g	INGR1_HUI	2	1.497037
48H 10MT/	Q9UI10	0.086805	0.013143	0.013687	EIF2B4	Translation	EI2BD_HUN	18	1.062016
48H 50MT/	P09874	0.034119	0.013145	0.013688	PARP1	Poly [ADP-r	PARP1_HUI	53	1.023931
48H 50MT/	Q9NY61	-0.18896	0.013152	0.013693	AATF	Protein AA	AATF_HUM	17	0.87724
48H 10MT/	Q2Q1W2	0.012904	0.013157	0.013698	TRIM71	E3 ubiquiti	LIN41_HUN	16	1.008984
48H 10MT/	Q96EK6	0.091936	0.013158	0.013698	GNPNAT1	Glucosamir	GNA1_HUN	11	1.0658
48H 50MT/	P35080	0.121561	0.013164	0.013703	PFN2	Profilin-2	PROF2_HUI	7	1.087912
24H 50MT/	Q9H5K3	0.241613	0.013177	0.013711	POMK	Protein O-n	SG196_HUI	5	1.182314
24H 50MT/	P23743	-0.02625	0.01319	0.013721	DGKA	Diacylglyce	DGKA_HUN	6	0.981968
24H 50MT/	Q99595	-0.19566	0.01319	0.013721	TIMM17A	Mitochond	TI17A_HUN	2	0.873171
24H 50MT/	Q04837	0.155173	0.013194	0.013724	SSBP1	Single-strar	SSBP_HUM	8	1.113555
48H 50MT/	O95445	0.651034	0.013197	0.013727	APOM	Apolipoprc	APOM_HUN	4	1.570293
24H 10MT/	Q9H8S9	0.139721	0.013201	0.01373	MOB1A	MOB kinase	MOB1A_HU	1	1.101692
24H 10MT/	Q14166	-0.09058	0.013203	0.013731	TTLL12	Tubulin--ty	TTL12_HUN	28	0.939145
24H 10MT/	Q16719	0.110451	0.013206	0.013733	KYNU	Kynurenina	KYNU_HUM	20	1.079565
48H 10MT/	P55735	0.108671	0.013208	0.013734	SEC13	Protein SEC	SEC13_HUN	11	1.078234
48H 50MT/	Q96KC8	0.093267	0.013215	0.01374	DNAJC1	DnaJ homo	DNJC1_HUI	10	1.066784
48H 50MT/	Q709C8	0.042716	0.013221	0.013744	VPS13C	Intermemb	VP13C_HUI	60	1.030051
48H 10MT/	Q9UJS0	-0.11909	0.013227	0.013749	SLC25A13	Electrogeni	S2513_HUI	29	0.920769
24H 50MT/	Q9UGP8	0.032086	0.013228	0.013749	SEC63	Translocati	SEC63_HUN	34	1.02249
24H 50MT/	Q9H6K5	0.03686	0.013231	0.013751	PRR36	Proline-rich	PRR36_HUI	7	1.025879
48H 50MT/	Q8IWJ2	-0.12218	0.013237	0.013755	GCC2	GRIP and co	GCC2_HUM	53	0.918799
24H 50MT/	Q96GQ7	-0.09404	0.013247	0.013762	DDX27	Probable A	DDX27_HU	31	0.936895
24H 10MT/	Q8IXK0	0.124517	0.013248	0.013763	PHC2	Polyhomec	PHC2_HUM	6	1.090143
48H 50MT/	P61970	0.17914	0.013257	0.013768	NUTF2	Nuclear tra	NTF2_HUM	6	1.132209
24H 50MT/	O43148	0.105305	0.013258	0.013769	RNMT	mRNA cap g	MCES_HUM	13	1.075722
24H 10MT/	Q12905	0.053102	0.013269	0.013778	ILF2	Interleukin	ILF2_HUMA	20	1.037493
24H 10MT/	Q8N5B7	-1.1027	0.013278	0.013784	CERS5	Ceramide s	CERS5_HUN	2	0.465645
48H 50MT/	Q9NX20	-0.26971	0.013283	0.013789	MRPL16	Large ribos	RM16_HUN	9	0.829487
48H 50MT/	A5YKK6	-0.00462	0.01329	0.013795	CNOT1	CCR4-NOT t	CNOT1_HU	73	0.996805
24H 50MT/	Q9UBN7	0.154295	0.013293	0.013797	HDAC6	Histone dea	HDAC6_HU	16	1.112878
48H 50MT/	Q8TCG1	-0.25609	0.013296	0.013799	CIP2A	Protein CIP	CIP2A_HUN	19	0.837356
48H 50MT/	Q9NYY8	-0.32931	0.013297	0.0138	FASTKD2	FAST kinase	FAKD2_HUI	17	0.795917
24H 50MT/	Q92766	0.090244	0.013307	0.013807	RREB1	Ras-respon:	RREB1_HUI	13	1.06455
48H 50MT/	Q99611	0.184004	0.013309	0.013808	SEPHS2	Selenide, w	SPS2_HUM	17	1.136032
24H 50MT/	P0CG39	-1.07278	0.013328	0.013825	POTEJ	POTE ankyr	POTEJ_HUN	1	0.475402
48H 10MT/	Q96N21	-0.77256	0.013337	0.013833	TEPSIN	AP-4 compl	AP4AT_HUI	1	0.585376
48H 50MT/	Q5J8M3	-0.06051	0.013344	0.013838	EMC4	ER membra	EMC4_HUN	6	0.958924
24H 50MT/	Q32P41	0.234074	0.013348	0.013841	TRMT5	tRNA (guan	TRM5_HUN	14	1.176152
24H 50MT/	P20290	-0.22308	0.013352	0.013843	BTF3	Transcripti	BTF3_HUM	11	0.856736
48H 10MT/	A8MW92	0.857763	0.013356	0.013846	PHF20L1	PHD finger	P20L1_HUI	1	1.812227
48H 50MT/	Q9NV35	0.227055	0.013359	0.013848	NUDT15	Nucleotide	NUD15_HU	7	1.170443
48H 10MT/	Q9Y375	-0.33707	0.013364	0.013852	NDUFAF1	Complex I i	CIA30_HUN	3	0.791646
48H 10MT/	Q9C0C4	0.176117	0.013366	0.013853	SEMA4C	Semaphori	SEM4C_HU	5	1.129839
48H 10MT/	Q15173	0.028488	0.013376	0.01386	PPP2R5B	Serine/thre	2A5B_HUM	2	1.019942
48H 50MT/	Q8TDJ6	-0.03277	0.013379	0.013862	DMXL2	DmX-like p	DMXL2_HU	15	0.97754
24H 50MT/	O00541	0.049135	0.013392	0.013874	PES1	Pescadillo I	PESC_HUM	21	1.034645
48H 50MT/	P19971	0.312075	0.013395	0.013877	TYMP	Thymidine	TYPH_HUM	4	1.241492

48H 50MTA	O43819	-0.25949	0.013403	0.013884	SCO2	Protein SCO	SCO2_HUM	6	0.835384
48H 50MTA	Q96PE7	0.12827	0.013405	0.013884	MCEE	Methylmal	MCEE_HUM	3	1.092982
24H 50MTA	Q9BXW9	0.081162	0.013413	0.013891	FANCD2	Fanconi an	FACD2_HU	34	1.05787
48H 50MTA	P52565	0.11456	0.01342	0.013896	ARHGDIA	Rho GDP-di	GDIR1_HU	9	1.082645
48H 10MTA	Q9NZJ9	-0.12698	0.013423	0.013898	NUDT4	Diphospho	NUDT4_HU	6	0.915747
24H 10MTA	Q9H4M9	-0.03812	0.013424	0.013898	EHD1	EH domain	EHD1_HUM	24	0.973922
48H 50MTA	Q6NUQ4	0.066846	0.013426	0.0139	TMEM214	Transmemb	TM214_HU	29	1.047424
48H 50MTA	Q13825	0.20641	0.013428	0.013901	AUH	Methylglut	AUHM_HU	8	1.153814
24H 10MTA	Q8TEX9	-0.0781	0.013429	0.013902	IPO4	Importin-4	IPO4_HUM	47	0.947303
48H 50MTA	P55795	0.021729	0.013435	0.013906	HNRNPH2	Heterogene	HNRH2_HU	9	1.015175
48H 10MTA	Q92609	0.037316	0.013436	0.013907	TBC1D5	TBC1 doma	TBCD5_HU	14	1.026203
48H 50MTA	P15735	-0.10199	0.013437	0.013907	PHKG2	Phosphoryl	PHKG2_HU	1	0.931744
48H 50MTA	Q86WX3	-0.11903	0.013437	0.013907	RPS19BP1	Active regu	AROS_HUM	5	0.920803
48H 10MTA	Q13151	0.147366	0.013442	0.013911	HNRNPA0	Heterogene	ROA0_HUM	8	1.107546
24H 50MTA	Q9HCM4	0.05841	0.013459	0.013925	EPB41L5	Band 4.1-lil	E41L5_HU	12	1.041318
48H 10MTA	P35658	0.027312	0.013459	0.013925	NUP214	Nuclear po	NU214_HU	48	1.019111
24H 50MTA	Q9Y3B3	0.213777	0.01346	0.013926	TMED7	Transmemb	TMED7_HU	7	1.15972
48H 10MTA	Q12872	-0.12221	0.013464	0.013928	SFSWAP	Splicing fac	SFSWA_HU	12	0.918778
24H 50MTA	O75683	0.068406	0.013472	0.013934	SURF6	Surfeit locu	SURF6_HU	7	1.048558
48H 10MTA	Q9UKV8	0.103625	0.013481	0.013942	ago-02	Protein arg	AGO2_HUM	14	1.07447
48H 10MTA	Q04637	0.033579	0.013484	0.013944	EIF4G1	Eukaryotic	IF4G1_HUM	55	1.023548
48H 10MTA	Q9BVW5	-0.28698	0.013485	0.013944	TIPIN	TIMELESS-ir	TIPIN_HUM	3	0.819618
48H 50MTA	P50552	0.116899	0.013494	0.013951	VASP	Vasodilator	VASP_HUM	16	1.084401
24H 10MTA	Q14240	-0.07729	0.013506	0.013959	EIF4A2	Eukaryotic	IF4A2_HUM	12	0.947838
24H 10MTA	Q9NUP9	-0.00337	0.013514	0.013966	LIN7C	Protein lin-	LIN7C_HUM	5	0.997667
48H 50MTA	P46199	-0.05849	0.013521	0.013971	MTIF2	Translation	IF2M_HUM	9	0.96027
48H 50MTA	Q9Y619	0.051729	0.013525	0.013973	SLC25A15	Mitochond	ORNT1_HU	15	1.036507
24H 10MTA	P26640	-0.06296	0.013528	0.013975	VARS1	Valine--tRN	SYVC_HUM	60	0.957297
48H 10MTA	P09874	0.016443	0.013531	0.013977	PARP1	Poly [ADP-r	PARP1_HU	53	1.011463
48H 10MTA	O60678	-0.01806	0.013536	0.013981	PRMT3	Protein arg	ANM3_HUM	20	0.98756
24H 50MTA	P53396	-0.06389	0.01354	0.013984	ACLY	ATP-citrate	ACLY_HUM	56	0.956678
48H 50MTA	Q8NDF8	-0.16145	0.013553	0.013997	TENT4B	Terminal nu	PAPD5_HU	11	0.894127
24H 50MTA	P02765	0.755081	0.013558	0.014001	AHSG	Alpha-2-HS	FETUA_HUM	5	1.687726
48H 10MTA	Q9BTX3	0.363196	0.01356	0.014001	TMEM208	Transmemb	TM208_HU	1	1.286273
24H 10MTA	O00170	-0.05605	0.013564	0.014003	AIP	AH recepto	AIP_HUMA	15	0.961893
24H 50MTA	O95630	0.003319	0.013563	0.014003	STAMBP	STAM-bindi	STABP_HUM	11	1.002303
24H 10MTA	P20340	0.104901	0.013565	0.014003	RAB6A	Ras-related	RAB6A_HU	11	1.07542
48H 10MTA	Q14677	0.044271	0.013564	0.014003	CLINT1	Clathrin int	EPN4_HUM	19	1.031162
24H 50MTA	O96018	1.818574	0.013564	0.014003	APBA3	Amyloid-be	APBA3_HU	1	3.527325
48H 50MTA	Q14165	0.203171	0.013566	0.014004	MLEC	Malectin	MLEC_HUM	10	1.151226
24H 10MTA	P05141	0.048994	0.013574	0.01401	SLC25A5	ADP/ATP tr	ADT2_HUM	20	1.034543
24H 50MTA	Q9UHR4	-0.08742	0.013578	0.014013	BAIAP2L1	BAR/IMD d	BI2L1_HUM	19	0.941204
24H 10MTA	Q6NXT1	-0.30316	0.013586	0.014018	ANKRD54	Ankyrin rep	ANR54_HU	4	0.810477
48H 10MTA	Q96MX6	0.003286	0.013596	0.014026	DNAAF10	Dynein axo	DAA10_HU	10	1.00228
48H 50MTA	O43747	0.037062	0.013599	0.014029	AP1G1	AP-1 compl	AP1G1_HU	28	1.026022
48H 10MTA	Q9BQ24	0.203019	0.013603	0.014032	ZFYVE21	Zinc finger	ZFY21_HUM	1	1.151105
24H 10MTA	P41252	0.030463	0.013617	0.014042	IARS1	Isoleucine-	SYIC_HUMA	60	1.02134
48H 50MTA	Q96HY6	0.004712	0.013625	0.014048	DDRGK1	DDRGK dor	DDRGK_HU	8	1.003271
48H 50MTA	Q8N6T7	-0.59777	0.013627	0.014049	SIRT6	NAD-depen	SIR6_HUMA	3	0.660777
48H 50MTA	Q9P0S2	0.207104	0.013634	0.014054	COX16	Cytochrom	COX16_HU	2	1.154369
48H 10MTA	Q9Y6N7	0.044084	0.013637	0.014057	ROBO1	Roundabou	ROBO1_HU	39	1.031028
24H 50MTA	P20339	0.054465	0.01364	0.014059	RAB5A	Ras-related	RAB5A_HU	8	1.038474

48H 10MT/	P37198	0.038925	0.01365	0.014066	NUP62	Nuclear po	NUP62_HU	11	1.027348
48H 50MT/	P08708	0.156553	0.013651	0.014067	RPS17	Small ribos	RS17_HUM	9	1.114621
24H 50MT/	Q15723	-1.40645	0.013652	0.014067	ELF2	ETS-related	ELF2_HUM/	1	0.37724
48H 50MT/	P98160	0.548468	0.013655	0.014068	HSPG2	Basement n	PGBM_HUI	19	1.462532
24H 50MT/	P37840	0.096938	0.013658	0.014069	SNCA	Alpha-synu	SYUA_HUM	6	1.069501
48H 50MT/	P61024	-0.24307	0.013662	0.014072	CKS1B	Cyclin-depe	CKS1_HUM	3	0.844944
48H 50MT/	Q3SXM5	-0.0332	0.013668	0.014077	HSDL1	Inactive hy	HSDL1_HU	7	0.977247
48H 50MT/	P61163	0.06173	0.013681	0.014083	ACTR1A	Alpha-centr	ACTZ_HUM	10	1.043716
48H 50MT/	Q9BXB4	-0.42071	0.013683	0.014084	OSBPL11	Oxysterol-b	OSB11_HU	12	0.747059
48H 10MT/	Q9BXC9	1.325102	0.013684	0.014084	BBS2	Bardet-Biec	BBS2_HUM	1	2.505507
48H 50MT/	Q8N999	-0.45651	0.013688	0.014087	RLIG1	RNA ligase :	RLIG1_HUN	6	0.728748
48H 50MT/	Q8WUX9	-0.11255	0.013691	0.014089	CHMP7	Charged mu	CHMP7_HU	9	0.924949
24H 50MT/	Q96GK7	0.180614	0.01369	0.014089	FAHD2A	Oxaloaceta	FAH2A_HU	10	1.133366
48H 50MT/	Q63HN8	0.082568	0.013691	0.014089	RNF213	E3 ubiquiti	RN213_HU	88	1.058901
24H 50MT/	Q9BTE3	-0.15322	0.013696	0.014092	MCMBP	Mini-chron	MCMBP_HU	26	0.89924
24H 10MT/	Q9BW92	-0.01125	0.013699	0.014094	TARS2	Threonine--	SYTM_HUM	20	0.992231
24H 10MT/	Q9NY26	0.289788	0.0137	0.014094	SLC39A1	Zinc transp	S39A1_HUI	1	1.222461
48H 50MT/	Q32P41	0.106267	0.01371	0.014102	TRMT5	tRNA (guan	TRM5_HUM	14	1.076439
24H 50MT/	Q99567	0.016737	0.01371	0.014102	NUP88	Nuclear po	NUP88_HU	20	1.011669
24H 10MT/	Q9H6K5	0.014621	0.01371	0.014102	PRR36	Proline-rich	PRR36_HUI	7	1.010186
48H 10MT/	O00629	0.026237	0.013719	0.014106	KPNA4	Importin su	IMA3_HUM	12	1.018353
24H 10MT/	P23396	0.013716	0.013718	0.014106	RPS3	Small ribos	RS3_HUMA	19	1.009553
24H 50MT/	P28347	-0.27705	0.013719	0.014106	TEAD1	Transcripti	TEAD1_HUI	4	0.825278
48H 10MT/	P69905	0.729565	0.01372	0.014106	HBA1	Hemoglobi	HBA_HUM/	5	1.658139
24H 50MT/	Q86X55	-0.02978	0.013723	0.014108	CARM1	Histone-arg	CARM1_HU	14	0.97957
48H 50MT/	Q9BXP2	0.129898	0.013723	0.014108	SLC12A9	Solute carri	S12A9_HUI	7	1.094216
48H 50MT/	Q6PD62	-0.11479	0.013735	0.014119	CTR9	RNA polym	CTR9_HUM	29	0.923514
24H 10MT/	P54577	-0.02857	0.013741	0.014124	YARS1	Tyrosine--t	SYYC_HUM/	36	0.980392
48H 10MT/	Q96NB2	0.123481	0.013754	0.014134	SFXN2	Sideroflexi	SFXN2_HUI	8	1.08936
24H 50MT/	Q9NVI7	0.004182	0.013763	0.014141	ATAD3A	ATPase fam	ATD3A_HUI	24	1.002903
24H 50MT/	Q5H8A4	0.211911	0.013766	0.014142	PIGG	GPI ethano	PIGG_HUM	6	1.158221
48H 10MT/	P50991	0.050438	0.013769	0.014145	CCT4	T-complex	TCPD_HUM	29	1.035579
24H 10MT/	P48739	-0.09358	0.013771	0.014146	PITPNB	Phosphatid	PIPNB_HUI	21	0.937191
24H 10MT/	Q9ULG6	0.015997	0.013773	0.014148	CCPG1	Cell cycle p	CCPG1_HU	4	1.01115
24H 50MT/	Q8N5L8	-0.30987	0.013774	0.014148	RPP25L	Ribonuclea	RP25L_HUI	1	0.806712
48H 10MT/	O00522	-0.05827	0.013787	0.014157	KRIT1	Krev intera	KRIT1_HUN	2	0.960414
24H 50MT/	Q8WXB1	0.07455	0.013787	0.014157	METTL21A	Protein N-l	MT21A_HU	3	1.053032
48H 10MT/	Q9HBG6	-0.0417	0.013786	0.014157	IFT122	Intraflagell	IF122_HUN	5	0.971508
24H 50MT/	Q9Y5B6	0.217997	0.013791	0.01416	PAXBP1	PAX3- and I	PAXB1_HUI	15	1.163118
48H 50MT/	P06213	0.086304	0.013793	0.014161	INSR	Insulin rece	INSR_HUM/	20	1.061647
24H 50MT/	Q9UIJ7	-0.04096	0.013804	0.014167	AK3	GTP:AMP p	KAD3_HUM	7	0.97201
24H 50MT/	A7E2V4	-0.19413	0.013807	0.01417	ZSWIM8	Zinc finger	ZSWM8_HU	9	0.874099
24H 10MT/	Q53EP0	0.037681	0.013816	0.014174	FNDC3B	Fibronectir	FND3B_HU	33	1.026463
48H 10MT/	P42345	0.009332	0.013832	0.014185	MTOR	Serine/thre	MTOR_HUN	62	1.006489
24H 50MT/	O00625	-0.13351	0.013846	0.014195	PIR	Pirin	PIR_HUMAI	13	0.911608
48H 50MT/	Q8NHP8	0.000496	0.013849	0.014198	PLBD2	Putative ph	PLBL2_HUN	10	1.000344
24H 50MT/	Q9GZT8	-0.15163	0.013863	0.014208	NIF3L1	NIF3-like pı	NIF3L_HUN	12	0.900232
48H 50MT/	Q9C0B1	0.053851	0.013866	0.014208	FTO	Alpha-keto	FTO_HUMA	13	1.038032
24H 50MT/	Q9UBS4	0.025961	0.013865	0.014208	DNAJB11	DnaJ homo	DJB11_HUI	15	1.018158
48H 10MT/	Q96A33	0.02952	0.013868	0.01421	CCDC47	PAT comple	CCD47_HU	20	1.020672
24H 50MT/	Q9UGM6	0.105742	0.01387	0.014211	WARS2	Tryptophar	SYWM_HUI	8	1.076047
24H 10MT/	P14866	0.071265	0.013873	0.014213	HNRNPL	Heterogene	HNRPL_HU	23	1.050637

24H 10MTA	Q9Y5Q9	0.085219	0.013874	0.014214	GTF3C3	General tra	TF3C3_HUI	24	1.060848
24H 10MTA	O43490	0.108773	0.013881	0.014219	PROM1	Prominin-1	PROM1_HU	21	1.078311
24H 10MTA	P63173	0.094724	0.013892	0.014229	RPL38	Large ribos	RL38_HUM	6	1.067861
48H 50MTA	Q13561	-0.01242	0.013893	0.01423	DCTN2	Dynactin su	DCTN2_HU	21	0.991425
24H 10MTA	Q9HAV7	-0.3179	0.013907	0.014241	GRPEL1	GrpE protei	GRPE1_HUI	8	0.802235
24H 50MTA	Q9BVJ6	0.033355	0.013909	0.014242	UTP14A	U3 small nu	UT14A_HU	18	1.023389
48H 10MTA	O14925	0.145306	0.013926	0.014253	TIMM23	Mitochond	TIM23_HUI	7	1.105965
24H 50MTA	P40616	0.120226	0.01394	0.014264	ARL1	ADP-ribosy	ARL1_HUM	10	1.086905
48H 50MTA	Q6P4F7	-0.68796	0.01394	0.014265	ARHGAP11	Rho GTPase	RHGBA_HU	4	0.620731
48H 10MTA	P02042	0.167295	0.013943	0.014266	HBD	Hemoglobi	HBD_HUMA	3	1.122951
24H 50MTA	Q9UGU0	0.359731	0.013961	0.01428	TCF20	Transcripti	TCF20_HUI	8	1.283187
48H 10MTA	Q9H3K2	0.076588	0.013967	0.014282	GHITM	Growth ho	GHITM_HU	8	1.054521
48H 50MTA	Q2T9J0	0.088637	0.013988	0.014299	TYSND1	Peroxisoma	TYSD1_HUI	15	1.063365
48H 50MTA	Q14160	-0.11497	0.013992	0.014302	SCRIB	Protein scri	SCRIB_HUM	40	0.923404
48H 10MTA	O14497	0.058094	0.014012	0.014315	ARID1A	AT-rich inte	ARI1A_HUM	34	1.041089
48H 10MTA	Q13363	-0.08772	0.014012	0.014315	CTBP1	C-terminal-	CTBP1_HUI	14	0.94101
48H 10MTA	P30153	0.032867	0.014017	0.014318	PPP2R1A	Serine/thre	2AAA_HUM	28	1.023043
24H 10MTA	O43676	0.244787	0.014022	0.014322	NDUFB3	NADH dehy	NDUB3_HU	3	1.184918
48H 50MTA	Q9H5Q4	-0.2403	0.014041	0.014336	TFB2M	Dimethylac	TFB2M_HU	19	0.846571
24H 50MTA	Q5M7Z0	0.263363	0.014054	0.014346	RNFT1	E3 ubiquiti	RNFT1_HUI	4	1.200274
24H 10MTA	Q9UEY8	0.105883	0.014061	0.01435	ADD3	Gamma-add	ADDG_HUM	16	1.076153
24H 10MTA	Q9Y2X9	0.107638	0.014061	0.01435	ZNF281	Zinc finger	ZN281_HUI	19	1.077463
24H 10MTA	Q9NVA4	-2.07684	0.014071	0.014357	TMEM184C	Transmemb	T184C_HUI	1	0.237032
48H 50MTA	Q9Y241	-0.24535	0.014071	0.014357	HIGD1A	HIG1 doma	HIG1A_HUI	3	0.843614
24H 10MTA	Q9HCL2	-1.09468	0.014075	0.01436	GPAM	Glycerol-3-	GPAT1_HUI	4	0.468241
24H 50MTA	Q16790	0.854775	0.014077	0.014361	CA9	Carbonic ar	CAH9_HUM	3	1.808476
24H 50MTA	P11172	-0.02793	0.014091	0.014372	UMPS	Uridine 5'-r	UMPS_HUM	24	0.980826
24H 50MTA	P55212	0.024481	0.014098	0.014378	CASP6	Caspase-6	CASP6_HUI	9	1.017114
24H 50MTA	Q96BW5	0.115886	0.0141	0.014379	PTER	Phosphotri	PTER_HUM	12	1.083641
48H 50MTA	Q9H9Y2	-0.24693	0.014104	0.014383	RPF1	Ribosome p	RPF1_HUM	10	0.842687
24H 50MTA	O94864	0.368198	0.014122	0.014397	SUPT7L	STAGA com	ST65G_HUI	2	1.29074
24H 10MTA	Q01658	0.153914	0.014122	0.014397	DR1	Protein Dr1	NC2B_HUM	6	1.112583
24H 50MTA	O00592	0.213825	0.01413	0.014404	PODXL	Podocalyxi	PODXL_HU	3	1.159759
24H 50MTA	Q9H9A6	-0.11095	0.014132	0.014405	LRRC40	Leucine-ric	LRC40_HUI	27	0.925976
24H 50MTA	Q9NVX2	0.066054	0.014137	0.01441	NLE1	Notchless p	NLE1_HUM	15	1.046849
48H 10MTA	O00217	0.017726	0.014166	0.014434	NDUFS8	NADH dehy	NDUS8_HU	6	1.012363
24H 10MTA	P11586	-0.07546	0.014168	0.014434	MTHFD1	C-1-tetrahy	C1TC_HUM	48	0.94904
24H 50MTA	Q9NV70	0.107071	0.014167	0.014434	EXOC1	Exocyst cor	EXOC1_HUI	19	1.077039
48H 50MTA	Q96FZ2	-0.51505	0.014171	0.014436	HMCES	Abasic site	HMCES_HU	11	0.699766
24H 10MTA	Q92522	0.166914	0.014179	0.01444	H1-10	Histone H1	H1X_HUMA	6	1.122655
48H 10MTA	Q16576	-0.25025	0.014179	0.01444	RBBP7	Histone-bir	RBBP7_HUI	12	0.840753
24H 10MTA	Q9GZU7	0.081967	0.014182	0.014443	CTDSP1	Carboxy-ter	CTDS1_HUI	3	1.05846
24H 10MTA	Q99543	0.009573	0.014185	0.014445	DNAJC2	DnaJ homo	DNJC2_HUI	21	1.006657
48H 50MTA	O95166	-0.23198	0.014191	0.014449	GABARAP	Gamma-am	GBRAP_HU	3	0.851465
24H 50MTA	Q96D42	2.345426	0.0142	0.014454	HAVCR1	Hepatitis A	HAVR1_HU	3	5.082105
24H 50MTA	Q7L099	0.207198	0.014203	0.014457	RUFY3	Protein RUI	RUFY3_HUI	6	1.154444
24H 50MTA	Q16629	0.162229	0.014212	0.014465	SRSF7	Serine/argi	SRSF7_HUM	6	1.119014
24H 50MTA	Q13425	0.105696	0.014218	0.014469	SNTB2	Beta-2-synt	SNTB2_HUI	13	1.076013
24H 50MTA	Q14739	0.132396	0.014226	0.014476	LBR	Delta(14)-st	LBR_HUMA	14	1.096113
48H 50MTA	Q96S55	0.2186	0.014226	0.014476	WRNIP1	ATPase WRI	WRIP1_HU	12	1.163604
24H 50MTA	Q9H4M9	-0.11121	0.014248	0.014493	EHD1	EH domain-	EHD1_HUM	24	0.925812
48H 50MTA	Q7Z2W9	-0.18053	0.01426	0.014502	MRPL21	Large ribos	RM21_HUM	10	0.88238

48H 10MTA	Q9P2K3	0.054839	0.014261	0.014502	RCOR3	REST corep	RCOR3_HU	10	1.038743
48H 10MTA	Q9Y3D5	0.374164	0.014262	0.014503	MRPS18C	Small ribos	RT18C_HU	3	1.296089
24H 10MTA	Q14919	0.19686	0.014264	0.014504	DRAP1	Dr1-associa	NC2A_HUM	6	1.146201
48H 50MTA	Q8IWT0	0.005063	0.014266	0.014505	ZBTB8OS	Protein arc	ARCH_HUM	2	1.003516
48H 50MTA	Q96GX9	0.311836	0.014275	0.014511	APIP	Methylthio	MTNB_HUM	8	1.241286
48H 50MTA	B5MCY1	-0.26078	0.014278	0.014513	TDRD15	Tudor dom	TDR15_HU	2	0.834638
48H 10MTA	P14923	0.041227	0.014292	0.014523	JUP	Junction pl	PLAK_HUM	30	1.028989
24H 50MTA	Q13129	0.197093	0.014291	0.014523	RLF	Zinc finger	RLF_HUMA	34	1.146386
24H 50MTA	P35575	0.454771	0.014303	0.01453	G6PC1	Glucose-6-p	G6PC1_HU	3	1.370566
48H 50MTA	Q9Y2K7	-0.30167	0.014303	0.01453	KDM2A	Lysine-spec	KDM2A_HU	26	0.811312
48H 50MTA	P53611	-0.12058	0.014307	0.014532	RABGGTB	Geranylgera	PGTB2_HU	14	0.919819
24H 10MTA	P55327	-0.07974	0.014321	0.014543	TPD52	Tumor prot	TPD52_HU	9	0.946225
48H 50MTA	P13716	0.10096	0.014332	0.014551	ALAD	Delta-amin	HEM2_HUM	8	1.072487
24H 50MTA	Q96I59	0.102913	0.014334	0.014552	NARS2	Asparaginyl	SYNM_HUM	15	1.073939
24H 50MTA	Q9BTC0	0.095386	0.014345	0.014559	DIDO1	Death-indu	DIDO1_HU	37	1.068352
24H 50MTA	Q13405	-0.16816	0.014347	0.014561	MRPL49	Large ribos	RM49_HUM	7	0.889977
24H 10MTA	P21333	0.048963	0.014351	0.014563	FLNA	Filamin-A	FLNA_HUM	104	1.034521
24H 50MTA	Q13325	0.193511	0.014351	0.014563	IFIT5	Interferon-i	IFIT5_HUM	12	1.143543
24H 50MTA	Q8IXB1	0.042446	0.014351	0.014563	DNAJC10	DnaJ homo	DJC10_HU	21	1.029858
48H 10MTA	Q04206	0.080026	0.014358	0.014568	RELA	Transcripti	TF65_HUM	15	1.057037
48H 50MTA	Q14684	-0.08436	0.014363	0.014571	RRP1B	Ribosomal	RRP1B_HU	30	0.943204
24H 50MTA	Q9NX62	0.169762	0.014363	0.014571	BPNT2	Golgi-resid	IMPA3_HU	12	1.124873
24H 10MTA	O75436	0.087976	0.014365	0.014573	VPS26A	Vacuolar pr	VP26A_HU	20	1.062878
48H 10MTA	Q9Y297	-0.1162	0.014378	0.014583	BTRC	F-box/WD r	FBW1A_HU	5	0.922615
24H 50MTA	Q8ND76	0.151827	0.014379	0.014583	CCNY	Cyclin-Y	CCNY_HUM	1	1.110976
24H 10MTA	Q15643	0.050022	0.014389	0.01459	TRIP11	Thyroid rec	TRIPB_HUM	35	1.035281
48H 50MTA	Q02878	-0.2076	0.014393	0.014592	RPL6	Large ribos	RL6_HUMA	14	0.865979
48H 10MTA	P19525	0.02142	0.014408	0.014603	EIF2AK2	Interferon-i	E2AK2_HU	18	1.014958
48H 10MTA	Q8IX12	0.032931	0.014408	0.014603	CCAR1	Cell divisio	CCAR1_HU	30	1.023088
24H 50MTA	Q9HBM0	0.266686	0.014416	0.014609	VEZT	Vezatin	VEZA_HUM	3	1.203041
24H 50MTA	Q9P2N5	0.143179	0.014416	0.014609	RBM27	RNA-bindir	RBM27_HU	25	1.104336
48H 10MTA	Q15459	0.029852	0.014418	0.01461	SF3A1	Splicing fac	SF3A1_HU	34	1.020907
24H 50MTA	Q9Y5A9	-0.05438	0.014421	0.014612	YTHDF2	YTH domai	YTHD2_HU	10	0.963006
48H 50MTA	Q96SB3	0.084257	0.014426	0.014615	PPP1R9B	Neurabin-2	NEB2_HUM	13	1.060141
24H 10MTA	Q2TAY7	0.03568	0.014428	0.014616	SMU1	WD40 repe	SMU1_HUM	20	1.02504
24H 10MTA	P15514	0.340721	0.014437	0.014623	AREG	Amphiregu	AREG_HUM	3	1.266389
24H 50MTA	Q9Y5U8	0.984436	0.014452	0.014635	MPC1	Mitochond	MPC1_HUM	2	1.97854
48H 10MTA	Q6P587	-0.09229	0.014455	0.014637	FAHD1	Oxaloaceta	FAHD1_HU	11	0.938035
48H 10MTA	O75400	0.069328	0.014463	0.014642	PRPF40A	Pre-mRNA-p	PR40A_HU	18	1.049228
48H 50MTA	P42025	0.084415	0.014463	0.014642	ACTR1B	Beta-centra	ACTY_HUM	15	1.060258
24H 50MTA	P00505	0.028604	0.014466	0.014644	GOT2	Aspartate a	AATM_HUM	26	1.020025
24H 10MTA	P50416	0.035112	0.014473	0.014649	CPT1A	Carnitine O	CPT1A_HU	28	1.024636
48H 50MTA	P20226	0.12208	0.014474	0.01465	TBP	TATA-box-b	TBP_HUMA	5	1.088303
24H 10MTA	Q8NHP8	0.031991	0.014474	0.01465	PLBD2	Putative ph	PLBL2_HUM	10	1.022422
24H 50MTA	P85298	1.160221	0.014484	0.014658	ARHGAP8	Rho GTPase	RHG08_HU	1	2.234916
24H 50MTA	Q96QC0	-0.06406	0.01449	0.014661	PPP1R10	Serine/thre	PP1RA_HU	22	0.956569
24H 10MTA	Q9BY50	-0.22013	0.014489	0.014661	SEC11C	Signal pept	SC11C_HU	5	0.858487
48H 10MTA	Q24JP5	0.733252	0.01449	0.014661	TMEM132A	Transmemb	T132A_HU	1	1.662382
24H 50MTA	Q9ULU4	0.20083	0.014491	0.014661	ZMYND8	MYND-type	ZMYD8_HU	23	1.149359
24H 10MTA	Q99700	-0.07755	0.014494	0.014663	ATXN2	Ataxin-2	ATX2_HUM	25	0.947666
48H 50MTA	Q8N8N7	0.244014	0.014497	0.014665	PTGR2	Prostagland	PTGR2_HU	9	1.184283
48H 50MTA	Q9P2I0	-0.02021	0.014498	0.014666	CPSF2	Cleavage ar	CPSF2_HUM	31	0.986087

48H 50MT/	Q9BRL6	0.486414	0.014499	0.014666	SRSF8	Serine/argi	SRSF8_HUN	1	1.400958
48H 10MT/	Q01415	-0.00805	0.01451	0.014675	GALK2	N-acetylgal	GALK2_HUI	10	0.994437
24H 10MT/	Q6P1J9	0.074278	0.014514	0.014677	CDC73	Parafibrom	CDC73_HU	27	1.052834
24H 10MT/	P02666	-0.20224	0.01452	0.014682	CSN2	Beta-casein	CASB_BOVI	4	0.869199
24H 50MT/	P15586	0.06476	0.01452	0.014682	GNS	N-acetylglu	GNS_HUM/	12	1.045911
24H 10MT/	O75390	0.086818	0.014526	0.014685	CS	Citrate synt	CISY_HUM/	18	1.062025
48H 10MT/	Q9Y6M9	0.008678	0.014524	0.014685	NDUFB9	NADH dehy	NDUB9_HU	11	1.006033
48H 10MT/	O15067	-0.00509	0.014527	0.014686	PFAS	Phosphorib	PUR4_HUN	39	0.996477
24H 10MT/	O95786	-0.119	0.014529	0.014687	RIGI	Antiviral in	RIGI_HUM/	16	0.920823
24H 10MT/	Q6RW13	0.02811	0.014544	0.014697	AGTRAP	Type-1 angi	ATRAP_HUI	4	1.019676
48H 10MT/	P42224	0.058257	0.014558	0.014708	STAT1	Signal trans	STAT1_HUN	37	1.041207
24H 50MT/	P46937	-0.23733	0.014558	0.014708	YAP1	Transcripti	YAP1_HUM	8	0.848317
48H 50MT/	O14763	-0.21876	0.014561	0.014708	TNFRSF10B	Tumor necr	TR10B_HUI	1	0.859304
24H 50MT/	Q15796	-0.18171	0.014563	0.014708	SMAD2	Mothers ag	SMAD2_HU	12	0.881659
48H 50MT/	Q86TX2	0.076624	0.014564	0.014708	ACOT1	Acyl-coenzy	ACOT1_HUI	19	1.054547
24H 50MT/	Q99497	-0.03572	0.01457	0.014713	PARK7	Parkinson c	PARK7_HUI	16	0.975545
48H 10MT/	P07942	0.053173	0.014581	0.01472	LAMB1	Laminin sul	LAMB1_HU	43	1.037544
48H 10MT/	P46013	0.001103	0.014581	0.01472	MKI67	Proliferatic	KI67_HUM/	119	1.000765
48H 50MT/	O60942	0.062744	0.014589	0.014726	RNGTT	mRNA-capp	MCE1_HUN	11	1.04445
48H 50MT/	P11498	0.062438	0.014593	0.014728	PC	Pyruvate ca	PYC_HUMA	45	1.044229
24H 50MT/	Q9UKS6	-0.08765	0.014592	0.014728	PACSIN3	Protein kin	PACN3_HU	15	0.941052
48H 50MT/	Q9Y584	-0.11226	0.0146	0.014733	TIMM22	Mitochond	TIM22_HUI	2	0.925138
48H 10MT/	Q8WWI1	0.019708	0.014617	0.014747	LMO7	LIM domair	LMO7_HUN	44	1.013755
48H 10MT/	Q8NBN3	0.147545	0.014618	0.014747	TMEM87A	Transmemk	TM87A_HU	10	1.107683
24H 50MT/	O75128	-0.31067	0.01463	0.014757	COBL	Protein cor	COBL_HUM	8	0.806269
48H 50MT/	Q13131	0.050827	0.014631	0.014757	PRKAA1	5'-AMP-acti	AAPK1_HUI	17	1.035859
24H 50MT/	P82675	-0.0021	0.014634	0.014759	MRPS5	Small ribos	RT05_HUM	13	0.998543
24H 10MT/	Q13206	0.082127	0.014647	0.014767	DDX10	Probable A	DDX10_HU	30	1.058577
24H 50MT/	Q92537	-0.50682	0.014656	0.014773	SUSD6	Sushi doma	SUSD6_HUI	1	0.703773
48H 50MT/	Q10472	0.067974	0.014664	0.01478	GALNT1	Polypeptid	GALT1_HUI	14	1.048243
48H 50MT/	Q9Y6G3	-0.40757	0.014665	0.01478	MRPL42	Large ribos	RM42_HUN	4	0.75389
48H 10MT/	Q8N5I9	-0.45833	0.014666	0.01478	NOPCHAP1	NOP protei	NOPC1_HU	2	0.727827
24H 10MT/	Q13813	0.013098	0.014671	0.014784	SPTAN1	Spectrin al	SPTN1_HUI	146	1.00912
48H 50MT/	P62913	-0.22504	0.014679	0.014789	RPL11	Large ribos	RL11_HUM	10	0.855572
48H 50MT/	P52294	0.065429	0.014685	0.014794	KPNA1	Importin su	IMA5_HUM	8	1.046396
24H 50MT/	Q9H0M0	1.20064	0.014689	0.014797	WWP1	NEDD4-like	WWP1_HU	2	2.298415
24H 50MT/	O75323	0.090187	0.014692	0.0148	NIPSNAP2	Protein Nip	NIPS2_HUN	7	1.064508
48H 50MT/	P47897	-0.08694	0.014698	0.014804	QARS1	Glutamine-	SYQ_HUMA	41	0.941518
24H 50MT/	Q16658	0.037772	0.014705	0.01481	FSCN1	Fascin	FSCN1_HUI	19	1.026527
24H 50MT/	P19838	-0.13432	0.014722	0.014823	NFKB1	Nuclear fac	NFKB1_HUI	18	0.911101
24H 50MT/	P52435;Q9	-0.35763	0.014733	0.014831	POLR2J;PO	DNA-direct	RPB11_HUI	3	0.780444
24H 10MT/	P04004	0.175217	0.014736	0.014833	VTN	Vitronectir	VTNC_HUM	9	1.129134
24H 50MT/	Q13884	0.067439	0.01474	0.014836	SNTB1	Beta-1-synt	SNTB1_HUI	18	1.047855
24H 50MT/	O43676	0.357417	0.014745	0.01484	NDUFB3	NADH dehy	NDUB3_HU	3	1.28113
48H 10MT/	Q13185	0.078876	0.01475	0.014844	CBX3	Chromobo:	CBX3_HUM	8	1.056195
48H 50MT/	Q96EZ8	-0.35207	0.014757	0.01485	MCRS1	Microspher	MCRS1_HU	3	0.783457
24H 50MT/	Q7Z7G8	0.239368	0.01476	0.014852	VPS13B	Intermemb	VP13B_HUI	7	1.180476
24H 50MT/	Q96K76	-0.12946	0.01476	0.014852	USP47	Ubiquitin c	UBP47_HU	34	0.914174
24H 50MT/	P19338	0.118991	0.014766	0.014856	NCL	Nucleolin	NUCL_HUN	27	1.085975
48H 50MT/	Q9NQ50	-0.18906	0.014771	0.01486	MRPL40	Large ribos	RM40_HUN	7	0.877175
24H 50MT/	Q96PU8	-0.05152	0.014776	0.014864	QKI	KH domain	QKI_HUMA	12	0.964922
48H 50MT/	P38919	0.023471	0.014791	0.014877	EIF4A3	Eukaryotic	IF4A3_HUN	24	1.016402

24H 10MT/	Q9H977	-0.01088	0.014792	0.014878	WDR54	WD repeat-	WDR54_HU	3	0.992486
24H 50MT/	Q8N138	-0.16119	0.014798	0.01488	ORMDL3	ORM1-like	ORML3_HU	1	0.894289
48H 50MT/	Q96PC5	0.106099	0.014811	0.01489	MIA2	Melanoma	MIA2_HUM	27	1.076314
24H 50MT/	Q9UHB9	0.040977	0.014811	0.01489	SRP68	Signal reco	SRP68_HU	31	1.02881
24H 10MT/	Q96AT9	-0.20604	0.014816	0.014893	RPE	Ribulose-pl	RPE_HUMA	7	0.866915
24H 10MT/	Q9UNH6	0.10401	0.014817	0.014894	SNX7	Sorting nex	SNX7_HUM	14	1.074757
24H 50MT/	P26358	-0.09553	0.014823	0.014898	DNMT1	DNA (cytosi	DNMT1_HU	51	0.935927
24H 50MT/	O00764	-0.15131	0.01483	0.014905	PDXK	Pyridoxal k	PDXK_HUM	16	0.900431
24H 10MT/	Q6JQN1	0.062755	0.014839	0.014912	ACAD10	Acyl-CoA de	ACD10_HU	25	1.044459
24H 50MT/	P40692	0.093492	0.014844	0.014916	MLH1	DNA misma	MLH1_HUM	31	1.06695
24H 10MT/	Q8N7R7	0.359063	0.014846	0.014917	CCNYL1	Cyclin-Y-lik	CCYL1_HUM	5	1.282593
24H 50MT/	Q4VC31	0.065155	0.014848	0.014917	MIX23	Protein MI)	MIX23_HU	6	1.046197
24H 10MT/	Q9BUH6	0.031317	0.014849	0.014917	PAXX	Protein PA)	PAXX_HUM	9	1.021945
24H 50MT/	Q9HB19	1.241193	0.014852	0.014919	PLEKHA2	Pleckstrin I	PKHA2_HU	2	2.363939
24H 50MT/	O14787	-0.22582	0.014888	0.014948	TNPO2	Transportir	TNPO2_HU	14	0.855108
48H 50MT/	Q96CN7	0.048222	0.01489	0.014949	ISOC1	Isochorism	ISOC1_HUM	9	1.03399
24H 10MT/	Q9H6T3	0.008169	0.014895	0.014953	RPAP3	RNA polym	RPAP3_HU	19	1.005678
24H 10MT/	Q9NXJ5	0.007162	0.014899	0.014956	PGPEP1	Pyroglutan	PGPI_HUM	7	1.004977
24H 10MT/	Q9H0A8	-0.08044	0.014914	0.014966	COMMD4	COMM don	COMD4_HU	7	0.945772
48H 10MT/	O75832	0.064386	0.014915	0.014967	PSMD10	26S protea	PSD10_HU	9	1.04564
48H 10MT/	Q99460	0.038616	0.014916	0.014967	PSMD1	26S protea	PSMD1_HU	42	1.027128
24H 10MT/	Q96C90	-0.25563	0.014919	0.014967	PPP1R14B	Protein pho	PP14B_HU	6	0.83762
24H 50MT/	Q9H3P2	0.118436	0.014919	0.014967	NELFA	Negative el	NELFA_HUM	10	1.085558
24H 10MT/	Q9Y4K4	0.162055	0.014919	0.014967	MAP4K5	Mitogen-ac	M4K5_HUM	5	1.11888
48H 50MT/	Q68CQ7	0.282926	0.014925	0.014972	GLT8D1	Glycosyltra	GL8D1_HU	9	1.21666
24H 50MT/	Q8TDJ6	-0.17326	0.014968	0.015001	DMXL2	DmX-like pi	DMXL2_HU	15	0.886837
48H 10MT/	O94826	0.066334	0.01497	0.015003	TOMM70	Mitochond	TOM70_HU	26	1.047053
24H 50MT/	O15127	0.031616	0.014975	0.015007	SCAMP2	Secretory c	SCAM2_HU	3	1.022157
48H 50MT/	Q7Z406	0.113121	0.014982	0.015014	MYH14	Myosin-14	MYH14_HU	42	1.081565
24H 50MT/	Q7L266	0.115826	0.014988	0.015019	ASRGL1	Isoaspartyl	ASGL1_HU	7	1.083595
48H 50MT/	Q9NX58	-0.08367	0.01499	0.01502	LYAR	Cell growth	LYAR_HUM	11	0.943655
48H 10MT/	Q9Y5U8	-0.33741	0.014991	0.01502	MPC1	Mitochond	MPC1_HUM	2	0.791459
24H 50MT/	Q9H252	-0.00924	0.014998	0.015024	KCNH6	Potassium	KCNH6_HU	2	0.993614
48H 10MT/	Q5T8D3	0.105966	0.015003	0.015027	ACBD5	Acyl-CoA-bi	ACBD5_HU	13	1.076214
48H 10MT/	Q92900	0.040951	0.01501	0.015031	UPF1	Regulator c	RENT1_HU	51	1.028792
24H 50MT/	Q7KZF4	0.063253	0.015014	0.015035	SND1	Staphyloco	SND1_HUM	50	1.044819
48H 50MT/	Q13085	-0.11746	0.015021	0.01504	ACACA	Acetyl-CoA	ACACA_HU	82	0.921812
48H 10MT/	Q15438	0.415713	0.015036	0.015052	CYTH1	Cytohesin-1	CYH1_HUM	3	1.333958
48H 50MT/	Q6NZY4	0.13598	0.015037	0.015052	ZCCHC8	Zinc finger	ZCHC8_HU	19	1.098839
24H 50MT/	P31749	0.088853	0.015041	0.015055	AKT1	RAC-alpha s	AKT1_HUM	8	1.063524
24H 10MT/	O15228	0.061374	0.015043	0.015056	GNPAT	Dihydroxya	GNPAT_HU	26	1.043459
48H 50MT/	Q16836	0.130201	0.01505	0.015061	HADH	Hydroxyacy	HCDH_HUM	10	1.094446
48H 50MT/	Q7Z2K6	0.004068	0.015055	0.015065	ERMP1	Endoplasm	ERMP1_HU	17	1.002824
24H 50MT/	Q9NXR5	0.967821	0.015055	0.015065	ANKRD10	Ankyrin rep	ANR10_HU	2	1.955885
24H 10MT/	Q9H7H0	-0.45686	0.015062	0.01507	METTL17	Ribosome a	MET17_HU	5	0.728569
48H 50MT/	Q9H4K7	-0.5569	0.015065	0.015073	MTG2	Mitochond	MTG2_HUM	5	0.679761
48H 50MT/	Q9UQN3	0.227703	0.015079	0.015084	CHMP2B	Charged mu	CHM2B_HU	2	1.170969
24H 50MT/	P09496	-0.36036	0.01509	0.015094	CLTA	Clathrin lig	CLCA_HUM	5	0.778972
48H 10MT/	A3KN83	0.073537	0.015091	0.015094	SBNO1	Protein stra	SBNO1_HU	24	1.052293
48H 10MT/	Q9BW60	-0.05677	0.015095	0.015097	ELOVL1	Very long cl	ELOV1_HU	4	0.961411
24H 10MT/	Q8N8S7	0.015299	0.015097	0.015098	ENAH	Protein ena	ENAH_HUM	19	1.010661
48H 50MT/	O75477	0.166195	0.015103	0.015102	ERLIN1	Erlin-1	ERLN1_HU	10	1.122095

24H 50MTA	O96007	0.03403	0.015108	0.015106	MOCS2	Molybdopt	MOC2B_HU	4	1.023868
48H 10MTA	P54132	-0.26892	0.015115	0.015112	BLM	RecQ-like D	BLM_HUMA	11	0.82994
48H 10MTA	Q6SJ93	-0.23861	0.015124	0.015119	FAM111B	Serine prot	F111B_HUI	8	0.847559
24H 10MTA	P17480	-0.02332	0.015134	0.015126	UBTF	Nucleolar t	UBF1_HUM	23	0.983967
24H 50MTA	Q9BVS4	0.105029	0.015135	0.015127	RIOK2	Serine/thre	RIOK2_HUI	11	1.075516
24H 50MTA	O43291	0.080844	0.015147	0.015136	SPINT2	Kunitz-type	SPIT2_HUM	3	1.057637
48H 50MTA	Q6ICG6	-0.11082	0.015153	0.015141	KIAA0930	Uncharacte	K0930_HUI	8	0.926063
48H 50MTA	Q13547	0.058534	0.015161	0.015147	HDAC1	Histone dea	HDAC1_HU	12	1.041407
48H 50MTA	Q9P2J9	-0.90547	0.015161	0.015147	PDP2	[Pyruvate d	PDP2_HUM	2	0.53386
24H 10MTA	Q15173	1.404131	0.015168	0.015151	PPP2R5B	Serine/thre	2A5B_HUM	2	2.646583
24H 50MTA	O15061	-0.24325	0.015181	0.015158	SYNM	Synemin	SYNEM_HU	3	0.844839
24H 10MTA	Q9NQW7	-0.08556	0.015184	0.01516	XPNPEP1	Xaa-Pro am	XPP1_HUM	28	0.94242
24H 10MTA	Q8N573	0.392586	0.015196	0.01517	OXR1	Oxidation r	OXR1_HUM	2	1.312745
24H 10MTA	P60660	-0.18146	0.015196	0.01517	MYL6	Myosin ligh	MYL6_HUM	7	0.881809
24H 50MTA	Q15181	0.045246	0.015202	0.015175	PPA1	Inorganic p	IPYR_HUMA	23	1.031859
48H 50MTA	P43251	-0.21999	0.015203	0.015175	BTD	Biotinidase	BTD_HUMA	1	0.85857
48H 50MTA	Q9Y265	0.062916	0.015204	0.015175	RUVBL1	RuvB-like 1	RUVB1_HU	25	1.044575
24H 50MTA	Q96RN5	-0.31825	0.015206	0.015176	MED15	Mediator o	MED15_HU	9	0.802041
48H 10MTA	Q96BI1	0.107227	0.015218	0.015186	SLC22A18	Solute carri	S22AI_HUM	4	1.077156
48H 10MTA	Q9BSY4	0.184156	0.015218	0.015186	CHCHD5	Coiled-coil-	CHCH5_HU	1	1.136152
24H 10MTA	O43822	-0.01307	0.01523	0.015195	CFAP410	Cilia- and fl	CF410_HUI	1	0.990981
48H 50MTA	Q14019	0.122526	0.015235	0.0152	COTL1	Coactosin-l	COTL1_HUI	8	1.088639
24H 10MTA	Q96RS6	0.08657	0.015237	0.0152	NUDCD1	NudC doma	NUDC1_HU	23	1.061843
48H 10MTA	Q86YP4	0.038058	0.015247	0.015207	GATAD2A	Transcripti	P66A_HUM	22	1.026731
48H 10MTA	Q99442	-0.11079	0.015248	0.015208	SEC62	Translocati	SEC62_HUI	9	0.92608
48H 50MTA	O75569	0.002122	0.015249	0.015208	PRKRA	Interferon-i	PRKRA_HUI	12	1.001472
48H 50MTA	Q12789	-0.07201	0.015258	0.015216	GTF3C1	General tra	TF3C1_HUI	59	0.95131
24H 50MTA	O76031	0.025543	0.015269	0.015222	CLPX	ATP-depenc	CLPX_HUM	28	1.017863
24H 10MTA	Q5EBL8	0.014845	0.015274	0.015225	PDZD11	PDZ domai	PDZ11_HUI	3	1.010343
24H 50MTA	Q13496	0.10522	0.015288	0.015236	MTM1	Myotubula	MTM1_HUI	15	1.075658
24H 10MTA	P43307	0.096176	0.01529	0.015237	SSR1	Translocon	SSRA_HUM	6	1.068936
48H 10MTA	Q12800	-0.02688	0.0153	0.015243	TFCP2	Alpha-globi	TFCP2_HUI	9	0.981543
48H 50MTA	O94855	0.238482	0.015301	0.015243	SEC24D	Protein tra	SC24D_HUI	27	1.179751
48H 10MTA	P04844	-0.10513	0.015312	0.015253	RPN2	Dolichyl-di	RPN2_HUM	29	0.929719
24H 10MTA	P62081	0.103851	0.015321	0.015259	RPS7	Small ribos	RS7_HUMA	10	1.074638
48H 10MTA	P02649	0.051193	0.015326	0.015263	APOE	Apolipoprc	APOE_HUM	17	1.036122
48H 10MTA	Q9H8H0	-0.10438	0.015327	0.015263	NOL11	Nucleolar p	NOL11_HU	24	0.930205
24H 10MTA	Q9Y2V7	0.10917	0.015329	0.015264	COG6	Conserved	COG6_HUM	20	1.078607
48H 10MTA	Q06547	0.061204	0.015335	0.015268	GABPB1	GA-binding	GABP1_HU	1	1.043336
48H 50MTA	Q15424	0.093288	0.01534	0.015273	SAFB	Scaffold att	SAFB1_HUI	14	1.066798
48H 50MTA	P19174	0.0543	0.015342	0.015274	PLCG1	1-phosphat	PLCG1_HUI	24	1.038355
24H 50MTA	Q8TEM1	0.074298	0.015357	0.015286	NUP210	Nuclear po	PO210_HU	45	1.052848
24H 50MTA	Q9NYV4	0.160521	0.01536	0.015288	CDK12	Cyclin-depe	CDK12_HU	16	1.117691
24H 10MTA	Q13685	-0.09949	0.015374	0.015298	AAMP	Angio-assoc	AAMP_HUM	12	0.933361
48H 10MTA	Q8N6H7	0.087436	0.015384	0.015306	ARFGAP2	ADP-ribosy	ARFG2_HUI	16	1.062481
24H 50MTA	Q9P2J5	-0.00792	0.015392	0.015312	LARS1	Leucine--tR	SYLC_HUMA	55	0.994523
48H 10MTA	P26583	0.27233	0.015399	0.015316	HMGB2	High mobil	HMGB2_HU	7	1.207757
48H 50MTA	O14646	-0.19129	0.0154	0.015316	CHD1	Chromodo	CHD1_HUM	20	0.875822
24H 10MTA	Q9P0U4	0.081134	0.0154	0.015316	CXXC1	CXXC-type	CXXC1_HUI	12	1.05785
48H 10MTA	Q9UBQ0	-0.38627	0.015399	0.015316	VPS29	Vacuolar pi	VPS29_HUI	10	0.765103
24H 50MTA	O75955	0.084811	0.015406	0.015321	FLOT1	Flotillin-1	FLOT1_HUM	23	1.060549
48H 10MTA	Q9NPJ6	0.066223	0.01541	0.015324	MED4	Mediator o	MED4_HUM	9	1.046972

24H 50MT/	Q9Y6K1	0.473366	0.015424	0.015336	DNMT3A	DNA (cytosi	DNM3A_HU	5	1.388345
24H 50MT/	Q9BZD2	0.697381	0.015425	0.015336	SLC29A3	Equilibrativ	S29A3_HU	5	1.621558
24H 10MT/	Q9NRR5	-0.15605	0.015428	0.015338	UBQLN4	Ubiquilin-4	UBQL4_HU	9	0.897477
48H 50MT/	P49842	-0.13595	0.015432	0.01534	STK19	Inactive ser	STK19_HUM	3	0.910073
24H 50MT/	Q14166	-0.12125	0.015436	0.015343	TTLL12	Tubulin--ty	TTL12_HUM	28	0.91939
24H 50MT/	P53814	-0.46851	0.015444	0.015348	SMTN	Smoothelir	SMTN_HUM	13	0.722711
48H 10MT/	Q9H840	0.600595	0.015449	0.01535	GEMIN7	Gem-associ	GEMI7_HU	1	1.516342
24H 10MT/	P52434	0.037156	0.015455	0.015355	POLR2H	DNA-direct	RPAB3_HU	7	1.026089
48H 10MT/	Q9Y6M1	0.06531	0.015467	0.015364	IGF2BP2	Insulin-like	IF2B2_HUM	20	1.04631
24H 50MT/	P16144	-0.21172	0.015469	0.015365	ITGB4	Integrin be	ITB4_HUM/	14	0.863507
48H 50MT/	O95674	0.070469	0.015476	0.01537	CDS2	Phosphatic	CDS2_HUM	5	1.050058
48H 50MT/	P62491	0.017592	0.015487	0.015378	RAB11A	Ras-related	RB11A_HU	13	1.012268
48H 50MT/	Q4G0F5	0.056202	0.0155	0.015389	VPS26B	Vacuolar pr	VP26B_HU	10	1.039725
24H 10MT/	Q56P03	0.84095	0.015501	0.015389	EAPP	E2F-associa	EAPP_HUM	2	1.79123
24H 50MT/	Q15836	0.035411	0.015503	0.015391	VAMP3	Vesicle-ass	VAMP3_HU	5	1.024849
24H 50MT/	P27361	0.054179	0.01551	0.015395	MAPK3	Mitogen-ac	MK03_HUM	15	1.038268
48H 50MT/	Q9ULD4	-0.48096	0.015522	0.015405	BRPF3	Bromodom	BRPF3_HU	4	0.716502
24H 50MT/	O15042	0.067993	0.015527	0.015407	U2SURP	U2 snRNP-a	SR140_HU	29	1.048258
24H 50MT/	P00390	0.024603	0.01553	0.015409	GSR	Glutathion	GSHR_HUM	21	1.0172
48H 10MT/	P07339	0.057723	0.01553	0.015409	CTSD	Cathepsin I	CATD_HUM	16	1.040822
24H 50MT/	P49354	-0.17029	0.015543	0.015419	FNTA	Protein fari	FNTA_HUM	14	0.888664
24H 10MT/	Q9Y547	0.103571	0.01557	0.015439	IFT25	Intraflagell	IFT25_HUM	4	1.07443
48H 10MT/	O95628	0.038176	0.015577	0.015445	CNOT4	CCR4-NOT t	CNOT4_HU	4	1.026815
48H 50MT/	P50225	0.884745	0.015585	0.01545	SULT1A1	Sulfotransf	ST1A1_HUM	2	1.846438
48H 50MT/	O94766	-0.01786	0.015589	0.015452	B3GAT3	Galactosylg	B3GA3_HU	8	0.987696
48H 50MT/	Q15643	0.121846	0.015592	0.015454	TRIP11	Thyroid rec	TRIPB_HUM	35	1.088127
48H 50MT/	O15164	0.031961	0.015602	0.015463	TRIM24	Transcripti	TIF1A_HUM	16	1.022401
24H 10MT/	P20839	0.112463	0.015621	0.015478	IMPDH1	Inosine-5'-r	IMDH1_HU	5	1.081072
48H 50MT/	P53999	0.149537	0.015627	0.015482	SUB1	Activated R	TCP4_HUM	8	1.109214
24H 50MT/	Q96GA7	-0.05322	0.01563	0.015485	SDSL	Serine dehy	SDSL_HUM	12	0.963786
24H 50MT/	P30530	0.29841	0.015634	0.015487	AXL	Tyrosine-pr	UFO_HUM/	8	1.229788
48H 50MT/	Q8TB96	-0.32283	0.015641	0.015493	ITFG1	T-cell immu	TIP_HUMAI	6	0.799498
24H 50MT/	P34949	0.213008	0.015642	0.015493	MPI	Mannose-6	MPI_HUMA	15	1.159102
24H 50MT/	Q8IUI8	0.06949	0.015648	0.015497	CRLF3	Cytokine re	CRLF3_HUM	9	1.049346
48H 10MT/	P78504	0.018502	0.015652	0.0155	JAG1	Protein jag	JAG1_HUM	4	1.012907
48H 50MT/	Q9BXY0	-0.15092	0.015652	0.0155	MAK16	Protein MA	MAK16_HU	11	0.900678
24H 50MT/	P23229	0.060367	0.015654	0.0155	ITGA6	Integrin alp	ITA6_HUM/	24	1.042731
24H 50MT/	P28715	0.220792	0.015656	0.015502	ERCC5	DNA excisic	ERCC5_HU	12	1.165373
24H 50MT/	Q9H3F6	0.569159	0.015657	0.015503	KCTD10	BTB/POZ dc	BACD3_HU	2	1.483658
48H 10MT/	P04049	0.281341	0.015661	0.015505	RAF1	RAF proto-c	RAF1_HUM	16	1.215324
24H 10MT/	Q06830	-0.06071	0.015707	0.015543	PRDX1	Peroxiredo	PRDX1_HU	19	0.958792
48H 10MT/	Q8N0V3	-0.40178	0.015717	0.01555	RBFA	Putative rik	RBFA_HUM	2	0.756923
24H 10MT/	Q9BTW9	-0.05061	0.015719	0.015552	TBCD	Tubulin-spe	TBCD_HUM	34	0.965526
24H 50MT/	P51571	0.063097	0.015721	0.015553	SSR4	Translocon	SSRD_HUM	4	1.044706
24H 50MT/	Q7L4I2	0.239961	0.015728	0.015558	RSRC2	Arginine/se	RSRC2_HU	8	1.18096
48H 50MT/	Q14202	0.060054	0.015731	0.015559	ZMYM3	Zinc finger	ZMYM3_HU	34	1.042505
48H 50MT/	Q9BPX3	-0.13309	0.015733	0.01556	NCAPG	Condensin	CND3_HUM	27	0.911876
48H 10MT/	Q9NSB2	0.504671	0.015735	0.015561	KRT84	Keratin, typ	KRT84_HU	2	1.418799
48H 10MT/	Q10570	-0.01334	0.01574	0.015565	CPSF1	Cleavage ar	CPSF1_HUM	43	0.990798
48H 50MT/	O15460	0.697495	0.015751	0.015574	P4HA2	Prolyl 4-hy	P4HA2_HU	16	1.621687
48H 50MT/	Q13356	-0.40174	0.015753	0.015575	PPIL2	RING-type I	PPIL2_HUM	11	0.756947
24H 10MT/	P69905	0.640762	0.015754	0.015575	HBA1	Hemoglobi	HBA_HUM/	5	1.559153

48H	10MT/	Q9BR76	0.063606	0.015757	0.015576	CORO1B	Coronin-1E	COR1B_HU	17	1.045075
48H	10MT/	Q99666	-1.0786	0.015767	0.015585	RGPD5	RANBP2-lik	RGPD5_HU	2	0.473488
48H	50MT/	Q9Y6G5	-0.03594	0.015775	0.015591	COMMD10	COMM don	COMDA_HU	5	0.975399
48H	50MT/	Q9UPX8	0.062393	0.015779	0.015593	SHANK2	SH3 and mu	SHAN2_HU	6	1.044196
24H	10MT/	P27540	0.133476	0.015797	0.015607	ARNT	Aryl hydroc	ARNT_HUM	6	1.096933
48H	10MT/	P30047	0.122635	0.015798	0.015607	GCHFR	GTP cycloh	GFRP_HUM	5	1.088721
24H	50MT/	Q9NS23	-0.64643	0.0158	0.015608	RASSF1	Ras associat	RASF1_HUI	1	0.638861
48H	10MT/	P22570	0.00308	0.0158	0.015608	FDXR	NADPH:adr	ADRO_HUM	21	1.002137
48H	10MT/	O15211	-0.01408	0.015802	0.015608	RGL2	Ral guanine	RGL2_HUM	3	0.990287
24H	10MT/	P43007	0.217506	0.015802	0.015608	SLC1A4	Neutral am	SATT_HUM/	5	1.162722
24H	50MT/	O43795	-0.08833	0.015803	0.015608	MYO1B	Unconvent	MYO1B_HU	43	0.940611
48H	50MT/	Q14671	0.028065	0.01581	0.015614	PUM1	Pumilio ho	PUM1_HUI	25	1.019644
48H	50MT/	Q8TCE6	0.005996	0.015813	0.015615	DENND10	DENN dom	DEN10_HU	1	1.004165
48H	10MT/	O95182	0.000415	0.015819	0.015619	NDUFA7	NADH dehy	NDUA7_HU	6	1.000287
48H	50MT/	Q8IVT2	-0.31424	0.015821	0.01562	MISP	Mitotic int	MISP_HUM	8	0.804276
24H	10MT/	Q13952	0.152446	0.015836	0.015632	NFYC	Nuclear tra	NFYC_HUM	6	1.111453
48H	50MT/	Q03135	0.618451	0.015845	0.015638	CAV1	Caveolin-1	CAV1_HUM	3	1.535226
24H	50MT/	Q9NWU5	-0.00594	0.01585	0.015642	MRPL22	Large ribos	RM22_HUM	8	0.995891
48H	50MT/	O43865	0.065806	0.015854	0.015644	AHCYL1	S-adenosyll	SAHH2_HU	12	1.04667
48H	50MT/	Q9BZD2	-0.51143	0.015867	0.015654	SLC29A3	Equilibrativ	S29A3_HUI	5	0.701529
24H	10MT/	Q9Y6R0	-0.93878	0.015875	0.015659	NUMBL	Numb-like	NUMBL_HU	1	0.521673
48H	10MT/	P10809	-0.11799	0.015879	0.015661	HSPD1	60 kDa heat	CH60_HUM	49	0.921472
24H	50MT/	Q7Z5L2	0.240895	0.015879	0.015661	R3HCC1L	Coiled-coil	R3HCL_HUI	2	1.181726
24H	50MT/	O14727	0.211715	0.015896	0.015674	APAF1	Apoptotic	APAF_HUM	22	1.158064
24H	50MT/	Q96EK5	0.104542	0.0159	0.015677	KIFBP	KIF-binding	KBP_HUMA	23	1.075153
48H	50MT/	Q8N584	0.077964	0.015902	0.015678	TTC39C	Tetratricop	TT39C_HUI	18	1.055527
48H	50MT/	Q9BSD7	0.074284	0.015904	0.015679	NTPCR	Cancer-rela	NTPCR_HUI	10	1.052838
48H	10MT/	Q8N5G2	-0.15367	0.015912	0.015685	MACO1	Macoilin	MACOI_HU	3	0.898959
24H	50MT/	Q86YD1	-0.40883	0.015913	0.015686	PTOV1	Prostate tu	PTOV1_HUI	3	0.753232
48H	50MT/	Q9H7X7	-0.58927	0.015914	0.015686	IFT22	Intraflagell	IFT22_HUM	1	0.664679
24H	10MT/	Q96RN5	-0.31857	0.015915	0.015687	MED15	Mediator o	MED15_HU	9	0.801862
48H	10MT/	Q9P021	-0.42969	0.015917	0.015687	CRIPT	Cysteine-ric	CRIPT_HUM	2	0.742423
24H	10MT/	Q8TED0	0.101315	0.015926	0.015695	UTP15	U3 small nu	UTP15_HUI	25	1.07275
24H	50MT/	Q9Y5X1	0.006792	0.01593	0.015698	SNX9	Sorting nex	SNX9_HUM	18	1.004719
48H	50MT/	Q15392	0.066994	0.015946	0.015711	DHCR24	Delta(24)-st	DHC24_HU	17	1.047531
48H	50MT/	Q6ZS30	-0.0096	0.015947	0.015712	NBEAL1	Neurobeacl	NBEL1_HUI	28	0.993366
24H	10MT/	Q13547	0.054022	0.015961	0.015723	HDAC1	Histone dea	HDAC1_HU	12	1.038155
48H	10MT/	P34741	0.483041	0.015967	0.015729	SDC2	Syndecan-2	SDC2_HUM	2	1.397687
24H	10MT/	Q92896	-0.04392	0.015972	0.015731	GLG1	Golgi appar	GSLG1_HUI	40	0.970016
48H	50MT/	Q9Y6M5	-0.22978	0.015974	0.015732	SLC30A1	Proton-cou	ZNT1_HUM	12	0.852763
24H	10MT/	P62249	0.043069	0.016006	0.015758	RPS16	Small ribos	RS16_HUM	10	1.030304
48H	50MT/	O75330	-0.12098	0.016037	0.015783	HMMR	Hyaluronar	HMMR_HU	17	0.919564
48H	50MT/	Q9H2H8	-0.12402	0.01604	0.015786	PPIL3	Peptidyl-pr	PPIL3_HUM	7	0.917626
24H	10MT/	O95486	-0.08455	0.016067	0.015807	SEC24A	Protein trai	SC24A_HUI	27	0.943078
48H	50MT/	O43795	-0.0965	0.016083	0.015821	MYO1B	Unconvent	MYO1B_HU	43	0.935298
24H	10MT/	P27144	-0.1264	0.016083	0.015821	AK4	Adenylate k	KAD4_HUM	14	0.916117
24H	50MT/	Q8N8Q8	0.2702	0.016089	0.015825	COX18	Cytochrom	COX18_HU	3	1.205975
24H	10MT/	Q9BUF5	-0.00238	0.016105	0.015836	TUBB6	Tubulin bet	TBB6_HUM	16	0.998353
24H	10MT/	Q15427	0.195143	0.016108	0.015838	SF3B4	Splicing fac	SF3B4_HUI	8	1.144838
48H	50MT/	P80404	0.374018	0.016119	0.015846	ABAT	4-aminobu	GABT_HUM	11	1.295957
48H	50MT/	O14949	0.307495	0.016121	0.015848	UQCRQ	Cytochrom	QCR8_HUM	3	1.237557
48H	50MT/	Q5JVF3	-0.01799	0.016124	0.015849	PCID2	PCI domair	PCID2_HUI	13	0.987605

24H 50MT/	P40939	0.04869	0.016125	0.01585	HADHA	Trifunction	ECHA_HUM	37	1.034325
24H 50MT/	O94876	0.327188	0.016127	0.015851	TMCC1	Transmemb	TMCC1_HU	2	1.254566
48H 50MT/	Q9BTC8	0.101062	0.016131	0.015853	MTA3	Metastasis-	MTA3_HUM	14	1.072563
48H 50MT/	P04264	0.247038	0.016142	0.01586	KRT1	Keratin, typ	K2C1_HUM	38	1.186768
48H 50MT/	Q8IV36	0.113994	0.016149	0.015866	HID1	Protein HID	HID1_HUM	9	1.08222
48H 10MT/	Q13332	0.36156	0.016152	0.015867	PTPRS	Receptor-ty	PTPRS_HUI	3	1.284815
24H 50MT/	Q8WUF8	-1.72818	0.016152	0.015867	ARB2A	Cotranscrip	ARB2A_HUI	1	0.301833
24H 50MT/	P24928	0.040693	0.016165	0.015876	POLR2A	DNA-direct	RPB1_HUM	63	1.028608
24H 50MT/	P33176	-0.09485	0.016174	0.015883	KIF5B	Kinesin-1 h	KINH_HUM	52	0.93637
24H 10MT/	P61923	-0.38268	0.016201	0.015905	COPZ1	Coatomer s	COPZ1_HUI	6	0.767012
24H 10MT/	Q13838	0.028242	0.016201	0.015905	DDX39B	Spliceosom	DX39B_HU	22	1.019769
24H 10MT/	P23142	0.440699	0.016208	0.015909	FBLN1	Fibulin-1	FBLN1_HUI	11	1.357262
24H 10MT/	Q5JPI3	0.018852	0.016222	0.015918	C3orf38	Uncharacte	CC038_HUI	1	1.013153
24H 10MT/	Q9NQE9	0.065291	0.016224	0.015919	HINT3	Adenosine	HINT3_HUI	7	1.046296
48H 50MT/	P04798	0.591801	0.016234	0.015926	CYP1A1	Cytochrom	CP1A1_HUI	10	1.507127
48H 10MT/	P20645	0.100657	0.016235	0.015926	M6PR	Cation-dep	MPRD_HUI	7	1.072262
24H 50MT/	Q7L0Y3	0.02792	0.016237	0.015928	TRMT10C	tRNA meth	TM10C_HU	24	1.019541
24H 50MT/	Q92665	0.049125	0.016239	0.01593	MRPS31	Small ribos	RT31_HUM	15	1.034637
48H 10MT/	P17655	0.063078	0.016245	0.015934	CAPN2	Calpain-2 c	CAN2_HUM	22	1.044692
48H 50MT/	Q9Y2H5	0.026124	0.016251	0.015939	PLEKHA6	Pleckstrin h	PKHA6_HU	4	1.018272
48H 50MT/	Q8WUY9	-0.56124	0.016266	0.01595	DEPDC1B	DEP domai	DEP1B_HUI	1	0.677721
24H 50MT/	P51553	0.111537	0.01627	0.015952	IDH3G	Isocitrate d	IDH3G_HUI	12	1.080379
24H 10MT/	Q96BT3	0.573941	0.016277	0.015957	CENPT	Centromer	CENPT_HUI	3	1.488584
48H 10MT/	P31321	-0.35524	0.016297	0.015973	PRKAR1B	cAMP-depe	KAP1_HUM	5	0.781742
24H 10MT/	P51116	0.032172	0.016298	0.015973	FXR2	RNA-bindir	FXR2_HUM	18	1.022551
48H 50MT/	Q8TEB1	0.063605	0.016315	0.015987	DCAF11	DDB1- and	DCA11_HU	11	1.045074
48H 50MT/	P19404	0.09437	0.016326	0.015994	NDUFV2	NADH dehy	NDUV2_HU	9	1.067599
48H 50MT/	Q5T2W1	0.122916	0.01633	0.015997	PDZK1	Na(+)/H(+) e	NHRF3_HU	26	1.088934
48H 50MT/	O15121	0.135632	0.016334	0.015999	DEGS1	Sphingolipi	DEGS1_HUI	3	1.098574
24H 50MT/	P46109	0.101369	0.016333	0.015999	CRKL	Crk-like prc	CRKL_HUM	14	1.072791
24H 10MT/	P51608	0.217385	0.016335	0.016001	MECP2	Methyl-CpC	MECP2_HU	12	1.162625
24H 50MT/	Q96JJ7	0.180925	0.016338	0.016003	TMX3	Protein dis	TMX3_HUM	12	1.133611
48H 50MT/	P67936	0.056028	0.016343	0.016006	TPM4	Tropomyos	TPM4_HUM	11	1.0396
48H 10MT/	Q8N490	0.089591	0.016345	0.016008	PNKD	Probable h	PNKD_HUM	4	1.064069
24H 10MT/	P13807	-0.00249	0.01635	0.01601	GYS1	Glycogen [s	GYS1_HUM	15	0.998276
48H 10MT/	Q08378	-0.0023	0.016354	0.016013	GOLGA3	Golgin subf	GOGA3_HU	46	0.998405
48H 50MT/	Q9UBM1	0.108048	0.016358	0.016015	PEMT	Phosphatid	PEMT_HUM	1	1.077769
48H 50MT/	Q5VV41	0.568992	0.016361	0.016017	ARHGEF16	Rho guanin	ARHGG_HU	3	1.483487
48H 10MT/	Q9UIJ7	-0.0235	0.016363	0.016018	AK3	GTP:AMP p	KAD3_HUM	7	0.983843
24H 10MT/	P12035	0.626522	0.016364	0.016018	KRT3	Keratin, typ	K2C3_HUM	3	1.543839
24H 10MT/	O43776	0.037235	0.016369	0.016023	NARS1	Asparagine	SYNC_HUM	26	1.026145
24H 10MT/	Q06330	0.122766	0.016385	0.016036	RBPJ	Recombinii	SUH_HUM/	5	1.08882
48H 50MT/	Q99717	-0.32352	0.016388	0.016038	SMAD5	Mothers ag	SMAD5_HU	2	0.799115
48H 50MT/	Q86W34	0.391287	0.016403	0.016051	AMZ2	Archaemet	AMZ2_HUM	3	1.311563
48H 10MT/	O75208	0.135489	0.01641	0.016056	COQ9	Ubiquinon	COQ9_HUM	7	1.098465
24H 50MT/	Q13526	-0.05282	0.01641	0.016056	PIN1	Peptidyl-pr	PIN1_HUM	6	0.964053
48H 10MT/	Q9UBN7	-0.02242	0.016421	0.016064	HDAC6	Histone dea	HDAC6_HU	16	0.984577
48H 10MT/	Q9BT17	-0.06867	0.016426	0.016066	MTG1	Mitochond	MTG1_HUM	5	0.953517
24H 50MT/	Q9HAU5	-0.14336	0.016427	0.016067	UPF2	Regulator c	RENT2_HUI	24	0.905409
48H 50MT/	P04066	0.098918	0.016429	0.016068	FUCA1	Tissue alph	FUCO_HUM	8	1.07097
48H 10MT/	Q9H5N1	0.175794	0.016433	0.01607	RABEP2	Rab GTPase	RABE2_HUI	11	1.129586
48H 50MT/	P17655	0.096417	0.016437	0.016074	CAPN2	Calpain-2 c	CAN2_HUM	22	1.069115

48H 50MTA	O60779	-0.07262	0.016454	0.016088	SLC19A2	Thiamine tr	S19A2_HUI	1	0.950909
48H 50MTA	P67870	0.067961	0.016458	0.016091	CSNK2B	Casein kina	CSK2B_HUI	10	1.048234
24H 10MTA	Q92888	-0.07873	0.016458	0.016091	ARHGEF1	Rho guanin	ARHG1_HU	20	0.946891
48H 50MTA	P14210	-0.12165	0.01646	0.016093	HGF	Hepatocyte	HGF_HUMA	1	0.919134
48H 50MTA	P50213	-0.11494	0.016465	0.016096	IDH3A	Isocitrate d	IDH3A_HUI	17	0.923422
48H 50MTA	Q63ZY3	-0.25466	0.016469	0.016098	KANK2	KN motif ar	KANK2_HU	14	0.838186
48H 10MTA	P46109	0.052377	0.016474	0.016101	CRKL	Crk-like prc	CRKL_HUM	14	1.036972
48H 10MTA	P20042	0.210305	0.016479	0.016106	EIF2S2	Eukaryotic	IF2B_HUMA	19	1.156933
48H 50MTA	Q8ND24	0.145669	0.016485	0.01611	RNF214	RING finger	RN214_HU	20	1.106243
48H 10MTA	Q5F1R6	0.135621	0.016499	0.016121	DNAJC21	DnaJ homo	DJC21_HUI	11	1.098566
24H 50MTA	P06454	1.109859	0.016505	0.016125	PTMA	Prothymos	PTMA_HUM	2	2.158246
48H 50MTA	P35813	0.132092	0.016529	0.01614	PPM1A	Protein phc	PPM1A_HU	11	1.095882
24H 50MTA	Q9NWF9	-0.14011	0.016529	0.01614	RNF216	E3 ubiquiti	RN216_HU	2	0.907449
48H 10MTA	Q86UW6	-0.05014	0.016531	0.016142	N4BP2	NEDD4-bin	N4BP2_HU	3	0.96584
24H 50MTA	O00462	0.130706	0.01654	0.016149	MANBA	Beta-mann	MANBA_HU	12	1.09483
48H 10MTA	P04183	0.091807	0.016543	0.016152	TK1	Thymidine	KITH_HUMA	12	1.065704
48H 10MTA	Q9GZN1	0.011435	0.01655	0.016155	ACTR6	Actin-relate	ARP6_HUM	5	1.007957
24H 50MTA	O95671	0.122314	0.016556	0.016159	ASMTL	Probable bi	ASML_HUM	20	1.088479
48H 50MTA	O00391	-1.61592	0.016581	0.016178	QSOX1	Sulfhydryl c	QSOX1_HU	1	0.326258
48H 50MTA	Q92547	-0.51689	0.01659	0.016186	TOPBP1	DNA topois	TOPB1_HUI	13	0.698876
48H 10MTA	P12814	0.01099	0.016594	0.016189	ACTN1	Alpha-actin	ACTN1_HUI	58	1.007647
48H 10MTA	Q6NZ67	0.054455	0.016605	0.016198	MZT2B	Mitotic-spi	MZT2B_HU	6	1.038467
48H 50MTA	Q9NUL5	0.319896	0.016606	0.016199	SHFL	Shiftless an	SHFL_HUM	4	1.248241
48H 50MTA	Q86UN3	1.395659	0.01663	0.016215	RTN4RL2	Reticulon-4	R4RL2_HUI	2	2.631087
24H 50MTA	Q9UHN6	0.036867	0.016641	0.016222	CEMIP2	Inactive cel	CEIP2_HUM	40	1.025884
24H 10MTA	Q05048	0.089044	0.016647	0.016226	CSTF1	Cleavage sti	CSTF1_HUM	14	1.063665
48H 10MTA	Q96I99	-0.01342	0.016654	0.016232	SUCLG2	Succinate--	SUCB2_HUI	19	0.990741
48H 50MTA	O95989	0.060687	0.016656	0.016234	NUDT3	Diphospho	NUDT3_HU	5	1.042963
48H 50MTA	E9PRG8	0.147783	0.016703	0.016269	C11orf98	Uncharacte	CK098_HUI	4	1.107866
48H 10MTA	Q13045	0.001989	0.016715	0.016278	FLII	Protein flig	FLII_HUMA	39	1.00138
48H 10MTA	Q01130	0.457142	0.016742	0.016298	SRSF2	Serine/argi	SRSF2_HUM	5	1.372819
48H 10MTA	P52565	0.076718	0.016748	0.016303	ARHGDIA	Rho GDP-di	GDIR1_HUI	9	1.054616
48H 50MTA	Q96CM3	-1.71581	0.016753	0.016307	RPUSD4	Pseudourid	RUSD4_HU	2	0.304432
48H 10MTA	O00425	0.048384	0.016753	0.016307	IGF2BP3	Insulin-like	IF2B3_HUM	23	1.034106
24H 50MTA	Q2TBE0	0.261111	0.016761	0.016311	CWF19L2	CWF19-like	C19L2_HUI	12	1.198401
48H 50MTA	Q9NVA2	0.013685	0.016779	0.016326	SEPTIN11	Septin-11	SEP11_HUM	20	1.009531
48H 10MTA	Q8IWE2	0.065078	0.016785	0.016331	FAM114A1	Protein NO	NXP20_HU	14	1.046142
48H 50MTA	Q9UK99	0.036621	0.016795	0.016338	FBXO3	F-box only	FBX3_HUM	10	1.025708
24H 50MTA	O76027	1.248887	0.016797	0.016339	ANXA9	Annexin A9	ANXA9_HU	3	2.376581
24H 50MTA	Q8N9M1	0.129774	0.016808	0.016346	C19orf47	Uncharacte	CS047_HUI	4	1.094122
48H 10MTA	P30041	0.057776	0.016817	0.016352	PRDX6	Peroxiredo	PRDX6_HU	19	1.04086
48H 10MTA	Q99595	0.124592	0.016818	0.016352	TIMM17A	Mitochond	TI17A_HUM	2	1.0902
24H 50MTA	Q96RE7	0.04454	0.016822	0.016354	NACC1	Nucleus acc	NACC1_HU	13	1.031355
48H 10MTA	Q06830	-0.1445	0.016833	0.016361	PRDX1	Peroxiredo	PRDX1_HU	19	0.904691
48H 50MTA	Q9H6Y2	0.057911	0.016836	0.016363	WDR55	WD repeat-	WDR55_HU	13	1.040957
48H 10MTA	Q9NW08	0.104453	0.016841	0.016366	POLR3B	DNA-direct	RPC2_HUM	17	1.075087
24H 10MTA	Q9UKG1	0.079046	0.016843	0.016366	APPL1	DCC-intera	DP13A_HU	20	1.056319
48H 10MTA	Q9UJC5	0.243531	0.016846	0.016368	SH3BGRL2	SH3 domai	SH3L2_HUI	5	1.183887
24H 50MTA	P78346	0.097044	0.016849	0.016369	RPP30	Ribonuclea	RPP30_HUI	14	1.06958
24H 50MTA	Q8NBU5	0.169596	0.016849	0.016369	ATAD1	Outer mito	ATAD1_HUI	12	1.124743
48H 10MTA	A5YKK6	0.013886	0.01686	0.016377	CNOT1	CCR4-NOT t	CNOT1_HU	73	1.009671
48H 50MTA	P13807	0.26574	0.016861	0.016378	GYS1	Glycogen [s	GYS1_HUM	15	1.202253

48H 10MT/	Q9NR19	0.037952	0.016872	0.016386	ACSS2	Acetyl-coer	ACSA_HUM	12	1.026655
24H 50MT/	O60870	-0.14281	0.016881	0.016392	KIN	DNA/RNA-b	KIN17_HUI	8	0.905753
48H 10MT/	P0DPH7;P(	0.158426	0.016904	0.016411	TUBA3C;TU	Tubulin alp	TBA3C_HUI	2	1.116069
48H 10MT/	O15347	0.116596	0.01691	0.016416	HMGB3	High mobil	HMGB3_HU	7	1.084174
24H 50MT/	Q02750	0.193632	0.016915	0.016419	MAP2K1	Dual specifi	MP2K1_HU	10	1.143639
48H 50MT/	Q96P11	-0.22096	0.016915	0.016419	NSUN5	28S rRNA (c	NSUN5_HU	17	0.857993
24H 50MT/	P27797	0.133278	0.016921	0.016424	CALR	Calreticulir	CALR_HUM	21	1.096783
24H 50MT/	Q9Y5Z7	0.253293	0.016931	0.01643	HCFC2	Host cell fa	HCFC2_HUI	4	1.191925
24H 50MT/	Q9Y289	0.169165	0.016934	0.016431	SLC5A6	Sodium-dep	SC5A6_HUI	4	1.124408
24H 10MT/	Q86UK7	0.019582	0.016948	0.016442	ZNF598	E3 ubiquiti	ZN598_HUI	22	1.013666
24H 50MT/	Q9UHQ9	0.078535	0.016963	0.016453	CYB5R1	NADH-cyto	NB5R1_HU	15	1.055945
48H 10MT/	P34947	-0.06032	0.016966	0.016454	GRK5	G protein-c	GRK5_HUM	9	0.959051
24H 50MT/	Q8NHG8	0.045715	0.016968	0.016455	ZNRF2	E3 ubiquiti	ZNRF2_HUI	3	1.032194
24H 10MT/	P07195	0.675051	0.016971	0.016456	LDHB	L-lactate de	LDHB_HUM	1	1.596653
48H 50MT/	P11766	0.062748	0.016973	0.016458	ADH5	Alcohol del	ADHX_HUM	19	1.044453
48H 50MT/	Q9H7N4	0.31683	0.016982	0.016465	SCAF1	Splicing fac	SFR19_HUI	7	1.245591
24H 50MT/	Q9UNX4	0.165017	0.016991	0.016473	WDR3	WD repeat-	WDR3_HUI	35	1.121179
24H 50MT/	Q96FC7	0.0067	0.016999	0.016479	PHYHIPL	Phytanoyl-(	PHIPL_HUM	9	1.004655
48H 50MT/	P40937	-0.10104	0.017006	0.016485	RFC5	Replicatior	RFC5_HUM	22	0.932363
24H 50MT/	Q9UMR5	0.189891	0.017011	0.016488	PPT2	Lysosomal 1	PPT2_HUM	4	1.140678
48H 10MT/	Q04756	0.536386	0.017015	0.01649	HGFAC	Hepatocyte	HGFA_HUM	4	1.450334
24H 50MT/	Q96RD7	-0.32325	0.017022	0.016494	PANX1	Pannexin-1	PANX1_HU	3	0.799267
24H 50MT/	P52815	-0.22742	0.017047	0.016512	MRPL12	Large ribos	RM12_HUM	5	0.854161
24H 50MT/	Q15061	0.122573	0.017047	0.016512	WDR43	WD repeat-	WDR43_HU	25	1.088675
48H 10MT/	Q9Y287	-0.07376	0.017058	0.016518	ITM2B	Integral me	ITM2B_HUI	6	0.95016
24H 50MT/	Q13232	0.028792	0.017064	0.016522	NME3	Nucleoside	NDK3_HUM	8	1.020157
24H 50MT/	P17096	0.060474	0.01707	0.016527	HMGA1	High mobil	HMGA1_HU	3	1.042808
24H 50MT/	Q9NRR4	0.175128	0.01707	0.016527	DROSHA	Ribonuclea	RNC_HUMA	9	1.129064
24H 10MT/	Q9Y5B6	0.083335	0.017074	0.01653	PAXBP1	PAX3- and I	PAXB1_HUI	15	1.059464
24H 50MT/	Q13310	0.155314	0.017086	0.016538	PABPC4	Polyadenyl	PABP4_HUI	21	1.113664
48H 10MT/	Q9Y2I7	0.088695	0.017085	0.016538	PIKFYVE	1-phosphat	FYV1_HUM	16	1.063408
24H 50MT/	O00762	-0.33168	0.017088	0.016538	UBE2C	Ubiquitin-c	UBE2C_HUI	8	0.79461
24H 10MT/	Q96GX9	-0.09296	0.017088	0.016538	APIP	Methylthio	MTNB_HUM	8	0.937599
48H 10MT/	Q96LR5	0.08988	0.017095	0.016542	UBE2E2	Ubiquitin-c	UB2E2_HUI	5	1.064281
48H 50MT/	Q9NYZ3	0.622272	0.017098	0.016544	GTSE1	G2 and S pl	GTSE1_HUI	2	1.539297
24H 50MT/	Q8WUA2	-0.0232	0.017126	0.016563	PPIL4	Peptidyl-pr	PPIL4_HUM	20	0.984051
48H 50MT/	Q7Z3T8	0.013741	0.017133	0.016569	ZFYVE16	Zinc finger I	ZFY16_HUM	28	1.00957
24H 50MT/	Q96BP3	0.151532	0.017148	0.016578	PPWD1	Peptidylpr(	PPWD1_HU	24	1.110749
24H 10MT/	P62330	0.106581	0.01715	0.016578	ARF6	ADP-ribosy	ARF6_HUM	8	1.076674
24H 50MT/	Q9BST9	-0.29401	0.017159	0.016584	RTKN	Rhotekin	RTKN_HUM	11	0.815633
48H 10MT/	Q5TH69	0.080899	0.017162	0.016586	ARFGEF3	Brefeldin A	BIG3_HUM	24	1.057677
24H 50MT/	Q16822	0.035442	0.017169	0.016589	PCK2	Phosphoen	PCKGM_HU	30	1.024871
48H 10MT/	Q9H1Z4	0.069719	0.017168	0.016589	WDR13	WD repeat-	WDR13_HU	13	1.049513
24H 10MT/	Q9Y6N5	0.097243	0.017169	0.016589	SQOR	Sulfide:qui	SQOR_HUM	30	1.069727
24H 50MT/	Q9UM54	0.061607	0.017173	0.016592	MYO6	Unconvent	MYO6_HUM	56	1.043628
48H 50MT/	Q7RTS9	-0.22082	0.017185	0.016601	DYM	Dymeclin	DYM_HUM/	7	0.858077
48H 10MT/	Q04917	0.002445	0.01719	0.016605	YWHAH	14-3-3 prot	1433F_HUI	13	1.001696
24H 10MT/	Q53HV7	-0.14882	0.017191	0.016605	SMUG1	Single-strar	SMUG1_HU	2	0.901985
24H 50MT/	Q96KQ7	0.200825	0.017196	0.016608	EHMT2	Histone-lys	EHMT2_HU	15	1.149355
24H 50MT/	O43818	0.004001	0.017208	0.016618	RRP9	U3 small nu	U3IP2_HUI	13	1.002777
48H 50MT/	P49589	0.059227	0.017208	0.016618	CARS1	Cysteine--tI	SYCC_HUM	34	1.041908
48H 50MT/	Q13136	0.157449	0.017208	0.016618	PPFIA1	Liprin-alph	LIPA1_HUM	28	1.115313

48H 10MTA	P17900	-0.03763	0.017212	0.01662	GM2A	Ganglioside	SAP3_HUM	4	0.974255
24H 10MTA	Q96D42	1.674369	0.017212	0.01662	HAVCR1	Hepatitis A	HAVR1_HU	3	3.191797
24H 10MTA	Q08334	-1.2032	0.017214	0.01662	IL10RB	Interleukin	I10R2_HUM	2	0.43431
48H 50MTA	Q6UB35	-0.09721	0.017218	0.016623	MTHFD1L	Monofunct	C1TM_HUM	29	0.934842
24H 10MTA	Q92558	-0.29728	0.017219	0.016623	WASF1	Actin-bindi	WASF1_HU	1	0.813785
48H 10MTA	Q9P2D0	0.10538	0.017233	0.016632	IBTK	Inhibitor o	IBTK_HUMA	8	1.075778
24H 10MTA	O00303	0.050354	0.017234	0.016633	EIF3F	Eukaryotic	EIF3F_HUM	11	1.035519
48H 10MTA	Q9NRR3	0.372213	0.017238	0.016635	CDC42SE2	CDC42 sma	C42S2_HUI	1	1.294336
24H 10MTA	P62937	0.007138	0.017247	0.016642	PPIA	Peptidyl-pr	PPIA_HUMA	11	1.00496
48H 50MTA	Q2TBE0	-0.21542	0.017249	0.016644	CWF19L2	CWF19-like	C19L2_HUI	12	0.861294
24H 10MTA	Q9P0P0	-0.4868	0.017255	0.016647	RNF181	E3 ubiquiti	RN181_HU	2	0.713608
48H 50MTA	Q9Y5V0	-0.25205	0.017256	0.016648	ZNF706	Zinc finger	ZN706_HUI	2	0.839701
24H 10MTA	O94903	-0.14498	0.01726	0.01665	PLPBP	Pyridoxal p	PLPHP_HUI	11	0.904395
48H 10MTA	P48444	0.004942	0.017265	0.016653	ARCN1	Coatomer s	COPD_HUM	32	1.003431
24H 50MTA	Q4ZIN3	-0.04426	0.01727	0.016657	TMEM259	Membralin	MBRL_HUM	5	0.969786
48H 50MTA	Q13586	0.007293	0.017275	0.016661	STIM1	Stromal int	STIM1_HUM	20	1.005068
24H 10MTA	Q92499	0.023596	0.017278	0.016663	DDX1	ATP-depenc	DDX1_HUM	42	1.01649
48H 50MTA	P05114	0.269754	0.01728	0.016664	HMGN1	Non-histon	HMGN1_HU	3	1.205603
48H 10MTA	O14795	-0.00812	0.017284	0.016665	UNC13B	Protein unc	UN13B_HU	13	0.99439
48H 50MTA	A0A0B4J2D	0.081665	0.017299	0.016676	GATD3B;GA	Putative glu	GAL3B_HUI	8	1.058238
24H 50MTA	Q9UNK9	0.483791	0.017312	0.016686	ANGEL1	Protein ang	ANGE1_HU	4	1.398414
24H 50MTA	Q96KC2	-0.08144	0.017315	0.016689	ARL5B	ADP-ribosy	ARL5B_HUI	2	0.945117
48H 10MTA	P53992	0.05257	0.017319	0.016691	SEC24C	Protein tra	SC24C_HUI	35	1.037111
48H 10MTA	Q8IUF8	-0.00598	0.01732	0.016691	RIOX2	Ribosomal	RIOX2_HUM	12	0.995863
48H 10MTA	P62913	-0.17455	0.01734	0.016705	RPL11	Large ribos	RL11_HUM	10	0.886043
24H 50MTA	Q14011	0.033305	0.017349	0.016712	CIRBP	Cold-induc	CIRBP_HUM	4	1.023354
48H 50MTA	Q8IV08	0.130783	0.017351	0.016713	PLD3	5'-3' exonu	PLD3_HUM	6	1.094888
24H 50MTA	Q13616	0.02594	0.017362	0.01672	CUL1	Cullin-1	CUL1_HUM	36	1.018143
48H 10MTA	P35637	0.036328	0.017367	0.016723	FUS	RNA-bindir	FUS_HUMA	12	1.0255
24H 10MTA	Q8N6T3	-0.02858	0.017368	0.016724	ARFGAP1	ADP-ribosy	ARFG1_HUI	15	0.980386
24H 50MTA	Q6ZMI0	0.024551	0.01737	0.016724	PPP1R21	Protein pho	PPR21_HUI	9	1.017163
48H 50MTA	Q9H1I8	0.069492	0.017374	0.016727	ASCC2	Activating s	ASCC2_HUI	14	1.049347
24H 50MTA	P13797	-0.09005	0.017395	0.016739	PLS3	Plastin-3	PLST_HUMA	38	0.939493
48H 50MTA	Q9UKY4	-0.16045	0.017404	0.016746	POMT2	Protein O-n	POMT2_HU	3	0.894747
48H 50MTA	Q9HBH1	-0.60209	0.017404	0.016746	PDF	Peptide def	DEFM_HUM	4	0.658797
48H 10MTA	P53621	0.025409	0.017406	0.016747	COPA	Coatomer s	COPA_HUM	68	1.017768
24H 50MTA	Q71RC2	-0.10047	0.017419	0.016756	LARP4	La-related p	LARP4_HUI	20	0.932727
48H 10MTA	P49207	0.046226	0.01742	0.016756	RPL34	Large ribos	RL34_HUM	2	1.03256
48H 50MTA	P55263	0.018382	0.01742	0.016756	ADK	Adenosine	ADK_HUMA	23	1.012823
48H 50MTA	O15031	-0.01684	0.017429	0.016762	PLXNB2	Plexin-B2	PLXB2_HUI	50	0.988395
24H 10MTA	O95400	0.025874	0.017432	0.016763	CD2BP2	CD2 antiger	CD2B2_HU	12	1.018096
24H 10MTA	Q92979	0.067309	0.017432	0.016763	EMG1	Ribosomal	NEP1_HUM	15	1.047761
48H 50MTA	O15269	-0.18495	0.017435	0.016764	SPTLC1	Serine palm	SPTC1_HUI	10	0.87968
24H 50MTA	Q9BX66	0.235218	0.017435	0.016764	SORBS1	Sorbin and	SRBS1_HUM	31	1.177085
48H 10MTA	Q15029	0.001927	0.017454	0.016781	EFTUD2	116 kDa U5	U5S1_HUM	51	1.001337
24H 50MTA	Q86X67	-0.71162	0.017454	0.016781	NUDT13	NAD(P)H py	NUD13_HU	1	0.610635
48H 50MTA	Q96FN4	0.315259	0.017468	0.016789	CPNE2	Copine-2	CPNE2_HUI	8	1.244235
48H 50MTA	Q8NHY2	0.216526	0.017467	0.016789	COP1	E3 ubiquiti	COP1_HUM	2	1.161932
24H 50MTA	Q9H9P8	0.189818	0.017467	0.016789	L2HGDH	L-2-hydroxy	L2HDH_HU	16	1.14062
48H 50MTA	Q96FF9	-0.56774	0.017479	0.016797	CDCA5	Sororin	CDCA5_HU	5	0.674671
24H 50MTA	P62829	0.098576	0.017483	0.016799	RPL23	Large ribos	RL23_HUM	8	1.070716
48H 50MTA	Q7L3S4	0.185102	0.017484	0.016799	ZNF771	Zinc finger	ZN771_HUI	3	1.136897

48H 50MT/	P23381	0.065146	0.017486	0.0168	WARS1	Tryptophar	SYWC_HUM	23	1.046191
48H 50MT/	P49137	0.094496	0.017486	0.0168	MAPKAPK2	MAP kinase	MAPK2_HU	5	1.067692
24H 50MT/	Q5JPE7	0.077352	0.017487	0.016801	NOMO2	BOS compl	NOMO2_HU	44	1.05508
48H 10MT/	Q05086	0.015677	0.017494	0.016806	UBE3A	Ubiquitin-p	UBE3A_HU	28	1.010925
48H 10MT/	O94925	-0.13147	0.017502	0.016811	GLS	Glutaminas	GLSK_HUM	21	0.912899
24H 50MT/	P78345	0.07155	0.017503	0.016811	RPP38	Ribonuclea	RPP38_HU	9	1.050845
24H 50MT/	P21580	-0.49698	0.017507	0.016814	TNFAIP3	Tumor necr	TNAP3_HU	6	0.70859
48H 50MT/	Q3KQU3	0.049334	0.017513	0.016817	MAP7D1	MAP7 dom	MA7D1_HU	10	1.034787
24H 50MT/	Q70CQ2	0.040434	0.017522	0.016822	USP34	Ubiquitin c	UBP34_HU	36	1.028423
48H 10MT/	Q9NPF4	0.025663	0.017559	0.016845	OSGEP	tRNA N6-ad	OSGEP_HU	14	1.017947
48H 10MT/	Q92783	0.012118	0.017567	0.016851	STAM	Signal trans	STAM1_HU	15	1.008435
48H 50MT/	Q9BZD4	-0.32719	0.017574	0.016856	NUF2	Kinetochor	NUF2_HUM	12	0.797089
48H 10MT/	P11172	-0.0273	0.01758	0.01686	UMPS	Uridine 5'-r	UMPS_HUM	24	0.981254
48H 50MT/	P49585	-0.01706	0.017586	0.016865	PCYT1A	Choline-ph	PCY1A_HU	14	0.988241
24H 10MT/	O75530	0.014672	0.017611	0.016886	EED	Polycomb p	EED_HUMA	15	1.010221
48H 50MT/	P60520	-0.15695	0.017612	0.016886	GABARAPL2	Gamma-am	GBRL2_HU	4	0.89692
24H 50MT/	Q9NYB9	-0.17598	0.017612	0.016886	ABI2	Abl interact	ABI2_HUM	13	0.885168
48H 50MT/	P49810	-0.28979	0.017623	0.016893	PSEN2	Presenilin-2	PSN2_HUM	2	0.818023
48H 50MT/	Q9BZE9	0.010788	0.017625	0.016894	ASPSCR1	Tether cont	ASPC1_HU	17	1.007506
48H 50MT/	O43301	0.322452	0.017627	0.016895	HSPA12A	Heat shock	HS12A_HU	7	1.250454
48H 10MT/	O60547	-0.064	0.017627	0.016895	GMDS	GDP-mann	GMDS_HUM	13	0.956611
24H 50MT/	P40337	0.404751	0.017639	0.016903	VHL	von Hippel-	VHL_HUMA	2	1.32386
24H 50MT/	O60832	0.013209	0.017644	0.016906	DKC1	H/ACA ribo	DKC1_HUM	27	1.009198
48H 10MT/	Q99613	0.055484	0.017656	0.016913	EIF3C	Eukaryotic	EIF3C_HUM	34	1.039208
24H 10MT/	Q6ZWJ1	0.077873	0.017658	0.016915	STXBP4	Syntaxin-bi	STXB4_HUM	10	1.055461
48H 50MT/	Q86UY8	-0.01093	0.017667	0.016921	NT5DC3	5'-nucleoti	NT5D3_HU	6	0.992451
24H 50MT/	P52298	0.105251	0.01767	0.016923	NCBP2	Nuclear cap	NCBP2_HU	4	1.075682
48H 50MT/	Q9BX40	-0.18321	0.017674	0.016927	LSM14B	Protein LSM	LS14B_HUM	13	0.880743
48H 10MT/	Q8IZ83	0.068372	0.017681	0.016931	ALDH16A1	Aldehyde d	A16A1_HU	20	1.048533
48H 50MT/	P13473	-0.09222	0.017687	0.016935	LAMP2	Lysosome-a	LAMP2_HU	3	0.938078
48H 10MT/	Q15024	0.052993	0.017686	0.016935	EXOSC7	Exosome co	EXOS7_HU	10	1.037415
48H 50MT/	Q8TBK6	-3.10842	0.017691	0.016937	ZCCHC10	Zinc finger	ZCH10_HU	1	0.11595
24H 10MT/	P25789	0.066975	0.017692	0.016938	PSMA4	Proteasom	PSA4_HUM	13	1.047518
24H 50MT/	Q8TAA5	0.021012	0.017699	0.016943	GRPEL2	GrpE protei	GRPE2_HU	7	1.014671
24H 10MT/	Q5TDH0	-0.07794	0.0177	0.016943	DDI2	Protein DD	DDI2_HUM	10	0.947412
48H 10MT/	P78540	-0.28073	0.017705	0.016946	ARG2	Arginase-2,	ARGI2_HUM	9	0.823176
48H 10MT/	O00148	0.157697	0.017713	0.016951	DDX39A	ATP-depend	DX39A_HU	12	1.115505
48H 50MT/	Q9Y3C1	-0.23384	0.01772	0.016956	NOP16	Nucleolar p	NOP16_HU	9	0.850368
24H 50MT/	Q86VR2	-0.07005	0.017738	0.016967	RETREG3	Reticuloph	RETR3_HUM	5	0.952604
24H 50MT/	Q9Y2H6	0.158269	0.01776	0.016984	FNDC3A	Fibronectir	FND3A_HU	27	1.115947
24H 50MT/	Q8NCR0	-0.3143	0.017761	0.016984	B3GALNT2	UDP-GalNA	B3GL2_HU	2	0.804244
24H 10MT/	O00458	0.177828	0.017765	0.016987	IFRD1	Interferon-	IFRD1_HUM	6	1.13118
48H 50MT/	A6NHL2	-0.51387	0.017769	0.016989	TUBAL3	Tubulin alp	TBAL3_HUM	2	0.700342
48H 50MT/	P06899;Q1	-0.14103	0.017777	0.016992	H2BC11;H2	Histone H2	H2B1J_HU	2	0.906871
48H 50MT/	P13804	0.065461	0.01778	0.016995	ETFA	Electron tra	ETFA_HUM	16	1.046419
48H 10MT/	O00244	0.227076	0.017785	0.016999	ATOX1	Copper trar	ATOX1_HU	6	1.17046
24H 50MT/	P04004	0.098964	0.017792	0.017004	VTN	Vitronectir	VTNC_HUM	9	1.071004
24H 10MT/	Q16394	0.161325	0.017793	0.017004	EXT1	Exostosin-1	EXT1_HUM	3	1.118314
24H 10MT/	Q13509	-0.31784	0.017797	0.017007	TUBB3	Tubulin bet	TBB3_HUM	4	0.802271
24H 50MT/	Q96DH6	0.112652	0.017806	0.017012	MSI2	RNA-bindir	MSI2H_HU	6	1.081214
48H 50MT/	Q9UI95	-0.25216	0.017812	0.017016	MAD2L2	Mitotic spi	MD2L2_HU	1	0.839637
48H 50MT/	Q7KZN9	0.144512	0.017816	0.017019	COX15	Heme A syn	COX15_HU	9	1.105357

24H 10MT/	O15126	0.05502	0.017831	0.017031	SCAMP1	Secretory c	SCAM1_HU	5	1.038873
24H 10MT/	Q01813	0.033547	0.017834	0.017032	PFKP	ATP-depen(	PFKAP_HUI	27	1.023526
24H 50MT/	Q9NZT2	0.071819	0.017835	0.017032	OGFR	Opioid gro\	OGFR_HUM	21	1.051041
48H 50MT/	Q8TCC3	-0.38064	0.017836	0.017032	MRPL30	Large ribos	RM30_HUM	2	0.768098
48H 10MT/	Q05209	0.053563	0.017837	0.017033	PTPN12	Tyrosine-pr	PTN12_HUI	15	1.037825
48H 50MT/	P35527	0.335314	0.017841	0.017036	KRT9	Keratin, typ	K1C9_HUM	31	1.261652
24H 10MT/	Q9H6U8	0.053236	0.017844	0.017037	ALG9	Alpha-1,2-r	ALG9_HUM	7	1.03759
48H 10MT/	P09651	-0.17033	0.017861	0.01705	HNRNPA1	Heterogene	ROA1_HUM	22	0.888637
24H 50MT/	Q96J01	0.10014	0.017867	0.017055	THOC3	THO compl	THOC3_HU	9	1.071878
48H 50MT/	Q9Y5B0	0.10188	0.017895	0.017076	CTDP1	RNA polym	CTDP1_HUI	13	1.073171
24H 50MT/	P12830	0.417811	0.017902	0.017081	CDH1	Cadherin-1	CADH1_HU	7	1.335899
24H 50MT/	O00151	-0.11161	0.017906	0.017081	PDLIM1	PDZ and LII	PDLI1_HUM	21	0.925555
24H 50MT/	P43251	-0.79422	0.017905	0.017081	BTD	Biotinidase	BTD_HUMA	1	0.576655
24H 50MT/	Q9H098	-0.38898	0.017906	0.017081	FAM107B	Protein FAI	F107B_HUI	7	0.763667
48H 50MT/	Q08379	0.009339	0.017914	0.017087	GOLGA2	Golgin subf	GOGA2_HU	25	1.006494
48H 50MT/	O14933	0.76911	0.017922	0.017091	UBE2L6	Ubiquitin/I	UB2L6_HUI	1	1.704218
48H 50MT/	Q9BRP8	0.047318	0.017945	0.017109	PYM1	Partner of Y	PYM1_HUM	9	1.033342
48H 10MT/	Q9BZX2	0.038674	0.01795	0.017114	UCK2	Uridine-cyt	UCK2_HUM	11	1.027169
24H 50MT/	Q9NZJ9	-0.18797	0.017971	0.01713	NUDT4	Diphospho	NUDT4_HU	6	0.877838
48H 50MT/	Q6N063	0.41512	0.017976	0.017133	OGFOD2	2-oxoglutaı	OGFD2_HU	3	1.333409
24H 10MT/	Q9UJX2	0.07501	0.017983	0.017137	CDC23	Cell divisio	CDC23_HU	24	1.053368
48H 50MT/	Q03013	0.301628	0.017984	0.017138	GSTM4	Glutathion	GSTM4_HU	9	1.232534
48H 10MT/	P56378	0.213644	0.017984	0.017138	ATP5MJ	ATP syntha	ATP68_HUI	4	1.159614
24H 10MT/	Q6UX04	-0.05556	0.017992	0.017143	CWC27	Spliceosom	CWC27_HU	6	0.962221
24H 50MT/	Q9GZT9	0.042556	0.018003	0.017152	EGLN1	Egl nine ho	EGLN1_HUI	11	1.029937
48H 50MT/	Q02086	-0.6853	0.018031	0.017172	SP2	Transcripti	SP2_HUMA	1	0.621878
24H 10MT/	P80723	-0.40945	0.018033	0.017173	BASP1	Brain acid s	BASP1_HUI	7	0.752911
24H 50MT/	P49736	0.045845	0.018034	0.017174	MCM2	DNA replica	MCM2_HUI	36	1.032288
48H 50MT/	O15372	-0.12093	0.018037	0.017175	EIF3H	Eukaryotic	EIF3H_HUM	21	0.919595
24H 50MT/	Q9H7B4	-0.09845	0.018038	0.017175	SMYD3	Histone-lys	SMYD3_HU	6	0.934034
24H 10MT/	P62318	0.043143	0.018057	0.017189	SNRPD3	Small nucle	SMD3_HUM	5	1.030356
48H 10MT/	P78560	-0.09444	0.018057	0.017189	CRADD	Death dom	CRADD_HU	6	0.936638
48H 50MT/	Q86UW6	-0.13195	0.018063	0.017193	N4BP2	NEDD4-bin	N4BP2_HU	3	0.912598
48H 10MT/	O95214	-0.00477	0.018064	0.017193	LEPROTL1	Leptin rece	LERL1_HUM	1	0.996702
24H 10MT/	Q7Z5L2	0.087268	0.018065	0.017194	R3HCC1L	Coiled-coil	R3HCL_HUI	2	1.062357
48H 50MT/	Q9NQG5	0.017536	0.018068	0.017195	RPRD1B	Regulation	RPR1B_HU	12	1.012229
24H 10MT/	Q9NRN9	0.238044	0.018074	0.0172	METTL5	rRNA N6-ac	METL5_HUI	5	1.179393
24H 10MT/	Q03426	-0.08564	0.018079	0.017202	MVK	Mevalonate	KIME_HUM	9	0.942364
48H 50MT/	P10109	0.163757	0.018084	0.017206	FDX1	Adrenodox	ADX_HUM/	5	1.1202
48H 50MT/	Q9BT78	0.019859	0.018091	0.01721	COPS4	COP9 signa	CSN4_HUM	25	1.01386
24H 10MT/	Q9H8X2	0.420911	0.018108	0.017223	IPPK	Inositol-pe	IPPK_HUM.	3	1.338773
24H 10MT/	O00214	-0.02732	0.01811	0.017224	LGALS8	Galectin-8	LEG8_HUM	8	0.981241
48H 50MT/	Q07812	0.162771	0.018112	0.017226	BAX	Apoptosis r	BAX_HUMA	8	1.119435
24H 10MT/	Q9H8G2	0.898838	0.018144	0.017249	CAAP1	Caspase act	CAAP1_HUI	2	1.864564
24H 50MT/	Q8IXW5	-0.08624	0.018162	0.017262	RPAP2	Putative RI	RPAP2_HUI	15	0.941972
48H 50MT/	Q8TCT8	-0.51039	0.018162	0.017262	SPPL2A	Signal pept	SPP2A_HUI	4	0.702034
48H 10MT/	Q14669	-0.04845	0.018165	0.017263	TRIP12	E3 ubiquiti	TRIPC_HUM	55	0.966973
48H 50MT/	Q9Y2Z2	-0.14889	0.018171	0.017267	MTO1	Protein MT	MTO1_HUM	18	0.901947
24H 50MT/	O43399	-0.15466	0.018177	0.017272	TPD52L2	Tumor prot	TPD54_HUI	11	0.898346
48H 10MT/	Q9Y448	0.27861	0.018182	0.017275	KNSTRN	Small kinet	SKAP_HUM	5	1.213026
48H 50MT/	Q9UBU8	-0.21314	0.018182	0.017275	MORF4L1	Mortality fa	MO4L1_HU	11	0.862657
24H 50MT/	P18031	0.00941	0.018198	0.017287	PTPN1	Tyrosine-pr	PTN1_HUM	21	1.006544

48H 10MTA	Q7L592	-0.08195	0.018202	0.01729	NDUFAF7	Protein arg	NDUF7_HU	14	0.944779
48H 50MTA	P12755	0.158102	0.018205	0.017292	SKI	Ski oncoger	SKI_HUMAI	2	1.115818
48H 10MTA	O15344	0.031013	0.018209	0.017294	MID1	E3 ubiquiti	TRI18_HUN	20	1.02173
24H 10MTA	P22102	-0.06818	0.018209	0.017294	GART	Trifunction	PUR2_HUN	42	0.953841
24H 50MTA	Q92973	-0.10996	0.018211	0.017295	TNPO1	Transportir	TNPO1_HU	31	0.926614
24H 50MTA	Q8TBA6	0.137216	0.018217	0.017297	GOLGA5	Golgin subf	GOGA5_HU	19	1.099781
48H 50MTA	Q9BVG9	-0.20785	0.018218	0.017297	PTDSS2	Phosphatid	PTSS2_HUN	7	0.865826
24H 50MTA	Q14247	-0.08097	0.018227	0.017305	CTTN	Src substrat	SRC8_HUM	19	0.945423
48H 50MTA	Q4KMQ2	0.1904	0.018239	0.017312	ANO6	Anoctamin	ANO6_HUN	16	1.14108
24H 50MTA	Q96GA3	0.114598	0.018244	0.017316	LTV1	Protein LTV	LTV1_HUM	17	1.082673
48H 50MTA	Q16513	0.039989	0.018254	0.017324	PKN2	Serine/thre	PKN2_HUN	42	1.028106
24H 50MTA	A0FGR8	0.027145	0.018268	0.017336	ESYT2	Extended sy	ESYT2_HUN	25	1.018993
48H 10MTA	Q8TCJ2	0.016954	0.018276	0.017341	STT3B	Dolichyl-di	STT3B_HUN	14	1.011821
48H 50MTA	P46063	0.061638	0.018282	0.017345	RECQL	ATP-depend	RECQ1_HUI	27	1.04365
24H 50MTA	P83876	-0.5276	0.018281	0.017345	TXNL4A	Thioredoxi	TXN4A_HUI	6	0.693706
48H 50MTA	Q9P275	-0.54807	0.018284	0.017346	USP36	Ubiquitin c	UBP36_HU	7	0.683934
48H 50MTA	Q96HE9	0.349605	0.018289	0.01735	PRR11	Proline-ricl	PRR11_HUI	2	1.274212
24H 50MTA	O94830	-0.17735	0.01829	0.017351	DDHD2	Phospholip	DDHD2_HU	7	0.884328
48H 10MTA	P52747	0.379719	0.018303	0.017361	ZNF143	Zinc finger	ZN143_HUI	2	1.301088
24H 50MTA	Q15370	0.132543	0.018308	0.017362	ELOB	Elongin-B	ELOB_HUM	10	1.096224
24H 10MTA	Q9NNW5	-0.16337	0.018308	0.017362	WDR6	tRNA (34-2'	WDR6_HUI	26	0.892935
24H 50MTA	Q8NFW8	0.130862	0.018328	0.017377	CMAS	N-acylneura	NEUA_HUM	14	1.094948
24H 50MTA	O75554	0.179759	0.018336	0.017384	WBP4	WW domai	WBP4_HUN	4	1.132695
24H 50MTA	Q03013	0.010848	0.018337	0.017384	GSTM4	Glutathion	GSTM4_HU	9	1.007547
48H 10MTA	Q86YD1	-0.04805	0.018339	0.017385	PTOV1	Prostate tu	PTOV1_HUI	3	0.967244
24H 10MTA	Q8IUH4	0.043715	0.01834	0.017386	ZDHHC13	Palmitoyltr	ZDH13_HU	2	1.030765
24H 50MTA	Q96KR1	0.038567	0.018341	0.017386	ZFR	Zinc finger	ZFR_HUMA	33	1.027093
24H 50MTA	A0AVT1	-0.05305	0.018349	0.017392	UBA6	Ubiquitin-l	UBA6_HUN	43	0.963894
48H 10MTA	Q9BVM2	0.073763	0.01836	0.017401	DPCD	Protein DP(	DPCD_HUN	7	1.052459
24H 50MTA	Q9NVH1	0.060189	0.018371	0.017405	DNAJC11	DnaJ homo	DJC11_HUI	18	1.042602
24H 50MTA	Q04828	0.08237	0.018377	0.017409	AKR1C1	Aldo-keto r	AK1C1_HUI	21	1.058756
48H 50MTA	P61254	0.135633	0.018387	0.017416	RPL26	Large ribos	RL26_HUM	8	1.098575
48H 10MTA	Q9H0D6	0.011913	0.018392	0.017419	XRN2	5'-3' exorib	XRN2_HUN	38	1.008292
48H 10MTA	Q9P2I0	-0.02733	0.018393	0.01742	CPSF2	Cleavage ar	CPSF2_HUN	31	0.981232
24H 50MTA	Q5HYI7	-0.05654	0.018405	0.017428	MTX3	Metaxin-3	MTX3_HUN	5	0.961569
24H 10MTA	P49427	-0.0114	0.018407	0.01743	CDC34	Ubiquitin-c	UB2R1_HU	5	0.992126
48H 10MTA	O75157	0.111949	0.018425	0.017444	TSC22D2	TSC22 dom	T22D2_HUI	2	1.080687
24H 10MTA	Q9Y5K6	-0.0705	0.018433	0.017449	CD2AP	CD2-associ	CD2AP_HU	27	0.952307
24H 10MTA	P49842	-0.34666	0.018438	0.017454	STK19	Inactive ser	STK19_HUN	3	0.7864
48H 10MTA	Q15287	0.068034	0.018441	0.017455	RNPS1	RNA-bindir	RNPS1_HUI	6	1.048287
48H 50MTA	Q9BYD6	-0.19485	0.018447	0.01746	MRPL1	Large ribos	RM01_HUN	13	0.873663
24H 10MTA	O60508	0.014913	0.018464	0.017472	CDC40	Pre-mRNA-p	PRP17_HUI	24	1.010391
48H 50MTA	O15318	-0.13326	0.018487	0.017487	POLR3G	DNA-direct	RPC7_HUM	3	0.911768
24H 50MTA	O15121	0.198749	0.018488	0.017487	DEGS1	Sphingolipi	DEGS1_HUI	3	1.147703
24H 50MTA	P56945	0.017231	0.018494	0.017492	BCAR1	Breast canc	BCAR1_HUI	10	1.012015
48H 50MTA	P35659	-0.01006	0.018497	0.017493	DEK	Protein DEI	DEK_HUMA	16	0.99305
24H 50MTA	P13716	-0.04891	0.018503	0.017497	ALAD	Delta-amin	HEM2_HUN	8	0.966669
24H 10MTA	Q99643	0.292722	0.01852	0.017509	SDHC	Succinate d	C560_HUM	3	1.224949
24H 50MTA	P30622	-0.17648	0.018538	0.017524	CLIP1	CAP-Gly do	CLIP1_HUN	44	0.88486
24H 50MTA	P16083	0.05185	0.018548	0.017531	NQO2	Ribosyldihy	NQO2_HUN	13	1.036594
48H 10MTA	Q16790	0.26573	0.018557	0.017537	CA9	Carbonic ar	CAH9_HUN	3	1.202244
48H 10MTA	Q9UJV9	0.006402	0.018558	0.017537	DDX41	Probable A	DDX41_HU	29	1.004447

48H 50MT/	Q93009	0.028951	0.018567	0.017544	USP7	Ubiquitin c	UBP7_HUM	54	1.02027
24H 10MT/	Q68EM7	-0.15834	0.018573	0.017547	ARHGAP17	Rho GTPase	RHG17_HU	16	0.896055
24H 10MT/	P49441	-0.15117	0.01858	0.017552	INPP1	Inositol pol	INPP_HUM	9	0.900519
48H 10MT/	P05387	0.198703	0.018581	0.017552	RPLP2	Large ribos	RLA2_HUM	7	1.147666
48H 10MT/	Q9BTL3	0.065996	0.018581	0.017552	RAMAC	RNA guanin	RAMAC_HU	5	1.046807
48H 50MT/	O75340	0.042819	0.018583	0.017553	PDCD6	Programme	PDCD6_HU	9	1.030125
24H 10MT/	Q8TEM1	-0.05322	0.018594	0.017559	NUP210	Nuclear po	PO210_HU	45	0.963782
48H 50MT/	Q9BZG1	0.0684	0.018595	0.01756	RAB34	Ras-related	RAB34_HU	9	1.048553
24H 10MT/	Q9Y2X3	0.006449	0.018605	0.017567	NOP58	Nucleolar p	NOP58_HU	24	1.00448
24H 50MT/	Q8IYL3	-0.14986	0.018605	0.017567	C1orf174	UPF0688 p	CA174_HU	1	0.901335
24H 50MT/	Q9NTN9	0.197326	0.018619	0.017575	SEMA4G	Semaphori	SEM4G_HU	19	1.146571
48H 10MT/	Q9P2K5	0.989932	0.018624	0.017578	MYEF2	Myelin exp	MYEF2_HU	1	1.986091
24H 50MT/	Q99538	0.219276	0.018633	0.017583	LGMN	Legumain	LGMN_HUM	6	1.164149
24H 50MT/	O14936	0.132777	0.018634	0.017583	CASK	Peripheral	CSKP_HUM	18	1.096402
48H 50MT/	P02768	-0.41929	0.018642	0.017588	ALB	Albumin	ALBU_HUM	22	0.74779
24H 50MT/	Q5VTB9	0.20489	0.018652	0.017597	RNF220	E3 ubiquiti	RN220_HU	5	1.152598
24H 50MT/	Q9UM13	-0.00501	0.018661	0.017603	ANAPC10	Anaphase-p	APC10_HU	3	0.996532
48H 10MT/	O75131	0.014061	0.018669	0.017607	CPNE3	Copine-3	CPNE3_HU	23	1.009794
24H 50MT/	P30626	-0.00247	0.018671	0.017608	SRI	Sorcin	SORCN_HU	8	0.998287
48H 50MT/	Q9C0A1	0.028327	0.018673	0.01761	ZFHX2	Zinc finger	ZFHX2_HU	1	1.019829
48H 50MT/	Q9NX38	0.688343	0.018686	0.017617	ABITRAM	Protein Abi	ABITM_HU	1	1.611432
24H 10MT/	Q9Y6M5	0.104626	0.018701	0.017627	SLC30A1	Proton-cou	ZNT1_HUM	12	1.075216
48H 10MT/	P16152	0.044578	0.01871	0.017634	CBR1	Carbonyl re	CBR1_HUM	17	1.031381
48H 50MT/	Q99571	0.400571	0.018718	0.017638	P2RX4	P2X purino	P2RX4_HU	5	1.32003
48H 50MT/	Q9H8Y5	0.401446	0.018723	0.017642	ANKZF1	tRNA endo	ANKZ1_HU	12	1.320831
24H 10MT/	P07225	-0.10836	0.018725	0.017642	PROS1	Vitamin K-c	PROS_HUM	9	0.927642
24H 10MT/	Q9NZ01	0.095912	0.018728	0.017644	TECR	Very-long-c	TECR_HUM	12	1.068741
48H 10MT/	P63000	0.104578	0.01875	0.017661	RAC1	Ras-related	RAC1_HUM	9	1.07518
24H 50MT/	Q8NC60	-0.09307	0.018773	0.017677	NOA1	Nitric oxide	NOA1_HUM	14	0.937524
24H 50MT/	P62861	0.230748	0.018774	0.017678	FAU	Ubiquitin-l	RS30_HUM	2	1.173443
48H 50MT/	P47755	0.048575	0.018776	0.017679	CAPZA2	F-actin-cap	CAZA2_HU	11	1.034243
48H 50MT/	Q96NB3	-0.08046	0.018778	0.01768	ZNF830	Zinc finger	ZN830_HU	4	0.945759
48H 50MT/	P53597	-0.11592	0.018783	0.017682	SUCLG1	Succinate--	SUCA_HUM	9	0.922791
48H 10MT/	Q7Z5L9	0.081003	0.018787	0.017684	IRF2BP2	Interferon	I2BP2_HUM	21	1.057753
48H 50MT/	P52732	-0.16902	0.018787	0.017684	KIF11	Kinesin-like	KIF11_HUM	30	0.889445
24H 50MT/	Q9H6T0	-0.39945	0.0188	0.017691	ESRP2	Epithelial s	ESRP2_HUM	10	0.758145
48H 50MT/	Q9UIQ6	-0.17773	0.018802	0.017692	LNPEP	Leucyl-cyst	LCAP_HUM	12	0.884091
48H 50MT/	Q6UWE0	0.032299	0.018815	0.017703	LRSAM1	E3 ubiquiti	LRSM1_HU	25	1.02264
48H 50MT/	P49069	0.14204	0.018817	0.017703	CAMLG	Guided ent	CAMLG_HU	4	1.103465
48H 50MT/	O43776	0.037933	0.018822	0.017706	NARS1	Asparagine-	SYNC_HUM	26	1.026642
48H 50MT/	Q9NQS1	-0.06267	0.018824	0.017707	AVEN	Cell death r	AVEN_HUM	7	0.957488
48H 10MT/	P63010	0.010966	0.018826	0.017708	AP2B1	AP-2 compl	AP2B1_HU	45	1.00763
48H 50MT/	P04114	0.077395	0.018827	0.017709	APOB	Apolipopro	APOB_HUM	89	1.055111
24H 50MT/	Q14320	-0.116	0.018832	0.017711	FAM50A	Protein FA	FA50A_HU	12	0.922741
24H 50MT/	O14933	-0.10957	0.018835	0.017713	UBE2L6	Ubiquitin/I	UB2L6_HU	1	0.926862
48H 10MT/	P67936	0.000113	0.018839	0.017716	TPM4	Tropomyos	TPM4_HUM	11	1.000078
24H 10MT/	P49792	-0.01611	0.01884	0.017716	RANBP2	E3 SUMO-p	RBP2_HUM	96	0.988895
48H 50MT/	Q96NB2	0.214094	0.018843	0.017718	SFXN2	Sideroflexi	SFXN2_HU	8	1.159975
24H 50MT/	Q8N9I0	3.498751	0.01885	0.017723	SYT2	Synaptotag	SYT2_HUM	1	11.30392
48H 50MT/	Q12846	0.110329	0.018852	0.017724	STX4	Syntaxin-4	STX4_HUM	11	1.079474
24H 10MT/	Q14691	0.051604	0.018852	0.017724	GINS1	DNA replica	PSF1_HUM	7	1.036416
24H 50MT/	O60762	0.068211	0.018857	0.017728	DPM1	Dolichol-pl	DPM1_HUM	13	1.048416

48H	10MT/	Q9Y315	0.049313	0.018862	0.01773	DERA	Deoxyribos	DEOC_HUM	17	1.034772
48H	10MT/	P17844	-0.00929	0.018867	0.017733	DDX5	Probable A	DDX5_HUM	33	0.993584
24H	10MT/	Q96C86	-0.01759	0.018883	0.017745	DCPS	m7GpppX (	DCPS_HUM	14	0.987885
24H	10MT/	Q9P2I0	0.052101	0.018888	0.017749	CPSF2	Cleavage ar	CPSF2_HUM	31	1.036774
24H	50MT/	Q14781	0.492289	0.018907	0.017763	CBX2	Chromobo:	CBX2_HUM	5	1.406675
24H	10MT/	Q96RL7	0.056038	0.018907	0.017763	VPS13A	Intermemb	VP13A_HU	7	1.039607
24H	50MT/	O95235	-0.14902	0.018916	0.017769	KIF20A	Kinesin-like	KI20A_HUM	22	0.901862
48H	10MT/	P52788	0.07389	0.018924	0.017775	SMS	Spermine s	SPSY_HUM/	19	1.052551
24H	10MT/	Q03111	0.022912	0.018926	0.017776	MLLT1	Protein ENI	ENL_HUMA	6	1.016008
48H	50MT/	Q6P3W7	0.09033	0.018929	0.017778	SCYL2	SCY1-like p	SCYL2_HUM	17	1.064614
24H	10MT/	Q92539	-0.17868	0.018929	0.017778	LPIN2	Phosphatid	LPIN2_HUM	11	0.883511
48H	50MT/	O95747	0.108252	0.018935	0.017782	OXSR1	Serine/thre	OXSR1_HU	16	1.077922
24H	50MT/	Q7Z794	0.21641	0.018949	0.017791	KRT77	Keratin, typ	K2C1B_HU	14	1.161839
48H	50MT/	Q99959	0.15983	0.018952	0.017793	PKP2	Plakophilir	PKP2_HUM	27	1.117156
48H	50MT/	Q53GS9	-0.10985	0.018956	0.017796	USP39	Ubiquitin c	UBP39_HU	15	0.926687
48H	50MT/	P51812	0.022318	0.01896	0.017798	RPS6KA3	Ribosomal	KS6A3_HU	34	1.01559
48H	10MT/	O95905	0.093292	0.018963	0.017799	ECD	Protein ecd	ECD_HUMA	18	1.066802
24H	50MT/	Q9BTY7	-0.65783	0.018981	0.017812	HGH1	Protein HG	HGH1_HUM	12	0.63383
24H	50MT/	Q7Z7K6	0.098341	0.018991	0.01782	CENPV	Centromer	CENPV_HU	6	1.070542
48H	50MT/	Q8IXQ4	0.104548	0.018992	0.017821	GPALPP1	GPALPP mc	GPAM1_HU	3	1.075157
24H	10MT/	Q13905	-0.50912	0.019	0.017827	RAPGEF1	Rap guanin	RPGF1_HU	2	0.702653
48H	10MT/	P48147	0.059158	0.019003	0.017829	PREP	Prolyl endc	PPCE_HUM	35	1.041858
48H	50MT/	O95235	-0.47193	0.019005	0.01783	KIF20A	Kinesin-like	KI20A_HUM	22	0.721002
24H	50MT/	Q16740	0.094785	0.019009	0.017832	CLPP	ATP-depend	CLPP_HUM	10	1.067907
48H	10MT/	P45973	0.06676	0.019011	0.017834	CBX5	Chromobo:	CBX5_HUM	9	1.047362
24H	50MT/	O14972	-1.14449	0.01903	0.017848	VPS26C	Vacuolar pr	VP26C_HU	1	0.452349
48H	50MT/	Q5JTJ3	0.131502	0.019031	0.017848	COA6	Cytochrom	COA6_HUM	4	1.095433
48H	10MT/	P62495	-0.09114	0.01905	0.017863	ETF1	Eukaryotic	ERF1_HUM	20	0.938782
24H	10MT/	Q9NU23	0.221649	0.01907	0.017877	LYRM2	LYR motif-c	LYRM2_HU	3	1.166066
48H	50MT/	Q9H6Z4	0.077066	0.019078	0.017881	RANBP3	Ran-bindin	RANB3_HU	15	1.05487
48H	10MT/	P02751	0.190992	0.019079	0.017882	FN1	Fibronectir	FINC_HUM/	77	1.141549
48H	50MT/	Q5JRA6	0.157163	0.019082	0.017884	MIA3	Transport a	TGO1_HUM	43	1.115092
24H	10MT/	Q5VTR2	0.067681	0.019082	0.017884	RNF20	E3 ubiquiti	BRE1A_HU	30	1.04803
24H	50MT/	Q9Y6C9	0.048513	0.019085	0.017885	MTCH2	Mitochond	MTCH2_HU	12	1.034198
48H	50MT/	Q96JM3	-0.02583	0.019087	0.017887	CHAMP1	Chromosor	CHAP1_HU	17	0.982257
24H	10MT/	Q96MX6	-0.21345	0.019112	0.017906	DNAAF10	Dynein axo	DAA10_HU	10	0.86247
24H	10MT/	Q7Z794	0.397974	0.019115	0.017908	KRT77	Keratin, typ	K2C1B_HU	14	1.317656
48H	50MT/	P20042	0.190808	0.01912	0.01791	EIF2S2	Eukaryotic	IF2B_HUM/	19	1.141403
24H	50MT/	Q9P0L2	-0.81343	0.019122	0.017911	MARK1	Serine/thre	MARK1_HU	1	0.569028
24H	50MT/	Q92995	0.191917	0.019127	0.017914	USP13	Ubiquitin c	UBP13_HU	6	1.14228
48H	50MT/	Q16740	0.086014	0.019141	0.017924	CLPP	ATP-depend	CLPP_HUM	10	1.061433
48H	50MT/	P0C7T5	0.018123	0.019148	0.017929	ATXN1L	Ataxin-1-lik	ATX1L_HUN	3	1.012641
48H	10MT/	Q9Y4R8	0.016463	0.01915	0.017931	TELO2	Telomere le	TELO2_HUN	24	1.011477
48H	50MT/	P98194	0.05877	0.019153	0.017933	ATP2C1	Calcium-tra	AT2C1_HU	16	1.041577
48H	50MT/	Q96P70	0.054066	0.019169	0.017943	IPO9	Importin-9	IPO9_HUM	28	1.038187
24H	50MT/	Q15393	0.086187	0.019171	0.017944	SF3B3	Splicing fac	SF3B3_HUN	48	1.061561
48H	50MT/	Q99439	0.009286	0.019185	0.017954	CNN2	Calponin-2	CNN2_HUM	14	1.006457
24H	10MT/	Q6PIW4	0.170322	0.019189	0.017957	FIGNL1	Fidgetin-lik	FIGL1_HUM	8	1.12531
48H	50MT/	Q9NSP4	-0.45499	0.019193	0.01796	CENPM	Centromer	CENPM_HU	3	0.729513
48H	50MT/	Q96TC7	-0.01928	0.019194	0.01796	RMDN3	Regulator c	RMD3_HUN	15	0.986728
24H	10MT/	P04035	-0.24866	0.019214	0.017975	HMGCR	3-hydroxy-:	HMDH_HU	5	0.841678
24H	50MT/	Q969M3	-0.25605	0.019215	0.017975	YIPF5	Protein YIP	YIPF5_HUM	2	0.837375

24H 50MT/	P13639	-0.10223	0.019221	0.017979	EEF2	Elongation	EF2_HUMA	57	0.931593
48H 10MT/	O95045	-0.62131	0.019222	0.01798	UPP2	Uridine pho	UPP2_HUM	1	0.650082
24H 10MT/	Q99426	0.048433	0.019226	0.017983	TBCB	Tubulin-fol	TBCB_HUM	13	1.034141
24H 50MT/	Q03701	0.043202	0.019228	0.017984	CEBPZ	CCAAT/enh	CEBPZ_HUM	36	1.030398
24H 10MT/	P31431	0.478583	0.019229	0.017984	SDC4	Syndecan-4	SDC4_HUM	5	1.393374
24H 50MT/	Q9UNF1	0.080105	0.019229	0.017984	MAGED2	Melanoma-	MAGD2_HU	21	1.057095
48H 10MT/	P82930	0.081559	0.019233	0.017986	MRPS34	Small ribos	RT34_HUM	12	1.058161
48H 50MT/	Q9NS56	-0.30328	0.019236	0.017988	TOPORS	E3 ubiquiti	TOPRS_HUI	7	0.810411
48H 10MT/	Q86XK2	-0.02356	0.019246	0.017993	FBXO11	F-box only	FBX11_HUI	3	0.983803
24H 10MT/	Q16643	0.096216	0.019251	0.017997	DBN1	Drebrin	DREB_HUM	23	1.068966
48H 10MT/	Q96ES7	-0.10007	0.019273	0.018012	SGF29	SAGA-assoc	SGF29_HUI	4	0.932988
24H 50MT/	Q9BWF3	0.029184	0.019284	0.018019	RBM4	RNA-bindir	RBM4_HUM	4	1.020435
24H 50MT/	P63167	0.220914	0.019301	0.018031	DYNLL1	Dynein ligh	DYL1_HUM	1	1.165472
24H 50MT/	Q9Y2R9	-0.07522	0.019302	0.018031	MRPS7	Small ribos	RT07_HUM	12	0.9492
24H 50MT/	Q53FT3	0.02619	0.019306	0.018035	HIKESHI	Protein Hik	HIKES_HUM	5	1.018319
48H 10MT/	P35579	0.010382	0.019308	0.018036	MYH9	Myosin-9	MYH9_HUM	102	1.007222
48H 10MT/	Q6Y2X3	0.216909	0.01931	0.018037	DNAJC14	DnaJ homo	DJC14_HUI	2	1.162241
24H 50MT/	Q96PM5	-0.10979	0.01931	0.018037	RCHY1	RING finger	ZN363_HUI	1	0.926721
24H 50MT/	Q8TCG2	0.391549	0.019312	0.018037	PI4K2B	Phosphatid	P4K2B_HUI	9	1.311802
48H 50MT/	P18846	1.388688	0.019317	0.01804	ATF1	Cyclic AMP	ATF1_HUM	1	2.618405
24H 10MT/	P12429	0.104962	0.019325	0.018045	ANXA3	Annexin A3	ANXA3_HU	15	1.075466
24H 50MT/	P62306	0.04688	0.019324	0.018045	SNRPF	Small nucle	RUXF_HUM	7	1.033029
48H 10MT/	Q96PE3	0.112061	0.019338	0.018057	INPP4A	Inositol pol	INP4A_HUI	6	1.080771
24H 50MT/	O15173	0.0844	0.019349	0.018065	PGRMC2	Membrane-	PGRC2_HU	11	1.060247
48H 10MT/	Q8NEB9	0.01624	0.019351	0.018065	PIK3C3	Phosphatid	PK3C3_HUI	20	1.011321
24H 50MT/	Q96AG3	1.107533	0.019351	0.018065	SLC25A46	Mitochond	S2546_HUI	2	2.154768
48H 50MT/	P46108	0.025508	0.019352	0.018066	CRK	Adapter mc	CRK_HUMA	13	1.017838
24H 50MT/	O75934	-0.30452	0.019371	0.01808	BCAS2	Pre-mRNA-:	SPF27_HUI	11	0.809711
24H 50MT/	Q8WWQ0	0.170564	0.019387	0.018093	PHIP	PH-interact	PHIP_HUM	38	1.125499
24H 10MT/	Q04206	0.004184	0.019394	0.018097	RELA	Transcripti	TF65_HUM	15	1.002904
24H 10MT/	Q9H1E5	0.169334	0.019398	0.018099	TMX4	Thioredoxi	TMX4_HUM	5	1.12454
48H 10MT/	P52701	0.015812	0.019399	0.0181	MSH6	DNA misma	MSH6_HUM	51	1.01102
24H 50MT/	Q9H0G5	0.724792	0.019412	0.01811	NSRP1	Nuclear spe	NSRP1_HUI	4	1.652662
48H 10MT/	Q9BU61	0.037146	0.019412	0.01811	NDUFAF3	NADH dehy	NDUF3_HU	6	1.026082
48H 50MT/	Q96ES7	-0.28768	0.019427	0.018119	SGF29	SAGA-assoc	SGF29_HUI	4	0.819218
24H 50MT/	Q8NDZ4	0.294119	0.019431	0.018122	DIPK2A	Divergent p	DIK2A_HUM	4	1.226136
24H 10MT/	Q17R98	0.389458	0.01943	0.018122	ZNF827	Zinc finger	ZN827_HUI	2	1.309901
48H 10MT/	P51991	-0.08827	0.019434	0.018124	HNRNPA3	Heterogene	ROA3_HUM	22	0.940649
24H 10MT/	A1A4S6	-0.22384	0.019454	0.018136	ARHGAP10	Rho GTPase	RHG10_HU	4	0.856281
48H 50MT/	Q9UIV1	0.032281	0.019455	0.018138	CNOT7	CCR4-NOT t	CNOT7_HU	7	1.022628
24H 50MT/	Q6PJT7	0.04589	0.019476	0.018152	ZC3H14	Zinc finger	ZC3HE_HUI	32	1.03232
48H 10MT/	Q96GR4	0.369454	0.01948	0.018153	ZDHHC12	Palmitoyltr	ZDH12_HU	2	1.291864
48H 50MT/	Q92572	0.113515	0.019481	0.018154	AP3S1	AP-3 compl	AP3S1_HUI	7	1.081861
24H 50MT/	Q96K37	0.183727	0.019487	0.018156	SLC35E1	Solute carri	S35E1_HUM	6	1.135814
48H 50MT/	O75064	-0.65362	0.019489	0.018157	DENND4B	DENN doma	DEN4B_HU	2	0.635685
48H 10MT/	Q9P0V9	0.200497	0.019502	0.018167	SEPTIN10	Septin-10	SEP10_HUM	15	1.149094
24H 50MT/	O60869	-0.15839	0.019508	0.018172	EDF1	Endothelial	EDF1_HUM	7	0.896022
48H 10MT/	Q6P3X3	-0.01145	0.019517	0.018177	TTC27	Tetratricop	TTC27_HUM	21	0.992097
48H 10MT/	Q9H479	0.042995	0.019547	0.018198	FN3K	Fructosami	FN3K_HUM	8	1.03025
48H 10MT/	Q15393	0.010475	0.01956	0.018209	SF3B3	Splicing fac	SF3B3_HUM	48	1.007287
24H 10MT/	P20073	0.024722	0.019568	0.018216	ANXA7	Annexin A7	ANXA7_HU	19	1.017283
24H 50MT/	O43583	-0.19469	0.01957	0.018218	DENR	Density-reg	DENR_HUM	9	0.873758

48H 50MTA	Q9BTX3	0.472144	0.01959	0.018232	TMEM208	Transmemb	TM208_HU	1	1.38717
24H 10MTA	O75167	-0.0496	0.019595	0.018236	PHACTR2	Phosphatas	PHAR2_HU	3	0.966202
48H 10MTA	Q709F0	0.084314	0.019607	0.018244	ACAD11	Acyl-CoA de	ACD11_HU	11	1.060184
48H 50MTA	Q9Y6X4	0.101309	0.019609	0.018245	FAM169A	Soluble lam	F169A_HUI	12	1.072747
48H 50MTA	Q13242	6.33E-05	0.019611	0.018247	SRSF9	Serine/argi	SRSF9_HUN	14	1.000044
24H 50MTA	O14617	-0.08753	0.019626	0.018254	AP3D1	AP-3 compl	AP3D1_HU	36	0.94113
24H 50MTA	P49773	-0.08184	0.019627	0.018254	HINT1	Adenosine	HINT1_HUN	9	0.944849
24H 50MTA	Q9UPQ3	0.801364	0.019626	0.018254	AGAP1	Arf-GAP wit	AGAP1_HU	2	1.742748
48H 50MTA	P53396	-0.10853	0.019628	0.018255	ACLY	ATP-citrate	ACLY_HUM	56	0.92753
24H 10MTA	O60683	-0.0566	0.01963	0.018256	PEX10	Peroxisome	PEX10_HUI	3	0.961525
24H 10MTA	P78324	0.765986	0.019637	0.018262	SIRPA	Tyrosine-pr	SHPS1_HUI	3	1.700532
24H 50MTA	Q96QD8	-0.13028	0.019645	0.018267	SLC38A2	Sodium-cou	S38A2_HUI	5	0.913655
48H 10MTA	O14744	0.021783	0.019651	0.018269	PRMT5	Protein arg	ANM5_HUN	28	1.015213
48H 50MTA	Q15642	0.046114	0.019651	0.018269	TRIP10	Cdc42-inte	CIP4_HUMA	12	1.03248
24H 50MTA	Q13409	0.050666	0.019654	0.018271	DYNC1I2	Cytoplasmi	DC1I2_HUN	19	1.035743
48H 50MTA	Q16881	-0.13636	0.019664	0.018277	TXNRD1	Thioredoxi	TRXR1_HUI	28	0.909812
48H 10MTA	Q96DC7	-0.40075	0.019663	0.018277	TMCO6	Transmemb	TMCO6_HU	2	0.757462
24H 50MTA	Q68CP4	0.208992	0.019667	0.018279	HGSNAT	Heparan-al	HGNAT_HU	4	1.15588
48H 50MTA	P54727	0.100965	0.019672	0.018282	RAD23B	UV excision	RD23B_HU	15	1.07249
24H 50MTA	Q9H5X1	0.390168	0.019725	0.018318	CIAO2A	Cytosolic ir	CIA2A_HUN	5	1.310546
48H 10MTA	Q9H008	-0.04812	0.019729	0.018321	LHPP	Phospholys	LHPP_HUM	5	0.967196
24H 50MTA	P11142	-0.12055	0.019735	0.018327	HSPA8	Heat shock	HSP7C_HUI	27	0.919839
48H 10MTA	Q13243	0.013817	0.01974	0.018329	SRSF5	Serine/argi	SRSF5_HUN	8	1.009623
24H 50MTA	Q9H8G2	1.07738	0.019742	0.01833	CAAP1	Caspase act	CAAP1_HUI	2	2.110201
48H 50MTA	P11274	0.053739	0.019752	0.018336	BCR	Breakpoint	BCR_HUMA	10	1.037951
24H 10MTA	Q9H254	0.142537	0.019755	0.018337	SPTBN4	Spectrin be	SPTN4_HUI	2	1.103844
48H 10MTA	P16144	-0.0666	0.019757	0.018339	ITGB4	Integrin be	ITB4_HUMA	14	0.954889
48H 10MTA	P30043	-0.10537	0.019765	0.018344	BLVRB	Flavin redu	BLVRB_HUI	9	0.929568
48H 10MTA	O95218	0.189018	0.019785	0.018358	ZRANB2	Zinc finger	ZRAB2_HUI	14	1.139987
24H 50MTA	Q96FZ7	0.038064	0.019789	0.01836	CHMP6	Charged mu	CHMP6_HU	6	1.026735
48H 50MTA	O43187	-0.23025	0.0198	0.018369	IRAK2	Interleukin	IRAK2_HUN	4	0.85249
48H 10MTA	Q6KC79	0.006668	0.019803	0.018371	NIPBL	Nipped-B-li	NIPBL_HUN	47	1.004633
24H 10MTA	Q8N4L2	-0.0526	0.019806	0.018373	PIP4P2	Type 2 phos	PP4P2_HUI	3	0.964194
24H 50MTA	Q02487	1.301816	0.01981	0.018375	DSC2	Desmocolli	DSC2_HUM	2	2.465389
48H 10MTA	P82663	-0.01486	0.019826	0.018387	MRPS25	Small ribos	RT25_HUM	10	0.98975
48H 50MTA	Q53H12	-0.11484	0.019826	0.018387	AGK	Acylglycerc	AGK_HUMA	15	0.923486
24H 50MTA	Q96A65	0.094147	0.019841	0.018398	EXOC4	Exocyst cor	EXOC4_HUI	32	1.067434
24H 50MTA	O95625	-0.4814	0.019843	0.018399	ZBTB11	Zinc finger	ZBT11_HUN	4	0.716281
24H 10MTA	Q9UIQ6	0.124821	0.019845	0.0184	LNPEP	Leucyl-cyst	LCAP_HUM	12	1.090372
48H 50MTA	P51858	0.164773	0.019851	0.018405	HDGF	Hepatoma-	HDGF_HUN	22	1.12099
48H 10MTA	O60784	0.07235	0.019854	0.018407	TOM1	Target of M	TOM1_HUN	13	1.051428
24H 50MTA	Q8NDF8	0.069331	0.019867	0.018419	TENT4B	Terminal nu	PAPD5_HU	11	1.04923
24H 50MTA	O75351	-0.14501	0.019878	0.018428	VPS4B	Vacuolar pr	VPS4B_HUI	16	0.904376
24H 10MTA	O75643	0.074845	0.019892	0.018436	SNRNP200	U5 small nu	U520_HUM	116	1.053248
48H 50MTA	Q8N183	0.096066	0.019894	0.018437	NDUFAF2	NADH dehy	NDUF2_HU	10	1.068855
24H 10MTA	Q2TAL8	0.139374	0.019907	0.018447	QRICH1	Transcripti	QRIC1_HUN	13	1.101427
24H 50MTA	Q9BUB7	0.223891	0.019925	0.018458	TMEM70	Transmemb	TMM70_HU	6	1.167879
48H 10MTA	O00423	0.072943	0.019925	0.018458	EML1	Echinoderm	EMAL1_HU	11	1.05186
48H 10MTA	Q9HBL7	0.380348	0.01995	0.018477	PLGRKT	Plasminoge	PLRKT_HUN	5	1.301656
24H 50MTA	O96000	0.207945	0.019959	0.018483	NDUFB10	NADH dehy	NDUBA_HU	11	1.155042
24H 50MTA	O95297	0.192007	0.019963	0.018485	MPZL1	Myelin prot	MPZL1_HU	4	1.142352
48H 50MTA	Q96SB4	-0.19535	0.019965	0.018485	SRPK1	SRSF protei	SRPK1_HUI	18	0.873359

48H 50MT/	Q8N5C6	-0.08327	0.019967	0.018486	SRBD1	S1 RNA-bin	SRBD1_HU	21	0.943916
24H 50MT/	Q5VW36	-0.00523	0.019969	0.018487	FOCAD	Focadhesin	FOCAD_HU	22	0.996381
48H 50MT/	Q9Y5W7	0.13485	0.019971	0.018488	SNX14	Sorting nex	SNX14_HU	7	1.097979
24H 50MT/	P52926	0.050567	0.019973	0.018488	HMGA2	High mobil	HMGA2_HU	4	1.035672
48H 50MT/	Q14204	0.047641	0.019978	0.018491	DYNC1H1	Cytoplasmi	DYHC1_HU	233	1.033574
24H 50MT/	P58546	-0.42931	0.019982	0.018493	MTPN	Myotrophi	MTPN_HUM	2	0.742617
48H 50MT/	Q9BUP3	-0.07725	0.019982	0.018493	HTATIP2	Oxidoreduc	HTAI2_HUM	16	0.947864
48H 50MT/	Q9NX74	-0.01306	0.019993	0.018502	DUS2	tRNA-dihyd	DUS2L_HU	23	0.990986
24H 50MT/	Q5JPI9	0.151473	0.020017	0.018519	EEF1AKMT2	EEF1A lysin	EFMT2_HU	4	1.110703
48H 10MT/	P35222	0.058734	0.020031	0.018526	CTNNB1	Catenin bet	CTNB1_HU	35	1.041551
48H 50MT/	O43156	-0.23832	0.020034	0.018528	TTI1	TELO2-inte	TTI1_HUMA	15	0.847735
48H 10MT/	P0C0L4	0.313039	0.020042	0.018533	C4A	Compleme	CO4A_HUM	10	1.242322
48H 50MT/	O60927	0.081624	0.020046	0.018534	PPP1R11	E3 ubiquiti	PP1RB_HU	4	1.058209
48H 50MT/	Q86YD1	-0.30791	0.020054	0.018538	PTOV1	Prostate tu	PTOV1_HU	3	0.807813
48H 50MT/	P40938	-0.07311	0.020056	0.018538	RFC3	Replication	RFC3_HUM	17	0.950585
48H 10MT/	Q8WUM9	-0.63252	0.020055	0.018538	SLC20A1	Sodium-de	S20A1_HU	1	0.645047
48H 10MT/	P08581	0.818884	0.020057	0.018538	MET	Hepatocyte	MET_HUMA	1	1.764041
24H 10MT/	Q93008	-0.03241	0.02007	0.018546	USP9X	Ubiquitin c	USP9X_HU	64	0.977784
24H 50MT/	P78362	-0.10307	0.020071	0.018546	SRPK2	SRSF protei	SRPK2_HU	16	0.93105
48H 50MT/	Q9UBN7	0.206209	0.020074	0.018548	HDAC6	Histone dea	HDAC6_HU	16	1.153653
24H 50MT/	Q96ME1	-0.00258	0.020079	0.018552	FBXL18	F-box/LRR-	FXL18_HUM	20	0.998214
24H 10MT/	P18031	0.035921	0.020081	0.018553	PTPN1	Tyrosine-pr	PTN1_HUM	21	1.025211
24H 50MT/	Q2QL34	0.000145	0.020081	0.018553	MPV17L	Mpv17-like	MP17L_HU	4	1.000101
24H 10MT/	P32456	-0.17449	0.020088	0.018557	GBP2	Guanylate-l	GBP2_HUM	11	0.886081
24H 10MT/	P61086	0.028186	0.020088	0.018557	UBE2K	Ubiquitin-c	UBE2K_HU	12	1.019729
48H 10MT/	Q9BPU6	-0.3871	0.020089	0.018557	DPYSL5	Dihydropyr	DPYL5_HU	1	0.764666
48H 10MT/	Q14376	0.079595	0.02009	0.018557	GALE	UDP-glucos	GALE_HUM	13	1.056721
24H 50MT/	Q96JB6	-0.33875	0.020091	0.018559	LOXL4	Lysyl oxida	LOXL4_HU	11	0.790727
48H 50MT/	Q13569	1.584889	0.020095	0.018561	TDG	G/T mismat	TDG_HUMA	1	2.999848
24H 10MT/	P00156	0.332026	0.020104	0.018566	MT-CYB	Cytochrom	CYB_HUMA	1	1.25878
48H 50MT/	Q9H2M9	-0.01211	0.020107	0.018568	RAB3GAP2	Rab3 GTPas	RBGPR_HU	45	0.991644
48H 10MT/	P20340	0.027438	0.020118	0.018576	RAB6A	Ras-related	RAB6A_HU	11	1.019201
48H 10MT/	O43148	-0.08669	0.020142	0.018595	RNMT	mRNA cap g	MCES_HUM	13	0.941678
24H 50MT/	Q6SJ93	-0.2845	0.020144	0.018596	FAM111B	Serine prot	F111B_HU	8	0.821025
48H 50MT/	Q02241	-0.04554	0.020148	0.018599	KIF23	Kinesin-like	KIF23_HUM	17	0.968928
24H 10MT/	Q99547	0.162977	0.02015	0.0186	MPHOSPH6	M-phase ph	MPH6_HUM	3	1.119595
24H 10MT/	P15144	-0.07345	0.020191	0.018628	ANPEP	Aminopept	AMPN_HUM	25	0.950366
24H 10MT/	P28799	-0.08155	0.020226	0.018653	GRN	Progranuli	GRN_HUMA	9	0.94504
48H 10MT/	Q6IBW4	0.262581	0.020237	0.018659	NCAPH2	Condensin-	CNDH2_HU	7	1.199623
24H 50MT/	Q9NW82	0.160408	0.020238	0.018659	WDR70	WD repeat-	WDR70_HU	18	1.117603
24H 10MT/	Q6ZT21	0.022525	0.020242	0.018662	TMPPE	Transmemb	TMPPE_HU	4	1.015736
24H 10MT/	O14933	0.086894	0.020259	0.018673	UBE2L6	Ubiquitin/I	UB2L6_HU	1	1.062081
24H 50MT/	Q86SQ9	0.122654	0.02028	0.018689	DHDDS	Dehydrodo	DHDDS_HU	5	1.088736
48H 10MT/	O95376	0.078367	0.020289	0.018695	ARIH2	E3 ubiquiti	ARI2_HUMA	13	1.055822
24H 50MT/	Q9NTW7	-0.69502	0.020291	0.018696	ZFP64	Zinc finger	ZF64B_HUM	2	0.617703
48H 10MT/	Q8WW33	0.043865	0.020294	0.018698	GTSF1	Gametocyt	GTSF1_HUM	5	1.030872
24H 50MT/	Q5J8M3	0.016691	0.0203	0.018702	EMC4	ER membra	EMC4_HUM	6	1.011637
48H 50MT/	Q13042	-0.10642	0.020315	0.018712	CDC16	Cell divisio	CDC16_HU	23	0.928887
48H 10MT/	Q96RU3	0.102903	0.020317	0.018714	FNBP1	Formin-bin	FNBP1_HU	16	1.073932
24H 10MT/	Q9Y613	-0.37797	0.020347	0.018737	FHOD1	FH1/FH2 do	FHOD1_HU	8	0.76952
24H 10MT/	P05362	0.056403	0.020351	0.01874	ICAM1	Intercellula	ICAM1_HU	17	1.03987
24H 10MT/	Q2TAZ0	-0.29122	0.02036	0.018744	ATG2A	Autophagy-	ATG2A_HU	15	0.817212

24H	50MTA	O75874	0.040395	0.020367	0.018748	IDH1	Isocitrate d	IDHC_HUM	23	1.028395
48H	50MTA	P13995	-0.23103	0.020375	0.018753	MTHFD2	Bifunctiona	MTDC_HUM	13	0.852024
48H	50MTA	Q9UGQ3	0.333343	0.020382	0.018756	SLC2A6	Solute carri	GTR6_HUM	1	1.259929
48H	10MTA	Q6I9Y2	0.10629	0.020381	0.018756	THOC7	THO compl	THOC7_HU	8	1.076457
48H	10MTA	Q5VYK3	0.002659	0.020389	0.018761	ECPAS	Proteasom	ECM29_HU	69	1.001845
48H	50MTA	O95497	-1.49868	0.020392	0.018763	VNN1	Pantethein	VNN1_HUM	2	0.353877
48H	50MTA	Q13153	0.148621	0.020398	0.018766	PAK1	Serine/thre	PAK1_HUM	13	1.108509
24H	10MTA	P00395	0.321413	0.020404	0.018772	MT-CO1	Cytochrom	COX1_HUM	4	1.249553
24H	50MTA	Q8N4T4	0.326367	0.020412	0.018778	ARHGEF39	Rho guanin	ARG39_HU	4	1.253852
48H	10MTA	Q99459	0.02433	0.020413	0.018778	CDC5L	Cell divisio	CDC5L_HU	33	1.017008
24H	50MTA	P24468	-0.35915	0.020427	0.018788	NR2F2	COUP trans	COT2_HUM	4	0.779626
48H	10MTA	Q9Y6Y8	0.031652	0.020429	0.018788	SEC23IP	SEC23-inte	S23IP_HUM	27	1.022182
24H	50MTA	Q15293	0.070619	0.020434	0.018791	RCN1	Reticulocal	RCN1_HUM	15	1.050167
24H	10MTA	O15460	0.198837	0.020438	0.018795	P4HA2	Prolyl 4-hy	P4HA2_HU	16	1.147773
48H	50MTA	Q5JPI3	-0.43645	0.020446	0.018801	C3orf38	Uncharacte	CC038_HU	1	0.738951
24H	10MTA	Q9NVP1	0.053755	0.020451	0.018804	DDX18	ATP-depen	DDX18_HU	29	1.037963
24H	50MTA	Q9H4D0	2.418155	0.020451	0.018804	CLSTN2	Calsynenir	CSTN2_HU	1	5.344871
48H	10MTA	Q7Z7A3	-0.03287	0.020458	0.018808	CTU1	Cytoplasmi	CTU1_HUM	2	0.977476
24H	50MTA	Q9H706	-0.9644	0.020463	0.018812	GAREM1	GRB2-assoc	GARE1_HU	2	0.512492
48H	10MTA	P18031	0.008033	0.020471	0.018817	PTPN1	Tyrosine-pr	PTN1_HUM	21	1.005584
48H	50MTA	P14678	0.092323	0.020488	0.018829	SNRPB	Small nucle	RSMB_HUM	12	1.066085
48H	50MTA	P16298	0.303913	0.020493	0.018833	PPP3CB	Serine/thre	PP2BB_HU	4	1.234488
48H	50MTA	Q9UNF0	-0.01553	0.020496	0.018835	PACSIN2	Protein kin	PACN2_HU	16	0.989293
48H	10MTA	Q5VTE0	-1.48809	0.020515	0.018848	EEF1A1P5	Putative el	EF1A3_HUM	1	0.356485
48H	10MTA	P49848	0.028168	0.02052	0.018851	TAF6	Transcripti	TAF6_HUM	17	1.019717
48H	10MTA	Q14692	-0.03875	0.020524	0.018854	BMS1	Ribosome b	BMS1_HUM	33	0.973496
48H	10MTA	Q8WYA0	-0.0346	0.020536	0.018863	IFT81	Intraflagell	IFT81_HUM	2	0.976306
48H	10MTA	Q04446	0.020721	0.02054	0.018865	GBE1	1,4-alpha-g	GLGB_HUM	27	1.014466
24H	10MTA	Q15904	0.07539	0.020541	0.018866	ATP6AP1	V-type prot	VAS1_HUM	12	1.053646
48H	50MTA	Q6NUS6	-0.32389	0.020543	0.018866	TCTN3	Tectonic-3	TECT3_HUM	6	0.798911
48H	10MTA	Q9HB19	-0.57814	0.020544	0.018866	PLEKHA2	Pleckstrin h	PKHA2_HU	2	0.669829
48H	10MTA	P11168	-0.32089	0.020546	0.018867	SLC2A2	Solute carri	GTR2_HUM	7	0.800578
48H	50MTA	O60447	0.024069	0.020553	0.01887	EVI5	Ecotropic v	EVI5_HUM	12	1.016823
24H	10MTA	Q9H2P9	0.061581	0.020557	0.018873	DPH5	Diphthine r	DPH5_HUM	11	1.043608
48H	50MTA	Q6UX53	-0.10651	0.020563	0.018878	TMT1B	Thiol S-met	TMT1B_HU	10	0.928834
48H	10MTA	P63165	0.12789	0.020583	0.018892	SUMO1	Small ubiq	SUMO1_HU	3	1.092694
24H	10MTA	Q8N201	-0.00618	0.020596	0.0189	INTS1	Integrator c	INT1_HUM	32	0.995723
24H	10MTA	Q9NVA2	0.041719	0.020597	0.018902	SEPTIN11	Septin-11	SEP11_HUM	20	1.029339
48H	50MTA	Q14728	-0.34715	0.020602	0.018904	MFSD10	Major facili	MFS10_HU	3	0.786138
24H	50MTA	O75431	0.178077	0.020618	0.018915	MTX2	Metaxin-2	MTX2_HUM	10	1.131375
24H	10MTA	Q9NWZ5	-0.14399	0.020623	0.018917	UCKL1	Uridine-cyt	UCKL1_HU	14	0.905015
48H	10MTA	P06737	0.017605	0.020629	0.018922	PYGL	Glycogen p	PYGL_HUM	37	1.012277
24H	50MTA	Q9BPX3	-0.07245	0.020631	0.018922	NCAPG	Condensin	CND3_HUM	27	0.951024
48H	50MTA	O00233	-0.13642	0.020636	0.018923	PSMD9	26S protea	PSMD9_HU	7	0.909773
48H	50MTA	P10412	-0.18442	0.020636	0.018923	H1-4	Histone H1	H14_HUM	9	0.880004
48H	50MTA	Q9NZU5	-0.00957	0.020636	0.018923	LMCD1	LIM and cys	LMCD1_HU	21	0.993389
24H	10MTA	Q86Y46	2.234516	0.020636	0.018923	KRT73	Keratin, typ	K2C73_HU	1	4.706049
48H	10MTA	P04746;P0	-0.66241	0.020646	0.018928	AMY2A;AM	Pancreatic	AMYP_HUM	1	0.631824
48H	10MTA	Q9NRX2	-0.10699	0.020649	0.018929	MRPL17	Large ribos	RM17_HUM	10	0.928522
48H	10MTA	Q9Y6B6	0.053091	0.020661	0.018937	SAR1B	Small COPII	SAR1B_HU	13	1.037485
48H	10MTA	Q96EU7	-0.02543	0.020684	0.018953	C1GALT1C1	C1GALT1-sp	C1GLC_HU	9	0.982526
48H	50MTA	Q8N1F7	-0.05661	0.02069	0.018957	NUP93	Nuclear po	NUP93_HU	39	0.961518

48H 10MTA	O94979	0.018797	0.020693	0.018959	SEC31A	Protein tra	SC31A_HUI	36	1.013114
24H 10MTA	Q15758	0.087683	0.020698	0.018961	SLC1A5	Neutral am	AAAT_HUM	11	1.062663
24H 50MTA	Q96EK9	0.077236	0.020705	0.018964	KTI12	Protein KTI	KTI12_HUM	11	1.054995
48H 50MTA	Q9NUQ6	-0.01247	0.020709	0.018966	SPATS2L	SPATS2-like	SPS2L_HUM	18	0.991393
24H 50MTA	Q16851	0.068296	0.020719	0.018974	UGP2	UTP--gluco	UGPA_HUM	25	1.048478
48H 10MTA	Q01433	-0.00577	0.02072	0.018974	AMPD2	AMP deami	AMPD2_HU	28	0.996007
48H 50MTA	Q86X27	0.211841	0.020723	0.018976	RALGPS2	Ras-specific	RGPS2_HUI	7	1.158165
24H 50MTA	Q9Y6A4	-0.07119	0.020734	0.018984	CFAP20	Cilia- and fl	CFA20_HUI	11	0.951854
48H 10MTA	Q66LE6	0.073417	0.020749	0.018996	PPP2R2D	Serine/thre	2ABD_HUM	3	1.052206
24H 10MTA	Q9UQE7	0.051501	0.020752	0.018998	SMC3	Structural r	SMC3_HUM	49	1.036343
48H 50MTA	Q8IUW5	0.322459	0.020768	0.019009	RELL1	RELT-like pr	RELL1_HUM	2	1.25046
24H 50MTA	O75367	0.064011	0.020773	0.019011	MACROH2A	Core histor	H2AY_HUM	15	1.045368
24H 10MTA	P60880	0.15414	0.020772	0.019011	SNAP25	Synaptosor	SNP25_HUI	9	1.112758
48H 50MTA	Q9NPA8	-0.25668	0.020775	0.019012	ENY2	Transcripti	ENY2_HUM	1	0.837012
48H 50MTA	O60566	-0.21562	0.020778	0.019014	BUB1B	Mitotic che	BUB1B_HU	25	0.861175
24H 50MTA	Q96DX4	-0.03029	0.020784	0.019018	RSPRY1	RING finger	RSPRY_HUM	10	0.979225
24H 10MTA	Q7L523	0.069063	0.020785	0.019019	RRAGA	Ras-related	RRAGA_HU	10	1.049035
48H 50MTA	Q9Y4E5	-0.236	0.020786	0.019019	ZNF451	E3 SUMO-p	ZN451_HUI	8	0.849099
48H 50MTA	O95336	0.123285	0.020795	0.019024	PGLS	6-phospho	6PGL_HUM	12	1.089212
48H 50MTA	Q30201	-0.00554	0.020795	0.019024	HFE	Hereditary	HFE_HUMA	3	0.996168
24H 50MTA	P05155	0.374037	0.020806	0.019031	SERPING1	Plasma pro	IC1_HUMA	8	1.295974
48H 10MTA	Q6P2E9	0.011682	0.020805	0.019031	EDC4	Enhancer o	EDC4_HUM	49	1.00813
48H 50MTA	O00170	0.023472	0.020814	0.019037	AIP	AH recepto	AIP_HUMA	15	1.016403
24H 50MTA	P27694	0.026991	0.02082	0.01904	RPA1	Replication	RFA1_HUM	30	1.018885
48H 50MTA	Q8IVS2	-0.10623	0.020828	0.019045	MCAT	Malonyl-Co	FABD_HUM	13	0.929015
48H 10MTA	P07814	0.024257	0.020837	0.019051	EPRS1	Bifunctiona	SYEP_HUMA	85	1.016956
24H 50MTA	Q9HCS7	0.112782	0.02084	0.019053	XAB2	Pre-mRNA-	SYF1_HUMA	30	1.081311
24H 10MTA	O00483	-0.19449	0.020884	0.019083	NDUFA4	Cytochrom	NDUA4_HU	4	0.873884
24H 50MTA	Q15361	-0.69333	0.020893	0.019089	TTF1	Transcripti	TTF1_HUMA	5	0.618426
48H 10MTA	Q08379	-0.00799	0.020898	0.019094	GOLGA2	Golgin subf	GOGA2_HU	25	0.994477
48H 50MTA	Q8WXA9	-0.01838	0.020923	0.019112	SREK1	Splicing reg	SREK1_HUM	3	0.987343
48H 50MTA	O14717	-0.33478	0.020935	0.019116	TRDMT1	tRNA (cytos	TRDMT_HU	1	0.792906
48H 50MTA	Q9NPQ8	-0.12179	0.020942	0.01912	RIC8A	Synembryn	RIC8A_HUM	17	0.919048
48H 10MTA	Q14257	0.326276	0.020942	0.01912	RCN2	Reticulocal	RCN2_HUM	13	1.253773
48H 10MTA	Q13126	0.135362	0.020945	0.019122	MTAP	S-methyl-5'	MTAP_HUM	18	1.098368
48H 50MTA	Q6KC79	0.021099	0.02098	0.019145	NIPBL	Nipped-B-li	NIPBL_HUM	47	1.014732
48H 50MTA	Q8N128	-0.14402	0.020979	0.019145	FAM177A1	Protein FAM	F177A_HUI	4	0.904995
48H 50MTA	Q9NUD5	-0.20429	0.020981	0.019146	ZCCHC3	Zinc finger	ZCHC3_HUI	8	0.867963
48H 10MTA	Q9UK58	0.032644	0.020986	0.019148	CCNL1	Cyclin-L1	CCNL1_HUI	4	1.022885
48H 50MTA	O75663	0.089508	0.020996	0.019155	TIPRL	TIP41-like p	TIPRL_HUM	15	1.064007
48H 10MTA	P28070	0.040577	0.020999	0.019157	PSMB4	Proteasom	PSB4_HUM	10	1.028525
24H 50MTA	Q9UKX3	0.930826	0.021004	0.01916	MYH13	Myosin-13	MYH13_HU	1	1.906368
48H 50MTA	P20962	0.243729	0.021032	0.019181	PTMS	Parathymo	PTMS_HUM	1	1.184049
48H 10MTA	Q92598	-0.00021	0.021036	0.019185	HSPH1	Heat shock	HS105_HUI	44	0.999857
48H 50MTA	P49711	-0.24194	0.021078	0.019214	CTCF	Transcripti	CTCF_HUM	14	0.84561
24H 10MTA	Q86SQ4	0.348631	0.021103	0.019229	ADGRG6	Adhesion G	AGRG6_HU	8	1.273352
48H 50MTA	Q8TCY9	-0.26446	0.021112	0.019237	URGCP	Up-regulat	URGCP_HU	22	0.832512
24H 10MTA	Q13564	0.035628	0.021128	0.019247	NAE1	NEDD8-acti	ULA1_HUM	18	1.025003
48H 10MTA	O00338	0.25274	0.021134	0.01925	SULT1C2	Sulfotransf	ST1C2_HUM	1	1.191468
24H 50MTA	P23434	0.254657	0.021137	0.019251	GCSH	Glycine cle	GCSH_HUM	4	1.193052
48H 10MTA	Q9BX66	-0.00642	0.021137	0.019251	SORBS1	Sorbin and	SRBS1_HUM	31	0.995559
48H 50MTA	Q8N5K1	0.039725	0.021151	0.01926	CISD2	CDGSH iror	CISD2_HUM	7	1.027918

24H 10MTA	Q86UE4	-0.00595	0.021159	0.019266	MTDH	Protein LYR	LYRIC_HUM	22	0.995881
48H 50MTA	Q15084	0.135884	0.021178	0.01928	PDIA6	Protein dis	PDIA6_HUM	18	1.098766
24H 10MTA	Q9H4G0	0.324064	0.021181	0.019282	EPB41L1	Band 4.1-lil	E41L1_HUM	4	1.251852
24H 50MTA	Q9NRA8	-0.18426	0.0212	0.019296	EIF4ENIF1	Eukaryotic	4ET_HUMA	15	0.880097
24H 10MTA	O75477	0.052024	0.021212	0.019302	ERLIN1	Erlin-1	ERLN1_HUI	10	1.036718
24H 50MTA	Q9BQ13	0.027952	0.02122	0.019308	KCTD14	BTB/POZ dc	KCD14_HU	9	1.019564
48H 10MTA	A0A0B4J2A	0.211548	0.021222	0.019309	PPIAL4C	Peptidyl-pr	PAL4C_HUI	4	1.15793
48H 50MTA	Q6ZSZ5	-0.07804	0.02123	0.019314	ARHGEF18	Rho guanin	ARHGI_HUI	15	0.947341
24H 50MTA	Q9H2P0	0.128893	0.021234	0.019316	ADNP	Activity-de	ADNP_HUM	32	1.093454
48H 10MTA	P60866	0.105235	0.021236	0.019317	RPS20	Small ribos	RS20_HUM	5	1.07567
48H 10MTA	Q7RTS9	-0.33366	0.021243	0.019321	DYM	Dymeclin	DYM_HUMA	7	0.79352
48H 50MTA	Q9BV57	0.196591	0.021246	0.019322	ADI1	Acireducto	MTND_HUI	10	1.145987
24H 10MTA	P15311	0.087719	0.021259	0.01933	EZR	Ezrin	EZRI_HUMA	25	1.062689
24H 50MTA	P60709	0.030722	0.021268	0.019336	ACTB	Actin, cyto	ACTB_HUM	18	1.021523
48H 50MTA	Q8TDX7	-0.18118	0.021279	0.019345	NEK7	Serine/thre	NEK7_HUM	10	0.881983
48H 10MTA	Q9Y6R1	0.104125	0.021297	0.019355	SLC4A4	Electrogeni	S4A4_HUM	3	1.074842
48H 10MTA	Q9Y5W9	0.831792	0.021302	0.019357	SNX11	Sorting nex	SNX11_HUI	1	1.779895
24H 10MTA	Q9BQA1	0.010263	0.021306	0.019359	WDR77	Methylosor	MEP50_HU	12	1.007139
24H 50MTA	Q6Y1H2	0.295031	0.02131	0.019361	HACD2	Very-long-c	HACD2_HU	4	1.226912
24H 50MTA	Q9NXS2	-0.07695	0.021311	0.019361	QPCTL	Glutaminyl	QPCTL_HUI	7	0.948062
48H 50MTA	Q96QR8	-0.10249	0.021312	0.019361	PURB	Transcripti	PURB_HUM	7	0.931422
24H 50MTA	Q15392	0.129062	0.021329	0.019374	DHCR24	Delta(24)-s	DHC24_HU	17	1.093583
48H 10MTA	P49327	-0.11036	0.021333	0.019376	FASN	Fatty acid s	FAS_HUMA	123	0.926355
48H 50MTA	Q86W42	-0.02504	0.021354	0.019393	THOC6	THO compl	THOC6_HU	16	0.982795
48H 10MTA	Q9NRY4	0.037921	0.021377	0.01941	ARHGAP35	Rho GTPase	RHG35_HU	27	1.026633
48H 50MTA	Q9NQE9	-0.06227	0.021379	0.019411	HINT3	Adenosine	HINT3_HUI	7	0.957755
48H 50MTA	Q68CZ6	-0.32053	0.02139	0.019419	HAUS3	HAUS augm	HAUS3_HU	6	0.800777
48H 10MTA	A6NHR9	-0.02764	0.021396	0.019423	SMCHD1	Structural	SMHD1_HU	74	0.981025
48H 10MTA	Q9P2N6	-0.25805	0.021405	0.019427	KANSL3	KAT8 regula	KANL3_HUI	6	0.836217
24H 50MTA	Q9H0P0	0.133162	0.021405	0.019427	NT5C3A	Cytosolic 5	5NT3A_HUI	15	1.096694
24H 50MTA	Q9H1K1	0.231336	0.021408	0.019428	ISCU	Iron-sulfur	ISCU_HUMA	6	1.173921
24H 10MTA	Q15046	0.033856	0.021418	0.019437	KARS1	Lysine--tRN	SYK_HUMA	29	1.023745
24H 10MTA	Q9NYA1	0.450159	0.021419	0.019437	SPHK1	Sphingosin	SPHK1_HUI	1	1.366191
24H 50MTA	Q86YS3	0.531864	0.021421	0.019438	RAB11FIP4	Rab11 fami	RFIP4_HUM	3	1.445796
48H 10MTA	Q9UQ90	-0.56729	0.021445	0.019455	SPG7	Mitochond	SPG7_HUM	4	0.674884
48H 50MTA	P08579	0.101782	0.021454	0.019459	SNRPB2	U2 small nu	RU2B_HUM	6	1.073098
24H 10MTA	Q99757	-0.18282	0.02146	0.019462	TXN2	Thioredoxi	THIOM_HU	3	0.880982
48H 50MTA	Q14678	0.154904	0.021463	0.019464	KANK1	KN motif ar	KANK1_HU	23	1.113347
24H 50MTA	Q709F0	0.262457	0.021464	0.019465	ACAD11	Acyl-CoA de	ACD11_HU	11	1.199519
48H 50MTA	Q99633	0.324175	0.021467	0.019466	PRPF18	Pre-mRNA-	PRP18_HUI	2	1.251948
24H 50MTA	Q8NHP8	0.085115	0.021466	0.019466	PLBD2	Putative ph	PLBL2_HUI	10	1.060773
24H 50MTA	Q7Z4S6	-0.16019	0.021472	0.019469	KIF21A	Kinesin-like	KI21A_HUM	40	0.89491
48H 10MTA	Q9NWX6	-0.07468	0.021483	0.019476	THG1L	Probable tF	THG1_HUM	5	0.94955
48H 50MTA	P62861	0.214101	0.02149	0.019482	FAU	Ubiquitin-l	RS30_HUM	2	1.159981
24H 10MTA	P47224	0.041068	0.021499	0.019487	RABIF	Guanine nu	MSS4_HUM	4	1.028875
24H 10MTA	Q13033	0.006703	0.021498	0.019487	STRN3	Striatin-3	STRN3_HUI	14	1.004657
24H 50MTA	Q8N392	0.22289	0.021498	0.019487	ARHGAP18	Rho GTPase	RHG18_HU	15	1.167069
48H 50MTA	P60709	-0.00194	0.021504	0.019491	ACTB	Actin, cyto	ACTB_HUM	18	0.998655
48H 10MTA	P55081	0.105927	0.021519	0.019502	MFAP1	Microfibril	MFAP1_HU	11	1.076185
48H 50MTA	Q96F46	0.083456	0.021526	0.019507	IL17RA	Interleukin	I17RA_HUM	7	1.059553
24H 10MTA	P36021	0.143086	0.021534	0.019513	SLC16A2	Monocarbo	MOT8_HUM	2	1.104265
48H 10MTA	Q9Y2A7	0.059184	0.021535	0.019513	NCKAP1	Nck-associa	NCKP1_HU	37	1.041877

24H 50MT/	Q9UJP4	-0.41365	0.021548	0.019521	KLHL21	Kelch-like p	KLH21_HU	1	0.750723
48H 50MT/	Q8N6R0	-0.20839	0.021551	0.019522	METTL13	eEF1A lysin	EFNMT_HU	17	0.865502
24H 10MT/	O00154	-0.00455	0.021577	0.019541	ACOT7	Cytosolic a	BACH_HUM	13	0.996852
48H 10MT/	O75822	0.075775	0.021576	0.019541	EIF3J	Eukaryotic	EIF3J_HUM	15	1.053927
24H 10MT/	Q92882	-0.16797	0.021578	0.019542	OSTF1	Osteoclast-	OSTF1_HUI	7	0.890092
24H 50MT/	O15067	-0.00458	0.021593	0.019555	PFAS	Phosphorib	PUR4_HUM	39	0.996833
24H 10MT/	Q9UIA9	0.003427	0.021602	0.01956	XPO7	Exportin-7	XPO7_HUM	37	1.002378
48H 10MT/	Q96HY7	-0.46082	0.021605	0.019562	DHTKD1	2-oxoadipa	DHTK1_HU	5	0.726571
48H 10MT/	Q3KRA9	-0.02064	0.021615	0.019569	ALKBH6	Alpha-keto	ALKB6_HUI	3	0.985799
24H 50MT/	Q7KZN9	0.119327	0.021625	0.019575	COX15	Heme A syn	COX15_HU	9	1.086228
48H 10MT/	Q9Y2S6	0.136839	0.021626	0.019575	TMA7	Translation	TMA7_HUM	2	1.099493
24H 10MT/	Q8TCS8	0.000349	0.021633	0.019579	PNPT1	Polyribonu	PNPT1_HU	40	1.000242
24H 10MT/	Q9H773	-0.26961	0.021633	0.019579	DCTPP1	dCTP pyrop	DCTP1_HUI	8	0.829543
48H 50MT/	Q9GZS1	-0.13684	0.021635	0.01958	POLR1E	DNA-direct	RPA49_HUI	10	0.909512
48H 50MT/	P08240	0.035809	0.021666	0.019603	SRPRA	Signal reco	SRPRA_HUI	28	1.025131
48H 10MT/	Q68CZ2	0.06316	0.021696	0.019625	TNS3	Tensin-3	TENS3_HUM	36	1.044751
24H 10MT/	Q53EZ4	0.097314	0.021709	0.019634	CEP55	Centrosom	CEP55_HUI	13	1.06978
24H 10MT/	Q9HBM0	0.467395	0.021712	0.019636	VEZT	Vezatin	VEZA_HUM	3	1.382611
48H 10MT/	P48163	0.032041	0.02172	0.019641	ME1	NADP-depe	MAOX_HUM	20	1.022458
24H 50MT/	Q4KMP7	-0.05716	0.021735	0.019651	TBC1D10B	TBC1 doma	TB10B_HUI	13	0.961157
24H 50MT/	O43657	0.070029	0.02174	0.019653	TSPAN6	Tetraspanir	TSN6_HUM	5	1.049738
48H 10MT/	P08865	-0.1072	0.021749	0.019661	RPSA	Small ribos	RSSA_HUM	18	0.928386
48H 50MT/	Q5TAP6	-0.13227	0.021756	0.019666	UTP14C	U3 small nu	UT14C_HUI	8	0.912392
24H 50MT/	O95071	-0.02079	0.021773	0.019677	UBR5	E3 ubiquiti	UBR5_HUM	47	0.98569
24H 50MT/	P28072	0.13209	0.021778	0.01968	PSMB6	Proteasom	PSB6_HUM	8	1.09588
24H 50MT/	O43663	-0.13117	0.021782	0.019682	PRC1	Protein reg	PRC1_HUM	16	0.913092
24H 50MT/	Q8IVS2	0.169994	0.021782	0.019682	MCAT	Malonyl-Co	FABD_HUM	13	1.125054
48H 50MT/	Q9UDR5	0.236972	0.021789	0.019687	AASS	Alpha-amir	AASS_HUM	13	1.178517
48H 10MT/	P41214	0.04074	0.021792	0.019688	EIF2D	Eukaryotic	EIF2D_HUM	10	1.028641
24H 10MT/	Q15269	0.091683	0.021803	0.019694	PWP2	Periodic try	PWP2_HUM	27	1.065612
24H 50MT/	Q8IWB7	0.162403	0.021801	0.019694	WDFY1	WD repeat	WDFY1_HU	15	1.119149
24H 50MT/	Q6P2H3	-0.13643	0.021806	0.019696	CEP85	Centrosom	CEP85_HUI	10	0.90977
24H 10MT/	O75152	-0.00275	0.021822	0.019707	ZC3H11A	Zinc finger	ZC11A_HUI	18	0.998096
48H 50MT/	Q9Y6D5	0.013782	0.02184	0.01972	ARFGEF2	Brefeldin A	BIG2_HUM	56	1.009599
48H 50MT/	Q15742	-0.10186	0.021858	0.019733	NAB2	NGFI-A-bin	NAB2_HUM	6	0.931828
24H 10MT/	Q9H999	-0.04913	0.021858	0.019733	PANK3	Pantothena	PANK3_HU	10	0.966518
48H 10MT/	Q6UWE0	-0.01357	0.021865	0.019738	LRSAM1	E3 ubiquiti	LRSM1_HU	25	0.990637
48H 50MT/	P52179	-2.42865	0.021868	0.019739	MYOM1	Myomesin-	MYOM1_HU	2	0.185739
48H 10MT/	Q9Y2Q5	0.009509	0.021872	0.019741	LAMTOR2	Ragulator c	LTOR2_HUI	6	1.006613
48H 10MT/	Q5JTJ3	0.110182	0.021881	0.019747	COA6	Cytochrom	COA6_HUM	4	1.079364
48H 10MT/	Q96BD8	-1.39246	0.021894	0.019754	SKA1	Spindle anc	SKA1_HUM	2	0.380914
48H 10MT/	Q9UKX3	-0.43466	0.021897	0.019756	MYH13	Myosin-13	MYH13_HU	1	0.739867
48H 50MT/	P0C5J1;Q8	-0.68515	0.021922	0.019775	FAM86B2;F	Putative pr	F86B2_HUI	1	0.621943
48H 50MT/	Q66GS9	0.23009	0.021983	0.01982	CEP135	Centrosom	CP135_HUI	1	1.172908
48H 10MT/	P19367	0.05885	0.021984	0.01982	HK1	Hexokinase	HXK1_HUM	35	1.041635
24H 10MT/	Q9BR61	0.219118	0.021995	0.019827	ACBD6	Acyl-CoA-bi	ACBD6_HU	9	1.164022
24H 10MT/	Q9BWD1	-0.13994	0.022	0.019831	ACAT2	Acetyl-CoA	THIC_HUM	17	0.907556
24H 10MT/	Q92688	-0.12219	0.022004	0.019833	ANP32B	Acidic leuci	AN32B_HU	4	0.918794
48H 50MT/	Q9UBT2	0.034493	0.022011	0.019838	UBA2	SUMO-activ	SAE2_HUM	30	1.024197
48H 50MT/	P51684	0.914332	0.022016	0.019841	CCR6	C-C chemol	CCR6_HUM	1	1.884696
24H 50MT/	Q5BKZ1	-0.03311	0.022021	0.019844	ZNF326	DBIRD com	ZN326_HU	19	0.977309
24H 50MT/	Q9Y296	-0.20174	0.02203	0.01985	TRAPPC4	Trafficking	TPPC4_HUI	13	0.869502

48H	50MT/	Q9Y3Q3	0.090082	0.022032	0.019852	TMED3	Transmemb	TMED3_HU	3	1.064431
24H	50MT/	Q99543	0.030161	0.022041	0.019856	DNAJC2	DnaJ homo	DNJC2_HU	21	1.021126
24H	10MT/	Q8TC12	-0.11133	0.022043	0.019857	RDH11	Retinol deh	RDH11_HU	10	0.925737
48H	10MT/	Q86V21	-0.07103	0.022048	0.01986	AACS	Acetoacety	AACS_HUM	15	0.951957
48H	50MT/	Q8WV74	-0.22274	0.022049	0.01986	NUDT8	Mitochond	NUDT8_HU	6	0.856937
48H	10MT/	O60518	0.082523	0.022064	0.019869	RANBP6	Ran-bindin	RNBP6_HU	9	1.058868
48H	50MT/	Q8NCM8	0.060572	0.022064	0.019869	DYNC2H1	Cytoplasmi	DYHC2_HU	33	1.042879
24H	50MT/	Q8IZ81	0.050668	0.022066	0.01987	ELMOD2	ELMO doma	ELMD2_HU	8	1.035745
24H	10MT/	Q6P5R6	0.722243	0.022084	0.01988	RPL22L1	Ribosomal	RL22L_HUI	1	1.649745
48H	10MT/	P19174	0.073428	0.022085	0.01988	PLCG1	1-phosphat	PLCG1_HU	24	1.052214
24H	50MT/	Q8IXT5	0.080821	0.022088	0.019882	RBM12B	RNA-bindir	RB12B_HU	22	1.05762
48H	10MT/	O43502	0.069113	0.022093	0.019885	RAD51C	DNA repair	RA51C_HU	6	1.049072
24H	50MT/	Q96ER9	0.172278	0.022109	0.019897	CCDC51	Mitochond	MITOK_HU	11	1.126837
48H	50MT/	P01111	0.091065	0.022124	0.019906	NRAS	GTPase NRa	RASN_HUM	8	1.065156
24H	50MT/	Q9NPR9	0.239324	0.022135	0.019914	GPR108	Protein GPI	GP108_HU	5	1.180439
48H	10MT/	P83916	0.159262	0.022139	0.019917	CBX1	Chromobo:	CBX1_HUM	5	1.116716
24H	10MT/	P48553	0.435563	0.022146	0.019922	TRAPPC10	Trafficking	TPC10_HUI	3	1.352438
24H	50MT/	Q96CV9	-0.24829	0.022154	0.019926	OPTN	Optineurin	OPTN_HUM	13	0.841895
24H	50MT/	P04049	0.013572	0.022169	0.019935	RAF1	RAF proto-(	RAF1_HUM	16	1.009452
48H	50MT/	Q92887	-0.1495	0.022182	0.019945	ABCC2	ATP-bindin	MRP2_HUM	32	0.901565
24H	10MT/	Q29RF7	0.067388	0.022184	0.019946	PDS5A	Sister chror	PDS5A_HU	51	1.047818
24H	10MT/	Q96Q89	0.12023	0.022191	0.01995	KIF20B	Kinesin-like	KI20B_HUM	22	1.086908
24H	50MT/	Q8WW59	0.147564	0.022193	0.019951	SPRYD4	SPRY doma	SPRY4_HUI	13	1.107698
48H	10MT/	Q9BZJ0	0.035876	0.022196	0.019953	CRNKL1	Crooked ne	CRNL1_HU	24	1.025179
24H	50MT/	Q562E7	0.199604	0.022219	0.019966	WDR81	WD repeat-	WDR81_HU	24	1.148383
48H	10MT/	Q9Y2L1	0.015963	0.02222	0.019966	DIS3	Exosome cc	RRP44_HU	38	1.011126
48H	50MT/	Q9NZ01	-0.15256	0.022246	0.019984	TECR	Very-long-c	TECR_HUM	12	0.89965
24H	50MT/	Q6P6B7	-0.49573	0.022292	0.020012	ANKRD16	Ankyrin rep	ANR16_HU	1	0.709201
48H	50MT/	Q4VC44	-0.43294	0.022296	0.020015	FLYWCH1	FLYWCH-ty	FWCH1_HU	6	0.740751
24H	50MT/	P43897	0.069665	0.022297	0.020015	TSFM	Elongation	EFTS_HUM/	12	1.049473
48H	10MT/	Q9NP72	0.028635	0.022309	0.020022	RAB18	Ras-related	RAB18_HU	12	1.020047
24H	50MT/	Q2TB90	0.026535	0.022313	0.020024	HKDC1	Hexokinase	HKDC1_HU	18	1.018563
48H	50MT/	P61009	0.024386	0.022321	0.020029	SPCS3	Signal pept	SPCS3_HUI	4	1.017046
48H	10MT/	P02760	0.062371	0.022326	0.020032	AMBP	Protein AM	AMBP_HUM	11	1.044181
24H	10MT/	P42694	0.010661	0.022328	0.020033	HELZ	Probable h	HELZ_HUM	13	1.007417
48H	50MT/	P02647	0.370958	0.022342	0.020042	APOA1	Apolipoprc	APOA1_HU	9	1.293212
24H	50MT/	Q6UWE0	-0.17706	0.022367	0.020055	LRSAM1	E3 ubiquiti	LRSM1_HU	25	0.884506
48H	50MT/	Q14146	-0.20641	0.022376	0.020063	URB2	Unhealthy	URB2_HUM	22	0.866689
48H	50MT/	Q8NCN4	-0.28358	0.022377	0.020063	RNF169	E3 ubiquiti	RN169_HU	8	0.821549
48H	10MT/	Q9BQE3	0.087574	0.022381	0.020065	TUBA1C	Tubulin alp	TBA1C_HUI	4	1.062582
24H	10MT/	Q15393	0.072018	0.022382	0.020066	SF3B3	Splicing fac	SF3B3_HUI	48	1.051186
48H	10MT/	Q99426	0.155071	0.022404	0.020082	TBCB	Tubulin-fol	TBCB_HUM	13	1.113476
24H	10MT/	Q9P0L0	-0.11704	0.022405	0.020082	VAPA	Vesicle-assc	VAPA_HUM	9	0.922077
48H	10MT/	Q9H1E5	-0.24925	0.022413	0.020087	TMX4	Thioredoxi	TMX4_HUM	5	0.841334
48H	50MT/	Q9Y2T3	0.062038	0.022447	0.020112	GDA	Guanine de	GUAD_HUM	11	1.04394
48H	10MT/	P15056	0.288367	0.022451	0.020115	BRAF	Serine/thre	BRAF_HUM	4	1.221258
24H	10MT/	P50748	0.046751	0.022456	0.020119	KNTC1	Kinetochor	KNTC1_HU	36	1.032936
48H	10MT/	Q5T2D3	-0.03119	0.022461	0.020122	OTUD3	OTU domai	OTUD3_HU	1	0.978613
24H	10MT/	Q9NQC8	0.549662	0.022462	0.020123	IFT46	Intraflagell	IFT46_HUM	1	1.463743
48H	50MT/	Q9UJX3	-0.12875	0.022463	0.020124	ANAPC7	Anaphase-p	APC7_HUM	27	0.914623
24H	50MT/	A0MZ66	-0.10726	0.022465	0.020124	SHTN1	Shootin-1	SHOT1_HU	28	0.928353
24H	50MT/	O94919	0.06395	0.022466	0.020124	ENDOD1	Endonuclea	ENDD1_HU	13	1.045324

24H 10MT/	O95251	0.152855	0.02247	0.020127	KAT7	Histone ace	KAT7_HUM	15	1.111768
24H 50MT/	O75356	0.069006	0.02248	0.020135	ENTPD5	Nucleoside	ENTP5_HUI	4	1.048994
24H 10MT/	P62873	0.104053	0.022486	0.02014	GNB1	Guanine nu	GBB1_HUM	14	1.074788
48H 50MT/	Q15006	-0.07038	0.022488	0.02014	EMC2	ER membra	EMC2_HUM	10	0.952386
48H 50MT/	Q9NW08	0.043253	0.022492	0.020143	POLR3B	DNA-direct	RPC2_HUM	17	1.030435
24H 50MT/	O75794	-0.19437	0.022492	0.020143	CDC123	Translation	CD123_HU	12	0.873954
24H 50MT/	O75191	0.106055	0.022498	0.020147	XYLB	Xylulose kir	XYLB_HUM	9	1.076281
48H 50MT/	O15254	0.26712	0.022529	0.020165	ACOX3	Peroxisoma	ACOX3_HU	15	1.203403
24H 10MT/	Q9NVA1	0.112146	0.022534	0.020169	UQCC1	Ubiquinol-(	UQCC1_HU	6	1.080835
24H 50MT/	Q9BZL1	-0.30987	0.02254	0.020172	UBL5	Ubiquitin-l	UBL5_HUM	4	0.806717
48H 10MT/	Q9P275	-0.33161	0.022545	0.020176	USP36	Ubiquitin c	UBP36_HU	7	0.794647
24H 10MT/	Q9BTX1	0.087325	0.022547	0.020177	NDC1	Nucleopori	NDC1_HUM	17	1.062399
48H 10MT/	O00764	0.05499	0.022581	0.020196	PDXK	Pyridoxal k	PDXK_HUM	16	1.038852
48H 10MT/	Q9H0H5	0.052943	0.02258	0.020196	RACGAP1	Rac GTPase	RGAP1_HU	16	1.037379
24H 10MT/	Q9Y277	0.051431	0.022578	0.020196	VDAC3	Voltage-dep	VDAC3_HU	13	1.036292
48H 10MT/	Q9UHQ4	-0.25202	0.022594	0.020205	BCAP29	B-cell recep	BAP29_HUI	5	0.839719
48H 10MT/	Q9H497	-0.26671	0.022607	0.020215	TOR3A	Torsin-3A	TOR3A_HUI	7	0.831213
48H 10MT/	P01034	0.684928	0.022614	0.020219	CST3	Cystatin-C	CYTC_HUM	1	1.607622
48H 10MT/	O43933	0.066733	0.022615	0.020219	PEX1	Peroxisoma	PEX1_HUM	15	1.047342
48H 10MT/	Q92551	0.156165	0.022619	0.020222	IP6K1	Inositol hex	IP6K1_HUM	3	1.114321
24H 50MT/	Q9NY35	0.637664	0.022622	0.020225	CLDND1	Claudin dor	CLDN1_HU	3	1.555808
24H 10MT/	P49768	0.119871	0.022625	0.020226	PSEN1	Presenilin-1	PSN1_HUM	4	1.086638
24H 10MT/	Q9NYL4	0.000888	0.022636	0.020233	FKBP11	Peptidyl-pr	FKB11_HUI	5	1.000616
48H 50MT/	Q93074	-0.12959	0.022641	0.020236	MED12	Mediator o	MED12_HU	19	0.914091
48H 50MT/	Q9NTZ6	-0.09577	0.022669	0.020258	RBM12	RNA-bindir	RBM12_HU	22	0.935772
48H 10MT/	Q9C086	-0.71305	0.022686	0.02027	INO80B	INO80 com	IN80B_HUI	2	0.61003
24H 10MT/	P17174	-0.06316	0.022689	0.02027	GOT1	Aspartate a	AATC_HUM	23	0.957165
24H 50MT/	Q15070	-0.05307	0.022705	0.020281	OXA1L	Mitochond	OXA1L_HUI	8	0.963886
24H 50MT/	Q96GG9	0.03307	0.02272	0.020292	DCUN1D1	DCN1-like p	DCNL1_HU	10	1.023187
48H 10MT/	Q15435	0.033729	0.022722	0.020293	PPP1R7	Protein pho	PP1R7_HUI	18	1.023654
24H 50MT/	P09327	0.052827	0.022724	0.020294	VIL1	Villin-1	VILI_HUMA	23	1.037295
48H 10MT/	Q9NZ09	0.136954	0.022725	0.020294	UBAP1	Ubiquitin-a	UBAP1_HU	6	1.099581
24H 50MT/	P28702	0.17444	0.022726	0.020294	RXRB	Retinoic ac	RXRB_HUM	5	1.128526
24H 50MT/	P54577	-0.0592	0.022731	0.020299	YARS1	Tyrosine--tI	SYYC_HUM/	36	0.959793
24H 10MT/	O95394	-0.06619	0.022752	0.020315	PGM3	Phosphoac	AGM1_HUI	21	0.955159
48H 10MT/	Q96HQ2	-0.29796	0.022757	0.020318	CDKN2AIPI	CDKN2AIP I	C2AIL_HUN	2	0.813402
24H 50MT/	Q86XN6	-0.20763	0.022758	0.020319	ZNF761	Zinc finger	ZN761_HUI	1	0.86596
24H 10MT/	Q9BWF3	-0.13302	0.022761	0.02032	RBM4	RNA-bindir	RBM4_HUN	4	0.911921
48H 50MT/	Q6UXV4	0.054987	0.022768	0.020324	APOOL	MICOS com	MIC27_HUI	12	1.03885
24H 10MT/	P61978	0.046649	0.02277	0.020325	HNRNPK	Heterogene	HNRPK_HU	27	1.032863
48H 50MT/	Q9BSJ8	-0.07038	0.02278	0.02033	ESYT1	Extended sy	ESYT1_HUN	45	0.952389
24H 10MT/	Q96IZ0	0.079131	0.022783	0.020331	PAWR	PRKC apopt	PAWR_HUI	12	1.056382
24H 50MT/	Q13428	0.101981	0.022786	0.020333	TCOF1	Treacle pro	TCOF_HUM	28	1.073246
24H 10MT/	P78318	-0.08317	0.022804	0.020343	IGBP1	Immunoglo	IGBP1_HUN	15	0.943983
48H 50MT/	Q5HYK3	0.080732	0.022822	0.020353	COQ5	2-methoxy-	COQ5_HUM	12	1.057555
24H 10MT/	O75533	0.044537	0.022839	0.020364	SF3B1	Splicing fac	SF3B1_HUI	66	1.031352
24H 10MT/	P39060	0.325672	0.02284	0.020365	COL18A1	Collagen al	COIA1_HUI	14	1.253248
24H 10MT/	P60510	-0.01882	0.022845	0.020368	PPP4C	Serine/thre	PP4C_HUM	13	0.987038
24H 10MT/	Q9NY27	0.030175	0.022851	0.020373	PPP4R2	Serine/thre	PP4R2_HUI	14	1.021136
24H 50MT/	Q05682	0.016826	0.022855	0.020375	CALD1	Caldesmon	CALD1_HUI	19	1.011731
24H 10MT/	Q9P0U3	0.013181	0.022862	0.02038	SENP1	Sentrin-spe	SENP1_HUI	4	1.009178
24H 10MT/	P52597	0.048971	0.022865	0.020382	HNRNPF	Heterogene	HNRPF_HU	18	1.034527

48H 50MTA	Q13126	0.172497	0.022871	0.020386	MTAP	S-methyl-5'	MTAP_HUN	18	1.127007
48H 10MTA	O43719	0.034852	0.022883	0.020395	HTATSF1	17S U2 SnR	HTSF1_HUI	20	1.024452
24H 50MTA	Q9NQ88	-0.17943	0.022901	0.020409	TIGAR	Fructose-2,	TIGAR_HUN	9	0.883052
48H 10MTA	Q13485	0.145631	0.022902	0.020409	SMAD4	Mothers ag	SMAD4_HU	10	1.106214
48H 10MTA	Q8IXM3	-0.00454	0.022905	0.020411	MRPL41	Large ribos	RM41_HUN	6	0.99686
24H 10MTA	Q09161	0.069829	0.022909	0.020414	NCBP1	Nuclear cap	NCBP1_HU	28	1.049592
24H 10MTA	P27816	-0.11678	0.022913	0.020417	MAP4	Microtubul	MAP4_HUN	41	0.922247
48H 50MTA	Q15021	-0.13222	0.022917	0.020418	NCAPD2	Condensin	CND1_HUN	53	0.912429
48H 10MTA	P20020	0.011071	0.022929	0.020428	ATP2B1	Plasma mer	AT2B1_HUI	37	1.007703
24H 50MTA	Q14444	-0.10956	0.022931	0.020429	CAPRIN1	Caprin-1	CAPR1_HUI	13	0.926872
48H 50MTA	P62979	0.068678	0.022938	0.020433	RPS27A	Ubiquitin-r	RS27A_HUI	8	1.048755
48H 10MTA	Q9Y312	0.111211	0.022949	0.02044	AAR2	Protein AAI	AAR2_HUN	12	1.080134
24H 50MTA	Q9C0C4	0.510433	0.022962	0.020448	SEMA4C	Semaphori	SEM4C_HU	5	1.424477
24H 50MTA	Q9UNZ2	0.088854	0.022968	0.020452	NSFL1C	NSFL1 cofa	NSF1C_HUI	17	1.063525
24H 50MTA	Q9NXG2	0.045859	0.022973	0.020456	THUMPD1	THUMP doi	THUM1_HU	17	1.032298
48H 10MTA	Q96M27	0.085145	0.022989	0.020466	PRRC1	Protein PRI	PRRC1_HUI	10	1.060794
48H 10MTA	P14174	0.29968	0.022994	0.020467	MIF	Macrophag	MIF_HUMA	5	1.230871
48H 10MTA	Q15120	0.065107	0.023002	0.020473	PDK3	[Pyruvate d	PDK3_HUN	13	1.046162
48H 10MTA	Q8WV44	-0.2455	0.023014	0.020479	TRIM41	E3 ubiquiti	TRI41_HUN	2	0.843524
24H 50MTA	P78318	-0.09222	0.023015	0.020479	IGBP1	Immunoglo	IGBP1_HUN	15	0.938081
24H 10MTA	Q96E39	0.158791	0.023034	0.020493	RBMXL1	RNA bindin	RMXL1_HU	3	1.116351
48H 50MTA	Q9NUJ7	0.105937	0.023077	0.020525	PLCXD1	PI-PLC X do	PLCX1_HUI	4	1.076193
24H 50MTA	Q8IWW6	-0.21015	0.023082	0.020528	ARHGAP12	Rho GTPase	RHG12_HU	13	0.864449
24H 50MTA	P03897	0.002237	0.023088	0.020532	MT-ND3	NADH-ubiq	NU3M_HUI	1	1.001552
48H 50MTA	Q8N3F8	-0.00761	0.023093	0.020534	MICALL1	MICAL-like	MILK1_HUI	4	0.994737
48H 10MTA	Q9P2R7	-0.08436	0.023094	0.020534	SUCLA2	Succinate--	SUCB1_HUI	20	0.943202
24H 50MTA	Q12874	-0.02042	0.0231	0.020538	SF3A3	Splicing fac	SF3A3_HUN	26	0.985946
48H 50MTA	Q9NZM3	0.111162	0.023113	0.020546	ITSN2	Intersectin	ITSN2_HUN	23	1.080098
24H 10MTA	Q96JB6	0.383207	0.023114	0.020547	LOXL4	Lysyl oxidas	LOXL4_HUI	11	1.304238
48H 50MTA	Q5D862	0.329291	0.023115	0.020547	FLG2	Filaggrin-2	FILA2_HUN	6	1.256396
24H 10MTA	Q9BV10	-0.08222	0.023123	0.020552	ALG12	Dol-P-Man:	ALG12_HUI	4	0.944603
24H 50MTA	O94776	0.133297	0.023126	0.020555	MTA2	Metastasis-	MTA2_HUN	24	1.096797
48H 50MTA	Q96RT1	0.054104	0.023136	0.020562	ERBIN	Erbin	ERBIN_HUN	16	1.038214
48H 10MTA	O60884	0.029875	0.02314	0.020563	DNAJA2	DnaJ homo	DNJA2_HUI	18	1.020923
48H 10MTA	Q53EL6	0.162948	0.023147	0.020568	PDCD4	Programme	PDCD4_HU	12	1.119572
48H 10MTA	P50148	-0.11757	0.023151	0.020571	GNAQ	Guanine nu	GNAQ_HUN	2	0.921736
48H 10MTA	Q96KB5	0.083607	0.023161	0.020579	PBK	Lymphokin	TOPK_HUN	15	1.059664
48H 10MTA	Q8NBL1	-0.07488	0.023162	0.020579	POGLUT1	Protein O-g	PGLT1_HUI	12	0.949419
48H 10MTA	Q9Y394	-0.03467	0.023172	0.020586	DHRS7	Dehydroge	DHRS7_HU	14	0.976257
48H 50MTA	O94776	-0.10977	0.023176	0.020589	MTA2	Metastasis-	MTA2_HUN	24	0.926738
48H 50MTA	Q8WUA7	0.246884	0.023178	0.020589	TBC1D22A	TBC1 doma	TB22A_HUI	9	1.186641
24H 50MTA	O14841	0.401619	0.02319	0.020598	OPLAH	5-oxoprolir	OPLA_HUN	7	1.320989
24H 10MTA	Q8N4V1	-0.28595	0.023196	0.020602	MMGT1	ER membra	EMC5_HUN	4	0.820199
24H 50MTA	P54105	0.160093	0.0232	0.020605	CLNS1A	Methylosor	ICLN_HUM	8	1.117359
48H 50MTA	Q96NT5	-1.43231	0.023201	0.020606	SLC46A1	Proton-cou	PCFT_HUM	3	0.370536
48H 10MTA	Q5BKT4;Q5	-0.37848	0.02321	0.020611	ALG10;ALG	Dol-P-Glc:G	AG10A_HU	3	0.769248
48H 50MTA	O75688	0.062163	0.023219	0.020617	PPM1B	Protein pho	PPM1B_HU	8	1.04403
48H 50MTA	Q9Y5U2	-0.47784	0.02324	0.02063	TSSC4	U5 small nu	TSSC4_HUN	3	0.71805
48H 10MTA	Q5VW32	0.02416	0.023255	0.020641	BROX	BRO1 doma	BROX_HUN	7	1.016887
24H 10MTA	Q01415	-0.0402	0.023258	0.020643	GALK2	N-acetylgal	GALK2_HUI	10	0.97252
24H 50MTA	Q6P6C2	0.224657	0.023274	0.020655	ALKBH5	RNA demet	ALKB5_HUI	12	1.168499
24H 10MTA	Q2TAA2	-0.09613	0.023289	0.020662	IAH1	Isoamyl ace	IAH1_HUM	8	0.93554

48H 10MTA	O43164	0.350095	0.023291	0.020663	PJA2	E3 ubiquiti	PJA2_HUM	5	1.274644
48H 10MTA	Q9Y2H2	-0.08395	0.023291	0.020663	INPP5F	Phosphatic	SAC2_HUM	5	0.943473
24H 50MTA	Q14493	-0.12869	0.023295	0.020666	SLBP	Histone RN	SLBP_HUM	4	0.914663
48H 10MTA	O76075	0.378015	0.02331	0.020676	DFFB	DNA fragme	DFFB_HUM	1	1.299552
24H 50MTA	Q9UNW1	0.169526	0.023313	0.020678	MINPP1	Multiple in	MINP1_HU	11	1.124689
48H 10MTA	Q8TAF3	0.017515	0.023326	0.020687	WDR48	WD repeat-	WDR48_HU	13	1.012214
48H 50MTA	P08123	0.319363	0.023329	0.020689	COL1A2	Collagen al	CO1A2_HU	3	1.247779
48H 50MTA	Q8IYU8	0.241269	0.023356	0.020709	MICU2	Calcium up	MICU2_HU	13	1.182032
24H 10MTA	P21741	0.432968	0.02336	0.020712	MDK	Midkine	MK_HUMA	2	1.350008
24H 10MTA	P01024	0.086919	0.023368	0.020717	C3	Compleme	CO3_HUMA	58	1.0621
24H 10MTA	P28370	0.048976	0.023369	0.020717	SMARCA1	Probable gl	SMCA1_HU	24	1.03453
48H 50MTA	Q86WW8	0.10706	0.023377	0.020721	COA5	Cytochrom	COA5_HUM	2	1.077031
48H 50MTA	Q9Y221	-0.06498	0.023385	0.020725	NIP7	60S ribosor	NIP7_HUM	9	0.955961
24H 50MTA	Q00587	-0.17181	0.023405	0.020738	CDC42EP1	Cdc42 effec	BORG5_HU	6	0.887726
48H 10MTA	Q8IWT6	0.004918	0.023412	0.020743	LRRC8A	Volume-reg	LRC8A_HU	15	1.003414
48H 10MTA	Q9UKY7	0.089123	0.023417	0.020746	CDV3	Protein CD'	CDV3_HUM	13	1.063723
48H 50MTA	Q9Y576	0.003886	0.023418	0.020747	ASB1	Ankyrin rep	ASB1_HUM	2	1.002697
48H 50MTA	P14324	-0.13556	0.023425	0.020751	FDPS	Farnesyl py	FPPS_HUM	10	0.910318
24H 10MTA	P11597	0.324782	0.023426	0.020752	CETP	Cholesteryl	CETP_HUM	1	1.252475
24H 10MTA	Q9H2C2	-0.1953	0.023433	0.020757	ARV1	Protein AR'	ARV1_HUM	1	0.87339
24H 10MTA	Q9UK61	0.031801	0.023436	0.020758	TASOR	Protein TAS	TASOR_HU	40	1.022287
24H 50MTA	Q99614	-0.15483	0.023455	0.020772	TTC1	Tetratricop	TTC1_HUM	13	0.898236
48H 10MTA	Q12913	-0.1336	0.023458	0.020772	PTPRJ	Receptor-ty	PTPRJ_HUM	7	0.911553
48H 50MTA	Q9Y282	0.145567	0.023457	0.020772	ERGIC3	Endoplasm	ERGI3_HUM	9	1.106165
24H 10MTA	Q86Y56	-0.02583	0.023462	0.020774	DNAAF5	Dynein axo	DAAF5_HU	35	0.982252
48H 10MTA	Q7L1V2	0.058511	0.023473	0.020782	MON1B	Vacuolar fu	MON1B_HU	4	1.04139
24H 10MTA	P11279	0.085926	0.023474	0.020783	LAMP1	Lysosome-a	LAMP1_HU	6	1.061369
48H 50MTA	O00499	-0.0488	0.023477	0.020785	BIN1	Myc box-de	BIN1_HUM	10	0.96674
48H 50MTA	O14976	0.037339	0.023477	0.020785	GAK	Cyclin-G-as	GAK_HUMA	26	1.026219
24H 50MTA	P51970	0.072308	0.023479	0.020785	NDUFA8	NADH dehy	NDUA8_HU	7	1.051397
24H 10MTA	O43684	0.054759	0.023488	0.020791	BUB3	Mitotic che	BUB3_HUM	12	1.038685
24H 10MTA	Q8WUD6	-0.01993	0.023488	0.020791	CHPT1	Cholinephc	CHPT1_HU	1	0.986278
24H 50MTA	P30049	0.315307	0.023491	0.020792	ATP5F1D	ATP syntha	ATPD_HUM	5	1.244276
48H 10MTA	Q8IYB1	0.05454	0.023491	0.020792	MB21D2	Nucleotidy	M21D2_HU	10	1.038528
48H 50MTA	Q13190	0.120001	0.023507	0.020803	STX5	Syntaxin-5	STX5_HUM	13	1.086736
24H 10MTA	Q9BU76	0.078348	0.023509	0.020805	MMTAG2	Multiple m	MMTA2_HU	5	1.055808
48H 50MTA	Q96DE5	-0.3577	0.023522	0.020814	ANAPC16	Anaphase-p	APC16_HU	4	0.780407
48H 10MTA	Q13555	0.218494	0.023525	0.020815	CAMK2G	Calcium/ca	KCC2G_HU	6	1.163519
24H 50MTA	A1A4S6	-0.04758	0.023529	0.020818	ARHGAP10	Rho GTPase	RHG10_HU	4	0.967559
48H 50MTA	P08047	0.227345	0.023534	0.020822	SP1	Transcripti	SP1_HUMA	3	1.170679
24H 50MTA	Q86VN1	-0.13345	0.02354	0.020826	VPS36	Vacuolar pr	VPS36_HU	17	0.911649
24H 10MTA	O95347	0.013495	0.023542	0.020826	SMC2	Structural r	SMC2_HUM	48	1.009398
24H 10MTA	Q8WYP5	0.043987	0.023561	0.020838	AHCTF1	Protein ELY	ELYS_HUMA	52	1.030959
48H 50MTA	Q96G25	0.886598	0.023564	0.02084	MED8	Mediator o	MED8_HUM	2	1.848812
48H 50MTA	Q96JB6	-0.25532	0.023577	0.020849	LOXL4	Lysyl oxida	LOXL4_HU	11	0.837803
48H 50MTA	Q14554	0.244919	0.02358	0.020849	PDIA5	Protein dis	PDIA5_HUM	14	1.185026
24H 50MTA	Q9Y371	-0.08427	0.023599	0.020864	SH3GLB1	Endophilin	SHLB1_HU	13	0.943259
24H 10MTA	O43852	0.047187	0.023606	0.020869	CALU	Calumenin	CALU_HUM	21	1.033248
48H 10MTA	O60934	0.009461	0.02361	0.020869	NBN	Nibrin	NBN_HUMA	22	1.006579
48H 50MTA	Q7Z7K0	-0.20737	0.023618	0.020874	CMC1	COX assem	COXM1_HU	3	0.866117
48H 50MTA	Q9BY49	0.173865	0.023618	0.020874	PECR	Peroxisoma	PECR_HUM	11	1.128076
48H 50MTA	Q9Y2X7	-0.0206	0.023623	0.020876	GIT1	ARF GTPase	GIT1_HUMA	11	0.985823

48H 50MT/	O14672	-0.02153	0.023633	0.020883	ADAM10	Disintegrin	ADA10_HU	22	0.985187
48H 10MT/	Q9Y261	0.164933	0.023641	0.020888	FOXA2	Hepatocyte	FOXA2_HU	3	1.121114
48H 10MT/	O43684	-0.04411	0.023662	0.020903	BUB3	Mitotic che	BUB3_HUM	12	0.969888
24H 50MT/	Q9Y6I4	0.147765	0.023663	0.020903	USP3	Ubiquitin c	UBP3_HUM	16	1.107852
24H 10MT/	P08238	-0.14443	0.023676	0.02091	HSP90AB1	Heat shock	HS90B_HU	50	0.904736
24H 10MT/	Q5VTB9	0.040354	0.023678	0.020911	RNF220	E3 ubiquiti	RN220_HU	5	1.028366
48H 50MT/	Q9UKE5	0.004697	0.023686	0.020918	TNIK	TRAF2 and	TNIK_HUM/	13	1.003261
24H 50MT/	Q9Y5W7	0.254129	0.023695	0.020924	SNX14	Sorting nex	SNX14_HU	7	1.192615
24H 50MT/	Q86XL3	0.021976	0.023701	0.020928	ANKLE2	Ankyrin rep	ANKL2_HU	21	1.015349
48H 50MT/	Q8IY37	-0.22401	0.023731	0.020949	DHX37	Probable A	DHX37_HU	32	0.85618
48H 10MT/	Q14116	0.123709	0.023733	0.02095	IL18	Interleukin	IL18_HUM/	10	1.089533
24H 50MT/	Q6GMV3	-0.30284	0.02375	0.020963	PTRHD1	Putative pe	PTRD1_HU	6	0.810655
24H 50MT/	Q96DB5	0.142453	0.023751	0.020963	RMDN1	Regulator c	RMD1_HUN	13	1.10378
48H 10MT/	Q03135	0.649207	0.023753	0.020965	CAV1	Caveolin-1	CAV1_HUM	3	1.568306
48H 50MT/	Q16763	-0.42354	0.023784	0.020986	UBE2S	Ubiquitin-c	UBE2S_HU	7	0.745592
48H 50MT/	P27797	0.041379	0.023802	0.020999	CALR	Calreticulir	CALR_HUM	21	1.029097
24H 50MT/	O43776	0.02307	0.02381	0.021005	NARS1	Asparagine-	SYNC_HUM	26	1.016119
24H 50MT/	Q96Q07	-0.94671	0.023836	0.021022	BTBD9	BTB/POZ dc	BTBD9_HU	1	0.518814
24H 50MT/	P84095	-0.05846	0.023838	0.021024	RHOG	Rho-relatec	RHOG_HUN	8	0.960287
48H 50MT/	Q6GQQ9	0.145626	0.023843	0.021026	OTUD7B	OTU domai	OTU7B_HU	10	1.106211
48H 10MT/	Q01628	-0.30001	0.023855	0.021034	IFITM3	Interferon-i	IFM3_HUM	3	0.812246
48H 50MT/	O75438	-0.86356	0.023858	0.021036	NDUFB1	NADH dehy	NDUB1_HU	1	0.549594
48H 50MT/	Q86WJ1	0.11996	0.023861	0.021038	CHD1L	Chromodo	CHD1L_HU	16	1.086705
24H 50MT/	Q6ZMG9	0.116988	0.023877	0.021049	CERS6	Ceramide s	CERS6_HUN	6	1.084469
48H 10MT/	O94822	-0.00181	0.023878	0.021049	LTN1	E3 ubiquiti	LTN1_HUM	40	0.998745
48H 10MT/	Q969S3	0.018889	0.023879	0.02105	ZNF622	Cytoplasmi	ZN622_HU	14	1.013179
24H 50MT/	Q9ULA0	0.044859	0.023881	0.021051	DNPEP	Aspartyl am	DNPEP_HU	16	1.031582
24H 50MT/	Q07157	0.035137	0.023889	0.021056	TJP1	Tight juncti	ZO1_HUMA	50	1.024654
24H 50MT/	Q7LBC6	0.117923	0.023893	0.021057	KDM3B	Lysine-spec	KDM3B_HU	31	1.085172
24H 10MT/	Q96AQ6	-0.08549	0.023896	0.021059	PBXIP1	Pre-B-cell le	PBIP1_HUN	19	0.942461
48H 50MT/	Q9NZN3	-0.05753	0.023903	0.021064	EHD3	EH domain-	EHD3_HUM	18	0.960909
24H 10MT/	Q15629	0.056834	0.023907	0.021066	TRAM1	Translocati	TRAM1_HU	6	1.04018
24H 50MT/	O95373	-0.11667	0.02392	0.021075	IPO7	Importin-7	IPO7_HUM	43	0.922312
48H 50MT/	P45973	0.029549	0.02393	0.021083	CBX5	Chromobo:	CBX5_HUM	9	1.020693
48H 10MT/	Q9H4M9	0.095765	0.023931	0.021083	EHD1	EH domain-	EHD1_HUM	24	1.068632
48H 50MT/	P15056	0.314669	0.023933	0.021085	BRAF	Serine/thre	BRAF_HUM	4	1.243726
48H 10MT/	Q9NZD8	-0.01651	0.023936	0.021086	SPG21	Maspardin	SPG21_HU	13	0.988625
24H 50MT/	Q14677	-0.00098	0.023939	0.021088	CLINT1	Clathrin int	EPN4_HUM	19	0.999319
24H 50MT/	Q15005	0.13494	0.023938	0.021088	SPCS2	Signal pept	SPCS2_HUN	10	1.098047
48H 50MT/	Q9H6H4	-0.18045	0.023947	0.021095	REEP4	Receptor e	REEP4_HUN	4	0.882428
24H 10MT/	Q14847	-0.13883	0.023952	0.021097	LASP1	LIM and SH	LASP1_HUN	12	0.908254
48H 50MT/	P04792	0.091837	0.023959	0.021102	HSPB1	Heat shock	HSPB1_HU	16	1.065726
24H 50MT/	Q9BVC6	0.445363	0.023967	0.021106	TMEM109	Voltage-gat	TM109_HU	2	1.361657
24H 50MT/	O60476	0.115082	0.023967	0.021106	MAN1A2	Mannosyl-c	MA1A2_HU	8	1.083036
48H 50MT/	Q9Y6X3	0.069795	0.023972	0.021109	MAU2	MAU2 chro	SCC4_HUM	10	1.049568
48H 10MT/	Q9H900	0.108983	0.023974	0.02111	ZWILCH	Protein zwi	ZWILC_HU	13	1.078468
24H 50MT/	Q5VT52	-0.02108	0.023982	0.021115	RPRD2	Regulation	RPRD2_HU	26	0.985493
48H 10MT/	O43865	0.10524	0.023985	0.021117	AHCYL1	S-adenosyll	SAHH2_HU	12	1.075673
48H 10MT/	Q9HAT2	0.071889	0.023996	0.021125	SIAE	Sialate O-ac	SIAE_HUM/	5	1.051092
48H 10MT/	Q9C0D3	-0.31092	0.024018	0.021141	ZYG11B	Protein zyg	ZY11B_HUN	12	0.806125
48H 50MT/	O43670	-0.02035	0.024034	0.021151	ZNF207	BUB3-inter	ZN207_HU	7	0.985994
24H 50MT/	Q9UKM7	0.010698	0.024034	0.021151	MAN1B1	Endoplasm	MA1B1_HU	13	1.007443

24H 50MTA	P06746	0.281597	0.024036	0.021151	POLB	DNA polym	DPOLB_HU	7	1.215539
48H 10MTA	P61158	0.02702	0.024042	0.021155	ACTR3	Actin-relate	ARP3_HUM	18	1.018905
48H 10MTA	O00479	-1.18219	0.024044	0.021156	HMGN4	High mobil	HMGN4_HU	1	0.440682
24H 10MTA	Q32P44	-0.03717	0.02406	0.021168	EML3	Echinoderm	EMAL3_HU	16	0.974565
24H 10MTA	Q9HAV4	0.006942	0.024061	0.021168	XPO5	Exportin-5	XPO5_HUM	47	1.004824
48H 50MTA	Q96BK5	-0.31255	0.024068	0.021171	PINX1	PIN2/TERF1	PINX1_HUM	7	0.805219
48H 50MTA	Q9Y2S2	0.923699	0.024072	0.021173	CRYL1	Lambda-cry	CRYL1_HUM	8	1.896973
48H 50MTA	O00291	0.168674	0.024076	0.021176	HIP1	Huntingtin	HIP1_HUM	22	1.124025
24H 50MTA	Q8WZ42	-0.23935	0.024077	0.021177	TTN	Titin	TITIN_HUM	10	0.847128
48H 10MTA	Q9H2J7	-1.00066	0.024078	0.021177	SLC6A15	Sodium-dep	S6A15_HUM	1	0.499772
48H 50MTA	P35579	-0.00888	0.024099	0.021191	MYH9	Myosin-9	MYH9_HUM	102	0.993866
24H 50MTA	Q9Y576	0.15138	0.024104	0.021195	ASB1	Ankyrin rep	ASB1_HUM	2	1.110631
48H 50MTA	P24278	0.232725	0.024107	0.021195	ZBTB25	Zinc finger a	ZBT25_HUM	1	1.175052
24H 50MTA	Q12933	-0.20348	0.024109	0.021195	TRAF2	TNF recepto	TRAF2_HUM	17	0.868455
24H 50MTA	Q9BZE2	0.036978	0.024108	0.021195	PUS3	tRNA pseud	PUS3_HUM	6	1.025962
24H 10MTA	Q9H840	-0.45181	0.024116	0.0212	GEMIN7	Gem-associ	GEMI7_HUM	1	0.731125
48H 50MTA	Q9UMY1	-0.23039	0.024128	0.02121	NOL7	U3 small nu	UTP16_HUM	5	0.852403
24H 10MTA	Q16851	0.003172	0.024149	0.021222	UGP2	UTP--glucos	UGPA_HUM	25	1.002201
24H 10MTA	Q9BW60	0.143378	0.024148	0.021222	ELOVL1	Very long ch	ELOV1_HUM	4	1.104488
48H 10MTA	Q96D46	0.008832	0.024151	0.021223	NMD3	60S riboson	NMD3_HUM	17	1.006141
24H 10MTA	P52209	-0.09132	0.024178	0.021241	PGD	6-phospho	6PGD_HUM	25	0.938664
48H 50MTA	Q86SX3	-0.70876	0.024195	0.021253	TEDC1	Tubulin eps	TEDC1_HUM	3	0.611844
24H 50MTA	Q13885	-0.09924	0.02422	0.021269	TUBB2A	Tubulin bet	TBB2A_HUM	7	0.933524
24H 50MTA	O75448	0.055455	0.024218	0.021269	MED24	Mediator o	MED24_HU	9	1.039187
24H 50MTA	Q8N8N7	0.108304	0.02422	0.021269	PTGR2	Prostagland	PTGR2_HUM	9	1.077961
48H 50MTA	O14732	-0.22773	0.024225	0.021271	IMPA2	Inositol mo	IMPA2_HUM	4	0.853977
48H 50MTA	O43347	-0.16447	0.024235	0.021278	MSI1	RNA-bindin	MSI1H_HUM	4	0.892258
24H 50MTA	O60684	0.060196	0.024237	0.021278	KPNA6	Importin su	IMA7_HUM	10	1.042607
24H 50MTA	Q4U2R6	-0.02613	0.024244	0.021282	MRPL51	Large ribos	RM51_HUM	2	0.982048
48H 10MTA	O15164	0.031593	0.024248	0.021284	TRIM24	Transcripti	TIF1A_HUM	16	1.02214
24H 10MTA	Q13107	0.175749	0.024274	0.021299	USP4	Ubiquitin c	UBP4_HUM	9	1.12955
48H 10MTA	P61966	0.115045	0.024278	0.021301	AP1S1	AP-1 compl	AP1S1_HUM	7	1.083009
24H 50MTA	P52272	0.028637	0.024294	0.021313	HNRNPM	Heterogene	HNRPM_HU	41	1.020048
24H 10MTA	Q9H0R4	0.156586	0.024309	0.021322	HDHD2	Haloacid de	HDHD2_HU	7	1.114646
48H 50MTA	P68104	-0.16837	0.024311	0.021324	EEF1A1	Elongation	EF1A1_HUM	30	0.889848
48H 10MTA	P38606	0.009699	0.024332	0.021337	ATP6V1A	V-type prot	VATA_HUM	36	1.006746
48H 50MTA	P00325	-0.25186	0.024346	0.021344	ADH1B	All-trans-re	ADH1B_HU	2	0.839811
24H 50MTA	P42345	0.020909	0.024345	0.021344	MTOR	Serine/thre	MTOR_HUM	62	1.014598
24H 10MTA	Q96RF0	-0.25627	0.024349	0.021346	SNX18	Sorting nex	SNX18_HUM	7	0.837251
24H 10MTA	Q9BTV4	0.025825	0.024355	0.021349	TMEM43	Transmemb	TMM43_HU	20	1.018062
48H 10MTA	Q8TBP6	-0.75989	0.024362	0.021353	SLC25A40	Mitochond	S2540_HUM	2	0.590543
48H 50MTA	P60842	-0.12587	0.024367	0.021356	EIF4A1	Eukaryotic	IF4A1_HUM	30	0.916448
48H 10MTA	Q08257	-0.10805	0.024369	0.021357	CRYZ	Quinone ox	QOR_HUMA	17	0.927842
48H 50MTA	Q7Z5J4	0.491652	0.024375	0.02136	RAI1	Retinoic ac	RAI1_HUMA	4	1.406054
24H 50MTA	Q09028	0.084583	0.024393	0.021372	RBBP4	Histone-bir	RBBP4_HUM	15	1.060381
24H 50MTA	O75170	0.096458	0.024397	0.021374	PPP6R2	Serine/thre	PP6R2_HUM	30	1.069145
48H 10MTA	P51684	2.683094	0.024403	0.021379	CCR6	C-C chemol	CCR6_HUM	1	6.422317
24H 50MTA	P57740	-0.01479	0.024415	0.021386	NUP107	Nuclear por	NU107_HU	41	0.989801
48H 10MTA	Q03701	-0.09427	0.024416	0.021386	CEBPZ	CCAAT/enh	CEBPZ_HUM	36	0.936747
48H 10MTA	O95229	-0.03536	0.024424	0.021392	ZWINT	ZW10 inter	ZWINT_HUM	7	0.975788
24H 50MTA	O95251	0.281955	0.024429	0.021395	KAT7	Histone ace	KAT7_HUM	15	1.215841
48H 10MTA	P31948	0.102297	0.024433	0.021397	STIP1	Stress-indu	STIP1_HUM	34	1.073481

24H 50MT/	Q9P2D3	-0.06912	0.024447	0.021406	HEATR5B	HEAT repea	HTR5B_HUI	31	0.953222
24H 50MT/	Q14847	-0.13438	0.024464	0.021416	LASP1	LIM and SH	LASP1_HUI	12	0.911063
24H 10MT/	Q9NWT1	0.086685	0.02447	0.02142	PAK1IP1	p21-activat	PK1IP_HUI	16	1.061928
48H 50MT/	Q8IYD1	-0.23151	0.024489	0.021433	GSPT2	Eukaryotic	ERF3B_HUI	13	0.851741
24H 10MT/	Q8ND56	0.078823	0.024496	0.021438	LSM14A	Protein LSI	LS14A_HUI	7	1.056156
48H 50MT/	Q9ULG6	-0.30575	0.024502	0.021441	CCPG1	Cell cycle p	CCPG1_HU	4	0.80902
48H 50MT/	Q96GQ5	0.214119	0.024505	0.021442	RUSF1	RUS family	RUSF1_HUI	7	1.159995
48H 50MT/	Q96BD8	-1.36631	0.024517	0.021451	SKA1	Spindle anc	SKA1_HUM	2	0.387881
48H 10MT/	Q9UBD5	-0.13575	0.024518	0.021452	ORC3	Origin reco	ORC3_HUM	16	0.910197
48H 10MT/	Q5MNZ9	0.212779	0.024523	0.021453	WIPI1	WD repeat	WIPI1_HUI	2	1.158918
48H 50MT/	Q96JB2	0.068133	0.024528	0.021456	COG3	Conserved	COG3_HUM	28	1.048359
48H 10MT/	Q96FV2	0.134923	0.024534	0.02146	SCRN2	Secernin-2	SCRN2_HUI	9	1.098034
48H 10MT/	Q9NP50	-0.56136	0.02457	0.021485	SINHCAF	SIN3-HDAC	SHCAF_HUI	2	0.677662
24H 10MT/	P17152	0.060597	0.024573	0.021486	TMEM11	Transmemb	TMM11_HU	3	1.042897
24H 50MT/	P31483	0.105309	0.024581	0.02149	TIA1	Cytotoxic g	TIA1_HUM/	10	1.075725
24H 50MT/	P63096	0.281171	0.024582	0.02149	GNAI1	Guanine nu	GNAI1_HUI	2	1.21518
24H 10MT/	Q5F1R6	-0.16209	0.024592	0.021497	DNAJC21	DnaJ homo	DJC21_HUI	11	0.893731
24H 50MT/	O43674	0.183182	0.024602	0.021504	NDUFB5	NADH dehy	NDUB5_HU	5	1.135385
24H 10MT/	P78540	-0.26059	0.024602	0.021504	ARG2	Arginase-2,	ARGI2_HUI	9	0.834749
48H 10MT/	P07948	0.046256	0.024605	0.021507	LYN	Tyrosine-pr	LYN_HUMA	19	1.032582
48H 10MT/	Q9BXS6	0.068635	0.024618	0.021516	NUSAP1	Nucleolar a	NUSAP_HU	11	1.048724
24H 50MT/	Q9Y2R5	0.096823	0.024628	0.021523	MRPS17	Small ribos	RT17_HUM	4	1.069416
48H 10MT/	Q14690	-0.08407	0.024641	0.021532	PDCD11	Protein RRI	RRP5_HUM	86	0.943393
48H 50MT/	Q9UN86	-0.16469	0.024642	0.021533	G3BP2	Ras GTPase-	G3BP2_HU	16	0.892118
48H 50MT/	Q9NP97	0.077851	0.024649	0.021539	DYNLRB1	Dynein ligh	DLRB1_HUI	5	1.055444
48H 50MT/	P15509	-0.44578	0.024657	0.021543	CSF2RA	Granulocyt	CSF2R_HUI	1	0.734187
48H 10MT/	Q9Y478	-0.00062	0.024664	0.021548	PRKAB1	5'-AMP-acti	AAKB1_HUI	9	0.999573
48H 10MT/	P56556	-0.14519	0.024666	0.021549	NDUFA6	NADH dehy	NDUA6_HU	6	0.904259
48H 50MT/	P84077	-0.46307	0.024675	0.021555	ARF1	ADP-ribosy	ARF1_HUM	3	0.725439
48H 10MT/	P48059	0.09182	0.024687	0.021563	LIMS1	LIM and ser	LIMS1_HUI	12	1.065714
24H 10MT/	O75475	0.107564	0.024691	0.021565	PSIP1	PC4 and SFI	PSIP1_HUM	14	1.077408
48H 10MT/	O14907	0.017842	0.024696	0.021567	TAX1BP3	Tax1-bindir	TX1B3_HUI	4	1.012444
24H 50MT/	Q7Z5L9	0.098686	0.024697	0.021567	IRF2BP2	Interferon r	I2BP2_HUI	21	1.070798
24H 10MT/	Q9Y2R5	0.075274	0.024697	0.021567	MRPS17	Small ribos	RT17_HUM	4	1.053561
48H 10MT/	O76003	0.071311	0.024705	0.021573	GLRX3	Glutaredox	GLRX3_HUI	17	1.050671
24H 50MT/	P20839	0.253614	0.024706	0.021573	IMPDH1	Inosine-5'-r	IMDH1_HU	5	1.19219
48H 10MT/	Q96QD8	-0.20788	0.024733	0.02159	SLC38A2	Sodium-cou	S38A2_HUI	5	0.865809
24H 50MT/	P61106	0.074429	0.024738	0.021593	RAB14	Ras-related	RAB14_HUI	13	1.052944
24H 50MT/	Q9HBH0	-0.01943	0.024752	0.021602	RHOF	Rho-relatec	RHOF_HUM	10	0.986624
24H 50MT/	Q9H307	0.245845	0.024759	0.021606	PNN	Pinin	PININ_HUM	21	1.185787
24H 10MT/	Q86XN6	-0.26333	0.024774	0.021615	ZNF761	Zinc finger	ZN761_HUI	1	0.833165
48H 50MT/	Q9NVP1	-0.06629	0.024776	0.021616	DDX18	ATP-depen	DDX18_HU	29	0.955093
48H 10MT/	Q9Y5P6	-0.04013	0.024792	0.021626	GMPPB	Mannose-1	GMPPB_HU	13	0.972569
48H 10MT/	Q12860	0.130211	0.024802	0.021635	CNTN1	Contactin-1	CNTN1_HU	37	1.094454
24H 50MT/	Q96CP7	0.318647	0.024815	0.021642	TLCD1	TLC domair	TLCD1_HUI	4	1.247161
24H 10MT/	Q9Y316	-0.15906	0.024822	0.021647	MEMO1	Protein ME	MEMO1_HU	14	0.895609
24H 10MT/	Q9Y6K5	-0.28873	0.024837	0.021658	OAS3	2'-5'-oligoa	OAS3_HUM	7	0.818623
48H 10MT/	Q9UKY4	-0.02975	0.02484	0.021659	POMT2	Protein O-n	POMT2_HU	3	0.979589
24H 10MT/	O95453	0.086324	0.024841	0.021659	PARN	Poly(A)-spe	PARN_HUM	22	1.061662
48H 50MT/	O95402	-0.42964	0.024846	0.021661	MED26	Mediator o	MED26_HU	1	0.742445
24H 10MT/	O75052	0.030504	0.024851	0.021664	NOS1AP	Carboxyl-te	CAPON_HU	3	1.021369
48H 50MT/	O95816	-0.07314	0.024858	0.02167	BAG2	BAG family	BAG2_HUM	12	0.950564

48H 10MTA	Q8N6M0	-0.01484	0.024867	0.021674	OTUD6B	Deubiquiti	OTU6B_HU	8	0.989769
24H 50MTA	Q96CN9	0.095003	0.024868	0.021674	GCC1	GRIP and co	GCC1_HUM	10	1.068068
48H 50MTA	P41208	0.053877	0.024871	0.021676	CETN2	Centrin-2	CETN2_HUI	5	1.038051
24H 50MTA	O75319	-0.04594	0.024873	0.021677	DUSP11	RNA/RNP c	DUS11_HU	8	0.968655
24H 50MTA	Q9Y4A5	0.095567	0.024878	0.02168	TRRAP	Transforma	TRRAP_HUI	41	1.068485
48H 10MTA	P23921	0.01078	0.024914	0.021705	RRM1	Ribonuclec	RIR1_HUMA	30	1.0075
24H 10MTA	P78346	0.089761	0.024915	0.021706	RPP30	Ribonuclea	RPP30_HUI	14	1.064194
48H 10MTA	Q01968	0.025118	0.024919	0.021708	OCRL	Inositol pol	OCRL_HUM	26	1.017563
24H 50MTA	Q9H4A5	0.199081	0.02492	0.021708	GOLPH3L	Golgi phosp	GLP3L_HUI	5	1.147967
24H 10MTA	Q12906	0.034382	0.024942	0.021724	ILF3	Interleukin	ILF3_HUMA	38	1.024118
24H 10MTA	Q6IQ22	0.148841	0.024967	0.02174	RAB12	Ras-related	RAB12_HUI	12	1.108678
24H 10MTA	Q92572	-0.10756	0.024973	0.021744	AP3S1	AP-3 compl	AP3S1_HUI	7	0.928157
48H 10MTA	P99999	0.068239	0.025	0.021761	CYCS	Cytochrom	CYC_HUMA	8	1.048436
24H 10MTA	Q9Y467	0.316753	0.025009	0.021765	SALL2	Sal-like pro	SALL2_HUM	4	1.245524
24H 50MTA	Q9UGV2	-0.10877	0.025041	0.021785	NDRG3	Protein ND	NDRG3_HU	12	0.927376
48H 10MTA	P51956	0.237672	0.025043	0.021787	NEK3	Serine/thre	NEK3_HUM	4	1.179089
24H 10MTA	P27635	-0.15979	0.025058	0.021797	RPL10	Large ribos	RL10_HUM	12	0.895156
48H 10MTA	P61960	0.071957	0.025059	0.021797	UFM1	Ubiquitin-f	UFM1_HUM	5	1.051141
48H 50MTA	Q9UBX3	-0.16381	0.02506	0.021797	SLC25A10	Mitochond	DIC_HUMA	13	0.892666
24H 10MTA	Q8IV50	-0.52925	0.025067	0.0218	LYSMD2	LysM and p	LYSM2_HUI	1	0.692914
48H 50MTA	P31321	-0.26052	0.025067	0.0218	PRKAR1B	cAMP-depe	KAP1_HUM	5	0.834785
48H 10MTA	P62873	0.09452	0.025068	0.0218	GNB1	Guanine nu	GBB1_HUM	14	1.06771
48H 10MTA	P52888	0.03738	0.025073	0.021803	THOP1	Thimet olig	THOP1_HU	27	1.026248
48H 50MTA	P62820	0.095511	0.025074	0.021803	RAB1A	Ras-related	RAB1A_HUI	3	1.068444
24H 50MTA	Q9NQV6	0.237007	0.025079	0.021807	PRDM10	PR domain	PRD10_HU	4	1.178545
24H 10MTA	Q92506	0.030907	0.025081	0.021808	HSD17B8	(3R)-3-hydr	DHB8_HUM	6	1.021654
48H 50MTA	O15084	-0.02116	0.025127	0.021839	ANKRD28	Serine/thre	ANR28_HU	15	0.985441
48H 10MTA	P39687	0.134803	0.02513	0.021839	ANP32A	Acidic leuci	AN32A_HU	10	1.097943
24H 50MTA	Q96AC1	-0.0095	0.025133	0.021841	FERMT2	Fermitin fai	FERM2_HU	27	0.993435
48H 50MTA	P23443	0.008728	0.025136	0.021842	RPS6KB1	Ribosomal	KS6B1_HUI	7	1.006068
48H 10MTA	Q9GZT4	0.26708	0.025138	0.021843	SRR	Serine racei	SRR_HUMA	4	1.203369
24H 50MTA	O00767	0.465729	0.025167	0.021863	SCD	Stearoyl-Cc	SCD_HUMA	6	1.381015
48H 10MTA	Q8WTV0	0.141449	0.025173	0.021868	SCARB1	Scavenger r	SCRB1_HUI	8	1.103012
24H 10MTA	P61803	-0.16912	0.025185	0.021876	DAD1	Dolichyl-di	DAD1_HUM	3	0.889385
24H 50MTA	O95602	0.010996	0.025191	0.021879	POLR1A	DNA-direct	RPA1_HUM	50	1.007651
24H 50MTA	Q9NYR9	-0.55603	0.025191	0.021879	NKIRAS2	NF-kappa-B	KBRS2_HUI	5	0.68017
24H 10MTA	Q14116	0.176385	0.025196	0.021881	IL18	Interleukin	IL18_HUMA	10	1.130049
24H 50MTA	Q6WKZ4	0.102587	0.025205	0.021887	RAB11FIP1	Rab11 fami	RFIP1_HUM	18	1.073697
24H 10MTA	O75113	-0.415	0.025206	0.021887	N4BP1	NEDD4-bin	N4BP1_HU	6	0.750021
24H 50MTA	Q8IVW6	0.043151	0.025211	0.02189	ARID3B	AT-rich inte	ARI3B_HUM	11	1.030362
48H 10MTA	Q8IVL5	0.135674	0.025226	0.021901	P3H2	Prolyl 3-hy	P3H2_HUM	13	1.098606
48H 50MTA	Q9Y2D2	0.050158	0.02523	0.021902	SLC35A3	UDP-N-acet	S35A3_HUI	2	1.035379
48H 10MTA	Q9NRW7	-0.02544	0.025234	0.021904	VPS45	Vacuolar pı	VPS45_HUI	21	0.982524
48H 50MTA	P62269	-0.10952	0.025247	0.02191	RPS18	Small ribos	RS18_HUM	11	0.926897
48H 10MTA	P49137	-0.02072	0.025265	0.021921	MAPKAPK2	MAP kinase	MAPK2_HU	5	0.985742
24H 10MTA	O75420	-0.62676	0.025291	0.021939	GIGYF1	GRB10-inte	GGYF1_HUI	2	0.64763
48H 10MTA	Q5VT25	-0.40271	0.025292	0.02194	CDC42BPA	Serine/thre	MRCKA_HU	7	0.756437
48H 50MTA	Q9Y2I1	-0.05686	0.025322	0.021956	NISCH	Nischarin	NISCH_HUI	10	0.961353
24H 50MTA	P49441	-0.18881	0.025336	0.021965	INPP1	Inositol pol	INPP_HUM	9	0.877328
48H 10MTA	P25054	0.123609	0.025342	0.021968	APC	Adenomatc	APC_HUMA	7	1.089457
24H 50MTA	Q9NZC3	0.162804	0.02535	0.021973	GDE1	Glycerophc	GDE1_HUM	6	1.119461
24H 10MTA	Q99942	-1.35933	0.025352	0.021973	RNF5	E3 ubiquiti	RNF5_HUM	1	0.389764

24H 50MT/	Q14919	0.123857	0.025366	0.021981	DRAP1	Dr1-associa	NC2A_HUM	6	1.089644
48H 50MT/	O94760	0.095184	0.02537	0.021983	DDAH1	N(G),N(G)-d	DDAH1_HU	16	1.068201
48H 50MT/	O43488	0.196697	0.025375	0.021984	AKR7A2	Aflatoxin B	ARK72_HU	10	1.146071
48H 50MT/	O75530	-0.1538	0.025374	0.021984	EED	Polycomb p	EED_HUMA	15	0.898883
48H 50MT/	P28749	-0.44224	0.025373	0.021984	RBL1	Retinoblast	RBL1_HUM	12	0.735992
24H 10MT/	P49585	0.089003	0.025374	0.021984	PCYT1A	Choline-ph	PCY1A_HU	14	1.063635
48H 50MT/	Q9H9G7	0.045827	0.02539	0.021994	ago-03	Protein arg	AGO3_HUM	10	1.032275
24H 50MT/	P13807	0.118969	0.025396	0.021998	GYS1	Glycogen [s	GYS1_HUM	15	1.085958
48H 10MT/	O75052	0.380853	0.025406	0.022006	NOS1AP	Carboxyl-te	CAPON_HU	3	1.302112
24H 50MT/	Q14558	0.03405	0.025411	0.02201	PRPSAP1	Phosphorib	KPRA_HUM	11	1.023882
48H 50MT/	Q96MF7	-0.09453	0.025418	0.022014	NSMCE2	E3 SUMO-p	NSE2_HUM	4	0.936579
48H 10MT/	Q9H633	0.97226	0.025418	0.022014	RPP21	Ribonuclea	RPP21_HU	2	1.961912
24H 10MT/	Q96EP5	0.082189	0.025436	0.022025	DAZAP1	DAZ-associa	DAZP1_HU	12	1.058623
48H 50MT/	P49720	0.129793	0.025446	0.022032	PSMB3	Proteasom	PSB3_HUM	12	1.094136
24H 10MT/	Q16401	-0.04173	0.025447	0.022032	PSMD5	26S protea	PSMD5_HU	24	0.971489
48H 10MT/	P07686	0.021724	0.025448	0.022032	HEXB	Beta-hexos	HEXB_HUM	22	1.015172
24H 50MT/	Q9NRN9	-0.07234	0.025451	0.022032	METTL5	rRNA N6-ad	METL5_HU	5	0.951092
48H 50MT/	O00567	-0.1067	0.025465	0.022043	NOP56	Nucleolar p	NOP56_HU	27	0.928707
24H 10MT/	Q8IVS8	-1.19313	0.025466	0.022043	GLYCTK	Glycerate k	GLCTK_HU	2	0.437354
48H 50MT/	Q6PJG2	-0.24572	0.025507	0.022075	MIDEAS	Mitotic dea	MDEAS_HU	7	0.843395
24H 10MT/	Q8IZL8	0.016645	0.025514	0.022079	PELP1	Proline-, gl	PELP1_HU	24	1.011604
48H 50MT/	Q9BRX5	-0.15135	0.025514	0.022079	GINS3	DNA replica	PSF3_HUM	9	0.900405
24H 50MT/	Q9NWU1	0.195748	0.025522	0.022084	OXSM	3-oxoacyl-[	OXSM_HUM	14	1.145318
48H 10MT/	P04920	-0.11006	0.025524	0.022085	SLC4A2	Anion exch	B3A2_HUM	21	0.926551
48H 10MT/	O43760	0.063282	0.025525	0.022086	SYNGR2	Synaptogyr	SNG2_HUM	4	1.04484
48H 50MT/	P63313	0.162316	0.025554	0.022102	TMSB10	Thymosin b	TYB10_HU	1	1.119082
48H 50MT/	O75489	-0.14927	0.025555	0.022103	NDUFS3	NADH dehy	NDUS3_HU	13	0.901706
48H 10MT/	Q9NVI1	-0.00323	0.025572	0.022113	FANCI	Fanconi an	FANCI_HUM	53	0.99776
48H 10MT/	Q92968	-0.04587	0.025582	0.022121	PEX13	Peroxisom	PEX13_HU	6	0.968704
48H 10MT/	Q9H074	0.137873	0.025597	0.022129	PAIP1	Polyadenyl	PAIP1_HUM	14	1.100282
48H 50MT/	O14802	-0.2741	0.025603	0.022133	POLR3A	DNA-direct	RPC1_HUM	36	0.826965
24H 50MT/	Q99547	0.25699	0.025606	0.022135	MPHOSPH6	M-phase ph	MPH6_HUM	3	1.194983
48H 50MT/	Q9H773	-0.22609	0.025648	0.022161	DCTPP1	dCTP pyrop	DCTP1_HU	8	0.854951
24H 10MT/	Q9BX46	0.169251	0.025657	0.022166	RBM24	RNA-bindir	RBM24_HU	6	1.124475
48H 50MT/	O95359	0.142157	0.025661	0.022168	TACC2	Transformi	TACC2_HU	40	1.103554
24H 10MT/	Q9BT09	0.129875	0.025676	0.022178	CNPY3	Protein can	CNPY3_HU	8	1.094199
48H 50MT/	P18206	0.026046	0.025677	0.022178	VCL	Vinculin	VINC_HUM	52	1.018218
48H 50MT/	Q9BW60	-0.15424	0.025687	0.022185	ELOVL1	Very long c	ELOV1_HU	4	0.898603
48H 10MT/	P24752	0.098599	0.025691	0.022187	ACAT1	Acetyl-CoA	THIL_HUMA	20	1.070733
48H 50MT/	Q6WKZ4	0.117905	0.025691	0.022187	RAB11FIP1	Rab11 fami	RFIP1_HUM	18	1.085158
24H 10MT/	P68371	-0.06104	0.025705	0.022195	TUBB4B	Tubulin bet	TBB4B_HU	30	0.958571
24H 50MT/	O96028	0.347151	0.025713	0.0222	NSD2	Histone-lys	NSD2_HUM	10	1.272046
48H 10MT/	Q92597	0.169935	0.025716	0.022201	NDRG1	Protein ND	NDRG1_HU	12	1.125007
24H 50MT/	P61009	0.336491	0.025747	0.022223	SPCS3	Signal pept	SPCS3_HUM	4	1.262682
24H 50MT/	O60427	0.06243	0.025749	0.022224	FADS1	Acyl-CoA (8	FADS1_HU	12	1.044223
24H 50MT/	Q96A49	0.159825	0.025766	0.022236	SYAP1	Synapse-as	SYAP1_HUM	18	1.117151
48H 50MT/	P11387	-0.12064	0.025781	0.022248	TOP1	DNA topois	TOP1_HUM	30	0.91978
48H 10MT/	Q86W34	0.255596	0.025784	0.022249	AMZ2	Archaemet	AMZ2_HUM	3	1.193829
24H 10MT/	Q96RE7	-0.00956	0.025789	0.022251	NACC1	Nucleus ac	NACC1_HU	13	0.993398
48H 10MT/	Q9HC98	0.596591	0.025801	0.022257	NEK6	Serine/thre	NEK6_HUM	1	1.512139
48H 50MT/	Q15291	-0.07676	0.025809	0.022259	RBBP5	Retinoblast	RBBP5_HU	15	0.948182
48H 50MT/	Q15582	0.145504	0.025808	0.022259	TGFBI	Transformi	BGH3_HUM	11	1.106117

48H 50MTA	P49748	0.044727	0.025837	0.022282	ACADVL	Very long-c	ACADV_HU	31	1.031488
48H 50MTA	P55884	-0.07543	0.025847	0.022288	EIF3B	Eukaryotic	EIF3B_HUM	47	0.94906
48H 10MTA	Q7L8L6	0.023572	0.025855	0.022293	FASTKD5	FAST kinase	FAKD5_HU	24	1.016473
48H 50MTA	Q8IXB1	-0.00504	0.025863	0.022299	DNAJC10	DnaJ homo	DJC10_HU	21	0.996513
24H 50MTA	P14209	-0.89598	0.025873	0.022304	CD99	CD99 antig	CD99_HUM	1	0.537382
48H 10MTA	P02461	0.461432	0.025878	0.022307	COL3A1	Collagen al	CO3A1_HU	4	1.376907
24H 10MTA	P40616	0.103578	0.025879	0.022308	ARL1	ADP-ribosy	ARL1_HUM	10	1.074435
48H 50MTA	Q9BSV6	-0.03549	0.025897	0.022319	TSEN34	tRNA-splici	SEN34_HU	7	0.9757
24H 10MTA	P13645	0.423624	0.025898	0.02232	KRT10	Keratin, typ	K1C10_HU	30	1.341293
48H 50MTA	Q9NWH2	-0.09221	0.025909	0.022328	TMEM242	Transmemb	TM242_HU	2	0.938085
48H 50MTA	Q9Y375	-0.2627	0.025911	0.022328	NDUFAF1	Complex I i	CIA30_HUM	3	0.833527
24H 50MTA	P52758	0.051216	0.025916	0.022331	RIDA	2-iminobut	RIDA_HUM	5	1.036138
24H 10MTA	O95825	0.015411	0.02592	0.022331	CRYZL1	Quinone ox	QORL1_HU	12	1.010739
48H 50MTA	O95340	0.51078	0.025922	0.022333	PAPSS2	Bifunctiona	PAPS2_HU	9	1.42482
24H 10MTA	Q9H6R3	0.016984	0.025925	0.022334	ACSS3	Acyl-CoA sy	ACSS3_HUM	17	1.011842
24H 10MTA	Q9UKZ1	-0.06065	0.025939	0.022344	CNOT11	CCR4-NOT t	CNO11_HU	9	0.958829
48H 50MTA	P49736	-0.07963	0.025951	0.022351	MCM2	DNA replica	MCM2_HU	36	0.946298
24H 10MTA	Q86XP3	0.056319	0.025964	0.022358	DDX42	ATP-depen	DDX42_HU	31	1.039809
48H 50MTA	O00142	0.037086	0.025973	0.022364	TK2	Thymidine	KITM_HUM	4	1.02604
48H 10MTA	O75394	-0.04898	0.025973	0.022364	MRPL33	Large ribos	RM33_HUM	2	0.96662
24H 10MTA	Q5VW36	0.020685	0.02598	0.022368	FOCAD	Focadhesin	FOCAD_HU	22	1.014441
48H 10MTA	P25685	-0.01391	0.025983	0.02237	DNAJB1	DnaJ homo	DNJB1_HU	17	0.990408
48H 50MTA	O15397	-0.30851	0.025999	0.022381	IPO8	Importin-8	IPO8_HUM	23	0.807475
48H 50MTA	Q15274	0.092355	0.026002	0.022383	QPRT	Nicotinate-	NADC_HUM	8	1.066109
48H 50MTA	P20020	0.027823	0.026005	0.022385	ATP2B1	Plasma mer	AT2B1_HU	37	1.019473
48H 10MTA	Q96BP2	0.053515	0.026021	0.022395	CHCHD1	Small ribos	CHCH1_HU	1	1.03779
48H 10MTA	Q9H1A4	0.091623	0.026027	0.0224	ANAPC1	Anaphase-p	APC1_HUM	38	1.065568
48H 50MTA	O15381	-0.17895	0.026036	0.022405	NVL	Nuclear val	NVL_HUMA	19	0.883345
24H 10MTA	Q99873	0.077134	0.026036	0.022405	PRMT1	Protein arg	ANM1_HUM	23	1.05492
48H 50MTA	O75317	0.454705	0.026043	0.022409	USP12	Ubiquitin c	UBP12_HU	2	1.370502
24H 50MTA	P08240	0.044499	0.026056	0.022416	SRPRA	Signal reco	SRPRA_HU	28	1.031325
48H 10MTA	Q6ZMG9	0.072553	0.026072	0.022426	CERS6	Ceramide s	CERS6_HUM	6	1.051576
24H 10MTA	Q8TCD5	-0.14865	0.026083	0.022434	NT5C	5'(3')-deoxy	NT5C_HUM	9	0.902097
24H 10MTA	P48651	0.210399	0.026102	0.022447	PTDSS1	Phosphatid	PTSS1_HUM	7	1.157008
48H 50MTA	Q13191	-0.14992	0.026119	0.022459	CBLB	E3 ubiquiti	CBLB_HUM	1	0.901301
48H 10MTA	P08069	0.013722	0.026124	0.022461	IGF1R	Insulin-like	IGF1R_HUM	16	1.009557
24H 50MTA	Q7Z2E3	0.085745	0.026128	0.022463	APTX	Aprataxin	APTX_HUM	7	1.061236
24H 10MTA	A0A024RB(	0.013257	0.02613	0.022463	NUDT4B	Diphospho	NUD4B_HU	1	1.009231
48H 10MTA	P48735	0.043418	0.026129	0.022463	IDH2	Isocitrate d	IDHP_HUM	28	1.030552
48H 50MTA	Q96FK6	0.002957	0.026133	0.022466	WDR89	WD repeat-	WDR89_HU	4	1.002051
24H 50MTA	P0DMV8;P(	-0.13045	0.026135	0.022466	HSPA1A;HS	Heat shock	HS71A_HU	25	0.913548
24H 50MTA	P41250	-0.00913	0.026135	0.022466	GARS1	Glycine--tR	GARS_HUM	35	0.993694
24H 10MTA	Q9H583	0.025329	0.026158	0.022481	HEATR1	HEAT repea	HEAT1_HU	76	1.017712
48H 10MTA	Q15796	0.068996	0.026167	0.022487	SMAD2	Mothers ag	SMAD2_HU	12	1.048986
24H 10MTA	P52298	0.112354	0.026169	0.022487	NCBP2	Nuclear cap	NCBP2_HU	4	1.080991
24H 10MTA	P49023	0.07417	0.02617	0.022488	PXN	Paxillin	PAXI_HUMA	18	1.052756
48H 50MTA	Q68CP9	-0.30883	0.026181	0.022496	ARID2	AT-rich inte	ARID2_HUM	10	0.807295
48H 10MTA	O00762	-0.04461	0.026196	0.022506	UBE2C	Ubiquitin-c	UBE2C_HU	8	0.969555
48H 10MTA	O75312	0.023951	0.026199	0.022508	ZPR1	Zinc finger	ZPR1_HUM	17	1.01674
24H 50MTA	Q13151	0.20485	0.026202	0.022509	HNRNPA0	Heterogene	ROA0_HUM	8	1.152567
24H 50MTA	P49336	0.268716	0.026204	0.02251	CDK8	Cyclin-depe	CDK8_HUM	6	1.204735
48H 50MTA	Q8TAA5	0.074592	0.026209	0.022511	GRPEL2	GrpE protei	GRPE2_HU	7	1.053063

48H 50MT/	Q96B45	-0.25731	0.026208	0.022511	BORCS7	BLOC-1-rela	BORC7_HU	3	0.836646
24H 10MT/	Q9BQ70	-0.04739	0.026208	0.022511	TCF25	Ribosome c	TCF25_HUI	16	0.967685
24H 50MT/	P42285	0.037794	0.026217	0.022517	MTREX	Exosome RI	MTREX_HU	47	1.026543
24H 50MT/	Q9UJC5	0.160557	0.02623	0.022526	SH3BGRL2	SH3 domai	SH3L2_HUI	5	1.117718
48H 10MT/	Q86XZ4	0.055055	0.02624	0.022532	SPATS2	Spermatog	SPAS2_HUI	11	1.038898
24H 10MT/	P21589	-1.21402	0.026242	0.022533	NT5E	5'-nucleoti	5NTD_HUM	1	0.431066
48H 10MT/	Q66K89	0.12295	0.026276	0.022556	E4F1	Transcripti	E4F1_HUM	1	1.088959
24H 10MT/	Q9Y237	-0.16373	0.026283	0.022561	PIN4	Peptidyl-pr	PIN4_HUM	6	0.892715
24H 50MT/	Q6NUK1	0.138346	0.026287	0.022563	SLC25A24	Mitochond	SCMC1_HU	20	1.100643
48H 50MT/	P61225	0.185752	0.026291	0.022566	RAP2B	Ras-related	RAP2B_HUI	5	1.137409
48H 10MT/	P23786	-0.02683	0.026299	0.02257	CPT2	Carnitine O	CPT2_HUM	21	0.981577
24H 10MT/	Q9BT22	-0.03892	0.026304	0.022573	ALG1	Chitobiosyl	ALG1_HUM	10	0.973382
24H 10MT/	Q8IWV8	-0.09668	0.026336	0.022593	UBR2	E3 ubiquiti	UBR2_HUM	24	0.935184
24H 10MT/	Q15008	0.073845	0.026349	0.022602	PSMD6	26S protea	PSMD6_HU	30	1.052518
48H 50MT/	P61160	0.130768	0.026355	0.022606	ACTR2	Actin-relate	ARP2_HUM	16	1.094876
48H 10MT/	P54578	0.064448	0.02636	0.02261	USP14	Ubiquitin c	UBP14_HU	24	1.045684
24H 50MT/	Q9Y6J9	0.184445	0.026361	0.02261	TAF6L	TAF6-like RI	TAF6L_HUI	14	1.13638
48H 50MT/	Q99741	-1.50087	0.026365	0.022612	CDC6	Cell divisio	CDC6_HUM	1	0.353341
24H 50MT/	P35580	0.033983	0.026367	0.022614	MYH10	Myosin-10	MYH10_HU	90	1.023835
24H 50MT/	Q14511	-0.64317	0.026369	0.022614	NEDD9	Enhancer o	CASL_HUM	1	0.640304
48H 10MT/	Q5SNT2	-0.0912	0.026378	0.02262	TMEM201	Transmemb	TM201_HU	9	0.938743
48H 50MT/	Q9NRX4	0.266262	0.026396	0.022632	PHPT1	14 kDa pho	PHP14_HU	6	1.202688
48H 50MT/	Q9NZB2	0.07702	0.026406	0.02264	FAM120A	Constitutiv	F120A_HUI	31	1.054837
24H 50MT/	Q9P003	0.164066	0.026407	0.02264	CNIH4	Protein cor	CNIH4_HUI	1	1.120441
48H 10MT/	Q86UX2	0.736659	0.026406	0.02264	ITIH5	Inter-alpha	ITIH5_HUM	1	1.666313
48H 10MT/	P12109	0.456445	0.026408	0.02264	COL6A1	Collagen al	CO6A1_HU	3	1.372156
48H 50MT/	Q9UK61	-0.06332	0.026412	0.022641	TASOR	Protein TAS	TASOR_HUI	40	0.957056
24H 10MT/	O14617	-0.05679	0.026413	0.022642	AP3D1	AP-3 compl	AP3D1_HU	36	0.961404
48H 10MT/	Q02040	0.087821	0.026428	0.02265	AKAP17A	A-kinase an	AK17A_HUI	12	1.062764
48H 10MT/	Q86YZ3	-0.65771	0.026431	0.022653	HRNR	Hornerin	HORN_HUM	3	0.633884
48H 10MT/	O15315	-0.04638	0.026433	0.022653	RAD51B	DNA repair	RA51B_HUI	1	0.96836
48H 10MT/	P12755	0.130358	0.026442	0.022657	SKI	Ski oncoger	SKI_HUMAI	2	1.094565
24H 10MT/	Q04837	0.019744	0.026442	0.022657	SSBP1	Single-strar	SSBP_HUM	8	1.01378
48H 10MT/	Q9Y6C9	-0.1102	0.02645	0.022663	MTCH2	Mitochond	MTCH2_HU	12	0.92646
24H 10MT/	Q12959	0.000394	0.026455	0.022665	DLG1	Disks large	DLG1_HUM	22	1.000273
24H 50MT/	O43187	-0.79825	0.026499	0.022694	IRAK2	Interleukin	IRAK2_HUM	4	0.575047
48H 10MT/	O43251	0.027391	0.026507	0.0227	RBFOX2	RNA bindin	RFOX2_HUI	7	1.019167
48H 50MT/	Q8N163	0.026201	0.026527	0.022713	CCAR2	Cell cycle a	CCAR2_HUI	34	1.018327
24H 50MT/	O15294	-0.12492	0.026548	0.022726	OGT	UDP-N-acet	OGT1_HUM	36	0.917057
48H 10MT/	Q9NPH2	-0.08473	0.026549	0.022726	ISYNA1	Inositol-3-p	INO1_HUM	14	0.942961
48H 50MT/	Q13322	0.997633	0.026552	0.022727	GRB10	Growth fac	GRB10_HU	1	1.996721
48H 50MT/	P31431	0.337997	0.026553	0.022727	SDC4	Syndecan-4	SDC4_HUM	5	1.264
24H 50MT/	O60568	0.06823	0.02657	0.022739	PLOD3	Multifuncti	PLOD3_HU	28	1.04843
24H 50MT/	Q9Y2R0	0.035464	0.026584	0.022751	COA3	Cytochrom	COA3_HUM	4	1.024886
24H 50MT/	Q5JPH6	0.092098	0.026602	0.022763	EARS2	Nondiscrim	SYEM_HUM	14	1.065919
24H 10MT/	P02649	-0.0768	0.026606	0.022766	APOE	Apolipoprc	APOE_HUM	17	0.948156
48H 10MT/	P10109	0.18223	0.026618	0.022774	FDX1	Adrenodox	ADX_HUM/	5	1.134636
24H 50MT/	P15291	0.121702	0.026628	0.022781	B4GALT1	Beta-1,4-ga	B4GT1_HUI	8	1.088018
24H 10MT/	P62714	-0.04062	0.026629	0.022782	PPP2CB	Serine/thre	PP2AB_HUI	12	0.972238
48H 50MT/	O15120	0.772077	0.02664	0.022788	AGPAT2	1-acyl-sn-gl	PLCB_HUM	4	1.707727
48H 50MT/	Q56NI9	-0.8929	0.026646	0.022789	ESCO2	N-acetyltra	ESCO2_HUI	2	0.538529
48H 50MT/	Q969Y2	-0.29658	0.026646	0.022789	GTPBP3	tRNA modi	GTPB3_HUI	7	0.814178

48H 10MT/	P50213	-0.15068	0.026666	0.022801	IDH3A	Isocitrate d	IDH3A_HUI	17	0.900825
48H 50MT/	Q9NVI7	-0.14391	0.026685	0.022812	ATAD3A	ATPase fam	ATD3A_HUI	24	0.905061
48H 10MT/	P0CG13	0.095505	0.02669	0.022815	CHTF8	Chromosor	CTF8_HUM	3	1.068439
24H 10MT/	P02794	-0.28016	0.026729	0.022844	FTH1	Ferritin hea	FRIH_HUM/	1	0.823498
48H 50MT/	Q9BSH4	-0.11594	0.026729	0.022844	TACO1	Translation	TACO1_HUI	12	0.922784
48H 10MT/	Q9Y3F4	0.101111	0.026769	0.022868	STRAP	Serine-thre	STRAP_HUI	19	1.072599
48H 10MT/	P22695	-0.16142	0.026771	0.022869	UQCRC2	Cytochrom	QCR2_HUM	12	0.894142
48H 50MT/	Q96GI7	0.054494	0.026774	0.022871	FAM89A	Protein FAI	FA89A_HUI	1	1.038495
48H 50MT/	Q9NTX7	-0.26748	0.026783	0.022877	RNF146	E3 ubiquiti	RN146_HU	1	0.830771
48H 10MT/	Q0VDF9	-0.01239	0.026799	0.022887	HSPA14	Heat shock	HSP7E_HUI	19	0.99145
48H 10MT/	Q15329	0.822782	0.0268	0.022887	E2F5	Transcripti	E2F5_HUM	1	1.768814
24H 10MT/	P22061	-0.03312	0.026804	0.02289	PCMT1	Protein-L-is	PIMT_HUM	15	0.977303
24H 50MT/	Q7Z3D4	0.564923	0.026806	0.02289	LYSMD3	LysM and p	LYSM3_HUI	2	1.479308
48H 50MT/	Q9Y6D6	0.056684	0.026816	0.022897	ARFGEF1	Brefeldin A	BIG1_HUM	27	1.040073
48H 50MT/	Q8N2Z9	-0.778	0.02682	0.022897	CENPS	Centromer	CENPS_HUI	2	0.583176
48H 10MT/	Q9UKF6	-0.03911	0.026825	0.022901	CPSF3	Cleavage ar	CPSF3_HUI	24	0.973257
48H 10MT/	Q8TA86	0.146386	0.02683	0.022904	RP9	Retinitis pi	RP9_HUMA	3	1.106794
24H 10MT/	O60264	0.055417	0.026834	0.022907	SMARCA5	SWI/SNF-re	SMCA5_HU	48	1.039159
24H 10MT/	Q99519	0.018276	0.026837	0.022908	NEU1	Sialidase-1	NEUR1_HU	14	1.012749
48H 10MT/	Q96AH0	-0.15217	0.026838	0.022908	NABP1	SOSS comp	SOSB2_HUI	2	0.899894
48H 50MT/	O75475	0.160703	0.026846	0.022913	PSIP1	PC4 and SFI	PSIP1_HUM	14	1.117831
24H 10MT/	Q63HN8	0.021795	0.026855	0.02292	RNF213	E3 ubiquiti	RN213_HU	88	1.015222
24H 50MT/	Q06520	1.075069	0.026873	0.022932	SULT2A1	Sulfotransf	ST2A1_HUI	2	2.106823
48H 10MT/	Q8NF37	-0.06873	0.026881	0.022936	LPCAT1	Lysophospl	PCAT1_HUI	14	0.953479
24H 50MT/	P05161	-0.13715	0.026895	0.022944	ISG15	Ubiquitin-l	ISG15_HUM	5	0.909311
48H 50MT/	Q8N1G2	0.009185	0.026901	0.022946	CMTR1	Cap-specifi	CMTR1_HU	33	1.006387
48H 50MT/	Q8TDR2	0.985306	0.026905	0.022947	STK35	Serine/thre	STK35_HUI	1	1.979733
48H 50MT/	Q15813	0.029391	0.026909	0.022949	TBCE	Tubulin-spe	TBCE_HUM	29	1.020581
24H 10MT/	O15431	0.338153	0.026913	0.022951	SLC31A1	High affinit	COPT1_HUI	1	1.264137
48H 10MT/	O75420	0.07474	0.026913	0.022951	GIGYF1	GRB10-inte	GGYF1_HUI	2	1.053171
24H 10MT/	Q2WGJ6	0.762369	0.026913	0.022951	KLHL38	Kelch-like p	KLH38_HUI	2	1.696274
48H 50MT/	O15145	-0.00322	0.026959	0.022983	ARPC3	Actin-relate	ARPC3_HUI	8	0.997773
48H 10MT/	Q00610	-0.11831	0.026961	0.022983	CLTC	Clathrin he	CLH1_HUM	79	0.921267
48H 10MT/	Q8WZA0	0.111156	0.026966	0.022986	LZIC	Protein LZI	LZIC_HUMA	9	1.080093
24H 50MT/	O14686	0.094385	0.026985	0.022999	KMT2D	Histone-lys	KMT2D_HU	9	1.06761
48H 10MT/	Q3B7T1	-0.1789	0.026987	0.023	EDRF1	Erythroid d	EDRF1_HUI	12	0.883374
48H 50MT/	O75925	-0.54252	0.027004	0.02301	PIAS1	E3 SUMO-p	PIAS1_HUM	4	0.686568
48H 50MT/	Q5VTR2	-0.14329	0.027003	0.02301	RNF20	E3 ubiquiti	BRE1A_HUI	30	0.905455
24H 10MT/	Q9UN86	0.08886	0.027011	0.023015	G3BP2	Ras GTPase	G3BP2_HU	16	1.063529
24H 50MT/	P31641	0.62253	0.027015	0.023017	SLC6A6	Sodium- an	SC6A6_HUI	2	1.539572
24H 10MT/	Q4ZIN3	-0.0663	0.027035	0.023029	TMEM259	Membralin	MBRL_HUM	5	0.955083
48H 10MT/	P82650	-0.10879	0.027047	0.023035	MRPS22	Small ribos	RT22_HUM	15	0.927367
48H 10MT/	Q9UBC2	0.060128	0.027049	0.023036	EPS15L1	Epidermal §	EP15R_HUI	27	1.042558
24H 10MT/	Q9NRY5	0.025077	0.027053	0.023039	FAM114A2	Protein FAI	F1142_HUI	15	1.017534
24H 10MT/	P30039	-0.32331	0.027054	0.023039	PBLD	Phenazine I	PBLD_HUM	2	0.799233
24H 10MT/	Q00534	0.096883	0.027062	0.023045	CDK6	Cyclin-depe	CDK6_HUM	12	1.069461
24H 50MT/	O94842	0.119182	0.027071	0.023051	TOX4	TOX high m	TOX4_HUM	10	1.086119
24H 50MT/	P03891	0.180462	0.027071	0.023051	MT-ND2	NADH-ubiq	NU2M_HUI	1	1.133247
48H 50MT/	P19784	-0.08083	0.027071	0.023051	CSNK2A2	Casein kina	CSK22_HUI	17	0.945511
24H 50MT/	Q9H251	0.641037	0.027088	0.023058	CDH23	Cadherin-2	CAD23_HU	2	1.55945
24H 50MT/	Q96NB2	0.096658	0.027109	0.023074	SFXN2	Sideroflexir	SFXN2_HUI	8	1.069293
48H 50MT/	Q13509	0.111605	0.027129	0.023087	TUBB3	Tubulin bet	TBB3_HUM	4	1.08043

48H 50MT/	Q9H223	-0.01145	0.027134	0.02309	EHD4	EH domain-	EHD4_HUM	21	0.992097
48H 10MT/	Q8WVK2	0.086167	0.027138	0.023092	SNRNP27	U4/U6.U5 s	SNR27_HU	2	1.061546
48H 10MT/	O00584	0.071164	0.027142	0.023094	RNASET2	Ribonuclea	RNT2_HUM	11	1.050564
48H 50MT/	P07996	0.266294	0.027143	0.023095	THBS1	Thrombosp	TSP1_HUM	9	1.202714
24H 50MT/	Q9ULP9	0.102354	0.027149	0.023097	TBC1D24	TBC1 doma	TBC24_HU	13	1.073524
48H 10MT/	P26641	-0.17314	0.027151	0.023098	EEF1G	Elongation	EF1G_HUM	27	0.886909
48H 10MT/	P56545	-0.05451	0.027155	0.023099	CTBP2	C-terminal-	CTBP2_HU	6	0.962921
24H 10MT/	O75676	0.028742	0.027159	0.023102	RPS6KA4	Ribosomal	KS6A4_HU	9	1.020122
24H 50MT/	Q9H974	0.013978	0.027178	0.023115	QTRT2	Queuine tR	QTRT2_HU	14	1.009736
48H 50MT/	P60891	0.117262	0.027186	0.023118	PRPS1	Ribose-pho	PRPS1_HU	14	1.084674
48H 10MT/	Q6P3W7	0.076139	0.027186	0.023118	SCYL2	SCY1-like p	SCYL2_HUM	17	1.054193
48H 50MT/	Q96M89	-0.41197	0.027192	0.023121	CCDC138	Coiled-coil	CC138_HU	4	0.751595
48H 50MT/	Q01844	0.089328	0.027198	0.023125	EWSR1	RNA-bindir	EWS_HUM/	9	1.063874
48H 10MT/	O15511	0.242167	0.027209	0.023132	ARPC5	Actin-relate	ARPC5_HU	5	1.182768
24H 50MT/	Q9NV88	0.230596	0.02723	0.023145	INTS9	Integrator c	INT9_HUM/	7	1.17332
24H 50MT/	O75380	0.07015	0.027233	0.023146	NDUFS6	NADH dehy	NDUS6_HU	5	1.049826
48H 10MT/	Q9BRP1	0.040338	0.027248	0.023155	PDCD2L	Programme	PDD2L_HU	7	1.028355
48H 50MT/	Q13610	-0.16085	0.027267	0.023167	PWP1	Periodic try	PWP1_HUM	22	0.894497
24H 10MT/	Q13177	-0.05399	0.027273	0.023171	PAK2	Serine/thre	PAK2_HUM	21	0.963268
24H 50MT/	Q9GZU8	-0.09429	0.02728	0.023174	PSME3IP1	PSME3-inte	PIP30_HUM	8	0.93673
48H 50MT/	Q9UPW5	-0.02159	0.027283	0.023176	AGTPBP1	Cytosolic ca	CBPC1_HU	1	0.985147
24H 50MT/	Q8IWI9	0.164389	0.02729	0.023181	MGA	MAX gene-a	MGAP_HUM	20	1.120691
24H 10MT/	P06730	0.110957	0.027312	0.023196	EIF4E	Eukaryotic	IF4E_HUM/	9	1.079944
24H 50MT/	Q9NQH7	0.225872	0.027323	0.0232	XPNPEP3	Xaa-Pro am	XPP3_HUM	12	1.169484
24H 50MT/	P04066	0.099623	0.02734	0.02321	FUCA1	Tissue alpha	FUCO_HUM	8	1.071494
48H 50MT/	Q13427	0.031665	0.027344	0.023212	PPIG	Peptidyl-pr	PPIG_HUM	8	1.022191
24H 10MT/	Q9BTY7	-0.45126	0.027344	0.023212	HGH1	Protein HG	HGH1_HUM	12	0.731406
24H 10MT/	Q96HA1	0.031783	0.027348	0.023214	POM121	Nuclear env	P121A_HU	3	1.022275
24H 10MT/	Q7LGA3	0.012211	0.027356	0.02322	HS2ST1	Heparan su	HS2ST_HUM	13	1.0085
24H 50MT/	O60306	0.148842	0.027358	0.02322	AQR	RNA helicas	AQR_HUM/	44	1.108679
48H 50MT/	Q14651	0.041821	0.027363	0.023223	PLS1	Plastin-1	PLSI_HUM/	22	1.029412
24H 50MT/	O14495	-0.40723	0.027364	0.023223	PLPP3	Phospholip	PLPP3_HU	5	0.754069
48H 10MT/	Q96KP1	-0.05169	0.027367	0.023225	EXOC2	Exocyst cor	EXOC2_HU	19	0.964807
48H 50MT/	P62942	0.06261	0.027371	0.023227	FKBP1A	Peptidyl-pr	FKB1A_HU	7	1.044353
24H 50MT/	O95219	-0.02228	0.027387	0.023235	SNX4	Sorting nex	SNX4_HUM	21	0.984674
48H 10MT/	P12830	0.066878	0.027387	0.023235	CDH1	Cadherin-1	CADH1_HU	7	1.047448
48H 50MT/	Q02978	-0.10362	0.027399	0.023239	SLC25A11	Mitochond	M2OM_HU	17	0.930695
48H 50MT/	Q93096	0.087548	0.027409	0.023248	PTP4A1	Protein tyr	TP4A1_HU	5	1.062562
48H 10MT/	Q13435	0.037056	0.027413	0.02325	SF3B2	Splicing fac	SF3B2_HUM	39	1.026018
24H 50MT/	Q6PD62	0.086391	0.027413	0.02325	CTR9	RNA polym	CTR9_HUM	29	1.061711
24H 50MT/	Q0VF96	-0.7311	0.027425	0.023258	CGNL1	Cingulin-lik	CGNL1_HU	1	0.602443
24H 50MT/	P62760	0.073577	0.027427	0.023259	VSNL1	Visinin-like	VISL1_HUM	7	1.052322
24H 10MT/	P15735	-0.07848	0.027437	0.023265	PHKG2	Phosphoryl	PHKG2_HU	1	0.947053
48H 10MT/	Q9BXP2	0.103609	0.02746	0.023279	SLC12A9	Solute carri	S12A9_HU	7	1.074458
24H 10MT/	Q96GD0	0.027093	0.027463	0.02328	PDXP	Chronophi	PLPP_HUM	9	1.018957
24H 50MT/	O75348	-0.10182	0.02747	0.023285	ATP6V1G1	V-type prot	VATG1_HU	4	0.931854
24H 50MT/	O43681	0.017506	0.02748	0.023291	GET3	ATPase GET	GET3_HUM	14	1.012208
24H 10MT/	Q96QK1	0.075395	0.027491	0.023296	VPS35	Vacuolar pr	VPS35_HU	32	1.053649
48H 50MT/	Q99643	0.185007	0.027503	0.023304	SDHC	Succinate c	C560_HUM	3	1.136822
24H 10MT/	P84022	-0.19823	0.027507	0.023306	SMAD3	Mothers ag	SMAD3_HU	5	0.871617
48H 10MT/	P57721	0.578542	0.027514	0.023311	PCBP3	Poly(rC)-bir	PCBP3_HU	1	1.493339
48H 50MT/	Q07960	0.094692	0.027514	0.023311	ARHGAP1	Rho GTPase	RHG01_HU	20	1.067837

24H 10MTA	Q9ULG1	0.372076	0.027522	0.023315	INO80	Chromatin	INO80_HUI	8	1.294214
24H 10MTA	O94766	-0.19138	0.027539	0.023325	B3GAT3	Galactosylg	B3GA3_HU	8	0.875768
48H 50MTA	P00740	0.709583	0.027541	0.023327	F9	Coagulatio	FA9_HUMA	1	1.635331
24H 10MTA	P11802	-0.06352	0.027543	0.023328	CDK4	Cyclin-depe	CDK4_HUM	12	0.956929
24H 50MTA	Q9NUJ3	-0.83701	0.027548	0.023331	TCP11L1	T-complex	T11L1_HUI	3	0.559801
48H 50MTA	Q8IZQ5	-0.52967	0.027567	0.023343	SELENOH	Selenoprot	SELH_HUM	2	0.692716
24H 50MTA	Q96F86	-0.12892	0.027571	0.023344	EDC3	Enhancer o	EDC3_HUM	17	0.914516
48H 10MTA	O43306	0.659719	0.027581	0.02335	ADCY6	Adenylate c	ADCY6_HUI	6	1.579775
24H 10MTA	Q53T94	-0.08134	0.02759	0.023356	TAF1B	TATA box-b	TAF1B_HUI	2	0.945177
24H 50MTA	A6NH11	0.037477	0.0276	0.023364	GLTPD2	Glycolipid	GLTD2_HUI	6	1.026318
48H 10MTA	Q9NRZ9	0.043906	0.027607	0.023368	HELLS	Lymphoid-s	HELLS_HUM	30	1.030901
24H 10MTA	P11310	0.029613	0.027625	0.02338	ACADM	Medium-ch	ACADM_HU	19	1.020738
48H 10MTA	Q16527	0.071698	0.027636	0.023385	CSRP2	Cysteine an	CSRP2_HUI	8	1.050953
24H 10MTA	Q9BTT6	-0.10902	0.027636	0.023385	LRRC1	Leucine-ric	LRRC1_HUI	16	0.927216
48H 50MTA	P49454	-0.35573	0.027639	0.023387	CENPF	Centromer	CENPF_HUI	46	0.781477
48H 50MTA	Q9P0L0	0.095717	0.027642	0.02339	VAPA	Vesicle-ass	VAPA_HUM	9	1.068596
24H 10MTA	P39019	-0.20239	0.027684	0.023412	RPS19	Small ribos	RS19_HUM	11	0.869111
24H 10MTA	Q13029	1.653709	0.027684	0.023412	PRDM2	PR domain	PRDM2_HU	2	3.146415
24H 10MTA	Q96RS0	0.145216	0.027686	0.023413	TGS1	Trimethylg	TGS1_HUM	8	1.105896
24H 10MTA	O14777	0.005113	0.027693	0.023415	NDC80	Kinetochor	NDC80_HU	23	1.00355
48H 50MTA	Q8N6T3	0.014209	0.027692	0.023415	ARFGAP1	ADP-ribosy	ARFG1_HUI	15	1.009897
48H 10MTA	Q7L1T6	0.199044	0.027697	0.023417	CYB5R4	Cytochrom	NB5R4_HU	5	1.147937
24H 50MTA	Q99584	-0.23349	0.027697	0.023417	S100A13	Protein S10	S10AD_HUI	5	0.850577
48H 10MTA	O00142	0.021772	0.027701	0.023418	TK2	Thymidine	KITM_HUM	4	1.015205
48H 50MTA	Q9H000	0.346229	0.027704	0.02342	MKRN2	E3 ubiquiti	MKRN2_HU	5	1.271234
24H 10MTA	O75179	-0.003	0.02771	0.023423	ANKRD17	Ankyrin rep	ANR17_HU	22	0.997926
48H 10MTA	P04181	-0.0862	0.027715	0.023427	OAT	Ornithine a	OAT_HUMA	21	0.941998
24H 50MTA	P50402	0.065937	0.027719	0.023429	EMD	Emerin	EMD_HUMA	12	1.046765
48H 10MTA	O00391	-0.99353	0.027727	0.023434	QSOX1	Sulfhydryl	QSOX1_HU	1	0.502247
24H 50MTA	O75131	0.08247	0.027729	0.023435	CPNE3	Copine-3	CPNE3_HUI	23	1.05883
24H 50MTA	Q93009	0.055895	0.027729	0.023435	USP7	Ubiquitin c	UBP7_HUM	54	1.039503
48H 50MTA	Q2VYF4	0.032081	0.027736	0.023439	LETM2	LETM1 dom	LETM2_HUI	1	1.022486
48H 50MTA	Q9UKK9	0.09046	0.027738	0.02344	NUDT5	ADP-sugar	NUDT5_HU	13	1.06471
24H 50MTA	Q13643	-0.70589	0.02775	0.023449	FHL3	Four and a	FHL3_HUM	3	0.613065
48H 50MTA	P61088	0.050934	0.027758	0.023453	UBE2N	Ubiquitin-c	UBE2N_HU	9	1.035936
24H 50MTA	Q7Z6E9	-0.09189	0.02776	0.023454	RBBP6	E3 ubiquiti	RBBP6_HUI	18	0.938292
48H 50MTA	Q86VX2	-0.25649	0.027766	0.023458	COMMD7	COMM dom	COMD7_HU	3	0.83712
48H 10MTA	P11586	0.003486	0.027787	0.023472	MTHFD1	C-1-tetrahy	C1TC_HUM	48	1.002419
48H 50MTA	Q8NF37	0.009393	0.027798	0.023479	LPCAT1	Lysophospl	PCAT1_HUI	14	1.006532
48H 50MTA	Q02447	-0.07342	0.027836	0.023502	SP3	Transcripti	SP3_HUMA	6	0.950386
24H 10MTA	O75947	-0.13854	0.027851	0.02351	ATP5PD	ATP syntha	ATP5H_HUI	12	0.908438
48H 50MTA	Q8NEL9	-0.74727	0.027852	0.02351	DDHD1	Phospholip	DDHD1_HU	4	0.595729
24H 10MTA	P68104	-0.0607	0.027855	0.023512	EEF1A1	Elongation	EF1A1_HUI	30	0.9588
48H 10MTA	Q96QK1	-0.10068	0.027876	0.023525	VPS35	Vacuolar pr	VPS35_HUI	32	0.932593
48H 10MTA	Q15269	0.046901	0.027879	0.023526	PWP2	Periodic try	PWP2_HUI	27	1.033043
48H 50MTA	A0A1B0GTU	0.157863	0.027881	0.023526	ZC3H11B	Zinc finger	ZC11B_HUI	1	1.115634
24H 10MTA	Q14997	-0.0055	0.027899	0.023538	PSME4	Proteasom	PSME4_HU	48	0.996198
24H 10MTA	O43390	0.072358	0.027901	0.02354	HNRNPR	Heterogene	HNRPR_HU	23	1.051434
48H 50MTA	Q9BS26	-0.02414	0.027903	0.023541	ERP44	Endoplasm	ERP44_HUI	17	0.983406
48H 50MTA	Q14004	0.05702	0.027911	0.023546	CDK13	Cyclin-depe	CDK13_HU	5	1.040314
48H 50MTA	Q13885	0.060158	0.027921	0.023552	TUBB2A	Tubulin bet	TBB2A_HUI	7	1.04258
24H 10MTA	P18621	-0.08109	0.027923	0.023553	RPL17	Large ribos	RL17_HUM	8	0.94534

24H 10MTA	O94925	0.011416	0.027927	0.023554	GLS	Glutaminas	GLSK_HUM	21	1.007944
48H 50MTA	Q12906	-0.01876	0.027933	0.023557	ILF3	Interleukin	ILF3_HUMA	38	0.987078
24H 50MTA	O94952	0.189252	0.02794	0.023562	FBXO21	F-box only	FBX21_HUI	8	1.140172
24H 10MTA	O95817	-0.06203	0.027958	0.023575	BAG3	BAG family	BAG3_HUM	18	0.957916
48H 50MTA	Q9UJC5	0.312703	0.027965	0.023578	SH3BGRL2	SH3 domai	SH3L2_HUI	5	1.242032
48H 10MTA	P54577	-0.00239	0.027965	0.023578	YARS1	Tyrosine--t	SYYC_HUMA	36	0.998347
24H 10MTA	P31944	0.58691	0.02799	0.023593	CASP14	Caspase-14	CASPE_HUI	3	1.502026
48H 10MTA	P54725	0.145932	0.027996	0.023595	RAD23A	UV excision	RD23A_HU	7	1.106445
24H 10MTA	Q9H0S4	0.029305	0.028014	0.023606	DDX47	Probable A	DDX47_HU	18	1.020521
24H 50MTA	Q92888	-0.11828	0.028027	0.023615	ARHGEF1	Rho guanin	ARHG1_HU	20	0.921282
24H 50MTA	Q9UPT8	0.12659	0.028044	0.023624	ZC3H4	Zinc finger	ZC3H4_HUI	14	1.091711
24H 10MTA	Q9C0B5	-0.09429	0.028051	0.023629	ZDHHC5	Palmitoyltr	ZDHC5_HU	5	0.936735
24H 50MTA	O00629	-0.1932	0.028056	0.023632	KPNA4	Importin su	IMA3_HUM	12	0.874662
24H 50MTA	Q92536	-0.26768	0.028066	0.023639	SLC7A6	Y+L amino a	YLAT2_HUI	3	0.830654
24H 10MTA	Q9GZT3	-0.17655	0.028066	0.023639	SLIRP	SRA stem-lo	SLIRP_HUM	9	0.884817
24H 10MTA	Q13263	0.069952	0.028072	0.023642	TRIM28	Transcripti	TIF1B_HUM	33	1.049682
24H 50MTA	Q00535	-0.1153	0.028074	0.023643	CDK5	Cyclin-depe	CDK5_HUM	13	0.923189
48H 10MTA	P52179	-2.29603	0.028136	0.023682	MYOM1	Myomesin-	MYOM1_HU	2	0.203622
24H 50MTA	Q02127	0.073286	0.028143	0.023686	DHODH	Dihydroorc	PYRD_HUM	19	1.05211
48H 50MTA	Q9UNS1	-0.44226	0.028154	0.023692	TIMELESS	Protein tim	TIM_HUMA	18	0.735981
48H 10MTA	O15243	-0.14765	0.028163	0.023697	LEPROT	Leptin rece	OBRG_HUM	2	0.902723
24H 10MTA	Q9UK73	0.151695	0.028171	0.023702	FEM1B	Protein fem	FEM1B_HU	3	1.110873
48H 10MTA	Q8WZ42	-0.33905	0.02818	0.023707	TTN	Titin	TITIN_HUM	10	0.79056
24H 50MTA	Q969H6	0.173994	0.028187	0.023712	POP5	Ribonuclea	POP5_HUM	5	1.128178
24H 50MTA	P10586	0.50684	0.028188	0.023712	PTPRF	Receptor-ty	PTPRF_HUI	25	1.420934
48H 10MTA	Q9Y4P3	-0.04544	0.02819	0.023713	TBL2	Transducin	TBL2_HUM	21	0.968993
24H 10MTA	Q9Y6K9	-0.21083	0.028191	0.023714	IKBKG	NF-kappa-B	NEMO_HUI	11	0.864043
48H 10MTA	Q8TAD7	0.989787	0.028192	0.023714	OCC1	Overexpres	OCC1_HUM	1	1.985892
24H 10MTA	P27708	-0.07836	0.028193	0.023715	CAD	Multifuncti	PYR1_HUM	79	0.947135
48H 10MTA	Q9BZ95	0.467741	0.028215	0.02373	NSD3	Histone-lys	NSD3_HUM	4	1.382942
48H 10MTA	Q9UL15	-0.13418	0.028219	0.023732	BAG5	BAG family	BAG5_HUM	10	0.911186
48H 10MTA	Q15833	0.043176	0.028222	0.023734	STXBP2	Syntaxin-bi	STXB2_HUI	25	1.03038
24H 10MTA	Q9Y4A5	0.122269	0.028227	0.023737	TRRAP	Transforma	TRRAP_HUI	41	1.088445
24H 10MTA	Q9H981	0.1313	0.028234	0.023741	ACTR8	Actin-relate	ARP8_HUM	13	1.09528
48H 10MTA	Q9BTE3	-0.00961	0.028236	0.023742	MCMBP	Mini-chron	MCMBP_HU	26	0.99336
24H 50MTA	O95685	-0.14497	0.028255	0.023756	PPP1R3D	Protein pho	PPR3D_HU	5	0.9044
24H 50MTA	O14950;P1	-0.10968	0.02826	0.023759	MYL12B;MY	Myosin regu	ML12B_HU	10	0.926794
24H 10MTA	A6NHR9	0.062301	0.028271	0.023767	SMCHD1	Structural	SMHD1_HU	74	1.044129
24H 50MTA	P12694	0.038955	0.028283	0.023775	BCKDHA	2-oxoisoval	ODBA_HUM	13	1.027369
24H 50MTA	P00813	-0.05071	0.028321	0.023799	ADA	Adenosine	ADA_HUMA	14	0.965458
24H 10MTA	Q9Y2X7	0.058731	0.028361	0.023829	GIT1	ARF GTPase	GIT1_HUMA	11	1.041549
48H 10MTA	O75530	-0.00655	0.028384	0.023843	EED	Polycomb	EED_HUMA	15	0.995469
24H 10MTA	Q9NZN8	-0.03568	0.028395	0.02385	CNOT2	CCR4-NOT t	CNOT2_HU	11	0.975574
24H 50MTA	Q96DP5	0.265943	0.028421	0.023866	MTFMT	Methionyl-	FMT_HUMA	7	1.202422
48H 10MTA	Q6P2P2	0.096564	0.028439	0.023876	PRMT9	Protein arg	ANM9_HUM	8	1.069224
48H 50MTA	P06756	-0.05826	0.028459	0.023887	ITGAV	Integrin alp	ITAV_HUMA	37	0.960425
24H 10MTA	Q8IYD1	-0.14956	0.028485	0.023907	GSPT2	Eukaryotic	ERF3B_HUI	13	0.901527
24H 50MTA	Q96I45	0.291602	0.028489	0.023908	TMEM141	Transmemb	TM141_HU	2	1.223999
24H 50MTA	P39019	-0.19273	0.028491	0.02391	RPS19	Small ribos	RS19_HUM	11	0.874951
24H 50MTA	Q9BY12	-0.17406	0.028497	0.023913	SCAPER	S phase cyc	SCAPE_HUI	7	0.886348
48H 50MTA	Q96KM6	-0.11618	0.02852	0.023927	ZNF512B	Zinc finger	Z512B_HUI	12	0.922624
48H 10MTA	Q9NPI1	0.072266	0.028523	0.023929	BRD7	Bromodom	BRD7_HUM	3	1.051367

48H 10MTA	O43688	-0.25615	0.028531	0.023933	PLPP2	Phospholip	PLPP2_HU	6	0.837322
24H 50MTA	O94855	0.085314	0.028531	0.023933	SEC24D	Protein tra	SC24D_HU	27	1.060918
24H 10MTA	Q14839	0.067915	0.028542	0.023937	CHD4	Chromodo	CHD4_HUM	68	1.048201
48H 50MTA	Q9NVZ3	0.015276	0.028543	0.023937	NECAP2	Adaptin ea	NECP2_HU	7	1.010644
48H 10MTA	Q9P013	-0.13475	0.028541	0.023937	CWC15	Spliceosom	CWC15_HU	9	0.910827
24H 10MTA	Q9Y3A2	0.268303	0.028545	0.023938	UTP11	Probable U	UTP11_HU	11	1.20439
48H 50MTA	Q96C57	-0.08747	0.028557	0.023944	CUSTOS	Protein CU	CSTOS_HUM	12	0.941174
24H 50MTA	Q9C0B9	-0.28577	0.028564	0.023949	ZCCHC2	Zinc finger	ZCHC2_HU	2	0.820304
24H 10MTA	Q6NUK1	0.086316	0.028571	0.023954	SLC25A24	Mitochond	SCMC1_HU	20	1.061656
48H 10MTA	P16298	0.236527	0.028581	0.023962	PPP3CB	Serine/thre	PP2BB_HU	4	1.178153
24H 50MTA	Q13630	-0.10347	0.028583	0.023962	GFUS	GDP-L-fuco	FCL_HUMA	12	0.930789
24H 50MTA	O75616	-0.15872	0.028589	0.023966	ERAL1	GTPase Era,	ERAL1_HUM	11	0.895819
48H 10MTA	P55039	0.009946	0.028594	0.023969	DRG2	Developme	DRG2_HUM	17	1.006918
24H 50MTA	Q15843	0.27851	0.028607	0.023977	NEDD8	NEDD8	NEDD8_HU	4	1.212941
24H 50MTA	P16989	0.082901	0.028612	0.02398	YBX3	Y-box-bindi	YBOX3_HU	8	1.059146
48H 10MTA	Q96P16	-0.17712	0.028614	0.023981	RPRD1A	Regulation	RPR1A_HU	14	0.884465
48H 10MTA	Q14191	-0.76216	0.028631	0.023991	WRN	Bifunctiona	WRN_HUM	3	0.589613
48H 50MTA	Q86YV5	-0.36012	0.028634	0.023993	PRAG1	Inactive tyr	PRAG1_HU	5	0.779101
48H 10MTA	P28072	-0.0409	0.028641	0.023996	PSMB6	Proteasom	PSB6_HUM	8	0.972045
24H 10MTA	Q13099	-0.04272	0.028643	0.023996	IFT88	Intraflagell	IFT88_HUM	2	0.970823
48H 10MTA	O15131	0.013648	0.028672	0.024015	KPNA5	Importin su	IMA6_HUM	15	1.009505
24H 10MTA	Q86UL3	0.069323	0.028715	0.024044	GPAT4	Glycerol-3-	GPAT4_HU	6	1.049224
48H 10MTA	Q13617	0.008223	0.028734	0.024056	CUL2	Cullin-2	CUL2_HUM	24	1.005716
24H 10MTA	P57737	-0.06932	0.028739	0.024059	CORO7	Coronin-7	CORO7_HU	23	0.953087
48H 50MTA	Q9UI09	0.063564	0.028751	0.024066	NDUFA12	NADH dehy	NDUAC_HU	9	1.045044
24H 10MTA	Q9UL54	-0.08527	0.028752	0.024066	TAOK2	Serine/thre	TAOK2_HU	9	0.942605
48H 10MTA	P33908	-0.16889	0.028759	0.024069	MAN1A1	Mannosyl-c	MA1A1_HU	16	0.889529
48H 50MTA	Q9HCD5	-0.04472	0.028759	0.024069	NCOA5	Nuclear rec	NCOA5_HU	19	0.969476
48H 50MTA	Q9Y5R8	-0.22609	0.028784	0.024085	TRAPPC1	Trafficking	TPPC1_HU	7	0.854948
48H 50MTA	P07711	0.245287	0.028812	0.024102	CTSL	Procatheps	CATL1_HUM	8	1.185328
48H 50MTA	Q9H6Q4	0.067238	0.028825	0.024111	CIAO3	Cytosolic ir	CIAO3_HUM	12	1.047709
24H 10MTA	P52788	0.011941	0.028835	0.024116	SMS	Spermine s	SPSY_HUMA	19	1.008311
24H 50MTA	Q53H54	2.173815	0.028837	0.024117	TRMT12	tRNA wybu	TYW2_HUM	1	4.512151
48H 50MTA	P57105	0.033963	0.028844	0.024121	SYNJ2BP	Synaptojan	SYJ2B_HUM	4	1.023821
24H 50MTA	Q9BRD0	0.045958	0.028846	0.024121	BUD13	BUD13 hon	BUD13_HU	9	1.032369
24H 50MTA	Q9C037	0.018012	0.028846	0.024121	TRIM4	E3 ubiquiti	TRIM4_HU	7	1.012563
24H 50MTA	Q9NWY4	0.24743	0.028849	0.024123	HPF1	Histone PA	HPF1_HUM	9	1.187091
24H 10MTA	Q92879	0.01685	0.028851	0.024123	CELF1	CUGBP Elav	CELF1_HUM	10	1.011748
24H 50MTA	Q5SSJ5	0.029826	0.028884	0.024147	HP1BP3	Heterochro	HP1B3_HU	22	1.020889
24H 10MTA	P08670	0.061471	0.028888	0.024148	VIM	Vimentin	VIME_HUM	34	1.043529
24H 10MTA	P15291	0.110108	0.0289	0.024154	B4GALT1	Beta-1,4-ga	B4GT1_HU	8	1.079309
48H 50MTA	Q96SK2	0.02428	0.028908	0.02416	TMEM209	Transmemb	TM209_HU	9	1.016972
48H 10MTA	Q9BW92	-0.1362	0.028912	0.024163	TARS2	Threonine--	SYTM_HUM	20	0.909911
48H 50MTA	Q7L7V1	-0.17616	0.028918	0.024166	DHX32	Putative pr	DHX32_HU	16	0.885058
24H 50MTA	Q9NXV2	-0.34057	0.028921	0.024167	KCTD5	BTB/POZ do	KCTD5_HU	5	0.789729
48H 50MTA	Q9HBI1	0.089472	0.028923	0.024167	PARVB	Beta-parvin	PARVB_HU	8	1.063981
48H 50MTA	P24928	-0.02143	0.028932	0.02417	POLR2A	DNA-direct	RPB1_HUM	63	0.985255
48H 50MTA	O15230	0.032102	0.028944	0.024178	LAMA5	Laminin sul	LAMA5_HU	57	1.022501
24H 50MTA	P52824	0.194679	0.028943	0.024178	DGKQ	Diacylglyce	DGKQ_HUM	2	1.144469
24H 50MTA	O95470	0.063942	0.028959	0.024189	SGPL1	Sphingosin	SGPL1_HU	17	1.045318
24H 10MTA	Q9NRA0	-0.15294	0.028961	0.02419	SPHK2	Sphingosin	SPHK2_HU	4	0.899413
48H 50MTA	Q8N5J2	0.247611	0.028966	0.024194	MINDY1	Ubiquitin c	MINY1_HU	2	1.18724

24H 10MTA	P26358	0.022554	0.028969	0.024194	DNMT1	DNA (cytosi	DNMT1_HU	51	1.015756
24H 50MTA	Q7RTS9	0.100312	0.028969	0.024194	DYM	Dymeclin	DYM_HUMA	7	1.072005
24H 10MTA	P51617	-0.70442	0.028971	0.024195	IRAK1	Interleukin	IRAK1_HUM	7	0.613688
48H 50MTA	P30622	-0.0467	0.028977	0.024199	CLIP1	CAP-Gly do	CLIP1_HUM	44	0.968151
24H 50MTA	Q9NXE4	0.019228	0.028982	0.024202	SMPD4	Sphingomy	NSMA3_HU	20	1.013417
48H 50MTA	Q9P1U1	0.108762	0.028991	0.02421	ACTR3B	Actin-relate	ARP3B_HUI	7	1.078302
48H 10MTA	Q8IZ69	-0.02437	0.029003	0.024216	TRMT2A	tRNA (uraci	TRM2A_HU	14	0.983247
24H 10MTA	Q9UL45	-0.19539	0.029007	0.024218	BLOC1S6	Biogenesis	BL1S6_HUM	2	0.873339
24H 10MTA	Q9Y5Z7	0.051503	0.029014	0.024223	HCFC2	Host cell fa	HCFC2_HUI	4	1.036344
24H 10MTA	Q92905	0.02566	0.029027	0.024231	COPS5	COP9 signa	CSN5_HUM	16	1.017945
48H 50MTA	P20936	0.092936	0.029049	0.024246	RASA1	Ras GTPase-	RASA1_HUI	19	1.066539
24H 50MTA	Q9NZI7	-0.02653	0.029069	0.024258	UBP1	Upstream-b	UBIP1_HUM	4	0.981778
24H 10MTA	Q9Y2K7	0.087568	0.029074	0.024261	KDM2A	Lysine-spec	KDM2A_HU	26	1.062578
48H 10MTA	O95785	-0.01294	0.029082	0.024265	WIZ	Protein Wi	WIZ_HUMA	14	0.991071
24H 50MTA	Q14331	0.60357	0.02909	0.02427	FRG1	Protein FRC	FRG1_HUM	3	1.519471
48H 10MTA	Q14651	0.023098	0.029089	0.02427	PLS1	Plastin-1	PLSI_HUMA	22	1.016139
24H 10MTA	Q8N1F8	0.279026	0.029111	0.024281	STK11IP	Serine/thre	S11IP_HUM	14	1.213375
24H 50MTA	Q13277	0.574079	0.029113	0.024282	STX3	Syntaxin-3	STX3_HUM	6	1.488726
48H 50MTA	Q9H467	0.226239	0.029118	0.024285	CUEDC2	CUE domai	CUED2_HU	4	1.169782
24H 50MTA	O95302	0.054193	0.029127	0.024291	FKBP9	Peptidyl-pr	FKBP9_HUI	14	1.038278
24H 10MTA	Q86W34	0.501335	0.029138	0.024298	AMZ2	Archaemet	AMZ2_HUM	3	1.415523
48H 10MTA	Q5T2W1	0.087036	0.029137	0.024298	PDZK1	Na(+)/H(+) e	NHRF3_HU	26	1.062185
48H 50MTA	Q15629	-0.0926	0.02915	0.024304	TRAM1	Translocati	TRAM1_HU	6	0.93783
48H 50MTA	P60484	0.415088	0.029169	0.024317	PTEN	Phosphatic	PTEN_HUM	2	1.33338
48H 50MTA	Q9UI15	0.39039	0.029175	0.02432	TAGLN3	Transgelin-	TAGL3_HUI	1	1.310748
24H 10MTA	P13674	0.005397	0.029178	0.024321	P4HA1	Prolyl 4-hy	P4HA1_HU	23	1.003748
48H 10MTA	Q8NCM8	-0.03064	0.029181	0.024322	DYNC2H1	Cytoplasmi	DYHC2_HU	33	0.978989
48H 10MTA	O75663	0.058284	0.029192	0.024329	TIPRL	TIP41-like p	TIPRL_HUM	15	1.041227
48H 50MTA	Q9BV86	0.241734	0.029199	0.024334	NTMT1	N-terminal	NTM1A_HU	8	1.182413
48H 50MTA	Q9HBL0	1.370987	0.029225	0.024351	TNS1	Tensin-1	TENS1_HUM	6	2.586475
48H 50MTA	Q9UJM3	-0.50684	0.029239	0.024361	ERRFI1	ERBB recep	ERRFI_HUM	1	0.703763
48H 10MTA	O95487	0.03102	0.029245	0.024365	SEC24B	Protein tra	SC24B_HUI	28	1.021734
48H 50MTA	Q9NSD9	0.003318	0.029249	0.024368	FARSB	Phenylalan	SYFB_HUMA	29	1.002302
48H 10MTA	P62304	-0.01317	0.029265	0.024378	SNRPE	Small nucle	RUXE_HUM	4	0.99091
24H 10MTA	Q13123	-0.04923	0.029274	0.024382	IK	Protein Rec	RED_HUMA	19	0.966455
24H 50MTA	Q8NE86	-0.06852	0.029279	0.024384	MCU	Calcium un	MCU_HUM	10	0.953619
48H 50MTA	O15235	-0.21588	0.029284	0.024387	MRPS12	Small ribos	RT12_HUM	5	0.861022
24H 50MTA	P10809	0.005148	0.029289	0.02439	HSPD1	60 kDa hea	CH60_HUM	49	1.003574
48H 10MTA	P35613	0.161796	0.029304	0.024399	BSG	Basigin	BASI_HUMA	9	1.118679
48H 10MTA	Q14517	-0.66038	0.029305	0.024399	FAT1	Protocadhe	FAT1_HUM	8	0.632713
48H 10MTA	Q9H6R0	0.292287	0.029307	0.024399	DHX33	ATP-depen	DHX33_HU	7	1.22458
24H 50MTA	Q9BT09	0.268622	0.029318	0.024404	CNPY3	Protein car	CNPY3_HUI	8	1.204656
24H 10MTA	Q9H223	0.098617	0.029336	0.024417	EHD4	EH domain-	EHD4_HUM	21	1.070747
24H 50MTA	Q6P1X6	0.138454	0.029375	0.024441	C8orf82	UPF0598 p	CH082_HU	5	1.100725
48H 50MTA	Q4V328	-0.00076	0.029389	0.02445	GRIPAP1	GRIP1-asso	GRAP1_HU	18	0.99947
48H 10MTA	O14817	-0.70406	0.029391	0.024452	TSPAN4	Tetraspanir	TSN4_HUM	2	0.613843
48H 50MTA	Q9NQH7	0.044069	0.029392	0.024452	XPNPEP3	Xaa-Pro am	XPP3_HUM	12	1.031017
48H 50MTA	Q12962	0.834065	0.029394	0.024452	TAF10	Transcripti	TAF10_HUM	2	1.782701
24H 10MTA	P60033	0.123092	0.02942	0.024469	CD81	CD81 antig	CD81_HUM	2	1.089066
24H 50MTA	Q9P2R3	-0.00955	0.029424	0.024472	ANKFY1	Rabankyrin	ANFY1_HUI	26	0.993399
48H 50MTA	P31150	0.104419	0.02943	0.024473	GDI1	Rab GDP di	GDIA_HUM	13	1.075061
48H 10MTA	Q6EMK4	0.164587	0.02943	0.024473	VASN	Vasorin	VASN_HUM	4	1.120845

24H 50MT/	Q9BW83	-0.03417	0.029431	0.024473	IFT27	Intraflagell	IFT27_HUM	8	0.976594
48H 50MT/	P02795	-0.37955	0.029444	0.024481	MT2A	Metallothi	MT2_HUM/	1	0.768679
24H 50MT/	Q9UMS4	-0.00405	0.029445	0.024482	PRPF19	Pre-mRNA-		PRP19_HU	17	0.997195
48H 10MT/	O95490	0.05748	0.029449	0.024484	ADGRL2	Adhesion G	AGRL2_HU	28	1.040646
24H 50MT/	Q9ULX9	-0.0876	0.029467	0.024491	MAFF	Transcripti	MAFF_HUM	4	0.941084
48H 50MT/	Q9BQA9	-0.32862	0.029477	0.024497	CYBC1	Cytochrom	CYBC1_HU	3	0.7963
24H 50MT/	P53365	-0.08293	0.02948	0.024499	ARFIP2	Arfaptin-2	ARFP2_HU	12	0.944135
48H 10MT/	Q9NRX4	0.112254	0.029496	0.024506	PHPT1	14 kDa pho	PHP14_HU	6	1.080916
24H 50MT/	Q13952	0.118062	0.02952	0.024522	NFYC	Nuclear tra	NFYC_HUM	6	1.085276
48H 10MT/	P52298	0.204058	0.029523	0.024524	NCBP2	Nuclear cap	NCBP2_HU	4	1.151934
48H 50MT/	Q8TBA6	0.10511	0.029532	0.024529	GOLGA5	Golgin subf	GOGA5_HU	19	1.075577
24H 10MT/	Q9GZN7	-1.30067	0.029542	0.024535	ROGDI	Protein rog	ROGDI_HU	2	0.405937
24H 10MT/	Q9UBK8	-0.17055	0.029549	0.02454	MTRR	Methionine	MTRR_HUM	10	0.888506
48H 50MT/	Q8WXW3	0.126868	0.029561	0.024548	PIBF1	Progestero	PIBF1_HUM	16	1.091921
48H 10MT/	O95551	0.060588	0.029565	0.024549	TDP2	Tyrosyl-DN	TYDP2_HU	11	1.04289
24H 10MT/	P00558	-0.08181	0.029565	0.024549	PGK1	Phosphogly	PGK1_HUM	37	0.944872
48H 50MT/	Q9UKZ1	-0.01974	0.029565	0.024549	CNOT11	CCR4-NOT t	CNO11_HU	9	0.986408
48H 10MT/	Q86VP6	0.017127	0.029568	0.02455	CAND1	Cullin-asso	CAND1_HU	58	1.011942
24H 10MT/	Q53RE8	-0.33975	0.029569	0.024551	ANKRD39	Ankyrin rep	ANR39_HU	3	0.790177
48H 10MT/	O75116	-0.00044	0.029575	0.024554	ROCK2	Rho-associa	ROCK2_HU	39	0.999698
48H 50MT/	Q9H6R3	0.255851	0.029574	0.024554	ACSS3	Acyl-CoA sy	ACSS3_HUI	17	1.19404
48H 50MT/	Q16719	0.110759	0.029587	0.024561	KYNU	Kynurenina	KYNU_HUM	20	1.079796
24H 10MT/	Q8N9N2	-0.09018	0.029602	0.02457	ASCC1	Activating s	ASCC1_HU	8	0.939408
48H 50MT/	Q709F0	0.252923	0.029627	0.024587	ACAD11	Acyl-CoA de	ACD11_HU	11	1.191619
24H 50MT/	Q9Y2K9	0.129249	0.029628	0.024587	STXBP5L	Syntaxin-bi	STB5L_HUM	4	1.093724
48H 50MT/	P33992	-0.07923	0.029633	0.024591	MCM5	DNA replica	MCM5_HU	41	0.946561
24H 10MT/	Q9UL15	0.0563	0.029648	0.0246	BAG5	BAG family	BAG5_HUM	10	1.039796
48H 50MT/	Q6IQ22	-0.18894	0.029653	0.024603	RAB12	Ras-related	RAB12_HU	12	0.877252
24H 50MT/	Q9NSY1	-0.37613	0.029655	0.024605	BMP2K	BMP-2-indu	BMP2K_HU	3	0.770502
24H 50MT/	Q9Y5P6	-0.13389	0.029661	0.024608	GMPPB	Mannose-1	GMPPB_HU	13	0.911369
24H 50MT/	P51153	0.134814	0.029667	0.024611	RAB13	Ras-related	RAB13_HU	6	1.097951
24H 10MT/	P60520	0.216094	0.029681	0.024622	GABARAPL2	Gamma-am	GBRL2_HU	4	1.161585
48H 10MT/	P23458	-0.01818	0.029702	0.024637	JAK1	Tyrosine-pr	JAK1_HUM	29	0.98748
24H 10MT/	Q9UM54	0.041351	0.029706	0.024639	MYO6	Unconvent	MYO6_HUM	56	1.029077
48H 50MT/	O15194	-0.30013	0.029714	0.024644	CTDSPL	CTD small p	CTDSL_HUM	1	0.812177
24H 50MT/	Q13445	0.019539	0.029721	0.024647	TMED1	Transmemb	TMED1_HU	5	1.013635
24H 50MT/	Q9NUL3	0.146321	0.029728	0.024651	STAU2	Double-stra	STAU2_HU	15	1.106743
48H 50MT/	P52735	0.186645	0.02973	0.024652	VAV2	Guanine nu	VAV2_HUM	25	1.138114
24H 50MT/	O60447	0.126551	0.029751	0.024664	EVI5	Ecotropic v	EVI5_HUM/	12	1.091681
48H 50MT/	P54819	0.037658	0.029763	0.024671	AK2	Adenylate k	KAD2_HUM	14	1.026447
24H 50MT/	P23468	0.690168	0.029779	0.024681	PTPRD	Receptor-ty	PTPRD_HU	8	1.613471
24H 50MT/	Q8NF91	0.589854	0.029791	0.024689	SYNE1	Nesprin-1	SYNE1_HUM	3	1.505094
48H 50MT/	P52306	-0.04896	0.029791	0.024689	RAP1GDS1	Rap1 GTPas	GDS1_HUM	14	0.966635
48H 50MT/	Q13200	0.022114	0.029797	0.024693	PSMD2	26S protea	PSMD2_HU	53	1.015447
48H 10MT/	Q7Z7C8	0.021172	0.0298	0.024695	TAF8	Transcripti	TAF8_HUM	5	1.014783
24H 50MT/	Q9Y3T6	-0.241	0.029801	0.024695	R3HCC1	R3H and co	R3HC1_HU	3	0.846158
24H 50MT/	Q9UBP6	0.121772	0.029806	0.024698	METTL1	tRNA (guan	TRMB_HUM	10	1.08807
24H 10MT/	Q8IVD9	-0.10449	0.029821	0.024708	NUDCD3	NudC doma	NUDC3_HU	11	0.930136
24H 50MT/	Q9Y2H0	0.084912	0.029828	0.024712	DLGAP4	Disks large-	DLGP4_HU	11	1.060623
24H 50MT/	Q8N5I2	-0.03408	0.029851	0.024729	ARRDC1	Arrestin do	ARRD1_HU	5	0.976655
48H 50MT/	P49754	-0.00636	0.029888	0.024757	VPS41	Vacuolar pr	VPS41_HU	11	0.9956
24H 10MT/	Q96QE5	0.117234	0.02989	0.024757	TEFM	Transcripti	TEFM_HUM	8	1.084653

48H 10MT/	O43670	0.05284	0.029915	0.024773	ZNF207	BUB3-inter	ZN207_HU	7	1.037305
24H 10MT/	Q6P996	-0.06041	0.029919	0.024775	PDXDC1	Pyridoxal-c	PDXD1_HU	33	0.958991
48H 50MT/	Q12851	0.547518	0.029922	0.024777	MAP4K2	Mitogen-ac	M4K2_HUN	4	1.461569
24H 50MT/	O43493	0.443315	0.029933	0.024784	TGOLN2	Trans-Golgi	TGON2_HU	7	1.359725
48H 50MT/	Q9H3R5	-0.21032	0.029933	0.024784	CENPH	Centromer	CENPH_HU	6	0.864343
48H 10MT/	Q9UHY1	-0.10635	0.029939	0.024788	NRBP1	Nuclear rec	NRBP_HUN	11	0.928936
24H 10MT/	Q15021	0.064632	0.029945	0.024793	NCAPD2	Condensin	CND1_HUN	53	1.045818
24H 50MT/	P56381;Q5	-0.13587	0.029968	0.02481	ATP5F1E;A	ATP synthas	ATP5E_HUN	1	0.910124
48H 10MT/	P51610	0.063181	0.029987	0.024821	HCFC1	Host cell fa	HCFC1_HU	40	1.044767
48H 50MT/	Q86V21	-0.09905	0.03	0.024828	AACS	Acetoacety	AACS_HUM	15	0.933651
48H 50MT/	Q9H0K1	0.271773	0.03002	0.024836	SIK2	Serine/thre	SIK2_HUM/	9	1.207291
48H 50MT/	O15027	0.077228	0.030015	0.024836	SEC16A	Protein tra	SC16A_HU	45	1.054989
24H 50MT/	Q3B7T1	0.030992	0.030019	0.024836	EDRF1	Erythroid d	EDRF1_HU	12	1.021714
24H 50MT/	Q9NP79	0.022793	0.030016	0.024836	VTA1	Vacuolar pr	VTA1_HUM	11	1.015925
24H 50MT/	E9PRG8	0.135616	0.030028	0.02484	C11orf98	Uncharacte	CK098_HU	4	1.098562
48H 10MT/	Q9BVC4	0.024209	0.030036	0.024845	MLST8	Target of ra	LST8_HUM/	7	1.016922
48H 10MT/	O94762	0.012963	0.030038	0.024846	RECQL5	ATP-depen	RECQ5_HU	9	1.009026
24H 10MT/	Q6MZP7	0.26569	0.030041	0.024848	LIN54	Protein lin-	LIN54_HUN	5	1.202211
48H 50MT/	P41440	-0.52045	0.030064	0.024862	SLC19A1	Reduced fo	S19A1_HU	3	0.697152
24H 50MT/	Q9NS00	0.00379	0.030079	0.024872	C1GALT1	Glycoprote	C1GLT_HU	9	1.002631
48H 10MT/	Q86VW0	0.09579	0.030102	0.024885	SESTD1	SEC14 dom	SESD1_HUN	14	1.068651
48H 50MT/	O00189	0.030022	0.030121	0.024897	AP4M1	AP-4 compl	AP4M1_HU	9	1.021027
24H 10MT/	Q9BX10	0.422138	0.030139	0.024908	GTPBP2	GTP-bindin	GTPB2_HU	4	1.339912
48H 10MT/	Q8TB61	0.024953	0.030138	0.024908	SLC35B2	Adenosine	S35B2_HU	11	1.017447
48H 50MT/	O60218	0.267943	0.030142	0.024909	AKR1B10	Aldo-keto r	AK1BA_HU	1	1.20409
48H 50MT/	P19338	0.059476	0.030142	0.024909	NCL	Nucleolin	NUCL_HUN	27	1.042087
24H 10MT/	O95625	-0.06963	0.03015	0.024913	ZBTB11	Zinc finger	ZBT11_HUN	4	0.952881
24H 10MT/	Q9H4I3	-0.21086	0.030173	0.024927	TRABD	TraB domai	TRABD_HU	10	0.864021
24H 50MT/	Q9H6Z4	0.033305	0.030176	0.024929	RANBP3	Ran-bindin	RANB3_HU	15	1.023354
48H 10MT/	O43293	-0.43019	0.030191	0.024938	DAPK3	Death-asso	DAPK3_HU	4	0.742162
48H 50MT/	Q96HD1	0.375709	0.030192	0.024938	CRELD1	Protein dis	CREL1_HUN	5	1.297477
48H 50MT/	Q96S59	-0.13362	0.030198	0.024942	RANBP9	Ran-bindin	RANB9_HU	15	0.911539
24H 50MT/	Q12788	0.006836	0.030205	0.024946	TBL3	Transducin	TBL3_HUM	35	1.004749
48H 50MT/	Q9H1B7	-0.11598	0.030215	0.024951	IRF2BPL	Probable E	I2BPL_HUN	6	0.922758
48H 50MT/	Q6N069	-0.06634	0.030249	0.024972	NAA16	N-alpha-ac	NAA16_HU	13	0.955055
24H 50MT/	Q15814	-0.10474	0.030251	0.024973	TBCC	Tubulin-spe	TBCC_HUM	12	0.929975
48H 10MT/	Q01973	0.064473	0.030252	0.024973	ROR1	Inactive tyr	ROR1_HUN	8	1.045703
24H 50MT/	Q9Y6K9	-0.29013	0.030259	0.024979	IKBKG	NF-kappa-B	NEMO_HUN	11	0.817829
24H 50MT/	Q9BRJ7	0.183037	0.030289	0.024998	NUDT16L1	Tudor-inter	TIRR_HUM/	11	1.135271
24H 50MT/	O75923	-0.00568	0.030293	0.025	DYSF	Dysferlin	DYSF_HUM	1	0.996074
24H 10MT/	Q7L592	-0.09229	0.030292	0.025	NDUFAF7	Protein arg	NDUF7_HU	14	0.938033
24H 50MT/	Q01201	0.226521	0.030293	0.025	RELB	Transcripti	RELB_HUM	5	1.17001
24H 50MT/	P0C0S8;P2	0.159966	0.030308	0.025009	H2AC11;H2	Histone H2	H2A1_HUM	8	1.117261
24H 10MT/	Q9P0B6	0.826346	0.030311	0.02501	CCDC167	Coiled-coil	CC167_HU	1	1.773189
24H 10MT/	P10768	-0.09575	0.030319	0.025015	ESD	S-formylglu	ESTD_HUM	15	0.935783
24H 10MT/	Q96QZ7	-0.12718	0.030342	0.025031	MAGI1	Membrane-	MAGI1_HU	19	0.915617
48H 10MT/	P18074	-0.00186	0.030345	0.025033	ERCC2	General tra	ERCC2_HU	15	0.998715
48H 50MT/	P40616	0.078567	0.030345	0.025033	ARL1	ADP-ribosy	ARL1_HUM	10	1.055968
24H 10MT/	Q14011	0.054965	0.030357	0.025039	CIRBP	Cold-induc	CIRBP_HUN	4	1.038834
48H 50MT/	Q96E52	0.313836	0.030364	0.025042	OMA1	Metalloend	OMA1_HUN	5	1.243009
24H 10MT/	Q96DB5	0.13291	0.030368	0.025045	RMDN1	Regulator c	RMD1_HUN	13	1.096503
48H 50MT/	Q969K3	-0.05117	0.030371	0.025046	RNF34	E3 ubiquiti	RNF34_HU	5	0.965153

48H 10MT/	Q13322	0.686769	0.030372	0.025047	GRB10	Growth fac	GRB10_HU	1	1.609675
48H 50MT/	Q9BTY2	-0.08834	0.030387	0.025056	FUCA2	Plasma alpl	FUCO2_HU	13	0.940606
24H 50MT/	Q9BUL5	-0.18895	0.030402	0.025067	PHF23	PHD finger	PHF23_HUI	6	0.877246
24H 50MT/	P83436	0.049899	0.030405	0.025069	COG7	Conserved (	COG7_HUN	23	1.035193
48H 50MT/	P35610	-0.22006	0.030413	0.025073	SOAT1	Sterol O-ac	SOAT1_HUI	11	0.858533
48H 10MT/	P42330	-0.2187	0.030413	0.025073	AKR1C3	Aldo-keto r	AK1C3_HUI	11	0.859337
24H 50MT/	Q8NEY4	0.736264	0.030417	0.025074	ATP6V1C2	V-type prot	VATC2_HUI	2	1.665856
24H 50MT/	Q9NS84	0.352504	0.030424	0.025079	CHST7	Carbohydra	CHST7_HUI	1	1.276775
24H 10MT/	Q96HR3	1.331972	0.030458	0.025101	MED30	Mediator o	MED30_HU	2	2.517466
24H 10MT/	Q96EY8	0.138962	0.030468	0.025106	MMAB	Corrinoid a	MMAB_HUI	7	1.101113
24H 10MT/	Q9BTU6	-0.01772	0.030468	0.025106	PI4K2A	Phosphatid	P4K2A_HUI	22	0.987791
48H 10MT/	O60361	-0.23401	0.030472	0.025108	NME2P1	Putative nu	NDK8_HUN	1	0.850266
24H 10MT/	P56381;Q5	-0.23008	0.030475	0.025109	ATP5F1E;A1	ATP synthas	ATP5E_HUN	1	0.852589
24H 50MT/	Q8WTS1	-0.28366	0.03048	0.025112	ABHD5	1-acylglyce	ABHD5_HU	3	0.821505
24H 10MT/	Q99986	0.129521	0.030481	0.025112	VRK1	Serine/thre	VRK1_HUM	13	1.093931
24H 10MT/	P46782	-0.22905	0.030482	0.025113	RPS5	Small ribos	RS5_HUMA	10	0.853197
24H 50MT/	O60487	-0.05455	0.030502	0.025126	MPZL2	Myelin prot	MPZL2_HU	4	0.962894
24H 50MT/	Q8N1G2	0.081316	0.030506	0.025127	CMTR1	Cap-specifi	CMTR1_HU	33	1.057983
48H 10MT/	Q15654	-0.24719	0.03051	0.025128	TRIP6	Thyroid rec	TRIP6_HUN	8	0.842538
24H 50MT/	Q4VC44	0.103651	0.030509	0.025128	FLYWCH1	FLYWCH-ty	FWCH1_HU	6	1.074489
24H 10MT/	P0C0S8;P2	0.191095	0.030528	0.025139	H2AC11;H2	Histone H2	H2A1_HUN	8	1.14163
48H 10MT/	P00558	0.068944	0.030531	0.02514	PGK1	Phosphogly	PGK1_HUM	37	1.048949
24H 50MT/	Q96AH0	-0.15365	0.03054	0.025145	NABP1	SOSS comp	SOSB2_HUI	2	0.898975
24H 50MT/	Q9NQZ2	-0.03451	0.03054	0.025145	UTP3	Something	SAS10_HUN	11	0.976364
48H 10MT/	Q07955	0.021074	0.030555	0.025154	SRSF1	Serine/argi	SRSF1_HUN	14	1.014715
24H 50MT/	Q6NUK4	0.180452	0.03056	0.025156	REEP3	Receptor e	REEP3_HUN	3	1.133239
48H 50MT/	Q9NZQ3	0.264748	0.030567	0.025161	NCKIPSD	NCK-intera	SPN90_HUI	8	1.201426
24H 10MT/	Q6ZRP7	-0.08552	0.030583	0.025169	QSOX2	Sulfhydryl (	QSOX2_HU	20	0.942446
48H 50MT/	Q15819	0.243165	0.030599	0.025179	UBE2V2	Ubiquitin-c	UB2V2_HU	5	1.183586
48H 50MT/	P48047	0.11982	0.030602	0.02518	ATP5PO	ATP synthas	ATPO_HUM	10	1.086599
48H 50MT/	P51531	0.403163	0.030613	0.025189	SMARCA2	Probable gl	SMCA2_HU	12	1.322404
24H 10MT/	P07947	0.055906	0.030619	0.025192	YES1	Tyrosine-pr	YES_HUMA	18	1.039512
24H 50MT/	P25398	-0.17607	0.030631	0.0252	RPS12	Small ribos	RS12_HUM	9	0.885108
24H 10MT/	Q10570	0.118975	0.030641	0.025206	CPSF1	Cleavage ar	CPSF1_HUN	43	1.085963
48H 50MT/	P61923	0.172457	0.030644	0.025207	COPZ1	Coatomer s	COPZ1_HUI	6	1.126976
48H 10MT/	Q9UPN7	-0.01334	0.030658	0.025217	PPP6R1	Serine/thre	PP6R1_HUI	14	0.990795
48H 50MT/	O75935	-0.17931	0.030659	0.025218	DCTN3	Dynactin su	DCTN3_HU	7	0.883126
24H 50MT/	Q7Z4W1	0.067286	0.030664	0.02522	DCXR	L-xylulose r	DCXR_HUM	10	1.047744
24H 50MT/	Q6UWP7	0.05255	0.03067	0.025222	LCLAT1	Lysocardiol	LCLT1_HUN	7	1.037096
48H 10MT/	Q9H1P3	0.080093	0.030676	0.025224	OSBPL2	Oxysterol-b	OSBL2_HUI	9	1.057086
24H 10MT/	Q9Y3B3	0.231465	0.030674	0.025224	TMED7	Transmemb	TMED7_HU	7	1.174026
48H 10MT/	Q09472	0.13716	0.030683	0.025228	EP300	Histone ace	EP300_HUI	7	1.099738
48H 10MT/	Q2PZI1	0.136535	0.030683	0.025228	DPY19L1	Protein C-m	D19L1_HUI	13	1.099261
24H 50MT/	Q6PIJ6	-0.25036	0.030684	0.025228	FBXO38	F-box only	FBX38_HUI	3	0.840689
48H 10MT/	Q8IZV5	-0.02016	0.030693	0.025235	RDH10	Retinol deh	RDH10_HU	9	0.986123
24H 10MT/	Q9UKD2	0.044661	0.030699	0.025238	MRTO4	mRNA turn	MRT4_HUN	15	1.031441
24H 50MT/	Q96BK5	-0.53661	0.0307	0.025238	PINX1	PIN2/TERF1	PINX1_HUN	7	0.689391
48H 10MT/	Q96JH7	0.085407	0.030701	0.025238	VCPIP1	Deubiquiti	VCIP1_HUN	30	1.060987
24H 50MT/	Q99439	-0.25312	0.030702	0.025238	CNN2	Calponin-2	CNN2_HUN	14	0.839083
24H 50MT/	Q66LE6	-0.01921	0.030705	0.02524	PPP2R2D	Serine/thre	2ABD_HUM	3	0.986773
48H 10MT/	O14773	-0.08841	0.030722	0.025251	TPP1	Tripeptidyl	TPP1_HUM	11	0.940557
24H 10MT/	Q99729	0.092939	0.030735	0.025259	HNRNPAB	Heterogene	ROAA_HUN	11	1.066541

48H 50MTA	L0R6Q1	-0.1988	0.030747	0.025266	SLC35A4	SLC35A4 up	S35U4_HUI		6	0.871274
48H 50MTA	P46776	-0.12831	0.030757	0.025273	RPL27A	Large ribos	RL27A_HUI		7	0.9149
24H 50MTA	Q6NYC1	0.107578	0.030761	0.025275	JMJD6	Bifunctiona	JMJD6_HUI		11	1.077418
24H 50MTA	P00403	-0.05706	0.030763	0.025275	MT-CO2	Cytochrom	COX2_HUM		7	0.961224
24H 50MTA	Q92930	0.142548	0.030774	0.025282	RAB8B	Ras-related	RAB8B_HUI		4	1.103853
24H 10MTA	Q99623	0.055029	0.030778	0.025285	PHB2	Prohibitin-	PHB2_HUM		19	1.03888
48H 50MTA	Q9NWH9	-0.08993	0.0308	0.025298	SLTM	SAFB-like tr	SLTM_HUM		17	0.939569
48H 10MTA	Q13242	0.028655	0.030809	0.025304	SRSF9	Serine/argi	SRSF9_HUM		14	1.020061
48H 10MTA	Q9BY32	-0.11555	0.030831	0.025321	ITPA	Inosine trip	ITPA_HUMA		12	0.923028
24H 10MTA	O95857	-0.1735	0.030833	0.025321	TSPAN13	Tetraspanir	TSN13_HUI		4	0.88669
48H 10MTA	Q14203	-0.00013	0.030838	0.025323	DCTN1	Dynactin su	DCTN1_HU		52	0.999907
48H 50MTA	Q9NTN9	0.129646	0.030838	0.025323	SEMA4G	Semaphori	SEM4G_HU		19	1.094025
24H 10MTA	Q9UKM7	-0.12611	0.030861	0.025336	MAN1B1	Endoplasm	MA1B1_HU		13	0.916302
48H 10MTA	Q9Y5N6	-0.56242	0.030862	0.025336	ORC6	Origin reco	ORC6_HUM		4	0.677164
48H 10MTA	Q68EM7	-0.02357	0.030865	0.025337	ARHGAP17	Rho GTPase	RHG17_HU		16	0.983793
24H 50MTA	Q96GD4	-0.19175	0.030872	0.025342	AURKB	Aurora kina	AURKB_HU		11	0.875543
48H 50MTA	Q6PJI9	0.057242	0.030885	0.02535	WDR59	GATOR2 co	WDR59_HU		15	1.040475
24H 50MTA	Q9P0K8	0.182149	0.030885	0.02535	FOXJ2	Forkhead b	FOXJ2_HUI		4	1.134573
48H 10MTA	O95630	0.009721	0.030896	0.025357	STAMBP	STAM-bindi	STABP_HUI		11	1.006761
48H 50MTA	Q96JB5	-0.00543	0.0309	0.025359	CDK5RAP3	CDK5 regul	CK5P3_HUI		18	0.996244
48H 50MTA	O14686	0.000653	0.030903	0.025362	KMT2D	Histone-lys	KMT2D_HU		9	1.000452
24H 50MTA	A2RUC4	0.290797	0.030916	0.02537	TYW5	tRNA wybu	TYW5_HUM		7	1.223316
24H 10MTA	Q6NS38	0.019286	0.03092	0.025373	ALKBH2	DNA oxidat	ALKB2_HUI		5	1.013458
24H 50MTA	O94761	0.780063	0.030944	0.025387	RECQL4	ATP-depen	RECQ4_HUI		1	1.717206
24H 10MTA	Q8NEZ2	-0.14635	0.030957	0.025394	VPS37A	Vacuolar pr	VP37A_HUI		10	0.903534
48H 50MTA	Q06203	0.106915	0.030961	0.025396	PPAT	Amidophos	PUR1_HUM		17	1.076923
24H 50MTA	Q13627	0.240472	0.030968	0.025398	DYRK1A	Dual specifi	DYR1A_HUI		7	1.181379
24H 10MTA	Q16625	0.109585	0.030967	0.025398	OCLN	Occludin	OCLN_HUM		8	1.078918
24H 50MTA	Q96S94	0.283592	0.030986	0.025408	CCNL2	Cyclin-L2	CCNL2_HUI		1	1.217222
48H 50MTA	P07205	0.295975	0.031017	0.025426	PGK2	Phosphogly	PGK2_HUM		3	1.227715
48H 10MTA	O43572	-0.04994	0.031028	0.025434	AKAP10	A-kinase an	AKA10_HUI		7	0.965977
24H 50MTA	Q9UJX6	0.12889	0.031031	0.025435	ANAPC2	Anaphase-p	ANC2_HUM		21	1.093452
48H 50MTA	Q92599	0.070309	0.031037	0.025439	SEPTIN8	Septin-8	SEPT8_HUM		7	1.049942
48H 50MTA	Q629K1	-0.26396	0.031044	0.025443	TRIQK	Triple QxxK	TRIQK_HUM		1	0.832798
48H 10MTA	Q9UBR2	0.01264	0.031046	0.025443	CTSZ	Cathepsin Z	CATZ_HUM		8	1.0088
24H 50MTA	O75208	0.156468	0.031065	0.025457	COQ9	Ubiquinon	COQ9_HUM		7	1.114555
48H 10MTA	Q9HBH0	0.006603	0.031073	0.025463	RHOF	Rho-relatec	RHOF_HUM		10	1.004587
24H 10MTA	Q9Y6A4	0.051439	0.031083	0.025467	CFAP20	Cilia- and fl	CFA20_HUI		11	1.036298
48H 50MTA	O95208	-0.06389	0.031095	0.025475	EPN2	Epsin-2	EPN2_HUM		4	0.956678
48H 50MTA	P16083	0.091254	0.031097	0.025476	NQO2	Ribosyldihy	NQO2_HUM		13	1.065296
48H 50MTA	P07948	0.039561	0.031105	0.025478	LYN	Tyrosine-pr	LYN_HUMA		19	1.027801
48H 50MTA	Q0P6H9	-0.49183	0.031105	0.025478	TMEM62	Transmemb	TMM62_HU		3	0.711123
24H 50MTA	Q9BU19	-0.13935	0.031104	0.025478	ZNF692	Zinc finger	ZN692_HUI		1	0.907927
24H 50MTA	P00761	-0.81392	0.03111	0.02548	NaN	Trypsin (Lal	TRYP_PIG		7	0.568834
24H 50MTA	P39687	-0.03315	0.031134	0.025497	ANP32A	Acidic leuci	AN32A_HU		10	0.977283
48H 10MTA	O75128	-0.28375	0.031147	0.025505	COBL	Protein cor	COBL_HUM		8	0.821452
24H 50MTA	P55795	0.031755	0.031165	0.025517	HNRNPH2	Heterogene	HNRH2_HU		9	1.022255
24H 50MTA	Q9ULT8	-0.03759	0.03117	0.025519	HECTD1	E3 ubiquiti	HECD1_HU		64	0.974281
48H 10MTA	Q9GZR2	0.02172	0.031176	0.025523	REXO4	RNA exonu	REXO4_HUI		13	1.015169
48H 10MTA	Q8NI22	1.084967	0.031193	0.025534	MCFD2	Multiple cc	MCFD2_HU		1	2.121327
24H 10MTA	Q9Y666	-0.02552	0.031203	0.02554	SLC12A7	Solute carri	S12A7_HUI		17	0.982468
48H 50MTA	Q8TDD1	-0.15602	0.031222	0.025552	DDX54	ATP-depen	DDX54_HU		37	0.897495

48H 10MT/	O75928	0.122279	0.031239	0.025564	PIAS2	E3 SUMO-p	PIAS2_HUN	4	1.088453
48H 50MT/	P51649	0.071322	0.031242	0.025565	ALDH5A1	Succinate-s	SSDH_HUN	15	1.050679
24H 50MT/	Q9BZX2	-0.23594	0.031246	0.025567	UCK2	Uridine-cyt	UCK2_HUN	11	0.849131
48H 10MT/	Q8N196	0.445702	0.031249	0.025567	SIX5	Homeobox	SIX5_HUM/	1	1.361977
24H 50MT/	Q9NSP4	-0.10329	0.031249	0.025567	CENPM	Centromer	CENPM_HU	3	0.930908
24H 50MT/	Q7Z3Y8	2.29537	0.03125	0.025567	KRT27	Keratin, typ	K1C27_HUI	2	4.9088
48H 50MT/	P14649	0.016686	0.031263	0.025575	MYL6B	Myosin ligh	MYL6B_HU	7	1.011633
24H 50MT/	Q9Y2T3	0.167084	0.031271	0.025579	GDA	Guanine de	GUAD_HUN	11	1.122787
24H 50MT/	Q9P0I2	0.047951	0.031281	0.025584	EMC3	ER membra	EMC3_HUN	11	1.033796
24H 50MT/	Q6NUM9	0.030825	0.031285	0.025586	RETSAT	All-trans-re	RETST_HUN	15	1.021596
24H 10MT/	Q8WVC6	0.370568	0.031299	0.025595	DCAKD	Dephospho	DCAKD_HU	3	1.292862
48H 10MT/	Q9NUN5	0.145878	0.0313	0.025595	LMBRD1	Lysosomal	LMBD1_HU	3	1.106404
48H 50MT/	O43542	-0.14345	0.031302	0.025597	XRCC3	DNA repair	XRCC3_HUI	6	0.905353
24H 10MT/	Q8N5M9	0.357116	0.031304	0.025598	JAGN1	Protein jag	JAGN1_HUI	2	1.280863
48H 50MT/	Q9Y487	-0.11605	0.031328	0.025614	ATP6V0A2	V-type prot	VPP2_HUM	15	0.922713
24H 10MT/	Q8N7H5	-0.00126	0.031378	0.025646	PAF1	RNA polym	PAF1_HUM	21	0.99913
24H 50MT/	Q16555	0.035348	0.031382	0.025648	DPYSL2	Dihydropyr	DPYL2_HUI	17	1.024804
48H 10MT/	Q12979	-0.18519	0.031384	0.02565	ABR	Active brea	ABR_HUMA	13	0.879535
48H 10MT/	Q96I24	-0.0149	0.031396	0.025658	FUBP3	Far upstrea	FUBP3_HUI	24	0.989723
24H 10MT/	P19256	0.079186	0.031397	0.025659	CD58	Lymphocyt	LFA3_HUM	2	1.056422
48H 10MT/	Q2KHT3	-0.44191	0.031399	0.025659	CLEC16A	Protein CLE	CL16A_HUI	1	0.736157
48H 10MT/	Q9UKD2	-0.01172	0.031402	0.025661	MRTO4	mRNA turn	MRT4_HUN	15	0.99191
24H 50MT/	O75027	0.043936	0.031417	0.025669	ABCB7	Iron-sulfur	ABCB7_HUI	17	1.030923
48H 50MT/	P49459	-0.15314	0.031427	0.025675	UBE2A	Ubiquitin-c	UBE2A_HUI	3	0.899288
48H 50MT/	Q7L273	-0.42178	0.031446	0.025686	KCTD9	BTB/POZ dc	KCTD9_HUI	4	0.746502
24H 10MT/	P49006	-0.22666	0.031451	0.025689	MARCKSL1	MARCKS-re	MRP_HUM.	2	0.85461
24H 50MT/	Q7Z401	-0.10648	0.031469	0.025698	DENND4A	C-myc pron	MYCPP_HU	6	0.928851
24H 10MT/	Q08188	0.596915	0.031472	0.025701	TGM3	Protein-glu	TGM3_HUN	2	1.512478
48H 10MT/	P15531	-0.19903	0.031473	0.025701	NME1	Nucleoside	NDKA_HUN	6	0.871133
24H 50MT/	Q14160	0.010287	0.031474	0.025701	SCRIB	Protein scri	SCRIB_HUN	40	1.007156
48H 50MT/	O75116	0.003048	0.031494	0.025713	ROCK2	Rho-associa	ROCK2_HU	39	1.002115
24H 50MT/	Q8NBJ7	0.068339	0.031513	0.025725	SUMF2	Inactive C-a	SUMF2_HU	10	1.048509
24H 10MT/	Q9UKY7	-0.03577	0.031515	0.025725	CDV3	Protein CD'	CDV3_HUN	13	0.975514
24H 10MT/	Q9Y2V2	-0.03781	0.031528	0.025735	CARHSP1	Calcium-re	CHSP1_HUI	7	0.974136
24H 50MT/	O75864	0.070524	0.031547	0.025747	PPP1R37	Protein phc	PPR37_HUI	2	1.050098
24H 50MT/	O95394	-0.06663	0.031548	0.025747	PGM3	Phosphoac	AGM1_HUN	21	0.954863
48H 10MT/	O95793	0.022465	0.031556	0.025752	STAU1	Double-stra	STAU1_HUI	28	1.015694
24H 10MT/	Q13158	-0.19911	0.031564	0.025756	FADD	FAS-associa	FADD_HUN	6	0.871085
48H 50MT/	Q99618	-0.35574	0.031574	0.025762	CDCA3	Cell divisio	CDCA3_HU	3	0.781468
24H 50MT/	Q9NYP7	-0.09474	0.03158	0.025765	ELOVL5	Very long cl	ELOV5_HUI	2	0.936439
48H 10MT/	Q9NVE7	0.024734	0.031601	0.025777	PANK4	4'-phospho	PANK4_HU	23	1.017292
48H 10MT/	Q8TCC3	-0.17611	0.031608	0.025782	MRPL30	Large ribos	RM30_HUN	2	0.885085
48H 10MT/	P60228	0.029261	0.031621	0.025791	EIF3E	Eukaryotic	EIF3E_HUN	25	1.020489
48H 10MT/	Q14145	0.021889	0.03163	0.025796	KEAP1	Kelch-like E	KEAP1_HUI	20	1.015288
48H 10MT/	Q9Y6A9	0.081619	0.031631	0.025796	SPCS1	Signal pept	SPCS1_HUN	2	1.058205
24H 50MT/	A6NDU8	0.017657	0.031636	0.0258	RIMOC1	RAB7A-inte	RIMC1_HUI	9	1.012314
48H 10MT/	Q9NQW7	0.063511	0.031639	0.025802	XPNPEP1	Xaa-Pro am	XPP1_HUM	28	1.045006
48H 10MT/	Q9H9Y2	-0.11966	0.031644	0.025803	RPF1	Ribosome p	RPF1_HUM	10	0.920407
48H 50MT/	Q76FK4	-0.13575	0.031656	0.025809	NOL8	Nucleolar p	NOL8_HUN	16	0.910195
48H 50MT/	Q9UQ16	0.473608	0.031662	0.025812	DNM3	Dynamin-3	DYN3_HUN	2	1.388578
24H 10MT/	Q9H1Y0	-0.13517	0.031661	0.025812	ATG5	Autophagy	ATG5_HUM	9	0.910559
24H 50MT/	P18615	0.035439	0.031687	0.025827	NELFE	Negative el	NELFE_HUN	15	1.024868

24H 50MT/	Q9BQ87	-0.29617	0.031688	0.025827	TBL1Y	F-box-like/\	TBL1Y_HUN	2	0.81441
24H 50MT/	Q9C0C7	0.179883	0.031697	0.025833	AMBRA1	Activating r	AMRA1_HU	9	1.132792
24H 10MT/	Q2TAA5	-0.06877	0.031705	0.025837	ALG11	GDP-Man:N	ALG11_HUI	8	0.953453
48H 50MT/	O00483	-0.16048	0.031723	0.025848	NDUFA4	Cytochrom	NDUA4_HU	4	0.89473
48H 50MT/	P10599	0.035571	0.031739	0.025858	TXN	Thioredoxi	THIO_HUM	7	1.024962
48H 10MT/	Q96NT0	0.106533	0.031752	0.025866	CCDC115	Coiled-coil	CC115_HUI	4	1.076638
48H 10MT/	Q9UKT4	0.471482	0.031757	0.025868	FBXO5	F-box only p	FBX5_HUM	2	1.386533
48H 10MT/	Q96PM5	-0.06693	0.031758	0.025868	RCHY1	RING finger	ZN363_HUI	1	0.954665
24H 10MT/	Q9NZ08	0.125389	0.031761	0.02587	ERAP1	Endoplasm	ERAP1_HUI	27	1.090802
48H 50MT/	P31946	0.062926	0.03177	0.025875	YWHAB	14-3-3 prot	1433B_HUI	17	1.044582
24H 50MT/	P04062	0.068415	0.031784	0.025881	GBA1	Lysosomal a	GBA1_HUM	16	1.048564
48H 50MT/	P43487	-0.15487	0.031783	0.025881	RANBP1	Ran-specifi	RANG_HUN	8	0.898214
48H 50MT/	Q6NXT4	0.067116	0.031784	0.025881	SLC30A6	Zinc transp	ZNT6_HUM	4	1.047621
24H 50MT/	Q12996	0.034497	0.031786	0.025881	CSTF3	Cleavage sti	CSTF3_HUN	24	1.024199
24H 10MT/	P78356	0.445928	0.031795	0.025885	PIP4K2B	Phosphatid	PI42B_HUN	2	1.36219
48H 10MT/	Q9H477	0.111533	0.031801	0.025889	RBKS	Ribokinase	RBSK_HUM	6	1.080376
48H 50MT/	Q9UBP6	-0.1295	0.03181	0.025893	METTL1	tRNA (guan	TRMB_HUN	10	0.914145
24H 10MT/	P15924	-0.06679	0.031813	0.025895	DSP	Desmoplak	DESP_HUM	135	0.954758
48H 10MT/	Q9Y6N5	0.005937	0.031818	0.025898	SQOR	Sulfide:qui	SQOR_HUN	30	1.004124
24H 50MT/	Q8WVC6	0.614233	0.031845	0.025918	DCAKD	Dephosphc	DCAKD_HU	3	1.530744
24H 10MT/	O60216	0.117991	0.031864	0.02593	RAD21	Double-stra	RAD21_HU	22	1.085223
24H 50MT/	Q8WUN7	0.38022	0.031868	0.025932	UBTD2	Ubiquitin d	UBTD2_HU	2	1.30154
24H 50MT/	Q96CM8	0.359574	0.031883	0.02594	ACSF2	Medium-ch	ACSF2_HUI	13	1.283047
48H 10MT/	O15033	0.425049	0.031889	0.025943	AREL1	Apoptosis-r	AREL1_HUN	1	1.342618
48H 50MT/	Q9BR61	-0.20073	0.031899	0.025949	ACBD6	Acyl-CoA-bi	ACBD6_HU	9	0.870109
24H 10MT/	Q96HA8	0.230555	0.0319	0.02595	NTAQ1	Protein N-t	NTAQ1_HU	2	1.173286
24H 50MT/	P46108	-0.11491	0.031902	0.02595	CRK	Adapter mc	CRK_HUMA	13	0.923437
48H 50MT/	Q9NYL4	-0.09057	0.031905	0.025951	FKBP11	Peptidyl-pr	FKB11_HUI	5	0.939151
24H 50MT/	P51884	-1.24875	0.031922	0.025962	LUM	Lumican	LUM_HUMA	4	0.420812
48H 10MT/	O95104	0.175757	0.031927	0.025964	SCAF4	SR-related a	SCAF4_HUN	14	1.129557
48H 50MT/	P01009	0.204598	0.031941	0.025972	SERPINA1	Alpha-1-ant	A1AT_HUM	31	1.152365
24H 10MT/	Q9P275	-0.00581	0.031963	0.025985	USP36	Ubiquitin c	UBP36_HU	7	0.995983
48H 10MT/	Q8TCY9	0.023478	0.031967	0.025988	URGCP	Up-regulatc	URGCP_HU	22	1.016407
48H 50MT/	P49441	-0.18879	0.031975	0.025992	INPP1	Inositol pol	INPP_HUM	9	0.877342
24H 50MT/	P12270	0.037215	0.031985	0.025997	TPR	Nucleoprot	TPR_HUMA	81	1.026131
48H 10MT/	P11717	0.017672	0.032006	0.02601	IGF2R	Cation-inde	MPRI_HUM	85	1.012325
48H 50MT/	Q9Y3A4	-0.24593	0.032007	0.02601	RRP7A	Ribosomal	RRP7A_HUI	11	0.843272
48H 50MT/	Q4LE39	0.035836	0.032023	0.02602	ARID4B	AT-rich inte	ARI4B_HUN	5	1.025151
48H 50MT/	P32119	0.119616	0.032025	0.02602	PRDX2	Peroxiredo	PRDX2_HU	13	1.086446
48H 50MT/	Q8IWF2	-0.32186	0.032035	0.026025	FOXRED2	FAD-depen	FXRD2_HUI	4	0.800039
24H 10MT/	O75363	0.035238	0.032042	0.026029	BCAS1	Breast carci	BCAS1_HUI	5	1.024726
24H 50MT/	P17568	0.222758	0.032043	0.026029	NDUFB7	NADH dehy	NDUB7_HU	5	1.166962
48H 10MT/	Q6P087	-0.07855	0.032062	0.026041	RPUSD3	Mitochond	RUSD3_HU	8	0.947007
24H 10MT/	P51148	0.037949	0.032067	0.026045	RAB5C	Ras-related	RAB5C_HUI	6	1.026654
48H 50MT/	P11171	0.185189	0.032072	0.026048	EPB41	Protein 4.1	EPB41_HUI	7	1.136966
24H 10MT/	Q9H9E3	0.047191	0.03208	0.026053	COG4	Conserved (	COG4_HUN	26	1.033251
48H 50MT/	Q13418	0.12136	0.032082	0.026054	ILK	Integrin-lin	ILK_HUMAI	17	1.08776
48H 10MT/	Q9UKZ1	0.063144	0.03209	0.026058	CNOT11	CCR4-NOT t	CNO11_HU	9	1.04474
48H 50MT/	Q96FW1	0.040845	0.03211	0.02607	OTUB1	Ubiquitin t	OTUB1_HU	14	1.028716
24H 50MT/	O15144	-0.01799	0.032122	0.026077	ARPC2	Actin-relate	ARPC2_HUI	19	0.987609
24H 50MT/	P08238	-0.14148	0.032126	0.026079	HSP90AB1	Heat shock	HS90B_HUI	50	0.906588
24H 10MT/	Q8NBJ5	0.054151	0.032131	0.026082	COLGALT1	Procollager	GT251_HUI	26	1.038248

48H	50MT/	Q7Z7K6	0.060832	0.032133	0.026083	CENPV	Centromer	CENPV_HU	6	1.043067
48H	10MT/	Q7KZI7	0.044191	0.032137	0.026086	MARK2	Serine/thre	MARK2_HU	19	1.031105
24H	50MT/	Q9Y303	-0.13485	0.032148	0.026092	AMDHD2	N-acetylglu	NAGA_HUM	4	0.910762
48H	10MT/	Q9Y3A2	-0.17717	0.032151	0.026093	UTP11	Probable U	UTP11_HU	11	0.884436
48H	10MT/	P09327	0.011699	0.032191	0.026116	VIL1	Villin-1	VILI_HUMA	23	1.008142
24H	50MT/	P35606	0.020924	0.032196	0.026118	COPB2	Coatomer s	COPB2_HU	43	1.014609
24H	50MT/	Q13085	-0.01589	0.032201	0.02612	ACACA	Acetyl-CoA	ACACA_HU	82	0.989046
48H	10MT/	Q9NYB0	0.018196	0.032217	0.026128	TERF2IP	Telomeric r	TE2IP_HUM	10	1.012693
48H	50MT/	Q14344	-0.09599	0.032231	0.026138	GNA13	Guanine nu	GNA13_HU	14	0.935631
48H	50MT/	Q99816	0.154033	0.032232	0.026139	TSG101	Tumor susc	TS101_HUM	16	1.112675
48H	10MT/	Q9NQ94	0.008778	0.03226	0.026158	A1CF	APOBEC1 c	A1CF_HUM	33	1.006103
48H	50MT/	Q9BXS5	0.019618	0.032274	0.026168	AP1M1	AP-1 compl	AP1M1_HU	23	1.013691
24H	50MT/	Q6IN84	-0.34485	0.032276	0.026168	MRM1	rRNA meth	MRM1_HU	6	0.787391
24H	10MT/	P14625	0.025986	0.032278	0.026169	HSP90B1	Endoplasm	ENPL_HUM	52	1.018175
24H	10MT/	O75909	0.062875	0.032296	0.026179	CCNK	Cyclin-K	CCNK_HUM	9	1.044545
24H	10MT/	P48729	0.06144	0.032301	0.026182	CSNK1A1	Casein kina	KC1A_HUM	13	1.043507
48H	50MT/	Q9NR56	-0.05551	0.032304	0.026183	MBNL1	Muscleblin	MBNL1_HU	7	0.962256
48H	50MT/	Q6ZNA5	-0.29956	0.032321	0.026193	FRRS1	Ferric-chel	FRRS1_HUM	8	0.812498
24H	10MT/	P05023	0.008469	0.03233	0.026199	ATP1A1	Sodium/po	AT1A1_HU	41	1.005887
48H	10MT/	Q13190	0.069001	0.032338	0.026204	STX5	Syntaxin-5	STX5_HUM	13	1.04899
48H	10MT/	Q9Y606	-0.07045	0.032346	0.026208	PUS1	Pseudourid	PUS1_HUM	16	0.952341
24H	50MT/	Q9BXY0	0.054045	0.032353	0.026212	MAK16	Protein MA	MAK16_HU	11	1.038171
48H	10MT/	P61244	0.059318	0.032371	0.026223	MAX	Protein ma	MAX_HUM	3	1.041973
24H	50MT/	Q969T3	-0.32677	0.032375	0.026225	SNX21	Sorting nex	SNX21_HU	4	0.797318
48H	50MT/	Q9UBQ7	0.090085	0.032388	0.026233	GRHPR	Glyoxylate	GRHPR_HU	14	1.064433
48H	10MT/	Q9UL54	-0.16688	0.032394	0.026235	TAOK2	Serine/thre	TAOK2_HU	9	0.890767
48H	10MT/	Q12830	0.038619	0.032401	0.026237	BPTF	Nucleosom	BPTF_HUM	26	1.02713
48H	10MT/	P61088	0.049129	0.032407	0.026241	UBE2N	Ubiquitin-c	UBE2N_HU	9	1.03464
48H	50MT/	P42285	-0.0639	0.032412	0.026243	MTREX	Exosome RI	MTREX_HU	47	0.956673
48H	50MT/	Q6P1Q9	-0.46963	0.032426	0.026252	METTL2B	tRNA N(3)-r	MET2B_HU	6	0.72215
24H	10MT/	Q96AE4	0.03753	0.032426	0.026252	FUBP1	Far upstrea	FUBP1_HU	28	1.026355
24H	10MT/	Q6P9B6	0.035906	0.032436	0.026259	MEAK7	MTOR-asso	MEAK7_HU	10	1.0252
24H	10MT/	O14737	-0.13324	0.032438	0.026259	PDCD5	Programme	PDCD5_HU	6	0.911784
24H	10MT/	P07996	0.66599	0.03245	0.026266	THBS1	Thrombosp	TSP1_HUM	9	1.586656
24H	50MT/	Q99700	0.027151	0.03245	0.026266	ATXN2	Ataxin-2	ATX2_HUM	25	1.018998
24H	10MT/	Q5TAX3	0.099123	0.032453	0.026267	TUT4	Terminal u	TUT4_HUM	9	1.071122
24H	10MT/	P83881;Q9	-0.13792	0.03246	0.02627	RPL36A;RP	Large ribos	RL36A_HU	4	0.908826
48H	10MT/	Q86Y82	0.105359	0.032482	0.026285	STX12	Syntaxin-1	STX12_HUM	9	1.075762
24H	50MT/	P28161	0.07173	0.032485	0.026286	GSTM2	Glutathion	GSTM2_HU	16	1.050976
48H	50MT/	P16144	-0.04617	0.032487	0.026287	ITGB4	Integrin be	ITB4_HUM	14	0.968507
48H	50MT/	Q13505	-0.01709	0.032488	0.026287	MTX1	Metaxin-1	MTX1_HUM	11	0.988226
48H	50MT/	Q9BTY7	-0.36692	0.032491	0.026289	HGH1	Protein HG	HGH1_HUM	12	0.775436
48H	10MT/	P62857	0.045039	0.032494	0.026289	RPS28	Small ribos	RS28_HUM	3	1.031711
24H	50MT/	Q15742	-0.03939	0.032497	0.026291	NAB2	NGFI-A-bin	NAB2_HUM	6	0.973066
48H	50MT/	Q9Y4L1	0.03142	0.032499	0.026292	HYOU1	Hypoxia up	HYOU1_HU	44	1.022017
24H	10MT/	P20962	-0.25242	0.0325	0.026292	PTMS	Parathymo	PTMS_HUM	1	0.83949
48H	50MT/	Q3ZAQ7	-1.00123	0.032503	0.026294	VMA21	Vacuolar A	VMA21_HU	2	0.499574
24H	50MT/	Q96BJ3	-0.0688	0.032518	0.026304	AIDA	Axin intera	AIDA_HUM	7	0.953434
48H	10MT/	Q9NVA4	0.286092	0.032559	0.026331	TMEM184C	Transmemb	T184C_HU	1	1.219333
48H	10MT/	P10321	0.095109	0.03256	0.026331	HLA-C	HLA class I	HLAC_HUM	3	1.068146
48H	50MT/	Q9NWU5	-0.18353	0.032565	0.026333	MRPL22	Large ribos	RM22_HUM	8	0.880549
24H	50MT/	P22087	0.078495	0.032569	0.026335	FBL	rRNA 2'-O-r	FBRL_HUM	15	1.055916

48H 10MTA	Q8N5C6	-0.08223	0.032575	0.026339	SRBD1	S1 RNA-bin	SRBD1_HUI	21	0.944599
48H 10MTA	Q8WTT2	-0.12885	0.032579	0.026341	NOC3L	Nucleolar c	NOC3L_HU	20	0.914561
24H 50MTA	Q96EK6	0.055281	0.032579	0.026341	GNPNAT1	Glucosamir	GNA1_HUM	11	1.039061
48H 10MTA	Q14790	0.083376	0.032588	0.026346	CASP8	Caspase-8	CASP8_HUI	17	1.059494
48H 10MTA	Q49A26	0.025178	0.032589	0.026346	GLYR1	Cytokine-lil	GLYR1_HUI	18	1.017605
48H 10MTA	Q8NBM4	-0.02195	0.0326	0.026353	UBAC2	Ubiquitin-a	UBAC2_HU	14	0.984903
48H 50MTA	Q9NZJ7	0.070897	0.032605	0.026355	MTCH1	Mitochond	MTCH1_HU	11	1.05037
24H 50MTA	Q9NRD1	0.118438	0.032606	0.026356	FBXO6	F-box only	FBX6_HUM	7	1.085559
48H 50MTA	O43709	-0.14407	0.032608	0.026357	BUD23	18S rRNA (g	BUD23_HU	5	0.904964
48H 10MTA	P25205	0.00231	0.032624	0.026367	MCM3	DNA replica	MCM3_HUI	37	1.001603
48H 10MTA	O75880	-0.11714	0.032639	0.026378	SCO1	Protein SCO	SCO1_HUM	8	0.922011
24H 50MTA	Q8WW12	-0.16348	0.032645	0.026381	PCNP	PEST prote	PCNP_HUM	6	0.89287
48H 50MTA	O43683	-0.17892	0.032649	0.026384	BUB1	Mitotic che	BUB1_HUM	21	0.883363
24H 10MTA	P11172	-0.04839	0.032656	0.026388	UMPS	Uridine 5'-r	UMPS_HUM	24	0.967016
48H 10MTA	Q5UIP0	-0.00521	0.032658	0.026388	RIF1	Telomere-a	RIF1_HUMA	48	0.996394
24H 50MTA	Q8N2H4	-0.46657	0.032691	0.026412	SYS1	Protein SYS	SYS1_HUMA	2	0.723683
48H 50MTA	Q07955	0.003286	0.032699	0.026417	SRSF1	Serine/argi	SRSF1_HUM	14	1.00228
24H 50MTA	Q9Y2V2	0.161754	0.032703	0.02642	CARHSP1	Calcium-reg	CHSP1_HUI	7	1.118646
24H 10MTA	Q6ZT62	0.051746	0.032723	0.026432	BARGIN	Bargin	BGIN_HUM	3	1.036519
48H 50MTA	Q92567	-0.16757	0.032725	0.026433	FAM168A	Protein FAM	F168A_HUI	1	0.890339
48H 10MTA	O96008	-0.13815	0.032737	0.026439	TOMM40	Mitochond	TOM40_HU	11	0.908687
48H 50MTA	Q5SY16	-0.13406	0.032742	0.02644	NOL9	Polynucleo	NOL9_HUM	21	0.911264
48H 10MTA	P53985	-0.07414	0.032754	0.026449	SLC16A1	Monocarbo	MOT1_HUM	11	0.949909
48H 10MTA	Q8NEN9	-0.46467	0.032755	0.02645	PDZD8	PDZ domai	PDZD8_HU	4	0.724636
48H 50MTA	Q99873	0.063754	0.032758	0.026451	PRMT1	Protein arg	ANM1_HUM	23	1.045182
48H 50MTA	Q6UW68	0.144146	0.032768	0.026458	TMEM205	Transmemb	TM205_HU	5	1.105076
48H 50MTA	O60427	-0.21579	0.032773	0.026461	FADS1	Acyl-CoA (8	FADS1_HUI	12	0.861075
48H 10MTA	Q12824	0.216415	0.032777	0.026463	SMARCB1	SWI/SNF-re	SNF5_HUM	12	1.161843
48H 10MTA	Q86X55	-0.01461	0.032782	0.026466	CARM1	Histone-arg	CARM1_HU	14	0.989921
24H 50MTA	Q96I76	-1.9455	0.032787	0.026469	GPATCH3	G patch do	GPTC3_HUI	1	0.259625
48H 50MTA	P42677	-0.15402	0.03279	0.026471	RPS27	Small ribos	RS27_HUM	2	0.898746
48H 50MTA	Q9Y483	-0.42109	0.032799	0.026477	MTF2	Metal-respo	MTF2_HUM	8	0.746859
24H 10MTA	Q9H5V9	0.044589	0.032804	0.02648	STEEP1	STING ER ex	STEEP_HUM	4	1.031389
24H 50MTA	Q8NI36	0.053801	0.032807	0.026481	WDR36	WD repeat-	WDR36_HU	30	1.037996
48H 10MTA	Q9UHA4	-0.1354	0.032837	0.026502	LAMTOR3	Ragulator c	LTOR3_HUI	5	0.91042
24H 50MTA	Q8TEQ6	0.003334	0.032842	0.026506	GEMIN5	Gem-associ	GEMI5_HUI	49	1.002314
24H 10MTA	Q9Y606	0.038005	0.032844	0.026507	PUS1	Pseudourid	PUS1_HUM	16	1.026693
24H 50MTA	P15336	-0.24401	0.032857	0.026513	ATF2	Cyclic AMP	ATF2_HUM	3	0.844397
24H 10MTA	Q6P2I3	-0.16583	0.032864	0.026515	FAHD2B	Oxaloaceta	FAH2B_HUI	1	0.891416
48H 10MTA	Q9H9A7	-2.21483	0.032866	0.026516	RMI1	RecQ-media	RMI1_HUM	1	0.215412
24H 10MTA	O75347	-0.21345	0.032867	0.026516	TBCA	Tubulin-spe	TBCA_HUM	10	0.86247
24H 50MTA	Q9UEG4	-0.27921	0.032871	0.026517	ZNF629	Zinc finger	ZN629_HUI	7	0.82404
48H 10MTA	P14324	-0.1153	0.032873	0.026518	FDPS	Farnesyl py	FPPS_HUM	10	0.923192
24H 50MTA	Q9BY77	0.173149	0.032885	0.026525	POLDIP3	Polymerase	PDIP3_HUM	17	1.127516
24H 10MTA	Q96PK6	0.027207	0.032901	0.026535	RBM14	RNA-bindir	RBM14_HU	25	1.019037
24H 50MTA	Q8N954	-0.34556	0.032911	0.026541	GPATCH11	G patch do	GPT11_HUI	4	0.787005
48H 50MTA	Q9Y3C0	1.067706	0.032917	0.026544	WASHC3	WASH com	WASC3_HU	4	2.096097
24H 10MTA	Q15397	-0.03315	0.032923	0.026547	PUM3	Pumilio ho	PUM3_HUM	24	0.977283
48H 10MTA	Q9UIW2	-0.34793	0.032961	0.026572	PLXNA1	Plexin-A1	PLXA1_HUI	8	0.785712
24H 50MTA	A4D1E9	-0.13805	0.032964	0.026574	GTPBP10	GTP-bindin	GTPBA_HUI	12	0.908748
48H 50MTA	Q9NVA4	2.64727	0.032967	0.026576	TMEM184C	Transmemb	T184C_HUI	1	6.264806
48H 50MTA	Q92581	0.118245	0.032975	0.026579	SLC9A6	Sodium/hy	SL9A6_HUM	5	1.085413

24H 50MT/	Q8IWQ3	-0.07703	0.032981	0.026583	BRSK2	Serine/thre	BRSK2_HUI	4	0.94801
48H 10MT/	P55347	0.332524	0.032984	0.026583	PKNOX1	Homeobox	PKNX1_HU	1	1.259215
48H 50MT/	Q9NUJ1	0.016361	0.032987	0.026584	ABHD10	Palmitoyl-p	ABHDA_HU	11	1.011405
24H 10MT/	Q96D46	0.131239	0.032993	0.026587	NMD3	60S riboso	NMD3_HUI	17	1.095234
48H 50MT/	P0CG39	-1.17867	0.033015	0.0266	POTEJ	POTE ankyr	POTEJ_HUN	1	0.441758
48H 10MT/	O00232	0.044385	0.033017	0.0266	PSMD12	26S protea	PSD12_HUI	26	1.031243
48H 50MT/	Q9BU02	0.044516	0.033022	0.026602	THTPA	Thiamine-tı	THTPA_HUI	5	1.031337
48H 50MT/	Q96I59	-0.1206	0.033026	0.026605	NARS2	Asparaginyl	SYNM_HUN	15	0.919808
24H 50MT/	P16452	3.284043	0.033027	0.026606	EPB42	Protein 4.2	EPB42_HUI	1	9.740819
48H 50MT/	P43357	-0.63879	0.03305	0.026622	MAGEA3	Melanoma-	MAGA3_HU	4	0.642253
48H 10MT/	P26368	0.100672	0.033068	0.026635	U2AF2	Splicing fac	U2AF2_HUI	13	1.072273
48H 10MT/	Q9NV56	0.04908	0.033072	0.026637	MRGBP	MRG/MORI	MRGBP_HU	3	1.034605
48H 50MT/	Q15434	-0.12902	0.033077	0.026639	RBMS2	RNA-bindir	RBMS2_HU	4	0.914451
24H 50MT/	Q16831	-0.03692	0.033086	0.026645	UPP1	Uridine pho	UPP1_HUN	16	0.974737
48H 10MT/	Q6ZWJ1	-0.0285	0.033094	0.02665	STXBP4	Syntaxin-bi	STXB4_HUN	10	0.980442
48H 50MT/	Q99732	0.375844	0.033104	0.026656	LITAF	Lipopolysa	LITAF_HUN	1	1.297598
48H 10MT/	Q7LBR1	0.219894	0.033103	0.026656	CHMP1B	Charged mu	CHM1B_HU	3	1.164648
24H 50MT/	O60281	0.747271	0.033128	0.026671	ZNF292	Zinc finger p	ZN292_HUI	6	1.678615
48H 50MT/	O15305	0.06796	0.033129	0.026671	PMM2	Phosphoma	PMM2_HUI	15	1.048233
48H 10MT/	Q9BUQ8	-0.03331	0.033134	0.026675	DDX23	Probable A	DDX23_HU	32	0.977173
48H 10MT/	Q9HCE1	0.006276	0.033139	0.026678	MOV10	Helicase M(	MOV10_HU	44	1.00436
48H 10MT/	P04843	-0.13214	0.033139	0.026678	RPN1	Dolichyl-di	RPN1_HUN	32	0.912476
48H 10MT/	Q9Y2T2	-0.00863	0.033147	0.026682	AP3M1	AP-3 compl	AP3M1_HU	17	0.994033
24H 50MT/	Q9H9J4	0.169169	0.033182	0.026701	USP42	Ubiquitin c	UBP42_HU	6	1.124411
24H 50MT/	Q6P4F2	-0.65963	0.03321	0.02672	FDX2	Ferredoxin-	FDX2_HUN	3	0.633041
48H 50MT/	Q6IA69	0.080426	0.033211	0.02672	NADSYN1	Glutamine-	NADE_HUN	10	1.05733
24H 50MT/	Q9H936	0.200253	0.033224	0.026726	SLC25A22	Mitochond	GHC1_HUN	11	1.1489
24H 50MT/	Q99640	-0.52769	0.033238	0.026733	PKMYT1	Membrane-	PMYT1_HU	3	0.693665
48H 10MT/	Q8TBZ6	-0.30597	0.03324	0.026734	TRMT10A	tRNA meth	TM10A_HU	3	0.808897
48H 10MT/	Q96RS6	0.048272	0.033258	0.026745	NUDCD1	NudC doma	NUDC1_HU	23	1.034026
24H 10MT/	P15151	-0.05143	0.033264	0.026748	PVR	Poliovirus r	PVR_HUMA	6	0.964981
48H 10MT/	Q6PJG2	-0.17938	0.033274	0.026755	MIDEAS	Mitotic dea	MDEAS_HU	7	0.883085
24H 10MT/	O75496	-0.10525	0.033276	0.026756	GMNN	Geminin	GEMI_HUN	6	0.929642
24H 50MT/	P08134	-0.01042	0.033278	0.026756	RHOC	Rho-relatec	RHOC_HUN	10	0.992801
24H 10MT/	Q9UKS6	0.029552	0.033279	0.026756	PACSIN3	Protein kin	PACN3_HU	15	1.020695
24H 50MT/	Q5JYT7	0.727681	0.033282	0.026757	KIAA1755	Uncharacte	K1755_HUI	1	1.655975
24H 50MT/	Q9Y4W2	0.007317	0.033318	0.026779	LAS1L	Ribosomal	LAS1L_HUN	29	1.005085
48H 50MT/	Q15056	0.168492	0.033322	0.026781	EIF4H	Eukaryotic	IF4H_HUM/	9	1.123883
48H 10MT/	Q9Y496	0.106847	0.033326	0.026784	KIF3A	Kinesin-like	KIF3A_HUN	11	1.076872
24H 50MT/	Q92845	-0.1931	0.033331	0.026787	KIFAP3	Kinesin-ass	KIFA3_HUN	11	0.874727
24H 50MT/	Q9NY93	0.02054	0.033339	0.02679	DDX56	Probable A	DDX56_HU	22	1.014339
48H 10MT/	P49810	-0.06146	0.033352	0.026797	PSEN2	Presenilin-2	PSN2_HUN	2	0.958294
48H 10MT/	O15037	0.221572	0.033364	0.026802	KHNYN	Protein KHI	KHNYN_HU	4	1.166004
48H 50MT/	Q8TBB5	-0.22451	0.033391	0.026817	KLHDC4	Kelch doma	KLDC4_HUI	11	0.855887
24H 10MT/	Q9HA77	0.065952	0.033396	0.026818	CARS2	Probable cy	SYCM_HUN	16	1.046776
24H 10MT/	Q9NP64	-0.15081	0.033436	0.026841	ZCCHC17	Zinc finger (	ZCC17_HUI	3	0.900746
48H 50MT/	Q9Y6J9	-0.38655	0.033436	0.026841	TAF6L	TAF6-like RI	TAF6L_HUN	14	0.764959
24H 10MT/	Q9H501	0.131797	0.03345	0.026849	ESF1	ESF1 homo	ESF1_HUM/	17	1.095657
48H 10MT/	Q8NEJ9	0.062961	0.033465	0.026858	NGDN	Neuroguidi	NGDN_HUN	11	1.044608
48H 10MT/	O15460	0.127249	0.033476	0.026861	P4HA2	Prolyl 4-hy	P4HA2_HU	16	1.092209
48H 50MT/	P83876	0.211742	0.033479	0.026863	TXNL4A	Thioredoxi	TXN4A_HUI	6	1.158086
24H 50MT/	O95573	0.03649	0.033491	0.026871	ACSL3	Fatty acid C	ACSL3_HUN	28	1.025616

24H 10MT/	P63172	-0.1867	0.033492	0.026872	DYNLT1	Dynein ligh	DYLT1_HUI	5	0.878612
48H 50MT/	O00148	0.171353	0.033503	0.026878	DDX39A	ATP-depen	DX39A_HU	12	1.126114
48H 10MT/	Q9NTN9	-0.05721	0.033503	0.026878	SEMA4G	Semaphori	SEM4G_HU	19	0.961119
48H 10MT/	Q68DH5	0.620126	0.033504	0.026878	LMBRD2	G-protein c	LMBD2_HU	3	1.53701
48H 10MT/	Q2TAL8	0.064291	0.033509	0.026881	QRICH1	Transcripti	QRIC1_HUI	13	1.045571
48H 10MT/	Q8N2K1	0.953566	0.033529	0.026895	UBE2J2	Ubiquitin-c	UB2J2_HUI	3	1.936653
24H 10MT/	O95563	0.150343	0.033545	0.026905	MPC2	Mitochond	MPC2_HUI	7	1.109834
48H 10MT/	Q9UJX3	-0.0016	0.033547	0.026906	ANAPC7	Anaphase-p	APC7_HUM	27	0.998888
24H 10MT/	Q9BSJ5	-0.61801	0.033548	0.026906	MTNAP1	Mitochond	CQ080_HU	3	0.651571
48H 50MT/	Q9NSE4	0.026301	0.033562	0.026915	IARS2	Isoleucine-	SYIM_HUM	36	1.018398
48H 10MT/	Q9GZN8	-0.04342	0.033599	0.026938	ADISSP	Adipose-se	ADSSP_HUI	9	0.970355
24H 50MT/	Q8IY33	0.472237	0.033636	0.026962	MICALL2	MICAL-like	MILK2_HUI	3	1.387259
24H 50MT/	Q9NX47	0.166497	0.033638	0.026963	MARCHF5	E3 ubiquiti	MARH5_HU	13	1.12233
48H 10MT/	P11047	0.027643	0.033643	0.026965	LAMC1	Laminin sul	LAMC1_HU	40	1.019345
24H 50MT/	O60749	-0.05098	0.033675	0.026987	SNX2	Sorting nex	SNX2_HUM	22	0.965277
24H 50MT/	Q6NUS6	0.133668	0.033677	0.026988	TCTN3	Tectonic-3	TECT3_HUI	6	1.097079
48H 50MT/	Q14192	0.610171	0.033685	0.026993	FHL2	Four and a	FHL2_HUM	1	1.526441
24H 10MT/	Q9UNX4	0.146872	0.033691	0.026998	WDR3	WD repeat-	WDR3_HUI	35	1.107166
24H 50MT/	O43402	0.02499	0.033716	0.027012	EMC8	ER membra	EMC8_HUI	5	1.017473
48H 10MT/	Q9BXJ9	0.012776	0.033716	0.027012	NAA15	N-alpha-ac	NAA15_HU	41	1.008895
24H 10MT/	Q66LE6	-0.03337	0.03372	0.027013	PPP2R2D	Serine/thre	2ABD_HUM	3	0.977132
48H 50MT/	Q9NZJ5	0.37964	0.033722	0.027014	EIF2AK3	Eukaryotic	E2AK3_HUI	6	1.301017
48H 10MT/	Q9NQ55	0.017352	0.033723	0.027014	PPAN	Suppressor	SSF1_HUM	12	1.0121
24H 10MT/	A7E2V4	-0.06133	0.033725	0.027015	ZSWIM8	Zinc finger	ZSWM8_HU	9	0.958378
24H 50MT/	P04406	-0.09459	0.033732	0.02702	GAPDH	Glyceraldel	G3P_HUM/	23	0.936541
24H 10MT/	Q8IWZ8	0.040724	0.033737	0.027022	SUGP1	SURP and G	SUGP1_HU	20	1.02863
24H 50MT/	Q9BXK1	-0.19641	0.033741	0.027023	KLF16	Krueppel-li	KLF16_HUI	2	0.872722
24H 50MT/	P61966	0.132983	0.033755	0.027031	AP1S1	AP-1 compl	AP1S1_HUI	7	1.096558
24H 10MT/	P08047	0.037133	0.033785	0.027049	SP1	Transcripti	SP1_HUMA	3	1.026073
24H 50MT/	Q9ULG1	0.273436	0.033787	0.02705	INO80	Chromatin	INO80_HUI	8	1.208683
48H 10MT/	P62253	-0.14226	0.033793	0.027052	UBE2G1	Ubiquitin-c	UB2G1_HU	7	0.906098
48H 50MT/	Q96EH3	-0.56215	0.033805	0.027058	MALSU1	Mitochond	MASU1_HU	3	0.677291
48H 10MT/	Q96HN2	-0.05802	0.033817	0.027065	AHCYL2	Adenosylhc	SAHH3_HU	12	0.960581
48H 10MT/	P52569	0.054511	0.033818	0.027065	SLC7A2	Cationic an	CTR2_HUM	8	1.038507
24H 10MT/	P60604	-0.11872	0.033822	0.027067	UBE2G2	Ubiquitin-c	UB2G2_HU	4	0.921005
48H 10MT/	Q9HAU4	0.499868	0.03383	0.027071	SMURF2	E3 ubiquiti	SMUF2_HU	1	1.414084
48H 50MT/	Q96G74	-0.43785	0.03383	0.027071	OTUD5	OTU domai	OTUD5_HU	4	0.738235
48H 50MT/	Q13444	-0.1271	0.033841	0.027077	ADAM15	Disintegrin	ADA15_HU	7	0.915669
48H 50MT/	Q13523	0.169826	0.033846	0.02708	PRP4K	Serine/thre	PRP4K_HUI	20	1.124923
48H 50MT/	O75886	-0.00358	0.033874	0.027097	STAM2	Signal trans	STAM2_HU	13	0.997521
48H 50MT/	Q7Z4F1	0.343759	0.03389	0.027108	LRP10	Low-densit	LRP10_HUI	4	1.269059
48H 50MT/	Q16891	0.011942	0.033895	0.02711	IMMT	MICOS com	MIC60_HUI	35	1.008312
24H 50MT/	Q9BV23	0.316574	0.033901	0.027113	ABHD6	Monoacylg	ABHD6_HU	5	1.24537
48H 10MT/	Q5XKP0	0.064279	0.03391	0.027119	MICOS13	MICOS com	MIC13_HUI	4	1.045562
24H 50MT/	P52788	0.007516	0.033911	0.027119	SMS	Spermine s	SPSY_HUM	19	1.005223
24H 50MT/	P82932	0.116013	0.033912	0.02712	MRPS6	Small ribos	RT06_HUM	4	1.083736
48H 10MT/	Q9H4B7	0.771378	0.033941	0.027138	TUBB1	Tubulin bet	TBB1_HUM	2	1.706899
24H 10MT/	Q9GZL7	0.042154	0.033961	0.027152	WDR12	Ribosome b	WDR12_HU	18	1.02965
24H 50MT/	O60841	0.030282	0.033966	0.027155	EIF5B	Eukaryotic	IF2P_HUM/	46	1.021212
24H 50MT/	Q9UHD1	0.053378	0.033967	0.027155	CHORDC1	Cysteine an	CHRD1_HU	20	1.037692
24H 50MT/	Q5JVS0	-0.25743	0.033968	0.027155	HABP4	Intracellula	HABP4_HU	5	0.836576
24H 50MT/	P48509	-0.13222	0.033995	0.027173	CD151	CD151 anti	CD151_HU	2	0.912427

24H	10MT/	Q8NCE2	0.118606	0.033996	0.027174	MTMR14	Myotubula	MTMRE_HU	10	1.085685
48H	50MT/	Q9Y692	0.0001	0.033998	0.027175	GMEB1	Glucocortic	GMEB1_HU	4	1.00007
24H	50MT/	Q96RK0	0.261521	0.034005	0.02718	CIC	Protein cap	CIC_HUMA	8	1.198742
48H	10MT/	Q16543	0.078612	0.03401	0.027182	CDC37	Hsp90 co-c	CDC37_HU	9	1.056002
48H	50MT/	P38117	0.012125	0.034043	0.027204	ETFB	Electron tra	ETFB_HUM/	17	1.00844
48H	10MT/	Q9Y6M5	-0.07149	0.034058	0.027213	SLC30A1	Proton-cou	ZNT1_HUM	12	0.951656
48H	10MT/	Q12959	-0.02385	0.034094	0.027236	DLG1	Disks large	DLG1_HUM	22	0.983607
24H	10MT/	Q9H3G5	-0.19566	0.034097	0.027237	CPVL	Probable se	CPVL_HUM	5	0.873172
24H	50MT/	Q9UN36	0.145466	0.034105	0.027242	NDRG2	Protein ND	NDRG2_HU	8	1.106088
24H	50MT/	Q96DI7	-0.06478	0.034118	0.02725	SNRNP40	U5 small nu	SNR40_HUI	17	0.95609
48H	50MT/	Q9GZU7	-0.10029	0.03413	0.027255	CTDSP1	Carboxy-ter	CTDS1_HUI	3	0.932846
24H	10MT/	Q86Y39	0.027528	0.034132	0.027256	NDUFA11	NADH dehy	NDUAB_HU	7	1.019264
24H	50MT/	Q9Y294	0.082674	0.034132	0.027256	ASF1A	Histone cha	ASF1A_HUI	5	1.058979
48H	10MT/	P16452	1.423745	0.03416	0.027273	EPB42	Protein 4.2	EPB42_HUI	1	2.68281
48H	10MT/	Q7Z401	0.058163	0.034163	0.027274	DENND4A	C-myc pron	MYCPP_HU	6	1.041139
24H	10MT/	Q9Y2C4	-0.06159	0.034173	0.027281	EXOG	Nuclease E>	EXOG_HUM	4	0.958205
48H	10MT/	Q13371	-0.04071	0.034179	0.027284	PDCL	Phosducin-	PHLP_HUM	2	0.972173
48H	10MT/	Q92562	-0.50957	0.034182	0.027285	FIG4	Polyphospl	FIG4_HUM/	4	0.702434
24H	10MT/	P38919	0.0582	0.034186	0.027286	EIF4A3	Eukaryotic	IF4A3_HUM	24	1.041166
48H	50MT/	Q8NDX6	-0.14799	0.034187	0.027286	ZNF740	Zinc finger	ZN740_HUI	1	0.902507
48H	50MT/	Q96HR8	-0.16321	0.034206	0.027298	NAF1	H/ACA ribo	NAF1_HUM	4	0.893035
24H	50MT/	Q9NSG2	-0.28824	0.034219	0.027305	FIRRM	FIGNL1-inte	FIRRM_HUI	9	0.818898
48H	50MT/	Q8N0Z6	-0.18527	0.034223	0.027307	TTC5	Tetratricop	TTC5_HUM	9	0.879487
48H	50MT/	Q9BT30	-0.07601	0.034247	0.027323	ALKBH7	Alpha-keto	ALKB7_HUI	5	0.94868
24H	50MT/	Q14690	0.002822	0.034264	0.027332	PDCD11	Protein RRI	RRP5_HUM	86	1.001958
48H	10MT/	P11940	0.022341	0.034319	0.027364	PABPC1	Polyadenyl	PABP1_HUI	36	1.015606
48H	50MT/	P51532	-1.15877	0.034321	0.027364	SMARCA4	Transcripti	SMCA4_HU	2	0.447895
24H	10MT/	Q3ZCW2	-0.21414	0.03432	0.027364	LGALSL	Galectin-re	LEGL_HUM	6	0.862062
24H	10MT/	P31749	0.121758	0.034362	0.02739	AKT1	RAC-alpha s	AKT1_HUM	8	1.08806
24H	50MT/	Q2M2I8	-0.07058	0.034361	0.02739	AAK1	AP2-associa	AAK1_HUM	18	0.952257
48H	10MT/	Q8NHM5	-0.51295	0.034378	0.027398	KDM2B	Lysine-spec	KDM2B_HU	4	0.700789
24H	10MT/	Q92696	-0.06578	0.034386	0.0274	RABGGTA	Geranylgera	PGTA_HUM	21	0.955429
48H	10MT/	Q06520	-0.66546	0.034405	0.027413	SULT2A1	Sulfotransfe	ST2A1_HUI	2	0.630488
24H	10MT/	Q6ZTW0	0.148553	0.034418	0.027421	TPGS1	Tubulin pol	TPGS1_HUI	6	1.108457
48H	50MT/	O14715	-0.17331	0.034426	0.027427	RGPD8	RANBP2-lik	RGPD8_HU	43	0.886807
48H	10MT/	Q8NHY2	0.17578	0.034428	0.027428	COP1	E3 ubiquiti	COP1_HUM	2	1.129575
24H	50MT/	P12814	-0.06041	0.034444	0.027439	ACTN1	Alpha-actir	ACTN1_HUI	58	0.958991
24H	10MT/	Q86UY8	0.273861	0.034451	0.02744	NT5DC3	5'-nucleoti	NT5D3_HU	6	1.209039
48H	10MT/	Q8N8A2	-0.29617	0.034452	0.02744	ANKRD44	Serine/thre	ANR44_HU	5	0.814413
48H	50MT/	Q9NZC3	-0.25297	0.034448	0.02744	GDE1	Glycerophc	GDE1_HUM	6	0.839169
48H	10MT/	Q9UHG0	0.098885	0.034448	0.02744	DCDC2	Doublecort	DCDC2_HU	10	1.070945
48H	10MT/	Q9GZL7	0.043973	0.034475	0.027454	WDR12	Ribosome k	WDR12_HU	18	1.030949
24H	50MT/	Q8IWL3	0.02625	0.034483	0.027459	HSCB	Iron-sulfur	HSC20_HUI	8	1.018362
24H	50MT/	O14545	0.438436	0.034491	0.027464	TRAFD1	TRAF-type z	TRAD1_HUI	9	1.355134
24H	10MT/	Q6YP21	-0.05441	0.034496	0.027467	KYAT3	Kynurenine	KAT3_HUM	14	0.96299
24H	10MT/	Q9BVL2	0.183564	0.034496	0.027467	NUP58	Nucleopori	NUP58_HU	6	1.135686
48H	10MT/	P46940	0.018522	0.034524	0.027485	IQGAP1	Ras GTPase-	IQGA1_HUI	83	1.012921
48H	10MT/	P26572	0.035542	0.03453	0.027488	MGAT1	Alpha-1,3-r	MGAT1_HU	7	1.024942
48H	50MT/	Q04323	-0.01995	0.034532	0.027489	UBXN1	UBX domai	UBXN1_HU	10	0.986264
24H	10MT/	P50895	0.042965	0.034549	0.027501	BCAM	Basal cell ac	BCAM_HUM	16	1.030229
24H	10MT/	Q9P2J5	0.011331	0.034561	0.027508	LARS1	Leucine--tR	SYLC_HUMA	55	1.007885
24H	50MT/	P50747	0.142999	0.034565	0.02751	HLCS	Biotin--pro	BPL1_HUM	9	1.104198

24H 50MT/	Q5TDH0	-0.13305	0.034565	0.02751	DDI2	Protein DD	DDI2_HUM	10	0.911903
48H 10MT/	Q96RL1	-0.18917	0.034569	0.02751	UIMC1	BRCA1-A cc	UIMC1_HU	7	0.877108
48H 10MT/	P52594	0.046107	0.034598	0.027528	AGFG1	Arf-GAP do	AGFG1_HU	11	1.032475
24H 50MT/	Q8TBZ3	-0.41435	0.034603	0.027531	WDR20	WD repeat-	WDR20_HU	3	0.750358
24H 10MT/	Q9ULD2	-0.0207	0.034605	0.027531	MTUS1	Microtubul	MTUS1_HU	32	0.985751
48H 50MT/	O43731	0.201201	0.034622	0.027542	KDELR3	ER lumen p	ERD23_HU	3	1.149655
24H 10MT/	Q9GZT8	-0.06353	0.034622	0.027542	NIF3L1	NIF3-like pr	NIF3L_HUN	12	0.95692
24H 50MT/	Q15382	-0.19423	0.034666	0.027567	RHEB	GTP-bindin	RHEB_HUM	7	0.874041
48H 50MT/	Q9NY27	0.058283	0.034669	0.027568	PPP4R2	Serine/thre	PP4R2_HU	14	1.041226
48H 50MT/	Q96I76	1.204732	0.034677	0.027572	GPATCH3	G patch do	GPTC3_HU	1	2.304945
24H 10MT/	Q9HD26	-0.08287	0.034683	0.027576	GOPC	Golgi-assoc	GOPC_HUN	17	0.944176
48H 50MT/	Q9UPT9	-0.37587	0.034698	0.027583	USP22	Ubiquitin c	UBP22_HU	6	0.770641
24H 50MT/	Q6VY07	-0.28492	0.034699	0.027583	PACS1	Phosphofu	PACS1_HU	16	0.820786
48H 10MT/	Q9Y5Z9	-0.21395	0.034703	0.027585	UBIAD1	UbiA preny	UBIA1_HUN	5	0.862175
48H 10MT/	Q9Y2Z0	0.04841	0.034727	0.0276	SUGT1	Protein SG	SGT1_HUM	24	1.034125
48H 10MT/	Q8WWH5	-0.05735	0.034733	0.027604	TRUB1	Pseudouric	TRUB1_HU	15	0.961027
24H 50MT/	Q9H3K2	0.086746	0.034732	0.027604	GHITM	Growth ho	GHITM_HU	8	1.061972
48H 10MT/	O75937	0.028434	0.034743	0.027608	DNAJC8	DnaJ homo	DNJC8_HU	12	1.019904
24H 10MT/	P49406	-0.01023	0.034752	0.027614	MRPL19	Large ribos	RM19_HUN	12	0.992937
48H 10MT/	O95571	-0.0103	0.034757	0.027616	ETHE1	Persulfide c	ETHE1_HUN	10	0.992883
48H 50MT/	Q9P219	-0.16602	0.034767	0.027622	CCDC88C	Protein Da	DAPLE_HU	2	0.891301
48H 50MT/	Q96CP2	0.163387	0.034779	0.02763	FLYWCH2	FLYWCH fa	FWCH2_HU	6	1.119913
48H 10MT/	Q9Y4Y9	-0.17106	0.0348	0.027642	LSM5	U6 snRNA-a	LSM5_HUN	2	0.888192
24H 50MT/	Q9UK45	-0.62277	0.034805	0.027644	LSM7	U6 snRNA-a	LSM7_HUN	5	0.649421
48H 10MT/	Q7Z6V5	0.203649	0.034821	0.027649	ADAT2	tRNA-speci	ADAT2_HU	4	1.151607
24H 50MT/	Q8TC27	-0.82328	0.034845	0.027663	ADAM32	Disintegrin	ADA32_HU	1	0.565154
48H 10MT/	P62314	-0.11044	0.034848	0.027665	SNRPD1	Small nucle	SMD1_HUN	5	0.926305
24H 50MT/	Q9NZM3	-0.09762	0.034859	0.027672	ITSN2	Intersectin	ITSN2_HUN	23	0.934571
48H 10MT/	O75367	-0.05918	0.034891	0.027692	MACROH2/	Core histor	H2AY_HUM	15	0.959812
48H 10MT/	Q99798	-0.00819	0.034897	0.027696	ACO2	Aconitate h	ACON_HUN	37	0.994336
24H 50MT/	Q9NX24	0.098098	0.034899	0.027696	NHP2	H/ACA ribo	NHP2_HUN	6	1.070362
48H 10MT/	O75608	0.017073	0.034908	0.027699	LYPLA1	Acyl-protei	LYPA1_HUN	9	1.011905
24H 10MT/	O94855	-0.13212	0.034906	0.027699	SEC24D	Protein tra	SC24D_HU	27	0.912491
48H 10MT/	Q9H4G0	0.353722	0.034916	0.027703	EPB41L1	Band 4.1-lil	E41L1_HUN	4	1.277853
24H 50MT/	Q9UIM3	-0.53244	0.034955	0.027723	FKBPL	FK506-binc	FKBPL_HUN	3	0.691384
48H 50MT/	Q96PY5	0.037614	0.034963	0.027727	FMNL2	Formin-like	FMNL2_HU	9	1.026415
48H 10MT/	Q12789	-0.0163	0.034965	0.027728	GTF3C1	General tra	TF3C1_HUN	59	0.988767
48H 10MT/	Q9UHE5	0.082845	0.035013	0.027759	NAT8	N-acetyltra	NAT8_HUN	7	1.059104
24H 50MT/	P63000	-0.13164	0.035014	0.027759	RAC1	Ras-related	RAC1_HUN	9	0.912793
24H 10MT/	P15428	-0.65939	0.035016	0.027759	HPGD	15-hydroxy	PGDH_HUN	1	0.633145
24H 50MT/	O95819	0.005333	0.035022	0.027763	MAP4K4	Mitogen-ac	M4K4_HUN	31	1.003703
24H 10MT/	Q6P1Q9	-0.18884	0.035027	0.027765	METTL2B	tRNA N(3)-r	MET2B_HU	6	0.877311
24H 50MT/	O00411	0.071867	0.035037	0.027772	POLRMT	DNA-direct	RPOM_HUN	32	1.051076
48H 10MT/	Q8NC44	0.279557	0.035042	0.027772	RETREG2	Reticuloph	RETR2_HUN	4	1.213822
48H 50MT/	P07951	0.083455	0.035041	0.027772	TPM2	Tropomyos	TPM2_HUN	10	1.059552
48H 10MT/	Q9UBP0	0.085531	0.035042	0.027772	SPAST	Spastin	SPAST_HUN	15	1.061078
48H 50MT/	Q00403	-0.00742	0.035044	0.027772	GTF2B	Transcripti	TF2B_HUM	17	0.994869
24H 10MT/	Q9UMS4	-0.13929	0.035048	0.027775	PRPF19	Pre-mRNA-|	PRP19_HU	17	0.907968
24H 50MT/	Q96AE7	-0.24465	0.03506	0.027781	TTC17	Tetratricop	TTC17_HUN	12	0.84402
48H 10MT/	Q9NX02	-0.02121	0.035063	0.027782	NLRP2	NACHT, LRF	NALP2_HU	46	0.985406
48H 50MT/	P63096	0.096963	0.035078	0.02779	GNAI1	Guanine nu	GNAI1_HU	2	1.06952
48H 50MT/	Q5VIR6	-0.0584	0.035077	0.02779	VPS53	Vacuolar pr	VPS53_HU	16	0.960331

24H 10MT/	Q969S9	0.004933	0.035083	0.027793	GFM2	Ribosome-r	RRF2M_HU	15	1.003425
48H 50MT/	Q9Y6I9	0.02279	0.035106	0.027809	TEX264	Testis-expre	TX264_HUI	10	1.015922
48H 50MT/	P59780	-0.24475	0.035129	0.027821	AP3S2	AP-3 compl	AP3S2_HUI	2	0.843963
24H 50MT/	Q5T5C0	0.061326	0.035131	0.027821	STXBP5	Syntaxin-bi	STXB5_HUI	12	1.043424
24H 50MT/	Q92804	0.281782	0.035134	0.027822	TAF15	TATA-bindi	RBP56_HUI	7	1.215695
24H 50MT/	P50135	0.047481	0.035165	0.027845	HNMT	Histamine I	HNMT_HUN	10	1.033459
24H 50MT/	Q7L9L4	-0.09247	0.035169	0.027847	MOB1B	MOB kinase	MOB1B_HU	7	0.937914
24H 10MT/	O95772	0.081934	0.035189	0.027859	STARD3NL	STARD3 N-t	STR3N_HUI	3	1.058436
24H 50MT/	P35998	-0.13405	0.035189	0.027859	PSMC2	26S protea	PRS7_HUM	30	0.911268
24H 50MT/	Q9UBK7;Q!	-0.17352	0.035214	0.027871	RABL2A;RA	Rab-like pr	RBL2A_HUI	3	0.886674
24H 50MT/	Q9Y547	0.115804	0.035217	0.027872	IFT25	Intraflagell	IFT25_HUM	4	1.083579
48H 10MT/	O60341	0.036995	0.035221	0.027874	KDM1A	Lysine-spec	KDM1A_HU	23	1.025975
24H 50MT/	Q6P2E9	-0.00317	0.035229	0.027878	EDC4	Enhancer o	EDC4_HUM	49	0.997802
48H 10MT/	Q8IWB7	0.13503	0.035233	0.027879	WDFY1	WD repeat	WDFY1_HU	15	1.098115
24H 10MT/	Q9NY97	0.392605	0.035235	0.027881	B3GNT2	N-acetyllac	B3GN2_HU	1	1.312761
24H 50MT/	P48051;P4	1.602396	0.035245	0.027887	KCNJ6;KCN	G protein-a	KCNJ6_HUI	1	3.036471
48H 10MT/	P51531	0.196727	0.035271	0.027902	SMARCA2	Probable gl	SMCA2_HU	12	1.146095
48H 50MT/	Q4G176	-0.00949	0.035273	0.027903	ACSF3	Malonate--	ACSF3_HUI	18	0.993446
48H 10MT/	P13073	-0.14968	0.035276	0.027904	COX4I1	Cytochrom	COX41_HU	10	0.901452
48H 50MT/	P21741	0.83789	0.035285	0.027911	MDK	Midkine	MK_HUMA	2	1.787433
48H 10MT/	Q16514	0.46854	0.035287	0.027912	TAF12	Transcripti	TAF12_HUI	2	1.383709
48H 10MT/	Q7L5Y1	0.106652	0.035301	0.02792	ENOSF1	Mitochond	ENOF1_HUI	13	1.076726
24H 10MT/	Q8TA86	0.22839	0.035328	0.027938	RP9	Retinitis pi	RP9_HUMA	3	1.171527
48H 50MT/	Q9NRD1	0.202642	0.035336	0.027943	FBXO6	F-box only	FBX6_HUM	7	1.150804
48H 10MT/	Q99623	-0.12112	0.03536	0.027957	PHB2	Prohibitin-	PHB2_HUM	19	0.919475
24H 10MT/	P36404	-0.07799	0.035366	0.027961	ARL2	ADP-ribosy	ARL2_HUM	8	0.947375
24H 50MT/	Q32MZ4	-0.06162	0.03537	0.027963	LRRFIP1	Leucine-ric	LRRF1_HUN	26	0.958191
48H 10MT/	Q8TDX6	-0.00911	0.035376	0.027967	CSGALNAC	Chondroiti	CGAT1_HUI	8	0.993707
24H 50MT/	O14737	-0.11859	0.035387	0.027974	PDCD5	Programme	PDCD5_HU	6	0.921086
48H 50MT/	Q9UNK0	0.182242	0.035401	0.027982	STX8	Syntaxin-8	STX8_HUM	5	1.134646
48H 10MT/	Q9UJC3	0.052601	0.035449	0.028011	HOOK1	Protein Ho	HOOK1_HU	22	1.037133
48H 50MT/	P11047	0.016097	0.035454	0.028013	LAMC1	Laminin sul	LAMC1_HU	40	1.01122
24H 10MT/	Q8N4W6	-0.52513	0.035452	0.028013	DNAJC22	DnaJ homo	DJC22_HUI	1	0.694896
24H 50MT/	Q9P0U1	-1.089	0.035453	0.028013	TOMM7	Mitochond	TOM7_HUN	2	0.470086
48H 10MT/	P07900	-0.12906	0.035462	0.028018	HSP90AA1	Heat shock	HS90A_HUI	30	0.914425
48H 50MT/	O00622	0.052479	0.035463	0.028018	CCN1	CCN family	CCN1_HUM	7	1.037045
48H 10MT/	O94927	-0.03512	0.03547	0.028022	HAUS5	HAUS augm	HAUS5_HU	10	0.975949
48H 50MT/	Q8IYB7	0.046419	0.03547	0.028022	DIS3L2	DIS3-like e	DI3L2_HUN	34	1.032698
24H 10MT/	O14980	0.045506	0.035483	0.02803	XPO1	Exportin-1	XPO1_HUM	44	1.032045
24H 10MT/	O75122	0.054062	0.035488	0.028031	CLASP2	CLIP-associ	CLAP2_HUI	34	1.038184
24H 10MT/	Q10472	0.068704	0.035493	0.028034	GALNT1	Polypeptid	GALT1_HUI	14	1.048774
48H 10MT/	P09497	0.114437	0.035497	0.028036	CLTB	Clathrin lig	CLCB_HUM	7	1.082553
48H 50MT/	Q7RTP0	-0.17653	0.0355	0.028037	NIPA1	Magnesium	NIPA1_HUI	3	0.884829
48H 50MT/	O43581	0.943064	0.03552	0.028049	SYT7	Synaptotag	SYT7_HUM	6	1.922608
48H 10MT/	P30044	0.018457	0.035531	0.028057	PRDX5	Peroxiredo	PRDX5_HU	11	1.012876
24H 10MT/	Q6V1X1	0.016218	0.03555	0.028068	DPP8	Dipeptidyl	DPP8_HUM	8	1.011305
24H 50MT/	Q15800	0.253702	0.035578	0.028084	MSMO1	Methylster	MSMO1_HU	4	1.192263
24H 10MT/	Q8NHU6	-0.00574	0.035583	0.028086	TDRD7	Tudor dom	TDRD7_HU	7	0.99603
48H 10MT/	Q6ZW31	0.105028	0.035644	0.028127	SYDE1	Rho GTPase	SYDE1_HUN	11	1.075515
24H 10MT/	P29401	-0.08568	0.035646	0.028128	TKT	Transketola	TKT_HUMA	29	0.942342
48H 50MT/	Q9BRQ0	-0.43464	0.035647	0.028128	PYGO2	Pygopus hc	PYGO2_HU	2	0.739878
48H 50MT/	P25815	-2.00828	0.035659	0.028135	S100P	Protein S1(	S100P_HUI	1	0.248569

48H 50MT/	Q15796	0.050185	0.035688	0.028155	SMAD2	Mothers ag	SMAD2_HU	12	1.035398
24H 10MT/	P30837	-0.08196	0.035691	0.028156	ALDH1B1	Aldehyde d	AL1B1_HUI	16	0.94477
48H 10MT/	Q92922	-0.01785	0.035692	0.028156	SMARCC1	SWI/SNF cc	SMRC1_HU	33	0.987706
48H 10MT/	P08493	0.135773	0.035694	0.028156	MGP	Matrix Gla	MGP_HUM	1	1.098682
48H 50MT/	Q01814	-0.24635	0.035713	0.028166	ATP2B2	Plasma mer	AT2B2_HUI	3	0.843027
48H 10MT/	Q96IZ0	0.172631	0.035713	0.028166	PAWR	PRKC apopt	PAWR_HUN	12	1.127112
24H 50MT/	Q9Y297	0.364629	0.035717	0.028168	BTRC	F-box/WD r	FBW1A_HU	5	1.287551
24H 10MT/	Q9BUR4	-0.00547	0.035727	0.028175	WRAP53	Telomerase	TCAB1_HUI	6	0.996214
48H 10MT/	Q93100	-0.03296	0.035754	0.028192	PHKB	Phosphoryl	KPBB_HUN	12	0.977412
48H 10MT/	Q9HD42	0.196973	0.035753	0.028192	CHMP1A	Charged mu	CHM1A_HU	4	1.14629
48H 50MT/	Q96A33	0.022686	0.035757	0.028194	CCDC47	PAT comple	CCD47_HU	20	1.015849
24H 10MT/	Q8IUX1	0.028762	0.03576	0.028196	TMEM126E	Complex I a	T126B_HUI	5	1.020136
24H 50MT/	Q9NQC8	1.325589	0.03577	0.028202	IFT46	Intraflagell	IFT46_HUN	1	2.506352
48H 10MT/	Q6UW63	-0.02304	0.035773	0.028203	POGLUT2	Protein O-g	PLGT2_HUI	18	0.984155
24H 10MT/	Q9Y2B0	-0.12118	0.035774	0.028204	CNPY2	Protein car	CNPY2_HUI	11	0.919437
24H 50MT/	Q05519	0.355434	0.035784	0.028209	SRSF11	Serine/argi	SRS11_HUN	7	1.279371
24H 50MT/	Q99941	-0.47076	0.035784	0.028209	ATF6B	Cyclic AMP	ATF6B_HUN	6	0.721586
24H 10MT/	P30050	-0.1195	0.035787	0.02821	RPL12	Large ribos	RL12_HUM	6	0.920504
48H 10MT/	Q9H845	-0.13643	0.035789	0.02821	ACAD9	Complex I a	ACAD9_HU	25	0.909767
48H 10MT/	Q9UM13	0.093835	0.035793	0.028212	ANAPC10	Anaphase-p	APC10_HUI	3	1.067203
48H 10MT/	Q9BWH2	-0.11453	0.035797	0.028214	FUNDC2	FUN14 don	FUND2_HU	5	0.923682
24H 50MT/	O95070	0.139278	0.035814	0.028224	YIF1A	Protein YIF	YIF1A_HUN	4	1.101354
24H 50MT/	P48553	0.13363	0.035823	0.028229	TRAPPC10	Trafficking	TPC10_HUI	3	1.09705
48H 10MT/	Q99575	0.058428	0.035827	0.028231	POP1	Ribonuclea	POP1_HUN	28	1.04133
48H 10MT/	Q9UJW0	0.056145	0.035828	0.028231	DCTN4	Dynactin su	DCTN4_HU	19	1.039684
48H 50MT/	Q9UL12	0.25923	0.035843	0.028237	SARDH	Sarcosine d	SARDH_HU	6	1.19684
48H 50MT/	Q6PCB0	0.242624	0.035878	0.02826	VWA1	von Willebr	VWA1_HUN	5	1.183143
24H 10MT/	O95749	-0.1616	0.035889	0.028266	GGPS1	Geranylger	GGPPS_HU	7	0.894036
24H 10MT/	O00479	0.454307	0.035904	0.028276	HMGN4	High mobil	HMGN4_HU	1	1.370125
48H 50MT/	Q8N9N5	-1.34787	0.03592	0.028286	BANP	Protein BAI	BANP_HUN	1	0.392873
24H 50MT/	P07199	0.126432	0.035924	0.028288	CENPB	Major centr	CENPB_HU	5	1.09159
24H 10MT/	O00567	0.019673	0.03594	0.028297	NOP56	Nucleolar p	NOP56_HU	27	1.01373
24H 50MT/	Q08752	0.123138	0.035952	0.028303	PPID	Peptidyl-pr	PPID_HUM	21	1.089101
48H 10MT/	Q86YH6	-0.05247	0.035965	0.028311	PDSS2	All trans-pc	DLP1_HUN	16	0.964285
24H 50MT/	Q9UEE9	-0.133	0.035964	0.028311	CFDP1	Craniofacia	CFDP1_HUI	5	0.911933
48H 50MT/	Q9ULF5	0.011205	0.035985	0.028324	SLC39A10	Zinc transp	S39AA_HUI	2	1.007797
48H 50MT/	Q9NRV9	-0.00035	0.035991	0.028328	HEBP1	Heme-bind	HEBP1_HU	8	0.999755
48H 10MT/	Q9UL40	0.051196	0.036014	0.028342	ZNF346	Zinc finger	ZN346_HUI	7	1.036123
24H 50MT/	Q9P000	-0.21763	0.036022	0.028347	COMMD9	COMM don	COMD9_HU	8	0.859977
24H 10MT/	Q8WUH2	-0.0065	0.036047	0.028362	TGFBRAP1	Transformi	TGFA1_HUI	13	0.995503
24H 50MT/	Q9BRP8	-0.08212	0.036056	0.028367	PYM1	Partner of Y	PYM1_HUN	9	0.944671
24H 50MT/	Q9H4B7	0.618347	0.036062	0.028371	TUBB1	Tubulin bet	TBB1_HUM	2	1.535115
48H 50MT/	Q9NW68	0.298711	0.03607	0.028377	BSDC1	BSD domai	BSDC1_HU	3	1.230045
24H 10MT/	O14772	0.073436	0.036083	0.028385	FPGT	Fucose-1-pl	FPGT_HUM	27	1.05222
24H 50MT/	P49959	0.043049	0.03609	0.02839	MRE11	Double-stra	MRE11_HU	20	1.030289
24H 10MT/	Q14CZ7	0.429647	0.036091	0.02839	FASTKD3	FAST kinase	FAKD3_HUI	6	1.346904
24H 50MT/	Q9BYD6	0.051227	0.036113	0.028403	MRPL1	Large ribos	RM01_HUN	13	1.036146
24H 10MT/	O14686	0.089645	0.036117	0.028406	KMT2D	Histone-lys	KMT2D_HU	9	1.064108
48H 50MT/	Q9BRQ8	-0.1879	0.03614	0.028421	AIFM2	Ferroptosis	FSP1_HUM	15	0.877881
48H 10MT/	Q9NZZ3	0.029532	0.036153	0.028427	CHMP5	Charged mu	CHMP5_HU	6	1.020681
24H 50MT/	Q8N4P3	-0.32864	0.036161	0.02843	HDDC3	Guanosine-	MESH1_HU	2	0.796284
48H 10MT/	Q99543	0.010907	0.036167	0.028433	DNAJC2	DnaJ homo	DNJC2_HUI	21	1.007589

48H 10MT/	Q9UBU6	-0.10389	0.036167	0.028433	FAM8A1	Protein FAI	FA8A1_HUI	10	0.930519
48H 50MT/	Q8TCS8	-0.08409	0.036178	0.028441	PNPT1	Polyribonu	PNPT1_HUI	40	0.943377
24H 50MT/	O95782	0.00586	0.036183	0.028443	AP2A1	AP-2 compl	AP2A1_HUI	40	1.00407
48H 10MT/	P55795	-0.03977	0.036185	0.028443	HNRNPH2	Heterogene	HNRH2_HU	9	0.972807
24H 10MT/	Q15048	-0.23995	0.036186	0.028443	LRRC14	Leucine-ric	LRC14_HUI	3	0.846772
48H 10MT/	Q96CB8	0.084695	0.036189	0.028443	INTS12	Integrator (	INT12_HUN	5	1.060463
24H 10MT/	Q96FV9	0.06095	0.036188	0.028443	THOC1	THO compl	THOC1_HU	24	1.043153
24H 50MT/	Q6ZUT9	-0.20999	0.036191	0.028444	DENND5B	DENN dom	DEN5B_HU	5	0.864545
24H 50MT/	P82650	0.036794	0.036206	0.028453	MRPS22	Small ribos	RT22_HUM	15	1.025831
24H 50MT/	Q709C8	0.047842	0.036227	0.028466	VPS13C	Intermemb	VP13C_HUI	60	1.033718
24H 50MT/	Q7Z7A4	0.048009	0.036239	0.028473	PXK	PX domain	PXK_HUMA	6	1.033837
48H 50MT/	Q9Y5V3	0.23819	0.036239	0.028473	MAGED1	Melanoma-	MAGD1_HU	10	1.179512
24H 50MT/	Q9NW81	-0.09561	0.03624	0.028473	DMAC2	Distal mem	DMAC2_HU	3	0.935875
48H 10MT/	Q86Y79	-0.42608	0.036244	0.028475	PTRH1	Peptidyl-tR	PTH_HUMA	5	0.744284
48H 10MT/	Q9H6Y2	0.027319	0.036245	0.028475	WDR55	WD repeat-	WDR55_HU	13	1.019116
48H 10MT/	Q9H4L5	0.100877	0.036254	0.028482	OSBPL3	Oxysterol-b	OSBL3_HUI	20	1.072425
48H 50MT/	Q9UKM9	-0.06578	0.036264	0.028488	RALY	RNA-bindir	RALY_HUM	11	0.955432
48H 10MT/	Q96QZ7	-0.01054	0.036267	0.028489	MAGI1	Membrane-	MAGI1_HU	19	0.992719
48H 10MT/	Q6DN90	-0.06832	0.036274	0.028491	IQSEC1	IQ motif an	IQEC1_HUN	13	0.953745
24H 10MT/	Q9BV86	0.022719	0.036273	0.028491	NTMT1	N-terminal	NTM1A_HU	8	1.015872
24H 50MT/	O95900	-0.27813	0.036277	0.028493	TRUB2	Pseudourid	TRUB2_HUI	6	0.824658
24H 50MT/	Q96PU4	1.355486	0.03628	0.028493	UHRF2	E3 ubiquiti	UHRF2_HU	1	2.558833
24H 50MT/	Q9HBL7	0.244018	0.036296	0.028499	PLGRKT	Plasminoge	PLRKT_HUN	5	1.184287
24H 10MT/	P52306	-0.03673	0.03631	0.028506	RAP1GDS1	Rap1 GTPa:	GDS1_HUN	14	0.974863
24H 50MT/	Q6NZ67	-0.18386	0.036317	0.028508	MZT2B	Mitotic-spi	MZT2B_HU	6	0.880342
24H 50MT/	O14735	0.056955	0.03633	0.028516	CDIPT	CDP-diacyl	CDIPT_HUN	6	1.040268
24H 50MT/	P78316	0.081676	0.036335	0.028519	NOP14	Nucleolar p	NOP14_HU	22	1.058247
24H 50MT/	P25815	2.201399	0.036347	0.028524	S100P	Protein S1(	S100P_HUI	1	4.599252
24H 50MT/	Q96AG4	0.059617	0.036365	0.028535	LRRC59	Leucine-ric	LRC59_HUI	13	1.042189
48H 10MT/	Q49AR2	0.062812	0.036379	0.028545	C5orf22	UPF0489 p	CE022_HUI	9	1.0445
48H 50MT/	Q9NPI1	-0.15657	0.03639	0.028551	BRD7	Bromodom	BRD7_HUN	3	0.897158
48H 10MT/	O76094	0.067431	0.036391	0.028551	SRP72	Signal reco	SRP72_HUI	28	1.047849
24H 50MT/	Q9BZQ6	0.122193	0.0364	0.028557	EDEM3	ER degrada	EDEM3_HU	9	1.088388
24H 50MT/	Q9BRT2	-0.03979	0.036428	0.028576	UQCC2	Ubiquinol-	UQCC2_HU	6	0.972796
48H 10MT/	O15031	-0.04371	0.036445	0.028589	PLXNB2	Plexin-B2	PLXB2_HUI	50	0.970158
24H 50MT/	Q9NZJ0	-0.21021	0.036461	0.028597	DTL	Denticleles	DTL_HUMA	4	0.864411
24H 10MT/	Q9NRA2	0.006303	0.036472	0.028604	SLC17A5	Sialin	S17A5_HUI	4	1.004379
24H 10MT/	Q0ZGT2	0.075241	0.036475	0.028605	NEXN	Nexilin	NEXN_HUN	9	1.053537
48H 50MT/	O60256	0.097256	0.036481	0.028608	PRPSAP2	Phosphorib	KPRB_HUN	14	1.069737
48H 50MT/	Q6P1M0	-0.13938	0.036487	0.028611	SLC27A4	Long-chain	S27A4_HUI	19	0.907909
48H 50MT/	P51687	0.081018	0.0365	0.028619	SUOX	Sulfite oxid	SUOX_HUN	11	1.057764
48H 10MT/	Q4G0X4	0.000171	0.036509	0.028625	KCTD21	BTB/POZ dc	KCD21_HU	6	1.000118
24H 50MT/	Q9BV44	-0.0532	0.036514	0.028626	THUMPD3	tRNA (guan	THUM3_HU	14	0.963794
24H 50MT/	Q9P0L0	0.03917	0.036524	0.02863	VAPA	Vesicle-assc	VAPA_HUN	9	1.027523
48H 10MT/	Q5TEU4	-0.05219	0.036531	0.028635	NDUFAF5	Arginine-hy	NDUF5_HU	3	0.964472
48H 10MT/	Q9HAF1	-0.16108	0.036538	0.02864	MEAF6	Chromatin	EAF6_HUM	2	0.894357
24H 10MT/	Q96FZ7	-0.03862	0.036565	0.028658	CHMP6	Charged mu	CHMP6_HU	6	0.973586
24H 10MT/	Q9BST9	-0.01353	0.036572	0.028662	RTKN	Rhotekin	RTKN_HUN	11	0.990666
24H 50MT/	O95777	0.04353	0.036585	0.028669	LSM8	U6 snRNA-a	LSM8_HUN	7	1.030633
24H 10MT/	O60422	-0.00939	0.036598	0.028677	ONECUT3	One cut doı	ONEC3_HU	3	0.99351
24H 10MT/	Q10713	0.058709	0.036599	0.028677	PMPCA	Mitochond	MPPA_HUN	24	1.041533
24H 10MT/	Q2PZI1	0.225515	0.036603	0.028679	DPY19L1	Protein C-n	D19L1_HUI	13	1.169194

24H 10MTA	Q9Y6X3	0.127879	0.036619	0.02869	MAU2	MAU2 chro	SCC4_HUM	10	1.092686
24H 50MTA	O00468	0.202993	0.036625	0.028691	AGRN	Agrin	AGRIN_HUI	42	1.151084
48H 50MTA	P50542	0.142091	0.036634	0.028694	PEX5	Peroxisoma	PEX5_HUM	22	1.103503
24H 10MTA	P51572	0.024239	0.036633	0.028694	BCAP31	B-cell recep	BAP31_HUI	11	1.016943
48H 50MTA	P63151	0.039855	0.036635	0.028694	PPP2R2A	Serine/thre	2ABA_HUM	14	1.028011
24H 50MTA	Q86YT6	-0.10263	0.036634	0.028694	MIB1	E3 ubiquiti	MIB1_HUM	10	0.931335
48H 50MTA	Q96DP5	-0.17335	0.036661	0.02871	MTFMT	Methionyl-	FMT_HUMA	7	0.88678
24H 10MTA	P34947	-0.07859	0.036674	0.028718	GRK5	G protein-c	GRK5_HUM	9	0.94698
24H 50MTA	Q8WUJ3	0.521938	0.036686	0.028723	CEMIP	Cell migrati	CEMIP_HUI	5	1.435883
48H 10MTA	Q14202	0.075067	0.036713	0.02874	ZMYM3	Zinc finger	ZMYM3_HU	34	1.05341
24H 50MTA	Q7LBR1	0.783123	0.036744	0.02876	CHMP1B	Charged mu	CHM1B_HU	3	1.720853
24H 10MTA	P11441	-0.21394	0.036748	0.02876	UBL4A	Ubiquitin-l	UBL4A_HUI	6	0.862179
24H 50MTA	Q13823	-0.05528	0.036747	0.02876	GNL2	Nucleolar G	NOG2_HUM	21	0.96241
48H 50MTA	Q8NEC7	0.085064	0.03675	0.028762	GSTCD	Glutathion	GSTCD_HUI	6	1.060735
48H 50MTA	O00213	0.33172	0.036765	0.028772	APBB1	Amyloid be	APBB1_HUI	3	1.258513
24H 50MTA	O95983	0.146636	0.036769	0.028774	MBD3	Methyl-CpG	MBD3_HUM	9	1.106985
24H 50MTA	Q16836	0.094841	0.03677	0.028774	HADH	Hydroxyacy	HCDH_HUM	10	1.067947
48H 10MTA	Q96PV6	0.533514	0.036781	0.028779	LENG8	Leukocyte r	LENG8_HUI	2	1.44745
24H 10MTA	P20783	-0.17194	0.036801	0.02879	NTF3	Neurotroph	NTF3_HUM	2	0.887647
48H 10MTA	Q9H4I9	0.338646	0.036807	0.028793	SMDT1	Essential M	EMRE_HUM	2	1.264569
24H 50MTA	O14777	-0.12386	0.03681	0.028795	NDC80	Kinetochor	NDC80_HU	23	0.917727
48H 10MTA	O94888	-0.04928	0.036833	0.028809	UBXN7	UBX domai	UBXN7_HU	15	0.966415
24H 10MTA	Q9NP66	-0.23266	0.036859	0.028823	HMG20A	High mobil	HM20A_HU	5	0.851066
48H 10MTA	Q13418	0.13208	0.036886	0.028838	ILK	Integrin-lin	ILK_HUMAI	17	1.095873
48H 10MTA	Q9UBM1	0.183705	0.036904	0.028846	PEMT	Phosphatid	PEMT_HUM	1	1.135797
24H 10MTA	Q16762	-0.08933	0.036912	0.02885	TST	Thiosulfate	THTR_HUM	15	0.939959
24H 50MTA	Q68CJ9	-0.38239	0.036946	0.028869	CREB3L3	Cyclic AMP	CR3L3_HUI	2	0.767166
24H 50MTA	P55268	0.113621	0.036948	0.028869	LAMB2	Laminin sul	LAMB2_HU	28	1.081941
24H 50MTA	O60858	0.135556	0.036969	0.02888	TRIM13	E3 ubiquiti	TRI13_HUM	4	1.098516
48H 10MTA	P14927	0.03303	0.036976	0.028884	UQCRB	Cytochrom	QCR7_HUM	8	1.023159
48H 10MTA	P37837	0.037557	0.036983	0.028888	TALDO1	Transaldola	TALDO_HUI	16	1.026375
48H 50MTA	Q9HCJ6	-0.24421	0.036985	0.028889	VAT1L	Synaptic ve	VAT1L_HUM	12	0.844277
24H 10MTA	Q9BVA1	-0.02854	0.036995	0.028895	TUBB2B	Tubulin bet	TBB2B_HUI	1	0.980411
48H 50MTA	Q8IXM6	-0.07978	0.037001	0.028899	NRM	Nurim	NRM_HUM	5	0.946203
48H 10MTA	P00441	-0.1866	0.037014	0.028908	SOD1	Superoxide	SODC_HUM	7	0.878675
24H 10MTA	Q92542	0.048262	0.037022	0.028913	NCSTN	Nicastrin	NICA_HUM	12	1.034018
24H 10MTA	Q96S99	0.034646	0.037035	0.028919	PLEKHF1	Pleckstrin h	PKHF1_HUI	3	1.024306
48H 50MTA	Q15910	-0.20519	0.037039	0.028921	EZH2	Histone-lys	EZH2_HUM	10	0.867424
48H 50MTA	P49407	-0.16212	0.037056	0.02893	ARRB1	Beta-arresti	ARRB1_HUI	13	0.893712
48H 10MTA	Q86WC4	0.042087	0.037075	0.028942	OSTM1	Osteopetro	OSTM1_HU	2	1.029602
48H 10MTA	A0AVI4	-0.76479	0.037079	0.028945	TMEM129	E3 ubiquiti	TM129_HU	1	0.588538
48H 50MTA	Q9NVN3	-0.08268	0.037109	0.028963	RIC8B	Synembryn	RIC8B_HUM	7	0.944302
48H 10MTA	Q9UHK0	0.098779	0.037112	0.028964	NUFIP1	FMR1-inter	NUFP1_HU	3	1.070867
24H 50MTA	Q9P1Y5	0.14798	0.037155	0.028989	CAMSAP3	Calmodulir	CAMP3_HU	17	1.108017
48H 10MTA	O60701	-0.03566	0.03716	0.02899	UGDH	UDP-glucos	UGDH_HUM	30	0.975587
48H 50MTA	Q9P1Z2	-0.76471	0.037164	0.028993	CALCOCO1	Calcium-bi	CACO1_HU	2	0.588573
48H 50MTA	Q96LT9	0.022906	0.037175	0.028999	RNPC3	RNA-bindir	RNPC3_HU	5	1.016004
48H 10MTA	Q8WU17	-0.41127	0.037193	0.02901	RNF139	E3 ubiquiti	RN139_HU	3	0.751963
48H 10MTA	O75063	-0.04552	0.037203	0.029016	FAM20B	Glycosamir	XYLK_HUM	7	0.968939
48H 50MTA	Q9BRJ6	-0.09711	0.037208	0.029018	C7orf50	Uncharacte	CG050_HU	9	0.934905
24H 50MTA	Q8N3R9	4.62E-05	0.037228	0.029028	PALS1	Protein PAI	PALS1_HUM	15	1.000032
24H 50MTA	Q9Y2X7	0.18973	0.037231	0.029029	GIT1	ARF GTPase	GIT1_HUMA	11	1.14055

24H 10MT/	P18850	0.604132	0.037248	0.029038	ATF6	Cyclic AMP	ATF6A_HUI	2	1.520063
48H 50MT/	Q9H9Y6	-0.214	0.037248	0.029038	POLR1B	DNA-direct	RPA2_HUN	31	0.862144
48H 50MT/	Q15059	0.320957	0.037272	0.029054	BRD3	Bromodom	BRD3_HUN	8	1.249159
48H 50MT/	P04183	-0.1944	0.03728	0.029058	TK1	Thymidine	KITH_HUM/	12	0.873936
24H 10MT/	Q92786	0.013387	0.0373	0.029073	PROX1	Prospero h	PROX1_HU	17	1.009322
48H 10MT/	Q9NRR5	0.039293	0.037303	0.029074	UBQLN4	Ubiquilin-4	UBQL4_HU	9	1.02761
48H 50MT/	O00203	0.029577	0.037306	0.029076	AP3B1	AP-3 compl	AP3B1_HUI	37	1.020713
48H 10MT/	Q9Y5B0	0.011462	0.037306	0.029076	CTDP1	RNA polym	CTDP1_HUI	13	1.007976
48H 50MT/	Q96GN5	-0.30914	0.037356	0.029109	CDCA7L	Cell divisio	CDA7L_HUI	2	0.807126
24H 10MT/	P35250	0.003948	0.037363	0.029113	RFC2	Replicatior	RFC2_HUM	19	1.00274
48H 10MT/	O75204	-0.06276	0.037373	0.029118	TMEM127	Transmemb	TM127_HU	1	0.957432
24H 50MT/	Q9UKK6	0.557815	0.03739	0.029126	NXT1	NTF2-relate	NXT1_HUN	3	1.472038
48H 50MT/	P53007	0.172418	0.037387	0.029126	SLC25A1	Tricarboxyl	TXTP_HUM	10	1.126946
24H 10MT/	Q9Y261	-0.23235	0.037395	0.029128	FOXA2	Hepatocyte	FOXA2_HUI	3	0.851244
48H 10MT/	Q6PHR2	-0.01496	0.03742	0.029141	ULK3	Serine/thre	ULK3_HUN	6	0.989687
48H 50MT/	P68036	0.133917	0.037422	0.029142	UBE2L3	Ubiquitin-c	UB2L3_HUI	9	1.097268
24H 50MT/	Q96BW9	0.043607	0.037422	0.029142	TAMM41	Phosphatic	TAM41_HU	6	1.030688
48H 10MT/	Q8N2W9	-0.01331	0.037432	0.029148	PIAS4	E3 SUMO-p	PIAS4_HUN	6	0.990814
48H 10MT/	O43504	-0.12975	0.037455	0.029161	LAMTOR5	Ragulator c	LTOR5_HUI	2	0.913991
24H 50MT/	O75391	0.073259	0.037489	0.029181	SPAG7	Sperm-asso	SPAG7_HUI	8	1.05209
24H 50MT/	P16333	-0.16822	0.03749	0.029181	NCK1	SH2/SH3 ac	NCK1_HUN	8	0.88994
24H 10MT/	Q9UNS2	-0.03263	0.037546	0.029213	COPS3	COP9 signa	CSN3_HUN	18	0.977639
24H 50MT/	P36954	-0.16053	0.037553	0.029216	POLR2I	DNA-direct	RPB9_HUN	6	0.894698
48H 10MT/	P07741	0.006931	0.037559	0.029219	APRT	Adenine ph	APT_HUMA	12	1.004816
24H 10MT/	O15446	0.042476	0.037561	0.029219	POLR1G	DNA-direct	RPA34_HUI	8	1.02988
24H 10MT/	Q504Q3	-0.39543	0.037562	0.029219	PAN2	PAN2-PAN3	PAN2_HUN	3	0.760261
24H 10MT/	Q15370	0.089174	0.037566	0.029222	ELOB	Elongin-B	ELOB_HUM	10	1.063761
48H 10MT/	Q13395	-0.0985	0.037571	0.029225	TARBP1	Probable m	TARB1_HUI	32	0.934005
48H 10MT/	Q7Z2Z2	0.051415	0.037573	0.029225	EFL1	Elongation	EFL1_HUM/	20	1.036281
24H 10MT/	Q99447	-0.13955	0.037577	0.029225	PCYT2	Ethanolami	PCY2_HUM	13	0.907801
24H 50MT/	Q5T6F2	-0.18436	0.037586	0.02923	UBAP2	Ubiquitin-a	UBAP2_HU	11	0.880037
24H 50MT/	P78406	0.068954	0.03759	0.029232	RAE1	mRNA expc	RAE1L_HUI	11	1.048956
24H 10MT/	P62256	0.041188	0.037608	0.029242	UBE2H	Ubiquitin-c	UBE2H_HU	3	1.028961
24H 50MT/	Q9BXJ9	-0.07272	0.037636	0.029259	NAA15	N-alpha-ace	NAA15_HU	41	0.950841
24H 50MT/	Q6QNY1	0.767775	0.03764	0.029261	BLOC1S2	Biogenesis	BL1S2_HUI	2	1.702642
24H 10MT/	Q06609	0.222295	0.037644	0.029262	RAD51	DNA repair	RAD51_HU	10	1.166588
48H 50MT/	Q92734	0.100113	0.037645	0.029262	TFG	Protein TFG	TFG_HUMA	12	1.071857
48H 50MT/	Q9UPU5	0.036399	0.037648	0.029262	USP24	Ubiquitin c	UBP24_HU	61	1.025551
48H 50MT/	Q9UL40	0.055453	0.037651	0.029264	ZNF346	Zinc finger	ZN346_HUI	7	1.039186
24H 50MT/	P50395	-0.06148	0.037665	0.029272	GDI2	Rab GDP di	GDIB_HUM	36	0.958282
24H 50MT/	Q9UHR6	0.169144	0.03767	0.029274	ZNHIT2	Zinc finger	ZNHI2_HUN	8	1.124391
24H 50MT/	Q14257	0.201394	0.037674	0.029275	RCN2	Reticulocal	RCN2_HUN	13	1.149809
24H 50MT/	Q96TA2	0.03223	0.037683	0.02928	YME1L1	ATP-depen	YMEL1_HUI	20	1.022592
48H 50MT/	P61011	0.0431	0.037684	0.02928	SRP54	Signal reco	SRP54_HUI	22	1.030326
48H 50MT/	Q9Y587	-0.59888	0.037689	0.029284	AP4S1	AP-4 compl	AP4S1_HUI	3	0.660267
48H 10MT/	Q9GZT6	0.522313	0.037697	0.029287	CCDC90B	Coiled-coil	CC90B_HUI	3	1.436256
48H 50MT/	Q9UGJ1	-0.11841	0.037718	0.029298	TUBGCP4	Gamma-tul	GCP4_HUN	12	0.921205
48H 50MT/	Q9H497	-0.33779	0.03773	0.029305	TOR3A	Torsin-3A	TOR3A_HUI	7	0.791253
24H 50MT/	Q8NEZ5	-0.00299	0.037734	0.029307	FBXO22	F-box only	FBX22_HUI	12	0.99793
48H 10MT/	P24390	-0.87422	0.037757	0.029322	KDELR1	ER lumen p	ERD21_HUI	2	0.545549
48H 50MT/	Q92871	0.413395	0.037769	0.02933	PMM1	Phosphom	PMM1_HUI	3	1.331816
24H 50MT/	Q86YZ3	-0.68413	0.037776	0.029332	HRNR	Hornerin	HORN_HUN	3	0.62238

24H 10MTA	Q8NF64	-0.23356	0.037793	0.029342	ZMIZ2	Zinc finger I	ZMIZ2_HUI	5	0.850535
24H 50MTA	Q9UNA4	1.54999	0.037797	0.029344	POLI	DNA polym	POLI_HUM	1	2.928152
24H 10MTA	P51532	0.745578	0.037816	0.029355	SMARCA4	Transcripti	SMCA4_HU	2	1.676646
48H 10MTA	Q5T5C0	-0.08108	0.03782	0.029355	STXBP5	Syntaxin-bi	STXB5_HUI	12	0.94535
24H 50MTA	Q6IN85	0.041703	0.037825	0.029357	PPP4R3A	Serine/thre	P4R3A_HUI	17	1.029328
48H 50MTA	Q9P2T1	0.235774	0.03783	0.02936	GMPR2	GMP reduc	GMPR2_HU	15	1.177538
24H 50MTA	Q9NWH2	0.189777	0.037833	0.029361	TMEM242	Transmemb	TM242_HU	2	1.140587
24H 50MTA	Q00613	-0.20339	0.03784	0.029364	HSF1	Heat shock	HSF1_HUM	3	0.868506
48H 10MTA	Q6GMR7	-0.48523	0.037839	0.029364	FAAH2	Fatty-acid a	FAAH2_HUI	1	0.714381
24H 10MTA	Q8N6S5	-0.04575	0.037839	0.029364	ARL6IP6	ADP-ribosy	AR6P6_HUI	1	0.968789
48H 50MTA	Q14C86	-0.06877	0.037842	0.029365	GAPVD1	GTPase-acti	GAPD1_HU	39	0.953449
24H 50MTA	Q9UHA4	0.126685	0.037845	0.029367	LAMTOR3	Ragulator c	LTOR3_HUI	5	1.091782
24H 10MTA	Q96D05	0.743401	0.037846	0.029367	FAM241B	Protein FAI	F241B_HUI	1	1.674118
48H 50MTA	O14925	0.021473	0.037848	0.029367	TIMM23	Mitochond	TIM23_HUI	7	1.014995
24H 10MTA	Q8WWC4	0.248856	0.037848	0.029367	MAIP1	m-AAA prot	MAIP1_HUI	7	1.188265
24H 50MTA	Q9Y6B6	0.027138	0.037851	0.029369	SAR1B	Small COPII	SAR1B_HUI	13	1.018988
48H 10MTA	Q9H0B6	0.019993	0.037863	0.029375	KLC2	Kinesin ligh	KLC2_HUM	22	1.013955
24H 10MTA	P40938	0.02339	0.037865	0.029375	RFC3	Replication	RFC3_HUM	17	1.016345
48H 10MTA	Q9NYJ1	0.094748	0.037883	0.029386	COA4	Cytochrom	COA4_HUM	1	1.067879
48H 50MTA	P11177	0.008905	0.037892	0.029391	PDHB	Pyruvate de	ODPB_HUM	17	1.006191
48H 50MTA	Q2NL82	-0.10183	0.037891	0.029391	TSR1	Pre-rRNA-p	TSR1_HUM	23	0.931848
48H 50MTA	Q9UNH7	0.087679	0.037922	0.029406	SNX6	Sorting nex	SNX6_HUM	19	1.062659
48H 10MTA	Q9Y281	-0.00015	0.037922	0.029406	CFL2	Cofilin-2	COF2_HUM	9	0.999896
48H 10MTA	Q969E8	0.364626	0.037995	0.02945	TSR2	Pre-rRNA-p	TSR2_HUM	2	1.287548
48H 50MTA	Q5JYT7	0.369163	0.037998	0.029452	KIAA1755	Uncharacte	K1755_HUI	1	1.291604
24H 10MTA	Q9H3P2	0.093805	0.038019	0.029467	NELFA	Negative el	NELFA_HUI	10	1.067181
24H 50MTA	Q13636	-0.31208	0.03803	0.029473	RAB31	Ras-related	RAB31_HUI	4	0.805482
24H 50MTA	Q7Z434	0.04134	0.038039	0.029478	MAVS	Mitochond	MAVS_HUM	14	1.029069
24H 10MTA	Q9NVC6	0.005857	0.038039	0.029478	MED17	Mediator o	MED17_HU	10	1.004068
48H 10MTA	Q9Y580	-0.04511	0.03805	0.029485	RBM7	RNA-bindir	RBM7_HUM	9	0.969214
48H 50MTA	A7KAX9	0.616738	0.038057	0.029489	ARHGAP32	Rho GTPase	RHG32_HU	3	1.533404
24H 50MTA	P29084	0.006015	0.038098	0.029513	GTF2E2	Transcripti	T2EB_HUM	9	1.004178
24H 10MTA	Q7Z2K6	0.021688	0.038099	0.029513	ERMP1	Endoplasm	ERMP1_HU	17	1.015146
24H 50MTA	O95801	-0.10359	0.038107	0.029517	TTC4	Tetratricop	TTC4_HUM	20	0.930717
24H 10MTA	Q9NW82	0.088319	0.038115	0.029522	WDR70	WD repeat-	WDR70_HU	18	1.063131
24H 10MTA	Q5T4S7	0.019715	0.038144	0.029542	UBR4	E3 ubiquiti	UBR4_HUM	145	1.013759
24H 10MTA	Q96N67	0.058333	0.038147	0.029543	DOCK7	Dedicator c	DOCK7_HU	56	1.041262
48H 50MTA	O00116	-0.02435	0.03816	0.029552	AGPS	Alkyldihydr	ADAS_HUM	32	0.983263
48H 50MTA	Q99733	0.02498	0.038167	0.029556	NAP1L4	Nucleosom	NP1L4_HUI	14	1.017465
24H 10MTA	Q9H0U3	0.050651	0.038175	0.02956	MAGT1	Magnesium	MAGT1_HU	7	1.035732
24H 10MTA	Q9BQ69	-0.12053	0.038189	0.029568	MACROD1	ADP-ribose	MACD1_HU	4	0.919848
24H 10MTA	Q9Y3Z3	-0.04716	0.038187	0.029568	SAMHD1	Deoxynucle	SAMH1_HU	31	0.967838
48H 50MTA	Q9H6S3	0.07822	0.038194	0.02957	EPS8L2	Epidermal g	ES8L2_HUM	23	1.055715
48H 50MTA	P62857	-0.15371	0.0382	0.029573	RPS28	Small ribos	RS28_HUM	3	0.898938
24H 50MTA	Q8TAF3	0.147047	0.038221	0.029584	WDR48	WD repeat-	WDR48_HU	13	1.107301
48H 50MTA	P21283	-0.12184	0.038225	0.029585	ATP6V1C1	V-type prot	VATC1_HUI	19	0.919016
24H 50MTA	P20823	0.030318	0.038228	0.029586	HNF1A	Hepatocyte	HNF1A_HU	6	1.021237
48H 10MTA	Q03154	0.097507	0.03824	0.029594	ACY1	Aminoacyla	ACY1_HUM	17	1.069923
48H 10MTA	Q8IWA4	0.08219	0.038255	0.029602	MFN1	Mitofusin-1	MFN1_HUM	10	1.058624
48H 10MTA	O00411	-0.02605	0.038276	0.029612	POLRMT	DNA-direct	RPOM_HUI	32	0.982105
24H 50MTA	O76024	0.089955	0.038276	0.029612	WFS1	Wolframin	WFS1_HUM	19	1.064337
48H 10MTA	P08670	0.034366	0.038297	0.029623	VIM	Vimentin	VIME_HUM	34	1.024107

48H 10MTA	Q8IX01	-0.02759	0.038325	0.02964	SUGP2	SURP and G	SUGP2_HU	30	0.981055
48H 50MTA	O75431	-0.17262	0.038338	0.029648	MTX2	Metaxin-2	MTX2_HUM	10	0.887228
48H 10MTA	P17174	-0.03953	0.038342	0.029651	GOT1	Aspartate a	AATC_HUM	23	0.972971
24H 10MTA	Q15633	0.147155	0.038376	0.029673	TARBP2	RISC-loadir	TRBP2_HU	6	1.107383
48H 10MTA	Q9H446	-0.1476	0.038377	0.029673	RWDD1	RWD doma	RWDD1_HU	4	0.902752
24H 50MTA	P52948	0.010958	0.03838	0.029674	NUP98	Nuclear po	NUP98_HU	54	1.007625
48H 10MTA	Q9UHR5	0.061902	0.038409	0.029694	SAP30BP	SAP30-binc	S30BP_HU	7	1.043841
24H 50MTA	Q13107	0.215156	0.038413	0.029695	USP4	Ubiquitin c	UBP4_HUM	9	1.160829
48H 10MTA	P17612	-0.1302	0.038416	0.029697	PRKACA	cAMP-depe	KAPCA_HU	5	0.913706
24H 50MTA	Q9NVA1	0.157153	0.038432	0.029708	UQCC1	Ubiquinol-	UQCC1_HU	6	1.115084
48H 50MTA	Q8WV07	-0.5782	0.038436	0.029709	LTO1	Protein LTC	LTO1_HUM	3	0.6698
48H 10MTA	Q9UBM7	-0.14728	0.038446	0.029714	DHCR7	7-dehydroc	DHCR7_HU	7	0.902952
48H 10MTA	Q9Y3D9	-0.12213	0.038466	0.029728	MRPS23	Small ribos	RT23_HUM	11	0.918831
48H 50MTA	P82675	-0.12313	0.038478	0.029734	MRPS5	Small ribos	RT05_HUM	13	0.918191
24H 50MTA	Q9NUQ6	-0.14325	0.038479	0.029734	SPATS2L	SPATS2-like	SPS2L_HUM	18	0.905476
24H 10MTA	Q12929	0.019756	0.038487	0.029738	EPS8	Epidermal	EPS8_HUM	32	1.013788
24H 50MTA	Q92783	-0.11203	0.038488	0.029738	STAM	Signal trans	STAM1_HU	15	0.925285
48H 50MTA	Q9GZZ1	-0.14926	0.038489	0.029738	NAA50	N-alpha-ace	NAA50_HU	13	0.901715
48H 50MTA	P41743	0.1325	0.0385	0.029746	PRKCI	Protein kin	KPCI_HUM	18	1.096192
48H 50MTA	Q8NAN2	-0.20477	0.038506	0.029749	MIGA1	Mitoguardi	MIGA1_HU	16	0.867676
48H 10MTA	Q8TDZ2	-0.01581	0.03852	0.029757	MICAL1	[F-actin]-m	MICA1_HU	22	0.989101
48H 50MTA	Q9NZZ3	0.045835	0.038524	0.029759	CHMP5	Charged mu	CHMP5_HU	6	1.03228
24H 50MTA	P08922	0.376172	0.038528	0.029761	ROS1	Proto-onco	ROS1_HUM	1	1.297893
24H 50MTA	Q9GZM5	0.093379	0.038532	0.029763	YIPF3	Protein YIP	YIPF3_HUM	8	1.066866
24H 10MTA	O14908	-0.07449	0.038547	0.029773	GIPC1	PDZ domai	GIPC1_HUM	13	0.949681
24H 50MTA	A0PJW6	0.096075	0.038561	0.029781	TMEM223	Transmemb	TM223_HU	4	1.068862
24H 50MTA	Q8N5F7	0.535788	0.038577	0.029792	NKAP	NF-kappa-B	NKAP_HUM	3	1.449734
48H 10MTA	Q9BRJ7	-0.05348	0.038586	0.029796	NUDT16L1	Tudor-inter	TIRR_HUMA	11	0.963612
24H 50MTA	Q14203	-0.05615	0.038594	0.0298	DCTN1	Dynactin su	DCTN1_HU	52	0.961828
48H 10MTA	Q92504	-0.50946	0.038595	0.029801	SLC39A7	Zinc transp	S39A7_HU	2	0.702487
48H 10MTA	Q9BUA3	0.402016	0.038608	0.029806	SPINDOC	Spindlin int	SPNDC_HU	3	1.321353
24H 50MTA	Q14149	0.018585	0.038608	0.029806	MORC3	MORC fami	MORC3_HU	17	1.012966
48H 10MTA	Q2T9J0	-0.00649	0.038606	0.029806	TYSND1	Peroxisoma	TYSD1_HUM	15	0.995515
48H 50MTA	O75496	-0.35951	0.038615	0.029809	GMNN	Geminin	GEMI_HUM	6	0.779427
24H 10MTA	Q9Y5P4	0.018975	0.038635	0.029821	CERT1	Ceramide t	CERT_HUM	13	1.01324
48H 50MTA	Q9P0T7	-0.15564	0.038636	0.029821	TMEM9	Proton-trar	TMEM9_HU	4	0.897734
48H 50MTA	Q9P0U4	0.003213	0.038648	0.029827	CXXC1	CXXC-type	CXXC1_HU	12	1.002229
48H 10MTA	Q9BZQ6	-0.0116	0.038649	0.029827	EDEM3	ER degrada	EDEM3_HU	9	0.991992
24H 50MTA	Q15276	-0.06969	0.038652	0.029827	RABEP1	Rab GTPase	RABE1_HU	12	0.952845
48H 10MTA	O95861	0.051392	0.038653	0.029828	BPNT1	3'(2'),5'-bis	BPNT1_HU	19	1.036264
48H 10MTA	Q8IUS5	0.051076	0.038654	0.029828	EPHX4	Epoxide hy	EPHX4_HU	4	1.036037
24H 50MTA	Q6PKC3	-0.07397	0.038661	0.029831	TXNDC11	Thioredoxi	TXD11_HU	9	0.950019
48H 10MTA	O76095	-0.06037	0.038663	0.029832	JTB	Protein JTB	JTB_HUMA	1	0.959016
24H 50MTA	P43155	0.113537	0.038669	0.029835	CRAT	Carnitine O	CACP_HUM	16	1.081877
24H 50MTA	O95639	0.078896	0.038674	0.029838	CPSF4	Cleavage ar	CPSF4_HUM	9	1.056209
48H 10MTA	Q99547	0.10294	0.038695	0.029849	MPHOSPH6	M-phase ph	MPH6_HUM	3	1.07396
48H 50MTA	Q8NBK3	0.339133	0.038704	0.029853	SUMF1	Formylglyc	SUMF1_HU	5	1.264996
48H 50MTA	Q9P1U0	-0.90563	0.038718	0.029859	POLR1H	DNA-direct	RPA12_HU	1	0.533799
48H 10MTA	P09211	0.065174	0.038731	0.029867	GSTP1	Glutathion	GSTP1_HU	16	1.046211
48H 10MTA	A6NHL2	-0.41457	0.038751	0.029879	TUBAL3	Tubulin alp	TBAL3_HUM	2	0.750241
24H 10MTA	P29558	0.070258	0.038756	0.029882	RBMS1	RNA-bindir	RBMS1_HU	1	1.049905
24H 50MTA	P04632	0.028967	0.038759	0.029884	CAPNS1	Calpain sm	CPNS1_HU	11	1.020281

24H 10MT/	P81877;Q9	0.824385	0.038763	0.029886	SSBP2;SSBF	Single-strar	SSBP2_HUI	1	1.77078
24H 10MT/	Q96BP3	0.119243	0.03877	0.029891	PPWD1	Peptidylprc	PPWD1_HU	24	1.086165
48H 10MT/	Q9Y276	-0.24511	0.03879	0.029903	BCS1L	Mitochond	BCS1_HUM	19	0.843753
24H 10MT/	Q8IUR0	0.042167	0.038807	0.029915	TRAPPC5	Trafficking	TPPC5_HUI	5	1.029659
48H 50MT/	Q969N2	-0.05189	0.038811	0.029917	PIGT	GPI transan	PIGT_HUM.	19	0.96467
48H 10MT/	O95140	0.1008	0.038827	0.029925	MFN2	Mitofusin-2	MFN2_HUN	22	1.072368
48H 50MT/	O75191	0.196349	0.038838	0.029931	XYLB	Xylulose kir	XYLB_HUM	9	1.145795
48H 50MT/	Q96GC5	-0.27097	0.038853	0.029938	MRPL48	Large ribos	RM48_HUN	4	0.828761
24H 50MT/	Q99615	-0.01353	0.038892	0.02996	DNAJC7	DnaJ homo	DNJC7_HUI	27	0.990667
48H 50MT/	Q9H7Z6	-0.07759	0.038924	0.029979	KAT8	Histone ace	KAT8_HUM	5	0.947641
48H 10MT/	Q969F1	0.307088	0.038926	0.029979	GTF3C6	General tra	TF3C6_HUN	1	1.237208
48H 10MT/	Q7Z2T5	0.015541	0.038926	0.029979	TRMT1L	TRMT1-like	TRM1L_HU	27	1.01083
48H 50MT/	P46782	0.115063	0.038932	0.029982	RPS5	Small ribos	RS5_HUMA	10	1.083022
24H 10MT/	Q12788	0.026797	0.038951	0.029993	TBL3	Transducin	TBL3_HUM	35	1.018748
48H 10MT/	Q9BZH6	0.026908	0.038958	0.029994	WDR11	WD repeat-	WDR11_HU	32	1.018826
24H 50MT/	Q96E52	0.171479	0.038983	0.030008	OMA1	Metalloenc	OMA1_HUN	5	1.126212
24H 10MT/	Q8IVM0	-0.09534	0.038997	0.030014	CCDC50	Coiled-coil	CCD50_HU	12	0.936052
48H 10MT/	Q9Y305	-0.01996	0.039012	0.03002	ACOT9	Acyl-coenzy	ACOT9_HUI	20	0.986263
48H 10MT/	Q9Y608	-0.04493	0.039014	0.03002	LRRFIP2	Leucine-ric	LRRF2_HUN	9	0.969334
48H 50MT/	P36578	-0.11495	0.039029	0.030025	RPL4	Large ribos	RL4_HUMA	22	0.923417
24H 10MT/	P61224	0.007065	0.03903	0.030025	RAP1B	Ras-related	RAP1B_HUI	10	1.004909
48H 10MT/	Q8IYD1	-0.18218	0.03903	0.030025	GSPT2	Eukaryotic	ERF3B_HUN	13	0.881369
24H 10MT/	Q8TF74	-0.18231	0.039026	0.030025	WIPF2	WAS/WASL	WIPF2_HUI	11	0.881291
24H 50MT/	Q8NFQ8	-0.02822	0.039034	0.030028	TOR1AIP2	Torsin-1A-ii	TOIP2_HUN	15	0.980627
48H 10MT/	Q14CB8	0.91442	0.039052	0.030039	ARHGAP19	Rho GTPase	RHG19_HU	1	1.884811
48H 50MT/	O95714	-0.11319	0.039072	0.030048	HERC2	E3 ubiquiti	HERC2_HUI	58	0.924544
48H 50MT/	Q9UHR6	-0.13983	0.039093	0.030059	ZNHIT2	Zinc finger	ZNHI2_HUN	8	0.907626
24H 10MT/	Q8TD16	0.09646	0.039113	0.03007	BICD2	Protein bic	BICD2_HUN	16	1.069147
48H 10MT/	O14526	1.235324	0.039128	0.030078	FCHO1	F-BAR dom	FCHO1_HU	1	2.354342
48H 10MT/	P48426	-0.22378	0.039132	0.03008	PIP4K2A	Phosphatid	PI42A_HUN	8	0.85632
48H 50MT/	Q8IZV5	-0.08003	0.039143	0.030087	RDH10	Retinol deh	RDH10_HU	9	0.946037
24H 10MT/	Q8IZN3	-0.05314	0.039152	0.030092	ZDHHC14	Palmitoyltr	ZDH14_HU	3	0.963838
24H 50MT/	Q9H9S3	0.050191	0.039159	0.030096	SEC61A2	Protein trai	S61A2_HUI	7	1.035402
48H 50MT/	Q13425	0.064626	0.039167	0.0301	SNTB2	Beta-2-synt	SNTB2_HUI	13	1.045814
48H 50MT/	P49642	-0.19391	0.03917	0.030101	PRIM1	DNA primas	PRI1_HUM.	18	0.874233
48H 50MT/	Q6NXE6	-0.10935	0.039177	0.030106	ARMC6	Armadillo r	ARMC6_HU	14	0.927007
24H 50MT/	Q9NX20	0.001789	0.039179	0.030106	MRPL16	Large ribos	RM16_HUN	9	1.001241
48H 10MT/	Q93015	0.509992	0.039187	0.03011	NAA80	N-alpha-ace	NAA80_HU	1	1.424042
48H 50MT/	Q96DG6	0.16328	0.039187	0.03011	CMBL	Carboxyme	CMBL_HUN	10	1.11983
24H 10MT/	Q03169	0.470902	0.03919	0.030111	TNFAIP2	Tumor necr	TNAP2_HUI	2	1.385976
24H 50MT/	Q15911	0.040086	0.039224	0.030132	ZFHX3	Zinc finger	ZFHX3_HUI	2	1.028175
24H 50MT/	Q8TCE6	-0.39033	0.03923	0.030134	DENND10	DENN dom	DEN10_HU	1	0.762955
48H 50MT/	Q9HCL2	-0.80568	0.039232	0.030134	GPAM	Glycerol-3-	GPAT1_HUI	4	0.572092
24H 50MT/	O00401	-0.24661	0.039241	0.030138	WASL	Actin nucle	WASL_HUN	8	0.842877
24H 50MT/	P00156	0.290076	0.039244	0.03014	MT-CYB	Cytochrom	CYB_HUMA	1	1.222704
48H 50MT/	O43432	-0.04546	0.039256	0.030147	EIF4G3	Eukaryotic	IF4G3_HUN	43	0.96898
48H 50MT/	O75157	0.132474	0.039257	0.030147	TSC22D2	TSC22 dom	T22D2_HUI	2	1.096171
48H 10MT/	O14910	0.052064	0.039259	0.030148	LIN7A	Protein lin-	LIN7A_HUN	9	1.036747
48H 50MT/	Q15418	0.104588	0.039274	0.030156	RPS6KA1	Ribosomal	KS6A1_HUI	15	1.075187
24H 50MT/	Q8NDI1	-0.02399	0.039276	0.030156	EHBP1	EH domain-	EHBP1_HUI	20	0.983508
24H 10MT/	O15270	-0.00351	0.039292	0.030163	SPTLC2	Serine palm	SPTC2_HUN	11	0.997569
24H 50MT/	P08574	0.144153	0.039293	0.030163	CYC1	Cytochrom	CY1_HUMA	8	1.105082

48H 50MTA	Q6Y2X3	-0.29923	0.039293	0.030163	DNAJC14	DnaJ homo	DJC14_HUI	2	0.812689
24H 50MTA	Q9UBV8	0.046613	0.039291	0.030163	PEF1	Peflin	PEF1_HUM	5	1.032837
48H 50MTA	P62851	-0.14226	0.039297	0.030165	RPS25	Small ribos	RS25_HUM	4	0.906099
24H 50MTA	Q96BT3	0.432035	0.039324	0.03018	CENPT	Centromer	CENPT_HUI	3	1.349135
48H 50MTA	Q9NS73	-0.17896	0.039334	0.030186	MBIP	MAP3K12-I	MBIP1_HUI	4	0.883338
24H 50MTA	Q9Y3A5	-0.11431	0.039347	0.030194	SBDS	Ribosome r	SBDS_HUM	18	0.923827
24H 50MTA	P61457	-0.11898	0.03937	0.030206	PCBD1	Pterin-4-al	PHS_HUMA	7	0.920837
24H 50MTA	O14523	0.190377	0.039373	0.030206	C2CD2L	Phospholip	C2C2L_HUI	5	1.141061
48H 50MTA	Q587I9	-0.08958	0.039375	0.030206	SFT2D3	Vesicle trar	SFT2C_HUM	3	0.939795
24H 50MTA	Q5EBL8	0.093479	0.039377	0.030207	PDZD11	PDZ domai	PDZ11_HUI	3	1.06694
48H 50MTA	Q9BYN8	-0.16273	0.039389	0.030215	MRPS26	Small ribos	RT26_HUM	9	0.893331
24H 50MTA	Q99805	0.1338	0.039398	0.03022	TM9SF2	Transmemb	TM9S2_HU	14	1.09718
48H 50MTA	Q6UX01;Q8	-0.0758	0.039419	0.030233	LMBR1L;LM	Protein LM	LMBRL_HU	1	0.948819
48H 10MTA	O00567	-0.0787	0.039435	0.030241	NOP56	Nucleolar p	NOP56_HU	27	0.946911
48H 50MTA	P31040	0.021667	0.039442	0.030246	SDHA	Succinate d	SDHA_HUM	24	1.015131
24H 50MTA	Q8IZ21	-0.05914	0.039462	0.030258	PHACTR4	Phosphatas	PHAR4_HU	10	0.959833
48H 50MTA	P49914	0.14461	0.039472	0.030264	MTHFS	5-formyltet	MTHFS_HU	8	1.105432
24H 10MTA	Q6PRD1	-0.04432	0.039475	0.030265	GPR179	Probable G	GP179_HU	1	0.96975
48H 50MTA	P36639	0.044726	0.039491	0.030274	NUDT1	Oxidized pu	8ODP_HUM	6	1.031487
48H 50MTA	Q13636	0.20557	0.039498	0.030278	RAB31	Ras-related	RAB31_HUI	4	1.153142
48H 50MTA	Q9NP77	-0.11786	0.039503	0.03028	SSU72	RNA polym	SSU72_HUI	5	0.921557
48H 10MTA	Q96HE9	0.154196	0.03951	0.030285	PRR11	Proline-rich	PRR11_HUI	2	1.112801
24H 10MTA	P06737	0.019002	0.039514	0.030286	PYGL	Glycogen p	PYGL_HUM	37	1.013258
24H 10MTA	O96008	-0.07944	0.039519	0.030287	TOMM40	Mitochond	TOM40_HU	11	0.946423
24H 50MTA	Q86U86	0.431078	0.039541	0.0303	PBRM1	Protein pol	PB1_HUMA	6	1.34824
24H 10MTA	P49643	0.01054	0.039551	0.030305	PRIM2	DNA primas	PRI2_HUMA	20	1.007332
48H 10MTA	Q9UNK0	0.051902	0.039564	0.030313	STX8	Syntaxin-8	STX8_HUM	5	1.036631
24H 10MTA	Q93096	0.059824	0.039565	0.030313	PTP4A1	Protein tyr	TP4A1_HUI	5	1.042339
24H 10MTA	Q01968	-0.13283	0.039573	0.030318	OCRL	Inositol pol	OCRL_HUM	26	0.912039
24H 50MTA	P07108	-0.1868	0.039593	0.030329	DBI	Acyl-CoA-bi	ACBP_HUM	4	0.878553
24H 10MTA	Q6I9Y2	0.073923	0.039598	0.03033	THOC7	THO compl	THOC7_HU	8	1.052575
24H 50MTA	Q9NTG7	0.019086	0.039598	0.03033	SIRT3	NAD-depen	SIR3_HUMA	6	1.013317
24H 10MTA	Q9Y2E8	-0.24917	0.039603	0.030333	SLC9A8	Sodium/hy	SL9A8_HUM	1	0.841379
48H 50MTA	O75054	0.148819	0.03961	0.030337	IGSF3	Immunoglo	IGSF3_HUM	12	1.108662
24H 50MTA	Q15262	0.073491	0.039611	0.030337	PTPRK	Receptor-ty	PTPRK_HUI	15	1.05226
24H 10MTA	P02668	-0.04879	0.039643	0.030353	CSN3	Kappa-case	CASK_BOVI	2	0.966749
48H 10MTA	Q9UDY4	0.012195	0.039641	0.030353	DNAJB4	DnaJ homo	DNJB4_HUI	11	1.008489
48H 10MTA	Q8IUC4	0.114048	0.039656	0.030355	RHPN2	Rhophilin-2	RHPN2_HU	21	1.082261
48H 50MTA	Q8NFH5	-0.00404	0.039658	0.030356	NUP35	Nucleopori	NUP35_HU	11	0.997205
48H 10MTA	P48449	-0.02297	0.039662	0.030358	LSS	Lanosterol	LSS_HUMA	22	0.984202
48H 50MTA	P11310	0.028976	0.039666	0.03036	ACADM	Medium-ch	ACADM_HU	19	1.020288
24H 50MTA	Q92538	0.05011	0.039668	0.030361	GBF1	Golgi-speci	GBF1_HUM	70	1.035344
48H 50MTA	P21953	0.159846	0.039691	0.030375	BCKDHB	2-oxoisoval	ODBB_HUM	8	1.117168
48H 50MTA	P17480	-0.1329	0.039709	0.030386	UBTF	Nucleolar t	UBF1_HUM	23	0.911998
24H 10MTA	P45880	0.052362	0.039724	0.030392	VDAC2	Voltage-de	VDAC2_HU	14	1.036961
24H 10MTA	Q9UJV9	0.053461	0.039725	0.030392	DDX41	Probable A	DDX41_HU	29	1.037752
48H 50MTA	Q13443	0.077139	0.039728	0.030394	ADAM9	Disintegrin	ADAM9_HU	13	1.054924
48H 50MTA	O60658	0.149341	0.039754	0.03041	PDE8A	High affinit	PDE8A_HUI	14	1.109063
48H 10MTA	Q93062	0.031691	0.039754	0.03041	RBPMS	RNA-bindir	RBPMS_HU	8	1.02221
24H 10MTA	O43148	0.016251	0.039765	0.030416	RNMT	mRNA cap g	MCES_HUM	13	1.011328
24H 50MTA	Q14165	0.0801	0.039767	0.030417	MLEC	Malectin	MLEC_HUM	10	1.057091
48H 50MTA	Q7L3B6	0.523541	0.039773	0.03042	CDC37L1	Hsp90 co-c	CD37L_HUI	2	1.437479

48H	10MT/	Q9HAV0	-0.00057	0.039795	0.030432	GNB4	Guanine nu	GBB4_HUM	6	0.999608
48H	50MT/	Q9Y2B0	0.036575	0.039802	0.030436	CNPY2	Protein car	CNPY2_HUI	11	1.025676
48H	50MT/	Q9UPN9	-0.11622	0.039808	0.030438	TRIM33	E3 ubiquiti	TRI33_HUM	21	0.9226
48H	50MT/	P49959	0.066428	0.039819	0.030444	MRE11	Double-stra	MRE11_HU	20	1.047121
24H	50MT/	P57764	-0.1553	0.039826	0.030448	GSDMD	Gasdermin-	GSDMD_HU	7	0.897948
24H	50MT/	O75886	0.040034	0.039831	0.030451	STAM2	Signal trans	STAM2_HU	13	1.028138
24H	50MT/	Q9Y4C2	-0.12657	0.039843	0.030458	TCAF1	TRPM8 cha	TCAF1_HUM	14	0.916008
48H	50MT/	Q9UKI8	-0.28643	0.039848	0.030461	TLK1	Serine/thre	TLK1_HUM	9	0.819926
24H	10MT/	O43164	-0.00933	0.03986	0.030468	PJA2	E3 ubiquiti	PJA2_HUM	5	0.993553
48H	10MT/	P23610	-0.05514	0.039874	0.030472	F8A1	40-kDa hur	HAP40_HU	5	0.9625
48H	10MT/	P63146	0.215915	0.039871	0.030472	UBE2B	Ubiquitin-c	UBE2B_HUI	1	1.161441
24H	10MT/	Q6EMK4	0.213019	0.039875	0.030472	VASN	Vasorin	VASN_HUM	4	1.159111
48H	10MT/	P48047	0.111661	0.03989	0.030481	ATP5PO	ATP synthas	ATPO_HUM	10	1.080471
48H	10MT/	P51649	0.06869	0.039899	0.030485	ALDH5A1	Succinate-s	SSDH_HUM	15	1.048764
24H	50MT/	Q15390	-0.10062	0.039939	0.030504	MTFR1	Mitochond	MTFR1_HU	3	0.932631
24H	50MT/	Q5BJH2	-0.1647	0.039939	0.030504	TMEM128	Transmemb	TM128_HU	1	0.892114
24H	10MT/	Q9BTC0	0.053534	0.039942	0.030505	DIDO1	Death-indu	DIDO1_HUI	37	1.037804
48H	10MT/	Q14694	0.01315	0.03996	0.030516	USP10	Ubiquitin c	UBP10_HU	25	1.009157
24H	10MT/	Q3KQZ1	-0.31196	0.039962	0.030517	SLC25A35	Solute carri	S2535_HUI	5	0.805549
48H	50MT/	O75348	-0.03567	0.039963	0.030517	ATP6V1G1	V-type prot	VATG1_HUI	4	0.975578
24H	10MT/	O95793	-0.08606	0.039965	0.030518	STAU1	Double-stra	STAU1_HUI	28	0.942093
24H	50MT/	P49448	0.173713	0.040003	0.030543	GLUD2	Glutamate	DHE4_HUM	8	1.127958
24H	50MT/	O60678	-0.18687	0.040018	0.030551	PRMT3	Protein arg	ANM3_HUM	20	0.878511
48H	50MT/	Q13595	-0.09353	0.04003	0.030557	TRA2A	Transforme	TRA2A_HUI	8	0.937228
24H	10MT/	Q5T0D9	-0.0706	0.040032	0.030557	TPRG1L	Tumor prot	TPRGL_HUI	4	0.952245
48H	10MT/	P11234	0.016223	0.040037	0.030559	RALB	Ras-related	RALB_HUM	10	1.011309
24H	10MT/	P42695	0.149698	0.040038	0.030559	NCAPD3	Condensin-	CNDD3_HU	22	1.109337
24H	50MT/	Q643R3	0.004893	0.040037	0.030559	LPCAT4	Lysophospl	LPCT4_HUM	4	1.003397
24H	50MT/	Q969S9	0.050491	0.040047	0.030564	GFM2	Ribosome-r	RRF2M_HU	15	1.035617
48H	50MT/	Q9Y5Y0	-0.16666	0.040048	0.030564	FLVCR1	Heme trans	FLVC1_HUM	4	0.8909
48H	50MT/	Q8IZP0	0.053528	0.040065	0.030575	ABI1	Abl interact	ABI1_HUMA	11	1.0378
48H	50MT/	Q9BQE5	0.138774	0.040071	0.030577	APOL2	Apolipoprc	APOL2_HUI	8	1.100969
48H	50MT/	Q13409	0.052219	0.040078	0.03058	DYNC1I2	Cytoplasmi	DC1I2_HUM	19	1.036859
24H	50MT/	Q8N4A0	0.232776	0.040077	0.03058	GALNT4	Polypeptid	GALT4_HUI	7	1.175094
48H	50MT/	Q92508	-0.20152	0.040088	0.030586	PIEZO1	Piezo-type	PIEZ1_HUM	3	0.869635
48H	50MT/	P62736;P6	-0.19643	0.040109	0.030599	ACTA2;ACT	Actin, aorti	ACTA_HUM	5	0.872705
48H	50MT/	Q5VW36	0.101942	0.04011	0.030599	FOCAD	Focadhesin	FOCAD_HU	22	1.073217
48H	10MT/	O75925	-0.40159	0.040145	0.030619	PIAS1	E3 SUMO-p	PIAS1_HUM	4	0.757026
24H	50MT/	Q86U44	-0.11114	0.040154	0.030625	METTL3	N6-adenosi	MTA70_HU	15	0.925854
48H	10MT/	P13473	-0.13335	0.040166	0.030631	LAMP2	Lysosome-a	LAMP2_HU	3	0.911711
24H	50MT/	Q8TB36	0.102448	0.040168	0.030633	GDAP1	Ganglioside	GDAP1_HU	11	1.073594
24H	10MT/	P42166	0.040224	0.040184	0.030641	TMPO	Lamina-ass	LAP2A_HUI	35	1.028274
48H	10MT/	P0C024	0.671016	0.040192	0.030645	NUDT7	Peroxisoma	NUDT7_HU	1	1.592193
48H	50MT/	Q96EQ0	1.005316	0.040213	0.030658	SGTB	Small gluta	SGTB_HUM	1	2.007384
48H	50MT/	Q8TCT7	0.891801	0.040229	0.030668	SPPL2B	Signal pept	SPP2B_HUI	1	1.855491
48H	10MT/	P62166	0.275068	0.040238	0.030674	NCS1	Neuronal ca	NCS1_HUM	3	1.210051
48H	50MT/	Q5JSZ5	-0.23105	0.040254	0.030685	PRRC2B	Protein PRI	PRC2B_HUI	18	0.852012
48H	50MT/	Q86YP4	-0.01902	0.040264	0.030691	GATAD2A	Transcripti	P66A_HUM	22	0.986902
24H	50MT/	Q9Y4C8	-0.02495	0.040279	0.0307	RBM19	Probable RI	RBM19_HU	15	0.982853
48H	10MT/	Q5T4S7	0.003863	0.040294	0.030708	UBR4	E3 ubiquiti	UBR4_HUM	145	1.002681
48H	50MT/	P53701	-0.13993	0.040322	0.030725	HCCS	Holocytoch	CCHL_HUM	12	0.907563
48H	10MT/	Q14432	0.07471	0.040333	0.03073	PDE3A	cGMP-inhil	PDE3A_HUI	10	1.05315

24H 50MT/	Q08188	0.263878	0.040374	0.030756	TGM3	Protein-glu	TGM3_HUN	2	1.200702
48H 10MT/	Q13868	0.015414	0.040381	0.030759	EXOSC2	Exosome cc	EXOS2_HUI	12	1.010742
24H 50MT/	Q96L92	0.158733	0.040383	0.03076	SNX27	Sorting nex	SNX27_HUI	16	1.116306
24H 10MT/	Q8TB61	0.138948	0.0404	0.030769	SLC35B2	Adenosine :	S35B2_HUI	11	1.101102
24H 10MT/	P53350	0.100163	0.040406	0.030773	PLK1	Serine/thre	PLK1_HUM	21	1.071895
48H 10MT/	P15151	0.055172	0.04043	0.030787	PVR	Poliovirus r	PVR_HUMA	6	1.038983
48H 50MT/	Q9NV88	-0.07643	0.040432	0.030787	INTS9	Integrator c	INT9_HUMA	7	0.948404
48H 50MT/	Q8WUD1	-0.49679	0.040441	0.030793	RAB2B	Ras-related	RAB2B_HUI	2	0.708683
48H 50MT/	P62241	-0.10917	0.040445	0.030795	RPS8	Small ribos	RS8_HUMA	11	0.927124
48H 50MT/	Q93008	0.088336	0.040473	0.030811	USP9X	Ubiquitin c	USP9X_HUI	64	1.063143
48H 50MT/	Q6ZNC8	0.460723	0.040485	0.030816	MBOAT1	Lysophospl	MBOA1_HU	1	1.376232
48H 10MT/	Q7L0Y3	0.020142	0.040484	0.030816	TRMT10C	tRNA methy	TM10C_HU	24	1.014059
48H 50MT/	Q9NQS7	-0.36705	0.040485	0.030816	INCENP	Inner centr	INCE_HUMA	12	0.775365
48H 50MT/	Q9NVC6	-0.09014	0.040501	0.030825	MED17	Mediator o	MED17_HU	10	0.939434
24H 50MT/	O60292	0.454501	0.040502	0.030825	SIPA1L3	Signal-indu	SI1L3_HUM	2	1.370309
24H 10MT/	O15164	0.1041	0.040509	0.030829	TRIM24	Transcripti	TIF1A_HUN	16	1.074823
48H 50MT/	Q96K21	0.11985	0.040513	0.030831	ZFYVE19	Abscission/	ANCHR_HU	6	1.086622
48H 50MT/	O95999	0.068721	0.040539	0.030846	BCL10	B-cell lymp	BCL10_HUI	5	1.048786
48H 10MT/	P07996	-0.37403	0.040554	0.030852	THBS1	Thrombosp	TSP1_HUM	9	0.771623
48H 10MT/	Q92552	-0.12525	0.040564	0.030858	MRPS27	Small ribos	RT27_HUM	19	0.916846
48H 50MT/	Q6UW63	-0.03545	0.040604	0.030883	POGLUT2	Protein O-g	PLGT2_HUI	18	0.97573
24H 10MT/	P80303	-0.1889	0.040618	0.030891	NUCB2	Nucleobinc	NUCB2_HU	14	0.877273
48H 10MT/	Q53EP0	0.03499	0.040625	0.030894	FNDC3B	Fibronectir	FND3B_HU	33	1.02455
48H 50MT/	P04350	0.258262	0.040635	0.0309	TUBB4A	Tubulin bet	TBB4A_HUI	3	1.196037
24H 50MT/	Q9Y2Q5	-0.12273	0.040645	0.030903	LAMTOR2	Ragulator c	LTOR2_HUI	6	0.918446
48H 10MT/	Q14157	0.024434	0.040653	0.030907	UBAP2L	Ubiquitin-a	UBP2L_HUI	21	1.01708
48H 50MT/	Q9UG63	-0.14721	0.040661	0.030912	ABCF2	ATP-bindin	ABCF2_HUI	24	0.902998
24H 50MT/	P52179	-1.19868	0.040676	0.03092	MYOM1	Myomesin-	MYOM1_HU	2	0.435672
48H 50MT/	Q9UK23	-0.17845	0.040697	0.030934	NAGPA	N-acetylglu	NAGPA_HU	10	0.883655
48H 10MT/	Q9NPI0	-0.64439	0.040698	0.030934	TMEM138	Transmemb	TM138_HU	1	0.639765
24H 10MT/	Q6ZS30	0.094121	0.040704	0.030938	NBEAL1	Neurobeacl	NBEL1_HUI	28	1.067415
48H 10MT/	Q6UX53	-0.00161	0.040705	0.030938	TMT1B	Thiol S-met	TMT1B_HU	10	0.998882
48H 50MT/	Q92636	-0.20376	0.040708	0.03094	NSMAF	Protein FAN	FAN_HUMA	4	0.868284
24H 50MT/	Q149N8	-0.05912	0.04072	0.030946	SHPRH	E3 ubiquiti	SHPRH_HU	12	0.95985
48H 10MT/	Q8TDX7	-0.02801	0.040722	0.030947	NEK7	Serine/thre	NEK7_HUM	10	0.980775
24H 10MT/	P36776	0.028532	0.040735	0.030955	LONP1	Lon protea:	LONM_HUN	45	1.019974
24H 50MT/	Q8WTW3	0.036132	0.040791	0.030991	COG1	Conserved (	COG1_HUM	26	1.025361
24H 50MT/	A6NGU5;P1	0.25376	0.040792	0.030991	GGT3P;GGT	Putative glu	GGT3_HUM	4	1.19231
24H 50MT/	P42858	0.003257	0.040795	0.030992	HTT	Huntingtin	HD_HUMAI	55	1.00226
24H 10MT/	Q13277	0.687386	0.040796	0.030992	STX3	Syntaxin-3	STX3_HUM	6	1.610363
24H 10MT/	P17655	0.034342	0.04081	0.030999	CAPN2	Calpain-2 c	CAN2_HUM	22	1.02409
48H 50MT/	Q9BUN8	0.029957	0.040825	0.031007	DERL1	Derlin-1	DERL1_HUI	7	1.020982
24H 50MT/	O00566	0.008818	0.040831	0.031009	MPHOSPH1	U3 small nu	MPP10_HU	16	1.006131
48H 10MT/	Q7L3B6	0.419049	0.04084	0.031015	CDC37L1	Hsp90 co-c	CD37L_HUI	2	1.337046
24H 50MT/	Q5VUA4	0.195644	0.040846	0.031017	ZNF318	Zinc finger	ZN318_HUI	27	1.145235
24H 10MT/	Q14683	0.054732	0.04085	0.031018	SMC1A	Structural r	SMC1A_HU	54	1.038666
48H 50MT/	O60645	0.062436	0.040866	0.031029	EXOC3	Exocyst cor	EXOC3_HUI	16	1.044228
24H 50MT/	O43464	-0.14353	0.040873	0.031032	HTRA2	Serine prot	HTRA2_HUI	8	0.905302
24H 10MT/	P49641	0.257238	0.040872	0.031032	MAN2A2	Alpha-mani	MA2A2_HU	10	1.195188
24H 10MT/	Q9UI26	0.07589	0.040876	0.031034	IPO11	Importin-1	IPO11_HUN	27	1.054011
48H 50MT/	P51946	-0.18945	0.040889	0.031043	CCNH	Cyclin-H	CCNH_HUN	10	0.876938
48H 50MT/	P30046	0.219535	0.040927	0.031065	DDT	D-dopachr(	DOPD_HUN	8	1.164358

48H 10MT/	Q9BRL6	0.692293	0.040938	0.031072	SRSF8	Serine/argi	SRSF8_HUM	1	1.61585
24H 10MT/	Q96B70	0.347646	0.040979	0.031096	LENG9	Leukocyte r	LENG9_HU	2	1.272482
48H 50MT/	Q15329	0.882195	0.04099	0.031103	E2F5	Transcripti	E2F5_HUM	1	1.843177
24H 10MT/	O43143	0.035869	0.041	0.03111	DHX15	ATP-depen	DHX15_HU	36	1.025174
24H 50MT/	Q6P1M0	0.121119	0.041008	0.031115	SLC27A4	Long-chain	S27A4_HU	19	1.087578
24H 50MT/	O75832	0.2867	0.041015	0.031118	PSMD10	26S protea	PSD10_HU	9	1.219847
24H 50MT/	Q96RY7	-0.48243	0.041018	0.03112	IFT140	Intraflagell	IF140_HUN	4	0.71577
24H 10MT/	P41250	-0.08985	0.041027	0.031125	GARS1	Glycine--tR	GARS_HUM	35	0.93962
48H 50MT/	Q86UU1	-0.24042	0.041028	0.031125	PHLDB1	Pleckstrin h	PHLB1_HU	12	0.846499
24H 50MT/	Q92896	0.074062	0.041053	0.031137	GLG1	Golgi appar	GSLG1_HU	40	1.052677
48H 50MT/	Q99676	-0.0791	0.04106	0.031142	ZNF184	Zinc finger	ZN184_HU	1	0.946646
48H 50MT/	Q14118	0.266785	0.041079	0.031151	DAG1	Dystroglyca	DAG1_HUM	12	1.203123
24H 10MT/	Q86WR0	0.009985	0.041083	0.031152	CCDC25	Coiled-coil	CCD25_HU	6	1.006945
48H 10MT/	Q9Y3L5	0.033616	0.041089	0.031156	RAP2C	Ras-related	RAP2C_HU	4	1.023574
24H 10MT/	Q9Y2S2	-0.01124	0.041102	0.031161	CRYL1	Lambda-cry	CRYL1_HUM	8	0.992236
48H 10MT/	Q9BY50	-0.45092	0.041123	0.031174	SEC11C	Signal pept	SC11C_HU	5	0.731577
48H 50MT/	O14980	0.015803	0.041146	0.031189	XPO1	Exportin-1	XPO1_HUM	44	1.011014
48H 10MT/	Q6ZSR9	0.062689	0.041186	0.031212	NaN	Uncharacte	YJ005_HUM	8	1.044411
48H 50MT/	O75928	0.146671	0.0412	0.031219	PIAS2	E3 SUMO-p	PIAS2_HUM	4	1.107012
48H 50MT/	Q9UN36	0.02087	0.041199	0.031219	NDRG2	Protein ND	NDRG2_HU	8	1.014571
48H 50MT/	P22681	0.094645	0.041212	0.031227	CBL	E3 ubiquiti	CBL_HUMA	15	1.067803
24H 50MT/	P18669	0.03001	0.041215	0.031227	PGAM1	Phosphogly	PGAM1_HU	20	1.021019
48H 10MT/	Q99536	0.004339	0.041214	0.031227	VAT1	Synaptic ve	VAT1_HUM	16	1.003012
24H 50MT/	Q6P4A8	0.13341	0.041244	0.031246	PLBD1	Phospholip	PLBL1_HUM	10	1.096883
24H 50MT/	Q9NYH9	0.132402	0.041253	0.031251	UTP6	U3 small nu	UTP6_HUM	22	1.096117
24H 10MT/	Q8IYT4	0.028641	0.04127	0.031261	KATNAL2	Katanin p60	KATL2_HUM	1	1.020051
24H 50MT/	P17029	0.322465	0.041325	0.031293	ZKSCAN1	Zinc finger	ZKSC1_HUM	4	1.250466
48H 10MT/	Q8WVI0	-0.12719	0.041333	0.031298	UQCC5	Ubiquinol-c	UQCC5_HU	1	0.915613
24H 50MT/	Q16352	-0.03484	0.041337	0.031299	INA	Alpha-inter	AINX_HUM	15	0.976141
24H 50MT/	P06748	0.088001	0.041351	0.031308	NPM1	Nucleopho	NPM_HUM	13	1.062896
48H 10MT/	Q8TBZ3	-0.11743	0.041377	0.03132	WDR20	WD repeat-	WDR20_HU	3	0.921827
48H 50MT/	Q9UPN4	-0.04614	0.041378	0.03132	CEP131	Centrosom	CP131_HU	14	0.968523
24H 10MT/	Q6ZSJ8	-0.29254	0.041379	0.03132	C1orf122	Uncharacte	CA122_HU	4	0.816463
48H 50MT/	Q8TEK3	-3.42364	0.041389	0.031326	DOT1L	Histone-lys	DOT1L_HU	2	0.093193
48H 10MT/	P13667	0.007709	0.041398	0.031329	PDIA4	Protein dis	PDIA4_HUM	41	1.005357
24H 10MT/	P00734	0.043595	0.041423	0.031343	F2	Prothromb	THRB_HUM	26	1.030679
24H 50MT/	Q86VQ1	-0.49411	0.041434	0.03135	GLCCI1	Glucocorti	GLCI1_HUM	2	0.710001
24H 50MT/	P27986	0.449024	0.041461	0.031365	PIK3R1	Phosphatid	P85A_HUM	2	1.365116
48H 10MT/	Q9HCM4	0.05109	0.041487	0.03138	EPB41L5	Band 4.1-lil	E41L5_HUM	12	1.036048
24H 10MT/	Q16531	0.068906	0.041491	0.031383	DDB1	DNA damag	DDB1_HUM	57	1.048921
24H 50MT/	P82663	0.095931	0.041512	0.031396	MRPS25	Small ribos	RT25_HUM	10	1.068755
48H 50MT/	Q9Y4G6	0.048739	0.04154	0.031413	TLN2	Talin-2	TLN2_HUM	64	1.034361
24H 50MT/	Q9UDY2	-0.02693	0.041542	0.031414	TJP2	Tight juncti	ZO2_HUMA	35	0.98151
48H 50MT/	Q68EM7	-0.03516	0.041558	0.031424	ARHGAP17	Rho GTPase	RHG17_HU	16	0.975924
48H 50MT/	Q15545	-0.63041	0.041576	0.031431	TAF7	Transcripti	TAF7_HUM	4	0.645992
48H 10MT/	Q9NWV4	-0.03323	0.041576	0.031431	CZIB	CXXC motif	CZIB_HUM	5	0.97723
48H 50MT/	O75937	0.016579	0.041595	0.031442	DNAJC8	DnaJ homo	DNJC8_HU	12	1.011558
24H 10MT/	Q8NE86	-0.17024	0.041594	0.031442	MCU	Calcium un	MCU_HUM	10	0.888693
48H 10MT/	Q8TEW0	0.009512	0.04161	0.031449	PARD3	Partitionin	PARD3_HU	26	1.006615
24H 50MT/	P27708	-0.05148	0.04162	0.031456	CAD	Multifuncti	PYR1_HUM	79	0.964944
24H 10MT/	Q96SQ9	0.063018	0.041625	0.031458	CYP2S1	Cytochrom	CP2S1_HU	10	1.044649
48H 10MT/	Q93063	-0.07297	0.041645	0.031471	EXT2	Exostosin-2	EXT2_HUM	10	0.950677

48H 10MT/	Q32P28	-0.01135	0.041662	0.031482	P3H1	Prolyl 3-hy	P3H1_HUN	19	0.992164
24H 10MT/	Q13061	-0.53931	0.041679	0.031487	TRDN	Triadin	TRDN_HUN	1	0.688102
24H 10MT/	Q9HCM9	-0.70122	0.041681	0.031487	TRIM39	E3 ubiquiti	TRI39_HUN	1	0.61505
24H 10MT/	P06493	-0.00845	0.041686	0.031489	CDK1	Cyclin-depe	CDK1_HUN	17	0.994157
24H 10MT/	P61106	0.023066	0.041718	0.031508	RAB14	Ras-related	RAB14_HUI	13	1.016117
48H 10MT/	Q8TBC4	0.068108	0.041718	0.031508	UBA3	NEDD8-acti	UBA3_HUN	19	1.048341
24H 50MT/	Q8WYP5	0.072207	0.041732	0.031515	AHCTF1	Protein ELY	ELYS_HUM/	52	1.051324
48H 50MT/	O75362	-0.1469	0.041738	0.031517	ZNF217	Zinc finger	ZN217_HUI	8	0.903191
48H 50MT/	P16885	0.043978	0.041753	0.031525	PLCG2	1-phosphat	PLCG2_HUI	5	1.030953
48H 10MT/	Q6NXE6	0.069834	0.041764	0.031533	ARMC6	Armadillo r	ARMC6_HU	14	1.049596
48H 50MT/	P49368	0.047298	0.041786	0.031542	CCT3	T-complex	TCPG_HUN	39	1.033328
24H 50MT/	Q15785	-0.12776	0.041786	0.031542	TOMM34	Mitochond	TOM34_HU	16	0.915251
24H 50MT/	Q9NZ32	-0.01738	0.041787	0.031542	ACTR10	Actin-relate	ARP10_HUI	18	0.988024
24H 50MT/	Q5VW38	0.162573	0.041805	0.031552	GPR107	Protein GPI	GP107_HU	6	1.119281
24H 50MT/	P61204	0.024785	0.041807	0.031554	ARF3	ADP-ribosy	ARF3_HUM	12	1.017328
48H 50MT/	Q99961	0.070006	0.041816	0.031559	SH3GL1	Endophilin	SH3G1_HU	12	1.049721
24H 10MT/	Q9BRR6	0.042782	0.041816	0.031559	ADPGK	ADP-depen	ADPGK_HU	11	1.030098
24H 50MT/	Q6IPR3	-0.02708	0.041827	0.031563	TYW3	tRNA wybu	TYW3_HUN	10	0.981404
24H 50MT/	Q6NZY4	0.232549	0.041884	0.031594	ZCCHC8	Zinc finger	ZCHC8_HUI	19	1.174909
24H 10MT/	Q96DF8	0.107664	0.041913	0.031608	ESS2	Splicing fac	ESS2_HUM/	7	1.077482
48H 50MT/	Q02833	-0.65028	0.041951	0.031626	RASSF7	Ras associa	RASF7_HUN	3	0.637157
24H 50MT/	Q9BZ67	-0.28054	0.041953	0.031628	FRMD8	FERM doma	FRMD8_HU	9	0.823283
24H 10MT/	P02795	0.590414	0.041964	0.031633	MT2A	Metallothi	MT2_HUM/	1	1.505679
48H 50MT/	Q5T5Y3	0.124464	0.041971	0.031635	CAMSAP1	Calmodulir	CAMP1_HU	4	1.090103
24H 10MT/	Q14019	0.089355	0.04198	0.031639	COTL1	Coactosin-l	COTL1_HUI	8	1.063894
48H 10MT/	Q9HAU5	-0.08143	0.041985	0.03164	UPF2	Regulator c	RENT2_HUI	24	0.945121
48H 10MT/	Q14BN4	0.029901	0.041995	0.031645	SLMAP	Sarcolemm	SLMAP_HU	9	1.020942
24H 10MT/	Q8N0X7	-0.49586	0.041997	0.031646	SPART	Spartin	SPART_HUN	3	0.709137
24H 10MT/	O14929	0.072605	0.042002	0.031649	HAT1	Histone ace	HAT1_HUM	13	1.051614
48H 10MT/	O14737	-0.01664	0.042008	0.031652	PDCD5	Programme	PDCD5_HU	6	0.988531
24H 50MT/	P36543	-0.16174	0.042017	0.031655	ATP6V1E1	V-type prot	VATE1_HUN	12	0.893945
24H 50MT/	Q9BXJ1	0.311836	0.04202	0.031656	C1QTNF1	Compleme	C1QT1_HUI	1	1.241287
24H 10MT/	Q8IUE6	0.032174	0.042032	0.031665	H2AC21	Histone H2	H2A2B_HU	3	1.022552
24H 10MT/	Q86W50	0.274277	0.04204	0.03167	METTL16	RNA N6-ad	MET16_HU	11	1.209388
24H 10MT/	Q70CQ3	-1.4631	0.04204	0.03167	USP30	Ubiquitin c	UBP30_HU	1	0.362713
24H 10MT/	Q96CP2	0.052548	0.042052	0.031676	FLYWCH2	FLYWCH fa	FWCH2_HU	6	1.037095
48H 50MT/	P17174	-0.09131	0.042077	0.031693	GOT1	Aspartate a	AATC_HUM	23	0.938667
24H 50MT/	Q5T2W1	0.039589	0.042094	0.0317	PDZK1	Na(+)/H(+) e	NHRF3_HU	26	1.027821
48H 50MT/	P11233	-0.13547	0.042097	0.031701	RALA	Ras-related	RALA_HUM	4	0.910372
48H 50MT/	Q96DE0	-0.14092	0.042121	0.031715	NUDT16	U8 snoRNA	NUD16_HU	13	0.906939
24H 50MT/	Q9H330	0.130567	0.042129	0.031719	TMEM245	Transmemb	TM245_HU	6	1.094724
48H 50MT/	Q96RU2	-0.10063	0.04213	0.03172	USP28	Ubiquitin c	UBP28_HU	20	0.932627
48H 10MT/	Q9HCS7	-0.02824	0.042141	0.031726	XAB2	Pre-mRNA-	SYF1_HUM/	30	0.980614
24H 50MT/	Q9H582	-0.25451	0.042147	0.031729	ZNF644	Zinc finger	ZN644_HUI	2	0.838273
24H 50MT/	P34913	-0.1983	0.042151	0.031732	EPHX2	Bifunctiona	HYES_HUM	14	0.871578
48H 10MT/	Q08357	-0.02144	0.04217	0.031743	SLC20A2	Sodium-de	S20A2_HUI	6	0.985245
48H 50MT/	Q06587	0.067764	0.042179	0.031748	RING1	E3 ubiquiti	RING1_HUI	8	1.048091
24H 10MT/	P50395	-0.07148	0.04218	0.031748	GDI2	Rab GDP di	GDIB_HUM	36	0.951663
24H 50MT/	P29317	-0.47305	0.042188	0.031753	EPHA2	Ephrin type	EPHA2_HUI	17	0.720442
24H 50MT/	O75083	0.049149	0.042189	0.031753	WDR1	WD repeat-	WDR1_HUI	28	1.034655
48H 10MT/	Q13838	0.034094	0.042195	0.031756	DDX39B	Spliceosom	DX39B_HU	22	1.023914
48H 10MT/	P48739	0.051705	0.042198	0.031758	PITPNB	Phosphatic	PIPNB_HUN	21	1.036489

48H 50MTA	Q15800	0.146272	0.0422	0.031758	MSMO1	Methylstero	MSMO1_HU	4	1.106706
48H 10MTA	O95825	0.072946	0.042213	0.031765	CRYZL1	Quinone ox	QORL1_HU	12	1.051863
48H 10MTA	P49914	0.140363	0.042218	0.031767	MTHFS	5-formyltet	MTHFS_HU	8	1.102183
24H 10MTA	Q8WXI9	0.013086	0.042218	0.031767	GATAD2B	Transcripti	P66B_HUM	12	1.009111
24H 10MTA	P16220	0.010559	0.042229	0.031774	CREB1	Cyclic AMP	CREB1_HU	3	1.007346
48H 50MTA	O00255	-0.20435	0.042243	0.031783	MEN1	Menin	MEN1_HUM	10	0.867928
24H 10MTA	O00469	0.09729	0.042252	0.031785	PLOD2	Procollager	PLOD2_HU	17	1.069762
24H 50MTA	Q96IF1	-0.17047	0.04225	0.031785	AJUBA	LIM domair	AJUBA_HU	16	0.888555
48H 50MTA	Q9NPF4	0.082053	0.042253	0.031785	OSGEP	tRNA N6-ad	OSGEP_HU	14	1.058524
48H 50MTA	Q9BRF8	-0.03152	0.042261	0.031789	CPPED1	Serine/thre	CPPED_HU	12	0.978388
24H 50MTA	Q5PRF9	0.052105	0.042266	0.031792	SAMD4B	Protein Sm	SMAG2_HU	3	1.036777
48H 10MTA	Q8N6G6	0.417763	0.042271	0.031795	ADAMTSL1	ADAMTS-lik	ATL1_HUM	2	1.335854
48H 50MTA	Q8TF74	-0.22773	0.042279	0.031798	WIPF2	WAS/WASL	WIPF2_HU	11	0.853977
24H 50MTA	Q9UK73	0.153456	0.042305	0.031813	FEM1B	Protein fem	FEM1B_HU	3	1.112231
48H 50MTA	Q86VR2	-0.19105	0.042307	0.031814	RETREG3	Reticuloph	RETR3_HUM	5	0.875969
48H 10MTA	Q9Y399	-0.13745	0.042327	0.031824	MRPS2	Small ribos	RT02_HUM	10	0.909127
24H 10MTA	Q9BUP3	0.037816	0.042334	0.031829	HTATIP2	Oxidoreduc	HTAI2_HUM	16	1.026559
24H 10MTA	Q12913	0.120297	0.042343	0.031833	PTPRJ	Receptor-ty	PTPRJ_HUM	7	1.086958
48H 50MTA	Q9UBV8	0.089122	0.042343	0.031833	PEF1	Peflin	PEF1_HUM	5	1.063723
48H 10MTA	O43657	-0.22037	0.042368	0.031849	TSPAN6	Tetraspanir	TSN6_HUM	5	0.858346
24H 10MTA	Q92536	-0.17379	0.042369	0.031849	SLC7A6	Y+L amino a	YLAT2_HUM	3	0.886512
24H 50MTA	Q9Y5K5	0.022448	0.042379	0.031855	UCHL5	Ubiquitin c	UCHL5_HU	16	1.015682
24H 50MTA	P04150	-0.1379	0.042417	0.031878	NR3C1	Glucocortic	GCR_HUMA	16	0.908843
48H 10MTA	Q00341	0.001376	0.042441	0.031893	HDLBP	Vigilin	VIGLN_HUM	61	1.000954
48H 50MTA	Q9NQC8	1.012292	0.042451	0.031898	IFT46	Intraflagell	IFT46_HUM	1	2.017113
48H 50MTA	P48634	-0.14697	0.042453	0.031898	PRRC2A	Protein PRI	PRC2A_HU	44	0.903144
48H 10MTA	Q13153	0.06997	0.04246	0.031902	PAK1	Serine/thre	PAK1_HUM	13	1.049695
24H 50MTA	O60701	-0.0074	0.042464	0.031904	UGDH	UDP-glucos	UGDH_HUM	30	0.99488
24H 10MTA	Q92820	0.074791	0.042474	0.031909	GGH	Gamma-glu	GGH_HUMA	11	1.053208
48H 50MTA	P14550	0.054903	0.042479	0.03191	AKR1A1	Aldo-keto r	AK1A1_HU	19	1.038789
24H 10MTA	P48735	-0.03779	0.042495	0.031918	IDH2	Isocitrate d	IDHP_HUM	28	0.974148
24H 50MTA	P34897	0.026087	0.04251	0.031926	SHMT2	Serine hydr	GLYM_HUM	24	1.018247
48H 10MTA	Q69YN2	0.024306	0.042509	0.031926	CWF19L1	CWF19-like	C19L1_HU	25	1.01699
48H 50MTA	Q9Y617	-0.00299	0.042509	0.031926	PSAT1	Phosphoser	SERC_HUM	20	0.997933
24H 10MTA	O15027	-0.10749	0.042523	0.031933	SEC16A	Protein trai	SC16A_HU	45	0.928199
24H 50MTA	Q02447	0.35059	0.042532	0.03194	SP3	Transcripti	SP3_HUMA	6	1.275082
24H 50MTA	Q16842	-1.20875	0.042539	0.031941	ST3GAL2	CMP-N-acet	SIA4B_HUM	1	0.432644
48H 10MTA	Q86TB9	0.026139	0.042539	0.031941	PATL1	Protein PAT	PATL1_HUM	20	1.018283
24H 50MTA	Q9UKY1	0.041691	0.042541	0.031941	ZHX1	Zinc fingers	ZHX1_HUM	1	1.02932
24H 10MTA	A6NDG6	-0.0983	0.042553	0.031947	PGP	Glycerol-3-	PGP_HUMA	14	0.934132
24H 50MTA	Q96H55	-0.13562	0.042575	0.03196	MYO19	Unconvent	MYO19_HU	6	0.910279
48H 50MTA	Q6P1M3	-0.00666	0.0426	0.031975	LLGL2	LLGL scribb	L2GL2_HU	10	0.995393
24H 50MTA	P19022	0.130526	0.04262	0.031987	CDH2	Cadherin-2	CADH2_HU	15	1.094693
48H 50MTA	Q58FG1	-0.53485	0.042622	0.031987	HSP90AA4I	Putative he	HS904_HU	4	0.690231
24H 50MTA	Q8N1G0	0.283882	0.042629	0.03199	ZNF687	Zinc finger	ZN687_HU	11	1.217466
48H 10MTA	Q15723	1.393962	0.042655	0.032006	ELF2	ETS-related	ELF2_HUMA	1	2.627994
48H 10MTA	Q12929	-0.0003	0.042679	0.03202	EPS8	Epidermal g	EPS8_HUM	32	0.999789
48H 50MTA	Q9BT40	-0.17005	0.042693	0.032027	INPP5K	Inositol pol	INP5K_HUM	2	0.888813
48H 10MTA	P00390	0.061008	0.042697	0.032027	GSR	Glutathion	GSHR_HUM	21	1.043194
24H 50MTA	Q9Y5V3	0.092307	0.042698	0.032027	MAGED1	Melanoma-	MAGD1_HU	10	1.066073
48H 10MTA	Q9BX95	-0.02763	0.042711	0.032034	SGPP1	Sphingosin	SGPP1_HU	1	0.98103
48H 10MTA	Q15652	-0.30211	0.042717	0.032034	JMJD1C	Probable Jr	JHD2C_HU	4	0.811066

48H 50MT/	Q5JUR7	-0.20382	0.042729	0.032041	TEX30	Testis-expre	TEX30_HUI	7	0.86825
48H 50MT/	Q7Z569	0.131066	0.042735	0.032044	BRAP	BRCA1-assc	BRAP_HUM	9	1.095103
48H 10MT/	Q9NX76	-0.32097	0.042743	0.032047	CMTM6	CKLF-like M	CKLF6_HUI	2	0.800531
24H 10MT/	P61163	0.050796	0.042749	0.032049	ACTR1A	Alpha-centr	ACTZ_HUM	10	1.035836
24H 50MT/	Q9HC36	-0.03783	0.04276	0.032055	MRM3	rRNA methy	MRM3_HUI	8	0.97412
48H 10MT/	Q9GZT9	0.108552	0.042767	0.03206	EGLN1	Egl nine hoi	EGLN1_HUI	11	1.078145
24H 50MT/	Q9BRT3	-0.11913	0.042778	0.032066	MIEN1	Migration a	MIEN1_HUI	1	0.920742
48H 10MT/	Q7Z4G1	0.555727	0.042786	0.032069	COMMD6	COMM don	COMD6_HU	1	1.469909
24H 50MT/	Q86X27	0.045868	0.042788	0.03207	RALGPS2	Ras-specific	RGPS2_HUI	7	1.032304
48H 50MT/	Q6ZMG9	0.10743	0.042795	0.032071	CERS6	Ceramide s	CERS6_HUI	6	1.077307
48H 10MT/	Q96BY9	-0.6353	0.04281	0.032078	SARAF	Store-opera	SARAF_HUI	1	0.643807
24H 50MT/	Q9NW15	0.036076	0.042824	0.032088	ANO10	Anoctamin	ANO10_HU	9	1.025322
24H 10MT/	P16401	-0.21979	0.042828	0.03209	H1-5	Histone H1	H15_HUM/	8	0.858688
48H 50MT/	Q9BT17	-0.3372	0.04283	0.032092	MTG1	Mitochond	MTG1_HUI	5	0.791578
48H 50MT/	Q9BQ24	0.275732	0.042836	0.032095	ZFYVE21	Zinc finger	ZFY21_HUI	1	1.210608
48H 10MT/	Q8IWW6	0.07763	0.04284	0.032098	ARHGAP12	Rho GTPase	RHG12_HU	13	1.055283
48H 50MT/	P08910	-0.96697	0.042877	0.032119	ABHD2	Monoacylg	ABHD2_HU	1	0.51158
24H 50MT/	O95140	-0.20143	0.042884	0.032123	MFN2	Mitofusin-2	MFN2_HUI	22	0.869689
24H 10MT/	P60981	0.081848	0.042886	0.032124	DSTN	Destrin	DEST_HUM	11	1.058373
24H 50MT/	O43707	-0.0443	0.042907	0.032134	ACTN4	Alpha-actir	ACTN4_HU	40	0.96976
24H 10MT/	Q96MV1	0.087879	0.042926	0.032146	TLCD4	TLC domair	TLCD4_HUI	4	1.062807
48H 10MT/	Q9NY12	-0.07092	0.042958	0.032166	GAR1	H/ACA ribo	GAR1_HUI	8	0.952028
48H 50MT/	O60934	-0.01014	0.042981	0.032177	NBN	Nibrin	NBN_HUM/	22	0.992998
24H 50MT/	Q96BP2	-0.02528	0.042984	0.032178	CHCHD1	Small ribos	CHCH1_HU	1	0.982627
48H 50MT/	O00559	0.743561	0.042995	0.032183	EBAG9	Receptor-b	RCAS1_HUI	2	1.674304
48H 10MT/	P63172	-0.07627	0.043001	0.032186	DYNLT1	Dynein ligh	DYLT1_HUI	5	0.948509
48H 50MT/	O00115	0.234875	0.043005	0.032187	DNASE2	Deoxyribor	DNS2A_HU	5	1.176805
48H 50MT/	P00390	0.04189	0.043007	0.032188	GSR	Glutathion	GSHR_HUM	21	1.029461
24H 10MT/	Q8N983	-0.03645	0.043021	0.032196	MRPL43	Large ribos	RM43_HUM	5	0.975054
24H 50MT/	Q5TAQ9	0.073099	0.043025	0.032199	DCAF8	DDB1- and	DCAF8_HUI	10	1.051974
48H 50MT/	P04439	0.321522	0.043028	0.0322	HLA-A	HLA class I	HLAA_HUM	2	1.249648
48H 10MT/	P18846	1.140419	0.043036	0.032204	ATF1	Cyclic AMP	ATF1_HUM	1	2.20445
24H 10MT/	O43913	-0.00429	0.043066	0.032224	ORC5	Origin reco	ORC5_HUM	12	0.997028
24H 50MT/	Q9Y3D3	-0.12732	0.043069	0.032225	MRPS16	Small ribos	RT16_HUM	4	0.915528
24H 50MT/	Q9Y597	0.447223	0.043076	0.032229	KCTD3	BTB/POZ dc	KCTD3_HUI	4	1.363413
24H 50MT/	P00883	0.456592	0.043097	0.032242	ALDOA	Fructose-bi	ALDOA_RAI	2	1.372296
24H 50MT/	Q8TCT7	-0.27585	0.043143	0.032269	SPPL2B	Signal pept	SPP2B_HUI	1	0.825961
48H 50MT/	Q9H4G0	0.460563	0.043149	0.032273	EPB41L1	Band 4.1-lil	E41L1_HUI	4	1.376078
24H 50MT/	Q8WUH6	0.51502	0.04316	0.03228	TMEM263	Transmemb	TM263_HU	4	1.429014
24H 50MT/	Q4KMQ1	-0.32392	0.043163	0.032281	TPRN	Taperin	TPRN_HUM	11	0.798896
48H 50MT/	Q96CU9	0.103823	0.043184	0.032294	FOXRED1	FAD-depen	FXRD1_HUI	12	1.074617
24H 10MT/	O14745	-0.18798	0.043193	0.032296	NHERF1	Na(+)/H(+) e	NHRF1_HU	23	0.877832
24H 50MT/	Q9UK41	-0.10622	0.0432	0.0323	VPS28	Vacuolar pi	VPS28_HUI	11	0.929022
48H 50MT/	P57721	0.655077	0.043209	0.032302	PCBP3	Poly(rC)-bir	PCBP3_HUI	1	1.5747
24H 50MT/	P81877;Q9	0.754307	0.04321	0.032303	SSBP2;SSBF	Single-strar	SSBP2_HUI	1	1.686821
24H 10MT/	P49903	0.061675	0.043213	0.032303	SEPHS1	Selenide, w	SPS1_HUM	11	1.043677
48H 50MT/	Q13347	-0.15771	0.043212	0.032303	EIF3I	Eukaryotic	EIF3I_HUM	19	0.896447
24H 10MT/	Q9H490	0.017848	0.043233	0.032315	PIGU	Phosphatid	PIGU_HUM	6	1.012448
24H 50MT/	Q7Z412	0.474938	0.043262	0.03233	PEX26	Peroxisome	PEX26_HUI	4	1.389859
48H 50MT/	Q6NZ67	-0.13176	0.043263	0.03233	MZT2B	Mitotic-spi	MZT2B_HU	6	0.912714
24H 10MT/	Q9BVK6	0.052436	0.043271	0.032332	TMED9	Transmemb	TMED9_HU	10	1.037015
24H 10MT/	Q15435	-0.0876	0.04328	0.032337	PPP1R7	Protein pho	PP1R7_HUI	18	0.941088

48H 10MT/	Q96S38	0.045028	0.043298	0.032349	RPS6KC1	Ribosomal	KS6C1_HUI	4	1.031703
48H 50MT/	Q96S52	0.051101	0.043299	0.032349	PIGS	GPI transan	PIGS_HUM/	16	1.036055
24H 10MT/	O95716	0.106733	0.043304	0.032352	RAB3D	Ras-related	RAB3D_HU	8	1.076787
48H 10MT/	P28676	0.203906	0.043306	0.032353	GCA	Grancalcin	GRAN_HUM	7	1.151813
48H 10MT/	P68400	-0.10594	0.043317	0.03236	CSNK2A1	Casein kina	CSK21_HUI	18	0.9292
48H 50MT/	Q9UKX7	-0.10287	0.043324	0.032364	NUP50	Nuclear po	NUP50_HU	18	0.931176
48H 10MT/	Q9NVS2	-0.1388	0.043334	0.03237	MRPS18A	Large ribos	RT18A_HUI	7	0.908276
24H 50MT/	P59998	-0.05691	0.043347	0.032378	ARPC4	Actin-relate	ARPC4_HU	8	0.961318
24H 50MT/	Q9P275	-0.0743	0.043378	0.032398	USP36	Ubiquitin c	UBP36_HU	7	0.949801
48H 10MT/	Q13601	-0.18998	0.043381	0.0324	KRR1	KRR1 small	KRR1_HUM	13	0.876615
24H 10MT/	O00400	-0.15814	0.043383	0.032401	SLC33A1	Acetyl-coer	ACATN_HUI	12	0.89618
48H 10MT/	Q86U44	-0.00862	0.043396	0.03241	METTL3	N6-adenosi	MTA70_HU	15	0.994041
24H 50MT/	Q9NRM6	-0.52439	0.043398	0.032411	IL17RB	Interleukin	I17RB_HUM	2	0.695253
48H 50MT/	Q9UBB4	0.081879	0.043421	0.032422	ATXN10	Ataxin-10	ATX10_HUI	22	1.058395
24H 50MT/	P46934	-0.05987	0.043439	0.032433	NEDD4	E3 ubiquiti	NEDD4_HU	9	0.959351
48H 50MT/	Q5MNZ9	0.27294	0.043444	0.032436	WIPI1	WD repeat	WIPI1_HUM	2	1.208268
48H 10MT/	Q7Z5W3	0.129517	0.043449	0.032438	BCDIN3D	RNA 5'-mor	BN3D2_HU	1	1.093928
48H 50MT/	O75665	-0.16679	0.043452	0.032439	OFD1	Centriole a	OFD1_HUM	11	0.890821
24H 50MT/	Q96FF7	-1.20811	0.043458	0.032442	MISP3	Uncharacte	MISP3_HUI	1	0.432834
48H 10MT/	O60293	0.015007	0.043483	0.032452	ZFC3H1	Zinc finger	ZC3H1_HU	20	1.010456
48H 50MT/	Q15154	0.014719	0.043483	0.032452	PCM1	Pericentrio	PCM1_HUM	73	1.010255
48H 50MT/	Q8TB36	0.212314	0.043481	0.032452	GDAP1	Ganglioside	GDAP1_HU	11	1.158545
48H 50MT/	Q96IZ5	-0.3628	0.043482	0.032452	RBM41	RNA-bindin	RBM41_HU	2	0.777652
48H 10MT/	Q9HCL2	-0.61658	0.043497	0.032458	GPAM	Glycerol-3-	GPAT1_HUI	4	0.652213
48H 10MT/	Q9Y6R4	-0.00569	0.043497	0.032458	MAP3K4	Mitogen-ac	M3K4_HUM	19	0.996061
48H 50MT/	Q8TAG9	0.121044	0.043504	0.032462	EXOC6	Exocyst cor	EXOC6_HU	21	1.087522
24H 10MT/	Q8IYB7	-0.02209	0.043522	0.032472	DIS3L2	DIS3-like ex	DI3L2_HUM	34	0.984808
24H 50MT/	Q9H9S5	0.203695	0.043528	0.032476	FKRP	Ribitol 5-pł	FKRP_HUM	5	1.151644
48H 50MT/	Q15003	-0.08895	0.043544	0.032485	NCAPH	Condensin	CND2_HUM	21	0.940205
48H 10MT/	P12956	0.017517	0.04356	0.032492	XRCC6	X-ray repair	XRCC6_HUI	43	1.012216
48H 50MT/	Q8IWL3	-0.22907	0.043569	0.032496	HSCB	Iron-sulfur	HSC20_HUI	8	0.853184
24H 50MT/	P07711	0.176807	0.043578	0.032499	CTSL	Procatheps	CATL1_HUM	8	1.130379
48H 50MT/	P49716	0.421976	0.043586	0.032503	CEBPD	CCAAT/enh	CEBPD_HUI	3	1.339761
24H 50MT/	O94822	0.073963	0.04359	0.032505	LTN1	E3 ubiquiti	LTN1_HUM	40	1.052604
24H 50MT/	O75503	0.241676	0.043604	0.032513	CLN5	Bis(monoac	CLN5_HUM	7	1.182365
48H 10MT/	Q12904	0.037834	0.043645	0.032535	AIMP1	Aminoacyl	AIMP1_HUI	18	1.026571
24H 10MT/	Q96FN4	0.323216	0.043653	0.032538	CPNE2	Copine-2	CPNE2_HUI	8	1.251117
48H 10MT/	O00469	0.141402	0.043677	0.032549	PLOD2	Procollager	PLOD2_HU	17	1.102977
48H 10MT/	Q9BVC5	-0.22104	0.043676	0.032549	C2orf49	Ashwin	ASHWN_HU	5	0.857947
24H 50MT/	P16298	0.489555	0.043684	0.032552	PPP3CB	Serine/thre	PP2BB_HUI	4	1.404012
48H 10MT/	O43325	-0.8648	0.0437	0.032562	LYRM1	LYR motif-c	LYRM1_HU	1	0.549122
48H 10MT/	P04406	-0.11579	0.043719	0.032573	GAPDH	Glyceraldel	G3P_HUM/	23	0.922876
24H 10MT/	Q9BVK2	0.395737	0.04374	0.032586	ALG8	Dolichyl py	ALG8_HUM	4	1.315615
48H 50MT/	Q16204	0.108113	0.043751	0.032592	CCDC6	Coiled-coil	CCDC6_HU	17	1.077818
24H 50MT/	O14732	-0.1913	0.043755	0.032594	IMPA2	Inositol mc	IMPA2_HU	4	0.875815
48H 50MT/	P20073	-0.0011	0.043763	0.032598	ANXA7	Annexin A7	ANXA7_HU	19	0.999235
48H 10MT/	Q9HB90	0.12584	0.043769	0.032602	RRAGC	Ras-related	RRAGC_HU	11	1.091143
24H 50MT/	P18858	-0.24744	0.043777	0.032607	LIG1	DNA ligase	DNLI1_HUM	11	0.842392
24H 10MT/	Q9NPA8	-0.33159	0.043784	0.032611	ENY2	Transcripti	ENY2_HUM	1	0.794659
24H 10MT/	P54105	0.12042	0.043822	0.032634	CLNS1A	Methylosor	ICLN_HUM/	8	1.087051
24H 10MT/	P49716	-0.62848	0.043826	0.032636	CEBPD	CCAAT/enh	CEBPD_HUI	3	0.646857
24H 50MT/	Q9NZJ6	0.086759	0.043827	0.032637	COQ3	Ubiquinone	COQ3_HUM	4	1.061982

24H 50MT/	Q13242	0.013676	0.043831	0.032639	SRSF9	Serine/argi	SRSF9_HUN	14	1.009524
48H 50MT/	P49590	0.032277	0.043832	0.032639	HARS2	Histidine--t	SYHM_HUN	15	1.022625
24H 50MT/	Q5VWZ2	-0.17969	0.043866	0.032657	LYPLAL1	Lysophospl	LYPL1_HUN	6	0.882892
48H 10MT/	Q92576	0.088022	0.043867	0.032657	PHF3	PHD finger	PHF3_HUM	36	1.062912
24H 50MT/	P00395	0.375036	0.043894	0.032674	MT-CO1	Cytochrom	COX1_HUM	4	1.296872
48H 10MT/	Q9Y333	0.140475	0.043899	0.032678	LSM2	U6 snRNA-a	LSM2_HUM	6	1.102268
48H 50MT/	Q9UIJ7	0.075921	0.04391	0.032682	AK3	GTP:AMP p	KAD3_HUM	7	1.054034
48H 10MT/	Q16644	-0.46524	0.043915	0.032684	MAPKAPK3	MAP kinase	MAPK3_HU	3	0.724349
48H 50MT/	Q7L1T6	0.162515	0.043922	0.032688	CYB5R4	Cytochrom	NB5R4_HU	5	1.119237
48H 10MT/	Q7LG56	0.000107	0.043922	0.032688	RRM2B	Ribonuclec	RIR2B_HUN	7	1.000074
24H 10MT/	Q6YHU6	0.023301	0.04394	0.032698	THADA	tRNA (32-2'	THADA_HU	36	1.016282
24H 50MT/	P61221	0.046604	0.043958	0.032708	ABCE1	ATP-bindin	ABCE1_HUI	28	1.032831
48H 10MT/	Q8N6T3	0.057253	0.043959	0.032708	ARFGAP1	ADP-ribosy	ARFG1_HUI	15	1.040483
48H 10MT/	Q4U2R6	0.136936	0.043987	0.032724	MRPL51	Large ribos	RM51_HUN	2	1.099568
48H 10MT/	Q969V6	0.010852	0.044001	0.032733	MRTFA	Myocardin-	MRTFA_HU	8	1.007551
48H 10MT/	Q9NS91	-0.00632	0.044005	0.032734	RAD18	E3 ubiquiti	RAD18_HU	15	0.995627
24H 50MT/	Q96P48	-0.18316	0.044024	0.032746	ARAP1	Arf-GAP wit	ARAP1_HUI	16	0.880769
48H 10MT/	Q9HBF4	-0.51866	0.044024	0.032746	ZFYVE1	Zinc finger I	ZFYV1_HUN	4	0.69802
48H 10MT/	Q12772	-0.7067	0.044026	0.032747	SREBF2	Sterol regul	SRBP2_HUI	3	0.612719
48H 10MT/	Q15493	0.204605	0.044056	0.032767	RGN	Regucalcin	RGN_HUM/	7	1.152371
48H 50MT/	Q8WU90	0.002561	0.044056	0.032767	ZC3H15	Zinc finger (	ZC3HF_HUI	15	1.001776
24H 10MT/	O60488	-0.05768	0.04408	0.03278	ACSL4	Long-chain	ACSL4_HUN	33	0.960809
48H 50MT/	Q8NEW0	0.033786	0.04408	0.03278	SLC30A7	Zinc transp	ZNT7_HUM	8	1.023695
48H 50MT/	O94919	0.0384	0.044091	0.032784	ENDOD1	Endonuclea	ENDD1_HU	13	1.026974
24H 10MT/	Q9NUY8	-0.01049	0.0441	0.03279	TBC1D23	TBC1 doma	TBC23_HUI	13	0.992757
24H 50MT/	Q6ZN54	-0.29483	0.044115	0.032796	DEF8	Differential	DEFI8_HUN	2	0.815171
24H 50MT/	P31943	-0.12744	0.044144	0.032812	HNRNPH1	Heterogene	HNRH1_HU	18	0.915456
48H 50MT/	O94876	0.342368	0.044191	0.03284	TMCC1	Transmemb	TMCC1_HU	2	1.267836
24H 50MT/	Q9HB20	0.184509	0.044199	0.032845	PLEKHA3	Pleckstrin h	PKHA3_HU	5	1.13643
48H 50MT/	Q9HC98	-0.47809	0.044201	0.032845	NEK6	Serine/thre	NEK6_HUM	1	0.717926
24H 10MT/	Q66PJ3	-0.37789	0.044207	0.032849	ARL6IP4	ADP-ribosy	AR6P4_HUI	5	0.769562
48H 10MT/	Q9Y6I9	0.032576	0.044213	0.032853	TEX264	Testis-expre	TX264_HUI	10	1.022837
48H 10MT/	P53680	0.103878	0.044223	0.032859	AP2S1	AP-2 compl	AP2S1_HUI	7	1.074658
24H 10MT/	P14859	0.063137	0.04423	0.032861	POU2F1	POU domai	PO2F1_HUI	9	1.044735
24H 10MT/	Q9BZJ0	0.050333	0.04423	0.032861	CRNKL1	Crooked ne	CRNL1_HUI	24	1.035504
48H 50MT/	P51808	0.138074	0.044276	0.03289	DYNLT3	Dynein ligh	DYLT3_HUN	3	1.100435
48H 50MT/	Q9UHK0	-0.77192	0.044287	0.032897	NUFIP1	FMR1-inter	NUFP1_HU	3	0.585637
48H 50MT/	Q6DD87	-0.17058	0.04429	0.032899	ZNF787	Zinc finger	ZN787_HUI	9	0.888484
48H 50MT/	Q96S94	-0.8018	0.044296	0.032902	CCNL2	Cyclin-L2	CCNL2_HUI	1	0.573631
24H 10MT/	P12883	0.169247	0.0443	0.032903	MYH7	Myosin-7	MYH7_HUM	1	1.124471
48H 50MT/	Q9H920	1.520358	0.044309	0.032907	RNF121	E3 ubiquiti	RN121_HU	1	2.868623
24H 10MT/	Q9Y2Q9	-0.05043	0.044319	0.032911	MRPS28	Small ribos	RT28_HUM	6	0.965651
48H 10MT/	P59998	-0.09574	0.044322	0.032912	ARPC4	Actin-relate	ARPC4_HUI	8	0.935794
48H 50MT/	P51668;Q9	0.097527	0.044335	0.032919	UBE2D1;UE	Ubiquitin-c	UB2D1_HU	1	1.069938
48H 50MT/	Q5RKV6	-0.08986	0.04435	0.032928	EXOSC6	Exosome cc	EXOS6_HUI	8	0.939616
48H 50MT/	P07947	0.059654	0.044394	0.032954	YES1	Tyrosine-pr	YES_HUMAI	18	1.042216
48H 50MT/	Q96BY7	0.037343	0.044394	0.032954	ATG2B	Autophagy-	ATG2B_HUI	12	1.026222
48H 10MT/	P22102	0.011195	0.044412	0.032962	GART	Trifunction	PUR2_HUM	42	1.00779
48H 50MT/	Q9UHY7	0.070326	0.044412	0.032962	ENOPH1	Enolase-ph	ENOPH_HU	8	1.049954
24H 50MT/	Q9Y314	-0.18355	0.044409	0.032962	NOSIP	Nitric oxide	NOSIP_HUN	12	0.880536
48H 10MT/	Q14493	-0.04463	0.044417	0.032965	SLBP	Histone RN	SLBP_HUM	4	0.969538
48H 50MT/	Q9C0I1	-0.04673	0.044426	0.032968	MTMR12	Myotubula	MTMRC_HU	11	0.968131

48H 10MTA	Q6S8J3	-0.14352	0.044455	0.032982	POTEE	POTE ankyr	POTEE_HUI	16	0.905307
48H 50MTA	O14880	0.098108	0.04446	0.032984	MGST3	Glutathion	MGST3_HU	6	1.070369
24H 50MTA	Q9Y4C1	0.273134	0.044462	0.032986	KDM3A	Lysine-spec	KDM3A_HU	19	1.20843
48H 10MTA	O14662	0.014549	0.044469	0.03299	STX16	Syntaxin-16	STX16_HUI	8	1.010136
24H 10MTA	Q96GC9	0.150036	0.044474	0.032992	VMP1	Vacuole me	VMP1_HUI	6	1.109597
24H 10MTA	Q15022	0.028348	0.044499	0.033007	SUZ12	Polycomb p	SUZ12_HUI	9	1.019844
48H 10MTA	Q7L8J4	0.050183	0.044509	0.033013	SH3BP5L	SH3 domai	3BP5L_HUI	4	1.035397
48H 50MTA	Q9P0W0	0.469169	0.044513	0.033015	IFNK	Interferon k	IFNK_HUMA	1	1.384312
48H 10MTA	P62136	0.025571	0.044517	0.033017	PPP1CA	Serine/thre	PP1A_HUM	7	1.017882
48H 10MTA	O15397	-0.21	0.044526	0.033022	IPO8	Importin-8	IPO8_HUM	23	0.864536
24H 10MTA	Q9P0V9	0.019656	0.044526	0.033022	SEPTIN10	Septin-10	SEP10_HUI	15	1.013718
48H 50MTA	P00491	0.023139	0.044535	0.033026	PNP	Purine nucl	PNPH_HUM	14	1.016168
48H 50MTA	Q9H0G5	0.523856	0.044545	0.033031	NSRP1	Nuclear spe	NSRP1_HUI	4	1.437793
24H 50MTA	Q12974	0.011018	0.044545	0.033031	PTP4A2	Protein tyr	TP4A2_HUI	7	1.007666
48H 10MTA	Q969K3	0.221409	0.04455	0.033034	RNF34	E3 ubiquiti	RNF34_HUI	5	1.165872
48H 10MTA	P49189	0.035967	0.044561	0.033041	ALDH9A1	4-trimethyl	AL9A1_HUI	21	1.025244
24H 10MTA	P24390	0.501267	0.044564	0.033041	KDELR1	ER lumen p	ERD21_HUI	2	1.415456
48H 10MTA	Q86V81	0.010295	0.04457	0.033045	ALYREF	THO compl	THOC4_HU	10	1.007162
24H 10MTA	Q15067	-0.07377	0.044572	0.033046	ACOX1	Peroxisoma	ACOX1_HU	26	0.950154
48H 10MTA	Q9NUM4	-0.12053	0.044613	0.033071	TMEM106B	Transmemb	T106B_HUI	9	0.91985
48H 50MTA	Q01628	-0.29347	0.044628	0.033079	IFITM3	Interferon-i	IFM3_HUM	3	0.815935
24H 50MTA	P62316	-0.00358	0.044639	0.033086	SNRPD2	Small nucle	SMD2_HUI	8	0.997522
24H 50MTA	P54277	0.084935	0.044642	0.033087	PMS1	PMS1 prote	PMS1_HUM	16	1.06064
48H 50MTA	Q6ZRS2	-0.0896	0.044671	0.033106	SRCAP	Helicase SR	SRCAP_HUI	17	0.939781
48H 50MTA	O95785	-0.15002	0.044679	0.033111	WIZ	Protein Wi:	WIZ_HUMA	14	0.901239
24H 50MTA	Q5R372	-0.13085	0.044685	0.033114	RABGAP1L	Rab GTPase	RBG1L_HUI	7	0.913296
48H 50MTA	Q9NWT6	-0.07928	0.044721	0.033135	HIF1AN	Hypoxia-ind	HIF1N_HUI	10	0.946527
48H 50MTA	Q9P260	0.095437	0.044721	0.033135	RELCH	RAB11-bind	RELCH_HUI	31	1.068389
48H 50MTA	Q9Y6A1	-0.2341	0.044731	0.03314	POMT1	Protein O-n	POMT1_HU	4	0.850215
48H 10MTA	O15173	0.041747	0.044744	0.033147	PGRMC2	Membrane-	PGRC2_HU	11	1.029359
48H 50MTA	O43422	-0.35539	0.044745	0.033147	THAP12	52 kDa repr	P52K_HUM	9	0.781659
48H 10MTA	Q92844	-0.57128	0.044761	0.033157	TANK	TRAF family	TANK_HUM	2	0.673018
24H 10MTA	Q1KMD3	0.087355	0.044786	0.033171	HNRNPUL2	Heterogene	HNRL2_HU	27	1.062421
24H 50MTA	Q9Y580	0.106612	0.044787	0.033171	RBM7	RNA-bindir	RBM7_HUM	9	1.076696
24H 50MTA	P07148	0.153247	0.044819	0.033188	FABP1	Fatty acid-b	FABPL_HUI	7	1.112069
48H 10MTA	P38935	-0.12059	0.044824	0.03319	IGHMBP2	DNA-bindir	SMBP2_HU	8	0.919814
24H 50MTA	Q96C01	-0.01755	0.044832	0.033195	FAM136A	Protein FAI	F136A_HUI	5	0.987909
48H 50MTA	Q9Y508	0.152765	0.044859	0.033212	RNF114	E3 ubiquiti	RN114_HU	6	1.111698
48H 50MTA	Q9NWT8	-0.28749	0.044876	0.033224	AURKAIP1	Small ribos	AKIP_HUMA	2	0.819325
48H 50MTA	A0MZ66	0.002661	0.044902	0.033236	SHTN1	Shootin-1	SHOT1_HUI	28	1.001846
48H 10MTA	P13284	0.227252	0.044902	0.033236	IFI30	Gamma-int	GILT_HUMA	3	1.170603
48H 10MTA	P35232	-0.10188	0.044913	0.03324	PHB1	Prohibitin 1	PHB1_HUM	20	0.931815
48H 10MTA	Q5HYJ3	0.08151	0.044932	0.033253	FAM76B	Protein FAI	FA76B_HUI	3	1.058125
48H 50MTA	Q6ZMI0	-0.01275	0.044955	0.033265	PPP1R21	Protein pho	PPR21_HUI	9	0.9912
48H 10MTA	P28288	-0.12247	0.04497	0.033274	ABCD3	ATP-bindin	ABCD3_HU	33	0.91861
48H 10MTA	P50570	0.008252	0.044975	0.033277	DNM2	Dynamin-2	DYN2_HUM	43	1.005736
24H 10MTA	Q8N398	-0.50779	0.044978	0.033278	VWA5B2	von Willebr	VW5B2_HU	1	0.703298
24H 10MTA	Q9ULX3	0.030198	0.044987	0.033283	NOB1	RNA-bindir	NOB1_HUM	9	1.021152
24H 50MTA	Q12849	-0.00576	0.045002	0.033289	GRSF1	G-rich sequ	GRSF1_HUI	23	0.996017
48H 10MTA	Q16643	0.040933	0.045011	0.033294	DBN1	Drebrin	DREB_HUM	23	1.028779
24H 10MTA	Q53FA7	0.026726	0.045014	0.033295	TP53I3	Quinone ox	QORX_HUM	13	1.018698
48H 10MTA	Q9UGV2	0.00929	0.045015	0.033295	NDRG3	Protein ND	NDRG3_HU	12	1.00646

24H	10MT/	P09327	0.003841	0.045047	0.033315	VIL1	Villin-1	VILI_HUMA	23	1.002666
48H	10MT/	P50552	0.041964	0.045073	0.03333	VASP	Vasodilator	VASP_HUM	16	1.029515
24H	50MT/	Q9HBU6	-0.11567	0.045078	0.033331	ETNK1	Ethanolami	EKI1_HUMA	8	0.922953
24H	50MT/	P00740	-1.24864	0.045083	0.033332	F9	Coagulatio	FA9_HUMA	1	0.420844
24H	50MT/	Q9Y672	0.0755	0.045085	0.033332	ALG6	Dolichyl py	ALG6_HUM	7	1.053726
24H	10MT/	P53701	0.10991	0.045088	0.033333	HCCS	Holocytoch	CCHL_HUM	12	1.079161
24H	50MT/	O95214	0.216263	0.045103	0.033341	LEPROTL1	Leptin rece	LERL1_HUM	1	1.16172
48H	50MT/	Q13641	0.139776	0.045114	0.033348	TPBG	Trophoblas	TPBG_HUM	3	1.101734
48H	50MT/	P62249	-0.09583	0.045119	0.033351	RPS16	Small ribos	RS16_HUM	10	0.935732
48H	10MT/	Q8IYS2	-0.02855	0.04514	0.033363	KIAA2013	Uncharacte	K2013_HUI	13	0.980403
48H	50MT/	Q96AQ6	-0.01454	0.045144	0.033366	PBXIP1	Pre-B-cell le	PBIP1_HUM	19	0.989972
24H	10MT/	Q9BV57	-0.19218	0.045156	0.033373	ADI1	Acireducto	MTND_HUM	10	0.875284
48H	10MT/	Q9UK23	0.005384	0.04516	0.033375	NAGPA	N-acetylglu	NAGPA_HU	10	1.003739
24H	50MT/	O75569	0.027215	0.045165	0.033377	PRKRA	Interferon-i	PRKRA_HUI	12	1.019043
24H	10MT/	Q9UHJ6	0.115104	0.045166	0.033377	SHPK	Sedoheptul	SHPK_HUM	11	1.083053
48H	10MT/	O95415	0.079444	0.045174	0.033383	BRI3	Membrane	BRI3_HUM/	1	1.056611
24H	50MT/	Q15311	0.015724	0.045178	0.033384	RALBP1	RalA-bindir	RBP1_HUM	6	1.010959
48H	10MT/	Q8N9E0	1.777747	0.04518	0.033384	FAM133A	Protein FAI	F133A_HUI	2	3.428904
48H	50MT/	Q9H173	0.135639	0.045182	0.033385	SIL1	Nucleotide	SIL1_HUMA	9	1.098579
48H	50MT/	P00966	0.191701	0.045186	0.033386	ASS1	Argininosu	ASSY_HUM/	27	1.14211
48H	50MT/	P62714	-0.08533	0.045214	0.033404	PPP2CB	Serine/thre	PP2AB_HUI	12	0.942567
24H	10MT/	Q9BW27	0.033672	0.045224	0.033409	NUP85	Nuclear poi	NUP85_HU	23	1.023614
48H	50MT/	P15374	0.094781	0.04527	0.033434	UCHL3	Ubiquitin c	UCHL3_HU	11	1.067903
48H	10MT/	Q6ZRQ5	0.189476	0.045292	0.033447	MMS22L	Protein MI	MMS22_HU	10	1.14035
24H	10MT/	Q96KP4	-0.07931	0.045292	0.033447	CNDP2	Cytosolic n	CNDP2_HU	26	0.946512
48H	50MT/	Q13405	-0.26016	0.045294	0.033447	MRPL49	Large ribos	RM49_HUM	7	0.834994
24H	10MT/	Q8TAQ2	0.031008	0.045297	0.033449	SMARCC2	SWI/SNF cc	SMRC2_HU	20	1.021725
48H	10MT/	P61024	0.106721	0.045325	0.033464	CKS1B	Cyclin-depe	CKS1_HUM	3	1.076778
48H	10MT/	Q6AI12	0.1941	0.045325	0.033464	ANKRD40	Ankyrin rep	ANR40_HU	3	1.14401
48H	10MT/	Q5JPI3	-0.26493	0.04534	0.033472	C3orf38	Uncharacte	CC038_HUI	1	0.832241
48H	50MT/	Q9Y3D8	-0.308	0.045359	0.033482	AK6	Adenylate k	KAD6_HUM	10	0.807759
48H	10MT/	Q9NZT1	0.849784	0.045365	0.033484	CALML5	Calmodulir	CALL5_HUI	1	1.802231
24H	50MT/	P54578	0.01972	0.045371	0.033485	USP14	Ubiquitin c	UBP14_HU	24	1.013763
24H	10MT/	Q8N5M4	-0.16141	0.045378	0.033489	TTC9C	Tetratricop	TTC9C_HUI	5	0.894154
24H	10MT/	Q92614	-0.02882	0.045378	0.033489	MYO18A	Unconvent	MY18A_HU	43	0.980222
48H	10MT/	P28749	0.011599	0.045385	0.033492	RBL1	Retinoblast	RBL1_HUM	12	1.008072
48H	10MT/	Q8NHP6	-0.08713	0.045407	0.033504	MOSPD2	Motile sper	MSPD2_HU	11	0.941393
48H	50MT/	O94762	0.027401	0.045426	0.033516	RECQL5	ATP-depen	RECQ5_HUI	9	1.019174
48H	50MT/	Q14258	-0.06847	0.045442	0.033522	TRIM25	E3 ubiquiti	TRI25_HUM	30	0.953647
24H	50MT/	P49848	0.166736	0.045444	0.033522	TAF6	Transcripti	TAF6_HUM	17	1.122516
48H	50MT/	Q9NQT4	-0.13891	0.045451	0.033525	EXOSC5	Exosome cc	EXOS5_HUI	7	0.908206
24H	10MT/	Q9UNF0	-0.10238	0.045452	0.033526	PACSIN2	Protein kin	PACN2_HU	16	0.931493
48H	10MT/	Q3KNV8	0.284794	0.045465	0.033534	PCGF3	Polycomb g	PCGF3_HUI	1	1.218236
24H	50MT/	A6NFI3	0.15214	0.045474	0.033539	ZNF316	Zinc finger	ZN316_HUI	5	1.111217
24H	50MT/	Q8TDP1	0.075143	0.045481	0.033541	RNASEH2C	Ribonuclea	RNH2C_HU	7	1.053466
24H	10MT/	P21291	0.082219	0.04551	0.033556	CSRP1	Cysteine an	CSRP1_HUI	8	1.058645
48H	10MT/	P31150	0.068658	0.045522	0.033561	GDI1	Rab GDP di	GDIA_HUM	13	1.048741
48H	10MT/	Q01459	-0.24321	0.045521	0.033561	CTBS	Di-N-acetyl	DIAC_HUM	7	0.844861
24H	50MT/	Q562F6	0.049862	0.045537	0.03357	SGO2	Shugoshin 2	SGO2_HUM	5	1.035166
48H	50MT/	Q02543	0.0356	0.045538	0.03357	RPL18A	Large ribos	RL18A_HUI	8	1.024983
48H	10MT/	Q14624	0.250226	0.045541	0.033571	ITIH4	Inter-alpha	ITIH4_HUM	1	1.189393
48H	50MT/	Q86WG5	-0.5808	0.045558	0.033582	SBF2	Myotubula	MTMRD_HU	5	0.668593

24H 50MTA	Q14534	0.222064	0.045562	0.033584	SQLE	Squalene m	ERG1_HUM	11	1.166401
24H 50MTA	Q6V1X1	0.175052	0.04557	0.033587	DPP8	Dipeptidyl	DPP8_HUM	8	1.129005
24H 10MTA	P21359	-0.06229	0.045587	0.033596	NF1	Neurofibro	NF1_HUMA	36	0.957742
48H 10MTA	Q8NCC3	-0.00221	0.045598	0.033602	PLA2G15	Lysosomal	PAG15_HU	8	0.998468
24H 10MTA	O60518	0.11026	0.045614	0.033613	RANBP6	Ran-bindin	RNBP6_HU	9	1.079423
48H 50MTA	Q03518	-0.16814	0.045628	0.03362	TAP1	Antigen pe	TAP1_HUM	8	0.889989
24H 50MTA	Q96AQ6	0.02103	0.045628	0.03362	PBXIP1	Pre-B-cell l	PBIP1_HUN	19	1.014684
48H 50MTA	Q12972	0.084344	0.045649	0.033633	PPP1R8	Nuclear inh	PP1R8_HU	13	1.060205
24H 50MTA	Q12972	0.027122	0.045697	0.033662	PPP1R8	Nuclear inh	PP1R8_HU	13	1.018977
48H 10MTA	Q7L273	-0.0776	0.045708	0.033668	KCTD9	BTB/POZ dc	KCTD9_HU	4	0.947632
48H 50MTA	Q86TN4	0.372991	0.045713	0.033669	TRPT1	tRNA 2'-ph	TRPT1_HUN	5	1.295035
24H 50MTA	P07902	-0.10226	0.045731	0.03368	GALT	Galactose-1	GALT_HUM	8	0.931571
24H 50MTA	Q5TC63	0.23446	0.045731	0.03368	GRTP1	Growth ho	GRTP1_HU	4	1.176466
48H 50MTA	Q8TEB7	0.049705	0.045749	0.033691	RNF128	E3 ubiquiti	RN128_HU	1	1.035053
48H 10MTA	Q9H977	0.063259	0.045751	0.033692	WDR54	WD repeat-	WDR54_HU	3	1.044823
24H 10MTA	Q7L4I2	0.079024	0.04576	0.033696	RSRC2	Arginine/se	RSRC2_HU	8	1.056303
24H 50MTA	P11717	0.023303	0.045765	0.033698	IGF2R	Cation-inde	MPRI_HUM	85	1.016284
24H 10MTA	O76054	0.099514	0.045791	0.033709	SEC14L2	SEC14-like	S14L2_HUN	10	1.071413
24H 50MTA	Q562R1	0.115985	0.045793	0.033709	ACTBL2	Beta-actin-l	ACTBL_HUN	3	1.083715
24H 10MTA	P10155	0.018734	0.045813	0.033721	RO60	RNA-bindir	RO60_HUM	20	1.01307
48H 50MTA	Q9H9F9	-0.25624	0.045812	0.033721	ACTR5	Actin-relate	ARP5_HUM	10	0.837266
48H 50MTA	Q8NHZ8	-0.53295	0.045831	0.033731	CDC26	Anaphase-p	CDC26_HU	1	0.69114
48H 10MTA	Q9H488	0.018594	0.045836	0.033734	POFUT1	GDP-fucose	OFUT1_HU	13	1.012972
48H 10MTA	P52926	0.045888	0.045849	0.033742	HMGA2	High mobil	HMGA2_HU	4	1.032318
24H 50MTA	P00374	0.062008	0.045853	0.033745	DHFR	Dihydrofol	DYR_HUMA	8	1.043917
48H 10MTA	Q9NRW1	-0.03645	0.045922	0.033786	RAB6B	Ras-related	RAB6B_HU	2	0.975051
48H 10MTA	Q93096	-0.02274	0.04594	0.033797	PTP4A1	Protein tyr	TP4A1_HU	5	0.98436
48H 10MTA	P28161	0.019297	0.04595	0.033805	GSTM2	Glutathion	GSTM2_HU	16	1.013465
48H 10MTA	O00178	0.201785	0.045967	0.033813	GTPBP1	GTP-bindin	GTPB1_HU	17	1.150121
24H 50MTA	Q9UNS2	-0.06648	0.045969	0.033813	COPS3	COP9 signa	CSN3_HUM	18	0.954968
48H 10MTA	P56181	1.272586	0.04597	0.033813	NDUFV3	NADH dehy	NDUV3_HU	1	2.415942
24H 10MTA	O00743	0.041125	0.045972	0.033814	PPP6C	Serine/thre	PPP6_HUM	14	1.028916
24H 10MTA	O14579	-0.26514	0.045996	0.033827	COPE	Coatomer s	COPE_HUM	13	0.832115
24H 10MTA	Q53H96	0.051051	0.046002	0.033831	PYCR3	Pyrroline-5	P5CR3_HU	9	1.036019
24H 50MTA	Q9Y692	-0.00393	0.046014	0.033837	GMEB1	Glucocortic	GMEB1_HU	4	0.99728
24H 50MTA	Q8WWI5	0.013784	0.046016	0.033838	SLC44A1	Choline tra	CTL1_HUM	2	1.0096
24H 10MTA	P04439	0.356002	0.046024	0.033842	HLA-A	HLA class I	HLAA_HUM	2	1.279874
24H 10MTA	Q05655	0.043818	0.046063	0.033865	PRKCD	Protein kin	KPCD_HUM	6	1.030839
48H 10MTA	P21281	-0.01645	0.046084	0.033878	ATP6V1B2	V-type prot	VATB2_HU	22	0.988665
48H 10MTA	Q04656	-0.00143	0.046092	0.033882	ATP7A	Copper-tra	ATP7A_HU	22	0.999008
48H 10MTA	Q86WQ0	0.058954	0.046106	0.033889	NR2C2AP	Nuclear rec	NR2CA_HU	5	1.04171
48H 10MTA	Q8NFD5	0.058429	0.046119	0.033894	ARID1B	AT-rich inte	ARI1B_HUN	26	1.041331
48H 10MTA	Q9BSQ5	-0.12522	0.046129	0.033899	CCM2	Cerebral ca	CCM2_HUN	3	0.916862
24H 50MTA	Q14161	-0.20986	0.046154	0.033913	GIT2	ARF GTPase	GIT2_HUMA	17	0.864623
24H 10MTA	Q9NRZ7	0.076652	0.046181	0.033929	AGPAT3	1-acyl-sn-gl	PLCC_HUM	9	1.054568
24H 50MTA	P36957	0.070045	0.046204	0.033943	DLST	Dihydrolip	ODO2_HUN	11	1.049749
24H 50MTA	Q9P2Y5	0.279816	0.046217	0.033949	UVRAG	UV radiatio	UVRAG_HU	7	1.21404
24H 50MTA	Q9NQP4	0.247955	0.046214	0.033949	PFDN4	Prefoldin s	PFD4_HUM	3	1.187523
24H 10MTA	Q9HC62	-0.04687	0.046236	0.033961	SENP2	Sentrin-spe	SENP2_HU	5	0.968033
48H 50MTA	O15173	0.074935	0.046269	0.03398	PGRMC2	Membrane-	PGRC2_HU	11	1.053314
48H 50MTA	Q6P9B9	-0.13291	0.046278	0.033985	INTS5	Integrator	INT5_HUMA	15	0.91199
24H 50MTA	O43252	-0.02547	0.046285	0.03399	PAPSS1	Bifunctiona	PAPS1_HU	21	0.982499

48H 10MTA	Q15041	0.160462	0.046318	0.034007	ARL6IP1	ADP-ribosy	AR6P1_HU	3	1.117645
48H 50MTA	Q9UMN6	-0.03321	0.046323	0.034009	KMT2B	Histone-lys	KMT2B_HU	12	0.977242
24H 10MTA	Q8N5V2	-0.17617	0.046346	0.034021	NGEF	Ephexin-1	NGEF_HUM	12	0.885049
48H 50MTA	Q7RTV5	-0.35072	0.046362	0.034027	PRXL2C	Peroxiredo	PXL2C_HU	3	0.78419
24H 10MTA	Q99805	0.122144	0.046362	0.034027	TM9SF2	Transmemb	TM9S2_HU	14	1.088351
24H 50MTA	O00559	0.12721	0.046365	0.034029	EBAG9	Receptor-b	RCAS1_HU	2	1.09218
24H 50MTA	P61970	-0.13443	0.046375	0.034035	NUTF2	Nuclear tra	NTF2_HUM	6	0.911032
24H 10MTA	Q9UBF8	-0.11761	0.046377	0.034035	PI4KB	Phosphatid	PI4KB_HUM	9	0.921716
48H 50MTA	P29401	0.059055	0.046414	0.034057	TKT	Transketola	TKT_HUMA	29	1.041783
24H 10MTA	Q8WV99	-0.40982	0.046429	0.034064	ZFAND2B	AN1-type zi	ZFN2B_HU	3	0.752716
24H 10MTA	O94806	-0.02192	0.046448	0.034076	PRKD3	Serine/thre	KPCD3_HU	2	0.98492
24H 10MTA	O76075	-0.16125	0.046452	0.034078	DFFB	DNA fragme	DFFB_HUM	1	0.894248
24H 10MTA	Q9BYW2	0.105266	0.046465	0.034085	SETD2	Histone-lys	SETD2_HUM	13	1.075693
24H 50MTA	Q9BSU3	0.784655	0.046478	0.034094	NAA11	N-alpha-ace	NAA11_HU	2	1.72268
24H 10MTA	P49848	0.062679	0.04648	0.034094	TAF6	Transcripti	TAF6_HUM	17	1.044403
48H 50MTA	O75052	0.313114	0.046509	0.03411	NOS1AP	Carboxyl-te	CAPON_HU	3	1.242387
48H 50MTA	Q9H2D1	-0.10479	0.046518	0.034115	SLC25A32	Solute carri	S2532_HU	8	0.92994
48H 50MTA	Q7Z333	-0.27741	0.04654	0.03413	SETX	Probable h	SETX_HUMA	17	0.825073
48H 50MTA	P45985	0.000456	0.046543	0.034131	MAP2K4	Dual specifi	MP2K4_HU	14	1.000316
24H 10MTA	P31146	0.190111	0.046546	0.034132	CORO1A	Coronin-1A	COR1A_HU	3	1.140852
48H 50MTA	P30825	-0.08039	0.046557	0.034139	SLC7A1	High affinit	CTR1_HUM	5	0.945804
48H 50MTA	Q02487	0.572591	0.046569	0.034144	DSC2	Desmocolli	DSC2_HUM	2	1.487192
24H 10MTA	Q8NEM2	0.146008	0.046569	0.034144	SHCBP1	SHC SH2 dc	SHCBP_HU	20	1.106503
48H 10MTA	Q96HR3	-0.66229	0.046568	0.034144	MED30	Mediator o	MED30_HU	2	0.631875
48H 10MTA	Q12778	0.476266	0.046589	0.034155	FOXO1	Forkhead b	FOXO1_HU	5	1.391138
48H 10MTA	P40939	-0.0029	0.046595	0.034158	HADHA	Trifunction	ECHA_HUM	37	0.99799
24H 10MTA	P36955	0.243902	0.046603	0.034162	SERPINF1	Pigment ep	PEDF_HUM	10	1.184191
24H 50MTA	P14373	0.078663	0.046618	0.03417	TRIM27	Zinc finger	TRI27_HUM	8	1.056039
24H 50MTA	Q7L7V1	0.094987	0.046639	0.034183	DHX32	Putative pr	DHX32_HU	16	1.068056
24H 50MTA	Q9UHB7	-0.11219	0.04666	0.034196	AFF4	AF4/FMR2 f	AFF4_HUM	8	0.925182
48H 10MTA	Q92665	-0.0293	0.046665	0.034198	MRPS31	Small ribos	RT31_HUM	15	0.979898
48H 10MTA	Q96G28	0.009849	0.046679	0.034207	CFAP36	Cilia- and fl	CFA36_HU	10	1.00685
24H 50MTA	O15431	0.289598	0.04669	0.034211	SLC31A1	High affinit	COPT1_HU	1	1.2223
48H 50MTA	P36021	0.198199	0.046694	0.034213	SLC16A2	Monocarbo	MOT8_HUM	2	1.147265
48H 50MTA	Q96L73	-0.29943	0.046701	0.034217	NSD1	Histone-lys	NSD1_HUM	3	0.812575
48H 50MTA	Q96N64	-0.50914	0.046704	0.034217	PWWP2A	PWWP don	PWP2A_HU	2	0.70264
24H 50MTA	Q9BT17	0.068737	0.04674	0.034237	MTG1	Mitochond	MTG1_HUM	5	1.048798
48H 50MTA	Q9HA77	0.068796	0.046754	0.034245	CARS2	Probable cy	SYCM_HUM	16	1.048841
24H 50MTA	Q9NTJ5	0.022613	0.046775	0.034257	SACM1L	Phosphatid	SAC1_HUM	28	1.015798
24H 10MTA	Q9NQ50	-0.17078	0.046777	0.034257	MRPL40	Large ribos	RM40_HUM	7	0.888362
48H 10MTA	Q86X27	0.137698	0.046779	0.034258	RALGPS2	Ras-specific	RGPS2_HU	7	1.100148
24H 10MTA	P84090	-0.13994	0.046788	0.034261	ERH	Enhancer o	ERH_HUMA	3	0.907555
48H 50MTA	Q8IXM3	-0.16783	0.046787	0.034261	MRPL41	Large ribos	RM41_HUM	6	0.890183
48H 10MTA	Q96F44	0.111145	0.046798	0.034267	TRIM11	E3 ubiquiti	TRI11_HUM	3	1.080085
24H 10MTA	P35659	0.035252	0.046806	0.034271	DEK	Protein DEI	DEK_HUMA	16	1.024736
48H 50MTA	O75396	0.059801	0.046857	0.034302	SEC22B	Vesicle-traf	SC22B_HU	11	1.042322
48H 50MTA	Q15785	0.028514	0.046859	0.034302	TOMM34	Mitochond	TOM34_HU	16	1.019961
48H 50MTA	Q8NE01	-0.26256	0.046859	0.034302	CNNM3	Metal trans	CNNM3_HU	19	0.833608
24H 50MTA	P14867;P4	0.384991	0.04689	0.034321	GABRA1;GA	Gamma-am	GBRA1_HU	1	1.305852
24H 50MTA	Q96DE0	-0.22233	0.04689	0.034321	NUDT16	U8 snoRNA	NUD16_HU	13	0.857182
24H 50MTA	Q99933	-0.0109	0.046902	0.034329	BAG1	BAG family	BAG1_HUM	1	0.992473
24H 50MTA	P04040	0.000831	0.04692	0.034338	CAT	Catalase	CATA_HUM	26	1.000576

24H 50MT/	P19971	0.27854	0.046963	0.034362	TYMP	Thymidine	TYPH_HUM	4	1.212967
24H 50MT/	O76075	-0.50247	0.046969	0.034366	DFFB	DNA fragm	DFFB_HUM	1	0.705897
24H 10MT/	Q96MB7	0.787556	0.046975	0.034368	HARBI1	Putative nu	HARB1_HU	1	1.726147
48H 50MT/	Q13137	-0.28613	0.046978	0.034369	CALCOCO2	Calcium-bi	CACO2_HU	14	0.820099
48H 50MT/	P10643	0.462905	0.046996	0.034378	C7	Compleme	CO7_HUM/	2	1.378315
24H 50MT/	Q5TBB1	0.073083	0.047	0.03438	RNASEH2B	Ribonuclea	RNH2B_HU	7	1.051963
48H 50MT/	Q641Q2	0.03627	0.047009	0.034385	WASHC2A	WASH com	WAC2A_HU	28	1.025459
48H 50MT/	O95406	0.800636	0.047021	0.03439	CNIH1	Protein cor	CNIH1_HUI	1	1.741868
24H 50MT/	Q9H6R4	-0.03136	0.047041	0.034403	NOL6	Nucleolar p	NOL6_HUM	26	0.978495
24H 10MT/	P02042	0.44598	0.047049	0.034408	HBD	Hemoglobi	HBD_HUM/	3	1.362239
24H 50MT/	O15084	-0.03807	0.047063	0.034417	ANKRD28	Serine/thre	ANR28_HU	15	0.973954
48H 10MT/	P35813	0.119978	0.047069	0.03442	PPM1A	Protein ph	PPM1A_HU	11	1.086718
24H 10MT/	Q6PIU2	0.05065	0.047069	0.03442	NCEH1	Neutral ch	NCEH1_HU	18	1.035732
24H 10MT/	P63096	0.273559	0.047077	0.034423	GNAI1	Guanine nu	GNAI1_HUI	2	1.208786
48H 50MT/	Q9H2D6	0.099196	0.047078	0.034423	TRIOBP	TRIO and F-	TARA_HUM	13	1.071177
24H 50MT/	P43358	-0.01704	0.047103	0.034437	MAGEA4	Melanoma-	MAGA4_HU	11	0.988261
24H 10MT/	Q629K1	-0.03418	0.047123	0.034448	TRIQK	Triple QxxK	TRIQK_HUN	1	0.976584
48H 10MT/	Q9NU19	-0.23293	0.047123	0.034448	TBC1D22B	TBC1 doma	TB22B_HUI	3	0.850905
24H 50MT/	Q14624	2.719008	0.047123	0.034448	ITIH4	Inter-alpha	ITIH4_HUM	1	6.5842
48H 10MT/	Q96BR5	-0.08683	0.047133	0.034455	COA7	Cytochrom	COA7_HUM	5	0.941587
48H 10MT/	Q96EU6	0.024444	0.047139	0.034457	RRP36	Ribosomal	RRP36_HUI	8	1.017088
48H 10MT/	O00471	0.016577	0.047161	0.034471	EXOC5	Exocyst cor	EXOC5_HUI	24	1.011557
48H 50MT/	P98173	0.109965	0.047175	0.034479	FAM3A	Protein FAI	FAM3A_HU	4	1.079202
48H 50MT/	Q8WW59	-0.07328	0.047197	0.034492	SPRYD4	SPRY doma	SPRY4_HUI	13	0.950473
48H 10MT/	Q9BRJ6	-0.01051	0.047197	0.034492	C7orf50	Uncharacte	CG050_HU	9	0.992744
48H 50MT/	Q9UKL6	0.712151	0.047203	0.034494	PCTP	Phosphatid	PPCT_HUM	2	1.638245
24H 10MT/	O43719	0.040527	0.047204	0.034494	HTATSF1	17S U2 SnR	HTSF1_HUI	20	1.02849
24H 50MT/	P78563	-1.42753	0.047211	0.034498	ADARB1	Double-stra	RED1_HUM	1	0.371768
48H 10MT/	P13807	-0.0266	0.047242	0.034515	GYS1	Glycogen [s	GYS1_HUM	15	0.981734
24H 10MT/	Q8N3Y1	0.011902	0.047249	0.034518	FBXW8	F-box/WD r	FBXW8_HU	2	1.008284
24H 10MT/	O75607	-0.12197	0.047256	0.034522	NPM3	Nucleoplas	NPM3_HUI	7	0.91893
24H 50MT/	Q53H12	-0.04101	0.047277	0.034532	AGK	Acylglycerc	AGK_HUM/	15	0.971976
24H 50MT/	Q9HBL8	-1.58E-05	0.047311	0.034553	NMRAL1	NmrA-like f	NMRL1_HU	4	0.999989
24H 10MT/	P43487	-0.21361	0.04733	0.034564	RANBP1	Ran-specifi	RANG_HUN	8	0.862378
48H 10MT/	O60347	0.013799	0.04734	0.034568	TBC1D12	TBC1 doma	TBC12_HUI	3	1.009611
24H 50MT/	Q96BR1	0.231046	0.047352	0.034574	SGK3	Serine/thre	SGK3_HUM	5	1.173686
48H 50MT/	Q9NVR0	-0.23745	0.047355	0.034575	KLHL11	Kelch-like p	KLH11_HU	10	0.848243
24H 10MT/	Q9Y365	-0.09138	0.047355	0.034575	STARD10	START dom	STA10_HUI	9	0.938626
24H 50MT/	O75414	-0.07518	0.047362	0.034578	NME6	Nucleoside	NDK6_HUM	4	0.949222
24H 50MT/	O15126	0.178721	0.047369	0.034581	SCAMP1	Secretory c	SCAM1_HU	5	1.13188
24H 10MT/	Q9Y5Y2	-0.01116	0.047386	0.034591	NUBP2	Cytosolic F	NUBP2_HU	11	0.992297
24H 50MT/	O00244	-0.00674	0.04739	0.034593	ATOX1	Copper tra	ATOX1_HUI	6	0.995336
24H 50MT/	P27540	0.06641	0.047427	0.034615	ARNT	Aryl hydroc	ARNT_HUM	6	1.047108
48H 10MT/	P07237	-0.12396	0.047431	0.034617	P4HB	Protein dis	PDIA1_HUI	35	0.917663
48H 10MT/	Q8TD16	-0.15226	0.04744	0.034621	BICD2	Protein bic	BICD2_HUI	16	0.899839
48H 10MT/	P83111	-0.01437	0.047443	0.034623	LACTB	Serine beta	LACTB_HUI	23	0.990088
24H 50MT/	O60884	-0.11424	0.047456	0.03463	DNAJA2	DnaJ homo	DNJA2_HUI	18	0.923867
48H 10MT/	Q14681	0.415925	0.047459	0.034631	KCTD2	BTB/POZ dc	KCTD2_HUI	1	1.334154
24H 10MT/	O76031	0.057741	0.047469	0.034637	CLPX	ATP-depen	CLPX_HUM	28	1.040835
24H 50MT/	Q9Y618	0.157969	0.047482	0.034643	NCOR2	Nuclear rec	NCOR2_HU	7	1.115716
48H 50MT/	Q96T51	0.062093	0.047512	0.034661	RUFY1	RUN and FY	RUFY1_HUI	20	1.043979
24H 10MT/	Q6P587	-0.07472	0.047516	0.034664	FAHD1	Oxaloaceta	FAHD1_HU	11	0.949525

24H 50MT/	P61163	0.010884	0.047522	0.034666	ACTR1A	Alpha-centı	ACTZ_HUM	10	1.007572
48H 10MT/	P08134	0.007141	0.047523	0.034666	RHOC	Rho-relatec	RHOC_HUM	10	1.004962
48H 10MT/	P98179	-0.26227	0.047535	0.034673	RBM3	RNA-bindir	RBM3_HUM	5	0.833773
24H 10MT/	Q9BSE4	-0.84737	0.047557	0.034684	HERPUD2	Homocyste	HERP2_HU	1	0.555796
48H 10MT/	Q9P219	-0.16408	0.047576	0.034693	CCDC88C	Protein Da	DAPLE_HU	2	0.892498
48H 10MT/	Q14728	-0.19155	0.04758	0.034694	MFSD10	Major facili	MFS10_HU	3	0.875665
24H 50MT/	Q92615	0.029673	0.047585	0.034696	LARP4B	La-related p	LAR4B_HU	17	1.020781
48H 50MT/	Q9H1E3	0.288528	0.047592	0.0347	NUCKS1	Nuclear ubi	NUCKS_HU	6	1.221394
24H 50MT/	Q12769	0.041506	0.047602	0.034706	NUP160	Nuclear po	NU160_HU	41	1.029188
48H 50MT/	O43353	-0.07435	0.047603	0.034707	RIPK2	Receptor-ir	RIPK2_HUM	12	0.949771
48H 10MT/	Q9NYU2	-0.0089	0.047606	0.034707	UGGT1	UDP-glucos	UGGG1_HU	67	0.993851
24H 50MT/	P54619	0.046973	0.047608	0.034708	PRKAG1	5'-AMP-acti	AAKG1_HU	11	1.033095
48H 10MT/	P29317	-0.17392	0.047621	0.034715	EPHA2	Ephrin type	EPHA2_HU	17	0.886432
24H 10MT/	Q8N511	-0.05582	0.047624	0.034716	TMEM199	Transmemb	TM199_HU	4	0.96205
24H 50MT/	Q8N4V1	-0.13018	0.047634	0.034721	MMGT1	ER membra	EMC5_HUM	4	0.91372
24H 10MT/	Q9HBM1	0.061619	0.04764	0.034724	SPC25	Kinetochor	SPC25_HU	8	1.043636
24H 10MT/	Q9UK45	-0.4021	0.047677	0.034744	LSM7	U6 snRNA-a	LSM7_HUM	5	0.756756
24H 10MT/	P08559	-0.08369	0.04771	0.034763	PDHA1	Pyruvate de	ODPA_HUM	21	0.94364
48H 50MT/	Q9P2N6	-0.44466	0.047712	0.034764	KANSL3	KAT8 regula	KANL3_HU	6	0.734756
24H 10MT/	Q9UKK6	0.358909	0.04772	0.034766	NXT1	NTF2-relate	NXT1_HUM	3	1.282456
24H 50MT/	Q8TF68	0.135293	0.04773	0.034771	ZNF384	Zinc finger	ZN384_HU	6	1.098316
24H 50MT/	Q9NRF9	-0.20098	0.047734	0.034773	POLE3	DNA polym	DPOE3_HU	4	0.869961
48H 10MT/	Q68CQ4	-0.12024	0.047757	0.034788	UTP25	U3 small nu	UTP25_HU	20	0.920034
24H 50MT/	Q9Y697	0.027139	0.047776	0.034798	NFS1	Cysteine de	NFS1_HUM	20	1.01899
48H 50MT/	O60568	-0.03959	0.047798	0.034808	PLOD3	Multifuncti	PLOD3_HU	28	0.972932
24H 50MT/	Q12767	-0.28042	0.047798	0.034808	TMEM94	Endoplasm	ERMA_HUM	2	0.823351
48H 10MT/	A1L0T0	-0.04188	0.047836	0.034831	ILVBL	2-hydroxya	HACL2_HU	22	0.971387
48H 50MT/	O60844	-0.25471	0.047835	0.034831	ZG16	Zymogen gr	ZG16_HUM	1	0.838155
48H 50MT/	Q9H7E9	-0.23232	0.047856	0.034842	C8orf33	UPF0488 p	CH033_HU	4	0.851266
48H 10MT/	Q5BJF2	-0.14655	0.047867	0.034846	TMEM97	Sigma intra	SGMR2_HU	2	0.903409
48H 10MT/	Q9Y2L5	0.011742	0.047898	0.034865	TRAPPC8	Trafficking	TPPC8_HU	12	1.008172
48H 10MT/	O75391	0.106411	0.047907	0.034869	SPAG7	Sperm-asso	SPAG7_HU	8	1.076547
48H 10MT/	P61224	-0.06088	0.047914	0.034873	RAP1B	Ras-related	RAP1B_HU	10	0.95868
24H 50MT/	Q58DX5	-0.06608	0.047936	0.034884	NAALADL2	Inactive N-a	NADL2_HU	1	0.955229
24H 50MT/	Q96BN2	0.23349	0.047937	0.034884	TADA1	Transcripti	TADA1_HU	2	1.175675
48H 10MT/	P30405	0.053786	0.047944	0.034888	PPIF	Peptidyl-pr	PPIF_HUM/	5	1.037985
48H 10MT/	P78362	-0.01828	0.047975	0.034904	SRPK2	SRSF protei	SRPK2_HU	16	0.987409
48H 10MT/	P16435	-0.01001	0.048004	0.03492	POR	NADPH--cy	NCPR_HUM	39	0.993088
48H 50MT/	P16402	0.376528	0.048019	0.034928	H1-3	Histone H1	H13_HUM/	1	1.298214
24H 10MT/	P07902	-0.2317	0.04802	0.034928	GALT	Galactose-1	GALT_HUM	8	0.851628
24H 10MT/	Q9HB40	0.041738	0.048018	0.034928	SCPEP1	Retinoid-in	RISC_HUM/	11	1.029353
48H 50MT/	P10398	0.099283	0.048023	0.034928	ARAF	Serine/thre	ARAF_HUM	10	1.071241
48H 10MT/	Q9UI30	-0.02435	0.048029	0.034932	TRMT112	Multifuncti	TR112_HU	4	0.983261
24H 50MT/	Q9NP61	0.039594	0.048031	0.034932	ARFGAP3	ADP-ribosy	ARFG3_HU	13	1.027825
24H 50MT/	P17900	-0.07136	0.04806	0.034946	GM2A	Ganglioside	SAP3_HUM	4	0.951738
48H 10MT/	P62736;P6	-0.31517	0.048064	0.034948	ACTA2;ACT	Actin, aorti	ACTA_HUM	5	0.803758
48H 50MT/	Q9H446	-0.05979	0.048105	0.034969	RWDD1	RWD doma	RWDD1_HU	4	0.959404
48H 50MT/	Q9NTW7	-0.10242	0.048126	0.034978	ZFP64	Zinc finger	ZF64B_HUI	2	0.931467
24H 50MT/	Q9H4B0	-0.04616	0.048131	0.034981	OSGEPL1	tRNA N6-ac	OSGL1_HU	5	0.968513
48H 10MT/	P55786	-0.01029	0.048137	0.034983	NPEPPS	Puromycin	PSA_HUMA	43	0.992895
48H 50MT/	Q9H1Y0	-0.04953	0.048135	0.034983	ATG5	Autophagy	ATG5_HUM	9	0.966251
24H 50MT/	Q9Y619	0.07679	0.048175	0.035007	SLC25A15	Mitochond	ORNT1_HU	15	1.054669

48H 50MTA	Q9NPR2	0.595069	0.04819	0.035014	SEMA4B	Semaphori	SEM4B_HU	7	1.510545
24H 50MTA	Q9H668	0.057005	0.048191	0.035014	STN1	CST comple	STN1_HUM	2	1.040304
48H 50MTA	Q86Y91	-0.79593	0.048196	0.035016	KIF18B	Kinesin-like	KI18B_HUN	5	0.575973
24H 50MTA	Q9BT73	-0.17496	0.048227	0.035034	PSMG3	Proteasom	PSMG3_HU	5	0.885791
24H 50MTA	Q9Y696	0.053449	0.048232	0.035035	CLIC4	Chloride in	CLIC4_HUN	17	1.037743
48H 50MTA	Q8TER5	0.076587	0.048236	0.035036	ARHGEF40	Rho guanin	ARH40_HU	15	1.05452
48H 10MTA	Q6NUQ1	0.139118	0.048284	0.035063	RINT1	RAD50-inte	RINT1_HUN	9	1.101232
24H 50MTA	P55735	0.064213	0.048326	0.03509	SEC13	Protein SEC	SEC13_HUN	11	1.045514
24H 10MTA	P51965	0.06524	0.04833	0.035092	UBE2E1	Ubiquitin-c	UB2E1_HU	1	1.046259
48H 10MTA	P28482	-0.02563	0.048345	0.035102	MAPK1	Mitogen-ac	MK01_HUN	25	0.982392
48H 50MTA	Q92841	0.012256	0.048357	0.035108	DDX17	Probable AT	DDX17_HU	27	1.008532
48H 50MTA	Q96BN2	-1.32046	0.048383	0.035122	TADA1	Transcripti	TADA1_HU	2	0.400408
24H 50MTA	Q15046	0.015175	0.048405	0.035134	KARS1	Lysine--tRN	SYK_HUMA	29	1.010574
48H 50MTA	Q9BV10	0.100011	0.048413	0.035138	ALG12	Dol-P-Man:	ALG12_HU	4	1.071781
24H 50MTA	P14923	0.049396	0.04842	0.035141	JUP	Junction pl	PLAK_HUM	30	1.034831
48H 10MTA	Q99470	0.17554	0.048419	0.035141	SDF2	Stromal cel	SDF2_HUM	5	1.129387
48H 50MTA	Q9Y5K5	0.04607	0.048433	0.035148	UCHL5	Ubiquitin c	UCHL5_HU	16	1.032449
24H 10MTA	P24539	0.077914	0.048444	0.035154	ATP5PB	ATP synthas	AT5F1_HUN	11	1.055491
24H 50MTA	Q8IZP0	-0.04831	0.048459	0.035162	ABI1	Abl interact	ABI1_HUMA	11	0.967069
48H 10MTA	Q9Y2H5	-0.16809	0.048471	0.035169	PLEKHA6	Pleckstrin h	PKHA6_HU	4	0.890021
24H 10MTA	Q9H078	0.030455	0.048487	0.035177	CLPB	Mitochond	CLPB_HUM	23	1.021334
24H 50MTA	P62249	-0.07558	0.048494	0.035178	RPS16	Small ribos	RS16_HUM	10	0.948962
24H 10MTA	Q15061	0.077303	0.0485	0.035178	WDR43	WD repeat-	WDR43_HU	25	1.055044
48H 50MTA	Q8N9N2	0.038566	0.048511	0.035186	ASCC1	Activating s	ASCC1_HUI	8	1.027093
24H 50MTA	Q14103	0.040623	0.048528	0.035192	HNRNPD	Heterogene	HNRPD_HU	15	1.028558
48H 50MTA	Q9H4I2	0.316458	0.048536	0.035197	ZHX3	Zinc fingers	ZHX3_HUM	2	1.245269
24H 50MTA	Q9UGP5	-0.81938	0.048549	0.035203	POLL	DNA polym	DPOLL_HUI	1	0.566685
48H 10MTA	Q96G74	0.084116	0.048551	0.035204	OTUD5	OTU domai	OTUD5_HU	4	1.060038
48H 50MTA	Q9H2P9	-0.09299	0.048554	0.035205	DPH5	Diphthine r	DPH5_HUN	11	0.937576
24H 50MTA	Q9H1Y0	-0.13294	0.048589	0.035225	ATG5	Autophagy	ATG5_HUM	9	0.911974
48H 10MTA	P62826	-0.14456	0.048612	0.03524	RAN	GTP-bindin	RAN_HUMA	11	0.904657
48H 50MTA	O43741	0.264757	0.048619	0.035244	PRKAB2	5'-AMP-acti	AAKB2_HU	4	1.201434
48H 10MTA	Q4LE39	0.339242	0.048635	0.035251	ARID4B	AT-rich inte	ARI4B_HUN	5	1.265092
24H 10MTA	P53582	-0.10103	0.04865	0.035257	METAP1	Methionine	MAP11_HU	18	0.932364
24H 50MTA	Q15125	0.120611	0.048653	0.035258	EBP	3-beta-hydr	EBP_HUMA	3	1.087196
48H 50MTA	Q9Y2K9	-0.06388	0.048653	0.035258	STXBP5L	Syntaxin-bi	STB5L_HUN	4	0.956688
48H 10MTA	Q9H7N4	0.029632	0.048654	0.035258	SCAF1	Splicing fac	SFR19_HUN	7	1.020751
24H 10MTA	Q07960	-0.08869	0.04868	0.035271	ARHGAP1	Rho GTPase	RHG01_HU	20	0.940378
24H 10MTA	P12955	-0.12522	0.048683	0.035273	PEPD	Xaa-Pro dip	PEPD_HUM	21	0.916864
24H 50MTA	P25789	0.026371	0.048711	0.03529	PSMA4	Proteasom	PSA4_HUM	13	1.018447
24H 10MTA	P48723	0.123672	0.048719	0.035295	HSPA13	Heat shock	HSP13_HUI	14	1.089504
48H 50MTA	Q13330	-0.1719	0.048738	0.035304	MTA1	Metastasis-	MTA1_HUN	12	0.887673
24H 50MTA	O75054	0.167083	0.048745	0.035307	IGSF3	Immunoglo	IGSF3_HUN	12	1.122786
24H 10MTA	Q9Y5R8	0.172424	0.048756	0.035312	TRAPPC1	Trafficking	TPPC1_HUI	7	1.12695
24H 10MTA	P15880	0.051343	0.048791	0.035329	RPS2	Small ribos	RS2_HUMA	17	1.036229
48H 10MTA	P61163	0.016777	0.048828	0.035351	ACTR1A	Alpha-centr	ACTZ_HUM	10	1.011697
24H 10MTA	Q96L92	0.056574	0.048829	0.035352	SNX27	Sorting nex	SNX27_HUI	16	1.039993
24H 10MTA	Q8IZ52	-0.25296	0.048861	0.03537	CHPF	Chondroiti	CHSS2_HUI	7	0.839171
48H 10MTA	P55201	-0.68832	0.048863	0.03537	BRPF1	Peregrin	BRPF1_HUI	4	0.620578
48H 10MTA	Q5VTL8	0.046392	0.048869	0.035374	PRPF38B	Pre-mRNA-s	PR38B_HU	11	1.032679
48H 10MTA	Q16763	0.165932	0.048876	0.035376	UBE2S	Ubiquitin-c	UBE2S_HUI	7	1.121891
48H 50MTA	P59190	0.483303	0.048878	0.035377	RAB15	Ras-related	RAB15_HUI	1	1.397941

24H 50MT/	Q13042	0.050236	0.048902	0.03539	CDC16	Cell divisio	CDC16_HU	23	1.035434
24H 50MT/	Q15165	0.097093	0.048912	0.035395	PON2	Serum para	PON2_HUI	16	1.069616
48H 50MT/	Q24JP5	0.496791	0.048935	0.035408	TMEM132A	Transmemb	T132A_HUI	1	1.411072
24H 10MT/	Q9UPY3	-0.06288	0.048938	0.035408	DICER1	Endoribonu	DICER_HUI	25	0.957352
48H 10MT/	Q8NFH4	0.068678	0.048971	0.035427	NUP37	Nucleopori	NUP37_HU	11	1.048755
48H 10MT/	O75152	-0.03804	0.04899	0.035437	ZC3H11A	Zinc finger	ZC11A_HUI	18	0.973974
48H 50MT/	Q5JRX3	0.002528	0.048995	0.035439	PITRM1	Presequenc	PREP_HUM	33	1.001754
24H 10MT/	Q92621	0.019238	0.049035	0.035462	NUP205	Nuclear po	NU205_HU	72	1.013424
48H 10MT/	P41440	-0.04115	0.049053	0.035473	SLC19A1	Reduced fo	S19A1_HUI	3	0.97188
24H 50MT/	Q9C0A1	-0.25475	0.049066	0.035478	ZFHX2	Zinc finger	ZFHX2_HUI	1	0.838135
48H 10MT/	Q7KZ85	0.029524	0.049072	0.035479	SUPT6H	Transcripti	SPT6H_HUI	55	1.020675
48H 10MT/	Q9C0B9	0.10397	0.049073	0.035479	ZCCHC2	Zinc finger	ZCHC2_HUI	2	1.074727
48H 10MT/	P31943	0.031337	0.049095	0.035489	HNRNPH1	Heterogene	HNRH1_HU	18	1.021959
48H 10MT/	P56270	-0.00956	0.049095	0.035489	MAZ	Myc-associ	MAZ_HUM/	6	0.993397
24H 50MT/	Q6L8Q7	0.011581	0.049107	0.035496	PDE12	2',5'-phosp	PDE12_HUI	23	1.00806
48H 10MT/	Q9BZG1	0.034892	0.04913	0.035509	RAB34	Ras-related	RAB34_HUI	9	1.02448
24H 10MT/	Q06481	0.040155	0.049149	0.035521	APLP2	Amyloid be	APLP2_HUI	16	1.028224
24H 50MT/	Q6EKJ0;Q8	-0.40087	0.04915	0.035521	GTF2IRD2B	General tra	GTD2B_HU	1	0.757403
24H 10MT/	P20674	-0.48368	0.049154	0.035523	COX5A	Cytochrom	COX5A_HU	6	0.71515
48H 10MT/	P48634	0.002564	0.049161	0.035526	PRRC2A	Protein PRI	PRC2A_HUI	44	1.001779
48H 50MT/	P53803	0.906162	0.049164	0.035527	POLR2K	DNA-direct	RPAB4_HUI	2	1.874054
48H 10MT/	Q6P1N0	-0.08924	0.04918	0.035535	CC2D1A	Coiled-coil	C2D1A_HU	21	0.940018
48H 10MT/	Q10713	-0.10558	0.049196	0.035545	PMPCA	Mitochond	MPPA_HUN	24	0.929431
24H 50MT/	Q99961	-0.06215	0.049199	0.035546	SH3GL1	Endophilin	SH3G1_HU	12	0.957838
48H 10MT/	Q9Y2K2	-0.0304	0.0492	0.035546	SIK3	Serine/thre	SIK3_HUM/	2	0.979148
48H 50MT/	P52747	0.132027	0.049206	0.035548	ZNF143	Zinc finger	ZN143_HUI	2	1.095832
48H 10MT/	Q99081	-0.26047	0.049227	0.035557	TCF12	Transcripti	HTF4_HUM	3	0.834816
48H 10MT/	Q13569	1.33683	0.049231	0.035559	TDG	G/T mismat	TDG_HUM/	1	2.525957
24H 10MT/	Q6QNY0	-0.00081	0.049254	0.035569	BLOC1S3	Biogenesis	BL1S3_HUI	7	0.999441
24H 50MT/	Q8IXQ4	-0.03238	0.049256	0.03557	GPALPP1	GPALPP mc	GPAM1_HU	3	0.977805
48H 50MT/	Q92791	0.097286	0.049289	0.035587	P3H4	Endoplasm	SC65_HUM	7	1.069759
48H 10MT/	Q00765	-0.01664	0.049294	0.03559	REEP5	Receptor e	REEP5_HUI	3	0.988533
48H 50MT/	O14975	0.155645	0.0493	0.035593	SLC27A2	Long-chain	S27A2_HUI	15	1.11392
48H 10MT/	P42695	-0.10308	0.049302	0.035594	NCAPD3	Condensin-	CNDD3_HU	22	0.93104
48H 50MT/	Q7LG56	0.042447	0.049312	0.0356	RRM2B	Ribonuclec	RIR2B_HUN	7	1.029859
24H 10MT/	Q9NVZ3	-0.05778	0.049321	0.035605	NECAP2	Adaptin ear	NECP2_HUI	7	0.960739
48H 10MT/	Q9NXD2	0.799243	0.049334	0.035613	MTMR10	Myotubula	MTMRA_HU	6	1.740187
48H 10MT/	Q9NXC5	0.035519	0.049349	0.035622	MIOS	GATOR2 co	MIOS_HUM	26	1.024925
24H 10MT/	Q9Y6R1	-0.43233	0.049352	0.035623	SLC4A4	Electrogeni	S4A4_HUM	3	0.741064
24H 10MT/	Q9H1H9	0.249067	0.049359	0.035627	KIF13A	Kinesin-like	KI13A_HUN	4	1.188438
24H 50MT/	Q13123	-0.06745	0.049374	0.035634	IK	Protein Rec	RED_HUMA	19	0.954326
24H 50MT/	Q9Y2U9	0.194913	0.049394	0.035644	KLHDC2	Kelch doma	KLDC2_HUI	4	1.144655
48H 10MT/	Q9BUE6	-0.01208	0.049398	0.035646	ISCA1	Iron-sulfur	ISCA1_HUN	3	0.991662
48H 10MT/	Q9UIV1	0.056658	0.049428	0.035665	CNOT7	CCR4-NOT t	CNOT7_HU	7	1.040054
48H 50MT/	Q6PML9	0.135968	0.049461	0.035682	SLC30A9	Proton-cou	ZNT9_HUM	6	1.09883
48H 10MT/	A6NCE7;Q9	-0.07519	0.049521	0.035717	MAP1LC3B	Microtubul	MP3B2_HU	1	0.949218
24H 50MT/	Q9Y5B0	0.097218	0.049524	0.035718	CTDP1	RNA polym	CTDP1_HUI	13	1.069709
24H 50MT/	P13984	0.107822	0.049536	0.035725	GTF2F2	General tra	T2FB_HUM	16	1.0776
48H 50MT/	Q96EA4	-0.13311	0.04954	0.035726	SPDL1	Protein Spi	SPDLY_HUN	9	0.911864
48H 50MT/	A6NIH7	0.007915	0.049576	0.035749	UNC119B	Protein unc	U119B_HU	7	1.005501
24H 50MT/	Q96HA7	-0.07813	0.049623	0.035779	TONSL	Tonsoku-lik	TONSL_HUI	11	0.947284
48H 50MT/	Q99523	-0.05499	0.049622	0.035779	SORT1	Sortilin	SORT_HUM	15	0.962598

24H	10MT/	Q8WX93	0.231056	0.049637	0.035787	PALLD	Palladin	PALLD_HUI	7	1.173694
24H	50MT/	P41240	-0.08618	0.049638	0.035787	CSK	Tyrosine-pr	CSK_HUMA	24	0.942016
24H	10MT/	Q12996	0.067062	0.049649	0.035792	CSTF3	Cleavage sti	CSTF3_HUN	24	1.047581
48H	50MT/	Q8NHG8	-0.13095	0.049649	0.035792	ZNRF2	E3 ubiquiti	ZNRF2_HUI	3	0.913233
48H	50MT/	P0DPH7;P(	0.275961	0.04966	0.035797	TUBA3C;TU	Tubulin alp	TBA3C_HUI	2	1.2108
24H	10MT/	Q96DE5	-0.11515	0.049671	0.035804	ANAPC16	Anaphase-p	APC16_HUI	4	0.923289
48H	50MT/	O95562	0.16619	0.049673	0.035804	SFT2D2	Vesicle trar	SFT2B_HUN	1	1.122091
48H	50MT/	Q9UPT5	0.030109	0.049685	0.035811	EXOC7	Exocyst cor	EXOC7_HUI	27	1.02109
24H	50MT/	Q15120	0.068675	0.049695	0.035816	PDK3	[Pyruvate d	PDK3_HUM	13	1.048753
24H	50MT/	Q8N697	-0.33613	0.049707	0.03582	SLC15A4	Solute carri	S15A4_HUI	2	0.792164
48H	50MT/	Q92613	0.185805	0.049723	0.035828	JADE3	Protein Jad	JADE3_HUN	2	1.137451
24H	50MT/	P05026	0.025501	0.049724	0.035829	ATP1B1	Sodium/po	AT1B1_HUI	10	1.017833
48H	50MT/	Q9H2U1	-0.17032	0.049755	0.035844	DHX36	ATP-depen	DHX36_HU	29	0.888645
48H	50MT/	P52298	0.155369	0.049756	0.035845	NCBP2	Nuclear cap	NCBP2_HU	4	1.113706
24H	10MT/	O75330	0.167997	0.049778	0.035857	HMMR	Hyaluronar	HMMR_HU	17	1.123498
48H	10MT/	P17481;P3	-1.07848	0.0498	0.035869	HOXB8;HO	Homeobox	HXB8_HUN	1	0.473529
48H	50MT/	Q9BWQ6	0.400429	0.049803	0.035869	YIPF2	Protein YIP	YIPF2_HUN	2	1.319901
48H	50MT/	Q06124	0.074486	0.049823	0.035879	PTPN11	Tyrosine-pr	PTN11_HUI	25	1.052986
48H	10MT/	P49750	-0.04245	0.049832	0.035884	YLPM1	YLP motif-c	YLPM1_HU	34	0.971002
48H	50MT/	O60701	-0.01011	0.049868	0.035906	UGDH	UDP-glucos	UGDH_HUN	30	0.993014
24H	50MT/	Q92530	-0.13084	0.049877	0.035909	PSMF1	Proteasom	PSMF1_HU	8	0.913299
24H	50MT/	Q9NYF3	-0.89387	0.049884	0.035912	FAM53C	Protein FAI	FA53C_HUI	1	0.538169
48H	10MT/	Q9H329	-0.01742	0.049914	0.03593	EPB41L4B	Band 4.1-lil	E41LB_HUN	3	0.988001
48H	50MT/	Q9BVK6	0.082644	0.049918	0.035931	TMED9	Transmemk	TMED9_HU	10	1.058957
24H	10MT/	Q9BTY2	0.028923	0.049924	0.035934	FUCA2	Plasma alpl	FUCO2_HU	13	1.02025
24H	10MT/	O94901	0.015681	0.049929	0.035935	SUN1	SUN domai	SUN1_HUN	30	1.010928
24H	10MT/	P11177	0.03944	0.049939	0.03594	PDHB	Pyruvate de	ODPB_HUN	17	1.027715
48H	50MT/	Q8WYP5	-0.13913	0.049947	0.035944	AHCTF1	Protein ELY	ELYS_HUM/	52	0.908067
48H	10MT/	O00410	0.021413	0.04996	0.035951	IPO5	Importin-5	IPO5_HUM	53	1.014953
48H	50MT/	Q9H993	0.076953	0.049966	0.035954	ARMT1	Damage-co	ARMT1_HU	16	1.054788
48H	50MT/	Q9UJ83	-0.06722	0.049978	0.03596	HACL1	2-hydroxya	HACL1_HUI	13	0.954474
24H	50MT/	Q9NR19	0.136463	0.049981	0.035961	ACSS2	Acetyl-coer	ACSA_HUM	12	1.099207
24H	10MT/	Q9BQC3	-0.14582	0.049985	0.035963	DPH2	2-(3-amino	DPH2_HUN	13	0.903862
24H	50MT/	Q8N2W9	0.127497	0.050021	0.035982	PIAS4	E3 SUMO-p	PIAS4_HUN	6	1.092397
24H	50MT/	P11441	-0.48387	0.050034	0.035989	UBL4A	Ubiquitin-l	UBL4A_HUI	6	0.715056
48H	50MT/	Q9BVG8	-0.19715	0.050056	0.036001	KIFC3	Kinesin-like	KIFC3_HUN	16	0.87227
48H	50MT/	Q9H488	0.01588	0.050068	0.036007	POFUT1	GDP-fucose	OFUT1_HUI	13	1.011068
48H	10MT/	Q9Y237	0.080716	0.05007	0.036007	PIN4	Peptidyl-pr	PIN4_HUM	6	1.057543
48H	50MT/	Q96IZ6	0.977833	0.050086	0.036016	METTL2A	tRNA N(3)-r	MET2A_HU	1	1.969504
24H	50MT/	Q9H910	-0.21214	0.050098	0.036023	JPT2	Jupiter mic	JUPI2_HUN	9	0.863255
48H	10MT/	Q86SQ4	0.033828	0.050114	0.036033	ADGRG6	Adhesion G	AGRG6_HU	8	1.023725
48H	50MT/	Q8TB72	0.099229	0.050132	0.036044	PUM2	Pumilio ho	PUM2_HUN	10	1.071201
24H	10MT/	O43399	-0.11839	0.050138	0.036048	TPD52L2	Tumor prot	TPD54_HUI	11	0.921216
48H	10MT/	Q96AE7	-0.25975	0.050142	0.03605	TTC17	Tetratricop	TTC17_HUN	12	0.835231
24H	10MT/	Q15645	0.079532	0.050188	0.036076	TRIP13	Pachytene	PCH2_HUM	20	1.056675
24H	50MT/	Q86UK7	0.021226	0.050213	0.03609	ZNF598	E3 ubiquiti	ZN598_HUI	22	1.014821
48H	50MT/	A1A4S6	0.047444	0.05025	0.03611	ARHGAP10	Rho GTPase	RHG10_HU	4	1.033432
24H	10MT/	Q9BRT2	-0.03476	0.050254	0.036112	UQCC2	Ubiquinol-	UQCC2_HU	6	0.976192
24H	50MT/	Q8TAP9	0.672178	0.050294	0.036134	MPLKIP	M-phase-sp	MPLKI_HUI	1	1.593476
48H	50MT/	P42574	0.129737	0.050305	0.036141	CASP3	Caspase-3	CASP3_HUI	7	1.094094
48H	50MT/	Q9NVH6	-0.11514	0.050312	0.036145	TMLHE	Trimethylly	TMLH_HUN	5	0.92329
24H	10MT/	Q15024	0.12319	0.050329	0.036153	EXOSC7	Exosome cc	EXOS7_HUI	10	1.08914

48H 50MT/	Q9NUQ9	0.069206	0.050356	0.036168	CYRIB	CYFIP-relat	CYRIB_HUN	15	1.049139
48H 10MT/	Q86V88	0.72636	0.050374	0.036177	MDP1	Magnesium	MGDP1_HU	2	1.654459
24H 10MT/	Q5VIR6	-0.09827	0.050392	0.036187	VPS53	Vacuolar pı	VPS53_HUI	16	0.934153
48H 10MT/	P48730	-0.04732	0.050436	0.036213	CSNK1D	Casein kina	KC1D_HUN	4	0.967732
48H 10MT/	Q9NY97	-0.70289	0.050438	0.036214	B3GNT2	N-acetyllac	B3GN2_HU	1	0.614342
48H 10MT/	Q9P283	0.077388	0.050447	0.036218	SEMA5B	Semaphori	SEM5B_HU	1	1.055106
24H 10MT/	P05783	0.038688	0.050462	0.036224	KRT18	Keratin, tyլ	K1C18_HUI	36	1.027179
48H 50MT/	Q9BZX2	-0.12854	0.050473	0.036229	UCK2	Uridine-cyt	UCK2_HUN	11	0.914757
24H 50MT/	P08962	0.071825	0.050475	0.036229	CD63	CD63 antig	CD63_HUN	2	1.051045
24H 50MT/	Q99661	-0.18016	0.050515	0.036252	KIF2C	Kinesin-like	KIF2C_HUN	22	0.882607
48H 10MT/	Q9NWT6	0.139612	0.050514	0.036252	HIF1AN	Hypoxia-in	HIF1N_HUN	10	1.101609
48H 50MT/	P31749	-0.01604	0.050543	0.036268	AKT1	RAC-alpha s	AKT1_HUM	8	0.988946
24H 10MT/	Q9UKN8	0.019195	0.050576	0.036285	GTF3C4	General tra	TF3C4_HUI	25	1.013394
48H 50MT/	P24468	0.536844	0.050594	0.036289	NR2F2	COUP trans	COT2_HUN	4	1.450796
24H 10MT/	Q08AE8	-0.08707	0.050609	0.036297	SPIRE1	Protein spi	SPIR1_HUN	5	0.941434
24H 10MT/	Q13425	0.026397	0.050634	0.036311	SNTB2	Beta-2-synt	SNTB2_HUI	13	1.018465
48H 10MT/	O95807	-0.06441	0.050645	0.036317	TMEM50A	Transmemk	TM50A_HU	1	0.956334
24H 50MT/	Q9H9Q2	-0.05078	0.050658	0.036325	COPS7B	COP9 signa	CSN7B_HUI	7	0.965415
24H 50MT/	Q96I24	0.014177	0.050665	0.036329	FUBP3	Far upstrea	FUBP3_HUI	24	1.009875
48H 50MT/	Q9P0J7	0.098824	0.050671	0.036331	KCMF1	E3 ubiquiti	KCMF1_HU	5	1.0709
24H 10MT/	P31271;P3	0.728132	0.050681	0.036338	HOXA13;H(	Homeobox	HXA13_HU	1	1.656493
48H 10MT/	Q9NXV6	-0.12549	0.0507	0.036348	CDKN2AIP	CDKN2A-in	CARF_HUM	8	0.916691
48H 10MT/	P49748	0.008158	0.05073	0.036363	ACADVL	Very long-c	ACADV_HU	31	1.00567
24H 10MT/	Q9Y490	-0.07228	0.05073	0.036363	TLN1	Talin-1	TLN1_HUM	110	0.951132
48H 50MT/	Q9H992	-0.35039	0.050731	0.036363	MARCHF7	E3 ubiquiti	MARH7_HU	4	0.784372
24H 10MT/	Q5SY16	0.046781	0.050733	0.036364	NOL9	Polynucleo	NOL9_HUN	21	1.032958
24H 10MT/	O75446	0.233902	0.050766	0.036382	SAP30	Histone dea	SAP30_HUI	3	1.176011
24H 50MT/	Q86UT6	0.109534	0.050767	0.036382	NLRX1	NLR family	NLRX1_HUI	10	1.078879
48H 10MT/	Q93074	-0.04221	0.050808	0.036402	MED12	Mediator o	MED12_HU	19	0.971166
48H 50MT/	P14406	-0.2032	0.050814	0.036406	COX7A2	Cytochrom	CX7A2_HUI	2	0.868622
24H 10MT/	Q6UX07	0.27011	0.050822	0.03641	DHRS13	Dehydroge	DHR13_HU	9	1.2059
48H 50MT/	Q12824	0.211938	0.050833	0.036415	SMARCB1	SWI/SNF-re	SNF5_HUM	12	1.158243
48H 10MT/	O00160	-0.18415	0.050835	0.036416	MYO1F	Unconvent	MYO1F_HU	7	0.88017
48H 10MT/	Q13557	0.014826	0.050846	0.036423	CAMK2D	Calcium/ca	KCC2D_HU	10	1.010329
24H 10MT/	P30086	-0.11508	0.050848	0.036423	PEBP1	Phosphatic	PEBP1_HUI	13	0.923334
48H 10MT/	Q15276	-0.0771	0.050848	0.036423	RABEP1	Rab GTPase	RABE1_HUI	12	0.94796
24H 10MT/	Q16831	0.073889	0.050893	0.03645	UPP1	Uridine ph(	UPP1_HUN	16	1.05255
48H 10MT/	Q8N543	0.021247	0.050905	0.036455	OGFOD1	Prolyl 3-hy	OGFD1_HU	15	1.014836
48H 10MT/	Q86SQ9	-0.04176	0.050919	0.036464	DHDDS	Dehydrodo	DHDDS_HU	5	0.971472
48H 10MT/	P53990	0.039109	0.050931	0.03647	IST1	IST1 homol	IST1_HUM/	11	1.027479
24H 50MT/	P03915	-0.35062	0.050941	0.036475	MT-ND5	NADH-ubiq	NU5M_HUI	5	0.784248
48H 10MT/	Q9NXR7	0.046176	0.050947	0.036479	BABAM2	BRISC and E	BABA2_HUI	7	1.032525
24H 50MT/	O96011	0.131603	0.050969	0.03649	PEX11B	Peroxisoma	PX11B_HUI	9	1.09551
24H 50MT/	B2RPK0	0.246752	0.050982	0.036497	HMGB1P1	Putative hi	HGB1A_HU	2	1.186533
48H 10MT/	P50452	-0.18914	0.051079	0.036554	SERPINB8	Serpin B8	SPB8_HUM	7	0.877129
24H 50MT/	Q96GX9	-0.02278	0.051108	0.036572	APIP	Methylthio	MTNB_HUN	8	0.984334
24H 10MT/	Q7Z5L9	0.016368	0.051162	0.036604	IRF2BP2	Interferon I	I2BP2_HUN	21	1.01141
48H 10MT/	Q3V6T2	-0.05411	0.051213	0.036634	CCDC88A	Girdin	GRDN_HUN	17	0.963187
24H 10MT/	Q9P258	0.016788	0.051239	0.036649	RCC2	Protein RC(	RCC2_HUN	24	1.011704
48H 50MT/	P23434	0.322928	0.051254	0.036657	GCSH	Glycine cle	GCSH_HUN	4	1.250867
24H 10MT/	Q04941	0.098703	0.051256	0.036658	PLP2	Proteolipic	PLP2_HUM	1	1.07081
48H 10MT/	Q5VUD6	0.420917	0.051261	0.03666	DIPK1B	Divergent p	DIK1B_HUN	2	1.338778

48H 50MTA	Q5C9Z4	-0.11507	0.051282	0.036673	NOM1	Nucleolar M	NOM1_HUI	11	0.923338
48H 50MTA	P02656	0.396083	0.051311	0.036689	APOC3	Apolipoprc	APOC3_HU	1	1.31593
48H 10MTA	Q9NU02	-0.13673	0.05132	0.036693	ANKEF1	Ankyrin rep	ANKE1_HUI	1	0.909581
24H 50MTA	Q8WTS6	-0.31974	0.051362	0.036719	SETD7	Histone-lys	SETD7_HUI	7	0.801215
24H 10MTA	Q9H9F9	0.023963	0.051371	0.036723	ACTR5	Actin-relate	ARP5_HUM	10	1.016748
48H 50MTA	P05362	0.032824	0.05138	0.036728	ICAM1	Intercellula	ICAM1_HUI	17	1.023013
48H 10MTA	Q9Y4B6	-0.08874	0.051396	0.036739	DCAF1	DDB1- and	DCAF1_HUI	27	0.940342
48H 50MTA	Q9UEG4	0.213054	0.051424	0.036755	ZNF629	Zinc finger	ZN629_HUI	7	1.159139
48H 10MTA	P15882	0.13497	0.051429	0.036756	CHN1	N-chimaeri	CHIN_HUM	2	1.09807
48H 50MTA	P16403	-0.43776	0.051429	0.036756	H1-2	Histone H1	H12_HUMA	1	0.738282
24H 10MTA	P62314	0.01258	0.051436	0.036759	SNRPD1	Small nucle	SMD1_HUM	5	1.008758
24H 50MTA	Q9H9V9	-1.01924	0.051441	0.036762	JMJD4	2-oxoglutar	JMJD4_HUI	2	0.493377
48H 50MTA	Q5VZK9	-0.03825	0.051457	0.036771	CARMIL1	F-actin-unc	CARL1_HUI	24	0.973832
24H 10MTA	Q03252	-0.00985	0.051483	0.036786	LMNB2	Lamin-B2	LMNB2_HU	27	0.993199
48H 50MTA	P27144	0.096994	0.051507	0.0368	AK4	Adenylate k	KAD4_HUM	14	1.069542
24H 10MTA	Q9H2F9	0.513371	0.051531	0.036812	CCDC68	Coiled-coil	CCD68_HU	1	1.427381
24H 50MTA	Q9HAU0	0.067192	0.051539	0.036817	PLEKHA5	Pleckstrin h	PKHA5_HU	27	1.047676
48H 10MTA	Q9BUR5	-0.14205	0.051564	0.036832	APOO	MICOS com	MIC26_HUI	5	0.906229
24H 10MTA	Q9HAD4	0.177014	0.051567	0.036833	WDR41	WD repeat-	WDR41_HU	7	1.130542
24H 50MTA	Q8WVY7	0.062114	0.05158	0.036839	UBLCP1	Ubiquitin-l	UBCP1_HU	14	1.043995
24H 10MTA	Q71UM5	-0.14262	0.051585	0.036842	RPS27L	Ribosomal	RS27L_HUI	4	0.905872
24H 50MTA	O75694	0.015082	0.051592	0.036846	NUP155	Nuclear po	NU155_HU	46	1.010509
48H 10MTA	P11597	1.220942	0.051603	0.036852	CETP	Cholesteryl	CETP_HUM	1	2.330988
24H 10MTA	O60825	0.612211	0.051612	0.036857	PFKFB2	6-phospho	F262_HUM	2	1.5286
48H 50MTA	Q93015	0.705022	0.051615	0.036859	NAA80	N-alpha-ac	NAA80_HU	1	1.63017
48H 50MTA	Q8N2G8	-0.02565	0.051618	0.03686	GHDC	GH3 domai	GHDC_HUM	16	0.982376
24H 10MTA	P56545	0.110141	0.051658	0.036882	CTBP2	C-terminal-	CTBP2_HUI	6	1.079334
24H 10MTA	Q13424	0.762428	0.051679	0.036895	SNTA1	Alpha-1-syr	SNTA1_HUI	1	1.696343
48H 10MTA	Q9BRT9	0.046034	0.051682	0.036897	GINS4	DNA replica	SLD5_HUM	9	1.032423
48H 50MTA	P16870	0.463813	0.051688	0.036899	CPE	Carboxype	CBPE_HUM	3	1.379182
24H 10MTA	O15067	-0.05688	0.051689	0.036899	PFAS	Phosphorik	PUR4_HUM	39	0.96134
48H 50MTA	Q96GX5	-0.16607	0.051743	0.036927	MASTL	Serine/thre	GWL_HUM	10	0.891267
24H 50MTA	Q9NQR4	0.015173	0.051746	0.036927	NIT2	Omega-ami	NIT2_HUM	16	1.010573
24H 50MTA	Q6P1N0	-0.21377	0.05178	0.036946	CC2D1A	Coiled-coil	C2D1A_HU	21	0.862281
24H 50MTA	P08708	-0.14264	0.051789	0.03695	RPS17	Small ribos	RS17_HUM	9	0.90586
48H 50MTA	O95273	0.68681	0.051847	0.036983	CCNDBP1	Cyclin-D1-k	CCDB1_HU	2	1.60972
24H 50MTA	Q5T3J3	-0.72653	0.051872	0.037	LRIF1	Ligand-dep	LRIF1_HUM	2	0.604356
48H 50MTA	Q9Y2Y1	-0.05322	0.051883	0.037006	POLR3K	DNA-direct	RPC10_HUI	3	0.963781
48H 50MTA	Q9Y6C9	0.059522	0.051898	0.037014	MTCH2	Mitochond	MTCH2_HU	12	1.04212
24H 50MTA	O75376	-0.17716	0.051939	0.037039	NCOR1	Nuclear rec	NCOR1_HU	14	0.884441
24H 10MTA	P49916	0.071185	0.051945	0.037043	LIG3	DNA ligase	DNLI3_HUM	20	1.050579
48H 10MTA	Q9H9J2	0.008097	0.051954	0.037046	MRPL44	Large ribos	RM44_HUM	14	1.005628
48H 50MTA	P07197	-0.25774	0.051959	0.037049	NEFM	Neurofilam	NFM_HUMA	1	0.836398
48H 50MTA	Q96EI5	0.167385	0.051974	0.037059	TCEAL4	Transcripti	TCAL4_HUM	7	1.123021
48H 50MTA	Q8NE86	-0.06362	0.051978	0.037062	MCU	Calcium un	MCU_HUM	10	0.956862
24H 50MTA	Q96M89	0.566994	0.051991	0.037069	CCDC138	Coiled-coil	CC138_HUI	4	1.481434
48H 50MTA	P49840	-0.13745	0.051998	0.037073	GSK3A	Glycogen sy	GSK3A_HUI	8	0.909126
48H 10MTA	Q8IY47	0.068679	0.052001	0.037074	KBTBD2	Kelch repea	KBTB2_HUI	4	1.048756
24H 50MTA	Q9H0H5	-0.06717	0.051999	0.037074	RACGAP1	Rac GTPase	RGAP1_HU	16	0.954508
48H 10MTA	Q02880	0.027467	0.05207	0.037107	TOP2B	DNA topois	TOP2B_HUI	42	1.019221
48H 50MTA	Q96RL1	-0.27749	0.052076	0.03711	UIMC1	BRCA1-A cc	UIMC1_HU	7	0.825027
48H 10MTA	O43852	4.15E-05	0.052079	0.037112	CALU	Calumenin	CALU_HUM	21	1.000029

24H 10MT/	P07737	0.03536	0.05211	0.037129	PFN1	Profilin-1	PROF1_HU	11	1.024812
48H 50MT/	O43157	0.112774	0.052126	0.037137	PLXNB1	Plexin-B1	PLXB1_HU	21	1.081305
24H 50MT/	Q9NNW7	0.08096	0.052135	0.037142	TXNRD2	Thioredoxi	TRXR2_HU	10	1.057722
48H 10MT/	O75521	-0.0355	0.052139	0.037143	ECI2	Enoyl-CoA	ECI2_HUM/	18	0.975694
48H 50MT/	Q9BYC8	-0.19947	0.052147	0.037145	MRPL32	Large ribos	RM32_HUN	3	0.870872
48H 50MT/	Q9HAZ1	-0.51018	0.052152	0.037148	CLK4	Dual specifi	CLK4_HUM	3	0.702137
24H 50MT/	Q9HBK9	0.037576	0.05219	0.037168	AS3MT	Arsenite m	AS3MT_HU	24	1.026388
48H 10MT/	Q32P44	-0.0824	0.052195	0.03717	EML3	Echinoderr	EMAL3_HU	16	0.944485
48H 50MT/	Q8IV48	-0.05531	0.052212	0.037179	ERI1	3'-5' exorib	ERI1_HUM/	4	0.96239
48H 50MT/	O00422	0.077749	0.052218	0.037182	SAP18	Histone dea	SAP18_HU	7	1.05537
48H 10MT/	Q9NWZ8	0.044259	0.052227	0.037186	GEMIN8	Gem-associ	GEMI8_HU	5	1.031153
48H 50MT/	Q8N9N8	-0.11287	0.052242	0.037193	EIF1AD	Probable R	EIF1A_HUN	7	0.924744
48H 10MT/	Q96BJ3	0.063531	0.052243	0.037193	AIDA	Axin intera	AIDA_HUM	7	1.04502
48H 50MT/	Q68CP4	-0.02976	0.052259	0.037201	HGSNAT	Heparan-al	HGNAT_HU	4	0.97958
48H 10MT/	Q9NTZ6	0.00778	0.052275	0.037209	RBM12	RNA-bindir	RBM12_HU	22	1.005407
48H 10MT/	Q86TU7	0.053274	0.052289	0.037215	SETD3	Actin-histic	SETD3_HUN	19	1.037617
48H 10MT/	Q96KR1	0.040613	0.052303	0.037222	ZFR	Zinc finger	ZFR_HUMA	33	1.028551
24H 50MT/	Q8IXQ5	-0.05781	0.052304	0.037222	KLHL7	Kelch-like p	KLHL7_HU	11	0.960722
24H 10MT/	P52435;Q9	-0.23751	0.052323	0.037232	POLR2J;PO	DNA-direct	RPB11_HU	3	0.848206
24H 10MT/	Q53SF7	0.01522	0.052326	0.037233	COBLL1	Cordon-ble	COBL1_HU	15	1.010606
24H 50MT/	Q9BUK0	0.11367	0.052334	0.037235	CHCHD7	Coiled-coil	CHCH7_HU	1	1.081977
24H 10MT/	Q96G75	0.317457	0.052348	0.037244	RMND5B	E3 ubiquiti	RMD5B_HU	2	1.246132
24H 10MT/	Q96I25	0.076859	0.052366	0.037256	RBM17	Splicing fac	SPF45_HUN	11	1.054719
24H 10MT/	P08621	-0.05533	0.052377	0.037262	SNRNP70	U1 small nu	RU17_HUM	14	0.962373
24H 50MT/	P50995	-0.07891	0.052387	0.037268	ANXA11	Annexin A1	ANX11_HU	19	0.946774
24H 10MT/	P00374	0.08712	0.052411	0.03728	DHFR	Dihydrofol	DYR_HUMA	8	1.062247
24H 10MT/	Q96I15	-0.07079	0.052426	0.037288	SCLY	Selenocyst	SCLY_HUM	18	0.952119
24H 50MT/	Q9BYJ9	0.025259	0.052427	0.037288	YTHDF1	YTH domair	YTHD1_HU	9	1.017663
48H 50MT/	O75964	0.018998	0.052428	0.037289	ATP5MG	ATP synthas	ATP5L_HUN	6	1.013256
48H 10MT/	Q9NVA2	0.007928	0.052432	0.03729	SEPTIN11	Septin-11	SEP11_HUN	20	1.00551
24H 50MT/	Q9NTK5	-0.07837	0.052462	0.037306	OLA1	Obg-like AT	OLA1_HUM	29	0.947128
48H 50MT/	O95985	-0.18833	0.052476	0.037314	TOP3B	DNA topois	TOP3B_HU	8	0.877623
24H 50MT/	Q5BJF6	-0.17925	0.052481	0.037317	ODF2	Outer dens	ODFP2_HU	6	0.883162
24H 50MT/	Q5T7M9	0.21301	0.052522	0.037341	DIPK1A	Divergent p	DIK1A_HUN	6	1.159104
24H 50MT/	Q13111	-0.05702	0.052524	0.037342	CHAF1A	Chromatin	CAF1A_HU	13	0.961246
24H 10MT/	Q96BH1	0.032337	0.052576	0.03737	RNF25	E3 ubiquiti	RNF25_HU	5	1.022668
48H 10MT/	Q7Z739	0.004426	0.052591	0.037377	YTHDF3	YTH domair	YTHD3_HU	14	1.003073
48H 10MT/	Q15397	-0.02195	0.052596	0.037378	PUM3	Pumilio ho	PUM3_HUN	24	0.984898
48H 50MT/	Q86Y82	0.10789	0.052642	0.037405	STX12	Syntaxin-1	STX12_HUN	9	1.077651
48H 50MT/	O95477	0.793352	0.052658	0.037412	ABCA1	Phospholip	ABCA1_HU	2	1.733096
24H 10MT/	P60842	0.024734	0.052668	0.037416	EIF4A1	Eukaryotic	IF4A1_HUN	30	1.017292
24H 10MT/	Q6P2Q9	0.027427	0.052668	0.037416	PRPF8	Pre-mRNA-	PRP8_HUM	107	1.019193
48H 10MT/	Q92890	0.066129	0.052668	0.037416	UFD1	Ubiquitin r	UFD1_HUM	13	1.046904
24H 50MT/	Q96EV8	0.350666	0.052696	0.037429	DTNBP1	Dysbindin	DTBP1_HU	4	1.275149
48H 50MT/	Q8NBZ7	-0.11106	0.052698	0.037429	UXS1	UDP-glucur	UXS1_HUM	11	0.925907
24H 10MT/	Q9Y2Z9	0.086169	0.052707	0.037433	COQ6	Ubiquinon	COQ6_HUN	11	1.061547
24H 50MT/	O75844	0.047729	0.052752	0.037462	ZMPSTE24	CAAX preny	FACE1_HUN	15	1.033636
48H 50MT/	Q6IN85	0.05166	0.052757	0.037464	PPP4R3A	Serine/thre	P4R3A_HU	17	1.036457
24H 10MT/	Q9HBU6	-0.12785	0.052768	0.037471	ETNK1	Ethanolami	EKI1_HUM/	8	0.915193
24H 50MT/	Q9UJF2	-0.26569	0.052774	0.037475	RASAL2	Ras GTPase	NGAP_HUN	13	0.831801
24H 50MT/	Q96A33	0.01885	0.052782	0.037479	CCDC47	PAT comple	CCD47_HU	20	1.013152
48H 10MT/	Q15363	-0.26049	0.052795	0.037483	TMED2	Transmemb	TMED2_HU	5	0.834802

48H 10MT/	P18084	0.045192	0.052799	0.037484	ITGB5	Integrin be	ITB5_HUM/	24	1.03182
24H 10MT/	O75915	-0.19429	0.052809	0.03749	ARL6IP5	PRA1 famil	PRAF3_HUI	6	0.874003
48H 50MT/	Q9Y2I9	1.090775	0.052835	0.037504	TBC1D30	TBC1 doma	TBC30_HUI	1	2.129884
48H 10MT/	Q9BYT8	0.047094	0.052834	0.037504	NLN	Neurolysin,	NEUL_HUM	19	1.033182
24H 50MT/	Q9Y5X3	-0.17511	0.05285	0.037513	SNX5	Sorting nex	SNX5_HUM	14	0.8857
24H 10MT/	Q6UW02	0.072383	0.052889	0.037532	CYP20A1	Cytochrom	CP20A_HUI	17	1.051452
48H 50MT/	P55209	0.065875	0.052896	0.037536	NAP1L1	Nucleosom	NP1L1_HUI	13	1.04672
48H 10MT/	Q9BUL9	-0.05877	0.052898	0.037536	RPP25	Ribonuclea	RPP25_HUI	8	0.960079
48H 50MT/	Q9BV20	-0.10067	0.052906	0.037538	MRI1	Methylthio	MTNA_HUM	15	0.932602
48H 10MT/	Q9NZJ5	0.311201	0.052912	0.037541	EIF2AK3	Eukaryotic	E2AK3_HUI	6	1.24074
48H 10MT/	Q8TEK3	-3.06119	0.052914	0.037542	DOT1L	Histone-lys	DOT1L_HUI	2	0.119809
24H 50MT/	P08123	-0.99947	0.05292	0.037545	COL1A2	Collagen al	CO1A2_HU	3	0.500184
48H 50MT/	Q9NQT5	-0.15367	0.052925	0.037546	EXOSC3	Exosome co	EXOS3_HUI	10	0.898962
48H 50MT/	Q9Y608	-0.08497	0.052926	0.037546	LRRFIP2	Leucine-ric	LRRF2_HUI	9	0.942807
48H 50MT/	P14618	0.22588	0.052945	0.037558	PKM	Pyruvate ki	KPYM_HUM	43	1.16949
24H 10MT/	Q68E01	0.089303	0.052953	0.037561	INTS3	Integrator	INT3_HUM/	30	1.063856
24H 10MT/	Q9H9Y6	0.067625	0.052959	0.037564	POLR1B	DNA-direct	RPA2_HUM	31	1.04799
24H 50MT/	Q9C091	-0.17708	0.052963	0.037565	GREB1L	GREB1-like	GRB1L_HUI	1	0.884488
48H 50MT/	Q96A73	0.470155	0.052968	0.037568	KIAA1191	Putative m	P33MX_HU	3	1.385258
48H 50MT/	Q712K3	-0.11987	0.052973	0.03757	UBE2R2	Ubiquitin-c	UB2R2_HU	4	0.920268
24H 50MT/	Q5SQH8	0.382838	0.052994	0.037582	C6orf136	Uncharacte	CF136_HUI	2	1.303905
24H 50MT/	Q7Z7N9	0.07478	0.053018	0.037594	TMEM179E	Transmemb	T179B_HUI	2	1.0532
48H 10MT/	Q96GW9	-0.13532	0.053019	0.037594	MARS2	Methionine	SYMM_HUI	13	0.910468
24H 50MT/	Q6ULP2	-0.03334	0.053026	0.037598	AFTPH	Aftiphilin	AFTIN_HUM	10	0.977153
24H 50MT/	Q9Y2R4	0.021076	0.053073	0.037628	DDX52	Probable A	DDX52_HU	18	1.014716
24H 50MT/	Q53HV7	-0.16469	0.05308	0.03763	SMUG1	Single-stra	SMUG1_HU	2	0.892117
48H 50MT/	O14828	-0.16293	0.053092	0.037637	SCAMP3	Secretory c	SCAM3_HU	9	0.893209
24H 10MT/	P62829	0.086694	0.053165	0.037682	RPL23	Large ribos	RL23_HUM	8	1.061934
24H 50MT/	Q9H5Q4	-0.10711	0.053208	0.037707	TFB2M	Dimethylac	TFB2M_HU	19	0.928447
48H 10MT/	Q9H6S3	0.011678	0.05321	0.037708	EPS8L2	Epidermal	ES8L2_HUI	23	1.008127
24H 10MT/	Q92990	0.016177	0.053216	0.037711	GLMN	Glomulin	GLMN_HUI	20	1.011276
48H 50MT/	P46777	-0.11804	0.053218	0.037711	RPL5	Large ribos	RL5_HUMA	14	0.921436
24H 50MT/	Q9Y365	-0.07082	0.053236	0.03772	STARD10	START dom	STA10_HUI	9	0.952095
48H 50MT/	P55327	0.073052	0.053239	0.03772	TPD52	Tumor prot	TPD52_HUI	9	1.051939
24H 10MT/	Q5VTL8	0.207687	0.053244	0.037723	PRPF38B	Pre-mRNA-	PR38B_HUI	11	1.154835
48H 10MT/	Q9BYB4	0.186063	0.053247	0.037724	GNB1L	Guanine nu	GNB1L_HU	9	1.137655
24H 50MT/	Q08945	0.040784	0.053309	0.037756	SSRP1	FACT comp	SSRP1_HUI	24	1.028672
24H 50MT/	Q9NWT1	0.031639	0.053309	0.037756	PAK1IP1	p21-activat	PK1IP_HUI	16	1.022173
48H 50MT/	O75962	0.022179	0.05333	0.037766	TRIO	Triple funct	TRIO_HUM/	29	1.015492
48H 50MT/	Q7Z3E5	-0.40735	0.053342	0.037773	ARMC9	LisH domai	ARMC9_HU	3	0.754005
48H 50MT/	Q96LI5	0.179132	0.053369	0.037788	CNOT6L	CCR4-NOT t	CNO6L_HU	2	1.132203
48H 50MT/	Q96C19	0.104249	0.053371	0.037789	EFHD2	EF-hand do	EFHD2_HUI	13	1.074935
48H 50MT/	Q9ULA0	-0.04242	0.053398	0.037804	DNPEP	Aspartyl am	DNPEP_HU	16	0.971022
24H 10MT/	Q13888;Q6	0.157673	0.053419	0.037813	GTF2H2;GT	General tra	TF2H2_HUI	11	1.115486
48H 50MT/	P61964	-0.08986	0.053449	0.037828	WDR5	WD repeat-	WDR5_HUI	12	0.939617
24H 10MT/	Q6ZRS2	0.068378	0.053451	0.037828	SRCAP	Helicase SR	SRCAP_HUI	17	1.048537
48H 10MT/	Q9UKD1	-0.00048	0.053475	0.03784	GMEB2	Glucocorti	GMEB2_HU	3	0.999665
24H 50MT/	P23588	0.074445	0.053516	0.037863	EIF4B	Eukaryotic	IF4B_HUM/	20	1.052956
48H 10MT/	O15084	-0.0064	0.053521	0.037866	ANKRD28	Serine/thre	ANR28_HU	15	0.995573
48H 50MT/	Q12981	-0.15493	0.053521	0.037866	BNIP1	Vesicle tran	SEC20_HUI	9	0.898176
24H 10MT/	Q9Y2S6	-0.36679	0.053547	0.03788	TMA7	Translation	TMA7_HUM	2	0.775505
48H 50MT/	P55081	0.048876	0.053556	0.037885	MFAP1	Microfibril	MFAP1_HU	11	1.034459

48H 50MT/	Q96E29	-0.25281	0.053562	0.037887	MTERF3	Transcripti	MTEF3_HUI	9	0.839259
24H 50MT/	Q8TF71	0.021985	0.053606	0.037911	SLC16A10	Monocarbo	MOT10_HU	1	1.015356
24H 50MT/	Q8IUH5	0.330863	0.053628	0.037923	ZDHHC17	Palmitoyltr	ZDH17_HU	3	1.257765
48H 10MT/	Q2VPB7	-0.14331	0.053649	0.037933	AP5B1	AP-5 compl	AP5B1_HUI	11	0.905439
48H 10MT/	P30039	-0.56948	0.053659	0.037938	PBLD	Phenazine I	PBLD_HUM	2	0.67386
24H 50MT/	Q13972	0.668056	0.053663	0.037939	RASGRF1	Ras-specific	RGRF1_HUI	1	1.588931
48H 10MT/	Q16629	0.151698	0.053694	0.037958	SRSF7	Serine/argi	SRSF7_HUM	6	1.110876
48H 10MT/	O15126	0.116528	0.053701	0.037961	SCAMP1	Secretory c	SCAM1_HU	5	1.084123
48H 10MT/	Q9Y5X1	0.016373	0.053725	0.037972	SNX9	Sorting nex	SNX9_HUM	18	1.011413
24H 10MT/	P11413	-0.08734	0.053742	0.037982	G6PD	Glucose-6-p	G6PD_HUM	34	0.941255
48H 10MT/	Q9H6Z4	0.037649	0.053764	0.037994	RANBP3	Ran-bindin	RANB3_HU	15	1.026439
48H 10MT/	Q9H1X3	-0.09656	0.053768	0.037996	DNAJC25	DnaJ homo	DJC25_HUI	3	0.935258
24H 50MT/	P62330	0.056665	0.053778	0.038	ARF6	ADP-ribosy	ARF6_HUM	8	1.040059
24H 50MT/	P49754	0.188008	0.05378	0.038001	VPS41	Vacuolar pr	VPS41_HUI	11	1.139189
24H 50MT/	Q9Y6J0	0.889587	0.053815	0.038021	CABIN1	Calcineurin	CABIN_HUN	1	1.852645
24H 10MT/	Q9Y692	0.040486	0.053824	0.038025	GMEB1	Glucocortio	GMEB1_HU	4	1.028461
48H 10MT/	Q8IUW5	0.204561	0.053838	0.038031	RELL1	RELT-like pr	RELL1_HUM	2	1.152336
48H 10MT/	Q8IWZ8	-0.09787	0.053843	0.038033	SUGP1	SURP and G	SUGP1_HU	20	0.934409
48H 10MT/	O43776	0.009826	0.053869	0.038046	NARS1	Asparagine-	SYNC_HUM	26	1.006834
24H 50MT/	Q9ULH7	-0.07807	0.053875	0.038049	MRTFB	Myocardin-	MRTFB_HU	9	0.947322
24H 10MT/	P09884	0.071772	0.053886	0.038054	POLA1	DNA polym	DPOLA_HU	35	1.051007
48H 50MT/	O95873	-0.14228	0.053889	0.038054	C6orf47	Uncharacte	CF047_HUI	1	0.906083
48H 50MT/	Q7Z2T5	0.011416	0.05389	0.038054	TRMT1L	TRMT1-like	TRM1L_HU	27	1.007944
24H 10MT/	P60468	-0.06685	0.053907	0.038064	SEC61B	Protein trai	SC61B_HUI	3	0.954722
24H 50MT/	Q6PJG2	0.22824	0.053923	0.038072	MIDEAS	Mitotic dea	MDEAS_HU	7	1.171405
48H 10MT/	Q8N6T7	-0.14882	0.05395	0.038086	SIRT6	NAD-depen	SIR6_HUM/	3	0.901989
48H 10MT/	Q9BQ95	0.03338	0.053966	0.038096	ECSIT	Evolutionar	ECSIT_HUM	11	1.023407
48H 10MT/	P16383	-0.17545	0.053968	0.038096	GCFC2	Intron Larg	GCFC2_HUI	15	0.885489
48H 10MT/	Q7L3T8	0.072043	0.053977	0.0381	PARS2	Probable pr	SYPM_HUM	11	1.051204
48H 50MT/	Q9NQ88	-0.07107	0.054005	0.038116	TIGAR	Fructose-2,	TIGAR_HUN	9	0.951933
24H 50MT/	P41227	-0.04926	0.054056	0.038148	NAA10	N-alpha-ace	NAA10_HU	9	0.966435
48H 50MT/	O95372	0.108338	0.05407	0.038154	LYPLA2	Acyl-protei	LYPA2_HUN	8	1.077985
24H 50MT/	P08579	0.041946	0.054081	0.038161	SNRPB2	U2 small nu	RU2B_HUM	6	1.029501
48H 50MT/	Q9GZU8	-0.08006	0.054093	0.038167	PSME3IP1	PSME3-inte	PIP30_HUN	8	0.94602
24H 50MT/	Q9NZN3	-0.03441	0.054102	0.038171	EHD3	EH domain-	EHD3_HUM	18	0.976431
24H 10MT/	Q9Y342	-0.217	0.05411	0.038175	PLLP	Plasmolipin	PLLP_HUM	2	0.86035
24H 10MT/	P15586	-0.06086	0.054128	0.038186	GNS	N-acetylglu	GNS_HUM/	12	0.958691
48H 50MT/	O00214	-0.1402	0.054141	0.038195	LGALS8	Galectin-8	LEG8_HUM	8	0.907392
24H 50MT/	Q8NBZ0	0.480998	0.054148	0.038197	INO80E	INO80 com	IN80E_HUN	1	1.395708
48H 50MT/	Q8N7H5	-0.06231	0.054147	0.038197	PAF1	RNA polym	PAF1_HUM	21	0.957732
24H 50MT/	Q96AJ9	-0.02243	0.054149	0.038197	VTI1A	Vesicle trar	VTI1A_HUN	5	0.984576
48H 50MT/	O76075	0.191571	0.054156	0.0382	DFFB	DNA fragme	DFFB_HUM	1	1.142007
24H 50MT/	Q9UBB6	-0.07012	0.054234	0.038244	NCDN	Neurochon	NCDN_HUM	15	0.952559
48H 50MT/	O60870	-0.1814	0.054236	0.038244	KIN	DNA/RNA-b	KIN17_HUN	8	0.881848
48H 50MT/	Q9Y6P5	-0.81922	0.054241	0.038247	SESN1	Sestrin-1	SESN1_HUN	1	0.566748
48H 50MT/	Q71RG4	-0.19534	0.054256	0.038254	TMUB2	Transmemb	TMUB2_HU	4	0.873365
48H 10MT/	Q9Y2X7	0.017148	0.054274	0.038262	GIT1	ARF GTPase	GIT1_HUM/	11	1.011957
24H 50MT/	P55039	-0.10778	0.054278	0.038263	DRG2	Developme	DRG2_HUN	17	0.928012
24H 10MT/	Q5VZL5	0.082091	0.054296	0.038273	ZMYM4	Zinc finger I	ZMYM4_HU	15	1.058551
24H 50MT/	Q9H444	-0.12674	0.05435	0.038302	CHMP4B	Charged mu	CHM4B_HU	8	0.915898
24H 10MT/	Q14204	-0.02517	0.054358	0.038307	DYNC1H1	Cytoplasmi	DYHC1_HU	233	0.982705
48H 10MT/	Q9BV20	-0.14522	0.054363	0.03831	MRI1	Methylthio	MTNA_HUN	15	0.904242

48H 50MT/	Q08554	0.404631	0.054375	0.038314	DSC1	Desmocolli	DSC1_HUM	3	1.323751
48H 10MT/	Q30201	-0.09087	0.054376	0.038314	HFE	Hereditary	HFE_HUMA	3	0.938953
48H 10MT/	O75494	-0.09862	0.054408	0.038331	SRSF10	Serine/argi	SRS10_HUI	6	0.933926
48H 50MT/	Q9UI30	-0.03021	0.054416	0.038336	TRMT112	Multifuncti	TR112_HUI	4	0.97928
48H 50MT/	Q16643	0.062365	0.054442	0.03835	DBN1	Drebrin	DREB_HUM	23	1.044176
24H 50MT/	Q9H875	-0.8786	0.054471	0.038366	PRKRIP1	PRKR-intera	PKRI1_HUI	1	0.543893
24H 10MT/	Q9P0J1	0.023341	0.054475	0.038368	PDP1	[Pyruvate d	PDP1_HUM	12	1.01631
24H 50MT/	Q04446	0.00263	0.054491	0.038379	GBE1	1,4-alpha-g	GLGB_HUM	27	1.001824
24H 10MT/	P82933	-0.12789	0.054535	0.038404	MRPS9	Small ribos	RT09_HUM	18	0.915171
24H 50MT/	P30740	-0.11238	0.054556	0.038416	SERPINB1	Leukocyte (	ILEU_HUMA	19	0.925062
48H 10MT/	Q9Y4B5	-0.00445	0.05458	0.03843	MTCL1	Microtubul	MTCL1_HU	42	0.996917
48H 10MT/	O95232	0.08235	0.054606	0.038443	LUC7L3	Luc7-like pı	LC7L3_HUI	10	1.058741
24H 50MT/	Q9BT25	-0.03323	0.054615	0.038447	HAUS8	HAUS augm	HAUS8_HU	4	0.977228
48H 50MT/	A6NKD9	-0.25265	0.054617	0.038447	CCDC85C	Coiled-coil	CC85C_HUI	4	0.839351
48H 10MT/	O14880	-0.12401	0.054639	0.038457	MGST3	Glutathion	MGST3_HU	6	0.917634
24H 10MT/	O75448	0.004401	0.054641	0.038457	MED24	Mediator o	MED24_HU	9	1.003055
48H 50MT/	Q8WWQ0	-0.08104	0.054653	0.038463	PHIP	PH-interact	PHIP_HUM	38	0.945377
24H 50MT/	Q8N5V2	-0.26694	0.054665	0.038471	NGEF	Ephexin-1	NGEF_HUM	12	0.831082
48H 50MT/	Q5W111	0.00271	0.054685	0.038481	SPRYD7	SPRY doma	SPRY7_HUI	5	1.001881
24H 50MT/	Q8NCH0	-0.07356	0.054694	0.038484	CHST14	Carbohydra	CHSTE_HUI	9	0.950289
48H 50MT/	O95219	0.02143	0.054716	0.038497	SNX4	Sorting nex	SNX4_HUM	21	1.014965
24H 50MT/	Q9Y4Y9	-0.36448	0.054781	0.038537	LSM5	U6 snRNA-a	LSM5_HUM	2	0.77675
48H 50MT/	O00429	0.036618	0.054798	0.038547	DNM1L	Dynamin-1	DNM1L_HU	44	1.025707
24H 10MT/	Q9H1B7	0.000389	0.054806	0.038552	IRF2BPL	Probable E3	I2BPL_HUM	6	1.00027
24H 10MT/	Q9NPF4	-0.00548	0.054809	0.038553	OSGEP	tRNA N6-ad	OSGEP_HUI	14	0.996207
24H 10MT/	Q02809	-0.00881	0.05483	0.038563	PLOD1	Procollager	PLOD1_HU	27	0.993914
24H 50MT/	Q8IVT5	-1.02172	0.054836	0.038566	KSR1	Kinase supp	KSR1_HUM	2	0.49253
24H 50MT/	Q68CP9	-0.04046	0.054857	0.03858	ARID2	AT-rich inte	ARID2_HUI	10	0.972344
24H 10MT/	P35237	-0.05197	0.054886	0.038598	SERPINB6	Serpin B6	SPB6_HUM	18	0.964619
24H 50MT/	Q6ZRV2	-0.12918	0.054889	0.038599	FAM83H	Protein FAI	FA83H_HUI	23	0.91435
48H 10MT/	Q04726	0.138506	0.054901	0.038605	TLE3	Transducin	TLE3_HUM/	11	1.100764
24H 10MT/	Q8WUH1	0.208151	0.054916	0.038614	CHURC1	Protein Chu	CHUR_HUM	3	1.155207
24H 50MT/	Q969X5	0.041433	0.054919	0.038614	ERGIC1	Endoplasm	ERGI1_HUM	9	1.029135
24H 10MT/	O95365	-0.53311	0.054927	0.038619	ZBTB7A	Zinc finger	ZBT7A_HUI	4	0.691063
48H 50MT/	O75503	0.115059	0.05493	0.03862	CLN5	Bis(monoac	CLN5_HUM	7	1.083019
48H 10MT/	Q9Y6W5	-0.02788	0.054937	0.038624	WASF2	Actin-bindi	WASF2_HU	9	0.980861
48H 10MT/	Q96EK7	0.101883	0.054976	0.038647	FAM120B	Constitutiv	F120B_HUI	24	1.073173
24H 50MT/	Q96KR6	0.115787	0.054986	0.038652	FAM210B	Protein FAI	F210B_HUI	3	1.083566
24H 10MT/	P55268	0.096389	0.054993	0.038653	LAMB2	Laminin sul	LAMB2_HU	28	1.069094
48H 50MT/	Q9BRX2	-0.06583	0.054991	0.038653	PELO	Protein pel	PELO_HUM	11	0.955395
48H 50MT/	P38435	-0.00207	0.055013	0.038664	GGCX	Vitamin K-c	VKGC_HUM	16	0.998564
24H 50MT/	Q9Y2U8	0.052445	0.055022	0.038669	LEMD3	Inner nucle	MAN1_HUI	25	1.037021
24H 50MT/	P52888	0.030272	0.055041	0.03868	THOP1	Thimet olig	THOP1_HU	27	1.021205
48H 10MT/	Q86W56	0.042661	0.055082	0.038698	PARG	Poly(ADP-ri	PARG_HUM	12	1.030012
24H 50MT/	Q96EN8	0.058122	0.055084	0.038699	MOCOS	Molybdenu	MOCOS_HU	9	1.041109
48H 10MT/	P00492	0.008081	0.05509	0.038702	HPRT1	Hypoxanth	HPRT_HUM	13	1.005617
48H 10MT/	Q96JN8	0.311103	0.055094	0.038702	NEURL4	Neuralized-	NEUL4_HUI	2	1.240656
24H 50MT/	Q6UN15	0.065537	0.055096	0.038702	FIP1L1	Pre-mRNA 3	FIP1_HUMA	10	1.046474
48H 10MT/	Q13443	0.044711	0.055102	0.038706	ADAM9	Disintegrin	ADAM9_HU	13	1.031477
24H 50MT/	Q9Y388	0.15007	0.055111	0.03871	RBMX2	RNA-bindir	RBMX2_HU	3	1.109623
24H 50MT/	Q9P2K5	0.406309	0.055124	0.038718	MYEF2	Myelin expı	MYEF2_HUI	1	1.325291
24H 50MT/	P55317	-0.33451	0.055128	0.03872	FOXA1	Hepatocyte	FOXA1_HUI	2	0.793056

24H 10MTA	Q9NUJ1	-0.01068	0.055137	0.038722	ABHD10	Palmitoyl-p	ABHDA_HU	11	0.992627
24H 50MTA	P12883	0.319997	0.055146	0.038723	MYH7	Myosin-7	MYH7_HUM	1	1.248328
48H 50MTA	O75436	0.076204	0.055148	0.038723	VPS26A	Vacuolar pr	VP26A_HUI	20	1.05424
48H 50MTA	Q68D10	-0.06613	0.055145	0.038723	SPTY2D1	Protein SPT	SPT2_HUM	13	0.9552
48H 50MTA	Q7Z2E3	-0.11724	0.055148	0.038723	APTX	Aprataxin	APTX_HUM	7	0.921948
24H 50MTA	O43768	-0.25316	0.05515	0.038724	ENSA	Alpha-endo	ENSA_HUM	7	0.839055
24H 10MTA	Q8N5U6	0.110433	0.055155	0.038727	RNF10	E3 ubiquiti	RNF10_HUI	8	1.079552
24H 50MTA	Q8WVQ1	0.128654	0.055156	0.038727	CANT1	Soluble cal	CANT1_HUI	13	1.093273
48H 10MTA	Q14241	0.026027	0.055169	0.038733	ELOA	Elongin-A	ELOA1_HUI	7	1.018204
24H 10MTA	Q6DKI1	0.02763	0.055174	0.038736	RPL7L1	Ribosomal	RL7L_HUM	12	1.019336
48H 50MTA	P51116	-0.02287	0.055193	0.038746	FXR2	RNA-bindir	FXR2_HUM	18	0.984273
24H 10MTA	Q9UFC0	0.087401	0.055195	0.038747	LRWD1	Leucine-ric	LRWD1_HU	13	1.062455
24H 50MTA	O95749	-0.1641	0.05521	0.038756	GGPS1	Geranylgera	GGPPS_HU	7	0.892484
24H 50MTA	Q04725	0.754153	0.055215	0.038758	TLE2	Transducin	TLE2_HUMA	1	1.686642
24H 50MTA	O96005	0.161998	0.055216	0.038758	CLPTM1	Putative lip	CLPT1_HUI	16	1.118835
48H 50MTA	P37837	0.064946	0.055226	0.038762	TALDO1	Transaldola	TALDO_HUI	16	1.046046
24H 10MTA	Q8NAN2	0.01726	0.055235	0.038768	MIGA1	Mitoguardi	MIGA1_HU	16	1.012035
24H 50MTA	P55769	0.055771	0.055249	0.038775	SNU13	NHP2-like p	NH2L1_HU	9	1.039415
24H 50MTA	Q8TEW0	-0.07702	0.055277	0.038791	PARD3	Partitionin	PARD3_HU	26	0.948016
48H 10MTA	O60749	0.010103	0.055293	0.038797	SNX2	Sorting nex	SNX2_HUM	22	1.007027
48H 10MTA	O15118	-0.1038	0.05531	0.038805	NPC1	NPC intrace	NPC1_HUM	16	0.930576
24H 50MTA	O75970	-0.01259	0.055312	0.038805	MPDZ	Multiple PD	MPDZ_HUM	13	0.99131
24H 10MTA	Q8TBC4	0.007667	0.055313	0.038805	UBA3	NEDD8-acti	UBA3_HUM	19	1.005328
24H 50MTA	Q9Y5W8	0.758505	0.055323	0.03881	SNX13	Sorting nex	SNX13_HUI	5	1.691736
48H 10MTA	Q9BRS2	0.16414	0.055326	0.038812	RIOK1	Serine/thre	RIOK1_HUM	11	1.120498
24H 10MTA	Q6P4F2	-0.55037	0.055334	0.038815	FDX2	Ferredoxin-	FDX2_HUM	3	0.682845
24H 10MTA	Q9NUW8	0.039957	0.055334	0.038815	TDP1	Tyrosyl-DNA	TYDP1_HUI	9	1.028083
24H 10MTA	Q9GZT4	-0.15905	0.055337	0.038816	SRR	Serine racei	SRR_HUMA	4	0.895614
48H 50MTA	Q14894	0.350164	0.055342	0.038818	CRYM	Ketimine re	CRYM_HUM	4	1.274706
24H 50MTA	O75306	0.014022	0.055372	0.038835	NDUFS2	NADH dehy	NDUS2_HU	20	1.009767
24H 10MTA	Q09666	0.029694	0.055394	0.038847	AHNAK	Neuroblast	AHNK_HUM	240	1.020795
24H 50MTA	Q9H4L7	0.02549	0.055393	0.038847	SMARCAD1	SWI/SNF-re	SMRCD_HU	19	1.017825
24H 50MTA	Q9Y385	0.213772	0.055399	0.038849	UBE2J1	Ubiquitin-c	UB2J1_HUI	6	1.159716
48H 50MTA	Q8N668	-0.21814	0.055416	0.038859	COMMD1	COMM dom	COMD1_HU	4	0.85967
24H 50MTA	O60683	0.210494	0.055427	0.038864	PEX10	Peroxisome	PEX10_HUI	3	1.157085
24H 10MTA	Q9C0C9	-0.03702	0.055444	0.038875	UBE2O	(E3-indeper	UBE2O_HU	30	0.974664
48H 50MTA	Q8WV41	-0.01125	0.05546	0.038883	SNX33	Sorting nex	SNX33_HUI	5	0.992232
24H 50MTA	Q86U38	0.031288	0.05552	0.038918	NOP9	Nucleolar p	NOP9_HUM	20	1.021924
48H 10MTA	Q9H3U1	0.019932	0.055535	0.038926	UNC45A	Protein unc	UN45A_HU	39	1.013912
48H 10MTA	Q99653	0.021746	0.055552	0.038936	CHP1	Calcineurin	CHP1_HUM	10	1.015187
24H 10MTA	Q9NR61	0.340274	0.055582	0.038952	DLL4	Delta-like p	DLL4_HUM	1	1.265997
24H 50MTA	Q9NYZ2	0.566252	0.055584	0.038952	SLC25A37	Mitoferrin-	MFRN1_HU	1	1.480672
48H 50MTA	Q16850	-0.14504	0.055586	0.038953	CYP51A1	Lanosterol	CP51A_HUI	23	0.904355
48H 10MTA	Q13163	0.539523	0.055597	0.038959	MAP2K5	Dual specifi	MP2K5_HU	2	1.453492
48H 10MTA	Q8TEY7	0.051749	0.055597	0.038959	USP33	Ubiquitin c	UBP33_HU	11	1.036521
48H 10MTA	O95059	-0.0126	0.055602	0.038961	RPP14	Ribonuclea	RPP14_HUI	2	0.991307
24H 10MTA	Q15287	0.134332	0.055612	0.038967	RNPS1	RNA-bindir	RNPS1_HUI	6	1.097584
48H 50MTA	Q8IUR0	-0.23906	0.055621	0.03897	TRAPPC5	Trafficking	TPPC5_HUI	5	0.847296
24H 10MTA	Q9BRK4	0.374174	0.055637	0.03898	LZTS2	Leucine zip	LZTS2_HUM	6	1.296097
24H 50MTA	Q8TA86	0.307311	0.055653	0.038987	RP9	Retinitis pi	RP9_HUMA	3	1.2374
48H 10MTA	Q9H501	0.004263	0.055652	0.038987	ESF1	ESF1 homo	ESF1_HUMA	17	1.00296
48H 50MTA	P53004	0.067628	0.055671	0.038998	BLVRA	Biliverdin r	BIEA_HUMA	15	1.047992

24H 50MT/	Q8IXH7	0.029607	0.055669	0.038998	NELFCD	Negative el	NELFD_HUI	14	1.020734
48H 10MT/	Q9NQX4	-0.10933	0.05567	0.038998	MYO5C	Unconvent	MYO5C_HU	13	0.927017
24H 50MT/	Q9BPX5	-0.10048	0.055687	0.039008	ARPC5L	Actin-relate	ARP5L_HUI	5	0.932723
48H 50MT/	Q8TE77	0.299423	0.055691	0.03901	SSH3	Protein pho	SSH3_HUM	8	1.230652
24H 10MT/	Q5RI15	-0.02234	0.055692	0.03901	COX20	Cytochrom	COX20_HU	6	0.984635
48H 50MT/	Q56P03	-0.20977	0.055711	0.039019	EAPP	E2F-associa	EAPP_HUM	2	0.864677
48H 50MT/	Q9NVF7	-0.43449	0.055712	0.039019	FBXO28	F-box only	FBX28_HUI	5	0.739956
48H 50MT/	O60911	-0.91534	0.055744	0.039037	CTSV	Cathepsin L	CATL2_HUI	2	0.53022
24H 10MT/	Q9P0T7	-0.00231	0.055746	0.039037	TMEM9	Proton-trai	TMEM9_HU	4	0.998399
24H 50MT/	P10321	0.206148	0.055752	0.039037	HLA-C	HLA class I	HLAC_HUM	3	1.153604
24H 50MT/	P52630	-0.49483	0.055751	0.039037	STAT2	Signal trans	STAT2_HUI	7	0.709644
48H 50MT/	Q9Y2Q3	0.093027	0.055755	0.039037	GSTK1	Glutathion	GSTK1_HUI	13	1.066606
48H 10MT/	Q8NEY8	-0.15005	0.055757	0.039038	PPHLN1	Periphilin-1	PPHLN_HU	8	0.901217
48H 50MT/	Q92558	0.108624	0.055763	0.039041	WASF1	Actin-bindi	WASF1_HU	1	1.0782
48H 10MT/	Q8NE71	0.072247	0.05577	0.039044	ABCF1	ATP-bindin	ABCF1_HUI	33	1.051353
24H 50MT/	Q8IYB3	0.248185	0.055789	0.039054	SRRM1	Serine/argi	SRRM1_HU	7	1.187712
48H 10MT/	O95782	0.010448	0.055808	0.039066	AP2A1	AP-2 compl	AP2A1_HUI	40	1.007268
24H 50MT/	P20585	0.10617	0.055808	0.039066	MSH3	DNA misma	MSH3_HUM	18	1.076367
48H 50MT/	Q7Z3C6	0.162185	0.055836	0.039082	ATG9A	Autophagy-	ATG9A_HUI	17	1.11898
24H 50MT/	Q14738	-0.1328	0.055839	0.039084	PPP2R5D	Serine/thre	2A5D_HUM	21	0.912059
24H 50MT/	Q13596	-0.07711	0.055847	0.039087	SNX1	Sorting nex	SNX1_HUM	17	0.947952
24H 10MT/	Q15493	-0.09727	0.055865	0.039094	RGN	Regucalcin	RGN_HUM/	7	0.934803
24H 50MT/	Q9NUQ9	-0.06945	0.055864	0.039094	CYRIB	CYFIP-relate	CYRIB_HUM	15	0.952999
24H 50MT/	P42167	-0.07184	0.055878	0.039102	TMPO	Lamina-ass	LAP2B_HUI	11	0.951425
48H 50MT/	O43159	-0.14343	0.055888	0.039106	RRP8	Ribosomal	RRP8_HUM	14	0.905365
48H 50MT/	Q92539	-0.19749	0.055889	0.039106	LPIN2	Phosphatid	LPIN2_HUM	11	0.872069
48H 10MT/	Q13367	-0.10401	0.055917	0.039122	AP3B2	AP-3 compl	AP3B2_HUI	1	0.930446
24H 10MT/	P49407	-0.03832	0.055918	0.039122	ARRB1	Beta-arresti	ARRB1_HUI	13	0.973787
48H 50MT/	Q6NZI2	-0.842	0.055929	0.039127	CAVIN1	Caveolae-as	CAVN1_HU	2	0.557869
24H 10MT/	Q92747	-0.11372	0.055938	0.039131	ARPC1A	Actin-relate	ARC1A_HUI	13	0.924204
24H 50MT/	Q5HYJ3	0.229616	0.055941	0.039132	FAM76B	Protein FAI	FA76B_HUI	3	1.172523
24H 50MT/	Q9H254	-0.10911	0.055952	0.039138	SPTBN4	Spectrin be	SPTN4_HUI	2	0.927161
24H 10MT/	O95470	0.026205	0.055958	0.039141	SGPL1	Sphingosin	SGPL1_HUI	17	1.01833
48H 10MT/	Q9GZT8	0.000923	0.055959	0.039141	NIF3L1	NIF3-like pı	NIF3L_HUM	12	1.00064
48H 10MT/	Q15386	0.022443	0.055965	0.039144	UBE3C	Ubiquitin-p	UBE3C_HUI	37	1.015678
48H 50MT/	P18031	-0.02735	0.05601	0.039171	PTPN1	Tyrosine-pr	PTN1_HUM	21	0.981221
24H 10MT/	Q9Y6G9	0.001137	0.056025	0.039179	DYNC1LI1	Cytoplasmi	DC1L1_HUI	27	1.000788
24H 10MT/	Q14667	0.572794	0.05603	0.039181	BLTP2	Bridge-like	BLTP2_HUI	2	1.487401
24H 50MT/	P78324	0.547543	0.056034	0.039183	SIRPA	Tyrosine-pr	SHPS1_HUI	3	1.461594
48H 10MT/	Q9UPT5	0.011412	0.056047	0.03919	EXOC7	Exocyst cor	EXOC7_HUI	27	1.007942
24H 10MT/	A6NHX0	-0.14089	0.056056	0.039193	CASTOR2	Cytosolic a	CAST2_HUI	9	0.906959
24H 10MT/	Q9NUQ6	0.044584	0.056058	0.039194	SPATS2L	SPATS2-like	SPS2L_HUM	18	1.031385
24H 50MT/	Q8NB78	-0.28618	0.056065	0.039198	KDM1B	Lysine-spec	KDM1B_HU	4	0.820071
24H 50MT/	Q96BI3	-0.20363	0.056082	0.039205	APH1A	Gamma-sec	APH1A_HU	2	0.868362
24H 10MT/	Q9Y266	-0.09806	0.056096	0.039213	NUDC	Nuclear mi	NUDC_HUM	19	0.934289
48H 10MT/	L0R6Q1	-0.07924	0.056115	0.039221	SLC35A4	SLC35A4 u	S35U4_HUI	6	0.946554
48H 10MT/	O43301	0.113486	0.056114	0.039221	HSPA12A	Heat shock	HS12A_HUI	7	1.081839
24H 50MT/	Q9NW13	0.070055	0.056124	0.039223	RBM28	RNA-bindir	RBM28_HU	24	1.049757
24H 50MT/	Q6UX04	-0.06776	0.056127	0.039225	CWC27	Spliceosom	CWC27_HU	6	0.95412
48H 50MT/	Q5TAQ9	0.08059	0.056144	0.039231	DCAF8	DDB1- and	DCAF8_HUI	10	1.057451
24H 50MT/	Q15434	-0.30594	0.056157	0.039236	RBMS2	RNA-bindir	RBMS2_HU	4	0.808917
24H 10MT/	Q5T4F4	-0.4489	0.056186	0.039252	ZFYVE27	Protrudin	ZFY27_HUM	3	0.732603

48H 50MTA	Q9UH17	-0.22259	0.056188	0.039252	APOBEC3B	DNA dC->dU	ABC3B_HUI	9	0.857023
24H 50MTA	Q13510	0.090779	0.056209	0.039264	ASAH1	Acid ceram	ASAH1_HUI	17	1.064945
48H 10MTA	Q9H993	0.036916	0.056214	0.039266	ARMT1	Damage-co	ARMT1_HU	16	1.025919
24H 50MTA	Q2TAA2	0.091509	0.056244	0.039286	IAH1	Isoamyl ace	IAH1_HUM	8	1.065484
48H 10MTA	Q68DC2	0.024149	0.056268	0.039297	ANKS6	Ankyrin rep	ANKS6_HUI	2	1.01688
24H 50MTA	Q6PCB7	-0.63357	0.056275	0.039301	SLC27A1	Long-chain	S27A1_HUI	1	0.64458
48H 10MTA	P61970	0.056484	0.056295	0.03931	NUTF2	Nuclear tra	NTF2_HUM	6	1.039928
24H 50MTA	L0R8F8	-0.14053	0.056307	0.039315	MIEF1	Mitochond	MIDUO_HU	3	0.907189
24H 50MTA	Q96A35	-0.02869	0.05631	0.039316	MRPL24	Large ribos	RM24_HUM	12	0.980309
48H 10MTA	Q9H7X7	-0.16436	0.056326	0.039323	IFT22	Intraflagell	IFT22_HUM	1	0.892327
48H 10MTA	O00291	0.040328	0.056334	0.039326	HIP1	Huntingtin	HIP1_HUM	22	1.028347
24H 10MTA	Q16877	0.160895	0.056347	0.039334	PFKFB4	6-phosphof	F264_HUM	9	1.11798
48H 10MTA	Q8IYB5	-0.00042	0.056352	0.039336	SMAP1	Stromal me	SMAP1_HU	4	0.999708
24H 50MTA	Q03167	0.393473	0.056354	0.039337	TGFBR3	Transformi	TGBR3_HUI	2	1.313552
24H 10MTA	Q4VC44	0.304946	0.056363	0.03934	FLYWCH1	FLYWCH-ty	FWCH1_HU	6	1.235372
24H 10MTA	Q9BV38	0.040044	0.056374	0.039345	WDR18	WD repeat-	WDR18_HU	13	1.028145
48H 50MTA	Q99828	-0.24407	0.056383	0.039349	CIB1	Calcium an	CIB1_HUMA	4	0.844358
24H 10MTA	Q13283	-0.03352	0.056389	0.03935	G3BP1	Ras GTPase-	G3BP1_HU	23	0.977034
48H 10MTA	O75175	0.053411	0.056391	0.039351	CNOT3	CCR4-NOT t	CNOT3_HU	8	1.037715
24H 10MTA	Q96B01	0.146791	0.056411	0.039363	RAD51AP1	RAD51-asso	R51A1_HUI	3	1.107104
48H 50MTA	P17612	0.012178	0.056431	0.039372	PRKACA	cAMP-depe	KAPCA_HUI	5	1.008477
24H 50MTA	O43301	0.125516	0.05644	0.039378	HSPA12A	Heat shock	HS12A_HUI	7	1.090898
48H 10MTA	Q7L211	-0.558	0.056443	0.039379	ABHD13	Protein ABI	ABHDD_HU	3	0.679243
48H 10MTA	Q9NWH9	-0.08332	0.056469	0.039394	SLTM	SAFB-like tr	SLTM_HUM	17	0.943881
48H 10MTA	Q9GZP9	0.025875	0.05647	0.039394	DERL2	Derlin-2	DERL2_HUI	5	1.018097
24H 50MTA	O60271	0.014101	0.056491	0.039406	SPAG9	C-Jun-amin	JIP4_HUMA	45	1.009822
24H 10MTA	Q6ZNW5	0.041029	0.056516	0.039418	GDPGP1	GDP-D-gluc	GDPP1_HU	5	1.028847
24H 50MTA	Q96BX8	0.46581	0.056532	0.039427	MOB3A	MOB kinase	MOB3A_HU	3	1.381093
24H 10MTA	Q13439	-0.06025	0.056542	0.039432	GOLGA4	Golgin subf	GOGA4_HU	41	0.959099
24H 10MTA	P40261	-0.13812	0.056563	0.039445	NNMT	Nicotinami	NNMT_HUM	11	0.908705
48H 10MTA	Q2TB90	0.181747	0.056571	0.039449	HKDC1	Hexokinase	HKDC1_HU	18	1.134256
48H 10MTA	O60503	-0.31254	0.056604	0.039462	ADCY9	Adenylate c	ADCY9_HUI	4	0.805226
48H 10MTA	Q96AX1	0.010357	0.056604	0.039462	VPS33A	Vacuolar pr	VP33A_HUI	25	1.007205
48H 10MTA	Q7Z2W4	-0.03627	0.05661	0.039465	ZC3HAV1	Zinc finger	ZCCHV_HUI	37	0.975176
24H 10MTA	Q13356	-0.16446	0.056614	0.039467	PPIL2	RING-type E	PPIL2_HUM	11	0.892263
24H 10MTA	P43361	-0.21719	0.056615	0.039467	MAGEA8	Melanoma-	MAGA8_HU	1	0.860237
24H 10MTA	Q8N4P2	0.56913	0.056637	0.039478	IFT70B	Intraflagell	IT70B_HUM	2	1.483629
24H 50MTA	P00742	-0.44402	0.056676	0.039501	F10	Coagulatio	FA10_HUM	4	0.735086
24H 50MTA	Q96SI9	0.071019	0.056687	0.039508	STRBP	Spermatid	STRBP_HUI	22	1.050459
48H 10MTA	Q2NL82	-0.00603	0.056689	0.039509	TSR1	Pre-rRNA-p	TSR1_HUM	23	0.995827
24H 50MTA	Q6IQ23	0.081102	0.056711	0.039519	PLEKHA7	Pleckstrin h	PKHA7_HU	12	1.057826
24H 50MTA	Q9P0P0	0.076581	0.056712	0.039519	RNF181	E3 ubiquiti	RN181_HU	2	1.054516
24H 10MTA	Q4J6C6	0.04136	0.056714	0.03952	PREPL	Prolyl endo	PPCEL_HUI	17	1.029083
24H 50MTA	O00193	0.169915	0.056724	0.039526	SMAP	Small acidi	SMAP_HUM	3	1.124993
24H 50MTA	O15111	-0.0434	0.056734	0.039532	CHUK	Inhibitor o	IKKA_HUMA	25	0.970368
48H 10MTA	Q9UPQ8	0.123779	0.056748	0.039539	DOLK	Dolichol ki	DOLK_HUM	4	1.089585
48H 10MTA	P47224	0.124667	0.056765	0.039551	RABIF	Guanine nu	MSS4_HUM	4	1.090256
48H 50MTA	Q9UHD1	-0.12365	0.05678	0.039559	CHORDC1	Cysteine an	CHRD1_HU	20	0.917861
48H 50MTA	O60739	0.066962	0.056792	0.039565	EIF1B	Eukaryotic	EIF1B_HUM	1	1.047508
48H 50MTA	Q969T9	-0.21578	0.056792	0.039565	WBP2	WW domai	WBP2_HUM	6	0.861078
24H 10MTA	P63244	0.02747	0.056802	0.03957	RACK1	Small ribos	RACK1_HUI	20	1.019223
48H 50MTA	Q9BWS9	0.268717	0.056831	0.039585	CHID1	Chitinase d	CHID1_HUI	9	1.204736

48H 10MT/	Q460N5	0.392297	0.056831	0.039585	PARP14	Protein mo	PAR14_HU	1	1.312482
24H 10MT/	Q8TDX7	0.01585	0.05683	0.039585	NEK7	Serine/thre	NEK7_HUM	10	1.011047
48H 50MT/	Q9NU23	-0.21685	0.056839	0.039589	LYRM2	LYR motif-c	LYRM2_HU	3	0.860443
24H 10MT/	Q9NRG7	-0.05337	0.056842	0.03959	SDR39U1	Epimerase f	D39U1_HU	2	0.963682
24H 10MT/	Q969X5	-0.1153	0.056869	0.039602	ERGIC1	Endoplasm	ERGI1_HUM	9	0.923193
24H 50MT/	Q9NVI1	-0.09777	0.056872	0.039602	FANCI	Fanconi an	FANCI_HUM	53	0.934475
24H 50MT/	Q0IIM8	0.344139	0.056875	0.039603	TBC1D8B	TBC1 doma	TBC8B_HU	11	1.269393
24H 10MT/	P28702	-0.06685	0.05688	0.039604	RXRB	Retinoic ac	RXRB_HUM	5	0.954718
24H 50MT/	Q8N4L2	0.03863	0.056907	0.039621	PIP4P2	Type 2 pho	PP4P2_HU	3	1.027138
48H 10MT/	Q6P1N9	0.093253	0.05693	0.039631	TATDN1	Deoxyribor	TATD1_HU	14	1.066773
48H 10MT/	O43264	0.035045	0.056948	0.03964	ZW10	Centromer	ZW10_HUM	30	1.024589
48H 50MT/	Q9BQ13	0.046211	0.056953	0.039642	KCTD14	BTB/POZ dc	KCD14_HU	9	1.032549
48H 10MT/	Q13643	0.417022	0.056955	0.039643	FHL3	Four and a	FHL3_HUM	3	1.335168
48H 50MT/	O00505	-0.05755	0.056994	0.039665	KPNA3	Importin su	IMA4_HUM	20	0.960893
24H 50MT/	Q6ZS30	-0.09947	0.057006	0.03967	NBEAL1	Neurobeacl	NBEL1_HU	28	0.933377
48H 50MT/	Q7L014	0.065682	0.057015	0.039674	DDX46	Probable A	DDX46_HU	42	1.04658
48H 50MT/	O14910	0.079512	0.057033	0.039685	LIN7A	Protein lin-	LIN7A_HUM	9	1.056661
24H 50MT/	Q99715	0.193799	0.057044	0.039691	COL12A1	Collagen al	COCA1_HU	2	1.143772
48H 50MT/	Q9BTL3	0.159614	0.057045	0.039691	RAMAC	RNA guanir	RAMAC_HU	5	1.116988
48H 10MT/	Q13636	0.020681	0.05705	0.039693	RAB31	Ras-related	RAB31_HU	4	1.014438
24H 10MT/	O75317	0.207989	0.057052	0.039694	USP12	Ubiquitin c	UBP12_HU	2	1.155077
24H 10MT/	Q8N9T8	0.017401	0.057057	0.039697	KRI1	Protein KRI	KRI1_HUM	12	1.012134
48H 10MT/	Q9UHV9	0.066878	0.057068	0.039701	PFDN2	Prefoldin su	PFD2_HUM	7	1.047447
24H 10MT/	P50542	-0.11464	0.057081	0.039707	PEX5	Peroxisoma	PEX5_HUM	22	0.923614
48H 10MT/	Q13191	-0.24849	0.057092	0.039713	CBLB	E3 ubiquiti	CBLB_HUM	1	0.841776
48H 10MT/	Q92520	0.083328	0.057138	0.039739	FAM3C	Protein FAI	FAM3C_HU	8	1.059459
48H 10MT/	Q14C86	-0.0117	0.057145	0.039742	GAPVD1	GTPase-acti	GAPD1_HU	39	0.99192
48H 10MT/	O60763	-0.00778	0.057153	0.039746	USO1	General ves	USO1_HUM	42	0.994621
48H 50MT/	P26640	-0.0942	0.057164	0.039752	VARS1	Valine--tRN	SYVC_HUM	60	0.936793
48H 50MT/	P62899	0.006323	0.057165	0.039752	RPL31	Large ribos	RL31_HUM	5	1.004393
24H 10MT/	O95758	-0.06153	0.057182	0.039762	PTBP3	Polypyrimi	PTBP3_HU	13	0.958247
24H 50MT/	Q9BZE4	0.043611	0.057187	0.039764	GTPBP4	GTP-bindin	GTPB4_HU	33	1.03069
48H 10MT/	Q00796	0.005133	0.057196	0.039768	SORD	Sorbitol de	DHSO_HUM	15	1.003565
48H 50MT/	O75150	-0.13472	0.057201	0.03977	RNF40	E3 ubiquiti	BRE1B_HU	26	0.910846
48H 50MT/	Q9NX46	0.125971	0.057221	0.039781	ADPRS	ADP-ribosy	ADPRS_HU	10	1.091242
48H 10MT/	Q6YHU6	0.043574	0.057229	0.039784	THADA	tRNA (32-2'	THADA_HU	36	1.030664
24H 10MT/	Q9Y6E2	0.037426	0.057226	0.039784	BZW2	eIF5-mimic	5MP1_HUM	22	1.026281
24H 10MT/	Q5TBB1	0.09496	0.057295	0.039819	RNASEH2B	Ribonuclea	RNH2B_HU	7	1.068036
24H 10MT/	Q9NQT5	0.082104	0.057317	0.039831	EXOSC3	Exosome cc	EXOS3_HU	10	1.058561
48H 50MT/	Q6P4I2	-0.28768	0.057327	0.039833	WDR73	WD repeat-	WDR73_HU	10	0.819219
48H 50MT/	Q7Z4V5	0.073575	0.057324	0.039833	HDGFL2	Hepatoma-	HDGR2_HU	11	1.052321
24H 10MT/	P43358	0.029497	0.057332	0.039836	MAGEA4	Melanoma-	MAGA4_HU	11	1.020657
24H 10MT/	Q9NX40	-0.07535	0.057337	0.039836	OCIAD1	OCIA doma	OCAD1_HU	9	0.949111
48H 10MT/	Q9H9L4	0.390721	0.057344	0.039838	KANSL2	KAT8 regula	KANL2_HU	2	1.311048
48H 50MT/	P48507	-0.07797	0.05736	0.039846	GCLM	Glutamate-	GSH0_HUM	11	0.94739
48H 10MT/	Q96IZ5	0.240189	0.05737	0.03985	RBM41	RNA-bindir	RBM41_HU	2	1.181147
48H 10MT/	O95573	-0.00607	0.057395	0.039862	ACSL3	Fatty acid C	ACSL3_HUI	28	0.995805
48H 10MT/	P20823	0.037586	0.057401	0.039865	HNF1A	Hepatocyte	HNF1A_HU	6	1.026395
24H 50MT/	P27105	0.086987	0.057405	0.039866	STOM	Stomatin	STOM_HUM	7	1.062149
48H 10MT/	Q96ND0	0.154488	0.057424	0.039878	FAM210A	Protein FAI	F210A_HU	3	1.113026
48H 50MT/	P07237	0.086101	0.057436	0.039885	P4HB	Protein dis	PDIA1_HUI	35	1.061497
48H 10MT/	O75396	-0.16157	0.057456	0.039897	SEC22B	Vesicle-traf	SC22B_HU	11	0.894054

24H 10MT/	Q9Y4P3	0.051767	0.057497	0.039923	TBL2	Transducin	TBL2_HUM	21	1.036534
24H 50MT/	O43759	0.064365	0.057499	0.039924	SYNGR1	Synaptogyr	SNG1_HUM	2	1.045625
48H 50MT/	Q96JG6	0.061544	0.057524	0.039937	VPS50	Syndetin	VPS50_HUI	20	1.043582
24H 10MT/	Q9H8H3	-0.08412	0.057538	0.039944	TMT1A	Thiol S-met	TMT1A_HU	7	0.943357
48H 50MT/	Q9NV92	0.228676	0.057581	0.039967	NDFIP2	NEDD4 fam	NFIP2_HUM	2	1.171759
48H 10MT/	O75947	0.058687	0.057593	0.039973	ATP5PD	ATP synthas	ATP5H_HUI	12	1.041517
48H 50MT/	Q9NVG8	-0.07218	0.057592	0.039973	TBC1D13	TBC1 doma	TBC13_HUI	15	0.951201
48H 50MT/	Q8IYB3	0.130763	0.057617	0.039987	SRRM1	Serine/argi	SRRM1_HU	7	1.094873
48H 10MT/	Q86WW8	-0.03429	0.057624	0.039988	COA5	Cytochrom	COA5_HUM	2	0.976511
48H 10MT/	Q8IYU8	0.215607	0.057628	0.039988	MICU2	Calcium up	MICU2_HU	13	1.161193
24H 10MT/	Q92947	-0.17123	0.05763	0.039989	GCDH	Glutaryl-Co	GCDH_HUM	9	0.888084
24H 50MT/	Q9UMY4	-0.02203	0.057632	0.03999	SNX12	Sorting nex	SNX12_HUI	9	0.984848
24H 50MT/	P78381	0.355573	0.057643	0.039994	SLC35A2	UDP-galact	S35A2_HUI	3	1.279494
24H 10MT/	P14618	-0.06649	0.057653	0.04	PKM	Pyruvate ki	KPYM_HUM	43	0.95496
24H 10MT/	Q9BRJ2	-0.03522	0.057669	0.040008	MRPL45	Large ribos	RM45_HUM	14	0.975883
24H 10MT/	Q9P2B2	0.211097	0.05769	0.040022	PTGFRN	Prostaglanc	FPRP_HUM	18	1.157568
24H 10MT/	Q9H4D0	1.886324	0.057692	0.040022	CLSTN2	Calsyntenir	CSTN2_HUI	1	3.696921
48H 10MT/	P43155	0.000171	0.057701	0.040026	CRAT	Carnitine O	CACP_HUM	16	1.000118
24H 10MT/	P36542	0.004676	0.057828	0.040101	ATP5F1C	ATP synthas	ATPG_HUM	10	1.003247
24H 10MT/	O95685	-0.00462	0.057848	0.040114	PPP1R3D	Protein pho	PPR3D_HU	5	0.9968
24H 50MT/	P82664	-0.01872	0.057861	0.040119	MRPS10	Small ribos	RT10_HUM	4	0.987111
48H 50MT/	Q8NEY4	-0.41917	0.05786	0.040119	ATP6V1C2	V-type prot	VATC2_HUI	2	0.747854
48H 10MT/	Q9BV10	-0.00015	0.057862	0.040119	ALG12	Dol-P-Man:	ALG12_HUI	4	0.999893
48H 10MT/	Q9Y3C0	0.93199	0.057869	0.040123	WASHC3	WASH com	WASC3_HU	4	1.907906
48H 10MT/	Q12962	1.135205	0.057875	0.040126	TAF10	Transcripti	TAF10_HUI	2	2.196497
48H 50MT/	Q06547	-0.1502	0.057877	0.040126	GABPB1	GA-binding	GABP1_HU	1	0.901128
48H 10MT/	Q96DX4	0.090748	0.057876	0.040126	RSPRY1	RING finger	RSPRY_HUI	10	1.064922
48H 10MT/	Q9BUK6	0.096848	0.057903	0.040141	MSTO1	Protein mis	MSTO1_HU	17	1.069435
24H 50MT/	O15347	0.105711	0.057936	0.040158	HMGB3	High mobil	HMGB3_HU	7	1.076024
48H 50MT/	Q13796	-0.0254	0.057952	0.040167	SHROOM2	Protein Shr	SHRM2_HU	16	0.982547
48H 50MT/	Q92794	-0.46082	0.057965	0.040174	KAT6A	Histone ace	KAT6A_HUI	3	0.726574
24H 10MT/	O15085	0.057712	0.05797	0.040176	ARHGEF11	Rho guanin	ARHGB_HU	12	1.040814
48H 10MT/	A6NKD9	-0.13227	0.057995	0.040189	CCDC85C	Coiled-coil	CC85C_HUI	4	0.912395
24H 50MT/	Q6UW02	0.080592	0.058002	0.040192	CYP20A1	Cytochrom	CP20A_HUI	17	1.057452
24H 10MT/	Q9UBD5	0.100988	0.058	0.040192	ORC3	Origin reco	ORC3_HUM	16	1.072507
24H 50MT/	Q9UHJ3	0.59363	0.058021	0.040202	SFMBT1	Scm-like wi	SMBT1_HU	1	1.509039
48H 10MT/	Q8WZA9	0.005298	0.058051	0.040217	IRGQ	Immunity-r	IRGQ_HUM	17	1.003679
24H 10MT/	Q66GS9	1.567352	0.058058	0.040221	CEP135	Centrosom	CP135_HUI	1	2.963602
24H 10MT/	Q9Y680	0.103481	0.05807	0.040227	FKBP7	Peptidyl-pr	FKBP7_HUI	6	1.074363
24H 10MT/	Q8N8A2	-0.1522	0.058091	0.040239	ANKRD44	Serine/thre	ANR44_HU	5	0.899879
24H 50MT/	P53985	0.170107	0.058119	0.040255	SLC16A1	Monocarbo	MOT1_HUM	11	1.125142
48H 10MT/	O75414	0.109148	0.058141	0.040266	NME6	Nucleoside	NDK6_HUM	4	1.078591
48H 50MT/	O43493	0.041004	0.058148	0.04027	TGOLN2	Trans-Golgi	TGON2_HU	7	1.028829
48H 10MT/	Q5VST6	-0.84106	0.058152	0.04027	ABHD17B	Alpha/beta	AB17B_HUI	2	0.558232
24H 10MT/	Q96C01	-0.09256	0.058155	0.040271	FAM136A	Protein FAN	F136A_HUI	5	0.937854
48H 50MT/	Q7L1V2	0.049488	0.058171	0.040281	MON1B	Vacuolar fu	MON1B_HU	4	1.034898
48H 50MT/	Q8WWH5	-0.11758	0.058182	0.040285	TRUB1	Pseudourid	TRUB1_HUI	15	0.921735
48H 10MT/	Q9BU02	0.085201	0.058257	0.040331	THTPA	Thiamine-tr	THTPA_HUI	5	1.060836
24H 50MT/	O96017	0.18856	0.058268	0.040334	CHEK2	Serine/thre	CHK2_HUM	14	1.139626
24H 50MT/	Q8NCM8	0.083819	0.058277	0.040339	DYNC2H1	Cytoplasmi	DYHC2_HU	33	1.05982
24H 50MT/	O00124	0.164128	0.058307	0.040359	UBXN8	UBX domai	UBXN8_HU	4	1.120489
24H 10MT/	P41223	-0.05906	0.05832	0.040366	BUD31	Protein BUI	BUD31_HU	8	0.959888

24H 10MTA	Q14103	-0.091	0.058323	0.040367	HNRNPD	Heterogene	HNRPD_HU	15	0.938874
24H 50MTA	O14894	-1.09094	0.05834	0.040377	TM4SF5	Transmemb	T4S5_HUM	1	0.469455
24H 50MTA	Q7RTV0	-0.06017	0.058344	0.040378	PHF5A	PHD finger-	PHF5A_HU	6	0.959151
48H 10MTA	Q9Y2X3	-0.03939	0.058368	0.040392	NOP58	Nucleolar p	NOP58_HU	24	0.973065
24H 10MTA	P46063	0.082866	0.058375	0.040396	RECQL	ATP-depend	RECQ1_HU	27	1.05912
48H 10MTA	Q9P1U1	0.079301	0.058404	0.040412	ACTR3B	Actin-relate	ARP3B_HU	7	1.056506
24H 50MTA	Q9UHK0	-0.21243	0.058412	0.040416	NUFIP1	FMR1-inter	NUFP1_HU	3	0.863084
48H 50MTA	Q0JRZ9	-0.11032	0.058417	0.040419	FCHO2	F-BAR dom	FCHO2_HU	6	0.926382
48H 10MTA	Q7Z422	0.039031	0.058431	0.040424	SZRD1	SUZ RNA-bi	SZRD1_HU	2	1.027423
48H 50MTA	Q9C0E8	0.001465	0.058499	0.040463	LNPK	Endoplasm	LNP_HUMA	12	1.001016
24H 50MTA	Q15345	0.551974	0.058508	0.040467	LRRC41	Leucine-ric	LRC41_HU	1	1.46609
24H 50MTA	Q15058	-0.03061	0.058521	0.040473	KIF14	Kinesin-like	KIF14_HUM	15	0.979005
24H 50MTA	Q5BJD5	0.280419	0.058524	0.040474	TMEM41B	Transmemb	TM41B_HU	3	1.214547
24H 10MTA	Q9Y6D9	-0.00395	0.058569	0.040497	MAD1L1	Mitotic spi	MD1L1_HU	22	0.997269
48H 10MTA	A9UHW6	-0.22254	0.058572	0.040497	MIF4GD	MIF4G dom	MI4GD_HU	1	0.857053
24H 10MTA	Q9UDY4	0.070495	0.058575	0.040498	DNAJB4	DnaJ homo	DNJB4_HU	11	1.050077
48H 50MTA	Q13155	-0.11012	0.058592	0.040507	AIMP2	Aminoacyl	AIMP2_HU	13	0.926509
24H 50MTA	Q9UKG9	-0.0574	0.058608	0.040513	CROT	Peroxisoma	OCTC_HUM	10	0.960992
48H 10MTA	Q9NQ34	0.325463	0.058617	0.040517	TMEM9B	Transmemb	TMM9B_HU	2	1.253067
24H 10MTA	Q9UKT4	0.353561	0.058619	0.040517	FBXO5	F-box only	FBX5_HUM	2	1.277711
24H 10MTA	Q7Z434	-0.06012	0.058642	0.040527	MAVS	Mitochond	MAVS_HUM	14	0.959184
24H 50MTA	Q9P0S2	0.223902	0.058646	0.040528	COX16	Cytochrom	COX16_HU	2	1.167888
24H 50MTA	Q92643	0.015411	0.05866	0.040537	PIGK	GPI-anchor	GPI8_HUM	13	1.01074
48H 50MTA	P15311	-0.09667	0.058669	0.04054	EZR	Ezrin	EZRI_HUMA	25	0.935191
48H 50MTA	Q9H2G2	0.001003	0.058682	0.040547	SLK	STE20-like s	SLK_HUMA	36	1.000695
48H 50MTA	Q8WXA3	0.006041	0.058693	0.040552	RUFY2	RUN and FY	RUFY2_HU	9	1.004196
24H 50MTA	O43314	-0.13358	0.058708	0.04056	PPIP5K2	Inositol he	VIP2_HUMA	10	0.911563
48H 10MTA	Q6UXN9	0.056835	0.058712	0.040561	WDR82	WD repeat-	WDR82_HU	13	1.040181
24H 10MTA	Q15428	0.099718	0.058722	0.040565	SF3A2	Splicing fac	SF3A2_HUM	9	1.071564
48H 10MTA	Q7RTS7	-0.48519	0.058734	0.040571	KRT74	Keratin, typ	K2C74_HU	3	0.714401
24H 10MTA	O94906	0.041236	0.058739	0.040573	PRPF6	Pre-mRNA-	PRP6_HUM	38	1.028995
24H 10MTA	P50990	0.014182	0.058758	0.040582	CCT8	T-complex	TCPQ_HUM	35	1.009878
48H 50MTA	Q7KZI7	0.00371	0.058756	0.040582	MARK2	Serine/thre	MARK2_HU	19	1.002575
48H 50MTA	P12955	0.053942	0.058774	0.040589	PEPD	Xaa-Pro dip	PEPD_HUM	21	1.038097
24H 10MTA	Q9NW15	0.061613	0.058789	0.040596	ANO10	Anoctamin	ANO10_HU	9	1.043632
24H 10MTA	Q9H4B7	0.84108	0.058791	0.040597	TUBB1	Tubulin bet	TBB1_HUM	2	1.79139
24H 50MTA	P50750	-0.00187	0.058801	0.040603	CDK9	Cyclin-depe	CDK9_HUM	10	0.998705
24H 10MTA	Q9BXJ8	-0.15949	0.058809	0.040607	TMEM120A	Ion channe	TACAN_HU	3	0.895341
24H 50MTA	Q9P260	-0.05259	0.058813	0.040609	RELCH	RAB11-bind	RELCH_HU	31	0.964206
24H 50MTA	Q12962	0.2699	0.058831	0.040615	TAF10	Transcripti	TAF10_HUM	2	1.205725
48H 50MTA	Q9H633	0.892106	0.058829	0.040615	RPP21	Ribonuclea	RPP21_HU	2	1.855883
24H 50MTA	P10074	0.10242	0.058842	0.040619	ZBTB48	Telomere zi	TZAP_HUM	3	1.073573
24H 10MTA	Q14296	0.118952	0.058843	0.040619	FASTK	Fas-activate	FASTK_HUM	2	1.085946
24H 50MTA	Q9NR77	-0.48103	0.058854	0.040623	PXMP2	Peroxisoma	PXMP2_HU	5	0.716465
48H 50MTA	Q5HYI7	-0.07079	0.058865	0.040629	MTX3	Metaxin-3	MTX3_HUM	5	0.952118
48H 10MTA	Q9NSD9	-0.02228	0.05887	0.040631	FARSB	Phenylalan	SYFB_HUMA	29	0.984672
48H 50MTA	P78332	-0.11906	0.058874	0.040632	RBM6	RNA-bindir	RBM6_HUM	23	0.920786
24H 50MTA	Q9C0B7	0.163942	0.058883	0.040635	TANGO6	Transport a	TNG6_HUM	11	1.120345
48H 10MTA	Q9Y3E5	-0.019	0.05889	0.040639	PTRH2	Peptidyl-tR	PTH2_HUM	7	0.986919
24H 10MTA	Q96BW1	-0.20179	0.058954	0.040676	UPRT	Uracil phos	UPP_HUMA	7	0.86947
48H 50MTA	Q5MNZ6	-0.01048	0.058972	0.040683	WDR45B	WD repeat	WIPI3_HUM	10	0.992762
48H 50MTA	Q86YQ8	-0.17994	0.058976	0.040685	CPNE8	Copine-8	CPNE8_HU	17	0.882742

48H 10MT/	P49756	0.039546	0.058999	0.040696	RBM25	RNA-bindir	RBM25_HU	27	1.027791
48H 10MT/	Q7Z6J9	0.103123	0.058998	0.040696	TSEN54	tRNA-splici	SEN54_HUI	7	1.074096
48H 50MT/	P32929	0.314755	0.059001	0.040697	CTH	Cystathioni	CGL_HUMA	12	1.243801
24H 10MT/	P55145	-0.20359	0.059004	0.040698	MANF	Mesenceph	MANF_HUN	7	0.868385
24H 10MT/	Q9BY32	-0.1613	0.059009	0.0407	ITPA	Inosine trip	ITPA_HUM/	12	0.894218
24H 50MT/	Q13614	0.19978	0.05905	0.040724	MTMR2	Myotubula	MTMR2_HU	5	1.148523
48H 50MT/	P02663	-1.14748	0.05905	0.040724	CSN1S2	Alpha-S2-ca	CASA2_BOV	3	0.451412
48H 10MT/	P19838	0.065117	0.059083	0.040741	NFKB1	Nuclear fac	NFKB1_HUI	18	1.04617
24H 10MT/	Q9BRX8	0.052222	0.059083	0.040741	PRXL2A	Peroxiredo	PXL2A_HUI	9	1.03686
24H 50MT/	P19793	0.173383	0.059085	0.040742	RXRA	Retinoic ac	RXRA_HUM	6	1.1277
48H 10MT/	P61086	0.078435	0.05909	0.040744	UBE2K	Ubiquitin-c	UBE2K_HUI	12	1.055872
24H 10MT/	O14639	0.119386	0.059094	0.040745	ABLIM1	Actin-bindi	ABLM1_HU	21	1.086273
24H 10MT/	Q12774	-0.09794	0.05912	0.040755	ARHGEF5	Rho guanin	ARHG5_HU	12	0.934366
24H 10MT/	Q99418	0.601644	0.059139	0.040765	CYTH2	Cytohesin-2	CYH2_HUM	3	1.517445
24H 10MT/	Q15059	0.16861	0.059165	0.040777	BRD3	Bromodom	BRD3_HUM	8	1.123975
48H 10MT/	Q9BTT6	0.061186	0.059167	0.040778	LRRC1	Leucine-ric	LRRC1_HUI	16	1.043323
48H 50MT/	P56378	0.1226	0.05917	0.040779	ATP5MJ	ATP synthas	ATP68_HUI	4	1.088695
48H 10MT/	Q5JS54	-0.20757	0.059203	0.040795	PSMG4	Proteasome	PSMG4_HU	5	0.865994
48H 10MT/	Q6DKK2	0.235978	0.059198	0.040795	TTC19	Tetratricop	TTC19_HUN	9	1.177705
24H 50MT/	O43760	0.032617	0.059239	0.040812	SYNGR2	Synaptogyr	SNG2_HUM	4	1.022866
24H 50MT/	Q9H6S0	-0.03566	0.059263	0.040826	YTHDC2	3'-5' RNA he	YTDC2_HUI	37	0.975582
24H 10MT/	O43861	0.181632	0.059271	0.040828	ATP9B	Probable pl	ATP9B_HUI	4	1.134166
48H 10MT/	P63279	0.046495	0.059276	0.04083	UBE2I	SUMO-conj	UBC9_HUM	12	1.032753
24H 10MT/	Q14232	0.032263	0.059282	0.040833	EIF2B1	Translation	EI2BA_HUN	15	1.022615
48H 50MT/	P16220	0.221945	0.05929	0.040835	CREB1	Cyclic AMP	CREB1_HUI	3	1.166305
24H 50MT/	P02749	-0.90514	0.059345	0.040868	APOH	Beta-2-glyc	APOH_HUN	1	0.533983
24H 10MT/	Q9NXH9	-0.11328	0.059346	0.040868	TRMT1	tRNA (guan	TRM1_HUN	14	0.924485
48H 10MT/	Q9Y277	0.009754	0.059354	0.040871	VDAC3	Voltage-dep	VDAC3_HU	13	1.006784
24H 50MT/	Q86XK3	-0.31877	0.059361	0.040874	SFR1	Swi5-deper	SFR1_HUM	3	0.801752
48H 10MT/	Q96C90	-0.01252	0.059368	0.040877	PPP1R14B	Protein pho	PP14B_HUI	6	0.991362
24H 10MT/	Q13576	-0.05156	0.059381	0.040885	IQGAP2	Ras GTPase-	IQGA2_HUI	66	0.964896
24H 10MT/	P23677	0.070108	0.059393	0.040889	ITPKA	Inositol-tri:	IP3KA_HUN	10	1.049795
24H 50MT/	P84243	0.666647	0.059453	0.040921	H3-3A	Histone H3	H33_HUMA	1	1.587379
48H 50MT/	Q96RN5	-0.16574	0.059518	0.040952	MED15	Mediator o	MED15_HU	9	0.891473
24H 10MT/	Q9BYC8	0.118596	0.059518	0.040952	MRPL32	Large ribos	RM32_HUN	3	1.085678
48H 10MT/	P54760	-0.02585	0.059547	0.040969	EPHB4	Ephrin type	EPHB4_HUI	10	0.982239
24H 50MT/	P50452	0.047602	0.059562	0.040977	SERPINB8	Serpin B8	SPB8_HUM	7	1.033546
48H 10MT/	Q9GZU7	0.105918	0.059567	0.040978	CTDSP1	Carboxy-te	CTDS1_HUI	3	1.076179
48H 10MT/	Q86VN1	0.064075	0.059586	0.040985	VPS36	Vacuolar pr	VPS36_HUI	17	1.045415
24H 10MT/	P49459	0.063625	0.059609	0.041	UBE2A	Ubiquitin-c	UBE2A_HUI	3	1.045089
48H 50MT/	Q7Z794	0.191638	0.059616	0.041001	KRT77	Keratin, typ	K2C1B_HUI	14	1.142059
48H 50MT/	Q9NU02	-0.20814	0.059613	0.041001	ANKEF1	Ankyrin rep	ANKE1_HUI	1	0.865654
24H 10MT/	P0C2W1	0.614448	0.059625	0.041005	FBXO45	F-box/SPRY	FBSP1_HUN	2	1.530972
24H 50MT/	Q8N9N7	-0.17794	0.059636	0.041011	LRRC57	Leucine-ric	LRC57_HUI	7	0.883967
24H 50MT/	Q9Y324	-0.02972	0.059655	0.041022	FCF1	rRNA-proce	FCF1_HUM	5	0.979612
48H 50MT/	P09958	-0.06651	0.059659	0.041023	FURIN	Furin	FURIN_HUN	7	0.954947
48H 50MT/	P62310	0.262771	0.059702	0.041048	LSM3	U6 snRNA-a	LSM3_HUM	3	1.199781
24H 50MT/	P13646	-0.68805	0.059707	0.041051	KRT13	Keratin, typ	K1C13_HUI	6	0.62069
48H 10MT/	Q14764	0.014132	0.05972	0.041056	MVP	Major vault	MVP_HUM/	44	1.009844
24H 50MT/	Q8NDX6	-0.06107	0.059727	0.04106	ZNF740	Zinc finger		ZN740_HUI	1	0.958555
24H 50MT/	P42684	-0.47872	0.059761	0.041078	ABL2	Tyrosine-pr	ABL2_HUM	3	0.717614
48H 50MT/	Q99640	-0.50121	0.059801	0.041101	PKMYT1	Membrane-	PMYT1_HU	3	0.706514

48H 50MT/	Q9H307	0.106151	0.059808	0.041105	PNN	Pinin	PININ_HUM	21	1.076353
24H 10MT/	Q8IVL5	0.1168	0.059827	0.041113	P3H2	Prolyl 3-hy	P3H2_HUM	13	1.084327
48H 50MT/	P57678	-0.14002	0.059837	0.041119	GEMIN4	Gem-associ	GEMI4_HU	26	0.907504
24H 10MT/	Q5T6V5	0.001804	0.059839	0.04112	QNG1	Queuosine	QNG1_HUM	15	1.001251
24H 50MT/	P07948	0.05877	0.059847	0.041123	LYN	Tyrosine-pr	LYN_HUMA	19	1.041577
48H 50MT/	Q99986	0.057933	0.059879	0.041139	VRK1	Serine/thre	VRK1_HUM	13	1.040973
48H 50MT/	Q96JH7	0.042056	0.059881	0.041139	VCPIP1	Deubiquiti	VCIP1_HUM	30	1.02958
48H 50MT/	Q96FV9	0.05611	0.059919	0.041158	THOC1	THO compl	THOC1_HU	24	1.039659
48H 50MT/	Q9H3P7	0.113658	0.059938	0.041169	ACBD3	Golgi resid	GCP60_HU	18	1.081968
48H 50MT/	Q6PI48	0.018186	0.059944	0.041171	DARS2	Aspartate--	SYDM_HUM	31	1.012685
24H 50MT/	P16118	0.335378	0.059978	0.04119	PFKFB1	6-phospho	F261_HUM	6	1.261708
24H 50MT/	P08048;P1	0.245698	0.059979	0.04119	ZFY;ZFX	Zinc finger	ZFY_HUMA	2	1.185666
24H 10MT/	Q5JTW2	-0.15283	0.060001	0.041204	CEP78	Centrosom	CEP78_HU	2	0.899486
48H 10MT/	O43187	-0.18633	0.060041	0.041225	IRAK2	Interleukin	IRAK2_HUM	4	0.878836
48H 50MT/	Q9NYJ1	0.173224	0.060105	0.04126	COA4	Cytochrom	COA4_HUM	1	1.127575
48H 50MT/	P14384	0.062228	0.060116	0.041266	CPM	Carboxype	CBPM_HUM	9	1.044077
24H 50MT/	Q9H8M7	-0.1468	0.060117	0.041266	MINDY3	Ubiquitin c	MINY3_HU	3	0.903249
48H 50MT/	P23921	-0.11481	0.060145	0.041281	RRM1	Ribonuclec	RIR1_HUM	30	0.923507
48H 10MT/	O60294	0.059534	0.060165	0.041293	LCMT2	tRNA wybu	TYW4_HUM	9	1.042129
24H 10MT/	Q9BQG2	0.335858	0.060179	0.041298	NUDT12	NAD-cappe	NUD12_HU	4	1.262128
24H 10MT/	Q92692	-0.08402	0.060227	0.041324	NECTIN2	Nectin-2	NECT2_HU	11	0.943423
24H 50MT/	Q2VPK5	-0.26234	0.06025	0.041338	CTU2	Cytoplasmi	CTU2_HUM	4	0.833732
48H 10MT/	Q7Z6J8	0.322498	0.060258	0.041343	UBE3D	E3 ubiquiti	UBE3D_HU	4	1.250494
48H 50MT/	P02760	0.015155	0.060269	0.041347	AMBP	Protein AM	AMBP_HUM	11	1.01056
24H 50MT/	Q15596	-0.06198	0.060281	0.041353	NCOA2	Nuclear rec	NCOA2_HU	12	0.957947
24H 10MT/	Q9NWL6	0.101613	0.060292	0.04136	ASNSD1	Asparagine	ASND1_HU	4	1.072972
48H 10MT/	Q9P2C4	-0.00345	0.060292	0.04136	TMEM181	Transmemb	TM181_HU	3	0.997614
48H 10MT/	A4D1P6	0.059994	0.0603	0.041364	WDR91	WD repeat-	WDR91_HU	20	1.042461
48H 50MT/	Q9NRX1	-0.14887	0.060313	0.04137	PNO1	RNA-bindir	PNO1_HUM	7	0.901959
48H 10MT/	Q9BRX8	0.004251	0.060322	0.041376	PRXL2A	Peroxiredo	PXL2A_HU	9	1.002951
24H 10MT/	P07311	-0.1922	0.060324	0.041377	ACYP1	Acylphospl	ACYP1_HU	5	0.87527
48H 10MT/	Q14353	0.148371	0.060338	0.041385	GAMT	Guanidinoa	GAMT_HUM	10	1.108317
24H 50MT/	Q86SR1	-0.05003	0.060341	0.041386	GALNT10	Polypeptid	GLT10_HU	5	0.965917
48H 10MT/	P09601	0.058937	0.060375	0.041406	HMOX1	Heme oxyg	HMOX1_HU	10	1.041698
24H 50MT/	Q96CQ1	-0.45507	0.06038	0.041409	SLC25A36	Solute carri	S2536_HU	2	0.729473
24H 50MT/	Q8IWD4	0.236621	0.060388	0.041412	CCDC117	Coiled-coil	CC117_HU	8	1.178229
24H 10MT/	P50402	-0.03697	0.060392	0.041412	EMD	Emerin	EMD_HUM	12	0.974697
24H 50MT/	O43414	0.117564	0.060399	0.041415	ERI3	ERI1 exorib	ERI3_HUM	4	1.084902
24H 50MT/	O60704	0.032358	0.060408	0.041418	TPST2	Protein-tyr	TPST2_HUM	1	1.022682
48H 50MT/	Q86Z14	-0.10172	0.060444	0.041437	KLB	Beta-klothc	KLOTB_HU	4	0.931921
48H 50MT/	P22087	-0.14338	0.060454	0.04144	FBL	rRNA 2'-O-r	FBRL_HUM	15	0.905393
48H 10MT/	Q96EI5	0.147178	0.060477	0.041451	TCEAL4	Transcripti	TCAL4_HUM	7	1.107401
24H 50MT/	P30047	0.091925	0.060492	0.041458	GCHFR	GTP cycloh	GFRP_HUM	5	1.065791
48H 10MT/	P12035	-0.34005	0.060525	0.041475	KRT3	Keratin, typ	K2C3_HUM	3	0.790014
48H 10MT/	P48637	0.009252	0.060523	0.041475	GSS	Glutathion	GSHB_HUM	26	1.006434
48H 10MT/	P40227	-0.08993	0.060537	0.041481	CCT6A	T-complex	TCPZ_HUM	27	0.93957
48H 50MT/	Q8N6S5	0.473027	0.06054	0.041483	ARL6IP6	ADP-ribosy	AR6P6_HU	1	1.388019
48H 10MT/	Q9P086	-0.01036	0.060597	0.041514	MED11	Mediator o	MED11_HU	1	0.992844
24H 10MT/	P33993	0.020451	0.060616	0.041524	MCM7	DNA replica	MCM7_HU	38	1.014276
24H 10MT/	O60784	-0.10496	0.06062	0.041526	TOM1	Target of M	TOM1_HUM	13	0.929829
48H 10MT/	Q9Y289	-0.35194	0.060652	0.041541	SLC5A6	Sodium-de	SC5A6_HU	4	0.783532
24H 10MT/	O60870	0.014749	0.060681	0.041555	KIN	DNA/RNA-b	KIN17_HUM	8	1.010276

48H 10MT/	P21675	0.178919	0.060682	0.041555	TAF1	Transcripti	TAF1_HUM	11	1.132035
24H 10MT/	A2RU67	-0.33449	0.060697	0.041564	FAM234B	Protein FAI	F234B_HUI	1	0.793066
48H 50MT/	Q8WXG6	-0.09053	0.060708	0.041568	MADD	MAP kinase	MADD_HUI	7	0.939177
24H 50MT/	Q9P107	-0.36375	0.060712	0.04157	GMIP	GEM-intera	GMIP_HUM	2	0.777141
48H 50MT/	Q9Y297	0.285381	0.060714	0.041571	BTRC	F-box/WD r	FBW1A_HU	5	1.218732
48H 50MT/	O60704	0.211675	0.06072	0.041574	TPST2	Protein-tyr	TPST2_HUM	1	1.158032
24H 50MT/	Q8TCA0	-0.00738	0.060729	0.041578	LRRC20	Leucine-ric	LRC20_HUI	10	0.994901
48H 10MT/	P62879	0.055785	0.06075	0.04159	GNB2	Guanine nu	GBB2_HUM	8	1.039425
48H 10MT/	P45877	-0.04639	0.060755	0.041593	PPIC	Peptidyl-pr	PPIC_HUM/	2	0.968353
48H 10MT/	Q15059	0.115213	0.060773	0.041604	BRD3	Bromodom	BRD3_HUM	8	1.083135
24H 10MT/	Q99627	0.044237	0.060783	0.04161	COPS8	COP9 signa	CSN8_HUM	7	1.031137
48H 10MT/	O95249	0.048729	0.06079	0.041613	GOSR1	Golgi SNAP	GOSR1_HU	12	1.034353
24H 50MT/	P14649	0.113578	0.060808	0.041622	MYL6B	Myosin ligh	MYL6B_HU	7	1.081908
48H 50MT/	Q8NBT0	-1.26712	0.060853	0.041647	POC1A	POC1 centr	POC1A_HU	1	0.415488
24H 50MT/	Q00341	0.004519	0.060864	0.041652	HDLBP	Vigilin	VIGLN_HUI	61	1.003137
24H 50MT/	Q9Y4P8	0.13458	0.060865	0.041652	WIPI2	WD repeat	WIPI2_HUI	8	1.097773
48H 50MT/	P25788	0.015156	0.060888	0.041665	PSMA3	Proteasom	PSA3_HUM	9	1.010561
24H 10MT/	O75306	-0.09994	0.06089	0.041666	NDUFS2	NADH dehy	NDUS2_HU	20	0.93307
24H 50MT/	Q9NVS2	0.015594	0.060905	0.041675	MRPS18A	Large ribos	RT18A_HUI	7	1.010868
24H 50MT/	Q7Z4H7	-0.00742	0.060921	0.041685	HAUS6	HAUS augm	HAUS6_HU	11	0.994873
48H 10MT/	Q96HC4	0.056957	0.060926	0.041688	PDLIM5	PDZ and LII	PDLI5_HUM	23	1.04027
24H 10MT/	P20823	-0.04975	0.060929	0.041689	HNF1A	Hepatocyte	HNF1A_HU	6	0.966104
48H 10MT/	Q96RD7	-0.62475	0.060938	0.041693	PANX1	Pannexin-1	PANX1_HU	3	0.648532
24H 10MT/	Q9UDR5	-0.02353	0.060944	0.041696	AASS	Alpha-amir	AASS_HUM	13	0.983826
48H 50MT/	Q99714	-0.01415	0.06095	0.041698	HSD17B10	3-hydroxya	HCD2_HUM	18	0.990237
24H 50MT/	Q9UIA9	-0.04411	0.060957	0.041701	XPO7	Exportin-7	XPO7_HUM	37	0.969886
48H 50MT/	Q6ZSR9	0.102012	0.060962	0.041703	NaN	Uncharacte	YJ005_HUM	8	1.073269
24H 10MT/	Q9NX14	-0.2963	0.060989	0.041717	NDUFB11	NADH dehy	NDUBB_HU	4	0.814338
24H 50MT/	Q13443	0.003752	0.060992	0.041718	ADAM9	Disintegrin	ADAM9_HU	13	1.002604
48H 10MT/	Q07812	0.089296	0.061	0.041722	BAX	Apoptosis r	BAX_HUMA	8	1.063851
24H 10MT/	O75396	0.043749	0.06103	0.041739	SEC22B	Vesicle-traf	SC22B_HUI	11	1.030789
24H 10MT/	Q96C19	0.001166	0.061043	0.041745	EFHD2	EF-hand do	EFHD2_HUI	13	1.000809
48H 50MT/	Q96RD7	-0.52095	0.061044	0.041745	PANX1	Pannexin-1	PANX1_HU	3	0.696914
24H 10MT/	Q03135	0.509407	0.061053	0.041749	CAV1	Caveolin-1	CAV1_HUM	3	1.423465
24H 50MT/	O60502	-0.09305	0.061099	0.04177	OGA	Protein O-G	OGA_HUM/	30	0.937542
24H 50MT/	Q7RTP0	-0.00163	0.061117	0.041779	NIPA1	Magnesium	NIPA1_HUI	3	0.998868
48H 50MT/	O15119	-0.34132	0.061149	0.041794	TBX3	T-box trans	TBX3_HUM	5	0.78932
48H 50MT/	Q96EV2	0.163873	0.061153	0.041796	RBM33	RNA-bindir	RBM33_HU	11	1.120291
48H 50MT/	Q96S44	-0.0551	0.061168	0.041804	TP53RK	EKC/KEOPS	PRPK_HUM	6	0.96253
48H 10MT/	Q96FK6	-0.01413	0.061211	0.041828	WDR89	WD repeat-	WDR89_HU	4	0.990255
24H 50MT/	Q9BWM7	0.068168	0.061211	0.041828	SFXN3	Sideroflexir	SFXN3_HUI	14	1.048385
48H 10MT/	Q9Y2I8	0.056535	0.061218	0.04183	WDR37	WD repeat-	WDR37_HU	6	1.039965
48H 50MT/	O14545	0.177586	0.061219	0.04183	TRAFD1	TRAF-type z	TRAD1_HUI	9	1.130989
24H 50MT/	P34931	-0.15701	0.061244	0.041842	HSPA1L	Heat shock	HS71L_HUI	3	0.896881
48H 10MT/	O00165	-0.16369	0.061259	0.041851	HAX1	HCLS1-asso	HAX1_HUM	10	0.892742
24H 10MT/	O43681	-0.04546	0.061325	0.041884	GET3	ATPase GET	GET3_HUM	14	0.968981
48H 50MT/	Q9HCS7	-0.13287	0.061348	0.041896	XAB2	Pre-mRNA-s	SYF1_HUM/	30	0.912017
24H 50MT/	Q9UPN6	0.100968	0.061367	0.041905	SCAF8	SR-related a	SCAF8_HUI	11	1.072493
48H 10MT/	Q9P0M6	0.194906	0.061379	0.041912	MACROH2/	Core histor	H2AW_HUI	8	1.144649
24H 50MT/	Q13099	0.181166	0.061383	0.041913	IFT88	Intraflagell	IFT88_HUM	2	1.1338
48H 50MT/	Q8NI60	-0.19972	0.061385	0.041914	COQ8A	Atypical kir	COQ8A_HU	9	0.870717
48H 10MT/	P23511	-0.28663	0.061428	0.041935	NFYA	Nuclear tra	NFYA_HUM	2	0.819817

24H 10MTA	O75381	0.231933	0.061433	0.041936	PEX14	Peroxisoma	PEX14_HUI	2	1.174407
48H 10MTA	O75410	-0.0878	0.061456	0.041948	TACC1	Transformi	TACC1_HUI	15	0.940955
48H 10MTA	P32969	-0.09731	0.06153	0.041993	RPL9	Large ribos	RL9_HUMA	12	0.934772
48H 10MTA	O75306	0.035496	0.06156	0.042008	NDUFS2	NADH dehy	NDUS2_HU	20	1.024909
24H 10MTA	Q6GMV2	-0.1136	0.061615	0.042039	SMYD5	Histone-lys	SMYD5_HU	11	0.924282
48H 50MTA	Q9Y5A9	-0.1605	0.061618	0.04204	YTHDF2	YTH domai	YTHD2_HUI	10	0.894718
24H 10MTA	A6NH11	0.042419	0.061623	0.042043	GLTPD2	Glycolipid	GLTD2_HUI	6	1.029839
24H 50MTA	Q9BQC6	0.211438	0.06163	0.042047	MRPL57	Large ribos	RT63_HUM	1	1.157842
24H 50MTA	Q92791	-0.00613	0.06164	0.042051	P3H4	Endoplasm	SC65_HUM	7	0.995762
24H 10MTA	P16949	-0.30971	0.061643	0.042053	STMN1	Stathmin	STMN1_HU	6	0.806806
48H 10MTA	P35240	0.069025	0.061649	0.042055	NF2	Merlin	MERL_HUM	10	1.049008
24H 10MTA	P19174	0.063206	0.061654	0.042058	PLCG1	1-phosphat	PLCG1_HUI	24	1.044785
24H 50MTA	P17028	0.091608	0.061667	0.042065	ZNF24	Zinc finger	ZNF24_HUI	7	1.065557
24H 10MTA	Q9BY49	-0.03935	0.061678	0.042072	PECR	Peroxisoma	PECR_HUM	11	0.973092
24H 50MTA	Q93050	-0.006	0.061691	0.042077	ATP6V0A1	V-type prot	VPP1_HUM	23	0.995847
48H 50MTA	Q9Y6X8	-0.03105	0.06169	0.042077	ZHX2	Zinc fingers	ZHX2_HUM	9	0.97871
24H 50MTA	Q9BRP4	0.325451	0.061756	0.042115	PAAF1	Proteasom	PAAF1_HUI	7	1.253056
48H 10MTA	P08651	0.249259	0.06176	0.042116	NFIC	Nuclear fac	NFIC_HUMA	2	1.188597
48H 10MTA	Q9Y483	-0.0038	0.061759	0.042116	MTF2	Metal-resp	MTF2_HUM	8	0.997366
24H 10MTA	Q9BTX7	-0.29775	0.061776	0.042126	TTPAL	Alpha-toco	TTPAL_HUN	9	0.813523
24H 10MTA	P53355	-0.05344	0.061777	0.042126	DAPK1	Death-asso	DAPK1_HU	8	0.963635
48H 50MTA	Q0ZGT2	0.278108	0.061799	0.042136	NEXN	Nexilin	NEXN_HUM	9	1.212604
48H 10MTA	Q9ULJ3	-0.05818	0.061813	0.042144	ZBTB21	Zinc finger	ZBT21_HUN	5	0.960473
24H 10MTA	Q9ULW2	0.206708	0.061826	0.042151	FZD10	Frizzled-10	FZD10_HUI	4	1.154052
24H 50MTA	Q9NYA4	1.288894	0.061833	0.042154	MTMR4	Myotubula	MTMR4_HU	1	2.443406
24H 50MTA	Q96T60	-0.08651	0.061843	0.042159	PNKP	Bifunctiona	PNKP_HUM	12	0.941799
48H 50MTA	O14979	-0.02689	0.06186	0.042169	HNRNPDL	Heterogene	HNRDL_HU	14	0.981536
24H 10MTA	Q86T24	-0.22657	0.06187	0.042173	ZBTB33	Transcripti	KAISO_HUN	1	0.854664
48H 50MTA	Q9UJP4	0.467512	0.061883	0.042181	KLHL21	Kelch-like p	KLH21_HUI	1	1.382722
48H 50MTA	P51159	-0.05343	0.061893	0.042185	RAB27A	Ras-related	RB27A_HUI	5	0.963641
24H 10MTA	Q8N1G2	0.068332	0.061906	0.042191	CMTR1	Cap-specifi	CMTR1_HU	33	1.048504
48H 10MTA	Q9UH62	-0.1029	0.061941	0.042212	ARMCX3	Armadillo r	ARMX3_HU	12	0.931157
48H 50MTA	Q15063	0.507218	0.061961	0.042217	POSTN	Periostin	POSTN_HUI	3	1.421306
24H 10MTA	O60664	-0.06848	0.061959	0.042217	PLIN3	Perilipin-3	PLIN3_HUN	20	0.953643
48H 50MTA	P40337	-0.11498	0.06196	0.042217	VHL	von Hippel-	VHL_HUMA	2	0.923395
48H 50MTA	Q76M96	0.341464	0.061978	0.042225	CCDC80	Coiled-coil	CCD80_HU	1	1.267042
24H 50MTA	O00159	0.090173	0.061991	0.042229	MYO1C	Unconvent	MYO1C_HU	43	1.064498
48H 10MTA	Q9BSD7	0.021468	0.062	0.042233	NTPCR	Cancer-rela	NTPCR_HUI	10	1.014992
48H 50MTA	Q330K2	0.101363	0.062004	0.042234	NDUFAF6	NADH dehy	NDUF6_HU	1	1.072787
48H 10MTA	O94973	-0.00632	0.062033	0.042251	AP2A2	AP-2 compl	AP2A2_HUI	29	0.995627
24H 10MTA	Q96G61	-0.01204	0.062048	0.042257	NUDT11	Diphospho	NUD11_HU	1	0.99169
48H 10MTA	P38646	-0.08875	0.062068	0.042265	HSPA9	Stress-70 p	GRP75_HU	32	0.94034
24H 50MTA	Q9NP81	0.039079	0.06207	0.042265	SARS2	Serine--tRN	SYSM_HUM	17	1.027457
24H 10MTA	Q9Y303	-0.03958	0.06207	0.042265	AMDHD2	N-acetylglu	NAGA_HUM	4	0.97294
24H 10MTA	Q13972	0.076848	0.062072	0.042266	RASGRF1	Ras-specific	RGRF1_HUI	1	1.054711
48H 10MTA	P98173	0.07576	0.062107	0.042286	FAM3A	Protein FAI	FAM3A_HU	4	1.053916
24H 10MTA	Q6IN84	-0.21291	0.062108	0.042286	MRM1	rRNA meth	MRM1_HUI	6	0.862795
24H 50MTA	Q92618	0.281758	0.062117	0.042291	ZNF516	Zinc finger	ZN516_HUI	8	1.215675
48H 50MTA	Q71DI3	-0.95388	0.062119	0.042292	H3C15	Histone H3	H32_HUMA	1	0.516243
24H 50MTA	Q92734	0.1568	0.062138	0.042301	TFG	Protein TFG	TFG_HUMA	12	1.114812
24H 10MTA	Q8NG27	0.4041	0.062155	0.042309	PJA1	E3 ubiquiti	PJA1_HUM	1	1.323263
24H 10MTA	Q9P0S9	0.154956	0.062161	0.042311	TMEM14C	Transmemb	TM14C_HU	3	1.113388

48H 10MT/	O75381	-0.75377	0.062162	0.042311	PEX14	Peroxisoma	PEX14_HUI	2	0.593051
48H 50MT/	Q15050	-0.10935	0.062183	0.042322	RRS1	Ribosome b	RRS1_HUM	16	0.927005
24H 10MT/	Q9UHR6	-0.031	0.062222	0.042343	ZNHIT2	Zinc finger	ZNHI2_HUN	8	0.978743
48H 10MT/	Q9NZM3	0.112398	0.062226	0.042344	ITSN2	Intersectin	ITSN2_HUN	23	1.081024
48H 10MT/	Q96F46	0.01742	0.062233	0.042348	IL17RA	Interleukin	I17RA_HUN	7	1.012148
24H 10MT/	Q8N5I9	-0.21047	0.062254	0.042357	NOPCHAP1	NOP protei	NOPC1_HU	2	0.864254
24H 50MT/	Q9BU23	-0.10475	0.062255	0.042357	LMF2	Lipase matu	LMF2_HUM	8	0.929967
48H 50MT/	Q9NVT9	0.08796	0.062256	0.042357	ARMC1	Armadillo r	ARMC1_HU	10	1.062866
24H 10MT/	Q9UHK6	-0.25135	0.062255	0.042357	AMACR	Alpha-meth	AMACR_HU	5	0.84011
48H 10MT/	Q9H0G5	0.512005	0.062276	0.042366	NSRP1	Nuclear spe	NSRP1_HUI	4	1.426031
24H 50MT/	O75818	0.097315	0.062328	0.042397	RPP40	Ribonuclea	RPP40_HUI	9	1.069781
48H 10MT/	Q12834	-0.09793	0.062331	0.042398	CDC20	Cell divisio	CDC20_HU	11	0.934375
48H 50MT/	Q04695	0.118125	0.062343	0.042403	KRT17	Keratin, typ	K1C17_HUI	15	1.085323
48H 10MT/	Q86WA8	0.047869	0.06235	0.042407	LONP2	Lon proteas	LONP2_HU	28	1.033737
24H 50MT/	Q92520	0.034257	0.062363	0.042413	FAM3C	Protein FAI	FAM3C_HU	8	1.024029
48H 50MT/	Q99459	-0.01457	0.062364	0.042413	CDC5L	Cell divisio	CDC5L_HUI	33	0.989952
48H 50MT/	Q9H3H5	0.121437	0.06237	0.042413	DPAGT1	UDP-N-acet	GPT_HUMA	2	1.087818
24H 10MT/	Q06323	-0.10388	0.062393	0.042425	PSME1	Proteasom	PSME1_HU	19	0.930525
24H 10MT/	Q9HC52	0.215446	0.062401	0.042429	CBX8	Chromobo:	CBX8_HUM	9	1.161063
24H 10MT/	P54578	0.060919	0.062403	0.042429	USP14	Ubiquitin c	UBP14_HU	24	1.04313
24H 50MT/	Q9BXB1	-0.10415	0.062403	0.042429	LGR4	Leucine-ric	LGR4_HUM	1	0.930352
48H 50MT/	Q8IYB1	0.258444	0.06241	0.042432	MB21D2	Nucleotidy	M21D2_HU	10	1.196188
48H 10MT/	Q14997	0.01803	0.06243	0.042444	PSME4	Proteasom	PSME4_HU	48	1.012576
24H 10MT/	Q96EX3	0.053469	0.062458	0.04246	DYNC2I2	Cytoplasmi	DC2I2_HUN	5	1.037757
48H 10MT/	Q9NYA1	-0.69759	0.062459	0.04246	SPHK1	Sphingosin	SPHK1_HUI	1	0.616603
24H 50MT/	Q92551	0.04506	0.062488	0.042478	IP6K1	Inositol he:	IP6K1_HUN	3	1.031726
48H 10MT/	O75534	0.005644	0.062512	0.04249	CSDE1	Cold shock	CSDE1_HUI	44	1.00392
24H 10MT/	Q9H4L7	0.005442	0.062513	0.04249	SMARCAD1	SWI/SNF-re	SMRCD_HU	19	1.003779
48H 50MT/	Q9Y3M8	-0.26577	0.062515	0.04249	STARD13	StAR-relate	STA13_HUN	2	0.831756
24H 10MT/	O75410	-0.01301	0.062521	0.042494	TACC1	Transformi	TACC1_HUI	15	0.991022
24H 50MT/	P62910	0.283234	0.062532	0.042499	RPL32	Large ribos	RL32_HUM	7	1.21692
48H 50MT/	P28838	-0.03288	0.062534	0.042499	LAP3	Cytosol am	AMPL_HUN	29	0.977464
24H 50MT/	P11802	-0.05207	0.062541	0.042502	CDK4	Cyclin-depe	CDK4_HUM	12	0.96455
24H 50MT/	Q15811	0.131369	0.062578	0.042521	ITSN1	Intersectin	ITSN1_HUN	8	1.095333
48H 50MT/	Q15012	-0.08902	0.06258	0.042521	LAPTM4A	Lysosomal-	LAP4A_HUI	1	0.940158
24H 50MT/	Q5HYI8	0.070817	0.062589	0.042526	RABL3	Rab-like pro	RABL3_HUI	6	1.050311
48H 50MT/	P17980	0.023415	0.062593	0.042527	PSMC3	26S proteas	PRS6A_HUI	28	1.016362
24H 10MT/	Q9P260	-0.08437	0.062623	0.042539	RELCH	RAB11-binc	RELCH_HUI	31	0.943196
48H 10MT/	Q9UHW9	-0.24059	0.062647	0.04255	SLC12A6	Solute carri	S12A6_HUI	7	0.846402
24H 10MT/	Q16891	-0.05019	0.06265	0.042552	IMMT	MICOS com	MIC60_HUI	35	0.965811
48H 50MT/	Q8TF05	-0.03169	0.062701	0.042578	PPP4R1	Serine/thre	PP4R1_HUI	32	0.978275
24H 10MT/	Q13362	-0.03254	0.062787	0.042622	PPP2R5C	Serine/thre	2A5G_HUM	15	0.977699
24H 50MT/	P35548	0.061803	0.06279	0.042623	MSX2	Homeobox	MSX2_HUM	1	1.043769
48H 10MT/	Q03167	0.407542	0.062803	0.042629	TGFBR3	Transformi	TGBR3_HUI	2	1.326424
48H 10MT/	Q09161	-0.01885	0.062804	0.042629	NCBP1	Nuclear cap	NCBP1_HU	28	0.98702
24H 10MT/	P20339	0.059517	0.062826	0.042637	RAB5A	Ras-related	RAB5A_HUI	8	1.042117
48H 50MT/	Q7Z4G1	0.068131	0.062824	0.042637	COMMD6	COMM dom	COMD6_HU	1	1.048357
48H 50MT/	O95243	-0.2426	0.062829	0.042638	MBD4	Methyl-CpG	MBD4_HUN	4	0.845218
48H 50MT/	P53384	0.207085	0.062843	0.042644	NUBP1	Cytosolic Fe	NUBP1_HU	9	1.154353
24H 50MT/	P02042	-0.52613	0.062912	0.042679	HBD	Hemoglobi	HBD_HUM/	3	0.694413
24H 50MT/	P45983	0.020179	0.06291	0.042679	MAPK8	Mitogen-ac	MK08_HUM	6	1.014085
24H 50MT/	Q96I25	0.064245	0.062935	0.04269	RBM17	Splicing fac	SPF45_HUN	11	1.045538

48H	10MT/	Q9Y6A5	0.01334	0.062945	0.042695	TACC3	Transformi	TACC3_HUI	15	1.009289
24H	50MT/	Q6W2J9	0.960569	0.062977	0.042713	BCOR	BCL-6 corep	BCOR_HUM	4	1.946078
48H	10MT/	Q9NXR1	0.087585	0.062986	0.042718	NDE1	Nuclear dis	NDE1_HUM	12	1.06259
48H	50MT/	P10768	0.034782	0.063021	0.042736	ESD	S-formylglu	ESTD_HUM	15	1.024402
48H	50MT/	P51003	0.029087	0.063037	0.042742	PAPOLA	Poly(A) poly	PAPOA_HU	22	1.020366
24H	50MT/	Q3ZCW2	-0.17658	0.063038	0.042742	LGALSL	Galectin-re	LEGL_HUM	6	0.884795
24H	10MT/	P23246	0.017343	0.063053	0.04275	SFPQ	Splicing fac	SFPQ_HUM	25	1.012094
48H	50MT/	P13646	-0.61172	0.063069	0.042759	KRT13	Keratin, typ	K1C13_HUI	6	0.654417
24H	10MT/	Q8N2U0	-0.04376	0.063123	0.04279	TMEM256	Transmemb	TM256_HU	2	0.970125
24H	10MT/	P32929	-0.0684	0.063145	0.042802	CTH	Cystathioni	CGL_HUMA	12	0.953697
48H	50MT/	P04899	0.048219	0.063149	0.042804	GNAI2	Guanine nu	GNAI2_HUI	13	1.033988
48H	10MT/	Q330K2	-0.06779	0.063172	0.042818	NDUFAF6	NADH dehy	NDUF6_HU	1	0.954098
24H	50MT/	A0A1B0GTU	-0.2295	0.063178	0.042821	ZC3H11B	Zinc finger	ZC11B_HUI	1	0.852929
24H	10MT/	Q96CS4	-0.16471	0.06319	0.042828	ZNF689	Zinc finger	ZN689_HUI	8	0.892105
48H	10MT/	P40855	-0.58541	0.063193	0.042829	PEX19	Peroxisoma	PEX19_HUI	1	0.66646
48H	10MT/	O43826	-0.08283	0.063197	0.042831	SLC37A4	Glucose-6-p	G6PT1_HUI	3	0.944206
48H	50MT/	P23458	-0.01943	0.063202	0.042834	JAK1	Tyrosine-pr	JAK1_HUM	29	0.986624
48H	50MT/	P30084	0.022656	0.063213	0.042838	ECHS1	Enoyl-CoA h	ECHM_HUM	18	1.015828
48H	50MT/	Q07864	-0.21373	0.063223	0.042843	POLE	DNA polym	DPOE1_HU	31	0.862308
48H	50MT/	Q13163	0.254024	0.063234	0.04285	MAP2K5	Dual specifi	MP2K5_HU	2	1.192528
48H	10MT/	Q5T5X7	0.100158	0.063239	0.042852	BEND3	BEN domai	BEND3_HU	10	1.071891
48H	10MT/	O15213	0.011345	0.063286	0.042875	WDR46	WD repeat-	WDR46_HU	18	1.007895
24H	10MT/	Q12888	0.005602	0.0633	0.042882	TP53BP1	TP53-bindi	TP53B_HUI	51	1.003891
24H	50MT/	O43731	0.120477	0.063309	0.042885	KDELR3	ER lumen p	ERD23_HUI	3	1.087094
48H	10MT/	P62917	-0.20452	0.063312	0.042887	RPL8	Large ribos	RL8_HUMA	14	0.867825
48H	50MT/	P54253	0.247086	0.063314	0.042888	ATXN1	Ataxin-1	ATX1_HUM	2	1.186808
24H	10MT/	Q9NVV0	-0.3668	0.063318	0.04289	TMEM38B	Trimeric int	TM38B_HU	3	0.7755
24H	10MT/	P29317	0.171967	0.063335	0.042898	EPHA2	Ephrin type	EPHA2_HUI	17	1.126593
24H	50MT/	Q9H3H5	0.181082	0.063352	0.042906	DPAGT1	UDP-N-acet	GPT_HUMA	2	1.133734
24H	10MT/	Q6ZN54	0.258848	0.063418	0.042943	DEF8	Differential	DEFI8_HUM	2	1.196523
48H	50MT/	Q8N2K1	-0.30138	0.063455	0.042963	UBE2J2	Ubiquitin-c	UB2J2_HUI	3	0.811473
24H	10MT/	P08574	-0.02523	0.06346	0.042963	CYC1	Cytochrom	CY1_HUMA	8	0.982663
24H	10MT/	P21796	0.037439	0.06346	0.042963	VDAC1	Non-selecti	VDAC1_HU	19	1.02629
48H	10MT/	P49760	-0.12048	0.063458	0.042963	CLK2	Dual specifi	CLK2_HUM	2	0.91988
24H	50MT/	P26583	-0.11764	0.063462	0.042964	HMGB2	High mobil	HMGB2_HU	7	0.921695
24H	10MT/	Q9BSB4	0.144314	0.063531	0.043002	ATG101	Autophagy-	ATGA1_HUI	5	1.105205
24H	10MT/	Q8N635	0.676655	0.063533	0.043003	MEIOB	Meiosis-spe	MEIOB_HUI	2	1.59843
24H	50MT/	Q8TF01	0.622306	0.063545	0.043007	PNISR	Arginine/se	PNISR_HUM	2	1.539334
48H	10MT/	Q9Y512	-0.03094	0.063581	0.043025	SAMM50	Sorting and	SAM50_HU	23	0.97878
48H	50MT/	Q9UHQ4	-0.27287	0.063606	0.043038	BCAP29	B-cell recep	BAP29_HUI	5	0.827673
24H	10MT/	Q08257	0.039126	0.063637	0.043055	CRYZ	Quinone ox	QOR_HUM/	17	1.027491
48H	50MT/	Q15464	-0.13915	0.063636	0.043055	SHB	SH2 domai	SHB_HUMA	5	0.908052
24H	10MT/	P19823	0.261226	0.063641	0.043055	ITIH2	Inter-alpha	ITIH2_HUM	20	1.198497
24H	10MT/	P16298	0.32258	0.063646	0.043055	PPP3CB	Serine/thre	PP2BB_HUI	4	1.250565
24H	10MT/	P51956	-0.36764	0.063646	0.043055	NEK3	Serine/thre	NEK3_HUM	4	0.77505
24H	50MT/	Q5UIP0	0.039665	0.063642	0.043055	RIF1	Telomere-a	RIF1_HUM/	48	1.027875
24H	10MT/	Q96AY4	-0.38722	0.063644	0.043055	TTC28	Tetratricop	TTC28_HUM	3	0.764601
48H	10MT/	Q969U7	0.078264	0.063674	0.043069	PSMG2	Proteasom	PSMG2_HU	8	1.055747
48H	10MT/	Q9NQZ2	-0.13515	0.063702	0.043083	UTP3	Something	SAS10_HUI	11	0.910574
48H	10MT/	Q92934	0.045065	0.063706	0.043085	BAD	Bcl2-associ	BAD_HUM/	4	1.031729
24H	10MT/	P78417	-0.04618	0.063717	0.04309	GSTO1	Glutathion	GSTO1_HUI	19	0.968497
48H	50MT/	Q9H3P2	0.027454	0.063721	0.043091	NELFA	Negative el	NELFA_HUI	10	1.019212

24H 50MTA	O60361	-0.83418	0.063755	0.043105	NME2P1	Putative nu	NDK8_HUM	1	0.560901
48H 10MTA	Q96CU9	0.061867	0.063759	0.043106	FOXRED1	FAD-depen	FXRD1_HU	12	1.043815
48H 10MTA	Q9H1Y0	0.029868	0.063759	0.043106	ATG5	Autophagy	ATG5_HUM	9	1.020919
24H 10MTA	Q13188	-0.02146	0.063762	0.043106	STK3	Serine/thre	STK3_HUM	7	0.985235
24H 10MTA	Q86SX6	-0.38715	0.063764	0.043107	GLRX5	Glutaredox	GLRX5_HU	4	0.764641
24H 50MTA	P98082	-0.83747	0.063771	0.043111	DAB2	Disabled ho	DAB2_HUM	1	0.559623
48H 10MTA	P50895	-0.00126	0.063791	0.043117	BCAM	Basal cell ac	BCAM_HUM	16	0.999129
48H 50MTA	Q13177	-0.00772	0.063817	0.04313	PAK2	Serine/thre	PAK2_HUM	21	0.994662
48H 10MTA	Q86UT6	-0.09815	0.063825	0.043133	NLRX1	NLR family	NLRX1_HU	10	0.934228
24H 10MTA	P61604	0.082239	0.063836	0.043139	HSPE1	10 kDa heat	CH10_HUM	13	1.05866
48H 50MTA	P54277	-0.26409	0.063851	0.043144	PMS1	PMS1 prote	PMS1_HUM	16	0.832722
24H 50MTA	Q96RU2	0.123338	0.06387	0.043151	USP28	Ubiquitin c	UBP28_HU	20	1.089252
24H 50MTA	Q9H6S3	-0.20137	0.063869	0.043151	EPS8L2	Epidermal g	ES8L2_HUM	23	0.869721
48H 50MTA	Q5R3I4	0.001777	0.06389	0.043161	TTC38	Tetratricop	TTC38_HUM	16	1.001232
48H 10MTA	P06865	-4.87E-05	0.063908	0.04317	HEXA	Beta-hexos	HEXA_HUM	18	0.999966
48H 50MTA	Q9NX07	-0.02549	0.063908	0.04317	TRNAU1AP	tRNA selen	TSAP1_HUM	4	0.982488
48H 10MTA	O14757	-0.02344	0.063918	0.043177	CHEK1	Serine/thre	CHK1_HUM	12	0.983885
48H 10MTA	O94855	0.066839	0.063927	0.043182	SEC24D	Protein tra	SC24D_HU	27	1.047419
48H 50MTA	Q9UPP1	-0.07536	0.063929	0.043182	PHF8	Histone lysi	PHF8_HUM	4	0.949107
48H 10MTA	P54652	0.016471	0.06394	0.043187	HSPA2	Heat shock	HSP72_HU	22	1.011482
24H 50MTA	Q53FV1	0.161274	0.063962	0.043201	ORMDL2	ORM1-like	ORML2_HU	2	1.118275
48H 50MTA	Q9H9E3	0.049567	0.064035	0.043237	COG4	Conserved	COG4_HUM	26	1.034954
48H 10MTA	Q8WXD5	0.064359	0.064044	0.043242	GEMIN6	Gem-associ	GEMI6_HU	5	1.04562
24H 10MTA	O15213	-0.01186	0.064063	0.043251	WDR46	WD repeat-	WDR46_HU	18	0.991816
48H 50MTA	P58511	-0.53546	0.064073	0.043256	SMIM11	Small integ	SIM11_HU	1	0.689937
48H 10MTA	P01111	0.033016	0.06409	0.043266	NRAS	GTPase NRa	RASN_HUM	8	1.023149
48H 10MTA	O00115	0.135625	0.06411	0.043277	DNASE2	Deoxyribor	DNS2A_HU	5	1.098568
48H 10MTA	Q4V328	-0.02763	0.064113	0.043278	GRIPAP1	GRIP1-asso	GRAP1_HU	18	0.981031
24H 10MTA	P12830	0.1986	0.06412	0.043281	CDH1	Cadherin-1	CADH1_HU	7	1.147584
48H 10MTA	Q86YS6	-0.10569	0.064123	0.043282	RAB43	Ras-related	RAB43_HU	8	0.929358
24H 50MTA	Q96JP5	0.08551	0.064126	0.043282	ZFP91	E3 ubiquiti	ZFP91_HUM	6	1.061063
24H 10MTA	Q96CW1	0.026224	0.064138	0.043289	AP2M1	AP-2 compl	AP2M1_HU	22	1.018344
24H 10MTA	Q8NCN5	-0.00451	0.064143	0.04329	PDPR	Pyruvate de	PDPR_HUM	24	0.996876
24H 50MTA	Q8IVD9	-0.05584	0.064146	0.04329	NUDCD3	NudC doma	NUDC3_HU	11	0.962035
24H 10MTA	Q7Z412	0.338126	0.064208	0.043324	PEX26	Peroxisome	PEX26_HU	4	1.264114
24H 50MTA	Q96ES7	0.128616	0.064209	0.043324	SGF29	SAGA-assoc	SGF29_HU	4	1.093244
48H 50MTA	Q9NVE7	0.120239	0.064214	0.043325	PANK4	4'-phospho	PANK4_HU	23	1.086915
48H 50MTA	P51991	0.025329	0.064226	0.043333	HNRNPA3	Heterogene	ROA3_HUM	22	1.017712
48H 50MTA	O43847	-0.06565	0.064243	0.043343	NRDC	Nardilysin	NRDC_HUM	37	0.955512
48H 50MTA	Q96RP9	-0.1207	0.064242	0.043343	GFM1	Elongation	EFGM_HUM	29	0.919743
48H 50MTA	Q9UKG1	0.086805	0.064256	0.04335	APPL1	DCC-intera	DP13A_HU	20	1.062016
24H 10MTA	Q9UI09	-0.04463	0.064265	0.043355	NDUFA12	NADH dehy	NDUAC_HU	9	0.969536
24H 10MTA	Q96GA3	0.073326	0.064294	0.043371	LTV1	Protein LTV	LTV1_HUM	17	1.05214
24H 50MTA	O75607	-0.1023	0.064297	0.043373	NPM3	Nucleoplas	NPM3_HUM	7	0.93155
48H 50MTA	Q9BXK1	-0.27413	0.064311	0.043378	KLF16	Krueppel-li	KLF16_HUM	2	0.826949
24H 50MTA	P13995	-0.03097	0.064327	0.043387	MTHFD2	Bifunctiona	MTDC_HUM	13	0.978762
24H 10MTA	Q9NQX6	0.029772	0.064339	0.043394	ZNF331	Zinc finger	ZN331_HU	4	1.020851
24H 50MTA	O00471	0.110686	0.064341	0.043394	EXOC5	Exocyst cor	EXOC5_HU	24	1.079742
48H 10MTA	Q8NB46	0.009322	0.064343	0.043394	ANKRD52	Serine/thre	ANR52_HU	17	1.006483
24H 50MTA	Q70IA6	-0.54162	0.06436	0.043404	MOB2	MOB kinase	MOB2_HUM	3	0.687001
24H 50MTA	Q8IV48	0.120906	0.06439	0.043421	ERI1	3'-5' exorib	ERI1_HUMA	4	1.087417
48H 10MTA	P27144	-0.11878	0.06441	0.043431	AK4	Adenylate k	KAD4_HUM	14	0.920967

24H 10MTA	O43488	-0.05744	0.064507	0.043485	AKR7A2	Aflatoxin B1	ARK72_HU	10	0.960966
24H 10MTA	Q9P2B4	0.057722	0.064521	0.043492	CTTNBP2NL	CTTNBP2 N	CT2NL_HU	11	1.040821
48H 50MTA	P49721	0.101393	0.064522	0.043492	PSMB2	Proteasome	PSB2_HUM	10	1.072809
48H 50MTA	Q9NUY8	0.102534	0.064565	0.043518	TBC1D23	TBC1 doma	TBC23_HU	13	1.073658
24H 10MTA	O60231	0.0479	0.064574	0.043522	DHX16	Pre-mRNA-s	DHX16_HU	28	1.033759
48H 10MTA	Q86VQ1	-0.19454	0.064574	0.043522	GLCCI1	Glucocortic	GLCI1_HUM	2	0.873851
48H 10MTA	P04350	0.053123	0.064577	0.043522	TUBB4A	Tubulin bet	TBB4A_HU	3	1.037508
24H 50MTA	Q86TB9	0.012549	0.064576	0.043522	PATL1	Protein PAT	PATL1_HUM	20	1.008736
24H 10MTA	P15104	-0.09957	0.064584	0.043525	GLUL	Glutamine	GLNA_HUM	7	0.933309
24H 50MTA	Q05209	-0.11297	0.064629	0.04355	PTPN12	Tyrosine-pr	PTN12_HU	15	0.92468
48H 50MTA	Q5VWJ9	0.502248	0.064652	0.043561	SNX30	Sorting nex	SNX30_HU	3	1.416419
48H 50MTA	Q08188	-0.00967	0.064658	0.043564	TGM3	Protein-glu	TGM3_HUM	2	0.993321
48H 10MTA	Q9NXN4	-0.11727	0.064663	0.043566	GDAP2	Ganglioside	GDAP2_HU	6	0.921932
24H 50MTA	Q9H2H8	0.127004	0.064669	0.043568	PPIL3	Peptidyl-pr	PPIL3_HUM	7	1.092024
48H 50MTA	Q13442	0.026966	0.064685	0.043575	PDAP1	28 kDa heat	HAP28_HU	6	1.018867
24H 10MTA	P31942	0.013034	0.064722	0.043595	HNRNPH3	Heterogene	HNRH3_HU	12	1.009075
24H 50MTA	Q14139	0.134853	0.064734	0.043602	UBE4A	Ubiquitin c	UBE4A_HU	30	1.097981
48H 50MTA	Q6PD74	0.013924	0.064767	0.04362	AAGAB	Alpha- and	AAGAB_HU	6	1.009698
24H 10MTA	Q32M88	0.350816	0.064814	0.043646	PGGHG	Protein-glu	PGGHG_HU	3	1.275282
48H 10MTA	P49959	-0.01016	0.064813	0.043646	MRE11	Double-stra	MRE11_HU	20	0.992981
48H 50MTA	Q9NRB3	0.221445	0.064814	0.043646	CHST12	Carbohydra	CHSTC_HU	1	1.165901
48H 50MTA	Q86YH6	-0.06865	0.064828	0.043651	PDSS2	All trans-po	DLP1_HUM	16	0.95353
48H 10MTA	Q9BZ23	-0.2134	0.064846	0.043661	PANK2	Pantothena	PANK2_HU	7	0.862499
48H 10MTA	P05198	0.01597	0.064856	0.043666	EIF2S1	Eukaryotic	IF2A_HUMA	25	1.011131
24H 10MTA	P07237	-0.05455	0.064872	0.043676	P4HB	Protein dis	PDIA1_HUM	35	0.962897
24H 50MTA	Q6GMR7	1.013918	0.064876	0.043677	FAAH2	Fatty-acid a	FAAH2_HU	1	2.019388
24H 10MTA	P61626	0.597768	0.064891	0.043687	LYZ	Lysozyme C	LYSC_HUMA	1	1.513374
48H 50MTA	Q86WQ0	-0.12204	0.064926	0.043705	NR2C2AP	Nuclear rec	NR2CA_HU	5	0.918884
24H 10MTA	Q9HBL7	0.233758	0.064927	0.043705	PLGRKT	Plasminoge	PLRKT_HUM	5	1.175894
24H 50MTA	Q9NYL4	-0.01026	0.064927	0.043705	FKBP11	Peptidyl-pr	FKB11_HU	5	0.992914
24H 50MTA	Q9BW19	-0.18482	0.064931	0.043707	KIFC1	Kinesin-like	KIFC1_HUM	21	0.879758
48H 50MTA	O95210	0.37397	0.064933	0.043707	STBD1	Starch-binc	STBD1_HU	5	1.295914
48H 50MTA	O94916	-0.30096	0.064944	0.043714	NFAT5	Nuclear fac	NFAT5_HU	10	0.811715
24H 10MTA	P26639	-0.06803	0.064958	0.04372	TARS1	Threonine--	SYTC_HUMA	37	0.953942
24H 10MTA	P62244	0.138859	0.064961	0.043721	RPS15A	Small ribos	RS15A_HU	6	1.101034
48H 10MTA	Q96EL3	-0.20629	0.06496	0.043721	MRPL53	Large ribos	RM53_HUM	5	0.866766
24H 10MTA	Q06520	0.2162	0.064979	0.043726	SULT2A1	Sulfotransf	ST2A1_HUM	2	1.16167
48H 10MTA	Q8IY67	0.033366	0.064972	0.043726	RAVER1	Ribonuclec	RAVR1_HU	17	1.023397
24H 50MTA	Q96P70	-0.07656	0.064976	0.043726	IPO9	Importin-9	IPO9_HUM	28	0.948318
24H 10MTA	Q9BVG8	0.069395	0.064973	0.043726	KIFC3	Kinesin-like	KIFC3_HUM	16	1.049276
24H 50MTA	O76054	-0.18593	0.064986	0.04373	SEC14L2	SEC14-like p	S14L2_HUM	10	0.879079
24H 50MTA	P49757	-0.01667	0.064993	0.043732	NUMB	Protein nur	NUMB_HU	11	0.988512
24H 50MTA	Q12979	-0.40399	0.065015	0.043743	ABR	Active brea	ABR_HUMA	13	0.755767
48H 50MTA	Q9NR50	0.004369	0.065018	0.043744	EIF2B3	Translation	EI2BG_HUM	21	1.003033
24H 50MTA	Q4G0F5	0.006511	0.065024	0.043748	VPS26B	Vacuolar pr	VP26B_HU	10	1.004524
48H 10MTA	O94760	0.028271	0.065034	0.043753	DDAH1	N(G),N(G)-d	DDAH1_HU	16	1.019789
24H 50MTA	P31321	-0.3112	0.065054	0.043764	PRKAR1B	cAMP-depe	KAP1_HUM	5	0.805969
48H 10MTA	Q9H6R6	0.148124	0.065117	0.043799	ZDHHC6	Palmitoyltr	ZDHC6_HU	2	1.108128
24H 10MTA	Q9Y4E8	0.041848	0.065116	0.043799	USP15	Ubiquitin c	UBP15_HU	40	1.029431
24H 50MTA	P17066	-0.0328	0.065125	0.043801	HSPA6	Heat shock	HSP76_HU	2	0.977521
48H 10MTA	Q8TDH9	0.815983	0.06514	0.043809	BLOC1S5	Biogenesis c	BL1S5_HUM	2	1.760497
48H 50MTA	Q7Z589	-0.25034	0.065166	0.043821	EMSY	BRCA2-inte	EMSY_HUM	7	0.840697

48H 50MTA	Q9H0R3	-0.45768	0.065167	0.043821	TMEM222	Transmemb	TM222_HU	4	0.728158
48H 10MTA	Q96BN2	-1.20964	0.065177	0.043824	TADA1	Transcripti	TADA1_HU	2	0.432375
24H 50MTA	Q5VUD6	0.602212	0.0652	0.043834	DIPK1B	Divergent p	DIK1B_HUN	2	1.518043
48H 50MTA	Q9Y3A2	-0.13864	0.0652	0.043834	UTP11	Probable U	UTP11_HU	11	0.908373
24H 50MTA	Q92785	0.101938	0.065234	0.043855	DPF2	Zinc finger	REQU_HUM	14	1.073214
24H 50MTA	Q9Y2I1	-0.23525	0.065249	0.043862	NISCH	Nischarin	NISCH_HUN	10	0.849537
48H 50MTA	Q96E22	-0.32777	0.065255	0.043865	NUS1	Dehydrodo	NGBR_HUN	2	0.796769
24H 10MTA	Q9NU02	0.164345	0.065281	0.04388	ANKEF1	Ankyrin rep	ANKE1_HU	1	1.120657
48H 10MTA	O14893	0.391424	0.065284	0.043881	GEMIN2	Gem-associ	GEMI2_HU	6	1.311688
24H 10MTA	B2RPK0	-0.68909	0.065302	0.04389	HMGB1P1	Putative hi	HGB1A_HU	2	0.620243
24H 10MTA	Q9NZI8	0.013441	0.065316	0.043898	IGF2BP1	Insulin-like	IF2B1_HUN	25	1.00936
48H 10MTA	P16402	0.124813	0.065319	0.043898	H1-3	Histone H1	H13_HUMA	1	1.090366
24H 10MTA	Q9UBV8	0.087472	0.065332	0.043903	PEF1	Peflin	PEF1_HUM	5	1.062507
48H 10MTA	P49674	-0.04255	0.065343	0.043909	CSNK1E	Casein kina	KC1E_HUM	11	0.970935
24H 10MTA	P52815	-0.15562	0.065358	0.043918	MRPL12	Large ribos	RM12_HUN	5	0.897749
24H 10MTA	Q12849	0.006406	0.065385	0.043931	GRSF1	G-rich sequ	GRSF1_HU	23	1.00445
24H 50MTA	Q92560	0.038196	0.065419	0.04395	BAP1	Ubiquitin c	BAP1_HUM	4	1.026829
48H 10MTA	Q96Q15	0.024523	0.065452	0.043966	SMG1	Serine/thre	SMG1_HUN	30	1.017143
24H 50MTA	Q8TBP6	0.554165	0.065456	0.043969	SLC25A40	Mitochond	S2540_HU	2	1.468319
24H 10MTA	P15056	-0.34804	0.065463	0.043971	BRAF	Serine/thre	BRAF_HUM	4	0.785649
48H 50MTA	Q9UH65	-0.04572	0.065464	0.043971	SWAP70	Switch-asso	SWP70_HU	26	0.968806
48H 10MTA	Q14457	-0.15694	0.065487	0.043984	BECN1	Beclin-1	BECN1_HU	7	0.896925
24H 50MTA	Q08ER8	-0.18971	0.065529	0.044006	ZNF543	Zinc finger	ZN543_HU	1	0.876782
24H 10MTA	Q96FV2	0.122353	0.06553	0.044006	SCRN2	Secernin-2	SCRN2_HU	9	1.088509
24H 50MTA	Q9BQ15	0.57644	0.065543	0.044014	NABP2	SOSS comp	SOSB1_HU	2	1.491165
24H 50MTA	P42694	0.041204	0.065568	0.044023	HELZ	Probable h	HELZ_HUM	13	1.028972
24H 50MTA	Q9GZY8	-0.23108	0.065568	0.044023	MFF	Mitochond	MFF_HUMA	9	0.851995
24H 50MTA	Q9Y450	-0.01133	0.065565	0.044023	HBS1L	HBS1-like p	HBS1L_HU	30	0.992177
48H 50MTA	Q13416	-0.14124	0.065613	0.044044	ORC2	Origin reco	ORC2_HUM	13	0.906736
24H 50MTA	Q99418	-0.19085	0.065625	0.044051	CYTH2	Cytohesin-2	CYH2_HUM	3	0.876088
48H 10MTA	P40189	-0.14526	0.06567	0.044074	IL6ST	Interleukin	IL6RB_HUN	5	0.904213
48H 10MTA	P19387	0.004238	0.065682	0.044081	POLR2C	DNA-direct	RPB3_HUM	12	1.002942
24H 10MTA	Q13232	-0.00716	0.065693	0.044086	NME3	Nucleoside	NDK3_HUN	8	0.99505
48H 10MTA	Q9H8Y5	0.061167	0.065701	0.044089	ANKZF1	tRNA endor	ANKZ1_HU	12	1.04331
48H 10MTA	O60869	0.035783	0.065709	0.044093	EDF1	Endothelial	EDF1_HUM	7	1.025113
24H 50MTA	Q9H4L4	0.042229	0.06572	0.044099	SENP3	Sentrin-spe	SENP3_HU	13	1.029704
24H 50MTA	Q9H9L4	0.201707	0.065724	0.044101	KANSL2	KAT8 regula	KANL2_HU	2	1.150058
24H 50MTA	Q13724	0.044838	0.065732	0.044103	MOGS	Mannosyl-c	MOGS_HUN	33	1.031567
24H 10MTA	Q96S52	0.073789	0.065732	0.044103	PIGS	GPI transan	PIGS_HUMA	16	1.052477
24H 10MTA	Q9BSL1	-0.10208	0.065777	0.044127	UBAC1	Ubiquitin-a	UBAC1_HU	3	0.931687
24H 10MTA	Q32P51	-0.41112	0.06578	0.044128	HNRNPA1L	Heterogene	RA1L2_HU	1	0.75204
48H 10MTA	Q8WUY8	0.185003	0.065789	0.044132	NAT14	Probable N	NAT14_HU	2	1.136819
24H 10MTA	Q15005	0.04101	0.065829	0.044154	SPCS2	Signal pept	SPCS2_HUN	10	1.028834
48H 10MTA	Q8IUH3	0.183497	0.065839	0.044157	RBM45	RNA-bindir	RBM45_HU	11	1.135633
48H 50MTA	Q2NKX8	-0.07186	0.065854	0.044166	ERCC6L	DNA excisic	ERC6L_HUN	38	0.951409
24H 50MTA	Q8NEF9	-0.10084	0.065872	0.044172	SRFBP1	Serum resp	SRFB1_HUN	7	0.932488
48H 10MTA	Q9NUT2	-0.14271	0.065872	0.044172	ABCB8	Mitochond	MITOS_HU	1	0.905813
48H 50MTA	Q6PK18	-0.73546	0.065912	0.044192	OGFOD3	2-oxoglutar	OGFD3_HU	2	0.600627
48H 50MTA	Q96D42	0.521824	0.065916	0.044193	HAVCR1	Hepatitis A	HAVR1_HU	3	1.43577
48H 50MTA	Q13485	-0.05376	0.06594	0.044205	SMAD4	Mothers ag	SMAD4_HU	10	0.963424
48H 10MTA	A0FGR8	-0.02922	0.065941	0.044205	ESYT2	Extended sy	ESYT2_HUN	25	0.979952
24H 50MTA	O75521	0.02623	0.065945	0.044206	ECI2	Enoyl-CoA (	ECI2_HUMA	18	1.018348

24H 10MTA	Q9H0C8	0.033463	0.065968	0.04422	ILKAP	Integrin-lin	ILKAP_HUM	13	1.023466
24H 10MTA	Q9Y4C1	-0.3394	0.065985	0.044229	KDM3A	Lysine-spec	KDM3A_HU	19	0.790372
24H 50MTA	P17152	0.03624	0.066038	0.044256	TMEM11	Transmemb	TMM11_HU	3	1.025438
24H 10MTA	Q96S55	0.076899	0.06607	0.04427	WRNIP1	ATPase WRI	WRIP1_HU	12	1.054748
24H 10MTA	O14966	-0.07288	0.066096	0.044281	RAB29	Ras-related	RAB7L_HUI	4	0.950738
24H 10MTA	O60447	0.183797	0.066104	0.044285	EVI5	Ecotropic v	EVI5_HUMA	12	1.13587
48H 10MTA	P41252	-0.04343	0.06611	0.044288	IARS1	Isoleucine--	SYIC_HUMA	60	0.970342
48H 50MTA	O14737	-0.12125	0.066114	0.044288	PDCD5	Programme	PDCD5_HU	6	0.919388
48H 50MTA	Q15477	0.057826	0.066119	0.044289	SKIC2	Superkiller	SKI2_HUMA	41	1.040896
24H 10MTA	Q9NY33	-0.08798	0.066141	0.044301	DPP3	Dipeptidyl	DPP3_HUM	31	0.940836
24H 10MTA	P23919	0.040771	0.066151	0.044304	DTYMK	Thymidylat	KTHY_HUM	19	1.028664
24H 10MTA	O00151	-0.04651	0.066181	0.04432	PDLIM1	PDZ and LIN	PDLI1_HUM	21	0.968279
24H 10MTA	Q9H6Q4	-0.11409	0.066192	0.044324	CIAO3	Cytosolic ir	CIAO3_HUM	12	0.923967
48H 10MTA	P35573	-0.02716	0.066201	0.044328	AGL	Glycogen d	GDE_HUMA	77	0.98135
48H 50MTA	P50502	-0.10203	0.06621	0.044333	ST13	Hsc70-inte	F10A1_HUI	11	0.931724
48H 50MTA	Q8IVT5	-0.36578	0.066211	0.044333	KSR1	Kinase supp	KSR1_HUM	2	0.776049
24H 10MTA	P55196	-0.00314	0.066234	0.044346	AFDN	Afadin	AFAD_HUM	58	0.997827
48H 50MTA	Q6PJT7	-0.08422	0.066239	0.044347	ZC3H14	Zinc finger	ZC3HE_HUI	32	0.943294
24H 10MTA	Q14681	-0.63885	0.066249	0.044352	KCTD2	BTB/POZ dc	KCTD2_HUI	1	0.642225
24H 50MTA	O60763	0.022715	0.066251	0.044353	USO1	General ves	USO1_HUM	42	1.015869
24H 50MTA	Q96E22	0.233621	0.066264	0.04436	NUS1	Dehydrodo	NGBR_HUM	2	1.175783
24H 10MTA	Q9UHA3	-0.18196	0.066285	0.044372	RSL24D1	Probable ri	RLP24_HUI	4	0.881504
24H 50MTA	A3KMH1	0.21627	0.066289	0.044373	VWA8	von Willebr	VWA8_HUM	43	1.161726
24H 50MTA	Q92879	0.009364	0.066292	0.044374	CELF1	CUGBP Elav	CELF1_HUM	10	1.006512
48H 10MTA	P68036	0.129406	0.066301	0.044378	UBE2L3	Ubiquitin-c	UB2L3_HUI	9	1.093844
24H 50MTA	Q68DC2	-0.37729	0.066306	0.04438	ANKS6	Ankyrin rep	ANKS6_HUI	2	0.769884
24H 10MTA	Q8IZ81	0.073728	0.066315	0.044384	ELMOD2	ELMO doma	ELMD2_HU	8	1.052433
24H 50MTA	Q6ZSB9	0.408811	0.066321	0.044385	ZBTB49	Zinc finger	ZBT49_HUM	1	1.327591
24H 50MTA	O95248	0.033766	0.06632	0.044385	SBF1	Myotubula	MTMR5_HU	16	1.023681
24H 50MTA	Q9NVV5	0.019617	0.06632	0.044385	AIG1	Androgen-i	AIG1_HUM	3	1.01369
48H 10MTA	Q9HCN8	-0.00778	0.06635	0.044398	SDF2L1	Stromal cel	SDF2L_HUM	6	0.99462
24H 50MTA	Q9H3R1	1.387743	0.066369	0.044408	NDST4	Bifunctiona	NDST4_HUI	1	2.616689
48H 10MTA	Q9Y5V3	0.109555	0.066382	0.044415	MAGED1	Melanoma-	MAGD1_HU	10	1.078895
24H 50MTA	Q9NSC2	0.390368	0.066402	0.044425	SALL1	Sal-like pro	SALL1_HUM	4	1.310728
48H 10MTA	O15550	0.202239	0.066436	0.044443	KDM6A	Lysine-spec	KDM6A_HU	2	1.150482
48H 10MTA	Q9BQ70	-0.16595	0.066437	0.044443	TCF25	Ribosome c	TCF25_HUM	16	0.891339
48H 10MTA	Q96T17	-0.08742	0.066452	0.044448	MAP7D2	MAP7 dom	MA7D2_HU	9	0.941202
48H 10MTA	Q9NVC6	-0.06589	0.06645	0.044448	MED17	Mediator o	MED17_HU	10	0.955354
48H 50MTA	Q9Y5X2	-0.17626	0.066451	0.044448	SNX8	Sorting nex	SNX8_HUM	18	0.884992
48H 10MTA	Q6P9B9	-0.08552	0.06646	0.044452	INTS5	Integrator c	INT5_HUMA	15	0.942445
48H 50MTA	P28066	0.019704	0.066499	0.044476	PSMA5	Proteasom	PSA5_HUM	13	1.013752
24H 50MTA	P07437	-0.14066	0.066526	0.04449	TUBB	Tubulin bet	TBB5_HUM	5	0.907102
48H 50MTA	Q9NW82	0.061029	0.066528	0.04449	WDR70	WD repeat-	WDR70_HU	18	1.04321
48H 50MTA	Q9ULC4	-0.0188	0.066604	0.044527	MCTS1	Malignant T	MCTS1_HU	11	0.987057
48H 50MTA	Q7Z4L5	0.024385	0.066626	0.04454	TTC21B	Tetratricop	TT21B_HUM	11	1.017046
48H 50MTA	Q15126	0.039114	0.066667	0.044559	PMVK	Phosphom	PMVK_HUM	11	1.027483
24H 50MTA	P09669	0.122361	0.066683	0.044568	COX6C	Cytochrom	COX6C_HU	8	1.088515
24H 50MTA	Q969G3	0.059846	0.066687	0.044569	SMARCE1	SWI/SNF-re	SMCE1_HU	12	1.042354
48H 10MTA	P31153	0.012554	0.066701	0.044576	MAT2A	S-adenosylr	METK2_HU	16	1.00874
24H 10MTA	Q96GG9	-0.03147	0.066748	0.044598	DCUN1D1	DCN1-like p	DCNL1_HU	10	0.978424
48H 10MTA	Q9UHI6	0.034394	0.066747	0.044598	DDX20	Probable A	DDX20_HU	21	1.024126
48H 10MTA	Q8IXW5	0.124939	0.066753	0.044601	RPAP2	Putative RN	RPAP2_HUI	15	1.090462

48H 10MT/	Q9Y617	-0.03828	0.066772	0.04461	PSAT1	Phosphoser	SERC_HUM	20	0.973813
48H 10MT/	Q99848	-0.07692	0.066792	0.044622	EBNA1BP2	Probable rF	EBP2_HUM	13	0.94808
24H 50MT/	O75064	-0.18415	0.066794	0.044623	DENND4B	DENN doma	DEN4B_HU	2	0.880169
48H 50MT/	O75410	-0.0232	0.066811	0.04463	TACC1	Transformi	TACC1_HUI	15	0.984045
48H 50MT/	Q3KRA6	-0.46303	0.066818	0.044633	C2orf76	UPF0538 p	CB076_HUI	3	0.725459
24H 10MT/	P05388	0.066618	0.066824	0.044636	RPLP0	Large ribos	RLA0_HUM	13	1.047259
48H 50MT/	Q8NG11	0.086021	0.066826	0.044636	TSPAN14	Tetraspanir	TSN14_HUI	2	1.061439
48H 50MT/	Q9Y426	0.206355	0.06684	0.044642	C2CD2	C2 domain-	C2CD2_HU	4	1.153769
24H 10MT/	Q9UKF6	0.065826	0.066844	0.044644	CPSF3	Cleavage ar	CPSF3_HUI	24	1.046684
48H 50MT/	P48051;P4	-0.77881	0.066857	0.044651	KCNJ6;KCN	G protein-a	KCNJ6_HUI	1	0.582849
48H 50MT/	P02461	2.079893	0.066857	0.044651	COL3A1	Collagen al	CO3A1_HU	4	4.227757
48H 10MT/	Q96G03	-0.00468	0.066875	0.04466	PGM2	Phosphope	PGM2_HUI	30	0.996761
24H 10MT/	Q14032	0.262811	0.066917	0.044681	BAAT	Bile acid-Cc	BAAT_HUM	3	1.199814
24H 10MT/	P78549	0.171296	0.066919	0.044681	NTHL1	Endonuclea	NTH_HUM/	2	1.12607
48H 10MT/	Q8NBN7	-0.06136	0.066919	0.044681	RDH13	Retinol deh	RDH13_HU	8	0.958358
48H 50MT/	Q96G23	-0.10544	0.066921	0.044681	CERS2	Ceramide s	CERS2_HUI	9	0.929524
24H 10MT/	Q9NQ35	-0.48465	0.066921	0.044681	NRIP3	Nuclear rec	NRIP3_HUI	1	0.714669
24H 10MT/	Q9NXU5	0.002208	0.06696	0.044701	ARL15	ADP-ribosy	ARL15_HUI	3	1.001532
48H 10MT/	Q9NX08	-0.5075	0.066964	0.044703	COMMD8	COMM don	COMD8_HU	1	0.703441
48H 50MT/	Q99807	0.10974	0.066975	0.044708	COQ7	5-demetho	COQ7_HUM	4	1.079034
48H 50MT/	Q9NXE4	-0.10057	0.066978	0.044709	SMPD4	Sphingomy	NSMA3_HU	20	0.932666
48H 50MT/	Q9NX55	-0.51997	0.066984	0.044712	HYPK	Huntingtin	HYPK_HUM	2	0.697387
24H 50MT/	Q9UH17	-0.20021	0.066988	0.044714	APOBEC3B	DNA dC->dU	ABC3B_HUI	9	0.870423
48H 10MT/	O14777	0.09485	0.067006	0.044721	NDC80	Kinetochor	NDC80_HU	23	1.067955
24H 10MT/	Q15025	0.096262	0.067034	0.044737	TNIP1	TNFAIP3-in	TNIP1_HUM	6	1.069
24H 10MT/	Q96T76	-0.05601	0.067034	0.044737	MMS19	MMS19 nu	MMS19_HU	37	0.96192
48H 10MT/	P46736	-0.02905	0.067043	0.04474	BRCC3	Lys-63-spec	BRCC3_HUI	10	0.980062
24H 50MT/	P61077	-0.11757	0.067042	0.04474	UBE2D3	Ubiquitin-c	UB2D3_HU	4	0.921736
48H 10MT/	Q92643	-0.17869	0.067041	0.04474	PIGK	GPI-anchor	GPI8_HUM	13	0.883506
24H 50MT/	Q9NQ34	-0.18255	0.067082	0.044764	TMEM9B	Transmemb	TMM9B_HU	2	0.881142
48H 10MT/	P43034	0.052392	0.067099	0.044774	PAFAH1B1	Platelet-act	LIS1_HUM/	20	1.036983
24H 10MT/	Q8IVW6	0.106212	0.067118	0.044785	ARID3B	AT-rich inte	ARI3B_HUM	11	1.076398
48H 10MT/	Q13464	0.026381	0.067134	0.044795	ROCK1	Rho-associa	ROCK1_HU	42	1.018454
48H 50MT/	Q9P258	-0.07117	0.067167	0.044811	RCC2	Protein RC(	RCC2_HUM	24	0.951864
48H 10MT/	Q9UJY4	0.109284	0.067179	0.044818	GGA2	ADP-ribosy	GGA2_HUM	13	1.078693
24H 50MT/	Q6PJI9	0.083855	0.067185	0.044821	WDR59	GATOR2 co	WDR59_HU	15	1.059847
48H 50MT/	O75608	-0.00419	0.067188	0.044822	LYPLA1	Acyl-protei	LYPA1_HUM	9	0.997098
24H 10MT/	Q9Y2R0	-0.02818	0.06726	0.04486	COA3	Cytochrom	COA3_HUM	4	0.98066
48H 50MT/	P33240	0.009446	0.067292	0.044877	CSTF2	Cleavage sti	CSTF2_HUM	17	1.006569
24H 10MT/	Q9NYA4	1.066788	0.067318	0.044886	MTMR4	Myotubula	MTMR4_HU	1	2.094765
24H 50MT/	Q13610	-0.026	0.06732	0.044886	PWP1	Periodic try	PWP1_HUM	22	0.982139
24H 50MT/	Q15417	-0.10974	0.067314	0.044886	CNN3	Calponin-3	CNN3_HUM	15	0.926757
48H 50MT/	Q8IXK0	0.179121	0.067319	0.044886	PHC2	Polyhomec	PHC2_HUM	6	1.132194
24H 10MT/	O60551	-0.08685	0.067328	0.04489	NMT2	Glycylpepti	NMT2_HUM	1	0.941577
48H 10MT/	Q16555	-0.04495	0.06733	0.04489	DPYSL2	Dihydropyr	DPYL2_HUI	17	0.969324
48H 10MT/	O60231	-0.00717	0.067332	0.04489	DHX16	Pre-mRNA-s	DHX16_HU	28	0.995042
24H 50MT/	Q15102	0.06233	0.06734	0.044895	PAFAH1B3	Platelet-act	PA1B3_HUI	10	1.044151
24H 10MT/	Q9UBX1	-0.22357	0.06735	0.044901	CTSF	Cathepsin F	CATF_HUM	3	0.856444
48H 50MT/	O43181	0.089828	0.067358	0.044905	NDUFS4	NADH dehy	NDUS4_HU	5	1.064243
24H 50MT/	Q8IUW5	-0.19441	0.067363	0.044908	RELL1	RELT-like pr	RELL1_HUM	2	0.87393
48H 50MT/	Q86TG7	-0.23301	0.067382	0.044916	PEG10	Retrotransp	PEG10_HUI	6	0.850859
48H 50MT/	P53985	0.049796	0.067386	0.044918	SLC16A1	Monocarbc	MOT1_HUM	11	1.035119

48H 50MT/	Q9C0D9	-0.43994	0.06739	0.044919	SELENOI	Ethanolami	EPT1_HUM	2	0.737164
24H 10MT/	Q13620	0.044852	0.067413	0.04493	CUL4B	Cullin-4B	CUL4B_HUI	42	1.031577
48H 50MT/	O43823	0.029546	0.067466	0.044956	AKAP8	A-kinase an	AKAP8_HUI	21	1.020691
48H 10MT/	P40337	-0.24959	0.067469	0.044956	VHL	von Hippel-	VHL_HUMA	2	0.841133
48H 10MT/	Q9BQ13	0.057029	0.06747	0.044957	KCTD14	BTB/POZ dc	KCD14_HU	9	1.040321
24H 50MT/	O95478	-0.06297	0.067483	0.044964	NSA2	Ribosome b	NSA2_HUM	8	0.957293
48H 50MT/	Q9Y680	0.203601	0.067487	0.044966	FKBP7	Peptidyl-pr	FKBP7_HUI	6	1.151569
24H 10MT/	Q71RG4	-0.03823	0.067509	0.044975	TMUB2	Transmemb	TMUB2_HU	4	0.973851
24H 50MT/	Q9Y4E6	0.172402	0.067517	0.044978	WDR7	WD repeat-	WDR7_HUI	11	1.126933
24H 10MT/	Q99471	-0.51263	0.06756	0.045	PFDN5	Prefoldin su	PFD5_HUM	4	0.700941
24H 50MT/	Q9H1C4	0.181472	0.067565	0.045002	UNC93B1	Protein unc	UN93B_HU	4	1.13404
24H 10MT/	Q504Y3	-1.02423	0.067573	0.045005	ZCWPW2	Zinc finger	ZCPW2_HU	1	0.491671
24H 50MT/	Q06830	-0.00845	0.067577	0.045007	PRDX1	Peroxiredo	PRDX1_HU	19	0.994162
48H 10MT/	Q9H832	0.13558	0.06758	0.045008	UBE2Z	Ubiquitin-c	UBE2Z_HUI	10	1.098534
48H 10MT/	Q9BSJ8	-0.0115	0.06761	0.045023	ESYT1	Extended sy	ESYT1_HUM	45	0.992057
48H 50MT/	P31943	0.036523	0.06764	0.045037	HNRNPH1	Heterogene	HNRH1_HU	18	1.025639
24H 10MT/	Q9H8T0	0.132922	0.067648	0.04504	AKTIP	AKT-interac	AKTIP_HUM	6	1.096512
24H 10MT/	Q8N9M1	0.090878	0.06765	0.045041	C19orf47	Uncharacte	CS047_HUI	4	1.065018
24H 50MT/	Q9Y2U5	-0.4055	0.067662	0.045045	MAP3K2	Mitogen-ac	M3K2_HUM	7	0.754972
24H 10MT/	Q6XD76	1.853755	0.067731	0.045082	ASCL4	Achaete-scu	ASCL4_HUI	1	3.614396
24H 10MT/	Q9GZY8	-0.2025	0.067795	0.045113	MFF	Mitochond	MFF_HUMA	9	0.869041
48H 50MT/	P07203	-0.01992	0.067832	0.045135	GPX1	Glutathion	GPX1_HUM	9	0.986286
24H 50MT/	P51398	-0.13784	0.067842	0.04514	DAP3	Small ribos	RT29_HUM	18	0.908876
24H 10MT/	Q86XI2	0.18472	0.067841	0.04514	NCAPG2	Condensin-	CNDG2_HU	15	1.136596
48H 50MT/	Q9BST9	0.033316	0.067855	0.045147	RTKN	Rhotekin	RTKN_HUM	11	1.023361
48H 10MT/	P22830	-0.04549	0.067873	0.045154	FECH	Ferrochelat	HEMH_HUI	12	0.968957
24H 50MT/	P54727	0.042708	0.067876	0.045154	RAD23B	UV excision	RD23B_HU	15	1.030046
24H 50MT/	Q7Z3E5	-0.57858	0.067884	0.045156	ARMC9	LisH domai	ARMC9_HU	3	0.669623
24H 10MT/	Q8WZA1	-0.10306	0.0679	0.045163	POMGNT1	Protein O-li	PMGT1_HU	7	0.931054
48H 50MT/	O43164	0.482401	0.067918	0.045172	PJA2	E3 ubiquiti	PJA2_HUM	5	1.397067
48H 10MT/	Q86TS9	-0.06633	0.067927	0.045177	MRPL52	Large ribos	RM52_HUM	2	0.955064
48H 50MT/	P83436	0.00755	0.06793	0.045177	COG7	Conserved	COG7_HUM	23	1.005247
48H 10MT/	Q96AY4	0.028439	0.067965	0.045192	TTC28	Tetratricop	TTC28_HUI	3	1.019908
48H 50MT/	O96006	-0.51856	0.067969	0.045193	ZBED1	E3 SUMO-p	ZBED1_HUI	3	0.698068
48H 10MT/	Q9H6U8	-0.09278	0.067975	0.045196	ALG9	Alpha-1,2-r	ALG9_HUM	7	0.937713
24H 50MT/	Q8WUA7	-0.07656	0.068001	0.045208	TBC1D22A	TBC1 doma	TB22A_HUI	9	0.948314
24H 50MT/	Q9H9S4	0.61726	0.068032	0.04522	CAB39L	Calcium-bi	CB39L_HUI	3	1.53396
24H 50MT/	Q8NEB5	0.228674	0.068037	0.045223	PLPP5	Phospholip	PLPP5_HUI	1	1.171757
48H 50MT/	P31785	-0.75329	0.06804	0.045223	IL2RG	Cytokine re	IL2RG_HUM	1	0.593247
48H 10MT/	Q9UPW6	-0.2533	0.068042	0.045223	SATB2	DNA-bindir	SATB2_HUI	5	0.838977
48H 10MT/	Q13823	-0.07174	0.068047	0.045224	GNL2	Nucleolar G	NOG2_HUM	21	0.951493
24H 50MT/	Q96GF1	-0.51783	0.068056	0.045227	RNF185	E3 ubiquiti	RN185_HU	2	0.69842
24H 50MT/	P19474	1.120026	0.068066	0.04523	TRIM21	E3 ubiquiti	RO52_HUM	1	2.173509
48H 10MT/	Q9NUL3	0.078748	0.068088	0.04524	STAU2	Double-stra	STAU2_HUI	15	1.056101
48H 10MT/	Q9NPF5	0.059853	0.068118	0.045256	DMAP1	DNA methy	DMAP1_HU	9	1.04236
24H 50MT/	P35573	0.017306	0.068198	0.045302	AGL	Glycogen d	GDE_HUMA	77	1.012068
24H 10MT/	Q00403	-0.12036	0.068229	0.045318	GTF2B	Transcripti	TF2B_HUM	17	0.919955
48H 50MT/	Q9GZZ9	0.006273	0.068249	0.045329	UBA5	Ubiquitin-l	UBA5_HUM	10	1.004357
24H 10MT/	Q15853	0.06844	0.068259	0.045333	USF2	Upstream s	USF2_HUM	4	1.048582
48H 10MT/	Q8IWU2	-0.21659	0.068262	0.045334	LMTK2	Serine/thre	LMTK2_HU	6	0.860599
24H 10MT/	Q96NB2	0.050964	0.068275	0.045341	SFXN2	Sideroflexir	SFXN2_HUI	8	1.035957
48H 10MT/	P35520	0.040255	0.068279	0.045342	CBS	Cystathioni	CBS_HUMA	22	1.028296

48H 10MT/	Q9BS26	-0.0546	0.068295	0.045349	ERP44	Endoplasm	ERP44_HUI	17	0.96286
48H 10MT/	Q96GF1	-0.48436	0.068329	0.045368	RNF185	E3 ubiquiti	RN185_HU	2	0.714816
24H 10MT/	P52564	0.092824	0.068353	0.045379	MAP2K6	Dual specifi	MP2K6_HU	2	1.066455
24H 50MT/	P61201	0.004705	0.068355	0.045379	COPS2	COP9 signa	CSN2_HUM	21	1.003267
48H 10MT/	Q7LGA3	-0.15805	0.06835	0.045379	HS2ST1	Heparan su	HS2ST_HUI	13	0.896233
24H 50MT/	Q9Y2T2	-0.01119	0.068351	0.045379	AP3M1	AP-3 compl	AP3M1_HU	17	0.992274
24H 10MT/	Q8WWY3	0.003819	0.068356	0.045379	PRPF31	U4/U6 sma	PRP31_HUI	17	1.002651
24H 50MT/	P21860	0.292587	0.068376	0.045389	ERBB3	Receptor ty	ERBB3_HUI	7	1.224834
24H 10MT/	P0C264	0.508036	0.068381	0.04539	SBK3	Uncharacte	SBK3_HUM	1	1.422113
24H 50MT/	P17987	-0.06554	0.068385	0.045391	TCP1	T-complex	TCPA_HUM	32	0.955588
48H 50MT/	O75787	-0.35792	0.068388	0.045392	ATP6AP2	Renin recep	RENR_HUM	11	0.780287
24H 10MT/	Q3MIR4	-0.01011	0.068422	0.045409	TMEM30B	Cell cycle c	CC50B_HUI	3	0.993017
48H 50MT/	Q9UJF2	-0.20943	0.068422	0.045409	RASAL2	Ras GTPase-	NGAP_HUM	13	0.864876
48H 50MT/	Q8TAP9	-0.93521	0.068439	0.045416	MPLKIP	M-phase-sp	MPLKI_HUI	1	0.522966
48H 50MT/	P50990	-0.07835	0.068458	0.045426	CCT8	T-complex	TCPQ_HUM	35	0.947138
24H 50MT/	P36639	-0.05023	0.068461	0.045427	NUDT1	Oxidized pu	8ODP_HUM	6	0.96578
48H 10MT/	O95136	-0.34145	0.068465	0.045429	S1PR2	Sphingosin	S1PR2_HUI	1	0.789247
48H 50MT/	Q32M88	0.344913	0.068471	0.045431	PGGHG	Protein-glu	PGGHG_HU	3	1.270074
48H 10MT/	P02533	-0.15452	0.068476	0.045433	KRT14	Keratin, typ	K1C14_HUI	33	0.898432
24H 10MT/	P0C025	0.37042	0.068479	0.045434	NUDT17	Nucleoside	NUD17_HU	1	1.292729
24H 10MT/	Q86Y82	-0.09502	0.06851	0.045451	STX12	Syntaxin-1.	STX12_HUI	9	0.936259
48H 10MT/	Q15637	0.073839	0.068532	0.045462	SF1	Splicing fac	SF01_HUM	17	1.052514
48H 10MT/	Q9H4I2	0.541985	0.068539	0.045465	ZHX3	Zinc fingers	ZHX3_HUM	2	1.455974
48H 10MT/	Q9NW13	-0.06475	0.068545	0.045467	RBM28	RNA-bindir	RBM28_HU	24	0.956111
24H 10MT/	P16422	0.172264	0.068547	0.045468	EPCAM	Epithelial c	EPCAM_HU	4	1.126825
24H 10MT/	Q96HU1	0.710002	0.068573	0.045479	SGSM3	Small G prc	SGSM3_HU	3	1.635806
48H 10MT/	O43318	0.197739	0.068589	0.045488	MAP3K7	Mitogen-ac	M3K7_HUM	8	1.1469
24H 10MT/	Q9P206	0.014432	0.068603	0.045495	NHSL3	NHS-like pr	NHSL3_HUI	6	1.010054
48H 50MT/	P09132	-0.05509	0.068615	0.045502	SRP19	Signal reco	SRP19_HUI	4	0.962534
48H 10MT/	Q96G61	-0.21471	0.068617	0.045502	NUDT11	Diphospho	NUD11_HU	1	0.861721
24H 50MT/	Q9BYK8	-0.19823	0.06865	0.045519	HELZ2	3'-5' exorib	HELZ2_HUI	17	0.87162
24H 10MT/	Q96BD8	0.294766	0.068654	0.04552	SKA1	Spindle anc	SKA1_HUM	2	1.226686
48H 10MT/	Q6NXT6	-0.40895	0.068692	0.045539	TAPT1	Transmemb	TAPT1_HUI	5	0.75317
24H 50MT/	Q7Z6V5	-0.07805	0.068706	0.045545	ADAT2	tRNA-specit	ADAT2_HUI	4	0.947338
48H 50MT/	Q6NXT6	-0.51388	0.06871	0.045546	TAPT1	Transmemb	TAPT1_HUI	5	0.700339
24H 50MT/	Q86VP1	-0.36824	0.068717	0.04555	TAX1BP1	Tax1-bindir	TAXB1_HUI	11	0.774729
48H 10MT/	Q9ULI0	-0.12346	0.068726	0.045555	ATAD2B	ATPase fam	ATD2B_HUI	8	0.917985
48H 50MT/	Q9ULP9	0.02187	0.068744	0.045566	TBC1D24	TBC1 doma	TBC24_HUI	13	1.015274
24H 50MT/	Q9UNF0	-0.14139	0.068747	0.045567	PACSIN2	Protein kin	PACN2_HU	16	0.906647
48H 10MT/	Q9HD45	-0.03794	0.068753	0.045569	TM9SF3	Transmemb	TM9S3_HU	13	0.974042
24H 10MT/	P31785	0.693041	0.068761	0.045572	IL2RG	Cytokine re	IL2RG_HUM	1	1.616688
24H 10MT/	Q9UM11	0.193541	0.068766	0.045573	FZR1	Fizzy-relate	FZR1_HUM	3	1.143567
48H 50MT/	Q9BZF9	0.10783	0.068854	0.045622	UACA	Uveal autoa	UACA_HUM	21	1.077606
24H 10MT/	Q7Z2W9	0.034262	0.068878	0.045633	MRPL21	Large ribos	RM21_HUM	10	1.024033
24H 10MT/	Q9NXK8	-0.27615	0.068906	0.045648	FBXL12	F-box/LRR-	FXL12_HUI	4	0.825789
48H 10MT/	Q96I76	0.95367	0.06899	0.04569	GPATCH3	G patch do	GPTC3_HUI	1	1.936794
48H 10MT/	P49257	-0.15018	0.068988	0.04569	LMAN1	Protein ER(	LMAN1_HU	19	0.90114
48H 10MT/	Q5JYT7	0.026367	0.068991	0.04569	KIAA1755	Uncharacte	K1755_HUI	1	1.018444
48H 10MT/	Q7L2E3	-0.03732	0.069001	0.045693	DHX30	ATP-depen	DHX30_HU	64	0.974465
24H 50MT/	Q9BQL6	0.798039	0.06901	0.045697	FERMT1	Fermitin fa	FERM1_HU	1	1.738736
48H 50MT/	Q9BSJ5	-0.56837	0.06901	0.045697	MTNAP1	Mitochond	CQ080_HU	3	0.674378
48H 10MT/	Q9Y221	-0.10706	0.069014	0.045698	NIP7	60S ribosor	NIP7_HUM	9	0.928479

48H 50MT/	P16452	1.352979	0.069021	0.045701	EPB42	Protein 4.2	EPB42_HUI	1	2.554391
24H 10MT/	Q9NRX2	-0.0787	0.069033	0.045706	MRPL17	Large ribos	RM17_HUN	10	0.946908
48H 10MT/	Q6ZUT9	0.095602	0.069048	0.045711	DENND5B	DENN dom	DEN5B_HU	5	1.068511
48H 50MT/	Q8NHV9	0.145894	0.069064	0.04572	RHOXF1	Rhox home	RHXF1_HUI	1	1.106416
24H 50MT/	Q9H633	0.455762	0.069129	0.045756	RPP21	Ribonuclea	RPP21_HUI	2	1.371507
24H 50MT/	Q8WZA0	0.103206	0.069157	0.045772	LZIC	Protein LZI	LZIC_HUM/	9	1.074158
48H 10MT/	Q14152	0.02125	0.06917	0.045779	EIF3A	Eukaryotic	EIF3A_HUN	68	1.014838
24H 50MT/	P30041	0.057638	0.069198	0.045792	PRDX6	Peroxiredo	PRDX6_HU	19	1.040761
24H 10MT/	Q06265	0.126125	0.069198	0.045792	EXOSC9	Exosome co	EXOS9_HUI	11	1.091358
24H 50MT/	Q9NVR2	0.035628	0.069195	0.045792	INTS10	Integrator	INT10_HUN	18	1.025003
24H 10MT/	Q5SGD2	0.112157	0.069217	0.045803	PPM1L	Protein ph	PPM1L_HU	1	1.080843
48H 50MT/	O60266	-0.25609	0.069225	0.045807	ADCY3	Adenylate	ADCY3_HUI	3	0.837352
48H 10MT/	Q00537	-1.79081	0.069255	0.045824	CDK17	Cyclin-dep	CDK17_HU	2	0.28901
24H 10MT/	Q96MS3	0.81462	0.069269	0.045826	GLT1D1	Glycosyltra	GL1D1_HU	1	1.758835
24H 50MT/	P24386	-0.10583	0.069266	0.045826	CHM	Rab protei	RAE1_HUM	16	0.929272
24H 10MT/	P82912	0.051258	0.069271	0.045826	MRPS11	Small ribos	RT11_HUM	5	1.036168
24H 50MT/	Q8IUX1	0.207918	0.069295	0.045836	TMEM126E	Complex I a	T126B_HUI	5	1.15502
48H 50MT/	P55786	-0.00305	0.069303	0.04584	NPEPPS	Puromycin	PSA_HUMA	43	0.997891
48H 50MT/	Q6NSJ5	-0.2954	0.069309	0.045844	LRRC8E	Volume-reg	LRC8E_HUN	16	0.814847
24H 50MT/	P49407	-0.09031	0.06932	0.045848	ARRB1	Beta-arresti	ARRB1_HUI	13	0.93932
24H 50MT/	Q9BU76	0.060998	0.069331	0.045854	MMTAG2	Multiple m	MMTA2_HU	5	1.043187
24H 10MT/	Q96ED9	0.371946	0.069343	0.045858	HOOK2	Protein Ho	HOOK2_HU	1	1.294097
48H 50MT/	Q5RI15	-0.14696	0.069348	0.045861	COX20	Cytochrom	COX20_HU	6	0.903152
48H 50MT/	Q14123	0.676624	0.069372	0.045873	PDE1C	Dual specifi	PDE1C_HUI	1	1.598395
48H 50MT/	Q7Z3Y8	1.096327	0.069381	0.045878	KRT27	Keratin, typ	K1C27_HUI	2	2.138096
48H 10MT/	P29590	0.005921	0.069392	0.045885	PML	Protein PM	PML_HUM/	11	1.004113
48H 10MT/	Q9Y3A6	-0.16685	0.069399	0.045886	TMED5	Transmemb	TMED5_HU	9	0.890787
24H 10MT/	Q14160	-0.01884	0.069401	0.045887	SCRIB	Protein scri	SCRIB_HUN	40	0.987027
48H 50MT/	O60306	-0.14281	0.069416	0.045895	AQR	RNA helicas	AQR_HUM/	44	0.905756
24H 50MT/	Q9H1E3	0.447227	0.069423	0.045899	NUCKS1	Nuclear ubi	NUCKS_HU	6	1.363417
24H 10MT/	Q8N3C0	-0.00217	0.069469	0.045927	ASCC3	Activating	ASCC3_HUI	63	0.998498
48H 50MT/	B7U540	0.758223	0.069482	0.045932	KCNJ18	Inward rect	KCJ18_HUN	1	1.691406
24H 10MT/	Q8IXH7	0.055784	0.069482	0.045932	NELFCD	Negative el	NELFD_HUI	14	1.039424
24H 50MT/	Q8TAG9	-0.03959	0.069498	0.04594	EXOC6	Exocyst cor	EXOC6_HUI	21	0.972934
24H 10MT/	Q9HBF4	0.265429	0.069502	0.045941	ZFYVE1	Zinc finger	ZFYV1_HUN	4	1.201993
48H 50MT/	Q86VZ5	-0.16811	0.069502	0.045941	SGMS1	Phosphatid	SMS1_HUM	3	0.89001
48H 50MT/	Q9BRP1	-0.06774	0.069505	0.045942	PDCD2L	Programme	PDD2L_HU	7	0.95413
24H 10MT/	P21980	-0.1143	0.069543	0.04596	TGM2	Protein-glu	TGM2_HUN	18	0.923829
48H 50MT/	Q96TA1	0.074128	0.069575	0.045974	NIBAN2	Protein Nib	NIBA2_HUN	13	1.052725
48H 50MT/	O75323	0.077536	0.069604	0.045986	NIPSNAP2	Protein Nip	NIPS2_HUN	7	1.055215
48H 50MT/	Q12905	0.008776	0.069612	0.045989	ILF2	Interleukin	ILF2_HUM/	20	1.006101
48H 10MT/	P61160	0.041939	0.069619	0.045991	ACTR2	Actin-relate	ARP2_HUM	16	1.029497
48H 10MT/	Q8IUD2	0.012838	0.069626	0.045994	ERC1	ELKS/Rab6-	RB6I2_HUN	26	1.008939
48H 10MT/	P62491	-0.05932	0.069633	0.045997	RAB11A	Ras-related	RB11A_HUI	13	0.959718
24H 50MT/	Q9NR61	1.115925	0.069651	0.046008	DLL4	Delta-like p	DLL4_HUM	1	2.167339
24H 50MT/	O94888	0.021703	0.069656	0.046008	UBXN7	UBX domai	UBXN7_HU	15	1.015157
48H 50MT/	P19447	-0.13202	0.069654	0.046008	ERCC3	General tra	ERCC3_HUI	19	0.912553
24H 10MT/	Q53H82	-0.08402	0.069656	0.046008	LACTB2	Endoribon	LACB2_HUI	9	0.943426
24H 10MT/	P49207	0.079577	0.069675	0.046019	RPL34	Large ribos	RL34_HUM	2	1.056708
48H 10MT/	Q9UPR3	0.044025	0.069678	0.04602	SMG5	Nonsense-r	SMG5_HUN	10	1.030986
24H 10MT/	P08247	0.121994	0.069701	0.04603	SYP	Synaptophy	SYPH_HUM	3	1.088238
48H 10MT/	Q969F9	0.036104	0.069711	0.046033	HPS3	BLOC-2 con	HPS3_HUM	21	1.025341

48H 50MT/	P08133	0.587013	0.069713	0.046034	ANXA6	Annexin A6	ANXA6_HU	6	1.502134
24H 50MT/	O75586	0.210827	0.069716	0.046035	MED6	Mediator o	MED6_HUI	2	1.157351
48H 10MT/	Q9HAD4	0.030426	0.069722	0.046037	WDR41	WD repeat-	WDR41_HU	7	1.021313
24H 10MT/	Q9H6R0	-0.47725	0.069726	0.046038	DHX33	ATP-depen	DHX33_HU	7	0.718343
48H 50MT/	Q5SXM2	-0.62372	0.069734	0.04604	SNAPC4	snRNA-acti	SNPC4_HUI	2	0.648997
24H 10MT/	Q12981	0.057186	0.069737	0.04604	BNIP1	Vesicle tran	SEC20_HUI	9	1.040435
24H 50MT/	P04920	-0.15493	0.069772	0.046054	SLC4A2	Anion exch	B3A2_HUM	21	0.898174
24H 50MT/	Q9NTZ6	-0.15982	0.069778	0.046056	RBM12	RNA-bindir	RBM12_HU	22	0.895134
48H 50MT/	Q9UBS0	0.245631	0.069783	0.046058	RPS6KB2	Ribosomal	KS6B2_HUI	4	1.185611
48H 10MT/	Q14676	0.018573	0.069796	0.046063	MDC1	Mediator o	MDC1_HUI	43	1.012957
48H 10MT/	Q14204	-0.00372	0.0698	0.046064	DYNC1H1	Cytoplasmi	DYHC1_HU	233	0.997428
24H 10MT/	O43865	-0.05919	0.069823	0.046076	AHCYL1	S-adenosyll	SAHH2_HU	12	0.959806
24H 10MT/	Q7Z333	0.045498	0.069845	0.046083	SETX	Probable h	SETX_HUM/	17	1.032039
48H 50MT/	Q8TBF4	0.157207	0.069868	0.046098	ZCRB1	Zinc finger	ZCRB1_HUI	4	1.115126
48H 10MT/	O95400	0.094115	0.069909	0.04612	CD2BP2	CD2 antiger	CD2B2_HU	12	1.067411
24H 10MT/	Q96GS4	-0.02232	0.06991	0.04612	BORCS6	BLOC-1-rela	BORC6_HU	11	0.984645
24H 10MT/	P55060	0.061702	0.069921	0.046126	CSE1L	Exportin-2	XPO2_HUM	52	1.043697
48H 10MT/	Q9UHJ3	0.843611	0.069934	0.046134	SFMBT1	Scm-like wi	SMBT1_HU	1	1.794536
24H 10MT/	Q9H3S7	-0.02479	0.069936	0.046134	PTPN23	Tyrosine-pr	PTN23_HUI	32	0.982966
48H 50MT/	Q92664	-2.0144	0.06994	0.046135	GTF3A	Transcripti	TF3A_HUM	1	0.247516
24H 50MT/	Q9Y248	-0.31583	0.06994	0.046135	GINS2	DNA replica	PSF2_HUM	3	0.803386
48H 50MT/	Q9NZP8	0.895696	0.069944	0.046136	C1RL	Complemer	C1RL_HUM	1	1.860508
24H 10MT/	P00367	-0.07694	0.069958	0.046142	GLUD1	Glutamate	DHE3_HUM	29	0.948069
24H 50MT/	Q9HCK4	0.398925	0.07003	0.046182	ROBO2	Roundabou	ROBO2_HU	9	1.318525
24H 50MT/	Q6PIW4	0.052534	0.07006	0.046196	FIGNL1	Fidgetin-lik	FIGL1_HUM	8	1.037085
48H 10MT/	P46060	-0.00598	0.070091	0.046209	RANGAP1	Ran GTPase	RAGP1_HU	33	0.995867
48H 10MT/	Q9NP66	0.111449	0.070111	0.046218	HMG20A	High mobil	HM20A_HU	5	1.080313
24H 50MT/	Q9UQE7	0.01795	0.070118	0.046222	SMC3	Structural	SMC3_HUM	49	1.01252
24H 10MT/	O95497	0.745085	0.070129	0.046228	VNN1	Pantethein	VNN1_HUM	2	1.676073
24H 10MT/	Q15291	0.172355	0.070142	0.046235	RBBP5	Retinoblast	RBBP5_HUI	15	1.126896
24H 50MT/	Q99470	0.129744	0.070182	0.046259	SDF2	Stromal cel	SDF2_HUM	5	1.094099
24H 50MT/	O75665	-0.18258	0.070214	0.046276	OFD1	Centriole a	OFD1_HUM	11	0.881125
24H 50MT/	Q9BTV6	0.028209	0.07023	0.046285	DPH7	Diphthine	DPH7_HUM	1	1.019746
24H 10MT/	Q9NVH1	0.093001	0.070243	0.046292	DNAJC11	DnaJ homo	DJC11_HUI	18	1.066586
48H 50MT/	Q969V6	-0.03396	0.070259	0.0463	MRTFA	Myocardin-	MRTFA_HU	8	0.976738
48H 10MT/	P51858	0.076151	0.070307	0.046324	HDGF	Hepatoma-	HDGF_HUM	22	1.054202
24H 10MT/	Q9HCK8	0.005297	0.070313	0.046328	CHD8	Chromodor	CHD8_HUM	28	1.003678
24H 10MT/	P15252	1.016065	0.070337	0.046339	NaN	Rubber elo	REF_HEVBR	5	2.022396
24H 10MT/	Q9NPD8	0.103516	0.070343	0.046342	UBE2T	Ubiquitin-c	UBE2T_HUI	7	1.074388
24H 50MT/	Q99570	-0.00012	0.070347	0.046344	PIK3R4	Phosphoin	PI3R4_HUM	24	0.99992
24H 10MT/	Q8NB15	-0.11178	0.07035	0.046345	ZNF511	Zinc finger	ZN511_HUI	5	0.925444
24H 50MT/	Q8WUA4	-0.03773	0.070381	0.04636	GTF3C2	General tra	TF3C2_HUI	18	0.974187
24H 10MT/	Q9NQ84	-0.13856	0.070403	0.046371	GPRC5C	G-protein c	GPC5C_HU	7	0.908423
48H 50MT/	Q9NVP2	-0.16486	0.070404	0.046371	ASF1B	Histone cha	ASF1B_HUI	4	0.892012
48H 50MT/	Q5VWX1	-0.08486	0.070422	0.04638	KHDRBS2	KH domain	KHDR2_HU	2	0.942873
24H 50MT/	O60291	-0.03743	0.070474	0.046407	MGRN1	E3 ubiquiti	MGRN1_HU	7	0.974391
48H 50MT/	Q9H6T3	-0.12323	0.070522	0.04643	RPAP3	RNA polym	RPAP3_HUI	19	0.918129
24H 50MT/	P62244	0.113165	0.070529	0.046433	RPS15A	Small ribos	RS15A_HUI	6	1.081599
24H 50MT/	O76071	-0.20942	0.070547	0.046441	CIAO1	Probable cy	CIAO1_HUI	14	0.864887
48H 50MT/	P33552	-0.4641	0.070554	0.046445	CKS2	Cyclin-depe	CKS2_HUM	2	0.724924
24H 50MT/	O15287	-0.1227	0.070561	0.046448	FANCG	Fanconi an	FANCG_HU	9	0.918466
24H 50MT/	P00568	0.070314	0.070568	0.04645	AK1	Adenylate k	KAD1_HUM	9	1.049945

48H 50MT/	P61626	1.061908	0.070599	0.046465	LYZ	Lysozyme C	LYSC_HUM/	1	2.08769
24H 50MT/	Q9Y2K2	0.389799	0.070637	0.046485	SIK3	Serine/thre	SIK3_HUM/	2	1.310211
48H 50MT/	P61086	0.054322	0.070659	0.046498	UBE2K	Ubiquitin-c	UBE2K_HUI	12	1.038371
48H 10MT/	Q99638	0.201999	0.070666	0.046502	RAD9A	Cell cycle cl	RAD9A_HU	4	1.150291
48H 10MT/	P51570	-0.03711	0.070754	0.046552	GALK1	Galactokin:	GALK1_HUI	14	0.974607
24H 50MT/	Q96ST2	0.211291	0.070803	0.046577	IWS1	Protein IWS	IWS1_HUM	13	1.157724
24H 50MT/	Q8N2U0	0.109075	0.07082	0.046582	TMEM256	Transmemb	TM256_HU	2	1.078536
24H 50MT/	Q96FQ6	-0.24036	0.070821	0.046582	S100A16	Protein S1(	S10AG_HUI	4	0.846535
48H 10MT/	Q14320	-0.00545	0.070827	0.046585	FAM50A	Protein FAN	FA50A_HUI	12	0.996229
48H 10MT/	Q5T160	-0.07694	0.070831	0.046586	RARS2	Probable ar	SYRM_HUM	21	0.948065
24H 10MT/	P48507	0.027477	0.070834	0.046588	GCLM	Glutamate-	GSH0_HUM	11	1.019228
24H 50MT/	Q92540	-0.17294	0.070846	0.046593	SMG7	Nonsense-n	SMG7_HUN	9	0.887032
48H 10MT/	Q9UJX2	-0.0003	0.070903	0.046626	CDC23	Cell divisior	CDC23_HU	24	0.99979
48H 10MT/	Q9Y3D6	-0.08106	0.070949	0.04665	FIS1	Mitochond	FIS1_HUM/	6	0.945362
48H 50MT/	Q9H0U3	-0.0279	0.070954	0.04665	MAGT1	Magnesium	MAGT1_HU	7	0.98085
48H 50MT/	Q96TA2	-0.17612	0.070964	0.046653	YME1L1	ATP-depen(	YMEL1_HUI	20	0.885081
24H 50MT/	O75190	-0.20154	0.070967	0.046654	DNAJB6	DnaJ homo	DNJB6_HUI	7	0.869624
48H 10MT/	Q9P1F3	-0.23652	0.070975	0.046656	ABRACL	Costars fam	ABRAL_HUI	2	0.84879
24H 50MT/	Q8N2G8	0.078505	0.071	0.046668	GHDC	GH3 domai	GHDC_HUN	16	1.055923
24H 10MT/	Q9BSE5	0.000212	0.071003	0.04667	AGMAT	Guanidino	GDAH_HUN	14	1.000147
24H 50MT/	Q5T9A4	-0.05408	0.07101	0.046672	ATAD3B	ATPase fam	ATD3B_HUI	15	0.963207
24H 50MT/	Q58FG1	-0.38616	0.071024	0.046679	HSP90AA4I	Putative he	HS904_HUI	4	0.765164
48H 50MT/	Q14681	0.103606	0.071055	0.046695	KCTD2	BTB/POZ dc	KCTD2_HUI	1	1.074456
24H 50MT/	Q9Y2X0	0.195632	0.071057	0.046695	MED16	Mediator o	MED16_HU	6	1.145226
48H 10MT/	A6NIZ1	0.130895	0.071062	0.046698	RAP1BL	Ras-related	RP1BL_HUI	1	1.094973
48H 50MT/	Q2TAA5	-0.11545	0.071086	0.04671	ALG11	GDP-Man:N	ALG11_HUI	8	0.923096
24H 50MT/	Q6AI08	-0.17357	0.071093	0.046714	HEATR6	HEAT repea	HEAT6_HUI	17	0.886645
48H 50MT/	Q9NV66	0.220247	0.071131	0.046735	TYW1	S-adenosyl-	TYW1_HUN	9	1.164933
48H 50MT/	P49326	0.603662	0.071145	0.046743	FMO5	Flavin-cont	FMO5_HUN	2	1.519569
24H 50MT/	Q15008	0.039528	0.071148	0.046744	PSMD6	26S protea:	PSMD6_HU	30	1.027777
48H 50MT/	Q9UFN0	0.027091	0.071191	0.046767	NIPSNAP3A	Protein Nip	NPS3A_HUI	9	1.018956
24H 50MT/	Q9UGJ1	-0.03238	0.071204	0.046772	TUBGCP4	Gamma-tul	GCP4_HUM	12	0.977807
24H 50MT/	Q9BQQ3	-0.01811	0.071208	0.046774	GORASP1	Golgi reasse	GORS1_HU	1	0.987527
48H 50MT/	Q14814	-0.37311	0.071237	0.046788	MEF2D	Myocyte-sp	MEF2D_HU	8	0.772117
48H 50MT/	O95630	-0.01305	0.071245	0.046792	STAMBP	STAM-bindi	STABP_HUN	11	0.990997
24H 10MT/	P41229	-0.29252	0.071248	0.046793	KDM5C	Lysine-spec	KDM5C_HU	15	0.816473
48H 10MT/	Q9BQ52	0.012714	0.071273	0.046807	ELAC2	Zinc phospl	RNZ2_HUM	31	1.008852
48H 10MT/	Q14980	-0.03309	0.071289	0.046815	NUMA1	Nuclear mi	NUMA1_HU	76	0.977327
48H 10MT/	P38159	-0.09719	0.071291	0.046816	RBMX	RNA-bindir	RBMX_HUN	12	0.934851
24H 50MT/	Q14978	0.035375	0.071295	0.046817	NOLC1	Nucleolar a	NOLC1_HU	10	1.024823
48H 10MT/	Q969J3	0.120444	0.071305	0.046823	BORCS5	BLOC-1-rela	BORC5_HU	5	1.087069
24H 50MT/	Q8IWP9	0.464921	0.071314	0.046826	CCDC28A	Coiled-coil	CC28A_HUI	1	1.380242
48H 10MT/	Q9H4A3	0.021928	0.071314	0.046826	WNK1	Serine/thre	WNK1_HUI	36	1.015315
24H 10MT/	Q8NDF8	0.056483	0.071319	0.046828	TENT4B	Terminal nu	PAPD5_HU	11	1.039927
48H 50MT/	Q96D31	-0.13372	0.071325	0.046831	ORAI1	Calcium rel	ORAI1_HUN	2	0.911477
48H 50MT/	P24386	0.018269	0.071342	0.046839	CHM	Rab proteir	RAE1_HUM	16	1.012744
48H 50MT/	O43149	0.013385	0.071355	0.046844	ZZEF1	Zinc finger	ZZEF1_HUN	18	1.009321
24H 50MT/	Q99986	0.100762	0.071368	0.04685	VRK1	Serine/thre	VRK1_HUM	13	1.072339
48H 10MT/	Q5MIZ7	0.01214	0.07138	0.046856	PPP4R3B	Serine/thre	P4R3B_HUI	18	1.00845
48H 10MT/	Q8NBP0	-0.224	0.071389	0.046859	TTC13	Tetratricop	TTC13_HUN	8	0.856185
24H 10MT/	P30622	0.031004	0.071402	0.046864	CLIP1	CAP-Gly do	CLIP1_HUM	44	1.021723
24H 10MT/	Q9BVS4	0.024189	0.071402	0.046864	RIOK2	Serine/thre	RIOK2_HUN	11	1.016908

48H 10MT/	Q9H467	0.224782	0.071415	0.04687	CUEDC2	CUE domai	CUED2_HU	4	1.1686
24H 50MT/	Q96RG2	-0.1685	0.071437	0.046883	PASK	PAS domai	PASK_HUM	8	0.889768
24H 10MT/	O95302	0.029274	0.071488	0.046912	FKBP9	Peptidyl-pr	FKBP9_HUI	14	1.020498
24H 50MT/	Q86UX6	0.137684	0.071488	0.046912	STK32C	Serine/thre	ST32C_HUI	5	1.100137
24H 10MT/	Q9BUJ2	0.041474	0.071523	0.04693	HNRNPUL1	Heterogene	HNRL1_HU	25	1.029164
48H 10MT/	Q14596	0.086648	0.071527	0.046931	NBR1	Next to BR(	NBR1_HUN	1	1.0619
24H 50MT/	Q9BTW9	-0.10391	0.071544	0.046939	TBCD	Tubulin-spe	TBCD_HUN	34	0.930507
48H 10MT/	Q15773	0.104701	0.071554	0.046941	MLF2	Myeloid leu	MLF2_HUN	6	1.075271
24H 50MT/	Q13825	0.229919	0.071577	0.046951	AUH	Methylglut	AUHM_HUI	8	1.172769
48H 50MT/	Q9Y376	-0.16168	0.071585	0.046956	CAB39	Calcium-bi	CAB39_HUI	13	0.893981
48H 10MT/	O15269	-0.03344	0.071595	0.04696	SPTLC1	Serine paln	SPTC1_HUN	10	0.977086
24H 50MT/	Q9BSB4	0.153864	0.071607	0.046966	ATG101	Autophagy-	ATGA1_HUI	5	1.112545
24H 10MT/	Q567V2	-0.03572	0.071615	0.046971	MPV17L2	Mpv17-like	M17L2_HU	4	0.975548
48H 50MT/	Q8N5A5	-0.32557	0.071632	0.046979	ZGPAT	Zinc finger (	ZGPAT_HUI	9	0.797984
24H 50MT/	Q8WUY9	-0.20484	0.071653	0.046991	DEPDC1B	DEP domai	DEP1B_HUI	1	0.867633
48H 10MT/	P46199	-0.10181	0.071655	0.046992	MTIF2	Translation	IF2M_HUM	9	0.931864
24H 10MT/	Q92995	0.1497	0.071658	0.046993	USP13	Ubiquitin c	UBP13_HU	6	1.109339
24H 50MT/	O75717	0.044411	0.071662	0.046993	WDHD1	WD repeat	WDHD1_HI	28	1.031262
48H 10MT/	Q8IXH7	0.03643	0.071662	0.046993	NELFCD	Negative el	NELFD_HUI	14	1.025573
48H 10MT/	Q8NHZ8	-0.33741	0.071682	0.047	CDC26	Anaphase-p	CDC26_HU	1	0.79146
48H 50MT/	Q92503	0.025921	0.071689	0.047003	SEC14L1	SEC14-like	S14L1_HUN	12	1.018129
48H 50MT/	P62277	0.031309	0.071708	0.047009	RPS13	Small ribos	RS13_HUM	10	1.021939
24H 50MT/	Q9BYT8	0.110069	0.071707	0.047009	NLN	Neurolysin,	NEUL_HUN	19	1.07928
48H 50MT/	Q5U5X0	-0.22591	0.071718	0.047014	LYRM7	Complex III	LYRM7_HU	3	0.855056
48H 10MT/	Q9Y2R5	-0.18195	0.071735	0.047021	MRPS17	Small ribos	RT17_HUM	4	0.881511
48H 10MT/	O95453	0.057372	0.071765	0.047038	PARN	Poly(A)-spe	PARN_HUN	22	1.040569
24H 50MT/	Q68D10	0.026733	0.071769	0.047039	SPTY2D1	Protein SPT	SPT2_HUM	13	1.018702
48H 50MT/	Q96HW7	0.051906	0.071803	0.047052	INTS4	Integrator (	INT4_HUM.	19	1.036633
24H 50MT/	Q16270	0.497027	0.071821	0.047062	IGFBP7	Insulin-like	IBP7_HUM.	6	1.411302
48H 10MT/	Q6ICG6	-0.29208	0.071822	0.047062	KIAA0930	Uncharacte	K0930_HUI	8	0.816723
48H 10MT/	O43913	0.058987	0.071825	0.047064	ORC5	Origin reco	ORC5_HUN	12	1.041734
24H 10MT/	Q6YN16	0.021744	0.071831	0.047065	HSDL2	Hydroxyste	HSDL2_HUI	27	1.015186
24H 10MT/	P43652	0.344263	0.07187	0.047085	AFM	Afamin	AFAM_HUN	1	1.269502
24H 10MT/	Q969H6	0.203474	0.071878	0.047088	POP5	Ribonuclea	POP5_HUN	5	1.151468
48H 10MT/	Q96A35	0.054475	0.071905	0.047101	MRPL24	Large ribos	RM24_HUN	12	1.038481
48H 50MT/	Q8IWV7	-0.01056	0.071937	0.047118	UBR1	E3 ubiquiti	UBR1_HUN	28	0.992705
48H 50MT/	O75165	-0.00792	0.071967	0.047129	DNAJC13	DnaJ homo	DJC13_HUI	62	0.994523
48H 10MT/	P82909	0.100789	0.071967	0.047129	KGD4	Alpha-keto	KGD4_HUN	6	1.07236
48H 50MT/	Q9GZS3	0.035289	0.071969	0.04713	SKIC8	Superkiller	SKI8_HUM/	15	1.024762
48H 10MT/	P05981	-0.42184	0.072025	0.047158	HPN	Serine prot	HEPS_HUM	6	0.746471
24H 10MT/	P26196	0.040173	0.07204	0.047165	DDX6	Probable A	DDX6_HUN	25	1.028237
24H 10MT/	Q9NTK5	-0.06824	0.072041	0.047165	OLA1	Obg-like AT	OLA1_HUN	29	0.953804
24H 50MT/	Q8IZ07	-0.18843	0.072048	0.047169	ANKRD13A	Ankyrin rep	AN13A_HU	6	0.877562
24H 50MT/	P21964	0.02622	0.072072	0.04718	COMT	Catechol O	COMT_HUN	14	1.018341
24H 10MT/	P04075	-0.07767	0.072075	0.047181	ALDOA	Fructose-bi	ALDOA_HU	29	0.947585
48H 10MT/	P16083	0.014042	0.072077	0.047181	NQO2	Ribosyldihy	NQO2_HUN	13	1.009781
48H 50MT/	P27986	0.618042	0.07208	0.047182	PIK3R1	Phosphatid	P85A_HUM	2	1.53479
48H 10MT/	Q96GC9	0.208349	0.072083	0.047183	VMP1	Vacuole me	VMP1_HUN	6	1.155365
24H 10MT/	Q9NYP3	-0.10295	0.072084	0.047183	DONSON	Protein dov	DONS_HUN	6	0.931124
48H 10MT/	P85037	-0.09009	0.072088	0.047185	FOXK1	Forkhead b	FOXK1_HUI	14	0.939465
24H 10MT/	Q9H0P0	0.089581	0.072092	0.047186	NT5C3A	Cytosolic 5	5NT3A_HUI	15	1.064061
24H 50MT/	P19827	-0.66872	0.072101	0.04719	ITIH1	Inter-alpha	ITIH1_HUN	1	0.629066

48H 50MTA	P19623	-0.03016	0.072142	0.047208	SRM	Spermidine	SPEE_HUMA	18	0.979309
48H 10MTA	Q13459	0.089006	0.072205	0.047237	MYO9B	Unconvent	MYO9B_HU	10	1.063637
48H 10MTA	Q8WXX5	0.035532	0.072207	0.047237	DNAJC9	DnaJ homo	DNJC9_HUI	15	1.024935
48H 50MTA	Q96RG2	-0.2179	0.072221	0.047244	PASK	PAS domair	PASK_HUM	8	0.859817
48H 10MTA	Q08170	0.083209	0.072228	0.047247	SRSF4	Serine/argi	SRSF4_HUN	4	1.059372
24H 50MTA	O15305	-0.0836	0.072235	0.047249	PMM2	Phosphoma	PMM2_HUI	15	0.9437
24H 10MTA	Q8N4T8	-0.16649	0.072243	0.047252	CBR4	3-oxoacyl-[	CBR4_HUM	5	0.891009
48H 50MTA	Q9NPJ6	-0.05327	0.07225	0.047254	MED4	Mediator o	MED4_HUN	9	0.963749
24H 50MTA	Q9NQW6	-0.16037	0.07225	0.047254	ANLN	Anillin	ANLN_HUM	37	0.894795
48H 50MTA	Q9C0D3	-0.18165	0.072273	0.047266	ZYG11B	Protein zyg	ZY11B_HUI	12	0.881692
24H 10MTA	Q86UW6	-0.02495	0.072291	0.047275	N4BP2	NEDD4-bin	N4BP2_HU	3	0.982856
24H 10MTA	Q9UHJ3	0.598327	0.072295	0.047277	SFMBT1	Scm-like wi	SMBT1_HU	1	1.51396
48H 50MTA	Q12805	0.583656	0.072302	0.047281	EFEMP1	EGF-contai	FBLN3_HUI	1	1.498642
24H 50MTA	O00165	0.080926	0.07231	0.047284	HAX1	HCLS1-asso	HAX1_HUM	10	1.057697
24H 10MTA	Q15750	-0.0869	0.072315	0.047286	TAB1	TGF-beta-ac	TAB1_HUM	17	0.941547
24H 50MTA	Q9Y262	-0.0558	0.072342	0.047302	EIF3L	Eukaryotic	EIF3L_HUM	32	0.962063
48H 10MTA	O95336	0.061217	0.072363	0.047311	PGLS	6-phospho	6PGL_HUM	12	1.043345
48H 50MTA	O95832	-0.23076	0.072363	0.047311	CLDN1	Claudin-1	CLD1_HUM	5	0.852189
48H 10MTA	Q9UGP4	-0.10214	0.072402	0.047331	LIMD1	LIM domair	LIMD1_HUI	14	0.93165
48H 50MTA	Q15750	0.025134	0.072406	0.047333	TAB1	TGF-beta-ac	TAB1_HUM	17	1.017574
24H 10MTA	P78344	0.019258	0.072423	0.04734	EIF4G2	Eukaryotic	IF4G2_HUN	46	1.013438
48H 10MTA	Q06787	0.081382	0.072431	0.047345	FMR1	Fragile X m	FMR1_HUN	11	1.058031
48H 50MTA	I3L3R5	-0.55642	0.072481	0.047371	CCER2	Coiled-coil	CCER2_HUI	1	0.679989
48H 50MTA	P84085	0.150779	0.072481	0.047371	ARF5	ADP-ribosy	ARF5_HUM	6	1.110169
24H 50MTA	Q8NBZ7	-0.21683	0.072498	0.04738	UXS1	UDP-glucur	UXS1_HUM	11	0.860456
24H 10MTA	Q86YS3	0.537189	0.072543	0.047404	RAB11FIP4	Rab11 fami	RFIP4_HUN	3	1.451142
48H 50MTA	P35232	-0.10037	0.072545	0.047404	PHB1	Prohibitin	PHB1_HUM	20	0.932792
24H 50MTA	Q13188	0.068324	0.072561	0.047409	STK3	Serine/thre	STK3_HUM	7	1.048498
48H 10MTA	O95302	0.035246	0.072565	0.047409	FKBP9	Peptidyl-pr	FKBP9_HUI	14	1.024732
24H 50MTA	Q86X76	-0.06742	0.072567	0.047409	NIT1	Deaminate	NIT1_HUMA	11	0.95434
48H 10MTA	Q9NZU5	0.007077	0.072568	0.047409	LMCD1	LIM and cys	LMCD1_HU	21	1.004918
24H 10MTA	O75880	0.087609	0.072575	0.047413	SCO1	Protein SC0	SCO1_HUM	8	1.062608
24H 50MTA	P07992	0.07158	0.072593	0.047421	ERCC1	DNA excisic	ERCC1_HUI	7	1.050867
48H 10MTA	Q14738	-0.01772	0.072593	0.047421	PPP2R5D	Serine/thre	2A5D_HUM	21	0.987791
24H 50MTA	Q9BQS8	0.145194	0.072599	0.047424	FYCO1	FYVE and cc	FYCO1_HUI	27	1.105879
48H 50MTA	P56945	-0.21584	0.072658	0.047453	BCAR1	Breast canc	BCAR1_HUI	10	0.861043
24H 50MTA	Q9H944	0.078664	0.072661	0.047455	MED20	Mediator o	MED20_HU	9	1.05604
24H 10MTA	O60437	0.207979	0.072677	0.047463	PPL	Periplakin	PEPL_HUM	11	1.155069
24H 10MTA	Q8IY26	0.038944	0.072679	0.047463	PLPP6	Polyisoprer	PLPP6_HUI	5	1.027361
24H 50MTA	Q9Y5L0	-0.098	0.072685	0.047464	TNPO3	Transportir	TNPO3_HU	23	0.934327
24H 10MTA	P61960	-0.26546	0.072696	0.047469	UFM1	Ubiquitin-f	UFM1_HUN	5	0.831933
24H 50MTA	P07355	-0.09775	0.072705	0.047473	ANXA2	Annexin A2	ANXA2_HU	28	0.934492
24H 10MTA	Q96DE0	-0.1719	0.07274	0.047494	NUDT16	U8 snoRNA	NUD16_HU	13	0.887675
24H 10MTA	Q9NV06	0.07922	0.072753	0.047502	DCAF13	DDB1- and	DCA13_HU	14	1.056447
24H 50MTA	Q9UK58	0.281415	0.072774	0.047513	CCNL1	Cyclin-L1	CCNL1_HUI	4	1.215387
24H 10MTA	Q9NXA8	0.201978	0.072781	0.047517	SIRT5	NAD-depen	SIR5_HUMA	8	1.150274
48H 50MTA	O95782	-0.01791	0.07279	0.047521	AP2A1	AP-2 compl	AP2A1_HUI	40	0.98766
24H 10MTA	O75197	-0.63089	0.072796	0.047524	LRP5	Low-densit	LRP5_HUM	2	0.645779
24H 10MTA	Q96G25	-0.2241	0.072811	0.04753	MED8	Mediator o	MED8_HUN	2	0.856132
48H 10MTA	P17568	0.219083	0.072815	0.047532	NDUFB7	NADH dehy	NDUB7_HU	5	1.163993
48H 50MTA	Q96JJ3	0.006599	0.072845	0.04755	ELMO2	Engulfment	ELMO2_HU	14	1.004584
24H 10MTA	Q15390	-0.13354	0.07288	0.04757	MTFR1	Mitochond	MTFR1_HU	3	0.911589

24H 10MT/	Q8IUF8	0.05382	0.072879	0.04757	RIOX2	Ribosomal	RIOX2_HUI	12	1.038009
48H 50MT/	O76031	-0.11139	0.072891	0.047575	CLPX	ATP-depend	CLPX_HUM	28	0.925698
24H 10MT/	Q8IXM6	0.069977	0.072892	0.047575	NRM	Nurim	NRM_HUM	5	1.0497
24H 10MT/	Q9NZW5	0.09074	0.072906	0.047584	PALS2	Protein PAI	PALS2_HUI	24	1.064917
48H 50MT/	P10620	-0.06434	0.072928	0.047595	MGST1	Microsoma	MGST1_HU	4	0.956383
24H 50MT/	Q96ST3	0.070934	0.072936	0.047598	SIN3A	Paired amp	SIN3A_HUM	31	1.050397
24H 50MT/	O95299	0.043309	0.072989	0.047626	NDUFA10	NADH dehy	NDUAA_HU	12	1.030475
48H 50MT/	Q9NP81	-0.08013	0.072995	0.04763	SARS2	Serine--tRN	SYSM_HUM	17	0.945972
48H 50MT/	Q8WU10	0.095728	0.073029	0.047649	PYROXD1	Pyridine nu	PYRD1_HUI	5	1.068604
24H 50MT/	Q8IWJ2	9.56E-05	0.073064	0.047666	GCC2	GRIP and co	GCC2_HUM	53	1.000066
24H 10MT/	Q86TX2	0.034531	0.073079	0.047672	ACOT1	Acyl-coenzy	ACOT1_HUI	19	1.024224
24H 10MT/	Q9BSK2	0.075761	0.073095	0.047679	SLC25A33	Solute carri	S2533_HUI	8	1.053917
48H 50MT/	Q15125	-0.13929	0.073099	0.047681	EBP	3-beta-hydr	EBP_HUMA	3	0.907965
48H 10MT/	P46778	0.121297	0.07313	0.047698	RPL21	Large ribos	RL21_HUM	8	1.087712
24H 10MT/	O75319	-0.06108	0.073134	0.0477	DUSP11	RNA/RNP c	DUS11_HU	8	0.958546
48H 50MT/	O14981	-0.01486	0.073145	0.047706	BTAF1	TATA-bindi	BTAF1_HUI	47	0.989752
24H 50MT/	P11586	-0.03856	0.073184	0.047727	MTHFD1	C-1-tetrahy	C1TC_HUM	48	0.973627
48H 50MT/	O94906	-0.05204	0.073186	0.047727	PRPF6	Pre-mRNA-p	PRP6_HUM	38	0.96457
24H 50MT/	Q96G46	0.098767	0.073189	0.047727	DUS3L	tRNA-dihyd	DUS3L_HUI	15	1.070858
24H 50MT/	Q9NVE7	-0.01591	0.07319	0.047727	PANK4	4'-phospho	PANK4_HU	23	0.989032
48H 50MT/	P05783	-0.08391	0.073205	0.047734	KRT18	Keratin, typ	K1C18_HUI	36	0.943496
48H 10MT/	Q9UNF0	-0.00327	0.073207	0.047734	PACSIN2	Protein kin	PACN2_HU	16	0.997736
48H 10MT/	Q6Y7W6	0.045499	0.073221	0.04774	GIGYF2	GRB10-inte	GGYF2_HUI	31	1.03204
48H 10MT/	Q58FF6	-0.30927	0.073234	0.047747	HSP90AB4I	Putative he	H90B4_HU	3	0.807049
48H 50MT/	Q9HBK9	0.045975	0.073296	0.047782	AS3MT	Arsenite me	AS3MT_HU	24	1.032381
48H 10MT/	A7MBM2	0.144885	0.073315	0.047791	DISP2	Protein disp	DISP2_HUM	2	1.105643
24H 10MT/	Q5TC12	-0.00317	0.073325	0.047793	ATPAF1	ATP syntha	ATPF1_HUI	9	0.997807
48H 10MT/	Q8NF64	-0.18733	0.073331	0.047795	ZMIZ2	Zinc finger	ZMIZ2_HUI	5	0.878231
24H 50MT/	P12532;P1	0.363414	0.073373	0.047818	CKMT1A;CK	Creatine kir	KCRU_HUM	1	1.286467
24H 10MT/	Q96FJ2	-0.11036	0.073372	0.047818	DYNLL2	Dynein ligh	DYL2_HUM	5	0.926359
24H 10MT/	Q03001	0.003105	0.073374	0.047818	DST	Dystonin	DYST_HUM	42	1.002154
48H 50MT/	Q96I45	0.64768	0.073381	0.047819	TMEM141	Transmemk	TM141_HU	2	1.566646
48H 50MT/	Q8WWM7	-0.11211	0.073407	0.047833	ATXN2L	Ataxin-2-lik	ATX2L_HUI	32	0.925237
48H 10MT/	Q5VWJ9	0.270313	0.073423	0.047839	SNX30	Sorting nex	SNX30_HUI	3	1.20607
48H 50MT/	Q96IZ7	-0.10719	0.073435	0.047846	RSRC1	Serine/Argi	RSRC1_HUI	2	0.928395
48H 50MT/	O94826	0.000918	0.073518	0.047891	TOMM70	Mitochond	TOM70_HU	26	1.000636
48H 50MT/	Q8IW41	0.320853	0.073536	0.047901	MAPKAPK5	MAP kinase	MAPK5_HU	1	1.249069
24H 50MT/	Q8IVP5	-0.71686	0.073572	0.047921	FUNDC1	FUN14 don	FUND1_HU	1	0.608422
24H 50MT/	Q9HBM6	0.115701	0.073581	0.047926	TAF9B	Transcripti	TAF9B_HUI	10	1.083501
24H 10MT/	P11021	-0.03123	0.073592	0.047931	HSPA5	Endoplasm	BIP_HUMA	35	0.978586
48H 50MT/	Q13315	0.045513	0.073593	0.047931	ATM	Serine-prot	ATM_HUM/	48	1.03205
48H 50MT/	Q9Y2T2	-0.03497	0.07359	0.047931	AP3M1	AP-3 compl	AP3M1_HU	17	0.97605
48H 10MT/	Q15007	0.073514	0.073655	0.047963	WTAP	Pre-mRNA-s	FL2D_HUM	8	1.052277
24H 50MT/	Q9UKT4	0.353516	0.073667	0.047968	FBXO5	F-box only	FBX5_HUM	2	1.27767
24H 10MT/	P10586	0.320536	0.073675	0.047971	PTPRF	Receptor-ty	PTPRF_HUI	25	1.248794
48H 10MT/	Q96MG7	-0.22678	0.073703	0.047986	NSMCE3	Non-structu	NSE3_HUM	4	0.85454
24H 50MT/	Q01650	0.214232	0.073728	0.047998	SLC7A5	Large neutr	LAT1_HUM	8	1.160086
48H 10MT/	Q9P032	-0.10766	0.073735	0.048001	NDUFAF4	NADH dehy	NDUF4_HU	5	0.928091
24H 10MT/	Q92567	0.121056	0.073738	0.048003	FAM168A	Protein FAI	F168A_HUI	1	1.08753
24H 50MT/	Q15067	-0.02678	0.073763	0.048014	ACOX1	Peroxisoma	ACOX1_HU	26	0.981612
24H 50MT/	P46060	-0.07141	0.073778	0.04802	RANGAP1	Ran GTPase	RAGP1_HU	33	0.951709
24H 10MT/	Q2T9J0	0.009124	0.073782	0.048021	TYSND1	Peroxisoma	TYSD1_HUI	15	1.006345

24H	10MTA	P29992	-0.01535	0.073795	0.048028	GNA11	Guanine nu	GNA11_HU	14	0.989419
24H	50MTA	Q9UFN0	0.178209	0.073796	0.048028	NIPSNAP3A	Protein Nip	NPS3A_HUI	9	1.131479
24H	10MTA	O14672	0.028343	0.073843	0.048055	ADAM10	Disintegrin	ADA10_HU	22	1.01984
48H	50MTA	Q8NBN7	-0.14058	0.073946	0.048107	RDH13	Retinol deh	RDH13_HU	8	0.907157
24H	10MTA	Q15121	-0.32007	0.073977	0.048123	PEA15	Astrocytic p	PEA15_HUI	6	0.801032
24H	50MTA	P0C0L4	-0.51164	0.073986	0.048128	C4A	Complemen	CO4A_HUM	10	0.701426
48H	50MTA	O43684	-0.08599	0.073996	0.048132	BUB3	Mitotic che	BUB3_HUM	12	0.94214
48H	10MTA	Q96RQ1	0.016719	0.073996	0.048132	ERGIC2	Endoplasm	ERGI2_HUM	6	1.011656
24H	10MTA	Q9ULD4	0.258144	0.074029	0.04815	BRPF3	Bromodom	BRPF3_HUI	4	1.195939
24H	50MTA	P08581	-0.52403	0.074097	0.048183	MET	Hepatocyte	MET_HUMA	1	0.695424
48H	10MTA	Q5VU43	-0.23127	0.074106	0.048187	PDE4DIP	Myomegali	MYOME_HU	6	0.851886
48H	10MTA	Q6QNY1	-0.67229	0.074109	0.048188	BLOC1S2	Biogenesis	BL1S2_HUM	2	0.627511
48H	50MTA	Q8IXL7	-0.3345	0.074125	0.048196	MSRB3	Methionine	MSRB3_HU	2	0.793058
24H	10MTA	O76080	-0.29171	0.074132	0.0482	ZFAND5	AN1-type zi	ZFAN5_HUI	3	0.816933
48H	50MTA	Q9NQX6	0.189652	0.074146	0.048206	ZNF331	Zinc finger	ZN331_HUI	4	1.140489
48H	50MTA	Q14697	0.056779	0.074152	0.048208	GANAB	Neutral alp	GANAB_HU	50	1.040141
24H	10MTA	Q7KYR7	-0.07432	0.074158	0.048211	BTN2A1	Butyrophili	BT2A1_HUI	10	0.949787
24H	10MTA	Q9Y2I9	1.458077	0.074162	0.048213	TBC1D30	TBC1 doma	TBC30_HUI	1	2.747418
48H	10MTA	Q7Z5J4	0.380837	0.074174	0.04822	RAI1	Retinoic ac	RAI1_HUMA	4	1.302097
48H	50MTA	Q13686	-0.20404	0.074192	0.048229	ALKBH1	Nucleic aci	ALKB1_HUI	4	0.868117
48H	50MTA	P35269	0.011093	0.074203	0.048233	GTF2F1	General tra	T2FA_HUM	15	1.007719
24H	10MTA	Q8IW41	-0.54226	0.074215	0.04824	MAPKAPK5	MAP kinase	MAPK5_HU	1	0.686695
24H	50MTA	Q9BUR4	-0.2218	0.074218	0.04824	WRAP53	Telomerase	TCAB1_HUI	6	0.857493
24H	50MTA	Q99613	-0.06562	0.074224	0.048241	EIF3C	Eukaryotic	EIF3C_HUM	34	0.955531
24H	50MTA	P49716	0.945304	0.074257	0.048258	CEBPD	CCAAT/enh	CEBPD_HUI	3	1.925595
48H	50MTA	O15078	-0.13522	0.074256	0.048258	CEP290	Centrosom	CE290_HUI	12	0.910528
24H	50MTA	Q6Y7W6	-0.028	0.074266	0.048261	GIGYF2	GRB10-inte	GGYF2_HUI	31	0.980777
24H	50MTA	Q13029	1.987398	0.074274	0.048265	PRDM2	PR domain	PRDM2_HU	2	3.965213
24H	50MTA	Q70Z53	0.194154	0.074284	0.04827	FRA10AC1	Protein FRA	F10C1_HUI	5	1.144053
24H	50MTA	P20674	-0.43975	0.07429	0.048273	COX5A	Cytochrom	COX5A_HU	6	0.737264
48H	10MTA	P53609	-0.05609	0.074349	0.048304	PGGT1B	Geranylger	PGTB1_HUI	5	0.961869
48H	10MTA	P32321	0.161281	0.074355	0.048306	DCTD	Deoxycytid	DCTD_HUM	3	1.118279
48H	10MTA	Q8TAD4	-0.18586	0.074381	0.048321	SLC30A5	Proton-cou	ZNT5_HUM	6	0.879128
48H	50MTA	Q8N4P2	0.556121	0.07439	0.048325	IFT70B	Intraflagell	IT70B_HUM	2	1.470311
48H	50MTA	Q9UM82	-0.56909	0.074404	0.04833	SPATA2	Spermatog	SPAT2_HUM	1	0.674043
24H	10MTA	O95232	0.10559	0.074426	0.04834	LUC7L3	Luc7-like p	LC7L3_HUM	10	1.075935
48H	50MTA	P35520	0.053873	0.074443	0.048348	CBS	Cystathioni	CBS_HUMA	22	1.038048
24H	10MTA	Q9Y3I1	-0.11205	0.074483	0.04837	FBXO7	F-box only	FBX7_HUM	11	0.925272
48H	10MTA	Q96KM6	0.024474	0.074488	0.048373	ZNF512B	Zinc finger	Z512B_HUI	12	1.017109
24H	10MTA	P68400	-0.03707	0.074496	0.048376	CSNK2A1	Casein kina	CSK21_HUI	18	0.974635
24H	50MTA	Q96MX6	-0.21669	0.074532	0.048396	DNAAF10	Dynein axo	DAA10_HU	10	0.860538
24H	50MTA	Q9H8X2	0.207911	0.074533	0.048396	IPPK	Inositol-pe	IPPK_HUMA	3	1.155014
48H	10MTA	Q9P000	0.001461	0.074535	0.048396	COMMD9	COMM don	COMD9_HU	8	1.001013
48H	50MTA	Q9BUR4	-0.05163	0.074554	0.048404	WRAP53	Telomerase	TCAB1_HUI	6	0.964847
48H	10MTA	Q6UN15	-0.16692	0.074559	0.048405	FIP1L1	Pre-mRNA 3	FIP1_HUMA	10	0.890744
24H	50MTA	Q13332	0.52085	0.074562	0.048406	PTPRS	Receptor-ty	PTPRS_HUM	3	1.4348
24H	50MTA	Q6UVJ0	-0.23117	0.074576	0.048413	SASS6	Spindle ass	SAS6_HUM	12	0.851945
48H	50MTA	P07954	0.021025	0.074594	0.048422	FH	Fumarate h	FUMH_HUM	20	1.01468
48H	10MTA	P82970	0.364092	0.074604	0.048424	HMGN5	High mobil	HMGN5_HU	5	1.287071
24H	50MTA	Q9H0E3	0.00695	0.074613	0.048426	SAP130	Histone dea	SP130_HUI	10	1.004829
24H	10MTA	P28827	0.135128	0.074617	0.048428	PTPRM	Receptor-ty	PTPRM_HU	11	1.09819
24H	10MTA	Q86TP1	-0.07526	0.074625	0.04843	PRUNE1	Exopolyph	PRUN1_HU	12	0.94917

48H 10MTA	Q70CQ3	0.137863	0.074645	0.048437	USP30	Ubiquitin c	UBP30_HU	1	1.100274
24H 10MTA	Q9UNZ2	0.03172	0.074644	0.048437	NSFL1C	NSFL1 cofa	NSF1C_HU	17	1.02223
24H 10MTA	Q9UEE9	-0.11681	0.07466	0.048445	CFDP1	Craniofacia	CFDP1_HU	5	0.922222
24H 50MTA	Q8WUB8	-0.24987	0.074673	0.048452	PHF10	PHD finger	PHF10_HU	6	0.840974
48H 10MTA	A1L390	0.08593	0.074695	0.048462	PLEKHG3	Pleckstrin h	PKHG3_HU	5	1.061372
24H 10MTA	Q9Y580	0.064055	0.074728	0.048478	RBM7	RNA-bindir	RBM7_HUM	9	1.0454
24H 10MTA	P19387	-0.04366	0.07473	0.048478	POLR2C	DNA-direct	RPB3_HUM	12	0.970191
24H 50MTA	Q58FF7	-0.21073	0.074732	0.048479	HSP90AB3I	Putative he	H90B3_HU	5	0.864102
24H 50MTA	Q9NNW5	-0.14699	0.074794	0.048507	WDR6	tRNA (34-2'	WDR6_HUI	26	0.903134
24H 10MTA	Q96D71	-0.0057	0.074799	0.048509	REPS1	RalBP1-asso	REPS1_HUI	11	0.996056
24H 10MTA	P36639	-0.02007	0.074847	0.048534	NUDT1	Oxidized pu	8ODP_HUM	6	0.986186
48H 10MTA	Q9Y217	-0.04426	0.074922	0.048574	MTMR6	Myotubula	MTMR6_HU	22	0.969786
48H 50MTA	Q96FJ2	0.082229	0.074929	0.048577	DYNLL2	Dynein ligh	DYL2_HUM	5	1.058653
48H 10MTA	P46937	0.171115	0.074967	0.048599	YAP1	Transcripti	YAP1_HUM	8	1.125928
24H 50MTA	P11182	0.075335	0.07497	0.0486	DBT	Lipoamide	ODB2_HUM	22	1.053606
48H 50MTA	P48551	0.525433	0.074982	0.048604	IFNAR2	Interferon a	INAR2_HUI	2	1.439365
48H 10MTA	Q6GPH6	-0.41802	0.074985	0.048605	ITPRIPL1	Inositol 1,4	IPIL1_HUM	1	0.74845
24H 50MTA	Q9NUP1	0.120123	0.075009	0.048618	BLOC1S4	Biogenesis	BL1S4_HUI	4	1.086828
24H 50MTA	Q5VZL5	0.128	0.075013	0.04862	ZMYM4	Zinc finger	ZMYM4_HU	15	1.092778
24H 50MTA	O43913	-0.06623	0.075023	0.048625	ORC5	Origin reco	ORC5_HUM	12	0.955129
24H 50MTA	Q9BV68	-0.16358	0.075071	0.048652	RNF126	E3 ubiquiti	RN126_HU	6	0.892809
48H 10MTA	Q9UHW5	0.13686	0.075083	0.048658	GPN3	GPN-loop G	GPN3_HUM	7	1.099509
24H 50MTA	Q86V21	-0.1748	0.075088	0.04866	AACS	Acetoacety	AACS_HUM	15	0.885888
48H 50MTA	Q5T4F4	0.532686	0.0751	0.048664	ZFYVE27	Protrudin	ZFY27_HUI	3	1.44662
48H 10MTA	Q01844	0.109792	0.075109	0.048669	EWSR1	RNA-bindir	EWS_HUMA	9	1.079072
48H 10MTA	Q96GG9	0.028379	0.07514	0.048684	DCUN1D1	DCN1-like p	DCNL1_HU	10	1.019866
48H 10MTA	Q6NT16	0.695515	0.075191	0.048713	SLC18B1	MFS-type tr	S18B1_HUI	2	1.619462
24H 50MTA	Q96JX3	0.104137	0.075195	0.048715	SERAC1	Protein SEF	SRAC1_HUI	5	1.074851
48H 50MTA	Q9NRZ7	0.235623	0.075223	0.048731	AGPAT3	1-acyl-sn-gl	PLCC_HUM	9	1.177415
24H 10MTA	Q96M96	0.054397	0.075227	0.048732	FGD4	FYVE, RhoG	FGD4_HUM	21	1.038425
24H 10MTA	O95905	-0.09994	0.075237	0.048737	ECD	Protein ecd	ECD_HUMA	18	0.933073
24H 50MTA	P28749	0.050147	0.075239	0.048738	RBL1	Retinoblast	RBL1_HUM	12	1.03537
48H 50MTA	Q9H082	-0.53559	0.075249	0.048742	RAB33B	Ras-related	RB33B_HUI	2	0.689877
48H 50MTA	Q9BRG1	0.152604	0.075265	0.048749	VPS25	Vacuolar pr	VPS25_HUI	11	1.111574
48H 10MTA	Q6P444	0.015788	0.075277	0.048754	MTFR2	Mitochond	MTFR2_HU	6	1.011003
48H 10MTA	Q5TC12	0.094609	0.07529	0.04876	ATPAF1	ATP syntha	ATPF1_HUI	9	1.067776
48H 50MTA	Q8N9E0	1.889152	0.075307	0.048768	FAM133A	Protein FAI	F133A_HUI	2	3.704175
48H 10MTA	Q9UNE7	0.019648	0.075381	0.04881	STUB1	E3 ubiquiti	CHIP_HUM	16	1.013712
24H 50MTA	Q9HBL0	0.470587	0.075399	0.048816	TNS1	Tensin-1	TENS1_HUI	6	1.385674
24H 50MTA	Q9BYC8	-0.04768	0.075403	0.048818	MRPL32	Large ribos	RM32_HUI	3	0.967493
24H 50MTA	Q96DF8	0.04134	0.075425	0.04883	ESS2	Splicing fac	ESS2_HUM	7	1.029069
24H 10MTA	Q5R372	-0.14163	0.075458	0.04885	RABGAP1L	Rab GTPase	RBG1L_HUI	7	0.906495
48H 50MTA	Q96EK7	0.19161	0.075482	0.048861	FAM120B	Constitutiv	F120B_HUI	24	1.142037
48H 10MTA	Q7Z4L5	0.071642	0.075485	0.048861	TTC21B	Tetratricop	TT21B_HUI	11	1.050912
24H 10MTA	Q99487	-0.2346	0.075487	0.048862	PAFAH2	Platelet-act	PAFA2_HUI	5	0.849918
48H 10MTA	Q8NCN5	-0.02162	0.0755	0.048867	PDPR	Pyruvate de	PDPR_HUM	24	0.985124
24H 50MTA	O75879	0.115646	0.075504	0.048868	GATB	Glutamyl-tl	GATB_HUM	15	1.08346
24H 10MTA	Q86UE8	-0.51463	0.075508	0.048868	TLK2	Serine/thre	TLK2_HUM	4	0.69997
24H 10MTA	Q8NBJ7	0.051158	0.075514	0.048871	SUMF2	Inactive C-a	SUMF2_HU	10	1.036096
24H 50MTA	Q8WUM4	-0.01144	0.07553	0.048879	PDCD6IP	Programme	PDC6I_HUI	48	0.9921
48H 10MTA	P46976	0.034936	0.075537	0.048881	GYG1	Glycogenin	GLYG_HUM	12	1.024512
48H 50MTA	O95059	0.146216	0.07554	0.048882	RPP14	Ribonuclea	RPP14_HUI	2	1.106663

24H 50MTA	P23025	0.1373	0.075592	0.048909	XPA	DNA repair	XPA_HUMA	6	1.099845
48H 10MTA	P14373	0.11472	0.07563	0.048926	TRIM27	Zinc finger	TRI27_HUM	8	1.082765
48H 10MTA	Q96EK5	0.044929	0.07565	0.048936	KIFBP	KIF-binding	KBP_HUMA	23	1.031632
24H 50MTA	Q13191	-0.3007	0.075657	0.048938	CBLB	E3 ubiquiti	CBLB_HUM	1	0.811858
48H 10MTA	Q9BZE4	-0.11776	0.075655	0.048938	GTPBP4	GTP-bindin	GTPB4_HU	33	0.921616
24H 50MTA	Q5VU43	0.087528	0.075659	0.048939	PDE4DIP	Myomegali	MYOME_HU	6	1.062548
24H 10MTA	O95425	0.068166	0.075723	0.048969	SVIL	Supervillin	SVIL_HUMA	34	1.048383
24H 50MTA	Q9BVM2	0.106575	0.07573	0.048973	DPCD	Protein DP	DPCD_HUM	7	1.076669
24H 50MTA	O00299	-0.06139	0.075748	0.048983	CLIC1	Chloride in	CLIC1_HUM	15	0.958341
24H 50MTA	O95870	-0.10936	0.075779	0.049	ABHD16A	Phosphatid	ABHGA_HU	12	0.927001
48H 10MTA	P43360	-0.54865	0.075798	0.049009	MAGEA6	Melanoma-	MAGA6_HU	2	0.683658
24H 50MTA	P14543	0.26222	0.075843	0.049031	NID1	Nidogen-1	NID1_HUM	17	1.199323
48H 50MTA	Q9BYK8	0.148704	0.07585	0.049033	HELZ2	3'-5' exorib	HELZ2_HUM	17	1.108573
48H 50MTA	Q9Y6B6	-0.01217	0.075873	0.049046	SAR1B	Small COPI	SAR1B_HUI	13	0.991598
48H 50MTA	O95983	-0.12853	0.075875	0.049047	MBD3	Methyl-CpG	MBD3_HUM	9	0.914765
24H 50MTA	Q15054	-0.23229	0.075887	0.049053	POLD3	DNA polym	DPOD3_HU	11	0.851283
24H 10MTA	Q86X29	-0.10147	0.075947	0.049084	LSR	Lipolysis-st	LSR_HUMA	10	0.932084
48H 10MTA	Q96T60	0.002329	0.075955	0.049089	PNKP	Bifunctiona	PNKP_HUM	12	1.001615
24H 50MTA	P54198	-0.03782	0.075959	0.04909	HIRA	Protein HIR	HIRA_HUM	8	0.974123
48H 10MTA	Q92794	-0.30696	0.075983	0.049104	KAT6A	Histone ace	KAT6A_HUI	3	0.808343
48H 10MTA	P13056	-0.64059	0.075989	0.049106	NR2C1	Nuclear rec	NR2C1_HU	2	0.64145
24H 50MTA	Q9H477	0.24351	0.075996	0.04911	RBKS	Ribokinase	RBSK_HUM	6	1.183869
24H 10MTA	Q96JG8	0.126773	0.076	0.049112	MAGED4	Melanoma-	MAGD4_HU	4	1.091849
24H 50MTA	P52747	0.143298	0.076005	0.049114	ZNF143	Zinc finger	ZN143_HUI	2	1.104427
24H 50MTA	Q9NV06	0.018905	0.076019	0.049116	DCAF13	DDB1- and	DCA13_HU	14	1.01319
24H 10MTA	Q9P270	0.018439	0.076031	0.049122	SLAIN2	SLAIN moti	SLAI2_HUM	11	1.012863
24H 50MTA	Q8TCS8	-0.01116	0.076048	0.04913	PNPT1	Polyribonu	PNPT1_HUI	40	0.992294
24H 50MTA	Q92997	-0.13854	0.076053	0.049132	DVL3	Segment po	DVL3_HUM	6	0.908436
24H 10MTA	Q9BPX3	0.102388	0.076064	0.049137	NCAPG	Condensin	CND3_HUM	27	1.073549
48H 10MTA	Q9UPW5	-0.55304	0.076066	0.049137	AGTPBP1	Cytosolic ca	CBPC1_HUI	1	0.68158
48H 50MTA	P61769	-0.29849	0.076082	0.049144	B2M	Beta-2-micr	B2MG_HUM	3	0.813101
48H 50MTA	P42356	-0.00684	0.0761	0.049152	PI4KA	Phosphatid	PI4KA_HUM	26	0.995269
48H 10MTA	Q9H000	0.369252	0.076107	0.049156	MKRN2	E3 ubiquiti	MKRN2_HU	5	1.291683
24H 10MTA	Q8WTP8	1.017637	0.076128	0.049164	AEN	Apoptosis-e	AEN_HUMA	1	2.024599
24H 50MTA	Q86TU7	0.024453	0.076176	0.04919	SETD3	Actin-histic	SETD3_HUM	19	1.017094
48H 50MTA	Q9UHW5	0.132066	0.076176	0.04919	GPN3	GPN-loop G	GPN3_HUM	7	1.095862
48H 10MTA	Q12805	0.411512	0.076227	0.049213	EFEMP1	EGF-contai	FBLN3_HUI	1	1.330079
24H 10MTA	Q14156	-0.01314	0.076244	0.049221	EFR3A	Protein EFF	EFR3A_HUM	11	0.990931
48H 10MTA	Q14019	-0.03651	0.076252	0.049225	COTL1	Coactosin-l	COTL1_HUI	8	0.975008
48H 50MTA	O00194	-0.10921	0.076277	0.049237	RAB27B	Ras-related	RB27B_HUI	4	0.927097
48H 10MTA	Q9NQS7	0.087402	0.076294	0.049247	INCENP	Inner centr	INCE_HUMA	12	1.062456
24H 10MTA	Q92997	-0.081	0.076304	0.04925	DVL3	Segment po	DVL3_HUM	6	0.945402
24H 50MTA	Q96EX3	-0.15652	0.076309	0.049251	DYNC2I2	Cytoplasmi	DC2I2_HUM	5	0.897188
24H 50MTA	A2RUB6	4.474025	0.076317	0.049253	CCDC66	Coiled-coil	CCD66_HU	1	22.22367
24H 10MTA	P11766	-0.03548	0.076319	0.049254	ADH5	Alcohol deh	ADHX_HUM	19	0.975709
24H 50MTA	Q04771	0.022523	0.076349	0.049268	ACVR1	Activin rece	ACVR1_HUI	2	1.015735
24H 10MTA	Q86Y79	-0.18575	0.076366	0.049277	PTRH1	Peptidyl-tR	PTH_HUMA	5	0.879194
24H 50MTA	O15160	-0.02863	0.076376	0.049281	POLR1C	DNA-direct	RPAC1_HUI	17	0.980348
24H 10MTA	P34932	-0.03083	0.076376	0.049281	HSPA4	Heat shock	HSP74_HUI	47	0.978854
24H 10MTA	Q13496	0.096995	0.076429	0.049307	MTM1	Myotubula	MTM1_HUI	15	1.069544
24H 50MTA	O95415	0.058099	0.076431	0.049307	BRI3	Membrane	BRI3_HUMA	1	1.041093
24H 10MTA	Q14966	0.045098	0.07645	0.049315	ZNF638	Zinc finger	ZN638_HUI	46	1.031753

24H 50MTA	Q86WR0	-0.23998	0.076463	0.04932	CCDC25	Coiled-coil	CCD25_HU	6	0.846756
48H 50MTA	P32780	-0.15641	0.076488	0.04933	GTF2H1	General tra	TF2H1_HUI	8	0.897258
24H 50MTA	Q8NC51	-0.0413	0.076487	0.04933	SERBP1	SERPINE1 n	SERB1_HUN	21	0.971782
24H 10MTA	Q9BQL6	0.596424	0.076495	0.049331	FERMT1	Fermitin fa	FERM1_HU	1	1.511964
24H 50MTA	Q96HQ2	-0.72611	0.076492	0.049331	CDKN2AIPN	CDKN2AIP I	C2AIL_HUN	2	0.604532
48H 10MTA	O14653	0.234738	0.07656	0.049367	GOSR2	Golgi SNAP	GOSR2_HU	5	1.176693
24H 50MTA	Q16513	-0.07299	0.076583	0.04938	PKN2	Serine/thre	PKN2_HUN	42	0.950666
24H 50MTA	Q8TEQ8	0.174833	0.07659	0.049382	PIGO	GPI ethano	PIGO_HUM	11	1.128834
48H 50MTA	O60422	0.028441	0.076594	0.049383	ONECUT3	One cut do	ONEC3_HU	3	1.019909
24H 50MTA	Q5SW79	-0.05348	0.076609	0.049389	CEP170	Centrosom	CE170_HUI	23	0.963607
48H 50MTA	P22695	-0.10975	0.076613	0.04939	UQCRC2	Cytochrom	QCR2_HUN	12	0.926747
24H 50MTA	Q9NZQ3	0.044322	0.076616	0.049391	NCKIPSD	NCK-intera	SPN90_HUI	8	1.031199
24H 50MTA	O43572	0.098214	0.076619	0.049392	AKAP10	A-kinase an	AKA10_HUI	7	1.070448
48H 10MTA	Q16740	0.049	0.076621	0.049393	CLPP	ATP-depenc	CLPP_HUM	10	1.034547
24H 50MTA	Q16531	0.035592	0.076624	0.049393	DDB1	DNA damag	DDB1_HUN	57	1.024977
48H 10MTA	P08123	0.277504	0.07664	0.049399	COL1A2	Collagen al	CO1A2_HU	3	1.212096
24H 10MTA	Q96ND0	0.070284	0.076676	0.04942	FAM210A	Protein FAI	F210A_HUI	3	1.049924
48H 50MTA	Q86U44	-0.03319	0.076679	0.04942	METTL3	N6-adenosi	MTA70_HU	15	0.977254
24H 50MTA	Q86VI3	-0.2907	0.076689	0.049425	IQGAP3	Ras GTPase-	IQGA3_HUI	9	0.817506
48H 50MTA	Q8IZA0	0.018136	0.076709	0.049434	KIAA0319L	Dyslexia-as	K319L_HUI	10	1.012651
48H 10MTA	Q96SZ6	-0.1725	0.076708	0.049434	CDK5RAP1	Mitochond	CK5P1_HUI	13	0.887307
24H 50MTA	Q9Y2E8	-0.25176	0.07671	0.049435	SLC9A8	Sodium/hy	SL9A8_HUN	1	0.839871
48H 50MTA	Q92804	0.317736	0.076773	0.049468	TAF15	TATA-bindi	RBP56_HUI	7	1.246373
48H 10MTA	P29350	-0.03426	0.076807	0.049485	PTPN6	Tyrosine-pr	PTN6_HUN	14	0.976534
48H 10MTA	Q9H1E3	0.211201	0.076831	0.049496	NUCKS1	Nuclear ubi	NUCKS_HU	6	1.157651
24H 50MTA	Q53S58	-0.27402	0.076867	0.049513	TMEM177	Transmemk	TM177_HU	2	0.827014
24H 50MTA	Q99618	-0.49662	0.076882	0.04952	CDCA3	Cell divisio	CDCA3_HU	3	0.708765
24H 10MTA	P35908	0.36869	0.076891	0.049523	KRT2	Keratin, typ	K22E_HUM	31	1.29118
48H 50MTA	Q9UGU5	0.050484	0.076984	0.04957	HMGXB4	HMG doma	HMGX4_HU	1	1.035612
48H 10MTA	O75781	0.049557	0.076986	0.049571	PALM	Paralemmi	PALM_HUN	2	1.034947
48H 10MTA	Q6NUQ4	0.032336	0.077018	0.049589	TMEM214	Transmemk	TM214_HU	29	1.022667
48H 10MTA	Q4UJ75;Q5	0.394848	0.077023	0.04959	ANKRD20A	Putative an	A20A4_HUI	1	1.314804
24H 50MTA	Q14527	-0.00513	0.077045	0.049599	HLTF	Helicase-lik	HLTF_HUM	28	0.99645
48H 50MTA	P82930	-0.01224	0.077048	0.049601	MRPS34	Small ribos	RT34_HUM	12	0.991553
24H 50MTA	O75379	0.715453	0.077054	0.049601	VAMP4	Vesicle-assc	VAMP4_HU	2	1.641999
24H 50MTA	P0C264	0.248057	0.077056	0.049601	SBK3	Uncharacte	SBK3_HUM	1	1.187607
48H 10MTA	Q14165	0.068704	0.077054	0.049601	MLEC	Malectin	MLEC_HUN	10	1.048774
48H 10MTA	Q15043	0.06687	0.077089	0.049619	SLC39A14	Metal catio	S39AE_HUN	4	1.047441
24H 50MTA	Q8WXG6	-0.41002	0.077098	0.049624	MADD	MAP kinase	MADD_HUI	7	0.752613
48H 50MTA	Q96AJ9	-0.01539	0.077129	0.049635	VTI1A	Vesicle trar	VTI1A_HUN	5	0.989388
24H 10MTA	Q8N3U4	0.012275	0.077154	0.049648	STAG2	Cohesin sul	STAG2_HUI	25	1.008545
48H 50MTA	O60476	0.10356	0.077179	0.049662	MAN1A2	Mannosyl-c	MA1A2_HU	8	1.074421
48H 10MTA	P51798	0.028744	0.077195	0.049672	CLCN7	H(+)/Cl(-) ex	CLCN7_HUI	6	1.020124
24H 50MTA	P13473	0.261387	0.077205	0.049677	LAMP2	Lysosome-a	LAMP2_HU	3	1.198631
24H 10MTA	O15439	0.061025	0.077239	0.049694	ABCC4	ATP-bindin	MRP4_HUN	14	1.043206
24H 50MTA	Q9NUI1	-0.27491	0.077251	0.049699	DECR2	Peroxisoma	DECR2_HUI	5	0.826503
24H 10MTA	Q99460	-0.01073	0.077274	0.049708	PSMD1	26S protea	PSMD1_HU	42	0.992587
24H 10MTA	Q15006	0.016925	0.077288	0.049713	EMC2	ER membra	EMC2_HUN	10	1.0118
24H 50MTA	Q9H2U1	-0.11461	0.077292	0.049713	DHX36	ATP-depenc	DHX36_HU	29	0.923629
24H 50MTA	Q9Y4I1	-0.03507	0.077323	0.049729	MYO5A	Unconvent	MYO5A_HU	25	0.975985
24H 10MTA	O75608	0.030288	0.077338	0.049736	LYPLA1	Acyl-protei	LYPA1_HUN	9	1.021216
24H 10MTA	O43157	0.105176	0.077374	0.049756	PLXNB1	Plexin-B1	PLXB1_HUI	21	1.075626

24H 10MT/	Q8WUA4	0.002469	0.077382	0.04976	GTF3C2	General tra	TF3C2_HUI	18	1.001713
24H 10MT/	Q96HP0	-0.17047	0.077397	0.049767	DOCK6	Dedicator c	DOCK6_HU	4	0.888556
48H 10MT/	Q9NVR2	-0.05427	0.077401	0.049768	INTS10	Integrator c	INT10_HUI	18	0.963084
24H 50MT/	Q16763	-0.45187	0.077458	0.049796	UBE2S	Ubiquitin-c	UBE2S_HUI	7	0.731093
48H 10MT/	Q9BV40	0.063659	0.07751	0.049824	VAMP8	Vesicle-assc	VAMP8_HU	4	1.045113
24H 10MT/	Q9NY61	0.047241	0.077517	0.049825	AATF	Protein AA	AATF_HUM	17	1.033287
24H 50MT/	Q92545	-0.06202	0.07752	0.049826	TMEM131	Transmemb	TM131_HU	7	0.957921
48H 10MT/	P61513	0.098956	0.077623	0.049877	RPL37A	Large ribos	RL37A_HUI	3	1.070999
24H 10MT/	Q96J42	0.112086	0.077639	0.049886	TXNDC15	Thioredoxi	TXD15_HUI	5	1.08079
48H 10MT/	Q06323	0.058535	0.077679	0.049906	PSME1	Proteasom	PSME1_HU	19	1.041408
24H 10MT/	Q12931	0.029321	0.077703	0.049917	TRAP1	Heat shock	TRAP1_HUI	34	1.020532
48H 10MT/	P12694	-0.01473	0.077709	0.049918	BCKDHA	2-oxoisoval	ODBA_HUM	13	0.989843
48H 10MT/	Q08334	0.686406	0.077711	0.049918	IL10RB	Interleukin	I10R2_HUI	2	1.60927
48H 50MT/	Q05513	0.315548	0.077713	0.049919	PRKCZ	Protein kin	KPCZ_HUM	5	1.244484
48H 10MT/	Q9UBE0	0.034596	0.077717	0.04992	SAE1	SUMO-activ	SAE1_HUM	23	1.02427
48H 10MT/	Q96CN7	0.011914	0.077725	0.049924	ISOC1	Isochorism	ISOC1_HUI	9	1.008293
48H 10MT/	O95562	-0.09014	0.077729	0.049926	SFT2D2	Vesicle tran	SFT2B_HUI	1	0.939431
24H 50MT/	Q8N9N8	-0.17903	0.077736	0.049929	EIF1AD	Probable R	EIF1A_HUM	7	0.883298
24H 10MT/	B2RXH8	-0.12486	0.077739	0.04993	HNRNPCL2	Heterogene	HNRC2_HU	1	0.917095
24H 10MT/	Q9H8Y8	-0.07828	0.077791	0.049956	GORASP2	Golgi reass	GORS2_HU	15	0.947187
48H 50MT/	O75648	-0.2825	0.07784	0.049983	TRMU	Mitochond	MTU1_HUI	13	0.822166
48H 10MT/	Q9BV36	-0.18055	0.077845	0.049985	MLPH	Melanophil	MELPH_HU	16	0.882368
48H 50MT/	P28290	-0.17282	0.077853	0.049988	ITPRID2	Protein ITP	ITPI2_HUM	16	0.887106
24H 50MT/	Q8WYQ5	0.202241	0.077855	0.049988	DGCR8	Microproc	DGCR8_HU	5	1.150484
24H 50MT/	Q9GZR1	-0.14709	0.077855	0.049988	SENP6	Sentrin-spe	SENP6_HUI	6	0.903068
48H 50MT/	Q8N5B7	0.158502	0.07787	0.049996	CERS5	Ceramide s	CERS5_HUI	2	1.116128
24H 10MT/	Q96SB4	0.054738	0.077886	0.050005	SRPK1	SRSF protei	SRPK1_HUI	18	1.038671
24H 10MT/	Q8TDZ2	0.138595	0.077906	0.050014	MICAL1	[F-actin]-m	MICA1_HUI	22	1.100833
48H 50MT/	O14893	0.320426	0.077915	0.050018	GEMIN2	Gem-associ	GEMI2_HUI	6	1.248699
24H 10MT/	Q86U42	0.057148	0.077997	0.050065	PABPN1	Polyadenyl	PABP2_HUI	8	1.040407
48H 10MT/	Q9NYV6	-0.07174	0.077999	0.050065	RRN3	RNA polym	RRN3_HUM	5	0.951491
48H 50MT/	Q13554	0.0919	0.07801	0.05007	CAMK2B	Calcium/ca	KCC2B_HUI	6	1.065773
48H 10MT/	Q6PID6	-0.09664	0.078061	0.050101	TTC33	Tetratricop	TTC33_HUI	4	0.935211
48H 10MT/	P06748	0.013255	0.078098	0.050117	NPM1	Nucleopho	NPM_HUM	13	1.00923
48H 50MT/	P26045	-0.20668	0.078112	0.050123	PTPN3	Tyrosine-pr	PTN3_HUM	7	0.86653
24H 50MT/	P01042	-0.75997	0.078145	0.05014	KNG1	Kininogen-	KNG1_HUM	1	0.590508
24H 10MT/	Q96JM3	0.08997	0.078151	0.050144	CHAMP1	Chromosor	CHAP1_HU	17	1.064348
48H 50MT/	Q6VN20	-0.16441	0.078153	0.050144	RANBP10	Ran-bindin	RBP10_HUI	14	0.892294
24H 50MT/	O60318	-0.09919	0.078159	0.050147	MCM3AP	Germinal-c	GANP_HUM	22	0.93356
24H 10MT/	Q8NEY4	0.803371	0.078161	0.050147	ATP6V1C2	V-type prot	VATC2_HUI	2	1.745174
48H 10MT/	Q86X95	-0.02223	0.078167	0.05015	CIR1	Corepresso	CIR1_HUM	1	0.984708
24H 50MT/	P06702	1.18776	0.078172	0.050152	S100A9	Protein S1(	S10A9_HUI	2	2.277988
48H 10MT/	Q8WX93	-0.07947	0.078216	0.050174	PALLD	Palladin	PALLD_HUI	7	0.946404
24H 50MT/	Q9UQ90	0.873545	0.078284	0.050206	SPG7	Mitochond	SPG7_HUM	4	1.832159
48H 50MT/	P46736	-0.10333	0.078306	0.050215	BRCC3	Lys-63-spec	BRCC3_HUI	10	0.930881
48H 50MT/	Q8N9F7	0.060456	0.078385	0.050255	GDPD1	Lysophospl	GDPD1_HU	9	1.042795
24H 10MT/	P43304	0.034419	0.078389	0.050256	GPD2	Glycerol-3-	GPDM_HUI	36	1.024144
48H 10MT/	Q9NVQ4	-0.15938	0.078442	0.050286	FAIM	Fas apopto	FAIM1_HUI	4	0.895411
24H 10MT/	Q5T5U3	0.394286	0.078462	0.050295	ARHGAP21	Rho GTPase	RHG21_HU	8	1.314292
48H 10MT/	Q9P0M9	-0.3714	0.078476	0.050302	MRPL27	Large ribos	RM27_HUM	5	0.773034
48H 10MT/	P23378	0.001938	0.078529	0.050331	GLDC	Glycine del	GCSP_HUM	33	1.001344
24H 10MT/	P38435	-0.03194	0.078537	0.050335	GGCX	Vitamin K-c	VKGC_HUM	16	0.978103

48H 50MT/	P01023	-0.00938	0.078602	0.050368	A2M	Alpha-2-ma	A2MG_HUI	57	0.993519
48H 50MT/	Q9Y3T6	-0.37461	0.078602	0.050368	R3HCC1	R3H and co	R3HC1_HU	3	0.771312
24H 10MT/	O60318	-0.07498	0.078651	0.050393	MCM3AP	Germinal-c	GANP_HUM	22	0.949357
24H 10MT/	Q15388	0.273445	0.078726	0.050434	TOMM20	Mitochond	TOM20_HU	3	1.208691
48H 10MT/	Q9UBB4	0.063631	0.078735	0.050437	ATXN10	Ataxin-10	ATX10_HUI	22	1.045093
24H 50MT/	Q7Z7E8	-0.16302	0.078743	0.050441	UBE2Q1	Ubiquitin-c	UB2Q1_HU	9	0.893155
48H 10MT/	Q9P107	-0.58375	0.078747	0.050442	GMIP	GEM-intera	GMIP_HUM	2	0.667226
48H 10MT/	Q6UVJ0	0.023988	0.07875	0.050443	SASS6	Spindle ass	SAS6_HUM	12	1.016766
48H 10MT/	Q92484	-0.17221	0.078753	0.050444	SMPDL3A	Cyclic GMP	ASM3A_HU	11	0.887482
48H 10MT/	Q9BZF3	-1.14887	0.078784	0.050461	OSBPL6	Oxysterol-b	OSBL6_HUI	2	0.450979
24H 50MT/	Q9Y4K4	0.155172	0.078788	0.050462	MAP4K5	Mitogen-ac	M4K5_HUM	5	1.113554
24H 50MT/	Q8NEZ3	0.021953	0.078794	0.050465	WDR19	WD repeat-	WDR19_HU	5	1.015333
24H 10MT/	Q8N6G6	-0.3066	0.078807	0.050471	ADAMTSL1	ADAMTS-lik	ATL1_HUM	2	0.808545
24H 10MT/	P35244	-0.14479	0.078825	0.05048	RPA3	Replication	RFA3_HUM	7	0.904512
48H 10MT/	Q9UDY8	0.079487	0.078834	0.050485	MALT1	Mucosa-ass	MALT1_HU	22	1.056642
48H 50MT/	Q8TEH3	0.661935	0.078845	0.05049	DENND1A	DENN doma	DEN1A_HU	3	1.582204
24H 50MT/	Q9NZN5	-0.00882	0.078847	0.05049	ARHGEF12	Rho guanin	ARHGC_HU	37	0.993904
48H 50MT/	Q58FF7	-0.71787	0.078899	0.050513	HSP90AB3I	Putative he	H90B3_HU	5	0.607994
24H 50MT/	P07602	0.077043	0.078939	0.050532	PSAP	Prosaposin	SAP_HUMA	17	1.054854
24H 10MT/	Q9BTE3	0.007424	0.078946	0.050535	MCMBP	Mini-chron	MCMBP_HU	26	1.005159
48H 50MT/	P43307	0.011829	0.078957	0.050537	SSR1	Translocon	SSRA_HUM	6	1.008233
24H 50MT/	Q9UK76	-0.30573	0.078963	0.05054	JPT1	Jupiter mic	JUPI1_HUM	7	0.809034
24H 10MT/	Q70UQ0	0.005806	0.078969	0.050543	IKBIP	Inhibitor o	IKIP_HUMA	14	1.004032
24H 50MT/	P09132	-0.05912	0.078984	0.05055	SRP19	Signal reco	SRP19_HUI	4	0.959851
24H 50MT/	P02788	-0.74557	0.078987	0.050551	LTF	Lactotransf	TRFL_HUMA	2	0.596432
48H 50MT/	Q9Y4Y9	0.2475	0.078992	0.050553	LSM5	U6 snRNA-a	LSM5_HUM	2	1.187148
48H 50MT/	Q9BVT8	-0.13438	0.079133	0.050624	TMUB1	Transmemb	TMUB1_HU	3	0.911064
48H 10MT/	Q8NEY4	-0.75944	0.079153	0.050634	ATP6V1C2	V-type prot	VATC2_HUI	2	0.590726
24H 50MT/	O95865	0.068815	0.079165	0.050639	DDAH2	Putative hy	DDAH2_HU	14	1.048855
24H 50MT/	O95785	-0.01006	0.07917	0.05064	WIZ	Protein Wi	WIZ_HUMA	14	0.993053
24H 50MT/	Q9HAT2	0.20199	0.079186	0.050647	SIAE	Sialate O-ac	SIAE_HUMA	5	1.150284
24H 50MT/	Q9HCE3	0.012219	0.079206	0.050657	ZNF532	Zinc finger	ZN532_HUI	2	1.008506
48H 10MT/	P26640	0.02595	0.079229	0.050668	VARS1	Valine--tRN	SYVC_HUM	60	1.01815
24H 50MT/	Q96DV4	0.016593	0.079231	0.050668	MRPL38	Large ribos	RM38_HUM	14	1.011568
24H 10MT/	Q7Z7F0	-2.50045	0.079236	0.050669	KHDC4	KH homolo	KHDC4_HU	2	0.176721
24H 10MT/	P61513	0.089264	0.079245	0.050674	RPL37A	Large ribos	RL37A_HUI	3	1.063828
24H 10MT/	P07741	-0.12182	0.07928	0.050691	APRT	Adenine ph	APT_HUMA	12	0.919026
24H 50MT/	O00443	-0.0176	0.079284	0.050693	PIK3C2A	Phosphatic	P3C2A_HUI	26	0.987874
24H 10MT/	P68363	0.026514	0.079289	0.050694	TUBA1B	Tubulin alp	TBA1B_HUI	30	1.018548
48H 50MT/	Q08945	-0.03922	0.079302	0.050698	SSRP1	FACT comp	SSRP1_HUI	24	0.973184
24H 10MT/	Q9BVC6	0.173242	0.079336	0.050712	TMEM109	Voltage-gat	TM109_HU	2	1.12759
24H 50MT/	Q9UMY1	0.176124	0.079364	0.050726	NOL7	U3 small nu	UTP16_HUI	5	1.129844
48H 50MT/	Q9H5X1	0.194806	0.079367	0.050727	CIAO2A	Cytosolic ir	CIA2A_HUM	5	1.14457
24H 10MT/	Q9NQ88	-0.1404	0.07937	0.050728	TIGAR	Fructose-2,	TIGAR_HUM	9	0.907267
48H 50MT/	Q9BZG8	-0.10529	0.079377	0.050732	DPH1	2-(3-amino	DPH1_HUM	10	0.929618
24H 10MT/	O75928	0.23036	0.079385	0.050734	PIAS2	E3 SUMO-p	PIAS2_HUM	4	1.173127
24H 10MT/	Q6DKK2	0.16906	0.079405	0.050742	TTC19	Tetratricop	TTC19_HUM	9	1.124326
48H 10MT/	Q96DV4	-0.08337	0.079421	0.050749	MRPL38	Large ribos	RM38_HUM	14	0.943847
48H 50MT/	P29353	0.049331	0.079435	0.050757	SHC1	SHC-transfc	SHC1_HUM	13	1.034785
24H 50MT/	P35052	0.663857	0.079469	0.050776	GPC1	Glypican-1	GPC1_HUM	2	1.584312
48H 50MT/	P40429	-0.13533	0.079476	0.05078	RPL13A	Large ribos	RL13A_HUI	10	0.910462
24H 10MT/	Q13442	-0.02369	0.079484	0.050784	PDAP1	28 kDa hea	HAP28_HU	6	0.983717

24H 10MT/	Q96SB8	0.267955	0.079498	0.050792	SMC6	Structural r	SMC6_HUM	9	1.2041
48H 50MT/	Q96CW5	-0.02579	0.079511	0.050799	TUBGCP3	Gamma-tul	GCP3_HUM	26	0.98228
48H 10MT/	P63272	-0.14048	0.079526	0.050803	SUPT4H1	Transcripti	SPT4H_HUI	5	0.90722
24H 10MT/	P09497	-0.23485	0.079532	0.050805	CLTB	Clathrin lig	CLCB_HUM	7	0.849773
48H 10MT/	P18858	0.121627	0.079534	0.050805	LIG1	DNA ligase	DNLI1_HUM	11	1.087961
48H 10MT/	Q8IZP0	0.067405	0.079532	0.050805	ABI1	Abl interact	ABI1_HUMA	11	1.04783
24H 50MT/	Q9UK59	0.029782	0.079539	0.050806	DBR1	Lariat debra	DBR1_HUM	9	1.020858
48H 50MT/	Q9HCP0	0.070347	0.079549	0.050811	CSNK1G1	Casein kina	KC1G1_HU	8	1.049969
48H 10MT/	Q9NX24	0.137567	0.079561	0.050815	NHP2	H/ACA ribo	NHP2_HUM	6	1.100048
48H 10MT/	Q8IZ81	0.04816	0.079564	0.050816	ELMOD2	ELMO doma	ELMD2_HU	8	1.033946
48H 10MT/	Q13642	-0.31684	0.07962	0.050848	FHL1	Four and a	FHL1_HUM	1	0.802827
48H 10MT/	Q92613	0.0198	0.079624	0.050849	JADE3	Protein Jad	JADE3_HUM	2	1.013819
24H 50MT/	O15344	0.092635	0.079644	0.050856	MID1	E3 ubiquiti	TRI18_HUM	20	1.066316
24H 10MT/	Q00973	0.058167	0.079644	0.050856	B4GALNT1	Beta-1,4 N-	B4GN1_HU	13	1.041142
24H 10MT/	Q5SWX8	0.098407	0.079642	0.050856	ODR4	Protein odr	ODR4_HUM	12	1.070591
24H 50MT/	Q9UL12	0.255487	0.079644	0.050856	SARDH	Sarcosine d	SARDH_HU	6	1.193739
24H 10MT/	Q9Y324	-0.04853	0.07964	0.050856	FCF1	rRNA-proce	FCF1_HUM	5	0.966924
48H 50MT/	Q8IZT6	-0.51097	0.079674	0.050872	ASPM	Abnormal s	ASPM_HUM	9	0.701752
48H 10MT/	O43422	-0.10488	0.079681	0.050875	THAP12	52 kDa repr	P52K_HUM	9	0.929883
48H 50MT/	P46459	0.008401	0.079692	0.050879	NSF	Vesicle-fusi	NSF_HUMA	43	1.00584
24H 50MT/	Q8N5C6	0.077938	0.079762	0.050915	SRBD1	S1 RNA-bin	SRBD1_HUI	21	1.055508
24H 50MT/	Q9NPG8	-0.07421	0.079793	0.05093	ZDHHC4	Palmitoyltr	ZDHC4_HU	1	0.949865
24H 50MT/	Q9Y6X4	0.029067	0.079791	0.05093	FAM169A	Soluble lam	F169A_HUI	12	1.020352
48H 50MT/	Q99447	-0.18487	0.079827	0.050946	PCYT2	Ethanolami	PCY2_HUM	13	0.879727
24H 50MT/	Q9GZZ9	-0.06401	0.079834	0.05095	UBA5	Ubiquitin-l	UBA5_HUM	10	0.956602
48H 10MT/	P04083	-0.12677	0.079842	0.050954	ANXA1	Annexin A1	ANXA1_HU	7	0.915877
24H 50MT/	Q9P1F3	-0.42506	0.079852	0.05096	ABRACL	Costars fam	ABRAL_HUI	2	0.744807
48H 10MT/	P15927	-0.10706	0.079916	0.050996	RPA2	Replication	RFA2_HUM	8	0.928479
48H 10MT/	Q5TC63	-0.12047	0.079926	0.051	GRTP1	Growth hor	GRTP1_HUI	4	0.919889
48H 10MT/	O94763	-0.04553	0.079947	0.051011	URI1	Unconvent	RMP_HUMA	5	0.968937
48H 10MT/	P41227	0.094938	0.079952	0.051013	NAA10	N-alpha-ace	NAA10_HU	9	1.06802
24H 10MT/	P50548	-0.50611	0.079959	0.051015	ERF	ETS domain	ERF_HUMA	3	0.704118
48H 10MT/	Q8WW01	0.085241	0.079973	0.051022	TSEN15	tRNA-splici	SEN15_HUI	1	1.060865
48H 50MT/	O15211	0.170422	0.080056	0.051064	RGL2	Ral guanine	RGL2_HUM	3	1.125387
24H 50MT/	P12931	0.063203	0.080056	0.051064	SRC	Proto-onco	SRC_HUMA	11	1.044783
24H 50MT/	Q9NQS1	0.164222	0.080062	0.051067	AVEN	Cell death r	AVEN_HUM	7	1.120562
24H 50MT/	P50990	-0.0613	0.080074	0.051069	CCT8	T-complex	TCPQ_HUM	35	0.9584
24H 50MT/	Q92604	0.079888	0.08007	0.051069	LPGAT1	Acyl-CoA:ly	LGAT1_HUI	12	1.056936
48H 10MT/	Q9Y2R4	-0.07182	0.080071	0.051069	DDX52	Probable A	DDX52_HU	18	0.951438
24H 50MT/	Q9Y4B6	0.047193	0.080075	0.051069	DCAF1	DDB1- and	DCAF1_HUI	27	1.033252
24H 50MT/	Q9NQ92	-0.42712	0.080078	0.05107	COPRS	Coordinato	COPRS_HUI	1	0.743747
24H 10MT/	Q9BVL4	0.059761	0.08009	0.051075	SELENOO	Protein ade	SELO_HUM	11	1.042293
24H 10MT/	P63313	-0.46604	0.080103	0.051083	TMSB10	Thymosin b	TYB10_HUM	1	0.723949
24H 10MT/	Q8TF01	0.306317	0.080117	0.051088	PNISR	Arginine/se	PNISR_HUM	2	1.236547
48H 50MT/	P49841	-0.14319	0.080132	0.051094	GSK3B	Glycogen sy	GSK3B_HUI	13	0.905517
48H 50MT/	Q9HC35	0.010688	0.080153	0.051104	EML4	Echinoderm	EMAL4_HU	37	1.007436
24H 50MT/	Q8NFH4	0.02375	0.08019	0.051123	NUP37	Nucleopori	NUP37_HU	11	1.016598
48H 10MT/	Q96RS0	0.152075	0.080236	0.051148	TGS1	Trimethylg	TGS1_HUM	8	1.111166
48H 10MT/	P33897	-0.14019	0.080251	0.051153	ABCD1	ATP-bindin	ABCD1_HU	7	0.907403
24H 10MT/	Q5VW32	-0.14282	0.080252	0.051153	BROX	BRO1 doma	BROX_HUM	7	0.905745
24H 10MT/	O14715	0.016238	0.080256	0.051155	RGPD8	RANBP2-lik	RGPD8_HU	43	1.011319
24H 10MT/	Q9NVQ4	-0.02733	0.080263	0.051158	FAIM	Fas apopto	FAIM1_HUI	4	0.981232

48H 10MT/	O75330	0.025016	0.080278	0.051165	HMMR	Hyaluronar	HMMR_HU	17	1.017491
48H 10MT/	Q9UM21	-0.15392	0.080295	0.051172	MGAT4A	Alpha-1,3-r	MGT4A_HU	2	0.898806
48H 50MT/	Q07820	-0.07012	0.080303	0.051176	MCL1	Induced my	MCL1_HUM	3	0.95256
24H 10MT/	Q9UPN6	0.095949	0.080313	0.051181	SCAF8	SR-related a	SCAF8_HUI	11	1.068768
24H 50MT/	P48729	-0.00594	0.080321	0.051185	CSNK1A1	Casein kina	KC1A_HUM	13	0.995888
24H 10MT/	P13798	-0.05608	0.080355	0.051201	APEH	Acylamino-	ACPH_HUM	23	0.961875
48H 50MT/	Q5VTE0	-0.75287	0.080358	0.051202	EEF1A1P5	Putative elc	EF1A3_HUI	1	0.593423
24H 50MT/	P28332	0.138415	0.080423	0.051233	ADH6	Alcohol deh	ADH6_HUM	11	1.100695
48H 50MT/	Q99943	0.090174	0.080446	0.051244	AGPAT1	1-acyl-sn-gl	PLCA_HUM	4	1.064498
24H 50MT/	Q14728	-0.10036	0.080449	0.051245	MFSD10	Major facili	MFS10_HU	3	0.932801
48H 50MT/	Q9NVU7	-0.15666	0.080465	0.051251	SDAD1	Protein SD/	SDA1_HUM	14	0.897101
48H 50MT/	O15243	0.066743	0.080504	0.051275	LEPROT	Leptin rece	OBRG_HUM	2	1.04735
48H 10MT/	Q9Y530	0.042617	0.080524	0.051285	OARD1	ADP-ribose	OARD1_HU	5	1.029981
24H 10MT/	Q6IBS0	-0.02446	0.080547	0.051298	TWF2	Twinfilin-2	TWF2_HUM	17	0.983191
24H 10MT/	P11182	-0.11346	0.080551	0.051299	DBT	Lipoamide	ODB2_HUM	22	0.924366
24H 10MT/	P52824	-0.53795	0.080571	0.051309	DGKQ	Diacylglyce	DGKQ_HUM	2	0.688749
48H 50MT/	Q6P1X5	-0.00619	0.08061	0.051327	TAF2	Transcripti	TAF2_HUM	11	0.995719
24H 10MT/	P51610	0.01972	0.080612	0.051327	HCFC1	Host cell fa	HCFC1_HUI	40	1.013763
24H 50MT/	Q93084	-0.17717	0.080618	0.05133	ATP2A3	Sarcoplasm	AT2A3_HUI	3	0.884434
48H 10MT/	O95166	-0.18988	0.080621	0.05133	GABARAP	Gamma-am	GBRAP_HU	3	0.876676
24H 10MT/	Q8IZQ1	-0.34973	0.080641	0.051341	WDFY3	WD repeat	WDFY3_HU	4	0.784731
24H 10MT/	Q6NW29	-0.20521	0.080645	0.051342	RWDD4	RWD doma	RWDD4_HU	3	0.86741
48H 10MT/	Q8WVJ2	0.056836	0.080671	0.051357	NUDCD2	NudC doma	NUDC2_HU	6	1.040182
24H 10MT/	Q7Z417	-0.02353	0.080673	0.051358	NUFIP2	FMR1-inter	NUFP2_HU	18	0.983825
48H 50MT/	O95685	0.244085	0.080688	0.051363	PPP1R3D	Protein pho	PPR3D_HU	5	1.184342
48H 10MT/	Q9BRP8	0.009894	0.080688	0.051363	PYM1	Partner of Y	PYM1_HUM	9	1.006882
24H 10MT/	O15305	-0.07011	0.080722	0.051375	PMM2	Phosphoma	PMM2_HUI	15	0.952568
48H 50MT/	Q8TA86	0.152449	0.080722	0.051375	RP9	Retinitis pi	RP9_HUMA	3	1.111455
48H 50MT/	Q96IJ6	0.033445	0.080745	0.051387	GMPPA	Mannose-1	GMPPA_HU	16	1.023453
48H 50MT/	Q6GMR7	0.384868	0.080761	0.051395	FAAH2	Fatty-acid a	FAAH2_HUI	1	1.305741
48H 50MT/	Q9UJW0	0.015132	0.080833	0.051431	DCTN4	Dynactin su	DCTN4_HU	19	1.010544
48H 50MT/	Q969X1	0.57682	0.080841	0.051434	TMBIM1	Protein life	LFG3_HUM	2	1.491558
24H 10MT/	Q13554	0.03097	0.080885	0.051458	CAMK2B	Calcium/ca	KCC2B_HUI	6	1.021699
24H 10MT/	A8CG34	0.068164	0.080887	0.051458	POM121C	Nuclear env	P121C_HUI	13	1.048381
48H 50MT/	Q9BZH6	0.024168	0.080927	0.05148	WDR11	WD repeat-	WDR11_HU	32	1.016893
24H 50MT/	Q9HAV4	-0.09972	0.080936	0.051483	XPO5	Exportin-5	XPO5_HUM	47	0.933214
24H 50MT/	Q9UKD1	-0.1943	0.080942	0.051486	GMEB2	Glucocortic	GMEB2_HU	3	0.873994
24H 50MT/	Q8IZ83	-0.01811	0.08095	0.051487	ALDH16A1	Aldehyde d	A16A1_HUI	20	0.987526
24H 50MT/	Q8NI22	-0.55169	0.080957	0.051489	MCFD2	Multiple cc	MCFD2_HU	1	0.682219
24H 10MT/	Q3KQV9	0.103046	0.080966	0.051493	UAP1L1	UDP-N-acet	UAP1L_HUI	10	1.074039
24H 10MT/	P14406	-0.14235	0.080971	0.051496	COX7A2	Cytochrom	CX7A2_HUI	2	0.906044
48H 10MT/	P35080	-0.01728	0.08098	0.0515	PFN2	Profilin-2	PROF2_HUI	7	0.988092
48H 50MT/	Q96DI7	-0.12332	0.080983	0.051501	SNRNP40	U5 small nu	SNR40_HUI	17	0.918072
48H 50MT/	Q9NTG7	0.145621	0.080999	0.051508	SIRT3	NAD-depen	SIR3_HUM/	6	1.106206
48H 50MT/	Q8N5F7	0.241165	0.081028	0.051523	NKAP	NF-kappa-B	NKAP_HUM	3	1.181947
48H 10MT/	Q9NVM6	-0.07723	0.081064	0.051543	DNAJC17	DnaJ homo	DJC17_HUI	5	0.947877
24H 10MT/	Q96KB5	0.078792	0.081067	0.051545	PBK	Lymphokin	TOPK_HUM	15	1.056133
24H 10MT/	Q5T653	0.00357	0.081084	0.051554	MRPL2	Large ribos	RM02_HUM	7	1.002477
48H 10MT/	Q99614	0.055927	0.081112	0.051567	TTC1	Tetratricop	TTC1_HUM	13	1.039527
24H 50MT/	Q9NQC1	0.522651	0.081168	0.051595	JADE2	E3 ubiquiti	JADE2_HUM	1	1.436593
24H 10MT/	Q96IJ6	-0.07401	0.0812	0.051609	GMPPA	Mannose-1	GMPPA_HU	16	0.949997
24H 10MT/	Q96CW5	0.055973	0.08121	0.051615	TUBGCP3	Gamma-tul	GCP3_HUM	26	1.03956

48H 10MT/	Q9BTV6	0.899979	0.081223	0.05162	DPH7	Diphthine r	DPH7_HUM	1	1.866039
48H 50MT/	Q13972	0.659013	0.081239	0.051628	RASGRF1	Ras-specific	RGRF1_HUI	1	1.579002
48H 50MT/	Q5F1R6	0.020418	0.081243	0.051629	DNAJC21	DnaJ homo	DJC21_HUI	11	1.014253
24H 10MT/	P48059	0.058813	0.081277	0.051644	LIMS1	LIM and ser	LIMS1_HUI	12	1.041608
24H 10MT/	Q969V6	0.100351	0.08128	0.051644	MRTFA	Myocardin-	MRTFA_HU	8	1.072034
48H 10MT/	P12004	0.004685	0.081325	0.051668	PCNA	Proliferatir	PCNA_HUM	13	1.003253
24H 50MT/	Q9Y2H1	-0.65792	0.081335	0.051674	STK38L	Serine/thre	ST38L_HUM	1	0.63379
48H 50MT/	Q96S99	-0.07263	0.081368	0.05169	PLEKHF1	Pleckstrin h	PKHF1_HUI	3	0.950903
24H 10MT/	P11234	0.021244	0.081377	0.051695	RALB	Ras-related	RALB_HUM	10	1.014834
24H 50MT/	P36873	-0.05082	0.081389	0.0517	PPP1CC	Serine/thre	PP1G_HUM	17	0.96539
48H 50MT/	P32519	0.057854	0.081397	0.051703	ELF1	ETS-related	ELF1_HUM/	7	1.040916
48H 50MT/	Q8TEA8	0.195541	0.081407	0.051707	DTD1	D-aminoac	DTD1_HUM	3	1.145154
24H 50MT/	Q5VIR6	-0.15459	0.081503	0.051758	VPS53	Vacuolar pr	VPS53_HUI	16	0.898385
48H 50MT/	Q5BJH2	0.085823	0.081524	0.051768	TMEM128	Transmemb	TM128_HU	1	1.061293
24H 50MT/	Q9H008	-0.14036	0.081537	0.051773	LHPP	Phospholys	LHPP_HUM	5	0.90729
24H 10MT/	Q7Z3B4	0.000132	0.081542	0.051775	NUP54	Nucleopori	NUP54_HU	19	1.000092
24H 10MT/	Q04726	0.012984	0.081597	0.051803	TLE3	Transducin	TLE3_HUM/	11	1.00904
48H 10MT/	P61289	0.023747	0.081614	0.051809	PSME3	Proteasom	PSME3_HU	17	1.016596
24H 10MT/	Q96NB3	-0.06033	0.081623	0.051812	ZNF830	Zinc finger	ZN830_HUI	4	0.959048
24H 10MT/	Q9H993	-0.13638	0.081626	0.051813	ARMT1	Damage-co	ARMT1_HU	16	0.909796
48H 10MT/	Q9H788	0.096637	0.081661	0.051829	SH2D4A	SH2 domai	SH24A_HUI	10	1.069278
24H 10MT/	P37287	0.007424	0.081681	0.051841	PIGA	Phosphatid	PIGA_HUM	3	1.005159
48H 10MT/	O95625	0.032724	0.081708	0.051855	ZBTB11	Zinc finger	ZBT11_HUI	4	1.022942
48H 10MT/	Q9UFC0	0.039038	0.081742	0.051873	LRWD1	Leucine-ric	LRWD1_HU	13	1.027428
24H 50MT/	Q96MS3	0.7104	0.081752	0.051878	GLT1D1	Glycosyltra	GL1D1_HU	1	1.636258
48H 50MT/	Q9NX01	-0.12486	0.08177	0.051888	TXNL4B	Thioredoxi	TXN4B_HUI	3	0.917091
24H 10MT/	P05165	0.069556	0.081803	0.051905	PCCA	Propionyl-(	PCCA_HUM	24	1.049394
48H 10MT/	Q69YH5	-0.34019	0.08182	0.051913	CDCA2	Cell divisio	CDCA2_HU	2	0.78994
48H 50MT/	Q01973	-0.05185	0.081845	0.051926	ROR1	Inactive tyr	ROR1_HUM	8	0.9647
48H 50MT/	Q9NYA1	-0.40181	0.081866	0.051938	SPHK1	Sphingosin	SPHK1_HUI	1	0.756907
24H 50MT/	O94817	-0.66058	0.081877	0.051943	ATG12	Ubiquitin-l	ATG12_HUI	2	0.632626
48H 50MT/	Q9UK22	0.058354	0.081876	0.051943	FBXO2	F-box only	FBX2_HUM	11	1.041277
24H 50MT/	Q8WWK9	-0.28744	0.081881	0.051944	CKAP2	Cytoskeleto	CKAP2_HUI	9	0.819353
48H 50MT/	Q7Z5W3	0.079426	0.08191	0.051958	BCDIN3D	RNA 5'-mor	BN3D2_HU	1	1.056598
24H 50MT/	Q9H981	0.006714	0.081935	0.051971	ACTR8	Actin-relate	ARP8_HUM	13	1.004665
48H 50MT/	O60487	-0.21772	0.081954	0.051982	MPZL2	Myelin prot	MPZL2_HU	4	0.859923
24H 10MT/	P12270	0.026638	0.081964	0.051987	TPR	Nucleoprot	TPR_HUMA	81	1.018635
24H 10MT/	Q8TDH9	0.708162	0.081981	0.051994	BLOC1S5	Biogenesis	BL1S5_HUI	2	1.633721
24H 50MT/	Q9NUQ2	0.054705	0.082009	0.052008	AGPAT5	1-acyl-sn-gl	PLCE_HUM	12	1.038647
48H 10MT/	Q9NPL8	-0.11392	0.082034	0.052019	TIMMDC1	Complex I a	TIDC1_HUI	10	0.924074
24H 10MT/	P82094	-0.0113	0.08205	0.052028	TMF1	TATA eleme	TMF1_HUM	34	0.992198
48H 10MT/	Q99418	-0.41906	0.082057	0.05203	CYTH2	Cytohesin-2	CYH2_HUM	3	0.747913
48H 10MT/	Q12874	-0.00857	0.082059	0.05203	SF3A3	Splicing fac	SF3A3_HUI	26	0.994081
48H 10MT/	P07737	-0.00491	0.082063	0.052031	PFN1	Profilin-1	PROF1_HUI	11	0.996604
24H 50MT/	Q99988	-0.07708	0.082072	0.052035	GDF15	Growth/dif	GDF15_HU	7	0.947972
48H 50MT/	Q8N1G1	-0.52818	0.082084	0.05204	REXO1	RNA exonu	REXO1_HUI	2	0.693431
24H 10MT/	A5PLN9	-0.05112	0.082119	0.052057	TRAPPC13	Trafficking	TPC13_HUI	5	0.965185
24H 50MT/	P61224	-0.0074	0.082172	0.052083	RAP1B	Ras-related	RAP1B_HUI	10	0.994884
24H 10MT/	P54253	-0.50949	0.082176	0.052083	ATXN1	Ataxin-1	ATX1_HUM	2	0.70247
48H 10MT/	P80723	-0.25215	0.082194	0.052093	BASP1	Brain acid s	BASP1_HUI	7	0.839647
48H 10MT/	Q16842	-1.35796	0.082196	0.052093	ST3GAL2	CMP-N-acet	SIA4B_HUM	1	0.390132
48H 10MT/	Q16222	0.036018	0.0822	0.052094	UAP1	UDP-N-acet	UAP1_HUM	9	1.02528

24H 50MT/	P62280	0.02199	0.082218	0.052103	RPS11	Small ribos	RS11_HUM	14	1.015359
24H 50MT/	P63220	-0.23136	0.082288	0.052139	RPS21	Small ribos	RS21_HUM	6	0.851832
24H 10MT/	Q9NVH0	-0.01024	0.082293	0.052141	EXD2	Exonucleas	EXD2_HUM	17	0.992924
24H 50MT/	P56937	0.045154	0.08231	0.052151	HSD17B7	3-keto-ster	DHB7_HUM	8	1.031793
48H 50MT/	Q14296	0.025182	0.082334	0.05216	FASTK	Fas-activate	FASTK_HUM	2	1.017608
48H 50MT/	P21333	-0.06468	0.082364	0.05217	FLNA	Filamin-A	FLNA_HUM	104	0.956159
48H 10MT/	Q9NS69	0.182133	0.082424	0.052199	TOMM22	Mitochond	TOM22_HU	5	1.13456
48H 10MT/	P36543	0.051594	0.082446	0.052211	ATP6V1E1	V-type prot	VATE1_HUM	12	1.036409
48H 10MT/	Q08188	-0.1761	0.082471	0.052221	TGM3	Protein-glu	TGM3_HUM	2	0.885093
48H 50MT/	P15144	0.074337	0.082472	0.052221	ANPEP	Aminopept	AMPN_HUM	25	1.052877
48H 50MT/	A1L3X0	-0.53297	0.082485	0.052224	ELOVL7	Very long cl	ELOV7_HUI	1	0.69113
24H 10MT/	Q96G28	-0.14958	0.0825	0.052231	CFAP36	Cilia- and fl	CFA36_HUI	10	0.90151
24H 10MT/	O60841	0.029449	0.082517	0.05224	EIF5B	Eukaryotic	IF2P_HUM/	46	1.020622
24H 10MT/	Q9P265	-0.02442	0.082546	0.052253	DIP2B	Disco-inter	DIP2B_HUM	34	0.983216
24H 10MT/	P36969	0.045501	0.082602	0.052285	GPX4	Phospholip	GPX4_HUM	6	1.032042
24H 10MT/	Q9Y2H1	-0.78364	0.082606	0.052287	STK38L	Serine/thre	ST38L_HUM	1	0.580898
48H 50MT/	Q5T280	-0.26227	0.082612	0.052289	SPOUT1	Putative m	CI114_HUM	6	0.833774
24H 50MT/	Q5T8I3	0.088005	0.082615	0.052289	EEIG2	EEIG family	EEIG2_HUM	11	1.0629
24H 50MT/	P60520	0.178866	0.08264	0.052302	GABARAPL2	Gamma-am	GBRL2_HUI	4	1.131994
48H 10MT/	Q16204	0.123139	0.08265	0.052306	CCDC6	Coiled-coil	CCDC6_HU	17	1.089102
24H 10MT/	Q6P4I2	0.215076	0.082653	0.052307	WDR73	WD repeat-	WDR73_HU	10	1.160765
24H 10MT/	Q9H0U6	0.082524	0.082664	0.052312	MRPL18	Large ribos	RM18_HUM	3	1.058869
24H 50MT/	P50542	-0.09124	0.082677	0.052318	PEX5	Peroxisom	PEX5_HUM	22	0.938714
48H 50MT/	Q9H299	0.175309	0.082678	0.052318	SH3BGRL3	SH3 domai	SH3L3_HUI	4	1.129207
24H 50MT/	Q969N2	0.121682	0.082705	0.052331	PIGT	GPI transan	PIGT_HUM/	19	1.088003
48H 10MT/	Q99714	-0.02339	0.082725	0.05234	HSD17B10	3-hydroxya	HCD2_HUM	18	0.983919
24H 10MT/	P11387	-0.01007	0.082753	0.052353	TOP1	DNA topois	TOP1_HUM	30	0.993042
24H 50MT/	Q8N398	-0.39753	0.082755	0.052353	VWA5B2	von Willeb	VW5B2_HU	1	0.759159
48H 10MT/	Q96KP4	-0.0016	0.082764	0.052357	CNDP2	Cytosolic n	CNDP2_HU	26	0.998891
48H 50MT/	Q92882	-0.04181	0.082772	0.05236	OSTF1	Osteoclast-	OSTF1_HUM	7	0.971436
48H 10MT/	Q15042	0.030121	0.082785	0.052367	RAB3GAP1	Rab3 GTPas	RB3GP_HU	40	1.021098
24H 10MT/	O43765	-0.10541	0.082788	0.052367	SGTA	Small gluta	SGTA_HUM	11	0.929538
24H 10MT/	Q9HD23	0.03748	0.082846	0.052396	MRS2	Magnesium	MRS2_HUM	15	1.026319
24H 50MT/	P50548	-0.73851	0.082854	0.052399	ERF	ETS domain	ERF_HUMA	3	0.599358
24H 10MT/	Q9BV20	-0.07568	0.082861	0.052402	MRI1	Methylthio	MTNA_HUM	15	0.948893
48H 10MT/	Q13136	0.125207	0.082902	0.052419	PPFIA1	Liprin-alph	LIPA1_HUM	28	1.090664
24H 50MT/	Q9Y5S9	-0.04517	0.082901	0.052419	RBM8A	RNA-bindir	RBM8A_HU	8	0.969176
24H 10MT/	P20810	-0.07235	0.082921	0.052429	CAST	Calpastatin	ICAL_HUM/	22	0.951086
24H 50MT/	Q8TDZ2	-0.05158	0.082927	0.05243	MICAL1	[F-actin]-m	MICA1_HUI	22	0.964877
48H 10MT/	Q9ULJ8	-0.21119	0.082936	0.052434	PPP1R9A	Neurabin-1	NEB1_HUM	3	0.863824
24H 10MT/	Q96EV2	-0.02795	0.082941	0.052436	RBM33	RNA-bindir	RBM33_HU	11	0.980814
24H 10MT/	Q9H8K7	-0.42722	0.082955	0.052444	PAAT	ATPase PAA	PAAT_HUM	3	0.743696
48H 50MT/	P14927	0.087509	0.082966	0.05245	UQCRB	Cytochrom	QCR7_HUM	8	1.062534
24H 50MT/	Q8WUH1	0.130532	0.083008	0.052472	CHURC1	Protein Chu	CHUR_HUM	3	1.094697
48H 10MT/	Q9BRT2	-0.01185	0.083018	0.052478	UQCC2	Ubiquinol-c	UQCC2_HU	6	0.991823
48H 10MT/	Q9NUQ9	0.059786	0.08303	0.052483	CYRIB	CYFIP-relat	CYRIB_HUM	15	1.042311
48H 10MT/	P35244	0.036931	0.083035	0.052484	RPA3	Replication	RFA3_HUM	7	1.025929
24H 50MT/	Q9P0J1	0.052134	0.083091	0.052513	PDP1	[Pyruvate d	PDP1_HUM	12	1.036797
48H 10MT/	Q9P2X3	0.058101	0.0831	0.052517	IMPACT	Protein IMI	IMPCT_HUI	6	1.041095
48H 50MT/	Q9GZV4	-0.1928	0.083104	0.052519	EIF5A2	Eukaryotic	IF5A2_HUM	7	0.874907
48H 10MT/	Q9H0E2	0.118781	0.083117	0.052524	TOLLIP	Toll-interac	TOLIP_HUM	7	1.085817
48H 10MT/	Q9Y6A1	-0.36093	0.083157	0.052541	POMT1	Protein O-n	POMT1_HU	4	0.778661

48H 50MTA	Q9BSQ5	0.17837	0.083176	0.052551	CCM2	Cerebral ca	CCM2_HUN	3	1.131605
48H 10MTA	Q8TCF1	0.028861	0.083183	0.052554	ZFAND1	AN1-type zi	ZFAN1_HUI	7	1.020206
24H 50MTA	P13987	0.377183	0.0832	0.05256	CD59	CD59 glyco	CD59_HUM	4	1.298803
48H 10MTA	P22415	0.24915	0.083221	0.052565	USF1	Upstream s	USF1_HUM	1	1.188507
24H 10MTA	Q9H8M5	0.01144	0.083245	0.052578	CNNM2	Metal trans	CNNM2_HU	12	1.007961
24H 10MTA	Q16718	-0.17987	0.083268	0.052589	NDUFA5	NADH dehy	NDUA5_HU	6	0.88278
48H 10MTA	O00267	0.024245	0.083277	0.052593	SUPT5H	Transcripti	SPT5H_HUI	38	1.016947
48H 50MTA	Q96SZ6	-0.11335	0.083279	0.052593	CDK5RAP1	Mitochond	CK5P1_HUI	13	0.924439
48H 10MTA	O95372	0.107318	0.083296	0.052601	LYPLA2	Acyl-protei	LYPA2_HUN	8	1.077224
48H 50MTA	Q9UBF6	0.37302	0.083328	0.052618	RNF7	RING-box p	RBX2_HUM	3	1.295061
48H 10MTA	P45974	-0.00388	0.083344	0.052627	USP5	Ubiquitin c	UBP5_HUM	35	0.997314
48H 10MTA	Q9UMR5	0.130055	0.083355	0.052631	PPT2	Lysosomal	PPT2_HUM	4	1.094335
48H 10MTA	Q9Y6M7	-0.06967	0.083364	0.052634	SLC4A7	Sodium bic	S4A7_HUM	15	0.952854
48H 50MTA	Q8N8A6	-0.23844	0.083398	0.052652	DDX51	ATP-depend	DDX51_HU	20	0.847664
48H 50MTA	Q53R41	0.166758	0.083541	0.05273	FASTKD1	FAST kinase	FAKD1_HUI	8	1.122533
24H 50MTA	Q504Q3	-0.19795	0.083551	0.052734	PAN2	PAN2-PAN3	PAN2_HUM	3	0.871791
24H 10MTA	Q14739	0.013636	0.083589	0.052752	LBR	Delta(14)-s	LBR_HUMA	14	1.009497
24H 50MTA	Q13574	-0.5949	0.083603	0.052759	DGKZ	Diacylglyce	DGKZ_HUM	1	0.662092
24H 10MTA	O15031	0.007523	0.083614	0.05276	PLXNB2	Plexin-B2	PLXB2_HUI	50	1.005228
24H 10MTA	P26583	-0.02522	0.083609	0.05276	HMGB2	High mobil	HMGB2_HU	7	0.982669
48H 50MTA	Q9H6K5	0.2129	0.083668	0.05279	PRR36	Proline-ricl	PRR36_HUI	7	1.159016
48H 50MTA	Q13395	-0.19948	0.083672	0.052791	TARBP1	Probable m	TARB1_HUI	32	0.870862
48H 10MTA	O95391	-0.11235	0.083686	0.052797	SLU7	Pre-mRNA-s	SLU7_HUM	7	0.925079
24H 50MTA	P47756	0.082703	0.083693	0.052798	CAPZB	F-actin-cap	CAPZB_HUI	19	1.059
24H 10MTA	O75044	0.883673	0.083757	0.052826	SRGAP2	SLIT-ROBO	SRGP2_HUI	2	1.845067
24H 10MTA	Q04323	-0.01982	0.083762	0.052829	UBXN1	UBX domai	UBXN1_HU	10	0.986359
48H 50MTA	B1AK53	1.3117	0.083766	0.052829	ESPN	Espin	ESPN_HUM	1	2.482339
24H 50MTA	P16383	0.061053	0.083789	0.052841	GCFC2	Intron Larg	GCFC2_HUI	15	1.043227
48H 10MTA	P61019	-0.11227	0.083793	0.052841	RAB2A	Ras-related	RAB2A_HUI	14	0.92513
24H 10MTA	Q9BXJ1	-0.05681	0.083816	0.05285	C1QTNF1	Complemer	C1QT1_HUI	1	0.961391
48H 50MTA	O14639	0.038901	0.08384	0.052862	ABLIM1	Actin-bindi	ABLM1_HU	21	1.027331
48H 50MTA	Q9BWE0	-0.2353	0.083854	0.05287	REPIN1	DNA-bindir	REPI1_HUN	11	0.849508
48H 50MTA	Q92945	-0.01245	0.083859	0.052872	KHSRP	Far upstrea	FUBP2_HUI	28	0.991409
24H 50MTA	Q9Y282	0.211122	0.083887	0.052886	ERGIC3	Endoplasm	ERGI3_HUN	9	1.157588
48H 10MTA	A1A4S6	-0.09823	0.083895	0.052889	ARHGAP10	Rho GTPase	RHG10_HU	4	0.934179
48H 50MTA	Q9Y6I4	-0.10365	0.083905	0.052893	USP3	Ubiquitin c	UBP3_HUM	16	0.930677
24H 10MTA	P50613	-0.00529	0.084012	0.052948	CDK7	Cyclin-depe	CDK7_HUM	14	0.996341
48H 50MTA	Q9BTT6	0.045802	0.084023	0.052953	LRRC1	Leucine-ric	LRRC1_HUI	16	1.032257
24H 50MTA	Q9BYE7	0.538533	0.084047	0.052965	PCGF6	Polycomb g	PCGF6_HUI	3	1.452495
48H 50MTA	O43299	-0.0688	0.0841	0.052992	AP5Z1	AP-5 compl	AP5Z1_HUI	8	0.953431
48H 10MTA	O75821	0.032247	0.084111	0.052997	EIF3G	Eukaryotic	EIF3G_HUN	19	1.022603
48H 50MTA	O95297	0.081532	0.084122	0.053001	MPZL1	Myelin prot	MPZL1_HU	4	1.058141
48H 10MTA	Q76EJ3	-0.25527	0.084164	0.05302	SLC35D2	Nucleotide	S35D2_HUI	3	0.837828
48H 10MTA	Q5HYI7	0.075875	0.084242	0.053058	MTX3	Metaxin-3	MTX3_HUN	5	1.054
48H 50MTA	Q15555	-0.06971	0.084257	0.053063	MAPRE2	Microtubul	MARE2_HU	11	0.952827
24H 50MTA	Q9UL40	-0.0124	0.084278	0.053075	ZNF346	Zinc finger	ZN346_HUI	7	0.991441
24H 10MTA	O14910	0.035635	0.084281	0.053076	LIN7A	Protein lin-	LIN7A_HUN	9	1.025008
48H 10MTA	Q96BT3	-0.03172	0.084289	0.053079	CENPT	Centromer	CENPT_HUI	3	0.978253
48H 10MTA	Q8WX92	0.046911	0.084306	0.053086	NELFB	Negative el	NELFB_HUN	13	1.033051
24H 50MTA	O75352	-0.02238	0.084362	0.053113	MPDU1	Mannose-P	MPU1_HUN	4	0.98461
48H 10MTA	Q9UNY4	-0.11147	0.084361	0.053113	TTF2	Transcripti	TTF2_HUMA	32	0.925645
24H 50MTA	P56545	-0.02485	0.08438	0.053121	CTBP2	C-terminal-	CTBP2_HUI	6	0.982925

48H 10MT/	Q96G23	-0.05003	0.084387	0.053124	CERS2	Ceramide s	CERS2_HUI	9	0.965917
24H 50MT/	Q00059	0.036391	0.084397	0.053128	TFAM	Transcripti	TFAM_HUM	12	1.025545
48H 50MT/	O43760	0.046541	0.084417	0.053137	SYNGR2	Synaptogyr	SNG2_HUM	4	1.032786
48H 10MT/	Q5VTE6	-0.27664	0.08444	0.053149	ANGEL2	Protein ang	ANGE2_HU	3	0.82551
24H 10MT/	P62913	0.072995	0.084453	0.053156	RPL11	Large ribos	RL11_HUM	10	1.051898
48H 10MT/	P78381	-0.11529	0.08447	0.053163	SLC35A2	UDP-galact	S35A2_HUI	3	0.923196
48H 10MT/	P55010	0.008062	0.084483	0.053168	EIF5	Eukaryotic	IF5_HUMAI	17	1.005604
48H 50MT/	Q53H54	-0.43946	0.084487	0.05317	TRMT12	tRNA wybu	TYW2_HUM	1	0.737408
48H 50MT/	Q9H3Z4	0.173189	0.084497	0.053175	DNAJC5	DnaJ homo	DNJC5_HUI	4	1.127548
48H 10MT/	P52789	0.104007	0.084498	0.053175	HK2	Hexokinase	HXK2_HUM	31	1.074754
24H 10MT/	O14907	0.073578	0.0845	0.053175	TAX1BP3	Tax1-bindir	TX1B3_HUI	4	1.052323
48H 10MT/	Q9UGJ1	-0.0832	0.084525	0.053188	TUBGCP4	Gamma-tul	GCP4_HUM	12	0.943964
24H 50MT/	Q13155	0.035323	0.084546	0.053198	AIMP2	Aminoacyl	AIMP2_HUI	13	1.024786
48H 50MT/	Q14155	0.116531	0.084573	0.053211	ARHGEF7	Rho guanin	ARHG7_HU	8	1.084125
24H 50MT/	P50914	-0.10271	0.0846	0.053224	RPL14	Large ribos	RL14_HUM	4	0.931282
24H 10MT/	Q9Y597	0.195183	0.084638	0.053237	KCTD3	BTB/POZ dc	KCTD3_HUI	4	1.14487
48H 50MT/	O94880	-0.13534	0.084664	0.053247	PHF14	PHD finger	PHF14_HUI	16	0.910456
24H 50MT/	Q9Y3A3	-0.15824	0.084693	0.053262	MOB4	MOB-like pi	PHOCN_HU	6	0.89612
48H 10MT/	Q01105	0.068545	0.084748	0.053289	SET	Protein SET	SET_HUMAI	10	1.048658
24H 50MT/	Q6PJG6	-0.06159	0.084784	0.053307	BRAT1	BRCA1-assc	BRAT1_HUI	37	0.958205
24H 10MT/	Q9BQP7	0.070055	0.084798	0.053312	MGME1	Mitochond	MGME1_HU	12	1.049757
24H 50MT/	Q6ZU35	0.521896	0.084812	0.053316	CRACD	Capping pr	CRACD_HU	2	1.435841
24H 50MT/	Q9NYJ8	-0.13502	0.084945	0.053384	TAB2	TGF-beta-ac	TAB2_HUM	5	0.910657
24H 10MT/	Q9H3C7	0.615117	0.084966	0.053394	GGNBP2	Gametogen	GGNB2_HU	2	1.531683
48H 10MT/	Q96DT6	-0.27565	0.084979	0.053398	ATG4C	Cysteine pr	ATG4C_HUI	1	0.826078
48H 10MT/	O96028	-0.00539	0.08499	0.053403	NSD2	Histone-lys	NSD2_HUM	10	0.996272
24H 10MT/	Q5HYJ3	0.129998	0.084994	0.053404	FAM76B	Protein FAI	FA76B_HUI	3	1.094292
48H 10MT/	Q86WX3	-0.048	0.085033	0.053421	RPS19BP1	Active regu	AROS_HUM	5	0.967274
48H 50MT/	P68431	-0.25785	0.085058	0.053432	H3C1	Histone H3	H31_HUMA	6	0.836334
48H 50MT/	Q6ZW31	0.054468	0.08509	0.053447	SYDE1	Rho GTPase	SYDE1_HUI	11	1.038476
24H 50MT/	Q96MF7	0.281077	0.0851	0.053452	NSMCE2	E3 SUMO-p	NSE2_HUM	4	1.215102
24H 10MT/	Q1MSJ5	0.150696	0.085116	0.053459	CSPP1	Centrosom	CSPP1_HUI	7	1.110105
48H 50MT/	Q8IY22	-0.04751	0.085146	0.053474	CMIP	C-Maf-indu	CMIP_HUM	12	0.967604
48H 50MT/	P04080	0.06884	0.085149	0.053475	CSTB	Cystatin-B	CYTB_HUM	6	1.048873
48H 50MT/	Q01650	-0.14522	0.085167	0.053483	SLC7A5	Large neutr	LAT1_HUM	8	0.904243
48H 50MT/	Q08J23	0.024526	0.085174	0.053484	NSUN2	RNA cytosir	NSUN2_HU	41	1.017146
24H 10MT/	Q9H5Z1	0.040905	0.085176	0.053484	DHX35	Probable A	DHX35_HU	15	1.028759
48H 50MT/	O95202	-0.09321	0.085179	0.053485	LETM1	Mitochond	LETM1_HUI	34	0.937436
24H 10MT/	P08648	0.095363	0.085213	0.053503	ITGA5	Integrin alp	ITA5_HUMA	19	1.068334
24H 10MT/	Q92733	-0.08171	0.085233	0.053514	PRCC	Proline-ricl	PRCC_HUM	5	0.944938
48H 10MT/	P39880	-0.0667	0.085241	0.053515	CUX1	Homeobox	CUX1_HUM	9	0.95482
48H 50MT/	P36969	0.044039	0.085248	0.053516	GPX4	Phospholip	GPX4_HUM	6	1.030996
48H 50MT/	P40818	0.125134	0.085248	0.053516	USP8	Ubiquitin c	UBP8_HUM	22	1.090609
24H 10MT/	Q15637	0.01183	0.085244	0.053516	SF1	Splicing fac	SF01_HUM	17	1.008234
24H 10MT/	Q53HL2	0.152662	0.085256	0.053519	CDCA8	Borealin	BOREA_HUI	4	1.111618
48H 50MT/	Q9NPR9	0.021458	0.08526	0.053519	GPR108	Protein GPI	GP108_HU	5	1.014985
48H 10MT/	P35249	-0.00496	0.085309	0.053538	RFC4	Replicatior	RFC4_HUM	18	0.996568
24H 10MT/	O94919	0.026791	0.085324	0.053543	ENDOD1	Endonuclea	ENDD1_HU	13	1.018744
48H 50MT/	O95749	0.039239	0.085323	0.053543	GGPS1	Geranylger	GGPPS_HU	7	1.027571
24H 50MT/	Q96EF6	0.087497	0.085352	0.053558	FBXO17	F-box only	FBX17_HUI	4	1.062525
48H 50MT/	O43399	-0.12817	0.085366	0.053563	TPD52L2	Tumor prot	TPD54_HUI	11	0.914989
24H 50MT/	Q96JK2	0.090921	0.085454	0.053612	DCAF5	DDB1- and	DCAF5_HUI	1	1.06505

24H 10MT/	Q8TBN0	-0.15337	0.085499	0.053632	RAB3IL1	Guanine nu	R3GEF_HU	6	0.899146
24H 50MT/	Q96C36	0.056278	0.0855	0.053632	PYCR2	Pyrroline-5	P5CR2_HU	14	1.03978
48H 50MT/	Q9UPZ3	-0.19474	0.085503	0.053632	HPS5	BLOC-2 con	HPS5_HUM	8	0.87373
48H 10MT/	Q8NEU8	0.117647	0.085531	0.053643	APPL2	DCC-intera	DP13B_HU	15	1.084964
48H 50MT/	Q9GZT8	-0.01524	0.085554	0.053652	NIF3L1	NIF3-like pr	NIF3L_HUM	12	0.989491
24H 50MT/	Q96C86	0.076153	0.085562	0.053654	DCPS	m7GpppX (	DCPS_HUM	14	1.054203
48H 50MT/	Q96KR6	0.138626	0.085567	0.053656	FAM210B	Protein FAM	F210B_HU	3	1.100856
24H 50MT/	Q9NZP8	0.626189	0.085585	0.053665	C1RL	Compleme	C1RL_HUM	1	1.543482
48H 50MT/	Q8N3E9	0.079762	0.085612	0.053677	PLCD3	1-phosphat	PLCD3_HU	2	1.056844
24H 10MT/	Q9UBB6	-0.04127	0.085611	0.053677	NCDN	Neurochon	NCDN_HUM	15	0.971802
48H 10MT/	P08579	0.081922	0.085623	0.053681	SNRPB2	U2 small nu	RU2B_HUM	6	1.058427
24H 10MT/	Q9H3R1	0.714542	0.08564	0.053689	NDST4	Bifunctiona	NDST4_HU	1	1.640962
48H 50MT/	Q7Z7H8	-0.15037	0.085652	0.053692	MRPL10	Large ribos	RM10_HUM	6	0.901018
24H 10MT/	Q8NF37	0.083421	0.085653	0.053692	LPCAT1	Lysophosph	PCAT1_HU	14	1.059527
48H 10MT/	A8CG34	-0.10135	0.085663	0.053696	POM121C	Nuclear env	P121C_HU	13	0.932158
48H 10MT/	Q8WUX9	-0.01244	0.085687	0.053706	CHMP7	Charged mu	CHMP7_HU	9	0.991415
48H 10MT/	Q7L9B9	-0.08569	0.085715	0.05372	EEPD1	Endonuclea	EEPD1_HU	15	0.942332
48H 50MT/	Q16143	0.587776	0.085727	0.053725	SNCB	Beta-synucl	SYUB_HUM	1	1.502928
48H 50MT/	Q9UNA4	0.618137	0.085727	0.053725	POLI	DNA polym	POLI_HUMA	1	1.534892
24H 10MT/	P35754	-0.56223	0.085734	0.053728	GLRX	Glutaredox	GLRX1_HU	3	0.677253
48H 50MT/	Q13415	-0.23428	0.085741	0.053731	ORC1	Origin reco	ORC1_HUM	4	0.850112
48H 50MT/	Q9H0C8	-0.10678	0.085771	0.053743	ILKAP	Integrin-lin	ILKAP_HUM	13	0.928657
48H 10MT/	O14523	-0.03392	0.085776	0.053745	C2CD2L	Phospholip	C2C2L_HU	5	0.976761
48H 50MT/	P51809	-0.1974	0.085791	0.05375	VAMP7	Vesicle-ass	VAMP7_HU	11	0.87212
48H 10MT/	P04798	0.153743	0.085805	0.053755	CYP1A1	Cytochrom	CP1A1_HU	10	1.112452
48H 10MT/	Q9BV94	0.000127	0.085811	0.053757	EDEM2	ER degrada	EDEM2_HU	6	1.000088
24H 50MT/	P52907	-0.11143	0.085863	0.053783	CAPZA1	F-actin-cap	CAZA1_HU	13	0.925667
48H 10MT/	O00159	-0.12272	0.085882	0.053792	MYO1C	Unconvent	MYO1C_HU	43	0.918455
24H 50MT/	Q9P0J7	-0.17099	0.085881	0.053792	KCMF1	E3 ubiquiti	KCMF1_HU	5	0.888231
24H 10MT/	Q9Y3I0	-0.0613	0.085887	0.053794	RTCB	RNA-splicin	RTCB_HUM	24	0.958403
24H 10MT/	Q3KQU3	-0.01278	0.085904	0.053801	MAP7D1	MAP7 dom	MA7D1_HU	10	0.991184
24H 50MT/	P36404	-0.13563	0.085908	0.053803	ARL2	ADP-ribosy	ARL2_HUM	8	0.910274
24H 10MT/	Q13011	0.041989	0.085933	0.053817	ECH1	Delta(3,5)-I	ECH1_HUM	16	1.029532
24H 50MT/	P78332	0.137988	0.085976	0.053839	RBM6	RNA-bindir	RBM6_HUM	23	1.100369
24H 50MT/	Q96EY4	0.163062	0.085976	0.053839	TMA16	Translation	TMA16_HU	8	1.119661
24H 10MT/	Q8TF42	0.014027	0.086002	0.053852	UBASH3B	Ubiquitin-a	UBS3B_HU	13	1.00977
48H 50MT/	Q96BP3	0.008243	0.086048	0.053873	PPWD1	Peptidylpr	PPWD1_HU	24	1.00573
24H 10MT/	Q9NXS2	-0.05039	0.086049	0.053873	QPCTL	Glutaminyl	QPCTL_HU	7	0.965676
24H 50MT/	Q9UKV5	-0.17391	0.086055	0.053877	AMFR	E3 ubiquiti	AMFR_HUM	7	0.88644
48H 10MT/	Q5EBL8	-0.21753	0.086062	0.053879	PDZD11	PDZ domai	PDZ11_HU	3	0.860036
48H 50MT/	Q7L2H7	-0.08271	0.086083	0.053891	EIF3M	Eukaryotic	EIF3M_HUM	19	0.944281
24H 10MT/	O76003	0.050497	0.086091	0.053893	GLRX3	Glutaredox	GLRX3_HU	17	1.035621
48H 50MT/	Q9BQP7	-0.18805	0.08609	0.053893	MGME1	Mitochond	MGME1_HU	12	0.877794
48H 10MT/	Q6P4A8	-0.02039	0.086094	0.053895	PLBD1	Phospholip	PLBL1_HUM	10	0.985966
24H 50MT/	O94906	0.016671	0.086155	0.053928	PRPF6	Pre-mRNA-p	PRP6_HUM	38	1.011622
48H 10MT/	Q9Y4G8	-0.00681	0.086159	0.053928	RAPGEF2	Rap guanin	RPGF2_HU	6	0.995288
24H 10MT/	Q96EK5	0.044348	0.086185	0.053943	KIFBP	KIF-binding	KBP_HUMA	23	1.031217
48H 10MT/	O15305	0.009018	0.086192	0.053946	PMM2	Phosphoma	PMM2_HU	15	1.006271
24H 50MT/	Q9Y613	0.040537	0.086195	0.053947	FHOD1	FH1/FH2 do	FHOD1_HU	8	1.028497
48H 10MT/	Q96T51	0.086087	0.086198	0.053947	RUFY1	RUN and FY	RUFY1_HU	20	1.061487
24H 10MT/	O94762	0.012365	0.0862	0.053948	RECQL5	ATP-depend	RECQ5_HU	9	1.008608
24H 10MT/	Q7Z7G8	0.305242	0.086209	0.053953	VPS13B	Intermemb	VP13B_HU	7	1.235626

48H	10MT/	Q9UH17	0.013543	0.086275	0.05399	APOBEC3B	DNA dC->dU	ABC3B_HUI	9	1.009432
48H	10MT/	Q6ZW49	0.345614	0.086291	0.053999	PAXIP1	PAX-interac	PAXI1_HUN	3	1.270692
48H	10MT/	P21397	-0.0775	0.086301	0.054004	MAOA	Amine oxid	AOFA_HUM	10	0.947696
24H	10MT/	P08579	0.078988	0.086416	0.054062	SNRPB2	U2 small nu	RU2B_HUM	6	1.056277
48H	50MT/	Q15942	-0.08022	0.086432	0.054068	ZYX	Zyxin	ZYX_HUMA	18	0.945914
48H	50MT/	Q7L5Y9	-0.24425	0.086432	0.054068	MAEA	E3 ubiquiti	MAEA_HUN	11	0.844253
24H	50MT/	O60779	0.939519	0.086448	0.054075	SLC19A2	Thiamine tr	S19A2_HUI	1	1.917888
48H	50MT/	Q9H9V9	0.360219	0.086447	0.054075	JMJD4	2-oxoglutar	JMJD4_HUI	2	1.283621
24H	50MT/	Q00688	-0.13696	0.086468	0.054084	FKBP3	Peptidyl-pr	FKBP3_HUI	11	0.909434
24H	50MT/	Q7Z392	-0.1853	0.086466	0.054084	TRAPPC11	Trafficking	TPC11_HUI	10	0.879467
24H	50MT/	P35542	0.099805	0.08648	0.05409	SAA4	Serum amy	SAA4_HUM	2	1.071628
24H	50MT/	O76080	-0.38882	0.086486	0.054093	ZFAND5	AN1-type zi	ZFAN5_HUI	3	0.763752
48H	50MT/	O60762	-0.01964	0.086506	0.054103	DPM1	Dolichol-pl	DPM1_HUN	13	0.986476
48H	10MT/	Q6ZNJ1	0.045392	0.086523	0.054107	NBEAL2	Neurobeacl	NBEL2_HUI	11	1.031963
24H	50MT/	P35869	0.008329	0.086528	0.054109	AHR	Aryl hydroc	AHR_HUMA	14	1.00579
48H	50MT/	P08670	0.01836	0.086535	0.054112	VIM	Vimentin	VIME_HUM	34	1.012808
24H	50MT/	Q92830	0.078317	0.086535	0.054112	KAT2A	Histone ace	KAT2A_HUI	11	1.055786
24H	10MT/	Q01459	-0.06727	0.086547	0.054118	CTBS	Di-N-acetyl	DIAC_HUM	7	0.954445
24H	50MT/	Q15047	0.16263	0.086679	0.054189	SETDB1	Histone-lys	SETB1_HUN	13	1.119326
24H	10MT/	Q7Z3C6	-0.02909	0.086719	0.054209	ATG9A	Autophagy-	ATG9A_HUI	17	0.980039
24H	50MT/	O43463	-0.26633	0.086731	0.054213	SUV39H1	Histone-lys	SUV91_HUI	2	0.831433
24H	10MT/	P07942	0.049284	0.086732	0.054213	LAMB1	Laminin sul	LAMB1_HU	43	1.034751
24H	50MT/	Q9Y2K7	-0.03189	0.086774	0.054234	KDM2A	Lysine-spec	KDM2A_HU	26	0.978138
48H	50MT/	P40205	0.218704	0.086787	0.054237	MYCNOS	N-cym prot	NCYM_HUN	1	1.163688
48H	50MT/	Q96NL8	0.577131	0.086787	0.054237	CFAP418	Cilia- and fl	CF418_HUI	1	1.491879
24H	10MT/	Q9NZ45	-0.13126	0.086793	0.054239	CISD1	CDGSH iror	CISD1_HUN	5	0.913032
24H	10MT/	Q6NUQ4	-0.10939	0.086809	0.054247	TMEM214	Transmemb	TM214_HU	29	0.926978
48H	10MT/	P16949	0.082331	0.086825	0.054255	STMN1	Stathmin	STMN1_HU	6	1.058728
48H	50MT/	Q7Z417	-0.07172	0.086833	0.054258	NUFIP2	FMR1-inter	NUFP2_HU	18	0.951501
48H	10MT/	Q5JTZ9	-0.01168	0.086875	0.054279	AARS2	Alanine--tR	SYAM_HUN	29	0.991938
24H	50MT/	Q9BZF9	-0.11041	0.087022	0.054355	UACA	Uveal autoa	UACA_HUN	21	0.926325
24H	10MT/	Q07864	0.131099	0.087048	0.054366	POLE	DNA polym	DPOE1_HU	31	1.095128
48H	50MT/	P21359	-0.12385	0.087065	0.054376	NF1	Neurofibro	NF1_HUMA	36	0.917738
24H	10MT/	Q9UMN6	0.048938	0.087075	0.05438	KMT2B	Histone-lys	KMT2B_HU	12	1.034503
48H	50MT/	Q9P032	-0.1822	0.087093	0.054389	NDUFAF4	NADH dehy	NDUF4_HU	5	0.88136
48H	50MT/	O43314	0.068714	0.087191	0.054436	PPIP5K2	Inositol he	VIP2_HUM	10	1.048782
24H	50MT/	Q8TCX1	0.368423	0.087193	0.054437	DYNC2LI1	Cytoplasmi	DC2L1_HUI	3	1.290941
24H	10MT/	P01130	0.046383	0.087228	0.054455	LDLR	Low-densit	LDLR_HUM	18	1.032673
48H	50MT/	P05452	-0.44533	0.087231	0.054456	CLEC3B	Tetranectir	TETN_HUM	3	0.734418
24H	10MT/	Q8NCH0	-0.02883	0.087306	0.054495	CHST14	Carbohydra	CHSTE_HUN	9	0.980214
24H	50MT/	Q9H6K4	0.046084	0.087312	0.054498	OPA3	Optic atrop	OPA3_HUM	3	1.032459
24H	10MT/	Q9H857	0.089627	0.087329	0.054507	NT5DC2	5'-nucleoti	NT5D2_HU	25	1.064095
24H	10MT/	P49588	-0.05344	0.08736	0.054521	AARS1	Alanine--tR	SYAC_HUM	44	0.963635
48H	50MT/	P63104	-0.00177	0.087389	0.054534	YWHAZ	14-3-3 prot	1433Z_HUI	13	0.998776
48H	10MT/	Q32MZ4	0.015157	0.087421	0.054551	LRRFIP1	Leucine-ric	LRRF1_HUN	26	1.010561
48H	10MT/	Q9P1U0	-0.72361	0.087449	0.054564	POLR1H	DNA-direct	RPA12_HUI	1	0.605579
24H	50MT/	Q5THJ4	0.461502	0.087486	0.054582	VPS13D	Intermemb	VP13D_HU	12	1.376974
48H	50MT/	O95801	-0.17079	0.087505	0.054592	TTC4	Tetratricop	TTC4_HUM	20	0.888358
24H	10MT/	Q6L8Q7	0.02284	0.087511	0.054594	PDE12	2',5'-phosp	PDE12_HUI	23	1.015957
24H	50MT/	O75044	1.293218	0.087535	0.054603	SRGAP2	SLIT-ROBO	SRGP2_HUI	2	2.450742
48H	10MT/	Q9UNM6	-0.00842	0.087538	0.054603	PSMD13	26S proteas	PSD13_HUI	27	0.994184
48H	50MT/	Q8WW01	0.038695	0.08754	0.054603	TSEN15	tRNA-splici	SEN15_HUI	1	1.027184

48H 50MTA	Q96EB1	0.07774	0.087569	0.054615	ELP4	Elongator c	ELP4_HUM	7	1.055364
48H 10MTA	Q5SRI9	0.472106	0.087594	0.054626	MANEA	Glycoprote	MANEA_HU	2	1.387133
48H 10MTA	Q96AJ9	-0.02284	0.087602	0.05463	VTI1A	Vesicle tran	VTI1A_HUM	5	0.984294
24H 10MTA	Q6DD87	0.005097	0.087611	0.054635	ZNF787	Zinc finger	ZN787_HUI	9	1.003539
24H 50MTA	Q8N131	0.232676	0.087631	0.054646	TMEM123	Porimin	PORIM_HU	1	1.175012
48H 50MTA	Q86Y56	0.037892	0.087665	0.05466	DNAAF5	Dynein axo	DAAF5_HUI	35	1.026613
48H 10MTA	Q96S59	-0.00241	0.087664	0.05466	RANBP9	Ran-bindin	RANB9_HU	15	0.998329
48H 50MTA	P56537	-0.05779	0.087683	0.05467	EIF6	Eukaryotic	IF6_HUMAI	10	0.960736
48H 50MTA	Q9NYP3	-0.25054	0.087707	0.05468	DONSON	Protein dov	DONS_HUM	6	0.840579
48H 10MTA	O14972	-0.02655	0.087711	0.054681	VPS26C	Vacuolar pr	VP26C_HUI	1	0.981766
24H 10MTA	Q9UBT7	0.226836	0.087726	0.05469	CTNNAL1	Alpha-catul	CTNL1_HUI	7	1.170266
48H 50MTA	Q9C0B7	-0.07078	0.087729	0.05469	TANGO6	Transport a	TNG6_HUM	11	0.952122
48H 10MTA	Q9BZE9	0.088724	0.087754	0.054702	ASPSCR1	Tether cont	ASPC1_HUI	17	1.063429
24H 10MTA	Q8WV93	0.057151	0.087789	0.05472	AFG1L	AFG1-like A	AFG1L_HUI	7	1.040409
24H 50MTA	Q99757	-0.09546	0.087815	0.054734	TXN2	Thioredoxi	THIOM_HU	3	0.935973
48H 50MTA	P51648	-0.06501	0.087829	0.054741	ALDH3A2	Aldehyde d	AL3A2_HUI	13	0.955937
24H 10MTA	O60281	0.485612	0.087851	0.054752	ZNF292	Zinc finger	ZN292_HUI	6	1.40018
24H 50MTA	Q8WXW3	0.120632	0.087872	0.054763	PIBF1	Progesteroi	PIBF1_HUM	16	1.087211
24H 10MTA	Q8IZ21	-0.01017	0.087924	0.054788	PHACTR4	Phosphatas	PHAR4_HU	10	0.992978
48H 50MTA	Q8IWV8	0.065243	0.087931	0.054791	UBR2	E3 ubiquiti	UBR2_HUM	24	1.046261
48H 50MTA	Q16514	-0.21955	0.087966	0.054803	TAF12	Transcripti	TAF12_HUI	2	0.858834
48H 10MTA	Q9Y256	0.057992	0.08798	0.054811	RCE1	CAAX preny	FACE2_HUI	2	1.041016
24H 50MTA	Q6NXT1	-0.20295	0.088007	0.054824	ANKRD54	Ankyrin rep	ANR54_HU	4	0.868771
48H 10MTA	Q9H6R4	-0.09614	0.088015	0.054828	NOL6	Nucleolar p	NOL6_HUM	26	0.935535
24H 10MTA	Q96BW9	0.0397	0.088059	0.054852	TAMM41	Phosphatid	TAM41_HU	6	1.0279
48H 50MTA	P17676	0.009729	0.08809	0.054865	CEBPB	CCAAT/enh	CEBPB_HUI	6	1.006767
48H 10MTA	Q7L576	-0.01295	0.088093	0.054865	CYFIP1	Cytoplasmi	CYFP1_HUI	46	0.991064
48H 50MTA	Q9Y4B4	-0.8661	0.088099	0.054866	RAD54L2	Helicase AR	ARIP4_HUM	2	0.548629
24H 10MTA	Q8N2F6	0.191018	0.08812	0.054875	ARMC10	Armadillo r	ARM10_HU	9	1.141569
48H 10MTA	P36578	0.018024	0.088123	0.054876	RPL4	Large ribos	RL4_HUMA	22	1.012571
24H 10MTA	P57076	0.064121	0.088163	0.054895	CFAP298	Cilia- and fl	CF298_HUI	8	1.045448
48H 10MTA	Q9C0J8	-0.03972	0.088162	0.054895	WDR33	pre-mRNA 3	WDR33_HU	16	0.972843
48H 50MTA	Q8IY57	-0.41158	0.08819	0.054906	YAF2	YY1-associa	YAF2_HUM	3	0.751799
48H 10MTA	Q0JRZ9	0.066609	0.088216	0.05492	FCHO2	F-BAR dom	FCHO2_HU	6	1.047252
48H 10MTA	Q9NVT9	0.121117	0.088223	0.054924	ARMC1	Armadillo r	ARMC1_HU	10	1.087577
48H 10MTA	Q6PJT7	0.01438	0.088279	0.054948	ZC3H14	Zinc finger	ZC3HE_HUI	32	1.010017
24H 50MTA	O15120	0.410354	0.088293	0.054952	AGPAT2	1-acyl-sn-gl	PLCB_HUM	4	1.329012
48H 50MTA	Q96HP0	0.508482	0.088328	0.054968	DOCK6	Dedicator c	DOCK6_HU	4	1.422553
24H 10MTA	P37802	0.052157	0.088345	0.054978	TAGLN2	Transgelin-	TAGL2_HUI	15	1.036814
24H 10MTA	Q9NRR4	0.015191	0.088354	0.054983	DROSHA	Ribonuclea	RNC_HUMA	9	1.010585
24H 50MTA	Q9ULD4	0.229029	0.088355	0.054983	BRPF3	Bromodom	BRPF3_HUI	4	1.172046
48H 50MTA	Q9NR77	-0.47501	0.08836	0.054984	PXMP2	Peroxisom	PXMP2_HU	5	0.719462
24H 10MTA	Q9BWS9	0.117704	0.088367	0.054987	CHID1	Chitinase d	CHID1_HUI	9	1.085007
24H 10MTA	Q14BN4	-0.08005	0.08837	0.054988	SLMAP	Sarcolemm	SLMAP_HU	9	0.946026
48H 50MTA	Q9H9L4	0.308119	0.088409	0.05501	KANSL2	KAT8 regul	KANL2_HUI	2	1.238092
48H 10MTA	Q03252	-0.01154	0.088417	0.055014	LMNB2	Lamin-B2	LMNB2_HU	27	0.992031
24H 10MTA	P49748	0.011857	0.088433	0.055021	ACADVL	Very long-c	ACADV_HU	31	1.008252
48H 10MTA	Q9UKN8	-0.08473	0.088439	0.055023	GTF3C4	General tra	TF3C4_HUI	25	0.942962
24H 50MTA	O15269	0.15809	0.088462	0.055036	SPTLC1	Serine palm	SPTC1_HUI	10	1.115809
24H 10MTA	Q9Y664	0.070223	0.088477	0.055043	KPTN	KICSTOR co	KPTN_HUM	2	1.049879
48H 50MTA	Q9UJT0	0.622155	0.088495	0.055049	TUBE1	Tubulin eps	TBE_HUMA	1	1.539173
24H 50MTA	Q8TDH9	0.702502	0.088497	0.05505	BLOC1S5	Biogenesis	BL1S5_HUI	2	1.627324

48H 10MT/	Q96QD9	-0.02011	0.088499	0.05505	FYTTD1	UAP56-inte	UIF_HUMA	7	0.986155
48H 50MT/	Q96S82	0.098572	0.088511	0.055056	UBL7	Ubiquitin-l	UBL7_HUM	8	1.070713
24H 10MT/	Q8N755	-0.13584	0.088555	0.055075	SLC66A3	Solute carri	S66A3_HUI	3	0.910138
24H 50MT/	Q96JM3	0.113015	0.088555	0.055075	CHAMP1	Chromosor	CHAP1_HU	17	1.081486
48H 10MT/	Q9BWQ6	0.500966	0.088572	0.055081	YIPF2	Protein YIP	YIPF2_HUM	2	1.415161
48H 10MT/	Q9ULW2	0.261283	0.088611	0.055104	FZD10	Frizzled-10	FZD10_HUI	4	1.198544
24H 10MT/	Q8NFF5	-0.12262	0.088619	0.055107	FLAD1	FAD syntha	FAD1_HUM	16	0.918516
24H 10MT/	Q8IUD2	0.024919	0.088657	0.055123	ERC1	ELKS/Rab6-	RB6I2_HUM	26	1.017423
24H 10MT/	Q8WUA7	-0.08666	0.08867	0.055129	TBC1D22A	TBC1 doma	TB22A_HUI	9	0.941701
24H 10MT/	Q13526	-0.014	0.088678	0.055133	PIN1	Peptidyl-pr	PIN1_HUM	6	0.99034
24H 10MT/	Q13795	-0.19202	0.088711	0.055147	ARFRP1	ADP-ribosy	ARFRP_HUI	2	0.875381
24H 10MT/	Q9BXI6	0.289669	0.088781	0.055179	TBC1D10A	TBC1 doma	TB10A_HUI	12	1.22236
48H 10MT/	Q15742	-0.08099	0.088786	0.055181	NAB2	NGFI-A-bin	NAB2_HUM	6	0.945406
24H 10MT/	Q6P1Q0	0.05659	0.088811	0.055191	LETMD1	LETM1 dom	LTMD1_HU	5	1.040004
48H 10MT/	Q15643	0.055442	0.088816	0.055193	TRIP11	Thyroid rec	TRIPB_HUM	35	1.039178
48H 10MT/	P54920	0.028483	0.088832	0.055198	NAPA	Alpha-solul	SNAA_HUM	18	1.019939
48H 10MT/	P23434	0.310812	0.088858	0.055208	GCSH	Glycine cle	GCSH_HUM	4	1.240406
48H 10MT/	O14715	-0.1354	0.088893	0.055225	RGPD8	RANBP2-lik	RGPD8_HU	43	0.910419
48H 10MT/	P13716	0.07101	0.088912	0.055233	ALAD	Delta-amin	HEM2_HUM	8	1.050452
48H 50MT/	Q9BX93	-0.22762	0.088919	0.055235	PLA2G12B	Group XIIB	PG12B_HU	2	0.854045
48H 10MT/	Q96EY5	-0.05598	0.088937	0.055245	MVB12A	Multivesicu	MB12A_HU	6	0.96194
48H 10MT/	Q96P47	-0.13555	0.088965	0.055257	AGAP3	Arf-GAP wit	AGAP3_HU	5	0.910321
48H 50MT/	Q9P031	-0.10935	0.088978	0.055262	CCDC59	Thyroid tra	TAP26_HUI	5	0.927007
24H 10MT/	Q9NRU3	-0.12729	0.089032	0.055287	CNNM1	Metal trans	CNNM1_HU	5	0.91555
48H 10MT/	Q8N4C8	0.111383	0.089039	0.055289	MINK1	Misshapen-	MINK1_HU	7	1.080263
24H 50MT/	O14818	0.048251	0.089055	0.055296	PSMA7	Proteasom	PSA7_HUM	15	1.034011
24H 10MT/	Q9UP95	0.071179	0.089059	0.055296	SLC12A4	Solute carri	S12A4_HUI	12	1.050575
24H 10MT/	Q2NKX8	0.07408	0.089099	0.055318	ERCC6L	DNA excisio	ERC6L_HUM	38	1.05269
24H 10MT/	Q08431	0.195736	0.089125	0.055331	MFGE8	Lactadherir	MFGM_HUI	4	1.145309
48H 10MT/	P33176	-0.02124	0.089131	0.055333	KIF5B	Kinesin-1 h	KINH_HUM	52	0.985386
48H 50MT/	P49761	0.303388	0.089159	0.055345	CLK3	Dual specifi	CLK3_HUM	5	1.234039
24H 10MT/	Q6DKJ4	-0.05347	0.089165	0.055347	NXN	Nucleoredo	NXN_HUMA	20	0.963613
24H 10MT/	Q9Y255	0.025901	0.089173	0.055351	PRELID1	PRELI doma	PRLD1_HUI	3	1.018115
48H 10MT/	P55210	-0.00244	0.089187	0.055355	CASP7	Caspase-7	CASP7_HUI	7	0.998312
48H 10MT/	Q9BPW8	-0.02537	0.089217	0.055361	NIPSNAP1	Protein Nip	NIPS1_HUM	11	0.982571
48H 10MT/	Q9BYK8	-0.10387	0.089247	0.055376	HELZ2	3'-5' exorib	HELZ2_HUM	17	0.930531
48H 50MT/	P37108	-0.02593	0.089255	0.055379	SRP14	Signal reco	SRP14_HUI	7	0.982187
24H 50MT/	O43633	-0.16353	0.089262	0.055383	CHMP2A	Charged mu	CHM2A_HU	7	0.892836
24H 10MT/	Q9NX38	-0.47491	0.089282	0.055392	ABITRAM	Protein Abi	ABITM_HUI	1	0.71951
48H 50MT/	Q9H081	-0.05367	0.089364	0.055435	MIS12	Protein MIS	MIS12_HUI	1	0.963484
48H 10MT/	P48382	-0.16371	0.089426	0.055464	RFX5	DNA-bindir	RFX5_HUM	6	0.892729
24H 50MT/	P60602	0.219146	0.089433	0.055464	ROMO1	Reactive ox	ROMO1_HU	2	1.164044
48H 50MT/	Q7Z7A4	0.083201	0.089442	0.055467	PXK	PX domain-	PXK_HUMA	6	1.059366
48H 10MT/	Q8NHS3	-0.24552	0.089448	0.05547	MFSD8	Major facili	MFSD8_HU	4	0.843512
24H 50MT/	Q08J23	0.016857	0.089469	0.055479	NSUN2	RNA cytosir	NSUN2_HU	41	1.011753
24H 50MT/	Q9Y3P9	0.148406	0.089477	0.055483	RABGAP1	Rab GTPase	RBGP1_HU	21	1.108345
24H 10MT/	Q9BYN8	-0.1431	0.089506	0.0555	MRPS26	Small ribos	RT26_HUM	9	0.90557
24H 10MT/	P16615	0.013439	0.08954	0.055518	ATP2A2	Sarcoplasm	AT2A2_HUI	45	1.009359
48H 10MT/	Q9NPR2	0.051543	0.089555	0.055526	SEMA4B	Semaphori	SEM4B_HU	7	1.036373
24H 50MT/	Q9BYG3	0.02759	0.089574	0.055535	NIFK	MKI67 FHA	MK67I_HUI	17	1.019308
24H 50MT/	Q9Y6X8	0.180794	0.089577	0.055535	ZHX2	Zinc fingers	ZHX2_HUM	9	1.133508
24H 50MT/	Q8NF64	-0.26592	0.08958	0.055536	ZMIZ2	Zinc finger I	ZMIZ2_HUM	5	0.831668

48H 50MT/	P46100	0.005324	0.08959	0.055539	ATRX	Transcripti	ATRX_HUM	44	1.003697
48H 50MT/	Q9NXV2	-0.13056	0.089595	0.055542	KCTD5	BTB/POZ dc	KCTD5_HUI	5	0.913474
24H 10MT/	O75348	-0.00831	0.089601	0.055544	ATP6V1G1	V-type prot	VATG1_HUI	4	0.994258
24H 50MT/	Q8WV24	-0.75525	0.089635	0.055562	PHLDA1	Pleckstrin I	PHLA1_HUI	3	0.592444
24H 10MT/	Q5MNZ6	0.047087	0.089644	0.055566	WDR45B	WD repeat	WIPI3_HUM	10	1.033177
24H 50MT/	Q86UY6	-4.98E-05	0.089652	0.05557	NAA40	N-alpha-ace	NAA40_HU	6	0.999966
24H 10MT/	Q96QD8	0.106065	0.089663	0.055575	SLC38A2	Sodium-coi	S38A2_HUI	5	1.076288
48H 10MT/	Q9Y3B2	-0.06577	0.089679	0.055583	EXOSC1	Exosome cc	EXOS1_HUI	6	0.955432
48H 50MT/	Q13049	-0.16509	0.089683	0.055584	TRIM32	E3 ubiquiti	TRI32_HUM	11	0.89187
24H 10MT/	O95071	0.006867	0.089704	0.055591	UBR5	E3 ubiquiti	UBR5_HUM	47	1.004771
48H 10MT/	P02768	-0.32952	0.089707	0.055591	ALB	Albumin	ALBU_HUM	22	0.795804
24H 50MT/	Q7Z6B7	-0.07405	0.089738	0.055605	SRGAP1	SLIT-ROBO I	SRGP1_HUI	13	0.949969
24H 10MT/	Q9BYD6	0.035815	0.089753	0.055612	MRPL1	Large ribos	RM01_HUM	13	1.025136
24H 50MT/	Q9Y285	-0.06797	0.089752	0.055612	FARSA	Phenylalan	SYFA_HUM/	20	0.953978
24H 10MT/	Q9H270	-0.01731	0.089762	0.055615	VPS11	Vacuolar pı	VPS11_HUI	28	0.988076
48H 50MT/	Q13107	0.04393	0.089776	0.055622	USP4	Ubiquitin c	UBP4_HUM	9	1.030918
24H 50MT/	Q8N201	0.014367	0.089796	0.055631	INTS1	Integrator (	INT1_HUM/	32	1.010008
24H 10MT/	Q13347	0.04285	0.089831	0.055646	EIF3I	Eukaryotic	EIF3I_HUM	19	1.030147
48H 50MT/	Q86TB9	-0.0238	0.089855	0.055659	PATL1	Protein PA	PATL1_HUM	20	0.983636
24H 50MT/	Q8N1F8	0.204109	0.089858	0.05566	STK11IP	Serine/thre	S11IP_HUM	14	1.151975
24H 50MT/	P50213	0.050817	0.089862	0.05566	IDH3A	Isocitrate d	IDH3A_HUI	17	1.035851
24H 50MT/	Q9H9A5	-0.13801	0.089877	0.055665	CNOT10	CCR4-NOT t	CNO10_HU	15	0.908772
24H 50MT/	Q8IY22	-0.17148	0.089916	0.055684	CMIP	C-Maf-indu	CMIP_HUM	12	0.887933
48H 50MT/	O14841	0.038784	0.089946	0.055699	OPLAH	5-oxoprolir	OPLA_HUM	7	1.027248
24H 10MT/	O94760	-0.06678	0.089967	0.055711	DDAH1	N(G),N(G)-c	DDAH1_HU	16	0.954769
24H 50MT/	Q6ZW49	0.325386	0.089995	0.055725	PAXIP1	PAX-interac	PAXI1_HUM	3	1.252999
48H 10MT/	Q9H0U6	0.009865	0.090001	0.055728	MRPL18	Large ribos	RM18_HUM	3	1.006861
48H 10MT/	Q8IX90	0.283441	0.090016	0.055734	SKA3	Spindle anc	SKA3_HUM	6	1.217094
24H 50MT/	Q9UHD9	-0.04182	0.090024	0.055736	UBQLN2	Ubiquilin-2	UBQL2_HU	10	0.971425
48H 50MT/	Q7RTV0	-0.12735	0.090031	0.055739	PHF5A	PHD finger-	PHF5A_HUI	6	0.915513
24H 10MT/	P28482	0.006488	0.090051	0.055749	MAPK1	Mitogen-ac	MK01_HUM	25	1.004507
24H 10MT/	Q9Y2T2	-0.00462	0.090066	0.055757	AP3M1	AP-3 compl	AP3M1_HU	17	0.996805
24H 50MT/	Q13505	0.020388	0.090133	0.055785	MTX1	Metaxin-1	MTX1_HUM	11	1.014232
48H 10MT/	Q86XN6	0.164349	0.090189	0.05581	ZNF761	Zinc finger	ZN761_HUI	1	1.120661
24H 50MT/	O75410	0.01466	0.090206	0.055818	TACC1	Transformi	TACC1_HUI	15	1.010213
24H 50MT/	Q7L211	0.41781	0.090208	0.055818	ABHD13	Protein ABI	ABHDD_HU	3	1.335898
48H 10MT/	Q96MH6	-0.23445	0.090249	0.055842	TMEM68	DGAT1/2-ir	DIESL_HUM	4	0.850012
48H 10MT/	O75369	-0.01908	0.090263	0.055849	FLNB	Filamin-B	FLNB_HUM	42	0.98686
48H 10MT/	Q7L2J0	-0.17365	0.090273	0.055853	MEPCE	7SK snRNA	MEPCE_HU	15	0.886597
48H 50MT/	Q9BW27	-0.10483	0.090275	0.055853	NUP85	Nuclear poi	NUP85_HU	23	0.929917
24H 50MT/	B7ZAQ6;P0	0.182894	0.09029	0.055861	GPR89A;GI	Golgi pH re	GPHRA_HU	5	1.135159
24H 50MT/	Q9NTN3	0.039287	0.090292	0.055861	SLC35D1	Nucleotide	S35D1_HUI	1	1.027606
24H 10MT/	O15194	0.449409	0.09031	0.05587	CTDSPL	CTD small p	CTDSL_HUI	1	1.365481
48H 50MT/	O60613	-0.02349	0.090358	0.055891	SELENOF	Selenoprot	SEP15_HUM	5	0.983852
48H 50MT/	O75954	0.074638	0.09036	0.055891	TSPAN9	Tetraspanir	TSN9_HUM	2	1.053097
48H 50MT/	Q8NE62	-0.22179	0.090393	0.055907	CHDH	Choline del	CHDH_HUM	19	0.857504
48H 50MT/	O75947	0.080852	0.090401	0.055911	ATP5PD	ATP syntha:	ATP5H_HUI	12	1.057642
48H 10MT/	Q9NRK6	-0.02199	0.090409	0.055915	ABCB10	ATP-bindin	ABCBA_HUI	15	0.984875
48H 50MT/	Q9UKB1	0.094099	0.090428	0.055925	FBXW11	F-box/WD r	FBW1B_HU	11	1.067399
48H 10MT/	Q3KQV9	-0.14003	0.090445	0.055932	UAP1L1	UDP-N-acet	UAP1L_HUI	10	0.907498
24H 10MT/	Q5D862	0.375864	0.090476	0.055947	FLG2	Filaggrin-2	FILA2_HUM	6	1.297616
24H 10MT/	Q96GY0	0.29745	0.090496	0.055954	ZC2HC1A	Zinc finger	ZC21A_HUI	4	1.22897

48H	10MT/	Q8IY22	0.024956	0.090504	0.055956	CMIP	C-Maf-indu	CMIP_HUM	12	1.017449
48H	10MT/	Q8WWQ0	-0.02017	0.090541	0.055977	PHIP	PH-interact	PHIP_HUM	38	0.986118
24H	50MT/	Q9BY32	-0.2335	0.09055	0.055981	ITPA	Inosine trip	ITPA_HUM/	12	0.850568
48H	50MT/	Q9UNS2	0.00316	0.090563	0.055986	COPS3	COP9 signa	CSN3_HUM	18	1.002193
24H	50MT/	O15530	-0.1803	0.090573	0.055989	PDPK1	3-phosphoi	PDPK1_HU	16	0.882519
48H	50MT/	Q9BZ67	0.082571	0.090663	0.056034	FRMD8	FERM doma	FRMD8_HU	9	1.058903
24H	10MT/	P03886	-0.13197	0.090675	0.056039	MT-ND1	NADH-ubiq	NU1M_HUI	4	0.912586
24H	50MT/	Q9P0S9	0.073328	0.090696	0.056049	TMEM14C	Transmemb	TM14C_HU	3	1.052141
48H	10MT/	O15504	-0.01792	0.090717	0.056057	NUP42	Nucleopori	NUP42_HU	5	0.987655
48H	10MT/	Q9H1K0	0.415213	0.090736	0.056067	RBSN	Rabenosyn-	RBNS5_HUI	1	1.333496
24H	50MT/	Q9BUP3	-0.07679	0.090745	0.05607	HTATIP2	Oxidoreduc	HTAI2_HUN	16	0.948164
48H	10MT/	Q9Y3P9	0.053092	0.090761	0.056078	RABGAP1	Rab GTPase	RBGP1_HU	21	1.037486
24H	50MT/	O00178	0.189245	0.090794	0.056091	GTPBP1	GTP-bindin	GTPB1_HUI	17	1.140167
24H	50MT/	Q86SF2	0.101509	0.090804	0.056094	GALNT7	N-acetylgal	GALT7_HUI	13	1.072895
24H	10MT/	Q15056	-0.20106	0.090826	0.056105	EIF4H	Eukaryotic	IF4H_HUM/	9	0.869911
48H	50MT/	Q00796	0.032906	0.090829	0.056106	SORD	Sorbitol de	DHSO_HUM	15	1.023071
24H	50MT/	Q8N0T1	0.263903	0.090842	0.056113	RBIS	Ribosomal	RBIS_HUM/	3	1.200722
48H	50MT/	Q14849	-0.24242	0.090843	0.056113	STARD3	StAR-relate	STAR3_HUI	5	0.845329
24H	10MT/	P30793	-0.08099	0.090954	0.056164	GCH1	GTP cycloh	GCH1_HUM	10	0.945411
48H	50MT/	Q6IAA8	-0.11087	0.090973	0.056174	LAMTOR1	Ragulator c	LTOR1_HUI	7	0.926032
24H	10MT/	Q9BZI7	-0.02769	0.090979	0.056177	UPF3B	Regulator c	REN3B_HUI	7	0.980988
48H	10MT/	Q9UK99	0.043116	0.090995	0.056183	FBXO3	F-box only	FBX3_HUM	10	1.030337
24H	50MT/	Q9Y4F1	0.130913	0.090995	0.056183	FARP1	FERM, ARH	FARP1_HUI	22	1.094987
24H	10MT/	O00189	0.019816	0.091005	0.056186	AP4M1	AP-4 compl	AP4M1_HU	9	1.01383
48H	50MT/	Q9BTE7	-0.15258	0.091029	0.056195	DCUN1D5	DCN1-like	DCNL5_HU	9	0.899643
24H	10MT/	Q9GZN8	-0.03545	0.091032	0.056196	ADISSP	Adipose-se	ADSSP_HUI	9	0.975726
48H	50MT/	P62760	0.173836	0.091038	0.056197	VSNL1	Visinin-like	VISL1_HUM	7	1.128054
24H	10MT/	P39687	0.029946	0.0911	0.05623	ANP32A	Acidic leuci	AN32A_HU	10	1.020974
24H	10MT/	Q9H1P3	-0.08301	0.091116	0.056238	OSBPL2	Oxysterol-b	OSBL2_HUI	9	0.944086
48H	50MT/	O75494	-0.08879	0.091118	0.056238	SRSF10	Serine/argi	SRS10_HUN	6	0.940311
24H	50MT/	Q8N1G4	-0.04271	0.09113	0.056242	LRRC47	Leucine-ric	LRC47_HUI	23	0.970827
48H	10MT/	P49448	0.087219	0.091176	0.056262	GLUD2	Glutamate	DHE4_HUM	8	1.06232
24H	10MT/	Q9BRT9	-0.05747	0.091176	0.056262	GINS4	DNA replica	SLD5_HUM	9	0.960946
48H	10MT/	Q9P2K2	0.002746	0.091197	0.056273	TXNDC16	Thioredoxi	TXD16_HUI	4	1.001905
24H	10MT/	Q8NCW5	-0.10547	0.0912	0.056274	NAXE	NAD(P)H-hy	NNRE_HUM	10	0.929503
48H	50MT/	Q9Y530	0.101751	0.091203	0.056275	OARD1	ADP-ribose	OARD1_HU	5	1.073075
48H	10MT/	Q13948	-0.08351	0.091207	0.056275	CUX1	Protein CA	CASP_HUM	3	0.943762
48H	10MT/	Q6VN20	-0.06571	0.09121	0.056275	RANBP10	Ran-bindin	RBP10_HUI	14	0.955472
48H	10MT/	Q9ULK4	0.013545	0.09121	0.056275	MED23	Mediator o	MED23_HU	28	1.009433
48H	10MT/	P31937	-0.02752	0.091218	0.056278	HIBADH	3-hydroxyi	3HIDH_HUI	12	0.981105
48H	10MT/	Q9NX07	0.060026	0.091227	0.056282	TRNAU1AP	tRNA selen	TSAP1_HUN	4	1.042484
48H	50MT/	Q9Y6A9	-0.01183	0.091243	0.05629	SPCS1	Signal pept	SPCS1_HUN	2	0.99183
48H	10MT/	Q96CS3	-0.09316	0.091248	0.056291	FAF2	FAS-associa	FAF2_HUM	18	0.93747
48H	50MT/	Q8NEB5	0.187454	0.091299	0.056316	PLPP5	Phospholip	PLPP5_HUI	1	1.138752
24H	10MT/	Q9H2J7	0.857928	0.091324	0.056327	SLC6A15	Sodium-de	S6A15_HUI	1	1.812433
24H	10MT/	Q9Y6K1	0.198146	0.091345	0.056339	DNMT3A	DNA (cytosi	DNM3A_HU	5	1.147223
48H	10MT/	Q5SGD2	-0.25974	0.091353	0.056343	PPM1L	Protein ph	PPM1L_HU	1	0.83524
24H	50MT/	Q8NCF5	-0.3003	0.091361	0.056347	NFATC2IP	NFATC2-int	NF2IP_HUN	5	0.812086
24H	10MT/	Q9BUA3	0.162555	0.09138	0.056356	SPINDOC	Spindlin in	SPNDC_HU	3	1.119268
48H	10MT/	A6ND36	-0.65351	0.091385	0.056357	FAM83G	Protein FAI	FA83G_HUI	1	0.635732
48H	50MT/	P62070	0.10263	0.091385	0.056357	RRAS2	Ras-related	RRAS2_HUI	7	1.073729
24H	10MT/	P09958	0.256094	0.091404	0.056364	FURIN	Furin	FURIN_HUN	7	1.194241

24H 10MT/	Q9NQC1	0.515198	0.091422	0.056374	JADE2	E3 ubiquiti	JADE2_HUI	1	1.42919
48H 50MT/	Q9UBW8	-0.07542	0.091435	0.056378	COPS7A	COP9 signa	CSN7A_HUI	10	0.949063
48H 10MT/	Q6ZS17	-0.18287	0.091451	0.056386	RIPOR1	Rho family-	RIPR1_HUN	7	0.88095
48H 10MT/	O60216	0.039901	0.091455	0.056387	RAD21	Double-stra	RAD21_HU	22	1.028044
24H 50MT/	Q9NR09	-0.09788	0.091457	0.056387	BIRC6	Baculoviral	BIRC6_HUN	65	0.934403
48H 50MT/	P23743	-0.13949	0.091483	0.056399	DGKA	Diacylglyce	DGKA_HUN	6	0.907841
48H 10MT/	O15119	-0.08272	0.091501	0.056407	TBX3	T-box trans	TBX3_HUM	5	0.944274
48H 50MT/	P07199	-0.19245	0.091529	0.05642	CENPB	Major centi	CENPB_HUI	5	0.87512
48H 50MT/	Q8N131	0.082921	0.09156	0.056433	TMEM123	Porimin	PORIM_HU	1	1.059161
24H 50MT/	Q9UN37	-0.10369	0.091572	0.056437	VPS4A	Vacuolar pı	VPS4A_HUI	13	0.930647
24H 50MT/	P09958	0.159318	0.091606	0.056453	FURIN	Furin	FURIN_HUN	7	1.116759
48H 10MT/	Q9Y618	0.367475	0.091632	0.056466	NCOR2	Nuclear rec	NCOR2_HU	7	1.290093
48H 50MT/	Q03113	-0.07579	0.091635	0.056467	GNA12	Guanine nu	GNA12_HU	5	0.94882
48H 10MT/	Q9Y2X9	0.068422	0.091641	0.056469	ZNF281	Zinc finger	ZN281_HUI	19	1.048569
24H 50MT/	Q9UHP3	-0.08903	0.091653	0.056475	USP25	Ubiquitin c	UBP25_HU	14	0.940155
48H 10MT/	Q8TBX8	-0.09923	0.091662	0.056478	PIP4K2C	Phosphatic	PI42C_HUN	17	0.93353
48H 10MT/	P07306	0.054417	0.091694	0.056493	ASGR1	Asialoglyco	ASGR1_HUI	14	1.03844
24H 50MT/	P24941	-0.16127	0.0917	0.056496	CDK2	Cyclin-depe	CDK2_HUN	17	0.894238
48H 10MT/	P04637	-0.26717	0.091704	0.056496	TP53	Cellular tur	P53_HUMA	3	0.830946
48H 10MT/	Q04724	-0.01901	0.091706	0.056497	TLE1	Transducin	TLE1_HUM.	7	0.986908
24H 10MT/	Q969Z0	0.048407	0.091725	0.056508	TBRG4	FAST kinase	FAKD4_HUI	19	1.034123
48H 50MT/	P11940	-0.05156	0.091755	0.056521	PABPC1	Polyadenyl	PABP1_HUI	36	0.96489
24H 10MT/	Q9BSU3	0.294457	0.091759	0.056523	NAA11	N-alpha-ace	NAA11_HU	2	1.226423
24H 50MT/	Q9P2K8	-0.08369	0.091783	0.056533	EIF2AK4	eIF-2-alpha	E2AK4_HUI	22	0.94364
24H 10MT/	O15417	-0.02858	0.091796	0.056536	TNRC18	Trinucleoti	TNC18_HUI	3	0.980382
48H 10MT/	Q14119	0.168523	0.091804	0.05654	VEZF1	Vascular en	VEZF1_HUN	4	1.123907
48H 10MT/	Q9Y2H6	0.003031	0.091841	0.056557	FNDC3A	Fibronectir	FND3A_HU	27	1.002103
48H 50MT/	Q92989	-0.24416	0.091866	0.056571	CLP1	Polyribonu	CLP1_HUM	12	0.844309
24H 10MT/	Q6UWE0	-0.09943	0.091873	0.056573	LRSAM1	E3 ubiquiti	LRSM1_HU	25	0.933402
48H 50MT/	O60826	0.038725	0.091881	0.056577	CCDC22	Coiled-coil	CCD22_HU	22	1.027206
24H 10MT/	E9PRG8	0.190232	0.091915	0.056596	C11orf98	Uncharacte	CK098_HUI	4	1.140947
24H 50MT/	Q9Y5T4	-0.3859	0.091931	0.056602	DNAJC15	DnaJ homo	DJC15_HUI	3	0.765302
24H 10MT/	Q969F9	-0.0408	0.09195	0.056611	HPS3	BLOC-2 con	HPS3_HUM	21	0.972115
48H 10MT/	O43847	-0.00084	0.091961	0.056613	NRDC	Nardilysin	NRDC_HUN	37	0.999421
48H 10MT/	P15291	-0.00799	0.091959	0.056613	B4GALT1	Beta-1,4-ga	B4GT1_HUI	8	0.994475
24H 10MT/	O75554	0.208955	0.091975	0.056619	WBP4	WW domai	WBP4_HUN	4	1.155851
48H 10MT/	Q9Y223	0.029258	0.091986	0.056623	GNE	Bifunctiona	GLCNE_HUI	24	1.020487
48H 50MT/	Q96BX8	0.655791	0.092002	0.056629	MOB3A	MOB kinase	MOB3A_HU	3	1.575479
24H 10MT/	P49756	0.001652	0.092028	0.056644	RBM25	RNA-bindir	RBM25_HU	27	1.001146
24H 10MT/	O75083	-0.0672	0.092099	0.056677	WDR1	WD repeat-	WDR1_HUI	28	0.95449
48H 50MT/	Q96S38	-0.12415	0.092115	0.056684	RPS6KC1	Ribosomal	KS6C1_HUI	4	0.917545
48H 10MT/	P49588	0.024807	0.092146	0.0567	AARS1	Alanine--tR	SYAC_HUM	44	1.017343
24H 50MT/	Q9UKX7	-0.02775	0.092155	0.056704	NUP50	Nuclear poı	NUP50_HU	18	0.980952
24H 10MT/	Q96EK4	-0.06972	0.092176	0.056713	THAP11	THAP doma	THA11_HUI	7	0.952823
24H 10MT/	Q8NET6	0.555556	0.092201	0.056725	CHST13	Carbohydra	CHSTD_HUI	5	1.469735
24H 50MT/	Q5VSL9	-0.14873	0.092244	0.056742	STRIP1	Striatin-int	STRP1_HUN	14	0.902041
24H 50MT/	O95857	0.074007	0.092254	0.056744	TSPAN13	Tetraspanir	TSN13_HUI	4	1.052637
48H 10MT/	P53701	-0.00643	0.092299	0.056764	HCCS	Holocytoch	CCHL_HUM	12	0.995553
24H 50MT/	Q16719	0.060198	0.092305	0.056766	KYNU	Kynurenina	KYNU_HUN	20	1.042609
48H 10MT/	P21399	0.035484	0.09232	0.056772	ACO1	Cytoplasmi	ACOHC_HU	41	1.0249
24H 10MT/	Q96EF6	-0.07361	0.092321	0.056772	FBXO17	F-box only	FBX17_HUI	4	0.950255
48H 10MT/	O94907	-0.03563	0.092333	0.056777	DKK1	Dickkopf-re	DKK1_HUN	2	0.975608

24H 50MT/	Q0D2I5;Q5	0.01231	0.09237	0.056796	IFFO1;IFFO	Non-homol	IFFO1_HUM	1	1.008569
24H 10MT/	Q9H4K7	-0.05624	0.092374	0.056797	MTG2	Mitochond	MTG2_HUM	5	0.96177
48H 10MT/	P49768	-0.24149	0.092463	0.056842	PSEN1	Presenilin-:	PSN1_HUM	4	0.845872
48H 10MT/	Q92766	0.059151	0.09246	0.056842	RREB1	Ras-respon:	RREB1_HUI	13	1.041853
24H 50MT/	Q9Y3E5	0.039675	0.092461	0.056842	PTRH2	Peptidyl-tR	PTH2_HUM	7	1.027882
24H 50MT/	P24534	0.144205	0.092468	0.056844	EEF1B2	Elongation	EF1B_HUM	10	1.105122
48H 50MT/	O15118	-0.10105	0.092472	0.056846	NPC1	NPC intrac	NPC1_HUM	16	0.932355
24H 50MT/	Q2TAZ0	-0.19999	0.092491	0.056855	ATG2A	Autophagy-	ATG2A_HUI	15	0.870559
24H 10MT/	Q9NV88	0.188823	0.092528	0.056874	INTS9	Integrator	INT9_HUM	7	1.139833
48H 50MT/	Q9UHA4	-0.11208	0.092543	0.056881	LAMTOR3	Ragulator c	LTOR3_HUI	5	0.925251
48H 10MT/	Q8TB37	-0.03953	0.092555	0.056886	NUBPL	Iron-sulfur	NUBPL_HU	6	0.972971
24H 50MT/	P34947	-0.15991	0.092564	0.056889	GRK5	G protein-c	GRK5_HUM	9	0.895083
24H 50MT/	Q8NFH3	-0.00309	0.092671	0.056944	NUP43	Nucleopori	NUP43_HU	11	0.997861
48H 50MT/	Q9P0U3	-0.22394	0.092704	0.05696	SENP1	Sentrin-spe	SENP1_HUI	4	0.856224
48H 10MT/	O75558	-0.12439	0.092724	0.05697	STX11	Syntaxin-1:	STX11_HUI	1	0.917389
48H 10MT/	Q9Y263	0.052662	0.092776	0.056995	PLAA	Phospholip	PLAP_HUM	26	1.037177
48H 10MT/	Q5TCZ1	-0.31331	0.092785	0.057	SH3PXD2A	SH3 and PX	SPD2A_HUI	14	0.804792
48H 10MT/	Q8NEB5	0.041704	0.09279	0.057002	PLPP5	Phospholip	PLPP5_HUI	1	1.029329
48H 10MT/	Q96EK4	0.023096	0.092797	0.057004	THAP11	THAP doma	THA11_HUI	7	1.016138
48H 50MT/	Q9Y2L9	-0.25125	0.092836	0.057019	LRCH1	Leucine-ric	LRCH1_HUI	5	0.840168
24H 10MT/	Q96DC7	-0.2094	0.092874	0.057038	TMCO6	Transmemb	TMCO6_HU	2	0.864895
24H 50MT/	O75496	-0.11736	0.092878	0.057039	GMNN	Geminin	GEMI_HUM	6	0.921876
24H 10MT/	Q9H6H4	0.061177	0.092877	0.057039	REEP4	Receptor e:	REEP4_HUI	4	1.043317
48H 10MT/	Q9P260	0.037425	0.092939	0.05707	RELCH	RAB11-bin	RELCH_HUI	31	1.026281
24H 10MT/	Q8WVM0	0.007352	0.092944	0.057072	TFB1M	Dimethylac	TFB1M_HU	15	1.005109
48H 50MT/	P10644	-0.00139	0.092974	0.057085	PRKAR1A	cAMP-depe	KAP0_HUM	21	0.999039
48H 50MT/	Q14203	-0.03159	0.092995	0.057094	DCTN1	Dynactin su	DCTN1_HU	52	0.978344
48H 10MT/	Q9P2W9	-0.09736	0.093027	0.057108	STX18	Syntaxin-1{	STX18_HUI	11	0.934739
24H 50MT/	O75150	-0.02883	0.093042	0.057115	RNF40	E3 ubiquiti	BRE1B_HUI	26	0.980212
24H 50MT/	P19387	0.039911	0.093044	0.057115	POLR2C	DNA-direct	RPB3_HUM	12	1.02805
24H 50MT/	Q8WX92	0.079921	0.093079	0.057134	NELFB	Negative el	NELFB_HUI	13	1.05696
48H 50MT/	A0A0B4J2A	0.129797	0.093083	0.057135	PPIAL4C	Peptidyl-pr	PAL4C_HUI	4	1.09414
24H 10MT/	Q00266	-0.12739	0.093118	0.057152	MAT1A	S-adenosyl	METK1_HU	3	0.915484
48H 10MT/	Q969Z0	-0.09124	0.093133	0.057159	TBRG4	FAST kinase	FAKD4_HUI	19	0.938715
48H 50MT/	Q96RL7	-0.28696	0.093136	0.05716	VPS13A	Intermemb	VP13A_HUI	7	0.819628
48H 50MT/	O95772	-0.05661	0.093143	0.057163	STARD3NL	STARD3 N-t	STR3N_HUI	3	0.961521
48H 50MT/	Q9BVC4	0.007	0.093149	0.057166	MLST8	Target of ra	LST8_HUM	7	1.004863
48H 50MT/	Q07020	-0.0288	0.093181	0.05718	RPL18	Large ribos	RL18_HUM	7	0.980236
48H 10MT/	Q9HC62	0.0223	0.093181	0.05718	SENP2	Sentrin-spe	SENP2_HUI	5	1.015577
48H 50MT/	Q92560	-0.41472	0.093188	0.057184	BAP1	Ubiquitin c	BAP1_HUM	4	0.750163
24H 10MT/	P30533	0.078022	0.093203	0.057189	LRPAP1	Alpha-2-ma	AMRP_HUM	14	1.05557
24H 50MT/	Q13530	0.295479	0.093203	0.057189	SERINC3	Serine inco	SERC3_HUI	4	1.227292
48H 10MT/	P24666	0.041552	0.093207	0.057189	ACP1	Low molec	PPAC_HUM	9	1.029221
48H 10MT/	P78371	-0.02368	0.093205	0.057189	CCT2	T-complex	TCPB_HUM	35	0.983723
48H 10MT/	Q8WWK9	0.01627	0.093273	0.057222	CKAP2	Cytoskelet	CKAP2_HUI	9	1.011342
48H 10MT/	Q6QNY0	-0.12777	0.093278	0.057223	BLOC1S3	Biogenesis	BL1S3_HUI	7	0.915242
24H 10MT/	Q9BTA9	-0.03902	0.093278	0.057223	WAC	WW domai	WAC_HUM	10	0.973319
24H 10MT/	Q9Y296	0.063587	0.093295	0.057231	TRAPPC4	Trafficking	TPPC4_HUI	13	1.045061
24H 50MT/	Q9HAF1	-0.21599	0.093322	0.057244	MEAF6	Chromatin	EAF6_HUM	2	0.860957
48H 10MT/	P40429	0.017945	0.093328	0.057247	RPL13A	Large ribos	RL13A_HUI	10	1.012516
24H 50MT/	P62424	0.066288	0.093398	0.057282	RPL7A	Large ribos	RL7A_HUM	20	1.047019
48H 50MT/	P23025	0.12747	0.09342	0.057294	XPA	DNA repair	XPA_HUM	6	1.092376

48H 50MT/	P62306	-0.01544	0.093434	0.0573	SNRPF	Small nucle	RUXF_HUM	7	0.989353
24H 10MT/	Q9NWU5	0.049122	0.093439	0.057301	MRPL22	Large ribos	RM22_HUM	8	1.034635
48H 50MT/	Q9Y316	-0.17045	0.093438	0.057301	MEMO1	Protein ME	MEMO1_HU	14	0.888564
24H 50MT/	P78549	0.21812	0.093477	0.057317	NTHL1	Endonuclea	NTH_HUMA	2	1.163216
48H 10MT/	Q8WUJ3	0.298654	0.093513	0.057337	CEMIP	Cell migrati	CEMIP_HUI	5	1.229997
48H 10MT/	P46100	-0.02146	0.09354	0.057351	ATRX	Transcripti	ATRX_HUM	44	0.985233
48H 50MT/	Q8WYH8	-0.51393	0.093555	0.057359	ING5	Inhibitor of	ING5_HUM	2	0.700313
48H 10MT/	Q8WYP5	0.01071	0.093567	0.057364	AHCTF1	Protein ELY	ELYS_HUMA	52	1.007451
48H 10MT/	Q9H9G7	0.033904	0.093571	0.057365	ago-03	Protein arg	AGO3_HUM	10	1.023778
24H 10MT/	O94964	-0.05638	0.093587	0.057373	MTCL2	Microtubul	MTCL2_HU	9	0.961675
24H 10MT/	P31943	0.030386	0.093615	0.057384	HNRNPH1	Heterogene	HNRH1_HU	18	1.021285
48H 50MT/	Q9HAC8	-0.00689	0.093659	0.057407	UBTD1	Ubiquitin d	UBTD1_HU	3	0.995235
24H 10MT/	O95155	-0.02186	0.093672	0.057413	UBE4B	Ubiquitin c	UBE4B_HUI	37	0.984963
24H 50MT/	Q9UBI1	-0.14579	0.093688	0.057421	COMMD3	COMM don	COMD3_HU	7	0.903881
48H 10MT/	Q9H967	-0.13005	0.093695	0.057424	WDR76	WD repeat-	WDR76_HU	10	0.913798
48H 10MT/	P30419	-0.00128	0.093703	0.057426	NMT1	Glycylpepti	NMT1_HUM	16	0.999112
24H 10MT/	Q9H1C7	0.142496	0.093753	0.05745	CYSTM1	Cysteine-ric	CYTM1_HU	1	1.103813
24H 50MT/	O14646	0.103115	0.093765	0.057455	CHD1	Chromodo	CHD1_HUM	20	1.07409
48H 50MT/	Q9NQX3	0.099737	0.093769	0.057456	GPHN	Gephyrin	GEPH_HUM	22	1.071578
24H 50MT/	Q9BU02	-0.0123	0.093776	0.057459	THTPA	Thiamine-tr	THTPA_HUI	5	0.991512
48H 10MT/	Q13613	0.04772	0.093798	0.05747	MTMR1	Myotubula	MTMR1_HU	10	1.03363
24H 10MT/	Q13505	-0.11443	0.09385	0.057497	MTX1	Metaxin-1	MTX1_HUM	11	0.923748
24H 50MT/	P35443	-0.59373	0.093866	0.057504	THBS4	Thrombosp	TSP4_HUM	2	0.662628
24H 10MT/	Q8NBI6	-0.11968	0.093872	0.057507	XXYLT1	Xyloside xy	XXLT1_HUM	8	0.920395
48H 10MT/	Q4G0N4	-0.01014	0.093894	0.057519	NADK2	NAD kinase	NAKD2_HU	18	0.992997
24H 10MT/	Q8N5A5	0.099001	0.093907	0.057524	ZGPAT	Zinc finger	ZGPAT_HUI	9	1.071031
24H 50MT/	Q9Y227	-0.70647	0.093934	0.057534	ENTPD4	Ectonucleo	ENTP4_HUI	1	0.61282
24H 50MT/	Q15397	-0.04634	0.093955	0.057545	PUM3	Pumilio ho	PUM3_HUM	24	0.968388
24H 50MT/	Q96RT8	0.109791	0.093983	0.057558	TUBGCP5	Gamma-tul	GCP5_HUM	3	1.079072
24H 10MT/	Q02224	0.091185	0.093986	0.05756	CENPE	Centromer	CENPE_HUI	13	1.065245
24H 50MT/	O60231	0.064985	0.094005	0.057569	DHX16	Pre-mRNA-	DHX16_HU	28	1.046074
48H 10MT/	O43920	0.115005	0.094014	0.057572	NDUFS5	NADH dehy	NDUS5_HU	4	1.082979
24H 10MT/	Q6P158	0.015458	0.094032	0.057582	DHX57	Putative AT	DHX57_HU	39	1.010773
48H 10MT/	O95365	0.096077	0.094051	0.057591	ZBTB7A	Zinc finger	ZBT7A_HUM	4	1.068863
48H 50MT/	P42694	0.069186	0.094055	0.057593	HELZ	Probable h	HELZ_HUM	13	1.049124
48H 10MT/	Q96PU4	0.068427	0.094094	0.057612	UHRF2	E3 ubiquiti	UHRF2_HU	1	1.048573
48H 50MT/	Q5SRI9	0.152128	0.094098	0.057613	MANEA	Glycoprote	MANEA_HU	2	1.111207
24H 10MT/	Q96LB3	-0.07789	0.094153	0.057639	IFT74	Intraflagell	IFT74_HUM	9	0.94744
48H 50MT/	Q8NF91	0.432804	0.094191	0.057657	SYNE1	Nesprin-1	SYNE1_HUM	3	1.349854
48H 10MT/	Q6UXV4	0.014363	0.094194	0.057658	APOOL	MICOS com	MIC27_HUI	12	1.010005
24H 50MT/	Q5JTH9	-0.06077	0.094227	0.057673	RRP12	RRP12-like	RRP12_HUI	45	0.958753
24H 10MT/	Q6NYC8	0.087259	0.094275	0.057697	PPP1R18	Phostensin	PPR18_HUI	8	1.06235
48H 10MT/	Q9UHG3	0.044885	0.094334	0.057728	PCYOX1	Prenylcyste	PCYOX_HUI	16	1.031601
24H 50MT/	Q9NVU7	-0.03895	0.09434	0.057731	SDAD1	Protein SDA	SDA1_HUM	14	0.973365
24H 10MT/	Q12986	0.041861	0.094363	0.057742	NFX1	Transcripti	NFX1_HUM	18	1.029441
24H 50MT/	Q9BZH6	-0.06851	0.09439	0.057757	WDR11	WD repeat-	WDR11_HU	32	0.95362
48H 50MT/	Q6Y1H2	-0.23871	0.094397	0.057758	HACD2	Very-long-c	HACD2_HU	4	0.847505
48H 10MT/	Q9H2J4	0.086016	0.094397	0.057758	PDCL3	Phosducin-	PDCL3_HUI	15	1.061435
48H 50MT/	Q8NCE2	-0.11337	0.094412	0.057766	MTMR14	Myotubula	MTMRE_HU	10	0.924427
48H 10MT/	Q14524	-0.08802	0.094423	0.057771	SCN5A	Sodium cha	SCN5A_HUI	1	0.94081
48H 50MT/	Q8N5N7	-0.22325	0.094429	0.057774	MRPL50	Large ribos	RM50_HUM	7	0.856634
48H 10MT/	Q99676	-0.21775	0.094448	0.057785	ZNF184	Zinc finger	ZN184_HUI	1	0.859903

24H 50MT/	Q9NXF1	0.077782	0.094461	0.05779	TEX10	Testis-expre	TEX10_HUI	29	1.055394
48H 50MT/	Q6P1N0	-0.12735	0.0945	0.057813	CC2D1A	Coiled-coil	C2D1A_HU	21	0.915514
48H 50MT/	Q92542	-0.09361	0.094502	0.057813	NCSTN	Nicastrin	NICA_HUM	12	0.937178
48H 10MT/	Q9UQ16	0.061863	0.09453	0.057826	DNM3	Dynamin-3	DYN3_HUM	2	1.043812
24H 50MT/	Q7Z4F1	0.484673	0.09454	0.05783	LRP10	Low-densit	LRP10_HUI	4	1.399268
48H 10MT/	Q92871	0.335403	0.094573	0.057846	PMM1	Phosphoma	PMM1_HUI	3	1.26173
24H 10MT/	Q69YN2	0.021161	0.094604	0.057862	CWF19L1	CWF19-like	C19L1_HUI	25	1.014776
48H 10MT/	Q8TER5	0.022543	0.094614	0.057867	ARHGEF40	Rho guanin	ARH40_HU	15	1.015749
24H 10MT/	O14832	0.291378	0.094627	0.057873	PHYH	Phytanoyl-(	PAHX_HUM	9	1.223809
48H 10MT/	Q8TAD8	0.018284	0.094653	0.057885	SNIP1	Smad nucle	SNIP1_HUM	5	1.012754
48H 10MT/	Q9BVA1	-0.01298	0.094677	0.057897	TUBB2B	Tubulin bet	TBB2B_HUI	1	0.991042
48H 10MT/	Q96EM0	0.02896	0.094681	0.057898	L3HYPDH	Trans-3-hyc	T3HPD_HU	7	1.020277
48H 10MT/	Q9BXI6	0.088305	0.094685	0.057899	TBC1D10A	TBC1 doma	TB10A_HUI	12	1.06312
24H 10MT/	Q9C0H2	-0.13	0.0947	0.057905	TTYH3	Protein twe	TTYH3_HUI	1	0.913833
24H 50MT/	P34059	0.045619	0.094727	0.05792	GALNS	N-acetylgal	GALNS_HUI	6	1.032126
24H 10MT/	P13284	-0.17275	0.094733	0.057921	IFI30	Gamma-int	GILT_HUM/	3	0.887149
48H 10MT/	Q15051	0.666112	0.094739	0.057924	IQCB1	IQ calmodu	IQCB1_HUI	2	1.586791
48H 10MT/	P02686	0.220167	0.094747	0.057927	MBP	Myelin basi	MBP_HUM/	2	1.164869
48H 10MT/	Q8IUX1	-0.09162	0.094759	0.057933	TMEM126E	Complex I a	T126B_HUI	5	0.938469
48H 10MT/	P33552	-0.01536	0.09478	0.057942	CKS2	Cyclin-depe	CKS2_HUM	2	0.989407
48H 10MT/	C4AMC7;Q	0.051842	0.09479	0.057945	WASH3P;W	Putative W	WASH3_HU	11	1.036588
48H 50MT/	P60981	-0.10968	0.0948	0.057949	DSTN	Destrin	DEST_HUM	11	0.926793
24H 50MT/	P62879	0.067073	0.094799	0.057949	GNB2	Guanine nu	GBB2_HUM	8	1.047589
24H 10MT/	P06733	-0.12203	0.094806	0.057952	ENO1	Alpha-enol	ENOA_HUM	28	0.918892
24H 50MT/	Q15633	0.007332	0.094813	0.057953	TARBP2	RISC-loadir	TRBP2_HUI	6	1.005095
24H 10MT/	Q8N6M0	0.045268	0.094812	0.057953	OTUD6B	Deubiquitii	OTU6B_HU	8	1.031875
24H 10MT/	Q8WXB1	0.02036	0.094848	0.057972	METTL21A	Protein N-l	MT21A_HU	3	1.014213
24H 10MT/	P49590	0.080079	0.094854	0.057974	HARS2	Histidine--t	SYHM_HUM	15	1.057076
24H 10MT/	P55884	0.005371	0.094854	0.057974	EIF3B	Eukaryotic	EIF3B_HUM	47	1.003729
48H 50MT/	Q4G0J3	-0.03006	0.094866	0.057979	LARP7	La-related p	LARP7_HUI	17	0.979379
48H 10MT/	Q9UJ41	-0.0057	0.0949	0.057997	RABGEF1	Rab5 GDP/(	RABX5_HUI	11	0.996054
48H 10MT/	P62899	0.040744	0.094906	0.058	RPL31	Large ribos	RL31_HUM	5	1.028644
24H 50MT/	Q9NUJ7	-0.25758	0.094929	0.058012	PLCXD1	PI-PLC X do	PLCX1_HUI	4	0.836492
24H 10MT/	A4D1E9	-0.05949	0.094939	0.058015	GTPBP10	GTP-bindin	GTPBA_HUI	12	0.959604
24H 10MT/	Q16563	0.163778	0.094941	0.058016	SYPL1	Synaptophy	SYPL1_HUM	4	1.120217
48H 50MT/	Q8WUB8	-0.12245	0.094947	0.058018	PHF10	PHD finger	PHF10_HUI	6	0.918625
48H 50MT/	Q9H5H4	0.022641	0.094954	0.058021	ZNF768	Zinc finger	ZN768_HUI	8	1.015818
48H 10MT/	Q9NXA8	-0.04863	0.094963	0.058026	SIRT5	NAD-depen	SIR5_HUM/	8	0.966851
24H 50MT/	Q02952	0.026027	0.094977	0.058031	AKAP12	A-kinase an	AKA12_HUI	79	1.018204
24H 50MT/	Q15121	-0.39743	0.094977	0.058031	PEA15	Astrocytic p	PEA15_HUI	6	0.759208
24H 10MT/	P56182	-0.21167	0.094993	0.058037	RRP1	Ribosomal	RRP1_HUM	13	0.863537
48H 10MT/	Q9C0H2	-0.24554	0.094995	0.058038	TTYH3	Protein twe	TTYH3_HUI	1	0.843497
24H 50MT/	Q96HR9	0.130232	0.095006	0.058044	REEP6	Receptor e	REEP6_HUI	6	1.09447
48H 50MT/	Q8IWZ8	-0.13857	0.095037	0.058058	SUGP1	SURP and G	SUGP1_HU	20	0.90842
48H 10MT/	Q6NUM9	-0.08335	0.095044	0.058061	RETSAT	All-trans-re	RETST_HUM	15	0.943863
48H 10MT/	O15392	-0.52662	0.095054	0.058066	BIRC5	Baculoviral	BIRC5_HUM	2	0.694181
48H 50MT/	Q5SGD2	-0.27546	0.095083	0.05808	PPM1L	Protein pho	PPM1L_HU	1	0.826187
24H 10MT/	P14635	0.087921	0.095086	0.058081	CCNB1	G2/mitotic	CCNB1_HU	10	1.062837
48H 50MT/	Q9Y5W9	0.605857	0.095113	0.058094	SNX11	Sorting nex	SNX11_HUI	1	1.521883
24H 50MT/	P57678	0.038513	0.095134	0.058105	GEMIN4	Gem-associ	GEMI4_HUI	26	1.027055
24H 10MT/	P61962	-0.19698	0.095199	0.058133	DCAF7	DDB1- and	DCAF7_HUI	8	0.872372
24H 10MT/	Q8WVZ9	-0.10582	0.095198	0.058133	KBTBD7	Kelch repea	KBTB7_HUI	1	0.929276

48H 50MT/	Q08AE8	0.325608	0.095216	0.058137	SPIRE1	Protein spi	SPIR1_HUM	5	1.253192
24H 50MT/	Q12830	0.067573	0.095216	0.058137	BPTF	Nucleosom	BPTF_HUM	26	1.047952
24H 50MT/	Q15493	-0.02363	0.095214	0.058137	RGN	Regucalcin	RGN_HUM/	7	0.983755
48H 50MT/	Q9UHD9	0.082672	0.095214	0.058137	UBQLN2	Ubiquilin-2	UBQL2_HU	10	1.058977
48H 10MT/	P25787	-0.05519	0.095262	0.058158	PSMA2	Proteasom	PSA2_HUM	14	0.962467
48H 10MT/	O95999	0.071544	0.095267	0.058158	BCL10	B-cell lymp	BCL10_HUI	5	1.050841
48H 10MT/	Q9NWX5	0.072562	0.095268	0.058158	ASB6	Ankyrin rep	ASB6_HUM	2	1.051583
48H 50MT/	Q9BR76	0.009878	0.095305	0.058176	CORO1B	Coronin-1E	COR1B_HU	17	1.006871
24H 50MT/	Q9NWR8	0.20925	0.095311	0.058178	MCUB	Calcium un	MCUB_HUI	4	1.156087
24H 10MT/	Q96T88	0.071407	0.095316	0.05818	UHRF1	E3 ubiquiti	UHRF1_HU	31	1.050741
48H 10MT/	Q9Y5Y0	-0.14716	0.095317	0.05818	FLVCR1	Heme trans	FLVC1_HUI	4	0.903029
48H 50MT/	Q96J42	-0.11435	0.095334	0.058189	TXNDC15	Thioredoxi	TXD15_HUI	5	0.923799
48H 10MT/	Q53EZ4	-0.09136	0.09536	0.058201	CEP55	Centrosom	CEP55_HUI	13	0.938639
24H 10MT/	A6NHL2	0.737824	0.095374	0.058209	TUBAL3	Tubulin alp	TBAL3_HUI	2	1.667659
48H 50MT/	Q13434;Q	-0.65969	0.095412	0.058225	MKRN4P;M	Putative E3	MKRN4_HL	1	0.633016
24H 50MT/	Q96EV2	0.057283	0.095429	0.058233	RBM33	RNA-bindir	RBM33_HL	11	1.040505
48H 50MT/	O14548	0.159047	0.095509	0.058275	COX7A2L	Cytochrom	COX7R_HU	4	1.116549
24H 10MT/	O75175	0.017077	0.095525	0.05828	CNOT3	CCR4-NOT t	CNOT3_HU	8	1.011907
24H 50MT/	O95155	-0.09658	0.095522	0.05828	UBE4B	Ubiquitin c	UBE4B_HUI	37	0.935247
24H 50MT/	Q0P6H9	-0.48827	0.09554	0.058286	TMEM62	Transmemb	TMM62_HL	3	0.712877
24H 50MT/	Q14667	0.225423	0.095543	0.058287	BLTP2	Bridge-like	BLTP2_HUI	2	1.16912
48H 10MT/	Q99538	0.12537	0.095555	0.05829	LGMN	Legumain	LGMN_HUI	6	1.090787
24H 10MT/	Q96HQ2	-0.58507	0.095596	0.058307	CDKN2AIPN	CDKN2AIP I	C2AIL_HUN	2	0.666618
48H 50MT/	Q9H3R0	-0.978	0.0956	0.058307	KDM4C	Lysine-spec	KDM4C_HL	2	0.507684
24H 50MT/	Q9NRR3	0.250759	0.095607	0.05831	CDC42SE2	CDC42 sma	C42S2_HUI	1	1.189833
48H 10MT/	Q5T7M9	0.357696	0.095676	0.058347	DIPK1A	Divergent p	DIK1A_HUI	6	1.281378
48H 50MT/	Q9GZR1	-0.16412	0.095681	0.058348	SENP6	Sentrin-spe	SENP6_HUI	6	0.892472
24H 10MT/	Q5JUR7	0.127368	0.095732	0.05837	TEX30	Testis-expr	TEX30_HUI	7	1.092299
24H 10MT/	Q99717	1.310947	0.095758	0.058384	SMAD5	Mothers ag	SMAD5_HL	2	2.481043
48H 10MT/	P49407	-0.11057	0.095763	0.058386	ARRB1	Beta-arresti	ARRB1_HUI	13	0.926224
24H 50MT/	O94766	-0.08039	0.095768	0.058388	B3GAT3	Galactosylg	B3GA3_HU	8	0.9458
48H 10MT/	P30049	-0.01272	0.095802	0.058403	ATP5F1D	ATP syntha	ATPD_HUM	5	0.991221
48H 10MT/	Q9H2A7	0.302838	0.095808	0.058405	CXCL16	C-X-C motif	CXL16_HUI	2	1.233568
48H 10MT/	Q27J81	0.03658	0.095835	0.058419	INF2	Inverted fo	INF2_HUM/	25	1.02568
24H 10MT/	Q8WXH0	0.061637	0.095845	0.058424	SYNE2	Nesprin-2	SYNE2_HUI	22	1.043649
48H 50MT/	Q9BVQ7	-0.19805	0.095926	0.058466	AFG2B	ATPase fam	AFG2B_HUI	21	0.871728
24H 50MT/	Q9NWX6	-0.04682	0.095939	0.058471	THG1L	Probable tF	THG1_HUM	5	0.968067
48H 10MT/	Q8NDX5	0.131593	0.095948	0.058475	PHC3	Polyhomec	PHC3_HUM	9	1.095502
24H 50MT/	P61586	-0.01768	0.095952	0.058476	RHOA	Transformi	RHOA_HUM	3	0.987822
24H 50MT/	P26639	-0.06901	0.095969	0.058484	TARS1	Threonine--	SYTC_HUM/	37	0.953291
24H 50MT/	Q8N5U6	-0.23532	0.095983	0.05849	RNF10	E3 ubiquiti	RNF10_HUI	8	0.849499
48H 50MT/	Q9H4M9	0.040866	0.095988	0.058491	EHD1	EH domain-	EHD1_HUM	24	1.028731
48H 10MT/	P53004	-0.09061	0.096032	0.058513	BLVRA	Biliverdin r	BIEA_HUM/	15	0.939123
24H 50MT/	Q9UI09	0.107322	0.096057	0.058524	NDUFA12	NADH dehy	NDUAC_HU	9	1.077226
24H 10MT/	Q9HCG8	0.094571	0.096067	0.058528	CWC22	Pre-mRNA-s	CWC22_HU	16	1.067748
24H 50MT/	Q9BSQ5	-0.23734	0.096069	0.058528	CCM2	Cerebral ca	CCM2_HUM	3	0.848306
48H 50MT/	Q99614	0.049878	0.096107	0.058547	TTC1	Tetratricop	TTC1_HUM	13	1.035177
48H 10MT/	P00403	-0.02834	0.096114	0.05855	MT-CO2	Cytochrom	COX2_HUM	7	0.980546
48H 10MT/	P19256	0.130854	0.096158	0.058564	CD58	Lymphocyt	LFA3_HUM	2	1.094941
48H 50MT/	O43929	-0.15934	0.096175	0.05857	ORC4	Origin reco	ORC4_HUM	17	0.895437
48H 10MT/	Q9UN30	0.049761	0.096185	0.058575	SCML1	Sex comb o	SCML1_HU	3	1.035094
24H 50MT/	P10606	-0.2028	0.096197	0.05858	COX5B	Cytochrom	COX5B_HU	6	0.868861

24H 50MT/	Q5JS37	0.004001	0.096199	0.05858	NHLRC3	NHL repeat	NHLC3_HU	5	1.002777
48H 10MT/	Q04837	-0.01217	0.096205	0.058582	SSBP1	Single-strar	SSBP_HUM	8	0.991602
24H 10MT/	Q9H6S0	0.013102	0.096217	0.058585	YTHDC2	3'-5' RNA he	YTDC2_HUI	37	1.009123
24H 10MT/	Q8N5L8	-0.23548	0.096236	0.058592	RPP25L	Ribonuclea	RP25L_HUI	1	0.8494
48H 10MT/	Q9P2N5	0.011646	0.096303	0.058625	RBM27	RNA-bindir	RBM27_HU	25	1.008105
48H 10MT/	Q8NB90	-0.03044	0.09635	0.058648	AFG2A	ATPase fam	AFG2A_HUI	21	0.979121
24H 50MT/	Q9HCU9	0.195481	0.096349	0.058648	BRMS1	Breast canc	BRMS1_HU	4	1.145106
24H 10MT/	Q8IWB7	0.165789	0.096352	0.058648	WDFY1	WD repeat	WDFY1_HU	15	1.121779
48H 50MT/	Q9UGI8	0.066235	0.096354	0.058649	TES	Testin	TES_HUMAI	22	1.046981
48H 50MT/	Q8NC51	-0.11772	0.096396	0.058667	SERBP1	SERPINE1 n	SERB1_HUI	21	0.92164
24H 10MT/	Q8IZ73	0.062596	0.096419	0.058675	RPUSD2	Pseudourid	RUSD2_HU	12	1.044343
24H 50MT/	Q9BRT9	0.066439	0.096433	0.05868	GINS4	DNA replica	SLD5_HUM	9	1.047129
48H 10MT/	P04899	-0.07053	0.096463	0.058697	GNAI2	Guanine nu	GNAI2_HUI	13	0.952289
48H 50MT/	Q9H799	0.156091	0.096479	0.058705	CPLANE1	Ciliogenesi	CPLN1_HU	1	1.114264
48H 10MT/	Q9H4H8	0.00207	0.096485	0.058706	FAM83D	Protein FAI	FA83D_HUI	12	1.001436
24H 10MT/	Q6P1K1	-0.00068	0.09654	0.058734	SLC48A1	Heme trans	HRG1_HUN	1	0.999527
48H 10MT/	P62633	0.042967	0.096545	0.058735	CNBP	CCHC-type	CNBP_HUM	6	1.03023
48H 10MT/	Q14137	-0.06579	0.09656	0.058741	BOP1	Ribosome b	BOP1_HUM	26	0.955419
24H 10MT/	Q2QL34	-0.00089	0.096558	0.058741	MPV17L	Mpv17-like	MP17L_HU	4	0.999385
24H 10MT/	P33908	0.060944	0.096595	0.058756	MAN1A1	Mannosyl-c	MA1A1_HU	16	1.043148
24H 50MT/	Q9Y2Y1	-0.14796	0.096616	0.058768	POLR3K	DNA-direct	RPC10_HUI	3	0.902527
48H 10MT/	Q99618	0.075619	0.096635	0.058779	CDCA3	Cell divisio	CDCA3_HU	3	1.053813
48H 10MT/	Q8WXA9	-0.11305	0.096661	0.05879	SREK1	Splicing reg	SREK1_HUI	3	0.924632
24H 50MT/	O43353	-0.15792	0.096667	0.058791	RIPK2	Receptor-ir	RIPK2_HUN	12	0.896319
24H 10MT/	Q96GM5	0.063386	0.096668	0.058791	SMARCD1	SWI/SNF-re	SMRD1_HU	17	1.044915
24H 50MT/	A6NCE7;Q9	-0.37517	0.096731	0.058825	MAP1LC3B	Microtubul	MP3B2_HU	1	0.771012
48H 50MT/	Q96EL3	-0.21889	0.096739	0.058829	MRPL53	Large ribos	RM53_HUN	5	0.859225
48H 10MT/	O75694	-0.0745	0.096749	0.058832	NUP155	Nuclear po	NU155_HU	46	0.949671
24H 10MT/	Q9BSG0	-0.16459	0.096757	0.058836	PRADC1	Protease-as	PADC1_HU	1	0.89218
24H 50MT/	P08236	0.065966	0.096792	0.058852	GUSB	Beta-glucur	BGLR_HUM	6	1.046786
24H 50MT/	Q12894	-0.37148	0.096833	0.05887	IFRD2	Interferon-i	IFRD2_HUN	7	0.772989
48H 10MT/	Q9NXV2	0.026823	0.09683	0.05887	KCTD5	BTB/POZ dc	KCTD5_HUI	5	1.018766
24H 50MT/	B2RXH8	-0.15022	0.096863	0.058884	HNRNPCL2	Heterogene	HNRC2_HU	1	0.901111
48H 10MT/	Q9Y294	0.059791	0.09691	0.058907	ASF1A	Histone cha	ASF1A_HUI	5	1.042315
48H 10MT/	O43447	0.027538	0.096939	0.058922	PPIH	Peptidyl-pr	PPIH_HUM	8	1.019272
48H 50MT/	Q9H3N1	-0.07129	0.096988	0.058947	TMX1	Thioredoxi	TMX1_HUN	14	0.951784
24H 10MT/	P15374	-0.15463	0.096996	0.058951	UCHL3	Ubiquitin c	UCHL3_HU	11	0.898363
48H 50MT/	O14497	0.051384	0.097007	0.058955	ARID1A	AT-rich inte	ARI1A_HUN	34	1.036258
48H 10MT/	P16930	0.016694	0.097047	0.058973	FAH	Fumarylace	FAAA_HUM	16	1.011638
24H 10MT/	P0C024	-0.62518	0.097065	0.058982	NUDT7	Peroxisoma	NUDT7_HU	1	0.648337
24H 50MT/	Q9NQ50	-0.1358	0.097079	0.05899	MRPL40	Large ribos	RM40_HUN	7	0.910167
48H 50MT/	O95299	-0.07182	0.097081	0.05899	NDUFA10	NADH dehy	NDUAA_HU	12	0.951439
48H 10MT/	P10620	-0.10902	0.097132	0.059016	MGST1	Microsoma	MGST1_HU	4	0.92722
48H 50MT/	Q13367	-0.0091	0.09714	0.05902	AP3B2	AP-3 compl	AP3B2_HUI	1	0.99371
48H 10MT/	O75197	-0.15876	0.097245	0.059069	LRP5	Low-densit	LRP5_HUM	2	0.895795
24H 10MT/	Q6ZNB6	-0.10463	0.09729	0.059091	NFXL1	NF-X1-type	NFXL1_HUI	18	0.930041
48H 10MT/	Q15819	0.149409	0.097294	0.059091	UBE2V2	Ubiquitin-c	UB2V2_HU	5	1.109115
48H 10MT/	Q06587	0.13048	0.097297	0.059092	RING1	E3 ubiquiti	RING1_HUI	8	1.094658
48H 50MT/	Q5TBB1	0.016528	0.097331	0.05911	RNASEH2B	Ribonuclea	RNH2B_HU	7	1.011523
48H 50MT/	Q5TFE4	-0.0007	0.097354	0.05912	NT5DC1	5'-nucleoti	NT5D1_HU	18	0.999515
24H 10MT/	Q9Y696	0.112631	0.09737	0.059127	CLIC4	Chloride in	CLIC4_HUN	17	1.081198
24H 50MT/	Q86UW6	-0.08474	0.097382	0.05913	N4BP2	NEDD4-bin	N4BP2_HU	3	0.942955

24H 50MTA	Q9BQC3	-0.02695	0.097389	0.059133	DPH2	2-(3-amino	DPH2_HUM	13	0.981492
24H 50MTA	Q6P3X3	-0.05907	0.097392	0.059134	TTC27	Tetratricop	TTC27_HUM	21	0.959882
48H 50MTA	O15127	-0.03325	0.097407	0.059141	SCAMP2	Secretory c	SCAM2_HU	3	0.977216
48H 50MTA	Q6QNY1	-0.37348	0.097419	0.059147	BLOC1S2	Biogenesis	BL1S2_HUM	2	0.771918
48H 10MTA	Q9BXK1	0.061037	0.097438	0.059156	KLF16	Krueppel-li	KLF16_HUM	2	1.043216
24H 10MTA	A8MWL7;C	0.62213	0.09745	0.05916	TMEM14DP	Transmemb	TM14D_HU	1	1.539146
24H 10MTA	Q9NS91	0.079912	0.097456	0.059162	RAD18	E3 ubiquiti	RAD18_HU	15	1.056954
24H 50MTA	Q969V5	-0.05971	0.097462	0.059165	MUL1	Mitochond	MUL1_HUM	2	0.959459
48H 50MTA	Q5T160	-0.11639	0.097473	0.059171	RARS2	Probable ar	SYRM_HUM	21	0.922494
24H 50MTA	Q5U3C3	0.485267	0.097524	0.059192	TMEM164	Transmemb	TM164_HU	1	1.399844
48H 10MTA	Q9H4B0	-0.08351	0.097539	0.059201	OSGEPL1	tRNA N6-ad	OSGL1_HUM	5	0.943761
24H 50MTA	Q99590	-0.02996	0.097542	0.059201	SCAF11	Protein SCA	SCAFB_HUM	22	0.979448
48H 50MTA	O00299	0.054108	0.097548	0.059203	CLIC1	Chloride in	CLIC1_HUM	15	1.038217
48H 50MTA	Q8NB16	0.094017	0.097549	0.059203	MLKL	Mixed linea	MLKL_HUM	11	1.067338
24H 10MTA	P24385	0.198471	0.097586	0.059219	CCND1	G1/S-specif	CCND1_HU	5	1.147481
24H 10MTA	P09110	-0.14132	0.097593	0.059221	ACAA1	3-ketoacyl-	THIK_HUMA	18	0.90669
24H 10MTA	P20248	0.067471	0.097595	0.059221	CCNA2	Cyclin-A2	CCNA2_HU	10	1.047878
24H 10MTA	Q96PE3	-0.06263	0.097592	0.059221	INPP4A	Inositol pol	INP4A_HUM	6	0.957515
24H 50MTA	Q969Z3	0.106734	0.097612	0.059231	MTARC2	Mitochond	MARC2_HU	4	1.076788
48H 50MTA	Q66LE6	-0.01406	0.097617	0.059232	PPP2R2D	Serine/thre	2ABD_HUM	3	0.990303
48H 10MTA	Q08431	0.120477	0.097665	0.059254	MFGE8	Lactadherin	MFGM_HUM	4	1.087094
48H 10MTA	P10114	-0.15716	0.09778	0.059314	RAP2A	Ras-related	RAP2A_HU	2	0.896787
48H 50MTA	Q9NVM9	0.11043	0.09778	0.059314	INTS13	Integrator	INT13_HUM	16	1.07955
24H 50MTA	Q9NV92	-0.04213	0.097786	0.059316	NDFIP2	NEDD4 fam	NFIP2_HUM	2	0.971222
24H 10MTA	Q96T51	0.023306	0.097788	0.059316	RUFY1	RUN and FY	RUFY1_HUM	20	1.016286
24H 50MTA	Q96HE9	-0.59884	0.097811	0.059324	PRR11	Proline-rich	PRR11_HUM	2	0.660285
24H 50MTA	Q8NB90	0.004598	0.097869	0.059351	AFG2A	ATPase fam	AFG2A_HUM	21	1.003192
48H 50MTA	Q9HAB8	0.034754	0.097881	0.059355	PPCS	Phosphopa	PPCS_HUM	15	1.024382
48H 50MTA	Q6BDS2	-0.30016	0.097945	0.059386	BLTP3A	Bridge-like	BLT3A_HUM	6	0.812161
24H 50MTA	Q9H2J4	-0.18902	0.097994	0.059415	PDCL3	Phosducin-	PDCL3_HUM	15	0.877198
24H 10MTA	Q14687	-0.09549	0.09803	0.059431	GSE1	Genetic sup	GSE1_HUM	5	0.935952
24H 50MTA	O75179	0.003306	0.098036	0.059432	ANKRD17	Ankyrin rep	ANR17_HU	22	1.002294
48H 10MTA	P49005	0.069819	0.098036	0.059432	POLD2	DNA polym	DPOD2_HU	16	1.049585
24H 10MTA	Q13153	-0.15192	0.098076	0.059452	PAK1	Serine/thre	PAK1_HUM	13	0.900051
48H 10MTA	P19784	-0.08008	0.098086	0.059455	CSNK2A2	Casein kina	CSK22_HUM	17	0.946006
48H 10MTA	Q9NR09	0.056973	0.098109	0.059467	BIRC6	Baculoviral	BIRC6_HUM	65	1.040281
48H 50MTA	Q6NT89	-0.56119	0.09812	0.059472	TRNP1	TMF-regula	TRNP1_HUM	2	0.677742
24H 50MTA	P13489	-0.01993	0.098141	0.059481	RNH1	Ribonuclea	RINI_HUMA	23	0.986281
48H 50MTA	P62166	0.076922	0.098147	0.059481	NCS1	Neuronal ca	NCS1_HUM	3	1.054766
24H 50MTA	Q9UBQ5	-0.07499	0.098163	0.059487	EIF3K	Eukaryotic	EIF3K_HUM	11	0.949351
24H 10MTA	P61421	-0.17381	0.098166	0.059488	ATP6V0D1	V-type prot	VA0D1_HU	10	0.886496
24H 50MTA	Q9BX93	-0.23046	0.098168	0.059488	PLA2G12B	Group XIIB	PG12B_HU	2	0.852362
48H 50MTA	Q92614	0.036306	0.09819	0.059499	MYO18A	Unconvent	MY18A_HU	43	1.025484
24H 10MTA	O75691	0.038824	0.098218	0.059513	UTP20	Small subu	UTP20_HUM	83	1.027276
24H 10MTA	Q53GL7	0.038068	0.098261	0.059538	PARP10	Protein mo	PAR10_HUM	1	1.026738
48H 50MTA	P51608	-0.01798	0.098266	0.05954	MECP2	Methyl-CpG	MECP2_HU	12	0.987614
48H 10MTA	Q9HB40	0.028221	0.098268	0.05954	SCPEP1	Retinoid-in	RISC_HUMA	11	1.019754
24H 10MTA	Q9Y520	-0.07267	0.098339	0.059574	PRRC2C	Protein PRR	PRC2C_HUM	62	0.950879
24H 10MTA	Q01831	-0.26473	0.098342	0.059575	XPC	DNA repair	XPC_HUMA	6	0.832354
24H 50MTA	Q2VPB7	-0.21589	0.098353	0.059579	AP5B1	AP-5 compl	AP5B1_HUM	11	0.861012
48H 10MTA	P08247	-0.0626	0.098404	0.059602	SYP	Synaptophy	SYPH_HUM	3	0.957539
24H 10MTA	Q99661	0.074798	0.09842	0.05961	KIF2C	Kinesin-like	KIF2C_HUM	22	1.053214

48H 50MT/	O43768	-0.21558	0.098436	0.059618	ENSA	Alpha-endc	ENSA_HUM	7	0.861199
24H 50MT/	Q8TC07	-0.10902	0.098438	0.059618	TBC1D15	TBC1 doma	TBC15_HUI	26	0.927216
24H 50MT/	Q9UBC3	0.031316	0.098442	0.059619	DNMT3B	DNA (cytosi	DNM3B_HU	1	1.021944
24H 10MT/	Q0D2I5;Q5	-0.06036	0.098473	0.059634	IFFO1;IFFO	Non-homol	IFFO1_HUN	1	0.959026
24H 50MT/	Q96Q45	0.112617	0.098481	0.059636	TMEM237	Transmemk	TM237_HU	6	1.081187
24H 10MT/	Q99798	0.010142	0.098486	0.059639	ACO2	Aconitate h	ACON_HUN	37	1.007055
48H 10MT/	O00506	-0.08651	0.098497	0.059642	STK25	Serine/thre	STK25_HUI	4	0.941801
24H 10MT/	Q9BZ23	-0.03421	0.098495	0.059642	PANK2	Pantothena	PANK2_HU	7	0.976568
48H 10MT/	P25815	0.885337	0.098504	0.059646	S100P	Protein S1(	S100P_HUI	1	1.847196
48H 10MT/	Q14155	0.099576	0.098522	0.05965	ARHGEF7	Rho guanin	ARHG7_HU	8	1.071458
48H 10MT/	Q14192	0.655394	0.098593	0.05968	FHL2	Four and a l	FHL2_HUM	1	1.575046
48H 50MT/	Q9Y383	0.021549	0.098593	0.05968	LUC7L2	Putative RN	LC7L2_HUI	8	1.015049
48H 50MT/	P32969	-0.101	0.098601	0.059681	RPL9	Large ribos	RL9_HUMA	12	0.932385
48H 50MT/	Q9Y256	0.079133	0.098613	0.059684	RCE1	CAAX preny	FACE2_HUI	2	1.056383
24H 50MT/	P61247	0.144914	0.098644	0.0597	RPS3A	Small ribos	RS3A_HUM	18	1.105665
48H 10MT/	Q8NG31	0.058003	0.09865	0.059701	KNL1	Kinetochor	KNL1_HUM	24	1.041023
24H 10MT/	Q9BUE6	-0.18753	0.098649	0.059701	ISCA1	Iron-sulfur	ISCA1_HUN	3	0.878109
48H 10MT/	Q9NPA0	0.009815	0.098663	0.059707	EMC7	Endoplasm	EMC7_HUN	7	1.006826
48H 50MT/	O94966	-0.20159	0.098673	0.059709	USP19	Ubiquitin c	UBP19_HU	25	0.869593
24H 10MT/	Q92545	-0.00591	0.098685	0.059714	TMEM131	Transmemk	TM131_HU	7	0.995915
24H 50MT/	O75436	-0.05692	0.098703	0.059723	VPS26A	Vacuolar pı	VP26A_HUI	20	0.961317
48H 50MT/	Q9Y2X9	-0.10547	0.098773	0.059756	ZNF281	Zinc finger	ZN281_HUI	19	0.929502
24H 50MT/	Q9BUI4	-0.12962	0.098776	0.059757	POLR3C	DNA-direct	RPC3_HUM	11	0.914071
48H 50MT/	Q9BXF6	0.219589	0.098793	0.059762	RAB11FIP5	Rab11 fami	RFIP5_HUN	12	1.164401
24H 10MT/	P16452	-0.59737	0.098818	0.059775	EPB42	Protein 4.2	EPB42_HUI	1	0.660959
48H 10MT/	O75794	-0.06587	0.098828	0.059778	CDC123	Translation	CD123_HU	12	0.955372
48H 50MT/	Q15554	-0.00065	0.098825	0.059778	TERF2	Telomeric r	TERF2_HUN	13	0.999551
24H 10MT/	Q7RTP0	-0.09158	0.09883	0.059778	NIPA1	Magnesium	NIPA1_HUN	3	0.938494
24H 10MT/	Q9BRA2	-0.07393	0.098827	0.059778	TXNDC17	Thioredoxi	TXD17_HUI	5	0.950043
24H 50MT/	Q330K2	-0.0306	0.098833	0.059778	NDUFAF6	NADH dehy	NDUF6_HU	1	0.97901
48H 10MT/	Q86UA1	-0.07427	0.098846	0.059784	PRPF39	Pre-mRNA-	PRP39_HUI	13	0.949825
48H 50MT/	Q6P4A7	-0.07332	0.098889	0.059806	SFXN4	Sideroflexir	SFXN4_HUI	2	0.950451
48H 10MT/	Q8TEP8	-0.02316	0.098907	0.059816	CEP192	Centrosom	CE192_HUI	10	0.984078
48H 50MT/	Q96K49	-0.0379	0.098917	0.05982	TMEM87B	Transmemk	TM87B_HU	3	0.974075
24H 10MT/	P12004	-0.00332	0.098962	0.059836	PCNA	Proliferatir	PCNA_HUN	13	0.997703
48H 50MT/	P0CG12	0.316606	0.098978	0.059841	DERPC	Decreased e	DERPC_HUI	12	1.245397
48H 10MT/	P60900	-0.0121	0.098988	0.059847	PSMA6	Proteasom	PSA6_HUM	14	0.991648
48H 10MT/	Q9ULD2	0.014737	0.099004	0.059855	MTUS1	Microtubul	MTUS1_HU	32	1.010267
24H 50MT/	P52655	0.535862	0.099017	0.05986	GTF2A1	Transcripti	TF2AA_HUI	2	1.449808
24H 10MT/	O95140	0.033079	0.099029	0.059862	MFN2	Mitofusin-2	MFN2_HUN	22	1.023193
24H 50MT/	Q9UNQ2	-0.0983	0.099027	0.059862	DIMT1	Dimethylac	DIM1_HUM	13	0.934134
24H 10MT/	Q92797	0.053976	0.099049	0.059873	SYMPK	Symplekin	SYMPK_HU	40	1.038122
24H 50MT/	Q96Q89	0.017189	0.099056	0.059875	KIF20B	Kinesin-like	KI20B_HUN	22	1.011986
48H 10MT/	Q969Z3	0.072057	0.099079	0.059887	MTARC2	Mitochond	MARC2_HU	4	1.051214
24H 50MT/	P49366	0.142471	0.099091	0.059892	DHPS	Deoxyhypu	DHYS_HUM	11	1.103794
24H 10MT/	P53990	-0.02324	0.099099	0.059896	IST1	IST1 homol	IST1_HUM/	11	0.984021
48H 10MT/	Q5SRE5	-0.05828	0.099162	0.059929	NUP188	Nucleopori	NU188_HU	51	0.960406
48H 10MT/	Q9NW81	0.017512	0.099171	0.059933	DMAC2	Distal mem	DMAC2_HU	3	1.012212
24H 10MT/	Q14114	-0.06021	0.09923	0.059959	LRP8	Low-densit	LRP8_HUM	5	0.959122
24H 10MT/	Q8IXT5	0.051006	0.099228	0.059959	RBM12B	RNA-bindir	RB12B_HUI	22	1.035987
48H 50MT/	O95352	0.048244	0.099255	0.059969	ATG7	Ubiquitin-l	ATG7_HUM	21	1.034006
24H 10MT/	Q9UJX3	0.001694	0.099255	0.059969	ANAPC7	Anaphase-p	APC7_HUM	27	1.001175

48H 10MT/	Q8WUF8	0.719019	0.099292	0.05998	ARB2A	Cotranscrip	ARB2A_HUI	1	1.646062
48H 50MT/	Q9UQ84	0.452917	0.099313	0.05999	EXO1	Exonucleas	EXO1_HUN	1	1.368805
48H 10MT/	Q5T447	-0.06693	0.099366	0.06002	HECTD3	E3 ubiquiti	HECD3_HU	17	0.954665
48H 50MT/	Q96RF0	0.035648	0.099371	0.060021	SNX18	Sorting nex	SNX18_HUI	7	1.025017
48H 50MT/	P01116	-0.21365	0.09938	0.060023	KRAS	GTPase KRa	RASK_HUM	1	0.862352
48H 10MT/	Q5RKV6	0.066091	0.09939	0.060027	EXOSC6	Exosome cc	EXOS6_HUI	8	1.046877
24H 10MT/	Q16740	-0.05785	0.099439	0.060053	CLPP	ATP-depen	CLPP_HUM	10	0.960694
24H 50MT/	O75317	0.249326	0.099467	0.060067	USP12	Ubiquitin c	UBP12_HU	2	1.188652
24H 10MT/	Q8TCZ2	0.640645	0.099477	0.060072	CD99L2	CD99 antig	C99L2_HUI	1	1.559026
48H 10MT/	Q9NVH1	-0.06484	0.09949	0.060075	DNAJC11	DnaJ homo	DJC11_HUI	18	0.956052
24H 50MT/	Q6PCD5	0.036999	0.099502	0.06008	RFWD3	E3 ubiquiti	RFWD3_HU	3	1.025978
24H 10MT/	Q9UHL0	0.942935	0.099516	0.060084	DDX25	ATP-depen	DDX25_HU	1	1.922435
48H 50MT/	Q9BQG2	0.157291	0.099532	0.060088	NUDT12	NAD-cappe	NUD12_HU	4	1.115191
48H 10MT/	Q9BSH3	-1.1297	0.099527	0.060088	NICN1	Nicolin-1	NICN1_HUI	1	0.45701
48H 10MT/	P37235	-0.18949	0.09955	0.060097	HPCAL1	Hippocalci	HPCL1_HUI	5	0.876914
48H 50MT/	P62314	-0.07126	0.099574	0.060108	SNRPD1	Small nucle	SMD1_HUN	5	0.951809
24H 10MT/	Q13595	0.127024	0.099626	0.060134	TRA2A	Transforme	TRA2A_HUI	8	1.092039
48H 10MT/	O15143	-0.00648	0.099641	0.060141	ARPC1B	Actin-relate	ARC1B_HUI	16	0.995519
24H 10MT/	Q9Y3D6	-0.14959	0.099662	0.06015	FIS1	Mitochond	FIS1_HUM/	6	0.901507
48H 10MT/	Q15008	-0.03183	0.099673	0.060155	PSMD6	26S protea:	PSMD6_HU	30	0.978179
48H 10MT/	Q96EB1	0.069256	0.099718	0.060178	ELP4	Elongator c	ELP4_HUM	7	1.049176
24H 10MT/	Q16881	0.084708	0.099722	0.060179	TXNRD1	Thioredoxi	TRXR1_HUI	28	1.060473
24H 10MT/	Q9Y4Y9	-0.5085	0.099744	0.060189	LSM5	U6 snRNA-a	LSM5_HUN	2	0.702951
48H 10MT/	P84077	0.032918	0.099749	0.06019	ARF1	ADP-ribosy	ARF1_HUM	3	1.023079
24H 50MT/	Q9UG63	-0.08085	0.099757	0.06019	ABCF2	ATP-bindin	ABCF2_HUI	24	0.945498
48H 50MT/	Q9NXK8	-0.07616	0.09978	0.060199	FBXL12	F-box/LRR-I	FXL12_HUI	4	0.948576
24H 10MT/	P54198	-0.13649	0.099809	0.060212	HIRA	Protein HIF	HIRA_HUM	8	0.909731
48H 50MT/	Q8N8Q8	-0.21147	0.099814	0.060213	COX18	Cytochrom	COX18_HU	3	0.863656
48H 10MT/	Q9NTG7	0.019577	0.099845	0.060229	SIRT3	NAD-depen	SIR3_HUM/	6	1.013662
24H 10MT/	P29279	0.179963	0.099897	0.060249	CCN2	CCN family	CCN2_HUN	5	1.132855
48H 10MT/	Q99627	0.090054	0.099898	0.060249	COPS8	COP9 signa	CSN8_HUM	7	1.06441
48H 50MT/	Q9NZT2	0.105645	0.099898	0.060249	OGFR	Opioid grov	OGFR_HUN	21	1.075975
48H 10MT/	P48553	-0.27922	0.099937	0.060264	TRAPPC10	Trafficking	TPC10_HUI	3	0.824037
48H 50MT/	Q99720	0.000877	0.100002	0.060297	SIGMAR1	Sigma non-	SGMR1_HU	5	1.000608
48H 50MT/	Q99538	0.145787	0.100018	0.060303	LGMN	Legumain	LGMN_HUI	6	1.106334
24H 50MT/	P55327	-0.00516	0.100027	0.060306	TPD52	Tumor prot	TPD52_HUI	9	0.996427
24H 50MT/	P37837	0.060308	0.100034	0.060308	TALDO1	Transaldola	TALDO_HUI	16	1.042688
48H 10MT/	P56377	-0.05451	0.100069	0.060326	AP1S2	AP-1 compl	AP1S2_HUI	5	0.96292
24H 10MT/	Q9BSJ2	0.017192	0.100067	0.060326	TUBGCP2	Gamma-tul	GCP2_HUN	35	1.011988
48H 50MT/	O75110	-0.00788	0.10009	0.060337	ATP9A	Probable pl	ATP9A_HUI	7	0.99455
48H 10MT/	Q9BUT9	-0.52854	0.1001	0.060341	MCRIP2	MAPK regul	MCRI2_HUI	2	0.693254
24H 10MT/	O43395	0.054559	0.100109	0.060345	PRPF3	U4/U6 sma	PRPF3_HUI	23	1.038541
24H 10MT/	P08962	0.187527	0.10011	0.060345	CD63	CD63 antig	CD63_HUN	2	1.13881
48H 10MT/	Q8WXW3	0.08025	0.100124	0.060349	PIBF1	Progesteroi	PIBF1_HUN	16	1.057201
48H 10MT/	Q9UGM6	0.047712	0.100154	0.060362	WARS2	Tryptophar	SYWM_HUI	8	1.033624
24H 10MT/	Q6PI26	-0.76131	0.100199	0.060386	SHQ1	Protein SH(	SHQ1_HUN	1	0.589959
24H 10MT/	Q9H936	0.107571	0.100205	0.060387	SLC25A22	Mitochond	GHC1_HUN	11	1.077413
48H 50MT/	Q8NEN9	-0.27976	0.100216	0.060393	PDZD8	PDZ domaii	PDZD8_HU	4	0.82373
24H 50MT/	Q9H0H0	0.210928	0.10023	0.0604	INTS2	Integrator (	INT2_HUM/	11	1.157432
24H 10MT/	Q9Y315	-0.00551	0.100234	0.060401	DERA	Deoxyribos	DEOC_HUN	17	0.996188
48H 10MT/	Q6P0N0	-0.17155	0.100247	0.060406	MIS18BP1	Mis18-binc	M18BP_HU	4	0.887891
24H 10MT/	Q5TEU4	-0.0032	0.100328	0.060443	NDUFAF5	Arginine-hy	NDUF5_HU	3	0.997782

24H 50MT/	O00212	-0.4555	0.100338	0.060445	RHOD	Rho-relatec	RHOD_HUM	3	0.729258
48H 10MT/	Q9NT62	0.022642	0.100336	0.060445	ATG3	Ubiquitin-l	ATG3_HUM	7	1.015818
48H 10MT/	O00214	-0.09273	0.100364	0.060457	LGALS8	Galectin-8	LEG8_HUM	8	0.937747
24H 10MT/	Q9Y2L1	0.047916	0.100366	0.060457	DIS3	Exosome cc	RRP44_HUI	38	1.033771
48H 10MT/	P20936	-0.00813	0.100379	0.060458	RASA1	Ras GTPase-	RASA1_HUI	19	0.99438
24H 50MT/	P29279	-0.07352	0.100373	0.060458	CCN2	CCN family	CCN2_HUM	5	0.950315
48H 50MT/	P81877;Q9	-1.02284	0.100374	0.060458	SSBP2;SSBF	Single-strar	SSBP2_HUI	1	0.492148
24H 50MT/	Q9H063	0.021559	0.100376	0.060458	MAF1	Repressor c	MAF1_HUM	2	1.015056
24H 50MT/	O60506	0.016561	0.100391	0.060461	SYNCRIP	Heterogene	HNRPQ_HU	27	1.011545
24H 10MT/	P56378	0.18972	0.100387	0.060461	ATP5MJ	ATP synthas	ATP68_HUI	4	1.140542
24H 10MT/	Q8TBE9	0.372735	0.100392	0.060461	NANP	N-acylneura	NANP_HUM	4	1.294805
24H 50MT/	Q9NRP0	0.184587	0.100385	0.060461	OSTC	Oligosacch	OSTC_HUM	3	1.136491
24H 10MT/	Q9GZZ1	-0.04473	0.100402	0.060462	NAA50	N-alpha-ace	NAA50_HU	13	0.969471
48H 50MT/	Q8NAF0	-0.02332	0.100428	0.060473	ZNF579	Zinc finger		ZN579_HUI	5	0.983967
24H 10MT/	Q96IW7	0.180166	0.100429	0.060473	SEC22A	Vesicle-traf	SC22A_HUI	4	1.133014
24H 50MT/	Q96S55	0.09233	0.100434	0.060474	WRNIP1	ATPase WRI	WRIP1_HU	12	1.066091
24H 50MT/	P04746;P0	-1.02481	0.1005	0.060508	AMY2A;AM	Pancreatic	AMYP_HUM	1	0.491474
48H 10MT/	Q96B70	0.385895	0.100516	0.060515	LENG9	Leukocyte r	LENG9_HUI	2	1.30667
48H 10MT/	Q6JQN1	-0.00837	0.100548	0.06053	ACAD10	Acyl-CoA de	ACD10_HU	25	0.994218
48H 50MT/	Q9UM13	0.056003	0.100551	0.060531	ANAPC10	Anaphase-p	APC10_HUI	3	1.039582
24H 10MT/	Q92599	-0.04191	0.100573	0.060539	SEPTIN8	Septin-8	SEPT8_HUM	7	0.971367
24H 50MT/	O43488	0.009835	0.100577	0.06054	AKR7A2	Aflatoxin B	ARK72_HUI	10	1.006841
48H 50MT/	Q8WTW3	-0.03523	0.100608	0.060553	COG1	Conserved	COG1_HUM	26	0.975877
48H 10MT/	Q9BWT3	-0.18564	0.100652	0.060577	PAPOLG	Poly(A) poly	PAPOG_HU	6	0.879261
24H 10MT/	Q9Y2L5	0.113463	0.100652	0.060577	TRAPPC8	Trafficking		TPPC8_HUI	12	1.081822
24H 10MT/	Q8WYQ5	0.359279	0.100695	0.060593	DGCR8	Microproce	DGCR8_HU	5	1.282784
48H 10MT/	Q96Q11	-0.01271	0.100696	0.060593	TRNT1	CCA tRNA n	TRNT1_HUI	12	0.99123
24H 10MT/	O15344	0.069983	0.100721	0.060606	MID1	E3 ubiquiti	TRI18_HUM	20	1.049704
48H 50MT/	P63172	-0.01142	0.100725	0.060606	DYNLT1	Dynein ligh	DYLT1_HUI	5	0.992117
48H 50MT/	P48382	0.304594	0.10077	0.060627	RFX5	DNA-bindir	RFX5_HUM	6	1.235071
48H 10MT/	Q4KMQ2	0.076001	0.100779	0.060631	ANO6	Anoctamin	ANO6_HUM	16	1.054092
48H 10MT/	Q16719	-0.00767	0.100795	0.060636	KYNU	Kynurenina	KYNU_HUM	20	0.994698
48H 10MT/	Q6PKC3	-0.20157	0.100793	0.060636	TXNDC11	Thioredoxi	TXD11_HUI	9	0.869605
48H 50MT/	Q9BPU6	0.208338	0.100851	0.060665	DPYSL5	Dihydropyr	DPYL5_HUI	1	1.155356
24H 10MT/	O43325	0.620424	0.100873	0.060675	LYRM1	LYR motif-c	LYRM1_HU	1	1.537327
24H 50MT/	Q8IU81	0.034365	0.100873	0.060675	IRF2BP1	Interferon r	I2BP1_HUM	18	1.024106
24H 10MT/	Q9NUQ3	0.061	0.100881	0.060677	TXLNG	Gamma-tax	TXLNG_HUI	17	1.043188
48H 50MT/	Q4U2R6	0.291968	0.100892	0.06068	MRPL51	Large ribos	RM51_HUM	2	1.224309
48H 10MT/	O75607	-0.07457	0.100902	0.060685	NPM3	Nucleoplas	NPM3_HUM	7	0.949622
24H 10MT/	P07355	0.021933	0.100909	0.060688	ANXA2	Annexin A2	ANXA2_HU	28	1.015319
24H 50MT/	Q12792	-0.04014	0.100923	0.060694	TWF1	Twinfilin-1	TWF1_HUM	15	0.972561
24H 50MT/	Q6ZRQ5	-0.00928	0.100931	0.060697	MMS22L	Protein MM	MMS22_HU	10	0.993586
24H 10MT/	P49411	-0.05117	0.100939	0.060701	TUFM	Elongation	EFTU_HUM	31	0.965155
48H 50MT/	O00506	0.091272	0.100957	0.060709	STK25	Serine/thre	STK25_HUM	4	1.065309
48H 10MT/	P21589	0.508879	0.100978	0.060718	NT5E	5'-nucleoti	5NTD_HUM	1	1.422944
48H 10MT/	Q92542	-0.12169	0.101016	0.060734	NCSTN	Nicastrin	NICA_HUM	12	0.91911
48H 50MT/	C9JLW8	0.108793	0.101044	0.060743	MCRIP1	Mapk-regul	MCRI1_HUI	4	1.078326
48H 50MT/	A6NDG6	-0.13979	0.101117	0.060775	PGP	Glycerol-3-	PGP_HUMA	14	0.90765
24H 10MT/	P78536	-0.01606	0.101142	0.060787	ADAM17	Disintegrin	ADA17_HU	8	0.988927
24H 50MT/	Q14686	0.214629	0.101148	0.060789	NCOA6	Nuclear rec	NCOA6_HU	1	1.160405
24H 50MT/	O15321	0.005601	0.101191	0.060811	TM9SF1	Transmemk	TM9S1_HU	8	1.00389
48H 50MT/	P04406	0.119273	0.101214	0.060822	GAPDH	Glyceraldel	G3P_HUMA	23	1.086188

24H	10MT/	Q96DX5	-0.2068	0.101218	0.060823	ASB9	Ankyrin rep	ASB9_HUM	3	0.866457
48H	50MT/	P78417	0.010784	0.10122	0.060823	GSTO1	Glutathion	GSTO1_HU	19	1.007503
24H	10MT/	Q9NRG4	0.110557	0.101233	0.060828	SMYD2	N-lysine me	SMYD2_HU	5	1.079645
24H	10MT/	Q9NZJ9	-0.12242	0.101232	0.060828	NUDT4	Diphospho	NUDT4_HU	6	0.918644
48H	10MT/	Q9NNW5	-0.0222	0.101239	0.06083	WDR6	tRNA (34-2'	WDR6_HU	26	0.98473
48H	10MT/	Q8WV99	0.038331	0.101265	0.060843	ZFAND2B	AN1-type zi	ZFN2B_HU	3	1.026925
48H	50MT/	Q16637	0.016465	0.101267	0.060843	SMN1	Survival mc	SMN_HUM/	8	1.011478
24H	10MT/	Q9Y450	0.001527	0.101282	0.060851	HBS1L	HBS1-like p	HBS1L_HU	30	1.001059
24H	50MT/	P01130	0.040174	0.101296	0.060858	LDLR	Low-densit	LDLR_HUM	18	1.028238
48H	10MT/	P35558	0.114104	0.101301	0.060858	PCK1	Phosphoen	PCKGC_HU	18	1.082303
24H	50MT/	Q96G25	-0.1664	0.101304	0.060859	MED8	Mediator o	MED8_HUM	2	0.891061
48H	50MT/	Q9H0N0	0.179876	0.101324	0.060866	RAB6C	Ras-related	RAB6C_HU	2	1.132787
24H	50MT/	A5PLL7	0.141995	0.101373	0.060886	PEDS1	Plasmanyle	PDES1_HU	4	1.10343
48H	10MT/	O00221	-0.48432	0.101389	0.060893	NFKBIE	NF-kappa-B	IKBE_HUM/	1	0.714835
48H	50MT/	Q9HD42	0.136967	0.101392	0.060893	CHMP1A	Charged mu	CHM1A_HU	4	1.099591
48H	10MT/	Q12851	-0.07577	0.101403	0.060897	MAP4K2	Mitogen-ac	M4K2_HUM	4	0.948836
24H	50MT/	Q8WXA9	0.461349	0.101406	0.060897	SREK1	Splicing reg	SREK1_HUM	3	1.376828
24H	50MT/	Q9H3C7	0.222651	0.101402	0.060897	GGNBP2	Gametogen	GGNB2_HU	2	1.166875
48H	50MT/	Q96EY5	-0.07177	0.10141	0.060898	MVB12A	Multivesicu	MB12A_HU	6	0.95147
48H	50MT/	Q8WZ42	-0.32519	0.101412	0.060899	TTN	Titin	TITIN_HUM	10	0.798192
48H	50MT/	Q7Z7G8	-0.14119	0.101458	0.06092	VPS13B	Intermemb	VP13B_HU	7	0.906771
24H	10MT/	P31150	0.014133	0.101559	0.060973	GDI1	Rab GDP di	GDIA_HUM	13	1.009844
48H	50MT/	O43264	-0.07297	0.101572	0.060979	ZW10	Centromer	ZW10_HUM	30	0.950679
24H	10MT/	Q9H2D6	0.055584	0.101578	0.060982	TRIOBP	TRIO and F-	TARA_HUM	13	1.03928
24H	10MT/	Q3B7T1	0.097753	0.101585	0.060982	EDRF1	Erythroid d	EDRF1_HU	12	1.070105
48H	10MT/	Q99988	-0.25008	0.101585	0.060982	GDF15	Growth/dif	GDF15_HU	7	0.840851
24H	50MT/	Q8NCN4	0.144879	0.101622	0.061002	RNF169	E3 ubiquiti	RN169_HU	8	1.105638
24H	10MT/	Q2NL82	0.047799	0.101654	0.061013	TSR1	Pre-rRNA-p	TSR1_HUM	23	1.033687
24H	10MT/	Q14520	-0.11552	0.1017	0.061034	HABP2	Hyaluronar	HABP2_HU	10	0.923046
24H	50MT/	O43815	0.042417	0.101716	0.061041	STRN	Striatin	STRN_HUM	18	1.029838
48H	50MT/	Q9BRA0	0.185653	0.101754	0.061062	NAA38	N-alpha-ace	NAA38_HU	4	1.137332
48H	50MT/	P13645	0.154068	0.101756	0.061062	KRT10	Keratin, typ	K1C10_HU	30	1.112702
24H	10MT/	Q9Y2S7	0.020322	0.101762	0.061062	POLDIP2	Polymerase	PDIP2_HUM	16	1.014186
24H	50MT/	P15252	0.817442	0.101772	0.061065	NaN	Rubber elo	REF_HEVBR	5	1.762279
24H	10MT/	Q05639	0.033594	0.101774	0.061065	EEF1A2	Elongation	EF1A2_HUM	8	1.023559
48H	10MT/	Q8NHG8	-0.16927	0.101785	0.06107	ZNRF2	E3 ubiquiti	ZNRF2_HU	3	0.889293
48H	50MT/	Q9BY89	0.410967	0.101785	0.06107	KIAA1671	Uncharacte	K1671_HU	7	1.329577
48H	10MT/	Q6ZW76	-0.44123	0.101798	0.061074	ANKS3	Ankyrin rep	ANKS3_HU	1	0.736505
24H	50MT/	Q8NCE0	0.070991	0.10181	0.061078	TSEN2	tRNA-splici	SEN2_HUM	7	1.050438
48H	50MT/	Q96GS4	0.151436	0.101808	0.061078	BORCS6	BLOC-1-rela	BORC6_HU	11	1.110675
48H	50MT/	O43293	-0.12023	0.101812	0.061078	DAPK3	Death-asso	DAPK3_HU	4	0.920043
48H	50MT/	O43759	0.077389	0.101815	0.061079	SYNGR1	Synaptogyr	SNG1_HUM	2	1.055107
48H	50MT/	P07311	0.010701	0.101819	0.061079	ACYP1	Acylphosph	ACYP1_HU	5	1.007445
24H	50MT/	P41214	-0.25125	0.101828	0.06108	EIF2D	Eukaryotic	EIF2D_HUM	10	0.84017
48H	10MT/	Q96JB5	-0.10695	0.101829	0.06108	CDK5RAP3	CDK5 regul	CK5P3_HU	18	0.928547
48H	50MT/	Q12986	-0.08747	0.101864	0.061096	NFX1	Transcripti	NFX1_HUM	18	0.941173
24H	10MT/	Q9Y5P6	-0.07403	0.101899	0.061113	GMPPB	Mannose-1	GMPPB_HU	13	0.94998
24H	50MT/	P61981	0.086302	0.101908	0.061116	YWHAG	14-3-3 prot	1433G_HU	11	1.061645
48H	10MT/	P14868	-0.0132	0.101935	0.06113	DARS1	Aspartate--	SYDC_HUM	36	0.990889
48H	50MT/	Q96N11	0.14739	0.101985	0.061155	INTS15	Integrator c	INT15_HUM	2	1.107564
24H	10MT/	O00622	0.258279	0.101989	0.061156	CCN1	CCN family	CCN1_HUM	7	1.196051
48H	50MT/	P07741	0.050212	0.102006	0.061163	APRT	Adenine ph	APT_HUMA	12	1.035417

48H 10MT/	Q7Z5G4	-0.01586	0.102002	0.061163	GOLGA7	Golgin subf	GOGA7_HU	4	0.989068
24H 50MT/	Q9Y6D5	-0.01852	0.102046	0.061178	ARFGEF2	Brefeldin A-	BIG2_HUM	56	0.987247
48H 50MT/	Q5SW96	-0.22529	0.102062	0.061184	LDLRAP1	Low density	ARH_HUMA	2	0.855425
48H 10MT/	Q01804	0.014121	0.102101	0.061202	OTUD4	OTU domai	OTUD4_HU	11	1.009836
48H 10MT/	Q96DH6	0.105425	0.102114	0.061208	MSI2	RNA-bindir	MSI2H_HUI	6	1.075811
48H 10MT/	Q14344	-0.0862	0.102123	0.061212	GNA13	Guanine nu	GNA13_HU	14	0.942001
24H 50MT/	O00483	0.67098	0.102169	0.061236	NDUFA4	Cytochrom	NDUA4_HU	4	1.592154
24H 10MT/	O14646	0.060478	0.10218	0.061237	CHD1	Chromodor	CHD1_HUM	20	1.042811
24H 50MT/	Q8WVM0	-0.05131	0.10218	0.061237	TFB1M	Dimethylac	TFB1M_HU	15	0.965057
48H 50MT/	Q96KG9	0.041286	0.102201	0.061247	SCYL1	N-terminal	SCYL1_HUM	26	1.029031
24H 10MT/	Q13627	0.206161	0.102223	0.061257	DYRK1A	Dual specifi	DYR1A_HUI	7	1.153614
48H 10MT/	Q16850	-0.01674	0.102234	0.061262	CYP51A1	Lanosterol	CP51A_HUI	23	0.988463
48H 10MT/	P13984	0.011527	0.102274	0.061279	GTF2F2	General tra	T2FB_HUM	16	1.008022
24H 10MT/	Q92901	0.234978	0.102306	0.061294	RPL3L	Ribosomal	RL3L_HUM	2	1.176889
24H 50MT/	Q96GW9	0.013972	0.102366	0.061316	MARS2	Methionine	SYMM_HUM	13	1.009732
48H 10MT/	Q5ST30	0.02407	0.102377	0.061319	VARS2	Valine--tRN	SYVM_HUM	22	1.016824
24H 10MT/	P46019	-0.1406	0.10239	0.061325	PHKA2	Phosphoryl	KPB2_HUM	17	0.907143
48H 50MT/	Q8WYN0	-0.11149	0.102395	0.061327	ATG4A	Cysteine pr	ATG4A_HUI	6	0.92563
24H 10MT/	Q9UPT9	0.355783	0.102399	0.061327	USP22	Ubiquitin c	UBP22_HU	6	1.27968
48H 50MT/	P54760	0.079368	0.102402	0.061328	EPHB4	Ephrin type	EPHB4_HUI	10	1.056555
48H 50MT/	Q9BPX7	-0.02658	0.102414	0.061333	C7orf25	UPF0415 p	CG025_HU	6	0.981747
48H 50MT/	Q9UGC7	-0.26524	0.102414	0.061333	MTRF1L	Peptide cha	RF1ML_HU	4	0.832059
48H 10MT/	Q7L266	0.072604	0.102472	0.061362	ASRGL1	Isoaspartyl	ASGL1_HUI	7	1.051613
48H 10MT/	Q13867	0.039861	0.102525	0.06139	BLMH	Bleomycin	BLMH_HUM	14	1.028015
24H 50MT/	Q8N1A6	0.370504	0.102529	0.061392	C4orf33	UPF0462 p	CD033_HU	1	1.292804
48H 10MT/	P48643	0.011529	0.102539	0.061395	CCT5	T-complex	TCPE_HUM	42	1.008023
48H 50MT/	Q14166	-0.08369	0.102558	0.061403	TTLL12	Tubulin--ty	TTL12_HUM	28	0.943642
48H 10MT/	Q14032	-0.19391	0.102574	0.061409	BAAT	Bile acid-Cc	BAAT_HUM	3	0.874233
24H 10MT/	Q9HAN9	0.214251	0.102591	0.061412	NMNAT1	Nicotinami	NMNA1_HU	5	1.160101
48H 50MT/	Q9Y548	0.470314	0.102592	0.061412	YIPF1	Protein YIP	YIPF1_HUM	3	1.385411
24H 50MT/	O95822	0.178147	0.102601	0.061416	MLYCD	Malonyl-Cc	DCMC_HUM	14	1.13143
24H 10MT/	Q9H2C0	0.046166	0.102649	0.061436	GAN	Gigaxonin	GAN_HUMA	15	1.032517
24H 10MT/	Q9UBF2	0.096406	0.102699	0.061458	COPG2	Coatomer s	COPG2_HU	19	1.069107
48H 10MT/	Q9UBV2	-0.07864	0.102724	0.06147	SEL1L	Protein sel-	SE1L1_HUM	24	0.946952
24H 10MT/	Q5T447	-0.03662	0.102728	0.061471	HECTD3	E3 ubiquiti	HECD3_HU	17	0.974936
24H 10MT/	Q9H974	0.012959	0.10278	0.061494	QTRT2	Queuine tR	QTRT2_HUI	14	1.009023
24H 50MT/	Q9NPF4	0.032914	0.10282	0.061514	OSGEP	tRNA N6-ad	OSGEP_HUI	14	1.023077
48H 10MT/	Q7Z3B4	-0.01028	0.102826	0.061516	NUP54	Nucleopori	NUP54_HU	19	0.992903
48H 50MT/	P19838	0.023794	0.102868	0.061535	NFKB1	Nuclear fac	NFKB1_HUI	18	1.016629
48H 10MT/	Q86SF2	-0.05173	0.10291	0.061557	GALNT7	N-acetylgal	GALT7_HUI	13	0.964776
48H 50MT/	P52907	0.037409	0.102946	0.061577	CAPZA1	F-actin-cap	CAZA1_HUI	13	1.026269
24H 10MT/	P11474	-0.34033	0.102952	0.061578	ESRRA	Steroid hor	ERR1_HUM	3	0.789863
48H 50MT/	O76094	0.040784	0.102992	0.061596	SRP72	Signal reco	SRP72_HUI	28	1.028673
48H 10MT/	O15431	-0.29188	0.103008	0.061603	SLC31A1	High affinit	COPT1_HUI	1	0.816838
24H 50MT/	Q6SPF0	-0.0613	0.103016	0.061606	SAMD1	Sterile alph	SAMD1_HU	5	0.958399
24H 50MT/	Q9NXE8	0.139302	0.103046	0.061621	CWC25	Pre-mRNA-	CWC25_HU	7	1.101372
48H 50MT/	Q15773	0.050201	0.103074	0.061635	MLF2	Myeloid leu	MLF2_HUM	6	1.035409
24H 10MT/	Q14CX7	0.01956	0.103106	0.061651	NAA25	N-alpha-ace	NAA25_HU	30	1.01365
48H 10MT/	Q6NXT1	-0.08104	0.103108	0.061651	ANKRD54	Ankyrin rep	ANR54_HU	4	0.945373
24H 50MT/	Q96LB3	-0.11205	0.103116	0.061655	IFT74	Intraflagell	IFT74_HUM	9	0.925275
48H 10MT/	P56589	0.048429	0.103147	0.061669	PEX3	Peroxisoma	PEX3_HUM	10	1.034138
48H 10MT/	Q12982	0.050762	0.103184	0.061687	BNIP2	BCL2/aden	BNIP2_HUM	3	1.035812

24H 50MT/	Q96G75	0.173945	0.103199	0.061694	RMND5B	E3 ubiquiti	RMD5B_HU	2	1.128139
48H 10MT/	O95084	0.408644	0.103203	0.061694	PRSS23	Serine prot	PRS23_HUI	4	1.327438
48H 50MT/	P20618	-0.0181	0.103236	0.061711	PSMB1	Proteasome	PSB1_HUM	13	0.987536
24H 10MT/	Q6UX53	-0.13103	0.10324	0.061712	TMT1B	Thiol S-met	TMT1B_HU	10	0.913176
48H 10MT/	P24863	-0.43351	0.103298	0.061744	CCNC	Cyclin-C	CCNC_HUM	2	0.740458
24H 50MT/	P38935	-0.09583	0.10335	0.061767	IGHMBP2	DNA-bindir	SMBP2_HU	8	0.935736
24H 50MT/	Q9NX74	0.023339	0.10338	0.061777	DUS2	tRNA-dihyd	DUS2L_HUI	23	1.016309
48H 50MT/	Q6PGP7	0.000698	0.103424	0.061794	SKIC3	Superkiller	SKI3_HUM/	45	1.000484
24H 50MT/	Q9NRX4	0.180547	0.103481	0.061821	PHPT1	14 kDa pho	PHP14_HU	6	1.133314
24H 50MT/	Q86SQ4	0.059078	0.103496	0.061826	ADGRG6	Adhesion G	AGRG6_HU	8	1.0418
48H 50MT/	P60900	0.005719	0.103502	0.061828	PSMA6	Proteasome	PSA6_HUM	14	1.003972
48H 50MT/	Q12778	0.4728	0.103529	0.061843	FOXO1	Forkhead b	FOXO1_HU	5	1.3878
48H 10MT/	O95685	0.224091	0.103542	0.061849	PPP1R3D	Protein pho	PPR3D_HU	5	1.168041
24H 10MT/	Q9Y3Q3	0.266324	0.103559	0.061857	TMED3	Transmemb	TMED3_HU	3	1.202739
48H 50MT/	P45974	0.019013	0.103587	0.061862	USP5	Ubiquitin c	UBP5_HUM	35	1.013266
24H 50MT/	P60059	-0.27432	0.103603	0.061869	SEC61G	Protein trai	SC61G_HUI	2	0.82684
24H 10MT/	Q15043	-0.17177	0.10364	0.061887	SLC39A14	Metal catio	S39AE_HUI	4	0.887754
24H 10MT/	Q14139	-0.03565	0.10369	0.061914	UBE4A	Ubiquitin c	UBE4A_HUI	30	0.975594
48H 50MT/	P15880	-0.10329	0.103714	0.061925	RPS2	Small ribos	RS2_HUMA	17	0.93091
48H 10MT/	Q9BQ67	0.073243	0.103713	0.061925	GRWD1	Glutamate-	GRWD1_HU	17	1.052079
24H 10MT/	Q9UKY1	0.064167	0.103723	0.06193	ZHX1	Zinc fingers	ZHX1_HUM	1	1.045481
24H 50MT/	P15924	-0.00042	0.103733	0.061932	DSP	Desmoplak	DESP_HUM	135	0.999706
48H 10MT/	Q96SL1	-0.10412	0.103777	0.061956	SLC49A4	Solute carri	DIRC2_HUI	1	0.93037
48H 10MT/	P11310	-0.07107	0.103795	0.061964	ACADM	Medium-ch	ACADM_HU	19	0.951932
24H 50MT/	O94811	0.113531	0.10382	0.061976	TPPP	Tubulin pol	TPPP_HUM	3	1.081873
48H 50MT/	Q8N755	-0.16938	0.103832	0.061982	SLC66A3	Solute carri	S66A3_HUI	3	0.889225
24H 50MT/	Q9BPX7	-0.02114	0.103841	0.061985	C7orf25	UPF0415 p	CG025_HU	6	0.985452
24H 10MT/	Q99081	0.014512	0.103877	0.062003	TCF12	Transcripti	HTF4_HUM	3	1.01011
24H 50MT/	Q96S82	-0.10267	0.103894	0.062007	UBL7	Ubiquitin-l	UBL7_HUM	8	0.931311
24H 50MT/	Q9UM00	-0.06699	0.103893	0.062007	TMCO1	Calcium loa	TMCO1_HU	3	0.954627
48H 10MT/	Q9BTX1	-0.0559	0.103896	0.062008	NDC1	Nucleopori	NDC1_HUM	17	0.961993
24H 10MT/	O14949	0.027577	0.103902	0.062009	UQCRQ	Cytochrom	QCR8_HUM	3	1.019299
24H 10MT/	Q14258	-0.04812	0.103909	0.062013	TRIM25	E3 ubiquiti	TRI25_HUM	30	0.967194
48H 10MT/	Q9NVM4	-0.10445	0.103938	0.062027	PRMT7	Protein arg	ANM7_HUI	8	0.930162
48H 10MT/	Q7KZN9	0.060469	0.104003	0.062059	COX15	Heme A syn	COX15_HU	9	1.042804
24H 10MT/	Q96DH6	0.017781	0.104051	0.062077	MSI2	RNA-bindir	MSI2H_HUI	6	1.012401
48H 50MT/	O00418	0.054394	0.104083	0.062093	EEF2K	Eukaryotic	EF2K_HUM	8	1.038423
48H 50MT/	Q9NYV6	-0.32364	0.104082	0.062093	RRN3	RNA polym	RRN3_HUM	5	0.799049
24H 10MT/	A0FGR8	0.019534	0.104105	0.062104	ESYT2	Extended sy	ESYT2_HUM	25	1.013632
48H 10MT/	Q6BDS2	-0.00605	0.10414	0.06212	BLTP3A	Bridge-like	BLT3A_HUI	6	0.995812
48H 50MT/	Q96ME7	-0.00629	0.104146	0.062123	ZNF512	Zinc finger	ZN512_HUI	12	0.995652
48H 10MT/	P07384	0.057015	0.104163	0.062132	CAPN1	Calpain-1 c	CAN1_HUM	38	1.040311
48H 10MT/	Q9Y692	-0.01535	0.104193	0.062145	GMEB1	Glucocorti	GMEB1_HU	4	0.989417
48H 10MT/	Q9H063	-0.43664	0.104197	0.062145	MAF1	Repressor c	MAF1_HUM	2	0.738854
48H 10MT/	P52429	0.011209	0.104203	0.062147	DGKE	Diacylglyce	DGKE_HUM	1	1.0078
48H 10MT/	Q9NZP8	0.015399	0.104206	0.062148	C1RL	Compleme	C1RL_HUM	1	1.010731
48H 50MT/	P42167	-0.16689	0.104281	0.062184	TMPO	Lamina-ass	LAP2B_HUI	11	0.890764
48H 50MT/	Q9NVH1	-0.10388	0.104311	0.062197	DNAJC11	DnaJ homo	DJC11_HUI	18	0.930525
48H 10MT/	Q96Q05	-0.14647	0.104317	0.062197	TRAPPC9	Trafficking	TPPC9_HUI	7	0.903458
24H 50MT/	Q8N5D0	0.315557	0.104332	0.062202	WDTC1	WD and tet	WDTC1_HU	1	1.244492
24H 50MT/	Q9BY89	0.192562	0.10433	0.062202	KIAA1671	Uncharacte	K1671_HUI	7	1.142792
24H 50MT/	Q9BQK8	-0.04842	0.104346	0.062208	LPIN3	Phosphatid	LPIN3_HUM	3	0.966997

24H 50MT/	Q9H0X4	0.259532	0.104354	0.062211	FAM234A	Protein FAI	F234A_HUI	6	1.19709
24H 50MT/	P35270	-0.15652	0.104363	0.062213	SPR	Sepiapterir	SPRE_HUM	15	0.897188
24H 10MT/	Q4G0N4	0.007778	0.104383	0.062222	NADK2	NAD kinase	NAKD2_HU	18	1.005406
48H 10MT/	Q9NQ35	-0.83701	0.104411	0.062236	NRIP3	Nuclear rec	NRIP3_HUI	1	0.559803
24H 50MT/	O43299	-0.20666	0.104437	0.062246	AP5Z1	AP-5 compl	AP5Z1_HUI	8	0.86654
24H 10MT/	Q9HDC9	0.037509	0.104441	0.062247	APMAP	Adipocyte p	APMAP_HU	21	1.02634
24H 10MT/	Q6UW63	0.028722	0.104478	0.062265	POGLUT2	Protein O-g	PLGT2_HUI	18	1.020108
24H 50MT/	P31785	0.737467	0.104489	0.06227	IL2RG	Cytokine re	IL2RG_HUI	1	1.667246
48H 10MT/	P04264	0.106865	0.104509	0.062279	KRT1	Keratin, typ	K2C1_HUM	38	1.076886
24H 50MT/	Q8IZE3	0.08213	0.104526	0.062287	SCYL3	Protein-ass	PACE1_HUI	6	1.05858
48H 10MT/	Q92544	0.01505	0.104546	0.062296	TM9SF4	Transmemb	TM9S4_HU	17	1.010487
24H 10MT/	P78316	0.084046	0.104552	0.062299	NOP14	Nucleolar p	NOP14_HU	22	1.059987
24H 10MT/	O00186	0.001465	0.104571	0.062306	STXBP3	Syntaxin-bi	STXB3_HUI	19	1.001016
48H 10MT/	Q9UPY8	-0.06546	0.104592	0.062316	MAPRE3	Microtubul	MARE3_HU	1	0.955639
24H 10MT/	Q96BP2	-0.00822	0.10464	0.062337	CHCHD1	Small ribos	CHCH1_HU	1	0.994316
48H 50MT/	Q12770	-0.65861	0.104666	0.062351	SCAP	Sterol regul	SCAP_HUM	2	0.633488
48H 10MT/	P04003	-0.06135	0.104673	0.062353	C4BPA	C4b-bindin	C4BPA_HUI	1	0.958366
48H 50MT/	Q13393	0.098825	0.104693	0.062362	PLD1	Phospholip	PLD1_HUM	24	1.070901
48H 10MT/	O95394	0.017087	0.104696	0.062363	PGM3	Phosphoac	AGM1_HUI	21	1.011914
24H 10MT/	P07951	0.030682	0.104704	0.062367	TPM2	Tropomyos	TPM2_HUM	10	1.021495
24H 10MT/	Q5VWX1	0.149471	0.104707	0.062368	KHDRBS2	KH domain	KHDR2_HU	2	1.109163
48H 10MT/	O00273	0.001534	0.104768	0.062398	DFFA	DNA fragme	DFFA_HUM	14	1.001064
48H 50MT/	O75446	-0.08056	0.104771	0.062398	SAP30	Histone dea	SAP30_HUI	3	0.945688
48H 10MT/	P08236	-0.0211	0.104791	0.062405	GUSB	Beta-glucur	BGLR_HUM	6	0.985483
24H 50MT/	Q06203	0.185942	0.104809	0.062414	PPAT	Amidophos	PUR1_HUM	17	1.13756
48H 10MT/	P13861	0.075877	0.104816	0.062417	PRKAR2A	cAMP-depe	KAP2_HUM	21	1.054001
48H 50MT/	P42696	-0.16829	0.104822	0.062419	RBM34	RNA-bindir	RBM34_HU	19	0.889894
48H 10MT/	Q9NPQ8	-0.1016	0.104838	0.062427	RIC8A	Synembryn	RIC8A_HUI	17	0.932001
48H 50MT/	Q96MH6	0.225358	0.104873	0.062441	TMEM68	DGAT1/2-ir	DIESL_HUM	4	1.169067
24H 10MT/	Q9Y281	0.054487	0.104882	0.062444	CFL2	Cofilin-2	COF2_HUM	9	1.038489
48H 50MT/	Q14449	-0.44401	0.104914	0.062461	GRB14	Growth fac	GRB14_HU	4	0.735087
48H 10MT/	Q9H1B7	-0.0015	0.104924	0.062465	IRF2BPL	Probable E3	I2BPL_HUM	6	0.99896
48H 10MT/	O43665	0.137517	0.104928	0.062466	RGS10	Regulator c	RGS10_HUI	4	1.10001
48H 50MT/	Q13555	0.144897	0.105018	0.062511	CAMK2G	Calcium/ca	KCC2G_HU	6	1.105652
48H 50MT/	Q2PZI1	0.140863	0.105019	0.062511	DPY19L1	Protein C-m	D19L1_HUI	13	1.102565
48H 50MT/	Q92782	0.219154	0.105025	0.062512	DPF1	Zinc finger	DPF1_HUM	1	1.164051
24H 50MT/	P47985	0.134365	0.105048	0.062523	UQCRFS1	Cytochrom	UCRI_HUM	11	1.09761
24H 50MT/	O75152	0.012134	0.105054	0.062524	ZC3H11A	Zinc finger	ZC11A_HUI	18	1.008446
48H 50MT/	P46976	0.031125	0.105104	0.062543	GYG1	Glycogenin	GLYG_HUM	12	1.021809
48H 50MT/	Q96K19	0.087161	0.105108	0.062544	RNF170	E3 ubiquiti	RN170_HU	6	1.062277
48H 50MT/	Q8IVP5	0.86721	0.105126	0.062554	FUNDC1	FUN14 don	FUND1_HU	1	1.824132
48H 50MT/	Q13769	0.060442	0.10515	0.062565	THOC5	THO compl	THOC5_HU	23	1.042785
24H 50MT/	O00115	0.10551	0.10516	0.062569	DNASE2	Deoxyribor	DNS2A_HU	5	1.075875
24H 10MT/	Q9UJ41	-0.05087	0.105183	0.06258	RABGEF1	Rab5 GDP/	RABX5_HUI	11	0.965352
48H 50MT/	Q8NEZ5	0.085127	0.105221	0.062599	FBXO22	F-box only	FBX22_HUI	12	1.060781
48H 50MT/	Q92805	-0.08576	0.105256	0.062618	GOLGA1	Golgin subf	GOGA1_HU	23	0.942288
24H 10MT/	Q9HBM6	0.075178	0.105283	0.062629	TAF9B	Transcripti	TAF9B_HUI	10	1.053491
48H 10MT/	P40205	0.311243	0.105288	0.062631	MYCNOS	N-cym prot	NCYM_HUM	1	1.240776
24H 10MT/	P60891	0.010839	0.105295	0.062632	PRPS1	Ribose-pho	PRPS1_HUI	14	1.007541
48H 10MT/	Q9H6S0	0.043181	0.105312	0.062641	YTHDC2	3'-5' RNA he	YTDC2_HUI	37	1.030383
24H 50MT/	Q8WU90	-0.01579	0.105317	0.062643	ZC3H15	Zinc finger	ZC3HF_HUI	15	0.989116
48H 50MT/	Q96EY1	-0.12599	0.105355	0.06266	DNAJA3	DnaJ homo	DNJA3_HUI	15	0.916373

48H 50MT/	Q5M775	-0.21872	0.105367	0.062665	SPECC1	Cytospin-B	CYTSB_HUN	17	0.859327
48H 50MT/	Q7Z4R8	0.277208	0.105393	0.062674	C6orf120	UPF0669 p	CF120_HUI	1	1.211847
24H 50MT/	Q9P032	0.167968	0.105433	0.062695	NDUFAF4	NADH dehy	NDUF4_HU	5	1.123475
48H 10MT/	Q9H6H4	-0.13398	0.105441	0.062699	REEP4	Receptor e	REEP4_HUN	4	0.911315
24H 50MT/	Q8IWZ8	0.03466	0.10546	0.062701	SUGP1	SURP and G	SUGP1_HU	20	1.024315
24H 50MT/	Q16773	-0.14276	0.105469	0.062705	KYAT1	Kynurenine	KAT1_HUM	3	0.905782
24H 50MT/	Q9H1Z4	0.012303	0.105522	0.062734	WDR13	WD repeat-	WDR13_HU	13	1.008565
48H 50MT/	O75391	-0.01142	0.10553	0.062738	SPAG7	Sperm-asso	SPAG7_HUI	8	0.992113
48H 50MT/	P51580	-0.00799	0.105545	0.062744	TPMT	Thiopurine	TPMT_HUM	11	0.994477
24H 50MT/	Q96TC7	0.063328	0.105561	0.062752	RMDN3	Regulator c	RMD3_HUN	15	1.044873
24H 50MT/	Q17RY0	-0.44608	0.105565	0.062754	CPEB4	Cytoplasmi	CPEB4_HUI	11	0.734036
24H 10MT/	Q96P70	0.04002	0.105568	0.062754	IPO9	Importin-9	IPO9_HUM	28	1.028128
48H 10MT/	O94876	0.082789	0.105586	0.062762	TMCC1	Transmemb	TMCC1_HU	2	1.059063
24H 10MT/	Q9H6U6	-0.23806	0.105586	0.062762	BCAS3	BCAS3 micr	BCAS3_HUI	3	0.847887
48H 10MT/	P35568	0.012131	0.105604	0.06277	IRS1	Insulin rece	IRS1_HUM/	5	1.008444
48H 10MT/	O95870	-0.03821	0.105613	0.062772	ABHD16A	Phosphatic	ABHGA_HU	12	0.973865
24H 10MT/	Q9UDT6	-0.04146	0.105612	0.062772	CLIP2	CAP-Gly do	CLIP2_HUN	31	0.971674
24H 10MT/	Q9Y4E5	0.044592	0.105617	0.062773	ZNF451	E3 SUMO-p	ZN451_HUI	8	1.031391
48H 10MT/	Q9NVN8	0.002337	0.105672	0.062801	GNL3L	Guanine nu	GNL3L_HUI	17	1.001621
48H 10MT/	Q6ZTW0	-0.02385	0.1057	0.062813	TPGS1	Tubulin pol	TPGS1_HUI	6	0.983607
48H 50MT/	Q9H3H3	0.129045	0.105746	0.062834	C11orf68	UPF0696 p	CK068_HUI	12	1.093569
24H 10MT/	P61326	0.078128	0.105768	0.062846	MAGOH	Protein ma	MGN_HUM	1	1.055647
24H 10MT/	P38935	0.054083	0.105788	0.062852	IGHMBP2	DNA-bindir	SMBP2_HU	8	1.038199
24H 50MT/	Q16630	-0.11124	0.105786	0.062852	CPSF6	Cleavage ar	CPSF6_HUN	11	0.925791
24H 50MT/	Q70E73	-0.00202	0.105786	0.062852	RAPH1	Ras-associa	RAPH1_HU	4	0.998601
48H 10MT/	Q9NQT8	-0.01988	0.105787	0.062852	KIF13B	Kinesin-like	KI13B_HUN	20	0.986313
24H 50MT/	Q96QF0	-0.44853	0.105797	0.062856	RAB3IP	Rab-3A-inte	RAB3I_HUN	4	0.732787
48H 50MT/	Q9H063	-0.35089	0.105825	0.062868	MAF1	Repressor c	MAF1_HUN	2	0.784098
48H 50MT/	Q9NYZ1	0.247995	0.105913	0.062911	TVP23B	Golgi appar	TV23B_HUI	4	1.187556
48H 50MT/	Q8NBF6	-0.18195	0.105939	0.062923	AVL9	Late secret	AVL9_HUM	11	0.881509
24H 50MT/	Q96N66	-0.02686	0.105956	0.06293	MBOAT7	Lysophospl	MBOA7_HU	7	0.981551
24H 10MT/	Q6UWZ7	0.21114	0.105978	0.062941	ABRAXAS1	BRCA1-A cc	ABRX1_HUI	3	1.157603
24H 50MT/	Q8TCT8	-0.01911	0.106061	0.062983	SPPL2A	Signal pept	SPP2A_HUI	4	0.986844
24H 50MT/	Q03426	-0.10341	0.106109	0.063008	MVK	Mevalonate	KIME_HUM	9	0.930832
24H 10MT/	Q969N2	-0.12612	0.106139	0.063024	PIGT	GPI transan	PIGT_HUM/	19	0.91629
24H 50MT/	A3KN83	-0.08123	0.106174	0.063039	SBNO1	Protein stra	SBNO1_HU	24	0.945248
48H 10MT/	Q00266	-0.25303	0.106174	0.063039	MAT1A	S-adenosylı	METK1_HU	3	0.839132
24H 10MT/	Q12834	0.110699	0.106176	0.063039	CDC20	Cell divisioı	CDC20_HU	11	1.079752
48H 10MT/	Q15311	0.020184	0.106183	0.063039	RALBP1	RalA-bindir	RBP1_HUM	6	1.014089
24H 10MT/	Q15836	0.075117	0.106182	0.063039	VAMP3	Vesicle-assc	VAMP3_HU	5	1.053446
24H 50MT/	Q6UB28	-0.04488	0.106183	0.063039	METAP1D	Methionine	MAP12_HU	2	0.96937
24H 50MT/	P48507	-0.1319	0.106195	0.063043	GCLM	Glutamate-	GSH0_HUM	11	0.912627
24H 50MT/	Q7Z569	-0.07789	0.106195	0.063043	BRAP	BRCA1-assc	BRAP_HUM	9	0.947445
48H 50MT/	Q969F1	0.255054	0.106222	0.063054	GTF3C6	General tra	TF3C6_HUN	1	1.193381
24H 50MT/	Q9BW85	-0.06016	0.106223	0.063054	YJU2	Splicing fac	YJU2_HUM	12	0.959157
48H 50MT/	Q16352	-0.04434	0.10623	0.063057	INA	Alpha-inter	AINX_HUM	15	0.96973
24H 10MT/	Q12830	0.018022	0.106272	0.063079	BPTF	Nucleosom	BPTF_HUM	26	1.01257
48H 10MT/	Q13523	0.057445	0.106291	0.063089	PRP4K	Serine/thre	PRP4K_HUI	20	1.040621
48H 10MT/	Q9Y3I1	0.162352	0.106306	0.063096	FBXO7	F-box only	FBX7_HUM	11	1.11911
24H 10MT/	Q9Y5L4	-0.08751	0.106406	0.063147	TIMM13	Mitochond	TIM13_HUI	7	0.941146
48H 50MT/	A9UHW6	0.333215	0.106465	0.063179	MIF4GD	MIF4G dom	MI4GD_HU	1	1.259818
24H 10MT/	P03891	0.085327	0.106469	0.06318	MT-ND2	NADH-ubiq	NU2M_HUI	1	1.060928

48H 10MT/	Q7Z4V5	-0.01693	0.106478	0.063184	HDGFL2	Hepatoma-	HDGR2_HU	11	0.988332
24H 10MT/	Q7Z6J9	0.124927	0.106484	0.063184	TSEN54	tRNA-splici	SEN54_HUI	7	1.090453
24H 10MT/	Q02790	-0.0262	0.106525	0.063203	FKBP4	Peptidyl-pr	FKBP4_HUI	34	0.982
24H 10MT/	O95873	0.010776	0.106551	0.063211	C6orf47	Uncharacte	CF047_HUI	1	1.007497
24H 50MT/	Q14241	-0.0224	0.106557	0.063211	ELOA	Elongin-A	ELOA1_HUI	7	0.984595
48H 50MT/	Q9NZV5	0.169241	0.106549	0.063211	SELENON	Selenoprot	SELN_HUM	4	1.124467
48H 10MT/	Q96LT9	0.099273	0.10656	0.063212	RNPC3	RNA-bindir	RNPC3_HU	5	1.071234
24H 50MT/	Q8TCY9	-0.11695	0.106571	0.063216	URGCP	Up-regulat	URGCP_HU	22	0.922133
24H 50MT/	Q99571	0.058153	0.106577	0.063219	P2RX4	P2X purino	P2RX4_HUI	5	1.041132
24H 50MT/	P20226	-0.02404	0.106584	0.063221	TBP	TATA-box-b	TBP_HUMA	5	0.983475
48H 50MT/	Q9UKX3	-1.0033	0.106586	0.063221	MYH13	Myosin-13	MYH13_HU	1	0.498857
24H 50MT/	P35244	-0.00964	0.106603	0.063227	RPA3	Replication	RFA3_HUM	7	0.993337
24H 50MT/	Q96AY4	0.002602	0.106621	0.063236	TTC28	Tetratricop	TTC28_HUI	3	1.001805
24H 50MT/	Q8NAV1	0.251483	0.106635	0.063241	PRPF38A	Pre-mRNA-:	PR38A_HUI	10	1.19043
48H 10MT/	P30040	-0.00761	0.106638	0.063242	ERP29	Endoplasm	ERP29_HUI	14	0.994739
24H 50MT/	O43306	0.147811	0.106645	0.063242	ADCY6	Adenylate c	ADCY6_HUI	6	1.107888
48H 50MT/	Q96B01	-0.31298	0.106646	0.063242	RAD51AP1	RAD51-assc	R51A1_HUI	3	0.804977
24H 10MT/	Q9H7C9	-0.11401	0.106668	0.063252	AAMDC	Mth938 do	AAMDC_HU	7	0.924013
48H 10MT/	Q01201	-0.07906	0.106737	0.063284	RELB	Transcripti	RELB_HUM	5	0.946675
48H 10MT/	Q7Z7A4	-0.00095	0.106825	0.063324	PXK	PX domain-	PXK_HUMA	6	0.999343
24H 10MT/	P35249	0.018235	0.106838	0.063327	RFC4	Replication	RFC4_HUM	18	1.01272
48H 10MT/	Q8NEC7	-0.0677	0.106835	0.063327	GSTCD	Glutathion	GSTCD_HUI	6	0.954158
48H 50MT/	Q9P086	0.160897	0.106842	0.063328	MED11	Mediator o	MED11_HU	1	1.117982
48H 50MT/	Q9H6U8	0.062225	0.106906	0.063358	ALG9	Alpha-1,2-r	ALG9_HUM	7	1.044075
48H 10MT/	O43678	-0.0489	0.106929	0.063367	NDUFA2	NADH dehy	NDUA2_HU	4	0.966671
48H 10MT/	P00813	-0.00662	0.106926	0.063367	ADA	Adenosine	ADA_HUMA	14	0.995425
48H 50MT/	P00505	-0.06305	0.106937	0.06337	GOT2	Aspartate a	AATM_HUM	26	0.957241
24H 50MT/	Q8NBL1	-0.00912	0.106942	0.063372	POGLUT1	Protein O-g	PGLT1_HUI	12	0.993696
48H 50MT/	P46783	-0.0645	0.106994	0.063396	RPS10	Small ribos	RS10_HUM	7	0.956276
48H 50MT/	Q14CX7	-0.0658	0.107025	0.063407	NAA25	N-alpha-ace	NAA25_HU	30	0.955412
24H 50MT/	Q8WWX9	-0.52995	0.10704	0.063413	SELENOM	Selenoprot	SELM_HUM	1	0.692578
48H 50MT/	Q8WX93	0.026313	0.107044	0.063414	PALLD	Palladin	PALLD_HUI	7	1.018406
48H 50MT/	P33897	-0.05638	0.107048	0.063415	ABCD1	ATP-bindin	ABCD1_HU	7	0.961675
48H 10MT/	Q9NZJ0	-0.20745	0.107049	0.063415	DTL	Denticleles	DTL_HUMA	4	0.866067
24H 10MT/	Q6VN20	0.028589	0.107065	0.063422	RANBP10	Ran-bindin	RBP10_HUI	14	1.020014
24H 50MT/	Q14145	-0.04911	0.107074	0.063425	KEAP1	Kelch-like E	KEAP1_HUI	20	0.966531
24H 50MT/	Q7RTV5	-0.05817	0.107095	0.063435	PRXL2C	Peroxiredo	PXL2C_HUI	3	0.960481
24H 10MT/	Q7KZI7	0.048457	0.10711	0.063441	MARK2	Serine/thre	MARK2_HU	19	1.034158
24H 50MT/	Q9NVV0	-0.54828	0.107113	0.063442	TMEM38B	Trimeric in	TM38B_HU	3	0.683834
48H 50MT/	Q7Z7F7	-0.18304	0.107146	0.063458	MRPL55	Large ribos	RM55_HUM	3	0.880848
48H 50MT/	Q9P253	-0.0927	0.107153	0.063461	VPS18	Vacuolar pi	VPS18_HUI	27	0.937764
24H 10MT/	P08133	-0.45073	0.107176	0.063471	ANXA6	Annexin A6	ANXA6_HU	6	0.73167
48H 10MT/	P52294	-0.00673	0.107175	0.063471	KPNA1	Importin su	IMA5_HUM	8	0.995348
48H 10MT/	Q8WUX2	0.115153	0.107208	0.063487	CHAC2	Glutathion	CHAC2_HU	3	1.08309
48H 10MT/	Q9BVG8	0.057008	0.10722	0.063492	KIFC3	Kinesin-like	KIFC3_HUM	16	1.040306
24H 10MT/	Q96CT7	-0.10414	0.107292	0.063529	CCDC124	Coiled-coil	CC124_HUI	10	0.930362
24H 10MT/	Q13148	0.05123	0.107301	0.063533	TARDBP	TAR DNA-bi	TADBP_HUI	14	1.036148
24H 50MT/	P26885	0.020692	0.10731	0.063537	FKBP2	Peptidyl-pr	FKBP2_HUI	6	1.014446
48H 10MT/	P78536	-0.13931	0.107347	0.063555	ADAM17	Disintegrin	ADA17_HU	8	0.907952
48H 50MT/	O14815	-0.27764	0.107366	0.063563	CAPN9	Calpain-9	CAN9_HUM	1	0.824939
48H 10MT/	P11274	-0.01871	0.107401	0.06358	BCR	Breakpoint	BCR_HUMA	10	0.987116
48H 10MT/	Q96HV5	-0.15959	0.107401	0.06358	TMEM41A	Transmemb	TM41A_HU	3	0.895282

48H 10MT/	Q15058	0.045733	0.107432	0.063597	KIF14	Kinesin-like	KIF14_HUM	15	1.032207
48H 50MT/	Q9UP83	-0.04003	0.107438	0.063597	COG5	Conserved	COG5_HUM	23	0.972633
48H 10MT/	Q96E52	0.211881	0.107459	0.063605	OMA1	Metalloend	OMA1_HUM	5	1.158198
48H 50MT/	O94964	-0.33003	0.107463	0.063606	MTCL2	Microtubul	MTCL2_HU	9	0.795519
24H 10MT/	Q86X95	-0.27176	0.10747	0.063609	CIR1	Corepresso	CIR1_HUM	1	0.828308
48H 10MT/	Q15365	-0.12336	0.107475	0.063611	PCBP1	Poly(rC)-bi	PCBP1_HU	14	0.918044
24H 50MT/	Q9Y5Y5	0.114137	0.107483	0.063615	PEX16	Peroxisoma	PEX16_HU	6	1.082328
24H 50MT/	Q96R05	0.04546	0.107524	0.063632	RBP7	Retinoid-bi	RET7_HUM	2	1.032012
24H 10MT/	B1AK53	0.159795	0.107578	0.063654	ESPN	Espin	ESPN_HUM	1	1.117128
48H 50MT/	O75197	1.137874	0.107603	0.063667	LRP5	Low-densit	LRP5_HUM	2	2.200565
48H 50MT/	Q96EV8	-0.01413	0.10763	0.06368	DTNBP1	Dysbindin	DTBP1_HU	4	0.990253
24H 50MT/	Q9NRD5	-0.15107	0.107661	0.063696	PICK1	PRKCA-bin	PICK1_HUM	3	0.90058
48H 10MT/	Q5SQH8	-0.4873	0.107682	0.063701	C6orf136	Uncharacte	CF136_HU	2	0.713359
48H 10MT/	Q9BT40	0.026908	0.107682	0.063701	INPP5K	Inositol pol	INP5K_HUM	2	1.018826
48H 50MT/	Q8N2M8	0.074105	0.10769	0.063705	CLASRP	CLK4-assoc	CLASR_HUM	2	1.052708
24H 50MT/	Q14376	-0.00184	0.107699	0.063708	GALE	UDP-glucos	GALE_HUM	13	0.998723
48H 10MT/	O60704	0.215958	0.107712	0.063715	TPST2	Protein-tyr	TPST2_HUM	1	1.161475
48H 50MT/	O95396	-0.04303	0.107713	0.063715	MOCS3	Adenylyltra	MOCS3_HU	13	0.970617
24H 50MT/	Q13895	-0.00324	0.107722	0.063718	BYSL	Bystin	BYST_HUM	20	0.997754
48H 50MT/	O76011	-0.18634	0.107748	0.063729	KRT34	Keratin, typ	KRT34_HU	1	0.878833
24H 50MT/	Q9Y3F4	-0.06147	0.107774	0.06374	STRAP	Serine-thre	STRAP_HUM	19	0.958289
48H 10MT/	P40424	0.12919	0.107787	0.063745	PBX1	Pre-B-cell le	PBX1_HUM	3	1.09368
48H 10MT/	Q9NY26	-0.06875	0.107806	0.063754	SLC39A1	Zinc transp	S39A1_HU	1	0.953466
48H 10MT/	O75648	-0.19984	0.107817	0.06376	TRMU	Mitochond	MTU1_HUM	13	0.870645
48H 10MT/	Q9C0B0	0.08022	0.107838	0.063769	UNK	RING finger	UNK_HUM	3	1.057179
24H 50MT/	Q13148	0.080837	0.107843	0.06377	TARDBP	TAR DNA-bi	TADBP_HU	14	1.057632
24H 10MT/	Q13426	0.117673	0.10785	0.063773	XRCC4	DNA repair	XRCC4_HU	9	1.084983
24H 10MT/	Q14558	0.085633	0.107852	0.063773	PRPSAP1	Phosphorib	KPRA_HUM	11	1.061153
48H 50MT/	P27635	-0.03164	0.107857	0.063775	RPL10	Large ribos	RL10_HUM	12	0.978308
48H 50MT/	P24666	0.031253	0.107888	0.063789	ACP1	Low molec	PPAC_HUM	9	1.021899
24H 10MT/	P25098	0.128598	0.107893	0.063791	GRK2	Beta-adren	ARBK1_HU	7	1.093231
48H 10MT/	Q9C075	-0.07601	0.107918	0.063804	KRT23	Keratin, typ	K1C23_HU	7	0.948679
48H 10MT/	Q9ULT8	0.040073	0.10793	0.063806	HECTD1	E3 ubiquiti	HECD1_HU	64	1.028166
48H 50MT/	O75312	-0.02146	0.107943	0.063812	ZPR1	Zinc finger	ZPR1_HUM	17	0.985238
24H 10MT/	Q9BY12	3.09E-05	0.107991	0.063837	SCAPER	S phase cyc	SCAPE_HUM	7	1.000021
24H 10MT/	Q96MW1	-0.06424	0.107995	0.063839	CCDC43	Coiled-coil	CCD43_HU	7	0.956451
24H 10MT/	P56556	0.110442	0.10802	0.063846	NDUFA6	NADH dehy	NDUA6_HU	6	1.079559
24H 10MT/	Q9UBX3	0.050312	0.10802	0.063846	SLC25A10	Mitochond	DIC_HUMA	13	1.035489
48H 50MT/	Q86UB9	-0.23879	0.108048	0.063861	TMEM135	Transmemb	TM135_HU	7	0.847458
24H 10MT/	Q9NRD5	-0.00064	0.108103	0.063885	PICK1	PRKCA-bin	PICK1_HUM	3	0.999557
48H 50MT/	Q92562	-0.38242	0.108106	0.063885	FIG4	Polyphosph	FIG4_HUM	4	0.767149
48H 50MT/	Q93073	0.125045	0.108118	0.063891	SECISBP2L	Selenocyst	SBP2L_HUM	5	1.090541
48H 50MT/	Q14524	0.09139	0.108141	0.063903	SCN5A	Sodium cha	SCN5A_HU	1	1.065396
24H 50MT/	P0C2W1	0.228617	0.108149	0.063907	FBXO45	F-box/SPRY	FBSP1_HUM	2	1.171711
48H 10MT/	Q96IV0	0.078141	0.108163	0.063914	NGLY1	Peptide-N(4	NGLY1_HU	11	1.055657
48H 50MT/	P32189	-0.01119	0.108186	0.063926	GK	Glycerol kir	GLPK_HUM	13	0.992277
24H 50MT/	Q13670	-0.10051	0.108192	0.063927	PMS2P11	Putative pc	PM2PB_HU	1	0.9327
48H 10MT/	Q8IVD9	0.075986	0.108192	0.063927	NUDCD3	NudC doma	NUDC3_HU	11	1.054081
48H 50MT/	Q9Y4D1	-1.61401	0.108197	0.063929	DAAM1	Disheveled	DAAM1_HU	1	0.326689
24H 10MT/	O76011	0.554756	0.108206	0.063933	KRT34	Keratin, typ	KRT34_HU	1	1.46892
48H 50MT/	Q6ZRQ5	0.021241	0.108241	0.063953	MMS22L	Protein MN	MMS22_HU	10	1.014832
24H 10MT/	P61204	0.072987	0.108257	0.06396	ARF3	ADP-ribosy	ARF3_HUM	12	1.051892

24H 50MT/	Q92620	0.022141	0.108298	0.063979	DHX38	Pre-mRNA-s	PRP16_HUI	33	1.015466
24H 50MT/	Q92696	-0.12979	0.108306	0.063983	RABGGTA	Geranylgera	PGTA_HUM	21	0.913962
48H 10MT/	Q8TEH3	0.212488	0.10833	0.063995	DENND1A	DENN doma	DEN1A_HU	3	1.158685
24H 10MT/	P18615	0.060214	0.108353	0.064005	NELFE	Negative ele	NELFE_HUM	15	1.042621
24H 50MT/	Q9H6S1	-0.11539	0.108402	0.064029	AZI2	5-azacytidi	AZI2_HUM/	5	0.923134
48H 10MT/	O00203	-0.00951	0.108484	0.06407	AP3B1	AP-3 compl	AP3B1_HUI	37	0.993433
24H 50MT/	Q8WUM0	-0.04685	0.108493	0.064073	NUP133	Nuclear po	NU133_HU	46	0.96805
24H 10MT/	Q92574	-0.48913	0.1085	0.064075	TSC1	Hamartin	TSC1_HUM	1	0.712454
48H 10MT/	Q16831	-0.06851	0.108504	0.064076	UPP1	Uridine pho	UPP1_HUM	16	0.953623
48H 10MT/	Q8NEM2	-0.08664	0.10854	0.064094	SHCBP1	SHC SH2 dc	SHCBP_HUI	20	0.941715
48H 50MT/	P30101	0.113945	0.108649	0.064145	PDIA3	Protein dis	PDIA3_HUM	40	1.082183
48H 10MT/	Q9Y5Q9	-0.07992	0.108657	0.064147	GTF3C3	General tra	TF3C3_HUI	24	0.946108
24H 10MT/	P45877	0.240834	0.108665	0.06415	PPIC	Peptidyl-pr	PPIC_HUM/	2	1.181675
48H 10MT/	P62888	0.012217	0.108671	0.064153	RPL30	Large ribos	RL30_HUM	5	1.008504
48H 50MT/	Q8NBX0	0.003915	0.108698	0.064167	SCCPDH	Saccharopi	SCPDL_HUI	12	1.002717
48H 50MT/	O75179	-0.15646	0.108729	0.064183	ANKRD17	Ankyrin rep	ANR17_HU	22	0.897224
48H 10MT/	P09619	-0.40935	0.108762	0.064195	PDGFRB	Platelet-der	PGFRB_HUI	2	0.752964
24H 10MT/	Q8IV48	0.020542	0.108764	0.064195	ERI1	3'-5' exorib	ERI1_HUM/	4	1.014341
24H 10MT/	Q99959	0.086856	0.108788	0.064203	PKP2	Plakophilin	PKP2_HUM	27	1.062053
24H 50MT/	Q9Y394	0.14431	0.108823	0.064219	DHRS7	Dehydroger	DHRS7_HU	14	1.105202
48H 10MT/	O15121	0.025551	0.108844	0.06423	DEGS1	Sphingolipi	DEGS1_HUI	3	1.017868
24H 50MT/	P41134	0.312259	0.108846	0.06423	ID1	DNA-bindir	ID1_HUMA	4	1.24165
24H 10MT/	P62760	-0.30676	0.108928	0.064265	VSNL1	Visinin-like	VISL1_HUM	7	0.808455
24H 50MT/	Q15599	0.021466	0.108968	0.064284	NHERF2	Na(+)/H(+) e	NHRF2_HU	6	1.014991
48H 50MT/	O75676	-0.14313	0.109039	0.064319	RPS6KA4	Ribosomal	KS6A4_HUI	9	0.905554
48H 10MT/	Q12923	-0.69525	0.109061	0.06433	PTPN13	Tyrosine-pr	PTN13_HUI	1	0.617602
48H 50MT/	O95232	0.037112	0.109082	0.064338	LUC7L3	Luc7-like pı	LC7L3_HUM	10	1.026058
24H 10MT/	A6NCE7;Q9	0.01843	0.109116	0.06435	MAP1LC3B	Microtubul	MP3B2_HU	1	1.012857
24H 50MT/	Q9UID3	0.004754	0.109121	0.064351	VPS51	Vacuolar pı	VPS51_HUI	23	1.003301
48H 10MT/	Q6ZNA5	-0.07426	0.109129	0.064354	FRRS1	Ferric-chela	FRRS1_HUM	8	0.949828
24H 10MT/	Q9Y312	0.010966	0.109133	0.064355	AAR2	Protein AAI	AAR2_HUM	12	1.00763
24H 10MT/	Q05086	0.067455	0.109143	0.064359	UBE3A	Ubiquitin-p	UBE3A_HUI	28	1.047867
24H 10MT/	Q9BSH5	0.057638	0.109155	0.064364	HDHD3	Haloacid de	HDHD3_HU	10	1.040761
24H 50MT/	O60921	0.04149	0.109163	0.064366	HUS1	Checkpoint	HUS1_HUM	9	1.029176
48H 10MT/	P50395	-0.00644	0.109167	0.064366	GDI2	Rab GDP di	GDIB_HUM	36	0.995549
24H 10MT/	Q9Y276	0.003015	0.109166	0.064366	BCS1L	Mitochond	BCS1_HUM	19	1.002092
48H 50MT/	Q9NV96	0.04613	0.109211	0.064385	TMEM30A	Cell cycle c	CC50A_HUI	6	1.032491
48H 10MT/	Q96DB5	0.056401	0.109219	0.064388	RMDN1	Regulator c	RMD1_HUM	13	1.039868
48H 10MT/	O60281	-0.09638	0.109238	0.064398	ZNF292	Zinc finger	ZN292_HUI	6	0.935378
24H 10MT/	P26038	-0.00853	0.109251	0.064403	MSN	Moesin	MOES_HUM	27	0.994103
24H 10MT/	Q49A26	0.043789	0.109264	0.064409	GLYR1	Cytokine-lil	GLYR1_HUI	18	1.030817
24H 50MT/	P51649	0.125826	0.10927	0.064411	ALDH5A1	Succinate-s	SSDH_HUM	15	1.091132
48H 10MT/	Q9Y4E8	0.035329	0.109303	0.064427	USP15	Ubiquitin c	UBP15_HU	40	1.024791
48H 50MT/	Q5T6F2	-0.07715	0.109312	0.064427	UBAP2	Ubiquitin-a	UBAP2_HU	11	0.947927
24H 50MT/	Q9C0C9	-0.02192	0.109311	0.064427	UBE2O	(E3-indeper	UBE2O_HU	30	0.984922
24H 10MT/	Q96QU8	0.045917	0.109317	0.064429	XPO6	Exportin-6	XPO6_HUM	15	1.032339
24H 10MT/	Q9NRN7	0.04136	0.10932	0.064429	AASDHPPT	L-aminoadi	ADPPT_HUI	11	1.029083
48H 10MT/	P11413	-0.05674	0.109346	0.06444	G6PD	Glucose-6-p	G6PD_HUM	34	0.961435
24H 10MT/	P61764	-0.04045	0.109342	0.06444	STXBP1	Syntaxin-bi	STXB1_HUM	13	0.972352
48H 50MT/	Q9BSY4	0.25533	0.109361	0.064444	CHCHD5	Coiled-coil-	CHCH5_HU	1	1.193609
24H 50MT/	Q6ZNC8	0.101284	0.109368	0.064447	MBOAT1	Lysophospl	MBOA1_HU	1	1.072728
48H 50MT/	Q9NW15	-0.21523	0.109403	0.064461	ANO10	Anoctamin	ANO10_HU	9	0.861411

24H 50MT/	P24390	0.370392	0.10948	0.064488	KDELR1	ER lumen p	ERD21_HUI	2	1.292704
48H 50MT/	Q6P4A8	-0.08673	0.109483	0.064488	PLBD1	Phospholip	PLBL1_HUN	10	0.941655
48H 50MT/	Q9Y2S6	0.106754	0.109478	0.064488	TMA7	Translation	TMA7_HUN	2	1.076803
24H 10MT/	Q9NUP7	0.060996	0.109521	0.064506	TRMT13	tRNA:m(4))	TRM13_HU	7	1.043185
48H 10MT/	P62341	-1.4031	0.109529	0.064507	SELENOT	Thioredoxi	SELT_HUM/	2	0.378116
24H 50MT/	Q9Y5L4	-0.17716	0.109539	0.064512	TIMM13	Mitochond	TIM13_HUI	7	0.884444
24H 50MT/	Q99598	-0.03148	0.109573	0.064529	TSNAX	Translin-ass	TSNAX_HUI	10	0.978413
48H 10MT/	P62241	0.026292	0.109612	0.064549	RPS8	Small ribos	RS8_HUMA	11	1.018391
48H 10MT/	Q13972	0.7095	0.10961	0.064549	RASGRF1	Ras-specific	RGRF1_HUI	1	1.635237
48H 10MT/	Q9UNQ2	-0.02976	0.109636	0.064562	DIMT1	Dimethylac	DIM1_HUN	13	0.979581
48H 10MT/	O14617	-0.02217	0.109654	0.064571	AP3D1	AP-3 compl	AP3D1_HU	36	0.984748
48H 10MT/	O75170	0.041611	0.109688	0.064588	PPP6R2	Serine/thre	PP6R2_HUI	30	1.029262
24H 50MT/	P30536	0.291226	0.109688	0.064588	TSPO	Translocatc	TSPO_HUM	2	1.22368
24H 10MT/	O43615	-0.00345	0.109716	0.064599	TIMM44	Mitochond	TIM44_HUI	22	0.99761
24H 50MT/	P62487	-0.05124	0.109738	0.064609	POLR2G	DNA-direct	RPB7_HUM	7	0.965108
48H 50MT/	O95807	-0.01427	0.109754	0.064617	TMEM50A	Transmemb	TM50A_HU	1	0.990159
24H 10MT/	P05198	0.001256	0.109767	0.064624	EIF2S1	Eukaryotic	IF2A_HUM/	25	1.000871
24H 10MT/	O43583	-0.15061	0.109778	0.064625	DENR	Density-reg	DENR_HUM	9	0.900873
48H 50MT/	P51688	-0.14799	0.109777	0.064625	SGSH	N-sulphogl	SPHM_HUN	6	0.902506
48H 50MT/	Q15181	0.028642	0.109821	0.064648	PPA1	Inorganic p	IPYR_HUM/	23	1.020052
24H 50MT/	Q8ND24	0.033209	0.10984	0.064657	RNF214	RING finger	RN214_HU	20	1.023286
24H 10MT/	Q06587	0.054524	0.109927	0.064698	RING1	E3 ubiquiti	RING1_HUI	8	1.038516
48H 10MT/	P00568	0.106886	0.109951	0.064707	AK1	Adenylate k	KAD1_HUN	9	1.076901
24H 10MT/	Q9HB07	-0.09543	0.109953	0.064707	MYG1	MYG1 exon	MYG1_HUN	15	0.935995
48H 10MT/	P63151	-0.0279	0.110038	0.064749	PPP2R2A	Serine/thre	2ABA_HUM	14	0.980847
24H 50MT/	Q9BUM1	-0.01425	0.110042	0.064751	G6PC3	Glucose-6-p	G6PC3_HU	1	0.990174
48H 50MT/	O00458	0.358371	0.110066	0.064761	IFRD1	Interferon-i	IFRD1_HUN	6	1.281978
24H 10MT/	Q86V48	0.00278	0.110088	0.064768	LUZP1	Leucine zip	LUZP1_HUI	22	1.001929
24H 50MT/	P23368	0.011011	0.110105	0.064776	ME2	NAD-depen	MAOM_HU	24	1.007662
48H 10MT/	Q9Y2B0	0.039896	0.110108	0.064776	CNPY2	Protein can	CNPY2_HUI	11	1.02804
24H 10MT/	O14681	0.184436	0.110137	0.064789	EI24	Etoposide-i	EI24_HUM/	5	1.136372
48H 50MT/	P36915	0.03857	0.110159	0.064799	GNL1	Guanine nu	GNL1_HUN	23	1.027095
24H 50MT/	Q96FS4	0.13715	0.110229	0.064834	SIPA1	Signal-indu	SIPA1_HUN	15	1.099731
48H 10MT/	O95239	0.039847	0.110232	0.064835	KIF4A	Chromosor	KIF4A_HUN	34	1.028005
24H 50MT/	Q9NX04	-0.65411	0.110238	0.064837	AIRIM	AFG2-inter	AIRIM_HUN	1	0.635468
48H 50MT/	Q6ZSS7	0.24015	0.110249	0.064839	MFSD6	Major facili	MFSD6_HU	2	1.181116
24H 50MT/	O00622	-0.52648	0.1103	0.064861	CCN1	CCN family	CCN1_HUN	7	0.694247
48H 10MT/	Q14103	0.021611	0.110305	0.064863	HNRNPD	Heterogene	HNRPD_HU	15	1.015092
24H 10MT/	Q9NQC3	0.040837	0.11033	0.064875	RTN4	Reticulon-4	RTN4_HUM	11	1.028711
48H 50MT/	Q8IZL8	-0.0703	0.110373	0.064892	PELP1	Proline-, gl	PELP1_HUN	24	0.952437
24H 50MT/	Q96QU8	-0.03383	0.110388	0.064897	XPO6	Exportin-6	XPO6_HUN	15	0.976827
48H 10MT/	Q9HCP0	0.054777	0.110403	0.064903	CSNK1G1	Casein kina	KC1G1_HU	8	1.038698
24H 10MT/	P35251	0.038139	0.110443	0.064923	RFC1	Replicatior	RFC1_HUM	26	1.026788
48H 50MT/	Q9NXJ5	0.077052	0.110469	0.064933	PGPEP1	Pyroglutan	PGPI_HUM	7	1.054861
24H 10MT/	Q8NC51	-0.01089	0.110474	0.064933	SERBP1	SERPINE1 n	SERB1_HUN	21	0.992477
48H 10MT/	Q8WVM0	-0.17288	0.110471	0.064933	TFB1M	Dimethylac	TFB1M_HU	15	0.887071
24H 50MT/	O95045	1.208212	0.110532	0.064958	UPP2	Uridine pho	UPP2_HUN	1	2.310511
24H 50MT/	Q8IZR5	0.038436	0.110589	0.064979	CMTM4	CKLF-like M	CKLF4_HUN	1	1.027
48H 10MT/	Q8NFA0	0.022298	0.110611	0.064991	USP32	Ubiquitin c	UBP32_HU	13	1.015576
48H 50MT/	Q6IQ49	-0.35411	0.110625	0.064998	SDE2	Splicing reg	SDE2_HUM	3	0.782351
24H 10MT/	Q69YQ0	0.066271	0.110678	0.065024	SPECC1L	Cytospin-A	CYTSA_HUN	23	1.047007
24H 50MT/	Q8WUY1	0.00276	0.110684	0.065026	THEM6	Protein THI	THEM6_HU	10	1.001915

48H 10MT/	Q9UPX8	-0.0802	0.110691	0.065028	SHANK2	SH3 and mu	SHAN2_HU	6	0.945928
48H 10MT/	P08697	0.009096	0.110709	0.065031	SERPINF2	Alpha-2-ant	A2AP_HUM	14	1.006325
48H 50MT/	Q9H2F3	0.150365	0.110708	0.065031	HSD3B7	3 beta-hydr	3BHS7_HU	9	1.10985
24H 10MT/	Q96FX7	0.044959	0.110726	0.065037	TRMT61A	tRNA (aden	TRM61_HU	7	1.031654
24H 10MT/	Q8NE71	0.006441	0.110743	0.065045	ABCF1	ATP-bindin	ABCF1_HU	33	1.004475
24H 50MT/	P35914	0.018186	0.110771	0.065059	HMGCL	Hydroxyme	HMGCL_HU	11	1.012686
48H 10MT/	Q9UIS9	-0.0474	0.110776	0.065061	MBD1	Methyl-CpG	MBD1_HUM	6	0.967676
48H 10MT/	Q6P1L5	-0.21359	0.110817	0.06508	FAM117B	Protein FAM	F117B_HU	3	0.86239
48H 50MT/	Q99933	-0.86536	0.11085	0.065095	BAG1	BAG family	BAG1_HUM	1	0.548908
24H 50MT/	P14406	-0.16734	0.110854	0.065096	COX7A2	Cytochrom	CX7A2_HU	2	0.890485
24H 50MT/	Q9C0E2	-0.05844	0.110875	0.065106	XPO4	Exportin-4	XPO4_HUM	25	0.960303
48H 10MT/	L0R8F8	-0.25922	0.110965	0.065151	MIEF1	Mitochond	MIDUO_HU	3	0.835542
24H 10MT/	P20645	0.017506	0.111029	0.065182	M6PR	Cation-dep	MPRD_HUM	7	1.012208
48H 50MT/	P28340	-0.10469	0.111037	0.065183	POLD1	DNA polym	DPOD1_HU	46	0.930006
24H 10MT/	Q9Y3D9	-0.08474	0.111045	0.065187	MRPS23	Small ribos	RT23_HUM	11	0.942953
24H 50MT/	Q00973	0.020823	0.111067	0.065195	B4GALNT1	Beta-1,4 N-	B4GN1_HU	13	1.014538
48H 50MT/	Q9H3S5	-0.37717	0.111079	0.065201	PIGM	GPI manno	PIGM_HUM	1	0.769949
24H 10MT/	Q9H6R6	-0.25798	0.111084	0.065203	ZDHHC6	Palmitoyltr	ZDHC6_HU	2	0.836256
24H 10MT/	O43747	0.009369	0.111101	0.065208	AP1G1	AP-1 compl	AP1G1_HU	28	1.006515
24H 10MT/	P62266	0.004512	0.111099	0.065208	RPS23	Small ribos	RS23_HUM	7	1.003132
48H 10MT/	A2RUC4	-0.10491	0.111111	0.065212	TYW5	tRNA wybu	TYW5_HUM	7	0.929862
48H 10MT/	Q6ZT62	0.07794	0.111114	0.065212	BARGIN	Bargin	BGIN_HUM	3	1.05551
24H 10MT/	P15531	0.017946	0.111125	0.065217	NME1	Nucleoside	NDKA_HUM	6	1.012517
24H 50MT/	P10301	0.006735	0.111139	0.065222	RRAS	Ras-related	RRAS_HUM	7	1.004679
48H 10MT/	Q99757	-0.02154	0.111148	0.065226	TXN2	Thioredoxi	THIOM_HU	3	0.985179
24H 50MT/	Q8TED0	0.086611	0.111164	0.065234	UTP15	U3 small nu	UTP15_HU	25	1.061873
24H 50MT/	A4D161	0.446436	0.111177	0.065237	FAM221A	Protein FAM	F221A_HU	3	1.36267
48H 50MT/	Q96RS6	0.036731	0.111176	0.065237	NUDCD1	NudC doma	NUDC1_HU	23	1.025787
48H 50MT/	P26639	0.00124	0.111184	0.06524	TARS1	Threonine--	SYTC_HUMA	37	1.00086
24H 10MT/	Q9Y224	-0.21462	0.111189	0.065242	RTRAF	RNA transcr	RTRAF_HUM	14	0.861775
24H 10MT/	Q9Y5M8	0.016673	0.111228	0.06526	SRPRB	Signal reco	SRPRB_HU	15	1.011624
48H 50MT/	O76080	-0.04824	0.111268	0.065278	ZFAND5	AN1-type zi	ZFAN5_HU	3	0.967116
24H 50MT/	Q12802	0.089933	0.111299	0.065291	AKAP13	A-kinase an	AKP13_HU	23	1.064321
48H 50MT/	O95721	0.028574	0.111339	0.06531	SNAP29	Synaptosor	SNP29_HU	10	1.020003
48H 50MT/	Q13045	-0.0263	0.111374	0.065329	FLII	Protein flig	FLII_HUMA	39	0.981936
24H 50MT/	P51148	0.030513	0.111385	0.065332	RAB5C	Ras-related	RAB5C_HU	6	1.021375
48H 10MT/	O95832	-0.2266	0.111394	0.065336	CLDN1	Claudin-1	CLD1_HUM	5	0.854649
48H 10MT/	A6NDB9	0.423504	0.111397	0.065337	PALM3	Paralemmi	PALM3_HU	1	1.341181
24H 50MT/	Q9NUP7	0.156991	0.111398	0.065337	TRMT13	tRNA:m(4)X	TRM13_HU	7	1.114959
48H 50MT/	Q15022	-0.2423	0.111422	0.065349	SUZ12	Polycomb p	SUZ12_HU	9	0.845396
24H 50MT/	Q5VYK3	-0.07362	0.111434	0.065355	ECPAS	Proteasome	ECM29_HU	69	0.95025
48H 10MT/	Q86TX2	-0.00934	0.111463	0.065369	ACOT1	Acyl-coenzy	ACOT1_HU	19	0.993546
48H 10MT/	Q9NVI7	-0.01662	0.11151	0.065394	ATAD3A	ATPase fam	ATD3A_HU	24	0.988549
48H 50MT/	P30566	-0.08077	0.111514	0.065395	ADSL	Adenylosuc	PUR8_HUM	23	0.945555
48H 50MT/	Q9BWM7	-0.0524	0.11155	0.065411	SFXN3	Sideroflexir	SFXN3_HU	14	0.964328
48H 10MT/	Q96NW4	0.881518	0.1116	0.065437	ANKRD27	Ankyrin rep	ANR27_HU	2	1.842313
48H 10MT/	P07954	-0.07814	0.111605	0.065439	FH	Fumarate h	FUMH_HUM	20	0.947278
24H 50MT/	Q32P51	0.158654	0.111627	0.06545	HNRNPA1L	Heterogene	RA1L2_HU	1	1.116246
48H 10MT/	P05067	-0.04948	0.111632	0.065452	APP	Amyloid-be	A4_HUMAN	10	0.966287
24H 10MT/	O14976	-0.06459	0.111648	0.065458	GAK	Cyclin-G-as	GAK_HUMA	26	0.956219
24H 10MT/	Q9BZF9	0.087203	0.111672	0.065471	UACA	Uveal autoa	UACA_HUM	21	1.062308
24H 10MT/	Q8IVH2	0.190893	0.111689	0.065475	FOXP4	Forkhead b	FOXP4_HU	1	1.14147

24H 50MT/	P62937	-0.0487	0.111704	0.065481	PPIA	Peptidyl-pr	PPIA_HUM/	11	0.966805
24H 50MT/	Q9ULJ8	-0.39733	0.111808	0.065524	PPP1R9A	Neurabin-1	NEB1_HUM	3	0.75926
48H 50MT/	Q13557	0.066083	0.111813	0.065525	CAMK2D	Calcium/ca	KCC2D_HU	10	1.04687
48H 50MT/	Q7Z7F0	1.555073	0.111836	0.065534	KHDC4	KH homolo	KHDC4_HU	2	2.938486
24H 10MT/	P10074	-0.03159	0.111848	0.06554	ZBTB48	Telomere zi	TZAP_HUM	3	0.978342
24H 10MT/	O43847	0.011585	0.111946	0.065591	NRDC	Nardilysin	NRDC_HUM	37	1.008062
24H 10MT/	Q53FT3	0.097611	0.111948	0.065591	HIKESHI	Protein Hik	HIKES_HUM	5	1.07
48H 50MT/	Q9BZ23	-0.20284	0.111984	0.065606	PANK2	Pantothena	PANK2_HU	7	0.868839
24H 10MT/	O43187	-0.10836	0.111986	0.065606	IRAK2	Interleukin	IRAK2_HUM	4	0.927642
48H 10MT/	P40692	0.005404	0.112026	0.065625	MLH1	DNA misma	MLH1_HUM	31	1.003753
24H 10MT/	Q9BVC4	0.004634	0.112029	0.065626	MLST8	Target of ra	LST8_HUM/	7	1.003217
48H 50MT/	O15042	0.039766	0.112035	0.065629	U2SURP	U2 snRNP-a	SR140_HUI	29	1.027947
48H 10MT/	O75110	-0.05359	0.11205	0.065634	ATP9A	Probable pl	ATP9A_HUI	7	0.963536
48H 10MT/	Q12873	0.40178	0.112108	0.06566	CHD3	Chromodo	CHD3_HUM	2	1.321137
48H 50MT/	P29323	0.700037	0.112111	0.065661	EPHB2	Ephrin type	EPHB2_HUI	1	1.624546
24H 50MT/	Q9Y2I7	0.06149	0.112114	0.065661	PIKFYVE	1-phosphat	FYV1_HUM	16	1.043543
24H 10MT/	P25208	0.079468	0.112197	0.065701	NFYB	Nuclear tra	NFYB_HUM	2	1.056629
24H 50MT/	P22607	-0.1304	0.112205	0.065704	FGFR3	Fibroblast g	FGFR3_HUI	5	0.913577
48H 50MT/	P52948	-0.08272	0.112206	0.065704	NUP98	Nuclear po	NUP98_HU	54	0.944276
48H 10MT/	Q9UNX4	-0.07699	0.11221	0.065705	WDR3	WD repeat-	WDR3_HUI	35	0.948032
24H 10MT/	Q8TDJ6	-0.13155	0.112221	0.065708	DMXL2	DmX-like pi	DMXL2_HU	15	0.91285
24H 50MT/	Q6R327	-0.03282	0.112268	0.06573	RICTOR	Rapamycin	RICTR_HUM	22	0.977508
48H 50MT/	Q96SI9	0.011305	0.112285	0.065739	STRBP	Spermatid	STRBP_HUI	22	1.007867
48H 50MT/	Q5VZE5	0.012626	0.112309	0.065751	NAA35	N-alpha-ac	NAA35_HU	20	1.00879
24H 50MT/	Q9Y520	-0.05039	0.112317	0.065752	PRRC2C	Protein PRI	PRC2C_HUI	62	0.965673
24H 50MT/	Q8NB37	-0.07401	0.112343	0.065764	GATD1	Glutamine	GALD1_HU	4	0.949993
24H 10MT/	Q9Y6W3	0.003496	0.112356	0.065769	CAPN7	Calpain-7	CAN7_HUM	18	1.002426
24H 10MT/	Q13686	0.093238	0.112363	0.065772	ALKBH1	Nucleic aci	ALKB1_HUI	4	1.066762
48H 10MT/	Q9Y624	0.001349	0.112366	0.065772	F11R	Junctional	JAM1_HUM	10	1.000936
24H 10MT/	Q9NX05	-0.68538	0.112373	0.065776	FAM120C	Constitutiv	F120C_HUI	3	0.621842
24H 10MT/	Q16875	0.36081	0.112378	0.065777	PFKFB3	6-phospho	F263_HUM	8	1.284147
48H 50MT/	Q5VT06	-0.14001	0.11238	0.065777	CEP350	Centrosom	CE350_HUI	11	0.90751
48H 50MT/	Q96T23	0.065716	0.112392	0.065781	RSF1	Remodelin	RSF1_HUM	27	1.046604
24H 50MT/	O15213	-0.03989	0.112418	0.065795	WDR46	WD repeat-	WDR46_HU	18	0.97273
24H 10MT/	Q8NB78	-0.21104	0.11243	0.0658	KDM1B	Lysine-spec	KDM1B_HU	4	0.863914
48H 50MT/	Q9Y696	-0.03813	0.112431	0.0658	CLIC4	Chloride in	CLIC4_HUM	17	0.973915
48H 10MT/	P98160	0.486801	0.112433	0.0658	HSPG2	Basement r	PGBM_HUI	19	1.401334
48H 50MT/	Q969H8	-0.02862	0.112441	0.065804	MYDGF	Myeloid-de	MYDGF_HU	7	0.980354
24H 10MT/	Q9Y6Y8	-0.02496	0.112458	0.065812	SEC23IP	SEC23-inte	S23IP_HUM	27	0.982846
48H 50MT/	Q9P2Y5	-0.0199	0.112491	0.065827	UVRAG	UV radiatio	UVRAG_HU	7	0.9863
48H 10MT/	A0PJW6	-0.20134	0.112505	0.065833	TMEM223	Transmemb	TM223_HU	4	0.869741
48H 10MT/	Q9NV35	0.108877	0.112512	0.065835	NUDT15	Nucleotide	NUD15_HU	7	1.078388
24H 10MT/	Q9Y2Z2	0.005454	0.112549	0.065849	MTO1	Protein MT	MTO1_HUM	18	1.003788
24H 50MT/	P11474	-0.40728	0.112566	0.065858	ESRRA	Steroid hor	ERR1_HUM	3	0.754044
48H 50MT/	Q8TDP1	-0.05118	0.11263	0.065887	RNASEH2C	Ribonuclea	RNH2C_HU	7	0.965149
48H 50MT/	Q9UHN1	-0.36569	0.112626	0.065887	POLG2	DNA polym	DPOG2_HU	4	0.776099
48H 50MT/	P13196	0.212228	0.112664	0.065902	ALAS1	5-aminolev	HEM1_HUM	7	1.158476
48H 10MT/	P07951	0.051667	0.112696	0.065914	TPM2	Tropomyos	TPM2_HUM	10	1.036462
48H 10MT/	Q9HB29	2.009466	0.112699	0.065914	IL1RL2	Interleukin	ILRL2_HUM	1	4.026331
48H 50MT/	P62312	0.092643	0.112722	0.065924	LSM6	U6 snRNA-a	LSM6_HUM	6	1.066322
24H 10MT/	P01034	-0.11058	0.112729	0.065926	CST3	Cystatin-C	CYTC_HUM	1	0.926216
48H 10MT/	Q9NX46	-0.05886	0.112733	0.065928	ADPRS	ADP-ribosy	ADPRS_HUI	10	0.960025

24H 10MTA	O15211	-0.06077	0.112746	0.065933	RGL2	Ral guanine	RGL2_HUM	3	0.958755
24H 50MTA	O15194	0.201799	0.112771	0.065945	CTDSPL	CTD small p	CTDSL_HUM	1	1.150132
24H 10MTA	Q96AH0	0.036995	0.112774	0.065945	NABP1	SOSS comp	SOSB2_HUM	2	1.025975
24H 10MTA	O43759	-0.1648	0.112811	0.065958	SYNGR1	Synaptogyr	SNG1_HUM	2	0.892053
48H 10MTA	Q00613	0.033719	0.112809	0.065958	HSF1	Heat shock	HSF1_HUM	3	1.023648
24H 50MTA	P36915	-0.0549	0.112823	0.065964	GNL1	Guanine nu	GNL1_HUM	23	0.96266
24H 10MTA	P36957	-0.02959	0.112863	0.065979	DLST	Dihydrolipo	ODO2_HUM	11	0.979697
48H 50MTA	Q53TQ3	-0.07121	0.112863	0.065979	INO80D	INO80 com	IN80D_HUM	1	0.951841
48H 10MTA	Q9UBU9	-0.06004	0.112861	0.065979	NXF1	Nuclear RN	NXF1_HUM	25	0.95924
24H 50MTA	O60507	0.552354	0.112874	0.065981	TPST1	Protein-tyr	TPST1_HUM	3	1.466476
48H 50MTA	O76071	0.044279	0.112871	0.065981	CIAO1	Probable cy	CIAO1_HUM	14	1.031168
24H 50MTA	P11171	0.011632	0.112871	0.065981	EPB41	Protein 4.1	EPB41_HUM	7	1.008096
48H 10MTA	P63241	-0.04401	0.112879	0.065982	EIF5A	Eukaryotic	IF5A1_HUM	14	0.969958
24H 10MTA	Q92552	-0.06819	0.112878	0.065982	MRPS27	Small ribos	RT27_HUM	19	0.953831
48H 10MTA	Q9P1Z2	-0.21594	0.112885	0.065983	CALCOCO1	Calcium-bi	CACO1_HU	2	0.860987
24H 10MTA	Q99735	0.093319	0.112888	0.065984	MGST2	Microsoma	MGST2_HU	2	1.066822
24H 50MTA	Q9BV73	0.136714	0.112915	0.065998	CEP250	Centrosom	CP250_HUM	18	1.099398
48H 10MTA	Q14894	-0.17451	0.112991	0.066033	CRYM	Ketimine re	CRYM_HUM	4	0.886068
48H 50MTA	Q9UJH8	-0.10394	0.113025	0.066051	METRN	Meteorin	METRN_HU	1	0.930489
24H 10MTA	Q13310	-0.07015	0.113045	0.066061	PABPC4	Polyadenyl	PABP4_HUM	21	0.952541
24H 10MTA	Q9BYN0	0.235385	0.113084	0.066082	SRXN1	Sulfiredoxi	SRXN1_HUM	5	1.177221
24H 50MTA	Q9BQ67	0.003934	0.113087	0.066082	GRWD1	Glutamate-	GRWD1_HU	17	1.002731
48H 10MTA	Q7L775	-0.17475	0.113091	0.066082	EPM2AIP1	EPM2A-inte	EPMIP_HUM	11	0.885922
24H 50MTA	Q9Y483	-0.10979	0.113102	0.066087	MTF2	Metal-respo	MTF2_HUM	8	0.926721
24H 10MTA	O60927	-0.08154	0.113148	0.066109	PPP1R11	E3 ubiquiti	PP1RB_HUM	4	0.945051
24H 10MTA	P52565	-0.00519	0.113149	0.066109	ARHGDIA	Rho GDP-di	GDIR1_HUM	9	0.99641
24H 10MTA	Q9NUL5	-0.26219	0.113163	0.066114	SHFL	Shiftless an	SHFL_HUM	4	0.833819
48H 10MTA	P22061	-0.02043	0.113175	0.066118	PCMT1	Protein-L-is	PIMT_HUM	15	0.985942
48H 10MTA	P53350	0.057809	0.113175	0.066118	PLK1	Serine/thre	PLK1_HUM	21	1.040884
48H 50MTA	Q5XPI4	-0.06514	0.113232	0.066144	RNF123	E3 ubiquiti	RN123_HU	7	0.955849
24H 10MTA	Q93009	0.034688	0.11323	0.066144	USP7	Ubiquitin c	UBP7_HUM	54	1.024335
48H 10MTA	Q8NG68	0.022014	0.113249	0.066151	TTL	Tubulin--ty	TTL_HUMA	5	1.015376
24H 50MTA	Q8TB03	-0.02225	0.113299	0.066172	CXorf38	Uncharacte	CX038_HUM	3	0.984695
24H 50MTA	Q16611	0.123241	0.113316	0.066176	BAK1	Bcl-2 homo	BAK_HUMA	4	1.089179
24H 10MTA	Q92674	0.03037	0.113328	0.066181	CENPI	Centromere	CENPI_HUM	6	1.021274
24H 50MTA	Q8IVH8	-0.10374	0.11336	0.066195	MAP4K3	Mitogen-ac	M4K3_HUM	7	0.930614
24H 50MTA	Q3ZCQ8	-0.03298	0.113364	0.066195	TIMM50	Mitochond	TIM50_HUM	11	0.977402
24H 10MTA	O60832	-0.00779	0.113409	0.066219	DKC1	H/ACA ribo	DKC1_HUM	27	0.994615
24H 10MTA	Q9UBF6	0.022646	0.113421	0.066224	RNF7	RING-box p	RBX2_HUM	3	1.015821
24H 50MTA	Q76FK4	0.09016	0.113435	0.066229	NOL8	Nucleolar p	NOL8_HUM	16	1.064488
24H 50MTA	Q6XZF7	-0.13109	0.113478	0.06625	DNMBP	Dynamin-b	DNMBP_HU	16	0.913144
48H 10MTA	O60830	0.058338	0.11349	0.066255	TIMM17B	Mitochond	TI17B_HUM	4	1.041265
48H 10MTA	P30520	-0.04467	0.113499	0.066259	ADSS2	Adenylosuc	PURA2_HU	17	0.969511
24H 50MTA	Q96GD0	0.059663	0.113512	0.066265	PDXP	Chronophi	PLPP_HUM	9	1.042222
24H 10MTA	Q9HBL8	-0.03468	0.113527	0.066272	NMRAL1	NmrA-like f	NMRL1_HU	4	0.976245
48H 50MTA	Q9H7Z7	0.100193	0.113535	0.066275	PTGES2	Prostagland	PGES2_HUM	14	1.071917
24H 10MTA	O43542	-0.00405	0.113619	0.06631	XRCC3	DNA repair	XRCC3_HUM	6	0.997197
48H 10MTA	P04114	0.001907	0.113641	0.066321	APOB	Apolipopro	APOB_HUM	89	1.001323
48H 50MTA	Q9NSB2	0.326107	0.113677	0.066338	KRT84	Keratin, typ	KRT84_HUM	2	1.253626
48H 10MTA	Q9HCK8	-0.01028	0.113797	0.066395	CHD8	Chromodo	CHD8_HUM	28	0.992898
48H 10MTA	Q9UL33	-0.01365	0.113802	0.066396	TRAPPC2L	Trafficking	TPC2L_HUM	6	0.990586
24H 50MTA	O14967	-0.06656	0.113825	0.066404	CLGN	Calmegin	CLGN_HUM	9	0.954914

24H 50MTA	Q9H7Z3	0.251251	0.113834	0.066407	NRDE2	Nuclear exc	NRDE2_HU	12	1.190239
24H 50MTA	P08034	0.240479	0.113866	0.066424	GJB1	Gap junctio	CXB1_HUM	4	1.181385
48H 10MTA	Q9NPE3	0.116729	0.113893	0.066434	NOP10	H/ACA ribo	NOP10_HU	4	1.084274
24H 10MTA	P52848	0.052979	0.113906	0.066439	NDST1	Bifunctiona	NDST1_HU	9	1.037405
48H 50MTA	Q8N684	-0.1321	0.11393	0.06645	CPSF7	Cleavage an	CPSF7_HUM	10	0.912504
48H 10MTA	Q9NRA8	0.214289	0.113937	0.066453	EIF4ENIF1	Eukaryotic	4ET_HUMA	15	1.160132
48H 10MTA	Q8WVV4	0.083248	0.113988	0.066476	POF1B	Protein PO	POF1B_HU	6	1.0594
48H 10MTA	Q6PML9	0.087912	0.113993	0.066478	SLC30A9	Proton-cou	ZNT9_HUM	6	1.062831
48H 50MTA	P02662	-1.49691	0.114015	0.066489	CSN1S1	Alpha-S1-ca	CASA1_BOV	4	0.35431
24H 50MTA	P30040	0.031304	0.11403	0.066494	ERP29	Endoplasm	ERP29_HU	14	1.021935
24H 10MTA	O43808	0.295802	0.114039	0.066498	SLC25A17	Peroxisoma	PM34_HUM	8	1.227567
24H 10MTA	P49448	-0.04798	0.114052	0.066503	GLUD2	Glutamate	DHE4_HUM	8	0.967292
24H 50MTA	Q6PML9	0.05028	0.11411	0.066526	SLC30A9	Proton-cou	ZNT9_HUM	6	1.035466
48H 10MTA	Q9BQL6	0.200938	0.114109	0.066526	FERMT1	Fermitin fa	FERM1_HU	1	1.149445
48H 50MTA	Q5T1V6	-0.03029	0.114143	0.066541	DDX59	Probable A	DDX59_HU	7	0.979225
48H 10MTA	P10588	-0.12108	0.114171	0.066554	NR2F6	Nuclear rec	NR2F6_HU	7	0.919501
48H 50MTA	P21675	0.127159	0.114186	0.06656	TAF1	Transcripti	TAF1_HUM	11	1.092141
24H 50MTA	P37198	0.050002	0.114187	0.06656	NUP62	Nuclear po	NUP62_HU	11	1.035266
24H 50MTA	O95406	-0.89861	0.114225	0.066577	CNIH1	Protein cor	CNIH1_HU	1	0.536405
24H 10MTA	Q96CD2	0.02042	0.114243	0.066583	PPCDC	Phosphopa	COAC_HUM	2	1.014255
24H 10MTA	P62277	-0.0653	0.114272	0.066596	RPS13	Small ribos	RS13_HUM	10	0.955749
48H 50MTA	Q8N6M0	-0.0697	0.114275	0.066596	OTUD6B	Deubiquiti	OTU6B_HU	8	0.952836
24H 10MTA	P16144	0.102775	0.114284	0.066601	ITGB4	Integrin be	ITB4_HUMA	14	1.073837
24H 50MTA	Q9BUT1	0.233634	0.11431	0.066612	BDH2	Dehydroge	DHRS6_HU	5	1.175793
24H 50MTA	Q13033	-0.04665	0.114318	0.066614	STRN3	Striatin-3	STRN3_HU	14	0.968184
24H 50MTA	O60493	-0.07822	0.114371	0.066642	SNX3	Sorting nex	SNX3_HUM	12	0.947223
24H 50MTA	Q9HAC8	0.198763	0.114374	0.066643	UBTD1	Ubiquitin c	UBTD1_HU	3	1.147714
24H 50MTA	P43652	-0.75003	0.114435	0.066673	AFM	Afamin	AFAM_HUM	1	0.594589
48H 50MTA	P26651	0.599117	0.114524	0.066721	ZFP36	mRNA deca	TTP_HUMA	2	1.514789
48H 50MTA	Q96A28	-0.96687	0.114566	0.066741	SLAMF9	SLAM famil	SLAF9_HUM	1	0.511613
24H 10MTA	Q6ZXV5	0.038864	0.114573	0.066742	TMTC3	Protein O-n	TMTC3_HU	17	1.027304
48H 50MTA	O14929	-0.08643	0.114594	0.06675	HAT1	Histone ace	HAT1_HUM	13	0.941852
48H 10MTA	Q9NRD1	0.105742	0.114643	0.066774	FBXO6	F-box only	FBX6_HUM	7	1.076047
24H 10MTA	Q9Y4W2	0.055811	0.11465	0.066776	LAS1L	Ribosomal	LAS1L_HUM	29	1.039443
48H 10MTA	O15440	-0.05028	0.114701	0.066796	ABCC5	ATP-bindin	MRP5_HUM	16	0.96575
48H 50MTA	Q643R3	0.32923	0.114719	0.066806	LPCAT4	Lysophosph	LPCT4_HUM	4	1.256343
24H 50MTA	Q14571	0.198026	0.114726	0.066808	ITPR2	Inositol 1,4	ITPR2_HUM	28	1.147128
24H 50MTA	Q5T8I9	-0.34657	0.114762	0.066826	HENMT1	Small RNA 2	HENMT_HU	5	0.786452
24H 10MTA	P55011	-0.06743	0.11477	0.066829	SLC12A2	Solute carri	S12A2_HU	31	0.954335
24H 50MTA	Q03933	-0.20686	0.114778	0.066831	HSF2	Heat shock	HSF2_HUM	1	0.866418
48H 50MTA	Q9NX08	0.083325	0.114778	0.066831	COMMD8	COMM dom	COMD8_HU	1	1.059457
48H 10MTA	Q13595	0.019423	0.114809	0.066843	TRA2A	Transforme	TRA2A_HU	8	1.013554
24H 10MTA	Q9HBI1	0.092071	0.114807	0.066843	PARVB	Beta-parvin	PARVB_HU	8	1.065899
48H 10MTA	Q9BR61	-0.03653	0.114816	0.066846	ACBD6	Acyl-CoA-bi	ACBD6_HU	9	0.974994
24H 10MTA	O15347	0.111542	0.114822	0.066849	HMGB3	High mobil	HMGB3_HU	7	1.080382
24H 10MTA	Q8WYA0	-0.08531	0.114837	0.066855	IFT81	Intraflagell	IFT81_HUM	2	0.942584
48H 10MTA	P31689	-0.10618	0.114924	0.066899	DNAJA1	DnaJ homo	DNJA1_HU	18	0.929042
48H 10MTA	P23528	-0.07121	0.114934	0.066902	CFL1	Cofilin-1	COF1_HUM	18	0.951841
48H 50MTA	Q9H9S4	0.251698	0.114991	0.066928	CAB39L	Calcium-bi	CB39L_HU	3	1.190608
48H 10MTA	P00966	-0.09117	0.115075	0.066965	ASS1	Argininosu	ASSY_HUMA	27	0.938762
24H 50MTA	Q8IWF2	0.241574	0.115077	0.066965	FOXRED2	FAD-depen	FXRD2_HU	4	1.182282
48H 10MTA	Q8WXI9	0.105309	0.11509	0.066971	GATAD2B	Transcripti	P66B_HUM	12	1.075725

24H 10MT/	O15533	-0.09494	0.115098	0.066972	TAPBP	Tapasin	TPSN_HUM	5	0.936311
48H 10MT/	P24928	-0.01764	0.115118	0.066974	POLR2A	DNA-direct	RPB1_HUM	63	0.987847
48H 10MT/	Q4G0F5	-0.02466	0.115115	0.066974	VPS26B	Vacuolar pr	VP26B_HUI	10	0.983054
24H 10MT/	Q6PCD5	0.203844	0.115116	0.066974	RFWD3	E3 ubiquiti	RFWD3_HU	3	1.151763
24H 10MT/	Q7L266	0.040888	0.115118	0.066974	ASRGL1	Isoaspartyl	ASGL1_HUI	7	1.028747
48H 10MT/	Q8WVV9	-0.04085	0.115114	0.066974	HNRNPLL	Heterogene	HNRLL_HUI	16	0.97208
48H 10MT/	P17931	-0.14823	0.115123	0.066974	LGALS3	Galectin-3	LEG3_HUM	6	0.902357
48H 10MT/	Q99487	-0.03477	0.115174	0.066997	PAFAH2	Platelet-act	PAFA2_HUI	5	0.976187
24H 50MT/	Q9NWZ8	0.198615	0.115191	0.067002	GEMIN8	Gem-associ	GEMI8_HUI	5	1.147596
48H 10MT/	P84085	0.119241	0.115235	0.067022	ARF5	ADP-ribosy	ARF5_HUM	6	1.086163
24H 10MT/	Q96CV9	-0.08499	0.11524	0.067023	OPTN	Optineurin	OPTN_HUM	13	0.942789
24H 50MT/	Q96GM5	0.010897	0.115246	0.067026	SMARCD1	SWI/SNF-re	SMRD1_HU	17	1.007582
48H 10MT/	Q9UJZ1	0.000268	0.115257	0.06703	STOML2	Stomatin-li	STML2_HUI	17	1.000185
48H 50MT/	P36873	0.027619	0.115297	0.067049	PPP1CC	Serine/thre	PP1G_HUM	17	1.019328
48H 10MT/	Q92615	0.031679	0.115376	0.067092	LARP4B	La-related p	LAR4B_HUI	17	1.022201
24H 10MT/	P51531	-0.13315	0.115424	0.067116	SMARCA2	Probable gl	SMCA2_HU	12	0.911839
24H 50MT/	Q93073	0.120973	0.115427	0.067116	SECISBP2L	Selenocyste	SBP2L_HUI	5	1.087468
48H 50MT/	Q9Y365	0.053036	0.115463	0.067131	STARD10	START dom	STA10_HUI	9	1.037446
48H 50MT/	O14727	-0.0773	0.11549	0.067146	APAF1	Apoptotic p	APAF_HUM	22	0.947832
24H 50MT/	P09429	0.057967	0.115497	0.067148	HMGB1	High mobil	HMGB1_HU	14	1.040998
48H 50MT/	Q9H410	-0.19111	0.115505	0.067151	DSN1	Kinetochor	DSN1_HUM	9	0.875935
24H 50MT/	Q92990	0.005213	0.115514	0.067154	GLMN	Glomulin	GLMN_HUI	20	1.00362
48H 10MT/	P34932	0.001215	0.115544	0.067167	HSPA4	Heat shock	HSP74_HUI	47	1.000842
24H 10MT/	Q6ZRQ5	-0.08334	0.115616	0.067195	MMS22L	Protein MM	MMS22_HU	10	0.943867
48H 50MT/	Q8NB37	0.16508	0.115622	0.067197	GATD1	Glutamine	GALD1_HU	4	1.121228
24H 10MT/	Q99732	0.078052	0.115631	0.067199	LITAF	Lipopolysac	LITAF_HUM	1	1.055591
24H 50MT/	Q9NRG4	0.02216	0.115728	0.067242	SMYD2	N-lysine me	SMYD2_HU	5	1.015479
48H 10MT/	Q9Y2G5	-0.03274	0.115759	0.067253	POFUT2	GDP-fucose	OFUT2_HUI	15	0.977564
24H 50MT/	Q5SW24	-0.52169	0.115766	0.067254	DACT2	Dapper hor	DACT2_HUI	7	0.696554
24H 50MT/	A8MW92	-0.58009	0.115859	0.067305	PHF20L1	PHD finger	P20L1_HUI	1	0.668924
48H 50MT/	Q86X10	0.080029	0.115877	0.067315	RALGAPB	Ral GTPase-	RLGPB_HUI	30	1.057039
48H 10MT/	P36955	0.035248	0.115933	0.067339	SERPINF1	Pigment ep	PEDF_HUM	10	1.024733
24H 50MT/	Q8WV99	-0.06413	0.115935	0.06734	ZFAND2B	AN1-type zi	ZFN2B_HUI	3	0.956524
48H 10MT/	Q9Y365	-0.09791	0.115994	0.067362	STARD10	START dom	STA10_HUI	9	0.934383
24H 10MT/	Q04721	0.506262	0.116008	0.067369	NOTCH2	Neurogenic	NOTC2_HU	8	1.420365
24H 50MT/	Q5ST30	0.072205	0.116087	0.067409	VARS2	Valine--tRN	SYVM_HUM	22	1.051323
24H 50MT/	Q9H2K8	-0.18299	0.116117	0.067421	TAOK3	Serine/thre	TAOK3_HUI	10	0.880877
24H 50MT/	P20073	-0.07614	0.11612	0.067422	ANXA7	Annexin A7	ANXA7_HU	19	0.948592
24H 50MT/	A6NIZ1	0.923379	0.116134	0.067426	RAP1BL	Ras-related	RP1BL_HUI	1	1.896551
48H 10MT/	O60716	-0.01883	0.116133	0.067426	CTNND1	Catenin del	CTND1_HU	40	0.987034
24H 10MT/	Q9P2R3	-0.02323	0.116153	0.067431	ANKFY1	Rabankyrin	ANFY1_HUI	26	0.98403
48H 10MT/	Q96EX1	-0.2376	0.116192	0.067451	SMIM12	Small integ	SIM12_HUI	2	0.848155
48H 50MT/	O95619	-0.05534	0.116203	0.067453	YEATS4	YEATS dom	YETS4_HUM	4	0.962369
48H 10MT/	Q8NAF0	-0.03171	0.116219	0.067461	ZNF579	Zinc finger p	ZN579_HUI	5	0.978257
48H 10MT/	Q08AM6	0.057916	0.116226	0.067464	VAC14	Protein VAC	VAC14_HUI	21	1.040961
24H 50MT/	Q99523	-0.00147	0.116231	0.067466	SORT1	Sortilin	SORT_HUM	15	0.998983
48H 10MT/	P32780	-0.03664	0.116243	0.067472	GTF2H1	General tra	TF2H1_HUI	8	0.974924
24H 50MT/	Q15326	-0.40959	0.116247	0.067473	ZMYND11	Zinc finger	ZMY11_HU	5	0.752837
48H 10MT/	O15042	0.021056	0.116278	0.067488	U2SURP	U2 snRNP-a	SR140_HUI	29	1.014702
24H 10MT/	Q96IX5	-0.11604	0.116287	0.067491	ATP5MK	ATP synthas	ATPMK_HU	3	0.922717
48H 10MT/	Q12849	-0.01104	0.116312	0.0675	GRSF1	G-rich sequ	GRSF1_HUI	23	0.992376
48H 10MT/	Q6P1Q9	-0.47131	0.116338	0.06751	METTL2B	tRNA N(3)-r	MET2B_HU	6	0.721308

48H 50MT/	P63279	0.02839	0.116398	0.067537	UBE2I	SUMO-conj	UBC9_HUM	12	1.019873
24H 10MT/	Q8TCX1	-0.1871	0.11641	0.067539	DYNC2LI1	Cytoplasmi	DC2L1_HU	3	0.878368
24H 50MT/	Q8WWY3	-0.02601	0.116432	0.067548	PRPF31	U4/U6 sma	PRP31_HU	17	0.982135
24H 10MT/	P07205	0.17659	0.11646	0.067553	PGK2	Phosphogl	PGK2_HUM	3	1.130209
48H 50MT/	P12273	0.511207	0.11646	0.067553	PIP	Prolactin-ir	PIP_HUMA	2	1.425242
48H 10MT/	Q9UGQ3	0.398391	0.116491	0.067566	SLC2A6	Solute carri	GTR6_HUM	1	1.318037
24H 10MT/	Q9C0B1	-0.10543	0.116496	0.067568	FTO	Alpha-keto	FTO_HUMA	13	0.929529
24H 50MT/	O00267	0.023763	0.116527	0.067581	SUPT5H	Transcripti	SPT5H_HU	38	1.016607
48H 50MT/	Q9GZT6	0.335531	0.116549	0.067589	CCDC90B	Coiled-coil	CC90B_HU	3	1.261842
48H 50MT/	P43358	0.000754	0.11657	0.0676	MAGEA4	Melanoma-	MAGA4_HU	11	1.000522
24H 10MT/	P48509	0.176369	0.116618	0.067623	CD151	CD151 anti	CD151_HU	2	1.130036
48H 50MT/	O75781	0.317617	0.116633	0.067625	PALM	Paralemmi	PALM_HUM	2	1.24627
48H 10MT/	Q13415	-0.00187	0.11663	0.067625	ORC1	Origin reco	ORC1_HUM	4	0.998702
24H 10MT/	Q9GZT6	-0.48163	0.116628	0.067625	CCDC90B	Coiled-coil	CC90B_HU	3	0.71617
24H 10MT/	Q5VU43	-0.01883	0.116645	0.067631	PDE4DIP	Myomegali	MYOME_HU	6	0.987036
24H 50MT/	Q9UDY8	-0.0899	0.116659	0.067635	MALT1	Mucosa-ass	MALT1_HU	22	0.939589
48H 50MT/	Q9UMS0	0.073066	0.11666	0.067635	NFU1	NFU1 iron-	NFU1_HUM	6	1.05195
24H 10MT/	Q5BJH7	0.057149	0.116679	0.067644	YIF1B	Protein YIF	YIF1B_HUM	5	1.040407
24H 10MT/	Q9NZT2	0.010337	0.116687	0.067646	OGFR	Opioid grov	OGFR_HUM	21	1.007191
24H 10MT/	O00505	0.055423	0.116709	0.067656	KPNA3	Importin su	IMA4_HUM	20	1.039164
24H 50MT/	Q8NEC7	-0.15581	0.116716	0.067658	GSTCD	Glutathion	GSTCD_HU	6	0.897628
48H 10MT/	O14579	0.110427	0.116801	0.067699	COPE	Coatomer s	COPE_HUM	13	1.079547
48H 10MT/	P13647	-0.19729	0.116818	0.067707	KRT5	Keratin, typ	K2C5_HUM	14	0.872188
48H 10MT/	Q04760	-0.01083	0.116827	0.067709	GLO1	Lactoylglut	LGUL_HUM	12	0.992519
24H 10MT/	Q9BRX5	-4.38E-06	0.116868	0.067727	GINS3	DNA replica	PSF3_HUM	9	0.999997
48H 10MT/	P51159	-0.07336	0.116874	0.067728	RAB27A	Ras-related	RB27A_HU	5	0.950422
48H 10MT/	P25789	-0.08556	0.116878	0.067729	PSMA4	Proteasom	PSA4_HUM	13	0.942421
48H 50MT/	Q9UBF8	0.168153	0.116894	0.067736	PI4KB	Phosphatid	PI4KB_HUN	9	1.123619
24H 50MT/	Q99707	-0.12167	0.116911	0.067744	MTR	Methionine	METH_HUM	15	0.919121
24H 50MT/	Q9Y2D2	-0.0026	0.116911	0.067744	SLC35A3	UDP-N-acet	S35A3_HU	2	0.998202
48H 10MT/	P56182	0.018336	0.116936	0.067753	RRP1	Ribosomal	RRP1_HUM	13	1.012791
24H 50MT/	Q9Y3C0	0.165983	0.116937	0.067753	WASHC3	WASH com	WASC3_HU	4	1.12193
24H 10MT/	P22392	-0.0566	0.116953	0.067759	NME2	Nucleoside	NDKB_HUM	14	0.961525
48H 50MT/	P34896	0.074319	0.116957	0.06776	SHMT1	Serine hydr	GLYC_HUM	17	1.052864
24H 10MT/	Q92598	-0.0664	0.116957	0.06776	HSPH1	Heat shock	HS105_HU	44	0.955019
24H 10MT/	Q96AB3	-0.19525	0.11709	0.067826	ISOC2	Isochorism	ISOC2_HUM	7	0.873419
48H 10MT/	P28370	0.018264	0.117174	0.067869	SMARCA1	Probable gl	SMCA1_HU	24	1.01274
48H 10MT/	Q8N9N7	-0.0409	0.117191	0.067877	LRRC57	Leucine-ric	LRC57_HU	7	0.972051
24H 50MT/	O96019	-0.02074	0.117224	0.067889	ACTL6A	Actin-like p	ACL6A_HU	15	0.985726
48H 50MT/	Q9UN30	0.082819	0.117235	0.067893	SCML1	Sex comb o	SCML1_HU	3	1.059086
48H 50MT/	Q9UDX5	0.044514	0.117272	0.067907	MTFP1	Mitochond	MTFP1_HU	8	1.031336
24H 10MT/	Q12797	0.071612	0.117294	0.067916	ASPH	Aspartyl/as	ASPH_HUM	23	1.050891
48H 50MT/	Q5PSV4	-0.0048	0.117295	0.067916	BRMS1L	Breast canc	BRM1L_HU	2	0.996681
48H 10MT/	P12270	-0.00948	0.11733	0.067926	TPR	Nucleoprot	TPR_HUMA	81	0.993448
24H 50MT/	Q96MG7	0.041301	0.11734	0.06793	NSMCE3	Non-struct	NSE3_HUM	4	1.029041
48H 10MT/	P60981	-0.06905	0.117376	0.067947	DSTN	Destrin	DEST_HUM	11	0.953262
48H 10MT/	Q6IN85	0.017301	0.117389	0.067951	PPP4R3A	Serine/thre	P4R3A_HU	17	1.012064
24H 10MT/	Q92734	-0.0916	0.117388	0.067951	TFG	Protein TFG	TFG_HUMA	12	0.93848
24H 10MT/	Q9Y4P1	-0.03116	0.117392	0.067951	ATG4B	Cysteine pr	ATG4B_HU	14	0.978634
24H 50MT/	A2RRP1	0.076141	0.117396	0.067951	NBAS	NBAS subur	NBAS_HUM	27	1.054194
24H 50MT/	Q9H6Y2	-0.16328	0.117405	0.067952	WDR55	WD repeat-	WDR55_HU	13	0.892995
48H 10MT/	P82664	-0.19104	0.117428	0.067963	MRPS10	Small ribos	RT10_HUM	4	0.875971

24H 50MTA	O00186	0.071446	0.117434	0.067964	STXBP3	Syntaxin-bi	STXB3_HUI	19	1.05077
48H 10MTA	Q03001	-0.01786	0.117451	0.067972	DST	Dystonin	DYST_HUM	42	0.987695
24H 50MTA	P33947	0.532158	0.117468	0.067981	KDELR2	ER lumen p	ERD22_HUI	6	1.446091
48H 10MTA	Q96C24	-0.12088	0.117479	0.067984	SYTL4	Synaptotag	SYTL4_HUM	7	0.919628
48H 50MTA	P38935	-0.24119	0.117511	0.068	IGHMBP2	DNA-bindir	SMBP2_HU	8	0.846049
48H 10MTA	Q8IY81	-0.03479	0.117538	0.06801	FTSJ3	pre-rRNA 2'	SPB1_HUM	27	0.976175
48H 10MTA	Q5GLZ8	-0.02262	0.117574	0.068025	HERC4	Probable E3	HERC4_HUI	27	0.984443
48H 50MTA	Q8WXH0	0.061951	0.117618	0.068045	SYNE2	Nesprin-2	SYNE2_HUI	22	1.043876
48H 50MTA	Q9NRU3	-0.39289	0.11764	0.068054	CNNM1	Metal trans	CNNM1_HU	5	0.761601
48H 10MTA	Q9NTW7	-0.24167	0.117646	0.068056	ZFP64	Zinc finger	ZF64B_HUI	2	0.845767
48H 50MTA	Q14185	0.110731	0.117676	0.06807	DOCK1	Dedicator c	DOCK1_HU	18	1.079775
24H 10MTA	Q9GZQ3	0.008777	0.117684	0.068073	COMMD5	COMM dom	COMD5_HU	3	1.006103
48H 10MTA	Q9UJ70	-0.04148	0.117709	0.068082	NAGK	N-acetyl-D-	NAGK_HUM	18	0.971657
48H 50MTA	Q9BQL6	-0.39703	0.117722	0.068088	FERMT1	Fermitin fa	FERM1_HU	1	0.759422
24H 10MTA	Q15738	-0.04793	0.117728	0.068091	NSDHL	Sterol-4-alp	NSDHL_HU	15	0.967321
48H 50MTA	O95292	-0.06757	0.117734	0.068092	VAPB	Vesicle-assc	VAPB_HUM	9	0.954245
24H 10MTA	Q9Y6A1	0.057451	0.117798	0.068118	POMT1	Protein O-n	POMT1_HU	4	1.040626
24H 10MTA	Q92538	0.053258	0.117812	0.068123	GBF1	Golgi-speci	GBF1_HUM	70	1.037606
24H 50MTA	Q2TAK8	-0.3558	0.117825	0.068128	PWWP3A	PWWP dor	PWP3A_HU	2	0.781436
24H 50MTA	Q96KA5	0.122512	0.117826	0.068128	CLPTM1L	Lipid scram	CLP1L_HUI	11	1.088629
24H 10MTA	O00255	-0.00609	0.117848	0.068138	MEN1	Menin	MEN1_HUI	10	0.995788
24H 50MTA	P43686	-0.05049	0.117875	0.068149	PSMC4	26S protea:	PRS6B_HUI	29	0.965609
48H 10MTA	Q9H583	-0.12093	0.117895	0.06816	HEATR1	HEAT repea	HEAT1_HUI	76	0.919597
48H 50MTA	Q6P2P2	0.087615	0.117922	0.068173	PRMT9	Protein arg	ANM9_HUI	8	1.062612
48H 10MTA	Q96HS1	-0.0505	0.117927	0.068175	PGAM5	Serine/thre	PGAM5_HU	14	0.965603
48H 10MTA	Q9UHJ6	0.059965	0.117948	0.068183	SHPK	Sedoheptul	SHPK_HUM	11	1.042441
48H 10MTA	Q9UH36	0.036141	0.117951	0.068184	SRRD	SRR1-like p	SRR1L_HUI	1	1.025367
24H 50MTA	P46976	0.023125	0.117953	0.068184	GYG1	Glycogenin	GLYG_HUM	12	1.016158
24H 50MTA	Q8NFZ5	-0.57956	0.117966	0.06819	TNIP2	TNFAIP3-in	TNIP2_HUI	1	0.669168
24H 10MTA	O15084	-0.03988	0.11797	0.068191	ANKRD28	Serine/thre	ANR28_HU	15	0.972734
48H 10MTA	Q96F63	-0.07406	0.11798	0.068194	CCDC97	Coiled-coil	CCD97_HU	8	0.949958
48H 50MTA	Q9Y625	0.188307	0.11798	0.068194	GPC6	Glypican-6	GPC6_HUM	3	1.139426
24H 50MTA	Q5SWX8	0.17583	0.117995	0.0682	ODR4	Protein odr	ODR4_HUM	12	1.129614
24H 50MTA	Q86V48	-0.04616	0.117997	0.0682	LUZP1	Leucine zip	LUZP1_HUI	22	0.96851
24H 10MTA	Q9HC36	0.097304	0.118036	0.068217	MRM3	rRNA meth	MRM3_HUI	8	1.069773
24H 50MTA	Q15274	0.040483	0.118064	0.068229	QPRT	Nicotinate-	NADC_HUM	8	1.028458
48H 10MTA	Q9H920	-0.25223	0.118068	0.068229	RNF121	E3 ubiquiti	RN121_HU	1	0.839598
24H 10MTA	Q9UQ13	0.02912	0.118125	0.068253	SHOC2	Leucine-ric	SHOC2_HU	9	1.020389
48H 10MTA	Q8WZA1	0.338964	0.118135	0.068256	POMGNT1	Protein O-li	PMGT1_HU	7	1.264848
24H 10MTA	Q8IUH5	0.021444	0.11814	0.068257	ZDHHC17	Palmitoyltr	ZDH17_HU	3	1.014975
48H 50MTA	Q9UNX4	-0.03359	0.118148	0.068261	WDR3	WD repeat-	WDR3_HUI	35	0.976987
48H 10MTA	P82932	0.021151	0.118155	0.068264	MRPS6	Small ribos	RT06_HUM	4	1.014769
48H 10MTA	P00533	0.01883	0.118172	0.068271	EGFR	Epidermal g	EGFR_HUM	40	1.013137
48H 10MTA	Q9NZM5	-0.12789	0.118201	0.06828	NOP53	Ribosome b	NOP53_HU	8	0.915167
24H 10MTA	Q9ULT8	-0.01199	0.118217	0.068288	HECTD1	E3 ubiquiti	HECD1_HU	64	0.991722
24H 10MTA	Q13155	0.087146	0.11826	0.068306	AIMP2	Aminoacyl	AIMP2_HUI	13	1.062267
48H 10MTA	O00746	-0.01182	0.118305	0.068328	NME4	Nucleoside	NDKM_HUI	11	0.991843
48H 10MTA	P29353	-0.00589	0.118306	0.068328	SHC1	SHC-transfc	SHC1_HUM	13	0.995924
48H 10MTA	Q92572	0.095839	0.118351	0.068348	AP3S1	AP-3 compl	AP3S1_HUI	7	1.068686
24H 50MTA	O15056	-0.19277	0.118402	0.06837	SYNJ2	Synaptojan	SYNJ2_HUM	11	0.874927
48H 10MTA	Q8IVF7	-0.0666	0.118446	0.06839	FMNL3	Formin-like	FMNL3_HU	1	0.954883
48H 10MTA	Q8NEZ2	0.180664	0.118458	0.068395	VPS37A	Vacuolar pi	VP37A_HUI	10	1.133405

48H 10MT/	P30740	0.02762	0.11846	0.068395	SERPINB1	Leukocyte e	ILEU_HUM/	19	1.019329
24H 50MT/	Q8IXU6	-0.89971	0.118483	0.068407	SLC35F2	Solute carri	S35F2_HUI	1	0.535993
24H 50MT/	Q9NXX6	0.082147	0.11851	0.068419	NSMCE4A	Non-struct	NSE4A_HUI	6	1.058593
24H 50MT/	Q96LI5	0.368069	0.118523	0.068423	CNOT6L	CCR4-NOT t	CNO6L_HU	2	1.290624
48H 50MT/	Q14999	0.353513	0.118556	0.068434	CUL7	Cullin-7	CUL7_HUM	11	1.277668
48H 50MT/	Q86W50	0.108073	0.118566	0.068438	METTL16	RNA N6-ade	MET16_HU	11	1.077788
24H 50MT/	Q8IWA5	0.601426	0.118572	0.068441	SLC44A2	Choline tra	CTL2_HUM	4	1.517216
24H 10MT/	Q5M7Z0	-0.00895	0.118595	0.068451	RNFT1	E3 ubiquiti	RNFT1_HUI	4	0.993818
24H 10MT/	P62899	0.017737	0.118654	0.068474	RPL31	Large ribos	RL31_HUM	5	1.01237
24H 50MT/	Q96S59	-0.05859	0.11866	0.068475	RANBP9	Ran-bindin	RANB9_HU	15	0.960201
24H 50MT/	Q6P4I2	0.000838	0.118684	0.068487	WDR73	WD repeat-	WDR73_HU	10	1.000581
24H 10MT/	Q9H633	0.438003	0.118705	0.068496	RPP21	Ribonuclea	RPP21_HUI	2	1.354728
48H 50MT/	Q9HCC0	-0.01988	0.118732	0.068508	MCCC2	Methylcrot	MCCB_HUM	24	0.986313
24H 10MT/	P19784	0.045569	0.11875	0.068517	CSNK2A2	Casein kina	CSK22_HUI	17	1.03209
24H 50MT/	Q14807	-0.12791	0.11878	0.06853	KIF22	Kinesin-like	KIF22_HUM	25	0.915158
48H 10MT/	Q8N5P1	0.425861	0.118899	0.068585	ZC3H8	Zinc finger	ZC3H8_HUI	4	1.343374
24H 10MT/	O00267	0.024249	0.118911	0.068588	SUPT5H	Transcripti	SPT5H_HUI	38	1.01695
48H 50MT/	Q68CJ9	0.543067	0.118924	0.068593	CREB3L3	Cyclic AMP	CR3L3_HUI	2	1.457067
48H 10MT/	O75787	-0.13424	0.118944	0.068602	ATP6AP2	Renin recep	RENR_HUM	11	0.911153
48H 10MT/	Q92990	-0.02681	0.11896	0.068609	GLMN	Glomulin	GLMN_HUM	20	0.98159
24H 10MT/	Q7L9L4	-0.0735	0.119031	0.068644	MOB1B	MOB kinase	MOB1B_HU	7	0.950332
48H 10MT/	Q9BZF9	-0.00574	0.119078	0.068662	UACA	Uveal auto	UACA_HUM	21	0.996029
24H 50MT/	P22570	-0.03838	0.119082	0.068662	FDXR	NADPH:adr	ADRO_HUM	21	0.973745
24H 10MT/	O00213	-0.47523	0.119123	0.068679	APBB1	Amyloid be	APBB1_HUI	3	0.719353
48H 10MT/	Q9NQZ5	-0.11187	0.119121	0.068679	STARD7	StAR-relate	STAR7_HUM	9	0.92539
48H 50MT/	Q53EZ4	-0.25749	0.119175	0.068699	CEP55	Centrosom	CEP55_HUI	13	0.836543
24H 50MT/	P60002	-0.03063	0.11921	0.068714	ELOF1	Transcripti	ELOF1_HUM	1	0.978993
48H 50MT/	Q8TF46	-0.27891	0.119235	0.068724	DIS3L	DIS3-like ex	DI3L1_HUM	7	0.824212
48H 50MT/	Q96MB7	0.518495	0.119238	0.068724	HARBI1	Putative nu	HARB1_HU	1	1.43246
48H 10MT/	Q9HAC8	-0.0281	0.119253	0.068732	UBTD1	Ubiquitin d	UBTD1_HU	3	0.980712
48H 10MT/	O15127	0.019619	0.119271	0.068741	SCAMP2	Secretory c	SCAM2_HU	3	1.013692
48H 50MT/	Q9BWT6	-0.33804	0.119294	0.068748	MND1	Meiotic nu	MND1_HUI	1	0.791116
24H 10MT/	O94886	-0.12465	0.119327	0.068765	TMEM63A	CSC1-like p	CSCL1_HUM	2	0.917225
24H 10MT/	Q9BZD2	0.22995	0.119356	0.06878	SLC29A3	Equilibrativ	S29A3_HUI	5	1.172795
48H 10MT/	P09960	0.054604	0.119396	0.068799	LTA4H	Leukotrien	LKHA4_HUI	33	1.038574
24H 10MT/	Q9Y6I4	0.199973	0.119399	0.068799	USP3	Ubiquitin c	UBP3_HUM	16	1.148677
48H 10MT/	Q9HB71	0.001523	0.11941	0.068803	CACYBP	Calcyclin-b	CYBP_HUM	18	1.001056
48H 10MT/	Q9NXF1	-0.06805	0.119421	0.068808	TEX10	Testis-expre	TEX10_HUM	29	0.953924
24H 10MT/	P50570	-0.03284	0.119462	0.068825	DNM2	Dynamin-2	DYN2_HUM	43	0.977495
24H 50MT/	Q96DR4	-0.51656	0.119542	0.068862	STARD4	StAR-relate	STAR4_HUM	2	0.699038
24H 10MT/	Q9Y5S9	0.133007	0.119578	0.068879	RBM8A	RNA-bindir	RBM8A_HU	8	1.096577
48H 50MT/	Q9BQS8	0.072756	0.119607	0.068892	FYCO1	FYVE and co	FYCO1_HUI	27	1.051724
48H 50MT/	Q5TCQ9	-0.22147	0.119637	0.068902	MAGI3	Membrane-	MAGI3_HU	6	0.857693
24H 10MT/	Q9HAZ1	0.263768	0.119638	0.068902	CLK4	Dual specifi	CLK4_HUM	3	1.20061
48H 10MT/	Q14746	-0.05149	0.119644	0.068904	COG2	Conserved	COG2_HUM	25	0.964939
48H 50MT/	Q8NHG7	-0.65485	0.119644	0.068904	SVIP	Small VCP/	SVIP_HUM/	1	0.635144
48H 50MT/	O14757	-0.39888	0.119654	0.068908	CHEK1	Serine/thre	CHK1_HUM	12	0.758445
24H 10MT/	Q8WY22	-0.00546	0.119731	0.068943	BRI3BP	BRI3-bindir	BRI3B_HUM	3	0.996221
48H 10MT/	Q9H3H3	-0.01802	0.119776	0.068963	C11orf68	UPF0696 p	CK068_HUI	12	0.987587
48H 10MT/	Q8IW41	0.262645	0.11978	0.068964	MAPKAPK5	MAP kinase	MAPK5_HU	1	1.199676
48H 50MT/	Q3KNV8	0.176384	0.119791	0.068969	PCGF3	Polycomb g	PCGF3_HUI	1	1.130048
24H 10MT/	P51003	0.03738	0.119815	0.068981	PAPOLA	Poly(A) pol	PAPOA_HU	22	1.026249

24H 10MT/	Q9UHP3	0.071671	0.119843	0.068994	USP25	Ubiquitin c	UBP25_HU	14	1.050933
24H 10MT/	Q9HCE5	0.097897	0.119866	0.069004	METTL14	N6-adenosi	MET14_HU	6	1.070212
48H 50MT/	Q5VUD6	0.922636	0.119872	0.069007	DIPK1B	Divergent p	DIK1B_HUI	2	1.895576
24H 10MT/	O60232	-0.18989	0.11989	0.069015	ZNRD2	Protein ZNI	ZNRD2_HU	5	0.876672
24H 50MT/	Q9H0U4	0.000531	0.119902	0.06902	RAB1B	Ras-related	RAB1B_HUI	9	1.000368
24H 50MT/	P31946	-0.10943	0.119934	0.069038	YWHAB	14-3-3 prot	1433B_HUI	17	0.926953
24H 10MT/	P30048	-0.09871	0.119968	0.06905	PRDX3	Thioredoxi	PRDX3_HU	11	0.933869
48H 50MT/	P09651	-0.09452	0.119971	0.069051	HNRNPA1	Heterogene	ROA1_HUM	22	0.936587
24H 10MT/	Q8NI27	0.035205	0.119976	0.069052	THOC2	THO compl	THOC2_HU	41	1.024702
48H 10MT/	Q5W111	-0.06082	0.119987	0.069056	SPRYD7	SPRY doma	SPRY7_HUI	5	0.958717
24H 50MT/	Q01082	-0.00283	0.120013	0.069069	SPTBN1	Spectrin be	SPTB2_HUI	127	0.998042
48H 50MT/	Q8N9M1	-0.22441	0.120021	0.06907	C19orf47	Uncharacte	CS047_HUI	4	0.855947
24H 10MT/	Q8IVB5	-0.19146	0.120032	0.069075	LIX1L	LIX1-like pr	LIX1L_HUN	1	0.875721
48H 50MT/	O00479	0.929081	0.120074	0.069092	HMGN4	High mobil	HMGN4_HU	1	1.904062
48H 50MT/	Q9H5U6	-0.14145	0.120073	0.069092	ZCCHC4	rRNA N6-ad	ZCHC4_HUI	3	0.906609
24H 50MT/	Q96HN2	-0.09875	0.120085	0.069097	AHCYL2	Adenosylhc	SAHH3_HU	12	0.933844
48H 50MT/	Q96GZ6	-0.3798	0.120095	0.069102	SLC41A3	Solute carri	S41A3_HUI	2	0.768544
48H 50MT/	Q12873	-0.21566	0.120175	0.069142	CHD3	Chromodoı	CHD3_HUN	2	0.86115
24H 50MT/	O75911	0.116996	0.120262	0.069185	DHRS3	Short-chair	DHRS3_HU	8	1.084474
48H 50MT/	Q6P1L5	-0.02502	0.12026	0.069185	FAM117B	Protein FAI	F117B_HUI	3	0.982805
48H 50MT/	A6NDB9	0.786044	0.120266	0.069186	PALM3	Paralemmi	PALM3_HU	1	1.72434
48H 10MT/	Q5MNZ6	0.014933	0.120326	0.069212	WDR45B	WD repeat	WIPI3_HUN	10	1.010405
24H 10MT/	Q9Y6D6	0.056572	0.120342	0.069219	ARFGEF1	Brefeldin A	BIG1_HUM	27	1.039992
48H 10MT/	P21980	-0.00071	0.120374	0.069236	TGM2	Protein-glu	TGM2_HUN	18	0.999509
24H 10MT/	Q96PZ0	-0.04965	0.120382	0.06924	PUS7	Pseudourid	PUS7_HUM	28	0.96617
48H 10MT/	P46779	0.039456	0.12039	0.069242	RPL28	Large ribos	RL28_HUM	5	1.027726
48H 50MT/	O14526	1.105951	0.120461	0.069274	FCHO1	F-BAR dom	FCHO1_HU	1	2.152407
24H 10MT/	O95363	0.325984	0.120466	0.069274	FARS2	Phenylalan	SYFM_HUM	4	1.253519
48H 50MT/	P10588	-0.05963	0.120485	0.069282	NR2F6	Nuclear rec	NR2F6_HUI	7	0.959507
48H 50MT/	Q15386	0.025457	0.120483	0.069282	UBE3C	Ubiquitin-p	UBE3C_HUI	37	1.017802
24H 10MT/	Q8N1N4	0.299378	0.120487	0.069282	KRT78	Keratin, typ	K2C78_HUI	8	1.230614
48H 10MT/	P35542	-0.22977	0.120489	0.069282	SAA4	Serum amy	SAA4_HUM	2	0.85277
48H 10MT/	P51452	-0.06279	0.120519	0.069296	DUSP3	Dual specifi	DUS3_HUN	8	0.957409
48H 50MT/	Q08431	0.773643	0.120543	0.069304	MFGE8	Lactadherii	MFGM_HUI	4	1.709582
48H 10MT/	Q96BX8	0.395604	0.120542	0.069304	MOB3A	MOB kinase	MOB3A_HU	3	1.315494
48H 10MT/	Q8NHP8	-0.02044	0.120578	0.06932	PLBD2	Putative ph	PLBL2_HUN	10	0.985932
24H 10MT/	Q9Y2Z4	-0.14183	0.120666	0.069363	YARS2	Tyrosine--tI	SYYM_HUM	18	0.906371
48H 50MT/	Q8NF64	-0.02786	0.120689	0.069372	ZMIZ2	Zinc finger I	ZMIZ2_HUN	5	0.980876
48H 10MT/	P54198	-0.04055	0.120764	0.069403	HIRA	Protein HIF	HIRA_HUM	8	0.972285
48H 10MT/	Q7Z5K2	0.021551	0.12078	0.069408	WAPL	Wings apar	WAPL_HUN	23	1.01505
48H 10MT/	Q14123	0.608077	0.120807	0.069421	PDE1C	Dual specifi	PDE1C_HUI	1	1.524226
24H 10MT/	Q69YH5	0.249383	0.120809	0.069421	CDCA2	Cell divisio	CDCA2_HU	2	1.188699
48H 50MT/	Q6IBW4	-0.13065	0.120816	0.069422	NCAPH2	Condensin-	CNDH2_HU	7	0.913422
48H 10MT/	Q9H4I3	0.097291	0.120823	0.069425	TRABD	TraB domai	TRABD_HUI	10	1.069763
24H 50MT/	O95825	-0.02795	0.120847	0.069436	CRYZL1	Quinone ox	QORL1_HU	12	0.980812
24H 50MT/	P35670	0.12469	0.120865	0.069442	ATP7B	Copper-tra	ATP7B_HUI	16	1.090273
48H 10MT/	Q8NEW0	0.04792	0.120923	0.069469	SLC30A7	Zinc transp	ZNT7_HUM	8	1.033774
24H 10MT/	P41091	0.002726	0.120941	0.069472	EIF2S3	Eukaryotic	IF2G_HUM.	24	1.001891
24H 50MT/	Q96AA3	0.274392	0.120952	0.069478	RFT1	Man(5)GlcI	RFT1_HUM	5	1.209484
24H 10MT/	P56377	0.016957	0.120963	0.069481	AP1S2	AP-1 compl	AP1S2_HUI	5	1.011823
24H 50MT/	A6NKD9	-0.23659	0.120989	0.069493	CCDC85C	Coiled-coil	CC85C_HUI	4	0.848751
24H 10MT/	Q9UHD9	-0.02544	0.120991	0.069493	UBQLN2	Ubiquilin-2	UBQL2_HU	10	0.98252

48H 50MT/	A2RUC4	0.084342	0.121014	0.069502	TYW5	tRNA wybu	TYW5_HUN	7	1.060204
48H 10MT/	Q9Y5W7	0.046294	0.121027	0.069509	SNX14	Sorting nex	SNX14_HUI	7	1.032609
48H 10MT/	P04179	-0.08558	0.121033	0.069511	SOD2	Superoxide	SODM_HUI	9	0.942406
24H 50MT/	O95461	0.542259	0.121037	0.069511	LARGE1	Xylosyl- and	LARG1_HUI	3	1.456251
24H 50MT/	P46926	0.009301	0.12108	0.069528	GNPDA1	Glucosamir	GNPI1_HUI	14	1.006468
24H 10MT/	P21964	-0.08757	0.121095	0.069534	COMT	Catechol O-	COMT_HUN	14	0.941105
24H 10MT/	P31153	-0.01514	0.12113	0.069548	MAT2A	S-adenosyl	METK2_HU	16	0.989564
48H 10MT/	Q99832	-0.04318	0.121138	0.069552	CCT7	T-complex	TCPH_HUM	36	0.970517
24H 50MT/	Q96HA1	0.346619	0.121191	0.069577	POM121	Nuclear env	P121A_HUI	3	1.271577
48H 10MT/	Q8TBM7	-0.2274	0.121235	0.069593	TMEM254	Transmemb	TM254_HU	1	0.854174
48H 10MT/	Q5T0D9	-0.13322	0.121245	0.069596	TPRG1L	Tumor prot	TPRGL_HUI	4	0.911796
48H 50MT/	Q99747	-0.11521	0.12129	0.069617	NAPG	Gamma-sol	SNAG_HUN	12	0.92325
24H 50MT/	P10515	0.044749	0.121328	0.069632	DLAT	Dihydrolip	ODP2_HUN	25	1.031503
24H 50MT/	P40261	0.037192	0.12136	0.069648	NNMT	Nicotinami	NNMT_HUN	11	1.026115
48H 10MT/	Q96S66	-0.11563	0.121401	0.069667	CLCC1	Chloride ch	CLCC1_HUI	20	0.922981
48H 10MT/	A6NIH7	0.047107	0.121404	0.069668	UNC119B	Protein unc	U119B_HU	7	1.033191
48H 10MT/	Q9BVP2	-0.05063	0.121418	0.069673	GNL3	Guanine nu	GNL3_HUN	24	0.965517
48H 50MT/	Q14562	-0.02732	0.121428	0.069677	DHX8	ATP-depend	DHX8_HUN	35	0.981245
48H 10MT/	Q96BP3	-0.00102	0.121427	0.069677	PPWD1	Peptidylpro	PPWD1_HU	24	0.999295
48H 50MT/	P61313	0.052216	0.121472	0.069698	RPL15	Large ribos	RL15_HUM	10	1.036856
48H 50MT/	Q5W0V3	-0.0144	0.12148	0.0697	FHIP2A	FHF comple	FHI2A_HUN	10	0.990069
48H 50MT/	Q8IY26	-0.13487	0.121489	0.069701	PLPP6	Polyisoprer	PLPP6_HUI	5	0.910755
48H 10MT/	Q93099	-0.10904	0.121497	0.069703	HGD	Homogenti	HGD_HUM/	17	0.927208
24H 10MT/	Q96IZ7	0.168728	0.121568	0.069735	RSRC1	Serine/Argi	RSRC1_HUI	2	1.124067
48H 10MT/	Q8WYH8	-0.12861	0.121612	0.069752	ING5	Inhibitor of	ING5_HUM	2	0.914714
48H 10MT/	Q9BUH6	0.042138	0.121621	0.069755	PAXX	Protein PA)	PAXX_HUM	9	1.029638
24H 50MT/	Q9H3H3	-0.14682	0.121649	0.069769	C11orf68	UPF0696 p	CK068_HUI	12	0.903239
48H 50MT/	O95613	-0.19751	0.121659	0.069771	PCNT	Pericentrin	PCNT_HUM	18	0.872052
24H 10MT/	P15170	-0.0229	0.121659	0.069771	GSPT1	Eukaryotic	ERF3A_HUI	24	0.984253
48H 50MT/	Q9BS34	-0.4712	0.121698	0.06979	ZNF670	Zinc finger	ZN670_HUI	4	0.721365
24H 10MT/	Q8WV24	-0.54884	0.1217	0.06979	PHLDA1	Pleckstrin h	PHLA1_HUI	3	0.683568
24H 50MT/	Q9P0U3	0.156612	0.121742	0.06981	SENP1	Sentrin-spe	SENP1_HUI	4	1.114667
24H 10MT/	Q9GZV4	0.173875	0.121807	0.069841	EIF5A2	Eukaryotic	IF5A2_HUN	7	1.128084
48H 50MT/	Q8TBM8	-0.47679	0.121823	0.069846	DNAJB14	DnaJ homo	DJB14_HUI	6	0.718574
24H 50MT/	P05543	-0.66755	0.121829	0.069849	SERPINA7	Thyroxine-l	THBG_HUN	3	0.629574
24H 10MT/	Q9Y3A4	0.053111	0.121835	0.069851	RRP7A	Ribosomal	RRP7A_HUI	11	1.037499
24H 50MT/	Q92506	0.06108	0.121911	0.069885	HSD17B8	(3R)-3-hydr	DHB8_HUN	6	1.043247
48H 50MT/	Q8IYB5	-0.01354	0.121958	0.069906	SMAP1	Stromal me	SMAP1_HU	4	0.990661
24H 10MT/	P49189	-0.04862	0.121975	0.069914	ALDH9A1	4-trimethyl	AL9A1_HUI	21	0.96686
24H 10MT/	Q9UK76	-0.1702	0.122067	0.069955	JPT1	Jupiter mic	JUPI1_HUN	7	0.888717
48H 50MT/	O14939	-0.03403	0.122071	0.069955	PLD2	Phospholip	PLD2_HUM	2	0.976686
24H 10MT/	Q9NZV5	-0.06112	0.12207	0.069955	SELENON	Selenoprot	SELN_HUM	4	0.958519
48H 10MT/	P04792	0.081788	0.122101	0.069967	HSPB1	Heat shock	HSPB1_HUI	16	1.058329
24H 10MT/	Q8NEC7	0.002837	0.122108	0.069969	GSTCD	Glutathion	GSTCD_HUI	6	1.001968
48H 50MT/	P62380	-0.1242	0.122164	0.069997	TBPL1	TATA box-b	TBPL1_HUN	7	0.917511
48H 50MT/	Q9NTJ4	-0.10714	0.122182	0.07	MAN2C1	Alpha-mani	MA2C1_HU	5	0.928424
48H 10MT/	Q9UL12	-0.16424	0.122181	0.07	SARDH	Sarcosine d	SARDH_HU	6	0.8924
24H 10MT/	Q9BXK1	-0.31155	0.122187	0.070002	KLF16	Krueppel-li	KLF16_HUN	2	0.805776
48H 50MT/	P41229	0.350793	0.12221	0.070012	KDM5C	Lysine-spec	KDM5C_HU	15	1.275262
24H 10MT/	P48551	0.522141	0.12227	0.07004	IFNAR2	Interferon a	INAR2_HUN	2	1.436084
48H 10MT/	Q13795	0.044825	0.12227	0.07004	ARFRP1	ADP-ribosy	ARFRP_HUI	2	1.031558
48H 50MT/	Q6P1A2	-0.08365	0.122284	0.070047	LPCAT3	Lysophospl	MBOA5_HU	8	0.943666

48H	10MT/	Q5VSG8	-0.08253	0.122295	0.070051	MANEAL	Glycoprote	MANEL_HU	1	0.9444
48H	10MT/	Q9NZ52	-0.18816	0.12234	0.070072	GGA3	ADP-ribosy	GGA3_HUN	13	0.877726
48H	10MT/	P20339	-0.03411	0.122424	0.070108	RAB5A	Ras-related	RAB5A_HU	8	0.976633
24H	10MT/	O14727	0.082678	0.122469	0.070128	APAF1	Apoptotic p	APAF_HUM	22	1.058982
24H	10MT/	Q9UPQ3	0.371143	0.12247	0.070128	AGAP1	Arf-GAP wit	AGAP1_HU	2	1.293377
48H	10MT/	P52735	0.085591	0.122481	0.070131	VAV2	Guanine nu	VAV2_HUM	25	1.061122
24H	10MT/	O95865	0.003526	0.122486	0.070133	DDAH2	Putative hy	DDAH2_HU	14	1.002447
48H	10MT/	Q9BUK0	0.172596	0.122566	0.070169	CHCHD7	Coiled-coil-	CHCH7_HU	1	1.127085
24H	50MT/	Q6PK04	-0.10879	0.122576	0.070172	CCDC137	Coiled-coil	CC137_HUI	6	0.927367
48H	10MT/	Q9UJ04	-0.09867	0.122577	0.070172	TSPYL4	Testis-speci	TSYL4_HUN	2	0.933893
24H	50MT/	P14672	-0.29546	0.122581	0.070173	SLC2A4	Solute carri	GLUT4_HUI	1	0.814813
24H	50MT/	Q10713	0.004023	0.122608	0.070185	PMPCA	Mitochond	MPPA_HUN	24	1.002793
24H	10MT/	Q96BJ3	-0.06657	0.12261	0.070186	AIDA	Axin intera	AIDA_HUM	7	0.954905
24H	10MT/	Q92544	0.043379	0.122615	0.070187	TM9SF4	Transmemb	TM9S4_HU	17	1.030525
48H	50MT/	Q9BUE6	0.122149	0.122632	0.070196	ISCA1	Iron-sulfur	ISCA1_HUN	3	1.088355
48H	10MT/	P47914	-0.34557	0.12265	0.070202	RPL29	Large ribos	RL29_HUM	2	0.786998
24H	50MT/	O94760	0.051544	0.122668	0.070209	DDAH1	N(G),N(G)-d	DDAH1_HU	16	1.036374
48H	10MT/	P17706	0.01514	0.122758	0.070252	PTPN2	Tyrosine-pr	PTN2_HUM	9	1.010549
24H	50MT/	O94874	0.004767	0.122763	0.070254	UFL1	E3 UFM1-pr	UFL1_HUM	37	1.00331
24H	50MT/	Q9NZ52	0.110786	0.12277	0.070256	GGA3	ADP-ribosy	GGA3_HUN	13	1.079816
24H	10MT/	P61026	0.0667	0.122811	0.070276	RAB10	Ras-related	RAB10_HUI	11	1.047318
48H	50MT/	Q5T4S7	-0.01634	0.12282	0.07028	UBR4	E3 ubiquiti	UBR4_HUM	145	0.988735
48H	10MT/	Q9UGC7	-0.19605	0.122853	0.070296	MTRF1L	Peptide cha	RF1ML_HU	4	0.872937
24H	10MT/	Q02487	0.738852	0.12286	0.070298	DSC2	Desmocolli	DSC2_HUM	2	1.668847
48H	10MT/	P08574	0.016217	0.122865	0.0703	CYC1	Cytochrom	CY1_HUMA	8	1.011304
48H	10MT/	Q641Q2	0.067206	0.122904	0.070317	WASHC2A	WASH com	WAC2A_HU	28	1.047685
24H	10MT/	Q9BXS9	-0.25714	0.122921	0.070321	SLC26A6	Solute carri	S26A6_HUI	3	0.836743
48H	10MT/	P51687	0.053126	0.122933	0.070327	SUOX	Sulfite oxid	SUOX_HUM	11	1.037511
24H	10MT/	P51398	-0.09445	0.122987	0.070348	DAP3	Small ribos	RT29_HUM	18	0.93663
48H	10MT/	Q92625	-0.11754	0.122993	0.070349	ANKS1A	Ankyrin rep	ANS1A_HUI	15	0.921755
24H	10MT/	Q15652	-0.32603	0.122997	0.07035	JMJD1C	Probable Jr	JHD2C_HUI	4	0.79773
24H	10MT/	P55735	0.050501	0.12301	0.070355	SEC13	Protein SEC	SEC13_HUN	11	1.035625
24H	50MT/	Q8TCT9	0.09258	0.123008	0.070355	HM13	Minor histo	HM13_HUN	10	1.066275
48H	10MT/	Q8N138	0.174834	0.123035	0.070366	ORMDL3	ORM1-like p	ORML3_HU	1	1.128835
24H	10MT/	O95273	0.091971	0.123074	0.070382	CCNDBP1	Cyclin-D1-b	CCDB1_HU	2	1.065825
48H	50MT/	Q9Y3E7	0.000617	0.123073	0.070382	CHMP3	Charged mu	CHMP3_HU	5	1.000428
48H	50MT/	P42766	-0.10548	0.123082	0.070386	RPL35	Large ribos	RL35_HUM	5	0.929495
48H	10MT/	O15258	0.179683	0.123111	0.070398	RER1	Protein REF	RER1_HUM	6	1.132635
48H	10MT/	Q9HD23	-0.03001	0.12312	0.070402	MRS2	Magnesium	MRS2_HUN	15	0.979414
24H	50MT/	Q8IZL8	0.015801	0.123126	0.070403	PELP1	Proline-, gl	PELP1_HUN	24	1.011012
48H	10MT/	Q9P209	0.257612	0.123138	0.070408	CEP72	Centrosom	CEP72_HUI	8	1.195498
24H	10MT/	P40227	0.042176	0.123168	0.07042	CCT6A	T-complex p	TCPZ_HUM	27	1.029665
24H	50MT/	Q9NX18	-0.90534	0.123211	0.070439	SDHAF2	Succinate d	SDHF2_HUI	3	0.533906
24H	10MT/	P52630	-0.40088	0.123217	0.07044	STAT2	Signal trans	STAT2_HUN	7	0.757398
48H	10MT/	Q96GX9	0.163066	0.123217	0.07044	APIP	Methylthio	MTNB_HUN	8	1.119664
48H	10MT/	P51608	-0.08882	0.123241	0.070447	MECP2	Methyl-CpG	MECP2_HU	12	0.940292
24H	10MT/	Q9UI14	0.202001	0.123239	0.070447	RABAC1	Prenylated	PRAF1_HUI	4	1.150293
24H	50MT/	Q8ND04	-0.01351	0.123253	0.07045	SMG8	Nonsense-n	SMG8_HUN	15	0.990679
24H	10MT/	P53816	0.413868	0.123262	0.070453	PLAAT3	Phospholip	PLAT3_HUN	2	1.332253
48H	10MT/	Q53EU6	-0.10774	0.123274	0.070456	GPAT3	Glycerol-3-	GPAT3_HUI	14	0.92804
48H	10MT/	P06733	-0.03033	0.123352	0.070491	ENO1	Alpha-enol	ENOA_HUM	28	0.979193
24H	50MT/	Q9H9C1	0.161435	0.123402	0.070513	VIPAS39	Spermatog	SPE39_HUN	14	1.118399

24H 10MTA	Q9Y4Z0	-0.33773	0.123404	0.070513	LSM4	U6 snRNA-a	LSM4_HUM	5	0.791283
24H 10MTA	Q10471	0.044229	0.123408	0.070514	GALNT2	Polypeptid	GALT2_HUI	26	1.031132
48H 10MTA	P53384	0.127247	0.123443	0.070529	NUBP1	Cytosolic Fe	NUBP1_HU	9	1.092207
48H 50MTA	A0JLT2	-0.50795	0.123461	0.070535	MED19	Mediator o	MED19_HU	3	0.703221
48H 50MTA	Q96GK7	0.054207	0.12346	0.070535	FAHD2A	Oxaloaceta	FAH2A_HUI	10	1.038288
24H 50MTA	Q8IZ13	-0.20323	0.123489	0.070548	FAM200C	Protein FAI	F200C_HUI	1	0.868605
24H 50MTA	Q9Y5U2	0.364085	0.123505	0.070555	TSSC4	U5 small nu	TSSC4_HUN	3	1.287065
48H 50MTA	P20930	0.347702	0.123519	0.070561	FLG	Filaggrin	FILA_HUMA	1	1.272532
24H 50MTA	Q5R3I4	0.01952	0.123602	0.0706	TTC38	Tetratricop	TTC38_HUI	16	1.013622
48H 50MTA	P62888	-0.08288	0.123605	0.070601	RPL30	Large ribos	RL30_HUM	5	0.94417
48H 10MTA	Q5JS37	-0.27262	0.123616	0.070606	NHLRC3	NHL repeat	NHLC3_HU	5	0.827815
48H 50MTA	Q9H6U6	0.549389	0.123643	0.070618	BCAS3	BCAS3 micr	BCAS3_HUI	3	1.463466
48H 10MTA	Q9Y4A5	0.019798	0.123653	0.070621	TRRAP	Transforma	TRRAP_HUI	41	1.013818
48H 50MTA	Q9Y6W3	0.035443	0.123689	0.070636	CAPN7	Calpain-7	CAN7_HUM	18	1.024871
48H 50MTA	Q96E39	0.029992	0.123699	0.07064	RBMXL1	RNA bindin	RMXL1_HU	3	1.021006
24H 50MTA	O43292	-0.06625	0.123725	0.070646	GPAA1	Glycosylph	GPAA1_HU	11	0.955115
24H 10MTA	P55212	0.034701	0.123725	0.070646	CASP6	Caspase-6	CASP6_HUI	9	1.024345
48H 10MTA	Q9Y320	-0.07515	0.123728	0.070646	TMX2	Thioredoxi	TMX2_HUN	13	0.949243
48H 10MTA	Q68D10	0.008361	0.123775	0.07067	SPTY2D1	Protein SPT	SPT2_HUM	13	1.005813
48H 50MTA	P11168	-0.32311	0.123781	0.07067	SLC2A2	Solute carri	GTR2_HUM	7	0.799345
48H 50MTA	P50443	-0.58807	0.123783	0.07067	SLC26A2	Sulfate tran	S26A2_HUI	2	0.665232
24H 50MTA	Q8NFV4	-0.19879	0.123801	0.070677	ABHD11	sn-1-specifi	ABHDB_HU	9	0.871282
48H 50MTA	Q6PKG0	-0.09344	0.123832	0.070691	LARP1	La-related p	LARP1_HUI	37	0.937287
24H 50MTA	Q96SN8	0.079036	0.123863	0.070704	CDK5RAP2	CDK5 regul	CK5P2_HUI	9	1.056312
48H 10MTA	O60684	0.061949	0.123886	0.070713	KPNA6	Importin su	IMA7_HUM	10	1.043875
24H 10MTA	Q9H4B0	-0.11958	0.123921	0.070725	OSGEPL1	tRNA N6-ad	OSGL1_HUI	5	0.920458
24H 50MTA	Q9NXW2	-0.16293	0.123932	0.070729	DNAJB12	DnaJ homo	DJB12_HUI	10	0.893209
48H 50MTA	Q9NRR5	0.023661	0.123955	0.070738	UBQLN4	Ubiquilin-4	UBQL4_HU	9	1.016536
48H 10MTA	Q9NXU5	-0.18216	0.123967	0.070742	ARL15	ADP-ribosy	ARL15_HUI	3	0.881381
48H 10MTA	Q8TCX1	-0.6743	0.124014	0.070764	DYNC2LI1	Cytoplasmi	DC2L1_HUI	3	0.626636
48H 10MTA	O15516	-0.25814	0.124019	0.070766	CLOCK	Circadian lo	CLOCK_HUI	1	0.836163
24H 50MTA	Q86Y37	0.022976	0.124025	0.070768	CACUL1	CDK2-assoc	CACL1_HUI	4	1.016053
48H 50MTA	P62304	0.027329	0.124049	0.070778	SNRPE	Small nucle	RUXE_HUM	4	1.019124
48H 10MTA	Q9ULA0	-0.00871	0.124176	0.070835	DNPEP	Aspartyl am	DNPEP_HU	16	0.993979
48H 50MTA	Q99627	0.084025	0.124195	0.070844	COPS8	COP9 signa	CSN8_HUM	7	1.059971
48H 50MTA	Q69YL0	-0.41052	0.124218	0.070851	NCBP2AS2	Protein NCI	NCAS2_HUI	3	0.752355
48H 10MTA	Q96IU4	-0.1044	0.124219	0.070851	ABHD14B	Putative pr	ABHEB_HUI	6	0.930193
24H 10MTA	Q9Y6H1	-0.02238	0.124217	0.070851	CHCHD2	Coiled-coil-	CHCH2_HU	4	0.98461
48H 10MTA	Q86VZ5	-0.21129	0.124234	0.070856	SGMS1	Phosphatid	SMS1_HUM	3	0.863763
24H 50MTA	Q96GM8	-0.07308	0.124241	0.070858	TOE1	Target of EG	TOE1_HUM	14	0.950603
24H 10MTA	P61964	0.025599	0.12425	0.070862	WDR5	WD repeat-	WDR5_HUI	12	1.017903
48H 10MTA	Q9H0P0	0.045209	0.124267	0.070867	NT5C3A	Cytosolic 5	5NT3A_HUI	15	1.031833
24H 50MTA	Q8N2Z9	0.566461	0.124288	0.070877	CENPS	Centromere	CENPS_HUI	2	1.480887
24H 10MTA	Q58FF6	0.239947	0.124293	0.070879	HSP90AB4P	Putative he	H90B4_HU	3	1.180949
24H 50MTA	P78344	-0.06325	0.1243	0.07088	EIF4G2	Eukaryotic	IF4G2_HUN	46	0.957105
24H 10MTA	Q9BXC9	-0.34567	0.124307	0.070881	BBS2	Bardet-Bied	BBS2_HUM	1	0.786942
48H 10MTA	P30260	-0.09471	0.124349	0.0709	CDC27	Cell divisio	CDC27_HU	20	0.936461
24H 10MTA	Q14978	-0.17929	0.12435	0.0709	NOLC1	Nucleolar a	NOLC1_HU	10	0.883136
24H 10MTA	Q99598	-0.08157	0.124347	0.0709	TSNAX	Translin-ass	TSNAX_HUI	10	0.94503
24H 10MTA	Q9NS00	0.088955	0.124354	0.070902	C1GALT1	Glycoprote	C1GLT_HUI	9	1.0636
48H 50MTA	P22607	0.158339	0.124369	0.070907	FGFR3	Fibroblast g	FGFR3_HUI	5	1.116002
48H 10MTA	Q8N0Z3	-0.27664	0.124385	0.070913	SPICE1	Spindle anc	SPICE_HUM	3	0.825509

24H 10MTA	O43837	0.079931	0.124427	0.070933	IDH3B	Isocitrate d	IDH3B_HUI	17	1.056968
48H 50MTA	Q5SW79	0.135788	0.124432	0.070935	CEP170	Centrosom	CE170_HUI	23	1.098693
48H 10MTA	Q15554	0.023635	0.12445	0.070944	TERF2	Telomeric r	TERF2_HUM	13	1.016517
24H 50MTA	O14795	0.139964	0.124468	0.070954	UNC13B	Protein unc	UN13B_HU	13	1.101877
48H 50MTA	Q9H8M1	0.020109	0.124489	0.070963	COQ10B	Coenzyme (	CQ10B_HU	4	1.014036
48H 50MTA	Q9Y371	-0.06593	0.124507	0.070969	SH3GLB1	Endophilin	SHLB1_HUI	13	0.955332
24H 10MTA	Q8NDI1	-0.00075	0.124525	0.070976	EHBP1	EH domain-	EHBP1_HUI	20	0.999479
48H 10MTA	P05423	-0.1413	0.124533	0.07098	POLR3D	DNA-direct	RPC4_HUM	6	0.906701
24H 10MTA	Q6ZUT9	0.216256	0.124566	0.070996	DENND5B	DENN doma	DEN5B_HU	5	1.161715
24H 10MTA	Q9UP83	-0.04909	0.124602	0.071013	COG5	Conserved (	COG5_HUM	23	0.966544
24H 10MTA	P0DMV1;P(	0.049029	0.124632	0.071024	CT45A8;CT4	Cancer/test	CT458_HUI	9	1.034568
48H 10MTA	Q9BQ04	-0.04266	0.124628	0.071024	RBM4B	RNA-bindin	RBM4B_HU	15	0.970866
48H 50MTA	O75683	-0.08078	0.124644	0.071027	SURF6	Surfeit locu	SURF6_HUI	7	0.945549
48H 50MTA	O95470	-0.02123	0.124675	0.07104	SGPL1	Sphingosin	SGPL1_HUI	17	0.985391
48H 10MTA	O43488	0.046812	0.124698	0.071047	AKR7A2	Aflatoxin B	ARK72_HUI	10	1.03298
24H 50MTA	P17812	-0.07151	0.124739	0.071065	CTPS1	CTP syntha	PYRG1_HUI	29	0.95164
24H 10MTA	Q92576	0.086633	0.124744	0.071067	PHF3	PHD finger	PHF3_HUM	36	1.061889
24H 50MTA	Q8N0U8	-0.05371	0.124783	0.071085	VKORC1L1	Vitamin K e	VKORL_HUI	5	0.963455
48H 50MTA	O95167	-0.28145	0.124795	0.071089	NDUFA3	NADH dehy	NDUA3_HU	2	0.822762
48H 50MTA	Q5VT52	-0.0544	0.124802	0.071092	RPRD2	Regulation	RPRD2_HU	26	0.962998
24H 10MTA	Q9C0E2	0.066685	0.124819	0.071098	XPO4	Exportin-4	XPO4_HUM	25	1.047307
24H 10MTA	Q96T21	0.320841	0.124829	0.071102	SECISBP2	Selenocyste	SEBP2_HUI	3	1.249059
48H 50MTA	Q9H252	-0.25627	0.12486	0.071117	KCNH6	Potassium v	KCNH6_HU	2	0.837251
24H 50MTA	Q8WU17	-1.54139	0.124874	0.071121	RNF139	E3 ubiquiti	RN139_HU	3	0.343555
24H 10MTA	Q99715	-0.02525	0.124893	0.071129	COL12A1	Collagen al	COCA1_HU	2	0.982648
24H 10MTA	Q8N0W3	-0.14022	0.12503	0.071186	FCSK	L-fucose kir	FCSK_HUM	5	0.907382
48H 50MTA	Q8WUM9	0.577909	0.125048	0.071193	SLC20A1	Sodium-de	S20A1_HUI	1	1.492684
24H 10MTA	Q4G0F5	0.001838	0.125086	0.071209	VPS26B	Vacuolar pr	VP26B_HUI	10	1.001275
48H 10MTA	Q66GS9	-0.07361	0.125084	0.071209	CEP135	Centrosom	CP135_HUI	1	0.950259
48H 50MTA	Q9HBG6	0.092412	0.125088	0.071209	IFT122	Intraflagell	IF122_HUM	5	1.066151
48H 10MTA	Q9NX38	0.287223	0.125086	0.071209	ABITRAM	Protein Abi	ABITM_HUI	1	1.220289
24H 50MTA	Q9UQ80	-0.03668	0.125089	0.071209	PA2G4	Proliferatic	PA2G4_HU	23	0.974898
48H 10MTA	P43897	0.011177	0.125102	0.071216	TSFM	Elongation	EFTS_HUM	12	1.007778
24H 50MTA	P10412	0.217273	0.125111	0.071219	H1-4	Histone H1	H14_HUMA	9	1.162534
24H 50MTA	P51798	0.00013	0.125123	0.071223	CLCN7	H(+)/Cl(-) ex	CLCN7_HUI	6	1.00009
48H 10MTA	Q86UN3	0.607798	0.125128	0.071223	RTN4RL2	Reticulon-4	R4RL2_HUI	2	1.523932
48H 50MTA	Q9UI26	0.033695	0.125128	0.071223	IPO11	Importin-1	IPO11_HUI	27	1.023631
24H 10MTA	Q9Y676	0.063137	0.125137	0.071227	MRPS18B	Small ribos	RT18B_HUI	9	1.044735
48H 10MTA	Q15121	-0.32534	0.125146	0.07123	PEA15	Astrocytic p	PEA15_HUI	6	0.798109
48H 50MTA	P56545	-0.01773	0.125148	0.07123	CTBP2	C-terminal-	CTBP2_HUI	6	0.987786
24H 50MTA	Q8N543	-0.10951	0.125192	0.071247	OGFOD1	Prolyl 3-hy	OGFD1_HU	15	0.9269
24H 50MTA	Q8TER5	-0.09563	0.125201	0.07125	ARHGEF40	Rho guanin	ARH40_HU	15	0.935862
24H 10MTA	P13196	-0.20048	0.125256	0.071278	ALAS1	5-aminolev	HEM1_HUM	7	0.87026
48H 10MTA	P40925	0.017914	0.125257	0.071278	MDH1	Malate deh	MDHC_HUI	13	1.012494
24H 50MTA	Q9C086	0.542448	0.12526	0.071278	INO80B	INO80 com	IN80B_HUI	2	1.456442
24H 50MTA	Q96IX5	-0.02058	0.125429	0.071362	ATP5MK	ATP syntha	ATPMK_HU	3	0.985835
48H 10MTA	Q8TDR2	0.569742	0.125483	0.071384	STK35	Serine/thre	STK35_HUI	1	1.484258
48H 50MTA	Q9UPS6	0.522611	0.125516	0.071397	SETD1B	Histone-lys	SET1B_HUI	3	1.436552
24H 10MTA	P17302	0.250225	0.125539	0.071404	GJA1	Gap junctic	CXA1_HUM	8	1.189393
48H 10MTA	Q9Y678	-0.0212	0.125561	0.071413	COPG1	Coatomer s	COPG1_HU	42	0.985414
24H 10MTA	Q13418	-0.09241	0.125611	0.071433	ILK	Integrin-lin	ILK_HUMAI	17	0.937957
24H 10MTA	Q14108	0.094845	0.125622	0.071436	SCARB2	Lysosome n	SCRB2_HUI	11	1.067951

48H	50MT/	A8MWD9;F	0.041741	0.125632	0.071441	SNRPGP15;	Putative sm	RUXGL_HU	3	1.029355
24H	50MT/	P62191	-0.07669	0.125671	0.071462	PSMC1	26S protea	PRS4_HUM	25	0.948227
24H	50MT/	P49589	-0.08693	0.125685	0.071467	CARS1	Cysteine--tI	SYCC_HUM	34	0.941524
24H	50MT/	O43639	0.275836	0.125714	0.071476	NCK2	Cytoplasmi	NCK2_HUM	4	1.210696
24H	10MT/	P51884	0.324661	0.125713	0.071476	LUM	Lumican	LUM_HUM/	4	1.25237
24H	10MT/	Q9NXE4	-0.00746	0.125753	0.071496	SMPD4	Sphingomy	NSMA3_HU	20	0.994841
24H	50MT/	Q96GC9	0.123935	0.125767	0.071503	VMP1	Vacuole me	VMP1_HUM	6	1.089703
24H	10MT/	P12273	0.18996	0.125817	0.071525	PIP	Prolactin-ir	PIP_HUMAI	2	1.140732
48H	10MT/	O75503	-0.00059	0.125829	0.07153	CLN5	Bis(monoac	CLN5_HUM	7	0.99959
48H	10MT/	O60783	0.088027	0.126007	0.071617	MRPS14	Small ribos	RT14_HUM	4	1.062915
24H	10MT/	Q5VW38	0.088564	0.126013	0.071619	GPR107	Protein GPI	GP107_HU	6	1.063311
24H	50MT/	Q14186	0.009712	0.126025	0.071624	TFDP1	Transcripti	TFDP1_HUI	3	1.006755
48H	10MT/	Q9NR56	-0.00755	0.126031	0.071625	MBNL1	Muscleblin	MBNL1_HU	7	0.99478
48H	10MT/	O75955	-0.02271	0.126074	0.071641	FLOT1	Flotillin-1	FLOT1_HUI	23	0.98438
48H	50MT/	Q9P2C4	0.229475	0.126074	0.071641	TMEM181	Transmemb	TM181_HU	3	1.172408
24H	50MT/	Q07866	-0.08717	0.126084	0.071644	KLC1	Kinesin ligh	KLC1_HUM	17	0.94137
48H	10MT/	O75380	-0.02423	0.126094	0.071647	NDUFS6	NADH dehy	NDUS6_HU	5	0.983347
48H	50MT/	P11802	-0.21207	0.126118	0.071658	CDK4	Cyclin-depe	CDK4_HUM	12	0.863295
24H	10MT/	O15382	0.128786	0.126149	0.071672	BCAT2	Branched-c	BCAT2_HUI	17	1.093373
24H	10MT/	Q9UL12	-0.14533	0.126209	0.071698	SARDH	Sarcosine d	SARDH_HU	6	0.90417
48H	10MT/	Q96JJ7	-0.11364	0.126293	0.071738	TMX3	Protein dis	TMX3_HUM	12	0.924253
48H	10MT/	Q9NWU1	-0.10536	0.126345	0.071762	OXSM	3-oxoacyl-[	OXSM_HUM	14	0.92957
24H	50MT/	P09497	-0.25046	0.126357	0.071766	CLTB	Clathrin lig	CLCB_HUM	7	0.840626
24H	50MT/	E9PAV3;Q1	-0.14529	0.126371	0.071773	NACA	Nascent po	NACAM_HU	5	0.904195
24H	10MT/	Q9Y4E6	0.135283	0.126385	0.071779	WDR7	WD repeat-	WDR7_HUI	11	1.098308
24H	10MT/	P24752	0.044137	0.12639	0.071781	ACAT1	Acetyl-CoA	THIL_HUM/	20	1.031066
24H	10MT/	P35813	0.002559	0.126396	0.071782	PPM1A	Protein pho	PPM1A_HU	11	1.001775
48H	50MT/	Q8WXB1	-0.05711	0.126456	0.071812	METTL21A	Protein N-l	MT21A_HU	3	0.961188
24H	10MT/	P22413	0.012565	0.126484	0.071823	ENPP1	Ectonucleo	ENPP1_HUI	13	1.008747
24H	50MT/	P02774	-0.5579	0.126503	0.071829	GC	Vitamin D-I	VTDB_HUM	5	0.67929
48H	50MT/	Q96Q07	-0.56041	0.126578	0.071864	BTBD9	BTB/POZ dc	BTBD9_HUI	1	0.678108
24H	50MT/	Q96DZ1	0.01126	0.126599	0.071871	ERLEC1	Endoplasm	ERLEC_HUM	14	1.007835
48H	50MT/	Q9NYB0	-0.10651	0.126605	0.071873	TERF2IP	Telomeric r	TE2IP_HUM	10	0.928835
24H	10MT/	Q5VWZ2	0.001309	0.126693	0.071914	LYPLAL1	Lysophospl	LYPL1_HUM	6	1.000908
24H	10MT/	Q96G03	0.058412	0.126736	0.071928	PGM2	Phosphope	PGM2_HUI	30	1.041319
24H	50MT/	Q99653	-0.02089	0.126752	0.071935	CHP1	Calcineurin	CHP1_HUM	10	0.985625
24H	50MT/	O95626	-0.202	0.126777	0.071946	ANP32D	Acidic leuci	AN32D_HU	1	0.869344
24H	50MT/	Q7L5Y1	0.095928	0.126789	0.071951	ENOSF1	Mitochond	ENOF1_HUI	13	1.068752
24H	50MT/	P51617	-0.22229	0.126809	0.071958	IRAK1	Interleukin	IRAK1_HUM	7	0.857206
24H	10MT/	Q8TAP9	-0.93421	0.126839	0.071973	MPLKIP	M-phase-sp	MPLKI_HUI	1	0.523327
48H	10MT/	P17987	-0.02171	0.126847	0.071975	TCP1	T-complex p	TCPA_HUM	32	0.985064
48H	50MT/	Q12996	-0.04576	0.126903	0.071993	CSTF3	Cleavage sti	CSTF3_HUM	24	0.968781
24H	50MT/	Q9Y312	-0.09093	0.126938	0.07201	AAR2	Protein AAI	AAR2_HUM	12	0.938921
48H	10MT/	Q9UMZ2	0.121793	0.126962	0.072019	SYNRG	Synergin ga	SYNRG_HUI	13	1.088086
24H	10MT/	O15371	-0.00395	0.127008	0.072039	EIF3D	Eukaryotic	EIF3D_HUM	24	0.997268
24H	50MT/	Q9P258	0.004919	0.127008	0.072039	RCC2	Protein RC(	RCC2_HUM	24	1.003415
48H	50MT/	Q9NY35	-0.00599	0.127013	0.072041	CLDND1	Claudin doi	CLDN1_HU	3	0.995854
24H	50MT/	P06493	-0.10438	0.12705	0.072059	CDK1	Cyclin-depe	CDK1_HUM	17	0.930203
48H	10MT/	Q9UNH7	0.016681	0.127052	0.072059	SNX6	Sorting nex	SNX6_HUM	19	1.011629
48H	50MT/	Q9H008	0.219956	0.127064	0.072064	LHPP	Phospholys	LHPP_HUM	5	1.164698
24H	10MT/	Q96BR1	0.272841	0.12707	0.072065	SGK3	Serine/thre	SGK3_HUM	5	1.208184
24H	10MT/	O60675	0.078944	0.12711	0.072083	MAFK	Transcripti	MAFK_HUM	4	1.056245

24H 50MT/	P42126	0.010271	0.127141	0.072096	ECI1	Enoyl-CoA (	ECI1_HUM/	9	1.007145
24H 10MT/	Q12769	0.03359	0.127149	0.072096	NUP160	Nuclear po	NU160_HU	41	1.023556
48H 50MT/	Q9Y3T9	-0.1254	0.127147	0.072096	NOC2L	Nucleolar c	NOC2L_HU	23	0.916751
24H 10MT/	P17028	0.077697	0.127217	0.072123	ZNF24	Zinc finger	ZNF24_HUI	7	1.055332
24H 50MT/	Q8TBK6	-0.19047	0.127246	0.072138	ZCCHC10	Zinc finger	ZCH10_HUI	1	0.876318
48H 10MT/	Q6WKZ4	0.033233	0.127262	0.072143	RAB11FIP1	Rab11 fami	RFIP1_HUM	18	1.023303
48H 10MT/	Q8TF71	-0.05539	0.127279	0.072149	SLC16A10	Monocarbo	MOT10_HU	1	0.962338
48H 10MT/	Q92738	-0.30936	0.127287	0.072152	USP6NL	USP6 N-ter	US6NL_HUI	4	0.807002
48H 50MT/	Q8NB78	-0.29347	0.127296	0.072156	KDM1B	Lysine-spec	KDM1B_HU	4	0.815937
48H 10MT/	Q8WXB1	-0.1079	0.127317	0.072157	METTL21A	Protein N-l	MT21A_HU	3	0.927937
48H 10MT/	Q9UEG4	0.099143	0.127318	0.072157	ZNF629	Zinc finger	ZN629_HUI	7	1.071137
24H 50MT/	Q9HCE5	0.199476	0.127349	0.072169	METTL14	N6-adenosi	MET14_HU	6	1.148281
48H 50MT/	Q96BZ8	0.050303	0.127362	0.072172	LENG1	Leukocyte r	LENG1_HUI	5	1.035483
48H 50MT/	P15529	-0.12413	0.127369	0.072175	CD46	Membrane	MCP_HUM/	7	0.917559
48H 10MT/	O14732	-0.09149	0.127377	0.072178	IMPA2	Inositol mc	IMPA2_HUI	4	0.938553
48H 10MT/	O00116	-0.03235	0.127457	0.072219	AGPS	Alkyldihydr	ADAS_HUM	32	0.977828
24H 50MT/	Q9P253	-0.05517	0.127472	0.072221	VPS18	Vacuolar pi	VPS18_HUI	27	0.962482
48H 50MT/	Q9NZ09	0.070435	0.127498	0.072231	UBAP1	Ubiquitin-a	UBAP1_HU	6	1.050033
48H 10MT/	A6NH11	-0.0165	0.12751	0.072235	GLTPD2	Glycolipid	GLTD2_HUI	6	0.988631
48H 10MT/	Q96A49	0.043365	0.127513	0.072235	SYAP1	Synapse-as	SYAP1_HUI	18	1.030514
24H 50MT/	A6NKF1	-0.23657	0.127547	0.072245	SAC3D1	SAC3 doma	SAC31_HUI	8	0.848759
24H 10MT/	Q9BYT8	0.025739	0.127546	0.072245	NLN	Neurolysin,	NEUL_HUM	19	1.018001
48H 10MT/	Q9HAV4	-0.01368	0.127643	0.072287	XPO5	Exportin-5	XPO5_HUM	47	0.990562
48H 10MT/	O75718	-0.00288	0.127676	0.0723	CRTAP	Cartilage-as	CRTAP_HUI	13	0.998003
24H 10MT/	Q14692	0.011563	0.127725	0.072322	BMS1	Ribosome b	BMS1_HUM	33	1.008047
24H 50MT/	O00410	-0.09562	0.127759	0.072338	IPO5	Importin-5	IPO5_HUM	53	0.935871
48H 10MT/	O60427	-0.15455	0.127762	0.072339	FADS1	Acyl-CoA (8	FADS1_HUI	12	0.89841
24H 10MT/	Q9Y508	0.080465	0.127818	0.072366	RNF114	E3 ubiquiti	RN114_HU	6	1.057359
24H 10MT/	A2RUB6	0.671893	0.127885	0.072397	CCDC66	Coiled-coil	CCD66_HU	1	1.593162
48H 50MT/	Q8N0T1	-0.09688	0.127904	0.072407	RBIS	Ribosomal	RBIS_HUM/	3	0.935054
24H 10MT/	Q96FQ6	-0.2824	0.127911	0.072408	S100A16	Protein S1(	S10AG_HUI	4	0.82222
24H 10MT/	Q9UNP9	-0.01522	0.127978	0.072439	PPIE	Peptidyl-pr	PPIE_HUM/	17	0.989507
48H 10MT/	Q8IYU2	-0.55932	0.128027	0.072462	HACE1	E3 ubiquiti	HACE1_HUI	2	0.678622
48H 10MT/	Q58FF8	-0.0276	0.128048	0.072469	HSP90AB2I	Putative he	H90B2_HU	2	0.981052
24H 10MT/	Q9UGV2	-0.10633	0.128071	0.072479	NDRG3	Protein ND	NDRG3_HU	12	0.928948
48H 10MT/	Q8NDD1	-0.59995	0.128107	0.072493	C1orf131	Uncharacte	CA131_HUI	2	0.659775
48H 50MT/	Q92930	-0.01995	0.12811	0.072493	RAB8B	Ras-related	RAB8B_HUI	4	0.98627
24H 10MT/	Q96RT8	0.249511	0.128153	0.072511	TUBGCP5	Gamma-tul	GCP5_HUM	3	1.188804
24H 50MT/	Q8IUF8	0.109041	0.128186	0.072525	RIOX2	Ribosomal	RIOX2_HUI	12	1.078511
24H 10MT/	Q8NFZ5	-0.39275	0.128185	0.072525	TNIP2	TNFAIP3-in	TNIP2_HUM	1	0.761675
48H 10MT/	Q96MF7	0.107508	0.128208	0.072534	NSMCE2	E3 SUMO-p	NSE2_HUM	4	1.077366
24H 50MT/	Q9NY12	0.009078	0.128208	0.072534	GAR1	H/ACA ribo	GAR1_HUM	8	1.006312
48H 10MT/	Q9H0U3	-0.03781	0.128231	0.072545	MAGT1	Magnesium	MAGT1_HU	7	0.974133
48H 50MT/	Q14457	-0.11604	0.128246	0.072551	BECN1	Beclin-1	BECN1_HUI	7	0.922718
24H 50MT/	P08670	-0.09475	0.128253	0.072552	VIM	Vimentin	VIME_HUM	34	0.936436
48H 50MT/	Q16644	-0.45184	0.128272	0.072561	MAPKAPK3	MAP kinase	MAPK3_HU	3	0.731112
24H 10MT/	Q96PU8	0.034655	0.128291	0.07257	QKI	KH domain	QKI_HUMA	12	1.024312
24H 50MT/	Q92625	-0.00324	0.12831	0.072579	ANKS1A	Ankyrin rep	ANS1A_HUI	15	0.997756
48H 50MT/	Q96BP2	-0.15647	0.128323	0.072581	CHCHD1	Small ribos	CHCH1_HU	1	0.89722
48H 10MT/	Q9P2T1	0.077366	0.128353	0.07259	GMPR2	GMP reduc	GMPR2_HU	15	1.05509
24H 10MT/	Q6PJ69	0.501617	0.128397	0.072607	TRIM65	E3 ubiquiti	TRI65_HUM	3	1.415799
48H 50MT/	P06400	-0.13527	0.128475	0.072643	RB1	Retinoblast	RB_HUMAN	24	0.910498

24H 10MT/	Q16706	0.118611	0.128476	0.072643	MAN2A1	Alpha-man	MA2A1_HU	13	1.085689
24H 50MT/	P47813	0.072751	0.128494	0.072649	EIF1AX	Eukaryotic	IF1AX_HUM	9	1.051721
24H 10MT/	Q04756	0.386643	0.128496	0.072649	HGFAC	Hepatocyte	HGFA_HUM	4	1.307348
24H 10MT/	A4D161	0.348943	0.128498	0.072649	FAM221A	Protein FAM	F221A_HUI	3	1.273627
24H 50MT/	Q9Y3C1	-0.03822	0.128508	0.072652	NOP16	Nucleolar p	NOP16_HU	9	0.973859
48H 50MT/	Q13308	0.29664	0.128565	0.072674	PTK7	Inactive tyr	PTK7_HUM	9	1.22828
48H 10MT/	Q8IXK0	0.067963	0.128595	0.072689	PHC2	Polyhomec	PHC2_HUM	6	1.048235
48H 10MT/	Q8NI36	0.001042	0.128684	0.072731	WDR36	WD repeat-	WDR36_HU	30	1.000722
48H 50MT/	Q9NR28	0.027621	0.128683	0.072731	DIABLO	Diablo IAP-	DBLOH_HU	9	1.01933
48H 10MT/	Q96Q89	-0.16289	0.128705	0.072741	KIF20B	Kinesin-like	KI20B_HUM	22	0.893233
48H 10MT/	Q9UK97	0.109566	0.128707	0.072741	FBXO9	F-box only	FBX9_HUM	4	1.078904
48H 10MT/	P35998	0.018976	0.128725	0.072746	PSMC2	26S protea	PRS7_HUM	30	1.01324
48H 50MT/	Q9NVN8	-0.22862	0.128724	0.072746	GNL3L	Guanine nu	GNL3L_HU	17	0.853454
24H 50MT/	Q9UBF6	0.073417	0.128728	0.072747	RNF7	RING-box p	RBX2_HUM	3	1.052206
48H 10MT/	Q9NZN5	-0.01731	0.128734	0.07275	ARHGEF12	Rho guanin	ARHGC_HU	37	0.988071
48H 10MT/	Q9UDT6	-0.05078	0.128861	0.072808	CLIP2	CAP-Gly do	CLIP2_HUM	31	0.965414
48H 50MT/	Q8N0Z3	-0.25406	0.128873	0.072812	SPICE1	Spindle anc	SPICE_HUM	3	0.838535
24H 10MT/	A6NJ88	0.853328	0.128885	0.072818	SAGE2P	Putative SA	SGE2P_HUI	1	1.806664
48H 50MT/	Q6IE81	-0.23664	0.128904	0.072828	JADE1	Protein Jad	JADE1_HUM	2	0.848722
24H 50MT/	Q7Z6J8	-0.33008	0.128916	0.072833	UBE3D	E3 ubiquiti	UBE3D_HU	4	0.795492
24H 10MT/	O43678	0.002342	0.128987	0.072863	NDUFA2	NADH dehy	NDUA2_HU	4	1.001625
48H 10MT/	Q86YV9	0.048309	0.129014	0.072874	HPS6	BLOC-2 con	HPS6_HUM	14	1.034052
24H 10MT/	O94905	0.002414	0.129017	0.072874	ERLIN2	Erlin-2	ERLN2_HUI	19	1.001675
48H 50MT/	P25098	0.073475	0.129036	0.072882	GRK2	Beta-adren	ARBK1_HUI	7	1.052248
24H 10MT/	Q5U4P2	0.032203	0.129082	0.072904	ASPHD1	Aspartate b	ASPH1_HUI	3	1.022572
24H 10MT/	O60502	0.008249	0.129085	0.072904	OGA	Protein O-G	OGA_HUM/	30	1.005734
24H 10MT/	Q8NEB5	-0.46216	0.129099	0.072911	PLPP5	Phospholip	PLPP5_HUI	1	0.725901
48H 10MT/	Q9NQ48	-0.3647	0.129116	0.072917	LZTFL1	Leucine zip	LZTL1_HUM	7	0.776629
24H 10MT/	O95402	0.113419	0.129129	0.072924	MED26	Mediator o	MED26_HU	1	1.081789
24H 10MT/	Q16543	-0.08351	0.129256	0.072981	CDC37	Hsp90 co-c	CDC37_HU	9	0.943761
24H 10MT/	P15529	-0.0539	0.129258	0.072981	CD46	Membrane	MCP_HUM/	7	0.963327
48H 10MT/	Q9H999	-0.05794	0.129266	0.072984	PANK3	Pantothena	PANK3_HU	10	0.960632
48H 10MT/	Q9UQR0	0.039151	0.129294	0.072996	SCML2	Sex comb o	SCML2_HU	15	1.027509
24H 10MT/	Q9Y2H0	0.081941	0.129293	0.072996	DLGAP4	Disks large-	DLGP4_HU	11	1.058441
24H 50MT/	P16220	0.054553	0.129315	0.073005	CREB1	Cyclic AMP	CREB1_HUI	3	1.038537
48H 10MT/	Q9Y6K9	-0.02656	0.129416	0.073055	IKBKG	NF-kappa-B	NEMO_HUM	11	0.981761
24H 50MT/	Q9BW27	0.000184	0.129442	0.073065	NUP85	Nuclear po	NUP85_HU	23	1.000128
48H 50MT/	Q6PI98	-0.27851	0.129464	0.073075	INO80C	INO80 com	IN80C_HUM	3	0.824444
24H 10MT/	Q13112	0.082671	0.129486	0.073084	CHAF1B	Chromatin	CAF1B_HUI	14	1.058977
24H 10MT/	Q15004	0.120632	0.129513	0.073097	PCLAF	PCNA-assoc	PAF15_HUI	8	1.087211
48H 10MT/	Q86YS7	-0.04373	0.129522	0.0731	C2CD5	C2 domain	C2CD5_HU	13	0.970142
24H 50MT/	P12081	-0.04836	0.129527	0.073101	HARS1	Histidine--t	HARS1_HUI	29	0.967038
48H 50MT/	Q9UMX5	-0.02939	0.12954	0.073104	NENF	Neudesin	NENF_HUM	7	0.979833
24H 10MT/	P04844	0.044167	0.129546	0.073106	RPN2	Dolichyl-di	RPN2_HUM	29	1.031088
24H 10MT/	Q9BV81	-0.06431	0.129553	0.073108	EMC6	ER membra	EMC6_HUM	2	0.956401
48H 10MT/	O75191	0.049212	0.129557	0.073109	XYLB	Xylulose kir	XYLB_HUM	9	1.0347
24H 10MT/	P18669	-0.08547	0.1296	0.073131	PGAM1	Phosphogly	PGAM1_HU	20	0.942478
48H 10MT/	Q9Y6G9	0.010345	0.129631	0.073143	DYNC1LI1	Cytoplasmi	DC1L1_HUI	27	1.007197
24H 10MT/	Q8TAF3	0.085166	0.129641	0.073145	WDR48	WD repeat-	WDR48_HU	13	1.06081
24H 50MT/	Q9Y5X2	-0.09281	0.129684	0.073166	SNX8	Sorting nex	SNX8_HUM	18	0.937693
48H 10MT/	O43427	-0.03724	0.129701	0.073175	FIBP	Acidic fibro	FIBP_HUM/	13	0.974517
24H 10MT/	P62841	0.093573	0.129768	0.0732	RPS15	Small ribos	RS15_HUM	6	1.06701

48H 50MTA	P01019	0.011276	0.129771	0.073201	AGT	Angiotensin	ANGT_HUM	12	1.007846
48H 10MTA	Q9NPR9	0.228123	0.129795	0.07321	GPR108	Protein GP	GP108_HU	5	1.17131
24H 50MTA	Q8WV92	-0.07855	0.129834	0.073229	MITD1	MIT domain	MITD1_HU	2	0.947012
48H 50MTA	Q13151	0.051549	0.12986	0.073243	HNRNPA0	Heterogene	ROA0_HUM	8	1.036377
24H 50MTA	Q9UPY8	0.1211	0.129887	0.073254	MAPRE3	Microtubul	MARE3_HU	1	1.087564
48H 10MTA	Q96PY5	-0.02696	0.129918	0.073267	FMNL2	Formin-like	FMNL2_HU	9	0.981487
48H 50MTA	Q13867	0.038775	0.129932	0.073273	BLMH	Bleomycin	BLMH_HUM	14	1.027241
24H 50MTA	P15735	0.0454	0.129936	0.073273	PHKG2	Phosphoryl	PHKG2_HU	1	1.031969
24H 10MTA	P33981	0.052938	0.129936	0.073273	TTK	Dual specifi	TTK_HUMA	19	1.037376
48H 10MTA	Q9UJK0	-0.21158	0.12994	0.073274	TSR3	18S rRNA a	TSR3_HUM	6	0.863592
24H 50MTA	P0CJ79;P1	-0.67608	0.129949	0.073277	ZNF888;ZN	Zinc finger	ZN888_HU	2	0.625864
24H 10MTA	P02771	0.423661	0.129964	0.073282	AFP	Alpha-fetop	FETA_HUM	3	1.341327
48H 50MTA	Q15042	-0.01615	0.129965	0.073282	RAB3GAP1	Rab3 GTPas	RB3GP_HU	40	0.988871
24H 10MTA	Q9UNX3	-0.30778	0.130019	0.073306	RPL26L1	Ribosomal	RL26L_HUM	2	0.807881
48H 50MTA	O15144	-0.03415	0.130047	0.073319	ARPC2	Actin-relate	ARPC2_HU	19	0.976606
48H 50MTA	Q7LGA3	0.057749	0.130056	0.073323	HS2ST1	Heparan su	HS2ST_HUM	13	1.04084
48H 50MTA	Q9NVW2	-0.25659	0.130072	0.07333	RLIM	E3 ubiquiti	RNF12_HU	5	0.837063
24H 50MTA	Q07812	0.054088	0.130087	0.073336	BAX	Apoptosis r	BAX_HUMA	8	1.038202
48H 50MTA	P53801	-0.2216	0.130104	0.073344	PTTG1IP	Pituitary tu	PTTG_HUM	1	0.857611
48H 10MTA	O14894	0.973115	0.130165	0.073372	TM4SF5	Transmemb	T4S5_HUM	1	1.963074
48H 10MTA	Q92547	-0.18391	0.130191	0.073382	TOPBP1	DNA topois	TOPB1_HU	13	0.880315
48H 10MTA	Q8TBQ9	-0.12634	0.130231	0.0734	TMEM167A	Protein kish	KISHA_HUM	3	0.916155
48H 10MTA	Q9NUJ7	-0.04981	0.130251	0.07341	PLCXD1	PI-PLC X do	PLCX1_HU	4	0.966066
24H 10MTA	P36915	-0.0397	0.130269	0.073419	GNL1	Guanine nu	GNL1_HUM	23	0.972855
24H 10MTA	Q15797	-0.06789	0.130297	0.073431	SMAD1	Mothers ag	SMAD1_HU	5	0.954031
24H 50MTA	P62906	0.005266	0.130333	0.073448	RPL10A	Large ribos	RL10A_HU	13	1.003657
48H 50MTA	P18440	-0.21734	0.130374	0.073465	NAT1	Arylamine I	ARY1_HUM	7	0.860149
48H 10MTA	Q93034	-0.04785	0.13041	0.073479	CUL5	Cullin-5	CUL5_HUM	35	0.96738
24H 10MTA	P52569	-0.05161	0.130412	0.07348	SLC7A2	Cationic an	CTR2_HUM	8	0.964857
24H 50MTA	O00170	-0.00601	0.130416	0.073481	AIP	AH recepto	AIP_HUMA	15	0.995845
48H 50MTA	Q9NQV6	-0.20793	0.130466	0.073505	PRDM10	PR domain	PRD10_HU	4	0.865777
48H 10MTA	Q969T7	0.449871	0.130499	0.073521	NT5C3B	7-methylgu	5NT3B_HU	4	1.365918
48H 10MTA	O60256	0.037455	0.130609	0.073571	PRPSAP2	Phosphorib	KPRB_HUM	14	1.026302
24H 10MTA	O60306	0.062775	0.130614	0.073573	AQR	RNA helicas	AQR_HUMA	44	1.044473
48H 50MTA	Q8NDV7	-0.53059	0.130627	0.073577	TNRC6A	Trinucleoti	TNR6A_HU	3	0.692272
48H 50MTA	Q7L0Y3	-0.04004	0.130631	0.073578	TRMT10C	tRNA methy	TM10C_HU	24	0.972626
48H 50MTA	P82664	-0.36492	0.130657	0.073589	MRPS10	Small ribos	RT10_HUM	4	0.77651
24H 50MTA	P43034	0.015576	0.130666	0.073592	PAFAH1B1	Platelet-act	LIS1_HUMA	20	1.010855
24H 50MTA	Q15021	-0.04918	0.130694	0.0736	NCAPD2	Condensin	CND1_HUM	53	0.966485
24H 50MTA	Q99741	-0.71271	0.130694	0.0736	CDC6	Cell divisio	CDC6_HUM	1	0.610171
24H 50MTA	P40189	-0.1085	0.1307	0.073602	IL6ST	Interleukin	IL6RB_HUM	5	0.927551
48H 50MTA	Q7Z2K8	0.032227	0.130706	0.073605	GPRIN1	G protein-r	GRIN1_HU	10	1.022589
24H 10MTA	P36873	0.010586	0.130761	0.073626	PPP1CC	Serine/thre	PP1G_HUM	17	1.007364
48H 10MTA	Q8IUE6	0.123534	0.130766	0.073628	H2AC21	Histone H2	H2A2B_HU	3	1.089401
24H 10MTA	Q02750	-0.02228	0.130775	0.073631	MAP2K1	Dual specifi	MP2K1_HU	10	0.984673
48H 10MTA	Q5JSH3	-0.02545	0.130819	0.073653	WDR44	WD repeat-	WDR44_HU	22	0.982516
48H 10MTA	O96018	-0.6758	0.130856	0.073665	APBA3	Amyloid-be	APBA3_HU	1	0.625984
24H 10MTA	Q6KB66	0.273277	0.130864	0.073665	KRT80	Keratin, typ	K2C80_HU	5	1.20855
48H 50MTA	Q04724	0.091547	0.130872	0.073668	TLE1	Transducin	TLE1_HUMA	7	1.065512
48H 10MTA	Q96KG9	0.044819	0.130949	0.073705	SCYL1	N-terminal	SCYL1_HUM	26	1.031554
48H 10MTA	P22033	-0.08779	0.131028	0.073741	MMUT	Methylmal	MUTA_HUM	24	0.94096
24H 10MTA	Q9BZK3	-0.35871	0.131028	0.073741	NACA4P	Putative na	NACP4_HU	1	0.779864

24H 10MT/	Q9C0B9	-0.15275	0.131059	0.073754	ZCCHC2	Zinc finger (	ZCHC2_HUI	2	0.899531
24H 10MT/	P30043	-0.00718	0.131079	0.073763	BLVRB	Flavin redu	BLVRB_HUI	9	0.995037
24H 10MT/	O75896	-0.15576	0.131114	0.07378	TUSC2	Tumor supp	TUSC2_HUI	4	0.897657
48H 50MT/	O14773	0.094719	0.131236	0.073833	TPP1	Tripeptidyl	TPP1_HUM	11	1.067858
24H 50MT/	Q01085	0.180229	0.131233	0.073833	TIAL1	Nucleolysir	TIAR_HUM/	9	1.133064
24H 10MT/	P51114	0.033373	0.131245	0.073835	FXR1	RNA-bindir	FXR1_HUM	21	1.023402
48H 10MT/	Q9NQ50	-0.03725	0.131247	0.073835	MRPL40	Large ribos	RM40_HUN	7	0.974511
24H 50MT/	Q53SF7	0.084708	0.131303	0.07386	COBLL1	Cordon-ble	COBL1_HUI	15	1.060473
48H 10MT/	O60218	0.217412	0.131336	0.073875	AKR1B10	Aldo-keto r	AK1BA_HUI	1	1.162646
48H 10MT/	Q71SY5	0.525668	0.131335	0.073875	MED25	Mediator o	MED25_HU	4	1.4396
48H 50MT/	Q8IUD2	0.027081	0.131342	0.073877	ERC1	ELKS/Rab6-	RB6I2_HUN	26	1.018948
48H 10MT/	P07195	4.926471	0.131351	0.073879	LDHB	L-lactate de	LDHB_HUN	1	30.40994
48H 10MT/	P62805	-0.12941	0.131353	0.073879	H4C1	Histone H4	H4_HUMAN	12	0.914207
24H 10MT/	Q9NVR2	0.095707	0.131364	0.073883	INTS10	Integrator (	INT10_HUN	18	1.068589
24H 10MT/	Q86UN3	0.827983	0.131451	0.073923	RTN4RL2	Reticulon-4	R4RL2_HUI	2	1.775202
48H 50MT/	Q9H7H0	-0.14311	0.131474	0.073931	METTL17	Ribosome a	MET17_HU	5	0.905568
24H 50MT/	Q96K21	-0.05204	0.131508	0.073949	ZFYVE19	Abscission/	ANCHR_HU	6	0.964572
24H 10MT/	O75153	-0.04676	0.131597	0.073991	CLUH	Clustered n	CLU_HUMA	45	0.968106
48H 50MT/	Q08170	0.233607	0.131613	0.073996	SRSF4	Serine/argi	SRSF4_HUN	4	1.175771
24H 50MT/	Q96A28	-0.30818	0.131632	0.074003	SLAMF9	SLAM famil	SLAF9_HUN	1	0.807661
48H 50MT/	Q8N3P4	-0.14313	0.131675	0.074022	VPS8	Vacuolar pr	VPS8_HUM	12	0.90555
48H 10MT/	Q6ZXV5	0.048322	0.131729	0.074049	TMTC3	Protein O-n	TMTC3_HU	17	1.034062
24H 10MT/	Q96D53	-0.06708	0.131728	0.074049	COQ8B	Atypical kir	COQ8B_HU	7	0.954567
48H 10MT/	Q9H8X2	-0.39503	0.131737	0.074052	IPPK	Inositol-pe	IPPK_HUM/	3	0.760471
24H 10MT/	Q9Y6E0	0.059269	0.131762	0.074062	STK24	Serine/thre	STK24_HUN	17	1.041938
24H 10MT/	Q15334	0.319074	0.131794	0.074077	LLGL1	Lethal(2) gi	L2GL1_HUI	8	1.24753
48H 10MT/	Q9BSK2	-0.11725	0.131796	0.074077	SLC25A33	Solute carri	S2533_HUI	8	0.921943
48H 10MT/	Q86V85	-0.11642	0.131804	0.074079	GPR180	Integral me	GP180_HU	4	0.922471
48H 10MT/	Q9NTI5	-0.02385	0.131807	0.074079	PDS5B	Sister chror	PDS5B_HUI	41	0.983607
48H 50MT/	P00742	0.82132	0.131816	0.074082	F10	Coagulatio	FA10_HUM	4	1.767022
48H 10MT/	O43676	0.035741	0.131842	0.074095	NDUFB3	NADH dehy	NDUB3_HU	3	1.025083
24H 10MT/	Q9H061	-0.01284	0.131851	0.074098	TMEM126A	Transmemb	T126A_HUI	10	0.991138
24H 10MT/	O95573	0.044688	0.131884	0.074112	ACSL3	Fatty acid C	ACSL3_HUN	28	1.03146
24H 10MT/	P63261	0.023358	0.131929	0.074129	ACTG1	Actin, cyto	ACTG_HUM	3	1.016322
48H 10MT/	Q8WWY3	0.024663	0.131958	0.074141	PRPF31	U4/U6 sma	PRP31_HUI	17	1.017242
24H 10MT/	Q96JC1	-0.03751	0.13198	0.074149	VPS39	Vam6/Vps3	VPS39_HUI	19	0.974335
48H 50MT/	Q16539	0.046511	0.131984	0.074149	MAPK14	Mitogen-ac	MK14_HUN	13	1.032764
24H 50MT/	Q9Y4L5	-0.40856	0.131997	0.074156	RNF115	E3 ubiquiti	RN115_HU	3	0.753375
48H 50MT/	Q8WUA4	-0.1585	0.132005	0.074158	GTF3C2	General tra	TF3C2_HUN	18	0.895954
48H 10MT/	Q9H9S5	-0.10139	0.132038	0.074174	FKRP	Ribitol 5-pl	FKRP_HUM	5	0.932132
48H 50MT/	Q9H0H5	-0.2039	0.132074	0.074188	RACGAP1	Rac GTPase	RGAP1_HU	16	0.868203
24H 10MT/	Q9NW08	0.102065	0.132109	0.074205	POLR3B	DNA-direct	RPC2_HUN	17	1.073309
48H 50MT/	Q9P270	-0.01549	0.132109	0.074205	SLAIN2	SLAIN moti	SLAI2_HUN	11	0.989319
24H 50MT/	Q13795	-0.30745	0.132132	0.074213	ARFRP1	ADP-ribosy	ARFRP_HUI	2	0.808069
48H 10MT/	O95273	0.432644	0.132152	0.074221	CCNDBP1	Cyclin-D1-k	CCDB1_HU	2	1.349705
24H 50MT/	O43708	-0.05346	0.132216	0.074248	GSTZ1	Maleylacet	MAAI_HUN	5	0.96362
48H 50MT/	Q8N2W9	-0.09118	0.132216	0.074248	PIAS4	E3 SUMO-p	PIAS4_HUN	6	0.938757
48H 50MT/	Q92900	-0.07077	0.132242	0.074261	UPF1	Regulator c	RENT1_HUI	51	0.952133
24H 50MT/	Q9Y6N1	0.107261	0.132242	0.074261	COX11	Cytochrom	COX11_HU	6	1.077181
24H 10MT/	Q9Y6M1	0.056593	0.132269	0.074271	IGF2BP2	Insulin-like	IF2B2_HUN	20	1.040007
24H 10MT/	P06746	0.040782	0.132362	0.074316	POLB	DNA polym	DPOLB_HU	7	1.028671
48H 50MT/	Q5T8I9	-0.17635	0.132374	0.074322	HENMT1	Small RNA 2	HENMT_HU	5	0.884937

48H 50MT/	Q9UJX2	-0.00249	0.132384	0.074327	CDC23	Cell divisio	CDC23_HU	24	0.998277
24H 50MT/	Q9P2E3	-0.14284	0.132433	0.074347	ZNFX1	NFX1-type	ZNFX1_HUI	17	0.905732
24H 10MT/	A0A0A0MS	0.556815	0.132461	0.074361	TRBV23-1	Probable n	TVB23_HUI	1	1.471018
24H 50MT/	Q96BY7	0.048364	0.132463	0.074361	ATG2B	Autophagy	ATG2B_HUI	12	1.034092
48H 50MT/	Q14247	-0.03699	0.132504	0.074376	CTTN	Src substra	SRC8_HUM	19	0.974684
24H 10MT/	Q14790	0.027985	0.132514	0.07438	CASP8	Caspase-8	CASP8_HUI	17	1.019587
48H 10MT/	Q13619	-0.07487	0.13255	0.074396	CUL4A	Cullin-4A	CUL4A_HUI	28	0.94943
48H 50MT/	P43304	-0.03625	0.132583	0.074411	GPD2	Glycerol-3-	GPDM_HUI	36	0.975185
24H 50MT/	Q6MZP7	0.338789	0.132587	0.074411	LIN54	Protein lin-	LIN54_HUN	5	1.264695
24H 10MT/	Q17RS7	-0.01865	0.132594	0.074413	GEN1	Flap endon	GEN_HUMA	3	0.987158
48H 50MT/	Q6NS38	-0.0647	0.132648	0.074438	ALKBH2	DNA oxidat	ALKB2_HUI	5	0.956143
48H 10MT/	Q2TAK8	0.721476	0.132659	0.074442	PWWP3A	PWWP don	PWP3A_HU	2	1.648868
48H 50MT/	P55211	-0.21769	0.132688	0.074455	CASP9	Caspase-9	CASP9_HUI	4	0.85994
48H 10MT/	P49411	-0.08268	0.132824	0.074518	TUFM	Elongation	EFTU_HUM	31	0.944302
24H 50MT/	O94966	-0.15713	0.132852	0.074532	USP19	Ubiquitin c	UBP19_HU	25	0.89681
24H 10MT/	Q5UCC4	0.019535	0.132924	0.074561	EMC10	ER membra	EMC10_HU	3	1.013633
24H 50MT/	Q86Z14	0.229099	0.13293	0.074564	KLB	Beta-kloth	KLOTB_HUI	4	1.172102
24H 10MT/	Q9BQK8	-0.30813	0.132945	0.07457	LPIN3	Phosphatid	LPIN3_HUN	3	0.80769
24H 10MT/	Q7Z3T8	-0.07004	0.132985	0.074588	ZFYVE16	Zinc finger	ZFY16_HUN	28	0.952609
24H 50MT/	Q96EX1	0.524193	0.133037	0.074613	SMIM12	Small integ	SIM12_HUI	2	1.438129
48H 50MT/	Q8NCF5	-0.2588	0.133084	0.074634	NFATC2IP	NFATC2-int	NF2IP_HUN	5	0.835781
48H 50MT/	P42566	-0.0245	0.133157	0.074671	EPS15	Epidermal	EPS15_HUN	22	0.98316
24H 50MT/	P55036	-0.05934	0.133162	0.074671	PSMD4	26S protea	PSMD4_HU	14	0.9597
24H 10MT/	O15498	0.03932	0.133174	0.074677	YKT6	Synaptobre	YKT6_HUM	11	1.02763
48H 50MT/	P02666	-0.87347	0.133184	0.07468	CSN2	Beta-casein	CASB_BOVI	4	0.545832
24H 10MT/	Q15020	-0.00345	0.133194	0.074684	SART3	Squamous	SART3_HUN	38	0.997612
24H 10MT/	Q6PJG2	0.16774	0.133195	0.074684	MIDEAS	Mitotic dea	MDEAS_HU	7	1.123298
24H 50MT/	O75223	-0.11768	0.133228	0.074698	GGCT	Gamma-glu	GGCT_HUM	8	0.921666
24H 50MT/	P55011	0.062342	0.133282	0.074722	SLC12A2	Solute carri	S12A2_HUI	31	1.04416
48H 10MT/	Q5VIR6	-0.00465	0.133283	0.074722	VPS53	Vacuolar pr	VPS53_HUI	16	0.996784
48H 50MT/	Q9NS84	-1.15464	0.133484	0.074817	CHST7	Carbohydra	CHST7_HUI	1	0.449177
24H 50MT/	Q9H4H8	-0.48138	0.133499	0.074823	FAM83D	Protein FAI	FA83D_HUI	12	0.716291
48H 10MT/	Q12846	0.012546	0.133557	0.074842	STX4	Syntaxin-4	STX4_HUM	11	1.008734
48H 10MT/	O94806	0.425184	0.13358	0.07485	PRKD3	Serine/thre	KPCD3_HU	2	1.342743
24H 10MT/	Q14241	-0.06281	0.133596	0.074858	ELOA	Elongin-A	ELOA1_HUI	7	0.957399
48H 10MT/	Q9Y3Q8	0.377987	0.133635	0.074874	TSC22D4	TSC22 dom	T22D4_HUI	2	1.299527
48H 10MT/	O15230	0.021763	0.13374	0.074923	LAMA5	Laminin sul	LAMA5_HU	57	1.015199
24H 50MT/	Q8NBN3	-0.03106	0.133833	0.074963	TMEM87A	Transmemb	TM87A_HU	10	0.978698
48H 10MT/	P07205	0.02394	0.13385	0.074972	PGK2	Phosphogly	PGK2_HUM	3	1.016732
48H 10MT/	Q9UHR6	0.022821	0.133926	0.075009	ZNHIT2	Zinc finger	ZNHI2_HUN	8	1.015944
24H 50MT/	O75164	0.721113	0.133968	0.075025	KDM4A	Lysine-spec	KDM4A_HU	4	1.648453
24H 10MT/	Q9P0K8	0.145166	0.133966	0.075025	FOXJ2	Forkhead b	FOXJ2_HUN	4	1.105858
24H 50MT/	Q9C0D3	0.012505	0.133979	0.075028	ZYG11B	Protein zyg	ZY11B_HUN	12	1.008705
24H 10MT/	Q9Y3C1	-0.02927	0.13398	0.075028	NOP16	Nucleolar p	NOP16_HU	9	0.979913
48H 50MT/	Q4VNC0	1.292686	0.134037	0.075053	ATP13A5	Probable ca	AT135_HUI	1	2.449837
48H 50MT/	O75153	-0.13961	0.13409	0.075075	CLUH	Clustered n	CLU_HUMA	45	0.907765
48H 50MT/	Q9UNX3	-0.11064	0.134088	0.075075	RPL26L1	Ribosomal	RL26L_HUN	2	0.92618
48H 10MT/	P15252	-1.45229	0.134097	0.075078	NaN	Rubber elo	REF_HEVBR	5	0.365441
48H 10MT/	O15160	-0.06656	0.134124	0.07509	POLR1C	DNA-direct	RPAC1_HUI	17	0.954914
24H 10MT/	O60783	-0.06536	0.134136	0.075093	MRPS14	Small ribos	RT14_HUM	4	0.955709
48H 10MT/	P35610	-0.19567	0.1342	0.075122	SOAT1	Sterol O-ac	SOAT1_HUI	11	0.873166
48H 10MT/	Q9H2U1	-0.05063	0.134217	0.075129	DHX36	ATP-depen	DHX36_HU	29	0.965513

24H 50MT/	Q8TCJ2	0.062184	0.134283	0.075157	STT3B	Dolichyl-di	STT3B_HUI	14	1.044045
24H 50MT/	Q9UI26	-0.0195	0.134284	0.075157	IPO11	Importin-1	IPO11_HUI	27	0.986576
48H 10MT/	Q9UP95	-0.1519	0.134306	0.075168	SLC12A4	Solute carri	S12A4_HUI	12	0.900066
24H 50MT/	Q8IZA0	-0.17764	0.134323	0.075174	KIAA0319L	Dyslexia-as:	K319L_HUI	10	0.884151
24H 10MT/	Q6IQ23	0.07429	0.134355	0.075187	PLEKHA7	Pleckstrin I	PKHA7_HU	12	1.052843
48H 10MT/	A0MZ66	0.027274	0.134397	0.075201	SHTN1	Shootin-1	SHOT1_HUI	28	1.019084
24H 10MT/	Q08945	0.009042	0.1344	0.075201	SSRP1	FACT comp	SSRP1_HUI	24	1.006287
48H 10MT/	P42898	-0.24137	0.134405	0.075202	MTHFR	Methylenet	MTHR_HUI	11	0.845944
24H 10MT/	Q8N5J2	0.389116	0.13441	0.075202	MINDY1	Ubiquitin c	MINY1_HUI	2	1.309591
48H 50MT/	Q9UKT4	0.62779	0.134435	0.075211	FBXO5	F-box only	FBX5_HUM	2	1.545196
48H 10MT/	Q8WU76	-0.17634	0.134459	0.075224	SCFD2	Sec1 family	SCFD2_HUI	9	0.884944
48H 50MT/	Q96CD2	0.063329	0.134486	0.075233	PPCDC	Phosphopa	COAC_HUM	2	1.044874
48H 50MT/	P62333	0.040419	0.134495	0.075237	PSMC6	26S protea:	PRS10_HUI	21	1.028413
48H 50MT/	Q9P246	-0.57352	0.134539	0.075257	STIM2	Stromal int	STIM2_HUI	5	0.671976
24H 10MT/	Q9P2K2	0.36854	0.134579	0.075269	TXNDC16	Thioredoxi	TXD16_HUI	4	1.291046
24H 10MT/	O60844	-0.56494	0.134588	0.075272	ZG16	Zymogen gi	ZG16_HUM	1	0.675985
48H 50MT/	Q9BZL4	-0.01291	0.134593	0.075274	PPP1R12C	Protein pho	PP12C_HUI	10	0.991092
48H 50MT/	Q8NI27	-0.01031	0.134608	0.075277	THOC2	THO compl	THOC2_HU	41	0.992881
48H 10MT/	Q3KQU3	-0.02876	0.134633	0.075288	MAP7D1	MAP7 dom	MA7D1_HU	10	0.980264
48H 10MT/	Q9NVU7	-0.06083	0.134635	0.075288	SDAD1	Protein SD/	SDA1_HUM	14	0.958711
24H 10MT/	P61916	-0.14709	0.134676	0.075306	NPC2	NPC intrace	NPC2_HUM	7	0.903071
24H 10MT/	Q96G23	0.063851	0.134682	0.075309	CERS2	Ceramide s	CERS2_HUI	9	1.045252
48H 10MT/	P25440	-0.02209	0.134793	0.075365	BRD2	Bromodom	BRD2_HUM	14	0.984804
24H 10MT/	Q9NRB3	-0.20915	0.134802	0.075368	CHST12	Carbohydra	CHSTC_HUI	1	0.865049
48H 50MT/	Q9Y2D4	-0.05906	0.13483	0.075378	EXOC6B	Exocyst cor	EXC6B_HUI	12	0.959887
24H 10MT/	P26373	-0.03303	0.134922	0.075418	RPL13	Large ribos	RL13_HUM	7	0.977366
24H 10MT/	O00308	0.219978	0.134978	0.075445	WWP2	NEDD4-like	WWP2_HU	5	1.164715
48H 10MT/	P23468	-0.04587	0.134998	0.075453	PTPRD	Receptor-ty	PTPRD_HUI	8	0.968704
48H 10MT/	Q4G176	-0.07106	0.135015	0.075461	ACSF3	Malonate--	ACSF3_HUI	18	0.951936
24H 50MT/	Q9ULW2	0.280043	0.135019	0.075461	FZD10	Frizzled-10	FZD10_HUI	4	1.214231
48H 50MT/	Q8N0X4	-0.07023	0.135024	0.075461	CLYBL	Citramalyl-	CLYBL_HUM	7	0.952483
48H 10MT/	P54278	0.066265	0.135033	0.075465	PMS2	Mismatch r	PMS2_HUM	23	1.047002
24H 10MT/	O00142	-0.57941	0.135038	0.075466	TK2	Thymidine	KITM_HUM	4	0.669236
24H 50MT/	Q9Y252	0.197825	0.135053	0.075472	RNF6	E3 ubiquiti	RNF6_HUM	1	1.146968
24H 50MT/	P19623	-0.00043	0.1351	0.075496	SRM	Spermidine	SPEE_HUM/	18	0.9997
48H 50MT/	Q99615	-0.14799	0.135106	0.075498	DNAJC7	DnaJ homo	DNJC7_HUI	27	0.902508
24H 10MT/	A0A0B4J2A	-0.15759	0.135116	0.075501	PPIAL4C	Peptidyl-pr	PAL4C_HUI	4	0.896523
24H 10MT/	P21580	-0.11043	0.135189	0.075533	TNFAIP3	Tumor necr	TNAP3_HUI	6	0.926313
24H 50MT/	Q8IUR0	-0.08026	0.135203	0.075536	TRAPPC5	Trafficking	TPPC5_HUI	5	0.945889
24H 50MT/	Q3ZCM7	-0.07227	0.135237	0.075551	TUBB8	Tubulin bet	TBB8_HUM	3	0.951143
24H 50MT/	Q9UJ70	-0.13077	0.135275	0.075569	NAGK	N-acetyl-D-	NAGK_HUM	18	0.913346
48H 10MT/	P42684	-0.36887	0.135292	0.075574	ABL2	Tyrosine-pr	ABL2_HUM	3	0.774389
24H 10MT/	Q15717	0.02549	0.13537	0.075615	ELAVL1	ELAV-like pi	ELAV1_HUI	11	1.017826
48H 10MT/	Q13362	-0.03178	0.135433	0.075642	PPP2R5C	Serine/thre	2A5G_HUM	15	0.97821
48H 10MT/	Q9BQA9	-0.20473	0.135432	0.075642	CYBC1	Cytochrom	CYBC1_HUI	3	0.867704
24H 10MT/	P00761	0.362864	0.135456	0.075652	NaN	Trypsin (Lal	TRYP_PIG	7	1.285976
48H 50MT/	Q6P2Q9	-0.056	0.135496	0.075665	PRPF8	Pre-mRNA-		PRP8_HUM	107	0.961925
24H 10MT/	P08236	0.058562	0.135503	0.075667	GUSB	Beta-glucui	BGLR_HUM	6	1.041427
24H 10MT/	Q96SN8	0.178342	0.135501	0.075667	CDK5RAP2	CDK5 regul	CK5P2_HUI	9	1.131583
24H 50MT/	P20783	0.158322	0.135519	0.075673	NTF3	Neurotropl	NTF3_HUM	2	1.115988
48H 10MT/	Q9UI95	0.452819	0.13553	0.075677	MAD2L2	Mitotic spi	MD2L2_HU	1	1.368712
24H 50MT/	Q9NSD9	0.002392	0.135578	0.075698	FARSB	Phenylalan	SYFB_HUM/	29	1.00166

48H 10MT/	P51571	-0.00424	0.135587	0.075701	SSR4	Translocon	SSRD_HUM	4	0.997065
24H 10MT/	Q01664	-0.00172	0.135601	0.075708	TFAP4	Transcripti	TFAP4_HUI	1	0.998812
24H 10MT/	Q9BRQ6	0.104343	0.135625	0.075719	CHCHD6	MICOS com	MIC25_HUI	7	1.075005
24H 10MT/	Q9NVG8	-0.06737	0.135638	0.075722	TBC1D13	TBC1 doma	TBC13_HUI	15	0.954377
24H 50MT/	Q6PI78	-0.19779	0.135658	0.07573	TMEM65	Transmemk	TMM65_HU	7	0.871887
48H 10MT/	Q9P0T7	-0.04178	0.135711	0.075753	TMEM9	Proton-trar	TMEM9_HU	4	0.971455
48H 10MT/	Q02750	0.019089	0.135739	0.075764	MAP2K1	Dual specifi	MP2K1_HU	10	1.013319
24H 50MT/	Q9BZI7	0.072287	0.135778	0.075777	UPF3B	Regulator c	REN3B_HUI	7	1.051382
24H 50MT/	Q12774	0.067523	0.135819	0.075792	ARHGEF5	Rho guanin	ARHG5_HU	12	1.047916
48H 50MT/	O75592	0.177602	0.135835	0.075798	MYCBP2	E3 ubiquiti	MYCB2_HU	11	1.131002
48H 10MT/	Q3B8N5	0.161842	0.13587	0.075813	PROX2	Prospero h	PROX2_HU	2	1.118714
48H 50MT/	Q6N075	0.213489	0.135873	0.075813	MFSD5	Molybdate-	MFSD5_HU	5	1.159489
48H 10MT/	P16220	0.302943	0.135887	0.075818	CREB1	Cyclic AMP	CREB1_HUI	3	1.233659
48H 10MT/	Q8N122	-0.1489	0.135925	0.075831	RPTOR	Regulatory-	RPTOR_HUI	16	0.901939
24H 50MT/	P41091	0.025118	0.135967	0.07585	EIF2S3	Eukaryotic	IF2G_HUM/	24	1.017563
24H 10MT/	O15372	0.042303	0.135981	0.075852	EIF3H	Eukaryotic	EIF3H_HUN	21	1.029756
24H 50MT/	Q92934	0.107848	0.135973	0.075852	BAD	Bcl2-associ	BAD_HUM/	4	1.07762
48H 10MT/	Q9Y548	0.16688	0.135981	0.075852	YIPF1	Protein YIP	YIPF1_HUN	3	1.122628
48H 10MT/	Q13427	-0.00032	0.13602	0.075868	PPIG	Peptidyl-pr	PPIG_HUM	8	0.99978
24H 10MT/	Q96CF2	-0.14504	0.136027	0.07587	CHMP4C	Charged mu	CHM4C_HU	3	0.904354
48H 50MT/	Q9Y4H2	-0.55689	0.136038	0.075875	IRS2	Insulin rece	IRS2_HUM/	3	0.679766
24H 10MT/	Q14894	-0.15321	0.136114	0.07591	CRYM	Ketimine re	CRYM_HUN	4	0.899244
24H 50MT/	O15143	0.01415	0.136168	0.075933	ARPC1B	Actin-relate	ARC1B_HUI	16	1.009856
48H 10MT/	Q1ED39	0.005362	0.136214	0.075955	KNOP1	Lysine-rich	KNOP1_HU	13	1.003724
48H 50MT/	Q1MSJ5	0.475399	0.136282	0.075985	CSPP1	Centrosom	CSPP1_HUI	7	1.390302
24H 10MT/	Q16352	0.112765	0.136325	0.076004	INA	Alpha-inter	AINX_HUM	15	1.081298
24H 10MT/	Q9GZS3	0.043757	0.136399	0.076039	SKIC8	Superkiller	SKI8_HUM/	15	1.030795
24H 50MT/	Q96K49	-0.10344	0.136402	0.076039	TMEM87B	Transmemk	TM87B_HU	3	0.930813
48H 10MT/	Q495W5	-0.04108	0.136426	0.07605	FUT11	Alpha-(1,3)	FUT11_HUI	5	0.971926
24H 10MT/	Q8TDQ7	0.025728	0.136515	0.076095	GNPDA2	Glucosamir	GNPI2_HUI	6	1.017993
24H 50MT/	Q8IYB5	-0.21001	0.136536	0.0761	SMAP1	Stromal me	SMAP1_HU	4	0.86453
24H 50MT/	Q6ZVM7	-0.17337	0.136558	0.07611	TOM1L2	TOM1-like p	TM1L2_HU	6	0.886772
48H 50MT/	Q13439	0.00324	0.136567	0.076112	GOLGA4	Golgin subf	GOGA4_HU	41	1.002248
24H 10MT/	Q9UPQ8	0.252528	0.136569	0.076113	DOLK	Dolichol ki	DOLK_HUN	4	1.191292
48H 50MT/	Q08209	0.095569	0.136596	0.076123	PPP3CA	Protein pho	PP2BA_HUI	6	1.068487
48H 10MT/	Q14CX7	0.015515	0.136691	0.076169	NAA25	N-alpha-ace	NAA25_HU	30	1.010812
24H 50MT/	Q9UBD5	-0.02569	0.136766	0.076199	ORC3	Origin reco	ORC3_HUN	16	0.982348
24H 10MT/	Q9NTI5	0.030205	0.136789	0.07621	PDS5B	Sister chror	PDS5B_HUI	41	1.021158
48H 10MT/	Q9BQ61	0.237649	0.136815	0.07622	TRIR	Telomerase	TRIR_HUM/	4	1.179069
48H 10MT/	P41235	0.049537	0.136842	0.076233	HNF4A	Hepatocyte	HNF4A_HU	11	1.034933
48H 10MT/	Q8IYI6	0.018917	0.136861	0.076242	EXOC8	Exocyst cor	EXOC8_HUI	19	1.013199
24H 50MT/	P48730	0.158632	0.136893	0.076257	CSNK1D	Casein kina	KC1D_HUN	4	1.116228
24H 10MT/	P35443	0.458845	0.136916	0.076264	THBS4	Thrombosp	TSP4_HUM	2	1.374441
48H 10MT/	Q15418	-0.04946	0.136914	0.076264	RPS6KA1	Ribosomal	KS6A1_HUI	15	0.966295
24H 10MT/	Q8N6T7	0.100205	0.136921	0.076265	SIRT6	NAD-depen	SIR6_HUM/	3	1.071926
24H 50MT/	Q9NQC3	0.00422	0.136944	0.076277	RTN4	Reticulon-4	RTN4_HUN	11	1.002929
24H 50MT/	Q9H089	0.044271	0.136961	0.076284	LSG1	Large subur	LSG1_HUM	18	1.031162
24H 50MT/	Q99633	-0.18857	0.136975	0.076291	PRPF18	Pre-mRNA-s	PRP18_HUI	2	0.877474
24H 10MT/	Q8N543	-0.00013	0.136989	0.076296	OGFOD1	Prolyl 3-hy	OGFD1_HU	15	0.99991
48H 10MT/	P08833	0.063474	0.137	0.076301	IGFBP1	Insulin-like	IBP1_HUM/	7	1.044979
24H 10MT/	Q14197	0.020354	0.137095	0.076347	MRPL58	Large ribos	ICT1_HUM/	6	1.014208
48H 10MT/	Q6R327	0.014451	0.137121	0.07636	RICTOR	Rapamycin	RICTR_HUN	22	1.010067

48H 10MT/	Q76M96	0.204302	0.137123	0.07636	CCDC80	Coiled-coil	CCD80_HU	1	1.152128
48H 10MT/	Q1MSJ5	0.324129	0.137215	0.076402	CSPP1	Centrosom	CSPP1_HUI	7	1.251909
48H 50MT/	Q14157	-0.0583	0.137238	0.076413	UBAP2L	Ubiquitin-a	UBP2L_HU	21	0.960398
24H 50MT/	Q9Y4Z0	-0.32175	0.137252	0.076417	LSM4	U6 snRNA-a	LSM4_HUN	5	0.800099
48H 10MT/	Q6XE24	0.264298	0.137268	0.076425	RBMS3	RNA-bindir	RBMS3_HU	2	1.201051
24H 10MT/	Q9NX18	-1.24412	0.137282	0.07643	SDHAF2	Succinate d	SDHF2_HU	3	0.422167
24H 50MT/	Q96PV6	0.760283	0.13731	0.076445	LENG8	Leukocyte r	LENG8_HUI	2	1.693822
48H 10MT/	O14908	0.033679	0.137419	0.076494	GIPC1	PDZ domai	GIPC1_HUN	13	1.023619
48H 10MT/	Q9HCK4	0.079167	0.137418	0.076494	ROBO2	Roundabou	ROBO2_HU	9	1.056408
48H 50MT/	Q5JPH6	0.039766	0.137441	0.076503	EARS2	Nondiscrim	SYEM_HUM	14	1.027947
24H 10MT/	O00410	0.002055	0.137449	0.076506	IPO5	Importin-5	IPO5_HUM	53	1.001425
24H 50MT/	P33121	-0.23223	0.137474	0.076516	ACSL1	Long-chain	ACSL1_HUN	2	0.851316
24H 50MT/	Q9BZK3	-0.41522	0.137474	0.076516	NACA4P	Putative na	NACP4_HU	1	0.749907
24H 10MT/	Q9NPE3	-0.1202	0.137511	0.076535	NOP10	H/ACA ribo	NOP10_HU	4	0.920057
24H 10MT/	P42858	-0.00521	0.137545	0.076549	HTT	Huntingtin	HD_HUMAI	55	0.996394
48H 10MT/	Q96CS4	-0.0744	0.137575	0.076554	ZNF689	Zinc finger	ZN689_HUI	8	0.949737
48H 50MT/	Q7Z4S6	0.005734	0.13767	0.076597	KIF21A	Kinesin-like	KI21A_HUN	40	1.003982
48H 10MT/	Q6P1X5	-0.04248	0.137675	0.076598	TAF2	Transcripti	TAF2_HUM	11	0.970983
24H 50MT/	Q9Y316	-0.20555	0.137697	0.076607	MEMO1	Protein ME	MEMO1_HU	14	0.867208
24H 10MT/	Q9NW64	0.009937	0.137749	0.076633	RBM22	Pre-mRNA-:	RBM22_HU	12	1.006912
24H 10MT/	Q8IZ83	-0.0602	0.137765	0.076639	ALDH16A1	Aldehyde d	A16A1_HUI	20	0.959133
24H 50MT/	P49755	-0.04033	0.137787	0.076648	TMED10	Transmemb	TMEDA_HU	7	0.972435
48H 10MT/	Q02790	0.016245	0.137801	0.076655	FKBP4	Peptidyl-pr	FKBP4_HUI	34	1.011324
48H 10MT/	Q9C037	-0.12444	0.137809	0.076657	TRIM4	E3 ubiquiti	TRIM4_HUI	7	0.917361
48H 10MT/	P0C025	0.041488	0.137861	0.076679	NUDT17	Nucleoside	NUD17_HU	1	1.029175
48H 10MT/	Q63HN8	-0.02226	0.137881	0.076688	RNF213	E3 ubiquiti	RN213_HU	88	0.984692
48H 50MT/	Q9H4Z3	0.16836	0.138001	0.076741	PCIF1	mRNA (2'-O	CAPAM_HU	7	1.12378
48H 10MT/	P13798	-0.00293	0.138045	0.076756	APEH	Acylamino-	ACPH_HUM	23	0.997969
24H 50MT/	O95168	-0.06993	0.138087	0.07677	NDUFB4	NADH dehy	NDUB4_HU	6	0.952684
24H 50MT/	Q9NU19	0.170457	0.138115	0.076781	TBC1D22B	TBC1 doma	TB22B_HUI	3	1.125415
48H 50MT/	Q06546	0.022027	0.138153	0.076799	GABPA	GA-binding	GABPA_HU	9	1.015385
24H 50MT/	P43361	-0.25037	0.138226	0.076834	MAGEA8	Melanoma-	MAGA8_HU	1	0.840679
24H 50MT/	Q86TP1	-0.04901	0.138324	0.076883	PRUNE1	Exopolyph	PRUN1_HU	12	0.966598
48H 50MT/	P00395	0.024006	0.138372	0.076903	MT-CO1	Cytochrom	COX1_HUN	4	1.016779
48H 50MT/	P83731	-0.01035	0.138416	0.076926	RPL24	Large ribos	RL24_HUM	6	0.992849
24H 10MT/	Q9C0C7	0.151516	0.138429	0.076931	AMBRA1	Activating r	AMRA1_HU	9	1.110736
48H 50MT/	Q9BXB1	-0.71674	0.138455	0.076941	LGR4	Leucine-ric	LGR4_HUN	1	0.608471
24H 10MT/	P43363	0.008276	0.138468	0.076945	MAGEA10	Melanoma-	MAGAA_HU	5	1.005753
48H 50MT/	Q13608	-0.00258	0.138468	0.076945	PEX6	Peroxisoma	PEX6_HUM	14	0.998214
48H 10MT/	Q8TAT6	0.00876	0.13848	0.076949	NPLOC4	Nuclear prc	NPL4_HUN	27	1.00609
24H 10MT/	P43246	0.001837	0.138487	0.076951	MSH2	DNA misma	MSH2_HUN	40	1.001274
24H 10MT/	Q5T1C6	-0.30819	0.138509	0.076961	THEM4	Acyl-coenzy	THEM4_HU	4	0.807655
24H 10MT/	Q9Y3A5	0.035712	0.138511	0.076961	SBDS	Ribosome r	SBDS_HUM	18	1.025063
48H 50MT/	Q6PIW4	-0.38253	0.138521	0.076966	FIGNL1	Fidgetin-lik	FIGL1_HUN	8	0.76709
48H 50MT/	Q9H2F9	0.123192	0.138532	0.076971	CCDC68	Coiled-coil	CCD68_HU	1	1.089142
48H 50MT/	Q5SW24	-0.52112	0.138557	0.076981	DACT2	Dapper hor	DACT2_HUI	7	0.69683
24H 10MT/	P09651	0.03445	0.13859	0.076996	HNRNPA1	Heterogene	ROA1_HUN	22	1.024166
24H 10MT/	O14965	-0.05986	0.138598	0.076999	AURKA	Aurora kina	AURKA_HU	15	0.95936
24H 10MT/	P51153	0.077752	0.13861	0.077002	RAB13	Ras-related	RAB13_HUI	6	1.055373
48H 50MT/	Q8NB15	0.011932	0.138626	0.077008	ZNF511	Zinc finger	ZN511_HUI	5	1.008305
24H 50MT/	P03905	0.172535	0.138761	0.077075	MT-ND4	NADH-ubiq	NU4M_HUI	4	1.127037
48H 50MT/	Q07617	0.11035	0.138781	0.07708	SPAG1	Sperm-asso	SPAG1_HUI	13	1.07949

48H 50MT/	Q8TF68	0.187946	0.138782	0.07708	ZNF384	Zinc finger	ZN384_HUI	6	1.139141
48H 50MT/	P46379	-0.07052	0.138841	0.077109	BAG6	Large prolir	BAG6_HUM	36	0.952292
24H 50MT/	Q8IWX8	0.03056	0.138859	0.077114	CHERP	Calcium ho	CHERP_HUI	17	1.021409
48H 50MT/	Q96P48	0.080908	0.138893	0.077129	ARAP1	Arf-GAP wit	ARAP1_HUI	16	1.057684
24H 10MT/	O60244	0.101157	0.138985	0.077167	MED14	Mediator o	MED14_HU	15	1.072633
48H 50MT/	Q58DX5	0.26268	0.138994	0.077169	NAALADL2	Inactive N-a	NADL2_HU	1	1.199705
24H 50MT/	O75954	0.598367	0.139012	0.077177	TSPAN9	Tetraspanir	TSN9_HUM	2	1.514002
24H 10MT/	P12236	0.068829	0.13902	0.077179	SLC25A6	ADP/ATP tr	ADT3_HUM	4	1.048865
48H 50MT/	P06454	0.498292	0.139026	0.077181	PTMA	Prothymos	PTMA_HUM	2	1.41254
24H 50MT/	P08758	-0.08546	0.139047	0.07719	ANXA5	Annexin A5	ANXA5_HU	23	0.942484
24H 50MT/	O43242	0.005616	0.139052	0.077191	PSMD3	26S protea:	PSMD3_HU	31	1.0039
48H 50MT/	P82912	-0.11653	0.139063	0.077194	MRPS11	Small ribos	RT11_HUM	5	0.922401
48H 50MT/	Q9UHB4	-0.16653	0.139063	0.077194	NDOR1	NADPH-dep	NDOR1_HU	12	0.890982
48H 10MT/	Q9Y2Y0	0.028414	0.139072	0.077198	ARL2BP	ADP-ribosy	AR2BP_HUI	6	1.01989
24H 10MT/	Q9UJC5	-0.0914	0.139088	0.077201	SH3BGRL2	SH3 domai	SH3L2_HUI	5	0.938613
48H 10MT/	Q53TQ3	-0.17091	0.139119	0.077214	INO80D	INO80 com	IN80D_HUI	1	0.888284
48H 10MT/	P19623	-0.04643	0.139169	0.077241	SRM	Spermidine	SPEE_HUM/	18	0.96833
48H 50MT/	Q9BSJ2	-0.09731	0.13918	0.077244	TUBGCP2	Gamma-tul	GCP2_HUM	35	0.934776
48H 10MT/	Q8WUY9	1.360813	0.139217	0.07726	DEPDC1B	DEP domai	DEP1B_HUI	1	2.568299
24H 10MT/	Q9Y6D5	-0.00139	0.139218	0.07726	ARFGEF2	Brefeldin A	BIG2_HUM	56	0.99904
24H 50MT/	Q8WVC0	0.023993	0.13928	0.077285	LEO1	RNA polym	LEO1_HUM	11	1.016769
48H 10MT/	Q9UPM8	-0.13952	0.139296	0.077293	AP4E1	AP-4 compl	AP4E1_HUI	8	0.907822
24H 10MT/	Q2Q1W2	0.027489	0.139303	0.077296	TRIM71	E3 ubiquiti	LIN41_HUM	16	1.019237
24H 10MT/	Q86YD1	-0.48983	0.139363	0.07732	PTOV1	Prostate tu	PTOV1_HUI	3	0.71211
24H 10MT/	Q8WXE0	0.055234	0.139376	0.077322	CASKIN2	Caskin-2	CSKI2_HUM	7	1.039027
48H 10MT/	Q9Y5T5	0.072892	0.139391	0.077328	USP16	Ubiquitin c	UBP16_HU	18	1.051823
24H 50MT/	P46063	0.082997	0.139437	0.077349	RECQL	ATP-depenc	RECQ1_HUI	27	1.059216
48H 50MT/	Q9Y3Q8	0.452606	0.139449	0.077353	TSC22D4	TSC22 dom	T22D4_HUI	2	1.36851
24H 50MT/	Q86XA9	-0.02673	0.139492	0.077367	HEATR5A	HEAT repea	HTR5A_HUI	23	0.981644
24H 10MT/	Q155Q3	-0.12775	0.139502	0.07737	DIXDC1	Dixin	DIXC1_HUM	4	0.915256
48H 10MT/	P78417	-0.00455	0.139514	0.077374	GSTO1	Glutathion	GSTO1_HUI	19	0.996852
24H 50MT/	O15379	-0.08612	0.139555	0.077386	HDAC3	Histone dea	HDAC3_HU	7	0.942053
24H 50MT/	P29558	0.001675	0.139553	0.077386	RBMS1	RNA-bindir	RBMS1_HU	1	1.001162
24H 10MT/	P56945	0.136353	0.13956	0.077386	BCAR1	Breast canc	BCAR1_HUI	10	1.099123
24H 50MT/	A6ZKI3	0.052762	0.139586	0.077392	RTL8C	Retrotransp	RTL8C_HUM	5	1.037249
48H 50MT/	Q92890	-0.01141	0.139596	0.077396	UFD1	Ubiquitin r	UFD1_HUM	13	0.99212
24H 10MT/	Q0P6H9	-0.27377	0.13962	0.077408	TMEM62	Transmemb	TMM62_HU	3	0.827155
24H 10MT/	P40939	-0.04962	0.139635	0.077414	HADHA	Trifunction	ECHA_HUM	37	0.966191
48H 50MT/	O15258	0.074049	0.139644	0.077414	RER1	Protein REF	RER1_HUM	6	1.052667
48H 10MT/	Q8N2M8	0.378607	0.13964	0.077414	CLASRP	CLK4-assoc	CLASR_HUM	2	1.300086
24H 10MT/	Q99436	-0.09762	0.139685	0.077428	PSMB7	Proteasom	PSB7_HUM	12	0.934571
48H 50MT/	Q9BSH5	0.017561	0.139687	0.077428	HDHD3	Haloacid de	HDHD3_HU	10	1.012247
24H 10MT/	Q14192	0.073252	0.139693	0.077429	FHL2	Four and a	FHL2_HUM	1	1.052086
48H 10MT/	P02452	-0.32907	0.139707	0.077435	COL1A1	Collagen al	CO1A1_HU	14	0.796049
24H 10MT/	Q9Y2P8	0.012406	0.139802	0.077482	RCL1	RNA 3'-tern	RCL1_HUM	13	1.008636
24H 10MT/	Q96CP6	0.0439	0.139805	0.077482	GRAMD1A	Protein Ast	ASTRA_HUM	8	1.030897
48H 10MT/	Q8WU10	0.103238	0.139825	0.077491	PYROXD1	Pyridine nu	PYRD1_HUI	5	1.074182
48H 50MT/	Q9NV31	-0.19213	0.13987	0.077505	IMP3	U3 small nu	IMP3_HUM	10	0.875314
48H 10MT/	Q5VW36	0.011482	0.139885	0.077513	FOCAD	Focadhesin	FOCAD_HU	22	1.007991
24H 50MT/	Q9NP64	-0.12876	0.13989	0.077514	ZCCHC17	Zinc finger	ZCC17_HUI	3	0.91462
48H 50MT/	Q6UXD5	0.205357	0.1399	0.077516	SEZ6L2	Seizure 6-lil	SE6L2_HUM	6	1.152972
48H 10MT/	Q92747	0.066787	0.139914	0.07752	ARPC1A	Actin-relate	ARC1A_HUI	13	1.047382

24H 50MT/	Q9P0M6	0.087629	0.139949	0.077532	MACROH2/	Core histor	H2AW_HUI	8	1.062622
48H 10MT/	O95445	0.233214	0.139994	0.077553	APOM	Apolipoprc	APOM_HUI	4	1.175451
48H 50MT/	P51956	0.180987	0.140043	0.077575	NEK3	Serine/thre	NEK3_HUM	4	1.133659
24H 10MT/	Q05397	-0.03676	0.140106	0.077603	PTK2	Focal adhe:	FAK1_HUM	20	0.974842
48H 50MT/	O75113	-0.21565	0.140122	0.077609	N4BP1	NEDD4-bin	N4BP1_HU	6	0.86116
48H 10MT/	P26373	0.037025	0.140126	0.077609	RPL13	Large ribos	RL13_HUM	7	1.025996
48H 50MT/	Q9NRR4	-0.24504	0.140126	0.077609	DROSHA	Ribonuclea	RNC_HUM/	9	0.843791
24H 10MT/	P42696	-0.01153	0.140167	0.077626	RBM34	RNA-bindir	RBM34_HU	19	0.992042
24H 10MT/	Q92918	-0.26904	0.140176	0.07763	MAP4K1	Mitogen-ac	M4K1_HUN	1	0.829871
48H 50MT/	Q14683	-0.09991	0.140198	0.077639	SMC1A	Structural r	SMC1A_HU	54	0.93309
48H 10MT/	P29323	0.532912	0.140215	0.077645	EPHB2	Ephrin type	EPHB2_HUI	1	1.446847
24H 10MT/	Q8WUX2	0.111112	0.140237	0.077652	CHAC2	Glutathion	CHAC2_HU	3	1.08006
24H 10MT/	Q9P0U1	-0.60681	0.140261	0.077662	TOMM7	Mitochond	TOM7_HUN	2	0.656647
48H 50MT/	Q6PI26	0.507667	0.140267	0.077664	SHQ1	Protein SH(	SHQ1_HUM	1	1.421749
48H 10MT/	Q96FQ6	-0.17224	0.140293	0.077674	S100A16	Protein S1(	S10AG_HUI	4	0.887463
24H 10MT/	Q9BRQ0	-0.16318	0.140305	0.077678	PYGO2	Pygopus hc	PYGO2_HU	2	0.893051
48H 10MT/	Q5VT06	-0.01096	0.140351	0.0777	CEP350	Centrosom	CE350_HUI	11	0.992433
24H 50MT/	Q8TDQ7	0.113051	0.140414	0.07773	GNPDA2	Glucosamir	GNPI2_HUI	6	1.081513
24H 50MT/	P10398	0.096641	0.140452	0.077744	ARAF	Serine/thre	ARAF_HUM	10	1.069281
24H 10MT/	P49356	-0.00893	0.140453	0.077744	FNTB	Protein farr	FNTB_HUM	7	0.993827
24H 10MT/	P46108	-0.05849	0.14046	0.077747	CRK	Adapter mc	CRK_HUMA	13	0.960267
48H 10MT/	Q9UDX5	-0.09198	0.14048	0.077757	MTFP1	Mitochond	MTFP1_HU	8	0.938237
48H 10MT/	Q9UJ83	-0.04354	0.140504	0.077768	HACL1	2-hydroxya	HACL1_HUI	13	0.970272
48H 50MT/	O95295	-1.62471	0.140551	0.077786	SNAPIN	SNARE-asso	SNAPN_HU	3	0.324276
48H 10MT/	Q16143	-0.08307	0.140554	0.077786	SNCB	Beta-synucl	SYUB_HUM	1	0.94405
48H 50MT/	Q9H3G5	0.006831	0.140555	0.077786	CPVL	Probable se	CPVL_HUM	5	1.004746
24H 50MT/	P53701	0.050829	0.140578	0.077794	HCCS	Holocytoch	CCHL_HUM	12	1.03586
48H 50MT/	P12081	-0.07016	0.140589	0.077797	HARS1	Histidine--t	HARS1_HUI	29	0.952535
48H 50MT/	Q8NCE0	-0.19386	0.140691	0.077839	TSEN2	tRNA-splici	SEN2_HUM	7	0.874265
24H 10MT/	Q96JP5	-0.02936	0.140691	0.077839	ZFP91	E3 ubiquiti	ZFP91_HUN	6	0.979858
48H 10MT/	Q96R05	0.118779	0.14071	0.077847	RBP7	Retinoid-bi	RET7_HUM	2	1.085816
24H 10MT/	Q96HE9	-0.4826	0.140713	0.077848	PRR11	Proline-ricl	PRR11_HUI	2	0.715687
24H 10MT/	Q9NYV4	0.029062	0.140748	0.077862	CDK12	Cyclin-depe	CDK12_HU	16	1.020349
24H 50MT/	Q6NSJ0	-0.08005	0.140754	0.077863	MYORG	Myogenesis	MYORG_HU	6	0.946022
48H 50MT/	Q0VG06	-0.4289	0.140758	0.077864	FAAP100	Fanconi an	FP100_HUI	2	0.742828
24H 10MT/	Q68CJ9	-0.0004	0.140769	0.077868	CREB3L3	Cyclic AMP	CR3L3_HUI	2	0.99972
48H 10MT/	Q9GZS3	0.010364	0.140772	0.077869	SKIC8	Superkiller	SKI8_HUM/	15	1.007209
24H 50MT/	Q96Q15	-0.06485	0.140831	0.077897	SMG1	Serine/thre	SMG1_HUN	30	0.956046
24H 10MT/	O96018	1.699644	0.140845	0.077903	APBA3	Amyloid-be	APBA3_HUI	1	3.248208
48H 50MT/	Q2QL34	-0.022	0.140857	0.077908	MPV17L	Mpv17-like	MP17L_HU	4	0.984868
24H 10MT/	Q9H9C1	-0.10908	0.140866	0.077913	VIPAS39	Spermatog	SPE39_HUN	14	0.927177
48H 50MT/	Q9UHJ6	-0.09982	0.140868	0.077913	SHPK	Sedoheptul	SHPK_HUM	11	0.933148
24H 50MT/	P53582	0.046845	0.140883	0.077918	METAP1	Methionine	MAP11_HU	18	1.033003
48H 10MT/	Q86WU2	0.456887	0.140898	0.077926	LDHD	Probable D	LDHD_HUN	3	1.372577
24H 10MT/	Q6P0N0	0.29241	0.140988	0.077964	MIS18BP1	Mis18-binc	M18BP_HU	4	1.224685
24H 10MT/	Q8N4A0	0.132913	0.140994	0.077965	GALNT4	Polypeptid	GALT4_HUI	7	1.096505
48H 50MT/	Q5XKP0	0.005404	0.141004	0.077969	MICOS13	MICOS com	MIC13_HUI	4	1.003753
24H 10MT/	Q9NQG5	-0.08449	0.141012	0.077972	RPRD1B	Regulation	RPR1B_HUI	12	0.94312
24H 10MT/	Q96S97	0.125895	0.141055	0.077995	MYADM	Myeloid-as	MYADM_HU	4	1.091184
24H 50MT/	O14744	0.022463	0.141063	0.077998	PRMT5	Protein arg	ANM5_HUN	28	1.015692
24H 10MT/	Q99615	0.017416	0.141064	0.077998	DNAJC7	DnaJ homo	DNJC7_HUI	27	1.012145
48H 50MT/	Q9BX70	0.128543	0.141095	0.078011	BTBD2	BTB/POZ dc	BTBD2_HUI	3	1.093189

48H 50MT/	Q99595	-0.31423	0.141106	0.078016	TIMM17A	Mitochond	TI17A_HUM	2	0.804283
24H 10MT/	Q9NSB2	-2.13514	0.141119	0.078021	KRT84	Keratin, typ	KRT84_HUI	2	0.227646
48H 10MT/	P21953	0.019704	0.141161	0.078042	BCKDHB	2-oxoisoval	ODBB_HUM	8	1.013752
24H 50MT/	Q5T9S5	-0.23125	0.141221	0.07807	CCDC18	Coiled-coil	CCD18_HU	6	0.851897
24H 10MT/	P16930	-0.07593	0.141231	0.078071	FAH	Fumarylace	FAAA_HUM	16	0.94873
24H 10MT/	Q9Y487	-0.09092	0.141241	0.078074	ATP6V0A2	V-type prot	VPP2_HUM	15	0.938927
24H 10MT/	Q4V339;Q5	0.079442	0.141274	0.078086	ZNG1F;ZNG	Zinc-regula	ZNG1F_HUI	5	1.056609
24H 10MT/	Q9ULC5	0.04055	0.141273	0.078086	ACSL5	Long-chain	ACSL5_HUI	20	1.028506
24H 10MT/	P42167	-0.03313	0.14131	0.078102	TMPO	Lamina-ass	LAP2B_HUI	11	0.977295
48H 10MT/	Q8NBT2	0.039709	0.141327	0.078107	SPC24	Kinetochor	SPC24_HUI	4	1.027906
48H 50MT/	Q9HBH5	-0.091	0.141337	0.078109	RDH14	Retinol deh	RDH14_HU	12	0.938869
24H 10MT/	P31751	-0.21128	0.141386	0.078132	AKT2	RAC-beta se	AKT2_HUM	9	0.86377
48H 10MT/	Q9NTJ5	-0.05937	0.1414	0.078137	SACM1L	Phosphatic	SAC1_HUM	28	0.959684
48H 50MT/	P55789	-0.17974	0.141414	0.078142	GFER	FAD-linked	ALR_HUMA	4	0.882863
48H 10MT/	Q96T37	-0.06523	0.141426	0.078146	RBM15	RNA-bindir	RBM15_HU	22	0.955793
24H 10MT/	Q6ZMG9	0.168389	0.141431	0.078147	CERS6	Ceramide s	CERS6_HUI	6	1.123803
24H 50MT/	Q92522	0.146209	0.141445	0.078153	H1-10	Histone H1	H1X_HUMA	6	1.106658
48H 50MT/	Q99653	-0.06621	0.141484	0.078171	CHP1	Calcineurir	CHP1_HUM	10	0.955145
48H 10MT/	Q7Z4H3	-0.0258	0.141513	0.078182	HDDC2	5'-deoxynu	HDDC2_HU	11	0.982275
48H 50MT/	P35548	-0.03242	0.141544	0.078195	MSX2	Homeobox	MSX2_HUM	1	0.977779
24H 50MT/	Q9Y4K3	0.555944	0.141585	0.078214	TRAF6	TNF recept	TRAF6_HUI	2	1.47013
24H 10MT/	Q9BXP2	-0.02243	0.141609	0.078225	SLC12A9	Solute carri	S12A9_HUI	7	0.984573
24H 10MT/	P62140	0.02873	0.141642	0.07824	PPP1CB	Serine/thre	PP1B_HUM	9	1.020114
48H 50MT/	Q86SR1	-0.03016	0.141661	0.078249	GALNT10	Polypeptid	GLT10_HUI	5	0.979314
24H 10MT/	Q96E11	0.118786	0.141671	0.078252	MRRF	Ribosome-r	RRFM_HUM	8	1.085821
48H 50MT/	O75843	-0.21174	0.141692	0.078262	AP1G2	AP-1 compl	AP1G2_HU	4	0.863498
24H 10MT/	P63272	0.055176	0.141705	0.078265	SUPT4H1	Transcripti	SPT4H_HUI	5	1.038986
48H 50MT/	Q13185	-0.05417	0.141713	0.078266	CBX3	Chromobo	CBX3_HUM	8	0.963148
48H 10MT/	Q13686	-0.10159	0.141712	0.078266	ALKBH1	Nucleic aci	ALKB1_HUI	4	0.932005
48H 50MT/	Q16611	-0.02114	0.141709	0.078266	BAK1	Bcl-2 homc	BAK_HUMA	4	0.985456
48H 50MT/	O15530	0.058457	0.141739	0.078272	PDPK1	3-phosphoi	PDPK1_HU	16	1.041352
48H 50MT/	Q13033	-0.05258	0.14176	0.078277	STRN3	Striatin-3	STRN3_HUI	14	0.964212
24H 10MT/	Q86VR2	-0.12421	0.14176	0.078277	RETREG3	Reticuloph	RETR3_HUI	5	0.917509
24H 10MT/	Q8N766	0.043005	0.141779	0.078285	EMC1	ER membra	EMC1_HUM	37	1.030257
24H 50MT/	Q9NYZ1	0.073941	0.141832	0.078311	TVP23B	Golgi appar	TV23B_HUI	4	1.052588
24H 50MT/	P54646	-0.17003	0.141863	0.078326	PRKAA2	5'-AMP-acti	AAPK2_HUI	11	0.888825
24H 50MT/	P13693	0.083787	0.141905	0.078344	TPT1	Translation	TCTP_HUM	7	1.059796
24H 10MT/	Q9P1F3	-0.24726	0.141983	0.078372	ABRACL	Costars fam	ABRAL_HUI	2	0.842497
24H 50MT/	Q6P3W7	0.022476	0.142006	0.078381	SCYL2	SCY1-like pi	SCYL2_HUN	17	1.015701
48H 50MT/	Q92785	-0.03929	0.142016	0.078384	DPF2	Zinc finger	REQU_HUM	14	0.973133
48H 50MT/	P32322	0.013737	0.142027	0.078386	PYCR1	Pyrroline-5	P5CR1_HUI	14	1.009567
24H 50MT/	Q676U5	-0.12092	0.142031	0.078387	ATG16L1	Autophagy	A16L1_HUI	13	0.919599
48H 10MT/	Q9NVN3	-0.01558	0.142045	0.078392	RIC8B	Synembryn	RIC8B_HUN	7	0.989257
48H 10MT/	Q8NHV4	0.049171	0.142056	0.078397	NEDD1	Protein NEI	NEDD1_HU	14	1.03467
24H 10MT/	Q9NVE5	0.088516	0.142098	0.078413	USP40	Ubiquitin c	UBP40_HU	4	1.063276
48H 10MT/	Q9NPA8	0.030804	0.142102	0.078414	ENY2	Transcripti	ENY2_HUM	1	1.021581
24H 10MT/	Q8IXQ4	0.005174	0.142121	0.078421	GPALPP1	GPALPP mc	GPAM1_HU	3	1.003593
24H 50MT/	Q15032	0.463994	0.142123	0.078421	R3HDM1	R3H domai	R3HD1_HU	1	1.379356
24H 50MT/	Q13617	-0.04021	0.142139	0.078425	CUL2	Cullin-2	CUL2_HUM	24	0.972513
24H 10MT/	O60921	0.073101	0.142181	0.078446	HUS1	Checkpoint	HUS1_HUM	9	1.051976
24H 10MT/	P62683;P6	0.866958	0.142202	0.078455	ERVK-21;HI	Endogenou	GAK21_HU	1	1.823814
24H 50MT/	Q8TBF4	0.093117	0.142221	0.078461	ZCRB1	Zinc finger	ZCRB1_HUI	4	1.066672

48H 10MT/	Q8IWQ3	0.062442	0.142238	0.078468	BRSK2	Serine/thre	BRSK2_HUI	4	1.044232
48H 50MT/	Q92934	-0.03247	0.142283	0.078488	BAD	Bcl2-associ	BAD_HUM/	4	0.977745
24H 10MT/	Q96C34	-0.18349	0.142282	0.078488	RUNDC1	RUN domai	RUND1_HU	1	0.880568
24H 10MT/	O15160	0.049684	0.142288	0.07849	POLR1C	DNA-direct	RPAC1_HUI	17	1.035038
48H 10MT/	Q68CZ6	-0.06638	0.142302	0.078495	HAUS3	HAUS augm	HAUS3_HU	6	0.955034
48H 50MT/	P35575	0.078353	0.142309	0.078498	G6PC1	Glucose-6-ı	G6PC1_HU	3	1.055812
48H 10MT/	P49593	-0.08666	0.142318	0.078502	PPM1F	Protein ph	PPM1F_HU	13	0.941699
48H 10MT/	Q9BX70	0.134678	0.142323	0.078503	BTBD2	BTB/POZ dc	BTBD2_HUI	3	1.097848
48H 10MT/	O14776	-0.015	0.142339	0.078511	TCERG1	Transcripti	TCRG1_HUI	32	0.989654
24H 10MT/	Q96EB1	-0.20078	0.142435	0.078557	ELP4	Elongator c	ELP4_HUM	7	0.870077
24H 10MT/	Q6WCQ1	0.131843	0.142457	0.078564	MPRIP	Myosin phc	MPRIP_HUI	13	1.095693
48H 50MT/	Q7LBC6	0.153613	0.142469	0.078566	KDM3B	Lysine-spec	KDM3B_HU	31	1.112352
24H 50MT/	P04035	-0.13244	0.1425	0.078578	HMGCR	3-hydroxy-:	HMDH_HUI	5	0.912286
48H 10MT/	O43759	-0.06807	0.142556	0.0786	SYNGR1	Synaptogyr	SNG1_HUM	2	0.953915
24H 10MT/	O43716	-0.25011	0.142601	0.078619	GATC	Glutamyl-tl	GATC_HUM	2	0.840831
24H 10MT/	P29083	0.016151	0.142621	0.078626	GTF2E1	General tra	T2EA_HUM	11	1.011258
24H 50MT/	O43924	-0.10811	0.142645	0.078633	PDE6D	Retinal rod	PDE6D_HU	4	0.927802
24H 50MT/	Q49A26	-0.00217	0.142643	0.078633	GLYR1	Cytokine-lil	GLYR1_HUI	18	0.998494
24H 10MT/	O43639	0.14565	0.142707	0.078663	NCK2	Cytoplasmi	NCK2_HUM	4	1.106229
24H 50MT/	Q9UDT6	-0.08831	0.142713	0.078665	CLIP2	CAP-Gly do	CLIP2_HUM	31	0.940621
24H 10MT/	P53634	-0.0033	0.142727	0.078669	CTSC	Dipeptidyl	CATC_HUM	11	0.997719
48H 10MT/	Q9BSJ5	-0.44386	0.142726	0.078669	MTNAP1	Mitochond	CQ080_HU	3	0.735163
48H 50MT/	Q8WWX9	-0.37913	0.142771	0.078685	SELENOM	Selenoprot	SELM_HUM	1	0.768902
48H 50MT/	Q9UPN7	-0.00744	0.14278	0.078688	PPP6R1	Serine/thre	PP6R1_HUI	14	0.99486
48H 50MT/	Q14119	-0.09832	0.14284	0.078714	VEZF1	Vascular en	VEZF1_HUN	4	0.934117
48H 50MT/	Q96L91	-0.12555	0.142901	0.07874	EP400	E1A-bindin	EP400_HUI	13	0.916654
48H 10MT/	Q03014	0.006346	0.142926	0.078751	HHEX	Hematopoi	HHEX_HUM	3	1.004408
48H 50MT/	Q96MX6	-0.1082	0.142942	0.078757	DNAAF10	Dynein axo	DAA10_HU	10	0.927746
48H 10MT/	P51812	-0.03345	0.142955	0.07876	RPS6KA3	Ribosomal	KS6A3_HUI	34	0.977083
24H 10MT/	Q8IWA0	0.076206	0.143005	0.078779	WDR75	WD repeat-	WDR75_HU	26	1.054242
24H 50MT/	O94880	-0.12089	0.143023	0.078787	PHF14	PHD finger	PHF14_HUI	16	0.919619
24H 50MT/	Q7Z5G4	0.126918	0.143053	0.078799	GOLGA7	Golgin subf	GOGA7_HU	4	1.091958
24H 50MT/	P61020	0.081962	0.143131	0.078833	RAB5B	Ras-related	RAB5B_HUI	4	1.058457
48H 50MT/	P47914	-0.34071	0.143149	0.078841	RPL29	Large ribos	RL29_HUM	2	0.789655
24H 10MT/	Q9NX58	0.054804	0.14319	0.078859	LYAR	Cell growth	LYAR_HUM	11	1.038718
24H 10MT/	Q13951	0.050448	0.143208	0.078864	CBFB	Core-bindir	PEBB_HUM	7	1.035586
48H 50MT/	O95258	-0.22499	0.143211	0.078864	SLC25A14	Brain mitoc	UCP5_HUM	3	0.8556
48H 50MT/	Q14241	-0.15571	0.143278	0.078898	ELOA	Elongin-A	ELOA1_HUI	7	0.897689
24H 50MT/	P40429	0.104738	0.143289	0.078901	RPL13A	Large ribos	RL13A_HUI	10	1.075299
48H 50MT/	Q16795	-0.0684	0.143299	0.078905	NDUFA9	NADH dehy	NDUA9_HU	17	0.953697
48H 10MT/	O95139	0.114389	0.143325	0.078912	NDUFB6	NADH dehy	NDUB6_HU	3	1.082516
48H 10MT/	Q9BYJ9	0.016629	0.143325	0.078912	YTHDF1	YTH domair	YTHD1_HUI	9	1.011593
24H 50MT/	Q9H814	-0.05334	0.143326	0.078912	PHAX	Phosphoryl	PHAX_HUM	11	0.963699
48H 10MT/	Q9NZI8	-0.00205	0.143355	0.07892	IGF2BP1	Insulin-like	IF2B1_HUN	25	0.998577
48H 50MT/	Q9BY50	-0.219	0.143462	0.078971	SEC11C	Signal pept	SC11C_HUI	5	0.85916
24H 50MT/	P04179	0.004416	0.143491	0.078983	SOD2	Superoxide	SODM_HUN	9	1.003065
24H 50MT/	Q9HB71	-0.08122	0.143533	0.078996	CACYBP	Calcyclin-b	CYBP_HUM	18	0.945261
24H 10MT/	Q04637	0.009771	0.143541	0.078999	EIF4G1	Eukaryotic	IF4G1_HUN	55	1.006795
48H 10MT/	Q9NTM9	0.142502	0.143551	0.079003	CUTC	Copper hor	CUTC_HUM	9	1.103818
24H 10MT/	P34913	-0.1501	0.143576	0.079016	EPHX2	Bifunctiona	HYES_HUM	14	0.901189
24H 10MT/	Q8NC54	0.209176	0.143616	0.079031	KCT2	Keratinocy	KCT2_HUM	3	1.156028
24H 10MT/	Q9UKL0	0.028036	0.143674	0.079055	RCOR1	REST corep	RCOR1_HU	9	1.019623

24H 50MT/	Q5T447	-0.07926	0.143681	0.079057	HECTD3	E3 ubiquiti	HECD3_HU	17	0.94654
48H 10MT/	Q5VT52	0.011839	0.14371	0.079069	RPRD2	Regulation	RPRD2_HU	26	1.00824
24H 10MT/	Q8N2Z9	0.856761	0.143733	0.079078	CENPS	Centromer	CENPS_HUI	2	1.810967
24H 10MT/	P09619	0.366394	0.14375	0.079082	PDGFRB	Platelet-de	PGFRB_HUI	2	1.289126
48H 50MT/	P09543	-0.01522	0.143753	0.079083	CNP	2',3'-cyclic-	CN37_HUM	20	0.989507
48H 50MT/	Q96BR1	-0.08675	0.143774	0.079091	SGK3	Serine/thre	SGK3_HUM	5	0.941644
48H 10MT/	Q9UNL2	0.095347	0.143845	0.079116	SSR3	Translocon	SSRG_HUM	2	1.068322
24H 10MT/	Q86VS8	0.003405	0.14389	0.079136	HOOK3	Protein Ho	HOOK3_HU	21	1.002363
24H 50MT/	Q9H6Q4	0.092588	0.143921	0.079149	CIAO3	Cytosolic ir	CIAO3_HUI	12	1.066281
24H 10MT/	Q99943	-0.09353	0.14394	0.079156	AGPAT1	1-acyl-sn-gl	PLCA_HUM	4	0.937229
48H 50MT/	Q9P0J0	-0.14659	0.143966	0.079167	NDUFA13	NADH dehy	NDUAD_HU	9	0.903384
24H 10MT/	Q13614	0.15787	0.143996	0.079181	MTMR2	Myotubula	MTMR2_HU	5	1.115639
48H 10MT/	O60613	-0.07377	0.144017	0.079189	SELENOF	Selenoprot	SEP15_HUI	5	0.950154
48H 50MT/	O00754	-0.20329	0.144025	0.07919	MAN2B1	Lysosomal	MA2B1_HU	18	0.868568
48H 10MT/	Q9UPU5	0.009317	0.144026	0.07919	USP24	Ubiquitin c	UBP24_HU	61	1.006479
24H 10MT/	Q8N806	0.117682	0.14403	0.079191	UBR7	Putative E3	UBR7_HUM	11	1.084991
48H 10MT/	Q9UHY7	-0.08733	0.144036	0.079193	ENOPH1	Enolase-ph	ENOPH_HU	8	0.941261
24H 50MT/	Q9BW61	-0.18157	0.144058	0.079201	DDA1	DET1- and D	DDA1_HUM	2	0.88174
24H 10MT/	Q96RR1	0.044798	0.14407	0.079205	TWNK	Twinkle mt	PEO1_HUM	13	1.031539
24H 50MT/	Q8WXI9	0.068436	0.144074	0.079206	GATAD2B	Transcripti	P66B_HUM	12	1.048579
48H 10MT/	Q3ZCQ8	-0.08038	0.144115	0.079223	TIMM50	Mitochond	TIM50_HUI	11	0.945809
48H 10MT/	Q8TAG9	0.085888	0.144168	0.079248	EXOC6	Exocyst cor	EXOC6_HUI	21	1.061341
48H 50MT/	P48556	-0.01778	0.144189	0.079257	PSMD8	26S protea	PSMD8_HU	13	0.987755
24H 50MT/	P53004	0.089699	0.144203	0.079258	BLVRA	Biliverdin r	BIEA_HUM/	15	1.064148
48H 50MT/	Q9UBF2	0.018048	0.144197	0.079258	COPG2	Coatomer s	COPG2_HU	19	1.012589
48H 50MT/	P62424	0.010632	0.144208	0.079259	RPL7A	Large ribos	RL7A_HUM	20	1.007397
24H 50MT/	Q16774	0.007495	0.144224	0.079264	GUK1	Guanylate l	KGUA_HUM	8	1.005208
48H 10MT/	Q92600	0.022716	0.144279	0.079287	CNOT9	CCR4-NOT t	CNOT9_HU	10	1.01587
24H 50MT/	Q6UXD5	0.087124	0.144304	0.079296	SEZ6L2	Seizure 6-lil	SE6L2_HUM	6	1.06225
48H 50MT/	Q6ZVF9	0.109724	0.144326	0.079306	GPRIN3	G protein-r	GRIN3_HUI	9	1.079022
24H 50MT/	Q6P1L8	0.038018	0.144424	0.07935	MRPL14	Large ribos	RM14_HUM	5	1.026702
48H 50MT/	Q9NTK5	0.007899	0.144436	0.079355	OLA1	Obg-like AT	OLA1_HUM	29	1.00549
24H 10MT/	P23381	-0.13385	0.144448	0.07936	WARS1	Tryptophar	SYWC_HUM	23	0.911396
48H 50MT/	O15315	-0.46082	0.144473	0.079373	RAD51B	DNA repair	RA51B_HUI	1	0.726573
24H 50MT/	P08237	-0.11409	0.144509	0.079385	PFKM	ATP-depen	PFKAM_HU	1	0.923965
24H 10MT/	P23511	-0.33627	0.144519	0.079388	NFYA	Nuclear tra	NFYA_HUM	2	0.792084
24H 10MT/	Q9BYG3	-0.01973	0.144521	0.079388	NIFK	MKI67 FHA	MK67I_HUI	17	0.986418
24H 50MT/	P50895	-0.01384	0.144537	0.079396	BCAM	Basal cell a	BCAM_HUM	16	0.990452
48H 50MT/	Q2VPK5	-0.20974	0.144552	0.079402	CTU2	Cytoplasmi	CTU2_HUM	4	0.864693
48H 50MT/	Q9ULH1	0.218896	0.144596	0.079424	ASAP1	Arf-GAP wit	ASAP1_HUI	1	1.163843
48H 10MT/	Q9Y2R0	0.146276	0.144639	0.07944	COA3	Cytochrom	COA3_HUM	4	1.106709
24H 10MT/	Q15398	0.02245	0.144672	0.079454	DLGAP5	Disks large-	DLGP5_HU	16	1.015683
24H 50MT/	Q14BN4	-0.35217	0.144813	0.079516	SLMAP	Sarcolemm	SLMAP_HU	9	0.783406
24H 10MT/	Q709C8	-0.06921	0.144834	0.079526	VPS13C	Intermemb	VP13C_HUI	60	0.953162
24H 10MT/	P30085	0.012266	0.14484	0.079527	CMPK1	UMP-CMP l	KCY_HUMA	15	1.008538
24H 50MT/	Q8TDX6	-0.05903	0.144862	0.079535	CSGALNAC	Chondroiti	CGAT1_HUI	8	0.959908
24H 10MT/	A6NJ78	0.004032	0.144874	0.079538	METTL15	12S rRNA N	MET15_HU	13	1.002798
48H 50MT/	Q9NRN9	-0.03802	0.144929	0.079563	METTL5	rRNA N6-ad	METL5_HUI	5	0.973993
48H 10MT/	O43708	0.152326	0.14497	0.07958	GSTZ1	Maleylacet	MAAI_HUM	5	1.11136
24H 10MT/	P55265	0.038626	0.145021	0.079602	ADAR	Double-stra	DSRAD_HU	33	1.027135
24H 10MT/	Q96CN7	-0.07884	0.145025	0.079602	ISOC1	Isochorism	ISOC1_HUM	9	0.946819
48H 50MT/	P35268	-0.18816	0.145044	0.079608	RPL22	Large ribos	RL22_HUM	4	0.877727

48H 10MT/	P08195	-0.07294	0.145087	0.079628	SLC3A2	Amino acid	4F2_HUMA	32	0.950696
48H 10MT/	Q9HAJ7	0.126967	0.145094	0.079629	SAP30L	Histone dea	SP30L_HUI	1	1.091996
24H 10MT/	Q96EQ0	0.863649	0.145114	0.079636	SGTB	Small gluta	SGTB_HUM	1	1.819635
48H 50MT/	Q8N5M9	0.071543	0.145121	0.079638	JAGN1	Protein jag	JAGN1_HUI	2	1.05084
48H 50MT/	P62854	-0.1209	0.145124	0.079639	RPS26	Small ribos	RS26_HUM	5	0.919612
48H 10MT/	Q7L523	-0.18594	0.145134	0.079643	RRAGA	Ras-related	RRAGA_HU	10	0.879076
24H 10MT/	Q96LT9	-0.16748	0.14516	0.079655	RNPC3	RNA-bindir	RNPC3_HU	5	0.890398
48H 10MT/	Q16401	-0.05242	0.145203	0.079673	PSMD5	26S protea:	PSMD5_HU	24	0.964316
24H 50MT/	Q96LR5	0.009802	0.14527	0.079701	UBE2E2	Ubiquitin-c	UB2E2_HUI	5	1.006818
48H 10MT/	O60831	-0.01682	0.145321	0.079727	PRAF2	PRA1 famil	PRAF2_HUI	1	0.988409
24H 50MT/	Q70CQ3	-0.63994	0.145339	0.079734	USP30	Ubiquitin c	UBP30_HU	1	0.64174
48H 10MT/	Q15014	-0.14005	0.145363	0.079745	MORF4L2	Mortality fa	MO4L2_HU	8	0.907485
24H 10MT/	Q969T7	-0.15256	0.145457	0.079785	NT5C3B	7-methylgu	5NT3B_HUI	4	0.899655
48H 50MT/	Q96SW2	-0.10794	0.145455	0.079785	CRBN	Protein cer	CRBN_HUM	1	0.927914
48H 10MT/	O94829	0.187594	0.145481	0.079795	IPO13	Importin-1	IPO13_HUI	7	1.138863
24H 10MT/	Q96BM9	-0.03932	0.145501	0.079801	ARL8A	ADP-ribosy	ARL8A_HUI	10	0.973116
24H 10MT/	Q9UK58	0.151744	0.145565	0.079829	CCNL1	Cyclin-L1	CCNL1_HUI	4	1.110911
24H 50MT/	Q9NPI0	0.040851	0.145627	0.079854	TMEM138	Transmemb	TM138_HU	1	1.02872
48H 10MT/	Q9H4A4	0.030781	0.14575	0.079909	RNPEP	Aminopept	AMPB_HUM	29	1.021565
24H 50MT/	Q9Y678	-0.06489	0.145756	0.079911	COPG1	Coatomer s	COPG1_HU	42	0.956018
48H 10MT/	P62310	0.369509	0.145772	0.079915	LSM3	U6 snRNA-a	LSM3_HUM	3	1.291913
24H 50MT/	Q9BVA1	-0.23197	0.145801	0.079927	TUBB2B	Tubulin bet	TBB2B_HUI	1	0.85147
24H 10MT/	P21281	-0.03174	0.145935	0.079985	ATP6V1B2	V-type prot	VATB2_HUI	22	0.978242
24H 50MT/	P61244	0.107187	0.145961	0.079996	MAX	Protein ma	MAX_HUM/	3	1.077126
24H 10MT/	Q9H5N1	-0.31498	0.145988	0.080009	RABEP2	Rab GTPase	RABE2_HUI	11	0.803864
24H 10MT/	Q92560	-0.44004	0.146008	0.080016	BAP1	Ubiquitin c	BAP1_HUM	4	0.737115
48H 10MT/	Q9H2F9	0.144959	0.146149	0.080083	CCDC68	Coiled-coil	CCD68_HU	1	1.105699
48H 50MT/	Q6R327	0.005878	0.146157	0.080085	RICTOR	Rapamycin	RICTR_HUM	22	1.004083
24H 10MT/	Q3KP66	0.389088	0.146185	0.080097	INAVA	Innate imm	INAVA_HUI	4	1.309565
24H 50MT/	Q9GZN7	-1.1395	0.146234	0.080113	ROGDI	Protein rog	ROGDI_HUI	2	0.453918
48H 10MT/	Q9NRY5	-0.0585	0.146234	0.080113	FAM114A2	Protein FAI	F1142_HUI	15	0.960259
48H 50MT/	O14617	-0.02175	0.146253	0.080122	AP3D1	AP-3 compl	AP3D1_HU	36	0.985037
48H 50MT/	Q8NBL1	-0.10905	0.146315	0.080147	POGLUT1	Protein O-g	PGLT1_HUI	12	0.9272
48H 50MT/	Q9BW83	0.038766	0.146342	0.08016	IFT27	Intraflagell	IFT27_HUM	8	1.027235
48H 10MT/	Q13257	-0.05283	0.146391	0.080179	MAD2L1	Mitotic spi	MD2L1_HU	7	0.96404
24H 50MT/	P01031	-0.93302	0.146395	0.08018	C5	Compleme	CO5_HUM/	2	0.523762
48H 10MT/	Q96EX3	0.052019	0.146415	0.08019	DYNC2I2	Cytoplasmi	DC2I2_HUI	5	1.036715
48H 50MT/	P10114	0.294578	0.146426	0.080193	RAP2A	Ras-related	RAP2A_HUI	2	1.226526
24H 10MT/	Q96KR6	-0.04703	0.146426	0.080193	FAM210B	Protein FAI	F210B_HUI	3	0.967929
24H 50MT/	O15211	-0.12844	0.146446	0.080202	RGL2	Ral guanine	RGL2_HUM	3	0.91482
48H 10MT/	P05388	-0.10654	0.146471	0.080212	RPLP0	Large ribos	RLA0_HUM	13	0.928814
48H 50MT/	Q86VI3	-0.70845	0.146494	0.08022	IQGAP3	Ras GTPase-	IQGA3_HUI	9	0.611978
24H 10MT/	Q6P3W7	0.043899	0.146524	0.080236	SCYL2	SCY1-like p	SCYL2_HUM	17	1.030896
24H 50MT/	Q6P587	0.061675	0.146564	0.08025	FAHD1	Oxaloaceta	FAHD1_HU	11	1.043677
24H 10MT/	Q01081	-0.10909	0.146605	0.080265	U2AF1	Splicing fac	U2AF1_HUI	11	0.927175
24H 10MT/	Q9H3N1	-0.03967	0.146632	0.080277	TMX1	Thioredoxi	TMX1_HUM	14	0.972874
24H 10MT/	Q9H8M7	-0.1953	0.146635	0.080277	MINDY3	Ubiquitin c	MINY3_HUI	3	0.873392
24H 10MT/	Q9P2Y4	0.029012	0.146642	0.080279	ZNF219	Zinc finger	ZN219_HUI	6	1.020313
24H 50MT/	Q96S97	-0.1275	0.146666	0.080287	MYADM	Myeloid-as:	MYADM_HU	4	0.915418
48H 10MT/	Q9UKG1	0.08407	0.146691	0.080298	APPL1	DCC-intera	DP13A_HU	20	1.060004
48H 50MT/	Q53S58	0.189577	0.146722	0.080311	TMEM177	Transmemb	TM177_HU	2	1.140429
48H 10MT/	Q8IWR0	0.046679	0.146738	0.080316	ZC3H7A	Zinc finger	Z3H7A_HUI	14	1.032884

48H 50MTA	Q9Y6K1	0.088937	0.146749	0.080319	DNMT3A	DNA (cytosi	DNM3A_HU	5	1.063586
48H 10MTA	Q7Z2W9	0.00462	0.146767	0.080327	MRPL21	Large ribos	RM21_HUM	10	1.003208
24H 10MTA	Q8WWQ0	0.068941	0.146784	0.080335	PHIP	PH-interact	PHIP_HUM	38	1.048946
24H 50MTA	Q06546	0.170232	0.146796	0.080339	GABPA	GA-binding	GABPA_HU	9	1.125239
24H 50MTA	Q96H78	0.079272	0.146794	0.080339	SLC25A44	Solute carri	S2544_HUI	4	1.056485
24H 10MTA	Q8N108	0.048814	0.146822	0.080352	MIER1	Mesoderm	MIER1_HUI	6	1.034414
48H 50MTA	Q6P087	-0.35103	0.146848	0.080363	RPUSD3	Mitochond	RUSD3_HU	8	0.784025
24H 50MTA	Q96PY6	0.435152	0.146875	0.080376	NEK1	Serine/thre	NEK1_HUM	2	1.352053
24H 50MTA	Q9HCP0	0.022636	0.146911	0.080394	CSNK1G1	Casein kina	KC1G1_HU	8	1.015814
48H 10MTA	Q8IWA0	-0.0754	0.146916	0.080394	WDR75	WD repeat-	WDR75_HU	26	0.949077
24H 50MTA	Q8WW01	-0.14222	0.146923	0.080397	TSEN15	tRNA-splici	SEN15_HUI	1	0.906125
24H 50MTA	P04156	0.09462	0.146937	0.0804	PRNP	Major prior	PRIO_HUM	4	1.067784
48H 10MTA	A6NDG6	-0.02616	0.146962	0.08041	PGP	Glycerol-3-	PGP_HUMA	14	0.982034
48H 50MTA	Q93063	-0.04263	0.146976	0.080416	EXT2	Exostosin-2	EXT2_HUM	10	0.970882
24H 50MTA	P08247	0.175781	0.146993	0.080423	SYP	Synaptophy	SYPH_HUM	3	1.129575
24H 10MTA	Q6UWP7	0.019011	0.147016	0.080434	LCLAT1	Lysocardiol	LCLT1_HUM	7	1.013264
24H 10MTA	P99999	0.046231	0.147019	0.080435	CYCS	Cytochrom	CYC_HUMA	8	1.032564
24H 10MTA	Q9P2T1	-0.00199	0.147031	0.080439	GMPR2	GMP reduc	GMPR2_HU	15	0.99862
48H 10MTA	Q15477	0.035426	0.147043	0.080443	SKIC2	Superkiller	SKI2_HUMA	41	1.02486
24H 10MTA	Q9NVM9	0.038864	0.147066	0.080452	INTS13	Integrator c	INT13_HUM	16	1.027305
24H 10MTA	O75369	0.101198	0.147072	0.080454	FLNB	Filamin-B	FLNB_HUM	42	1.072664
48H 10MTA	P31939	-0.00797	0.147081	0.080455	ATIC	Bifunctiona	PUR9_HUM	41	0.994492
48H 10MTA	Q9H6E5	0.031909	0.147078	0.080455	TUT1	Speckle targ	STPAP_HUM	9	1.022364
48H 10MTA	O60563	-0.11015	0.147104	0.080465	CCNT1	Cyclin-T1	CCNT1_HUI	1	0.926493
48H 10MTA	O14640	-0.01741	0.147154	0.080484	DVL1	Segment pc	DVL1_HUM	7	0.988002
48H 10MTA	Q9H871	-0.00864	0.147201	0.080504	RMND5A	E3 ubiquiti	RMD5A_HU	8	0.994026
24H 10MTA	P15559	0.019448	0.147204	0.080504	NQO1	NAD(P)H de	NQO1_HUM	9	1.013572
48H 10MTA	Q9UKT5	-0.10978	0.147348	0.080574	FBXO4	F-box only	FBX4_HUM	3	0.926727
48H 10MTA	Q9Y597	-0.31816	0.147372	0.080582	KCTD3	BTB/POZ dc	KCTD3_HUI	4	0.802094
24H 50MTA	Q9Y6P5	0.840495	0.147373	0.080582	SESN1	Sestrin-1	SESN1_HUM	1	1.790664
24H 10MTA	Q9H6Z4	-0.04494	0.147405	0.080596	RANBP3	Ran-bindin	RANB3_HU	15	0.96933
48H 50MTA	P26572	-0.01582	0.14742	0.080601	MGAT1	Alpha-1,3-r	MGAT1_HU	7	0.989098
24H 10MTA	Q676U5	-0.09969	0.147444	0.080609	ATG16L1	Autophagy-	A16L1_HUI	13	0.933231
24H 10MTA	P30047	-0.18017	0.147468	0.080621	GCHFR	GTP cycloh	GFRP_HUM	5	0.882601
48H 10MTA	Q9NQE9	-0.09988	0.147505	0.08064	HINT3	Adenosine !	HINT3_HUM	7	0.93311
48H 10MTA	O95049	0.032006	0.147509	0.080641	TJP3	Tight juncti	ZO3_HUMA	2	1.022433
24H 50MTA	Q9UFC0	0.02182	0.147527	0.080647	LRWD1	Leucine-ric	LRWD1_HU	13	1.015239
48H 10MTA	Q9BQ15	-0.16432	0.147542	0.080653	NABP2	SOSS comp	SOSB1_HUI	2	0.892347
24H 50MTA	Q96M96	-0.25373	0.147567	0.080666	FGD4	FYVE, RhoG	FGD4_HUM	21	0.838725
48H 10MTA	Q92688	-0.02993	0.147596	0.080678	ANP32B	Acidic leuci	AN32B_HU	4	0.97947
48H 10MTA	Q9BW83	0.059204	0.147594	0.080678	IFT27	Intraflagell	IFT27_HUM	8	1.041891
48H 10MTA	Q9NWR8	-0.1069	0.147602	0.080679	MCUB	Calcium un	MCUB_HUM	4	0.92858
48H 10MTA	P12081	-0.00555	0.147678	0.080715	HARS1	Histidine--t	HARS1_HUI	29	0.996162
24H 10MTA	Q5VZE5	0.000177	0.147683	0.080716	NAA35	N-alpha-ace	NAA35_HU	20	1.000123
48H 10MTA	Q15836	0.042372	0.147704	0.080724	VAMP3	Vesicle-assc	VAMP3_HU	5	1.029806
48H 10MTA	Q14126	0.019333	0.147714	0.080726	DSG2	Desmoglein	DSG2_HUM	25	1.013491
24H 10MTA	Q9UL46	0.05856	0.147768	0.080749	PSME2	Proteasom	PSME2_HU	14	1.041425
24H 10MTA	Q14517	0.25169	0.147772	0.08075	FAT1	Protocadhe	FAT1_HUM	8	1.190601
48H 50MTA	Q9NSC2	-0.48579	0.147781	0.080754	SALL1	Sal-like pro	SALL1_HUM	4	0.714108
24H 50MTA	Q9BW92	0.029847	0.14784	0.080779	TARS2	Threonine--	SYTM_HUM	20	1.020904
24H 50MTA	Q9H8H2	0.085471	0.147847	0.080781	DDX31	ATP-depen	DDX31_HU	18	1.061034
24H 10MTA	O15400	0.038536	0.14786	0.080785	STX7	Syntaxin-7	STX7_HUM	10	1.027071

24H 50MT/	Q13615	0.047759	0.147896	0.0808	MTMR3	Myotubula	MTMR3_HU	4	1.033658
48H 10MT/	P08238	0.010728	0.147901	0.080801	HSP90AB1	Heat shock	HS90B_HUI	50	1.007463
24H 10MT/	O95479	-0.06377	0.147942	0.080821	H6PD	GDH/6PGL	G6PE_HUM	25	0.956762
48H 50MT/	Q7Z6K5	0.15279	0.147997	0.080847	ARPIN	Arpin	ARPIN_HUI	5	1.111717
48H 10MT/	O43493	-0.0017	0.148006	0.080851	TGOLN2	Trans-Golgi	TGON2_HU	7	0.998822
24H 10MT/	P19013	0.717923	0.148035	0.080864	KRT4	Keratin, typ	K2C4_HUM	9	1.644813
24H 50MT/	Q9BSF4	0.100957	0.148086	0.080885	TIMM29	Mitochond	TIM29_HUI	6	1.072485
48H 10MT/	O75083	-0.00232	0.148104	0.080893	WDR1	WD repeat-	WDR1_HUI	28	0.998393
48H 10MT/	P50443	-0.31521	0.148114	0.080898	SLC26A2	Sulfate tran	S26A2_HUI	2	0.803732
24H 50MT/	Q8WYA6	0.09852	0.148153	0.080909	CTNNBL1	Beta-cateni	CTBL1_HUI	21	1.070675
24H 50MT/	Q9NVC3	-0.11651	0.148173	0.080916	SLC38A7	Sodium-co	S38A7_HUI	1	0.922419
48H 10MT/	P42696	-0.10542	0.148188	0.080921	RBM34	RNA-bindir	RBM34_HU	19	0.929537
48H 50MT/	P30043	0.086003	0.148356	0.080996	BLVRB	Flavin redu	BLVRB_HUI	9	1.061425
24H 50MT/	Q96B54	0.101076	0.148398	0.081013	ZNF428	Zinc finger	ZN428_HUI	4	1.072573
48H 50MT/	O60858	-0.42875	0.148417	0.081021	TRIM13	E3 ubiquiti	TRI13_HUM	4	0.742907
24H 10MT/	Q9NS84	-0.29468	0.148463	0.08104	CHST7	Carbohydra	CHST7_HUI	1	0.815252
48H 10MT/	P61026	-0.02162	0.148614	0.081102	RAB10	Ras-related	RAB10_HUI	11	0.985124
48H 10MT/	Q8TAP9	0.192993	0.148748	0.081156	MPLKIP	M-phase-sp	MPLKI_HUI	1	1.143133
24H 10MT/	Q9P2K3	0.10948	0.148828	0.081188	RCOR3	REST corep	RCOR3_HU	10	1.078839
48H 10MT/	O15551	0.030734	0.148934	0.081236	CLDN3	Claudin-3	CLD3_HUM	1	1.021532
24H 50MT/	Q4G148	-0.12884	0.14895	0.081243	GXYLT1	Glucoside x	GXLT1_HUI	6	0.914565
48H 10MT/	Q9Y3M8	-0.00711	0.148984	0.08126	STARD13	StAR-relate	STA13_HUI	2	0.995083
48H 50MT/	O14894	1.005131	0.149035	0.081282	TM4SF5	Transmemb	T4S5_HUM	1	2.007126
48H 50MT/	Q7Z6M1	0.071675	0.149036	0.081282	RABEPK	Rab9 effect	RABEK_HUI	13	1.050936
48H 50MT/	Q96ED9	0.666781	0.149036	0.081282	HOOK2	Protein Ho	HOOK2_HU	1	1.587526
48H 10MT/	Q9ULX3	-0.00832	0.149039	0.081282	NOB1	RNA-bindir	NOB1_HUM	9	0.99425
24H 50MT/	Q9BT67	-0.6606	0.149051	0.081288	NDFIP1	NEDD4 fam	NFIP1_HUN	1	0.632615
24H 10MT/	Q6DN90	-0.06599	0.149098	0.081305	IQSEC1	IQ motif an	IQEC1_HUN	13	0.955289
48H 10MT/	Q9BZE2	0.040995	0.149098	0.081305	PUS3	tRNA pseud	PUS3_HUM	6	1.028823
48H 10MT/	Q14680	-0.03774	0.149108	0.081308	MELK	Maternal er	MELK_HUM	13	0.974178
24H 10MT/	Q14511	-0.2603	0.149117	0.08131	NEDD9	Enhancer o	CASL_HUM	1	0.834917
24H 50MT/	Q6P2P2	-0.04884	0.149117	0.08131	PRMT9	Protein arg	ANM9_HUN	8	0.966717
24H 10MT/	O00330	0.000507	0.149219	0.081357	PDHX	Pyruvate de	ODPX_HUM	18	1.000351
48H 50MT/	P48651	0.067619	0.149255	0.081371	PTDSS1	Phosphatic	PTSS1_HUN	7	1.047986
24H 10MT/	Q9BUN8	0.049616	0.149333	0.081409	DERL1	Derlin-1	DERL1_HUI	7	1.034989
48H 50MT/	Q6UWU4	-0.64871	0.149388	0.081435	C6orf89	Bombesin r	CF089_HUI	2	0.637852
48H 10MT/	Q6NZI2	-0.90902	0.149393	0.081437	CAVIN1	Caveolae-as	CAVN1_HU	2	0.532546
24H 50MT/	O15182	-0.70063	0.14943	0.081456	CETN3	Centrin-3	CETN3_HUI	1	0.615305
24H 10MT/	P62736;P6	0.060972	0.149453	0.081466	ACTA2;ACT	Actin, aorti	ACTA_HUM	5	1.043168
24H 10MT/	Q8NBF6	-0.00063	0.149499	0.081488	AVL9	Late secret	AVL9_HUM	11	0.999561
48H 10MT/	Q8IWZ3	0.007327	0.149522	0.081496	ANKHD1	Ankyrin rep	ANKH1_HU	33	1.005091
24H 50MT/	Q8N6H7	-0.2042	0.149533	0.081501	ARFGAP2	ADP-ribosy	ARFG2_HUI	16	0.86802
24H 50MT/	Q8N1B4	-0.03652	0.149538	0.081503	VPS52	Vacuolar pr	VPS52_HUI	18	0.975005
48H 50MT/	P68400	-0.05769	0.149587	0.081522	CSNK2A1	Casein kina	CSK21_HUI	18	0.960798
48H 10MT/	Q7RTV5	-0.11051	0.149583	0.081522	PRXL2C	Peroxiredo	PXL2C_HUI	3	0.926263
24H 10MT/	Q9GZP4	-0.01009	0.149609	0.081532	PITHD1	PITH domai	PITH1_HUN	10	0.993031
24H 50MT/	Q9H6E5	0.219589	0.149644	0.081548	TUT1	Speckle tar	STPAP_HUI	9	1.164402
24H 50MT/	Q5MNZ6	0.032353	0.149694	0.081566	WDR45B	WD repeat	WIPI3_HUN	10	1.022679
24H 10MT/	Q32P41	-0.00584	0.149734	0.081583	TRMT5	tRNA (guan	TRM5_HUN	14	0.995961
24H 50MT/	Q9UKT5	-0.40697	0.149746	0.081588	FBXO4	F-box only	FBX4_HUM	3	0.754204
24H 10MT/	Q9UNQ0	0.021406	0.149758	0.081592	ABCG2	Broad subs	ABCG2_HU	9	1.014948
48H 50MT/	Q8NI37	0.098488	0.149764	0.081593	PPTC7	Protein ph	PPTC7_HUI	7	1.070651

24H 10MT/	Q9P2R7	-0.00813	0.149775	0.081597	SUCLA2	Succinate--	SUCB1_HUI	20	0.994379
48H 10MT/	Q6VMQ6	-0.02601	0.149826	0.081619	ATF7IP	Activating t	MCAF1_HU	7	0.98213
48H 10MT/	P09382	-0.01181	0.149843	0.081626	LGALS1	Galectin-1	LEG1_HUM	9	0.991844
48H 10MT/	Q8TDW0	-0.05928	0.149847	0.081627	LRRC8C	Volume-reg	LRC8C_HUI	12	0.959742
24H 50MT/	O95835	-0.08396	0.149928	0.081665	LATS1	Serine/thre	LATS1_HUN	8	0.943462
48H 50MT/	Q9BYE7	0.28342	0.150008	0.081702	PCGF6	Polycomb g	PCGF6_HUI	3	1.217077
48H 10MT/	P43246	0.006637	0.150015	0.081704	MSH2	DNA misma	MSH2_HUN	40	1.004611
48H 50MT/	Q96E11	0.0306	0.150018	0.081704	MRRF	Ribosome-r	RRFM_HUN	8	1.021437
48H 50MT/	Q04837	0.032353	0.150038	0.08171	SSBP1	Single-strar	SSBP_HUM	8	1.022678
24H 50MT/	Q8IXM6	0.046601	0.150072	0.081725	NRM	Nurim	NRM_HUM	5	1.032829
48H 50MT/	P08185	0.631893	0.150095	0.081736	SERPINA6	Corticoster	CBG_HUM/	1	1.549597
24H 50MT/	Q9UK39	0.209665	0.150121	0.081747	NOCT	Nocturnin	NOCT_HUN	1	1.15642
48H 50MT/	Q9NTI5	-0.02775	0.150164	0.081768	PDS5B	Sister chror	PDS5B_HUI	41	0.980952
24H 50MT/	O14734	0.233829	0.150188	0.081778	ACOT8	Acyl-coenzy	ACOT8_HUI	6	1.175952
48H 50MT/	Q96DR7	0.158173	0.150187	0.081778	ARHGEF26	Rho guanin	ARHGQ_HL	1	1.115873
48H 10MT/	Q7Z3V4	-0.37901	0.150245	0.081801	UBE3B	Ubiquitin-p	UBE3B_HUI	4	0.768965
24H 10MT/	O15551	-0.47913	0.150279	0.081818	CLDN3	Claudin-3	CLD3_HUM	1	0.717408
24H 10MT/	P24468	-0.40385	0.15029	0.081823	NR2F2	COUP trans	COT2_HUM	4	0.755838
48H 50MT/	Q9UGN5	-0.16034	0.150335	0.081843	PARP2	Poly [ADP-r	PARP2_HUI	10	0.894817
24H 50MT/	Q8NC54	0.313234	0.150351	0.081849	KCT2	Keratinocy	KCT2_HUM	3	1.24249
48H 50MT/	Q9NX02	-0.0703	0.150372	0.081858	NLRP2	NACHT, LRI	NALP2_HUI	46	0.95244
48H 10MT/	O00429	0.01206	0.150385	0.081864	DNM1L	Dynamin-1	DNM1L_HL	44	1.008395
24H 50MT/	Q14116	-0.06619	0.150423	0.081876	IL18	Interleukin	IL18_HUM/	10	0.955155
24H 10MT/	Q8NB37	-0.02756	0.150453	0.08189	GATD1	Glutamine	GALD1_HU	4	0.981077
24H 50MT/	Q9UHN1	0.180177	0.150463	0.081895	POLG2	DNA polym	DPOG2_HL	4	1.133023
24H 10MT/	A6ZKI3	-0.26417	0.150483	0.081903	RTL8C	Retrotransp	RTL8C_HUI	5	0.832676
48H 10MT/	Q9NUG6	0.169494	0.150485	0.081903	PDRG1	p53 and DN	PDRG1_HU	2	1.124664
24H 10MT/	Q9H6Y2	-0.08887	0.150491	0.081904	WDR55	WD repeat-	WDR55_HL	13	0.940258
48H 10MT/	O60437	0.056741	0.150538	0.081924	PPL	Periplakin	PEPL_HUM	11	1.040113
24H 10MT/	Q9Y4L1	-0.00838	0.15055	0.081924	HYOU1	Hypoxia up	HYOU1_HU	44	0.994206
48H 50MT/	P52597	0.014643	0.150627	0.081962	HNRNPF	Heterogene	HNRPF_HU	18	1.010202
48H 50MT/	P31944	-0.14908	0.150662	0.081977	CASP14	Caspase-14	CASPE_HUI	3	0.901824
24H 10MT/	Q9BRK5	-0.10868	0.150741	0.082017	SDF4	45 kDa calc	CAB45_HUI	13	0.927436
48H 50MT/	Q13061	0.338175	0.150762	0.082026	TRDN	Triadin	TRDN_HUN	1	1.264157
48H 10MT/	O60306	-0.08457	0.150791	0.082035	AQR	RNA helicas	AQR_HUM/	44	0.943068
48H 50MT/	Q96CV9	0.061608	0.150842	0.082055	OPTN	Optineurin	OPTN_HUN	13	1.043628
24H 50MT/	P18433	0.012807	0.15085	0.082056	PTPRA	Receptor-ty	PTPRA_HUI	7	1.008917
48H 10MT/	Q02878	-0.08887	0.15085	0.082056	RPL6	Large ribos	RL6_HUMA	14	0.940262
24H 10MT/	Q99523	0.038265	0.150876	0.082066	SORT1	Sortilin	SORT_HUM	15	1.026878
48H 50MT/	P22061	0.031723	0.150898	0.082071	PCMT1	Protein-L-is	PIMT_HUM	15	1.022232
24H 10MT/	P24386	0.042964	0.150891	0.082071	CHM	Rab proteir	RAE1_HUM	16	1.030228
48H 10MT/	P56199	0.020958	0.150893	0.082071	ITGA1	Integrin alp	ITA1_HUM/	12	1.014633
48H 10MT/	Q08752	0.095428	0.150896	0.082071	PPID	Peptidyl-pr	PPID_HUM	21	1.068382
48H 10MT/	Q9UM11	0.076278	0.15092	0.08208	FZR1	Fizzy-relate	FZR1_HUM	3	1.054294
48H 50MT/	P02749	-0.08469	0.15093	0.082083	APOH	Beta-2-glyc	APOH_HUN	1	0.942988
24H 50MT/	Q71SY5	0.238794	0.150933	0.082083	MED25	Mediator o	MED25_HL	4	1.180006
48H 10MT/	O14981	-0.04031	0.150959	0.082093	BTAF1	TATA-bindi	BTAF1_HUI	47	0.972446
24H 10MT/	Q15126	0.003177	0.15096	0.082093	PMVK	Phosphom	PMVK_HUN	11	1.002204
48H 10MT/	Q13564	0.021604	0.150982	0.082096	NAE1	NEDD8-acti	ULA1_HUN	18	1.015088
24H 10MT/	Q01518	-0.01253	0.151047	0.082121	CAP1	Adenylyl cy	CAP1_HUM	30	0.99135
48H 50MT/	Q15070	-0.06408	0.151057	0.082121	OXA1L	Mitochond	OXA1L_HUI	8	0.956556
48H 50MT/	Q17RS7	-0.1009	0.151055	0.082121	GEN1	Flap endon	GEN_HUM/	3	0.932451

24H 10MT/	Q03519	0.169762	0.151159	0.082165	TAP2	Antigen pe|	TAP2_HUM	9	1.124873
48H 50MT/	Q9H9P8	-0.01839	0.151165	0.082168	L2HGDH	L-2-hydrox	L2HDH_HU	16	0.987336
24H 10MT/	P51665	0.022339	0.151168	0.082168	PSMD7	26S protea	PSMD7_HU	12	1.015604
24H 50MT/	P07311	-0.08345	0.1512	0.082179	ACYP1	Acylphosph	ACYP1_HUI	5	0.943798
48H 10MT/	Q9P0K8	0.161263	0.151201	0.082179	FOXJ2	Forkhead b	FOXJ2_HUN	4	1.118265
24H 10MT/	Q8IXM3	-0.06651	0.15122	0.082187	MRPL41	Large ribos	RM41_HUN	6	0.954942
48H 10MT/	Q96K49	-0.09405	0.151239	0.082195	TMEM87B	Transmemb	TM87B_HU	3	0.93689
48H 10MT/	O95067	0.130465	0.151285	0.082214	CCNB2	G2/mitotic	CCNB2_HU	5	1.094647
24H 10MT/	Q9UKV8	0.030204	0.151305	0.08222	ago-02	Protein arg	AGO2_HUN	14	1.021157
24H 50MT/	Q92613	-0.22785	0.151337	0.082232	JADE3	Protein Jad	JADE3_HUN	2	0.853909
24H 10MT/	Q9UL25	0.048877	0.151433	0.082272	RAB21	Ras-related	RAB21_HUI	11	1.034459
48H 10MT/	O95989	-0.07978	0.151454	0.082281	NUDT3	Diphospho	NUDT3_HU	5	0.946204
48H 10MT/	P36405	-0.02578	0.15147	0.082284	ARL3	ADP-ribosy	ARL3_HUM	11	0.982287
24H 10MT/	Q6UW68	0.108854	0.151475	0.082285	TMEM205	Transmemb	TM205_HU	5	1.078371
24H 10MT/	Q6GMV3	-0.25042	0.151511	0.082298	PTRHD1	Putative pe	PTRD1_HUI	6	0.840653
48H 10MT/	Q9NUQ7	-0.06472	0.151509	0.082298	UFSP2	Ufm1-speci	UFSP2_HUI	12	0.956132
24H 50MT/	O95149	-0.03592	0.151523	0.082302	SNUPN	Snurportin	SPN1_HUN	6	0.975412
48H 10MT/	Q9NVR5	0.05587	0.15154	0.082309	DNAAF2	Protein kin	KTU_HUMA	5	1.039486
48H 10MT/	O43567	0.642829	0.151544	0.082311	RNF13	E3 ubiquiti	RNF13_HUI	2	1.561388
48H 10MT/	Q8IYH5	0.36666	0.151553	0.082314	ZZZ3	ZZ-type zinc	ZZZ3_HUM/	1	1.289364
48H 10MT/	Q6ICB0	-0.33123	0.151574	0.082322	DESI1	Desumoyla	DESI1_HUN	1	0.794859
24H 50MT/	O00232	0.008771	0.151579	0.082323	PSMD12	26S protea	PSD12_HUI	26	1.006098
48H 10MT/	P82933	-0.08381	0.151627	0.082343	MRPS9	Small ribos	RT09_HUM	18	0.943563
24H 10MT/	Q9H910	-0.08312	0.151629	0.082343	JPT2	Jupiter mic	JUPI2_HUN	9	0.944012
48H 10MT/	Q13433	-0.21642	0.151663	0.08236	SLC39A6	Zinc transp	S39A6_HUI	1	0.860696
24H 10MT/	Q9NTN3	0.189415	0.151731	0.082392	SLC35D1	Nucleotide	S35D1_HUI	1	1.140301
24H 50MT/	Q92738	-0.30692	0.151797	0.082423	USP6NL	USP6 N-ter	US6NL_HUI	4	0.808367
24H 50MT/	Q15388	0.313671	0.151841	0.082443	TOMM20	Mitochond	TOM20_HU	3	1.242867
48H 10MT/	Q9H2U2	-0.0607	0.151866	0.082453	PPA2	Inorganic p	IPYR2_HUN	14	0.958798
48H 50MT/	Q9NS87	-0.28697	0.151863	0.082453	KIF15	Kinesin-like	KIF15_HUN	14	0.81962
24H 50MT/	Q9UJX5	0.010287	0.151921	0.082472	ANAPC4	Anaphase-p	APC4_HUM	18	1.007156
24H 10MT/	Q9BZL6	-0.27408	0.15197	0.08249	PRKD2	Serine/thre	KPCD2_HU	7	0.826978
24H 50MT/	Q15366	0.05191	0.151991	0.082498	PCBP2	Poly(rC)-bi	PCBP2_HUI	20	1.036637
24H 10MT/	Q14119	-0.35809	0.152031	0.08251	VEZF1	Vascular en	VEZF1_HUN	4	0.780197
24H 10MT/	Q96A35	-0.02784	0.152034	0.08251	MRPL24	Large ribos	RM24_HUN	12	0.980888
24H 10MT/	Q86Z14	0.116722	0.152043	0.082511	KLB	Beta-klotho	KLOTB_HUI	4	1.084269
24H 10MT/	P46736	0.023446	0.152053	0.082513	BRCC3	Lys-63-spec	BRCC3_HUI	10	1.016384
48H 10MT/	P62280	0.029008	0.152051	0.082513	RPS11	Small ribos	RS11_HUM	14	1.02031
24H 10MT/	Q14781	0.157928	0.15205	0.082513	CBX2	Chromobo:	CBX2_HUM	5	1.115683
48H 50MT/	Q9H1Z4	0.04713	0.152075	0.082522	WDR13	WD repeat-	WDR13_HU	13	1.033207
48H 50MT/	Q13435	-0.00768	0.15209	0.082528	SF3B2	Splicing fac	SF3B2_HUN	39	0.994694
24H 10MT/	Q9UM21	-0.03324	0.152117	0.082541	MGAT4A	Alpha-1,3-r	MGT4A_HU	2	0.977223
48H 10MT/	O95822	-0.09726	0.152155	0.082559	MLYCD	Malonyl-Cc	DCMC_HUN	14	0.934809
24H 50MT/	P49326	0.464432	0.15216	0.08256	FMO5	Flavin-cont	FMO5_HUN	2	1.379774
24H 10MT/	P49247	-0.06252	0.152167	0.082562	RPIA	Ribose-5-pl	RPIA_HUM/	5	0.957592
48H 50MT/	P13051	-0.36529	0.152195	0.082574	UNG	Uracil-DNA	UNG_HUM/	6	0.776312
24H 10MT/	P20290	-0.11049	0.152211	0.082582	BTF3	Transcripti	BTF3_HUM	11	0.926273
48H 50MT/	Q9Y6M9	-0.02037	0.152286	0.082616	NDUFB9	NADH dehy	NDUB9_HU	11	0.985976
24H 50MT/	Q8NB46	-0.04447	0.152295	0.08262	ANKRD52	Serine/thre	ANR52_HU	17	0.969646
48H 50MT/	P21281	-0.01719	0.152305	0.082623	ATP6V1B2	V-type prot	VATB2_HUI	22	0.988156
48H 50MT/	O14569	-0.01698	0.15232	0.082629	CYB561D2	Transmemb	C56D2_HU	1	0.988301
48H 50MT/	Q8WU17	0.338687	0.152382	0.082653	RNF139	E3 ubiquiti	RN139_HU	3	1.264605

48H 10MTA	O14975	-0.02907	0.152389	0.082654	SLC27A2	Long-chain	S27A2_HUI	15	0.980053
48H 50MTA	Q86SF2	-0.05384	0.152405	0.08266	GALNT7	N-acetylgal	GALT7_HUI	13	0.963367
24H 50MTA	Q9Y3D6	-0.16996	0.152412	0.082662	FIS1	Mitochond	FIS1_HUMA	6	0.888865
24H 10MTA	Q9H3L0	-0.58736	0.152423	0.082667	MMADHC	Cobalamin	MMAD_HU	2	0.665561
48H 10MTA	P51149	-0.04138	0.152456	0.082681	RAB7A	Ras-related	RAB7A_HUI	17	0.971725
24H 10MTA	Q9NRK6	-0.00264	0.152469	0.082686	ABCB10	ATP-bindin	ABCBA_HUI	15	0.998175
48H 50MTA	Q6PID6	0.013853	0.152518	0.082711	TTC33	Tetratricop	TTC33_HUN	4	1.009649
24H 10MTA	Q12894	0.066368	0.152534	0.082716	IFRD2	Interferon-i	IFRD2_HUN	7	1.047077
48H 50MTA	Q14596	-0.07805	0.152535	0.082716	NBR1	Next to BR(	NBR1_HUN	1	0.947336
48H 50MTA	O75144	0.42093	0.152555	0.082721	ICOSLG	ICOS ligand	ICOSL_HUN	1	1.33879
48H 50MTA	Q96GC9	0.147615	0.15257	0.082726	VMP1	Vacuole me	VMP1_HUN	6	1.107737
24H 10MTA	Q9NRR3	0.309704	0.152641	0.082758	CDC42SE2	CDC42 sma	C42S2_HUI	1	1.239454
48H 50MTA	P49641	-0.06335	0.152682	0.082773	MAN2A2	Alpha-mani	MA2A2_HU	10	0.957037
24H 10MTA	Q08357	0.162805	0.152688	0.082775	SLC20A2	Sodium-de	S20A2_HUI	6	1.119461
24H 10MTA	P07437	-0.09637	0.152715	0.082788	TUBB	Tubulin bet	TBB5_HUM	5	0.935382
24H 10MTA	Q9NTX7	-0.612	0.152746	0.082801	RNF146	E3 ubiquiti	RN146_HU	1	0.654291
48H 10MTA	Q9HBM6	0.023618	0.152798	0.082824	TAF9B	Transcripti	TAF9B_HUN	10	1.016506
24H 50MTA	O75592	0.041249	0.152845	0.082843	MYCBP2	E3 ubiquiti	MYCB2_HU	11	1.029005
48H 10MTA	P51809	-0.14349	0.152857	0.082848	VAMP7	Vesicle-asso	VAMP7_HU	11	0.905326
48H 10MTA	Q9NVC3	-0.12854	0.152861	0.082848	SLC38A7	Sodium-co	S38A7_HUI	1	0.914758
48H 50MTA	Q68CZ2	-0.08658	0.152868	0.082849	TNS3	Tensin-3	TENS3_HUN	36	0.941753
24H 50MTA	Q86VZ5	-0.06673	0.15287	0.082849	SGMS1	Phosphatic	SMS1_HUN	3	0.954799
48H 10MTA	O75489	-0.09384	0.152961	0.082885	NDUFS3	NADH dehy	NDUS3_HU	13	0.937027
48H 10MTA	Q92620	0.03485	0.152987	0.082894	DHX38	Pre-mRNA-!	PRP16_HUI	33	1.02445
48H 50MTA	Q96DX5	0.132696	0.152994	0.082897	ASB9	Ankyrin rep	ASB9_HUM	3	1.096341
24H 50MTA	Q3V6T2	-0.11626	0.153035	0.082913	CCDC88A	Girdin	GRDN_HUN	17	0.922575
24H 10MTA	P20226	0.087025	0.153048	0.082919	TBP	TATA-box-b	TBP_HUMA	5	1.062177
48H 50MTA	Q6PHR2	-0.06037	0.153058	0.082923	ULK3	Serine/thre	ULK3_HUN	6	0.95902
24H 10MTA	Q9BUL8	0.114849	0.153087	0.082935	PDCD10	Programme	PDC10_HU	9	1.082861
24H 10MTA	Q14566	0.023871	0.153228	0.083005	MCM6	DNA replica	MCM6_HUI	43	1.016684
48H 10MTA	Q96I45	-0.18207	0.153231	0.083006	TMEM141	Transmemb	TM141_HU	2	0.881438
24H 10MTA	O95292	-0.00194	0.153278	0.083024	VAPB	Vesicle-asso	VAPB_HUN	9	0.998657
24H 10MTA	P07307	0.051275	0.153308	0.083037	ASGR2	Asialoglyco	ASGR2_HUI	10	1.03618
24H 10MTA	Q6P444	0.029542	0.153312	0.083037	MTFR2	Mitochond	MTFR2_HU	6	1.020688
24H 10MTA	Q2VIR3	0.13233	0.153466	0.083108	EIF2S3B	Eukaryotic	IF2GL_HUN	1	1.096062
48H 10MTA	Q6IA69	0.055731	0.153509	0.083126	NADSYN1	Glutamine-	NADE_HUN	10	1.039385
24H 10MTA	O43402	0.007709	0.153548	0.083139	EMC8	ER membra	EMC8_HUN	5	1.005358
24H 50MTA	Q6ZS17	-0.22564	0.153549	0.083139	RIPOR1	Rho family-	RIPR1_HUN	7	0.855217
24H 10MTA	O15541	-0.00649	0.15357	0.083145	RNF113A	E3 ubiquiti	R113A_HUI	6	0.995512
48H 50MTA	Q9BV40	-0.04807	0.153605	0.08316	VAMP8	Vesicle-asso	VAMP8_HU	4	0.967227
24H 10MTA	P22695	0.05114	0.153664	0.083183	UQCRC2	Cytochrom	QCR2_HUN	12	1.036083
48H 10MTA	O14647	-0.01776	0.153694	0.083197	CHD2	Chromodoi	CHD2_HUN	14	0.987768
48H 10MTA	P57678	0.008776	0.1537	0.083199	GEMIN4	Gem-associ	GEMI4_HUI	26	1.006101
48H 50MTA	Q29RF7	-0.03946	0.153716	0.083204	PDS5A	Sister chroi	PDS5A_HUI	51	0.973022
24H 50MTA	Q14669	-0.04073	0.15374	0.083215	TRIP12	E3 ubiquiti	TRIPC_HUN	55	0.972164
24H 10MTA	P48426	-0.0473	0.153752	0.083218	PIP4K2A	Phosphatic	PI42A_HUN	8	0.967744
24H 10MTA	Q13352	0.969146	0.153826	0.083249	ITGB3BP	Centromer	CENPR_HUI	1	1.957681
24H 50MTA	Q8NAT1	-0.03107	0.153824	0.083249	POMGNT2	Protein O-li	PMGT2_HU	8	0.978692
48H 10MTA	Q8WUK0	-0.14963	0.153901	0.083281	PTPMT1	Phosphatic	PTPM1_HU	7	0.901479
48H 50MTA	Q9Y285	-0.05869	0.15393	0.083293	FARSA	Phenylalan	SYFA_HUM	20	0.960139
24H 50MTA	Q9UBX3	0.002799	0.153933	0.083293	SLC25A10	Mitochond	DIC_HUMA	13	1.001942
48H 50MTA	Q8NEF9	-0.15507	0.15397	0.083308	SRFBP1	Serum resp	SRFB1_HUN	7	0.89809

24H	10MT/	Q5SW24	-0.26108	0.153985	0.08331	DACT2	Dapper hor	DACT2_HUI	7	0.834462
24H	10MT/	P46776	0.028498	0.154022	0.083327	RPL27A	Large ribos	RL27A_HUI	7	1.019949
48H	10MT/	Q8NE62	-0.14699	0.15403	0.08333	CHDH	Choline del	CHDH_HUN	19	0.903135
48H	10MT/	Q8IY17	-0.04773	0.154043	0.083334	PNPLA6	Patatin-like	PLPL6_HUN	22	0.967454
48H	50MT/	Q66K74	0.085134	0.154087	0.083354	MAP1S	Microtubul	MAP1S_HU	20	1.060786
48H	10MT/	P13646	0.486405	0.154105	0.083361	KRT13	Keratin, typ	K1C13_HUI	6	1.400949
24H	10MT/	P35579	0.026989	0.154142	0.083377	MYH9	Myosin-9	MYH9_HUN	102	1.018883
48H	50MT/	Q9UBV7	0.200218	0.154161	0.083386	B4GALT7	Beta-1,4-ga	B4GT7_HUI	3	1.148872
24H	10MT/	P09001	-0.08323	0.154176	0.083392	MRPL3	Large ribos	RM03_HUN	11	0.943944
24H	10MT/	O60583	0.11109	0.154185	0.083395	CCNT2	Cyclin-T2	CCNT2_HUI	2	1.080044
24H	10MT/	Q13126	-0.00174	0.154192	0.083395	MTAP	S-methyl-5'	MTAP_HUN	18	0.998797
48H	10MT/	P14550	0.019395	0.154228	0.08341	AKR1A1	Aldo-keto r	AK1A1_HUI	19	1.013534
24H	50MT/	Q9UKR5	-0.11275	0.154234	0.083412	ERG28	Ergosterol I	ERG28_HUI	1	0.924821
24H	10MT/	Q9Y639	0.062726	0.154249	0.083418	NPTN	Neuroplast	NPTN_HUN	7	1.044438
24H	50MT/	P63261	-0.54282	0.154261	0.083422	ACTG1	Actin, cyto	ACTG_HUM	3	0.68643
48H	10MT/	P18621	-0.00328	0.154317	0.083448	RPL17	Large ribos	RL17_HUM	8	0.997727
48H	10MT/	O95433	0.057272	0.154372	0.083473	AHSA1	Activator o	AHSA1_HUI	18	1.040497
24H	10MT/	P49326	-0.10799	0.154386	0.08348	FMO5	Flavin-cont	FMO5_HUN	2	0.92788
24H	10MT/	Q9BVV7	-0.37646	0.154399	0.083483	TIMM21	Mitochond	TIM21_HUI	3	0.770323
24H	10MT/	O75439	-0.03197	0.154445	0.083504	PMPCB	Mitochond	MPPB_HUN	22	0.978081
24H	10MT/	Q9H4M3	-0.2746	0.154451	0.083506	FBXO44	F-box only	FBX44_HUI	3	0.826681
24H	10MT/	P53992	-0.05925	0.154522	0.083539	SEC24C	Protein trai	SC24C_HUI	35	0.959764
48H	10MT/	Q5TBB1	0.047246	0.154527	0.083539	RNASEH2B	Ribonuclea	RNH2B_HU	7	1.033291
24H	10MT/	Q96EY7	-0.06626	0.154554	0.08355	PTCD3	Small ribos	PTCD3_HUI	26	0.955112
48H	50MT/	Q9NRL3	-0.05689	0.154602	0.083571	STRN4	Striatin-4	STRN4_HUI	14	0.961336
48H	10MT/	P24386	0.064836	0.154649	0.083587	CHM	Rab proteir	RAE1_HUM	16	1.045966
24H	50MT/	Q13158	-0.22839	0.15465	0.083587	FADD	FAS-associa	FADD_HUN	6	0.853589
24H	10MT/	Q3B8N5	-0.58259	0.154743	0.083629	PROX2	Prospero h	PROX2_HU	2	0.667766
48H	50MT/	Q9BTV6	0.558505	0.154751	0.083632	DPH7	Diphthine r	DPH7_HUN	1	1.472742
24H	10MT/	O15173	-0.05112	0.154799	0.083652	PGRMC2	Membrane-	PGRC2_HU	11	0.965185
48H	10MT/	Q8TCT8	-0.24512	0.154807	0.083655	SPPL2A	Signal pept	SPP2A_HUI	4	0.843744
24H	10MT/	Q14669	0.01895	0.154817	0.083656	TRIP12	E3 ubiquiti	TRIPC_HUN	55	1.013222
48H	50MT/	Q96CW1	0.013033	0.15483	0.08366	AP2M1	AP-2 compl	AP2M1_HU	22	1.009075
24H	10MT/	P07814	-0.00273	0.154989	0.083725	EPRS1	Bifunctiona	SYEP_HUM/	85	0.998112
24H	10MT/	Q03014	-0.19576	0.155012	0.083732	HHEX	Hematopoi	HHEX_HUN	3	0.873114
48H	10MT/	Q9GZU8	0.038851	0.155012	0.083732	PSME3IP1	PSME3-inte	PIP30_HUN	8	1.027295
24H	10MT/	P35573	-0.06523	0.15505	0.083748	AGL	Glycogen d	GDE_HUM/	77	0.955792
48H	50MT/	Q9H469	0.066447	0.155097	0.083771	FBXL15	F-box/LRR-i	FXL15_HUN	8	1.047135
48H	10MT/	P80303	-0.07678	0.155154	0.083796	NUCB2	Nucleobinc	NUCB2_HU	14	0.948169
24H	50MT/	Q13555	0.128975	0.155189	0.083809	CAMK2G	Calcium/ca	KCC2G_HU	6	1.093517
48H	10MT/	Q9H4A5	0.221975	0.155189	0.083809	GOLPH3L	Golgi phos	GLP3L_HUI	5	1.166329
24H	10MT/	P54098	0.006116	0.155232	0.083827	POLG	DNA polym	DPOG1_HU	17	1.004248
24H	10MT/	P55899	-0.30568	0.155282	0.08385	FCGRT	IgG recepto	FCGRN_HU	2	0.809061
48H	50MT/	Q96A72	-0.02931	0.155285	0.083851	MAGOHB	Protein ma	MGN2_HUI	10	0.97989
48H	10MT/	Q7Z589	-0.14014	0.155313	0.083864	EMSY	BRCA2-inte	EMSY_HUN	7	0.907431
24H	50MT/	P40938	-0.06534	0.155343	0.083875	RFC3	Replication	RFC3_HUM	17	0.955723
24H	10MT/	Q9Y5U8	0.042283	0.155354	0.083879	MPC1	Mitochond	MPC1_HUN	2	1.029742
24H	50MT/	P19174	-0.10459	0.155465	0.083931	PLCG1	1-phosphat	PLCG1_HUI	24	0.930072
48H	10MT/	Q6P1L8	-0.1242	0.155463	0.083931	MRPL14	Large ribos	RM14_HUN	5	0.91751
24H	50MT/	P02769	-0.67352	0.155479	0.083935	ALB	Serum albu	ALBU_BOVI	56	0.626975
24H	10MT/	O43731	0.085276	0.155501	0.083943	KDELR3	ER lumen p	ERD23_HUI	3	1.060891
24H	50MT/	Q86W42	0.056959	0.155543	0.083957	THOC6	THO compl	THOC6_HU	16	1.040271

24H 50MT/	P61619	0.0543	0.155552	0.083958	SEC61A1	Protein tra	S61A1_HU	7	1.038355
24H 10MT/	Q9UL26	0.199736	0.15555	0.083958	RAB22A	Ras-related	RB22A_HU	8	1.148488
48H 50MT/	Q9BUL8	0.003703	0.155564	0.083959	PDCD10	Programme	PDC10_HU	9	1.00257
24H 10MT/	Q9Y4P8	0.12257	0.155563	0.083959	WIPI2	WD repeat	WIPI2_HU	8	1.088673
48H 50MT/	Q6P5R6	-0.65646	0.155608	0.083982	RPL22L1	Ribosomal	RL22L_HU	1	0.634433
48H 10MT/	Q13144	-0.03731	0.155666	0.084006	EIF2B5	Translation	EI2BE_HUM	18	0.974473
24H 50MT/	Q8N5C7	0.407631	0.155699	0.084023	DTWD1	tRNA-uridi	DTWD1_HU	1	1.326505
48H 50MT/	Q9BWJ5	-0.21616	0.155705	0.084025	SF3B5	Splicing fac	SF3B5_HUI	4	0.860851
48H 10MT/	Q9NV64	0.828548	0.155715	0.084029	TMEM39A	Transmemb	TM39A_HU	1	1.775897
24H 50MT/	P17844	-0.02167	0.155722	0.08403	DDX5	Probable A	DDX5_HUN	33	0.985088
48H 10MT/	Q96AE4	-0.01053	0.155728	0.084032	FUBP1	Far upstrea	FUBP1_HU	28	0.992729
48H 50MT/	P51571	-0.00371	0.155756	0.084043	SSR4	Translocon	SSRD_HUM	4	0.997433
24H 10MT/	O95208	-0.47296	0.155777	0.084048	EPN2	Epsin-2	EPN2_HUM	4	0.720486
48H 50MT/	Q9HC36	-0.11333	0.155814	0.084066	MRM3	rRNA meth	MRM3_HU	8	0.924449
24H 10MT/	O43663	0.126935	0.15583	0.084074	PRC1	Protein reg	PRC1_HUM	16	1.091971
24H 10MT/	Q9H0E2	-0.06461	0.155893	0.084104	TOLLIP	Toll-intera	TOLIP_HUN	7	0.956207
24H 50MT/	Q8WXF1	0.165596	0.155955	0.084132	PSPC1	Paraspeckl	PSPC1_HU	14	1.12163
48H 10MT/	Q9H9P8	-0.05061	0.156026	0.084168	L2HGDH	L-2-hydrox	L2HDH_HU	16	0.965527
48H 50MT/	Q9P2K3	-0.05792	0.156054	0.084182	RCOR3	REST corep	RCOR3_HU	10	0.960645
24H 10MT/	Q8IYB8	-0.02966	0.156145	0.084224	SUPV3L1	ATP-depen	SUV3_HUM	25	0.979651
48H 10MT/	P45954	-0.06356	0.15617	0.084235	ACADSB	Short/bran	ACDSB_HU	17	0.956899
24H 50MT/	Q5MNZ9	0.048116	0.156233	0.084261	WIPI1	WD repeat	WIPI1_HUN	2	1.033914
24H 10MT/	Q9UGH3	0.021356	0.156252	0.084269	SLC23A2	Solute carr	S23A2_HU	1	1.014913
48H 10MT/	Q8IXU6	0.201303	0.156267	0.084276	SLC35F2	Solute carr	S35F2_HUN	1	1.149737
24H 50MT/	Q99996	0.182654	0.156274	0.084279	AKAP9	A-kinase an	AKAP9_HU	32	1.13497
48H 10MT/	P08621	-0.00627	0.156287	0.084282	SNRNP70	U1 small n	RU17_HUM	14	0.995662
24H 10MT/	Q9NUL3	0.05558	0.156323	0.084297	STAU2	Double-str	STAU2_HU	15	1.039277
48H 50MT/	Q01804	0.002122	0.156377	0.084316	OTUD4	OTU domai	OTUD4_HU	11	1.001472
24H 10MT/	Q9Y4K3	0.264472	0.156371	0.084316	TRAF6	TNF recept	TRAF6_HUN	2	1.201196
24H 50MT/	Q99638	-0.05131	0.156411	0.08433	RAD9A	Cell cycle c	RAD9A_HU	4	0.965057
24H 10MT/	P10109	-0.47872	0.156457	0.084347	FDX1	Adrenodox	ADX_HUM/	5	0.717616
24H 50MT/	Q86WB0	0.085805	0.156477	0.084355	ZC3HC1	Zinc finger	ZC3C1_HU	16	1.06128
24H 50MT/	Q5SNT2	-0.28649	0.156488	0.08436	TMEM201	Transmemb	TM201_HU	9	0.819895
24H 50MT/	Q96JI7	-0.16447	0.1565	0.084362	SPG11	Spatacsin	SPTCS_HUN	4	0.892255
24H 10MT/	O00462	0.124803	0.156553	0.084384	MANBA	Beta-mann	MANBA_HU	12	1.090359
24H 10MT/	O94876	-0.34766	0.15664	0.084425	TMCC1	Transmemb	TMCC1_HU	2	0.785859
24H 10MT/	Q3KRA9	-0.01892	0.156647	0.084428	ALKBH6	Alpha-keto	ALKB6_HU	3	0.986972
48H 50MT/	Q9H4G4	0.459051	0.15667	0.084437	GLIPR2	Golgi-assoc	GAPR1_HU	4	1.374637
24H 10MT/	P84095	-0.04972	0.156706	0.084452	RHOG	Rho-relate	RHOG_HUN	8	0.966125
24H 50MT/	Q9HA92	0.600485	0.156757	0.084476	RSAD1	Radical S-a	RSAD1_HU	3	1.516226
48H 10MT/	Q99569	-0.06578	0.15677	0.084481	PKP4	Plakophili	PKP4_HUM	20	0.955428
24H 50MT/	P61353	-0.05178	0.156809	0.084498	RPL27	Large ribos	RL27_HUM	7	0.964743
24H 10MT/	Q86SX3	0.144011	0.15682	0.084501	TEDC1	Tubulin ep	TEDC1_HU	3	1.104973
48H 10MT/	Q9NXK8	0.13113	0.15683	0.084505	FBXL12	F-box/LRR-	FXL12_HUN	4	1.095151
24H 50MT/	P51692	0.002964	0.156873	0.084523	STAT5B	Signal tran	STA5B_HU	13	1.002057
48H 10MT/	Q9BX40	-0.06698	0.15688	0.084525	LSM14B	Protein LSN	LS14B_HUN	13	0.954635
48H 50MT/	Q9NP50	-0.25953	0.156897	0.084528	SINHCAF	SIN3-HDAC	SHCAF_HU	2	0.835362
24H 10MT/	O75144	0.445138	0.156906	0.084531	ICOSLG	ICOS ligand	ICOSL_HUN	1	1.361444
24H 50MT/	Q9NPI6	0.074189	0.156981	0.084568	DCP1A	mRNA-deca	DCP1A_HU	14	1.052769
48H 10MT/	O00139	-0.04513	0.157049	0.084598	KIF2A	Kinesin-lik	KIF2A_HUN	14	0.969204
48H 10MT/	Q9NR31	0.013856	0.15705	0.084598	SAR1A	Small COPI	SAR1A_HU	8	1.009651
48H 50MT/	Q7Z401	0.155981	0.157084	0.084611	DENND4A	C-myc pron	MYCPP_HU	6	1.114179

48H 10MT/	Q9BTD8	-0.01976	0.157105	0.084622	RBM42	RNA-bindir	RBM42_HU	11	0.9864
48H 50MT/	Q6ZUJ8	-0.12516	0.157142	0.084638	PIK3AP1	Phosphoin	BCAP_HUM	12	0.916902
48H 10MT/	Q96JB6	0.05898	0.157204	0.084659	LOXL4	Lysyl oxida	LOXL4_HUI	11	1.041729
48H 50MT/	P52888	0.031555	0.157248	0.084677	THOP1	Thimet olig	THOP1_HU	27	1.022113
48H 50MT/	Q5BJD5	-0.27722	0.157284	0.084691	TMEM41B	Transmemb	TM41B_HU	3	0.825178
48H 10MT/	Q96AY3	-0.01945	0.157286	0.084691	FKBP10	Peptidyl-pr	FKB10_HUI	27	0.986609
48H 10MT/	Q9NY61	-0.00769	0.157331	0.084708	AATF	Protein AA	AATF_HUM	17	0.994685
48H 10MT/	Q6V1X1	0.221978	0.157348	0.084714	DPP8	Dipeptidyl	DPP8_HUM	8	1.166331
24H 50MT/	Q9NV96	0.127012	0.157415	0.084748	TMEM30A	Cell cycle c	CC50A_HUI	6	1.09203
48H 10MT/	Q9C0B1	0.005732	0.157579	0.084816	FTO	Alpha-keto	FTO_HUMA	13	1.003981
24H 10MT/	P46087	0.000453	0.157589	0.084819	NOP2	28S rRNA (c	NOP2_HUM	25	1.000314
48H 10MT/	O75923	-0.20482	0.157633	0.084836	DYSF	Dysferlin	DYSF_HUM	1	0.867649
24H 10MT/	Q8IU81	0.028983	0.15769	0.084846	IRF2BP1	Interferon r	I2BP1_HUM	18	1.020293
24H 10MT/	Q9UHK0	-0.00975	0.15771	0.084856	NUFIP1	FMR1-inter	NUFP1_HU	3	0.993267
24H 50MT/	Q96FX7	0.057955	0.157716	0.084858	TRMT61A	tRNA (aden	TRM61_HU	7	1.040989
48H 50MT/	Q9NRF8	0.057904	0.157737	0.084867	CTPS2	CTP synthas	PYRG2_HUI	24	1.040952
24H 10MT/	P62333	-0.0108	0.157801	0.084894	PSMC6	26S protea	PRS10_HUI	21	0.992539
24H 50MT/	Q13686	-0.07868	0.1578	0.084894	ALKBH1	Nucleic aci	ALKB1_HUI	4	0.946921
24H 10MT/	Q9BUF7	-0.27785	0.1578	0.084894	CRB3	Protein cru	CRUM3_HU	1	0.824821
48H 50MT/	A6ND36	4.37047	0.157833	0.084907	FAM83G	Protein FAI	FA83G_HUI	1	20.68438
24H 50MT/	P02771	-0.49427	0.157867	0.084922	AFP	Alpha-fetop	FETA_HUMA	3	0.70992
48H 50MT/	Q9HAV4	-0.07216	0.15795	0.084961	XPO5	Exportin-5	XPO5_HUM	47	0.951214
48H 10MT/	O15254	0.057022	0.158004	0.084981	ACOX3	Peroxisoma	ACOX3_HU	15	1.040316
48H 50MT/	Q96L58	0.011076	0.158016	0.084986	B3GALT6	Beta-1,3-ga	B3GT6_HUI	6	1.007707
48H 50MT/	Q06265	-0.05353	0.15804	0.084996	EXOSC9	Exosome cc	EXOS9_HUI	11	0.963575
48H 10MT/	Q13442	0.032724	0.158101	0.085025	PDAP1	28 kDa hea	HAP28_HU	6	1.022942
48H 10MT/	Q96J42	-0.10197	0.158113	0.085031	TXNDC15	Thioredoxi	TXD15_HUI	5	0.931759
24H 10MT/	Q99856	-0.01683	0.158177	0.08506	ARID3A	AT-rich inte	ARI3A_HUM	17	0.988401
48H 10MT/	Q9BQ48	-0.07996	0.158196	0.085069	MRPL34	Large ribos	RM34_HUM	2	0.946086
24H 10MT/	P62851	-0.09067	0.158203	0.08507	RPS25	Small ribos	RS25_HUM	4	0.939089
48H 50MT/	Q9NXR5	-0.53642	0.158207	0.08507	ANKRD10	Ankyrin rep	ANR10_HU	2	0.689482
48H 10MT/	Q2NKX8	-0.00464	0.158214	0.085071	ERCC6L	DNA excisic	ERC6L_HUM	38	0.996792
48H 50MT/	Q9BTX7	-0.30411	0.158215	0.085071	TTPAL	Alpha-toco	TTPAL_HUM	9	0.809939
48H 10MT/	P23142	0.178822	0.158243	0.085082	FBLN1	Fibulin-1	FBLN1_HUI	11	1.131959
24H 50MT/	P49593	0.106655	0.158247	0.085082	PPM1F	Protein pho	PPM1F_HU	13	1.076729
48H 50MT/	Q9BTA9	-0.13983	0.158241	0.085082	WAC	WW domai	WAC_HUM	10	0.907624
48H 50MT/	Q8TDH9	0.45307	0.158273	0.085093	BLOC1S5	Biogenesis	BL1S5_HUM	2	1.368951
24H 50MT/	Q5T1V6	-0.15555	0.158293	0.085101	DDX59	Probable A	DDX59_HU	7	0.897792
48H 50MT/	Q7Z6K3	-0.18914	0.158295	0.085101	PTAR1	Protein pre	PTAR1_HUI	12	0.877131
24H 50MT/	P14210	0.905092	0.158323	0.085114	HGF	Hepatocyte	HGF_HUMA	1	1.872664
48H 10MT/	Q68CP4	-0.36078	0.158406	0.085152	HGSNAT	Heparan-al	HGNAT_HU	4	0.778744
48H 50MT/	Q96B26	-0.13623	0.158487	0.085185	EXOSC8	Exosome cc	EXOS8_HUI	9	0.909891
24H 50MT/	Q15041	-0.60509	0.158491	0.085185	ARL6IP1	ADP-ribosy	AR6P1_HUI	3	0.65743
48H 50MT/	Q16718	-0.14455	0.158497	0.085186	NDUFA5	NADH dehy	NDUA5_HU	6	0.904659
48H 10MT/	Q9BVQ7	-0.06723	0.15851	0.085188	AFG2B	ATPase fam	AFG2B_HUI	21	0.954469
24H 50MT/	Q96TA1	-0.10291	0.158573	0.085213	NIBAN2	Protein Nib	NIBA2_HUM	13	0.931154
24H 10MT/	P35222	-0.00241	0.158635	0.08524	CTNNB1	Catenin bet	CTNB1_HUI	35	0.998329
24H 10MT/	P50213	-0.04811	0.158686	0.085263	IDH3A	Isocitrate d	IDH3A_HUI	17	0.967205
24H 50MT/	Q6DD87	-0.05283	0.158706	0.085273	ZNF787	Zinc finger	ZN787_HUI	9	0.96404
48H 50MT/	B2RXH8	0.043219	0.15871	0.085274	HNRNPCL2	Heterogene	HNRC2_HU	1	1.030411
48H 10MT/	Q7L1W4	-0.37736	0.158715	0.085274	LRRC8D	Volume-reg	LRC8D_HUI	3	0.769844
24H 10MT/	Q9P2E5	0.269565	0.158765	0.085291	CHPF2	Chondroiti	CHPF2_HUI	1	1.205445

48H 10MT/	Q58FG1	-0.32582	0.158791	0.085302	HSP90AA4I	Putative he	HS904_HUI	4	0.797842
24H 10MT/	Q9HCE1	-0.03683	0.158805	0.085307	MOV10	Helicase M(	MOV10_HU	44	0.974794
24H 50MT/	Q92600	-0.18375	0.158841	0.08532	CNOT9	CCR4-NOT t	CNOT9_HU	10	0.88041
48H 10MT/	P41229	0.137404	0.158844	0.085321	KDM5C	Lysine-spec	KDM5C_HU	15	1.099924
24H 10MT/	P40189	0.101567	0.15887	0.085327	IL6ST	Interleukin	IL6RB_HUN	5	1.072939
24H 50MT/	Q14657	0.508279	0.158882	0.085332	LAGE3	EKC/KEOPS	LAGE3_HUI	2	1.422353
48H 50MT/	P43361	0.378559	0.158886	0.085333	MAGEA8	Melanoma-	MAGA8_HU	1	1.300042
48H 50MT/	Q6EKJ0;Q8	-0.29544	0.158939	0.085358	GTF2IRD2B	General tra	GTD2B_HU	1	0.814827
24H 50MT/	Q9UJZ1	0.024524	0.158987	0.085382	STOML2	Stomatin-li	STML2_HUI	17	1.017144
48H 50MT/	Q5HYK7	-0.02946	0.158996	0.085386	SH3D19	SH3 domai	SH319_HUI	1	0.979784
24H 10MT/	Q8N5N7	-0.2281	0.159111	0.085442	MRPL50	Large ribos	RM50_HUN	7	0.85376
48H 50MT/	P25686	0.13667	0.159144	0.085456	DNAJB2	DnaJ homo	DNJB2_HUI	6	1.099365
24H 10MT/	Q659C4	0.063219	0.159178	0.085472	LARP1B	La-related p	LAR1B_HUI	5	1.044794
24H 50MT/	P68363	-0.09747	0.159202	0.085483	TUBA1B	Tubulin alp	TBA1B_HUI	30	0.93467
48H 10MT/	Q12792	0.00323	0.159258	0.085503	TWF1	Twinfilin-1	TWF1_HUN	15	1.002241
48H 50MT/	P40424	0.016248	0.159286	0.085516	PBX1	Pre-B-cell le	PBX1_HUM	3	1.011326
48H 50MT/	Q9UMZ2	0.007175	0.15929	0.085517	SYNRG	Synergin ga	SYNRG_HUI	13	1.004986
48H 10MT/	Q9UNZ2	-0.01466	0.159308	0.085523	NSFL1C	NSFL1 cofa	NSF1C_HUI	17	0.989892
24H 10MT/	Q15596	-0.0517	0.159353	0.085543	NCOA2	Nuclear rec	NCOA2_HU	12	0.964801
48H 10MT/	Q6PJG6	-0.0054	0.159403	0.085564	BRAT1	BRCA1-assc	BRAT1_HUI	37	0.996265
24H 50MT/	Q9UGJ0	-0.12626	0.159424	0.085571	PRKAG2	5'-AMP-acti	AAKG2_HU	4	0.916206
48H 50MT/	Q96FS4	0.093433	0.159428	0.085572	SIPA1	Signal-indu	SIPA1_HUN	15	1.066906
48H 50MT/	Q52LW3	0.085406	0.159501	0.085601	ARHGAP29	Rho GTPase	RHG29_HU	43	1.060986
24H 10MT/	Q7RTV5	-0.08887	0.159514	0.085604	PRXL2C	Peroxiredo	PXL2C_HUI	3	0.940258
48H 50MT/	Q9UHD2	-0.00257	0.159516	0.085605	TBK1	Serine/thre	TBK1_HUM	19	0.99822
48H 10MT/	Q07866	-0.03364	0.159568	0.085629	KLC1	Kinesin ligh	KLC1_HUM	17	0.976953
48H 10MT/	Q15599	-0.05199	0.159604	0.08564	NHERF2	Na(+)/H(+) e	NHRF2_HU	6	0.964605
24H 10MT/	Q96PY5	-0.03124	0.159603	0.08564	FMNL2	Formin-like	FMNL2_HU	9	0.97858
24H 50MT/	P16455	-0.00119	0.15966	0.085666	MGMT	Methylated	MGMT_HUI	5	0.999177
24H 10MT/	Q658Y4	0.070355	0.159677	0.085673	FAM91A1	Protein FAI	F91A1_HUI	17	1.049975
48H 50MT/	P17706	0.131377	0.159701	0.085681	PTPN2	Tyrosine-pr	PTN2_HUM	9	1.095339
24H 10MT/	Q9NVC3	-0.04316	0.159721	0.085691	SLC38A7	Sodium-co	S38A7_HUI	1	0.970525
24H 10MT/	Q96A49	-0.19599	0.159759	0.085703	SYAP1	Synapse-ass	SYAP1_HUI	18	0.872971
48H 50MT/	Q9H668	0.186654	0.159761	0.085703	STN1	CST comple	STN1_HUM	2	1.138121
48H 50MT/	Q5TC63	0.121794	0.159764	0.085703	GRTP1	Growth ho	GRTP1_HUI	4	1.088087
48H 50MT/	Q8N2U0	-0.19832	0.159766	0.085703	TMEM256	Transmemb	TM256_HU	2	0.871564
24H 50MT/	P98179	0.011981	0.1598	0.085717	RBM3	RNA-bindin	RBM3_HUN	5	1.008339
48H 10MT/	Q9C0C2	0.063997	0.159796	0.085717	TNKS1BP1	182 kDa tar	TB182_HUI	35	1.045358
48H 10MT/	Q13428	-0.09359	0.15983	0.085731	TCOF1	Treacle pro	TCOF_HUM	28	0.937186
48H 10MT/	Q6P1J9	0.015676	0.159856	0.085741	CDC73	Parafibrom	CDC73_HU	27	1.010925
48H 50MT/	Q9NUN5	0.059378	0.159945	0.085779	LMBRD1	Lysosomal	LMBD1_HU	3	1.042016
48H 10MT/	Q8N511	-0.31666	0.159955	0.085779	TMEM199	Transmemb	TM199_HU	4	0.802927
48H 10MT/	Q13286	-0.25658	0.159976	0.085788	CLN3	Battenin	CLN3_HUM	3	0.837068
48H 10MT/	O43432	-0.00286	0.159988	0.085789	EIF4G3	Eukaryotic	IF4G3_HUN	43	0.998016
48H 50MT/	Q9NZ08	0.034127	0.160022	0.085803	ERAP1	Endoplasm	ERAP1_HUI	27	1.023937
24H 50MT/	Q9BQA1	-0.02559	0.160053	0.085815	WDR77	Methylosor	MEP50_HU	12	0.982417
24H 10MT/	Q7L3B6	-0.4887	0.160083	0.085826	CDC37L1	Hsp90 co-c	CD37L_HUI	2	0.712665
48H 50MT/	Q15393	-0.01088	0.160216	0.085888	SF3B3	Splicing fac	SF3B3_HUN	48	0.992486
24H 10MT/	P58511	0.21619	0.160225	0.08589	SMIM11	Small integ	SIM11_HUI	1	1.161662
24H 10MT/	Q8IYL3	0.128789	0.160234	0.085894	C1orf174	UPF0688 p	CA174_HUI	1	1.093376
24H 10MT/	Q8ND04	-0.03234	0.160258	0.085903	SMG8	Nonsense-n	SMG8_HUN	15	0.977832
24H 10MT/	P54709	0.037447	0.160264	0.085905	ATP1B3	Sodium/po	AT1B3_HUI	12	1.026296

24H 50MT/	P28070	0.05413	0.160267	0.085905	PSMB4	Proteasom	PSB4_HUM	10	1.038233
24H 50MT/	Q15645	-0.03968	0.160328	0.08593	TRIP13	Pachytene	PCH2_HUM	20	0.972874
48H 10MT/	Q9NS23	0.04223	0.160341	0.085936	RASSF1	Ras associa	RASF1_HUI	1	1.029704
48H 10MT/	Q9BYN0	-0.11493	0.160358	0.085943	SRXN1	Sulfiredoxi	SRXN1_HUI	5	0.923428
48H 50MT/	Q9NRA2	-0.00516	0.160466	0.08599	SLC17A5	Sialin	S17A5_HUI	4	0.996429
24H 10MT/	Q13200	0.012916	0.160512	0.086012	PSMD2	26S protea	PSMD2_HU	53	1.008993
24H 10MT/	P78504	0.56985	0.160614	0.086062	JAG1	Protein jag	JAG1_HUM	4	1.484369
48H 50MT/	Q96RS0	0.041114	0.160648	0.086073	TGS1	Trimethylg	TGS1_HUM	8	1.028908
48H 10MT/	Q6PL18	-0.05165	0.160668	0.086083	ATAD2	ATPase fam	ATAD2_HUI	28	0.96483
24H 50MT/	Q13554	0.100652	0.160694	0.086094	CAMK2B	Calcium/ca	KCC2B_HUI	6	1.072258
48H 50MT/	Q96C86	-0.00981	0.160727	0.086105	DCPS	m7GpppX	DCPS_HUM	14	0.993223
48H 10MT/	O14966	-0.09942	0.160791	0.086131	RAB29	Ras-related	RAB7L_HUI	4	0.933405
24H 10MT/	Q8NBJ9	0.076916	0.16079	0.086131	SIDT2	SID1 transn	SIDT2_HUN	4	1.054761
48H 10MT/	Q96D42	0.247882	0.160807	0.086138	HAVCR1	Hepatitis A	HAVR1_HU	3	1.187463
24H 50MT/	O15417	0.001896	0.160868	0.086168	TNRC18	Trinucleoti	TNC18_HUI	3	1.001315
24H 10MT/	Q8TE76	-0.05611	0.160884	0.086172	MORC4	MORC fami	MORC4_HU	3	0.961851
48H 10MT/	Q9HBH1	-0.22476	0.160892	0.086172	PDF	Peptide def	DEFM_HUM	4	0.855736
48H 50MT/	O14776	-0.01045	0.160943	0.086192	TCERG1	Transcripti	TCRG1_HUI	32	0.992786
48H 50MT/	Q96H22	-0.61084	0.160938	0.086192	CENPN	Centromer	CENPN_HU	1	0.654816
48H 10MT/	Q8IY95	-0.08006	0.160983	0.086206	TMEM192	Transmemb	TM192_HU	3	0.946018
24H 10MT/	Q9H790	1.01147	0.160991	0.086209	EXO5	Exonucleas	EXO5_HUM	1	2.015963
24H 50MT/	P57775	-0.23256	0.160996	0.086211	FBXW4	F-box/WD r	FBXW4_HU	2	0.851123
48H 10MT/	P62330	-0.01696	0.161029	0.086227	ARF6	ADP-ribosy	ARF6_HUM	8	0.988312
24H 50MT/	P51948	-0.15163	0.161038	0.086228	MNAT1	CDK-activa	MAT1_HUN	11	0.90023
24H 50MT/	Q8WYN0	-0.05398	0.161037	0.086228	ATG4A	Cysteine pr	ATG4A_HUI	6	0.963277
24H 10MT/	P30046	-0.12959	0.161155	0.086278	DDT	D-dopachr	DOPD_HUN	8	0.914089
24H 10MT/	C4AMC7;Q	-0.1455	0.161162	0.086281	WASH3P;W	Putative W	WASH3_HU	11	0.904065
24H 50MT/	O00194	0.090031	0.161195	0.08629	RAB27B	Ras-related	RB27B_HUI	4	1.064393
48H 50MT/	Q9Y6R1	0.210472	0.161198	0.08629	SLC4A4	Electrogeni	S4A4_HUM	3	1.157067
48H 10MT/	Q9P0S2	0.173169	0.161218	0.086298	COX16	Cytochrom	COX16_HU	2	1.127532
24H 10MT/	Q9NZP8	-0.36472	0.161236	0.086305	C1RL	Complemer	C1RL_HUM	1	0.776621
24H 10MT/	Q9BW61	-0.51004	0.161307	0.086332	DDA1	DET1- and I	DDA1_HUN	2	0.702204
24H 10MT/	Q5VWG9	-0.33138	0.161413	0.086376	TAF3	Transcripti	TAF3_HUM	1	0.794775
48H 50MT/	Q8NB90	-0.1231	0.161425	0.08638	AFG2A	ATPase fam	AFG2A_HUI	21	0.918214
24H 10MT/	P00505	-0.06988	0.161434	0.086383	GOT2	Aspartate a	AATM_HUN	26	0.952717
24H 50MT/	O14618	-0.01424	0.161512	0.086421	CCS	Copper cha	CCS_HUMA	8	0.990182
24H 10MT/	Q8NHM5	0.477326	0.161524	0.086426	KDM2B	Lysine-spec	KDM2B_HU	4	1.392161
48H 10MT/	Q9NYM9	0.397078	0.161532	0.086427	BET1L	BET1-like p	BET1L_HUN	3	1.316838
24H 50MT/	O43615	-0.03412	0.161659	0.086489	TIMM44	Mitochond	TIM44_HUI	22	0.976627
24H 10MT/	P17301	0.097307	0.161666	0.08649	ITGA2	Integrin alp	ITA2_HUM/	10	1.069775
24H 10MT/	Q8WXD5	-0.09873	0.161705	0.086509	GEMIN6	Gem-associ	GEMI6_HUI	5	0.933856
24H 10MT/	P53985	0.079863	0.161771	0.086539	SLC16A1	Monocarbo	MOT1_HUN	11	1.056917
48H 50MT/	P54619	-0.1191	0.161787	0.086545	PRKAG1	5'-AMP-acti	AAKG1_HU	11	0.920762
48H 10MT/	Q53HC9	0.079219	0.161793	0.086547	EIPR1	EARP and G	EIPR1_HUN	9	1.056446
48H 50MT/	Q0PNE2	-0.15566	0.161818	0.086557	ELP6	Elongator c	ELP6_HUM	7	0.897721
48H 10MT/	Q8NI37	0.026449	0.161825	0.086559	PPTC7	Protein ph	PPTC7_HUI	7	1.018502
48H 10MT/	P52824	-0.50968	0.161838	0.086564	DGKQ	Diacylglyce	DGKQ_HUN	2	0.702377
48H 10MT/	Q9BT22	-0.08416	0.161868	0.086579	ALG1	Chitobiosyl	ALG1_HUM	10	0.943333
24H 50MT/	P21589	-0.59907	0.161879	0.086583	NT5E	5'-nucleoti	5NTD_HUM	1	0.66018
48H 10MT/	O75592	-0.11373	0.161891	0.086588	MYCBP2	E3 ubiquiti	MYCB2_HU	11	0.924193
24H 10MT/	Q07020	0.115155	0.16196	0.08662	RPL18	Large ribos	RL18_HUM	7	1.083091
48H 10MT/	P62701	-0.01309	0.162043	0.086658	RPS4X	Small ribos	RS4X_HUM	17	0.99097

24H 10MT/	Q9BRD0	0.01911	0.162056	0.086663	BUD13	BUD13 hor	BUD13_HU	9	1.013334
48H 50MT/	Q9Y3A0	0.146398	0.162057	0.086663	COQ4	Ubiquinon	COQ4_HUN	3	1.106803
24H 10MT/	Q92968	0.004017	0.16209	0.086677	PEX13	Peroxisoma	PEX13_HUI	6	1.002789
48H 50MT/	Q9Y6I8	-0.12333	0.162101	0.086682	PXMP4	Peroxisoma	PXMP4_HU	5	0.918063
48H 10MT/	Q9ULR0	-0.08871	0.162121	0.086689	ISY1	Pre-mRNA-:	ISY1_HUM/	6	0.940362
24H 10MT/	Q9Y3C0	-0.26912	0.162174	0.086704	WASHC3	WASH com	WASC3_HU	4	0.829823
48H 10MT/	Q03169	0.330284	0.1622	0.086712	TNFAIP2	Tumor necr	TNAP2_HUI	2	1.257261
48H 10MT/	Q5VWX1	-0.08972	0.162211	0.086717	KHDRBS2	KH domain	KHDR2_HU	2	0.939703
24H 10MT/	Q9NZB2	-0.01376	0.162267	0.086742	FAM120A	Constitutiv	F120A_HUI	31	0.990509
24H 50MT/	P12955	-0.02906	0.162318	0.086761	PEPD	Xaa-Pro dip	PEPD_HUM	21	0.980056
24H 10MT/	Q14353	-0.03688	0.162317	0.086761	GAMT	Guanidinoa	GAMT_HUN	10	0.974757
24H 50MT/	Q8TBM7	0.535188	0.162352	0.086775	TMEM254	Transmemb	TM254_HU	1	1.449131
48H 10MT/	P53634	-0.01181	0.162371	0.086781	CTSC	Dipeptidyl	CATC_HUM	11	0.991848
24H 10MT/	Q99996	0.108314	0.162372	0.086781	AKAP9	A-kinase an	AKAP9_HUI	32	1.077968
48H 50MT/	P20336	-0.02276	0.162376	0.086782	RAB3A	Ras-related	RAB3A_HUI	1	0.984349
24H 10MT/	Q8WUB8	-0.14702	0.162402	0.086793	PHF10	PHD finger	PHF10_HUI	6	0.903117
48H 10MT/	Q9BS34	-0.16696	0.162466	0.086823	ZNF670	Zinc finger	ZN670_HUI	4	0.890719
24H 10MT/	P14324	-0.16838	0.162468	0.086823	FDPS	Farnesyl py	FPPS_HUM	10	0.889843
48H 10MT/	Q9BTX7	-0.28789	0.162524	0.086842	TTPAL	Alpha-toco	TTPAL_HUN	9	0.819098
24H 50MT/	P62263	-0.08611	0.162549	0.086845	RPS14	Small ribos	RS14_HUM	9	0.942063
48H 10MT/	Q15019	0.038554	0.162548	0.086845	SEPTIN2	Septin-2	SEPT2_HUN	16	1.027084
24H 50MT/	Q96A57	0.442131	0.162549	0.086845	TMEM230	Transmemb	TM230_HU	3	1.35861
24H 10MT/	Q9Y5X1	0.012223	0.162554	0.086846	SNX9	Sorting nex	SNX9_HUM	18	1.008508
24H 10MT/	Q96PY6	0.230121	0.162559	0.086848	NEK1	Serine/thre	NEK1_HUM	2	1.172934
24H 10MT/	Q9Y6G5	0.036103	0.162586	0.086859	COMMD10	COMM dor	COMDA_HU	5	1.02534
24H 50MT/	Q9C0D9	-0.17033	0.162589	0.08686	SELENOI	Ethanolami	EPT1_HUM	2	0.888639
48H 10MT/	Q13641	-0.09753	0.162621	0.086871	TPBG	Trophoblas	TPBG_HUM	3	0.934631
24H 10MT/	Q7Z3Y8	-0.26424	0.162642	0.086877	KRT27	Keratin, typ	K1C27_HUI	2	0.832638
24H 50MT/	Q9NZE8	-0.06135	0.162654	0.086882	MRPL35	Large ribos	RM35_HUN	1	0.95837
48H 10MT/	O75691	-0.0823	0.162728	0.086916	UTP20	Small subu	UTP20_HUI	83	0.94455
24H 50MT/	Q86UV5	0.118691	0.162725	0.086916	USP48	Ubiquitin c	UBP48_HU	15	1.08575
24H 10MT/	Q14562	-0.01739	0.162745	0.086921	DHX8	ATP-depen	DHX8_HUN	35	0.988017
48H 50MT/	Q99729	0.067297	0.16276	0.086925	HNRNPAB	Heterogene	ROAA_HUN	11	1.047752
24H 10MT/	Q9Y2I7	-0.10819	0.162759	0.086925	PIKFYVE	1-phosphat	FYV1_HUM	16	0.927754
24H 50MT/	Q8WV74	-0.20103	0.162789	0.086936	NUDT8	Mitochond	NUDT8_HU	6	0.86993
24H 10MT/	O14893	0.265767	0.162801	0.086941	GEMIN2	Gem-associ	GEMI2_HUI	6	1.202275
48H 50MT/	P06753	-0.02277	0.162816	0.086948	TPM3	Tropomyos	TPM3_HUN	14	0.984338
24H 10MT/	Q6ZSZ5	0.142756	0.162848	0.086961	ARHGEF18	Rho guanin	ARHGI_HUI	15	1.104012
48H 10MT/	O15235	-0.0091	0.162868	0.086966	MRPS12	Small ribos	RT12_HUM	5	0.993709
48H 10MT/	P15374	0.041116	0.162865	0.086966	UCHL3	Ubiquitin c	UCHL3_HU	11	1.028909
48H 50MT/	Q86X95	-0.34107	0.162867	0.086966	CIR1	Corepresso	CIR1_HUM/	1	0.789454
24H 10MT/	P09382	-0.058	0.162887	0.08697	LGALS1	Galectin-1	LEG1_HUM	9	0.960594
48H 10MT/	Q5VTB9	0.017536	0.1629	0.086974	RNF220	E3 ubiquiti	RN220_HU	5	1.01223
24H 10MT/	Q93074	0.041983	0.162912	0.086979	MED12	Mediator o	MED12_HU	19	1.029528
48H 50MT/	O95625	-0.27845	0.162993	0.087016	ZBTB11	Zinc finger	ZBT11_HUN	4	0.824474
24H 50MT/	Q96GQ5	0.118289	0.162993	0.087016	RUSF1	RUS family	RUSF1_HUI	7	1.085447
24H 50MT/	P01008	-0.69275	0.163	0.087017	SERPINC1	Antithroml	ANT3_HUM	6	0.618676
24H 50MT/	Q9P0M2	0.286313	0.163077	0.087052	AKAP7	A-kinase an	AKA7G_HU	3	1.21952
48H 10MT/	Q5D862	0.458971	0.163126	0.087075	FLG2	Filaggrin-2	FILA2_HUN	6	1.374561
24H 50MT/	Q12800	0.003017	0.163152	0.087082	TFCP2	Alpha-globi	TFCP2_HUI	9	1.002093
48H 50MT/	Q8N8A2	-0.21654	0.163181	0.08709	ANKRD44	Serine/thre	ANR44_HU	5	0.860624
48H 10MT/	Q9NW68	0.142444	0.163212	0.087102	BSDC1	BSD domai	BSDC1_HUI	3	1.103773

24H 10MT/	Q14764	0.015488	0.163268	0.087131	MVP	Major vault	MVP_HUM/	44	1.010794
24H 50MT/	O95486	-0.02662	0.163282	0.087133	SEC24A	Protein trai	SC24A_HUI	27	0.98172
48H 50MT/	Q8WW33	-0.05879	0.16328	0.087133	GTSF1	Gametocyt	GTSF1_HUI	5	0.960068
24H 10MT/	P02788	0.464891	0.16331	0.087144	LTF	Lactotransf	TRFL_HUM/	2	1.380213
48H 10MT/	Q9UBC3	0.10331	0.163311	0.087144	DNMT3B	DNA (cytosi	DNM3B_HU	1	1.074235
48H 10MT/	P39210	-0.15071	0.163341	0.087159	MPV17	Protein Mp	MPV17_HU	1	0.900805
24H 10MT/	O15269	0.165889	0.16339	0.087181	SPTLC1	Serine palm	SPTC1_HUI	10	1.121857
24H 50MT/	Q9BX95	-0.09777	0.163411	0.087189	SGPP1	Sphingosin	SGPP1_HUI	1	0.934479
48H 10MT/	Q16864	-0.36334	0.163423	0.087192	ATP6V1F	V-type prot	VATF_HUM	3	0.777365
48H 50MT/	A0AV96	-0.13556	0.163462	0.087207	RBM47	RNA-bindir	RBM47_HU	16	0.910317
24H 10MT/	Q9BX40	0.071615	0.163461	0.087207	LSM14B	Protein LSI	LS14B_HUI	13	1.050892
24H 10MT/	Q9ULK4	0.033687	0.163481	0.087215	MED23	Mediator o	MED23_HU	28	1.023625
48H 50MT/	Q6MZT1	0.267892	0.163485	0.087216	RGS7BP	Regulator c	R7BP_HUM	1	1.204047
48H 50MT/	Q9UNW1	-0.01862	0.163542	0.08724	MINPP1	Multiple in	MINP1_HU	11	0.987178
48H 50MT/	Q92771	-0.01051	0.163556	0.087245	DDX12P	Putative AT	DDX12_HU	8	0.99274
24H 10MT/	Q9Y4E1	-0.01069	0.163587	0.087259	WASHC2C	WASH com	WAC2C_HU	1	0.992619
48H 50MT/	O43752	0.098451	0.163596	0.087259	STX6	Syntaxin-6	STX6_HUM	7	1.070624
48H 10MT/	Q15334	-0.09781	0.163606	0.08726	LLGL1	Lethal(2) gi	L2GL1_HUI	8	0.934452
48H 10MT/	Q15398	0.090834	0.163605	0.08726	DLGAP5	Disks large-	DLGP5_HU	16	1.064986
48H 50MT/	Q15397	-0.15027	0.163648	0.087278	PUM3	Pumilio ho	PUM3_HUI	24	0.901081
48H 10MT/	O43741	0.061038	0.163653	0.087279	PRKAB2	5'-AMP-acti	AAKB2_HUI	4	1.043216
24H 10MT/	P48051;P4	1.26546	0.163668	0.087285	KCNJ6;KCN	G protein-a	KCNJ6_HUI	1	2.404039
24H 10MT/	Q9UGM6	0.016366	0.163676	0.087287	WARS2	Tryptophar	SYWM_HUI	8	1.011409
24H 50MT/	Q96FF9	-0.19046	0.163682	0.087289	CDCA5	Sororin	CDCA5_HU	5	0.876328
24H 10MT/	Q86UX2	0.800963	0.163699	0.087295	ITIH5	Inter-alpha	ITIH5_HUM	1	1.742263
24H 10MT/	Q9BRP8	-0.08173	0.163728	0.087307	PYM1	Partner of \	PYM1_HUM	9	0.944924
48H 10MT/	O75676	0.0698	0.163751	0.087314	RPS6KA4	Ribosomal	KS6A4_HUI	9	1.049571
48H 50MT/	P54646	0.027905	0.16375	0.087314	PRKAA2	5'-AMP-acti	AAPK2_HUI	11	1.01953
24H 50MT/	P62913	-0.05681	0.163824	0.087347	RPL11	Large ribos	RL11_HUM	10	0.961391
48H 10MT/	Q92604	0.023056	0.163823	0.087347	LPGAT1	Acyl-CoA:ly	LGAT1_HUI	12	1.016109
48H 50MT/	Q9C0C7	-0.17003	0.163831	0.087349	AMBRA1	Activating r	AMRA1_HU	9	0.888826
48H 10MT/	Q9NZL4	0.084022	0.163863	0.087363	HSPBP1	Hsp70-binc	HPBP1_HU	16	1.059969
48H 50MT/	Q9NX05	-0.20198	0.163867	0.087364	FAM120C	Constitutiv	F120C_HUI	3	0.869356
24H 10MT/	Q58WW2	0.040787	0.163915	0.087381	DCAF6	DDB1- and	DCAF6_HUI	14	1.028675
24H 10MT/	O00311	0.264251	0.163927	0.087385	CDC7	Cell divisio	CDC7_HUM	1	1.201012
48H 10MT/	Q13308	0.226112	0.163939	0.087389	PTK7	Inactive tyr	PTK7_HUM	9	1.169678
24H 10MT/	Q9H7D7	0.043141	0.164008	0.087416	WDR26	WD repeat-	WDR26_HU	15	1.030354
24H 50MT/	Q69YH5	-0.30753	0.16402	0.087421	CDCA2	Cell divisio	CDCA2_HU	2	0.808023
48H 10MT/	P49642	0.014846	0.164026	0.087422	PRIM1	DNA prima	PRI1_HUM/	18	1.010343
24H 10MT/	Q13459	-0.07047	0.164069	0.087439	MYO9B	Unconvent	MYO9B_HU	10	0.95233
24H 50MT/	Q96HD1	0.153284	0.164092	0.087449	CRELD1	Protein dis	CREL1_HUI	5	1.112098
24H 50MT/	Q9BPX6	0.190292	0.164108	0.087455	MICU1	Calcium up	MICU1_HU	8	1.140995
24H 10MT/	P00813	0.009472	0.164143	0.087471	ADA	Adenosine	ADA_HUM/	14	1.006587
24H 10MT/	P29353	-0.0092	0.164145	0.087471	SHC1	SHC-transfc	SHC1_HUM	13	0.993645
48H 10MT/	P47897	-0.04996	0.164158	0.087476	QARS1	Glutamine-	SYQ_HUMA	41	0.965961
24H 50MT/	Q14119	-0.70387	0.164191	0.087492	VEZF1	Vascular en	VEZF1_HUI	4	0.613923
48H 10MT/	Q68CP9	-0.10648	0.16421	0.087499	ARID2	AT-rich inte	ARID2_HUI	10	0.928851
48H 50MT/	Q8TEB9	-0.14218	0.164255	0.087522	RHBDD1	Rhomboid-	RHBL4_HUI	4	0.906151
48H 50MT/	O95214	-0.23227	0.164257	0.087522	LEPROTL1	Leptin rece	LERL1_HUI	1	0.851297
48H 10MT/	Q9NZB2	0.051117	0.164278	0.08753	FAM120A	Constitutiv	F120A_HUI	31	1.036067
24H 50MT/	Q13546	-0.1947	0.164288	0.087533	RIPK1	Receptor-ir	RIPK1_HUI	20	0.873756
24H 10MT/	Q15075	-0.04413	0.164298	0.087537	EEA1	Early endos	EEA1_HUM	63	0.969877

24H 10MT/	Q9HB21	0.021474	0.16432	0.087545	PLEKHA1	Pleckstrin h	PKHA1_HU	10	1.014996
48H 50MT/	Q13643	0.062617	0.164343	0.087556	FHL3	Four and a h	FHL3_HUM	3	1.044359
48H 10MT/	Q9BTM9	0.280229	0.164363	0.087564	URM1	Ubiquitin-r	URM1_HUN	1	1.214387
24H 10MT/	P37173	0.061585	0.164428	0.08759	TGFBR2	TGF-beta re	TGFR2_HUI	12	1.043612
48H 50MT/	Q86TI2	-0.00324	0.164428	0.08759	DPP9	Dipeptidyl	DPP9_HUM	32	0.997757
24H 10MT/	Q5VWJ9	-0.51804	0.164435	0.087592	SNX30	Sorting nex	SNX30_HUI	3	0.698318
24H 50MT/	A9UHW6	-0.42168	0.164446	0.087595	MIF4GD	MIF4G dom	MI4GD_HU	1	0.746553
48H 10MT/	O95747	-0.00016	0.164471	0.087605	OXSR1	Serine/thre	OXSR1_HUI	16	0.999892
24H 10MT/	O95297	0.051002	0.164501	0.087619	MPZL1	Myelin prot	MPZL1_HU	4	1.035984
48H 10MT/	O95696	-0.5137	0.164569	0.087645	BRD1	Bromodom	BRD1_HUM	2	0.700425
24H 10MT/	Q15036	0.073897	0.164575	0.087646	SNX17	Sorting nex	SNX17_HUI	17	1.052556
48H 10MT/	Q9UIF9	0.104764	0.164582	0.087647	BAZ2A	Bromodom	BAZ2A_HUI	26	1.075319
24H 10MT/	P53778	-0.23432	0.164605	0.087656	MAPK12	Mitogen-ac	MK12_HUM	5	0.850085
48H 50MT/	P52630	-0.01911	0.164636	0.087671	STAT2	Signal trans	STAT2_HUM	7	0.986844
48H 10MT/	Q13637	-0.04315	0.164693	0.087697	RAB32	Ras-related	RAB32_HUI	13	0.97053
48H 10MT/	Q05397	-0.01632	0.16471	0.087703	PTK2	Focal adhes	FAK1_HUM	20	0.988753
48H 10MT/	Q14642	-0.30618	0.164716	0.087705	INPP5A	Inositol pol	I5P1_HUM/	6	0.808782
24H 10MT/	O60513	-0.51299	0.164739	0.087712	B4GALT4	Beta-1,4-ga	B4GT4_HUI	2	0.700768
24H 10MT/	A6NIZ1	0.688505	0.164776	0.08773	RAP1BL	Ras-related	RP1BL_HUI	1	1.611613
48H 50MT/	Q86V88	1.139283	0.164782	0.087732	MDP1	Magnesium	MGDP1_HU	2	2.202715
24H 10MT/	Q9BVX2	0.143164	0.16479	0.087734	TMEM106C	Transmemb	T106C_HUI	1	1.104325
48H 10MT/	O75446	-0.09723	0.164794	0.087734	SAP30	Histone dea	SAP30_HUI	3	0.934827
24H 10MT/	O75884	-0.17864	0.164806	0.087739	RBBP9	Serine hydr	RBBP9_HUI	8	0.883535
48H 50MT/	Q6PIJ6	-0.03169	0.164818	0.087744	FBXO38	F-box only p	FBX38_HUI	3	0.978273
24H 10MT/	Q8IYU2	-0.43827	0.164853	0.087758	HACE1	E3 ubiquiti	HACE1_HUI	2	0.73802
48H 10MT/	Q07889	-0.04916	0.164862	0.087761	SOS1	Son of sever	SOS1_HUM	15	0.966498
24H 50MT/	P34932	-0.04776	0.16487	0.087763	HSPA4	Heat shock	HSP74_HUI	47	0.96744
48H 50MT/	P0CG38	-0.03533	0.16489	0.087771	POTEI	POTE ankyr	POTEI_HUM	3	0.975807
48H 10MT/	Q3MHD2	-0.04942	0.164892	0.087771	LSM12	Protein LSM	LSM12_HU	5	0.966323
24H 10MT/	O75362	0.018947	0.164922	0.087782	ZNF217	Zinc finger p	ZN217_HUI	8	1.01322
24H 10MT/	P11171	-0.0093	0.164947	0.087792	EPB41	Protein 4.1	EPB41_HUI	7	0.993578
48H 50MT/	P19793	-0.05073	0.165061	0.087845	RXRA	Retinoic ac	RXRA_HUM	6	0.965445
48H 10MT/	Q8NC51	0.028009	0.165084	0.087851	SERBP1	SERPINE1 m	SERB1_HUN	21	1.019604
24H 50MT/	O60566	-0.14263	0.165098	0.087855	BUB1B	Mitotic che	BUB1B_HU	25	0.905866
48H 50MT/	Q9C0K7	0.419131	0.165165	0.087889	STRADB	STE20-relat	STRAB_HUN	1	1.337122
24H 50MT/	O60547	0.020894	0.165176	0.087893	GMDS	GDP-mann	GMDS_HUN	13	1.014588
48H 10MT/	Q14686	0.031338	0.165186	0.087898	NCOA6	Nuclear rec	NCOA6_HU	1	1.021959
24H 10MT/	Q5JPE7	0.04899	0.165191	0.087899	NOMO2	BOS compl	NOMO2_HU	44	1.034541
24H 10MT/	Q9Y3A3	-0.01186	0.165213	0.087908	MOB4	MOB-like p	PHOCN_HU	6	0.991813
48H 10MT/	Q9NR46	-0.06474	0.165217	0.087908	SH3GLB2	Endophilin	SHLB2_HUI	11	0.956116
24H 10MT/	Q96A73	-0.14118	0.165267	0.08793	KIAA1191	Putative m	P33MX_HU	3	0.906779
24H 50MT/	Q8N8Y2	0.319046	0.165273	0.087932	ATP6V0D2	V-type prot	VA0D2_HU	1	1.247506
48H 10MT/	Q9UKL6	0.494423	0.165297	0.087941	PCTP	Phosphatid	PPCT_HUM	2	1.408757
24H 50MT/	Q5U4P2	-0.27201	0.165313	0.087948	ASPHD1	Aspartate b	ASPH1_HUI	3	0.828167
24H 10MT/	Q9UBW8	0.004118	0.16532	0.087948	COPS7A	COP9 signa	CSN7A_HUI	10	1.002858
24H 50MT/	P80217	-0.13589	0.165329	0.087952	IFI35	Interferon-i	IN35_HUM	6	0.910111
24H 50MT/	Q9UPZ3	-0.27217	0.165346	0.087956	HPS5	BLOC-2 con	HPS5_HUM	8	0.828073
24H 10MT/	P29320	0.260161	0.16546	0.088009	EPHA3	Ephrin type	EPHA3_HUI	1	1.197613
24H 10MT/	Q96FS4	0.040037	0.165488	0.088021	SIPA1	Signal-indu	SIPA1_HUN	15	1.02814
24H 10MT/	A0AVI4	-0.82782	0.165516	0.088031	TMEM129	E3 ubiquiti	TM129_HU	1	0.563379
24H 10MT/	Q9H0H5	0.139548	0.165515	0.088031	RACGAP1	Rac GTPase	RGAP1_HU	16	1.10156
48H 50MT/	Q14686	-0.19857	0.165529	0.088036	NCOA6	Nuclear rec	NCOA6_HU	1	0.871415

48H 50MT/	P28799	0.008865	0.165553	0.088045	GRN	Progranulir	GRN_HUM/	9	1.006164
24H 50MT/	P55081	0.101021	0.165557	0.088045	MFAP1	Microfibrill	MFAP1_HU	11	1.072532
48H 10MT/	P56211	0.12741	0.165557	0.088045	ARPP19	cAMP-regul	ARP19_HUI	6	1.092331
24H 50MT/	P08047	-0.00232	0.165584	0.088057	SP1	Transcripti	SP1_HUMA	3	0.998394
24H 50MT/	Q9C0C2	0.0222	0.165587	0.088057	TNKS1BP1	182 kDa tar	TB182_HUI	35	1.015507
48H 10MT/	Q8TDY2	-0.02322	0.165659	0.088085	RB1CC1	RB1-induci	RBCC1_HUI	23	0.984037
24H 50MT/	Q9H446	-0.01179	0.165707	0.088106	RWDD1	RWD doma	RWDD1_HU	4	0.991859
48H 50MT/	Q8TCA0	0.133368	0.165744	0.088122	LRRC20	Leucine-ric	LRC20_HUI	10	1.096851
48H 50MT/	O75970	0.034924	0.165808	0.088149	MPDZ	Multiple PI	MPDZ_HUN	13	1.024503
48H 10MT/	P60842	0.030618	0.165808	0.088149	EIF4A1	Eukaryotic	IF4A1_HUN	30	1.02145
48H 10MT/	Q8N668	-0.02855	0.165809	0.088149	COMMD1	COMM don	COMD1_HU	4	0.980405
48H 10MT/	Q8NHQ9	-0.07046	0.165802	0.088149	DDX55	ATP-depen	DDX55_HU	16	0.952333
48H 50MT/	Q96I15	0.0764	0.165802	0.088149	SCLY	Selenocyst	SCLY_HUM/	18	1.054383
48H 10MT/	Q96CV9	0.015514	0.165857	0.08817	OPTN	Optineurin	OPTN_HUN	13	1.010811
24H 10MT/	Q9Y2I8	-0.30081	0.165866	0.088173	WDR37	WD repeat-	WDR37_HU	6	0.811799
48H 10MT/	O96011	-0.19646	0.165879	0.088179	PEX11B	Peroxisom	PX11B_HUI	9	0.872692
24H 50MT/	Q5VST6	0.122256	0.165904	0.088189	ABHD17B	Alpha/beta	AB17B_HUI	2	1.088435
48H 50MT/	Q9HB58	-0.7442	0.165926	0.088197	SP110	Sp110 nucl	SP110_HUI	2	0.596997
24H 10MT/	P49711	-0.09053	0.166028	0.088239	CTCF	Transcripti	CTCF_HUM	14	0.939174
24H 50MT/	P14550	-0.09255	0.166087	0.088266	AKR1A1	Aldo-keto r	AK1A1_HUI	19	0.937864
24H 10MT/	Q9H0K6	0.105216	0.166088	0.088266	PUS7L	Pseudouric	PUS7L_HUI	6	1.075655
48H 50MT/	O14521	0.291607	0.166132	0.088285	SDHD	Succinate c	DHSD_HUN	2	1.224003
48H 10MT/	P55769	0.02128	0.166133	0.088285	SNU13	NHP2-like p	NH2L1_HU	9	1.014859
48H 10MT/	P15735	-0.06901	0.166152	0.088289	PHKG2	Phosphoryl	PHKG2_HU	1	0.953293
48H 10MT/	P20290	-0.03014	0.166154	0.088289	BTF3	Transcripti	BTF3_HUM	11	0.979324
48H 10MT/	Q9Y314	0.047692	0.166169	0.088295	NOSIP	Nitric oxid	NOSIP_HUN	12	1.03361
24H 50MT/	O94927	-0.15459	0.166185	0.088299	HAUS5	HAUS augm	HAUS5_HU	10	0.898388
24H 50MT/	Q7L5N1	-0.01787	0.166197	0.088304	COPS6	COP9 signa	CSN6_HUN	13	0.98769
48H 10MT/	Q5T1C6	0.087104	0.166217	0.088311	THEM4	Acyl-coenz	THEM4_HU	4	1.062236
48H 10MT/	O14672	0.009165	0.166255	0.088328	ADAM10	Disintegrin	ADA10_HU	22	1.006373
24H 50MT/	Q15642	-0.01563	0.166271	0.088333	TRIP10	Cdc42-inte	CIP4_HUM/	12	0.989227
48H 10MT/	P16333	-0.01322	0.166317	0.08835	NCK1	SH2/SH3 ac	NCK1_HUN	8	0.990878
48H 10MT/	Q96GQ5	-0.00746	0.166336	0.088358	RUSF1	RUS family	RUSF1_HUI	7	0.994843
48H 50MT/	P35244	0.070201	0.16635	0.088362	RPA3	Replicatior	RFA3_HUM	7	1.049863
24H 10MT/	Q9NVV5	0.001405	0.166353	0.088363	AIG1	Androgen-i	AIG1_HUM	3	1.000974
48H 50MT/	O60684	0.058835	0.166376	0.088373	KPNA6	Importin su	IMA7_HUM	10	1.041624
24H 10MT/	Q6PI78	-0.05467	0.166421	0.088385	TMEM65	Transmemb	TMM65_HU	7	0.962813
48H 50MT/	Q9UBC3	0.096683	0.166469	0.088406	DNMT3B	DNA (cytosi	DNM3B_HU	1	1.069312
48H 10MT/	Q9UBW8	-0.03982	0.166467	0.088406	COPS7A	COP9 signa	CSN7A_HUI	10	0.972774
48H 10MT/	Q9H992	-0.53608	0.166472	0.088406	MARCHF7	E3 ubiquiti	MARH7_HU	4	0.689644
24H 10MT/	O75865	0.269626	0.166512	0.088425	TRAPPC6A	Trafficking	TPC6A_HUI	1	1.205495
24H 10MT/	P56557	-0.27115	0.166577	0.088452	TMEM50B	Transmemb	TM50B_HU	1	0.828662
48H 10MT/	Q9Y4X5	0.136264	0.166589	0.088457	ARIH1	E3 ubiquiti	ARI1_HUM/	12	1.099056
48H 10MT/	Q9BXW6	0.070359	0.166608	0.088463	OSBPL1A	Oxysterol-b	OSBL1_HUI	7	1.049978
24H 10MT/	Q96EB6	-0.0409	0.166639	0.088477	SIRT1	NAD-depen	SIR1_HUM/	8	0.972051
48H 10MT/	Q15907	-0.11262	0.166817	0.088557	RAB11B	Ras-related	RB11B_HUI	2	0.924904
48H 10MT/	Q96MW5	-0.09892	0.16684	0.088567	COG8	Conserved	COG8_HUN	18	0.933732
24H 10MT/	Q7Z478	0.045252	0.166869	0.088581	DHX29	ATP-depen	DHX29_HU	37	1.031863
24H 10MT/	P16383	-0.00287	0.166887	0.088589	GCFC2	Intron Larg	GCFC2_HUI	15	0.998013
48H 10MT/	O43149	-0.01872	0.166917	0.088596	ZZEF1	Zinc finger	ZZEF1_HUN	18	0.987107
24H 50MT/	Q9NQ35	0.688044	0.166914	0.088596	NRIP3	Nuclear rec	NRIP3_HUN	1	1.611097
48H 10MT/	Q96M89	-0.18805	0.16693	0.088601	CCDC138	Coiled-coil	CC138_HUI	4	0.877792

24H 50MT/	P10746	0.107526	0.166951	0.088608	UROS	Uroporphy	HEM4_HUN	5	1.077379
24H 50MT/	Q8N122	-0.08584	0.166973	0.088618	RPTOR	Regulatory	RPTOR_HUI	16	0.942238
48H 10MT/	Q86UU1	0.028197	0.167027	0.088643	PHLDB1	Pleckstrin I	PHLB1_HUI	12	1.019737
24H 10MT/	Q16656	0.037196	0.167054	0.088654	NRF1	Nuclear res	NRF1_HUM	3	1.026118
48H 50MT/	P07902	0.22339	0.167083	0.088661	GALT	Galactose-1	GALT_HUM	8	1.167473
24H 10MT/	P04062	0.019274	0.1671	0.088665	GBA1	Lysosomal	GBA1_HUM	16	1.013449
24H 50MT/	Q9H6D7	-0.18066	0.167123	0.088675	HAUS4	HAUS augm	HAUS4_HU	10	0.882302
24H 50MT/	Q8N5C8	-0.9518	0.167174	0.088699	TAB3	TGF-beta-ac	TAB3_HUM	2	0.516986
48H 50MT/	O96028	-0.1724	0.1672	0.088709	NSD2	Histone-lys	NSD2_HUM	10	0.887368
48H 50MT/	Q8WXE0	-0.12468	0.167199	0.088709	CASKIN2	Caskin-2	CSKI2_HUN	7	0.917206
48H 10MT/	P05455	-0.01006	0.167214	0.088715	SSB	Lupus La pr	LA_HUMAN	29	0.993052
24H 50MT/	P32456	-0.00439	0.167306	0.088754	GBP2	Guanylate-I	GBP2_HUM	11	0.996964
24H 10MT/	Q9H2J4	0.015731	0.167385	0.088789	PDCL3	Phosducin-	PDCL3_HUI	15	1.010963
48H 10MT/	P49917	0.018978	0.167417	0.0888	LIG4	DNA ligase	DNLI4_HUN	11	1.013241
48H 10MT/	Q5PSV4	-0.37265	0.167417	0.0888	BRMS1L	Breast canc	BRM1L_HU	2	0.772362
24H 10MT/	Q9Y4D1	0.945132	0.167413	0.0888	DAAM1	Disheveled	DAAM1_HU	1	1.925365
48H 10MT/	Q8N357	-0.01138	0.167438	0.088809	SLC35F6	Solute carri	S35F6_HUN	2	0.992146
48H 10MT/	Q9H8T0	-0.12794	0.167449	0.088812	AKTIP	AKT-interac	AKTIP_HUN	6	0.915136
24H 10MT/	Q13867	-0.05099	0.167467	0.088818	BLMH	Bleomycin	BLMH_HUN	14	0.965274
24H 50MT/	Q2M1Z3	-0.84185	0.167508	0.088836	ARHGAP31	Rho GTPase	RHG31_HU	2	0.557926
48H 10MT/	Q8TCT9	-0.1882	0.167534	0.088848	HM13	Minor histc	HM13_HUN	10	0.8777
48H 10MT/	P26045	-0.075	0.167583	0.088867	PTPN3	Tyrosine-pr	PTN3_HUM	7	0.949344
48H 10MT/	P02679	-0.0917	0.167633	0.088886	FGG	Fibrinogen	FIBG_HUM/	20	0.938417
48H 10MT/	P26651	0.328649	0.167724	0.088931	ZFP36	mRNA deca	TTP_HUMA	2	1.255837
48H 50MT/	P48506	0.008892	0.167748	0.088939	GCLC	Glutamate-	GSH1_HUN	23	1.006182
48H 50MT/	O95139	0.007023	0.167798	0.088957	NDUFB6	NADH dehy	NDUB6_HU	3	1.00488
24H 50MT/	Q13485	0.026925	0.16782	0.088963	SMAD4	Mothers ag	SMAD4_HU	10	1.018838
48H 50MT/	P30876	0.000295	0.167825	0.088964	POLR2B	DNA-direct	RPB2_HUM	41	1.000205
48H 50MT/	Q13043	-0.06846	0.167844	0.088973	STK4	Serine/thre	STK4_HUM	22	0.953656
24H 50MT/	Q8WVV4	0.252347	0.167869	0.088981	POF1B	Protein POI	POF1B_HUI	6	1.191144
24H 10MT/	Q99676	-0.5025	0.167875	0.088983	ZNF184	Zinc finger	ZN184_HUI	1	0.705883
24H 10MT/	Q9H4I2	-0.63877	0.167889	0.088988	ZHX3	Zinc fingers	ZHX3_HUM	2	0.642259
24H 10MT/	Q9NQX7	0.145067	0.167893	0.088988	ITM2C	Integral me	ITM2C_HUI	5	1.105782
48H 50MT/	Q9UET6	-0.02032	0.16793	0.089002	FTSJ1	tRNA (cytid	TRM7_HUN	6	0.986013
48H 10MT/	Q8IXJ6	-0.52567	0.168013	0.089041	SIRT2	NAD-depen	SIR2_HUM/	2	0.694636
48H 50MT/	Q9BPX5	-0.05817	0.168048	0.089057	ARPC5L	Actin-relate	ARP5L_HUI	5	0.960479
24H 10MT/	Q9C035	-0.01747	0.168049	0.089057	TRIM5	Tripartite n	TRIM5_HUI	7	0.98796
24H 10MT/	Q8TAD4	-0.00726	0.168146	0.089094	SLC30A5	Proton-cou	ZNT5_HUM	6	0.99498
24H 10MT/	Q15771	-0.77073	0.168171	0.089102	RAB30	Ras-related	RAB30_HUI	1	0.586121
48H 10MT/	Q9H6D7	-0.08597	0.168192	0.089112	HAUS4	HAUS augm	HAUS4_HU	10	0.942153
48H 10MT/	Q9H8V3	-0.08952	0.168213	0.089121	ECT2	Protein EC1	ECT2_HUM	28	0.939835
24H 10MT/	P37837	0.01565	0.168257	0.089141	TALDO1	Transaldola	TALDO_HUI	16	1.010907
24H 10MT/	O14526	1.108448	0.168262	0.089141	FCHO1	F-BAR dom	FCHO1_HU	1	2.156136
24H 10MT/	P16870	-0.73227	0.168271	0.089142	CPE	Carboxypej	CBPE_HUM	3	0.601957
24H 50MT/	Q7Z5J4	-0.34062	0.168271	0.089142	RAI1	Retinoic ac	RAI1_HUM/	4	0.789701
48H 10MT/	Q8IV38	0.122088	0.168304	0.089156	ANKMY2	Ankyrin rep	ANKY2_HUI	6	1.088308
48H 50MT/	Q9Y4D8	0.098045	0.168317	0.08916	HECTD4	Probable E3	HECD4_HU	15	1.070322
48H 50MT/	Q92990	0.005177	0.168332	0.089163	GLMN	Glomulin	GLMN_HUI	20	1.003595
48H 10MT/	Q49B96	-0.05589	0.168368	0.089177	COX19	Cytochrom	COX19_HU	1	0.962004
48H 10MT/	Q53S58	0.174793	0.168386	0.089185	TMEM177	Transmemt	TM177_HU	2	1.128803
24H 50MT/	P50336	0.050195	0.168392	0.089187	PPOX	Protoporpl	PPOX_HUM	12	1.035405
48H 10MT/	Q15797	-0.1028	0.16841	0.089195	SMAD1	Mothers ag	SMAD1_HU	5	0.931222

24H 10MT/	O95834	-0.00343	0.168469	0.08922	EML2	Echinoderm	EMAL2_HU	7	0.997629
24H 10MT/	O43324	0.077832	0.1685	0.089233	EEF1E1	Eukaryotic	MCA3_HUM	10	1.055431
24H 10MT/	Q96ER3	-0.00047	0.16851	0.089235	SAAL1	Protein SA/	SAAL1_HUM	6	0.999676
48H 10MT/	Q92499	-0.01294	0.16862	0.089287	DDX1	ATP-depend	DDX1_HUM	42	0.991073
24H 50MT/	Q04721	0.446625	0.168629	0.089291	NOTCH2	Neurogenic	NOTC2_HU	8	1.362848
24H 50MT/	Q8TEB9	0.049579	0.168634	0.089291	RHBDD1	Rhomboid-	RHBL4_HUI	4	1.034963
48H 50MT/	Q96F10	0.033742	0.168711	0.089327	SAT2	Thialysine N	SAT2_HUM	1	1.023664
48H 50MT/	O14733	-0.10397	0.168723	0.089333	MAP2K7	Dual specifi	MP2K7_HU	11	0.930471
48H 10MT/	Q9NV96	0.079325	0.168813	0.089375	TMEM30A	Cell cycle c	CC50A_HUI	6	1.056524
24H 50MT/	P61225	-0.03803	0.168817	0.089376	RAP2B	Ras-related	RAP2B_HUI	5	0.973987
48H 10MT/	Q02818	0.015015	0.168835	0.089385	NUCB1	Nucleobind	NUCB1_HU	18	1.010462
24H 10MT/	Q86V21	-0.13185	0.168855	0.089393	AACS	Acetoacety	AACS_HUM	15	0.912662
24H 10MT/	P46783	0.014775	0.168969	0.089445	RPS10	Small ribos	RS10_HUM	7	1.010294
48H 10MT/	Q5VTR2	-0.00898	0.168971	0.089445	RNF20	E3 ubiquiti	BRE1A_HUI	30	0.993794
48H 10MT/	Q8NFQ8	-0.08946	0.169022	0.089467	TOR1AIP2	Torsin-1A-i	TOIP2_HUM	15	0.939875
24H 50MT/	O15040	-0.2354	0.169027	0.089468	TECPR2	Tectonin be	TCPR2_HUI	1	0.849447
24H 50MT/	O94907	-0.00317	0.169112	0.089508	DKK1	Dickkopf-re	DKK1_HUM	2	0.997808
24H 50MT/	Q5JTZ9	0.071179	0.169124	0.08951	AARS2	Alanine--tR	SYAM_HUM	29	1.050575
48H 10MT/	Q9HCM9	0.394604	0.169135	0.089515	TRIM39	E3 ubiquiti	TRI39_HUM	1	1.314582
48H 10MT/	P60709	-0.10752	0.169141	0.089517	ACTB	Actin, cyto	ACTB_HUM	18	0.928185
48H 50MT/	Q9H2J7	-0.59416	0.169183	0.089534	SLC6A15	Sodium-de	S6A15_HUI	1	0.662431
48H 10MT/	Q9NQH7	-0.02427	0.169185	0.089534	XPNPEP3	Xaa-Pro am	XPP3_HUM	12	0.983317
24H 10MT/	Q9H9S3	0.081992	0.169242	0.08956	SEC61A2	Protein tra	S61A2_HUI	7	1.058479
24H 10MT/	Q969T9	-0.20285	0.169252	0.089563	WBP2	WW domai	WBP2_HUM	6	0.86883
48H 50MT/	P0DI83	-0.28913	0.169275	0.08957	RAB34	Ras-related	NARR_HUM	3	0.818398
24H 50MT/	Q16576	0.01237	0.169278	0.08957	RBBP7	Histone-bir	RBBP7_HUI	12	1.008611
24H 10MT/	P38398	0.449444	0.169313	0.089584	BRCA1	Breast canc	BRCA1_HUI	3	1.365514
24H 10MT/	Q9UKX3	0.480881	0.169314	0.089584	MYH13	Myosin-13	MYH13_HU	1	1.395596
48H 50MT/	Q3MIT2	-0.32768	0.16936	0.089603	PUS10	tRNA pseud	PUS10_HUI	4	0.796818
24H 10MT/	Q14690	0.010974	0.169375	0.089609	PDCD11	Protein RRI	RRP5_HUM	86	1.007636
48H 50MT/	Q9NRK6	-0.06849	0.169485	0.089659	ABCB10	ATP-bindin	ABCBA_HUI	15	0.953633
48H 10MT/	O95757	0.020668	0.169509	0.089666	HSPA4L	Heat shock	HS74L_HUI	36	1.014429
48H 10MT/	O14639	0.018928	0.169547	0.089683	ABLIM1	Actin-bindi	ABLM1_HU	21	1.013206
48H 10MT/	Q8N0U8	0.016165	0.169545	0.089683	VKORC1L1	Vitamin K e	VKORL_HUI	5	1.011267
48H 10MT/	Q969V3	-0.14693	0.169561	0.089688	NCLN	BOS compl	NCLN_HUM	22	0.903169
24H 10MT/	Q08378	0.006898	0.169604	0.089704	GOLGA3	Golgin subf	GOGA3_HU	46	1.004793
24H 50MT/	Q9NS91	0.056137	0.16961	0.089705	RAD18	E3 ubiquiti	RAD18_HU	15	1.039678
24H 50MT/	Q15048	-0.06505	0.169679	0.089736	LRRC14	Leucine-ric	LRC14_HUI	3	0.95591
24H 10MT/	Q8WUM9	-0.1943	0.169689	0.08974	SLC20A1	Sodium-de	S20A1_HUI	1	0.873998
48H 10MT/	P10586	0.1707	0.169702	0.089746	PTPRF	Receptor-ty	PTPRF_HUM	25	1.125604
24H 10MT/	Q6P1L5	0.034801	0.169737	0.089761	FAM117B	Protein FAM	F117B_HUI	3	1.024416
24H 50MT/	Q8IYT4	0.047194	0.169747	0.089764	KATNAL2	Katanin p6	KATL2_HUM	1	1.033254
24H 50MT/	O95067	0.551997	0.169784	0.089781	CCNB2	G2/mitotic	CCNB2_HU	5	1.466114
48H 50MT/	Q15654	-0.26076	0.169787	0.089782	TRIP6	Thyroid rec	TRIP6_HUM	8	0.83465
48H 10MT/	O00400	-0.23245	0.169814	0.089793	SLC33A1	Acetyl-coer	ACATN_HUI	12	0.851187
48H 10MT/	Q14697	-0.08873	0.169817	0.089793	GANAB	Neutral alp	GANAB_HU	50	0.94035
48H 50MT/	Q01459	0.174739	0.169838	0.0898	CTBS	Di-N-acetyl	DIAC_HUM	7	1.12876
24H 50MT/	O60610	-0.00388	0.169898	0.089821	DIAPH1	Protein dia	DIAP1_HUM	44	0.997316
24H 50MT/	O60930	-0.08248	0.169905	0.089823	RNASEH1	Ribonuclea	RNH1_HUM	7	0.944432
24H 50MT/	Q8N9N2	0.105035	0.169939	0.08983	ASCC1	Activating s	ASCC1_HUI	8	1.07552
24H 50MT/	O60337	0.232997	0.169972	0.089843	MARCHF6	E3 ubiquiti	MARH6_HU	1	1.175274
24H 10MT/	A2RTX5	0.093265	0.170034	0.089872	TARS3	Threonine-	SYTC2_HUM	9	1.066781

24H 10MT/	Q9Y512	0.09398	0.170098	0.089898	SAMM50	Sorting and	SAM50_HU	23	1.06731
24H 10MT/	Q14728	-0.28818	0.170129	0.089912	MFSD10	Major facili	MFS10_HU	3	0.818937
24H 50MT/	O15164	0.053092	0.170166	0.089929	TRIM24	Transcripti	TIF1A_HUM	16	1.037486
24H 10MT/	Q5T8I3	-0.02682	0.170178	0.089933	EEIG2	EEIG family	EEIG2_HUM	11	0.981584
24H 10MT/	Q6ZU35	0.294172	0.170193	0.08994	CRACD	Capping pr	CRACD_HU	2	1.226181
24H 10MT/	P20337	0.073645	0.170244	0.089963	RAB3B	Ras-related	RAB3B_HU	3	1.052372
24H 50MT/	Q9H993	-0.10735	0.170251	0.089965	ARMT1	Damage-co	ARMT1_HU	16	0.928293
48H 50MT/	Q9Y5A7	0.0509	0.170366	0.090016	NUB1	NEDD8 ulti	NUB1_HUM	8	1.035911
48H 10MT/	Q9NYK5	0.015451	0.170424	0.090039	MRPL39	Large ribos	RM39_HUM	17	1.010768
24H 50MT/	Q6PKG0	-0.09766	0.170472	0.090059	LARP1	La-related p	LARP1_HUI	37	0.934549
24H 10MT/	Q9H446	0.029809	0.170472	0.090059	RWDD1	RWD doma	RWDD1_HU	4	1.020877
24H 10MT/	Q9NZL9	-0.02073	0.170507	0.090073	MAT2B	Methionine	MAT2B_HU	20	0.985731
48H 10MT/	P35226	-0.05271	0.170575	0.090101	BMI1	Polycomb c	BMI1_HUM	4	0.964125
48H 10MT/	A0A096LPC	0.085853	0.170604	0.090111	SMIM26	Small integ	SIM26_HUI	1	1.061315
48H 10MT/	Q16540	-0.14185	0.170607	0.090111	MRPL23	Large ribos	RM23_HUM	9	0.906353
48H 50MT/	Q7Z5R6	-0.31734	0.170605	0.090111	APBB1IP	Amyloid be	AB1IP_HUM	1	0.802548
48H 50MT/	Q04828	0.070112	0.170621	0.090115	AKR1C1	Aldo-keto r	AK1C1_HUI	21	1.049798
48H 50MT/	Q6Y7W6	-0.09939	0.170627	0.090117	GIGYF2	GRB10-inte	GGYF2_HUI	31	0.933425
24H 50MT/	Q6PF05	0.507983	0.17063	0.090118	TTC23L	Tetratricop	TT23L_HUM	1	1.42206
48H 50MT/	Q9H270	-0.03161	0.170642	0.09012	VPS11	Vacuolar pr	VPS11_HUI	28	0.978331
48H 50MT/	O14734	0.120225	0.1707	0.090137	ACOT8	Acyl-coenzy	ACOT8_HUI	6	1.086904
48H 50MT/	P79522	0.410168	0.1707	0.090137	PRR3	Proline-rich	PRR3_HUM	2	1.328841
48H 10MT/	Q13107	-0.01569	0.170713	0.090141	USP4	Ubiquitin c	UBP4_HUM	9	0.989181
24H 50MT/	Q92499	-0.02233	0.170772	0.090164	DDX1	ATP-depend	DDX1_HUM	42	0.984644
48H 10MT/	O14979	-0.03041	0.170784	0.090168	HNRNPDL	Heterogene	HNRDL_HU	14	0.979141
48H 10MT/	Q8NHG7	-0.62864	0.170828	0.090189	SVIP	Small VCP/	SVIP_HUM/	1	0.646784
48H 50MT/	P12236	-0.1324	0.17093	0.090234	SLC25A6	ADP/ATP tr	ADT3_HUM	4	0.912309
24H 50MT/	Q658P3	-0.23694	0.170942	0.090237	STEAP3	Metallored	STEA3_HUM	5	0.848546
24H 10MT/	Q7L1V2	-0.03127	0.17096	0.090244	MON1B	Vacuolar fu	MON1B_HU	4	0.978555
48H 10MT/	Q9UQE7	0.002506	0.171106	0.090304	SMC3	Structural r	SMC3_HUM	49	1.001738
24H 10MT/	P78395	-0.32178	0.171129	0.090311	PRAME	Melanoma	PRAME_HU	2	0.800081
48H 10MT/	Q10472	-0.04133	0.171132	0.090311	GALNT1	Polypeptid	GALT1_HUI	14	0.971758
48H 50MT/	Q9BV73	0.073519	0.171129	0.090311	CEP250	Centrosom	CP250_HUI	18	1.052281
48H 10MT/	Q16270	0.23838	0.171162	0.090322	IGFBP7	Insulin-like	IBP7_HUM/	6	1.179667
24H 10MT/	P30536	0.370361	0.17118	0.090329	TSPO	Translocato	TSPO_HUM	2	1.292676
24H 50MT/	Q9BXR0	-0.14292	0.171215	0.090345	QTRT1	Queuine tR	TGT_HUMA	10	0.905685
48H 50MT/	O43290	-0.00822	0.17125	0.090363	SART1	U4/U6.U5 t	SNUT1_HUI	30	0.99432
48H 10MT/	Q8WUA7	0.047719	0.171286	0.090374	TBC1D22A	TBC1 doma	TB22A_HUI	9	1.033629
48H 10MT/	O94851	-0.182	0.171294	0.090377	MICAL2	[F-actin]-m	MICA2_HUI	2	0.881479
24H 10MT/	Q9BZF1	-0.06969	0.171328	0.090393	OSBPL8	Oxysterol-b	OSBL8_HUI	17	0.952843
24H 10MT/	A5PLL7	0.061759	0.171343	0.0904	PEDS1	Plasmanyle	PDES1_HUI	4	1.043737
48H 10MT/	P30046	0.012651	0.171371	0.090412	DDT	D-dopachro	DOPD_HUM	8	1.008808
48H 50MT/	Q92466	0.023625	0.171384	0.090413	DDB2	DNA damag	DDB2_HUM	5	1.01651
24H 10MT/	O60361	-0.55838	0.171417	0.090428	NME2P1	Putative nu	NDK8_HUM	1	0.679064
24H 50MT/	P43357	-0.3152	0.171427	0.09043	MAGEA3	Melanoma-	MAGA3_HU	4	0.803742
48H 10MT/	O14686	0.013054	0.171434	0.090431	KMT2D	Histone-lys	KMT2D_HU	9	1.009089
48H 10MT/	P26639	0.009192	0.171542	0.090476	TARS1	Threonine--	SYTC_HUM/	37	1.006392
24H 50MT/	O95613	-0.07036	0.171566	0.090482	PCNT	Pericentrin	PCNT_HUM	18	0.952397
48H 50MT/	Q07666	-0.02862	0.171664	0.090528	KHDRBS1	KH domain	KHDR1_HU	9	0.980358
48H 10MT/	O43813	-0.04779	0.171692	0.090539	LANCL1	Glutathion	LANC1_HUI	15	0.967418
48H 10MT/	Q14653	0.0646	0.171745	0.090563	IRF3	Interferon r	IRF3_HUM/	9	1.045795
48H 50MT/	Q9BSE4	-0.32875	0.171778	0.090574	HERPUD2	Homocyste	HERP2_HUI	1	0.796224

48H 10MT/	Q5JTH9	-0.06332	0.171789	0.090578	RRP12	RRP12-like	RRP12_HUI	45	0.957057
24H 50MT/	Q6P4A7	-0.2324	0.171793	0.090578	SFXN4	Sideroflexii	SFXN4_HUI	2	0.851215
48H 10MT/	P47985	0.056756	0.171828	0.090591	UQCRFS1	Cytochrom	UCRI_HUM	11	1.040124
48H 10MT/	Q9Y5A7	-0.00939	0.171829	0.090591	NUB1	NEDD8 ulti	NUB1_HUN	8	0.993516
48H 50MT/	O94927	-0.19564	0.171862	0.090606	HAUS5	HAUS augm	HAUS5_HU	10	0.873183
24H 10MT/	Q9NR30	0.010949	0.171873	0.090611	DDX21	Nucleolar F	DDX21_HU	43	1.007618
48H 10MT/	O60826	0.017983	0.171975	0.090653	CCDC22	Coiled-coil	CCD22_HU	22	1.012543
48H 10MT/	Q96DZ1	0.000307	0.172013	0.090669	ERLEC1	Endoplasm	ERLEC_HUN	14	1.000213
24H 10MT/	Q01130	0.036178	0.17205	0.090685	SRSF2	Serine/argi	SRSF2_HUN	5	1.025394
24H 10MT/	Q0VDG4	-0.04306	0.172055	0.090685	SCRN3	Secernin-3	SCRN3_HUI	9	0.970594
24H 10MT/	Q9BX70	-0.25127	0.172057	0.090685	BTBD2	BTB/POZ dc	BTBD2_HUI	3	0.840158
24H 10MT/	Q9GZY4	-0.05582	0.172119	0.090716	COA1	Cytochrom	COA1_HUN	6	0.962046
48H 50MT/	Q03188	-0.39027	0.172135	0.090722	CENPC	Centromer	CENPC_HUI	5	0.762987
24H 50MT/	Q9BVI4	0.071772	0.172143	0.090722	NOC4L	Nucleolar c	NOC4L_HU	23	1.051007
48H 50MT/	Q9H2C2	0.098336	0.172143	0.090722	ARV1	Protein AR\	ARV1_HUM	1	1.070538
24H 10MT/	Q96L58	-0.00989	0.172211	0.09075	B3GALT6	Beta-1,3-ga	B3GT6_HUI	6	0.993165
24H 50MT/	Q13432	0.119067	0.172232	0.090759	UNC119	Protein unc	U119A_HU	3	1.086032
48H 10MT/	Q58DX5	-0.41064	0.172265	0.090772	NAALADL2	Inactive N-a	NADL2_HU	1	0.752288
24H 10MT/	O14562	-0.04303	0.172296	0.090783	UBFD1	Ubiquitin d	UBFD1_HU	7	0.970612
48H 50MT/	P03897	0.098711	0.172312	0.090786	MT-ND3	NADH-ubiq	NU3M_HUI	1	1.070816
48H 10MT/	Q5JTW2	0.067923	0.172371	0.090811	CEP78	Centrosom	CEP78_HUI	2	1.048206
48H 10MT/	O75317	0.409399	0.172393	0.09082	USP12	Ubiquitin c	UBP12_HU	2	1.328132
24H 10MT/	Q96GQ5	0.033449	0.172415	0.090829	RUSF1	RUS family	RUSF1_HUI	7	1.023456
24H 10MT/	Q96KG9	-0.06546	0.17244	0.090838	SCYL1	N-terminal	SCYL1_HUN	26	0.955641
24H 10MT/	Q9BQE4	-0.13872	0.172452	0.090842	SELENOS	Selenoprot	SELS_HUM/	6	0.908326
48H 10MT/	Q6P1X6	0.110411	0.172456	0.090843	C8orf82	UPF0598 p	CH082_HU	5	1.079536
24H 50MT/	P51648	-0.06803	0.172513	0.090864	ALDH3A2	Aldehyde d	AL3A2_HUI	13	0.95394
48H 50MT/	Q9NXG2	-0.03178	0.172514	0.090864	THUMPD1	THUMP doi	THUM1_HU	17	0.978211
24H 10MT/	P22415	0.021865	0.17254	0.090866	USF1	Upstream s	USF1_HUM	1	1.015271
24H 10MT/	Q86WA8	0.029323	0.172539	0.090866	LONP2	Lon protea:	LONP2_HU	28	1.020533
48H 50MT/	Q13247	-0.01243	0.17258	0.090881	SRSF6	Serine/argi	SRSF6_HUN	9	0.991422
48H 50MT/	Q8NCR0	-0.26695	0.172578	0.090881	B3GALNT2	UDP-GalNA	B3GL2_HUI	2	0.831077
48H 10MT/	Q96PY6	0.219257	0.172623	0.090894	NEK1	Serine/thre	NEK1_HUM	2	1.164134
48H 10MT/	O95299	-0.02751	0.172625	0.090894	NDUFA10	NADH dehy	NDUAA_HU	12	0.981112
24H 10MT/	P06400	0.087145	0.172632	0.090897	RB1	Retinoblast	RB_HUMAN	24	1.062266
48H 10MT/	Q7Z2E3	-0.05442	0.172644	0.090902	APTX	Aprataxin	APTX_HUM	7	0.962981
48H 50MT/	P08397	-0.07237	0.172666	0.090912	HMBS	Porphobilii	HEM3_HUN	17	0.951075
24H 50MT/	Q96Q11	0.008774	0.172707	0.090929	TRNT1	CCA tRNA n	TRNT1_HUI	12	1.0061
48H 10MT/	Q8TCE6	-0.06878	0.172729	0.090937	DENND10	DENN dom	DEN10_HU	1	0.953442
24H 10MT/	Q8IVP5	-0.33956	0.172732	0.090938	FUNDC1	FUN14 don	FUND1_HU	1	0.790281
48H 50MT/	Q9NUI1	0.15078	0.17277	0.090952	DECR2	Peroxisom	DECR2_HUI	5	1.110169
24H 10MT/	Q15437	-0.06442	0.172816	0.090968	SEC23B	Protein tra	SC23B_HUI	29	0.956329
48H 10MT/	Q96A73	0.145778	0.172846	0.090981	KIAA1191	Putative m	P33MX_HU	3	1.106327
48H 10MT/	Q9BW27	0.034705	0.172857	0.090984	NUP85	Nuclear po	NUP85_HU	23	1.024347
48H 10MT/	Q15006	-0.01461	0.172869	0.090988	EMC2	ER membra	EMC2_HUN	10	0.989921
48H 10MT/	Q8TEQ6	-0.04924	0.172875	0.09099	GEMIN5	Gem-associ	GEMI5_HUI	49	0.966443
24H 10MT/	Q6P6C2	0.105369	0.17288	0.090991	ALKBH5	RNA demet	ALKB5_HUI	12	1.07577
24H 50MT/	P04264	-0.30329	0.17291	0.091004	KRT1	Keratin, typ	K2C1_HUM	38	0.810402
48H 10MT/	Q8NBI6	0.034492	0.172932	0.091013	XXYLT1	Xyloside xy	XXLT1_HUN	8	1.024196
24H 10MT/	Q07617	0.046054	0.172968	0.091026	SPAG1	Sperm-asso	SPAG1_HUI	13	1.032437
48H 10MT/	Q15758	-0.09099	0.173145	0.091097	SLC1A5	Neutral am	AAAT_HUM	11	0.93888
24H 50MT/	Q9Y333	0.028666	0.173287	0.091156	LSM2	U6 snRNA-a	LSM2_HUN	6	1.020068

24H 50MT/	Q9Y664	-0.00471	0.173285	0.091156	KPTN	KICSTOR co	KPTN_HUM	2	0.996742
24H 50MT/	P23526	0.031628	0.173377	0.091193	AHCY	Adenosylhc	SAHH_HUM	25	1.022165
24H 50MT/	Q9UER7	-0.03747	0.173396	0.091201	DAXX	Death dom	DAXX_HUM	7	0.974359
48H 50MT/	Q9BRN9	-0.09811	0.173411	0.091204	TM2D3	TM2 domai	TM2D3_HU	3	0.934258
24H 50MT/	Q9BZ29	0.17846	0.17341	0.091204	DOCK9	Dedicator c	DOCK9_HU	3	1.131675
48H 10MT/	A6NGU5;P1	-0.1764	0.173462	0.091226	GGT3P;GGT	Putative glu	GGT3_HUM	4	0.884908
24H 50MT/	Q5T0N5	-0.0579	0.173508	0.091241	FNBP1L	Formin-bin	FBP1L_HUM	17	0.960659
24H 50MT/	O14641	-0.06497	0.173645	0.091299	DVL2	Segment pc	DVL2_HUM	7	0.955966
24H 50MT/	P68402	0.124455	0.173645	0.091299	PAFAH1B2	Platelet-act	PA1B2_HUI	8	1.090095
24H 10MT/	Q8TB03	0.07952	0.173674	0.091306	CXorf38	Uncharacte	CX038_HUI	3	1.056666
24H 50MT/	Q9NUQ8	-0.10127	0.173682	0.091306	ABCF3	ATP-bindin	ABCF3_HUI	34	0.932213
48H 10MT/	P05023	-0.0546	0.173771	0.091349	ATP1A1	Sodium/po	AT1A1_HUI	41	0.962864
48H 50MT/	Q16401	-0.03336	0.173773	0.091349	PSMD5	26S proteas	PSMD5_HU	24	0.977142
24H 10MT/	Q9GZR2	-0.22412	0.173786	0.091355	REXO4	RNA exonu	REXO4_HUI	13	0.856119
24H 50MT/	Q2PPJ7	0.243562	0.17379	0.091355	RALGAPA2	Ral GTPase-	RGPA2_HU	8	1.183912
48H 10MT/	Q8IVB5	-0.43645	0.173801	0.091356	LIX1L	LIX1-like pr	LIX1L_HUM	1	0.73895
48H 50MT/	P15586	0.071781	0.173841	0.091372	GNS	N-acetylglu	GNS_HUMA	12	1.051013
24H 10MT/	P28072	0.067388	0.173916	0.091406	PSMB6	Proteasom	PSB6_HUM	8	1.047818
48H 10MT/	Q16775	0.017534	0.173941	0.091418	HAGH	Hydroxyacy	GLO2_HUM	8	1.012228
24H 50MT/	Q9NZ43	0.11794	0.173949	0.091421	USE1	Vesicle tran	USE1_HUM	4	1.085184
48H 10MT/	P10253	0.043816	0.173967	0.091425	GAA	Lysosomal a	LYAG_HUM	15	1.030837
48H 50MT/	Q9NS86	-0.07036	0.173965	0.091425	LANCL2	LanC-like pi	LANC2_HUI	14	0.952403
24H 10MT/	Q9UFN0	0.042842	0.173979	0.091428	NIPSNAP3A	Protein Nip	NPS3A_HUI	9	1.030141
24H 10MT/	O43463	-0.06315	0.173982	0.091428	SUV39H1	Histone-lys	SUV91_HUI	2	0.95717
48H 50MT/	Q8N9I0	0.632078	0.173996	0.091431	SYT2	Synaptotag	SYT2_HUMA	1	1.549796
48H 10MT/	Q15286	-0.11722	0.174029	0.091446	RAB35	Ras-related	RAB35_HUI	9	0.921961
48H 10MT/	Q8TAE8	0.06224	0.174056	0.091458	GADD45GII	Large ribos	G45IP_HUM	7	1.044086
48H 50MT/	Q7L2J0	-0.21083	0.174101	0.091477	MEPCE	7SK snRNA	MEPCE_HU	15	0.86404
48H 50MT/	Q8N3D4	-0.01252	0.17412	0.091483	EHBP1L1	EH domain-	EH1L1_HUI	17	0.991358
48H 10MT/	Q9NY93	-0.07631	0.174122	0.091483	DDX56	Probable AT	DDX56_HU	22	0.948479
48H 50MT/	P49006	-0.02226	0.174128	0.091485	MARCKSL1	MARCKS-re	MRP_HUMA	2	0.984687
24H 10MT/	Q9BUB7	0.18189	0.174148	0.09149	TMEM70	Transmemb	TMM70_HU	6	1.134369
24H 50MT/	P19447	0.011754	0.174179	0.091502	ERCC3	General tra	ERCC3_HUI	19	1.008181
24H 10MT/	Q9NYB9	-0.04594	0.17418	0.091502	ABI2	Abl interact	ABI2_HUMA	13	0.968657
24H 10MT/	P53609	0.050551	0.17422	0.091519	PGGT1B	Geranylgera	PGTB1_HUI	5	1.03566
24H 50MT/	Q9UQR0	0.11361	0.174269	0.091538	SCML2	Sex comb o	SCML2_HU	15	1.081932
48H 10MT/	Q9NUW8	-0.02423	0.174277	0.091538	TDP1	Tyrosyl-DNA	TYDP1_HUI	9	0.983345
48H 10MT/	O95429	-0.10071	0.174331	0.091558	BAG4	BAG family	BAG4_HUM	4	0.932571
24H 50MT/	O94806	-0.14059	0.174391	0.091584	PRKD3	Serine/thre	KPCD3_HU	2	0.907148
48H 50MT/	Q12933	-0.07097	0.174389	0.091584	TRAF2	TNF recepto	TRAF2_HUM	17	0.951995
24H 10MT/	Q8IY33	0.432754	0.174409	0.091591	MICALL2	MICAL-like	MILK2_HUI	3	1.349808
48H 10MT/	Q6PRD1	0.196582	0.174531	0.091637	GPR179	Probable G	GP179_HU	1	1.14598
48H 10MT/	P15104	-0.17749	0.174551	0.091644	GLUL	Glutamine	GLNA_HUM	7	0.884241
24H 50MT/	Q96B70	-0.10915	0.174656	0.091684	LENG9	Leukocyte r	LENG9_HUI	2	0.927137
48H 10MT/	P48551	0.52662	0.174665	0.091685	IFNAR2	Interferon a	INAR2_HUM	2	1.440551
48H 10MT/	P53803	0.709398	0.174818	0.091752	POLR2K	DNA-direct	RPAB4_HUI	2	1.635121
48H 10MT/	Q96I51	-0.07001	0.174866	0.091774	RCC1L	RCC1-like G	RCC1L_HUI	13	0.952634
24H 50MT/	Q9UK97	-0.22432	0.174876	0.091777	FBXO9	F-box only p	FBX9_HUM	4	0.855999
24H 10MT/	O60547	-0.06024	0.174884	0.091779	GMDS	GDP-manno	GMDS_HUM	13	0.959105
24H 10MT/	Q9UI12	-0.01255	0.174884	0.091779	ATP6V1H	V-type prot	VATH_HUM	20	0.991337
48H 10MT/	Q15434	-0.01464	0.174891	0.091781	RBMS2	RNA-bindir	RBMS2_HU	4	0.989901
48H 50MT/	O75879	-0.03798	0.174954	0.091809	GATB	Glutamyl-tI	GATB_HUM	15	0.974018

48H 10MT/	Q9UHD8	-0.00417	0.174961	0.091811	SEPTIN9	Septin-9	SEPT9_HUI	28	0.997115
48H 10MT/	Q9H2P9	-0.05488	0.175	0.091828	DPH5	Diphthine i	DPH5_HUI	11	0.962675
48H 50MT/	Q9NUP7	-0.22885	0.175014	0.091833	TRMT13	tRNA:m(4))	TRM13_HU	7	0.853315
48H 10MT/	Q9Y6K0	-0.04243	0.175022	0.091835	CEPT1	Choline/etl	CEPT1_HUI	6	0.971017
24H 50MT/	Q13459	-0.11606	0.175033	0.091838	MYO9B	Unconvent	MYO9B_HU	10	0.922706
48H 50MT/	P61960	0.018967	0.175073	0.091855	UFM1	Ubiquitin-f	UFM1_HUI	5	1.013234
24H 50MT/	Q03518	-0.10203	0.175078	0.091856	TAP1	Antigen pe	TAP1_HUM	8	0.931722
24H 50MT/	P09601	0.141598	0.17512	0.091873	HMOX1	Heme oxyg	HMOX1_HU	10	1.103127
24H 10MT/	Q96B26	0.045515	0.175171	0.091892	EXOSC8	Exosome cc	EXOS8_HUI	9	1.032052
48H 10MT/	O60506	-0.00773	0.175185	0.091895	SYNCRIP	Heterogene	HNRPQ_HU	27	0.994659
24H 10MT/	Q9NVX2	0.045166	0.175185	0.091895	NLE1	Notchless p	NLE1_HUM	15	1.031802
24H 50MT/	P49459	-0.05535	0.175225	0.091912	UBE2A	Ubiquitin-c	UBE2A_HUI	3	0.962359
48H 50MT/	Q9H1H9	-0.21305	0.17524	0.091915	KIF13A	Kinesin-like	KI13A_HUI	4	0.862711
48H 50MT/	Q15075	-0.05583	0.175284	0.091931	EEA1	Early endos	EEA1_HUM	63	0.962042
24H 50MT/	Q9P2E5	-0.20909	0.175284	0.091931	CHPF2	Chondroiti	CHPF2_HUI	1	0.865082
48H 50MT/	P19022	-0.07801	0.175289	0.091933	CDH2	Cadherin-2	CADH2_HU	15	0.947361
24H 10MT/	Q8TE67	-0.13569	0.175291	0.091933	EPS8L3	Epidermal ε	ES8L3_HUI	16	0.910238
24H 10MT/	Q8ND76	0.075486	0.175309	0.09194	CCNY	Cyclin-Y	CCNY_HUM	1	1.053716
24H 10MT/	P50579	0.017933	0.175329	0.091946	METAP2	Methionine	MAP2_HUM	22	1.012508
48H 10MT/	Q7RTP0	-0.00144	0.175359	0.091959	NIPA1	Magnesium	NIPA1_HUI	3	0.999002
24H 50MT/	Q7Z6K5	-0.11803	0.175392	0.091974	ARPIN	Arpin	ARPIN_HUI	5	0.921445
24H 10MT/	P82930	0.04328	0.17545	0.092001	MRPS34	Small ribos	RT34_HUM	12	1.030454
48H 50MT/	Q9Y276	-0.23216	0.175464	0.092002	BCS1L	Mitochond	BCS1_HUM	19	0.85136
48H 10MT/	Q9BSV6	0.079692	0.175472	0.092003	TSEN34	tRNA-splici	SEN34_HUI	7	1.056792
24H 50MT/	Q5BJH7	0.065723	0.175482	0.092005	YIF1B	Protein YIF	YIF1B_HUI	5	1.046609
24H 10MT/	Q15181	-0.06432	0.175531	0.092023	PPA1	Inorganic p	IPYR_HUM/	23	0.956394
24H 10MT/	O94776	0.081333	0.175549	0.09203	MTA2	Metastasis-	MTA2_HUM	24	1.057995
24H 50MT/	Q92503	0.118346	0.175588	0.092047	SEC14L1	SEC14-like	S14L1_HUI	12	1.08549
48H 50MT/	Q8N806	-0.12011	0.175596	0.092049	UBR7	Putative E3	UBR7_HUM	11	0.920118
48H 50MT/	O60333	-0.0273	0.175611	0.092055	KIF1B	Kinesin-like	KIF1B_HUI	29	0.981255
48H 10MT/	P09661	-0.06554	0.175621	0.092056	SNRPA1	U2 small nu	RU2A_HUM	13	0.955584
48H 50MT/	Q8NBT2	-0.32904	0.175663	0.092074	SPC24	Kinetochor	SPC24_HUI	4	0.796064
24H 10MT/	Q9H173	0.091003	0.175689	0.092084	SIL1	Nucleotide	SIL1_HUM/	9	1.065111
48H 50MT/	O60671	-0.26655	0.17573	0.0921	RAD1	Cell cycle cl	RAD1_HUM	7	0.831306
48H 10MT/	O43169	-0.15083	0.175792	0.092122	CYB5B	Cytochrom	CYB5B_HUI	6	0.900731
24H 10MT/	Q14746	-0.02823	0.175855	0.092149	COG2	Conserved	COG2_HUM	25	0.980623
24H 50MT/	Q9Y6I8	-0.11563	0.175847	0.092149	PXMP4	Peroxisoma	PXMP4_HU	5	0.922979
48H 50MT/	Q9P206	-0.0152	0.175876	0.092157	NHSL3	NHS-like pr	NHSL3_HUI	6	0.989519
48H 50MT/	O95707	-0.24116	0.176015	0.092221	POP4	Ribonuclea	RPP29_HUI	6	0.846066
24H 10MT/	P84077	-0.25603	0.176016	0.092221	ARF1	ADP-ribosy	ARF1_HUM	3	0.837389
48H 50MT/	Q6ICB0	-0.40482	0.176025	0.092222	DESI1	Desumoyla	DESI1_HUI	1	0.75533
24H 50MT/	Q9BY50	0.001495	0.17602	0.092222	SEC11C	Signal pept	SC11C_HUI	5	1.001037
24H 10MT/	Q9NWR8	0.028192	0.176023	0.092222	MCUB	Calcium un	MCUB_HUI	4	1.019733
48H 10MT/	P50897	-0.07861	0.176095	0.092252	PPT1	Palmitoyl-p	PPT1_HUM	11	0.946973
48H 50MT/	P61586	-0.11058	0.176148	0.092273	RHOA	Transformi	RHOA_HUI	3	0.926218
24H 50MT/	O75787	0.038712	0.176173	0.092284	ATP6AP2	Renin rece	RENR_HUM	11	1.027197
24H 10MT/	P30038	-0.16341	0.176192	0.092292	ALDH4A1	Delta-1-pyr	AL4A1_HUI	13	0.892911
48H 50MT/	Q96BQ5	0.021559	0.176195	0.092292	CCDC127	Coiled-coil	CC127_HUI	7	1.015056
24H 50MT/	Q15910	0.005963	0.176232	0.092311	EZH2	Histone-lys	EZH2_HUM	10	1.004142
24H 10MT/	Q5M775	0.04041	0.176291	0.092336	SPECC1	Cytospin-B	CYTSB_HUI	17	1.028406
24H 10MT/	Q58DX5	-0.03847	0.176299	0.092339	NAALADL2	Inactive N-a	NADL2_HU	1	0.973687
48H 10MT/	P54098	0.05763	0.17632	0.092345	POLG	DNA polym	DPOG1_HU	17	1.040755

48H	50MT/	Q9NQZ7	-0.0533	0.176328	0.092348	ENTPD7	Ectonucleo	ENTP7_HUI	6	0.963728
24H	10MT/	P05452	0.248951	0.176339	0.092352	CLEC3B	Tetranectir	TETN_HUM	3	1.188342
48H	10MT/	Q5U4P2	0.08473	0.176368	0.092364	ASPHD1	Aspartate b	ASPH1_HUI	3	1.060489
48H	10MT/	P10909	-0.03125	0.176413	0.092386	CLU	Clusterin	CLUS_HUM	5	0.978574
48H	10MT/	P45985	-0.02514	0.176456	0.092402	MAP2K4	Dual specifi	MP2K4_HU	14	0.982723
48H	10MT/	Q9NUJ3	0.03692	0.176461	0.092403	TCP11L1	T-complex		T11L1_HUI	3	1.025921
48H	50MT/	A2RTX5	0.04865	0.176474	0.092405	TARS3	Threonine--	SYTC2_HUI	9	1.034296
48H	50MT/	Q53LP3	-0.12917	0.176503	0.092415	SOWAHC	Ankyrin rep	SWAHC_HU	16	0.914357
24H	10MT/	O95248	-0.06907	0.176545	0.092427	SBF1	Myotubula	MTMR5_HU	16	0.953254
48H	50MT/	P35226	-0.26391	0.176544	0.092427	BMI1	Polycomb (	BMI1_HUM	4	0.83283
24H	10MT/	Q7Z5G4	-0.00167	0.17654	0.092427	GOLGA7	Golgin subf	GOGA7_HU	4	0.998842
24H	10MT/	Q8IWD4	0.118067	0.176545	0.092427	CCDC117	Coiled-coil	CC117_HUI	8	1.085279
24H	50MT/	Q9H1K0	-1.10163	0.176563	0.092435	RBSN	Rabenosyn-	RBNS5_HUI	1	0.465989
24H	10MT/	Q8NBZ0	-0.00711	0.176577	0.092441	INO80E	INO80 com	IN80E_HUN	1	0.995085
48H	10MT/	Q8N9I0	0.684094	0.176625	0.092459	SYT2	Synaptotag	SYT2_HUM/	1	1.606692
24H	10MT/	Q9UJ04	-0.01246	0.176669	0.092478	TSPYL4	Testis-speci	TSYL4_HUN	2	0.9914
48H	10MT/	O95857	-0.16303	0.176708	0.092493	TSPAN13	Tetraspanir	TSN13_HUI	4	0.893145
48H	50MT/	Q9Y4P8	0.072719	0.176706	0.092493	WIPI2	WD repeat	WIPI2_HUN	8	1.051697
24H	50MT/	Q9HD42	0.163113	0.176767	0.092516	CHMP1A	Charged mu	CHM1A_HU	4	1.119701
48H	10MT/	Q9H792	-0.62422	0.176797	0.092526	PEAK1	Inactive tyr	PEAK1_HUI	1	0.648772
48H	50MT/	O14653	0.173776	0.176812	0.092528	GOSR2	Golgi SNAP	GOSR2_HU	5	1.128007
24H	50MT/	Q14694	-0.06186	0.17691	0.092571	USP10	Ubiquitin c	UBP10_HU	25	0.958029
48H	10MT/	Q8TC07	0.000112	0.176939	0.092581	TBC1D15	TBC1 doma	TBC15_HUI	26	1.000078
24H	10MT/	P34896	-0.04237	0.176996	0.092607	SHMT1	Serine hydr	GLYC_HUM	17	0.971058
24H	10MT/	Q01973	-0.1634	0.176994	0.092607	ROR1	Inactive tyr	ROR1_HUM	8	0.892915
48H	10MT/	Q712K3	-0.00425	0.177001	0.092608	UBE2R2	Ubiquitin-c	UB2R2_HU	4	0.99706
24H	10MT/	Q9NUU6	-0.00753	0.17702	0.092617	OTULINL	Inactive ub	OTULL_HUI	3	0.994791
48H	10MT/	Q96JJ3	0.022126	0.177026	0.092619	ELMO2	Engulfment	ELMO2_HU	14	1.015454
48H	50MT/	Q15233	-0.09262	0.177037	0.092622	NONO	Non-POU d	NONO_HUI	22	0.937817
48H	50MT/	Q9H8E8	-0.02444	0.177102	0.092645	KAT14	Cysteine-ric	CSR2B_HUI	2	0.983203
48H	10MT/	Q6ZWT7	-1.13114	0.177123	0.092655	MBOAT2	Lysophospl	MBOA2_HU	1	0.456555
48H	50MT/	Q9Y4A5	-0.14549	0.177173	0.092677	TRRAP	Transforma	TRRAP_HUI	41	0.904074
48H	10MT/	Q9BX93	-0.39534	0.177205	0.092688	PLA2G12B	Group XIIB	PG12B_HU	2	0.760308
48H	10MT/	P17028	-0.06777	0.177242	0.092702	ZNF24	Zinc finger		ZNF24_HUI	7	0.954114
24H	50MT/	Q96SW2	0.091769	0.177254	0.092703	CRBN	Protein cer	CRBN_HUN	1	1.065676
48H	10MT/	O75165	-0.04336	0.177317	0.092731	DNAJC13	DnaJ homo	DJC13_HUI	62	0.970392
48H	50MT/	O43920	-0.02327	0.177343	0.092743	NDUFS5	NADH dehy	NDUS5_HU	4	0.983997
24H	50MT/	Q5VTE0	0.380418	0.177348	0.092743	EEF1A1P5	Putative elo	EF1A3_HUN	1	1.301719
24H	10MT/	Q7Z5R6	0.209217	0.177397	0.092765	APBB1IP	Amyloid be	AB1IP_HUN	1	1.156061
48H	10MT/	Q9C0I1	-0.06058	0.177414	0.092772	MTMR12	Myotubula	MTMRC_HU	11	0.958879
24H	50MT/	Q9BWD1	-0.06171	0.177461	0.092784	ACAT2	Acetyl-CoA	THIC_HUM/	17	0.958127
24H	50MT/	O75052	0.227973	0.177554	0.09282	NOS1AP	Carboxyl-te	CAPON_HU	3	1.171188
24H	10MT/	P62280	0.033949	0.177595	0.092837	RPS11	Small ribos	RS11_HUM	14	1.023811
24H	50MT/	P50991	-0.08011	0.177646	0.092857	CCT4	T-complex		TCPD_HUM	29	0.945988
48H	10MT/	P15144	-0.06371	0.177673	0.092868	ANPEP	Aminopept	AMPN_HUN	25	0.956798
24H	10MT/	P17707	-0.65272	0.177678	0.092869	AMD1	S-adenosyl	DCAM_HUN	4	0.63608
48H	10MT/	Q8WVM7	-0.10126	0.177826	0.092936	STAG1	Cohesin sul	STAG1_HUI	13	0.932218
24H	50MT/	Q96EB1	-0.19599	0.177878	0.09296	ELP4	Elongator c	ELP4_HUM	7	0.872973
48H	50MT/	Q6DKK2	0.28139	0.177899	0.092965	TTC19	Tetratricop	TTC19_HUN	9	1.215365
48H	50MT/	Q9UJS0	-0.06128	0.177907	0.092968	SLC25A13	Electrogeni	S2513_HUI	29	0.958416
24H	50MT/	Q69YN2	-0.05027	0.177919	0.092973	CWF19L1	CWF19-like	C19L1_HUI	25	0.965759
24H	50MT/	Q9H8M1	0.378729	0.177946	0.092982	COQ10B	Coenzyme (	CQ10B_HU	4	1.300196

48H 10MT/	O95149	0.068815	0.17797	0.092991	SNUPN	Snurportin	SPN1_HUM	6	1.048855
48H 10MT/	Q12972	0.009392	0.178037	0.093024	PPP1R8	Nuclear inh	PP1R8_HUI	13	1.006531
48H 10MT/	Q7Z794	1.716106	0.178069	0.093038	KRT77	Keratin, typ	K2C1B_HUI	14	3.285485
24H 50MT/	Q9BZE9	0.021935	0.178094	0.093048	ASPSCR1	Tether cont	ASPC1_HUI	17	1.01532
48H 10MT/	O75323	-0.15729	0.178117	0.093058	NIPSNAP2	Protein Nip	NIPS2_HUM	7	0.896706
24H 10MT/	Q9BWH6	-0.03352	0.17814	0.093068	RPAP1	RNA polym	RPAP1_HUI	32	0.977032
24H 10MT/	Q6P4E1	-0.08333	0.178175	0.093083	GOLM2	Protein GO	GOLM2_HU	9	0.943878
24H 10MT/	P50502	-0.00108	0.178217	0.093098	ST13	Hsc70-inte	F10A1_HUI	11	0.999251
48H 50MT/	O60524	-0.04953	0.178226	0.093102	NEMF	Ribosome c	NEMF_HUM	19	0.966251
48H 50MT/	O96000	0.032407	0.178249	0.093111	NDUFB10	NADH dehy	NDUBA_HU	11	1.022717
48H 10MT/	P48723	-0.03427	0.178296	0.093132	HSPA13	Heat shock	HSP13_HUI	14	0.976523
48H 50MT/	O15260	0.093147	0.17832	0.093142	SURF4	Surfeit locu	SURF4_HUI	6	1.066695
48H 10MT/	Q96JB2	-0.0279	0.178356	0.093157	COG3	Conserved	COG3_HUM	28	0.980849
24H 50MT/	Q9NQX7	0.215599	0.178412	0.09318	ITM2C	Integral me	ITM2C_HUI	5	1.161186
48H 10MT/	Q9NS15	0.179397	0.178469	0.093206	LTBP3	Latent-tran	LTBP3_HUI	1	1.13241
24H 50MT/	Q86YS7	0.103457	0.178534	0.093235	C2CD5	C2 domain-	C2CD5_HU	13	1.074345
24H 50MT/	Q641Q2	-0.04024	0.178566	0.093246	WASHC2A	WASH com	WAC2A_HU	28	0.972496
48H 50MT/	P42575	-1.16065	0.178576	0.09325	CASP2	Caspase-2	CASP2_HUI	2	0.447312
48H 10MT/	Q8TCT7	0.624774	0.178607	0.093263	SPPL2B	Signal pept	SPP2B_HUI	1	1.541969
48H 10MT/	Q9BVK2	-0.1614	0.178609	0.093263	ALG8	Dolichyl py	ALG8_HUM	4	0.894156
48H 50MT/	Q9NVX7	-0.25639	0.178612	0.093263	KBTBD4	Kelch repea	KBTB4_HUI	8	0.837179
24H 50MT/	P43360	-0.05996	0.17864	0.093271	MAGEA6	Melanoma-	MAGA6_HU	2	0.959293
24H 10MT/	Q9UK59	-0.04703	0.178684	0.093287	DBR1	Lariat debr	DBR1_HUM	9	0.967928
24H 10MT/	O75663	-0.06904	0.178709	0.093295	TIPRL	TIP41-like p	TIPRL_HUM	15	0.953275
48H 50MT/	Q9BV81	-0.25267	0.178711	0.093295	EMC6	ER membra	EMC6_HUM	2	0.839344
24H 10MT/	Q96NC0	-0.05555	0.178789	0.093327	ZMAT2	Zinc finger	ZMAT2_HU	4	0.962227
48H 50MT/	P29084	0.066393	0.178795	0.093327	GTF2E2	Transcripti	T2EB_HUM	9	1.047095
48H 50MT/	Q0D2I5;Q5	-0.29971	0.178797	0.093327	IFFO1;IFFO	Non-homol	IFFO1_HUM	1	0.812418
48H 50MT/	Q13526	-0.0194	0.17883	0.093342	PIN1	Peptidyl-pr	PIN1_HUM	6	0.986642
24H 10MT/	Q14696	-0.00936	0.178898	0.093372	MESD	LRP chaper	MESD_HUM	12	0.99353
24H 10MT/	P02686	-0.03608	0.178907	0.093374	MBP	Myelin basi	MBP_HUM/	2	0.9753
24H 10MT/	Q9UKE5	-0.12186	0.178905	0.093374	TNIK	TRAF2 and	TNIK_HUM/	13	0.919
48H 50MT/	Q86UK7	-0.08619	0.178925	0.093381	ZNF598	E3 ubiquiti	ZN598_HUI	22	0.942004
48H 50MT/	O95573	-0.04132	0.178929	0.093382	ACSL3	Fatty acid C	ACSL3_HUI	28	0.971762
48H 10MT/	Q99496	-0.02905	0.17894	0.093385	RNF2	E3 ubiquiti	RING2_HUI	10	0.980065
48H 50MT/	O14662	0.068071	0.178971	0.093397	STX16	Syntaxin-16	STX16_HUI	8	1.048314
24H 50MT/	Q00765	-0.06526	0.17897	0.093397	REEP5	Receptor e	REEP5_HUI	3	0.95577
24H 10MT/	Q6UN15	0.056932	0.178981	0.093401	FIP1L1	Pre-mRNA 3	FIP1_HUM/	10	1.040251
48H 10MT/	P50402	0.00149	0.179031	0.09342	EMD	Emerin	EMD_HUM/	12	1.001033
24H 10MT/	Q15154	-0.03039	0.179146	0.093467	PCM1	Pericentrio	PCM1_HUM	73	0.979157
48H 10MT/	Q5XPI4	-0.21941	0.179167	0.093475	RNF123	E3 ubiquiti	RN123_HU	7	0.858918
24H 10MT/	O60493	0.007529	0.179204	0.093487	SNX3	Sorting nex	SNX3_HUM	12	1.005232
48H 50MT/	P30519	-0.02954	0.179257	0.09351	HMOX2	Heme oxyg	HMOX2_HU	15	0.979736
24H 10MT/	P12235	0.030997	0.179278	0.09352	SLC25A4	ADP/ATP tr	ADT1_HUM	8	1.021718
48H 50MT/	P63244	0.020613	0.179286	0.093523	RACK1	Small ribos	RACK1_HUI	20	1.014391
24H 10MT/	P08582	-0.2453	0.179297	0.093527	MELTF	Melanotrar	TRFM_HUM	3	0.843643
48H 10MT/	Q6ZVM7	0.179182	0.179363	0.093554	TOM1L2	TOM1-like p	TM1L2_HU	6	1.132242
48H 10MT/	O75143	-0.01102	0.179398	0.093569	ATG13	Autophagy-	ATG13_HUI	10	0.992392
48H 10MT/	Q86UE4	-0.04415	0.179426	0.093579	MTDH	Protein LYF	LYRIC_HUM	22	0.96986
24H 50MT/	Q9NRK6	-0.04276	0.179478	0.093599	ABCB10	ATP-bindin	ABCBA_HUI	15	0.970796
24H 50MT/	Q9Y3Q3	0.358666	0.179485	0.093602	TMED3	Transmemb	TMED3_HU	3	1.28224
48H 50MT/	Q8N5U6	-0.07286	0.179514	0.093614	RNF10	E3 ubiquiti	RNF10_HUI	8	0.950753

48H 10MT/	Q9UHD2	0.01316	0.179604	0.093658	TBK1	Serine/thre	TBK1_HUM	19	1.009163
48H 10MT/	Q12965	-0.00587	0.179627	0.093669	MYO1E	Unconvent	MYO1E_HU	35	0.995942
24H 50MT/	P60983	-0.02013	0.179635	0.093672	GMFB	Glia matur	GMFB_HUM	8	0.986143
24H 50MT/	Q7Z4V5	-0.06489	0.179687	0.093692	HDGFL2	Hepatoma-	HDGR2_HU	11	0.956019
48H 50MT/	P00883	-0.22262	0.179706	0.0937	ALDOA	Fructose-bi	ALDOA_RAI	2	0.857007
48H 50MT/	Q9BX68	0.113694	0.179759	0.093723	HINT2	Adenosine	HINT2_HUM	4	1.081995
24H 50MT/	P51003	0.071328	0.179828	0.09375	PAPOLA	Poly(A) pol	PAPOA_HU	22	1.050683
24H 10MT/	Q15070	-0.09178	0.179894	0.093779	OXA1L	Mitochond	OXA1L_HUI	8	0.938367
24H 10MT/	Q13724	-0.08905	0.180124	0.09388	MOGS	Mannosyl-c	MOGS_HUM	33	0.94014
48H 50MT/	Q5QP82	0.722418	0.180158	0.093896	DCAF10	DDB1- and	DCA10_HU	1	1.649945
48H 10MT/	O94919	0.004103	0.180181	0.093905	ENDOD1	Endonucle	ENDD1_HU	13	1.002848
24H 50MT/	Q9Y6Q9	0.120404	0.180182	0.093905	NCOA3	Nuclear rec	NCOA3_HU	16	1.087039
24H 50MT/	Q16775	0.043009	0.180192	0.093909	HAGH	Hydroxyacy	GLO2_HUM	8	1.03026
48H 50MT/	P18074	0.030875	0.180215	0.093916	ERCC2	General tra	ERCC2_HUI	15	1.021631
24H 50MT/	Q9NX14	-0.11376	0.180227	0.093921	NDUFB11	NADH dehy	NDUBB_HU	4	0.924177
24H 50MT/	Q9NRX6	0.70063	0.180267	0.093939	TMEM167E	Protein kisl	KISHB_HUM	3	1.625214
48H 10MT/	Q5BJD5	-0.3157	0.180286	0.093942	TMEM41B	Transmemb	TM41B_HU	3	0.803458
48H 10MT/	Q99598	0.002432	0.18031	0.093954	TSNAX	Translin-ass	TSNAX_HUI	10	1.001687
48H 50MT/	Q6P2H3	-0.22659	0.180317	0.093955	CEP85	Centrosom	CEP85_HUI	10	0.85465
24H 50MT/	Q9Y5N6	0.072939	0.180317	0.093955	ORC6	Origin reco	ORC6_HUM	4	1.051858
48H 10MT/	Q96DF8	0.123001	0.180333	0.093959	ESS2	Splicing fac	ESS2_HUM/	7	1.088998
48H 10MT/	Q96CP6	-0.13204	0.180419	0.093991	GRAMD1A	Protein Ast	ASTRA_HUM	8	0.912541
24H 10MT/	P57678	0.077633	0.180448	0.093999	GEMIN4	Gem-associ	GEMI4_HUI	26	1.055285
48H 10MT/	Q9UJH8	-0.08928	0.180444	0.093999	METRN	Meteorin	METRN_HU	1	0.939995
24H 10MT/	Q9UJX4	0.024748	0.180512	0.094031	ANAPC5	Anaphase-p	APC5_HUM	25	1.017302
48H 10MT/	O95295	-1.79811	0.180536	0.094039	SNAPIN	SNARE-asso	SNAPN_HU	3	0.287552
48H 50MT/	Q15637	0.067245	0.180599	0.094068	SF1	Splicing fac	SF01_HUM	17	1.047714
48H 50MT/	Q8TBN0	-0.1235	0.180602	0.094069	RAB3IL1	Guanine nu	R3GEF_HUI	6	0.917958
48H 10MT/	Q5T200	-0.00067	0.180608	0.09407	ZC3H13	Zinc finger	ZC3HD_HU	14	0.999535
24H 10MT/	O95835	0.043872	0.180637	0.094081	LATS1	Serine/thre	LATS1_HUM	8	1.030877
24H 10MT/	P35670	0.027568	0.18069	0.094099	ATP7B	Copper-tra	ATP7B_HUI	16	1.019293
24H 10MT/	Q96A72	0.035402	0.180719	0.094111	MAGOHB	Protein ma	MGN2_HUI	10	1.024843
48H 10MT/	Q9BTY2	-0.03596	0.180735	0.094118	FUCA2	Plasma alpl	FUCO2_HU	13	0.975383
24H 50MT/	Q96EM0	0.032465	0.180763	0.09413	L3HYPDH	Trans-3-hyc	T3HPD_HU	7	1.022758
24H 10MT/	Q01085	0.078383	0.180875	0.094179	TIAL1	Nucleolysir	TIAR_HUM/	9	1.055834
48H 10MT/	O15439	0.015589	0.180922	0.094197	ABCC4	ATP-bindin	MRP4_HUM	14	1.010864
24H 10MT/	Q96SU4	-0.02411	0.180959	0.094211	OSBPL9	Oxysterol-b	OSBL9_HUI	10	0.983425
48H 50MT/	Q13630	-0.03231	0.180968	0.094214	GFUS	GDP-L-fuco	FCL_HUMA	12	0.977852
24H 10MT/	Q07817	0.199665	0.181064	0.094261	BCL2L1	Bcl-2-like p	B2CL1_HUI	5	1.148432
48H 10MT/	O95373	-0.05462	0.181081	0.094268	IPO7	Importin-7	IPO7_HUM	43	0.962849
24H 10MT/	Q86YH6	0.093973	0.181169	0.094307	PDSS2	All trans-pc	DLP1_HUM	16	1.067305
48H 50MT/	Q6ZT21	-0.07613	0.18119	0.094315	TMPPE	Transmemb	TMPPE_HU	4	0.948602
48H 50MT/	Q6PJ69	-0.12671	0.181257	0.094339	TRIM65	E3 ubiquiti	TRI65_HUM	3	0.915919
24H 50MT/	Q9UGC7	0.419744	0.181276	0.094348	MTRF1L	Peptide cha	RF1ML_HU	4	1.33769
48H 10MT/	P45880	0.003897	0.181295	0.094352	VDAC2	Voltage-de	VDAC2_HU	14	1.002705
48H 50MT/	Q86YS3	0.459793	0.181314	0.094355	RAB11FIP4	Rab11 fami	RFIP4_HUM	3	1.375344
48H 10MT/	Q6ULP2	-0.02694	0.181327	0.094361	AFTPH	Aftiphilin	AFTIN_HUM	10	0.981497
24H 50MT/	Q9H4M3	0.145282	0.181402	0.094392	FBXO44	F-box only	FBX44_HUI	3	1.105946
24H 50MT/	Q155Q3	0.124931	0.181416	0.094398	DIXDC1	Dixin	DIXC1_HUM	4	1.090455
48H 10MT/	Q9NVU0	-0.08063	0.181425	0.094401	POLR3E	DNA-direct	RPC5_HUM	14	0.945646
24H 10MT/	Q8IUS5	-0.04778	0.181444	0.094405	EPHX4	Epoxide hy	EPHX4_HUI	4	0.967426
48H 10MT/	P15848	-0.03358	0.181452	0.094406	ARSB	Arylsulfata	ARSB_HUM	9	0.976995

48H 50MT/	Q14692	-0.0453	0.181479	0.094417	BMS1	Ribosome b	BMS1_HUM	33	0.969085
48H 50MT/	Q9UBK8	-0.20966	0.18153	0.094433	MTRR	Methionine	MTRR_HUM	10	0.864742
48H 10MT/	Q9UMN6	-0.10834	0.181531	0.094433	KMT2B	Histone-lys	KMT2B_HU	12	0.927652
24H 10MT/	P46109	-0.04389	0.181541	0.094436	CRKL	Crk-like pro	CRKL_HUM	14	0.970039
24H 10MT/	Q96CP7	0.08281	0.181571	0.094448	TLCD1	TLC domain	TLCD1_HUI	4	1.059079
24H 10MT/	Q96S66	0.005584	0.181625	0.094471	CLCC1	Chloride ch	CLCC1_HUI	20	1.003878
48H 50MT/	Q96EK5	0.04858	0.181635	0.094475	KIFBP	KIF-binding	KBP_HUMA	23	1.034247
24H 10MT/	Q16775	-0.0467	0.181705	0.094504	HAGH	Hydroxyacy	GLO2_HUM	8	0.96815
24H 10MT/	P50440	0.005163	0.181729	0.094509	GATM	Glycine am	GATM_HUM	19	1.003585
48H 10MT/	P83876	0.17513	0.181724	0.094509	TXNL4A	Thioredoxi	TXN4A_HUI	6	1.129066
24H 10MT/	Q8WW12	-0.01021	0.181724	0.094509	PCNP	PEST protec	PCNP_HUM	6	0.992951
48H 10MT/	Q7Z6M1	0.07561	0.181744	0.094512	RABEPK	Rab9 effect	RABEK_HUI	13	1.053806
24H 50MT/	Q93052	-0.04774	0.18176	0.094518	LPP	Lipoma-pre	LPP_HUMA	15	0.967449
48H 50MT/	Q96QF0	-0.00155	0.181819	0.094544	RAB3IP	Rab-3A-inte	RAB3I_HUM	4	0.998923
24H 50MT/	Q8IX03	-0.05864	0.181854	0.094554	WWC1	Protein KIB	KIBRA_HUM	6	0.960167
24H 50MT/	Q13619	0.035417	0.181861	0.094555	CUL4A	Cullin-4A	CUL4A_HUI	28	1.024853
24H 10MT/	O14735	0.009903	0.18187	0.094559	CDIPT	CDP-diacylg	CDIPT_HUM	6	1.006888
48H 50MT/	Q9UJY5	0.101572	0.181938	0.094586	GGA1	ADP-ribosy	GGA1_HUM	10	1.072942
24H 50MT/	P11766	0.024712	0.181997	0.094611	ADH5	Alcohol deh	ADHX_HUM	19	1.017277
48H 50MT/	P09211	0.034716	0.182038	0.094626	GSTP1	Glutathion	GSTP1_HUI	16	1.024355
48H 10MT/	Q9Y680	-0.07335	0.182046	0.094627	FKBP7	Peptidyl-pr	FKBP7_HUI	6	0.950432
24H 10MT/	Q9BPX6	-0.41096	0.182073	0.09464	MICU1	Calcium up	MICU1_HU	8	0.752124
24H 10MT/	Q9Y385	0.210766	0.182089	0.094644	UBE2J1	Ubiquitin-c	UB2J1_HUI	6	1.157302
48H 50MT/	Q5T5X7	-0.56331	0.182165	0.094671	BEND3	BEN domain	BEND3_HU	10	0.67675
24H 10MT/	P31321	-0.30162	0.182182	0.094678	PRKAR1B	cAMP-depe	KAP1_HUM	5	0.811338
48H 50MT/	O15155	-0.08572	0.182214	0.094692	BET1	BET1 homo	BET1_HUM	2	0.942314
24H 10MT/	Q5VSL9	0.056248	0.182217	0.094692	STRIP1	Striatin-int	STRP1_HUM	14	1.039758
24H 50MT/	Q13153	-0.1194	0.182258	0.094708	PAK1	Serine/thre	PAK1_HUM	13	0.920572
24H 50MT/	Q86VS8	-0.09256	0.182375	0.094758	HOOK3	Protein Ho	HOOK3_HU	21	0.937859
48H 50MT/	P53609	0.013768	0.182405	0.094771	PGGT1B	Geranylger	PGTB1_HUI	5	1.009589
48H 10MT/	Q9NRG7	0.039973	0.182493	0.09481	SDR39U1	Epimerase f	D39U1_HU	2	1.028094
48H 10MT/	O15056	-0.08196	0.182522	0.094818	SYNJ2	Synaptojan	SYNJ2_HUM	11	0.944771
24H 50MT/	P61289	-0.02931	0.182631	0.094857	PSME3	Proteasom	PSME3_HU	17	0.979887
48H 10MT/	O75934	-0.05482	0.18266	0.094865	BCAS2	Pre-mRNA-	SPF27_HUM	11	0.962712
24H 50MT/	P00747	-0.52367	0.18272	0.094891	PLG	Plasminoge	PLMN_HUM	3	0.695599
24H 10MT/	Q16186	0.133226	0.182739	0.094897	ADRM1	Proteasom	ADRM1_HU	9	1.096744
48H 50MT/	Q9UHY8	-0.19486	0.18274	0.094897	FEZ2	Fasciculatic	FEZ2_HUM	5	0.87366
24H 10MT/	Q96BI1	-0.11699	0.182794	0.09492	SLC22A18	Solute carri	S22AI_HUM	4	0.922109
24H 50MT/	O75382	-0.19109	0.182827	0.094932	TRIM3	Tripartite m	TRIM3_HUI	5	0.875942
24H 50MT/	Q9H0E9	0.135679	0.182873	0.09495	BRD8	Bromodom	BRD8_HUM	7	1.09861
48H 10MT/	Q2TAA5	-0.1151	0.182882	0.094953	ALG11	GDP-Man:M	ALG11_HUI	8	0.923315
24H 10MT/	Q96P48	-0.08294	0.182912	0.094964	ARAP1	Arf-GAP wit	ARAP1_HUI	16	0.944133
48H 50MT/	Q6ZVX9	0.017107	0.182924	0.094968	PAQR9	Membrane	PAQR9_HU	1	1.011928
24H 50MT/	Q12824	0.073797	0.182941	0.094976	SMARCB1	SWI/SNF-re	SNF5_HUM	12	1.052483
24H 10MT/	Q9UHR4	0.092593	0.182973	0.09499	BAIAP2L1	BAR/IMD do	BI2L1_HUM	19	1.066285
48H 50MT/	Q9P000	-0.15212	0.18298	0.094991	COMMD9	COMM dom	COMD9_HU	8	0.899928
48H 10MT/	Q9BZL1	0.091128	0.183029	0.095008	UBL5	Ubiquitin-l	UBL5_HUM	4	1.065203
48H 10MT/	Q9NR30	-0.07938	0.183133	0.095056	DDX21	Nucleolar R	DDX21_HU	43	0.946464
48H 10MT/	P50225	0.295358	0.183149	0.095061	SULT1A1	Sulfotransf	ST1A1_HUM	2	1.22719
48H 50MT/	Q8N4Q0	0.035808	0.183222	0.09509	PTGR3	Prostagland	PTGR3_HUI	3	1.02513
24H 50MT/	Q12872	0.175265	0.183242	0.095096	SFSWAP	Splicing fac	SFSWA_HU	12	1.129172
48H 50MT/	Q9UPM8	-0.06785	0.183297	0.095123	AP4E1	AP-4 compl	AP4E1_HUI	8	0.954061

24H 50MT/	O95363	0.511507	0.183321	0.095131	FARS2	Phenylalan	SYFM_HUM	4	1.425538
48H 50MT/	P06746	0.000833	0.183318	0.095131	POLB	DNA polym	DPOLB_HU	7	1.000578
24H 10MT/	O00193	0.037099	0.183341	0.095139	SMAP	Small acidi	SMAP_HUM	3	1.026048
48H 50MT/	Q04864	-0.14789	0.183404	0.095166	REL	Proto-onco	REL_HUMA	4	0.902567
48H 10MT/	Q02127	-0.06252	0.183429	0.095175	DHODH	Dihydroorc	PYRD_HUM	19	0.957592
48H 10MT/	Q8IYT4	0.096268	0.183541	0.095222	KATNAL2	Katanin p6	KATL2_HUM	1	1.069005
24H 10MT/	Q92889	0.003462	0.183545	0.095223	ERCC4	DNA repair	XPF_HUMA	14	1.002403
24H 10MT/	Q92845	-0.05109	0.183579	0.095235	KIFAP3	Kinesin-ass	KIFA3_HUM	11	0.965206
48H 10MT/	Q96GZ6	-0.07813	0.183598	0.095242	SLC41A3	Solute carri	S41A3_HUI	2	0.947287
48H 10MT/	Q9BT09	-0.00253	0.183596	0.095242	CNPY3	Protein car	CNPY3_HUI	8	0.99825
48H 10MT/	Q53R41	0.172561	0.183615	0.095247	FASTKD1	FAST kinase	FAKD1_HUI	8	1.127058
48H 10MT/	Q9BRA0	0.17161	0.183633	0.095252	NAA38	N-alpha-ace	NAA38_HU	4	1.126315
24H 50MT/	O75865	0.594879	0.183695	0.09528	TRAPPC6A	Trafficking	TPC6A_HUI	1	1.510346
48H 50MT/	P46977	-0.07223	0.183697	0.09528	STT3A	Dolichyl-di	STT3A_HUM	16	0.951165
24H 10MT/	Q8N3X1	0.06108	0.1837	0.09528	FNBP4	Formin-bin	FNBP4_HUI	17	1.043247
48H 10MT/	Q6W2J9	0.190282	0.183748	0.0953	BCOR	BCL-6 core	BCOR_HUM	4	1.140986
48H 50MT/	P35443	-0.19296	0.183764	0.095303	THBS4	Thrombosp	TSP4_HUM	2	0.874811
48H 50MT/	Q9Y697	-0.00489	0.183789	0.095312	NFS1	Cysteine de	NFS1_HUM	20	0.996617
24H 50MT/	Q96DM3	-0.0816	0.183801	0.095314	RMC1	Regulator c	RMC1_HUM	11	0.945006
24H 50MT/	Q99735	0.02388	0.183873	0.095344	MGST2	Microsoma	MGST2_HU	2	1.01669
24H 50MT/	P30043	-0.02848	0.183904	0.095357	BLVRB	Flavin redu	BLVRB_HUI	9	0.980453
24H 50MT/	Q969R5	0.202942	0.183965	0.095386	L3MBTL2	Lethal(3)m	LMBL2_HU	3	1.151044
24H 10MT/	O15440	-0.08081	0.183996	0.095397	ABCC5	ATP-bindin	MRP5_HUM	16	0.945529
24H 10MT/	Q9NYY8	0.101893	0.183999	0.095397	FASTKD2	FAST kinase	FAKD2_HUI	17	1.07318
48H 50MT/	P11117	-0.03128	0.184112	0.095444	ACP2	Lysosomal	PPAL_HUM	11	0.978554
24H 10MT/	Q8NFD5	0.030692	0.184117	0.095445	ARID1B	AT-rich inte	ARI1B_HUM	26	1.021502
24H 10MT/	Q96AJ9	0.035663	0.184122	0.095446	VTI1A	Vesicle trar	VTI1A_HUM	5	1.025028
24H 50MT/	Q96N67	-0.04738	0.184153	0.095455	DOCK7	Dedicator c	DOCK7_HU	56	0.96769
48H 50MT/	P57772	-0.0014	0.184222	0.095483	EEFSEC	Selenocyste	SELB_HUM/	15	0.999032
48H 50MT/	Q15633	-0.0879	0.184253	0.095494	TARBP2	RISC-loadir	TRBP2_HUI	6	0.940889
24H 50MT/	Q96RS0	0.073537	0.184254	0.095494	TGS1	Trimethylg	TGS1_HUM	8	1.052293
48H 10MT/	Q9UBQ7	0.047548	0.184299	0.095513	GRHPR	Glyoxylate	GRHPR_HU	14	1.033507
24H 10MT/	P82979	0.00661	0.184418	0.095565	SARNP	SAP domair	SARNP_HUI	8	1.004592
48H 50MT/	Q9UMS4	0.009732	0.184419	0.095565	PRPF19	Pre-mRNA-	PRP19_HUI	17	1.006768
24H 50MT/	Q9Y5Q8	-0.01243	0.184442	0.095573	GTF3C5	General tra	TF3C5_HUM	18	0.991421
24H 10MT/	Q9H0E3	-0.01658	0.184525	0.09561	SAP130	Histone dea	SP130_HUI	10	0.988577
24H 50MT/	P00441	-0.06108	0.184537	0.095615	SOD1	Superoxide	SODC_HUM	7	0.958545
48H 10MT/	O43598	0.000841	0.184576	0.095631	DNPH1	5-hydroxyn	DNPH1_HU	10	1.000583
48H 50MT/	P02668	-0.62028	0.184576	0.095631	CSN3	Kappa-case	CASK_BOVI	2	0.650544
24H 50MT/	Q06033	-0.66559	0.184621	0.095648	ITIH3	Inter-alpha	ITIH3_HUM	4	0.63043
48H 10MT/	Q9NTJ4	-0.16661	0.184655	0.095659	MAN2C1	Alpha-man	MA2C1_HU	5	0.890934
48H 10MT/	P51116	0.022163	0.184676	0.095666	FXR2	RNA-bindir	FXR2_HUM	18	1.01548
48H 50MT/	Q9BQC3	0.111589	0.184706	0.095678	DPH2	2-(3-amino	DPH2_HUM	13	1.080418
24H 10MT/	Q6ZNA5	-0.0304	0.184743	0.095692	FRRS1	Ferric-chel	FRRS1_HUM	8	0.979149
48H 10MT/	Q8N201	-0.09454	0.184749	0.095694	INTS1	Integrator	INT1_HUM/	32	0.936574
48H 50MT/	Q68DH5	0.661756	0.184797	0.095712	LMBRD2	G-protein c	LMBD2_HU	3	1.582007
24H 10MT/	P06702	0.442623	0.184861	0.095742	S100A9	Protein S1	S10A9_HUI	2	1.359073
48H 10MT/	Q9H8P0	-0.27812	0.184863	0.095742	SRD5A3	Polyprenol	PORED_HU	2	0.824665
24H 50MT/	P60484	0.24729	0.184887	0.095752	PTEN	Phosphatic	PTEN_HUM	2	1.186976
48H 10MT/	P31146	-0.21817	0.184955	0.095781	CORO1A	Coronin-1A	COR1A_HU	3	0.859657
24H 10MT/	Q8IWJ2	0.019624	0.184954	0.095781	GCC2	GRIP and c	GCC2_HUM	53	1.013695
24H 50MT/	O95905	-0.09634	0.18498	0.095792	ECD	Protein ecd	ECD_HUMA	18	0.935406

24H 50MT/	Q6ZUM4	-0.2025	0.185012	0.095805	ARHGAP27	Rho GTPase	RHG27_HU	1	0.869046
48H 50MT/	P57081	0.03163	0.185041	0.095817	WDR4	tRNA (guan	WDR4_HUI	11	1.022167
48H 10MT/	Q8NAN2	-0.13721	0.185079	0.095831	MIGA1	Mitoguardi	MIGA1_HU	16	0.909278
24H 10MT/	Q9UPN9	0.03979	0.185107	0.095842	TRIM33	E3 ubiquiti	TRI33_HUN	21	1.027964
24H 10MT/	O15155	-0.02028	0.185125	0.095846	BET1	BET1 homo	BET1_HUM	2	0.986038
24H 50MT/	Q9UJ14	0.215306	0.185147	0.095856	GGT7	Glutathion	GGT7_HUN	2	1.16095
48H 50MT/	Q9ULH0	-0.07331	0.185149	0.095856	KIDINS220	Kinase D-in	KDIS_HUM/	17	0.950456
48H 50MT/	O14519	-2.84542	0.185224	0.095885	CDK2AP1	Cyclin-depe	CDKA1_HU	1	0.139137
48H 50MT/	Q96AT9	0.06206	0.185231	0.095887	RPE	Ribulose-pl	RPE_HUMA	7	1.043955
24H 50MT/	Q53F19	0.109028	0.18524	0.09589	NCBP3	Nuclear cap	NCBP3_HU	11	1.078502
48H 50MT/	A0FGR8	-0.03506	0.185365	0.095938	ESYT2	Extended sy	ESYT2_HUN	25	0.975993
48H 50MT/	Q16656	-0.1146	0.185368	0.095938	NRF1	Nuclear res	NRF1_HUN	3	0.923638
24H 50MT/	Q8IUD2	-0.0636	0.185403	0.095952	ERC1	ELKS/Rab6-	RB6I2_HUN	26	0.95687
48H 10MT/	Q8N3C0	-0.03083	0.185509	0.095999	ASCC3	Activating s	ASCC3_HUI	63	0.978858
24H 10MT/	Q9Y2K9	0.214795	0.185521	0.096	STXBP5L	Syntaxin-bi	STB5L_HUN	4	1.160539
24H 50MT/	Q9H8H0	0.088908	0.185548	0.096011	NOL11	Nucleolar p	NOL11_HU	24	1.063565
48H 10MT/	P62937	-0.06995	0.185569	0.096018	PPIA	Peptidyl-pr	PPIA_HUM/	11	0.952671
24H 50MT/	Q9UIV1	0.068433	0.185606	0.096033	CNOT7	CCR4-NOT t	CNOT7_HU	7	1.048577
48H 10MT/	Q96GM8	-0.04416	0.1857	0.096071	TOE1	Target of EG	TOE1_HUM	14	0.969857
48H 50MT/	P82094	0.057245	0.185703	0.096071	TMF1	TATA eleme	TMF1_HUN	34	1.040477
24H 50MT/	Q5MIZ7	0.045826	0.185725	0.09608	PPP4R3B	Serine/thre	P4R3B_HUI	18	1.032274
24H 10MT/	P62253	0.011876	0.185748	0.09609	UBE2G1	Ubiquitin-c	UB2G1_HU	7	1.008266
48H 10MT/	Q9ULQ1	-0.16218	0.185793	0.09611	TPCN1	Two pore cl	TPC1_HUM	9	0.893672
24H 10MT/	Q8WU17	-1.05683	0.185867	0.096141	RNF139	E3 ubiquiti	RN139_HU	3	0.480686
48H 10MT/	Q9UBK8	-0.04664	0.185943	0.096176	MTRR	Methionine	MTRR_HUN	10	0.968191
48H 50MT/	Q5T7M9	0.346709	0.185989	0.096194	DIPK1A	Divergent p	DIK1A_HUI	6	1.271656
24H 10MT/	Q8N9N5	-0.02437	0.186095	0.096235	BANP	Protein BAI	BANP_HUN	1	0.983248
48H 10MT/	O95900	-0.03972	0.186108	0.096238	TRUB2	Pseudourid	TRUB2_HUI	6	0.972844
48H 10MT/	Q53RE8	0.200194	0.186128	0.096245	ANKRD39	Ankyrin rep	ANR39_HU	3	1.148853
24H 50MT/	Q9NYB0	-0.13867	0.18615	0.096252	TERF2IP	Telomeric r	TE2IP_HUN	10	0.908359
24H 10MT/	O94782	0.428806	0.186338	0.096336	USP1	Ubiquitin c	UBP1_HUN	4	1.346119
24H 10MT/	Q96CN9	0.113438	0.186367	0.096345	GCC1	GRIP and cc	GCC1_HUN	10	1.081803
48H 50MT/	Q9H7L9	-0.01819	0.186391	0.096352	SUDS3	Sin3 histon	SDS3_HUM	8	0.98747
24H 50MT/	Q9Y5Y0	0.121206	0.186393	0.096352	FLVCR1	Heme trans	FLVC1_HUN	4	1.087644
48H 50MT/	Q96N67	-0.01295	0.186406	0.096357	DOCK7	Dedicator c	DOCK7_HU	56	0.991064
48H 50MT/	Q7Z7E8	-0.05585	0.18642	0.096362	UBE2Q1	Ubiquitin-c	UB2Q1_HU	9	0.962025
24H 50MT/	Q13464	0.046297	0.186425	0.096363	ROCK1	Rho-associa	ROCK1_HU	42	1.032611
24H 10MT/	Q12805	0.606458	0.186455	0.096372	EFEMP1	EGF-contai	FBLN3_HUI	1	1.522517
24H 10MT/	Q7Z7K6	0.06657	0.186507	0.096391	CENPV	Centromere	CENPV_HUI	6	1.047224
24H 10MT/	Q9H307	0.085729	0.186535	0.096401	PNN	Pinin	PININ_HUN	21	1.061224
48H 10MT/	P54646	0.024158	0.186553	0.096406	PRKAA2	5'-AMP-acti	AAPK2_HUI	11	1.016886
48H 10MT/	P00352	0.032313	0.186734	0.096485	ALDH1A1	Aldehyde d	AL1A1_HUI	32	1.022651
24H 10MT/	P45984	-0.07421	0.186749	0.096492	MAPK9	Mitogen-ac	MK09_HUN	9	0.949859
48H 50MT/	P29590	0.020774	0.186843	0.096529	PML	Protein PM	PML_HUM/	11	1.014504
24H 10MT/	Q9H9B1	0.053526	0.186942	0.096567	EHMT1	Histone-lys	EHMT1_HU	11	1.037798
48H 10MT/	Q8IY57	0.018188	0.186952	0.096571	YAF2	YY1-associa	YAF2_HUM	3	1.012687
48H 50MT/	O75223	-0.03542	0.186955	0.096571	GGCT	Gamma-glu	GGCT_HUN	8	0.97575
24H 50MT/	Q32P44	-0.10557	0.187029	0.096606	EML3	Echinoderm	EMAL3_HU	16	0.929437
24H 10MT/	Q0IIM8	0.119464	0.187046	0.096612	TBC1D8B	TBC1 doma	TBC8B_HUI	11	1.086331
24H 10MT/	Q5SYC1	0.25964	0.187047	0.096612	CLVS2	Clavesin-2	CLVS2_HUN	4	1.19718
48H 50MT/	Q2M3G4	-0.35056	0.187057	0.096616	SHROOM1	Protein Shr	SHRM1_HU	2	0.784277
48H 10MT/	Q9NY35	-0.03782	0.187072	0.096622	CLDND1	Claudin doı	CLDN1_HU	3	0.974123

48H 10MT/	Q15424	0.048144	0.187084	0.096627	SAFB	Scaffold att	SAFB1_HUI	14	1.033934
48H 10MT/	Q9H490	0.086277	0.187097	0.096632	PIGU	Phosphatic	PIGU_HUM	6	1.061627
48H 50MT/	P62829	-0.08444	0.187109	0.096636	RPL23	Large ribos	RL23_HUM	8	0.943151
48H 50MT/	Q9NQ35	-0.56903	0.187134	0.096648	NRIP3	Nuclear rec	NRIP3_HUI	1	0.674069
48H 10MT/	Q6ZRY4	0.080775	0.187281	0.096701	RBPMS2	RNA-bindir	RBPS2_HUI	1	1.057586
48H 10MT/	Q9H4L4	0.037606	0.187342	0.096724	SENP3	Sentrin-spe	SENP3_HUI	13	1.026409
24H 50MT/	P37287	0.095909	0.187397	0.09675	PIGA	Phosphatic	PIGA_HUM	3	1.068739
48H 10MT/	Q9BWS9	0.123066	0.18749	0.096784	CHID1	Chitinase d	CHID1_HUI	9	1.089046
48H 50MT/	Q9HCM4	0.07682	0.187483	0.096784	EPB41L5	Band 4.1-lil	E41L5_HUI	12	1.05469
48H 50MT/	Q9H8K7	0.748851	0.187523	0.096793	PAAT	ATPase PAA	PAAT_HUM	3	1.680454
24H 10MT/	O95295	-0.27172	0.187527	0.096794	SNAPIN	SNARE-asso	SNAPN_HU	3	0.828332
48H 50MT/	Q9H6K4	-0.03291	0.1876	0.096822	OPA3	Optic atrop	OPA3_HUM	3	0.977449
48H 50MT/	O43395	-0.1356	0.187604	0.096822	PRPF3	U4/U6 sma	PRPF3_HUI	23	0.910294
48H 10MT/	Q13017	0.010458	0.187608	0.096823	ARHGAP5	Rho GTPase	RHG05_HU	15	1.007275
48H 10MT/	O60499	0.159625	0.18762	0.096825	STX10	Syntaxin-1(	STX10_HUI	5	1.116997
48H 10MT/	Q9UPQ9	0.109852	0.18762	0.096825	TNRC6B	Trinucleoti	TNR6B_HUI	17	1.079118
24H 10MT/	Q12899	-0.01994	0.187624	0.096826	TRIM26	Tripartite n	TRI26_HUM	14	0.986277
24H 10MT/	Q9NRX4	-0.04778	0.187656	0.096838	PHPT1	14 kDa pho	PHP14_HU	6	0.967426
48H 10MT/	P30291	-0.1253	0.187664	0.09684	WEE1	Wee1-like p	WEE1_HUM	5	0.916812
24H 10MT/	Q9Y2G3	0.069266	0.187701	0.096856	ATP11B	Phospholip	AT11B_HUI	11	1.049183
48H 50MT/	Q3MIR4	-0.25373	0.187707	0.096858	TMEM30B	Cell cycle c	CC50B_HUI	3	0.838726
24H 10MT/	Q9Y619	-0.00539	0.187718	0.096862	SLC25A15	Mitochond	ORNT1_HU	15	0.996273
48H 10MT/	Q13546	0.092175	0.187734	0.096866	RIPK1	Receptor-ir	RIPK1_HUI	20	1.065976
24H 50MT/	Q96GC5	-0.05668	0.187796	0.096891	MRPL48	Large ribos	RM48_HUM	4	0.961477
48H 10MT/	Q9UHV7	-0.10981	0.187809	0.096894	MED13	Mediator o	MED13_HU	4	0.926708
24H 50MT/	Q04656	0.101022	0.187884	0.096925	ATP7A	Copper-trai	ATP7A_HUI	22	1.072533
24H 50MT/	Q8WV93	-0.01815	0.187971	0.096959	AFG1L	AFG1-like A	AFG1L_HUI	7	0.987496
48H 50MT/	Q13952	-0.08054	0.187996	0.096969	NFYC	Nuclear tra	NFYC_HUM	6	0.945702
48H 10MT/	Q13217	-0.05245	0.188056	0.096992	DNAJC3	DnaJ homo	DNJC3_HUI	21	0.964295
24H 10MT/	Q5TGL8	0.238992	0.188071	0.096997	PXDC1	PX domain-	PXDC1_HU	2	1.180168
48H 50MT/	Q99715	0.495673	0.188136	0.097025	COL12A1	Collagen al	COCA1_HU	2	1.409979
48H 10MT/	Q9BY78	-0.06844	0.18821	0.097056	RNF26	E3 ubiquiti	RNF26_HUI	1	0.953665
48H 50MT/	P31948	0.050186	0.188283	0.097084	STIP1	Stress-indu	STIP1_HUM	34	1.035399
48H 50MT/	O96005	-0.06454	0.188307	0.097093	CLPTM1	Putative lip	CLPT1_HUI	16	0.956248
24H 50MT/	O95081	-0.03286	0.188348	0.097107	AGFG2	Arf-GAP do	AGFG2_HU	7	0.977482
48H 10MT/	Q9NVH0	0.00091	0.188392	0.097129	EXD2	Exonucleas	EXD2_HUM	17	1.000631
48H 50MT/	Q12899	-0.15766	0.188413	0.097137	TRIM26	Tripartite n	TRI26_HUM	14	0.896479
48H 10MT/	P20742	0.165804	0.188443	0.09715	PZP	Pregnancy	PZP_HUMA	3	1.121791
24H 10MT/	O43708	-0.00125	0.188463	0.097157	GSTZ1	Maleylacet	MAAI_HUM	5	0.999134
24H 10MT/	P22087	0.054925	0.188519	0.097181	FBL	rRNA 2'-O-n	FBRL_HUM	15	1.038805
48H 10MT/	Q9H082	-0.36921	0.188596	0.097216	RAB33B	Ras-related	RB33B_HUI	2	0.774206
24H 10MT/	Q13907	0.056282	0.18869	0.097259	IDI1	Isopenteny	IDI1_HUM/	10	1.039783
24H 50MT/	Q9H799	-0.08946	0.188712	0.097265	CPLANE1	Ciliogenesis	CPLN1_HUI	1	0.939877
48H 10MT/	Q9NRG4	-0.18923	0.188751	0.097284	SMYD2	N-lysine me	SMYD2_HU	5	0.877073
24H 50MT/	Q13426	-0.07454	0.188771	0.09729	XRCC4	DNA repair	XRCC4_HUI	9	0.949648
48H 50MT/	Q6UX07	0.122117	0.188789	0.097295	DHRS13	Dehydroge	DHR13_HU	9	1.088331
24H 10MT/	Q86X55	0.044088	0.188794	0.097295	CARM1	Histone-arg	CARM1_HU	14	1.031031
24H 10MT/	Q96FW1	0.012694	0.1888	0.097295	OTUB1	Ubiquitin t	OTUB1_HU	14	1.008837
24H 50MT/	O14893	0.24315	0.188817	0.097303	GEMIN2	Gem-associ	GEMI2_HUI	6	1.183574
24H 50MT/	Q4LE39	0.227665	0.188896	0.097338	ARID4B	AT-rich inte	ARI4B_HUM	5	1.170938
24H 10MT/	Q96QF0	-0.29984	0.188931	0.097352	RAB3IP	Rab-3A-inte	RAB3I_HUM	4	0.81234
48H 50MT/	P0DPB6	-0.08309	0.188963	0.097364	POLR1D	DNA-direct	RPAC2_HUI	5	0.944036

24H	50MT/	Q9NRR5	0.018834	0.188965	0.097364	UBQLN4	Ubiquilin-4	UBQL4_HU	9	1.013141
48H	10MT/	P25325	-0.06045	0.188996	0.097377	MPST	3-mercapto	THTM_HUN	15	0.958968
48H	10MT/	P02656	0.339015	0.189029	0.097387	APOC3	Apolipopro	APOC3_HU	1	1.264892
24H	10MT/	P61981	0.04433	0.189044	0.097393	YWHAG	14-3-3 prot	1433G_HU	11	1.031204
24H	10MT/	Q17RN3	-0.16785	0.189084	0.097409	FAM98C	Protein FAN	FA98C_HUI	3	0.890166
48H	50MT/	Q8IZ13	0.000463	0.189131	0.097429	FAM200C	Protein FAN	F200C_HUI	1	1.000321
24H	50MT/	Q8NHV9	-0.52971	0.189222	0.097468	RHOXF1	Rhox home	RHXF1_HUI	1	0.692694
48H	10MT/	Q9UFW8	-0.08394	0.189268	0.097488	CGGBP1	CGG triplet	CGBP1_HU	6	0.943478
48H	10MT/	P60520	-0.19664	0.189293	0.097498	GABARAPL2	Gamma-am	GBRL2_HUI	4	0.872578
24H	10MT/	Q8IWU2	-0.41396	0.189293	0.097498	LMTK2	Serine/thre	LMTK2_HU	6	0.75056
48H	10MT/	Q9BRJ2	-0.09362	0.189337	0.097518	MRPL45	Large ribos	RM45_HUN	14	0.937168
48H	50MT/	Q6AI08	-0.03197	0.189375	0.097535	HEATR6	HEAT repea	HEAT6_HUI	17	0.978086
48H	10MT/	P29218	0.031521	0.189448	0.097564	IMPA1	Inositol mc	IMPA1_HUI	14	1.022089
48H	50MT/	Q86TM6	-0.05765	0.189485	0.097582	SYVN1	E3 ubiquiti	SYVN1_HUI	12	0.96083
48H	50MT/	Q9UGL1	0.186765	0.189498	0.097586	KDM5B	Lysine-spec	KDM5B_HU	6	1.138208
24H	50MT/	P62891;Q5	0.084163	0.189509	0.097587	RPL39;RPL3	Large ribos	RL39_HUM	2	1.060073
48H	10MT/	Q6ZUJ8	0.005165	0.189507	0.097587	PIK3AP1	Phosphoin	BCAP_HUM	12	1.003586
24H	50MT/	O60333	-0.05142	0.189611	0.097625	KIF1B	Kinesin-like	KIF1B_HUN	29	0.964983
48H	10MT/	P47813	-0.11299	0.189692	0.097657	EIF1AX	Eukaryotic	IF1AX_HUN	9	0.924667
24H	50MT/	Q96F44	-0.11247	0.189777	0.097695	TRIM11	E3 ubiquiti	TRI11_HUN	3	0.925006
48H	10MT/	Q9UBX3	-0.09581	0.189843	0.097727	SLC25A10	Mitochond	DIC_HUMA	13	0.935749
48H	10MT/	P62244	-0.07724	0.189864	0.097732	RPS15A	Small ribos	RS15A_HUI	6	0.947871
24H	10MT/	O95166	-0.10249	0.189909	0.097748	GABARAP	Gamma-am	GBRAP_HU	3	0.931423
24H	50MT/	Q9GZY4	0.254083	0.189969	0.097776	COA1	Cytochrom	COA1_HUN	6	1.192577
24H	50MT/	Q8N3V7	-0.33621	0.190022	0.097794	SYNPO	Synaptopo	SYNPO_HUI	6	0.792122
48H	50MT/	Q96Q15	-0.02097	0.190065	0.097811	SMG1	Serine/thre	SMG1_HUN	30	0.985571
24H	50MT/	Q7Z406	-0.00532	0.190146	0.09784	MYH14	Myosin-14	MYH14_HU	42	0.996319
48H	50MT/	Q0VDF9	-0.06443	0.190191	0.097862	HSPA14	Heat shock	HSP7E_HUI	19	0.956321
24H	50MT/	Q9NP97	-0.09315	0.190216	0.097872	DYNLRB1	Dynein ligh	DLRB1_HUI	5	0.937474
24H	50MT/	Q92917	0.035015	0.190348	0.097928	GPKOW	G-patch do	GPKOW_HU	19	1.024568
24H	50MT/	Q9H583	-0.00098	0.190417	0.097954	HEATR1	HEAT repea	HEAT1_HUI	76	0.999321
24H	10MT/	Q5BJF6	0.061966	0.19052	0.097999	ODF2	Outer dens	ODFP2_HU	6	1.043887
48H	50MT/	Q96R05	0.380818	0.190531	0.098003	RBP7	Retinoid-bi	RET7_HUM	2	1.302079
48H	50MT/	Q96IX5	-0.10524	0.190537	0.098005	ATP5MK	ATP synthas	ATPMK_HU	3	0.929648
48H	50MT/	Q99471	-0.10947	0.190626	0.098045	PFDN5	Prefoldin su	PFD5_HUM	4	0.926931
24H	50MT/	Q6NXE6	-0.08005	0.190645	0.098052	ARMC6	Armadillo r	ARMC6_HU	14	0.946025
24H	50MT/	P86790;P8	0.053019	0.190701	0.09807	CCZ1B;CCZ1	Vacuolar fu	CCZ1B_HUI	21	1.037434
48H	50MT/	Q14738	-0.01344	0.1907	0.09807	PPP2R5D	Serine/thre	2A5D_HUN	21	0.990726
24H	50MT/	Q9ULI0	-0.03795	0.190754	0.09809	ATAD2B	ATPase fam	ATD2B_HUI	8	0.974035
48H	50MT/	Q15723	1.053066	0.190798	0.098112	ELF2	ETS-related	ELF2_HUM/	1	2.074935
48H	10MT/	Q659A1	0.377973	0.190812	0.098117	ICE2	Little elong	ICE2_HUM/	1	1.299515
24H	10MT/	Q9UHD8	-0.05467	0.190908	0.098157	SEPTIN9	Septin-9	SEPT9_HUN	28	0.962816
48H	50MT/	Q15019	0.062035	0.190921	0.098161	SEPTIN2	Septin-2	SEPT2_HUN	16	1.043937
24H	50MT/	P09110	0.049148	0.190942	0.098171	ACAA1	3-ketoacyl-	THIK_HUM/	18	1.034654
48H	10MT/	P62760	-0.0392	0.191022	0.098202	VSNL1	Visinin-like	VISL1_HUN	7	0.973195
48H	50MT/	P78395	-0.70673	0.19106	0.098218	PRAME	Melanoma	PRAME_HU	2	0.612706
24H	10MT/	P02461	0.253905	0.191121	0.098245	COL3A1	Collagen al	CO3A1_HU	4	1.192431
48H	50MT/	P48426	-0.0712	0.191136	0.09825	PIP4K2A	Phosphatid	PI42A_HUN	8	0.951849
24H	10MT/	Q06033	0.48247	0.191147	0.098254	ITIH3	Inter-alpha	ITIH3_HUN	4	1.397133
48H	10MT/	Q7Z6E9	0.020378	0.191167	0.098258	RBBP6	E3 ubiquiti	RBBP6_HUI	18	1.014225
48H	10MT/	O14920	-0.04411	0.191178	0.098258	IKBKB	Inhibitor o	IKKB_HUM/	10	0.969889
24H	10MT/	Q7L099	0.257423	0.191183	0.098258	RUFY3	Protein RU	RUFY3_HUI	6	1.195342

24H 50MT/	Q9Y2I9	1.340589	0.191175	0.098258	TBC1D30	TBC1 doma	TBC30_HUI	1	2.532546
48H 10MT/	O94811	0.124315	0.1912	0.098263	TPPP	Tubulin pol	TPPP_HUM	3	1.08999
48H 50MT/	Q567U6	0.016955	0.191223	0.098273	CCDC93	Coiled-coil	CCD93_HU	11	1.011822
48H 50MT/	P62633	-0.07309	0.191262	0.098287	CNBP	CCHC-type	CNBP_HUM	6	0.950602
48H 50MT/	Q99613	-0.05991	0.191294	0.098298	EIF3C	Eukaryotic	EIF3C_HUM	34	0.959323
48H 10MT/	Q7Z5R6	-0.70815	0.191321	0.098309	APBB1IP	Amyloid be	AB1IP_HUM	1	0.612103
48H 50MT/	O15446	-0.07813	0.191327	0.09831	POLR1G	DNA-direct	RPA34_HUI	8	0.947282
48H 50MT/	Q9H9J4	-0.00924	0.191328	0.09831	USP42	Ubiquitin c	UBP42_HU	6	0.993614
48H 50MT/	Q9NXA8	0.026937	0.191362	0.098324	SIRT5	NAD-depen	SIR5_HUM/	8	1.018847
24H 50MT/	Q53EU6	-0.10167	0.191378	0.098329	GPAT3	Glycerol-3-	GPAT3_HUI	14	0.931954
24H 50MT/	O60825	0.714392	0.191396	0.098334	PFKFB2	6-phospho	F262_HUM	2	1.640792
24H 10MT/	Q16254	-0.60001	0.191399	0.098335	E2F4	Transcripti	E2F4_HUM	2	0.65975
24H 50MT/	Q8TAT6	-0.05934	0.191427	0.098346	NPLOC4	Nuclear prc	NPL4_HUM	27	0.959703
48H 50MT/	Q96HR3	0.764724	0.191438	0.098347	MED30	Mediator o	MED30_HU	2	1.699045
48H 50MT/	P34931	0.595403	0.191506	0.098374	HSPA1L	Heat shock	HS71L_HUI	3	1.510894
24H 50MT/	P49815	0.009071	0.191539	0.098388	TSC2	Tuberin	TSC2_HUM	2	1.006307
48H 10MT/	P61758	-0.03756	0.191544	0.09839	VBP1	Prefoldin su	PFD3_HUM	10	0.974301
24H 10MT/	Q70Z53	0.136226	0.191556	0.098395	FRA10AC1	Protein FR/	F10C1_HUI	5	1.099027
48H 50MT/	O75439	-0.05015	0.19161	0.098418	PMPCB	Mitochond	MPPB_HUM	22	0.965833
24H 50MT/	Q7Z478	0.10152	0.191615	0.098419	DHX29	ATP-depend	DHX29_HU	37	1.072903
48H 10MT/	O75874	0.004347	0.191637	0.098424	IDH1	Isocitrate d	IDHC_HUM	23	1.003018
24H 10MT/	Q96R06	0.093213	0.191731	0.098459	SPAG5	Sperm-asso	SPAG5_HUI	24	1.066744
24H 50MT/	P26641	-0.08555	0.191753	0.098468	EEF1G	Elongation	EF1G_HUM	27	0.942422
24H 50MT/	Q8IXI2	0.237492	0.191827	0.098495	RHOT1	Mitochond	MIRO1_HU	8	1.178941
24H 50MT/	Q9GZN8	-0.10882	0.191854	0.098505	ADISSP	Adipose-sec	ADSSP_HUI	9	0.927347
48H 10MT/	Q5T2R2	-0.44487	0.191864	0.098509	PDSS1	All trans-pc	DPS1_HUM	3	0.734649
24H 10MT/	Q6P2P2	0.025117	0.19189	0.098521	PRMT9	Protein arg	ANM9_HUM	8	1.017562
48H 10MT/	Q13618	0.021169	0.192076	0.098598	CUL3	Cullin-3	CUL3_HUM	35	1.014781
24H 50MT/	Q5TCQ9	-0.05438	0.192071	0.098598	MAGI3	Membrane-	MAGI3_HU	6	0.963007
48H 50MT/	Q9NWX6	-0.02576	0.192076	0.098598	THG1L	Probable tF	THG1_HUM	5	0.9823
24H 50MT/	P40227	0.028273	0.192095	0.098607	CCT6A	T-complex	TCPZ_HUM	27	1.019791
48H 10MT/	Q9UKK9	-0.02095	0.1921	0.098608	NUDT5	ADP-sugar p	NUDT5_HU	13	0.985581
24H 10MT/	P17020	0.590929	0.192108	0.098608	ZNF16	Zinc finger	ZNF16_HUI	1	1.506216
24H 10MT/	Q9BV36	-0.15992	0.192126	0.098616	MLPH	Melanophil	MELPH_HU	16	0.895077
48H 10MT/	Q96PE7	-0.10561	0.192161	0.098628	MCEE	Methylmal	MCEE_HUM	3	0.929411
24H 50MT/	Q96MW5	0.062603	0.19218	0.098636	COG8	Conserved	COG8_HUM	18	1.044348
48H 10MT/	Q96SZ5	0.049158	0.192194	0.098638	ADO	2-aminoeth	AEDO_HUM	5	1.034661
24H 50MT/	Q9BRA2	0.006484	0.192196	0.098638	TXNDC17	Thioredoxi	TXD17_HUI	5	1.004504
48H 10MT/	O60512	-0.24394	0.192236	0.098653	B4GALT3	Beta-1,4-ga	B4GT3_HUI	3	0.844438
24H 50MT/	P36871	0.018933	0.192374	0.09871	PGM1	Phosphoglu	PGM1_HUM	28	1.01321
24H 10MT/	P60006	-0.13545	0.192401	0.098722	ANAPC15	Anaphase-p	APC15_HUI	2	0.910387
48H 10MT/	Q7Z4R8	-0.00214	0.192406	0.098722	C6orf120	UPF0669 p	CF120_HUI	1	0.998519
48H 50MT/	Q9NRY5	-0.06146	0.192423	0.098725	FAM114A2	Protein FAM	F1142_HUI	15	0.958294
24H 10MT/	P13646	1.011734	0.192464	0.098742	KRT13	Keratin, typ	K1C13_HUI	6	2.016333
24H 50MT/	O43933	-0.06092	0.192532	0.098768	PEX1	Peroxisoma	PEX1_HUM	15	0.958654
24H 10MT/	Q32P28	0.012178	0.192635	0.098813	P3H1	Prolyl 3-hy	P3H1_HUM	19	1.008477
48H 50MT/	P50748	-0.15148	0.192644	0.098814	KNTC1	Kinetochor	KNTC1_HUI	36	0.900323
24H 10MT/	Q9H467	0.008408	0.192664	0.098819	CUEDC2	CUE domai	CUED2_HU	4	1.005845
48H 10MT/	Q8N0Y2	-0.16456	0.192681	0.098826	ZNF444	Zinc finger	ZN444_HUI	3	0.892199
24H 10MT/	P47914	0.123007	0.192756	0.098854	RPL29	Large ribos	RL29_HUM	2	1.089002
24H 50MT/	Q8N5M9	0.325618	0.192768	0.098859	JAGN1	Protein jag	JAGN1_HUI	2	1.253201
48H 50MT/	Q9HCE5	-0.37821	0.192815	0.098879	METTL14	N6-adenosi	MET14_HU	6	0.769391

24H	10MT/	Q9Y6I3	-0.06405	0.192882	0.098906	EPN1	Epsin-1	EPN1_HUM	10	0.956577
48H	10MT/	P29372	0.000716	0.192906	0.098917	MPG	DNA-3-met	3MG_HUM	11	1.000497
24H	10MT/	Q9BUE0	-0.06661	0.192986	0.098948	MED18	Mediator o	MED18_HU	3	0.954879
24H	10MT/	Q9ULW3	-0.05998	0.19303	0.098965	ABT1	Activator o	ABT1_HUM	8	0.959279
24H	10MT/	Q96IU4	-0.11475	0.193037	0.098967	ABHD14B	Putative pr	ABHEB_HU	6	0.923541
24H	10MT/	P17706	0.056833	0.193073	0.098983	PTPN2	Tyrosine-pr	PTN2_HUM	9	1.04018
24H	10MT/	Q8NEF9	0.045272	0.193073	0.098983	SRFBP1	Serum resp	SRFB1_HU	7	1.031878
24H	50MT/	O76021	0.024315	0.1931	0.098992	RSL1D1	Ribosomal	RL1D1_HU	22	1.016997
24H	50MT/	O75113	0.13865	0.193111	0.098995	N4BP1	NEDD4-bin	N4BP1_HU	6	1.100875
48H	50MT/	Q13625	-0.18094	0.193135	0.099003	TP53BP2	Apoptosis-s	ASPP2_HU	3	0.882125
24H	10MT/	Q13488	0.033259	0.193184	0.099023	TCIRG1	V-type prot	VPP3_HUM	3	1.023321
48H	10MT/	Q9H0W8	0.055459	0.193233	0.099038	SMG9	Nonsense-n	SMG9_HUN	10	1.03919
48H	10MT/	P41091	-0.00144	0.193262	0.099052	EIF2S3	Eukaryotic	IF2G_HUM/	24	0.998999
48H	10MT/	Q96ST2	0.007619	0.19327	0.099054	IWS1	Protein IWS	IWS1_HUM	13	1.005295
48H	10MT/	P15170	-0.06962	0.193312	0.099072	GSPT1	Eukaryotic	ERF3A_HU	24	0.952889
24H	10MT/	Q8IZH2	0.014034	0.193354	0.099085	XRN1	5'-3' exorib	XRN1_HUM	13	1.009775
48H	10MT/	P02769	-0.00219	0.193358	0.099086	ALB	Serum albu	ALBU_BOVI	56	0.998484
24H	50MT/	O00220	0.207281	0.193379	0.099094	TNFRSF10A	Tumor necr	TR10A_HU	3	1.15451
24H	10MT/	Q9UMZ2	0.06041	0.193417	0.099106	SYNRG	Synergin ga	SYNRG_HU	13	1.042762
48H	50MT/	Q6GYQ0	0.116397	0.193427	0.09911	RALGAPA1	Ral GTPase-	RGPA1_HU	17	1.084024
24H	10MT/	P61158	-0.07573	0.193455	0.099116	ACTR3	Actin-relate	ARP3_HUM	18	0.948859
48H	10MT/	Q9BY12	-0.07733	0.193469	0.099122	SCAPER	S phase cyc	SCAPE_HU	7	0.947812
24H	50MT/	P16422	0.202767	0.193499	0.099135	EPCAM	Epithelial c	EPCAM_HU	4	1.150904
24H	50MT/	Q9UH65	-0.11358	0.193529	0.099147	SWAP70	Switch-asso	SWP70_HU	26	0.924291
24H	10MT/	P36507	-0.07689	0.193537	0.09915	MAP2K2	Dual specifi	MP2K2_HU	29	0.948101
48H	10MT/	P11498	-0.10937	0.19358	0.099169	PC	Pyruvate ca	PYC_HUMA	45	0.926991
48H	10MT/	Q9NVD7	-0.11368	0.193611	0.099182	PARVA	Alpha-parvi	PARVA_HU	4	0.924228
48H	10MT/	Q01664	-0.28342	0.193633	0.099188	TFAP4	Transcripti	TFAP4_HU	1	0.821642
24H	10MT/	O15315	0.292238	0.19364	0.09919	RAD51B	DNA repair	RA51B_HU	1	1.224538
24H	10MT/	Q9H9A6	-0.07758	0.193642	0.09919	LRRC40	Leucine-ric	LRC40_HU	27	0.947644
48H	50MT/	Q15427	-0.16988	0.193655	0.099194	SF3B4	Splicing fac	SF3B4_HU	8	0.888915
48H	50MT/	Q9P2B4	-0.06438	0.193653	0.099194	CTTNBP2NI	CTTNBP2 N	CT2NL_HU	11	0.956355
48H	50MT/	O60499	0.088468	0.193669	0.0992	STX10	Syntaxin-1(	STX10_HU	5	1.06324
48H	10MT/	O75896	-0.14107	0.193681	0.0992	TUSC2	Tumor supp	TUSC2_HU	4	0.906844
24H	50MT/	O14757	-0.02626	0.193714	0.099212	CHEK1	Serine/thre	CHK1_HUM	12	0.981961
48H	10MT/	Q9H1I8	0.025568	0.193727	0.099216	ASCC2	Activating s	ASCC2_HU	14	1.017881
48H	50MT/	O43681	0.027184	0.193738	0.099218	GET3	ATPase GET	GET3_HUM	14	1.019021
48H	50MT/	Q2M2I8	0.035208	0.193737	0.099218	AAK1	AP2-associa	AAK1_HUM	18	1.024705
24H	10MT/	P62854	0.06177	0.193754	0.099224	RPS26	Small ribos	RS26_HUM	5	1.043746
24H	50MT/	P11940	0.022783	0.193807	0.099243	PABPC1	Polyadenyl	PABP1_HU	36	1.015917
24H	10MT/	Q8WV22	0.186011	0.193883	0.099276	NSMCE1	Non-structu	NSE1_HUM	5	1.137614
24H	50MT/	Q15717	0.030618	0.193923	0.099289	ELAVL1	ELAV-like pr	ELAV1_HU	11	1.02145
48H	50MT/	Q5T0F9	-0.06389	0.193954	0.0993	CC2D1B	Coiled-coil	C2D1B_HU	11	0.956679
48H	50MT/	A4D161	0.048562	0.193988	0.099315	FAM221A	Protein FAM	F221A_HU	3	1.034234
24H	50MT/	Q6Y2X3	0.36519	0.194013	0.099326	DNAJC14	DnaJ homo	DJC14_HU	2	1.288052
24H	10MT/	P47985	-0.05954	0.194048	0.099341	UQCRFS1	Cytochrom	UCRI_HUM	11	0.959573
24H	10MT/	P30613	-0.01201	0.194118	0.099365	PKLR	Pyruvate ki	KPYR_HUM	4	0.991712
24H	50MT/	Q8IUC4	-0.00609	0.194125	0.099366	RHPN2	Rhophilin-2	RHPN2_HU	21	0.995791
24H	10MT/	P63162	0.311212	0.194139	0.09937	SNRPN	Small nucle	RSMN_HUN	1	1.24075
24H	50MT/	Q8WV41	0.225773	0.194163	0.09938	SNX33	Sorting nex	SNX33_HU	5	1.169404
48H	50MT/	Q9P0P0	-0.2157	0.194175	0.099385	RNF181	E3 ubiquiti	RN181_HU	2	0.861127
48H	10MT/	A0A1B0GTU	0.033764	0.194224	0.099403	ZC3H11B	Zinc finger (	ZC11B_HU	1	1.02368

24H	10MT/	Q0JRZ9	-0.0619	0.19425	0.099415	FCHO2	F-BAR dom:	FCHO2_HU	6	0.958003
24H	10MT/	P0C0L4	0.332924	0.194259	0.099418	C4A	Complemer	CO4A_HUM	10	1.259564
24H	10MT/	Q8IZ13	-0.13503	0.194283	0.099429	FAM200C	Protein FAI	F200C_HUI	1	0.910649
48H	10MT/	Q0P6H9	-0.21669	0.194333	0.09945	TMEM62	Transmemk	TMM62_HU	3	0.860537
48H	50MT/	P43246	-0.07377	0.1944	0.09948	MSH2	DNA misma	MSH2_HUM	40	0.950154
48H	10MT/	Q9UBF8	0.014985	0.194454	0.099495	PI4KB	Phosphatid	PI4KB_HUM	9	1.010441
48H	50MT/	Q6ZWJ1	-0.04584	0.194508	0.099515	STXBP4	Syntaxin-bi	STXB4_HUM	10	0.968722
24H	50MT/	Q6IQ22	0.068163	0.194512	0.099516	RAB12	Ras-related	RAB12_HUI	12	1.048381
48H	10MT/	O95758	-0.00889	0.194548	0.099531	PTBP3	Polypyrimi	PTBP3_HUI	13	0.993857
24H	50MT/	O15533	0.009065	0.194694	0.099585	TAPBP	Tapasin	TPSN_HUM	5	1.006303
48H	10MT/	Q9P253	-0.08738	0.194724	0.099599	VPS18	Vacuolar pı	VPS18_HUI	27	0.941231
24H	10MT/	Q8NG68	0.086802	0.194728	0.0996	TTL	Tubulin--ty	TTL_HUMAI	5	1.062013
24H	10MT/	Q9NPF5	0.00321	0.194751	0.09961	DMAP1	DNA methy	DMAP1_HU	9	1.002228
48H	50MT/	Q9UQ53	-0.42025	0.194812	0.099637	MGAT4B	Alpha-1,3-r	MGT4B_HU	4	0.747293
48H	10MT/	Q504Y3	-0.53355	0.194841	0.099648	ZCWPW2	Zinc finger	ZCPW2_HU	1	0.690851
48H	50MT/	O94967	0.108587	0.195075	0.099749	WDR47	WD repeat-	WDR47_HU	10	1.078172
48H	10MT/	P38398	0.159644	0.195125	0.099771	BRCA1	Breast canc	BRCA1_HUI	3	1.117012
24H	50MT/	Q13330	-0.11944	0.19513	0.099771	MTA1	Metastasis-	MTA1_HUM	12	0.920545
48H	10MT/	Q9UEE9	0.021282	0.195129	0.099771	CFDP1	Craniofacia	CFDP1_HUI	5	1.014861
48H	10MT/	Q9UKM7	-0.07	0.195151	0.099774	MAN1B1	Endoplasm	MA1B1_HU	13	0.952637
24H	10MT/	Q9NUM4	0.058725	0.195164	0.099779	TMEM106E	Transmemk	T106B_HUI	9	1.041545
48H	10MT/	P19013	0.042853	0.195211	0.099798	KRT4	Keratin, tyı	K2C4_HUM	9	1.030149
24H	50MT/	Q86Y79	-0.16762	0.195263	0.09982	PTRH1	Peptidyl-tR	PTH_HUMA	5	0.890311
24H	10MT/	O75208	-0.05077	0.195304	0.099834	COQ9	Ubiquinon	COQ9_HUM	7	0.96542
48H	50MT/	Q13619	-0.093	0.195309	0.099835	CUL4A	Cullin-4A	CUL4A_HUI	28	0.937571
48H	10MT/	Q99571	-0.00493	0.195315	0.099837	P2RX4	P2X purino	P2RX4_HUI	5	0.996589
24H	10MT/	Q9Y294	0.103254	0.195319	0.099837	ASF1A	Histone ch	ASF1A_HUI	5	1.074194
48H	50MT/	O95571	-0.03123	0.195348	0.09985	ETHE1	Persulfide	ETHE1_HUI	10	0.978583
48H	10MT/	O14967	-0.06436	0.195394	0.099869	CLGN	Calmegin	CLGN_HUM	9	0.956367
48H	50MT/	Q9P2D3	-0.12486	0.195392	0.099869	HEATR5B	HEAT repea	HTR5B_HUI	31	0.917096
48H	50MT/	Q9UJ14	0.015927	0.195398	0.099869	GGT7	Glutathion	GGT7_HUM	2	1.011101
48H	10MT/	P78332	-0.09428	0.195434	0.099882	RBM6	RNA-bindir	RBM6_HUM	23	0.936737
24H	10MT/	Q13596	-0.059	0.195473	0.099899	SNX1	Sorting nex	SNX1_HUM	17	0.959926
24H	50MT/	Q9GZT4	0.131561	0.195487	0.099902	SRR	Serine race	SRR_HUMA	4	1.095478
48H	10MT/	Q9BQC6	0.013049	0.195505	0.09991	MRPL57	Large ribos	RT63_HUM	1	1.009086
48H	50MT/	P08621	0.018871	0.195527	0.099917	SNRNP70	U1 small nu	RU17_HUM	14	1.013166
48H	50MT/	Q9H074	0.084172	0.19554	0.09992	PAIP1	Polyadenyl	PAIP1_HUM	14	1.060079
24H	50MT/	Q9HD26	0.07163	0.195593	0.099941	GOPC	Golgi-assoc	GOPC_HUM	17	1.050903
48H	50MT/	Q9UDY8	-0.0276	0.19559	0.099941	MALT1	Mucosa-ass	MALT1_HU	22	0.98105
24H	50MT/	Q8N5A5	0.053459	0.195608	0.099948	ZGPAT	Zinc finger	ZGPAT_HUI	9	1.03775
24H	10MT/	Q9UHB9	0.007423	0.195643	0.099957	SRP68	Signal reco	SRP68_HUI	31	1.005159
48H	50MT/	Q96SB8	0.032504	0.195929	0.100069	SMC6	Structural	SMC6_HUM	9	1.022785
24H	50MT/	Q96A72	0.052368	0.195959	0.100081	MAGOHB	Protein ma	MGN2_HUI	10	1.036966
24H	10MT/	Q9BZQ6	0.105332	0.195967	0.100082	EDEM3	ER degrada	EDEM3_HU	9	1.075742
48H	10MT/	Q92692	0.038164	0.195975	0.100085	NECTIN2	Nectin-2	NECT2_HUI	11	1.026806
48H	10MT/	Q8N0T1	0.001975	0.196025	0.100106	RBIS	Ribosomal	RBIS_HUM/	3	1.00137
48H	10MT/	Q7L5D6	-0.06411	0.19603	0.100108	GET4	Golgi to ER	GET4_HUM	12	0.956537
48H	10MT/	O43861	0.016261	0.196052	0.100118	ATP9B	Probable pl	ATP9B_HUI	4	1.011335
24H	50MT/	Q9Y6M4	0.724985	0.196075	0.100128	CSNK1G3	Casein kina	KC1G3_HU	2	1.652884
24H	50MT/	Q9BQA9	0.020796	0.196111	0.10014	CYBC1	Cytochrom	CYBC1_HUI	3	1.014519
48H	50MT/	Q00325	-0.03526	0.196126	0.100144	SLC25A3	Solute carri	S25A3_HUI	15	0.975858
48H	50MT/	P14209	0.436	0.196144	0.100152	CD99	CD99 antig	CD99_HUM	1	1.352849

24H	10MT/	Q658P3	-0.237	0.196249	0.100195	STEAP3	Metallored	STEA3_HUM	5	0.848507
24H	50MT/	Q9HAC7	-0.56164	0.19627	0.100203	SUGCT	Succinate--	SUCHY_HUI	1	0.67753
48H	10MT/	Q8WUA2	-0.03745	0.196344	0.10023	PPIL4	Peptidyl-pr	PPIL4_HUM	20	0.974375
24H	10MT/	P38159	0.127484	0.196357	0.100234	RBMX	RNA-bindir	RBMX_HUM	12	1.092387
24H	50MT/	Q15628	-0.38024	0.196418	0.10026	TRADD	Tumor necr	TRADD_HU	6	0.768308
24H	10MT/	Q9UBC2	-0.04777	0.196438	0.100265	EPS15L1	Epidermal g	EP15R_HUI	27	0.967432
24H	50MT/	Q14568	-0.08643	0.196458	0.100269	HSP90AA2I	Heat shock	HS902_HUI	1	0.941851
24H	10MT/	P20618	-0.01438	0.196555	0.100308	PSMB1	Proteasom	PSB1_HUM	13	0.990084
24H	10MT/	O75400	0.073696	0.196651	0.100347	PRPF40A	Pre-mRNA-|	PR40A_HUI	18	1.052409
48H	50MT/	Q16629	0.077697	0.196727	0.10038	SRSF7	Serine/argi	SRSF7_HUM	6	1.055332
48H	50MT/	Q92889	-0.13167	0.196731	0.10038	ERCC4	DNA repair	XPF_HUMA	14	0.912773
48H	50MT/	P27105	0.144624	0.196802	0.100409	STOM	Stomatin	STOM_HUM	7	1.105442
48H	50MT/	Q9Y6W5	0.011295	0.196846	0.100427	WASF2	Actin-bindi	WASF2_HU	9	1.00786
24H	10MT/	Q9NYF3	-0.90445	0.196878	0.100442	FAM53C	Protein FAI	FA53C_HUI	1	0.534237
48H	50MT/	Q86SE9	0.308401	0.196913	0.100456	PCGF5	Polycomb g	PCGF5_HUI	1	1.238334
24H	50MT/	Q3KP66	0.190491	0.196936	0.100465	INAVA	Innate imm	INAVA_HUM	4	1.141152
48H	50MT/	P61018	0.410712	0.196947	0.100468	RAB4B	Ras-related	RAB4B_HUI	3	1.329342
24H	10MT/	O76024	0.028839	0.196975	0.100478	WFS1	Wolframin	WFS1_HUM	19	1.020191
24H	50MT/	Q86VW0	-0.09658	0.196982	0.100479	SESTD1	SEC14 dom	SESD1_HUI	14	0.93525
48H	10MT/	Q6PD62	-0.06054	0.197032	0.100501	CTR9	RNA polym	CTR9_HUM	29	0.958906
24H	50MT/	O95544	-0.30391	0.197056	0.100508	NADK	NAD kinase	NADK_HUM	3	0.810056
48H	10MT/	Q96C12	-0.1274	0.197056	0.100508	ARMC5	Armadillo r	ARMC5_HU	4	0.915479
24H	50MT/	Q9BSL1	0.017298	0.197054	0.100508	UBAC1	Ubiquitin-a	UBAC1_HU	3	1.012062
48H	50MT/	Q14691	-0.21966	0.197157	0.100551	GINS1	DNA replica	PSF1_HUM	7	0.858767
48H	10MT/	Q96RT8	0.056993	0.197197	0.100569	TUBGCP5	Gamma-tul	GCP5_HUM	3	1.040295
48H	50MT/	Q8NFF5	-0.16578	0.197301	0.100615	FLAD1	FAD syntha	FAD1_HUM	16	0.891447
24H	50MT/	Q9UJ04	0.07374	0.19735	0.100633	TSPYL4	Testis-speci	TSYL4_HUM	2	1.052442
24H	10MT/	Q9H1I8	0.070734	0.197547	0.100712	ASCC2	Activating s	ASCC2_HUI	14	1.050251
48H	50MT/	Q13371	-0.10026	0.197571	0.100721	PDCL	Phosducin-	PHLP_HUM	2	0.932865
24H	50MT/	Q0VG06	0.537333	0.197584	0.100726	FAAP100	Fanconi an	FP100_HUI	2	1.451287
48H	10MT/	Q13162	-0.02193	0.197592	0.100728	PRDX4	Peroxiredo	PRDX4_HU	13	0.984917
24H	10MT/	Q9H6S1	0.077566	0.197617	0.10074	AZI2	5-azacytidi	AZI2_HUM/	5	1.055236
48H	50MT/	O15234	0.323981	0.197628	0.100744	CASC3	Protein CAS	CASC3_HUI	2	1.25178
24H	50MT/	Q15648	0.070314	0.197726	0.100786	MED1	Mediator o	MED1_HUM	13	1.049946
48H	50MT/	Q5W0B1	0.003022	0.197725	0.100786	OBI1	ORC ubiqui	OBI1_HUM	3	1.002097
24H	10MT/	Q6ZT12	-0.04881	0.197728	0.100786	UBR3	E3 ubiquiti	UBR3_HUM	23	0.966732
48H	10MT/	Q8NCN4	0.003833	0.197723	0.100786	RNF169	E3 ubiquiti	RN169_HU	8	1.00266
24H	10MT/	O43688	-0.09942	0.197736	0.100787	PLPP2	Phospholip	PLPP2_HUI	6	0.933408
24H	10MT/	Q96A28	-0.18594	0.197764	0.100797	SLAMF9	SLAM famil	SLAF9_HUM	1	0.879079
24H	10MT/	Q8NBU5	0.044657	0.197771	0.100798	ATAD1	Outer mito	ATAD1_HUI	12	1.031438
48H	10MT/	Q96B26	-0.0458	0.197795	0.100808	EXOSC8	Exosome cc	EXOS8_HUI	9	0.968752
24H	50MT/	Q92526	0.558803	0.197802	0.10081	CCT6B	T-complex		TCPW_HUM	2	1.473046
48H	10MT/	Q13148	-0.0369	0.197817	0.100811	TARDBP	TAR DNA-bi	TADBP_HUI	14	0.974748
24H	50MT/	Q8IV50	-0.10242	0.197811	0.100811	LYSMD2	LysM and p	LYSM2_HUI	1	0.931466
48H	10MT/	Q13131	0.008422	0.197835	0.100816	PRKAA1	5'-AMP-acti	AAPK1_HUI	17	1.005855
48H	10MT/	Q14739	-0.08208	0.197838	0.100816	LBR	Delta(14)-st	LBR_HUMA	14	0.944694
48H	50MT/	Q9Y2Y0	-0.02945	0.197965	0.100871	ARL2BP	ADP-ribosy	AR2BP_HUI	6	0.979791
48H	10MT/	Q6NSJ5	-0.08803	0.197989	0.100882	LRRC8E	Volume-reg	LRC8E_HUI	16	0.940805
24H	10MT/	O75586	0.284358	0.198013	0.100889	MED6	Mediator o	MED6_HUM	2	1.217868
48H	50MT/	P11279	-0.01208	0.198034	0.100896	LAMP1	Lysosome-a	LAMP1_HU	6	0.991662
48H	10MT/	P53041	0.033671	0.198051	0.100901	PPP5C	Serine/thre	PPP5_HUM	17	1.023614
24H	50MT/	Q9BZF3	-0.90105	0.19806	0.100905	OSBPL6	Oxysterol-b	OSBL6_HUI	2	0.535497

24H 10MT/	P14867;P4	0.21045	0.198115	0.100927	GABRA1;G/	Gamma-am	GBRA1_HU	1	1.157049
48H 10MT/	P50749;Q9	0.899027	0.198169	0.100949	RASSF2;RAS	Ras associat	RASF2_HUM	1	1.864808
24H 50MT/	Q9BT22	0.011565	0.198191	0.100955	ALG1	Chitobiosyl	ALG1_HUM	10	1.008048
48H 10MT/	Q8TF74	-0.05332	0.198264	0.100982	WIPF2	WAS/WASL	WIPF2_HUI	11	0.963716
24H 10MT/	P02675	0.019866	0.198308	0.101	FGB	Fibrinogen	FIBB_HUM/	22	1.013865
24H 10MT/	Q9BRR8	0.194247	0.19832	0.101005	GPATCH1	G patch do	GPTC1_HUI	11	1.144127
24H 10MT/	P78382	-0.46246	0.198358	0.101019	SLC35A1	CMP-sialic a	S35A1_HUI	2	0.72575
48H 50MT/	Q15029	-0.03352	0.198402	0.101038	EFTUD2	116 kDa U5	U5S1_HUM	51	0.977034
24H 10MT/	O95620	0.219965	0.198462	0.101059	DUS4L	tRNA-dihyd	DUS4L_HUI	1	1.164705
48H 10MT/	Q6XD76	-0.54467	0.198494	0.10107	ASCL4	Achaete-scu	ASCL4_HUM	1	0.68555
48H 10MT/	Q9BSL1	0.33905	0.198554	0.101088	UBAC1	Ubiquitin-a	UBAC1_HU	3	1.264923
24H 50MT/	P56199	0.179512	0.198728	0.101154	ITGA1	Integrin alp	ITA1_HUM/	12	1.132501
48H 10MT/	P35270	-0.03683	0.198741	0.101159	SPR	Sepiapterin	SPRE_HUM	15	0.974793
24H 50MT/	Q8NHG7	-0.30694	0.198745	0.101159	SVIP	Small VCP/	SVIP_HUM/	1	0.808353
48H 50MT/	Q9UGU0	-0.06975	0.19877	0.101167	TCF20	Transcripti	TCF20_HUM	8	0.952803
48H 50MT/	P82932	-0.10746	0.19879	0.101173	MRPS6	Small ribos	RT06_HUM	4	0.92822
24H 50MT/	Q8IY26	0.247182	0.198824	0.101186	PLPP6	Polyisoprer	PLPP6_HUI	5	1.186887
48H 10MT/	Q5JVF3	-0.06376	0.198831	0.101188	PCID2	PCI domain	PCID2_HUM	13	0.956769
24H 10MT/	O60524	0.039053	0.199031	0.101281	NEMF	Ribosome c	NEMF_HUM	19	1.027439
24H 10MT/	O43826	0.062028	0.199056	0.101289	SLC37A4	Glucose-6-p	G6PT1_HUI	3	1.043932
24H 50MT/	P49184	1.401884	0.199146	0.101317	DNASE1L1	Deoxyribor	DNSL1_HUI	1	2.642465
48H 50MT/	Q96NT0	0.075097	0.199147	0.101317	CCDC115	Coiled-coil	CC115_HUI	4	1.053432
24H 50MT/	O95429	0.020425	0.199161	0.101322	BAG4	BAG family	BAG4_HUM	4	1.014258
24H 10MT/	P61254	0.071699	0.19916	0.101322	RPL26	Large ribos	RL26_HUM	8	1.050954
24H 10MT/	Q14573	-0.05358	0.199165	0.101322	ITPR3	Inositol 1,4	ITPR3_HUM	23	0.963543
24H 50MT/	Q9HCE6	0.256473	0.199169	0.101323	ARHGEF10I	Rho guanin	ARGAL_HUI	10	1.194555
48H 10MT/	P55011	-0.10665	0.19932	0.101383	SLC12A2	Solute carri	S12A2_HUI	31	0.928744
24H 10MT/	P18887	-8.53E-05	0.199367	0.101402	XRCC1	DNA repair	XRCC1_HUI	11	0.999941
48H 50MT/	P49902	-0.1141	0.199375	0.101405	NT5C2	Cytosolic p	5NTC_HUM	26	0.923959
48H 50MT/	Q96D46	-0.0848	0.199382	0.101406	NMD3	60S ribosor	NMD3_HUI	17	0.942912
24H 50MT/	A7KAX9	0.410941	0.199447	0.101432	ARHGAP32	Rho GTPase	RHG32_HU	3	1.329553
24H 50MT/	Q9UGU5	0.280513	0.199468	0.101439	HMGXB4	HMG doma	HMGX4_HU	1	1.214627
24H 10MT/	O75832	0.151187	0.199599	0.101494	PSMD10	26S proteas	PSD10_HUI	9	1.110482
24H 10MT/	Q9BQ15	0.253052	0.199739	0.101551	NABP2	SOSS comp	SOSB1_HUI	2	1.191725
24H 50MT/	Q9C0H2	-0.21625	0.199885	0.101611	TTYH3	Protein twe	TTYH3_HUM	1	0.860797
48H 50MT/	C4AMC7;Q	0.083813	0.199978	0.101653	WASH3P;W	Putative W	WASH3_HU	11	1.059815
24H 50MT/	Q13126	0.01863	0.200033	0.101672	MTAP	S-methyl-5'	MTAP_HUM	18	1.012997
48H 50MT/	P39019	-0.15711	0.200039	0.101674	RPS19	Small ribos	RS19_HUM	11	0.896821
24H 10MT/	O00268	0.103104	0.200054	0.101679	TAF4	Transcripti	TAF4_HUM	13	1.074082
48H 50MT/	Q9UBK9	-0.25486	0.200071	0.101685	UXT	Protein UXT	UXT_HUMA	2	0.838068
24H 10MT/	P49917	-0.05616	0.200169	0.101726	LIG4	DNA ligase	DNLI4_HUM	11	0.96182
24H 50MT/	Q8NC56	0.029468	0.200195	0.101733	LEMD2	LEM domai	LEMD2_HU	23	1.020636
24H 10MT/	Q9Y4I1	-0.05611	0.200222	0.101741	MYO5A	Unconvent	MYO5A_HU	25	0.961853
48H 10MT/	Q9UBK9	-0.08767	0.200256	0.101752	UXT	Protein UXT	UXT_HUMA	2	0.941044
24H 50MT/	P60981	0.032512	0.200274	0.101759	DSTN	Destrin	DEST_HUM	11	1.022791
48H 50MT/	Q96PY6	0.460099	0.200301	0.10177	NEK1	Serine/thre	NEK1_HUM	2	1.375636
24H 50MT/	Q96JB3	-0.12639	0.200311	0.101773	HIC2	Hypermeth	HIC2_HUM	5	0.916124
24H 10MT/	P54132	0.019717	0.200368	0.101796	BLM	RecQ-like D	BLM_HUM/	11	1.01376
24H 10MT/	O14964	-0.0538	0.200413	0.101813	HGS	Hepatocyte	HGS_HUM/	17	0.963392
48H 10MT/	P51398	-0.1259	0.200503	0.101847	DAP3	Small ribos	RT29_HUM	18	0.916431
24H 10MT/	Q96CU9	0.074	0.200545	0.101864	FOXRED1	FAD-depen	FXRD1_HUI	12	1.052631
24H 10MT/	Q86SE9	-0.69519	0.200554	0.101867	PCGF5	Polycomb g	PCGF5_HUI	1	0.617628

48H 10MT/	P59190	0.245763	0.200561	0.101868	RAB15	Ras-related	RAB15_HU	1	1.18572
24H 10MT/	Q9H8H0	0.095015	0.200577	0.101872	NOL11	Nucleolar p	NOL11_HU	24	1.068076
24H 10MT/	P52756	0.067695	0.200732	0.10194	RBM5	RNA-bindir	RBM5_HUM	14	1.048041
48H 10MT/	O15145	-0.00759	0.200805	0.101972	ARPC3	Actin-relate	ARPC3_HU	8	0.994753
24H 50MT/	P27635	0.017828	0.200838	0.101985	RPL10	Large ribos	RL10_HUM	12	1.012434
48H 50MT/	Q8TF42	-0.08649	0.200837	0.101985	UBASH3B	Ubiquitin-a	UBS3B_HU	13	0.941814
24H 10MT/	Q13409	0.076703	0.200864	0.101989	DYNC1I2	Cytoplasmi	DC1I2_HUN	19	1.054606
48H 10MT/	Q13885	-0.03745	0.200863	0.101989	TUBB2A	Tubulin bet	TBB2A_HU	7	0.974376
24H 10MT/	Q14432	-0.01295	0.200859	0.101989	PDE3A	cGMP-inhil	PDE3A_HU	10	0.991065
48H 50MT/	Q5VW38	-0.02099	0.200856	0.101989	GPR107	Protein GP	GP107_HU	6	0.985554
24H 50MT/	Q9HBH1	-0.15534	0.200854	0.101989	PDF	Peptide def	DEFM_HUM	4	0.897921
48H 10MT/	Q92830	-0.05804	0.200886	0.101995	KAT2A	Histone ace	KAT2A_HU	11	0.960566
48H 50MT/	Q6P4E1	0.02647	0.200924	0.102011	GOLM2	Protein GO	GOLM2_HU	9	1.018517
24H 10MT/	Q76M96	-0.08243	0.200929	0.102011	CCDC80	Coiled-coil	CCD80_HU	1	0.944464
48H 10MT/	Q2VIR3	-0.06108	0.200957	0.102023	EIF2S3B	Eukaryotic	IF2GL_HUM	1	0.958543
24H 10MT/	Q8N8Y2	0.305084	0.200968	0.102025	ATP6V0D2	V-type prot	VA0D2_HU	1	1.235491
24H 10MT/	Q9H9T3	-0.0786	0.200981	0.102028	ELP3	Elongator c	ELP3_HUM	18	0.946973
48H 50MT/	Q8IWX8	-0.02337	0.200985	0.102029	CHERP	Calcium ho	CHERP_HU	17	0.983932
24H 10MT/	Q96BT7	-0.03231	0.200992	0.102031	ALKBH8	Alkylated D	ALKB8_HU	7	0.977857
24H 50MT/	O95359	-0.02442	0.201028	0.102046	TACC2	Transformi	TACC2_HU	40	0.983219
48H 10MT/	Q15291	-0.03	0.201053	0.102057	RBBP5	Retinoblast	RBBP5_HU	15	0.979423
24H 50MT/	Q96T21	0.486474	0.201099	0.102077	SECISBP2	Selenocyste	SEBP2_HUN	3	1.401017
48H 10MT/	P04066	-0.07269	0.201136	0.102094	FUCA1	Tissue alph	FUCO_HUM	8	0.950866
24H 50MT/	Q9BQ24	-0.41336	0.201185	0.102116	ZFYVE21	Zinc finger	ZFY21_HUN	1	0.750872
48H 10MT/	Q9C091	-1.19836	0.201261	0.102146	GREB1L	GREB1-like	GRB1L_HU	1	0.435771
48H 50MT/	Q9BQ04	-0.02385	0.201265	0.102147	RBM4B	RNA-bindir	RBM4B_HU	15	0.983604
48H 10MT/	Q9Y4C8	-0.11422	0.20127	0.102148	RBM19	Probable R	RBM19_HU	15	0.923881
24H 10MT/	O94967	0.178548	0.201317	0.102166	WDR47	WD repeat-	WDR47_HU	10	1.131745
24H 50MT/	P47224	-0.19695	0.201321	0.102167	RABIF	Guanine nu	MSS4_HUM	4	0.872393
48H 50MT/	Q9NZD2	-0.15246	0.201347	0.102177	GLTP	Glycolipid	GLTP_HUM	8	0.899717
24H 50MT/	A0AV96	-0.10503	0.201389	0.102192	RBM47	RNA-bindir	RBM47_HU	16	0.929788
48H 10MT/	Q9NUD5	-0.01513	0.20143	0.102207	ZCCHC3	Zinc finger	ZCHC3_HU	8	0.98957
48H 50MT/	P84098	0.0013	0.201471	0.102222	RPL19	Large ribos	RL19_HUM	6	1.000902
48H 50MT/	Q9Y6M7	0.007976	0.20148	0.102225	SLC4A7	Sodium bic	S4A7_HUM	15	1.005544
48H 10MT/	P42338	-0.06344	0.201494	0.102231	PIK3CB	Phosphatic	PK3CB_HU	10	0.95698
48H 10MT/	Q9C0C7	-0.05513	0.201507	0.102235	AMBRA1	Activating	AMRA1_HU	9	0.962505
24H 50MT/	Q2TAY7	-0.01262	0.201541	0.102248	SMU1	WD40 repe	SMU1_HUN	20	0.991293
48H 10MT/	P11021	-0.03581	0.201664	0.102302	HSPA5	Endoplasm	BIP_HUMA	35	0.975483
24H 10MT/	Q96KQ7	0.057918	0.2017	0.102311	EHMT2	Histone-lys	EHMT2_HU	15	1.040963
48H 50MT/	P39656	0.00179	0.201726	0.102318	DDOST	Dolichyl-di	OST48_HU	16	1.001242
24H 50MT/	Q16584	0.154674	0.201735	0.102318	MAP3K11	Mitogen-ac	M3K11_HU	2	1.11317
24H 50MT/	Q9H4G4	0.302096	0.201723	0.102318	GLIPR2	Golgi-assoc	GAPR1_HU	4	1.232934
48H 50MT/	O94913	-0.09737	0.201783	0.102334	PCF11	Pre-mRNA	PCF11_HU	19	0.934738
48H 50MT/	Q13325	0.064664	0.201794	0.102337	IFIT5	Interferon-i	IFIT5_HUM	12	1.045841
24H 10MT/	Q6P1K2	0.054132	0.201794	0.102337	PMF1	Polyamine-	PMF1_HUN	4	1.038234
48H 10MT/	O14717	-0.07966	0.201841	0.102351	TRDMT1	tRNA (cytos	TRDMT_HU	1	0.946279
48H 10MT/	O00212	-0.35263	0.201854	0.102356	RHOD	Rho-relatec	RHOD_HUN	3	0.783152
48H 50MT/	O75865	1.220418	0.201893	0.102369	TRAPPC6A	Trafficking	TPC6A_HU	1	2.330143
48H 10MT/	Q9UJT0	0.76062	0.201888	0.102369	TUBE1	Tubulin eps	TBE_HUMA	1	1.694218
24H 10MT/	Q9UGP4	-0.04388	0.201924	0.102379	LIMD1	LIM domair	LIMD1_HU	14	0.970046
24H 10MT/	Q9NZ52	-0.03594	0.201945	0.102386	GGA3	ADP-ribosy	GGA3_HUM	13	0.975395
24H 10MT/	Q14203	-0.0441	0.201985	0.102401	DCTN1	Dynactin su	DCTN1_HU	52	0.969897

24H 10MT/	Q9UIM3	-0.06686	0.202017	0.102415	FKBPL	FK506-binc	FKBPL_HUI	3	0.954711
48H 50MT/	P30533	0.000531	0.202036	0.102419	LRPAP1	Alpha-2-ma	AMRP_HUM	14	1.000368
24H 10MT/	Q9Y262	0.029088	0.202032	0.102419	EIF3L	Eukaryotic	EIF3L_HUM	32	1.020367
24H 50MT/	O43181	0.230077	0.202041	0.10242	NDUFS4	NADH dehy	NDUS4_HU	5	1.172898
48H 50MT/	Q9NPF5	-0.20129	0.202054	0.102423	DMAP1	DNA methy	DMAP1_HU	9	0.869771
24H 50MT/	Q13434;Q9	0.047972	0.202061	0.102425	MKRN4P;M	Putative E3	MKRN4_HU	1	1.03381
24H 10MT/	Q96C24	0.2557	0.202146	0.102458	SYTL4	Synaptotag	SYTL4_HUM	7	1.193915
48H 10MT/	P61923	0.10507	0.202152	0.102459	COPZ1	Coatomer s	COPZ1_HUI	6	1.075547
24H 10MT/	Q01804	-0.0394	0.202173	0.102464	OTUD4	OTU domai	OTUD4_HU	11	0.973062
24H 50MT/	Q9GZS1	-0.01038	0.20227	0.102502	POLR1E	DNA-direct	RPA49_HUI	10	0.99283
24H 10MT/	Q8WZ42	-0.00493	0.202295	0.102511	TTN	Titin	TITIN_HUM	10	0.996589
48H 10MT/	P36957	-0.0358	0.202309	0.102516	DLST	Dihydrolip	ODO2_HUM	11	0.975494
48H 10MT/	P06454	0.005025	0.20238	0.102548	PTMA	Prothymos	PTMA_HUM	2	1.003489
24H 50MT/	Q9Y2Y0	-0.02993	0.202421	0.102565	ARL2BP	ADP-ribosy	AR2BP_HUI	6	0.97947
48H 50MT/	P18827	0.555317	0.202468	0.102585	SDC1	Syndecan-1	SDC1_HUM	3	1.469492
48H 10MT/	Q9H6T0	-0.00099	0.202469	0.102585	ESRP2	Epithelial s	ESRP2_HUI	10	0.999315
48H 10MT/	Q96N06	-0.02847	0.20256	0.102615	SPATA33	Spermatog	SPT33_HUI	1	0.98046
24H 10MT/	Q7L5N1	-0.04568	0.202565	0.102616	COPS6	COP9 signa	CSN6_HUM	13	0.968834
24H 10MT/	Q6PF05	0.486628	0.202594	0.102628	TTC23L	Tetratricop	TT23L_HUM	1	1.401167
48H 10MT/	P14625	-0.07775	0.202616	0.102638	HSP90B1	Endoplasm	ENPL_HUM	52	0.947535
48H 50MT/	Q8IV61	0.221799	0.202649	0.10265	RASGRP3	Ras guanyl-	GRP3_HUM	2	1.166187
24H 10MT/	Q9Y4B5	-0.00536	0.202657	0.102653	MTCL1	Microtubul	MTCL1_HU	42	0.996295
48H 10MT/	Q8N9N5	-0.71266	0.202705	0.102675	BANP	Protein BAI	BANP_HUM	1	0.610193
48H 50MT/	Q02040	0.012762	0.202712	0.102677	AKAP17A	A-kinase an	AK17A_HUI	12	1.008885
24H 10MT/	O75394	-0.08373	0.20273	0.102681	MRPL33	Large ribos	RM33_HUM	2	0.943613
48H 10MT/	Q96MB7	0.174634	0.202787	0.102705	HARBI1	Putative nu	HARB1_HU	1	1.128678
24H 10MT/	Q86U28	-0.02787	0.202846	0.102732	ISCA2	Iron-sulfur	ISCA2_HUM	8	0.980871
48H 50MT/	Q9BSG0	-0.48799	0.202911	0.10276	PRADC1	Protease-as	PADC1_HU	1	0.713015
48H 10MT/	Q9Y4D8	-0.0548	0.202931	0.102766	HECTD4	Probable E3	HECD4_HU	15	0.962726
24H 10MT/	Q9UBS0	-0.05934	0.202934	0.102767	RPS6KB2	Ribosomal	KS6B2_HUI	4	0.959702
24H 50MT/	Q9H082	0.066168	0.203014	0.102804	RAB33B	Ras-related	RB33B_HUI	2	1.046932
24H 10MT/	P49902	0.004564	0.20303	0.102807	NT5C2	Cytosolic p	5NTC_HUM	26	1.003169
24H 50MT/	P22694	0.150473	0.20304	0.102809	PRKACB	cAMP-depe	KAPCB_HUI	12	1.109933
24H 10MT/	Q9NRY4	-0.10147	0.20307	0.102822	ARHGAP35	Rho GTPase	RHG35_HU	27	0.932083
48H 50MT/	Q8TE67	0.106286	0.203075	0.102822	EPS8L3	Epidermal g	ES8L3_HUM	16	1.076453
24H 50MT/	P21359	-0.03916	0.203084	0.102824	NF1	Neurofibro	NF1_HUMA	36	0.973222
24H 50MT/	P18074	-0.05616	0.203118	0.102838	ERCC2	General tra	ERCC2_HUI	15	0.961818
24H 50MT/	Q13637	0.109415	0.203186	0.102866	RAB32	Ras-related	RAB32_HUI	13	1.078791
48H 50MT/	A1L390	-0.01808	0.203211	0.102877	PLEKHG3	Pleckstrin h	PKHG3_HU	5	0.987544
48H 50MT/	Q6UXH1	0.059749	0.203254	0.102896	CRELD2	Protein dis	CREL2_HUI	5	1.042284
48H 10MT/	A4D1E9	-0.15556	0.203278	0.102905	GTPBP10	GTP-bindin	GTPBA_HUI	12	0.897784
24H 10MT/	Q9H788	-0.07635	0.203318	0.10292	SH2D4A	SH2 domai	SH24A_HUI	10	0.948456
24H 10MT/	O14967	0.016207	0.203357	0.102934	CLGN	Calmegin	CLGN_HUM	9	1.011297
24H 50MT/	Q8N128	0.026858	0.203364	0.102936	FAM177A1	Protein FAI	F177A_HUI	4	1.018791
24H 50MT/	P78382	0.387776	0.203381	0.102942	SLC35A1	CMP-sialic a	S35A1_HUI	2	1.308375
48H 10MT/	P29966	-0.07773	0.203467	0.102966	MARCKS	Myristoylat	MARCS_HU	13	0.947544
48H 10MT/	Q9P2Y4	-0.22583	0.203453	0.102966	ZNF219	Zinc finger	ZN219_HUI	6	0.855101
48H 10MT/	O75962	0.006223	0.203523	0.102988	TRIO	Triple funct	TRIO_HUM	29	1.004323
24H 50MT/	O14715	-0.00315	0.203603	0.103025	RGPD8	RANBP2-lik	RGPD8_HU	43	0.997821
24H 10MT/	O95372	-0.09426	0.203607	0.103026	LYPLA2	Acyl-protei	LYPA2_HUM	8	0.93675
24H 10MT/	Q6R327	0.003939	0.2037	0.103058	RICTOR	Rapamycin	RICTR_HUM	22	1.002734
48H 50MT/	P17568	0.086136	0.203766	0.103086	NDUFB7	NADH dehy	NDUB7_HU	5	1.061523

24H	10MT/	P26885	-0.00418	0.20384	0.103117	FKBP2	Peptidyl-pr	FKBP2_HUI	6	0.997109
24H	50MT/	Q9NUY8	-0.0939	0.203849	0.103117	TBC1D23	TBC1 doma	TBC23_HUI	13	0.936986
24H	10MT/	Q9Y265	0.021659	0.203857	0.10312	RUVBL1	RuvB-like 1	RUVB1_HU	25	1.015126
24H	10MT/	P00748	-0.27102	0.203893	0.103132	F12	Coagulatio	FA12_HUM	5	0.828735
24H	10MT/	P32969	0.076312	0.203934	0.103149	RPL9	Large ribos	RL9_HUMA	12	1.054319
48H	10MT/	Q6ZT21	-0.08363	0.203933	0.103149	TMPPE	Transmemb	TMPPE_HU	4	0.943683
48H	10MT/	Q96MH2	-0.00335	0.204001	0.103175	HEXIM2	Protein HE	HEXI2_HUI	3	0.997678
24H	50MT/	O14776	0.021512	0.204044	0.103194	TCERG1	Transcripti	TCRG1_HUI	32	1.015023
48H	10MT/	Q9Y4K3	-0.03292	0.204137	0.10323	TRAF6	TNF recept	TRAF6_HUI	2	0.977442
48H	50MT/	P36542	-0.03358	0.204164	0.103239	ATP5F1C	ATP synthas	ATPG_HUM	10	0.976992
24H	50MT/	Q8NBF6	0.008512	0.204165	0.103239	AVL9	Late secret	AVL9_HUM	11	1.005917
48H	50MT/	Q15326	-0.14058	0.204178	0.103244	ZMYND11	Zinc finger	ZMY11_HU	5	0.907152
24H	10MT/	Q9NR19	-0.06781	0.204225	0.103262	ACSS2	Acetyl-coer	ACSA_HUM	12	0.954083
24H	50MT/	Q13573	0.008453	0.204232	0.103264	SNW1	SNW doma	SNW1_HUI	24	1.005877
24H	10MT/	Q86UP2	0.008666	0.204255	0.10327	KTN1	Kinectin	KTN1_HUM	68	1.006025
48H	10MT/	Q9NU22	-0.05597	0.204255	0.10327	MDN1	Midasin	MDN1_HUI	115	0.96195
48H	50MT/	Q96PK6	-0.06416	0.2043	0.103288	RBM14	RNA-bindir	RBM14_HU	25	0.956501
48H	10MT/	Q7RTV0	-0.04907	0.204402	0.10333	PHF5A	PHD finger-	PHF5A_HUI	6	0.966557
48H	50MT/	Q9H9Q2	0.079582	0.204473	0.10336	COPS7B	COP9 signa	CSN7B_HUI	7	1.056712
48H	10MT/	P07437	-0.07601	0.204704	0.103462	TUBB	Tubulin bet	TBB5_HUM	5	0.94868
24H	50MT/	Q5T1M5	-0.07895	0.204751	0.103482	FKBP15	FK506-binc	FKB15_HUI	25	0.946749
24H	10MT/	O75521	-0.07769	0.204788	0.103497	ECI2	Enoyl-CoA	ECI2_HUM/	18	0.947572
24H	10MT/	Q9BQ39	0.037991	0.204824	0.103512	DDX50	ATP-depend	DDX50_HU	25	1.026683
24H	50MT/	P51668;Q9	-0.30168	0.204944	0.103555	UBE2D1;UE	Ubiquitin-c	UB2D1_HU	1	0.811304
48H	50MT/	P42858	0.083988	0.205038	0.103592	HTT	Huntingtin	HD_HUMAI	55	1.059944
48H	50MT/	Q9Y2V2	0.097001	0.205088	0.103614	CARHSP1	Calcium-re	CHSP1_HUI	7	1.069548
24H	10MT/	P24863	-0.32534	0.20519	0.103652	CCNC	Cyclin-C	CCNC_HUM	2	0.798112
24H	10MT/	Q14191	-0.27274	0.20519	0.103652	WRN	Bifunction	WRN_HUM	3	0.827747
24H	10MT/	Q8N6R0	-0.04034	0.205228	0.103668	METTL13	eEF1A lysin	EFNMT_HU	17	0.972428
48H	10MT/	Q9BQA1	-0.01707	0.205265	0.10368	WDR77	Methylosor	MEP50_HU	12	0.988238
24H	50MT/	O15439	0.181599	0.20536	0.103718	ABCC4	ATP-bindin	MRP4_HUM	14	1.13414
48H	10MT/	Q8N1G1	-0.54866	0.205407	0.103737	REXO1	RNA exonu	REXO1_HUI	2	0.683655
24H	10MT/	Q13191	-0.22322	0.205443	0.10375	CBLB	E3 ubiquiti	CBLB_HUM	1	0.856653
24H	50MT/	Q9BT78	-0.02313	0.205476	0.103764	COPS4	COP9 signa	CSN4_HUM	25	0.984098
48H	10MT/	Q9UHN1	-0.18159	0.205574	0.1038	POLG2	DNA polym	DPOG2_HU	4	0.881734
24H	50MT/	Q9Y4R8	-0.24755	0.205583	0.103804	TELO2	Telomere le	TELO2_HUI	24	0.842325
24H	10MT/	Q86VU5	-0.31556	0.205703	0.103855	COMTD1	Catechol O	CMTD1_HU	1	0.803541
24H	10MT/	O00418	0.004749	0.205721	0.103862	EEF2K	Eukaryotic	EF2K_HUM	8	1.003297
24H	10MT/	P26641	0.009947	0.205741	0.103868	EEF1G	Elongation	EF1G_HUM	27	1.006919
48H	50MT/	Q9UNK9	-0.19638	0.20574	0.103868	ANGEL1	Protein ang	ANGE1_HU	4	0.872741
48H	10MT/	Q9NS56	-0.06607	0.205776	0.103884	TOPORS	E3 ubiquiti	TOPRS_HUI	7	0.955234
24H	50MT/	Q01105	0.011715	0.205796	0.10389	SET	Protein SET	SET_HUMAI	10	1.008153
48H	10MT/	Q8TB52	0.082451	0.2058	0.10389	FBXO30	F-box only	FBX30_HUI	6	1.058816
48H	10MT/	Q9Y5K8	-0.03494	0.205811	0.103893	ATP6V1D	V-type prot	VATD_HUM	7	0.976073
48H	10MT/	Q96C19	0.053013	0.205824	0.103898	EFHD2	EF-hand do	EFHD2_HUI	13	1.03743
24H	10MT/	P16435	0.001364	0.205889	0.103928	POR	NADPH--cy	NCPR_HUM	39	1.000946
48H	10MT/	Q0D2I5;Q5	0.58097	0.205943	0.103947	IFFO1;IFFO	Non-homol	IFFO1_HUM	1	1.495855
48H	10MT/	Q9UGL1	0.079091	0.206001	0.103972	KDM5B	Lysine-spec	KDM5B_HU	6	1.056352
48H	10MT/	P55317	-0.09785	0.206005	0.103973	FOXA1	Hepatocyte	FOXA1_HUI	2	0.934424
24H	50MT/	O15116	-0.04763	0.206015	0.103976	LSM1	U6 snRNA-a	LSM1_HUM	5	0.967523
24H	10MT/	Q14554	0.059868	0.206021	0.103978	PDIA5	Protein dis	PDIA5_HUI	14	1.04237
48H	50MT/	Q15631	-0.0594	0.2061	0.104014	TSN	Translin	TSN_HUMA	12	0.959664

24H 10MTA	Q9BQT8	-0.15383	0.206115	0.104019	SLC25A21	Mitochond	ODC_HUMA	7	0.898862
24H 50MTA	Q9H299	0.263976	0.206133	0.104027	SH3BGRL3	SH3 domai	SH3L3_HUI	4	1.200784
48H 10MTA	Q9NVE5	-0.01182	0.206143	0.104029	USP40	Ubiquitin c	UBP40_HU	4	0.991838
24H 50MTA	Q9H3L0	-0.82549	0.20615	0.10403	MMADHC	Cobalamin	MMAD_HU	2	0.56429
24H 50MTA	P53384	0.083245	0.206238	0.104066	NUBP1	Cytosolic F	NUBP1_HU	9	1.059398
24H 10MTA	Q14980	0.014622	0.206304	0.104095	NUMA1	Nuclear mi	NUMA1_HU	76	1.010187
24H 10MTA	O15042	-0.00503	0.20639	0.104131	U2SURP	U2 snRNP-a	SR140_HUI	29	0.996519
48H 10MTA	P29558	-0.02784	0.20642	0.104144	RBMS1	RNA-bindir	RBMS1_HU	1	0.980888
48H 50MTA	Q9NYS0	-0.45498	0.206509	0.104181	NKIRAS1	NF-kappa-B	KBRS1_HUI	2	0.729522
24H 10MTA	Q13643	-0.21589	0.206515	0.104183	FHL3	Four and a l	FHL3_HUM	3	0.861016
24H 50MTA	O43920	0.010013	0.206546	0.104193	NDUFS5	NADH dehy	NDUS5_HU	4	1.006964
48H 50MTA	Q07157	0.017271	0.206562	0.104197	TJP1	Tight juncti	ZO1_HUMA	50	1.012043
24H 10MTA	Q8IX01	0.017982	0.206562	0.104197	SUGP2	SURP and G	SUGP2_HU	30	1.012542
48H 10MTA	Q5JUR7	-0.03339	0.206576	0.104201	TEX30	Testis-expr	TEX30_HUI	7	0.977125
24H 10MTA	O14653	-0.0466	0.206597	0.104206	GOSR2	Golgi SNAP	GOSR2_HU	5	0.968213
48H 50MTA	P15927	-0.08809	0.206612	0.104211	RPA2	Replication	RFA2_HUM	8	0.940767
24H 50MTA	P49770	-0.13816	0.20662	0.104213	EIF2B2	Translation	EI2BB_HUN	8	0.908675
48H 10MTA	P02647	-0.09495	0.206668	0.104229	APOA1	Apolipoprc	APOA1_HU	9	0.936308
24H 50MTA	P39210	0.062958	0.206661	0.104229	MPV17	Protein Mp	MPV17_HL	1	1.044605
48H 50MTA	Q13573	0.000754	0.206667	0.104229	SNW1	SNW doma	SNW1_HUI	24	1.000523
48H 10MTA	P38571	0.125324	0.206693	0.104237	LIPA	Lysosomal a	LICH_HUMA	5	1.090753
48H 50MTA	Q99832	-0.04517	0.206766	0.104268	CCT7	T-complex p	TCPH_HUM	36	0.969175
48H 10MTA	P08133	0.035138	0.206802	0.104281	ANXA6	Annexin A6	ANXA6_HU	6	1.024655
48H 50MTA	Q12800	-0.14922	0.206801	0.104281	TFCP2	Alpha-globi	TFCP2_HUI	9	0.901735
24H 10MTA	Q9BY89	-0.04428	0.206807	0.104282	KIAA1671	Uncharacte	K1671_HUI	7	0.969775
48H 50MTA	Q969M7	0.596092	0.206812	0.104283	UBE2F	NEDD8-cor	UBE2F_HUI	3	1.511616
48H 10MTA	Q9BUP3	-0.04529	0.20684	0.104293	HTATIP2	Oxidoreduc	HTAI2_HUI	16	0.969092
48H 50MTA	Q8WVL7	-0.14401	0.20687	0.104305	ANKRD49	Ankyrin rep	ANR49_HU	4	0.904998
48H 10MTA	P06280	-0.0256	0.206924	0.104328	GLA	Alpha-galac	AGAL_HUM	13	0.982413
24H 50MTA	Q14894	0.312056	0.206927	0.104328	CRYM	Ketimine re	CRYM_HUN	4	1.241476
48H 10MTA	P28838	-0.03021	0.206949	0.104336	LAP3	Cytosol am	AMPL_HUN	29	0.979276
48H 10MTA	Q8N5M9	0.152233	0.206947	0.104336	JAGN1	Protein jag	JAGN1_HUI	2	1.111288
24H 50MTA	Q15166	0.123974	0.206991	0.104356	PON3	Serum para	PON3_HUN	10	1.089733
48H 50MTA	Q9H7B4	0.106431	0.20701	0.104363	SMYD3	Histone-lys	SMYD3_HU	6	1.076561
24H 10MTA	Q96GI7	-0.23977	0.207182	0.104434	FAM89A	Protein FAI	FA89A_HUI	1	0.846881
24H 10MTA	Q9Y2G5	-0.02434	0.20721	0.104446	POFUT2	GDP-fucose	OFUT2_HUI	15	0.983272
48H 10MTA	Q96DR7	-0.20493	0.207292	0.104479	ARHGEF26	Rho guanin	ARHGQ_HL	1	0.86758
48H 10MTA	P57060	-0.17764	0.207334	0.104495	RWDD2B	RWD doma	RWD2B_HL	1	0.884146
48H 10MTA	O15212	0.13519	0.207381	0.104514	PFDN6	Prefoldin sı	PFD6_HUM	7	1.098238
48H 10MTA	Q9NRM6	-0.93496	0.207461	0.104545	IL17RB	Interleukin	I17RB_HUN	2	0.523056
24H 10MTA	Q9Y6B6	-0.0751	0.207462	0.104545	SAR1B	Small COPII	SAR1B_HUI	13	0.949274
48H 50MTA	Q5TC12	-0.07372	0.207512	0.104564	ATPAF1	ATP syntha	ATPF1_HUI	9	0.950184
24H 10MTA	P36578	-0.15472	0.207538	0.104573	RPL4	Large ribos	RL4_HUMA	22	0.898309
48H 10MTA	Q9NPG3	-0.3122	0.207563	0.104583	UBN1	Ubinuclein	UBN1_HUN	3	0.805414
24H 10MTA	Q8TCT9	0.083665	0.207574	0.104587	HM13	Minor histo	HM13_HUI	10	1.059707
24H 50MTA	Q9UGL1	0.402072	0.207606	0.104602	KDM5B	Lysine-spec	KDM5B_HL	6	1.321405
24H 10MTA	Q8N122	-0.05725	0.207622	0.104609	RPTOR	Regulatory-	RPTOR_HUI	16	0.961094
24H 10MTA	O95352	-0.12652	0.207683	0.104629	ATG7	Ubiquitin-l	ATG7_HUM	21	0.916037
24H 50MTA	Q5TKA1	0.061904	0.207754	0.104655	LIN9	Protein lin-	LIN9_HUMA	2	1.043843
24H 50MTA	Q96K19	0.253608	0.20775	0.104655	RNF170	E3 ubiquiti	RN170_HU	6	1.192185
48H 10MTA	Q01780	-0.05707	0.2078	0.104674	EXOSC10	Exosome cc	EXOSX_HUI	38	0.961214
24H 50MTA	Q8NDD1	0.833809	0.207808	0.104674	C1orf131	Uncharacte	CA131_HUI	2	1.782385

24H 10MT/	O95487	-0.01692	0.208036	0.104773	SEC24B	Protein tra	SC24B_HU	28	0.98834
24H 10MT/	Q8NC60	0.037219	0.208106	0.1048	NOA1	Nitric oxid	NOA1_HUN	14	1.026134
48H 10MT/	Q8N5F7	0.086896	0.208138	0.104812	NKAP	NF-kappa-B	NKAP_HUN	3	1.062083
24H 10MT/	Q96BX8	0.611735	0.208233	0.104855	MOB3A	MOB kinase	MOB3A_HU	3	1.528096
24H 10MT/	Q9GZM5	0.028397	0.20828	0.104876	YIPF3	Protein YIP	YIPF3_HUN	8	1.019879
48H 10MT/	P30084	0.005221	0.20832	0.104886	ECHS1	Enoyl-CoA I	ECHM_HUN	18	1.003625
48H 50MT/	Q9UJZ1	-0.00921	0.208319	0.104886	STOML2	Stomatin-li	STML2_HU	17	0.993636
48H 50MT/	Q16635	-0.28603	0.208373	0.104904	TAFAZZIN	Tafazzin	TAZ_HUMA	2	0.820157
48H 50MT/	Q8N6N3	-0.20378	0.208371	0.104904	C1orf52	UPF0690 p	CA052_HU	7	0.868274
24H 50MT/	Q8NHV4	0.121489	0.208371	0.104904	NEDD1	Protein NE	NEDD1_HU	14	1.087857
24H 50MT/	Q9NRS6	0.027767	0.208382	0.104904	SNX15	Sorting nex	SNX15_HU	7	1.019433
24H 50MT/	P54725	-0.02636	0.208478	0.104945	RAD23A	UV excision	RD23A_HU	7	0.981895
48H 10MT/	Q9NZ01	-0.08525	0.208508	0.104957	TECR	Very-long-c	TECR_HUM	12	0.942623
48H 50MT/	Q9H6R7	0.023448	0.208568	0.10498	WDCP	WD repeat	WDCP_HU	3	1.016386
48H 50MT/	Q8N442	-0.23024	0.208595	0.104987	GUF1	Translation	GUF1_HUN	10	0.852494
48H 10MT/	Q70HW3	-0.25672	0.208632	0.105	SLC25A26	Mitochond	SAMC_HUN	3	0.836986
48H 50MT/	Q9UNL2	-0.21517	0.208647	0.105006	SSR3	Translocon	SSRG_HUM	2	0.861443
24H 50MT/	Q9Y4E8	0.069645	0.208703	0.105031	USP15	Ubiquitin c	UBP15_HU	40	1.049458
24H 10MT/	P16083	0.00804	0.208711	0.105032	NQO2	Ribosyldihy	NQO2_HUN	13	1.005589
24H 10MT/	Q9Y3D0	-0.28682	0.208719	0.105032	CIAO2B	Cytosolic ir	CIA2B_HUN	4	0.819707
48H 50MT/	Q9Y5K6	0.001253	0.208717	0.105032	CD2AP	CD2-associ	CD2AP_HU	27	1.000869
24H 10MT/	O94874	-0.02031	0.208838	0.105082	UFL1	E3 UFM1-p	UFL1_HUM	37	0.986019
48H 10MT/	P49281	-0.04053	0.208843	0.105083	SLC11A2	Natural resi	NRAM2_HU	2	0.972298
48H 10MT/	Q86YS3	0.232876	0.208905	0.105112	RAB11FIP4	Rab11 fami	RFIP4_HUN	3	1.175175
24H 50MT/	P26038	-0.02545	0.208958	0.105132	MSN	Moesin	MOES_HUN	27	0.982515
24H 10MT/	Q5TC63	0.149006	0.208958	0.105132	GRTP1	Growth ho	GRTP1_HU	4	1.108805
48H 10MT/	Q14678	0.047588	0.20899	0.105144	KANK1	KN motif ar	KANK1_HU	23	1.033535
48H 50MT/	Q96C12	-0.26665	0.209007	0.105151	ARMC5	Armadillo r	ARMC5_HU	4	0.831248
48H 50MT/	Q05469	-0.97621	0.209032	0.105162	LIPE	Hormone-s	LIPS_HUMA	1	0.508314
24H 10MT/	Q8IWB9	0.04088	0.209038	0.105164	TEX2	Testis-expr	TEX2_HUM	12	1.028741
24H 50MT/	P62273	-0.05716	0.209069	0.105177	RPS29	Small ribos	RS29_HUM	4	0.961156
48H 50MT/	P04179	0.085373	0.209098	0.105188	SOD2	Superoxide	SODM_HUN	9	1.060962
24H 10MT/	Q8NHV9	-0.74928	0.209109	0.105193	RHOXF1	Rhox home	RHXF1_HU	1	0.594901
48H 10MT/	Q9NUU6	-0.25008	0.209162	0.105211	OTULINL	Inactive ub	OTULL_HU	3	0.840848
24H 50MT/	P61086	-0.04936	0.209227	0.105236	UBE2K	Ubiquitin-c	UBE2K_HU	12	0.966367
48H 50MT/	Q9NXC5	-0.0157	0.209285	0.10526	MIOS	GATOR2 co	MIOS_HUM	26	0.98918
24H 10MT/	O75695	0.03479	0.209305	0.105267	RP2	Protein XRI	XRP2_HUM	10	1.024408
48H 50MT/	Q15051	0.582641	0.209405	0.105313	IQCB1	IQ calmodu	IQCB1_HUN	2	1.497588
24H 10MT/	P21127	0.017844	0.209414	0.105315	CDK11B	Cyclin-depe	CD11B_HU	21	1.012446
48H 10MT/	Q13888;Q6	-0.04253	0.209417	0.105315	GTF2H2;GT	General tra	TF2H2_HU	11	0.970953
24H 50MT/	Q08431	-0.053	0.209474	0.10534	MFGE8	Lactadherir	MFGM_HU	4	0.963928
24H 50MT/	P62736;P6	-0.16812	0.209479	0.105341	ACTA2;ACT	Actin, aorti	ACTA_HUM	5	0.89
48H 10MT/	Q96ED9	0.498905	0.209501	0.105349	HOOK2	Protein Ho	HOOK2_HU	1	1.413141
48H 10MT/	Q9BUB7	-0.14362	0.209508	0.105349	TMEM70	Transmemb	TMM70_HU	6	0.905247
24H 10MT/	Q9H6E4	0.050143	0.209523	0.105353	CCDC134	Coiled-coil	CC134_HU	7	1.035368
24H 50MT/	Q9UKA9	0.068457	0.209546	0.105361	PTBP2	Polypyrimi	PTBP2_HU	9	1.048594
24H 10MT/	Q712K3	-0.05153	0.209681	0.105421	UBE2R2	Ubiquitin-c	UB2R2_HU	4	0.964911
24H 50MT/	Q9UNS1	0.030416	0.209714	0.105433	TIMELESS	Protein tim	TIM_HUMA	18	1.021307
48H 50MT/	P25705	0.036062	0.209737	0.105442	ATP5F1A	ATP syntha	ATPA_HUM	30	1.025311
24H 50MT/	P63313	-0.29415	0.209775	0.105459	TMSB10	Thymosin b	TYB10_HUN	1	0.815554
48H 50MT/	Q05086	-0.00485	0.209776	0.105459	UBE3A	Ubiquitin-p	UBE3A_HU	28	0.996644
48H 10MT/	P62318	-0.07895	0.209815	0.105476	SNRPD3	Small nucle	SMD3_HUN	5	0.946748

24H	10MT/	Q9ULW0	0.06143	0.20985	0.105491	TPX2	Targeting p	TPX2_HUM	31	1.043499
24H	10MT/	Q13322	0.660149	0.209856	0.105491	GRB10	Growth fac	GRB10_HU	1	1.580246
24H	10MT/	Q9H553	0.065869	0.209874	0.105498	ALG2	Alpha-1,3/1	ALG2_HUM	13	1.046715
48H	10MT/	P49406	-0.1467	0.209966	0.10553	MRPL19	Large ribos	RM19_HUM	12	0.903313
24H	10MT/	Q96AC1	-0.00546	0.209964	0.10553	FERMT2	Fermitin fa	FERM2_HU	27	0.996221
24H	10MT/	Q9HCU5	-0.09109	0.20998	0.105536	PREB	Guanine nu	SEC12_HUM	14	0.938815
48H	10MT/	Q99436	0.054017	0.209989	0.105538	PSMB7	Proteasom	PSB7_HUM	12	1.038151
48H	50MT/	Q9NYA4	0.010119	0.209991	0.105538	MTMR4	Myotubula	MTMR4_HU	1	1.007039
48H	50MT/	Q6ZNJ1	-0.11683	0.210027	0.10555	NBEAL2	Neurobeacl	NBEL2_HUI	11	0.922209
24H	50MT/	Q0VDG4	0.058275	0.210062	0.105565	SCRN3	Secernin-3	SCRN3_HUI	9	1.04122
24H	50MT/	Q8NC44	0.068432	0.210169	0.10561	RETREG2	Reticuloph	RETR2_HUM	4	1.048576
24H	50MT/	Q8N4W6	-0.56359	0.210229	0.105631	DNAJC22	DnaJ homo	DJC22_HUI	1	0.676616
24H	50MT/	Q8N511	0.001251	0.210255	0.105641	TMEM199	Transmemb	TM199_HU	4	1.000867
48H	50MT/	O94953	0.31505	0.210286	0.105651	KDM4B	Lysine-spec	KDM4B_HU	8	1.244055
24H	10MT/	Q7Z6K5	-0.15741	0.210311	0.105661	ARPIN	Arpin	ARPIN_HUM	5	0.896631
24H	10MT/	Q8TB72	-0.00347	0.2104	0.105701	PUM2	Pumilio ho	PUM2_HUM	10	0.997599
48H	10MT/	P00505	-0.08171	0.210458	0.105727	GOT2	Aspartate a	AATM_HUM	26	0.94494
48H	50MT/	P52756	-0.14475	0.210522	0.105751	RBM5	RNA-bindir	RBM5_HUM	14	0.904534
24H	10MT/	P22059	-0.03629	0.210529	0.105751	OSBP	Oxysterol-b	OSBP1_HUI	26	0.975162
48H	10MT/	Q5VUA4	-0.04715	0.210528	0.105751	ZNF318	Zinc finger	ZN318_HUI	27	0.967844
48H	50MT/	Q8ND30	0.177023	0.210571	0.105767	PPFIBP2	Liprin-beta	LIPB2_HUM	2	1.130548
24H	50MT/	Q96RP9	0.037051	0.210635	0.105793	GFM1	Elongation	EFGM_HUM	29	1.026014
24H	10MT/	Q9UK39	0.04758	0.21068	0.105805	NOCT	Nocturnin	NOCT_HUM	1	1.03353
24H	10MT/	P43360	0.052122	0.210686	0.105807	MAGEA6	Melanoma-	MAGA6_HU	2	1.036789
24H	10MT/	Q9NPD3	0.027987	0.210688	0.105807	EXOSC4	Exosome co	EXOS4_HUI	8	1.019588
48H	10MT/	O95985	-0.12459	0.21074	0.105826	TOP3B	DNA topois	TOP3B_HUI	8	0.917266
48H	10MT/	Q9UHY8	-0.08946	0.210761	0.105833	FEZ2	Fasciculatio	FEZ2_HUM/	5	0.939874
24H	10MT/	Q9H1Z4	0.019383	0.210814	0.105853	WDR13	WD repeat-	WDR13_HU	13	1.013526
48H	10MT/	O43414	0.001012	0.210864	0.105876	ERI3	ERI1 exorib	ERI3_HUM/	4	1.000701
24H	50MT/	Q9H1I8	0.119298	0.210895	0.105889	ASCC2	Activating s	ASCC2_HUI	14	1.086206
24H	10MT/	A8MXV4	-0.07757	0.210938	0.105905	NUDT19	Acyl-coenzy	NUD19_HU	16	0.947652
48H	10MT/	P11216	-0.02954	0.210972	0.105918	PYGB	Glycogen p	PYGB_HUM	30	0.979734
24H	50MT/	O95858	0.20033	0.210985	0.105923	TSPAN15	Tetraspanir	TSN15_HUI	3	1.148961
48H	10MT/	B1AK53	0.656141	0.211012	0.105932	ESPN	Espin	ESPN_HUM	1	1.575862
24H	10MT/	Q6ZSB9	-0.01752	0.21107	0.105957	ZBTB49	Zinc finger	ZBT49_HUM	1	0.987928
48H	50MT/	Q9UK45	-0.02043	0.211097	0.105967	LSM7	U6 snRNA-a	LSM7_HUM	5	0.985937
48H	50MT/	Q9H840	0.299851	0.211198	0.106011	GEMIN7	Gem-associ	GEMI7_HUI	1	1.231018
24H	50MT/	Q13322	0.464123	0.211214	0.106016	GRB10	Growth fac	GRB10_HU	1	1.379478
48H	10MT/	Q96PC5	0.038209	0.211239	0.106027	MIA2	Melanoma	MIA2_HUM	27	1.026838
48H	50MT/	Q92540	-0.25944	0.211313	0.106062	SMG7	Nonsense-n	SMG7_HUM	9	0.835412
48H	50MT/	Q05BQ5	-0.36719	0.211346	0.106075	MBTD1	MBT domai	MBTD1_HU	1	0.77529
24H	50MT/	Q9NY26	-0.09379	0.211362	0.106081	SLC39A1	Zinc transp	S39A1_HUI	1	0.937055
48H	10MT/	P36871	0.060764	0.211374	0.106086	PGM1	Phosphoglu	PGM1_HUM	28	1.043018
24H	50MT/	Q9BYD3	0.006471	0.211405	0.106095	MRPL4	Large ribos	RM04_HUM	12	1.004495
24H	10MT/	Q00587	0.009577	0.211417	0.106098	CDC42EP1	Cdc42 effec	BORG5_HU	6	1.006661
48H	10MT/	Q13111	0.127853	0.211434	0.106105	CHAF1A	Chromatin	CAF1A_HUI	13	1.092666
24H	50MT/	P53350	-0.12659	0.211474	0.10612	PLK1	Serine/thre	PLK1_HUM	21	0.915997
24H	50MT/	Q96P11	0.017561	0.211476	0.10612	NSUN5	28S rRNA (c	NSUN5_HU	17	1.012247
24H	10MT/	B7ZAQ6;P0	0.055085	0.211526	0.106132	GPR89A;GF	Golgi pH re	GPHRA_HU	5	1.03892
24H	10MT/	Q9BSF4	-0.05847	0.211588	0.106156	TIMM29	Mitochond	TIM29_HUI	6	0.960283
24H	50MT/	Q9BX74	0.248537	0.211655	0.106184	TM2D1	TM2 domai	TM2D1_HU	1	1.188002
24H	50MT/	Q2NKX8	0.038853	0.211691	0.106197	ERCC6L	DNA excisio	ERC6L_HUM	38	1.027297

48H 50MTA	P23528	-0.03626	0.211698	0.106197	CFL1	Cofilin-1	COF1_HUM	18	0.975177
48H 50MTA	P49281	0.101195	0.211699	0.106197	SLC11A2	Natural resi	NRAM2_HU	2	1.072662
48H 50MTA	P17707	-0.62817	0.211709	0.1062	AMD1	S-adenosylr	DCAM_HUM	4	0.646996
24H 10MTA	Q5QP82	0.728942	0.211736	0.106209	DCAF10	DDB1- and	DCA10_HU	1	1.657423
24H 10MTA	P28347	0.053328	0.21174	0.106209	TEAD1	Transcripti	TEAD1_HUI	4	1.037656
24H 10MTA	Q9NPI0	-0.00555	0.211753	0.106214	TMEM138	Transmemb	TM138_HU	1	0.996162
48H 50MTA	Q96B36	-0.05528	0.211758	0.106215	AKT1S1	Proline-ricl	AKTS1_HUM	4	0.962411
24H 50MTA	Q13416	-0.07381	0.211804	0.106231	ORC2	Origin reco	ORC2_HUM	13	0.950128
48H 10MTA	P05543	-0.44593	0.211829	0.106242	SERPINA7	Thyroxine-l	THBG_HUM	3	0.734111
48H 10MTA	Q15070	-0.05822	0.211846	0.106247	OXA1L	Mitochond	OXA1L_HUI	8	0.960451
24H 50MTA	Q7Z5R6	-0.02198	0.211843	0.106247	APBB1IP	Amyloid be	AB1IP_HUM	1	0.984881
48H 10MTA	Q9H444	-0.01722	0.211862	0.106252	CHMP4B	Charged mu	CHM4B_HU	8	0.988137
48H 50MTA	Q96CP6	-0.07359	0.211866	0.106253	GRAMD1A	Protein Ast	ASTRA_HUM	8	0.950269
24H 50MTA	P98196	0.044769	0.211887	0.106262	ATP11A	Phospholip	AT11A_HUI	6	1.031518
48H 50MTA	Q96AG4	-0.02379	0.211905	0.106268	LRRC59	Leucine-ric	LRC59_HUI	13	0.983645
24H 50MTA	Q96EA4	0.07121	0.211919	0.10627	SPDL1	Protein Spi	SPDLY_HUM	9	1.050597
24H 10MTA	Q9BYG5	-0.05627	0.211923	0.106271	PARD6B	Partitionin	PAR6B_HUI	9	0.961748
48H 50MTA	Q6VY07	-0.02543	0.211941	0.106276	PACS1	Phosphofu	PACS1_HUI	16	0.982529
24H 50MTA	Q8WVB6	-0.14744	0.211942	0.106276	CHTF18	Chromosor	CTF18_HUM	22	0.902849
48H 10MTA	Q07954	0.047539	0.211987	0.106294	LRP1	Prolow-der	LRP1_HUM	40	1.033501
24H 10MTA	P05161	-0.09911	0.212004	0.1063	ISG15	Ubiquitin-l	ISG15_HUM	5	0.933608
48H 10MTA	Q9H8M1	-0.1292	0.212064	0.106326	COQ10B	Coenzyme (	CQ10B_HU	4	0.914337
48H 50MTA	Q13948	0.058672	0.212145	0.106355	CUX1	Protein CAS	CASP_HUM	3	1.041507
48H 50MTA	Q9UPY8	0.18369	0.212156	0.106357	MAPRE3	Microtubul	MARE3_HU	1	1.135785
24H 50MTA	P0C7T5	0.12573	0.212186	0.106368	ATXN1L	Ataxin-1-lik	ATX1L_HUM	3	1.091059
48H 50MTA	P56192	-0.024	0.212237	0.106389	MARS1	Methionine	SYMC_HUM	43	0.983502
24H 50MTA	Q9UMX5	-0.05323	0.212257	0.106397	NENF	Neudesin	NENF_HUM	7	0.963775
48H 10MTA	Q9H019	0.277058	0.212276	0.106403	MTFR1L	Mitochond	MFR1L_HU	4	1.211721
24H 50MTA	Q96F46	0.135568	0.212282	0.106405	IL17RA	Interleukin	I17RA_HUM	7	1.098525
48H 10MTA	Q99956	0.030972	0.212499	0.106499	DUSP9	Dual specifi	DUS9_HUM	12	1.0217
48H 10MTA	Q9BY44	0.009155	0.212546	0.106518	EIF2A	Eukaryotic	EIF2A_HUM	32	1.006366
48H 50MTA	P16949	-0.07813	0.21255	0.106519	STMN1	Stathmin	STMN1_HU	6	0.947286
48H 10MTA	Q9NX74	0.003126	0.212553	0.106519	DUS2	tRNA-dihyd	DUS2L_HUI	23	1.002169
48H 10MTA	Q14807	-0.0312	0.212561	0.106521	KIF22	Kinesin-like	KIF22_HUM	25	0.978605
48H 10MTA	Q9BQD3	-0.10948	0.212596	0.106537	KXD1	KxDL motif	KXDL1_HUI	2	0.92692
48H 50MTA	P23526	0.008446	0.212644	0.106554	AHCY	Adenosylhc	SAHH_HUM	25	1.005871
24H 50MTA	Q8NCE2	0.057197	0.212704	0.106577	MTMR14	Myotubula	MTMRE_HU	10	1.040442
48H 50MTA	Q3KP66	-0.29152	0.212718	0.106583	INAVA	Innate imm	INAVA_HUM	4	0.81704
48H 50MTA	Q9UL54	-0.18911	0.212762	0.106599	TAOK2	Serine/thre	TAOK2_HUI	9	0.877148
48H 10MTA	P47755	-0.02225	0.212802	0.106613	CAPZA2	F-actin-cap	CAZA2_HUI	11	0.984696
48H 10MTA	Q9Y5W8	0.2437	0.2129	0.106648	SNX13	Sorting nex	SNX13_HUI	5	1.184025
48H 50MTA	Q9BTC0	-0.02553	0.212991	0.106689	DIDO1	Death-indu	DIDO1_HUI	37	0.982462
24H 50MTA	Q9UPW5	-0.15519	0.212992	0.106689	AGTPBP1	Cytosolic ca	CBPC1_HUI	1	0.898013
24H 50MTA	Q6UX01;Q8	-0.02406	0.213	0.106692	LMBR1L;LM	Protein LM	LMBRL_HU	1	0.983461
48H 10MTA	Q6UW02	-0.08358	0.21315	0.106763	CYP20A1	Cytochrom	CP20A_HU	17	0.943712
24H 50MTA	P61421	-0.17245	0.213171	0.10677	ATP6V0D1	V-type prot	VA0D1_HU	10	0.887332
24H 50MTA	Q96JG6	-0.08949	0.213192	0.10678	VPS50	Syndetin	VPS50_HUI	20	0.939853
48H 10MTA	P42785	-0.07043	0.2132	0.106782	PRCP	Lysosomal	PCP_HUMA	12	0.952357
48H 50MTA	P07737	-0.02921	0.213239	0.106793	PFN1	Profilin-1	PROF1_HUI	11	0.97996
24H 50MTA	Q8N806	0.02465	0.213237	0.106793	UBR7	Putative E3	UBR7_HUM	11	1.017233
24H 50MTA	O60244	0.141955	0.213349	0.106835	MED14	Mediator o	MED14_HU	15	1.103399
24H 50MTA	P84077	-0.43623	0.213366	0.106841	ARF1	ADP-ribosy	ARF1_HUM	3	0.739065

24H 10MT/	P32780	0.18977	0.213404	0.106854	GTF2H1	General tra	TF2H1_HUI	8	1.140582
48H 10MT/	Q9Y2S2	0.01503	0.213415	0.106857	CRYL1	Lambda-cry	CRYL1_HUI	8	1.010472
24H 50MT/	P35611	0.028579	0.21347	0.106875	ADD1	Alpha-addu	ADDA_HUM	16	1.020007
24H 10MT/	Q9P2K8	0.011328	0.213468	0.106875	EIF2AK4	eIF-2-alpha	E2AK4_HUI	22	1.007883
48H 10MT/	Q8N5V2	0.075161	0.213506	0.106886	NGEF	Ephexin-1	NGEF_HUM	12	1.053479
48H 10MT/	Q16891	-0.01971	0.213558	0.106908	IMMT	MICOS com	MIC60_HUI	35	0.986433
48H 50MT/	Q12802	0.045435	0.213598	0.106925	AKAP13	A-kinase an	AKP13_HUI	23	1.031994
48H 50MT/	Q7Z4G4	-0.13431	0.213643	0.106943	TRMT11	tRNA (guan	TRM11_HU	8	0.911106
48H 10MT/	P05026	-0.08387	0.213696	0.106961	ATP1B1	Sodium/po	AT1B1_HUI	10	0.943523
48H 10MT/	Q02543	-0.01227	0.213721	0.106972	RPL18A	Large ribos	RL18A_HUI	8	0.991533
48H 10MT/	P0DPD7	-0.09959	0.213724	0.106972	EEF1AKMT4	EEF1A lysin	EFMT4_HUI	10	0.9333
48H 10MT/	Q9Y4P1	-0.06312	0.213755	0.106982	ATG4B	Cysteine pr	ATG4B_HUI	14	0.957193
24H 10MT/	Q13530	0.118793	0.213798	0.107002	SERINC3	Serine inco	SERC3_HUI	4	1.085826
24H 10MT/	Q86TN4	-0.12539	0.213857	0.107026	TRPT1	tRNA 2'-pho	TRPT1_HUI	5	0.916756
24H 50MT/	Q13137	-0.12287	0.21391	0.107042	CALCOCO2	Calcium-bi	CACO2_HU	14	0.918356
48H 10MT/	Q15800	0.001797	0.213939	0.107053	MSMO1	Methylster	MSMO1_HU	4	1.001246
48H 10MT/	Q96AQ8	0.495248	0.21394	0.107053	MCUR1	Mitochond	MCUR1_HU	1	1.409563
48H 10MT/	P43361	0.366753	0.21398	0.107064	MAGEA8	Melanoma-	MAGA8_HU	1	1.289447
48H 50MT/	P49411	-0.06689	0.214002	0.107073	TUFM	Elongation	EFTU_HUM	31	0.954691
48H 10MT/	Q9NP81	-0.01958	0.214013	0.107077	SARS2	Serine--tRN	SYSM_HUM	17	0.986523
24H 50MT/	Q96EY5	-0.1542	0.214081	0.107106	MVB12A	Multivesicu	MB12A_HU	6	0.89863
24H 50MT/	Q04760	0.049208	0.214089	0.107107	GLO1	Lactoylglut	LGUL_HUM	12	1.034696
48H 50MT/	Q9NWS0	-0.05101	0.214089	0.107107	PIH1D1	PIH1 doma	PIHD1_HUI	9	0.965259
48H 10MT/	Q8NBF6	-0.20417	0.21413	0.107124	AVL9	Late secret	AVL9_HUM	11	0.868036
48H 10MT/	Q9H061	0.024639	0.214187	0.107146	TMEM126A	Transmemb	T126A_HUI	10	1.017225
48H 50MT/	Q9H4B7	0.250388	0.214255	0.107167	TUBB1	Tubulin bet	TBB1_HUM	2	1.189527
24H 10MT/	P83876	-0.00549	0.214277	0.107176	TXNL4A	Thioredoxi	TXN4A_HUI	6	0.996204
24H 50MT/	Q9BVX2	0.17719	0.214282	0.107177	TMEM106C	Transmemb	T106C_HUI	1	1.130679
24H 50MT/	Q8NFC6	0.118187	0.214337	0.1072	BOD1L1	Biorientati	BD1L1_HUI	24	1.08537
48H 10MT/	Q8IUR7	0.092371	0.214357	0.107208	ARMC8	Armadillo r	ARMC8_HU	19	1.066121
24H 10MT/	P61313	0.112056	0.214424	0.10724	RPL15	Large ribos	RL15_HUM	10	1.080767
48H 50MT/	Q9BT67	-0.19391	0.214433	0.107243	NDFIP1	NEDD4 fam	NFIP1_HUI	1	0.874231
24H 10MT/	P02647	-0.39097	0.214475	0.107259	APOA1	Apolipopro	APOA1_HU	9	0.762618
24H 50MT/	Q9Y3D5	0.295945	0.214511	0.107276	MRPS18C	Small ribos	RT18C_HUI	3	1.227689
24H 10MT/	Q49AR2	0.022355	0.214592	0.107312	C5orf22	UPF0489 p	CE022_HUI	9	1.015616
48H 50MT/	O95453	-0.02959	0.214611	0.107317	PARN	Poly(A)-spe	PARN_HUM	22	0.979702
24H 50MT/	O00418	-0.23165	0.21463	0.107324	EEF2K	Eukaryotic	EF2K_HUM	8	0.851659
48H 50MT/	Q7Z6J8	-0.11719	0.214671	0.10734	UBE3D	E3 ubiquiti	UBE3D_HU	4	0.921985
24H 50MT/	P46778	-0.01115	0.214714	0.107358	RPL21	Large ribos	RL21_HUM	8	0.992298
48H 10MT/	Q8N1G4	0.01389	0.214722	0.10736	LRRC47	Leucine-ric	LRC47_HUI	23	1.009674
24H 10MT/	P55211	-0.29474	0.214733	0.107363	CASP9	Caspase-9	CASP9_HUI	4	0.815221
48H 50MT/	P78382	0.015251	0.214734	0.107363	SLC35A1	CMP-sialic	S35A1_HUI	2	1.010627
24H 10MT/	Q96JB5	0.010085	0.214762	0.107375	CDK5RAP3	CDK5 regul	CK5P3_HUI	18	1.007015
24H 10MT/	O95376	-0.07203	0.21487	0.107419	ARIH2	E3 ubiquiti	ARI2_HUM/	13	0.951298
48H 10MT/	Q9H5H4	0.082334	0.214907	0.107433	ZNF768	Zinc finger	ZN768_HUI	8	1.058729
48H 10MT/	O75581	0.558615	0.214953	0.107452	LRP6	Low-densit	LRP6_HUM	4	1.472855
48H 10MT/	Q53H12	-0.08372	0.215003	0.107475	AGK	Acylglycerc	AGK_HUM/	15	0.94362
24H 10MT/	Q8TAD7	0.096199	0.215017	0.10748	OCC1	Overexpres	OCC1_HUM	1	1.068953
48H 10MT/	P17707	-0.55939	0.215029	0.107481	AMD1	S-adenosyl	DCAM_HUI	4	0.678591
24H 10MT/	Q8NHG8	-0.10314	0.215109	0.107518	ZNRF2	E3 ubiquiti	ZNRF2_HUI	3	0.931005
48H 10MT/	Q8IX03	0.042279	0.215144	0.107524	WWC1	Protein KIB	KIBRA_HUI	6	1.029739
24H 50MT/	P42696	-0.0274	0.215166	0.107533	RBM34	RNA-bindir	RBM34_HU	19	0.981185

24H	10MT/	Q15329	-0.1157	0.215174	0.107534	E2F5	Transcripti	E2F5_HUM	1	0.922934
24H	10MT/	Q9H814	0.008856	0.21518	0.107535	PHAX	Phosphoryl	PHAX_HUM	11	1.006158
24H	10MT/	Q8N5C6	0.039527	0.215404	0.107626	SRBD1	S1 RNA-bin	SRBD1_HU	21	1.027777
48H	50MT/	Q9H910	-0.12578	0.21548	0.107658	JPT2	Jupiter mic	JUPI2_HUM	9	0.916508
24H	50MT/	Q9HC07	0.002636	0.215482	0.107658	TMEM165	Putative di	TM165_HU	5	1.001829
24H	50MT/	Q9UPN9	-0.05632	0.215563	0.107693	TRIM33	E3 ubiquiti	TRI33_HUM	21	0.961717
48H	50MT/	Q9H0R6	-0.05272	0.215577	0.107697	QRSL1	Glutamyl-t	GATA_HUM	16	0.964116
48H	10MT/	O95155	0.05168	0.215654	0.107732	UBE4B	Ubiquitin c	UBE4B_HU	37	1.036471
24H	50MT/	Q9BTX3	0.287474	0.215715	0.107758	TMEM208	Transmemb	TM208_HU	1	1.220501
24H	10MT/	P27695	-0.01599	0.215763	0.107779	APEX1	DNA repair	APEX1_HU	16	0.988977
24H	10MT/	Q13885	0.103927	0.21584	0.107814	TUBB2A	Tubulin bet	TBB2A_HU	7	1.074695
48H	10MT/	P55884	0.021005	0.215845	0.107814	EIF3B	Eukaryotic	EIF3B_HUM	47	1.014666
24H	10MT/	Q08554	-0.54523	0.215848	0.107814	DSC1	Desmocolli	DSC1_HUM	3	0.685284
24H	10MT/	Q8TCT7	0.031337	0.215861	0.107816	SPPL2B	Signal pept	SPP2B_HU	1	1.021959
24H	50MT/	O15315	0.270829	0.215906	0.107833	RAD51B	DNA repair	RA51B_HU	1	1.206501
24H	10MT/	Q8WYH8	0.274478	0.215906	0.107833	ING5	Inhibitor of	ING5_HUM	2	1.209556
48H	50MT/	Q9Y5J9	-0.293	0.215952	0.107855	TIMM8B	Mitochond	TIM8B_HU	3	0.816205
48H	10MT/	Q9H936	-0.07369	0.215998	0.107875	SLC25A22	Mitochond	GHC1_HUM	11	0.950206
48H	50MT/	Q2Q1W2	0.002517	0.21601	0.107879	TRIM71	E3 ubiquiti	LIN41_HUM	16	1.001746
48H	50MT/	Q8ND76	0.135455	0.21602	0.107881	CCNY	Cyclin-Y	CCNY_HUM	1	1.098439
48H	50MT/	Q14156	0.231053	0.216032	0.107886	EFR3A	Protein EFR	EFR3A_HUM	11	1.173691
24H	10MT/	P02663	-0.02238	0.21605	0.107893	CSN1S2	Alpha-S2-ca	CASA2_BOV	3	0.98461
24H	50MT/	P02452	-0.23205	0.216058	0.107894	COL1A1	Collagen alp	CO1A1_HU	14	0.851421
24H	10MT/	Q86XX4	0.071183	0.216254	0.107972	FRAS1	Extracellula	FRAS1_HUM	31	1.050578
48H	50MT/	Q96JK2	-0.08428	0.216267	0.107977	DCAF5	DDB1- and	DCAF5_HU	1	0.943258
48H	50MT/	Q6NYC1	0.058304	0.216309	0.107992	JMJD6	Bifunctiona	JMJD6_HU	11	1.041241
48H	50MT/	Q9Y324	-0.27202	0.21631	0.107992	FCF1	rRNA-proce	FCF1_HUM	5	0.828161
48H	10MT/	Q00577	0.174174	0.216382	0.10802	PURA	Transcripti	PURA_HUM	5	1.128318
48H	50MT/	Q96A49	0.062397	0.216397	0.108024	SYAP1	Synapse-ass	SYAP1_HUM	18	1.044199
48H	50MT/	Q15628	0.236317	0.21643	0.108037	TRADD	Tumor necr	TRADD_HU	6	1.177982
24H	10MT/	P05156	0.308086	0.216434	0.108037	CFI	Complemen	CFAI_HUM/	2	1.238064
24H	10MT/	Q5J8M3	-0.05993	0.216456	0.108046	EMC4	ER membra	EMC4_HUM	6	0.959309
24H	10MT/	Q13569	1.264571	0.21647	0.108049	TDG	G/T mismat	TDG_HUMA	1	2.402557
48H	10MT/	Q9NRU3	-0.31587	0.2165	0.108059	CNNM1	Metal trans	CNNM1_HU	5	0.803367
48H	50MT/	A6NKF1	-0.18318	0.216508	0.108059	SAC3D1	SAC3 doma	SAC31_HU	8	0.88076
48H	50MT/	P29218	0.040487	0.2166	0.108098	IMPA1	Inositol mo	IMPA1_HU	14	1.028461
48H	10MT/	Q9NZJ7	0.006912	0.216618	0.108104	MTCH1	Mitochond	MTCH1_HU	11	1.004803
24H	10MT/	Q53GA4	0.035539	0.216671	0.108127	PHLDA2	Pleckstrin h	PHLA2_HU	1	1.02494
24H	50MT/	Q969X6	-0.01035	0.216717	0.108143	UTP4	U3 small nu	UTP4_HUM	22	0.99285
48H	10MT/	Q9Y6H1	0.022863	0.216725	0.108146	CHCHD2	Coiled-coil-	CHCH2_HU	4	1.015974
48H	50MT/	O15083	0.004622	0.216773	0.108167	ERC2	ERC protein	ERC2_HUM	1	1.003209
24H	50MT/	Q9BY78	-0.07911	0.216795	0.108172	RNF26	E3 ubiquiti	RNF26_HU	1	0.946638
24H	10MT/	P13051	-0.06746	0.21687	0.108204	UNG	Uracil-DNA	UNG_HUMA	6	0.954318
48H	10MT/	P62266	0.052228	0.217059	0.108279	RPS23	Small ribos	RS23_HUM	7	1.036865
24H	50MT/	P32121	-0.111	0.217104	0.108295	ARRB2	Beta-arresti	ARRB2_HU	10	0.925945
24H	10MT/	O43172	-0.01012	0.217157	0.108313	PRPF4	U4/U6 sma	PRP4_HUM	19	0.993013
24H	10MT/	Q9BUT9	0.341548	0.217181	0.108319	MCRIP2	MAPK regul	MCRI2_HU	2	1.267116
24H	10MT/	Q9H3H1	-0.0159	0.217238	0.108338	TRIT1	tRNA dimet	MOD5_HU	17	0.989038
48H	10MT/	Q9Y388	0.075298	0.217307	0.10837	RBMX2	RNA-bindir	RBMX2_HU	3	1.053579
24H	50MT/	P42892	0.066435	0.21732	0.108373	ECE1	Endothelin	ECE1_HUM	8	1.047126
48H	10MT/	Q9NVS9	-0.09928	0.217357	0.108386	PNPO	Pyridoxine-	PNPO_HUM	13	0.933496
24H	10MT/	O43504	-0.30493	0.21744	0.108419	LAMTOR5	Ragulator c	LTOR5_HU	2	0.809484

24H 50MT/	Q7L273	-0.42143	0.217509	0.108448	KCTD9	BTB/POZ dc	KCTD9_HUI	4	0.746683
48H 10MT/	Q6IAN0	-0.0133	0.217692	0.108517	DHRS7B	Dehydroge	DRS7B_HUI	9	0.990825
48H 50MT/	Q99547	-0.04736	0.217711	0.108522	MPHOSPH6	M-phase ph	MPH6_HUI	3	0.967709
48H 10MT/	P41240	-0.02497	0.217762	0.108541	CSK	Tyrosine-pr	CSK_HUMA	24	0.982839
48H 10MT/	P62487	-0.0248	0.217802	0.108559	POLR2G	DNA-direct	RPB7_HUM	7	0.982959
48H 50MT/	Q13670	0.403977	0.217812	0.108562	PMS2P11	Putative pc	PM2PB_HU	1	1.32315
24H 10MT/	O94916	-0.09236	0.21786	0.108582	NFAT5	Nuclear fac	NFAT5_HUI	10	0.937984
24H 10MT/	O94952	0.08262	0.2179	0.108597	FBXO21	F-box only	FBX21_HUI	8	1.05894
48H 50MT/	Q9Y3D5	0.16374	0.217928	0.10861	MRPS18C	Small ribos	RT18C_HUI	3	1.120187
48H 10MT/	Q13049	0.017859	0.218019	0.108649	TRIM32	E3 ubiquiti	TRI32_HUM	11	1.012456
48H 50MT/	O14657	-0.16365	0.218084	0.108676	TOR1B	Torsin-1B	TOR1B_HUI	8	0.892762
48H 50MT/	P55199	0.0756	0.218091	0.108676	ELL	RNA polym	ELL_HUMAI	8	1.053799
48H 10MT/	P62333	0.005897	0.218136	0.108691	PSMC6	26S protea:	PRS10_HUI	21	1.004096
24H 10MT/	Q96RQ1	0.128539	0.218165	0.108697	ERGIC2	Endoplasm	ERGI2_HUM	6	1.093186
48H 10MT/	Q9NXW2	-0.034	0.218164	0.108697	DNAJB12	DnaJ homo	DJB12_HUI	10	0.976711
24H 50MT/	P23142	-0.39813	0.21818	0.108703	FBLN1	Fibulin-1	FBLN1_HUI	11	0.758842
24H 50MT/	O00754	0.040639	0.218209	0.108713	MAN2B1	Lysosomal	MA2B1_HU	18	1.028569
24H 10MT/	Q16611	0.002226	0.21828	0.108742	BAK1	Bcl-2 homc	BAK_HUMA	4	1.001544
48H 50MT/	Q8WVV9	0.005266	0.218292	0.108746	HNRNPLL	Heterogene	HNRLL_HUI	16	1.003657
48H 10MT/	Q14674	0.008984	0.218303	0.108749	ESPL1	Separin	ESPL1_HUM	15	1.006247
24H 10MT/	Q16584	0.14526	0.218306	0.108749	MAP3K11	Mitogen-ac	M3K11_HU	2	1.10593
48H 50MT/	Q6UB28	-0.403	0.218396	0.108785	METAP1D	Methionine	MAP12_HU	2	0.756284
24H 50MT/	Q9NV31	0.020629	0.218427	0.108797	IMP3	U3 small nu	IMP3_HUM	10	1.014402
24H 50MT/	Q5UCC4	0.09525	0.218504	0.108826	EMC10	ER membra	EMC10_HU	3	1.06825
24H 10MT/	P31327	0.011006	0.218525	0.108835	CPS1	Carbamoyl-	CPSM_HUM	84	1.007658
24H 10MT/	Q13404	-0.08794	0.218544	0.108843	UBE2V1	Ubiquitin-c	UB2V1_HU	9	0.940867
24H 50MT/	P57076	0.019221	0.218578	0.108856	CFAP298	Cilia- and fl	CF298_HUI	8	1.013412
24H 50MT/	P63244	-0.0291	0.218573	0.108856	RACK1	Small ribos	RACK1_HUI	20	0.980033
24H 10MT/	P82650	0.006186	0.218594	0.108859	MRPS22	Small ribos	RT22_HUM	15	1.004297
48H 10MT/	Q6Y288	-0.14901	0.218614	0.108865	B3GLCT	Beta-1,3-gl	B3GLT_HUI	5	0.901867
48H 50MT/	Q9Y2K2	-0.09894	0.218657	0.108879	SIK3	Serine/thre	SIK3_HUM/	2	0.933717
24H 10MT/	P00742	-0.37504	0.218674	0.10888	F10	Coagulatio	FA10_HUM	4	0.771084
24H 10MT/	P17066	0.019274	0.218674	0.10888	HSPA6	Heat shock	HSP76_HUI	2	1.013449
48H 50MT/	Q8N3X1	-0.05859	0.218674	0.10888	FNBP4	Formin-bin	FNBP4_HUI	17	0.960204
24H 10MT/	Q9BSH4	0.027334	0.218662	0.10888	TACO1	Translation	TACO1_HUI	12	1.019127
48H 10MT/	Q9NWB6	-0.07807	0.218673	0.10888	ARGLU1	Arginine an	ARGL1_HUI	2	0.947324
24H 50MT/	Q9H490	0.027106	0.218709	0.108891	PIGU	Phosphatic	PIGU_HUM	6	1.018966
48H 10MT/	Q9Y2D2	-0.2844	0.21873	0.108899	SLC35A3	UDP-N-acet	S35A3_HUI	2	0.821083
24H 10MT/	Q9UPU5	0.015541	0.218746	0.108904	USP24	Ubiquitin c	UBP24_HU	61	1.010831
24H 10MT/	P17861	-0.364	0.218789	0.108921	XBP1	X-box-bind	XBP1_HUM	1	0.777009
24H 10MT/	Q99848	-0.07889	0.21882	0.108935	EBNA1BP2	Probable rF	EBP2_HUM	13	0.946789
48H 50MT/	P30086	0.098279	0.218869	0.108955	PEBP1	Phosphatic	PEBP1_HUI	13	1.070496
48H 50MT/	Q5UCC4	0.054882	0.218929	0.108981	EMC10	ER membra	EMC10_HU	3	1.038774
48H 50MT/	Q5R372	0.001087	0.218937	0.108983	RABGAP1L	Rab GTPase	RBG1L_HUI	7	1.000754
24H 50MT/	Q969V6	-0.38638	0.21901	0.109011	MRTFA	Myocardin-	MRTFA_HU	8	0.765045
48H 10MT/	Q643R3	0.153834	0.219056	0.109023	LPCAT4	Lysophospl	LPCT4_HUM	4	1.112522
48H 10MT/	P28827	-0.0136	0.219076	0.109029	PTPRM	Receptor-ty	PTPRM_HU	11	0.990615
48H 10MT/	P06756	-0.00876	0.219098	0.109038	ITGAV	Integrin alp	ITAV_HUM/	37	0.993945
24H 10MT/	Q9H7N4	0.11157	0.219105	0.109039	SCAF1	Splicing fac	SFR19_HUM	7	1.080403
24H 10MT/	Q8IXU6	-0.52449	0.219157	0.109056	SLC35F2	Solute carri	S35F2_HUM	1	0.695204
48H 50MT/	Q9BVK2	-0.13341	0.219231	0.109085	ALG8	Dolichyl py	ALG8_HUM	4	0.911671
48H 50MT/	Q8N5Y8	-0.39324	0.219244	0.109089	PARP16	Protein mo	PAR16_HUI	3	0.761419

48H 50MTA	Q71SY5	0.510628	0.219247	0.109089	MED25	Mediator o	MED25_HU	4	1.42467
24H 10MTA	P00747	0.373249	0.219488	0.109196	PLG	Plasminoge	PLMN_HUM	3	1.295266
48H 10MTA	Q4V339;Q5	-0.08372	0.219537	0.109215	ZNG1F;ZNG	Zinc-regula	ZNG1F_HU	5	0.94362
48H 50MTA	P13798	-0.01524	0.219713	0.109277	APEH	Acylamino-	ACPH_HUM	23	0.989493
48H 50MTA	Q96AQ8	-0.66699	0.219714	0.109277	MCUR1	Mitochond	MCUR1_HU	1	0.629819
24H 50MTA	Q9H6V9	0.141054	0.219728	0.109279	LDAH	Lipid dropl	LDAH_HUM	6	1.102711
24H 10MTA	Q9UPT5	0.020368	0.219767	0.109293	EXOC7	Exocyst cor	EXOC7_HU	27	1.014218
24H 10MTA	P35914	-0.06588	0.219823	0.109318	HMGCL	Hydroxyme	HMGCL_HU	11	0.955365
48H 50MTA	P23142	0.053665	0.219955	0.109372	FBLN1	Fibulin-1	FBLN1_HU	11	1.037898
24H 50MTA	Q8WUQ7	0.185268	0.220115	0.109443	CACTIN	Splicing fac	CATIN_HUM	6	1.137028
24H 10MTA	Q8WVK2	0.660033	0.220151	0.109456	SNRNP27	U4/U6.U5 s	SNR27_HU	2	1.580118
48H 10MTA	Q9Y383	0.044794	0.220166	0.109462	LUC7L2	Putative RN	LC7L2_HUM	8	1.031536
24H 10MTA	Q86XZ4	-0.00075	0.220194	0.109473	SPATS2	Spermatog	SPAS2_HUM	11	0.999477
48H 10MTA	P24278	-0.00533	0.220326	0.10952	ZBTB25	Zinc finger	ZBT25_HUM	1	0.996309
24H 50MTA	Q7L1T6	-0.16692	0.220356	0.109527	CYB5R4	Cytochrom	NB5R4_HU	5	0.890739
48H 10MTA	Q8TBE9	0.03376	0.22046	0.109568	NANP	N-acylneur	NANP_HUM	4	1.023677
48H 50MTA	Q14534	0.067151	0.220597	0.109624	SQLE	Squalene m	ERG1_HUM	11	1.047646
24H 10MTA	P26572	0.086356	0.220812	0.10972	MGAT1	Alpha-1,3-r	MGAT1_HU	7	1.061685
24H 10MTA	P49915	-0.04339	0.221036	0.109812	GMPS	GMP synth	GUAA_HUM	35	0.970375
24H 10MTA	P24928	0.01819	0.221051	0.109815	POLR2A	DNA-direct	RPB1_HUM	63	1.012688
24H 10MTA	Q9Y6I9	0.097829	0.221084	0.10983	TEX264	Testis-expre	TX264_HU	10	1.070162
48H 50MTA	O60502	-0.01856	0.221099	0.109834	OGA	Protein O-G	OGA_HUMA	30	0.987216
24H 50MTA	Q9UGN5	0.190789	0.221121	0.109842	PARP2	Poly [ADP-r	PARP2_HU	10	1.141388
24H 10MTA	P30041	0.026147	0.221165	0.109863	PRDX6	Peroxiredo	PRDX6_HU	19	1.018289
48H 10MTA	O43390	-0.0048	0.221198	0.109876	HNRNPR	Heterogene	HNRPR_HU	23	0.996679
48H 50MTA	Q96SY0	-0.09896	0.221217	0.109884	INTS14	Integrator	INT14_HUM	10	0.933708
48H 50MTA	P49356	-0.02628	0.221293	0.109916	FNTB	Protein far	FNTB_HUM	7	0.981946
48H 50MTA	Q86Y79	-0.21747	0.221317	0.109926	PTRH1	Peptidyl-tR	PTH_HUMA	5	0.860074
48H 10MTA	Q9UK73	0.011784	0.221353	0.109939	FEM1B	Protein fem	FEM1B_HU	3	1.008202
48H 10MTA	Q6PCB0	-0.03048	0.221388	0.109954	VWA1	von Willebr	VWA1_HUM	5	0.979092
24H 10MTA	Q96A19	0.155067	0.221419	0.109961	CCDC102A	Coiled-coil	C102A_HU	7	1.113473
24H 50MTA	O00268	0.145077	0.221438	0.109966	TAF4	Transcripti	TAF4_HUM	13	1.10579
48H 50MTA	Q9ULL5	-0.33994	0.221446	0.109968	PRR12	Proline-rich	PRR12_HU	3	0.790072
24H 50MTA	O43865	-0.07432	0.221476	0.109975	AHCYL1	S-adenosyll	SAHH2_HU	12	0.94979
24H 10MTA	Q6NUM9	-0.03244	0.221512	0.109989	RETSAT	All-trans-re	RETST_HUM	15	0.977762
48H 10MTA	Q96KC8	-0.1927	0.221532	0.109997	DNAJC1	DnaJ homo	DNJC1_HU	10	0.874968
24H 10MTA	Q96GR4	-0.20684	0.221584	0.110014	ZDHHC12	Palmitoyltr	ZDH12_HU	2	0.866434
48H 50MTA	Q9BYJ9	0.014256	0.221602	0.11002	YTHDF1	YTH domair	YTHD1_HU	9	1.00993
48H 10MTA	P32189	-0.06008	0.221608	0.110021	GK	Glycerol kir	GLPK_HUM	13	0.959214
48H 10MTA	P27348	-0.04679	0.221702	0.110055	YWHAQ	14-3-3 prot	1433T_HU	13	0.968085
24H 10MTA	Q9BT25	0.018971	0.221698	0.110055	HAUS8	HAUS augm	HAUS8_HU	4	1.013236
48H 50MTA	Q9BZZ5	0.001639	0.221721	0.110062	API5	Apoptosis i	API5_HUM	25	1.001137
24H 10MTA	P38117	0.040982	0.22173	0.110063	ETFB	Electron tra	ETFB_HUM	17	1.028814
48H 10MTA	Q8IX04	-0.04441	0.221727	0.110063	UEVLD	Ubiquitin-c	UEVLD_HU	13	0.969684
48H 10MTA	Q9NWS8	-0.04178	0.221748	0.110071	RMND1	Required fc	RMND1_HU	9	0.971458
48H 50MTA	Q8NAT1	-0.05867	0.221857	0.110108	POMGNT2	Protein O-li	PMGT2_HU	8	0.960149
24H 50MTA	Q6XD76	1.665776	0.221867	0.110109	ASCL4	Achaete-scu	ASCL4_HUM	1	3.172843
24H 10MTA	P28288	0.017686	0.221908	0.110127	ABCD3	ATP-bindin	ABCD3_HU	33	1.012334
48H 10MTA	Q12905	-0.01122	0.221948	0.110142	ILF2	Interleukin	ILF2_HUMA	20	0.992253
48H 50MTA	P67809	-0.05477	0.221965	0.110147	YBX1	Y-box-bindi	YBOX1_HU	17	0.96275
24H 50MTA	P33527	0.07153	0.221995	0.110158	ABCC1	Multidrug r	MRP1_HUM	23	1.05083
24H 50MTA	O60563	-0.67112	0.222005	0.110161	CCNT1	Cyclin-T1	CCNT1_HU	1	0.628017

24H 50MT/	P51784	0.145797	0.22205	0.110178	USP11	Ubiquitin c	UBP11_HU	23	1.106341
24H 10MT/	Q9GZZ9	-0.03301	0.222088	0.110195	UBA5	Ubiquitin-l	UBA5_HUM	10	0.977379
48H 10MT/	Q86TI0	0.104213	0.222117	0.110207	TBC1D1	TBC1 doma	TBCD1_HU	2	1.074908
48H 10MT/	Q86WG5	-0.61119	0.222127	0.110209	SBF2	Myotubula	MTMRD_HU	5	0.654656
48H 50MT/	O94952	0.265916	0.222161	0.11022	FBXO21	F-box only	FBX21_HU	8	1.202399
48H 50MT/	Q13510	0.025607	0.222244	0.110256	ASAH1	Acid ceram	ASAH1_HU	17	1.017908
48H 10MT/	Q9Y5S2	0.014543	0.222247	0.110256	CDC42BPB	Serine/thre	MRCKB_HU	29	1.010132
24H 10MT/	Q8IXJ6	-0.4541	0.222251	0.110256	SIRT2	NAD-depen	SIR2_HUM/	2	0.729963
48H 10MT/	P61599	-0.13685	0.22229	0.11027	NAA20	N-alpha-ac	NAA20_HU	7	0.909504
24H 50MT/	Q9H2A7	-0.81986	0.222298	0.110272	CXCL16	C-X-C motif	CXL16_HU	2	0.566497
24H 50MT/	P42025	-0.08685	0.22232	0.110277	ACTR1B	Beta-centra	ACTY_HUM	15	0.941577
24H 50MT/	Q8TAD4	-0.02596	0.222373	0.110296	SLC30A5	Proton-cou	ZNT5_HUM	6	0.982167
48H 50MT/	Q96BI3	-0.49227	0.222379	0.110298	APH1A	Gamma-sec	APH1A_HU	2	0.710907
48H 10MT/	Q5JVS0	0.126642	0.222532	0.110362	HABP4	Intracellula	HABP4_HU	5	1.09175
48H 50MT/	Q15208	-0.00043	0.222666	0.110421	STK38	Serine/thre	STK38_HU	9	0.9997
48H 10MT/	Q1XH10	-0.44116	0.222664	0.110421	SKIDA1	SKI/DACH c	SKDA1_HU	2	0.73654
24H 50MT/	P45984	-0.07553	0.222752	0.110455	MAPK9	Mitogen-ac	MK09_HUM	9	0.948993
48H 50MT/	P46778	0.029369	0.222756	0.110455	RPL21	Large ribos	RL21_HUM	8	1.020565
24H 10MT/	Q9H2U2	-0.11335	0.222779	0.110463	PPA2	Inorganic p	IPYR2_HUM	14	0.92444
24H 50MT/	Q7L576	0.026868	0.222787	0.110465	CYFIP1	Cytoplasmi	CYFP1_HUM	46	1.018798
24H 10MT/	Q15375	0.252337	0.222796	0.110468	EPHA7	Ephrin type	EPHA7_HU	5	1.191135
24H 10MT/	Q53HC9	0.209818	0.222804	0.11047	EIPR1	EARP and G	EIPR1_HUM	9	1.156542
24H 10MT/	Q9BTD8	-0.07294	0.222807	0.11047	RBM42	RNA-bindir	RBM42_HU	11	0.950699
24H 10MT/	Q15047	0.066992	0.222845	0.110486	SETDB1	Histone-lys	SETB1_HUM	13	1.047531
48H 50MT/	Q9UBB6	-0.12839	0.222875	0.110498	NCDN	Neurochon	NCDN_HUM	15	0.914851
48H 10MT/	Q709C8	-0.02436	0.222891	0.110501	VPS13C	Intermemb	VP13C_HU	60	0.983259
48H 50MT/	Q96CF2	-0.08447	0.222896	0.110501	CHMP4C	Charged mu	CHM4C_HU	3	0.94313
24H 50MT/	Q96F07	0.195965	0.222894	0.110501	CYFIP2	Cytoplasmi	CYFP2_HUM	10	1.14549
48H 10MT/	Q9UKM9	-0.04084	0.222993	0.110546	RALY	RNA-bindir	RALY_HUM	11	0.972087
48H 10MT/	P31749	-0.05162	0.223064	0.110575	AKT1	RAC-alpha s	AKT1_HUM	8	0.964853
48H 50MT/	Q7Z3B4	-0.00384	0.223112	0.110595	NUP54	Nucleopori	NUP54_HU	19	0.997341
24H 10MT/	Q8WV74	-0.20187	0.223269	0.110656	NUDT8	Mitochond	NUDT8_HU	6	0.869425
48H 10MT/	Q0PNE2	0.068781	0.223302	0.11067	ELP6	Elongator c	ELP6_HUM	7	1.04883
24H 50MT/	P35250	-0.08978	0.223344	0.110687	RFC2	Replicatior	RFC2_HUM	19	0.939668
48H 10MT/	Q9H6K5	-0.20609	0.223402	0.110709	PRR36	Proline-ricl	PRR36_HU	7	0.866881
48H 10MT/	Q6P4A7	-0.00513	0.223418	0.110715	SFXN4	Sideroflexir	SFXN4_HU	2	0.996449
48H 10MT/	Q9GZZ9	-0.00761	0.223441	0.110722	UBA5	Ubiquitin-l	UBA5_HUM	10	0.994739
24H 10MT/	P28332	-0.07552	0.223461	0.110731	ADH6	Alcohol del	ADH6_HUM	11	0.948997
48H 50MT/	Q6PI78	0.045362	0.223569	0.110769	TMEM65	Transmemb	TMM65_HU	7	1.031942
48H 50MT/	Q9UHA3	-0.46872	0.223608	0.110782	RSL24D1	Probable ri	RLP24_HU	4	0.722605
24H 50MT/	O43709	0.028978	0.223622	0.110783	BUD23	18S rRNA (g	BUD23_HU	5	1.020289
48H 10MT/	Q14966	-0.00976	0.223619	0.110783	ZNF638	Zinc finger	ZN638_HU	46	0.993259
48H 50MT/	Q8WVZ9	-0.33157	0.223619	0.110783	KBTBD7	Kelch repea	KBTB7_HU	1	0.794674
24H 10MT/	P09234	0.314239	0.223638	0.110788	SNRPC	U1 small nu	RU1C_HUM	2	1.243355
24H 10MT/	Q86VP6	0.015507	0.22364	0.110788	CAND1	Cullin-asso	CAND1_HU	58	1.010807
24H 10MT/	Q6P2E9	-0.01622	0.22366	0.110795	EDC4	Enhancer o	EDC4_HUM	49	0.988819
48H 50MT/	Q96BH1	-0.2877	0.223726	0.110819	RNF25	E3 ubiquiti	RNF25_HU	5	0.819209
24H 50MT/	P07197	-0.98683	0.22374	0.110823	NEFM	Neurofilam	NFM_HUM/	1	0.504586
24H 10MT/	O60704	-0.05705	0.223811	0.110849	TPST2	Protein-tyr	TPST2_HUM	1	0.961225
24H 50MT/	Q9NR12	-0.07077	0.223822	0.110851	PDLIM7	PDZ and LIN	PDLI7_HUM	12	0.95213
48H 50MT/	Q05209	0.088094	0.223941	0.110897	PTPN12	Tyrosine-pr	PTN12_HU	15	1.062965
24H 50MT/	Q7L7X3	0.018538	0.224011	0.11092	TAOK1	Serine/thre	TAOK1_HU	11	1.012932

24H 50MT/	Q5VZK9	-0.03641	0.224025	0.110925	CARMIL1	F-actin-unc	CARL1_HUI	24	0.975079
48H 50MT/	P25325	0.043625	0.224078	0.110944	MPST	3-mercapto	THTM_HUN	15	1.030701
24H 10MT/	P48506	0.013139	0.224111	0.110959	GCLC	Glutamate-	GSH1_HUN	23	1.009149
24H 10MT/	Q9ULJ3	0.137042	0.224126	0.110962	ZBTB21	Zinc finger a	ZBT21_HUI	5	1.099648
48H 10MT/	Q9H974	-0.05683	0.224175	0.110977	QTRT2	Queuine tR	QTRT2_HUI	14	0.961375
48H 50MT/	Q9UER7	-0.11795	0.224172	0.110977	DAXX	Death dom	DAXX_HUM	7	0.921496
24H 10MT/	P0C0S5;Q7	-0.00308	0.224209	0.110991	H2AZ1;H2A	Histone H2	H2AZ_HUM	3	0.997864
48H 10MT/	P33527	-0.057	0.224232	0.110997	ABCC1	Multidrug r	MRP1_HUN	23	0.961264
24H 10MT/	P57775	-0.44309	0.224237	0.110997	FBXW4	F-box/WD r	FBXW4_HU	2	0.735559
24H 50MT/	P30793	0.024095	0.224315	0.11103	GCH1	GTP cycloh	GCH1_HUN	10	1.016842
48H 50MT/	Q9GZP4	-0.14864	0.224327	0.111033	PITHD1	PITH domai	PITH1_HUN	10	0.902103
48H 50MT/	O75674	-0.02628	0.224335	0.111035	TOM1L1	TOM1-like p	TM1L1_HU	7	0.981951
24H 50MT/	Q86UE8	-0.28275	0.224336	0.111035	TLK2	Serine/thre	TLK2_HUM	4	0.82202
24H 50MT/	Q92782	-0.87183	0.224363	0.111045	DPF1	Zinc finger p	DPF1_HUM	1	0.546454
24H 10MT/	Q9BZE9	-0.02489	0.22438	0.11105	ASPSCR1	Tether cont	ASPC1_HUI	17	0.982893
24H 10MT/	O75146	-0.04558	0.224403	0.11106	HIP1R	Huntingtin	HIP1R_HUN	19	0.968903
24H 50MT/	Q13356	-0.05082	0.224411	0.111062	PPIL2	RING-type E	PPIL2_HUN	11	0.965386
24H 10MT/	Q9NVU7	0.047721	0.224475	0.111089	SDAD1	Protein SD/	SDA1_HUM	14	1.033631
24H 50MT/	Q8N635	-0.02712	0.224479	0.111089	MEIOB	Meiosis-spe	MEIOB_HUI	2	0.981375
24H 50MT/	P51530	0.072432	0.224487	0.111091	DNA2	DNA replica	DNA2_HUN	18	1.051488
24H 50MT/	Q15208	-0.10047	0.224507	0.111095	STK38	Serine/thre	STK38_HUN	9	0.932728
24H 50MT/	Q5JPI3	-0.15566	0.224507	0.111095	C3orf38	Uncharacte	CC038_HUI	1	0.897725
24H 10MT/	Q9UPQ9	-0.04524	0.224538	0.111109	TNRC6B	Trinucleoti	TNR6B_HUI	17	0.969126
24H 10MT/	Q15208	-0.09119	0.224562	0.111115	STK38	Serine/thre	STK38_HUN	9	0.938749
24H 50MT/	Q9Y3Z3	0.017768	0.22456	0.111115	SAMHD1	Deoxynucle	SAMH1_HU	31	1.012392
24H 10MT/	O43505	-0.04099	0.224579	0.111122	B4GAT1	Beta-1,4-gl	B4GA1_HU	13	0.971989
48H 50MT/	Q6ZNW5	-0.08445	0.224637	0.111148	GDPGP1	GDP-D-gluc	GDPP1_HU	5	0.943145
24H 10MT/	O15318	-0.05474	0.224683	0.111163	POLR3G	DNA-direct	RPC7_HUM	3	0.962769
48H 10MT/	Q96K76	-0.05266	0.224679	0.111163	USP47	Ubiquitin c	UBP47_HU	34	0.964155
24H 50MT/	P81605	0.189735	0.224696	0.111168	DCD	Dermcidin	DCD_HUM/	2	1.140554
24H 10MT/	Q9UF12	-0.19351	0.224713	0.111173	PRODH2	Hydroxypro	HYPDH_HU	10	0.874473
24H 50MT/	Q96IZ6	-0.57394	0.224719	0.111175	METTL2A	tRNA N(3)-r	MET2A_HU	1	0.67178
24H 10MT/	Q9NZD8	0.026025	0.224727	0.111177	SPG21	Maspardin	SPG21_HUI	13	1.018203
48H 50MT/	P30039	0.175421	0.224828	0.11122	PBLD	Phenazine b	PBLD_HUM	2	1.129294
24H 10MT/	Q9Y289	0.00461	0.224854	0.111228	SLC5A6	Sodium-dep	SC5A6_HUI	4	1.003201
48H 10MT/	Q9H0A8	0.083718	0.224894	0.111247	COMMD4	COMM dom	COMD4_HU	7	1.059746
48H 10MT/	Q9UL18	0.274558	0.224969	0.111273	ago-01	Protein arg	AGO1_HUN	2	1.209623
24H 10MT/	Q7Z7A4	-0.11381	0.224994	0.11128	PXK	PX domain-	PXK_HUMA	6	0.924143
48H 50MT/	P55212	0.103614	0.22502	0.111285	CASP6	Caspase-6	CASP6_HUI	9	1.074462
24H 50MT/	Q15751	-0.11114	0.22502	0.111285	HERC1	Probable E3	HERC1_HUI	24	0.925854
24H 10MT/	Q9UNF1	-0.00153	0.225019	0.111285	MAGED2	Melanoma-	MAGD2_HU	21	0.99894
48H 50MT/	P46059	0.360607	0.225029	0.111288	SLC15A1	Solute carri	S15A1_HUI	4	1.283966
48H 50MT/	Q6P2C8	-0.07336	0.225093	0.111312	MED27	Mediator o	MED27_HU	7	0.950424
24H 50MT/	O60645	-0.01799	0.225109	0.111319	EXOC3	Exocyst cor	EXOC3_HUI	16	0.987611
48H 50MT/	Q3KRA9	-0.02269	0.225185	0.111347	ALKBH6	Alpha-keto	ALKB6_HUI	3	0.984395
48H 10MT/	Q5SXM8	0.238375	0.225236	0.111368	DNLZ	DNL-type zi	DNLZ_HUM	1	1.179663
24H 10MT/	Q92562	0.048378	0.225283	0.111388	FIG4	Polyphosph	FIG4_HUM/	4	1.034102
24H 50MT/	Q05655	-0.10382	0.225356	0.111417	PRKCD	Protein kin	KPCD_HUM	6	0.930568
48H 10MT/	O00566	0.00258	0.225378	0.111421	MPHOSPH1	U3 small nu	MPP10_HU	16	1.00179
24H 50MT/	Q9Y6R4	-0.19815	0.225406	0.111426	MAP3K4	Mitogen-ac	M3K4_HUN	19	0.871666
48H 10MT/	Q9H0R4	-0.15092	0.225474	0.111453	HDHD2	Haloacid de	HDHD2_HU	7	0.900676
24H 50MT/	Q8NBT2	-0.54374	0.22553	0.111474	SPC24	Kinetochor	SPC24_HUI	4	0.685989

48H 10MT/	Q13426	0.042538	0.225579	0.111491	XRCC4	DNA repair	XRCC4_HUI	9	1.029924
24H 50MT/	P23396	-0.07898	0.225596	0.111496	RPS3	Small ribos	RS3_HUMA	19	0.946729
48H 50MT/	Q96RY7	0.349826	0.225615	0.111503	IFT140	Intraflagell	IF140_HUN	4	1.274407
48H 10MT/	P62841	-0.26271	0.225639	0.111513	RPS15	Small ribos	RS15_HUM	6	0.833524
24H 10MT/	Q6P1M0	0.107053	0.225648	0.111516	SLC27A4	Long-chain	S27A4_HUI	19	1.077026
48H 50MT/	Q9UKN8	-0.14133	0.225713	0.111541	GTF3C4	General tra	TF3C4_HUN	25	0.906684
48H 10MT/	Q9UGU5	-0.02458	0.225741	0.11155	HMGXB4	HMG doma	HMGX4_HU	1	0.983109
48H 10MT/	Q9NSP4	-0.18309	0.225748	0.111552	CENPM	Centromer	CENPM_HU	3	0.880814
48H 10MT/	O75815	0.076371	0.225772	0.111561	BCAR3	Breast canc	BCAR3_HUI	11	1.054363
24H 50MT/	Q8N0X4	0.07677	0.225944	0.111627	CLYBL	Citramalyl-	CLYBL_HUN	7	1.054654
24H 10MT/	O75204	-0.10455	0.225965	0.111633	TMEM127	Transmemb	TM127_HU	1	0.930094
24H 10MT/	Q9UBM1	0.186997	0.225969	0.111633	PEMT	Phosphatid	PEMT_HUN	1	1.138392
24H 10MT/	P09972	0.214791	0.225995	0.111643	ALDOC	Fructose-bi	ALDOC_HU	8	1.160536
48H 10MT/	Q629K1	-0.15751	0.226158	0.111703	TRIQK	Triple QxxK	TRIQK_HUN	1	0.896569
48H 10MT/	O15381	-0.05433	0.226181	0.11171	NVL	Nuclear val	NVL_HUMA	19	0.963043
48H 50MT/	P38571	0.098904	0.226202	0.111716	LIPA	Lysosomal a	LICH_HUM.	5	1.07096
24H 10MT/	Q5TEC6	-0.03889	0.226296	0.111753	H3-7	Histone H3	H37_HUMA	1	0.973407
48H 50MT/	Q01201	0.128455	0.226331	0.111767	RELB	Transcripti	RELB_HUM	5	1.093123
48H 10MT/	Q96KR6	-0.06687	0.226372	0.11178	FAM210B	Protein FAI	F210B_HUI	3	0.954707
24H 10MT/	Q15545	-0.41512	0.226379	0.111782	TAF7	Transcripti	TAF7_HUM	4	0.749958
24H 50MT/	Q9BSG0	-0.12302	0.226433	0.111791	PRADC1	Protease-as	PADC1_HU	1	0.918265
48H 50MT/	Q9HAD4	-0.04666	0.226475	0.111807	WDR41	WD repeat-	WDR41_HU	7	0.968173
24H 10MT/	Q9H8P0	-0.15093	0.22649	0.111808	SRD5A3	Polyprenol	PORED_HU	2	0.90067
24H 10MT/	Q9Y5K8	0.030143	0.226514	0.111819	ATP6V1D	V-type prot	VATD_HUM	7	1.021114
48H 10MT/	Q15013	-0.00137	0.226517	0.111819	MAD2L1BP	MAD2L1-bi	MD2BP_HU	3	0.99905
48H 10MT/	Q9Y2Z9	-0.03222	0.226521	0.111819	COQ6	Ubiquinon	COQ6_HUN	11	0.977913
48H 10MT/	O75348	-0.07754	0.226541	0.111827	ATP6V1G1	V-type prot	VATG1_HUI	4	0.947671
48H 50MT/	P09622	-0.08063	0.22655	0.111827	DLD	Dihydrolip	DLDH_HUN	14	0.945643
48H 10MT/	P13378	-0.1233	0.226553	0.111827	HOXD8	Homeobox	HXD8_HUN	2	0.918087
48H 10MT/	Q9Y484	-0.15637	0.226615	0.11185	WDR45	WD repeat	WIPI4_HUN	4	0.897279
24H 10MT/	Q9H497	0.176603	0.226677	0.111877	TOR3A	Torsin-3A	TOR3A_HUI	7	1.130219
48H 10MT/	Q16718	-0.07755	0.226728	0.111898	NDUFA5	NADH dehy	NDUA5_HU	6	0.947669
24H 50MT/	Q13257	0.027999	0.226741	0.111902	MAD2L1	Mitotic spi	MD2L1_HU	7	1.019597
24H 50MT/	Q07864	0.111615	0.226767	0.111909	POLE	DNA polym	DPOE1_HU	31	1.080437
24H 50MT/	Q9NRX2	0.014026	0.22678	0.111914	MRPL17	Large ribos	RM17_HUN	10	1.00977
24H 10MT/	P62263	-0.08686	0.226838	0.111932	RPS14	Small ribos	RS14_HUM	9	0.941569
24H 10MT/	P49674	-0.08712	0.226847	0.111932	CSNK1E	Casein kina	KC1E_HUM	11	0.941403
24H 10MT/	Q15262	0.066613	0.226847	0.111932	PTPRK	Receptor-ty	PTPRK_HUI	15	1.047255
24H 10MT/	Q9UH99	-0.01903	0.226846	0.111932	SUN2	SUN domai	SUN2_HUN	19	0.986897
48H 10MT/	Q5R372	-0.01031	0.226872	0.111942	RABGAP1L	Rab GTPase	RBG1L_HUI	7	0.992878
48H 10MT/	Q9UBB6	-0.03932	0.227102	0.112037	NCDN	Neurochon	NCDN_HUN	15	0.973116
24H 10MT/	Q9Y6Q9	0.026572	0.227109	0.112039	NCOA3	Nuclear rec	NCOA3_HU	16	1.018589
24H 50MT/	Q14296	0.072288	0.227114	0.11204	FASTK	Fas-activate	FASTK_HUN	2	1.051383
24H 10MT/	Q9Y3T9	0.001833	0.227147	0.112055	NOC2L	Nucleolar c	NOC2L_HU	23	1.001272
48H 50MT/	Q9Y2H1	0.200182	0.227163	0.112061	STK38L	Serine/thre	ST38L_HUN	1	1.148843
24H 50MT/	Q9H7P9	0.331271	0.227187	0.112065	PLEKHG2	Pleckstrin h	PKHG2_HU	1	1.258121
24H 10MT/	Q86YV5	-0.03768	0.227199	0.112067	PRAG1	Inactive tyr	PRAG1_HU	5	0.974222
24H 50MT/	Q0PNE2	0.251849	0.22722	0.112074	ELP6	Elongator c	ELP6_HUM	7	1.190733
24H 10MT/	Q709F0	0.029394	0.227251	0.112087	ACAD11	Acyl-CoA de	ACD11_HU	11	1.020583
48H 10MT/	Q9UKU7	-0.03652	0.227272	0.112094	ACAD8	Isobutyryl-	ACAD8_HU	10	0.975005
24H 10MT/	P22626	-0.01092	0.227284	0.112095	HNRNPA2B	Heterogene	ROA2_HUN	24	0.992462
48H 50MT/	Q9Y295	0.016046	0.2274	0.112141	DRG1	Developme	DRG1_HUN	23	1.011184

48H 10MT/	Q86YV5	-0.12818	0.227417	0.112143	PRAG1	Inactive tyr	PRAG1_HU	5	0.914984
48H 50MT/	P61457	0.110139	0.227456	0.11216	PCBD1	Pterin-4-alp	PHS_HUMA	7	1.079332
48H 10MT/	Q96CW6	-0.34481	0.227461	0.11216	SLC7A6OS	Probable RI	S7A6O_HUI	3	0.787412
48H 50MT/	Q9HB29	2.351442	0.227477	0.112167	IL1RL2	Interleukin	ILRL2_HUM	1	5.10334
24H 10MT/	Q86W56	0.159135	0.227482	0.112167	PARG	Poly(ADP-ri	PARG_HUM	12	1.116618
48H 50MT/	P82909	0.120907	0.227561	0.112203	KGD4	Alpha-keto	KGD4_HUM	6	1.087419
24H 10MT/	Q9Y371	0.035193	0.227566	0.112205	SH3GLB1	Endophilin	SHLB1_HUI	13	1.024694
24H 50MT/	O15498	-0.01364	0.227612	0.112223	YKT6	Synaptobre	YKT6_HUM	11	0.990588
48H 50MT/	Q8IUS5	0.143527	0.227629	0.11223	EPHX4	Epoxide hy	EPHX4_HUI	4	1.104602
24H 10MT/	Q5H9R7	0.035251	0.227653	0.112237	PPP6R3	Serine/thre	PP6R3_HUI	35	1.024735
48H 10MT/	P43357	-0.14366	0.227734	0.11227	MAGEA3	Melanoma-	MAGA3_HU	4	0.90522
24H 10MT/	Q14186	0.058777	0.22776	0.112279	TFDP1	Transcripti	TFDP1_HUI	3	1.041583
24H 50MT/	Q8N5B7	-0.41808	0.227826	0.112306	CERS5	Ceramide s	CERS5_HUI	2	0.748419
24H 50MT/	Q04323	0.008567	0.227871	0.112322	UBXN1	UBX domai	UBXN1_HU	10	1.005956
48H 10MT/	Q9P0U3	-0.22653	0.227871	0.112322	SENP1	Sentrin-spe	SENP1_HUI	4	0.854685
24H 10MT/	Q86TI2	0.007279	0.227893	0.112328	DPP9	Dipeptidyl	DPP9_HUM	32	1.005058
24H 50MT/	Q15329	-0.04012	0.228007	0.112372	E2F5	Transcripti	E2F5_HUM	1	0.972575
24H 50MT/	Q9Y5N5	-0.38133	0.228038	0.112385	N6AMT1	Methyltran	N6MT1_HU	3	0.767732
24H 50MT/	P01034	0.211415	0.228072	0.112397	CST3	Cystatin-C	CYTC_HUM	1	1.157823
24H 10MT/	Q03113	0.022569	0.228073	0.112397	GNA12	Guanine nu	GNA12_HU	5	1.015767
24H 50MT/	Q9NXB9	0.152254	0.228078	0.112399	ELOVL2	Very long c	ELOV2_HUI	4	1.111305
48H 10MT/	Q8WVK7	0.058087	0.228166	0.112433	SKA2	Spindle anc	SKA2_HUM	1	1.041084
48H 50MT/	Q9Y5Q9	-0.09161	0.228188	0.112441	GTF3C3	General tra	TF3C3_HUI	24	0.938472
48H 10MT/	Q9H0M0	-0.17929	0.228245	0.112466	WWP1	NEDD4-like	WWP1_HU	2	0.88314
48H 10MT/	O75865	0.829507	0.228257	0.11247	TRAPPC6A	Trafficking	TPC6A_HUI	1	1.777078
24H 10MT/	P82664	0.091937	0.228377	0.112523	MRPS10	Small ribos	RT10_HUM	4	1.0658
24H 10MT/	Q96RT1	-0.01161	0.228441	0.112547	ERBIN	Erbin	ERBIN_HUI	16	0.991987
24H 10MT/	Q9UL40	0.020806	0.228486	0.112564	ZNF346	Zinc finger	ZN346_HUI	7	1.014526
48H 10MT/	A1X283	-0.00985	0.228574	0.112598	SH3PXD2B	SH3 and PX	SPD2B_HUI	13	0.993194
24H 10MT/	O60762	-0.03423	0.228621	0.112618	DPM1	Dolichol-pl	DPM1_HUI	13	0.976552
48H 10MT/	P19012	0.214687	0.228666	0.112632	KRT15	Keratin, typ	K1C15_HUI	1	1.160452
24H 50MT/	Q7Z7F0	0.543179	0.228718	0.112652	KHDC4	KH homolo	KHDC4_HU	2	1.45718
48H 50MT/	Q9UHN6	-0.07042	0.228722	0.112652	CEMIP2	Inactive cel	CEIP2_HUM	40	0.952358
48H 10MT/	Q9Y5K6	-0.00638	0.228756	0.112665	CD2AP	CD2-associ	CD2AP_HU	27	0.995591
48H 10MT/	Q9Y6D5	-0.04168	0.228781	0.112676	ARFGEF2	Brefeldin A	BIG2_HUM	56	0.971526
24H 10MT/	Q4V328	0.025061	0.228837	0.112696	GRIPAP1	GRIP1-asso	GRAP1_HU	18	1.017522
48H 50MT/	Q8WZA1	0.215254	0.228854	0.112703	POMGNT1	Protein O-li	PMGT1_HU	7	1.160908
24H 10MT/	Q9NYM9	-0.16897	0.228895	0.112717	BET1L	BET1-like pi	BET1L_HUM	3	0.889475
48H 10MT/	Q9NUQ2	0.08942	0.228977	0.11275	AGPAT5	1-acyl-sn-gl	PLCE_HUM	12	1.063942
48H 50MT/	Q9Y224	-0.10386	0.228977	0.11275	RTRAF	RNA transci	RTRAF_HUI	14	0.930537
24H 10MT/	Q9NSP4	0.194605	0.228997	0.112758	CENPM	Centromer	CENPM_HU	3	1.144411
24H 10MT/	Q13574	-0.33678	0.229012	0.112761	DGKZ	Diacylglyce	DGKZ_HUM	1	0.791808
24H 10MT/	Q7Z392	-0.13402	0.229131	0.112811	TRAPPC11	Trafficking	TPC11_HUI	10	0.911287
24H 10MT/	Q9H2G4	-0.06549	0.229138	0.112812	TSPYL2	Testis-speci	TSYL2_HUM	5	0.955623
24H 50MT/	Q9BVG9	0.071458	0.229172	0.112824	PTDSS2	Phosphatid	PTSS2_HUM	7	1.050778
24H 10MT/	Q9NY12	-0.02474	0.229227	0.112849	GAR1	H/ACA ribo	GAR1_HUM	8	0.982995
24H 50MT/	Q5RI15	0.001357	0.229266	0.112863	COX20	Cytochrom	COX20_HU	6	1.000941
48H 10MT/	Q15149	-0.0343	0.229296	0.112875	PLEC	Plectin	PLEC_HUM	199	0.976503
48H 50MT/	Q9BVC5	-0.08275	0.229338	0.112893	C2orf49	Ashwin	ASHWN_HU	5	0.944253
24H 10MT/	P22234	-0.0077	0.229347	0.112894	PAICS	Bifunction	PUR6_HUM	23	0.994674
24H 10MT/	O95049	0.559229	0.229367	0.112901	TJP3	Tight juncti	ZO3_HUMA	2	1.473482
48H 10MT/	Q08426	-0.0415	0.229382	0.112907	EHHADH	Peroxisom	ECHP_HUM	31	0.971646

48H 10MT/	Q5U3C3	-0.41615	0.229568	0.112976	TMEM164	Transmemb	TM164_HU	1	0.749424
24H 50MT/	Q32M88	0.087237	0.229586	0.112982	PGGHG	Protein-glu	PGGHG_HU	3	1.062334
24H 10MT/	Q9NTU7	0.055922	0.229611	0.112993	CBLN4	Cerebellin-4	CBLN4_HU	1	1.039523
48H 10MT/	Q13206	-0.04069	0.229651	0.113009	DDX10	Probable AT	DDX10_HU	30	0.972192
24H 10MT/	P39023	-0.06594	0.229725	0.11304	RPL3	Large ribos	RL3_HUMA	21	0.955322
24H 10MT/	Q6ZVX9	-0.00255	0.229735	0.113043	PAQR9	Membrane	PAQR9_HU	1	0.998231
48H 50MT/	Q3V6T2	-0.02848	0.229742	0.113044	CCDC88A	Girdin	GRDN_HUM	17	0.980452
48H 10MT/	P08709	-0.01613	0.229757	0.113048	F7	Coagulation	FA7_HUMA	4	0.988881
48H 50MT/	P42330	-0.01385	0.229766	0.113051	AKR1C3	Aldo-keto r	AK1C3_HU	11	0.990443
24H 50MT/	Q9BSC4	-0.00756	0.229853	0.113084	NOL10	Nucleolar p	NOL10_HU	25	0.994773
24H 50MT/	Q8TB96	0.079409	0.229878	0.113095	ITFG1	T-cell immu	TIP_HUMA	6	1.056585
48H 10MT/	Q15545	-0.29841	0.229929	0.113114	TAF7	Transcriptio	TAF7_HUM	4	0.813148
24H 10MT/	P42356	0.057612	0.229954	0.113124	PI4KA	Phosphatid	PI4KA_HUM	26	1.040741
48H 10MT/	Q15102	-0.07582	0.229994	0.113142	PAFAH1B3	Platelet-act	PA1B3_HU	10	0.948802
48H 10MT/	Q16563	-0.13797	0.230021	0.113152	SYPL1	Synaptophy	SYPL1_HUM	4	0.908795
48H 50MT/	Q8IVD9	0.069977	0.230093	0.113182	NUDCD3	NudC doma	NUDC3_HU	11	1.0497
24H 50MT/	Q8IWB9	0.134647	0.230151	0.113198	TEX2	Testis-expre	TEX2_HUM	12	1.097824
24H 50MT/	P52732	-0.08362	0.230167	0.113201	KIF11	Kinesin-like	KIF11_HUM	30	0.943689
48H 10MT/	O75616	-0.09307	0.230184	0.113207	ERAL1	GTPase Era,	ERAL1_HUM	11	0.937524
48H 50MT/	Q9NQ34	-0.18833	0.230208	0.113215	TMEM9B	Transmemb	TMM9B_HU	2	0.877622
24H 50MT/	Q13796	0.171487	0.230215	0.113217	SHROOM2	Protein Shr	SHRM2_HU	16	1.126219
24H 10MT/	O00401	-0.18086	0.230379	0.113286	WASL	Actin nucle	WASL_HUM	8	0.882177
48H 50MT/	Q7L099	0.014846	0.230396	0.113291	RUFY3	Protein RU	RUFY3_HU	6	1.010344
24H 10MT/	Q8NAT1	-0.03083	0.230418	0.113296	POMGNT2	Protein O-li	PMGT2_HU	8	0.978856
24H 50MT/	Q9NUD5	-0.12694	0.230417	0.113296	ZCCHC3	Zinc finger	ZCHC3_HU	8	0.915769
48H 50MT/	O96018	-0.34408	0.230428	0.113299	APBA3	Amyloid-be	APBA3_HU	1	0.787812
48H 50MT/	Q9NSI6	-0.01438	0.23052	0.113336	BRWD1	Bromodom	BRWD1_HU	1	0.990079
48H 50MT/	O43169	-0.09702	0.230549	0.113346	CYB5B	Cytochrom	CYB5B_HU	6	0.934962
24H 10MT/	P29597	-0.2748	0.230554	0.113346	TYK2	Non-recept	TYK2_HUM	4	0.826565
48H 10MT/	P20962	0.100109	0.230564	0.113348	PTMS	Parathymo	PTMS_HUM	1	1.071854
24H 50MT/	Q8WX93	0.042232	0.23058	0.113355	PALLD	Palladin	PALLD_HU	7	1.029706
24H 50MT/	P28074	-0.02298	0.230623	0.113371	PSMB5	Proteasom	PSB5_HUM	15	0.984199
48H 10MT/	Q9BWH6	0.007968	0.230638	0.113377	RPAP1	RNA polym	RPAP1_HU	32	1.005538
24H 10MT/	Q15811	-0.03086	0.230694	0.1134	ITSN1	Intersectin	ITSN1_HUM	8	0.978835
48H 10MT/	Q14573	0.042514	0.230699	0.113401	ITPR3	Inositol 1,4	ITPR3_HUM	23	1.029907
24H 50MT/	Q9Y530	-0.01524	0.230712	0.113406	OARD1	ADP-ribose	OARD1_HU	5	0.98949
48H 50MT/	Q9NY33	-0.00269	0.230753	0.113417	DPP3	Dipeptidyl	DPP3_HUM	31	0.998139
48H 10MT/	P55145	0.083722	0.230914	0.113485	MANF	Mesenceph	MANF_HUM	7	1.059749
48H 10MT/	Q3ZCQ3	0.023195	0.230928	0.113489	FAM174B	Membrane	F174B_HU	1	1.016208
48H 50MT/	P27540	0.195807	0.230965	0.1135	ARNT	Aryl hydroc	ARNT_HUM	6	1.145365
48H 50MT/	Q7Z412	0.04433	0.231002	0.113515	PEX26	Peroxisome	PEX26_HU	4	1.031204
24H 10MT/	Q9BQ75	-0.0872	0.231012	0.113518	CMSS1	Protein CM	CMS1_HUM	8	0.941346
48H 50MT/	Q9H1P3	0.117129	0.23103	0.113526	OSBPL2	Oxysterol-b	OSBL2_HU	9	1.084575
24H 10MT/	Q8IX90	0.020511	0.231066	0.113539	SKA3	Spindle and	SKA3_HUM	6	1.014319
48H 10MT/	P22681	0.084351	0.231113	0.113556	CBL	E3 ubiquiti	CBL_HUMA	15	1.060211
24H 10MT/	Q5JTZ9	-0.01753	0.231129	0.113559	AARS2	Alanine--tR	SYAM_HUM	29	0.987925
48H 10MT/	Q8WXH0	-0.15158	0.231148	0.113567	SYNE2	Nesprin-2	SYNE2_HUM	22	0.900264
24H 50MT/	Q96IK1	0.087738	0.231172	0.113573	BOD1	Biorientatio	BOD1_HUM	4	1.062702
48H 50MT/	O00160	-0.17804	0.231175	0.113573	MYO1F	Unconvent	MYO1F_HU	7	0.883901
24H 10MT/	P00325	-2.14319	0.231237	0.113594	ADH1B	All-trans-re	ADH1B_HU	2	0.226379
24H 50MT/	Q9H3Z4	-0.05508	0.231287	0.113616	DNAJC5	DnaJ homo	DNJC5_HU	4	0.962544
24H 50MT/	Q9H3R0	0.537116	0.231399	0.113665	KDM4C	Lysine-spec	KDM4C_HU	2	1.451069

48H 10MT/	Q9BXY0	-0.08322	0.231414	0.113671	MAK16	Protein MA	MAK16_HU	11	0.943951
48H 10MT/	Q3T8J9	0.361416	0.231426	0.113673	GON4L	GON-4-like	GON4L_HU	2	1.284686
24H 10MT/	Q86YQ8	0.068362	0.231478	0.113693	CPNE8	Copine-8	CPNE8_HU	17	1.048526
48H 10MT/	Q07065	-0.01409	0.2315	0.113703	CKAP4	Cytoskeleto	CKAP4_HU	29	0.990279
48H 50MT/	O75911	-0.17011	0.23151	0.113706	DHRS3	Short-chair	DHRS3_HU	8	0.888776
24H 10MT/	Q12789	0.054185	0.231519	0.113707	GTF3C1	General tra	TF3C1_HUI	59	1.038272
24H 10MT/	P09622	-0.08169	0.231553	0.113719	DLD	Dihydrolip	DLDH_HUN	14	0.944953
48H 50MT/	Q9Y6Y0	0.440345	0.231691	0.113778	IVNS1ABP	Influenza vi	NS1BP_HU	6	1.356929
24H 50MT/	P56282	0.102351	0.231699	0.113781	POLE2	DNA polym	DPOE2_HU	7	1.073522
48H 10MT/	Q96Q07	-0.69286	0.231772	0.113813	BTBD9	BTB/POZ dc	BTBD9_HU	1	0.618627
48H 10MT/	P56381;Q5	0.728287	0.231815	0.113833	ATP5F1E;A	ATP synthas	ATP5E_HUI	1	1.656671
24H 50MT/	Q96NT5	-0.14292	0.23182	0.113834	SLC46A1	Proton-cou	PCFT_HUM	3	0.905686
24H 10MT/	Q15651	-0.35544	0.231889	0.113856	HMGN3	High mobil	HMGN3_HU	3	0.781632
48H 50MT/	Q8N108	0.088027	0.231915	0.113865	MIER1	Mesoderm	MIER1_HUI	6	1.062915
24H 50MT/	Q5H9R7	0.017155	0.231977	0.113886	PPP6R3	Serine/thre	PP6R3_HU	35	1.011962
48H 50MT/	Q9BUB7	-0.09015	0.232005	0.113894	TMEM70	Transmemb	TMM70_HU	6	0.939423
48H 10MT/	Q96BW5	0.041136	0.232032	0.113903	PTER	Phosphotri	PTER_HUM	12	1.028924
48H 10MT/	Q70UQ0	0.029715	0.232058	0.113914	IKBIP	Inhibitor of	IKIP_HUMA	14	1.02081
24H 50MT/	Q16394	0.123025	0.232131	0.113944	EXT1	Exostosin-1	EXT1_HUM	3	1.089016
24H 10MT/	Q9UHQ9	0.013786	0.232147	0.113951	CYB5R1	NADH-cyto	NB5R1_HU	15	1.009601
24H 50MT/	Q9NV56	0.049513	0.232158	0.113951	MRGBP	MRG/MORF	MRGBP_HU	3	1.034915
48H 10MT/	P05165	0.016582	0.232227	0.113974	PCCA	Propionyl-(	PCCA_HUM	24	1.01156
48H 50MT/	Q9H4A5	0.173118	0.23235	0.114023	GOLPH3L	Golgi phosp	GLP3L_HUI	5	1.127493
48H 50MT/	O95835	-0.14118	0.232403	0.114048	LATS1	Serine/thre	LATS1_HUN	8	0.906779
24H 10MT/	Q8N2K1	0.028866	0.232493	0.114079	UBE2J2	Ubiquitin-c	UB2J2_HUI	3	1.02021
48H 50MT/	Q9Y4L5	-0.33218	0.232516	0.114089	RNF115	E3 ubiquiti	RN115_HU	3	0.794335
48H 50MT/	Q92625	0.022908	0.23256	0.114105	ANKS1A	Ankyrin rep	ANS1A_HUI	15	1.016005
24H 10MT/	A0AVT1	-0.02171	0.232577	0.11411	UBA6	Ubiquitin-l	UBA6_HUN	43	0.985062
48H 10MT/	Q8N3Y1	0.198743	0.232777	0.114202	FBXW8	F-box/WD r	FBXW8_HU	2	1.147698
48H 50MT/	Q9Y2W6	-0.05528	0.232788	0.114205	TDRKH	Tudor and I	TDRKH_HU	4	0.96241
24H 10MT/	Q10469	0.049913	0.232793	0.114205	MGAT2	Alpha-1,6-r	MGAT2_HU	11	1.035202
48H 50MT/	Q2KHT3	0.369394	0.232826	0.114216	CLEC16A	Protein CLE	CL16A_HUI	1	1.29181
24H 50MT/	Q3LXA3	-0.13358	0.232926	0.114254	TKFC	Triokinase/	TKFC_HUM	18	0.911564
24H 10MT/	Q13137	-0.02642	0.233046	0.114301	CALCOCO2	Calcium-bi	CACO2_HU	14	0.981854
24H 50MT/	Q9HCU5	0.022812	0.233045	0.114301	PREB	Guanine nu	SEC12_HUI	14	1.015938
48H 10MT/	P53814	0.065003	0.23309	0.114318	SMTN	Smoothelir	SMTN_HUN	13	1.046087
48H 50MT/	Q92685	-0.24574	0.233089	0.114318	ALG3	Dol-P-Man:	ALG3_HUM	3	0.843383
24H 10MT/	Q9UIF9	0.059372	0.233132	0.114336	BAZ2A	Bromodom	BAZ2A_HUI	26	1.042012
48H 10MT/	Q5T0F9	-0.07436	0.233177	0.114354	CC2D1B	Coiled-coil	C2D1B_HU	11	0.949762
48H 50MT/	Q8N4T8	0.045183	0.2332	0.114358	CBR4	3-oxoacyl-[	CBR4_HUM	5	1.031814
24H 50MT/	P52895	-0.09568	0.233216	0.114362	AKR1C2	Aldo-keto r	AK1C2_HUI	3	0.935828
48H 50MT/	Q86SQ9	-0.13771	0.233229	0.114366	DHDDS	Dehydrodo	DHDDS_HU	5	0.908963
24H 10MT/	P49757	0.019736	0.233234	0.114367	NUMB	Protein nur	NUMB_HUI	11	1.013774
24H 10MT/	O00273	-0.14059	0.233271	0.114376	DFFA	DNA fragme	DFFA_HUM	14	0.907145
48H 10MT/	Q8WUD1	-0.21411	0.23327	0.114376	RAB2B	Ras-related	RAB2B_HUI	2	0.862077
48H 10MT/	Q8TEB1	0.097778	0.233277	0.114377	DCAF11	DDB1- and	DCA11_HU	11	1.070124
24H 10MT/	Q9BVA0	0.107778	0.233334	0.114396	KATNB1	Katanin p8	KTNB1_HUI	10	1.077568
24H 10MT/	O43301	0.049263	0.233355	0.114405	HSPA12A	Heat shock	HS12A_HUI	7	1.034737
24H 50MT/	Q15126	-0.02824	0.23338	0.114413	PMVK	Phosphom	PMVK_HUN	11	0.980614
48H 50MT/	Q8WXD5	-0.14662	0.233446	0.114437	GEMIN6	Gem-associ	GEMI6_HUI	5	0.903365
24H 50MT/	P29218	-0.07866	0.23346	0.114443	IMPA1	Inositol mc	IMPA1_HUI	14	0.946939
48H 10MT/	O75964	-0.01161	0.233474	0.114447	ATP5MG	ATP synthas	ATP5L_HUN	6	0.991982

48H 10MT/	P23743	-0.18238	0.233513	0.114463	DGKA	Diacylglyce	DGKA_HUM	6	0.881251
48H 50MT/	Q15032	0.385379	0.23353	0.114466	R3HDM1	R3H domai	R3HD1_HU	1	1.306203
24H 10MT/	Q8IV61	-0.63229	0.233565	0.114478	RASGRP3	Ras guanyl-	GRP3_HUM	2	0.645153
24H 50MT/	O15085	-0.11414	0.233608	0.114496	ARHGEF11	Rho guanin	ARHGB_HU	12	0.92393
48H 10MT/	Q9UJX6	-0.01664	0.233671	0.114523	ANAPC2	Anaphase-p	ANC2_HUM	21	0.988534
24H 10MT/	Q9Y6J0	-0.34051	0.233712	0.114539	CABIN1	Calcineurin	CABIN_HUI	1	0.789761
24H 10MT/	P98175	0.014163	0.233723	0.114542	RBM10	RNA-bindir	RBM10_HU	20	1.009866
48H 10MT/	Q53S33	0.114577	0.233723	0.114542	BOLA3	BolA-like pr	BOLA3_HUI	3	1.082658
48H 10MT/	Q9UJX4	-0.08414	0.233757	0.114557	ANAPC5	Anaphase-p	APC5_HUM	25	0.943346
48H 10MT/	Q9BYD3	-0.07341	0.233789	0.114564	MRPL4	Large ribos	RM04_HUM	12	0.950388
24H 10MT/	Q9H4A3	-0.08374	0.233806	0.114567	WNK1	Serine/thre	WNK1_HUI	36	0.943606
24H 50MT/	Q08334	-0.45628	0.233816	0.11457	IL10RB	Interleukin	I10R2_HUM	2	0.728865
48H 50MT/	Q6NX49	-0.71157	0.233825	0.114571	ZNF544	Zinc finger	ZN544_HUI	1	0.610654
24H 10MT/	P43243	-0.05547	0.233838	0.114573	MATR3	Matrin-3	MATR3_HU	31	0.962281
24H 50MT/	Q9H6R7	-0.03051	0.233916	0.114604	WDCP	WD repeat	WDCP_HUI	3	0.979071
24H 10MT/	P22307	-0.0586	0.23393	0.114608	SCP2	Sterol carri	SCP2_HUM	24	0.960193
24H 50MT/	Q8WUD4	-0.06649	0.234052	0.114652	CCDC12	Coiled-coil	CCD12_HU	7	0.954958
24H 50MT/	Q02086	-0.23058	0.234061	0.114655	SP2	Transcripti	SP2_HUMA	1	0.85229
24H 10MT/	Q8TD19	-0.06736	0.234084	0.114665	NEK9	Serine/thre	NEK9_HUM	18	0.95438
24H 10MT/	O00499	-0.14744	0.234097	0.11467	BIN1	Myc box-de	BIN1_HUM	10	0.902851
24H 50MT/	Q7RTS7	0.028129	0.23412	0.11468	KRT74	Keratin, typ	K2C74_HUI	3	1.019689
48H 50MT/	Q9H3C7	-0.06863	0.23414	0.114686	GGNBP2	Gametogen	GGNB2_HU	2	0.953545
24H 10MT/	O14495	0.123614	0.234161	0.114695	PLPP3	Phospholip	PLPP3_HUI	5	1.08946
24H 50MT/	Q9P086	0.394093	0.234183	0.114705	MED11	Mediator o	MED11_HU	1	1.314116
24H 10MT/	Q96KP1	-0.08356	0.234301	0.114747	EXOC2	Exocyst cor	EXOC2_HUI	19	0.943728
48H 50MT/	Q96RE7	-0.01099	0.234349	0.114768	NACC1	Nucleus acc	NACC1_HU	13	0.992412
24H 50MT/	A0JNW5	0.001202	0.234361	0.114772	BLTP3B	Bridge-like	BLT3B_HUI	14	1.000834
48H 10MT/	Q9BVN2	0.387675	0.234433	0.114799	RUSC1	AP-4 compl	RUSC1_HUI	3	1.308283
48H 50MT/	Q96HQ2	-0.24801	0.234493	0.114819	CDKN2AIPI	CDKN2AIP I	C2AIL_HUM	2	0.842058
24H 50MT/	O95834	-0.0573	0.234499	0.11482	EML2	Echinoderm	EMAL2_HU	7	0.961061
48H 50MT/	Q8TAT6	-0.02283	0.234644	0.114882	NPLOC4	Nuclear prc	NPL4_HUM	27	0.984301
48H 10MT/	Q13490	0.115529	0.234719	0.114915	BIRC2	Baculoviral	BIRC2_HUM	10	1.083372
48H 10MT/	O00189	0.132502	0.234737	0.114921	AP4M1	AP-4 compl	AP4M1_HU	9	1.096193
48H 10MT/	Q9UL46	-0.04373	0.23478	0.114937	PSME2	Proteasom	PSME2_HU	14	0.970141
48H 50MT/	Q9C0J8	-0.03262	0.234879	0.114981	WDR33	pre-mRNA 3	WDR33_HU	16	0.977642
24H 50MT/	Q9NTJ4	-0.26003	0.234991	0.115024	MAN2C1	Alpha-mani	MA2C1_HU	5	0.835073
48H 10MT/	O75223	0.031839	0.235008	0.115029	GGCT	Gamma-glu	GGCT_HUM	8	1.022314
48H 50MT/	Q8WTP8	-0.59624	0.23503	0.115037	AEN	Apoptosis-e	AEN_HUMA	1	0.661476
48H 50MT/	Q8N5C8	0.397438	0.235038	0.115037	TAB3	TGF-beta-ac	TAB3_HUM	2	1.317166
48H 10MT/	Q2TBE0	-0.12932	0.235102	0.115061	CWF19L2	CWF19-like	C19L2_HUI	12	0.914261
24H 10MT/	Q86U38	-0.06195	0.235115	0.115066	NOP9	Nucleolar p	NOP9_HUM	20	0.957971
48H 10MT/	Q9H081	-0.03411	0.235163	0.115082	MIS12	Protein MIS	MIS12_HUI	1	0.976632
24H 10MT/	Q9BZ67	-0.30896	0.2352	0.115096	FRMD8	FERM doma	FRMD8_HU	9	0.807225
24H 10MT/	Q8TEK3	-1.33099	0.235282	0.11513	DOT1L	Histone-lys	DOT1L_HUI	2	0.397495
24H 50MT/	P21283	-0.07895	0.235428	0.115188	ATP6V1C1	V-type prot	VATC1_HUI	19	0.946748
48H 10MT/	Q9UQ53	0.001965	0.235441	0.115191	MGAT4B	Alpha-1,3-r	MGT4B_HU	4	1.001363
24H 10MT/	P62633	-0.11948	0.235616	0.115254	CNBP	CCHC-type	CNBP_HUM	6	0.920523
48H 50MT/	Q9P2Y4	-0.27189	0.235616	0.115254	ZNF219	Zinc finger	ZN219_HUI	6	0.828235
48H 10MT/	Q8N1I0	-0.06628	0.235659	0.115267	DOCK4	Dedicator c	DOCK4_HU	37	0.955095
48H 10MT/	P03886	0.099783	0.235702	0.11528	MT-ND1	NADH-ubiq	NU1M_HUI	4	1.071612
48H 10MT/	Q13467	-0.21863	0.235726	0.115289	FZD5	Frizzled-5	FZD5_HUM	1	0.859381
24H 10MT/	Q9P1U0	-0.24999	0.235849	0.115338	POLR1H	DNA-direct	RPA12_HUI	1	0.8409

24H 10MT/	P62879	0.016756	0.235942	0.115376	GNB2	Guanine nu	GBB2_HUM	8	1.011682
48H 50MT/	Q9BUM1	0.046768	0.236128	0.115444	G6PC3	Glucose-6-p	G6PC3_HU	1	1.032949
48H 10MT/	Q96A46	0.1777	0.236132	0.115445	SLC25A28	Mitoferrin-	MFRN2_HU	5	1.131079
24H 50MT/	Q8WV44	0.158118	0.236145	0.11545	TRIM41	E3 ubiquiti	TRI41_HUM	2	1.11583
48H 10MT/	Q9NQX3	-0.00813	0.236163	0.115457	GPHN	Gephyrin	GEPH_HUM	22	0.994382
48H 10MT/	O76011	-1.41989	0.236311	0.115515	KRT34	Keratin, typ	KRT34_HUI	1	0.373741
24H 10MT/	Q9NX74	0.007352	0.236433	0.115563	DUS2	tRNA-dihyd	DUS2L_HUI	23	1.005109
24H 50MT/	P62857	-0.11908	0.236444	0.115565	RPS28	Small ribos	RS28_HUM	3	0.920777
24H 50MT/	Q7Z6K3	-0.12352	0.23646	0.115568	PTAR1	Protein pre	PTAR1_HUI	12	0.917942
48H 10MT/	Q93008	0.017231	0.236472	0.115572	USP9X	Ubiquitin c	USP9X_HUI	64	1.012015
48H 50MT/	Q8N7X0	-0.24966	0.236486	0.115576	ADGB	Androglobi	ADGB_HUM	1	0.841095
48H 10MT/	Q13572	-0.052	0.236504	0.115584	ITPK1	Inositol-tet	ITPK1_HUM	3	0.964598
24H 50MT/	O43264	-0.00322	0.23656	0.115608	ZW10	Centromer	ZW10_HUM	30	0.997772
24H 10MT/	Q92609	0.186221	0.236565	0.115609	TBC1D5	TBC1 doma	TBCD5_HUI	14	1.13778
24H 10MT/	Q09472	0.146551	0.236576	0.115613	EP300	Histone ace	EP300_HUI	7	1.10692
48H 50MT/	Q86V48	-0.03911	0.236608	0.115624	LUZP1	Leucine zip	LUZP1_HUI	22	0.973257
48H 10MT/	Q9Y620	-0.21798	0.236665	0.115644	RAD54B	DNA repair	RA54B_HUI	12	0.859769
24H 10MT/	Q68CP9	0.02049	0.236706	0.115659	ARID2	AT-rich inte	ARID2_HUM	10	1.014304
48H 50MT/	Q8WYQ5	-0.36602	0.236792	0.115688	DGCR8	Microproc	DGCR8_HU	5	0.77592
24H 50MT/	Q9Y261	-0.21395	0.236893	0.115714	FOXA2	Hepatocyte	FOXA2_HUI	3	0.862172
48H 10MT/	Q92930	-0.06942	0.236898	0.115715	RAB8B	Ras-related	RAB8B_HUI	4	0.953023
24H 50MT/	O43716	-0.00866	0.23694	0.11573	GATC	Glutamyl-tl	GATC_HUM	2	0.994015
24H 10MT/	Q9NTZ6	0.021676	0.236957	0.115733	RBM12	RNA-bindir	RBM12_HU	22	1.015138
24H 10MT/	Q9C0E8	0.112121	0.237047	0.115773	LNPK	Endoplasm	LNP_HUMA	12	1.080816
24H 10MT/	Q9Y263	0.03	0.237077	0.115783	PLAA	Phospholip	PLAP_HUM	26	1.021012
24H 10MT/	Q99653	-0.11478	0.237082	0.115783	CHP1	Calcineurir	CHP1_HUM	10	0.923525
48H 10MT/	Q15118	0.129271	0.237095	0.115787	PDK1	[Pyruvate d	PDK1_HUM	8	1.093741
48H 10MT/	P51153	0.019193	0.237201	0.115831	RAB13	Ras-related	RAB13_HUI	6	1.013392
48H 10MT/	Q8IZT6	-0.26925	0.237213	0.115833	ASPM	Abnormal s	ASPM_HUM	9	0.829749
48H 50MT/	O00308	-0.70221	0.237235	0.115841	WWP2	NEDD4-like	WWP2_HU	5	0.614629
24H 10MT/	Q15906	0.161263	0.23727	0.115855	VPS72	Vacuolar pı	VPS72_HUI	2	1.118266
48H 10MT/	P62312	0.030121	0.23729	0.115863	LSM6	U6 snRNA-a	LSM6_HUM	6	1.021098
24H 10MT/	Q92615	0.121655	0.237294	0.115864	LARP4B	La-related p	LAR4B_HUI	17	1.087983
48H 50MT/	Q96MS3	0.029177	0.237312	0.115868	GLT1D1	Glycosyltra	GL1D1_HU	1	1.02043
48H 10MT/	Q53FV1	0.154511	0.237327	0.115874	ORMDL2	ORM1-like p	ORML2_HU	2	1.113044
24H 50MT/	O94763	0.572038	0.237341	0.115879	URI1	Unconvent	RMP_HUM/	5	1.486622
24H 50MT/	Q9H1B7	-0.06987	0.237374	0.115886	IRF2BPL	Probable E3	I2BPL_HUM	6	0.952724
24H 10MT/	Q6ZW49	0.257079	0.237417	0.115903	PAXIP1	PAX-interac	PAXI1_HUM	3	1.195057
24H 50MT/	Q9NY33	-0.0484	0.237421	0.115903	DPP3	Dipeptidyl	DPP3_HUM	31	0.967011
24H 10MT/	Q08AM6	-0.09177	0.237491	0.115934	VAC14	Protein VA(	VAC14_HUI	21	0.938372
24H 50MT/	Q5XKL5	0.382934	0.237491	0.115934	BTBD8	BTB/POZ dc	BTBD8_HUI	4	1.303991
24H 10MT/	Q6ZRV2	0.038619	0.237504	0.115939	FAM83H	Protein FAI	FA83H_HUI	23	1.02713
24H 10MT/	O14802	-0.01572	0.237574	0.115969	POLR3A	DNA-direct	RPC1_HUM	36	0.989163
48H 10MT/	Q86VD7	-0.21731	0.23761	0.115978	SLC25A42	Mitochond	S2542_HUI	4	0.860168
24H 10MT/	Q12979	-0.06255	0.237689	0.116006	ABR	Active brea	ABR_HUMA	13	0.957569
24H 10MT/	Q9H8V3	0.075446	0.237771	0.116042	ECT2	Protein ECT	ECT2_HUM	28	1.053687
24H 50MT/	Q8IV63	-0.08912	0.237779	0.116044	VRK3	Serine/thre	VRK3_HUM	3	0.940095
48H 50MT/	P34913	0.021761	0.23781	0.116058	EPHX2	Bifunctiona	HYES_HUM	14	1.015198
48H 10MT/	P10301	-0.05481	0.237907	0.116102	RRAS	Ras-related	RRAS_HUM	7	0.962718
24H 10MT/	Q6IAA8	-0.13013	0.237917	0.116105	LAMTOR1	Ragulator c	LTOR1_HUI	7	0.913752
24H 50MT/	P40818	0.111316	0.237924	0.116105	USP8	Ubiquitin c	UBP8_HUM	22	1.080213
24H 10MT/	Q9H469	-0.23673	0.237921	0.116105	FBXL15	F-box/LRR-I	FXL15_HUI	8	0.848665

48H 10MTA	Q9NZ32	-0.11388	0.237931	0.116105	ACTR10	Actin-relate	ARP10_HUI	18	0.924097
48H 50MTA	Q92747	0.047502	0.238065	0.116165	ARPC1A	Actin-relate	ARC1A_HUI	13	1.033474
48H 50MTA	P29320	-0.15595	0.238077	0.11617	EPHA3	Ephrin type	EPHA3_HUI	1	0.897538
24H 50MTA	P48163	-0.07182	0.238187	0.116219	ME1	NADP-depe	MAOX_HUI	20	0.95144
24H 50MTA	O95340	0.025529	0.23826	0.116243	PAPSS2	Bifunctiona	PAPS2_HUI	9	1.017853
24H 50MTA	Q6ZMR3	0.419902	0.23829	0.116255	LDHAL6A	L-lactate de	LDH6A_HU	1	1.337837
24H 10MTA	P05166	0.009323	0.238308	0.116262	PCCB	Propionyl-(	PCCB_HUM	19	1.006483
48H 10MTA	Q9NQ29	0.002728	0.238314	0.116264	LUC7L	Putative RN	LUC7L_HUI	7	1.001892
48H 10MTA	Q6ZRP7	-0.07071	0.238325	0.116267	QSOX2	Sulfhydryl c	QSOX2_HU	20	0.952172
24H 10MTA	Q9NPI1	0.101053	0.238337	0.11627	BRD7	Bromodom	BRD7_HUM	3	1.072556
24H 10MTA	Q03167	0.15774	0.238388	0.116291	TGFBR3	Transformi	TGBR3_HUI	2	1.115538
48H 10MTA	P0DMV8;P(	-0.03121	0.238438	0.116309	HSPA1A;HS	Heat shock	HS71A_HUI	25	0.978596
24H 10MTA	Q8NBM4	0.024679	0.238441	0.116309	UBAC2	Ubiquitin-a	UBAC2_HU	14	1.017253
24H 50MTA	P67809	0.00064	0.238464	0.116318	YBX1	Y-box-bindi	YBOX1_HUI	17	1.000444
48H 50MTA	Q99570	-0.11252	0.238506	0.116333	PIK3R4	Phosphoin	PI3R4_HUM	24	0.924971
48H 10MTA	Q9NNW7	-0.11128	0.238503	0.116333	TXNRD2	Thioredoxi	TRXR2_HUI	10	0.925765
24H 10MTA	Q9BT67	-0.48479	0.238534	0.116343	NDFIP1	NEDD4 fam	NFIP1_HUM	1	0.714599
24H 10MTA	P46976	-0.06778	0.238553	0.11635	GYG1	Glycogenin	GLYG_HUM	12	0.954104
48H 10MTA	O75879	0.060282	0.238564	0.116353	GATB	Glutamyl-tl	GATB_HUM	15	1.04267
24H 50MTA	Q9BVW5	0.359888	0.238584	0.116362	TIPIN	TIMELESS-ir	TIPIN_HUM	3	1.283326
24H 50MTA	P53992	-0.09683	0.238638	0.116385	SEC24C	Protein trai	SC24C_HUI	35	0.935087
48H 10MTA	O14730	0.18777	0.238657	0.11639	RIOK3	Serine/thre	RIOK3_HUM	7	1.139002
24H 50MTA	P51809	0.109241	0.238657	0.11639	VAMP7	Vesicle-assc	VAMP7_HU	11	1.078661
24H 50MTA	P29320	-0.07645	0.238693	0.1164	EPHA3	Ephrin type	EPHA3_HUI	1	0.948389
48H 50MTA	Q9UKG9	-0.04923	0.238696	0.1164	CROT	Peroxisoma	OCTC_HUM	10	0.96645
48H 50MTA	P60002	0.093879	0.238746	0.116418	ELOF1	Transcripti	ELOF1_HUI	1	1.067236
48H 50MTA	Q13423	-0.009	0.238788	0.116435	NNT	NAD(P) trar	NNTM_HUI	37	0.993779
48H 50MTA	Q8N4V1	-0.12492	0.238786	0.116435	MMGT1	ER membra	EMC5_HUM	4	0.917056
24H 10MTA	Q9P2N5	0.055779	0.238823	0.116446	RBM27	RNA-bindir	RBM27_HU	25	1.03942
24H 50MTA	Q9UHR5	-0.02741	0.238823	0.116446	SAP30BP	SAP30-binc	S30BP_HUI	7	0.981179
48H 10MTA	Q9H0H0	0.034725	0.238878	0.116466	INTS2	Integrator (	INT2_HUM	11	1.024362
48H 10MTA	Q9UBF6	0.271446	0.238905	0.116477	RNF7	RING-box p	RBX2_HUM	3	1.207017
24H 10MTA	Q9UNN5	-0.09401	0.238951	0.116496	FAF1	FAS-associa	FAF1_HUM	16	0.936917
24H 50MTA	Q9ULV3	0.003125	0.23902	0.116525	CIZ1	Cip1-intera	CIZ1_HUM	7	1.002169
24H 10MTA	Q7LBR1	-0.20114	0.239046	0.116531	CHMP1B	Charged mu	CHM1B_HU	3	0.86986
24H 50MTA	Q96IZ0	-0.11155	0.239171	0.116583	PAWR	PRKC apop	PAWR_HUI	12	0.925592
24H 50MTA	Q6NXT4	0.199796	0.239201	0.116595	SLC30A6	Zinc transp	ZNT6_HUM	4	1.148536
24H 50MTA	Q9HC62	0.187918	0.239229	0.116605	SENP2	Sentrin-spe	SENP2_HUI	5	1.139119
24H 50MTA	Q96D71	-0.17234	0.239238	0.116606	REPS1	RalBP1-assc	REPS1_HUI	11	0.887402
48H 10MTA	Q9NY33	-0.07454	0.239239	0.116606	DPP3	Dipeptidyl	DPP3_HUM	31	0.949645
48H 10MTA	P28290	-0.12383	0.239278	0.116621	ITPRID2	Protein ITP	ITPI2_HUM	16	0.917749
24H 10MTA	Q9Y4C2	-0.0148	0.239297	0.116628	TCAF1	TRPM8 cha	TCAF1_HUI	14	0.989794
48H 50MTA	P18433	0.196926	0.239386	0.11666	PTPRA	Receptor-ty	PTPRA_HUI	7	1.146253
48H 50MTA	Q5T447	-0.15899	0.23941	0.11667	HECTD3	E3 ubiquiti	HECD3_HU	17	0.895649
24H 50MTA	P50453	0.021589	0.239416	0.116672	SERPINB9	Serpin B9	SPB9_HUM	16	1.015077
24H 10MTA	Q96C23	-0.08602	0.239473	0.116693	GALM	Galactose n	GALM_HUM	8	0.942121
24H 50MTA	Q9BYV8	-0.01341	0.239515	0.116709	CEP41	Centrosom	CEP41_HUI	8	0.990746
48H 50MTA	O43567	0.603111	0.239619	0.116751	RNF13	E3 ubiquiti	RNF13_HUI	2	1.518989
48H 10MTA	Q9Y6A4	-0.06201	0.239652	0.116762	CFAP20	Cilia- and fl	CFA20_HUI	11	0.957932
24H 10MTA	Q9NP92	0.006722	0.239669	0.116767	MRPS30	Large ribos	RT30_HUM	12	1.00467
24H 10MTA	Q13510	0.063468	0.239894	0.116869	ASAH1	Acid ceram	ASAH1_HUI	17	1.044975
48H 10MTA	Q5T440	-0.05884	0.239914	0.116877	IBA57	Putative tra	CAF17_HUI	8	0.960035

48H 50MTA	Q92499	-0.00097	0.239945	0.116888	DDX1	ATP-depend	DDX1_HUM	42	0.999331
24H 10MTA	Q14493	-0.17239	0.239978	0.116901	SLBP	Histone RN	SLBP_HUM	4	0.887369
48H 50MTA	Q0VGL1	-0.07319	0.239986	0.116902	LAMTOR4	Ragulator c	LTOR4_HUI	3	0.950532
24H 50MTA	Q96PE7	-0.00912	0.23999	0.116902	MCEE	Methylmal	MCEE_HUM	3	0.993697
48H 10MTA	Q9NRP0	-0.09065	0.240026	0.116911	OSTC	Oligosacch	OSTC_HUM	3	0.9391
24H 10MTA	Q01814	0.24683	0.240085	0.116933	ATP2B2	Plasma mer	AT2B2_HUI	3	1.186597
24H 50MTA	Q9HBR0	0.011776	0.24009	0.116934	SLC38A10	Solute carri	S38AA_HUI	13	1.008196
48H 10MTA	Q9H3L0	0.228913	0.240111	0.116941	MMADHC	Cobalamin	MMAD_HU	2	1.171951
24H 10MTA	Q9NYL2	0.029031	0.240149	0.116955	MAP3K20	Mitogen-ac	M3K20_HU	8	1.020326
48H 50MTA	P24390	-0.22457	0.240169	0.116963	KDELR1	ER lumen p	ERD21_HUI	2	0.855849
48H 10MTA	P49589	-0.02484	0.240193	0.116972	CARS1	Cysteine--tI	SYCC_HUM	34	0.982928
24H 50MTA	Q6Y288	0.131897	0.240231	0.116985	B3GLCT	Beta-1,3-gl	B3GLT_HUI	5	1.095733
24H 50MTA	P02533	-1.03979	0.240274	0.117002	KRT14	Keratin, typ	K1C14_HUI	33	0.486399
48H 50MTA	P62263	-0.08213	0.240282	0.117004	RPS14	Small ribos	RS14_HUM	9	0.944662
48H 50MTA	Q9BVL4	0.142841	0.240287	0.117005	SELENOO	Protein ade	SELO_HUM	11	1.104077
24H 50MTA	O95376	-0.05189	0.240293	0.117006	ARIH2	E3 ubiquiti	ARI2_HUM	13	0.964669
48H 10MTA	P48729	0.093184	0.240308	0.117008	CSNK1A1	Casein kina	KC1A_HUM	13	1.066722
24H 10MTA	P31483	0.037542	0.240367	0.117032	TIA1	Cytotoxic g	TIA1_HUM	10	1.026364
24H 50MTA	Q7Z6Z7	-0.0357	0.240382	0.117036	HUWE1	E3 ubiquiti	HUWE1_HU	111	0.975559
24H 50MTA	O15235	-0.01153	0.240386	0.117037	MRPS12	Small ribos	RT12_HUM	5	0.992039
48H 10MTA	P16403	-0.09182	0.240418	0.117049	H1-2	Histone H1	H12_HUM	1	0.938338
48H 10MTA	Q13033	-0.08033	0.240422	0.117049	STRN3	Striatin-3	STRN3_HUI	14	0.945843
48H 10MTA	Q9Y2I9	0.79548	0.240445	0.117057	TBC1D30	TBC1 doma	TBC30_HUI	1	1.735655
24H 50MTA	Q5U5X0	-0.09327	0.240455	0.117059	LYRM7	Complex III	LYRM7_HU	3	0.937398
48H 50MTA	Q04725	-0.59683	0.240465	0.117062	TLE2	Transducin	TLE2_HUM	1	0.661206
48H 10MTA	O43674	-0.05565	0.240492	0.11707	NDUFB5	NADH dehy	NDUB5_HU	5	0.962158
48H 50MTA	Q9NU22	-0.13574	0.240492	0.11707	MDN1	Midasin	MDN1_HUI	115	0.910201
48H 10MTA	Q8IUI8	-0.09216	0.240539	0.117089	CRLF3	Cytokine re	CRLF3_HUM	9	0.938114
24H 10MTA	P49755	-0.01647	0.240552	0.117093	TMED10	Transmemb	TMEDA_HU	7	0.988649
24H 10MTA	Q8TCG2	0.149772	0.240577	0.1171	PI4K2B	Phosphatid	P4K2B_HUI	9	1.109394
48H 50MTA	Q96C34	-0.41947	0.24061	0.117111	RUNDC1	RUN domai	RUND1_HU	1	0.747699
24H 50MTA	Q9Y3U8	-0.04497	0.24064	0.117123	RPL36	Large ribos	RL36_HUM	5	0.969307
24H 50MTA	Q9UH62	0.08288	0.240665	0.117134	ARMCX3	Armadillo r	ARMX3_HU	12	1.05913
48H 10MTA	O95983	-0.04195	0.240679	0.117137	MBD3	Methyl-CpG	MBD3_HUM	9	0.971338
48H 50MTA	P30085	0.005611	0.240787	0.117179	CMPK1	UMP-CMP k	KCY_HUMA	15	1.003897
48H 50MTA	Q9Y5W8	0.355241	0.240985	0.117258	SNX13	Sorting nex	SNX13_HUI	5	1.279199
24H 10MTA	Q13617	-0.00024	0.240989	0.117259	CUL2	Cullin-2	CUL2_HUM	24	0.999833
24H 10MTA	Q9BUL9	-0.02791	0.241055	0.117283	RPP25	Ribonuclea	RPP25_HUI	8	0.980838
48H 50MTA	P63208	-0.02105	0.241129	0.11731	SKP1	S-phase kin	SKP1_HUM	6	0.985513
48H 50MTA	Q15149	-0.03075	0.241142	0.117315	PLEC	Plectin	PLEC_HUM	199	0.97891
24H 50MTA	O75663	-0.08017	0.241159	0.117321	TIPRL	TIP41-like p	TIPRL_HUM	15	0.945944
24H 50MTA	Q9NP92	0.006317	0.241188	0.117331	MRPS30	Large ribos	RT30_HUM	12	1.004388
48H 50MTA	Q9BSU3	-0.05378	0.241201	0.117336	NAA11	N-alpha-ac	NAA11_HU	2	0.96341
48H 10MTA	Q4LDG9	0.516237	0.241276	0.117366	DNAL1	Dynein axo	DNAL1_HU	2	1.43022
24H 10MTA	Q9BVC5	-0.33541	0.241484	0.117453	C2orf49	Ashwin	ASHWN_HU	5	0.792562
48H 10MTA	Q8N5C7	0.204224	0.241557	0.117483	DTWD1	tRNA-uridir	DTWD1_HU	1	1.152067
24H 10MTA	O14618	-0.12291	0.241577	0.117489	CCS	Copper cha	CCS_HUMA	8	0.918332
24H 10MTA	Q9NWV8	0.037269	0.241624	0.117507	BABAM1	BRISC and E	BABA1_HUI	6	1.026169
24H 50MTA	Q9H000	0.203764	0.241655	0.117516	MKRN2	E3 ubiquiti	MKRN2_HU	5	1.151699
24H 50MTA	Q27J81	-0.0804	0.241682	0.117526	INF2	Inverted for	INF2_HUM	25	0.945793
48H 50MTA	O60503	0.089382	0.241711	0.117537	ADCY9	Adenylate c	ADCY9_HUI	4	1.063914
24H 50MTA	Q8WWH5	0.016831	0.241786	0.117568	TRUB1	Pseudourid	TRUB1_HUI	15	1.011735

48H 50MT/	Q96IK1	0.01524	0.241783	0.117568	BOD1	Biorientati	BOD1_HUN	4	1.010619
48H 50MT/	Q9BTE1	0.233162	0.241814	0.117578	DCTN5	Dynactin su	DCTN5_HU	3	1.175408
48H 50MT/	P00167	0.105748	0.241824	0.117581	CYB5A	Cytochrom	CYB5_HUM	4	1.076052
24H 10MT/	P10321	0.033919	0.241881	0.117603	HLA-C	HLA class I I	HLAC_HUM	3	1.02379
48H 10MT/	Q9NUL7	-0.07399	0.241896	0.117607	DDX28	Probable A	DDX28_HU	11	0.950007
24H 50MT/	Q9NYS0	-0.55473	0.241924	0.117618	NKIRAS1	NF-kappa-B	KBRS1_HUI	2	0.680783
24H 10MT/	Q562R1	0.282039	0.241948	0.117626	ACTBL2	Beta-actin-l	ACTBL_HUN	3	1.215912
48H 50MT/	P62995	0.007127	0.241989	0.117645	TRA2B	Transforme	TRA2B_HUI	6	1.004952
24H 50MT/	P61006	0.052207	0.242002	0.117648	RAB8A	Ras-related	RAB8A_HUI	5	1.03685
48H 50MT/	P51970	-0.09377	0.242016	0.117652	NDUFA8	NADH dehy	NDUA8_HU	7	0.937072
48H 50MT/	Q8IXU6	-0.32954	0.242056	0.117665	SLC35F2	Solute carri	S35F2_HUN	1	0.795792
24H 50MT/	Q9Y232	-0.11158	0.242102	0.117681	CDYL	Chromodo	CDYL_HUM	6	0.925574
24H 10MT/	Q8N8N7	-0.12762	0.242112	0.117684	PTGR2	Prostaglanc	PTGR2_HUI	9	0.915341
24H 50MT/	Q8N108	-0.31964	0.242124	0.117685	MIER1	Mesoderm	MIER1_HUI	6	0.801269
24H 10MT/	O43318	-0.10388	0.242184	0.117707	MAP3K7	Mitogen-ac	M3K7_HUN	8	0.930527
48H 10MT/	P26440	-0.0052	0.242218	0.117719	IVD	Isovaleryl-C	IVD_HUMA	12	0.996402
24H 10MT/	Q9Y3A6	0.065886	0.242264	0.117737	TMED5	Transmemb	TMED5_HU	9	1.046728
48H 10MT/	Q96S44	-0.08074	0.242382	0.117781	TP53RK	EKC/KEOPS	PRPK_HUN	6	0.94557
48H 50MT/	Q9HAU4	0.345978	0.242412	0.11779	SMURF2	E3 ubiquiti	SMUF2_HU	1	1.271013
24H 50MT/	Q9Y6R0	-0.65524	0.242464	0.117806	NUMBL	Numb-like	NUMBL_HU	1	0.634971
24H 10MT/	O95639	0.048282	0.242518	0.117827	CPSF4	Cleavage ar	CPSF4_HUN	9	1.034033
48H 50MT/	Q13490	0.065877	0.242542	0.117836	BIRC2	Baculoviral	BIRC2_HUN	10	1.046721
24H 50MT/	Q9NQG5	-0.07474	0.242542	0.117836	RPRD1B	Regulation	RPR1B_HUI	12	0.949513
48H 50MT/	O75821	-0.11931	0.242589	0.117854	EIF3G	Eukaryotic	EIF3G_HUN	19	0.92063
24H 10MT/	Q5VV42	0.073399	0.242716	0.117905	CDKAL1	Threonylca	CDKAL_HUI	15	1.052193
24H 10MT/	Q8N3F8	0.115107	0.242714	0.117905	MICALL1	MICAL-like	MILK1_HUI	4	1.083056
48H 50MT/	Q9Y312	-0.02137	0.242747	0.117914	AAR2	Protein AAI	AAR2_HUN	12	0.985297
24H 10MT/	Q8NFH4	0.030324	0.242866	0.117958	NUP37	Nucleopori	NUP37_HU	11	1.021241
24H 10MT/	P12109	0.769267	0.242879	0.117962	COL6A1	Collagen al	CO6A1_HU	3	1.704403
48H 10MT/	Q9Y5R8	-0.0728	0.242924	0.117981	TRAPPC1	Trafficking	TPPC1_HUI	7	0.950792
24H 10MT/	P51684	-0.90481	0.24302	0.118018	CCR6	C-C chemol	CCR6_HUN	1	0.534103
24H 50MT/	Q9UKV8	0.102688	0.243022	0.118018	ago-02	Protein arg	AGO2_HUN	14	1.073772
48H 10MT/	Q9BV38	0.002323	0.243069	0.118035	WDR18	WD repeat-	WDR18_HU	13	1.001611
48H 50MT/	Q9BXK5	-0.01726	0.243071	0.118035	BCL2L13	Bcl-2-like p	B2L13_HUI	12	0.988108
24H 10MT/	Q99595	-0.06813	0.243094	0.118043	TIMM17A	Mitochond	TI17A_HUN	2	0.953873
24H 50MT/	Q8WWB7	0.055075	0.243175	0.118074	GLMP	Glycosylate	GLMP_HUN	2	1.038913
48H 50MT/	Q969Q5	-0.0884	0.243184	0.118075	RAB24	Ras-related	RAB24_HUI	5	0.940567
48H 10MT/	Q9UBP6	-0.04092	0.24327	0.118106	METTL1	tRNA (guan	TRMB_HUN	10	0.972034
24H 50MT/	Q9H1C7	0.019041	0.24345	0.118171	CYSTM1	Cysteine-ric	CYTM1_HU	1	1.013286
48H 50MT/	Q9Y2H2	-0.04114	0.243472	0.118179	INPP5F	Phosphatid	SAC2_HUM	5	0.971889
24H 10MT/	P00488	0.579447	0.24352	0.118195	F13A1	Coagulatio	F13A_HUM	1	1.494276
48H 10MT/	P38432	-0.0267	0.243529	0.118197	COIL	Coilin	COIL_HUM	11	0.981663
48H 10MT/	Q9NX01	0.327531	0.243535	0.118198	TXNL4B	Thioredoxi	TXN4B_HUI	3	1.254864
24H 50MT/	Q8N9N5	0.023759	0.243541	0.118199	BANP	Protein BAI	BANP_HUN	1	1.016605
48H 10MT/	Q14999	0.053004	0.243576	0.118212	CUL7	Cullin-7	CUL7_HUN	11	1.037423
24H 50MT/	P09884	-0.07287	0.243652	0.118242	POLA1	DNA polym	DPOLA_HU	35	0.950744
48H 10MT/	Q9NSK0	-0.00453	0.243842	0.118321	KLC4	Kinesin ligh	KLC4_HUM	16	0.996862
48H 10MT/	Q9P1Q0	0.041986	0.243858	0.118327	VPS54	Vacuolar pi	VPS54_HUI	5	1.02953
24H 10MT/	Q9NP61	-0.0895	0.243878	0.118335	ARFGAP3	ADP-ribosy	ARFG3_HUI	13	0.939845
24H 50MT/	Q8IWY9	-0.52534	0.243924	0.118352	CDAN1	Codanin-1	CDAN1_HU	6	0.694795
48H 10MT/	Q9BVC6	-0.04991	0.243957	0.118361	TMEM109	Voltage-gat	TM109_HU	2	0.965995
24H 10MT/	P85037	0.003091	0.243998	0.118377	FOXK1	Forkhead b	FOXK1_HUI	14	1.002145

24H 50MT/	Q9UJY4	-0.0593	0.244048	0.118396	GGA2	ADP-ribosy	GGA2_HUM	13	0.959729
48H 10MT/	Q96EY9	-0.04572	0.244058	0.1184	ADAT3	Probable ir	ADAT3_HUI	5	0.968805
48H 50MT/	Q9H0B6	-0.11763	0.244157	0.118435	KLC2	Kinesin ligh	KLC2_HUM	22	0.921698
24H 10MT/	P33240	-0.00233	0.244168	0.118438	CSTF2	Cleavage sti	CSTF2_HUM	17	0.998383
48H 50MT/	Q9Y666	0.020418	0.24417	0.118438	SLC12A7	Solute carri	S12A7_HUI	17	1.014253
24H 10MT/	P48668	-1.00973	0.244182	0.118441	KRT6C	Keratin, typ	K2C6C_HUI	32	0.496639
48H 50MT/	O15131	-0.1056	0.244193	0.118445	KPNA5	Importin su	IMA6_HUM	15	0.929419
24H 10MT/	Q9H4G4	0.24529	0.244196	0.118445	GLIPR2	Golgi-assoc	GAPR1_HU	4	1.185331
48H 50MT/	Q96PU5	0.002673	0.244211	0.118447	NEDD4L	E3 ubiquiti	NED4L_HUI	27	1.001854
24H 50MT/	Q9NXC5	0.128469	0.244314	0.118489	MIOS	GATOR2 co	MIOS_HUM	26	1.093133
48H 50MT/	P06748	-0.01703	0.244356	0.118507	NPM1	Nucleopho	NPM_HUM	13	0.988262
48H 50MT/	Q9Y2R0	0.102488	0.24436	0.118507	COA3	Cytochrom	COA3_HUM	4	1.073623
24H 50MT/	Q9ULM6	0.130902	0.244381	0.118514	CNOT6	CCR4-NOT t	CNOT6_HU	4	1.094978
48H 50MT/	P23588	0.006155	0.244458	0.118545	EIF4B	Eukaryotic	IF4B_HUM/	20	1.004276
24H 50MT/	Q9ULW0	-0.1278	0.244494	0.118558	TPX2	Targeting p	TPX2_HUM	31	0.915224
48H 10MT/	Q8N335	-0.02874	0.244518	0.118568	GPD1L	Glycerol-3-	GPD1L_HU	12	0.980277
48H 10MT/	Q9Y5Z7	0.220828	0.244561	0.118581	HCFC2	Host cell fa	HCFC2_HUI	4	1.165402
48H 50MT/	O60678	-0.13276	0.244626	0.118605	PRMT3	Protein arg	ANM3_HUM	20	0.912083
24H 10MT/	P05981	-0.1255	0.244677	0.118622	HPN	Serine prot	HEPS_HUM	6	0.916686
24H 50MT/	Q9NWM8	0.124071	0.244705	0.118631	FKBP14	Peptidyl-pr	FKB14_HUI	5	1.089805
24H 50MT/	P42575	-0.14596	0.244723	0.118636	CASP2	Caspase-2	CASP2_HUI	2	0.903777
48H 10MT/	Q15035	-0.29997	0.244725	0.118636	TRAM2	Translocati	TRAM2_HU	2	0.812271
24H 10MT/	Q96SL1	0.073572	0.244731	0.118637	SLC49A4	Solute carri	DIRC2_HUM	1	1.052319
48H 10MT/	P02795	-0.5981	0.244833	0.118679	MT2A	Metallothi	MT2_HUM/	1	0.660625
24H 10MT/	P56199	-0.01061	0.244875	0.118696	ITGA1	Integrin alp	ITA1_HUM/	12	0.992671
24H 50MT/	Q9NRM2	-0.27974	0.24488	0.118697	ZNF277	Zinc finger	ZN277_HUI	3	0.823739
48H 50MT/	Q86XA9	-0.05609	0.24489	0.118697	HEATR5A	HEAT repea	HTR5A_HUI	23	0.961869
48H 50MT/	P50395	0.002009	0.24496	0.118728	GDI2	Rab GDP di	GDIB_HUM	36	1.001393
48H 10MT/	Q96SB8	-0.04474	0.244975	0.118731	SMC6	Structural	SMC6_HUM	9	0.969461
48H 10MT/	P54727	0.064611	0.244993	0.118738	RAD23B	UV excision	RD23B_HU	15	1.045803
24H 50MT/	Q7L3S4	-0.00507	0.24501	0.118743	ZNF771	Zinc finger	ZN771_HUI	3	0.996494
48H 50MT/	Q8NHQ9	-0.30557	0.24501	0.118743	DDX55	ATP-depen	DDX55_HU	16	0.809122
48H 10MT/	O14569	-0.05264	0.245044	0.118753	CYB561D2	Transmemb	C56D2_HU	1	0.964171
48H 10MT/	Q9BSG0	-0.06837	0.245079	0.118768	PRADC1	Protease-as	PADC1_HU	1	0.953713
48H 10MT/	Q8NFZ5	0.115255	0.245088	0.118771	TNIP2	TNFAIP3-in	TNIP2_HUM	1	1.083166
48H 10MT/	P50750	-0.04383	0.245163	0.118796	CDK9	Cyclin-depe	CDK9_HUM	10	0.970076
24H 10MT/	P08922	-0.26956	0.245203	0.11881	ROS1	Proto-oncc	ROS1_HUM	1	0.829572
24H 50MT/	Q15906	0.019395	0.245229	0.118821	VPS72	Vacuolar pr	VPS72_HUI	2	1.013534
48H 10MT/	O00458	0.166391	0.245241	0.118823	IFRD1	Interferon-	IFRD1_HUM	6	1.122248
24H 10MT/	Q96AQ8	0.123797	0.245279	0.118837	MCUR1	Mitochond	MCUR1_HU	1	1.089599
48H 50MT/	Q9BV94	-0.09367	0.24532	0.118852	EDEM2	ER degrada	EDEM2_HU	6	0.937137
24H 50MT/	Q9Y3C6	0.016586	0.245383	0.118879	PPIL1	Peptidyl-pr	PPIL1_HUM	7	1.011563
24H 10MT/	O95613	-0.02577	0.2454	0.118885	PCNT	Pericentrin	PCNT_HUM	18	0.982297
24H 10MT/	Q8N999	0.273485	0.245425	0.118896	RLIG1	RNA ligase	RLIG1_HUM	6	1.208724
48H 10MT/	Q96FS4	-0.01459	0.245504	0.118924	SIPA1	Signal-indu	SIPA1_HUM	15	0.989941
24H 10MT/	Q5TH69	0.02575	0.245566	0.11895	ARFGEF3	Brefeldin A	BIG3_HUM	24	1.018009
24H 10MT/	Q9C0I1	-0.02222	0.245604	0.118965	MTMR12	Myotubula	MTMRC_HU	11	0.984716
48H 10MT/	P63098	-0.15494	0.245688	0.11899	PPP3R1	Calcineurin	CANB1_HU	5	0.898169
48H 50MT/	Q15286	-0.10915	0.245688	0.11899	RAB35	Ras-related	RAB35_HUI	9	0.927135
24H 50MT/	Q9NV39	0.766032	0.245679	0.11899	PRR34	Proline-rich	PRR34_HUI	1	1.700586
24H 50MT/	Q00534	-0.24518	0.245715	0.119002	CDK6	Cyclin-depe	CDK6_HUM	12	0.84371
24H 10MT/	Q9HAW4	-0.17102	0.245726	0.119005	CLSPN	Claspin	CLSPN_HUI	6	0.888216

48H 50MTA	Q8NBP0	-0.31286	0.245744	0.119012	TTC13	Tetratricop	TTC13_HUI	8	0.805045
24H 10MTA	Q2M1Z3	-0.70765	0.245827	0.11905	ARHGAP31	Rho GTPase	RHG31_HU	2	0.612317
48H 50MTA	Q86X83	-0.17336	0.245868	0.119062	COMMD2	COMM dom	COMD2_HU	8	0.886774
24H 10MTA	Q9BVM2	0.07154	0.245887	0.119069	DPCD	Protein DP	DPCD_HUM	7	1.050838
48H 50MTA	Q9Y613	0.058492	0.245931	0.119084	FHOD1	FH1/FH2 do	FHOD1_HU	8	1.041377
24H 50MTA	P08582	-0.13302	0.245995	0.119109	MELTF	Melanotran	TRFM_HUM	3	0.911922
24H 50MTA	P49810	0.079336	0.246013	0.119109	PSEN2	Presenilin-2	PSN2_HUM	2	1.056531
24H 50MTA	P68104	-0.12112	0.246003	0.119109	EEF1A1	Elongation	EF1A1_HUI	30	0.919473
24H 10MTA	Q9NPR9	0.149844	0.246013	0.119109	GPR108	Protein GP	GP108_HU	5	1.10945
24H 50MTA	Q9NR50	-0.05431	0.246012	0.119109	EIF2B3	Translation	EI2BG_HUI	21	0.963052
24H 10MTA	Q7RTV0	-0.12316	0.246052	0.119124	PHF5A	PHD finger-	PHF5A_HUI	6	0.918174
48H 50MTA	P29692	-0.05178	0.246103	0.119143	EEF1D	Elongation	EF1D_HUM	18	0.964744
48H 10MTA	Q587I9	-0.22725	0.24614	0.119158	SFT2D3	Vesicle tran	SFT2C_HUI	3	0.854259
24H 10MTA	Q96Q45	0.077962	0.24621	0.119185	TMEM237	Transmemb	TM237_HU	6	1.055526
24H 50MTA	Q9NYJ1	0.117292	0.246296	0.119213	COA4	Cytochrom	COA4_HUM	1	1.084697
24H 10MTA	Q9NQR4	0.006952	0.246322	0.119222	NIT2	Omega-ami	NIT2_HUM	16	1.00483
48H 50MTA	Q8N635	-0.30493	0.246382	0.119244	MEIOB	Meiosis-spe	MEIOB_HUI	2	0.809479
48H 50MTA	P56557	-0.31197	0.246403	0.119251	TMEM50B	Transmemb	TM50B_HU	1	0.805539
24H 10MTA	Q12770	0.052536	0.246402	0.119251	SCAP	Sterol regul	SCAP_HUM	2	1.037086
24H 10MTA	Q8IYQ7	0.206613	0.246424	0.119256	THNSL1	Threonine s	THNS1_HUI	1	1.153976
24H 50MTA	Q969M7	-0.06766	0.246504	0.119287	UBE2F	NEDD8-con	UBE2F_HUI	3	0.954186
24H 50MTA	Q92575	0.0328	0.246784	0.119396	UBXN4	UBX domai	UBXN4_HU	9	1.022996
24H 50MTA	P17661	0.341007	0.246802	0.119401	DES	Desmin	DESM_HUM	2	1.26664
48H 10MTA	Q9Y5A9	0.046324	0.246827	0.11941	YTHDF2	YTH domain	YTHD2_HUI	10	1.03263
48H 50MTA	Q02790	0.018375	0.246844	0.119417	FKBP4	Peptidyl-pr	FKBP4_HUI	34	1.012818
48H 50MTA	O95870	-0.02398	0.246866	0.119424	ABHD16A	Phosphatid	ABHGA_HU	12	0.983518
24H 10MTA	Q5QJ74	-0.0794	0.246885	0.119432	TBCEL	Tubulin-spe	TBCEL_HUM	6	0.946454
48H 50MTA	Q9BQD3	-0.29538	0.246919	0.119444	KXD1	KxDL motif	KXDL1_HUI	2	0.814857
48H 50MTA	P55210	-0.12907	0.247053	0.119501	CASP7	Caspase-7	CASP7_HUI	7	0.91442
48H 10MTA	P20073	-0.0405	0.247085	0.119513	ANXA7	Annexin A7	ANXA7_HU	19	0.972315
48H 10MTA	Q16877	0.049399	0.24711	0.119524	PFKFB4	6-phosphof	F264_HUM	9	1.034834
24H 50MTA	Q49B96	-0.21382	0.247142	0.119534	COX19	Cytochrom	COX19_HU	1	0.86225
48H 50MTA	P50148	-0.0095	0.247241	0.119578	GNAQ	Guanine nu	GNAQ_HUM	2	0.99344
24H 50MTA	O43164	0.070129	0.247309	0.1196	PJA2	E3 ubiquiti	PJA2_HUM	5	1.04981
48H 50MTA	Q8N954	-0.42647	0.247333	0.119607	GPATCH11	G patch do	GPT11_HUI	4	0.744078
48H 10MTA	Q9Y342	-0.0947	0.247441	0.119646	PLLP	Plasmolipin	PLLP_HUM	2	0.936468
48H 50MTA	Q8IZ52	-0.18141	0.247445	0.119647	CHPF	Chondroiti	CHSS2_HUI	7	0.881843
24H 50MTA	Q9P1Z2	-1.01045	0.247449	0.119647	CALCOCO1	Calcium-bi	CACO1_HU	2	0.496392
24H 10MTA	Q1ED39	0.023278	0.247492	0.119657	KNOP1	Lysine-rich	KNOP1_HU	13	1.016266
48H 10MTA	P49336	0.015483	0.247498	0.119658	CDK8	Cyclin-depe	CDK8_HUM	6	1.01079
48H 10MTA	O60318	-0.08086	0.247505	0.11966	MCM3AP	Germinal-c	GANP_HUM	22	0.945494
48H 50MTA	Q32MZ4	-0.00311	0.247518	0.119664	LRRFIP1	Leucine-ric	LRRF1_HUI	26	0.997848
24H 50MTA	Q8N302	-0.24714	0.247522	0.119665	AGGF1	Angiogenic	AGGF1_HU	2	0.842563
48H 10MTA	Q04323	0.041866	0.247527	0.119666	UBXN1	UBX domai	UBXN1_HU	10	1.029444
48H 50MTA	Q9BTV4	-0.05061	0.247539	0.119667	TMEM43	Transmemb	TMM43_HU	20	0.965526
48H 10MTA	Q9BZL4	-0.01036	0.247562	0.119674	PPP1R12C	Protein pho	PP12C_HUI	10	0.992848
48H 10MTA	Q9UBT2	0.018195	0.2476	0.119684	UBA2	SUMO-activ	SAE2_HUM	30	1.012692
48H 50MTA	Q8IX90	-0.08709	0.24767	0.119707	SKA3	Spindle and	SKA3_HUM	6	0.941418
24H 10MTA	Q9BZV1	-0.08867	0.247667	0.119707	UBXN6	UBX domai	UBXN6_HU	12	0.940388
48H 50MTA	Q96CS4	-0.29829	0.247712	0.119724	ZNF689	Zinc finger	ZN689_HUI	8	0.813213
48H 10MTA	Q8WUH6	0.01787	0.247715	0.119724	TMEM263	Transmemb	TM263_HU	4	1.012464
48H 50MTA	O95857	-0.22318	0.247812	0.119761	TSPAN13	Tetraspanin	TSN13_HUI	4	0.856675

24H 10MT/	Q8N128	0.035903	0.247833	0.119768	FAM177A1	Protein FAI	F177A_HUI	4	1.025199
48H 50MT/	Q00535	-0.00688	0.247836	0.119768	CDK5	Cyclin-depe	CDK5_HUN	13	0.995241
24H 10MT/	Q9NR12	0.102509	0.247864	0.11978	PDLIM7	PDZ and LII	PDLI7_HUI	12	1.073639
48H 10MT/	O75943	0.080595	0.247886	0.119788	RAD17	Cell cycle cl	RAD17_HU	7	1.057454
24H 10MT/	Q96JI7	-0.15291	0.247992	0.119826	SPG11	Spatacsin	SPTCS_HUN	4	0.899436
48H 10MT/	Q9BRR8	-0.06455	0.248016	0.119835	GPATCH1	G patch doı	GPTC1_HUI	11	0.956242
48H 10MT/	Q9UGY1	-0.2757	0.248039	0.119844	NOL12	Nucleolar p	NOL12_HU	4	0.82605
48H 10MT/	Q08211	-0.06299	0.248069	0.119856	DHX9	ATP-depend	DHX9_HUN	64	0.95728
48H 10MT/	Q9H7B4	0.023598	0.248086	0.119862	SMYD3	Histone-lys	SMYD3_HU	6	1.016492
24H 50MT/	P55854;P6	0.675606	0.24814	0.119881	SUMO3;SUI	Small ubiqu	SUMO3_HU	1	1.597268
24H 10MT/	Q96T23	0.024213	0.248141	0.119881	RSF1	Remodeling	RSF1_HUM	27	1.016925
48H 10MT/	Q9NP77	-0.07352	0.248173	0.119895	SSU72	RNA polym	SSU72_HUI	5	0.950319
24H 10MT/	Q86X67	-0.07457	0.248309	0.119948	NUDT13	NAD(P)H py	NUD13_HU	1	0.949627
48H 10MT/	Q9H6R7	-0.08182	0.248324	0.119954	WDCP	WD repeat	WDCP_HUI	3	0.944868
24H 50MT/	Q9BVK2	0.049446	0.248337	0.119959	ALG8	Dolichyl py	ALG8_HUM	4	1.034867
24H 10MT/	Q7Z401	-0.02313	0.248378	0.11997	DENND4A	C-myc pron	MYCPP_HU	6	0.984095
48H 10MT/	O75884	0.034971	0.248384	0.119972	RBBP9	Serine hydr	RBBP9_HUI	8	1.024536
48H 50MT/	Q15811	0.01047	0.24843	0.119991	ITSN1	Intersectin-	ITSN1_HUN	8	1.007284
48H 10MT/	Q96S15	-0.21004	0.248504	0.120016	WDR24	GATOR2 co	WDR24_HU	5	0.864513
24H 10MT/	Q8IY47	-0.0105	0.248526	0.120025	KBTBD2	Kelch repea	KBTB2_HUI	4	0.992748
24H 10MT/	Q9H9S4	0.239235	0.248559	0.120036	CAB39L	Calcium-bii	CB39L_HUI	3	1.180367
24H 10MT/	Q14257	-0.11498	0.248568	0.120036	RCN2	Reticulocal	RCN2_HUN	13	0.923397
24H 10MT/	P51648	-0.06549	0.248673	0.120075	ALDH3A2	Aldehyde d	AL3A2_HUI	13	0.955618
48H 10MT/	Q96C23	-0.02633	0.24873	0.120101	GALM	Galactose n	GALM_HUN	8	0.981915
48H 50MT/	P78524	-0.37264	0.248749	0.120107	DENND2B	DENN dom	DEN2B_HU	1	0.772366
24H 10MT/	Q9C004	-0.03139	0.248884	0.120163	SPRY4	Protein spr	SPY4_HUM	4	0.978476
48H 50MT/	P61916	0.033688	0.248925	0.12018	NPC2	NPC intrace	NPC2_HUN	7	1.023625
48H 50MT/	P78347	0.033629	0.248975	0.120199	GTF2I	General tra	GTF2I_HUN	45	1.023583
24H 10MT/	Q9BSH3	-0.1318	0.249004	0.120212	NICN1	Nicolin-1	NICN1_HUI	1	0.912692
24H 50MT/	P60604	-0.16915	0.249044	0.120227	UBE2G2	Ubiquitin-c	UB2G2_HU	4	0.889364
24H 10MT/	P41567	0.030801	0.249072	0.120235	EIF1	Eukaryotic	EIF1_HUM/	10	1.021579
24H 10MT/	Q99590	-0.01996	0.249167	0.120276	SCAF11	Protein SC/	SCAFB_HUI	22	0.986259
24H 10MT/	O95619	0.021309	0.249204	0.120286	YEATS4	YEATS dom	YETS4_HUN	4	1.01488
24H 50MT/	Q9UDY4	-0.09863	0.249205	0.120286	DNAJB4	DnaJ homo	DNJB4_HUI	11	0.933921
48H 50MT/	Q8TF71	-0.16945	0.249235	0.120293	SLC16A10	Monocarbc	MOT10_HU	1	0.889183
48H 50MT/	Q9Y639	0.127923	0.249279	0.120311	NPTN	Neuroplast	NPTN_HUN	7	1.092719
24H 10MT/	Q14671	-0.07152	0.249289	0.120315	PUM1	Pumilio ho	PUM1_HUN	25	0.951637
48H 50MT/	O60869	0.00183	0.249374	0.12034	EDF1	Endothelial	EDF1_HUM	7	1.001269
48H 10MT/	P41743	0.022966	0.249416	0.120354	PRKCI	Protein kin	KPCI_HUM/	18	1.016046
48H 50MT/	Q9BT25	-0.11337	0.249457	0.120371	HAUS8	HAUS augm	HAUS8_HU	4	0.924425
48H 10MT/	P27708	-0.00909	0.249486	0.120383	CAD	Multifuncti	PYR1_HUM	79	0.993716
24H 10MT/	O75460	-0.10724	0.249572	0.120421	ERN1	Serine/thre	ERN1_HUN	3	0.928363
48H 10MT/	P01023	-0.11188	0.249691	0.120469	A2M	Alpha-2-ma	A2MG_HUN	57	0.92538
24H 50MT/	Q99081	0.022505	0.249724	0.120479	TCF12	Transcripti	HTF4_HUM	3	1.015721
48H 10MT/	P31629	-0.02996	0.249742	0.120483	HIVEP2	Transcripti	ZEP2_HUM	4	0.979447
48H 10MT/	Q07020	-0.02622	0.249871	0.120534	RPL18	Large ribos	RL18_HUM	7	0.98199
48H 50MT/	P37235	-0.14257	0.249937	0.120559	HPCAL1	Hippocalci	HPCL1_HUI	5	0.905905
48H 50MT/	Q8NDX5	0.152214	0.250169	0.120649	PHC3	Polyhomec	PHC3_HUN	9	1.111274
24H 10MT/	P62979	-0.04203	0.250177	0.120651	RPS27A	Ubiquitin-r	RS27A_HUI	8	0.971286
48H 50MT/	Q9UIC8	0.038688	0.250224	0.120667	LCMT1	Leucine car	LCMT1_HU	10	1.027179
48H 50MT/	P35052	-0.07485	0.250229	0.120667	GPC1	Glypican-1	GPC1_HUN	2	0.949442
24H 10MT/	P46781	-0.09236	0.250248	0.120675	RPS9	Small ribos	RS9_HUMA	14	0.937985

24H 10MT/	Q86W92	-0.00247	0.250266	0.120677	PPFIBP1	Liprin-beta	LIPB1_HUM	28	0.998286
48H 50MT/	Q8N680	0.110825	0.250261	0.120677	ZBTB2	Zinc finger	ZBTB2_HUM	4	1.079846
48H 10MT/	Q9NY27	0.003735	0.25029	0.120686	PPP4R2	Serine/thre	PP4R2_HUM	14	1.002592
24H 50MT/	P48637	-0.04261	0.250315	0.120696	GSS	Glutathion	GSHB_HUM	26	0.970896
48H 10MT/	O00443	-0.04748	0.250487	0.120762	PIK3C2A	Phosphatic	P3C2A_HUM	26	0.967624
24H 50MT/	P55884	-0.05743	0.250509	0.120769	EIF3B	Eukaryotic	EIF3B_HUM	47	0.960975
48H 10MT/	Q96NU7	0.004621	0.250507	0.120769	AMDHD1	Probable in	HUTI_HUM	8	1.003208
24H 50MT/	Q02413	-0.39544	0.250521	0.120773	DSG1	Desmoglein	DSG1_HUM	6	0.760257
24H 10MT/	Q9BZE4	0.041088	0.250552	0.120782	GTPBP4	GTP-bindin	GTPB4_HUM	33	1.02889
24H 10MT/	Q16822	-0.06684	0.250578	0.120788	PCK2	Phosphoen	PCKGM_HU	30	0.954729
24H 50MT/	Q9NRA0	-0.21502	0.250591	0.120793	SPHK2	Sphingosin	SPHK2_HUM	4	0.861535
48H 50MT/	O75379	-0.06187	0.250596	0.120794	VAMP4	Vesicle-assc	VAMP4_HU	2	0.958021
48H 50MT/	P18084	0.01452	0.250621	0.120801	ITGB5	Integrin bet	ITB5_HUMA	24	1.010115
48H 10MT/	Q99541	-0.02582	0.250619	0.120801	PLIN2	Perilipin-2	PLIN2_HUM	13	0.982263
24H 50MT/	Q9H2J7	0.663817	0.250642	0.120808	SLC6A15	Sodium-dep	S6A15_HUM	1	1.584268
48H 10MT/	P42356	-0.04481	0.250665	0.120814	PI4KA	Phosphatic	PI4KA_HUM	26	0.969418
24H 10MT/	P13984	0.073474	0.250685	0.120822	GTF2F2	General tra	T2FB_HUM	16	1.052247
48H 10MT/	Q13247	-0.00796	0.250766	0.120851	SRSF6	Serine/argi	SRSF6_HUM	9	0.994499
24H 10MT/	Q13884	0.017673	0.250759	0.120851	SNTB1	Beta-1-synt	SNTB1_HUM	18	1.012325
24H 50MT/	Q8WWM7	-0.09669	0.250775	0.120851	ATXN2L	Ataxin-2-lik	ATX2L_HUM	32	0.935175
48H 50MT/	Q9H0E9	0.113006	0.250777	0.120851	BRD8	Bromodom	BRD8_HUM	7	1.081479
24H 10MT/	P41235	-0.01004	0.250804	0.120859	HNF4A	Hepatocyte	HNF4A_HU	11	0.993065
24H 10MT/	Q13423	0.015528	0.250808	0.120859	NNT	NAD(P) trar	NNTM_HUM	37	1.010821
24H 10MT/	O60610	-0.00406	0.250943	0.120911	DIAPH1	Protein dia	DIAP1_HUM	44	0.99719
48H 10MT/	Q6PCB7	-0.24016	0.25094	0.120911	SLC27A1	Long-chain	S27A1_HUM	1	0.846653
24H 10MT/	Q9H6T0	-0.00512	0.250954	0.120914	ESRP2	Epithelial s	ESRP2_HUM	10	0.996461
24H 50MT/	Q8N6M3	0.018703	0.250974	0.120921	FITM2	Acyl-coenzy	FITM2_HUM	5	1.013048
24H 50MT/	Q6N069	-0.02406	0.251034	0.120943	NAA16	N-alpha-ace	NAA16_HU	13	0.98346
24H 10MT/	P17900	-0.02797	0.251057	0.120951	GM2A	Ganglioside	SAP3_HUM	4	0.980797
24H 50MT/	P16930	-0.05254	0.251067	0.120954	FAH	Fumarylace	FAAA_HUM	16	0.96424
24H 50MT/	Q6ZNA5	0.248713	0.251077	0.120955	FRRS1	Ferric-chela	FRRS1_HUM	8	1.188146
24H 10MT/	A6NIH7	-0.03222	0.251095	0.12096	UNC119B	Protein unc	U119B_HU	7	0.977912
48H 50MT/	Q14739	-0.08801	0.251095	0.12096	LBR	Delta(14)-st	LBR_HUMA	14	0.940822
24H 50MT/	Q14674	-0.00834	0.251162	0.120984	ESPL1	Separin	ESPL1_HUM	15	0.994239
48H 50MT/	Q4J6C6	-0.19484	0.25117	0.120987	PREPL	Prolyl endc	PPCEL_HUM	17	0.873672
24H 50MT/	Q9H7E9	-0.10451	0.251215	0.121001	C8orf33	UPF0488 p	CH033_HU	4	0.930124
48H 10MT/	Q8N3R9	-0.0799	0.251233	0.121007	PALS1	Protein PAI	PALS1_HUM	15	0.946126
48H 10MT/	Q96JB3	-0.18293	0.251241	0.121009	HIC2	Hypermeth	HIC2_HUM	5	0.880913
24H 50MT/	Q96DT6	0.197756	0.251269	0.121019	ATG4C	Cysteine pr	ATG4C_HUM	1	1.146913
24H 10MT/	Q9NR77	-0.29407	0.251283	0.121022	PXMP2	Peroxisoma	PXMP2_HU	5	0.815599
24H 50MT/	O60499	0.299975	0.25129	0.121024	STX10	Syntaxin-10	STX10_HUM	5	1.231123
24H 10MT/	O43660	-0.00029	0.251314	0.121031	PLRG1	Pleiotropic	PLRG1_HUM	22	0.9998
48H 50MT/	Q9NQX4	-0.07529	0.251348	0.121041	MYO5C	Unconvent	MYO5C_HU	13	0.949151
24H 50MT/	Q9NVC6	-0.02608	0.251352	0.121041	MED17	Mediator o	MED17_HU	10	0.982082
24H 10MT/	Q9BT78	-0.059	0.251413	0.121069	COPS4	COP9 signa	CSN4_HUM	25	0.959932
48H 10MT/	Q15582	-0.00962	0.25144	0.121077	TGFBI	Transformi	BGH3_HUM	11	0.993351
24H 10MT/	P51991	0.004106	0.251516	0.121105	HNRNPA3	Heterogene	ROA3_HUM	22	1.00285
24H 50MT/	Q9GZT6	-0.14828	0.251571	0.121123	CCDC90B	Coiled-coil	CC90B_HUM	3	0.902323
24H 10MT/	Q5VYS8	-0.05077	0.251584	0.121126	TUT7	Terminal ur	TUT7_HUM	7	0.965421
24H 10MT/	O60888	-0.65231	0.25161	0.121133	CUTA	Protein Cut	CUTA_HUM	3	0.63626
24H 50MT/	Q03111	0.065959	0.251606	0.121133	MLLT1	Protein ENI	ENL_HUMA	6	1.04678
48H 10MT/	P40926	-0.08953	0.251718	0.121179	MDH2	Malate deh	MDHM_HU	15	0.939831

24H 10MTA	Q7Z4L5	0.033544	0.251725	0.12118	TTC21B	Tetratricop	TT21B_HUI	11	1.023523
24H 50MTA	Q567V2	-0.04942	0.251747	0.121189	MPV17L2	Mpv17-like	M17L2_HU	4	0.966324
48H 50MTA	O60814;P5	0.057157	0.25187	0.121238	H2BC12;H2	Histone H2	H2B1K_HU	8	1.040413
48H 50MTA	P09234	-0.23673	0.251918	0.121257	SNRPC	U1 small nu	RU1C_HUM	2	0.848664
48H 10MTA	Q8WWC4	-0.05213	0.251925	0.121259	MAIP1	m-AAA prot	MAIP1_HUI	7	0.964514
48H 50MTA	Q9UPQ3	0.032343	0.25207	0.121321	AGAP1	Arf-GAP wit	AGAP1_HU	2	1.022671
48H 10MTA	Q13277	0.238575	0.252117	0.121337	STX3	Syntaxin-3	STX3_HUM	6	1.179827
24H 50MTA	Q9Y620	0.024147	0.252127	0.121337	RAD54B	DNA repair	RA54B_HUI	12	1.016878
48H 10MTA	O60832	-0.05991	0.25216	0.121346	DKC1	H/ACA ribo	DKC1_HUM	27	0.959323
48H 10MTA	Q96DX5	-0.2797	0.252163	0.121346	ASB9	Ankyrin rep	ASB9_HUM	3	0.82376
24H 10MTA	Q01201	0.14862	0.252191	0.121354	RELB	Transcripti	RELB_HUM	5	1.108508
24H 10MTA	Q9HCS7	0.007533	0.25219	0.121354	XAB2	Pre-mRNA-	SYF1_HUM	30	1.005235
48H 50MTA	P41247	-0.515	0.252221	0.121366	PNPLA4	Patatin-like	PLPL4_HUI	2	0.699795
24H 50MTA	Q9H9J2	-0.11258	0.252267	0.121385	MRPL44	Large ribos	RM44_HUM	14	0.924934
24H 10MTA	P82921	0.028252	0.252295	0.121389	MRPS21	Small ribos	RT21_HUM	3	1.019776
24H 10MTA	Q96T60	-0.03404	0.252288	0.121389	PNKP	Bifunction	PNKP_HUM	12	0.976679
24H 50MTA	O95757	-0.06479	0.252303	0.121392	HSPA4L	Heat shock	HS74L_HUI	36	0.956083
24H 50MTA	Q6KB66	0.348674	0.252338	0.121399	KRT80	Keratin, typ	K2C80_HUI	5	1.27339
24H 10MTA	Q7L1W4	0.356922	0.252345	0.121399	LRRC8D	Volume-reg	LRC8D_HUI	3	1.28069
24H 50MTA	P08779	-2.88499	0.252438	0.121439	KRT16	Keratin, typ	K1C16_HUI	23	0.135372
48H 10MTA	Q6IN84	-0.04611	0.252469	0.121449	MRM1	rRNA meth	MRM1_HUI	6	0.968543
24H 10MTA	P49281	-0.05303	0.25249	0.121456	SLC11A2	Natural resi	NRAM2_HU	2	0.963908
24H 50MTA	Q3KRA9	-0.25366	0.252533	0.121474	ALKBH6	Alpha-keto	ALKB6_HUI	3	0.838768
48H 50MTA	P62993	-0.01692	0.252666	0.12153	GRB2	Growth fac	GRB2_HUM	13	0.988341
24H 50MTA	Q9NVR0	-0.11203	0.252663	0.12153	KLHL11	Kelch-like p	KLH11_HUI	10	0.925286
48H 50MTA	Q9Y5B9	-0.00047	0.252683	0.121535	SUPT16H	FACT comp	SP16H_HUI	43	0.999676
48H 10MTA	A3KMH1	-0.05339	0.252735	0.12155	VWA8	von Willebr	VWA8_HUM	43	0.963671
48H 50MTA	Q8N1S5	0.018144	0.252781	0.121569	SLC39A11	Zinc transp	S39AB_HUI	3	1.012656
24H 50MTA	Q9UKF6	0.065805	0.252807	0.121578	CPSF3	Cleavage ar	CPSF3_HUI	24	1.046669
24H 50MTA	Q96S15	-0.28012	0.252902	0.12162	WDR24	GATOR2 co	WDR24_HU	5	0.823525
48H 10MTA	Q58WW2	0.006302	0.252914	0.121622	DCAF6	DDB1- and	DCAF6_HUI	14	1.004378
24H 10MTA	A6NDU8	-0.08977	0.252926	0.121626	RIMOC1	RAB7A-inte	RIMC1_HUI	9	0.93967
24H 10MTA	Q9UJF2	0.038803	0.252929	0.121626	RASAL2	Ras GTPase	NGAP_HUM	13	1.027261
48H 50MTA	P14373	0.053868	0.252938	0.121629	TRIM27	Zinc finger	TRI27_HUM	8	1.038044
24H 50MTA	P48506	-0.05112	0.253013	0.121659	GCLC	Glutamate-	GSH1_HUM	23	0.965184
24H 10MTA	Q17RY0	-0.06888	0.25307	0.121682	CPEB4	Cytoplasmi	CPEB4_HUI	11	0.953376
48H 50MTA	Q13620	-0.00266	0.253083	0.121684	CUL4B	Cullin-4B	CUL4B_HUI	42	0.998161
24H 10MTA	Q9C0D3	-0.03344	0.253086	0.121684	ZYG11B	Protein zyg	ZY11B_HUI	12	0.977086
24H 50MTA	Q8N999	0.352425	0.253115	0.121694	RLIG1	RNA ligase	RLIG1_HUM	6	1.276705
48H 50MTA	Q9P2E3	-0.17548	0.253139	0.121702	ZNFX1	NFX1-type	ZNFX1_HUI	17	0.885471
24H 10MTA	Q05469	-0.16382	0.253173	0.121714	LIPE	Hormone-s	LIPS_HUMA	1	0.892658
48H 50MTA	Q2PPJ7	0.314473	0.25318	0.121714	RALGAPA2	Ral GTPase-	RGPA2_HU	8	1.243557
24H 10MTA	Q9UNM6	0.031102	0.253186	0.121715	PSMD13	26S protea	PSD13_HUI	27	1.021792
48H 10MTA	O60292	0.790085	0.253303	0.121758	SIPA1L3	Signal-indu	SI1L3_HUM	2	1.729176
48H 10MTA	Q13670	0.489253	0.253325	0.121764	PMS2P11	Putative pc	PM2PB_HU	1	1.403718
48H 10MTA	Q96MX3	-0.34046	0.253342	0.121769	ZNF48	Zinc finger	ZNF48_HUI	1	0.789789
24H 10MTA	Q04695	-2.19452	0.253373	0.121781	KRT17	Keratin, typ	K1C17_HUI	15	0.218466
24H 10MTA	Q9H0H0	0.163512	0.253389	0.121785	INTS2	Integrator	INT2_HUM	11	1.12001
48H 50MTA	P35250	-0.04013	0.253404	0.121791	RFC2	Replication	RFC2_HUM	19	0.97257
24H 10MTA	Q9UQN3	0.08207	0.253462	0.121808	CHMP2B	Charged mu	CHM2B_HU	2	1.058535
24H 10MTA	P56192	-0.01282	0.25347	0.12181	MARS1	Methionine	SYMC_HUM	43	0.991153
48H 50MTA	P06132	-0.03549	0.253508	0.121824	UROD	Uroporphy	DCUP_HUM	15	0.975703

24H 10MT/	Q14457	0.062688	0.253542	0.121835	BECN1	Beclin-1	BECN1_HUI	7	1.04441
24H 10MT/	P46777	-0.01943	0.253707	0.121902	RPL5	Large ribos	RL5_HUMA	14	0.986624
48H 10MT/	O95298	-0.19178	0.253724	0.121907	NDUFC2	NADH dehy	NDUC2_HU	4	0.875523
48H 10MT/	O75864	0.282656	0.253829	0.121953	PPP1R37	Protein pho	PPR37_HUI	2	1.216432
48H 50MT/	Q16625	-0.03732	0.253866	0.121965	OCLN	Occludin	OCLN_HUM	8	0.974466
48H 10MT/	A6NHX0	0.017639	0.253881	0.12197	CASTOR2	Cytosolic a	CAST2_HUI	9	1.012301
24H 10MT/	Q12800	0.023766	0.254041	0.122026	TFCP2	Alpha-globi	TFCP2_HUI	9	1.01661
48H 50MT/	O15020	-0.08142	0.254057	0.12203	SPTBN2	Spectrin be	SPTN2_HUI	13	0.945127
24H 10MT/	P49257	-0.02207	0.254087	0.122039	LMAN1	Protein ER(	LMAN1_HU	19	0.984817
24H 10MT/	Q9NZU5	-0.0271	0.25415	0.122061	LMCD1	LIM and cys	LMCD1_HU	21	0.981389
24H 10MT/	P42575	0.545838	0.254172	0.122068	CASP2	Caspase-2	CASP2_HUI	2	1.459868
24H 10MT/	Q9Y2Q3	0.021654	0.254197	0.122077	GSTK1	Glutathion	GSTK1_HUI	13	1.015123
24H 50MT/	P27707	-0.10627	0.254202	0.122078	DCK	Deoxycytid	DCK_HUM/	11	0.928988
48H 10MT/	Q9UPN6	-0.01226	0.254275	0.122102	SCAF8	SR-related	SCAF8_HUI	11	0.99154
48H 10MT/	P54619	-0.10076	0.254299	0.122109	PRKAG1	5'-AMP-acti	AAKG1_HU	11	0.932539
48H 10MT/	P24385	-0.17236	0.254334	0.122121	CCND1	G1/S-specif	CCND1_HU	5	0.887389
48H 10MT/	O00257	0.067153	0.254392	0.122144	CBX4	E3 SUMO-p	CBX4_HUM	6	1.047647
48H 50MT/	Q9Y5S2	0.069363	0.25444	0.12216	CDC42BPB	Serine/thre	MRCKB_HU	29	1.049253
48H 10MT/	P25398	-0.02409	0.254459	0.122166	RPS12	Small ribos	RS12_HUM	9	0.983444
48H 10MT/	P06400	-0.01386	0.254508	0.122181	RB1	Retinoblast	RB_HUMAN	24	0.990437
24H 50MT/	Q8NEU8	-0.05471	0.254512	0.122181	APPL2	DCC-intera	DP13B_HU	15	0.96279
24H 10MT/	Q9NP79	-0.10879	0.254507	0.122181	VTA1	Vacuolar pı	VTA1_HUM	11	0.927364
24H 10MT/	Q86XK2	0.031355	0.254532	0.122187	FBXO11	F-box only	FBX11_HUI	3	1.021972
48H 50MT/	O00443	0.046758	0.254568	0.122198	PIK3C2A	Phosphatid	P3C2A_HUI	26	1.032941
24H 50MT/	Q96RT1	-0.07445	0.254565	0.122198	ERBIN	Erbin	ERBIN_HUN	16	0.949701
24H 10MT/	Q07889	0.019123	0.254586	0.1222	SOS1	Son of seve	SOS1_HUM	15	1.013343
48H 10MT/	O95816	-0.04044	0.254639	0.122219	BAG2	BAG family	BAG2_HUM	12	0.972357
24H 10MT/	O14787	-0.07637	0.254667	0.122231	TNPO2	Transportir	TNPO2_HU	14	0.94844
24H 50MT/	O43379	-0.16369	0.254687	0.122237	WDR62	WD repeat-	WDR62_HU	9	0.892742
48H 50MT/	Q13574	0.447189	0.254684	0.122237	DGKZ	Diacylglyce	DGKZ_HUM	1	1.363381
24H 10MT/	O75391	0.103007	0.254702	0.122243	SPAG7	Sperm-asso	SPAG7_HUI	8	1.07401
48H 50MT/	Q96AX1	-0.08299	0.25472	0.12225	VPS33A	Vacuolar pı	VP33A_HUI	25	0.944101
24H 50MT/	Q9BWT6	-0.24769	0.25473	0.122254	MND1	Meiotic nu	MND1_HUI	1	0.842243
48H 10MT/	P18440	-0.3058	0.254762	0.122267	NAT1	Arylamine I	ARY1_HUM	7	0.808995
48H 10MT/	Q13884	0.007006	0.254915	0.122326	SNTB1	Beta-1-synt	SNTB1_HUI	18	1.004868
48H 50MT/	O00459	0.265726	0.25493	0.122331	PIK3R2	Phosphatid	P85B_HUM	7	1.202241
48H 10MT/	Q6XZF7	-0.00148	0.25498	0.122353	DNMBP	Dynamin-b	DNMBP_HU	16	0.998973
48H 10MT/	Q6P4I2	-0.1225	0.255002	0.12236	WDR73	WD repeat-	WDR73_HU	10	0.918597
24H 50MT/	Q9NVD7	-0.0786	0.255	0.12236	PARVA	Alpha-parvi	PARVA_HUI	4	0.946979
48H 50MT/	Q9H9Q4	0.078433	0.255014	0.122364	NHEJ1	Non-homol	NHEJ1_HUI	5	1.05587
24H 10MT/	O14734	-0.02877	0.255108	0.122395	ACOT8	Acyl-coenz	ACOT8_HUI	6	0.980254
48H 50MT/	Q8WVQ1	0.128495	0.255282	0.122455	CANT1	Soluble cal	CANT1_HUI	13	1.093152
24H 10MT/	Q8NBZ7	9.31E-05	0.255302	0.122462	UXS1	UDP-glucur	UXS1_HUM	11	1.000065
48H 10MT/	Q96CF2	-0.02719	0.255316	0.122466	CHMP4C	Charged mı	CHM4C_HU	3	0.981332
24H 10MT/	Q9Y6J9	0.046738	0.255343	0.122478	TAF6L	TAF6-like R	TAF6L_HUN	14	1.032927
24H 50MT/	Q7L8J4	0.002382	0.255471	0.122529	SH3BP5L	SH3 domai	3BP5L_HUI	4	1.001653
48H 50MT/	Q8TEW0	-0.0513	0.25552	0.122546	PARD3	Partitionin	PARD3_HU	26	0.965064
48H 50MT/	Q9Y4C2	-0.19783	0.255525	0.122546	TCAF1	TRPM8 cha	TCAF1_HUI	14	0.871859
48H 10MT/	Q96EH3	-0.21538	0.255563	0.122562	MALSU1	Mitochond	MASU1_HU	3	0.861318
24H 50MT/	Q9P219	0.211437	0.255584	0.122569	CCDC88C	Protein Da	DAPLE_HUI	2	1.157841
24H 10MT/	O95373	0.006145	0.255681	0.12261	IPO7	Importin-7	IPO7_HUM	43	1.004268
48H 10MT/	Q9H330	0.024255	0.255699	0.122613	TMEM245	Transmemt	TM245_HU	6	1.016955

48H 50MT/	Q9UIW2	-0.32519	0.255808	0.122657	PLXNA1	Plexin-A1	PLXA1_HUI	8	0.798196
48H 10MT/	Q15390	-0.31096	0.255821	0.122662	MTFR1	Mitochond	MTFR1_HU	3	0.806103
48H 50MT/	A8MW92	0.041075	0.25585	0.122672	PHF20L1	PHD finger	P20L1_HUI	1	1.02888
48H 50MT/	Q16254	-0.68518	0.255862	0.122674	E2F4	Transcripti	E2F4_HUM	2	0.621928
48H 10MT/	Q9H089	-0.04669	0.256032	0.122741	LSG1	Large subur	LSG1_HUM	18	0.968156
24H 50MT/	Q92552	-0.0637	0.256087	0.12276	MRPS27	Small ribos	RT27_HUM	19	0.956809
48H 50MT/	P17661	0.108226	0.25613	0.122777	DES	Desmin	DESM_HUN	2	1.077902
24H 50MT/	P10620	0.053289	0.256146	0.122781	MGST1	Microsoma	MGST1_HU	4	1.037628
24H 50MT/	Q9GZQ3	-0.18356	0.256151	0.122781	COMMD5	COMM don	COMD5_HU	3	0.880526
24H 10MT/	Q9NVR5	0.13704	0.256149	0.122781	DNAAF2	Protein kin	KTU_HUMA	5	1.099647
24H 10MT/	Q9H477	0.086708	0.256208	0.1228	RBKS	Ribokinase	RBSK_HUM	6	1.061944
48H 50MT/	P07437	0.091572	0.256216	0.122802	TUBB	Tubulin bet	TBB5_HUM	5	1.06553
48H 50MT/	P29372	0.044687	0.256221	0.122803	MPG	DNA-3-met	3MG_HUM	11	1.031459
24H 50MT/	O95997;Q9	-0.19771	0.256291	0.122829	PTTG1;PTT(	Securin;Sec	PTTG1_HUI	1	0.871931
24H 10MT/	Q8N3R9	-0.0108	0.256357	0.122855	PALS1	Protein PAI	PALS1_HUI	15	0.992545
48H 10MT/	Q96PU8	-0.08592	0.256367	0.122859	QKI	KH domain	QKI_HUMA	12	0.942182
24H 10MT/	Q9NXE8	0.008872	0.256379	0.122863	CWC25	Pre-mRNA-:	CWC25_HU	7	1.006169
24H 50MT/	Q9ULJ3	-0.0015	0.256426	0.122881	ZBTB21	Zinc finger :	ZBT21_HUI	5	0.998957
48H 50MT/	Q969T7	0.322973	0.256476	0.122903	NT5C3B	7-methylgu	5NT3B_HUI	4	1.250906
24H 10MT/	Q99538	-0.03737	0.256524	0.122925	LGMN	Legumain	LGMN_HUI	6	0.97443
24H 10MT/	Q562F6	0.130648	0.256571	0.122937	SGO2	Shugoshin :	SGO2_HUN	5	1.094785
48H 10MT/	Q8N6N3	0.016418	0.256605	0.122951	C1orf52	UPF0690 p	CA052_HUI	7	1.011445
24H 50MT/	O75907	-0.17724	0.256801	0.12303	DGAT1	Diacylglyce	DGAT1_HU	5	0.884395
48H 10MT/	Q9UBQ6	0.007087	0.256801	0.12303	EXTL2	Exostosin-li	EXTL2_HUN	6	1.004924
48H 10MT/	O00622	-0.16401	0.256856	0.123054	CCN1	CCN family	CCN1_HUN	7	0.892539
48H 10MT/	Q96ME7	-0.09491	0.256968	0.123097	ZNF512	Zinc finger		ZN512_HUI	12	0.936329
48H 10MT/	Q8TC27	-0.85845	0.257084	0.123146	ADAM32	Disintegrin	ADA32_HU	1	0.551545
24H 50MT/	O95497	-0.52188	0.257108	0.123155	VNN1	Pantethein	VNN1_HUN	2	0.696463
48H 10MT/	Q86U28	-0.05374	0.25715	0.123169	ISCA2	Iron-sulfur	ISCA2_HUN	8	0.963434
48H 50MT/	P46934	-0.07994	0.257199	0.123187	NEDD4	E3 ubiquiti	NEDD4_HU	9	0.946096
24H 10MT/	P13056	0.189367	0.257229	0.1232	NR2C1	Nuclear rec	NR2C1_HU	2	1.140264
48H 50MT/	P26885	-0.1342	0.257316	0.123225	FKBP2	Peptidyl-pr	FKBP2_HUI	6	0.911177
48H 50MT/	Q96EB6	-0.20969	0.257385	0.123244	SIRT1	NAD-depen	SIR1_HUM/	8	0.864723
48H 10MT/	P52848	-0.09248	0.257451	0.123268	NDST1	Bifunctiona	NDST1_HUI	9	0.937908
48H 10MT/	Q9Y227	0.191639	0.257457	0.123269	ENTPD4	Ectonucleo	ENTP4_HUI	1	1.142061
48H 10MT/	Q70E73	0.306062	0.257598	0.123321	RAPH1	Ras-associa	RAPH1_HU	4	1.236329
48H 10MT/	Q9NPD3	-0.11387	0.257669	0.123343	EXOSC4	Exosome cc	EXOS4_HUI	8	0.924105
24H 50MT/	P48643	-0.06725	0.257701	0.123357	CCT5	T-complex		TCPE_HUM	42	0.954458
24H 50MT/	Q96IY1	0.21797	0.257705	0.123357	NSL1	Kinetochor	NSL1_HUM	5	1.163096
48H 50MT/	O00257	-0.00194	0.257827	0.123408	CBX4	E3 SUMO-p	CBX4_HUM	6	0.998656
48H 10MT/	Q15208	-0.02508	0.257838	0.123408	STK38	Serine/thre	STK38_HUI	9	0.982769
48H 10MT/	Q14689	0.016959	0.257863	0.123414	DIP2A	Disco-inter	DIP2A_HUI	9	1.011824
24H 10MT/	P08651	0.326881	0.25791	0.123431	NFIC	Nuclear fac	NFIC_HUM/	2	1.254299
48H 10MT/	Q02338	-0.01431	0.257913	0.123431	BDH1	D-beta-hyd	BDH_HUM/	5	0.990127
48H 50MT/	Q9H788	-0.09455	0.257928	0.123434	SH2D4A	SH2 domai	SH24A_HUI	10	0.936568
24H 10MT/	O94992	-0.05756	0.25794	0.123436	HEXIM1	Protein HE)	HEXI1_HUN	8	0.960887
24H 10MT/	Q96EI5	-0.25185	0.257947	0.123436	TCEAL4	Transcripti	TCAL4_HUN	7	0.839818
48H 50MT/	Q9NQT8	0.002504	0.257947	0.123436	KIF13B	Kinesin-like	KI13B_HUN	20	1.001737
24H 50MT/	P53611	-0.0947	0.258029	0.123468	RABGGTB	Geranylger:	PGTB2_HUI	14	0.93647
48H 10MT/	O95219	-0.06171	0.258082	0.123489	SNX4	Sorting nex	SNX4_HUN	21	0.958129
24H 50MT/	Q9BVL2	0.094585	0.258084	0.123489	NUP58	Nucleopori	NUP58_HU	6	1.067758
24H 10MT/	Q16342	-0.02888	0.25812	0.123503	PDCD2	Programme	PDCD2_HU	7	0.980179

24H 10MT/	Q9UJ70	-0.02661	0.258153	0.123514	NAGK	N-acetyl-D-	NAGK_HUN	18	0.981722
24H 50MT/	Q9P2L0	-0.12296	0.258161	0.123516	WDR35	WD repeat-	WDR35_HU	5	0.918302
48H 10MT/	O94964	-0.12593	0.258294	0.12357	MTCL2	Microtubul	MTCL2_HU	9	0.916411
48H 50MT/	Q7L590	0.082277	0.258298	0.123571	MCM10	Protein MC	MCM10_HU	1	1.058687
48H 10MT/	Q3SXM5	-0.04475	0.258352	0.123593	HSDL1	Inactive hy	HSDL1_HUI	7	0.969459
48H 10MT/	P04062	-0.00683	0.258359	0.123595	GBA1	Lysosomal	GBA1_HUM	16	0.99528
24H 10MT/	P25490	0.106064	0.258427	0.123623	YY1	Transcripti	TYY1_HUM/	7	1.076288
48H 10MT/	P30050	0.044155	0.258465	0.123636	RPL12	Large ribos	RL12_HUM	6	1.031079
48H 10MT/	Q9GZS1	-0.09776	0.258489	0.123646	POLR1E	DNA-direct	RPA49_HUI	10	0.934482
48H 10MT/	Q4G0J3	-0.0347	0.258497	0.123648	LARP7	La-related p	LARP7_HUI	17	0.976238
48H 50MT/	Q9Y5N5	-0.49489	0.258606	0.123693	N6AMT1	Methyltran	N6MT1_HU	3	0.709616
48H 50MT/	Q7Z6E9	-0.09714	0.258766	0.123759	RBBP6	E3 ubiquiti	RBBP6_HUI	18	0.934884
48H 10MT/	Q03113	-0.1182	0.258783	0.123765	GNA12	Guanine nu	GNA12_HU	5	0.921338
48H 10MT/	Q96RN5	-0.02612	0.25886	0.123796	MED15	Mediator o	MED15_HU	9	0.982057
48H 10MT/	P27824	-0.02061	0.258971	0.123838	CANX	Calnexin	CALX_HUM	36	0.985816
24H 10MT/	O60271	0.011636	0.259038	0.123859	SPAG9	C-Jun-amin	JIP4_HUMA	45	1.008098
24H 50MT/	Q9P021	0.213856	0.259052	0.123864	CRIPT	Cysteine-ric	CRIPT_HUN	2	1.159784
48H 10MT/	Q155Q3	-0.02911	0.25907	0.123868	DIXDC1	Dixin	DIXC1_HUN	4	0.980025
24H 50MT/	Q9NVU0	-0.08712	0.25909	0.123876	POLR3E	DNA-direct	RPC5_HUM	14	0.941399
24H 10MT/	O75382	-0.04309	0.25914	0.123893	TRIM3	Tripartite n	TRIM3_HUI	5	0.970571
24H 10MT/	Q5SYE7	-0.08212	0.25917	0.123904	NHSL1	NHS-like pr	NHSL1_HUI	14	0.944668
24H 50MT/	Q9BRR8	0.26306	0.259237	0.123933	GPATCH1	G patch do	GPTC1_HUI	11	1.200021
24H 50MT/	Q92685	-0.0398	0.259261	0.123943	ALG3	Dol-P-Man:	ALG3_HUM	3	0.972791
24H 50MT/	Q9Y281	0.029134	0.259426	0.124001	CFL2	Cofilin-2	COF2_HUM	9	1.020399
48H 10MT/	Q03426	0.02182	0.259443	0.124003	MVK	Mevalonate	KIME_HUM	9	1.01524
24H 50MT/	Q6P996	0.018142	0.259439	0.124003	PDXDC1	Pyridoxal-d	PDXD1_HU	33	1.012655
48H 50MT/	P84243	-0.52293	0.259486	0.124014	H3-3A	Histone H3	H33_HUMA	1	0.695958
48H 10MT/	P50914	-0.0905	0.259522	0.124021	RPL14	Large ribos	RL14_HUM	4	0.9392
24H 10MT/	P49354	-0.0414	0.259554	0.124035	FNTA	Protein farn	FNTA_HUM	14	0.971709
24H 10MT/	Q9P0W0	0.36721	0.259619	0.124061	IFNK	Interferon k	IFNK_HUMA	1	1.289856
24H 50MT/	Q96JC1	-0.0071	0.259652	0.124069	VPS39	Vam6/Vps3	VPS39_HUI	19	0.99509
48H 10MT/	O14841	-0.0124	0.259695	0.124088	OPLAH	5-oxoprolir	OPLA_HUM	7	0.991441
48H 50MT/	Q9NZD8	-0.16053	0.259755	0.124107	SPG21	Maspardin	SPG21_HUI	13	0.894699
24H 10MT/	O15243	0.15021	0.259776	0.124114	LEPROT	Leptin rece	OBRG_HUN	2	1.109731
48H 10MT/	P23470	0.020526	0.259812	0.124128	PTPRG	Receptor-ty	PTPRG_HUI	14	1.014329
24H 50MT/	Q7L1V2	0.024996	0.259853	0.124141	MON1B	Vacuolar fu	MON1B_HU	4	1.017477
24H 50MT/	P15121	0.023075	0.259968	0.124189	AKR1B1	Aldo-keto r	ALDR_HUM	19	1.016123
48H 50MT/	Q8IZ81	-0.01145	0.259979	0.124191	ELMOD2	ELMO doma	ELMD2_HU	8	0.992094
24H 50MT/	Q13185	-0.05006	0.259983	0.124192	CBX3	Chromobo:	CBX3_HUM	8	0.965896
48H 10MT/	Q9UPY6	0.307133	0.26007	0.124227	WASF3	Actin-bindi	WASF3_HU	3	1.237247
24H 10MT/	P04179	-0.01484	0.260092	0.124231	SOD2	Superoxide	SODM_HUN	9	0.989768
48H 50MT/	Q9BVN2	-0.00944	0.260093	0.124231	RUSC1	AP-4 compl	RUSC1_HUI	3	0.993475
48H 50MT/	P05387	0.045493	0.260163	0.124258	RPLP2	Large ribos	RLA2_HUM	7	1.032036
24H 10MT/	P38432	-0.05061	0.260176	0.124262	COIL	Coilin	COIL_HUMA	11	0.965525
48H 10MT/	P31350	0.031387	0.260197	0.124268	RRM2	Ribonuclec	RIR2_HUMA	19	1.021994
48H 10MT/	Q9NX04	-0.29193	0.260222	0.124272	AIRIM	AFG2-intera	AIRIM_HUN	1	0.81681
24H 50MT/	Q8TB72	-0.03314	0.260349	0.124323	PUM2	Pumilio ho	PUM2_HUN	10	0.977288
24H 50MT/	Q96F63	-0.02076	0.260364	0.124327	CCDC97	Coiled-coil	CCD97_HU	8	0.985715
48H 10MT/	Q86TG7	0.003761	0.260438	0.124353	PEG10	Retrotransp	PEG10_HUI	6	1.00261
24H 10MT/	Q02127	-0.16379	0.260516	0.124382	DHODH	Dihydroorc	PYRD_HUM	19	0.892676
24H 10MT/	O75419	0.122727	0.260607	0.124421	CDC45	Cell divisio	CDC45_HU	6	1.088791
24H 10MT/	Q8NHH9	0.051527	0.260662	0.124442	ATL2	Atlastin-2	ATLA2_HUN	17	1.036361

48H 50MT/	Q96N06	-0.54098	0.260683	0.124451	SPATA33	Spermatog	SPT33_HUI	1	0.687303
48H 10MT/	Q8N8Q8	-0.1347	0.26069	0.124452	COX18	Cytochrom	COX18_HU	3	0.91086
48H 10MT/	O14965	-0.00768	0.260861	0.124516	AURKA	Aurora kina	AURKA_HU	15	0.994692
24H 10MT/	Q9BWE0	-0.24167	0.260892	0.124527	REPIN1	DNA-bindir	REPI1_HUM	11	0.845764
48H 10MT/	Q6FI81	-0.02697	0.260913	0.124533	CIAPIN1	Anamorsin	CPIN1_HUI	20	0.981481
24H 10MT/	Q13506	0.077077	0.260966	0.124549	NAB1	NGFI-A-bin	NAB1_HUM	5	1.054878
48H 50MT/	P02452	1.982835	0.260992	0.124554	COL1A1	Collagen al	CO1A1_HU	14	3.952691
48H 50MT/	P05556	-0.0025	0.260995	0.124554	ITGB1	Integrin be	ITB1_HUM/	25	0.998271
24H 10MT/	Q4VC31	-0.01379	0.26099	0.124554	MIX23	Protein MI	MIX23_HU	6	0.990489
24H 10MT/	P16219	-0.06121	0.26103	0.124565	ACADS	Short-chair	ACADS_HU	9	0.958459
48H 10MT/	Q13496	-0.01805	0.261125	0.124607	MTM1	Myotubula	MTM1_HUI	15	0.987568
48H 10MT/	Q9NYU1	0.073753	0.261161	0.124618	UGGT2	UDP-glucos	UGGG2_HU	7	1.052451
48H 10MT/	Q9BSU3	-0.5993	0.261206	0.124632	NAA11	N-alpha-ac	NAA11_HU	2	0.660074
48H 50MT/	Q9BSK2	-0.13885	0.26127	0.12466	SLC25A33	Solute carr	S2533_HUI	8	0.90824
48H 50MT/	P07919	-0.20891	0.261295	0.124668	UQCRH	Cytochrom	QCR6_HUM	3	0.865189
24H 50MT/	Q6GPH6	0.333222	0.261301	0.124668	ITPRIPL1	Inositol 1,4	IPIL1_HUM	1	1.259824
24H 10MT/	Q9H252	-0.017	0.261353	0.12469	KCNH6	Potassium	KCNH6_HU	2	0.988288
24H 10MT/	Q8NBN7	-0.11323	0.261423	0.124714	RDH13	Retinol deh	RDH13_HU	8	0.924516
24H 50MT/	O75534	0.026632	0.2616	0.124787	CSDE1	Cold shock	CSDE1_HUI	44	1.018631
24H 10MT/	Q5TBA9	0.494024	0.261611	0.12479	FRY	Protein fur	FRY_HUMA	4	1.408368
48H 10MT/	Q9NX05	-0.15492	0.261716	0.124833	FAM120C	Constitutiv	F120C_HUI	3	0.898182
48H 50MT/	Q9HBM6	-0.14937	0.261766	0.124852	TAF9B	Transcripti	TAF9B_HUI	10	0.901644
24H 10MT/	Q15166	0.082075	0.2618	0.124864	PON3	Serum para	PON3_HUM	10	1.058539
24H 10MT/	P42898	-0.00911	0.261843	0.124881	MTHFR	Methylenet	MTHR_HUM	11	0.993703
48H 50MT/	P62745	-0.42168	0.261839	0.124881	RHOB	Rho-relatec	RHOB_HUM	4	0.746555
24H 10MT/	Q9NV96	0.074893	0.261848	0.124882	TMEM30A	Cell cycle c	CC50A_HUI	6	1.053283
48H 50MT/	O43678	-0.02699	0.261885	0.124895	NDUFA2	NADH dehy	NDUA2_HU	4	0.981468
24H 50MT/	Q9H6K1	-0.26431	0.261915	0.124908	ILRUN	Protein ILR	ILRUN_HUI	1	0.832599
24H 10MT/	Q6GMR7	-0.33607	0.261953	0.124923	FAAH2	Fatty-acid a	FAAH2_HUI	1	0.792196
48H 50MT/	Q9Y4W6	-0.04716	0.262088	0.124977	AFG3L2	Mitochond	AFG32_HUI	34	0.967836
48H 10MT/	Q9Y619	-0.03626	0.262223	0.125035	SLC25A15	Mitochond	ORNT1_HU	15	0.975179
48H 10MT/	P30793	-0.08708	0.262241	0.125041	GCH1	GTP cycloh	GCH1_HUM	10	0.941428
48H 50MT/	P16401	-0.26423	0.262259	0.125048	H1-5	Histone H1	H15_HUMA	8	0.832646
24H 50MT/	P22392	-0.01333	0.262281	0.125055	NME2	Nucleoside	NDKB_HUM	14	0.990805
48H 10MT/	P28066	-0.00766	0.262327	0.12507	PSMA5	Proteasom	PSA5_HUM	13	0.994702
24H 10MT/	Q00610	0.038619	0.262388	0.125092	CLTC	Clathrin he	CLH1_HUM	79	1.02713
48H 10MT/	Q32NB8	-0.09225	0.26244	0.125114	PGS1	CDP-diacyl	PGPS1_HUI	6	0.938062
48H 50MT/	Q6AI12	0.078816	0.262443	0.125114	ANKRD40	Ankyrin rep	ANR40_HU	3	1.056151
24H 10MT/	Q7Z7E8	-0.03384	0.26247	0.125118	UBE2Q1	Ubiquitin-c	UB2Q1_HU	9	0.976818
48H 50MT/	Q8IUH3	0.143749	0.262481	0.125119	RBM45	RNA-bindir	RBM45_HU	11	1.104772
48H 50MT/	Q9UK76	-0.1485	0.26253	0.125135	JPT1	Jupiter mic	JUPI1_HUM	7	0.90219
24H 10MT/	Q8N5K1	-0.03063	0.262561	0.125144	CISD2	CDGSH iror	CISD2_HUM	7	0.97899
48H 10MT/	Q06210	-0.03794	0.262614	0.125164	GFPT1	Glutamine-	GFPT1_HUI	39	0.974043
48H 10MT/	Q86UU0	0.040014	0.262696	0.125196	BCL9L	B-cell CLL/l	BCL9L_HUI	12	1.028124
48H 50MT/	P11597	0.673184	0.262719	0.125201	CETP	Cholesteryl	CETP_HUM	1	1.594588
24H 10MT/	Q969R5	0.094722	0.262728	0.125201	L3MBTL2	Lethal(3)m	LMBL2_HU	3	1.06786
24H 50MT/	P33240	0.036325	0.262814	0.125233	CSTF2	Cleavage st	CSTF2_HUI	17	1.025498
48H 10MT/	O75352	-0.10374	0.262851	0.125243	MPDU1	Mannose-P	MPU1_HUI	4	0.930617
48H 10MT/	Q8IUH5	-0.42093	0.262857	0.125244	ZDHHC17	Palmitoyltr	ZDH17_HU	3	0.746945
48H 50MT/	Q96IZ0	0.013659	0.262869	0.125247	PAWR	PRKC apop	PAWR_HUM	12	1.009513
24H 50MT/	Q7L3B6	-0.08704	0.263016	0.125307	CDC37L1	Hsp90 co-c	CD37L_HUI	2	0.941455
24H 50MT/	P49023	-0.03633	0.263033	0.125312	PXN	Paxillin	PAXI_HUM/	18	0.975133

48H 10MTA	Q9NYL4	-0.07108	0.263041	0.125314	FKBP11	Peptidyl-pr	FKB11_HUI	5	0.951927
24H 10MTA	Q15063	0.674536	0.263126	0.125347	POSTN	Periostin	POSTN_HUI	3	1.596084
24H 10MTA	O43567	0.293463	0.263138	0.125349	RNF13	E3 ubiquiti	RNF13_HUI	2	1.225579
48H 10MTA	P52292	0.005492	0.263151	0.125352	KPNA2	Importin su	IMA1_HUM	24	1.003814
48H 50MTA	Q5VTE6	-0.78802	0.263194	0.125369	ANGEL2	Protein ang	ANGE2_HU	3	0.579137
48H 10MTA	O43505	-0.07468	0.263203	0.125372	B4GAT1	Beta-1,4-gl	B4GA1_HU	13	0.949552
24H 10MTA	O76021	-0.13421	0.263289	0.125398	RSL1D1	Ribosomal	RL1D1_HUI	22	0.911169
24H 10MTA	P49458	-0.06869	0.263334	0.12541	SRP9	Signal reco	SRP09_HUI	3	0.953502
24H 10MTA	P09417	-0.03598	0.263343	0.125412	QDPR	Dihydropte	DHPR_HUM	10	0.975367
48H 50MTA	P61247	-0.0346	0.263356	0.125414	RPS3A	Small ribos	RS3A_HUM	18	0.976301
48H 10MTA	P42892	-0.13275	0.263509	0.125474	ECE1	Endothelin	ECE1_HUM	8	0.912092
48H 50MTA	Q96GM8	-0.18046	0.263523	0.125479	TOE1	Target of EG	TOE1_HUM	14	0.88242
24H 10MTA	O14763	-0.06504	0.263541	0.125486	TNFRSF10B	Tumor necr	TR10B_HUI	1	0.955918
48H 50MTA	Q8NBQ5	-0.05212	0.26359	0.125504	HSD17B11	Estradiol 17	DHB11_HU	14	0.964518
48H 10MTA	Q9H553	-0.09643	0.263655	0.125528	ALG2	Alpha-1,3/1	ALG2_HUM	13	0.935342
24H 10MTA	P04259	-3.19755	0.263715	0.125552	KRT6B	Keratin, typ	K2C6B_HUI	2	0.109003
24H 50MTA	Q14191	0.152106	0.263726	0.125554	WRN	Bifunctiona	WRN_HUM	3	1.11119
48H 10MTA	Q7Z406	0.002235	0.263726	0.125554	MYH14	Myosin-14	MYH14_HU	42	1.00155
24H 50MTA	Q96T23	0.003289	0.263731	0.125555	RSF1	Remodeling	RSF1_HUM	27	1.002282
24H 10MTA	Q9UHB6	-0.0632	0.263831	0.125594	LIMA1	LIM domain	LIMA1_HUI	22	0.957141
48H 50MTA	P19474	0.213357	0.263924	0.125629	TRIM21	E3 ubiquiti	RO52_HUM	1	1.159383
24H 50MTA	P16885	-0.3156	0.263942	0.125635	PLCG2	1-phosphat	PLCG2_HUI	5	0.803517
48H 10MTA	P08034	-0.12093	0.263946	0.125635	GJB1	Gap junctio	CXB1_HUM	4	0.919596
24H 10MTA	Q92636	0.072933	0.263999	0.12565	NSMAF	Protein FAN	FAN_HUMA	4	1.051853
48H 50MTA	Q86X40	-0.58446	0.264024	0.125657	LRRC28	Leucine-ric	LRC28_HUI	2	0.6669
24H 10MTA	Q5VZF2	-0.22359	0.264059	0.125672	MBNL2	Muscleblin	MBNL2_HU	3	0.856434
24H 10MTA	Q9UBU6	-0.02895	0.264061	0.125672	FAM8A1	Protein FAN	FA8A1_HUI	10	0.980131
48H 10MTA	O75027	-0.04868	0.264086	0.125678	ABCB7	Iron-sulfur	ABCB7_HUI	17	0.966818
48H 50MTA	O95229	-0.29906	0.264078	0.125678	ZWINT	ZW10 inter	ZWINT_HUI	7	0.812784
24H 10MTA	P09874	0.004317	0.264092	0.125678	PARP1	Poly [ADP-r	PARP1_HUI	53	1.002997
48H 10MTA	Q9H9L3	0.010423	0.264084	0.125678	ISG20L2	Interferon-s	I20L2_HUM	5	1.007251
24H 50MTA	P05783	-0.01533	0.2641	0.125679	KRT18	Keratin, typ	K1C18_HUI	36	0.989432
24H 50MTA	O43678	-0.00521	0.264173	0.125701	NDUFA2	NADH dehy	NDUA2_HU	4	0.996393
24H 10MTA	Q9UJP4	-0.26419	0.26421	0.125711	KLHL21	Kelch-like p	KLH21_HUI	1	0.832669
24H 10MTA	Q9Y3B8	-0.08531	0.26424	0.125721	REXO2	Oligoribonu	ORN_HUMA	10	0.94258
24H 50MTA	Q9Y2G3	0.088907	0.264268	0.12573	ATP11B	Phospholip	AT11B_HUI	11	1.063564
24H 10MTA	O95081	-0.14655	0.264272	0.12573	AGFG2	Arf-GAP do	AGFG2_HU	7	0.903411
24H 50MTA	Q96RU3	-0.05449	0.264297	0.125739	FNBP1	Formin-bin	FNBP1_HUI	16	0.962935
24H 10MTA	Q96DT6	0.283842	0.264303	0.12574	ATG4C	Cysteine pr	ATG4C_HUI	1	1.217433
24H 10MTA	Q8TAD8	-0.02243	0.264363	0.125764	SNIP1	Smad nucle	SNIP1_HUM	5	0.98457
48H 10MTA	Q9ULP9	-0.04911	0.264393	0.125773	TBC1D24	TBC1 doma	TBC24_HUI	13	0.966529
24H 50MTA	G2XKQ0	-0.07378	0.264439	0.12579	SUMO1P1	Small ubiqu	SUMO5_HU	3	0.950148
24H 50MTA	Q504Y3	-0.03878	0.264448	0.125792	ZCWPW2	Zinc finger	ZCPW2_HU	1	0.973478
24H 50MTA	Q9C0B1	-0.17776	0.26445	0.125792	FTO	Alpha-keto	FTO_HUMA	13	0.884074
48H 10MTA	Q8WV22	-0.15742	0.264558	0.125838	NSMCE1	Non-struct	NSE1_HUM	5	0.896624
24H 10MTA	Q86V81	0.055383	0.264582	0.125848	ALYREF	THO compl	THOC4_HU	10	1.039135
24H 50MTA	Q52LJ0	0.018267	0.264629	0.125866	FAM98B	Protein FAN	FA98B_HUI	12	1.012742
48H 50MTA	P13073	-0.12989	0.264654	0.125874	COX4I1	Cytochrom	COX41_HU	10	0.913898
24H 50MTA	Q9UBK9	-0.30237	0.264654	0.125874	UXT	Protein UXT	UXT_HUMA	2	0.810917
24H 50MTA	O14974	-0.01286	0.264686	0.125888	PPP1R12A	Protein pho	MYPT1_HU	29	0.991124
24H 10MTA	P54259	-0.29965	0.264713	0.125896	ATN1	Atrophin-1	ATN1_HUM	1	0.812449
48H 10MTA	Q96RE7	-0.00302	0.264826	0.125937	NACC1	Nucleus acc	NACC1_HU	13	0.997908

48H 10MT/	Q6ZRV2	-0.03606	0.264863	0.125953	FAM83H	Protein FAI	FA83H_HUI	23	0.975316
24H 10MT/	Q6ZWT7	-0.12034	0.264927	0.12598	MBOAT2	Lysophospl	MBOA2_HU	1	0.919969
24H 10MT/	Q9UJA5	-0.0117	0.265155	0.126074	TRMT6	tRNA (aden	TRM6_HUM	19	0.99192
48H 50MT/	Q14032	-0.35439	0.265188	0.126088	BAAT	Bile acid-Cc	BAAT_HUM	3	0.782198
48H 10MT/	O95297	-0.02651	0.265195	0.12609	MPZL1	Myelin pro	MPZL1_HU	4	0.981791
48H 10MT/	Q8TEV9	-0.12501	0.265377	0.126152	SMCR8	Guanine nu	SMCR8_HU	1	0.916999
48H 50MT/	Q96BY9	0.428662	0.265378	0.126152	SARAF	Store-opera	SARAF_HUI	1	1.345985
48H 10MT/	O95997;Q9	0.090537	0.265528	0.126214	PTTG1;PTT(	Securin;Sec	PTTG1_HUI	1	1.064766
24H 10MT/	Q9UJS0	-0.04275	0.265529	0.126214	SLC25A13	Electrogeni	S2513_HUI	29	0.970804
24H 10MT/	Q8NAV1	0.142609	0.265535	0.126215	PRPF38A	Pre-mRNA-:	PR38A_HUI	10	1.1039
24H 50MT/	O94913	-0.11021	0.265568	0.12623	PCF11	Pre-mRNA (	PCF11_HUI	19	0.926452
48H 50MT/	Q9BZ29	0.035804	0.265628	0.126253	DOCK9	Dedicator c	DOCK9_HU	3	1.025128
24H 50MT/	Q86UX2	0.384496	0.265663	0.126268	ITIH5	Inter-alpha	ITIH5_HUM	1	1.305404
24H 50MT/	Q8N4C8	0.013999	0.265682	0.126275	MINK1	Misshapen-	MINK1_HU	7	1.00975
24H 10MT/	O75503	0.09561	0.265795	0.126321	CLN5	Bis(monoac	CLN5_HUM	7	1.068517
48H 50MT/	Q96FF7	0.797699	0.265848	0.126341	MISP3	Uncharacte	MISP3_HUI	1	1.738327
24H 50MT/	Q9UKJ3	0.023383	0.265859	0.126343	GPATCH8	G patch do	GPTC8_HUI	12	1.01634
48H 10MT/	Q8NFV4	-0.10512	0.265977	0.126385	ABHD11	sn-1-specifi	ABHDB_HU	9	0.929727
24H 50MT/	A6NHL2	0.165816	0.26599	0.126387	TUBAL3	Tubulin alp	TBAL3_HUI	2	1.1218
24H 10MT/	O15379	0.067994	0.266026	0.1264	HDAC3	Histone dea	HDAC3_HU	7	1.048258
48H 50MT/	Q11206	-0.17803	0.266189	0.126465	ST3GAL4	CMP-N-ace	SIA4C_HUI	3	0.883909
48H 50MT/	P53778	0.064549	0.266248	0.126488	MAPK12	Mitogen-ac	MK12_HUM	5	1.045758
48H 50MT/	P54259	0.664108	0.266282	0.126502	ATN1	Atrophin-1	ATN1_HUM	1	1.584588
24H 10MT/	O14657	-0.05944	0.266322	0.126513	TOR1B	Torsin-1B	TOR1B_HUI	8	0.959639
48H 10MT/	P18669	0.052438	0.266325	0.126513	PGAM1	Phosphogly	PGAM1_HU	20	1.037016
48H 10MT/	Q8N108	0.002037	0.266322	0.126513	MIER1	Mesoderm	MIER1_HUI	6	1.001413
48H 10MT/	Q8N1A6	0.563664	0.266329	0.126513	C4orf33	UPF0462 p	CD033_HU	1	1.478018
48H 10MT/	Q86TM6	-0.15102	0.26635	0.126518	SYVN1	E3 ubiquiti	SYVN1_HUI	12	0.900612
24H 10MT/	Q8N612	-0.10612	0.266359	0.126521	FHIP1B	FHF comple	FHI1B_HUM	7	0.929082
48H 10MT/	Q75QN2	0.070629	0.266429	0.126547	INTS8	Integrator (	INT8_HUM/	15	1.050174
48H 10MT/	Q7L9L4	-0.02454	0.266431	0.126547	MOB1B	MOB kinase	MOB1B_HU	7	0.983136
48H 10MT/	Q9Y467	0.133479	0.266435	0.126547	SALL2	Sal-like pro	SALL2_HUM	4	1.096936
24H 50MT/	Q9P2Y4	-0.0164	0.266515	0.12658	ZNF219	Zinc finger	ZN219_HUI	6	0.988697
24H 10MT/	Q9BXB5	-0.10882	0.266601	0.126611	OSBPL10	Oxysterol-b	OSB10_HUI	14	0.927344
24H 10MT/	O43768	-0.12328	0.266614	0.126616	ENSA	Alpha-endc	ENSA_HUM	7	0.918101
24H 10MT/	Q8IUC4	-0.05563	0.266617	0.126616	RHPN2	Rhophilin-2	RHPN2_HU	21	0.962175
48H 10MT/	P61916	-0.01162	0.266893	0.126733	NPC2	NPC intrace	NPC2_HUM	7	0.991981
24H 50MT/	Q02224	-0.2306	0.266891	0.126733	CENPE	Centromer	CENPE_HUI	13	0.852282
48H 10MT/	Q9Y2X0	0.073645	0.266916	0.126738	MED16	Mediator o	MED16_HU	6	1.052372
24H 50MT/	O60725	0.103555	0.266944	0.126746	ICMT	Protein-S-is	ICMT_HUM	3	1.074418
24H 10MT/	Q8IYA6	0.113557	0.266968	0.126756	CKAP2L	Cytoskeleto	CKP2L_HUI	5	1.081892
24H 10MT/	P40763	-0.03212	0.26703	0.126782	STAT3	Signal trans	STAT3_HUM	37	0.977983
48H 10MT/	P61201	-0.03919	0.267046	0.126788	COPS2	COP9 signa	CSN2_HUM	21	0.973199
48H 10MT/	O95865	-0.01816	0.267266	0.126878	DDAH2	Putative hy	DDAH2_HU	14	0.987489
24H 50MT/	Q96AB3	-0.03083	0.267279	0.126881	ISOC2	Isochorism	ISOC2_HUM	7	0.978858
48H 50MT/	Q15843	0.005878	0.26735	0.12691	NEDD8	NEDD8	NEDD8_HU	4	1.004083
48H 50MT/	Q92918	-0.42219	0.267391	0.126921	MAP4K1	Mitogen-ac	M4K1_HUM	1	0.746293
48H 10MT/	Q9NP73	0.152378	0.267441	0.126938	ALG13	UDP-N-acet	ALG13_HUI	6	1.1114
48H 50MT/	Q86SZ2	-0.0948	0.267562	0.126978	TRAPPC6B	Trafficking	TPC6B_HUI	6	0.936404
48H 10MT/	Q14232	0.021354	0.267606	0.126989	EIF2B1	Translation	EI2BA_HUM	15	1.014912
48H 50MT/	Q8NCN5	-0.03763	0.267653	0.127004	PDPR	Pyruvate de	PDPR_HUM	24	0.974256
48H 10MT/	Q96DR4	-0.13901	0.267672	0.127009	STARD4	StAR-relate	STAR4_HUM	2	0.908142

48H 50MT/	Q6IN84	-0.25064	0.267678	0.12701	MRM1	rRNA meth	MRM1_HU	6	0.840522
24H 50MT/	Q8WVM7	0.052181	0.267695	0.127013	STAG1	Cohesin sul	STAG1_HU	13	1.036831
48H 50MT/	Q96DX4	-0.03497	0.267694	0.127013	RSPRY1	RING finger	RSPRY_HUI	10	0.97605
48H 10MT/	Q9H9Q2	-0.02432	0.267752	0.12703	COPS7B	COP9 signa	CSN7B_HU	7	0.983283
24H 10MT/	Q8NHP6	0.079616	0.267902	0.127095	MOSPD2	Motile sper	MSPD2_HU	11	1.056737
24H 50MT/	Q00610	0.034525	0.267914	0.127099	CLTC	Clathrin he	CLH1_HUM	79	1.02422
24H 10MT/	Q12778	-0.23374	0.26795	0.127114	FOXO1	Forkhead b	FOXO1_HU	5	0.850425
48H 50MT/	Q96Q05	-0.07255	0.267976	0.127122	TRAPPC9	Trafficking	TPPC9_HU	7	0.950955
24H 10MT/	Q8IXI2	-0.05317	0.268126	0.127182	RHOT1	Mitochond	MIRO1_HU	8	0.963819
48H 50MT/	Q14CZ7	-0.11719	0.268137	0.127186	FASTKD3	FAST kinase	FAKD3_HU	6	0.921979
24H 10MT/	P02538	-1.43773	0.26815	0.12719	KRT6A	Keratin, typ	K2C6A_HU	2	0.369147
48H 10MT/	Q8IZE3	-0.06735	0.268203	0.12721	SCYL3	Protein-ass	PACE1_HU	6	0.954387
48H 10MT/	Q6NW34	-0.07233	0.268247	0.127226	NEPRO	Nucleolus a	NEPRO_HU	4	0.951098
24H 10MT/	Q5BJD5	0.025457	0.268258	0.12723	TMEM41B	Transmemb	TM41B_HU	3	1.017802
24H 10MT/	Q5JPH6	0.029672	0.26843	0.127288	EARS2	Nondiscrim	SYEM_HUM	14	1.02078
48H 10MT/	P60006	-0.05852	0.268446	0.127291	ANAPC15	Anaphase-p	APC15_HU	2	0.960246
48H 50MT/	Q9NNW7	0.084257	0.268477	0.127301	TXNRD2	Thioredoxi	TRXR2_HU	10	1.060142
24H 50MT/	O14730	-0.25294	0.268485	0.127301	RIOK3	Serine/thre	RIOK3_HUI	7	0.839186
24H 10MT/	Q96DV4	0.006947	0.268586	0.127335	MRPL38	Large ribos	RM38_HUM	14	1.004827
24H 50MT/	Q8NHU6	0.003218	0.268674	0.127375	TDRD7	Tudor dom	TDRD7_HU	7	1.002233
48H 50MT/	Q9UPN6	-0.01228	0.268697	0.127379	SCAF8	SR-related	SCAF8_HUI	11	0.991526
48H 10MT/	O95834	0.009077	0.268722	0.127387	EML2	Echinoderm	EMAL2_HU	7	1.006312
48H 10MT/	P16118	0.107004	0.268728	0.127388	PFKFB1	6-phospho	F261_HUM	6	1.07699
24H 10MT/	Q5VWQ0	-0.02618	0.268759	0.127394	RSBN1	Lysine-spec	RSBN1_HU	13	0.982018
24H 10MT/	P48436	-0.15606	0.268771	0.127398	SOX9	Transcripti	SOX9_HUM	1	0.897471
48H 50MT/	P01031	-1.00787	0.268883	0.127439	C5	Compleme	CO5_HUM/	2	0.49728
24H 10MT/	P07992	-0.011	0.268883	0.127439	ERCC1	DNA excisic	ERCC1_HU	7	0.992406
24H 50MT/	Q96CM3	-0.17793	0.268892	0.12744	RPUSD4	Pseudourid	RUSD4_HU	2	0.883972
48H 10MT/	P0C5J1;Q8	-0.22008	0.268972	0.127473	FAM86B2;F	Putative pr	F86B2_HU	1	0.858515
24H 50MT/	Q13608	-0.10703	0.268988	0.127478	PEX6	Peroxisom	PEX6_HUM	14	0.928497
24H 10MT/	Q14202	0.045922	0.268997	0.12748	ZMYM3	Zinc finger	ZMYM3_HU	34	1.032343
48H 10MT/	Q86XA9	-0.06436	0.269042	0.127498	HEATR5A	HEAT repea	HTR5A_HU	23	0.956369
48H 10MT/	Q9H773	-0.09269	0.269056	0.127504	DCTPP1	dCTP pyrop	DCTP1_HU	8	0.937775
48H 10MT/	O60476	0.020317	0.26912	0.127526	MAN1A2	Mannosyl-c	MA1A2_HU	8	1.014182
48H 50MT/	Q14161	-0.09991	0.269158	0.12754	GIT2	ARF GTPase	GIT2_HUM/	17	0.933093
24H 10MT/	P15313	0.080682	0.269179	0.127547	ATP6V1B1	V-type prot	VATB1_HU	1	1.057518
48H 50MT/	Q96PM5	-0.64791	0.269239	0.127564	RCHY1	RING finger	ZN363_HU	1	0.638202
24H 50MT/	P83731	-0.00826	0.269244	0.127565	RPL24	Large ribos	RL24_HUM	6	0.99429
24H 10MT/	Q99633	0.148798	0.269261	0.127571	PRPF18	Pre-mRNA-	PRP18_HU	2	1.108645
24H 10MT/	Q96F07	0.136644	0.269399	0.127623	CYFIP2	Cytoplasmi	CYFP2_HUI	10	1.099345
24H 50MT/	Q13200	-0.0703	0.269427	0.12763	PSMD2	26S protea	PSMD2_HU	53	0.952439
24H 50MT/	Q96CP2	0.085896	0.269495	0.127651	FLYWCH2	FLYWCH fa	FWCH2_HU	6	1.061346
48H 10MT/	O95486	-0.06014	0.269535	0.127665	SEC24A	Protein tra	SC24A_HU	27	0.959168
24H 10MT/	O95671	0.031509	0.269654	0.127713	ASMTL	Probable bi	ASML_HUM	20	1.022081
24H 10MT/	Q53LP3	-0.20218	0.269662	0.127715	SOWAHC	Ankyrin rep	SWAHC_HU	16	0.869236
24H 10MT/	Q9NRF2	-0.67025	0.26975	0.127752	SH2B1	SH2B adapt	SH2B1_HU	5	0.628399
24H 10MT/	Q15542	0.082267	0.26978	0.127761	TAF5	Transcripti	TAF5_HUM	14	1.05868
24H 50MT/	P53367	-0.02239	0.269842	0.127786	ARFIP1	Arfaptin-1	ARFP1_HU	14	0.984603
48H 50MT/	Q8IVF7	-0.28697	0.269867	0.127794	FMNL3	Formin-like	FMNL3_HU	1	0.81962
48H 10MT/	Q15596	0.111221	0.269915	0.127812	NCOA2	Nuclear rec	NCOA2_HU	12	1.080142
24H 50MT/	Q99611	0.060075	0.269978	0.127834	SEPHS2	Selenide, w	SPS2_HUM	17	1.04252
24H 50MT/	P47929	0.843369	0.269985	0.127835	LGALS7	Galectin-7	LEG7_HUM	4	1.794235

48H 10MT/	Q9H0C8	-0.00157	0.270009	0.127842	ILKAP	Integrin-lin	ILKAP_HUM	13	0.998914
48H 50MT/	O60513	-0.10495	0.270061	0.127863	B4GALT4	Beta-1,4-ga	B4GT4_HUI	2	0.929837
24H 10MT/	P19447	0.073159	0.270085	0.127866	ERCC3	General tra	ERCC3_HUI	19	1.052018
24H 50MT/	Q9NPE3	0.052995	0.270082	0.127866	NOP10	H/ACA ribo	NOP10_HU	4	1.037416
48H 10MT/	Q9NWH2	0.174986	0.27008	0.127866	TMEM242	Transmemb	TM242_HU	2	1.128954
48H 50MT/	Q567V2	-0.28849	0.270113	0.127877	MPV17L2	Mpv17-like	M17L2_HU	4	0.818758
24H 50MT/	Q9C035	-0.06741	0.270134	0.127881	TRIM5	Tripartite n	TRIM5_HUI	7	0.954348
48H 10MT/	Q96N67	-0.0249	0.27018	0.127898	DOCK7	Dedicator c	DOCK7_HU	56	0.982886
24H 10MT/	P25440	-0.00761	0.270235	0.127919	BRD2	Bromodom	BRD2_HUM	14	0.994737
24H 10MT/	Q96DA6	-0.112	0.270264	0.12793	DNAJC19	Mitochond	TIM14_HUI	3	0.925305
24H 10MT/	Q13501	-0.00071	0.270367	0.12797	SQSTM1	Sequestoso	SQSTM_HU	18	0.999505
48H 10MT/	Q6SZW1	-0.02563	0.27037	0.12797	SARM1	NAD(+) hyd	SARM1_HU	28	0.982389
48H 10MT/	O43896	-0.00133	0.270384	0.127973	KIF1C	Kinesin-like	KIF1C_HUM	4	0.999078
48H 50MT/	P27695	-0.02716	0.270386	0.127973	APEX1	DNA repair	APEX1_HUI	16	0.981353
24H 10MT/	Q7KZF4	-0.00355	0.270389	0.127973	SND1	Staphyloco	SND1_HUM	50	0.997541
24H 50MT/	Q17RS7	0.232818	0.270419	0.127983	GEN1	Flap endon	GEN_HUMA	3	1.175128
48H 50MT/	Q96QZ7	-0.03738	0.270487	0.12801	MAGI1	Membrane-	MAGI1_HU	19	0.974423
48H 10MT/	Q14568	-0.93461	0.27053	0.128026	HSP90AA2I	Heat shock	HS902_HUI	1	0.523184
48H 50MT/	Q06136	-0.06044	0.270596	0.128052	KDSR	3-ketodihy	KDSR_HUM	5	0.958974
48H 50MT/	P04156	-0.06731	0.270663	0.128074	PRNP	Major prior	PRIO_HUM	4	0.954413
48H 50MT/	Q96EK9	-0.17938	0.270666	0.128074	KTI12	Protein KTI	KTI12_HUM	11	0.883085
48H 50MT/	Q4ZIN3	0.119444	0.270672	0.128075	TMEM259	Membralin	MBRL_HUM	5	1.086316
48H 10MT/	Q6PL24	0.152542	0.270689	0.12808	TMED8	Protein TM	TMED8_HU	1	1.111526
24H 10MT/	Q8IWE2	-0.10888	0.270698	0.128083	FAM114A1	Protein NO	NXP20_HU	14	0.927309
48H 10MT/	Q06481	0.077329	0.270715	0.128087	APLP2	Amyloid be	APLP2_HUI	16	1.055063
48H 10MT/	Q9BT73	0.008006	0.270893	0.128158	PSMG3	Proteasom	PSMG3_HU	5	1.005564
24H 50MT/	Q96CB8	0.087769	0.270904	0.128162	INTS12	Integrator	INT12_HUM	5	1.062725
24H 50MT/	A6NNA2	-0.20304	0.270957	0.128182	SRRM3	Serine/argi	SRRM3_HU	1	0.868716
24H 10MT/	Q6IA69	-0.11816	0.270955	0.128182	NADSYN1	Glutamine-	NADE_HUM	10	0.921363
24H 50MT/	Q7Z6J9	0.186278	0.271006	0.128202	TSEN54	tRNA-splici	SEN54_HUI	7	1.137825
48H 50MT/	P19012	0.369188	0.271114	0.128246	KRT15	Keratin, typ	K1C15_HUI	1	1.291625
24H 10MT/	Q8TEW6	-0.1301	0.271196	0.12828	DOK4	Docking pr	DOK4_HUM	4	0.913766
48H 10MT/	Q9UHJ9	-0.17887	0.271232	0.128294	PGAP2	Post-GPI at	PGAP2_HU	1	0.883397
24H 10MT/	Q4U2R6	-0.20891	0.271238	0.128295	MRPL51	Large ribos	RM51_HUM	2	0.865188
48H 10MT/	Q8WXF1	-0.02422	0.271249	0.128299	PSPC1	Paraspeckle	PSPC1_HUI	14	0.983349
48H 10MT/	Q15555	-0.12261	0.271258	0.1283	MAPRE2	Microtubul	MARE2_HU	11	0.918522
24H 10MT/	Q9UHA4	0.115554	0.27129	0.128312	LAMTOR3	Ragulator c	LTOR3_HUI	5	1.083391
48H 50MT/	Q03164	-0.11348	0.271317	0.128319	KMT2A	Histone-lys	KMT2A_HU	32	0.924353
48H 50MT/	O95716	-0.09959	0.271442	0.128369	RAB3D	Ras-related	RAB3D_HU	8	0.933298
24H 50MT/	Q9UQB8	-0.11809	0.27145	0.128371	BAIAP2	BAR/IMD d	BAIP2_HUM	11	0.921406
24H 10MT/	Q9UBW7	0.107791	0.271479	0.12838	ZMYM2	Zinc finger	ZMYM2_HU	16	1.077577
24H 50MT/	Q9H0R6	0.007453	0.271594	0.128428	QRSL1	Glutamyl-tl	GATA_HUM	16	1.00518
48H 10MT/	Q9NRM2	0.194251	0.271701	0.128474	ZNF277	Zinc finger	ZN277_HUI	3	1.14413
48H 50MT/	Q8N6G6	-0.09643	0.271717	0.128479	ADAMTSL1	ADAMTS-lik	ATL1_HUM	2	0.935342
24H 10MT/	P09493	0.099023	0.271766	0.128497	TPM1	Tropomyos	TPM1_HUM	6	1.071048
24H 10MT/	O60671	0.157835	0.271813	0.128517	RAD1	Cell cycle c	RAD1_HUM	7	1.115611
24H 50MT/	Q92887	-0.06251	0.271848	0.12853	ABCC2	ATP-bindin	MRP2_HUM	32	0.957597
48H 10MT/	P0CG39	-0.76909	0.271881	0.128535	POTEJ	POTE ankyr	POTEJ_HUM	1	0.586788
48H 50MT/	O75387	-0.00102	0.271948	0.128559	SLC43A1	Large neutr	LAT3_HUM	3	0.999291
24H 50MT/	P48668	-0.98763	0.271944	0.128559	KRT6C	Keratin, typ	K2C6C_HUI	32	0.504306
48H 10MT/	Q969H6	-0.13464	0.271988	0.128573	POP5	Ribonuclea	POP5_HUM	5	0.910897
48H 10MT/	P56537	0.018494	0.272044	0.128589	EIF6	Eukaryotic	IF6_HUMAI	10	1.012902

24H 50MT/	Q15084	0.018065	0.272144	0.128632	PDIA6	Protein dis	PDIA6_HUI	18	1.0126
24H 10MT/	Q9Y5S2	0.018442	0.272173	0.128642	CDC42BPB	Serine/thre	MRCKB_HU	29	1.012865
48H 10MT/	O15234	0.334697	0.272207	0.128656	CASC3	Protein CAS	CASC3_HUI	2	1.261112
24H 50MT/	Q9GZR7	-0.01549	0.27226	0.128678	DDX24	ATP-depend	DDX24_HU	23	0.989319
24H 10MT/	Q9H1A4	-0.00852	0.27229	0.128684	ANAPC1	Anaphase-p	APC1_HUM	38	0.994109
48H 50MT/	Q5TGZ0	-0.01268	0.272372	0.128716	MICOS10	MICOS com	MIC10_HUI	2	0.991248
48H 10MT/	P61221	-0.03408	0.272391	0.12872	ABCE1	ATP-bindin	ABCE1_HUI	28	0.976652
48H 50MT/	Q16342	-0.17731	0.272394	0.12872	PDCD2	Programme	PDCD2_HU	7	0.884351
24H 50MT/	P48449	-0.09759	0.272448	0.128741	LSS	Lanosterol	LSS_HUMA	22	0.934592
48H 50MT/	Q7Z478	-0.03307	0.272475	0.128749	DHX29	ATP-depend	DHX29_HU	37	0.977338
48H 50MT/	O15213	-0.14228	0.272489	0.128754	WDR46	WD repeat-	WDR46_HU	18	0.906086
24H 10MT/	O60814;P5	0.026178	0.272562	0.12878	H2BC12;H2	Histone H2	H2B1K_HU	8	1.018311
24H 50MT/	Q13371	-0.27652	0.27259	0.12879	PDCL	Phosducin-	PHLP_HUM	2	0.82558
48H 10MT/	P16870	-0.12731	0.272675	0.128815	CPE	Carboxypep	CBPE_HUM	3	0.915537
24H 10MT/	Q15631	0.04099	0.272674	0.128815	TSN	Translin	TSN_HUMA	12	1.02882
48H 10MT/	Q8NBK3	0.129893	0.272673	0.128815	SUMF1	Formylglyc	SUMF1_HU	5	1.094213
24H 50MT/	Q96D53	-0.1109	0.27268	0.128815	COQ8B	Atypical kir	COQ8B_HU	7	0.926009
24H 50MT/	O75347	-0.10459	0.272751	0.128845	TBCA	Tubulin-spe	TBCA_HUM	10	0.93007
24H 10MT/	O95714	0.001982	0.27278	0.128857	HERC2	E3 ubiquiti	HERC2_HUI	58	1.001375
24H 10MT/	O00160	0.047531	0.272811	0.128867	MYO1F	Unconvent	MYO1F_HU	7	1.033495
24H 50MT/	Q8IZ73	0.121477	0.272815	0.128867	RPUSD2	Pseudourid	RUSD2_HU	12	1.087848
48H 10MT/	Q8N4A0	-0.02535	0.27282	0.128867	GALNT4	Polypeptid	GALT4_HUI	7	0.982583
24H 50MT/	Q9HCE1	-0.02692	0.272848	0.128879	MOV10	Helicase MO	MOV10_HU	44	0.981516
48H 50MT/	A6NHX0	-0.12702	0.272918	0.128903	CASTOR2	Cytosolic a	CAST2_HUI	9	0.915718
48H 50MT/	Q8N9T8	-0.14233	0.272958	0.128921	KRI1	Protein KRI	KRI1_HUM	12	0.906054
24H 10MT/	O75822	-0.04435	0.272971	0.128922	EIF3J	Eukaryotic	EIF3J_HUM	15	0.969723
48H 10MT/	Q5T8I9	-0.04711	0.272971	0.128922	HENMT1	Small RNA 2	HENMT_HU	5	0.967875
24H 10MT/	Q96PC5	-0.07006	0.272987	0.128926	MIA2	Melanoma	MIA2_HUM	27	0.952598
48H 50MT/	Q9BVS4	-0.07637	0.273018	0.128936	RIOK2	Serine/thre	RIOK2_HUI	11	0.94844
24H 10MT/	Q9H098	-0.02706	0.273079	0.128956	FAM107B	Protein FAI	F107B_HUI	7	0.981418
24H 10MT/	Q9NR50	-0.04554	0.273155	0.128982	EIF2B3	Translation	EI2BG_HUM	21	0.968925
24H 10MT/	Q9NYU2	0.029367	0.273148	0.128982	UGGT1	UDP-glucos	UGGG1_HU	67	1.020564
24H 50MT/	Q9H1A3	0.42608	0.273176	0.128991	METTL9	Protein-L-h	METL9_HUI	4	1.343578
48H 50MT/	O00629	-0.0678	0.27318	0.128991	KPNA4	Importin su	IMA3_HUM	12	0.954094
24H 10MT/	Q86VM9	-0.14292	0.27326	0.12902	ZC3H18	Zinc finger	ZCH18_HUI	12	0.905687
24H 10MT/	P15121	0.020177	0.273302	0.129034	AKR1B1	Aldo-keto r	ALDR_HUM	19	1.014084
24H 50MT/	Q15056	-0.09417	0.273335	0.129046	EIF4H	Eukaryotic	IF4H_HUM	9	0.936809
48H 10MT/	Q9NSI6	0.113825	0.273334	0.129046	BRWD1	Bromodom	BRWD1_HU	1	1.082093
48H 10MT/	Q96N64	0.270983	0.273476	0.129089	PWWP2A	PWWP dor	PWP2A_HU	2	1.20663
24H 50MT/	Q9BTT6	-0.07821	0.273537	0.129108	LRRC1	Leucine-ric	LRRC1_HUI	16	0.947231
48H 50MT/	Q92667	-0.02203	0.273594	0.129133	AKAP1	A-kinase an	AKAP1_HUI	21	0.984844
48H 10MT/	O60513	-0.31523	0.273635	0.129148	B4GALT4	Beta-1,4-ga	B4GT4_HUI	2	0.803724
24H 10MT/	P42892	4.26E-05	0.273712	0.129173	ECE1	Endothelin	ECE1_HUM	8	1.000029
24H 10MT/	Q92771	0.143996	0.273803	0.129206	DDX12P	Putative AT	DDX12_HU	8	1.104961
24H 10MT/	P40818	-0.01218	0.273844	0.12922	USP8	Ubiquitin c	UBP8_HUM	22	0.991595
48H 10MT/	O94885	0.033701	0.273938	0.129258	SASH1	SAM and SH	SASH1_HUI	5	1.023635
24H 10MT/	Q8WVM7	-0.02569	0.273958	0.129261	STAG1	Cohesin sul	STAG1_HUI	13	0.982351
48H 50MT/	Q69YH5	-0.21916	0.274092	0.129304	CDCA2	Cell divisio	CDCA2_HU	2	0.859065
24H 10MT/	P51809	0.11107	0.274144	0.129325	VAMP7	Vesicle-ass	VAMP7_HU	11	1.080029
24H 10MT/	Q9Y3D7	-0.00523	0.27415	0.129325	PAM16	Mitochond	TIM16_HUI	3	0.996381
24H 10MT/	Q92551	0.059982	0.274171	0.129333	IP6K1	Inositol he	IP6K1_HUM	3	1.042453
24H 10MT/	Q9Y388	0.020044	0.274185	0.129338	RBMX2	RNA-bindir	RBMX2_HU	3	1.013991

24H 10MT/	P52272	0.009722	0.274286	0.129379	HNRNPM	Heterogene	HNRPM_HU	41	1.006761
48H 50MT/	Q8N1I0	-0.02112	0.274385	0.129418	DOCK4	Dedicator c	DOCK4_HU	37	0.985465
24H 10MT/	Q5T8I9	-0.00841	0.27444	0.12944	HENMT1	Small RNA 2	HENMT_HU	5	0.994188
48H 10MT/	O15040	-0.44048	0.27446	0.129446	TECPR2	Tectonin be	TCPR2_HUI	1	0.73689
24H 50MT/	Q8NHM5	-0.21832	0.274533	0.129476	KDM2B	Lysine-spec	KDM2B_HU	4	0.859568
48H 10MT/	P08237	0.091011	0.274554	0.129479	PFKM	ATP-depend	PFKAM_HU	1	1.065117
48H 10MT/	Q96P63	-0.42204	0.274647	0.129514	SERPINB12	Serpin B12	SPB12_HUI	3	0.746368
24H 10MT/	Q96S94	0.333088	0.274728	0.129548	CCNL2	Cyclin-L2	CCNL2_HUI	1	1.259707
48H 50MT/	Q16875	0.183552	0.274754	0.129557	PFKFB3	6-phosphof	F263_HUM	8	1.135676
48H 10MT/	O43716	0.484113	0.274783	0.129567	GATC	Glutamyl-tl	GATC_HUM	2	1.398726
48H 10MT/	P42677	0.010314	0.274835	0.129587	RPS27	Small ribos	RS27_HUM	2	1.007175
48H 10MT/	Q01850	0.052947	0.274855	0.129595	CDR2	Cerebellar c	CDR2_HUM	2	1.037382
24H 10MT/	Q7Z739	-0.03949	0.274929	0.129618	YTHDF3	YTH domair	YTHD3_HUI	14	0.972999
24H 50MT/	Q01813	-0.08186	0.274945	0.129622	PFKP	ATP-depend	PFKAP_HUI	27	0.944839
48H 10MT/	O15294	-0.04677	0.275037	0.129657	OGT	UDP-N-acet	OGT1_HUM	36	0.968101
24H 50MT/	P54652	-0.03259	0.275063	0.129666	HSPA2	Heat shock	HSP72_HUI	22	0.977666
48H 10MT/	Q8WXE1	0.10438	0.275133	0.129694	ATRIP	ATR-interac	ATRIP_HUM	1	1.075032
48H 50MT/	Q8NBZ0	-0.26333	0.275152	0.129701	INO80E	INO80 com	IN80E_HUM	1	0.833162
48H 10MT/	Q9NTK5	-0.0944	0.275194	0.129713	OLA1	Obg-like AT	OLA1_HUM	29	0.936662
24H 50MT/	P05423	0.033898	0.27522	0.129724	POLR3D	DNA-direct	RPC4_HUM	6	1.023774
24H 50MT/	O15541	0.119095	0.275235	0.129728	RNF113A	E3 ubiquiti	R113A_HUI	6	1.086053
24H 50MT/	Q96QE3	-0.05266	0.275285	0.129746	ATAD5	ATPase fam	ATAD5_HUI	5	0.964157
48H 50MT/	Q8WUQ7	-0.12892	0.27529	0.129747	CACTIN	Splicing fac	CATIN_HUM	6	0.914517
24H 10MT/	P45983	0.098249	0.275316	0.129757	MAPK8	Mitogen-ac	MK08_HUM	6	1.070474
24H 10MT/	P08779	-2.71139	0.275343	0.129767	KRT16	Keratin, typ	K1C16_HUI	23	0.152683
48H 10MT/	Q86SX3	-0.28439	0.275437	0.129804	TEDC1	Tubulin eps	TEDC1_HUI	3	0.821087
24H 50MT/	Q9NUQ7	-0.04202	0.275442	0.129805	UFSP2	Ufm1-speci	UFSP2_HUI	12	0.971291
24H 50MT/	Q9UPY6	0.463928	0.275444	0.129805	WASF3	Actin-bindi	WASF3_HU	3	1.379292
48H 10MT/	Q14004	0.041846	0.275528	0.129831	CDK13	Cyclin-depe	CDK13_HU	5	1.02943
24H 50MT/	Q9H1P3	0.077732	0.275527	0.129831	OSBPL2	Oxysterol-b	OSBL2_HUI	9	1.055358
48H 10MT/	Q96ER9	-0.10252	0.275546	0.129836	CCDC51	Mitochond	MITOK_HUI	11	0.931404
24H 50MT/	Q8IV36	0.124963	0.275616	0.129863	HID1	Protein HID	HID1_HUM	9	1.09048
24H 10MT/	Q9UHQ1	-0.30164	0.275619	0.129863	NARF	Nuclear pre	NARF_HUM	3	0.811332
24H 50MT/	P16949	0.071863	0.275697	0.129885	STMN1	Stathmin	STMN1_HU	6	1.051073
24H 50MT/	Q9NQX4	0.03343	0.27569	0.129885	MYO5C	Unconvent	MYO5C_HU	13	1.023442
48H 10MT/	P22413	-0.11978	0.275726	0.129896	ENPP1	Ectonucleo	ENPP1_HUI	13	0.920326
48H 50MT/	Q9BZQ6	-0.03484	0.275792	0.12992	EDEM3	ER degrada	EDEM3_HU	9	0.976138
24H 10MT/	Q9P2C4	-0.36385	0.275859	0.129942	TMEM181	Transmemb	TM181_HU	3	0.777089
24H 10MT/	P78524	-0.30457	0.275881	0.129951	DENND2B	DENN doma	DEN2B_HU	1	0.809682
48H 10MT/	Q9H2W6	-0.08612	0.276048	0.130018	MRPL46	Large ribos	RM46_HUM	11	0.942051
24H 50MT/	Q15758	0.036993	0.276087	0.130031	SLC1A5	Neutral am	AAAT_HUM	11	1.025973
24H 10MT/	P42338	-0.09761	0.276121	0.130041	PIK3CB	Phosphatid	PK3CB_HUI	10	0.934582
48H 50MT/	Q6SPF0	-0.12165	0.276119	0.130041	SAMD1	Sterile alph	SAMD1_HU	5	0.919135
24H 10MT/	Q8WUK0	-0.02751	0.276281	0.130097	PTPMT1	Phosphatid	PTPM1_HU	7	0.98111
24H 50MT/	Q5VWG9	-0.00953	0.276295	0.130101	TAF3	Transcripti	TAF3_HUM	1	0.993417
48H 50MT/	Q6PCD5	-0.24741	0.276461	0.130167	RFWD3	E3 ubiquiti	RFWD3_HU	3	0.842405
24H 10MT/	Q15628	-0.13564	0.276467	0.130169	TRADD	Tumor necr	TRADD_HU	6	0.910263
48H 10MT/	P24534	-0.10345	0.276752	0.130276	EEF1B2	Elongation	EF1B_HUM	10	0.930806
48H 50MT/	Q9NVH0	0.004053	0.276759	0.130278	EXD2	Exonucleas	EXD2_HUM	17	1.002813
24H 10MT/	Q96EH3	0.069283	0.276773	0.130279	MALSU1	Mitochond	MASU1_HU	3	1.049195
24H 50MT/	O15357	-0.02258	0.276835	0.130302	INPPL1	Phosphatid	SHIP2_HUM	32	0.984469
24H 50MT/	P63272	0.079462	0.276861	0.130312	SUPT4H1	Transcripti	SPT4H_HUI	5	1.056624

48H 10MT/	Q13509	-0.20056	0.27696	0.130352	TUBB3	Tubulin bet	TBB3_HUM	4	0.870214
24H 10MT/	P55072	-0.01132	0.27701	0.130373	VCP	Transitiona	TERA_HUM	57	0.992185
24H 10MT/	Q9NXG2	-0.00125	0.277031	0.130381	THUMPD1	THUMP dor	THUM1_HU	17	0.999134
48H 10MT/	Q8N6L1	-0.1456	0.277044	0.130386	KRTCAP2	Keratinocy	KTAP2_HUI	1	0.904004
24H 10MT/	Q9BZL4	-0.14456	0.277061	0.13039	PPP1R12C	Protein phc	PP12C_HUI	10	0.904655
24H 10MT/	P08910	-0.0307	0.277091	0.130397	ABHD2	Monoacylg	ABHD2_HU	1	0.978948
24H 10MT/	Q9NTJ4	-0.2073	0.277214	0.130443	MAN2C1	Alpha-mani	MA2C1_HU	5	0.866158
24H 50MT/	Q9BRP1	-0.04547	0.277258	0.130461	PDCD2L	Programme	PDD2L_HU	7	0.968975
24H 50MT/	O14653	-0.13504	0.277281	0.13047	GOSR2	Golgi SNAP	GOSR2_HU	5	0.910643
48H 50MT/	Q8IWU2	0.167927	0.277294	0.130475	LMTK2	Serine/thre	LMTK2_HU	6	1.123443
48H 10MT/	P43250	-0.11224	0.277376	0.130502	GRK6	G protein-c	GRK6_HUM	8	0.925152
24H 50MT/	Q14432	0.0429	0.277397	0.130508	PDE3A	cGMP-inhil	PDE3A_HUI	10	1.030182
24H 50MT/	Q9H3S7	-0.12812	0.277475	0.130542	PTPN23	Tyrosine-pr	PTN23_HUI	32	0.915025
48H 10MT/	Q6ZVF9	0.02611	0.277524	0.130558	GPRIN3	G protein-r	GRIN3_HUI	9	1.018263
24H 10MT/	P50135	-0.03007	0.277548	0.130564	HNMT	Histamine I	HNMT_HUI	10	0.979369
48H 10MT/	Q9Y6Y0	0.279212	0.277609	0.130586	IVNS1ABP	Influenza vi	NS1BP_HUI	6	1.213532
48H 10MT/	P31327	-0.00181	0.277621	0.13059	CPS1	Carbamoyl-	CPSM_HUM	84	0.998746
24H 50MT/	P43694	0.214226	0.277643	0.130594	GATA4	Transcripti	GATA4_HUI	5	1.160081
48H 50MT/	O14647	0.082858	0.277667	0.130602	CHD2	Chromodo	CHD2_HUM	14	1.059114
48H 10MT/	Q9BTV4	-0.05674	0.277766	0.130635	TMEM43	Transmemk	TMM43_HU	20	0.961433
24H 50MT/	Q8ND56	-0.00685	0.277776	0.130638	LSM14A	Protein LSN	LS14A_HUI	7	0.995263
48H 50MT/	Q96C24	0.156673	0.277892	0.130691	SYTL4	Synaptotag	SYTL4_HUM	7	1.114714
24H 50MT/	P29692	0.004166	0.278011	0.130735	EEF1D	Elongation	EF1D_HUM	18	1.002892
24H 10MT/	Q8WWV3	0.009781	0.278041	0.130746	RTN4IP1	Reticulon-4	RT4I1_HUM	13	1.006803
48H 50MT/	Q6P1J9	-0.07208	0.278075	0.130754	CDC73	Parafibrom	CDC73_HU	27	0.951265
24H 10MT/	Q9NVM4	-0.08059	0.278073	0.130754	PRMT7	Protein arg	ANM7_HUI	8	0.94567
24H 10MT/	O43181	0.036251	0.278085	0.130757	NDUFS4	NADH dehy	NDUS4_HU	5	1.025446
24H 50MT/	Q96NW4	-0.24502	0.278125	0.130766	ANKRD27	Ankyrin rep	ANR27_HU	2	0.843802
48H 50MT/	Q9ULG1	-0.16588	0.278125	0.130766	INO80	Chromatin-	INO80_HUI	8	0.891387
24H 50MT/	Q96EB6	-0.01419	0.278153	0.130777	SIRT1	NAD-depen	SIR1_HUM/	8	0.990212
48H 50MT/	Q9NYV4	-0.16148	0.278325	0.130843	CDK12	Cyclin-depe	CDK12_HU	16	0.894107
48H 50MT/	O43306	0.362862	0.278379	0.130859	ADCY6	Adenylate c	ADCY6_HUI	6	1.285975
24H 10MT/	Q70HW3	-0.23369	0.278381	0.130859	SLC25A26	Mitochond	SAMC_HUM	3	0.850457
48H 50MT/	Q8WUY1	-0.19896	0.278679	0.130978	THEM6	Protein THI	THEM6_HU	10	0.87118
24H 10MT/	P61011	-0.02708	0.2787	0.130986	SRP54	Signal reco	SRP54_HUI	22	0.981401
24H 10MT/	P00441	-0.0652	0.27877	0.131011	SOD1	Superoxide	SODC_HUM	7	0.95581
24H 50MT/	Q96IJ6	-0.0243	0.278819	0.131029	GMPPA	Mannose-1	GMPPA_HU	16	0.983297
48H 50MT/	Q14517	-0.32346	0.278858	0.131045	FAT1	Protocadhe	FAT1_HUM	8	0.799151
24H 10MT/	Q9UQ84	-0.04645	0.278865	0.131047	EXO1	Exonucleas	EXO1_HUM	1	0.968318
24H 10MT/	Q9UHY1	-0.05279	0.278901	0.131059	NRBP1	Nuclear rec	NRBP_HUM	11	0.96407
24H 50MT/	Q9BY42	-0.0781	0.279143	0.131159	RTF2	Replication	RTF2_HUM	8	0.947304
48H 10MT/	Q9NQZ7	0.158513	0.279186	0.131173	ENTPD7	Ectonucleo	ENTP7_HUI	6	1.116136
24H 10MT/	Q15050	-0.03635	0.279259	0.131201	RRS1	Ribosome k	RRS1_HUM	16	0.975122
24H 50MT/	Q5M775	-0.18098	0.279279	0.131208	SPECC1	Cytospin-B	CYTSB_HUM	17	0.882103
24H 10MT/	Q32MZ4	0.022106	0.279324	0.131223	LRRFIP1	Leucine-ric	LRRF1_HUI	26	1.015441
24H 10MT/	Q9Y3X0	0.075134	0.279365	0.131239	CCDC9	Coiled-coil	CCDC9_HU	4	1.053459
24H 50MT/	Q9BQ69	-0.10139	0.27937	0.131239	MACROD1	ADP-ribose	MACD1_HU	4	0.932137
24H 10MT/	Q9Y2Q5	0.01516	0.279422	0.131259	LAMTOR2	Ragulator c	LTOR2_HUI	6	1.010563
24H 10MT/	Q9NQ92	-0.30657	0.279449	0.131269	COPRS	Coordinato	COPRS_HUI	1	0.808563
24H 50MT/	Q9P206	-0.17622	0.279552	0.131305	NHSL3	NHS-like pr	NHSL3_HUI	6	0.885019
48H 10MT/	Q9H0E3	-0.06845	0.279583	0.131313	SAP130	Histone dea	SP130_HUI	10	0.953665
48H 50MT/	Q9BUF7	-0.22842	0.279592	0.131315	CRB3	Protein cru	CRUM3_HU	1	0.853567

24H 10MT/	Q8N1B4	-0.07967	0.279628	0.131328	VPS52	Vacuolar pı	VPS52_HUI	18	0.946272
24H 50MT/	Q9BTE7	-0.02037	0.279664	0.131339	DCUN1D5	DCN1-like p	DCNL5_HU	9	0.985981
24H 50MT/	O15156	0.056766	0.279713	0.131352	ZBTB7B	Zinc finger a	ZBT7B_HUI	3	1.040131
48H 10MT/	Q8NF50	-0.33099	0.279766	0.131372	DOCK8	Dedicator c	DOCK8_HU	3	0.794991
48H 50MT/	O60231	-0.03672	0.279885	0.131417	DHX16	Pre-mRNA-s	DHX16_HU	28	0.974867
24H 10MT/	Q96F46	0.119583	0.27992	0.131432	IL17RA	Interleukin	I17RA_HUM	7	1.086421
48H 50MT/	P50579	-0.08423	0.279969	0.131449	METAP2	Methionine	MAP2_HUM	22	0.943288
24H 10MT/	Q15125	-0.08742	0.280012	0.131458	EBP	3-beta-hydı	EBP_HUMA	3	0.941204
48H 10MT/	Q96FF9	-0.08243	0.280013	0.131458	CDCA5	Sororin	CDCA5_HU	5	0.944468
48H 10MT/	O95470	-0.12047	0.280048	0.131472	SGPL1	Sphingosin	SGPL1_HUI	17	0.919889
48H 10MT/	P03897	0.210991	0.280096	0.131488	MT-ND3	NADH-ubiq	NU3M_HUI	1	1.157483
48H 50MT/	Q9BV38	-0.01401	0.280192	0.131523	WDR18	WD repeat-	WDR18_HU	13	0.990336
48H 50MT/	Q7L1Q6	-0.06578	0.280208	0.131528	BZW1	eIF5-mimic	5MP2_HUM	19	0.955427
48H 10MT/	Q14240	-0.01237	0.280256	0.131544	EIF4A2	Eukaryotic	IF4A2_HUM	12	0.99146
24H 50MT/	Q9UK55	-0.83597	0.280258	0.131544	SERPINA10	Protein Z-d	ZPI_HUMAI	1	0.560206
24H 50MT/	Q07817	-0.01675	0.280335	0.131574	BCL2L1	Bcl-2-like p	B2CL1_HUI	5	0.988455
24H 50MT/	P54132	0.165363	0.280383	0.131594	BLM	RecQ-like D	BLM_HUM/	11	1.121448
48H 10MT/	O14646	-0.16342	0.280391	0.131595	CHD1	Chromodoı	CHD1_HUM	20	0.892908
24H 10MT/	P51159	0.115595	0.280399	0.131597	RAB27A	Ras-related	RB27A_HUI	5	1.083422
24H 10MT/	P42025	-0.07574	0.280408	0.1316	ACTR1B	Beta-centra	ACTY_HUM	15	0.948857
48H 50MT/	Q93052	0.082946	0.280428	0.131604	LPP	Lipoma-pre	LPP_HUMA	15	1.059179
24H 10MT/	Q96BW5	0.015996	0.280437	0.131605	PTER	Phosphotri	PTER_HUM	12	1.011149
24H 50MT/	Q9P270	-0.04807	0.280551	0.131646	SLAIN2	SLAIN moti	SLAI2_HUM	11	0.967226
24H 10MT/	Q9UGU5	-0.11233	0.280578	0.131654	HMGXB4	HMG doma	HMGX4_HU	1	0.925094
24H 10MT/	Q9NRG9	0.070507	0.280609	0.131663	AAAS	Aladin	AAAS_HUM	26	1.050086
24H 10MT/	Q9P0M6	0.060034	0.280639	0.131673	MACROH2/	Core histor	H2AW_HUI	8	1.042491
24H 10MT/	Q9BRT6	-0.05195	0.280668	0.13168	LLPH	Protein LLF	LLPH_HUM	4	0.964632
48H 10MT/	O75717	0.075943	0.280844	0.13175	WDHD1	WD repeat	WDHD1_HI	28	1.05405
48H 50MT/	Q92537	-0.45406	0.280886	0.131765	SUSD6	Sushi doma	SUSD6_HUI	1	0.729983
24H 10MT/	P46199	0.054809	0.28097	0.131799	MTIF2	Translation	IF2M_HUM	9	1.038721
24H 10MT/	Q9UQ35	0.08427	0.281	0.13181	SRRM2	Serine/argi	SRRM2_HU	45	1.060151
48H 10MT/	P56282	0.051415	0.281183	0.131882	POLE2	DNA polym	DPOE2_HU	7	1.036281
24H 10MT/	O75683	-0.1469	0.281192	0.131882	SURF6	Surfeit locu	SURF6_HUI	7	0.903191
48H 50MT/	P12883	-0.03702	0.2812	0.131884	MYH7	Myosin-7	MYH7_HUM	1	0.974665
48H 10MT/	Q9NUQ3	-0.09049	0.281221	0.131892	TXLNG	Gamma-tax	TXLNG_HUI	17	0.939204
24H 50MT/	P23511	-0.21497	0.281231	0.131892	NFYA	Nuclear tra	NFYA_HUM	2	0.861564
48H 10MT/	Q9ULF5	-0.19185	0.281437	0.13197	SLC39A10	Zinc transp	S39AA_HUI	2	0.875483
48H 50MT/	P28072	-0.06546	0.281537	0.132012	PSMB6	Proteasom	PSB6_HUM	8	0.955644
48H 50MT/	Q969S9	0.070279	0.281546	0.132015	GFM2	Ribosome-r	RRF2M_HU	15	1.049919
48H 10MT/	P49753	-0.24711	0.281557	0.132018	ACOT2	Acyl-coenzy	ACOT2_HUI	1	0.84258
48H 10MT/	Q8N5L8	0.015034	0.281563	0.132019	RPP25L	Ribonuclea	RP25L_HUI	1	1.010475
48H 50MT/	Q8IYT4	0.012516	0.281619	0.132039	KATNAL2	Katanin p6	KATL2_HUM	1	1.008713
48H 10MT/	Q8TE67	-0.10039	0.281632	0.132043	EPS8L3	Epidermal g	ES8L3_HUM	16	0.932781
24H 50MT/	Q12834	-0.00718	0.281899	0.132151	CDC20	Cell divisioı	CDC20_HU	11	0.995034
48H 50MT/	Q8WXX5	-0.06381	0.281964	0.132179	DNAJC9	DnaJ homo	DNJC9_HUI	15	0.956736
24H 50MT/	Q92793	-0.10619	0.282096	0.132222	CREBBP	CREB-bindi	CBP_HUMA	14	0.929035
48H 10MT/	P62861	0.078693	0.282144	0.132239	FAU	Ubiquitin-l	RS30_HUM	2	1.056061
24H 10MT/	Q8NEU8	0.004691	0.282141	0.132239	APPL2	DCC-intera	DP13B_HU	15	1.003257
24H 10MT/	Q9BXS5	0.009114	0.282162	0.132246	AP1M1	AP-1 compl	AP1M1_HU	23	1.006338
24H 10MT/	Q9NTW7	0.271184	0.282221	0.132269	ZFP64	Zinc finger	ZF64B_HUI	2	1.206798
24H 10MT/	Q9UJ83	0.010344	0.282237	0.132273	HACL1	2-hydroxya	HACL1_HUI	13	1.007196
24H 50MT/	Q86XI2	0.041867	0.282263	0.132281	NCAPG2	Condensin-	CNDG2_HU	15	1.029445

48H 50MT/	O43665	0.362758	0.282276	0.132285	RGS10	Regulator c	RGS10_HUI	4	1.285882
48H 10MT/	Q96JP5	0.063663	0.2823	0.132294	ZFP91	E3 ubiquiti	ZFP91_HUN	6	1.045116
48H 10MT/	Q13630	-0.02287	0.282345	0.132307	GFUS	GDP-L-fuco	FCL_HUMA	12	0.984275
24H 50MT/	P35226	0.029996	0.282424	0.132333	BMI1	Polycomb c	BMI1_HUM	4	1.021009
24H 50MT/	Q7Z589	0.113517	0.282466	0.132346	EMSY	BRCA2-inte	EMSY_HUM	7	1.081862
48H 10MT/	Q8TEX9	-0.03907	0.282584	0.132387	IPO4	Importin-4	IPO4_HUM	47	0.973285
48H 10MT/	Q8WVZ9	-0.26739	0.282584	0.132387	KBTBD7	Kelch repea	KBTB7_HUI	1	0.830819
48H 10MT/	Q9Y2Q9	-0.08266	0.282611	0.132394	MRPS28	Small ribos	RT28_HUM	6	0.944317
48H 10MT/	Q8WVC0	-0.05956	0.282699	0.132426	LEO1	RNA polym	LEO1_HUM	11	0.959558
24H 50MT/	Q14644	0.884223	0.282712	0.132428	RASA3	Ras GTPase-	RASA3_HUI	1	1.84577
24H 50MT/	Q5T5X7	-0.16369	0.282744	0.132442	BEND3	BEN domai	BEND3_HU	10	0.892739
48H 50MT/	Q9NWF9	-0.16797	0.28278	0.132455	RNF216	E3 ubiquiti	RN216_HU	2	0.890093
24H 10MT/	O94763	0.311497	0.282807	0.132466	URI1	Unconvent	RMP_HUM/	5	1.240994
24H 10MT/	Q969T3	0.053015	0.282855	0.132485	SNX21	Sorting nex	SNX21_HUI	4	1.037431
48H 10MT/	O60573	-0.05759	0.282879	0.132493	EIF4E2	Eukaryotic	IF4E2_HUN	12	0.960868
48H 50MT/	O15226	-0.15339	0.282928	0.13251	NKRF	NF-kappa-B	NKRF_HUM	23	0.899138
24H 10MT/	P51687	-0.10594	0.282974	0.132528	SUOX	Sulfite oxid	SUOX_HUN	11	0.9292
24H 50MT/	P46782	-0.14079	0.283008	0.132537	RPS5	Small ribos	RS5_HUMA	10	0.907024
24H 50MT/	P49915	-0.08431	0.283069	0.132559	GMPS	GMP synth	GUAA_HUN	35	0.943236
24H 50MT/	P49247	0.036691	0.283145	0.132589	RPIA	Ribose-5-pl	RPIA_HUM/	5	1.025759
48H 10MT/	Q96EK9	-0.06827	0.283175	0.132593	KTI12	Protein KTI	KTI12_HUN	11	0.953778
24H 50MT/	Q13283	-0.04653	0.283187	0.132595	G3BP1	Ras GTPase-	G3BP1_HU	23	0.968261
48H 50MT/	O75943	-0.09037	0.28327	0.132624	RAD17	Cell cycle cl	RAD17_HU	7	0.939281
24H 10MT/	Q9Y6Y0	0.002038	0.283265	0.132624	IVNS1ABP	Influenza vi	NS1BP_HUI	6	1.001413
24H 50MT/	Q96D46	0.025196	0.283292	0.132633	NMD3	60S riboso	NMD3_HUI	17	1.017618
24H 10MT/	Q15835	0.175596	0.283334	0.132649	GRK1	Rhodopsin	GRK1_HUM	1	1.129431
48H 10MT/	Q9UPY3	0.014021	0.283398	0.132676	DICER1	Endoribon	DICER_HUN	25	1.009766
24H 50MT/	Q9BUA3	0.043854	0.283455	0.13269	SPINDOC	Spindlin in	SPNDC_HU	3	1.030864
48H 50MT/	Q9H8T0	0.020654	0.283467	0.132691	AKTIP	AKT-interac	AKTIP_HUN	6	1.01442
24H 10MT/	Q6UXH1	0.030176	0.283472	0.132691	CRELD2	Protein dis	CREL2_HUN	5	1.021136
24H 10MT/	P16070	0.469105	0.283586	0.132733	CD44	CD44 antig	CD44_HUM	1	1.38425
24H 50MT/	Q99436	0.055444	0.283589	0.132733	PSMB7	Proteasom	PSB7_HUM	12	1.039179
48H 10MT/	Q9UNW1	-0.02968	0.283653	0.132755	MINPP1	Multiple in	MINP1_HU	11	0.979638
48H 10MT/	Q8NCR0	-0.31765	0.283739	0.132785	B3GALNT2	UDP-GalNA	B3GL2_HUI	2	0.802378
24H 10MT/	P42566	0.064752	0.283901	0.132842	EPS15	Epidermal	EPS15_HUN	22	1.045905
48H 10MT/	Q9NUP1	-0.03512	0.283908	0.132844	BLOC1S4	Biogenesis	BL1S4_HUN	4	0.97595
48H 50MT/	Q96RU3	-0.00374	0.283934	0.132849	FNBP1	Formin-bin	FNBP1_HUI	16	0.997413
48H 50MT/	Q96LJ7	-0.13871	0.283956	0.132854	DHRS1	Dehydroge	DHRS1_HU	10	0.90833
48H 50MT/	O43674	-0.0297	0.284034	0.132886	NDUFB5	NADH dehy	NDUB5_HU	5	0.979625
24H 50MT/	Q96HY7	0.386898	0.284048	0.132889	DHTKD1	2-oxoadipa	DHTK1_HU	5	1.307579
48H 50MT/	Q9BXW6	0.002193	0.284067	0.132896	OSBPL1A	Oxysterol-k	OSBL1_HUI	7	1.001521
24H 10MT/	O43379	-0.0584	0.284096	0.132907	WDR62	WD repeat-	WDR62_HU	9	0.960329
24H 50MT/	Q8TAA9	-0.06219	0.284104	0.132907	VANGL1	Vang-like p	VANG1_HU	8	0.95781
48H 10MT/	Q9BXS4	0.006668	0.284103	0.132907	TMEM59	Transmemb	TMM59_HU	4	1.004633
48H 10MT/	P62910	-0.0883	0.284158	0.132927	RPL32	Large ribos	RL32_HUM	7	0.940633
48H 10MT/	Q16394	-0.00174	0.284186	0.132937	EXT1	Exostosin-1	EXT1_HUM	3	0.998792
48H 10MT/	Q9H9A6	-0.01145	0.28419	0.132937	LRRC40	Leucine-ric	LRC40_HUI	27	0.992098
24H 10MT/	P06748	0.037862	0.284212	0.132942	NPM1	Nucleopho	NPM_HUM	13	1.026592
24H 50MT/	Q9BRL6	0.635583	0.284238	0.132952	SRSF8	Serine/argi	SRSF8_HUN	1	1.553566
48H 50MT/	P56134	-0.05178	0.284323	0.132979	ATP5MF	ATP syntha	ATPK_HUM	4	0.964743
48H 50MT/	P27448	-0.06222	0.284327	0.132979	MARK3	MAP/micro	MARK3_HU	16	0.957788
48H 10MT/	Q9P2E5	-0.0552	0.284361	0.132986	CHPF2	Chondroiti	CHPF2_HUI	1	0.96246

48H	10MT/	P37287	0.062846	0.284365	0.132987	PIGA	Phosphatic	PIGA_HUM	3	1.044525
24H	50MT/	P57081	0.021711	0.284486	0.133035	WDR4	tRNA (guan	WDR4_HUI	11	1.015163
24H	50MT/	O75530	0.088921	0.284551	0.133059	EED	Polycomb	EED_HUMA	15	1.063575
24H	50MT/	Q13838	-0.01646	0.28457	0.133064	DDX39B	Spliceosom	DX39B_HU	22	0.988659
48H	10MT/	O00462	-0.1791	0.284642	0.133094	MANBA	Beta-mann	MANBA_HU	12	0.883254
24H	10MT/	Q8WVK7	-0.06512	0.284701	0.133119	SKA2	Spindle and	SKA2_HUM	1	0.955865
24H	10MT/	P62241	-0.00831	0.284722	0.13312	RPS8	Small ribos	RS8_HUMA	11	0.994258
24H	50MT/	Q5W0V3	0.079772	0.284721	0.13312	FHIP2A	FHF comple	FHI2A_HUN	10	1.056851
48H	50MT/	P61019	-0.02879	0.284733	0.133123	RAB2A	Ras-related	RAB2A_HUI	14	0.980239
48H	10MT/	Q9UL03	0.10568	0.284742	0.133124	INTS6	Integrator	INT6_HUMA	11	1.076001
48H	50MT/	Q9Y606	-0.05233	0.284754	0.133128	PUS1	Pseudourid	PUS1_HUM	16	0.964379
48H	50MT/	P23246	0.006589	0.284792	0.13314	SFPQ	Splicing fac	SFPQ_HUM	25	1.004577
24H	50MT/	Q8ND82	0.17485	0.284793	0.13314	ZNF280C	Zinc finger	Z280C_HUI	2	1.128847
48H	10MT/	O75674	-0.00652	0.284825	0.13315	TOM1L1	TOM1-like	TM1L1_HU	7	0.995488
48H	10MT/	Q9P015	-0.14104	0.284857	0.133159	MRPL15	Large ribos	RM15_HUN	17	0.906867
48H	10MT/	Q13232	-0.06543	0.284962	0.133192	NME3	Nucleoside	NDK3_HUN	8	0.955659
48H	10MT/	Q9NXX6	-0.06009	0.284953	0.133192	NSMCE4A	Non-struct	NSE4A_HUI	6	0.959206
24H	50MT/	P16402	0.445045	0.285001	0.133206	H1-3	Histone H1	H13_HUMA	1	1.361356
48H	50MT/	Q8N1A6	0.800023	0.285	0.133206	C4orf33	UPF0462 p	CD033_HU	1	1.741129
48H	50MT/	O15212	-0.21041	0.285015	0.133211	PFDN6	Prefoldin s	PFD6_HUM	7	0.864292
24H	50MT/	Q9Y4E1	0.259337	0.285063	0.133225	WASHC2C	WASH com	WAC2C_HU	1	1.196928
24H	50MT/	Q8TAE8	-0.05758	0.285068	0.133226	GADD45GI	Large ribos	G45IP_HUN	7	0.960874
48H	10MT/	Q9BW72	-0.0982	0.285114	0.133241	HIGD2A	HIG1 doma	HIG2A_HUI	3	0.934195
48H	10MT/	Q8N5C8	-0.06863	0.285178	0.133259	TAB3	TGF-beta-a	TAB3_HUM	2	0.953546
48H	10MT/	Q969H8	-0.1195	0.285215	0.133268	MYDGF	Myeloid-de	MYDGF_HU	7	0.920504
48H	10MT/	P11117	-0.04933	0.285317	0.133307	ACP2	Lysosomal	PPAL_HUM	11	0.966385
48H	10MT/	Q9P0W0	0.347299	0.285321	0.133307	IFNK	Interferon	IFNK_HUMA	1	1.272177
24H	10MT/	P22694	0.078352	0.285397	0.133334	PRKACB	cAMP-depe	KAPCB_HUI	12	1.055811
48H	50MT/	Q99797	-0.06079	0.285511	0.13338	MIPEP	Mitochond	MIPEP_HUI	20	0.958736
24H	50MT/	Q9Y3I0	-0.06568	0.285596	0.133413	RTCB	RNA-splicin	RTCB_HUM	24	0.955495
24H	10MT/	Q9UJX5	0.03674	0.285618	0.133419	ANAPC4	Anaphase-p	APC4_HUM	18	1.025793
48H	50MT/	P16333	0.024116	0.285639	0.133422	NCK1	SH2/SH3 ac	NCK1_HUN	8	1.016857
48H	50MT/	Q9UJA2	0.11305	0.285709	0.133445	CRLS1	Cardiolipin	CRLS1_HUN	1	1.081512
48H	10MT/	Q92771	0.095828	0.285784	0.133478	DDX12P	Putative AT	DDX12_HU	8	1.068679
24H	50MT/	Q04637	0.001915	0.285794	0.133481	EIF4G1	Eukaryotic	IF4G1_HUN	55	1.001329
48H	50MT/	P48739	-0.04025	0.285847	0.133497	PITPNB	Phosphatic	PIPNB_HUN	21	0.972483
24H	10MT/	Q99417	-0.41092	0.285847	0.133497	MYCBP	c-Myc-bind	MYCBP_HU	1	0.752145
48H	50MT/	Q8WVE0	0.046071	0.285876	0.133509	EEF1AKMT1	EEF1A lysin	EFMT1_HUI	1	1.032449
48H	50MT/	O15550	-0.38375	0.28591	0.13352	KDM6A	Lysine-spec	KDM6A_HU	2	0.766443
48H	10MT/	O60583	-0.02893	0.285914	0.13352	CCNT2	Cyclin-T2	CCNT2_HUI	2	0.980147
48H	50MT/	P78563	0.819388	0.285939	0.133528	ADARB1	Double-stra	RED1_HUM	1	1.764657
24H	50MT/	P02538	-1.40822	0.28613	0.133597	KRT6A	Keratin, typ	K2C6A_HUI	2	0.376777
48H	50MT/	Q658P3	-0.1886	0.286129	0.133597	STEAP3	Metallored	STEA3_HUN	5	0.877458
48H	50MT/	Q8WTS6	-0.1982	0.28616	0.13361	SETD7	Histone-lys	SETD7_HUN	7	0.871635
48H	10MT/	Q9BRK4	-0.17832	0.286211	0.133632	LZTS2	Leucine zip	LZTS2_HUN	6	0.883734
48H	10MT/	Q17RS7	0.011764	0.286238	0.133639	GEN1	Flap endon	GEN_HUMA	3	1.008187
48H	10MT/	Q9ULM3	-0.10139	0.286313	0.13367	YEATS2	YEATS dom	YETS2_HUN	9	0.932137
24H	50MT/	Q8IX01	0.002371	0.286328	0.133673	SUGP2	SURP and G	SUGP2_HU	30	1.001645
48H	10MT/	Q6KB66	-0.11606	0.286378	0.133689	KRT80	Keratin, typ	K2C80_HUI	5	0.922703
48H	10MT/	P49754	-0.11072	0.286412	0.1337	VPS41	Vacuolar pr	VPS41_HUI	11	0.926128
24H	50MT/	Q8TBB5	0.03393	0.286423	0.133702	KLHDC4	Kelch doma	KLDC4_HUI	11	1.023797
48H	10MT/	Q9BT67	0.002043	0.286438	0.133707	NDFIP1	NEDD4 fam	NFIP1_HUN	1	1.001417

24H 50MTA	O95873	0.217349	0.286451	0.133711	C6orf47	Uncharacte	CF047_HUI	1	1.162595
24H 10MTA	P18858	-0.01757	0.28646	0.133714	LIG1	DNA ligase	DNLI1_HUN	11	0.987898
48H 10MTA	Q9NYJ8	0.069748	0.286483	0.13372	TAB2	TGF-beta-ac	TAB2_HUM	5	1.049534
48H 50MTA	O75251	0.144052	0.286552	0.133734	NDUFS7	NADH dehy	NDUS7_HU	5	1.105004
24H 50MTA	Q9NVG8	-0.18423	0.286592	0.133749	TBC1D13	TBC1 doma	TBC13_HUI	15	0.880117
24H 50MTA	O95832	0.064291	0.286608	0.133754	CLDN1	Claudin-1	CLD1_HUM	5	1.045571
48H 50MTA	O14920	0.043345	0.286671	0.133781	IKBKB	Inhibitor of	IKKB_HUMA	10	1.0305
24H 50MTA	Q9BUF7	-1.08426	0.286714	0.133797	CRB3	Protein cru	CRUM3_HU	1	0.471635
48H 50MTA	Q5VWZ2	0.170044	0.286744	0.133805	LYPLAL1	Lysophospl	LYPL1_HUN	6	1.125093
24H 50MTA	O14817	0.277902	0.286792	0.133823	TSPAN4	Tetraspanir	TSN4_HUM	2	1.21243
48H 10MTA	Q06136	-0.11779	0.286805	0.133828	KDSR	3-ketodihy	KDSR_HUM	5	0.921599
48H 50MTA	Q15005	0.016898	0.286886	0.133859	SPCS2	Signal pept	SPCS2_HUN	10	1.011782
48H 50MTA	Q53T59	-0.00737	0.286895	0.133861	HS1BP3	HCLS1-binc	H1BP3_HU	17	0.994903
48H 50MTA	Q9HD34	-0.17373	0.286903	0.133863	LYRM4	LYR motif-c	LYRM4_HU	4	0.886549
24H 50MTA	Q9Y3B7	0.009652	0.286906	0.133863	MRPL11	Large ribos	RM11_HUN	10	1.006713
24H 50MTA	P02768	-0.65825	0.286917	0.133865	ALB	Albumin	ALBU_HUM	22	0.633648
48H 50MTA	Q8NBS9	-0.05249	0.286963	0.133883	TXNDC5	Thioredoxi	TXND5_HU	21	0.964273
24H 50MTA	Q8TCF1	0.055892	0.287017	0.133905	ZFAND1	AN1-type zi	ZFAN1_HUI	7	1.039501
48H 10MTA	Q07960	0.011194	0.287037	0.133912	ARHGAP1	Rho GTPase	RHG01_HU	20	1.007789
24H 10MTA	Q9Y5B8	0.073163	0.28705	0.133917	NME7	Nucleoside	NDK7_HUN	8	1.05202
24H 10MTA	O14656	0.079189	0.287065	0.133922	TOR1A	Torsin-1A	TOR1A_HUI	11	1.056424
24H 50MTA	P21953	-0.02212	0.287135	0.133948	BCKDHB	2-oxoisoval	ODBB_HUN	8	0.984787
24H 10MTA	Q9P2S5	0.224329	0.287167	0.133959	WRAP73	WD repeat-	WRP73_HU	5	1.168234
48H 50MTA	Q00577	0.025122	0.28718	0.13396	PURA	Transcripti	PURA_HUM	5	1.017566
24H 50MTA	P14621	-0.31162	0.287189	0.133963	ACYP2	Acylphosph	ACYP2_HUI	2	0.805737
24H 50MTA	O95136	0.707941	0.287263	0.133989	S1PR2	Sphingosin	S1PR2_HUI	1	1.633472
24H 50MTA	P42356	0.059685	0.287274	0.133989	PI4KA	Phosphatic	PI4KA_HUN	26	1.042238
48H 50MTA	P62891;Q5	0.078677	0.287341	0.134015	RPL39;RPL	Large ribos	RL39_HUM	2	1.056049
24H 10MTA	O14523	0.118901	0.287369	0.134025	C2CD2L	Phospholip	C2C2L_HUI	5	1.085907
48H 10MTA	Q9UGU0	-0.07169	0.287423	0.134042	TCF20	Transcripti	TCF20_HUN	8	0.951524
24H 50MTA	Q9Y490	-0.06492	0.287424	0.134042	TLN1	Talin-1	TLN1_HUM	110	0.955995
48H 50MTA	Q9H078	-0.10421	0.287429	0.134043	CLPB	Mitochond	CLPB_HUM	23	0.930313
48H 50MTA	Q96F86	-0.15603	0.287529	0.134086	EDC3	Enhancer o	EDC3_HUM	17	0.897493
48H 10MTA	Q9BQG0	-0.03187	0.287558	0.134098	MYBBP1A	Myb-bindir	MBB1A_HU	48	0.978152
24H 50MTA	P49840	-0.00411	0.287562	0.134098	GSK3A	Glycogen sy	GSK3A_HUI	8	0.997155
48H 10MTA	Q9UKL0	-0.01007	0.287571	0.134098	RCOR1	REST corepi	RCOR1_HU	9	0.993043
24H 50MTA	P51812	-0.02589	0.287583	0.134099	RPS6KA3	Ribosomal	KS6A3_HUI	34	0.982213
48H 10MTA	Q86UX6	0.090158	0.287647	0.134124	STK32C	Serine/thre	ST32C_HUN	5	1.064487
24H 50MTA	Q9UM82	0.248679	0.287722	0.134156	SPATA2	Spermatog	SPAT2_HUN	1	1.188119
48H 10MTA	P30876	-0.03466	0.287763	0.134172	POLR2B	DNA-direct	RPB2_HUM	41	0.976264
48H 10MTA	Q9UQ80	0.001397	0.287792	0.134182	PA2G4	Proliferatic	PA2G4_HU	23	1.000969
24H 10MTA	Q9UBI1	-0.18346	0.287814	0.134187	COMMD3	COMM don	COMD3_HU	7	0.880586
24H 50MTA	Q5NDL2	0.083666	0.287825	0.13419	EOGT	EGF domair	EOGT_HUM	8	1.059708
48H 50MTA	Q5T5U3	0.000728	0.28783	0.134191	ARHGAP21	Rho GTPase	RHG21_HU	8	1.000505
24H 50MTA	O94903	-0.05575	0.28793	0.134228	PLPBP	Pyridoxal p	PLPHP_HUI	11	0.962097
48H 50MTA	Q15645	-0.05768	0.287935	0.134228	TRIP13	Pachytene	PCH2_HUM	20	0.960811
48H 10MTA	Q09028	0.003719	0.287981	0.134248	RBBP4	Histone-bir	RBBP4_HUI	15	1.002581
24H 10MTA	O14818	-0.01657	0.288171	0.134323	PSMA7	Proteasom	PSA7_HUM	15	0.988582
48H 50MTA	Q9H4L5	-0.01521	0.288184	0.134325	OSBPL3	Oxysterol-b	OSBL3_HUI	20	0.989511
24H 10MTA	Q9NWX6	-0.03787	0.288244	0.134347	THG1L	Probable tF	THG1_HUM	5	0.974093
48H 10MTA	Q96BD5	0.30561	0.288262	0.134352	PHF21A	PHD finger	PF21A_HUI	5	1.235941
24H 10MTA	P27707	0.023548	0.288285	0.13436	DCK	Deoxycytid	DCK_HUMA	11	1.016456

48H	10MT/	Q9Y5B8	-0.07484	0.288292	0.13436	NME7	Nucleoside	NDK7_HUM	8	0.949444
24H	10MT/	Q9BWU0	-0.08596	0.288315	0.134368	SLC4A1AP	Kanadaptir	NADAP_HU	18	0.942157
24H	10MT/	A7KAX9	0.298969	0.288362	0.134383	ARHGAP32	Rho GTPase	RHG32_HU	3	1.230265
48H	10MT/	Q6N063	0.138227	0.288424	0.134403	OGFOD2	2-oxoglutai	OGFD2_HU	3	1.100552
24H	10MT/	Q8WVI0	0.150459	0.288449	0.134413	UQCC5	Ubiquinol-c	UQCC5_HU	1	1.109923
48H	10MT/	Q9H3Z4	0.084603	0.288469	0.134419	DNAJC5	DnaJ homo	DNJC5_HUI	4	1.060396
48H	10MT/	Q5JTD0	-0.01594	0.288501	0.134426	TJAP1	Tight juncti	TJAP1_HUM	9	0.989014
24H	50MT/	Q02040	0.086621	0.288676	0.134476	AKAP17A	A-kinase an	AK17A_HUI	12	1.06188
24H	10MT/	Q96K49	0.156269	0.288673	0.134476	TMEM87B	Transmemb	TM87B_HU	3	1.114401
48H	10MT/	P08397	-0.03294	0.288686	0.134478	HMBS	Porphobilii	HEM3_HUM	17	0.977423
48H	50MT/	O94886	0.036165	0.28873	0.134493	TMEM63A	CSC1-like p	CSCL1_HUM	2	1.025384
24H	10MT/	Q16774	-0.09226	0.288743	0.134498	GUK1	Guanylate I	KGUA_HUM	8	0.938051
48H	50MT/	P61006	-0.12492	0.288756	0.134502	RAB8A	Ras-related	RAB8A_HUI	5	0.917052
24H	10MT/	O14545	0.081054	0.288773	0.134508	TRAFD1	TRAF-type z	TRAD1_HUI	9	1.05779
48H	10MT/	Q9BX74	-0.47973	0.288814	0.134521	TM2D1	TM2 domai	TM2D1_HU	1	0.717114
24H	50MT/	Q9Y2D4	-0.16894	0.288859	0.134538	EXOC6B	Exocyst cor	EXC6B_HUI	12	0.889496
24H	10MT/	P42677	0.046589	0.288933	0.134566	RPS27	Small ribos	RS27_HUM	2	1.03282
24H	10MT/	Q6UX01;Q8	0.11143	0.288965	0.134576	LMBR1L;LM	Protein LM	LMBRL_HU	1	1.080298
48H	50MT/	Q9H4H8	-0.27576	0.289023	0.134594	FAM83D	Protein FAI	FA83D_HUI	12	0.826018
48H	10MT/	Q8TE77	0.12398	0.289067	0.134609	SSH3	Protein pho	SSH3_HUM	8	1.089737
48H	10MT/	Q96IK1	-0.01931	0.289127	0.134631	BOD1	Biorientatio	BOD1_HUM	4	0.986704
24H	10MT/	Q86WW8	-0.04812	0.289222	0.134668	COA5	Cytochrom	COA5_HUM	2	0.967199
48H	10MT/	Q96PZ0	-0.02781	0.289337	0.13471	PUS7	Pseudourid	PUS7_HUM	28	0.980911
48H	50MT/	P54098	0.04632	0.289353	0.134716	POLG	DNA polym	DPOG1_HU	17	1.032628
24H	10MT/	O00541	-0.00446	0.289378	0.134725	PES1	Pescadillo I	PESC_HUM	21	0.996916
24H	50MT/	Q969L4	-0.33141	0.289383	0.134726	LSM10	U7 snRNA-a	LSM10_HU	1	0.794759
24H	50MT/	Q15750	-0.20832	0.289416	0.134736	TAB1	TGF-beta-ac	TAB1_HUM	17	0.865546
24H	10MT/	Q16763	0.073232	0.289414	0.134736	UBE2S	Ubiquitin-c	UBE2S_HUI	7	1.052071
24H	10MT/	Q16222	-0.12773	0.28947	0.134756	UAP1	UDP-N-acet	UAP1_HUM	9	0.91527
48H	10MT/	Q969R5	-0.0126	0.289493	0.134762	L3MBTL2	Lethal(3)ma	LMBL2_HU	3	0.991304
24H	50MT/	Q96QD5	0.018927	0.289546	0.13478	DEPDC7	DEP domai	DEPD7_HU	3	1.013206
24H	10MT/	Q8IX03	0.143445	0.289575	0.134787	WWC1	Protein KIB	KIBRA_HUM	6	1.10454
48H	50MT/	Q8WWB7	-0.12929	0.289632	0.13481	GLMP	Glycosylate	GLMP_HUM	2	0.914282
24H	10MT/	O75815	-0.17535	0.289661	0.134817	BCAR3	Breast canc	BCAR3_HUI	11	0.885553
48H	10MT/	Q9BY76	-0.34295	0.289702	0.13483	ANGPTL4	Angiopoiet	ANGL4_HU	1	0.788427
48H	10MT/	Q969T3	-0.15892	0.289766	0.134855	SNX21	Sorting nex	SNX21_HUI	4	0.895696
48H	10MT/	Q02218	-0.01217	0.289828	0.134882	OGDH	2-oxoglutai	ODO1_HUM	41	0.991597
48H	50MT/	P62877	-0.08466	0.289939	0.134929	RBX1	E3 ubiquiti	RBX1_HUM	2	0.943004
48H	50MT/	P42126	0.018619	0.289952	0.134933	ECI1	Enoyl-CoA c	ECI1_HUM/	9	1.01299
24H	50MT/	Q12981	0.133658	0.290017	0.134958	BNIP1	Vesicle trar	SEC20_HUM	9	1.097072
48H	10MT/	P19793	-0.15379	0.290047	0.134971	RXRA	Retinoic ac	RXRA_HUM	6	0.898887
24H	10MT/	A1L0T0	-0.00856	0.290068	0.134977	ILVBL	2-hydroxya	HACL2_HUI	22	0.994085
48H	50MT/	Q96NW4	0.917383	0.290174	0.135016	ANKRD27	Ankyrin rep	ANR27_HU	2	1.888686
24H	10MT/	P56282	0.073997	0.290241	0.135045	POLE2	DNA polym	DPOE2_HU	7	1.052629
24H	50MT/	Q15014	0.01839	0.290286	0.135061	MORF4L2	Mortality fa	MO4L2_HU	8	1.012829
24H	50MT/	P62491	0.008112	0.290297	0.135063	RAB11A	Ras-related	RB11A_HUI	13	1.005639
48H	10MT/	Q96BT7	-0.31522	0.290422	0.135107	ALKBH8	Alkylated D	ALKB8_HUI	7	0.803727
48H	10MT/	Q9BPY3	0.045767	0.290428	0.135108	FAM118B	Protein FAI	F118B_HUI	6	1.032232
48H	10MT/	P27797	-0.02546	0.290595	0.13517	CALR	Calreticulir	CALR_HUM	21	0.982504
48H	50MT/	P18887	0.00817	0.290651	0.135194	XRCC1	DNA repair	XRCC1_HUI	11	1.005679
48H	50MT/	Q13188	0.135269	0.290667	0.135199	STK3	Serine/thre	STK3_HUM	7	1.098297
24H	10MT/	Q9BQ04	-0.02611	0.290795	0.13525	RBM4B	RNA-bindir	RBM4B_HU	15	0.982066

24H 10MT/	Q9P0M2	-0.58865	0.290797	0.13525	AKAP7	A-kinase an	AKA7G_HU	3	0.664965
48H 10MT/	P49902	-0.11336	0.290815	0.135255	NT5C2	Cytosolic p	5NTC_HUM	26	0.92443
24H 50MT/	Q9NS86	-0.05714	0.290846	0.135267	LANCL2	LanC-like p	LANC2_HU	14	0.96117
24H 10MT/	Q9UBV7	0.062752	0.290863	0.135272	B4GALT7	Beta-1,4-ga	B4GT7_HU	3	1.044456
24H 10MT/	P09668	-0.22903	0.290911	0.135289	CTSH	Pro-cathep	CATH_HUM	2	0.853207
48H 10MT/	Q8TF46	0.033027	0.291034	0.135341	DIS3L	DIS3-like ex	DI3L1_HUM	7	1.023157
48H 50MT/	P41223	-0.1214	0.291051	0.135346	BUD31	Protein BU	BUD31_HU	8	0.919296
24H 50MT/	Q96A73	-0.03221	0.291072	0.135354	KIAA1191	Putative m	P33MX_HU	3	0.977921
48H 50MT/	Q13596	-0.03462	0.291088	0.13536	SNX1	Sorting nex	SNX1_HUM	17	0.976292
24H 50MT/	Q92664	-0.32666	0.291111	0.135367	GTF3A	Transcripti	TF3A_HUM	1	0.79738
24H 10MT/	Q8IVF7	-0.06539	0.291173	0.135386	FMNL3	Formin-like	FMNL3_HU	1	0.955688
48H 50MT/	Q9NZN5	-0.00039	0.291229	0.135406	ARHGEF12	Rho guanin	ARHGC_HU	37	0.99973
24H 10MT/	Q9Y5T4	0.056798	0.291246	0.135409	DNAJC15	DnaJ homo	DJC15_HU	3	1.040155
24H 10MT/	Q9UKK9	-0.06211	0.291264	0.135414	NUDT5	ADP-sugar	NUDT5_HU	13	0.957865
48H 10MT/	P36873	0.002229	0.291308	0.135431	PPP1CC	Serine/thre	PP1G_HUM	17	1.001546
24H 10MT/	Q9BV23	-0.27618	0.291361	0.135449	ABHD6	Monoacylg	ABHD6_HU	5	0.825775
48H 50MT/	Q9Y2X3	-0.08006	0.291362	0.135449	NOP58	Nucleolar p	NOP58_HU	24	0.946017
24H 10MT/	Q12846	-0.00773	0.291434	0.135481	STX4	Syntaxin-4	STX4_HUM	11	0.994655
24H 10MT/	Q9BZG1	0.052818	0.291486	0.135498	RAB34	Ras-related	RAB34_HU	9	1.037289
48H 10MT/	Q8TED0	-0.13144	0.291522	0.135512	UTP15	U3 small nu	UTP15_HU	25	0.91292
48H 10MT/	Q8NI35	0.478801	0.291569	0.135528	PATJ	InaD-like p	INADL_HUM	2	1.393585
24H 50MT/	P30291	0.138368	0.291584	0.13553	WEE1	Wee1-like p	WEE1_HUM	5	1.100659
24H 50MT/	O14578	-0.11981	0.291599	0.135535	CIT	Citron Rho-	CTRO_HUM	26	0.920311
24H 10MT/	O43432	-0.06038	0.29161	0.135536	EIF4G3	Eukaryotic	IF4G3_HUM	43	0.959014
24H 10MT/	O00220	0.242412	0.291617	0.135537	TNFRSF10A	Tumor necr	TR10A_HU	3	1.182969
48H 10MT/	Q96RT1	0.009154	0.291621	0.135537	ERBIN	Erbin	ERBIN_HUM	16	1.006365
24H 10MT/	Q9HA65	-0.11557	0.291642	0.135545	TBC1D17	TBC1 doma	TBC17_HU	8	0.923015
24H 50MT/	Q13620	0.013061	0.291681	0.135559	CUL4B	Cullin-4B	CUL4B_HU	42	1.009095
24H 10MT/	P07711	0.102893	0.291687	0.135559	CTSL	Procatheps	CATL1_HUM	8	1.073925
24H 10MT/	Q13042	0.015423	0.291705	0.135564	CDC16	Cell divisio	CDC16_HU	23	1.010748
24H 10MT/	P04066	-0.02077	0.291712	0.135565	FUCA1	Tissue alph	FUCO_HUM	8	0.985707
48H 50MT/	O95429	0.028983	0.291735	0.135573	BAG4	BAG family	BAG4_HUM	4	1.020293
48H 50MT/	Q8IWB9	0.053164	0.291755	0.135581	TEX2	Testis-expr	TEX2_HUM	12	1.037538
24H 10MT/	Q86TG7	-0.15313	0.291829	0.135604	PEG10	Retrotrans	PEG10_HU	6	0.899294
48H 10MT/	Q9UJA2	0.048789	0.291832	0.135604	CRLS1	Cardiolipin	CRLS1_HUM	1	1.034397
24H 50MT/	Q14974	-0.06405	0.291903	0.135631	KPNB1	Importin su	IMB1_HUM	39	0.956575
24H 10MT/	Q9P107	-0.37102	0.291944	0.135646	GMIP	GEM-intera	GMIP_HUM	2	0.773235
48H 50MT/	P28330	0.24923	0.292009	0.135671	ACADL	Long-chain	ACADL_HU	4	1.188573
48H 50MT/	P29083	0.052992	0.292055	0.135688	GTF2E1	General tra	T2EA_HUM	11	1.037414
48H 10MT/	Q14444	-0.09256	0.292077	0.135694	CAPRIN1	Caprin-1	CAPR1_HU	13	0.937856
24H 10MT/	Q9NTG7	-0.10558	0.292098	0.135699	SIRT3	NAD-depen	SIR3_HUM	6	0.929428
24H 10MT/	Q9P2Y5	0.261018	0.292103	0.1357	UVRAG	UV radiatio	UVRAG_HU	7	1.198324
24H 50MT/	Q8NBN7	-0.0115	0.292117	0.135705	RDH13	Retinol deh	RDH13_HU	8	0.99206
48H 10MT/	P02794	-0.26132	0.292161	0.135718	FTH1	Ferritin hea	FRIH_HUM	1	0.834327
48H 50MT/	P10606	-0.13793	0.292194	0.13573	COX5B	Cytochrom	COX5B_HU	6	0.908824
24H 10MT/	Q00536	0.159484	0.292214	0.135736	CDK16	Cyclin-depe	CDK16_HU	7	1.116887
48H 10MT/	Q15653	0.136646	0.292228	0.135738	NFKBIB	NF-kappa-B	IKBB_HUM	4	1.099347
48H 10MT/	Q4VNC0	1.358488	0.292235	0.135739	ATP13A5	Probable ca	AT135_HU	1	2.564163
48H 50MT/	Q14642	-0.02924	0.292305	0.135767	INPP5A	Inositol pol	I5P1_HUM	6	0.979938
24H 10MT/	Q14C86	0.000597	0.292302	0.135767	GAPVD1	GTPase-acti	GAPD1_HU	39	1.000414
48H 50MT/	Q9UNH6	-0.0558	0.292333	0.135774	SNX7	Sorting nex	SNX7_HUM	14	0.962059
48H 50MT/	Q9H089	-0.14957	0.292405	0.135794	LSG1	Large subu	LSG1_HUM	18	0.901517

48H 50MT/	Q9P0M2	0.358686	0.292419	0.135796	AKAP7	A-kinase an	AKA7G_HU	3	1.282257
24H 10MT/	Q9H0U4	-0.0155	0.2925	0.135828	RAB1B	Ras-related	RAB1B_HU	9	0.989311
24H 50MT/	Q5JTJ3	0.245923	0.29253	0.13584	COA6	Cytochrom	COA6_HUM	4	1.185851
48H 50MT/	O43822	-0.03653	0.29256	0.135849	CFAP410	Cilia- and fl	CF410_HU	1	0.974996
48H 10MT/	A1L3X0	-0.05847	0.292588	0.135857	ELOVL7	Very long cl	ELOV7_HU	1	0.960283
48H 10MT/	Q96S99	0.15323	0.292594	0.135858	PLEKHF1	Pleckstrin h	PKHF1_HU	3	1.112056
24H 50MT/	Q5TGZ0	-0.01578	0.292614	0.135862	MICOS10	MICOS com	MIC10_HU	2	0.989123
24H 10MT/	P49638	-0.16774	0.292637	0.135871	TTPA	Alpha-toco	TTPA_HUM	5	0.890238
48H 50MT/	Q9UL15	-0.30599	0.292653	0.135876	BAG5	BAG family	BAG5_HUM	10	0.808886
48H 10MT/	Q9NQ84	-0.08482	0.292772	0.13592	GPRC5C	G-protein c	GPC5C_HU	7	0.942902
48H 10MT/	Q69YL0	-0.16826	0.292803	0.135931	NCBP2AS2	Protein NC	NCAS2_HU	3	0.889916
48H 50MT/	Q9UID3	0.004266	0.292802	0.135931	VPS51	Vacuolar pr	VPS51_HU	23	1.002961
48H 10MT/	Q6UX04	0.083059	0.292902	0.13597	CWC27	Spliceosom	CWC27_HU	6	1.059262
48H 50MT/	Q7Z6B7	-0.0809	0.293056	0.13603	SRGAP1	SLIT-ROBO	SRGP1_HU	13	0.945469
48H 10MT/	Q8N567	-0.1055	0.293107	0.136048	ZCCHC9	Zinc finger	ZCHC9_HU	3	0.929481
24H 10MT/	P55157	-0.03096	0.293121	0.136053	MTTP	Microsoma	MTP_HUM/	36	0.978768
24H 50MT/	Q8N584	0.036828	0.293142	0.136059	TTC39C	Tetratricop	TT39C_HU	18	1.025856
24H 10MT/	Q14320	-0.08418	0.293155	0.136064	FAM50A	Protein FAI	FA50A_HU	12	0.943324
48H 10MT/	P41236	0.005855	0.293238	0.136098	PPP1R2	Protein ph	IPP2_HUM/	9	1.004067
24H 10MT/	Q13535	-0.03389	0.293257	0.136104	ATR	Serine/thre	ATR_HUMA	28	0.976785
24H 10MT/	Q9P209	-0.06049	0.293269	0.136107	CEP72	Centrosom	CEP72_HU	8	0.958936
48H 10MT/	Q96F24	0.169715	0.293287	0.136113	NRBF2	Nuclear rec	NRBF2_HU	4	1.124836
48H 10MT/	Q15172	0.010819	0.293301	0.136118	PPP2R5A	Serine/thre	2A5A_HUM	14	1.007527
48H 50MT/	Q9NV56	-0.041	0.293315	0.136121	MRGBP	MRG/MORI	MRGBP_HU	3	0.971982
24H 10MT/	Q5T0N5	-0.06784	0.293337	0.136124	FNBP1L	Formin-bin	FBP1L_HU	17	0.954068
48H 50MT/	Q9Y3B2	-0.20434	0.293342	0.136125	EXOSC1	Exosome co	EXOS1_HU	6	0.867938
48H 50MT/	Q9NXR1	-0.04561	0.293346	0.136125	NDE1	Nuclear dis	NDE1_HUM	12	0.968878
48H 50MT/	Q8N1A0	0.215598	0.293387	0.13614	KRT222	Keratin-like	KT222_HU	3	1.161185
48H 50MT/	Q8WV99	0.024727	0.293487	0.136177	ZFAND2B	AN1-type zi	ZFN2B_HU	3	1.017287
24H 10MT/	Q99807	0.003737	0.293598	0.13622	COQ7	5-demetho	COQ7_HUM	4	1.002593
24H 50MT/	P43363	-0.0768	0.293672	0.136249	MAGEA10	Melanoma-	MAGAA_HU	5	0.948158
48H 50MT/	Q9NZ52	-0.00534	0.293703	0.136261	GGA3	ADP-ribosy	GGA3_HUM	13	0.996305
24H 50MT/	Q13506	-0.29248	0.29373	0.136271	NAB1	NGFI-A-bin	NAB1_HUM	5	0.816495
24H 10MT/	Q96CB8	0.078332	0.293733	0.136271	INTS12	Integrator	INT12_HUM	5	1.055797
24H 10MT/	O00233	-0.16052	0.293979	0.136369	PSMD9	26S protea	PSMD9_HU	7	0.894705
48H 10MT/	O60313	-0.02033	0.293995	0.136375	OPA1	Dynamin-li	OPA1_HUM	46	0.986004
24H 10MT/	P46934	-0.03514	0.294023	0.136386	NEDD4	E3 ubiquiti	NEDD4_HU	9	0.97594
48H 50MT/	P58004	0.153122	0.294065	0.136404	SESN2	Sestrin-2	SESN2_HUI	2	1.111973
48H 50MT/	Q8WV92	-0.12111	0.294072	0.136404	MITD1	MIT domai	MITD1_HU	2	0.919479
48H 10MT/	Q9H0A0	-0.03243	0.294096	0.136409	NAT10	RNA cytidir	NAT10_HU	48	0.977774
24H 10MT/	Q13098	-0.00584	0.294112	0.136412	GPS1	COP9 signa	CSN1_HUM	17	0.995957
48H 10MT/	Q8N3P4	-0.1238	0.294128	0.136414	VPS8	Vacuolar pr	VPS8_HUM	12	0.917767
48H 10MT/	Q5VZF2	0.088522	0.294154	0.136424	MBNL2	Muscleblin	MBNL2_HU	3	1.06328
48H 50MT/	Q5T8I3	0.103082	0.294207	0.136444	EEIG2	EEIG family	EEIG2_HUM	11	1.074065
24H 10MT/	O75688	-0.04415	0.294349	0.136494	PPM1B	Protein ph	PPM1B_HU	8	0.969863
48H 10MT/	P23396	-0.0098	0.294369	0.136498	RPS3	Small ribos	RS3_HUMA	19	0.993233
24H 10MT/	Q14008	-0.00017	0.294417	0.136514	CKAP5	Cytoskeleto	CKAP5_HU	75	0.999884
48H 10MT/	Q6ZT12	-0.04114	0.29447	0.136533	UBR3	E3 ubiquiti	UBR3_HUM	23	0.971886
24H 10MT/	Q9NXD2	-0.10676	0.294505	0.136547	MTMR10	Myotubula	MTMRA_HU	6	0.928674
24H 10MT/	Q14678	-0.06121	0.294512	0.136548	KANK1	KN motif ar	KANK1_HU	23	0.958459
48H 50MT/	O95793	-0.0226	0.294559	0.136562	STAU1	Double-stra	STAU1_HU	28	0.984457
48H 10MT/	Q8WV92	-0.08968	0.29457	0.136564	MITD1	MIT domai	MITD1_HU	2	0.939731

24H 50MT/	Q9NTX7	-0.65507	0.294751	0.136629	RNF146	E3 ubiquiti	RN146_HU	1	0.635046
24H 10MT/	P33316	0.033141	0.294934	0.136696	DUT	Deoxyuridi	DUT_HUM/	9	1.023238
24H 10MT/	Q7L5D6	0.02895	0.294931	0.136696	GET4	Golgi to ER	GET4_HUM	12	1.02027
24H 50MT/	Q9H7D7	0.012934	0.294935	0.136696	WDR26	WD repeat-	WDR26_HU	15	1.009006
24H 10MT/	Q14674	0.017458	0.295025	0.13673	ESPL1	Separin	ESPL1_HUN	15	1.012174
48H 50MT/	Q9BWT3	-0.54477	0.295032	0.136732	PAPOLG	Poly(A) pol	PAPOG_HU	6	0.6855
48H 10MT/	Q9P2K8	0.008081	0.295108	0.136762	EIF2AK4	eIF-2-alpha	E2AK4_HUI	22	1.005617
48H 10MT/	P19022	-0.01069	0.295165	0.136784	CDH2	Cadherin-2	CADH2_HU	15	0.992616
48H 10MT/	O14530	0.024908	0.295239	0.136809	TXNDC9	Thioredoxi	TXND9_HU	10	1.017415
24H 50MT/	P04350	0.076924	0.295295	0.136832	TUBB4A	Tubulin bet	TBB4A_HUI	3	1.054767
48H 10MT/	P04150	0.064649	0.295321	0.136842	NR3C1	Glucocorti	GCR_HUM/	16	1.045831
24H 10MT/	Q969S3	-0.09008	0.295397	0.13687	ZNF622	Cytoplasmi	ZN622_HUI	14	0.939473
24H 10MT/	Q13619	0.079795	0.295437	0.136884	CUL4A	Cullin-4A	CUL4A_HUI	28	1.056868
48H 50MT/	O95365	-0.03078	0.29547	0.136896	ZBTB7A	Zinc finger	ZBT7A_HUN	4	0.978892
48H 50MT/	Q92504	-0.52451	0.295477	0.136897	SLC39A7	Zinc transp	S39A7_HUI	2	0.695195
24H 10MT/	Q04917	-0.02208	0.295564	0.13693	YWHAH	14-3-3 prot	1433F_HUI	13	0.984813
48H 50MT/	O15047	-0.08855	0.295585	0.136933	SETD1A	Histone-lys	SET1A_HUN	12	0.940469
24H 50MT/	Q8N8A2	-0.29134	0.295607	0.136939	ANKRD44	Serine/thre	ANR44_HU	5	0.817143
48H 10MT/	Q969M7	0.511289	0.295616	0.136939	UBE2F	NEDD8-cor	UBE2F_HUI	3	1.425323
24H 50MT/	Q15257	-0.02413	0.295642	0.136949	PTPA	Serine/thre	PTPA_HUM	10	0.983412
48H 50MT/	Q9UM22	0.228665	0.295653	0.136951	EPDR1	Mammaliar	EPDR1_HUI	2	1.17175
48H 50MT/	Q9Y3P9	-0.01606	0.295682	0.136961	RABGAP1	Rab GTPase	RBGP1_HU	21	0.988927
24H 50MT/	Q9UPU7	-0.03676	0.295691	0.136962	TBC1D2B	TBC1 doma	TBD2B_HUI	6	0.974839
48H 10MT/	Q15020	-0.00221	0.295752	0.136987	SART3	Squamous	SART3_HUN	38	0.998467
24H 10MT/	Q9HCD5	0.031169	0.295766	0.13699	NCOA5	Nuclear rec	NCOA5_HU	19	1.02184
24H 10MT/	O60313	0.010845	0.295804	0.137006	OPA1	Dynamin-li	OPA1_HUN	46	1.007546
48H 10MT/	O75935	-0.03693	0.295849	0.137021	DCTN3	Dynactin su	DCTN3_HU	7	0.974726
24H 10MT/	Q96DI7	-0.08822	0.295872	0.13703	SNRNP40	U5 small nu	SNR40_HUI	17	0.940684
48H 50MT/	Q9UL03	-0.01592	0.295964	0.137068	INTS6	Integrator	INT6_HUM/	11	0.989024
48H 50MT/	P01137	-0.03753	0.296009	0.137085	TGFB1	Transformi	TGFB1_HUI	2	0.974323
24H 10MT/	O75529	-1.2324	0.296029	0.137089	TAF5L	TAF5-like R	TAF5L_HUN	1	0.42561
24H 10MT/	Q02338	-0.10264	0.296048	0.137096	BDH1	D-beta-hyd	BDH_HUM/	5	0.931328
48H 50MT/	Q9UQ80	-0.06846	0.296065	0.137102	PA2G4	Proliferatic	PA2G4_HU	23	0.953658
48H 10MT/	P40818	0.047732	0.296099	0.137116	USP8	Ubiquitin c	UBP8_HUN	22	1.033639
48H 50MT/	O15516	-0.24923	0.296116	0.137123	CLOCK	Circadian lc	CLOCK_HUI	1	0.841346
48H 50MT/	O00151	0.039372	0.296142	0.13713	PDLIM1	PDZ and LII	PDLI1_HUN	21	1.027667
48H 50MT/	P04844	-0.00107	0.296143	0.13713	RPN2	Dolichyl-di	RPN2_HUN	29	0.999257
24H 10MT/	Q9HC98	0.426796	0.296202	0.137149	NEK6	Serine/thre	NEK6_HUN	1	1.344245
24H 10MT/	Q9UBU9	0.013106	0.296257	0.137162	NXF1	Nuclear RN	NXF1_HUN	25	1.009126
24H 10MT/	P05543	0.558774	0.296346	0.137196	SERPINA7	Thyroxine-l	THBG_HUN	3	1.473017
24H 10MT/	O95571	-0.09226	0.296443	0.137231	ETHE1	Persulfide	ETHE1_HUN	10	0.938051
24H 10MT/	Q6IA86	0.086088	0.296457	0.137236	ELP2	Elongator c	ELP2_HUM	20	1.061488
48H 50MT/	Q6ZXV5	0.008685	0.296484	0.137245	TMTC3	Protein O-n	TMTC3_HU	17	1.006038
48H 10MT/	Q00013	-0.12989	0.296493	0.137245	MPP1	55 kDa eryt	EM55_HUN	10	0.913902
48H 50MT/	Q96HP4	-0.05327	0.296671	0.137323	OXNAD1	Oxidoreduc	OXND1_HU	4	0.963746
24H 10MT/	Q9UH17	0.170937	0.296755	0.137352	APOBEC3B	DNA dC->dl	ABC3B_HUI	9	1.125789
24H 50MT/	P06733	-0.0944	0.296874	0.137396	ENO1	Alpha-enol	ENOA_HUN	28	0.93666
48H 10MT/	P11177	-0.06199	0.296898	0.137398	PDHB	Pyruvate de	ODPB_HUN	17	0.957941
24H 10MT/	Q8TEB7	0.041257	0.296896	0.137398	RNF128	E3 ubiquiti	RN128_HU	1	1.02901
48H 10MT/	Q92947	-0.00754	0.296889	0.137398	GCDH	Glutaryl-Cc	GCDH_HUN	9	0.994785
48H 50MT/	P42898	-0.12451	0.296946	0.137414	MTHFR	Methylenet	MTHR_HUN	11	0.917313
24H 50MT/	Q5XPI4	-0.42467	0.296937	0.137414	RNF123	E3 ubiquiti	RN123_HU	7	0.745007

24H 10MT/	Q96S59	0.019214	0.297018	0.137439	RANBP9	Ran-bindin	RANB9_HU	15	1.013407
24H 50MT/	Q9P246	-0.00019	0.297039	0.137445	STIM2	Stromal int	STIM2_HUI	5	0.999872
24H 10MT/	P84098	-0.03621	0.297061	0.137451	RPL19	Large ribos	RL19_HUM	6	0.975216
48H 10MT/	Q56P03	-0.17961	0.297103	0.137468	EAPP	E2F-associa	EAPP_HUM	2	0.882939
48H 10MT/	Q9HBM0	-0.36615	0.297125	0.137476	VEZT	Vezatin	VEZA_HUM	3	0.775853
48H 10MT/	Q9H307	0.010179	0.29722	0.137513	PNN	Pinin	PININ_HUM	21	1.00708
48H 10MT/	Q9Y3B4	-0.09377	0.297223	0.137513	SF3B6	Splicing fac	SF3B6_HUI	6	0.937068
48H 50MT/	Q6VMQ6	-0.15275	0.297249	0.137523	ATF7IP	Activating t	MCAF1_HU	7	0.899537
48H 50MT/	Q9BXJ1	-1.37994	0.297293	0.137542	C1QTNF1	Complemer	C1QT1_HUI	1	0.384235
48H 50MT/	Q9NX18	-0.30884	0.297314	0.13755	SDHAF2	Succinate d	SDHF2_HUI	3	0.807293
24H 10MT/	Q9P035	-0.041	0.297381	0.137577	HACD3	Very-long-c	HACD3_HU	11	0.971979
48H 50MT/	Q9H2A7	-0.39717	0.297408	0.137588	CXCL16	C-X-C motif	CXL16_HUI	2	0.759344
24H 50MT/	Q969S3	-0.10124	0.297533	0.137635	ZNF622	Cytoplasmi	ZN622_HUI	14	0.932233
24H 50MT/	P08243	-0.06523	0.297537	0.137636	ASNS	Asparagine	ASNS_HUM	26	0.955792
24H 10MT/	P78362	-0.0527	0.29762	0.137666	SRPK2	SRSF protei	SRPK2_HUI	16	0.964128
24H 10MT/	P01008	0.353334	0.297642	0.137672	SERPINC1	Antithroml	ANT3_HUM	6	1.27751
48H 10MT/	Q53GL7	0.04095	0.297659	0.137678	PARP10	Protein mo	PAR10_HUI	1	1.028791
48H 10MT/	Q9H2V7	-0.01181	0.297665	0.137679	SPNS1	Protein spi	SPNS1_HUI	8	0.991844
48H 10MT/	Q8NEL9	-0.02603	0.297738	0.137697	DDHD1	Phospholip	DDHD1_HU	4	0.982123
24H 10MT/	Q9H7E2	-0.0605	0.297736	0.137697	TDRD3	Tudor dom	TDRD3_HU	3	0.958932
24H 10MT/	P08243	-0.03261	0.297803	0.137722	ASNS	Asparagine	ASNS_HUM	26	0.97765
24H 50MT/	Q96S38	-0.40638	0.297812	0.137723	RPS6KC1	Ribosomal	KS6C1_HUI	4	0.754514
24H 10MT/	P53367	-0.05901	0.297968	0.137781	ARFIP1	Arfaptin-1	ARFP1_HUI	14	0.959925
24H 10MT/	Q9UBQ7	-0.03818	0.298012	0.137795	GRHPR	Glyoxylate	GRHPR_HU	14	0.973884
48H 10MT/	A0A0K0K1/	0.464631	0.298071	0.137817	TRBV6-5	T cell recep	TVB65_HUI	1	1.379964
48H 10MT/	Q9H4Z3	-0.02763	0.298097	0.137827	PCIF1	mRNA (2'-O	CAPAM_HU	7	0.981027
48H 50MT/	Q9NXS2	-0.11221	0.298104	0.137829	QPCTL	Glutaminyl	QPCTL_HUI	7	0.925171
48H 10MT/	Q96CM3	0.149598	0.298172	0.137855	RPUSD4	Pseudouric	RUSD4_HU	2	1.10926
24H 10MT/	Q14527	-0.02055	0.298194	0.13786	HLTF	Helicase-lik	HLTF_HUM	28	0.985855
48H 50MT/	Q9BY32	-0.10104	0.298207	0.137864	ITPA	Inosine trip	ITPA_HUM/	12	0.932359
24H 10MT/	P02533	-0.8877	0.298231	0.137872	KRT14	Keratin, typ	K1C14_HUI	33	0.540476
24H 10MT/	Q8N4C8	-0.26778	0.298322	0.137909	MINK1	Misshapen-	MINK1_HU	7	0.830596
24H 50MT/	P83916	-0.0784	0.29835	0.137918	CBX1	Chromobo:	CBX1_HUM	5	0.947106
24H 50MT/	Q9NX63	0.036946	0.298366	0.137924	CHCHD3	MICOS com	MIC19_HUI	10	1.02594
24H 10MT/	P19793	-0.34151	0.298423	0.137943	RXRA	Retinoic ac	RXRA_HUM	6	0.789212
48H 50MT/	Q9H1X3	-0.06233	0.298492	0.137969	DNAJC25	DnaJ homo	DJC25_HUI	3	0.957718
24H 10MT/	Q9H6R4	-0.00088	0.298503	0.137972	NOL6	Nucleolar p	NOL6_HUM	26	0.999391
24H 10MT/	P59998	0.052465	0.298536	0.137983	ARPC4	Actin-relate	ARPC4_HUI	8	1.037035
48H 10MT/	P84022	-0.01421	0.298555	0.13799	SMAD3	Mothers ag	SMAD3_HU	5	0.9902
48H 10MT/	Q9H2C2	0.261226	0.298561	0.137992	ARV1	Protein AR\	ARV1_HUM	1	1.198497
48H 10MT/	Q9UM54	-0.07705	0.29863	0.138015	MYO6	Unconvent	MYO6_HUM	56	0.947995
48H 50MT/	Q8WXF0	-0.42252	0.29864	0.138018	SRSF12	Serine/argi	SRS12_HUI	2	0.746118
48H 50MT/	P12270	-0.02938	0.29875	0.138056	TPR	Nucleoprot	TPR_HUMA	81	0.979844
48H 50MT/	O75306	-0.03283	0.298804	0.138078	NDUFS2	NADH dehy	NDUS2_HU	20	0.9775
24H 10MT/	Q13136	0.022675	0.298832	0.138087	PPFIA1	Liprin-alph	LIPA1_HUM	28	1.015841
24H 50MT/	Q15904	0.00369	0.298957	0.138133	ATP6AP1	V-type prot	VAS1_HUM	12	1.002561
24H 10MT/	Q68CQ7	0.000163	0.299123	0.138192	GLT8D1	Glycosyltra	GL8D1_HU	9	1.000113
48H 50MT/	Q16584	0.040368	0.299139	0.138196	MAP3K11	Mitogen-ac	M3K11_HU	2	1.028376
24H 10MT/	O95235	0.162861	0.299153	0.138201	KIF20A	Kinesin-like	KI20A_HUM	22	1.119505
48H 10MT/	Q6ZRS2	-0.00268	0.299216	0.138223	SRCAP	Helicase SR	SRCAP_HUI	17	0.998143
48H 10MT/	O60927	0.120141	0.29922	0.138224	PPP1R11	E3 ubiquiti	PP1RB_HUI	4	1.086841
48H 10MT/	O95858	0.207528	0.299342	0.138271	TSPAN15	Tetraspanir	TSN15_HUI	3	1.154708

24H 50MTA	Q92636	0.052358	0.299381	0.138281	NSMAF	Protein FAN	FAN_HUMA	4	1.036959
24H 10MTA	Q8NE01	-0.09673	0.299421	0.138297	CNNM3	Metal trans	CNNM3_HU	19	0.935152
24H 50MTA	P00491	0.026403	0.299556	0.138351	PNP	Purine nucl	PNPH_HUM	14	1.018469
24H 50MTA	P53816	-0.30967	0.299577	0.138358	PLAAT3	Phospholip	PLAT3_HUM	2	0.806826
24H 50MTA	P36955	-0.53389	0.299583	0.138359	SERPINF1	Pigment ep	PEDF_HUM	10	0.690689
48H 50MTA	P51530	-0.34501	0.299595	0.138362	DNA2	DNA replica	DNA2_HUM	18	0.7873
48H 50MTA	Q9NUU7	0.016535	0.299665	0.138388	DDX19A	ATP-depend	DD19A_HU	26	1.011527
24H 10MTA	P49368	0.008949	0.299752	0.138424	CCT3	T-complex	TCPG_HUM	39	1.006222
48H 10MTA	Q9NRH1	-0.14004	0.299784	0.138434	YAE1	Protein YAE	YAE1_HUM	2	0.907497
24H 10MTA	Q9Y5A7	0.183516	0.299965	0.138507	NUB1	NEDD8 ulti	NUB1_HUM	8	1.135648
24H 50MTA	Q9Y6W5	-0.05552	0.300006	0.138521	WASF2	Actin-bindi	WASF2_HU	9	0.96225
24H 50MTA	Q8WYH8	0.609105	0.300056	0.138535	ING5	Inhibitor of	ING5_HUM	2	1.525313
24H 10MTA	Q9ULC4	-0.05685	0.300126	0.138564	MCTS1	Malignant T	MCTS1_HU	11	0.961363
24H 50MTA	P05109	-1.52224	0.300136	0.138567	S100A8	Protein S10	S10A8_HUM	1	0.348144
48H 10MTA	Q8N4P2	0.263266	0.300226	0.138596	IFT70B	Intraflagella	IT70B_HUM	2	1.200192
48H 10MTA	Q8N697	-0.31319	0.30028	0.138618	SLC15A4	Solute carri	S15A4_HUM	2	0.804862
48H 10MTA	Q9UI15	0.090767	0.300296	0.138622	TAGLN3	Transgelin-3	TAGL3_HUM	1	1.064937
24H 10MTA	O00767	-0.05273	0.300328	0.138629	SCD	Stearoyl-Co	SCD_HUMA	6	0.96411
24H 50MTA	Q9BX63	0.251044	0.300357	0.138636	BRIP1	Fanconi an	FANCJ_HUM	1	1.190068
48H 10MTA	Q13315	0.014233	0.30037	0.13864	ATM	Serine-prot	ATM_HUMA	48	1.009914
24H 10MTA	Q8N9E0	0.840391	0.300376	0.138641	FAM133A	Protein FAM	F133A_HUM	2	1.790536
24H 50MTA	P68366	-0.12767	0.300408	0.138651	TUBA4A	Tubulin alp	TBA4A_HUM	4	0.915308
24H 10MTA	Q14596	-0.03896	0.300435	0.138662	NBR1	Next to BRC	NBR1_HUM	1	0.973354
48H 10MTA	Q9Y672	-0.23273	0.300459	0.13867	ALG6	Dolichyl py	ALG6_HUM	7	0.851024
48H 50MTA	P43897	-0.01304	0.300564	0.138711	TSFM	Elongation	EFTS_HUMA	12	0.991004
48H 10MTA	Q9GZY4	-0.33676	0.300567	0.138711	COA1	Cytochrom	COA1_HUM	6	0.791819
48H 50MTA	Q5TDH0	-0.06767	0.300608	0.138728	DDI2	Protein DD	DDI2_HUM	10	0.954178
48H 50MTA	Q9ULQ1	0.032913	0.300617	0.13873	TPCN1	Two pore ch	TPC1_HUM	9	1.023076
24H 10MTA	Q9BVT8	-0.08775	0.300627	0.138733	TMUB1	Transmemb	TMUB1_HU	3	0.940991
24H 10MTA	Q9P1Z2	-0.81951	0.300653	0.138742	CALCOCO1	Calcium-bin	CACO1_HU	2	0.566636
24H 10MTA	Q96BN2	0.063562	0.300771	0.138789	TADA1	Transcriptio	TADA1_HUM	2	1.045043
24H 50MTA	Q9UGR2	-0.17983	0.300787	0.138793	ZC3H7B	Zinc finger	Z3H7B_HUM	9	0.882807
24H 10MTA	Q8IWR0	-0.02628	0.300835	0.13881	ZC3H7A	Zinc finger	Z3H7A_HUM	14	0.981948
24H 10MTA	Q9ULJ8	-0.35476	0.300877	0.138824	PPP1R9A	Neurabin-1	NEB1_HUM	3	0.781998
24H 10MTA	Q9BQS8	0.058254	0.300903	0.138831	FYCO1	FYVE and co	FYCO1_HUM	27	1.041205
24H 10MTA	Q13049	0.076056	0.300939	0.138841	TRIM32	E3 ubiquiti	TRI32_HUM	11	1.054133
24H 10MTA	Q5HYI8	0.028868	0.301095	0.138899	RABL3	Rab-like pro	RABL3_HUM	6	1.020211
24H 50MTA	Q04695	-2.02455	0.301114	0.138906	KRT17	Keratin, typ	K1C17_HUM	15	0.245782
24H 50MTA	Q6NXR4	-0.1106	0.301188	0.138929	TTI2	TELO2-inter	TTI2_HUMA	9	0.9262
24H 50MTA	O60583	0.307527	0.301262	0.138953	CCNT2	Cyclin-T2	CCNT2_HUM	2	1.237585
48H 10MTA	Q14008	0.012564	0.301262	0.138953	CKAP5	Cytoskeleto	CKAP5_HUM	75	1.008747
24H 10MTA	P10398	-0.03264	0.301295	0.138964	ARAF	Serine/thre	ARAF_HUM	10	0.977631
24H 10MTA	Q9Y2D2	-0.13539	0.301345	0.138978	SLC35A3	UDP-N-acet	S35A3_HUM	2	0.910424
48H 10MTA	Q96FW1	-0.04809	0.301359	0.138982	OTUB1	Ubiquitin t	OTUB1_HU	14	0.967217
24H 50MTA	O95620	0.369841	0.301384	0.13899	DUS4L	tRNA-dihyd	DUS4L_HUM	1	1.29221
48H 10MTA	Q86W42	-0.08867	0.301478	0.139025	THOC6	THO compl	THOC6_HU	16	0.940388
24H 50MTA	Q9ULC5	0.04991	0.30152	0.139037	ACSL5	Long-chain	ACSL5_HUM	20	1.0352
48H 50MTA	P14672	-0.44095	0.301573	0.139055	SLC2A4	Solute carri	GLUT4_HUM	1	0.73665
24H 50MTA	P03886	-0.17123	0.301607	0.139065	MT-ND1	NADH-ubiq	NU1M_HUM	4	0.888086
48H 50MTA	P61106	0.007361	0.301611	0.139065	RAB14	Ras-related	RAB14_HU	13	1.005115
24H 10MTA	Q9NWS0	-0.07314	0.301618	0.139065	PIH1D1	PIH1 doma	PIHD1_HUM	9	0.950566
24H 50MTA	Q96BF6	0.207728	0.301749	0.139105	NACC2	Nucleus acc	NACC2_HU	3	1.154868

24H 10MT/	Q5VTE6	-0.77239	0.30181	0.139123	ANGEL2	Protein ang	ANGE2_HU	3	0.585447
24H 10MT/	Q8TDN6	0.029708	0.301801	0.139123	BRIX1	Ribosome b	BRX1_HUM	20	1.020805
48H 50MT/	P22455	0.001126	0.301881	0.139148	FGFR4	Fibroblast g	FGFR4_HUI	12	1.000781
24H 50MT/	Q96QK1	-0.01835	0.301985	0.139186	VPS35	Vacuolar pr	VPS35_HUI	32	0.987363
48H 10MT/	O43824	-0.05502	0.302053	0.139207	GTPBP6	Putative GT	GTPB6_HUI	2	0.962579
48H 50MT/	P17066	-0.29812	0.302129	0.139236	HSPA6	Heat shock	HSP76_HUI	2	0.813313
48H 10MT/	P22670	-0.10363	0.302161	0.139248	RFX1	MHC class I	RFX1_HUM	9	0.930689
24H 10MT/	P62136	-0.02625	0.302165	0.139248	PPP1CA	Serine/thre	PP1A_HUM	7	0.981968
24H 50MT/	Q9NQE9	-0.01022	0.302206	0.139265	HINT3	Adenosine 5	HINT3_HUI	7	0.992939
48H 50MT/	Q8TCG2	0.07613	0.302222	0.139269	PI4K2B	Phosphatid	P4K2B_HUI	9	1.054186
48H 50MT/	P80723	-0.15346	0.302248	0.139276	BASP1	Brain acid s	BASP1_HUI	7	0.89909
48H 10MT/	Q9Y2V2	0.002323	0.302282	0.139288	CARHSP1	Calcium-reg	CHSP1_HUI	7	1.001611
24H 10MT/	P78509	0.022902	0.302341	0.13931	RELN	Reelin	RELN_HUM	48	1.016001
24H 10MT/	P40855	0.534904	0.30237	0.139321	PEX19	Peroxisoma	PEX19_HUI	1	1.448845
48H 10MT/	Q96C34	0.20254	0.302453	0.139355	RUNDC1	RUN domai	RUND1_HU	1	1.150723
24H 50MT/	P49748	0.011842	0.30253	0.139386	ACADVL	Very long-c	ACADV_HU	31	1.008242
48H 50MT/	Q9UJX6	-0.11047	0.302534	0.139387	ANAPC2	Anaphase-p	ANC2_HUM	21	0.926289
24H 50MT/	Q01167	0.068719	0.302545	0.13939	FOXK2	Forkhead b	FOXK2_HUI	3	1.048785
24H 10MT/	P16403	-0.1796	0.302549	0.13939	H1-2	Histone H1	H12_HUMA	1	0.882947
24H 10MT/	Q15796	-0.10757	0.302583	0.1394	SMAD2	Mothers ag	SMAD2_HU	12	0.928153
48H 10MT/	P48200	0.025663	0.302651	0.13943	IREB2	Iron-respor	IREB2_HUM	27	1.017948
48H 50MT/	O75164	0.151475	0.302789	0.139473	KDM4A	Lysine-spec	KDM4A_HU	4	1.110704
24H 50MT/	P60468	0.063542	0.302775	0.139473	SEC61B	Protein trai	SC61B_HUI	3	1.045029
48H 10MT/	O94901	-0.04324	0.302808	0.13948	SUN1	SUN domai	SUN1_HUM	30	0.970473
24H 50MT/	Q9Y376	0.004476	0.302817	0.139482	CAB39	Calcium-bi	CAB39_HUI	13	1.003107
24H 10MT/	P50148	-0.10704	0.30285	0.139492	GNAQ	Guanine nu	GNAQ_HUM	2	0.928491
24H 50MT/	P00167	-0.09031	0.302866	0.139498	CYB5A	Cytochrom	CYB5_HUM	4	0.93932
48H 10MT/	O60422	0.013218	0.302897	0.139506	ONECUT3	One cut do	ONEC3_HU	3	1.009204
48H 50MT/	Q96G46	-0.1679	0.302909	0.139509	DUS3L	tRNA-dihyd	DUS3L_HUI	15	0.890136
48H 50MT/	Q16706	-0.07724	0.302917	0.139511	MAN2A1	Alpha-mani	MA2A1_HU	13	0.947871
24H 10MT/	Q8NFW8	0.061528	0.302932	0.139513	CMAS	N-acylneura	NEUA_HUM	14	1.04357
24H 50MT/	Q86YV5	-0.2879	0.303011	0.139545	PRAG1	Inactive tyr	PRAG1_HU	5	0.819092
24H 50MT/	O95260	-0.06898	0.30305	0.139558	ATE1	Arginyl-tRN	ATE1_HUM	12	0.953314
48H 10MT/	Q9P0L2	0.568782	0.303053	0.139558	MARK1	Serine/thre	MARK1_HU	1	1.483271
24H 10MT/	Q96RT7	-0.05983	0.303109	0.139578	TUBGCP6	Gamma-tul	GCP6_HUM	5	0.959374
24H 50MT/	Q969J3	-0.00883	0.303113	0.139579	BORCS5	BLOC-1-rela	BORC5_HU	5	0.993898
24H 50MT/	Q3B726	0.056322	0.303147	0.139592	POLR1F	DNA-direct	RPA43_HUI	5	1.039811
24H 10MT/	Q8IVS2	0.027068	0.303157	0.139595	MCAT	Malonyl-Co	FABD_HUM	13	1.01894
24H 10MT/	P40424	0.003539	0.303215	0.139619	PBX1	Pre-B-cell le	PBX1_HUM	3	1.002456
48H 10MT/	P51825	-0.05111	0.303221	0.139619	AFF1	AF4/FMR2 f	AFF1_HUM	3	0.965195
24H 50MT/	Q8N5P1	-0.10413	0.303221	0.139619	ZC3H8	Zinc finger	ZC3H8_HUI	4	0.930364
48H 50MT/	Q96QE3	-0.03884	0.303298	0.139648	ATAD5	ATPase fam	ATAD5_HUI	5	0.973436
24H 10MT/	P18583	0.074858	0.303362	0.139669	SON	Protein SOI	SON_HUMA	39	1.053257
24H 10MT/	Q9NRZ9	0.063303	0.303427	0.13969	HELLS	Lymphoid-s	HELLS_HUM	30	1.044855
48H 10MT/	Q6UWU4	-0.71711	0.303496	0.139715	C6orf89	Bombesin r	CF089_HUI	2	0.608313
24H 10MT/	Q3MIT2	0.180183	0.303545	0.139732	PUS10	tRNA pseud	PUS10_HUI	4	1.133028
24H 10MT/	P18077	-0.02642	0.303561	0.139738	RPL35A	Large ribos	RL35A_HUI	4	0.981856
24H 10MT/	Q68CP4	-0.01992	0.303687	0.13978	HGSNAT	Heparan-al	HGNAT_HU	4	0.986289
24H 50MT/	Q8NI37	0.023756	0.303716	0.139788	PPTC7	Protein pho	PPTC7_HUI	7	1.016603
48H 50MT/	P52657	-0.16075	0.303722	0.139789	GTF2A2	Transcripti	T2AG_HUM	2	0.894562
48H 50MT/	Q96MU7	0.027854	0.303737	0.139794	YTHDC1	YTH domair	YTDC1_HUI	7	1.019495
24H 10MT/	Q7L5Y9	0.057592	0.304001	0.139899	MAEA	E3 ubiquiti	MAEA_HUM	11	1.040727

24H 50MT/	O95674	-0.07041	0.304008	0.1399	CDS2	Phosphatic	CDS2_HUM	5	0.952369
24H 10MT/	Q49AN0	-0.14187	0.304019	0.139901	CRY2	Cryptochrc	CRY2_HUM	2	0.906343
48H 50MT/	Q96D53	-0.28174	0.30407	0.13992	COQ8B	Atypical kir	COQ8B_HU	7	0.822596
24H 50MT/	Q5T3F8	-0.06898	0.304156	0.139954	TMEM63B	CSC1-like p	CSCL2_HUI	8	0.953314
48H 50MT/	P02765	0.330107	0.304257	0.139992	AHSG	Alpha-2-HS	FETUA_HUI	5	1.257107
24H 50MT/	Q99733	-0.08084	0.304262	0.139992	NAP1L4	Nucleosom	NP1L4_HUI	14	0.945509
48H 10MT/	Q92785	-0.00107	0.304289	0.140001	DPF2	Zinc finger	REQU_HUM	14	0.999257
24H 10MT/	O15145	-0.07103	0.304305	0.140005	ARPC3	Actin-relate	ARPC3_HUI	8	0.951958
48H 50MT/	O15287	-0.31331	0.304326	0.140011	FANCG	Fanconi an	FANCG_HU	9	0.804796
24H 50MT/	Q5VVQ6	0.160893	0.304439	0.140053	YOD1	Ubiquitin t	OTU1_HUM	2	1.117979
24H 10MT/	Q9Y6X2	0.051216	0.304447	0.140055	PIAS3	E3 SUMO-p	PIAS3_HUM	1	1.036138
24H 10MT/	Q9NRM2	0.132848	0.304509	0.14008	ZNF277	Zinc finger	ZN277_HUI	3	1.096456
24H 10MT/	Q8IY17	-0.02385	0.304538	0.140088	PNPLA6	Patatin-like	PLPL6_HUI	22	0.983602
24H 10MT/	Q8N6M3	-0.29564	0.304579	0.140101	FITM2	Acyl-coenz	FITM2_HUI	5	0.814713
24H 50MT/	P49257	0.013774	0.304636	0.140124	LMAN1	Protein ER(	LMAN1_HU	19	1.009593
48H 50MT/	Q5MIZ7	0.013884	0.304743	0.140163	PPP4R3B	Serine/thre	P4R3B_HUI	18	1.00967
24H 10MT/	Q6ZW76	0.718647	0.304744	0.140163	ANKS3	Ankyrin rep	ANKS3_HUI	1	1.645638
48H 50MT/	Q92738	-0.26944	0.304747	0.140163	USP6NL	USP6 N-ter	US6NL_HUI	4	0.829643
48H 50MT/	Q9Y618	0.184439	0.304746	0.140163	NCOR2	Nuclear rec	NCOR2_HU	7	1.136375
24H 10MT/	Q8IVT2	0.207562	0.304816	0.14019	MISP	Mitotic int	MISP_HUM	8	1.154735
48H 10MT/	P27448	-0.10306	0.304827	0.140191	MARK3	MAP/micrc	MARK3_HU	16	0.931058
24H 10MT/	O60333	0.012329	0.304872	0.14021	KIF1B	Kinesin-like	KIF1B_HUM	29	1.008582
24H 10MT/	Q96GA7	-0.13206	0.304879	0.14021	SDSL	Serine dehy	SDSL_HUM	12	0.912527
48H 10MT/	P35527	0.113146	0.304921	0.140226	KRT9	Keratin, typ	K1C9_HUM	31	1.081584
48H 50MT/	Q13099	0.251354	0.304999	0.140258	IFT88	Intraflagell	IFT88_HUM	2	1.190323
48H 50MT/	Q96DT7	0.030123	0.305103	0.140299	ZBTB10	Zinc finger	ZBT10_HUI	6	1.021099
24H 10MT/	Q9NP97	-0.0559	0.305114	0.140301	DYNLRB1	Dynein ligh	DLRB1_HUI	5	0.961993
24H 10MT/	Q99961	-0.0211	0.305133	0.140306	SH3GL1	Endophilin	SH3G1_HU	12	0.985478
48H 10MT/	P52209	-0.02617	0.30516	0.140314	PGD	6-phospho	6PGD_HUM	25	0.982023
48H 10MT/	Q96CW5	-0.05561	0.30521	0.14033	TUBGCP3	Gamma-tul	GCP3_HUM	26	0.962191
24H 10MT/	Q9BRZ2	0.046262	0.305213	0.14033	TRIM56	E3 ubiquiti	TRI56_HUM	16	1.032586
48H 10MT/	Q8IZ13	-0.60239	0.30534	0.14038	FAM200C	Protein FAI	F200C_HUI	1	0.65866
24H 10MT/	Q9UKD1	-0.31092	0.305353	0.140383	GMEB2	Glucocorti	GMEB2_HU	3	0.80613
24H 50MT/	P05362	-0.06299	0.305371	0.140386	ICAM1	Intercellula	ICAM1_HUI	17	0.957278
48H 50MT/	P58557	-0.15541	0.305406	0.1404	YBEY	Endoribon	YBEY_HUM	2	0.897877
48H 50MT/	Q53FV1	0.690041	0.305447	0.140413	ORMDL2	ORM1-like	ORML2_HU	2	1.613329
24H 50MT/	Q8WXE0	-0.28792	0.305532	0.140444	CASKIN2	Caskin-2	CSKI2_HUM	7	0.819083
24H 10MT/	A5YKK6	0.005417	0.305543	0.140447	CNOT1	CCR4-NOT t	CNOT1_HU	73	1.003762
24H 10MT/	Q9BQ48	-0.34628	0.305577	0.140457	MRPL34	Large ribos	RM34_HUM	2	0.786612
24H 10MT/	Q9UBN7	0.058386	0.305597	0.140465	HDAC6	Histone de	HDAC6_HU	16	1.0413
24H 50MT/	P07951	-0.02543	0.305649	0.14048	TPM2	Tropomyos	TPM2_HUM	10	0.982527
24H 50MT/	Q8TAD8	0.229666	0.305713	0.140508	SNIP1	Smad nucle	SNIP1_HUM	5	1.172563
24H 10MT/	Q9NP72	-0.01439	0.305782	0.140536	RAB18	Ras-related	RAB18_HUI	12	0.990077
24H 10MT/	Q9H2H9	0.148466	0.305829	0.140554	SLC38A1	Sodium-co	S38A1_HUI	6	1.10839
48H 10MT/	Q9HAB8	-0.00634	0.305856	0.140561	PPCS	Phosphopa	PPCS_HUM	15	0.995615
48H 50MT/	O60244	-0.17017	0.305977	0.140612	MED14	Mediator o	MED14_HU	15	0.88874
48H 50MT/	Q9UKK6	-0.25314	0.305974	0.140612	NXT1	NTF2-relate	NXT1_HUM	3	0.839067
48H 10MT/	Q8N1N4	-0.02859	0.305983	0.140612	KRT78	Keratin, typ	K2C78_HUI	8	0.980375
24H 50MT/	P48426	-0.14536	0.306126	0.140673	PIP4K2A	Phosphatic	PI42A_HUM	8	0.904156
48H 50MT/	Q99996	0.04674	0.306139	0.140677	AKAP9	A-kinase an	AKAP9_HUI	32	1.032928
48H 50MT/	Q8WY22	-0.07512	0.306157	0.140684	BRI3BP	BRI3-bindir	BRI3B_HUM	3	0.949264
24H 10MT/	P02656	0.285287	0.306192	0.140696	APOC3	Apolipoprc	APOC3_HU	1	1.218652

48H 10MT/	Q9NRD5	0.30217	0.306194	0.140696	PICK1	PRKCA-binc	PICK1_HUM	3	1.232997
24H 50MT/	O75154	0.50884	0.306261	0.140718	RAB11FIP3	Rab11 fami	RFIP3_HUM	1	1.422905
48H 10MT/	Q969X5	-0.00699	0.306261	0.140718	ERGIC1	Endoplasm	ERGI1_HUM	9	0.995163
48H 10MT/	Q6P1A2	-0.18192	0.306327	0.140743	LPCAT3	Lysophospl	MBOA5_HU	8	0.881527
48H 10MT/	Q13492	-0.00595	0.306467	0.140798	PICALM	Phosphatic	PICAL_HUM	17	0.995883
48H 50MT/	Q8TB37	-0.12985	0.306527	0.140815	NUBPL	Iron-sulfur	NUBPL_HU	6	0.913925
24H 10MT/	O43683	-0.00645	0.306541	0.140816	BUB1	Mitotic che	BUB1_HUM	21	0.995536
24H 50MT/	Q6BDS2	0.048308	0.306555	0.140821	BLTP3A	Bridge-like	BLT3A_HUM	6	1.034051
24H 10MT/	Q9H1D9	-0.03121	0.30663	0.140845	POLR3F	DNA-direct	RPC6_HUM	6	0.978597
48H 50MT/	Q02818	0.024695	0.30664	0.140848	NUCB1	Nucleobinc	NUCB1_HU	18	1.017264
48H 10MT/	P51178	0.224531	0.306673	0.14086	PLCD1	1-phosphat	PLCD1_HU	2	1.168397
24H 10MT/	Q13257	0.238694	0.306674	0.14086	MAD2L1	Mitotic spi	MD2L1_HU	7	1.179924
24H 10MT/	Q13428	0.0328	0.306728	0.140878	TCOF1	Treacle pro	TCOF_HUM	28	1.022996
24H 10MT/	O43237	0.039594	0.30676	0.140887	DYNC1LI2	Cytoplasmi	DC1L2_HU	17	1.027825
24H 50MT/	A6NCS6	0.22608	0.306805	0.140902	C2orf72	Uncharacte	CB072_HU	7	1.169652
24H 50MT/	P60866	-0.08074	0.306864	0.140928	RPS20	Small ribos	RS20_HUM	5	0.945574
24H 10MT/	Q9Y3B7	0.037282	0.306915	0.140948	MRPL11	Large ribos	RM11_HUM	10	1.026179
48H 10MT/	P60484	-0.38408	0.306998	0.140977	PTEN	Phosphatic	PTEN_HUM	2	0.766266
24H 10MT/	P08069	0.046481	0.307032	0.140986	IGF1R	Insulin-like	IGF1R_HUM	16	1.032743
24H 10MT/	P49753	-0.44471	0.307029	0.140986	ACOT2	Acyl-coenzy	ACOT2_HU	1	0.734733
48H 10MT/	P38919	-0.0137	0.307044	0.140988	EIF4A3	Eukaryotic	IF4A3_HUM	24	0.990552
24H 50MT/	Q9H6T3	-0.07741	0.307057	0.14099	RPAP3	RNA polym	RPAP3_HU	19	0.947756
[truncated: 1,065,118 more chars]
