# Supplementary material for: Decoding the Cellular Response to Methylthioadenosine Uncovers Novel Targets for Chronic Liver Disease
Source: Cells. 2026 Sep 22;15(19):1720. doi: 10.3390/cells15191720 (PMC13664110; doi:10.3390/cells15191720)
Supplement: Supplementary file 1 [file cells-15-01720-s001.zip › Table S2_PISA_IntactCells.pdf]

Compariso	ProteinGro	AVG Log2 F	Pvalue	Qvalue	Genes	ProteinDes	ProteinNar	# Unique To	FC
15_MTA / C	Q9UBC9	-1.81285	3.08E-10	1.02E-06	SPRR3	Small proli	SPRR3_HUI	4	0.284628
15_MTA / C	P04083	0.763656	1.00E-07	0.00012	ANXA1	Annexin A1	ANXA1_HU	5	1.697787
15_MTA / C	P17812	-0.54041	1.17E-07	0.00012	CTPS1	CTP synthas	PYRG1_HUI	9	0.687575
15_MTA / C	P55060	-0.87299	1.45E-07	0.00012	CSE1L	Exportin-2	XPO2_HUM	10	0.546014
15_MTA / C	P11586	-0.5246	1.14E-06	0.000757	MTHFD1	C-1-tetrahy	C1TC_HUM	18	0.695152
30_MTA / C	Q15070	-1.84533	3.16E-06	0.001396	OXA1L	Mitochond	OXA1L_HUI	1	0.278291
30_MTA / C	Q8N2U0	-5.21458	7.49E-06	0.002256	TMEM256	Transmemb	TM256_HU	1	0.026931
15_MTA / C	O15067	-0.67429	1.98E-05	0.005044	PFAS	Phosphorib	PUR4_HUM	3	0.626641
15_MTA / C	P26639	-0.48478	1.87E-05	0.005044	TARS1	Threonine--	SYTC_HUMA	15	0.714606
15_MTA / C	O00487	-0.64742	4.48E-05	0.005934	PSMD14	26S protea	PSDE_HUM	3	0.638419
15_MTA / C	O76021	1.48983	3.05E-05	0.005934	RSL1D1	Ribosomal	RL1D1_HUI	5	2.808558
15_MTA / C	P06703	-4.11211	4.52E-05	0.005934	S100A6	Protein S1C	S10A6_HUI	1	0.057827
15_MTA / C	P0DMV8;P(	-0.2245	4.66E-05	0.005934	HSPA1A;HS	Heat shock	HS71A_HUI	20	0.855889
15_MTA / C	P20073	1.067753	4.00E-05	0.005934	ANXA7	Annexin A7	ANXA7_HU	7	2.096166
15_MTA / C	P46087	2.005671	4.38E-05	0.005934	NOP2	28S rRNA (c	NOP2_HUM	4	4.015753
15_MTA / C	P49588	-0.73644	3.72E-05	0.005934	AARS1	Alanine--tR	SYAC_HUM	11	0.600217
30_MTA / C	Q9C0E8	-0.95077	3.70E-05	0.005934	LNPK	Endoplasm	LNP_HUMA	5	0.517358
15_MTA / C	O60547	-1.16536	5.01E-05	0.006029	GMDS	GDP-mann	GMDS_HUM	2	0.445853
15_MTA / C	O14950;P1	-1.91411	5.80E-05	0.006195	MYL12B;MY	Myosin reg	ML12B_HU	1	0.265336
15_MTA / C	P08758	-0.80909	6.78E-05	0.006572	ANXA5	Annexin A5	ANXA5_HU	11	0.570743
15_MTA / C	P50995	1.626132	7.45E-05	0.006572	ANXA11	Annexin A1	ANX11_HU	5	3.086842
30_MTA / C	Q8TEX9	-1.3465	7.58E-05	0.006572	IPO4	Importin-4	IPO4_HUM	7	0.393244
30_MTA / C	Q96DA6	-0.72417	7.71E-05	0.006572	DNAJC19	Mitochond	TIM14_HUI	1	0.605346
15_MTA / C	P62937	-0.53143	8.66E-05	0.007003	PPIA	Peptidyl-pr	PPIA_HUMA	10	0.691867
15_MTA / C	P05090	2.412484	0.000111	0.007732	APOD	Apolipoprc	APOD_HUM	5	5.323903
15_MTA / C	P30041	-0.47364	0.00011	0.007732	PRDX6	Peroxiredo	PRDX6_HU	11	0.720147
30_MTA / C	P55011	-0.65765	0.000113	0.007732	SLC12A2	Solute carri	S12A2_HUI	7	0.63391
30_MTA / C	Q676U5	-0.94984	0.000108	0.007732	ATG16L1	Autophagy-	A16L1_HUI	1	0.517692
15_MTA / C	Q7Z794	-2.51921	0.000115	0.007732	KRT77	Keratin, typ	K2C1B_HUI	4	0.174438
30_MTA / C	Q9BRT3	-3.16103	0.000117	0.007732	MIEN1	Migration a	MIEN1_HUI	1	0.111799
15_MTA / C	P51570	-1.84708	0.000127	0.008273	GALK1	Galactokina	GALK1_HUI	2	0.277953
30_MTA / C	O95857;Q1	-0.5679	0.000132	0.008388	TSPAN13;TS	Tetraspanir	TSN13_HUI	1	0.674597
30_MTA / C	P02675	-1.76458	0.000142	0.00887	FGB	Fibrinogen	FIBB_HUMA	2	0.294312
15_MTA / C	P00966	-0.50996	0.000166	0.010169	ASS1	Argininosu	ASSY_HUMA	15	0.70224
30_MTA / C	P12830	-0.42396	0.000192	0.010801	CDH1	Cadherin-1	CADH1_HU	1	0.745375
15_MTA / C	P14735	-0.50086	0.000217	0.01089	IDE	Insulin-deg	IDE_HUMAI	5	0.706685
30_MTA / C	Q96GD0	-0.93225	0.000214	0.01089	PDXP	Chronophir	PLPP_HUM	4	0.52404
30_MTA / C	Q9HCE1	-1.63409	0.000223	0.011024	MOV10	Helicase MC	MOV10_HU	2	0.322174
15_MTA / C	O95394	-0.78024	0.000229	0.01114	PGM3	Phosphoac	AGM1_HUM	6	0.582269
30_MTA / C	P35269	2.553642	0.000251	0.011555	GTF2F1	General tra	T2FA_HUM	1	5.871144
15_MTA / C	Q8N4P3	0.261486	0.000246	0.011555	HDDC3	Guanosine-	MESH1_HU	2	1.198713
30_MTA / C	Q8NCC3	-1.53374	0.000247	0.011555	PLA2G15	Lysosomal	PAG15_HU	2	0.345381
15_MTA / C	Q14444	0.590156	0.000259	0.011761	CAPRIN1	Caprin-1	CAPR1_HUI	6	1.505409
30_MTA / C	Q99614	-2.4722	0.000264	0.01184	TTC1	Tetratricop	TTC1_HUM	2	0.180216
15_MTA / C	P08238	-0.39365	0.000274	0.011919	HSP90AB1	Heat shock	HS90B_HUI	24	0.761204
30_MTA / C	Q9BW83	-2.53987	0.000293	0.011984	IFT27	Intraflagell	IFT27_HUM	1	0.171958
15_MTA / C	P00533	1.679899	0.00032	0.012796	EGFR	Epidermal g	EGFR_HUM	3	3.204056
15_MTA / C	O75874	-0.21043	0.000386	0.013423	IDH1	Isocitrate d	IDHC_HUM	8	0.864277
15_MTA / C	P00568	-1.01975	0.000378	0.013423	AK1	Adenylate k	KAD1_HUM	2	0.493202
15_MTA / C	P07737	-0.49578	0.000364	0.013423	PFN1	Profilin-1	PROF1_HUI	8	0.709178
30_MTA / C	P22059	-1.62664	0.000375	0.013423	OSBP	Oxysterol-b	OSBP1_HUI	2	0.323842

15_MTA / C	P46459	-1.12092	0.000369	0.013423	NSF	Vesicle-fusi	NSF_HUMA	1	0.459799
30_MTA / C	Q9H845	-2.46174	0.00036	0.013423	ACAD9	Complex I a	ACAD9_HU	2	0.181527
30_MTA / C	Q9Y5L4	0.673599	0.000355	0.013423	TIMM13	Mitochond	TIM13_HUI	4	1.595047
15_MTA / C	P53611	-0.62716	0.000415	0.01348	RABGGTB	Geranylgera	PGTB2_HUI	2	0.647448
30_MTA / C	Q14108	-0.01646	0.000407	0.01348	SCARB2	Lysosome n	SCRB2_HUI	1	0.988657
30_MTA / C	Q969V3	-0.69926	0.000431	0.01348	NCLN	BOS comple	NCLN_HUM	5	0.615888
30_MTA / C	Q9UIJ7	-2.56213	0.000407	0.01348	AK3	GTP:AMP p	KAD3_HUM	2	0.169325
30_MTA / C	Q15843	-4.42095	0.000441	0.01365	NEDD8	NEDD8	NEDD8_HU	1	0.046683
30_MTA / C	Q8NBL1	-0.97204	0.000446	0.013685	POGLUT1	Protein O-g	PGLT1_HUI	1	0.509786
30_MTA / C	Q13423	-0.61076	0.000462	0.01392	NNT	NAD(P) trar	NNTM_HUI	8	0.654849
30_MTA / C	Q01085	-1.65191	0.000468	0.013986	TIAL1	Nucleolysir	TIAR_HUMA	2	0.318218
15_MTA / C	P09211	-0.53851	0.000492	0.014443	GSTP1	Glutathion	GSTP1_HUI	11	0.688483
15_MTA / C	P52209	-0.41323	0.000504	0.014511	PGD	6-phospho	6PGD_HUM	9	0.75094
30_MTA / C	P42167	0.251678	0.000513	0.014527	TMPO	Lamina-ass	LAP2B_HUI	13	1.190591
30_MTA / C	Q6KCM7;Q	-1.33613	0.000512	0.014527	SLC25A25;S	Mitochond	SCMC2_HU	1	0.396081
15_MTA / C	P11234	0.857422	0.000518	0.014551	RALB	Ras-related	RALB_HUM	1	1.811798
15_MTA / C	Q01469	-1.08608	0.000576	0.015214	FABP5	Fatty acid-b	FABP5_HUI	3	0.471039
15_MTA / C	Q14974	-0.56183	0.000579	0.015214	KPNB1	Importin su	IMB1_HUM	10	0.677442
15_MTA / C	Q96HS1	0.978929	0.000588	0.015214	PGAM5	Serine/thre	PGAM5_HU	4	1.971002
15_MTA / C	Q9BZE4	1.517123	0.00057	0.015214	GTPBP4	GTP-bindin	GTPB4_HUI	1	2.862197
30_MTA / C	Q96BI1	-0.52692	0.000621	0.015963	SLC22A18	Solute carri	S22AI_HUM	1	0.694037
15_MTA / C	P14324	-1.40589	0.000645	0.016077	FDPS	Farnesyl py	FPPS_HUM	1	0.377386
15_MTA / C	Q96BI1	0.520582	0.000643	0.016077	SLC22A18	Solute carri	S22AI_HUM	1	1.434534
15_MTA / C	P08670	-0.34453	0.000687	0.016629	VIM	Vimentin	VIME_HUM	19	0.787565
30_MTA / C	Q9C005	-1.14402	0.000707	0.016828	DPY30	Protein dpy	DPY30_HUI	2	0.452497
15_MTA / C	P48634	2.545106	0.000731	0.017191	PRRC2A	Protein PRI	PRC2A_HUI	1	5.83651
30_MTA / C	P82970	-1.57932	0.000738	0.017232	HMGN5	High mobil	HMGN5_HU	1	0.334639
15_MTA / C	O95825	-0.03666	0.000761	0.017291	CRYZL1	Quinone ox	QORL1_HU	3	0.974909
30_MTA / C	Q99733	-0.78762	0.000755	0.017291	NAP1L4	Nucleosom	NP1L4_HUI	3	0.579299
30_MTA / C	O00505	-1.43132	0.00077	0.017294	KPNA3	Importin su	IMA4_HUM	5	0.370791
30_MTA / C	P23229	-2.34463	0.000778	0.017294	ITGA6	Integrin alp	ITA6_HUMA	1	0.196877
30_MTA / C	P15848	-1.47287	0.000798	0.017512	ARSB	Arylsulfatas	ARSB_HUM	2	0.360265
15_MTA / C	O15260	0.537223	0.000826	0.017667	SURF4	Surfeit locu	SURF4_HUI	3	1.451177
15_MTA / C	P04844	0.226878	0.000823	0.017667	RPN2	Dolichyl-di	RPN2_HUM	7	1.1703
30_MTA / C	P84101	-3.8926	0.000837	0.017772	SERF2	Small EDRK	SERF2_HUM	2	0.06733
30_MTA / C	Q9NX76	-0.83141	0.000848	0.017818	CMTM6	CKLF-like M	CKLF6_HUM	1	0.561981
30_MTA / C	Q86Y39	-2.9817	0.000886	0.017872	NDUFA11	NADH dehy	NDUAB_HU	1	0.126596
15_MTA / C	Q92598	-0.62284	0.000888	0.017872	HSPH1	Heat shock	HS105_HUI	10	0.649392
30_MTA / C	Q9H0U3	-0.90227	0.000887	0.017872	MAGT1	Magnesium	MAGT1_HU	3	0.535044
30_MTA / C	Q13011	0.537179	0.000908	0.018018	ECH1	Delta(3,5)-I	ECH1_HUM	7	1.451132
15_MTA / C	P62195	-0.34851	0.000975	0.018624	PSMC5	26S protea	PRS8_HUM	7	0.785396
15_MTA / C	Q8WUH6	-1.8312	0.001019	0.019179	TMEM263	Transmemb	TM263_HU	1	0.281031
30_MTA / C	P53611	-0.91219	0.001043	0.019207	RABGGTB	Geranylgera	PGTB2_HUI	2	0.531376
15_MTA / C	Q14103	-0.34154	0.001039	0.019207	HNRNPD	Heterogene	HNRPD_HU	3	0.7892
30_MTA / C	Q8WU90	2.769842	0.001026	0.019207	ZC3H15	Zinc finger (	ZC3HF_HUI	1	6.820331
15_MTA / C	O15212	-2.06722	0.001081	0.019791	PFDN6	Prefoldin su	PFD6_HUM	2	0.238618
30_MTA / C	O95295	-1.56164	0.001097	0.019791	SNAPIN	SNARE-asso	SNAPN_HU	1	0.338766
15_MTA / C	Q01844	1.035114	0.0011	0.019791	EWSR1	RNA-bindir	EWS_HUMA	4	2.049275
15_MTA / C	Q16836	1.047232	0.00109	0.019791	HADH	Hydroxyacy	HCDH_HUM	1	2.066562
30_MTA / C	Q9NX47	-1.59261	0.001105	0.019791	MARCHF5	E3 ubiquiti	MARH5_HU	3	0.331572
15_MTA / C	P14868	-0.89828	0.001131	0.020048	DARS1	Aspartate--t	SYDC_HUM	8	0.536527
15_MTA / C	P34932	-0.49254	0.001146	0.020087	HSPA4	Heat shock	HSP74_HUI	13	0.710772

30_MTA / C	Q9NZ08	-1.21908	0.001142	0.020087	ERAP1	Endoplasm	ERAP1_HUI	1	0.429556
30_MTA / C	Q13347	-1.48632	0.001157	0.020168	EIF3I	Eukaryotic	EIF3I_HUM	4	0.356921
15_MTA / C	Q9NSB2	0.631787	0.001162	0.020168	KRT84	Keratin, typ	KRT84_HUI	3	1.549483
30_MTA / C	Q2TAA2	-1.12607	0.001173	0.020241	IAH1	Isoamyl ace	IAH1_HUM	2	0.458164
30_MTA / C	Q8TCA0	-1.01384	0.001216	0.020659	LRRC20	Leucine-ric	LRC20_HUI	4	0.495225
30_MTA / C	Q15819	-0.92321	0.001254	0.021101	UBE2V2	Ubiquitin-c	UB2V2_HU	2	0.527333
15_MTA / C	Q9P2T1	-0.56392	0.001295	0.021349	GMPR2	GMP reduc	GMPR2_HU	5	0.67646
15_MTA / C	Q9UBQ7	-0.20846	0.001324	0.021647	GRHPR	Glyoxylate	GRHPR_HU	1	0.865462
30_MTA / C	Q14118	1.867475	0.001363	0.02215	DAG1	Dystroglyca	DAG1_HUM	2	3.648934
15_MTA / C	P49748	0.567263	0.001441	0.022989	ACADVL	Very long-c	ACADV_HU	11	1.48171
15_MTA / C	P61077;P6	-0.57193	0.001453	0.022989	UBE2D3;UE	Ubiquitin-c	UB2D3_HU	1	0.672716
30_MTA / C	Q96M27	-0.83981	0.001448	0.022989	PRRC1	Protein PRI	PRRC1_HUI	3	0.558717
30_MTA / C	Q9UI14	-0.78982	0.001457	0.022989	RABAC1	Prenylated	PRAF1_HUI	2	0.578414
30_MTA / C	P10599	0.695777	0.001488	0.023257	TXN	Thioredoxi	THIO_HUM	4	1.619756
30_MTA / C	Q8IVS2	-1.85162	0.001521	0.023472	MCAT	Malonyl-Co	FABD_HUM	2	0.277081
15_MTA / C	Q8NBQ5	0.777315	0.001523	0.023472	HSD17B11	Estradiol 17	DHB11_HU	4	1.713938
15_MTA / C	A6NHQ2	1.282838	0.001531	0.023494	FBLL1	rRNA/tRNA	FBLL1_HUM	1	2.433171
30_MTA / C	Q9NZZ3	-1.89685	0.001633	0.024487	CHMP5	Charged mu	CHMP5_HU	1	0.26853
30_MTA / C	P60602	2.359458	0.001652	0.024493	ROMO1	Reactive ox	ROMO1_HU	1	5.131774
30_MTA / C	Q16576	-1.00451	0.001643	0.024493	RBBP7	Histone-bir	RBBP7_HUI	1	0.498439
15_MTA / C	Q9NQX3	0.846055	0.001669	0.024578	GPHN	Gephyrin	GEPH_HUM	5	1.797579
15_MTA / C	Q96FX7	-0.66608	0.001676	0.024583	TRMT61A	tRNA (aden	TRM61_HU	1	0.630217
15_MTA / C	P50402	0.925539	0.00169	0.024678	EMD	Emerin	EMD_HUMA	1	1.899394
30_MTA / C	P01019	-0.83566	0.001707	0.02481	AGT	Angiotensir	ANGT_HUM	6	0.560326
30_MTA / C	Q96A57	-0.83316	0.00173	0.024924	TMEM230	Transmemb	TM230_HU	1	0.561298
15_MTA / C	P21333	-0.36481	0.001745	0.024932	FLNA	Filamin-A	FLNA_HUM	27	0.776569
30_MTA / C	Q6UW63	-0.59665	0.001806	0.02547	POGLUT2	Protein O-g	PLGT2_HUI	5	0.661289
15_MTA / C	E9PRG8	2.392338	0.001878	0.026153	C11orf98	Uncharacte	CK098_HUI	1	5.250075
15_MTA / C	Q92734	0.858222	0.001903	0.026387	TFG	Protein TFG	TFG_HUMA	9	1.812803
15_MTA / C	P0DPB6	-1.56464	0.001948	0.026425	POLR1D	DNA-direct	RPAC2_HUI	1	0.338062
15_MTA / C	P22102	-0.34895	0.001944	0.026425	GART	Trifunction	PUR2_HUM	9	0.785153
15_MTA / C	Q7Z739	1.098236	0.001954	0.026425	YTHDF3	YTH domair	YTHD3_HUI	4	2.140928
15_MTA / C	P62891;Q5	1.311311	0.001974	0.026598	RPL39;RPL3	Large ribos	RL39_HUM	1	2.48167
30_MTA / C	Q9NXH9	-1.55114	0.001998	0.026808	TRMT1	tRNA (guan	TRM1_HUM	3	0.34124
15_MTA / C	Q08945	1.617466	0.002112	0.027725	SSRP1	FACT comp	SSRP1_HUI	2	3.068357
30_MTA / C	Q9H9B4	-0.43616	0.002171	0.028099	SFXN1	Sideroflexir	SFXN1_HUI	6	0.739097
30_MTA / C	Q8N1G4	-0.9805	0.002225	0.028358	LRRC47	Leucine-ric	LRC47_HUI	4	0.506803
15_MTA / C	O14672	1.029983	0.002254	0.028618	ADAM10	Disintegrin	ADA10_HU	2	2.042
15_MTA / C	O75223	-0.31701	0.002313	0.028927	GGCT	Gamma-glu	GGCT_HUM	7	0.80273
30_MTA / C	P21397	-0.6429	0.002311	0.028927	MAOA	Amine oxid	AOFA_HUM	4	0.640423
30_MTA / C	Q9UK76	1.227393	0.002329	0.028979	JPT1	Jupiter mic	JUPI1_HUM	3	2.341434
15_MTA / C	Q12797	1.502105	0.002369	0.029076	ASPH	Aspartyl/as	ASPH_HUM	3	2.832557
30_MTA / C	O60547	-0.77327	0.002395	0.029179	GMDS	GDP-mann	GMDS_HUM	2	0.58509
30_MTA / C	Q15024	-2.49786	0.002418	0.029264	EXOSC7	Exosome cc	EXOS7_HUI	1	0.177039
30_MTA / C	O00468	-3.16156	0.00244	0.029408	AGRN	Agrin	AGRIN_HUI	1	0.111757
30_MTA / C	P62877	-2.07205	0.002508	0.029677	RBX1	E3 ubiquiti	RBX1_HUM	2	0.237821
30_MTA / C	Q96AA3	-0.63101	0.00252	0.029677	RFT1	Man(5)GlcN	RFT1_HUM	2	0.645725
30_MTA / C	Q9P0I2	-1.17513	0.002561	0.029851	EMC3	ER membra	EMC3_HUM	2	0.442842
15_MTA / C	P00846	1.749038	0.002613	0.030044	MT-ATP6	ATP synthas	ATP6_HUM	1	3.361344
30_MTA / C	P13646	1.460875	0.002595	0.030044	KRT13	Keratin, typ	K1C13_HUI	15	2.752753
30_MTA / C	P23258;Q9	-0.98507	0.002614	0.030044	TUBG1;TUE	Tubulin gar	TBG1_HUM	2	0.5052
30_MTA / C	Q12905	0.390659	0.002626	0.030044	ILF2	Interleukin	ILF2_HUMA	5	1.310992

15_MTA / C	P51610	1.673756	0.002658	0.030265	HCFC1	Host cell fa	HCFC1_HU	10	3.190442
30_MTA / C	O15067	-1.11179	0.00291	0.031065	PFAS	Phosphorib	PUR4_HUM	3	0.462719
15_MTA / C	P02538	-0.76391	0.002938	0.031065	KRT6A	Keratin, typ	K2C6A_HU	25	0.588897
15_MTA / C	P07741	-0.53322	0.002884	0.031065	APRT	Adenine ph	APT_HUMA	6	0.691012
15_MTA / C	P35611	1.006505	0.002907	0.031065	ADD1	Alpha-addu	ADDA_HUM	5	2.009039
15_MTA / C	Q00610	-0.26367	0.002921	0.031065	CLTC	Clathrin he	CLH1_HUM	15	0.832965
30_MTA / C	Q01844	0.952632	0.002879	0.031065	EWSR1	RNA-bindir	EWS_HUMA	4	1.935401
30_MTA / C	Q14240	-0.77922	0.002933	0.031065	EIF4A2	Eukaryotic	IF4A2_HUM	3	0.582681
30_MTA / C	Q15041	-2.91702	0.002821	0.031065	ARL6IP1	ADP-ribosy	AR6P1_HU	1	0.132401
30_MTA / C	Q5JTV8	-0.86252	0.002916	0.031065	TOR1AIP1	Torsin-1A-i	TOIP1_HUM	7	0.549993
30_MTA / C	Q5R3I4	-0.74643	0.002938	0.031065	TTC38	Tetratricop	TTC38_HUM	3	0.596076
30_MTA / C	Q9NVX2	-0.4871	0.00282	0.031065	NLE1	Notchless p	NLE1_HUM	2	0.713457
30_MTA / C	O43493	-3.33584	0.002976	0.031206	TGOLN2	Trans-Golgi	TGON2_HU	2	0.09904
15_MTA / C	O60701	-0.18595	0.002999	0.031348	UGDH	UDP-glucos	UGDH_HUM	14	0.879071
15_MTA / C	P41587	-1.19909	0.00302	0.031375	VIPR2	Vasoactive	VIPR2_HUM	1	0.435551
30_MTA / C	Q99808	-1.58607	0.00302	0.031375	SLC29A1	Equilibrativ	S29A1_HU	2	0.333077
30_MTA / C	P49281	1.437726	0.003046	0.031417	SLC11A2	Natural resi	NRAM2_HU	1	2.708936
15_MTA / C	P37837	-0.29404	0.003087	0.031483	TALDO1	Transaldola	TALDO_HU	14	0.815616
15_MTA / C	Q9BRP8	-0.98887	0.00308	0.031483	PYM1	Partner of Y	PYM1_HUM	1	0.503873
30_MTA / C	O43396	-2.00325	0.003159	0.031628	TXNL1	Thioredoxir	TXNL1_HU	4	0.249438
15_MTA / C	O75439	0.898909	0.003144	0.031628	PMPCB	Mitochond	MPPB_HUM	5	1.864656
30_MTA / C	P84095	-1.50759	0.003121	0.031628	RHOG	Rho-relatec	RHOG_HUM	2	0.351697
30_MTA / C	Q969T9	2.271702	0.003181	0.031753	WBP2	WW domai	WBP2_HUM	1	4.828924
15_MTA / C	Q9Y6C9	1.157307	0.003282	0.032272	MTCH2	Mitochond	MTCH2_HU	3	2.230407
15_MTA / C	P42167	0.299727	0.003301	0.032361	TMPO	Lamina-ass	LAP2B_HU	13	1.230911
15_MTA / C	P47985	0.603122	0.003327	0.032428	UQCRFS1	Cytochrom	UCRI_HUM	7	1.519001
15_MTA / C	O95793	1.153019	0.003399	0.03258	STAU1	Double-stra	STAU1_HU	3	2.223788
30_MTA / C	P07306	-1.52658	0.003375	0.03258	ASGR1	Asialoglyco	ASGR1_HU	3	0.3471
15_MTA / C	Q12765	-0.624	0.003402	0.03258	SCRN1	Secernin-1	SCRN1_HU	3	0.648871
30_MTA / C	Q8N5M9	-2.37992	0.003544	0.033461	JAGN1	Protein jag	JAGN1_HU	1	0.19212
30_MTA / C	Q96GF1	-0.85988	0.003606	0.033661	RNF185	E3 ubiquiti	RN185_HU	1	0.550998
30_MTA / C	Q9H3G5	-1.7856	0.003598	0.033661	CPVL	Probable se	CPVL_HUM	1	0.290056
15_MTA / C	P81605	-2.1983	0.003646	0.033937	DCD	Dermcidin	DCD_HUMA	4	0.217894
30_MTA / C	Q6UX53	-0.67262	0.003696	0.034211	TMT1B	Thiol S-met	TMT1B_HU	1	0.627365
30_MTA / C	P20290	-0.95267	0.003746	0.034378	BTF3	Transcripti	BTF3_HUM	3	0.516676
30_MTA / C	P62913	0.178162	0.003818	0.034476	RPL11	Large ribos	RL11_HUM	5	1.131441
15_MTA / C	Q16658	-0.29038	0.00386	0.034476	FSCN1	Fascin	FSCN1_HU	2	0.817685
15_MTA / C	Q9GZZ8	-1.03422	0.003839	0.034476	LACRT	Extracellula	LACRT_HUM	1	0.48828
15_MTA / C	O95249	1.668853	0.003902	0.034666	GOSR1	Golgi SNAP	GOSR1_HU	2	3.179618
15_MTA / C	Q9NZT1	-1.19912	0.004063	0.035718	CALML5	Calmodulir	CALL5_HUM	3	0.435541
15_MTA / C	P02649	0.361564	0.004355	0.037113	APOE	Apolipoprc	APOE_HUM	13	1.284818
15_MTA / C	P53004	-0.21275	0.00435	0.037113	BLVRA	Biliverdin r	BIEA_HUMA	4	0.862892
30_MTA / C	O60568	-0.5562	0.004408	0.037218	PLOD3	Multifuncti	PLOD3_HU	6	0.680091
15_MTA / C	Q9H0D6	1.049647	0.004386	0.037218	XRN2	5'-3' exorib	XRN2_HUM	6	2.070023
15_MTA / C	O00299	-0.51858	0.004503	0.037496	CLIC1	Chloride in	CLIC1_HUM	8	0.69806
15_MTA / C	P12277	-1.04913	0.004503	0.037496	CKB	Creatine kir	KCRB_HUM	6	0.483261
30_MTA / C	Q9NQW7	-1.61685	0.004485	0.037496	XPNPEP1	Xaa-Pro am	XPP1_HUM	2	0.326047
15_MTA / C	P09525	-0.58648	0.004562	0.037594	ANXA4	Annexin A4	ANXA4_HU	12	0.665965
30_MTA / C	Q13505	-2.04565	0.004554	0.037594	MTX1	Metaxin-1	MTX1_HUM	1	0.242213
15_MTA / C	Q5T6F2	2.877196	0.004554	0.037594	UBAP2	Ubiquitin-a	UBAP2_HU	6	7.347205
15_MTA / C	Q9HAV7	0.473594	0.004572	0.037594	GRPEL1	GrpE protei	GRPE1_HU	1	1.388564
15_MTA / C	P16435	0.361363	0.004619	0.03777	POR	NADPH--cyt	NCPR_HUM	7	1.284639

15_MTA / C	P46736	1.406821	0.004683	0.03777	BRCC3	Lys-63-spec	BRCC3_HU	4	2.651523
30_MTA / C	P50995	1.288309	0.004684	0.03777	ANXA11	Annexin A1	ANX11_HU	5	2.442416
30_MTA / C	Q8NBJ7	-0.55471	0.004642	0.03777	SUMF2	Inactive C-a	SUMF2_HU	4	0.680796
30_MTA / C	Q9NZ45	0.103579	0.00466	0.03777	CISD1	CDGSH iror	CISD1_HUM	2	1.074435
30_MTA / C	O60437	0.75836	0.004833	0.037775	PPL	Periplakin	PEPL_HUM	2	1.691567
15_MTA / C	O94925	1.638515	0.004867	0.037775	GLS	Glutaminas	GLSK_HUM	2	3.113452
30_MTA / C	P04818	-2.25116	0.004766	0.037775	TYMS	Thymidylat	TYSY_HUMA	1	0.210055
15_MTA / C	P09669	0.566701	0.004706	0.037775	COX6C	Cytochrom	COX6C_HU	5	1.481133
30_MTA / C	P46937	1.265082	0.004721	0.037775	YAP1	Transcripti	YAP1_HUM	2	2.403409
15_MTA / C	Q14157	1.022694	0.004838	0.037775	UBAP2L	Ubiquitin-a	UBP2L_HU	8	2.031709
30_MTA / C	Q15084	0.42907	0.004825	0.037775	PDIA6	Protein dis	PDIA6_HUM	12	1.346365
30_MTA / C	Q92945	0.480624	0.004839	0.037775	KHSRP	Far upstrea	FUBP2_HU	17	1.395347
30_MTA / C	Q9BQT8	-4.27615	0.004763	0.037775	SLC25A21	Mitochond	ODC_HUMA	1	0.051612
15_MTA / C	Q9UNW1	0.516879	0.004862	0.037775	MINPP1	Multiple in	MINP1_HU	4	1.430856
30_MTA / C	Q9Y5K8	-0.98336	0.004803	0.037775	ATP6V1D	V-type prot	VATD_HUM	4	0.505801
15_MTA / C	P00813	-0.42223	0.004958	0.03796	ADA	Adenosine	ADA_HUMA	5	0.746272
30_MTA / C	P61163	-1.09201	0.004923	0.03796	ACTR1A	Alpha-centr	ACTZ_HUM	4	0.469109
15_MTA / C	O43583	1.032943	0.005085	0.03856	DENR	Density-reg	DENR_HUM	2	2.046194
15_MTA / C	P31930	0.37993	0.005079	0.03856	UQCRC1	Cytochrom	QCR1_HUM	7	1.301279
30_MTA / C	Q96GC9	-0.64694	0.005135	0.038832	VMP1	Vacuole me	VMP1_HUM	1	0.638635
30_MTA / C	P15104	-1.98782	0.005229	0.03923	GLUL	Glutamine	GLNA_HUM	2	0.252119
30_MTA / C	Q9NQE9	3.285302	0.005232	0.03923	HINT3	Adenosine	HINT3_HUM	1	9.749323
15_MTA / C	P35269	2.676317	0.005282	0.039272	GTF2F1	General tra	T2FA_HUM	1	6.39222
15_MTA / C	Q14739	0.495147	0.005285	0.039272	LBR	Delta(14)-st	LBR_HUMA	2	1.409464
15_MTA / C	P42330;Q0	-0.47209	0.00537	0.039632	AKR1C3;AK	Aldo-keto r	AK1C3_HU	2	0.720918
15_MTA / C	Q04760	-0.33602	0.005368	0.039632	GLO1	Lactoylglut	LGUL_HUM	9	0.792225
30_MTA / C	Q9Y287	-0.12912	0.005473	0.040035	ITM2B	Integral me	ITM2B_HU	1	0.914389
30_MTA / C	O95372	-0.88299	0.005583	0.040307	LYPLA2	Acyl-protei	LYPA2_HUM	1	0.542241
30_MTA / C	O00330	2.904294	0.00564	0.040389	PDHX	Pyruvate de	ODPX_HUM	2	7.486512
30_MTA / C	O95684	-1.18493	0.005631	0.040389	CEP43	Centrosom	CEP43_HU	1	0.439846
15_MTA / C	P26641	-0.2854	0.005643	0.040389	EEF1G	Elongation	EF1G_HUM	13	0.820515
15_MTA / C	P31483	0.963386	0.005608	0.040389	TIA1	Cytotoxic g	TIA1_HUMA	3	1.949881
30_MTA / C	P61165	-1.05534	0.005676	0.040442	TMEM258	Transmemb	TM258_HU	1	0.481183
15_MTA / C	Q86U42	-0.73459	0.005712	0.040449	PABPN1	Polyadenyl	PABP2_HU	1	0.600989
30_MTA / C	O95168	-0.28527	0.00585	0.041084	NDUFB4	NADH dehy	NDUB4_HU	1	0.820588
15_MTA / C	Q9UBT2	-0.23957	0.005851	0.041084	UBA2	SUMO-activ	SAE2_HUM	5	0.847
15_MTA / C	O00400	0.970943	0.005916	0.04145	SLC33A1	Acetyl-coer	ACATN_HU	3	1.960122
30_MTA / C	Q01581	-0.37343	0.005955	0.04148	HMGCS1	Hydroxyme	HMCS1_HU	4	0.771943
30_MTA / C	Q92734	0.72475	0.005958	0.04148	TFG	Protein TFG	TFG_HUMA	9	1.652614
15_MTA / C	P49281	2.696373	0.006013	0.04153	SLC11A2	Natural resi	NRAM2_HU	1	6.481705
15_MTA / C	Q8IVM0	-0.26189	0.006015	0.04153	CCDC50	Coiled-coil	CCD50_HU	2	0.833996
30_MTA / C	Q13217	-0.85439	0.00612	0.042059	DNAJC3	DnaJ homo	DNJC3_HU	5	0.553099
15_MTA / C	Q9Y4Z0	-1.3848	0.006105	0.042059	LSM4	U6 snRNA-a	LSM4_HUM	1	0.382944
15_MTA / C	Q8WVM8	-0.2924	0.006147	0.042092	SCFD1	Sec1 family	SCFD1_HU	1	0.816543
15_MTA / C	P16070	-3.90765	0.006252	0.042543	CD44	CD44 antig	CD44_HUM	3	0.066632
15_MTA / C	P13489	-0.34856	0.006267	0.042562	RNH1	Ribonuclea	RINI_HUMA	4	0.785368
15_MTA / C	P24534	-0.42298	0.006291	0.042637	EEF1B2	Elongation	EF1B_HUM	2	0.745882
30_MTA / C	P22102	-0.48311	0.006358	0.043001	GART	Trifunction	PUR2_HUM	9	0.715435
15_MTA / C	Q9Y520	1.182782	0.006425	0.04328	PRRC2C	Protein PRI	PRC2C_HU	8	2.270141
15_MTA / C	Q9H1E5	1.688115	0.006488	0.043527	TMX4	Thioredoxi	TMX4_HUM	1	3.222354
30_MTA / C	O94905	-0.85123	0.006627	0.043748	ERLIN2	Erlin-2	ERLN2_HU	2	0.554314
30_MTA / C	P61009	-0.95042	0.006614	0.043748	SPCS3	Signal pept	SPCS3_HUM	2	0.517483

15_MTA / C	Q9UNP9	0.962256	0.00659	0.043748	PPIE	Peptidyl-pr	PPIE_HUMA	2	1.948354
15_MTA / C	Q8NEW0	0.494612	0.006644	0.043776	SLC30A7	Zinc transp	ZNT7_HUM	4	1.408942
30_MTA / C	Q01469	-0.85092	0.006737	0.044123	FABP5	Fatty acid-b	FABP5_HUI	3	0.554431
15_MTA / C	P07900	-0.46852	0.00686	0.044436	HSP90AA1	Heat shock	HS90A_HUI	12	0.722706
15_MTA / C	O43772	0.328335	0.006909	0.044551	SLC25A20	Mitochond	MCAT_HUM	7	1.255563
30_MTA / C	P10253	-0.4289	0.006922	0.044551	GAA	Lysosomal a	LYAG_HUM	5	0.742826
30_MTA / C	P04080	0.786433	0.007089	0.044937	CSTB	Cystatin-B	CYTB_HUM	2	1.724805
30_MTA / C	P53602	-0.68412	0.007066	0.044937	MVD	Diphospho	MVD1_HUM	2	0.622387
15_MTA / C	Q15233	-0.39646	0.007057	0.044937	NONO	Non-POU d	NONO_HUM	10	0.75972
30_MTA / C	Q92990	1.949704	0.007059	0.044937	GLMN	Glomulin	GLMN_HUM	1	3.862952
15_MTA / C	Q9Y295	1.74323	0.007038	0.044937	DRG1	Developme	DRG1_HUM	4	3.347839
15_MTA / C	P08833	2.134044	0.007134	0.045064	IGFBP1	Insulin-like	IBP1_HUMA	3	4.389463
30_MTA / C	Q9Y394	-1.18154	0.007193	0.045064	DHRS7	Dehydroger	DHRS7_HU	3	0.440881
30_MTA / C	P36776	-0.48215	0.007219	0.045138	LONP1	Lon proteas	LONM_HUM	9	0.715908
15_MTA / C	P43358	-0.27256	0.007252	0.04526	MAGEA4	Melanoma-	MAGA4_HU	5	0.827847
30_MTA / C	Q8TAF3	-0.81231	0.007309	0.045358	WDR48	WD repeat-	WDR48_HU	2	0.569471
30_MTA / C	P02654	2.77745	0.007577	0.046331	APOC1	Apolipopro	APOC1_HU	1	6.856392
30_MTA / C	P53634	-0.67695	0.007722	0.046396	CTSC	Dipeptidyl	CATC_HUM	4	0.625485
15_MTA / C	P56385	0.681515	0.007728	0.046396	ATP5ME	ATP synthas	ATP5I_HUM	4	1.603823
30_MTA / C	Q49AR2	0.778753	0.007681	0.046396	C5orf22	UPF0489 p	CE022_HUI	1	1.715647
30_MTA / C	Q8IZA0	-0.98075	0.007613	0.046396	KIAA0319L	Dyslexia-as	K319L_HUI	1	0.506715
15_MTA / C	Q9HC38	-0.48077	0.007695	0.046396	GLOD4	Glyoxalase	GLOD4_HU	3	0.716596
15_MTA / C	P14550	-1.23476	0.007801	0.046583	AKR1A1	Aldo-keto r	AK1A1_HUI	2	0.424913
15_MTA / C	P62136	-0.28412	0.007846	0.046599	PPP1CA	Serine/thre	PP1A_HUM	6	0.82124
15_MTA / C	Q14240	-0.64883	0.00784	0.046599	EIF4A2	Eukaryotic	IF4A2_HUM	3	0.637798
30_MTA / C	Q13310	-1.40101	0.007911	0.046814	PABPC4	Polyadenyl	PABP4_HUI	2	0.378664
15_MTA / C	O43865	0.759832	0.007944	0.046927	AHCYL1	S-adenosyll	SAHH2_HU	7	1.693293
15_MTA / C	O60830	0.162256	0.007979	0.047051	TIMM17B	Mitochond	TI17B_HUM	3	1.119036
15_MTA / C	P12004	-0.22904	0.008007	0.04709	PCNA	Proliferatir	PCNA_HUM	12	0.853202
15_MTA / C	Q9UQ80	-0.32709	0.008014	0.04709	PA2G4	Proliferatic	PA2G4_HU	13	0.797142
15_MTA / C	O14602;P4	-1.00887	0.008202	0.047238	EIF1AY;EIF1	Eukaryotic	IF1AY_HUM	1	0.496935
15_MTA / C	O75131	-0.52668	0.008239	0.047238	CPNE3	Copine-3	CPNE3_HUI	7	0.69415
30_MTA / C	P46736	2.66275	0.008182	0.047238	BRCC3	Lys-63-spec	BRCC3_HUI	4	6.332388
15_MTA / C	Q5JTV8	1.081505	0.008095	0.047238	TOR1AIP1	Torsin-1A-ir	TOIP1_HUM	7	2.116243
30_MTA / C	Q8NFH3	-0.61107	0.008172	0.047238	NUP43	Nucleopori	NUP43_HU	4	0.65471
30_MTA / C	Q99471	-0.50686	0.008142	0.047238	PFDN5	Prefoldin su	PFD5_HUM	3	0.703755
30_MTA / C	Q5T6F2	1.029261	0.008287	0.047351	UBAP2	Ubiquitin-a	UBAP2_HU	6	2.040978
15_MTA / C	Q5T6V5	-1.11315	0.008321	0.047461	QNG1	Queuosine	QNG1_HUM	1	0.462282
15_MTA / C	P48507	-1.80541	0.008349	0.047538	GCLM	Glutamate-	GSH0_HUM	3	0.286101
15_MTA / C	Q6P1N9	-0.20179	0.008364	0.047547	TATDN1	Deoxyribor	TATD1_HUI	7	0.86947
15_MTA / C	Q13838	-0.12429	0.008407	0.047707	DDX39B	Spliceosom	DX39B_HU	11	0.917458
15_MTA / C	O60762	2.064431	0.008435	0.047777	DPM1	Dolichol-pl	DPM1_HUM	1	4.182689
15_MTA / C	P04843	0.377117	0.008469	0.047777	RPN1	Dolichyl-di	RPN1_HUM	16	1.298744
15_MTA / C	P27694	0.7164	0.008495	0.047777	RPA1	Replicatior	RFA1_HUM	1	1.643077
30_MTA / C	P67809	0.817485	0.008506	0.047777	YBX1	Y-box-bindi	YBOX1_HUI	4	1.762332
15_MTA / C	Q9BT78	0.711381	0.008609	0.048276	COPS4	COP9 signa	CSN4_HUM	7	1.63737
30_MTA / C	P07099	-0.47095	0.008632	0.048324	EPHX1	Epoxide hy	HYEP_HUM	10	0.721489
15_MTA / C	O43242	-0.28745	0.008683	0.048362	PSMD3	26S proteas	PSMD3_HU	11	0.819346
15_MTA / C	Q9H3K2	0.279266	0.008783	0.048484	GHITM	Growth hor	GHITM_HU	5	1.213577
30_MTA / C	Q86UE4	0.277428	0.009122	0.049838	MTDH	Protein LYF	LYRIC_HUM	9	1.212032
30_MTA / C	Q9Y3E0	-2.44285	0.009128	0.049838	GOLT1B	Vesicle trar	GOT1B_HU	1	0.18392
30_MTA / C	O95571	-0.76508	0.009228	0.049886	ETHE1	Persulfide c	ETHE1_HUM	2	0.588419

30_MTA / C	P14324	-1.76966	0.00919	0.049886	FDPS	Farnesyl py	FPPS_HUM	1	0.293277
30_MTA / C	Q7Z4H8	-1.18144	0.009217	0.049886	POGLUT3	Protein O-g	PLGT3_HUI	2	0.440912
15_MTA / C	P22392	-0.45245	0.009284	0.050108	NME2	Nucleoside	NDKB_HUN	10	0.730801
15_MTA / C	O75083	-0.30808	0.009358	0.050114	WDR1	WD repeat-	WDR1_HUI	13	0.807717
30_MTA / C	P05166	-1.62673	0.009366	0.050114	PCCB	Propionyl-(	PCCB_HUM	1	0.323821
30_MTA / C	P10301;P6	-0.33713	0.00931	0.050114	RRAS;RRAS.	Ras-related	RRAS_HUM	1	0.791617
30_MTA / C	O14562	0.568793	0.00941	0.050172	UBFD1	Ubiquitin d	UBFD1_HU	1	1.483282
15_MTA / C	P11310	0.319593	0.009435	0.050172	ACADM	Medium-ch	ACADM_HU	12	1.247978
15_MTA / C	Q13564	-0.91221	0.009462	0.050172	NAE1	NEDD8-acti	ULA1_HUM	3	0.53137
15_MTA / C	P01857	-1.46949	0.009552	0.050458	IGHG1	Immunoglo	IGHG1_HUI	1	0.361111
15_MTA / C	P35221	0.423997	0.009547	0.050458	CTNNA1	Catenin alp	CTNA1_HUI	9	1.341639
15_MTA / C	O14735	0.980742	0.009672	0.050936	CDIPT	CDP-diacyl;	CDIPT_HUN	2	1.97348
15_MTA / C	P26885	-0.3464	0.009714	0.050936	FKBP2	Peptidyl-pr	FKBP2_HUI	1	0.786542
30_MTA / C	Q9BY44	-0.26805	0.009779	0.051036	EIF2A	Eukaryotic	EIF2A_HUN	13	0.830441
15_MTA / C	Q08211	0.925926	0.009809	0.05111	DHX9	ATP-depen(	DHX9_HUM	8	1.899904
30_MTA / C	P06454	-3.11042	0.009862	0.05115	PTMA	Prothymos	PTMA_HUM	3	0.11579
30_MTA / C	P54577	-0.78885	0.009863	0.05115	YARS1	Tyrosine--tI	SYYC_HUM/	6	0.578805
15_MTA / C	P29692	-0.75981	0.009978	0.051589	EEF1D	Elongation	EF1D_HUM	7	0.590575
30_MTA / C	A6NHQ2	0.850282	0.010149	0.052139	FBLL1	rRNA/tRNA	FBLL1_HUN	1	1.802854
15_MTA / C	O14818	-0.48122	0.010163	0.052139	PSMA7	Proteasom	PSA7_HUM	13	0.716371
15_MTA / C	P01871	-2.92595	0.010209	0.052139	IGHM	Immunoglo	IGHM_HUM	2	0.131584
15_MTA / C	P49419	-0.32247	0.010194	0.052139	ALDH7A1	Alpha-amir	AL7A1_HUI	7	0.799699
15_MTA / C	P55786	-0.21578	0.010226	0.052139	NPEPPS	Puromycin	PSA_HUMA	17	0.861079
30_MTA / C	P60953	-0.69964	0.010223	0.052139	CDC42	Cell divisior	CDC42_HU	3	0.615726
15_MTA / C	P17844	-0.52265	0.010252	0.05219	DDX5	Probable A	DDX5_HUN	7	0.696094
30_MTA / C	Q02543	-0.89165	0.010372	0.052469	RPL18A	Large ribos	RL18A_HUI	6	0.538997
30_MTA / C	Q08357	-1.70801	0.01036	0.052469	SLC20A2	Sodium-de	S20A2_HUI	1	0.306081
15_MTA / C	Q7L1Q6	-0.54557	0.01039	0.052469	BZW1	eIF5-mimic	5MP2_HUN	9	0.685121
30_MTA / C	Q9HC07	-0.63041	0.010514	0.052669	TMEM165	Putative di	TM165_HU	3	0.645994
30_MTA / C	Q9BX68	2.470645	0.010575	0.052728	HINT2	Adenosine !	HINT2_HUI	1	5.542915
15_MTA / C	P15924	-0.85556	0.010709	0.053049	DSP	Desmoplak	DESP_HUM	32	0.552653
15_MTA / C	Q9H9B4	0.418906	0.010702	0.053049	SFXN1	Sideroflexi	SFXN1_HUI	6	1.336913
15_MTA / C	Q16629	-0.49496	0.010764	0.053241	SRSF7	Serine/argi	SRSF7_HUN	3	0.709583
15_MTA / C	P45954	1.35982	0.01093	0.0539	ACADSB	Short/bran	ACDSB_HUI	4	2.566531
30_MTA / C	P24539	-0.61461	0.011046	0.05416	ATP5PB	ATP syntha	AT5F1_HUN	9	0.653107
15_MTA / C	P54577	-0.65164	0.011146	0.05416	YARS1	Tyrosine--tI	SYYC_HUM/	6	0.636554
15_MTA / C	P57088	0.656157	0.011132	0.05416	TMEM33	Transmemb	TMM33_HU	2	1.575879
30_MTA / C	Q8TEA8	0.597038	0.011081	0.05416	DTD1	D-aminoac	DTD1_HUM	1	1.512608
30_MTA / C	Q9NZM1	-0.36063	0.0111	0.05416	MYOF	Myoferlin	MYOF_HUM	9	0.778825
15_MTA / C	O15145	0.436725	0.011167	0.054184	ARPC3	Actin-relate	ARPC3_HUI	3	1.353528
15_MTA / C	Q14919	-1.66997	0.011233	0.054346	DRAP1	Dr1-associa	NC2A_HUM	2	0.31426
15_MTA / C	O95070	0.721223	0.011342	0.054633	YIF1A	Protein YIF	YIF1A_HUM	2	1.648579
30_MTA / C	Q96P70	-0.33415	0.01134	0.054633	IPO9	Importin-9	IPO9_HUM	4	0.793254
30_MTA / C	P62820	-0.54514	0.011405	0.054779	RAB1A	Ras-related	RAB1A_HUI	6	0.685326
15_MTA / C	P69849;Q1	0.611637	0.011446	0.054861	NOMO3;NC	BOS comple	NOMO3_HU	9	1.527992
15_MTA / C	Q7KZF4	-0.35897	0.011456	0.054861	SND1	Staphyloco	SND1_HUM	16	0.779724
30_MTA / C	Q9UHW5	-1.75165	0.011589	0.055183	GPN3	GPN-loop G	GPN3_HUM	1	0.296961
15_MTA / C	P61978	-0.18378	0.011617	0.055237	HNRNPK	Heterogene	HNRPK_HU	9	0.880395
15_MTA / C	P25787	-0.35991	0.011762	0.055801	PSMA2	Proteasom	PSA2_HUM	11	0.779212
15_MTA / C	Q8NBK3	1.247669	0.011803	0.055801	SUMF1	Formylglyc	SUMF1_HU	1	2.374574
15_MTA / C	Q96KP4	-0.37406	0.011824	0.055819	CNDP2	Cytosolic n	CNDP2_HU	9	0.771609
30_MTA / C	P46108	1.607123	0.011842	0.055822	CRK	Adapter mc	CRK_HUMA	1	3.046437

15_MTA / C	O95777	-0.34698	0.011914	0.056039	LSM8	U6 snRNA-a	LSM8_HUM	3	0.78623
15_MTA / C	Q9BXR0	0.593713	0.011921	0.056039	QTRT1	Queuine tR	TGT_HUMA	3	1.509126
30_MTA / C	Q9Y2B0	0.837787	0.011975	0.056052	CNPY2	Protein car	CNPY2_HU	4	1.787306
30_MTA / C	O43390	0.114416	0.012087	0.056182	HNRNPR	Heterogene	HNRPR_HU	5	1.082537
30_MTA / C	Q15904	-0.52081	0.012072	0.056182	ATP6AP1	V-type prot	VAS1_HUM	3	0.696981
30_MTA / C	Q9UKU7	-1.19169	0.012046	0.056182	ACAD8	Isobutyryl-	ACAD8_HU	2	0.437789
15_MTA / C	O95373	-0.42103	0.012248	0.05653	IPO7	Importin-7	IPO7_HUM	8	0.746893
30_MTA / C	Q14116	0.585003	0.012218	0.05653	IL18	Interleukin	IL18_HUM	6	1.500042
30_MTA / C	Q9H299	1.551324	0.012216	0.05653	SH3BGRL3	SH3 domai	SH3L3_HU	2	2.930859
30_MTA / C	Q14978	0.252083	0.012396	0.056975	NOLC1	Nucleolar a	NOLC1_HU	14	1.190925
30_MTA / C	Q9Y5J7	-1.00651	0.01253	0.057355	TIMM9	Mitochond	TIM9_HUM	3	0.497748
15_MTA / C	P10909	-0.25465	0.012598	0.057509	CLU	Clusterin	CLUS_HUM	7	0.83819
30_MTA / C	Q9HDC9	-0.292	0.012804	0.057967	APMAP	Adipocyte	APMAP_HU	10	0.816769
30_MTA / C	O14744	-0.44014	0.012824	0.05798	PRMT5	Protein arg	ANM5_HUM	7	0.737062
30_MTA / C	P62891;Q5	2.23083	0.012912	0.058138	RPL39;RPL	Large ribos	RL39_HUM	1	4.69404
30_MTA / C	Q9H6E4	-1.02388	0.01289	0.058138	CCDC134	Coiled-coil	CC134_HU	1	0.491793
15_MTA / C	P10599	-0.33558	0.01293	0.058141	TXN	Thioredoxi	THIO_HUM	4	0.792467
15_MTA / C	Q06830	-0.35901	0.012978	0.058246	PRDX1	Peroxiredo	PRDX1_HU	13	0.779697
15_MTA / C	P0DP23;P0	-0.94448	0.013027	0.058342	CALM1;CAL	Calmodulin	CALM1_HU	6	0.519617
30_MTA / C	P55769	0.987296	0.01311	0.058633	SNU13	NHP2-like	NH2L1_HU	4	1.982466
15_MTA / C	O43399	-0.41523	0.013204	0.058816	TPD52L2	Tumor prot	TPD54_HU	9	0.749902
30_MTA / C	Q9NWX6	-0.92728	0.013185	0.058816	THG1L	Probable tF	THG1_HUM	1	0.52585
15_MTA / C	P17900	-2.14216	0.013232	0.05886	GM2A	Ganglioside	SAP3_HUM	1	0.226541
15_MTA / C	P60891	-0.23081	0.013354	0.05909	PRPS1	Ribose-pho	PRPS1_HU	8	0.852157
30_MTA / C	Q9NQ84	-1.10489	0.013501	0.05909	GPRC5C	G-protein c	GPC5C_HU	5	0.464937
15_MTA / C	Q9Y241	-1.52729	0.013477	0.05909	HIGD1A	HIG1 doma	HIG1A_HU	1	0.346928
15_MTA / C	P01008	-1.66821	0.013634	0.059253	SERPINC1	Antithromb	ANT3_HUM	1	0.314643
15_MTA / C	P05362	-0.6268	0.013613	0.059253	ICAM1	Intercellula	ICAM1_HU	10	0.647612
30_MTA / C	Q9HCN8	1.271727	0.013642	0.059253	SDF2L1	Stromal cel	SDF2L_HU	2	2.414504
15_MTA / C	Q9NR31	2.191988	0.013611	0.059253	SAR1A	Small COPII	SAR1A_HU	2	4.569347
15_MTA / C	O95292	-0.41084	0.013712	0.059425	VAPB	Vesicle-ass	VAPB_HUM	2	0.752183
30_MTA / C	Q9Y281	0.853756	0.013717	0.059425	CFL2	Cofilin-2	COF2_HUM	4	1.807199
15_MTA / C	P29218	-0.31015	0.013868	0.059469	IMPA1	Inositol mo	IMPA1_HU	7	0.806558
15_MTA / C	P33316	-0.50036	0.013755	0.059469	DUT	Deoxyuridi	DUT_HUMA	4	0.706929
15_MTA / C	P60602	2.986222	0.013871	0.059469	ROMO1	Reactive ox	ROMO1_HU	1	7.923965
30_MTA / C	Q86YZ3	-1.76406	0.013834	0.059469	HRNR	Hornerin	HORN_HUM	11	0.294419
15_MTA / C	Q9P0S9	1.338161	0.013852	0.059469	TMEM14C	Transmemb	TM14C_HU	1	2.528288
30_MTA / C	P16401	0.453396	0.013979	0.059663	H1-5	Histone H1	H15_HUMA	6	1.36926
30_MTA / C	P20020	-0.64774	0.014027	0.059663	ATP2B1	Plasma mer	AT2B1_HU	7	0.638281
15_MTA / C	P31944	-0.93471	0.014043	0.059663	CASP14	Caspase-14	CASPE_HUM	2	0.523147
15_MTA / C	Q13541	4.015303	0.013946	0.059663	EIF4EBP1	Eukaryotic	4EBP1_HU	1	16.17061
30_MTA / C	P09496	0.190212	0.014208	0.060211	CLTA	Clathrin lig	CLCA_HUM	3	1.140931
30_MTA / C	Q96I36	-1.71676	0.014231	0.06023	COX14	Cytochrom	COX14_HU	1	0.304231
15_MTA / C	O95445	0.626733	0.014295	0.060347	APOM	Apolipopro	APOM_HUM	1	1.544064
15_MTA / C	P08243	-0.58364	0.014291	0.060347	ASNS	Asparagine	ASNS_HUM	3	0.66728
30_MTA / C	Q6IQ22	-0.85039	0.014456	0.060795	RAB12	Ras-related	RAB12_HU	3	0.554635
30_MTA / C	O76071	-0.43679	0.014504	0.060898	CIAO1	Probable c	CIAO1_HUM	3	0.738776
15_MTA / C	Q9H6Z4	1.986366	0.014517	0.060898	RANBP3	Ran-bindin	RANB3_HU	2	3.962376
15_MTA / C	O00232	-0.19998	0.014726	0.060939	PSMD12	26S protea	PSD12_HU	10	0.870565
30_MTA / C	P16403	-1.39444	0.014604	0.060939	H1-2	Histone H1	H12_HUMA	1	0.380394
15_MTA / C	P31025;Q5	-1.43154	0.014723	0.060939	LCN1;LCN1	Lipocalin-1	LCN1_HUM	1	0.370735
15_MTA / C	Q9BUL8	-1.05791	0.01465	0.060939	PDCD10	Programme	PDC10_HU	4	0.480326

15_MTA / C	Q15006	1.66622	0.014781	0.061042	EMC2	ER membra	EMC2_HUN	2	3.173818
15_MTA / C	O95302	0.422932	0.014842	0.061151	FKBP9	Peptidyl-pr	FKBP9_HUI	7	1.34065
30_MTA / C	Q96CQ1;Q9	-0.60693	0.014872	0.061151	SLC25A36;S	Solute carri	S2536_HUI	1	0.656593
30_MTA / C	O00487	-0.1415	0.014991	0.061409	PSMD14	26S protea:	PSDE_HUM	3	0.906578
30_MTA / C	P28482	-0.26451	0.014985	0.061409	MAPK1	Mitogen-ac	MK01_HUN	8	0.832482
30_MTA / C	P38117	0.288059	0.015181	0.061784	ETFB	Electron tra	ETFB_HUMA	9	1.220997
15_MTA / C	Q00796	-0.31744	0.015162	0.061784	SORD	Sorbitol de	DHSO_HUN	7	0.802493
15_MTA / C	Q96EP5	1.922533	0.01525	0.061784	DAZAP1	DAZ-associa	DAZP1_HUI	2	3.790882
15_MTA / C	Q9HD23	1.015712	0.015232	0.061784	MRS2	Magnesium	MRS2_HUN	3	2.021901
30_MTA / C	Q9P258	-0.22779	0.015237	0.061784	RCC2	Protein RC(	RCC2_HUN	3	0.853944
15_MTA / C	Q15637	0.493756	0.015278	0.06182	SF1	Splicing fac	SF01_HUM	3	1.408106
30_MTA / C	Q6Y1H2	-1.31908	0.015332	0.061965	HACD2	Very-long-c	HACD2_HU	2	0.40079
15_MTA / C	P05161	-0.53351	0.015419	0.062089	ISG15	Ubiquitin-l	ISG15_HUN	1	0.69087
15_MTA / C	Q12792	0.339837	0.015401	0.062089	TWF1	Twinfilin-1	TWF1_HUN	6	1.265614
15_MTA / C	P01040	-1.76836	0.015506	0.062213	CSTA	Cystatin-A	CYTA_HUM	2	0.293542
30_MTA / C	P14735	-0.69028	0.015646	0.062473	IDE	Insulin-deg	IDE_HUMAI	5	0.619733
30_MTA / C	Q96EK6	0.980055	0.016074	0.06372	GNPNAT1	Glucosamir	GNA1_HUN	4	1.972541
30_MTA / C	Q15365	0.370682	0.016182	0.063993	PCBP1	Poly(rC)-bir	PCBP1_HUI	6	1.292964
15_MTA / C	Q9NQT5	0.117866	0.01621	0.064027	EXOSC3	Exosome cc	EXOS3_HUI	1	1.085129
15_MTA / C	P68363	-0.43632	0.016323	0.06432	TUBA1B	Tubulin alp	TBA1B_HUI	18	0.739017
15_MTA / C	Q9NX46	0.789331	0.01632	0.06432	ADPRS	ADP-ribosy	ADPRS_HUI	4	1.728273
30_MTA / C	Q96DI7	2.833727	0.016536	0.06474	SNRNP40	U5 small nu	SNR40_HUI	2	7.129133
15_MTA / C	Q96PK6	1.334358	0.016499	0.06474	RBM14	RNA-bindir	RBM14_HU	4	2.521632
30_MTA / C	Q9NUM4	1.3977	0.016546	0.06474	TMEM106E	Transmemk	T106B_HUI	2	2.634812
15_MTA / C	P35613	0.336252	0.016587	0.064824	BSG	Basigin	BASI_HUMA	6	1.262473
15_MTA / C	Q92945	0.496396	0.016662	0.064961	KHSRP	Far upstrea	FUBP2_HUI	17	1.410685
30_MTA / C	O75223	0.431011	0.016766	0.065143	GGCT	Gamma-glu	GGCT_HUN	7	1.348178
15_MTA / C	O75947	0.409392	0.016786	0.065143	ATP5PD	ATP synthas	ATP5H_HUI	4	1.328126
30_MTA / C	Q9Y678	-0.27662	0.016739	0.065143	COPG1	Coatomer s	COPG1_HU	7	0.82552
15_MTA / C	P63279	-0.5495	0.017021	0.065295	UBE2I	SUMO-conj	UBC9_HUN	1	0.683258
15_MTA / C	Q15008	-0.92651	0.016946	0.065295	PSMD6	26S protea:	PSMD6_HU	5	0.526128
15_MTA / C	Q16134	1.234166	0.017023	0.065295	ETFDH	Electron tra	ETFD_HUM	1	2.352453
30_MTA / C	Q14061	0.436957	0.017119	0.065299	COX17	Cytochrom	COX17_HU	1	1.353746
30_MTA / C	Q99720	-0.69097	0.017095	0.065299	SIGMAR1	Sigma non-	SGMR1_HU	2	0.619439
30_MTA / C	Q9UNP9	0.671419	0.017123	0.065299	PPIE	Peptidyl-pr	PPIE_HUMA	2	1.592639
30_MTA / C	O95772	-0.55768	0.017283	0.065301	STARD3NL	STARD3 N-t	STR3N_HUI	1	0.679395
15_MTA / C	P00558	-0.3774	0.017322	0.065301	PGK1	Phosphogly	PGK1_HUN	33	0.769822
30_MTA / C	P19623	-1.04544	0.017184	0.065301	SRM	Spermidine	SPEE_HUMA	5	0.484497
30_MTA / C	Q14558	-1.12585	0.017182	0.065301	PRPSAP1	Phosphorib	KPRA_HUM	1	0.458231
15_MTA / C	Q7Z434	0.56272	0.017304	0.065301	MAVS	Mitochond	MAVS_HUN	6	1.477051
15_MTA / C	Q9BQA1	-0.13448	0.017208	0.065301	WDR77	Methylosor	MEP50_HU	2	0.911
30_MTA / C	P02751	0.427446	0.01742	0.065362	FN1	Fibronectir	FINC_HUMA	15	1.344851
30_MTA / C	Q12797	-0.50228	0.017382	0.065362	ASPH	Aspartyl/as	ASPH_HUN	3	0.705988
15_MTA / C	Q9H330	2.225317	0.017411	0.065362	TMEM245	Transmemk	TM245_HU	3	4.676137
15_MTA / C	Q9HB40	1.085548	0.017435	0.065362	SCPEP1	Retinoid-in	RISC_HUMA	2	2.122181
15_MTA / C	Q99714	0.496359	0.017489	0.065491	HSD17B10	3-hydroxya	HCD2_HUN	6	1.410649
15_MTA / C	P78527	-0.41259	0.017544	0.065548	PRKDC	DNA-depen	PRKDC_HU	4	0.751276
30_MTA / C	O14672	1.290148	0.017628	0.065656	ADAM10	Disintegrin	ADA10_HU	2	2.445531
15_MTA / C	P11142	-0.2589	0.017652	0.065656	HSPA8	Heat shock	HSP7C_HUI	28	0.835723
15_MTA / C	P14618	-0.31417	0.017595	0.065656	PKM	Pyruvate ki	KPYM_HUN	30	0.804313
15_MTA / C	O15305	-0.3201	0.017731	0.065872	PMM2	Phosphoma	PMM2_HUI	6	0.801013
15_MTA / C	P50440	2.960754	0.017766	0.065872	GATM	Glycine am	GATM_HUN	1	7.785305

15_MTA / C	Q9Y289	0.638587	0.01777	0.065872	SLC5A6	Sodium-de	SC5A6_HU	1	1.556804
15_MTA / C	P78381	0.636961	0.017932	0.066398	SLC35A2	UDP-galact	S35A2_HU	1	1.55505
30_MTA / C	O75822	1.275346	0.017963	0.06644	EIF3J	Eukaryotic	EIF3J_HUM	2	2.420568
15_MTA / C	Q9P0T7	0.569538	0.018009	0.066461	TMEM9	Proton-tra	TMEM9_HU	1	1.484048
15_MTA / C	Q14764	-0.14027	0.01805	0.066464	MVP	Major vault	MVP_HUM	11	0.907351
30_MTA / C	Q9Y2Z0	-0.71014	0.018034	0.066464	SUGT1	Protein SG	SGT1_HUM	7	0.611259
15_MTA / C	P13647	-0.64661	0.018155	0.06663	KRT5	Keratin, typ	K2C5_HUM	18	0.638779
30_MTA / C	O00410	-0.49815	0.018315	0.067068	IPO5	Importin-5	IPO5_HUM	8	0.708014
15_MTA / C	P04075	-0.20914	0.018306	0.067068	ALDOA	Fructose-bi	ALDOA_HU	29	0.865052
30_MTA / C	O75884	-0.59022	0.018398	0.067114	RBBP9	Serine hydr	RBBP9_HU	1	0.664242
15_MTA / C	P30086	-0.4154	0.018403	0.067114	PEBP1	Phosphatid	PEBP1_HU	6	0.749811
30_MTA / C	Q9NPA0	-0.14842	0.018396	0.067114	EMC7	Endoplasm	EMC7_HUM	3	0.902238
30_MTA / C	Q53HC9	-1.66242	0.018458	0.067147	EIPR1	EARP and G	EIPR1_HUN	1	0.315908
15_MTA / C	Q9ULA0	-0.32713	0.018513	0.067197	DNPEP	Aspartyl am	DNPEP_HU	4	0.797118
30_MTA / C	Q9Y570	-1.9195	0.018666	0.067604	PPME1	Protein pho	PPME1_HU	1	0.264346
30_MTA / C	P35637	0.564603	0.018726	0.06775	FUS	RNA-bindir	FUS_HUMA	7	1.478981
30_MTA / C	P00966	-0.25197	0.018781	0.067874	ASS1	Argininosu	ASSY_HUM	15	0.83975
30_MTA / C	O76021	0.657437	0.018996	0.068575	RSL1D1	Ribosomal	RL1D1_HU	5	1.577278
15_MTA / C	O75390	0.508815	0.019252	0.069197	CS	Citrate synt	CISY_HUM	8	1.422881
15_MTA / C	Q8WWM7	2.786519	0.019272	0.069197	ATXN2L	Ataxin-2-lik	ATX2L_HUN	1	6.899628
15_MTA / C	Q9NZJ7	2.321472	0.019331	0.069332	MTCH1	Mitochond	MTCH1_HU	2	4.998418
15_MTA / C	P30101	-0.25891	0.019366	0.069368	PDIA3	Protein dis	PDIA3_HUN	29	0.835717
30_MTA / C	Q9P2B2	-0.51618	0.019424	0.069441	PTGFRN	Prostaglan	FPRP_HUM	4	0.699222
30_MTA / C	P45954	0.819579	0.019448	0.069452	ACADSB	Short/bran	ACDSB_HU	4	1.76489
15_MTA / C	O14562	0.578904	0.019587	0.069666	UBFD1	Ubiquitin d	UBFD1_HU	1	1.493714
30_MTA / C	P18084	-0.22725	0.019616	0.069666	ITGB5	Integrin be	ITB5_HUM	1	0.854262
15_MTA / C	P28066	-0.4682	0.019709	0.069723	PSMA5	Proteasom	PSA5_HUM	5	0.722865
15_MTA / C	P61086	-0.40693	0.019734	0.069723	UBE2K	Ubiquitin-c	UBE2K_HU	5	0.754226
30_MTA / C	Q5W111	-0.1062	0.019729	0.069723	SPRYD7	SPRY doma	SPRY7_HUN	1	0.929031
15_MTA / C	Q96EC8	0.704	0.019773	0.069784	YIPF6	Protein YIP	YIPF6_HUM	2	1.629015
30_MTA / C	O00625	0.379248	0.019914	0.070132	PIR	Pirin	PIR_HUMA	7	1.300664
30_MTA / C	P49768;P4	-1.0234	0.019999	0.070282	PSEN1;PSE	Presenilin-	PSN1_HUM	1	0.491955
15_MTA / C	P56211	-2.17844	0.020042	0.070287	ARPP19	cAMP-regul	ARP19_HU	1	0.220914
15_MTA / C	Q9NZL9	-0.27072	0.02012	0.07041	MAT2B	Methionine	MAT2B_HU	7	0.828909
30_MTA / C	Q9H7C9	0.709192	0.020232	0.070577	AAMDC	Mth938 do	AAMDC_HU	4	1.634888
30_MTA / C	O15127	-1.23907	0.020297	0.070655	SCAMP2	Secretory c	SCAM2_HU	4	0.423646
15_MTA / C	Q92692	0.990437	0.020281	0.070655	NECTIN2	Nectin-2	NECT2_HU	3	1.986787
30_MTA / C	P11233	-0.2536	0.020445	0.070787	RALA	Ras-related	RALA_HUM	4	0.838801
15_MTA / C	P63244	-0.13061	0.020438	0.070787	RACK1	Small ribos	RACK1_HU	17	0.913444
15_MTA / C	Q8NFH4	-0.34079	0.020367	0.070787	NUP37	Nucleopori	NUP37_HU	2	0.789611
15_MTA / C	Q9H299	0.727283	0.0204	0.070787	SH3BGRL3	SH3 domai	SH3L3_HU	2	1.655519
30_MTA / C	Q9UBC9	6.866383	0.020475	0.070787	SPRR3	Small proli	SPRR3_HU	4	116.6775
30_MTA / C	P39687	0.479367	0.020581	0.071049	ANP32A	Acidic leuc	AN32A_HU	4	1.394132
30_MTA / C	Q92930	-0.56664	0.020612	0.071082	RAB8B	Ras-related	RAB8B_HU	1	0.675188
15_MTA / C	Q13200	-0.1634	0.020672	0.07114	PSMD2	26S protea	PSMD2_HU	14	0.89292
15_MTA / C	P48444	0.454138	0.020733	0.071272	ARCN1	Coatomer s	COPD_HUM	7	1.369964
15_MTA / C	Q14126	0.714592	0.020869	0.071521	DSG2	Desmogleir	DSG2_HUM	2	1.641019
15_MTA / C	P35914	1.661697	0.020991	0.071864	HMGCL	Hydroxyme	HMGCL_HU	1	3.163885
30_MTA / C	P04424	-1.07003	0.021019	0.071886	ASL	Argininosu	ARLY_HUM	1	0.476309
15_MTA / C	Q99575	1.536374	0.021176	0.072273	POP1	Ribonuclea	POP1_HUM	2	2.900646
15_MTA / C	P04275	-1.90751	0.021225	0.072291	VWF	von Willeb	VWF_HUM	10	0.266552
30_MTA / C	P14550	-1.0147	0.021416	0.072568	AKR1A1	Aldo-keto r	AK1A1_HU	2	0.494931

15_MTA / C	P04114	-1.21301	0.021487	0.072623	APOB	Apolipopro	APOB_HUM	27	0.431367
15_MTA / C	Q15165	0.966835	0.021498	0.072623	PON2	Serum para	PON2_HUM	1	1.954548
15_MTA / C	Q9NQE9	3.386612	0.021522	0.072632	HINT3	Adenosine	HINT3_HUM	1	10.45856
30_MTA / C	P04083	6.472418	0.021584	0.072636	ANXA1	Annexin A1	ANXA1_HU	5	88.79569
30_MTA / C	P13929	0.537002	0.021578	0.072636	ENO3	Beta-enolas	ENOB_HUM	2	1.450954
15_MTA / C	P15954	0.349487	0.021589	0.072636	COX7C	Cytochrom	COX7C_HU	1	1.274107
30_MTA / C	Q9UM00	-1.41766	0.021674	0.072848	TMCO1	Calcium loa	TMCO1_HU	3	0.374318
30_MTA / C	O43670	1.442916	0.021821	0.073142	ZNF207	BUB3-inter	ZN207_HU	3	2.718697
30_MTA / C	P02768	0.999129	0.021828	0.073142	ALB	Albumin	ALBU_HUM	4	1.998793
15_MTA / C	O43674	0.735039	0.022227	0.07433	NDUFB5	NADH dehy	NDUB5_HU	2	1.664442
30_MTA / C	P69905	-2.21102	0.02256	0.07529	HBA1	Hemoglobi	HBA_HUMA	2	0.215981
15_MTA / C	P49902	0.486838	0.022628	0.075441	NT5C2	Cytosolic p	5NTC_HUM	8	1.40137
30_MTA / C	P35998	-0.29913	0.022656	0.07546	PSMC2	26S proteas	PRS7_HUM	11	0.812741
15_MTA / C	Q07065	0.362221	0.022681	0.075466	CKAP4	Cytoskeleto	CKAP4_HU	13	1.285403
15_MTA / C	P16949	-0.30026	0.022904	0.075978	STMN1	Stathmin	STMN1_HU	6	0.812105
30_MTA / C	Q969X5	-0.10273	0.022957	0.07608	ERGIC1	Endoplasm	ERGI1_HUM	5	0.931267
15_MTA / C	O75940	1.528162	0.023325	0.076867	SMNDC1	Survival of	SPF30_HUM	1	2.884181
30_MTA / C	P52926	-1.75471	0.023339	0.076867	HMGA2	High mobil	HMGA2_HU	1	0.296334
15_MTA / C	Q96B26	1.048703	0.023357	0.076867	EXOSC8	Exosome co	EXOS8_HUI	3	2.06867
30_MTA / C	Q15517	-2.67092	0.023564	0.0771	CDSN	Corneodesr	CDSN_HUM	3	0.157027
30_MTA / C	Q12874	1.96232	0.023686	0.077335	SF3A3	Splicing fac	SF3A3_HUM	2	3.896881
30_MTA / C	Q92598	-0.40258	0.023748	0.07746	HSPH1	Heat shock	HS105_HUI	10	0.756503
15_MTA / C	O15127	-0.9577	0.023826	0.077641	SCAMP2	Secretory c	SCAM2_HU	4	0.514876
30_MTA / C	O75915	-0.62049	0.023912	0.077842	ARL6IP5	PRA1 family	PRAF3_HUI	1	0.650451
15_MTA / C	P04040	-0.37518	0.024266	0.078344	CAT	Catalase	CATA_HUM	12	0.771009
15_MTA / C	P04264	-1.23598	0.024141	0.078344	KRT1	Keratin, typ	K2C1_HUM	32	0.424553
30_MTA / C	P63104	0.223594	0.024155	0.078344	YWHAZ	14-3-3 prot	1433Z_HUI	11	1.167639
30_MTA / C	P81605	-1.41235	0.024321	0.078344	DCD	Dermcidin	DCD_HUMA	4	0.375699
15_MTA / C	Q15181	-0.27595	0.024244	0.078344	PPA1	Inorganic p	IPYR_HUMA	10	0.825905
30_MTA / C	Q92616	2.568605	0.024322	0.078344	GCN1	Stalled ribo	GCN1_HUM	2	5.932357
15_MTA / C	Q9UKD2	0.925027	0.024326	0.078344	MRTO4	mRNA turn	MRT4_HUM	1	1.89872
30_MTA / C	Q13630	-0.49562	0.024462	0.078552	GFUS	GDP-L-fuco	FCL_HUMA	5	0.709256
30_MTA / C	Q9NYU2	-0.39325	0.024459	0.078552	UGGT1	UDP-glucos	UGGG1_HU	17	0.761413
15_MTA / C	Q16891	0.676443	0.024608	0.07887	IMMT	MICOS com	MIC60_HUI	7	1.598195
15_MTA / C	P09493	-1.52645	0.024718	0.079069	TPM1	Tropomyos	TPM1_HUM	2	0.347131
15_MTA / C	Q16775	-0.23216	0.024758	0.079081	HAGH	Hydroxyacy	GLO2_HUM	3	0.851359
30_MTA / C	P61978	0.478429	0.024823	0.079162	HNRNPK	Heterogene	HNRPK_HU	9	1.393226
30_MTA / C	Q13098	-0.72647	0.024843	0.079162	GPS1	COP9 signa	CSN1_HUM	6	0.604382
30_MTA / C	Q9NXR7	-0.95474	0.024866	0.079162	BABAM2	BRISC and B	BABA2_HUI	1	0.515933
15_MTA / C	P06733	-0.38046	0.024999	0.079507	ENO1	Alpha-enola	ENOA_HUM	28	0.768193
15_MTA / C	P33993	-0.82771	0.02506	0.079549	MCM7	DNA replica	MCM7_HUI	6	0.563421
15_MTA / C	P16278	0.810291	0.025086	0.079554	GLB1	Beta-galact	BGAL_HUM	1	1.753565
15_MTA / C	Q9GZP9	1.056225	0.025195	0.079694	DERL2	Derlin-2	DERL2_HUI	1	2.079484
15_MTA / C	Q9UBV2	1.229733	0.025202	0.079694	SEL1L	Protein sel-	SE1L1_HUM	5	2.345235
15_MTA / C	Q99442	1.449706	0.025549	0.080483	SEC62	Translocati	SEC62_HUM	4	2.731524
15_MTA / C	P78417	-0.30127	0.025705	0.080898	GSTO1	Glutathion	GSTO1_HUI	14	0.811535
15_MTA / C	Q8TCA0	0.788872	0.025754	0.080977	LRRC20	Leucine-ric	LRC20_HUI	4	1.727723
15_MTA / C	P02671	0.423927	0.025816	0.081093	FGA	Fibrinogen	FIBA_HUMA	10	1.341575
30_MTA / C	O60927	-0.95307	0.025857	0.081146	PPP1R11	E3 ubiquiti	PP1RB_HUI	3	0.516533
15_MTA / C	P13645	-0.5472	0.02593	0.081222	KRT10	Keratin, typ	K1C10_HUI	23	0.684347
30_MTA / C	P61086	-0.43916	0.025955	0.081222	UBE2K	Ubiquitin-c	UBE2K_HUI	5	0.737562
15_MTA / C	P00387	0.238633	0.026111	0.081269	CYB5R3	NADH-cyto	NB5R3_HU	4	1.179874

30_MTA / C	P13489	-0.60106	0.026117	0.081269	RNH1	Ribonuclea	RINI_HUMA	4	0.659268
30_MTA / C	P51649	-1.37602	0.026108	0.081269	ALDH5A1	Succinate-s	SSDH_HUM	1	0.38528
30_MTA / C	Q9H173	-0.64011	0.026094	0.081269	SIL1	Nucleotide	SIL1_HUMA	1	0.641662
15_MTA / C	Q9Y584	-0.44831	0.026072	0.081269	TIMM22	Mitochond	TIM22_HUI	1	0.7329
30_MTA / C	P00846	0.392761	0.026256	0.08155	MT-ATP6	ATP syntha	ATP6_HUM	1	1.312904
15_MTA / C	P08962	-0.54338	0.026414	0.081629	CD63	CD63 antig	CD63_HUM	2	0.686159
15_MTA / C	P49755	0.725975	0.026355	0.081629	TMED10	Transmemb	TMEDA_HU	5	1.654018
30_MTA / C	P67812	-0.12694	0.026454	0.081629	SEC11A	Signal pept	SC11A_HUI	3	0.915773
15_MTA / C	Q15459	-0.7603	0.026378	0.081629	SF3A1	Splicing fac	SF3A1_HUM	1	0.590375
15_MTA / C	Q9BRF8	-0.25045	0.026387	0.081629	CPPED1	Serine/thre	CPPED_HUI	5	0.840635
15_MTA / C	P54886	-0.10532	0.026649	0.081927	ALDH18A1	Delta-1-pyr	P5CS_HUM	4	0.9296
15_MTA / C	Q14116	-0.4041	0.026606	0.081927	IL18	Interleukin	IL18_HUMA	6	0.755706
15_MTA / C	Q93034	1.123672	0.02665	0.081927	CUL5	Cullin-5	CUL5_HUM	5	2.179009
30_MTA / C	P31150	-0.82303	0.026773	0.08212	GDI1	Rab GDP di	GDIA_HUM	3	0.565252
15_MTA / C	P61165	1.549842	0.026812	0.08212	TMEM258	Transmemb	TM258_HU	1	2.92785
15_MTA / C	P53007	0.322429	0.026922	0.082228	SLC25A1	Tricarboxyl	TXTP_HUM	5	1.250434
15_MTA / C	Q96G03	-0.32578	0.027089	0.082513	PGM2	Phosphope	PGM2_HUM	17	0.797868
15_MTA / C	Q15154	1.366562	0.027183	0.082647	PCM1	Pericentrio	PCM1_HUM	4	2.578554
15_MTA / C	Q9NPH2	-0.39314	0.027267	0.082749	ISYNA1	Inositol-3-p	INO1_HUM	7	0.761469
30_MTA / C	Q7Z7H5	-0.71469	0.027476	0.083032	TMED4	Transmemb	TMED4_HU	2	0.609335
30_MTA / C	Q07955	0.541349	0.027566	0.083127	SRSF1	Serine/argi	SRSF1_HUM	5	1.455333
30_MTA / C	P00533	2.460869	0.027643	0.083207	EGFR	Epidermal g	EGFR_HUM	3	5.505481
15_MTA / C	P00918	-0.40688	0.027693	0.08328	CA2	Carbonic ar	CAH2_HUM	7	0.754253
15_MTA / C	P04792	-0.37214	0.027801	0.083529	HSPB1	Heat shock	HSPB1_HUI	6	0.772636
15_MTA / C	P25311	-0.99977	0.027826	0.083529	AZGP1	Zinc-alpha-	ZA2G_HUM	1	0.50008
15_MTA / C	Q12874	1.053946	0.028119	0.083872	SF3A3	Splicing fac	SF3A3_HUM	2	2.0762
15_MTA / C	Q15365	0.299849	0.028143	0.083872	PCBP1	Poly(rC)-bir	PCBP1_HUI	6	1.231016
15_MTA / C	O43684	0.294188	0.028203	0.083948	BUB3	Mitotic che	BUB3_HUM	7	1.226194
15_MTA / C	P24666	-0.84615	0.028284	0.083991	ACP1	Low molecu	PPAC_HUM	3	0.556267
30_MTA / C	Q7Z2K6	-0.70434	0.028272	0.083991	ERMP1	Endoplasm	ERMP1_HU	3	0.613725
15_MTA / C	P35222	0.454797	0.028834	0.085392	CTNNB1	Catenin bet	CTNB1_HUI	7	1.37059
15_MTA / C	P10109	0.301569	0.02917	0.086005	FDX1	Adrenodox	ADX_HUMA	1	1.232484
15_MTA / C	Q02413	-1.02095	0.029247	0.086154	DSG1	Desmoglein	DSG1_HUM	12	0.492793
30_MTA / C	P19338	0.469906	0.029386	0.086164	NCL	Nucleolin	NUCL_HUM	17	1.38502
15_MTA / C	P36639	-0.75233	0.029412	0.086164	NUDT1	Oxidized pu	8ODP_HUM	2	0.593644
15_MTA / C	Q96FQ6	-2.23255	0.029394	0.086164	S100A16	Protein S10	S10AG_HUI	1	0.212782
15_MTA / C	P08247	1.034735	0.029579	0.08629	SYP	Synaptophy	SYPH_HUM	1	2.048737
30_MTA / C	O95825	0.71919	0.029653	0.086374	CRYZL1	Quinone ox	QORL1_HU	3	1.646257
30_MTA / C	O14548	-0.85736	0.029688	0.086375	COX7A2L	Cytochrom	COX7R_HU	2	0.55196
15_MTA / C	Q01105	-0.72956	0.029713	0.086375	SET	Protein SET	SET_HUMAI	4	0.603087
15_MTA / C	Q07812	-0.06913	0.029853	0.086525	BAX	Apoptosis r	BAX_HUMA	5	0.953216
15_MTA / C	Q12931	0.416567	0.029838	0.086525	TRAP1	Heat shock	TRAP1_HUI	9	1.334748
30_MTA / C	Q8NBJ5	-0.38457	0.030121	0.087029	COLGALT1	Procollager	GT251_HUI	10	0.766006
15_MTA / C	P18621	-0.22279	0.030321	0.087454	RPL17	Large ribos	RL17_HUM	4	0.856907
30_MTA / C	Q02952	0.214086	0.030463	0.087788	AKAP12	A-kinase an	AKA12_HUI	25	1.159969
30_MTA / C	P84098	-0.41981	0.030653	0.088028	RPL19	Large ribos	RL19_HUM	5	0.747524
30_MTA / C	P38919	-0.446	0.03071	0.088116	EIF4A3	Eukaryotic	IF4A3_HUM	8	0.734078
15_MTA / C	P50453	-0.26651	0.030778	0.088158	SERPINB9	Serpin B9	SPB9_HUM	7	0.831328
15_MTA / C	Q9NY33	-0.27298	0.030765	0.088158	DPP3	Dipeptidyl	DPP3_HUM	11	0.827611
15_MTA / C	O15118	0.646704	0.030873	0.088201	NPC1	NPC intrace	NPC1_HUM	2	1.565588
15_MTA / C	O94903	-0.82799	0.030859	0.088201	PLPBP	Pyridoxal p	PLPHP_HUI	3	0.563312
15_MTA / C	Q9NRF9	-0.30571	0.030937	0.088233	POLE3	DNA polym	DPOE3_HU	1	0.809044

30_MTA / C	P01023	0.459487	0.031053	0.088486	A2M	Alpha-2-ma	A2MG_HUM	23	1.375053
15_MTA / C	P11766	-0.38227	0.03113	0.08863	ADH5	Alcohol deh	ADHX_HUM	12	0.76723
15_MTA / C	Q99426	-0.25998	0.031163	0.088647	TBCB	Tubulin-fol	TBCB_HUM	1	0.8351
30_MTA / C	O95749	-0.94161	0.031257	0.088737	GGPS1	Geranylgera	GGPPS_HU	2	0.520652
30_MTA / C	Q13247	0.637226	0.031275	0.088737	SRSF6	Serine/argi	SRSF6_HUM	7	1.555336
15_MTA / C	P21397	0.487108	0.031383	0.088816	MAOA	Amine oxid	AOFA_HUM	4	1.401633
15_MTA / C	P32119	-0.26415	0.031454	0.08894	PRDX2	Peroxiredo	PRDX2_HU	7	0.832686
30_MTA / C	P04843	-0.269	0.031641	0.089393	RPN1	Dolichyl-di	RPN1_HUM	16	0.829893
15_MTA / C	Q8N2C7	-0.46233	0.031793	0.089747	UNC80	Protein unc	UNC80_HU	1	0.725812
30_MTA / C	Q08623	-1.31892	0.031875	0.089825	PUDP	Pseudourid	HDHD1_HU	2	0.400836
15_MTA / C	Q14696	0.513676	0.031862	0.089825	MESD	LRP chaper	MESD_HUM	4	1.427684
15_MTA / C	O00410	-0.14245	0.032057	0.090109	IPO5	Importin-5	IPO5_HUM	8	0.905978
15_MTA / C	Q629K1	-0.33533	0.032025	0.090109	TRIQK	Triple QxxK	TRIQK_HUM	1	0.792604
30_MTA / C	Q96FQ6	-1.50274	0.032038	0.090109	S100A16	Protein S10	S10AG_HU	1	0.352883
30_MTA / C	O15118	0.069925	0.032178	0.090204	NPC1	NPC intrace	NPC1_HUM	2	1.049662
30_MTA / C	P61221	0.065512	0.0322	0.090204	ABCE1	ATP-bindin	ABCE1_HU	13	1.046456
15_MTA / C	P51148	0.575219	0.032245	0.090254	RAB5C	Ras-related	RAB5C_HU	4	1.489904
15_MTA / C	Q00839	-0.081	0.032367	0.09052	HNRNPU	Heterogene	HNRPU_HU	7	0.945403
30_MTA / C	Q15067	-0.04998	0.032431	0.09062	ACOX1	Peroxisoma	ACOX1_HU	2	0.965948
15_MTA / C	P02533	-0.54856	0.032485	0.090664	KRT14	Keratin, typ	K1C14_HU	22	0.6837
30_MTA / C	P30622	-0.67841	0.032732	0.090925	CLIP1	CAP-Gly do	CLIP1_HUM	4	0.624853
30_MTA / C	Q86TM6	-0.62209	0.032679	0.090925	SYVN1	E3 ubiquiti	SYVN1_HU	2	0.649731
15_MTA / C	P02662	-3.77467	0.032786	0.090998	CSN1S1	Alpha-S1-ca	CASA1_BOV	2	0.073065
15_MTA / C	P11117	0.798762	0.032832	0.091051	ACP2	Lysosomal a	PPAL_HUM	4	1.739608
15_MTA / C	P63261	-1.68476	0.032864	0.091062	ACTG1	Actin, cyto	ACTG_HUM	1	0.311054
15_MTA / C	O00231	-0.34784	0.032992	0.091264	PSMD11	26S protea	PSD11_HU	6	0.78576
15_MTA / C	Q5JS54	-0.40465	0.03306	0.091264	PSMG4	Proteasom	PSMG4_HU	1	0.755422
30_MTA / C	Q9UDX5	-0.79686	0.032997	0.091264	MTFP1	Mitochond	MTFP1_HU	3	0.575599
30_MTA / C	Q9NR30	0.662634	0.03313	0.091267	DDX21	Nucleolar R	DDX21_HU	10	1.58297
30_MTA / C	O75131	-0.48582	0.033205	0.091357	CPNE3	Copine-3	CPNE3_HU	7	0.71409
15_MTA / C	Q15005	-0.99058	0.033246	0.091357	SPCS2	Signal pept	SPCS2_HUM	4	0.503277
30_MTA / C	P61088;Q5	0.511676	0.033477	0.091764	UBE2N;UBE	Ubiquitin-c	UBE2N_HU	5	1.425706
15_MTA / C	Q13442	-0.29548	0.033458	0.091764	PDAP1	28 kDa hea	HAP28_HU	4	0.8148
30_MTA / C	P51812	-0.88449	0.033591	0.091845	RPS6KA3	Ribosomal	KS6A3_HU	1	0.541678
15_MTA / C	P61803	0.325186	0.033581	0.091845	DAD1	Dolichyl-di	DAD1_HUM	4	1.252826
15_MTA / C	Q6FI81	-0.49381	0.033617	0.091845	CIAPIN1	Anamorsin	CPIN1_HUM	3	0.710149
30_MTA / C	Q14739	-0.30378	0.033675	0.091925	LBR	Delta(14)-st	LBR_HUMA	2	0.810128
30_MTA / C	P05090	6.120186	0.033798	0.092187	APOD	Apolipopro	APOD_HUM	5	69.56002
30_MTA / C	P05089	-0.80819	0.033843	0.092201	ARG1	Arginase-1	ARGI1_HUM	3	0.571099
30_MTA / C	P01009	0.316012	0.033905	0.092206	SERPINA1	Alpha-1-ant	A1AT_HUM	10	1.244885
30_MTA / C	Q7Z5H4	0.693859	0.033971	0.092279	VN1R5	Vomeronas	VN1R5_HU	1	1.617604
30_MTA / C	Q13641	2.067659	0.034189	0.092774	TPBG	Trophoblas	TPBG_HUM	1	4.192059
15_MTA / C	O95865	-0.08779	0.034362	0.093111	DDAH2	Putative hy	DDAH2_HU	6	0.940966
30_MTA / C	P35080	-1.32544	0.034428	0.093214	PFN2	Profilin-2	PROF2_HU	1	0.399028
15_MTA / C	Q13153	0.38559	0.0345	0.093332	PAK1	Serine/thre	PAK1_HUM	1	1.306394
15_MTA / C	P41250	-0.14931	0.034724	0.093658	GARS1	Glycine--tR	GARS_HUM	19	0.901679
15_MTA / C	Q9Y223	-0.98666	0.034835	0.093779	GNE	Bifunctiona	GLCNE_HU	7	0.504646
15_MTA / C	P04424	-0.44869	0.035183	0.094501	ASL	Argininosu	ARLY_HUM	1	0.732709
15_MTA / C	Q9Y619	0.26815	0.035313	0.094758	SLC25A15	Mitochond	ORNT1_HU	7	1.204263
30_MTA / C	P20339	-0.16562	0.035475	0.095039	RAB5A	Ras-related	RAB5A_HU	5	0.891547
30_MTA / C	Q9HAB8	-0.61592	0.035597	0.09529	PPCS	Phosphopa	PPCS_HUM	4	0.652514
15_MTA / C	Q9BV81	0.948629	0.035644	0.095339	EMC6	ER membra	EMC6_HUM	1	1.930037

15_MTA / C	O60884	0.881833	0.035694	0.095356	DNAJA2	DnaJ homo	DNJA2_HU	1	1.842714
15_MTA / C	P32929	-0.11896	0.035708	0.095356	CTH	Cystathioni	CGL_HUMA	3	0.920851
15_MTA / C	P08195	-0.1847	0.035875	0.095647	SLC3A2	Amino acid	4F2_HUMA	16	0.879833
30_MTA / C	Q5T6V5	-0.92905	0.035914	0.095676	QNG1	Queuosine	QNG1_HUN	1	0.525204
15_MTA / C	Q9H0W9	-0.5952	0.036364	0.096486	C11orf54	Ester hydro	CK054_HU	4	0.66195
15_MTA / C	O43324	-1.58713	0.036742	0.097256	EEF1E1	Eukaryotic	MCA3_HUN	1	0.332834
30_MTA / C	P49748	-0.32856	0.036839	0.097433	ACADVL	Very long-c	ACADV_HU	11	0.796331
15_MTA / C	O43809	1.100766	0.037001	0.097525	NUDT21	Cleavage ar	CPSF5_HUN	7	2.144686
15_MTA / C	O95749	-0.56824	0.037236	0.097525	GGPS1	Geranylger	GGPPS_HU	2	0.674438
15_MTA / C	P07148	-0.48157	0.037154	0.097525	FABP1	Fatty acid-b	FABPL_HUN	2	0.716197
15_MTA / C	P11172	-0.47827	0.037125	0.097525	UMPS	Uridine 5'-r	UMPS_HUN	7	0.71784
15_MTA / C	P30419	1.069667	0.037284	0.097525	NMT1	Glycylpepti	NMT1_HUN	3	2.098948
15_MTA / C	P51665	-0.59162	0.037255	0.097525	PSMD7	26S protea	PSMD7_HU	2	0.663597
15_MTA / C	P60468	0.116477	0.037179	0.097525	SEC61B	Protein trai	SC61B_HU	3	1.084084
15_MTA / C	P62995	-0.55381	0.036942	0.097525	TRA2B	Transforme	TRA2B_HU	3	0.681219
15_MTA / C	Q969H8	-0.63935	0.037285	0.097525	MYDGF	Myeloid-de	MYDGF_HU	6	0.642002
15_MTA / C	Q9NR30	0.735925	0.037152	0.097525	DDX21	Nucleolar R	DDX21_HU	10	1.665465
15_MTA / C	Q9ULC4	-0.11798	0.037269	0.097525	MCTS1	Malignant T	MCTS1_HU	2	0.921476
15_MTA / C	Q92820	-0.25152	0.037556	0.098001	GGH	Gamma-glu	GGH_HUMA	7	0.840013
15_MTA / C	O14745	0.279834	0.037636	0.098078	NHERF1	Na(+)/H(+) e	NHRF1_HU	11	1.214055
15_MTA / C	P46781	-0.19859	0.037772	0.098254	RPS9	Small ribos	RS9_HUMA	14	0.8714
15_MTA / C	Q13435	-0.41057	0.03776	0.098254	SF3B2	Splicing fac	SF3B2_HUN	3	0.752325
30_MTA / C	Q9H330	1.285488	0.038068	0.098715	TMEM245	Transmemb	TM245_HU	3	2.437646
30_MTA / C	Q9Y282	-0.593	0.038145	0.098837	ERGIC3	Endoplasm	ERGI3_HUN	2	0.662962
15_MTA / C	P30048	-0.39392	0.038193	0.098879	PRDX3	Thioredoxi	PRDX3_HU	6	0.76106
15_MTA / C	O94919	1.41603	0.038916	0.098879	ENDOD1	Endonuclea	ENDD1_HU	3	2.668502
30_MTA / C	P06132	0.316505	0.038286	0.098879	UROD	Uroporphy	DCUP_HUN	4	1.24531
15_MTA / C	P08134	0.317397	0.0383	0.098879	RHOC	Rho-relatec	RHOC_HUN	3	1.24608
15_MTA / C	P15927	0.826926	0.038704	0.098879	RPA2	Replicatior	RFA2_HUM	2	1.773901
15_MTA / C	P18669	-0.41754	0.038751	0.098879	PGAM1	Phosphogly	PGAM1_HU	15	0.748699
15_MTA / C	P35241	-0.23552	0.038835	0.098879	RDX	Radixin	RADI_HUM	25	0.84938
15_MTA / C	P49257	0.38511	0.038495	0.098879	LMAN1	Protein ERC	LMAN1_HU	6	1.305959
30_MTA / C	P49257	-0.3346	0.038831	0.098879	LMAN1	Protein ERC	LMAN1_HU	6	0.793006
15_MTA / C	P52272	-0.36515	0.038689	0.098879	HNRNPM	Heterogene	HNRPM_HU	12	0.77639
15_MTA / C	P53701	-1.74999	0.038393	0.098879	HCCS	Holocytoch	CCHL_HUM	2	0.297303
30_MTA / C	P61586	0.106615	0.038509	0.098879	RHOA	Transformi	RHOA_HUN	3	1.076699
15_MTA / C	P61981	-0.20891	0.038909	0.098879	YWHAG	14-3-3 prot	1433G_HU	14	0.865194
15_MTA / C	P68431;P8	-0.74042	0.038997	0.098879	H3C1;H3-3	Histone H3	H31_HUMA	5	0.598564
15_MTA / C	Q02952	0.257986	0.038805	0.098879	AKAP12	A-kinase an	AKA12_HU	25	1.195808
15_MTA / C	Q13257	-0.29797	0.038356	0.098879	MAD2L1	Mitotic spi	MD2L1_HU	1	0.813397
30_MTA / C	Q14847	0.501814	0.038599	0.098879	LASP1	LIM and SH	LASP1_HUN	10	1.415993
15_MTA / C	Q5VW32	-1.15621	0.038993	0.098879	BROX	BRO1 doma	BROX_HUN	1	0.44869
30_MTA / C	Q8N2C7	1.129432	0.038596	0.098879	UNC80	Protein unc	UNC80_HU	1	2.187726
30_MTA / C	Q92520	-0.71514	0.03863	0.098879	FAM3C	Protein FAN	FAM3C_HU	2	0.609148
30_MTA / C	Q9HCU5	-0.27126	0.038756	0.098879	PREB	Guanine nu	SEC12_HUN	1	0.828593
15_MTA / C	O00330	0.810892	0.039065	0.0989	PDHX	Pyruvate de	ODPX_HUN	2	1.754296
15_MTA / C	O43670	0.962113	0.039267	0.099261	ZNF207	BUB3-inter	ZN207_HU	3	1.948161
15_MTA / C	O95168	1.087143	0.03925	0.099261	NDUFB4	NADH dehy	NDUB4_HU	1	2.124528
15_MTA / C	Q16831	-0.18956	0.039344	0.099378	UPP1	Uridine pho	UPP1_HUN	5	0.87687
30_MTA / C	P02787	0.116999	0.039509	0.099645	TF	Serotransfe	TRFE_HUMA	4	1.084477
30_MTA / C	P33240	-0.31871	0.039573	0.099731	CSTF2	Cleavage sti	CSTF2_HUN	7	0.801785
15_MTA / C	O95757	-0.69174	0.039757	0.100041	HSPA4L	Heat shock	HS74L_HU	6	0.619105

30_MTA / C	O60884	1.551196	0.039907	0.100268	DNAJA2	DnaJ homo	DNJA2_HUI	1	2.9306
15_MTA / C	Q13641	2.235365	0.039885	0.100268	TPBG	Trophoblas	TPBG_HUM	1	4.70882
30_MTA / C	O14737	0.664782	0.040017	0.100391	PDCD5	Programme	PDCD5_HU	8	1.585328
15_MTA / C	Q92990	0.38789	0.040183	0.10073	GLMN	Glomulin	GLMN_HUM	1	1.308478
30_MTA / C	Q96S66	-0.45	0.040305	0.100885	CLCC1	Chloride ch	CLCC1_HUI	4	0.732041
30_MTA / C	Q49B96	-0.57423	0.040374	0.100982	COX19	Cytochrom	COX19_HU	1	0.671643
30_MTA / C	Q9BVA1	-2.10981	0.040421	0.101023	TUBB2B	Tubulin bet	TBB2B_HUI	1	0.231677
30_MTA / C	E9PRG8	2.017153	0.04064	0.101493	C11orf98	Uncharacte	CK098_HUI	1	4.047841
30_MTA / C	O75414	-0.70666	0.04068	0.101517	NME6	Nucleoside	NDK6_HUM	1	0.612737
15_MTA / C	P06744	-0.44144	0.041199	0.102329	GPI	Glucose-6-p	G6PI_HUM	16	0.7364
30_MTA / C	Q13564	-0.75563	0.041253	0.102329	NAE1	NEDD8-acti	ULA1_HUM	3	0.592286
15_MTA / C	P04080	0.796057	0.041486	0.102649	CSTB	Cystatin-B	CYTB_HUM	2	1.736349
30_MTA / C	Q9UBT2	-0.47291	0.041534	0.102649	UBA2	SUMO-activ	SAE2_HUM	5	0.720511
15_MTA / C	P09382	-0.74746	0.04167	0.102827	LGALS1	Galectin-1	LEG1_HUM	5	0.59565
15_MTA / C	P16401	0.446657	0.041726	0.102888	H1-5	Histone H1	H15_HUMA	6	1.362879
30_MTA / C	P60174	0.529216	0.041846	0.103104	TPI1	Triosephos	TPIS_HUMA	18	1.443145
30_MTA / C	O94973;O9	-0.85959	0.042018	0.103377	AP2A2;AP2	AP-2 compl	AP2A2_HUI	1	0.55111
15_MTA / C	P00491	-0.88267	0.042153	0.10355	PNP	Purine nucl	PNPH_HUM	5	0.542363
30_MTA / C	P51580	-0.84481	0.042313	0.103793	TPMT	Thiopurine	TPMT_HUM	2	0.556785
15_MTA / C	P22314	-0.2664	0.042382	0.103809	UBA1	Ubiquitin-l	UBA1_HUM	9	0.831393
15_MTA / C	P26640	-0.71669	0.04236	0.103809	VARS1	Valine--tRN	SYVC_HUM	3	0.60849
15_MTA / C	P62241	-0.25027	0.042417	0.103818	RPS8	Small ribos	RS8_HUMA	7	0.840741
30_MTA / C	O14745	0.199474	0.042951	0.103893	NHERF1	Na(+)/H(+) e	NHRF1_HU	11	1.148279
30_MTA / C	P07108	0.37415	0.042905	0.103893	DBI	Acyl-CoA-bi	ACBP_HUM	4	1.296076
30_MTA / C	P07910	2.708154	0.04272	0.103893	HNRNPC	Heterogene	HNRPC_HU	5	6.534849
15_MTA / C	P24539	0.597522	0.042888	0.103893	ATP5PB	ATP synthas	AT5F1_HUM	9	1.513115
30_MTA / C	Q07065	-0.49854	0.042853	0.103893	CKAP4	Cytoskeleto	CKAP4_HUI	13	0.707822
15_MTA / C	Q8NCW5	-0.18367	0.04257	0.103893	NAXE	NAD(P)H-hy	NNRE_HUM	6	0.880459
15_MTA / C	Q92530	1.101174	0.042519	0.103893	PSMF1	Proteasom	PSMF1_HU	3	2.145292
15_MTA / C	Q969V3	0.73347	0.042709	0.103893	NCLN	BOS comple	NCLN_HUM	5	1.662634
30_MTA / C	Q9UH65	0.497751	0.042998	0.103893	SWAP70	Switch-asso	SWP70_HU	1	1.412011
15_MTA / C	P19256	-0.44297	0.04373	0.105211	CD58	Lymphocyt	LFA3_HUM	1	0.735619
30_MTA / C	Q9Y6G9	0.623211	0.04371	0.105211	DYNC1LI1	Cytoplasmi	DC1L1_HUI	2	1.5403
30_MTA / C	Q7LG56	-0.55846	0.043785	0.105224	RRM2B	Ribonuclec	RIR2B_HUM	1	0.679027
15_MTA / C	P31327	0.400485	0.043847	0.105227	CPS1	Carbamoyl-	CPSM_HUM	38	1.319952
15_MTA / C	Q9HCN4	-0.30397	0.04385	0.105227	GPN1	GPN-loop G	GPN1_HUM	3	0.810018
30_MTA / C	Q9GZP9	0.766675	0.044247	0.105814	DERL2	Derlin-2	DERL2_HUI	1	1.701344
30_MTA / C	Q9NQX3	0.786242	0.044225	0.105814	GPHN	Gephyrin	GEPH_HUM	5	1.724577
30_MTA / C	Q9H1E3	0.754482	0.044336	0.105861	NUCKS1	Nuclear ubi	NUCKS_HU	1	1.687026
30_MTA / C	Q9NSB2	5.670739	0.04442	0.105982	KRT84	Keratin, typ	KRT84_HUI	3	50.94041
30_MTA / C	P61421	-0.59607	0.044595	0.106245	ATP6V0D1	V-type prot	VA0D1_HU	2	0.661555
30_MTA / C	O75821	-0.49873	0.045365	0.107614	EIF3G	Eukaryotic	EIF3G_HUM	6	0.707732
15_MTA / C	O15144	-0.23004	0.045591	0.107997	ARPC2	Actin-relate	ARPC2_HUI	9	0.852611
15_MTA / C	Q15286	0.457684	0.04571	0.108202	RAB35	Ras-related	RAB35_HUI	2	1.373336
15_MTA / C	P43304	0.673173	0.045758	0.108239	GPD2	Glycerol-3-	GPDM_HUI	4	1.594576
15_MTA / C	Q96A49	0.457451	0.046142	0.108758	SYAP1	Synapse-ass	SYAP1_HUM	3	1.373114
15_MTA / C	P23368	0.536942	0.046311	0.10908	ME2	NAD-depen	MAOM_HU	5	1.450894
30_MTA / C	P36639	-0.57868	0.046372	0.109145	NUDT1	Oxidized pu	8ODP_HUM	2	0.669576
30_MTA / C	O43707	0.342739	0.046756	0.109349	ACTN4	Alpha-actir	ACTN4_HUI	33	1.268162
15_MTA / C	O76003	-0.61158	0.046737	0.109349	GLRX3	Glutaredox	GLRX3_HUI	4	0.654478
30_MTA / C	O94760	0.261427	0.046612	0.109349	DDAH1	N(G),N(G)-d	DDAH1_HU	8	1.198664
30_MTA / C	Q15257	-0.65898	0.046664	0.109349	PTPA	Serine/thre	PTPA_HUM	6	0.633328

30_MTA / C	P24311	-1.60052	0.046886	0.109423	COX7B	Cytochrom	COX7B_HU	1	0.329759
30_MTA / C	Q9Y5K5	-0.61526	0.046879	0.109423	UCHL5	Ubiquitin c	UCHL5_HU	1	0.652813
15_MTA / C	P02666	-2.42139	0.047076	0.109711	CSN2	Beta-casein	CASB_BOVI	3	0.186676
15_MTA / C	P55769	0.889923	0.04706	0.109711	SNU13	NHP2-like p	NH2L1_HU	4	1.853078
30_MTA / C	P46977	-0.66769	0.047174	0.109864	STT3A	Dolichyl-di	STT3A_HUN	5	0.629515
30_MTA / C	P02452	-4.82699	0.047393	0.11006	COL1A1	Collagen al	CO1A1_HU	3	0.035231
30_MTA / C	P46087	1.810873	0.047448	0.11006	NOP2	28S rRNA (c	NOP2_HUN	4	3.508545
15_MTA / C	P55157	0.460802	0.047474	0.11006	MTTP	Microsoma	MTP_HUMA	7	1.376307
15_MTA / C	P61221	0.188977	0.047445	0.11006	ABCE1	ATP-bindin	ABCE1_HUI	13	1.139955
15_MTA / C	O95372	0.871436	0.047651	0.110277	LYPLA2	Acyl-protei	LYPA2_HUN	1	1.829483
15_MTA / C	P06280	-0.3716	0.047628	0.110277	GLA	Alpha-galac	AGAL_HUM	3	0.772925
30_MTA / C	P35573	-0.95385	0.047807	0.110483	AGL	Glycogen d	GDE_HUMA	1	0.516252
30_MTA / C	Q9Y5V0	-1.76552	0.047861	0.11053	ZNF706	Zinc finger	ZN706_HUI	1	0.294121
30_MTA / C	P49354	2.356022	0.047976	0.110565	FNTA	Protein farr	FNTA_HUM	1	5.119566
15_MTA / C	Q13423	0.912501	0.048092	0.110755	NNT	NAD(P) trar	NNTM_HUN	8	1.882306
30_MTA / C	Q9H488	-0.41111	0.048329	0.111146	POFUT1	GDP-fucose	OFUT1_HUI	3	0.752043
30_MTA / C	Q08554	-1.7432	0.048406	0.111247	DSC1	Desmocolli	DSC1_HUM	5	0.298706
15_MTA / C	O75884	-0.2941	0.048553	0.111429	RBBP9	Serine hydr	RBBP9_HUI	1	0.815579
15_MTA / C	P02751	0.574501	0.048778	0.111715	FN1	Fibronectir	FINC_HUMA	15	1.489162
15_MTA / C	O94760	-0.25337	0.048846	0.111792	DDAH1	N(G),N(G)-d	DDAH1_HU	8	0.838933
15_MTA / C	P62805	-0.66862	0.048991	0.112047	H4C1	Histone H4	H4_HUMAN	8	0.629109
30_MTA / C	P07951	0.564509	0.049267	0.112524	TPM2	Tropomyos	TPM2_HUM	5	1.478884
30_MTA / C	P54819	0.404419	0.049319	0.112565	AK2	Adenylate k	KAD2_HUM	6	1.323555
15_MTA / C	Q9NX62	0.320387	0.049405	0.112683	BPNT2	Golgi-resid	IMPA3_HUI	3	1.248666
30_MTA / C	P14854	0.398929	0.049545	0.112924	COX6B1	Cytochrom	CX6B1_HUI	2	1.318528
15_MTA / C	P46976	-0.3439	0.049635	0.113053	GYG1	Glycogenin	GLYG_HUM	5	0.78791
15_MTA / C	P10155	-0.35676	0.049737	0.113098	RO60	RNA-bindir	RO60_HUM	5	0.780918
15_MTA / C	Q9H0V9	-0.74016	0.049964	0.113411	LMAN2L	VIP36-like p	LMA2L_HU	1	0.598674
15_MTA / C	P11717	0.47255	0.050106	0.113655	IGF2R	Cation-inde	MPRI_HUM	20	1.38756
30_MTA / C	Q9BUL9	1.027526	0.050156	0.113678	RPP25	Ribonuclea	RPP25_HUI	1	2.038525
30_MTA / C	Q8TCD1	-0.67027	0.050279	0.113736	C18orf32	UPF0729 p	CR032_HUI	1	0.628387
15_MTA / C	P19623	-0.73355	0.050547	0.114266	SRM	Spermidine	SPEE_HUMA	5	0.601422
15_MTA / C	P13995	-0.0332	0.050733	0.114451	MTHFD2	Bifunctiona	MTDC_HUN	2	0.977253
30_MTA / C	P47897	-0.82132	0.050955	0.11464	QARS1	Glutamine-	SYQ_HUMA	4	0.565924
15_MTA / C	Q92973	-0.96818	0.051097	0.114847	TNPO1	Transportir	TNPO1_HU	3	0.511152
15_MTA / C	P48735	0.927406	0.051265	0.115077	IDH2	Isocitrate d	IDHP_HUM	4	1.901853
30_MTA / C	Q9Y5J9	0.457437	0.051458	0.11538	TIMM8B	Mitochond	TIM8B_HUI	3	1.3731
30_MTA / C	P14635	0.531968	0.051535	0.115396	CCNB1	G2/mitotic	CCNB1_HU	1	1.4459
15_MTA / C	P61020	0.828752	0.05152	0.115396	RAB5B	Ras-related	RAB5B_HUI	2	1.776148
30_MTA / C	P07741	-0.25978	0.051936	0.115903	APRT	Adenine ph	APT_HUMA	6	0.835218
15_MTA / C	Q6NUK1	0.486064	0.051924	0.115903	SLC25A24	Mitochond	SCMC1_HU	11	1.400618
30_MTA / C	Q6ZVX7	-2.23062	0.051901	0.115903	NCCRP1	F-box only	FBX50_HUI	1	0.213067
30_MTA / C	Q99611	-0.27927	0.051855	0.115903	SEPHS2	Selenide, w	SPS2_HUM	6	0.82401
15_MTA / C	P36551	0.813929	0.052428	0.116374	CPOX	Oxygen-dep	HEM6_HUN	4	1.757993
30_MTA / C	P54578	-0.83359	0.052347	0.116374	USP14	Ubiquitin c	UBP14_HU	2	0.561132
15_MTA / C	P63173	-1.11206	0.052418	0.116374	RPL38	Large ribos	RL38_HUM	2	0.462633
30_MTA / C	Q4G0N4	-0.69654	0.052302	0.116374	NADK2	NAD kinase	NAKD2_HU	3	0.617049
15_MTA / C	Q9UL46	-1.04068	0.052397	0.116374	PSME2	Proteasom	PSME2_HU	6	0.486097
30_MTA / C	Q9HA64	-0.8978	0.052478	0.116406	FN3KRP	Ketosamine	KT3K_HUM	3	0.536704
15_MTA / C	Q9UKY7	-1.09349	0.052775	0.116831	CDV3	Protein CD	CDV3_HUM	4	0.468628
30_MTA / C	Q8NC51	0.313314	0.052899	0.116871	SERBP1	SERPINE1 m	SERB1_HUN	6	1.242558
15_MTA / C	Q9NNW7	0.547297	0.052871	0.116871	TXNRD2	Thioredoxi	TRXR2_HUI	5	1.461345

15_MTA / C	A0A0B4J2D	-0.16751	0.052999	0.117013	GATD3B;GA	Putative glu	GAL3B_HUI	5	0.890379
30_MTA / C	P12004	0.364125	0.053088	0.117132	PCNA	Proliferatin	PCNA_HUM	12	1.287101
15_MTA / C	P02545	-0.18263	0.053182	0.117244	LMNA	Prelamin-A	LMNA_HUM	18	0.881098
30_MTA / C	Q9Y224	1.854315	0.053209	0.117244	RTRAF	RNA transcr	RTRAF_HUI	2	3.615799
30_MTA / C	O95139	-1.15372	0.053264	0.117288	NDUFB6	NADH dehy	NDUB6_HU	3	0.449465
30_MTA / C	Q9P0P0	-0.13606	0.053327	0.117349	RNF181	E3 ubiquiti	RN181_HU	1	0.910002
15_MTA / C	P22059	0.835622	0.053479	0.117454	OSBP	Oxysterol-b	OSBP1_HUI	2	1.784627
30_MTA / C	O43813	-0.22806	0.053626	0.117615	LANCL1	Glutathion	LANC1_HUI	5	0.85378
15_MTA / C	P48163	-0.30473	0.053756	0.117666	ME1	NADP-depe	MAOX_HUM	7	0.809594
15_MTA / C	Q9H9S3	0.171596	0.05373	0.117666	SEC61A2	Protein trai	S61A2_HUI	7	1.126304
15_MTA / C	Q9P287	-0.73864	0.053805	0.117696	BCCIP	BRCA2 and	BCCIP_HUM	1	0.599303
15_MTA / C	Q969T9	3.279244	0.053854	0.117726	WBP2	WW domai	WBP2_HUM	1	9.70847
15_MTA / C	Q9BVK6	0.30207	0.053998	0.117758	TMED9	Transmemb	TMED9_HU	3	1.232912
30_MTA / C	Q9NRR5	-0.81129	0.054024	0.117758	UBQLN4	Ubiquilin-4	UBQL4_HU	2	0.569872
30_MTA / C	Q9NYJ1	1.149166	0.054005	0.117758	COA4	Cytochrom	COA4_HUM	1	2.217856
15_MTA / C	Q5XKE5	-0.15918	0.054155	0.117841	KRT79	Keratin, typ	K2C79_HUI	1	0.895535
30_MTA / C	Q9H0U4	-0.3357	0.054381	0.118176	RAB1B	Ras-related	RAB1B_HUI	6	0.7924
15_MTA / C	P23284	-0.32971	0.05451	0.118302	PPIB	Peptidyl-pr	PPIB_HUM/	14	0.795695
15_MTA / C	P60709	-0.30175	0.054687	0.118354	ACTB	Actin, cyto	ACTB_HUM	23	0.811265
30_MTA / C	Q13868	0.905567	0.054625	0.118354	EXOSC2	Exosome cc	EXOS2_HUI	4	1.873281
30_MTA / C	Q9HB07	0.24661	0.054631	0.118354	MYG1	MYG1 exon	MYG1_HUM	8	1.186416
15_MTA / C	P31948	0.463508	0.054894	0.118669	STIP1	Stress-indu	STIP1_HUM	16	1.37889
30_MTA / C	P19013	4.530165	0.054977	0.118694	KRT4	Keratin, typ	K2C4_HUM	22	23.10552
30_MTA / C	P01111	0.387571	0.055137	0.118806	NRAS	GTPase NRa	RASN_HUM	5	1.308189
15_MTA / C	Q676U5	-0.61461	0.055325	0.119057	ATG16L1	Autophagy-	A16L1_HUI	1	0.653108
15_MTA / C	P29508;P4	-1.32734	0.055385	0.119107	SERPINB3;S	Serpin B3;S	SPB3_HUM	2	0.398503
15_MTA / C	O95716	1.032647	0.055479	0.119174	RAB3D	Ras-related	RAB3D_HU	2	2.045774
30_MTA / C	Q96D05	0.19803	0.055487	0.119174	FAM241B	Protein FAM	F241B_HUI	1	1.147131
15_MTA / C	P35249	0.427097	0.055559	0.119194	RFC4	Replication	RFC4_HUM	4	1.344525
15_MTA / C	Q96CT7	-0.44144	0.055917	0.119787	CCDC124	Coiled-coil	CC124_HUI	5	0.736399
30_MTA / C	P0DP23;PC	0.589737	0.056204	0.120123	CALM1;CAL	Calmodulin	CALM1_HU	6	1.504973
15_MTA / C	P61758	0.629187	0.056235	0.120123	VBP1	Prefoldin su	PFD3_HUM	5	1.546693
30_MTA / C	Q07960	-0.6085	0.056255	0.120123	ARHGAP1	Rho GTPase	RHG01_HU	2	0.65588
15_MTA / C	O14495	1.241172	0.056368	0.120286	PLPP3	Phospholip	PLPP3_HUI	2	2.363904
30_MTA / C	Q08722	-0.51921	0.056554	0.120527	CD47	Leukocyte s	CD47_HUM	3	0.697756
15_MTA / C	Q96C90	0.368018	0.056668	0.120693	PPP1R14B	Protein pho	PP14B_HUI	3	1.290578
15_MTA / C	P09417	-0.343	0.056759	0.120798	QDPR	Dihydropte	DHPR_HUM	4	0.788402
15_MTA / C	P21399	1.312207	0.05679	0.120798	ACO1	Cytoplasmi	ACOHC_HU	5	2.483211
15_MTA / C	P55263	0.501422	0.056868	0.120886	ADK	Adenosine I	ADK_HUMA	3	1.415608
15_MTA / C	Q01581	-0.19407	0.057057	0.121133	HMGCS1	Hydroxyme	HMCS1_HU	4	0.874137
30_MTA / C	Q32P28	-0.3303	0.057115	0.121178	P3H1	Prolyl 3-hy	P3H1_HUM	6	0.795371
15_MTA / C	Q9BV20	-0.15757	0.057423	0.121752	MRI1	Methylthio	MTNA_HUM	4	0.896535
15_MTA / C	Q16643	0.816376	0.057547	0.121861	DBN1	Drebrin	DREB_HUM	3	1.760977
15_MTA / C	P67936	-0.19208	0.057868	0.122383	TPM4	Tropomyos	TPM4_HUM	14	0.875342
30_MTA / C	P35249	0.978412	0.058044	0.12252	RFC4	Replication	RFC4_HUM	4	1.970296
15_MTA / C	Q9HA64	0.718492	0.058043	0.12252	FN3KRP	Ketosamine	KT3K_HUM	3	1.645461
15_MTA / C	Q9UI14	0.265989	0.058009	0.12252	RABAC1	Prenylated	PRAF1_HUI	2	1.20246
30_MTA / C	Q5RI15	-2.4293	0.058134	0.122555	COX20	Cytochrom	COX20_HU	2	0.185655
30_MTA / C	Q96JJ7	-0.21417	0.058124	0.122555	TMX3	Protein disu	TMX3_HUM	1	0.86204
15_MTA / C	O75436	-0.30322	0.058384	0.122602	VPS26A	Vacuolar pr	VP26A_HUI	7	0.810441
15_MTA / C	P08754	0.359124	0.058418	0.122602	GNAI3	Guanine nu	GNAI3_HUI	4	1.282647
15_MTA / C	P49903	-0.31738	0.058434	0.122602	SEPHS1	Selenide, w	SPS1_HUM	2	0.802528

15_MTA / C	P56381;Q5	0.474983	0.058266	0.122602	ATP5F1E;A7	ATP synthas	ATP5E_HUM	1	1.389902
30_MTA / C	P61964	-0.51738	0.058531	0.122602	WDR5	WD repeat-	WDR5_HUI	5	0.69864
15_MTA / C	Q9GZS3	-0.88579	0.05855	0.122602	SKIC8	Superkiller	SKI8_HUMA	1	0.541192
15_MTA / C	P02769	-1.5995	0.058842	0.122852	ALB	Serum albu	ALBU_BOVI	26	0.329991
30_MTA / C	Q15366	0.351408	0.058868	0.122852	PCBP2	Poly(rC)-bir	PCBP2_HUI	7	1.275805
15_MTA / C	Q99523	0.733382	0.058847	0.122852	SORT1	Sortilin	SORT_HUM	3	1.662532
15_MTA / C	P60174	-0.19748	0.058926	0.122896	TPI1	Triosephos	TPIS_HUMA	18	0.872074
15_MTA / C	P22626	-0.56639	0.058998	0.122968	HNRNPA2B	Heterogene	ROA2_HUM	10	0.675302
15_MTA / C	O96000	0.606424	0.059206	0.123247	NDUFB10	NADH dehy	NDUBA_HU	2	1.52248
30_MTA / C	P17655	-0.53777	0.059175	0.123247	CAPN2	Calpain-2 c	CAN2_HUM	1	0.688836
30_MTA / C	P27824	0.225022	0.059256	0.123273	CANX	Calnexin	CALX_HUM	13	1.168795
30_MTA / C	Q86V81	1.447085	0.059435	0.12349	ALYREF	THO compl	THOC4_HU	2	2.726565
30_MTA / C	P49419	-0.43725	0.059481	0.123509	ALDH7A1	Alpha-amir	AL7A1_HUI	7	0.738539
15_MTA / C	Q9Y2R0	0.684351	0.059663	0.12381	COA3	Cytochrom	COA3_HUM	5	1.606979
30_MTA / C	Q8IVM0	0.618024	0.059724	0.123859	CCDC50	Coiled-coil	CCD50_HU	2	1.534772
30_MTA / C	Q9H0D6	2.422032	0.059848	0.124038	XRN2	5'-3' exorib	XRN2_HUM	6	5.359253
30_MTA / C	P11172	-0.34675	0.059977	0.124227	UMPS	Uridine 5'-r	UMPS_HUM	7	0.786352
15_MTA / C	O43617	0.242337	0.060323	0.124247	TRAPPC3	Trafficking	TPPC3_HUI	1	1.182908
30_MTA / C	P10768	-0.28087	0.060361	0.124247	ESD	S-formylglu	ESTD_HUM	4	0.823096
30_MTA / C	P56385	0.607631	0.0602	0.124247	ATP5ME	ATP synthas	ATP5I_HUM	4	1.523755
30_MTA / C	P61604	0.444598	0.060192	0.124247	HSPE1	10 kDa heat	CH10_HUM	11	1.360935
15_MTA / C	P62847	0.505481	0.060287	0.124247	RPS24	Small ribos	RS24_HUM	4	1.419596
30_MTA / C	P84103	0.313715	0.060347	0.124247	SRSF3	Serine/argi	SRSF3_HUM	3	1.242904
15_MTA / C	Q86SE5	-1.72964	0.060181	0.124247	RALYL	RNA-bindir	RALYL_HUM	1	0.301528
30_MTA / C	Q9GZZ1	-0.41559	0.060256	0.124247	NAA50	N-alpha-ace	NAA50_HU	4	0.749713
30_MTA / C	Q86X29	-0.21962	0.060422	0.124295	LSR	Lipolysis-st	LSR_HUMA	5	0.858791
15_MTA / C	O43776	-0.62331	0.060708	0.124516	NARS1	Asparagine-	SYNC_HUM	5	0.649178
15_MTA / C	P02663	-4.23564	0.060845	0.124516	CSN1S2	Alpha-S2-ca	CASA2_BOV	3	0.053082
30_MTA / C	P02666	-2.3365	0.060798	0.124516	CSN2	Beta-casein	CASB_BOVI	3	0.19799
15_MTA / C	P12814	-0.12703	0.06081	0.124516	ACTN1	Alpha-actir	ACTN1_HUI	19	0.915713
15_MTA / C	Q96QK1	-0.38597	0.060868	0.124516	VPS35	Vacuolar pr	VPS35_HUI	7	0.765264
15_MTA / C	Q9Y224	0.633251	0.060745	0.124516	RTRAF	RNA transc	RTRAF_HUM	2	1.551056
15_MTA / C	P01023	0.301922	0.061116	0.124882	A2M	Alpha-2-ma	A2MG_HUM	23	1.232786
30_MTA / C	P08123	-3.80524	0.061197	0.124882	COL1A2	Collagen al	CO1A2_HU	3	0.071533
30_MTA / C	Q5T750	0.756256	0.061169	0.124882	KPLCE	Protein KPI	KPLCE_HUM	1	1.689102
15_MTA / C	Q9Y3F4	-0.19256	0.06119	0.124882	STRAP	Serine-thre	STRAP_HUM	10	0.875053
15_MTA / C	P20042	0.451711	0.061309	0.125032	EIF2S2	Eukaryotic	IF2B_HUMA	4	1.367661
15_MTA / C	O75607	0.293837	0.061348	0.125035	NPM3	Nucleoplas	NPM3_HUM	1	1.225896
15_MTA / C	P61457	-0.26929	0.061598	0.125314	PCBD1	Pterin-4-alp	PHS_HUMA	5	0.829726
30_MTA / C	P53396	-1.53429	0.062088	0.126074	ACLY	ATP-citrate	ACLY_HUM	3	0.34525
15_MTA / C	P60842	-0.20921	0.062124	0.126074	EIF4A1	Eukaryotic	IF4A1_HUM	15	0.865009
15_MTA / C	P00352	-0.23907	0.062437	0.126168	ALDH1A1	Aldehyde d	AL1A1_HUI	19	0.847289
15_MTA / C	P40925	-0.27443	0.062251	0.126168	MDH1	Malate deh	MDHC_HUI	13	0.826777
15_MTA / C	Q14978	0.26624	0.062346	0.126168	NOLC1	Nucleolar a	NOLC1_HU	14	1.202669
15_MTA / C	P15121	-0.05388	0.062562	0.126344	AKR1B1	Aldo-keto r	ALDR_HUM	6	0.963343
30_MTA / C	P08833	0.957054	0.062759	0.126553	IGFBP1	Insulin-like	IBP1_HUMA	3	1.941342
30_MTA / C	P67936	0.371752	0.06278	0.126553	TPM4	Tropomyos	TPM4_HUM	14	1.293923
15_MTA / C	P02452	-4.44009	0.063018	0.126956	COL1A1	Collagen al	CO1A1_HU	3	0.046068
30_MTA / C	Q6PIU2	-0.40022	0.06306	0.126962	NCEH1	Neutral cho	NCEH1_HU	7	0.757742
15_MTA / C	Q9UJA5	0.676068	0.063139	0.127045	TRMT6	tRNA (aden	TRM6_HUM	2	1.597779
15_MTA / C	P32189	0.821218	0.063255	0.127158	GK	Glycerol kir	GLPK_HUM	1	1.766897
15_MTA / C	Q96IJ6	-0.39935	0.063272	0.127158	GMPPA	Mannose-1	GMPPA_HU	5	0.7582

30_MTA / C P20962	0.570348	0.063356	0.127172	PTMS	Parathymo	PTMS_HUN	1	1.484882
15_MTA / C Q15046	-0.00142	0.063329	0.127172	KARS1	Lysine--tRN	SYK_HUMA	7	0.999018
15_MTA / C Q9BZG8	1.170438	0.063402	0.127188	DPH1	2-(3-amino	DPH1_HUN	1	2.250801
15_MTA / C P01834	-0.63235	0.063456	0.127218	IGKC	Immunoglc	IGKC_HUM	1	0.645127
15_MTA / C O60828	-1.16916	0.063908	0.12748	PQBP1	Polyglutam	PQBP1_HU	3	0.44468
30_MTA / C P05787	0.447586	0.064009	0.12748	KRT8	Keratin, typ	K2C8_HUN	18	1.363756
15_MTA / C P08621	0.454723	0.063926	0.12748	SNRNP70	U1 small nu	RU17_HUN	4	1.37052
15_MTA / C P46937	1.435891	0.063675	0.12748	YAP1	Transcripti	YAP1_HUM	2	2.705493
30_MTA / C Q13838	0.365156	0.063854	0.12748	DDX39B	Spliceosom	DX39B_HU	11	1.288021
30_MTA / C Q16643	1.568655	0.063754	0.12748	DBN1	Drebrin	DREB_HUN	3	2.966281
15_MTA / C Q9BV40	-1.18592	0.063806	0.12748	VAMP8	Vesicle-assc	VAMP8_HU	2	0.439546
15_MTA / C Q9NYJ1	0.439197	0.063707	0.12748	COA4	Cytochrom	COA4_HUN	1	1.35585
30_MTA / C P62829	0.394038	0.064061	0.127505	RPL23	Large ribos	RL23_HUM	6	1.314067
30_MTA / C P10109	0.309848	0.064359	0.127716	FDX1	Adrenodox	ADX_HUMA	1	1.239577
30_MTA / C Q9Y6G5	-0.87815	0.064299	0.127716	COMMD10	COMM don	COMDA_HU	1	0.544064
30_MTA / C P06865	-0.4892	0.064459	0.127839	HEXA	Beta-hexos	HEXA_HUN	3	0.712418
30_MTA / C Q14764	-0.15922	0.064541	0.127923	MVP	Major vault	MVP_HUMA	11	0.89551
15_MTA / C O60841	0.745304	0.064639	0.127966	EIF5B	Eukaryotic	IF2P_HUMA	3	1.676327
15_MTA / C P78347	-0.84614	0.064839	0.128208	GTF2I	General tra	GTF2I_HUN	1	0.55627
30_MTA / C P06737	-0.73659	0.064939	0.128253	PYGL	Glycogen p	PYGL_HUM	9	0.600155
15_MTA / C Q5T1J5;Q9	1.048424	0.064994	0.128284	CHCHD2P9	Putative co	CHCH9_HU	2	2.068269
30_MTA / C P78371	-0.16845	0.065064	0.128346	CCT2	T-complex	TCPB_HUM	18	0.8898
15_MTA / C Q99598	-0.42957	0.065138	0.128361	TSNAX	Translin-ass	TSNAX_HUI	4	0.742481
15_MTA / C P16403	3.86258	0.065271	0.128449	H1-2	Histone H1	H12_HUMA	1	14.5463
15_MTA / C Q9Y5M8	0.409795	0.065451	0.128727	SRPRB	Signal reco	SRPRB_HUI	4	1.328497
30_MTA / C O60664	-0.20395	0.065954	0.129059	PLIN3	Perilipin-3	PLIN3_HUN	13	0.868171
15_MTA / C Q99829	-0.21716	0.066004	0.129059	CPNE1	Copine-1	CPNE1_HUI	7	0.860259
15_MTA / C Q9UHR4	0.979394	0.065941	0.129059	BAIAP2L1	BAR/IMD d	BI2L1_HUN	5	1.971637
30_MTA / C P51991	1.961849	0.066259	0.12947	HNRNPA3	Heterogene	ROA3_HUN	2	3.89561
15_MTA / C O43847	-0.75961	0.066465	0.129666	NRDC	Nardilysin	NRDC_HUN	3	0.590657
30_MTA / C P46459	1.648576	0.066534	0.129666	NSF	Vesicle-fusi	NSF_HUMA	1	3.13524
30_MTA / C P61224	-0.986	0.066496	0.129666	RAP1B	Ras-related	RAP1B_HUI	3	0.504876
30_MTA / C Q14103	0.315863	0.066555	0.129666	HNRNPD	Heterogene	HNRPD_HU	3	1.244756
15_MTA / C P12955	-0.39218	0.06683	0.13005	PEPD	Xaa-Pro dip	PEPD_HUN	12	0.761975
15_MTA / C P19338	-0.19572	0.066991	0.130191	NCL	Nucleolin	NUCL_HUN	17	0.873139
15_MTA / C P41440	0.958633	0.06702	0.130191	SLC19A1	Reduced fo	S19A1_HUI	3	1.943467
15_MTA / C P47897	-0.53785	0.066955	0.130191	QARS1	Glutamine-	SYQ_HUMA	4	0.688798
15_MTA / C Q9Y6N7	-0.21579	0.067116	0.130242	ROBO1	Roundabou	ROBO1_HU	8	0.861073
30_MTA / C Q96EC8	-1.51865	0.067196	0.130303	YIPF6	Protein YIP	YIPF6_HUN	2	0.349013
15_MTA / C Q96P63	-1.56099	0.067238	0.130307	SERPINB12	Serpin B12	SPB12_HUI	7	0.338919
15_MTA / C Q15102	-0.57835	0.067314	0.13038	PAFAH1B3	Platelet-act	PA1B3_HUI	4	0.669728
15_MTA / C O75821	-0.09377	0.067464	0.130531	EIF3G	Eukaryotic	EIF3G_HUN	6	0.937068
15_MTA / C Q15517	-2.16449	0.067711	0.130842	CDSN	Corneodesr	CDSN_HUN	3	0.223061
15_MTA / C Q96GA7	0.882937	0.067688	0.130842	SDSL	Serine dehy	SDSL_HUM	4	1.844126
30_MTA / C P01876	1.208238	0.067883	0.130945	IGHA1	Immunoglc	IGHA1_HUI	2	2.310553
30_MTA / C Q14696	0.482816	0.067806	0.130945	MESD	LRP chaper	MESD_HUN	4	1.397469
15_MTA / C Q9HA77	1.511607	0.067995	0.131008	CARS2	Probable cy	SYCM_HUN	1	2.851274
30_MTA / C Q9Y3C6	-0.75205	0.068206	0.131186	PPIL1	Peptidyl-pr	PPIL1_HUN	3	0.593759
30_MTA / C P27816	0.391709	0.068247	0.13119	MAP4	Microtubul	MAP4_HUN	22	1.311947
30_MTA / C Q13153	0.65298	0.068337	0.131191	PAK1	Serine/thre	PAK1_HUN	1	1.572412
15_MTA / C P46063	0.78565	0.068584	0.131303	RECQL	ATP-depenc	RECQ1_HUI	4	1.723869
30_MTA / C O95721	-0.673	0.068864	0.131764	SNAP29	Synaptosor	SNP29_HUI	1	0.627203

15_MTA / C	P46108	1.57633	0.068982	0.131838	CRK	Adapter mc	CRK_HUMA	1	2.982102
15_MTA / C	Q6RW13	0.429069	0.068978	0.131838	AGTRAP	Type-1 angi	ATRAP_HUI	3	1.346364
15_MTA / C	O43826	1.100759	0.069135	0.131904	SLC37A4	Glucose-6-p	G6PT1_HUI	3	2.144674
15_MTA / C	Q07955	-0.24699	0.069117	0.131904	SRSF1	Serine/argi	SRSF1_HUN	5	0.842653
30_MTA / C	Q8TCT9	-0.43914	0.069136	0.131904	HM13	Minor histc	HM13_HUN	6	0.737573
30_MTA / C	Q9H061	-0.12478	0.069224	0.131996	TMEM126A	Transmemb	T126A_HUI	2	0.917143
30_MTA / C	P56381;Q5	-0.07135	0.069268	0.132004	ATP5F1E;AT	ATP synthas	ATP5E_HUN	1	0.951745
15_MTA / C	P06748	-0.31728	0.069663	0.132452	NPM1	Nucleopho	NPM_HUM	6	0.80258
30_MTA / C	Q96I24	0.706793	0.070116	0.132932	FUBP3	Far upstrea	FUBP3_HUI	3	1.632171
30_MTA / C	P55854;P6	0.417524	0.070187	0.13299	SUMO3;SUI	Small ubiqu	SUMO3_HU	1	1.335633
15_MTA / C	P17174	-0.36236	0.070236	0.133006	GOT1	Aspartate a	AATC_HUM	17	0.77789
15_MTA / C	Q06265	0.317034	0.0707	0.133732	EXOSC9	Exosome cc	EXOS9_HUI	3	1.245767
15_MTA / C	P05155	0.463649	0.071078	0.134159	SERPING1	Plasma pro	IC1_HUMAI	1	1.379025
30_MTA / C	O14979	1.027031	0.071209	0.134235	HNRNPDL	Heterogene	HNRDL_HU	3	2.037827
30_MTA / C	Q16531	0.473235	0.071182	0.134235	DDB1	DNA damag	DDB1_HUN	30	1.388219
15_MTA / C	Q9HC07	0.512955	0.071396	0.134513	TMEM165	Putative di	TM165_HU	3	1.42697
30_MTA / C	Q5D862	-1.27436	0.071644	0.134903	FLG2	Filaggrin-2	FILA2_HUN	2	0.413408
30_MTA / C	P46783;Q9	-0.33131	0.07174	0.134942	RPS10;RPS	Small ribos	RS10_HUM	3	0.794813
30_MTA / C	Q99442	0.590881	0.071747	0.134942	SEC62	Translocati	SEC62_HUN	4	1.506166
15_MTA / C	P49959	0.612574	0.071888	0.134998	MRE11	Double-stra	MRE11_HU	3	1.528984
30_MTA / C	P54920	2.209273	0.071835	0.134998	NAPA	Alpha-solul	SNAA_HUN	2	4.624421
30_MTA / C	P31483	0.038535	0.072126	0.135272	TIA1	Cytotoxic g	TIA1_HUMA	3	1.02707
15_MTA / C	P51659	0.195823	0.07211	0.135272	HSD17B4	Peroxisoma	DHB4_HUN	10	1.145377
15_MTA / C	L0R6Q1	0.300666	0.072625	0.135814	SLC35A4	SLC35A4 u	S35U4_HUI	4	1.231713
15_MTA / C	P17096	-0.39908	0.072626	0.135814	HMGA1	High mobil	HMGA1_HU	4	0.758339
30_MTA / C	Q9BZE4	1.042454	0.072707	0.135824	GTPBP4	GTP-bindin	GTPB4_HUI	1	2.059729
15_MTA / C	P55854;P6	-0.23737	0.072887	0.136084	SUMO3;SUI	Small ubiqu	SUMO3_HU	1	0.848289
15_MTA / C	Q13867	-0.3036	0.073039	0.136219	BLMH	Bleomycin	BLMH_HUN	3	0.810229
15_MTA / C	P05455	-0.46893	0.073367	0.136501	SSB	Lupus La pr	LA_HUMAN	9	0.722499
15_MTA / C	P30044	-0.3537	0.073399	0.136501	PRDX5	Peroxiredo	PRDX5_HU	4	0.782573
15_MTA / C	Q8N1N4	-1.39847	0.073388	0.136501	KRT78	Keratin, typ	K2C78_HUI	5	0.379331
30_MTA / C	Q9H936	-0.3559	0.073535	0.136639	SLC25A22	Mitochond	GHC1_HUN	4	0.781383
15_MTA / C	P00761	-0.34502	0.073772	0.136657	NaN	Trypsin (Lal	TRYP_PIG	3	0.787296
30_MTA / C	P18206	0.303447	0.073637	0.136657	VCL	Vinculin	VINC_HUM	53	1.23409
15_MTA / C	P54920	1.070289	0.073753	0.136657	NAPA	Alpha-solul	SNAA_HUN	2	2.099854
30_MTA / C	P62195	-0.09598	0.073663	0.136657	PSMC5	26S protea	PRS8_HUM	7	0.935636
30_MTA / C	Q01650	-0.2827	0.073766	0.136657	SLC7A5	Large neutr	LAT1_HUM	6	0.822049
15_MTA / C	P18206	-0.29701	0.073932	0.136838	VCL	Vinculin	VINC_HUM	53	0.81394
30_MTA / C	Q92947	-0.59014	0.073981	0.136838	GCDH	Glutaryl-Co	GCDH_HUN	3	0.664277
15_MTA / C	Q96G23	1.393017	0.074035	0.136838	CERS2	Ceramide sy	CERS2_HUN	1	2.626274
30_MTA / C	P42704	2.280746	0.07429	0.137233	LRPPRC	Leucine-ric	LPPRC_HUI	7	4.859292
30_MTA / C	P35606	-0.29212	0.074367	0.137299	COPB2	Coatomer s	COPB2_HU	9	0.816701
15_MTA / C	P12081	-0.14061	0.074518	0.137502	HARS1	Histidine--t	HARS1_HUI	11	0.907136
15_MTA / C	P08865	-0.23579	0.074565	0.137511	RPSA	Small ribos	RSSA_HUM	9	0.849218
30_MTA / C	P22695	-0.14515	0.074783	0.137684	UQCRC2	Cytochrom	QCR2_HUN	7	0.904284
15_MTA / C	P31040	0.278449	0.074825	0.137684	SDHA	Succinate c	SDHA_HUN	10	1.21289
15_MTA / C	P31943	-0.48352	0.074805	0.137684	HNRNPH1	Heterogene	HNRH1_HU	5	0.71523
30_MTA / C	P08708	2.004119	0.074922	0.13771	RPS17	Small ribos	RS17_HUM	3	4.011436
30_MTA / C	Q96EP5	0.047101	0.074885	0.13771	DAZAP1	DAZ-associa	DAZP1_HUI	2	1.033187
15_MTA / C	P35527	-1.03411	0.075067	0.137749	KRT9	Keratin, typ	K1C9_HUM	25	0.488317
30_MTA / C	P38646	0.297063	0.07511	0.137749	HSPA9	Stress-70 pi	GRP75_HU	27	1.228641
30_MTA / C	Q7Z7K0	0.389029	0.07508	0.137749	CMC1	COX assemI	COXM1_HU	1	1.309512

30_MTA / C	Q9NR45	0.951817	0.075231	0.137896	NANS	Sialic acid s	SIAS_HUMA	2	1.934307
15_MTA / C	P60660	-0.60209	0.075285	0.137919	MYL6	Myosin ligh	MYL6_HUM	2	0.6588
15_MTA / C	Q8TDQ7	1.559823	0.075375	0.138006	GNPDA2	Glucosamir	GNPI2_HUI	1	2.948176
15_MTA / C	Q06323	-0.7723	0.075692	0.138345	PSME1	Proteasom	PSME1_HU	6	0.585484
15_MTA / C	Q07817	0.509053	0.075735	0.138345	BCL2L1	Bcl-2-like p	B2CL1_HUI	1	1.423116
30_MTA / C	Q15075	0.678405	0.07581	0.138345	EEA1	Early endos	EEA1_HUM	19	1.600369
30_MTA / C	P12814	0.29161	0.076266	0.138947	ACTN1	Alpha-actir	ACTN1_HUI	19	1.224006
30_MTA / C	P50897	-0.34459	0.076398	0.138958	PPT1	Palmitoyl-p	PPT1_HUM	3	0.78753
30_MTA / C	Q9NWV8	-2.14543	0.07636	0.138958	BABAM1	BRISC and E	BABA1_HUI	2	0.226027
15_MTA / C	P37802	-0.17912	0.076447	0.138971	TAGLN2	Transgelin-:	TAGL2_HUI	6	0.88324
15_MTA / C	P62306	-0.76967	0.076703	0.139208	SNRPF	Small nucle	RUXF_HUM	3	0.586552
30_MTA / C	O95716	0.628923	0.076919	0.139299	RAB3D	Ras-related	RAB3D_HU	2	1.54641
30_MTA / C	O43674	1.385877	0.077067	0.139331	NDUFB5	NADH dehy	NDUB5_HU	2	2.613307
15_MTA / C	Q5JS37	-0.28449	0.077107	0.139331	NHLRC3	NHL repeat	NHLC3_HU	1	0.821034
30_MTA / C	Q9H6Z4	-0.25973	0.07708	0.139331	RANBP3	Ran-bindin	RANB3_HU	2	0.835244
15_MTA / C	Q9H845	0.983487	0.077183	0.139392	ACAD9	Complex I a	ACAD9_HU	2	1.977239
30_MTA / C	O96005	-0.42151	0.077432	0.139765	CLPTM1	Putative lip	CLPT1_HUI	3	0.746642
15_MTA / C	P51649	1.196684	0.077895	0.140362	ALDH5A1	Succinate-s	SSDH_HUM	1	2.292122
30_MTA / C	Q9UHQ9	-0.50737	0.078065	0.140373	CYB5R1	NADH-cyto	NB5R1_HU	3	0.703503
15_MTA / C	Q9Y281	0.796533	0.078023	0.140373	CFL2	Cofilin-2	COF2_HUM	4	1.736922
30_MTA / C	O60739;P4	-0.50207	0.07818	0.140448	EIF1B;EIF1	Eukaryotic	EIF1B_HUM	1	0.706091
15_MTA / C	P14923	-0.5034	0.078205	0.140448	JUP	Junction pl	PLAK_HUM	13	0.705442
30_MTA / C	P35613	0.347317	0.078234	0.140448	BSG	Basigin	BASI_HUMA	6	1.272192
15_MTA / C	Q9Y3U8	0.419425	0.078421	0.140709	RPL36	Large ribos	RL36_HUM	3	1.337395
30_MTA / C	P20042	0.399896	0.07864	0.141025	EIF2S2	Eukaryotic	IF2B_HUMA	4	1.319413
30_MTA / C	P50395	0.292879	0.07924	0.141797	GDI2	Rab GDP di	GDIB_HUM	15	1.225083
15_MTA / C	Q969X5	0.231506	0.079242	0.141797	ERGIC1	Endoplasm	ERGI1_HUM	5	1.17406
30_MTA / C	Q8N1N4	-1.60573	0.079413	0.141949	KRT78	Keratin, typ	K2C78_HUI	5	0.32857
30_MTA / C	Q96G23	-1.0846	0.079545	0.142109	CERS2	Ceramide s	CERS2_HUM	1	0.471523
15_MTA / C	P0CG47;P0	-1.04408	0.079738	0.142223	UBB;UBC;R	Polyubiqui	UBB_HUMA	5	0.484954
15_MTA / C	P53384	-0.67883	0.079715	0.142223	NUBP1	Cytosolic F	NUBP1_HU	1	0.624672
15_MTA / C	O95336	-0.46917	0.079921	0.142285	PGLS	6-phospho	6PGL_HUM	5	0.722382
15_MTA / C	P15151	0.602638	0.079839	0.142285	PVR	Poliovirus r	PVR_HUMA	3	1.518491
15_MTA / C	Q14019	-0.3816	0.079868	0.142285	COTL1	Coactosin-l	COTL1_HUI	2	0.767585
30_MTA / C	Q86U90	0.127099	0.079944	0.142285	YRDC	Threonylca	YRDC_HUM	1	1.092096
15_MTA / C	Q9BTD8	1.768309	0.079991	0.142292	RBM42	RNA-bindir	RBM42_HU	1	3.406544
30_MTA / C	O00629	-0.21692	0.080176	0.142467	KPNA4	Importin su	IMA3_HUM	1	0.860398
15_MTA / C	Q9Y305	1.536565	0.08014	0.142467	ACOT9	Acyl-coenz	ACOT9_HUI	4	2.90103
30_MTA / C	P07900	-0.33877	0.080454	0.142729	HSP90AA1	Heat shock	HS90A_HUI	12	0.790718
15_MTA / C	Q9UMY4	-0.53655	0.080683	0.142729	SNX12	Sorting nex	SNX12_HUI	1	0.689418
15_MTA / C	Q9UN36	1.203928	0.0806	0.142729	NDRG2	Protein ND	NDRG2_HU	2	2.303661
30_MTA / C	Q9NZI8	-0.30577	0.081324	0.143737	IGF2BP1	Insulin-like	IF2B1_HUM	4	0.809011
30_MTA / C	P33316	0.493651	0.081553	0.144066	DUT	Deoxyuridi	DUT_HUMA	4	1.408003
30_MTA / C	P62310	0.621905	0.081756	0.144271	LSM3	U6 snRNA-a	LSM3_HUM	2	1.538906
15_MTA / C	Q05682	-0.54423	0.081747	0.144271	CALD1	Caldesmon	CALD1_HUI	4	0.685759
30_MTA / C	O75347	0.218791	0.082027	0.144517	TBCA	Tubulin-spe	TBCA_HUM	4	1.163758
30_MTA / C	P84085	-0.33097	0.082021	0.144517	ARF5	ADP-ribosy	ARF5_HUM	1	0.795001
15_MTA / C	P23528	-0.44687	0.082531	0.145098	CFL1	Cofilin-1	COF1_HUM	10	0.733635
15_MTA / C	P68104;Q5	-0.19933	0.082529	0.145098	EEF1A1;EEF	Elongation	EF1A1_HUM	18	0.870957
15_MTA / C	P01111	0.111565	0.082851	0.1455	NRAS	GTPase NRa	RASN_HUM	5	1.0804
30_MTA / C	P15144	-0.36108	0.082936	0.1455	ANPEP	Aminopept	AMPN_HUM	3	0.778582
15_MTA / C	Q86UE4	0.277671	0.082902	0.1455	MTDH	Protein LYF	LYRIC_HUM	9	1.212236

30_MTA / C	P24534	0.269465	0.083282	0.145924	EEF1B2	Elongation	EF1B_HUM	2	1.20536
30_MTA / C	O43847	-0.51399	0.0834	0.145957	NRDC	Nardilysin	NRDC_HUM	3	0.700281
30_MTA / C	P02663	-3.76651	0.08372	0.145957	CSN1S2	Alpha-S2-ca	CASA2_BOV	3	0.07348
15_MTA / C	P20962	-0.22098	0.083457	0.145957	PTMS	Parathymo	PTMS_HUM	1	0.857983
30_MTA / C	P48506	-0.10563	0.083725	0.145957	GCLC	Glutamate-	GSH1_HUM	1	0.929397
15_MTA / C	Q12907	0.348007	0.083553	0.145957	LMAN2	Vesicular ir	LMAN2_HU	5	1.272801
30_MTA / C	Q7Z434	-0.31002	0.083637	0.145957	MAVS	Mitochond	MAVS_HUM	6	0.806632
30_MTA / C	P08962	0.958107	0.083835	0.146072	CD63	CD63 antig	CD63_HUM	2	1.942758
15_MTA / C	P12270	0.842356	0.084018	0.146315	TPR	Nucleoprot	TPR_HUMA	2	1.792976
30_MTA / C	O43633	-1.40633	0.084596	0.146704	CHMP2A	Charged mu	CHM2A_HU	1	0.37727
30_MTA / C	P55157	-0.44379	0.0844	0.146704	MTTP	Microsoma	MTP_HUMA	7	0.735202
15_MTA / C	Q04837	-0.27025	0.084482	0.146704	SSBP1	Single-strar	SSBP_HUM	3	0.829179
15_MTA / C	Q7Z5H4	-0.61519	0.084593	0.146704	VN1R5	Vomeronas	VN1R5_HU	1	0.652842
30_MTA / C	O15347	0.43118	0.084688	0.146753	HMGB3	High mobil	HMGB3_HU	1	1.348336
30_MTA / C	P04179	0.381949	0.084849	0.146912	SOD2	Superoxide	SODM_HUM	8	1.303101
30_MTA / C	Q96A26	-0.1641	0.08498	0.147062	FAM162A	Protein FAM	F162A_HUI	2	0.892482
30_MTA / C	P06899;P2	0.460597	0.085304	0.14711	H2BC11;H2	Histone H2	H2B1J_HUI	1	1.376111
15_MTA / C	P17980	-0.197	0.085096	0.14711	PSMC3	26S protea:	PRS6A_HUI	8	0.872365
15_MTA / C	P26368	-0.69815	0.08533	0.14711	U2AF2	Splicing fac	U2AF2_HUI	1	0.616361
15_MTA / C	P26599	-0.06936	0.08534	0.14711	PTBP1	Polypyrimi	PTBP1_HUI	2	0.953063
30_MTA / C	P30153	-0.33871	0.085278	0.14711	PPP2R1A	Serine/thre	2AAA_HUM	7	0.79075
15_MTA / C	Q9HCU5	0.379715	0.085101	0.14711	PREB	Guanine nu	SEC12_HUM	1	1.301085
30_MTA / C	A0A0C4DH	1.017009	0.085625	0.147408	IGKV3D-20	Immunoglo	KVD20_HU	1	2.023719
15_MTA / C	Q92499	1.076505	0.085817	0.147509	DDX1	ATP-depend	DDX1_HUM	2	2.108921
15_MTA / C	P36871	-0.28315	0.085971	0.14762	PGM1	Phosphoglu	PGM1_HUM	17	0.821794
30_MTA / C	Q69YN2	-1.21676	0.085961	0.14762	CWF19L1	CWF19-like	C19L1_HUI	1	0.430248
30_MTA / C	Q96HE7	-0.42238	0.086433	0.148108	ERO1A	ERO1-like p	ERO1A_HUI	6	0.746192
30_MTA / C	Q8WVX3	-0.46437	0.086505	0.148153	C4orf3	Uncharacte	CD003_HU	1	0.724786
15_MTA / C	Q96B49	-0.40286	0.086591	0.148224	TOMM6	Mitochond	TOM6_HUM	3	0.756355
15_MTA / C	Q9H074	-0.52885	0.086782	0.148398	PAIP1	Polyadenyl	PAIP1_HUM	3	0.693107
15_MTA / C	O75822	1.532649	0.087227	0.148888	EIF3J	Eukaryotic	EIF3J_HUM	2	2.893166
15_MTA / C	P10768	-0.29169	0.087283	0.148888	ESD	S-formylglu	ESTD_HUM	4	0.816944
15_MTA / C	Q96AE4	0.514377	0.087293	0.148888	FUBP1	Far upstrea	FUBP1_HUI	24	1.428377
15_MTA / C	Q15691	-0.07211	0.087639	0.149049	MAPRE1	Microtubul	MARE1_HU	6	0.951243
15_MTA / C	Q8TAF3	0.668137	0.087519	0.149049	WDR48	WD repeat-	WDR48_HU	2	1.589019
15_MTA / C	P26440	0.818032	0.087828	0.149151	IVD	Isovaleryl-C	IVD_HUMA	2	1.763
15_MTA / C	Q9NWT1	1.07195	0.087942	0.149151	PAK1IP1	p21-activat	PK1IP_HUM	2	2.102273
30_MTA / C	Q9NZJ7	1.694535	0.088255	0.149604	MTCH1	Mitochond	MTCH1_HU	2	3.236725
30_MTA / C	P62993	0.261056	0.088434	0.149831	GRB2	Growth fac	GRB2_HUM	4	1.198355
15_MTA / C	P07237	-0.16732	0.088569	0.149908	P4HB	Protein dis	PDIA1_HUM	29	0.890495
30_MTA / C	Q9BTD8	2.602461	0.088653	0.149958	RBM42	RNA-bindin	RBM42_HU	1	6.073217
15_MTA / C	P25786	-0.33982	0.088757	0.149996	PSMA1	Proteasom	PSA1_HUM	16	0.790141
30_MTA / C	P51858	0.45025	0.089039	0.150394	HDGF	Hepatoma-	HDGF_HUM	3	1.366277
15_MTA / C	E9PAV3;Q1	-0.32743	0.08932	0.150475	NACA	Nascent po	NACAM_HU	4	0.796956
15_MTA / C	P09960	-0.11367	0.089231	0.150475	LTA4H	Leukotrien	LKHA4_HUI	18	0.924231
15_MTA / C	P22234	-0.05454	0.089221	0.150475	PAICS	Bifunctiona	PUR6_HUM	15	0.9629
15_MTA / C	P49321	0.438194	0.089404	0.150475	NASP	Nuclear aut	NASP_HUM	9	1.354907
30_MTA / C	P53999	0.648367	0.089135	0.150475	SUB1	Activated R	TCP4_HUM	5	1.567393
15_MTA / C	Q99735	0.753683	0.089303	0.150475	MGST2	Microsoma	MGST2_HU	1	1.686091
30_MTA / C	P31350	0.578248	0.089466	0.150503	RRM2	Ribonuclec	RIR2_HUMA	7	1.493035
30_MTA / C	Q01130	0.317374	0.089721	0.150856	SRSF2	Serine/argi	SRSF2_HUM	5	1.24606
15_MTA / C	Q99798	0.257661	0.089787	0.15089	ACO2	Aconitate h	ACON_HUM	1	1.195539

15_MTA / C	O60832	1.077014	0.089838	0.150898	DKC1	H/ACA ribo	DKC1_HUM	3	2.109665
15_MTA / C	P13797	-0.31825	0.090162	0.151212	PLS3	Plastin-3	PLST_HUM	11	0.80204
15_MTA / C	Q8TB61	0.965165	0.090287	0.151345	SLC35B2	Adenosine	S35B2_HUI	5	1.952287
15_MTA / C	P55072	-0.36692	0.09035	0.151361	VCP	Transitiona	TERA_HUM	37	0.775434
30_MTA / C	Q9NTJ4	-0.41864	0.090387	0.151361	MAN2C1	Alpha-mani	MA2C1_HU	1	0.748128
30_MTA / C	O95551	-1.18994	0.090613	0.151555	TDP2	Tyrosyl-DN	TYDP2_HUI	1	0.43832
30_MTA / C	P50402	0.417741	0.090777	0.151555	EMD	Emerin	EMD_HUM	1	1.335834
15_MTA / C	Q01658	0.597804	0.090778	0.151555	DR1	Protein Dr1	NC2B_HUM	2	1.513411
30_MTA / C	Q92890	-0.41947	0.090633	0.151555	UFD1	Ubiquitin r	UFD1_HUM	4	0.747701
15_MTA / C	P63241	-0.38625	0.090942	0.151753	EIF5A	Eukaryotic	IF5A1_HUM	3	0.765114
30_MTA / C	P60900	0.19886	0.091465	0.152396	PSMA6	Proteasom	PSA6_HUM	9	1.147791
30_MTA / C	O60830	-0.29143	0.091562	0.15248	TIMM17B	Mitochond	TI17B_HUM	3	0.817091
30_MTA / C	P84090	-1.81854	0.091814	0.152824	ERH	Enhancer o	ERH_HUMA	1	0.283507
15_MTA / C	Q16740	0.572234	0.092233	0.153297	CLPP	ATP-depen	CLPP_HUM	6	1.486824
30_MTA / C	P15586	0.429942	0.092891	0.154228	GNS	N-acetylglu	GNS_HUMA	7	1.347179
30_MTA / C	Q15006	0.752815	0.092863	0.154228	EMC2	ER membra	EMC2_HUM	2	1.685077
15_MTA / C	Q7Z2K6	0.2099	0.09347	0.154958	ERMP1	Endoplasm	ERMP1_HU	3	1.156608
15_MTA / C	P30084	0.422042	0.093924	0.155322	ECHS1	Enoyl-CoA	ECHM_HUM	6	1.339822
15_MTA / C	P62258	-0.18772	0.093891	0.155322	YWHAE	14-3-3 prot	1433E_HUI	14	0.877994
15_MTA / C	O00571	-0.22049	0.094071	0.155459	DDX3X	ATP-depen	DDX3X_HU	5	0.858272
30_MTA / C	O75874	-0.17888	0.094101	0.155459	IDH1	Isocitrate d	IDHC_HUM	8	0.883391
30_MTA / C	P17812	-0.17288	0.0946	0.156146	CTPS1	CTP syntha	PYRG1_HUI	9	0.887072
15_MTA / C	Q96CQ1;Q	0.396083	0.094642	0.156146	SLC25A36;	Solute carri	S2536_HUI	1	1.31593
30_MTA / C	P12277	-0.35721	0.094821	0.156337	CKB	Creatine kir	KCRB_HUM	6	0.780674
15_MTA / C	P05556	-0.53622	0.094878	0.156353	ITGB1	Integrin be	ITB1_HUM	10	0.689577
15_MTA / C	P23246	-0.11387	0.095027	0.156501	SFPQ	Splicing fac	SFPQ_HUM	6	0.924107
15_MTA / C	P60059	0.206991	0.095109	0.156501	SEC61G	Protein tra	SC61G_HUI	2	1.154278
30_MTA / C	Q9BVK2	-0.51702	0.095163	0.156511	ALG8	Dolichyl py	ALG8_HUM	1	0.698812
30_MTA / C	Q9HB71	-0.4469	0.095272	0.156613	CACYBP	Calcyclin-b	CYBP_HUM	2	0.733619
30_MTA / C	O75787	-0.55981	0.095486	0.156887	ATP6AP2	Renin recep	RENR_HUM	1	0.67839
30_MTA / C	P08243	-0.62189	0.095691	0.157068	ASNS	Asparagine	ASNS_HUM	3	0.649819
30_MTA / C	P31948	0.226159	0.095901	0.157335	STIP1	Stress-indu	STIP1_HUM	16	1.169716
15_MTA / C	P49915	-0.12923	0.095964	0.15736	GMPS	GMP syntha	GUAA_HUM	4	0.914321
15_MTA / C	Q9BX68	3.835063	0.096077	0.157467	HINT2	Adenosine	HINT2_HUM	1	14.27148
15_MTA / C	P14635	0.75634	0.096253	0.157599	CCNB1	G2/mitotic	CCNB1_HU	1	1.6892
15_MTA / C	Q13162	-0.12787	0.096494	0.157837	PRDX4	Peroxiredo	PRDX4_HU	3	0.915185
15_MTA / C	Q9H7C9	0.667081	0.09654	0.157837	AAMDC	Mth938 do	AAMDC_HU	4	1.587857
30_MTA / C	Q96C90	0.600329	0.096653	0.157942	PPP1R14B	Protein ph	PP14B_HUI	3	1.516063
15_MTA / C	Q6P4E1	0.371125	0.096701	0.157944	GOLM2	Protein GO	GOLM2_HU	2	1.293361
15_MTA / C	P61626	-0.83808	0.096932	0.158087	LYZ	Lysozyme C	LYSC_HUM	3	0.559389
30_MTA / C	P27694	0.670283	0.097124	0.158322	RPA1	Replication	RFA1_HUM	1	1.591385
15_MTA / C	Q9H8S9	0.728268	0.097469	0.158806	MOB1A	MOB kinase	MOB1A_HU	3	1.656649
15_MTA / C	P16083	-0.4243	0.097942	0.15942	NQO2	Ribosyldihy	NQO2_HUM	7	0.745199
15_MTA / C	Q9Y5T4	1.115968	0.098216	0.159788	DNAJC15	DnaJ homo	DJC15_HUI	1	2.167404
15_MTA / C	Q9UBX3	0.260112	0.098658	0.160271	SLC25A10	Mitochond	DIC_HUMA	7	1.197572
30_MTA / C	P24752	-0.25909	0.098723	0.160298	ACAT1	Acetyl-CoA	THIL_HUM	13	0.835614
15_MTA / C	O00505	-0.67449	0.098968	0.160538	KPNA3	Importin su	IMA4_HUM	5	0.626552
15_MTA / C	Q92542	-0.38508	0.099045	0.160552	NCSTN	Nicastrin	NICA_HUM	4	0.765737
30_MTA / C	Q9NR31	1.752768	0.099074	0.160552	SAR1A	Small COPII	SAR1A_HUI	2	3.370046
30_MTA / C	O95249	1.34285	0.099335	0.160585	GOSR1	Golgi SNAP	GOSR1_HU	2	2.536519
15_MTA / C	P08123	-3.27046	0.099384	0.160585	COL1A2	Collagen al	CO1A2_HU	3	0.103632
30_MTA / C	P13804	-0.43867	0.099332	0.160585	ETFA	Electron tra	ETFA_HUM	5	0.737817

30_MTA / C	P35908	0.457249	0.099198	0.160585	KRT2	Keratin, typ	K22E_HUM	30	1.372921
15_MTA / C	Q08623	-1.50331	0.099358	0.160585	PUDP	Pseudouric	HDHD1_HU	2	0.352743
15_MTA / C	Q9UHX1	0.532597	0.099569	0.160727	PUF60	Poly(U)-bin	PUF60_HU	2	1.44653
15_MTA / C	P25325	-0.19245	0.099937	0.160854	MPST	3-mercapto	THTM_HUM	5	0.875116
30_MTA / C	P62487	-0.19501	0.09996	0.160854	POLR2G	DNA-direct	RPB7_HUM	3	0.873569
30_MTA / C	P63218	-0.70362	0.099988	0.160854	GNG5	Guanine nu	GBG5_HUM	3	0.614028
30_MTA / C	P35270	-0.64369	0.100142	0.161024	SPR	Sepiapterin	SPRE_HUM	2	0.640076
15_MTA / C	P62244	0.163472	0.100235	0.161079	RPS15A	Small ribos	RS15A_HU	5	1.11998
15_MTA / C	P67870	-1.23255	0.100273	0.161079	CSNK2B	Casein kina	CSK2B_HU	1	0.425566
15_MTA / C	P05026	-0.15817	0.100551	0.16129	ATP1B1	Sodium/po	AT1B1_HU	4	0.896159
15_MTA / C	P11169	-0.26508	0.100808	0.161624	SLC2A3	Solute carri	GTR3_HUM	7	0.832154
15_MTA / C	Q8N766	0.364913	0.100909	0.161708	EMC1	ER membra	EMC1_HUM	9	1.287804
15_MTA / C	P25788	-0.35022	0.101125	0.161897	PSMA3	Proteasom	PSA3_HUM	11	0.784467
30_MTA / C	Q92688	0.44788	0.101206	0.16195	ANP32B	Acidic leuci	AN32B_HU	5	1.364034
30_MTA / C	P07355	0.293772	0.101332	0.161994	ANXA2	Annexin A2	ANXA2_HU	20	1.225841
30_MTA / C	Q9BSE5	0.38958	0.101298	0.161994	AGMAT	Guanidino	GDAH_HUM	8	1.310012
30_MTA / C	P21266	-0.37504	0.101499	0.162105	GSTM3	Glutathion	GSTM3_HU	4	0.771084
15_MTA / C	P14678;P6	-0.23667	0.101553	0.162113	SNRPB;SNR	Small nucle	RSMB_HUM	5	0.848705
30_MTA / C	P23246	0.841234	0.10173	0.162318	SFPQ	Splicing fac	SFPQ_HUM	6	1.791582
15_MTA / C	O15126	1.519535	0.102137	0.162681	SCAMP1	Secretory c	SCAM1_HU	2	2.866987
15_MTA / C	Q9UH65	0.252597	0.102154	0.162681	SWAP70	Switch-assc	SWP70_HU	1	1.19135
30_MTA / C	P07237	0.236032	0.102452	0.163076	P4HB	Protein dis	PDIA1_HUM	29	1.177749
30_MTA / C	O43598	-0.79046	0.102622	0.163244	DNPH1	5-hydroxym	DNPH1_HU	4	0.578158
15_MTA / C	P84098	-0.19522	0.102725	0.163276	RPL19	Large ribos	RL19_HUM	5	0.873437
30_MTA / C	O95793	0.277666	0.102815	0.163341	STAU1	Double-stra	STAU1_HU	3	1.212232
15_MTA / C	P49721	-0.26807	0.103047	0.163405	PSMB2	Proteasom	PSB2_HUM	5	0.830429
30_MTA / C	Q9GZT3	0.795573	0.10308	0.163405	SLIRP	SRA stem-lo	SLIRP_HUM	2	1.735767
15_MTA / C	P31939	-0.38881	0.103312	0.163425	ATIC	Bifunctiona	PUR9_HUM	23	0.763762
15_MTA / C	Q8NHG7	-0.59549	0.103217	0.163425	SVIP	Small VCP/	SVIP_HUM	1	0.661819
15_MTA / C	O43396	-0.75332	0.103378	0.163452	TXNL1	Thioredoxi	TXNL1_HU	4	0.593236
30_MTA / C	P49591	0.609798	0.103624	0.163633	SARS1	Serine--tRN	SYSC_HUM	1	1.526046
15_MTA / C	P10606	0.454092	0.103768	0.163645	COX5B	Cytochrom	COX5B_HU	5	1.369921
30_MTA / C	Q8NHG7	-0.53647	0.103786	0.163645	SVIP	Small VCP/	SVIP_HUM	1	0.689458
30_MTA / C	Q9UI30	-0.24927	0.103797	0.163645	TRMT112	Multifuncti	TR112_HU	2	0.841321
15_MTA / C	P45880	0.225943	0.104055	0.163897	VDAC2	Voltage-de	VDAC2_HU	13	1.169541
15_MTA / C	P53602	-0.55551	0.10418	0.164015	MVD	Diphospho	MVD1_HUM	2	0.680416
15_MTA / C	Q16630	1.180366	0.10442	0.164238	CPSF6	Cleavage ar	CPSF6_HU	2	2.266343
30_MTA / C	Q96B49	-0.32121	0.104643	0.16451	TOMM6	Mitochond	TOM6_HUM	3	0.800401
15_MTA / C	Q8WTV0	0.361643	0.105039	0.164819	SCARB1	Scavenger r	SCRB1_HU	2	1.284888
30_MTA / C	Q00765	-0.33967	0.10514	0.164901	REEP5	Receptor e	REEP5_HU	6	0.790221
15_MTA / C	P13639	-0.13309	0.105323	0.164939	EEF2	Elongation	EF2_HUMA	38	0.911875
30_MTA / C	Q15233	0.431694	0.105301	0.164939	NONO	Non-POU d	NONO_HUM	10	1.348816
15_MTA / C	Q99436	-0.24412	0.105502	0.164999	PSMB7	Proteasom	PSB7_HUM	4	0.844333
15_MTA / C	P15586	-0.26564	0.105568	0.165025	GNS	N-acetylglu	GNS_HUM	7	0.831827
30_MTA / C	P26885	0.411895	0.105645	0.16505	FKBP2	Peptidyl-pr	FKBP2_HU	1	1.330433
30_MTA / C	P27797	0.24523	0.105684	0.16505	CALR	Calreticulir	CALR_HUM	11	1.185282
30_MTA / C	P36543	-0.94113	0.105809	0.165091	ATP6V1E1	V-type prot	VATE1_HU	2	0.520824
15_MTA / C	Q96HV5	0.35208	0.105805	0.165091	TMEM41A	Transmemb	TM41A_HU	1	1.276399
30_MTA / C	P30408	-0.6711	0.10607	0.165419	TM4SF1	Transmemb	T4S1_HUM	1	0.628028
15_MTA / C	O60487	-0.83783	0.106784	0.166142	MPZL2	Myelin prot	MPZL2_HU	1	0.559486
30_MTA / C	Q9P0J0	1.108004	0.106746	0.166142	NDUFA13	NADH dehy	NDUAD_HU	2	2.155472
15_MTA / C	P62249	-0.15716	0.106938	0.166186	RPS16	Small ribos	RS16_HUM	9	0.89679

30_MTA / C	Q16836	0.216255	0.106963	0.166186	HADH	Hydroxyacy	HCDH_HUM	1	1.161714
30_MTA / C	P10606	-0.12238	0.107041	0.166204	COX5B	Cytochrom	COX5B_HU	5	0.918671
30_MTA / C	P51610	0.346955	0.107257	0.166409	HCFC1	Host cell fa	HCFC1_HU	10	1.271874
15_MTA / C	O14548	0.440415	0.107403	0.166557	COX7A2L	Cytochrom	COX7R_HU	2	1.356995
15_MTA / C	O60232	-0.01562	0.10781	0.167028	ZNRD2	Protein ZNI	ZNRD2_HU	2	0.989233
15_MTA / C	Q9Y5Z4	-0.29714	0.107858	0.167028	HEBP2	Heme-bind	HEBP2_HU	5	0.813863
15_MTA / C	P49368	-0.18089	0.108169	0.167276	CCT3	T-complex	TCPG_HUM	20	0.882162
15_MTA / C	Q9NTK5	-0.39604	0.10812	0.167276	OLA1	Obg-like AT	OLA1_HUM	5	0.759942
30_MTA / C	P55263	-0.20064	0.108459	0.167646	ADK	Adenosine	ADK_HUMA	3	0.870163
15_MTA / C	Q13347	-0.36424	0.108809	0.168109	EIF3I	Eukaryotic	EIF3I_HUM	4	0.77688
15_MTA / C	Q15024	2.390678	0.109041	0.168389	EXOSC7	Exosome cc	EXOS7_HU	1	5.244037
15_MTA / C	P08708	0.352271	0.109121	0.16841	RPS17	Small ribos	RS17_HUM	3	1.276568
15_MTA / C	Q02978	0.239429	0.10953	0.168594	SLC25A11	Mitochond	M2OM_HU	7	1.180526
15_MTA / C	Q9C005	1.193908	0.10949	0.168594	DPY30	Protein dpy	DPY30_HU	2	2.287716
30_MTA / C	Q15392	-0.94277	0.10985	0.168851	DHCR24	Delta(24)-s	DHC24_HU	1	0.520232
15_MTA / C	P43490	-0.42547	0.110407	0.169628	NAMPT	Nicotinami	NAMPT_HU	7	0.744596
15_MTA / C	O14980	-1.1874	0.11062	0.169651	XPO1	Exportin-1	XPO1_HUM	1	0.439093
30_MTA / C	P08579	-0.5503	0.110709	0.169651	SNRPB2	U2 small nu	RU2B_HUM	4	0.682877
30_MTA / C	Q9HCN4	-0.06086	0.110588	0.169651	GPN1	GPN-loop G	GPN1_HUM	3	0.958692
15_MTA / C	Q9NQ88	-0.57409	0.110657	0.169651	TIGAR	Fructose-2,	TIGAR_HUM	1	0.671709
15_MTA / C	Q16719	-0.32282	0.111053	0.169911	KYNU	Kynurenina	KYNU_HUM	1	0.799504
15_MTA / C	P61106	0.215418	0.111163	0.169922	RAB14	Ras-related	RAB14_HU	6	1.16104
30_MTA / C	Q9UGV2	-0.62425	0.111213	0.169922	NDRG3	Protein ND	NDRG3_HU	1	0.648756
30_MTA / C	Q9BVC6	-0.44825	0.11138	0.170098	TMEM109	Voltage-gat	TM109_HU	4	0.732932
30_MTA / C	Q92499	1.079447	0.111587	0.170337	DDX1	ATP-depen	DDX1_HUM	2	2.113226
30_MTA / C	O95394	-0.49536	0.111768	0.170377	PGM3	Phosphoac	AGM1_HUM	6	0.709386
30_MTA / C	P15954	0.38936	0.11168	0.170377	COX7C	Cytochrom	COX7C_HU	1	1.309812
15_MTA / C	Q7Z7K0	0.105517	0.11176	0.170377	CMC1	COX assem	COXM1_HU	1	1.07588
30_MTA / C	O60671	1.972922	0.11221	0.170521	RAD1	Cell cycle c	RAD1_HUM	1	3.925623
30_MTA / C	O75955	1.864178	0.112326	0.170521	FLOT1	Flotillin-1	FLOT1_HUM	2	3.640605
15_MTA / C	P10515	0.459986	0.112286	0.170521	DLAT	Dihydrolip	ODP2_HUM	6	1.375529
30_MTA / C	P14927	-0.96351	0.112251	0.170521	UQCRB	Cytochrom	QCR7_HUM	1	0.512806
30_MTA / C	P21281	-0.24461	0.112062	0.170521	ATP6V1B2	V-type prot	VATB2_HU	10	0.844046
30_MTA / C	P55060	-0.31598	0.112286	0.170521	CSE1L	Exportin-2	XPO2_HUM	10	0.803304
30_MTA / C	P63165	0.420667	0.112289	0.170521	SUMO1	Small ubiq	SUMO1_HU	2	1.338546
30_MTA / C	P68871	1.61982	0.112039	0.170521	HBB	Hemoglobi	HBB_HUM	2	3.073368
15_MTA / C	P54578	-0.73072	0.112696	0.170927	USP14	Ubiquitin c	UBP14_HU	2	0.602604
15_MTA / C	O95857;Q1	0.327467	0.113136	0.171286	TSPAN13;T	Tetraspanir	TSN13_HU	1	1.254808
30_MTA / C	Q15046	-0.0884	0.11314	0.171286	KARS1	Lysine--tRN	SYK_HUMA	7	0.940565
15_MTA / C	O43598	0.232094	0.113329	0.171417	DNPH1	5-hydroxyn	DNPH1_HU	4	1.174539
15_MTA / C	O75794	-1.84188	0.113537	0.171491	CDC123	Translation	CD123_HU	2	0.278958
30_MTA / C	Q8IYT8	2.485	0.113488	0.171491	ULK2	Serine/thre	ULK2_HUM	1	5.598344
30_MTA / C	Q08188	-0.93238	0.113759	0.171518	TGM3	Protein-glu	TGM3_HUM	3	0.523992
30_MTA / C	Q03154	0.245264	0.113879	0.171622	ACY1	Aminoacyla	ACY1_HUM	5	1.185309
15_MTA / C	P31431	-0.57594	0.11406	0.171737	SDC4	Syndecan-4	SDC4_HUM	1	0.670849
30_MTA / C	O14925	-0.56037	0.114396	0.172047	TIMM23	Mitochond	TIM23_HU	2	0.67813
30_MTA / C	O43776	-0.51744	0.114473	0.172047	NARS1	Asparagine	SYNC_HUM	5	0.698612
15_MTA / C	P05091	-0.04948	0.11443	0.172047	ALDH2	Aldehyde d	ALDH2_HU	15	0.966288
30_MTA / C	O15260	-0.03429	0.115269	0.172929	SURF4	Surfeit locu	SURF4_HU	3	0.976511
30_MTA / C	O43681	-0.09611	0.116165	0.174118	GET3	ATPase GET	GET3_HUM	1	0.935555
15_MTA / C	P06753	-0.27432	0.116166	0.174118	TPM3	Tropomyos	TPM3_HUM	4	0.82684
15_MTA / C	O14744	-0.20568	0.116741	0.174393	PRMT5	Protein arg	ANM5_HUM	7	0.867128

15_MTA / C	O43813	-0.17344	0.116593	0.174393	LANCL1	Glutathion	LANC1_HU	5	0.886728
15_MTA / C	P38159;Q9	-0.99737	0.116771	0.174393	RBMX;RBM	RNA-bindir	RBMX_HUN	1	0.500914
30_MTA / C	Q15291	0.094095	0.11649	0.174393	RBBP5	Retinoblast	RBBP5_HU	5	1.067396
15_MTA / C	Q99471	-0.13637	0.116876	0.174471	PFDN5	Prefoldin su	PFD5_HUM	3	0.909803
30_MTA / C	Q12860	-0.62863	0.117323	0.174981	CNTN1	Contactin-1	CNTN1_HU	9	0.646788
30_MTA / C	Q7LGA3	-0.3057	0.117379	0.174986	HS2ST1	Heparan su	HS2ST_HUN	1	0.809051
30_MTA / C	P06733	0.415408	0.118003	0.175758	ENO1	Alpha-enola	ENOA_HUN	28	1.333676
30_MTA / C	O43772	-0.41202	0.118358	0.175993	SLC25A20	Mitochond	MCAT_HUN	7	0.751569
15_MTA / C	P61019	0.448904	0.118288	0.175993	RAB2A	Ras-related	RAB2A_HU	8	1.365003
30_MTA / C	Q08211	1.037391	0.118283	0.175993	DHX9	ATP-depend	DHX9_HUN	8	2.052512
15_MTA / C	Q13610	-0.34965	0.118438	0.176011	PWP1	Periodic try	PWP1_HUN	2	0.784774
15_MTA / C	Q9UK45	-0.16993	0.118767	0.176266	LSM7	U6 snRNA-a	LSM7_HUN	1	0.888885
30_MTA / C	Q9ULC4	0.306475	0.118769	0.176266	MCTS1	Malignant T	MCTS1_HU	2	1.236682
15_MTA / C	O60568	0.353741	0.119207	0.176836	PLOD3	Multifuncti	PLOD3_HU	6	1.27787
15_MTA / C	P12830	0.15631	0.119556	0.177244	CDH1	Cadherin-1	CADH1_HU	1	1.114433
15_MTA / C	Q53FA7	-0.43371	0.119589	0.177244	TP53I3	Quinone ox	QORX_HUN	6	0.740357
15_MTA / C	Q08257	-0.20355	0.120138	0.17774	CRYZ	Quinone ox	QOR_HUMA	15	0.868414
30_MTA / C	Q16629	0.466866	0.120537	0.178091	SRSF7	Serine/argi	SRSF7_HUN	3	1.382104
30_MTA / C	Q9Y4Z0	1.07505	0.120528	0.178091	LSM4	U6 snRNA-a	LSM4_HUN	1	2.106795
30_MTA / C	P02662	-2.5326	0.12072	0.178282	CSN1S1	Alpha-S1-ca	CASA1_BOV	2	0.172826
15_MTA / C	P06396	-0.12081	0.121064	0.178711	GSN	Gelsolin	GELS_HUM	1	0.91967
30_MTA / C	P07902	-2.20056	0.121251	0.178859	GALT	Galactose-1	GALT_HUM	1	0.217554
15_MTA / C	Q13232	0.372603	0.121273	0.178859	NME3	Nucleoside	NDK3_HUN	3	1.294687
30_MTA / C	O60841	0.688663	0.12154	0.178894	EIF5B	Eukaryotic	IF2P_HUMA	3	1.611789
30_MTA / C	P09110	1.061605	0.121431	0.178894	ACAA1	3-ketoacyl-	THIK_HUMA	2	2.087253
30_MTA / C	P11177	-0.53195	0.121674	0.178894	PDHB	Pyruvate de	ODPB_HUN	2	0.691621
30_MTA / C	Q13835	-2.67743	0.12161	0.178894	PKP1	Plakophilin	PKP1_HUN	2	0.156319
15_MTA / C	Q9HBK9	0.517634	0.121802	0.179003	AS3MT	Arsenite me	AS3MT_HU	5	1.431606
30_MTA / C	Q13257	-0.49155	0.121877	0.179034	MAD2L1	Mitotic spin	MD2L1_HU	1	0.71126
30_MTA / C	P60228	-0.80018	0.122657	0.179844	EIF3E	Eukaryotic	EIF3E_HUM	5	0.574278
15_MTA / C	P67809	0.270263	0.122836	0.179844	YBX1	Y-box-bindi	YBOX1_HU	4	1.206028
30_MTA / C	Q8IXB1	-0.25179	0.122709	0.179844	DNAJC10	DnaJ homo	DJC10_HU	4	0.839851
30_MTA / C	P05204	0.968672	0.123046	0.17987	HMGN2	Non-histon	HMGN2_HU	3	1.957038
30_MTA / C	Q9HA77	-0.79011	0.123202	0.179944	CARS2	Probable cy	SYCM_HUM	1	0.578301
15_MTA / C	Q13409	0.318023	0.123289	0.179991	DYNC1I2	Cytoplasmi	DC1I2_HUN	1	1.246621
30_MTA / C	P10909	3.53506	0.123617	0.180004	CLU	Clusterin	CLUS_HUM	7	11.59202
30_MTA / C	P12956	0.194578	0.123624	0.180004	XRCC6	X-ray repair	XRCC6_HU	11	1.14439
30_MTA / C	P49321	0.390323	0.123532	0.180004	NASP	Nuclear aut	NASP_HUM	9	1.310687
15_MTA / C	P61224	-0.79641	0.123489	0.180004	RAP1B	Ras-related	RAP1B_HU	3	0.57578
15_MTA / C	Q96ER9	1.531484	0.123426	0.180004	CCDC51	Mitochond	MITOK_HU	2	2.89083
30_MTA / C	Q9H3K2	-0.21234	0.123357	0.180004	GHITM	Growth hor	GHITM_HU	5	0.863135
15_MTA / C	P00390	-0.24202	0.123894	0.180057	GSR	Glutathion	GSHR_HUM	13	0.84556
30_MTA / C	P16278	1.097002	0.123844	0.180057	GLB1	Beta-galact	BGAL_HUM	1	2.139098
15_MTA / C	Q02818	-0.80668	0.12391	0.180057	NUCB1	Nucleobind	NUCB1_HU	3	0.571696
15_MTA / C	Q15291	0.614958	0.123829	0.180057	RBBP5	Retinoblast	RBBP5_HU	5	1.531513
30_MTA / C	P62333	-0.72717	0.124282	0.180329	PSMC6	26S protea	PRS10_HU	4	0.604089
30_MTA / C	O60220	0.671862	0.124483	0.180428	TIMM8A	Mitochond	TIM8A_HU	2	1.593128
30_MTA / C	Q15758	-0.73672	0.124514	0.180428	SLC1A5	Neutral am	AAAT_HUM	4	0.600104
15_MTA / C	Q9Y678	0.247692	0.124487	0.180428	COPG1	Coatomer s	COPG1_HU	7	1.187306
30_MTA / C	P04259	1.35662	0.124859	0.180644	KRT6B	Keratin, typ	K2C6B_HU	4	2.560844
15_MTA / C	P15311	0.002978	0.124881	0.180644	EZR	Ezrin	EZRI_HUMA	10	1.002066
30_MTA / C	P13645	0.504987	0.125038	0.18075	KRT10	Keratin, typ	K1C10_HU	23	1.41911

30_MTA / C	P38606	-0.18119	0.125247	0.180779	ATP6V1A	V-type prot	VATA_HUM	8	0.881973
30_MTA / C	P46379	-0.12722	0.125221	0.180779	BAG6	Large prolir	BAG6_HUM	4	0.915596
30_MTA / C	P02679	-0.66872	0.125802	0.181301	FGG	Fibrinogen	FIBG_HUM	4	0.629067
30_MTA / C	Q9BQE4	-0.35548	0.125785	0.181301	SELENOS	Selenoprot	SELS_HUM	2	0.78161
30_MTA / C	Q9P2T1	0.379922	0.12583	0.181301	GMPR2	GMP reduc	GMPR2_HU	5	1.301272
30_MTA / C	P05141	0.347044	0.12597	0.181349	SLC25A5	ADP/ATP tr	ADT2_HUM	5	1.271952
30_MTA / C	P62081	0.209238	0.126087	0.181438	RPS7	Small ribos	RS7_HUMA	6	1.156077
30_MTA / C	Q9UNM6	-0.52911	0.126222	0.181553	PSMD13	26S protea	PSD13_HU	6	0.692981
30_MTA / C	P36551	0.141985	0.126524	0.181719	CPOX	Oxygen-dep	HEM6_HUM	4	1.103422
15_MTA / C	Q04917	-0.24313	0.126469	0.181719	YWHAH	14-3-3 prot	1433F_HU	6	0.844908
15_MTA / C	Q96FW1	-0.26736	0.126556	0.181719	OTUB1	Ubiquitin t	OTUB1_HU	2	0.830838
30_MTA / C	Q93034	0.608298	0.126817	0.182014	CUL5	Cullin-5	CUL5_HUM	5	1.52446
15_MTA / C	P31153	-0.30142	0.127082	0.182237	MAT2A	S-adenosylr	METK2_HU	9	0.811454
15_MTA / C	Q9Y6N5	-0.17895	0.127064	0.182237	SQOR	Sulfide:qui	SQOR_HUM	16	0.883344
15_MTA / C	Q14558	-0.41938	0.127201	0.182329	PRPSAP1	Phosphorib	KPRA_HUM	1	0.747744
30_MTA / C	O14818	0.262033	0.127421	0.182548	PSMA7	Proteasom	PSA7_HUM	13	1.199167
15_MTA / C	P62701	-0.16789	0.127464	0.182548	RPS4X	Small ribos	RS4X_HUM	13	0.890144
15_MTA / C	Q15392	-0.18443	0.127774	0.182913	DHCR24	Delta(24)-s	DHC24_HU	1	0.879999
30_MTA / C	P51665	-0.07637	0.128011	0.183173	PSMD7	26S protea	PSMD7_HU	2	0.948444
15_MTA / C	P46779	-0.07544	0.128169	0.18332	RPL28	Large ribos	RL28_HUM	9	0.949052
30_MTA / C	P06748	0.356334	0.128689	0.183911	NPM1	Nucleopho	NPM_HUM	6	1.280169
30_MTA / C	Q9NQT5	1.980815	0.128722	0.183911	EXOSC3	Exosome cc	EXOS3_HU	1	3.947159
30_MTA / C	O43175	-0.1128	0.128887	0.183938	PHGDH	D-3-phosph	SERA_HUM	11	0.924789
15_MTA / C	P04066	0.383099	0.128831	0.183938	FUCA1	Tissue alph	FUCO_HUM	3	1.30414
15_MTA / C	P15531	-0.43354	0.128934	0.183938	NME1	Nucleoside	NDKA_HUM	4	0.740444
30_MTA / C	Q9UI12	-0.9058	0.12924	0.184296	ATP6V1H	V-type prot	VATH_HUM	2	0.533737
15_MTA / C	P34896	0.441417	0.129524	0.184541	SHMT1	Serine hydr	GLYC_HUM	3	1.357938
15_MTA / C	Q8NBS9	-0.24394	0.129471	0.184541	TXNDC5	Thioredoxi	TXND5_HU	11	0.844436
15_MTA / C	P50213	0.177476	0.129724	0.184746	IDH3A	Isocitrate d	IDH3A_HU	4	1.130903
30_MTA / C	O15031	-0.13081	0.129794	0.184767	PLXNB2	Plexin-B2	PLXB2_HU	3	0.913317
15_MTA / C	P08648	-0.03186	0.129868	0.184793	ITGA5	Integrin alp	ITA5_HUM	9	0.97816
30_MTA / C	P61201	-0.26461	0.130135	0.184892	COPS2	COP9 signa	CSN2_HUM	1	0.832426
15_MTA / C	P00338	-0.20964	0.130705	0.185464	LDHA	L-lactate de	LDHA_HUM	16	0.864751
15_MTA / C	O43759	0.730453	0.131265	0.186061	SYNGR1	Synaptogyr	SNG1_HUM	2	1.65916
15_MTA / C	P40429	-0.219	0.131414	0.186194	RPL13A	Large ribos	RL13A_HU	8	0.859161
30_MTA / C	O75940	2.034296	0.131504	0.186227	SMNDC1	Survival of	SPF30_HU	1	4.096228
15_MTA / C	P02675	0.956407	0.13155	0.186227	FGB	Fibrinogen	FIBB_HUM	2	1.940471
15_MTA / C	Q86WQ0	-0.29817	0.131724	0.186234	NR2C2AP	Nuclear rec	NR2CA_HU	1	0.813285
15_MTA / C	Q9BVK2	0.534408	0.131631	0.186234	ALG8	Dolichyl py	ALG8_HUM	1	1.448348
15_MTA / C	P39023	-0.14555	0.131968	0.18642	RPL3	Large ribos	RL3_HUMA	16	0.904035
15_MTA / C	P46926	-0.48767	0.132187	0.18657	GNPDA1	Glucosamir	GNPI1_HU	7	0.713177
30_MTA / C	Q9UJ70	-0.09815	0.132131	0.18657	NAGK	N-acetyl-D-	NAGK_HUM	7	0.93423
15_MTA / C	O00115	-0.1895	0.132442	0.186693	DNASE2	Deoxyribor	DNS2A_HU	4	0.876911
15_MTA / C	Q08188	-0.91323	0.132443	0.186693	TGM3	Protein-glu	TGM3_HUM	3	0.530995
30_MTA / C	Q9BY32	-0.83436	0.132514	0.186715	ITPA	Inosine trip	ITPA_HUM	3	0.560833
30_MTA / C	O15145	0.499613	0.132917	0.187168	ARPC3	Actin-relate	ARPC3_HU	3	1.413834
30_MTA / C	P55010	-0.20074	0.132949	0.187168	EIF5	Eukaryotic	IF5_HUMA	2	0.870106
15_MTA / C	P23588	-0.29316	0.133073	0.187262	EIF4B	Eukaryotic	IF4B_HUM	8	0.816113
30_MTA / C	P35527	-0.7723	0.133241	0.187419	KRT9	Keratin, typ	K1C9_HUM	25	0.585482
30_MTA / C	O96008	0.415007	0.133463	0.187572	TOMM40	Mitochond	TOM40_HU	7	1.333305
30_MTA / C	Q99519	-0.28417	0.133413	0.187572	NEU1	Sialidase-1	NEUR1_HU	3	0.821217
30_MTA / C	P62266	-0.19645	0.134432	0.188431	RPS23	Small ribos	RS23_HUM	6	0.872696

15_MTA / C	Q9H936	0.499332	0.134252	0.188431	SLC25A22	Mitochond	GHC1_HUN	4	1.413558
30_MTA / C	Q9NX63	-0.50025	0.13428	0.188431	CHCHD3	MICOS com	MIC19_HUI	3	0.706984
30_MTA / C	Q04760	0.359729	0.135005	0.189098	GLO1	Lactoylglut	LGUL_HUM	9	1.283185
15_MTA / C	Q9UBE0	-0.08144	0.135125	0.189187	SAE1	SUMO-activ	SAE1_HUM	7	0.945112
15_MTA / C	Q9Y3D6	0.448558	0.13539	0.189344	FIS1	Mitochond	FIS1_HUMA	1	1.364676
15_MTA / C	Q99536	-0.17128	0.13559	0.189518	VAT1	Synaptic ve	VAT1_HUM	10	0.888056
30_MTA / C	O75607	-0.03942	0.135691	0.189578	NPM3	Nucleoplas	NPM3_HUN	1	0.973044
30_MTA / C	O00764	0.382545	0.13584	0.189706	PDXK	Pyridoxal k	PDXK_HUM	4	1.30364
15_MTA / C	Q9Y3A5	-0.26351	0.135916	0.189733	SBDS	Ribosome r	SBDS_HUM	4	0.833057
30_MTA / C	P48509	-0.12651	0.136219	0.189836	CD151	CD151 anti	CD151_HU	2	0.916047
15_MTA / C	Q9BWD1	-0.14535	0.136082	0.189836	ACAT2	Acetyl-CoA	THIC_HUMA	10	0.904161
30_MTA / C	Q9NS86	0.954861	0.136126	0.189836	LANCL2	LanC-like p	LANC2_HUI	3	1.938393
30_MTA / C	Q13510	-0.56609	0.136316	0.189891	ASAH1	Acid ceram	ASAH1_HUI	2	0.675446
30_MTA / C	O43684	0.10513	0.136659	0.190169	BUB3	Mitotic che	BUB3_HUM	7	1.075591
30_MTA / C	Q9BTV4	-0.41645	0.136745	0.190169	TMEM43	Transmemb	TMM43_HU	1	0.749264
15_MTA / C	Q9Y6D0	-2.23544	0.136737	0.190169	SELENOK	Selenoprot	SELK_HUMA	1	0.212357
30_MTA / C	Q13442	0.488604	0.136996	0.190358	PDAP1	28 kDa hea	HAP28_HU	4	1.403087
15_MTA / C	Q09028	-0.11268	0.137072	0.190384	RBBP4	Histone-bir	RBBP4_HUI	9	0.924869
15_MTA / C	P33176	1.079913	0.137337	0.190673	KIF5B	Kinesin-1 h	KINH_HUM	1	2.113908
30_MTA / C	O95757	-0.41492	0.137471	0.190698	HSPA4L	Heat shock	HS74L_HUI	6	0.750062
15_MTA / C	Q6UXV4	0.449547	0.137416	0.190698	APOOL	MICOS com	MIC27_HUI	5	1.365611
15_MTA / C	Q6ZVX7	-1.3002	0.137564	0.190748	NCCRP1	F-box only	FBX50_HUI	1	0.406071
15_MTA / C	P28070	-0.2606	0.13799	0.191179	PSMB4	Proteasom	PSB4_HUM	5	0.834738
15_MTA / C	P00367	-0.16618	0.138051	0.191183	GLUD1	Glutamate	DHE3_HUM	18	0.891199
30_MTA / C	Q9UGP8	-0.36204	0.138201	0.191311	SEC63	Translocati	SEC63_HUN	8	0.778065
15_MTA / C	Q86X76	0.657545	0.138422	0.191508	NIT1	Deaminate	NIT1_HUMA	2	1.577396
30_MTA / C	Q13232	0.623833	0.138609	0.191635	NME3	Nucleoside	NDK3_HUM	3	1.540964
15_MTA / C	O43681	-0.26695	0.138771	0.191779	GET3	ATPase GET	GET3_HUM	1	0.831077
30_MTA / C	P16152	0.18958	0.139067	0.191948	CBR1	Carbonyl re	CBR1_HUM	8	1.140431
30_MTA / C	P29508;P4	0.532178	0.139248	0.192045	SERPINB3;S	Serpin B3;S	SPB3_HUM	2	1.446111
30_MTA / C	Q6P1N9	-0.69062	0.139253	0.192045	TATDN1	Deoxyribor	TATD1_HUI	7	0.619588
15_MTA / C	P28072	-0.3536	0.139461	0.192171	PSMB6	Proteasom	PSB6_HUM	6	0.782627
15_MTA / C	P49411	-0.36193	0.139403	0.192171	TUFM	Elongation	EFTU_HUM	4	0.778121
30_MTA / C	Q96IU4	1.131156	0.139773	0.192522	ABHD14B	Putative pr	ABHEB_HUI	2	2.190342
15_MTA / C	Q16531	-0.21153	0.139866	0.19257	DDB1	DNA damag	DDB1_HUM	30	0.86362
15_MTA / C	P13693	-0.7733	0.140415	0.192789	TPT1	Translation	TCTP_HUM	4	0.58508
15_MTA / C	P61586	0.3932	0.140379	0.192789	RHOA	Transformi	RHOA_HUM	3	1.313303
30_MTA / C	P68036	1.285808	0.140223	0.192789	UBE2L3	Ubiquitin-c	UB2L3_HUI	3	2.438185
30_MTA / C	Q8IZ83	-0.39999	0.140488	0.192789	ALDH16A1	Aldehyde d	A16A1_HUI	11	0.757863
15_MTA / C	Q8N5R6	-0.33632	0.140491	0.192789	CCDC33	Coiled-coil	CCD33_HU	1	0.792061
15_MTA / C	O75027	0.503303	0.14082	0.192925	ABCB7	Iron-sulfur	ABCB7_HUI	3	1.417456
30_MTA / C	P06753	0.299658	0.140873	0.192925	TPM3	Tropomyos	TPM3_HUM	4	1.230852
30_MTA / C	P21291	-0.55341	0.140881	0.192925	CSRP1	Cysteine an	CSRP1_HUI	3	0.681405
15_MTA / C	Q9UHR5	1.644627	0.141056	0.193086	SAP30BP	SAP30-binc	S30BP_HUI	1	3.126671
15_MTA / C	Q9BY44	0.368649	0.14143	0.193517	EIF2A	Eukaryotic	EIF2A_HUM	13	1.291143
30_MTA / C	O43252	-1.23485	0.141537	0.193583	PAPSS1	Bifunctiona	PAPS1_HUI	1	0.424887
30_MTA / C	P46777	-0.28088	0.141857	0.193941	RPL5	Large ribos	RL5_HUMA	11	0.823091
15_MTA / C	Q99497	-0.61992	0.142066	0.194147	PARK7	Parkinson c	PARK7_HUI	13	0.650705
30_MTA / C	P46781	-0.15916	0.142138	0.194165	RPS9	Small ribos	RS9_HUMA	14	0.895543
30_MTA / C	Q8IZL8	-0.51895	0.142682	0.194619	PELP1	Proline-, gl	PELP1_HUN	1	0.697878
30_MTA / C	P35611	1.267245	0.142767	0.194623	ADD1	Alpha-addu	ADDA_HUM	5	2.407015
15_MTA / C	P08779	-0.4817	0.142828	0.194626	KRT16	Keratin, typ	K1C16_HUI	9	0.716132

30_MTA / C	O00231	-0.18155	0.142909	0.194657	PSMD11	26S protea:	PSD11_HUI	6	0.881756
15_MTA / C	P62424	-0.28787	0.142996	0.194695	RPL7A	Large ribos	RL7A_HUM	15	0.81911
15_MTA / C	Q9NRF8	0.254468	0.143158	0.194756	CTPS2	CTP syntha:	PYRG2_HUI	3	1.192896
15_MTA / C	Q9UMX5	0.439661	0.143121	0.194756	NENF	Neudesin	NENF_HUM	4	1.356285
15_MTA / C	P46109	-0.3349	0.143229	0.194773	CRKL	Crk-like prc	CRKL_HUM	1	0.792841
15_MTA / C	Q5BJF2	0.351836	0.143526	0.195016	TMEM97	Sigma intra	SGMR2_HU	1	1.276184
15_MTA / C	P22695	0.275067	0.144468	0.196055	UQCRC2	Cytochrom	QCR2_HUM	7	1.21005
15_MTA / C	P32969	-0.39932	0.144643	0.196212	RPL9	Large ribos	RL9_HUMA	3	0.758216
15_MTA / C	P68871	1.048376	0.144905	0.196487	HBB	Hemoglobi	HBB_HUMA	2	2.0682
30_MTA / C	P61026	-0.05811	0.145167	0.196531	RAB10	Ras-related	RAB10_HUI	4	0.960523
15_MTA / C	P69905	-0.97606	0.145229	0.196531	HBA1	Hemoglobi	HBA_HUMA	2	0.508366
15_MTA / C	Q16611	0.552346	0.145234	0.196531	BAK1	Bcl-2 homc	BAK_HUMA	2	1.466468
30_MTA / C	Q6PKG0	-0.74862	0.145176	0.196531	LARP1	La-related p	LARP1_HUI	3	0.595171
30_MTA / C	Q5RKV6	0.185899	0.145699	0.197077	EXOSC6	Exosome cc	EXOS6_HUI	4	1.137526
15_MTA / C	Q92979	0.592833	0.145756	0.197077	EMG1	Ribosomal	NEP1_HUM	4	1.508205
15_MTA / C	Q6IAA8	-0.29555	0.145951	0.19718	LAMTOR1	Ragulator c	LTOR1_HUI	2	0.814764
15_MTA / C	Q5T749	-1.57894	0.146197	0.197351	KPRP	Keratinocy	KPRP_HUM	5	0.334728
30_MTA / C	O43242	-0.41972	0.146341	0.197465	PSMD3	26S protea:	PSMD3_HU	11	0.74757
15_MTA / C	P39656	0.177359	0.146566	0.197608	DDOST	Dolichyl-di	OST48_HUI	7	1.130812
15_MTA / C	Q96IZ0	-1.13453	0.146545	0.197608	PAWR	PRKC apop	PAWR_HUM	2	0.455482
30_MTA / C	P11169	-0.11709	0.147038	0.198015	SLC2A3	Solute carri	GTR3_HUM	7	0.922044
30_MTA / C	Q9UBV2	0.426399	0.147048	0.198015	SEL1L	Protein sel-	SE1L1_HUN	5	1.343875
15_MTA / C	P48637	-0.22821	0.147193	0.19805	GSS	Glutathion	GSHB_HUM	20	0.853695
30_MTA / C	Q01658	0.469406	0.147291	0.198082	DR1	Protein Dr1	NC2B_HUM	2	1.384539
15_MTA / C	Q2QD12;Q	0.609997	0.147337	0.198082	RPEL1;RPE	Ribulose-pl	RPEL1_HUN	1	1.526256
30_MTA / C	O43752	0.849383	0.147849	0.198278	STX6	Syntaxin-6	STX6_HUM	1	1.801731
15_MTA / C	P16930	-0.28689	0.147901	0.198278	FAH	Fumarylace	FAAA_HUM	4	0.819669
30_MTA / C	Q92484	0.195716	0.147811	0.198278	SMPDL3A	Cyclic GMP	ASM3A_HU	5	1.145292
30_MTA / C	Q9Y285	-0.38942	0.147644	0.198278	FARSA	Phenylalan	SYFA_HUMA	2	0.763438
15_MTA / C	Q13112	0.36199	0.148095	0.198378	CHAF1B	Chromatin	CAF1B_HUI	3	1.285198
15_MTA / C	Q15257	-0.11707	0.148189	0.198423	PTPA	Serine/thre	PTPA_HUM	6	0.922061
15_MTA / C	P62310	1.134736	0.148441	0.198681	LSM3	U6 snRNA-a	LSM3_HUM	2	2.195784
30_MTA / C	P62258	0.246602	0.148727	0.198903	YWHAE	14-3-3 prot	1433E_HUI	14	1.186409
30_MTA / C	P30086	0.437948	0.148951	0.198978	PEBP1	Phosphatid	PEBP1_HUI	6	1.354676
15_MTA / C	Q96GD0	-0.29138	0.148963	0.198978	PDXP	Chronophi	PLPP_HUM	4	0.817121
15_MTA / C	O75608	0.283608	0.149152	0.199149	LYPLA1	Acyl-protei	LYPA1_HUN	3	1.217235
15_MTA / C	P61158	-0.10471	0.149273	0.199151	ACTR3	Actin-relate	ARP3_HUM	10	0.929993
15_MTA / C	P02654	1.785696	0.149433	0.199284	APOC1	Apolipoprc	APOC1_HU	1	3.447847
15_MTA / C	O43252	0.466405	0.150175	0.199817	PAPSS1	Bifunctiona	PAPS1_HUI	1	1.381663
15_MTA / C	P41236	0.460054	0.150022	0.199817	PPP1R2	Protein phc	IPP2_HUMA	5	1.375593
30_MTA / C	Q92692	0.562145	0.150161	0.199817	NECTIN2	Nectin-2	NECT2_HUI	3	1.476462
30_MTA / C	Q92820	0.240752	0.150146	0.199817	GGH	Gamma-glu	GGH_HUMA	7	1.181608
15_MTA / C	Q96EY1	1.953578	0.150195	0.199817	DNAJA3	DnaJ homo	DNJA3_HUI	1	3.87334
30_MTA / C	Q9NSD9	-0.56432	0.150225	0.199817	FARSB	Phenylalan	SYFB_HUMA	3	0.676272
30_MTA / C	Q15637	0.270012	0.150392	0.199919	SF1	Splicing fac	SF01_HUM	3	1.205818
30_MTA / C	P00761	0.469155	0.150696	0.200028	NaN	Trypsin (Lal	TRYP_PIG	3	1.384299
15_MTA / C	Q92520	0.501866	0.150544	0.200028	FAM3C	Protein FAI	FAM3C_HU	2	1.416044
30_MTA / C	P53007	0.190213	0.151089	0.200386	SLC25A1	Tricarboxyl	TXTP_HUM	5	1.140932
30_MTA / C	Q96P63	-1.37165	0.151105	0.200386	SERPINB12	Serpin B12	SPB12_HUI	7	0.386448
15_MTA / C	P15104	-0.89576	0.151302	0.200566	GLUL	Glutamine	GLNA_HUM	2	0.537464
30_MTA / C	Q9HBL7	-0.90258	0.151727	0.200969	PLGRKT	Plasminoge	PLRKT_HUI	2	0.534927
15_MTA / C	O95881	-0.26496	0.15191	0.200986	TXNDC12	Thioredoxi	TXD12_HUI	3	0.832221

15_MTA / C	Q9BVT8	0.54704	0.151922	0.200986	TMUB1	Transmemb	TMUB1_HU	2	1.461085
15_MTA / C	Q9UHN6	-0.01188	0.151836	0.200986	CEMIP2	Inactive cel	CEIP2_HUM	12	0.991797
30_MTA / C	P05388	-0.13573	0.152058	0.201005	RPLP0	Large ribos	RLA0_HUM	10	0.910209
15_MTA / C	P54725	-0.14543	0.152045	0.201005	RAD23A	UV excision	RD23A_HU	3	0.904113
15_MTA / C	P27708	-1.29081	0.152124	0.201012	CAD	Multifuncti	PYR1_HUM	4	0.408721
15_MTA / C	Q8N1S5	0.352033	0.15224	0.201085	SLC39A11	Zinc transp	S39AB_HU	1	1.276358
30_MTA / C	P08754	-0.27428	0.152586	0.201202	GNAI3	Guanine nu	GNAI3_HU	4	0.82686
15_MTA / C	P29401	-0.32148	0.152552	0.201202	TKT	Transketola	TKT_HUMA	24	0.80025
30_MTA / C	P48556	-0.6208	0.152415	0.201202	PSMD8	26S protea	PSMD8_HU	4	0.650308
15_MTA / C	Q6UX53	1.075068	0.152632	0.201202	TMT1B	Thiol S-met	TMT1B_HU	1	2.106821
30_MTA / C	P62304	0.58359	0.152814	0.201362	SNRPE	Small nucle	RUXE_HUM	2	1.498574
30_MTA / C	Q9UGI8	-0.21321	0.153329	0.20188	TES	Testin	TES_HUMA	2	0.862616
15_MTA / C	A6NDG6	-0.10296	0.153445	0.201952	PGP	Glycerol-3-	PGP_HUMA	8	0.931118
30_MTA / C	P45974	-0.31274	0.153506	0.201953	USP5	Ubiquitin c	UBP5_HUM	2	0.80511
15_MTA / C	Q96JA4	-0.27014	0.15362	0.202023	MS4A14	Membrane-	M4A14_HU	1	0.829239
15_MTA / C	Q5J8M3	1.239412	0.153818	0.202086	EMC4	ER membra	EMC4_HUM	2	2.361023
15_MTA / C	Q8WVX3	0.272483	0.153852	0.202086	C4orf3	Uncharacte	CD003_HU	1	1.207885
15_MTA / C	Q9BS26	0.430303	0.154087	0.202316	ERP44	Endoplasm	ERP44_HU	4	1.347517
15_MTA / C	A0A0C4DH	-0.42431	0.154275	0.202331	IGKV3D-20	Immunoglo	KVD20_HU	1	0.745195
30_MTA / C	Q15435	-0.44227	0.154625	0.202541	PPP1R7	Protein pho	PP1R7_HU	3	0.735978
30_MTA / C	P46063	-0.40602	0.155027	0.202906	RECQL	ATP-depend	RECQ1_HU	4	0.754705
15_MTA / C	P50897	0.224187	0.155329	0.202917	PPT1	Palmitoyl-p	PPT1_HUM	3	1.168119
15_MTA / C	P51812	-1.03033	0.1554	0.202917	RPS6KA3	Ribosomal	KS6A3_HU	1	0.489597
15_MTA / C	Q14566	0.004411	0.155459	0.202917	MCM6	DNA replica	MCM6_HU	1	1.003062
30_MTA / C	Q53GS9	-0.37431	0.155472	0.202917	USP39	Ubiquitin c	UBP39_HU	1	0.771472
15_MTA / C	Q99807	0.23716	0.155525	0.202917	COQ7	5-demetho	COQ7_HUM	2	1.17867
30_MTA / C	O43809	-0.3838	0.155763	0.203068	NUDT21	Cleavage ar	CPSF5_HUM	7	0.766415
30_MTA / C	Q9HBK9	0.560028	0.155903	0.20309	AS3MT	Arsenite me	AS3MT_HU	5	1.474298
30_MTA / C	Q9UJW0	-0.09123	0.155842	0.20309	DCTN4	Dynactin su	DCTN4_HU	6	0.938723
30_MTA / C	O00303	-0.4098	0.156013	0.203155	EIF3F	Eukaryotic	EIF3F_HUM	2	0.752729
15_MTA / C	P30046	-0.15558	0.156097	0.203184	DDT	D-dopachro	DOPD_HUM	5	0.897771
30_MTA / C	O15511	-0.16682	0.15622	0.203264	ARPC5	Actin-relate	ARPC5_HU	2	0.890804
15_MTA / C	Q9UBU6	0.42597	0.156338	0.203337	FAM8A1	Protein FAI	FA8A1_HU	2	1.343475
30_MTA / C	O75083	-0.27701	0.156697	0.203645	WDR1	WD repeat-	WDR1_HU	13	0.8253
30_MTA / C	Q9NYF8	0.513871	0.156795	0.203693	BCLAF1	Bcl-2-assoc	BCLF1_HUI	1	1.427877
15_MTA / C	O00264	0.186689	0.156963	0.20383	PGRMC1	Membrane-	PGRC1_HU	4	1.138149
15_MTA / C	P35573	-1.04946	0.157133	0.203919	AGL	Glycogen d	GDE_HUMA	1	0.483149
15_MTA / C	Q92930	0.287751	0.157198	0.203919	RAB8B	Ras-related	RAB8B_HU	1	1.220736
15_MTA / C	Q7Z4H8	0.873085	0.157436	0.204124	POGLUT3	Protein O-g	PLGT3_HU	2	1.831575
30_MTA / C	P21399	0.561012	0.157733	0.20435	ACO1	Cytoplasmi	ACOHC_HU	5	1.475304
30_MTA / C	O00442	-0.29272	0.15812	0.204631	RTCA	RNA 3'-tern	RTCA_HUM	5	0.816359
30_MTA / C	P02769	-0.98539	0.158099	0.204631	ALB	Serum albu	ALBU_BOVI	26	0.505091
30_MTA / C	P04439	0.456341	0.158135	0.204631	HLA-A	HLA class I	HLAA_HUM	3	1.372057
15_MTA / C	P46060	0.502744	0.158626	0.204973	RANGAP1	Ran GTPase	RAGP1_HU	7	1.416906
15_MTA / C	Q13885	-0.51791	0.158915	0.20524	TUBB2A	Tubulin bet	TBB2A_HU	4	0.698382
15_MTA / C	O95490	0.548463	0.159224	0.205399	ADGRL2	Adhesion G	AGRL2_HU	4	1.462527
30_MTA / C	P20810	0.337884	0.159296	0.205411	CAST	Calpastatin	ICAL_HUMA	4	1.263902
30_MTA / C	O14980	-0.98526	0.159425	0.205417	XPO1	Exportin-1	XPO1_HUM	1	0.505134
15_MTA / C	O43852	-0.33168	0.159368	0.205417	CALU	Calumenin	CALU_HUM	3	0.794611
30_MTA / C	P42126	-0.19622	0.159655	0.205634	ECI1	Enoyl-CoA	ECI1_HUMA	7	0.872836
30_MTA / C	P78381	-0.24485	0.159744	0.205669	SLC35A2	UDP-galact	S35A2_HU	1	0.843902
15_MTA / C	Q53GQ0	0.569752	0.159823	0.20569	HSD17B12	Very-long-c	DHB12_HU	3	1.484269

15_MTA / C	Q04941	-0.45449	0.160783	0.206846	PLP2	Proteolipid	PLP2_HUM	1	0.72977
15_MTA / C	P30740	0.057789	0.161198	0.207145	SERPINB1	Leukocyte e	ILEU_HUMA	6	1.040869
15_MTA / C	O00483	0.148374	0.161992	0.207274	NDUFA4	Cytochrom	NDUA4_HU	3	1.10832
15_MTA / C	O00746	0.265272	0.161812	0.207274	NME4	Nucleoside	NDKM_HUI	4	1.201863
30_MTA / C	O95292	-0.81523	0.161986	0.207274	VAPB	Vesicle-asso	VAPB_HUM	2	0.568319
15_MTA / C	Q9UGP8	0.298188	0.161648	0.207274	SEC63	Translocati	SEC63_HUI	8	1.229599
15_MTA / C	Q9Y277	0.213378	0.161378	0.207274	VDAC3	Voltage-dep	VDAC3_HU	9	1.1594
30_MTA / C	Q9Y3B3	1.333745	0.161929	0.207274	TMED7	Transmemb	TMED7_HU	1	2.520562
30_MTA / C	P26641	-0.14633	0.162662	0.207971	EEF1G	Elongation	EF1G_HUM	13	0.903547
30_MTA / C	P52597	0.232046	0.162865	0.208121	HNRNPF	Heterogene	HNRPF_HU	3	1.1745
15_MTA / C	Q9Y3C6	-0.7719	0.162905	0.208121	PPIL1	Peptidyl-pr	PPIL1_HUM	3	0.585647
30_MTA / C	Q13724	-0.14802	0.163501	0.208641	MOGS	Mannosyl-o	MOGS_HUI	4	0.902485
15_MTA / C	Q8IYT8	0.859651	0.163498	0.208641	ULK2	Serine/thre	ULK2_HUM	1	1.8146
30_MTA / C	P43358	0.273349	0.164153	0.209117	MAGEA4	Melanoma-	MAGA4_HU	5	1.20861
30_MTA / C	Q9UKK9	0.270638	0.164252	0.209117	NUDT5	ADP-sugar p	NUDT5_HU	4	1.206341
30_MTA / C	P04062	-0.39786	0.164518	0.209294	GBA1	Lysosomal a	GBA1_HUM	4	0.758984
15_MTA / C	P52888	-0.23779	0.164493	0.209294	THOP1	Thimet olig	THOP1_HU	12	0.848044
15_MTA / C	P49366	0.588777	0.164676	0.209391	DHPS	Deoxyhypu	DHYS_HUM	3	1.503972
15_MTA / C	Q9BV44	1.52457	0.165354	0.209794	THUMPD3	tRNA (guan	THUM3_HU	3	2.877009
15_MTA / C	Q9H173	0.40338	0.165329	0.209794	SIL1	Nucleotide	SIL1_HUMA	1	1.322603
30_MTA / C	Q14232	1.114994	0.165581	0.210002	EIF2B1	Translation	EI2BA_HUM	2	2.165941
30_MTA / C	Q15005	0.942549	0.165645	0.210003	SPCS2	Signal pept	SPCS2_HUI	4	1.921921
30_MTA / C	Q9NXG2	0.397829	0.16572	0.210017	THUMPD1	THUMP dor	THUM1_HU	6	1.317524
15_MTA / C	Q9BPX5	-0.63089	0.16592	0.210191	ARPC5L	Actin-relate	ARP5L_HUI	2	0.645776
30_MTA / C	P55786	-0.19257	0.166023	0.210241	NPEPPS	Puromycin	PSA_HUMA	17	0.875048
30_MTA / C	Q13867	-0.57423	0.166277	0.210482	BLMH	Bleomycin	BLMH_HUM	3	0.671643
30_MTA / C	Q9NX46	1.076731	0.166535	0.210728	ADPRS	ADP-ribosy	ADPRS_HUI	4	2.109252
30_MTA / C	P09661	0.28552	0.166782	0.21096	SNRPA1	U2 small nu	RU2A_HUM	7	1.21885
30_MTA / C	Q9UQ35	0.135471	0.166899	0.211028	SRRM2	Serine/argi	SRRM2_HU	4	1.098452
30_MTA / C	P99999	0.384577	0.167052	0.211106	CYCS	Cytochrom	CYC_HUMA	5	1.305477
15_MTA / C	Q9GZZ1	-0.51942	0.167088	0.211106	NAA50	N-alpha-ace	NAA50_HU	4	0.697651
15_MTA / C	P20700	0.134453	0.167203	0.21117	LMNB1	Lamin-B1	LMNB1_HU	7	1.097677
15_MTA / C	O96005	1.781244	0.167448	0.211399	CLPTM1	Putative lip	CLPT1_HUI	3	3.437223
30_MTA / C	Q10567	-0.21497	0.167747	0.211493	AP1B1	AP-1 compl	AP1B1_HUI	7	0.861565
15_MTA / C	Q93099	-0.11539	0.167777	0.211493	HGD	Homogenti	HGD_HUMA	3	0.923132
15_MTA / C	Q9NTX5	-0.11113	0.167733	0.211493	ECHDC1	Ethylmalor	ECHD1_HU	7	0.925861
30_MTA / C	P14868	-0.32357	0.16814	0.21158	DARS1	Aspartate--t	SYDC_HUM	8	0.799091
15_MTA / C	P50395	-0.17602	0.168111	0.21158	GDI2	Rab GDP di	GDIB_HUM	15	0.885141
15_MTA / C	Q9C0E8	0.773484	0.168166	0.21158	LNPK	Endoplasm	LNP_HUMA	5	1.709393
15_MTA / C	O60925	0.91381	0.168431	0.211833	PFDN1	Prefoldin su	PFD1_HUM	4	1.884015
30_MTA / C	Q9Y639	0.237061	0.168577	0.211936	NPTN	Neuroplast	NPTN_HUM	2	1.178589
30_MTA / C	Q13162	0.475567	0.168984	0.212286	PRDX4	Peroxiredo	PRDX4_HU	3	1.390465
15_MTA / C	P25789	-0.16812	0.169229	0.212374	PSMA4	Proteasome	PSA4_HUM	10	0.890004
30_MTA / C	Q16611	-0.47142	0.169246	0.212374	BAK1	Bcl-2 homo	BAK_HUMA	2	0.721257
15_MTA / C	O00442	-0.31286	0.169453	0.212554	RTCA	RNA 3'-term	RTCA_HUM	5	0.805047
15_MTA / C	O95563	-0.61252	0.16981	0.21276	MPC2	Mitochond	MPC2_HUM	1	0.654052
15_MTA / C	P47756	-0.09199	0.169734	0.21276	CAPZB	F-actin-cap	CAPZB_HUI	13	0.938227
15_MTA / C	Q8IV08	0.531024	0.170625	0.21362	PLD3	5'-3' exonu	PLD3_HUM	2	1.444955
30_MTA / C	P16989	0.698795	0.170832	0.213798	YBX3	Y-box-bindi	YBOX3_HUI	6	1.623148
15_MTA / C	P36873	-0.53049	0.170915	0.213821	PPP1CC	Serine/thre	PP1G_HUM	2	0.69232
30_MTA / C	O60763	-0.92599	0.171664	0.214187	USO1	General ves	USO1_HUM	4	0.52632
30_MTA / C	O75534	0.130697	0.171405	0.214187	CSDE1	Cold shock	CSDE1_HUI	8	1.094822

30_MTA / C	P54886	1.047884	0.171757	0.214187	ALDH18A1	Delta-1-pyr	P5CS_HUM	4	2.067495
15_MTA / C	P61160	-0.09778	0.171672	0.214187	ACTR2	Actin-relate	ARP2_HUM	6	0.934472
15_MTA / C	P62854	-0.11453	0.171516	0.214187	RPS26	Small ribos	RS26_HUM	3	0.923684
15_MTA / C	Q9UKK9	-0.09623	0.171902	0.21419	NUDT5	ADP-sugar p	NUDT5_HU	4	0.935472
15_MTA / C	Q9Y2Z0	0.175587	0.172063	0.214209	SUGT1	Protein SG	SGT1_HUM	7	1.129424
15_MTA / C	Q15067	0.758128	0.172363	0.214234	ACOX1	Peroxisoma	ACOX1_HU	2	1.691295
15_MTA / C	Q99733	0.55806	0.172409	0.214234	NAP1L4	Nucleosom	NP1L4_HU	3	1.472288
30_MTA / C	Q9Y241	-0.56635	0.172227	0.214234	HIGD1A	HIG1 doma	HIG1A_HU	1	0.675325
15_MTA / C	P30040	0.058067	0.172474	0.214235	ERP29	Endoplasm	ERP29_HU	7	1.04107
15_MTA / C	Q9UHG3	0.469557	0.17288	0.214421	PCYOX1	Prenylcyste	PCYOX_HU	5	1.384684
30_MTA / C	Q9UNQ0	-0.44195	0.17286	0.214421	ABCG2	Broad subs	ABCG2_HU	1	0.73614
15_MTA / C	P25398	-0.11841	0.173102	0.214452	RPS12	Small ribos	RS12_HUM	4	0.921202
15_MTA / C	Q8IZL8	-0.38263	0.173101	0.214452	PELP1	Proline-, gl	PELP1_HU	1	0.767037
15_MTA / C	O95218	-0.04372	0.17331	0.214517	ZRANB2	Zinc finger	ZRAB2_HU	3	0.970153
15_MTA / C	Q99729	-0.56787	0.173713	0.214727	HNRNPAB	Heterogene	ROAA_HUM	3	0.674613
15_MTA / C	P04406	-0.44841	0.17381	0.214768	GAPDH	Glyceraldel	G3P_HUMA	21	0.73285
30_MTA / C	P02545	0.242819	0.173943	0.214852	LMNA	Prelamin-A	LMNA_HUM	18	1.183302
15_MTA / C	Q9P2B2	0.754616	0.174143	0.215018	PTGFRN	Prostagland	FPRP_HUM	4	1.687182
30_MTA / C	Q9UHD9	-1.43524	0.174589	0.215329	UBQLN2	Ubiquilin-2	UBQL2_HU	1	0.369785
30_MTA / C	Q02127	-0.30947	0.175028	0.215549	DHODH	Dihydroor	PYRD_HUM	8	0.80694
15_MTA / C	Q8WUM4	0.586766	0.175093	0.215549	PDCD6IP	Programme	PDC6I_HU	7	1.501876
15_MTA / C	P48556	-0.30808	0.175329	0.21576	PSMD8	26S protea	PSMD8_HU	4	0.807714
15_MTA / C	P62993	0.19646	0.175589	0.216	GRB2	Growth fac	GRB2_HUM	4	1.145883
15_MTA / C	Q9H2P9	-0.04371	0.175787	0.216162	DPH5	Diphthine	DPH5_HUM	2	0.970155
30_MTA / C	P41440	-0.29505	0.176124	0.216416	SLC19A1	Reduced fo	S19A1_HU	3	0.815046
30_MTA / C	Q96IJ6	-0.29462	0.176201	0.216431	GMPPA	Mannose-1	GMPPA_HU	5	0.815284
15_MTA / C	P27144	0.582664	0.176286	0.216455	AK4	Adenylate k	KAD4_HUM	2	1.497613
15_MTA / C	P42574	-0.2014	0.176368	0.216475	CASP3	Caspase-3	CASP3_HU	3	0.869708
15_MTA / C	P11166	-0.19647	0.1767	0.216722	SLC2A1	Solute carri	GTR1_HUM	1	0.872682
15_MTA / C	Q07960	0.782938	0.176879	0.216861	ARHGAP1	Rho GTPase	RHG01_HU	2	1.720632
15_MTA / C	P09429	-0.53256	0.177037	0.216893	HMGB1	High mobil	HMGB1_HU	2	0.691327
30_MTA / C	Q00534	-0.24167	0.177165	0.216893	CDK6	Cyclin-depe	CDK6_HUM	3	0.845768
30_MTA / C	P32119	0.250383	0.177655	0.217131	PRDX2	Peroxiredo	PRDX2_HU	7	1.189523
30_MTA / C	P42771	0.856311	0.177689	0.217131	CDKN2A	Cyclin-depe	CDN2A_HU	1	1.810404
30_MTA / C	P55072	0.366297	0.17751	0.217131	VCP	Transitiona	TERA_HUM	37	1.28904
30_MTA / C	Q9BUN8	-0.78567	0.177634	0.217131	DERL1	Derlin-1	DERL1_HU	4	0.580084
15_MTA / C	O15143	-0.09997	0.177813	0.217203	ARPC1B	Actin-relate	ARC1B_HU	6	0.933054
15_MTA / C	P21796	0.194662	0.178152	0.217334	VDAC1	Non-selecti	VDAC1_HU	17	1.144456
15_MTA / C	P51153	0.452386	0.178313	0.217334	RAB13	Ras-related	RAB13_HU	4	1.368301
15_MTA / C	Q9UK76	0.843576	0.178248	0.217334	JPT1	Jupiter mic	JUPI1_HUM	3	1.794493
15_MTA / C	P83731	-0.35869	0.178453	0.217382	RPL24	Large ribos	RL24_HUM	5	0.779874
15_MTA / C	Q9H832	0.831787	0.178484	0.217382	UBE2Z	Ubiquitin-c	UBE2Z_HU	1	1.779889
15_MTA / C	Q8TBQ9	0.169467	0.178758	0.217556	TMEM167A	Protein kis	KISHA_HUM	1	1.124643
15_MTA / C	P82979	-0.15686	0.178973	0.217737	SARNP	SAP domai	SARNP_HU	7	0.896975
30_MTA / C	P08397	0.233993	0.17938	0.218006	HMBS	Porphobili	HEM3_HUM	6	1.176085
30_MTA / C	P48634	0.552243	0.179537	0.218104	PRRC2A	Protein PR	PRC2A_HU	1	1.466364
15_MTA / C	Q9NZ45	2.306102	0.179675	0.218191	CISD1	CDGSH iror	CISD1_HUM	2	4.94545
30_MTA / C	Q15018	-0.38698	0.180052	0.218488	ABRAXAS2	BRISC comp	ABRX2_HU	5	0.764728
15_MTA / C	Q15274	-0.2276	0.180068	0.218488	QPRT	Nicotinate-	NADC_HUM	7	0.854055
30_MTA / C	Q3ZCQ8	-0.3036	0.180536	0.218915	TIMM50	Mitochond	TIM50_HU	1	0.810225
15_MTA / C	P63092;Q5	0.099948	0.180868	0.218953	GNAS	Guanine nu	GNAS2_HU	1	1.071735
15_MTA / C	Q92841	0.067907	0.180795	0.218953	DDX17	Probable A	DDX17_HU	6	1.048195

15_MTA / C	Q9BW83	0.243068	0.180897	0.218953	IFT27	Intraflagell	IFT27_HUM	1	1.183507
15_MTA / C	P18124	-0.35184	0.181283	0.219223	RPL7	Large ribos	RL7_HUMA	15	0.783587
15_MTA / C	Q13438	0.8708	0.181318	0.219223	OS9	Protein OS-	OS9_HUMA	1	1.828676
15_MTA / C	P05387	-0.40625	0.182144	0.219842	RPLP2	Large ribos	RLA2_HUM	4	0.75458
30_MTA / C	Q99807	-0.15132	0.182144	0.219842	COQ7	5-demetho	COQ7_HUM	2	0.900423
30_MTA / C	Q9UMS0	0.265693	0.182163	0.219842	NFU1	NFU1 iron-	NFU1_HUM	2	1.202213
15_MTA / C	O00233	-0.44998	0.182335	0.219953	PSMD9	26S protea	PSMD9_HU	2	0.732053
30_MTA / C	Q96AE4	0.269586	0.182387	0.219953	FUBP1	Far upstrea	FUBP1_HU	24	1.205462
30_MTA / C	O94979	0.265185	0.182618	0.220151	SEC31A	Protein tra	SC31A_HU	3	1.20179
15_MTA / C	P23258;Q9	1.146031	0.183544	0.221026	TUBG1;TUE	Tubulin gar	TBG1_HUM	2	2.213043
30_MTA / C	P60891	-0.12197	0.183793	0.221166	PRPS1	Ribose-pho	PRPS1_HU	8	0.918934
30_MTA / C	P22061	0.250343	0.184045	0.221309	PCMT1	Protein-L-is	PIMT_HUM	6	1.18949
30_MTA / C	Q86X76	0.456673	0.184164	0.221371	NIT1	Deaminate	NIT1_HUM	2	1.372374
15_MTA / C	Q9NS86	0.658132	0.18447	0.221612	LANCL2	LanC-like p	LANC2_HU	3	1.578038
15_MTA / C	Q9BZX2	-0.4854	0.184785	0.221876	UCK2	Uridine-cyt	UCK2_HUM	4	0.714298
30_MTA / C	Q3ZAQ7	-0.01142	0.185019	0.221996	VMA21	Vacuolar A	VMA21_HU	2	0.992119
30_MTA / C	P04264	-0.60559	0.185129	0.222048	KRT1	Keratin, typ	K2C1_HUM	32	0.657203
15_MTA / C	P68371	-0.26975	0.185206	0.22206	TUBB4B	Tubulin bet	TBB4B_HU	18	0.829464
15_MTA / C	O00154	-0.12901	0.185351	0.222063	ACOT7	Cytosolic a	BACH_HUM	5	0.914461
15_MTA / C	Q13595	0.133885	0.185406	0.222063	TRA2A	Transforme	TRA2A_HU	1	1.097244
15_MTA / C	P56134	0.338319	0.185727	0.222283	ATP5MF	ATP syntha	ATPK_HUM	1	1.264283
30_MTA / C	Q02818	0.713901	0.185981	0.222506	NUCB1	Nucleobind	NUCB1_HU	3	1.640233
15_MTA / C	P11021	-0.13895	0.186462	0.22292	HSPA5	Endoplasm	BIP_HUMA	28	0.908182
15_MTA / C	Q53GS9	1.040263	0.18646	0.22292	USP39	Ubiquitin c	UBP39_HU	1	2.056602
15_MTA / C	Q9Y333	-0.61747	0.187251	0.223681	LSM2	U6 snRNA-a	LSM2_HUM	3	0.651813
15_MTA / C	P02768	0.844149	0.187488	0.223718	ALB	Albumin	ALBU_HUM	4	1.795206
30_MTA / C	Q9Y3I0	-0.12548	0.187534	0.223718	RTCB	RNA-splicir	RTCB_HUM	9	0.916696
30_MTA / C	Q96PK6	1.747936	0.188275	0.224441	RBM14	RNA-bindir	RBM14_HU	4	3.358776
15_MTA / C	Q9BUH6	0.163406	0.188429	0.224543	PAXX	Protein PA	PAXX_HUM	5	1.119928
15_MTA / C	Q96I99	-0.00634	0.188503	0.224551	SUCLG2	Succinate--	SUCB2_HU	1	0.995613
30_MTA / C	P62857	0.215672	0.189407	0.225465	RPS28	Small ribos	RS28_HUM	2	1.161245
15_MTA / C	P48059	0.19335	0.189483	0.225474	LIMS1	LIM and ser	LIMS1_HU	1	1.143416
15_MTA / C	Q16881	-0.22788	0.189571	0.225498	TXNRD1	Thioredoxi	TRXR1_HU	12	0.85389
15_MTA / C	P49354	1.401259	0.189879	0.225689	FNTA	Protein far	FNTA_HUM	1	2.641319
15_MTA / C	Q9BQ95	1.382739	0.189935	0.225689	ECSIT	Evolutiona	ECSIT_HUM	1	2.60763
30_MTA / C	Q13112	-0.27155	0.190149	0.225804	CHAF1B	Chromatin	CAF1B_HU	3	0.828431
15_MTA / C	O43464	0.220034	0.190297	0.225876	HTRA2	Serine prot	HTRA2_HU	3	1.164761
15_MTA / C	Q96JJ7	0.555143	0.190387	0.225901	TMX3	Protein dis	TMX3_HUM	1	1.469314
15_MTA / C	O95721	0.202931	0.190509	0.225966	SNAP29	Synaptosor	SNP29_HU	1	1.151035
15_MTA / C	P59998	-0.31257	0.191216	0.226491	ARPC4	Actin-relate	ARPC4_HU	6	0.805204
15_MTA / C	Q9NQG5	0.618869	0.191282	0.226491	RPRD1B	Regulation	RPR1B_HU	2	1.535671
30_MTA / C	Q9UNL2	0.880715	0.191294	0.226491	SSR3	Translocon	SSRG_HUM	1	1.841288
30_MTA / C	P62491;Q1	-0.10845	0.191897	0.226962	RAB11A;RA	Ras-related	RB11A_HU	11	0.927586
15_MTA / C	P30050	-0.30461	0.191997	0.226999	RPL12	Large ribos	RL12_HUM	5	0.80966
15_MTA / C	Q13618	1.272634	0.1921	0.22704	CUL3	Cullin-3	CUL3_HUM	3	2.416023
30_MTA / C	Q15369	0.585906	0.192553	0.227413	ELOC	Elongin-C	ELOC_HUM	1	1.500982
15_MTA / C	Q02878	-0.05616	0.192874	0.227711	RPL6	Large ribos	RL6_HUMA	11	0.961817
15_MTA / C	P60510	-0.09509	0.193327	0.228083	PPP4C	Serine/thre	PP4C_HUM	2	0.936213
30_MTA / C	Q9UHG3	-0.01816	0.193755	0.228506	PCYOX1	Prenylcyste	PCYOX_HU	5	0.987488
15_MTA / C	P35270	0.516961	0.193913	0.228547	SPR	Sepiapterin	SPRE_HUM	2	1.430937
15_MTA / C	P10412	1.188089	0.194308	0.228819	H1-4	Histone H1	H14_HUMA	8	2.278508
30_MTA / C	P00352	0.329112	0.194692	0.22896	ALDH1A1	Aldehyde d	AL1A1_HU	19	1.25624

15_MTA / C	Q06210	0.342414	0.194675	0.22896	GFPT1	Glutamine-	GFPT1_HUI	5	1.267877
15_MTA / C	O60814;P5	-0.20116	0.19501	0.229252	H2BC12;H2	Histone H2	H2B1K_HU	6	0.869849
15_MTA / C	P48775	-0.33577	0.195133	0.229316	TDO2	Tryptophar	T23O_HUM	1	0.792361
15_MTA / C	P60900	-0.31667	0.195408	0.229558	PSMA6	Proteasom	PSA6_HUM	9	0.802924
30_MTA / C	Q96EY1	0.375821	0.1963	0.230035	DNAJA3	DnaJ homo	DNJA3_HUI	1	1.297578
15_MTA / C	Q9UBW8	0.34593	0.196211	0.230035	COPS7A	COP9 signa	CSN7A_HUI	5	1.27097
30_MTA / C	P62937	0.306093	0.196521	0.230153	PPIA	Peptidyl-pr	PPIA_HUM.	10	1.236355
15_MTA / C	Q8WU90	0.261486	0.19654	0.230153	ZC3H15	Zinc finger	ZC3HF_HUI	1	1.198713
30_MTA / C	P42785	-0.2307	0.197016	0.230629	PRCP	Lysosomal	PCP_HUMA	3	0.852218
30_MTA / C	P17096	0.437118	0.197365	0.230956	HMGA1	High mobil	HMGA1_HU	4	1.353897
15_MTA / C	P23396	-0.04966	0.197831	0.231256	RPS3	Small ribos	RS3_HUMA	17	0.966161
30_MTA / C	P61619	-0.08778	0.197798	0.231256	SEC61A1	Protein tra	S61A1_HUI	3	0.94097
30_MTA / C	Q6KB66	-1.21717	0.197745	0.231256	KRT80	Keratin, typ	K2C80_HUI	3	0.430127
30_MTA / C	Q9H773	0.620974	0.197926	0.231285	DCTPP1	dCTP pyrop	DCTP1_HUI	6	1.537914
15_MTA / C	P0CG38	-0.34896	0.198229	0.231479	POTEI	POTE ankyr	POTEI_HUN	2	0.785148
15_MTA / C	Q9NP72	0.42476	0.198511	0.231479	RAB18	Ras-related	RAB18_HUI	5	1.342349
15_MTA / C	Q9UMX0	0.643673	0.198455	0.231479	UBQLN1	Ubiquilin-1	UBQL1_HU	8	1.562302
30_MTA / C	Q99460	0.031473	0.199016	0.231906	PSMD1	26S protea	PSMD1_HU	8	1.022055
15_MTA / C	P51991	-0.24561	0.199161	0.231993	HNRNPA3	Heterogene	ROA3_HUN	2	0.843461
15_MTA / C	P52657	-0.55818	0.199913	0.232461	GTF2A2	Transcripti	T2AG_HUN	1	0.679158
30_MTA / C	P07384	-0.11593	0.200191	0.232702	CAPN1	Calpain-1 c	CAN1_HUN	16	0.922787
30_MTA / C	Q9BT73	-0.74104	0.200573	0.232983	PSMG3	Proteasom	PSMG3_HU	3	0.598309
30_MTA / C	Q6P587	0.862792	0.200981	0.233375	FAHD1	Oxaloaceta	FAHD1_HU	1	1.818554
30_MTA / C	O95490	-0.12307	0.201487	0.23388	ADGRL2	Adhesion G	AGRL2_HUI	4	0.918233
30_MTA / C	Q9UBQ7	-0.18631	0.202489	0.234797	GRHPR	Glyoxylate	GRHPR_HU	1	0.878851
15_MTA / C	P21291	-0.34688	0.203013	0.235199	CSRP1	Cysteine an	CSRP1_HUI	3	0.786281
30_MTA / C	P48507	-0.74843	0.203049	0.235199	GCLM	Glutamate-	GSH0_HUN	3	0.595252
15_MTA / C	P53396	-0.36158	0.202918	0.235199	ACLY	ATP-citrate	ACLY_HUM	3	0.778309
30_MTA / C	Q5BJF2	-0.38293	0.203187	0.235277	TMEM97	Sigma intra	SGMR2_HU	1	0.76688
30_MTA / C	P09960	-0.14986	0.20355	0.235321	LTA4H	Leukotrien	LKHA4_HUI	18	0.90134
30_MTA / C	Q9UKD2	0.895209	0.203498	0.235321	MRTO4	mRNA turn	MRT4_HUN	1	1.85988
15_MTA / C	Q9NXR7	1.315141	0.203738	0.235339	BABAM2	BRISC and E	BABA2_HUI	1	2.488267
30_MTA / C	Q92506	-0.29656	0.203974	0.23553	HSD17B8	(3R)-3-hydr	DHB8_HUN	3	0.81419
15_MTA / C	P13798	-0.27238	0.204128	0.235543	APEH	Acylamino-	ACPH_HUN	12	0.827952
30_MTA / C	P37108	0.34552	0.204252	0.235604	SRP14	Signal reco	SRP14_HUI	5	1.270609
30_MTA / C	P22392	0.341883	0.204672	0.235685	NME2	Nucleoside	NDKB_HUN	10	1.26741
15_MTA / C	Q99832	-0.05869	0.204785	0.235727	CCT7	T-complex	TCPH_HUM	23	0.960132
30_MTA / C	Q9BV20	-0.19338	0.205325	0.236266	MRI1	Methylthio	MTNA_HUN	4	0.874555
15_MTA / C	Q07666	0.300503	0.205425	0.236291	KHDRBS1	KH domain	KHDR1_HU	1	1.231573
30_MTA / C	Q9P2X0	-0.3425	0.205961	0.236751	DPM3	Dolichol-pl	DPM3_HUN	1	0.788673
30_MTA / C	Q9H2V7	-0.16124	0.206151	0.236888	SPNS1	Protein spi	SPNS1_HUI	1	0.894254
15_MTA / C	Q92900	0.93807	0.206325	0.237005	UPF1	Regulator c	RENT1_HUI	6	1.915963
15_MTA / C	P38571	1.019361	0.206542	0.237173	LIPA	Lysosomal	LICH_HUM.	1	2.02702
15_MTA / C	P84103	0.024504	0.206625	0.237185	SRSF3	Serine/argi	SRSF3_HUN	3	1.01713
15_MTA / C	Q8NBL1	0.527625	0.207125	0.237595	POGLUT1	Protein O-g	PGLT1_HUI	1	1.441554
30_MTA / C	Q96QK1	-0.13502	0.207091	0.237595	VPS35	Vacuolar pr	VPS35_HUI	7	0.910658
30_MTA / C	O43148	-0.32075	0.207425	0.237775	RNMT	mRNA cap	MCES_HUN	3	0.800651
15_MTA / C	P38919	-0.05007	0.207675	0.237979	EIF4A3	Eukaryotic	IF4A3_HUN	8	0.965888
30_MTA / C	O75947	-0.44174	0.207934	0.237993	ATP5PD	ATP synthas	ATP5H_HUI	4	0.736247
30_MTA / C	P78540	-0.17251	0.208097	0.237993	ARG2	Arginase-2,	ARGI2_HUN	1	0.887299
30_MTA / C	Q13541	1.382076	0.208122	0.237993	EIF4EBP1	Eukaryotic	4EBP1_HUI	1	2.606432
30_MTA / C	Q9UBW8	0.759555	0.208161	0.237993	COPS7A	COP9 signa	CSN7A_HUI	5	1.692969

30_MTA / C	P78417	0.234303	0.208387	0.238136	GSTO1	Glutathion	GSTO1_HUI	14	1.176338
30_MTA / C	Q99627	-0.41783	0.20869	0.238304	COPS8	COP9 signa	CSN8_HUM	3	0.748551
15_MTA / C	P98179	-0.12677	0.209651	0.239168	RBM3	RNA-bindir	RBM3_HUM	2	0.91588
30_MTA / C	Q13685	-0.4002	0.209582	0.239168	AAMP	Angio-asso	AAMP_HUM	2	0.757756
30_MTA / C	Q8NBS9	0.165366	0.209761	0.239211	TXNDC5	Thioredoxi	TXND5_HU	11	1.12145
15_MTA / C	Q8TEX9	-0.32555	0.209875	0.239259	IPO4	Importin-4	IPO4_HUM	7	0.797997
30_MTA / C	P00568	-0.74444	0.21053	0.23984	AK1	Adenylate k	KAD1_HUM	2	0.596898
30_MTA / C	Q9NNW7	-0.32166	0.21068	0.23993	TXNRD2	Thioredoxi	TRXR2_HUI	5	0.800147
30_MTA / C	P42766	-0.57626	0.211167	0.239989	RPL35	Large ribos	RL35_HUM	2	0.670698
15_MTA / C	P62910	-0.01741	0.21097	0.239989	RPL32	Large ribos	RL32_HUM	7	0.988006
15_MTA / C	Q9HBL7	0.695691	0.211153	0.239989	PLGRKT	Plasminoge	PLRKT_HUI	2	1.61966
30_MTA / C	Q9NPH2	0.329022	0.211068	0.239989	ISYNA1	Inositol-3-p	INO1_HUM	7	1.256161
15_MTA / C	P0C0S8;Q1	-0.72354	0.21148	0.240179	H2AC11;H2	Histone H2	H2A1_HUM	4	0.605611
30_MTA / C	Q9H078	1.009603	0.211471	0.240179	CLPB	Mitochond	CLPB_HUM	2	2.013357
30_MTA / C	P41587	0.192736	0.21209	0.240708	VIPR2	Vasoactive	VIPR2_HUI	1	1.142929
15_MTA / C	P09651	-0.42167	0.212996	0.241405	HNRNPA1	Heterogene	ROA1_HUM	4	0.74656
15_MTA / C	P30049	-0.08053	0.21295	0.241405	ATP5F1D	ATP syntha	ATPD_HUM	3	0.945713
15_MTA / C	Q9UGI8	0.385544	0.214341	0.242764	TES	Testin	TES_HUMA	2	1.306352
15_MTA / C	Q06124	0.378809	0.214579	0.24295	PTPN11	Tyrosine-pr	PTN11_HUI	2	1.300268
30_MTA / C	P11940	0.215894	0.215021	0.243165	PABPC1	Polyadenyl	PABP1_HUI	15	1.161423
15_MTA / C	P49327	0.266006	0.214934	0.243165	FASN	Fatty acid s	FAS_HUMA	23	1.202474
15_MTA / C	P50502;Q8	-0.13811	0.214983	0.243165	ST13;ST13F	Hsc70-inte	F10A1_HUI	2	0.908707
30_MTA / C	Q8N4P3	1.919293	0.215063	0.243165	HDDC3	Guanosine-	MESH1_HU	2	3.782377
30_MTA / C	P05783	0.404346	0.215184	0.24322	KRT18	Keratin, typ	K1C18_HUI	18	1.323489
15_MTA / C	P16615	0.304178	0.2156	0.243441	ATP2A2	Sarcoplasm	AT2A2_HUI	16	1.234715
15_MTA / C	Q86X55	0.52547	0.215941	0.243602	CARM1	Histone-arg	CARM1_HU	4	1.439403
30_MTA / C	Q9NY33	-0.29442	0.216037	0.243602	DPP3	Dipeptidyl	DPP3_HUM	11	0.8154
30_MTA / C	P48643	0.194333	0.216957	0.244307	CCT5	T-complex	TCPE_HUM	20	1.144195
15_MTA / C	O75477	0.866418	0.217136	0.244359	ERLIN1	Erlin-1	ERLN1_HUI	5	1.82313
15_MTA / C	Q13510	-0.24204	0.21715	0.244359	ASAH1	Acid ceram	ASAH1_HUI	2	0.845549
30_MTA / C	O15258	-0.82798	0.217313	0.244376	RER1	Protein REF	RER1_HUM	4	0.563316
15_MTA / C	Q3ZCQ8	0.637574	0.217282	0.244376	TIMM50	Mitochond	TIM50_HUI	1	1.555711
15_MTA / C	Q13616	-0.03619	0.217442	0.244426	CUL1	Cullin-1	CUL1_HUM	10	0.975226
30_MTA / C	Q15691	-0.14753	0.217579	0.244426	MAPRE1	Microtubul	MARE1_HU	6	0.902794
30_MTA / C	P61923	0.481227	0.21766	0.244434	COPZ1	Coatomer s	COPZ1_HUI	4	1.39593
15_MTA / C	P07384	-0.05936	0.218362	0.244973	CAPN1	Calpain-1 c	CAN1_HUM	16	0.959689
30_MTA / C	Q15493	0.271631	0.218543	0.245093	RGN	Regucalcin	RGN_HUM/	2	1.207172
15_MTA / C	P09497	-0.41693	0.218756	0.245245	CLTB	Clathrin lig	CLCB_HUM	4	0.749017
30_MTA / C	P62888	0.201937	0.219155	0.24553	RPL30	Large ribos	RL30_HUM	6	1.150242
30_MTA / C	O14602;P4	1.042265	0.219427	0.245753	EIF1AY;EIF1	Eukaryotic	IF1AY_HUM	1	2.059458
15_MTA / C	P27797	-0.1426	0.219645	0.245831	CALR	Calreticulir	CALR_HUM	11	0.905883
15_MTA / C	P61970	-0.16192	0.219583	0.245831	NUTF2	Nuclear tra	NTF2_HUM	2	0.893837
15_MTA / C	Q9NWT6	0.386068	0.220478	0.246429	HIF1AN	Hypoxia-in	HIF1N_HUI	1	1.306827
15_MTA / C	P30408	-0.2116	0.220842	0.246587	TM4SF1	Transmemb	T4S1_HUM	1	0.86358
15_MTA / C	Q9H2U2	-0.70777	0.220713	0.246587	PPA2	Inorganic p	IPYR2_HUN	3	0.612268
30_MTA / C	Q9NZT1	0.485656	0.220768	0.246587	CALML5	Calmodulir	CALL5_HUI	3	1.400222
30_MTA / C	Q8NHP8	-0.19729	0.221019	0.246701	PLBD2	Putative ph	PLBL2_HUI	1	0.872187
15_MTA / C	Q9BSR8	0.542121	0.221603	0.247271	YIPF4	Protein YIP	YIPF4_HUM	2	1.456111
15_MTA / C	Q96S66	0.024188	0.222075	0.247714	CLCC1	Chloride ch	CLCC1_HUI	4	1.016907
30_MTA / C	P10809	0.283355	0.222195	0.247764	HSPD1	60 kDa heat	CH60_HUM	37	1.217022
30_MTA / C	Q9BT78	-0.12651	0.222667	0.248123	COPS4	COP9 signa	CSN4_HUM	7	0.916047
30_MTA / C	P78346	-0.34735	0.223129	0.248471	RPP30	Ribonuclea	RPP30_HUI	2	0.786025

15_MTA / C	P35908	-0.23515	0.223381	0.248668	KRT2	Keratin, typ	K22E_HUM	30	0.849597
15_MTA / C	P23526	-0.09042	0.22352	0.248739	AHCY	Adenosylhc	SAHH_HUM	21	0.939249
30_MTA / C	Q13561	0.287181	0.224692	0.249792	DCTN2	Dynactin su	DCTN2_HU	8	1.220254
15_MTA / C	P30520	-0.15503	0.224796	0.249824	ADSS2	Adenylosuc	PURA2_HU	8	0.898116
30_MTA / C	P11142	0.207527	0.22528	0.249992	HSPA8	Heat shock	HSP7C_HU	28	1.154707
30_MTA / C	P27348	0.249474	0.225278	0.249992	YWHAQ	14-3-3 prot	1433T_HU	4	1.188774
30_MTA / C	Q13404	0.208176	0.225325	0.249992	UBE2V1	Ubiquitin-c	UB2V1_HU	9	1.155226
15_MTA / C	Q9Y6M1	-0.27975	0.225152	0.249992	IGF2BP2	Insulin-like	IF2B2_HUM	2	0.823734
30_MTA / C	P04040	0.227985	0.225413	0.250006	CAT	Catalase	CATA_HUM	12	1.171198
30_MTA / C	Q8WZA0	0.287993	0.225785	0.250295	LZIC	Protein LZI	LZIC_HUMA	2	1.22094
15_MTA / C	Q96I24	0.760032	0.225824	0.250295	FUBP3	Far upstrea	FUBP3_HU	3	1.693529
15_MTA / C	Q9Y697	0.719985	0.226131	0.250551	NFS1	Cysteine de	NFS1_HUM	2	1.647164
15_MTA / C	P30622	0.550494	0.226426	0.250794	CLIP1	CAP-Gly do	CLIP1_HUM	4	1.464587
15_MTA / C	Q10567	0.316561	0.226546	0.250844	AP1B1	AP-1 compl	AP1B1_HU	7	1.245358
15_MTA / C	P28074	-0.225	0.226707	0.250854	PSMB5	Proteasom	PSB5_HUM	12	0.855596
30_MTA / C	Q13813	-0.04163	0.226685	0.250854	SPTAN1	Spectrin alp	SPTN1_HU	24	0.97156
30_MTA / C	Q9NRF8	1.340424	0.226869	0.250949	CTPS2	CTP synthas	PYRG2_HU	3	2.532258
30_MTA / C	P16435	-0.14557	0.227265	0.251304	POR	NADPH--cy	NCPR_HUM	7	0.904021
15_MTA / C	P14625	-0.15059	0.227619	0.251548	HSP90B1	Endoplasm	ENPL_HUM	30	0.900885
30_MTA / C	Q92900	0.591165	0.227638	0.251548	UPF1	Regulator c	RENT1_HU	6	1.506463
15_MTA / C	P26358	-0.4193	0.228646	0.252409	DNMT1	DNA (cytosi	DNMT1_HU	2	0.747786
15_MTA / C	P62873	0.412167	0.228608	0.252409	GNB1	Guanine nu	GBB1_HUM	7	1.330683
30_MTA / C	Q15424	1.481929	0.228642	0.252409	SAFB	Scaffold att	SAFB1_HUM	2	2.793219
15_MTA / C	Q86YZ3	-0.8951	0.228893	0.252514	HRNR	Hornerin	HORN_HUM	11	0.537708
30_MTA / C	Q9Y6M5	-0.05928	0.228881	0.252514	SLC30A1	Proton-cou	ZNT1_HUM	4	0.959744
15_MTA / C	Q8N6L1	1.39515	0.229148	0.252711	KRTCAP2	Keratinocy	KTAP2_HU	1	2.630159
15_MTA / C	O60749	0.664611	0.229262	0.252753	SNX2	Sorting nex	SNX2_HUM	2	1.585141
30_MTA / C	Q9BWD1	0.168906	0.229476	0.252904	ACAT2	Acetyl-CoA	THIC_HUMA	10	1.124206
30_MTA / C	P20700	1.41237	0.229745	0.252965	LMNB1	Lamin-B1	LMNB1_HU	7	2.661741
30_MTA / C	P62136	-0.19699	0.22976	0.252965	PPP1CA	Serine/thre	PP1A_HUM	6	0.87237
30_MTA / C	Q00688	0.923425	0.229948	0.253088	FKBP3	Peptidyl-pr	FKBP3_HU	2	1.896612
15_MTA / C	Q15435	0.64147	0.230281	0.253285	PPP1R7	Protein pho	PP1R7_HU	3	1.559917
30_MTA / C	Q9Y5T4	1.058393	0.230309	0.253285	DNAJC15	DnaJ homo	DJC15_HU	1	2.082611
15_MTA / C	Q7Z4H3	0.368409	0.231097	0.253874	HDDC2	5'-deoxynu	HDDC2_HU	2	1.290928
30_MTA / C	Q96CT7	0.11508	0.231122	0.253874	CCDC124	Coiled-coil	CC124_HU	5	1.083035
30_MTA / C	P26639	-0.17976	0.231474	0.254008	TARS1	Threonine--	SYTC_HUMA	15	0.88285
30_MTA / C	P50453	-0.1324	0.23141	0.254008	SERPINB9	Serpin B9	SPB9_HUM	7	0.912315
15_MTA / C	P63010	-0.37459	0.231398	0.254008	AP2B1	AP-2 compl	AP2B1_HU	3	0.771327
30_MTA / C	Q14141	-0.74364	0.231863	0.25435	SEPTIN6	Septin-6	SEPT6_HUM	4	0.59723
30_MTA / C	P41250	-0.13091	0.232218	0.254487	GARS1	Glycine--tR	GARS_HUM	19	0.913256
15_MTA / C	Q01130	-0.10571	0.232172	0.254487	SRSF2	Serine/argi	SRSF2_HUM	5	0.929345
30_MTA / C	Q7Z794	1.441843	0.232102	0.254487	KRT77	Keratin, typ	K2C1B_HU	4	2.716676
15_MTA / C	Q9NTJ4	-1.05344	0.232528	0.254744	MAN2C1	Alpha-man	MA2C1_HU	1	0.481818
30_MTA / C	Q8N4V1	-1.481	0.232632	0.254772	MMGT1	ER membra	EMC5_HUM	1	0.35824
15_MTA / C	Q9P283	-0.19623	0.233381	0.255255	SEMA5B	Semaphori	SEM5B_HU	1	0.87283
30_MTA / C	Q9Y266	-0.26862	0.233318	0.255255	NUDC	Nuclear mi	NUDC_HUM	7	0.830113
30_MTA / C	Q6PI78	0.084159	0.233517	0.255315	TMEM65	Transmemb	TMM65_HU	2	1.060069
30_MTA / C	Q8N766	-0.13249	0.233735	0.255315	EMC1	ER membra	EMC1_HUM	9	0.912257
30_MTA / C	Q9BXS5	-0.58123	0.234184	0.255628	AP1M1	AP-1 compl	AP1M1_HU	2	0.668396
30_MTA / C	O95989	0.410677	0.234539	0.25589	NUDT3	Diphospho	NUDT3_HU	1	1.32931
15_MTA / C	P62314	0.037917	0.234703	0.25589	SNRPD1	Small nucle	SMD1_HUM	3	1.02663
30_MTA / C	Q00839	0.425782	0.234698	0.25589	HNRNPU	Heterogene	HNRPU_HU	7	1.3433

15_MTA / C	Q9HAB8	-0.2028	0.234733	0.25589	PPCS	Phosphopa	PPCS_HUM	4	0.868862
30_MTA / C	Q6IAA8	-0.25645	0.234922	0.256012	LAMTOR1	Ragulator c	LTOR1_HUI	2	0.837147
30_MTA / C	P46778	-0.3905	0.235012	0.256025	RPL21	Large ribos	RL21_HUM	4	0.762864
30_MTA / C	Q05682	0.541624	0.235252	0.256203	CALD1	Caldesmon	CALD1_HUI	4	1.45561
15_MTA / C	P07108	-0.31602	0.235573	0.256224	DBI	Acyl-CoA-bi	ACBP_HUM	4	0.803282
15_MTA / C	P36578	-0.00085	0.235629	0.256224	RPL4	Large ribos	RL4_HUMA	17	0.999409
15_MTA / C	Q00973	0.792721	0.235866	0.256366	B4GALNT1	Beta-1,4 N-	B4GN1_HU	1	1.732338
30_MTA / C	Q9BV40	-0.92055	0.236027	0.256373	VAMP8	Vesicle-ass	VAMP8_HU	2	0.528306
30_MTA / C	Q9NUQ9	-0.0701	0.236001	0.256373	CYRIB	CYFIP-relat	CYRIB_HUM	2	0.95257
30_MTA / C	P06702	0.800751	0.236413	0.25654	S100A9	Protein S10	S10A9_HUI	1	1.742007
30_MTA / C	Q96GK7	0.798043	0.236511	0.256562	FAHD2A	Oxaloaceta	FAH2A_HUI	1	1.738741
30_MTA / C	Q05048	-0.12374	0.236706	0.256689	CSTF1	Cleavage st	CSTF1_HUM	2	0.917805
30_MTA / C	O43143	-0.01594	0.236899	0.256795	DHX15	ATP-depen	DHX15_HU	14	0.989012
30_MTA / C	Q14974	-0.17111	0.236958	0.256795	KPNB1	Importin su	IMB1_HUM	10	0.88816
30_MTA / C	Q9BUP3	-0.53533	0.237095	0.256859	HTATIP2	Oxidoreduc	HTAI2_HUM	1	0.690001
30_MTA / C	P53384	0.675046	0.237197	0.256886	NUBP1	Cytosolic F	NUBP1_HU	1	1.596647
15_MTA / C	Q4G0F5	-0.09593	0.237551	0.256933	VPS26B	Vacuolar pr	VP26B_HUI	1	0.935666
30_MTA / C	O60701	-0.04082	0.23814	0.257402	UGDH	UDP-gluco	UGDH_HUM	14	0.972104
30_MTA / C	P08621	0.285226	0.238129	0.257402	SNRNP70	U1 small nu	RU17_HUM	4	1.218601
15_MTA / C	Q9UNL2	0.484637	0.238284	0.257474	SSR3	Translocon	SSRG_HUM	1	1.399233
15_MTA / C	P50579	0.10849	0.238604	0.257568	METAP2	Methionine	MAP2_HUM	1	1.078099
30_MTA / C	P52209	-0.10311	0.238584	0.257568	PGD	6-phospho	6PGD_HUM	9	0.931024
30_MTA / C	P51659	-0.12786	0.238737	0.257627	HSD17B4	Peroxisoma	DHB4_HUM	10	0.915187
30_MTA / C	P29692	-0.29106	0.239165	0.257922	EEF1D	Elongation	EF1D_HUM	7	0.817299
15_MTA / C	Q9NWU1	0.399882	0.239326	0.258011	OXSM	3-oxoacyl-[	OXSM_HUM	3	1.3194
30_MTA / C	O75976	-0.13713	0.239467	0.258055	CPD	Carboxype	CBPD_HUM	9	0.909327
30_MTA / C	Q8NFQ8	-0.54858	0.239522	0.258055	TOR1AIP2	Torsin-1A-i	TOIP2_HUM	1	0.683694
15_MTA / C	O00567	0.518654	0.240014	0.258249	NOP56	Nucleolar p	NOP56_HU	1	1.432618
30_MTA / C	Q96IZ0	0.054028	0.240918	0.259053	PAWR	PRKC apop	PAWR_HUM	2	1.038159
15_MTA / C	P31350	0.334177	0.242336	0.260324	RRM2	Ribonucle	RIR2_HUM	7	1.260658
15_MTA / C	P10253	0.847661	0.242612	0.260418	GAA	Lysosomal	LYAG_HUM	5	1.799581
15_MTA / C	P20618	-0.13983	0.242895	0.260418	PSMB1	Proteasom	PSB1_HUM	8	0.907627
15_MTA / C	P40227	-0.04002	0.242755	0.260418	CCT6A	T-complex	TCPZ_HUM	17	0.972643
15_MTA / C	P42771	1.791732	0.242812	0.260418	CDKN2A	Cyclin-dep	CDN2A_HU	1	3.462304
15_MTA / C	Q96GX9	0.073638	0.24287	0.260418	APIP	Methylthio	MTNB_HUM	2	1.052367
30_MTA / C	O94919	1.119816	0.243488	0.260665	ENDOD1	Endonucle	ENDD1_HU	3	2.173192
15_MTA / C	P78371	-0.04116	0.243516	0.260665	CCT2	T-complex	TCPB_HUM	18	0.971875
15_MTA / C	Q9UBQ0	-0.61999	0.243659	0.260732	VPS29	Vacuolar pr	VPS29_HUI	2	0.650675
30_MTA / C	Q5XKE5	0.357676	0.243803	0.260801	KRT79	Keratin, typ	K2C79_HUI	1	1.28136
30_MTA / C	P02671	-0.25897	0.243912	0.260834	FGA	Fibrinogen	FIBA_HUM	10	0.835685
15_MTA / C	P63027	-0.40437	0.244071	0.26092	VAMP2	Vesicle-ass	VAMP2_HU	4	0.755566
30_MTA / C	P11586	-0.11388	0.244229	0.260963	MTHFD1	C-1-tetrahy	C1TC_HUM	18	0.9241
15_MTA / C	Q9UM00	0.644615	0.244398	0.260963	TMCO1	Calcium loa	TMCO1_HU	3	1.563322
15_MTA / C	Q9H008	-0.22485	0.244932	0.261349	LHPP	Phospholys	LHPP_HUM	1	0.855681
15_MTA / C	Q9NVJ2	0.117004	0.245103	0.261432	ARL8B	ADP-ribosy	ARL8B_HUI	2	1.08448
15_MTA / C	P11908	0.485898	0.245274	0.261447	PRPS2	Ribose-pho	PRPS2_HUI	1	1.400457
15_MTA / C	Q8NC51	0.540071	0.245267	0.261447	SERBP1	SERPINE1 m	SERB1_HUM	6	1.454044
30_MTA / C	Q14247	0.12809	0.245687	0.261803	CTTN	Src substra	SRC8_HUM	16	1.092846
15_MTA / C	P07954	0.419339	0.246162	0.262084	FH	Fumarate h	FUMH_HUM	4	1.337315
30_MTA / C	P18669	0.194555	0.246239	0.262084	PGAM1	Phosphogly	PGAM1_HU	15	1.144371
15_MTA / C	P61923	0.184536	0.246267	0.262084	COPZ1	Coatomer s	COPZ1_HUI	4	1.136451
30_MTA / C	Q9Y262	-0.35885	0.246245	0.262084	EIF3L	Eukaryotic	EIF3L_HUM	8	0.779784

15_MTA / C	Q9UHV9	0.492346	0.246402	0.262143	PFDN2	Prefoldin su	PFD2_HUM	6	1.406731
15_MTA / C	P62841	0.464753	0.246768	0.262365	RPS15	Small ribos	RS15_HUM	1	1.380081
30_MTA / C	Q5T749	-1.4814	0.246691	0.262365	KPRP	Keratinocyt	KPRP_HUM	5	0.358141
30_MTA / C	Q9BV44	0.187762	0.247371	0.262837	THUMPD3	tRNA (guan	THUM3_HU	3	1.138996
30_MTA / C	O00584	0.859795	0.24765	0.262846	RNASET2	Ribonuclea	RNT2_HUM	1	1.81478
15_MTA / C	Q9H773	0.868676	0.247524	0.262846	DCTPP1	dCTP pyrop	DCTP1_HU	6	1.825986
15_MTA / C	Q9UJ70	-0.05258	0.247811	0.262846	NAGK	N-acetyl-D-	NAGK_HUM	7	0.964213
30_MTA / C	P20073	0.205644	0.248147	0.262902	ANXA7	Annexin A7	ANXA7_HU	7	1.153201
15_MTA / C	Q587I9	0.474229	0.249571	0.264158	SFT2D3	Vesicle tran	SFT2C_HUM	1	1.389175
15_MTA / C	Q96AY3	0.186191	0.249545	0.264158	FKBP10	Peptidyl-pr	FKB10_HU	7	1.137756
30_MTA / C	P25789	0.180714	0.249818	0.264166	PSMA4	Proteasom	PSA4_HUM	10	1.133445
30_MTA / C	Q15437	-0.14056	0.249684	0.264166	SEC23B	Protein tra	SC23B_HU	4	0.907166
15_MTA / C	Q6KB66	-0.35786	0.249741	0.264166	KRT80	Keratin, typ	K2C80_HU	3	0.780321
30_MTA / C	Q6UXV4	-0.09058	0.250357	0.264567	APOOL	MICOS com	MIC27_HU	5	0.939143
15_MTA / C	P51858	0.027655	0.250451	0.264582	HDGF	Hepatoma-	HDGF_HUM	3	1.019354
30_MTA / C	P08238	-0.1046	0.250711	0.264603	HSP90AB1	Heat shock	HS90B_HU	24	0.930064
15_MTA / C	Q9NRP0	0.560151	0.25097	0.264755	OSTC	Oligosacch	OSTC_HUM	1	1.474424
15_MTA / C	Q9UKR5	-0.2236	0.251014	0.264755	ERG28	Ergosterol	ERG28_HU	1	0.856429
15_MTA / C	Q3ZAQ7	0.354061	0.251677	0.265201	VMA21	Vacuolar A	VMA21_HU	2	1.278153
15_MTA / C	P28288	0.540835	0.25263	0.266121	ABCD3	ATP-bindin	ABCD3_HU	2	1.454814
15_MTA / C	Q9NS69	0.180165	0.253068	0.266243	TOMM22	Mitochond	TOM22_HU	3	1.133014
30_MTA / C	P00918	0.300617	0.253693	0.266732	CA2	Carbonic a	CAH2_HUM	7	1.231671
15_MTA / C	P04632	-0.328	0.25438	0.267251	CAPNS1	Calpain sm	CPNS1_HU	3	0.796639
30_MTA / C	P30084	0.243016	0.254429	0.267251	ECHS1	Enoyl-CoA	ECHM_HUM	6	1.183464
15_MTA / C	Q9UNM6	-0.04806	0.254718	0.26747	PSMD13	26S protea	PSD13_HU	6	0.967237
15_MTA / C	Q15404	-0.13687	0.255072	0.267757	RSU1	Ras suppres	RSU1_HUM	2	0.909492
15_MTA / C	Q4G0N4	0.764544	0.255455	0.268074	NADK2	NAD kinase	NAKD2_HU	3	1.698833
15_MTA / C	Q15018	0.560955	0.255703	0.268249	ABRAXAS2	BRISC comp	ABRX2_HU	5	1.475245
30_MTA / C	P54652	0.54319	0.256453	0.268835	HSPA2	Heat shock	HSP72_HU	1	1.457191
30_MTA / C	Q9UHD8	-0.16032	0.256504	0.268835	SEPTIN9	Septin-9	SEPT9_HUM	10	0.894824
15_MTA / C	P42126	-0.002	0.257243	0.269491	ECI1	Enoyl-CoA	ECI1_HUM	7	0.998614
15_MTA / C	Q9P258	-0.12744	0.258011	0.270039	RCC2	Protein RC	RCC2_HUM	3	0.915453
30_MTA / C	O43583	-0.65862	0.258552	0.270295	DENR	Density-reg	DENR_HUM	2	0.633484
15_MTA / C	Q96A26	-0.21987	0.258528	0.270295	FAM162A	Protein FAI	F162A_HU	2	0.858644
30_MTA / C	Q96IX5	0.045649	0.258632	0.270295	ATP5MK	ATP syntha	ATPMK_HU	3	1.032148
30_MTA / C	Q96RS6	-0.42587	0.258841	0.270343	NUDCD1	NudC doma	NUDC1_HU	1	0.744391
30_MTA / C	Q969H8	0.419258	0.258958	0.270381	MYDGF	Myeloid-de	MYDGF_HU	6	1.33724
15_MTA / C	P62333	-0.24369	0.2596	0.270965	PSMC6	26S protea	PRS10_HU	4	0.844583
15_MTA / C	P26373	-0.14666	0.259953	0.271249	RPL13	Large ribos	RL13_HUM	7	0.903341
30_MTA / C	P63279	-0.66754	0.260542	0.271692	UBE2I	SUMO-conj	UBC9_HUM	1	0.629578
30_MTA / C	Q9NRP0	-0.5603	0.26094	0.271935	OSTC	Oligosacch	OSTC_HUM	1	0.678161
15_MTA / C	Q9Y287	1.186228	0.261301	0.272187	ITM2B	Integral me	ITM2B_HU	1	2.275569
30_MTA / C	P11117	0.366224	0.261515	0.272278	ACP2	Lysosomal	PPAL_HUM	4	1.288975
30_MTA / C	Q9NX40	0.100902	0.261949	0.272644	OCIAD1	OCIA doma	OCAD1_HU	4	1.072443
30_MTA / C	A6NGU5;P1	0.052103	0.262273	0.272895	GGT3P;GG	Putative gl	GGT3_HUM	3	1.036775
30_MTA / C	Q9UMX5	0.389578	0.262565	0.273114	NENF	Neudesin	NENF_HUM	4	1.31001
15_MTA / C	Q09666	0.124854	0.26271	0.273156	AHNAK	Neuroblast	AHNK_HUM	50	1.090397
30_MTA / C	P61081	-0.56423	0.263	0.273309	UBE2M	NEDD8-con	UBC12_HU	2	0.676315
15_MTA / C	Q9UGV2	0.421979	0.263131	0.27336	NDRG3	Protein ND	NDRG3_HU	1	1.339764
15_MTA / C	P55084	1.54722	0.263379	0.273419	HADHB	Trifunction	ECHB_HUM	2	2.922535
30_MTA / C	Q13200	-0.37963	0.263294	0.273419	PSMD2	26S protea	PSMD2_HU	14	0.768636
15_MTA / C	Q13561	-0.18571	0.263436	0.273419	DCTN2	Dynactin su	DCTN2_HU	8	0.879213

15_MTA / C	P30566	-0.11931	0.263818	0.27373	ADSL	Adenylosuc	PUR8_HUM	8	0.920627
30_MTA / C	O43617	0.40571	0.264352	0.274179	TRAPPC3	Trafficking	TPPC3_HUI	1	1.324741
15_MTA / C	P33992	0.777843	0.264597	0.274281	MCM5	DNA replica	MCM5_HUI	5	1.714565
15_MTA / C	P61964	-0.18757	0.264964	0.274575	WDR5	WD repeat-	WDR5_HUI	5	0.878086
30_MTA / C	Q16822	-0.13558	0.265327	0.274865	PCK2	Phosphoen	PCKGM_HU	4	0.910305
30_MTA / C	P29218	0.247739	0.265528	0.274987	IMPA1	Inositol mc	IMPA1_HUI	7	1.187345
15_MTA / C	Q9NVX2	0.101099	0.265682	0.275061	NLE1	Notchless p	NLE1_HUM	2	1.07259
30_MTA / C	P32189	0.560177	0.265855	0.275154	GK	Glycerol kir	GLPK_HUM	1	1.47445
15_MTA / C	Q86Y82	0.517797	0.266065	0.275285	STX12	Syntaxin-12	STX12_HUI	5	1.431768
30_MTA / C	Q5VW32	-1.03085	0.266259	0.2754	BROX	BRO1 doma	BROX_HUM	1	0.489423
30_MTA / C	P35244	-0.83917	0.266903	0.275895	RPA3	Replicatior	RFA3_HUM	2	0.558965
30_MTA / C	Q8WWM7	1.111167	0.267601	0.276346	ATXN2L	Ataxin-2-lik	ATX2L_HUI	1	2.160204
30_MTA / C	P04181	0.298202	0.267776	0.276365	OAT	Ornithine a	OAT_HUMA	7	1.229611
30_MTA / C	Q96MV1	0.444518	0.267958	0.276381	TLCD4	TLC domair	TLCD4_HUI	2	1.360859
15_MTA / C	Q08554	-0.86296	0.268147	0.27649	DSC1	Desmocolli	DSC1_HUM	5	0.549824
30_MTA / C	P30040	-0.15391	0.268549	0.276818	ERP29	Endoplasm	ERP29_HUI	7	0.898814
15_MTA / C	O95861	-0.07672	0.269	0.277111	BPNT1	3'(2'),5'-bis	BPNT1_HUI	11	0.948213
30_MTA / C	Q04323	0.183045	0.268929	0.277111	UBXN1	UBX domai	UBXN1_HU	1	1.135277
15_MTA / C	P08397	-0.10219	0.269647	0.277646	HMBS	Porphobilir	HEM3_HUM	6	0.93162
30_MTA / C	P50213	-0.23951	0.269687	0.277646	IDH3A	Isocitrate d	IDH3A_HUI	4	0.847031
30_MTA / C	Q9H490	-1.33082	0.27012	0.277919	PIGU	Phosphatic	PIGU_HUM	1	0.397542
30_MTA / C	P30793	-0.17511	0.270293	0.278011	GCH1	GTP cycloh	GCH1_HUM	3	0.885699
30_MTA / C	Q08945	0.193966	0.270655	0.278145	SSRP1	FACT comp	SSRP1_HUI	2	1.143904
30_MTA / C	Q9H444	0.101989	0.270675	0.278145	CHMP4B	Charged mu	CHM4B_HU	3	1.073252
30_MTA / C	Q9P2J5	0.229572	0.270546	0.278145	LARS1	Leucine--tR	SYLC_HUMA	2	1.172487
15_MTA / C	Q96IX5	0.245939	0.270888	0.278191	ATP5MK	ATP synthas	ATPMK_HU	3	1.185865
30_MTA / C	P09211	0.186695	0.270997	0.278217	GSTP1	Glutathion	GSTP1_HUI	11	1.138154
30_MTA / C	P01834	0.446883	0.271489	0.278463	IGKC	Immunoglc	IGKC_HUM	1	1.363092
15_MTA / C	P28332	-0.11471	0.271409	0.278463	ADH6	Alcohol del	ADH6_HUM	6	0.923565
15_MTA / C	Q6PI78	1.079709	0.271483	0.278463	TMEM65	Transmemb	TMM65_HU	2	2.11361
15_MTA / C	Q13177	0.292954	0.27231	0.279219	PAK2	Serine/thre	PAK2_HUM	13	1.225147
15_MTA / C	O75844	0.142759	0.272664	0.279495	ZMPSTE24	CAAX preny	FACE1_HUI	7	1.104015
30_MTA / C	P51571	-0.19632	0.272993	0.2795	SSR4	Translocon	SSRD_HUM	4	0.872777
15_MTA / C	Q00765	0.078577	0.272958	0.2795	REEP5	Receptor ex	REEP5_HUI	6	1.055976
30_MTA / C	Q13618	1.41604	0.273006	0.2795	CUL3	Cullin-3	CUL3_HUM	3	2.66852
15_MTA / C	Q15717	-0.10758	0.273286	0.279614	ELAVL1	ELAV-like pr	ELAV1_HUI	1	0.928141
15_MTA / C	P82970	1.224175	0.273586	0.279731	HMGN5	High mobil	HMGN5_HU	1	2.336218
30_MTA / C	Q8WUM4	-0.36737	0.273653	0.279731	PDCD6IP	Programme	PDC6I_HUI	7	0.775196
15_MTA / C	Q96HY6	0.499208	0.274007	0.279789	DDRGK1	DDRGK dor	DDRGK_HU	2	1.413437
30_MTA / C	Q9H2H9	-0.35488	0.274048	0.279789	SLC38A1	Sodium-co	S38A1_HUI	2	0.781937
30_MTA / C	Q93099	0.195358	0.274365	0.279928	HGD	Homogenti	HGD_HUMA	3	1.145008
30_MTA / C	P23588	0.272992	0.274674	0.280083	EIF4B	Eukaryotic	IF4B_HUMA	8	1.208311
30_MTA / C	Q9H1C7	0.169872	0.27483	0.280156	CYSTM1	Cysteine-ric	CYTM1_HU	1	1.124958
30_MTA / C	Q15029	0.238032	0.275607	0.280689	EFTUD2	116 kDa U5	U5S1_HUM	18	1.179383
15_MTA / C	P51149	0.160229	0.275908	0.280909	RAB7A	Ras-related	RAB7A_HUI	10	1.117464
30_MTA / C	P08670	0.225941	0.276745	0.281318	VIM	Vimentin	VIME_HUM	19	1.16954
15_MTA / C	Q53H96	0.009975	0.276628	0.281318	PYCR3	Pyrroline-5	P5CR3_HUI	3	1.006938
30_MTA / C	Q9BRG1	0.085795	0.276819	0.281318	VPS25	Vacuolar pr	VPS25_HUI	1	1.061272
15_MTA / C	P26038	-0.04545	0.277721	0.282128	MSN	Moesin	MOES_HUM	13	0.968989
15_MTA / C	O60869	0.037901	0.278014	0.282273	EDF1	Endothelial	EDF1_HUM	5	1.026619
30_MTA / C	Q6P4E1	0.398134	0.279432	0.283295	GOLM2	Protein GO	GOLM2_HU	2	1.317802
30_MTA / C	Q6UWP7	-0.08998	0.279448	0.283295	LCLAT1	Lysocardiol	LCLT1_HUM	3	0.939539

30_MTA / C	P36873	-0.44276	0.279935	0.283371	PPP1CC	Serine/thre	PP1G_HUM	2	0.735725
30_MTA / C	P38571	1.020894	0.279946	0.283371	LIPA	Lysosomal a	LICH_HUMA	1	2.029175
30_MTA / C	P50991	-0.02736	0.27995	0.283371	CCT4	T-complex p	TCPD_HUM	21	0.981214
30_MTA / C	Q9BWH2	-0.31724	0.279771	0.283371	FUNDC2	FUN14 dom	FUND2_HU	2	0.802604
15_MTA / C	Q9Y6G9	0.409024	0.280239	0.283489	DYNC1LI1	Cytoplasmi	DC1L1_HUI	2	1.327787
30_MTA / C	O43768	0.843638	0.280574	0.283623	ENSA	Alpha-endo	ENSA_HUM	2	1.79457
30_MTA / C	Q9NTK5	-0.30025	0.280627	0.283623	OLA1	Obg-like AT	OLA1_HUM	5	0.812112
15_MTA / C	P61619	0.146789	0.280798	0.283686	SEC61A1	Protein tra	S61A1_HUI	3	1.107103
15_MTA / C	P14927	-0.79348	0.281061	0.283801	UQCRB	Cytochrom	QCR7_HUM	1	0.57695
30_MTA / C	P59998	-0.36886	0.281192	0.283847	ARPC4	Actin-relate	ARPC4_HUI	6	0.774394
15_MTA / C	Q15904	0.371186	0.281621	0.284107	ATP6AP1	V-type prot	VAS1_HUM	3	1.293416
15_MTA / C	P61009	0.337291	0.282166	0.28457	SPCS3	Signal pept	SPCS3_HUM	2	1.263382
30_MTA / C	P12270	1.561079	0.282501	0.284795	TPR	Nucleoprot	TPR_HUMA	2	2.950745
30_MTA / C	P35221	-0.24067	0.282833	0.284897	CTNNA1	Catenin alp	CTNA1_HUI	9	0.846349
30_MTA / C	P15927	-0.38001	0.283317	0.28512	RPA2	Replication	RFA2_HUM	2	0.768435
30_MTA / C	P49588	-0.24537	0.283532	0.28512	AARS1	Alanine--tR	SYAC_HUM	11	0.8436
15_MTA / C	Q06203	0.030116	0.283434	0.28512	PPAT	Amidophos	PUR1_HUM	11	1.021094
30_MTA / C	Q4KWH8	-0.64822	0.283572	0.28512	PLCH1	1-phosphat	PLCH1_HUI	1	0.638069
15_MTA / C	Q9Y3I0	-0.02509	0.283747	0.285124	RTCB	RNA-splicin	RTCB_HUM	9	0.982761
15_MTA / C	O75976	0.256737	0.284461	0.28568	CPD	Carboxypep	CBPD_HUM	9	1.194773
15_MTA / C	Q9H3N1	0.238289	0.284474	0.28568	TMX1	Thioredoxi	TMX1_HUM	5	1.179593
15_MTA / C	Q14061	0.0284	0.284996	0.286027	COX17	Cytochrom	COX17_HU	1	1.01988
30_MTA / C	P11234	-0.11317	0.285457	0.286321	RALB	Ras-related	RALB_HUM	1	0.924556
15_MTA / C	Q9BQ67	-0.44114	0.285586	0.286363	GRWD1	Glutamate-	GRWD1_HU	2	0.73655
15_MTA / C	Q9UBS4	-0.00527	0.285897	0.286503	DNAJB11	DnaJ homo	DJB11_HUI	5	0.996353
30_MTA / C	O75608	-0.11195	0.286634	0.287061	LYPLA1	Acyl-protei	LYPA1_HUM	3	0.925335
30_MTA / C	Q9Y320	0.432627	0.286715	0.287061	TMX2	Thioredoxi	TMX2_HUM	2	1.349689
30_MTA / C	Q9BZG8	0.055392	0.28702	0.287193	DPH1	2-(3-amino	DPH1_HUM	1	1.039142
15_MTA / C	Q9UHD8	0.140629	0.287014	0.287193	SEPTIN9	Septin-9	SEPT9_HUM	10	1.102386
30_MTA / C	P17050	-0.1688	0.287526	0.287393	NAGA	Alpha-N-ac	NAGAB_HU	1	0.889581
15_MTA / C	Q9NYU2	0.146052	0.287566	0.287393	UGGT1	UDP-glucos	UGGG1_HU	17	1.106537
15_MTA / C	Q96S97	0.352069	0.287681	0.287421	MYADM	Myeloid-as	MYADM_HU	2	1.27639
30_MTA / C	Q9UJS0	-0.07537	0.287769	0.287422	SLC25A13	Electrogeni	S2513_HUI	19	0.9491
15_MTA / C	Q9UNS2	0.455835	0.287995	0.287561	COPS3	COP9 signa	CSN3_HUM	4	1.371576
30_MTA / C	P31025;Q5	1.070711	0.288345	0.287824	LCN1;LCN1	Lipocalin-1	LCN1_HUM	1	2.100468
15_MTA / C	Q01650	-0.13667	0.288643	0.288035	SLC7A5	Large neutr	LAT1_HUM	6	0.909617
30_MTA / C	P39023	-0.23006	0.289213	0.288517	RPL3	Large ribos	RL3_HUMA	16	0.852601
30_MTA / C	P27105	-0.17925	0.289421	0.288637	STOM	Stomatin	STOM_HUM	6	0.883164
30_MTA / C	P31040	-0.15172	0.289854	0.288895	SDHA	Succinate d	SDHA_HUM	10	0.900175
30_MTA / C	P43487	0.029226	0.290165	0.289065	RANBP1	Ran-specific	RANG_HUM	2	1.020465
15_MTA / C	P84085	-0.6978	0.290198	0.289065	ARF5	ADP-ribosy	ARF5_HUM	1	0.61651
30_MTA / C	Q9BUF5	-0.76221	0.290562	0.289252	TUBB6	Tubulin bet	TBB6_HUM	2	0.589594
15_MTA / C	Q9H061	-0.11074	0.290535	0.289252	TMEM126A	Transmemb	T126A_HUI	2	0.926114
30_MTA / C	P05161	0.27043	0.290851	0.289291	ISG15	Ubiquitin-l	ISG15_HUM	1	1.206168
30_MTA / C	Q96QR8	0.872835	0.290817	0.289291	PURB	Transcripti	PURB_HUM	4	1.831258
15_MTA / C	P52434	2.31588	0.290967	0.289308	POLR2H	DNA-direct	RPAB3_HUI	2	4.979084
15_MTA / C	P35998	-0.19805	0.291606	0.289857	PSMC2	26S protea	PRS7_HUM	11	0.871728
15_MTA / C	Q9NXG2	-0.26475	0.292483	0.290641	THUMPD1	THUMP dor	THUM1_HU	6	0.832341
30_MTA / C	Q9Y624	-0.35066	0.293039	0.291106	F11R	Junctional	JAM1_HUM	4	0.784227
30_MTA / C	P07602	-1.49761	0.293756	0.291333	PSAP	Prosaposin	SAP_HUMA	2	0.35414
15_MTA / C	P19013	0.044236	0.293717	0.291333	KRT4	Keratin, typ	K2C4_HUM	22	1.031137
15_MTA / C	Q15370	-0.1906	0.293648	0.291333	ELOB	Elongin-B	ELOB_HUM	3	0.876239

15_MTA / C	P06702	-0.15934	0.29425	0.291481	S100A9	Protein S1(	S10A9_HUI	1	0.895436
30_MTA / C	P56211	-0.68374	0.294295	0.291481	ARPP19	cAMP-regul	ARP19_HUI	1	0.622548
15_MTA / C	Q9BQ52	0.162244	0.294251	0.291481	ELAC2	Zinc phospl	RNZ2_HUM	3	1.119026
15_MTA / C	P35637	0.251806	0.294558	0.291654	FUS	RNA-bindir	FUS_HUMA	7	1.190697
15_MTA / C	Q13724	-0.22497	0.294898	0.291669	MOGS	Mannosyl-c	MOGS_HUI	4	0.855611
30_MTA / C	Q9NRF9	-0.00518	0.295098	0.291669	POLE3	DNA polym	DPOE3_HU	1	0.996415
15_MTA / C	Q02127	0.565414	0.295442	0.291745	DHODH	Dihydroorc	PYRD_HUM	8	1.479812
15_MTA / C	P46778	0.613345	0.296953	0.292974	RPL21	Large ribos	RL21_HUM	4	1.529802
30_MTA / C	P49458	0.318542	0.29695	0.292974	SRP9	Signal reco	SRP09_HUI	2	1.24707
15_MTA / C	Q15056	0.364269	0.296816	0.292974	EIF4H	Eukaryotic	IF4H_HUMA	3	1.287229
30_MTA / C	P20645	0.037748	0.297471	0.293311	M6PR	Cation-dep	MPRD_HUM	4	1.02651
15_MTA / C	P21281	0.095674	0.297467	0.293311	ATP6V1B2	V-type prot	VATB2_HUI	10	1.068565
15_MTA / C	P49458	-0.01084	0.297747	0.293364	SRP9	Signal reco	SRP09_HUI	2	0.992513
15_MTA / C	Q96HC4	1.079068	0.297761	0.293364	PDLIM5	PDZ and LIM	PDLI5_HUM	3	2.112671
15_MTA / C	Q9NQW7	-0.65423	0.29779	0.293364	XPNPEP1	Xaa-Pro am	XPP1_HUM	2	0.635416
15_MTA / C	Q9UJS0	0.276207	0.29795	0.293434	SLC25A13	Electrogeni	S2513_HUI	19	1.211007
30_MTA / C	P10515	-0.21502	0.298982	0.293511	DLAT	Dihydrolip	ODP2_HUM	6	0.861531
30_MTA / C	P52434	0.749724	0.298583	0.293511	POLR2H	DNA-direct	RPAB3_HUI	2	1.681471
15_MTA / C	P61289	-0.22962	0.29818	0.293511	PSME3	Proteasom	PSME3_HU	8	0.852857
30_MTA / C	Q12906	0.227019	0.299002	0.293511	ILF3	Interleukin	ILF3_HUMA	14	1.170414
15_MTA / C	Q15019	-0.20582	0.298656	0.293511	SEPTIN2	Septin-2	SEPT2_HUM	9	0.867048
15_MTA / C	Q16563	-0.09816	0.298934	0.293511	SYPL1	Synaptophy	SYPL1_HUM	3	0.934225
30_MTA / C	Q629K1	-0.22417	0.298707	0.293511	TRIQK	Triple QxxK	TRIQK_HUM	1	0.85609
15_MTA / C	Q96M27	0.41694	0.298715	0.293511	PRRC1	Protein PRI	PRRC1_HUI	3	1.335093
15_MTA / C	O95400	-1.42113	0.29974	0.293974	CD2BP2	CD2 antiger	CD2B2_HU	1	0.37342
15_MTA / C	P28838	0.034316	0.30007	0.294114	LAP3	Cytosol am	AMPL_HUM	7	1.024071
15_MTA / C	Q9NUJ1	-0.12336	0.300237	0.294114	ABHD10	Palmitoyl-p	ABHDA_HU	1	0.918047
15_MTA / C	Q15942	0.304149	0.300624	0.294405	ZYX	Zyxin	ZYX_HUMA	10	1.23469
15_MTA / C	Q15075	0.362832	0.301199	0.294882	EEA1	Early endos	EEA1_HUM	19	1.285947
30_MTA / C	P10114;P6	0.189809	0.301667	0.29524	RAP2A;RAP	Ras-related	RAP2A_HUI	1	1.140612
15_MTA / C	P53999	0.141384	0.301743	0.29524	SUB1	Activated R	TCP4_HUM	5	1.102962
30_MTA / C	Q9Y5P6	-0.13678	0.302048	0.295451	GMPPB	Mannose-1	GMPPB_HU	4	0.909548
30_MTA / C	Q00341	0.647755	0.302891	0.2961	HDLBP	Vigilin	VIGLN_HUM	5	1.566728
15_MTA / C	Q6UXN9	-0.43642	0.303032	0.296151	WDR82	WD repeat-	WDR82_HU	3	0.738968
30_MTA / C	O15212	0.851092	0.303342	0.296283	PFDN6	Prefoldin su	PFD6_HUM	2	1.803865
15_MTA / C	P35244	0.148101	0.303572	0.296417	RPA3	Replication	RFA3_HUM	2	1.10811
30_MTA / C	Q15382	0.028481	0.303883	0.296633	RHEB	GTP-bindin	RHEB_HUM	1	1.019938
30_MTA / C	P62318	0.214765	0.304044	0.296702	SNRPD3	Small nucle	SMD3_HUM	3	1.160515
30_MTA / C	Q7Z739	-0.07086	0.304291	0.296769	YTHDF3	YTH domair	YTHD3_HUI	4	0.95207
30_MTA / C	Q9H3U1	-0.48983	0.304254	0.296769	UNC45A	Protein unc	UN45A_HU	1	0.71211
15_MTA / C	P83881	0.162031	0.304723	0.29684	RPL36A	Large ribos	RL36A_HUI	5	1.118861
30_MTA / C	Q16881	0.210647	0.304696	0.29684	TXNRD1	Thioredoxi	TRXR1_HUI	12	1.157207
30_MTA / C	O75489	0.68561	0.305026	0.297049	NDUFS3	NADH dehy	NDUS3_HU	2	1.608382
15_MTA / C	P28482	0.113435	0.305995	0.297588	MAPK1	Mitogen-ac	MK01_HUM	8	1.081801
30_MTA / C	Q4G0F5	-0.77108	0.305691	0.297588	VPS26B	Vacuolar pı	VP26B_HUI	1	0.585978
30_MTA / C	Q9NQG5	0.984704	0.305802	0.297588	RPRD1B	Regulation	RPR1B_HUI	2	1.978907
15_MTA / C	P35268	0.0979	0.306291	0.297668	RPL22	Large ribos	RL22_HUM	2	1.070214
30_MTA / C	O95302	0.075084	0.306969	0.298195	FKBP9	Peptidyl-pr	FKBP9_HUI	7	1.053422
30_MTA / C	O95070	0.39465	0.307826	0.298634	YIF1A	Protein YIF	YIF1A_HUM	2	1.314624
15_MTA / C	P34897	-0.12173	0.307722	0.298634	SHMT2	Serine hydr	GLYM_HUM	15	0.919087
15_MTA / C	P09110	-0.15866	0.308079	0.298667	ACAA1	3-ketoacyl-	THIK_HUMA	2	0.895854
30_MTA / C	P11717	0.208902	0.308114	0.298667	IGF2R	Cation-inde	MPRI_HUM	20	1.155808

15_MTA / C	Q01085	1.047812	0.308338	0.298762	TIAL1	Nucleolysir	TIAR_HUM/	2	2.067392
30_MTA / C	Q9H3N1	-0.17557	0.308408	0.298762	TMX1	Thioredoxi	TMX1_HUN	5	0.885419
30_MTA / C	P69849;Q1	-0.21972	0.309135	0.299116	NOMO3;NC	BOS compl	NOMO3_HU	9	0.85873
15_MTA / C	P07355	-0.09962	0.30939	0.299275	ANXA2	Annexin A2	ANXA2_HU	20	0.93328
30_MTA / C	P06280	-0.30721	0.309598	0.29939	GLA	Alpha-galac	AGAL_HUM	3	0.808202
30_MTA / C	Q6P6C2	-0.3433	0.309985	0.299589	ALKBH5	RNA demet	ALKB5_HUI	1	0.788236
15_MTA / C	Q9BSH5	0.622553	0.30994	0.299589	HDHD3	Haloacid de	HDHD3_HU	1	1.539597
30_MTA / C	P52758	0.226657	0.310111	0.299623	RIDA	2-iminobut	RIDA_HUM	2	1.170121
15_MTA / C	P13674	0.461432	0.31059	0.299999	P4HA1	Prolyl 4-hy	P4HA1_HU	4	1.376908
30_MTA / C	Q15286	0.53136	0.31084	0.300065	RAB35	Ras-related	RAB35_HUI	2	1.445291
15_MTA / C	P62312	-0.11687	0.311744	0.30085	LSM6	U6 snRNA-a	LSM6_HUM	6	0.922184
15_MTA / C	Q86U90	0.151539	0.312117	0.301035	YRDC	Threonylca	YRDC_HUM	1	1.110754
15_MTA / C	Q8WXE9	-0.60305	0.312072	0.301035	STON2	Stonin-2	STON2_HUI	1	0.658363
15_MTA / C	Q9NRW1	-0.03085	0.312356	0.301178	RAB6B	Ras-related	RAB6B_HUI	1	0.978846
30_MTA / C	P17980	-0.44714	0.312564	0.301203	PSMC3	26S protea:	PRS6A_HUI	8	0.733497
15_MTA / C	Q9P2X0	0.916909	0.312486	0.301203	DPM3	Dolichol-pl	DPM3_HUN	1	1.888066
30_MTA / C	P25311	-0.70021	0.312773	0.301317	AZGP1	Zinc-alpha-	ZA2G_HUM	1	0.615482
30_MTA / C	P33908	-0.37973	0.313599	0.301948	MAN1A1	Mannosyl-c	MA1A1_HU	2	0.768581
15_MTA / C	P54652	-0.30819	0.31366	0.301948	HSPA2	Heat shock	HSP72_HUI	1	0.807654
15_MTA / C	P56537	-0.13669	0.313701	0.301948	EIF6	Eukaryotic	IF6_HUMAI	4	0.909606
15_MTA / C	P43686	-0.19574	0.314404	0.30236	PSMC4	26S protea:	PRS6B_HUI	6	0.873124
30_MTA / C	Q14554	-0.92286	0.314494	0.30236	PDIA5	Protein dis	PDIA5_HUN	1	0.527461
15_MTA / C	Q96DI7	0.261486	0.314395	0.30236	SNRNP40	U5 small nu	SNR40_HUI	2	1.198713
15_MTA / C	O60671	1.30251	0.314825	0.30254	RAD1	Cell cycle cl	RAD1_HUM	1	2.466577
15_MTA / C	Q15424	0.074272	0.315037	0.302618	SAFB	Scaffold att	SAFB1_HUN	2	1.05283
15_MTA / C	Q6UWP7	0.228039	0.315382	0.302862	LCLAT1	Lysocardiol	LCLT1_HUN	3	1.171242
15_MTA / C	O15498	0.378683	0.315593	0.302977	YKT6	Synaptobre	YKT6_HUM	4	1.300154
15_MTA / C	O94905	0.048027	0.31705	0.304111	ERLIN2	Erlin-2	ERLN2_HUI	2	1.03385
15_MTA / C	P62906	-0.08835	0.317038	0.304111	RPL10A	Large ribos	RL10A_HUI	7	0.940596
30_MTA / C	Q9Y584	0.405246	0.31737	0.30433	TIMM22	Mitochond	TIM22_HUI	1	1.324315
15_MTA / C	P30043	-0.21336	0.317544	0.304408	BLVRB	Flavin redu	BLVRB_HUI	3	0.862524
30_MTA / C	P54136	-0.23831	0.318918	0.305461	RARS1	Arginine--tl	SYRC_HUM	4	0.847736
15_MTA / C	Q15493	0.26922	0.319458	0.305713	RGN	Regucalcin	RGN_HUM/	2	1.205156
15_MTA / C	O75643	-0.08357	0.319973	0.306029	SNRNP200	U5 small nu	U520_HUM	6	0.943719
30_MTA / C	P61626	0.850474	0.320277	0.306232	LYZ	Lysozyme C	LYSC_HUM/	3	1.803094
30_MTA / C	O00115	-0.16702	0.321069	0.306812	DNASE2	Deoxyribor	DNS2A_HU	4	0.89068
15_MTA / C	O43818	-0.10121	0.321331	0.306821	RRP9	U3 small nu	U3IP2_HUN	5	0.932251
30_MTA / C	P30049	-0.07669	0.321449	0.306821	ATP5F1D	ATP syntha:	ATPD_HUM	3	0.948228
15_MTA / C	P63313	-0.23041	0.321404	0.306821	TMSB10	Thymosin b	TYB10_HUN	3	0.852394
30_MTA / C	Q9UJZ1	0.120216	0.321646	0.30692	STOML2	Stomatin-li	STML2_HUI	8	1.086897
30_MTA / C	Q16555	0.360769	0.32203	0.30711	DPYSL2	Dihydropyr	DPYL2_HUI	3	1.28411
15_MTA / C	Q86Y39	0.373183	0.322188	0.307172	NDUFA11	NADH dehy	NDUAB_HU	1	1.295207
15_MTA / C	Q13740	-0.21052	0.323963	0.308333	ALCAM	CD166 anti	CD166_HU	4	0.864228
15_MTA / C	P31689	0.421069	0.325271	0.309311	DNAJA1	DnaJ homo	DNJA1_HUI	3	1.338919
30_MTA / C	P51572	-0.15606	0.325367	0.309313	BCAP31	B-cell recep	BAP31_HUI	9	0.897474
15_MTA / C	P45974	0.014059	0.325699	0.309452	USP5	Ubiquitin c	UBP5_HUM	2	1.009793
15_MTA / C	Q92947	0.845761	0.326231	0.309868	GCDH	Glutaryl-Co	GCDH_HUN	3	1.797213
30_MTA / C	Q9Y4Y9	1.297709	0.3265	0.310035	LSM5	U6 snRNA-a	LSM5_HUM	1	2.458382
15_MTA / C	Q9NZZ3	0.791397	0.326676	0.310113	CHMP5	Charged mu	CHMP5_HU	1	1.73075
30_MTA / C	Q9BPX5	1.408495	0.327121	0.310447	ARPC5L	Actin-relate	ARP5L_HUI	2	2.654602
30_MTA / C	P13010	0.229422	0.327241	0.310472	XRCC5	X-ray repair	XRCC5_HUI	10	1.172365
30_MTA / C	P13797	0.081835	0.327916	0.310667	PLS3	Plastin-3	PLST_HUM/	11	1.058363

15_MTA / C	Q9GZT3	0.889712	0.327715	0.310667	SLIRP	SRA stem-lo	SLIRP_HUM	2	1.852806
15_MTA / C	Q9UBM7	0.701012	0.327905	0.310667	DHCR7	7-dehydroc	DHCR7_HU	1	1.625644
15_MTA / C	Q96GK7	0.398118	0.328157	0.310806	FAHD2A	Oxaloaceta	FAH2A_HU	1	1.317788
30_MTA / C	P08195	0.285772	0.328472	0.310839	SLC3A2	Amino acid	4F2_HUMA	16	1.219062
15_MTA / C	P48509	-0.15642	0.328696	0.310961	CD151	CD151 anti	CD151_HU	2	0.89725
15_MTA / C	P49585	0.291376	0.328819	0.31099	PCYT1A	Choline-ph	PCY1A_HU	3	1.223807
15_MTA / C	Q9P035	0.758115	0.329567	0.311518	HACD3	Very-long-c	HACD3_HU	2	1.691279
30_MTA / C	Q8N5R6	-0.17936	0.329701	0.31152	CCDC33	Coiled-coil	CCD33_HU	1	0.883094
15_MTA / C	Q8NFQ8	0.050213	0.329944	0.31152	TOR1AIP2	Torsin-1A-i	TOIP2_HUM	1	1.035418
30_MTA / C	P11279	0.203909	0.330551	0.311872	LAMP1	Lysosome-a	LAMP1_HU	4	1.151815
30_MTA / C	P21912	-0.22438	0.3306	0.311872	SDHB	Succinate d	SDHB_HUM	5	0.855965
15_MTA / C	O15511	-0.17886	0.330712	0.31189	ARPC5	Actin-relate	ARPC5_HU	2	0.883398
15_MTA / C	O43505	0.392806	0.33322	0.313986	B4GAT1	Beta-1,4-gl	B4GA1_HU	1	1.312944
30_MTA / C	P63027	0.273551	0.333325	0.313996	VAMP2	Vesicle-ass	VAMP2_HU	4	1.20878
30_MTA / C	Q8IV08	-0.1099	0.333437	0.314013	PLD3	5'-3' exonu	PLD3_HUM	2	0.926654
30_MTA / C	P10619	-0.38805	0.334584	0.314439	CTSA	Lysosomal	PPGB_HUM	2	0.764162
30_MTA / C	P14866	0.466719	0.33426	0.314439	HNRNPL	Heterogene	HNRPL_HU	3	1.381963
30_MTA / C	P35754	-0.9727	0.334649	0.314439	GLRX	Glutaredox	GLRX1_HU	1	0.50955
15_MTA / C	P62495	0.503814	0.334027	0.314439	ETF1	Eukaryotic	ERF1_HUM	3	1.417957
15_MTA / C	Q9UEU0	0.360037	0.334565	0.314439	VTI1B	Vesicle tran	VTI1B_HUM	3	1.283459
30_MTA / C	O94903	-0.4576	0.334761	0.314456	PLPBP	Pyridoxal p	PLPHP_HU	3	0.728195
15_MTA / C	Q96DA6	0.985184	0.335209	0.314698	DNAJC19	Mitochond	TIM14_HU	1	1.979566
15_MTA / C	P14174	-0.45915	0.335421	0.314808	MIF	Macrophag	MIF_HUMA	3	0.727414
15_MTA / C	O95197	0.370526	0.335762	0.314949	RTN3	Reticulon-3	RTN3_HUM	2	1.292824
30_MTA / C	Q14677	-0.10448	0.335748	0.314949	CLINT1	Clathrin int	EPN4_HUM	3	0.930139
30_MTA / C	P51148	-0.04298	0.336157	0.31523	RAB5C	Ras-related	RAB5C_HU	4	0.970648
15_MTA / C	Q8TCJ2	0.501864	0.336359	0.315331	STT3B	Dolichyl-di	STT3B_HUM	3	1.416042
30_MTA / C	P43686	-0.20184	0.336646	0.315421	PSMC4	26S protea	PRS6B_HU	6	0.869442
15_MTA / C	P49773	0.66244	0.337273	0.315656	HINT1	Adenosine	HINT1_HUM	3	1.582757
15_MTA / C	Q16555	0.755381	0.3371	0.315656	DPYSL2	Dihydropyr	DPYL2_HU	3	1.688078
30_MTA / C	O00161	-0.19548	0.338121	0.316267	SNAP23	Synaptosor	SNP23_HU	6	0.873281
30_MTA / C	O00483	-0.15752	0.338729	0.316656	NDUFA4	Cytochrom	NDUA4_HU	3	0.896565
15_MTA / C	Q9H490	-0.59943	0.339359	0.316977	PIGU	Phosphatid	PIGU_HUM	1	0.660013
30_MTA / C	Q9UHR5	-0.07369	0.33927	0.316977	SAP30BP	SAP30-binc	S30BP_HU	1	0.950208
30_MTA / C	Q9NPF0	0.090144	0.33949	0.31701	CD320	CD320 anti	CD320_HU	1	1.064476
15_MTA / C	P20290	1.256527	0.340197	0.317313	BTF3	Transcripti	BTF3_HUM	3	2.389198
30_MTA / C	P61981	0.190763	0.340193	0.317313	YWHAG	14-3-3 prot	1433G_HU	14	1.141367
30_MTA / C	Q6UW68	-0.13436	0.340585	0.317586	TMEM205	Transmemb	TM205_HU	4	0.911072
30_MTA / C	P49366	0.45447	0.341853	0.318588	DHPS	Deoxyhypu	DHYS_HUM	3	1.370279
30_MTA / C	Q9H8S9	1.072262	0.342348	0.31887	MOB1A	MOB kinase	MOB1A_HU	3	2.102728
15_MTA / C	O43707	-0.00826	0.342768	0.318993	ACTN4	Alpha-actin	ACTN4_HU	33	0.994288
15_MTA / C	Q9Y490	0.329778	0.342697	0.318993	TLN1	Talin-1	TLN1_HUM	20	1.25682
15_MTA / C	P15848	1.528572	0.342916	0.31904	ARSB	Arylsulfatas	ARSB_HUM	2	2.885001
30_MTA / C	P46060	-0.21622	0.343081	0.319104	RANGAP1	Ran GTPase	RAGP1_HU	7	0.860816
30_MTA / C	Q9Y5M8	0.023591	0.34328	0.3192	SRPRB	Signal reco	SRPRB_HU	4	1.016486
15_MTA / C	P61313	-0.08673	0.343442	0.319262	RPL15	Large ribos	RL15_HUM	11	0.941657
15_MTA / C	P21266	-0.37994	0.343752	0.319281	GSTM3	Glutathion	GSTM3_HU	4	0.768471
15_MTA / C	O60664	0.193304	0.344054	0.319388	PLIN3	Perilipin-3	PLIN3_HUM	13	1.143379
15_MTA / C	P12236	0.103393	0.34406	0.319388	SLC25A6	ADP/ATP tr	ADT3_HUM	15	1.074297
15_MTA / C	P43487	-0.03876	0.344724	0.319839	RANBP1	Ran-specifi	RANG_HUM	2	0.973493
30_MTA / C	Q9BRF8	0.131093	0.345028	0.319839	CPPED1	Serine/thre	CPPED_HU	5	1.095123
15_MTA / C	Q9NZI8	-0.09949	0.344865	0.319839	IGF2BP1	Insulin-like	IF2B1_HUM	4	0.933363

15_MTA / C	Q01813	0.035168	0.345455	0.320145	PFKP	ATP-depen(	PFKAP_HUI	12	1.024676
30_MTA / C	O75718	0.502965	0.345731	0.320311	CRTAP	Cartilage-as	CRTAP_HUI	3	1.417123
30_MTA / C	P62736;P6	0.517161	0.346012	0.320482	ACTA2;ACT(	Actin, aorti	ACTA_HUM	1	1.431136
15_MTA / C	P55010	0.191218	0.346649	0.320982	EIF5	Eukaryotic	IF5_HUMAI	2	1.141727
30_MTA / C	Q13885	-0.43193	0.34727	0.321288	TUBB2A	Tubulin bet	TBB2A_HUI	4	0.741267
15_MTA / C	P27105	0.426789	0.347533	0.32138	STOM	Stomatin	STOM_HUM	6	1.344239
30_MTA / C	P42574	0.587012	0.347889	0.321501	CASP3	Caspase-3	CASP3_HUI	3	1.502132
15_MTA / C	Q9BXS5	0.650116	0.349129	0.322288	AP1M1	AP-1 compl	AP1M1_HU	2	1.569294
15_MTA / C	P07602	-1.37035	0.349672	0.322609	PSAP	Prosaposin	SAP_HUMA	2	0.386798
30_MTA / C	P23919	-0.42939	0.349612	0.322609	DTYMK	Thymidylat	KTHY_HUM	6	0.742577
30_MTA / C	P63313	0.362579	0.350187	0.322905	TMSB10	Thymosin b	TYB10_HUN	3	1.285722
30_MTA / C	Q9P0U1	0.274057	0.350092	0.322905	TOMM7	Mitochond	TOM7_HUN	1	1.209203
30_MTA / C	O43826	0.249497	0.350782	0.32331	SLC37A4	Glucose-6-p	G6PT1_HUI	3	1.188792
15_MTA / C	P60983	0.158351	0.35131	0.32331	GMFB	Glia matura	GMFB_HUN	3	1.116011
15_MTA / C	Q00341	0.45583	0.351242	0.32331	HDLBP	Vigilin	VIGLN_HUI	5	1.371571
15_MTA / C	Q6PIU2	-0.16701	0.35129	0.32331	NCEH1	Neutral cho	NCEH1_HU	7	0.890689
30_MTA / C	Q8WVM8	0.056298	0.350993	0.32331	SCFD1	Sec1 family	SCFD1_HUI	1	1.039794
30_MTA / C	Q9BVT8	-1.12286	0.351207	0.32331	TMUB1	Transmemb	TMUB1_HU	2	0.459184
30_MTA / C	Q9NRA2	0.278749	0.351048	0.32331	SLC17A5	Sialin	S17A5_HUI	2	1.213142
30_MTA / C	Q04941	-0.35555	0.352385	0.32385	PLP2	Proteolipic	PLP2_HUM	1	0.781572
15_MTA / C	Q9Y4Y9	0.166827	0.352199	0.32385	LSM5	U6 snRNA-a	LSM5_HUN	1	1.122587
30_MTA / C	P54105	-0.09465	0.353267	0.324571	CLNS1A	Methylosor	ICLN_HUM/	2	0.936497
30_MTA / C	P04075	0.179592	0.353428	0.324629	ALDOA	Fructose-bi	ALDOA_HU	29	1.132564
15_MTA / C	O95989	0.070918	0.353738	0.324729	NUDT3	Diphospho	NUDT3_HU	1	1.050385
30_MTA / C	P20340	0.17244	0.35366	0.324729	RAB6A	Ras-related	RAB6A_HUI	10	1.126963
15_MTA / C	P26196	-0.23128	0.354123	0.324734	DDX6	Probable A	DDX6_HUN	2	0.851876
30_MTA / C	P30405	0.663347	0.354155	0.324734	PPIF	Peptidyl-pr	PPIF_HUM/	2	1.583752
30_MTA / C	Q9UBU6	0.373521	0.353971	0.324734	FAM8A1	Protein FAI	FA8A1_HUI	2	1.295511
30_MTA / C	P10155	-0.51344	0.354451	0.324848	RO60	RNA-bindir	RO60_HUN	5	0.700548
30_MTA / C	P16930	-0.18438	0.354565	0.324863	FAH	Fumarylace	FAAA_HUM	4	0.88003
30_MTA / C	P62873	-0.07837	0.355371	0.32535	GNB1	Guanine nu	GBB1_HUN	7	0.947127
30_MTA / C	P35232	-0.09256	0.355548	0.325404	PHB1	Prohibitin	PHB1_HUN	17	0.937858
30_MTA / C	P02538	0.47894	0.356418	0.326057	KRT6A	Keratin, typ	K2C6A_HUI	25	1.393719
30_MTA / C	P56537	0.0406	0.356459	0.326057	EIF6	Eukaryotic	IF6_HUMAI	4	1.028542
30_MTA / C	P50914	0.17342	0.35736	0.326611	RPL14	Large ribos	RL14_HUM	4	1.127729
15_MTA / C	Q9NX47	0.240482	0.357318	0.326611	MARCHF5	E3 ubiquiti	MARH5_HU	3	1.181387
30_MTA / C	P78310	-0.2117	0.358026	0.326859	CXADR	Coxsackievi	CXAR_HUM	8	0.863517
30_MTA / C	Q01813	-0.16155	0.358023	0.326859	PFKP	ATP-depen(	PFKAP_HUI	12	0.894066
30_MTA / C	Q03252	1.637362	0.358003	0.326859	LMNB2	Lamin-B2	LMNB2_HU	3	3.110964
15_MTA / C	P38646	0.177167	0.35834	0.327056	HSPA9	Stress-70 p	GRP75_HU	27	1.130662
30_MTA / C	E9PAV3;Q1	0.183227	0.358987	0.327226	NACA	Nascent po	NACAM_HU	4	1.135421
15_MTA / C	P07858	-0.30718	0.358945	0.327226	CTSB	Cathepsin E	CATB_HUM	2	0.808218
30_MTA / C	P21796	0.201887	0.358969	0.327226	VDAC1	Non-selecti	VDAC1_HU	17	1.150201
15_MTA / C	Q14232	0.691346	0.359514	0.327226	EIF2B1	Translation	EI2BA_HUN	2	1.614789
30_MTA / C	Q14657	-0.35393	0.359121	0.327226	LAGE3	EKC/KEOPS	LAGE3_HUI	1	0.782448
15_MTA / C	Q9H1C7	0.030237	0.359293	0.327226	CYSTM1	Cysteine-ric	CYTM1_HU	1	1.02118
30_MTA / C	Q9NP72	0.052491	0.359176	0.327226	RAB18	Ras-related	RAB18_HUI	5	1.037054
30_MTA / C	Q9Y289	0.817167	0.358862	0.327226	SLC5A6	Sodium-dep	SC5A6_HUI	1	1.761943
15_MTA / C	P36543	-0.2499	0.359653	0.327262	ATP6V1E1	V-type prot	VATE1_HUN	2	0.840952
30_MTA / C	Q00577	3.971816	0.359751	0.327262	PURA	Transcripti	PURA_HUN	1	15.69046
15_MTA / C	P22061	0.336901	0.360125	0.327422	PCMT1	Protein-L-is	PIMT_HUM	6	1.263041
30_MTA / C	Q14011	0.527017	0.360108	0.327422	CIRBP	Cold-induc	CIRBP_HUN	1	1.440947

15_MTA / C	P60866	0.666315	0.360503	0.327453	RPS20	Small ribos	RS20_HUM	4	1.587015
15_MTA / C	P62269	-0.0101	0.360555	0.327453	RPS18	Small ribos	RS18_HUM	10	0.993022
15_MTA / C	Q9Y617	-0.12253	0.360449	0.327453	PSAT1	Phosphose	SERC_HUM	12	0.918577
30_MTA / C	O00244	0.064871	0.360697	0.327493	ATOX1	Copper tra	ATOX1_HU	2	1.045991
15_MTA / C	P55735	0.308564	0.361301	0.327744	SEC13	Protein SEC	SEC13_HU	1	1.238475
15_MTA / C	O43488	0.764214	0.363172	0.328981	AKR7A2	Aflatoxin B	ARK72_HU	3	1.698444
15_MTA / C	P55011	-0.13385	0.363086	0.328981	SLC12A2	Solute carri	S12A2_HU	7	0.911399
30_MTA / C	Q96FX7	0.502418	0.363328	0.328981	TRMT61A	tRNA (aden	TRM61_HU	1	1.416586
30_MTA / C	Q9H3Z4	0.408274	0.363313	0.328981	DNAJC5	DnaJ homo	DNJC5_HU	1	1.327097
15_MTA / C	O76071	-0.63364	0.36366	0.329011	CIAO1	Probable cy	CIAO1_HU	3	0.644547
30_MTA / C	P31930	0.037048	0.363613	0.329011	UQCRC1	Cytochrom	QCR1_HUM	7	1.026012
15_MTA / C	P63165	0.20136	0.364103	0.329322	SUMO1	Small ubiq	SUMO1_HU	2	1.149782
15_MTA / C	P01024	0.210191	0.364864	0.329741	C3	Complemen	CO3_HUM	6	1.156842
30_MTA / C	P53621	-0.10407	0.364845	0.329741	COPA	Coatomer s	COPA_HUM	13	0.930403
15_MTA / C	B5ME19;Q	0.270426	0.365438	0.330169	EIF3CL;EIF3	Eukaryotic	EIFCL_HUM	3	1.206164
15_MTA / C	Q12860	-0.49315	0.365657	0.330277	CNTN1	Contactin-	CNTN1_HU	9	0.710471
15_MTA / C	P24311	-0.56733	0.365908	0.330414	COX7B	Cytochrom	COX7B_HU	1	0.674867
15_MTA / C	P55327	-0.0619	0.366115	0.330421	TPD52	Tumor prot	TPD52_HU	6	0.958004
15_MTA / C	Q9Y282	0.312656	0.366515	0.330601	ERGIC3	Endoplasm	ERGI3_HU	2	1.241992
30_MTA / C	Q06830	0.263756	0.36686	0.330823	PRDX1	Peroxiredo	PRDX1_HU	13	1.2006
30_MTA / C	P53985	-0.16558	0.367063	0.330916	SLC16A1	Monocarbo	MOT1_HUM	3	0.891569
15_MTA / C	Q6L8Q7	0.482745	0.368211	0.331685	PDE12	2',5'-phosp	PDE12_HU	4	1.3974
30_MTA / C	O00151	-0.56815	0.368743	0.331889	PDLIM1	PDZ and LI	PDLI1_HUM	8	0.674481
30_MTA / C	P26196	-0.31575	0.368669	0.331889	DDX6	Probable A	DDX6_HUM	2	0.803434
30_MTA / C	Q13501	0.023969	0.368642	0.331889	SQSTM1	Sequestoso	SQSTM_HU	3	1.016753
15_MTA / C	P61081	0.679245	0.368878	0.331921	UBE2M	NEDD8-con	UBC12_HU	2	1.601301
15_MTA / C	Q2TAA2	-0.07314	0.369262	0.332176	IAH1	Isoamyl ace	IAH1_HUM	2	0.950567
15_MTA / C	P39748	-0.21406	0.370431	0.332956	FEN1	Flap endon	FEN1_HUM	1	0.862106
15_MTA / C	P43307	-0.26847	0.370404	0.332956	SSR1	Translocon	SSRA_HUM	2	0.830202
30_MTA / C	P25787	0.203029	0.370742	0.333054	PSMA2	Proteasom	PSA2_HUM	11	1.151113
30_MTA / C	Q96A49	0.242295	0.370672	0.333054	SYAP1	Synapse-as	SYAP1_HU	3	1.182872
30_MTA / C	P11021	0.208194	0.371207	0.333383	HSPA5	Endoplasm	BIP_HUMA	28	1.155241
30_MTA / C	Q8WWC4	1.139423	0.372222	0.333862	MAIP1	m-AAA prot	MAIP1_HU	1	2.202929
15_MTA / C	P01130	-0.2679	0.372424	0.333932	LDLR	Low-densit	LDLR_HUM	3	0.830528
15_MTA / C	O75955	0.368455	0.372766	0.334149	FLOT1	Flotillin-1	FLOT1_HU	2	1.29097
30_MTA / C	Q9UK45	0.221287	0.372995	0.334263	LSM7	U6 snRNA-a	LSM7_HUM	1	1.165773
15_MTA / C	P60228	-0.06216	0.373227	0.334381	EIF3E	Eukaryotic	EIF3E_HUM	5	0.957831
30_MTA / C	P10412	0.101078	0.373755	0.334673	H1-4	Histone H1	H14_HUMA	8	1.072575
30_MTA / C	P0DMV8;P	0.119828	0.374256	0.334917	HSPA1A;HS	Heat shock	HS71A_HU	20	1.086605
15_MTA / C	P27707	-0.72007	0.374391	0.334917	DCK	Deoxycytid	DCK_HUMA	2	0.60707
30_MTA / C	P52272	1.245262	0.374432	0.334917	HNRNPM	Heterogene	HNRPM_HU	12	2.370617
15_MTA / C	Q9HCJ6	-0.29939	0.374408	0.334917	VAT1L	Synaptic ve	VAT1L_HU	2	0.812596
30_MTA / C	P16083	-0.17196	0.374721	0.334995	NQO2	Ribosyldihy	NQO2_HUM	7	0.887634
15_MTA / C	Q96K76	-0.2202	0.374643	0.334995	USP47	Ubiquitin c	UBP47_HU	1	0.858447
15_MTA / C	P17931	0.042387	0.375849	0.33546	LGALS3	Galectin-3	LEG3_HUM	1	1.029816
15_MTA / C	Q6QNY1	-0.17209	0.375666	0.33546	BLOC1S2	Biogenesis	BL1S2_HU	1	0.887554
30_MTA / C	Q9NQR4	0.198653	0.37569	0.33546	NIT2	Omega-ami	NIT2_HUM	6	1.147626
15_MTA / C	Q9UBR2	-0.14888	0.375794	0.33546	CTSZ	Cathepsin Z	CATZ_HUM	3	0.901953
30_MTA / C	P51687	0.077729	0.376285	0.335668	SUOX	Sulfite oxid	SUOX_HUM	1	1.055356
30_MTA / C	P46976	0.311342	0.376659	0.335911	GYG1	Glycogenin	GLYG_HUM	5	1.240861
15_MTA / C	Q6P587	1.661279	0.377061	0.335999	FAHD1	Oxaloaceta	FAHD1_HU	1	3.162969
30_MTA / C	P25398	0.329915	0.377471	0.336097	RPS12	Small ribos	RS12_HUM	4	1.256939

15_MTA / C	P14314	0.161432	0.378189	0.336625	PRKCSH	Glucosidas	GLU2B_HU	15	1.118397
30_MTA / C	P62314	0.203965	0.378391	0.336641	SNRPD1	Small nucle	SMD1_HUM	3	1.15186
15_MTA / C	Q13144	1.125193	0.378623	0.336757	EIF2B5	Translation	EI2BE_HUM	1	2.181308
30_MTA / C	Q16719	0.09447	0.379188	0.337078	KYNU	Kynurenina	KYNU_HUM	1	1.067673
30_MTA / C	Q8NBK3	0.313867	0.379185	0.337078	SUMF1	Formylglyc	SUMF1_HU	1	1.243035
15_MTA / C	Q9Y5J7	0.386971	0.379712	0.337454	TIMM9	Mitochond	TIM9_HUM	3	1.307645
30_MTA / C	Q9P0S9	-0.48208	0.379912	0.337541	TMEM14C	Transmemb	TM14C_HU	1	0.715943
15_MTA / C	O15031	-0.09429	0.383373	0.339927	PLXNB2	Plexin-B2	PLXB2_HUI	3	0.936733
30_MTA / C	P00441	0.515851	0.383239	0.339927	SOD1	Superoxide	SODC_HUM	2	1.429837
15_MTA / C	P07437	0.088606	0.3833	0.339927	TUBB	Tubulin bet	TBB5_HUM	3	1.063343
15_MTA / C	P29966	-0.14309	0.383418	0.339927	MARCKS	Myristoylat	MARCS_HU	6	0.905579
15_MTA / C	Q9UIJ7	0.775209	0.383223	0.339927	AK3	GTP:AMP p	KAD3_HUM	2	1.711438
15_MTA / C	Q13685	-0.6945	0.383863	0.34023	AAMP	Angio-asso	AAMP_HUM	2	0.617922
30_MTA / C	P13984	0.634983	0.384039	0.340296	GTF2F2	General tra	T2FB_HUM	1	1.552919
30_MTA / C	P98179	0.328471	0.384174	0.340324	RBM3	RNA-bindir	RBM3_HUM	2	1.255682
15_MTA / C	P42765	-0.13588	0.384303	0.340347	ACAA2	3-ketoacyl-	THIM_HUM	9	0.910118
30_MTA / C	P06703	0.557382	0.384835	0.340546	S100A6	Protein S1(	S10A6_HUI	1	1.471596
15_MTA / C	P07099	0.434365	0.384747	0.340546	EPHX1	Epoxide hy	HYEP_HUM	10	1.351316
30_MTA / C	P62906	-0.05335	0.384774	0.340546	RPL10A	Large ribos	RL10A_HUI	7	0.963699
30_MTA / C	P00390	0.297742	0.385136	0.340548	GSR	Glutathion	GSHR_HUM	13	1.229219
30_MTA / C	P63173	-0.17986	0.385028	0.340548	RPL38	Large ribos	RL38_HUM	2	0.882791
15_MTA / C	O75312	0.558935	0.385481	0.340572	ZPR1	Zinc finger	ZPR1_HUM	1	1.473182
15_MTA / C	P05388	-0.05054	0.385468	0.340572	RPLP0	Large ribos	RLA0_HUM	10	0.965573
30_MTA / C	P78330	0.30401	0.385672	0.340649	PSPH	Phosphose	SERB_HUM	9	1.234571
15_MTA / C	Q01433	0.225834	0.386324	0.340942	AMPD2	AMP deami	AMPD2_HU	11	1.169453
30_MTA / C	Q09666	0.234761	0.386325	0.340942	AHNAK	Neuroblast	AHNK_HUM	50	1.176712
15_MTA / C	Q15631	-0.09626	0.38636	0.340942	TSN	Translin	TSN_HUMA	11	0.935454
15_MTA / C	Q49AR2	-0.00227	0.386415	0.340942	C5orf22	UPF0489 p	CE022_HUI	1	0.998427
15_MTA / C	Q92616	0.080264	0.387117	0.34138	GCN1	Stalled ribo	GCN1_HUM	2	1.057211
15_MTA / C	P80723	-0.19268	0.387532	0.341655	BASP1	Brain acid s	BASP1_HUI	4	0.874976
30_MTA / C	O14495	0.74266	0.388004	0.341661	PLPP3	Phospholip	PLPP3_HUI	2	1.673258
15_MTA / C	P18077	-0.04882	0.387782	0.341661	RPL35A	Large ribos	RL35A_HUI	7	0.966728
30_MTA / C	P23381	-0.14537	0.387934	0.341661	WARS1	Tryptophar	SYWC_HUM	9	0.90415
15_MTA / C	Q4VC31	0.039676	0.387652	0.341661	MIX23	Protein MI)	MIX23_HUI	1	1.027883
15_MTA / C	Q9H0U3	0.163635	0.388055	0.341661	MAGT1	Magnesium	MAGT1_HU	3	1.120106
30_MTA / C	Q9BU61	0.138474	0.388201	0.341699	NDUFAF3	NADH dehy	NDUF3_HU	2	1.10074
30_MTA / C	Q86WQ0	0.321647	0.388606	0.341965	NR2C2AP	Nuclear rec	NR2CA_HU	1	1.249757
30_MTA / C	P39019	-0.13591	0.388948	0.342175	RPS19	Small ribos	RS19_HUM	7	0.910093
15_MTA / C	P09972	-0.11804	0.390393	0.343093	ALDOC	Fructose-bi	ALDOC_HU	2	0.921438
30_MTA / C	Q9P283	0.233525	0.390405	0.343093	SEMA5B	Semaphori	SEM5B_HU	1	1.175704
15_MTA / C	P20339	0.360358	0.390661	0.343136	RAB5A	Ras-related	RAB5A_HUI	5	1.283745
30_MTA / C	Q9Y520	0.389674	0.390885	0.343242	PRRC2C	Protein PRI	PRC2C_HUI	8	1.310097
15_MTA / C	O75347	-0.08988	0.391091	0.343331	TBCA	Tubulin-spe	TBCA_HUM	4	0.939602
30_MTA / C	Q9HC35	0.729485	0.391636	0.343719	EML4	Echinoderm	EMAL4_HU	2	1.658047
30_MTA / C	O60762	0.949376	0.391999	0.343743	DPM1	Dolichol-pl	DPM1_HUM	1	1.931037
15_MTA / C	P46379	0.336971	0.392286	0.343743	BAG6	Large prolir	BAG6_HUM	4	1.263102
15_MTA / C	Q7Z4V5	0.361409	0.392178	0.343743	HDGFL2	Hepatoma-	HDGR2_HU	1	1.28468
15_MTA / C	O43491	0.09493	0.393749	0.343934	EPB41L2	Band 4.1-lil	E41L2_HUM	8	1.068014
30_MTA / C	P20618	0.537059	0.393263	0.343934	PSMB1	Proteasom	PSB1_HUM	8	1.451012
30_MTA / C	P62269	0.132275	0.392973	0.343934	RPS18	Small ribos	RS18_HUM	10	1.096021
15_MTA / C	Q02809	0.444812	0.393708	0.343934	PLOD1	Procollage	PLOD1_HU	3	1.361137
30_MTA / C	Q3SY84	0.044309	0.393388	0.343934	KRT71	Keratin, typ	K2C71_HUI	1	1.031189

15_MTA / C	Q6GMV3	-0.27905	0.393109	0.343934	PTRHD1	Putative pe	PTRD1_HUI	3	0.824134
30_MTA / C	Q9UMX0	0.463691	0.393066	0.343934	UBQLN1	Ubiquilin-1	UBQL1_HU	8	1.379066
30_MTA / C	O75436	-0.00377	0.394102	0.344061	VPS26A	Vacuolar pr	VP26A_HUI	7	0.997389
15_MTA / C	P21912	0.014288	0.394288	0.344133	SDHB	Succinate d	SDHB_HUM	5	1.009953
30_MTA / C	Q9NUJ1	0.467438	0.395405	0.344744	ABHD10	Palmitoyl-p	ABHDA_HU	1	1.382652
15_MTA / C	Q13126	-0.30755	0.396212	0.345357	MTAP	S-methyl-5'	MTAP_HUM	6	0.808013
30_MTA / C	P68366	0.395965	0.396922	0.345885	TUBA4A	Tubulin alp	TBA4A_HUI	2	1.315822
15_MTA / C	O43768	0.545185	0.397069	0.345895	ENSA	Alpha-endo	ENSA_HUM	2	1.459208
15_MTA / C	Q9H8Y8	0.165633	0.397142	0.345895	GORASP2	Golgi reass	GORS2_HU	1	1.121658
15_MTA / C	P48047	0.151468	0.397356	0.34599	ATP5PO	ATP synthas	ATPO_HUM	8	1.110699
30_MTA / C	P18124	-0.22509	0.39748	0.346007	RPL7	Large ribos	RL7_HUMA	15	0.855542
15_MTA / C	Q07020	-0.10879	0.399479	0.347565	RPL18	Large ribos	RL18_HUM	8	0.927367
30_MTA / C	Q7Z4W1	-0.4251	0.399785	0.347739	DCXR	L-xylulose r	DCXR_HUM	2	0.744785
30_MTA / C	P60866	-0.15551	0.400222	0.347872	RPS20	Small ribos	RS20_HUM	4	0.897816
30_MTA / C	Q92979	0.22619	0.400293	0.347872	EMG1	Ribosomal	NEP1_HUM	4	1.169741
30_MTA / C	Q9Y265	-0.01302	0.400357	0.347872	RUVBL1	RuvB-like 1	RUVB1_HU	10	0.991018
15_MTA / C	Q9Y5K5	-0.04494	0.40012	0.347872	UCHL5	Ubiquitin c	UCHL5_HU	1	0.969333
15_MTA / C	O75533	0.07192	0.400916	0.347977	SF3B1	Splicing fac	SF3B1_HUI	6	1.051115
15_MTA / C	P04259	0.559616	0.401003	0.347977	KRT6B	Keratin, typ	K2C6B_HUI	4	1.473877
15_MTA / C	Q13263	0.005701	0.400866	0.347977	TRIM28	Transcripti	TIF1B_HUM	11	1.00396
15_MTA / C	P35606	0.25256	0.401701	0.3484	COPB2	Coatomer s	COPB2_HU	9	1.191319
30_MTA / C	P08779	0.630512	0.402179	0.348723	KRT16	Keratin, typ	K1C16_HUI	9	1.548114
15_MTA / C	Q9Y3C8	-0.53392	0.402408	0.348831	UFC1	Ubiquitin-f	UFC1_HUM	3	0.690676
15_MTA / C	Q96HR9	0.096783	0.402697	0.34899	REEP6	Receptor ex	REEP6_HUI	3	1.069386
30_MTA / C	P31946	-0.00578	0.403349	0.349463	YWHAB	14-3-3 prot	1433B_HUI	5	0.996001
30_MTA / C	Q9Y617	0.120903	0.403673	0.349653	PSAT1	Phosphoser	SERC_HUM	12	1.087415
15_MTA / C	P02760	0.329833	0.403962	0.349693	AMBP	Protein AM	AMBP_HUM	1	1.256868
15_MTA / C	Q9UI30	0.450848	0.40403	0.349693	TRMT112	Multifuncti	TR112_HUI	2	1.366843
15_MTA / C	P17858	-0.15565	0.40419	0.349735	PFKL	ATP-depend	PFKAL_HUI	8	0.897727
15_MTA / C	Q01082	0.021746	0.405486	0.350582	SPTBN1	Spectrin be	SPTB2_HUI	23	1.015187
30_MTA / C	Q7L1Q6	-0.36952	0.405475	0.350582	BZW1	eIF5-mimic	5MP2_HUM	9	0.774038
15_MTA / C	Q9NXB9	0.695079	0.406542	0.351312	ELOVL2	Very long cl	ELOV2_HUI	1	1.618974
15_MTA / C	Q9UHD1	0.435645	0.406481	0.351312	CHORDC1	Cysteine an	CHRD1_HU	3	1.352516
30_MTA / C	Q6FI81	-0.44774	0.407195	0.351785	CIAPIN1	Anamorsin	CPIN1_HUI	3	0.733192
15_MTA / C	O75352	0.231777	0.407794	0.352118	MPDU1	Mannose-P	MPU1_HUM	2	1.174281
30_MTA / C	P54725	0.566063	0.408168	0.352238	RAD23A	UV excision	RD23A_HU	3	1.480478
30_MTA / C	Q9Y6M1	-0.2143	0.408252	0.352238	IGF2BP2	Insulin-like	IF2B2_HUM	2	0.861962
15_MTA / C	P60953	0.282969	0.408892	0.352607	CDC42	Cell divisio	CDC42_HU	3	1.216696
30_MTA / C	Q9NVS9	0.580477	0.40973	0.353146	PNPO	Pyridoxine-	PNPO_HUM	2	1.495343
30_MTA / C	P52815	0.002136	0.410556	0.353582	MRPL12	Large ribos	RM12_HUM	4	1.001482
15_MTA / C	Q15382	0.069527	0.410735	0.353585	RHEB	GTP-bindin	RHEB_HUM	1	1.049373
30_MTA / C	Q5ZPR3	-1.08633	0.410773	0.353585	CD276	CD276 anti	CD276_HU	1	0.470958
30_MTA / C	P36871	-0.13413	0.411106	0.35376	PGM1	Phosphoglu	PGM1_HUM	17	0.911222
30_MTA / C	Q96A72	-0.16994	0.41119	0.35376	MAGOHB	Protein ma	MGN2_HUI	2	0.888882
30_MTA / C	Q9Y2R0	-0.39209	0.412433	0.354645	COA3	Cytochrom	COA3_HUM	5	0.762024
30_MTA / C	P17987	-0.15722	0.413354	0.35516	TCP1	T-complex p	TCPA_HUM	18	0.89675
15_MTA / C	Q9NVA2	0.01294	0.41331	0.35516	SEPTIN11	Septin-11	SEP11_HUM	12	1.00901
15_MTA / C	P62942	0.522289	0.41371	0.355171	FKBP1A	Peptidyl-pr	FKB1A_HUI	2	1.436232
30_MTA / C	Q92530	-0.29328	0.413762	0.355171	PSMF1	Proteasom	PSMF1_HU	3	0.816047
15_MTA / C	Q9Y624	-0.13004	0.413768	0.355171	F11R	Junctional	JAM1_HUM	4	0.913804
30_MTA / C	P05387	0.28204	0.415076	0.355778	RPLP2	Large ribos	RLA2_HUM	4	1.215913
15_MTA / C	P13861	0.459327	0.415115	0.355778	PRKAR2A	cAMP-depe	KAP2_HUM	5	1.3749

30_MTA / C	P28288	0.831898	0.415361	0.355778	ABCD3	ATP-bindin	ABCD3_HU	2	1.780026
30_MTA / C	P47756	0.051774	0.415299	0.355778	CAPZB	F-actin-cap	CAPZB_HUI	13	1.036538
15_MTA / C	Q15369	-0.1447	0.414947	0.355778	ELOC	Elongin-C	ELOC_HUM	1	0.904569
30_MTA / C	Q9UBE0	-0.37314	0.414789	0.355778	SAE1	SUMO-activ	SAE1_HUM	7	0.7721
15_MTA / C	O75746	0.422998	0.415565	0.355785	SLC25A12	Electrogeni	S2512_HUI	4	1.34071
30_MTA / C	P53992	0.505028	0.415663	0.355785	SEC24C	Protein trai	SC24C_HUI	2	1.419151
30_MTA / C	O43169	-0.43598	0.416699	0.356062	CYB5B	Cytochrom	CYB5B_HUI	6	0.739192
30_MTA / C	O95373	-0.51639	0.416757	0.356062	IPO7	Importin-7	IPO7_HUM	8	0.699117
30_MTA / C	P26358	-0.28551	0.416731	0.356062	DNMT1	DNA (cytosi	DNMT1_HU	2	0.820451
30_MTA / C	P30566	-0.10932	0.416767	0.356062	ADSL	Adenylosuc	PUR8_HUM	8	0.927024
30_MTA / C	Q14919	-0.77561	0.417152	0.356234	DRAP1	Dr1-associa	NC2A_HUM	2	0.584144
15_MTA / C	Q9ULV4	0.094775	0.417291	0.356234	CORO1C	Coronin-1C	COR1C_HU	5	1.067899
30_MTA / C	Q9UNN5	-0.20245	0.418007	0.356754	FAF1	FAS-associa	FAF1_HUM	2	0.869075
30_MTA / C	P01130	-0.18761	0.41812	0.356759	LDLR	Low-densit	LDLR_HUM	3	0.878057
15_MTA / C	P00441	-0.26776	0.418808	0.357254	SOD1	Superoxide	SODC_HUM	2	0.830609
30_MTA / C	Q16831	-0.19541	0.420148	0.358238	UPP1	Uridine pho	UPP1_HUM	5	0.873326
30_MTA / C	Q01518	-0.09271	0.420437	0.358366	CAP1	Adenylyl cy	CAP1_HUM	24	0.937762
30_MTA / C	P13995	0.700911	0.421179	0.358853	MTHFD2	Bifunctiona	MTDC_HUM	2	1.625531
30_MTA / C	Q86SE5	-1.10353	0.421572	0.359028	RALYL	RNA-bindir	RALYL_HUM	1	0.465377
30_MTA / C	Q9NYL4	0.228915	0.421647	0.359028	FKBP11	Peptidyl-pr	FKB11_HUI	2	1.171953
15_MTA / C	P62753	0.013065	0.421817	0.359081	RPS6	Small ribos	RS6_HUMA	9	1.009097
30_MTA / C	P51149	0.214888	0.423182	0.360151	RAB7A	Ras-related	RAB7A_HUI	10	1.160614
15_MTA / C	P00439	0.499823	0.423654	0.360459	PAH	Phenylalan	PH4H_HUM	3	1.41404
30_MTA / C	Q587I9	-0.54043	0.42525	0.361725	SFT2D3	Vesicle tran	SFT2C_HUM	1	0.687568
15_MTA / C	O00303	0.029745	0.425891	0.362177	EIF3F	Eukaryotic	EIF3F_HUM	2	1.020832
30_MTA / C	P62424	-0.27895	0.427185	0.36309	RPL7A	Large ribos	RL7A_HUM	15	0.82419
30_MTA / C	Q06124	-0.93019	0.427162	0.36309	PTPN11	Tyrosine-pr	PTN11_HUI	2	0.524788
15_MTA / C	P02787	0.11513	0.428511	0.363624	TF	Serotransfe	TRFE_HUMA	4	1.083073
30_MTA / C	P29144	-0.89844	0.42858	0.363624	TPP2	Tripeptidyl	TPP2_HUM	9	0.536465
15_MTA / C	P47914	0.305511	0.42845	0.363624	RPL29	Large ribos	RL29_HUM	2	1.235856
30_MTA / C	P62191	0.070656	0.428488	0.363624	PSMC1	26S protea	PRS4_HUM	4	1.050194
15_MTA / C	P02786	-0.0298	0.428915	0.363815	TFRC	Transferrin	TFR1_HUM	25	0.979555
15_MTA / C	A8MWD9;F	-0.03603	0.429443	0.363914	SNRPGP15;	Putative sm	RUXGL_HU	3	0.975334
15_MTA / C	P13716	0.169693	0.429582	0.363914	ALAD	Delta-amin	HEM2_HUM	2	1.124819
15_MTA / C	Q96GF1	-0.03556	0.429348	0.363914	RNF185	E3 ubiquiti	RN185_HU	1	0.975655
30_MTA / C	Q9HB40	0.503624	0.429312	0.363914	SCPEP1	Retinoid-in	RISC_HUMA	2	1.417771
30_MTA / C	Q9Y3A6	-0.09624	0.429528	0.363914	TMED5	Transmemb	TMED5_HU	4	0.93547
15_MTA / C	Q08722	0.559179	0.430326	0.364359	CD47	Leukocyte s	CD47_HUM	3	1.473431
15_MTA / C	Q9HB07	0.103174	0.430218	0.364359	MYG1	MYG1 exon	MYG1_HUM	8	1.074134
30_MTA / C	P31937	-0.11939	0.430546	0.364427	HIBADH	3-hydroxyis	3HIDH_HUI	5	0.920577
15_MTA / C	Q8WZA0	0.038043	0.430808	0.36448	LZIC	Protein LZIC	LZIC_HUMA	2	1.02672
15_MTA / C	Q96BW5	-0.09017	0.431013	0.36448	PTER	Phosphotri	PTER_HUM	6	0.939411
15_MTA / C	P04179	-0.16669	0.431837	0.364985	SOD2	Superoxide	SODM_HUM	8	0.890882
15_MTA / C	P15170	-0.41016	0.431735	0.364985	GSPT1	Eukaryotic	ERF3A_HUM	5	0.752539
30_MTA / C	P46776	0.072525	0.432322	0.365254	RPL27A	Large ribos	RL27A_HUI	4	1.051555
15_MTA / C	P99999	-0.34272	0.432375	0.365254	CYCS	Cytochrom	CYC_HUMA	5	0.788553
30_MTA / C	Q01433	0.118683	0.43275	0.365477	AMPD2	AMP deami	AMPD2_HU	11	1.085743
30_MTA / C	P45880	0.188947	0.433078	0.365605	VDAC2	Voltage-de	VDAC2_HU	13	1.139932
15_MTA / C	P78346	0.250064	0.433232	0.365605	RPP30	Ribonuclea	RPP30_HUI	2	1.189259
30_MTA / C	Q9HCC0	-0.07315	0.433192	0.365605	MCCC2	Methylcrot	MCCB_HUM	7	0.950563
15_MTA / C	O43447	-0.17092	0.433574	0.365698	PPIH	Peptidyl-pr	PPIH_HUM	1	0.888277
30_MTA / C	P23396	-0.01871	0.433685	0.365698	RPS3	Small ribos	RS3_HUMA	17	0.987113

15_MTA / C	P30626	0.423421	0.434916	0.366328	SRI	Sorcin	SORCN_HU		4	1.341104
30_MTA / C	P48735	1.241555	0.43476	0.366328	IDH2	Isocitrate d	IDHP_HUM		4	2.364533
15_MTA / C	P78330	-0.15072	0.434654	0.366328	PSPH	Phosphosei	SERB_HUM		9	0.900799
30_MTA / C	P35222	-0.33107	0.435983	0.366805	CTNNB1	Catenin bet	CTNB1_HU		7	0.794949
15_MTA / C	P62263	0.372144	0.435981	0.366805	RPS14	Small ribos	RS14_HUM		4	1.294275
15_MTA / C	Q7Z5J4	0.270827	0.436402	0.3669	RAI1	Retinoic ac	RAI1_HUM		1	1.206499
30_MTA / C	Q99829	-0.05837	0.43626	0.3669	CPNE1	Copine-1	CPNE1_HU		7	0.960347
15_MTA / C	Q9Y237	0.338735	0.436427	0.3669	PIN4	Peptidyl-pr	PIN4_HUM		2	1.264648
30_MTA / C	P01857	0.540097	0.436731	0.366969	IGHG1	Immunoglo	IGHG1_HU		1	1.454071
15_MTA / C	Q6UW68	0.38333	0.436647	0.366969	TMEM205	Transmemb	TM205_HU		4	1.304349
15_MTA / C	P11168	0.504724	0.436862	0.366986	SLC2A2	Solute carri	GTR2_HUM		2	1.418852
30_MTA / C	P32322	0.294687	0.437158	0.367142	PYCR1	Pyrroline-5	P5CR1_HU		10	1.226618
30_MTA / C	P62263	-0.09728	0.437567	0.367299	RPS14	Small ribos	RS14_HUM		4	0.934792
15_MTA / C	P53992	0.781125	0.43825	0.367779	SEC24C	Protein trai	SC24C_HU		2	1.71847
15_MTA / C	Q9BQE4	0.462758	0.438499	0.367895	SELENOS	Selenoprot	SELS_HUM		2	1.378174
15_MTA / C	P49720	0.012322	0.438693	0.367965	PSMB3	Proteasom	PSB3_HUM		7	1.008578
15_MTA / C	Q99460	0.153342	0.438882	0.368031	PSMD1	26S protea	PSMD1_HU		8	1.112143
15_MTA / C	Q96P70	0.385814	0.43946	0.368329	IPO9	Importin-9	IPO9_HUM		4	1.306597
30_MTA / C	P50454	0.089793	0.43992	0.368435	SERPINH1	Serpin H1	SERPH_HU		5	1.064217
30_MTA / C	Q9NPF4	-0.18852	0.439852	0.368435	OSGEP	tRNA N6-ad	OSGEP_HU		5	0.877503
30_MTA / C	P29966	-0.13514	0.440255	0.368529	MARCKS	Myristoylat	MARCS_HU		6	0.910579
15_MTA / C	P68036	-0.12813	0.440145	0.368529	UBE2L3	Ubiquitin-c	UB2L3_HU		3	0.915014
30_MTA / C	Q8N5K1	0.036979	0.440795	0.368887	CISD2	CDGSH iror	CISD2_HUN		5	1.025963
15_MTA / C	P13667	-0.06613	0.440981	0.36895	PDIA4	Protein dis	PDIA4_HUN		28	0.955196
30_MTA / C	P43243	1.540003	0.441739	0.369052	MATR3	Matrin-3	MATR3_HU		2	2.907951
15_MTA / C	Q96HE7	-0.1562	0.441537	0.369052	ERO1A	ERO1-like p	ERO1A_HU		6	0.897386
15_MTA / C	Q9UHD9	0.446755	0.441771	0.369052	UBQLN2	Ubiquilin-2	UBQL2_HU		1	1.362971
15_MTA / C	P05141	0.113722	0.44259	0.36927	SLC25A5	ADP/ATP tr	ADT2_HUM		5	1.082016
15_MTA / C	P06737	0.243869	0.442404	0.36927	PYGL	Glycogen p	PYGL_HUM		9	1.184164
15_MTA / C	P51580	-0.34424	0.442559	0.36927	TPMT	Thiopurine	TPMT_HUM		2	0.787721
15_MTA / C	P63218	-0.15007	0.442577	0.36927	GNG5	Guanine nu	GBG5_HUM		3	0.901209
30_MTA / C	O00232	-0.1125	0.442932	0.36943	PSMD12	26S protea	PSD12_HU		10	0.924984
30_MTA / C	P14314	0.184759	0.443383	0.36956	PRKCSH	Glucosidas	GLU2B_HU		15	1.136627
15_MTA / C	P35754	-0.71735	0.443303	0.36956	GLRX	Glutaredox	GLRX1_HU		1	0.608213
30_MTA / C	P14678;P6	0.158071	0.443883	0.369884	SNRPB;SNR	Small nucle	RSMB_HUM		5	1.115794
30_MTA / C	P14406	0.230505	0.444242	0.370047	COX7A2	Cytochrom	CX7A2_HU		1	1.173246
15_MTA / C	P35080	1.076742	0.444545	0.370091	PFN2	Profilin-2	PROF2_HU		1	2.109267
30_MTA / C	Q5T1J5;Q9	-0.0038	0.444607	0.370091	CHCHD2P9	Putative co	CHCH9_HU		2	0.997367
30_MTA / C	P0C0S8;Q1	-0.27532	0.444926	0.370194	H2AC11;H2	Histone H2	H2A1_HUM		4	0.826266
30_MTA / C	Q9UNS2	-0.03697	0.445633	0.37069	COPS3	COP9 signa	CSN3_HUM		4	0.974699
15_MTA / C	P01876	-0.09472	0.446483	0.371024	IGHA1	Immunoglo	IGHA1_HU		2	0.936456
30_MTA / C	P49903	0.408289	0.446477	0.371024	SEPHS1	Selenide, w	SPS1_HUM		2	1.327111
30_MTA / C	P05362	0.239823	0.446854	0.37114	ICAM1	Intercellula	ICAM1_HU		10	1.180848
30_MTA / C	P11908	0.014206	0.446969	0.37114	PRPS2	Ribose-pho	PRPS2_HU		1	1.009895
15_MTA / C	P61604	-0.07054	0.447139	0.37114	HSPE1	10 kDa heat	CH10_HUM		11	0.952283
30_MTA / C	Q8IYS2	0.011568	0.447377	0.371209	KIAA2013	Uncharacte	K2013_HU		2	1.00805
30_MTA / C	P13716	-0.33769	0.447997	0.371351	ALAD	Delta-amin	HEM2_HUM		2	0.79131
30_MTA / C	P63220	0.242264	0.447915	0.371351	RPS21	Small ribos	RS21_HUM		5	1.182847
15_MTA / C	Q14141	0.24864	0.44865	0.371707	SEPTIN6	Septin-6	SEPT6_HUN		4	1.188087
15_MTA / C	P09012	0.142711	0.448939	0.37176	SNRPA	U1 small nu	SNRPA_HU		6	1.103978
30_MTA / C	O75746	0.06691	0.450008	0.372273	SLC25A12	Electrogeni	S2512_HU		4	1.04747
30_MTA / C	P18621	-0.14834	0.449966	0.372273	RPL17	Large ribos	RL17_HUM		4	0.902286

30_MTA / C	Q96GX9	-0.00281	0.44995	0.372273	APIP	Methylthio	MTNB_HUN	2	0.998052
15_MTA / C	Q9Y3A6	0.325702	0.450674	0.372638	TMED5	Transmemb	TMED5_HU	4	1.253274
15_MTA / C	P00492	-0.06318	0.451284	0.37305	HPRT1	Hypoxanth	HPRT_HUM	6	0.957155
15_MTA / C	Q9UHQ9	0.572653	0.451745	0.373151	CYB5R1	NADH-cyto	NB5R1_HU	3	1.487256
30_MTA / C	O14732	-0.24811	0.452944	0.373955	IMPA2	Inositol mc	IMPA2_HUI	2	0.842
15_MTA / C	P56199	-0.25136	0.454758	0.375359	ITGA1	Integrin alp	ITA1_HUMA	1	0.840107
30_MTA / C	Q16181	0.282553	0.455998	0.376289	SEPTIN7	Septin-7	SEPT7_HUN	5	1.216345
30_MTA / C	O15400	-0.07843	0.456944	0.376468	STX7	Syntaxin-7	STX7_HUM	6	0.94709
30_MTA / C	P05026	-0.12575	0.457143	0.376468	ATP1B1	Sodium/po	AT1B1_HUI	4	0.916529
15_MTA / C	P37840	-0.19452	0.45735	0.376468	SNCA	Alpha-synu	SYUA_HUM	6	0.873867
15_MTA / C	Q13242	0.279853	0.457328	0.376468	SRSF9	Serine/argi	SRSF9_HUN	2	1.214071
30_MTA / C	Q92973	0.304232	0.457236	0.376468	TNPO1	Transportir	TNPO1_HU	3	1.234761
15_MTA / C	Q9H3G5	-0.08135	0.456963	0.376468	CPVL	Probable se	CPVL_HUM	1	0.945171
30_MTA / C	O43818	-0.36468	0.458459	0.376754	RRP9	U3 small nu	U3IP2_HUN	5	0.776643
30_MTA / C	O60814;P5	0.244611	0.458809	0.376754	H2BC12;H2	Histone H2	H2B1K_HU	6	1.184773
30_MTA / C	P35241	0.131165	0.458835	0.376754	RDX	Radixin	RADI_HUM	25	1.095178
15_MTA / C	Q12906	-0.00259	0.458403	0.376754	ILF3	Interleukin	ILF3_HUMA	14	0.998204
15_MTA / C	Q9UJZ1	0.108912	0.459887	0.377431	STOML2	Stomatin-li	STML2_HUI	8	1.078415
30_MTA / C	Q9NVJ2	-0.33768	0.460234	0.377583	ARL8B	ADP-ribosy	ARL8B_HUI	2	0.791314
30_MTA / C	Q8TBQ9	0.203981	0.460456	0.377617	TMEM167A	Protein kish	KISHA_HUN	1	1.151873
30_MTA / C	Q13144	0.381082	0.460964	0.377847	EIF2B5	Translation	EI2BE_HUN	1	1.302318
15_MTA / C	Q14677	0.177319	0.460928	0.377847	CLINT1	Clathrin int	EPN4_HUM	3	1.13078
30_MTA / C	Q9BUH6	0.108573	0.461732	0.378383	PAXX	Protein PAX	PAXX_HUM	5	1.078161
15_MTA / C	Q96A57	0.049253	0.461998	0.378414	TMEM230	Transmemb	TM230_HU	1	1.034729
30_MTA / C	P00558	-0.07493	0.462576	0.378419	PGK1	Phosphogly	PGK1_HUM	33	0.949391
15_MTA / C	Q13501	0.484492	0.462528	0.378419	SQSTM1	Sequestoso	SQSTM_HU	3	1.399093
15_MTA / C	Q5ZPR3	-0.71329	0.462359	0.378419	CD276	CD276 anti	CD276_HU	1	0.609927
15_MTA / C	Q96RS6	0.181495	0.463891	0.379308	NUDCD1	NudC doma	NUDC1_HU	1	1.134058
15_MTA / C	Q13098	0.271299	0.464409	0.379318	GPS1	COP9 signa	CSN1_HUM	6	1.206894
30_MTA / C	Q9Y5U8	-0.33422	0.464476	0.379318	MPC1	Mitochond	MPC1_HUN	1	0.793211
15_MTA / C	P08579	-0.14178	0.465197	0.379706	SNRPB2	U2 small nu	RU2B_HUM	4	0.906403
15_MTA / C	P61204;P8	-0.14973	0.465309	0.379706	ARF3;ARF1	ADP-ribosy	ARF3_HUM	6	0.901417
15_MTA / C	Q93050	0.422308	0.46539	0.379706	ATP6V0A1	V-type prot	VPP1_HUM	4	1.34007
15_MTA / C	A6PVC2	1.048941	0.465691	0.379749	TTLL8	Protein mo	TTLL8_HUN	2	2.06901
15_MTA / C	P09622	-0.20194	0.465579	0.379749	DLD	Dihydrolipo	DLDH_HUM	14	0.869382
30_MTA / C	Q92747	-0.12895	0.46637	0.380115	ARPC1A	Actin-relate	ARC1A_HUI	5	0.914495
15_MTA / C	Q14118	-0.31895	0.466911	0.380463	DAG1	Dystroglyca	DAG1_HUM	2	0.801653
15_MTA / C	Q8IVS2	-0.43291	0.467964	0.381227	MCAT	Malonyl-Co	FABD_HUM	2	0.740764
15_MTA / C	Q9HCE1	0.807863	0.469015	0.381734	MOV10	Helicase MO	MOV10_HU	2	1.750616
15_MTA / C	P62081	0.051204	0.469896	0.382331	RPS7	Small ribos	RS7_HUMA	6	1.036129
15_MTA / C	Q9NZM1	0.510749	0.470141	0.382437	MYOF	Myoferlin	MYOF_HUN	9	1.42479
30_MTA / C	P26440	1.250166	0.470607	0.382722	IVD	Isovaleryl-C	IVD_HUMA	2	2.378689
30_MTA / C	P07686	-0.13848	0.47105	0.382989	HEXB	Beta-hexosa	HEXB_HUM	8	0.908474
30_MTA / C	Q9UL46	-0.41064	0.471332	0.383124	PSME2	Proteasom	PSME2_HU	6	0.752292
30_MTA / C	Q5BJH7	-0.14431	0.471486	0.383155	YIF1B	Protein YIF	YIF1B_HUM	1	0.904813
30_MTA / C	O00754	-0.16173	0.472807	0.383663	MAN2B1	Lysosomal	MA2B1_HU	6	0.893954
15_MTA / C	O95456	-0.23106	0.472759	0.383663	PSMG1	Proteasom	PSMG1_HU	6	0.852009
15_MTA / C	Q14657	-0.02051	0.472529	0.383663	LAGE3	EKC/KEOPS	LAGE3_HUI	1	0.985886
30_MTA / C	Q16630	0.388059	0.472517	0.383663	CPSF6	Cleavage ar	CPSF6_HUN	2	1.308631
15_MTA / C	Q8WW12	-0.06282	0.472798	0.383663	PCNP	PEST protec	PCNP_HUM	5	0.957388
15_MTA / C	Q9NYL4	-0.21766	0.473377	0.384032	FKBP11	Peptidyl-pr	FKB11_HUI	2	0.859962
30_MTA / C	Q9Y3D6	-0.08826	0.473727	0.384222	FIS1	Mitochond	FIS1_HUMA	1	0.940657

30_MTA / C	O75533	0.626314	0.473922	0.384286	SF3B1	Splicing fac	SF3B1_HUI	6	1.543616
30_MTA / C	Q7Z4H3	-0.08436	0.474657	0.3846	HDDC2	5'-deoxynu	HDDC2_HU	2	0.943204
30_MTA / C	Q16134	0.226713	0.474906	0.384708	ETFDH	Electron tra	ETFD_HUM	1	1.170166
30_MTA / C	P62140	-0.21552	0.475188	0.384748	PPP1CB	Serine/thre	PP1B_HUM	6	0.861238
30_MTA / C	P52565	0.394115	0.475335	0.384773	ARHGDIA	Rho GDP-di	GDIR1_HUI	3	1.314136
15_MTA / C	Q9NYF8	0.29088	0.475572	0.384871	BCLAF1	Bcl-2-assoc	BCLF1_HUM	1	1.223386
30_MTA / C	P04632	0.478093	0.476438	0.385289	CAPNS1	Calpain sma	CPNS1_HUI	3	1.392901
15_MTA / C	Q13835	-1.67189	0.476578	0.385309	PKP1	Plakophilin	PKP1_HUM	2	0.313843
30_MTA / C	P18077	0.112501	0.476809	0.385401	RPL35A	Large ribos	RL35A_HUI	7	1.081101
15_MTA / C	P07951	0.217964	0.476954	0.385425	TPM2	Tropomyos	TPM2_HUM	5	1.163091
30_MTA / C	P61457	0.236849	0.47761	0.38586	PCBD1	Pterin-4-alp	PHS_HUMA	5	1.178416
30_MTA / C	Q02978	-0.0674	0.477962	0.385997	SLC25A11	Mitochond	M2OM_HU	7	0.954355
30_MTA / C	O75312	-0.32555	0.478323	0.38606	ZPR1	Zinc finger	ZPR1_HUM	1	0.797993
15_MTA / C	P48723	0.119944	0.478815	0.386347	HSPA13	Heat shock	HSP13_HUI	5	1.086693
30_MTA / C	Q8TC12	-0.1317	0.478912	0.386347	RDH11	Retinol deh	RDH11_HU	1	0.912758
30_MTA / C	O15126	-0.06408	0.479481	0.386618	SCAMP1	Secretory c	SCAM1_HU	2	0.956554
30_MTA / C	Q9BXJ9	-0.06994	0.47989	0.38676	NAA15	N-alpha-ace	NAA15_HU	6	0.95268
30_MTA / C	P17174	-0.1382	0.480467	0.387036	GOT1	Aspartate a	AATC_HUM	17	0.90865
15_MTA / C	P62899	0.139396	0.480665	0.387102	RPL31	Large ribos	RL31_HUM	6	1.101444
30_MTA / C	Q96ER9	-0.10945	0.480872	0.387175	CCDC51	Mitochond	MITOK_HUI	2	0.926944
15_MTA / C	Q14145	0.19052	0.481978	0.387971	KEAP1	Kelch-like E	KEAP1_HUI	2	1.141175
30_MTA / C	P06744	-0.11703	0.482402	0.388175	GPI	Glucose-6-p	G6PI_HUM	16	0.922085
15_MTA / C	Q96QR8	0.071297	0.482467	0.388175	PURB	Transcripti	PURB_HUM	4	1.050661
30_MTA / C	Q96C36	-0.17962	0.482695	0.388265	PYCR2	Pyrroline-5	P5CR2_HUI	5	0.882934
30_MTA / C	Q9H3K6	0.550731	0.484022	0.389237	BOLA2	BolA-like pr	BOLA2_HUI	2	1.464827
30_MTA / C	P22626	0.290782	0.484571	0.389374	HNRNPA2B	Heterogene	ROA2_HUM	10	1.223303
30_MTA / C	Q7Z5J4	1.62304	0.484401	0.389374	RAI1	Retinoic ac	RAI1_HUMA	1	3.080233
30_MTA / C	Q96JA4	0.204113	0.485	0.389551	MS4A14	Membrane-	M4A14_HU	1	1.151978
15_MTA / C	O00629	0.031863	0.486264	0.390283	KPNA4	Importin su	IMA3_HUM	1	1.022331
30_MTA / C	P23526	0.151596	0.486954	0.390459	AHCY	Adenosylho	SAHH_HUM	21	1.110798
15_MTA / C	P50991	0.030408	0.486887	0.390459	CCT4	T-complex	TCPD_HUM	21	1.021301
15_MTA / C	Q9H078	0.579733	0.486805	0.390459	CLPB	Mitochond	CLPB_HUM	2	1.494573
15_MTA / C	O60506	-0.00343	0.487298	0.390477	SYNCRIP	Heterogene	HNRPQ_HU	4	0.997623
30_MTA / C	O75439	0.106401	0.487331	0.390477	PMPCB	Mitochond	MPPB_HUM	5	1.076539
30_MTA / C	P83881	-0.03734	0.487132	0.390477	RPL36A	Large ribos	RL36A_HUI	5	0.974451
15_MTA / C	P42785	-0.13624	0.487707	0.390684	PRCP	Lysosomal	PCP_HUMA	3	0.909888
30_MTA / C	Q6RW13	-0.12473	0.488115	0.390822	AGTRAP	Type-1 angi	ATRAP_HUI	3	0.917175
15_MTA / C	P78310	0.021636	0.488796	0.391257	CXADR	Coxsackievi	CXAR_HUM	8	1.01511
30_MTA / C	P30041	-0.06534	0.489459	0.391447	PRDX6	Peroxiredo	PRDX6_HU	11	0.955719
30_MTA / C	P61254	0.042319	0.489639	0.391447	RPL26	Large ribos	RL26_HUM	9	1.029768
15_MTA / C	P62829	0.206966	0.489723	0.391447	RPL23	Large ribos	RL23_HUM	6	1.154258
15_MTA / C	Q02790	0.376762	0.489685	0.391447	FKBP4	Peptidyl-pr	FKBP4_HUI	7	1.298424
30_MTA / C	Q13148	0.815854	0.489353	0.391447	TARDBP	TAR DNA-bi	TADBP_HUI	1	1.76034
30_MTA / C	P63000	0.102887	0.490165	0.391706	RAC1	Ras-related	RAC1_HUM	5	1.07392
15_MTA / C	P07902	0.711698	0.490378	0.391782	GALT	Galactose-1	GALT_HUM	1	1.63773
15_MTA / C	Q99873	-0.24975	0.490917	0.392118	PRMT1	Protein arg	ANM1_HUI	2	0.841043
30_MTA / C	P37802	0.021854	0.491147	0.392207	TAGLN2	Transgelin-	TAGL2_HUI	6	1.015263
30_MTA / C	Q96S97	-0.12354	0.491765	0.392512	MYADM	Myeloid-as	MYADM_HU	2	0.917931
30_MTA / C	P06730	-0.40686	0.49234	0.392708	EIF4E	Eukaryotic	IF4E_HUMA	2	0.754261
15_MTA / C	Q969M3	0.20896	0.492366	0.392708	YIPF5	Protein YIP	YIPF5_HUM	1	1.155854
30_MTA / C	Q13595	0.061114	0.493119	0.393119	TRA2A	Transforme	TRA2A_HUI	1	1.043271
15_MTA / C	Q9BQ13	0.069448	0.493879	0.39363	KCTD14	BTB/POZ dc	KCD14_HU	1	1.049315

30_MTA / C	O00746	0.040699	0.494249	0.393648	NME4	Nucleoside	NDKM_HUI	4	1.028612
15_MTA / C	P18084	0.472107	0.494127	0.393648	ITGB5	Integrin be	ITB5_HUMA	1	1.387134
15_MTA / C	P51452	-0.60227	0.494376	0.393648	DUSP3	Dual specifi	DUS3_HUM	2	0.658715
15_MTA / C	P07306	-0.185	0.494724	0.393688	ASGR1	Asialoglyco	ASGR1_HUI	3	0.879649
15_MTA / C	P20340	-0.10726	0.495377	0.393688	RAB6A	Ras-related	RAB6A_HUI	10	0.928347
15_MTA / C	P46777	0.148396	0.494665	0.393688	RPL5	Large ribos	RL5_HUMA	11	1.108337
30_MTA / C	P53701	-0.06734	0.495226	0.393688	HCCS	Holocytoch	CCHL_HUM	2	0.954395
15_MTA / C	P55884	-0.08303	0.49491	0.393688	EIF3B	Eukaryotic	EIF3B_HUM	8	0.944073
30_MTA / C	P68104;Q5	0.129757	0.494783	0.393688	EEF1A1;EEF	Elongation	EF1A1_HUI	18	1.094109
30_MTA / C	Q15459	-0.35681	0.495131	0.393688	SF3A1	Splicing fac	SF3A1_HUI	1	0.78089
30_MTA / C	O43464	0.488625	0.495844	0.393805	HTRA2	Serine prot	HTRA2_HUI	3	1.403107
15_MTA / C	O95297	0.654757	0.495987	0.393805	MPZL1	Myelin prot	MPZL1_HU	2	1.57435
15_MTA / C	Q9Y512	0.164479	0.495999	0.393805	SAMM50	Sorting and	SAM50_HU	6	1.120761
30_MTA / C	P27707	-0.5274	0.496496	0.394076	DCK	Deoxycytid	DCK_HUMA	2	0.693806
15_MTA / C	Q99611	-0.00841	0.496579	0.394076	SEPHS2	Selenide, w	SPS2_HUM	6	0.994189
15_MTA / C	Q53HC9	-0.13883	0.497251	0.394436	EIPR1	EARP and G	EIPR1_HUN	1	0.908256
30_MTA / C	Q9NP79	0.383346	0.497384	0.394436	VTA1	Vacuolar pr	VTA1_HUM	2	1.304364
15_MTA / C	Q9Y262	0.153646	0.497389	0.394436	EIF3L	Eukaryotic	EIF3L_HUM	8	1.112377
30_MTA / C	Q5JSH3	0.143857	0.497585	0.394497	WDR44	WD repeat-	WDR44_HU	3	1.104855
30_MTA / C	P61106	-0.01733	0.497869	0.394533	RAB14	Ras-related	RAB14_HUI	6	0.988059
15_MTA / C	O14925	0.044124	0.498428	0.394568	TIMM23	Mitochond	TIM23_HUI	2	1.031057
15_MTA / C	Q15125	-0.163	0.498394	0.394568	EBP	3-beta-hydr	EBP_HUMA	1	0.893167
15_MTA / C	Q8N5G0	0.149319	0.498508	0.394568	SMIM20	Small integ	SIM20_HUI	1	1.109046
15_MTA / C	Q9Y5U9	0.185359	0.498303	0.394568	IER3IP1	Immediate	IR3IP_HUM	1	1.1371
15_MTA / C	Q9NZ08	0.709817	0.498671	0.394602	ERAP1	Endoplasm	ERAP1_HUI	1	1.635597
30_MTA / C	Q13263	0.214884	0.498826	0.394631	TRIM28	Transcripti	TIF1B_HUM	11	1.16061
30_MTA / C	P11168	0.167379	0.499078	0.394737	SLC2A2	Solute carri	GTR2_HUM	2	1.123017
15_MTA / C	Q32P28	0.495708	0.499287	0.394807	P3H1	Prolyl 3-hy	P3H1_HUM	6	1.410013
15_MTA / C	P10809	-0.13039	0.499467	0.394855	HSPD1	60 kDa hea	CH60_HUM	37	0.913583
30_MTA / C	O14944	0.764526	0.500007	0.395052	EREG	Proepiregu	EREG_HUM	2	1.698812
30_MTA / C	P05091	0.157141	0.500828	0.39522	ALDH2	Aldehyde d	ALDH2_HU	15	1.115075
30_MTA / C	P31939	-0.16419	0.500882	0.39522	ATIC	Bifunctiona	PUR9_HUM	23	0.892427
15_MTA / C	Q9NVS9	-0.27825	0.500741	0.39522	PNPO	Pyridoxine-	PNPO_HUM	2	0.824592
15_MTA / C	P15144	0.150335	0.501488	0.395509	ANPEP	Aminopept	AMPN_HUN	3	1.109827
15_MTA / C	P06493	-0.33306	0.502297	0.395959	CDK1	Cyclin-depe	CDK1_HUM	2	0.793852
15_MTA / C	Q08357	-0.77403	0.502528	0.396047	SLC20A2	Sodium-dep	S20A2_HUI	1	0.584783
30_MTA / C	Q2QD12;Q	-0.76722	0.503165	0.396266	RPEL1;RPE	Ribulose-pl	RPEL1_HUN	1	0.587547
30_MTA / C	Q01082	-0.08006	0.504154	0.396951	SPTBN1	Spectrin be	SPTB2_HUN	23	0.946021
15_MTA / C	Q9NP79	0.526101	0.504698	0.397285	VTA1	Vacuolar pr	VTA1_HUM	2	1.440032
30_MTA / C	P62995	0.825438	0.505376	0.397553	TRA2B	Transforme	TRA2B_HUI	3	1.772073
15_MTA / C	Q0VGL1	0.911597	0.505355	0.397553	LAMTOR4	Ragulator c	LTOR4_HUI	2	1.881127
30_MTA / C	P26599	0.568096	0.505618	0.397631	PTBP1	Polypyrimi	PTBP1_HUI	2	1.482566
30_MTA / C	P15880	-0.05174	0.506529	0.398064	RPS2	Small ribos	RS2_HUMA	11	0.964772
30_MTA / C	P43034	0.0804	0.50674	0.398136	PAFAH1B1	Platelet-act	LIS1_HUMA	12	1.057311
15_MTA / C	Q9Y230	0.123382	0.506981	0.39823	RUVBL2	RuvB-like 2	RUVB2_HU	18	1.089286
15_MTA / C	Q13620	-0.14882	0.507539	0.398332	CUL4B	Cullin-4B	CUL4B_HUI	3	0.90199
30_MTA / C	Q9ULV4	0.331684	0.507498	0.398332	CORO1C	Coronin-1C	COR1C_HU	5	1.258481
30_MTA / C	Q13438	0.07813	0.50791	0.398488	OS9	Protein OS-	OS9_HUMA	1	1.055649
30_MTA / C	Q02413	-0.83659	0.50824	0.398653	DSG1	Desmogleir	DSG1_HUM	12	0.559966
30_MTA / C	P62917	-0.13743	0.508691	0.398831	RPL8	Large ribos	RL8_HUMA	10	0.90914
30_MTA / C	Q16698	-0.08324	0.508709	0.398831	DECR1	2,4-dienoyl	DECR_HUM	9	0.943937
15_MTA / C	P53985	0.390032	0.511269	0.40046	SLC16A1	Monocarbo	MOT1_HUN	3	1.310423

15_MTA / C	Q9NRA2	-0.24741	0.511176	0.40046	SLC17A5	Sialin	S17A5_HUI	2	0.842405
15_MTA / C	P25205	0.457515	0.51159	0.400617	MCM3	DNA replica	MCM3_HUI	5	1.373174
15_MTA / C	Q99614	0.491605	0.511725	0.400627	TTC1	Tetratricop	TTC1_HUM	2	1.406008
15_MTA / C	Q9Y5K8	-0.14566	0.511972	0.400684	ATP6V1D	V-type prot	VATD_HUM	4	0.903966
30_MTA / C	P08758	0.167175	0.512752	0.401053	ANXA5	Annexin A5	ANXA5_HU	11	1.122857
30_MTA / C	P40925	0.118055	0.513199	0.401238	MDH1	Malate deh	MDHC_HUI	13	1.085271
30_MTA / C	Q9Y3C8	-0.25909	0.513555	0.401396	UFC1	Ubiquitin-f	UFC1_HUM	3	0.835614
15_MTA / C	P15289	0.749939	0.514042	0.401451	ARSA	Arylsulfata:	ARSA_HUM	1	1.681722
30_MTA / C	P60842	0.142569	0.51428	0.401451	EIF4A1	Eukaryotic	IF4A1_HUM	15	1.103869
30_MTA / C	P61289	0.156397	0.514351	0.401451	PSME3	Proteasom	PSME3_HU	8	1.1145
15_MTA / C	Q96C86	0.02659	0.514135	0.401451	DCPS	m7GpppX (	DCPS_HUM	5	1.018602
30_MTA / C	Q86VP6	-0.0747	0.514618	0.40147	CAND1	Cullin-asso	CAND1_HU	26	0.94954
15_MTA / C	Q9H3U5	0.098537	0.514786	0.401507	MFSD1	Lysosomal (	MFSD1_HU	1	1.070687
30_MTA / C	P31689	0.404904	0.515225	0.401731	DNAJA1	DnaJ homo	DNJA1_HUI	3	1.324001
15_MTA / C	Q3I5F7;Q8(	0.121972	0.515625	0.401877	ACOT6;ACC	Acyl-coenzy	ACOT6_HUI	2	1.088222
30_MTA / C	Q99735	-0.07157	0.516637	0.402571	MGST2	Microsoma	MGST2_HU	1	0.951604
30_MTA / C	P02786	-0.06241	0.51708	0.402727	TFRC	Transferrin	TFR1_HUM	25	0.957662
15_MTA / C	P14854	0.439418	0.517057	0.402727	COX6B1	Cytochrom	CX6B1_HUI	2	1.356057
15_MTA / C	Q9P0L0	-0.04797	0.517241	0.402758	VAPA	Vesicle-asso	VAPA_HUM	8	0.967297
30_MTA / C	P51452	-0.653	0.517908	0.403087	DUSP3	Dual specifi	DUS3_HUM	2	0.635956
30_MTA / C	P40926	0.154317	0.518451	0.403415	MDH2	Malate deh	MDHM_HU	17	1.112894
15_MTA / C	P0C221	-0.06424	0.518832	0.403427	CCDC175	Coiled-coil	CC175_HUI	1	0.956452
30_MTA / C	P35268	0.207057	0.518827	0.403427	RPL22	Large ribos	RL22_HUM	2	1.154331
15_MTA / C	Q9Y285	-0.23493	0.518965	0.403436	FARSA	Phenylalan	SYFA_HUM	2	0.849727
15_MTA / C	O75915	0.022665	0.520275	0.404265	ARL6IP5	PRA1 famil	PRAF3_HUI	1	1.015834
30_MTA / C	Q9NUU7;Q	0.070785	0.520183	0.404265	DDX19A;DI	ATP-depend	DD19A_HU	2	1.050288
15_MTA / C	Q8NBJ5	-0.09283	0.521312	0.404881	COLGALT1	Procollager	GT251_HUI	10	0.937684
15_MTA / C	O43633	-0.53183	0.521722	0.40501	CHMP2A	Charged mu	CHM2A_HU	1	0.691675
15_MTA / C	P00505	-0.1287	0.52172	0.40501	GOT2	Aspartate a	AATM_HUM	24	0.914653
15_MTA / C	O43676	-0.19172	0.522325	0.405383	NDUFB3	NADH dehy	NDUB3_HU	2	0.875561
30_MTA / C	P30626	-0.16119	0.522642	0.405534	SRI	Sorcin	SORCN_HU	4	0.894288
30_MTA / C	P24666	-0.17778	0.522819	0.405576	ACP1	Low molec	PPAC_HUM	3	0.884062
30_MTA / C	P53004	0.107365	0.523338	0.405694	BLVRA	Biliverdin r	BIEA_HUM	4	1.077259
15_MTA / C	Q9NQC3	-0.03932	0.523221	0.405694	RTN4	Reticulon-4	RTN4_HUM	7	0.973113
30_MTA / C	P07203	-0.35285	0.523748	0.405727	GPX1	Glutathion	GPX1_HUM	2	0.783036
30_MTA / C	Q9BV81	0.010243	0.523649	0.405727	EMC6	ER membra	EMC6_HUM	1	1.007125
15_MTA / C	P62277	0.000926	0.523996	0.405799	RPS13	Small ribos	RS13_HUM	7	1.000642
30_MTA / C	Q9BXP5	0.553038	0.524576	0.405799	SRRT	Serrate RN/	SRRT_HUM	1	1.467172
15_MTA / C	A6NGU5;P1	0.049973	0.524853	0.405824	GGT3P;GG	Putative glu	GGT3_HUM	3	1.035246
15_MTA / C	Q8TC12	-0.20932	0.52481	0.405824	RDH11	Retinol deh	RDH11_HU	1	0.864946
30_MTA / C	P13164;Q(	0.10534	0.525108	0.40583	IFITM1;IFIT	Interferon-i	IFM1_HUM	1	1.075748
15_MTA / C	P46776	-0.0105	0.525227	0.40583	RPL27A	Large ribos	RL27A_HUI	4	0.992747
30_MTA / C	Q15942	0.076129	0.525215	0.40583	ZYX	Zyxin	ZYX_HUMA	10	1.054186
30_MTA / C	P22314	-0.29978	0.525867	0.406135	UBA1	Ubiquitin-l	UBA1_HUM	9	0.812374
30_MTA / C	Q9NTM9	-0.0906	0.527064	0.406964	CUTC	Copper hor	CUTC_HUM	1	0.939133
30_MTA / C	P09382	-0.30092	0.527523	0.407179	LGALS1	Galectin-1	LEG1_HUM	5	0.811733
15_MTA / C	P17987	-0.10788	0.527965	0.407297	TCP1	T-complex (	TCPA_HUM	18	0.927953
30_MTA / C	Q15393	0.064813	0.528356	0.407297	SF3B3	Splicing fac	SF3B3_HUI	20	1.045949
15_MTA / C	Q8NHP8	0.19191	0.528139	0.407297	PLBD2	Putative ph	PLBL2_HUI	1	1.142275
30_MTA / C	P02533	0.18003	0.528502	0.407315	KRT14	Keratin, typ	K1C14_HUI	22	1.132907
30_MTA / C	P30050	0.068047	0.529058	0.407554	RPL12	Large ribos	RL12_HUM	5	1.048296
30_MTA / C	O60869	0.117026	0.529789	0.408022	EDF1	Endothelial	EDF1_HUM	5	1.084497

30_MTA / C	P11166	-0.30632	0.530088	0.408148	SLC2A1	Solute carri	GTR1_HUM	1	0.808703
15_MTA / C	Q13247	-0.04963	0.531279	0.40879	SRSF6	Serine/argi	SRSF6_HUM	7	0.966187
15_MTA / C	P31150	-0.17646	0.531558	0.408824	GDI1	Rab GDP di	GDIA_HUM	3	0.884873
15_MTA / C	P23381	0.01807	0.532633	0.409547	WARS1	Tryptophar	SYWC_HUM	9	1.012604
30_MTA / C	P48047	0.098891	0.532766	0.409554	ATP5PO	ATP synthas	ATPO_HUM	8	1.07095
15_MTA / C	P27816	0.239879	0.533567	0.410075	MAP4	Microtubul	MAP4_HUM	22	1.180894
30_MTA / C	Q5JS37	0.14607	0.53374	0.410076	NHLRC3	NHL repeat	NHLC3_HU	1	1.106551
15_MTA / C	Q9NZL4	0.126172	0.534049	0.410076	HSPBP1	Hsp70-binc	HPBP1_HU	1	1.091394
30_MTA / C	Q07812	0.059588	0.535711	0.410917	BAX	Apoptosis r	BAX_HUMA	5	1.042168
15_MTA / C	Q9BU61	0.522542	0.535496	0.410917	NDUFAF3	NADH dehy	NDUF3_HU	2	1.436484
15_MTA / C	Q03252	0.504334	0.536043	0.410929	LMNB2	Lamin-B2	LMNB2_HU	3	1.418469
30_MTA / C	L0R6Q1	-0.12217	0.538252	0.412199	SLC35A4	SLC35A4 up	S35U4_HU	4	0.918807
15_MTA / C	P17655	0.255756	0.538214	0.412199	CAPN2	Calpain-2 c	CAN2_HUM	1	1.193961
30_MTA / C	P27708	-0.92633	0.538326	0.412199	CAD	Multifuncti	PYR1_HUM	4	0.526194
30_MTA / C	P61758	0.369389	0.538446	0.412199	VBP1	Prefoldin su	PFD3_HUM	5	1.291806
15_MTA / C	P48506	0.685453	0.539027	0.412549	GCLC	Glutamate-	GSH1_HUM	1	1.608207
15_MTA / C	Q9BRA2	0.074503	0.539195	0.412582	TXNDC17	Thioredoxi	TXD17_HU	3	1.052998
15_MTA / C	P13010	0.05556	0.539628	0.412723	XRCC5	X-ray repair	XRCC5_HU	10	1.039263
15_MTA / C	Q86VP6	0.020619	0.540063	0.412865	CAND1	Cullin-asso	CAND1_HU	26	1.014395
30_MTA / C	Q96C86	-0.16286	0.540818	0.413251	DCPS	m7GpppX d	DCPS_HUM	5	0.893255
30_MTA / C	P82979	-0.18057	0.541479	0.413661	SARNP	SAP domair	SARNP_HU	7	0.882354
30_MTA / C	P56199	-0.30478	0.541627	0.413679	ITGA1	Integrin alp	ITA1_HUMA	1	0.809568
30_MTA / C	Q9NR28	-0.09747	0.541985	0.413723	DIABLO	Diablo IAP-	DBLOH_HU	5	0.934671
15_MTA / C	Q14697	0.009542	0.543186	0.414296	GANAB	Neutral alp	GANAB_HU	25	1.006636
15_MTA / C	P07942	0.28868	0.544293	0.414883	LAMB1	Laminin sul	LAMB1_HU	4	1.221522
30_MTA / C	Q9Y3U8	0.028317	0.544221	0.414883	RPL36	Large ribos	RL36_HUM	3	1.019821
15_MTA / C	Q9Y570	0.008004	0.54433	0.414883	PPME1	Protein pho	PPME1_HU	1	1.005563
30_MTA / C	P48637	-0.08608	0.54497	0.415084	GSS	Glutathion	GSHB_HUM	20	0.942078
15_MTA / C	Q5JSH3	0.326251	0.544908	0.415084	WDR44	WD repeat-	WDR44_HU	3	1.253751
15_MTA / C	P47755	0.259446	0.545564	0.415346	CAPZA2	F-actin-cap	CAZA2_HU	4	1.197019
15_MTA / C	Q16543	0.319545	0.546087	0.415553	CDC37	Hsp90 co-c	CDC37_HU	2	1.247937
30_MTA / C	P49736	-0.46861	0.546232	0.415567	MCM2	DNA replica	MCM2_HU	5	0.722663
15_MTA / C	Q3SY84	-0.45317	0.546423	0.415617	KRT71	Keratin, typ	K2C71_HU	1	0.730438
30_MTA / C	P04114	0.37328	0.546726	0.415753	APOB	Apolipopro	APOB_HUM	27	1.295295
30_MTA / C	Q96FW1	-0.26632	0.5471	0.415904	OTUB1	Ubiquitin t	OTUB1_HU	2	0.831438
30_MTA / C	O43399	0.235575	0.547726	0.41618	TPD52L2	Tumor prot	TPD54_HU	9	1.177376
15_MTA / C	P40926	-0.14827	0.548477	0.416415	MDH2	Malate deh	MDHM_HU	17	0.902329
15_MTA / C	Q15070	0.844645	0.548407	0.416415	OXA1L	Mitochond	OXA1L_HU	1	1.795822
15_MTA / C	O15400	-0.10734	0.548921	0.416657	STX7	Syntaxin-7	STX7_HUM	6	0.928295
30_MTA / C	P26373	-0.08255	0.549157	0.416741	RPL13	Large ribos	RL13_HUM	7	0.944384
30_MTA / C	O75348;O9	1.133445	0.550865	0.417655	ATP6V1G1;	V-type prot	VATG1_HU	1	2.19382
30_MTA / C	P36957	-0.19405	0.551257	0.417655	DLST	Dihydrolip	ODO2_HUM	9	0.874149
30_MTA / C	P46109	1.036159	0.55133	0.417655	CRKL	Crk-like pro	CRKL_HUM	1	2.050761
30_MTA / C	P49589	0.410926	0.550947	0.417655	CARS1	Cysteine--tI	SYCC_HUM	3	1.329539
30_MTA / C	Q93050	0.13465	0.551496	0.417655	ATP6V0A1	V-type prot	VPP1_HUM	4	1.097827
15_MTA / C	Q9H3K6	-0.41503	0.55106	0.417655	BOLA2	BolA-like pr	BOLA2_HU	2	0.750005
30_MTA / C	Q9NQC3	-0.16349	0.550695	0.417655	RTN4	Reticulon-4	RTN4_HUM	7	0.892864
15_MTA / C	Q15043	0.452119	0.552061	0.417898	SLC39A14	Metal catio	S39AE_HUI	1	1.368048
15_MTA / C	Q9Y5P6	-0.07436	0.552069	0.417898	GMPPB	Mannose-1	GMPPB_HU	4	0.949761
15_MTA / C	P62820	0.701807	0.553239	0.418628	RAB1A	Ras-related	RAB1A_HU	6	1.626541
30_MTA / C	P40227	0.01467	0.554088	0.418948	CCT6A	T-complex	TCPZ_HUM	17	1.01022
15_MTA / C	P54136	0.184393	0.554069	0.418948	RARS1	Arginine--tI	SYRC_HUM	4	1.136338

15_MTA / C	P31942	-0.95714	0.554515	0.419175	HNRNPH3	Heterogene	HNRH3_HU	2	0.515077
15_MTA / C	P41091	0.056694	0.555837	0.419983	EIF2S3	Eukaryotic	IF2G_HUMA	16	1.04008
15_MTA / C	Q00325	0.195491	0.556222	0.420178	SLC25A3	Solute carri	S25A3_HUI	13	1.145114
30_MTA / C	Q8WTV0	0.141812	0.556906	0.420486	SCARB1	Scavenger r	SCRB1_HUI	2	1.10329
30_MTA / C	P78347	0.701006	0.557776	0.420776	GTF2I	General tra	GTF2I_HUN	1	1.625638
15_MTA / C	Q9H2H9	-0.04537	0.557694	0.420776	SLC38A1	Sodium-co	S38A1_HUI	2	0.969041
15_MTA / C	O14732	0.117587	0.558172	0.420825	IMPA2	Inositol mc	IMPA2_HUI	2	1.084919
30_MTA / C	P15924	-0.29101	0.558232	0.420825	DSP	Desmoplak	DESP_HUM	32	0.817328
30_MTA / C	Q9NWT1	-0.17454	0.558299	0.420825	PAK1IP1	p21-activat	PK1IP_HUN	2	0.88605
30_MTA / C	Q9Y6B6	0.53183	0.558349	0.420825	SAR1B	Small COPII	SAR1B_HUI	1	1.445762
30_MTA / C	P13667	0.089078	0.558644	0.420952	PDIA4	Protein dis	PDIA4_HUN	28	1.06369
30_MTA / C	Q8NFH4	-0.03379	0.559284	0.421339	NUP37	Nucleopori	NUP37_HU	2	0.976847
15_MTA / C	O95684	0.36699	0.559561	0.421452	CEP43	Centrosom	CEP43_HUI	1	1.28966
30_MTA / C	P33992	-0.13066	0.560143	0.421794	MCM5	DNA replica	MCM5_HUI	5	0.913413
15_MTA / C	Q9NXH9	-0.26416	0.560427	0.421912	TRMT1	tRNA (guan	TRM1_HUN	3	0.832683
15_MTA / C	P20810	0.194207	0.561135	0.422253	CAST	Calpastatin	ICAL_HUMA	4	1.144095
15_MTA / C	P36776	0.345895	0.561291	0.422274	LONP1	Lon protea	LONM_HUN	9	1.270939
30_MTA / C	Q9BTL3	0.018034	0.561625	0.422429	RAMAC	RNA guanir	RAMAC_HL	1	1.012579
15_MTA / C	Q16698	0.199522	0.561955	0.422582	DECR1	2,4-dienoyl	DECR_HUM	9	1.148318
15_MTA / C	O14828	-0.01873	0.562165	0.422616	SCAMP3	Secretory c	SCAM3_HU	6	0.987098
30_MTA / C	Q9BRA2	-0.37665	0.562256	0.422616	TXNDC17	Thioredoxi	TXD17_HUI	3	0.770227
30_MTA / C	Q9GZM5	-0.24655	0.563277	0.423288	YIPF3	Protein YIP	YIPF3_HUN	3	0.842908
30_MTA / C	Q13740	0.253964	0.563589	0.423345	ALCAM	CD166 anti	CD166_HU	4	1.192479
15_MTA / C	Q9NRR5	-0.19129	0.564161	0.423568	UBQLN4	Ubiquilin-4	UBQL4_HU	2	0.875823
15_MTA / C	Q9HCC0	0.221304	0.564656	0.423844	MCCC2	Methylcrot	MCCB_HUN	7	1.165787
15_MTA / C	P06454	0.77983	0.565183	0.42412	PTMA	Prothymos	PTMA_HUN	3	1.716928
30_MTA / C	Q13907	-0.04831	0.565281	0.42412	IDI1	Isopenteny	IDI1_HUMA	1	0.967066
15_MTA / C	P14406	0.14219	0.566033	0.424493	COX7A2	Cytochrom	CX7A2_HUI	1	1.103579
30_MTA / C	Q0VGL1	0.692394	0.566192	0.424516	LAMTOR4	Ragulator c	LTOR4_HUI	2	1.615963
15_MTA / C	Q15084	-0.11119	0.566322	0.424517	PDIA6	Protein dis	PDIA6_HUN	12	0.925824
30_MTA / C	Q9NS69	-0.17903	0.567937	0.425439	TOMM22	Mitochond	TOM22_HU	3	0.883294
30_MTA / C	Q8NBQ5	-0.03051	0.568295	0.425563	HSD17B11	Estradiol 1	DHB11_HU	4	0.979077
30_MTA / C	Q9NVA2	0.074435	0.568616	0.425563	SEPTIN11	Septin-11	SEP11_HUN	12	1.052948
30_MTA / C	Q9NWU1	0.020679	0.568448	0.425563	OXSM	3-oxoacyl-[	OXSM_HUN	3	1.014437
30_MTA / C	P48059	-0.47748	0.569233	0.425832	LIMS1	LIM and ser	LIMS1_HUN	1	0.718232
30_MTA / C	Q96HC4	0.007407	0.569116	0.425832	PDLIM5	PDZ and LIN	PDLI5_HUN	3	1.005147
15_MTA / C	Q92896	0.326786	0.570218	0.426089	GLG1	Golgi appar	GSLG1_HUI	8	1.254216
30_MTA / C	P0DPB6	-0.02699	0.570701	0.42622	POLR1D	DNA-direct	RPAC2_HUI	1	0.981467
15_MTA / C	P15880	-0.01412	0.571013	0.42622	RPS2	Small ribos	RS2_HUMA	11	0.990259
30_MTA / C	P49902	-0.113	0.571294	0.42622	NT5C2	Cytosolic p	5NTC_HUN	8	0.924662
15_MTA / C	P60903	0.261486	0.571151	0.42622	S100A10	Protein S1(	S10AA_HUI	1	1.198713
30_MTA / C	P83731	-0.14452	0.571659	0.426396	RPL24	Large ribos	RL24_HUM	5	0.904678
30_MTA / C	P47755	0.309175	0.572699	0.426788	CAPZA2	F-actin-cap	CAZA2_HUI	4	1.238999
15_MTA / C	P54709	0.006639	0.572431	0.426788	ATP1B3	Sodium/po	AT1B3_HUI	6	1.004612
30_MTA / C	Q14126	-0.14178	0.572553	0.426788	DSG2	Desmoglein	DSG2_HUN	2	0.906402
30_MTA / C	Q15404	-0.07361	0.572903	0.426843	RSU1	Ras suppres	RSU1_HUN	2	0.950257
15_MTA / C	P05089	-0.47491	0.573523	0.427209	ARG1	Arginase-1	ARGI1_HUN	3	0.719511
15_MTA / C	Q7L5Y1	0.955851	0.57401	0.427476	ENOSF1	Mitochond	ENOF1_HUI	2	1.939724
30_MTA / C	P39748	-0.04459	0.5743	0.4275	FEN1	Flap endon	FEN1_HUM	1	0.969564
30_MTA / C	Q6L8Q7	-0.06498	0.574713	0.427616	PDE12	2',5'-phosp	PDE12_HUI	4	0.955961
30_MTA / C	Q96KP4	-0.11891	0.575263	0.427929	CNDP2	Cytosolic n	CNDP2_HU	9	0.920885
30_MTA / C	P09651	-0.19203	0.575589	0.427933	HNRNPA1	Heterogene	ROA1_HUN	4	0.875371

30_MTA / C	P31942	-1.00178	0.575816	0.427933	HNRNPH3	Heterogene	HNRH3_HU	2	0.499383
30_MTA / C	Q00325	-0.02945	0.576044	0.427933	SLC25A3	Solute carri	S25A3_HUI	13	0.979791
15_MTA / C	Q9NUQ9	0.333177	0.575723	0.427933	CYRIB	CYFIP-relat	CYRIB_HUM	2	1.259785
15_MTA / C	Q16822	0.103249	0.576575	0.428136	PCK2	Phosphoen	PCKGM_HU	4	1.07419
15_MTA / C	Q5RKV6	0.03903	0.57649	0.428136	EXOSC6	Exosome co	EXOS6_HUI	4	1.027423
15_MTA / C	Q8NE71	0.153803	0.57691	0.428288	ABCF1	ATP-bindin	ABCF1_HUI	5	1.112498
30_MTA / C	Q96GA7	-0.10267	0.577203	0.428393	SDSL	Serine dehy	SDSL_HUM	4	0.931309
15_MTA / C	Q9BT73	-0.31908	0.577439	0.428393	PSMG3	Proteasom	PSMG3_HU	3	0.80158
15_MTA / C	O75414	0.667009	0.578343	0.428872	NME6	Nucleoside	NDK6_HUM	1	1.587778
30_MTA / C	P02753	0.095159	0.580263	0.42952	RBP4	Retinol-bin	RET4_HUM	2	1.068183
15_MTA / C	P20338	0.44774	0.580053	0.42952	RAB4A	Ras-related	RAB4A_HUI	4	1.363902
15_MTA / C	Q6YHK3	-0.36809	0.580119	0.42952	CD109	CD109 anti	CD109_HU	2	0.774807
15_MTA / C	Q92688	-0.02614	0.580336	0.42952	ANP32B	Acidic leuci	AN32B_HU	5	0.982047
30_MTA / C	Q9H0W9	-0.03182	0.579756	0.42952	C11orf54	Ester hydro	CK054_HUI	4	0.978187
30_MTA / C	Q9Y6D0	-0.68474	0.579448	0.42952	SELENOK	Selenoprot	SELK_HUM	1	0.622117
30_MTA / C	O00233	-0.4807	0.580732	0.429564	PSMD9	26S protea	PSMD9_HU	2	0.716631
30_MTA / C	Q13126	-0.29144	0.580832	0.429564	MTAP	S-methyl-5'	MTAP_HUM	6	0.817084
15_MTA / C	P62280	-0.01839	0.580979	0.429577	RPS11	Small ribos	RS11_HUM	12	0.987335
15_MTA / C	P17612;P2	-0.23617	0.582093	0.430208	PRKACA;PR	cAMP-depe	KAPCA_HUI	1	0.848996
30_MTA / C	Q9H074	0.20607	0.582092	0.430208	PAIP1	Polyadenyl	PAIP1_HUM	3	1.153541
30_MTA / C	O60256	-0.03344	0.582342	0.43025	PRPSAP2	Phosphorik	KPRB_HUM	3	0.977087
30_MTA / C	Q9NVA1	0.209603	0.582408	0.43025	UQCC1	Ubiquinol-(	UQCC1_HU	2	1.15637
30_MTA / C	Q9Y3F4	0.061092	0.583747	0.431046	STRAP	Serine-thre	STRAP_HUM	10	1.043255
30_MTA / C	Q96I99	-0.42563	0.583988	0.431129	SUCLG2	Succinate--	SUCB2_HUI	1	0.744514
15_MTA / C	Q9H1E3	0.079584	0.585459	0.432022	NUCKS1	Nuclear ubi	NUCKS_HU	1	1.056713
15_MTA / C	Q9Y266	0.143826	0.585873	0.432135	NUDC	Nuclear mi	NUDC_HUM	7	1.104831
30_MTA / C	P13073	0.164894	0.5863	0.432296	COX4I1	Cytochrom	COX41_HU	9	1.121083
30_MTA / C	O75477	0.961882	0.586882	0.432494	ERLIN1	Erlin-1	ERLN1_HUI	5	1.947849
15_MTA / C	Q9Y639	-0.15487	0.587205	0.432636	NPTN	Neuroplast	NPTN_HUM	2	0.898216
15_MTA / C	P38606	-0.06415	0.587499	0.432757	ATP6V1A	V-type prot	VATA_HUM	8	0.956507
30_MTA / C	O95218	-0.07983	0.587734	0.432833	ZRANB2	Zinc finger	ZRAB2_HUI	3	0.946169
30_MTA / C	O95297	-0.21286	0.588316	0.433099	MPZL1	Myelin prot	MPZL1_HU	2	0.862826
30_MTA / C	Q9BZX2	0.355729	0.588356	0.433099	UCK2	Uridine-cyt	UCK2_HUM	4	1.279632
15_MTA / C	P33991	1.061263	0.588612	0.433191	MCM4	DNA replica	MCM4_HUI	2	2.086758
30_MTA / C	Q9Y6N7	-0.05272	0.590756	0.434383	ROBO1	Roundabou	ROBO1_HU	8	0.96412
15_MTA / C	Q99627	0.101008	0.591008	0.434465	COPS8	COP9 signa	CSN8_HUM	3	1.072523
30_MTA / C	Q15631	0.243064	0.591335	0.434519	TSN	Translin	TSN_HUMA	11	1.183503
30_MTA / C	P61020	-0.39817	0.592711	0.435145	RAB5B	Ras-related	RAB5B_HUI	2	0.75882
30_MTA / C	P07954	-0.19824	0.593289	0.43547	FH	Fumarate h	FUMH_HUM	4	0.871614
30_MTA / C	Q8NEW0	-0.08895	0.593417	0.43547	SLC30A7	Zinc transp	ZNT7_HUM	4	0.940209
15_MTA / C	O95295	0.878778	0.59413	0.435861	SNAPIN	SNARE-asso	SNAPN_HU	1	1.838817
30_MTA / C	P68363	-0.11715	0.594857	0.436237	TUBA1B	Tubulin alp	TBA1B_HUI	18	0.922007
30_MTA / C	P49959	-0.25938	0.595111	0.436327	MRE11	Double-stra	MRE11_HU	3	0.835444
30_MTA / C	P07339	0.057946	0.595551	0.436553	CTSD	Cathepsin I	CATD_HUM	7	1.040982
30_MTA / C	A6PVC2	-0.48915	0.596488	0.43659	TTLL8	Protein mo	TTLL8_HUM	2	0.712443
30_MTA / C	P02760	0.456268	0.596297	0.43659	AMBP	Protein AM	AMBP_HUM	1	1.371989
30_MTA / C	P15289	0.171859	0.596655	0.43659	ARSA	Arylsulfata	ARSA_HUM	1	1.126509
30_MTA / C	P61313	-0.01032	0.595777	0.43659	RPL15	Large ribos	RL15_HUM	11	0.992871
30_MTA / C	Q9H3U5	-0.90459	0.59627	0.43659	MFSD1	Lysosomal	MFSD1_HU	1	0.534183
30_MTA / C	Q9UBM7	1.105341	0.596334	0.43659	DHCR7	7-dehydroc	DHCR7_HU	1	2.151497
15_MTA / C	O95772	0.16628	0.597183	0.436879	STARD3NL	STARD3 N-t	STR3N_HUI	1	1.122161
30_MTA / C	Q9NX14	0.090722	0.597529	0.437036	NDUFB11	NADH dehy	NDUBB_HU	1	1.064903

30_MTA / C	P49773	-0.1602	0.598114	0.437321	HINT1	Adenosine !	HINT1_HUI		3	0.894899
30_MTA / C	P52907	0.041313	0.598239	0.437321	CAPZA1	F-actin-cap	CAZA1_HUI		3	1.02905
30_MTA / C	Q07666	0.073386	0.598534	0.437385	KHDRBS1	KH domain	KHDR1_HU		1	1.052183
30_MTA / C	O95400	-0.27569	0.599296	0.437749	CD2BP2	CD2 antiger	CD2B2_HU		1	0.826053
15_MTA / C	Q15417	0.295664	0.599499	0.4378	CNN3	Calponin-3	CNN3_HUN		4	1.227449
15_MTA / C	P11940	-0.02548	0.599719	0.437865	PABPC1	Polyadenyl	PABP1_HU		15	0.982494
15_MTA / C	O15258	0.420383	0.600182	0.438106	RER1	Protein REF	RER1_HUM		4	1.338283
15_MTA / C	O00754	0.10415	0.600771	0.438172	MAN2B1	Lysosomal a	MA2B1_HU		6	1.074861
30_MTA / C	O95445	-1.21103	0.600922	0.438172	APOM	Apolipoprc	APOM_HUN		1	0.43196
30_MTA / C	P16949	0.207012	0.60076	0.438172	STMN1	Stathmin	STMN1_HU		6	1.154295
15_MTA / C	Q9Y5J9	-0.11	0.600933	0.438172	TIMM8B	Mitochond	TIM8B_HUI		3	0.926586
15_MTA / C	P62266	-0.08861	0.602433	0.439072	RPS23	Small ribos	RS23_HUM		6	0.940429
15_MTA / C	Q16851	-0.00216	0.602846	0.43918	UGP2	UTP--glucos	UGPA_HUN		11	0.998507
30_MTA / C	P11413	-0.10021	0.60419	0.439768	G6PD	Glucose-6-p	G6PD_HUN		11	0.932899
30_MTA / C	P35237	-0.00335	0.604042	0.439768	SERPINB6	Serpin B6	SPB6_HUM		7	0.997683
30_MTA / C	P52788	0.140908	0.60406	0.439768	SMS	Spermine sy	SPSY_HUM/		3	1.102599
30_MTA / C	Q9NZL4	-0.47553	0.603884	0.439768	HSPBP1	Hsp70-binc	HPBP1_HU		1	0.719201
15_MTA / C	P39019	-0.05533	0.606786	0.44126	RPS19	Small ribos	RS19_HUM		7	0.962376
30_MTA / C	P55884	0.023386	0.606899	0.44126	EIF3B	Eukaryotic	EIF3B_HUN		8	1.016342
30_MTA / C	Q9Y5U9	-0.90632	0.60659	0.44126	IER3IP1	Immediate	IR3IP_HUN		1	0.533542
30_MTA / C	P16615	-0.09103	0.607715	0.441659	ATP2A2	Sarcoplasm	AT2A2_HUI		16	0.938853
15_MTA / C	P50454	0.247283	0.607695	0.441659	SERPINH1	Serpin H1	SERPH_HUI		5	1.18697
15_MTA / C	Q9GZT8	0.173877	0.608396	0.442058	NIF3L1	NIF3-like pr	NIF3L_HUN		6	1.128086
15_MTA / C	P16220	0.713909	0.608736	0.442111	CREB1	Cyclic AMP	CREB1_HUI		3	1.640242
15_MTA / C	Q9H3U1	-0.65823	0.608709	0.442111	UNC45A	Protein unc	UN45A_HU		1	0.633655
15_MTA / C	P46782	0.131058	0.609048	0.442237	RPS5	Small ribos	RS5_HUMA		8	1.095097
30_MTA / C	Q16658	-0.11446	0.609177	0.442237	FSCN1	Fascin	FSCN1_HUI		2	0.923726
30_MTA / C	P27695	-0.40917	0.609475	0.44226	APEX1	DNA repair	APEX1_HUI		1	0.753058
15_MTA / C	P49189	-0.04012	0.609401	0.44226	ALDH9A1	4-trimethyl	AL9A1_HUI		12	0.972572
30_MTA / C	Q7L5D6	-0.24407	0.609768	0.442376	GET4	Golgi to ER	GET4_HUM		2	0.844359
15_MTA / C	P31937	0.131287	0.610601	0.442883	HIBADH	3-hydroxyis	3HIDH_HUI		5	1.095271
30_MTA / C	O43505	-0.31245	0.611113	0.443061	B4GAT1	Beta-1,4-gl	B4GA1_HU		1	0.805273
30_MTA / C	Q15717	0.551519	0.611451	0.443208	ELAVL1	ELAV-like pr	ELAV1_HUN		1	1.465628
30_MTA / C	O15144	0.117021	0.612534	0.443392	ARPC2	Actin-relate	ARPC2_HUI		9	1.084493
15_MTA / C	O43390	0.128767	0.612464	0.443392	HNRNPR	Heterogene	HNRPR_HU		5	1.093359
15_MTA / C	O95832	-0.08585	0.612238	0.443392	CLDN1	Claudin-1	CLD1_HUM		3	0.94223
30_MTA / C	P0C221	0.102173	0.612171	0.443392	CCDC175	Coiled-coil	CC175_HUI		1	1.073389
15_MTA / C	P11177	-0.31278	0.61264	0.443392	PDHB	Pyruvate de	ODPB_HUN		2	0.80509
15_MTA / C	Q5W111	0.184056	0.612259	0.443392	SPRYD7	SPRY doma	SPRY7_HUI		1	1.136073
30_MTA / C	P41236	-0.09114	0.613338	0.443466	PPP1R2	Protein pho	IPP2_HUM/		5	0.938781
15_MTA / C	Q00534	0.032098	0.613948	0.443466	CDK6	Cyclin-depe	CDK6_HUN		3	1.022498
15_MTA / C	Q14847	0.114243	0.613495	0.443466	LASP1	LIM and SH	LASP1_HUI		10	1.082407
30_MTA / C	Q969M3	-0.2133	0.613566	0.443466	YIPF5	Protein YIP	YIPF5_HUN		1	0.862562
15_MTA / C	Q96EK6	0.132718	0.613032	0.443466	GNPNAT1	Glucosamir	GNA1_HUN		4	1.096357
15_MTA / C	Q9H444	-0.17311	0.613363	0.443466	CHMP4B	Charged mu	CHM4B_HU		3	0.886929
30_MTA / C	Q9NZL9	0.178244	0.613871	0.443466	MAT2B	Methionine	MAT2B_HU		7	1.131506
15_MTA / C	Q9Y6M5	0.069861	0.615353	0.444287	SLC30A1	Proton-cou	ZNT1_HUM		4	1.049616
15_MTA / C	P05198	0.084054	0.616079	0.444715	EIF2S1	Eukaryotic	IF2A_HUM/		11	1.059992
30_MTA / C	P30101	0.082027	0.616556	0.444866	PDIA3	Protein dis	PDIA3_HUN		29	1.058504
30_MTA / C	Q9UHN6	0.208304	0.616484	0.444866	CEMIP2	Inactive cel	CEIP2_HUN		12	1.15533
30_MTA / C	Q12765	0.050325	0.616845	0.444958	SCRN1	Secernin-1	SCRN1_HUI		3	1.035498
30_MTA / C	Q9GZZ8	0.536018	0.618168	0.44564	LACRT	Extracellula	LACRT_HUN		1	1.449965

15_MTA / C	Q8NFH3	0.249331	0.618884	0.445865	NUP43	Nucleopori	NUP43_HU	4	1.188656
30_MTA / C	P47914	0.373623	0.619289	0.445891	RPL29	Large ribos	RL29_HUM	2	1.295603
30_MTA / C	Q53FA7	0.136057	0.619323	0.445891	TP53I3	Quinone ox	QORX_HUM	6	1.098897
15_MTA / C	P24752	0.065959	0.619802	0.446139	ACAT1	Acetyl-CoA	THIL_HUMA	13	1.046781
15_MTA / C	Q8TEA8	-0.17214	0.62056	0.446491	DTD1	D-aminoac	DTD1_HUM	1	0.887528
15_MTA / C	P09601	0.297584	0.62093	0.446567	HMOX1	Heme oxyg	HMOX1_HU	4	1.229084
30_MTA / C	P54727	0.07232	0.620936	0.446567	RAD23B	UV excision	RD23B_HU	11	1.051406
30_MTA / C	Q86Y82	-0.0076	0.621889	0.447076	STX12	Syntaxin-12	STX12_HUM	5	0.994745
30_MTA / C	Q96B26	0.370039	0.621913	0.447076	EXOSC8	Exosome cc	EXOS8_HUI	3	1.292387
30_MTA / C	P26368	-0.08258	0.622839	0.447451	U2AF2	Splicing fac	U2AF2_HUI	1	0.94437
30_MTA / C	P33991	0.728375	0.622633	0.447451	MCM4	DNA replica	MCM4_HUI	2	1.656772
30_MTA / C	P60981	-0.1376	0.622792	0.447451	DSTN	Destrin	DEST_HUM	3	0.909032
15_MTA / C	Q16401	0.436328	0.623324	0.447702	PSMD5	26S proteas	PSMD5_HU	4	1.353156
30_MTA / C	P15170	-0.25024	0.623545	0.447748	GSPT1	Eukaryotic	ERF3A_HUM	5	0.840757
15_MTA / C	P61513	0.299408	0.623659	0.447748	RPL37A	Large ribos	RL37A_HUI	1	1.23064
30_MTA / C	Q9H2J4	0.135198	0.623943	0.447758	PDCL3	Phosducin-	PDCL3_HUI	3	1.098244
15_MTA / C	P18085	-0.0528	0.62455	0.447903	ARF4	ADP-ribosy	ARF4_HUM	4	0.964063
30_MTA / C	P61513	0.007299	0.624401	0.447903	RPL37A	Large ribos	RL37A_HUI	1	1.005072
15_MTA / C	Q02543	0.300402	0.624472	0.447903	RPL18A	Large ribos	RL18A_HUI	6	1.231487
30_MTA / C	P17844	0.256019	0.624761	0.447957	DDX5	Probable AT	DDX5_HUM	7	1.194179
15_MTA / C	Q9NTM9	-0.09213	0.625242	0.448205	CUTC	Copper hor	CUTC_HUM	1	0.938136
30_MTA / C	Q07817	0.323363	0.625734	0.448448	BCL2L1	Bcl-2-like p	B2CL1_HUI	1	1.251244
15_MTA / C	Q4KWH8	-0.21705	0.625987	0.448448	PLCH1	1-phosphat	PLCH1_HUI	1	0.86032
15_MTA / C	Q7Z7H5	-0.2437	0.625938	0.448448	TMED4	Transmemb	TMED4_HU	2	0.844578
30_MTA / C	P06756	0.148959	0.626879	0.448893	ITGAV	Integrin alp	ITAV_HUMA	1	1.108769
15_MTA / C	P62316	0.021255	0.627562	0.449091	SNRPD2	Small nucle	SMD2_HUM	6	1.014842
30_MTA / C	P15311	-0.09117	0.628013	0.449263	EZR	Ezrin	EZRI_HUMA	10	0.938764
15_MTA / C	P49207	0.052723	0.628073	0.449263	RPL34	Large ribos	RL34_HUM	5	1.037221
30_MTA / C	P06396	-0.06309	0.628434	0.449332	GSN	Gelsolin	GELS_HUM	1	0.957214
30_MTA / C	P49368	0.075179	0.628442	0.449332	CCT3	T-complex p	TCPG_HUM	20	1.053492
30_MTA / C	P62312	0.215442	0.628904	0.449566	LSM6	U6 snRNA-a	LSM6_HUM	6	1.161059
15_MTA / C	Q9P0I2	0.699321	0.629237	0.449707	EMC3	ER membra	EMC3_HUM	2	1.62374
30_MTA / C	Q06323	-0.47419	0.629733	0.449964	PSME1	Proteasom	PSME1_HU	6	0.719869
15_MTA / C	Q9NPF0	-0.06359	0.631099	0.450843	CD320	CD320 anti	CD320_HU	1	0.956881
15_MTA / C	Q9NP97	-0.15113	0.631641	0.451133	DYNLRB1	Dynein ligh	DLRB1_HUI	3	0.900543
15_MTA / C	P31946	-0.08436	0.633218	0.45187	YWHAB	14-3-3 prot	1433B_HUI	5	0.9432
15_MTA / C	P06865	0.232052	0.633789	0.45218	HEXA	Beta-hexos	HEXA_HUM	3	1.174505
15_MTA / C	Q5R3I4	-0.0348	0.634916	0.452887	TTC38	Tetratricop	TTC38_HUM	3	0.976166
30_MTA / C	P16220	-0.32249	0.635183	0.45298	CREB1	Cyclic AMP	CREB1_HUI	3	0.799691
15_MTA / C	Q13907	-0.20085	0.635465	0.453083	IDI1	Isopenteny	IDI1_HUMA	1	0.870038
15_MTA / C	P09661	0.071023	0.635898	0.453294	SNRPA1	U2 small nu	RU2A_HUM	7	1.050462
30_MTA / C	P55327	0.265611	0.636165	0.453346	TPD52	Tumor prot	TPD52_HUI	6	1.202145
15_MTA / C	Q49B96	-0.74778	0.636313	0.453346	COX19	Cytochrom	COX19_HU	1	0.595519
30_MTA / C	Q9BQ13	-0.3204	0.637062	0.453632	KCTD14	BTB/POZ dc	KCD14_HU	1	0.800849
15_MTA / C	Q9BRT3	0.16369	0.637192	0.453632	MIEN1	Migration a	MIEN1_HUI	1	1.120149
15_MTA / C	Q9UKU7	0.016329	0.636932	0.453632	ACAD8	Isobutyryl-	ACAD8_HU	2	1.011383
15_MTA / C	P05787	-0.08163	0.638596	0.454369	KRT8	Keratin, typ	K2C8_HUM	18	0.944989
30_MTA / C	P33176	-0.01313	0.638639	0.454369	KIF5B	Kinesin-1 h	KINH_HUM	1	0.990941
30_MTA / C	Q8N1S5	-0.30303	0.63863	0.454369	SLC39A11	Zinc transp	S39AB_HUI	1	0.810546
15_MTA / C	Q9Y6G5	0.670402	0.638838	0.454413	COMMD10	COMM dom	COMDA_HU	1	1.591516
30_MTA / C	Q7L5Y1	0.177213	0.639567	0.454638	ENOSF1	Mitochond	ENOF1_HUI	2	1.130698
30_MTA / C	P48163	-0.07175	0.640032	0.454774	ME1	NADP-depe	MAOX_HUM	7	0.951483

15_MTA / C	Q14554	0.551562	0.639977	0.454774	PDIA5	Protein dis	PDIA5_HUM	1	1.465672
30_MTA / C	P08648	0.043055	0.640836	0.455053	ITGA5	Integrin alp	ITA5_HUMA	9	1.030293
30_MTA / C	P13639	0.074547	0.640731	0.455053	EEF2	Elongation	EF2_HUMA	38	1.05303
30_MTA / C	P43307	0.255219	0.640835	0.455053	SSR1	Translocon	SSRA_HUM	2	1.193517
15_MTA / C	Q9GZM5	0.427572	0.641782	0.455529	YIPF3	Protein YIP	YIPF3_HUM	3	1.344968
30_MTA / C	O95777	0.065675	0.643077	0.456155	LSM8	U6 snRNA-a	LSM8_HUM	3	1.046574
30_MTA / C	Q9BQ95	-0.87655	0.643047	0.456155	ECSIT	Evolutional	ECSIT_HUM	1	0.54467
30_MTA / C	P09497	0.211663	0.643335	0.456235	CLTB	Clathrin lig	CLCB_HUM	4	1.158022
30_MTA / C	P18085	0.146737	0.643878	0.456235	ARF4	ADP-ribosy	ARF4_HUM	4	1.107063
30_MTA / C	Q8WW12	0.127336	0.643678	0.456235	PCNP	PEST protec	PCNP_HUM	5	1.092275
30_MTA / C	Q9BZZ5	-0.16364	0.643833	0.456235	API5	Apoptosis i	API5_HUMA	11	0.892767
15_MTA / C	Q9HB71	0.235758	0.644069	0.456246	CACYBP	Calcyclin-b	CYBP_HUM	2	1.177525
30_MTA / C	P42677	0.013944	0.644325	0.456247	RPS27	Small ribos	RS27_HUM	3	1.009712
30_MTA / C	P31153	-0.12899	0.644817	0.456412	MAT2A	S-adenosylr	METK2_HU	9	0.914474
15_MTA / C	Q13217	0.165271	0.645194	0.456484	DNAJC3	DnaJ homo	DNJC3_HU	5	1.121377
15_MTA / C	Q9BSE5	-0.05425	0.645887	0.456878	AGMAT	Guanidino	GDAH_HUM	8	0.963096
30_MTA / C	P23284	0.120979	0.646567	0.457163	PPIB	Peptidyl-pr	PPIB_HUMA	14	1.087473
30_MTA / C	P60660	0.17464	0.646961	0.457249	MYL6	Myosin ligh	MYL6_HUM	2	1.128683
30_MTA / C	P78527	1.097988	0.646965	0.457249	PRKDC	DNA-depen	PRKDC_HU	4	2.14056
15_MTA / C	Q69YN2	0.138872	0.647399	0.45729	CWF19L1	CWF19-like	C19L1_HU	1	1.101044
15_MTA / C	Q70UQ0	0.844592	0.647437	0.45729	IKBIP	Inhibitor o	IKIP_HUMA	2	1.795757
30_MTA / C	O14735	-0.19472	0.647871	0.457499	CDIPT	CDP-diacyl	CDIPT_HUM	2	0.873745
15_MTA / C	Q13404	-0.07512	0.648201	0.457635	UBE2V1	Ubiquitin-c	UB2V1_HU	9	0.949265
30_MTA / C	P62942	0.649416	0.648351	0.457643	FKBP1A	Peptidyl-pr	FKB1A_HU	2	1.568533
30_MTA / C	P28066	0.142494	0.648794	0.457858	PSMA5	Proteasom	PSA5_HUM	5	1.103812
30_MTA / C	Q99436	0.047416	0.649291	0.458112	PSMB7	Proteasom	PSB7_HUM	4	1.033412
15_MTA / C	Q9BY32	-0.82409	0.650677	0.458699	ITPA	Inosine trip	ITPA_HUMA	3	0.56484
15_MTA / C	Q5D862	0.005858	0.650998	0.458827	FLG2	Filaggrin-2	FILA2_HUM	2	1.004069
30_MTA / C	Q9Y4L1	-0.06872	0.651596	0.459054	HYOU1	Hypoxia up	HYOU1_HU	27	0.953483
30_MTA / C	P17900	0.370113	0.651862	0.459144	GM2A	Ganglioside	SAP3_HUM	1	1.292454
30_MTA / C	P48723	0.050297	0.652093	0.459209	HSPA13	Heat shock	HSP13_HU	5	1.035478
15_MTA / C	O96008	0.35917	0.652707	0.459348	TOMM40	Mitochond	TOM40_HU	7	1.282687
15_MTA / C	P06899;P2	-0.12561	0.652682	0.459348	H2BC11;H2	Histone H2	H2B1J_HU	1	0.916615
30_MTA / C	P62861	0.33593	0.652572	0.459348	FAU	Ubiquitin-l	RS30_HUM	1	1.262191
30_MTA / C	Q9UKR5	-0.23223	0.652988	0.459379	ERG28	Ergosterol l	ERG28_HU	1	0.851319
30_MTA / C	Q9UMS4	0.114139	0.653546	0.459646	PRPF19	Pre-mRNA-p	PRP19_HU	6	1.082329
30_MTA / C	P61247	-0.00416	0.653986	0.459858	RPS3A	Small ribos	RS3A_HUM	17	0.997121
15_MTA / C	Q96CN7	-0.0557	0.654523	0.460138	ISOC1	Isochorism	ISOC1_HUM	5	0.962126
30_MTA / C	P49720	0.103678	0.655392	0.460279	PSMB3	Proteasom	PSB3_HUM	7	1.07451
15_MTA / C	P51572	-0.00104	0.655363	0.460279	BCAP31	B-cell recep	BAP31_HU	9	0.999279
30_MTA / C	Q13610	0.090444	0.655398	0.460279	PWP1	Periodic try	PWP1_HUM	2	1.064698
30_MTA / C	Q9GZS3	-0.43835	0.655419	0.460279	SKIC8	Superkiller	SKI8_HUMA	1	0.73798
30_MTA / C	Q9BXR0	-0.27183	0.655696	0.460376	QTRT1	Queuine tR	TGT_HUMA	3	0.828271
30_MTA / C	P62851	-0.04812	0.656153	0.4606	RPS25	Small ribos	RS25_HUM	4	0.967196
30_MTA / C	P35579	-0.13323	0.656683	0.460776	MYH9	Myosin-9	MYH9_HUM	12	0.911789
30_MTA / C	P61204;P8	0.043217	0.657452	0.461127	ARF3;ARF1	ADP-ribosy	ARF3_HUM	6	1.030409
30_MTA / C	Q9NXB9	-1.04858	0.657461	0.461127	ELOVL2	Very long c	ELOV2_HU	1	0.483445
30_MTA / C	P62854	-0.09614	0.657672	0.461178	RPS26	Small ribos	RS26_HUM	3	0.935534
30_MTA / C	P07437	0.207775	0.657968	0.461288	TUBB	Tubulin bet	TBB5_HUM	3	1.154906
30_MTA / C	O15173	0.143353	0.658497	0.461366	PGRMC2	Membrane-	PGRC2_HU	4	1.104469
15_MTA / C	O94979	-0.24923	0.658531	0.461366	SEC31A	Protein tra	SC31A_HU	3	0.841343
30_MTA / C	P28838	0.096656	0.658637	0.461366	LAP3	Cytosol am	AMPL_HUM	7	1.069292

30_MTA / C	Q15019	-0.03021	0.658434	0.461366	SEPTIN2	Septin-2	SEPT2_HUN	9	0.97928
15_MTA / C	P07339	0.010087	0.659033	0.461528	CTSD	Cathepsin D	CATD_HUN	7	1.007016
30_MTA / C	P52657	-0.11582	0.659146	0.461528	GTF2A2	Transcripti	T2AG_HUN	1	0.922857
15_MTA / C	P68366	0.814643	0.659858	0.461831	TUBA4A	Tubulin alp	TBA4A_HUI	2	1.758863
30_MTA / C	Q16851	-0.003	0.659811	0.461831	UGP2	UTP--gluco:	UGPA_HUN	11	0.99792
30_MTA / C	Q7Z4V5	-0.01295	0.66021	0.46198	HDGFL2	Hepatoma-	HDGR2_HU	1	0.991063
30_MTA / C	Q13616	-0.19811	0.66165	0.462728	CUL1	Cullin-1	CUL1_HUN	10	0.871693
15_MTA / C	Q9BQT8	-0.91461	0.661698	0.462728	SLC25A21	Mitochond	ODC_HUMA	1	0.530487
15_MTA / C	Q9Y3E0	0.092559	0.661871	0.462751	GOLT1B	Vesicle trar	GOT1B_HU	1	1.06626
15_MTA / C	P05023	0.036537	0.662289	0.462945	ATP1A1	Sodium/po	AT1A1_HUI	28	1.025649
30_MTA / C	P68371	-0.0311	0.662449	0.46296	TUBB4B	Tubulin bet	TBB4B_HUI	18	0.978671
15_MTA / C	Q9UNQ0	-0.14061	0.662613	0.462977	ABCG2	Broad subst	ABCG2_HU	1	0.907135
15_MTA / C	O75718	-0.10043	0.663322	0.463277	CRTAP	Cartilage-as	CRTAP_HUI	3	0.932754
30_MTA / C	Q7KZF4	-0.03659	0.66423	0.463716	SND1	Staphyloco	SND1_HUN	16	0.974954
15_MTA / C	P30153	0.048644	0.665076	0.464111	PPP2R1A	Serine/thre	2AAA_HUN	7	1.034292
30_MTA / C	Q9UJA5	-0.24484	0.665519	0.464322	TRMT6	tRNA (aden	TRM6_HUN	2	0.843907
15_MTA / C	P08240	-0.15579	0.665904	0.464333	SRPRA	Signal reco	SRPRA_HUI	3	0.89764
30_MTA / C	P62241	0.037677	0.665744	0.464333	RPS8	Small ribos	RS8_HUMA	7	1.02646
30_MTA / C	Q13283	-0.16411	0.666235	0.464333	G3BP1	Ras GTPase-	G3BP1_HU	6	0.89248
15_MTA / C	Q9Y6B6	0.248205	0.666168	0.464333	SAR1B	Small COPII	SAR1B_HUI	1	1.187728
15_MTA / C	O43752	-0.21065	0.666655	0.464492	STX6	Syntaxin-6	STX6_HUM	1	0.86415
30_MTA / C	Q9Y6N5	0.018165	0.667033	0.464596	SQOR	Sulfide:quir	SQOR_HUN	16	1.01267
30_MTA / C	Q96HR9	-0.07123	0.667824	0.464951	REEP6	Receptor e>	REEP6_HUN	3	0.951827
30_MTA / C	O14828	-0.15027	0.668527	0.465156	SCAMP3	Secretory c	SCAM3_HU	6	0.901083
30_MTA / C	Q9Y230	0.095687	0.668539	0.465156	RUVBL2	RuvB-like 2	RUVB2_HU	18	1.068574
15_MTA / C	Q8IZA0	0.350624	0.668986	0.465369	KIAA0319L	Dyslexia-as:	K319L_HUI	1	1.275112
30_MTA / C	P57088	0.015913	0.669774	0.465819	TMEM33	Transmemb	TMM33_HU	2	1.011091
15_MTA / C	O15347	0.13862	0.67012	0.46591	HMGB3	High mobil	HMGB3_HU	1	1.100852
30_MTA / C	P00505	0.044966	0.670634	0.466027	GOT2	Aspartate a	AATM_HUN	24	1.031659
15_MTA / C	Q9NQH7	0.208961	0.671686	0.46666	XPNPEP3	Xaa-Pro am	XPP3_HUN	2	1.155856
30_MTA / C	P34897	-0.0245	0.671845	0.466672	SHMT2	Serine hydr	GLYM_HUN	15	0.983163
30_MTA / C	O75396	0.004092	0.672191	0.466815	SEC22B	Vesicle-traf	SC22B_HUI	3	1.00284
30_MTA / C	O15498	0.56165	0.673196	0.467121	YKT6	Synaptobre	YKT6_HUM	4	1.475957
15_MTA / C	Q13868	0.179472	0.67314	0.467121	EXOSC2	Exosome cc	EXOS2_HUI	4	1.132469
30_MTA / C	Q9P2E9	0.227018	0.673187	0.467121	RRBP1	Ribosome-l	RRBP1_HUI	16	1.170413
30_MTA / C	P27144	0.397835	0.673566	0.46728	AK4	Adenylate k	KAD4_HUN	2	1.317529
15_MTA / C	O00625	-0.1339	0.674318	0.467467	PIR	Pirin	PIR_HUMA	7	0.911365
15_MTA / C	P05166	-0.01096	0.674399	0.467467	PCCB	Propionyl-(	PCCB_HUN	1	0.992435
15_MTA / C	P54105	0.35419	0.674225	0.467467	CLNS1A	Methylosor	ICLN_HUMA	2	1.278267
30_MTA / C	P58546	-0.05985	0.674094	0.467467	MTPN	Myotrophi	MTPN_HUN	1	0.959366
15_MTA / C	P52907	0.263113	0.675666	0.468201	CAPZA1	F-actin-cap	CAZA1_HUI	3	1.200065
15_MTA / C	Q8WWC4	-0.26149	0.675882	0.468201	MAIP1	m-AAA prot	MAIP1_HUI	1	0.834228
30_MTA / C	O43760	-0.06799	0.676569	0.468254	SYNGR2	Synaptogyr	SNG2_HUN	4	0.953969
30_MTA / C	O60832	0.475844	0.676418	0.468254	DKC1	H/ACA ribo	DKC1_HUN	3	1.390731
30_MTA / C	P13674	-0.06058	0.67661	0.468254	P4HA1	Prolyl 4-hy	P4HA1_HU	4	0.958882
30_MTA / C	Q9UBS4	-0.06143	0.676553	0.468254	DNAJB11	DnaJ homo	DJB11_HUI	5	0.958316
30_MTA / C	Q9Y295	0.492465	0.676665	0.468254	DRG1	Developme	DRG1_HUN	4	1.406847
30_MTA / C	P0CG47;P0	-0.01615	0.677516	0.468425	UBB;UBC;R	Polyubiqui	UBB_HUMA	5	0.988867
30_MTA / C	P39656	0.089711	0.677325	0.468425	DDOST	Dolichyl-di	OST48_HUI	7	1.064157
15_MTA / C	Q5T750	0.185422	0.677186	0.468425	KPLCE	Protein KPI	KPLCE_HUN	1	1.13715
15_MTA / C	Q9BVC6	0.065228	0.677868	0.468425	TMEM109	Voltage-gat	TM109_HU	4	1.04625
30_MTA / C	Q9H2U2	-0.1321	0.677646	0.468425	PPA2	Inorganic p	IPYR2_HUN	3	0.912504

15_MTA / C	Q15836	0.14972	0.678577	0.468696	VAMP3	Vesicle-asso	VAMP3_HU		2	1.109354
30_MTA / C	Q06203	0.168769	0.679203	0.469031	PPAT	Amidophos	PUR1_HUM		11	1.124099
15_MTA / C	O14944	1.097806	0.681155	0.469565	EREG	Proepiregu	EREG_HUM		2	2.140289
30_MTA / C	O95881	-0.03536	0.680651	0.469565	TXNDC12	Thioredoxi	TXD12_HU		3	0.975786
15_MTA / C	P04062	0.122564	0.681068	0.469565	GBA1	Lysosomal	GBA1_HUM		4	1.088668
30_MTA / C	P11766	0.082952	0.681523	0.469565	ADH5	Alcohol del	ADHX_HUM		12	1.059183
15_MTA / C	Q13445	-0.00027	0.680176	0.469565	TMED1	Transmemb	TMED1_HU		1	0.999813
30_MTA / C	Q6YHK3	-0.48144	0.681225	0.469565	CD109	CD109 anti	CD109_HU		2	0.71626
30_MTA / C	Q6YP21	-0.03657	0.681535	0.469565	KYAT3	Kynurenine	KAT3_HUM		8	0.97497
30_MTA / C	Q9H008	-0.07785	0.68056	0.469565	LHPP	Phospholys	LHPP_HUM		1	0.947471
15_MTA / C	Q9H6E4	-0.30184	0.681449	0.469565	CCDC134	Coiled-coil	CC134_HU		1	0.811219
15_MTA / C	P05204	-0.02105	0.682101	0.469656	HMGN2	Non-histon	HMGN2_HU		3	0.985517
15_MTA / C	P32322	0.014816	0.682057	0.469656	PYCR1	Pyrroline-5	P5CR1_HU		10	1.010322
15_MTA / C	P61353	0.270166	0.68247	0.469721	RPL27	Large ribos	RL27_HUM		4	1.205947
30_MTA / C	P63010	0.030042	0.683051	0.470023	AP2B1	AP-2 compl	AP2B1_HU		3	1.021042
15_MTA / C	Q15843	-0.15613	0.684437	0.470879	NEDD8	NEDD8	NEDD8_HU		1	0.897431
30_MTA / C	P46782	0.108738	0.684701	0.470963	RPS5	Small ribos	RS5_HUMA		8	1.078285
15_MTA / C	P27348	-0.26441	0.685012	0.47101	YWHAQ	14-3-3 prot	1433T_HU		4	0.832537
30_MTA / C	Q8TCJ2	0.060035	0.686607	0.471784	STT3B	Dolichyl-di	STT3B_HUM		3	1.042491
15_MTA / C	Q9UJW0	0.383452	0.687064	0.471902	DCTN4	Dynactin su	DCTN4_HU		6	1.304459
15_MTA / C	P36542	-0.01133	0.687228	0.471918	ATP5F1C	ATP syntha	ATPG_HUM		8	0.992175
15_MTA / C	P53618	0.127006	0.687683	0.472034	COPB1	Coatomer s	COPB_HUM		6	1.092025
15_MTA / C	P62861	0.188337	0.687918	0.47206	FAU	Ubiquitin-l	RS30_HUM		1	1.13945
30_MTA / C	Q06265	0.177188	0.688006	0.47206	EXOSC9	Exosome cc	EXOS9_HU		3	1.130678
15_MTA / C	O95551	0.181363	0.688171	0.472076	TDP2	Tyrosyl-DN	TYDP2_HU		1	1.133955
15_MTA / C	P50990	0.003	0.688331	0.472088	CCT8	T-complex	TCPQ_HUM		25	1.002082
15_MTA / C	O00161	0.005146	0.690332	0.47307	SNAP23	Synaptosor	SNP23_HU		6	1.003573
15_MTA / C	P62191	-0.06601	0.690297	0.47307	PSMC1	26S protea	PRS4_HUM		4	0.955277
30_MTA / C	Q92841	-0.19508	0.690004	0.47307	DDX17	Probable A	DDX17_HU		6	0.873524
15_MTA / C	P52788	0.075001	0.690666	0.473102	SMS	Spermine s	SPSY_HUM		3	1.053362
15_MTA / C	Q9Y394	-0.14368	0.69059	0.473102	DHRS7	Dehydroge	DHRS7_HU		3	0.905205
30_MTA / C	O14950;P1	0.451318	0.691947	0.473686	MYL12B;M	Myosin reg	ML12B_HU		1	1.367289
30_MTA / C	P62495	0.955397	0.691877	0.473686	ETF1	Eukaryotic	ERF1_HUM		3	1.939113
15_MTA / C	Q9UL25	0.129523	0.691733	0.473686	RAB21	Ras-related	RAB21_HU		5	1.093932
30_MTA / C	O95865	-0.07874	0.69222	0.473775	DDAH2	Putative hy	DDAH2_HU		6	0.946884
15_MTA / C	P49589	0.02388	0.692422	0.473785	CARS1	Cysteine--t	SYCC_HUM		3	1.01669
30_MTA / C	Q9BQ67	-0.22083	0.692521	0.473785	GRWD1	Glutamate-	GRWD1_HU		2	0.858069
15_MTA / C	P10301;P6	-0.13907	0.692911	0.473815	RRAS;RRAS	Ras-related	RRAS_HUM		1	0.908105
30_MTA / C	P62750	0.071629	0.693137	0.473815	RPL23A	Large ribos	RL23A_HU		4	1.050903
15_MTA / C	Q9Y2B0	0.008298	0.692862	0.473815	CNPY2	Protein car	CNPY2_HU		4	1.005769
15_MTA / C	Q9Y5U8	0.052563	0.693328	0.473848	MPC1	Mitochond	MPC1_HUM		1	1.037106
30_MTA / C	P22234	-0.01917	0.693789	0.473858	PAICS	Bifunctiona	PUR6_HUM		15	0.986802
30_MTA / C	P55036	-0.10457	0.694057	0.473858	PSMD4	26S protea	PSMD4_HU		5	0.93008
15_MTA / C	Q6YP21	-0.15633	0.693884	0.473858	KYAT3	Kynurenine	KAT3_HUM		8	0.897302
30_MTA / C	Q99439	0.065097	0.693966	0.473858	CNN2	Calponin-2	CNN2_HUM		1	1.046155
15_MTA / C	Q9NWX6	-0.14877	0.693827	0.473858	THG1L	Probable tF	THG1_HUM		1	0.902021
30_MTA / C	Q9Y305	0.016147	0.694225	0.473874	ACOT9	Acyl-coenzy	ACOT9_HU		4	1.011255
30_MTA / C	P15151	-0.02807	0.694566	0.473882	PVR	Poliovirus r	PVR_HUMA		3	0.980729
15_MTA / C	Q9NVA1	0.418378	0.694664	0.473882	UQCC1	Ubiquinol-	UQCC1_HU		2	1.336424
30_MTA / C	P48444	-0.07868	0.69495	0.473979	ARCN1	Coatomer s	COPD_HUM		7	0.946923
15_MTA / C	P36957	-0.03007	0.696266	0.474584	DLST	Dihydrolip	ODO2_HUM		9	0.979376
15_MTA / C	Q9H3Z4	-0.00576	0.696696	0.474779	DNAJC5	DnaJ homo	DNJC5_HU		1	0.996015

15_MTA / C	Q9NX40	-0.04345	0.696904	0.474823	OCIAD1	OCIA doma	OCAD1_HU	4	0.970331
30_MTA / C	P55735	-0.61435	0.697775	0.475115	SEC13	Protein SEC	SEC13_HUI	1	0.653223
15_MTA / C	P84090	0.036757	0.69765	0.475115	ERH	Enhancer o	ERH_HUMA	1	1.025805
30_MTA / C	Q14254	-0.12053	0.698049	0.475115	FLOT2	Flotillin-2	FLOT2_HUI	3	0.919849
30_MTA / C	Q53GQ0	-0.00112	0.69791	0.475115	HSD17B12	Very-long-c	DHB12_HU	3	0.999224
30_MTA / C	Q9Y3A5	-0.62833	0.698245	0.475151	SBDS	Ribosome r	SBDS_HUM	4	0.646924
30_MTA / C	P05455	0.128672	0.698881	0.475291	SSB	Lupus La pr	LA_HUMAN	9	1.093287
30_MTA / C	Q9HD23	-0.00507	0.698855	0.475291	MRS2	Magnesium	MRS2_HUM	3	0.99649
30_MTA / C	Q6QNY1	0.143367	0.699225	0.475427	BLOC1S2	Biogenesis	BL1S2_HUI	1	1.10448
30_MTA / C	P07148	0.358367	0.700358	0.475921	FABP1	Fatty acid-k	FABPL_HUI	2	1.281974
30_MTA / C	Q13445	-0.16324	0.700369	0.475921	TMED1	Transmemk	TMED1_HU	1	0.893015
15_MTA / C	O75534	-0.1205	0.700689	0.476032	CSDE1	Cold shock	CSDE1_HUI	8	0.919866
15_MTA / C	P42766	-0.23821	0.701532	0.476507	RPL35	Large ribos	RL35_HUM	2	0.847796
30_MTA / C	P20338	0.195391	0.701868	0.476638	RAB4A	Ras-related	RAB4A_HUI	4	1.145035
30_MTA / C	P04406	-0.04285	0.702892	0.47723	GAPDH	Glyceraldel	G3P_HUMA	21	0.970737
30_MTA / C	P04844	-0.05594	0.703029	0.47723	RPN2	Dolichyl-di	RPN2_HUM	7	0.96197
30_MTA / C	O00571	0.320266	0.704286	0.477536	DDX3X	ATP-depenc	DDX3X_HU	5	1.248561
30_MTA / C	O15305	-0.10086	0.703799	0.477536	PMM2	Phosphoma	PMM2_HUI	6	0.932479
30_MTA / C	P07814	-0.18656	0.704168	0.477536	EPRS1	Bifunctiona	SYEP_HUM	8	0.878699
15_MTA / C	P23919	-0.12589	0.705143	0.477536	DTYMK	Thymidylat	KTHY_HUM	6	0.916436
15_MTA / C	P37108	0.362445	0.704677	0.477536	SRP14	Signal reco	SRP14_HUI	5	1.285603
30_MTA / C	P60468	-0.04611	0.704814	0.477536	SEC61B	Protein tra	SC61B_HUI	3	0.968547
15_MTA / C	P78540	0.491405	0.705287	0.477536	ARG2	Arginase-2,	ARGI2_HUI	1	1.405813
30_MTA / C	Q09028	-0.01438	0.70547	0.477536	RBBP4	Histone-bir	RBBP4_HUI	9	0.990085
30_MTA / C	Q15287	-0.31216	0.705496	0.477536	RNPS1	RNA-bindir	RNPS1_HUI	2	0.805435
30_MTA / C	Q9H9S3	-0.04088	0.705025	0.477536	SEC61A2	Protein tra	S61A2_HUI	7	0.972059
30_MTA / C	Q9NQ88	0.26149	0.70524	0.477536	TIGAR	Fructose-2,	TIGAR_HUI	1	1.198716
15_MTA / C	P33908	0.300759	0.705949	0.477651	MAN1A1	Mannosyl-c	MA1A1_HU	2	1.231792
15_MTA / C	Q05048	0.138252	0.706098	0.477651	CSTF1	Cleavage sti	CSTF1_HUI	2	1.100571
30_MTA / C	Q9UQ80	-0.06496	0.7061	0.477651	PA2G4	Proliferatic	PA2G4_HU	13	0.95597
30_MTA / C	Q12931	0.365475	0.707211	0.478137	TRAP1	Heat shock	TRAP1_HUI	9	1.288306
15_MTA / C	Q13011	0.118944	0.707034	0.478137	ECH1	Delta(3,5)-I	ECH1_HUM	7	1.08594
30_MTA / C	Q9P287	-0.20012	0.70738	0.478137	BCCIP	BRCA2 and	BCCIP_HUI	1	0.870478
30_MTA / C	P61160	-0.10129	0.709117	0.478765	ACTR2	Actin-relate	ARP2_HUM	6	0.932198
30_MTA / C	P62841	0.10198	0.708831	0.478765	RPS15	Small ribos	RS15_HUM	1	1.073245
30_MTA / C	Q04837	0.073428	0.709191	0.478765	SSBP1	Single-strar	SSBP_HUM	3	1.052214
15_MTA / C	P13987	-0.37662	0.71054	0.479481	CD59	CD59 glyco	CD59_HUM	2	0.77024
30_MTA / C	P63261	-0.17845	0.710466	0.479481	ACTG1	Actin, cyto	ACTG_HUM	1	0.883653
15_MTA / C	P25705	0.01117	0.710911	0.479633	ATP5F1A	ATP synthas	ATPA_HUM	26	1.007772
30_MTA / C	O43447	0.237833	0.711893	0.479694	PPIH	Peptidyl-pr	PPIH_HUM	1	1.17922
30_MTA / C	P00338	0.145883	0.712015	0.479694	LDHA	L-lactate de	LDHA_HUM	16	1.106408
30_MTA / C	P27635	-0.0849	0.711966	0.479694	RPL10	Large ribos	RL10_HUM	4	0.942847
30_MTA / C	P07942	-0.03998	0.71258	0.479977	LAMB1	Laminin sul	LAMB1_HU	4	0.972666
15_MTA / C	Q13283	0.177738	0.714028	0.48066	G3BP1	Ras GTPase-	G3BP1_HU	6	1.131109
15_MTA / C	Q9NX14	0.081293	0.714651	0.480982	NDUFB11	NADH dehy	NDUBB_HU	1	1.057966
15_MTA / C	P62851	-0.02588	0.715012	0.481127	RPS25	Small ribos	RS25_HUM	4	0.982219
30_MTA / C	O60232	-0.08077	0.715478	0.481245	ZNRD2	Protein ZNI	ZNRD2_HU	2	0.945556
15_MTA / C	O75964	0.041142	0.715442	0.481245	ATP5MG	ATP synthas	ATP5L_HUI	6	1.028928
15_MTA / C	O60220	-0.09575	0.71617	0.481443	TIMM8A	Mitochond	TIM8A_HUI	2	0.935785
30_MTA / C	P04066	-0.16565	0.71634	0.481443	FUCA1	Tissue alph	FUCO_HUM	3	0.891529
15_MTA / C	P05783	-0.00141	0.715986	0.481443	KRT18	Keratin, typ	K1C18_HUI	18	0.999021
15_MTA / C	P20020	0.092304	0.716644	0.481443	ATP2B1	Plasma mer	AT2B1_HUI	7	1.066071

30_MTA / C	P62316	0.06101	0.71663	0.481443	SNRPD2	Small nucle	SMD2_HUN	6	1.043196
30_MTA / C	Q96HY6	-0.34529	0.716495	0.481443	DDRGK1	DDRGK dor	DDRGK_HU	2	0.787148
30_MTA / C	O00116	0.95088	0.717254	0.481541	AGPS	Alkyldihydr	ADAS_HUN	3	1.933051
15_MTA / C	P52292	0.279467	0.717382	0.481541	KPNA2	Importin su	IMA1_HUN	4	1.213746
30_MTA / C	Q15417	-0.2845	0.717708	0.481541	CNN3	Calponin-3	CNN3_HUN	4	0.821027
15_MTA / C	Q96I15	-0.34606	0.717792	0.481541	SCLY	Selenocyste	SCLY_HUMA	1	0.786728
30_MTA / C	Q9UNW1	-0.3179	0.71774	0.481541	MINPP1	Multiple in	MINP1_HU	4	0.802237
15_MTA / C	P35520	0.035565	0.718195	0.481624	CBS	Cystathioni	CBS_HUMA	7	1.024958
30_MTA / C	O75643	0.461785	0.719925	0.482001	SNRNP200	U5 small nu	U520_HUN	6	1.377244
15_MTA / C	P43034	0.160737	0.719297	0.482001	PAFAH1B1	Platelet-act	LIS1_HUMA	12	1.117858
15_MTA / C	Q01518	0.040401	0.719947	0.482001	CAP1	Adenylyl cy	CAP1_HUN	24	1.028399
30_MTA / C	Q8N6L1	1.374014	0.719866	0.482001	KRTCAP2	Keratinocy	KTAP2_HUI	1	2.591907
30_MTA / C	Q9UHR4	0.075289	0.719806	0.482001	BAIAP2L1	BAR/IMD d	BI2L1_HUN	5	1.053572
15_MTA / C	P00403	0.072848	0.720239	0.482099	MT-CO2	Cytochrom	COX2_HUN	6	1.051791
30_MTA / C	Q9NPD3	0.123877	0.721091	0.482572	EXOSC4	Exosome co	EXOS4_HUI	5	1.089659
30_MTA / C	Q86X55	-0.26745	0.721376	0.482573	CARM1	Histone-arg	CARM1_HU	4	0.830788
15_MTA / C	P11216	0.340433	0.721856	0.482694	PYGB	Glycogen p	PYGB_HUN	2	1.266136
15_MTA / C	Q5VZM2;Q	0.798032	0.722125	0.482776	RRAGB;RRA	Ras-related	RRAGB_HU	1	1.738728
15_MTA / C	Q9HCN8	0.721113	0.722315	0.482806	SDF2L1	Stromal cel	SDF2L_HUN	2	1.648453
15_MTA / C	O00584	-0.22484	0.722699	0.482965	RNASET2	Ribonuclea	RNT2_HUN	1	0.85569
30_MTA / C	A0A0B4J2D	0.085865	0.723202	0.483107	GATD3B;GA	Putative glu	GAL3B_HUI	5	1.061324
15_MTA / C	Q99519	0.257572	0.723155	0.483107	NEU1	Sialidase-1	NEUR1_HU	3	1.195465
30_MTA / C	O95832	0.028936	0.725587	0.484602	CLDN1	Claudin-1	CLD1_HUN	3	1.020259
15_MTA / C	P54819	-0.02636	0.726398	0.484655	AK2	Adenylate k	KAD2_HUN	6	0.981893
30_MTA / C	P60903	1.191159	0.726324	0.484655	S100A10	Protein S10	S10AA_HUI	1	2.283361
30_MTA / C	P61077;P6	0.090555	0.72631	0.484655	UBE2D3;UE	Ubiquitin-c	UB2D3_HU	1	1.064779
30_MTA / C	Q04695	-0.08313	0.726242	0.484655	KRT17	Keratin, typ	K1C17_HUI	4	0.944006
30_MTA / C	Q9BVK6	0.018201	0.725959	0.484655	TMED9	Transmemb	TMED9_HU	3	1.012696
30_MTA / C	P00439	0.105739	0.726695	0.484756	PAH	Phenylalan	PH4H_HUN	3	1.076046
30_MTA / C	Q00796	0.023546	0.726887	0.484786	SORD	Sorbitol de	DHSO_HUN	7	1.016455
15_MTA / C	O75489	0.904451	0.728856	0.485604	NDUFS3	NADH dehy	NDUS3_HU	2	1.871833
30_MTA / C	O95202	0.577263	0.728737	0.485604	LETM1	Mitochond	LETM1_HUI	4	1.492016
30_MTA / C	P08247	-0.04968	0.728725	0.485604	SYP	Synaptophy	SYPH_HUM	1	0.966149
15_MTA / C	Q9P0U1	-0.1912	0.728992	0.485604	TOMM7	Mitochond	TOM7_HUN	1	0.875878
15_MTA / C	P13164;Q0	0.180653	0.72953	0.485815	IFITM1;IFIT	Interferon-i	IFM1_HUM	1	1.133397
30_MTA / C	P54687	-0.22988	0.729602	0.485815	BCAT1	Branched-c	BCAT1_HUI	3	0.852704
30_MTA / C	P49585	0.242896	0.731351	0.486784	PCYT1A	Choline-ph	PCY1A_HUI	3	1.183366
15_MTA / C	A0A024R1I	0.267519	0.731686	0.486909	TMA7B;TM	Translation	TMA7B_HU	2	1.203736
30_MTA / C	P43304	0.262595	0.732341	0.487149	GPD2	Glycerol-3-	GPDM_HUI	4	1.199635
30_MTA / C	Q5JS54	-0.62476	0.732316	0.487149	PSMG4	Proteasom	PSMG4_HU	1	0.648528
15_MTA / C	P18859	0.232526	0.733188	0.487521	ATP5PF	ATP synthas	ATP5J_HUN	3	1.174891
30_MTA / C	P35520	-0.17238	0.733376	0.487544	CBS	Cystathioni	CBS_HUMA	7	0.887379
15_MTA / C	P62487	0.427713	0.733557	0.487546	POLR2G	DNA-direct	RPB7_HUN	3	1.345099
30_MTA / C	Q70UQ0	0.885102	0.733674	0.487546	IKBIP	Inhibitor of	IKIP_HUMA	2	1.846895
15_MTA / C	P46783;Q9	0.291628	0.734332	0.487886	RPS10;RPS	Small ribos	RS10_HUM	3	1.224021
30_MTA / C	Q96BW5	-0.11901	0.735178	0.488155	PTER	Phosphotri	PTER_HUM	6	0.920822
15_MTA / C	Q9NSE4	0.130847	0.736192	0.488632	IARS2	Isoleucine-	SYIM_HUM	7	1.094937
30_MTA / C	O60749	-0.2324	0.736465	0.488716	SNX2	Sorting nex	SNX2_HUN	2	0.851215
30_MTA / C	P56134	-0.06083	0.736809	0.488846	ATP5MF	ATP synthas	ATPK_HUM	1	0.958711
15_MTA / C	O95433	0.096846	0.737214	0.488885	AHSA1	Activator o	AHSA1_HUI	3	1.069433
30_MTA / C	P06576	0.043117	0.737311	0.488885	ATP5F1B	ATP synthas	ATPB_HUM	18	1.030338
15_MTA / C	Q7L5D6	0.268025	0.737266	0.488885	GET4	Golgi to ER	GET4_HUM	2	1.204158

15_MTA / C	P13073	-0.00024	0.738899	0.489274	COX4I1	Cytochrom	COX41_HU	9	0.999831
30_MTA / C	P30520	-0.05371	0.738649	0.489274	ADSS2	Adenylosuc	PURA2_HU	8	0.963455
30_MTA / C	Q9H8Y8	-0.85701	0.73893	0.489274	GORASP2	Golgi reass	GORS2_HU	1	0.552097
30_MTA / C	Q16543	0.061267	0.739131	0.489309	CDC37	Hsp90 co-c	CDC37_HU	2	1.043382
15_MTA / C	Q9P0P0	-0.32064	0.740146	0.489883	RNF181	E3 ubiquiti	RN181_HU	1	0.800716
15_MTA / C	Q92506	0.513432	0.740316	0.489897	HSD17B8	(3R)-3-hydr	DHB8_HUM	3	1.427442
30_MTA / C	Q86U42	-0.01004	0.740492	0.489917	PABPN1	Polyadenyl	PABP2_HU	1	0.993065
15_MTA / C	Q9BXJ9	0.327584	0.740882	0.490076	NAA15	N-alpha-ac	NAA15_HU	6	1.25491
30_MTA / C	O95197	0.131767	0.743134	0.490414	RTN3	Reticulon-3	RTN3_HUM	2	1.095635
30_MTA / C	P01040	0.018587	0.742284	0.490414	CSTA	Cystatin-A	CYTA_HUM	2	1.012967
15_MTA / C	P10619	0.119472	0.742173	0.490414	CTSA	Lysosomal	PPGB_HUM	2	1.086338
30_MTA / C	P23368	-0.02223	0.743458	0.490414	ME2	NAD-depen	MAOM_HU	5	0.984707
15_MTA / C	P40616	0.395013	0.742413	0.490414	ARL1	ADP-ribosy	ARL1_HUM	1	1.314954
30_MTA / C	P47985	0.055939	0.743465	0.490414	UQCRFS1	Cytochrom	UCRI_HUM	7	1.039536
30_MTA / C	P49207	-0.11571	0.743244	0.490414	RPL34	Large ribos	RL34_HUM	5	0.922931
15_MTA / C	P52758	0.046388	0.74267	0.490414	RIDA	2-iminobut	RIDA_HUM	2	1.032676
15_MTA / C	P62888	0.378136	0.743357	0.490414	RPL30	Large ribos	RL30_HUM	6	1.299661
30_MTA / C	P00387	0.01913	0.744592	0.4907	CYB5R3	NADH-cyto	NB5R3_HU	4	1.013348
15_MTA / C	P61163	0.055899	0.74454	0.4907	ACTR1A	Alpha-cent	ACTZ_HUM	4	1.039507
15_MTA / C	Q6UW63	0.248287	0.744482	0.4907	POGLUT2	Protein O-g	PLGT2_HU	5	1.187796
15_MTA / C	Q9NX76	0.118407	0.744638	0.4907	CMTM6	CKLF-like M	CKLF6_HU	1	1.085535
30_MTA / C	Q13409	0.138096	0.745917	0.491273	DYNC1I2	Cytoplasmi	DC1I2_HU	1	1.100452
30_MTA / C	P62244	-5.33E-05	0.748336	0.492647	RPS15A	Small ribos	RS15A_HU	5	0.999963
15_MTA / C	P07686	0.054817	0.748579	0.492709	HEXB	Beta-hexos	HEXB_HUM	8	1.038727
30_MTA / C	P41227;Q9	0.210292	0.749141	0.492981	NAA10;NA	N-alpha-ac	NAA10_HU	1	1.156922
30_MTA / C	O95831	-0.04891	0.75043	0.493437	AIFM1	Apoptosis-i	AIFM1_HU	12	0.966664
15_MTA / C	Q96I36	0.545367	0.750703	0.493519	COX14	Cytochrom	COX14_HU	1	1.459392
30_MTA / C	P02649	0.076086	0.75122	0.493682	APOE	Apolipopro	APOE_HUM	13	1.054154
30_MTA / C	P52292	0.115803	0.751248	0.493682	KPNA2	Importin su	IMA1_HUM	4	1.083578
15_MTA / C	Q96HQ2	0.682982	0.752039	0.494104	CDKN2AIPN	CDKN2AIP	C2AIL_HUM	1	1.605455
30_MTA / C	P11216	0.255581	0.752343	0.494134	PYGB	Glycogen p	PYGB_HUM	2	1.193816
30_MTA / C	P23528	-0.01472	0.752383	0.494134	CFL1	Cofilin-1	COF1_HUM	10	0.989847
15_MTA / C	Q9BUR5	0.444033	0.75258	0.494165	APOO	MICOS com	MIC26_HU	2	1.360402
30_MTA / C	Q9UNZ2	-0.06057	0.75282	0.494225	NSFL1C	NSFL1 cofa	NSF1C_HU	2	0.958886
30_MTA / C	Q9NP97	0.380607	0.753166	0.494354	DYNLRB1	Dynein ligh	DLRB1_HU	3	1.301889
30_MTA / C	Q9NX62	-0.21283	0.753744	0.494635	BPNT2	Golgi-resid	IMPA3_HU	3	0.862843
15_MTA / C	P53621	0.045134	0.754139	0.494698	COPA	Coatomer s	COPA_HUM	13	1.031779
30_MTA / C	Q99714	0.081965	0.754014	0.494698	HSD17B10	3-hydroxya	HCD2_HUM	6	1.058459
15_MTA / C	P07203	0.103826	0.754696	0.494884	GPX1	Glutathion	GPX1_HUM	2	1.074619
15_MTA / C	P58546	0.069151	0.754879	0.494884	MTPN	Myotrophi	MTPN_HUM	1	1.049099
30_MTA / C	Q15370	0.128352	0.754867	0.494884	ELOB	Elongin-B	ELOB_HUM	3	1.093044
15_MTA / C	Q9BXP5	-0.04687	0.755019	0.494884	SRRT	Serrate RN	SRRT_HUM	1	0.968032
15_MTA / C	P04818	-0.39508	0.755475	0.494987	TYMS	Thymidylat	TYSY_HUM	1	0.760445
30_MTA / C	P49327	0.069864	0.755403	0.494987	FASN	Fatty acid s	FAS_HUMA	23	1.049618
15_MTA / C	P84095	0.189203	0.755954	0.495203	RHOG	Rho-relate	RHOG_HUM	2	1.140134
30_MTA / C	P34932	-0.04323	0.756264	0.495308	HSPA4	Heat shock	HSP74_HU	13	0.970477
30_MTA / C	P05155	-0.01845	0.756857	0.495403	SERPING1	Plasma pro	IC1_HUMA	1	0.98729
30_MTA / C	Q01105	0.310596	0.75675	0.495403	SET	Protein SET	SET_HUMA	4	1.24022
30_MTA / C	P09417	-0.08867	0.758824	0.496494	QDPR	Dihydropte	DHPR_HUM	4	0.940391
30_MTA / C	Q9NRW1	-0.36696	0.758685	0.496494	RAB6B	Ras-related	RAB6B_HU	1	0.775415
30_MTA / C	Q96AG4	0.052936	0.759026	0.496528	LRRC59	Leucine-ric	LRC59_HU	6	1.037374
30_MTA / C	Q9ULA0	0.204262	0.759768	0.496916	DNPEP	Aspartyl am	DNPEP_HU	4	1.152097

15_MTA / C	O75396	0.499729	0.760405	0.49694	SEC22B	Vesicle-traf	SC22B_HUI	3	1.413948
15_MTA / C	P62913	0.024052	0.760194	0.49694	RPL11	Large ribos	RL11_HUM	5	1.016811
30_MTA / C	Q02809	0.230583	0.759957	0.49694	PLOD1	Procollager	PLOD1_HU	3	1.173309
15_MTA / C	Q8N4V1	0.025167	0.760312	0.49694	MMGT1	ER membra	EMC5_HUM	1	1.017598
30_MTA / C	O75844	0.177003	0.760849	0.497067	ZMPSTE24	CAAX preny	FACE1_HUI	7	1.130533
15_MTA / C	P16989	-0.16626	0.761167	0.497067	YBX3	Y-box-bindi	YBOX3_HU	6	0.891152
30_MTA / C	P52888	-0.11924	0.761946	0.497083	THOP1	Thimet olig	THOP1_HU	12	0.920676
30_MTA / C	Q8TDQ7	-0.45181	0.761974	0.497083	GNPDA2	Glucosamir	GNPI2_HUI	1	0.731127
30_MTA / C	P05198	-0.02534	0.762805	0.497527	EIF2S1	Eukaryotic	IF2A_HUM	11	0.982587
30_MTA / C	A8MWD9;F	0.018488	0.763402	0.497721	SNRPGP15;	Putative sm	RUXGL_HU	3	1.012898
15_MTA / C	Q92890	-0.00935	0.763254	0.497721	UFD1	Ubiquitin r	UFD1_HUM	4	0.993537
15_MTA / C	P63000	0.163557	0.76372	0.497732	RAC1	Ras-related	RAC1_HUM	5	1.120045
30_MTA / C	Q9UL25	-0.26598	0.76405	0.497849	RAB21	Ras-related	RAB21_HUI	5	0.831634
15_MTA / C	P61247	0.03223	0.764243	0.497877	RPS3A	Small ribos	RS3A_HUM	17	1.022592
15_MTA / C	O00244	0.208699	0.767372	0.498117	ATOX1	Copper trai	ATOX1_HUI	2	1.155646
15_MTA / C	O43493	0.336224	0.765891	0.498117	TGOLN2	Trans-Golgi	TGON2_HU	2	1.262448
30_MTA / C	P21333	0.044055	0.767147	0.498117	FLNA	Filamin-A	FLNA_HUM	27	1.031008
30_MTA / C	P28070	0.063325	0.766059	0.498117	PSMB4	Proteasome	PSB4_HUM	5	1.044871
15_MTA / C	P30793	0.182284	0.767267	0.498117	GCH1	GTP cycloh	GCH1_HUM	3	1.134679
30_MTA / C	P36542	0.10287	0.767453	0.498117	ATP5F1C	ATP synthas	ATPG_HUM	8	1.073907
30_MTA / C	Q00973	0.330142	0.766195	0.498117	B4GALNT1	Beta-1,4 N-	B4GN1_HU	1	1.257137
15_MTA / C	Q13148	0.476023	0.764929	0.498117	TARDBP	TAR DNA-bi	TADBP_HUI	1	1.390904
30_MTA / C	Q15125	0.122672	0.766355	0.498117	EBP	3-beta-hydr	EBP_HUMA	1	1.08875
30_MTA / C	Q15165	-0.25063	0.766602	0.498117	PON2	Serum para	PON2_HUM	1	0.840531
15_MTA / C	Q5MNZ6	0.14991	0.767643	0.498117	WDR45B	WD repeat	WIPI3_HUI	2	1.1095
30_MTA / C	Q96G03	0.058383	0.765268	0.498117	PGM2	Phosphope	PGM2_HUI	17	1.041298
30_MTA / C	Q9H2P9	0.379872	0.765589	0.498117	DPH5	Diphthine r	DPH5_HUM	2	1.301226
15_MTA / C	Q9UMS4	0.158073	0.766172	0.498117	PRPF19	Pre-mRNA-p	PRP19_HUI	6	1.115796
30_MTA / C	Q9NWT6	-0.25623	0.768121	0.498249	HIF1AN	Hypoxia-in	HIF1N_HUI	1	0.837272
30_MTA / C	O75352	0.14525	0.768668	0.498469	MPDU1	Mannose-P	MPU1_HUM	2	1.105923
15_MTA / C	P42704	0.22705	0.768808	0.498469	LRPPRC	Leucine-ric	LPPRC_HUI	7	1.17044
30_MTA / C	Q9BQ52	-0.67275	0.768911	0.498469	ELAC2	Zinc phospl	RNZ2_HUM	3	0.627308
30_MTA / C	P61353	0.200271	0.770006	0.498788	RPL27	Large ribos	RL27_HUM	4	1.148914
15_MTA / C	Q9NR28	0.332081	0.769712	0.498788	DIABLO	Diablo IAP-	DBLOH_HU	5	1.258828
30_MTA / C	P31431	1.187291	0.771455	0.499629	SDC4	Syndecan-4	SDC4_HUM	1	2.277248
30_MTA / C	O43491	-0.06205	0.772621	0.500093	EPB41L2	Band 4.1-lil	E41L2_HUI	8	0.957899
30_MTA / C	P63241	-0.13156	0.772775	0.500093	EIF5A	Eukaryotic	IF5A1_HUM	3	0.912842
30_MTA / C	Q08209	0.312621	0.772575	0.500093	PPP3CA	Protein pho	PP2BA_HUI	2	1.241962
15_MTA / C	Q86X29	0.079263	0.772695	0.500093	LSR	Lipolysis-st	LSR_HUMA	5	1.056478
30_MTA / C	P60059	-0.17154	0.773202	0.500271	SEC61G	Protein trai	SC61G_HUI	2	0.887897
30_MTA / C	Q00610	-0.0029	0.773596	0.500429	CLTC	Clathrin he	CLH1_HUM	15	0.997993
30_MTA / C	P62306	-0.23214	0.77508	0.501291	SNRPF	Small nucle	RUXF_HUM	3	0.851372
30_MTA / C	P62328	0.477941	0.775342	0.501291	TMSB4X	Thymosin b	TYB4_HUM	5	1.392755
15_MTA / C	Q7LGA3	0.103567	0.775873	0.501412	HS2ST1	Heparan su	HS2ST_HUI	1	1.074426
15_MTA / C	P24390;P3	0.296824	0.77638	0.501434	KDELR1;KD	ER lumen p	ERD21_HUI	1	1.228437
15_MTA / C	P41227;Q9	0.24834	0.776463	0.501434	NAA10;NAA	N-alpha-ace	NAA10_HU	1	1.187839
15_MTA / C	P49768;P4	0.153957	0.776511	0.501434	PSEN1;PSEI	Presenilin-1	PSN1_HUM	1	1.112617
15_MTA / C	P62328	0.135296	0.776456	0.501434	TMSB4X	Thymosin b	TYB4_HUM	5	1.098318
15_MTA / C	Q8IZ83	0.130151	0.776835	0.501447	ALDH16A1	Aldehyde d	A16A1_HUI	11	1.094408
30_MTA / C	Q9Y333	0.270908	0.776742	0.501447	LSM2	U6 snRNA-a	LSM2_HUM	3	1.206567
15_MTA / C	P06756	0.151651	0.777289	0.501545	ITGAV	Integrin alp	ITAV_HUMA	1	1.11084
15_MTA / C	Q9NQR4	0.037073	0.777253	0.501545	NIT2	Omega-ami	NIT2_HUMA	6	1.02603

30_MTA / C	P62899	0.035914	0.777801	0.501778	RPL31	Large ribos	RL31_HUM	6	1.025206
30_MTA / C	P11310	-0.01383	0.778168	0.501917	ACADM	Medium-ch	ACADM_HU	12	0.990457
15_MTA / C	P62877	-0.41576	0.77887	0.502077	RBX1	E3 ubiquiti	RBX1_HUM	2	0.749623
15_MTA / C	Q08209	0.707395	0.77874	0.502077	PPP3CA	Protein pho	PP2BA_HU	2	1.632853
30_MTA / C	Q9UKY7	0.078599	0.779362	0.502101	CDV3	Protein CD	CDV3_HUM	4	1.055992
15_MTA / C	O95139	-0.37936	0.780723	0.502564	NDUFB6	NADH dehy	NDUB6_HU	3	0.768776
15_MTA / C	P14866	0.043857	0.78103	0.502564	HNRNPL	Heterogene	HNRPL_HU	3	1.030866
30_MTA / C	Q9BSR8	0.496976	0.781143	0.502564	YIPF4	Protein YIP	YIPF4_HUM	2	1.411252
15_MTA / C	Q9UNZ2	-0.08354	0.781015	0.502564	NSFL1C	NSFL1 cofa	NSF1C_HU	2	0.943741
30_MTA / C	Q9Y697	0.351235	0.780476	0.502564	NFS1	Cysteine de	NFS1_HUM	2	1.275652
15_MTA / C	P36405	-0.00877	0.781943	0.502981	ARL3	ADP-ribosy	ARL3_HUM	4	0.993937
30_MTA / C	P48775	0.119194	0.783209	0.503502	TDO2	Tryptophar	T23O_HUM	1	1.086128
15_MTA / C	Q03154	0.038296	0.783168	0.503502	ACY1	Aminoacyla	ACY1_HUM	5	1.0269
15_MTA / C	Q9Y4L1	0.001887	0.783075	0.503502	HYOU1	Hypoxia up	HYOU1_HU	27	1.001309
30_MTA / C	Q16775	-0.00977	0.783649	0.503688	HAGH	Hydroxyacy	GLO2_HUM	3	0.99325
15_MTA / C	Q04695	0.777608	0.784202	0.503848	KRT17	Keratin, typ	K1C17_HU	4	1.714286
15_MTA / C	Q9H910	-0.0314	0.784189	0.503848	JPT2	Jupiter mic	JUPI2_HUM	6	0.978472
30_MTA / C	P62847	-0.38583	0.784642	0.504032	RPS24	Small ribos	RS24_HUM	4	0.76534
30_MTA / C	O95433	0.076689	0.785657	0.504402	AHSA1	Activator o	AHSA1_HU	3	1.054595
30_MTA / C	P61803	0.067827	0.785674	0.504402	DAD1	Dolichyl-di	DAD1_HUM	4	1.048137
30_MTA / C	Q9BUR5	0.218044	0.78589	0.504443	APOO	MICOS com	MIC26_HU	2	1.163155
30_MTA / C	P30043	-0.04955	0.786496	0.504637	BLVRB	Flavin redu	BLVRB_HU	3	0.966236
15_MTA / C	Q16181	0.051084	0.786941	0.504824	SEPTIN7	Septin-7	SEPT7_HUM	5	1.036043
15_MTA / C	Q16576	0.414861	0.787095	0.504825	RBBP7	Histone-bir	RBBP7_HU	1	1.33317
15_MTA / C	O15173	0.260303	0.787262	0.504835	PGRMC2	Membrane-	PGRC2_HU	4	1.19773
30_MTA / C	P68431;P8	-0.12657	0.787685	0.505009	H3C1;H3-3	Histone H3	H31_HUMA	5	0.916008
15_MTA / C	Q14152	-0.11152	0.787977	0.505098	EIF3A	Eukaryotic	EIF3A_HUM	5	0.925613
30_MTA / C	O60506	0.165676	0.788472	0.505317	SYNCRIP	Heterogene	HNRPQ_HU	4	1.121692
30_MTA / C	Q9UBR2	-0.02574	0.78867	0.505347	CTSZ	Cathepsin Z	CATZ_HUM	3	0.982314
30_MTA / C	O75027	0.20034	0.789589	0.505545	ABCB7	Iron-sulfur	ABCB7_HU	3	1.148969
15_MTA / C	P13804	0.212204	0.789514	0.505545	ETFA	Electron tra	ETFA_HUMA	5	1.158457
30_MTA / C	P26640	0.799487	0.789492	0.505545	VARS1	Valine--tRN	SYVC_HUM	3	1.740482
15_MTA / C	P11279	-0.07554	0.790833	0.506002	LAMP1	Lysosome-a	LAMP1_HU	4	0.94899
15_MTA / C	Q96C01	-0.02067	0.790914	0.506002	FAM136A	Protein FAI	F136A_HU	3	0.985773
15_MTA / C	P62750	0.220588	0.791855	0.506311	RPL23A	Large ribos	RL23A_HU	4	1.165208
30_MTA / C	Q96EY8	0.818132	0.791805	0.506311	MMAB	Corrinoid a	MMAB_HU	1	1.763122
15_MTA / C	O60739;P4	0.33491	0.792523	0.50643	EIF1B;EIF1	Eukaryotic	EIF1B_HUM	1	1.261299
30_MTA / C	Q14204	-0.35979	0.792653	0.50643	DYNC1H1	Cytoplasmi	DYHC1_HU	13	0.779277
30_MTA / C	Q14697	0.124857	0.792528	0.50643	GANAB	Neutral alp	GANAB_HU	25	1.090399
30_MTA / C	Q9UMY4	-0.2593	0.793798	0.506966	SNX12	Sorting nex	SNX12_HU	1	0.835492
15_MTA / C	O60927	-0.03148	0.794261	0.507066	PPP1R11	E3 ubiquiti	PP1RB_HU	3	0.978415
30_MTA / C	P38159;Q9	-0.10154	0.794184	0.507066	RBMX;RBM	RNA-bindir	RBMX_HUM	1	0.932036
30_MTA / C	P14923	-0.16352	0.794597	0.507183	JUP	Junction pl	PLAK_HUM	13	0.892845
15_MTA / C	Q9H2V7	0.001402	0.79477	0.507196	SPNS1	Protein spi	SPNS1_HU	1	1.000973
15_MTA / C	P62736;P6	-0.20973	0.795176	0.507357	ACTA2;ACT	Actin, aorti	ACTA_HUM	1	0.864701
30_MTA / C	Q15008	0.403843	0.795514	0.507475	PSMD6	26S protea	PSMD6_HU	5	1.323028
30_MTA / C	O15143	-0.05537	0.796684	0.507985	ARPC1B	Actin-relate	ARC1B_HU	6	0.962347
30_MTA / C	P53618	-0.23649	0.797014	0.507985	COPB1	Coatomer s	COPB_HUM	6	0.848807
30_MTA / C	P60709	-0.02084	0.79708	0.507985	ACTB	Actin, cyto	ACTB_HUM	23	0.985659
15_MTA / C	P62304	0.157158	0.796494	0.507985	SNRPE	Small nucle	RUXE_HUM	2	1.115088
30_MTA / C	P43490	-0.23921	0.79758	0.508027	NAMPT	Nicotinami	NAMPT_HU	7	0.84721
30_MTA / C	Q9Y490	0.169865	0.797508	0.508027	TLN1	Talin-1	TLN1_HUM	20	1.124953

15_MTA / C	P62140	0.11306	0.798418	0.508447	PPP1CB	Serine/thre	PP1B_HUM	6	1.08152
30_MTA / C	O00264	0.083913	0.800071	0.508718	PGRMC1	Membrane-	PGRC1_HU	4	1.059889
15_MTA / C	Q00688	-0.09423	0.800072	0.508718	FKBP3	Peptidyl-pr	FKBP3_HUI	2	0.936774
15_MTA / C	Q7Z6M1	-0.15713	0.79988	0.508718	RABEPK	Rab9 effect	RABEK_HUI	2	0.896808
30_MTA / C	Q99426	-0.11899	0.799867	0.508718	TBCB	Tubulin-fol	TBCB_HUM	1	0.92083
15_MTA / C	Q99720	0.038599	0.799625	0.508718	SIGMAR1	Sigma non-	SGMR1_HU	2	1.027116
15_MTA / C	O00468	-0.12336	0.801424	0.508855	AGRN	Agrin	AGRIN_HUI	1	0.918049
30_MTA / C	O43865	-0.02465	0.801148	0.508855	AHCYL1	S-adenosylI	SAHH2_HU	7	0.983057
30_MTA / C	P00403	-0.04192	0.800738	0.508855	MT-CO2	Cytochrom	COX2_HUM	6	0.971359
30_MTA / C	Q16891	-0.12444	0.80141	0.508855	IMMT	MICOS com	MIC60_HUI	7	0.917359
30_MTA / C	Q99598	-0.10055	0.801412	0.508855	TSNAX	Translin-ass	TSNAX_HUI	4	0.932678
15_MTA / C	P63220	0.097093	0.801677	0.50886	RPS21	Small ribos	RS21_HUM	5	1.069616
30_MTA / C	Q07020	-0.00061	0.802281	0.509146	RPL18	Large ribos	RL18_HUM	8	0.99958
30_MTA / C	Q14157	-0.12567	0.803188	0.509429	UBAP2L	Ubiquitin-a	UBP2L_HUI	8	0.916579
30_MTA / C	Q9Y277	-0.01795	0.803146	0.509429	VDAC3	Voltage-de	VDAC3_HU	9	0.987637
30_MTA / C	Q9Y237	-0.45575	0.803574	0.509538	PIN4	Peptidyl-pr	PIN4_HUM	2	0.729129
30_MTA / C	Q13620	0.217978	0.804691	0.50996	CUL4B	Cullin-4B	CUL4B_HUI	3	1.163103
15_MTA / C	Q9BSY4	-0.3291	0.806042	0.510653	CHCHD5	Coiled-coil-	CHCH5_HU	1	0.796032
30_MTA / C	Q9NPJ3	0.123427	0.806569	0.510825	ACOT13	Acyl-coenzy	ACO13_HU	5	1.08932
15_MTA / C	P60981	-0.01568	0.807929	0.511359	DSTN	Destrin	DEST_HUM	3	0.989189
30_MTA / C	Q5VZM2;Q	-0.11252	0.807893	0.511359	RRAGB;RRA	Ras-related	RRAGB_HU	1	0.924974
30_MTA / C	P37840	-0.07391	0.808109	0.511375	SNCA	Alpha-synu	SYUA_HUM	6	0.95006
15_MTA / C	P35579	-0.02967	0.80827	0.51138	MYH9	Myosin-9	MYH9_HUM	12	0.979647
15_MTA / C	P01019	0.053009	0.808787	0.511383	AGT	Angiotensi	ANGT_HUM	6	1.037426
15_MTA / C	Q8NBJ7	-0.17982	0.809007	0.511383	SUMF2	Inactive C-a	SUMF2_HU	4	0.882812
15_MTA / C	Q96AA3	-0.10377	0.808508	0.511383	RFT1	Man(5)GlcN	RFT1_HUM	2	0.930596
30_MTA / C	P04792	-0.05379	0.80921	0.511388	HSPB1	Heat shock	HSPB1_HUI	6	0.963405
15_MTA / C	P02679	-0.00548	0.810328	0.51151	FGG	Fibrinogen	FIBG_HUMA	4	0.996211
15_MTA / C	P27635	0.036336	0.810158	0.51151	RPL10	Large ribos	RL10_HUM	4	1.025506
30_MTA / C	Q14566	-0.00416	0.810075	0.51151	MCM6	DNA replica	MCM6_HUI	1	0.99712
30_MTA / C	Q9BSY4	-0.22839	0.810209	0.51151	CHCHD5	Coiled-coil-	CHCH5_HU	1	0.853584
15_MTA / C	Q8TCD1	0.248025	0.810686	0.511638	C18orf32	UPF0729 p	CR032_HUI	1	1.18758
30_MTA / C	Q13242	0.305688	0.811794	0.512143	SRSF9	Serine/argi	SRSF9_HUM	2	1.236008
15_MTA / C	P53634	0.381266	0.812054	0.512209	CTSC	Dipeptidyl	CATC_HUM	4	1.302484
30_MTA / C	Q7L266	0.175693	0.812343	0.512294	ASRGL1	Isoaspartyl	ASGL1_HUI	1	1.129507
30_MTA / C	Q9HCJ6	0.282954	0.813038	0.512459	VAT1L	Synaptic ve	VAT1L_HUM	2	1.216684
30_MTA / C	Q9UEU0	0.146958	0.813068	0.512459	VTI1B	Vesicle tran	VTI1B_HUM	3	1.107232
30_MTA / C	P14174	-0.23443	0.813717	0.51277	MIF	Macrophag	MIF_HUMA	3	0.85002
15_MTA / C	P46977	0.179312	0.814579	0.512946	STT3A	Dolichyl-di	STT3A_HUM	5	1.132344
30_MTA / C	Q99873	0.095386	0.814616	0.512946	PRMT1	Protein arg	ANM1_HUM	2	1.068351
15_MTA / C	Q9H2J4	-0.03385	0.815427	0.51336	PDCL3	Phosducin-	PDCL3_HUI	3	0.976807
15_MTA / C	P11233	0.125808	0.817508	0.514466	RALA	Ras-related	RALA_HUM	4	1.091119
30_MTA / C	P60983	0.147111	0.81765	0.514466	GMFB	Glia matura	GMFB_HUM	3	1.107349
30_MTA / C	Q9UBV8	0.71225	0.819673	0.515543	PEF1	Peflin	PEF1_HUM	1	1.638357
30_MTA / C	P00492	0.270116	0.820329	0.515698	HPRT1	Hypoxanth	HPRT_HUM	6	1.205905
30_MTA / C	P25705	-0.01608	0.820498	0.515698	ATP5F1A	ATP syntha	ATPA_HUM	26	0.988918
30_MTA / C	Q16401	0.742789	0.820541	0.515698	PSMD5	26S protea	PSMD5_HU	4	1.673407
30_MTA / C	P16070	-0.42781	0.820906	0.515731	CD44	CD44 antig	CD44_HUM	3	0.743387
30_MTA / C	O43488	0.256339	0.821398	0.515845	AKR7A2	Aflatoxin B	ARK72_HUI	3	1.194444
30_MTA / C	P09525	-0.05663	0.822588	0.516382	ANXA4	Annexin A4	ANXA4_HU	12	0.961507
30_MTA / C	Q8NCW5	0.01516	0.82272	0.516382	NAXE	NAD(P)H-hy	NNRE_HUM	6	1.010564
30_MTA / C	Q9Y619	-0.07127	0.822662	0.516382	SLC25A15	Mitochond	ORNT1_HU	7	0.9518

30_MTA / C	P62910	-0.05001	0.823482	0.516664	RPL32	Large ribos	RL32_HUM	7	0.965929
15_MTA / C	Q99808	0.533071	0.82371	0.516709	SLC29A1	Equilibrativ	S29A1_HU	2	1.447006
15_MTA / C	P04439	0.293182	0.824565	0.516773	HLA-A	HLA class I	HLAA_HUM	3	1.22534
30_MTA / C	P09669	-0.00557	0.824	0.516773	COX6C	Cytochrom	COX6C_HU	5	0.996144
15_MTA / C	P78318	0.087308	0.824432	0.516773	IGBP1	Immunoglo	IGBP1_HU	3	1.062386
15_MTA / C	Q9BRG1	0.113672	0.824747	0.516773	VPS25	Vacuolar pr	VPS25_HU	1	1.081978
15_MTA / C	Q9NSD9	-0.11437	0.825096	0.516894	FARSB	Phenylalan	SYFB_HUM	3	0.923787
30_MTA / C	P09972	-0.06872	0.825657	0.517147	ALDOC	Fructose-bi	ALDOC_HU	2	0.953485
30_MTA / C	P08865	0.073069	0.825961	0.51724	RPSA	Small ribos	RSSA_HUM	9	1.051952
30_MTA / C	P25786	0.034451	0.827435	0.517968	PSMA1	Proteasom	PSA1_HUM	16	1.024167
30_MTA / C	Q7Z6M1	0.063164	0.827814	0.518009	RABEPK	Rab9 effect	RABEK_HU	2	1.044754
30_MTA / C	Q9BRP8	0.568026	0.828268	0.518195	PYM1	Partner of Y	PYM1_HUM	1	1.482493
30_MTA / C	A0A024R1	0.067635	0.828764	0.518408	TMA7B;TM	Translation	TMA7B_HU	2	1.047997
30_MTA / C	O95861	-0.02671	0.830264	0.51915	BPNT1	3'(2'),5'-bis	BPNT1_HU	11	0.981653
15_MTA / C	O95831	0.013128	0.830791	0.519267	AIFM1	Apoptosis-i	AIFM1_HU	12	1.009141
30_MTA / C	P18859	0.232708	0.831078	0.519267	ATP5PF	ATP syntha	ATP5J_HUM	3	1.175038
30_MTA / C	P63092;Q5	-0.0721	0.83103	0.519267	GNAS	Guanine nu	GNAS2_HU	1	0.951252
30_MTA / C	Q9GZL7	-0.08402	0.831051	0.519267	WDR12	Ribosome b	WDR12_HU	5	0.943425
30_MTA / C	O94925	0.063741	0.832392	0.519865	GLS	Glutamina	GLSK_HUM	2	1.045172
30_MTA / C	P0CG38	0.074643	0.832506	0.519865	POTEI	POTE ankyr	POTEI_HUM	2	1.053101
30_MTA / C	P00491	-0.0925	0.833831	0.52049	PNP	Purine nucl	PNPH_HUM	5	0.937897
30_MTA / C	P13987	0.041543	0.834179	0.52049	CD59	CD59 glyco	CD59_HUM	2	1.029214
30_MTA / C	P63244	0.035617	0.834264	0.52049	RACK1	Small ribos	RACK1_HU	17	1.024995
30_MTA / C	Q16740	-0.10438	0.834293	0.52049	CLPP	ATP-depen	CLPP_HUM	6	0.930206
15_MTA / C	Q9NPD3	0.232795	0.833908	0.52049	EXOSC4	Exosome co	EXOS4_HU	5	1.175109
15_MTA / C	Q13630	0.084309	0.835237	0.520884	GFUS	GDP-L-fuco	FCL_HUMA	5	1.06018
15_MTA / C	O43143	0.136778	0.836627	0.521006	DHX15	ATP-depen	DHX15_HU	14	1.099447
30_MTA / C	O43852	0.03137	0.836885	0.521006	CALU	Calumenin	CALU_HUM	3	1.021982
30_MTA / C	P61019	0.159138	0.836065	0.521006	RAB2A	Ras-related	RAB2A_HU	8	1.11662
15_MTA / C	Q14204	0.39581	0.836391	0.521006	DYNC1H1	Cytoplasmi	DYHC1_HU	13	1.315681
30_MTA / C	Q16563	-0.27382	0.83716	0.521006	SYPL1	Synaptophy	SYPL1_HUM	3	0.827125
30_MTA / C	Q8TB61	-0.21079	0.836507	0.521006	SLC35B2	Adenosine	S35B2_HU	5	0.864066
15_MTA / C	Q6Y1H2	-0.27587	0.837414	0.521065	HACD2	Very-long-c	HACD2_HU	2	0.825954
15_MTA / C	Q14247	-0.0069	0.837694	0.521134	CTTN	Src substra	SRC8_HUM	16	0.995231
15_MTA / C	Q7L266	0.410219	0.83784	0.521134	ASRGL1	Isoaspartyl	ASGL1_HU	1	1.328888
15_MTA / C	P63151	0.177083	0.839301	0.521749	PPP2R2A	Serine/thre	2ABA_HUM	3	1.130596
30_MTA / C	O95456	0.099534	0.839871	0.521908	PSMG1	Proteasom	PSMG1_HU	6	1.071427
15_MTA / C	P49736	0.054798	0.840807	0.522293	MCM2	DNA replica	MCM2_HU	5	1.038714
15_MTA / C	P09496	-0.02151	0.841623	0.522416	CLTA	Clathrin lig	CLCA_HUM	3	0.985204
30_MTA / C	P14618	-0.00389	0.841186	0.522416	PKM	Pyruvate ki	KPYM_HUM	30	0.997308
30_MTA / C	P80723	-0.11532	0.841335	0.522416	BASP1	Brain acid s	BASP1_HU	4	0.92318
30_MTA / C	Q8N5G0	-0.03814	0.841792	0.522416	SMIM20	Small integ	SIM20_HU	1	0.973908
30_MTA / C	Q9HC38	-0.14106	0.841686	0.522416	GLOD4	Glyoxalase	GLOD4_HU	3	0.906851
30_MTA / C	P09012	-0.16708	0.842228	0.522491	SNRPA	U1 small nu	SNRPA_HU	6	0.890645
30_MTA / C	A6NDG6	-0.03102	0.842826	0.52265	PGP	Glycerol-3-	PGP_HUMA	8	0.978726
30_MTA / C	Q15154	0.320995	0.842911	0.52265	PCM1	Pericentrio	PCM1_HUM	4	1.249191
15_MTA / C	Q9GZL7	0.239232	0.842959	0.52265	WDR12	Ribosome b	WDR12_HU	5	1.180364
30_MTA / C	P49411	0.362854	0.843564	0.522928	TUFM	Elongation	EFTU_HUM	4	1.285967
15_MTA / C	Q8N5M9	0.394671	0.843823	0.52299	JAGN1	Protein jag	JAGN1_HU	1	1.314643
15_MTA / C	P07814	0.107358	0.844718	0.523251	EPRS1	Bifunction	SYEP_HUM	8	1.077254
30_MTA / C	P42330;Q0	-0.01003	0.846665	0.52436	AKR1C3;AK	Aldo-keto r	AK1C3_HU	2	0.993072
15_MTA / C	Q86TM6	-0.02059	0.847437	0.524739	SYVN1	E3 ubiquiti	SYVN1_HU	2	0.985829

30_MTA / C	Q14165	0.738219	0.847841	0.524794	MLEC	Malectin	MLEC_HUM	2	1.668116
15_MTA / C	O14949	0.03881	0.849932	0.52499	UQCRQ	Cytochrom	QCR8_HUM	2	1.027266
15_MTA / C	O60763	-0.05503	0.848527	0.52499	USO1	General ves	USO1_HUM	4	0.962576
30_MTA / C	P05556	-0.01003	0.848693	0.52499	ITGB1	Integrin be	ITB1_HUM	10	0.993072
30_MTA / C	P35914	-0.06056	0.848346	0.52499	HMGCL	Hydroxyme	HMGCL_HU	1	0.958892
30_MTA / C	P37837	0.043141	0.850011	0.52499	TALDO1	Transaldola	TALDO_HU	14	1.030355
30_MTA / C	P50440	1.0332	0.849916	0.52499	GATM	Glycine am	GATM_HUM	1	2.046558
30_MTA / C	Q04917	0.022126	0.84884	0.52499	YWHAH	14-3-3 prot	1433F_HU	6	1.015455
15_MTA / C	Q15758	-0.02854	0.849179	0.52499	SLC1A5	Neutral am	AAAT_HUM	4	0.980409
15_MTA / C	Q6IQ22	0.102083	0.849254	0.52499	RAB12	Ras-related	RAB12_HU	3	1.073322
30_MTA / C	Q969U7	-0.19916	0.850059	0.52499	PSMG2	Proteasom	PSMG2_HU	3	0.871057
15_MTA / C	O43148	0.150662	0.851316	0.525571	RNMT	mRNA cap g	MCES_HUM	3	1.110079
15_MTA / C	Q9NPF4	-0.05508	0.851583	0.525637	OSGEP	tRNA N6-ac	OSGEP_HU	5	0.962543
30_MTA / C	P12268	-0.156	0.852667	0.525914	IMPDH2	Inosine-5'-r	IMDH2_HU	7	0.897509
30_MTA / C	O96000	0.201561	0.852877	0.525946	NDUFB10	NADH dehy	NDUBA_HU	2	1.149942
30_MTA / C	P62805	0.036274	0.853529	0.526251	H4C1	Histone H4	H4_HUMAN	8	1.025462
15_MTA / C	Q8NCC3	0.14091	0.854406	0.526595	PLA2G15	Lysosomal p	PAG15_HU	2	1.1026
15_MTA / C	Q9Y2V2	0.194009	0.856059	0.527319	CARHSP1	Calcium-re	CHSP1_HU	2	1.143938
15_MTA / C	Q5RI15	-0.0162	0.856557	0.527528	COX20	Cytochrom	COX20_HU	2	0.988834
30_MTA / C	P15531	0.227891	0.85851	0.528436	NME1	Nucleoside	NDKA_HUM	4	1.171122
30_MTA / C	P31944	0.108179	0.858452	0.528436	CASP14	Caspase-14	CASPE_HUM	2	1.077867
30_MTA / C	P00813	0.057613	0.858845	0.528545	ADA	Adenosine d	ADA_HUMA	5	1.040742
15_MTA / C	O00764	0.192525	0.859057	0.528577	PDXK	Pyridoxal k	PDXK_HUM	4	1.142762
15_MTA / C	O14737	0.300843	0.859225	0.528582	PDCD5	Programme	PDCD5_HU	8	1.231864
15_MTA / C	Q6PKG0	0.011224	0.85985	0.528783	LARP1	La-related p	LARP1_HU	3	1.00781
15_MTA / C	O43169	0.23007	0.860468	0.529029	CYB5B	Cytochrom	CYB5B_HU	6	1.172892
30_MTA / C	P09429	0.346311	0.86059	0.529029	HMGB1	High mobil	HMGB1_HU	2	1.271306
30_MTA / C	Q12907	0.095322	0.863214	0.530249	LMAN2	Vesicular in	LMAN2_HU	5	1.068304
30_MTA / C	O43676	0.471453	0.863583	0.530377	NDUFB3	NADH dehy	NDUB3_HU	2	1.386506
15_MTA / C	Q9BR76	0.037576	0.86393	0.530411	CORO1B	Coronin-1B	COR1B_HU	3	1.026388
30_MTA / C	Q9GZT8	0.048892	0.863976	0.530411	NIF3L1	NIF3-like pr	NIF3L_HUM	6	1.03447
30_MTA / C	Q9UBQ0	-0.09661	0.864279	0.530411	VPS29	Vacuolar pr	VPS29_HU	2	0.935228
30_MTA / C	Q9Y6C9	-0.10147	0.864678	0.530558	MTCH2	Mitochond	MTCH2_HU	3	0.932082
30_MTA / C	Q9NSE4	0.210837	0.865586	0.530918	IARS2	Isoleucine-	SYIM_HUM	7	1.15736
15_MTA / C	P27695	-0.07222	0.86743	0.531205	APEX1	DNA repair	APEX1_HU	1	0.951171
30_MTA / C	P51570	-0.35093	0.867308	0.531205	GALK1	Galactokin	GALK1_HU	2	0.784079
15_MTA / C	P54687	0.013004	0.867341	0.531205	BCAT1	Branched-c	BCAT1_HU	3	1.009055
30_MTA / C	Q02790	-0.33314	0.867495	0.531205	FKBP4	Peptidyl-pr	FKBP4_HU	7	0.793805
30_MTA / C	Q9BUL8	0.299209	0.86742	0.531205	PDCD10	Programme	PDC10_HU	4	1.230469
15_MTA / C	Q9HDC9	-0.02564	0.867131	0.531205	APMAP	Adipocyte p	APMAP_HU	10	0.982382
15_MTA / C	O00116	-0.18484	0.869884	0.531258	AGPS	Alkyldihydr	ADAS_HUM	3	0.879747
30_MTA / C	P12955	-0.06554	0.870712	0.531258	PEPD	Xaa-Pro dip	PEPD_HUM	12	0.955589
30_MTA / C	P13798	-0.01048	0.870328	0.531258	APEH	Acylamino-	ACPH_HUM	12	0.992763
30_MTA / C	P24390;P3	-0.46994	0.868546	0.531258	KDELR1;KD	ER lumen p	ERD21_HU	1	0.721994
30_MTA / C	P25325	0.005303	0.869922	0.531258	MPST	3-mercapto	THTM_HUM	5	1.003683
15_MTA / C	P29144	0.038872	0.869109	0.531258	TPP2	Tripeptidyl	TPP2_HUM	9	1.027311
15_MTA / C	P54727	0.106965	0.868691	0.531258	RAD23B	UV excision	RD23B_HU	11	1.07696
15_MTA / C	Q15041	0.221431	0.868285	0.531258	ARL6IP1	ADP-ribosy	AR6P1_HU	1	1.16589
30_MTA / C	Q15274	0.037175	0.870837	0.531258	QPRT	Nicotinate-	NADC_HUM	7	1.026103
30_MTA / C	Q15836	0.063507	0.867959	0.531258	VAMP3	Vesicle-ass	VAMP3_HU	2	1.045003
15_MTA / C	Q8N2U0	-0.13437	0.870686	0.531258	TMEM256	Transmemb	TM256_HU	1	0.911071
15_MTA / C	Q8N5K1	0.000614	0.870278	0.531258	CISD2	CDGSH iron	CISD2_HUM	5	1.000426

30_MTA / C	Q99798	-0.18548	0.869754	0.531258	ACO2	Aconitate h	ACON_HUM	1	0.879358
15_MTA / C	Q9BUF5	0.273082	0.869896	0.531258	TUBB6	Tubulin bet	TBB6_HUM	2	1.208387
15_MTA / C	Q9BWH2	0.1136	0.869394	0.531258	FUNDC2	FUN14 don	FUND2_HU	2	1.081925
30_MTA / C	Q9UHV9	0.062559	0.870812	0.531258	PFDN2	Prefoldin su	PFD2_HUM	6	1.044317
15_MTA / C	Q9Y320	0.067122	0.869821	0.531258	TMX2	Thioredoxi	TMX2_HUM	2	1.047624
15_MTA / C	Q9Y5L4	0.075851	0.87143	0.531258	TIMM13	Mitochond	TIM13_HUI	4	1.053983
30_MTA / C	P13861	0.261956	0.872233	0.531644	PRKAR2A	cAMP-depe	KAP2_HUM	5	1.199104
15_MTA / C	Q9NPJ3	0.00154	0.872384	0.531644	ACOT13	Acyl-coenzy	ACO13_HU	5	1.001068
15_MTA / C	P52815	0.119134	0.872928	0.531878	MRPL12	Large ribos	RM12_HUM	4	1.086083
15_MTA / C	Q14254	0.218979	0.874318	0.532333	FLOT2	Flotillin-2	FLOT2_HUI	3	1.16391
30_MTA / C	Q6GMV3	0.022678	0.874131	0.532333	PTRHD1	Putative pe	PTRD1_HUI	3	1.015843
30_MTA / C	Q9HAV7	-0.10184	0.873979	0.532333	GRPEL1	GrpE protei	GRPE1_HUI	1	0.931841
30_MTA / C	P25788	-0.04812	0.875216	0.532782	PSMA3	Proteasom	PSA3_HUM	11	0.967196
30_MTA / C	P01008	0.242296	0.876256	0.533031	SERPINC1	Antithroml	ANT3_HUM	1	1.182874
30_MTA / C	P30419	-0.48937	0.876574	0.533031	NMT1	Glycylpepti	NMT1_HUM	3	0.712334
15_MTA / C	Q9BTL3	-0.00789	0.876084	0.533031	RAMAC	RNA guanir	RAMAC_HU	1	0.994546
15_MTA / C	P16152	0.007908	0.87808	0.533479	CBR1	Carbonyl re	CBR1_HUM	8	1.005496
15_MTA / C	P50914	0.083258	0.878613	0.533576	RPL14	Large ribos	RL14_HUM	4	1.059408
30_MTA / C	Q15043	-0.24444	0.878601	0.533576	SLC39A14	Metal catio	S39AE_HUI	1	0.844144
15_MTA / C	P22307	0.143137	0.879508	0.53387	SCP2	Sterol carri	SCP2_HUM	7	1.104304
30_MTA / C	P25205	0.128602	0.879581	0.53387	MCM3	DNA replica	MCM3_HUI	5	1.093234
15_MTA / C	P52565	0.179305	0.880363	0.534149	ARHGDIA	Rho GDP-di	GDIR1_HUI	3	1.132338
30_MTA / C	P09601	-0.08447	0.880853	0.534349	HMOX1	Heme oxyg	HMOX1_HU	4	0.943131
15_MTA / C	Q96MV1	-0.21075	0.881874	0.53487	TLCD4	TLC domair	TLCD4_HUI	2	0.86409
30_MTA / C	Q12792	-0.08858	0.882502	0.535088	TWF1	Twinfilin-1	TWF1_HUM	6	0.940449
15_MTA / C	Q9UMS0	0.263434	0.882657	0.535088	NFU1	NFU1 iron-s	NFU1_HUM	2	1.200332
15_MTA / C	Q96D05	0.227326	0.883082	0.535121	FAM241B	Protein FAN	F241B_HUI	1	1.170663
15_MTA / C	Q9NPA0	0.336438	0.88316	0.535121	EMC7	Endoplasm	EMC7_HUM	3	1.262636
30_MTA / C	P34896	0.248855	0.883623	0.535245	SHMT1	Serine hydr	GLYC_HUM	3	1.188264
30_MTA / C	P01871	-0.01823	0.884116	0.53525	IGHM	Immunoglo	IGHM_HUM	2	0.987445
15_MTA / C	Q99623	-0.00117	0.884095	0.53525	PHB2	Prohibitin-	PHB2_HUM	17	0.999192
15_MTA / C	P17050	0.092586	0.884641	0.535445	NAGA	Alpha-N-ac	NAGAB_HU	1	1.06628
15_MTA / C	P62857	0.078933	0.885352	0.53559	RPS28	Small ribos	RS28_HUM	2	1.056237
30_MTA / C	Q6NUK1	-0.00763	0.885594	0.53559	SLC25A24	Mitochond	SCMC1_HU	11	0.994728
15_MTA / C	Q96A72	-0.13263	0.885647	0.53559	MAGOHB	Protein ma	MGN2_HUI	2	0.912169
30_MTA / C	O43759	0.367195	0.88659	0.53603	SYNGR1	Synaptogyr	SNG1_HUM	2	1.289843
30_MTA / C	P07737	0.03115	0.886698	0.53603	PFN1	Profilin-1	PROF1_HUI	8	1.021826
15_MTA / C	P13646	0.134428	0.887208	0.536241	KRT13	Keratin, typ	K1C13_HUI	15	1.097657
30_MTA / C	P00367	-0.01565	0.887722	0.536356	GLUD1	Glutamate	DHE3_HUM	18	0.989208
15_MTA / C	P62318	-0.0066	0.887614	0.536356	SNRPD3	Small nucle	SMD3_HUM	3	0.995439
15_MTA / C	Q9Y3B8	-0.11032	0.888151	0.536423	REXO2	Oligoribon	ORN_HUMA	4	0.926381
15_MTA / C	Q13813	0.050819	0.888671	0.536459	SPTAN1	Spectrin alp	SPTN1_HUI	24	1.035853
30_MTA / C	Q9P0T7	-0.10035	0.888703	0.536459	TMEM9	Proton-trar	TMEM9_HU	1	0.932808
30_MTA / C	P08134	0.159891	0.888881	0.536469	RHOC	Rho-relatec	RHOC_HUM	3	1.117203
30_MTA / C	Q9Y3B8	-0.08791	0.889076	0.536489	REXO2	Oligoribon	ORN_HUMA	4	0.940883
15_MTA / C	P04181	0.060915	0.889481	0.536636	OAT	Ornithine a	OAT_HUMA	7	1.043127
15_MTA / C	P10114;P6	0.193742	0.890233	0.536796	RAP2A;RAP	Ras-related	RAP2A_HUI	1	1.143727
30_MTA / C	P50579	0.200845	0.890109	0.536796	METAP2	Methionine	MAP2_HUM	1	1.149372
30_MTA / C	Q96HQ2	0.18186	0.891583	0.537512	CDKN2AIPN	CDKN2AIP	C2AIL_HUM	1	1.134345
30_MTA / C	P32969	0.175324	0.891942	0.537631	RPL9	Large ribos	RL9_HUMA	3	1.129218
15_MTA / C	P35232	-0.00942	0.892597	0.537928	PHB1	Prohibitin	PHB1_HUM	17	0.99349
30_MTA / C	Q9UHX1	-0.34933	0.893737	0.538419	PUF60	Poly(U)-bin	PUF60_HUI	2	0.784946

15_MTA / C	Q14108	-0.01283	0.894121	0.538553	SCARB2	Lysosome m	SCRB2_HUI	1	0.991146
30_MTA / C	P01024	-0.154	0.894284	0.538553	C3	Compleme	CO3_HUM/	6	0.898755
30_MTA / C	P31943	0.061596	0.895032	0.538861	HNRNPH1	Heterogene	HNRH1_HU	5	1.043619
30_MTA / C	P53041	0.249183	0.895121	0.538861	PPP5C	Serine/thre	PPP5_HUM	1	1.188534
15_MTA / C	P42677	0.04688	0.895344	0.538871	RPS27	Small ribos	RS27_HUM	3	1.033029
15_MTA / C	O95571	0.113851	0.897433	0.539474	ETHE1	Persulfide d	ETHE1_HUI	2	1.082113
15_MTA / C	P23229	0.141162	0.896981	0.539474	ITGA6	Integrin alp	ITA6_HUM/	1	1.102793
15_MTA / C	Q5TFE4	-0.06238	0.897928	0.539474	NT5DC1	5'-nucleoti	NT5D1_HU	3	0.957683
30_MTA / C	Q8NE71	-0.11411	0.897678	0.539474	ABCF1	ATP-bindin	ABCF1_HUI	5	0.92395
15_MTA / C	Q9UNN5	-0.02939	0.897925	0.539474	FAF1	FAS-associa	FAF1_HUM	2	0.979834
30_MTA / C	Q9Y512	0.054981	0.897174	0.539474	SAMM50	Sorting and	SAM50_HU	6	1.038845
15_MTA / C	Q96EY8	-0.05699	0.899041	0.539946	MMAB	Corrinoid a	MMAB_HUI	1	0.961269
15_MTA / C	P67812	0.550907	0.899337	0.540026	SEC11A	Signal pept	SC11A_HUI	3	1.465007
30_MTA / C	P54709	0.057725	0.900253	0.540065	ATP1B3	Sodium/po	AT1B3_HUI	6	1.040823
30_MTA / C	P62753	0.085003	0.90038	0.540065	RPS6	Small ribos	RS6_HUMA	9	1.06069
30_MTA / C	Q15102	0.1016	0.900084	0.540065	PAFAH1B3	Platelet-act	PA1B3_HUI	4	1.072963
30_MTA / C	Q96CN7	0.133457	0.900252	0.540065	ISOC1	Isochorism	ISOC1_HUM	5	1.096919
15_MTA / C	Q9BVA1	-0.16378	0.900295	0.540065	TUBB2B	Tubulin bet	TBB2B_HUI	1	0.892685
15_MTA / C	Q9NWV8	-0.55965	0.901069	0.540217	BABAM1	BRISC and E	BABA1_HUI	2	0.678468
15_MTA / C	Q9UDX5	0.196862	0.900991	0.540217	MTFP1	Mitochond	MTFP1_HU	3	1.146202
15_MTA / C	O00151	0.064462	0.902931	0.540661	PDLIM1	PDZ and LIM	PDLI1_HUM	8	1.045695
30_MTA / C	O00567	0.307164	0.902069	0.540661	NOP56	Nucleolar p	NOP56_HU	1	1.237273
15_MTA / C	O43760	-0.03532	0.90277	0.540661	SYNGR2	Synaptogyr	SNG2_HUM	4	0.975815
30_MTA / C	O95336	0.116487	0.902698	0.540661	PGLS	6-phospho	6PGL_HUM	5	1.084092
30_MTA / C	P50502;Q8	0.129539	0.902705	0.540661	ST13;ST13F	Hsc70-inter	F10A1_HUI	2	1.093944
30_MTA / C	Q96I15	0.614526	0.903006	0.540661	SCLY	Selenocyste	SCLY_HUMA	1	1.531055
15_MTA / C	Q15819	0.140158	0.904267	0.541319	UBE2V2	Ubiquitin-c	UB2V2_HU	2	1.102026
30_MTA / C	P05023	0.013486	0.90491	0.541349	ATP1A1	Sodium/po	AT1A1_HUI	28	1.009392
30_MTA / C	P55084	-0.07356	0.904854	0.541349	HADHB	Trifunction	ECHB_HUM	2	0.95029
30_MTA / C	P67870	-0.23437	0.904516	0.541349	CSNK2B	Casein kina	CSK2B_HUI	1	0.850055
30_MTA / C	Q96K76	-0.09059	0.905135	0.541349	USP47	Ubiquitin c	UBP47_HU	1	0.939136
30_MTA / C	Q99575	0.526899	0.905116	0.541349	POP1	Ribonuclea	POP1_HUM	2	1.440829
30_MTA / C	O60487	0.15093	0.905389	0.541403	MPZL2	Myelin prot	MPZL2_HU	1	1.110285
15_MTA / C	P02753	0.074992	0.905551	0.541403	RBP4	Retinol-bin	RET4_HUM	2	1.053355
30_MTA / C	P14625	0.028251	0.906345	0.541589	HSP90B1	Endoplasm	ENPL_HUM	30	1.019775
30_MTA / C	P28332	-0.12693	0.906287	0.541589	ADH6	Alcohol deh	ADH6_HUM	6	0.915778
30_MTA / C	Q9UN36	0.47906	0.906526	0.541595	NDRG2	Protein ND	NDRG2_HU	2	1.393835
15_MTA / C	P61254	0.048293	0.908558	0.542499	RPL26	Large ribos	RL26_HUM	9	1.034041
30_MTA / C	Q99729	-0.11588	0.908448	0.542499	HNRNPAB	Heterogene	ROAA_HUM	3	0.922817
30_MTA / C	Q9BS26	0.30542	0.908497	0.542499	ERP44	Endoplasm	ERP44_HUI	4	1.235778
15_MTA / C	Q9P0J0	0.137625	0.908694	0.542499	NDUFA13	NADH dehy	NDUAD_HU	2	1.100093
15_MTA / C	O15372	0.176628	0.909145	0.542605	EIF3H	Eukaryotic	EIF3H_HUM	4	1.130239
30_MTA / C	O43324	-0.50645	0.909199	0.542605	EEF1E1	Eukaryotic	MCA3_HUM	1	0.703953
15_MTA / C	O60256	0.042344	0.909483	0.542643	PRPSAP2	Phosphorib	KPRB_HUM	3	1.029786
30_MTA / C	P09493	0.318173	0.910015	0.542643	TPM1	Tropomyos	TPM1_HUM	2	1.246751
30_MTA / C	P15121	-0.01758	0.90982	0.542643	AKR1B1	Aldo-keto r	ALDR_HUM	6	0.987887
30_MTA / C	P28074	0.068482	0.910818	0.542984	PSMB5	Proteasom	PSB5_HUM	12	1.048613
30_MTA / C	P49915	-0.12553	0.911295	0.543073	GMPS	GMP synth	GUAA_HUM	4	0.916667
15_MTA / C	O43175	-0.01859	0.911889	0.543094	PHGDH	D-3-phosph	SERA_HUM	11	0.987196
30_MTA / C	P17858	-0.09852	0.911527	0.543094	PFKL	ATP-depend	PFKAL_HUI	8	0.93399
15_MTA / C	Q7LG56	-0.04781	0.911985	0.543094	RRM2B	Ribonuclec	RIR2B_HUI	1	0.967404
15_MTA / C	Q7Z4W1	-0.53721	0.912206	0.543128	DCXR	L-xylulose r	DCXR_HUM	2	0.689102

15_MTA / C	P06730	0.021335	0.913219	0.543633	EIF4E	Eukaryotic	IF4E_HUMA	2	1.014898
15_MTA / C	P12956	0.138174	0.913481	0.543692	XRCC6	X-ray repair	XRCC6_HU	11	1.100511
30_MTA / C	Q9BQA1	-0.03549	0.914015	0.543814	WDR77	Methylosor	MEP50_HU	2	0.975698
15_MTA / C	Q9BUN8	0.113331	0.914002	0.543814	DERL1	Derlin-1	DERL1_HU	4	1.081723
15_MTA / C	Q9Y3B3	1.249863	0.91484	0.544012	TMED7	Transmemb	TMED7_HU	1	2.378189
30_MTA / C	P36405	0.056154	0.915208	0.544134	ARL3	ADP-ribosy	ARL3_HUM	4	1.03969
30_MTA / C	P09622	-0.08509	0.916284	0.544382	DLD	Dihydrolip	DLDH_HUM	14	0.942729
15_MTA / C	Q13505	0.197921	0.916176	0.544382	MTX1	Metaxin-1	MTX1_HUM	1	1.147044
15_MTA / C	P01009	0.019368	0.91674	0.544458	SERPINA1	Alpha-1-ant	A1AT_HUM	10	1.013515
30_MTA / C	P06493	-0.1171	0.917472	0.544503	CDK1	Cyclin-depe	CDK1_HUM	2	0.922038
15_MTA / C	Q9NR45	0.411916	0.91708	0.544503	NANS	Sialic acid s	SIAS_HUMA	2	1.330452
30_MTA / C	P22307	-0.02899	0.918456	0.544706	SCP2	Sterol carri	SCP2_HUM	7	0.980107
15_MTA / C	Q96GC9	0.492675	0.91858	0.544706	VMP1	Vacuole me	VMP1_HUM	1	1.407052
15_MTA / C	Q9BZZ5	0.062235	0.918637	0.544706	API5	Apoptosis i	API5_HUMA	11	1.044082
30_MTA / C	P26038	-0.01173	0.91909	0.544787	MSN	Moesin	MOES_HUM	13	0.991899
30_MTA / C	Q15056	0.259637	0.919469	0.54481	EIF4H	Eukaryotic	IF4H_HUMA	3	1.197178
15_MTA / C	Q8IXB1	0.084946	0.920284	0.545097	DNAJC10	DnaJ homo	DJC10_HU	4	1.060648
30_MTA / C	Q06210	0.218342	0.921579	0.545669	GFPT1	Glutamine-	GFPT1_HU	5	1.163396
15_MTA / C	Q86V81	-0.13333	0.922103	0.545785	ALYREF	THO compl	THOC4_HU	2	0.911727
30_MTA / C	P30048	0.23105	0.922752	0.545959	PRDX3	Thioredoxi	PRDX3_HU	6	1.173689
30_MTA / C	P62277	-0.00299	0.922892	0.545959	RPS13	Small ribos	RS13_HUM	7	0.997932
15_MTA / C	Q15029	0.019619	0.923505	0.546224	EFTUD2	116 kDa U5	U5S1_HUM	18	1.013692
15_MTA / C	P12268	0.043242	0.924792	0.546888	IMPDH2	Inosine-5'-r	IMDH2_HU	7	1.030427
30_MTA / C	O00299	0.04764	0.925618	0.547279	CLIC1	Chloride in	CLIC1_HUM	8	1.033573
15_MTA / C	P35237	0.068442	0.925947	0.547376	SERPINB6	Serpin B6	SPB6_HUM	7	1.048584
15_MTA / C	P52597	0.176888	0.926116	0.547378	HNRNPF	Heterogene	HNRPF_HU	3	1.130443
30_MTA / C	P46779	0.197585	0.927817	0.548155	RPL28	Large ribos	RL28_HUM	9	1.146777
30_MTA / C	P49721	-0.09702	0.927926	0.548155	PSMB2	Proteasom	PSB2_HUM	5	0.93496
15_MTA / C	P06576	-0.03313	0.928641	0.548479	ATP5F1B	ATP syntha	ATPB_HUM	18	0.977298
30_MTA / C	B5ME19;Q	0.003638	0.929098	0.548649	EIF3CL;EIF3	Eukaryotic	EIFCL_HUM	3	1.002525
30_MTA / C	O00400	0.23499	0.929825	0.548787	SLC33A1	Acetyl-coer	ACATN_HU	3	1.176898
15_MTA / C	P30405	0.087533	0.930155	0.548884	PPIF	Peptidyl-pr	PPIF_HUMA	2	1.062551
30_MTA / C	O75794	-0.20915	0.930555	0.548925	CDC123	Translation	CD123_HU	2	0.865046
15_MTA / C	Q14011	0.333726	0.930419	0.548925	CIRBP	Cold-induc	CIRBP_HUM	1	1.260264
30_MTA / C	Q14019	-0.19596	0.930772	0.548926	COTL1	Coactosin-l	COTL1_HU	2	0.872993
15_MTA / C	Q96C36	-0.02995	0.930888	0.548926	PYCR2	Pyrroline-5	P5CR2_HU	5	0.979455
15_MTA / C	P49591	-0.29258	0.931875	0.549214	SARS1	Serine--tRN	SYSC_HUMA	1	0.816443
30_MTA / C	P36578	-0.01319	0.932554	0.549517	RPL4	Large ribos	RL4_HUMA	17	0.990902
15_MTA / C	O14979	-0.00308	0.933959	0.549575	HNRNPDL	Heterogene	HNRDL_HU	3	0.99787
30_MTA / C	P04275	0.223569	0.934435	0.549575	VWF	von Willebr	VWF_HUMA	10	1.167618
30_MTA / C	P33993	0.083882	0.934045	0.549575	MCM7	DNA replica	MCM7_HU	6	1.059866
30_MTA / C	P40429	-0.01261	0.934733	0.549575	RPL13A	Large ribos	RL13A_HU	8	0.9913
15_MTA / C	P53041	-0.14601	0.934421	0.549575	PPP5C	Serine/thre	PPP5_HUM	1	0.903747
30_MTA / C	Q92896	0.34113	0.934673	0.549575	GLG1	Golgi appar	GSLG1_HU	8	1.266748
15_MTA / C	Q96AG4	0.017343	0.934809	0.549575	LRRC59	Leucine-ric	LRC59_HU	6	1.012094
15_MTA / C	Q99439	0.041786	0.934457	0.549575	CNN2	Calponin-2	CNN2_HUM	1	1.029387
15_MTA / C	Q9BUP3	0.285373	0.93367	0.549575	HTATIP2	Oxidoreduc	HTAI2_HUM	1	1.218725
30_MTA / C	Q9H832	0.109178	0.933114	0.549575	UBE2Z	Ubiquitin-c	UBE2Z_HU	1	1.078614
15_MTA / C	Q9UBQ5	0.052946	0.933915	0.549575	EIF3K	Eukaryotic	EIF3K_HUM	1	1.037381
30_MTA / C	Q8WXE9	0.484737	0.935207	0.549712	STON2	Stonin-2	STON2_HU	1	1.399331
30_MTA / C	P07858	-0.21917	0.938269	0.550054	CTSB	Cathepsin E	CATB_HUM	2	0.859062
30_MTA / C	P08240	0.378999	0.9385	0.550054	SRPRA	Signal reco	SRPRA_HU	3	1.300439

15_MTA / C	P08559	-0.56185	0.937485	0.550054	PDHA1	Pyruvate de	ODPA_HUM	2	0.677435
30_MTA / C	P13693	-0.04253	0.937522	0.550054	TPT1	Translation	TCTP_HUM	4	0.970949
15_MTA / C	P38117	0.060846	0.936892	0.550054	ETFB	Electron tra	ETFB_HUMA	9	1.043077
15_MTA / C	P48643	0.021643	0.936792	0.550054	CCT5	T-complex	TCPE_HUM	20	1.015115
30_MTA / C	P61158	0.096043	0.937084	0.550054	ACTR3	Actin-relate	ARP3_HUM	10	1.068838
15_MTA / C	P61201	0.153194	0.936613	0.550054	COPS2	COP9 signa	CSN2_HUM	1	1.112028
30_MTA / C	P61970	-0.10556	0.937631	0.550054	NUTF2	Nuclear tra	NTF2_HUM	2	0.929444
15_MTA / C	P63104	0.006067	0.937995	0.550054	YWHAZ	14-3-3 prot	1433Z_HU	11	1.004214
15_MTA / C	Q16186	-0.13769	0.937845	0.550054	ADRM1	Proteasom	ADRM1_HU	2	0.908975
30_MTA / C	Q99832	0.052905	0.93861	0.550054	CCT7	T-complex	TCPH_HUM	23	1.037352
15_MTA / C	Q9NQ84	0.296739	0.935959	0.550054	GPRC5C	G-protein c	GPC5C_HU	5	1.228365
30_MTA / C	Q9UBQ5	-0.03777	0.938611	0.550054	EIF3K	Eukaryotic	EIF3K_HUM	1	0.97416
15_MTA / C	Q9Y265	-0.0458	0.936362	0.550054	RUVBL1	RuvB-like 1	RUVB1_HU	10	0.96875
15_MTA / C	Q9Y5V0	0.727254	0.937654	0.550054	ZNF706	Zinc finger	ZN706_HU	1	1.655485
30_MTA / C	P40616	-0.13827	0.939586	0.550431	ARL1	ADP-ribosy	ARL1_HUM	1	0.908608
15_MTA / C	O95202	0.066073	0.940711	0.550993	LETM1	Mitochond	LETM1_HU	4	1.046863
30_MTA / C	P31327	-0.01157	0.940991	0.551059	CPS1	Carbamoyl-	CPSM_HUM	38	0.992013
15_MTA / C	P61088;Q5	-0.00332	0.941499	0.551199	UBE2N;UBE	Ubiquitin-c	UBE2N_HU	5	0.997699
15_MTA / C	P62491;Q1	-0.01091	0.942023	0.551199	RAB11A;RA	Ras-related	RB11A_HU	11	0.992465
15_MTA / C	Q9NUM4	0.07206	0.942061	0.551199	TMEM106E	Transmemb	T106B_HU	2	1.051216
15_MTA / C	Q9NX63	-0.05241	0.941673	0.551199	CHCHD3	MICOS com	MIC19_HU	3	0.964322
30_MTA / C	Q96HS1	0.116653	0.942382	0.551289	PGAM5	Serine/thre	PGAM5_HU	4	1.084217
15_MTA / C	P20674	0.265657	0.942616	0.551329	COX5A	Cytochrom	COX5A_HU	2	1.202184
30_MTA / C	P08559	0.566456	0.943636	0.55146	PDHA1	Pyruvate de	ODPA_HUM	2	1.480881
30_MTA / C	P62280	0.034585	0.94419	0.55146	RPS11	Small ribos	RS11_HUM	12	1.024262
30_MTA / C	Q96C01	0.17808	0.943959	0.55146	FAM136A	Protein FAN	F136A_HU	3	1.131377
30_MTA / C	Q9BR76	0.133125	0.943401	0.55146	CORO1B	Coronin-1E	COR1B_HU	3	1.096667
30_MTA / C	Q96HV5	-0.71484	0.94484	0.551656	TMEM41A	Transmemb	TM41A_HU	1	0.609271
15_MTA / C	P06132	-0.02516	0.945368	0.551753	UROD	Uroporphy	DCUP_HUM	4	0.982713
30_MTA / C	Q3I5F7;Q8	-0.09946	0.945401	0.551753	ACOT6;ACC	Acyl-coenzy	ACOT6_HU	2	0.93338
30_MTA / C	Q5J8M3	-0.45912	0.945673	0.551753	EMC4	ER membra	EMC4_HUM	2	0.72743
30_MTA / C	Q99623	-0.00813	0.945518	0.551753	PHB2	Prohibitin-	PHB2_HUM	17	0.994382
30_MTA / C	Q4VC31	0.131002	0.947771	0.552491	MIX23	Protein MI	MIX23_HU	1	1.095054
15_MTA / C	Q9P2J5	0.086384	0.948295	0.552602	LARS1	Leucine--tR	SYLC_HUMA	2	1.061706
30_MTA / C	P49189	-0.03055	0.948764	0.552681	ALDH9A1	4-trimethyl	AL9A1_HU	12	0.979044
15_MTA / C	Q8TCT9	0.042986	0.94919	0.552832	HM13	Minor histo	HM13_HUN	6	1.030244
30_MTA / C	P17612;P2	0.070677	0.950232	0.55304	PRKACA;PR	cAMP-depe	KAPCA_HU	1	1.050209
15_MTA / C	P20645	0.081199	0.950601	0.55304	M6PR	Cation-dep	MPRD_HUN	4	1.057897
30_MTA / C	P49755	0.21355	0.950748	0.55304	TMED10	Transmemb	TMEDA_HU	5	1.159538
30_MTA / C	Q99536	-0.00232	0.950635	0.55304	VAT1	Synaptic ve	VAT1_HUM	10	0.998395
15_MTA / C	P33240	0.055159	0.951926	0.55355	CSTF2	Cleavage sti	CSTF2_HUM	7	1.038974
30_MTA / C	O75390	-0.15042	0.952615	0.553563	CS	Citrate synt	CISY_HUMA	8	0.900986
15_MTA / C	Q00577	-0.13563	0.952314	0.553563	PURA	Transcripti	PURA_HUM	1	0.910269
15_MTA / C	P13984	0.051208	0.953329	0.553686	GTF2F2	General tra	T2FB_HUM	1	1.036132
15_MTA / C	Q92484	0.588944	0.953315	0.553686	SMPDL3A	Cyclic GMP	ASM3A_HU	5	1.504145
30_MTA / C	Q9P035	0.656816	0.953274	0.553686	HACD3	Very-long-c	HACD3_HU	2	1.5766
30_MTA / C	Q14444	-0.06623	0.953572	0.55373	CAPRIN1	Caprin-1	CAPR1_HU	6	0.955129
30_MTA / C	O15372	-0.47019	0.954054	0.553913	EIF3H	Eukaryotic	EIF3H_HUM	4	0.721868
15_MTA / C	Q5BJH7	0.268984	0.954476	0.554061	YIF1B	Protein YIF	YIF1B_HUM	1	1.204959
30_MTA / C	Q9Y2V2	0.196461	0.954752	0.554124	CARHSP1	Calcium-re	CHSP1_HU	2	1.145884
15_MTA / C	O75348;O	0.785347	0.95554	0.55429	ATP6V1G1;	V-type prot	VATG1_HU	1	1.723506
15_MTA / C	P61421	0.124766	0.955434	0.55429	ATP6V0D1	V-type prot	VA0D1_HU	2	1.090331

15_MTA / C	Q9P2E9	0.392845	0.955911	0.554316	RRBP1	Ribosome-b	RRBP1_HU	16	1.31298
30_MTA / C	Q9UBX3	0.076463	0.955919	0.554316	SLC25A10	Mitochond	DIC_HUMA	7	1.054429
15_MTA / C	Q15437	0.268696	0.956212	0.554389	SEC23B	Protein tra	SC23B_HU	4	1.204719
30_MTA / C	P17931	-0.01814	0.958233	0.555379	LGALS3	Galectin-3	LEG3_HUM	1	0.987502
15_MTA / C	P51687	-0.04966	0.959558	0.555379	SUOX	Sulfite oxid	SUOX_HUM	1	0.966162
15_MTA / C	P60880	-0.27408	0.958954	0.555379	SNAP25	Synaptosor	SNP25_HU	1	0.826976
30_MTA / C	Q9BSH5	0.257135	0.959486	0.555379	HDHD3	Haloacid de	HDHD3_HU	1	1.195103
30_MTA / C	Q9H0V9	0.925205	0.959586	0.555379	LMAN2L	VIP36-like p	LMA2L_HU	1	1.898954
30_MTA / C	Q9H910	-0.01652	0.958965	0.555379	JPT2	Jupiter mic	JUPI2_HUM	6	0.988616
30_MTA / C	Q99497	0.003695	0.9602	0.555554	PARK7	Parkinson c	PARK7_HU	13	1.002565
15_MTA / C	Q9UBV8	0.264165	0.960234	0.555554	PEF1	Peflin	PEF1_HUM	1	1.20094
30_MTA / C	Q13435	0.404859	0.960444	0.555579	SF3B2	Splicing fac	SF3B2_HUM	3	1.323959
30_MTA / C	Q6UXN9	-0.57378	0.960772	0.555671	WDR82	WD repeat-	WDR82_HU	3	0.671852
30_MTA / C	P51153	-0.08345	0.961251	0.555852	RAB13	Ras-related	RAB13_HU	4	0.9438
30_MTA / C	P30046	-0.01559	0.961694	0.556011	DDT	D-dopachro	DOPD_HUM	5	0.989249
30_MTA / C	P29401	0.008235	0.962888	0.556604	TKT	Transketola	TKT_HUMA	24	1.005725
15_MTA / C	P62917	0.033938	0.964449	0.557409	RPL8	Large ribos	RL8_HUMA	10	1.023803
30_MTA / C	P20674	0.487913	0.965076	0.55748	COX5A	Cytochrom	COX5A_HU	2	1.402415
30_MTA / C	P30740	0.146407	0.964985	0.55748	SERPINB1	Leukocyte e	ILEU_HUM	6	1.106809
30_MTA / C	P12236	0.068594	0.965479	0.557518	SLC25A6	ADP/ATP tr	ADT3_HUM	15	1.048694
30_MTA / C	Q04637	-0.02724	0.965316	0.557518	EIF4G1	Eukaryotic	IF4G1_HUM	8	0.981293
15_MTA / C	Q13310	0.239356	0.96645	0.5579	PABPC4	Polyadenyl	PABP4_HU	2	1.180465
30_MTA / C	Q9H1E5	0.239438	0.966954	0.558078	TMX4	Thioredoxi	TMX4_HUM	1	1.180533
30_MTA / C	O95563	0.199134	0.967992	0.558191	MPC2	Mitochond	MPC2_HUM	1	1.148009
30_MTA / C	Q02878	0.071001	0.967347	0.558191	RPL6	Large ribos	RL6_HUMA	11	1.050445
15_MTA / C	Q6P6C2	0.192077	0.967837	0.558191	ALKBH5	RNA demet	ALKB5_HU	1	1.142407
15_MTA / C	Q9NUU7;Q	-0.02122	0.967846	0.558191	DDX19A;DI	ATP-depend	DD19A_HU	2	0.985402
15_MTA / C	Q9UI12	-0.40815	0.967663	0.558191	ATP6V1H	V-type prot	VATH_HUM	2	0.753591
30_MTA / C	P28072	0.029304	0.968743	0.55843	PSMB6	Proteasom	PSB6_HUM	6	1.02052
30_MTA / C	O60925	-0.07861	0.969369	0.558694	PFDN1	Prefoldin su	PFD1_HUM	4	0.946973
30_MTA / C	O76003	-0.0752	0.971076	0.558787	GLRX3	Glutaredox	GLRX3_HU	4	0.949213
30_MTA / C	P42765	-0.11062	0.971321	0.558787	ACAA2	3-ketoacyl-	THIM_HUM	9	0.926191
15_MTA / C	P52926	0.408889	0.971197	0.558787	HMGA2	High mobil	HMGA2_HU	1	1.327663
15_MTA / C	Q14165	0.198402	0.970646	0.558787	MLEC	Malectin	MLEC_HUM	2	1.147427
30_MTA / C	Q15181	0.019232	0.970481	0.558787	PPA1	Inorganic p	IPYR_HUM	10	1.01342
30_MTA / C	Q16186	0.041063	0.971118	0.558787	ADRM1	Proteasom	ADRM1_HU	2	1.028871
15_MTA / C	Q8N1G4	0.505614	0.969798	0.558787	LRRC47	Leucine-ric	LRC47_HU	4	1.419727
30_MTA / C	Q92542	-0.16127	0.971216	0.558787	NCSTN	Nicastrin	NICA_HUM	4	0.894238
15_MTA / C	Q92747	0.200185	0.971386	0.558787	ARPC1A	Actin-relate	ARC1A_HU	5	1.148846
15_MTA / C	Q96IU4	0.113189	0.972562	0.559367	ABHD14B	Putative pr	ABHEB_HU	2	1.081617
15_MTA / C	O94973;O	0.24734	0.972964	0.559501	AP2A2;AP2	AP-2 compl	AP2A2_HU	1	1.187016
30_MTA / C	Q99523	0.165863	0.973182	0.559529	SORT1	Sortilin	SORT_HUM	3	1.121837
30_MTA / C	Q9UHD1	0.118348	0.973563	0.559651	CHORDC1	Cysteine an	CHRD1_HU	3	1.085492
30_MTA / C	Q9Y5Z4	0.045927	0.974123	0.559779	HEBP2	Heme-bind	HEBP2_HU	5	1.032346
30_MTA / C	P46926	-0.05507	0.975518	0.559894	GNPDA1	Glucosamir	GNPI1_HU	7	0.962545
15_MTA / C	P51571	0.105683	0.97584	0.559894	SSR4	Translocon	SSRD_HUM	4	1.076004
30_MTA / C	P60880	-0.07089	0.976351	0.559894	SNAP25	Synaptosor	SNP25_HU	1	0.952051
15_MTA / C	Q15393	0.051121	0.975366	0.559894	SF3B3	Splicing fac	SF3B3_HUM	20	1.036069
30_MTA / C	Q9NTX5	0.012569	0.976195	0.559894	ECHDC1	Ethylmalor	ECHD1_HU	7	1.00875
30_MTA / C	Q9P0L0	-0.06044	0.97471	0.559894	VAPA	Vesicle-assc	VAPA_HUM	8	0.958971
15_MTA / C	Q9UHW5	0.035493	0.975544	0.559894	GPN3	GPN-loop G	GPN3_HUM	1	1.024907
30_MTA / C	P62701	-0.02856	0.977373	0.560286	RPS4X	Small ribos	RS4X_HUM	13	0.980397

15_MTA / C	Q04323	-0.08592	0.978022	0.560534	UBXN1	UBX domai	UBXN1_HU	1	0.942181
15_MTA / C	Q6KCM7;Q	0.421187	0.978725	0.56077	SLC25A25;:	Mitochond	SCMC2_HU	1	1.339029
15_MTA / C	P27824	0.06829	0.979806	0.561098	CANX	Calnexin	CALX_HUM	13	1.048474
15_MTA / C	Q9HC35	0.226279	0.979759	0.561098	EML4	Echinoderr	EMAL4_HU	2	1.169814
15_MTA / C	P84101	-0.2613	0.980461	0.561376	SERF2	Small EDRK	SERF2_HUM	2	0.834336
30_MTA / C	Q13177	0.002803	0.98069	0.56141	PAK2	Serine/thre	PAK2_HUM	13	1.001945
30_MTA / C	Q9NQH7	0.139013	0.980858	0.56141	XPNPEP3	Xaa-Pro am	XPP3_HUM	2	1.101152
30_MTA / C	P30044	0.198883	0.981838	0.561727	PRDX5	Peroxiredo	PRDX5_HU	4	1.147809
30_MTA / C	O60828	-0.00774	0.982235	0.56181	PQBP1	Polyglutam	PQBP1_HU	3	0.994649
30_MTA / C	P19256	-0.09544	0.982444	0.561833	CD58	Lymphocyt	LFA3_HUM	1	0.935989
15_MTA / C	Q9H0U4	0.089652	0.982916	0.561908	RAB1B	Ras-related	RAB1B_HUI	6	1.064114
15_MTA / C	Q8IYS2	0.405189	0.984408	0.562664	KIAA2013	Uncharacte	K2013_HUI	2	1.324263
30_MTA / C	P63151	0.397657	0.984962	0.562884	PPP2R2A	Serine/thre	2ABA_HUM	3	1.317366
15_MTA / C	P13929	0.018142	0.98516	0.562894	ENO3	Beta-enolas	ENOB_HUM	2	1.012655
30_MTA / C	O75964	-0.02245	0.987344	0.563096	ATP5MG	ATP syntha:	ATP5L_HUI	6	0.984562
30_MTA / C	P12081	0.072025	0.986795	0.563096	HARS1	Histidine--t	HARS1_HUI	11	1.051191
30_MTA / C	P13647	0.035892	0.986189	0.563096	KRT5	Keratin, tyr	K2C5_HUM	18	1.02519
30_MTA / C	P32929	0.030388	0.986713	0.563096	CTH	Cystathioni	CGL_HUMA	3	1.021287
15_MTA / C	P55036	0.098879	0.987371	0.563096	PSMD4	26S protea:	PSMD4_HU	5	1.070941
15_MTA / C	P61026	0.137189	0.987063	0.563096	RAB10	Ras-related	RAB10_HUI	4	1.09976
30_MTA / C	P62826	0.068696	0.98699	0.563096	RAN	GTP-bindin	RAN_HUMA	6	1.048768
30_MTA / C	Q08257	0.012974	0.987166	0.563096	CRYZ	Quinone ox	QOR_HUMA	15	1.009033
15_MTA / C	Q9UQ35	0.353124	0.986229	0.563096	SRRM2	Serine/argi	SRRM2_HU	4	1.277323
15_MTA / C	P39687	0.053577	0.987591	0.563124	ANP32A	Acidic leuci	AN32A_HU	4	1.037835
30_MTA / C	Q8WUH6	0.150583	0.9878	0.563146	TMEM263	Transmemb	TM263_HU	1	1.110018
15_MTA / C	Q15366	0.042087	0.988114	0.563229	PCBP2	Poly(rC)-bir	PCBP2_HUI	7	1.029602
15_MTA / C	Q12905	0.04362	0.989443	0.563792	ILF2	Interleukin	ILF2_HUMA	5	1.030697
15_MTA / C	Q969U7	0.167438	0.989946	0.563955	PSMG2	Proteasom	PSMG2_HU	3	1.123062
30_MTA / C	P78318	-0.27427	0.990652	0.564093	IGBP1	Immunoglo	IGBP1_HUI	3	0.826867
15_MTA / C	Q9BTV4	-0.14109	0.990602	0.564093	TMEM43	Transmemb	TMM43_HU	1	0.906835
15_MTA / C	P62826	-0.0476	0.991178	0.564198	RAN	GTP-bindin	RAN_HUMA	6	0.967548
30_MTA / C	Q14145	-0.01834	0.991047	0.564198	KEAP1	Kelch-like E	KEAP1_HUI	2	0.987365
15_MTA / C	Q04637	0.178948	0.991513	0.564201	EIF4G1	Eukaryotic	IF4G1_HUM	8	1.132058
30_MTA / C	O14949	-0.04282	0.992896	0.564519	UQCRQ	Cytochrom	QCR8_HUM	2	0.970753
15_MTA / C	P11413	0.078588	0.992949	0.564519	G6PD	Glucose-6-r	G6PD_HUM	11	1.055984
30_MTA / C	P41091	-0.02531	0.992316	0.564519	EIF2S3	Eukaryotic	IF2G_HUMA	16	0.982611
30_MTA / C	P62249	0.031461	0.993104	0.564519	RPS16	Small ribos	RS16_HUM	9	1.022046
30_MTA / C	Q5TFE4	-0.27725	0.992807	0.564519	NT5DC1	5'-nucleoti	NT5D1_HU	3	0.825162
30_MTA / C	Q96AY3	0.02263	0.99356	0.564681	FKBP10	Peptidyl-pr	FKB10_HUI	7	1.01581
30_MTA / C	O00154	0.107428	0.994612	0.564796	ACOT7	Cytosolic a	BACH_HUM	5	1.077306
30_MTA / C	P60510	-0.05192	0.994448	0.564796	PPP4C	Serine/thre	PP4C_HUM	2	0.964651
30_MTA / C	Q14152	-0.05806	0.994047	0.564796	EIF3A	Eukaryotic	EIF3A_HUM	5	0.960554
30_MTA / C	Q53H96	-0.30998	0.99447	0.564796	PYCR3	Pyrroline-5	P5CR3_HUI	3	0.806655
30_MTA / C	Q9Y223	0.055145	0.994613	0.564796	GNE	Bifunctiona	GLCNE_HUI	7	1.038964
15_MTA / C	O60437	0.259968	0.996382	0.565509	PPL	Periplakin	PEPL_HUM	2	1.197452
15_MTA / C	Q9H488	0.146502	0.996345	0.565509	POFUT1	GDP-fucose	OFUT1_HUI	3	1.106883
15_MTA / C	Q15287	0.453228	0.996946	0.565636	RNPS1	RNA-bindir	RNPS1_HUI	2	1.3691
15_MTA / C	O75787	-0.08518	0.997556	0.565885	ATP6AP2	Renin recep	RENR_HUM	1	0.94267
15_MTA / C	P07910	-0.05813	0.998086	0.565895	HNRNPC	Heterogene	HNRPC_HU	5	0.960507
30_MTA / C	Q5MNZ6	0.137826	0.998001	0.565895	WDR45B	WD repeat	WIPI3_HUM	2	1.100246
15_MTA / C	Q9BUL9	-0.01269	0.997901	0.565895	RPP25	Ribonuclea	RPP25_HUI	1	0.991242
30_MTA / C	P50990	0.017959	0.999125	0.566193	CCT8	T-complex	TCPQ_HUM	25	1.012526

15_MTA / C	P43243	-1.19E-07	1	0.566496	MATR3	Matrin-3	MATR3_HU	2	1
