# Supplementary material for: Decoding the Cellular Response to Methylthioadenosine Uncovers Novel Targets for Chronic Liver Disease
Source: Cells. 2026 Sep 22;15(19):1720. doi: 10.3390/cells15191720 (PMC13664110; doi:10.3390/cells15191720)
Supplement: Supplementary file 1 [file cells-15-01720-s001.zip › Table S3_PISA_Extracts_PLC.pdf]

Compariso	ProteinGro	AVG Log2 R	Pvalue	Qvalue	Genes	ProteinDes	ProteinNar	# Unique Tc	Ratio
15min_MT.	Q14525	0.722244	3.65E-20	2.86E-16	KRT33B	Keratin, typ	KT33B_HUI	2	1.649746
15min_MT.	Q9H2P9	2.02241	3.79E-12	1.48E-08	DPH5	Diphthine r	DPH5_HUN	8	4.062618
15min_MT.	P23526	1.143826	1.36E-11	3.54E-08	AHCY	Adenosylhc	SAHH_HUN	25	2.209662
30min_MT.	P52788	1.17861	6.76E-11	1.32E-07	SMS	Spermine sy	SPSY_HUM.	11	2.263586
30min_MT.	P23526	1.276905	9.14E-11	1.43E-07	AHCY	Adenosylhc	SAHH_HUN	25	2.423185
15min_MT.	Q9H479	1.110152	1.40E-10	1.82E-07	FN3K	Fructosami	FN3K_HUN	9	2.158684
30min_MT.	Q9H479	1.061995	3.90E-10	4.36E-07	FN3K	Fructosami	FN3K_HUN	9	2.087817
15min_MT.	P78385	0.807687	1.08E-09	1.06E-06	KRT83	Keratin, typ	KRT83_HUI	17	1.750403
15min_MT.	P19623	1.125089	3.30E-09	2.86E-06	SRM	Spermidine	SPEE_HUMA	8	2.18115
15min_MT.	Q99714	0.613952	1.55E-08	1.22E-05	HSD17B10	3-hydroxya	HCD2_HUN	14	1.530446
30min_MT.	P00439	0.984718	3.00E-08	2.13E-05	PAH	Phenylalan	PH4H_HUN	22	1.978926
15min_MT.	Q52LA3	1.408928	5.28E-08	3.14E-05	LIN52	Protein lin-	LIN52_HUN	1	2.655398
15min_MT.	Q96Q89	0.718918	5.62E-08	3.14E-05	KIF20B	Kinesin-like	KI20B_HUN	1	1.645947
30min_MT.	Q99714	0.573952	5.54E-08	3.14E-05	HSD17B10	3-hydroxya	HCD2_HUN	14	1.488596
15min_MT.	P20042	-0.40371	6.17E-08	3.22E-05	EIF2S2	Eukaryotic	IF2B_HUMA	23	0.755911
30min_MT.	Q9H2P9	1.988377	8.71E-08	4.26E-05	DPH5	Diphthine r	DPH5_HUN	8	3.967903
30min_MT.	Q52LA3	1.555052	1.23E-07	5.66E-05	LIN52	Protein lin-	LIN52_HUN	1	2.938442
15min_MT.	Q9H074	0.36495	2.05E-07	8.43E-05	PAIP1	Polyadenyl	PAIP1_HUN	17	1.287837
15min_MT.	P00439	1.10653	2.37E-07	9.28E-05	PAH	Phenylalan	PH4H_HUN	22	2.153271
15min_MT.	P47914	-0.67065	2.76E-07	9.94E-05	RPL29	Large ribos	RL29_HUM	2	0.628224
30min_MT.	P55263	0.881952	2.80E-07	9.94E-05	ADK	Adenosine l	ADK_HUMA	16	1.842867
15min_MT.	O76009	0.693106	3.12E-07	0.000106	KRT33A	Keratin, typ	KT33A_HUI	14	1.616761
15min_MT.	Q53EL6	0.3702	3.63E-07	0.000118	PDCD4	Programme	PDCD4_HU	17	1.292532
15min_MT.	Q8IWE2	0.409018	5.31E-07	0.000166	FAM114A1	Protein NO	NXP20_HU	14	1.327781
15min_MT.	P28482	0.391638	5.63E-07	0.000169	MAPK1	Mitogen-ac	MK01_HUN	15	1.311882
30min_MT.	P19013	0.571261	9.24E-07	0.000256	KRT4	Keratin, typ	K2C4_HUM	19	1.485822
15min_MT.	Q15257	0.334866	9.51E-07	0.000256	PTPA	Serine/thre	PTPA_HUM	11	1.261261
30min_MT.	P22102	0.293413	1.04E-06	0.000271	GART	Trifunction	PUR2_HUM	31	1.225536
30min_MT.	P19623	1.148518	1.13E-06	0.000277	SRM	Spermidine	SPEE_HUMA	8	2.216861
30min_MT.	Q9NPJ3	0.331594	1.13E-06	0.000277	ACOT13	Acyl-coenzy	ACO13_HU	5	1.258403
15min_MT.	Q9Y570	0.220595	1.75E-06	0.000414	PPME1	Protein phc	PPME1_HU	13	1.165214
15min_MT.	Q05397	0.339411	1.89E-06	0.000423	PTK2	Focal adhes	FAK1_HUM	17	1.26524
30min_MT.	P07148	0.292315	2.30E-06	0.000499	FABP1	Fatty acid-b	FABPL_HUN	6	1.224604
15min_MT.	P25691	0.950864	2.63E-06	0.000556	NaN	Keratin, typ	K2M3_SHEI	10	1.93303
30min_MT.	O95470	-4.32483	3.64E-06	0.00072	SGPL1	Sphingosin	SGPL1_HUI	1	0.0499
15min_MT.	P55263	0.676207	3.67E-06	0.00072	ADK	Adenosine l	ADK_HUMA	16	1.597933
15min_MT.	Q15014	0.524322	3.68E-06	0.00072	MORF4L2	Mortality fa	MO4L2_HU	7	1.438258
30min_MT.	O75369	0.143463	3.81E-06	0.000727	FLNB	Filamin-B	FLNB_HUM	67	1.104553
30min_MT.	Q9NPD3	0.255292	3.91E-06	0.000729	EXOSC4	Exosome cc	EXOS4_HUI	10	1.193577
30min_MT.	P07355	0.201699	4.44E-06	0.000801	ANXA2	Annexin A2	ANXA2_HU	27	1.150052
30min_MT.	Q15650	-0.6218	4.50E-06	0.000801	TRIP4	Activating s	TRIP4_HUN	2	0.649859
30min_MT.	P99999	0.513961	5.30E-06	0.000902	CYCS	Cytochrom	CYC_HUMA	6	1.427965
30min_MT.	Q9HD40	0.198996	5.29E-06	0.000902	SEPSECS	O-phospho	SPCS_HUM	9	1.147899
15min_MT.	Q13526	-1.26174	6.36E-06	0.001059	PIN1	Peptidyl-pr	PIN1_HUM	5	0.41704
30min_MT.	P05155	0.1728	6.92E-06	0.001083	SERPING1	Plasma pro	IC1_HUMAI	13	1.127244
15min_MT.	P32929	-0.30704	6.80E-06	0.001083	CTH	Cystathioni	CGL_HUMA	16	0.808297
15min_MT.	O43660	-0.34446	8.01E-06	0.001205	PLRG1	Pleiotropic	PLRG1_HUI	19	0.787604
30min_MT.	Q96DA0	0.704454	8.32E-06	0.001229	ZG16B	Pancreatic	PAUF_HUM	6	1.629527
15min_MT.	P83881	-0.57741	9.24E-06	0.001338	RPL36A	Large ribos	RL36A_HUI	4	0.670165
15min_MT.	Q9H173	0.401598	9.86E-06	0.001402	SIL1	Nucleotide	SIL1_HUMA	11	1.320971
30min_MT.	Q9UBC9	-0.82717	1.01E-05	0.001416	SPRR3	Small proli	SPRR3_HUI	8	0.563632

15min_MT.	P02534	0.418334	1.30E-05	0.001786	NaN	Keratin, typ	K1M1_SHEI	10	1.336383
15min_MT.	Q00765	-0.29602	1.35E-05	0.001801	REEP5	Receptor ex	REEP5_HUN	5	0.814498
15min_MT.	Q9UHD1	-0.28012	1.36E-05	0.001801	CHORDC1	Cysteine an	CHRD1_HU	16	0.823525
15min_MT.	Q9UBQ5	0.435355	1.46E-05	0.00181	EIF3K	Eukaryotic	EIF3K_HUM	7	1.352243
30min_MT.	Q9HA64	0.392479	1.55E-05	0.001897	FN3KRP	Ketosamine	KT3K_HUM	9	1.312647
15min_MT.	Q15287	-0.39949	1.64E-05	0.001971	RNPS1	RNA-bindir	RNPS1_HU	4	0.758126
15min_MT.	Q9HBK9	0.243639	1.79E-05	0.002085	AS3MT	Arsenite me	AS3MT_HU	20	1.183975
15min_MT.	P62750	-0.63706	2.02E-05	0.002273	RPL23A	Large ribos	RL23A_HUI	7	0.643023
15min_MT.	P84098	-0.49097	2.05E-05	0.002273	RPL19	Large ribos	RL19_HUM	5	0.711547
15min_MT.	Q9Y394	-0.98907	2.06E-05	0.002273	DHRS7	Dehydroger	DHRS7_HU	2	0.503802
30min_MT.	Q99426	0.194871	2.27E-05	0.002431	TBCB	Tubulin-fol	TBCB_HUM	12	1.144621
15min_MT.	Q14839	-0.22886	2.33E-05	0.002464	CHD4	Chromodor	CHD4_HUM	24	0.853307
30min_MT.	P01876	0.820586	2.61E-05	0.002622	IGHA1	Immunoglo	IGHA1_HUI	4	1.766123
15min_MT.	Q9NRN7	-0.83358	2.67E-05	0.002643	AASDHPPT	L-aminoadi	ADPPT_HUI	9	0.561134
15min_MT.	P40925	0.284431	2.72E-05	0.002663	MDH1	Malate deh	MDHC_HUI	17	1.21793
15min_MT.	P53367	0.29418	2.88E-05	0.002748	ARFIP1	Arfaptin-1	ARFP1_HUI	9	1.226188
15min_MT.	Q8N668	0.38759	2.85E-05	0.002748	COMMD1	COMM don	COMD1_HU	1	1.308207
15min_MT.	Q53FA7	0.206931	3.06E-05	0.002814	TP53I3	Quinone ox	QORX_HUM	16	1.15423
15min_MT.	Q8WTX7	-1.17329	3.22E-05	0.002893	CASTOR1	Cytosolic ar	CAST1_HUN	2	0.443409
15min_MT.	Q13033	0.19786	3.36E-05	0.002988	STRN3	Striatin-3	STRN3_HUI	16	1.146995
30min_MT.	P50570	-0.35029	3.67E-05	0.003187	DNM2	Dynamin-2	DYN2_HUM	22	0.784426
15min_MT.	Q86TP1	0.294662	3.80E-05	0.003231	PRUNE1	Exopolypho	PRUN1_HU	9	1.226597
15min_MT.	Q96GA7	0.246947	3.79E-05	0.003231	SDSL	Serine dehy	SDSL_HUM	9	1.186694
15min_MT.	Q6PD62	-0.38413	4.13E-05	0.003436	CTR9	RNA polym	CTR9_HUM	6	0.76624
15min_MT.	Q9GZQ3	0.627836	4.44E-05	0.003546	COMMD5	COMM don	COMD5_HU	6	1.545245
30min_MT.	P40925	0.346499	4.82E-05	0.003768	MDH1	Malate deh	MDHC_HUI	17	1.271471
15min_MT.	O96000	1.299318	4.88E-05	0.003778	NDUFB10	NADH dehy	NDUBA_HU	1	2.461125
15min_MT.	P13164;QC	-0.78229	5.16E-05	0.003806	IFITM1;IFIT	Interferon-i	IFM1_HUM	1	0.581443
30min_MT.	P43243	-0.27576	5.15E-05	0.003806	MATR3	Matrin-3	MATR3_HU	14	0.826018
30min_MT.	P48637	0.190945	5.12E-05	0.003806	GSS	Glutathion	GSHB_HUM	26	1.141511
15min_MT.	P51668	0.32117	5.09E-05	0.003806	UBE2D1	Ubiquitin-c	UB2D1_HU	2	1.249343
15min_MT.	Q9P1U1	0.2473	5.30E-05	0.003816	ACTR3B	Actin-relate	ARP3B_HUI	7	1.186983
30min_MT.	Q9Y639	0.763471	5.32E-05	0.003816	NPTN	Neuroplast	NPTN_HUM	2	1.69757
15min_MT.	P41743	0.439123	5.43E-05	0.003858	PRKCI	Protein kin	KPCI_HUMA	9	1.35578
15min_MT.	P43034	-0.15702	5.85E-05	0.003976	PAFAH1B1	Platelet-act	LIS1_HUMA	19	0.896875
15min_MT.	P52788	1.188796	5.86E-05	0.003976	SMS	Spermine sy	SPSY_HUMA	11	2.279625
15min_MT.	Q8TD08	0.456534	5.80E-05	0.003976	MAPK15	Mitogen-ac	MK15_HUM	1	1.372242
15min_MT.	Q96CD2	0.389796	5.90E-05	0.003976	PPCDC	Phosphopa	COAC_HUM	5	1.310208
15min_MT.	P54619	0.321227	6.28E-05	0.004142	PRKAG1	5'-AMP-acti	AAKG1_HU	13	1.249392
15min_MT.	P07355	0.161949	6.47E-05	0.004159	ANXA2	Annexin A2	ANXA2_HU	27	1.118798
15min_MT.	P20073	0.207955	6.54E-05	0.004159	ANXA7	Annexin A7	ANXA7_HU	19	1.15505
15min_MT.	P22102	0.318966	6.45E-05	0.004159	GART	Trifunction	PUR2_HUM	31	1.247436
30min_MT.	P00505	0.195933	6.78E-05	0.004227	GOT2	Aspartate a	AATM_HUM	30	1.145464
15min_MT.	Q9NVG8	0.366228	6.71E-05	0.004227	TBC1D13	TBC1 doma	TBC13_HUI	16	1.288979
15min_MT.	Q9NQ29	-0.40125	6.96E-05	0.004286	LUC7L	Putative RN	LUC7L_HUI	8	0.757202
15min_MT.	A8MXV4	-0.28108	7.26E-05	0.004325	NUDT19	Acyl-coenzy	NUD19_HU	11	0.822976
15min_MT.	O95825	0.149339	7.23E-05	0.004325	CRYZL1	Quinone ox	QORL1_HU	10	1.109061
30min_MT.	P01012	1.221014	7.18E-05	0.004325	SERPINB14	Ovalbumin	OVAL_CHIC	1	2.331105
15min_MT.	P40261	0.16914	7.35E-05	0.004325	NNMT	Nicotinami	NNMT_HUN	10	1.124388
30min_MT.	Q8WTX7	-1.01293	7.54E-05	0.004366	CASTOR1	Cytosolic ar	CAST1_HUN	2	0.495538
15min_MT.	Q8N543	0.460784	7.59E-05	0.004368	OGFOD1	Prolyl 3-hy	OGFD1_HU	3	1.37629
30min_MT.	Q96SI1	-0.2397	7.85E-05	0.004447	KCTD15	BTB/POZ dc	KCD15_HU	1	0.846919

15min_MT.	Q9BWS9	-0.97149	7.94E-05	0.004469	CHID1	Chitinase d	CHID1_HUI	3	0.509979
15min_MT.	O14744	0.476442	8.16E-05	0.004562	PRMT5	Protein arg	ANM5_HUI	15	1.391308
30min_MT.	O43252	-0.37701	8.79E-05	0.004676	PAPSS1	Bifunctiona	PAPS1_HUI	5	0.770029
15min_MT.	P02539	0.083882	8.78E-05	0.004676	NaN	Keratin, typ	K2M1_SHEI	2	1.059866
15min_MT.	P12081	0.210735	8.61E-05	0.004676	HARS1	Histidine--t	HARS1_HUI	22	1.157278
15min_MT.	P51580	0.275707	8.63E-05	0.004676	TPMT	Thiopurine	TPMT_HUM	12	1.210587
15min_MT.	Q13098	0.179091	8.76E-05	0.004676	GPS1	COP9 signa	CSN1_HUM	13	1.13217
15min_MT.	Q15008	-0.12226	8.67E-05	0.004676	PSMD6	26S protea	PSMD6_HU	22	0.91875
30min_MT.	Q86U86	1.002909	9.25E-05	0.004827	PBRM1	Protein pol	PB1_HUMA	1	2.004036
15min_MT.	P50583	-0.51366	9.37E-05	0.004855	NUDT2	Bis(5'-nucle	AP4A_HUM	6	0.700445
30min_MT.	Q86U28	0.924784	9.45E-05	0.004864	ISCA2	Iron-sulfur	ISCA2_HUM	5	1.8984
30min_MT.	P41743	0.590982	9.75E-05	0.004914	PRKCI	Protein kin	KPCI_HUMA	9	1.506271
30min_MT.	P51580	0.31043	9.83E-05	0.004914	TPMT	Thiopurine	TPMT_HUM	12	1.240077
30min_MT.	Q8TDL5	0.43	9.95E-05	0.004914	BPIFB1	BPI fold-coi	BPIB1_HUM	4	1.347234
30min_MT.	Q9P260	-0.43966	9.95E-05	0.004914	RELCH	RAB11-binc	RELCH_HUI	20	0.737311
15min_MT.	Q9Y6G9	0.177015	9.95E-05	0.004914	DYNC1LI1	Cytoplasmi	DC1L1_HUI	20	1.130543
15min_MT.	Q13595	-0.58053	0.000102	0.004937	TRA2A	Transforme	TRA2A_HUI	5	0.668718
30min_MT.	Q8N4T8	0.286084	0.000108	0.00517	CBR4	3-oxoacyl-[	CBR4_HUM	10	1.219326
30min_MT.	P34896	0.118067	0.00011	0.005173	SHMT1	Serine hydr	GLYC_HUM	15	1.08528
30min_MT.	P49354	0.278271	0.00011	0.005173	FNTA	Protein farı	FNTA_HUM	10	1.21274
30min_MT.	P22694	0.402195	0.000114	0.005265	PRKACB	cAMP-depe	KAPCB_HUI	7	1.321517
15min_MT.	Q05048	-0.1451	0.000113	0.005265	CSTF1	Cleavage sti	CSTF1_HUM	13	0.90432
15min_MT.	Q8NHP8	0.196857	0.000116	0.005327	PLBD2	Putative ph	PLBL2_HUM	12	1.146199
15min_MT.	Q6P1J9	0.277286	0.000118	0.005356	CDC73	Parafibrom	CDC73_HU	10	1.211913
30min_MT.	P14618	-0.24303	0.000126	0.005591	PKM	Pyruvate ki	KPYM_HUM	35	0.844971
30min_MT.	P81605	1.222597	0.000126	0.005591	DCD	Dermcidin	DCD_HUMA	3	2.333665
15min_MT.	O60678	0.230502	0.000129	0.005645	PRMT3	Protein arg	ANM3_HUM	9	1.173243
15min_MT.	Q9Y3L5	-1.07307	0.000128	0.005645	RAP2C	Ras-related	RAP2C_HUI	3	0.475306
30min_MT.	Q8IZ73	0.218092	0.00013	0.005658	RPUSD2	Pseudourid	RUSD2_HU	14	1.163194
15min_MT.	P30047	-1.40172	0.000131	0.005674	GCHFR	GTP cycloh	GFRP_HUM	4	0.378479
15min_MT.	Q86VX2	0.60978	0.000138	0.005898	COMMD7	COMM don	COMD7_HU	5	1.526026
30min_MT.	Q01469	0.315558	0.000139	0.005916	FABP5	Fatty acid-k	FABP5_HUI	11	1.244493
15min_MT.	P29401	-0.15384	0.000141	0.005946	TKT	Transketola	TKT_HUMA	25	0.898852
30min_MT.	P61626	1.433864	0.000142	0.005958	LYZ	Lysozyme C	LYSC_HUMA	4	2.701693
15min_MT.	P52434	-0.24247	0.000144	0.005993	POLR2H	DNA-direct	RPAB3_HUI	5	0.845297
15min_MT.	Q9HB71	-0.17656	0.000146	0.006003	CACYBP	Calcyclin-b	CYBP_HUM	20	0.88481
15min_MT.	Q8TE77	0.174714	0.000147	0.006033	SSH3	Protein pho	SSH3_HUM	8	1.128741
30min_MT.	Q8NHP8	0.324021	0.00015	0.006064	PLBD2	Putative ph	PLBL2_HUM	12	1.251815
30min_MT.	Q9NXH9	0.237441	0.000149	0.006064	TRMT1	tRNA (guan	TRM1_HUM	15	1.1789
15min_MT.	Q6UXN9	-0.16238	0.000152	0.006078	WDR82	WD repeat-	WDR82_HU	11	0.893548
15min_MT.	O95571	0.189153	0.000154	0.006086	ETHE1	Persulfide c	ETHE1_HUM	8	1.140094
15min_MT.	P36952	1.350032	0.000164	0.006287	SERPINB5	Serpin B5	SPB5_HUM	1	2.549179
30min_MT.	P36952	1.689687	0.000161	0.006287	SERPINB5	Serpin B5	SPB5_HUM	1	3.225867
15min_MT.	Q96AB3	0.591603	0.000165	0.006287	ISOC2	Isochorism	ISOC2_HUM	6	1.50692
15min_MT.	Q96H20	0.378015	0.000163	0.006287	SNF8	Vacuolar-sc	SNF8_HUM	6	1.299553
30min_MT.	P16422	-0.17172	0.000168	0.006314	EPCAM	Epithelial c	EPCAM_HU	2	0.887782
15min_MT.	A0A087WV	2.572941	0.000172	0.006412	SPEGNB	SPEG neigh	SPEGN_HUI	1	5.950214
30min_MT.	O14744	0.406819	0.000177	0.006412	PRMT5	Protein arg	ANM5_HUM	15	1.325759
30min_MT.	P04083	0.346434	0.000178	0.006412	ANXA1	Annexin A1	ANXA1_HU	13	1.271414
15min_MT.	P41235	0.404708	0.000172	0.006412	HNF4A	Hepatocyte	HNF4A_HU	6	1.323821
15min_MT.	Q969Q5	-0.41394	0.000175	0.006412	RAB24	Ras-related	RAB24_HUI	1	0.75057
15min_MT.	Q9NXG2	0.195493	0.000174	0.006412	THUMPD1	THUMP dor	THUM1_HU	20	1.145115

15min_MT.	Q9P260	-0.37044	0.000177	0.006412	RELCH	RAB11-binc	RELCH_HUI	20	0.773546
30min_MT.	Q15257	0.246735	0.000187	0.006642	PTPA	Serine/thre	PTPA_HUM	11	1.186519
15min_MT.	Q15366	0.163333	0.000187	0.006642	PCBP2	Poly(rC)-bir	PCBP2_HU	15	1.119871
15min_MT.	P62851	-0.50831	0.000191	0.006769	RPS25	Small ribos	RS25_HUM	4	0.703044
30min_MT.	P80404	0.102996	0.000193	0.006772	ABAT	4-aminobu	GABT_HUM	23	1.074002
15min_MT.	Q9P000	0.547331	0.000193	0.006772	COMMD9	COMM dom	COMD9_HU	10	1.46138
30min_MT.	P50135	0.301181	0.000195	0.006792	HNMT	Histamine I	HNMT_HUI	11	1.232153
15min_MT.	P40763	-0.38568	0.000198	0.006811	STAT3	Signal trans	STAT3_HUI	18	0.765416
15min_MT.	P50135	0.339617	0.000199	0.006811	HNMT	Histamine I	HNMT_HUI	11	1.26542
30min_MT.	Q9BYT8	0.106118	0.000204	0.006911	NLN	Neurolysin,	NEUL_HUM	20	1.076328
15min_MT.	Q9UPQ9	0.608254	0.000204	0.006911	TNRC6B	Trinucleoti	TNR6B_HUI	8	1.524413
15min_MT.	Q15437	-0.16778	0.000211	0.00707	SEC23B	Protein trai	SC23B_HUI	29	0.890213
30min_MT.	Q9UBG3	0.272951	0.000214	0.007157	CRNN	Cornulin	CRNN_HUM	7	1.208277
15min_MT.	P22694	0.435327	0.000217	0.007208	PRKACB	cAMP-depe	KAPCB_HUI	7	1.352218
30min_MT.	P40123	-0.12337	0.000217	0.007208	CAP2	Adenylyl cy	CAP2_HUM	13	0.918041
30min_MT.	P28482	0.326132	0.000219	0.007228	MAPK1	Mitogen-ac	MK01_HUM	15	1.253648
15min_MT.	O60356	-1.76678	0.000226	0.007254	NUPR1	Nuclear prc	NUPR1_HU	1	0.293864
30min_MT.	P08758	0.173091	0.00023	0.007254	ANXA5	Annexin A5	ANXA5_HU	23	1.127472
15min_MT.	P09429	-0.45261	0.000229	0.007254	HMGB1	High mobil	HMGB1_HU	12	0.730719
30min_MT.	P30041	0.116187	0.000228	0.007254	PRDX6	Peroxiredo	PRDX6_HU	21	1.083866
30min_MT.	P67870	0.285002	0.000224	0.007254	CSNK2B	Casein kina	CSK2B_HUI	5	1.218412
15min_MT.	Q66PJ3	-0.44895	0.000227	0.007254	ARL6IP4	ADP-ribosy	AR6P4_HUI	5	0.732575
30min_MT.	Q9HBK9	0.172703	0.000229	0.007254	AS3MT	Arsenite me	AS3MT_HU	20	1.127168
15min_MT.	Q9Y2U5	0.649644	0.000228	0.007254	MAP3K2	Mitogen-ac	M3K2_HUM	1	1.568781
30min_MT.	Q13510	0.178516	0.000233	0.007256	ASAH1	Acid ceram	ASAH1_HU	14	1.131719
15min_MT.	Q96GS4	0.454038	0.000236	0.007256	BORCS6	BLOC-1-rela	BORC6_HU	12	1.369869
15min_MT.	Q9Y678	-0.17316	0.000234	0.007256	COPG1	Coatomer s	COPG1_HU	40	0.886896
30min_MT.	O75351	-0.36878	0.00024	0.00729	VPS4B	Vacuolar pr	VPS4B_HUI	5	0.774439
15min_MT.	P35790	0.645608	0.00024	0.00729	CHKA	Choline kin	CHKA_HUM	2	1.564399
30min_MT.	Q15907	0.152177	0.00024	0.00729	RAB11B	Ras-related	RB11B_HUI	13	1.111245
30min_MT.	P02766	-1.1433	0.000245	0.007403	TTR	Transthyret	TTHY_HUM	10	0.452722
15min_MT.	Q99447	0.101015	0.000247	0.007438	PCYT2	Ethanolami	PCY2_HUM	12	1.072528
15min_MT.	A6NCE7;Q9	0.173999	0.000249	0.007448	MAP1LC3B	Microtubul	MP3B2_HU	1	1.128181
30min_MT.	O95630	0.116224	0.000254	0.007448	STAMBP	STAM-bindi	STABP_HUI	17	1.083894
15min_MT.	P09651	-0.36069	0.000252	0.007448	HNRNPA1	Heterogene	ROA1_HUM	19	0.778793
15min_MT.	P11279	0.276992	0.000252	0.007448	LAMP1	Lysosome-a	LAMP1_HU	5	1.211666
30min_MT.	Q92544	0.690017	0.000254	0.007448	TM9SF4	Transmemb	TM9S4_HU	2	1.613303
30min_MT.	Q9ULC4	0.20099	0.000251	0.007448	MCTS1	Malignant T	MCTS1_HU	7	1.149487
30min_MT.	P0C0L5	-0.96124	0.000255	0.007454	C4B	Complemer	CO4B_HUM	3	0.513616
15min_MT.	Q9NVH6	-0.17098	0.000257	0.007475	TMLHE	Trimethylly	TMLH_HUM	11	0.888239
30min_MT.	P01008	-1.15299	0.000263	0.007594	SERPINC1	Antithroml	ANT3_HUM	6	0.449693
30min_MT.	P49459	0.843232	0.000265	0.007594	UBE2A	Ubiquitin-c	UBE2A_HUI	1	1.794065
15min_MT.	Q9UNF1	-0.23375	0.000266	0.007594	MAGED2	Melanoma-	MAGD2_HU	15	0.85042
15min_MT.	P13639	-0.22467	0.000274	0.007723	EEF2	Elongation	EF2_HUMA	51	0.855793
30min_MT.	P14854	0.492877	0.000279	0.0078	COX6B1	Cytochrom	CX6B1_HUI	5	1.407248
15min_MT.	Q13838	-0.18253	0.000279	0.0078	DDX39B	Spliceosom	DX39B_HU	18	0.881156
15min_MT.	P15086	1.233165	0.000284	0.007821	CPB1	Carboxypep	CBPB1_HUI	1	2.350821
15min_MT.	P34096	-0.70668	0.000283	0.007821	RNASE4	Ribonuclea	RNAS4_HUI	5	0.612727
15min_MT.	Q8IVD9	0.21584	0.000283	0.007821	NUDCD3	NudC doma	NUDC3_HU	13	1.16138
30min_MT.	P00352	0.264905	0.000287	0.007886	ALDH1A1	Aldehyde d	AL1A1_HUI	31	1.201557
15min_MT.	Q9BQA1	0.355463	0.000295	0.007999	WDR77	Methylosor	MEP50_HU	7	1.279396
30min_MT.	P13716	0.26469	0.000299	0.008006	ALAD	Delta-amin	HEM2_HUM	16	1.201378

30min_MT.	P30740	0.192486	0.0003	0.008006	SERPINB1	Leukocyte e	ILEU_HUMA	17	1.142731
30min_MT.	Q9H074	0.207646	0.000299	0.008006	PAIP1	Polyadenyl	PAIP1_HUM	17	1.154803
15min_MT.	Q9NXH9	0.169894	0.000297	0.008006	TRMT1	tRNA (guan	TRM1_HUM	15	1.124976
15min_MT.	O00418	-0.29394	0.000305	0.008097	EEF2K	Eukaryotic	EF2K_HUM	5	0.815669
15min_MT.	Q8NCC3	0.194496	0.000305	0.008097	PLA2G15	Lysosomal	PAG15_HU	10	1.144324
15min_MT.	Q8TE04	-1.44901	0.000308	0.008142	PANK1	Pantothena	PANK1_HU	2	0.366272
30min_MT.	P09601	0.537655	0.000312	0.008174	HMOX1	Heme oxyge	HMOX1_HU	2	1.451611
30min_MT.	P01591	1.843622	0.000317	0.008225	JCHAIN	Immunoglo	IGJ_HUMAI	2	3.589099
30min_MT.	P08238	0.225107	0.000319	0.008256	HSP90AB1	Heat shock	HS90B_HUI	48	1.168864
15min_MT.	Q5VTL8	-0.34507	0.000324	0.008357	PRPF38B	Pre-mRNA-	PR38B_HUI	14	0.78727
30min_MT.	O60678	0.235747	0.000334	0.008404	PRMT3	Protein arg	ANM3_HUM	9	1.177517
30min_MT.	P04637	-1.30427	0.000332	0.008404	TP53	Cellular tur	P53_HUMA	1	0.404926
15min_MT.	P08621	-0.29256	0.000343	0.008404	SNRNP70	U1 small nu	RU17_HUM	13	0.816454
15min_MT.	P31025	0.87226	0.00034	0.008404	LCN1	Lipocalin-1	LCN1_HUM	2	1.830529
30min_MT.	P51003	-0.16606	0.000339	0.008404	PAPOLA	Poly(A) poly	PAPOA_HU	18	0.891275
30min_MT.	Q12996	-0.38299	0.000337	0.008404	CSTF3	Cleavage sti	CSTF3_HUM	16	0.766847
15min_MT.	Q13630	0.175606	0.00034	0.008404	GFUS	GDP-L-fuco	FCL_HUMA	9	1.129439
30min_MT.	Q53H82	0.124451	0.000338	0.008404	LACTB2	Endoribonu	LACB2_HUI	12	1.090093
30min_MT.	Q8TD08	0.343716	0.000342	0.008404	MAPK15	Mitogen-ac	MK15_HUM	1	1.269021
30min_MT.	Q96AX1	-0.27955	0.000338	0.008404	VPS33A	Vacuolar pr	VP33A_HUI	1	0.823848
15min_MT.	Q96IZ7	-0.40947	0.000343	0.008404	RSRC1	Serine/Argi	RSRC1_HUI	5	0.752899
15min_MT.	Q9NYF8	-0.30312	0.000336	0.008404	BCLAF1	Bcl-2-assoc	BCLF1_HUM	16	0.810495
15min_MT.	Q9BRJ6	-0.9945	0.000347	0.008453	C7orf50	Uncharacte	CG050_HU	2	0.50191
15min_MT.	P52657	0.488622	0.000367	0.008858	GTF2A2	Transcriptio	T2AG_HUM	3	1.403105
15min_MT.	P35520	-0.13218	0.000371	0.008934	CBS	Cystathioni	CBS_HUMA	22	0.912452
30min_MT.	Q9Y285	-0.31152	0.000377	0.009057	FARSA	Phenylalan	SYFA_HUMA	10	0.805795
30min_MT.	Q8IX07	-1.58532	0.000391	0.009349	ZFPM1	Zinc finger	FOG1_HUM	1	0.333251
30min_MT.	O95302	0.135658	0.000405	0.009393	FKBP9	Peptidyl-pr	FKBP9_HUI	14	1.098594
30min_MT.	P09960	0.204845	0.0004	0.009393	LTA4H	Leukotriene	LKHA4_HUI	31	1.152562
30min_MT.	P30520	0.09289	0.000413	0.009393	ADSS2	Adenylosuc	PURA2_HU	17	1.066505
30min_MT.	Q15291	0.203179	0.0004	0.009393	RBBP5	Retinoblast	RBBP5_HUI	20	1.151232
15min_MT.	Q5JPI9	0.510517	0.000412	0.009393	EEF1AKMT2	EEF1A lysin	EFMT2_HUI	3	1.424561
15min_MT.	Q8IX12	-0.10743	0.000409	0.009393	CCAR1	Cell divisio	CCAR1_HUI	26	0.928242
15min_MT.	Q96B97	0.272487	0.000412	0.009393	SH3KBP1	SH3 domair	SH3K1_HUI	9	1.207888
15min_MT.	Q96G03	-0.15234	0.000408	0.009393	PGM2	Phosphope	PGM2_HUM	26	0.899792
30min_MT.	Q9H3S7	-0.2773	0.000407	0.009393	PTPN23	Tyrosine-pr	PTN23_HUI	16	0.825137
30min_MT.	Q9NZT1	1.261764	0.000401	0.009393	CALML5	Calmodulir	CALL5_HUM	7	2.397888
15min_MT.	Q9Y383	-0.2882	0.000399	0.009393	LUC7L2	Putative RN	LC7L2_HUM	13	0.818926
15min_MT.	Q15276	0.280499	0.000434	0.009818	RABEP1	Rab GTPase	RABE1_HUI	13	1.214615
30min_MT.	P16083	0.38146	0.000438	0.00987	NQO2	Ribosyldihy	NQO2_HUM	10	1.302659
30min_MT.	P30626	0.220619	0.000439	0.009871	SRI	Sorcin	SORCN_HU	10	1.165234
15min_MT.	Q9BR76	-0.26912	0.000441	0.009876	CORO1B	Coronin-1E	COR1B_HU	15	0.829825
15min_MT.	Q02750	0.294235	0.000447	0.009986	MAP2K1	Dual specifi	MP2K1_HU	7	1.226234
15min_MT.	Q16576	-0.62124	0.000465	0.010333	RBBP7	Histone-bir	RBBP7_HUI	4	0.650114
30min_MT.	P17174	0.221623	0.000468	0.010365	GOT1	Aspartate a	AATC_HUM	24	1.166045
15min_MT.	P49354	0.264304	0.000471	0.010415	FNTA	Protein farr	FNTA_HUM	10	1.201056
15min_MT.	Q5T2E6	-0.1683	0.000475	0.010475	ARMH3	Armadillo-l	ARMD3_HU	11	0.889889
30min_MT.	P49721	0.219786	0.000478	0.010503	PSMB2	Proteasom	PSB2_HUM	10	1.164561
30min_MT.	O14672	-1.3988	0.000487	0.010638	ADAM10	Disintegrin	ADA10_HU	2	0.379243
30min_MT.	P34913	0.253431	0.00049	0.010639	EPHX2	Bifunctiona	HYES_HUM	19	1.192038
15min_MT.	Q05519	-0.76935	0.00049	0.010639	SRSF11	Serine/argi	SRS11_HUM	10	0.586681
30min_MT.	P02753	0.325922	0.000507	0.010833	RBP4	Retinol-bin	RET4_HUM	8	1.253465

30min_MT.	P04424	0.223578	0.000504	0.010833	ASL	Argininosu	ARLY_HUM		19	1.167626
15min_MT.	P16298	0.277256	0.000508	0.010833	PPP3CB	Serine/thre	PP2BB_HU		10	1.211888
15min_MT.	Q13510	0.285198	0.000502	0.010833	ASAH1	Acid ceram	ASAH1_HU		14	1.218577
15min_MT.	Q9BXR0	0.188962	0.000506	0.010833	QTRT1	Queuine tR	TGT_HUMA		8	1.139943
15min_MT.	Q9UHX1	-0.18766	0.000505	0.010833	PUF60	Poly(U)-bin	PUF60_HU		23	0.87803
15min_MT.	Q8IUI8	-0.30078	0.000512	0.010876	CRLF3	Cytokine re	CRLF3_HUN		8	0.811813
30min_MT.	Q16658	0.140884	0.000517	0.010961	FSCN1	Fascin	FSCN1_HU		23	1.102581
15min_MT.	Q9Y3P9	0.080402	0.000538	0.01134	RABGAP1	Rab GTPase	RBGP1_HU		28	1.057313
15min_MT.	Q8N163	-0.10022	0.000549	0.011478	CCAR2	Cell cycle a	CCAR2_HU		35	0.932893
30min_MT.	Q96CD2	0.184585	0.000548	0.011478	PPCDC	Phosphopa	COAC_HUM		5	1.13649
15min_MT.	P31947	0.522492	0.000553	0.0115	SFN	14-3-3 prot	1433S_HU		7	1.436434
30min_MT.	P61129	0.507939	0.000564	0.011667	ZC3H6	Zinc finger	ZC3H6_HU		1	1.422017
30min_MT.	O95861	0.176673	0.000575	0.011795	BPNT1	3'(2'),5'-bis	BPNT1_HU		14	1.130274
30min_MT.	C4AMC7;Q	-0.35078	0.000583	0.011811	WASH3P;W	Putative W	WASH3_HU		4	0.784161
15min_MT.	O75607	-1.23877	0.00058	0.011811	NPM3	Nucleoplas	NPM3_HUN		3	0.423734
15min_MT.	P49593	-0.15009	0.000582	0.011811	PPM1F	Protein pho	PPM1F_HU		7	0.901192
30min_MT.	Q16798	0.207459	0.000581	0.011811	ME3	NADP-depe	MAON_HU		3	1.154653
30min_MT.	P19012	-0.66659	0.000587	0.011815	KRT15	Keratin, typ	K1C15_HU		7	0.629996
30min_MT.	Q86X67	0.374239	0.000588	0.011815	NUDT13	NAD(P)H py	NUD13_HU		5	1.296156
15min_MT.	Q92544	0.874134	0.000587	0.011815	TM9SF4	Transmemb	TM9S4_HU		2	1.832908
30min_MT.	P62312	0.180994	0.000591	0.011832	LSM6	U6 snRNA-a	LSM6_HUM		5	1.133665
30min_MT.	Q9UHY7	0.58524	0.000595	0.011865	ENOPH1	Enolase-ph	ENOPH_HU		6	1.500289
15min_MT.	Q9NZD2	0.424207	0.000598	0.011899	GLTP	Glycolipid	GLTP_HUM		8	1.341835
15min_MT.	P55212	-0.42975	0.000604	0.01196	CASP6	Caspase-6	CASP6_HU		9	0.742392
30min_MT.	O60488	-0.48051	0.000611	0.011961	ACSL4	Long-chain	ACSL4_HUN		15	0.716723
15min_MT.	P50213	0.268813	0.000612	0.011961	IDH3A	Isocitrate d	IDH3A_HU		11	1.204816
30min_MT.	Q02790	0.088223	0.000609	0.011961	FKBP4	Peptidyl-pr	FKBP4_HU		28	1.06306
15min_MT.	Q9GZP4	-0.30222	0.000608	0.011961	PITHD1	PITH domai	PITH1_HUN		11	0.811002
30min_MT.	Q9H008	0.378974	0.000609	0.011961	LHPP	Phospholys	LHPP_HUM		7	1.300417
30min_MT.	P20645	-0.61224	0.000618	0.011979	M6PR	Cation-dep	MPRD_HUN		1	0.654181
15min_MT.	P62753	-0.13381	0.000619	0.011979	RPS6	Small ribos	RS6_HUMA		5	0.911422
30min_MT.	Q7Z7K0	-0.25542	0.000618	0.011979	CMC1	COX assemb	COXM1_HU		2	0.837742
30min_MT.	C9JRZ8	-1.12956	0.000626	0.012065	AKR1B15	Aldo-keto r	AK1BF_HU		1	0.457055
30min_MT.	P43363	-0.19384	0.000633	0.012174	MAGEA10	Melanoma-	MAGAA_HU		3	0.874273
15min_MT.	O75688	0.18786	0.000646	0.012349	PPM1B	Protein pho	PPM1B_HU		9	1.139072
15min_MT.	P21741	-0.25983	0.000645	0.012349	MDK	Midkine	MK_HUMAI		2	0.835186
15min_MT.	O00303	0.375116	0.000651	0.012382	EIF3F	Eukaryotic	EIF3F_HUM		12	1.296943
30min_MT.	P12035	-0.34644	0.000657	0.012391	KRT3	Keratin, typ	K2C3_HUM		8	0.786524
30min_MT.	P04066	0.195809	0.000671	0.012416	FUCA1	Tissue alph	FUCO_HUM		9	1.145366
30min_MT.	P05166	0.089614	0.000667	0.012416	PCCB	Propionyl-	PCCB_HUM		22	1.064085
30min_MT.	P31327	0.231517	0.000666	0.012416	CPS1	Carbamoyl-	CPSM_HUM		83	1.174069
15min_MT.	Q9UJC3	0.213708	0.000663	0.012416	HOOK1	Protein Ho	HOOK1_HU		19	1.159665
30min_MT.	Q8NCC3	0.208745	0.000681	0.012554	PLA2G15	Lysosomal	PAG15_HU		10	1.155683
15min_MT.	Q9BUL8	0.298927	0.000683	0.012563	PDCD10	Programme	PDC10_HU		7	1.230229
15min_MT.	Q9H008	0.285175	0.000688	0.012601	LHPP	Phospholys	LHPP_HUM		7	1.218558
30min_MT.	P07900	0.269217	0.000693	0.012644	HSP90AA1	Heat shock	HS90A_HU		25	1.205153
30min_MT.	Q13126	0.312736	0.0007	0.012698	MTAP	S-methyl-5'	MTAP_HUM		18	1.242061
15min_MT.	Q7Z4V5	-0.2815	0.000699	0.012698	HDGFL2	Hepatoma-	HDGR2_HU		19	0.822737
30min_MT.	Q5JPI9	0.354385	0.000704	0.012738	EEF1AKMT2	EEF1A lysin	EFMT2_HU		3	1.27844
30min_MT.	P35914	0.295296	0.000708	0.012757	HMGCL	Hydroxyme	HMGCL_HU		10	1.227137
30min_MT.	Q9H1Z4	0.206587	0.000723	0.012877	WDR13	WD repeat-	WDR13_HU		10	1.153955
30min_MT.	P52657	0.351965	0.000726	0.012905	GTF2A2	Transcripti	T2AG_HUM		3	1.276298

30min_MT.	Q9NVX2	0.24589	0.000735	0.013041	NLE1	Notchless p	NLE1_HUM	13	1.185824
15min_MT.	P05155	0.279872	0.000739	0.013049	SERPING1	Plasma pro	IC1_HUMA	13	1.214088
15min_MT.	Q9UHY7	0.659951	0.000741	0.013056	ENOPH1	Enolase-ph	ENOPH_HU	6	1.580029
15min_MT.	O94817	0.860845	0.000751	0.013095	ATG12	Ubiquitin-l	ATG12_HU	1	1.816102
30min_MT.	Q5TAQ9	0.201165	0.000752	0.013095	DCAF8	DDB1- and	DCAF8_HU	10	1.149626
15min_MT.	Q9HC38	0.194751	0.000747	0.013095	GLOD4	Glyoxalase	GLOD4_HU	12	1.144527
30min_MT.	P25788	0.264262	0.00076	0.013184	PSMA3	Proteasom	PSA3_HUM	14	1.201021
30min_MT.	P19823	-0.85509	0.000777	0.013444	ITIH2	Inter-alpha	ITIH2_HUM	24	0.552829
15min_MT.	P27482	1.129862	0.000779	0.013445	CALML3	Calmodulir	CALL3_HUN	2	2.188379
30min_MT.	Q92636	0.161997	0.00078	0.013445	NSMAF	Protein FAN	FAN_HUMA	1	1.118835
30min_MT.	Q05655	1.112352	0.000782	0.013448	PRKCD	Protein kin	KPCD_HUM	3	2.161978
30min_MT.	Q8WYN0	0.525778	0.000796	0.013536	ATG4A	Cysteine pr	ATG4A_HU	4	1.43971
30min_MT.	Q9BR76	-0.18588	0.000794	0.013536	CORO1B	Coronin-1E	COR1B_HU	15	0.879111
15min_MT.	Q96BW5	0.243157	0.000805	0.013633	PTER	Phosphotri	PTER_HUM	14	1.18358
30min_MT.	Q53HC9	0.182253	0.000823	0.013842	EIPR1	EARP and G	EIPR1_HUM	7	1.134655
30min_MT.	Q96FC7	0.07768	0.000826	0.013863	PHYHIPL	Phytanoyl-	PHIPL_HUM	14	1.055319
30min_MT.	P15086	1.023228	0.000831	0.013877	CPB1	Carboxyper	CBPB1_HU	1	2.032462
15min_MT.	Q86VM9	-0.20221	0.000832	0.013877	ZC3H18	Zinc finger	ZCH18_HU	13	0.86922
30min_MT.	Q96DR8	1.251842	0.000829	0.013877	MUCL1	Mucin-like	MUCL1_HU	2	2.381453
15min_MT.	Q969H8	-0.68485	0.000845	0.014048	MYDGF	Myeloid-de	MYDGF_HU	7	0.62207
15min_MT.	P47929	0.931648	0.000864	0.014313	LGALS7	Galectin-7	LEG7_HUM	7	1.907454
30min_MT.	Q8N3A8	0.392119	0.000867	0.014331	PARP8	Protein mo	PARP8_HU	1	1.312319
15min_MT.	Q96BX8	-0.45409	0.000885	0.014572	MOB3A	MOB kinase	MOB3A_HU	5	0.729969
30min_MT.	Q6P4A8	0.267878	0.000906	0.014761	PLBD1	Phospholip	PLBL1_HUM	11	1.204036
15min_MT.	Q86U86	1.037193	0.000911	0.014781	PBRM1	Protein pol	PB1_HUMA	1	2.052231
15min_MT.	Q9HA64	0.387623	0.00092	0.014903	FN3KRP	Ketosamine	KT3K_HUM	9	1.308236
15min_MT.	P16403	-0.76111	0.000935	0.015079	H1-2	Histone H1	H12_HUMA	1	0.590041
15min_MT.	Q9UGV2	0.33628	0.000944	0.015167	NDRG3	Protein ND	NDRG3_HU	12	1.262497
15min_MT.	Q9UN36	0.402005	0.000947	0.015187	NDRG2	Protein ND	NDRG2_HU	8	1.321343
15min_MT.	Q03426	0.533135	0.000957	0.015201	MVK	Mevalonate	KIME_HUM	4	1.44707
15min_MT.	Q9H9Q2	0.463664	0.000953	0.015201	COPS7B	COP9 signa	CSN7B_HU	5	1.379039
15min_MT.	O14772	-0.08002	0.00097	0.015321	FPGT	Fucose-1-p	FPGT_HUM	14	0.946044
15min_MT.	P00738	0.705089	0.000977	0.015321	HP	Haptoglobi	HPT_HUMA	3	1.630246
30min_MT.	P26440	0.161627	0.000979	0.015321	IVD	Isovaleryl-C	IVD_HUMA	11	1.118548
15min_MT.	Q01484	-0.21177	0.000977	0.015321	ANK2	Ankyrin-2	ANK2_HUM	17	0.86348
15min_MT.	Q9Y6E2	0.218302	0.000979	0.015321	BZW2	eIF5-mimic	5MP1_HUM	21	1.163364
15min_MT.	Q9H446	0.460074	0.000985	0.015381	RWDD1	RWD doma	RWDD1_HU	3	1.375613
15min_MT.	Q13427	-0.27855	0.000996	0.015482	PPIG	Peptidyl-pr	PPIG_HUM	6	0.824421
30min_MT.	Q92947	-0.46178	0.000994	0.015482	GCDH	Glutaryl-Co	GCDH_HUM	2	0.726092
30min_MT.	P50453	0.195972	0.001008	0.015646	SERPINB9	Serpin B9	SPB9_HUM	19	1.145495
15min_MT.	Q16719	-0.1882	0.001012	0.015646	KYNU	Kynurenina	KYNU_HUM	18	0.877698
30min_MT.	P62979	0.503012	0.001026	0.015774	RPS27A	Ubiquitin-r	RS27A_HU	7	1.417169
30min_MT.	Q9GZL7	0.126543	0.001027	0.015774	WDR12	Ribosome b	WDR12_HU	15	1.091675
30min_MT.	Q14849	0.864659	0.001043	0.015872	STARD3	StAR-relate	STAR3_HUM	1	1.820909
15min_MT.	P46060	0.204598	0.001046	0.015881	RANGAP1	Ran GTPase	RAGP1_HU	21	1.152366
30min_MT.	Q6YHK3	0.249394	0.00105	0.015883	CD109	CD109 anti	CD109_HU	24	1.188708
15min_MT.	Q04917	0.125484	0.001062	0.015927	YWHAH	14-3-3 prot	1433F_HU	11	1.090874
30min_MT.	Q15024	0.245637	0.001062	0.015927	EXOSC7	Exosome cc	EXOS7_HU	11	1.185617
15min_MT.	O94829	-0.55861	0.001075	0.01598	IPO13	Importin-1	IPO13_HUM	2	0.678955
30min_MT.	P01877	0.856592	0.001073	0.01598	IGHA2	Immunoglo	IGHA2_HU	5	1.810756
30min_MT.	P61457	0.308709	0.001074	0.01598	PCBD1	Pterin-4-alp	PHS_HUMA	5	1.238599
15min_MT.	Q92466	-0.41773	0.001074	0.01598	DDB2	DNA damag	DDB2_HUM	9	0.748601

15min_MT.	Q9H3G5	-0.04277	0.001082	0.016053	CPVL	Probable se	CPVL_HUM	8	0.970787
15min_MT.	P06702	1.204164	0.001088	0.016071	S100A9	Protein S10	S10A9_HUI	7	2.304037
30min_MT.	Q147X3	0.334621	0.001091	0.016071	NAA30	N-alpha-ace	NAA30_HU	4	1.261046
15min_MT.	Q9NYU2	-0.12552	0.001094	0.01608	UGGT1	UDP-glucos	UGGG1_HU	46	0.916672
30min_MT.	P19827	-0.80975	0.001099	0.016125	ITIH1	Inter-alpha	ITIH1_HUM	17	0.570479
30min_MT.	Q8N129	0.990145	0.001101	0.016125	CNPY4	Protein car	CNPY4_HU	1	1.986384
30min_MT.	Q9BRF8	0.254645	0.001115	0.016298	CPPED1	Serine/thre	CPPED_HUI	14	1.193042
30min_MT.	Q9H5X1	0.239404	0.001137	0.016528	CIAO2A	Cytosolic ir	CIA2A_HUM	3	1.180505
15min_MT.	P25208	0.588648	0.00115	0.016663	NFYB	Nuclear tra	NFYB_HUM	2	1.503836
15min_MT.	P62140	0.20853	0.001162	0.016768	PPP1CB	Serine/thre	PP1B_HUM	4	1.15551
15min_MT.	O75683	-1.24941	0.001168	0.016799	SURF6	Surfeit locu	SURF6_HUI	2	0.420621
15min_MT.	Q9H5X1	0.367695	0.001168	0.016799	CIAO2A	Cytosolic ir	CIA2A_HUM	3	1.29029
30min_MT.	Q14031	-1.00497	0.001192	0.017108	COL4A6	Collagen al	CO4A6_HU	1	0.498282
15min_MT.	O60763	-0.08643	0.001199	0.017148	USO1	General ves	USO1_HUM	36	0.941851
30min_MT.	P37837	0.212679	0.001202	0.017148	TALDO1	Transaldola	TALDO_HUI	19	1.158838
15min_MT.	P53999	-0.47867	0.001206	0.017148	SUB1	Activated R	TCP4_HUM	10	0.717637
15min_MT.	Q13867	-0.29666	0.001205	0.017148	BLMH	Bleomycin	BLMH_HUM	18	0.814133
30min_MT.	Q5T750	0.99246	0.001201	0.017148	KPLCE	Protein KPI	KPLCE_HUN	1	1.989574
30min_MT.	O15460	-0.19532	0.001211	0.017182	P4HA2	Prolyl 4-hy	P4HA2_HU	9	0.873379
30min_MT.	Q15459	-0.14843	0.001213	0.017182	SF3A1	Splicing fac	SF3A1_HUN	30	0.902234
15min_MT.	P78345	-0.16502	0.00122	0.017201	RPP38	Ribonuclea	RPP38_HUI	7	0.891916
30min_MT.	Q16342	0.325188	0.001229	0.017201	PDCD2	Programme	PDCD2_HU	3	1.252828
15min_MT.	Q6P4A8	0.095422	0.001227	0.017201	PLBD1	Phospholip	PLBL1_HUN	11	1.068378
15min_MT.	Q9NPE3	0.92324	0.001223	0.017201	NOP10	H/ACA ribo	NOP10_HU	1	1.89637
15min_MT.	P59998	0.240505	0.001232	0.017215	ARPC4	Actin-relate	ARPC4_HUI	8	1.181406
15min_MT.	Q9ULV4	-0.16462	0.001245	0.017328	CORO1C	Coronin-1C	COR1C_HU	20	0.892164
15min_MT.	O00442	0.100016	0.001253	0.017351	RTCA	RNA 3'-term	RTCA_HUM	8	1.071785
30min_MT.	Q8WVM8	-0.30738	0.001253	0.017351	SCFD1	Sec1 family	SCFD1_HUI	14	0.808109
30min_MT.	Q9Y646	0.312866	0.001251	0.017351	CPQ	Carboxypep	CBPQ_HUM	8	1.242173
30min_MT.	P13861	0.153606	0.001257	0.01736	PRKAR2A	cAMP-depe	KAP2_HUM	17	1.112347
15min_MT.	Q9H6F5	-0.60394	0.001276	0.017512	CCDC86	Coiled-coil	CCD86_HU	4	0.657955
30min_MT.	P80217	0.611677	0.001296	0.017661	IFI35	Interferon-i	IN35_HUM	5	1.528035
30min_MT.	P46527	-0.29067	0.001305	0.017748	CDKN1B	Cyclin-depe	CDN1B_HU	5	0.817524
15min_MT.	P20933	-0.4073	0.001311	0.017801	AGA	N(4)-(beta-I	ASPG_HUM	7	0.754034
30min_MT.	O75688	0.135816	0.001316	0.01784	PPM1B	Protein pho	PPM1B_HU	9	1.098714
15min_MT.	P63167	-0.75382	0.001321	0.017878	DYNLL1	Dynein ligh	DYL1_HUM	4	0.593032
30min_MT.	P05156	0.611008	0.00133	0.017895	CFI	Compleme	CFAI_HUM	1	1.527326
30min_MT.	P59998	0.239571	0.001331	0.017895	ARPC4	Actin-relate	ARPC4_HUI	8	1.180641
15min_MT.	Q96KG9	-0.14328	0.001332	0.017895	SCYL1	N-terminal	SCYL1_HUM	13	0.905457
15min_MT.	Q9H4A4	-0.11363	0.001329	0.017895	RNPEP	Aminopept	AMPB_HUM	23	0.92426
30min_MT.	A0A087WV	2.525051	0.001343	0.017957	SPEGNB	SPEG neigh	SPEGN_HUI	1	5.755938
30min_MT.	P11766	0.173326	0.001359	0.018107	ADH5	Alcohol deł	ADHX_HUM	16	1.127656
15min_MT.	O75531	0.395514	0.001377	0.018316	BANF1	Barrier-to-a	BAF_HUMA	6	1.315411
15min_MT.	P01876	1.597524	0.001385	0.018356	IGHA1	Immunoglo	IGHA1_HUI	4	3.026235
15min_MT.	P34913	0.179053	0.001384	0.018356	EPHX2	Bifunctiona	HYES_HUM	19	1.132141
15min_MT.	P13861	0.255335	0.001388	0.018365	PRKAR2A	cAMP-depe	KAP2_HUM	17	1.193613
30min_MT.	Q8WWV3	-0.4345	0.001395	0.018381	RTN4IP1	Reticulon-4	RT4I1_HUN	6	0.739948
15min_MT.	P13693	-0.43867	0.001409	0.018524	TPT1	Translation	TCTP_HUM	9	0.737814
15min_MT.	P61026	-0.58336	0.001416	0.018587	RAB10	Ras-related	RAB10_HUI	6	0.667407
15min_MT.	P13284	-0.78083	0.001445	0.018775	IFI30	Gamma-int	GILT_HUM	1	0.582034
30min_MT.	Q6ZNJ1	0.768459	0.001451	0.018814	NBEAL2	Neurobeacl	NBEL2_HUI	1	1.70345
30min_MT.	Q969E8	0.508076	0.001453	0.018814	TSR2	Pre-rRNA-p	TSR2_HUM	1	1.422152

30min_MT.	O14772	-0.16998	0.001456	0.018819	FPGT	Fucose-1-pl	FPGT_HUM	14	0.888856
30min_MT.	Q15276	0.191922	0.001466	0.018922	RABEP1	Rab GTPase	RABE1_HUI	13	1.142284
15min_MT.	Q9UK61	-0.41387	0.001475	0.018981	TASOR	Protein TAS	TASOR_HUI	1	0.750605
15min_MT.	O75909	0.244896	0.001483	0.018984	CCNK	Cyclin-K	CCNK_HUM	8	1.185007
30min_MT.	P14621	0.283756	0.001483	0.018984	ACYP2	Acylphospl	ACYP2_HUI	5	1.21736
30min_MT.	Q15833	-0.15362	0.00148	0.018984	STXBP2	Syntaxin-bi	STXB2_HUM	18	0.898991
30min_MT.	P39880	-0.19944	0.001487	0.018994	CUX1	Homeobox	CUX1_HUM	1	0.870887
30min_MT.	P05091	0.296273	0.001494	0.019026	ALDH2	Aldehyde d	ALDH2_HU	26	1.227968
30min_MT.	P08133	0.149058	0.00153	0.019393	ANXA6	Annexin A6	ANXA6_HU	20	1.108846
30min_MT.	P06702	1.43467	0.001546	0.019564	S100A9	Protein S1(	S10A9_HUI	7	2.703203
15min_MT.	P35270	0.433828	0.001548	0.019564	SPR	Sepiapterin	SPRE_HUM	10	1.350813
15min_MT.	Q01130	-0.39414	0.001558	0.019652	SRSF2	Serine/argi	SRSF2_HUM	10	0.760944
30min_MT.	P10155	0.186156	0.001575	0.019812	RO60	RNA-bindir	RO60_HUM	21	1.137728
30min_MT.	Q15843	0.204398	0.00159	0.019962	NEDD8	NEDD8	NEDD8_HU	4	1.152205
30min_MT.	O00625	0.201951	0.001596	0.019981	PIR	Pirin	PIR_HUMAI	11	1.150253
30min_MT.	P62140	0.145408	0.001597	0.019981	PPP1CB	Serine/thre	PP1B_HUM	4	1.106043
30min_MT.	P07686	0.231985	0.001626	0.020312	HEXB	Beta-hexos	HEXB_HUM	19	1.174449
15min_MT.	Q8N5F7	-0.12345	0.001632	0.020361	NKAP	NF-kappa-B	NKAP_HUM	4	0.917989
15min_MT.	Q147X3	0.385424	0.001637	0.020387	NAA30	N-alpha-ac	NAA30_HU	4	1.306244
15min_MT.	Q8N129	1.343458	0.001651	0.020499	CNPY4	Protein can	CNPY4_HUI	1	2.537589
30min_MT.	P12081	0.125464	0.001693	0.020796	HARS1	Histidine--t	HARS1_HUI	22	1.090858
15min_MT.	Q15291	0.17452	0.001699	0.020796	RBBP5	Retinoblast	RBBP5_HUI	20	1.128589
15min_MT.	Q86TU7	-0.06469	0.001699	0.020796	SETD3	Actin-histic	SETD3_HUM	7	0.956153
15min_MT.	P50452	0.189534	0.001709	0.020854	SERPINB8	Serpin B8	SPB8_HUM	13	1.140395
15min_MT.	Q12929	0.166869	0.001717	0.020917	EPS8	Epidermal g	EPS8_HUM	8	1.122619
15min_MT.	O75179	0.195338	0.001736	0.021011	ANKRD17	Ankyrin rep	ANR17_HU	8	1.144992
30min_MT.	P23610	-2.08776	0.001746	0.021011	F8A1	40-kDa hur	HAP40_HU	1	0.235245
30min_MT.	P28074	0.200642	0.001736	0.021011	PSMB5	Proteasome	PSB5_HUM	12	1.14921
30min_MT.	P63104	0.16748	0.001747	0.021011	YWHAZ	14-3-3 prot	1433Z_HUI	14	1.123095
30min_MT.	Q8N543	0.426711	0.001745	0.021011	OGFOD1	Prolyl 3-hy	OGFD1_HU	3	1.344166
30min_MT.	Q9BXB5	-0.18132	0.001748	0.021011	OSBPL10	Oxysterol-b	OSB10_HUI	4	0.881897
15min_MT.	Q7L1Q6	0.233569	0.001778	0.021231	BZW1	eIF5-mimic	5MP2_HUM	14	1.17574
15min_MT.	O75400	-0.17355	0.001792	0.021344	PRPF40A	Pre-mRNA-|	PR40A_HUI	18	0.88666
15min_MT.	Q5VZE5	-0.71503	0.001796	0.021344	NAA35	N-alpha-ac	NAA35_HU	2	0.609193
15min_MT.	Q9BRT6	-1.58922	0.001797	0.021344	LLPH	Protein LLF	LLPH_HUM	2	0.332352
15min_MT.	Q15493	0.188513	0.001801	0.021345	RGN	Regucalcin	RGN_HUM/	13	1.139589
15min_MT.	P22413	-1.25418	0.001825	0.021471	ENPP1	Ectonucleo	ENPP1_HUI	2	0.419231
15min_MT.	P35237	-0.26895	0.001824	0.021471	SERPINB6	Serpin B6	SPB6_HUM	16	0.829924
15min_MT.	Q8WUR7	-1.03643	0.001822	0.021471	C15orf40	UPF0235 p	CO040_HU	2	0.487532
30min_MT.	P13646	0.040061	0.001836	0.021494	KRT13	Keratin, typ	K1C13_HUI	10	1.028157
30min_MT.	Q8WU90	-0.29326	0.001835	0.021494	ZC3H15	Zinc finger (	ZC3HF_HUI	13	0.816057
15min_MT.	O43242	-0.16918	0.001866	0.021543	PSMD3	26S proteas	PSMD3_HU	27	0.889349
15min_MT.	P27144	0.2436	0.001847	0.021543	AK4	Adenylate k	KAD4_HUM	8	1.183943
30min_MT.	Q05397	0.291578	0.001852	0.021543	PTK2	Focal adhes	FAK1_HUM	17	1.223978
15min_MT.	Q86VP6	-0.07505	0.001861	0.021543	CAND1	Cullin-asso	CAND1_HU	47	0.949308
15min_MT.	Q9H3U5	0.443455	0.001865	0.021543	MFSD1	Lysosomal (	MFSD1_HU	1	1.359857
30min_MT.	O15084	-0.46341	0.001878	0.021607	ANKRD28	Serine/thre	ANR28_HU	5	0.725271
15min_MT.	P15531	0.216468	0.001882	0.021617	NME1	Nucleoside	NDKA_HUM	6	1.161886
30min_MT.	Q92692	0.241573	0.00189	0.021674	NECTIN2	Nectin-2	NECT2_HUI	8	1.182281
30min_MT.	O75223	0.166476	0.001903	0.021762	GGCT	Gamma-glu	GGCT_HUM	9	1.122313
30min_MT.	Q8TD30	-0.24721	0.001935	0.022033	GPT2	Alanine am	ALAT2_HUM	7	0.842524
15min_MT.	Q14997	-0.24676	0.001944	0.022105	PSME4	Proteasome	PSME4_HU	8	0.842787

15min_MT.	Q99536	0.172467	0.001957	0.022207	VAT1	Synaptic ve	VAT1_HUM	16	1.126984
30min_MT.	Q9BXK5	0.031063	0.001959	0.022207	BCL2L13	Bcl-2-like p	B2L13_HUI	6	1.021764
30min_MT.	P30050	-0.46926	0.001968	0.022273	RPL12	Large ribos	RL12_HUM	5	0.722337
15min_MT.	Q96P48	0.278823	0.00197	0.022273	ARAP1	Arf-GAP wit	ARAP1_HUI	4	1.213204
30min_MT.	Q96IJ6	-0.21937	0.001974	0.022283	GMPPA	Mannose-1	GMPPA_HU	9	0.858943
15min_MT.	P09525	0.13208	0.001981	0.022329	ANXA4	Annexin A4	ANXA4_HU	24	1.095873
30min_MT.	Q15181	0.217249	0.001986	0.022354	PPA1	Inorganic p	IPYR_HUM	19	1.162515
30min_MT.	Q99460	-0.08987	0.00199	0.022369	PSMD1	26S protea	PSMD1_HU	38	0.939607
30min_MT.	P01009	0.293396	0.002013	0.022523	SERPINA1	Alpha-1-ant	A1AT_HUM	25	1.225522
30min_MT.	Q92506	0.202105	0.002028	0.022625	HSD17B8	(3R)-3-hydr	DHB8_HUM	10	1.150376
15min_MT.	Q7RTP6	-0.27929	0.002036	0.022682	MICAL3	[F-actin]-m	MICA3_HUI	2	0.823997
15min_MT.	Q8IWB7	-0.10815	0.002039	0.022684	WDFY1	WD repeat	WDFY1_HU	11	0.927777
15min_MT.	O75044	-0.34763	0.002049	0.0227	SRGAP2	SLIT-ROBO	SRGP2_HUI	1	0.785874
15min_MT.	O95232	-0.33567	0.002048	0.0227	LUC7L3	Luc7-like p	LC7L3_HUI	13	0.792416
30min_MT.	P35558	0.249724	0.002046	0.0227	PCK1	Phosphoen	PCKGC_HU	22	1.18898
15min_MT.	P53675	-1.01184	0.002054	0.022725	CLTCL1	Clathrin he	CLH2_HUM	2	0.495915
30min_MT.	O43396	0.114707	0.002068	0.022744	TXNL1	Thioredoxi	TXNL1_HUI	13	1.082755
30min_MT.	P26640	-0.24652	0.002068	0.022744	VARS1	Valine--tRN	SYVC_HUM	35	0.84293
15min_MT.	P60866	-0.26259	0.00207	0.022744	RPS20	Small ribos	RS20_HUM	3	0.833588
30min_MT.	Q8WW59	0.142076	0.002091	0.022937	SPRYD4	SPRY doma	SPRY4_HUI	8	1.103492
30min_MT.	P04259	1.078243	0.002102	0.022962	KRT6B	Keratin, typ	K2C6B_HUI	5	2.111463
15min_MT.	Q9ULP0	0.375535	0.0021	0.022962	NDRG4	Protein ND	NDRG4_HU	3	1.297321
15min_MT.	P30511	-1.83869	0.002113	0.023056	HLA-F	HLA class I	HLAF_HUM	1	0.279576
15min_MT.	P07437	0.52879	0.002132	0.023225	TUBB	Tubulin bet	TBB5_HUM	4	1.442719
30min_MT.	A2RUC4	-0.82221	0.002146	0.02331	TYW5	tRNA wybu	TYW5_HUM	2	0.565576
30min_MT.	O15083	-0.85724	0.002144	0.02331	ERC2	ERC protei	ERC2_HUM	1	0.552008
30min_MT.	O60518	0.211162	0.002156	0.023381	RANBP6	Ran-bindin	RNBP6_HU	4	1.157621
15min_MT.	Q01518	-0.11151	0.00216	0.023381	CAP1	Adenylyl cy	CAP1_HUM	31	0.925621
30min_MT.	Q8IXI1	-0.42133	0.002171	0.023454	RHOT2	Mitochond	MIRO2_HU	1	0.746738
30min_MT.	Q53EL6	0.205494	0.00218	0.023454	PDCD4	Programme	PDCD4_HU	17	1.153081
15min_MT.	Q8WW59	0.136121	0.00218	0.023454	SPRYD4	SPRY doma	SPRY4_HUI	8	1.098947
30min_MT.	P52655	0.586959	0.002212	0.023636	GTF2A1	Transcripti	TF2AA_HUI	2	1.502078
30min_MT.	Q13426	0.115129	0.002208	0.023636	XRCC4	DNA repair	XRCC4_HUI	12	1.083072
30min_MT.	Q9H0R4	0.350165	0.002203	0.023636	HDHD2	Haloacid de	HDHD2_HU	7	1.274706
15min_MT.	Q9H9Q4	0.57348	0.002206	0.023636	NHEJ1	Non-homol	NHEJ1_HUI	1	1.488109
15min_MT.	Q13616	-0.14158	0.002218	0.023655	CUL1	Cullin-1	CUL1_HUM	34	0.906525
15min_MT.	Q5XKE5	0.937264	0.00222	0.023655	KRT79	Keratin, typ	K2C79_HUI	3	1.914893
15min_MT.	P68032	-0.74926	0.002248	0.023893	ACTC1	Actin, alph	ACTC_HUM	3	0.594909
15min_MT.	Q9NZP8	0.20712	0.002251	0.023893	C1RL	Compleme	C1RL_HUM	1	1.154382
30min_MT.	Q86YM7	0.461587	0.00226	0.023925	HOMER1	Homer pro	HOME1_HU	6	1.377056
15min_MT.	Q12906	-0.11877	0.002274	0.02404	ILF3	Interleukin	ILF3_HUM	32	0.920972
15min_MT.	P12956	-0.13158	0.002278	0.02405	XRCC6	X-ray repai	XRCC6_HUI	30	0.91283
30min_MT.	O95551	-0.20837	0.002291	0.02412	TDP2	Tyrosyl-DN	TYDP2_HUI	7	0.865517
15min_MT.	P35221	0.17526	0.002361	0.024627	CTNNA1	Catenin alp	CTNA1_HUI	36	1.129168
30min_MT.	Q8N567	0.960638	0.002354	0.024627	ZCCHC9	Zinc finger	ZCHC9_HUI	1	1.946171
30min_MT.	Q9NVG8	0.234873	0.002374	0.024729	TBC1D13	TBC1 doma	TBC13_HUI	16	1.176803
30min_MT.	Q9BTC0	-0.18183	0.002386	0.024818	DIDO1	Death-indu	DIDO1_HUI	17	0.881584
15min_MT.	Q15392	-0.67482	0.002409	0.025027	DHCR24	Delta(24)-s	DHC24_HU	2	0.626408
30min_MT.	P10163	0.805607	0.002438	0.025238	PRB4	Basic saliva	PRB4_HUM	2	1.747881
15min_MT.	Q9NPH0	0.191223	0.002439	0.025238	ACP6	Lysophospl	PPA6_HUM	8	1.141731
15min_MT.	O75865	0.628139	0.002446	0.025275	TRAPPC6A	Trafficking	TPC6A_HUI	1	1.54557
30min_MT.	P53675	-1.60964	0.002482	0.025549	CLTCL1	Clathrin he	CLH2_HUM	2	0.327679

30min_MT.	P02649	0.158964	0.002488	0.025571	APOE	Apolipopro	APOE_HUM		20	1.116485
30min_MT.	P40926	0.150799	0.002492	0.02558	MDH2	Malate deh	MDHM_HU		20	1.110184
15min_MT.	P00338	-0.33113	0.002519	0.025786	LDHA	L-lactate de	LDHA_HUM		21	0.794912
30min_MT.	P43490	0.114431	0.002527	0.025834	NAMPT	Nicotinami	NAMPT_HU		25	1.082548
15min_MT.	Q86U42	0.724293	0.002541	0.025943	PABPN1	Polyadenyl	PABP2_HU		3	1.652091
15min_MT.	P21333	-0.04843	0.002545	0.025955	FLNA	Filamin-A	FLNA_HUM		110	0.96699
30min_MT.	Q96G61	0.357887	0.002557	0.02604	NUDT11	Diphospho	NUD11_HU		1	1.281548
30min_MT.	Q14894	0.277861	0.002568	0.026119	CRYM	Ketimine re	CRYM_HUM		11	1.212396
30min_MT.	P28065	0.266793	0.002571	0.026119	PSMB9	Proteasom	PSB9_HUM		6	1.203131
30min_MT.	Q17R98	0.36999	0.002582	0.02619	ZNF827	Zinc finger	ZN827_HU		2	1.292344
30min_MT.	P42574	0.35657	0.002609	0.026205	CASP3	Caspase-3	CASP3_HU		6	1.280378
15min_MT.	P48556	0.310353	0.002611	0.026205	PSMD8	26S protea	PSMD8_HU		9	1.240011
30min_MT.	P49720	0.266588	0.002601	0.026205	PSMB3	Proteasom	PSB3_HUM		10	1.20296
30min_MT.	Q14694	-0.24155	0.00261	0.026205	USP10	Ubiquitin c	UBP10_HU		14	0.845836
15min_MT.	Q15750	0.322939	0.002615	0.026205	TAB1	TGF-beta-a	TAB1_HUM		12	1.250876
15min_MT.	Q92542	0.562808	0.002595	0.026205	NCSTN	Nicastrin	NICA_HUM		4	1.477141
30min_MT.	Q06481	0.282539	0.00262	0.02621	APLP2	Amyloid be	APLP2_HU		8	1.216333
30min_MT.	Q7Z5L9	-0.3572	0.002625	0.026225	IRF2BP2	Interferon	I2BP2_HUM		14	0.780679
30min_MT.	P55769	0.134094	0.002643	0.026282	SNU13	NHP2-like	NH2L1_HU		6	1.097404
15min_MT.	Q9HB40	0.702631	0.00264	0.026282	SCPEP1	Retinoid-in	RISC_HUM		5	1.627471
30min_MT.	Q14011	0.696056	0.002653	0.026338	CIRBP	Cold-induc	CIRBP_HUM		3	1.620069
15min_MT.	P01877	2.729473	0.002664	0.026378	IGHA2	Immunoglo	IGHA2_HU		5	6.632135
30min_MT.	P12694	-0.29228	0.002664	0.026378	BCKDHA	2-oxoisoval	ODBA_HUM		7	0.816612
30min_MT.	P32189	0.162999	0.002687	0.026462	GK	Glycerol kir	GLPK_HUM		12	1.119612
15min_MT.	P62891;Q5	-1.59956	0.002679	0.026462	RPL39;RPL	Large ribos	RL39_HUM		1	0.329978
30min_MT.	Q8TDQ7	0.307959	0.00269	0.026462	GNPDA2	Glucosamir	GNPI2_HU		4	1.237955
30min_MT.	Q9BWS9	-0.41465	0.002688	0.026462	CHID1	Chitinase d	CHID1_HU		3	0.750203
15min_MT.	Q86X83	0.429728	0.002694	0.026474	COMMD2	COMM dom	COMD2_HU		1	1.34698
30min_MT.	P46782	-0.24106	0.0027	0.026499	RPS5	Small ribos	RS5_HUMA		6	0.846121
15min_MT.	P27797	-0.34779	0.002704	0.026506	CALR	Calreticulir	CALR_HUM		24	0.785784
15min_MT.	O15305	0.097918	0.002726	0.026616	PMM2	Phosphoma	PMM2_HU		15	1.070228
30min_MT.	P51648	0.341376	0.002733	0.026616	ALDH3A2	Aldehyde d	AL3A2_HU		5	1.266965
30min_MT.	Q5JYT7	0.493792	0.002726	0.026616	KIAA1755	Uncharacte	K1755_HU		1	1.408141
15min_MT.	Q8TE67	0.085191	0.002732	0.026616	EPS8L3	Epidermal	ES8L3_HUM		22	1.060828
30min_MT.	Q9Y3F4	0.142708	0.002736	0.026616	STRAP	Serine-thre	STRAP_HUM		20	1.103975
30min_MT.	O15511	0.333352	0.00274	0.026623	ARPC5	Actin-relate	ARPC5_HU		5	1.259937
30min_MT.	Q13610	0.229391	0.002763	0.026727	PWP1	Periodic try	PWP1_HUM		9	1.17234
30min_MT.	P20618	0.271244	0.002771	0.026755	PSMB1	Proteasom	PSB1_HUM		9	1.206848
15min_MT.	Q9BPX5	0.655873	0.00281	0.027036	ARPC5L	Actin-relate	ARP5L_HU		2	1.57557
30min_MT.	O60749	-0.14833	0.002826	0.027092	SNX2	Sorting nex	SNX2_HUM		18	0.902297
15min_MT.	P13804	0.151734	0.002825	0.027092	ETFA	Electron tra	ETFA_HUM		15	1.110904
30min_MT.	P53041	0.121119	0.002826	0.027092	PPP5C	Serine/thre	PPP5_HUM		18	1.087578
15min_MT.	Q9BX93	0.687689	0.002833	0.027106	PLA2G12B	Group XIIB	PG12B_HU		3	1.610701
15min_MT.	Q9UPN6	-0.86467	0.002835	0.027106	SCAF8	SR-related	SCAF8_HUM		3	0.54917
15min_MT.	P53602	0.176106	0.002846	0.027177	MVD	Diphospho	MVD1_HUM		12	1.12983
15min_MT.	P54577	0.153572	0.002851	0.027197	YARS1	Tyrosine--t	SYYC_HUM		34	1.11232
30min_MT.	Q14980	-0.09836	0.00286	0.02725	NUMA1	Nuclear mi	NUMA1_HU		32	0.934096
30min_MT.	Q06033	-0.82799	0.002881	0.027352	ITIH3	Inter-alpha	ITIH3_HUM		7	0.563313
30min_MT.	Q13685	0.340915	0.002894	0.027439	AAMP	Angio-asso	AAMP_HUM		11	1.266559
15min_MT.	Q15102	0.141734	0.002908	0.027473	PAFAH1B3	Platelet-act	PA1B3_HU		7	1.10323
30min_MT.	Q96JB3	0.814843	0.002906	0.027473	HIC2	Hypermeth	HIC2_HUM		1	1.759107
15min_MT.	O15498	-0.21422	0.002916	0.027478	YKT6	Synaptobre	YKT6_HUM		11	0.86201

30min_MT.	O75503	0.293739	0.002913	0.027478	CLN5	Bis(monoac	CLN5_HUM	4	1.225813
30min_MT.	Q8N5A5	-0.74602	0.002976	0.027906	ZGPAT	Zinc finger	ZGPAT_HU	3	0.596245
15min_MT.	Q8WYA6	-0.32976	0.002973	0.027906	CTNNBL1	Beta-cateni	CTBL1_HUN	10	0.79567
15min_MT.	P52565	-0.14839	0.002988	0.027961	ARHGDIA	Rho GDP-di	GDIR1_HUI	9	0.902254
30min_MT.	P38646	0.132939	0.003002	0.028053	HSPA9	Stress-70 p	GRP75_HU	38	1.096525
15min_MT.	Q9UKK9	0.118482	0.003006	0.02806	NUDT5	ADP-sugar p	NUDT5_HU	12	1.085592
15min_MT.	P04183	-1.1185	0.003032	0.028224	TK1	Thymidine	KITH_HUM.	1	0.460572
30min_MT.	P12004	0.257065	0.003049	0.028224	PCNA	Proliferatir	PCNA_HUM	13	1.195045
15min_MT.	P61626	2.384365	0.003064	0.028282	LYZ	Lysozyme C	LYSC_HUM.	4	5.221141
30min_MT.	Q13045	-0.23994	0.003066	0.028282	FLII	Protein flig	FLII_HUMA	14	0.846779
30min_MT.	P00558	0.206506	0.003083	0.0284	PGK1	Phosphogly	PGK1_HUM	32	1.153891
15min_MT.	P04843	-0.63566	0.003091	0.028443	RPN1	Dolichyl-di	RPN1_HUM	2	0.643647
30min_MT.	O95831	0.082222	0.003109	0.028512	AIFM1	Apoptosis-i	AIFM1_HUI	22	1.058647
30min_MT.	P47756	0.195243	0.003107	0.028512	CAPZB	F-actin-cap	CAPZB_HUI	18	1.144917
15min_MT.	Q12860	0.1363	0.003109	0.028512	CNTN1	Contactin-1	CNTN1_HU	28	1.099083
15min_MT.	P27361	0.371878	0.003116	0.028539	MAPK3	Mitogen-ac	MK03_HUN	8	1.294036
30min_MT.	O00299	0.25471	0.003127	0.028604	CLIC1	Chloride in	CLIC1_HUN	15	1.193095
15min_MT.	Q9H1E3	-0.79061	0.003151	0.028761	NUCKS1	Nuclear ubi	NUCKS_HU	5	0.578101
30min_MT.	P04179	0.322317	0.003156	0.028768	SOD2	Superoxide	SODM_HUN	11	1.250337
15min_MT.	P16401	-0.33065	0.00316	0.028778	H1-5	Histone H1	H15_HUMA	6	0.795181
15min_MT.	O43314	0.931319	0.00317	0.028813	PPIP5K2	Inositol he	VIP2_HUMA	1	1.907019
30min_MT.	Q99447	0.065319	0.003188	0.028893	PCYT2	Ethanolami	PCY2_HUM	12	1.046316
15min_MT.	P03973	0.602454	0.003204	0.028922	SLPI	Antileukop	SLPI_HUMA	1	1.518297
15min_MT.	P34932	-0.058	0.003199	0.028922	HSPA4	Heat shock	HSP74_HUI	49	0.960592
15min_MT.	Q9C0C4	1.21708	0.003203	0.028922	SEMA4C	Semaphori	SEM4C_HU	2	2.324757
30min_MT.	Q9HB07	0.296845	0.003206	0.028922	MYG1	MYG1 exon	MYG1_HUN	12	1.228455
30min_MT.	P07311	0.523493	0.003221	0.028929	ACYP1	Acylphosph	ACYP1_HUI	4	1.437432
15min_MT.	Q8IYB3	-0.30684	0.003219	0.028929	SRRM1	Serine/argi	SRRM1_HU	14	0.808409
30min_MT.	Q8NE62	-0.38846	0.003221	0.028929	CHDH	Choline del	CHDH_HUN	4	0.763945
15min_MT.	Q96L46	0.318742	0.003221	0.028929	CAPNS2	Calpain sma	CPNS2_HUI	4	1.247243
15min_MT.	Q9Y6B7	-0.77095	0.003228	0.028955	AP4B1	AP-4 compl	AP4B1_HUI	2	0.586033
15min_MT.	P48729	0.928275	0.003252	0.029068	CSNK1A1	Casein kina	KC1A_HUN	3	1.902999
15min_MT.	P23588	0.274202	0.003257	0.029086	EIF4B	Eukaryotic	IF4B_HUMA	23	1.209325
15min_MT.	Q92506	0.200412	0.003264	0.029115	HSD17B8	(3R)-3-hydr	DHB8_HUN	10	1.149027
15min_MT.	P35249	0.140145	0.003278	0.029205	RFC4	Replication	RFC4_HUM	6	1.102016
30min_MT.	P01833	0.691003	0.003284	0.029224	PIGR	Polymeric i	PIGR_HUM	6	1.614405
15min_MT.	Q13011	0.174104	0.0033	0.029303	ECH1	Delta(3,5)-I	ECH1_HUM	10	1.128264
15min_MT.	O14910	0.201363	0.003311	0.029362	LIN7A	Protein lin-	LIN7A_HUN	4	1.149784
30min_MT.	O00154	0.204847	0.003321	0.029378	ACOT7	Cytosolic a	BACH_HUN	13	1.152564
30min_MT.	O60220	0.140836	0.003319	0.029378	TIMM8A	Mitochond	TIM8A_HUI	3	1.102544
15min_MT.	P01591	2.900904	0.003327	0.029378	JCHAIN	Immunoglo	IGJ_HUMAI	2	7.468941
15min_MT.	P17655	-0.1344	0.003335	0.029378	CAPN2	Calpain-2 c	CAN2_HUM	16	0.911049
15min_MT.	Q9BXK5	0.270816	0.003335	0.029378	BCL2L13	Bcl-2-like p	B2L13_HUI	6	1.20649
15min_MT.	P61020	0.37905	0.003344	0.029387	RAB5B	Ras-related	RAB5B_HUI	3	1.300485
15min_MT.	Q9H488	-0.1857	0.003344	0.029387	POFUT1	GDP-fucose	OFUT1_HUI	9	0.879225
15min_MT.	O95218	-0.19723	0.003387	0.029664	ZRANB2	Zinc finger	ZRAB2_HUI	17	0.872226
15min_MT.	P78371	0.082695	0.003385	0.029664	CCT2	T-complex	TCPB_HUM	27	1.058994
15min_MT.	Q9UJC5	-0.65124	0.003383	0.029664	SH3BGRL2	SH3 domai	SH3L2_HUI	5	0.636735
15min_MT.	P38571	0.253066	0.003415	0.029846	LIPA	Lysosomal	LICH_HUM.	3	1.191737
15min_MT.	P62837	0.125616	0.003431	0.029919	UBE2D2	Ubiquitin-c	UB2D2_HU	2	1.090973
15min_MT.	P0DOY2;PC	1.25857	0.003437	0.029938	IGLC2;IGLC	Immunoglo	IGLC2_HUN	2	2.392585
30min_MT.	Q587I9	-1.01615	0.003477	0.03025	SFT2D3	Vesicle trar	SFT2C_HUN	1	0.494436

15min_MT.	Q16798	0.22062	0.003493	0.030322	ME3	NADP-depe	MAON_HUI		3	1.165234
15min_MT.	Q9HCC0	-0.11621	0.00349	0.030322	MCCC2	Methylcrot	MCCB_HUN		21	0.922605
15min_MT.	P31040	-0.28995	0.003559	0.03044	SDHA	Succinate d	SDHA_HUM		22	0.817932
15min_MT.	P35244	0.121327	0.003551	0.03044	RPA3	Replication	RFA3_HUM		6	1.087735
15min_MT.	P49756	-0.34785	0.003575	0.03044	RBM25	RNA-bindir	RBM25_HU		16	0.785757
30min_MT.	Q13404	0.12549	0.00355	0.03044	UBE2V1	Ubiquitin-c	UB2V1_HU		9	1.090878
15min_MT.	Q96SI1	0.334944	0.003526	0.03044	KCTD15	BTB/POZ dc	KCD15_HU		1	1.261329
30min_MT.	Q99519	0.194155	0.003574	0.03044	NEU1	Sialidase-1	NEUR1_HU		11	1.144054
30min_MT.	Q9UBE0	0.124149	0.003565	0.03044	SAE1	SUMO-activ	SAE1_HUM		17	1.089865
15min_MT.	Q9UGM6	0.19872	0.003576	0.03044	WARS2	Tryptophar	SYWM_HUI		5	1.14768
30min_MT.	Q9UHX1	-0.15409	0.003528	0.03044	PUF60	Poly(U)-bin	PUF60_HUI		23	0.898698
30min_MT.	Q9Y2Z9	-0.65507	0.00353	0.03044	COQ6	Ubiquinon	COQ6_HUN		1	0.635044
30min_MT.	P31947	0.547223	0.003588	0.030508	SFN	14-3-3 prot	1433S_HUI		7	1.46127
15min_MT.	P16435	-0.08763	0.003609	0.03052	POR	NADPH--cyt	NCPR_HUM		14	0.941067
15min_MT.	P25788	0.14531	0.003596	0.03052	PSMA3	Proteasom	PSA3_HUM		14	1.105969
30min_MT.	P60709	-0.22449	0.003601	0.03052	ACTB	Actin, cyto	ACTB_HUM		23	0.855899
15min_MT.	Q7L099	0.525847	0.003609	0.03052	RUFY3	Protein RU	RUFY3_HUI		7	1.439779
30min_MT.	Q9NUM4	0.160557	0.003607	0.03052	TMEM106E	Transmemk	T106B_HUI		3	1.117719
30min_MT.	Q14814	-0.33609	0.003636	0.030678	MEF2D	Myocyte-sp	MEF2D_HU		6	0.792187
15min_MT.	P02649	0.263935	0.003645	0.030721	APOE	Apolipopro	APOE_HUM		20	1.200749
30min_MT.	Q92614	-0.3539	0.003674	0.030901	MYO18A	Unconvent	MY18A_HU		11	0.782465
15min_MT.	O00410	-0.13568	0.003686	0.030939	IPO5	Importin-5	IPO5_HUM		38	0.91024
30min_MT.	P26038	0.169415	0.003685	0.030939	MSN	Moesin	MOES_HUN		33	1.124602
15min_MT.	P15924	0.426126	0.0037	0.030944	DSP	Desmoplak	DESP_HUM		91	1.34362
30min_MT.	Q8WVC0	0.456057	0.003712	0.030991	LEO1	RNA polym	LEO1_HUM		10	1.371787
30min_MT.	Q13835	1.432394	0.003732	0.031089	PKP1	Plakophilir	PKP1_HUM		11	2.698942
15min_MT.	Q5JPH6	0.132575	0.003759	0.031215	EARS2	Nondiscrim	SYEM_HUM		9	1.096248
15min_MT.	Q9Y5L0	-0.20476	0.003757	0.031215	TNPO3	Transportir	TNPO3_HU		13	0.867684
30min_MT.	P18206	0.142609	0.003796	0.031297	VCL	Vinculin	VINC_HUM		74	1.1039
30min_MT.	P29034	1.06488	0.003788	0.031297	S100A2	Protein S10	S10A2_HUI		1	2.091996
15min_MT.	Q14457	0.316643	0.003789	0.031297	BECN1	Beclin-1	BECN1_HUI		3	1.245429
30min_MT.	Q92922	-0.2581	0.003786	0.031297	SMARCC1	SWI/SNF cc	SMRC1_HU		12	0.836188
30min_MT.	O94817	0.527382	0.003812	0.031361	ATG12	Ubiquitin-l	ATG12_HUI		1	1.441311
15min_MT.	P09601	0.963196	0.003813	0.031361	HMOX1	Heme oxyg	HMOX1_HU		2	1.949624
30min_MT.	P50993	-0.39381	0.00384	0.031555	ATP1A2	Sodium/po	AT1A2_HUI		1	0.761118
30min_MT.	Q15493	0.318073	0.003874	0.031763	RGN	Regucalcin	RGN_HUM/		13	1.246665
30min_MT.	Q96BJ3	-0.40549	0.003893	0.031821	AIDA	Axin intera	AIDA_HUM		7	0.75498
15min_MT.	P53618	-0.0794	0.003907	0.031899	COPB1	Coatomer s	COPB_HUN		39	0.946448
15min_MT.	P51531	-0.34558	0.003922	0.031951	SMARCA2	Probable gl	SMCA2_HU		4	0.786989
15min_MT.	Q00839	-0.23198	0.003919	0.031951	HNRNPU	Heterogene	HNRPU_HU		27	0.851463
15min_MT.	Q8N335	0.304001	0.003927	0.031951	GPD1L	Glycerol-3-	GPD1L_HU		12	1.234563
15min_MT.	P35914	0.259981	0.003937	0.031975	HMGCL	Hydroxyme	HMGCL_HU		10	1.197463
15min_MT.	Q9NZ71	-0.42269	0.003958	0.032081	RTEL1	Regulator c	RTEL1_HUN		1	0.746035
15min_MT.	Q15650	-0.59271	0.003974	0.032101	TRIP4	Activating s	TRIP4_HUN		2	0.663098
15min_MT.	Q9BW85	-0.27323	0.003977	0.032101	YJU2	Splicing fac	YJU2_HUM		4	0.827467
30min_MT.	Q9P2T1	0.198698	0.003966	0.032101	GMPR2	GMP reduc	GMPR2_HU		17	1.147662
30min_MT.	O60684	-0.11377	0.004005	0.032296	KPNA6	Importin su	IMA7_HUM		8	0.924168
30min_MT.	O60256	0.14388	0.004031	0.032472	PRPSAP2	Phosphorik	KPRB_HUM		13	1.104872
15min_MT.	P20839	0.210278	0.004056	0.03261	IMPDH1	Inosine-5'-r	IMDH1_HU		6	1.156911
15min_MT.	P42357	0.438497	0.004081	0.032739	HAL	Histidine ar	HUTH_HUN		8	1.355192
15min_MT.	P30613	0.258211	0.004091	0.03279	PKLR	Pyruvate ki	KPYR_HUM		10	1.195994
30min_MT.	Q13362	-0.31088	0.004102	0.032812	PPP2R5C	Serine/thre	2A5G_HUM		6	0.806149

15min_MT.	Q96EH3	-0.82625	0.004107	0.032812	MALSU1	Mitochond	MASU1_HU	1	0.563995
15min_MT.	P40123	-0.14131	0.004143	0.032885	CAP2	Adenylyl cy	CAP2_HUM	13	0.906693
15min_MT.	Q8WUQ7	-0.14834	0.00414	0.032885	CACTIN	Splicing fac	CATIN_HUM	2	0.90229
30min_MT.	Q9Y244	0.967712	0.004138	0.032885	POMP	Proteasom	POMP_HUM	5	1.955737
15min_MT.	O75427	-0.81109	0.004165	0.032968	LRCH4	Leucine-ric	LRCH4_HU	2	0.569951
30min_MT.	Q86U42	0.759114	0.004168	0.032968	PABPN1	Polyadenyl	PABP2_HU	3	1.692451
15min_MT.	Q6AI08	0.16194	0.004173	0.032971	HEATR6	HEAT repea	HEAT6_HU	1	1.11879
15min_MT.	O00488	-0.65253	0.004226	0.033198	ZNF593	Zinc finger	ZN593_HU	3	0.636166
30min_MT.	P20042	-0.25863	0.004222	0.033198	EIF2S2	Eukaryotic	IF2B_HUM	23	0.835883
30min_MT.	Q96B26	0.106375	0.004215	0.033198	EXOSC8	Exosome co	EXOS8_HU	6	1.07652
15min_MT.	Q5RKV6	0.472854	0.004232	0.033202	EXOSC6	Exosome co	EXOS6_HU	9	1.387852
30min_MT.	O14558	-0.70071	0.004265	0.033424	HSPB6	Heat shock	HSPB6_HU	1	0.615268
30min_MT.	Q9NPR2	0.059974	0.00428	0.033495	SEMA4B	Semaphori	SEM4B_HU	4	1.042447
30min_MT.	Q13630	0.234933	0.004307	0.033656	GFUS	GDP-L-fuco	FCL_HUMA	9	1.176852
30min_MT.	P02748	-0.87124	0.004361	0.033864	C9	Compleme	CO9_HUM	4	0.546676
15min_MT.	P49914	0.30456	0.004373	0.033864	MTHFS	5-formyltet	MTHFS_HU	1	1.235042
30min_MT.	P63279	0.310458	0.004348	0.033864	UBE2I	SUMO-conj	UBC9_HUM	9	1.240101
15min_MT.	Q8WUA7	0.028638	0.004365	0.033864	TBC1D22A	TBC1 doma	TB22A_HU	16	1.020049
30min_MT.	Q9BT73	0.277731	0.004354	0.033864	PSMG3	Proteasom	PSMG3_HU	6	1.212286
15min_MT.	Q9HAB8	0.170175	0.004378	0.033864	PPCS	Phosphopa	PPCS_HUM	13	1.125195
15min_MT.	Q9P2R7	0.190357	0.004375	0.033864	SUCLA2	Succinate--	SUCB1_HU	12	1.141046
15min_MT.	Q9NUJ1	-0.17034	0.004388	0.033882	ABHD10	Palmitoyl-p	ABHDA_HU	14	0.888634
15min_MT.	Q9NX58	-1.4975	0.004436	0.034189	LYAR	Cell growth	LYAR_HUM	2	0.354166
30min_MT.	O00221	0.385577	0.00445	0.034223	NFKBIE	NF-kappa-B	IKBE_HUM	1	1.306382
30min_MT.	O15488	-0.66221	0.004448	0.034223	GYG2	Glycogenin	GLYG2_HU	3	0.631911
30min_MT.	Q9NX08	-0.72766	0.004466	0.034226	COMMD8	COMM don	COMD8_HU	2	0.603881
15min_MT.	Q9Y3B2	-0.14748	0.004462	0.034226	EXOSC1	Exosome co	EXOS1_HU	6	0.902828
30min_MT.	Q9Y5A7	-0.33355	0.004456	0.034226	NUB1	NEDD8 ulti	NUB1_HUM	3	0.793582
15min_MT.	P80217	0.446594	0.004487	0.034339	IFI35	Interferon-i	IN35_HUM	5	1.362819
30min_MT.	Q08945	0.205446	0.004534	0.034602	SSRP1	FACT comp	SSRP1_HUM	13	1.153042
30min_MT.	P83436	-0.10544	0.00455	0.034691	COG7	Conserved	COG7_HUM	11	0.929523
30min_MT.	Q06265	0.062118	0.004564	0.03473	EXOSC9	Exosome co	EXOS9_HU	10	1.043997
15min_MT.	Q13835	0.801445	0.004562	0.03473	PKP1	Plakophilir	PKP1_HUM	11	1.742846
15min_MT.	P83876	-0.18214	0.004623	0.035041	TXNL4A	Thioredoxi	TXN4A_HU	5	0.881397
15min_MT.	Q06546	0.256553	0.004638	0.035074	GABPA	GA-binding	GABPA_HU	9	1.194621
15min_MT.	O15042	-0.18985	0.00465	0.035075	U2SURP	U2 snRNP-a	SR140_HU	17	0.876698
30min_MT.	P51688	0.15393	0.004648	0.035075	SGSH	N-sulphogl	SPHM_HUM	7	1.112596
15min_MT.	Q9NQ94	0.224684	0.004663	0.035109	A1CF	APOBEC1 c	A1CF_HUM	15	1.168521
15min_MT.	Q9NXR7	0.186563	0.004662	0.035109	BABAM2	BRISC and E	BABA2_HU	11	1.138049
30min_MT.	Q8NFD5	-0.24616	0.004674	0.035122	ARID1B	AT-rich inte	ARI1B_HUM	11	0.84314
30min_MT.	P02663	1.472886	0.004685	0.035133	CSN1S2	Alpha-S2-ca	CASA2_BOV	3	2.775766
15min_MT.	Q9NY61	-0.86807	0.004683	0.035133	AATF	Protein AA	AATF_HUM	2	0.54788
30min_MT.	O95456	0.170593	0.004695	0.035175	PSMG1	Proteasom	PSMG1_HU	13	1.125521
30min_MT.	P01040	1.121215	0.004727	0.035318	CSTA	Cystatin-A	CYTA_HUM	7	2.175301
15min_MT.	Q9C0D3	-0.5862	0.004727	0.035318	ZYG11B	Protein zyg	ZY11B_HUM	6	0.666095
15min_MT.	O96017	0.163652	0.004752	0.035469	CHEK2	Serine/thre	CHK2_HUM	10	1.120119
15min_MT.	Q9Y2X3	-0.20362	0.004772	0.035546	NOP58	Nucleolar p	NOP58_HU	9	0.868371
15min_MT.	O95989	-0.09115	0.004783	0.035562	NUDT3	Diphospho	NUDT3_HU	6	0.938776
30min_MT.	Q16637	-0.30029	0.00478	0.035562	SMN1	Survival mo	SMN_HUM	1	0.812092
30min_MT.	Q14790	0.308921	0.004881	0.036153	CASP8	Caspase-8	CASP8_HU	11	1.238781
30min_MT.	Q9NRN7	-0.48022	0.004887	0.036166	AASDHPPT	L-aminoadi	ADPPT_HU	9	0.71687
15min_MT.	P01833	1.744387	0.004931	0.036406	PIGR	Polymeric i	PIGR_HUM	6	3.350524

30min_MT.	Q15018	0.121429	0.004926	0.036406	ABRAXAS2	BRISC comp	ABRX2_HUI	17	1.087812
30min_MT.	Q16531	0.17822	0.004945	0.036456	DDB1	DNA damag	DDB1_HUN	56	1.131487
30min_MT.	Q16576	-0.44588	0.004959	0.036528	RBBP7	Histone-bir	RBBP7_HU	4	0.734135
15min_MT.	O14787	-0.16455	0.004978	0.036593	TNPO2	Transportir	TNPO2_HU	5	0.892205
30min_MT.	O95219	-0.16853	0.005001	0.03673	SNX4	Sorting nex	SNX4_HUM	10	0.889748
15min_MT.	O60610	0.105508	0.005033	0.03686	DIAPH1	Protein dia	DIAP1_HUI	32	1.075873
30min_MT.	P52758	0.095346	0.005032	0.03686	RIDA	2-iminobut	RIDA_HUM	6	1.068321
30min_MT.	O95825	0.177926	0.00505	0.036889	CRYZL1	Quinone ox	QORL1_HU	10	1.131256
30min_MT.	P14625	0.113986	0.005048	0.036889	HSP90B1	Endoplasm	ENPL_HUM	46	1.082214
15min_MT.	Q9BYN0	0.198695	0.00506	0.036889	SRXN1	Sulfiredoxi	SRXN1_HUI	3	1.14766
30min_MT.	P60981	-0.21659	0.005103	0.037029	DSTN	Destrin	DEST_HUM	11	0.860598
15min_MT.	P31483	0.164292	0.005124	0.037042	TIA1	Cytotoxic g	TIA1_HUM/	6	1.120616
30min_MT.	P62877	0.101398	0.005119	0.037042	RBX1	E3 ubiquiti	RBX1_HUM	2	1.072813
15min_MT.	Q13131	0.292083	0.005119	0.037042	PRKAA1	5'-AMP-acti	AAPK1_HUI	10	1.224407
15min_MT.	Q16513	0.238018	0.005134	0.037051	PKN2	Serine/thre	PKN2_HUM	20	1.179371
30min_MT.	Q8N335	0.116422	0.005154	0.037155	GPD1L	Glycerol-3-	GPD1L_HU	12	1.084043
15min_MT.	Q8IXT5	-0.1552	0.00517	0.0372	RBM12B	RNA-bindir	RB12B_HUI	8	0.898009
30min_MT.	O43818	0.18019	0.005216	0.037466	RRP9	U3 small nu	U3IP2_HUN	10	1.133033
30min_MT.	Q8TE02	-0.34006	0.005221	0.037466	ELP5	Elongator c	ELP5_HUM	3	0.790009
30min_MT.	Q04917	0.221027	0.005229	0.037492	YWHAH	14-3-3 prot	1433F_HUI	11	1.165563
15min_MT.	P05156	0.573375	0.005235	0.0375	CFI	Complemer	CFAI_HUM/	1	1.488001
15min_MT.	P07602	0.19463	0.005258	0.037563	PSAP	Prosaposin	SAP_HUMA	14	1.14443
30min_MT.	P35080	0.355241	0.005362	0.038121	PFN2	Profilin-2	PROF2_HUI	5	1.279199
30min_MT.	Q96RP9	-0.19296	0.005366	0.038121	GFM1	Elongation	EFGM_HUN	19	0.874811
30min_MT.	P46926	0.289093	0.005373	0.038139	GNPDA1	Glucosamir	GNPI1_HUI	14	1.221872
15min_MT.	O76021	-0.14831	0.005398	0.038178	RSL1D1	Ribosomal	RL1D1_HUI	9	0.902304
15min_MT.	Q06547	-1.28965	0.005412	0.038178	GABPB1	GA-binding	GABP1_HU	1	0.409051
30min_MT.	Q8WVV4	0.949646	0.005394	0.038178	POF1B	Protein POI	POF1B_HUI	11	1.931399
30min_MT.	Q96KG9	-0.32267	0.005404	0.038178	SCYL1	N-terminal	SCYL1_HUN	13	0.799589
15min_MT.	Q9UK41	0.178122	0.005413	0.038178	VPS28	Vacuolar pr	VPS28_HUI	5	1.13141
30min_MT.	O15116	0.722493	0.005435	0.038293	LSM1	U6 snRNA-a	LSM1_HUM	3	1.650031
30min_MT.	P22735	1.056191	0.005457	0.038384	TGM1	Protein-glu	TGM1_HUN	13	2.079433
15min_MT.	P00492	0.25729	0.005507	0.038524	HPRT1	Hypoxanth	HPRT_HUM	12	1.195231
30min_MT.	P00761	0.267838	0.005493	0.038524	NaN	Trypsin (Lal	TRYP_PIG	5	1.204002
30min_MT.	P06737	0.08371	0.005498	0.038524	PYGL	Glycogen p	PYGL_HUM	38	1.059739
30min_MT.	Q92542	0.709572	0.005504	0.038524	NCSTN	Nicastrin	NICA_HUM	4	1.635319
30min_MT.	P51687	0.160542	0.005553	0.038642	SUOX	Sulfite oxid	SUOX_HUM	14	1.117707
30min_MT.	Q15008	-0.06744	0.00558	0.03876	PSMD6	26S proteas	PSMD6_HU	22	0.954329
15min_MT.	Q14240	-0.05993	0.005598	0.038851	EIF4A2	Eukaryotic	IF4A2_HUM	11	0.959311
30min_MT.	P42677	-0.16825	0.00564	0.039041	RPS27	Small ribos	RS27_HUM	2	0.88992
15min_MT.	P01024	-0.02971	0.005702	0.039117	C3	Complemer	CO3_HUM/	76	0.979615
30min_MT.	P11586	-0.26583	0.005667	0.039117	MTHFD1	C-1-tetrahy	C1TC_HUM	23	0.83172
15min_MT.	P30793	0.090007	0.005692	0.039117	GCH1	GTP cycloh	GCH1_HUN	8	1.064375
30min_MT.	P36639	0.401032	0.005689	0.039117	NUDT1	Oxidized pu	8ODP_HUN	3	1.320452
30min_MT.	Q14019	0.162719	0.005674	0.039117	COTL1	Coactosin-l	COTL1_HUI	11	1.119394
30min_MT.	Q8N668	0.098917	0.005707	0.039117	COMMD1	COMM don	COMD1_HU	1	1.070969
30min_MT.	Q96HE7	0.130497	0.005703	0.039117	ERO1A	ERO1-like p	ERO1A_HUI	24	1.09467
15min_MT.	Q99816	0.246386	0.005702	0.039117	TSG101	Tumor susc	TS101_HUN	7	1.186232
30min_MT.	Q9NRY2	-0.66263	0.005666	0.039117	INIP	SOSS comp	SOSSC_HUN	3	0.631727
30min_MT.	P06744	0.186227	0.005781	0.039303	GPI	Glucose-6-p	G6PI_HUM	22	1.137784
15min_MT.	Q08257	-0.29392	0.005771	0.039303	CRYZ	Quinone ox	QOR_HUM/	18	0.81568
15min_MT.	Q14254	-0.05939	0.005786	0.039303	FLOT2	Flotillin-2	FLOT2_HUN	4	0.959669

15min_MT.	Q92688	0.293991	0.005788	0.039303	ANP32B	Acidic leuci	AN32B_HU		7	1.226027
30min_MT.	Q9UHR5	-0.23088	0.00575	0.039303	SAP30BP	SAP30-binc	S30BP_HUI		6	0.852113
30min_MT.	Q9Y5X3	-0.17905	0.005783	0.039303	SNX5	Sorting nex	SNX5_HUM		10	0.883283
15min_MT.	P05362	0.217964	0.005804	0.039339	ICAM1	Intercellula	ICAM1_HU		10	1.163091
15min_MT.	Q92692	0.262382	0.005825	0.039418	NECTIN2	Nectin-2	NECT2_HUI		8	1.199457
30min_MT.	Q92747	0.133715	0.005824	0.039418	ARPC1A	Actin-relate	ARC1A_HU		15	1.097115
15min_MT.	Q5R3I4	0.052188	0.005848	0.039487	TTC38	Tetratricop	TTC38_HUI		10	1.036836
15min_MT.	P49721	0.168919	0.00588	0.039516	PSMB2	Proteasom	PSB2_HUM		10	1.124216
30min_MT.	Q96D46	-0.24375	0.005871	0.039516	NMD3	60S ribosor	NMD3_HUI		10	0.844544
30min_MT.	Q9BXI6	-0.18451	0.00587	0.039516	TBC1D10A	TBC1 doma	TB10A_HUI		7	0.879951
15min_MT.	Q68D20	-0.95799	0.005887	0.039529	PMS2CL	Protein PM	PMS2L_HU		1	0.514772
15min_MT.	O60921	0.351349	0.005894	0.03954	HUS1	Checkpoint	HUS1_HUM		5	1.275753
15min_MT.	Q16718	0.186629	0.005906	0.039586	NDUFA5	NADH dehy	NDUA5_HU		3	1.138101
30min_MT.	P61764	-0.32141	0.005924	0.039641	STXBP1	Syntaxin-bi	STXB1_HUI		5	0.80029
30min_MT.	P62805	0.546266	0.005923	0.039641	H4C1	Histone H4	H4_HUMAN		7	1.460301
30min_MT.	Q9UNW1	0.126657	0.005933	0.039665	MINPP1	Multiple in	MINP1_HU		13	1.091761
30min_MT.	P00492	0.229617	0.00594	0.039677	HPRT1	Hypoxanth	HPRT_HUM		12	1.172523
15min_MT.	P51991	-0.21335	0.005951	0.03972	HNRNPA3	Heterogene	ROA3_HUM		14	0.86253
30min_MT.	P62318	0.27441	0.005964	0.039736	SNRPD3	Small nucle	SMD3_HUM		5	1.209499
15min_MT.	Q13243	-0.29558	0.005963	0.039736	SRSF5	Serine/argi	SRSF5_HUM		6	0.814742
30min_MT.	Q8N1F7	-0.19637	0.005978	0.039776	NUP93	Nuclear poi	NUP93_HU		28	0.872742
15min_MT.	Q8WVE0	0.063065	0.005989	0.039776	EEF1AKMT1	EEF1A lysin	EFMT1_HUI		1	1.044683
30min_MT.	Q9NP73	-0.86791	0.005983	0.039776	ALG13	UDP-N-acet	ALG13_HUI		1	0.547941
30min_MT.	Q8N465	0.444389	0.006004	0.039833	D2HGDH	D-2-hydrox	D2HDH_HU		3	1.360738
15min_MT.	Q99729	0.39593	0.006035	0.039968	HNRNPAB	Heterogene	ROAA_HUM		4	1.31579
30min_MT.	P20711	0.110874	0.006055	0.040069	DDC	Aromatic-L	DDC_HUMA		14	1.079882
15min_MT.	O75436	-0.10047	0.006091	0.040205	VPS26A	Vacuolar pi	VP26A_HUI		17	0.932732
15min_MT.	Q6PD74	0.229041	0.006089	0.040205	AAGAB	Alpha- and	AAGAB_HU		4	1.172056
15min_MT.	Q9Y5S9	-0.44224	0.006088	0.040205	RBM8A	RNA-bindir	RBM8A_HU		5	0.73599
15min_MT.	P62888	-0.22457	0.006109	0.040288	RPL30	Large ribos	RL30_HUM		5	0.855848
15min_MT.	P68366	0.247217	0.006166	0.040566	TUBA4A	Tubulin alp	TBA4A_HUI		2	1.186915
15min_MT.	Q13363	0.166582	0.006162	0.040566	CTBP1	C-terminal-	CTBP1_HUI		6	1.122396
30min_MT.	Q99627	0.411307	0.006178	0.040608	COPS8	COP9 signa	CSN8_HUM		4	1.32989
15min_MT.	P24863	1.042732	0.006211	0.040776	CCNC	Cyclin-C	CCNC_HUM		1	2.060125
15min_MT.	Q01650	-0.27995	0.00622	0.040783	SLC7A5	Large neutr	LAT1_HUM		4	0.823622
30min_MT.	O95571	0.055404	0.006238	0.040858	ETHE1	Persulfide c	ETHE1_HUI		8	1.03915
15min_MT.	P63208	-0.21734	0.006242	0.040858	SKP1	S-phase kin	SKP1_HUM		9	0.860147
15min_MT.	Q9Y2A7	-0.14815	0.006279	0.040926	NCKAP1	Nck-associa	NCKP1_HU		24	0.902405
15min_MT.	O00764	0.01592	0.006304	0.040941	PDXK	Pyridoxal k	PDXK_HUM		10	1.011096
30min_MT.	P62913	-0.16744	0.006298	0.040941	RPL11	Large ribos	RL11_HUM		3	0.890422
30min_MT.	Q6P6C2	-0.10429	0.006293	0.040941	ALKBH5	RNA demet	ALKB5_HUI		4	0.93026
15min_MT.	Q92673	0.371071	0.006297	0.040941	SORL1	Sortilin-rel	SORL_HUM		4	1.293312
15min_MT.	P21281	0.136557	0.006338	0.041052	ATP6V1B2	V-type prot	VATB2_HUI		14	1.099279
30min_MT.	Q8NI08	-0.62436	0.00634	0.041052	NCOA7	Nuclear rec	NCOA7_HU		1	0.648709
30min_MT.	P10253	0.276466	0.006378	0.041199	GAA	Lysosomal	LYAG_HUM		16	1.211224
30min_MT.	Q9UN36	0.209069	0.006377	0.041199	NDRG2	Protein ND	NDRG2_HU		8	1.155942
30min_MT.	O15357	-0.36921	0.006433	0.041399	INPPL1	Phosphatid	SHIP2_HUM		4	0.774207
15min_MT.	P05166	0.059986	0.006446	0.041399	PCCB	Propionyl-(	PCCB_HUM		22	1.042456
15min_MT.	P35659	-0.16804	0.006446	0.041399	DEK	Protein DEI	DEK_HUMA		15	0.890052
15min_MT.	Q7L5Y1	0.179345	0.00644	0.041399	ENOSF1	Mitochond	ENOF1_HUI		10	1.13237
15min_MT.	Q9NXJ5	0.057279	0.006426	0.041399	PGPEP1	Pyroglutam	PGPI_HUM		3	1.040502
15min_MT.	Q9UNN5	0.170441	0.006422	0.041399	FAF1	FAS-associa	FAF1_HUM		16	1.125402

30min_MT.	Q32P28	0.033808	0.006454	0.041411	P3H1	Prolyl 3-hy	P3H1_HUM	18	1.023711
30min_MT.	Q9H223	-0.61195	0.006459	0.041413	EHD4	EH domain	EHD4_HUM	6	0.654312
15min_MT.	P00761	0.201573	0.006489	0.041571	NaN	Trypsin (Lal	TRYP_PIG	5	1.149951
30min_MT.	Q01459	0.245988	0.006521	0.041705	CTBS	Di-N-acetyl	DIAC_HUM	10	1.185905
15min_MT.	Q96CD0	0.084301	0.006563	0.041907	FBXL8	F-box/LRR-r	FBXL8_HUM	3	1.060174
30min_MT.	O94855	-0.17556	0.006612	0.041914	SEC24D	Protein tra	SC24D_HUM	25	0.885423
15min_MT.	P02750	0.55688	0.006583	0.041914	LRG1	Leucine-ric	A2GL_HUM	1	1.471084
15min_MT.	P14625	-0.11654	0.006617	0.041914	HSP90B1	Endoplasm	ENPL_HUM	46	0.922398
30min_MT.	P25311	0.933261	0.006606	0.041914	AZGP1	Zinc-alpha-	ZA2G_HUM	11	1.909587
30min_MT.	P32322	-0.31543	0.006577	0.041914	PYCR1	Pyrroline-5	P5CR1_HUM	10	0.803611
30min_MT.	P35527	1.116026	0.006609	0.041914	KRT9	Keratin, typ	K1C9_HUM	32	2.167491
15min_MT.	Q13464	0.103418	0.006571	0.041914	ROCK1	Rho-associa	ROCK1_HU	46	1.074316
30min_MT.	Q9NYK5	-0.56147	0.006611	0.041914	MRPL39	Large ribos	RM39_HUM	2	0.677611
30min_MT.	Q9P016	-2.44673	0.006599	0.041914	THYN1	Thymocyte	THYN1_HUM	1	0.183426
15min_MT.	P01834	1.086267	0.006651	0.041973	IGKC	Immunoglo	IGKC_HUM	5	2.123239
30min_MT.	P02668	2.047752	0.006707	0.041973	CSN3	Kappa-case	CASK_BOVI	1	4.134611
30min_MT.	P04406	0.294192	0.006699	0.041973	GAPDH	Glyceraldeh	G3P_HUMA	20	1.226198
30min_MT.	P09104	0.775899	0.006691	0.041973	ENO2	Gamma-eno	ENOG_HUM	2	1.712257
15min_MT.	P61966	-0.32598	0.006675	0.041973	AP1S1	AP-1 compl	AP1S1_HUM	4	0.797758
15min_MT.	Q06203	-0.18171	0.006645	0.041973	PPAT	Amidophos	PUR1_HUM	18	0.88166
30min_MT.	Q16363	1.008009	0.00668	0.041973	LAMA4	Laminin sul	LAMA4_HU	1	2.011133
15min_MT.	Q5BKY9	-1.03628	0.006671	0.041973	FAM133B	Protein FAI	F133B_HUM	2	0.487584
30min_MT.	Q5RKV6	0.204866	0.006693	0.041973	EXOSC6	Exosome cc	EXOS6_HUM	9	1.152579
15min_MT.	Q8NEX9	0.348633	0.006666	0.041973	SDR9C7	Short-chair	DR9C7_HU	1	1.273354
15min_MT.	Q92934	0.172211	0.006677	0.041973	BAD	Bcl2-associ	BAD_HUMA	1	1.126784
15min_MT.	Q6ZT12	-0.68353	0.006732	0.042057	UBR3	E3 ubiquiti	UBR3_HUM	4	0.622638
15min_MT.	Q9Y6W5	0.264489	0.00674	0.042076	WASF2	Actin-bindi	WASF2_HU	7	1.20121
15min_MT.	Q8NBF2	0.123835	0.006752	0.042118	NHLRC2	NHL repeat	NHLC2_HU	17	1.089628
30min_MT.	O75940	0.225698	0.006768	0.042164	SMNDC1	Survival of	SPF30_HUM	7	1.169343
15min_MT.	P62306	-1.43935	0.00677	0.042164	SNRPF	Small nucle	RUXF_HUM	6	0.368732
15min_MT.	Q86TX2	0.093498	0.00678	0.042185	ACOT1	Acyl-coenzy	ACOT1_HUM	18	1.066954
30min_MT.	Q4G176	-0.32377	0.006797	0.042231	ACSF3	Malonate--	ACSF3_HUM	2	0.798979
15min_MT.	P62861	-0.65324	0.006895	0.042668	FAU	Ubiquitin-l	RS30_HUM	1	0.635852
30min_MT.	P15924	0.826536	0.006918	0.042742	DSP	Desmoplak	DESP_HUM	91	1.773422
30min_MT.	Q9HC84	0.35735	0.006938	0.042803	MUC5B	Mucin-5B	MUC5B_HU	5	1.281071
30min_MT.	Q04695	1.157213	0.006963	0.042889	KRT17	Keratin, typ	K1C17_HUM	11	2.230261
30min_MT.	Q9NZB2	-0.03527	0.006991	0.042989	FAM120A	Constitutiv	F120A_HUM	14	0.975846
15min_MT.	P26885	-0.29903	0.007	0.043015	FKBP2	Peptidyl-pr	FKBP2_HUM	6	0.812798
30min_MT.	Q8N163	-0.15169	0.007029	0.043057	CCAR2	Cell cycle ar	CCAR2_HUM	35	0.900193
15min_MT.	Q96B45	0.42675	0.007024	0.043057	BORCS7	BLOC-1-rela	BORC7_HU	3	1.344202
15min_MT.	O43819	0.201498	0.00704	0.043085	SCO2	Protein SCO	SCO2_HUM	1	1.149892
15min_MT.	P61163	-0.597	0.007048	0.043085	ACTR1A	Alpha-centr	ACTZ_HUM	4	0.661128
30min_MT.	P78330	0.122244	0.007092	0.043259	PSPH	Phosphose	SERB_HUM	12	1.088427
15min_MT.	Q08623	0.281698	0.007085	0.043259	PUDP	Pseudourid	HDHD1_HU	4	1.215625
15min_MT.	Q9BQS8	-0.07525	0.007096	0.043259	FYCO1	FYVE and cc	FYCO1_HUM	21	0.949176
15min_MT.	P50453	-0.05808	0.007103	0.043272	SERPINB9	Serpin B9	SPB9_HUM	19	0.960544
30min_MT.	O00116	-0.50903	0.007122	0.043355	AGPS	Alkyldihydr	ADAS_HUM	2	0.702696
15min_MT.	P09488	-0.20685	0.007144	0.043386	GSTM1	Glutathion	GSTM1_HU	11	0.86643
30min_MT.	Q8NEW0	-0.69517	0.007134	0.043386	SLC30A7	Zinc transp	ZNT7_HUM	2	0.617636
15min_MT.	Q92484	0.203386	0.007144	0.043386	SMPDL3A	Cyclic GMP	ASM3A_HU	12	1.151398
30min_MT.	O14776	-0.19691	0.007153	0.043407	TCERG1	Transcripti	TCRG1_HUM	14	0.872414
15min_MT.	P07942	0.078413	0.007162	0.043428	LAMB1	Laminin sul	LAMB1_HU	26	1.055856

15min_MT.	Q15019	-0.12593	0.00718	0.043443	SEPTIN2	Septin-2	SEPT2_HUM	14	0.916416
30min_MT.	Q96GA3	-0.25452	0.007203	0.043443	LTV1	Protein LTV	LTV1_HUM	7	0.838267
15min_MT.	Q9H9A6	0.195513	0.007215	0.043444	LRRC40	Leucine-ric	LRC40_HUI	14	1.145131
30min_MT.	O43865	-0.11272	0.007242	0.043486	AHCYL1	S-adenosyll	SAHH2_HU	5	0.92484
30min_MT.	P61604	0.457672	0.007263	0.043486	HSPE1	10 kDa heat	CH10_HUM	9	1.373324
15min_MT.	P63151	-0.10565	0.007263	0.043486	PPP2R2A	Serine/thre	2ABA_HUM	11	0.929388
30min_MT.	Q96FX7	0.098543	0.007245	0.043486	TRMT61A	tRNA (aden	TRM61_HU	6	1.070692
15min_MT.	Q96T51	0.231661	0.007249	0.043486	RUFY1	RUN and FY	RUFY1_HUI	22	1.174186
30min_MT.	Q9BX67	1.534523	0.007266	0.043486	JAM3	Junctional	JAM3_HUM	1	2.896927
15min_MT.	Q9BYD3	-0.50238	0.007256	0.043486	MRPL4	Large ribos	RM04_HUM	1	0.705942
30min_MT.	Q9UKK9	0.185988	0.007252	0.043486	NUDT5	ADP-sugar p	NUDT5_HU	12	1.137596
30min_MT.	Q92499	-0.33204	0.007281	0.043539	DDX1	ATP-depenc	DDX1_HUM	19	0.794412
30min_MT.	Q8NEZ2	-0.28705	0.007309	0.043677	VPS37A	Vacuolar pr	VP37A_HUI	7	0.819578
15min_MT.	P07741	0.237493	0.007378	0.043887	APRT	Adenine ph	APT_HUMA	5	1.178942
30min_MT.	P15586	0.35511	0.007375	0.043887	GNS	N-acetylglu	GNS_HUMA	11	1.279083
15min_MT.	Q9BRT3	0.249913	0.007374	0.043887	MIEN1	Migration a	MIEN1_HUI	4	1.189135
30min_MT.	P16930	0.195151	0.007402	0.04394	FAH	Fumarylace	FAAA_HUM	12	1.144844
15min_MT.	P18669	-0.19774	0.007428	0.04394	PGAM1	Phosphogly	PGAM1_HU	20	0.871918
15min_MT.	Q6P587	0.149348	0.007426	0.04394	FAHD1	Oxaloaceta	FAHD1_HU	11	1.109068
30min_MT.	Q8TF01	0.10885	0.007416	0.04394	PNISR	Arginine/se	PNISR_HUM	8	1.078368
15min_MT.	Q9BW92	0.354829	0.007406	0.04394	TARS2	Threonine--	SYTM_HUM	9	1.278834
30min_MT.	Q9P0R6	0.695001	0.007412	0.04394	GSKIP	GSK3B-inte	GSKIP_HUM	2	1.618886
15min_MT.	P00167	-0.21105	0.00745	0.044013	CYB5A	Cytochrom	CYB5_HUM	4	0.863907
15min_MT.	P02668	1.747996	0.007459	0.044032	CSN3	Kappa-case	CASK_BOVI	1	3.358917
30min_MT.	Q96IK1	0.082853	0.00747	0.044065	BOD1	Biorientati	BOD1_HUM	1	1.05911
30min_MT.	P09661	0.151668	0.007488	0.044071	SNRPA1	U2 small nu	RU2A_HUM	12	1.110853
15min_MT.	P11216	-0.07139	0.007481	0.044071	PYGB	Glycogen p	PYGB_HUM	25	0.951723
30min_MT.	P14923	0.619244	0.007484	0.044071	JUP	Junction pl	PLAK_HUM	27	1.53607
15min_MT.	Q15814	0.130941	0.0075	0.044071	TBCC	Tubulin-spe	TBCC_HUM	10	1.095008
30min_MT.	Q8WVN8	0.56307	0.007502	0.044071	UBE2Q2	Ubiquitin-c	UB2Q2_HU	2	1.47741
30min_MT.	Q15813	-0.7664	0.007518	0.044082	TBCE	Tubulin-spe	TBCE_HUM	8	0.587884
30min_MT.	P30101	0.260368	0.007539	0.044161	PDIA3	Protein disu	PDIA3_HUM	34	1.197784
30min_MT.	P62857	0.325343	0.007551	0.044161	RPS28	Small ribos	RS28_HUM	3	1.252962
15min_MT.	Q96RR4	1.17996	0.007543	0.044161	CAMKK2	Calcium/ca	KKCC2_HUI	1	2.265706
30min_MT.	P53992	-0.17037	0.007582	0.044294	SEC24C	Protein tra	SC24C_HUI	23	0.888613
15min_MT.	Q99470	-0.49779	0.007593	0.044321	SDF2	Stromal cel	SDF2_HUM	4	0.708192
15min_MT.	Q13426	0.115057	0.007657	0.044632	XRCC4	DNA repair	XRCC4_HUI	12	1.083018
30min_MT.	Q9Y6N7	0.071004	0.007652	0.044632	ROBO1	Roundabou	ROBO1_HU	24	1.050448
15min_MT.	O00148	-0.26065	0.007672	0.044656	DDX39A	ATP-depenc	DX39A_HU	5	0.83471
15min_MT.	Q8TB72	-0.12907	0.007673	0.044656	PUM2	Pumilio ho	PUM2_HUM	3	0.914422
15min_MT.	P42126	0.164327	0.007709	0.044659	ECI1	Enoyl-CoA c	ECI1_HUMA	8	1.120643
30min_MT.	P84022	-0.7513	0.007679	0.044659	SMAD3	Mothers ag	SMAD3_HU	4	0.594069
30min_MT.	Q15819	0.186968	0.007713	0.044659	UBE2V2	Ubiquitin-c	UB2V2_HU	5	1.138369
15min_MT.	Q9Y230	0.129245	0.007713	0.044659	RUVBL2	RuvB-like 2	RUVB2_HU	23	1.093721
30min_MT.	Q96G28	0.393989	0.007763	0.044812	CFAP36	Cilia- and fl	CFA36_HUI	5	1.314022
15min_MT.	Q5T750	0.517899	0.00778	0.044843	KPLCE	Protein KPI	KPLCE_HUM	1	1.431868
30min_MT.	Q9Y3B2	-0.23458	0.007793	0.044861	EXOSC1	Exosome cc	EXOS1_HUI	6	0.849931
15min_MT.	P02787	-0.17562	0.007821	0.044914	TF	Serotransfe	TRFE_HUMA	41	0.885386
30min_MT.	P16152	0.125735	0.007816	0.044914	CBR1	Carbonyl re	CBR1_HUM	16	1.091064
30min_MT.	Q9UJ70	0.110104	0.007809	0.044914	NAGK	N-acetyl-D-	NAGK_HUM	15	1.079306
15min_MT.	P36969	0.064479	0.007842	0.044972	GPX4	Phospholip	GPX4_HUM	8	1.045707
30min_MT.	Q15828	1.70453	0.007856	0.044982	CST6	Cystatin-M	CYTM_HUM	2	3.259227

30min_MT.	Q14692	1.025683	0.00789	0.045113	BMS1	Ribosome b	BMS1_HUM	2	2.035923
15min_MT.	Q9UIV1	0.327411	0.007885	0.045113	CNOT7	CCR4-NOT t	CNOT7_HU	3	1.25476
30min_MT.	P35790	0.540984	0.007902	0.045148	CHKA	Choline kin	CHKA_HUM	2	1.454965
30min_MT.	O75531	0.414725	0.007927	0.045261	BANF1	Barrier-to-a	BAF_HUMA	6	1.333045
15min_MT.	Q05655	0.438629	0.007941	0.045272	PRKCD	Protein kin	KPCD_HUM	3	1.355316
30min_MT.	Q9H446	0.29023	0.007939	0.045272	RWDD1	RWD doma	RWDD1_HU	3	1.222835
15min_MT.	P14209	0.02271	0.007966	0.045348	CD99	CD99 antig	CD99_HUM	4	1.015866
15min_MT.	Q9NPA8	0.335677	0.007963	0.045348	ENY2	Transcripti	ENY2_HUM	4	1.26197
30min_MT.	P13637	1.352767	0.008008	0.045497	ATP1A3	Sodium/po	AT1A3_HUI	1	2.554015
15min_MT.	Q5TDH0	-0.29351	0.008009	0.045497	DDI2	Protein DD	DDI2_HUM	16	0.815912
30min_MT.	P11274	-0.02938	0.008028	0.045535	BCR	Breakpoint	BCR_HUMA	3	0.97984
15min_MT.	P24386	-0.08163	0.008042	0.045584	CHM	Rab proteir	RAE1_HUM	14	0.944988
30min_MT.	Q96P48	0.429724	0.008075	0.045604	ARAP1	Arf-GAP wit	ARAP1_HUI	4	1.346976
15min_MT.	Q9P2X3	0.267583	0.008067	0.045604	IMPACT	Protein IMI	IMPCT_HUI	9	1.203789
15min_MT.	O15344	-0.17916	0.008108	0.045698	MID1	E3 ubiquiti	TRI18_HUM	17	0.883215
15min_MT.	Q53GS9	-0.24359	0.008109	0.045698	USP39	Ubiquitin c	UBP39_HU	10	0.844641
30min_MT.	O43819	0.11123	0.008185	0.045947	SCO2	Protein SC(	SCO2_HUM	1	1.080149
15min_MT.	P01111	0.219623	0.008174	0.045947	NRAS	GTPase NRa	RASN_HUM	6	1.164429
15min_MT.	P07225	0.090399	0.008188	0.045947	PROS1	Vitamin K-c	PROS_HUM	20	1.064664
30min_MT.	Q8NBF2	0.127122	0.008181	0.045947	NHLRC2	NHL repeat	NHLC2_HU	17	1.092113
15min_MT.	A4FU69	-1.48196	0.008208	0.046023	EFCAB5	EF-hand cal	EFCB5_HUI	1	0.358002
15min_MT.	Q8IV36	-0.17211	0.008261	0.046148	HID1	Protein HIC	HID1_HUM	11	0.887541
15min_MT.	Q8N567	0.835827	0.008242	0.046148	ZCCHC9	Zinc finger (	ZCHC9_HUI	1	1.78488
30min_MT.	Q9NTK5	-0.21142	0.008257	0.046148	OLA1	Obg-like AT	OLA1_HUM	14	0.863687
30min_MT.	Q9Y2X3	-0.11824	0.008266	0.046148	NOP58	Nucleolar p	NOP58_HU	9	0.921312
30min_MT.	Q52LJ0	-0.24707	0.008298	0.046298	FAM98B	Protein FAI	FA98B_HUI	5	0.842606
15min_MT.	Q8N3U4	-0.40026	0.008306	0.046308	STAG2	Cohesin sul	STAG2_HUI	14	0.75772
30min_MT.	Q92615	-0.16798	0.00836	0.046541	LARP4B	La-related p	LAR4B_HUI	8	0.890087
15min_MT.	P68371	0.236583	0.008404	0.046643	TUBB4B	Tubulin bet	TBB4B_HUI	18	1.178199
15min_MT.	Q8NI08	-0.82142	0.008408	0.046643	NCOA7	Nuclear rec	NCOA7_HU	1	0.565886
15min_MT.	Q9BX46;Q9	0.023569	0.008401	0.046643	RBM24;RBI	RNA-bindir	RBM24_HU	3	1.016471
15min_MT.	Q9ULJ6	0.314159	0.008385	0.046643	ZMIZ1	Zinc finger I	ZMIZ1_HUI	6	1.243287
30min_MT.	P47914	-0.1985	0.008428	0.046654	RPL29	Large ribos	RL29_HUM	2	0.871454
15min_MT.	Q96NC0	-1.0102	0.008423	0.046654	ZMAT2	Zinc finger	ZMAT2_HU	1	0.496478
15min_MT.	Q93099	-0.13604	0.008455	0.046727	HGD	Homogenti	HGD_HUMA	19	0.910011
15min_MT.	O75934	0.586034	0.008477	0.046791	BCAS2	Pre-mRNA-:	SPF27_HUI	6	1.501114
30min_MT.	P07205	1.133843	0.008515	0.046837	PGK2	Phosphogly	PGK2_HUM	5	2.194424
15min_MT.	P35241	-0.08118	0.00851	0.046837	RDX	Radixin	RADI_HUM	38	0.945286
30min_MT.	Q7L1Q6	0.114013	0.0085	0.046837	BZW1	eIF5-mimic	5MP2_HUM	14	1.082234
30min_MT.	Q9H0E9	0.688219	0.008492	0.046837	BRD8	Bromodom	BRD8_HUM	1	1.611293
15min_MT.	P37108	-0.13698	0.008535	0.046885	SRP14	Signal reco	SRP14_HUI	7	0.909424
30min_MT.	Q96KP4	0.228566	0.008534	0.046885	CNDP2	Cytosolic n	CNDP2_HU	18	1.17167
30min_MT.	P26639	0.141468	0.008549	0.046925	TARS1	Threonine--	SYTC_HUMA	29	1.103027
30min_MT.	Q9Y371	-0.40837	0.008568	0.046961	SH3GLB1	Endophilin	SHLB1_HUI	4	0.753476
15min_MT.	Q9NXC5	-0.21618	0.008583	0.046982	MIOS	GATOR2 co	MIOS_HUM	25	0.860842
30min_MT.	P25208	0.304636	0.008627	0.047124	NFYB	Nuclear tra	NFYB_HUM	2	1.235107
15min_MT.	P10619	0.294	0.008635	0.047136	CTSA	Lysosomal	PPGB_HUM	8	1.226034
30min_MT.	Q15031	-0.76231	0.008652	0.047195	LARS2	Leucine--tR	SYLM_HUM	4	0.589551
30min_MT.	P15848	0.318122	0.008687	0.047329	ARSB	Arylsulfata:	ARSB_HUM	11	1.246706
30min_MT.	Q9NXR7	0.165046	0.008715	0.047406	BABAM2	BRISC and E	BABA2_HUI	11	1.121201
15min_MT.	P61421	0.349691	0.008764	0.047605	ATP6V0D1	V-type prot	VA0D1_HU	3	1.274288
15min_MT.	O60841	-0.22634	0.008783	0.047642	EIF5B	Eukaryotic	IF2P_HUMA	45	0.854804

30min_MT.	P05165	-0.11003	0.008846	0.047862	PCCA	Propionyl-(	PCCA_HUM	17	0.926566
15min_MT.	P61224	-0.14124	0.008848	0.047862	RAP1B	Ras-related	RAP1B_HUI	7	0.90674
30min_MT.	O00418	-0.12815	0.008893	0.048014	EEF2K	Eukaryotic	EF2K_HUM	5	0.915003
30min_MT.	O14618	0.213203	0.008886	0.048014	CCS	Copper cha	CCS_HUMA	6	1.159259
15min_MT.	P60520	0.179652	0.008895	0.048014	GABARAPL2	Gamma-am	GBRL2_HUI	6	1.13261
30min_MT.	Q6P1J9	-0.02332	0.008905	0.048039	CDC73	Parafibrom	CDC73_HU	10	0.983964
30min_MT.	P25786	0.235105	0.008918	0.048075	PSMA1	Proteasom	PSA1_HUM	20	1.176992
15min_MT.	P13727	0.74158	0.008949	0.048142	PRG2	Bone marro	PRG2_HUM	2	1.672006
30min_MT.	Q13546	-0.2045	0.008961	0.048142	RIPK1	Receptor-ir	RIPK1_HUN	11	0.867837
30min_MT.	Q14566	-0.09631	0.008958	0.048142	MCM6	DNA replica	MCM6_HUI	23	0.935422
30min_MT.	Q15363	-1.20816	0.008946	0.048142	TMED2	Transmemb	TMED2_HU	1	0.43282
30min_MT.	Q9BUP3	-0.2216	0.008957	0.048142	HTATIP2	Oxidoreduc	HTAI2_HUN	3	0.857616
30min_MT.	P11279	0.377921	0.008988	0.048218	LAMP1	Lysosome-a	LAMP1_HU	5	1.299468
15min_MT.	P13489	0.106592	0.008994	0.048218	RNH1	Ribonuclea	RINI_HUMA	21	1.076682
30min_MT.	P63173	-0.53002	0.00899	0.048218	RPL38	Large ribos	RL38_HUM	3	0.692547
30min_MT.	P30047	-0.64423	0.009017	0.048274	GCHFR	GTP cycloh	GFRP_HUM	4	0.639833
15min_MT.	P29992	-0.55201	0.009046	0.048363	GNA11	Guanine nu	GNA11_HU	5	0.682069
15min_MT.	Q9BXW7	0.253059	0.009053	0.04837	HDHD5	Haloacid de	HDHD5_HU	5	1.191731
15min_MT.	O43148	0.068287	0.009065	0.048373	RNMT	mRNA cap g	MCES_HUM	17	1.048471
30min_MT.	P05089	0.881072	0.009102	0.048533	ARG1	Arginase-1	ARGI1_HUN	8	1.841744
15min_MT.	Q9BXS5	-0.10247	0.009114	0.048561	AP1M1	AP-1 compl	AP1M1_HU	18	0.931436
15min_MT.	Q13228	0.567918	0.009143	0.048652	SELENBP1	Methaneth	SBP1_HUM	4	1.482383
15min_MT.	A6NDU8	0.255731	0.009166	0.048676	RIMOC1	RAB7A-inte	RIMC1_HUI	6	1.193941
30min_MT.	P25774	0.179449	0.009216	0.048676	CTSS	Cathepsin S	CATS_HUM	7	1.132451
15min_MT.	P50995	0.19631	0.009234	0.048676	ANXA11	Annexin A1	ANX11_HU	15	1.145764
15min_MT.	Q16854	-0.44132	0.009208	0.048676	DGUOK	Deoxyguan	DGUOK_HU	4	0.73646
15min_MT.	Q5MNZ9	-0.02142	0.009237	0.048676	WIPI1	WD repeat	WIPI1_HUN	8	0.985262
30min_MT.	Q7RTS9	-1.11503	0.009179	0.048676	DYM	Dymeclin	DYM_HUMA	1	0.461681
30min_MT.	Q8IWV8	0.54072	0.009228	0.048676	UBR2	E3 ubiquiti	UBR2_HUM	5	1.454698
30min_MT.	Q8NEB9	-0.0934	0.009224	0.048676	PIK3C3	Phosphatid	PK3C3_HUI	4	0.937311
15min_MT.	Q8WVC0	0.33379	0.009195	0.048676	LEO1	RNA polym	LEO1_HUM	10	1.26032
30min_MT.	Q92688	0.417412	0.009169	0.048676	ANP32B	Acidic leuci	AN32B_HU	7	1.33553
30min_MT.	Q9BQA1	0.214303	0.009241	0.048676	WDR77	Methylosor	MEP50_HU	7	1.160143
15min_MT.	Q9BY32	-0.43043	0.009189	0.048676	ITPA	Inosine trip	ITPA_HUMA	7	0.74204
30min_MT.	P22413	-1.16096	0.009255	0.048699	ENPP1	Ectonucleo	ENPP1_HUI	2	0.447216
30min_MT.	P42224	-0.14863	0.00927	0.048699	STAT1	Signal trans	STAT1_HUN	24	0.902104
30min_MT.	Q6P587	0.273407	0.009264	0.048699	FAHD1	Oxaloaceta	FAHD1_HU	11	1.208659
30min_MT.	Q5HYK3	-1.74471	0.009283	0.04871	COQ5	2-methoxy-	COQ5_HUM	1	0.298394
30min_MT.	Q8WXA3	0.244805	0.009291	0.04871	RUFY2	RUN and FY	RUFY2_HUI	5	1.184932
15min_MT.	Q9Y639	0.758088	0.00929	0.04871	NPTN	Neuroplast	NPTN_HUM	2	1.691248
15min_MT.	Q86XE5	-1.01097	0.009305	0.048719	HOGA1	4-hydroxy-2	HOGA1_HU	1	0.496213
30min_MT.	Q9HCE6	-0.11167	0.009336	0.048845	ARHGEF10L	Rho guanin	ARGAL_HUI	12	0.925515
30min_MT.	P25789	0.191747	0.009356	0.048921	PSMA4	Proteasom	PSA4_HUM	11	1.142146
15min_MT.	P53365	0.234793	0.009392	0.049043	ARFIP2	Arfaptin-2	ARFP2_HUI	10	1.176738
30min_MT.	Q15542	-0.48586	0.009401	0.049057	TAF5	Transcripti	TAF5_HUM	6	0.714074
30min_MT.	P15529	0.225153	0.00942	0.049091	CD46	Membrane	MCP_HUMA	5	1.168901
30min_MT.	Q14432	-0.26472	0.009431	0.049114	PDE3A	cGMP-inhil	PDE3A_HUI	4	0.832362
30min_MT.	Q9Y312	-0.60515	0.009446	0.049159	AAR2	Protein AAI	AAR2_HUM	5	0.657405
30min_MT.	P62258	0.161198	0.009468	0.049242	YWHAE	14-3-3 prot	1433E_HUI	22	1.118215
15min_MT.	P24298	0.178323	0.00948	0.04927	GPT	Alanine am	ALAT1_HUN	13	1.131568
15min_MT.	Q96FW1	-0.12689	0.0095	0.049288	OTUB1	Ubiquitin t	OTUB1_HU	12	0.915801
30min_MT.	P05109	1.481882	0.009583	0.049442	S100A8	Protein S1(	S10A8_HUI	5	2.793129

30min_MT.	P55072	0.110002	0.009577	0.049442	VCP	Transitiona	TERA_HUM	60	1.07923
30min_MT.	Q6QNY1	-0.60534	0.009588	0.049442	BLOC1S2	Biogenesis	BL1S2_HUM	4	0.657316
15min_MT.	P62877	0.172439	0.009609	0.049514	RBX1	E3 ubiquiti	RBX1_HUM	2	1.126962
30min_MT.	P36551	0.113913	0.009651	0.049565	CPOX	Oxygen-dep	HEM6_HUM	18	1.08216
30min_MT.	Q9GZT8	0.210882	0.009641	0.049565	NIF3L1	NIF3-like pr	NIF3L_HUM	15	1.157396
30min_MT.	Q9NRS6	-0.41263	0.009645	0.049565	SNX15	Sorting nex	SNX15_HUM	2	0.751251
30min_MT.	P07858	0.045032	0.009683	0.049665	CTSB	Cathepsin B	CATB_HUM	12	1.031706
15min_MT.	P05089	0.29459	0.009704	0.049706	ARG1	Arginase-1	ARGI1_HUM	8	1.226537
30min_MT.	Q12792	0.083423	0.009701	0.049706	TWF1	Twinfilin-1	TWF1_HUM	14	1.059529
15min_MT.	Q8WZA9	0.36876	0.009724	0.049745	IRGQ	Immunity-r	IRGQ_HUM	15	1.291243
30min_MT.	P00813	0.135022	0.009804	0.04992	ADA	Adenosine	ADA_HUMA	19	1.09811
15min_MT.	P06576	0.082096	0.009832	0.04992	ATP5F1B	ATP syntha	ATPB_HUM	17	1.058555
15min_MT.	P35913	0.214048	0.009826	0.04992	PDE6B	Rod cGMP-	PDE6B_HUM	1	1.159938
15min_MT.	Q13330	-0.37414	0.009835	0.04992	MTA1	Metastasis-	MTA1_HUM	2	0.771568
15min_MT.	Q14790	0.306863	0.009783	0.04992	CASP8	Caspase-8	CASP8_HUM	11	1.237015
15min_MT.	Q53FT3	0.32044	0.009822	0.04992	HIKESHI	Protein Hik	HIKES_HUM	5	1.248711
30min_MT.	Q8WVE0	0.086787	0.009819	0.04992	EEF1AKMT1	EEF1A lysin	EFMT1_HUM	1	1.062003
30min_MT.	Q96PZ0	0.094973	0.009831	0.04992	PUS7	Pseudourid	PUS7_HUM	23	1.068046
30min_MT.	Q9BYW2	0.170905	0.00979	0.04992	SETD2	Histone-lys	SETD2_HUM	3	1.125764
30min_MT.	P11177	-0.11753	0.009847	0.049953	PDHB	Pyruvate de	ODPB_HUM	11	0.921766
30min_MT.	P17050	0.23888	0.009878	0.050042	NAGA	Alpha-N-ac	NAGAB_HU	9	1.180076
15min_MT.	P62841	0.572633	0.009899	0.050053	RPS15	Small ribos	RS15_HUM	1	1.487236
15min_MT.	P02790	0.950426	0.009913	0.050059	HPX	Hemopexin	HEMO_HUM	3	1.932443
30min_MT.	Q15393	0.075993	0.009907	0.050059	SF3B3	Splicing fac	SF3B3_HUM	41	1.054086
30min_MT.	P57740	-0.34087	0.009938	0.050153	NUP107	Nuclear po	NU107_HU	8	0.789566
30min_MT.	Q14525	7.799109	0.009969	0.050253	KRT33B	Keratin, typ	KT33B_HU	2	222.7233
30min_MT.	Q15287	-0.29181	0.010057	0.050444	RNPS1	RNA-bindir	RNPS1_HU	4	0.816876
15min_MT.	Q96EE3	-0.1837	0.010036	0.050444	SEH1L	Nucleopori	SEH1_HUM	8	0.880441
30min_MT.	Q9C075	0.852306	0.010041	0.050444	KRT23	Keratin, typ	K1C23_HU	8	1.805385
30min_MT.	P01130	0.205652	0.010068	0.050448	LDLR	Low-densit	LDLR_HUM	7	1.153207
30min_MT.	Q15404	0.087733	0.010092	0.050505	RSU1	Ras suppres	RSU1_HUM	9	1.062699
15min_MT.	O60447	0.369257	0.010151	0.05052	EVI5	Ecotropic v	EVI5_HUMA	11	1.291687
30min_MT.	P22830	-0.23668	0.010153	0.05052	FECH	Ferrochelat	HEMH_HUM	8	0.848694
30min_MT.	P31025	1.788196	0.010124	0.05052	LCN1	Lipocalin-1	LCN1_HUM	2	3.453827
15min_MT.	P78344	0.186276	0.010114	0.05052	EIF4G2	Eukaryotic	IF4G2_HUM	26	1.137823
30min_MT.	Q13228	0.815458	0.01015	0.05052	SELENBP1	Methaneth	SBP1_HUM	4	1.759857
15min_MT.	Q86WU2	0.148113	0.010138	0.05052	LDHD	Probable D	LDHD_HUM	9	1.108119
15min_MT.	Q92597	0.356641	0.010153	0.05052	NDRG1	Protein ND	NDRG1_HU	7	1.280441
15min_MT.	O00534	-0.17506	0.01016	0.050522	VWA5A	von Willebr	VMA5A_HU	14	0.885728
30min_MT.	P22570	-0.3602	0.01017	0.05054	FDXR	NADPH:adr	ADRO_HUM	7	0.779059
15min_MT.	Q96DH6	-0.85564	0.01018	0.050558	MSI2	RNA-bindir	MSI2H_HU	2	0.55262
30min_MT.	P00390	0.190341	0.010241	0.05067	GSR	Glutathion	GSHR_HUM	19	1.141034
30min_MT.	P06865	0.183113	0.010256	0.050709	HEXA	Beta-hexos	HEXA_HUM	15	1.135331
30min_MT.	Q7L4I2	-0.02084	0.010289	0.050782	RSRC2	Arginine/se	RSRC2_HU	6	0.985656
30min_MT.	Q02447	-0.5233	0.010302	0.050809	SP3	Transcripti	SP3_HUMA	2	0.695777
15min_MT.	Q9Y5V3	0.475276	0.010324	0.050857	MAGED1	Melanoma-	MAGD1_HU	1	1.390184
30min_MT.	P06493	-0.13386	0.010341	0.050858	CDK1	Cyclin-depe	CDK1_HUM	3	0.911389
15min_MT.	Q15773	0.195044	0.010339	0.050858	MLF2	Myeloid leu	MLF2_HUM	2	1.144759
30min_MT.	Q9HD34	0.198151	0.010345	0.050858	LYRM4	LYR motif-c	LYRM4_HU	3	1.147227
30min_MT.	Q9NWU1	0.180692	0.010355	0.050876	OXSM	3-oxoacyl-[	OXSM_HUM	10	1.133427
15min_MT.	P61764	-0.25838	0.010369	0.050914	STXBP1	Syntaxin-bi	STXB1_HUM	5	0.836025
30min_MT.	O00592	0.049502	0.010395	0.05096	PODXL	Podocalyxi	PODXL_HU	1	1.034908

30min_MT.	P07711	0.171169	0.010397	0.05096	CTSL	Procatheps	CATL1_HUI	3	1.125971
15min_MT.	O95260	-0.2943	0.010433	0.051095	ATE1	Arginyl-tRN	ATE1_HUM	8	0.815471
30min_MT.	Q02413	1.026118	0.010438	0.051095	DSG1	Desmoglein	DSG1_HUN	20	2.036537
30min_MT.	A6NDG6	0.258514	0.010503	0.051343	PGP	Glycerol-3-	PGP_HUMA	14	1.196246
30min_MT.	O75607	-0.96803	0.010525	0.051343	NPM3	Nucleoplas	NPM3_HUN	3	0.511204
15min_MT.	P28340	-0.08366	0.010528	0.051343	POLD1	DNA polym	DPOD1_HU	31	0.943664
15min_MT.	Q9NUP1	-1.34283	0.010573	0.05148	BLOC1S4	Biogenesis	BL1S4_HUN	1	0.394247
30min_MT.	Q9NU19	-0.0475	0.010623	0.051642	TBC1D22B	TBC1 doma	TB22B_HUI	1	0.967609
30min_MT.	Q9Y6E2	0.139767	0.01062	0.051642	BZW2	eIF5-mimic	5MP1_HUN	21	1.101727
15min_MT.	Q53HC9	0.083381	0.010654	0.051694	EIPR1	EARP and G	EIPR1_HUN	7	1.059498
30min_MT.	Q86TJ2	1.210716	0.010647	0.051694	TADA2B	Transcripti	TAD2B_HUI	1	2.314525
15min_MT.	Q96IK1	0.264698	0.010655	0.051694	BOD1	Biorientati	BOD1_HUN	1	1.201385
15min_MT.	Q5ZPR3	0.124659	0.010667	0.051698	CD276	CD276 anti	CD276_HU	2	1.09025
15min_MT.	Q9Y5K5	0.12377	0.010682	0.051737	UCHL5	Ubiquitin c	UCHL5_HU	13	1.089578
15min_MT.	Q9UQB8	-0.48275	0.010724	0.051876	BAIAP2	BAR/IMD de	BAIP2_HUN	2	0.715613
15min_MT.	O60333	-0.18505	0.010838	0.052331	KIF1B	Kinesin-like	KIF1B_HUN	4	0.879617
30min_MT.	Q13363	-0.11589	0.010846	0.052335	CTBP1	C-terminal-	CTBP1_HUI	6	0.92281
30min_MT.	O75832	-0.06994	0.010904	0.05252	PSMD10	26S protea:	PSD10_HUI	6	0.952679
15min_MT.	Q6UWP8	-0.47331	0.010899	0.05252	SBSN	Suprabasin	SBSN_HUM	5	0.720311
15min_MT.	P21291	-0.339	0.010926	0.052591	CSRP1	Cysteine an	CSRP1_HUI	10	0.79059
30min_MT.	P55786	0.195475	0.010934	0.052599	NPEPPS	Puromycin	PSA_HUMA	38	1.145101
15min_MT.	P02751	-0.06275	0.010951	0.052651	FN1	Fibronectir	FINC_HUM	84	0.95744
15min_MT.	O95490	0.234544	0.010973	0.052697	ADGRL2	Adhesion G	AGRL2_HUI	9	1.176535
30min_MT.	P01857	1.099579	0.010975	0.052697	IGHG1	Immunoglo	IGHG1_HUI	3	2.142921
30min_MT.	Q4VC31	0.379524	0.011031	0.052903	MIX23	Protein MI	MIX23_HUI	6	1.300913
30min_MT.	P15374	0.049497	0.011053	0.052904	UCHL3	Ubiquitin c	UCHL3_HU	10	1.034904
30min_MT.	Q12882	-0.06418	0.011058	0.052904	DPYD	Dihydropyr	DPYD_HUN	15	0.95649
30min_MT.	Q04446	-0.27712	0.011129	0.053148	GBE1	1,4-alpha-g	GLGB_HUN	18	0.825234
30min_MT.	Q13136	-0.15741	0.01113	0.053148	PPFIA1	Liprin-alph	LIPA1_HUN	14	0.896634
15min_MT.	Q9H0R4	0.248338	0.011129	0.053148	HDHD2	Haloacid de	HDHD2_HU	7	1.187838
30min_MT.	Q86UU0	-0.43426	0.011155	0.053206	BCL9L	B-cell CLL/l	BCL9L_HUN	2	0.740072
30min_MT.	Q12864	0.28566	0.011195	0.053266	CDH17	Cadherin-1	CAD17_HU	4	1.218968
30min_MT.	Q8N1N4	0.986983	0.011178	0.053266	KRT78	Keratin, typ	K2C78_HUI	12	1.982035
30min_MT.	P02765	-0.17129	0.011244	0.053327	AHSG	Alpha-2-HS	FETUA_HUN	2	0.88805
30min_MT.	P06733	0.286093	0.011231	0.053327	ENO1	Alpha-enol	ENOA_HUN	24	1.219334
15min_MT.	P10109	0.244222	0.011249	0.053327	FDX1	Adrenodox	ADX_HUMA	2	1.184454
15min_MT.	P23025	-1.88409	0.011232	0.053327	XPA	DNA repair	XPA_HUMA	1	0.270915
15min_MT.	Q9Y316	0.164612	0.011298	0.053463	MEMO1	Protein ME	MEMO1_HU	3	1.120865
30min_MT.	Q9P0P0	0.452311	0.011307	0.05347	RNF181	E3 ubiquiti	RN181_HU	3	1.368231
30min_MT.	P68032	-0.65758	0.011315	0.053476	ACTC1	Actin, alpha	ACTC_HUM	3	0.63394
15min_MT.	Q86U28	0.893045	0.011329	0.053512	ISCA2	Iron-sulfur	ISCA2_HUN	5	1.857092
30min_MT.	Q86WU2	0.18642	0.011346	0.05356	LDHD	Probable D	LDHD_HUN	9	1.137936
15min_MT.	Q9H832	0.142035	0.011362	0.053605	UBE2Z	Ubiquitin-c	UBE2Z_HUI	8	1.103461
30min_MT.	Q6PCE3	0.070325	0.011387	0.053655	PGM2L1	Glucose 1,6	PGM2L_HU	17	1.049953
30min_MT.	Q15014	0.25716	0.011447	0.05384	MORF4L2	Mortality fa	MO4L2_HU	7	1.195124
30min_MT.	P04264	1.014972	0.011512	0.054084	KRT1	Keratin, typ	K2C1_HUM	42	2.020863
15min_MT.	P00352	0.15599	0.011547	0.054168	ALDH1A1	Aldehyde d	AL1A1_HUI	31	1.114186
15min_MT.	Q9UJ70	0.10748	0.011551	0.054168	NAGK	N-acetyl-D-	NAGK_HUN	15	1.077345
15min_MT.	Q13126	0.148686	0.011561	0.05418	MTAP	S-methyl-5'	MTAP_HUN	18	1.10856
30min_MT.	P08779	0.712463	0.011568	0.054181	KRT16	Keratin, typ	K1C16_HUI	21	1.638599
15min_MT.	P31949	0.787165	0.011596	0.054207	S100A11	Protein S10	S10AB_HUI	4	1.72568
30min_MT.	Q9NUQ9	0.191176	0.01159	0.054207	CYRIB	CYFIP-relat	CYRIB_HUN	14	1.141694

15min_MT.	P62995	-0.30193	0.011638	0.054347	TRA2B	Transforme	TRA2B_HUI	8	0.811166
15min_MT.	P36955	0.25118	0.011659	0.054413	SERPINF1	Pigment ep	PEDF_HUM	7	1.19018
15min_MT.	P40926	0.12768	0.011712	0.05453	MDH2	Malate deh	MDHM_HU	20	1.092535
30min_MT.	O76031	-0.35849	0.011732	0.054561	CLPX	ATP-depen(	CLPX_HUM	7	0.77998
30min_MT.	Q6YN16	-0.20331	0.011726	0.054561	HSDL2	Hydroxyste	HSDL2_HUI	9	0.868558
15min_MT.	O96013	0.711674	0.011756	0.054639	PAK4	Serine/thre	PAK4_HUM	1	1.637703
30min_MT.	O75521	-0.1804	0.011838	0.0549	ECI2	Enoyl-CoA (	ECI2_HUM/	11	0.882458
30min_MT.	Q96P63	1.087434	0.011841	0.0549	SERPINB12	Serpin B12	SPB12_HUI	8	2.124957
30min_MT.	P05090	1.524191	0.011861	0.054962	APOD	Apolipoprc	APOD_HUM	2	2.876254
15min_MT.	O15164	-0.19514	0.011904	0.055066	TRIM24	Transcripti	TIF1A_HUM	4	0.873489
15min_MT.	Q9HCK4	-0.64	0.011903	0.055066	ROBO2	Roundabou	ROBO2_HU	4	0.641713
15min_MT.	O94880	-0.4468	0.01192	0.055106	PHF14	PHD finger	PHF14_HUI	1	0.73367
30min_MT.	P27482	1.045608	0.011976	0.055333	CALML3	Calmodulir	CALL3_HUI	2	2.064236
15min_MT.	Q6NXE6	0.204898	0.011991	0.055336	ARMC6	Armadillo r	ARMC6_HU	10	1.152605
15min_MT.	O15372	0.14001	0.012012	0.055358	EIF3H	Eukaryotic	EIF3H_HUM	13	1.101913
15min_MT.	Q14919	-0.72855	0.01202	0.055358	DRAP1	Dr1-associa	NC2A_HUM	3	0.60351
15min_MT.	Q7L4I2	-0.12041	0.012031	0.055358	RSRC2	Arginine/se	RSRC2_HUI	6	0.919924
15min_MT.	P17050	0.100463	0.012054	0.055396	NAGA	Alpha-N-ac	NAGAB_HU	9	1.072118
30min_MT.	O43251;Q9	0.766517	0.012174	0.055824	RBFOX2;RB	RNA bindin	RFOX2_HUI	2	1.701158
30min_MT.	O95425	-0.27469	0.012173	0.055824	SVIL	Supervillin	SVIL_HUM/	4	0.826628
30min_MT.	Q13247	0.158112	0.012183	0.055824	SRSF6	Serine/argi	SRSF6_HUM	15	1.115826
15min_MT.	P62910	-2.89447	0.012219	0.055893	RPL32	Large ribos	RL32_HUM	1	0.134486
15min_MT.	Q04637	-0.14592	0.012217	0.055893	EIF4G1	Eukaryotic	IF4G1_HUM	50	0.9038
15min_MT.	P07305	-0.21394	0.012293	0.056144	H1-0	Histone H1	H10_HUM/	6	0.862179
15min_MT.	Q9P0M2	0.528435	0.012295	0.056144	AKAP7	A-kinase an	AKA7G_HU	3	1.442363
15min_MT.	O75818	-0.10323	0.012326	0.056162	RPP40	Ribonuclea	RPP40_HUI	9	0.930947
30min_MT.	P42765	0.133639	0.012338	0.056162	ACAA2	3-ketoacyl-	THIM_HUM	16	1.097057
15min_MT.	P49588	-0.1275	0.01234	0.056162	AARS1	Alanine--tR	SYAC_HUM	43	0.915418
30min_MT.	P52209	0.17862	0.012333	0.056162	PGD	6-phospho	6PGD_HUM	23	1.131801
30min_MT.	P52306	0.147112	0.012336	0.056162	RAP1GDS1	Rap1 GTPas	GDS1_HUM	21	1.10735
30min_MT.	Q9HBL0	-0.09198	0.012342	0.056162	TNS1	Tensin-1	TENS1_HUM	11	0.938234
15min_MT.	Q9ULD2	0.067932	0.012369	0.056219	MTUS1	Microtubul	MTUS1_HU	4	1.048213
30min_MT.	P00738	0.558677	0.012461	0.05638	HP	Haptoglobi	HPT_HUMA	3	1.472917
30min_MT.	Q14574	0.791406	0.012448	0.05638	DSC3	Desmocolli	DSC3_HUM	5	1.73076
30min_MT.	Q92785	-0.25274	0.012462	0.05638	DPF2	Zinc finger	REQU_HUM	7	0.8393
15min_MT.	Q9P013	0.170028	0.012454	0.05638	CWC15	Spliceosom	CWC15_HU	7	1.12508
30min_MT.	Q15233	-0.10111	0.012511	0.056469	NONO	Non-POU d	NONO_HUM	25	0.932318
30min_MT.	O43157	0.577765	0.012557	0.056646	PLXNB1	Plexin-B1	PLXB1_HUI	3	1.492535
15min_MT.	P53611	0.176687	0.012568	0.05666	RABGGTB	Geranylger	PGTB2_HUI	8	1.130286
30min_MT.	Q86WR0	0.161536	0.012601	0.056691	CCDC25	Coiled-coil	CCD25_HU	6	1.118478
30min_MT.	Q9HAY6	-0.60316	0.012604	0.056691	BCO1	Beta,beta-c	BCDO1_HU	1	0.658309
30min_MT.	Q9UIC8	0.143897	0.01259	0.056691	LCMT1	Leucine car	LCMT1_HU	10	1.104885
30min_MT.	Q9ULR3	-0.08911	0.012617	0.056718	PPM1H	Protein pho	PPM1H_HU	1	0.9401
30min_MT.	P0C0L4	-0.2311	0.012644	0.056741	C4A	Compleme	CO4A_HUM	62	0.851983
30min_MT.	Q9HC35	-0.12719	0.012633	0.056741	EML4	Echinoderm	EMAL4_HU	34	0.915614
30min_MT.	P12273	1.165975	0.012714	0.05696	PIP	Prolactin-ir	PIP_HUMA	4	2.243848
15min_MT.	Q6UB28	-0.57016	0.01273	0.056963	METAP1D	Methionine	MAP12_HU	4	0.673544
15min_MT.	Q9H9S4	0.175509	0.012726	0.056963	CAB39L	Calcium-bi	CB39L_HUI	9	1.129363
15min_MT.	O00116	-0.23704	0.012742	0.056987	AGPS	Alkyldihydr	ADAS_HUM	2	0.848486
15min_MT.	P56181	-0.82778	0.012791	0.057105	NDUFV3	NADH dehy	NDUV3_HU	1	0.563396
15min_MT.	Q5SRH9	-0.98205	0.012839	0.057192	TTC39A	Tetratricop	TT39A_HUI	1	0.50626
30min_MT.	Q9UKF6	-0.1911	0.012821	0.057192	CPSF3	Cleavage ar	CPSF3_HUI	20	0.875935

15min_MT.	Q96EM0	-0.24984	0.012851	0.057211	L3HYPDH	Trans-3-hyc	T3HPD_HU		3	0.84099
15min_MT.	Q7L3B6	0.306129	0.01288	0.057306	CDC37L1	Hsp90 co-c	CD37L_HUI		4	1.236386
30min_MT.	P51610	-0.21816	0.012924	0.057426	HCFC1	Host cell fa	HCFC1_HUI		39	0.859659
30min_MT.	Q9NUP1	-1.30267	0.01292	0.057426	BLOC1S4	Biogenesis	BL1S4_HUI		1	0.405375
15min_MT.	Q9H3L0	0.58385	0.012938	0.057437	MMADHC	Cobalamin	MMAD_HU		2	1.498844
15min_MT.	P52895	-0.93598	0.013	0.057645	AKR1C2	Aldo-keto r	AK1C2_HUI		5	0.522687
15min_MT.	P12273	0.816124	0.013025	0.057683	PIP	Prolactin-ir	PIP_HUMA		4	1.76067
15min_MT.	P45954	0.189827	0.013031	0.057683	ACADSB	Short/bran	ACDSB_HUI		13	1.140627
30min_MT.	Q8TBZ6	-0.91189	0.013048	0.057696	TRMT10A	tRNA meth	TM10A_HU		1	0.531489
30min_MT.	P68106	-0.99566	0.013081	0.057775	FKBP1B	Peptidyl-pr	FKB1B_HUI		1	0.501506
15min_MT.	Q9Y6G3	-0.7418	0.013115	0.057894	MRPL42	Large ribos	RM42_HUN		1	0.597991
30min_MT.	Q9BX46;Q	0.014204	0.01314	0.057971	RBM24;RBI	RNA-bindir	RBM24_HU		3	1.009894
15min_MT.	P05109	0.592241	0.013149	0.057976	S100A8	Protein S1C	S10A8_HUI		5	1.507587
30min_MT.	P09972	0.17481	0.01316	0.057995	ALDOC	Fructose-bi	ALDOC_HU		15	1.128816
15min_MT.	O60488	-0.33305	0.013265	0.058224	ACSL4	Long-chain	ACSL4_HUI		15	0.793859
15min_MT.	Q7RTV0	-0.547	0.013255	0.058224	PHF5A	PHD finger-	PHF5A_HUI		6	0.684442
30min_MT.	Q86XI2	0.547154	0.013255	0.058224	NCAPG2	Condensin-	CNDG2_HU		1	1.461201
15min_MT.	Q9UJ41	0.123189	0.013259	0.058224	RABGEF1	Rab5 GDP/	RABX5_HUI		11	1.08914
15min_MT.	P10412	-0.30456	0.01334	0.058357	H1-4	Histone H1	H14_HUMA		8	0.809689
30min_MT.	Q6KB66	1.086563	0.013333	0.058357	KRT80	Keratin, typ	K2C80_HUI		19	2.123675
30min_MT.	Q93009	-0.11605	0.013323	0.058357	USP7	Ubiquitin c	UBP7_HUN		38	0.922713
30min_MT.	Q96J01	-0.22079	0.013331	0.058357	THOC3	THO compl	THOC3_HU		7	0.858098
15min_MT.	P02663	1.749799	0.013356	0.058398	CSN1S2	Alpha-S2-ca	CASA2_BOV		3	3.363117
15min_MT.	Q9NR19	-0.15988	0.013396	0.058505	ACSS2	Acetyl-coer	ACSA_HUM		16	0.895099
30min_MT.	Q12756	-0.23559	0.013421	0.058549	KIF1A	Kinesin-like	KIF1A_HUN		1	0.849335
15min_MT.	P81605	0.364743	0.013449	0.058639	DCD	Dermcidin	DCD_HUMA		3	1.287652
15min_MT.	Q7L5N1	0.139729	0.013476	0.058726	COPS6	COP9 signa	CSN6_HUN		10	1.101698
30min_MT.	Q8NEX9	0.8985	0.013484	0.058728	SDR9C7	Short-chair	DR9C7_HU		1	1.864127
30min_MT.	Q5JTV8	0.171537	0.013511	0.05881	TOR1AIP1	Torsin-1A-ir	TOIP1_HUN		4	1.126258
30min_MT.	O43592	0.176343	0.013563	0.058937	XPOT	Exportin-T	XPOT_HUM		17	1.130016
15min_MT.	P49419	0.082262	0.013558	0.058937	ALDH7A1	Alpha-amir	AL7A1_HUI		24	1.058677
30min_MT.	Q96GD0	0.13097	0.013551	0.058937	PDXP	Chronophi	PLPP_HUM		16	1.09503
15min_MT.	Q13242	0.278365	0.013593	0.059038	SRSF9	Serine/argi	SRSF9_HUN		10	1.212819
15min_MT.	P51808	-1.00063	0.013648	0.059178	DYNLT3	Dynein ligh	DYLT3_HUN		3	0.499783
30min_MT.	Q9H4M9	-0.29431	0.013661	0.059202	EHD1	EH domain-	EHD1_HUN		11	0.81546
30min_MT.	Q5T2W1	0.100818	0.013673	0.059221	PDZK1	Na(+)/H(+) e	NHRF3_HU		24	1.072382
15min_MT.	P28328	0.369007	0.013691	0.059239	PEX2	Peroxisome	PEX2_HUM		1	1.291463
15min_MT.	Q07065	0.605536	0.013697	0.059239	CKAP4	Cytoskeleto	CKAP4_HUI		4	1.521544
30min_MT.	Q9Y3E7	0.169948	0.0137	0.059239	CHMP3	Charged mu	CHMP3_HU		3	1.125018
30min_MT.	P35579	-0.23611	0.013721	0.059294	MYH9	Myosin-9	MYH9_HUN		74	0.84903
30min_MT.	O94966	-0.04684	0.013774	0.059428	USP19	Ubiquitin c	UBP19_HU		13	0.968051
30min_MT.	Q9H8H0	-0.26015	0.013773	0.059428	NOL11	Nucleolar p	NOL11_HU		10	0.835003
15min_MT.	P50750	0.477198	0.013784	0.059437	CDK9	Cyclin-depe	CDK9_HUN		5	1.392038
30min_MT.	P22307	0.081407	0.013816	0.059531	SCP2	Sterol carri	SCP2_HUM		14	1.058049
15min_MT.	Q15024	0.171538	0.013821	0.059531	EXOSC7	Exosome co	EXOS7_HUI		11	1.126259
15min_MT.	Q96DT7	-0.91022	0.013831	0.059541	ZBTB10	Zinc finger	ZBT10_HUN		2	0.532103
30min_MT.	Q9BTY2	0.218057	0.013841	0.05955	FUCA2	Plasma alpl	FUCO2_HU		15	1.163166
15min_MT.	P52566	0.790581	0.013906	0.059732	ARHGDIB	Rho GDP-di	GDIR2_HUI		1	1.729771
30min_MT.	P32321	0.279684	0.013931	0.059748	DCTD	Deoxycytid	DCTD_HUM		4	1.213929
15min_MT.	P14866	-0.16818	0.013954	0.059749	HNRNPL	Heterogene	HNRPL_HU		15	0.889963
15min_MT.	P51688	0.067213	0.013947	0.059749	SGSH	N-sulphogl	SPHM_HUN		7	1.047691
15min_MT.	Q9HAC7	0.873046	0.013956	0.059749	SUGCT	Succinate--	SUCHY_HUI		1	1.831525

15min_MT.	P00558	0.154642	0.014049	0.060051	PGK1	Phosphogly	PGK1_HUM	32	1.113146
30min_MT.	O75342	0.979206	0.014078	0.06007	ALOX12B	Arachidona	LX12B_HUI	6	1.971381
30min_MT.	Q8N9N8	0.046785	0.014062	0.06007	EIF1AD	Probable RI	EIF1A_HUM	5	1.032961
30min_MT.	Q9H9S4	0.17972	0.014072	0.06007	CAB39L	Calcium-bi	CB39L_HUI	9	1.132664
15min_MT.	O00469	0.11287	0.014135	0.060221	PLOD2	Procollager	PLOD2_HU	17	1.081377
30min_MT.	Q9NZL4	0.092101	0.014193	0.060358	HSPBP1	Hsp70-binc	HPBP1_HU	11	1.065921
15min_MT.	Q96BN8	0.074483	0.014249	0.06051	OTULIN	Ubiquitin t	OTUL_HUM	8	1.052984
15min_MT.	Q8IZ21	-0.24101	0.014291	0.060655	PHACTR4	Phosphatas	PHAR4_HU	3	0.846153
30min_MT.	P17655	-0.19601	0.014315	0.060721	CAPN2	Calpain-2 c	CAN2_HUM	16	0.872961
30min_MT.	Q96BW5	0.24655	0.014361	0.060851	PTER	Phosphotri	PTER_HUM	14	1.186367
30min_MT.	Q9NXA8	-0.17203	0.0144	0.060916	SIRT5	NAD-depen	SIR5_HUM/	5	0.88759
30min_MT.	P35658	-0.13897	0.014409	0.060925	NUP214	Nuclear poi	NU214_HU	36	0.908169
30min_MT.	P02538	0.802272	0.014455	0.061051	KRT6A	Keratin, typ	K2C6A_HUI	37	1.743845
15min_MT.	P59665;P5	-0.49027	0.014448	0.061051	DEFA1;DEF/	Neutrophil	DEF1_HUM	1	0.711891
30min_MT.	P52888	0.152399	0.014467	0.061061	THOP1	Thimet olig	THOP1_HU	32	1.111416
15min_MT.	Q92539	-0.01366	0.014473	0.061061	LPIN2	Phosphatic	LPIN2_HUM	8	0.99058
30min_MT.	P14209	-0.17319	0.014484	0.061073	CD99	CD99 antig	CD99_HUM	4	0.886877
15min_MT.	Q8NI22	-1.40096	0.014512	0.06113	MCFD2	Multiple cc	MCFD2_HL	2	0.378677
30min_MT.	Q9BXW6	-0.20278	0.014513	0.06113	OSBPL1A	Oxysterol-k	OSBL1_HUI	7	0.868875
30min_MT.	Q9NPH0	0.071716	0.014525	0.06115	ACP6	Lysophospl	PPA6_HUM	8	1.050966
15min_MT.	Q5HYK3	-1.76813	0.014543	0.061191	COQ5	2-methoxy-	COQ5_HUM	1	0.293589
15min_MT.	P29508	0.55624	0.014557	0.061216	SERPINB3	Serpin B3	SPB3_HUM	9	1.470432
15min_MT.	P62917	-0.16401	0.014587	0.061312	RPL8	Large ribos	RL8_HUMA	4	0.892539
30min_MT.	P29508	1.081355	0.0146	0.061332	SERPINB3	Serpin B3	SPB3_HUM	9	2.116023
30min_MT.	Q96C36	-0.3337	0.014639	0.061431	PYCR2	Pyrroline-5	P5CR2_HUI	9	0.793501
15min_MT.	Q8TDQ7	0.204464	0.01467	0.061528	GNPDA2	Glucosamir	GNPI2_HUI	4	1.152258
15min_MT.	Q96FJ2	-1.22996	0.014692	0.061554	DYNLL2	Dynein ligh	DYL2_HUM	2	0.42633
15min_MT.	P43358	0.093626	0.014747	0.06175	MAGEA4	Melanoma-	MAGA4_HL	11	1.067049
30min_MT.	O75083	0.16024	0.014762	0.061781	WDR1	WD repeat-	WDR1_HUI	25	1.117473
15min_MT.	P45973	-0.32389	0.01479	0.06183	CBX5	Chromobo:	CBX5_HUM	6	0.798915
15min_MT.	O00629	-0.46911	0.01488	0.062026	KPNA4	Importin su	IMA3_HUM	5	0.722412
15min_MT.	O75976	0.206392	0.014866	0.062026	CPD	Carboxypep	CBPD_HUM	28	1.153799
15min_MT.	Q01085	0.153382	0.014881	0.062026	TIAL1	Nucleolysir	TIAR_HUM/	7	1.112173
30min_MT.	P14174	0.277931	0.014951	0.062174	MIF	Macrophag	MIF_HUMA	4	1.212455
30min_MT.	Q96PU8	-0.22619	0.014944	0.062174	QKI	KH domain	QKI_HUMA	6	0.854891
30min_MT.	O00115	0.318076	0.014979	0.062256	DNASE2	Deoxyribor	DNS2A_HU	6	1.246667
15min_MT.	O95816	0.210235	0.015027	0.06242	BAG2	BAG family	BAG2_HUM	8	1.156877
15min_MT.	P11274	0.235297	0.015049	0.062446	BCR	Breakpoint	BCR_HUMA	3	1.177149
30min_MT.	P22392	0.13566	0.015057	0.062447	NME2	Nucleoside	NDKB_HUM	14	1.098595
30min_MT.	P29372	-0.3826	0.0151	0.062592	MPG	DNA-3-met	3MG_HUM	2	0.767055
15min_MT.	Q9UIJ7	0.168034	0.015143	0.062739	AK3	GTP:AMP p	KAD3_HUM	11	1.123527
30min_MT.	Q8NFH5	-0.88346	0.015228	0.063012	NUP35	Nucleopori	NUP35_HU	1	0.542065
30min_MT.	O15541	0.053057	0.015258	0.063047	RNF113A	E3 ubiquiti	R113A_HUI	5	1.037461
30min_MT.	Q9NXJ5	0.091826	0.01526	0.063047	PGPEP1	Pyroglutam	PGPI_HUM	3	1.065718
30min_MT.	O95486	-0.23293	0.015369	0.063407	SEC24A	Protein tra	SC24A_HUI	9	0.850906
30min_MT.	P29218	0.100092	0.015434	0.063542	IMPA1	Inositol mc	IMPA1_HUI	14	1.071842
30min_MT.	Q9NXR1	-0.39027	0.015435	0.063542	NDE1	Nuclear dis	NDE1_HUM	7	0.762986
30min_MT.	Q9BRT2	0.38287	0.015461	0.06359	UQCC2	Ubiquinol-(	UQCC2_HU	2	1.303933
15min_MT.	P17844	0.233918	0.015502	0.063718	DDX5	Probable A	DDX5_HUM	15	1.176025
30min_MT.	P31944	0.963456	0.015525	0.063747	CASP14	Caspase-14	CASPE_HUI	9	1.949975
30min_MT.	P15311	0.104451	0.015569	0.063893	EZR	Ezrin	EZRI_HUM/	21	1.075085
30min_MT.	Q9Y617	0.180204	0.015585	0.063926	PSAT1	Phosphosei	SERC_HUM	18	1.133044

15min_MT.	P53621	-0.0989	0.015626	0.064005	COPA	Coatomer s	COPA_HUM	54	0.933746
30min_MT.	Q9Y2U5	-0.01339	0.015637	0.064005	MAP3K2	Mitogen-ac	M3K2_HUM	1	0.990759
15min_MT.	Q8TDL5	2.087117	0.015672	0.064114	BPIFB1	BPI fold-co	BPIB1_HUM	4	4.248981
30min_MT.	Q9UHD8	-0.18089	0.015733	0.064295	SEPTIN9	Septin-9	SEPT9_HUM	23	0.882156
15min_MT.	Q9NXV6	0.123188	0.015763	0.064387	CDKN2AIP	CDKN2A-in	CARF_HUM	15	1.089139
15min_MT.	O43175	0.222418	0.015801	0.064504	PHGDH	D-3-phosph	SERA_HUM	17	1.166688
15min_MT.	O95049	-0.46344	0.015817	0.064504	TJP3	Tight juncti	ZO3_HUMA	2	0.725255
30min_MT.	Q9UJV9	-0.14082	0.015814	0.064504	DDX41	Probable A	DDX41_HU	1	0.907005
15min_MT.	P62487	-0.12951	0.015854	0.064612	POLR2G	DNA-direct	RPB7_HUM	5	0.914141
15min_MT.	Q9NUL5	0.220349	0.015868	0.064612	SHFL	Shiftless an	SHFL_HUM	6	1.165015
30min_MT.	P02814	0.618798	0.015894	0.064682	SMR3B	Submaxilla	SMR3B_HU	1	1.535595
30min_MT.	P49792	-0.14221	0.015931	0.064682	RANBP2	E3 SUMO-p	RBP2_HUM	68	0.906133
30min_MT.	Q13480	0.196321	0.015906	0.064682	GAB1	GRB2-assoc	GAB1_HUM	2	1.145773
15min_MT.	Q86WW8	-0.24696	0.015943	0.064682	COA5	Cytochrom	COA5_HUM	1	0.842668
15min_MT.	P22735	0.244728	0.015954	0.064691	TGM1	Protein-glu	TGM1_HUM	13	1.184869
15min_MT.	P48723	0.333744	0.015967	0.064713	HSPA13	Heat shock	HSP13_HU	9	1.26028
30min_MT.	Q14678	-0.0134	0.015999	0.064767	KANK1	KN motif ar	KANK1_HU	6	0.990757
15min_MT.	Q9HCE7	0.427076	0.015991	0.064767	SMURF1	E3 ubiquiti	SMUF1_HU	2	1.344506
15min_MT.	Q9UBV8	0.379728	0.016055	0.064901	PEF1	Peflin	PEF1_HUM	4	1.301096
15min_MT.	P09972	0.144772	0.016095	0.064981	ALDOC	Fructose-bi	ALDOC_HU	15	1.105556
15min_MT.	P30626	0.399478	0.016101	0.064981	SRI	Sorcin	SORCN_HU	10	1.319031
30min_MT.	P36873	0.503672	0.016098	0.064981	PPP1CC	Serine/thre	PP1G_HUM	2	1.417818
30min_MT.	P84085	-0.19277	0.016115	0.064981	ARF5	ADP-ribosy	ARF5_HUM	4	0.874927
30min_MT.	Q70IA6	-0.38917	0.01613	0.064981	MOB2	MOB kinase	MOB2_HUM	1	0.763567
30min_MT.	P49327	-0.17527	0.016176	0.065119	FASN	Fatty acid s	FAS_HUMA	73	0.885605
15min_MT.	P48643	0.09977	0.016236	0.065262	CCT5	T-complex	TCPE_HUM	31	1.071603
15min_MT.	Q9HC35	-0.10258	0.016234	0.065262	EML4	Echinoderm	EMAL4_HU	34	0.931368
15min_MT.	Q96SB4	-0.66381	0.016254	0.0653	SRPK1	SRSF protei	SRPK1_HU	3	0.63121
15min_MT.	P61604	0.321713	0.016292	0.065353	HSPE1	10 kDa hea	CH10_HUM	9	1.249813
15min_MT.	Q8WWX9	-1.36879	0.016304	0.065368	SELENOM	Selenoprot	SELM_HUM	1	0.387216
30min_MT.	Q7Z5H4	0.350681	0.016332	0.065444	VN1R5	Vomeronas	VN1R5_HU	1	1.275162
30min_MT.	Q99729	0.413811	0.016347	0.065472	HNRNPAB	Heterogene	ROAA_HUM	4	1.3322
30min_MT.	Q15020	-0.1045	0.016373	0.065508	SART3	Squamous	SART3_HUM	24	0.93013
15min_MT.	O15143	-0.15573	0.016411	0.065626	ARPC1B	Actin-relate	ARC1B_HU	19	0.897676
30min_MT.	Q07954	0.172308	0.016503	0.065862	LRP1	Prolow-der	LRP1_HUM	33	1.12686
30min_MT.	Q9GZM5	0.340254	0.016564	0.065972	YIPF3	Protein YIP	YIPF3_HUM	3	1.26598
30min_MT.	Q9NVM9	-0.21107	0.016565	0.065972	INTS13	Integrator	INT13_HUM	9	0.863895
15min_MT.	Q8N5V2	0.222537	0.016621	0.066131	NGEF	Ephexin-1	NGEF_HUM	6	1.166783
15min_MT.	Q9BVL2	-0.83966	0.016668	0.066283	NUP58	Nucleopori	NUP58_HU	3	0.558774
30min_MT.	Q9UGM6	-0.33831	0.016696	0.066361	WARS2	Tryptophar	SYWM_HU	5	0.790969
30min_MT.	P28066	0.183685	0.016782	0.066399	PSMA5	Proteasom	PSA5_HUM	12	1.135781
15min_MT.	Q8IWX8	0.255974	0.016715	0.066399	CHERP	Calcium ho	CHERP_HU	9	1.194142
30min_MT.	Q96CN7	0.326226	0.016739	0.066399	ISOC1	Isochorism	ISOC1_HUM	7	1.253729
30min_MT.	Q96DT7	-0.38192	0.016783	0.066399	ZBTB10	Zinc finger	ZBT10_HUM	2	0.767417
15min_MT.	Q96RU2	0.089092	0.016752	0.066399	USP28	Ubiquitin c	UBP28_HU	14	1.063701
15min_MT.	Q96TA1	0.049591	0.016808	0.066399	NIBAN2	Protein Nib	NIBA2_HUM	8	1.034971
30min_MT.	Q92841	-0.24968	0.016835	0.066405	DDX17	Probable A	DDX17_HU	15	0.841086
30min_MT.	Q96S59	-0.06491	0.016819	0.066405	RANBP9	Ran-bindin	RANB9_HU	10	0.956005
30min_MT.	P13647	0.885331	0.0169	0.066528	KRT5	Keratin, typ	K2C5_HUM	23	1.847188
15min_MT.	Q93009	-0.095	0.016894	0.066528	USP7	Ubiquitin c	UBP7_HUM	38	0.936274
15min_MT.	P13716	0.196559	0.016927	0.066601	ALAD	Delta-amin	HEM2_HUM	16	1.145962
15min_MT.	O75503	0.368889	0.017009	0.06682	CLN5	Bis(monoac	CLN5_HUM	4	1.291358

30min_MT.	O94829	-0.38068	0.017017	0.06682	IPO13	Importin-1	IPO13_HUI	2	0.768074
30min_MT.	Q00688	0.218267	0.017028	0.06682	FKBP3	Peptidyl-pr	FKBP3_HUI	8	1.163335
15min_MT.	Q8WV93	0.062787	0.017034	0.06682	AFG1L	AFG1-like A	AFG1L_HUI	1	1.044482
30min_MT.	O60356	-0.89897	0.017048	0.066841	NUPR1	Nuclear prc	NUPR1_HU	1	0.53627
15min_MT.	Q7Z4W1	0.20857	0.017071	0.066887	DCXR	L-xylulose r	DCXR_HUM	10	1.155542
30min_MT.	Q96RQ3	-0.33706	0.017077	0.066887	MCCC1	Methylcrot	MCCA_HUN	11	0.791653
30min_MT.	O95819	0.02739	0.017209	0.067104	MAP4K4	Mitogen-ac	M4K4_HUN	7	1.019167
30min_MT.	P42357	0.711169	0.017187	0.067104	HAL	Histidine ar	HUTH_HUN	8	1.63713
30min_MT.	Q9UI42	0.979706	0.017206	0.067104	CPA4	Carboxypep	CBPA4_HUI	3	1.972064
30min_MT.	O15212	0.371656	0.017224	0.06712	PFDN6	Prefoldin su	PFD6_HUM	7	1.293837
30min_MT.	O43314	0.316277	0.017244	0.06712	PPIP5K2	Inositol hex	VIP2_HUMA	1	1.245113
30min_MT.	P23368	0.178707	0.017259	0.06712	ME2	NAD-depen	MAOM_HU	19	1.131869
15min_MT.	P62266	-0.26502	0.017253	0.06712	RPS23	Small ribos	RS23_HUM	3	0.832186
15min_MT.	Q96I51	-0.37511	0.017252	0.06712	RCC1L	RCC1-like G	RCC1L_HUI	9	0.771049
15min_MT.	Q8NE71	-0.26006	0.0173	0.067188	ABCF1	ATP-bindin	ABCF1_HUI	15	0.835052
30min_MT.	Q8IZ69	0.074216	0.017379	0.067265	TRMT2A	tRNA (uraci	TRM2A_HU	3	1.052789
30min_MT.	Q10570	-0.02961	0.017427	0.067316	CPSF1	Cleavage ar	CPSF1_HUN	18	0.979688
30min_MT.	Q96BS2	0.296774	0.017419	0.067316	TESC	Calcineurin	CHP3_HUN	7	1.228394
15min_MT.	Q9NSI2	-0.39299	0.017451	0.06737	SLX9	Ribosome b	SLX9_HUM	6	0.76155
30min_MT.	Q16610	1.021323	0.017493	0.067471	ECM1	Extracellula	ECM1_HUN	1	2.02978
15min_MT.	P04066	0.115921	0.017526	0.067532	FUCA1	Tissue alpha	FUCO_HUN	9	1.083666
30min_MT.	P62917	0.450761	0.017522	0.067532	RPL8	Large ribos	RL8_HUMA	4	1.366761
30min_MT.	P01834	0.967788	0.017566	0.067552	IGKC	Immunoglo	IGKC_HUM	5	1.95584
30min_MT.	P45880	0.198378	0.017554	0.067552	VDAC2	Voltage-dep	VDAC2_HU	1	1.147408
15min_MT.	Q4VC31	0.209087	0.017563	0.067552	MIX23	Protein MIX	MIX23_HUI	6	1.155956
15min_MT.	Q9Y5X2	-0.07592	0.017554	0.067552	SNX8	Sorting nex	SNX8_HUM	13	0.948734
30min_MT.	P01023	0.115295	0.01763	0.067764	A2M	Alpha-2-ma	A2MG_HUN	58	1.083197
15min_MT.	P55884	-0.03138	0.017691	0.067916	EIF3B	Eukaryotic	EIF3B_HUN	30	0.978484
30min_MT.	Q13137	0.792028	0.017695	0.067916	CALCOCO2	Calcium-bi	CACO2_HU	4	1.731507
15min_MT.	O95210	-0.04589	0.017727	0.067972	STBD1	Starch-binc	STBD1_HUI	7	0.968695
15min_MT.	Q9P0P0	0.289443	0.017744	0.068004	RNF181	E3 ubiquiti	RN181_HU	3	1.222169
30min_MT.	P12110	0.169764	0.01782	0.068093	COL6A2	Collagen al	CO6A2_HU	3	1.124875
30min_MT.	P62829	-0.10467	0.017794	0.068093	RPL23	Large ribos	RL23_HUM	6	0.930019
15min_MT.	Q9UHN6	-0.08588	0.017815	0.068093	CEMIP2	Inactive cel	CEIP2_HUN	6	0.942209
15min_MT.	P53004	0.318276	0.017831	0.068102	BLVRA	Biliverdin r	BIEA_HUMA	11	1.24684
30min_MT.	P14866	-0.1501	0.017903	0.068278	HNRNPL	Heterogene	HNRPL_HU	15	0.901189
15min_MT.	P05091	0.148672	0.017919	0.068305	ALDH2	Aldehyde d	ALDH2_HU	26	1.108549
30min_MT.	O76003	0.129087	0.017976	0.06833	GLRX3	Glutaredox	GLRX3_HUI	12	1.093601
15min_MT.	P60510	0.138222	0.017971	0.06833	PPP4C	Serine/thre	PP4C_HUM	10	1.100548
30min_MT.	Q04118	0.828622	0.017958	0.06833	PRB3	Basic saliva	PRB3_HUN	3	1.775989
30min_MT.	Q05519	-0.41468	0.017978	0.06833	SRSF11	Serine/argi	SRS11_HUN	10	0.750185
15min_MT.	Q14683	-0.06436	0.017971	0.06833	SMC1A	Structural r	SMC1A_HU	46	0.956368
30min_MT.	Q92598	0.150947	0.018019	0.068353	HSPH1	Heat shock	HS105_HUI	33	1.110298
15min_MT.	Q15404	0.292385	0.018068	0.068474	RSU1	Ras suppres	RSU1_HUM	9	1.224663
15min_MT.	P55072	-0.04865	0.018099	0.068557	VCP	Transitiona	TERA_HUM	60	0.966843
30min_MT.	P02534	6.672205	0.018118	0.068596	NaN	Keratin, typ	K1M1_SHEI	10	101.9844
30min_MT.	Q08554	1.029282	0.018169	0.068756	DSC1	Desmocolli	DSC1_HUM	9	2.041008
30min_MT.	P61916	0.262758	0.018245	0.068875	NPC2	NPC intrace	NPC2_HUM	6	1.19977
30min_MT.	Q9P2X3	0.219082	0.018233	0.068875	IMPACT	Protein IMI	IMPCT_HUI	9	1.163993
30min_MT.	B1AK53	0.487272	0.01831	0.069054	ESPN	Espin	ESPN_HUM	3	1.401792
15min_MT.	Q9Y6G5	0.280461	0.018305	0.069054	COMMD10	COMM don	COMDA_HU	5	1.214583
30min_MT.	Q9BQ69	-0.32412	0.018333	0.06911	MACROD1	ADP-ribose	MACD1_HU	2	0.798785

15min_MT.	Q9P021	0.223691	0.018373	0.069225	CRIPT	Cysteine-ric	CRIPT_HUM	1	1.167717
15min_MT.	Q9GZV4	-0.51318	0.018391	0.069261	EIF5A2	Eukaryotic	IF5A2_HUM	3	0.700676
15min_MT.	O00221	0.639598	0.01842	0.069272	NFKBIE	NF-kappa-B	IKBE_HUMA	1	1.557895
30min_MT.	Q9NX58	-0.85043	0.018416	0.069272	LYAR	Cell growth	LYAR_HUM	2	0.554619
15min_MT.	P02652	0.540121	0.01846	0.069322	APOA2	Apolipopro	APOA2_HU	1	1.454094
30min_MT.	P83876	-0.24276	0.018481	0.06936	TXNL4A	Thioredoxi	TXN4A_HU	5	0.845126
30min_MT.	Q9Y259	0.002094	0.018488	0.06936	CHKB	Choline/eth	CHKB_HUM	8	1.001453
15min_MT.	Q9BZZ5	0.146556	0.018551	0.06943	API5	Apoptosis i	API5_HUMA	20	1.106924
30min_MT.	Q9Y5Z4	0.271443	0.018551	0.06943	HEBP2	Heme-bind	HEBP2_HU	8	1.207015
15min_MT.	Q8WVM7	-0.3578	0.018565	0.069448	STAG1	Cohesin sul	STAG1_HU	6	0.780354
15min_MT.	P10155	-0.12516	0.018619	0.069585	RO60	RNA-bindir	RO60_HUM	21	0.916903
15min_MT.	P01023	-0.10804	0.018658	0.069653	A2M	Alpha-2-ma	A2MG_HUM	58	0.927848
15min_MT.	P36873	0.454442	0.018697	0.069707	PPP1CC	Serine/thre	PP1G_HUM	2	1.370253
30min_MT.	Q9NPH2	0.149768	0.018692	0.069707	ISYNA1	Inositol-3-p	INO1_HUM	15	1.109391
30min_MT.	Q96AB3	0.41895	0.018708	0.069715	ISOC2	Isochorism	ISOC2_HUM	6	1.336954
30min_MT.	P07954	-0.11072	0.018745	0.069799	FH	Fumarate h	FUMH_HUM	13	0.926123
15min_MT.	Q7Z401	-0.37211	0.018748	0.069799	DENND4A	C-myc pron	MYCPP_HU	2	0.772652
30min_MT.	Q9NR28	0.160573	0.018793	0.069934	DIABLO	Diablo IAP-	DBLOH_HU	7	1.117731
15min_MT.	P38570	0.305927	0.018864	0.070063	ITGAE	Integrin alp	ITAE_HUMA	2	1.236212
30min_MT.	Q6P1R3	-0.16484	0.01889	0.070105	MSANTD2	Myb/SANT-	MSD2_HUM	4	0.892028
30min_MT.	P10301	0.329403	0.018958	0.070312	RRAS	Ras-related	RRAS_HUM	9	1.256493
15min_MT.	Q6P2P2	0.480583	0.019044	0.07055	PRMT9	Protein arg	ANM9_HUM	6	1.395308
15min_MT.	Q9Y5X1	0.085831	0.019039	0.07055	SNX9	Sorting nex	SNX9_HUM	10	1.061299
15min_MT.	Q5H9R7	-0.10691	0.01917	0.070797	PPP6R3	Serine/thre	PP6R3_HU	30	0.928575
15min_MT.	Q5JS54	-0.28912	0.019166	0.070797	PSMG4	Proteasom	PSMG4_HU	5	0.818401
15min_MT.	Q9Y265	0.103923	0.019154	0.070797	RUVBL1	RuvB-like 1	RUVB1_HU	19	1.074692
15min_MT.	Q6FI81	0.16983	0.019185	0.07082	CIAPIN1	Anamorsin	CPIN1_HUM	18	1.124926
15min_MT.	P01137	-0.44935	0.019343	0.07084	TGFB1	Transformi	TGFB1_HU	3	0.732371
15min_MT.	P02538	0.662709	0.019292	0.07084	KRT6A	Keratin, typ	K2C6A_HU	37	1.583052
30min_MT.	P02545	0.15244	0.019325	0.07084	LMNA	Prelamin-A	LMNA_HUM	36	1.111447
15min_MT.	P04439	0.345775	0.019301	0.07084	HLA-A	HLA class I	HLAA_HUM	3	1.270833
30min_MT.	P30153	0.122255	0.019345	0.07084	PPP2R1A	Serine/thre	2AAA_HUM	24	1.088435
30min_MT.	Q08188	0.924381	0.01928	0.07084	TGM3	Protein-glu	TGM3_HUM	16	1.89787
30min_MT.	Q13547	-0.56918	0.019224	0.07084	HDAC1	Histone dea	HDAC1_HU	2	0.674
15min_MT.	Q8N684	0.18086	0.019218	0.07084	CPSF7	Cleavage ar	CPSF7_HUM	13	1.13356
15min_MT.	Q99627	0.643352	0.019244	0.07084	COPS8	COP9 signa	CSN8_HUM	4	1.561954
30min_MT.	Q9H3P2	-0.23159	0.019267	0.07084	NELFA	Negative el	NELFA_HUM	4	0.851697
30min_MT.	Q9NQH7	0.292287	0.019337	0.07084	XPNPEP3	Xaa-Pro am	XPP3_HUM	6	1.22458
15min_MT.	Q9NWZ3	0.510352	0.019321	0.07084	IRAK4	Interleukin	IRAK4_HUM	6	1.424397
15min_MT.	Q969Q0	-0.4171	0.019355	0.070845	RPL36AL	Ribosomal	RL36L_HUM	1	0.748926
15min_MT.	P07307	-1.20174	0.019367	0.070857	ASGR2	Asialoglyco	ASGR2_HU	2	0.434751
30min_MT.	Q7Z794	0.838984	0.019387	0.070894	KRT77	Keratin, typ	K2C1B_HU	18	1.78879
15min_MT.	P60484	0.136035	0.019437	0.070951	PTEN	Phosphatid	PTEN_HUM	2	1.098881
30min_MT.	Q02252	-0.20114	0.019466	0.070951	ALDH6A1	Methylmal	MMSA_HU	15	0.869863
15min_MT.	Q08554	-0.28265	0.019436	0.070951	DSC1	Desmocolli	DSC1_HUM	9	0.822082
15min_MT.	Q8NEW0	-0.70611	0.019435	0.070951	SLC30A7	Zinc transp	ZNT7_HUM	2	0.61297
15min_MT.	Q8NHV4	-0.10773	0.019447	0.070951	NEDD1	Protein NEI	NEDD1_HU	9	0.928049
30min_MT.	Q96QR8	0.095744	0.019475	0.070951	PURB	Transcripti	PURB_HUM	8	1.068617
15min_MT.	Q9UHR5	-0.20767	0.019457	0.070951	SAP30BP	SAP30-binc	S30BP_HU	6	0.865936
15min_MT.	Q9UHR4	0.22765	0.019534	0.071102	BAIAP2L1	BAR/IMD d	BI2L1_HUM	13	1.170926
30min_MT.	P20700	0.190689	0.019577	0.071186	LMNB1	Lamin-B1	LMNB1_HU	10	1.141308
30min_MT.	Q00653	-0.12376	0.019601	0.071186	NFKB2	Nuclear fac	NFKB2_HU	14	0.91779

30min_MT.	Q99956	-0.28339	0.019588	0.071186	DUSP9	Dual specifi	DUS9_HUM	4	0.82166
15min_MT.	Q9P016	-1.82544	0.019603	0.071186	THYN1	Thymocyte	THYN1_HUI	1	0.282155
30min_MT.	Q13263	-0.07625	0.019614	0.071194	TRIM28	Transcripti	TIF1B_HUM	27	0.948518
30min_MT.	P07476	0.576838	0.01964	0.071222	IVL	Involucrin	INVO_HUM	5	1.491577
30min_MT.	Q68CZ2	-0.23759	0.019656	0.071225	TNS3	Tensin-3	TENS3_HUI	11	0.848163
15min_MT.	Q9UL33	0.061338	0.019659	0.071225	TRAPPC2L	Trafficking	TPC2L_HUI	5	1.043433
30min_MT.	P52292	-0.20494	0.019672	0.071237	KPNA2	Importin su	IMA1_HUM	6	0.867572
15min_MT.	Q01459	0.123797	0.019691	0.071272	CTBS	Di-N-acetyl	DIAC_HUM	10	1.089599
30min_MT.	Q09013	0.994065	0.019705	0.071284	DMPK	Myotonin-p	DMPK_HUI	1	1.991789
15min_MT.	Q9BTX7	0.849346	0.019726	0.0713	TTPAL	Alpha-toco	TTPAL_HUI	1	1.801684
15min_MT.	Q8NBL1	0.155087	0.019747	0.071305	POGLUT1	Protein O-g	PGLT1_HUI	7	1.113489
15min_MT.	Q9Y6I3	0.321918	0.019759	0.071305	EPN1	Epsin-1	EPN1_HUM	9	1.249991
30min_MT.	Q96E11	0.079992	0.019797	0.071333	MRRF	Ribosome-r	RRFM_HUM	7	1.057012
15min_MT.	Q99519	0.172094	0.019798	0.071333	NEU1	Sialidase-1	NEUR1_HU	11	1.126693
30min_MT.	Q9NQX3	-0.12112	0.019808	0.071333	GPHN	Gephyrin	GEPH_HUM	23	0.919476
15min_MT.	P19388	0.193696	0.019829	0.071344	POLR2E	DNA-direct	RPAB1_HUI	5	1.14369
15min_MT.	Q9NWS0	-0.5961	0.01982	0.071344	PIH1D1	PIH1 doma	PIHD1_HUI	1	0.661539
15min_MT.	Q8WXA3	-0.06426	0.019842	0.071356	RUFY2	RUN and FY	RUFY2_HUI	5	0.956433
30min_MT.	Q9NPE3	0.559343	0.01988	0.071461	NOP10	H/ACA ribo	NOP10_HU	1	1.473598
15min_MT.	Q8N0X4	0.16085	0.019911	0.071508	CLYBL	Citramalyl-	CLYBL_HUM	14	1.117945
30min_MT.	P61158	0.096015	0.019922	0.071514	ACTR3	Actin-relate	ARP3_HUM	15	1.068817
15min_MT.	Q13232	0.133974	0.019944	0.071559	NME3	Nucleoside	NDK3_HUM	8	1.097312
15min_MT.	O95551	-0.22755	0.019991	0.071632	TDP2	Tyrosyl-DN	TYDP2_HUI	7	0.854083
15min_MT.	P61088	0.216858	0.01998	0.071632	UBE2N	Ubiquitin-c	UBE2N_HU	9	1.1622
30min_MT.	Q5T1M5	-0.14197	0.019985	0.071632	FKBP15	FK506-binc	FKB15_HUI	22	0.906279
30min_MT.	P0DMV8;P(	0.182374	0.020033	0.07175	HSPA1A;HS	Heat shock	HS71A_HUI	28	1.134749
15min_MT.	P29590	0.235695	0.020084	0.071833	PML	Protein PM	PML_HUM/	14	1.177474
30min_MT.	Q7Z4H3	0.184209	0.020074	0.071833	HDDC2	5'-deoxynu	HDDC2_HU	5	1.136194
15min_MT.	P42025	0.288972	0.020099	0.071853	ACTR1B	Beta-centra	ACTY_HUM	6	1.221769
30min_MT.	P19338	0.345916	0.02014	0.071938	NCL	Nucleolin	NUCL_HUM	30	1.270958
15min_MT.	P20711	-0.08119	0.020142	0.071938	DDC	Aromatic-L	DDC_HUM/	14	0.945277
15min_MT.	Q96J01	-0.15017	0.02015	0.071938	THOC3	THO compl	THOC3_HU	7	0.901141
15min_MT.	Q96KR1	0.148956	0.020166	0.071962	ZFR	Zinc finger	ZFR_HUMA	22	1.108767
30min_MT.	Q8WVY7	-0.24293	0.020191	0.071986	UBLCP1	Ubiquitin-l	UBCP1_HU	8	0.845027
15min_MT.	O75832	-0.38313	0.020236	0.072113	PSMD10	26S protea	PSD10_HUI	6	0.766772
15min_MT.	Q96MX6	-0.13557	0.020297	0.072263	DNAAF10	Dynein axo	DAA10_HU	8	0.910309
30min_MT.	Q06323	0.259284	0.020367	0.072474	PSME1	Proteasom	PSME1_HU	19	1.196884
30min_MT.	Q86V35	0.196444	0.020375	0.072474	CABP7	Calcium-bi	CABP7_HUI	1	1.145871
15min_MT.	Q9ULC3	0.016026	0.020392	0.072502	RAB23	Ras-related	RAB23_HUI	8	1.011171
15min_MT.	O75608	0.138255	0.020418	0.072529	LYPLA1	Acyl-protei	LYPA1_HUI	5	1.100573
30min_MT.	Q5TFE4	0.062456	0.020416	0.072529	NT5DC1	5'-nucleoti	NT5D1_HU	12	1.044242
15min_MT.	P04908;P0	0.346328	0.020471	0.072633	H2AC4;H2/	Histone H2	H2A1B_HU	4	1.271321
30min_MT.	Q14957	-0.99115	0.020461	0.072633	GRIN2C	Glutamate	NMDE3_HU	1	0.503077
30min_MT.	P14859	-0.11678	0.020533	0.072774	POU2F1	POU domai	PO2F1_HUI	4	0.922244
15min_MT.	Q9NSY0	-0.16737	0.020558	0.072796	NRBP2	Nuclear rec	NRBP2_HU	1	0.890465
30min_MT.	Q9BV44	0.042122	0.020604	0.072821	THUMPD3	tRNA (guan	THUM3_HU	13	1.029627
30min_MT.	Q9H2D6	-0.0824	0.020586	0.072821	TRIOBP	TRIO and F-	TARA_HUM	12	0.944482
15min_MT.	Q04446	-0.21697	0.020649	0.072826	GBE1	1,4-alpha-g	GLGB_HUM	18	0.860369
30min_MT.	Q9BRQ0	0.15524	0.020635	0.072826	PYGO2	Pygopus hc	PYGO2_HU	3	1.113607
15min_MT.	Q9NQH7	0.317091	0.02066	0.072826	XPNPEP3	Xaa-Pro am	XPP3_HUM	6	1.245816
15min_MT.	Q9Y5B8	0.22378	0.02065	0.072826	NME7	Nucleoside	NDK7_HUM	3	1.167789
30min_MT.	P02812	-0.41314	0.020686	0.072844	PRB2	Basic saliva	PRB2_HUM	1	0.750988

15min_MT.	Q16595	0.150287	0.020682	0.072844	FXN	Frataxin, m	FRDA_HUM	4	1.109791
15min_MT.	Q5QJE6	-0.09209	0.020693	0.072844	DNTTIP2	Deoxynucle	TDIF2_HUM	3	0.938161
15min_MT.	P00505	0.077018	0.020739	0.072909	GOT2	Aspartate a	AATM_HUM	30	1.054836
15min_MT.	Q8IYL3	0.245606	0.020739	0.072909	C1orf174	UPF0688 p	CA174_HU	3	1.18559
15min_MT.	P12814	-0.08294	0.020754	0.072918	ACTN1	Alpha-actin	ACTN1_HU	57	0.944132
30min_MT.	Q8IUZ5	0.081068	0.020761	0.072918	PHYKPL	5-phosphol	AT2L2_HUM	12	1.057801
15min_MT.	Q13523	-0.19414	0.020798	0.073016	PRP4K	Serine/thre	PRP4K_HU	7	0.874094
15min_MT.	P29692	0.315991	0.020835	0.073094	EEF1D	Elongation	EF1D_HUM	11	1.244866
30min_MT.	Q9NZ71	-0.31986	0.020838	0.073094	RTEL1	Regulator c	RTEL1_HUM	1	0.801147
15min_MT.	Q96FN4	-0.22229	0.020859	0.073127	CPNE2	Copine-2	CPNE2_HU	4	0.857205
30min_MT.	Q96H79	-0.15952	0.020867	0.073127	ZC3HAV1L	Zinc finger	ZCCHL_HU	4	0.895323
15min_MT.	Q9UQ13	0.807852	0.020888	0.073168	SHOC2	Leucine-ric	SHOC2_HU	2	1.750603
15min_MT.	O60832	-0.06786	0.020922	0.073186	DKC1	H/ACA ribo	DKC1_HUM	17	0.954052
30min_MT.	P63272	0.218462	0.020938	0.073186	SUPT4H1	Transcripti	SPT4H_HU	5	1.163492
30min_MT.	Q8TAE8	0.478633	0.02093	0.073186	GADD45GI	Large ribos	G45IP_HUM	2	1.393423
30min_MT.	Q92878	-0.18272	0.02094	0.073186	RAD50	DNA repair	RAD50_HU	39	0.881042
15min_MT.	O75940	0.30946	0.020969	0.073256	SMNDC1	Survival of	SPF30_HUM	7	1.239243
15min_MT.	Q15021	-0.23844	0.021016	0.073386	NCAPD2	Condensin	CND1_HUM	10	0.847662
15min_MT.	P08865	0.240451	0.021078	0.073486	RPSA	Small ribos	RSSA_HUM	11	1.181362
30min_MT.	Q9NY33	0.112774	0.021065	0.073486	DPP3	Dipeptidyl	DPP3_HUM	25	1.081305
30min_MT.	Q15436	-0.20797	0.021092	0.07349	SEC23A	Protein tra	SC23A_HU	21	0.865754
15min_MT.	P08708	-0.16607	0.021135	0.073605	RPS17	Small ribos	RS17_HUM	5	0.89127
30min_MT.	Q32M88	0.256422	0.021153	0.073636	PGGHG	Protein-glu	PGGHG_HU	10	1.194512
15min_MT.	O60931	1.310755	0.021174	0.073675	CTNS	Cystinosin	CTNS_HUM	1	2.480713
30min_MT.	Q53FT3	0.29033	0.021223	0.07378	HIKESHI	Protein Hik	HIKES_HUM	5	1.22292
15min_MT.	Q9NXA8	-0.21074	0.021228	0.07378	SIRT5	NAD-depen	SIR5_HUM	5	0.864093
15min_MT.	Q14978	-0.18929	0.021302	0.073956	NOLC1	Nucleolar a	NOLC1_HU	18	0.877036
15min_MT.	Q14657	-0.54546	0.021319	0.073957	LAGE3	EKC/KEOPS	LAGE3_HU	2	0.685171
15min_MT.	P02769	-1.55162	0.021462	0.074181	ALB	Serum albu	ALBU_BOVI	39	0.341126
15min_MT.	P05198	0.051884	0.021575	0.07448	EIF2S1	Eukaryotic	IF2A_HUM	22	1.036618
30min_MT.	Q9Y600	0.40503	0.021665	0.074719	CSAD	Cysteine su	CSAD_HUM	1	1.324116
15min_MT.	Q9BTE3	-0.08766	0.021685	0.074756	MCMBP	Mini-chron	MCMBP_HU	5	0.94105
15min_MT.	O75083	-0.13163	0.021712	0.074784	WDR1	WD repeat-	WDR1_HU	25	0.912803
15min_MT.	P01012	2.277868	0.021864	0.074935	SERPINB14	Ovalbumin	OVAL_CHIC	1	4.849609
30min_MT.	P05362	0.12637	0.021846	0.074935	ICAM1	Intercellula	ICAM1_HU	10	1.091544
15min_MT.	P07737	-0.24529	0.021827	0.074935	PFN1	Profilin-1	PROF1_HU	10	0.843648
30min_MT.	P08236	0.180567	0.021863	0.074935	GUSB	Beta-glucur	BGLR_HUM	13	1.13333
15min_MT.	P20674	0.341083	0.021871	0.074935	COX5A	Cytochrom	COX5A_HU	3	1.266707
15min_MT.	Q96HQ2	-0.51436	0.021853	0.074935	CDKN2AIPN	CDKN2AIP	C2AIL_HUM	2	0.700101
15min_MT.	O15460	-0.10517	0.02199	0.075309	P4HA2	Prolyl 4-hy	P4HA2_HU	9	0.929697
15min_MT.	P60981	-0.24523	0.022009	0.075335	DSTN	Destrin	DEST_HUM	11	0.843681
30min_MT.	P30793	0.057189	0.022076	0.075437	GCH1	GTP cycloh	GCH1_HUM	8	1.040436
15min_MT.	P07738	0.312717	0.02214	0.075524	BPGM	Bisphosphc	PMGE_HUM	6	1.242045
15min_MT.	P36507	0.258196	0.02213	0.075524	MAP2K2	Dual specifi	MP2K2_HU	17	1.195982
15min_MT.	Q8WU90	-0.24354	0.022125	0.075524	ZC3H15	Zinc finger	ZC3HF_HU	13	0.844671
15min_MT.	Q9BYG5	0.36488	0.022119	0.075524	PARD6B	Partitionin	PAR6B_HU	4	1.287775
30min_MT.	P02788	0.812014	0.022186	0.075582	LTF	Lactotransf	TRFL_HUM	7	1.755661
15min_MT.	O15212	0.485874	0.022236	0.075654	PFDN6	Prefoldin su	PFD6_HUM	7	1.400434
15min_MT.	O75844	-0.23149	0.022223	0.075654	ZMPSTE24	CAAX preny	FACE1_HUM	4	0.851753
15min_MT.	Q01415	0.123912	0.02226	0.075704	GALK2	N-acetylgal	GALK2_HU	15	1.089686
15min_MT.	O75600	-0.5761	0.022296	0.075727	GCAT	2-amino-3-	KBL_HUMA	1	0.670775
15min_MT.	Q8N6H7	0.14437	0.022294	0.075727	ARFGAP2	ADP-ribosy	ARFG2_HU	13	1.105248

15min_MT.	P22059	-0.08218	0.022332	0.075729	OSBP	Oxysterol-b	OSBP1_HUI		28	0.944632
30min_MT.	P23284	0.180872	0.022332	0.075729	PPIB	Peptidyl-pr	PPIB_HUM.		12	1.133569
15min_MT.	P57076	0.237246	0.022335	0.075729	CFAP298	Cilia- and fl	CF298_HUI		1	1.178741
15min_MT.	P14923	0.238056	0.022377	0.075801	JUP	Junction pl	PLAK_HUM		27	1.179402
30min_MT.	Q14103	0.162113	0.022386	0.075801	HNRNPD	Heterogene	HNRPD_HU		14	1.118925
15min_MT.	P01009	0.183868	0.022411	0.075829	SERPINA1	Alpha-1-ant	A1AT_HUM		25	1.135926
15min_MT.	Q6NXT1	-0.97933	0.022413	0.075829	ANKRD54	Ankyrin rep	ANR54_HU		1	0.507215
30min_MT.	Q9UHV9	0.268541	0.022428	0.075846	PFDN2	Prefoldin sı	PFD2_HUM		6	1.204589
30min_MT.	Q92575	-0.30184	0.022465	0.075938	UBXN4	UBX domai	UBXN4_HU		4	0.811215
30min_MT.	O00203	-0.14005	0.022484	0.075958	AP3B1	AP-3 compl	AP3B1_HUI		19	0.907489
30min_MT.	P20933	-0.25539	0.02249	0.075958	AGA	N(4)-(beta-I	ASPG_HUM		7	0.837762
15min_MT.	Q0ZGT2	0.150664	0.022538	0.076019	NEXN	Nexilin	NEXN_HUM		9	1.11008
30min_MT.	P54727	0.169011	0.022583	0.076141	RAD23B	UV excision	RD23B_HU		15	1.124288
15min_MT.	P19387	0.359011	0.022607	0.076185	POLR2C	DNA-direct	RPB3_HUM		3	1.282546
15min_MT.	P50613	0.23002	0.022635	0.076185	CDK7	Cyclin-depe	CDK7_HUM		10	1.172851
30min_MT.	Q14978	0.143381	0.022618	0.076185	NOLC1	Nucleolar a	NOLC1_HU		18	1.104491
30min_MT.	Q9Y2T3	0.123099	0.022632	0.076185	GDA	Guanine de	GUAD_HUM		16	1.089072
30min_MT.	P78385	6.035982	0.022652	0.076207	KRT83	Keratin, typ	KRT83_HUI		17	65.61627
30min_MT.	Q9NWB6	-0.16621	0.022682	0.076242	ARGLU1	Arginine an	ARGL1_HUI		6	0.891178
15min_MT.	Q9UH77	-0.61217	0.022723	0.076316	KLHL3	Kelch-like p	KLHL3_HUI		1	0.654211
30min_MT.	Q8TB72	-0.43045	0.022785	0.076458	PUM2	Pumilio ho	PUM2_HUM		3	0.742031
30min_MT.	Q16831	0.099836	0.022822	0.076533	UPP1	Uridine pho	UPP1_HUM		13	1.071652
30min_MT.	Q9Y697	-0.19687	0.022827	0.076533	NFS1	Cysteine de	NFS1_HUM		6	0.872443
15min_MT.	Q4G0F5	-0.04214	0.022884	0.076657	VPS26B	Vacuolar pr	VP26B_HUI		10	0.971211
15min_MT.	Q9H845	-0.1515	0.02288	0.076657	ACAD9	Complex I a	ACAD9_HU		7	0.900313
15min_MT.	P51648	0.068789	0.022961	0.07685	ALDH3A2	Aldehyde d	AL3A2_HUI		5	1.048836
30min_MT.	Q96CT7	0.284572	0.023018	0.076977	CCDC124	Coiled-coil	CC124_HUI		9	1.218049
30min_MT.	Q96S82	0.136725	0.023042	0.077023	UBL7	Ubiquitin-l	UBL7_HUM		6	1.099407
15min_MT.	Q12996	-0.23334	0.023054	0.077029	CSTF3	Cleavage sti	CSTF3_HUM		16	0.850664
15min_MT.	Q86YM7	0.486098	0.023073	0.077029	HOMER1	Homer prot	HOME1_HU		6	1.400652
30min_MT.	Q8WWY3	-0.17632	0.023072	0.077029	PRPF31	U4/U6 sma	PRP31_HUI		6	0.884959
15min_MT.	P20020	-0.40088	0.023112	0.077124	ATP2B1	Plasma mer	AT2B1_HUI		9	0.757397
30min_MT.	Q9UNF1	-0.1778	0.023125	0.077136	MAGED2	Melanoma-	MAGD2_HU		15	0.884051
30min_MT.	O75506	0.513277	0.023227	0.077167	HSBP1	Heat shock	HSBP1_HUI		2	1.427288
30min_MT.	O76009	5.621926	0.02322	0.077167	KRT33A	Keratin, typ	KT33A_HUI		14	49.24571
15min_MT.	O95302	0.151286	0.02316	0.077167	FKBP9	Peptidyl-pr	FKBP9_HUI		14	1.110559
15min_MT.	P06454	-0.69877	0.023171	0.077167	PTMA	Prothymos	PTMA_HUM		8	0.616096
30min_MT.	P29350	-1.03445	0.023148	0.077167	PTPN6	Tyrosine-pr	PTN6_HUM		2	0.488202
30min_MT.	P51884	-1.54859	0.023215	0.077167	LUM	Lumican	LUM_HUM.		1	0.341844
30min_MT.	Q5XKE5	1.619249	0.023233	0.077167	KRT79	Keratin, typ	K2C79_HUI		3	3.07215
30min_MT.	Q9H9Q2	0.136142	0.023217	0.077167	COPS7B	COP9 signa	CSN7B_HUI		5	1.098963
15min_MT.	P48147	-0.06942	0.023247	0.07718	PREP	Prolyl endc	PPCE_HUM		28	0.953024
15min_MT.	Q9NUM4	0.110279	0.023322	0.077395	TMEM106E	Transmemb	T106B_HUI		3	1.079437
30min_MT.	Q96G74	-0.75162	0.023396	0.077577	OTUD5	OTU domai	OTUD5_HU		1	0.593937
15min_MT.	P19525	0.032002	0.023407	0.07758	EIF2AK2	Interferon-i	E2AK2_HUI		3	1.02243
30min_MT.	O43660	-0.19154	0.023466	0.077661	PLRG1	Pleiotropic	PLRG1_HUI		19	0.875671
30min_MT.	Q4LE39	0.224978	0.023483	0.077661	ARID4B	AT-rich inte	ARI4B_HUM		6	1.168759
30min_MT.	Q5D862	0.869489	0.023491	0.077661	FLG2	Filaggrin-2	FILA2_HUM		16	1.827015
15min_MT.	Q15020	-0.12052	0.023592	0.077896	SART3	Squamous	SART3_HUM		24	0.919859
30min_MT.	Q9Y6R4	-0.58304	0.02363	0.077989	MAP3K4	Mitogen-ac	M3K4_HUM		1	0.667557
15min_MT.	Q9Y547	-0.54562	0.023666	0.078074	IFT25	Intraflagell	IFT25_HUM		2	0.685096
15min_MT.	Q99829	0.154446	0.023692	0.078126	CPNE1	Copine-1	CPNE1_HUI		12	1.112994

15min_MT.	Q9UKL0	0.596172	0.023701	0.078126	RCOR1	REST corep	RCOR1_HU	4	1.5117
30min_MT.	Q9H6Z4	0.0849	0.023713	0.078131	RANBP3	Ran-bindin	RANB3_HU	22	1.060614
15min_MT.	P60709	-0.18986	0.023736	0.07817	ACTB	Actin, cyto	ACTB_HUM	23	0.876692
30min_MT.	Q16186	-0.11428	0.023755	0.07817	ADRM1	Proteasom	ADRM1_HU	5	0.923841
30min_MT.	P02675	-0.1701	0.023794	0.078256	FGB	Fibrinogen	FIBB_HUM	13	0.888783
15min_MT.	Q8N2M8	-0.47635	0.023801	0.078256	CLASRP	CLK4-assoc	CLASR_HUI	3	0.718794
15min_MT.	Q14678	0.071991	0.02382	0.078285	KANK1	KN motif ar	KANK1_HU	6	1.051167
15min_MT.	Q15811	-0.1257	0.023948	0.078555	ITSN1	Intersectin	ITSN1_HUN	14	0.91656
30min_MT.	Q7Z7E8	-0.1637	0.023925	0.078555	UBE2Q1	Ubiquitin-c	UB2Q1_HU	9	0.892732
15min_MT.	Q53H96	0.299481	0.023993	0.078624	PYCR3	Pyrroline-5	P5CR3_HUI	3	1.230702
15min_MT.	Q9NR28	0.198136	0.024167	0.079028	DIABLO	Diablo IAP-	DBLOH_HU	7	1.147215
15min_MT.	Q9H7D7	0.117554	0.024182	0.079029	WDR26	WD repeat-	WDR26_HU	9	1.084894
15min_MT.	Q16864	-0.27981	0.024204	0.079034	ATP6V1F	V-type prot	VATF_HUM	4	0.823698
15min_MT.	Q49B96	0.209123	0.024296	0.07915	COX19	Cytochrom	COX19_HU	2	1.155985
30min_MT.	Q6NVY1	0.184108	0.024291	0.07915	HIBCH	3-hydroxyi	HIBCH_HUI	15	1.136114
30min_MT.	Q14839	-0.13799	0.024316	0.079154	CHD4	Chromodo	CHD4_HUN	24	0.908784
30min_MT.	P50452	0.172615	0.024415	0.07944	SERPINB8	Serpin B8	SPB8_HUM	13	1.127099
15min_MT.	P15104	0.20922	0.024457	0.07951	GLUL	Glutamine	GLNA_HUM	8	1.156063
30min_MT.	Q9UK55	-1.00643	0.024497	0.079575	SERPINA10	Protein Z-d	ZPI_HUMAI	2	0.497775
30min_MT.	Q9UL33	0.187731	0.024641	0.079876	TRAPPC2L	Trafficking	TPC2L_HUN	5	1.138971
30min_MT.	Q2TAY7	-0.15019	0.02467	0.079918	SMU1	WD40 repe	SMU1_HUN	14	0.901135
15min_MT.	P24941	0.312693	0.024745	0.079986	CDK2	Cyclin-depe	CDK2_HUN	8	1.242024
15min_MT.	Q9UBF6	-0.03297	0.024785	0.079986	RNF7	RING-box p	RBX2_HUM	3	0.977404
15min_MT.	Q9H2C0	-0.16024	0.024813	0.080035	GAN	Gigaxonin	GAN_HUMA	15	0.894874
30min_MT.	P30530	-0.41478	0.024899	0.080209	AXL	Tyrosine-pr	UFO_HUMA	1	0.750134
15min_MT.	P41223	-0.54325	0.024908	0.080209	BUD31	Protein BU	BUD31_HU	5	0.686226
15min_MT.	Q9NNW7	-0.08495	0.024924	0.080228	TXNRD2	Thioredoxi	TRXR2_HUI	15	0.942816
30min_MT.	P02533	0.865935	0.024976	0.080331	KRT14	Keratin, typ	K1C14_HUI	33	1.82252
15min_MT.	Q5JS37	0.209524	0.025087	0.080588	NHLRC3	NHL repeat	NHLC3_HU	5	1.156306
15min_MT.	Q9NPR2	-0.03019	0.025111	0.080598	SEMA4B	Semaphori	SEM4B_HU	4	0.979288
15min_MT.	Q14103	0.24123	0.025158	0.080686	HNRNPD	Heterogene	HNRPD_HU	14	1.182
30min_MT.	Q9NU23	0.193126	0.025207	0.080774	LYRM2	LYR motif-c	LYRM2_HU	2	1.143238
15min_MT.	Q12981	-0.59811	0.025219	0.080778	BNIP1	Vesicle trar	SEC20_HUI	1	0.66062
30min_MT.	Q13576	-0.15032	0.02523	0.080782	IQGAP2	Ras GTPase	IQGA2_HUI	48	0.90105
30min_MT.	P41223	-0.28887	0.025256	0.080798	BUD31	Protein BU	BUD31_HU	5	0.818544
15min_MT.	P49407	-0.14889	0.025255	0.080798	ARRB1	Beta-arresti	ARRB1_HUI	13	0.901946
15min_MT.	Q8N8R5	-0.07262	0.025326	0.080957	C2orf69	Mitochond	CB069_HU	8	0.95091
30min_MT.	P47929	0.936498	0.025373	0.081073	LGALS7	Galectin-7	LEG7_HUM	7	1.913877
30min_MT.	O75449	-0.6964	0.025401	0.081096	KATNA1	Katanin p6	KTNA1_HUI	2	0.617109
30min_MT.	P11908	0.298804	0.025492	0.081155	PRPS2	Ribose-pho	PRPS2_HUI	4	1.230124
15min_MT.	P35250	0.273917	0.025459	0.081155	RFC2	Replicatior	RFC2_HUM	7	1.209086
15min_MT.	P50993	-0.32787	0.025481	0.081155	ATP1A2	Sodium/po	AT1A2_HUI	1	0.79671
30min_MT.	P51452	0.05105	0.025461	0.081155	DUSP3	Dual specifi	DUS3_HUN	7	1.036018
30min_MT.	Q06547	0.035453	0.025472	0.081155	GABPB1	GA-binding	GABP1_HU	1	1.024879
30min_MT.	Q9H173	0.183277	0.025433	0.081155	SIL1	Nucleotide	SIL1_HUMA	11	1.13546
15min_MT.	Q9Y2W1	-0.18407	0.025487	0.081155	THRAP3	Thyroid hor	TR150_HUI	23	0.880217
30min_MT.	Q9HC38	0.203736	0.025515	0.081195	GLOD4	Glyoxalase	GLOD4_HU	12	1.151677
15min_MT.	P23610	-1.05644	0.025553	0.08125	F8A1	40-kDa hur	HAP40_HU	1	0.480818
15min_MT.	Q7Z4H3	0.259863	0.025549	0.08125	HDDC2	5'-deoxynu	HDDC2_HU	5	1.197365
30min_MT.	Q01433	0.081002	0.025576	0.08129	AMPD2	AMP deami	AMPD2_HU	35	1.057752
30min_MT.	Q12765	0.185119	0.025589	0.081299	SCRN1	Secernin-1	SCRN1_HUI	14	1.136911
30min_MT.	Q9Y6G3	-0.38955	0.02561	0.081333	MRPL42	Large ribos	RM42_HUN	1	0.76337

30min_MT.	O94903	0.346782	0.025631	0.081334	PLPBP	Pyridoxal p	PLPHP_HUI		7	1.271721
30min_MT.	Q8IWE2	0.164731	0.025625	0.081334	FAM114A1	Protein NO	NXP20_HU		14	1.120957
15min_MT.	P07910	0.229701	0.02568	0.081402	HNRNPC	Heterogene	HNRPC_HU		16	1.172592
15min_MT.	Q93096	0.058489	0.025664	0.081402	PTP4A1	Protein tyr	TP4A1_HUI		7	1.041374
15min_MT.	Q96B26	0.093876	0.025697	0.08141	EXOSC8	Exosome cc	EXOS8_HUI		6	1.067233
15min_MT.	Q08170	-0.3981	0.025717	0.081442	SRSF4	Serine/argi	SRSF4_HUN		12	0.758858
15min_MT.	O15488	-0.69014	0.02577	0.081509	GYG2	Glycogenin	GLYG2_HUI		3	0.619794
15min_MT.	P20338	-0.05294	0.025769	0.081509	RAB4A	Ras-related	RAB4A_HUI		6	0.96397
30min_MT.	Q9HCY8	0.752666	0.02575	0.081509	S100A14	Protein S1C	S10AE_HUN		3	1.684904
15min_MT.	Q92764	0.188819	0.025804	0.081585	KRT35	Keratin, typ	KRT35_HUI		8	1.13983
30min_MT.	P48556	0.189187	0.025867	0.08168	PSMD8	26S proteas	PSMD8_HU		9	1.140121
30min_MT.	Q13277	0.061516	0.025876	0.08168	STX3	Syntaxin-3	STX3_HUM		3	1.043562
30min_MT.	P41252	-0.2134	0.025962	0.081818	IARS1	Isoleucine--	SYIC_HUMA		33	0.862503
30min_MT.	P53004	0.269903	0.025948	0.081818	BLVRA	Biliverdin r	BIEA_HUMA		11	1.205727
30min_MT.	Q9UNH7	-0.18524	0.025947	0.081818	SNX6	Sorting nex	SNX6_HUM		7	0.879503
30min_MT.	Q16775	0.17585	0.025993	0.081827	HAGH	Hydroxyacy	GLO2_HUM		10	1.12963
15min_MT.	Q9UH65	0.124431	0.025989	0.081827	SWAP70	Switch-assc	SWP70_HU		22	1.090078
15min_MT.	Q99549	-0.57409	0.026019	0.081867	MPHOSPH8	M-phase ph	MPP8_HUN		1	0.671712
15min_MT.	P26583	-0.19269	0.026067	0.081985	HMGB2	High mobil	HMGB2_HU		5	0.874971
30min_MT.	P35908	0.77779	0.026107	0.081999	KRT2	Keratin, typ	K22E_HUM		40	1.714503
30min_MT.	Q96EI5	-0.01931	0.026114	0.081999	TCEAL4	Transcripti	TCAL4_HUN		5	0.986701
15min_MT.	Q9UJ83	-0.06635	0.026124	0.081999	HACL1	2-hydroxya	HACL1_HUI		7	0.955052
30min_MT.	P18887	0.24911	0.026184	0.08208	XRCC1	DNA repair	XRCC1_HUI		5	1.188474
30min_MT.	Q03001	-0.12859	0.026179	0.08208	DST	Dystonin	DYST_HUM		33	0.914722
15min_MT.	Q9UKJ3	-0.28369	0.026208	0.082099	GPATCH8	G patch do	GPTC8_HUI		17	0.821486
30min_MT.	P60900	0.143839	0.026327	0.082145	PSMA6	Proteasom	PSA6_HUM		13	1.104841
30min_MT.	Q7Z3T8	-0.17538	0.026289	0.082145	ZFYVE16	Zinc finger	ZFY16_HUN		24	0.885534
30min_MT.	Q9BSB4	0.893087	0.026236	0.082145	ATG101	Autophagy-	ATGA1_HUI		2	1.857145
30min_MT.	Q9NUY8	-0.13396	0.026298	0.082145	TBC1D23	TBC1 doma	TBC23_HUI		10	0.911326
15min_MT.	Q9NZT1	0.345476	0.026336	0.082145	CALML5	Calmodulir	CALL5_HUN		7	1.27057
30min_MT.	Q9UL12	-0.27378	0.026339	0.082145	SARDH	Sarcosine d	SARDH_HU		9	0.82715
15min_MT.	Q8N1B4	-0.45289	0.02636	0.082179	VPS52	Vacuolar pr	VPS52_HUI		4	0.730578
30min_MT.	Q9UKU7	0.126087	0.026388	0.082233	ACAD8	Isobutyryl-	ACAD8_HU		13	1.091329
30min_MT.	P09382	0.258602	0.026449	0.082391	LGALS1	Galectin-1	LEG1_HUM		11	1.196319
30min_MT.	P51531	-0.03982	0.026499	0.08248	SMARCA2	Probable gl	SMCA2_HU		4	0.972779
30min_MT.	Q86VP6	0.127716	0.026549	0.082603	CAND1	Cullin-assoc	CAND1_HU		47	1.092563
30min_MT.	P11216	0.055249	0.026573	0.082612	PYGB	Glycogen p	PYGB_HUM		25	1.039038
30min_MT.	P30876	-0.25075	0.026563	0.082612	POLR2B	DNA-direct	RPB2_HUM		17	0.840461
15min_MT.	O14618	0.237228	0.026646	0.082692	CCS	Copper cha	CCS_HUMA		6	1.178725
30min_MT.	P07948;P0	0.196436	0.026633	0.082692	LYN;HCK	Tyrosine-pr	LYN_HUMA		1	1.145864
30min_MT.	P38570	0.024083	0.026651	0.082692	ITGAE	Integrin alp	ITAE_HUMA		2	1.016833
15min_MT.	Q9BY49	-0.38038	0.026639	0.082692	PECR	Peroxisoma	PECR_HUM		4	0.768235
30min_MT.	Q9H6E4	-0.46823	0.026641	0.082692	CCDC134	Coiled-coil	CC134_HUI		7	0.72285
30min_MT.	Q14696	0.140179	0.026704	0.082823	MESD	LRP chaper	MESD_HUN		11	1.102042
15min_MT.	Q16539	0.251664	0.026823	0.083059	MAPK14	Mitogen-ac	MK14_HUN		7	1.19058
15min_MT.	O43715	0.231905	0.026945	0.083372	TRIAP1	TP53-regul	TRIA1_HUN		5	1.174384
15min_MT.	O00401	0.118792	0.02698	0.083381	WASL	Actin nucle	WASL_HUN		8	1.085825
15min_MT.	P47755	-0.06758	0.026972	0.083381	CAPZA2	F-actin-cap	CAZA2_HUI		13	0.954236
15min_MT.	Q96CM8	0.235323	0.026972	0.083381	ACSF2	Medium-ch	ACSF2_HUN		15	1.17717
15min_MT.	O14967	-0.11335	0.026996	0.083396	CLGN	Calmegin	CLGN_HUN		1	0.924437
30min_MT.	Q96FJ2	-0.89857	0.027049	0.083529	DYNLL2	Dynein ligh	DYL2_HUM		2	0.536419
30min_MT.	P49711	-0.62248	0.027062	0.083535	CTCF	Transcripti	CTCF_HUM		2	0.649554

15min_MT.	P01019	0.266874	0.027128	0.083581	AGT	Angiotensir	ANGT_HUM	15	1.203198
30min_MT.	Q09028	0.129979	0.027117	0.083581	RBBP4	Histone-bir	RBBP4_HUI	12	1.094278
15min_MT.	Q3B726	-0.1998	0.02713	0.083581	POLR1F	DNA-direct	RPA43_HUI	2	0.87067
15min_MT.	Q96JG6	-0.93241	0.027118	0.083581	VPS50	Syndetin	VPS50_HUI	3	0.523981
30min_MT.	Q96S38	-0.12186	0.027116	0.083581	RPS6KC1	Ribosomal	KS6C1_HUI	3	0.919001
30min_MT.	P60174	0.194665	0.027155	0.083626	TPI1	Triosephos	TPIS_HUMA	21	1.144458
15min_MT.	P54819	0.171795	0.027183	0.083676	AK2	Adenylate k	KAD2_HUM	13	1.126459
30min_MT.	Q00796	0.221963	0.027203	0.083707	SORD	Sorbitol de	DHSO_HUM	14	1.166319
30min_MT.	Q9BRA2	0.257427	0.027262	0.083855	TXNDC17	Thioredoxi	TXD17_HUI	6	1.195345
15min_MT.	P13987	-0.15648	0.027313	0.083912	CD59	CD59 glyco	CD59_HUM	2	0.89721
15min_MT.	Q15435	0.227942	0.027306	0.083912	PPP1R7	Protein pho	PP1R7_HUI	11	1.171163
15min_MT.	Q9Y6X5	0.857182	0.027365	0.084007	ENPP4	Bis(5'-aden	ENPP4_HUI	2	1.811496
30min_MT.	P11171	0.114359	0.027386	0.084039	EPB41	Protein 4.1	EPB41_HUI	7	1.082494
30min_MT.	P35268	0.110125	0.027398	0.084043	RPL22	Large ribos	RL22_HUM	2	1.079322
15min_MT.	Q96D53	0.044246	0.027413	0.084056	COQ8B	Atypical kir	COQ8B_HU	7	1.031144
15min_MT.	Q9Y2Q3	0.199305	0.027445	0.084121	GSTK1	Glutathion	GSTK1_HUI	5	1.148145
30min_MT.	O60701	-0.20794	0.027478	0.084155	UGDH	UDP-glucos	UGDH_HUM	17	0.865772
15min_MT.	P21860	0.066354	0.027477	0.084155	ERBB3	Receptor ty	ERBB3_HUI	5	1.047067
15min_MT.	P08648	0.150792	0.027544	0.084286	ITGA5	Integrin alp	ITA5_HUMA	9	1.110178
15min_MT.	P42677	-0.11851	0.027593	0.084377	RPS27	Small ribos	RS27_HUM	2	0.921136
30min_MT.	P15104	0.128822	0.027682	0.084412	GLUL	Glutamine	GLNA_HUM	8	1.093401
30min_MT.	P48163	0.11622	0.02766	0.084412	ME1	NADP-depe	MAOX_HUM	18	1.083892
15min_MT.	Q7Z794	0.266286	0.027651	0.084412	KRT77	Keratin, typ	K2C1B_HUI	18	1.202708
30min_MT.	Q9H0E2	0.544793	0.027701	0.084412	TOLLIP	Toll-interac	TOLIP_HUM	7	1.458811
30min_MT.	O43924	0.594422	0.027742	0.084436	PDE6D	Retinal rod	PDE6D_HU	4	1.509868
15min_MT.	Q9BTY7	0.66164	0.027742	0.084436	HGH1	Protein HG	HGH1_HUM	3	1.58188
15min_MT.	Q9BZX2	0.442702	0.027733	0.084436	UCK2	Uridine-cyt	UCK2_HUM	3	1.359148
30min_MT.	Q00403	-0.43344	0.027772	0.084461	GTF2B	Transcripti	TF2B_HUM	3	0.740495
30min_MT.	Q92616	-0.31223	0.0278	0.08451	GCN1	Stalled ribc	GCN1_HUM	40	0.805397
30min_MT.	P13645	0.793866	0.027911	0.084687	KRT10	Keratin, typ	K1C10_HUI	30	1.733714
15min_MT.	P56545	0.244321	0.027929	0.084708	CTBP2	C-terminal-	CTBP2_HUI	4	1.184535
30min_MT.	O43493	0.546357	0.02803	0.084849	TGOLN2	Trans-Golgi	TGON2_HU	3	1.460393
30min_MT.	P86790;P8	-0.21167	0.02802	0.084849	CCZ1B;CCZ	Vacuolar fu	CCZ1B_HUI	17	0.863538
15min_MT.	P55157	-0.06088	0.028061	0.084911	MTTP	Microsoma	MTP_HUMA	35	0.958678
15min_MT.	P10114	0.192572	0.028078	0.08492	RAP2A	Ras-related	RAP2A_HUI	1	1.142799
30min_MT.	Q8IXL7	-0.49744	0.028086	0.08492	MSRB3	Methionine	MSRB3_HU	2	0.708363
15min_MT.	P24864	0.643094	0.028129	0.085018	CCNE1	G1/S-specif	CCNE1_HUI	1	1.561675
30min_MT.	P55795	0.172666	0.028244	0.085201	HNRNPH2	Heterogene	HNRH2_HU	5	1.127139
30min_MT.	Q16822	0.178985	0.028279	0.085239	PCK2	Phosphoen	PCKGM_HU	30	1.132087
30min_MT.	Q99623	0.443147	0.028318	0.08532	PHB2	Prohibitin-	PHB2_HUM	1	1.359567
15min_MT.	P48730;P4	2.261794	0.028349	0.085352	CSNK1D;CS	Casein kina	KC1D_HUM	1	4.795876
15min_MT.	Q13895	-0.25063	0.028363	0.085363	BYSL	Bystin	BYST_HUMA	8	0.840527
15min_MT.	P53990	0.189068	0.028499	0.085575	IST1	IST1 homol	IST1_HUMA	8	1.140027
30min_MT.	Q9NY12	0.661719	0.028472	0.085575	GAR1	H/ACA ribo	GAR1_HUM	2	1.581967
30min_MT.	Q9H4A5	-1.2347	0.028529	0.085632	GOLPH3L	Golgi phos	GLP3L_HUI	1	0.42493
15min_MT.	Q15785	0.67425	0.028611	0.085812	TOMM34	Mitochond	TOM34_HU	2	1.595767
30min_MT.	Q9BZH6	-0.21686	0.028603	0.085812	WDR11	WD repeat-	WDR11_HU	26	0.860434
30min_MT.	Q8NFZ8	0.357896	0.028645	0.085879	CADM4	Cell adhesi	CADM4_HU	4	1.281556
15min_MT.	P19013	4.042692	0.028737	0.086058	KRT4	Keratin, typ	K2C4_HUM	19	16.48054
30min_MT.	Q9P000	0.280395	0.028736	0.086058	COMMD9	COMM dom	COMD9_HU	10	1.214527
15min_MT.	Q9BYJ9	-0.84091	0.028768	0.086084	YTHDF1	YTH domair	YTHD1_HUI	1	0.558292
15min_MT.	Q92530	0.076508	0.028808	0.086138	PSMF1	Proteasom	PSMF1_HU	7	1.054462

15min_MT.	Q9HB07	0.209277	0.028808	0.086138	MYG1	MYG1 exon	MYG1_HUI	12	1.156108
15min_MT.	O60936	0.177394	0.028852	0.086203	NOL3	Nucleolar p	NOL3_HUM	1	1.130839
15min_MT.	O76003	0.268808	0.028878	0.08622	GLRX3	Glutaredox	GLRX3_HUI	12	1.204812
15min_MT.	P04075	-0.20454	0.028901	0.08622	ALDOA	Fructose-bi	ALDOA_HU	35	0.867814
30min_MT.	P12956	-0.13906	0.028906	0.08622	XRCC6	X-ray repair	XRCC6_HUI	30	0.908113
15min_MT.	Q9UI14	-0.18591	0.028889	0.08622	RABAC1	Prenylated	PRAF1_HUI	4	0.879095
15min_MT.	Q01082	-0.07629	0.029009	0.086442	SPTBN1	Spectrin be	SPTB2_HUM	124	0.948495
30min_MT.	P61086	0.113344	0.029047	0.08649	UBE2K	Ubiquitin-c	UBE2K_HUI	10	1.081733
30min_MT.	P02794	0.273729	0.029076	0.086542	FTH1	Ferritin hea	FRIH_HUM.	8	1.208929
30min_MT.	Q9Y376	0.131962	0.029089	0.086548	CAB39	Calcium-bi	CAB39_HUI	13	1.095783
15min_MT.	P06753	0.779148	0.02921	0.086645	TPM3	Tropomyos	TPM3_HUM	4	1.716117
30min_MT.	P31153	0.108065	0.029209	0.086645	MAT2A	S-adenosylr	METK2_HU	15	1.077782
30min_MT.	P54105	0.116241	0.029196	0.086645	CLNS1A	Methylosor	ICLN_HUM.	4	1.083907
30min_MT.	Q9BVC6	0.024728	0.029155	0.086645	TMEM109	Voltage-gat	TM109_HU	2	1.017288
15min_MT.	Q9UQ35	-0.17019	0.029169	0.086645	SRRM2	Serine/argi	SRRM2_HU	68	0.888726
15min_MT.	Q9HAS0	0.112909	0.029238	0.086696	C17orf75	Protein Njn	NJMU_HUM	13	1.081406
15min_MT.	Q9H5N1	0.206173	0.029262	0.086731	RABEP2	Rab GTPase	RABE2_HUI	14	1.153624
30min_MT.	Q9NZ32	-0.24151	0.029274	0.086736	ACTR10	Actin-relate	ARP10_HUI	11	0.845858
15min_MT.	P10586	0.104611	0.02933	0.086803	PTPRF	Receptor-ty	PTPRF_HUM	24	1.075204
15min_MT.	Q16363	0.732854	0.029321	0.086803	LAMA4	Laminin sul	LAMA4_HU	1	1.661924
30min_MT.	Q86UX2	-0.51569	0.029329	0.086803	ITIH5	Inter-alpha	ITIH5_HUM	1	0.699458
30min_MT.	P04150	0.067454	0.029414	0.087019	NR3C1	Glucocortic	GCR_HUMA	5	1.047866
15min_MT.	Q9UHV9	0.263245	0.029449	0.087059	PFDN2	Prefoldin su	PFD2_HUM	6	1.200176
15min_MT.	P04259	1.469103	0.02955	0.087256	KRT6B	Keratin, typ	K2C6B_HUI	5	2.768498
30min_MT.	P0DMV1;P(	-0.27514	0.02955	0.087256	CT45A8;CT4	Cancer/test	CT458_HUI	7	0.826373
15min_MT.	P46776	-0.09168	0.029581	0.087314	RPL27A	Large ribos	RL27A_HUI	3	0.938429
15min_MT.	Q15517	-0.24225	0.029605	0.087351	CDSN	Corneodesr	CDSN_HUM	3	0.845426
15min_MT.	O95470	1.79094	0.029658	0.087459	SGPL1	Sphingosin	SGPL1_HUI	1	3.460402
15min_MT.	P19022	0.086147	0.029704	0.08748	CDH2	Cadherin-2	CADH2_HU	4	1.061531
15min_MT.	P05090	1.722992	0.029789	0.087572	APOD	Apolipoprc	APOD_HUM	2	3.301204
15min_MT.	P83436	0.12158	0.029784	0.087572	COG7	Conserved	COG7_HUM	11	1.087926
15min_MT.	Q08AM6	-0.03251	0.029774	0.087572	VAC14	Protein VA(	VAC14_HUI	14	0.977721
30min_MT.	Q8IZ83	-0.04012	0.029814	0.087572	ALDH16A1	Aldehyde d	A16A1_HUI	15	0.972576
30min_MT.	Q93062	-0.26259	0.029802	0.087572	RBPMS	RNA-bindir	RBPMS_HU	2	0.833591
30min_MT.	Q96QA5	0.645205	0.029798	0.087572	GSDMA	Gasdermin-	GSDMA_HU	5	1.563962
30min_MT.	Q2M2I8	0.031085	0.029846	0.087634	AAK1	AP2-associ	AAK1_HUM	13	1.02178
15min_MT.	P46777	-0.19088	0.02991	0.087755	RPL5	Large ribos	RL5_HUMA	13	0.876071
15min_MT.	Q2M2D7	0.212375	0.029933	0.087758	TBC1D28	TBC1 doma	TBC28_HUI	1	1.158594
15min_MT.	Q5T8I3	-0.49462	0.030006	0.087939	EEIG2	EEIG family	EEIG2_HUM	3	0.709747
15min_MT.	O43815	0.051423	0.030028	0.087969	STRN	Striatin	STRN_HUM	9	1.036287
15min_MT.	P30041	0.091743	0.030105	0.088128	PRDX6	Peroxiredo	PRDX6_HU	21	1.065657
15min_MT.	Q96RS6	0.009956	0.030178	0.088311	NUDCD1	NudC doma	NUDC1_HU	16	1.006925
15min_MT.	A0FGR8	-0.18107	0.030416	0.088353	ESYT2	Extended sy	ESYT2_HUM	10	0.882048
30min_MT.	O00560	0.486267	0.030253	0.088353	SDCBP	Syntenin-1	SDCB1_HUI	1	1.400815
15min_MT.	O43823	-0.53791	0.030316	0.088353	AKAP8	A-kinase an	AKAP8_HUI	4	0.688769
15min_MT.	P00813	0.173797	0.03031	0.088353	ADA	Adenosine	ADA_HUMA	19	1.128024
30min_MT.	P40763	-0.19941	0.03043	0.088353	STAT3	Signal trans	STAT3_HUM	18	0.870907
15min_MT.	P63165	0.118934	0.030288	0.088353	SUMO1	Small ubiqu	SUMO1_HU	6	1.085932
15min_MT.	Q4KMP7	-0.33423	0.030326	0.088353	TBC1D10B	TBC1 doma	TB10B_HUI	8	0.793209
30min_MT.	Q7LBR1	0.184195	0.03027	0.088353	CHMP1B	Charged mu	CHM1B_HU	4	1.136183
15min_MT.	Q8N1Q1	0.123043	0.030274	0.088353	CA13	Carbonic ar	CAH13_HU	4	1.08903
30min_MT.	Q96DH6	-0.396	0.030441	0.088353	MSI2	RNA-bindir	MSI2H_HUI	2	0.759963

30min_MT.	Q96DI7	-0.22491	0.030427	0.088353	SNRNP40	U5 small nu	SNR40_HUI	11	0.855646
15min_MT.	Q96MU7	-0.1623	0.030427	0.088353	YTHDC1	YTH domair	YTDC1_HUI	2	0.893602
15min_MT.	Q99460	-0.07567	0.030394	0.088353	PSMD1	26S proteas	PSMD1_HU	38	0.948901
30min_MT.	Q9ULA0	0.105339	0.030364	0.088353	DNPEP	Aspartyl am	DNPEP_HU	13	1.075747
30min_MT.	Q9Y2H2	-0.42513	0.03036	0.088353	INPP5F	Phosphatic	SAC2_HUM	1	0.744771
15min_MT.	O43237	0.081887	0.030486	0.088418	DYNC1LI2	Cytoplasmi	DC1L2_HUI	12	1.058402
30min_MT.	Q96SZ5	0.166608	0.030511	0.088425	ADO	2-aminoeth	AEDO_HUM	5	1.122417
30min_MT.	Q9HAT2	0.118316	0.030504	0.088425	SIAE	Sialate O-ac	SIAE_HUMA	12	1.085467
15min_MT.	P28065	0.122277	0.030527	0.088438	PSMB9	Proteasom	PSB9_HUM	6	1.088452
30min_MT.	P28072	0.253126	0.030543	0.08845	PSMB6	Proteasom	PSB6_HUM	5	1.191787
15min_MT.	O95757	0.111551	0.030608	0.08854	HSPA4L	Heat shock	HS74L_HUI	25	1.080389
15min_MT.	P14649	0.222035	0.030619	0.08854	MYL6B	Myosin ligh	MYL6B_HU	6	1.166378
30min_MT.	O75427	-0.47613	0.030701	0.088679	LRCH4	Leucine-ric	LRCH4_HUI	2	0.718905
15min_MT.	Q13795	0.200068	0.030697	0.088679	ARFRP1	ADP-ribosy	ARFRP_HUI	1	1.148752
30min_MT.	Q96GA7	0.114939	0.030696	0.088679	SDSL	Serine dehy	SDSL_HUM	9	1.082929
15min_MT.	P01036	2.923434	0.030775	0.08885	CST4	Cystatin-S	CYTS_HUMA	3	7.586499
15min_MT.	P02788	0.492847	0.03084	0.08895	LTF	Lactotransf	TRFL_HUMA	7	1.407219
15min_MT.	Q96EK6	-0.09757	0.030866	0.08899	GNPNAT1	Glucosamir	GNA1_HUM	10	0.934609
15min_MT.	Q96BS2	0.076155	0.030941	0.089109	TESC	Calcineurir	CHP3_HUM	7	1.054205
30min_MT.	Q9NXC5	-0.19905	0.030926	0.089109	MIOS	GATOR2 co	MIOS_HUM	25	0.871126
30min_MT.	P30405	0.312443	0.031028	0.089261	PPIF	Peptidyl-pr	PPIF_HUMA	7	1.241809
30min_MT.	O96000	-0.73581	0.031135	0.089536	NDUFB10	NADH dehy	NDUBA_HU	1	0.600483
15min_MT.	B1AK53	0.637122	0.031197	0.089644	ESPN	Espin	ESPN_HUM	3	1.555223
15min_MT.	P13984	0.180563	0.031232	0.089684	GTF2F2	General tra	T2FB_HUM	11	1.133326
30min_MT.	Q9BVL2	-0.52154	0.031273	0.089768	NUP58	Nucleopori	NUP58_HU	3	0.696627
15min_MT.	P51610	-0.06048	0.031373	0.089989	HCFC1	Host cell fa	HCFC1_HUI	39	0.958946
30min_MT.	Q9UPX8	-0.13753	0.031521	0.090281	SHANK2	SH3 and mu	SHAN2_HU	3	0.909076
30min_MT.	O75533	-0.14064	0.031558	0.090288	SF3B1	Splicing fac	SF3B1_HUI	29	0.907118
15min_MT.	Q9H944	0.168099	0.031574	0.090299	MED20	Mediator o	MED20_HU	5	1.123577
30min_MT.	Q16774	-0.05988	0.031623	0.090341	GUK1	Guanylate l	KGUA_HUM	6	0.959347
30min_MT.	P25705	-0.24603	0.03169	0.090347	ATP5F1A	ATP syntha	ATPA_HUM	17	0.843216
15min_MT.	Q13409	-0.08244	0.031648	0.090347	DYNC1I2	Cytoplasmi	DC1I2_HUI	15	0.944462
15min_MT.	Q8TCA0	0.218251	0.031682	0.090347	LRRC20	Leucine-ric	LRC20_HUI	7	1.163322
15min_MT.	Q96GX9	-0.28799	0.031684	0.090347	APIP	Methylthio	MTNB_HUM	12	0.819044
30min_MT.	O75865	0.369565	0.03178	0.090558	TRAPPC6A	Trafficking	TPC6A_HUI	1	1.291963
30min_MT.	Q96N67	-0.97751	0.031809	0.090574	DOCK7	Dedicator c	DOCK7_HU	2	0.507855
30min_MT.	O75348	0.562502	0.031878	0.090738	ATP6V1G1	V-type prot	VATG1_HUI	3	1.476828
30min_MT.	Q14204	-0.24525	0.031934	0.090791	DYNC1H1	Cytoplasmi	DYHC1_HU	101	0.843671
15min_MT.	Q9HAV4	-0.07735	0.031941	0.090791	XPO5	Exportin-5	XPO5_HUM	20	0.947795
15min_MT.	O14561	-0.20579	0.032135	0.090842	NDUFAB1	Acyl carrier	ACPM_HUM	4	0.867066
30min_MT.	O60925	0.311564	0.032102	0.090842	PFDN1	Prefoldin su	PFD1_HUM	6	1.241052
15min_MT.	Q02790	0.0551	0.03205	0.090842	FKBP4	Peptidyl-pr	FKBP4_HUI	28	1.038931
15min_MT.	Q08380	0.220854	0.032115	0.090842	LGALS3BP	Galectin-3-	LG3BP_HUI	5	1.165423
30min_MT.	Q8IZL8	0.079388	0.032069	0.090842	PELP1	Proline-, glu	PELP1_HUI	9	1.05657
15min_MT.	Q8TBB5	-0.06217	0.032103	0.090842	KLHDC4	Kelch doma	KLDC4_HUI	9	0.957822
30min_MT.	Q93099	0.154473	0.032132	0.090842	HGD	Homogenti	HGD_HUMA	19	1.113015
15min_MT.	Q96CN7	0.281062	0.032015	0.090842	ISOC1	Isochorism	ISOC1_HUM	7	1.215089
15min_MT.	Q9BY44	-0.07414	0.032132	0.090842	EIF2A	Eukaryotic	EIF2A_HUM	21	0.949907
30min_MT.	Q8TCF1	0.602418	0.032283	0.091162	ZFAND1	AN1-type zi	ZFAN1_HUI	2	1.51826
15min_MT.	Q9Y263	-0.07712	0.03228	0.091162	PLAA	Phospholip	PLAP_HUM	29	0.94795
30min_MT.	P61221	-0.15153	0.032331	0.091174	ABCE1	ATP-bindin	ABCE1_HUI	18	0.900293
30min_MT.	Q92541	0.126738	0.032314	0.091174	RTF1	RNA polym	RTF1_HUM	17	1.091822

15min_MT.	Q96EN8	-0.10391	0.032338	0.091174	MOCOS	Molybdenu	MOCOS_HU	21	0.930508
15min_MT.	Q9NU19	0.099325	0.032346	0.091174	TBC1D22B	TBC1 doma	TB22B_HUI	1	1.071272
15min_MT.	P15252	0.120777	0.03237	0.091211	NaN	Rubber elo	REF_HEVBR	4	1.087321
15min_MT.	Q9UPY6	0.059575	0.032387	0.091213	WASF3	Actin-bindi	WASF3_HU	2	1.042158
15min_MT.	Q9HD42	0.259082	0.032447	0.091295	CHMP1A	Charged mu	CHM1A_HU	6	1.196717
30min_MT.	Q8ND24	-0.11843	0.03247	0.091327	RNF214	RING finger	RN214_HU	7	0.921188
30min_MT.	O94929	-0.1117	0.032534	0.091421	ABLIM3	Actin-bindi	ABLM3_HU	5	0.925499
15min_MT.	Q92889	0.087089	0.032538	0.091421	ERCC4	DNA repair	XPF_HUMA	4	1.062225
30min_MT.	Q9NXG2	0.130491	0.032515	0.091421	THUMPD1	THUMP dor	THUM1_HU	20	1.094666
30min_MT.	Q5NDL2	0.416782	0.032628	0.091609	EOGT	EGF domair	EOGT_HUM	7	1.334946
15min_MT.	Q6ZMI0	0.203403	0.032629	0.091609	PPP1R21	Protein pho	PPR21_HUI	9	1.151411
30min_MT.	P56199	0.12234	0.032665	0.091676	ITGA1	Integrin alp	ITA1_HUMA	5	1.088499
30min_MT.	P14543	0.068298	0.032715	0.091785	NID1	Nidogen-1	NID1_HUM	15	1.048479
15min_MT.	P62273	-1.15407	0.032892	0.092178	RPS29	Small ribos	RS29_HUM	1	0.449356
30min_MT.	Q96SU4	-0.11657	0.032894	0.092178	OSBPL9	Oxysterol-b	OSBL9_HUI	6	0.922378
30min_MT.	Q5VZ89	-0.25418	0.032919	0.092193	DENND4C	DENN doma	DEN4C_HU	12	0.838466
30min_MT.	P68104	-0.19837	0.032938	0.092212	EEF1A1	Elongation	EF1A1_HUI	25	0.871536
30min_MT.	O14737	0.299535	0.0331	0.092363	PDCD5	Programme	PDCD5_HU	10	1.230748
30min_MT.	P09622	0.160412	0.03309	0.092363	DLD	Dihydrolip	DLDH_HUM	17	1.117606
30min_MT.	P13489	0.094526	0.03308	0.092363	RNH1	Ribonuclea	RINI_HUMA	21	1.067715
15min_MT.	P28799	-0.20722	0.033088	0.092363	GRN	Progranulir	GRN_HUMA	6	0.866203
30min_MT.	Q08J23	0.080294	0.033006	0.092363	NSUN2	RNA cytosir	NSUN2_HU	37	1.057233
30min_MT.	Q86Y56	-0.23779	0.033107	0.092363	DNAAF5	Dynein axo	DAAF5_HUI	7	0.848044
30min_MT.	Q8IW75	1.077287	0.03313	0.092363	SERPINA12	Serpin A12	SPA12_HUI	5	2.110064
30min_MT.	Q9HCU8	-0.72559	0.033071	0.092363	POLD4	DNA polym	DPOD4_HU	2	0.604749
15min_MT.	P15170	0.127143	0.033174	0.092444	GSPT1	Eukaryotic	ERF3A_HUI	18	1.092129
30min_MT.	P63261	-1.79079	0.033252	0.092562	ACTG1	Actin, cyto	ACTG_HUM	1	0.289014
15min_MT.	Q6YP21	-0.12718	0.033235	0.092562	KYAT3	Kynurenine	KAT3_HUM	19	0.91562
15min_MT.	P51553	0.160484	0.033279	0.09257	IDH3G	Isocitrate d	IDH3G_HUI	3	1.117662
15min_MT.	Q9Y3B4	-0.47136	0.033271	0.09257	SF3B6	Splicing fac	SF3B6_HUI	1	0.721284
15min_MT.	Q9NWH9	-0.1371	0.033376	0.092774	SLTM	SAFB-like tr	SLTM_HUM	6	0.909345
30min_MT.	Q8TAQ2	0.041556	0.033395	0.092795	SMARCC2	SWI/SNF cc	SMRC2_HU	7	1.029223
30min_MT.	Q15811	-0.08439	0.033465	0.092957	ITSN1	Intersectin	ITSN1_HUI	14	0.943186
15min_MT.	O43617	0.150253	0.033493	0.092986	TRAPPC3	Trafficking	TPPC3_HUI	9	1.109764
15min_MT.	O95749	0.278109	0.033521	0.093012	GGPS1	Geranylger	GGPPS_HU	7	1.212605
30min_MT.	Q4KMP7	-0.37645	0.03355	0.093059	TBC1D10B	TBC1 doma	TB10B_HUI	8	0.770333
30min_MT.	Q9UIA9	0.034495	0.033659	0.093296	XPO7	Exportin-7	XPO7_HUM	30	1.024198
30min_MT.	P46736	0.106244	0.033709	0.093401	BRCC3	Lys-63-spec	BRCC3_HUI	8	1.076423
30min_MT.	Q9H6S3	-0.08345	0.033734	0.093437	EPS8L2	Epidermal	ES8L2_HUI	18	0.943795
15min_MT.	O00743	-0.10611	0.03382	0.093519	PPP6C	Serine/thre	PPP6_HUM	13	0.929089
15min_MT.	P67870	0.406551	0.033823	0.093519	CSNK2B	Casein kina	CSK2B_HUI	5	1.325513
30min_MT.	Q9NP77	-0.21076	0.033814	0.093519	SSU72	RNA polym	SSU72_HUI	4	0.864085
15min_MT.	O94903	0.297313	0.033849	0.093548	PLPBP	Pyridoxal p	PLPHP_HUI	7	1.228854
15min_MT.	P04792	0.25967	0.033898	0.09356	HSPB1	Heat shock	HSPB1_HUI	12	1.197204
15min_MT.	Q96B36	0.288026	0.033931	0.093586	AKT1S1	Proline-rich	AKTS1_HUI	2	1.220969
30min_MT.	O00767	-0.36022	0.033963	0.09362	SCD	Stearoyl-Cc	SCD_HUMA	1	0.779047
15min_MT.	Q8N2F6	0.196206	0.033979	0.09362	ARMC10	Armadillo r	ARM10_HU	1	1.145681
15min_MT.	Q9UI30	0.006582	0.033968	0.09362	TRMT112	Multifuncti	TR112_HUI	2	1.004572
30min_MT.	O60306	-0.62243	0.034115	0.093729	AQR	RNA helicas	AQR_HUMA	2	0.649577
15min_MT.	Q15628	0.275986	0.034083	0.093729	TRADD	Tumor necr	TRADD_HU	3	1.210821
30min_MT.	Q96IV0	-0.39324	0.034127	0.093729	NGLY1	Peptide-N(4	NGLY1_HUI	6	0.761418
30min_MT.	Q96PU5	-0.0418	0.034088	0.093729	NEDD4L	E3 ubiquiti	NED4L_HUI	4	0.971441

15min_MT.	Q9BPX1	0.336653	0.034086	0.093729	HSD17B14	17-beta-hy	DHB14_HU	2	1.262824
15min_MT.	Q96R05	-0.11709	0.034264	0.09404	RBP7	Retinoid-bi	RET7_HUM	3	0.922046
30min_MT.	Q9NTN9	0.061368	0.034314	0.094145	SEMA4G	Semaphori	SEM4G_HU	14	1.043455
30min_MT.	Q9Y3D8	-0.14754	0.034417	0.094373	AK6	Adenylate k	KAD6_HUM	4	0.902786
15min_MT.	P01037	0.777285	0.034501	0.094558	CST1	Cystatin-SN	CYTN_HUM	1	1.713902
15min_MT.	Q92817	0.472987	0.034548	0.094652	EVPL	Envoplakin	EVPL_HUM	1	1.38798
15min_MT.	O43765	0.200116	0.03462	0.094784	SGTA	Small gluta	SGTA_HUM	10	1.14879
15min_MT.	Q9UL18	-0.20609	0.034614	0.094784	ago-01	Protein arg	AGO1_HUM	12	0.866881
15min_MT.	Q13620	0.02099	0.034687	0.094868	CUL4B	Cullin-4B	CUL4B_HU	37	1.014655
15min_MT.	Q16762	-0.16488	0.034664	0.094868	TST	Thiosulfate	THTR_HUM	12	0.892004
15min_MT.	Q3ZCW2	0.288613	0.034826	0.095173	LGALSL	Galectin-re	LEGL_HUM	5	1.221465
15min_MT.	Q6P5S2	1.450823	0.034833	0.095173	LEG1	Protein LEG	LEG1H_HU	1	2.73364
30min_MT.	P17812	-0.16517	0.035004	0.09546	CTPS1	CTP synthas	PYRG1_HU	18	0.891822
30min_MT.	P18615	-0.15814	0.035025	0.09546	NELFE	Negative el	NELFE_HUM	8	0.896181
15min_MT.	P62857	0.362335	0.035019	0.09546	RPS28	Small ribos	RS28_HUM	3	1.285505
30min_MT.	Q8IV48	-0.27716	0.035039	0.095464	ERI1	3'-5' exorib	ERI1_HUM	4	0.825213
30min_MT.	P49736	-0.17881	0.035067	0.095506	MCM2	DNA replica	MCM2_HU	17	0.883429
15min_MT.	P04083	1.012955	0.035124	0.095507	ANXA1	Annexin A1	ANXA1_HU	13	2.01804
30min_MT.	Q14676	0.341349	0.035088	0.095507	MDC1	Mediator o	MDC1_HUM	9	1.26694
15min_MT.	Q9UIC8	0.156344	0.035128	0.095507	LCMT1	Leucine car	LCMT1_HU	10	1.114459
15min_MT.	P14927	-0.94763	0.035317	0.095821	UQCRB	Cytochrom	QCR7_HUM	1	0.518485
15min_MT.	P43897	0.167434	0.035278	0.095821	TSFM	Elongation	EFTS_HUM	8	1.123059
15min_MT.	P50454	-0.09312	0.035301	0.095821	SERPINH1	Serpin H1	SERPH_HU	11	0.93749
30min_MT.	Q9BUJ2	0.367892	0.035299	0.095821	HNRNPUL1	Heterogene	HNRL1_HU	14	1.290466
15min_MT.	Q9H0E2	0.569424	0.035328	0.095821	TOLLIP	Toll-interac	TOLIP_HUM	7	1.483931
15min_MT.	Q9UNH6	-0.0751	0.035297	0.095821	SNX7	Sorting nex	SNX7_HUM	5	0.949277
15min_MT.	Q06265	0.072449	0.03535	0.095844	EXOSC9	Exosome co	EXOS9_HU	10	1.0515
15min_MT.	P51784	0.186169	0.035484	0.096007	USP11	Ubiquitin c	UBP11_HU	6	1.137739
30min_MT.	Q16270	0.426407	0.03548	0.096007	IGFBP7	Insulin-like	IBP7_HUM	4	1.343883
30min_MT.	Q86YP4;Q8	-0.12836	0.035437	0.096007	GATAD2A;G	Transcripti	P66A_HUM	1	0.914871
15min_MT.	O75348	0.650801	0.035503	0.096024	ATP6V1G1	V-type prot	VATG1_HU	3	1.57004
30min_MT.	O75683	-0.54846	0.035565	0.096126	SURF6	Surfeit locu	SURF6_HU	2	0.68375
15min_MT.	Q13637	-0.11378	0.03564	0.096189	RAB32	Ras-related	RAB32_HU	6	0.924161
15min_MT.	Q9BPY3	0.173711	0.03565	0.096189	FAM118B	Protein FAM	F118B_HU	5	1.127956
30min_MT.	Q9BW85	-0.34538	0.035649	0.096189	YJU2	Splicing fac	YJU2_HUM	4	0.787102
15min_MT.	Q96BW1	0.295208	0.035663	0.096192	UPRT	Uracil phos	UPP_HUM	6	1.227062
15min_MT.	Q9Y597	0.200011	0.03571	0.096252	KCTD3	BTB/POZ dc	KCTD3_HU	5	1.148707
30min_MT.	P61326	-0.40048	0.035809	0.096352	MAGOH	Protein ma	MGN_HUM	1	0.757605
15min_MT.	Q9HCY8	0.489533	0.035837	0.096394	S100A14	Protein S10	S10AE_HUM	3	1.40399
30min_MT.	Q5TH69	-0.12455	0.035866	0.096439	ARFGEF3	Brefeldin A	BIG3_HUM	18	0.917288
15min_MT.	L0R819	-0.43717	0.035932	0.096485	ASDURF	ASNSD1 up	ASURF_HU	3	0.738582
30min_MT.	P02749	1.052491	0.035932	0.096485	APOH	Beta-2-glyc	APOH_HUM	1	2.074107
30min_MT.	P09525	0.246395	0.035922	0.096485	ANXA4	Annexin A4	ANXA4_HU	24	1.186239
30min_MT.	O96013	0.439743	0.035963	0.096514	PAK4	Serine/thre	PAK4_HUM	1	1.356363
15min_MT.	O43172	0.128958	0.036044	0.09662	PRPF4	U4/U6 sma	PRP4_HUM	9	1.093504
15min_MT.	P02647	0.259043	0.036078	0.096676	APOA1	Apolipoprc	APOA1_HU	13	1.196685
30min_MT.	P19525	-0.30524	0.03609	0.096676	EIF2AK2	Interferon-i	E2AK2_HU	3	0.809306
30min_MT.	Q6ZVF9	-0.06201	0.036134	0.096694	GPRIN3	G protein-r	GRIN3_HU	4	0.957929
15min_MT.	Q86V35	0.217172	0.036146	0.096694	CABP7	Calcium-bi	CABP7_HU	1	1.162453
30min_MT.	Q969U7	0.108929	0.036117	0.096694	PSMG2	Proteasom	PSMG2_HU	10	1.078427
15min_MT.	O94915	0.106701	0.036172	0.096698	FRYL	Protein fur	FRYL_HUM	11	1.076763
15min_MT.	Q99766	-0.44431	0.036171	0.096698	DMAC2L	ATP synthas	ATP5S_HUM	2	0.734937

15min_MT.	Q92541	-0.02965	0.036186	0.096702	RTF1	RNA polym	RTF1_HUM		17	0.979659
30min_MT.	O43148	0.044209	0.036255	0.096809	RNMT	mRNA cap g	MCES_HUM		17	1.031118
30min_MT.	P30613	-0.00034	0.036263	0.096809	PKLR	Pyruvate ki	KPYR_HUM		10	0.999762
30min_MT.	Q9HAV4	-0.0841	0.036353	0.096951	XPO5	Exportin-5	XPO5_HUM		20	0.943372
30min_MT.	O94915	-0.12828	0.036427	0.097049	FRYL	Protein furi	FRYL_HUM.		11	0.914919
15min_MT.	Q00653	0.171908	0.036505	0.097189	NFKB2	Nuclear fac	NFKB2_HUI		14	1.126548
15min_MT.	B5ME19;Q9	-0.10655	0.036561	0.097272	EIF3CL;EIF3	Eukaryotic	EIFCL_HUM		23	0.928808
15min_MT.	Q9Y244	0.658928	0.036647	0.097436	POMP	Proteasom	POMP_HUM		5	1.578909
15min_MT.	Q16881	-0.13452	0.036675	0.097475	TXNRD1	Thioredoxi	TRXR1_HUI		22	0.91097
30min_MT.	Q01081	-0.06303	0.036764	0.097646	U2AF1	Splicing fac	U2AF1_HUI		5	0.957251
30min_MT.	P03973	1.25789	0.036842	0.09781	SLPI	Antileukop	SLPI_HUMA		1	2.391458
15min_MT.	P35813	0.172209	0.036863	0.09781	PPM1A	Protein pho	PPM1A_HU		11	1.126783
15min_MT.	Q13308	0.139566	0.036873	0.09781	PTK7	Inactive tyr	PTK7_HUM		4	1.101573
30min_MT.	Q86X10	-0.12585	0.036919	0.097867	RALGAPB	Ral GTPase-	RLGPB_HUI		10	0.916464
15min_MT.	Q5VT52	0.360681	0.036953	0.097916	RPRD2	Regulation	RPRD2_HU		8	1.284032
30min_MT.	Q02750	0.143945	0.037001	0.097977	MAP2K1	Dual specifi	MP2K1_HU		7	1.104922
30min_MT.	P55039	-0.35744	0.037112	0.098199	DRG2	Developme	DRG2_HUM		4	0.780547
15min_MT.	P62805	0.286587	0.037116	0.098199	H4C1	Histone H4	H4_HUMAN		7	1.219751
30min_MT.	Q6UN15	-0.303	0.03715	0.098199	FIP1L1	Pre-mRNA 3	FIP1_HUMA		3	0.810568
15min_MT.	Q96DR8	1.557034	0.037173	0.098199	MUCL1	Mucin-like	MUCL1_HU		2	2.942483
30min_MT.	Q9Y2W2	-0.19602	0.037155	0.098199	WBP11	WW domai	WBP11_HU		15	0.872954
15min_MT.	O60504	-0.02437	0.03724	0.098311	SORBS3	Vinexin	VINEX_HUM		1	0.98325
30min_MT.	Q14203	-0.06429	0.037256	0.098318	DCTN1	Dynactin su	DCTN1_HU		45	0.956418
15min_MT.	Q8N465	0.108241	0.037277	0.09834	D2HGDH	D-2-hydrox	D2HDH_HU		3	1.077913
30min_MT.	P52926	0.269145	0.037308	0.098359	HMGA2	High mobil	HMGA2_HU		5	1.205093
15min_MT.	Q86YB7	0.400748	0.037309	0.098359	ECHDC2	Enoyl-CoA h	ECHD2_HU		3	1.320192
15min_MT.	Q96F07	-0.06833	0.037324	0.098365	CYFIP2	Cytoplasmi	CYFP2_HUM		18	0.953738
30min_MT.	Q9NUU7	-0.11883	0.03734	0.098375	DDX19A	ATP-depenc	DD19A_HU		16	0.920932
30min_MT.	P09110	0.160121	0.037387	0.098399	ACAA1	3-ketoacyl-	THIK_HUMA		9	1.117381
30min_MT.	Q8N392	-0.09598	0.037376	0.098399	ARHGAP18	Rho GTPase	RHG18_HU		14	0.935639
30min_MT.	P50991	0.090354	0.03741	0.098408	CCT4	T-complex p	TCPD_HUM		32	1.064632
15min_MT.	Q86VW0	0.119436	0.037423	0.098408	SESTD1	SEC14 dom	SESD1_HUM		9	1.08631
30min_MT.	P30414	-1.17683	0.037479	0.098474	NKTR	NK-tumor r	NKTR_HUM		1	0.442321
30min_MT.	Q14318	-0.05354	0.037527	0.098509	FKBP8	Peptidyl-pr	FKBP8_HUI		2	0.963572
30min_MT.	Q9NQT5	-0.32043	0.03753	0.098509	EXOSC3	Exosome cc	EXOS3_HUI		2	0.800834
30min_MT.	P00450	-0.09355	0.037622	0.098685	CP	Ceruloplasr	CERU_HUM		27	0.937214
15min_MT.	Q13541	-0.39029	0.037653	0.098733	EIF4EBP1	Eukaryotic	4EBP1_HUI		5	0.762977
15min_MT.	P21589	-1.07	0.037674	0.098756	NT5E	5'-nucleoti	5NTD_HUM		2	0.47632
15min_MT.	P07203	0.30325	0.037722	0.098848	GPX1	Glutathion	GPX1_HUM		3	1.233921
15min_MT.	Q9UQ80	0.085252	0.037794	0.09895	PA2G4	Proliferatic	PA2G4_HU		23	1.060873
30min_MT.	Q9Y394	-0.31642	0.037799	0.09895	DHRS7	Dehydroger	DHRS7_HU		2	0.803063
30min_MT.	Q86UN3	0.268402	0.037852	0.098955	RTN4RL2	Reticulon-4	R4RL2_HUI		1	1.204473
15min_MT.	Q8WZ82	-0.50177	0.037833	0.098955	OVCA2	Esterase OV	OVCA2_HU		3	0.706242
15min_MT.	Q9GZS3	0.071348	0.037817	0.098955	SKIC8	Superkiller	SKI8_HUMA		8	1.050698
15min_MT.	O60573	0.236872	0.037949	0.098992	EIF4E2	Eukaryotic	IF4E2_HUM		2	1.178435
15min_MT.	P16422	-0.0789	0.037922	0.098992	EPCAM	Epithelial c	EPCAM_HU		2	0.946782
30min_MT.	Q6UX04	0.353396	0.037954	0.098992	CWC27	Spliceosom	CWC27_HU		3	1.277564
15min_MT.	Q8TBX8	0.281246	0.037925	0.098992	PIP4K2C	Phosphatid	PI42C_HUM		7	1.215244
30min_MT.	Q8WXB1	0.032005	0.03791	0.098992	METTL21A	Protein N-l	MT21A_HU		4	1.022432
15min_MT.	O00391	-0.55866	0.037992	0.099015	QSOX1	Sulfhydryl c	QSOX1_HU		2	0.678933
15min_MT.	P62330	-0.30127	0.038039	0.099015	ARF6	ADP-ribosy	ARF6_HUM		2	0.81154
15min_MT.	Q15427	0.33354	0.038008	0.099015	SF3B4	Splicing fac	SF3B4_HUM		3	1.260102

30min_MT.	Q9BUL8	0.145314	0.038033	0.099015	PDCD10	Programme	PDC10_HU	7	1.105971
15min_MT.	Q9Y608	0.206946	0.037995	0.099015	LRRFIP2	Leucine-ric	LRRF2_HUI	10	1.154242
15min_MT.	P30046	0.196166	0.038077	0.099043	DDT	D-dopachro	DOPD_HUI	9	1.14565
30min_MT.	Q5T8I3	-0.43216	0.038088	0.099043	EEIG2	EEIG family	EEIG2_HUI	3	0.741151
15min_MT.	Q9GZL7	-0.14005	0.03808	0.099043	WDR12	Ribosome b	WDR12_HU	15	0.907487
30min_MT.	Q15003	-0.17659	0.03811	0.099069	NCAPH	Condensin	CND2_HUI	4	0.884794
15min_MT.	O43592	0.201548	0.03816	0.099148	XPOT	Exportin-T	XPOT_HUI	17	1.149932
30min_MT.	O60234	-0.00533	0.038166	0.099148	GMFG	Glia matura	GMFG_HUI	2	0.99631
15min_MT.	Q15041	-0.03353	0.038217	0.099182	ARL6IP1	ADP-ribosy	AR6P1_HU	1	0.977026
30min_MT.	Q8NBJ5	-0.11194	0.03821	0.099182	COLGALT1	Procollager	GT251_HU	13	0.925345
15min_MT.	Q9BUQ8	-0.04583	0.038196	0.099182	DDX23	Probable A	DDX23_HU	16	0.968731
15min_MT.	P19367	0.139577	0.038243	0.099217	HK1	Hexokinase	HXK1_HUI	21	1.101582
15min_MT.	P07954	0.253452	0.038314	0.099334	FH	Fumarate h	FUMH_HUI	13	1.192056
30min_MT.	P52597	-0.1489	0.038354	0.099371	HNRNPF	Heterogene	HNRPF_HU	13	0.901936
30min_MT.	Q9P013	0.148559	0.038443	0.099536	CWC15	Spliceosom	CWC15_HU	7	1.108462
15min_MT.	P30520	0.04987	0.038498	0.099609	ADSS2	Adenylosuc	PURA2_HU	17	1.035172
15min_MT.	Q9Y5B0	0.174786	0.038509	0.099609	CTDP1	RNA polym	CTDP1_HU	13	1.128797
30min_MT.	Q6Y7W6	-0.22758	0.038574	0.099711	GIGYF2	GRB10-inte	GGYF2_HU	14	0.854068
15min_MT.	Q8WTW3	-0.26736	0.038571	0.099711	COG1	Conserved	COG1_HUI	7	0.830836
15min_MT.	P02794	0.280395	0.038632	0.09973	FTH1	Ferritin hea	FRIH_HUM.	8	1.214527
30min_MT.	P69849;Q1	0.149101	0.038622	0.09973	NOMO3;NC	BOS compl	NOMO3_HU	7	1.108879
30min_MT.	Q16181	-0.12765	0.03862	0.09973	SEPTIN7	Septin-7	SEPT7_HUI	12	0.915323
30min_MT.	P49862	1.729398	0.038701	0.09984	KLK7	Kallikrein-7	KLK7_HUM	1	3.315894
30min_MT.	P61026	-0.29526	0.038692	0.09984	RAB10	Ras-related	RAB10_HU	6	0.814926
30min_MT.	Q9BTL3	0.358151	0.038797	0.09992	RAMAC	RNA guanir	RAMAC_HU	5	1.281782
15min_MT.	Q8IX07	-0.89905	0.038902	0.10003	ZFPM1	Zinc finger	FOG1_HUM	1	0.53624
15min_MT.	P0DMV1;P	-0.3183	0.039	0.1001	CT45A8;CT	Cancer/test	CT458_HU	7	0.802012
15min_MT.	P28838	0.070657	0.03906	0.100173	LAP3	Cytosol am	AMPL_HUI	18	1.050195
15min_MT.	Q15828	-0.22864	0.039052	0.100173	CST6	Cystatin-M	CYTM_HUI	2	0.853441
15min_MT.	Q63HM1	0.20846	0.039096	0.100232	AFMID	Kynurenine	KFA_HUMA	3	1.155454
15min_MT.	Q9BW71	-0.14108	0.039165	0.100376	HIRIP3	HIRA-intera	HIRP3_HUI	5	0.906839
30min_MT.	Q9UQE7	-0.03898	0.039241	0.100504	SMC3	Structural	SMC3_HUI	48	0.973343
15min_MT.	O00487	0.101827	0.039287	0.100524	PSMD14	26S protea	PSDE_HUM	7	1.073131
15min_MT.	Q6DKJ4	0.129374	0.039264	0.100524	NXN	Nucleoredo	NXN_HUM	12	1.093819
15min_MT.	Q70IA6	0.015503	0.03944	0.100849	MOB2	MOB kinase	MOB2_HUI	1	1.010804
15min_MT.	Q9P1Y5	0.187251	0.039462	0.100873	CAMSAP3	Calmodulir	CAMP3_HU	8	1.138592
30min_MT.	Q86YZ3	0.810094	0.039497	0.100897	HRNR	Hornerin	HORN_HUI	28	1.753326
30min_MT.	Q6SZW1	-0.30079	0.039558	0.101019	SARM1	NAD(+) hyd	SARM1_HU	4	0.811806
15min_MT.	O95197	-0.22119	0.039609	0.101087	RTN3	Reticulon-3	RTN3_HUI	4	0.857859
30min_MT.	P49366	0.172625	0.039623	0.101087	DHPS	Deoxyhypu	DHYS_HUI	10	1.127108
15min_MT.	Q06124	-0.00249	0.039689	0.101172	PTPN11	Tyrosine-pr	PTN11_HU	17	0.998275
15min_MT.	Q86WB0	-0.15037	0.039695	0.101172	ZC3HC1	Zinc finger	ZC3C1_HU	11	0.901019
30min_MT.	Q86X83	0.19776	0.039671	0.101172	COMMD2	COMM don	COMD2_HU	1	1.146916
15min_MT.	Q6GMV2	-0.18391	0.039785	0.101336	SMYD5	Histone-lys	SMYD5_HU	8	0.880316
15min_MT.	Q9UJA5	-0.06829	0.039776	0.101336	TRMT6	tRNA (aden	TRM6_HUI	15	0.953766
15min_MT.	P26599	0.061617	0.039917	0.101341	PTBP1	Polypyrimi	PTBP1_HU	9	1.043634
30min_MT.	P28838	-0.03567	0.039884	0.101341	LAP3	Cytosol am	AMPL_HUI	18	0.975577
15min_MT.	P49770	-0.11411	0.039873	0.101341	EIF2B2	Translation	EI2BB_HUI	10	0.923955
15min_MT.	Q7Z3T8	0.001912	0.039855	0.101341	ZFYVE16	Zinc finger	ZFY16_HUI	24	1.001326
15min_MT.	Q8IWV8	0.217405	0.039867	0.101341	UBR2	E3 ubiquiti	UBR2_HUI	5	1.16264
30min_MT.	Q969G3	-0.06809	0.039914	0.101341	SMARCE1	SWI/SNF-re	SMCE1_HU	7	0.953901
15min_MT.	Q9Y600	0.192345	0.039836	0.101341	CSAD	Cysteine su	CSAD_HUI	1	1.14262

15min_MT.	Q9BWJ5	0.229227	0.040094	0.101695	SF3B5	Splicing fac	SF3B5_HUM	2	1.172207
15min_MT.	Q9NQ84	0.249264	0.040095	0.101695	GPRC5C	G-protein c	GPC5C_HU	4	1.1886
15min_MT.	Q92974	-0.31646	0.040114	0.10171	ARHGEF2	Rho guanin	ARHG2_HU	4	0.803038
15min_MT.	Q13492	0.08144	0.040198	0.101857	PICALM	Phosphatid	PICAL_HUM	14	1.058073
15min_MT.	Q9UIA9	-0.10179	0.040241	0.10193	XPO7	Exportin-7	XPO7_HUM	30	0.931875
30min_MT.	P78356	-0.00444	0.040273	0.101961	PIP4K2B	Phosphatid	PI42B_HUM	3	0.996925
15min_MT.	P11172	0.158359	0.04033	0.101965	UMPS	Uridine 5'-r	UMPS_HUM	8	1.116017
30min_MT.	Q96DR5	-0.40166	0.040332	0.101965	BPIFA2	BPI fold-co	BPIA2_HUM	3	0.756986
30min_MT.	O75934	-0.19004	0.040382	0.102058	BCAS2	Pre-mRNA-	SPF27_HUM	6	0.876582
15min_MT.	O00231	0.136881	0.040452	0.102155	PSMD11	26S protea	PSD11_HUI	22	1.099525
30min_MT.	P17980	-0.07984	0.040473	0.102155	PSMC3	26S protea	PRS6A_HUI	21	0.946165
30min_MT.	Q8IX03	0.034529	0.040446	0.102155	WWC1	Protein KIB	KIBRA_HUM	4	1.024222
15min_MT.	Q9H857	-0.05937	0.040465	0.102155	NT5DC2	5'-nucleoti	NT5D2_HU	16	0.959681
15min_MT.	Q14697	-0.19699	0.040683	0.10249	GANAB	Neutral alp	GANAB_HU	36	0.872367
15min_MT.	Q5JTH9	0.13723	0.040697	0.10249	RRP12	RRP12-like	RRP12_HUI	5	1.099791
30min_MT.	Q8IYB7	0.062815	0.040658	0.10249	DIS3L2	DIS3-like ex	DI3L2_HUM	23	1.044502
30min_MT.	P41227	-0.29081	0.040769	0.102604	NAA10	N-alpha-ace	NAA10_HU	8	0.817442
30min_MT.	Q86VM9	-0.08101	0.040784	0.10261	ZC3H18	Zinc finger	ZCH18_HUI	13	0.945394
15min_MT.	Q9BU02	0.247735	0.040849	0.10274	THTPA	Thiamine-tı	THTPA_HUI	4	1.187341
30min_MT.	Q9Y6D5	-0.14332	0.0409	0.102836	ARFGEF2	Brefeldin A	BIG2_HUM	25	0.905435
30min_MT.	Q9UBR2	0.146882	0.040971	0.102948	CTSZ	Cathepsin Z	CATZ_HUM	7	1.107174
30min_MT.	P42126	0.106932	0.040986	0.102953	ECI1	Enoyl-CoA d	ECI1_HUMA	8	1.076936
30min_MT.	A8MXV4	-0.12359	0.041019	0.102959	NUDT19	Acyl-coenzy	NUD19_HU	11	0.917899
30min_MT.	P34059	0.098388	0.04104	0.102959	GALNS	N-acetylgal	GALNS_HUI	8	1.070576
30min_MT.	P53609	0.17648	0.041085	0.102986	PGGT1B	Geranylger	PGTB1_HUI	9	1.130123
15min_MT.	Q12797	0.404353	0.041087	0.102986	ASPH	Aspartyl/as	ASPH_HUM	3	1.323496
15min_MT.	Q7Z4H8	0.172411	0.041092	0.102986	POGLUT3	Protein O-g	PLGT3_HUI	9	1.12694
30min_MT.	Q13868	0.513618	0.041138	0.10307	EXOSC2	Exosome cc	EXOS2_HUI	6	1.427626
30min_MT.	P17931	0.287845	0.041255	0.103278	LGALS3	Galectin-3	LEG3_HUM	9	1.220816
30min_MT.	P62888	-0.20079	0.041301	0.103278	RPL30	Large ribos	RL30_HUM	5	0.870075
30min_MT.	Q14320	-0.06296	0.041287	0.103278	FAM50A	Protein FAI	FA50A_HUI	11	0.9573
30min_MT.	Q15751	0.138399	0.041295	0.103278	HERC1	Probable E	HERC1_HUI	5	1.100683
30min_MT.	Q5SW79	0.034882	0.0414	0.103427	CEP170	Centrosom	CE170_HUI	7	1.024473
15min_MT.	A0A024R1I	-0.3886	0.041489	0.103549	TMA7B;TM.	Translation	TMA7B_HU	1	0.763873
30min_MT.	Q9UK45	0.162023	0.041511	0.103573	LSM7	U6 snRNA-a	LSM7_HUM	3	1.118855
30min_MT.	Q04837	0.164734	0.04159	0.103736	SSBP1	Single-strar	SSBP_HUM	6	1.120959
15min_MT.	Q4J6C6	-0.24449	0.041656	0.103801	PREPL	Prolyl endc	PPCEL_HUM	4	0.844114
30min_MT.	P22629	-0.23405	0.041738	0.103939	NaN	Streptavidi	SAV_STRAV	2	0.850247
30min_MT.	P37802	0.268009	0.041765	0.103973	TAGLN2	Transgelin-	TAGL2_HUI	14	1.204145
30min_MT.	P27708	-0.26921	0.041785	0.103981	CAD	Multifuncti	PYR1_HUM	23	0.829776
30min_MT.	P11021	0.186468	0.041813	0.103993	HSPA5	Endoplasm	BIP_HUMA	36	1.137974
15min_MT.	P28715	-0.24806	0.041933	0.104018	ERCC5	DNA excisic	ERCC5_HUI	6	0.842025
15min_MT.	P41208;Q1	-0.42209	0.041865	0.104018	CETN2;CETI	Centrin-2;C	CETN2_HUI	1	0.746344
30min_MT.	P51148	0.220336	0.041931	0.104018	RAB5C	Ras-related	RAB5C_HUI	4	1.165005
15min_MT.	Q06033	0.780791	0.041942	0.104018	ITIH3	Inter-alpha	ITIH3_HUM	7	1.718073
30min_MT.	Q5TDH0	-0.25324	0.041897	0.104018	DDI2	Protein DD	DDI2_HUM	16	0.839011
30min_MT.	Q8IYT4	0.789894	0.041851	0.104018	KATNAL2	Katanin p6	KATL2_HUM	1	1.728948
30min_MT.	Q8TAG9	-0.19147	0.041923	0.104018	EXOC6	Exocyst cor	EXOC6_HUI	1	0.875712
15min_MT.	Q9BV40	0.179543	0.041839	0.104018	VAMP8	Vesicle-assc	VAMP8_HU	3	1.132525
15min_MT.	Q9Y2R5	-0.83	0.041942	0.104018	MRPS17	Small ribos	RT17_HUM	1	0.56253
30min_MT.	O43237	-0.08365	0.042	0.104062	DYNC1LI2	Cytoplasmi	DC1L2_HUI	12	0.943665
30min_MT.	P20674	0.234915	0.041997	0.104062	COX5A	Cytochrom	COX5A_HU	3	1.176837

30min_MT.	P25691	3.987782	0.041974	0.104062	NaN	Keratin, typ	K2M3_SHEI	10	15.86507
15min_MT.	P16930	0.139307	0.042031	0.104107	FAH	Fumarylace	FAAA_HUM	12	1.101376
15min_MT.	O60361	-0.34765	0.042291	0.104318	NME2P1	Putative nu	NDK8_HUM	1	0.785861
30min_MT.	P02750	0.438324	0.042192	0.104318	LRG1	Leucine-ric	A2GL_HUM	1	1.35503
15min_MT.	P06703	1.113422	0.042341	0.104318	S100A6	Protein S1(	S10A6_HUI	4	2.163582
15min_MT.	P13637	0.64381	0.042268	0.104318	ATP1A3	Sodium/po	AT1A3_HUI	1	1.562449
15min_MT.	Q12962	-0.55504	0.042333	0.104318	TAF10	Transcripti	TAF10_HUI	2	0.680638
15min_MT.	Q6P1N9	0.22939	0.042329	0.104318	TATDN1	Deoxyribor	TATD1_HUI	12	1.172339
30min_MT.	Q8N6T3	-0.19583	0.042303	0.104318	ARFGAP1	ADP-ribosy	ARFG1_HUI	9	0.87307
15min_MT.	Q96N67	-0.23088	0.042263	0.104318	DOCK7	Dedicator c	DOCK7_HU	2	0.852115
30min_MT.	Q96RR4	0.920382	0.042225	0.104318	CAMKK2	Calcium/ca	KKCC2_HUI	1	1.892616
15min_MT.	Q9H3P2	-0.17689	0.042184	0.104318	NELFA	Negative el	NELFA_HUI	4	0.884608
30min_MT.	Q9NWH9	0.059243	0.042357	0.104318	SLTM	SAFB-like tr	SLTM_HUM	6	1.041919
30min_MT.	Q9UL15	0.12953	0.042356	0.104318	BAG5	BAG family	BAG5_HUM	8	1.093937
15min_MT.	P40429;Q6	0.066455	0.042461	0.104398	RPL13A;RP	Large ribos	RL13A_HUI	3	1.04714
15min_MT.	Q9NQ48	0.343128	0.042471	0.104398	LZTFL1	Leucine zip	LZTL1_HUM	7	1.268504
15min_MT.	Q9NTG7	-0.49763	0.042537	0.104497	SIRT3	NAD-depen	SIR3_HUMA	2	0.708268
30min_MT.	P04040	0.143942	0.042676	0.104535	CAT	Catalase	CATA_HUM	23	1.10492
30min_MT.	P09651	-0.13515	0.04269	0.104535	HNRNPA1	Heterogene	ROA1_HUM	19	0.910574
30min_MT.	P36955	0.307684	0.042689	0.104535	SERPINF1	Pigment ep	PEDF_HUM	7	1.237719
30min_MT.	Q15517	0.607216	0.042621	0.104535	CDSN	Corneodesı	CDSN_HUM	3	1.523317
30min_MT.	Q16656	0.456109	0.042694	0.104535	NRF1	Nuclear res	NRF1_HUM	4	1.371837
15min_MT.	Q8N1G2	-0.76777	0.042583	0.104535	CMTR1	Cap-specifi	CMTR1_HU	2	0.587326
15min_MT.	Q9H7L9	0.237526	0.042633	0.104535	SUDS3	Sin3 histon	SDS3_HUM	3	1.178969
15min_MT.	Q15653	0.414763	0.042744	0.104581	NFKBIB	NF-kappa-B	IKBB_HUMA	3	1.33308
15min_MT.	P15121	-0.12716	0.042789	0.10465	AKR1B1	Aldo-keto r	ALDR_HUM	15	0.915633
15min_MT.	Q8WVV4	0.440336	0.042799	0.10465	POF1B	Protein POI	POF1B_HUI	11	1.35692
30min_MT.	Q92820	0.174953	0.042841	0.104719	GGH	Gamma-glu	GGH_HUMA	10	1.128928
30min_MT.	Q15386	-0.50068	0.042862	0.104739	UBE3C	Ubiquitin-p	UBE3C_HUI	4	0.706773
15min_MT.	Q5TH69	-0.2261	0.042905	0.104811	ARFGEF3	Brefeldin A	BIG3_HUM	18	0.854946
15min_MT.	P15586	0.134206	0.042951	0.104889	GNS	N-acetylglu	GNS_HUMA	11	1.097489
30min_MT.	P36969	0.138637	0.043	0.104974	GPX4	Phospholip	GPX4_HUM	8	1.100865
30min_MT.	Q14232	0.174696	0.043012	0.104974	EIF2B1	Translation	EI2BA_HUM	17	1.128727
15min_MT.	A5YM69;Q:	0.516039	0.043068	0.105007	ARHGEF35;	Rho guanin	ARG35_HU	3	1.430023
15min_MT.	O94830	-0.01551	0.043058	0.105007	DDHD2	Phospholip	DDHD2_HU	9	0.989308
15min_MT.	P52298	0.319208	0.043081	0.105007	NCBP2	Nuclear cap	NCBP2_HU	2	1.247646
30min_MT.	Q15185	0.201217	0.043155	0.105126	PTGES3	Prostagland	TEBP_HUM	7	1.149668
15min_MT.	Q8TC12	-0.10694	0.043174	0.105138	RDH11	Retinol deh	RDH11_HU	3	0.928553
15min_MT.	Q96HP4	0.158381	0.043216	0.105159	OXNAD1	Oxidoreduc	OXND1_HU	1	1.116034
30min_MT.	Q13526	-0.43575	0.043276	0.105191	PIN1	Peptidyl-pr	PIN1_HUM	5	0.73931
30min_MT.	Q53SF7	-0.11689	0.043276	0.105191	COBLL1	Cordon-ble	COBL1_HUI	1	0.922176
30min_MT.	P35237	0.151942	0.043346	0.105262	SERPINB6	Serpin B6	SPB6_HUM	16	1.111064
15min_MT.	P62913	0.084275	0.043323	0.105262	RPL11	Large ribos	RL11_HUM	3	1.060155
15min_MT.	Q9NWT6	-0.36746	0.04334	0.105262	HIF1AN	Hypoxia-in	HIF1N_HUI	5	0.775144
15min_MT.	Q14692	0.582715	0.043393	0.105344	BMS1	Ribosome b	BMS1_HUM	2	1.497665
30min_MT.	A6NCE7;Q9	0.235113	0.043439	0.105394	MAP1LC3B	Microtubul	MP3B2_HU	1	1.176999
30min_MT.	Q8IZ21	-0.01568	0.04344	0.105394	PHACTR4	Phosphatas	PHAR4_HU	3	0.989189
15min_MT.	O94919	-0.27014	0.043589	0.105428	ENDOD1	Endonuclea	ENDD1_HU	3	0.829241
15min_MT.	P10163	1.677068	0.043643	0.105428	PRB4	Basic saliva	PRB4_HUM	2	3.197774
15min_MT.	P32969	-0.23949	0.043707	0.105428	RPL9	Large ribos	RL9_HUMA	3	0.847045
30min_MT.	P56545	0.133231	0.043682	0.105428	CTBP2	C-terminal-	CTBP2_HUI	4	1.096747
15min_MT.	Q08209	0.113588	0.043704	0.105428	PPP3CA	Protein ph	PP2BA_HUI	7	1.081916

30min_MT.	Q8IYX8	-0.58095	0.043518	0.105428	CEP57L1	Centrosom	CE57L_HUI	1	0.668525
15min_MT.	Q8N612	-0.24336	0.043653	0.105428	FHIP1B	FHF comple	FHI1B_HUI	2	0.844776
15min_MT.	Q96SU4	0.102402	0.043644	0.105428	OSBPL9	Oxysterol-b	OSBL9_HUI	6	1.073559
15min_MT.	Q9BUE0	0.115973	0.043595	0.105428	MED18	Mediator o	MED18_HU	2	1.083706
15min_MT.	O43493	0.388066	0.043855	0.105668	TGOLN2	Trans-Golgi	TGON2_HU	3	1.308638
15min_MT.	Q6ZUJ8	-0.14982	0.043875	0.105668	PIK3AP1	Phosphoino	BCAP_HUM	6	0.901362
30min_MT.	Q9BW83	0.064448	0.04385	0.105668	IFT27	Intraflagell	IFT27_HUM	5	1.045685
15min_MT.	Q9Y5Z4	0.226842	0.044	0.105897	HEBP2	Heme-bind	HEBP2_HUI	8	1.17027
30min_MT.	A0FGR8	-0.20723	0.044067	0.105914	ESYT2	Extended sy	ESYT2_HUM	10	0.8662
15min_MT.	Q13155	0.049856	0.044094	0.105914	AIMP2	Aminoacyl	AIMP2_HUI	4	1.035161
30min_MT.	Q15582	0.217419	0.044086	0.105914	TGFBI	Transformi	BGH3_HUM	11	1.162652
30min_MT.	Q53FA7	0.190558	0.044092	0.105914	TP53I3	Quinone ox	QORX_HUM	16	1.141205
15min_MT.	Q92572	0.184179	0.044049	0.105914	AP3S1	AP-3 compl	AP3S1_HUI	5	1.136171
15min_MT.	P02748	0.584091	0.044174	0.106024	C9	Complemer	CO9_HUMA	4	1.499094
15min_MT.	P02671	-0.07991	0.044233	0.10612	FGA	Fibrinogen	FIBA_HUMA	20	0.946117
30min_MT.	P62899	0.2677	0.044241	0.10612	RPL31	Large ribos	RL31_HUM	5	1.203887
15min_MT.	P62875	0.175537	0.044323	0.106283	POLR2L	DNA-direct	RPAB5_HUI	1	1.129385
30min_MT.	P67775	0.402969	0.044367	0.106323	PPP2CA	Serine/thre	PP2AA_HUI	2	1.322226
15min_MT.	Q96DA0	3.411391	0.044358	0.106323	ZG16B	Pancreatic	PAUF_HUM	6	10.63974
30min_MT.	P0DPB6	0.133606	0.044448	0.106468	POLR1D	DNA-direct	RPAC2_HUI	2	1.097032
30min_MT.	Q96R05	0.002802	0.044455	0.106468	RBP7	Retinoid-bi	RET7_HUM	3	1.001944
30min_MT.	P50213	-0.0698	0.044517	0.106519	IDH3A	Isocitrate d	IDH3A_HUI	11	0.952767
15min_MT.	P22061	0.200421	0.044655	0.106668	PCMT1	Protein-L-is	PIMT_HUM	10	1.149034
15min_MT.	P28676	0.261181	0.044661	0.106668	GCA	Grancalcin	GRAN_HUM	4	1.198459
30min_MT.	Q9P0P8	0.415306	0.044644	0.106668	MTRES1	Mitochond	MRES1_HU	1	1.333581
15min_MT.	Q9UKF6	-0.15234	0.044656	0.106668	CPSF3	Cleavage ar	CPSF3_HUI	20	0.899788
15min_MT.	Q8IXQ4	0.170266	0.044776	0.106813	GPALPP1	GPALPP mc	GPAM1_HU	4	1.125266
15min_MT.	Q8N9N5	0.572401	0.044867	0.106931	BANP	Protein BAI	BANP_HUM	1	1.486996
30min_MT.	Q9BUH6	-0.24265	0.044841	0.106931	PAXX	Protein PA)	PAXX_HUM	8	0.845191
30min_MT.	Q6FI81	0.162	0.044953	0.107077	CIAPIN1	Anamorsin	CPIN1_HUI	18	1.118837
30min_MT.	P55084	-0.10249	0.045047	0.10717	HADHB	Trifunction	ECHB_HUM	17	0.931422
30min_MT.	Q96EP5	0.123903	0.045049	0.10717	DAZAP1	DAZ-associa	DAZP1_HUI	7	1.089679
30min_MT.	Q13131	0.193441	0.045103	0.107266	PRKAA1	5'-AMP-acti	AAPK1_HUI	10	1.143488
15min_MT.	P35611	0.136356	0.0452	0.107372	ADD1	Alpha-addu	ADDA_HUM	17	1.099126
30min_MT.	P48059	-0.352	0.045186	0.107372	LIMS1	LIM and ser	LIMS1_HUI	8	0.783498
15min_MT.	Q9H307	-0.17583	0.045191	0.107372	PNN	Pinin	PININ_HUM	9	0.885261
30min_MT.	Q9UP83	-0.19192	0.045216	0.107372	COG5	Conserved (	COG5_HUM	8	0.875439
30min_MT.	Q96EY7	-0.05659	0.045267	0.107394	PTCD3	Small ribos	PTCD3_HUI	2	0.961537
15min_MT.	Q9ULC4	0.035902	0.045377	0.107624	MCTS1	Malignant 1	MCTS1_HU	7	1.025197
15min_MT.	O15258	-0.28782	0.045487	0.107715	RER1	Protein REI	RER1_HUM	3	0.819137
15min_MT.	P37837	0.120877	0.045488	0.107715	TALDO1	Transaldola	TALDO_HUI	19	1.087396
15min_MT.	Q13144	0.16366	0.045448	0.107715	EIF2B5	Translation	EI2BE_HUM	11	1.120125
30min_MT.	Q96B23	0.407438	0.045458	0.107715	ARK2N	Protein ARI	ARK2N_HU	6	1.326328
15min_MT.	Q96G28	0.31257	0.045513	0.107715	CFAP36	Cilia- and fl	CFA36_HUI	5	1.241918
30min_MT.	Q9HAU5	-1.44579	0.045489	0.107715	UPF2	Regulator c	RENT2_HUI	2	0.367091
15min_MT.	O75506	0.590245	0.045606	0.107839	HSBP1	Heat shock	HSBP1_HUI	2	1.505503
15min_MT.	O75663	-0.09664	0.045716	0.107866	TIPRL	TIP41-like p	TIPRL_HUM	9	0.93521
15min_MT.	P63272	0.352589	0.045737	0.107866	SUPT4H1	Transcripti	SPT4H_HUI	5	1.27685
15min_MT.	Q15819	0.253575	0.045742	0.107866	UBE2V2	Ubiquitin-c	UB2V2_HU	5	1.192158
30min_MT.	Q8WYA6	-0.17245	0.045714	0.107866	CTNNBL1	Beta-cateni	CTBL1_HUI	10	0.887335
15min_MT.	Q9BRV8	0.424959	0.045648	0.107866	SIKE1	Suppressor	SIKE1_HUM	3	1.342534
15min_MT.	Q9BVC6	-0.0389	0.045728	0.107866	TMEM109	Voltage-gat	TM109_HU	2	0.973396

30min_MT.	Q9H0C8	-0.27772	0.045709	0.107866	ILKAP	Integrin-lin	ILKAP_HUM	11	0.824893
30min_MT.	Q9NZ08	0.157916	0.045635	0.107866	ERAP1	Endoplasm	ERAP1_HUI	19	1.115675
15min_MT.	Q9Y259	0.054046	0.045717	0.107866	CHKB	Choline/etl	CHKB_HUM	8	1.038172
15min_MT.	Q16656	0.617494	0.045766	0.10789	NRF1	Nuclear res	NRF1_HUM	4	1.534208
30min_MT.	P41214	-0.26886	0.045895	0.108086	EIF2D	Eukaryotic	EIF2D_HUM	1	0.829977
30min_MT.	P43246	-0.20909	0.045871	0.108086	MSH2	DNA misma	MSH2_HUM	18	0.86508
15min_MT.	P49459	0.816237	0.045904	0.108086	UBE2A	Ubiquitin-c	UBE2A_HUI	1	1.760808
15min_MT.	Q12792	0.069836	0.045879	0.108086	TWF1	Twinfilin-1	TWF1_HUM	14	1.049597
15min_MT.	Q9BZM5	0.106072	0.045925	0.108103	ULBP2	UL16-bindi	ULBP2_HUI	5	1.076294
15min_MT.	Q13371	0.645758	0.046	0.108246	PDCL	Phosducin-	PHLP_HUM	4	1.564561
30min_MT.	Q2M2D7	0.098904	0.046037	0.1083	TBC1D28	TBC1 doma	TBC28_HUI	1	1.07096
30min_MT.	O00442	0.148162	0.0461	0.108416	RTCA	RNA 3'-tern	RTCA_HUM	8	1.108157
30min_MT.	P0DTE7;P0	-1.53137	0.046239	0.108539	AMY1B;AM	Alpha-amyl	AMY1B_HU	15	0.345948
30min_MT.	P26358	0.096635	0.046277	0.108539	DNMT1	DNA (cytosi	DNMT1_HU	24	1.069276
30min_MT.	P49902	0.088112	0.046276	0.108539	NT5C2	Cytosolic p	5NTC_HUM	15	1.062978
15min_MT.	Q9NRY2	-0.03274	0.046261	0.108539	INIP	SOSS comp	SOSSC_HUI	3	0.977562
30min_MT.	Q96NT1	0.657777	0.046303	0.108567	NAP1L5	Nucleosom	NP1L5_HUI	1	1.57765
30min_MT.	P04114	0.050432	0.046323	0.108581	APOB	Apolipoprc	APOB_HUM	97	1.035575
15min_MT.	Q5VT25	-0.51745	0.046418	0.108772	CDC42BPA	Serine/thre	MRCKA_HU	2	0.698605
30min_MT.	P13995	0.197549	0.046488	0.108773	MTHFD2	Bifunctiona	MTDC_HUM	5	1.146749
15min_MT.	Q12765	0.178325	0.046466	0.108773	SCRN1	Secernin-1	SCRN1_HUI	14	1.13157
15min_MT.	Q96I15	-0.20264	0.046466	0.108773	SCLY	Selenocyste	SCLY_HUM.	13	0.868959
15min_MT.	P06396	-0.01648	0.046712	0.1092	GSN	Gelsolin	GELS_HUM	22	0.988643
15min_MT.	P61923	0.187833	0.046739	0.1092	COPZ1	Coatomer s	COPZ1_HUI	5	1.139051
30min_MT.	Q15654	-0.63125	0.046754	0.1092	TRIP6	Thyroid rec	TRIP6_HUM	3	0.645617
30min_MT.	Q86TU7	-0.13223	0.046743	0.1092	SETD3	Actin-histic	SETD3_HUM	7	0.912422
15min_MT.	O00425	-0.0911	0.046837	0.109262	IGF2BP3	Insulin-like	IF2B3_HUM	8	0.938808
30min_MT.	P55884	-0.06021	0.046878	0.109262	EIF3B	Eukaryotic	EIF3B_HUM	30	0.959122
15min_MT.	Q15651	0.389103	0.046879	0.109262	HMGN3	High mobil	HMGN3_HU	1	1.309579
15min_MT.	P43243	-0.13375	0.046903	0.109286	MATR3	Matrin-3	MATR3_HU	14	0.911461
15min_MT.	P11171	0.128006	0.046972	0.109402	EPB41	Protein 4.1	EPB41_HUI	7	1.092783
15min_MT.	Q9UK59	-0.12779	0.046981	0.109402	DBR1	Lariat debr	DBR1_HUM	10	0.915235
15min_MT.	O15511	0.360145	0.047003	0.109422	ARPC5	Actin-relate	ARPC5_HUI	5	1.283555
15min_MT.	O95273	0.299786	0.047051	0.109447	CCNDBP1	Cyclin-D1-k	CCDB1_HU	5	1.230961
30min_MT.	P0C0S5;Q7	-0.31819	0.047273	0.109854	H2AZ1;H2A	Histone H2	H2AZ_HUM	1	0.802075
15min_MT.	Q06481	0.25563	0.047288	0.109856	APLP2	Amyloid be	APLP2_HUI	8	1.193857
15min_MT.	P16083	0.245566	0.047307	0.109867	NQO2	Ribosyldihy	NQO2_HUM	10	1.185558
15min_MT.	Q96QR8	0.042057	0.047375	0.109896	PURB	Transcripti	PURB_HUM	8	1.029581
15min_MT.	Q9Y6Q9	0.045787	0.047363	0.109896	NCOA3	Nuclear rec	NCOA3_HU	6	1.032246
15min_MT.	Q9H8H0	-0.16823	0.047511	0.11017	NOL11	Nucleolar p	NOL11_HU	10	0.889934
30min_MT.	Q9P2N5	-0.04674	0.047522	0.11017	RBM27	RNA-bindir	RBM27_HU	7	0.968123
30min_MT.	P02662	1.112043	0.047599	0.110307	CSN1S1	Alpha-S1-ca	CASA1_BOV	3	2.161515
30min_MT.	Q9H8M7	-0.03886	0.047609	0.110307	MINDY3	Ubiquitin c	MINY3_HUI	5	0.973427
30min_MT.	Q9NQR4	-0.08025	0.047746	0.110559	NIT2	Omega-ami	NIT2_HUM.	14	0.945897
30min_MT.	Q15651	0.410794	0.047774	0.110592	HMGN3	High mobil	HMGN3_HU	1	1.329417
15min_MT.	Q6P9B6	0.35512	0.047804	0.110628	MEAK7	MTOR-asso	MEAK7_HU	8	1.279092
15min_MT.	P04626	-0.64065	0.047906	0.110746	ERBB2	Receptor ty	ERBB2_HUI	1	0.641425
30min_MT.	P31946	0.082707	0.047981	0.110808	YWHAB	14-3-3 prot	1433B_HUI	13	1.059003
15min_MT.	P49368	0.131027	0.047977	0.110808	CCT3	T-complex	TCPG_HUM	36	1.095073
30min_MT.	Q9NSK0	-0.08377	0.047996	0.11081	KLC4	Kinesin ligh	KLC4_HUM	18	0.943589
15min_MT.	Q0PNE2	0.060301	0.048056	0.110916	ELP6	Elongator c	ELP6_HUM	10	1.042683
30min_MT.	P36871	0.095649	0.04808	0.110939	PGM1	Phosphoglu	PGM1_HUM	25	1.068546

30min_MT.	O75436	0.07386	0.0481	0.110952	VPS26A	Vacuolar pr	VP26A_HUI	17	1.052529
30min_MT.	Q8NEZ5	0.165379	0.048115	0.110954	FBXO22	F-box only	FBX22_HUI	10	1.121461
15min_MT.	Q8WXA9	-0.50261	0.048197	0.11111	SREK1	Splicing reg	SREK1_HUI	5	0.70583
30min_MT.	P02786	0.111061	0.048292	0.111243	TFRC	Transferrin	TFR1_HUM	26	1.080022
30min_MT.	P62841	0.065845	0.048271	0.111243	RPS15	Small ribos	RS15_HUM	1	1.046698
15min_MT.	P63261	-1.5443	0.048297	0.111243	ACTG1	Actin, cyto	ACTG_HUM	1	0.342863
15min_MT.	Q99873	0.11512	0.048447	0.111456	PRMT1	Protein arg	ANM1_HUI	13	1.083065
30min_MT.	Q9BUT1	0.158439	0.048561	0.111686	BDH2	Dehydroge	DHRS6_HU	4	1.116079
30min_MT.	P48723	0.210334	0.048678	0.111855	HSPA13	Heat shock	HSP13_HUI	9	1.156956
30min_MT.	P62942	0.234739	0.048726	0.111933	FKBP1A	Peptidyl-pr	FKB1A_HUI	7	1.176694
30min_MT.	P82664	-0.06153	0.048744	0.111943	MRPS10	Small ribos	RT10_HUM	3	0.95825
30min_MT.	P08727	0.693372	0.048778	0.111987	KRT19	Keratin, typ	K1C19_HUI	4	1.617059
30min_MT.	Q7LBC6	-0.10554	0.048821	0.112052	KDM3B	Lysine-spec	KDM3B_HU	7	0.92946
30min_MT.	Q96EK6	0.124874	0.048929	0.112235	GNPNAT1	Glucosamir	GNA1_HUM	10	1.090413
30min_MT.	Q9Y3D6	-0.21683	0.049029	0.112431	FIS1	Mitochond	FIS1_HUMA	2	0.860457
15min_MT.	P52655	0.580604	0.049059	0.112436	GTF2A1	Transcripti	TF2AA_HUI	2	1.495475
30min_MT.	P04183	-0.18241	0.049166	0.112479	TK1	Thymidine	KITH_HUMA	1	0.881227
15min_MT.	Q587I9	-0.60929	0.049178	0.112479	SFT2D3	Vesicle trar	SFT2C_HUI	1	0.65552
30min_MT.	Q8WTS6	-0.03624	0.049217	0.112479	SETD7	Histone-lys	SETD7_HUI	5	0.975193
30min_MT.	Q96EK5	-0.24584	0.049138	0.112479	KIFBP	KIF-binding	KBP_HUMA	11	0.843323
15min_MT.	Q9BSU1	-0.34727	0.049217	0.112479	PHAF1	Phagosome	PHAF1_HUI	3	0.786068
15min_MT.	Q9UPY3	0.118538	0.049098	0.112479	DICER1	Endoribonu	DICER_HUI	5	1.085634
15min_MT.	Q8N6N3	0.028728	0.049283	0.112585	C1orf52	UPF0690 p	CA052_HUI	5	1.020112
15min_MT.	P07711	0.179903	0.049368	0.112714	CTSL	Procatheps	CATL1_HUI	3	1.132808
15min_MT.	O95747	0.119945	0.049526	0.112909	OXSR1	Serine/thre	OXSR1_HUI	12	1.086693
30min_MT.	P02768	0.489011	0.049524	0.112909	ALB	Albumin	ALBU_HUM	28	1.403482
15min_MT.	P23921	-0.18911	0.049525	0.112909	RRM1	Ribonuclec	RIR1_HUMA	7	0.877144
30min_MT.	P61020	0.368121	0.04952	0.112909	RAB5B	Ras-related	RAB5B_HUI	3	1.290671
15min_MT.	P31153	-0.12509	0.049695	0.113229	MAT2A	S-adenosyl	METK2_HU	15	0.916946
15min_MT.	Q8N5M4	-0.55693	0.049745	0.113309	TTC9C	Tetratricop	TTC9C_HUI	1	0.679748
30min_MT.	Q8WTW3	-0.38705	0.049781	0.113348	COG1	Conserved	COG1_HUM	7	0.764692
15min_MT.	P14678	-0.10092	0.04981	0.113358	SNRPB	Small nucle	RSMB_HUM	9	0.932437
30min_MT.	P07305	0.244177	0.049841	0.113396	H1-0	Histone H1	H10_HUMA	6	1.184417
30min_MT.	O75663	-0.1197	0.049858	0.1134	TIPRL	TIP41-like	TIPRL_HUM	9	0.92038
15min_MT.	Q96GK7	0.140079	0.049946	0.11347	FAHD2A	Oxaloaceta	FAH2A_HUI	8	1.101965
15min_MT.	Q9H8T0	-0.23594	0.049918	0.11347	AKTIP	AKT-interac	AKTIP_HUM	2	0.849132
15min_MT.	Q86XP3	0.181424	0.050011	0.113548	DDX42	ATP-depenc	DDX42_HU	6	1.134002
15min_MT.	Q9BYT8	0.083855	0.050024	0.113548	NLN	Neurolysin,	NEUL_HUM	20	1.059846
15min_MT.	Q9H4A5	-1.18728	0.049997	0.113548	GOLPH3L	Golgi phos	GLP3L_HUI	1	0.43913
15min_MT.	P30086	-0.19269	0.050134	0.113568	PEBP1	Phosphatid	PEBP1_HUI	13	0.874974
30min_MT.	P31942	0.172715	0.050123	0.113568	HNRNPH3	Heterogene	HNRH3_HU	6	1.127178
30min_MT.	P55157	0.095024	0.050072	0.113568	MTTP	Microsoma	MTP_HUMA	35	1.068083
30min_MT.	Q15435	0.156218	0.050071	0.113568	PPP1R7	Protein ph	PP1R7_HUI	11	1.114362
30min_MT.	Q16625	0.100971	0.050134	0.113568	OCLN	Occludin	OCLN_HUM	5	1.072495
30min_MT.	Q8TAT6	-0.14279	0.05008	0.113568	NPLOC4	Nuclear pro	NPL4_HUM	15	0.905764
30min_MT.	Q9UNZ2	0.14435	0.050224	0.113705	NSFL1C	NSFL1 cofa	NSF1C_HUI	18	1.105232
15min_MT.	O43598	-0.16384	0.050289	0.113752	DNPH1	5-hydroxyn	DNPH1_HU	6	0.892647
15min_MT.	O94927	-0.28211	0.050361	0.113752	HAUS5	HAUS augm	HAUS5_HU	1	0.822387
15min_MT.	O95219	-0.08245	0.050311	0.113752	SNX4	Sorting nex	SNX4_HUM	10	0.94445
30min_MT.	P48729	0.873496	0.050331	0.113752	CSNK1A1	Casein kina	KC1A_HUM	3	1.832097
15min_MT.	Q14011	0.624514	0.050357	0.113752	CIRBP	Cold-induc	CIRBP_HUM	3	1.541692
15min_MT.	Q8N8S7	0.135197	0.05039	0.113752	ENAH	Protein ena	ENAH_HUM	15	1.098243

30min_MT.	Q8WUR7	-0.56268	0.050352	0.113752	C15orf40	UPF0235 p	CO040_HU	2	0.677042
15min_MT.	P38117	0.106679	0.050435	0.11382	ETFB	Electron tra	ETFB_HUMA	18	1.076747
15min_MT.	Q96J02	-0.2301	0.050497	0.113841	ITCH	E3 ubiquiti	ITCH_HUMA	4	0.852578
15min_MT.	Q9H2G2	0.145981	0.050555	0.113841	SLK	STE20-like s	SLK_HUMA	31	1.106482
30min_MT.	Q9UPQ9	0.124395	0.050579	0.113841	TNRC6B	Trinucleoti	TNR6B_HUI	8	1.09005
30min_MT.	Q14919	-0.42401	0.050664	0.113944	DRAP1	Dr1-associa	NC2A_HUM	3	0.745348
15min_MT.	Q9GZZ1	0.021569	0.050733	0.114032	NAA50	N-alpha-ace	NAA50_HU	8	1.015063
15min_MT.	Q9NV96	-0.38356	0.050796	0.114142	TMEM30A	Cell cycle co	CC50A_HUI	1	0.766545
15min_MT.	P46778	-0.8811	0.050835	0.114196	RPL21	Large ribos	RL21_HUM	4	0.542953
30min_MT.	Q9H477	0.152413	0.050959	0.114442	RBKS	Ribokinase	RBSK_HUM	10	1.111427
15min_MT.	Q9NZL4	0.10237	0.051033	0.11451	HSPBP1	Hsp70-binc	HPBP1_HU	11	1.073535
15min_MT.	Q92879	0.220015	0.051072	0.114565	CELF1	CUGBP Elav	CELF1_HUM	4	1.164746
15min_MT.	Q9UFN0	-0.24929	0.051265	0.114832	NIPSNAP3A	Protein Nip	NPS3A_HUI	10	0.841312
15min_MT.	Q7Z460	-0.12817	0.051326	0.114903	CLASP1	CLIP-associ	CLAP1_HUI	22	0.91499
30min_MT.	P23588	0.337159	0.051348	0.11492	EIF4B	Eukaryotic	IF4B_HUMA	23	1.263267
15min_MT.	O00767	-0.51529	0.051363	0.114921	SCD	Stearoyl-Co	SCD_HUMA	1	0.699651
30min_MT.	O43143	-0.08027	0.051428	0.114934	DHX15	ATP-depenc	DHX15_HU	28	0.94588
15min_MT.	P50395	-0.08476	0.051487	0.115034	GDI2	Rab GDP di	GDIB_HUM	31	0.942942
15min_MT.	P33316	0.249936	0.051511	0.11504	DUT	Deoxyuridi	DUT_HUMA	6	1.189155
15min_MT.	Q96P63	0.369382	0.051552	0.115079	SERPINB12	Serpin B12	SPB12_HUI	8	1.2918
30min_MT.	Q9UPN6	-0.45798	0.051569	0.115086	SCAF8	SR-related a	SCAF8_HUM	3	0.728004
30min_MT.	P60866	-0.22298	0.051586	0.115091	RPS20	Small ribos	RS20_HUM	3	0.856797
15min_MT.	Q5VVQ6	0.127539	0.051653	0.115162	YOD1	Ubiquitin t	OTU1_HUM	4	1.092429
15min_MT.	Q99426	0.129976	0.051649	0.115162	TBCB	Tubulin-fol	TBCB_HUM	12	1.094275
15min_MT.	P09874	-0.0848	0.051679	0.115165	PARP1	Poly [ADP-r	PARP1_HUI	25	0.942914
15min_MT.	P09012	-0.14386	0.051743	0.115183	SNRPA	U1 small nu	SNRPA_HUI	10	0.905095
15min_MT.	P13646	2.165698	0.051709	0.115183	KRT13	Keratin, typ	K1C13_HUI	10	4.486833
15min_MT.	Q4VC05;Q8	0.358752	0.05176	0.115183	BCL7A;BCL7	B-cell CLL/l	BCL7A_HUI	1	1.282316
30min_MT.	Q8N4J0	0.00475	0.051756	0.115183	CARNMT1	Carnosine N	CARME_HU	4	1.003298
30min_MT.	Q99873	0.1218	0.051718	0.115183	PRMT1	Protein arg	ANM1_HUM	13	1.088091
30min_MT.	O00148	-0.35077	0.051813	0.115234	DDX39A	ATP-depenc	DX39A_HU	5	0.784166
15min_MT.	P24752	0.051455	0.051807	0.115234	ACAT1	Acetyl-CoA	THIL_HUMA	19	1.03631
30min_MT.	P62910	-1.71808	0.05188	0.115273	RPL32	Large ribos	RL32_HUM	1	0.303953
15min_MT.	Q9NRW1	-0.67315	0.051868	0.115273	RAB6B	Ras-related	RAB6B_HUI	2	0.627135
15min_MT.	O75695	-0.01863	0.05193	0.115331	RP2	Protein XRI	XRP2_HUM	5	0.98717
15min_MT.	O75489	0.238353	0.051966	0.115346	NDUFS3	NADH dehy	NDUS3_HU	5	1.179646
15min_MT.	Q00266	-0.04599	0.05196	0.115346	MAT1A	S-adenosylr	METK1_HU	8	0.968626
15min_MT.	Q04695	0.917456	0.052047	0.115359	KRT17	Keratin, typ	K1C17_HUI	11	1.888781
30min_MT.	Q15643	0.179383	0.052036	0.115359	TRIP11	Thyroid rec	TRIPB_HUM	17	1.1324
30min_MT.	Q5VVQ6	0.195529	0.052047	0.115359	YOD1	Ubiquitin t	OTU1_HUM	4	1.145144
30min_MT.	Q9BTW9	-0.12386	0.052041	0.115359	TBCD	Tubulin-spe	TBCD_HUM	15	0.917729
15min_MT.	P06733	0.147576	0.052202	0.115503	ENO1	Alpha-enola	ENOA_HUM	24	1.107707
30min_MT.	Q13219	-1.15968	0.052198	0.115503	PAPPA	Pappalysin-	PAPP1_HUI	5	0.447612
15min_MT.	Q15061	0.192708	0.052218	0.115503	WDR43	WD repeat-	WDR43_HU	8	1.142907
15min_MT.	Q52LW3	0.078138	0.052237	0.115503	ARHGAP29	Rho GTPase	RHG29_HU	29	1.055655
15min_MT.	Q9H8Y8	0.198647	0.052258	0.115503	GORASP2	Golgi reasse	GORS2_HU	9	1.147621
15min_MT.	P11169	-0.32959	0.052534	0.116046	SLC2A3	Solute carri	GTR3_HUM	6	0.795762
30min_MT.	P38117	0.041052	0.052645	0.116162	ETFB	Electron tra	ETFB_HUMA	18	1.028864
15min_MT.	Q9NX62	0.893927	0.052801	0.116374	BPNT2	Golgi-reside	IMPA3_HUI	2	1.858227
30min_MT.	P31151	1.41956	0.052871	0.116382	S100A7	Protein S10	S10A7_HUI	2	2.675039
30min_MT.	P47897	-0.21955	0.052837	0.116382	QARS1	Glutamine-	SYQ_HUMA	24	0.858834
15min_MT.	P62318	0.224925	0.052879	0.116382	SNRPD3	Small nucle	SMD3_HUM	5	1.168717

15min_MT.	Q9BWU0	-0.0738	0.052864	0.116382	SLC4A1AP	Kanadaptir	NADAP_HU	12	0.950129
15min_MT.	Q9UKL6	0.090317	0.052951	0.116421	PCTP	Phosphatic	PPCT_HUM	8	1.064604
30min_MT.	Q9UQ80	0.133067	0.052942	0.116421	PA2G4	Proliferatic	PA2G4_HU	23	1.096623
30min_MT.	O43617	0.127716	0.052975	0.116428	TRAPPC3	Trafficking	TPPC3_HUI	9	1.092563
15min_MT.	Q15717	0.270546	0.053074	0.116558	ELAVL1	ELAV-like pı	ELAV1_HUI	8	1.206264
15min_MT.	Q6RW13	-0.16856	0.053153	0.116656	AGTRAP	Type-1 angi	ATRAP_HUI	3	0.88973
15min_MT.	P05455	-0.11093	0.053198	0.116656	SSB	Lupus La pr	LA_HUMAN	29	0.925993
30min_MT.	P32754	0.040298	0.053185	0.116656	HPD	4-hydroxyp	HPPD_HUN	8	1.028326
30min_MT.	Q9Y4Y9	-1.05316	0.053174	0.116656	LSM5	U6 snRNA-a	LSM5_HUN	1	0.481911
30min_MT.	P23025	-1.19661	0.053246	0.116718	XPA	DNA repair	XPA_HUMA	1	0.436299
15min_MT.	Q9BT73	0.207405	0.053451	0.117048	PSMG3	Proteasom	PSMG3_HU	6	1.154609
30min_MT.	Q92621	-0.34284	0.05353	0.117188	NUP205	Nuclear po	NU205_HU	17	0.788486
30min_MT.	P14868	-0.16629	0.0536	0.117308	DARS1	Aspartate--	SYDC_HUM	28	0.89113
30min_MT.	P04275	-0.45206	0.053643	0.117312	VWF	von Willeb	VWF_HUMA	2	0.730999
30min_MT.	Q2VPK5	0.020119	0.053677	0.117312	CTU2	Cytoplasmi	CTU2_HUM	3	1.014043
15min_MT.	Q8IXI1	0.254372	0.053642	0.117312	RHOT2	Mitochond	MIRO2_HU	1	1.192816
15min_MT.	P13473	0.395431	0.053766	0.117343	LAMP2	Lysosome-a	LAMP2_HU	2	1.315335
30min_MT.	Q96Q89	3.842894	0.05376	0.117343	KIF20B	Kinesin-like	KI20B_HUN	1	14.34916
15min_MT.	Q99747	0.238912	0.053724	0.117343	NAPG	Gamma-sol	SNAG_HUN	11	1.180102
30min_MT.	Q9NZL9	0.150896	0.053835	0.117428	MAT2B	Methionine	MAT2B_HU	11	1.110258
15min_MT.	Q8WUF8	-0.36069	0.053908	0.117556	ARB2A	Cotranscrip	ARB2A_HUI	1	0.778793
30min_MT.	O43164	0.070572	0.053947	0.117607	PJA2	E3 ubiquiti	PJA2_HUM	4	1.050133
15min_MT.	P56377	0.01899	0.053999	0.117654	AP1S2	AP-1 compl	AP1S2_HUI	4	1.01325
15min_MT.	Q3SY84	0.473463	0.053993	0.117654	KRT71	Keratin, typ	K2C71_HUI	12	1.388439
30min_MT.	P02539	4.106932	0.054052	0.117737	NaN	Keratin, typ	K2M1_SHEI	2	17.23097
15min_MT.	Q6PKC3	0.29916	0.054071	0.117745	TXNDC11	Thioredoxi	TXD11_HUI	3	1.230427
15min_MT.	Q92667	-0.54698	0.054192	0.117976	AKAP1	A-kinase an	AKAP1_HUI	2	0.684451
15min_MT.	Q96AG4	0.241427	0.054269	0.118083	LRRC59	Leucine-ric	LRC59_HUI	6	1.182161
15min_MT.	P0C0S5;Q7	-0.24275	0.054372	0.118203	H2AZ1;H2A	Histone H2	H2AZ_HUN	1	0.845135
30min_MT.	Q9UBQ0	0.205317	0.054496	0.118408	VPS29	Vacuolar pı	VPS29_HUI	6	1.15294
30min_MT.	Q5TC12	0.269625	0.054514	0.118415	ATPAF1	ATP synthas	ATPF1_HUN	8	1.205494
15min_MT.	Q96E11	0.060902	0.054612	0.118594	MRRF	Ribosome-r	RRFM_HUN	7	1.043118
30min_MT.	Q9C0B1	-0.0281	0.054646	0.118634	FTO	Alpha-keto	FTO_HUMA	9	0.98071
15min_MT.	Q3T906	0.020822	0.054736	0.118641	GNPTAB	N-acetylglu	GNPTA_HU	1	1.014537
15min_MT.	Q9UMS4	-0.01705	0.054678	0.118641	PRPF19	Pre-mRNA-	PRP19_HUI	15	0.988253
15min_MT.	Q9Y312	-0.43192	0.054753	0.118641	AAR2	Protein AAI	AAR2_HUN	5	0.741275
30min_MT.	Q6ZMI0	-0.005	0.054815	0.118739	PPP1R21	Protein pho	PPR21_HUI	9	0.996541
30min_MT.	O94967	-0.09841	0.054938	0.118939	WDR47	WD repeat-	WDR47_HU	6	0.934065
30min_MT.	O60232	0.07801	0.055069	0.119168	ZNRD2	Protein ZNI	ZNRD2_HU	9	1.055561
15min_MT.	Q99574	0.410333	0.055193	0.119358	SERPINI1	Neuroserpi	NEUS_HUN	8	1.328992
30min_MT.	P27348	0.181306	0.055226	0.119363	YWHAQ	14-3-3 prot	1433T_HUI	12	1.13391
30min_MT.	Q13371	0.418503	0.055272	0.119431	PDCL	Phosducin-	PHLP_HUN	4	1.33654
30min_MT.	Q8TCS8	-0.11956	0.055374	0.119617	PNPT1	Polyribonu	PNPT1_HUI	29	0.920466
30min_MT.	Q8N6V4	-0.29388	0.055431	0.119642	C10orf53	UPF0728 p	CJ053_HUN	1	0.815705
30min_MT.	Q9Y608	0.155931	0.055418	0.119642	LRRFIP2	Leucine-ric	LRRF2_HUN	10	1.114141
30min_MT.	P42566	0.105587	0.055541	0.119683	EPS15	Epidermal	EPS15_HUN	26	1.075932
15min_MT.	Q04118	1.110703	0.055541	0.119683	PRB3	Basic saliva	PRB3_HUM	3	2.159508
15min_MT.	Q0JRZ9	0.250031	0.055537	0.119683	FCHO2	F-BAR dom	FCHO2_HU	8	1.189233
30min_MT.	Q6ZSR9	-0.6253	0.055542	0.119683	NaN	Uncharacte	YJ005_HUN	6	0.648284
30min_MT.	Q712K3	0.153649	0.055483	0.119683	UBE2R2	Ubiquitin-c	UB2R2_HU	6	1.112379
15min_MT.	P41250	-0.0482	0.055566	0.119703	GARS1	Glycine--tR	GARS_HUN	36	0.967143
30min_MT.	P10109	0.272553	0.055647	0.119806	FDX1	Adrenodox	ADX_HUMA	2	1.207944

15min_MT.	Q8TDR0	0.117266	0.055688	0.119833	TRAF3IP1	TRAF3-inte	MIPT3_HUI	2	1.084677
15min_MT.	Q96F46	-0.33274	0.055712	0.119851	IL17RA	Interleukin	I17RA_HUN	5	0.794026
15min_MT.	Q9H3K6	-0.24084	0.055895	0.120079	BOLA2	BolA-like pr	BOLA2_HUI	6	0.846252
30min_MT.	O43813	0.132627	0.05597	0.120209	LANCL1	Glutathion	LANC1_HUI	12	1.096288
15min_MT.	Q86SZ2	0.125963	0.056007	0.120255	TRAPPC6B	Trafficking	TPC6B_HUI	10	1.091236
15min_MT.	Q8NFQ8	-0.15619	0.056141	0.120411	TOR1AIP2	Torsin-1A-i	TOIP2_HUN	5	0.897389
15min_MT.	P29218	0.073086	0.056225	0.120557	IMPA1	Inositol mc	IMPA1_HUI	14	1.051965
30min_MT.	Q5VWZ2	0.132909	0.056288	0.120658	LYPLAL1	Lysophospl	LYPL1_HUN	7	1.096502
15min_MT.	O60271	0.059978	0.056326	0.120673	SPAG9	C-Jun-amin	JIP4_HUMA	52	1.04245
15min_MT.	Q96S82	0.304426	0.056442	0.120791	UBL7	Ubiquitin-l	UBL7_HUM	6	1.234927
30min_MT.	Q9H6R3	-0.1788	0.056421	0.120791	ACSS3	Acyl-CoA sy	ACSS3_HUN	17	0.88344
15min_MT.	Q7Z7K0	0.170643	0.056481	0.12082	CMC1	COX asseml	COXM1_HU	2	1.12556
15min_MT.	Q9UI26	-0.08963	0.056487	0.12082	IPO11	Importin-1	IPO11_HUN	14	0.939764
30min_MT.	P56181	-0.59507	0.056541	0.120904	NDUFV3	NADH dehy	NDUV3_HU	1	0.662011
30min_MT.	P29401	0.097877	0.056566	0.120923	TKT	Transketola	TKT_HUMA	25	1.070198
15min_MT.	P29350	-0.78997	0.056625	0.120984	PTPN6	Tyrosine-pr	PTN6_HUM	2	0.578355
30min_MT.	P50583	-0.22853	0.056622	0.120984	NUDT2	Bis(5'-nucle	AP4A_HUM	6	0.853506
30min_MT.	O00754	0.11834	0.056712	0.121098	MAN2B1	Lysosomal	MA2B1_HU	19	1.085485
30min_MT.	P60891	0.169715	0.056739	0.121098	PRPS1	Ribose-pho	PRPS1_HUI	16	1.124836
30min_MT.	Q8N3U4	-0.22858	0.056848	0.121212	STAG2	Cohesin sul	STAG2_HUI	14	0.853472
30min_MT.	Q9HB40	0.443328	0.056891	0.12122	SCPEP1	Retinoid-in	RISC_HUMA	5	1.359737
30min_MT.	Q15631	0.238636	0.05693	0.121271	TSN	Translin	TSN_HUMA	14	1.179877
15min_MT.	Q86WJ1	0.146788	0.056974	0.121331	CHD1L	Chromodo	CHD1L_HU	2	1.107102
15min_MT.	P13807	0.25683	0.057037	0.121387	GYS1	Glycogen [s	GYS1_HUM	7	1.19485
15min_MT.	Q15386	-0.7088	0.057074	0.121387	UBE3C	Ubiquitin-p	UBE3C_HUI	4	0.611829
15min_MT.	Q96CW1	-0.19793	0.057064	0.121387	AP2M1	AP-2 compl	AP2M1_HU	16	0.871798
15min_MT.	Q9NZM3	0.470546	0.057056	0.121387	ITSN2	Intersectin	ITSN2_HUN	8	1.385634
30min_MT.	Q9H5N1	0.078767	0.057116	0.121435	RABEP2	Rab GTPase	RABE2_HUI	14	1.056115
15min_MT.	P30039	0.888058	0.057209	0.121563	PBLD	Phenazine	PBLD_HUM	1	1.850684
15min_MT.	P30049	0.229855	0.057249	0.121563	ATP5F1D	ATP syntha	ATPD_HUM	3	1.172717
30min_MT.	P52815	0.143164	0.057244	0.121563	MRPL12	Large ribos	RM12_HUN	5	1.104325
15min_MT.	Q00610	-0.21353	0.057254	0.121563	CLTC	Clathrin he	CLH1_HUM	64	0.862424
15min_MT.	P48553	0.032709	0.057355	0.121713	TRAPPC10	Trafficking	TPC10_HUI	4	1.022931
15min_MT.	Q08752	0.229184	0.057347	0.121713	PPID	Peptidyl-pr	PPID_HUM	14	1.172172
30min_MT.	Q14684	0.114758	0.057438	0.121841	RRP1B	Ribosomal	RRP1B_HUI	2	1.082794
30min_MT.	Q5BKZ1	-0.18042	0.057447	0.121841	ZNF326	DBIRD com	ZN326_HUI	5	0.882449
30min_MT.	Q8N139	0.080504	0.057527	0.121977	ABCA6	ATP-bindin	ABCA6_HUI	1	1.057387
15min_MT.	Q16543	-0.11845	0.057591	0.12208	CDC37	Hsp90 co-c	CDC37_HU	12	0.921175
15min_MT.	Q13596	0.109556	0.057627	0.122124	SNX1	Sorting nex	SNX1_HUM	16	1.078896
15min_MT.	P78356	0.11315	0.057716	0.122247	PIP4K2B	Phosphatic	PI42B_HUN	3	1.081587
15min_MT.	O43790	0.57134	0.057799	0.12228	KRT86	Keratin, typ	KRT86_HUI	2	1.485903
30min_MT.	P24863	0.524016	0.057771	0.12228	CCNC	Cyclin-C	CCNC_HUM	1	1.437952
30min_MT.	Q96B97	0.16966	0.057783	0.12228	SH3KBP1	SH3 domai	SH3K1_HUI	9	1.124794
15min_MT.	Q9C0B1	0.185706	0.057821	0.12228	FTO	Alpha-keto	FTO_HUMA	9	1.137373
30min_MT.	Q9Y5B8	0.038328	0.057826	0.12228	NME7	Nucleoside	NDK7_HUN	3	1.026923
30min_MT.	Q53T59	0.008038	0.057879	0.12236	HS1BP3	HCLS1-binc	H1BP3_HU	12	1.005587
30min_MT.	O00629	-0.29199	0.057942	0.122446	KPNA4	Importin su	IMA3_HUM	5	0.816777
15min_MT.	P42704	-0.08652	0.058027	0.122503	LRPPRC	Leucine-ric	LPPRC_HUI	67	0.941791
15min_MT.	P46100	-0.06077	0.058065	0.122504	ATRX	Transcripti	ATRX_HUM	29	0.958751
15min_MT.	Q7Z2W4	0.073641	0.058088	0.122504	ZC3HAV1	Zinc finger	ZCCHV_HUI	5	1.052369
15min_MT.	P53370	0.093184	0.058194	0.122595	NUDT6	Nucleoside	NUDT6_HU	4	1.066722
30min_MT.	Q5JVF3	-0.30347	0.058283	0.122733	PCID2	PCI domair	PCID2_HUN	5	0.810299

30min_MT.	Q92979	0.174332	0.058307	0.122733	EMG1	Ribosomal	NEP1_HUM	4	1.128442
30min_MT.	P42229;P5	-0.24437	0.058373	0.122749	STAT5A;STA	Signal trans	STA5A_HUI	1	0.844186
30min_MT.	Q8NFH4	0.130389	0.058365	0.122749	NUP37	Nucleopori	NUP37_HU	9	1.094588
30min_MT.	Q9NQC3	-0.24171	0.058382	0.122749	RTN4	Reticulon-4	RTN4_HUM	9	0.845743
15min_MT.	Q15007	0.132411	0.058426	0.122772	WTAP	Pre-mRNA-s	FL2D_HUM	6	1.096124
30min_MT.	P40394	-2.8415	0.058518	0.122879	ADH7	All-trans-re	ADH7_HUM	1	0.139515
30min_MT.	Q9UHA4	0.52034	0.058554	0.122922	LAMTOR3	Ragulator c	LTOR3_HUI	1	1.434293
15min_MT.	P51884	0.997362	0.058605	0.122996	LUM	Lumican	LUM_HUMA	1	1.996346
15min_MT.	Q70E73	-0.33864	0.058639	0.123035	RAPH1	Ras-associa	RAPH1_HU	4	0.790784
30min_MT.	Q6DKJ4	0.112798	0.058711	0.123143	NXN	Nucleoredo	NXN_HUMA	12	1.081323
30min_MT.	Q9NRF9	-0.09022	0.058731	0.123143	POLE3	DNA polym	DPOE3_HU	2	0.939378
30min_MT.	Q9HAC7	0.227963	0.058776	0.12319	SUGCT	Succinate--	SUCHY_HUI	1	1.17118
30min_MT.	P07910	0.226479	0.058825	0.123226	HNRNPC	Heterogene	HNRPC_HU	16	1.169976
15min_MT.	Q99942	0.178356	0.058923	0.123399	RNF5	E3 ubiquiti	RNF5_HUM	2	1.131594
15min_MT.	Q15631	0.191144	0.058942	0.123405	TSN	Translin	TSN_HUMA	14	1.141669
30min_MT.	Q8TAA5	0.192128	0.059054	0.123541	GRPEL2	GrpE protei	GRPE2_HUI	6	1.142447
30min_MT.	Q9NZD2	0.149289	0.059101	0.123606	GLTP	Glycolipid t	GLTP_HUM	8	1.109022
30min_MT.	O14818	0.123172	0.059244	0.123714	PSMA7	Proteasom	PSA7_HUM	12	1.089127
30min_MT.	Q6P1N9	0.14814	0.059227	0.123714	TATDN1	Deoxyribor	TATD1_HUI	12	1.10814
15min_MT.	Q96TC7	0.133857	0.059247	0.123714	RMDN3	Regulator c	RMD3_HUM	3	1.097223
15min_MT.	Q9NTX7	-0.75886	0.059194	0.123714	RNF146	E3 ubiquiti	RN146_HU	2	0.590964
15min_MT.	Q9NW82	0.036428	0.059221	0.123714	WDR70	WD repeat-	WDR70_HU	11	1.025572
15min_MT.	O43395	-0.0222	0.0593	0.123771	PRPF3	U4/U6 sma	PRPF3_HUI	6	0.984727
15min_MT.	Q9Y6D6	-0.04665	0.059306	0.123771	ARFGEF1	Brefeldin A	BIG1_HUM	29	0.968182
15min_MT.	Q13617	-0.14711	0.059354	0.123806	CUL2	Cullin-2	CUL2_HUM	18	0.903055
15min_MT.	Q99417	0.218349	0.0594	0.123807	MYCBP	c-Myc-bind	MYCBP_HU	4	1.163402
15min_MT.	Q9NVT9	0.234598	0.059403	0.123807	ARMC1	Armadillo r	ARMC1_HU	5	1.176579
15min_MT.	Q15527	-0.18737	0.059525	0.123964	SURF2	Surfeit locu	SURF2_HUI	2	0.878204
30min_MT.	Q9NZ09	-0.24295	0.059505	0.123964	UBAP1	Ubiquitin-a	UBAP1_HU	1	0.845017
30min_MT.	Q9GZZ1	-0.20883	0.059814	0.124432	NAA50	N-alpha-ace	NAA50_HU	8	0.865236
30min_MT.	Q8TBC3	-0.24948	0.059961	0.124611	SHKBP1	SH3KBP1-b	SHKB1_HUI	8	0.841202
15min_MT.	Q9UNM6	0.118781	0.059964	0.124611	PSMD13	26S protea	PSD13_HUI	21	1.085817
15min_MT.	P06737	-0.05565	0.059981	0.124614	PYGL	Glycogen p	PYGL_HUM	38	0.962164
15min_MT.	P51572	-0.1133	0.060168	0.124761	BCAP31	B-cell recep	BAP31_HUI	5	0.924472
30min_MT.	P51784	0.147897	0.06012	0.124761	USP11	Ubiquitin c	UBP11_HU	6	1.107953
30min_MT.	Q14562	0.283405	0.060151	0.124761	DHX8	ATP-depen	DHX8_HUM	5	1.217064
15min_MT.	Q9UHA4	0.488549	0.06018	0.124761	LAMTOR3	Ragulator c	LTOR3_HUI	1	1.403033
30min_MT.	O95394	0.088975	0.060291	0.124958	PGM3	Phosphoac	AGM1_HUI	21	1.063615
30min_MT.	Q07817	0.291378	0.06039	0.125097	BCL2L1	Bcl-2-like p	B2CL1_HUI	1	1.223809
30min_MT.	Q15274	0.132547	0.060381	0.125097	QPRT	Nicotinate-	NADC_HUM	10	1.096227
15min_MT.	P52630	0.571322	0.060455	0.1252	STAT2	Signal trans	STAT2_HUM	2	1.485884
15min_MT.	Q5EBL8	0.590847	0.060642	0.125487	PDZD11	PDZ domai	PDZ11_HUI	2	1.50613
15min_MT.	P14621	0.04761	0.060797	0.125656	ACYP2	Acylphosph	ACYP2_HUI	5	1.033551
30min_MT.	Q6IBS0	0.091569	0.060823	0.125656	TWF2	Twinfilin-2	TWF2_HUM	15	1.065529
15min_MT.	Q96EK5	-0.07767	0.060836	0.125656	KIFBP	KIF-binding	KBP_HUMA	11	0.947589
30min_MT.	Q9UKX7	0.011593	0.06082	0.125656	NUP50	Nuclear po	NUP50_HU	10	1.008068
15min_MT.	Q9Y3Y2	-0.44512	0.060816	0.125656	CHTOP	Chromatin	CHTOP_HU	3	0.734525
15min_MT.	P04637	-0.82119	0.061038	0.125844	TP53	Cellular tur	P53_HUMA	1	0.565974
15min_MT.	P07476	0.650161	0.060987	0.125844	IVL	Involucrin	INVO_HUM	5	1.569343
30min_MT.	P13674	0.403739	0.061088	0.125844	P4HA1	Prolyl 4-hy	P4HA1_HU	15	1.322932
15min_MT.	P48059	-0.19494	0.061021	0.125844	LIMS1	LIM and ser	LIMS1_HUI	8	0.873609
30min_MT.	P62306	-0.7854	0.06103	0.125844	SNRPF	Small nucle	RUXF_HUM	6	0.580191

15min_MT.	Q13137	0.405191	0.061059	0.125844	CALCOCO2	Calcium-bi	CACO2_HU	4	1.324265
15min_MT.	Q96BY7	-0.08724	0.061065	0.125844	ATG2B	Autophagy-	ATG2B_HU	14	0.941319
30min_MT.	P11182	-0.24304	0.061173	0.125853	DBT	Lipoamide	ODB2_HUM	15	0.844961
15min_MT.	P20962	-0.21343	0.061245	0.125901	PTMS	Parathymo	PTMS_HUM	5	0.862486
15min_MT.	Q6GMV3	0.190234	0.061293	0.125969	PTRHD1	Putative pe	PTRD1_HU	5	1.140949
15min_MT.	P82970	-0.4567	0.061334	0.125986	HMGN5	High mobil	HMGN5_HU	10	0.728652
30min_MT.	Q9ULC3	-0.1934	0.061326	0.125986	RAB23	Ras-related	RAB23_HU	8	0.874544
30min_MT.	P12429	0.120499	0.061376	0.126006	ANXA3	Annexin A3	ANXA3_HU	13	1.087111
30min_MT.	O95813	0.645485	0.061431	0.126021	CER1	Cerberus	CER1_HUM	1	1.564266
30min_MT.	Q92673	0.255255	0.061422	0.126021	SORL1	Sortilin-rel	SORL_HUM	4	1.193547
30min_MT.	Q12860	0.16318	0.061481	0.126024	CNTN1	Contactin-1	CNTN1_HU	28	1.119752
15min_MT.	Q6P3W7	0.021948	0.061453	0.126024	SCYL2	SCY1-like pr	SCYL2_HUM	12	1.01533
30min_MT.	Q9NY27	0.130986	0.061495	0.126024	PPP4R2	Serine/thre	PP4R2_HU	13	1.095042
30min_MT.	Q8TBX8	0.21296	0.06152	0.126037	PIP4K2C	Phosphatid	PI42C_HUM	7	1.159064
15min_MT.	P55010	-0.16195	0.061553	0.126039	EIF5	Eukaryotic	IF5_HUMAI	19	0.893818
15min_MT.	P80404	0.047346	0.06154	0.126039	ABAT	4-aminobu	GABT_HUM	23	1.033362
30min_MT.	Q9Y2I1	-0.21187	0.061569	0.126039	NISCH	Nischarin	NISCH_HUM	2	0.863417
30min_MT.	O75152	-0.13199	0.061604	0.126077	ZC3H11A	Zinc finger	ZC11A_HU	7	0.912574
30min_MT.	P13987	-0.06541	0.061713	0.126235	CD59	CD59 glyco	CD59_HUM	2	0.955673
30min_MT.	P55212	-0.11275	0.061765	0.126283	CASP6	Caspase-6	CASP6_HU	9	0.924824
30min_MT.	Q8WZ82	-0.05234	0.061771	0.126283	OVCA2	Esterase OV	OVCA2_HU	3	0.964374
15min_MT.	Q8IUZ5	0.091556	0.06182	0.126288	PHYKPL	5-phosphol	AT2L2_HUM	12	1.065519
15min_MT.	P27824	-0.15109	0.062062	0.126749	CANX	Calnexin	CALX_HUM	26	0.900571
15min_MT.	P18583	-0.36954	0.062094	0.126781	SON	Protein SOI	SON_HUMA	8	0.774028
30min_MT.	P62263	-0.17429	0.062168	0.1269	RPS14	Small ribos	RS14_HUM	2	0.886201
30min_MT.	Q9NYY3	-0.31799	0.062196	0.126924	PLK2	Serine/thre	PLK2_HUM	1	0.802186
15min_MT.	P45880	0.55825	0.06222	0.12694	VDAC2	Voltage-de	VDAC2_HU	1	1.472482
15min_MT.	P30419	-0.06001	0.062346	0.12703	NMT1	Glycylpepti	NMT1_HUM	11	0.959256
15min_MT.	Q5VZK9	0.042076	0.062332	0.12703	CARMIL1	F-actin-unc	CARL1_HU	27	1.029594
15min_MT.	Q8NFV4	0.279504	0.062335	0.12703	ABHD11	sn-1-specifi	ABHDB_HU	6	1.213777
30min_MT.	Q9Y3D9	-0.26839	0.062316	0.12703	MRPS23	Small ribos	RT23_HUM	5	0.830243
30min_MT.	Q9BQE5	-0.15719	0.062382	0.12707	APOL2	Apolipopro	APOL2_HU	7	0.896768
30min_MT.	Q9UKV3	0.163475	0.06248	0.127236	ACIN1	Apoptotic	ACINU_HU	31	1.119982
15min_MT.	Q7L3T8	0.119235	0.062549	0.127344	PARS2	Probable pr	SYPM_HUM	3	1.086159
15min_MT.	Q9NYB0	0.269571	0.06264	0.127464	TERF2IP	Telomeric r	TE2IP_HUM	6	1.20545
15min_MT.	P01857	0.32211	0.062714	0.127581	IGHG1	Immunoglo	IGHG1_HU	3	1.250157
30min_MT.	P02671	-0.11155	0.062811	0.127709	FGA	Fibrinogen	FIBA_HUMA	20	0.925594
15min_MT.	P68106	-0.80301	0.062819	0.127709	FKBP1B	Peptidyl-pr	FKB1B_HU	1	0.573152
15min_MT.	Q8WVK2	-0.39611	0.062875	0.127775	SNRNP27	U4/U6.U5 s	SNR27_HU	4	0.759903
15min_MT.	Q96ER3	-0.2045	0.063047	0.128059	SAAL1	Protein SA	SAAL1_HUM	12	0.86784
30min_MT.	Q15392	-0.49639	0.063091	0.128115	DHCR24	Delta(24)-s	DHC24_HU	2	0.708878
15min_MT.	P02749	0.752035	0.063178	0.128129	APOH	Beta-2-glyc	APOH_HUM	1	1.684166
30min_MT.	P48730;P4	1.755952	0.063176	0.128129	CSNK1D;CS	Casein kina	KC1D_HUM	1	3.377491
15min_MT.	Q96AE4	0.268251	0.063232	0.128134	FUBP1	Far upstrea	FUBP1_HU	29	1.204347
30min_MT.	Q9GZS3	0.069229	0.063207	0.128134	SKIC8	Superkiller	SKI8_HUMA	8	1.049156
15min_MT.	Q9H7C9	0.231258	0.063231	0.128134	AAMDC	Mth938 do	AAMDC_HU	7	1.173858
30min_MT.	P25685	-0.07196	0.063322	0.128251	DNAJB1	DnaJ homo	DNJB1_HU	9	0.951348
30min_MT.	Q5VWQ8;C	-0.0962	0.063416	0.128375	DAB2IP;RA	Disabled ho	DAB2P_HU	1	0.935496
15min_MT.	Q5T653	0.269094	0.063461	0.128375	MRPL2	Large ribos	RM02_HUM	1	1.20505
30min_MT.	P12931	0.171197	0.063516	0.128445	SRC	Proto-onco	SRC_HUMA	10	1.125992
15min_MT.	P02656	0.763132	0.063695	0.12874	APOC3	Apolipopro	APOC3_HU	1	1.697171
15min_MT.	P38646	0.186685	0.063757	0.128767	HSPA9	Stress-70 pı	GRP75_HU	38	1.138145

30min_MT.	Q9UKK3	-0.12491	0.063758	0.128767	PARP4	Protein mo	PARP4_HUI	24	0.917058
30min_MT.	O95295	-0.04329	0.063813	0.128793	SNAPIN	SNARE-asso	SNAPN_HU	4	0.970442
30min_MT.	Q16698	-0.07868	0.063796	0.128793	DECR1	2,4-dienoyl	DECR_HUM	13	0.946927
15min_MT.	Q8IWE4	0.910903	0.063837	0.128793	DCUN1D3	DCN1-like p	DCNL3_HU	1	1.880222
15min_MT.	Q5NDL2	0.357202	0.063914	0.128882	EOGT	EGF domair	EOGT_HUM	7	1.280939
15min_MT.	Q6P3X3	-0.3223	0.063936	0.128895	TTC27	Tetratricop	TTC27_HUN	5	0.799792
30min_MT.	Q15056	0.289715	0.064075	0.129108	EIF4H	Eukaryotic	IF4H_HUM.	9	1.222398
15min_MT.	O60784	0.186214	0.064116	0.129137	TOM1	Target of M	TOM1_HUN	8	1.137774
30min_MT.	P46940	-0.1154	0.064169	0.129137	IQGAP1	Ras GTPase-	IQGA1_HUI	51	0.923124
30min_MT.	P63165	0.269493	0.064172	0.129137	SUMO1	Small ubiqu	SUMO1_HU	6	1.205385
15min_MT.	P68431;P8	-0.08806	0.064151	0.129137	H3C1;H3-3	Histone H3	H31_HUMA	4	0.940784
15min_MT.	Q53F19	-0.09738	0.064361	0.129391	NCBP3	Nuclear cap	NCBP3_HU	6	0.934726
30min_MT.	Q9BW91	0.169852	0.064356	0.129391	NUDT9	ADP-ribose	NUDT9_HU	12	1.124943
30min_MT.	P84098	-0.11232	0.064393	0.129415	RPL19	Large ribos	RL19_HUM	5	0.925097
15min_MT.	P23378	-0.03854	0.064441	0.129467	GLDC	Glycine deh	GCSP_HUM	15	0.973639
30min_MT.	Q13435	-0.13567	0.064452	0.129467	SF3B2	Splicing fac	SF3B2_HUN	29	0.910247
30min_MT.	O95352	-0.14284	0.06466	0.129529	ATG7	Ubiquitin-l	ATG7_HUM	10	0.905732
30min_MT.	P49756	-0.12211	0.064755	0.129529	RBM25	RNA-bindir	RBM25_HU	16	0.918842
15min_MT.	P82979	0.149101	0.064526	0.129529	SARNP	SAP domair	SARNP_HUI	9	1.108878
30min_MT.	Q08623	0.229	0.064814	0.129529	PUDP	Pseudouric	HDHD1_HU	4	1.172022
30min_MT.	Q09161	-0.0466	0.064539	0.129529	NCBP1	Nuclear cap	NCBP1_HU	9	0.968212
15min_MT.	Q13395	-0.2944	0.064603	0.129529	TARBP1	Probable m	TARB1_HUI	11	0.815409
30min_MT.	Q5R3I4	0.031933	0.064778	0.129529	TTC38	Tetratricop	TTC38_HUN	10	1.022381
15min_MT.	Q86UV5	0.305777	0.064674	0.129529	USP48	Ubiquitin c	UBP48_HU	2	1.236084
30min_MT.	Q86X76	0.106205	0.06467	0.129529	NIT1	Deaminate	NIT1_HUMA	13	1.076393
15min_MT.	Q8IYS1	0.261744	0.064639	0.129529	PM20D2	Xaa-Arg dip	P20D2_HU	4	1.198927
15min_MT.	Q8TAF3	0.125763	0.064655	0.129529	WDR48	WD repeat-	WDR48_HU	17	1.091085
15min_MT.	Q96JY6	0.242881	0.064605	0.129529	PDLIM2	PDZ and LIN	PDLI2_HUN	4	1.183353
30min_MT.	Q9UDT6	-0.10884	0.064769	0.129529	CLIP2	CAP-Gly do	CLIP2_HUN	26	0.927333
15min_MT.	Q8IV50	0.14435	0.064871	0.129609	LYSMD2	LysM and p	LYSM2_HUI	2	1.105232
30min_MT.	P51665	0.13294	0.064945	0.12967	PSMD7	26S proteas	PSMD7_HU	7	1.096526
30min_MT.	Q8WUA7	-0.00761	0.064995	0.12967	TBC1D22A	TBC1 doma	TB22A_HUI	16	0.994741
30min_MT.	Q969T4	-0.16779	0.064992	0.12967	UBE2E3	Ubiquitin-c	UB2E3_HUI	4	0.890205
30min_MT.	Q9NWM8	-0.52525	0.065013	0.12967	FKBP14	Peptidyl-pr	FKB14_HUI	2	0.694838
30min_MT.	Q9P2Y5	-0.37116	0.065017	0.12967	UVRAG	UV radiatio	UVRAG_HU	1	0.773161
30min_MT.	P17612	0.705498	0.065098	0.129764	PRKACA	cAMP-depe	KAPCA_HUI	3	1.630707
15min_MT.	Q9NQG5	0.152974	0.065114	0.129764	RPRD1B	Regulation	RPR1B_HUI	10	1.111859
15min_MT.	O95926	5.30E-05	0.065164	0.12983	SYF2	Pre-mRNA-:	SYF2_HUMA	1	1.000037
15min_MT.	Q9Y6Y8	-0.07629	0.065201	0.129837	SEC23IP	SEC23-inter	S23IP_HUN	26	0.948497
15min_MT.	Q86UA1	0.296876	0.06523	0.129863	PRPF39	Pre-mRNA-|	PRP39_HUI	2	1.228481
30min_MT.	P11142	0.134238	0.065395	0.129934	HSPA8	Heat shock	HSP7C_HUI	24	1.097513
15min_MT.	P41214	0.103735	0.065482	0.129934	EIF2D	Eukaryotic	EIF2D_HUN	1	1.074552
15min_MT.	Q02318	0.089342	0.065392	0.129934	CYP27A1	Sterol 26-h	CP27A_HUI	7	1.063885
15min_MT.	Q12789	-0.58552	0.065331	0.129934	GTF3C1	General tra	TF3C1_HUN	3	0.666408
15min_MT.	Q16186	0.138737	0.065448	0.129934	ADRM1	Proteasom	ADRM1_HU	5	1.100941
15min_MT.	Q6YHK3	0.141675	0.065329	0.129934	CD109	CD109 anti	CD109_HU	24	1.103185
15min_MT.	Q9NUU7	-0.01747	0.06542	0.129934	DDX19A	ATP-depen	DD19A_HU	16	0.987962
30min_MT.	P54819	0.173813	0.06555	0.130036	AK2	Adenylate k	KAD2_HUM	13	1.128036
30min_MT.	P60468	-0.4987	0.065618	0.130044	SEC61B	Protein tra	SC61B_HUI	1	0.707742
30min_MT.	Q9BXR0	0.052099	0.065588	0.130044	QTRT1	Queuine tR	TGT_HUMA	8	1.036772
30min_MT.	Q9BZF9	0.161869	0.065594	0.130044	UACA	Uveal autoa	UACA_HUM	3	1.118735
15min_MT.	O76013	-0.28564	0.065705	0.130113	KRT36	Keratin, typ	KRT36_HUI	6	0.820375

15min_MT.	P52758	0.186935	0.065691	0.130113	RIDA	2-iminobut	RIDA_HUM		6	1.138343
15min_MT.	Q96FN9	0.213175	0.065672	0.130113	DTD2	D-aminoac	DTD2_HUM		3	1.159236
15min_MT.	Q9BXJ9	-0.15312	0.065729	0.130118	NAA15	N-alpha-ace	NAA15_HU		23	0.899304
15min_MT.	Q8IW45	0.123094	0.065815	0.13022	NAXD	ATP-depend	NNRD_HUM		11	1.089068
15min_MT.	P09884	0.14605	0.065985	0.130371	POLA1	DNA polym	DPOLA_HU		3	1.106536
30min_MT.	P13798	0.092747	0.065963	0.130371	APEH	Acylamino-	ACPH_HUM		20	1.066399
30min_MT.	Q5EBL8	0.558644	0.065946	0.130371	PDZD11	PDZ domai	PDZ11_HU		2	1.472884
15min_MT.	Q13769	-0.14118	0.066219	0.130747	THOC5	THO compl	THOC5_HU		7	0.906778
30min_MT.	Q562R1	-1.07063	0.066283	0.130794	ACTBL2	Beta-actin-l	ACTBL_HUM		3	0.476111
15min_MT.	Q13627	-0.00036	0.066378	0.130948	DYRK1A	Dual specifi	DYR1A_HU		5	0.999752
15min_MT.	P11802	0.645871	0.066414	0.130954	CDK4	Cyclin-depe	CDK4_HUM		2	1.564684
30min_MT.	Q8NHH9	-0.25562	0.066406	0.130954	ATL2	Atlastin-2	ATLA2_HUM		7	0.837626
30min_MT.	Q92696	0.110763	0.066482	0.130974	RABGGTA	Geranylger	PGTA_HUM		20	1.079799
15min_MT.	Q92769	-0.26841	0.066493	0.130974	HDAC2	Histone dea	HDAC2_HU		8	0.830232
30min_MT.	Q9NX02	-0.41714	0.066508	0.130974	NLRP2	NACHT, LRF	NALP2_HU		6	0.74891
30min_MT.	P06753	0.505606	0.066574	0.131071	TPM3	Tropomyos	TPM3_HUM		4	1.41972
30min_MT.	O15042	-0.14722	0.066651	0.131163	U2SURP	U2 snRNP-a	SR140_HU		17	0.902986
15min_MT.	P02760	-0.21928	0.066722	0.131163	AMBP	Protein AM	AMBP_HUM		11	0.858991
15min_MT.	P28070	-0.13328	0.066806	0.131207	PSMB4	Proteasom	PSB4_HUM		7	0.911754
30min_MT.	Q9UPR3	-0.00891	0.06679	0.131207	SMG5	Nonsense-n	SMG5_HUM		1	0.993843
15min_MT.	P26038	-0.03801	0.066839	0.131218	MSN	Moesin	MOES_HUM		33	0.973999
15min_MT.	Q9UNZ2	0.148886	0.066951	0.131383	NSFL1C	NSFL1 cofa	NSF1C_HU		18	1.108713
15min_MT.	Q9HD34	0.21684	0.067019	0.13145	LYRM4	LYR motif-c	LYRM4_HU		3	1.162186
15min_MT.	Q8IV08	0.215263	0.067073	0.131504	PLD3	5'-3' exonu	PLD3_HUM		6	1.160915
30min_MT.	Q9UHJ6	-0.22508	0.06708	0.131504	SHPK	Sedoheptul	SHPK_HUM		6	0.855548
15min_MT.	P08962	-0.13867	0.067197	0.131602	CD63	CD63 antig	CD63_HUM		2	0.908358
15min_MT.	P02810	2.697579	0.067252	0.131675	PRH1	Salivary aci	PRPC_HUM		3	6.487123
15min_MT.	P25685	0.225197	0.067363	0.131794	DNAJB1	DnaJ homo	DNJB1_HU		9	1.168937
15min_MT.	P42574	0.206235	0.067348	0.131794	CASP3	Caspase-3	CASP3_HU		6	1.153673
30min_MT.	P49407	-0.14082	0.067403	0.13184	ARRB1	Beta-arresti	ARRB1_HU		13	0.907003
30min_MT.	Q9UNN5	0.091848	0.067534	0.131963	FAF1	FAS-associa	FAF1_HUM		16	1.065734
15min_MT.	O00193	-0.30804	0.067733	0.132031	SMAP	Small acidi	SMAP_HUM		3	0.807741
30min_MT.	O43847	0.131723	0.067754	0.132031	NRDC	Nardilysin	NRDC_HUM		26	1.095601
30min_MT.	Q13615	-0.36104	0.067681	0.132031	MTMR3	Myotubula	MTMR3_HU		6	0.778604
30min_MT.	Q8TB03	0.383242	0.067718	0.132031	CXorf38	Uncharacte	CX038_HU		5	1.30427
30min_MT.	Q9BY77	-0.3387	0.067687	0.132031	POLDIP3	Polymerase	PDIP3_HUM		4	0.790754
15min_MT.	Q9NQC3	-0.26536	0.067587	0.132031	RTN4	Reticulon-4	RTN4_HUM		9	0.831991
30min_MT.	Q9ULW0	0.007686	0.067662	0.132031	TPX2	Targeting p	TPX2_HUM		1	1.005342
15min_MT.	P84103	-0.22705	0.067904	0.132225	SRSF3	Serine/argi	SRSF3_HUM		7	0.854379
30min_MT.	Q9H6K1	0.202032	0.067899	0.132225	ILRUN	Protein ILR	ILRUN_HUM		1	1.150317
15min_MT.	P55854	0.062349	0.067976	0.132313	SUMO3	Small ubiq	SUMO3_HU		2	1.044164
15min_MT.	Q9BRT2	0.7064	0.068075	0.132426	UQCC2	Ubiquinol-	UQCC2_HU		2	1.631727
15min_MT.	P30876	-0.21367	0.068237	0.132708	POLR2B	DNA-direct	RPB2_HUM		17	0.862338
30min_MT.	Q8IV36	-0.23405	0.068255	0.132709	HID1	Protein HID	HID1_HUM		11	0.850246
15min_MT.	P62979	0.189229	0.068335	0.132799	RPS27A	Ubiquitin-r	RS27A_HU		7	1.140154
30min_MT.	Q13443	-1.56755	0.068322	0.132799	ADAM9	Disintegrin	ADAM9_HU		1	0.33738
30min_MT.	P61923	-0.17028	0.068393	0.132866	COPZ1	Coatomer s	COPZ1_HU		5	0.888672
30min_MT.	Q5JRX3	0.007983	0.068442	0.132909	PITRM1	Presequenc	PREP_HUM		30	1.005548
15min_MT.	Q9BTD8	-1.30688	0.068531	0.133047	RBM42	RNA-bindir	RBM42_HU		1	0.404194
15min_MT.	O14727	0.125278	0.068798	0.133434	APAF1	Apoptotic p	APAF_HUM		5	1.090718
30min_MT.	P39019	-0.2525	0.068793	0.133434	RPS19	Small ribos	RS19_HUM		4	0.839442
30min_MT.	Q8IYB3	-0.08211	0.068869	0.133539	SRRM1	Serine/argi	SRRM1_HU		14	0.944675

30min_MT.	P11233	0.197804	0.06893	0.133574	RALA	Ras-related	RALA_HUM	3	1.146951
15min_MT.	Q14376	0.233485	0.068982	0.133574	GALE	UDP-glucos	GALE_HUM	3	1.175672
30min_MT.	Q9BUE0	0.277676	0.068957	0.133574	MED18	Mediator o	MED18_HU	2	1.212241
30min_MT.	Q9UPY3	-0.05998	0.06899	0.133574	DICER1	Endoribonu	DICER_HUM	5	0.959279
30min_MT.	P46778	-0.61581	0.069044	0.133646	RPL21	Large ribos	RL21_HUM	4	0.652562
15min_MT.	O60437	0.497191	0.069185	0.133775	PPL	Periplakin	PEPL_HUM	23	1.411463
30min_MT.	P53602	0.065724	0.069134	0.133775	MVD	Diphospho	MVD1_HUM	12	1.04661
15min_MT.	Q52LJ0	0.084269	0.069192	0.133775	FAM98B	Protein FAM	FA98B_HUI	5	1.06015
15min_MT.	Q9P2Y5	-0.75685	0.069197	0.133775	UVRAG	UV radiatio	UVRAG_HU	1	0.591787
15min_MT.	Q8NCA5	0.412132	0.069289	0.133888	FAM98A	Protein FAM	FA98A_HUI	2	1.330651
15min_MT.	O14933	0.156243	0.069346	0.133964	UBE2L6	Ubiquitin/I	UB2L6_HUI	1	1.114381
15min_MT.	P19652	-0.36077	0.069496	0.134221	ORM2	Alpha-1-aci	A1AG2_HU	2	0.778748
30min_MT.	Q9NZQ3	-0.20671	0.069547	0.134254	NCKIPSD	NCK-intera	SPN90_HUI	2	0.866509
15min_MT.	P02666	0.947359	0.069604	0.134331	CSN2	Beta-casein	CASB_BOVI	1	1.928339
30min_MT.	Q8IYB8	-0.18085	0.069628	0.134345	SUPV3L1	ATP-depen	SUV3_HUM	22	0.882186
15min_MT.	P00450	0.041914	0.069646	0.134345	CP	Ceruloplas	CERU_HUM	27	1.029479
15min_MT.	P02662	0.863566	0.069819	0.134513	CSN1S1	Alpha-S1-ca	CASA1_BOV	3	1.81953
15min_MT.	Q9Y217	-0.62008	0.069874	0.134587	MTMR6	Myotubula	MTMR6_HU	2	0.650635
30min_MT.	O14979	0.227002	0.069926	0.134644	HNRNPDL	Heterogene	HNRDL_HU	7	1.1704
30min_MT.	O43708	-0.38856	0.070025	0.134644	GSTZ1	Maleylacet	MAAI_HUM	4	0.763893
30min_MT.	P16870	-0.07593	0.069957	0.134644	CPE	Carboxypep	CBPE_HUM	8	0.948732
30min_MT.	Q92625	0.232044	0.070023	0.134644	ANKS1A	Ankyrin rep	ANS1A_HUI	2	1.174498
30min_MT.	Q70Z53	0.107597	0.070103	0.134728	FRA10AC1	Protein FRA	F10C1_HUI	5	1.077432
15min_MT.	Q02878	-0.24907	0.070121	0.13473	RPL6	Large ribos	RL6_HUMA	9	0.84144
30min_MT.	O60671	0.077199	0.070189	0.134754	RAD1	Cell cycle c	RAD1_HUM	5	1.054968
15min_MT.	O95425	0.242639	0.070182	0.134754	SVIL	Supervillin	SVIL_HUMA	4	1.183155
30min_MT.	P18846	-1.01406	0.070202	0.134754	ATF1	Cyclic AMP	ATF1_HUM	1	0.495151
15min_MT.	P49720	0.129956	0.070202	0.134754	PSMB3	Proteasom	PSB3_HUM	10	1.094261
30min_MT.	Q96QZ7	-0.03766	0.070296	0.134867	MAGI1	Membrane	MAGI1_HU	7	0.974236
15min_MT.	Q14258	-0.06885	0.070385	0.134971	TRIM25	E3 ubiquiti	TRI25_HUM	20	0.9534
15min_MT.	Q6ZNJ1	0.151152	0.070372	0.134971	NBEAL2	Neurobeac	NBEL2_HUI	1	1.110456
30min_MT.	P20020	-0.02327	0.070436	0.135037	ATP2B1	Plasma me	AT2B1_HUI	9	0.983998
15min_MT.	P78324	-0.59439	0.07062	0.135356	SIRPA	Tyrosine-pr	SHPS1_HUI	2	0.662324
30min_MT.	Q96GC5	-0.29328	0.070688	0.135429	MRPL48	Large ribos	RM48_HUM	4	0.816047
15min_MT.	Q9HAY6	0.153308	0.070727	0.135429	BCO1	Beta,beta-c	BCDO1_HU	1	1.112117
30min_MT.	Q9UPY6	0.284702	0.070724	0.135429	WASF3	Actin-bindi	WASF3_HU	2	1.218158
30min_MT.	Q3SY84	0.713109	0.07076	0.135433	KRT71	Keratin, typ	K2C71_HUI	12	1.639333
30min_MT.	Q9Y3L3	0.477957	0.070764	0.135433	SH3BP1	SH3 domai	3BP1_HUM	3	1.39277
30min_MT.	P50454	-0.15122	0.070804	0.135476	SERPINH1	Serpin H1	SERPH_HUI	11	0.900487
15min_MT.	O14558	-0.69452	0.071086	0.135949	HSPB6	Heat shock	HSPB6_HUI	1	0.617915
30min_MT.	P0DOY2;PC	0.368805	0.07119	0.135949	IGLC2;IGLC	Immunoglo	IGLC2_HUM	2	1.291283
30min_MT.	P13473	0.364208	0.071133	0.135949	LAMP2	Lysosome-a	LAMP2_HU	2	1.287174
30min_MT.	P62714	0.062285	0.071174	0.135949	PPP2CB	Serine/thre	PP2AB_HUI	13	1.044118
30min_MT.	Q6EEV4	-0.93659	0.071158	0.135949	POLR2M	DNA-direct	GL1AD_HU	2	0.522468
15min_MT.	Q9NRW7	0.16505	0.071116	0.135949	VPS45	Vacuolar pr	VPS45_HUI	4	1.121205
30min_MT.	O00429	-0.13781	0.071281	0.136023	DNM1L	Dynamin-1	DNM1L_HU	28	0.908896
15min_MT.	Q9P2D3	-0.50074	0.071246	0.136023	HEATR5B	HEAT repea	HTR5B_HUI	2	0.706743
30min_MT.	Q9BV40	0.215041	0.071314	0.136054	VAMP8	Vesicle-ass	VAMP8_HU	3	1.160737
30min_MT.	Q8TAF3	0.18208	0.071377	0.136134	WDR48	WD repeat-	WDR48_HU	17	1.134518
30min_MT.	Q9BUK6	-0.33607	0.071391	0.136134	MSTO1	Protein mis	MSTO1_HU	3	0.792198
30min_MT.	Q96GX9	-0.20699	0.071466	0.136243	APIP	Methylthio	MTNB_HUM	12	0.866344
15min_MT.	O95486	-0.12987	0.071552	0.136275	SEC24A	Protein tra	SC24A_HUI	9	0.913913

30min_MT.	P29144	-0.09663	0.071542	0.136275	TPP2	Tripeptidyl	TPP2_HUM		34	0.935216
30min_MT.	Q13740	0.218427	0.071539	0.136275	ALCAM	CD166 anti	CD166_HU		7	1.163464
30min_MT.	Q6MZQ0	0.653346	0.071537	0.136275	PRR5L	Proline-rich	PRR5L_HU		1	1.572811
30min_MT.	P23381	0.090726	0.071634	0.136364	WARS1	Tryptophar	SYWC_HUM		27	1.064906
30min_MT.	P35222	0.083678	0.071711	0.136445	CTNNB1	Catenin bet	CTNB1_HU		26	1.059717
30min_MT.	Q16401	0.16922	0.071936	0.136693	PSMD5	26S proteas	PSMD5_HU		22	1.12445
30min_MT.	P20073	0.08677	0.071992	0.136746	ANXA7	Annexin A7	ANXA7_HU		19	1.06199
30min_MT.	P51668	0.20159	0.072052	0.136794	UBE2D1	Ubiquitin-c	UB2D1_HU		2	1.149965
15min_MT.	Q86W42	-0.11173	0.072037	0.136794	THOC6	THO compl	THOC6_HU		4	0.925479
30min_MT.	Q15717	0.120948	0.072099	0.136849	ELAVL1	ELAV-like pı	ELAV1_HUI		8	1.08745
30min_MT.	P43034	0.094333	0.072231	0.137067	PAFAH1B1	Platelet-act	LIS1_HUMA		19	1.067572
30min_MT.	Q9Y6Y8	-0.10486	0.072329	0.137154	SEC23IP	SEC23-inter	S23IP_HUM		26	0.929898
30min_MT.	Q16630	0.157774	0.072414	0.137207	CPSF6	Cleavage ar	CPSF6_HUI		13	1.115564
15min_MT.	Q96CT7	0.227319	0.072413	0.137207	CCDC124	Coiled-coil	CC124_HUI		9	1.170657
30min_MT.	Q9BXV9	-0.32374	0.072467	0.137215	GON7	EKC/KEOPS	GON7_HUM		2	0.798997
30min_MT.	P32119	0.193824	0.07255	0.13734	PRDX2	Peroxiredo	PRDX2_HU		12	1.143792
15min_MT.	Q7Z2K8	-0.19972	0.072584	0.13737	GPRIN1	G protein-r	GRIN1_HUI		2	0.870721
15min_MT.	P46940	-0.08239	0.072631	0.137425	IQGAP1	Ras GTPase-	IQGA1_HUI		51	0.944493
15min_MT.	Q7Z5H4	0.281546	0.072714	0.137489	VN1R5	Vomeronas	VN1R5_HU		1	1.215497
30min_MT.	P32121	0.194431	0.072809	0.137497	ARRB2	Beta-arresti	ARRB2_HUI		5	1.144273
15min_MT.	Q96NT1	0.934578	0.072766	0.137497	NAP1L5	Nucleosom	NP1L5_HUI		1	1.911331
15min_MT.	Q9H9T3	-0.01056	0.072796	0.137497	ELP3	Elongator c	ELP3_HUM		9	0.992709
30min_MT.	Q9UHD1	-0.15567	0.072792	0.137497	CHORDC1	Cysteine an	CHRD1_HU		16	0.897713
15min_MT.	Q9Y617	0.125145	0.072785	0.137497	PSAT1	Phosphoser	SERC_HUM		18	1.090617
15min_MT.	P30530	-0.43649	0.072981	0.137756	AXL	Tyrosine-pr	UFO_HUMA		1	0.738933
30min_MT.	Q9NYF8	-0.05928	0.073004	0.137766	BCLAF1	Bcl-2-assoc	BCLF1_HUI		16	0.95974
30min_MT.	P39687	0.255007	0.073045	0.137785	ANP32A	Acidic leuci	AN32A_HU		10	1.193342
30min_MT.	O15231	0.326556	0.07317	0.137922	ZNF185	Zinc finger	ZN185_HUI		1	1.254016
15min_MT.	P10301	0.139273	0.073193	0.137922	RRAS	Ras-related	RRAS_HUM		9	1.10135
30min_MT.	Q9Y2A7	-0.10292	0.073178	0.137922	NCKAP1	Nck-associa	NCKP1_HU		24	0.931146
30min_MT.	Q9Y3E0	-0.65672	0.073242	0.137982	GOLT1B	Vesicle trar	GOT1B_HU		1	0.634319
15min_MT.	Q96MW1	0.287777	0.073417	0.138245	CCDC43	Coiled-coil	CCD43_HU		7	1.220758
30min_MT.	Q8TEB1	-0.00995	0.073448	0.13827	DCAF11	DDB1- and	DCA11_HU		2	0.993128
30min_MT.	Q86UP2	0.143078	0.073473	0.138283	KTN1	Kinectin	KTN1_HUM		30	1.104258
30min_MT.	Q9UL18	-0.17328	0.073531	0.138359	ago-01	Protein arg	AGO1_HUM		12	0.886823
15min_MT.	O43255	-0.33773	0.073572	0.138404	SIAH2	E3 ubiquiti	SIAH2_HUM		2	0.791284
15min_MT.	Q99933	0.052407	0.073696	0.138604	BAG1	BAG family	BAG1_HUM		3	1.036993
15min_MT.	P00533	0.225022	0.07383	0.13882	EGFR	Epidermal g	EGFR_HUM		13	1.168795
15min_MT.	P56211	0.153228	0.073986	0.139048	ARPP19	cAMP-regul	ARP19_HUI		4	1.112055
15min_MT.	P05121	1.086355	0.074072	0.139135	SERPINE1	Plasminoge	PAI1_HUMA		1	2.123368
30min_MT.	P54578	0.097625	0.074138	0.1392	USP14	Ubiquitin c	UBP14_HU		27	1.070011
15min_MT.	Q495W5	0.546339	0.074205	0.139292	FUT11	Alpha-(1,3)	FUT11_HUI		1	1.460375
15min_MT.	O60502	0.048267	0.074292	0.139322	OGA	Protein O-G	OGA_HUMA		30	1.034022
30min_MT.	Q12797	0.195483	0.074244	0.139322	ASPH	Aspartyl/as	ASPH_HUM		3	1.145107
15min_MT.	Q16555	0.071286	0.074288	0.139322	DPYSL2	Dihydropyr	DPYL2_HUI		14	1.050653
15min_MT.	Q8NDV3	-0.50459	0.074278	0.139322	SMC1B	Structural r	SMC1B_HU		2	0.70486
15min_MT.	Q16698	0.086189	0.074697	0.139981	DECR1	2,4-dienoyl	DECR_HUM		13	1.061562
30min_MT.	Q9H6Y2	0.173674	0.074775	0.140094	WDR55	WD repeat-	WDR55_HU		10	1.127928
30min_MT.	P29083	-0.4619	0.07486	0.140219	GTF2E1	General tra	T2EA_HUM		6	0.726029
15min_MT.	Q01780	0.168898	0.075178	0.140767	EXOSC10	Exosome cc	EXOSX_HUI		1	1.1242
30min_MT.	P61978	-0.11513	0.075252	0.140851	HNRNPK	Heterogene	HNRPK_HU		23	0.923296
15min_MT.	P08779	1.157824	0.075307	0.140888	KRT16	Keratin, typ	K1C16_HUI		21	2.231206

30min_MT.	O75400	-0.11099	0.07546	0.140972	PRPF40A	Pre-mRNA-|	PR40A_HUI	18	0.925951
15min_MT.	O75764	0.529687	0.075584	0.140972	TCEA3	Transcripti	TCEA3_HUI	1	1.443616
15min_MT.	P15516	3.43402	0.075522	0.140972	HTN3	Histatin-3	HIS3_HUM.	1	10.80794
15min_MT.	P53778	0.095395	0.075605	0.140972	MAPK12	Mitogen-ac	MK12_HUI	6	1.068358
15min_MT.	Q9BVJ6	0.445672	0.075581	0.140972	UTP14A	U3 small nu	UT14A_HUI	1	1.361948
15min_MT.	Q9UBC9	2.48342	0.075459	0.140972	SPRR3	Small proli	SPRR3_HUI	8	5.592214
30min_MT.	Q9Y383	0.112929	0.075514	0.140972	LUC7L2	Putative RI	LC7L2_HUI	13	1.081422
15min_MT.	Q9Y5B9	0.115132	0.075451	0.140972	SUPT16H	FACT comp	SP16H_HUI	11	1.083074
30min_MT.	Q96C11	-0.36843	0.075749	0.141162	FGGY	FGGY carbo	FGGY_HUM	2	0.774625
30min_MT.	Q9NSY0	-0.2494	0.075779	0.141162	NRBP2	Nuclear rec	NRBP2_HU	1	0.841245
15min_MT.	Q14318	0.344654	0.075818	0.141202	FKBP8	Peptidyl-pr	FKBP8_HUI	2	1.269847
15min_MT.	O60524	-0.01243	0.075953	0.14142	NEMF	Ribosome c	NEMF_HUM	6	0.991422
30min_MT.	O43765	0.147228	0.076199	0.141582	SGTA	Small gluta	SGTA_HUM	10	1.10744
30min_MT.	Q9Y3L5	-0.44006	0.076206	0.141582	RAP2C	Ras-related	RAP2C_HUI	3	0.737102
15min_MT.	O60568	0.026337	0.076375	0.141801	PLOD3	Multifuncti	PLOD3_HU	22	1.018423
15min_MT.	P09104	0.610329	0.076437	0.141847	ENO2	Gamma-en	ENOG_HUM	2	1.526607
15min_MT.	P10606	0.240365	0.076419	0.141847	COX5B	Cytochrom	COX5B_HU	4	1.181291
30min_MT.	P33176	-0.05048	0.076514	0.141903	KIF5B	Kinesin-1 h	KINH_HUM	43	0.965615
15min_MT.	Q9NXR1	0.028587	0.07654	0.141903	NDE1	Nuclear dis	NDE1_HUM	7	1.020012
15min_MT.	Q9Y266	-0.04784	0.076557	0.141903	NUDC	Nuclear mi	NUDC_HUM	18	0.967382
15min_MT.	P32121	0.189443	0.076582	0.141916	ARRB2	Beta-arresti	ARRB2_HUI	5	1.140323
30min_MT.	Q7Z4W1	0.113543	0.07664	0.141989	DCXR	L-xylulose r	DCXR_HUM	10	1.081882
30min_MT.	Q96IZ7	-0.19216	0.076664	0.141999	RSRC1	Serine/Argi	RSRC1_HUI	5	0.875295
15min_MT.	Q712K3	0.136443	0.076699	0.142031	UBE2R2	Ubiquitin-c	UB2R2_HU	6	1.099192
15min_MT.	Q13885	0.179368	0.076749	0.142056	TUBB2A	Tubulin bet	TBB2A_HUI	5	1.132388
30min_MT.	Q9BYM8	-0.22479	0.076799	0.142116	RBCK1	RanBP-type	HOIL1_HUI	7	0.855717
30min_MT.	Q9UPT5	-0.18323	0.076818	0.142117	EXOC7	Exocyst cor	EXOC7_HUI	8	0.880726
30min_MT.	Q04637	-0.20042	0.076932	0.142261	EIF4G1	Eukaryotic	IF4G1_HUM	50	0.870297
15min_MT.	A1L170	0.246911	0.077035	0.142327	C1orf226	Uncharacte	CA226_HUI	2	1.186663
30min_MT.	A6NDB9	-0.16845	0.077059	0.142327	PALM3	Paralemmi	PALM3_HU	4	0.889799
15min_MT.	Q9H0A8	0.397468	0.077091	0.142327	COMMD4	COMM don	COMD4_HU	2	1.317194
30min_MT.	Q9NUL5	0.285581	0.077095	0.142327	SHFL	Shiftless an	SHFL_HUM	6	1.218901
30min_MT.	Q9Y5B0	0.119033	0.077088	0.142327	CTDP1	RNA polym	CTDP1_HUI	13	1.086007
30min_MT.	P10599	0.150702	0.077149	0.142378	TXN	Thioredoxi	THIO_HUM	7	1.110109
30min_MT.	Q9Y4K3	-0.51114	0.077159	0.142378	TRAF6	TNF recept	TRAF6_HUI	3	0.701669
30min_MT.	Q9H1P3	-0.47609	0.077212	0.142408	OSBPL2	Oxysterol-b	OSBL2_HUI	2	0.718922
30min_MT.	O43464	0.198688	0.077283	0.142472	HTRA2	Serine prot	HTRA2_HUI	6	1.147654
15min_MT.	P09960	-0.10786	0.077275	0.142472	LTA4H	Leukotrien	LKHA4_HUI	31	0.927964
30min_MT.	O15382	0.071491	0.077326	0.142517	BCAT2	Branched-c	BCAT2_HUI	18	1.050802
15min_MT.	P01871	-2.0929	0.077435	0.142634	IGHM	Immunoglo	IGHM_HUM	3	0.234408
15min_MT.	Q9UBF8	0.281131	0.077444	0.142634	PI4KB	Phosphatid	PI4KB_HUM	4	1.215147
30min_MT.	P49790	-0.206	0.077477	0.142661	NUP153	Nuclear po	NU153_HU	7	0.866939
15min_MT.	P52597	-0.11705	0.077573	0.142804	HNRNPF	Heterogene	HNRPF_HU	13	0.92207
30min_MT.	Q5VT25	-0.63324	0.077664	0.142905	CDC42BPA	Serine/thre	MRCKA_HU	2	0.644729
30min_MT.	Q9H7C9	0.27724	0.077646	0.142905	AAMDC	Mth938 do	AAMDC_HU	7	1.211874
15min_MT.	P17812	-0.14449	0.077754	0.143003	CTPS1	CTP syntha	PYRG1_HUI	18	0.9047
15min_MT.	O00330	0.124527	0.077785	0.143027	PDHX	Pyruvate de	ODPX_HUM	16	1.09015
15min_MT.	P32189	0.000471	0.078138	0.143608	GK	Glycerol kir	GLPK_HUM	12	1.000327
30min_MT.	O15372	-0.09696	0.078239	0.143676	EIF3H	Eukaryotic	EIF3H_HUM	13	0.935001
30min_MT.	O43837	-0.31071	0.078301	0.143676	IDH3B	Isocitrate d	IDH3B_HUI	7	0.806245
15min_MT.	Q13952	0.3075	0.078284	0.143676	NFYC	Nuclear tra	NFYC_HUM	3	1.237561
15min_MT.	Q8NFH4	-0.08091	0.078259	0.143676	NUP37	Nucleopori	NUP37_HU	9	0.945461

15min_MT.	Q9P0R6	0.515667	0.078322	0.143676	GSKIP	GSK3B-inte	GSKIP_HUM	2	1.429655
30min_MT.	Q9UIJ7	0.057266	0.078383	0.14372	AK3	GTP:AMP p	KAD3_HUM	11	1.040492
15min_MT.	O60232	0.24241	0.078431	0.143749	ZNRD2	Protein ZNI	ZNRD2_HU	9	1.182967
15min_MT.	Q969E8	0.199386	0.078476	0.143749	TSR2	Pre-rRNA-p	TSR2_HUM	1	1.14821
30min_MT.	Q96DG6	0.107036	0.07849	0.143749	CMBL	Carboxyme	CMBL_HUM	12	1.077014
30min_MT.	Q9C0D3	-0.2805	0.078487	0.143749	ZYG11B	Protein zyg	ZY11B_HUI	6	0.823308
15min_MT.	A4D126	-0.01568	0.078512	0.143754	CRPPA	D-ribitol-5-	ISPD_HUM.	6	0.989192
30min_MT.	Q9NP72	0.052241	0.078587	0.143859	RAB18	Ras-related	RAB18_HUI	8	1.036874
15min_MT.	Q9UBQ0	-0.15169	0.078699	0.143907	VPS29	Vacuolar pı	VPS29_HUI	6	0.900198
30min_MT.	Q14258	-0.1483	0.078751	0.143957	TRIM25	E3 ubiquiti	TRI25_HUM	20	0.902314
15min_MT.	A9UHW6	0.183295	0.079012	0.144202	MIF4GD	MIF4G dom	MI4GD_HU	6	1.135474
30min_MT.	P52630	0.340568	0.078972	0.144202	STAT2	Signal trans	STAT2_HUM	2	1.266255
15min_MT.	Q96FC7	0.012564	0.079014	0.144202	PHYHIPL	Phytanoyl-(	PHIPL_HUM	14	1.008747
30min_MT.	Q96SB3	0.11381	0.078933	0.144202	PPP1R9B	Neurabin-2	NEB2_HUM	8	1.082082
15min_MT.	Q9C0I1	-0.23064	0.078995	0.144202	MTMR12	Myotubula	MTMRC_HU	9	0.852258
30min_MT.	Q9NTM9	0.255069	0.078962	0.144202	CUTC	Copper hor	CUTC_HUM	8	1.193393
30min_MT.	Q58FG1	0.206186	0.079065	0.14426	HSP90AA4I	Putative he	HS904_HUI	2	1.153634
30min_MT.	Q8N5M4	-0.46595	0.07921	0.144391	TTC9C	Tetratricop	TTC9C_HUI	1	0.723994
30min_MT.	P24666	0.217121	0.079364	0.144544	ACP1	Low molecı	PPAC_HUM	4	1.162411
15min_MT.	Q6IBS0	-0.11056	0.079383	0.144544	TWF2	Twinfilin-2	TWF2_HUM	15	0.926231
15min_MT.	Q9P287	0.136273	0.079386	0.144544	BCCIP	BRCA2 and	BCCIP_HUM	14	1.099062
30min_MT.	Q01484	-0.10907	0.079413	0.144559	ANK2	Ankyrin-2	ANK2_HUM	17	0.927185
15min_MT.	C4AMC7;Q(	-0.32275	0.07948	0.144626	WASH3P;W	Putative W	WASH3_HU	4	0.799542
30min_MT.	O60936	0.242505	0.07957	0.144631	NOL3	Nucleolar p	NOL3_HUM	1	1.183045
15min_MT.	P25789	0.138225	0.079582	0.144631	PSMA4	Proteasom	PSA4_HUM	11	1.10055
15min_MT.	Q16531	0.081565	0.079581	0.144631	DDB1	DNA damag	DDB1_HUM	56	1.058165
15min_MT.	Q96P20	1.305139	0.079582	0.144631	NLRP3	NACHT, LRI	NLRP3_HUI	1	2.471075
15min_MT.	Q9H0U4	-0.08043	0.079532	0.144631	RAB1B	Ras-related	RAB1B_HUI	8	0.945776
15min_MT.	P25815	0.123148	0.079744	0.144892	S100P	Protein S1(	S100P_HUI	2	1.089108
15min_MT.	Q9BSR8	0.246479	0.079792	0.144898	YIPF4	Protein YIP	YIPF4_HUM	2	1.186308
30min_MT.	Q9BTD8	-1.2836	0.079783	0.144898	RBM42	RNA-bindir	RBM42_HU	1	0.410769
15min_MT.	P16220	0.235153	0.079849	0.144947	CREB1	Cyclic AMP	CREB1_HUI	3	1.177032
15min_MT.	Q9HCN4	0.166419	0.079897	0.144968	GPN1	GPN-loop G	GPN1_HUM	5	1.122269
15min_MT.	O43681	0.132914	0.080204	0.145389	GET3	ATPase GET	GET3_HUM	11	1.096506
30min_MT.	P05546	-0.48183	0.080334	0.14549	SERPIND1	Heparin co	HEP2_HUM	3	0.71607
15min_MT.	Q96D71	0.188141	0.080372	0.1455	REPS1	RalBP1-ass	REPS1_HUM	15	1.139295
15min_MT.	Q9BRQ0	0.245216	0.080376	0.1455	PYGO2	Pygopus hc	PYGO2_HU	3	1.18527
30min_MT.	A0AVT1	-0.10631	0.080422	0.145515	UBA6	Ubiquitin-l	UBA6_HUM	32	0.928963
30min_MT.	Q9H0W9	0.265942	0.080432	0.145515	C11orf54	Ester hydro	CK054_HUI	10	1.202421
30min_MT.	Q9NX24	-0.07728	0.080638	0.145772	NHP2	H/ACA ribo	NHP2_HUM	4	0.947844
15min_MT.	P23193	0.119858	0.080741	0.145827	TCEA1	Transcripti	TCEA1_HUI	16	1.086628
30min_MT.	P51808	-0.73425	0.080743	0.145827	DYNLT3	Dynein ligh	DYLT3_HUM	3	0.60113
15min_MT.	Q8IZ73	0.187984	0.080695	0.145827	RPUSD2	Pseudourid	RUSD2_HU	14	1.139171
15min_MT.	Q96D46	-0.23973	0.080724	0.145827	NMD3	60S riboso	NMD3_HUI	10	0.846903
15min_MT.	Q8IYB8	-0.02531	0.080847	0.145912	SUPV3L1	ATP-depen	SUV3_HUM	22	0.982608
15min_MT.	O43865	-0.06698	0.080885	0.145948	AHCYL1	S-adenosyl	SAHH2_HU	5	0.954633
30min_MT.	O15258	-0.56114	0.081134	0.146043	RER1	Protein REI	RER1_HUM	3	0.677767
30min_MT.	O60235;Q8	-0.3137	0.081143	0.146043	TMPRSS11I	Transmemb	TM11D_HU	1	0.804577
30min_MT.	Q16595	0.157205	0.081103	0.146043	FXN	Frataxin, m	FRDA_HUM	4	1.115125
30min_MT.	Q7Z478	-0.13259	0.080986	0.146043	DHX29	ATP-depen	DHX29_HU	7	0.912193
15min_MT.	Q99590	-0.16998	0.081099	0.146043	SCAF11	Protein SC/	SCAFB_HUI	11	0.888857
15min_MT.	O75369	0.056553	0.081233	0.146138	FLNB	Filamin-B	FLNB_HUM	67	1.039978

15min_MT.	P09110	0.105479	0.081225	0.146138	ACAA1	3-ketoacyl-	THIK_HUM.	9	1.075851
30min_MT.	Q96MK2	-0.32525	0.081278	0.146185	RIPOR3	RIPOR fami	RIPR3_HUN	2	0.798162
30min_MT.	P16298	0.162062	0.081435	0.146366	PPP3CB	Serine/thre	PP2BB_HUI	10	1.118885
30min_MT.	Q9Y3C4	0.443767	0.081428	0.146366	TPRKB	EKC/KEOPS	TPRKB_HUI	2	1.360151
15min_MT.	P16333	0.125482	0.081522	0.146421	NCK1	SH2/SH3 ac	NCK1_HUM	6	1.090872
15min_MT.	Q53T59	0.171504	0.081516	0.146421	HS1BP3	HCLS1-binc	H1BP3_HU	12	1.126232
15min_MT.	Q96TA2	0.049887	0.081582	0.146477	YME1L1	ATP-depen(	YMEL1_HUI	5	1.035184
15min_MT.	Q8WX93	-0.05703	0.081648	0.14648	PALLD	Palladin	PALLD_HUI	8	0.961238
15min_MT.	Q8WZ42	0.117701	0.081637	0.14648	TTN	Titin	TITIN_HUM	8	1.085004
30min_MT.	P12532;P1	0.551755	0.08176	0.146647	CKMT1A;CK	Creatine kiı	KCRU_HUM	1	1.465868
15min_MT.	Q14152	-0.13143	0.081859	0.146707	EIF3A	Eukaryotic	EIF3A_HUM	39	0.912923
15min_MT.	Q9BQ52	-0.10062	0.081866	0.146707	ELAC2	Zinc phospl	RNZ2_HUM	25	0.93263
15min_MT.	Q9NT62	0.408044	0.081868	0.146707	ATG3	Ubiquitin-l	ATG3_HUM	6	1.326886
15min_MT.	P30040	0.13601	0.081943	0.14674	ERP29	Endoplasm	ERP29_HUI	10	1.098862
30min_MT.	P61106	0.076475	0.081924	0.14674	RAB14	Ras-related	RAB14_HUI	13	1.054438
30min_MT.	Q9NV96	0.187836	0.08193	0.14674	TMEM30A	Cell cycle c	CC50A_HUI	1	1.139054
30min_MT.	Q9Y262	-0.08241	0.082185	0.14714	EIF3L	Eukaryotic	EIF3L_HUM	24	0.94448
15min_MT.	Q7L576	-0.19333	0.082272	0.147261	CYFIP1	Cytoplasmi	CYFP1_HUI	13	0.874586
30min_MT.	P49419	0.054812	0.082347	0.147295	ALDH7A1	Alpha-amir	AL7A1_HUI	24	1.038724
30min_MT.	P53384	-0.21438	0.082345	0.147295	NUBP1	Cytosolic F	NUBP1_HU	5	0.861916
15min_MT.	Q969Z0	-0.3558	0.082331	0.147295	TBRG4	FAST kinase	FAKD4_HUI	8	0.781435
15min_MT.	O95456	0.059364	0.082434	0.147349	PSMG1	Proteasom	PSMG1_HU	13	1.042006
30min_MT.	Q9BRT6	-0.95797	0.082523	0.14744	LLPH	Protein LLF	LLPH_HUM	2	0.514781
30min_MT.	Q5T6J7	0.167907	0.082601	0.147545	IDNK	Probable gl	GNTK_HUM	1	1.123427
15min_MT.	O95562	-0.06283	0.082743	0.147665	SFT2D2	Vesicle trar	SFT2B_HUN	1	0.957387
15min_MT.	P54727	0.159312	0.082729	0.147665	RAD23B	UV excision	RD23B_HU	15	1.116754
30min_MT.	Q9BXJ9	-0.16925	0.082742	0.147665	NAA15	N-alpha-ace	NAA15_HU	23	0.889303
15min_MT.	O94973	-0.04995	0.082809	0.147749	AP2A2	AP-2 compl	AP2A2_HUI	19	0.965971
30min_MT.	P07942	0.071037	0.082866	0.147782	LAMB1	Laminin sul	LAMB1_HU	26	1.050472
15min_MT.	Q9Y333	-0.08936	0.082865	0.147782	LSM2	U6 snRNA-a	LSM2_HUM	5	0.939938
30min_MT.	Q8IW45	0.040246	0.082908	0.147796	NAXD	ATP-depen(	NNRD_HUM	11	1.028289
15min_MT.	O43852	-0.26348	0.082938	0.147797	CALU	Calumenin	CALU_HUM	14	0.833074
15min_MT.	P82909	-0.10373	0.082954	0.147797	KGD4	Alpha-keto	KGD4_HUM	2	0.930624
30min_MT.	Q9H857	-0.06017	0.082968	0.147797	NT5DC2	5'-nucleoti	NT5D2_HU	16	0.95915
15min_MT.	Q7L014	-0.04333	0.083062	0.147896	DDX46	Probable A	DDX46_HU	27	0.970415
15min_MT.	P11166	-0.23466	0.083092	0.147916	SLC2A1	Solute carri	GTR1_HUM	1	0.849887
30min_MT.	Q14498	-0.16071	0.083215	0.148102	RBM39	RNA-bindir	RBM39_HU	9	0.894586
30min_MT.	Q8IV50	0.218457	0.083245	0.148122	LYSMD2	LysM and p	LYSM2_HUI	2	1.163489
15min_MT.	Q8IWT0	-0.24354	0.083266	0.148125	ZBTB8OS	Protein arc	ARCH_HUM	6	0.844671
15min_MT.	P60468	-0.47768	0.08329	0.148134	SEC61B	Protein trai	SC61B_HUI	1	0.718131
15min_MT.	P04181	0.134422	0.083376	0.148167	OAT	Ornithine a	OAT_HUMA	12	1.097653
30min_MT.	P15252	3.728183	0.083384	0.148167	NaN	Rubber elo	REF_HEVBR	4	13.25241
30min_MT.	Q9Y6N5	-0.06061	0.083362	0.148167	SQOR	Sulfide:qui	SQOR_HUM	17	0.958857
30min_MT.	O00762	1.47401	0.083503	0.148276	UBE2C	Ubiquitin-c	UBE2C_HUI	2	2.77793
30min_MT.	Q9UNH6	-0.19485	0.083535	0.1483	SNX7	Sorting nex	SNX7_HUM	5	0.873664
15min_MT.	P34741	-0.34546	0.083576	0.148323	SDC2	Syndecan-2	SDC2_HUM	3	0.787055
30min_MT.	Q0JRZ9	0.428361	0.083586	0.148323	FCHO2	F-BAR dom	FCHO2_HU	8	1.345704
30min_MT.	O95104	-0.20176	0.083648	0.148344	SCAF4	SR-related	SCAF4_HUN	8	0.869487
15min_MT.	Q15054	-0.5143	0.083655	0.148344	POLD3	DNA polym	DPOD3_HU	1	0.700131
30min_MT.	Q92667	0.088931	0.083641	0.148344	AKAP1	A-kinase an	AKAP1_HUI	2	1.063581
15min_MT.	O95159	0.036064	0.083726	0.14835	ZFPL1	Zinc finger	ZFPL1_HUN	3	1.025313
30min_MT.	Q86UY8	0.098265	0.083701	0.14835	NT5DC3	5'-nucleoti	NT5D3_HU	9	1.070485

15min_MT.	Q8IXH7	0.277227	0.083771	0.148382	NELFCD	Negative el	NELFD_HU	8	1.211863
30min_MT.	Q96P47	-0.44492	0.083796	0.148393	AGAP3	Arf-GAP wit	AGAP3_HU	1	0.734623
30min_MT.	Q8TBN0	0.153823	0.083863	0.148477	RAB3IL1	Guanine nu	R3GEF_HU	4	1.112513
15min_MT.	P01040	0.410291	0.084043	0.148763	CSTA	Cystatin-A	CYTA_HUM	7	1.328954
15min_MT.	O75190	0.012318	0.084196	0.148966	DNAJB6	DnaJ homo	DNJB6_HU	3	1.008574
15min_MT.	Q15532	0.708361	0.084195	0.148966	SS18	Protein SSX	SSXT_HUM	1	1.633947
15min_MT.	Q86XA9	-0.33482	0.084224	0.148983	HEATR5A	HEAT repea	HTR5A_HU	2	0.792882
15min_MT.	Q6IA86	-0.0307	0.084286	0.149024	ELP2	Elongator c	ELP2_HUM	14	0.978947
30min_MT.	Q96FV2	0.218666	0.084285	0.149024	SCRN2	Secernin-2	SCRN2_HU	7	1.163657
15min_MT.	Q9Y520	0.149983	0.084372	0.14911	PRRC2C	Protein PR	PRC2C_HU	32	1.109556
30min_MT.	Q99496	-0.44862	0.08445	0.149214	RNF2	E3 ubiquiti	RING2_HU	2	0.732741
30min_MT.	Q7Z739	-0.12501	0.084515	0.149227	YTHDF3	YTH domai	YTHD3_HU	12	0.916996
30min_MT.	Q9UJA5	0.117492	0.0845	0.149227	TRMT6	tRNA (aden	TRM6_HUM	15	1.084847
30min_MT.	Q96IZ0	-0.27548	0.084637	0.149409	PAWR	PRKC apop	PAWR_HUM	10	0.826173
30min_MT.	P52594	-0.0819	0.084708	0.149467	AGFG1	Arf-GAP do	AGFG1_HU	9	0.944812
15min_MT.	Q9NZL9	0.292179	0.084766	0.149468	MAT2B	Methionine	MAT2B_HU	11	1.224488
15min_MT.	P62304	0.087143	0.08491	0.149621	SNRPE	Small nucle	RUXE_HUM	2	1.062264
15min_MT.	Q96GM5	-0.43573	0.084907	0.149621	SMARCD1	SWI/SNF-re	SMRD1_HU	2	0.739318
15min_MT.	Q86UK7	0.333467	0.085046	0.149826	ZNF598	E3 ubiquiti	ZN598_HU	4	1.260038
15min_MT.	Q16836	0.166284	0.08511	0.149872	HADH	Hydroxyacy	HCDH_HUM	9	1.122164
15min_MT.	Q96FX2	-1.06397	0.085137	0.149886	DPH3	Diphthami	DPH3_HUM	1	0.478314
15min_MT.	Q8TCF1	0.267204	0.08516	0.149893	ZFAND1	AN1-type zi	ZFAN1_HU	2	1.203473
30min_MT.	P16989	0.249944	0.085309	0.149986	YBX3	Y-box-bindi	YBOX3_HU	10	1.189161
15min_MT.	Q9Y6X3	0.348202	0.085308	0.149986	MAU2	MAU2 chro	SCC4_HUM	3	1.272973
30min_MT.	Q8TEV9	0.012256	0.085373	0.150064	SMCR8	Guanine nu	SMCR8_HU	2	1.008531
15min_MT.	Q13496	-0.00582	0.0855	0.150165	MTM1	Myotubula	MTM1_HU	11	0.995972
30min_MT.	Q96C23	0.196922	0.085507	0.150165	GALM	Galactose n	GALM_HUM	5	1.14625
30min_MT.	Q9BUT9	-0.39999	0.085458	0.150165	MCRIP2	MAPK regul	MCRI2_HU	1	0.757862
15min_MT.	P09661	0.122412	0.08553	0.150172	SNRPA1	U2 small nu	RU2A_HUM	12	1.088553
15min_MT.	Q13685	0.150464	0.085594	0.150251	AAMP	Angio-asso	AAMP_HUM	11	1.109926
30min_MT.	P08240	-0.02618	0.085715	0.150295	SRPRA	Signal reco	SRPRA_HU	6	0.982016
30min_MT.	P35913	0.042073	0.085688	0.150295	PDE6B	Rod cGMP-	PDE6B_HU	1	1.029592
15min_MT.	Q15126	0.019921	0.085741	0.150295	PMVK	Phosphom	PMVK_HUM	9	1.013904
30min_MT.	Q96MX6	-0.08227	0.085654	0.150295	DNAAF10	Dynein axo	DAA10_HU	8	0.944568
15min_MT.	Q9BV94	0.014301	0.085739	0.150295	EDEM2	ER degrada	EDEM2_HU	4	1.009962
15min_MT.	Q9GZM5	0.171702	0.085727	0.150295	YIPF3	Protein YIP	YIPF3_HUM	3	1.126386
15min_MT.	Q2NL82	-0.10637	0.085847	0.15039	TSR1	Pre-rRNA-p	TSR1_HUM	3	0.92892
30min_MT.	Q9Y305	-0.16168	0.086012	0.150646	ACOT9	Acyl-coenz	ACOT9_HU	11	0.893984
15min_MT.	P13010	-0.13921	0.086105	0.150741	XRCC5	X-ray repair	XRCC5_HU	26	0.908016
30min_MT.	P22061	0.113715	0.086176	0.150798	PCMT1	Protein-L-is	PIMT_HUM	10	1.082011
30min_MT.	Q16719	0.11263	0.086157	0.150798	KYNU	Kynurenina	KYNU_HUM	18	1.081197
30min_MT.	P63098	-0.05293	0.086202	0.150811	PPP3R1	Calcineurin	CANB1_HU	2	0.963977
15min_MT.	Q5T6J7	0.166446	0.086252	0.150815	IDNK	Probable gl	GNTK_HUM	1	1.12229
30min_MT.	Q9Y666	0.007495	0.086228	0.150815	SLC12A7	Solute carri	S12A7_HU	2	1.005209
15min_MT.	P62195	0.16263	0.086361	0.15092	PSMC5	26S protea	PRS8_HUM	21	1.119326
15min_MT.	O75356	-0.41105	0.0864	0.150921	ENTPD5	Nucleoside	ENTP5_HU	5	0.752076
30min_MT.	Q9NRV9	0.18478	0.086389	0.150921	HEBP1	Heme-bind	HEBP1_HU	9	1.136644
15min_MT.	Q15120	0.086256	0.086508	0.151041	PDK3	[Pyruvate d	PDK3_HUM	6	1.061612
30min_MT.	P09543	-0.1062	0.08659	0.151083	CNP	2',3'-cyclic-	CN37_HUM	15	0.929031
15min_MT.	P62820	-0.08136	0.08658	0.151083	RAB1A	Ras-related	RAB1A_HU	6	0.945166
15min_MT.	Q8IZP0	0.25094	0.086579	0.151083	ABI1	Abl interac	ABI1_HUM	6	1.189983
15min_MT.	Q96M96	0.099152	0.086662	0.151175	FGD4	FYVE, RhoG	FGD4_HUM	10	1.071143

15min_MT.	Q7Z7A4	-0.41772	0.086929	0.151606	PXK	PX domain	PXK_HUMA	3	0.748608
15min_MT.	P23434	-0.3055	0.087155	0.15183	GCSH	Glycine cle	GCSH_HUM	4	0.809163
15min_MT.	Q09028	0.129884	0.087173	0.15183	RBBP4	Histone-bir	RBBP4_HUI	12	1.094206
30min_MT.	P00491	0.14546	0.087221	0.15188	PNP	Purine nucl	PNPH_HUM	16	1.106084
30min_MT.	O60884	-0.06922	0.087295	0.151931	DNAJA2	DnaJ homo	DNJA2_HUI	12	0.953151
15min_MT.	P08133	0.086827	0.087379	0.151931	ANXA6	Annexin A6	ANXA6_HU	20	1.062032
30min_MT.	Q92600	-0.17097	0.087301	0.151931	CNOT9	CCR4-NOT t	CNOT9_HU	7	0.888248
15min_MT.	Q9BTL3	0.242159	0.08738	0.151931	RAMAC	RNA guanin	RAMAC_HU	5	1.182762
15min_MT.	Q96I24	0.19336	0.087529	0.152145	FUBP3	Far upstrea	FUBP3_HUI	16	1.143423
30min_MT.	P35580	-0.1397	0.087604	0.152173	MYH10	Myosin-10	MYH10_HU	55	0.907709
15min_MT.	Q6ZSJ8	0.104536	0.08759	0.152173	C1orf122	Uncharacte	CA122_HUI	2	1.075149
30min_MT.	Q14764	-0.0984	0.087684	0.152244	MVP	Major vault	MVP_HUMA	33	0.934066
15min_MT.	Q9Y2Q5	0.095188	0.087758	0.15234	LAMTOR2	Ragulator c	LTOR2_HUI	3	1.068204
15min_MT.	Q9NVX2	0.094819	0.087829	0.152428	NLE1	Notchless p	NLE1_HUM	13	1.067931
30min_MT.	Q7L8L6	-0.47109	0.087848	0.152428	FASTKD5	FAST kinase	FAKD5_HUI	6	0.721419
15min_MT.	Q12882	-0.03616	0.088053	0.152683	DPYD	Dihydropyr	DPYD_HUM	15	0.975248
30min_MT.	Q0IIM8	-0.14618	0.088212	0.152859	TBC1D8B	TBC1 doma	TBC8B_HUI	11	0.903637
15min_MT.	Q9P258	-0.11001	0.088213	0.152859	RCC2	Protein RC(	RCC2_HUM	15	0.926581
15min_MT.	Q9NUQ9	0.084288	0.088283	0.152911	CYRIB	CYFIP-relat	CYRIB_HUN	14	1.060165
30min_MT.	P04439	0.28373	0.08841	0.152942	HLA-A	HLA class I	HLAA_HUM	3	1.217338
30min_MT.	P07384	-0.15476	0.088482	0.152942	CAPN1	Calpain-1 c	CAN1_HUM	29	0.898282
30min_MT.	P38432	0.088689	0.088463	0.152942	COIL	Coilin	COIL_HUMA	1	1.063404
30min_MT.	Q12849	0.226562	0.088442	0.152942	GRSF1	G-rich sequ	GRSF1_HUI	6	1.170043
30min_MT.	Q9BSE5	0.08233	0.088406	0.152942	AGMAT	Guanidino	GDAH_HUM	14	1.058727
15min_MT.	Q9NPF4	0.138588	0.088496	0.152942	OSGEP	tRNA N6-ad	OSGEP_HUI	8	1.100827
15min_MT.	Q9NTM9	0.107304	0.088443	0.152942	CUTC	Copper hor	CUTC_HUM	8	1.077214
30min_MT.	Q9NZU5	-0.07956	0.088395	0.152942	LMCD1	LIM and cys	LMCD1_HU	14	0.946348
15min_MT.	Q9UBE0	0.083451	0.088477	0.152942	SAE1	SUMO-activ	SAE1_HUM	17	1.059549
15min_MT.	Q6IQ23	1.660933	0.088528	0.152964	PLEKHA7	Pleckstrin I	PKHA7_HU	1	3.162209
30min_MT.	P09417	0.032238	0.088684	0.152994	QDPR	Dihydropte	DHPR_HUM	7	1.022597
15min_MT.	P45974	0.059554	0.088698	0.152994	USP5	Ubiquitin c	UBP5_HUM	27	1.042143
30min_MT.	Q03252	-0.02325	0.088702	0.152994	LMNB2	Lamin-B2	LMNB2_HU	9	0.984015
15min_MT.	Q9NUP9	0.330831	0.088628	0.152994	LIN7C	Protein lin-	LIN7C_HUM	4	1.257737
15min_MT.	Q9NVA2	-0.03251	0.088685	0.152994	SEPTIN11	Septin-11	SEP11_HUM	14	0.97772
15min_MT.	Q9NX74	0.56416	0.088567	0.152994	DUS2	tRNA-dihyd	DUS2L_HUI	2	1.478526
30min_MT.	O14727	-0.29178	0.088847	0.153092	APAF1	Apoptotic p	APAF_HUM	5	0.816894
30min_MT.	O43149	0.272839	0.088876	0.153092	ZZEF1	Zinc finger	ZZEF1_HUM	6	1.208183
30min_MT.	P38571	0.062002	0.088851	0.153092	LIPA	Lysosomal	LICH_HUMA	3	1.043914
15min_MT.	Q13162	-0.27208	0.088869	0.153092	PRDX4	Peroxiredo	PRDX4_HU	11	0.828123
30min_MT.	Q9UMY4	0.036846	0.088875	0.153092	SNX12	Sorting nex	SNX12_HUI	6	1.025868
15min_MT.	O60220	0.085749	0.088909	0.153115	TIMM8A	Mitochond	TIM8A_HUI	3	1.061239
15min_MT.	Q14849	0.549497	0.08894	0.153135	STARD3	StAR-relate	STAR3_HUM	1	1.463575
30min_MT.	P23396	-0.1615	0.089024	0.153245	RPS3	Small ribos	RS3_HUMA	15	0.894093
30min_MT.	Q96BM9	0.245744	0.089126	0.153353	ARL8A	ADP-ribosy	ARL8A_HUI	3	1.185704
30min_MT.	O76061	0.453049	0.089241	0.153517	STC2	Stanniocal	STC2_HUM	1	1.368931
15min_MT.	P26368	-0.15227	0.08939	0.153673	U2AF2	Splicing fac	U2AF2_HUI	12	0.899833
15min_MT.	Q5VWQ0	0.210091	0.089375	0.153673	RSBN1	Lysine-spec	RSBN1_HUI	9	1.156761
30min_MT.	Q13287	0.090598	0.089474	0.153783	NMI	N-myc-inte	NMI_HUMA	10	1.064812
30min_MT.	Q9P0V9	-0.20451	0.089501	0.153796	SEPTIN10	Septin-10	SEP10_HUM	5	0.867833
15min_MT.	Q96P16	0.278306	0.089547	0.153841	RPRD1A	Regulation	RPR1A_HUI	5	1.21277
15min_MT.	Q4V328	0.198408	0.089582	0.153868	GRIPAP1	GRIP1-asso	GRAP1_HU	22	1.147432
15min_MT.	O15260	0.051201	0.089676	0.153994	SURF4	Surfeit locu	SURF4_HUI	1	1.036127

15min_MT.	P27635	-0.30981	0.089778	0.154044	RPL10	Large ribos	RL10_HUM	4	0.806749
30min_MT.	Q9Y5S9	-0.28894	0.089784	0.154044	RBM8A	RNA-bindir	RBM8A_HU	5	0.818503
15min_MT.	Q9UJX6	0.312759	0.090018	0.154413	ANAPC2	Anaphase-p	ANC2_HUM	9	1.242081
30min_MT.	P60520	0.147642	0.090062	0.154421	GABARAPL2	Gamma-am	GBRL2_HU	6	1.107757
15min_MT.	P42766	-0.20822	0.090102	0.154455	RPL35	Large ribos	RL35_HUM	2	0.865604
30min_MT.	Q9Y2L1	-0.12428	0.090184	0.154561	DIS3	Exosome cc	RRP44_HU	22	0.917464
15min_MT.	O95819	0.204586	0.090246	0.154634	MAP4K4	Mitogen-ac	M4K4_HUM	7	1.152356
30min_MT.	P01031	-0.04844	0.090305	0.154702	C5	Complemen	CO5_HUMA	16	0.96698
30min_MT.	P15927	0.031743	0.090581	0.154923	RPA2	Replication	RFA2_HUM	6	1.022246
30min_MT.	P28332	-0.15056	0.090543	0.154923	ADH6	Alcohol del	ADH6_HUM	12	0.900902
15min_MT.	P56962	-0.64264	0.090589	0.154923	STX17	Syntaxin-17	STX17_HUM	1	0.640538
30min_MT.	Q7L014	-0.02684	0.090498	0.154923	DDX46	Probable AT	DDX46_HU	27	0.981569
15min_MT.	O76094	-0.23072	0.090693	0.154998	SRP72	Signal reco	SRP72_HUI	7	0.852208
30min_MT.	P11940	0.067498	0.090696	0.154998	PABPC1	Polyadenyl	PABP1_HUI	23	1.047898
15min_MT.	Q9HBI1	-0.23744	0.090932	0.155368	PARVB	Beta-parvin	PARVB_HUI	5	0.848252
30min_MT.	P19367	0.116924	0.090982	0.155413	HK1	Hexokinase	HXK1_HUM	21	1.08442
30min_MT.	O15230	0.04558	0.091328	0.15562	LAMA5	Laminin sul	LAMA5_HU	41	1.032098
30min_MT.	P10586	-0.07165	0.091329	0.15562	PTPRF	Receptor-ty	PTPRF_HUN	24	0.95155
30min_MT.	P49773	0.147553	0.091358	0.15562	HINT1	Adenosine 5	HINT1_HUN	4	1.107689
30min_MT.	Q8IYQ7	-0.16878	0.091348	0.15562	THNSL1	Threonine s	THNS1_HUI	3	0.889597
15min_MT.	Q92625	0.078099	0.091256	0.15562	ANKS1A	Ankyrin rep	ANS1A_HUI	2	1.055626
30min_MT.	Q9BQ04	-0.05268	0.091265	0.15562	RBM4B	RNA-bindir	RBM4B_HU	13	0.964141
15min_MT.	Q9H6K1	0.317393	0.091359	0.15562	ILRUN	Protein ILR	ILRUN_HUN	1	1.246077
15min_MT.	Q9Y2T2	0.135665	0.091336	0.15562	AP3M1	AP-3 compl	AP3M1_HU	5	1.098599
30min_MT.	P53621	-0.06385	0.091486	0.155726	COPA	Coatomer s	COPA_HUM	54	0.956707
15min_MT.	Q13268	0.197118	0.0915	0.155726	DHRS2	Dehydroger	DHRS2_HU	1	1.146406
15min_MT.	Q16352	0.294523	0.091478	0.155726	INA	Alpha-inter	AINX_HUM	2	1.22648
30min_MT.	Q96MW1	0.331221	0.091527	0.155737	CCDC43	Coiled-coil	CCD43_HU	7	1.258078
30min_MT.	P83881	-0.14903	0.09159	0.15581	RPL36A	Large ribos	RL36A_HUI	4	0.901858
30min_MT.	P49257	-0.17901	0.091654	0.155853	LMAN1	Protein ERC	LMAN1_HU	4	0.883309
15min_MT.	Q9BZ67	0.194736	0.091655	0.155853	FRMD8	FERM doma	FRMD8_HU	6	1.144514
15min_MT.	Q9BRP8	0.129834	0.091778	0.156026	PYM1	Partner of Y	PYM1_HUM	8	1.094168
15min_MT.	Q9BS26	0.125179	0.091797	0.156026	ERP44	Endoplasm	ERP44_HUI	13	1.090643
15min_MT.	P19827	0.630636	0.091828	0.156047	ITIH1	Inter-alpha	ITIH1_HUM	17	1.548248
15min_MT.	Q86UA6	-0.00156	0.09185	0.156049	RPAIN	RPA-interac	RIP_HUMAI	1	0.998916
30min_MT.	P47224	0.360413	0.091895	0.156092	RABIF	Guanine nu	MSS4_HUM	2	1.283793
30min_MT.	O95678	1.33136	0.09195	0.156117	KRT75	Keratin, typ	K2C75_HUI	1	2.516399
30min_MT.	P50747	-0.08752	0.091936	0.156117	HLCS	Biotin--pro	BPL1_HUM	8	0.941136
30min_MT.	P02795;P0	-0.15448	0.092178	0.156302	MT2A;MT1I	Metallothic	MT2_HUMA	1	0.898455
30min_MT.	Q5T2E6	-0.1852	0.092147	0.156302	ARMH3	Armadillo-l	ARMD3_HU	11	0.879529
15min_MT.	Q6P179	-0.26889	0.09216	0.156302	ERAP2	Endoplasm	ERAP2_HUI	5	0.829955
30min_MT.	Q86TB9	-0.4625	0.092125	0.156302	PATL1	Protein PAT	PATL1_HUN	1	0.725727
15min_MT.	Q9BRJ2	0.844577	0.092248	0.156385	MRPL45	Large ribos	RM45_HUM	1	1.795739
15min_MT.	O95848	0.246851	0.092462	0.156579	NUDT14	Uridine dip	NUD14_HU	2	1.186614
30min_MT.	Q9BPX5	0.295605	0.092423	0.156579	ARPC5L	Actin-relate	ARP5L_HUI	2	1.227399
15min_MT.	Q16143	0.200774	0.092587	0.156704	SNCB	Beta-synucl	SYUB_HUM	1	1.149315
15min_MT.	Q86YZ3	-0.2803	0.092577	0.156704	HRNR	Hornerin	HORN_HUM	28	0.823421
15min_MT.	O14979	0.284567	0.09264	0.156744	HNRNPDL	Heterogene	HNRDL_HU	7	1.218044
15min_MT.	Q6P1N0	-0.09404	0.092689	0.156794	CC2D1A	Coiled-coil	C2D1A_HU	2	0.936894
30min_MT.	Q5JPH6	0.009498	0.092719	0.156806	EARS2	Nondiscrim	SYEM_HUM	9	1.006605
15min_MT.	O15357	-0.47106	0.092787	0.156824	INPPL1	Phosphatid	SHIP2_HUM	4	0.721434
15min_MT.	P48681	0.931411	0.09287	0.156896	NES	Nestin	NEST_HUM	1	1.90714

15min_MT.	Q00403	0.0897	0.092902	0.156917	GTF2B	Transcripti	TF2B_HUM	3	1.064149
30min_MT.	P06730	0.114978	0.092965	0.156988	EIF4E	Eukaryotic	IF4E_HUM/	8	1.082958
30min_MT.	P40429;Q6	0.155897	0.09313	0.157234	RPL13A;RP	Large ribos	RL13A_HUI	3	1.114114
30min_MT.	O95210	-0.03754	0.093162	0.15724	STBD1	Starch-binc	STBD1_HUI	7	0.974316
15min_MT.	Q96C19	0.161755	0.093215	0.157275	EFHD2	EF-hand do	EFHD2_HUI	13	1.118647
30min_MT.	P62851	-0.22907	0.093483	0.157592	RPS25	Small ribos	RS25_HUM	4	0.853185
30min_MT.	P04004	-0.28094	0.093631	0.157611	VTN	Vitronectir	VTNC_HUM	11	0.823053
30min_MT.	Q06546	0.186766	0.093522	0.157611	GABPA	GA-binding	GABPA_HU	9	1.138209
15min_MT.	Q96EV2	-0.48857	0.093573	0.157611	RBM33	RNA-bindir	RBM33_HU	5	0.712733
15min_MT.	Q9BV20	0.04134	0.093578	0.157611	MRI1	Methylthio	MTNA_HUM	19	1.029069
15min_MT.	P05154	0.036542	0.093699	0.157684	SERPINA5	Plasma seri	IPSP_HUM/	2	1.025652
30min_MT.	P05771;P1	0.807611	0.09389	0.15794	PRKCB;PRK	Protein kin	KPCB_HUM	1	1.75031
30min_MT.	Q9BR11	0.164787	0.093892	0.15794	ZSWIM1	Zinc finger	ZSWM1_HU	1	1.121001
30min_MT.	P07738	0.168661	0.093923	0.157959	BPGM	Bisphosphc	PMGE_HUM	6	1.124015
15min_MT.	P16219	0.110256	0.09396	0.157987	ACADS	Short-chair	ACADS_HUI	10	1.07942
15min_MT.	O95292	0.155238	0.094025	0.158062	VAPB	Vesicle-assc	VAPB_HUM	3	1.113605
15min_MT.	Q7Z2Z2	-0.13431	0.094055	0.158078	EFL1	Elongation	EFL1_HUM/	14	0.911105
30min_MT.	Q96HS1	-0.03812	0.094213	0.158276	PGAM5	Serine/thre	PGAM5_HU	5	0.973923
15min_MT.	Q96MK2	-0.63506	0.094203	0.158276	RIPOR3	RIPOR fami	RIPR3_HUM	2	0.643912
15min_MT.	Q96EB1	0.165397	0.094502	0.158659	ELP4	Elongator c	ELP4_HUM	8	1.121475
15min_MT.	Q9NZ08	0.015853	0.094499	0.158659	ERAP1	Endoplasm	ERAP1_HUI	19	1.011049
30min_MT.	O75976	0.178562	0.094565	0.158732	CPD	Carboxypep	CBPD_HUM	28	1.131756
30min_MT.	Q9NR50	-0.14063	0.094706	0.15889	EIF2B3	Translation	EI2BG_HUM	16	0.907121
15min_MT.	P07858	0.085195	0.094749	0.158904	CTSB	Cathepsin E	CATB_HUM	12	1.060831
15min_MT.	P29034	0.616087	0.09478	0.158922	S100A2	Protein S1(	S10A2_HUI	1	1.532712
15min_MT.	O60884	0.069364	0.094872	0.159008	DNAJA2	DnaJ homo	DNJA2_HUI	12	1.049254
15min_MT.	P55345	0.284945	0.094868	0.159008	PRMT2	Protein arg	ANM2_HUM	1	1.218364
15min_MT.	Q96JB5	0.103794	0.094933	0.159075	CDK5RAP3	CDK5 regul	CK5P3_HUI	13	1.074596
30min_MT.	P17066	-0.01478	0.095076	0.159281	HSPA6	Heat shock	HSP76_HUI	3	0.989808
30min_MT.	O60313	-0.23992	0.095219	0.159453	OPA1	Dynamin-li	OPA1_HUM	6	0.846791
15min_MT.	Q9UKS6	0.335917	0.095263	0.159457	PACSIN3	Protein kin	PACN3_HU	4	1.26218
15min_MT.	P62829	-0.03767	0.095402	0.159589	RPL23	Large ribos	RL23_HUM	6	0.97423
15min_MT.	Q96GA3	-0.15176	0.095473	0.159674	LTV1	Protein LTV	LTV1_HUM	7	0.900154
30min_MT.	Q03013	0.180096	0.095529	0.159702	GSTM4	Glutathion	GSTM4_HU	5	1.132959
15min_MT.	Q13573	-0.14548	0.095531	0.159702	SNW1	SNW doma	SNW1_HUM	9	0.90408
15min_MT.	Q8WXI7	0.939543	0.095784	0.16009	MUC16	Mucin-16	MUC16_HU	3	1.91792
15min_MT.	Q86UX2	0.263135	0.095828	0.160095	ITIH5	Inter-alpha	ITIH5_HUM	1	1.200084
30min_MT.	P62750	-0.1926	0.095999	0.160131	RPL23A	Large ribos	RL23A_HUI	7	0.875027
30min_MT.	Q13595	-0.17678	0.095972	0.160131	TRA2A	Transforme	TRA2A_HUI	5	0.884676
30min_MT.	Q969Z0	-0.15213	0.095902	0.160131	TBRG4	FAST kinase	FAKD4_HUI	8	0.89992
30min_MT.	Q99541	-0.07304	0.096013	0.160131	PLIN2	Perilipin-2	PLIN2_HUM	7	0.950634
15min_MT.	Q9NZP5	0.413281	0.095914	0.160131	OR5AC2	Olfactory re	O5AC2_HU	1	1.331711
30min_MT.	Q9UBK8	0.065472	0.095998	0.160131	MTRR	Methionine	MTRR_HUM	10	1.046427
30min_MT.	Q9NR56	-0.25759	0.096175	0.160325	MBNL1	Muscleblin	MBNL1_HU	4	0.836482
30min_MT.	Q9NVA1	-0.20962	0.096184	0.160325	UQCC1	Ubiquinol-(	UQCC1_HU	5	0.864763
15min_MT.	P63098	0.240549	0.09629	0.160457	PPP3R1	Calcineurir	CANB1_HU	2	1.181442
30min_MT.	P38919	-0.15869	0.096418	0.16046	EIF4A3	Eukaryotic	IF4A3_HUM	12	0.89584
15min_MT.	P55789	0.030359	0.096373	0.16046	GFER	FAD-linked	ALR_HUMA	4	1.021266
30min_MT.	P60983	0.211405	0.096365	0.16046	GMFB	Glia matura	GMFB_HUM	5	1.157815
30min_MT.	Q7Z6M1	0.100538	0.096436	0.16046	RABEPK	Rab9 effect	RABEK_HUI	15	1.072173
30min_MT.	Q9BPX7	0.248285	0.096319	0.16046	C7orf25	UPF0415 p	CG025_HU	3	1.187794
15min_MT.	Q9H871	0.064928	0.096392	0.16046	RMND5A	E3 ubiquiti	RMD5A_HU	4	1.046033

30min_MT.	Q9NQG6	-0.46916	0.09647	0.160482	MIEF1	Mitochond	MID51_HU	2	0.722386
15min_MT.	Q13043	0.410824	0.096572	0.160617	STK4	Serine/thre	STK4_HUM	7	1.329445
30min_MT.	Q9NZP5	0.579375	0.096635	0.160689	OR5AC2	Olfactory re	O5AC2_HU	1	1.494202
15min_MT.	Q6P4F2	-0.63157	0.096685	0.160738	FDX2	Ferredoxin-	FDX2_HUM	1	0.645474
15min_MT.	Q96GF1	0.40601	0.096772	0.160848	RNF185	E3 ubiquiti	RN185_HU	1	1.325016
15min_MT.	Q96H79	-0.26499	0.096881	0.160914	ZC3HAV1L	Zinc finger	ZCCHL_HUI	4	0.832207
30min_MT.	Q96HR8	0.523903	0.096895	0.160914	NAF1	H/ACA ribo	NAF1_HUM	1	1.43784
15min_MT.	Q9NZT2	-0.10725	0.096893	0.160914	OGFR	Opioid grov	OGFR_HUM	15	0.928359
15min_MT.	Q9UJK0	0.167619	0.096915	0.160914	TSR3	18S rRNA a	TSR3_HUM	1	1.123203
30min_MT.	Q6NXT1	0.101069	0.09696	0.160921	ANKRD54	Ankyrin rep	ANR54_HU	1	1.072568
15min_MT.	Q9Y376	0.153407	0.09695	0.160921	CAB39	Calcium-bi	CAB39_HUI	13	1.112193
15min_MT.	P05161	-0.2434	0.097021	0.160988	ISG15	Ubiquitin-l	ISG15_HUM	8	0.844753
30min_MT.	P62241	0.137949	0.097166	0.161127	RPS8	Small ribos	RS8_HUMA	8	1.10034
30min_MT.	P46379	-0.20187	0.097321	0.16127	BAG6	Large prolir	BAG6_HUM	30	0.869421
30min_MT.	Q9Y5U2	0.128775	0.097313	0.16127	TSSC4	U5 small nu	TSSC4_HUM	4	1.093365
15min_MT.	P54578	-0.03767	0.09737	0.161294	USP14	Ubiquitin c	UBP14_HU	27	0.974226
15min_MT.	O95573	-0.64469	0.097432	0.161331	ACSL3	Fatty acid C	ACSL3_HUI	2	0.639629
15min_MT.	Q9UNS2	-0.08255	0.097603	0.161543	COPS3	COP9 signa	CSN3_HUM	14	0.94439
15min_MT.	Q7Z6K3	0.224621	0.097655	0.161595	PTAR1	Protein pre	PTAR1_HUI	9	1.16847
15min_MT.	P01034	0.318183	0.097723	0.161673	CST3	Cystatin-C	CYTC_HUM	2	1.246759
15min_MT.	Q16831	0.108369	0.097768	0.16169	UPP1	Uridine pho	UPP1_HUM	13	1.078009
30min_MT.	P02763	0.158873	0.097838	0.161761	ORM1	Alpha-1-aci	A1AG1_HU	3	1.116415
30min_MT.	P63208	-0.12518	0.097919	0.161861	SKP1	S-phase kin	SKP1_HUM	9	0.916891
30min_MT.	A5YKK6	-0.28308	0.097989	0.161942	CNOT1	CCR4-NOT t	CNOT1_HU	20	0.821834
30min_MT.	P48634	-0.14714	0.098083	0.161971	PRRC2A	Protein PRI	PRC2A_HUI	20	0.903039
30min_MT.	O15047	0.096061	0.098124	0.161989	SETD1A	Histone-lys	SET1A_HUM	1	1.068851
30min_MT.	Q02543	0.237133	0.098162	0.161989	RPL18A	Large ribos	RL18A_HUI	2	1.178648
30min_MT.	Q9UQN3	0.044832	0.098154	0.161989	CHMP2B	Charged mu	CHM2B_HU	3	1.031563
30min_MT.	Q8WUH6	0.072655	0.098272	0.162135	TMEM263	Transmemb	TM263_HU	2	1.05165
15min_MT.	Q8WUD4	-0.03197	0.09837	0.162263	CCDC12	Coiled-coil	CCD12_HU	6	0.978083
15min_MT.	O95396	-0.6878	0.098428	0.162324	MOCS3	Adenylyltra	MOCS3_HU	2	0.620801
30min_MT.	O43678	-0.63094	0.098532	0.162382	NDUFA2	NADH dehy	NDUA2_HU	3	0.645754
15min_MT.	P30044	0.114921	0.09856	0.162382	PRDX5	Peroxiredo	PRDX5_HU	11	1.082916
30min_MT.	Q13596	-0.04107	0.098667	0.162382	SNX1	Sorting nex	SNX1_HUM	16	0.971932
30min_MT.	Q92769	-0.13135	0.098599	0.162382	HDAC2	Histone dea	HDAC2_HU	8	0.912979
15min_MT.	Q9H6R3	-0.06521	0.098586	0.162382	ACSS3	Acyl-CoA sy	ACSS3_HUI	17	0.955808
15min_MT.	Q9NZI8	0.08532	0.098626	0.162382	IGF2BP1	Insulin-like	IF2B1_HUM	11	1.060923
15min_MT.	Q9UK45	0.150949	0.098671	0.162382	LSM7	U6 snRNA-a	LSM7_HUM	3	1.1103
15min_MT.	Q16777;Q6	-0.67345	0.098818	0.162527	H2AC20;H2	Histone H2	H2A2C_HU	1	0.627004
15min_MT.	Q9Y4Z0	0.103335	0.098919	0.162596	LSM4	U6 snRNA-a	LSM4_HUM	5	1.074254
30min_MT.	O95429	-0.25378	0.098995	0.162643	BAG4	BAG family	BAG4_HUM	1	0.838694
30min_MT.	O95391	-1.65148	0.099183	0.162856	SLU7	Pre-mRNA-s	SLU7_HUM	1	0.318314
15min_MT.	P25686	0.128952	0.099188	0.162856	DNAJB2	DnaJ homo	DNJB2_HUI	6	1.093499
15min_MT.	Q9H2M9	-0.05073	0.099228	0.162888	RAB3GAP2	Rab3 GTPas	RBGPR_HU	17	0.965448
30min_MT.	Q99622	-0.16224	0.099483	0.163272	C12orf57	Protein C10	C10_HUMA	3	0.893634
15min_MT.	P23919	0.162265	0.099542	0.163334	DTYMK	Thymidylat	KTHY_HUM	12	1.119043
30min_MT.	Q13541	-0.21092	0.099698	0.163503	EIF4EBP1	Eukaryotic	4EBP1_HUI	5	0.863988
15min_MT.	P22033	-0.05004	0.099812	0.163572	MMUT	Methylmal	MUTA_HUM	22	0.965907
15min_MT.	P51452	0.301942	0.09981	0.163572	DUSP3	Dual specifi	DUS3_HUM	7	1.232803
15min_MT.	Q9Y2X7	-0.52886	0.099789	0.163572	GIT1	ARF GTPase	GIT1_HUMA	1	0.693104
30min_MT.	Q9Y5K5	-0.02009	0.099834	0.163573	UCHL5	Ubiquitin c	UCHL5_HU	13	0.986173
30min_MT.	P31751	-0.25186	0.099987	0.163711	AKT2	RAC-beta se	AKT2_HUM	1	0.839811

30min_MT.	Q8IWX8	-0.04648	0.100002	0.163711	CHERP	Calcium ho	CHERP_HU	9	0.968296
15min_MT.	Q96CW6	0.287697	0.100101	0.163805	SLC7A6OS	Probable R	S7A6O_HU	3	1.22069
30min_MT.	P36507	-0.03962	0.100147	0.163846	MAP2K2	Dual specifi	MP2K2_HU	17	0.972909
30min_MT.	Q7RTS7	0.407364	0.100345	0.164102	KRT74	Keratin, typ	K2C74_HU	2	1.32626
30min_MT.	Q9NR19	-0.10326	0.100372	0.164112	ACSS2	Acetyl-coer	ACSA_HUM	16	0.930929
15min_MT.	P07384	-0.04893	0.100401	0.164125	CAPN1	Calpain-1 c	CAN1_HUM	29	0.966652
30min_MT.	Q9UQ13	0.820991	0.100558	0.164347	SHOC2	Leucine-ric	SHOC2_HU	2	1.766619
15min_MT.	P61353	-0.23626	0.100652	0.16444	RPL27	Large ribos	RL27_HUM	5	0.848942
30min_MT.	Q96C86	0.077835	0.100657	0.16444	DCPS	m7GpppX c	DCPS_HUM	11	1.055433
15min_MT.	O94874	0.165267	0.10072	0.164508	UFL1	E3 UFM1-p	UFL1_HUM	13	1.121374
30min_MT.	O14732	0.194689	0.100971	0.164815	IMPA2	Inositol mc	IMPA2_HU	3	1.144477
15min_MT.	P47895	-0.39822	0.101139	0.165055	ALDH1A3	Retinaldeh	AL1A3_HU	2	0.758794
15min_MT.	P32754	-0.05451	0.101372	0.165263	HPD	4-hydroxyp	HPPD_HUM	8	0.962921
15min_MT.	P46937	0.126945	0.101315	0.165263	YAP1	Transcripti	YAP1_HUM	7	1.091979
30min_MT.	Q13442	0.242824	0.101296	0.165263	PDAP1	28 kDa hea	HAP28_HU	7	1.183307
30min_MT.	Q96RS6	0.10779	0.101356	0.165263	NUDCD1	NudC doma	NUDC1_HU	16	1.077576
15min_MT.	Q92600	0.158955	0.101427	0.165301	CNOT9	CCR4-NOT t	CNOT9_HU	7	1.116478
15min_MT.	Q9UBG3	1.205641	0.101446	0.165301	CRNN	Cornulin	CRNN_HUM	7	2.306397
30min_MT.	Q5VW36	-0.41088	0.101488	0.165314	FOCAD	Focadhesin	FOCAD_HU	7	0.752165
15min_MT.	Q86VR2	-0.11917	0.101545	0.165373	RETREG3	Reticuloph	RETR3_HUM	1	0.920719
15min_MT.	Q8IWW6	-0.08197	0.101579	0.165393	ARHGAP12	Rho GTPase	RHG12_HU	4	0.94477
15min_MT.	Q9H4B0	-0.39485	0.101692	0.165474	OSGEPL1	tRNA N6-ad	OSGL1_HU	2	0.760569
15min_MT.	Q9NS18	0.431856	0.101852	0.1657	GLRX2	Glutaredox	GLRX2_HU	1	1.348968
30min_MT.	Q01780	0.024759	0.102121	0.165884	EXOSC10	Exosome cc	EXOSX_HU	1	1.01731
15min_MT.	Q7L5Y9	-0.0844	0.102124	0.165884	MAEA	E3 ubiquiti	MAEA_HUM	2	0.943176
30min_MT.	Q8TCT9	-0.1219	0.102111	0.165884	HM13	Minor histc	HM13_HUM	3	0.918978
15min_MT.	Q96EI5	-0.11201	0.102129	0.165884	TCEAL4	Transcripti	TCAL4_HUM	5	0.9253
15min_MT.	Q9H8K7	0.015954	0.102135	0.165884	PAAT	ATPase PAA	PAAT_HUM	5	1.01112
15min_MT.	P24928	0.027104	0.10219	0.165939	POLR2A	DNA-direct	RPB1_HUM	16	1.018964
30min_MT.	Q5ZPR3	0.028952	0.102272	0.166038	CD276	CD276 anti	CD276_HU	2	1.02027
30min_MT.	Q9Y6D6	-0.13041	0.102313	0.166071	ARFGEF1	Brefeldin A	BIG1_HUM	29	0.913572
15min_MT.	Q96DC7	0.633905	0.102448	0.166254	TMCO6	Transmemb	TMCO6_HU	1	1.551759
15min_MT.	Q9UK99	0.003481	0.102581	0.166437	FBXO3	F-box only	FBX3_HUM	4	1.002416
15min_MT.	P62314	-0.41141	0.102654	0.16652	SNRPD1	Small nucle	SMD1_HUM	4	0.751888
15min_MT.	Q7KZI7	-0.89596	0.102681	0.166524	MARK2	Serine/thre	MARK2_HU	1	0.537388
15min_MT.	Q8TBF4	-0.55038	0.102699	0.166524	ZCRB1	Zinc finger	ZCRB1_HU	1	0.68284
15min_MT.	P39023	-0.17389	0.10278	0.166552	RPL3	Large ribos	RL3_HUMA	13	0.886452
15min_MT.	P02814	2.325021	0.102873	0.166633	SMR3B	Submaxilla	SMR3B_HU	1	5.010731
15min_MT.	P52926	0.17327	0.103048	0.166796	HMGA2	High mobil	HMGA2_HU	5	1.127612
15min_MT.	Q9HC84	2.508183	0.103059	0.166796	MUC5B	Mucin-5B	MUC5B_HU	5	5.68903
15min_MT.	Q9UI12	0.026659	0.103191	0.166976	ATP6V1H	V-type prot	VATH_HUM	10	1.01865
15min_MT.	Q8WVV9	-0.74049	0.103283	0.16709	HNRNPLL	Heterogene	HNRLL_HU	2	0.598535
15min_MT.	O95391	-1.35926	0.103478	0.167211	SLU7	Pre-mRNA-	SLU7_HUM	1	0.389782
15min_MT.	Q12857	-0.44403	0.103486	0.167211	NFIA	Nuclear fac	NFIA_HUM	1	0.735079
15min_MT.	Q96PE7	-0.2946	0.103475	0.167211	MCEE	Methylmal	MCEE_HUM	3	0.815297
30min_MT.	Q9Y5R8	0.067801	0.103447	0.167211	TRAPPC1	Trafficking	TPPC1_HU	4	1.048118
30min_MT.	P47813	0.11429	0.103558	0.167267	EIF1AX	Eukaryotic	IF1AX_HUM	5	1.082442
30min_MT.	Q9Y316	0.121996	0.103564	0.167267	MEMO1	Protein ME	MEMO1_HU	3	1.08824
15min_MT.	Q96EP5	0.234891	0.103618	0.16732	DAZAP1	DAZ-associ	DAZP1_HU	7	1.176818
15min_MT.	Q86SX6	-0.39119	0.103659	0.167353	GLRX5	Glutaredox	GLRX5_HU	3	0.762503
30min_MT.	Q96A72	-0.14414	0.103708	0.167397	MAGOHB	Protein ma	MGN2_HU	7	0.904921
15min_MT.	O60739	-0.90839	0.103775	0.167413	EIF1B	Eukaryotic	EIF1B_HUM	1	0.532781

30min_MT.	P40261	0.129349	0.103794	0.167413	NNMT	Nicotinami	NNMT_HUM	10	1.0938
30min_MT.	Q3ZCW2	0.167438	0.103804	0.167413	LGALSL	Galectin-re	LEGL_HUM	5	1.123063
30min_MT.	Q86WQ0	0.069489	0.103745	0.167413	NR2C2AP	Nuclear rec	NR2CA_HU	4	1.049345
15min_MT.	P15529	0.064397	0.103868	0.167482	CD46	Membrane	MCP_HUM.	5	1.045648
30min_MT.	Q13112	-0.10437	0.103894	0.16749	CHAF1B	Chromatin	CAF1B_HUI	3	0.930214
30min_MT.	O95400	0.154143	0.103956	0.167555	CD2BP2	CD2 antiger	CD2B2_HU	3	1.11276
15min_MT.	Q9BRG1	0.295205	0.104078	0.167683	VPS25	Vacuolar pı	VPS25_HUI	8	1.227059
15min_MT.	P05787	-0.13393	0.104126	0.167725	KRT8	Keratin, typ	K2C8_HUM	29	0.911348
30min_MT.	Q68D20	-0.30387	0.104175	0.16777	PMS2CL	Protein PM	PMS2L_HU	1	0.810075
30min_MT.	O60763	-0.11246	0.104263	0.167877	USO1	General ves	USO1_HUM	36	0.925008
15min_MT.	O60869	0.119291	0.104318	0.167931	EDF1	Endothelial	EDF1_HUM	10	1.086201
15min_MT.	Q86XI2	0.724097	0.104366	0.167974	NCAPG2	Condensin-	CNDG2_HU	1	1.651866
15min_MT.	P38432	0.432351	0.104395	0.167985	COIL	Coilin	COIL_HUM.	1	1.349431
30min_MT.	Q5JS37	0.199916	0.104509	0.168105	NHLRC3	NHL repeat	NHLC3_HU	5	1.148632
15min_MT.	P12259	-0.51733	0.104567	0.168124	F5	Coagulatio	FA5_HUMA	3	0.698665
30min_MT.	Q7Z6B0	0.144914	0.104623	0.168144	CCDC91	Coiled-coil	CCD91_HU	2	1.105665
15min_MT.	Q9NP77	-0.18575	0.104613	0.168144	SSU72	RNA polym	SSU72_HUI	4	0.879194
30min_MT.	Q8TEQ6	-0.07955	0.104662	0.168173	GEMIN5	Gem-associ	GEMI5_HUI	22	0.946352
30min_MT.	Q9NUP7	-0.52974	0.104709	0.168215	TRMT13	tRNA:m(4)X	TRM13_HU	3	0.692682
30min_MT.	Q03393	0.221911	0.104754	0.168252	PTS	6-pyruvoyl	PTPS_HUM	4	1.166278
15min_MT.	P02765	-0.24375	0.10485	0.168294	AHSG	Alpha-2-HS	FETUA_HUI	2	0.844547
30min_MT.	P63167	-0.31086	0.104875	0.168294	DYNLL1	Dynein ligh	DYL1_HUM	4	0.806159
15min_MT.	Q9Y6X4	-0.00103	0.104954	0.168366	FAM169A	Soluble lam	F169A_HUI	7	0.999283
15min_MT.	Q9UGP4	0.069921	0.105112	0.16855	LIMD1	LIM domair	LIMD1_HUI	14	1.049659
15min_MT.	P15848	0.081778	0.10534	0.168765	ARSB	Arylsulfatas	ARSB_HUM	11	1.058321
15min_MT.	P55327	0.094124	0.105338	0.168765	TPD52	Tumor prot	TPD52_HUI	7	1.067417
30min_MT.	Q08752	0.123907	0.10535	0.168765	PPID	Peptidyl-pr	PPID_HUM	14	1.089682
30min_MT.	Q9P258	0.11642	0.105415	0.168794	RCC2	Protein RC(	RCC2_HUM	15	1.084041
30min_MT.	P10412	0.177595	0.105543	0.168835	H1-4	Histone H1	H14_HUMA	8	1.130997
15min_MT.	Q09666	-0.15733	0.105509	0.168835	AHNAK	Neuroblast	AHNK_HUM	207	0.896681
15min_MT.	Q99439	0.150272	0.105549	0.168835	CNN2	Calponin-2	CNN2_HUM	9	1.109779
15min_MT.	Q9NP73	-0.72198	0.105528	0.168835	ALG13	UDP-N-acet	ALG13_HUI	1	0.606265
30min_MT.	Q07866	-0.09309	0.105599	0.16888	KLC1	Kinesin ligh	KLC1_HUM	11	0.937515
30min_MT.	P78386	1.299281	0.105695	0.168988	KRT85	Keratin, typ	KRT85_HUI	1	2.461061
30min_MT.	Q8IV08	0.151467	0.105887	0.169168	PLD3	5'-3' exonu	PLD3_HUM	6	1.110698
30min_MT.	P08833	0.114577	0.106262	0.169733	IGFBP1	Insulin-like	IBP1_HUMA	11	1.082657
30min_MT.	Q86SZ2	0.129979	0.106446	0.169958	TRAPPC6B	Trafficking	TPC6B_HUI	10	1.094278
15min_MT.	Q9H900	-0.10454	0.106603	0.170167	ZWILCH	Protein zwi	ZWILC_HUI	2	0.930101
15min_MT.	Q13554	0.484997	0.106672	0.170179	CAMK2B	Calcium/ca	KCC2B_HUI	3	1.399583
30min_MT.	Q8WXI7	1.262313	0.106816	0.170339	MUC16	Mucin-16	MUC16_HU	3	2.3988
15min_MT.	Q9UDY8	0.121253	0.106794	0.170339	MALT1	Mucosa-ass	MALT1_HU	12	1.087679
15min_MT.	Q9Y6M1	0.136778	0.106924	0.170442	IGF2BP2	Insulin-like	IF2B2_HUM	11	1.099447
30min_MT.	Q12789	-0.25213	0.106998	0.170465	GTF3C1	General tra	TF3C1_HUI	3	0.839658
15min_MT.	Q9UBW8	0.245101	0.107248	0.170749	COPS7A	COP9 signa	CSN7A_HUI	10	1.185176
15min_MT.	P50336	0.149568	0.107535	0.171102	PPOX	Protoporpl	PPOX_HUM	6	1.109237
30min_MT.	Q14966	-0.16136	0.107589	0.171154	ZNF638	Zinc finger	ZN638_HUI	13	0.894181
30min_MT.	O76013	1.611682	0.107628	0.171181	KRT36	Keratin, typ	KRT36_HUI	6	3.056079
30min_MT.	P63172	0.450891	0.107699	0.171223	DYNLT1	Dynein ligh	DYLT1_HUM	4	1.366884
30min_MT.	O00469	0.096364	0.107894	0.17136	PLOD2	Procollager	PLOD2_HU	17	1.069076
15min_MT.	Q8IYT4	0.918904	0.107962	0.171433	KATNAL2	Katanin p6(	KATL2_HUM	1	1.890678
15min_MT.	P46199	0.095846	0.107989	0.17144	MTIF2	Translation	IF2M_HUM	10	1.068692
15min_MT.	Q92905	0.111009	0.10803	0.171471	COPS5	COP9 signa	CSN5_HUM	12	1.079984

15min_MT.	O00203	-0.0996	0.108285	0.171819	AP3B1	AP-3 compl	AP3B1_HUI	19	0.933291
15min_MT.	Q9BXW6	0.172268	0.108293	0.171819	OSBPL1A	Oxysterol-b	OSBL1_HUI	7	1.126829
30min_MT.	P08243	-0.27409	0.108541	0.172108	ASNS	Asparagine	ASNS_HUM	11	0.826974
30min_MT.	P62266	-0.37263	0.108602	0.172169	RPS23	Small ribos	RS23_HUM	3	0.772371
30min_MT.	Q00839	-0.13233	0.10874	0.172258	HNRNPU	Heterogene	HNRPU_HU	27	0.912356
15min_MT.	Q92896	0.174706	0.108707	0.172258	GLG1	Golgi appar	GSLG1_HUI	20	1.128735
15min_MT.	Q9P035	-0.27589	0.108746	0.172258	HACD3	Very-long-c	HACD3_HU	4	0.825939
15min_MT.	O15127	-0.64777	0.108887	0.172425	SCAMP2	Secretory c	SCAM2_HU	1	0.638267
30min_MT.	O75882	0.237748	0.108924	0.172425	ATRN	Attractin	ATRN_HUM	11	1.179151
30min_MT.	Q5T013	0.240199	0.108936	0.172425	HYI	Putative hy	HYI_HUMAI	3	1.181155
30min_MT.	P27797	-0.11012	0.108983	0.172457	CALR	Calreticulir	CALR_HUM	24	0.926513
15min_MT.	Q6NVY1	0.126046	0.109004	0.172457	HIBCH	3-hydroxyi:	HIBCH_HUI	15	1.091299
30min_MT.	P61254	0.16802	0.10907	0.172461	RPL26	Large ribos	RL26_HUM	6	1.123516
15min_MT.	Q14566	0.046521	0.109114	0.172461	MCM6	DNA replica	MCM6_HUI	23	1.032771
15min_MT.	Q9Y535	-0.10532	0.109117	0.172461	POLR3H	DNA-direct	RPC8_HUM	2	0.9296
15min_MT.	Q01546	1.002034	0.109357	0.172666	KRT76	Keratin, typ	K22O_HUM	6	2.002822
15min_MT.	Q9NRL3	0.071868	0.109387	0.172679	STRN4	Striatin-4	STRN4_HUI	10	1.051076
30min_MT.	P49915	-0.13726	0.109441	0.172695	GMPS	GMP synth	GUAA_HUM	18	0.909247
30min_MT.	Q9BWU0	-0.07463	0.109498	0.17275	SLC4A1AP	Kanadaptir	NADAP_HU	12	0.949583
30min_MT.	P38159	0.19599	0.109654	0.172958	RBMX	RNA-bindir	RBMX_HUM	13	1.14551
15min_MT.	Q6KB66	0.317724	0.109761	0.173025	KRT80	Keratin, typ	K2C80_HUI	19	1.246363
30min_MT.	O75884	0.156109	0.109842	0.173049	RBBP9	Serine hydr	RBBP9_HUI	7	1.114278
15min_MT.	P23284	0.054777	0.109818	0.173049	PPIB	Peptidyl-pr	PPIB_HUMA	12	1.038698
30min_MT.	P61981	0.108453	0.109822	0.173049	YWHAG	14-3-3 prot	1433G_HU	11	1.078071
30min_MT.	P29353	-0.16385	0.109953	0.173154	SHC1	SHC-transfc	SHC1_HUM	11	0.892637
30min_MT.	Q3LXA3	-0.17797	0.109938	0.173154	TKFC	Triokinase/	TKFC_HUM	11	0.883946
15min_MT.	Q9Y3L3	0.600889	0.110093	0.173269	SH3BP1	SH3 domai	3BP1_HUM	3	1.516651
30min_MT.	Q9UKS6	0.164711	0.110136	0.173302	PACSIN3	Protein kin	PACN3_HU	4	1.120942
30min_MT.	Q9UHN6	0.016785	0.110234	0.173363	CEMIP2	Inactive cel	CEIP2_HUM	6	1.011703
30min_MT.	Q9Y678	-0.1131	0.110263	0.173363	COPG1	Coatomer s	COPG1_HU	40	0.924598
30min_MT.	P78527	-0.19488	0.110339	0.173412	PRKDC	DNA-depen	PRKDC_HU	77	0.873643
15min_MT.	P48449	0.675287	0.110405	0.173481	LSS	Lanosterol	LSS_HUMAI	1	1.596915
30min_MT.	Q6IQ23	2.924169	0.110555	0.173647	PLEKHA7	Pleckstrin I	PKHA7_HU	1	7.590362
30min_MT.	Q9BVM2	-0.20055	0.110553	0.173647	DPCD	Protein DP	DPCD_HUM	7	0.870217
15min_MT.	P63172	-0.11535	0.11077	0.173819	DYNLT1	Dynein ligh	DYLT1_HUI	4	0.92316
15min_MT.	P82932	0.724044	0.110776	0.173819	MRPS6	Small ribos	RT06_HUM	1	1.651806
30min_MT.	Q9NQ48	0.00376	0.110811	0.173839	LZTFL1	Leucine zip	LZTL1_HUM	7	1.00261
15min_MT.	Q5VUM1	-0.12487	0.110889	0.173927	SDHAF4	Succinate c	SDHF4_HUI	3	0.917084
30min_MT.	O43598	-0.12742	0.110961	0.174005	DNPH1	5-hydroxyn	DNPH1_HU	6	0.91547
30min_MT.	Q01518	0.054461	0.111129	0.174129	CAP1	Adenylyl cy	CAP1_HUM	31	1.038471
30min_MT.	Q8NCW5	0.138112	0.111112	0.174129	NAXE	NAD(P)H-hy	NNRE_HUM	13	1.100464
30min_MT.	P21266	0.077545	0.111217	0.174164	GSTM3	Glutathion	GSTM3_HU	16	1.055221
30min_MT.	P49789	0.066688	0.111185	0.174164	FHIT	Bis(5'-aden	FHIT_HUMA	5	1.04731
30min_MT.	P01024	0.061744	0.111294	0.174171	C3	Compleme	CO3_HUMA	76	1.043727
15min_MT.	P07339	0.026826	0.111359	0.174171	CTSD	Cathepsin I	CATD_HUM	14	1.018768
30min_MT.	P13667	0.07756	0.111445	0.174171	PDIA4	Protein dis	PDIA4_HUI	41	1.055232
15min_MT.	P19012	2.420971	0.111435	0.174171	KRT15	Keratin, typ	K1C15_HUI	7	5.355315
15min_MT.	P24666	0.170738	0.111365	0.174171	ACP1	Low molec	PPAC_HUM	4	1.125634
30min_MT.	Q01968	-0.12394	0.111433	0.174171	OCRL	Inositol pol	OCRL_HUM	13	0.917675
15min_MT.	Q9UNE7	0.258586	0.111358	0.174171	STUB1	E3 ubiquiti	CHIP_HUM	9	1.196306
30min_MT.	Q5T749	0.198102	0.111889	0.174655	KPRP	Keratinocy	KPRP_HUM	9	1.147188
30min_MT.	Q96QK1	-0.0507	0.112044	0.174758	VPS35	Vacuolar p	VPS35_HUI	27	0.965466

15min_MT.	P08727	0.672855	0.112087	0.17479	KRT19	Keratin, typ	K1C19_HUI	4	1.594225
15min_MT.	P50914	-0.45536	0.112173	0.174854	RPL14	Large ribos	RL14_HUM	3	0.729327
30min_MT.	Q9UBV8	0.089386	0.11217	0.174854	PEF1	Peflin	PEF1_HUM	4	1.063917
15min_MT.	Q9HC21	0.190674	0.112239	0.1749	SLC25A19	Mitochond	TPC_HUMA	1	1.141297
15min_MT.	Q9NWB6	-0.24592	0.112265	0.1749	ARGLU1	Arginine an	ARGL1_HUI	6	0.843277
15min_MT.	Q92947	0.122381	0.112437	0.175056	GCDH	Glutaryl-Cc	GCDH_HUM	2	1.08853
30min_MT.	O43719	0.25273	0.112517	0.175114	HTATSF1	17S U2 SnR	HTSF1_HUI	25	1.191459
30min_MT.	P78345	-0.17546	0.112541	0.175114	RPP38	Ribonuclea	RPP38_HUI	7	0.885484
15min_MT.	O00754	0.110471	0.112585	0.175116	MAN2B1	Lysosomal	MA2B1_HU	19	1.07958
15min_MT.	Q8TAT6	0.117946	0.112641	0.175117	NPLOC4	Nuclear prc	NPL4_HUM	15	1.085189
30min_MT.	Q9H6T3	-0.21497	0.112655	0.175117	RPAP3	RNA polym	RPAP3_HUI	12	0.861563
30min_MT.	O43790	0.688241	0.112735	0.175207	KRT86	Keratin, typ	KRT86_HUI	2	1.611318
15min_MT.	P53634	-0.21178	0.11287	0.17535	CTSC	Dipeptidyl	CATC_HUM	11	0.863473
15min_MT.	P25786	0.121766	0.112919	0.175387	PSMA1	Proteasom	PSA1_HUM	20	1.088066
15min_MT.	Q8N392	0.045483	0.1131	0.175598	ARHGAP18	Rho GTPase	RHG18_HU	14	1.032028
15min_MT.	Q9H5H4	-1.05877	0.113092	0.175598	ZNF768	Zinc finger	ZN768_HUI	1	0.48004
30min_MT.	Q8N9N7	-0.21948	0.113231	0.175732	LRRC57	Leucine-ric	LRC57_HUI	5	0.858872
30min_MT.	Q9BVJ6	0.340072	0.1133	0.175805	UTP14A	U3 small nu	UT14A_HUI	1	1.26582
30min_MT.	Q96GF1	0.418493	0.113496	0.176004	RNF185	E3 ubiquiti	RN185_HU	1	1.336531
15min_MT.	P78330	0.037379	0.113549	0.176051	PSPH	Phosphose	SERB_HUM	12	1.026248
30min_MT.	O60870	-0.16351	0.113679	0.176163	KIN	DNA/RNA-b	KIN17_HUI	3	0.892853
15min_MT.	O75116	0.060433	0.113733	0.176197	ROCK2	Rho-associ	ROCK2_HU	40	1.042779
15min_MT.	O94966	0.123854	0.113897	0.176315	USP19	Ubiquitin c	UBP19_HU	13	1.089642
15min_MT.	Q9UHB6	0.088357	0.113931	0.176324	LIMA1	LIM domair	LIMA1_HUI	18	1.063159
30min_MT.	P56192	-0.15016	0.11419	0.176486	MARS1	Methionine	SYMC_HUM	27	0.901152
30min_MT.	P62995	0.148729	0.114167	0.176486	TRA2B	Transforme	TRA2B_HUI	8	1.108592
15min_MT.	Q15751	0.022251	0.114187	0.176486	HERC1	Probable E3	HERC1_HUI	5	1.015543
15min_MT.	Q8NFF5	-0.22191	0.114152	0.176486	FLAD1	FAD syntha	FAD1_HUM	2	0.857432
30min_MT.	Q9UBB6	-0.1055	0.114277	0.176551	NCDN	Neurochon	NCDN_HUM	4	0.929482
30min_MT.	Q09666	-0.10825	0.114382	0.176643	AHNAK	Neuroblast	AHNK_HUM	207	0.927714
15min_MT.	P00387	-0.20352	0.114583	0.176884	CYB5R3	NADH-cyto	NB5R3_HU	6	0.868429
30min_MT.	Q9Y5B9	0.16464	0.114788	0.177096	SUPT16H	FACT comp	SP16H_HUI	11	1.120886
15min_MT.	P62333	0.156625	0.114946	0.177268	PSMC6	26S protea	PRS10_HUI	15	1.114676
15min_MT.	O60518	0.164394	0.11526	0.177546	RANBP6	Ran-bindin	RNBP6_HU	4	1.120695
30min_MT.	P68366	-0.05663	0.115285	0.177546	TUBA4A	Tubulin alp	TBA4A_HUI	2	0.96151
15min_MT.	Q8WWH5	-0.21311	0.115235	0.177546	TRUB1	Pseudourid	TRUB1_HUI	5	0.862676
15min_MT.	Q8N4J0	0.121814	0.115408	0.177701	CARNMT1	Carnosine N	CARME_HU	4	1.088102
30min_MT.	O15371	-0.09434	0.115659	0.178052	EIF3D	Eukaryotic	EIF3D_HUM	8	0.936701
15min_MT.	P84077	-0.10013	0.115817	0.17812	ARF1	ADP-ribosy	ARF1_HUM	8	0.932948
15min_MT.	Q8WUM4	0.084626	0.115749	0.17812	PDCD6IP	Programme	PDC6I_HUI	38	1.060413
15min_MT.	Q99615	0.28176	0.115846	0.17813	DNAJC7	DnaJ homo	DNJC7_HUI	5	1.215677
15min_MT.	O15116	0.36496	0.116104	0.178373	LSM1	U6 snRNA-a	LSM1_HUM	3	1.287846
30min_MT.	P35637	0.202754	0.116152	0.178373	FUS	RNA-bindir	FUS_HUMA	10	1.150893
30min_MT.	P51608	0.545129	0.116178	0.178373	MECP2	Methyl-CpC	MECP2_HU	4	1.45915
15min_MT.	Q14562	0.190897	0.1161	0.178373	DHX8	ATP-depenc	DHX8_HUM	5	1.141473
30min_MT.	Q68EM7	-0.22323	0.116135	0.178373	ARHGAP17	Rho GTPase	RHG17_HU	7	0.856644
15min_MT.	Q9UJZ1	0.261472	0.11619	0.178373	STOML2	Stomatin-li	STML2_HUI	4	1.198701
15min_MT.	Q9Y5Q8	0.127852	0.11609	0.178373	GTF3C5	General tra	TF3C5_HUI	5	1.092666
30min_MT.	P08185	0.449392	0.116419	0.17852	SERPINA6	Corticoster	CBG_HUMA	4	1.365465
30min_MT.	P18077	0.162132	0.116395	0.17852	RPL35A	Large ribos	RL35A_HUI	3	1.118939
30min_MT.	Q5VIR6	-0.50963	0.11641	0.17852	VPS53	Vacuolar pr	VPS53_HUI	8	0.702404
15min_MT.	Q8WZB0	-0.37681	0.116411	0.17852	ERCC6L2-A	Putative ur	CI130_HUM	1	0.770139

15min_MT.	Q92747	-0.00414	0.116375	0.17852	ARPC1A	Actin-relate	ARC1A_HUI	15	0.997136
15min_MT.	Q9Y2Y0	0.352765	0.116453	0.178537	ARL2BP	ADP-ribosy	AR2BP_HUI	3	1.277006
15min_MT.	Q9NYB9	0.244034	0.116503	0.178547	ABI2	Abl interact	ABI2_HUM.	6	1.1843
30min_MT.	P22792	-0.16791	0.116532	0.178553	CPN2	Carboxypep	CPN2_HUM	1	0.890133
15min_MT.	P40394	-2.44387	0.116594	0.178579	ADH7	All-trans-re	ADH7_HUM	1	0.18379
15min_MT.	Q9Y680	-0.03926	0.116784	0.178764	FKBP7	Peptidyl-pr	FKBP7_HUI	5	0.973156
15min_MT.	Q7RTS7	0.346244	0.116807	0.178764	KRT74	Keratin, typ	K2C74_HUI	2	1.271247
15min_MT.	Q13158	-0.93198	0.11687	0.178825	FADD	FAS-associa	FADD_HUM	1	0.524139
15min_MT.	Q96HR8	-0.07283	0.117025	0.178957	NAF1	H/ACA ribo	NAF1_HUM	1	0.950769
30min_MT.	Q92609	-0.24722	0.117195	0.179112	TBC1D5	TBC1 doma	TBCD5_HUI	15	0.842516
30min_MT.	Q14008	-0.10754	0.117218	0.179113	CKAP5	Cytoskeleto	CKAP5_HUI	33	0.928171
15min_MT.	Q96ST2	0.014842	0.11739	0.179306	IWS1	Protein IWS	IWS1_HUM	17	1.010341
30min_MT.	Q6UB35	-0.25121	0.117444	0.179318	MTHFD1L	Monofunct	C1TM_HUM	14	0.840191
30min_MT.	Q8NE71	-0.05043	0.117518	0.179361	ABCF1	ATP-bindin	ABCF1_HUI	15	0.965648
15min_MT.	P28331	0.116935	0.117564	0.179365	NDUFS1	NADH-ubiq	NDUS1_HU	19	1.084429
15min_MT.	P61758	0.173107	0.117566	0.179365	VBP1	Prefoldin su	PFD3_HUM	8	1.127484
30min_MT.	P35241	0.095104	0.117811	0.179439	RDX	Radixin	RADI_HUM	38	1.068142
30min_MT.	P50750	-0.07808	0.117844	0.179439	CDK9	Cyclin-depe	CDK9_HUM	5	0.947317
15min_MT.	Q04656	0.168387	0.117696	0.179439	ATP7A	Copper-trai	ATP7A_HUI	11	1.123801
30min_MT.	Q13838	-0.10092	0.117723	0.179439	DDX39B	Spliceosom	DX39B_HU	18	0.932439
30min_MT.	Q7L523	0.138469	0.117651	0.179439	RRAGA	Ras-related	RRAGA_HU	5	1.100737
15min_MT.	Q9H9E3	0.263528	0.117785	0.179439	COG4	Conserved	COG4_HUM	12	1.20041
15min_MT.	Q9HA65	0.085891	0.117824	0.179439	TBC1D17	TBC1 doma	TBC17_HUI	6	1.061343
30min_MT.	Q9NP61	-0.05563	0.117923	0.179488	ARFGAP3	ADP-ribosy	ARFG3_HUI	15	0.962174
30min_MT.	P07814	-0.15155	0.117989	0.179517	EPRS1	Bifunctiona	SYEP_HUM.	59	0.900284
15min_MT.	O15400	0.116584	0.118284	0.179681	STX7	Syntaxin-7	STX7_HUM	6	1.084165
30min_MT.	O43310	0.651705	0.118262	0.179681	CTIF	CBP80/20-	CTIF_HUM/	3	1.571024
15min_MT.	P35670	0.164262	0.11818	0.179681	ATP7B	Copper-trai	ATP7B_HUI	3	1.120593
15min_MT.	P50570	-0.10578	0.118302	0.179681	DNM2	Dynamin-2	DYN2_HUM	22	0.929302
30min_MT.	Q6UWP8	0.905902	0.118197	0.179681	SBSN	Suprabasin	SBSN_HUM	5	1.873716
30min_MT.	Q8NHW5	-1.12548	0.11815	0.179681	RPLP0P6	Putative rib	RLA0L_HUI	1	0.458351
30min_MT.	P61218	-0.03648	0.118367	0.179745	POLR2F	DNA-direct	RPAB2_HUI	2	0.975034
15min_MT.	Q99650	0.245718	0.118423	0.179795	OSMR	Oncostatin	OSMR_HUM	2	1.185682
30min_MT.	Q9BTE1	-0.06723	0.11846	0.179816	DCTN5	Dynactin su	DCTN5_HU	3	0.95447
30min_MT.	Q7Z5Q5	0.265693	0.118583	0.179934	POLN	DNA polym	DPOLN_HU	1	1.202213
15min_MT.	Q86VX9	-0.21303	0.118649	0.179999	MON1A	Vacuolar fu	MON1A_HU	1	0.862722
30min_MT.	P26373	0.248716	0.118716	0.180065	RPL13	Large ribos	RL13_HUM	8	1.188149
15min_MT.	O75935	0.163085	0.118901	0.180277	DCTN3	Dynactin su	DCTN3_HU	8	1.119679
30min_MT.	Q92973	0.064254	0.118947	0.180288	TNPO1	Transportin	TNPO1_HU	25	1.045544
30min_MT.	Q99942	-0.08107	0.118968	0.180288	RNF5	E3 ubiquiti	RNF5_HUM	2	0.945355
15min_MT.	Q9Y2H5	-0.55603	0.119007	0.180288	PLEKHA6	Pleckstrin h	PKHA6_HU	2	0.680173
30min_MT.	Q8IXQ4	0.164267	0.1191	0.180299	GPALPP1	GPALPP mo	GPAM1_HU	4	1.120597
15min_MT.	Q8WVT3	0.510815	0.119097	0.180299	TRAPPC12	Trafficking	TPC12_HUI	1	1.424855
30min_MT.	Q92917	0.010882	0.119098	0.180299	GPKOW	G-patch do	GPKOW_HU	8	1.007571
30min_MT.	Q01546	0.569372	0.11915	0.180339	KRT76	Keratin, typ	K22O_HUM	6	1.483877
30min_MT.	Q9UKD1	0.410192	0.119234	0.180396	GMEB2	Glucocortic	GMEB2_HU	1	1.328863
30min_MT.	Q8IX12	-0.03642	0.119335	0.180442	CCAR1	Cell divisio	CCAR1_HUI	26	0.975075
15min_MT.	Q96EL3	0.333153	0.119321	0.180442	MRPL53	Large ribos	RM53_HUM	2	1.259763
30min_MT.	Q9H845	-0.24597	0.11938	0.180442	ACAD9	Complex I a	ACAD9_HU	7	0.843251
30min_MT.	O75494	0.280177	0.119571	0.180619	SRSF10	Serine/argi	SRS10_HUM	4	1.214344
30min_MT.	Q8TE04	-0.22344	0.119573	0.180619	PANK1	Pantothena	PANK1_HU	2	0.856522
15min_MT.	Q9UBB4	0.123733	0.119563	0.180619	ATXN10	Ataxin-10	ATX10_HUI	18	1.08955

30min_MT.	Q9ULP0	0.126557	0.119589	0.180619	NDRG4	Protein ND	NDRG4_HU	3	1.091685
30min_MT.	Q96CW6	0.427134	0.119676	0.180716	SLC7A6OS	Probable R	S7A6O_HU	3	1.34456
30min_MT.	P46063	-0.10781	0.119716	0.18074	RECQL	ATP-depend	RECQ1_HU	19	0.927993
30min_MT.	Q9NWV4	0.143939	0.119776	0.180762	CZIB	CXXC motif	CZIB_HUM	9	1.104918
30min_MT.	Q96FX2	-1.21159	0.120006	0.181038	DPH3	Diphthami	DPH3_HUM	1	0.431794
30min_MT.	Q9P2J5	-0.2606	0.120073	0.181105	LARS1	Leucine--tR	SYLC_HUM	21	0.834742
30min_MT.	P21912	0.007668	0.120121	0.181142	SDHB	Succinate d	SDHB_HUM	4	1.005329
30min_MT.	Q9UBF8	0.186726	0.120201	0.181229	PI4KB	Phosphatid	PI4KB_HUM	4	1.138178
15min_MT.	O00299	0.204793	0.120679	0.181703	CLIC1	Chloride in	CLIC1_HUM	15	1.152521
15min_MT.	P02545	0.156443	0.120639	0.181703	LMNA	Prelamin-A	LMNA_HUM	36	1.114536
30min_MT.	P20338	0.170509	0.120668	0.181703	RAB4A	Ras-related	RAB4A_HU	6	1.125455
30min_MT.	P61421	0.106273	0.120604	0.181703	ATP6V0D1	V-type prot	VA0D1_HU	3	1.076444
30min_MT.	P43686	-0.09594	0.120839	0.181805	PSMC4	26S protea	PRS6B_HU	19	0.93566
30min_MT.	Q9NXN4	-0.63751	0.120805	0.181805	GDAP2	Ganglioside	GDAP2_HU	1	0.642823
15min_MT.	Q9Y4E8	-0.0026	0.120832	0.181805	USP15	Ubiquitin c	UBP15_HU	26	0.998201
15min_MT.	O75915	-0.96878	0.120915	0.181849	ARL6IP5	PRA1 famil	PRAF3_HU	1	0.510939
15min_MT.	Q96IV0	-0.32851	0.120961	0.181849	NGLY1	Peptide-N(4	NGLY1_HU	6	0.796358
30min_MT.	Q9BYD6	0.082034	0.120942	0.181849	MRPL1	Large ribos	RM01_HUM	2	1.05851
30min_MT.	O75153	-0.06108	0.121049	0.181911	CLUH	Clustered m	CLU_HUMA	29	0.958549
15min_MT.	P27707	0.258777	0.121035	0.181911	DCK	Deoxycytid	DCK_HUMA	5	1.196464
30min_MT.	Q86WJ1	-0.12212	0.121191	0.182055	CHD1L	Chromodon	CHD1L_HU	2	0.918838
15min_MT.	P49840	0.369921	0.121221	0.182065	GSK3A	Glycogen sy	GSK3A_HU	6	1.292282
15min_MT.	Q04323	0.115677	0.121282	0.182121	UBXN1	UBX domai	UBXN1_HU	12	1.083483
15min_MT.	P25325	0.107556	0.121401	0.18223	MPST	3-mercapto	THTM_HUM	13	1.077401
30min_MT.	Q8NCE2	-0.13252	0.121396	0.18223	MTMR14	Myotubula	MTMRE_HU	7	0.91224
30min_MT.	P51153	-0.01289	0.121426	0.182232	RAB13	Ras-related	RAB13_HU	1	0.991104
15min_MT.	O75342	0.343366	0.121559	0.182341	ALOX12B	Arachidona	LX12B_HU	6	1.268713
15min_MT.	P07686	0.104978	0.121569	0.182341	HEXB	Beta-hexos	HEXB_HUM	19	1.075478
15min_MT.	Q9NTZ6	0.436383	0.121555	0.182341	RBM12	RNA-bindir	RBM12_HU	18	1.353207
15min_MT.	Q9UI17	-0.18898	0.121647	0.182425	DMGDH	Dimethylgl	M2GD_HU	4	0.877225
15min_MT.	Q13185	-0.24548	0.121743	0.182495	CBX3	Chromobo	CBX3_HUM	7	0.843533
15min_MT.	Q92797	-0.2595	0.121818	0.182495	SYMPK	Symplekin	SYMPK_HU	11	0.835375
30min_MT.	O75143	-0.07669	0.12189	0.182543	ATG13	Autophagy-	ATG13_HU	6	0.948228
30min_MT.	P50895	0.108396	0.122182	0.182876	BCAM	Basal cell a	BCAM_HUM	12	1.078029
30min_MT.	P06756	-0.42692	0.122365	0.18308	ITGAV	Integrin alp	ITAV_HUM	2	0.743846
30min_MT.	P35611	-0.12763	0.122428	0.183113	ADD1	Alpha-addu	ADDA_HUM	17	0.915331
30min_MT.	Q92844	-0.17985	0.122537	0.183232	TANK	TRAF family	TANK_HUM	2	0.882794
30min_MT.	Q05639	-0.2419	0.122727	0.183342	EEF1A2	Elongation	EF1A2_HUM	5	0.845629
30min_MT.	Q9H900	-0.18075	0.122702	0.183342	ZWILCH	Protein zwi	ZWILC_HU	2	0.882245
15min_MT.	P43251	0.22504	0.122816	0.183392	BTD	Biotinidase	BTD_HUMA	4	1.16881
15min_MT.	Q8WXE9	0.910115	0.122832	0.183392	STON2	Stonin-2	STON2_HU	1	1.879196
30min_MT.	P28799	0.00846	0.123024	0.183609	GRN	Progranulir	GRN_HUMA	6	1.005881
30min_MT.	P52789	0.080175	0.123163	0.183734	HK2	Hexokinase	HXK2_HUM	20	1.057146
30min_MT.	Q15714	0.085942	0.123223	0.183734	TSC22D1	TSC22 dom	T22D1_HU	3	1.061381
30min_MT.	Q9NX01	0.117026	0.123249	0.183734	TXNL4B	Thioredoxi	TXN4B_HU	1	1.084497
30min_MT.	Q7L576	-0.18137	0.123289	0.183758	CYFIP1	Cytoplasmi	CYFP1_HUM	13	0.881864
30min_MT.	Q13795	0.78389	0.123336	0.183794	ARFRP1	ADP-ribosy	ARFRP_HU	1	1.721767
15min_MT.	O75150	0.170914	0.123483	0.183908	RNF40	E3 ubiquiti	BRE1B_HU	7	1.125771
15min_MT.	Q8NE62	0.357384	0.12354	0.183957	CHDH	Choline del	CHDH_HUM	4	1.281101
30min_MT.	Q5T440	-0.06797	0.123692	0.183986	IBA57	Putative tra	CAF17_HU	4	0.953981
30min_MT.	Q96RE7	-0.05831	0.123717	0.183986	NACC1	Nucleus acc	NACC1_HU	7	0.960388
15min_MT.	Q99623	-0.45825	0.123724	0.183986	PHB2	Prohibitin-	PHB2_HUM	1	0.72787

15min_MT.	Q9Y4Y9	-0.79915	0.123722	0.183986	LSM5	U6 snRNA-a	LSM5_HUM	1	0.574687
15min_MT.	Q13435	-0.0778	0.123785	0.183996	SF3B2	Splicing fac	SF3B2_HUI	29	0.947501
15min_MT.	Q16629	-0.10721	0.123796	0.183996	SRSF7	Serine/argi	SRSF7_HUM	7	0.928384
15min_MT.	Q8TBQ9	0.476161	0.123938	0.184164	TMEM167A	Protein kisl	KISHA_HUM	1	1.391038
15min_MT.	P31749	-0.33084	0.12408	0.184271	AKT1	RAC-alpha s	AKT1_HUM	5	0.795076
30min_MT.	Q9BRP4	0.084589	0.124276	0.184492	PAAF1	Proteasom	PAAF1_HUI	12	1.060386
15min_MT.	Q9H7Z6	-0.15054	0.124367	0.184592	KAT8	Histone ace	KAT8_HUM	2	0.900912
15min_MT.	O00142	0.367439	0.124453	0.184684	TK2	Thymidine	KITM_HUM	4	1.290061
30min_MT.	O43752	0.479723	0.124555	0.184696	STX6	Syntaxin-6	STX6_HUM	2	1.394475
30min_MT.	P46100	0.022427	0.124518	0.184696	ATRX	Transcripti	ATRX_HUM	29	1.015667
30min_MT.	P61956	0.201285	0.124536	0.184696	SUMO2	Small ubiq	SUMO2_HU	1	1.149722
30min_MT.	Q8NI22	-0.81025	0.124509	0.184696	MCFD2	Multiple cc	MCFD2_HU	2	0.570283
15min_MT.	P46527	-0.15794	0.124662	0.184819	CDKN1B	Cyclin-depe	CDN1B_HU	5	0.896304
30min_MT.	Q14974	0.169968	0.124729	0.184849	KPNB1	Importin su	IMB1_HUM	30	1.125034
15min_MT.	P30837	0.188483	0.124865	0.184999	ALDH1B1	Aldehyde d	AL1B1_HUI	14	1.139565
15min_MT.	P49427	0.252043	0.124902	0.184999	CDC34	Ubiquitin-c	UB2R1_HU	3	1.190893
30min_MT.	Q13043	0.338039	0.125106	0.185268	STK4	Serine/thre	STK4_HUM	7	1.264037
30min_MT.	Q99567	-0.16246	0.125136	0.185276	NUP88	Nuclear po	NUP88_HU	16	0.893502
15min_MT.	Q5TC12	0.212997	0.125179	0.185294	ATPAF1	ATP synthas	ATPF1_HUI	8	1.159094
15min_MT.	Q9UKM9	0.195004	0.125196	0.185294	RALY	RNA-bindir	RALY_HUM	5	1.144727
30min_MT.	P52907	0.041025	0.125266	0.185299	CAPZA1	F-actin-cap	CAZA1_HUI	14	1.028845
30min_MT.	Q9H488	0.079651	0.12523	0.185299	POFUT1	GDP-fucose	OFUT1_HUI	9	1.056763
15min_MT.	Q96CP2	-0.1995	0.125328	0.185315	FLYWCH2	FLYWCH far	FWCH2_HU	7	0.870853
30min_MT.	P13284	-0.28802	0.125358	0.185325	IFI30	Gamma-int	GILT_HUM/	1	0.819024
30min_MT.	Q5MIZ7	0.105648	0.125401	0.185353	PPP4R3B	Serine/thre	P4R3B_HUI	10	1.075978
15min_MT.	Q9BW91	0.127281	0.125648	0.185648	NUDT9	ADP-ribose	NUDT9_HU	12	1.092233
15min_MT.	Q9Y6A4	-0.07076	0.125838	0.185859	CFAP20	Cilia- and fl	CFA20_HUI	5	0.952139
30min_MT.	P10768	0.044262	0.125869	0.185869	ESD	S-formylglu	ESTD_HUM	10	1.031156
30min_MT.	Q96ST2	0.063797	0.125916	0.185869	IWS1	Protein IWS	IWS1_HUM	17	1.045213
30min_MT.	Q12788	-0.24068	0.125944	0.185874	TBL3	Transducin	TBL3_HUM	1	0.846348
15min_MT.	P13995	0.103572	0.126008	0.185934	MTHFD2	Bifunctiona	MTDC_HUM	5	1.07443
15min_MT.	Q9Y3C4	0.422509	0.12605	0.185961	TPRKB	EKC/KEOPS	TPRKB_HUI	2	1.340257
30min_MT.	O00462	0.14977	0.126395	0.186159	MANBA	Beta-mann	MANBA_HU	16	1.109392
30min_MT.	P49207	0.136353	0.126275	0.186159	RPL34	Large ribos	RL34_HUM	3	1.099123
30min_MT.	Q7Z6J8	0.515202	0.126273	0.186159	UBE3D	E3 ubiquiti	UBE3D_HU	2	1.429194
15min_MT.	Q8NHG7	-0.99364	0.126398	0.186159	SVIP	Small VCP/	SVIP_HUM/	1	0.502209
15min_MT.	Q96FX7	0.168406	0.126365	0.186159	TRMT61A	tRNA (aden	TRM61_HU	6	1.123816
15min_MT.	Q9H974	0.118202	0.126367	0.186159	QTRT2	Queuine tR	QTRT2_HUI	1	1.085382
15min_MT.	P62328	-0.40008	0.126535	0.186185	TMSB4X	Thymosin b	TYB4_HUM	2	0.757819
15min_MT.	Q14511	0.046491	0.126471	0.186185	NEDD9	Enhancer o	CASL_HUM	1	1.03275
15min_MT.	P12035	2.336244	0.126642	0.186236	KRT3	Keratin, typ	K2C3_HUM	8	5.049861
30min_MT.	P13984	-0.16207	0.126629	0.186236	GTF2F2	General tra	T2FB_HUM	11	0.893745
15min_MT.	O43181	-0.3533	0.126837	0.186488	NDUFS4	NADH dehy	NDUS4_HU	2	0.78279
30min_MT.	Q96EC8	-0.11556	0.126915	0.186569	YIPF6	Protein YIP	YIPF6_HUM	2	0.923024
30min_MT.	Q9BQ67	0.324868	0.127174	0.186879	GRWD1	Glutamate-	GRWD1_HU	3	1.25255
30min_MT.	Q96DX5	0.123144	0.127202	0.186885	ASB9	Ankyrin rep	ASB9_HUM	2	1.089106
30min_MT.	P22891	-1.00423	0.127243	0.186909	PROZ	Vitamin K-c	PROZ_HUM	1	0.498535
15min_MT.	Q96C01	0.107092	0.127373	0.18703	FAM136A	Protein FAM	F136A_HUI	9	1.077055
15min_MT.	O00560	0.632259	0.12756	0.187234	SDCBP	Syntenin-1	SDCB1_HUI	1	1.54999
30min_MT.	O43681	0.126162	0.127939	0.187275	GET3	ATPase GET	GET3_HUM	11	1.091386
15min_MT.	P01130	0.199684	0.127785	0.187275	LDLR	Low-densit	LDLR_HUM	7	1.148446
30min_MT.	P16278	0.187104	0.127775	0.187275	GLB1	Beta-galact	BGAL_HUM	11	1.138476

15min_MT.	P36915	0.161486	0.12792	0.187275	GNL1	Guanine nu	GNL1_HUM		11	1.118439
15min_MT.	Q5W111	0.297503	0.127668	0.187275	SPRYD7	SPRY doma	SPRY7_HUI		1	1.229015
30min_MT.	Q86VX2	0.144462	0.127916	0.187275	COMMD7	COMM dom	COMD7_HU		5	1.105318
30min_MT.	Q92538	-0.09522	0.127947	0.187275	GBF1	Golgi-speci	GBF1_HUM		48	0.936129
15min_MT.	Q96S59	0.146382	0.127926	0.187275	RANBP9	Ran-bindin	RANB9_HU		10	1.106791
30min_MT.	Q9BQG0	0.315698	0.12793	0.187275	MYBBP1A	Myb-bindir	MBB1A_HU		5	1.244614
15min_MT.	Q9UKD1	0.506955	0.127615	0.187275	GMEB2	Glucocorti	GMEB2_HU		1	1.421047
30min_MT.	Q9UKG1	-0.05121	0.127718	0.187275	APPL1	DCC-intera	DP13A_HU		20	0.965128
15min_MT.	O60664	0.10391	0.128145	0.187496	PLIN3	Perilipin-3	PLIN3_HUM		25	1.074682
30min_MT.	P19652	0.003402	0.128182	0.187515	ORM2	Alpha-1-aci	A1AG2_HU		2	1.002361
15min_MT.	Q9BZE1	-0.0634	0.12825	0.187543	MRPL37	Large ribos	RM37_HUM		4	0.957005
30min_MT.	O75718	-0.01307	0.1284	0.187668	CRTAP	Cartilage-as	CRTAP_HUI		8	0.990978
15min_MT.	O95487	0.067833	0.128578	0.187848	SEC24B	Protein trai	SC24B_HUI		3	1.048141
15min_MT.	Q05209	0.200504	0.128616	0.187868	PTPN12	Tyrosine-pr	PTN12_HUI		7	1.149099
15min_MT.	Q9HBL0	-0.04724	0.128664	0.187904	TNS1	Tensin-1	TENS1_HUI		11	0.967783
15min_MT.	Q06587	-0.38564	0.128739	0.187942	RING1	E3 ubiquiti	RING1_HUI		4	0.765438
15min_MT.	Q96IZ0	-0.35562	0.128737	0.187942	PAWR	PRKC apopt	PAWR_HUM		10	0.781536
30min_MT.	P42167	-0.07722	0.128771	0.187954	TMPO	Lamina-ass	LAP2B_HUI		3	0.947886
15min_MT.	Q5VV43	0.065042	0.128915	0.188059	KIAA0319	Dyslexia-as	K0319_HUI		2	1.046116
30min_MT.	Q8N2F6	-0.19788	0.12889	0.188059	ARMC10	Armadillo r	ARM10_HU		1	0.871829
30min_MT.	O94811	2.254865	0.128973	0.188074	TPPP	Tubulin pol	TPPP_HUM		2	4.772896
30min_MT.	Q9BYN0	0.143439	0.128967	0.188074	SRXN1	Sulfiredoxi	SRXN1_HUI		3	1.104535
15min_MT.	A6NHX0	-0.2892	0.129158	0.188234	CASTOR2	Cytosolic a	CAST2_HUM		6	0.818354
15min_MT.	P52735	-0.33521	0.129346	0.188337	VAV2	Guanine nu	VAV2_HUM		5	0.792671
30min_MT.	Q7Z460	-0.25703	0.129346	0.188337	CLASP1	CLIP-associ	CLAP1_HUI		22	0.836808
15min_MT.	Q9NPD3	0.055524	0.129332	0.188337	EXOSC4	Exosome cc	EXOS4_HUI		10	1.039236
15min_MT.	Q9Y3U8	-0.37696	0.12929	0.188337	RPL36	Large ribos	RL36_HUM		3	0.770058
30min_MT.	Q08380	0.14248	0.12951	0.188471	LGALS3BP	Galectin-3-	LG3BP_HUI		5	1.103801
15min_MT.	Q96F44	-0.52167	0.129542	0.188482	TRIM11	E3 ubiquiti	TRI11_HUM		2	0.696567
15min_MT.	Q969T4	-0.2157	0.129612	0.188549	UBE2E3	Ubiquitin-c	UB2E3_HUI		4	0.861126
15min_MT.	Q9BV38	-0.07886	0.129684	0.188583	WDR18	WD repeat-	WDR18_HU		6	0.946804
30min_MT.	Q9H6U6	-0.28801	0.129765	0.18863	BCAS3	BCAS3 micr	BCAS3_HUI		5	0.819033
30min_MT.	Q96M27	0.259151	0.130013	0.188922	PRRC1	Protein PRI	PRRC1_HUI		8	1.196774
15min_MT.	P18621	-0.05894	0.130056	0.188948	RPL17	Large ribos	RL17_HUM		2	0.959971
30min_MT.	P08559	-0.11473	0.130119	0.188966	PDHA1	Pyruvate de	ODPA_HUM		15	0.923555
30min_MT.	P26196	-0.09385	0.130139	0.188966	DDX6	Probable AT	DDX6_HUM		12	0.937022
15min_MT.	Q15003	0.009543	0.130199	0.189016	NCAPH	Condensin	CND2_HUM		4	1.006637
30min_MT.	P22695	-0.11837	0.130266	0.189077	UQCRC2	Cytochrom	QCR2_HUM		2	0.921225
15min_MT.	Q96C57	-0.11646	0.130297	0.189087	CUSTOS	Protein CUS	CSTOS_HUM		5	0.922447
30min_MT.	Q99574	0.01455	0.13045	0.189239	SERPINI1	Neuroserpi	NEUS_HUM		8	1.010136
15min_MT.	O43657	0.071959	0.130584	0.189399	TSPAN6	Tetraspanir	TSN6_HUM		1	1.051143
15min_MT.	P18077	0.148547	0.130761	0.189599	RPL35A	Large ribos	RL35A_HUI		3	1.108452
30min_MT.	Q8WVK2	0.194813	0.13077	0.189599	SNRNP27	U4/U6.U5 s	SNR27_HUI		4	1.144576
30min_MT.	P20226	-0.26636	0.130808	0.189619	TBP	TATA-box-b	TBP_HUMA		1	0.831413
30min_MT.	O96006	-0.6744	0.130944	0.189745	ZBED1	E3 SUMO-p	ZBED1_HUI		1	0.626593
30min_MT.	Q8WXF1	-0.15024	0.131158	0.18995	PSPC1	Paraspeckle	PSPC1_HUI		13	0.9011
30min_MT.	Q99933	-0.25178	0.131123	0.18995	BAG1	BAG family	BAG1_HUM		3	0.839857
30min_MT.	P49914	-0.67398	0.131272	0.190024	MTHFS	5-formyltet	MTHFS_HU		1	0.626777
30min_MT.	Q92889	-0.14998	0.131254	0.190024	ERCC4	DNA repair	XPF_HUMA		4	0.90126
30min_MT.	Q9UI08	0.051916	0.131282	0.190024	EVL	Ena/VASP-li	EVL_HUMA		6	1.03664
15min_MT.	Q96RQ3	-0.08966	0.13131	0.190029	MCCC1	Methylcrot	MCCA_HUM		11	0.939746
30min_MT.	Q9Y365	0.05428	0.131371	0.190081	STARD10	START dom	STA10_HUM		6	1.03834

30min_MT.	Q9NQP4	0.240737	0.131453	0.190166	PFDN4	Prefoldin su	PFD4_HUM	3	1.181596
30min_MT.	Q92597	0.052099	0.131626	0.190354	NDRG1	Protein ND	NDRG1_HU	7	1.036772
30min_MT.	Q96R27	-1.07524	0.131634	0.190354	OR2M4	Olfactory re	OR2M4_HU	1	0.474591
15min_MT.	Q6DHV7	0.266924	0.131799	0.190525	ADAL	Adenosine	ADAL_HUM	7	1.203239
30min_MT.	P14735	0.061	0.131958	0.190685	IDE	Insulin-deg	IDE_HUMA	37	1.043189
30min_MT.	P10644	-0.04264	0.132212	0.191016	PRKAR1A	cAMP-depe	KAP0_HUM	7	0.970877
30min_MT.	Q8TE67	0.032025	0.132292	0.191065	EPS8L3	Epidermal	ES8L3_HUM	22	1.022446
15min_MT.	Q96Q11	0.19103	0.132294	0.191065	TRNT1	CCA tRNA n	TRNT1_HU	9	1.141579
15min_MT.	Q4G0J3	-0.16621	0.132379	0.191143	LARP7	La-related	LARP7_HU	10	0.891179
30min_MT.	Q8N5N7	-0.66268	0.132418	0.191143	MRPL50	Large ribos	RM50_HUM	2	0.631703
30min_MT.	P61970	0.229273	0.132472	0.191145	NUTF2	Nuclear tra	NTF2_HUM	5	1.172244
15min_MT.	P49711	-0.44226	0.132504	0.191156	CTCF	Transcripti	CTCF_HUM	2	0.735979
15min_MT.	P30050	-0.27694	0.132536	0.191166	RPL12	Large ribos	RL12_HUM	5	0.825339
15min_MT.	Q12872	-0.27966	0.13262	0.191182	SFSWAP	Splicing fac	SFSWA_HU	6	0.823787
15min_MT.	Q13257	0.041715	0.132611	0.191182	MAD2L1	Mitotic spi	MD2L1_HU	5	1.029336
15min_MT.	Q9UL40	0.054641	0.132725	0.191227	ZNF346	Zinc finger	ZN346_HU	1	1.038601
30min_MT.	P54886	-0.1113	0.132864	0.191316	ALDH18A1	Delta-1-pyr	P5CS_HUM	33	0.925755
30min_MT.	Q8WV93	-0.34599	0.132825	0.191316	AFG1L	AFG1-like A	AFG1L_HU	1	0.786767
30min_MT.	Q9BXY0	0.189788	0.132884	0.191316	MAK16	Protein MA	MAK16_HU	5	1.140596
15min_MT.	P42858	-0.33433	0.133021	0.19147	HTT	Huntingtin	HD_HUMA	6	0.793154
30min_MT.	Q04726	-0.05886	0.13304	0.19147	TLE3	Transducin	TLE3_HUM	10	0.960025
15min_MT.	A0AVT1	-0.08307	0.133191	0.191486	UBA6	Ubiquitin-l	UBA6_HUM	32	0.944048
30min_MT.	O95081	-0.13773	0.133163	0.191486	AGFG2	Arf-GAP do	AGFG2_HU	5	0.908945
30min_MT.	Q96PE3	-0.10675	0.133084	0.191486	INPP4A	Inositol pol	INP4A_HU	8	0.928675
15min_MT.	Q9Y2V7	0.035049	0.133203	0.191486	COG6	Conserved	COG6_HUM	10	1.024592
30min_MT.	Q92484	0.129038	0.133388	0.191653	SMPDL3A	Cyclic GMP	ASM3A_HU	12	1.093564
15min_MT.	Q10570	0.072965	0.133572	0.19175	CPSF1	Cleavage ar	CPSF1_HU	18	1.051877
30min_MT.	Q9BQC3	-0.02714	0.13356	0.19175	DPH2	2-(3-amino	DPH2_HUM	4	0.981364
15min_MT.	Q13547	-0.38809	0.133725	0.191821	HDAC1	Histone dea	HDAC1_HU	2	0.764143
15min_MT.	P15428	0.267226	0.133873	0.191927	HPGD	15-hydroxy	PGDH_HUM	5	1.203492
30min_MT.	Q12769	-0.21928	0.133859	0.191927	NUP160	Nuclear po	NU160_HU	6	0.858994
15min_MT.	O43143	-0.0431	0.134135	0.192087	DHX15	ATP-depen	DHX15_HU	28	0.97057
15min_MT.	P07311	0.247199	0.134156	0.192087	ACYP1	Acylphosph	ACYP1_HU	4	1.1869
15min_MT.	Q8N806	0.236297	0.134117	0.192087	UBR7	Putative E3	UBR7_HUM	9	1.177966
30min_MT.	Q5T0N5	-0.04816	0.134373	0.192286	FNBP1L	Formin-bin	FBP1L_HU	10	0.967168
15min_MT.	Q9Y508	0.157086	0.134513	0.192422	RNF114	E3 ubiquiti	RN114_HU	8	1.115033
15min_MT.	O94811	0.647639	0.134606	0.192479	TPPP	Tubulin pol	TPPP_HUM	2	1.566602
15min_MT.	Q06323	0.070092	0.134613	0.192479	PSME1	Proteasom	PSME1_HU	19	1.049783
30min_MT.	Q9HCN4	-0.09908	0.134627	0.192479	GPN1	GPN-loop G	GPN1_HUM	5	0.933628
15min_MT.	Q9P289	0.025829	0.134814	0.192676	STK26	Serine/thre	STK26_HU	11	1.018065
30min_MT.	Q9H0A8	0.162194	0.13504	0.192928	COMMD4	COMM don	COMD4_HU	2	1.118987
30min_MT.	Q9BV86	-0.43372	0.135089	0.192963	NTMT1	N-terminal	NTM1A_HU	2	0.74035
30min_MT.	O60610	-0.11234	0.135358	0.193242	DIAPH1	Protein dia	DIAP1_HU	32	0.925088
15min_MT.	P19823	0.487439	0.1355	0.193341	ITIH2	Inter-alpha	ITIH2_HUM	24	1.401954
15min_MT.	Q14498	-0.16279	0.135546	0.193341	RBM39	RNA-bindir	RBM39_HU	9	0.893294
15min_MT.	Q8IYQ7	-0.229	0.135573	0.193341	THNSL1	Threonine	THNS1_HU	3	0.853229
30min_MT.	Q8N9V6	-0.19936	0.135556	0.193341	ANKRD53	Ankyrin rep	ANR53_HU	1	0.870934
30min_MT.	Q96EV2	-0.26629	0.135603	0.193344	RBM33	RNA-bindir	RBM33_HU	5	0.831457
15min_MT.	P78562	-0.53755	0.135649	0.193358	PHEX	Phosphate-	PHEX_HUM	1	0.68894
30min_MT.	Q12802	-0.0835	0.135662	0.193358	AKAP13	A-kinase an	AKP13_HU	17	0.943763
30min_MT.	P62937	0.180525	0.135752	0.193439	PPIA	Peptidyl-pr	PPIA_HUMA	13	1.133296
15min_MT.	Q9UHD9	0.269886	0.135768	0.193439	UBQLN2	Ubiquilin-2	UBQL2_HU	6	1.205713

30min_MT.	Q96C01	0.141391	0.135803	0.193453	FAM136A	Protein FAI	F136A_HUI	9	1.102968
15min_MT.	A6NH11	0.090816	0.136072	0.193695	GLTPD2	Glycolipid	GLTD2_HUI	3	1.064972
30min_MT.	P82932	-0.07431	0.136068	0.193695	MRPS6	Small ribos	RT06_HUM	1	0.949794
15min_MT.	Q8N1F7	-0.14521	0.136039	0.193695	NUP93	Nuclear poi	NUP93_HU	28	0.90425
30min_MT.	P35998	-0.02642	0.13612	0.193728	PSMC2	26S protea:	PRS7_HUM	24	0.981852
15min_MT.	P49902	-0.02359	0.136365	0.194006	NT5C2	Cytosolic p	5NTC_HUM	15	0.98378
30min_MT.	P15531	0.102233	0.136467	0.194011	NME1	Nucleoside	NDKA_HUM	6	1.073434
30min_MT.	P43251	0.307282	0.136451	0.194011	BTD	Biotinidase	BTD_HUMA	4	1.237375
30min_MT.	Q16790	0.17305	0.136446	0.194011	CA9	Carbonic ai	CAH9_HUM	5	1.12744
30min_MT.	Q96F46	-0.35314	0.136465	0.194011	IL17RA	Interleukin	I17RA_HUM	5	0.782876
15min_MT.	Q5T200	-0.09995	0.136556	0.194101	ZC3H13	Zinc finger	ZC3HD_HU	18	0.933067
30min_MT.	P01111	0.060337	0.136716	0.194113	NRAS	GTPase NRa	RASN_HUM	6	1.042709
15min_MT.	P02675	-0.03368	0.136682	0.194113	FGB	Fibrinogen	FIBB_HUMA	13	0.976924
15min_MT.	Q6IN84	-0.009	0.136594	0.194113	MRM1	rRNA meth	MRM1_HUI	7	0.993783
15min_MT.	Q8NC96	0.091501	0.136738	0.194113	NECAP1	Adaptin ear	NECP1_HUI	4	1.065478
15min_MT.	Q9BVK6	0.357456	0.13672	0.194113	TMED9	Transmemb	TMED9_HU	1	1.281165
15min_MT.	Q9P1Y6	-0.23958	0.136641	0.194113	PHRF1	PHD and RI	PHRF1_HUI	6	0.846992
15min_MT.	P20248	1.108428	0.136788	0.194114	CCNA2	Cyclin-A2	CCNA2_HU	2	2.156106
30min_MT.	Q9UK99	0.167806	0.13684	0.194152	FBXO3	F-box only	FBX3_HUM	4	1.123349
15min_MT.	Q96S55	0.105835	0.136865	0.194153	WRNIP1	ATPase WRI	WRIP1_HU	5	1.076117
15min_MT.	P62899	-0.08497	0.137069	0.19423	RPL31	Large ribos	RL31_HUM	5	0.942807
30min_MT.	Q96LA8	0.505195	0.137028	0.19423	PRMT6	Protein arg	ANM6_HUI	3	1.419315
30min_MT.	Q9H7D7	0.05794	0.137039	0.19423	WDR26	WD repeat-	WDR26_HU	9	1.040978
30min_MT.	P49023	-0.20484	0.137145	0.194287	PXN	Paxillin	PAXI_HUMA	9	0.867634
15min_MT.	Q7Z3Y9	-0.27212	0.137278	0.194351	KRT26	Keratin, typ	K1C26_HUI	1	0.828101
15min_MT.	Q96HS1	0.140843	0.137276	0.194351	PGAM5	Serine/thre	PGAM5_HU	5	1.102549
15min_MT.	P62253	0.433832	0.137432	0.194419	UBE2G1	Ubiquitin-c	UB2G1_HU	4	1.350817
30min_MT.	Q08AI8	0.106201	0.137361	0.194419	MAB21L4	Protein ma	MB214_HU	3	1.07639
30min_MT.	Q8TBQ9	0.425729	0.137408	0.194419	TMEM167A	Protein kisl	KISHA_HUI	1	1.343251
30min_MT.	Q9NP55	1.102911	0.137384	0.194419	BPIFA1	BPI fold-coi	BPIA1_HUI	2	2.147876
30min_MT.	Q9Y2B0	0.174794	0.13745	0.194419	CNPY2	Protein can	CNPY2_HUI	7	1.128803
15min_MT.	Q96DM3	-0.46814	0.137537	0.194471	RMC1	Regulator c	RMC1_HUI	4	0.722898
15min_MT.	P43246	0.004521	0.137564	0.194475	MSH2	DNA misma	MSH2_HUI	18	1.003139
30min_MT.	P07602	0.186686	0.137901	0.19488	PSAP	Prosaposin	SAP_HUMA	14	1.138147
30min_MT.	Q96DX4	0.239952	0.138052	0.194988	RSPRY1	RING finger	RSPRY_HUI	3	1.180954
30min_MT.	Q96EH3	-0.17895	0.138003	0.194988	MALSU1	Mitochond	MASU1_HU	1	0.883346
30min_MT.	P78509	-0.03654	0.138228	0.195101	RELN	Reelin	RELN_HUM	23	0.974993
30min_MT.	Q8IY47	-0.2217	0.138184	0.195101	KBTBD2	Kelch repea	KBTB2_HUI	3	0.857552
15min_MT.	Q9NSK0	0.184728	0.138232	0.195101	KLC4	Kinesin ligh	KLC4_HUM	18	1.136602
15min_MT.	O60547	-0.16993	0.138526	0.195117	GMDS	GDP-mann	GMDS_HUI	11	0.888884
30min_MT.	O95926	0.219717	0.138339	0.195117	SYF2	Pre-mRNA-	SYF2_HUMA	1	1.164505
30min_MT.	P21860	0.015481	0.138349	0.195117	ERBB3	Receptor ty	ERBB3_HUI	5	1.010788
15min_MT.	P49757	0.051272	0.138485	0.195117	NUMB	Protein nui	NUMB_HUI	7	1.036178
30min_MT.	Q86VP1	-0.12485	0.138452	0.195117	TAX1BP1	Tax1-bindir	TAXB1_HUI	6	0.917102
15min_MT.	Q92945	0.254822	0.138528	0.195117	KHSRP	Far upstrea	FUBP2_HUI	28	1.193189
30min_MT.	Q9UBT7	-0.26814	0.138284	0.195117	CTNNAL1	Alpha-catul	CTNL1_HUI	1	0.830391
30min_MT.	Q9Y3I0	-0.15982	0.138543	0.195117	RTCB	RNA-splicir	RTCB_HUM	16	0.895136
30min_MT.	Q9Y6I3	0.020514	0.138629	0.195203	EPN1	Epsin-1	EPN1_HUM	9	1.014321
15min_MT.	P57088	-0.36549	0.138746	0.195334	TMEM33	Transmemb	TMM33_HU	4	0.776206
15min_MT.	P78417	0.105828	0.138914	0.195429	GSTO1	Glutathion	GSTO1_HUI	17	1.076112
15min_MT.	P47224	0.281889	0.138973	0.195477	RABIF	Guanine nu	MSS4_HUM	2	1.215786
30min_MT.	Q9UM54	-0.08708	0.139022	0.19551	MYO6	Unconvent	MYO6_HUM	14	0.941425

30min_MT.	A9UHW6	0.443589	0.139163	0.195568	MIF4GD	MIF4G dom	MI4GD_HU	6	1.359984
30min_MT.	Q13907	0.068816	0.139101	0.195568	IDI1	Isopenteny	IDI1_HUMA	8	1.048855
15min_MT.	O60927	-0.52172	0.139196	0.195579	PPP1R11	E3 ubiquiti	PP1RB_HU	5	0.696542
30min_MT.	Q99584	0.192009	0.139348	0.195653	S100A13	Protein S10	S10AD_HU	7	1.142354
30min_MT.	Q99611	0.026398	0.139333	0.195653	SEPHS2	Selenide, w	SPS2_HUM	14	1.018466
15min_MT.	A6NED2	-0.13247	0.139439	0.19571	RCCD1	RCC1 doma	RCCD1_HU	4	0.912271
15min_MT.	O95453	-0.0199	0.139524	0.195783	PARN	Poly(A)-spe	PARN_HUM	20	0.986304
15min_MT.	P62906	-0.6585	0.139541	0.195783	RPL10A	Large ribos	RL10A_HU	6	0.633536
15min_MT.	O94776	-0.09804	0.139597	0.195798	MTA2	Metastasis-	MTA2_HUM	1	0.9343
30min_MT.	P08621	0.091863	0.139668	0.195798	SNRNP70	U1 small nu	RU17_HUM	13	1.065746
30min_MT.	Q96Q11	0.222786	0.139658	0.195798	TRNT1	CCA tRNA n	TRNT1_HU	9	1.166985
30min_MT.	Q9BZX2	0.266256	0.139626	0.195798	UCK2	Uridine-cyt	UCK2_HUM	3	1.202682
15min_MT.	P20930	-0.38243	0.139838	0.195905	FLG	Filaggrin	FILA_HUMA	16	0.767146
15min_MT.	Q96RU3	0.296287	0.139861	0.195905	FNBP1	Formin-bin	FNBP1_HU	5	1.22798
15min_MT.	Q99807	0.56044	0.139903	0.195905	COQ7	5-demetho	COQ7_HUM	2	1.474719
15min_MT.	O95671	-0.05618	0.139936	0.195916	ASMTL	Probable bi	ASML_HUM	22	0.961809
15min_MT.	O95630	0.046737	0.140121	0.196031	STAMBP	STAM-bindi	STABP_HU	17	1.032926
15min_MT.	P07992	0.188083	0.140169	0.196031	ERCC1	DNA excisio	ERCC1_HU	4	1.139249
30min_MT.	P68400	-0.06059	0.140146	0.196031	CSNK2A1	Casein kina	CSK21_HU	9	0.958872
30min_MT.	Q15370	0.055545	0.140156	0.196031	ELOB	Elongin-B	ELOB_HUM	8	1.039251
15min_MT.	P09958	0.518188	0.14026	0.196124	FURIN	Furin	FURIN_HU	4	1.432155
30min_MT.	Q9H7L9	0.134441	0.140326	0.196181	SUDS3	Sin3 histon	SDS3_HUM	3	1.097668
30min_MT.	A8K2U0	0.438113	0.14046	0.196333	A2ML1	Alpha-2-ma	A2ML1_HU	4	1.354831
15min_MT.	P30101	0.143159	0.140864	0.19669	PDIA3	Protein dis	PDIA3_HU	34	1.10432
15min_MT.	P49336;Q9	0.713144	0.140796	0.19669	CDK8;CDK1	Cyclin-depe	CDK8_HUM	1	1.639373
30min_MT.	Q5MNZ6	-0.10053	0.140774	0.19669	WDR45B	WD repeat	WIPI3_HUM	5	0.932694
15min_MT.	Q6IQ22	-0.41213	0.140838	0.19669	RAB12	Ras-related	RAB12_HU	1	0.751512
15min_MT.	Q7Z3B4	0.055495	0.140854	0.19669	NUP54	Nucleopori	NUP54_HU	3	1.039216
15min_MT.	Q9UNF0	0.154392	0.140901	0.19669	PACSIN2	Protein kin	PACN2_HU	14	1.112952
30min_MT.	Q96NL8	0.253108	0.140971	0.196695	CFAP418	Cilia- and fl	CF418_HU	4	1.191771
30min_MT.	O15111	-0.263	0.1411	0.196773	CHUK	Inhibitor o	IKKA_HUM	1	0.833355
15min_MT.	O75146	0.019075	0.141116	0.196773	HIP1R	Huntingtin	HIP1R_HU	13	1.01331
15min_MT.	P16615	0.216852	0.141152	0.196773	ATP2A2	Sarcoplasm	AT2A2_HU	3	1.162194
30min_MT.	P48426	0.101936	0.141062	0.196773	PIP4K2A	Phosphatid	PI42A_HUM	8	1.073213
30min_MT.	P62807	0.127504	0.141209	0.196816	H2BC4	Histone H2	H2B1C_HU	8	1.092402
15min_MT.	Q13057	-0.02353	0.141295	0.196866	COASY	Bifunction	COASY_HU	7	0.983823
15min_MT.	P36639	0.30768	0.141344	0.196899	NUDT1	Oxidized pu	8ODP_HUM	3	1.237716
30min_MT.	Q8IXT5	-0.17079	0.141383	0.19692	RBM12B	RNA-bindin	RB12B_HU	8	0.888355
30min_MT.	Q00610	-0.16073	0.141459	0.19699	CLTC	Clathrin he	CLH1_HUM	64	0.894569
30min_MT.	O60739	-1.11788	0.141491	0.197	EIF1B	Eukaryotic	EIF1B_HUM	1	0.460771
15min_MT.	P14854	0.433666	0.141592	0.19707	COX6B1	Cytochrom	CX6B1_HU	5	1.350661
15min_MT.	P30414	-0.72211	0.141579	0.19707	NKTR	NK-tumor r	NKTR_HUM	1	0.606208
30min_MT.	Q15836	0.168556	0.141647	0.197111	VAMP3	Vesicle-ass	VAMP3_HU	4	1.123933
30min_MT.	P07339	0.125054	0.141686	0.197131	CTSD	Cathepsin D	CATD_HUM	14	1.090549
15min_MT.	A0A0B4J2D	0.133604	0.141785	0.197198	GATD3B;GA	Putative gl	GAL3B_HU	12	1.097031
15min_MT.	Q15424	-0.18476	0.141901	0.197294	SAFB	Scaffold att	SAFB1_HU	15	0.879798
15min_MT.	Q9NX01	0.361582	0.141905	0.197294	TXNL4B	Thioredoxi	TXN4B_HU	1	1.284834
30min_MT.	O14561	0.304088	0.141939	0.197307	NDUFAB1	Acyl carrier	ACPM_HUM	4	1.234638
30min_MT.	O94925	-0.09594	0.142122	0.197527	GLS	Glutamina	GLSK_HUM	4	0.93566
30min_MT.	P47895	0.248631	0.142196	0.197559	ALDH1A3	Retinaldeh	AL1A3_HU	2	1.188079
30min_MT.	Q7L3B6	0.166154	0.142193	0.197559	CDC37L1	Hsp90 co-c	CD37L_HU	4	1.122063
15min_MT.	P32456	0.083647	0.142378	0.197566	GBP2	Guanylate-	GBP2_HUM	17	1.059694

15min_MT.	P49750	0.222671	0.14236	0.197566	YLPM1	YLP motif-c	YLPM1_HU	17	1.166892
30min_MT.	Q96QG7	-0.24896	0.142297	0.197566	MTMR9	Myotubula	MTMR9_HU	3	0.8415
15min_MT.	Q9Y5Q9	-0.27535	0.142324	0.197566	GTF3C3	General tra	TF3C3_HUI	6	0.82625
30min_MT.	P52294	-0.14873	0.142409	0.197575	KPNA1	Importin su	IMA5_HUM	4	0.902046
15min_MT.	P50053	-0.0784	0.142505	0.197622	KHK	Ketohexoki	KHK_HUMA	10	0.947105
15min_MT.	Q12800	0.560775	0.142506	0.197622	TFCP2	Alpha-globi	TFCP2_HUI	2	1.475062
15min_MT.	Q3YEC7	-0.11269	0.142519	0.197622	RABL6	Rab-like pro	RABL6_HUI	13	0.924865
30min_MT.	P23528	0.116361	0.142571	0.197658	CFL1	Cofilin-1	COF1_HUM	20	1.083997
15min_MT.	Q9Y666	0.165204	0.142824	0.197917	SLC12A7	Solute carri	S12A7_HUI	2	1.121325
30min_MT.	Q676U5	-0.04963	0.142878	0.197944	ATG16L1	Autophagy-	A16L1_HUI	10	0.966182
15min_MT.	Q9Y6A5	0.123014	0.142966	0.198031	TACC3	Transformi	TACC3_HUI	2	1.089008
30min_MT.	P02771	0.063796	0.143231	0.198258	AFP	Alpha-fetop	FETA_HUMA	2	1.045212
15min_MT.	P42773	2.184865	0.143327	0.19831	CDKN2C	Cyclin-depe	CDN2C_HU	1	4.546841
15min_MT.	Q8WW01	0.09088	0.143345	0.19831	TSEN15	tRNA-splici	SEN15_HUI	3	1.06502
30min_MT.	Q9H2H8	0.113539	0.143324	0.19831	PPIL3	Peptidyl-pr	PPIL3_HUM	5	1.081879
30min_MT.	A6NHX0	0.05019	0.143694	0.198586	CASTOR2	Cytosolic ar	CAST2_HUI	6	1.035401
30min_MT.	Q86SQ4	0.185997	0.143666	0.198586	ADGRG6	Adhesion G	AGRG6_HU	1	1.137603
30min_MT.	P10619	0.213653	0.143799	0.198693	CTSA	Lysosomal p	PPGB_HUM	8	1.15962
30min_MT.	P62195	-0.07642	0.144097	0.198886	PSMC5	26S protea	PRS8_HUM	21	0.948407
15min_MT.	P82930	-0.54722	0.144043	0.198886	MRPS34	Small ribos	RT34_HUM	3	0.684339
15min_MT.	Q676U5	0.041199	0.144117	0.198886	ATG16L1	Autophagy-	A16L1_HUI	10	1.028969
30min_MT.	P46939	-0.05997	0.144319	0.198989	UTRN	Utrophin	UTRN_HUM	89	0.959286
30min_MT.	Q9NTX5	-0.14063	0.144379	0.199036	ECHDC1	Ethylmalon	ECHD1_HU	13	0.907123
30min_MT.	Q66K14	-0.13782	0.144421	0.19906	TBC1D9B	TBC1 doma	TBC9B_HUI	14	0.908893
30min_MT.	Q9BUE6	0.11282	0.144463	0.199081	ISCA1	Iron-sulfur	ISCA1_HUM	4	1.08134
30min_MT.	Q7Z2W4	-0.13093	0.144575	0.199202	ZC3HAV1	Zinc finger	ZCCHV_HUI	5	0.91324
15min_MT.	P12429	0.096859	0.14463	0.199223	ANXA3	Annexin A3	ANXA3_HU	13	1.069443
30min_MT.	Q9HDC9	-0.05158	0.144642	0.199223	APMAP	Adipocyte p	APMAP_HU	1	0.964879
30min_MT.	Q9H1K1	0.191298	0.144694	0.199259	ISCU	Iron-sulfur	ISCU_HUMA	7	1.141791
15min_MT.	P15336	0.213413	0.144831	0.199343	ATF2	Cyclic AMP	ATF2_HUM	4	1.159428
30min_MT.	P84090	0.265683	0.144933	0.199343	ERH	Enhancer o	ERH_HUMA	4	1.202205
15min_MT.	Q8WXD5	-0.29733	0.14486	0.199343	GEMIN6	Gem-associ	GEMI6_HUI	3	0.813755
15min_MT.	P49848	-0.20088	0.144974	0.199365	TAF6	Transcripti	TAF6_HUM	1	0.870022
15min_MT.	Q8IW75	-0.57155	0.145013	0.199384	SERPINA12	Serpin A12	SPA12_HUI	5	0.672895
30min_MT.	Q9H3L0	0.498296	0.14541	0.199859	MMADHC	Cobalamin	MMAD_HU	2	1.412544
30min_MT.	O75794	-0.37981	0.145488	0.199931	CDC123	Translation	CD123_HU	2	0.768536
15min_MT.	Q13951	0.132851	0.145619	0.200076	CBFB	Core-bindir	PEBB_HUM	5	1.096459
30min_MT.	Q99766	0.038994	0.145769	0.200212	DMAC2L	ATP synthas	ATP5S_HUI	2	1.027397
30min_MT.	P62847	-0.05034	0.145918	0.200328	RPS24	Small ribos	RS24_HUM	4	0.965712
30min_MT.	Q6DHV7	0.115201	0.145956	0.200328	ADAL	Adenosine	ADAL_HUM	7	1.083126
30min_MT.	Q9UH65	0.081913	0.145945	0.200328	SWAP70	Switch-asso	SWP70_HU	22	1.058421
15min_MT.	Q13283	0.027918	0.146269	0.200651	G3BP1	Ras GTPase-	G3BP1_HU	10	1.01954
30min_MT.	Q05209	0.147242	0.146383	0.200702	PTPN12	Tyrosine-pr	PTN12_HUI	7	1.107451
30min_MT.	Q15796	-0.23684	0.146362	0.200702	SMAD2	Mothers ag	SMAD2_HU	1	0.848603
15min_MT.	P08240	-0.30524	0.146428	0.200728	SRPRA	Signal reco	SRPRA_HUI	6	0.80931
15min_MT.	Q99442	0.316174	0.146673	0.20103	SEC62	Translocati	SEC62_HUI	2	1.245024
30min_MT.	P30511	-0.75366	0.147245	0.201555	HLA-F	HLA class I	HLAF_HUM	1	0.593097
30min_MT.	Q9C0B7	-0.36242	0.147314	0.201555	TANGO6	Transport a	TNG6_HUM	2	0.777859
15min_MT.	Q9Y5B6	-0.22214	0.147288	0.201555	PAXBP1	PAX3- and I	PAXB1_HUI	1	0.857294
30min_MT.	P31943	0.054759	0.147361	0.201584	HNRNPH1	Heterogene	HNRH1_HU	15	1.038685
30min_MT.	Q7KZ85	-0.07753	0.147512	0.201694	SUPT6H	Transcripti	SPT6H_HUI	17	0.947679
30min_MT.	Q15120	-0.13147	0.147611	0.201785	PDK3	[Pyruvate d	PDK3_HUM	6	0.912898

15min_MT.	Q92552	0.045447	0.147735	0.201919	MRPS27	Small ribos	RT27_HUM	5	1.032003
30min_MT.	Q8WW12	0.169619	0.147952	0.20211	PCNP	PEST protec	PCNP_HUM	8	1.124762
30min_MT.	Q86V81	0.195511	0.148009	0.202117	ALYREF	THO compl	THOC4_HU	6	1.14513
30min_MT.	Q8TBC5	-0.56097	0.148007	0.202117	ZSCAN18	Zinc finger a	ZSC18_HUN	1	0.677848
15min_MT.	Q9Y4W6	0.558336	0.148057	0.202143	AFG3L2	Mitochond	AFG32_HUI	2	1.47257
15min_MT.	Q93008	-0.04873	0.148151	0.202205	USP9X	Ubiquitin c	USP9X_HUI	47	0.966787
30min_MT.	Q9BPW8	-0.19706	0.14821	0.202251	NIPSNAP1	Protein Nip	NIPS1_HUN	6	0.872323
30min_MT.	Q8IU81	-0.26958	0.148308	0.202313	IRF2BP1	Interferon r	I2BP1_HUN	1	0.829561
15min_MT.	P49005	0.100047	0.148519	0.202334	POLD2	DNA polym	DPOD2_HU	11	1.071808
15min_MT.	P53396	0.095727	0.148571	0.202334	ACLY	ATP-citrate	ACLY_HUM	30	1.068604
15min_MT.	P54105	0.26665	0.148407	0.202334	CLNS1A	Methylosor	ICLN_HUM.	4	1.203012
30min_MT.	Q00341	-0.0804	0.148546	0.202334	HDLBP	Vigilin	VIGLN_HUN	48	0.945796
30min_MT.	Q13330	-0.22036	0.148425	0.202334	MTA1	Metastasis-	MTA1_HUN	2	0.858352
30min_MT.	Q6NXE6	-0.04114	0.148566	0.202334	ARMC6	Armadillo r	ARMC6_HU	10	0.971884
15min_MT.	Q13217	0.073785	0.148632	0.202367	DNAJC3	DnaJ homo	DNJC3_HUI	14	1.052475
15min_MT.	O75410	0.156604	0.148758	0.202442	TACC1	Transformi	TACC1_HUI	7	1.11466
30min_MT.	P62826	-0.08249	0.14879	0.202442	RAN	GTP-bindin	RAN_HUMA	9	0.944426
15min_MT.	P41252	-0.12728	0.148916	0.202475	IARS1	Isoleucine--	SYIC_HUMA	33	0.915556
15min_MT.	Q9NRY5	0.125259	0.148874	0.202475	FAM114A2	Protein FAN	F1142_HUI	21	1.090704
30min_MT.	Q9UBN7	-0.1029	0.148902	0.202475	HDAC6	Histone dea	HDAC6_HU	2	0.931162
15min_MT.	P07306	-0.1281	0.149055	0.202555	ASGR1	Asialoglyco	ASGR1_HUI	10	0.915038
15min_MT.	O60522	0.914226	0.149087	0.202563	TDRD6	Tudor dom	TDRD6_HU	1	1.884558
15min_MT.	P24534	0.265612	0.149163	0.202615	EEF1B2	Elongation	EF1B_HUM	9	1.202146
30min_MT.	Q9H0N0	0.186004	0.149197	0.202615	RAB6C	Ras-related	RAB6C_HUI	1	1.137608
15min_MT.	Q14676	0.172465	0.149306	0.20272	MDC1	Mediator o	MDC1_HUN	9	1.126982
15min_MT.	P39060	0.29287	0.149375	0.202754	COL18A1	Collagen al	COIA1_HUN	7	1.225075
15min_MT.	O43353	-0.04651	0.149513	0.202819	RIPK2	Receptor-ir	RIPK2_HUN	15	0.968277
30min_MT.	P36405	0.109885	0.149534	0.202819	ARL3	ADP-ribosy	ARL3_HUM	8	1.079142
15min_MT.	Q9H4A6	0.141733	0.149631	0.202916	GOLPH3	Golgi phos	GOLP3_HU	9	1.10323
15min_MT.	Q8TEW0	0.241955	0.149766	0.203063	PARD3	Partitionin	PARD3_HU	3	1.182595
15min_MT.	P52292	0.076827	0.149868	0.203166	KPNA2	Importin su	IMA1_HUM	6	1.054696
30min_MT.	Q9NS18	0.231475	0.149945	0.203235	GLRX2	Glutaredox	GLRX2_HUI	1	1.174035
15min_MT.	P14550	0.105771	0.150096	0.20337	AKR1A1	Aldo-keto r	AK1A1_HUI	13	1.076069
15min_MT.	P11586	0.209534	0.150182	0.203446	MTHFD1	C-1-tetrahy	C1TC_HUM	23	1.156315
15min_MT.	Q86W34	-0.00737	0.150218	0.203446	AMZ2	Archaemet	AMZ2_HUN	1	0.994905
30min_MT.	Q9UID3	-0.25906	0.150231	0.203446	VPS51	Vacuolar pr	VPS51_HUI	9	0.835633
15min_MT.	O43633	0.229424	0.15031	0.203448	CHMP2A	Charged mu	CHM2A_HU	5	1.172367
30min_MT.	P11413	-0.14099	0.150286	0.203448	G6PD	Glucose-6-p	G6PD_HUN	19	0.906899
15min_MT.	P22792	0.223591	0.150262	0.203448	CPN2	Carboxypep	CPN2_HUM	1	1.167636
30min_MT.	O95373	0.081135	0.150421	0.203469	IPO7	Importin-7	IPO7_HUM	23	1.05785
15min_MT.	P35527	0.246649	0.150451	0.203469	KRT9	Keratin, typ	K1C9_HUM	32	1.186448
30min_MT.	P46934	-0.02569	0.150455	0.203469	NEDD4	E3 ubiquiti	NEDD4_HU	3	0.982348
30min_MT.	Q8IVD9	0.129604	0.150532	0.203502	NUDCD3	NudC doma	NUDC3_HU	13	1.093993
15min_MT.	Q96EY1	0.011832	0.150509	0.203502	DNAJA3	DnaJ homo	DNJA3_HUI	5	1.008235
15min_MT.	Q01433	-0.02034	0.150675	0.203519	AMPD2	AMP deami	AMPD2_HU	35	0.985998
15min_MT.	Q8IU81	-0.75978	0.150714	0.203519	IRF2BP1	Interferon r	I2BP1_HUN	1	0.590587
30min_MT.	Q8NFW8	-0.1195	0.150697	0.203519	CMAS	N-acylneura	NEUA_HUM	5	0.920506
15min_MT.	Q8WVM8	-0.05026	0.150624	0.203519	SCFD1	Sec1 family	SCFD1_HUI	14	0.965761
30min_MT.	Q16555	0.06297	0.15076	0.203529	DPYSL2	Dihydropyr	DPYL2_HUI	14	1.044614
15min_MT.	Q9Y2B0	0.14695	0.150993	0.203772	CNPY2	Protein can	CNPY2_HUI	7	1.107226
30min_MT.	P49427	-0.03694	0.151227	0.204001	CDC34	Ubiquitin-c	UB2R1_HU	3	0.974722
30min_MT.	Q9H6F5	-0.45533	0.151404	0.204151	CCDC86	Coiled-coil	CCD86_HU	4	0.729341

15min_MT.	P13796	0.17046	0.151536	0.204216	LCP1	Plastin-2	PLSL_HUM.	10	1.125418
15min_MT.	P51812	0.145039	0.151509	0.204216	RPS6KA3	Ribosomal	KS6A3_HUI	19	1.105761
30min_MT.	Q01844	0.169647	0.151557	0.204216	EWSR1	RNA-bindir	EWS_HUM/	6	1.124783
30min_MT.	Q14999;Q8	-0.62215	0.151549	0.204216	CUL7;CUL9	Cullin-7;Cu	CUL7_HUM	2	0.6497
30min_MT.	O00743	0.064181	0.151635	0.204262	PPP6C	Serine/thre	PPP6_HUM	13	1.045491
30min_MT.	Q7RTV0	-0.1941	0.151643	0.204262	PHF5A	PHD finger-	PHF5A_HU	6	0.874116
15min_MT.	Q12933	-0.02299	0.151676	0.204272	TRAF2	TNF recepto	TRAF2_HUI	18	0.984191
15min_MT.	P61289	0.122982	0.151832	0.204405	PSME3	Proteasom	PSME3_HU	13	1.088983
15min_MT.	Q01581	0.094018	0.151853	0.204405	HMGCS1	Hydroxyme	HMCS1_HU	13	1.067339
15min_MT.	Q15172	-0.37242	0.15185	0.204405	PPP2R5A	Serine/thre	2A5A_HUM	4	0.772487
15min_MT.	Q15365	0.162346	0.151924	0.204464	PCBP1	Poly(rC)-bir	PCBP1_HUI	9	1.119106
15min_MT.	Q5T6F2	0.162188	0.151993	0.204497	UBAP2	Ubiquitin-a	UBAP2_HU	11	1.118983
15min_MT.	P28072	0.13451	0.152375	0.204931	PSMB6	Proteasom	PSB6_HUM	5	1.09772
15min_MT.	P61247	-0.10983	0.15241	0.204943	RPS3A	Small ribos	RS3A_HUM	11	0.926697
15min_MT.	P13647	0.206659	0.152457	0.204949	KRT5	Keratin, typ	K2C5_HUM	23	1.154013
15min_MT.	Q9NV56	-0.0781	0.152467	0.204949	MRGBP	MRG/MORI	MRGBP_HU	1	0.947303
30min_MT.	P54253	-0.54798	0.152554	0.205031	ATXN1	Ataxin-1	ATX1_HUM	1	0.683978
15min_MT.	Q6IA69	-0.08866	0.152591	0.205046	NADSYN1	Glutamine-	NADE_HUM	10	0.940396
30min_MT.	Q969X5	-0.27422	0.152635	0.205051	ERGIC1	Endoplasm	ERGI1_HUN	5	0.8269
30min_MT.	O60921	0.235758	0.152976	0.205104	HUS1	Checkpoint	HUS1_HUM	5	1.177525
30min_MT.	Q27J81	-0.02893	0.152919	0.205104	INF2	Inverted fo	INF2_HUM.	4	0.980144
15min_MT.	Q5SRE5	0.04619	0.152908	0.205104	NUP188	Nucleopori	NU188_HU	9	1.032535
15min_MT.	Q8IX04	0.017556	0.152856	0.205104	UEVLD	Ubiquitin-c	UEVLD_HUI	11	1.012243
30min_MT.	Q96T76	-0.09761	0.152963	0.205104	MMS19	MMS19 nu	MMS19_HU	8	0.934583
30min_MT.	Q9NWX6	-0.1439	0.15291	0.205104	THG1L	Probable tF	THG1_HUM	1	0.905072
30min_MT.	Q9Y5Q9	-0.1141	0.152924	0.205104	GTF3C3	General tra	TF3C3_HUN	6	0.923955
30min_MT.	Q9H4A6	0.203457	0.153206	0.205378	GOLPH3	Golgi phos	GOLP3_HU	9	1.151454
15min_MT.	O95813	0.343997	0.153342	0.205489	CER1	Cerberus	CER1_HUM	1	1.269268
15min_MT.	Q9BTM9	-0.29834	0.153441	0.205554	URM1	Ubiquitin-r	URM1_HUN	2	0.813186
30min_MT.	Q70UQ0	0.284619	0.153709	0.20577	IKBIP	Inhibitor o	IKIP_HUMA	1	1.218088
15min_MT.	Q96ME1	0.042468	0.153708	0.20577	FBXL18	F-box/LRR-	FXL18_HUN	10	1.029874
15min_MT.	P69849;Q1	0.113216	0.153809	0.205869	NOMO3;NC	BOS compl	NOMO3_HU	7	1.081636
30min_MT.	P54619	0.088702	0.153856	0.205896	PRKAG1	5'-AMP-acti	AAKG1_HU	13	1.063413
15min_MT.	P37802	0.159733	0.153915	0.205905	TAGLN2	Transgelin-	TAGL2_HUI	14	1.117081
15min_MT.	P78318	0.182469	0.153912	0.205905	IGBP1	Immunoglo	IGBP1_HUN	11	1.134824
30min_MT.	O43301	-0.24691	0.154362	0.206255	HSPA12A	Heat shock	HS12A_HUI	8	0.842701
30min_MT.	O95628	0.143654	0.154289	0.206255	CNOT4	CCR4-NOT t	CNOT4_HU	4	1.1047
15min_MT.	P45985	0.252104	0.154297	0.206255	MAP2K4	Dual specifi	MP2K4_HU	4	1.190943
15min_MT.	Q9C010	-0.35859	0.154579	0.206439	PKIB	cAMP-depe	IPKB_HUM.	3	0.779925
15min_MT.	P42898	0.111443	0.154607	0.206442	MTHFR	Methylenet	MTHR_HUN	7	1.080308
30min_MT.	P30048	0.143342	0.154823	0.206695	PRDX3	Thioredoxi	PRDX3_HU	9	1.104461
15min_MT.	Q9NVE7	0.395569	0.154959	0.2068	PANK4	4'-phospho	PANK4_HU	2	1.315461
15min_MT.	Q9UKY7	-0.23433	0.154975	0.2068	CDV3	Protein CD	CDV3_HUM	15	0.850081
15min_MT.	Q9H2W6	0.212692	0.155075	0.20689	MRPL46	Large ribos	RM46_HUN	2	1.158849
30min_MT.	Q8N108	0.063914	0.155342	0.206999	MIER1	Mesoderm	MIER1_HUI	5	1.045298
30min_MT.	Q96DM3	-0.25087	0.155337	0.206999	RMC1	Regulator c	RMC1_HUN	4	0.840389
15min_MT.	Q9H1K1	0.157086	0.155326	0.206999	ISCU	Iron-sulfur	ISCU_HUM.	7	1.115033
15min_MT.	O60925	0.119612	0.155432	0.207071	PFDN1	Prefoldin s	PFD1_HUM	6	1.086443
30min_MT.	O75475	-0.03893	0.155449	0.207071	PSIP1	PC4 and SF	PSIP1_HUN	7	0.973374
30min_MT.	P40227	0.107345	0.155591	0.20719	CCT6A	T-complex	TCPZ_HUM	21	1.077244
30min_MT.	L0R819	-0.1728	0.155723	0.207295	ASDURF	ASNSD1 up	ASURF_HUI	3	0.887121
30min_MT.	P31937	0.157046	0.156052	0.207697	HIBADH	3-hydroxyi	3HIDH_HUI	10	1.115002

15min_MT.	Q8IZ07	0.148472	0.156213	0.207841	ANKRD13A	Ankyrin rep	AN13A_HU	4	1.108395
15min_MT.	Q86Y56	-0.2658	0.156398	0.208051	DNAAF5	Dynein axo	DAAF5_HUI	7	0.831737
15min_MT.	P61086	0.218328	0.156558	0.208193	UBE2K	Ubiquitin-c	UBE2K_HUI	10	1.163384
15min_MT.	P61129	0.032008	0.156665	0.2083	ZC3H6	Zinc finger	ZC3H6_HUI	1	1.022434
15min_MT.	Q8N3A8	0.00961	0.156718	0.2083	PARP8	Protein mo	PARP8_HUI	1	1.006683
30min_MT.	Q9NPA8	0.115453	0.156857	0.208449	ENY2	Transcripti	ENY2_HUM	4	1.083315
15min_MT.	O00567	-0.10852	0.157018	0.208593	NOP56	Nucleolar p	NOP56_HU	7	0.927539
15min_MT.	Q9BTW9	-0.06678	0.157064	0.208604	TBCD	Tubulin-spe	TBCD_HUM	15	0.954767
30min_MT.	Q15750	0.160495	0.157352	0.208859	TAB1	TGF-beta-ac	TAB1_HUM	12	1.11767
30min_MT.	Q6P9B6	-0.13017	0.157347	0.208859	MEAK7	MTOR-asso	MEAK7_HU	8	0.913725
15min_MT.	P15880	-0.07998	0.157439	0.208882	RPS2	Small ribos	RS2_HUMA	10	0.946073
30min_MT.	Q13627	0.077077	0.157463	0.208882	DYRK1A	Dual specifi	DYR1A_HUI	5	1.054878
15min_MT.	Q5GLZ8	-0.10586	0.157455	0.208882	HERC4	Probable E3	HERC4_HUI	14	0.929251
15min_MT.	P48637	0.07285	0.157539	0.208931	GSS	Glutathion	GSHB_HUM	26	1.051792
30min_MT.	A4D2B0	0.412162	0.157622	0.209003	MBLAC1	Metallo-bet	MBLC1_HU	1	1.330678
30min_MT.	Q96EY8	0.08263	0.157694	0.209003	MMAB	Corrinoid a	MMAB_HUI	6	1.058947
30min_MT.	Q9NVE7	-0.01415	0.157701	0.209003	PANK4	4'-phospho	PANK4_HU	2	0.990238
15min_MT.	Q9Y6N7	0.091464	0.157662	0.209003	ROBO1	Roundabou	ROBO1_HU	24	1.065451
15min_MT.	O95716	0.472434	0.1578	0.209063	RAB3D	Ras-related	RAB3D_HU	3	1.387448
30min_MT.	O95249	0.737203	0.157924	0.209157	GOSR1	Golgi SNAP	GOSR1_HU	2	1.666941
30min_MT.	P10321	0.040732	0.158088	0.209303	HLA-C	HLA class I	HLAC_HUM	5	1.028636
30min_MT.	Q99807	0.461865	0.158345	0.209537	COQ7	5-demetho	COQ7_HUM	2	1.377321
15min_MT.	Q9C005	0.189628	0.158321	0.209537	DPY30	Protein dpy	DPY30_HUI	1	1.140469
15min_MT.	P27487	-0.03502	0.158544	0.209545	DPP4	Dipeptidyl	DPP4_HUM	8	0.976018
15min_MT.	Q8N4P3	-0.04439	0.158433	0.209545	HDDC3	Guanosine-	MESH1_HU	5	0.969703
15min_MT.	Q9NXN4	-0.43297	0.158464	0.209545	GDAP2	Ganglioside	GDAP2_HU	1	0.740733
30min_MT.	Q86X29	0.056628	0.158704	0.209586	LSR	Lipolysis-st	LSR_HUMA	7	1.040032
30min_MT.	Q99757	0.487458	0.158692	0.209586	TXN2	Thioredoxi	THIOM_HU	2	1.401973
15min_MT.	O14976	-0.1897	0.158908	0.209734	GAK	Cyclin-G-as	GAK_HUMA	6	0.876785
30min_MT.	O95834	0.134104	0.158957	0.209734	EML2	Echinoderm	EMAL2_HU	14	1.097411
30min_MT.	P62820	0.156496	0.159012	0.209734	RAB1A	Ras-related	RAB1A_HUI	6	1.114577
30min_MT.	Q13177	-0.06639	0.159022	0.209734	PAK2	Serine/thre	PAK2_HUM	18	0.955026
15min_MT.	Q96MG8	-0.06133	0.159015	0.209734	PCMTD1	Protein-L-is	PCMD1_HU	1	0.95838
30min_MT.	Q9NYJ1	0.308759	0.158925	0.209734	COA4	Cytochrom	COA4_HUM	6	1.238642
30min_MT.	P06454	-0.27597	0.15913	0.209743	PTMA	Prothymos	PTMA_HUM	8	0.825894
30min_MT.	Q8N1A0	-0.23778	0.159206	0.20979	KRT222	Keratin-like	KT222_HUI	1	0.848049
15min_MT.	Q96M27	0.219542	0.159262	0.209828	PRRC1	Protein PRI	PRRC1_HUI	8	1.164364
15min_MT.	Q86YS7	0.016255	0.159476	0.210039	C2CD5	C2 domain	C2CD5_HU	15	1.011331
30min_MT.	P51659	0.15972	0.159538	0.210085	HSD17B4	Peroxisoma	DHB4_HUM	12	1.117071
30min_MT.	Q96I15	-0.04346	0.159676	0.210165	SCLY	Selenocyste	SCLY_HUMA	13	0.970326
15min_MT.	A6NK58	-0.30824	0.159874	0.210221	LIPT2	Octanoyl-[a	LIPT2_HUM	4	0.807624
30min_MT.	P00533	0.152786	0.159853	0.210221	EGFR	Epidermal g	EGFR_HUM	13	1.111714
30min_MT.	Q5VTE6	0.11527	0.160087	0.210453	ANGEL2	Protein ang	ANGE2_HU	2	1.083178
15min_MT.	P08833	0.157082	0.160195	0.210526	IGFBP1	Insulin-like	IBP1_HUMA	11	1.11503
30min_MT.	P30046	-0.07824	0.160189	0.210526	DDT	D-dopachro	DOPD_HUM	9	0.947212
30min_MT.	O15040	0.415632	0.160501	0.210545	TECPR2	Tectonin be	TCPR2_HUI	5	1.333883
15min_MT.	P11498	0.223672	0.16049	0.210545	PC	Pyruvate ca	PYC_HUMA	24	1.167702
15min_MT.	P31751	-0.27743	0.160331	0.210545	AKT2	RAC-beta se	AKT2_HUM	1	0.825058
15min_MT.	P41236	0.163393	0.160387	0.210545	PPP1R2	Protein pho	IPP2_HUMA	10	1.119918
30min_MT.	Q7Z3Y7	-0.63773	0.160408	0.210545	KRT28	Keratin, typ	K1C28_HUI	2	0.642724
15min_MT.	Q92575	0.216273	0.160268	0.210545	UBXN4	UBX domai	UBXN4_HU	4	1.161729
15min_MT.	Q92734	-0.18285	0.160533	0.210545	TFG	Protein TFG	TFG_HUMA	10	0.880962

15min_MT.	Q96CB8	-0.38281	0.160572	0.210545	INTS12	Integrator (	INT12_HUN	3	0.766942
15min_MT.	Q96NU7	0.207215	0.160565	0.210545	AMDHD1	Probable in	HUTI_HUM	6	1.154458
15min_MT.	Q9NX18	0.214014	0.160372	0.210545	SDHAF2	Succinate d	SDHF2_HUI	3	1.159911
30min_MT.	Q9Y4Z0	0.1477	0.160444	0.210545	LSM4	U6 snRNA-a	LSM4_HUM	5	1.107802
30min_MT.	Q92882	-0.02122	0.160616	0.210548	OSTF1	Osteoclast-	OSTF1_HUN	4	0.985396
15min_MT.	O00748	-0.24001	0.160836	0.210633	CES2	Cocaine est	EST2_HUMA	7	0.846742
30min_MT.	P15170	0.041826	0.160842	0.210633	GSPT1	Eukaryotic	ERF3A_HUN	18	1.029416
15min_MT.	P54259	0.275524	0.160826	0.210633	ATN1	Atrophin-1	ATN1_HUM	1	1.210434
30min_MT.	Q13409	-0.04828	0.160799	0.210633	DYNC1I2	Cytoplasmi	DC1I2_HUN	15	0.967091
15min_MT.	Q14031	-0.32963	0.160823	0.210633	COL4A6	Collagen al	CO4A6_HU	1	0.795739
30min_MT.	Q9NRN9	-0.18656	0.160899	0.210672	METTL5	rRNA N6-ad	METL5_HUI	8	0.878697
15min_MT.	Q9NY33	0.040413	0.160991	0.210756	DPP3	Dipeptidyl	DPP3_HUM	25	1.028408
15min_MT.	Q9H0A9	-0.53532	0.16104	0.210757	SPATC1L	Speriolin-li	SPC1L_HUN	1	0.690004
30min_MT.	Q9NZJ9	0.156757	0.161045	0.210757	NUDT4	Diphospho	NUDT4_HU	5	1.114778
30min_MT.	Q15369	0.068437	0.161081	0.210768	ELOC	Elongin-C	ELOC_HUM	7	1.04858
30min_MT.	P60228	-0.13695	0.161323	0.211014	EIF3E	Eukaryotic	EIF3E_HUM	19	0.909439
15min_MT.	Q9Y223	0.299781	0.161464	0.211128	GNE	Bifunctiona	GLCNE_HUI	10	1.230958
15min_MT.	P98082	0.097556	0.161532	0.211153	DAB2	Disabled hc	DAB2_HUM	1	1.06996
30min_MT.	P98175	-0.09362	0.161627	0.211236	RBM10	RNA-bindir	RBM10_HU	11	0.937171
30min_MT.	Q9P253	-0.32539	0.161755	0.211368	VPS18	Vacuolar pr	VPS18_HUI	4	0.798082
30min_MT.	Q9NZI8	-0.16179	0.161823	0.211387	IGF2BP1	Insulin-like	IF2B1_HUN	11	0.893913
15min_MT.	Q9H6U6	-0.25823	0.1621	0.211713	BCAS3	BCAS3 micr	BCAS3_HUI	5	0.836115
15min_MT.	Q5TEC6	-0.24008	0.162242	0.211792	H3-7	Histone H3	H37_HUMA	1	0.846696
15min_MT.	Q9BY77	-0.30663	0.162296	0.211826	POLDIP3	Polymerase	PDIP3_HUN	4	0.808528
15min_MT.	Q03013	0.129302	0.162406	0.211908	GSTM4	Glutathion	GSTM4_HU	5	1.093765
15min_MT.	Q9BSL1	-0.10109	0.162435	0.211908	UBAC1	Ubiquitin-a	UBAC1_HU	3	0.932329
15min_MT.	Q9UKV8	-0.00559	0.162556	0.212025	ago-02	Protein arg	AGO2_HUN	11	0.996132
30min_MT.	Q96S44	0.029062	0.162676	0.212076	TP53RK	EKC/KEOPS	PRPK_HUM	2	1.020349
30min_MT.	Q9UPU5	-0.18933	0.162677	0.212076	USP24	Ubiquitin c	UBP24_HU	28	0.877013
30min_MT.	Q9HA77	0.065283	0.162912	0.2123	CARS2	Probable cy	SYCM_HUM	16	1.04629
15min_MT.	O75340	0.215806	0.162994	0.212348	PDCD6	Programme	PDCD6_HU	5	1.161352
15min_MT.	Q13219	0.639384	0.163094	0.212377	PAPPA	Pappalysin-	PAPP1_HUI	5	1.557664
15min_MT.	Q6PIJ6	0.104462	0.163098	0.212377	FBXO38	F-box only	FBX38_HUI	1	1.075093
30min_MT.	O60547	-0.10321	0.16332	0.212596	GMDS	GDP-mann	GMDS_HUN	11	0.930962
30min_MT.	P55209	0.262299	0.163351	0.212601	NAP1L1	Nucleosom	NP1L1_HUI	7	1.199389
30min_MT.	O00571	-0.11583	0.16366	0.212811	DDX3X	ATP-depen	DDX3X_HU	13	0.922852
30min_MT.	O95816	-0.02958	0.163778	0.212811	BAG2	BAG family	BAG2_HUM	8	0.979706
15min_MT.	P57086	-0.06555	0.163714	0.212811	SCAND1	SCAN doma	SCND1_HU	1	0.955583
30min_MT.	Q96JP5	0.132831	0.163757	0.212811	ZFP91	E3 ubiquiti	ZFP91_HUN	2	1.096443
30min_MT.	Q9H444	0.209008	0.163744	0.212811	CHMP4B	Charged mu	CHM4B_HU	10	1.155893
30min_MT.	Q9H4A4	-0.0277	0.163677	0.212811	RNPEP	Aminopept	AMPB_HUN	23	0.980982
30min_MT.	Q9UEY8	-0.1027	0.163741	0.212811	ADD3	Gamma-add	ADDG_HUN	16	0.931286
30min_MT.	O43707	0.070344	0.163818	0.212819	ACTN4	Alpha-actir	ACTN4_HUI	46	1.049967
30min_MT.	O00499	-0.19124	0.163853	0.212822	BIN1	Myc box-de	BIN1_HUM	5	0.875854
15min_MT.	P35269	-0.05234	0.163882	0.212822	GTF2F1	General tra	T2FA_HUM	15	0.964374
15min_MT.	Q8TEB1	-0.10912	0.164022	0.212942	DCAF11	DDB1- and	DCA11_HU	2	0.927154
30min_MT.	O60927	-0.26776	0.164105	0.212944	PPP1R11	E3 ubiquiti	PP1RB_HUI	5	0.830608
30min_MT.	P55265	0.027417	0.164082	0.212944	ADAR	Double-stra	DSRAD_HU	14	1.019186
15min_MT.	O75157	-0.13222	0.164319	0.213045	TSC22D2	TSC22 dom	T22D2_HUI	4	0.912426
30min_MT.	P31689	-0.19471	0.164315	0.213045	DNAJA1	DnaJ homo	DNJA1_HUI	11	0.873746
15min_MT.	P54709	-0.09381	0.164352	0.213053	ATP1B3	Sodium/po	AT1B3_HUI	6	0.937046
30min_MT.	Q8N1Q1	0.120492	0.16444	0.213097	CA13	Carbonic ar	CAH13_HU	4	1.087105

15min_MT.	Q9NSE4	-0.08628	0.164414	0.213097	IARS2	Isoleucine-	SYIM_HUM	28	0.941945
15min_MT.	P11047	0.070358	0.164595	0.213238	LAMC1	Laminin sul	LAMC1_HU	27	1.049977
30min_MT.	O60716	-0.13691	0.164769	0.213276	CTNND1	Catenin del	CTND1_HU	20	0.909462
30min_MT.	Q9NVT9	0.059209	0.16494	0.21342	ARMC1	Armadillo r	ARMC1_HU	5	1.041894
15min_MT.	Q9Y315	0.138302	0.164963	0.21342	DERA	Deoxyribos	DEOC_HUM	11	1.100609
15min_MT.	P46019	0.27474	0.165257	0.213659	PHKA2	Phosphoryl	KPB2_HUM	4	1.209776
30min_MT.	Q8TDH9	0.152267	0.165204	0.213659	BLOC1S5	Biogenesis	BL1S5_HUI	1	1.111315
30min_MT.	P57081	0.052193	0.165358	0.213719	WDR4	tRNA (guan	WDR4_HUI	8	1.03684
30min_MT.	P62380	0.0589	0.165706	0.214063	TBPL1	TATA box-b	TBPL1_HUI	3	1.041671
30min_MT.	P40222	-0.10315	0.165946	0.214303	TXLNA	Alpha-taxili	TXLNA_HUI	18	0.930997
30min_MT.	P55036	0.09396	0.16604	0.21431	PSMD4	26S proteas	PSMD4_HU	13	1.067295
15min_MT.	P61962	-0.02359	0.166019	0.21431	DCAF7	DDB1- and	DCAF7_HUI	6	0.983784
30min_MT.	Q9UBK9	0.249641	0.166048	0.21431	UXT	Protein UX	UXT_HUMA	2	1.188911
15min_MT.	Q9Y450	-0.08248	0.166062	0.21431	HBS1L	HBS1-like p	HBS1L_HUI	15	0.944435
15min_MT.	Q96C23	0.095587	0.166107	0.214334	GALM	Galactose n	GALM_HUM	5	1.0685
15min_MT.	P16989	0.141309	0.166281	0.214461	YBX3	Y-box-bindi	YBOX3_HUI	10	1.102906
30min_MT.	Q92934	0.18813	0.166252	0.214461	BAD	Bcl2-associ	BAD_HUMA	1	1.139286
30min_MT.	Q16629	0.136871	0.166408	0.214545	SRSF7	Serine/argi	SRSF7_HUM	7	1.099518
15min_MT.	Q14165	-0.44241	0.166477	0.214563	MLEC	Malectin	MLEC_HUM	2	0.735906
30min_MT.	P62191	-0.11791	0.166506	0.214565	PSMC1	26S proteas	PRS4_HUM	18	0.921519
15min_MT.	Q8NBQ5	-0.21474	0.166735	0.214754	HSD17B11	Estradiol 17	DHB11_HU	4	0.861702
15min_MT.	Q8TEA8	0.087552	0.166787	0.214786	DTD1	D-aminoac	DTD1_HUM	6	1.062566
30min_MT.	Q96IU4	0.141331	0.166844	0.214823	ABHD14B	Putative pr	ABHEB_HUI	9	1.102922
30min_MT.	O43432	-0.10876	0.167137	0.21513	EIF4G3	Eukaryotic	IF4G3_HUM	33	0.927382
15min_MT.	P22670	-0.0035	0.167127	0.21513	RFX1	MHC class I	RFX1_HUM	11	0.997576
15min_MT.	P52594	0.192453	0.167178	0.215148	AGFG1	Arf-GAP do	AGFG1_HU	9	1.142705
30min_MT.	Q6P1N0	0.251173	0.167335	0.215314	CC2D1A	Coiled-coil	C2D1A_HU	2	1.190174
15min_MT.	P0DMV8;P	0.070495	0.167577	0.215378	HSPA1A;HS	Heat shock	HS71A_HUI	28	1.050077
30min_MT.	P25490	-0.32277	0.167542	0.215378	YY1	Transcripti	TYY1_HUMA	2	0.799532
30min_MT.	P30085	0.128456	0.16744	0.215378	CMPK1	UMP-CMP k	KCY_HUMA	10	1.093123
15min_MT.	Q27J81	0.108544	0.167555	0.215378	INF2	Inverted fo	INF2_HUMA	4	1.07814
15min_MT.	Q5SYC1	0.226127	0.167549	0.215378	CLVS2	Clavesin-2	CLVS2_HUI	9	1.169691
30min_MT.	Q8NFP9	0.229851	0.167523	0.215378	NBEA	Neurobeacl	NBEA_HUM	1	1.172713
30min_MT.	Q9P287	0.110351	0.167571	0.215378	BCCIP	BRCA2 and	BCCIP_HUM	14	1.079491
15min_MT.	Q9UBL3	-0.04742	0.167728	0.215501	ASH2L	Set1/Ash2 l	ASH2L_HUI	3	0.967663
15min_MT.	Q9BZE9	0.119777	0.167841	0.21561	ASPSCR1	Tether cont	ASPC1_HUI	7	1.086567
30min_MT.	Q99829	0.073486	0.16806	0.215857	CPNE1	Copine-1	CPNE1_HUI	12	1.052256
15min_MT.	P55196	-0.03817	0.168188	0.21595	AFDN	Afadin	AFAD_HUM	59	0.973888
15min_MT.	Q13200	-0.03892	0.16817	0.21595	PSMD2	26S proteas	PSMD2_HU	45	0.973385
15min_MT.	Q96S44	-0.05254	0.16834	0.21611	TP53RK	EKC/KEOPS	PRPK_HUM	2	0.96424
30min_MT.	P05121	0.339203	0.168413	0.21615	SERPINE1	Plasminoge	PAI1_HUMA	1	1.265057
30min_MT.	P18583	-0.0166	0.168427	0.21615	SON	Protein SOI	SON_HUMA	8	0.988557
30min_MT.	P29590	0.122183	0.168798	0.216471	PML	Protein PM	PML_HUMA	14	1.088381
15min_MT.	P39748	-0.28703	0.168901	0.216471	FEN1	Flap endon	FEN1_HUM	4	0.819587
30min_MT.	Q13884	-0.24106	0.168912	0.216471	SNTB1	Beta-1-synt	SNTB1_HUI	10	0.846124
30min_MT.	Q3KQV9	-0.48779	0.168904	0.216471	UAP1L1	UDP-N-acet	UAP1L_HUI	1	0.713115
30min_MT.	Q6ZN17	0.075663	0.168926	0.216471	LIN28B	Protein lin-	LN28B_HUI	10	1.053845
30min_MT.	Q7Z3Y9	0.732715	0.16877	0.216471	KRT26	Keratin, typ	K1C26_HUI	1	1.661763
15min_MT.	O60826	0.12876	0.169004	0.2165	CCDC22	Coiled-coil	CCD22_HU	2	1.093354
30min_MT.	Q9Y6X5	0.672885	0.168979	0.2165	ENPP4	Bis(5'-aden	ENPP4_HUI	2	1.594258
30min_MT.	O75179	-0.09434	0.169278	0.216573	ANKRD17	Ankyrin rep	ANR17_HU	8	0.936703
15min_MT.	O75533	-0.02033	0.169218	0.216573	SF3B1	Splicing fac	SF3B1_HUI	29	0.986006

15min_MT.	P14859	0.589256	0.169197	0.216573	POU2F1	POU domai	PO2F1_HUI	4	1.504471
30min_MT.	Q969S3	0.312117	0.169141	0.216573	ZNF622	Cytoplasmi	ZN622_HUI	5	1.241528
15min_MT.	Q96A49	0.118785	0.169295	0.216573	SYAP1	Synapse-ass	SYAP1_HUN	8	1.08582
15min_MT.	Q96LD4	0.205677	0.169313	0.216573	TRIM47	E3 ubiquiti	TRI47_HUN	1	1.153228
15min_MT.	Q9H6Y2	0.076405	0.169338	0.216573	WDR55	WD repeat-	WDR55_HU	10	1.054388
15min_MT.	Q9UNW1	0.09829	0.169218	0.216573	MINPP1	Multiple in	MINP1_HU	13	1.070504
15min_MT.	O43583	-0.14119	0.169451	0.216683	DENR	Density-reg	DENR_HUN	7	0.906772
30min_MT.	Q01415	0.106401	0.169488	0.216694	GALK2	N-acetylgal	GALK2_HUI	15	1.076539
15min_MT.	P34896	0.063984	0.169611	0.216802	SHMT1	Serine hydr	GLYC_HUM	15	1.045348
15min_MT.	Q9P2N5	0.078845	0.169628	0.216802	RBM27	RNA-bindir	RBM27_HU	7	1.056172
30min_MT.	P40939	-0.11472	0.170037	0.217078	HADHA	Trifunction	ECHA_HUM	20	0.923562
15min_MT.	P49638	-0.20903	0.169956	0.217078	TTPA	Alpha-toco	TTPA_HUM	2	0.865118
30min_MT.	P78332	-0.28131	0.170021	0.217078	RBM6	RNA-bindir	RBM6_HUN	5	0.822841
15min_MT.	Q13507;Q9	-0.39731	0.170038	0.217078	TRPC3;TRP(	Short trans	TRPC3_HUI	1	0.759274
15min_MT.	P36776	-0.06948	0.17011	0.217135	LONP1	Lon protea:	LONM_HUN	28	0.952981
15min_MT.	P26374	-0.30506	0.170189	0.217199	CHML	Rab proteir	RAE2_HUM	2	0.809407
30min_MT.	Q15773	0.12195	0.170237	0.217226	MLF2	Myeloid leu	MLF2_HUN	2	1.088205
30min_MT.	Q86U44	-0.11761	0.170266	0.217227	METTL3	N6-adenosi	MTA70_HU	10	0.921711
30min_MT.	Q9H1I8	-0.23427	0.170337	0.217282	ASCC2	Activating s	ASCC2_HUI	1	0.850115
30min_MT.	Q9BRP8	0.216354	0.170444	0.217384	PYM1	Partner of Y	PYM1_HUN	8	1.161794
15min_MT.	O75122	-0.00473	0.170549	0.217386	CLASP2	CLIP-associ	CLAP2_HUI	14	0.996724
15min_MT.	Q92621	-0.21848	0.170557	0.217386	NUP205	Nuclear poi	NU205_HU	17	0.859472
30min_MT.	Q9H1E1	0.557869	0.170545	0.217386	RNASE7	Ribonuclea	RNAS7_HUI	1	1.472093
30min_MT.	O60443	0.174471	0.170788	0.217495	GSDME	Gasdermin-	GSDME_HU	4	1.128551
15min_MT.	Q14118	0.189066	0.170682	0.217495	DAG1	Dystroglyca	DAG1_HUN	5	1.140025
30min_MT.	Q5T1C6	0.18324	0.17078	0.217495	THEM4	Acyl-coenzy	THEM4_HU	5	1.135431
15min_MT.	Q6PID6	0.103363	0.170791	0.217495	TTC33	Tetratricop	TTC33_HUN	3	1.074275
30min_MT.	Q96D53	0.02992	0.17075	0.217495	COQ8B	Atypical kir	COQ8B_HU	7	1.020955
15min_MT.	Q9Y5Y2	0.567989	0.171025	0.217733	NUBP2	Cytosolic Fe	NUBP2_HU	2	1.482456
15min_MT.	Q8WUM0	-0.15326	0.171429	0.218036	NUP133	Nuclear poi	NU133_HU	9	0.899219
30min_MT.	P30622	0.071092	0.171531	0.218117	CLIP1	CAP-Gly do	CLIP1_HUN	65	1.050512
15min_MT.	Q8WUW1	0.22294	0.171557	0.218117	BRK1	Protein BRI	BRK1_HUN	5	1.16711
15min_MT.	Q14257	0.186738	0.171974	0.218586	RCN2	Reticulocal	RCN2_HUN	5	1.138187
15min_MT.	P49841	0.520687	0.172094	0.218668	GSK3B	Glycogen sy	GSK3B_HUI	6	1.434638
30min_MT.	O14908	-0.31355	0.172163	0.218684	GIPC1	PDZ domaii	GIPC1_HUN	5	0.804661
15min_MT.	Q5VW32	0.10771	0.172287	0.218807	BROX	BRO1 doma	BROX_HUN	5	1.077516
15min_MT.	O15541	0.082114	0.172347	0.218811	RNF113A	E3 ubiquiti	R113A_HUN	5	1.058568
30min_MT.	Q6ZVX7	0.747541	0.172401	0.218844	NCCRP1	F-box only	FBX50_HUI	8	1.678929
30min_MT.	Q9Y2Q3	-0.12785	0.172548	0.218995	GSTK1	Glutathion	GSTK1_HUI	5	0.915194
30min_MT.	O00410	0.087543	0.172693	0.219145	IPO5	Importin-5	IPO5_HUM	38	1.062559
30min_MT.	P54753	0.286005	0.172769	0.219205	EPHB3	Ephrin type	EPHB3_HUI	2	1.219259
15min_MT.	Q6IAA8	-0.11803	0.172878	0.219308	LAMTOR1	Ragulator c	LTOR1_HUI	3	0.921446
30min_MT.	P51397	0.40133	0.172944	0.219355	DAP	Death-asso	DAP1_HUN	2	1.320725
30min_MT.	P49247	0.033806	0.173104	0.219523	RPIA	Ribose-5-pl	RPIA_HUM.	8	1.02371
15min_MT.	Q13362	-0.01235	0.173378	0.219799	PPP2R5C	Serine/thre	2A5G_HUN	6	0.991478
15min_MT.	O60347	0.356644	0.173684	0.219974	TBC1D12	TBC1 doma	TBC12_HUI	2	1.280444
15min_MT.	P27540	-0.31433	0.173651	0.219974	ARNT	Aryl hydroc	ARNT_HUN	3	0.804224
30min_MT.	P41236	0.210914	0.173649	0.219974	PPP1R2	Protein pho	IPP2_HUM.	10	1.157422
15min_MT.	Q9NWT1	-0.15848	0.173643	0.219974	PAK1IP1	p21-activat	PK1IP_HUN	6	0.895966
15min_MT.	P61081	0.087771	0.173869	0.220173	UBE2M	NEDD8-cor	UBC12_HU	7	1.062727
30min_MT.	P67809	0.140585	0.173917	0.220197	YBX1	Y-box-bindi	YBOX1_HUI	16	1.102352
30min_MT.	Q99700	-0.0909	0.173945	0.220197	ATXN2	Ataxin-2	ATX2_HUM	14	0.938934

15min_MT.	Q70Z53	0.108344	0.174028	0.220231	FRA10AC1	Protein FR/	F10C1_HUI	5	1.07799
30min_MT.	P24941	0.061689	0.174186	0.220395	CDK2	Cyclin-depe	CDK2_HUM	8	1.043687
30min_MT.	P62081	-0.06517	0.174327	0.220451	RPS7	Small ribos	RS7_HUMA	8	0.95583
15min_MT.	Q8N584	-0.06761	0.174343	0.220451	TTC39C	Tetratricop	TT39C_HUI	18	0.954219
15min_MT.	Q709C8	-0.08298	0.174466	0.220501	VPS13C	Intermemb	VP13C_HUI	34	0.944102
15min_MT.	Q8IWV7	0.020215	0.174467	0.220501	UBR1	E3 ubiquiti	UBR1_HUM	7	1.014111
15min_MT.	Q12874	0.255902	0.174523	0.220537	SF3A3	Splicing fac	SF3A3_HUI	13	1.194082
30min_MT.	P49755	-0.37191	0.174608	0.220608	TMED10	Transmemb	TMEDA_HU	3	0.772758
30min_MT.	Q8IWW6	-0.23418	0.174784	0.220795	ARHGAP12	Rho GTPase	RHG12_HU	4	0.850166
30min_MT.	Q99856	-0.10455	0.174852	0.220812	ARID3A	AT-rich inte	ARI3A_HUI	10	0.930094
30min_MT.	Q9UL25	0.04598	0.174854	0.220812	RAB21	Ras-related	RAB21_HUI	7	1.032384
30min_MT.	P16219	0.147421	0.175038	0.221009	ACADS	Short-chair	ACADS_HUI	10	1.107588
30min_MT.	Q13427	-0.0826	0.175344	0.2213	PPIG	Peptidyl-pr	PPIG_HUM	6	0.944355
15min_MT.	Q9H501	-0.12705	0.1755	0.221449	ESF1	ESF1 homo	ESF1_HUM/	12	0.915699
30min_MT.	P60842	0.113409	0.175538	0.221461	EIF4A1	Eukaryotic	IF4A1_HUI	25	1.081782
30min_MT.	P13804	0.059311	0.175676	0.221524	ETFA	Electron tra	ETFA_HUM/	15	1.041968
15min_MT.	Q15075	0.143064	0.175701	0.221524	EEA1	Early endos	EEA1_HUM	65	1.104248
30min_MT.	Q9Y6D9	-0.17822	0.175657	0.221524	MAD1L1	Mitotic spi	MD1L1_HU	5	0.883795
30min_MT.	O00186	-0.1662	0.176039	0.22182	STXBP3	Syntaxin-bi	STXB3_HUI	12	0.891185
30min_MT.	Q14137	-0.56351	0.175971	0.22182	BOP1	Ribosome k	BOP1_HUM	3	0.676654
15min_MT.	Q9Y303	0.20898	0.176024	0.22182	AMDHD2	N-acetylglu	NAGA_HUM	4	1.155871
30min_MT.	P30040	0.163944	0.176137	0.221844	ERP29	Endoplasm	ERP29_HUI	10	1.120345
15min_MT.	P17706	0.264487	0.176248	0.221916	PTPN2	Tyrosine-pr	PTN2_HUM	1	1.201209
15min_MT.	P43686	0.041721	0.176268	0.221916	PSMC4	26S protea:	PRS6B_HUI	19	1.029341
30min_MT.	Q92576	-0.16576	0.176357	0.221993	PHF3	PHD finger	PHF3_HUM	25	0.891462
15min_MT.	Q13148	-0.07005	0.176543	0.222155	TARDBP	TAR DNA-bi	TADBP_HUI	7	0.952605
15min_MT.	Q9Y646	0.101821	0.176521	0.222155	CPQ	Carboxype|	CBPQ_HUM	8	1.073127
30min_MT.	Q10713	-0.12837	0.176652	0.222244	PMPCA	Mitochond	MPPA_HUM	13	0.914864
15min_MT.	Q9NUQ8	-0.02051	0.17667	0.222244	ABCF3	ATP-bindin	ABCF3_HUI	5	0.985882
15min_MT.	Q12849	0.257496	0.176718	0.22225	GRSF1	G-rich sequ	GRSF1_HUI	6	1.195402
30min_MT.	Q6GQQ9	-0.4909	0.176732	0.22225	OTUD7B	OTU domai	OTU7B_HU	2	0.711582
15min_MT.	O43504	-0.45188	0.176766	0.222257	LAMTOR5	Ragulator c	LTOR5_HUI	2	0.731088
30min_MT.	O95786	-0.20421	0.176933	0.222329	RIGI	Antiviral in	RIGI_HUMA	6	0.868011
15min_MT.	P12268	0.046004	0.176965	0.222329	IMPDH2	Inosine-5'-r	IMDH2_HU	26	1.032401
30min_MT.	P52272	-0.14513	0.176944	0.222329	HNRNPM	Heterogene	HNRPM_HU	20	0.904298
15min_MT.	Q86X29	0.10313	0.176907	0.222329	LSR	Lipolysis-st	LSR_HUMA	7	1.074101
30min_MT.	Q9Y5V3	0.324889	0.176883	0.222329	MAGED1	Melanoma-	MAGD1_HU	1	1.252568
30min_MT.	O00193	0.150042	0.177032	0.222347	SMAP	Small acidi	SMAP_HUM	3	1.109602
15min_MT.	O00762	1.00457	0.177037	0.222347	UBE2C	Ubiquitin-c	UBE2C_HUI	2	2.006345
30min_MT.	O43583	-0.07659	0.177121	0.222347	DENR	Density-reg	DENR_HUM	7	0.948293
30min_MT.	Q96NU7	0.12864	0.177101	0.222347	AMDHD1	Probable in	HUTI_HUM	6	1.093263
30min_MT.	Q9UHY1	-0.06194	0.17707	0.222347	NRBP1	Nuclear rec	NRBP_HUM	8	0.957974
15min_MT.	O43747	-0.06761	0.177387	0.222553	AP1G1	AP-1 compl	AP1G1_HU	22	0.954214
15min_MT.	Q96A35	-0.28808	0.177399	0.222553	MRPL24	Large ribos	RM24_HUM	1	0.818993
30min_MT.	P25787	0.114557	0.177448	0.222567	PSMA2	Proteasom	PSA2_HUM	11	1.082642
15min_MT.	O00267	0.080883	0.178062	0.223063	SUPT5H	Transcripti	SPT5H_HUI	25	1.057665
15min_MT.	P04899	-0.21317	0.178057	0.223063	GNAI2	Guanine nu	GNAI2_HUI	3	0.862639
30min_MT.	P19784	0.128043	0.178054	0.223063	CSNK2A2	Casein kina	CSK22_HUI	8	1.09281
15min_MT.	P0DPB6	-0.22118	0.178147	0.223133	POLR1D	DNA-direct	RPAC2_HUI	2	0.857861
30min_MT.	P27635	-0.31905	0.178291	0.223242	RPL10	Large ribos	RL10_HUM	4	0.801596
15min_MT.	P62316	-0.11307	0.178281	0.223242	SNRPD2	Small nucle	SMD2_HUM	9	0.924615
15min_MT.	Q9Y5H5	-0.82775	0.17844	0.223393	PCDHA9	Protocadh	PCDA9_HU	1	0.563406

15min_MT.	O76031	0.047742	0.178639	0.223427	CLPX	ATP-depend	CLPX_HUM	7	1.033646
30min_MT.	Q8N6H7	0.114225	0.178621	0.223427	ARFGAP2	ADP-ribosy	ARFG2_HU	13	1.082393
30min_MT.	Q8N612	-0.20957	0.178677	0.223439	FHIP1B	FHF comple	FHI1B_HUN	2	0.864792
30min_MT.	Q9NP97	0.080918	0.178907	0.223691	DYNLRB1	Dynein ligh	DLRB1_HU	7	1.057691
30min_MT.	O43795	-0.05558	0.179141	0.22384	MYO1B	Unconvent	MYO1B_HU	19	0.962206
30min_MT.	P55957	0.293288	0.179399	0.22402	BID	BH3-intera	BID_HUMA	4	1.22543
30min_MT.	P28827	0.006512	0.179517	0.224095	PTPRM	Receptor-ty	PTPRM_HU	11	1.004524
30min_MT.	Q9HCE1	0.094533	0.179574	0.224095	MOV10	Helicase M(	MOV10_HU	20	1.06772
15min_MT.	Q9NX40	0.123661	0.179609	0.224104	OCIAD1	OCIA doma	OCAD1_HU	1	1.089496
30min_MT.	P36776	0.064702	0.18001	0.22446	LONP1	Lon protea	LONM_HUN	28	1.045869
15min_MT.	Q6PII5	-0.42711	0.180068	0.224497	HAGHL	Hydroxyacy	HAGHL_HU	2	0.74375
15min_MT.	O43896	0.617968	0.180207	0.224529	KIF1C	Kinesin-like	KIF1C_HUM	2	1.534712
30min_MT.	O60563	-0.08751	0.18044	0.224529	CCNT1	Cyclin-T1	CCNT1_HU	1	0.941147
15min_MT.	O94888	0.153818	0.180351	0.224529	UBXN7	UBX domai	UBXN7_HU	12	1.11251
15min_MT.	P34059	0.227875	0.180489	0.224529	GALNS	N-acetylgal	GALNS_HU	8	1.171109
30min_MT.	P43358	0.038897	0.180525	0.224529	MAGEA4	Melanoma-	MAGA4_HU	11	1.027328
30min_MT.	P49840	0.306362	0.180194	0.224529	GSK3A	Glycogen sy	GSK3A_HU	6	1.236586
30min_MT.	P54136	-0.09429	0.180358	0.224529	RARS1	Arginine--tl	SYRC_HUM	31	0.936734
30min_MT.	Q13888;Q6	-0.11142	0.180433	0.224529	GTF2H2;GT	General tra	TF2H2_HUI	4	0.925678
15min_MT.	Q6MZQ0	0.571497	0.180133	0.224529	PRR5L	Proline-rich	PRR5L_HUI	1	1.486064
30min_MT.	Q9BRZ2	0.524311	0.180417	0.224529	TRIM56	E3 ubiquiti	TRI56_HUN	2	1.438247
30min_MT.	Q9Y2Z4	-0.01382	0.180335	0.224529	YARS2	Tyrosine--tl	SYYM_HUM	8	0.990469
30min_MT.	O15144	0.065234	0.180626	0.224609	ARPC2	Actin-relate	ARPC2_HU	15	1.046255
30min_MT.	Q6IQ49	0.290358	0.180704	0.224609	SDE2	Splicing reg	SDE2_HUM	4	1.222944
15min_MT.	Q9Y692	0.348268	0.180689	0.224609	GMEB1	Glucocorti	GMEB1_HU	4	1.273031
15min_MT.	Q8NFP9	0.180268	0.180945	0.224873	NBEA	Neurobeacl	NBEA_HUM	1	1.133094
30min_MT.	O00488	-0.58332	0.181026	0.224938	ZNF593	Zinc finger	ZN593_HUI	3	0.667426
15min_MT.	P06400	-0.37677	0.181246	0.225115	RB1	Retinoblast	RB_HUMAN	3	0.770161
15min_MT.	P61916	0.152258	0.181284	0.225115	NPC2	NPC intrace	NPC2_HUM	6	1.111308
15min_MT.	Q9BZH6	-0.13268	0.181262	0.225115	WDR11	WD repeat-	WDR11_HU	26	0.912134
30min_MT.	Q9UHD9	0.185578	0.181273	0.225115	UBQLN2	Ubiquilin-2	UBQL2_HU	6	1.137272
30min_MT.	O95071	-0.0505	0.181331	0.225139	UBR5	E3 ubiquiti	UBR5_HUM	28	0.965603
30min_MT.	O75175	0.038532	0.181873	0.225597	CNOT3	CCR4-NOT t	CNOT3_HU	8	1.027068
30min_MT.	P05386	0.312653	0.181931	0.225597	RPLP1	Large ribos	RLA1_HUM	2	1.241989
30min_MT.	P25815	0.173576	0.181881	0.225597	S100P	Protein S10	S100P_HUI	2	1.127851
15min_MT.	Q15056	0.077503	0.181963	0.225601	EIF4H	Eukaryotic	IF4H_HUM	9	1.05519
30min_MT.	P11717	0.055632	0.181998	0.225608	IGF2R	Cation-inde	MPRI_HUM	64	1.039314
15min_MT.	Q15059	-0.06497	0.182084	0.225679	BRD3	Bromodom	BRD3_HUM	6	0.955963
15min_MT.	Q9Y221	0.54734	0.182234	0.225794	NIP7	60S riboso	NIP7_HUM	1	1.461389
15min_MT.	P14543	0.100471	0.182273	0.225795	NID1	Nidogen-1	NID1_HUM	15	1.072123
30min_MT.	Q9BY89	-0.04416	0.182325	0.225799	KIAA1671	Uncharacte	K1671_HUI	2	0.969855
30min_MT.	P54577	0.074804	0.182459	0.225895	YARS1	Tyrosine--tl	SYYC_HUM	34	1.053218
15min_MT.	Q6BDS2	0.071262	0.182461	0.225895	BLTP3A	Bridge-like	BLT3A_HUI	3	1.050635
30min_MT.	P12955	0.081501	0.182564	0.225916	PEPD	Xaa-Pro dip	PEPD_HUM	19	1.058118
30min_MT.	Q0PNE2	-0.10206	0.18269	0.226001	ELP6	Elongator c	ELP6_HUM	10	0.931702
30min_MT.	Q5MNZ9	0.094758	0.182935	0.226232	WIPI1	WD repeat	WIPI1_HUI	8	1.067886
30min_MT.	Q9GZZ9	-0.05787	0.183003	0.226246	UBA5	Ubiquitin-l	UBA5_HUM	6	0.960683
15min_MT.	Q8NHW5	-1.51582	0.183128	0.226364	RPLP0P6	Putative rib	RLA0L_HUI	1	0.349698
30min_MT.	O95292	0.078364	0.183256	0.226486	VAPB	Vesicle-ass	VAPB_HUM	3	1.05582
30min_MT.	Q96G03	0.014949	0.183341	0.226555	PGM2	Phosphope	PGM2_HUN	26	1.010416
15min_MT.	Q5T5C0	0.141726	0.183495	0.22671	STXBP5	Syntaxin-bi	STXB5_HUI	4	1.103224
15min_MT.	Q9BRA2	0.108147	0.183663	0.226882	TXNDC17	Thioredoxi	TXD17_HUI	6	1.077843

15min_MT.	Q99735	0.610957	0.183719	0.226914	MGST2	Microsoma	MGST2_HU	1	1.527272
30min_MT.	Q7L8W6	0.543313	0.183845	0.227031	DPH6	Diphthine-	DPH6_HUN	1	1.457315
15min_MT.	Q9C0E8	0.16639	0.183919	0.227031	LNPK	Endoplasm	LNP_HUMA	6	1.122247
15min_MT.	Q9Y2Z4	-0.01591	0.183886	0.227031	YARS2	Tyrosine--tI	SYYM_HUM	8	0.989034
30min_MT.	O75323	-0.3652	0.184044	0.227137	NIPSNAP2	Protein Nip	NIPS2_HUN	4	0.776362
15min_MT.	P52815	0.063679	0.184124	0.227193	MRPL12	Large ribos	RM12_HUN	5	1.045127
15min_MT.	Q96SB3	0.160412	0.184176	0.227193	PPP1R9B	Neurabin-2	NEB2_HUN	8	1.117606
30min_MT.	Q9Y281	0.15513	0.184176	0.227193	CFL2	Cofilin-2	COF2_HUN	11	1.113522
30min_MT.	P51965	-0.28797	0.184291	0.227298	UBE2E1	Ubiquitin-c	UB2E1_HUI	1	0.819056
30min_MT.	Q5SRE5	-0.08544	0.18434	0.227322	NUP188	Nucleopori	NU188_HU	9	0.942499
15min_MT.	P19404	0.141148	0.184483	0.227427	NDUFV2	NADH dehy	NDUV2_HU	7	1.102782
30min_MT.	Q9H944	-0.09895	0.184467	0.227427	MED20	Mediator o	MED20_HU	5	0.93371
30min_MT.	Q5SYE7	-0.22103	0.184681	0.227599	NHSL1	NHS-like pr	NHSL1_HUI	7	0.857954
15min_MT.	Q9BQ69	0.107317	0.184664	0.227599	MACROD1	ADP-ribose	MACD1_HU	2	1.077223
15min_MT.	Q15714	0.039267	0.184728	0.227621	TSC22D1	TSC22 dom	T22D1_HUI	3	1.027592
30min_MT.	Q969H8	-0.22051	0.184909	0.227773	MYDGF	Myeloid-de	MYDGF_HU	7	0.858264
15min_MT.	Q9UBT2	-0.04578	0.1849	0.227773	UBA2	SUMO-activ	SAE2_HUM	24	0.968768
15min_MT.	Q99614	0.091366	0.18495	0.227788	TTC1	Tetratricop	TTC1_HUM	10	1.065378
15min_MT.	Q07954	0.229695	0.185015	0.227795	LRP1	Prolow-der	LRP1_HUM	33	1.172587
15min_MT.	Q92499	-0.13152	0.185011	0.227795	DDX1	ATP-depenc	DDX1_HUN	19	0.91287
15min_MT.	Q0VGL1	0.153451	0.185119	0.227852	LAMTOR4	Ragulator c	LTOR4_HUI	1	1.112227
30min_MT.	Q5VTR2	0.049425	0.185149	0.227853	RNF20	E3 ubiquiti	BRE1A_HUI	22	1.034852
15min_MT.	Q9H3H3	0.221813	0.185453	0.228192	C11orf68	UPF0696 p	CK068_HUI	9	1.166198
30min_MT.	O60361	-0.19524	0.185666	0.22838	NME2P1	Putative nu	NDK8_HUN	1	0.87343
15min_MT.	Q969T9	0.119062	0.185724	0.22838	WBP2	WW domai	WBP2_HUN	5	1.086029
30min_MT.	Q9BX66	-0.07896	0.185704	0.22838	SORBS1	Sorbin and	SRBS1_HUI	30	0.946737
30min_MT.	Q16836	0.152159	0.186011	0.2286	HADH	Hydroxyacy	HCDH_HUN	9	1.111231
30min_MT.	Q86VR2	-0.10546	0.186025	0.2286	RETREG3	Reticuloph	RETR3_HUI	1	0.929508
30min_MT.	Q92797	-0.21752	0.186074	0.2286	SYMPK	Symplekin	SYMPK_HU	11	0.860044
15min_MT.	Q9Y2H0	0.102815	0.186101	0.2286	DLGAP4	Disks large-	DLGP4_HU	9	1.073866
15min_MT.	Q14247	0.117825	0.186192	0.228669	CTTN	Src substra	SRC8_HUM	24	1.085097
15min_MT.	P0C7T5	-0.3153	0.186276	0.2287	ATXN1L	Ataxin-1-lik	ATX1L_HUN	3	0.803683
30min_MT.	P54687	0.031256	0.186269	0.2287	BCAT1	Branched-c	BCAT1_HUI	10	1.021902
15min_MT.	Q6UWZ7	-0.03711	0.186336	0.228739	ABRAXAS1	BRCA1-A cc	ABRX1_HUI	2	0.974604
30min_MT.	P46459	-0.12975	0.186653	0.229055	NSF	Vesicle-fusi	NSF_HUMA	26	0.913989
30min_MT.	Q9NSA3	0.080745	0.186735	0.22912	CTNNBIP1	Beta-cateni	CNBP1_HU	1	1.057564
15min_MT.	O14497	0.257262	0.186869	0.229193	ARID1A	AT-rich inte	ARI1A_HUN	17	1.195208
15min_MT.	Q8NDX5	-0.25097	0.186835	0.229193	PHC3	Polyhomec	PHC3_HUM	1	0.840333
30min_MT.	Q5T160	0.124913	0.187012	0.229281	RARS2	Probable ar	SYRM_HUM	6	1.090442
30min_MT.	Q8IUD2	0.003224	0.187081	0.229293	ERC1	ELKS/Rab6-	RB6I2_HUN	32	1.002237
15min_MT.	P50502	0.136055	0.187165	0.229324	ST13	Hsc70-inter	F10A1_HUI	10	1.098896
30min_MT.	Q9Y263	-0.08085	0.187165	0.229324	PLAA	Phospholip	PLAP_HUM	29	0.945501
30min_MT.	P33316	0.109699	0.18765	0.229811	DUT	Deoxyuridi	DUT_HUMA	6	1.079003
30min_MT.	Q9P2D3	-0.5442	0.18761	0.229811	HEATR5B	HEAT repea	HTR5B_HUI	2	0.68577
30min_MT.	Q9H0U4	-0.0308	0.187758	0.229907	RAB1B	Ras-related	RAB1B_HUI	8	0.978878
30min_MT.	Q8TDY2	-0.23166	0.18779	0.22991	RB1CC1	RB1-induci	RBCC1_HUI	17	0.851654
15min_MT.	P17405	-0.65666	0.187848	0.229944	SMPD1	Sphingomy	ASM_HUMA	4	0.634345
15min_MT.	O95400	0.31328	0.187933	0.229977	CD2BP2	CD2 antige	CD2B2_HU	3	1.242529
15min_MT.	Q14145	0.134849	0.187977	0.229994	KEAP1	Kelch-like E	KEAP1_HUI	7	1.097978
30min_MT.	Q8WW01	0.176197	0.188081	0.230049	TSEN15	tRNA-splici	SEN15_HUI	3	1.129901
15min_MT.	Q9Y3D6	0.180509	0.188055	0.230049	FIS1	Mitochond	FIS1_HUMA	2	1.133284
30min_MT.	P42704	0.082676	0.188201	0.230125	LRPPRC	Leucine-ric	LPPRC_HUI	67	1.05898

15min_MT.	Q96MH2	0.502789	0.188264	0.230166	HEXIM2	Protein HE	HEXI2_HUM	1	1.41695
15min_MT.	O75368	0.168244	0.188409	0.230235	SH3BGRL	Adapter SH	SH3L1_HUI	7	1.12369
15min_MT.	Q16630	0.08825	0.188405	0.230235	CPSF6	Cleavage ar	CPSF6_HUM	13	1.06308
30min_MT.	Q7Z6Z7	0.027008	0.188367	0.230235	HUWE1	E3 ubiquiti	HUWE1_HU	109	1.018897
15min_MT.	P47756	-0.0225	0.188523	0.230288	CAPZB	F-actin-cap	CAPZB_HUI	18	0.984522
30min_MT.	Q9BRJ6	-0.09328	0.188492	0.230288	C7orf50	Uncharacte	CG050_HU	2	0.937388
15min_MT.	Q96RE7	0.102352	0.188742	0.230391	NACC1	Nucleus acc	NACC1_HU	7	1.073522
15min_MT.	Q9GZT9	-0.15227	0.188731	0.230391	EGLN1	Egl nine ho	EGLN1_HUI	5	0.899834
30min_MT.	P16035	-0.116	0.188806	0.230433	TIMP2	Metallopro	TIMP2_HUI	2	0.922742
30min_MT.	Q6ZT12	-0.43724	0.188858	0.23046	UBR3	E3 ubiquiti	UBR3_HUM	4	0.738548
30min_MT.	Q5SSJ5	0.497463	0.188906	0.230464	HP1BP3	Heterochrc	HP1B3_HU	3	1.411728
15min_MT.	O60749	-0.00725	0.189312	0.230752	SNX2	Sorting nex	SNX2_HUM	18	0.994986
30min_MT.	P23786	-0.37489	0.189333	0.230752	CPT2	Carnitine O	CPT2_HUM	5	0.771164
15min_MT.	P68871	-0.82904	0.189331	0.230752	HBB	Hemoglobi	HBB_HUM/	3	0.562904
15min_MT.	P07948;P0	0.14634	0.189371	0.230761	LYN;HCK	Tyrosine-pr	LYN_HUMA	1	1.106758
30min_MT.	Q7Z401	-0.18552	0.189499	0.230882	DENND4A	C-myc pron	MYCPP_HU	2	0.879333
30min_MT.	O75122	-0.03918	0.189592	0.230924	CLASP2	CLIP-associ	CLAP2_HUI	14	0.973207
30min_MT.	P07306	-0.13134	0.189583	0.230924	ASGR1	Asialoglyco	ASGR1_HUI	10	0.91298
15min_MT.	O60645	-0.00923	0.189813	0.231125	EXOC3	Exocyst cor	EXOC3_HUI	2	0.993625
15min_MT.	Q03252	0.04904	0.189817	0.231125	LMNB2	Lamin-B2	LMNB2_HU	9	1.034576
15min_MT.	Q5MIZ7	0.01788	0.189877	0.231138	PPP4R3B	Serine/thre	P4R3B_HUI	10	1.012471
30min_MT.	Q8TF74	-0.09195	0.189914	0.231138	WIPF2	WAS/WASL	WIPF2_HUI	6	0.938254
30min_MT.	Q9NPI6	-0.2913	0.189916	0.231138	DCP1A	mRNA-deca	DCP1A_HU	4	0.817165
30min_MT.	Q6UWZ7	-0.37764	0.189946	0.231139	ABRAXAS1	BRCA1-A cc	ABRX1_HUI	2	0.769696
30min_MT.	Q12872	-0.30022	0.190044	0.231221	SFSWAP	Splicing fac	SFSWA_HU	6	0.812127
15min_MT.	O14562	0.156636	0.190211	0.23126	UBFD1	Ubiquitin d	UBFD1_HU	10	1.114685
30min_MT.	O60504	0.256419	0.190182	0.23126	SORBS3	Vinexin	VINEX_HUN	1	1.19451
30min_MT.	O75363	-0.04141	0.19029	0.23126	BCAS1	Breast carci	BCAS1_HUI	3	0.971708
15min_MT.	P17931	0.162102	0.190315	0.23126	LGALS3	Galectin-3	LEG3_HUM	9	1.118917
30min_MT.	Q8IUI8	-0.16418	0.190342	0.23126	CRLF3	Cytokine re	CRLF3_HUN	8	0.892435
15min_MT.	Q8NC56	-0.32357	0.19024	0.23126	LEMD2	LEM domai	LEMD2_HU	1	0.799093
15min_MT.	Q9NWY4	0.078515	0.190257	0.23126	HPF1	Histone PA	HPF1_HUM	2	1.05593
15min_MT.	P08397	0.125963	0.190433	0.231335	HMBS	Porphobili	HEM3_HUN	13	1.091236
30min_MT.	Q7L2H7	0.045491	0.190748	0.231646	EIF3M	Eukaryotic	EIF3M_HUN	13	1.032035
30min_MT.	Q9BYG5	0.281205	0.191028	0.231914	PARD6B	Partitionin	PAR6B_HUI	4	1.21521
30min_MT.	P35221	0.06854	0.191185	0.232069	CTNNA1	Catenin alp	CTNA1_HUI	36	1.048655
30min_MT.	Q0ZGT2	0.178765	0.191453	0.232119	NEXN	Nexilin	NEXN_HUM	9	1.131914
30min_MT.	Q13308	0.257912	0.191326	0.232119	PTK7	Inactive tyr	PTK7_HUM	4	1.195746
30min_MT.	Q86VS8	-0.0565	0.191385	0.232119	HOOK3	Protein Ho	HOOK3_HU	14	0.961593
15min_MT.	Q8WW33	0.211412	0.191464	0.232119	GTSF1	Gametocyt	GTSF1_HUN	6	1.157821
15min_MT.	Q96HE7	0.031423	0.191458	0.232119	ERO1A	ERO1-like p	ERO1A_HUI	24	1.02202
15min_MT.	Q9Y6Q2	-0.26034	0.191586	0.232211	STON1	Stonin-1	STON1_HUI	1	0.834893
15min_MT.	Q08379	0.031953	0.191842	0.232433	GOLGA2	Golgin subf	GOGA2_HU	12	1.022395
30min_MT.	O96017	0.014194	0.191874	0.232436	CHEK2	Serine/thre	CHK2_HUM	10	1.009887
15min_MT.	Q32P44	0.116105	0.192227	0.232792	EML3	Echinoderm	EMAL3_HU	12	1.083805
15min_MT.	P49589	0.110633	0.192388	0.2329	CARS1	Cysteine--tI	SYCC_HUM	25	1.079702
15min_MT.	Q9NQ88	-0.08405	0.192406	0.2329	TIGAR	Fructose-2,	TIGAR_HUN	5	0.943403
15min_MT.	P62633	-0.35037	0.192572	0.232993	CNBP	CCHC-type	CNBP_HUM	3	0.784385
15min_MT.	P05771;P1	0.549132	0.192669	0.233052	PRKCB;PRK	Protein kin	KPCB_HUM	1	1.463205
15min_MT.	P55060	-0.05272	0.19268	0.233052	CSE1L	Exportin-2	XPO2_HUM	34	0.964114
30min_MT.	Q96AH0;Q	0.552573	0.192731	0.233077	NABP1;NAE	SOSS comp	SOSB2_HUI	1	1.466699
30min_MT.	Q07020	0.245328	0.192832	0.233147	RPL18	Large ribos	RL18_HUM	4	1.185362

15min_MT.	O43318	0.676925	0.192929	0.233172	MAP3K7	Mitogen-ac	M3K7_HUM	2	1.598728
15min_MT.	P55769	0.074721	0.192927	0.233172	SNU13	NHP2-like p	NH2L1_HU	6	1.053157
30min_MT.	P61769	-0.06127	0.19312	0.233215	B2M	Beta-2-mic	B2MG_HUM	2	0.958419
30min_MT.	Q12931	-0.12799	0.193198	0.233215	TRAP1	Heat shock	TRAP1_HUI	29	0.915108
15min_MT.	Q13501	0.111642	0.193217	0.233215	SQSTM1	Sequestoso	SQSTM_HU	3	1.080457
15min_MT.	Q6P158	0.044242	0.19317	0.233215	DHX57	Putative AT	DHX57_HU	4	1.031141
30min_MT.	Q9BV94	-0.20335	0.193233	0.233215	EDEM2	ER degrada	EDEM2_HU	4	0.868531
15min_MT.	Q8TEM1	-0.15127	0.193327	0.233293	NUP210	Nuclear po	PO210_HU	4	0.900456
30min_MT.	P52566	-0.02111	0.193538	0.233415	ARHGDIB	Rho GDP-di	GDIR2_HUI	1	0.985473
30min_MT.	Q12962	-0.40313	0.193548	0.233415	TAF10	Transcripti	TAF10_HUI	2	0.756216
30min_MT.	Q9NWU2	-0.21221	0.193522	0.233415	GID8	Glucose-in	GID8_HUM	3	0.863211
30min_MT.	P02760	-0.17899	0.194015	0.233767	AMBP	Protein AM	AMBP_HUM	11	0.883324
15min_MT.	Q7LBR1	0.212582	0.194023	0.233767	CHMP1B	Charged mu	CHM1B_HU	4	1.15876
30min_MT.	Q96MW5	-0.15826	0.194064	0.233767	COG8	Conserved	COG8_HUM	3	0.896108
15min_MT.	Q96PZ0	-0.01508	0.194092	0.233767	PUS7	Pseudourid	PUS7_HUM	23	0.9896
15min_MT.	P20339	-0.1034	0.194221	0.233831	RAB5A	Ras-related	RAB5A_HUI	5	0.930835
30min_MT.	O75843	0.278816	0.194594	0.233914	AP1G2	AP-1 compl	AP1G2_HU	1	1.213199
15min_MT.	Q86V48	-0.0301	0.194619	0.233914	LUZP1	Leucine zip	LUZP1_HUI	10	0.979353
15min_MT.	Q9BXV9	-0.10322	0.194498	0.233914	GON7	EKC/KEOPS	GON7_HUM	2	0.930952
15min_MT.	Q9Y624	0.084089	0.19452	0.233914	F11R	Junctional	JAM1_HUM	8	1.060018
30min_MT.	O94874	-0.21859	0.194928	0.234233	UFL1	E3 UFM1-p	UFL1_HUM	13	0.859404
15min_MT.	Q9H9A5	-0.49366	0.194945	0.234233	CNOT10	CCR4-NOT t	CNO10_HU	4	0.71022
30min_MT.	Q8NHV4	-0.10522	0.195027	0.23426	NEDD1	Protein NE	NEDD1_HU	9	0.929666
15min_MT.	Q13542	-0.05898	0.195295	0.234371	EIF4EBP2	Eukaryotic	4EBP2_HUI	2	0.95994
15min_MT.	Q4G176	-0.05766	0.195268	0.234371	ACSF3	Malonate--	ACSF3_HUM	2	0.960824
30min_MT.	P27816	-0.16823	0.195363	0.234411	MAP4	Microtubul	MAP4_HUM	55	0.889936
15min_MT.	P62136	-0.03184	0.195406	0.234427	PPP1CA	Serine/thre	PP1A_HUM	15	0.978172
15min_MT.	Q07021	-0.17671	0.195529	0.234432	C1QBP	Compleme	C1QBP_HU	5	0.884719
15min_MT.	Q12846	-0.08546	0.19545	0.234432	STX4	Syntaxin-4	STX4_HUM	7	0.942487
15min_MT.	Q5U5X0	-0.38547	0.19559	0.234468	LYRM7	Complex III	LYRM7_HU	3	0.765532
30min_MT.	Q14457	-0.1624	0.195712	0.234506	BECN1	Beclin-1	BECN1_HUI	3	0.893538
15min_MT.	P25787	0.114565	0.195748	0.234514	PSMA2	Proteasom	PSA2_HUM	11	1.082648
15min_MT.	P06899	-0.31603	0.195883	0.234603	H2BC11	Histone H2	H2B1J_HUI	2	0.803276
30min_MT.	Q9UJW0	-0.12339	0.195882	0.234603	DCTN4	Dynactin su	DCTN4_HU	10	0.91803
30min_MT.	P43155	-0.06431	0.196047	0.234765	CRAT	Carnitine O	CACP_HUM	17	0.956406
30min_MT.	P01019	0.125942	0.196325	0.234809	AGT	Angiotensi	ANGT_HUM	15	1.09122
15min_MT.	P11717	0.04311	0.196276	0.234809	IGF2R	Cation-ind	MPRI_HUM	64	1.030333
15min_MT.	P20936	-0.66884	0.196275	0.234809	RASA1	Ras GTPase	RASA1_HUI	2	0.629014
30min_MT.	Q96CS3	-0.09226	0.196209	0.234809	FAF2	FAS-associa	FAF2_HUM	4	0.938049
30min_MT.	Q9UI17	-0.2723	0.196308	0.234809	DMGDH	Dimethylgl	M2GD_HUI	4	0.827999
30min_MT.	P02652	0.697098	0.196542	0.235033	APOA2	Apolipopro	APOA2_HU	1	1.62124
15min_MT.	P24385	0.101002	0.196613	0.235082	CCND1	G1/S-specif	CCND1_HU	3	1.072518
15min_MT.	Q8N9N7	0.049538	0.196676	0.235098	LRRC57	Leucine-ric	LRC57_HUI	5	1.034933
30min_MT.	Q92539	-0.08458	0.196686	0.235098	LPIN2	Phosphatid	LPIN2_HUM	8	0.943056
30min_MT.	Q8TF05	-0.02549	0.196841	0.235176	PPP4R1	Serine/thre	PP4R1_HUI	11	0.982486
30min_MT.	P18621	0.08588	0.197123	0.23544	RPL17	Large ribos	RL17_HUM	2	1.061335
30min_MT.	P07237	0.128872	0.19728	0.235519	P4HB	Protein dis	PDIA1_HUM	40	1.093438
15min_MT.	Q86UP2	0.145911	0.197278	0.235519	KTN1	Kinectin	KTN1_HUM	30	1.106429
30min_MT.	Q9UNP9	0.134736	0.197267	0.235519	PPIE	Peptidyl-pr	PPIE_HUMA	10	1.097892
15min_MT.	Q9BRX2	0.226055	0.197532	0.235749	PELO	Protein pel	PELO_HUM	7	1.169632
30min_MT.	Q9P2B4	0.055516	0.197619	0.235816	CTTNBP2NI	CTTNBP2 N	CT2NL_HUI	4	1.039231
30min_MT.	Q10567	0.068699	0.197688	0.235863	AP1B1	AP-1 compl	AP1B1_HUI	20	1.048771

30min_MT.	P61313	0.477201	0.197722	0.235868	RPL15	Large ribos	RL15_HUM	7	1.39204
15min_MT.	Q16790	0.250049	0.197994	0.236156	CA9	Carbonic ar	CAH9_HUM	5	1.189247
15min_MT.	Q15303	0.104341	0.198034	0.236167	ERBB4	Receptor ty	ERBB4_HUI	1	1.075003
15min_MT.	Q8WV22	0.409658	0.198121	0.236222	NSMCE1	Non-struct	NSE1_HUM	1	1.328371
15min_MT.	Q96EL2	0.265499	0.198163	0.236222	MRPS24	Small ribos	RT24_HUM	1	1.202052
15min_MT.	O75152	-0.11356	0.198232	0.23626	ZC3H11A	Zinc finger	ZC11A_HUI	7	0.924306
15min_MT.	P56199	0.107455	0.198325	0.236334	ITGA1	Integrin alp	ITA1_HUM/	5	1.077326
15min_MT.	P34897	0.143165	0.198389	0.236375	SHMT2	Serine hydr	GLYM_HUM	18	1.104326
15min_MT.	Q96K21	0.136383	0.198924	0.236832	ZFYVE19	Abscission/	ANCHR_HU	6	1.099146
15min_MT.	P0C0L5	0.396334	0.199133	0.237044	C4B	Complemei	CO4B_HUM	3	1.316159
30min_MT.	Q6VY07	-0.06072	0.199343	0.237223	PACS1	Phosphofu	PACS1_HUI	19	0.958789
30min_MT.	P21291	-0.06774	0.199533	0.237304	CSRP1	Cysteine an	CSRP1_HUI	10	0.954131
15min_MT.	Q08945	-0.03678	0.199561	0.237304	SSRP1	FACT comp	SSRP1_HUI	13	0.974831
30min_MT.	Q9NR09	-0.00831	0.19948	0.237304	BIRC6	Baculoviral	BIRC6_HUM	47	0.994254
15min_MT.	P21283	0.146894	0.199743	0.237446	ATP6V1C1	V-type prot	VATC1_HUI	21	1.107183
15min_MT.	Q9P0V9	-0.18654	0.200291	0.238038	SEPTIN10	Septin-10	SEP10_HUI	5	0.878709
15min_MT.	O60264	-0.0517	0.200361	0.238071	SMARCA5	SWI/SNF-re	SMCA5_HU	7	0.964798
15min_MT.	Q6XD76	0.139793	0.200466	0.23816	ASCL4	Achaete-sc	ASCL4_HUI	2	1.101747
30min_MT.	P11441	-0.11218	0.200684	0.238274	UBL4A	Ubiquitin-l	UBL4A_HUI	8	0.925187
15min_MT.	Q8IV38	0.037361	0.200625	0.238274	ANKMY2	Ankyrin rep	ANKY2_HUI	4	1.026235
30min_MT.	Q9Y5L0	-0.13505	0.200674	0.238274	TNPO3	Transportir	TNPO3_HU	13	0.910636
15min_MT.	P05067	0.197446	0.200986	0.238533	APP	Amyloid-be	A4_HUMAN	5	1.146667
30min_MT.	P62277	0.118249	0.200993	0.238533	RPS13	Small ribos	RS13_HUM	5	1.085417
15min_MT.	Q96C11	-0.37941	0.200963	0.238533	FGGY	FGGY carbc	FGGY_HUM	2	0.76875
15min_MT.	Q96HN2	0.111348	0.201031	0.238541	AHCYL2	Adenosylhc	SAHH3_HU	9	1.080237
30min_MT.	Q96FS4	-0.20539	0.201107	0.238595	SIPA1	Signal-indu	SIPA1_HUM	3	0.867306
15min_MT.	P35222	-0.06584	0.201329	0.238683	CTNNB1	Catenin bet	CTNB1_HUI	26	0.955386
30min_MT.	Q7L3T8	0.167741	0.201247	0.238683	PARS2	Probable pi	SYPM_HUM	3	1.123299
15min_MT.	Q9BWD1	0.123366	0.201333	0.238683	ACAT2	Acetyl-CoA	THIC_HUM/	13	1.089274
30min_MT.	Q99543	0.156441	0.201369	0.238689	DNAJC2	DnaJ homo	DNJC2_HUI	7	1.114534
30min_MT.	Q6PI48	-0.19804	0.201406	0.238696	DARS2	Aspartate--	SYDM_HUM	15	0.871736
15min_MT.	Q9Y6B6	-0.30824	0.201512	0.238749	SAR1B	Small COPII	SAR1B_HUI	2	0.807629
15min_MT.	O95786	-0.13424	0.201549	0.238757	RIGI	Antiviral in	RIGI_HUMA	6	0.911149
15min_MT.	P61326	-0.0689	0.20158	0.238759	MAGOH	Protein ma	MGN_HUM	1	0.953367
30min_MT.	P31949	0.532221	0.201675	0.238793	S100A11	Protein S1C	S10AB_HUI	4	1.446154
30min_MT.	Q6PD62	-0.17914	0.201701	0.238793	CTR9	RNA polym	CTR9_HUM	6	0.88323
30min_MT.	Q9NXV6	-0.17997	0.201657	0.238793	CDKN2AIP	CDKN2A-in	CARF_HUM	15	0.882721
15min_MT.	O00273	0.142319	0.201955	0.238804	DFFA	DNA fragm	DFFA_HUM	11	1.103678
15min_MT.	P15151	-0.03796	0.20193	0.238804	PVR	Poliovirus	PVR_HUMA	2	0.974029
30min_MT.	P53370	-0.04618	0.201774	0.238804	NUDT6	Nucleoside	NUDT6_HU	4	0.9685
15min_MT.	Q8TB61	0.832797	0.201953	0.238804	SLC35B2	Adenosine	S35B2_HUI	2	1.781135
30min_MT.	Q9UNI6	-0.12403	0.201914	0.238804	DUSP12	Dual specifi	DUS12_HU	8	0.917623
30min_MT.	Q8WVT3	-0.26726	0.202491	0.239294	TRAPPC12	Trafficking	TPC12_HUI	1	0.830898
30min_MT.	Q8WWC4	-0.27718	0.202454	0.239294	MAIP1	m-AAA prot	MAIP1_HUI	1	0.825203
30min_MT.	Q9BX68	0.249432	0.202488	0.239294	HINT2	Adenosine	HINT2_HUM	5	1.188739
15min_MT.	Q8TEV9	-0.68727	0.202655	0.239379	SMCR8	Guanine nu	SMCR8_HU	2	0.621028
15min_MT.	P02795;P0	-0.16922	0.202714	0.239389	MT2A;MT1	Metallothi	MT2_HUM/	1	0.88932
30min_MT.	Q9Y333	-0.04308	0.202727	0.239389	LSM2	U6 snRNA-a	LSM2_HUM	5	0.970581
30min_MT.	O95202	0.030981	0.202871	0.239428	LETM1	Mitochond	LETM1_HUI	7	1.021707
15min_MT.	Q8TEX9	-0.04699	0.20291	0.239428	IPO4	Importin-4	IPO4_HUM	23	0.967953
15min_MT.	Q96CV9	0.115649	0.202831	0.239428	OPTN	Optineurin	OPTN_HUM	16	1.083462
30min_MT.	P61758	0.092748	0.203044	0.239524	VBP1	Prefoldin s	PFD3_HUM	8	1.0664

30min_MT.	Q9UHK6	0.272874	0.203054	0.239524	AMACR	Alpha-meth	AMACR_HU	4	1.208212
15min_MT.	Q8TBN0	0.340625	0.203129	0.239572	RAB3IL1	Guanine nu	R3GEF_HUI	4	1.266305
15min_MT.	Q9Y294	0.025348	0.203156	0.239572	ASF1A	Histone cha	ASF1A_HUN	1	1.017725
30min_MT.	P11117	0.042638	0.203516	0.239923	ACP2	Lysosomal a	PPAL_HUM	11	1.029995
30min_MT.	P50336	0.078325	0.203545	0.239923	PPOX	Protoporpl	PPOX_HUM	6	1.055792
15min_MT.	P36957	0.096541	0.203789	0.240138	DLST	Dihydrolipo	ODO2_HUM	16	1.069207
15min_MT.	P55036	0.136613	0.204058	0.240418	PSMD4	26S proteas	PSMD4_HU	13	1.099321
30min_MT.	P22626	0.106903	0.204283	0.240648	HNRNPA2B	Heterogene	ROA2_HUM	19	1.076914
15min_MT.	O75521	0.120816	0.204428	0.240782	ECI2	Enoyl-CoA d	ECI2_HUMA	11	1.08735
15min_MT.	P00367	-0.06984	0.204648	0.24083	GLUD1	Glutamate	DHE3_HUM	27	0.952746
15min_MT.	P08238	-0.06279	0.204807	0.24083	HSP90AB1	Heat shock	HS90B_HUI	48	0.957411
30min_MT.	P09488	0.016479	0.204589	0.24083	GSTM1	Glutathion	GSTM1_HU	11	1.011488
30min_MT.	P31930	-0.22647	0.204657	0.24083	UQCRC1	Cytochrom	QCR1_HUM	4	0.854722
30min_MT.	P82979	0.11397	0.204704	0.24083	SARNP	SAP domair	SARNP_HUI	9	1.082202
15min_MT.	Q9C0A0	0.23979	0.204791	0.24083	CNTNAP4	Contactin-a	CNTP4_HUI	1	1.18082
30min_MT.	Q9C0B5	0.376113	0.204566	0.24083	ZDHHC5	Palmitoyltr	ZDHC5_HU	1	1.297841
15min_MT.	Q9ULW0	0.57096	0.20473	0.24083	TPX2	Targeting p	TPX2_HUM	1	1.485511
15min_MT.	Q9Y2Z0	0.051919	0.204763	0.24083	SUGT1	Protein SGT	SGT1_HUM	23	1.036643
30min_MT.	Q9Y6X4	-0.36583	0.204546	0.24083	FAM169A	Soluble lam	F169A_HUI	7	0.776023
30min_MT.	O43633	-0.13408	0.204914	0.240883	CHMP2A	Charged mu	CHM2A_HU	5	0.911249
15min_MT.	P26358	0.019762	0.205191	0.240926	DNMT1	DNA (cytosi	DNMT1_HU	24	1.013793
30min_MT.	P49406	0.072407	0.205076	0.240926	MRPL19	Large ribos	RM19_HUM	2	1.05147
15min_MT.	Q7RTS9	-0.60253	0.2053	0.240926	DYM	Dymeclin	DYM_HUMA	1	0.658599
15min_MT.	Q8WWC4	0.201818	0.205222	0.240926	MAIP1	m-AAA prot	MAIP1_HUI	1	1.150147
30min_MT.	Q9H8S9	-0.24867	0.204987	0.240926	MOB1A	MOB kinase	MOB1A_HU	1	0.841671
15min_MT.	Q9HCN8	-0.16646	0.20532	0.240926	SDF2L1	Stromal cel	SDF2L_HUM	6	0.891024
15min_MT.	P98170	0.166702	0.205413	0.240998	XIAP	E3 ubiquiti	XIAP_HUMA	12	1.12249
15min_MT.	O75223	-0.03682	0.205805	0.241314	GGCT	Gamma-glu	GGCT_HUM	9	0.974798
15min_MT.	Q9H6Q4	0.253878	0.205943	0.241404	CIAO3	Cytosolic ir	CIAO3_HUN	5	1.192408
15min_MT.	Q9BUK6	0.088669	0.206131	0.241588	MSTO1	Protein mis	MSTO1_HU	3	1.063389
15min_MT.	Q99611	0.051759	0.206183	0.241593	SEPHS2	Selenide, w	SPS2_HUM	14	1.036528
30min_MT.	O14936	0.024876	0.206446	0.241774	CASK	Peripheral p	CSKP_HUM	23	1.017392
30min_MT.	O75643	-0.15755	0.206563	0.241774	SNRNP200	U5 small nu	U520_HUM	40	0.896547
30min_MT.	P61081	0.013734	0.206557	0.241774	UBE2M	NEDD8-con	UBC12_HU	7	1.009565
15min_MT.	Q8WUA2	-0.07673	0.206466	0.241774	PPIL4	Peptidyl-pr	PPIL4_HUM	13	0.948203
30min_MT.	Q9HAB8	0.051079	0.206475	0.241774	PPCS	Phosphopa	PPCS_HUM	13	1.036039
15min_MT.	Q9NR56	0.139413	0.206568	0.241774	MBNL1	Musclebin	MBNL1_HU	4	1.101457
15min_MT.	Q9NUY8	0.013381	0.206781	0.241915	TBC1D23	TBC1 doma	TBC23_HUI	10	1.009318
30min_MT.	Q96C57	0.073004	0.206891	0.242007	CUSTOS	Protein CUS	CSTOS_HUN	5	1.051904
30min_MT.	Q86SQ0	0.280436	0.207029	0.24206	PHLDB2	Pleckstrin h	PHLB2_HUI	2	1.214562
15min_MT.	P48163	0.041496	0.207275	0.242239	ME1	NADP-depe	MAOX_HUN	18	1.02918
15min_MT.	Q8N8N7	-0.23	0.207248	0.242239	PTGR2	Prostagland	PTGR2_HUI	7	0.852632
30min_MT.	Q7Z7C8	-0.49347	0.207367	0.24231	TAF8	Transcripti	TAF8_HUM	1	0.710314
15min_MT.	P07947	-0.02362	0.207464	0.242313	YES1	Tyrosine-pr	YES_HUMA	10	0.983758
15min_MT.	P30153	0.094605	0.207562	0.242313	PPP2R1A	Serine/thre	2AAA_HUM	24	1.067773
30min_MT.	Q12982	0.268408	0.207677	0.242313	BNIP2	BCL2/aden	BNIP2_HUN	3	1.204478
30min_MT.	Q13033	0.027566	0.207702	0.242313	STRN3	Striatin-3	STRN3_HUI	16	1.019291
15min_MT.	Q86X55	0.094514	0.207636	0.242313	CARM1	Histone-arg	CARM1_HU	11	1.067705
30min_MT.	Q9BRK5	-0.33322	0.207584	0.242313	SDF4	45 kDa calc	CAB45_HUI	5	0.793765
15min_MT.	Q9HD40	0.073541	0.207739	0.242313	SEPSECS	O-phospho	SPCS_HUM	9	1.052296
30min_MT.	Q9NX46	-0.09404	0.207772	0.242313	ADPRS	ADP-ribosy	ADPRS_HUI	5	0.936894
30min_MT.	Q9UMX5	0.258286	0.207564	0.242313	NENF	Neudesin	NENF_HUM	7	1.196057

15min_MT.	Q9Y5A7	-0.05396	0.207749	0.242313	NUB1	NEDD8 ulti	NUB1_HUM	3	0.963289
15min_MT.	Q9UM22	0.1458	0.207889	0.242413	EPDR1	Mammaliar	EPDR1_HUI	4	1.106344
15min_MT.	Q02952	-0.12592	0.20793	0.242425	AKAP12	A-kinase an	AKA12_HUI	70	0.916421
15min_MT.	P06280	-0.20849	0.208005	0.242476	GLA	Alpha-galac	AGAL_HUM	7	0.865445
30min_MT.	O60907	0.015588	0.208125	0.242543	TBL1X	F-box-like/\	TBL1X_HUI	9	1.010863
30min_MT.	Q8WUX2	-0.14842	0.208123	0.242543	CHAC2	Glutathion	CHAC2_HU	1	0.902239
15min_MT.	Q8TDZ2	-0.0191	0.208244	0.24261	MICAL1	[F-actin]-m	MICA1_HUI	11	0.986848
15min_MT.	Q9BW27	0.045564	0.20823	0.24261	NUP85	Nuclear poi	NUP85_HU	6	1.032086
15min_MT.	Q5XKL5	0.326103	0.208369	0.242633	BTBD8	BTB/POZ dc	BTBD8_HUI	2	1.253622
15min_MT.	Q8WYQ3	0.183215	0.208424	0.242639	CHCHD10	Coiled-coil-	CHC10_HU	1	1.135411
15min_MT.	P21399	-0.10927	0.208532	0.242693	ACO1	Cytoplasmi	ACOHC_HU	30	0.927057
15min_MT.	Q14244	0.366707	0.2087	0.242852	MAP7	Ensconsin	MAP7_HUM	1	1.289406
15min_MT.	Q7L775	0.14981	0.209038	0.243173	EPM2AIP1	EPM2A-inte	EPMIP_HUI	2	1.109423
15min_MT.	Q00796	0.106396	0.209171	0.243291	SORD	Sorbitol de	DHSO_HUM	14	1.076536
30min_MT.	Q8N806	0.092528	0.209272	0.243337	UBR7	Putative E3	UBR7_HUM	9	1.066237
15min_MT.	Q12802	0.087643	0.209365	0.243409	AKAP13	A-kinase an	AKP13_HUI	17	1.062632
30min_MT.	P09874	-0.07969	0.209529	0.243527	PARP1	Poly [ADP-r	PARP1_HUI	25	0.946262
30min_MT.	P30408	0.015137	0.209683	0.24367	TM4SF1	Transmemk	T4S1_HUM	1	1.010547
30min_MT.	O14950;P1	-0.18607	0.209721	0.243678	MYL12B;M'	Myosin reg	ML12B_HU	6	0.878994
15min_MT.	Q8IXW5	0.003013	0.210243	0.244211	RPAP2	Putative RI	RPAP2_HUI	2	1.002091
15min_MT.	P52907	-0.04313	0.210352	0.244249	CAPZA1	F-actin-cap	CAZA1_HUI	14	0.970545
30min_MT.	Q9C0A0	0.230722	0.21034	0.244249	CNTNAP4	Contactin-a	CNTP4_HUI	1	1.173422
30min_MT.	Q9NX18	0.423635	0.2104	0.244249	SDHAF2	Succinate c	SDHF2_HUI	3	1.341303
30min_MT.	P61586	0.033713	0.210562	0.244365	RHOA	Transformi	RHOA_HUM	2	1.023643
15min_MT.	Q96GD0	-0.10062	0.210809	0.244573	PDXP	Chronophi	PLPP_HUM	16	0.932635
30min_MT.	O75131	-0.07474	0.211242	0.244936	CPNE3	Copine-3	CPNE3_HUI	18	0.949513
15min_MT.	Q0IIM8	0.039881	0.211311	0.244942	TBC1D8B	TBC1 doma	TBC8B_HUI	11	1.028029
30min_MT.	P00338	0.165551	0.211366	0.244971	LDHA	L-lactate de	LDHA_HUM	21	1.121594
30min_MT.	Q01813	-0.12912	0.211589	0.245037	PFKP	ATP-depen	PFKAP_HUI	13	0.914389
30min_MT.	Q8TDR0	0.00033	0.211587	0.245037	TRAF3IP1	TRAF3-inte	MIPT3_HUI	2	1.000229
15min_MT.	Q9BQG0	0.133066	0.211612	0.245037	MYBBP1A	Myb-bindir	MBB1A_HU	5	1.096622
30min_MT.	Q9UGI8	-0.05579	0.211842	0.24516	TES	Testin	TES_HUMA	21	0.962067
30min_MT.	Q9Y266	-0.03097	0.211843	0.24516	NUDC	Nuclear mi	NUDC_HUM	18	0.978762
15min_MT.	Q9H5Q4	-0.06427	0.212133	0.245397	TFB2M	Dimethylac	TFB2M_HU	2	0.956429
30min_MT.	Q6RW13	-0.14106	0.212227	0.245459	AGTRAP	Type-1 angi	ATRAP_HUI	3	0.906849
30min_MT.	Q96CU9	-0.11926	0.212462	0.245622	FOXRED1	FAD-depen	FXRD1_HUI	1	0.92066
30min_MT.	P07992	0.131686	0.212808	0.245796	ERCC1	DNA excisic	ERCC1_HUI	4	1.095573
15min_MT.	P08185	0.24001	0.212824	0.245796	SERPINA6	Corticoster	CBG_HUMA	4	1.181
15min_MT.	P83111	-0.12014	0.212895	0.245796	LACTB	Serine beta	LACTB_HUI	2	0.920101
15min_MT.	Q00341	0.071794	0.212855	0.245796	HDLBP	Vigilin	VIGLN_HUI	48	1.051023
30min_MT.	Q86YB7	0.374376	0.212763	0.245796	ECHDC2	Enoyl-CoA I	ECHD2_HU	3	1.296279
15min_MT.	P41227	-0.02547	0.213016	0.245891	NAA10	N-alpha-ac	NAA10_HU	8	0.982498
30min_MT.	Q9UI12	-0.12719	0.213644	0.246479	ATP6V1H	V-type prot	VATH_HUM	10	0.915611
15min_MT.	Q58FF6	0.176393	0.213797	0.246538	HSP90AB4I	Putative he	H90B4_HU	1	1.130055
30min_MT.	Q9C0E2	0.080868	0.213899	0.246591	XPO4	Exportin-4	XPO4_HUM	17	1.057654
30min_MT.	Q75N03	-0.11659	0.21408	0.246726	CBLL1	E3 ubiquiti	HAKAI_HUI	2	0.922366
30min_MT.	O43766	0.301042	0.214173	0.246797	LIAS	Lipoyl syntl	LIAS_HUMA	4	1.232034
30min_MT.	Q13200	-0.02838	0.214289	0.246894	PSMD2	26S proteas	PSMD2_HU	45	0.980519
30min_MT.	P17858	-0.07544	0.2144	0.246914	PFKL	ATP-depen	PFKAL_HUI	14	0.949054
15min_MT.	P68400	-0.03412	0.214343	0.246914	CSNK2A1	Casein kina	CSK21_HUI	9	0.976625
15min_MT.	Q8TCS8	-0.00337	0.214369	0.246914	PNPT1	Polyribonu	PNPT1_HUI	29	0.997664
15min_MT.	Q13136	-0.07142	0.214593	0.247027	PPFIA1	Liprin-alph	LIPA1_HUM	14	0.9517

15min_MT.	Q9UBP0	0.338861	0.214726	0.247144	SPAST	Spastin	SPAST_HUM	1	1.264758
15min_MT.	Q8TBC4	0.116504	0.214834	0.247175	UBA3	NEDD8-acti	UBA3_HUM	14	1.084105
15min_MT.	Q9Y6N5	-0.06727	0.214888	0.247175	SQOR	Sulfide:qui	SQOR_HUM	17	0.954445
30min_MT.	Q5BJH7	-0.04158	0.215007	0.247231	YIF1B	Protein YIF	YIF1B_HUM	1	0.971588
15min_MT.	P23368	0.146398	0.215096	0.247278	ME2	NAD-depen	MAOM_HU	19	1.106803
15min_MT.	P05386	-0.30526	0.215169	0.247289	RPLP1	Large ribos	RLA1_HUM	2	0.809297
30min_MT.	Q6UXH1	0.2029	0.215149	0.247289	CRELD2	Protein dis	CREL2_HUM	7	1.15101
30min_MT.	O94760	0.111726	0.215226	0.247318	DDAH1	N(G),N(G)-d	DDAH1_HU	15	1.08052
30min_MT.	P49448	-0.23712	0.215385	0.247429	GLUD2	Glutamate	DHE4_HUM	4	0.848436
30min_MT.	Q14527	-0.07343	0.215428	0.247442	HLTF	Helicase-lik	HLTF_HUM	1	0.950378
30min_MT.	Q00266	-0.05528	0.215472	0.247455	MAT1A	S-adenosyl	METK1_HU	8	0.962406
15min_MT.	Q9BT78	0.112392	0.215524	0.247479	COPS4	COP9 signa	CSN4_HUM	20	1.081019
30min_MT.	O00584	0.159821	0.215691	0.247635	RNASET2	Ribonuclea	RNT2_HUM	9	1.117149
15min_MT.	P08754	-0.18773	0.215856	0.247751	GNAI3	Guanine nu	GNAI3_HU	8	0.877988
30min_MT.	P23142	0.029305	0.215843	0.247751	FBLN1	Fibulin-1	FBLN1_HU	6	1.02052
15min_MT.	Q6ZVM7	0.148393	0.215987	0.247837	TOM1L2	TOM1-like	TM1L2_HU	3	1.108334
30min_MT.	Q8NDC0	-0.50662	0.215994	0.247837	MAPK1IP1L	MAPK-inter	MISSL_HUM	1	0.703868
30min_MT.	P51649	0.264358	0.216074	0.247848	ALDH5A1	Succinate-s	SSDH_HUM	4	1.201101
15min_MT.	Q9UHB4	-0.32097	0.216099	0.247848	NDOR1	NADPH-dep	NDOR1_HU	5	0.80053
15min_MT.	Q9UQE7	-0.01885	0.216062	0.247848	SMC3	Structural	SMC3_HUM	48	0.98702
15min_MT.	Q71UM5	-0.14348	0.216234	0.247967	RPS27L	Ribosomal	RS27L_HUM	3	0.905333
15min_MT.	O43490	0.204118	0.216482	0.248142	PROM1	Prominin-1	PROM1_HU	2	1.151982
30min_MT.	Q5T0F9	0.288064	0.216434	0.248142	CC2D1B	Coiled-coil	C2D1B_HU	1	1.221001
15min_MT.	Q8N139	1.062944	0.216476	0.248142	ABCA6	ATP-bindin	ABCA6_HU	1	2.08919
30min_MT.	P16615	-0.04671	0.216603	0.248208	ATP2A2	Sarcoplasm	AT2A2_HU	3	0.968141
30min_MT.	P78368;Q9	-0.04406	0.216577	0.248208	CSNK1G2;C	Casein kina	KC1G2_HU	2	0.969921
30min_MT.	Q2NL82	-0.04041	0.216644	0.248219	TSR1	Pre-rRNA-p	TSR1_HUM	3	0.97238
30min_MT.	Q6IN85	0.014495	0.216842	0.248337	PPP4R3A	Serine/thre	P4R3A_HU	15	1.010098
30min_MT.	O96033	-0.02022	0.216986	0.248461	MOCS2	Molybdopt	MOC2A_HU	4	0.986081
30min_MT.	P54709	-0.15025	0.217124	0.248477	ATP1B3	Sodium/po	AT1B3_HU	6	0.901096
30min_MT.	Q16543	-0.10542	0.21709	0.248477	CDC37	Hsp90 co-c	CDC37_HU	12	0.929536
15min_MT.	Q8NHZ8	-0.53739	0.217121	0.248477	CDC26	Anaphase-p	CDC26_HU	1	0.689019
30min_MT.	P18031	-0.20196	0.217179	0.248504	PTPN1	Tyrosine-pr	PTN1_HUM	8	0.869371
15min_MT.	Q8N7Z5	0.847866	0.217273	0.248575	ANKRD31	Ankyrin rep	ANR31_HU	1	1.799836
15min_MT.	P30408	-0.13291	0.21733	0.248604	TM4SF1	Transmemb	T4S1_HUM	1	0.911988
30min_MT.	P54652	0.072929	0.217409	0.248658	HSPA2	Heat shock	HSP72_HU	24	1.05185
30min_MT.	O15400	0.19929	0.217579	0.248762	STX7	Syntaxin-7	STX7_HUM	6	1.148133
15min_MT.	Q6P1Q9	0.139647	0.217596	0.248762	METTL2B	tRNA N(3)-r	MET2B_HU	1	1.101636
30min_MT.	Q9HB21	-0.11851	0.217665	0.248806	PLEKHA1	Pleckstrin	PKHA1_HU	5	0.921141
15min_MT.	Q15459	0.063318	0.217818	0.248944	SF3A1	Splicing fac	SF3A1_HUM	30	1.044866
30min_MT.	Q9UBW8	0.112017	0.218214	0.249324	COPS7A	COP9 signa	CSN7A_HU	10	1.080738
30min_MT.	P52298	0.218292	0.218294	0.249379	NCBP2	Nuclear cap	NCBP2_HU	2	1.163356
15min_MT.	P68036	0.162841	0.218532	0.249615	UBE2L3	Ubiquitin-c	UB2L3_HU	7	1.119489
30min_MT.	Q7L5D6	-0.11602	0.219032	0.250039	GET4	Golgi to ER	GET4_HUM	7	0.922733
30min_MT.	Q9UKD2	0.027856	0.219031	0.250039	MRTO4	mRNA turn	MRT4_HUM	9	1.019496
15min_MT.	Q9Y3A3	0.077893	0.219308	0.25026	MOB4	MOB-like p	PHOCN_HU	5	1.055475
30min_MT.	Q9UL63	-0.04558	0.219547	0.250407	MKLN1	Muskelin	MKLN1_HU	7	0.968901
30min_MT.	Q9Y6K9	-0.04413	0.219519	0.250407	IKBKG	NF-kappa-B	NEMO_HUM	6	0.969872
30min_MT.	Q14CN4	0.071421	0.219648	0.250485	KRT72	Keratin, typ	K2C72_HU	2	1.050751
15min_MT.	Q9Y6D5	-0.0512	0.219679	0.250485	ARFGEF2	Brefeldin A	BIG2_HUM	25	0.965135
30min_MT.	Q6PD74	0.047646	0.219746	0.250525	AAGAB	Alpha- and	AAGAB_HU	4	1.033577
30min_MT.	O00291	-0.0756	0.219853	0.250538	HIP1	Huntingtin	HIP1_HUM	25	0.948949

15min_MT.	Q86VS8	0.150297	0.219829	0.250538	HOOK3	Protein Ho	HOOK3_HU	14	1.109798
15min_MT.	O43432	0.022917	0.219967	0.250595	EIF4G3	Eukaryotic	IF4G3_HUN	33	1.016012
30min_MT.	Q96L46	0.368433	0.22027	0.250875	CAPNS2	Calpain sm	CPNS2_HU	4	1.29095
15min_MT.	Q9NUS5	0.129915	0.220278	0.250875	AP5S1	AP-5 compl	AP5S1_HUI	1	1.094229
30min_MT.	Q6XD76	0.098083	0.22052	0.251079	ASCL4	Achaete-sc	ASCL4_HUN	2	1.07035
15min_MT.	Q99856	0.13061	0.220638	0.251176	ARID3A	AT-rich inte	ARI3A_HUN	10	1.094757
30min_MT.	Q6P996	-0.10592	0.220705	0.251216	PDXDC1	Pyridoxal-d	PDXD1_HU	17	0.929211
15min_MT.	O00291	0.10057	0.220901	0.251319	HIP1	Huntingtin	HIP1_HUM	25	1.072197
15min_MT.	O75317	0.393392	0.220832	0.251319	USP12	Ubiquitin c	UBP12_HU	2	1.313478
30min_MT.	P01137	-0.12553	0.220883	0.251319	TGFB1	Transformi	TGFB1_HUI	3	0.916665
30min_MT.	Q9H307	-0.09721	0.220956	0.251319	PNN	Pinin	PININ_HUN	9	0.934842
15min_MT.	O95104	-0.00416	0.221114	0.251389	SCAF4	SR-related	SCAF4_HUN	8	0.997122
30min_MT.	P57737	-0.09678	0.221232	0.251486	CORO7	Coronin-7	CORO7_HU	12	0.935115
30min_MT.	O00233	0.09794	0.221294	0.25152	PSMD9	26S protea	PSMD9_HU	9	1.070244
30min_MT.	P00918	0.084055	0.221494	0.251564	CA2	Carbonic ar	CAH2_HUM	12	1.059993
15min_MT.	P62380	0.07479	0.22145	0.251564	TBPL1	TATA box-b	TBPL1_HUN	3	1.053207
15min_MT.	P62699	0.242595	0.221479	0.251564	YPEL5	Protein yip	YPEL5_HUN	4	1.183119
15min_MT.	Q6EEV4	-0.74264	0.221417	0.251564	POLR2M	DNA-direct	GL1AD_HU	2	0.597646
30min_MT.	Q12768	-0.26209	0.221654	0.251696	WASHC5	WASH com	WASC5_HU	12	0.833882
15min_MT.	Q6ZSR9	-0.44497	0.221674	0.251696	NaN	Uncharacte	YJ005_HUN	6	0.734597
15min_MT.	Q92686	-0.21706	0.22174	0.251698	NRGN	Neurograni	NEUG_HUN	1	0.860315
15min_MT.	P30519	0.219093	0.221812	0.251744	HMOX2	Heme oxyg	HMOX2_HU	5	1.164001
15min_MT.	O75817	-0.35049	0.221962	0.251804	POP7	Ribonuclea	POP7_HUN	1	0.784319
30min_MT.	Q6P1Q9	-0.14155	0.221938	0.251804	METTL2B	tRNA N(3)-r	MET2B_HU	1	0.906544
30min_MT.	Q86WP2	1.253869	0.222056	0.251874	GPBP1	Vasculin	GPBP1_HU	1	2.384802
15min_MT.	Q92538	-0.03863	0.22254	0.252152	GBF1	Golgi-speci	GBF1_HUM	48	0.973581
30min_MT.	Q9UJZ1	-0.07975	0.222417	0.252152	STOML2	Stomatin-li	STML2_HUI	4	0.946224
30min_MT.	P24298	0.114848	0.222694	0.252232	GPT	Alanine am	ALAT1_HUN	13	1.082861
15min_MT.	Q9NWU1	-0.03895	0.222812	0.252329	OXSM	3-oxoacyl-[	OXSM_HUN	10	0.97336
30min_MT.	P11047	0.094256	0.22296	0.252428	LAMC1	Laminin sul	LAMC1_HU	27	1.067515
30min_MT.	Q5TGL8	-0.28172	0.223044	0.252428	PXDC1	PX domain-	PXDC1_HU	1	0.822612
30min_MT.	Q8IWE4	0.402511	0.223061	0.252428	DCUN1D3	DCN1-like p	DCNL3_HU	1	1.321807
30min_MT.	Q8TEU7;Q9	-0.16271	0.22305	0.252428	RAPGEF6;R	Rap guanin	RPGF6_HUI	1	0.893347
30min_MT.	P63220	0.167627	0.223102	0.252438	RPS21	Small ribos	RS21_HUM	4	1.123209
15min_MT.	P49862	0.37134	0.223191	0.252502	KLK7	Kallikrein-7	KLK7_HUM	1	1.293554
30min_MT.	A5PLN9	0.490887	0.223296	0.252582	TRAPPC13	Trafficking	TPC13_HUI	2	1.405308
30min_MT.	Q9Y535	-0.28472	0.223373	0.252599	POLR3H	DNA-direct	RPC8_HUM	2	0.820902
30min_MT.	Q9Y2B9	0.036683	0.223515	0.25268	PKIG	cAMP-depe	IPKG_HUM	1	1.025753
30min_MT.	Q8NC69	0.042621	0.223736	0.25279	KCTD6	BTB/POZ dc	KCTD6_HUI	3	1.029984
30min_MT.	P48553	-0.06578	0.223919	0.252923	TRAPPC10	Trafficking	TPC10_HUI	4	0.955429
30min_MT.	Q7L1V2	-0.36137	0.224244	0.253181	MON1B	Vacuolar fu	MON1B_HU	3	0.778427
30min_MT.	Q12888	0.011987	0.224335	0.253207	TP53BP1	TP53-bindi	TP53B_HUI	55	1.008343
30min_MT.	Q13614	0.265611	0.22432	0.253207	MTMR2	Myotubula	MTMR2_HU	4	1.202145
15min_MT.	Q92804	0.230079	0.224475	0.253259	TAF15	TATA-bindi	RBP56_HUI	10	1.172899
15min_MT.	Q32M88	0.062595	0.224557	0.253315	PGGHG	Protein-glu	PGGHG_HU	10	1.044343
30min_MT.	O00268	0.295078	0.224882	0.253645	TAF4	Transcripti	TAF4_HUM	1	1.226951
15min_MT.	Q5VTR2	0.091408	0.225049	0.253796	RNF20	E3 ubiquiti	BRE1A_HUI	22	1.065409
15min_MT.	Q6ZN17	0.099973	0.225165	0.253888	LIN28B	Protein lin-	LN28B_HUI	10	1.071754
15min_MT.	Q9Y2T3	0.090144	0.225195	0.253888	GDA	Guanine de	GUAD_HUN	16	1.064477
15min_MT.	Q5JYT7	0.293751	0.225332	0.254006	KIAA1755	Uncharacte	K1755_HUI	1	1.225823
15min_MT.	Q13443	-0.90587	0.225413	0.25406	ADAM9	Disintegrin	ADAM9_HU	1	0.533712
30min_MT.	P29992	-0.38548	0.225693	0.254252	GNA11	Guanine nu	GNA11_HU	5	0.765527

15min_MT.	Q8N3D4	-0.0965	0.225778	0.254252	EHBP1L1	EH domain·	EH1L1_HUI	9	0.935298
30min_MT.	Q96GG9	0.109225	0.225741	0.254252	DCUN1D1	DCN1-like p	DCNL1_HU	8	1.078649
30min_MT.	Q9H1Y0	0.035148	0.225653	0.254252	ATG5	Autophagy	ATG5_HUM	10	1.024662
30min_MT.	Q8N9V7	-0.36253	0.22602	0.254415	TOPAZ1	Protein TOI	TOPZ1_HUI	1	0.777798
30min_MT.	Q9H0X4	0.694974	0.226001	0.254415	FAM234A	Protein FAI	F234A_HUI	1	1.618855
30min_MT.	Q8WX93	-0.05505	0.226225	0.254535	PALLD	Palladin	PALLD_HUI	8	0.962562
15min_MT.	Q99638	0.054193	0.226206	0.254535	RAD9A	Cell cycle cl	RAD9A_HU	4	1.038278
30min_MT.	Q9UJY5	-0.07062	0.226201	0.254535	GGA1	ADP-ribosy	GGA1_HUM	8	0.952226
30min_MT.	Q14697	-0.1147	0.226422	0.254684	GANAB	Neutral alp	GANAB_HU	36	0.923576
30min_MT.	Q13573	-0.01956	0.226616	0.254793	SNW1	SNW doma	SNW1_HUM	9	0.986536
30min_MT.	Q9BZZ5	0.078297	0.226588	0.254793	API5	Apoptosis i	API5_HUMA	20	1.055771
15min_MT.	P0DPD7;P(	0.29838	0.226673	0.254805	EEF1AKMT4	EEF1A lysin	EFMT4_HUI	4	1.229763
30min_MT.	Q9H299	0.17392	0.226693	0.254805	SH3BGRL3	SH3 domaii	SH3L3_HUI	4	1.12812
30min_MT.	Q6AI08	-0.33487	0.226794	0.254818	HEATR6	HEAT repea	HEAT6_HUI	1	0.792855
30min_MT.	Q9P2R3	0.014241	0.226791	0.254818	ANKFY1	Rabankyrin	ANFY1_HUI	7	1.00992
30min_MT.	P24390;P3	-0.78066	0.227046	0.254978	KDELR1;KD	ER lumen p	ERD21_HUI	1	0.582099
30min_MT.	P61289	0.131838	0.227128	0.254978	PSME3	Proteasom	PSME3_HU	13	1.095688
30min_MT.	Q13011	0.112792	0.227138	0.254978	ECH1	Delta(3,5)-I	ECH1_HUM	10	1.081319
30min_MT.	Q9UHR4	-0.106	0.22714	0.254978	BAIAP2L1	BAR/IMD d	BI2L1_HUM	13	0.929163
15min_MT.	Q9H6S3	0.111861	0.227192	0.255	EPS8L2	Epidermal g	ES8L2_HUM	18	1.080621
15min_MT.	Q9UKK3	-0.07377	0.227231	0.255008	PARP4	Protein mo	PARP4_HUI	24	0.950151
15min_MT.	Q9NX46	0.077693	0.227421	0.255147	ADPRS	ADP-ribosy	ADPRS_HUI	5	1.055329
15min_MT.	P20645	-0.39117	0.227552	0.255258	M6PR	Cation-dep	MPRD_HUM	1	0.76251
15min_MT.	O15231	0.821486	0.227637	0.255279	ZNF185	Zinc finger p	ZN185_HUI	1	1.767225
15min_MT.	Q99961	0.130577	0.227636	0.255279	SH3GL1	Endophilin	SH3G1_HU	11	1.094731
30min_MT.	O94973	-0.09452	0.227747	0.255366	AP2A2	AP-2 compl	AP2A2_HUI	19	0.936582
30min_MT.	P24386	-0.02951	0.227929	0.255389	CHM	Rab proteir	RAE1_HUM	14	0.979755
30min_MT.	P26927	-0.08676	0.227947	0.255389	MST1	Hepatocyte	HGFL_HUM	8	0.941637
30min_MT.	Q13158	0.551245	0.227905	0.255389	FADD	FAS-associa	FADD_HUM	1	1.46535
15min_MT.	Q15654	-0.45488	0.227995	0.255389	TRIP6	Thyroid rec	TRIP6_HUM	3	0.729571
30min_MT.	Q8WUW1	0.092623	0.227924	0.255389	BRK1	Protein BRI	BRK1_HUM	5	1.066307
15min_MT.	Q92623	0.193873	0.227972	0.255389	TTC9	Tetratricop	TTC9A_HUI	2	1.14383
30min_MT.	O43776	-0.12114	0.228164	0.255541	NARS1	Asparagine·	SYNC_HUM	15	0.919463
15min_MT.	P08697	0.165229	0.228581	0.255899	SERPINF2	Alpha-2-ant	A2AP_HUM	15	1.121344
30min_MT.	P48507	0.0374	0.228855	0.25608	GCLM	Glutamate-	GSH0_HUM	11	1.026262
15min_MT.	Q9UJW0	-0.06006	0.228853	0.25608	DCTN4	Dynactin su	DCTN4_HU	10	0.959227
15min_MT.	P14174	0.233889	0.228985	0.256151	MIF	Macrophag	MIF_HUMA	4	1.176001
15min_MT.	P23142	0.003133	0.22905	0.256151	FBLN1	Fibulin-1	FBLN1_HUI	6	1.002174
15min_MT.	Q96CB9	-0.14558	0.229007	0.256151	NSUN4	5-methylcy	NSUN4_HU	3	0.904019
30min_MT.	P10746	-0.05513	0.229248	0.256314	UROS	Uroporphy	HEM4_HUM	5	0.962511
15min_MT.	O75054	-0.46534	0.229661	0.25674	IGSF3	Immunoglo	IGSF3_HUM	4	0.7243
30min_MT.	P06576	0.033606	0.230095	0.257188	ATP5F1B	ATP synthas	ATPB_HUM	17	1.023568
30min_MT.	O75695	-0.16718	0.230162	0.257226	RP2	Protein XRI	XRP2_HUM	5	0.890581
30min_MT.	Q8IWZ3	-0.13087	0.230356	0.257406	ANKHD1	Ankyrin rep	ANKH1_HU	18	0.913278
30min_MT.	Q86WB0	-0.10985	0.23058	0.257583	ZC3HC1	Zinc finger (	ZC3C1_HUI	11	0.926683
15min_MT.	O75955	0.080368	0.230833	0.257738	FLOT1	Flotillin-1	FLOT1_HUM	4	1.057288
30min_MT.	Q15596	-0.27678	0.230791	0.257738	NCOA2	Nuclear rec	NCOA2_HU	5	0.825433
15min_MT.	Q9Y2L1	-0.03378	0.230851	0.257738	DIS3	Exosome cc	RRP44_HUI	22	0.976855
30min_MT.	P82650	-0.10847	0.230946	0.257771	MRPS22	Small ribos	RT22_HUM	5	0.927568
15min_MT.	Q96F24	0.052963	0.230928	0.257771	NRBF2	Nuclear rec	NRBF2_HUI	4	1.037393
30min_MT.	P07951	0.187647	0.231096	0.257829	TPM2	Tropomyos	TPM2_HUM	6	1.138905
30min_MT.	P08582	0.01846	0.231094	0.257829	MELTF	Melanotrar	TRFM_HUM	7	1.012878

15min_MT.	P63173	-0.17606	0.231065	0.257829	RPL38	Large ribos	RL38_HUM	3	0.88512
30min_MT.	P05067	0.333345	0.23164	0.258109	APP	Amyloid-be	A4_HUMAN	5	1.259931
15min_MT.	P11310	0.129702	0.231711	0.258109	ACADM	Medium-ch	ACADM_HU	15	1.094068
30min_MT.	P48739	0.07317	0.231682	0.258109	PITPNB	Phosphatid	PIPNB_HUI	16	1.052026
30min_MT.	P57772	-0.0925	0.231456	0.258109	EEFSEC	Selenocyste	SELB_HUMA	6	0.937897
15min_MT.	Q14651	-0.0121	0.231684	0.258109	PLS1	Plastin-1	PLSI_HUMA	19	0.99165
30min_MT.	Q16254	0.459048	0.23146	0.258109	E2F4	Transcripti	E2F4_HUM	3	1.374634
15min_MT.	P30038	0.364616	0.231748	0.258112	ALDH4A1	Delta-1-pyr	AL4A1_HUI	4	1.287539
30min_MT.	Q00765	-0.19421	0.231779	0.258112	REEP5	Receptor ex	REEP5_HUI	5	0.87405
30min_MT.	Q12974	-0.2963	0.231987	0.258265	PTP4A2	Protein tyr	TP4A2_HUI	3	0.814338
30min_MT.	Q9Y3D0	0.246501	0.231963	0.258265	CIAO2B	Cytosolic ir	CIA2B_HUN	3	1.186326
15min_MT.	P31942	0.190644	0.232173	0.258367	HNRNPH3	Heterogene	HNRH3_HU	6	1.141273
15min_MT.	P25398	0.154814	0.232347	0.258487	RPS12	Small ribos	RS12_HUM	8	1.113278
15min_MT.	Q9H6V9	0.533626	0.232497	0.25858	LDAH	Lipid dropl	LDAH_HUM	1	1.447562
15min_MT.	Q96FK6	-0.41683	0.232544	0.258596	WDR89	WD repeat-	WDR89_HU	3	0.749067
30min_MT.	O00170	-0.12398	0.232777	0.258744	AIP	AH recepto	AIP_HUMA	10	0.917653
15min_MT.	P27348	0.092608	0.232915	0.258859	YWHAQ	14-3-3 prot	1433T_HUI	12	1.066296
30min_MT.	Q8IWW8	0.150646	0.233221	0.259127	ADHFE1	Hydroxyaci	HOT_HUMA	7	1.110066
15min_MT.	Q07955	-0.10138	0.233412	0.259146	SRSF1	Serine/argi	SRSF1_HUN	14	0.932139
15min_MT.	Q92890	0.079408	0.233285	0.259146	UFD1	Ubiquitin r	UFD1_HUM	10	1.056585
30min_MT.	P80303	0.005703	0.233501	0.259149	NUCB2	Nucleobinc	NUCB2_HU	12	1.003961
15min_MT.	Q13610	0.123356	0.233702	0.25933	PWP1	Periodic try	PWP1_HUN	9	1.089266
15min_MT.	Q86UU0	-0.02613	0.234032	0.259622	BCL9L	B-cell CLL/l	BCL9L_HUN	2	0.98205
30min_MT.	O76021	0.238656	0.234249	0.259753	RSL1D1	Ribosomal	RL1D1_HUI	9	1.179893
30min_MT.	P04843	0.490068	0.234241	0.259753	RPN1	Dolichyl-di	RPN1_HUM	2	1.404511
30min_MT.	Q9Y6A5	-0.05903	0.234223	0.259753	TACC3	Transformi	TACC3_HUI	2	0.959907
15min_MT.	P04156	-0.23395	0.234621	0.260018	PRNP	Major prior	PRIO_HUM	3	0.850304
15min_MT.	Q15813	-0.35083	0.23462	0.260018	TBCE	Tubulin-spe	TBCE_HUM	8	0.784135
15min_MT.	Q9BU61	0.068727	0.234803	0.260182	NDUFAF3	NADH dehy	NDUF3_HU	4	1.048791
30min_MT.	Q9H773	-0.01254	0.234849	0.260197	DCTPP1	dCTP pyrop	DCTP1_HUI	10	0.991349
30min_MT.	P13164;QC	-0.03971	0.235186	0.260386	IFITM1;IFIT	Interferon-i	IFM1_HUM	1	0.972852
30min_MT.	Q96AY3	0.075177	0.235378	0.260525	FKBP10	Peptidyl-pr	FKB10_HUI	18	1.053491
30min_MT.	Q6P2Q9	-0.07117	0.235493	0.260605	PRPF8	Pre-mRNA-p	PRP8_HUM	46	0.951869
30min_MT.	P49411	-0.09786	0.235555	0.26061	TUFM	Elongation	EFTU_HUM	23	0.934421
30min_MT.	O60832	0.07637	0.235661	0.260653	DKC1	H/ACA ribo	DKC1_HUM	17	1.054362
15min_MT.	P49257	0.29316	0.236049	0.26092	LMAN1	Protein ERC	LMAN1_HU	4	1.225321
30min_MT.	Q13042	-0.06127	0.236039	0.26092	CDC16	Cell divisio	CDC16_HU	11	0.958419
15min_MT.	Q9UHN1	0.056139	0.236069	0.26092	POLG2	DNA polym	DPOG2_HU	6	1.03968
15min_MT.	P50552	0.061717	0.236142	0.260927	VASP	Vasodilator	VASP_HUM	12	1.043707
30min_MT.	O00746	-0.17103	0.236294	0.261058	NME4	Nucleoside	NDKM_HUI	3	0.888206
30min_MT.	Q8WZA9	0.15516	0.236439	0.261144	IRGQ	Immunity-r	IRGQ_HUM	15	1.113545
15min_MT.	Q6P2E9	0.047827	0.236517	0.261194	EDC4	Enhancer o	EDC4_HUM	31	1.033707
30min_MT.	O75367	0.440868	0.236659	0.261196	MACROH2A	Core histon	H2AY_HUM	2	1.357421
30min_MT.	P42773	0.154251	0.236583	0.261196	CDKN2C	Cyclin-depe	CDN2C_HU	1	1.112844
15min_MT.	P55957	0.251535	0.236624	0.261196	BID	BH3-intera	BID_HUMA	4	1.190473
15min_MT.	Q9BUT1	0.159141	0.236631	0.261196	BDH2	Dehydroger	DHRS6_HU	4	1.116622
30min_MT.	O95218	0.097925	0.236914	0.261267	ZRANB2	Zinc finger	ZRAB2_HUI	17	1.070233
15min_MT.	P22234	0.065484	0.236825	0.261267	PAICS	Bifunction	PUR6_HUM	20	1.046436
30min_MT.	P55345	0.257088	0.23689	0.261267	PRMT2	Protein arg	ANM2_HUN	1	1.195064
15min_MT.	Q9BV44	-0.05613	0.23688	0.261267	THUMPD3	tRNA (guan	THUM3_HU	13	0.961837
15min_MT.	Q9UPY8	0.027676	0.236918	0.261267	MAPRE3	Microtubul	MARE3_HU	4	1.019369
15min_MT.	Q9UM13	0.060247	0.237328	0.261646	ANAPC10	Anaphase-p	APC10_HUI	3	1.042644

30min_MT.	Q96F07	-0.05185	0.237401	0.261689	CYFIP2	Cytoplasmi	CYFP2_HUI	18	0.964701
15min_MT.	P22681	-0.05252	0.237595	0.261793	CBL	E3 ubiquiti	CBL_HUMA	22	0.964249
15min_MT.	P0DP23;P0	-0.11921	0.237739	0.261878	CALM1;CAL	Calmodulir	CALM1_HU	10	0.920691
30min_MT.	Q9NYB9	-0.1341	0.23772	0.261878	ABI2	Abl interact	ABI2_HUMA	6	0.911238
30min_MT.	Q9Y2T2	-0.16599	0.237811	0.261895	AP3M1	AP-3 compl	AP3M1_HU	5	0.891316
30min_MT.	O75717	-0.14098	0.23809	0.261973	WDHD1	WD repeat	WDHD1_HU	11	0.9069
15min_MT.	Q86YP4;Q8	0.03295	0.238093	0.261973	GATAD2A;G	Transcripti	P66A_HUM	1	1.023102
15min_MT.	Q9Y3D8	-0.05597	0.238029	0.261973	AK6	Adenylate k	KAD6_HUM	4	0.961946
30min_MT.	Q9Y6Q9	-0.03193	0.237987	0.261973	NCOA3	Nuclear rec	NCOA3_HU	6	0.97811
30min_MT.	Q08211	0.061129	0.238306	0.262109	DHX9	ATP-depend	DHX9_HUM	36	1.043282
30min_MT.	Q14139	-0.02707	0.238317	0.262109	UBE4A	Ubiquitin c	UBE4A_HUI	13	0.98141
30min_MT.	Q16527	0.146541	0.238316	0.262109	CSRP2	Cysteine an	CSRP2_HUI	7	1.106912
30min_MT.	Q01082	0.042077	0.238637	0.26235	SPTBN1	Spectrin be	SPTB2_HUM	124	1.029595
15min_MT.	Q92917	0.110838	0.23862	0.26235	GPKOW	G-patch do	GPKOW_HU	8	1.079855
30min_MT.	O60869	0.072174	0.238707	0.26236	EDF1	Endothelial	EDF1_HUM	10	1.0513
30min_MT.	O76054	0.031685	0.238747	0.26236	SEC14L2	SEC14-like	S14L2_HUM	11	1.022206
30min_MT.	P46783	-0.14241	0.238908	0.262463	RPS10	Small ribos	RS10_HUM	4	0.906006
30min_MT.	P00734	-0.08991	0.238962	0.262486	F2	Prothromb	THRB_HUM	22	0.939584
15min_MT.	P62873	-0.04002	0.239079	0.262578	GNB1	Guanine nu	GBB1_HUM	3	0.97264
15min_MT.	P28370	-0.2472	0.239481	0.262866	SMARCA1	Probable gl	SMCA1_HU	4	0.842533
15min_MT.	Q9NZB2	-0.10829	0.23951	0.262866	FAM120A	Constitutiv	F120A_HUI	14	0.927689
15min_MT.	Q15833	-0.0464	0.23958	0.262876	STXBP2	Syntaxin-bi	STXB2_HUM	18	0.968348
15min_MT.	Q5VTE6	-0.30684	0.239614	0.262876	ANGEL2	Protein ang	ANGE2_HU	2	0.808408
30min_MT.	O94826	-0.21241	0.239864	0.262923	TOMM70	Mitochond	TOM70_HU	1	0.863095
30min_MT.	O95793	0.230143	0.239848	0.262923	STAU1	Double-stra	STAU1_HUI	7	1.172952
15min_MT.	Q8WY54	-0.23434	0.239752	0.262923	PPM1E	Protein pho	PPM1E_HU	3	0.850073
30min_MT.	Q9HCE5	-0.17765	0.23982	0.262923	METTL14	N6-adenosi	MET14_HU	2	0.884142
30min_MT.	Q9Y5A9	0.012363	0.239839	0.262923	YTHDF2	YTH domair	YTHD2_HUI	7	1.008606
15min_MT.	O95628	0.120802	0.240301	0.263364	CNOT4	CCR4-NOT t	CNOT4_HU	4	1.087339
30min_MT.	Q9UJC3	-0.08346	0.24037	0.263365	HOOK1	Protein Ho	HOOK1_HU	19	0.94379
30min_MT.	Q8TDY4	0.148409	0.240523	0.263387	ASAP3	Arf-GAP wit	ASAP3_HUI	2	1.108346
30min_MT.	Q9NVN3	0.49328	0.240498	0.263387	RIC8B	Synembryn	RIC8B_HUM	2	1.407641
15min_MT.	Q9UPX8	0.181681	0.240717	0.263562	SHANK2	SH3 and mu	SHAN2_HU	3	1.134205
15min_MT.	Q9Y5X3	0.080098	0.240775	0.263589	SNX5	Sorting nex	SNX5_HUM	10	1.05709
15min_MT.	P21980	0.036398	0.24098	0.263705	TGM2	Protein-glu	TGM2_HUM	24	1.02555
15min_MT.	Q13546	0.051903	0.240982	0.263705	RIPK1	Receptor-ir	RIPK1_HUM	11	1.036631
30min_MT.	Q86UV5	0.380123	0.240955	0.263705	USP48	Ubiquitin c	UBP48_HU	2	1.301453
30min_MT.	O14910	0.054911	0.241168	0.263712	LIN7A	Protein lin-	LIN7A_HUM	4	1.038795
30min_MT.	O75177	-0.188	0.241258	0.263712	SS18L1	Calcium-res	CREST_HUM	1	0.877823
15min_MT.	P14384	-0.51746	0.241224	0.263712	CPM	Carboxypep	CBPM_HUM	1	0.698599
30min_MT.	Q16740	-0.04116	0.24117	0.263712	CLPP	ATP-depend	CLPP_HUM	10	0.971871
30min_MT.	Q6GMV2	-0.12281	0.241146	0.263712	SMYD5	Histone-lys	SMYD5_HU	8	0.9184
30min_MT.	Q7KZI7	-0.03518	0.241221	0.263712	MARK2	Serine/thre	MARK2_HU	1	0.975912
30min_MT.	Q03426	0.116966	0.241475	0.263885	MVK	Mevalonate	KIME_HUM	4	1.084452
30min_MT.	Q9Y5X1	-0.06626	0.241809	0.264092	SNX9	Sorting nex	SNX9_HUM	10	0.955113
30min_MT.	O00534	-0.04727	0.241979	0.264158	VWA5A	von Willebr	VMA5A_HU	14	0.967765
30min_MT.	P46776	-0.0894	0.242038	0.264158	RPL27A	Large ribos	RL27A_HUI	3	0.939915
15min_MT.	Q8N5K1	-0.26512	0.242031	0.264158	CISD2	CDGSH iror	CISD2_HUM	2	0.832128
30min_MT.	Q96FN4	-0.08085	0.242128	0.26422	CPNE2	Copine-2	CPNE2_HUI	4	0.945501
30min_MT.	Q14181	-0.00591	0.242232	0.264296	POLA2	DNA polym	DPOA2_HU	8	0.995911
15min_MT.	P04179	0.201459	0.242328	0.26434	SOD2	Superoxide	SODM_HUM	11	1.14986
30min_MT.	Q9Y2Q5	0.093744	0.242456	0.264411	LAMTOR2	Ragulator c	LTOR2_HUI	3	1.067136

15min_MT.	Q9UBQ7	0.037982	0.242651	0.264532	GRHPR	Glyoxylate	GRHPR_HU	12	1.026677
15min_MT.	P12111	-0.07305	0.242965	0.264727	COL6A3	Collagen al	CO6A3_HU	1	0.950624
30min_MT.	Q9NY93	-0.24179	0.242923	0.264727	DDX56	Probable A	DDX56_HU	1	0.845698
30min_MT.	Q14254	0.035552	0.243062	0.264776	FLOT2	Flotillin-2	FLOT2_HUI	4	1.024949
30min_MT.	Q9UEE9	0.209276	0.243078	0.264776	CFDP1	Craniofacia	CFDP1_HUI	5	1.156108
30min_MT.	Q99497	0.128115	0.243139	0.264783	PARK7	Parkinson c	PARK7_HUI	14	1.092865
15min_MT.	O60234	-0.00293	0.24336	0.264825	GMFG	Glia matura	GMFG_HUI	2	0.997969
15min_MT.	P12532;P1	-0.26062	0.243346	0.264825	CKMT1A;Ck	Creatine kir	KCRU_HUM	1	0.83473
30min_MT.	Q96CD0	0.057585	0.243348	0.264825	FBXL8	F-box/LRR-i	FBXL8_HUI	3	1.040722
30min_MT.	O75909	0.074783	0.243475	0.264848	CCNK	Cyclin-K	CCNK_HUM	8	1.053203
15min_MT.	Q03111	-0.06649	0.243548	0.264848	MLLT1	Protein ENI	ENL_HUMA	3	0.954959
30min_MT.	Q96CF2	0.141019	0.243515	0.264848	CHMP4C	Charged mu	CHM4C_HU	3	1.102684
15min_MT.	Q96F63	0.596581	0.243551	0.264848	CCDC97	Coiled-coil	CCD97_HU	2	1.512129
30min_MT.	Q9Y696	0.02224	0.243674	0.264909	CLIC4	Chloride in	CLIC4_HUM	15	1.015535
30min_MT.	Q13242	0.128409	0.243901	0.265119	SRSF9	Serine/argi	SRSF9_HUM	10	1.093088
15min_MT.	O76014	-1.20492	0.243943	0.265128	KRT37	Keratin, typ	KRT37_HUI	2	0.433793
30min_MT.	Q9UNF0	0.078642	0.244028	0.265183	PACSIN2	Protein kin	PACN2_HU	14	1.056023
30min_MT.	E9PRG8	0.151637	0.244283	0.26535	C11orf98	Uncharacte	CK098_HUI	2	1.110829
30min_MT.	P56537	0.056796	0.244256	0.26535	EIF6	Eukaryotic	IF6_HUMAI	8	1.040153
30min_MT.	Q5JVS0	0.271902	0.244481	0.265527	HABP4	Intracellula	HABP4_HU	2	1.207399
15min_MT.	P46821	-0.03728	0.244608	0.265629	MAP1B	Microtubul	MAP1B_HU	3	0.974492
15min_MT.	Q9BVS5	0.132825	0.245065	0.266014	TRMT61B	tRNA (aden	TR61B_HUI	5	1.096439
15min_MT.	Q9Y4E5	0.282993	0.245253	0.266144	ZNF451	E3 SUMO-p	ZN451_HUI	3	1.216716
15min_MT.	Q2M389	-0.27668	0.245503	0.266341	WASHC4	WASH com	WASC4_HU	6	0.825489
15min_MT.	P98160	0.044696	0.245571	0.266378	HSPG2	Basement n	PGBM_HUI	19	1.031466
15min_MT.	Q658Y4	-0.10959	0.245608	0.266382	FAM91A1	Protein FAI	F91A1_HUI	17	0.926854
30min_MT.	P50440	0.119812	0.245702	0.266431	GATM	Glycine am	GATM_HUM	19	1.086594
30min_MT.	Q8NFF5	-0.35083	0.245722	0.266431	FLAD1	FAD syntha	FAD1_HUM	2	0.784136
30min_MT.	Q7Z2Z2	-0.14049	0.245781	0.266459	EFL1	Elongation	EFL1_HUM.	14	0.907211
30min_MT.	P35573	-0.10865	0.245956	0.266595	AGL	Glycogen d	GDE_HUMA	29	0.927458
15min_MT.	Q14432	-0.08261	0.245975	0.266595	PDE3A	cGMP-inhil	PDE3A_HUI	4	0.944347
15min_MT.	Q96F86	0.199559	0.246136	0.266699	EDC3	Enhancer o	EDC3_HUM	9	1.148347
30min_MT.	P04062	0.016646	0.246201	0.26671	GBA1	Lysosomal	GBA1_HUM	7	1.011605
30min_MT.	Q9NS91	-0.13129	0.246814	0.267208	RAD18	E3 ubiquiti	RAD18_HU	6	0.913012
30min_MT.	P34897	-0.10433	0.246928	0.267221	SHMT2	Serine hydr	GLYM_HUM	18	0.930239
30min_MT.	Q9NQ92	0.160548	0.246902	0.267221	COPRS	Coordinatc	COPRS_HUI	1	1.117712
30min_MT.	P61966	-0.05111	0.247086	0.267292	AP1S1	AP-1 compl	AP1S1_HUI	4	0.965193
15min_MT.	Q14894	0.120813	0.247034	0.267292	CRYM	Ketimine re	CRYM_HUM	11	1.087348
30min_MT.	Q9H3U5	0.452136	0.247096	0.267292	MFSD1	Lysosomal	MFSD1_HU	1	1.368064
15min_MT.	P53680	-0.12016	0.247172	0.267299	AP2S1	AP-2 compl	AP2S1_HUI	3	0.920084
15min_MT.	O95208	0.761964	0.247407	0.267487	EPN2	Epsin-2	EPN2_HUM	1	1.695798
30min_MT.	Q9Y3A3	0.066452	0.247466	0.26749	MOB4	MOB-like pi	PHOCN_HU	5	1.047138
15min_MT.	P40937	-0.01008	0.247608	0.267549	RFC5	Replicatior	RFC5_HUM	6	0.993041
30min_MT.	Q15652	0.66059	0.247721	0.267634	JMJD1C	Probable Jr	JHD2C_HUI	1	1.580729
15min_MT.	P19784	0.123534	0.247804	0.267688	CSNK2A2	Casein kina	CSK22_HUI	8	1.0894
15min_MT.	Q9BSJ8	-0.0774	0.24826	0.268032	ESYT1	Extended sy	ESYT1_HUM	16	0.947764
30min_MT.	P08397	0.113842	0.248502	0.268108	HMBS	Porphobilir	HEM3_HUM	13	1.082106
30min_MT.	P13639	0.062522	0.248438	0.268108	EEF2	Elongation	EF2_HUMA	51	1.04429
15min_MT.	Q9GZZ9	0.123173	0.248599	0.268157	UBA5	Ubiquitin-l	UBA5_HUM	6	1.089128
15min_MT.	O43491	-0.0475	0.248699	0.26821	EPB41L2	Band 4.1-lil	E41L2_HUI	51	0.967608
15min_MT.	Q8NCW5	0.166909	0.248809	0.268291	NAXE	NAD(P)H-hy	NNRE_HUM	13	1.12265
30min_MT.	Q8TBB5	0.165557	0.24885	0.268298	KLHDC4	Kelch doma	KLDC4_HUI	9	1.121599

15min_MT.	P30048	0.172459	0.249009	0.268322	PRDX3	Thioredoxi	PRDX3_HU	9	1.126978
15min_MT.	O14617	-0.09443	0.249208	0.268371	AP3D1	AP-3 compl	AP3D1_HU	18	0.936641
30min_MT.	P12277	0.067093	0.249209	0.268371	CKB	Creatine kii	KCRB_HUM	18	1.047604
15min_MT.	Q99700	-0.03394	0.249261	0.268371	ATXN2	Ataxin-2	ATX2_HUM	14	0.976749
30min_MT.	Q9UJK0	0.323862	0.249181	0.268371	TSR3	18S rRNA ai	TSR3_HUM	1	1.251677
15min_MT.	Q15542	-0.28326	0.249389	0.268472	TAF5	Transcripti	TAF5_HUM	6	0.821735
30min_MT.	Q5ST30	0.030122	0.249429	0.268478	VARS2	Valine--tRN	SYVM_HUM	5	1.021098
30min_MT.	A6NDU8	0.089146	0.249473	0.268489	RIMOC1	RAB7A-inte	RIMC1_HUI	6	1.06374
30min_MT.	P16455	0.058189	0.249531	0.268515	MGMT	Methylatec	MGMT_HUI	5	1.041158
30min_MT.	Q86UK7	0.142313	0.249843	0.268684	ZNF598	E3 ubiquiti	ZN598_HUI	4	1.103673
15min_MT.	Q9NUB1	0.042503	0.2498	0.268684	ACSS1	Acetyl-coer	ACS2L_HUI	5	1.029899
30min_MT.	Q8NB37	0.025511	0.249939	0.268694	GATD1	Glutamine	GALD1_HU	3	1.01784
30min_MT.	Q5T200	0.215344	0.250107	0.268839	ZC3H13	Zinc finger	ZC3HD_HU	18	1.160981
30min_MT.	P53582	-0.06761	0.250337	0.269012	METAP1	Methionine	MAP11_HU	5	0.954216
30min_MT.	O00425	-0.19852	0.250568	0.269075	IGF2BP3	Insulin-like	IF2B3_HUM	8	0.871447
30min_MT.	Q15042	-0.12603	0.250517	0.269075	RAB3GAP1	Rab3 GTPas	RB3GP_HU	18	0.916353
15min_MT.	Q8TAA5	0.168814	0.25054	0.269075	GRPEL2	GrpE protei	GRPE2_HUI	6	1.124134
30min_MT.	O75113	-0.07272	0.250662	0.269139	N4BP1	NEDD4-bin	N4BP1_HU	5	0.950841
30min_MT.	Q8N8S7	-0.1326	0.250905	0.269216	ENAH	Protein ena	ENAH_HUM	15	0.912189
15min_MT.	P06865	0.070919	0.251182	0.269439	HEXA	Beta-hexos	HEXA_HUM	15	1.050386
15min_MT.	Q96EY4	0.110236	0.251379	0.269612	TMA16	Translation	TMA16_HU	3	1.079404
15min_MT.	Q96DT6	0.164748	0.251623	0.269838	ATG4C	Cysteine pr	ATG4C_HUI	1	1.12097
30min_MT.	O43823	-0.26462	0.251701	0.269847	AKAP8	A-kinase an	AKAP8_HUI	4	0.832421
30min_MT.	O95994	0.078362	0.251813	0.269847	AGR2	Anterior gr	AGR2_HUM	4	1.055819
15min_MT.	Q06210	-0.05056	0.251839	0.269847	GFPT1	Glutamine-	GFPT1_HUI	30	0.965564
15min_MT.	Q15738	-0.06504	0.251823	0.269847	NSDHL	Sterol-4-alp	NSDHL_HU	1	0.955917
15min_MT.	Q53H82	0.050638	0.251713	0.269847	LACTB2	Endoribonu	LACB2_HUI	12	1.035723
30min_MT.	P41567	0.084821	0.25193	0.269886	EIF1	Eukaryotic	EIF1_HUMA	4	1.060556
15min_MT.	Q99733	0.099494	0.251949	0.269886	NAP1L4	Nucleosom	NP1L4_HUI	8	1.071398
15min_MT.	Q8N3X1	-0.02016	0.252111	0.26999	FNBP4	Formin-bin	FNBP4_HUI	11	0.986125
30min_MT.	Q8WYQ3	0.159311	0.252218	0.270068	CHCHD10	Coiled-coil-	CHC10_HU	1	1.116754
15min_MT.	P17980	0.090058	0.252343	0.270128	PSMC3	26S proteas	PRS6A_HUI	21	1.064413
15min_MT.	Q9UBP6	0.500938	0.252555	0.270318	METTL1	tRNA (guan	TRMB_HUM	3	1.415133
30min_MT.	P09884	0.313753	0.252694	0.270356	POLA1	DNA polym	DPOLA_HU	3	1.242937
30min_MT.	P10606	0.147408	0.252692	0.270356	COX5B	Cytochrom	COX5B_HU	4	1.107577
30min_MT.	Q86V48	-0.09817	0.252732	0.27036	LUZP1	Leucine zip	LUZP1_HUI	10	0.934216
15min_MT.	P09327	0.03683	0.252843	0.270368	VIL1	Villin-1	VILI_HUMA	21	1.025857
30min_MT.	O14867	-0.01323	0.252976	0.270473	BACH1	Transcripti	BACH1_HU	6	0.990869
30min_MT.	O75347	0.203887	0.253062	0.270519	TBCA	Tubulin-spe	TBCA_HUM	9	1.151797
30min_MT.	Q15059	0.037045	0.253088	0.270519	BRD3	Bromodom	BRD3_HUM	6	1.02601
30min_MT.	P49593	-0.15971	0.253404	0.27082	PPM1F	Protein phc	PPM1F_HU	7	0.895204
30min_MT.	P62837	0.049336	0.253499	0.270847	UBE2D2	Ubiquitin-c	UB2D2_HU	2	1.034789
15min_MT.	O43488	0.155357	0.253553	0.270868	AKR7A2	Aflatoxin B	ARK72_HUI	6	1.113697
15min_MT.	Q9H2H8	0.031276	0.253646	0.270929	PPIL3	Peptidyl-pr	PPIL3_HUM	5	1.021916
15min_MT.	O00115	0.087703	0.253804	0.270946	DNASE2	Deoxyribor	DNS2A_HU	6	1.062677
30min_MT.	P52895	-0.43057	0.253751	0.270946	AKR1C2	Aldo-keto r	AK1C2_HUI	5	0.74197
15min_MT.	Q12972	-0.01229	0.253789	0.270946	PPP1R8	Nuclear inh	PP1R8_HUI	7	0.991521
30min_MT.	Q96JM3	-0.18553	0.253802	0.270946	CHAMP1	Chromosor	CHAP1_HU	9	0.879324
30min_MT.	Q96KR1	0.036688	0.253834	0.270946	ZFR	Zinc finger	ZFR_HUMA	22	1.025756
30min_MT.	O95989	0.030761	0.254044	0.271032	NUDT3	Diphospho	NUDT3_HU	6	1.021551
30min_MT.	P28331	-0.07596	0.254019	0.271032	NDUFS1	NADH-ubiq	NDUS1_HU	19	0.948712
30min_MT.	P55268	0.048031	0.254027	0.271032	LAMB2	Laminin sul	LAMB2_HU	21	1.033853

15min_MT.	Q9H2U1	-0.08933	0.254054	0.271032	DHX36	ATP-depend	DHX36_HU	10	0.939957
30min_MT.	P49336;Q9	0.26594	0.254162	0.271074	CDK8;CDK1	Cyclin-depe	CDK8_HUM	1	1.202419
30min_MT.	Q9Y4B6	0.15793	0.254209	0.271087	DCAF1	DDB1- and	DCAF1_HU	2	1.115685
15min_MT.	O60907	-0.06422	0.25448	0.2712	TBL1X	F-box-like/	TBL1X_HUI	9	0.956459
30min_MT.	P09668	0.124227	0.254489	0.2712	CTSH	Pro-cathep	CATH_HUM	5	1.089923
30min_MT.	Q8WV22	0.259689	0.254452	0.2712	NSMCE1	Non-structu	NSE1_HUM	1	1.19722
15min_MT.	O00170	0.040098	0.25467	0.271356	AIP	AH recepto	AIP_HUMA	10	1.028184
30min_MT.	Q7Z2T5	-0.34163	0.254728	0.271381	TRMT1L	TRMT1-like	TRM1L_HU	5	0.789148
15min_MT.	P51948	0.184781	0.254984	0.271581	MNAT1	CDK-activat	MAT1_HUM	3	1.136645
30min_MT.	O15027	-0.26742	0.255104	0.271671	SEC16A	Protein tra	SC16A_HUI	16	0.830803
15min_MT.	Q9Y5P4	-0.0641	0.255173	0.271671	CERT1	Ceramide tr	CERT_HUM	14	0.956542
30min_MT.	P27824	0.137838	0.255226	0.27169	CANX	Calnexin	CALX_HUM	26	1.100255
15min_MT.	Q9Y5J7	0.189053	0.25552	0.271929	TIMM9	Mitochond	TIM9_HUM	6	1.140015
30min_MT.	P35900	-1.20283	0.255605	0.271983	KRT20	Keratin, typ	K1C20_HU	1	0.434423
30min_MT.	Q9BXW7	-0.04348	0.255814	0.272141	HDHD5	Haloacid de	HDHD5_HU	5	0.970314
15min_MT.	O60231	0.163422	0.256019	0.272275	DHX16	Pre-mRNA-	DHX16_HU	4	1.119941
15min_MT.	O43752	0.391813	0.256147	0.272337	STX6	Syntaxin-6	STX6_HUM	2	1.312041
30min_MT.	Q96MU7	0.097214	0.256137	0.272337	YTHDC1	YTH domair	YTDC1_HUI	2	1.069706
30min_MT.	P62701	0.081598	0.256273	0.27243	RPS4X	Small ribos	RS4X_HUM	10	1.05819
15min_MT.	Q9NTN9	0.053217	0.256305	0.27243	SEMA4G	Semaphori	SEM4G_HU	14	1.037576
15min_MT.	P80303	0.018097	0.256385	0.272479	NUCB2	Nucleobinc	NUCB2_HU	12	1.012623
15min_MT.	O60493	0.060045	0.256511	0.272522	SNX3	Sorting nex	SNX3_HUM	8	1.042498
30min_MT.	Q9H5H4	0.906581	0.256565	0.272522	ZNF768	Zinc finger	ZN768_HUI	1	1.874597
15min_MT.	Q5VWZ2	0.099198	0.256792	0.272652	LYPLAL1	Lysophospl	LYPL1_HUN	7	1.071178
15min_MT.	Q9NZ63	-0.18496	0.256908	0.272701	C9orf78	Splicing fac	TLS1_HUM	2	0.879676
15min_MT.	Q8TAG9	0.172598	0.257316	0.272986	EXOC6	Exocyst cor	EXOC6_HUI	1	1.127086
15min_MT.	P62241	-0.05663	0.25769	0.273205	RPS8	Small ribos	RS8_HUMA	8	0.961509
15min_MT.	Q8TDH9	0.297196	0.257908	0.273391	BLOC1S5	Biogenesis	BL1S5_HUN	1	1.228754
30min_MT.	Q9NZP8	-0.03419	0.257958	0.273408	C1RL	Compleme	C1RL_HUM	1	0.976579
15min_MT.	Q8N6N6	-0.03197	0.25821	0.273601	NATD1	Protein NA	NATD1_HU	2	0.978086
30min_MT.	O75150	-0.24365	0.258311	0.273645	RNF40	E3 ubiquiti	BRE1B_HUI	7	0.844606
15min_MT.	Q8IYB1	-0.14187	0.258351	0.273645	MB21D2	Nucleotidy	M21D2_HU	10	0.906344
15min_MT.	P09543	0.013497	0.258596	0.273824	CNP	2',3'-cyclic-	CN37_HUM	15	1.0094
15min_MT.	Q5VZ89	0.061758	0.259102	0.274153	DENND4C	DENN dom	DEN4C_HU	12	1.043737
30min_MT.	Q9NZN5	-0.17297	0.259349	0.274324	ARHGEF12	Rho guanin	ARHGC_HU	33	0.887018
30min_MT.	O75818	0.129797	0.259461	0.274369	RPP40	Ribonuclea	RPP40_HUI	9	1.09414
30min_MT.	P09132	0.270248	0.259543	0.274418	SRP19	Signal reco	SRP19_HUI	2	1.206015
30min_MT.	P18669	0.174799	0.259643	0.274475	PGAM1	Phosphogly	PGAM1_HU	20	1.128807
15min_MT.	Q15149	0.063193	0.259942	0.274683	PLEC	Plectin	PLEC_HUM	144	1.044775
30min_MT.	Q9Y6G9	-0.01358	0.259969	0.274683	DYNC1LI1	Cytoplasmi	DC1L1_HUI	20	0.990635
30min_MT.	P60484	-0.03644	0.260065	0.274701	PTEN	Phosphatid	PTEN_HUM	2	0.975059
30min_MT.	Q9BV20	0.006909	0.260098	0.274701	MRI1	Methylthio	MTNA_HUN	19	1.004801
15min_MT.	Q9H0C8	-0.22637	0.260106	0.274701	ILKAP	Integrin-lin	ILKAP_HUN	11	0.854786
15min_MT.	O76054	-0.04173	0.260194	0.274713	SEC14L2	SEC14-like	S14L2_HUN	11	0.971488
30min_MT.	Q8N8A2	0.047648	0.260215	0.274713	ANKRD44	Serine/thre	ANR44_HU	3	1.033579
30min_MT.	Q9ULX6	-0.11216	0.260244	0.274713	AKAP8L	A-kinase an	AKP8L_HUI	3	0.925201
15min_MT.	P61254	-0.07193	0.260531	0.27491	RPL26	Large ribos	RL26_HUM	6	0.951362
30min_MT.	P31431	0.43071	0.260582	0.274922	SDC4	Syndecan-4	SDC4_HUM	2	1.347897
15min_MT.	Q15428	0.166358	0.260733	0.27501	SF3A2	Splicing fac	SF3A2_HUN	5	1.122222
30min_MT.	Q9H974	0.04413	0.260736	0.27501	QTRT2	Queuine tR	QTRT2_HUI	1	1.031062
30min_MT.	O60231	0.152281	0.260947	0.275159	DHX16	Pre-mRNA-	DHX16_HU	4	1.111325
30min_MT.	Q9H5V9	-0.41696	0.260939	0.275159	STEEP1	STING ER ex	STEEP_HUN	1	0.749003

15min_MT.	P62879	-0.19806	0.261333	0.275507	GNB2	Guanine nu	GBB2_HUM		9	0.871723
15min_MT.	Q13310	0.079759	0.261347	0.275507	PABPC4	Polyadenyl	PABP4_HUI		8	1.056841
15min_MT.	Q6DKK2	0.096759	0.261548	0.275643	TTC19	Tetratricop	TTC19_HUI		5	1.069368
30min_MT.	P62328	0.299224	0.261695	0.27575	TMSB4X	Thymosin b	TYB4_HUM		2	1.230483
30min_MT.	Q9BXB4	-0.34295	0.261719	0.27575	OSBPL11	Oxysterol-b	OSB11_HUI		2	0.788426
30min_MT.	P39748	-0.24282	0.261806	0.275767	FEN1	Flap endon	FEN1_HUM		4	0.845094
30min_MT.	Q9NQ29	-0.02781	0.261787	0.275767	LUC7L	Putative RN	LUC7L_HUI		8	0.980909
15min_MT.	P15927	0.044264	0.261851	0.275778	RPA2	Replication	RFA2_HUM		6	1.031157
30min_MT.	Q15477	-0.22742	0.261977	0.275873	SKIC2	Superkiller	SKI2_HUM/		3	0.854163
15min_MT.	Q9BQC3	0.26063	0.262324	0.275987	DPH2	2-(3-amino	DPH2_HUM		4	1.198002
15min_MT.	Q9BSB4	0.436082	0.262332	0.275987	ATG101	Autophagy-	ATGA1_HUI		2	1.352926
30min_MT.	Q9BTX7	0.452615	0.262292	0.275987	TTPAL	Alpha-toco	TTPAL_HUM		1	1.368518
15min_MT.	Q9UMY1	0.011218	0.262281	0.275987	NOL7	U3 small nu	UTP16_HUI		2	1.007806
15min_MT.	P04424	0.046277	0.26259	0.27611	ASL	Argininosu	ARLY_HUM		19	1.032597
15min_MT.	P51397	-0.10705	0.2628	0.276256	DAP	Death-asso	DAP1_HUM		2	0.928487
15min_MT.	Q96NL8	0.237454	0.263089	0.276522	CFAP418	Cilia- and fl	CF418_HUI		4	1.178911
15min_MT.	P17676	0.767557	0.263171	0.276548	CEBPB	CCAAT/enh	CEBPB_HUI		1	1.702385
15min_MT.	Q9Y5M8	-0.53097	0.263184	0.276548	SRPRB	Signal reco	SRPRB_HUI		5	0.692088
15min_MT.	P31327	0.051754	0.263266	0.276597	CPS1	Carbamoyl-	CPSM_HUM		83	1.036524
15min_MT.	Q14137	-0.77001	0.263436	0.276739	BOP1	Ribosome b	BOP1_HUM		3	0.586412
15min_MT.	P09497	0.126428	0.263515	0.276785	CLTB	Clathrin lig	CLCB_HUM		10	1.091588
30min_MT.	P51116	-0.81886	0.263694	0.276935	FXR2	RNA-bindir	FXR2_HUM		4	0.566888
30min_MT.	Q13616	0.021307	0.264038	0.277124	CUL1	Cullin-1	CUL1_HUM		34	1.014878
15min_MT.	Q5TFE4	-0.00071	0.263959	0.277124	NT5DC1	5'-nucleoti	NT5D1_HU		12	0.999508
15min_MT.	Q96FQ6	0.07249	0.264017	0.277124	S100A16	Protein S1(	S10AG_HUI		6	1.05153
15min_MT.	Q9NSK7	0.349762	0.264105	0.277143	C19orf12	Protein C1(	CS012_HUI		3	1.27435
30min_MT.	P08865	0.087188	0.264334	0.277196	RPSA	Small ribos	RSSA_HUM		11	1.062297
15min_MT.	P56537	0.044646	0.26427	0.277196	EIF6	Eukaryotic	IF6_HUMA		8	1.03143
30min_MT.	Q8TC12	-0.40347	0.264367	0.277196	RDH11	Retinol deh	RDH11_HU		3	0.756037
30min_MT.	Q96PK6	-0.1416	0.264329	0.277196	RBM14	RNA-bindir	RBM14_HU		15	0.906513
30min_MT.	Q9NX55	0.263054	0.264308	0.277196	HYPK	Huntingtin	HYPK_HUM		2	1.200017
15min_MT.	A4D1P6	0.125093	0.264557	0.277285	WDR91	WD repeat-	WDR91_HU		10	1.090578
15min_MT.	Q9NS91	0.095986	0.264594	0.277285	RAD18	E3 ubiquiti	RAD18_HU		6	1.068796
30min_MT.	Q13895	-0.19386	0.264853	0.277482	BYSL	Bystin	BYST_HUM/		8	0.874261
30min_MT.	P59780	-0.12518	0.265021	0.277584	AP3S2	AP-3 compl	AP3S2_HUI		1	0.91689
15min_MT.	O00541	-0.26154	0.265131	0.277603	PES1	Pescadillo I	PESC_HUM		4	0.834199
15min_MT.	Q02543	0.030701	0.265165	0.277603	RPL18A	Large ribos	RL18A_HUI		2	1.021508
15min_MT.	Q5JVF3	-0.23118	0.265077	0.277603	PCID2	PCI domair	PCID2_HUI		5	0.851937
30min_MT.	Q8WUH1	-0.14612	0.265182	0.277603	CHURC1	Protein Chu	CHUR_HUM		2	0.903676
15min_MT.	Q9H1H9	-0.08504	0.26527	0.277659	KIF13A	Kinesin-like	KI13A_HUM		9	0.94276
15min_MT.	P00403	0.509953	0.26545	0.277809	MT-CO2	Cytochrom	COX2_HUM		1	1.424003
15min_MT.	P05204	0.345511	0.265578	0.277869	HMGN2	Non-histon	HMGN2_HU		2	1.270601
15min_MT.	Q02809	-0.07515	0.26557	0.277869	PLOD1	Procollager	PLOD1_HU		15	0.949241
30min_MT.	P21333	0.039365	0.265705	0.277965	FLNA	Filamin-A	FLNA_HUM		110	1.027662
30min_MT.	O14745	0.136765	0.265904	0.277992	NHERF1	Na(+)/H(+) e	NHRF1_HU		23	1.099437
30min_MT.	P26599	-0.09437	0.265814	0.277992	PTBP1	Polypyrimi	PTBP1_HUI		9	0.936684
15min_MT.	P32320	-0.09548	0.265908	0.277992	CDA	Cytidine de	CDD_HUM/		2	0.935964
15min_MT.	Q01658	0.208873	0.265895	0.277992	DR1	Protein Dr1	NC2B_HUM		4	1.155785
15min_MT.	Q9H6Z4	0.079807	0.265811	0.277992	RANBP3	Ran-bindin	RANB3_HU		22	1.056876
15min_MT.	Q9UK22	0.062908	0.266022	0.278073	FBXO2	F-box only	FBX2_HUM		8	1.044569
15min_MT.	Q8TF05	0.127836	0.266117	0.278129	PPP4R1	Serine/thre	PP4R1_HUI		11	1.092653
30min_MT.	Q96F44	-0.05708	0.266146	0.278129	TRIM11	E3 ubiquiti	TRI11_HUM		2	0.961209

15min_MT.	P86790;P8	-0.00348	0.266185	0.278132	CCZ1B;CCZ	Vacuolar fu	CCZ1B_HU	17	0.997588
30min_MT.	Q13148	-0.05822	0.266328	0.278208	TARDBP	TAR DNA-bi	TADBP_HU	7	0.960449
30min_MT.	O43353	-0.07392	0.266391	0.278236	RIPK2	Receptor-ir	RIPK2_HUN	15	0.950054
30min_MT.	O95822	0.068667	0.266957	0.278679	MLYCD	Malonyl-Cc	DCMC_HUN	11	1.048747
15min_MT.	Q13480	-0.13187	0.266896	0.278679	GAB1	GRB2-assoc	GAB1_HUM	2	0.912645
15min_MT.	Q92990	0.128219	0.266945	0.278679	GLMN	Glomulin	GLMN_HUN	10	1.092944
30min_MT.	P55854	0.153623	0.267493	0.279089	SUMO3	Small ubiqu	SUMO3_HU	2	1.112359
30min_MT.	P23921	-0.09413	0.26762	0.27911	RRM1	Ribonuclec	RIR1_HUM.	7	0.936838
15min_MT.	Q96B23	0.04939	0.26757	0.27911	ARK2N	Protein ARI	ARK2N_HU	6	1.034828
30min_MT.	O75157	-0.21085	0.267769	0.279228	TSC22D2	TSC22 dom	T22D2_HU	4	0.864027
30min_MT.	Q06210	-0.03853	0.26791	0.2793	GFPT1	Glutamine-	GFPT1_HU	30	0.973644
30min_MT.	P06703	-0.5088	0.268116	0.279416	S100A6	Protein S1(	S10A6_HU	4	0.702805
15min_MT.	P42566	0.16075	0.268127	0.279416	EPS15	Epidermal ε	EPS15_HUN	26	1.117868
15min_MT.	Q7Z3Y7	-0.50123	0.268219	0.279454	KRT28	Keratin, typ	K1C28_HU	2	0.706503
30min_MT.	Q9Y3Y2	-0.30441	0.268235	0.279454	CHTOP	Chromatin	CHTOP_HU	3	0.809771
30min_MT.	O43488	-0.13617	0.268468	0.27951	AKR7A2	Aflatoxin B	ARK72_HU	6	0.909929
15min_MT.	P30085	0.106871	0.268428	0.27951	CMPK1	UMP-CMP I	KCY_HUMA	10	1.07689
15min_MT.	Q16563	-0.09528	0.268383	0.27951	SYPL1	Synaptoph	SYPL1_HUN	2	0.936089
15min_MT.	P35908	-0.13907	0.268577	0.279549	KRT2	Keratin, typ	K22E_HUM	40	0.908102
30min_MT.	Q9Y5P6	0.000485	0.268553	0.279549	GMPPB	Mannose-1	GMPPB_HU	9	1.000336
30min_MT.	Q15286	-0.0438	0.268754	0.279659	RAB35	Ras-related	RAB35_HU	4	0.970098
30min_MT.	Q495W5	-0.67475	0.268744	0.279659	FUT11	Alpha-(1,3)	FUT11_HU	1	0.626441
30min_MT.	Q02818	0.265777	0.268879	0.279677	NUCB1	Nucleobinc	NUCB1_HU	8	1.202284
30min_MT.	Q9H2W6	0.495304	0.268868	0.279677	MRPL46	Large ribos	RM46_HUN	2	1.409617
15min_MT.	O43903	0.082873	0.26893	0.279693	GAS2	Growth arr	GAS2_HUM	8	1.059125
30min_MT.	Q13617	-0.07219	0.269012	0.279704	CUL2	Cullin-2	CUL2_HUM	18	0.951192
15min_MT.	Q9H777	0.325981	0.268986	0.279704	ELAC1	Zinc phosp	RNZ1_HUM	3	1.253516
30min_MT.	Q5XPI4	-0.29416	0.269131	0.279761	RNF123	E3 ubiquiti	RN123_HU	2	0.815547
15min_MT.	Q9NZW5	0.186982	0.269138	0.279761	PALS2	Protein PA	PALS2_HUN	9	1.13838
30min_MT.	O00764	0.165471	0.269387	0.279923	PDXK	Pyridoxal k	PDXK_HUM	10	1.121532
30min_MT.	P50225	-0.08599	0.269402	0.279923	SULT1A1	Sulfotransf	ST1A1_HUN	5	0.94214
15min_MT.	Q15233	-0.04998	0.2699	0.280292	NONO	Non-POU d	NONO_HUN	25	0.96595
15min_MT.	Q9UPN3	-0.02405	0.269879	0.280292	MACF1	Microtubul	MACF1_HU	142	0.983465
30min_MT.	Q8IY31	-0.26817	0.270235	0.280528	IFT20	Intraflagell	IFT20_HUM	1	0.830374
30min_MT.	Q86YB8	-0.30023	0.270292	0.28055	ERO1B	ERO1-like p	ERO1B_HU	6	0.812122
30min_MT.	Q9Y5X2	-0.20897	0.270407	0.280595	SNX8	Sorting nex	SNX8_HUM	13	0.865155
30min_MT.	Q12965	-0.06152	0.270536	0.280691	MYO1E	Unconvent	MYO1E_HU	12	0.958251
15min_MT.	Q00059	0.032867	0.270728	0.280817	TFAM	Transcripti	TFAM_HUM	7	1.023043
30min_MT.	Q96BN8	0.033173	0.270826	0.280843	OTULIN	Ubiquitin t	OTUL_HUM	8	1.02326
15min_MT.	P04406	-0.08953	0.270958	0.280906	GAPDH	Glyceraldel	G3P_HUMA	20	0.939827
15min_MT.	Q99956	-0.05875	0.271034	0.280945	DUSP9	Dual specifi	DUS9_HUM	4	0.960094
15min_MT.	Q9H299	0.176353	0.271068	0.280945	SH3BGRL3	SH3 domai	SH3L3_HUI	4	1.130024
15min_MT.	P37840	-0.12378	0.271655	0.281441	SNCA	Alpha-synu	SYUA_HUM	5	0.917779
15min_MT.	O95994	0.16691	0.271886	0.281532	AGR2	Anterior gr	AGR2_HUM	4	1.122651
30min_MT.	P30049	0.07476	0.272011	0.281575	ATP5F1D	ATP synthas	ATPD_HUM	3	1.053186
30min_MT.	P53618	-0.0581	0.271975	0.281575	COPB1	Coatomer s	COPB_HUM	39	0.960528
15min_MT.	P20700	0.158265	0.272232	0.281703	LMNB1	Lamin-B1	LMNB1_HU	10	1.115944
30min_MT.	Q9UGP4	-0.12239	0.272275	0.281711	LIMD1	LIM domair	LIMD1_HU	14	0.918664
15min_MT.	Q8TAE8	0.749621	0.272317	0.281717	GADD45GI	Large ribos	G45IP_HUN	2	1.681351
30min_MT.	Q15365	-0.08965	0.272452	0.281783	PCBP1	Poly(rC)-bir	PCBP1_HU	9	0.939749
15min_MT.	Q5VYK3	-0.0654	0.272727	0.281917	ECPAS	Proteasom	ECM29_HU	17	0.95568
30min_MT.	P11172	-0.07789	0.27316	0.282328	UMPS	Uridine 5'-r	UMPS_HUN	8	0.947444

15min_MT.	O00186	-0.07234	0.273361	0.282461	STXBP3	Syntaxin-bi	STXB3_HUM	12	0.951097
30min_MT.	Q66PJ3	-0.05492	0.27336	0.282461	ARL6IP4	ADP-ribosy	AR6P4_HUI	5	0.96265
30min_MT.	P25205	-0.07437	0.273441	0.282506	MCM3	DNA replica	MCM3_HUI	27	0.949757
30min_MT.	Q9H7Z6	-0.15178	0.27353	0.282532	KAT8	Histone ace	KAT8_HUM	2	0.900139
15min_MT.	O00625	0.047449	0.273748	0.282535	PIR	Pirin	PIR_HUMAI	11	1.033436
15min_MT.	P56192	-0.06237	0.273758	0.282535	MARS1	Methionine	SYMC_HUM	27	0.957691
30min_MT.	Q14116	0.133627	0.273625	0.282535	IL18	Interleukin	IL18_HUM/	10	1.097048
15min_MT.	Q8N9N2	0.28404	0.273723	0.282535	ASCC1	Activating s	ASCC1_HUI	3	1.2176
15min_MT.	Q6UX53	-0.0376	0.273921	0.282597	TMT1B	Thiol S-met	TMT1B_HU	2	0.974276
30min_MT.	O94916	-0.34947	0.274325	0.282811	NFAT5	Nuclear fac	NFAT5_HUI	6	0.784874
30min_MT.	P08134	0.019888	0.274307	0.282811	RHOC	Rho-relatec	RHOC_HUM	5	1.013881
15min_MT.	P48739	0.040794	0.274357	0.282811	PITPNB	Phosphatid	PIPNB_HUM	16	1.02868
15min_MT.	P61956	0.107659	0.274495	0.282811	SUMO2	Small ubiqu	SUMO2_HU	1	1.077479
30min_MT.	Q96FK6	-0.36711	0.274257	0.282811	WDR89	WD repeat-	WDR89_HU	3	0.775333
30min_MT.	Q9NPQ8	-0.05187	0.27446	0.282811	RIC8A	Synembryn	RIC8A_HUM	13	0.964686
30min_MT.	Q8TC07	-0.16771	0.274645	0.282891	TBC1D15	TBC1 doma	TBC15_HUI	19	0.890254
30min_MT.	Q15025	0.338143	0.274719	0.282929	TNIP1	TNFAIP3-in	TNIP1_HUM	2	1.264128
30min_MT.	P62424	0.330137	0.274897	0.282984	RPL7A	Large ribos	RL7A_HUM	8	1.257133
30min_MT.	Q9NV06	0.110447	0.274916	0.282984	DCAF13	DDB1- and	DCA13_HU	1	1.079563
30min_MT.	P01037	-0.17905	0.275139	0.282986	CST1	Cystatin-SN	CYTN_HUM	1	0.883283
15min_MT.	Q5D862	-0.13547	0.275171	0.282986	FLG2	Filaggrin-2	FILA2_HUM	16	0.910374
30min_MT.	Q96NC0	-0.55221	0.27516	0.282986	ZMAT2	Zinc finger	ZMAT2_HU	1	0.681974
30min_MT.	Q9NS86	0.032329	0.27512	0.282986	LANCL2	LanC-like pi	LANC2_HUI	13	1.022662
30min_MT.	Q9Y6G5	0.08203	0.275131	0.282986	COMMD10	COMM don	COMDA_HU	5	1.058507
15min_MT.	P08559	0.178245	0.275264	0.283007	PDHA1	Pyruvate de	ODPA_HUM	15	1.131507
30min_MT.	Q9H7N4	0.113765	0.275378	0.283012	SCAF1	Splicing fac	SFR19_HUM	1	1.082048
30min_MT.	Q9Y4L1	-0.02783	0.275516	0.283042	HYOU1	Hypoxia up	HYOU1_HU	44	0.980896
15min_MT.	P49736	-0.10481	0.275594	0.283048	MCM2	DNA replica	MCM2_HUI	17	0.929926
15min_MT.	Q92598	0.093387	0.275644	0.283063	HSPH1	Heat shock	HS105_HUI	33	1.066872
15min_MT.	P49247	-0.12897	0.275772	0.283083	RPIA	Ribose-5-pl	RPIA_HUM/	8	0.914482
15min_MT.	P50225	0.141151	0.275773	0.283083	SULT1A1	Sulfotransf	ST1A1_HUM	5	1.102784
30min_MT.	P29558	-0.44913	0.275956	0.283197	RBMS1	RNA-bindir	RBMS1_HU	2	0.732483
15min_MT.	Q5T0N5	0.185065	0.276477	0.283509	FNBP1L	Formin-bin	FBP1L_HUM	10	1.136868
30min_MT.	Q9NZ52	-0.17137	0.276465	0.283509	GGA3	ADP-ribosy	GGA3_HUM	10	0.887998
15min_MT.	O15111	0.822541	0.277067	0.283964	CHUK	Inhibitor o	IKKA_HUM/	1	1.768518
30min_MT.	Q9H3S4	0.096573	0.277207	0.284071	TPK1	Thiamine p	TPK1_HUM	3	1.069231
15min_MT.	Q99622	-0.07289	0.277532	0.284309	C12orf57	Protein C1(	C10_HUMA	3	0.950729
30min_MT.	P22033	0.006958	0.277706	0.284359	MMUT	Methylmal	MUTA_HUM	22	1.004835
30min_MT.	Q13952	0.162882	0.277654	0.284359	NFYC	Nuclear tra	NFYC_HUM	3	1.119521
15min_MT.	Q14181	-0.04803	0.277691	0.284359	POLA2	DNA polym	DPOA2_HU	8	0.967257
30min_MT.	Q9NQ88	-0.01716	0.27799	0.284613	TIGAR	Fructose-2,	TIGAR_HUM	5	0.988173
30min_MT.	Q969T9	0.07491	0.278104	0.284691	WBP2	WW domai	WBP2_HUM	5	1.053295
30min_MT.	Q9P2E9	-0.07391	0.27832	0.284817	RRBP1	Ribosome-l	RRBP1_HUI	57	0.950057
30min_MT.	Q9UMY1	0.256852	0.278429	0.284866	NOL7	U3 small nu	UTP16_HUI	2	1.194869
30min_MT.	P62633	-0.29573	0.278669	0.285009	CNBP	CCHC-type	CNBP_HUM	3	0.814659
30min_MT.	P06280	-0.00986	0.278775	0.285043	GLA	Alpha-galac	AGAL_HUM	7	0.993189
15min_MT.	P31946	0.061433	0.278855	0.285087	YWHAB	14-3-3 prot	1433B_HUI	13	1.043502
30min_MT.	Q8ND56	0.216964	0.279126	0.28529	LSM14A	Protein LSN	LS14A_HUM	4	1.162285
15min_MT.	Q9HAN9	0.471105	0.27909	0.28529	NMNAT1	Nicotinami	NMNA1_HU	2	1.386171
30min_MT.	Q9Y4E8	0.007457	0.279309	0.285402	USP15	Ubiquitin c	UBP15_HU	26	1.005182
15min_MT.	O43813	-0.03661	0.279516	0.285459	LANCL1	Glutathion	LANC1_HUI	12	0.974942
30min_MT.	P09497	0.14192	0.279569	0.285459	CLTB	Clathrin lig	CLCB_HUM	10	1.103373

30min_MT.	P10809	0.065979	0.27959	0.285459	HSPD1	60 kDa hea	CH60_HUM	40	1.046795
30min_MT.	P62070	0.256769	0.27948	0.285459	RRAS2	Ras-related	RRAS2_HU	6	1.1948
30min_MT.	Q9H8H3	-0.24496	0.27962	0.285459	TMT1A	Thiol S-met	TMT1A_HU	3	0.843836
15min_MT.	Q5VW36	-0.30966	0.279701	0.285467	FOCAD	Focadhesin	FOCAD_HU	7	0.806832
30min_MT.	Q9BUI4	0.115851	0.279675	0.285467	POLR3C	DNA-direct	RPC3_HUM	7	1.083614
15min_MT.	P07900	0.054298	0.279738	0.285468	HSP90AA1	Heat shock	HS90A_HU	25	1.038354
30min_MT.	Q12846	0.034629	0.279803	0.285497	STX4	Syntaxin-4	STX4_HUM	7	1.024294
15min_MT.	Q02252	0.084331	0.279844	0.285501	ALDH6A1	Methylmal	MMSA_HU	15	1.060196
15min_MT.	O76075	0.338417	0.280136	0.285686	DFFB	DNA fragm	DFFB_HUM	2	1.264369
15min_MT.	P46063	-0.02843	0.280163	0.285686	RECQL	ATP-depen	RECQ1_HU	19	0.980487
15min_MT.	P78310	-0.09214	0.280171	0.285686	CXADR	Coxsackiev	CXAR_HUM	7	0.938131
15min_MT.	P19971	0.057703	0.280317	0.285691	TYMP	Thymidine	TYPH_HUM	16	1.040807
15min_MT.	Q9BTC0	-0.12361	0.280235	0.285691	DIDO1	Death-indu	DIDO1_HU	17	0.917886
30min_MT.	Q9NRW1	-0.28926	0.280322	0.285691	RAB6B	Ras-related	RAB6B_HU	2	0.818321
15min_MT.	P13645	-0.14047	0.280421	0.285717	KRT10	Keratin, typ	K1C10_HU	30	0.907226
15min_MT.	Q9H467	0.411132	0.280555	0.285782	CUEDC2	CUE domai	CUED2_HU	2	1.329729
30min_MT.	P10909	0.144917	0.280743	0.285822	CLU	Clusterin	CLUS_HUM	5	1.105667
30min_MT.	P57721	-0.00427	0.28073	0.285822	PCBP3	Poly(rC)-bi	PCBP3_HU	2	0.997042
30min_MT.	Q14247	0.119123	0.28069	0.285822	CTTN	Src substra	SRC8_HUM	24	1.086074
15min_MT.	Q13177	0.126259	0.280891	0.285936	PAK2	Serine/thre	PAK2_HUM	18	1.09146
30min_MT.	Q567U6	-0.23354	0.280934	0.285943	CCDC93	Coiled-coil	CCD93_HU	2	0.850545
30min_MT.	Q3MIT2	0.091798	0.281002	0.285974	PUS10	tRNA pseud	PUS10_HU	3	1.065698
15min_MT.	B7ZAP0	-0.08289	0.281076	0.285998	RABGAP1L	Rab GTPase	RBG10_HU	5	0.944166
30min_MT.	P14927	-0.82242	0.281152	0.285998	UQCRB	Cytochrom	QCR7_HUM	1	0.565493
15min_MT.	P61586	0.00995	0.281214	0.285998	RHOA	Transformi	RHOA_HUM	2	1.006921
30min_MT.	Q7Z417	-0.05842	0.281258	0.285998	NUFIP2	FMR1-inter	NUFP2_HU	11	0.960316
30min_MT.	Q9BRT3	0.144108	0.281119	0.285998	MIEN1	Migration a	MIEN1_HU	4	1.105047
30min_MT.	P52948	-0.00859	0.281576	0.28626	NUP98	Nuclear po	NUP98_HU	18	0.994064
30min_MT.	Q96I24	-0.13286	0.281657	0.286305	FUBP3	Far upstrea	FUBP3_HU	16	0.912025
15min_MT.	P53992	-0.03633	0.281771	0.286384	SEC24C	Protein tra	SC24C_HU	23	0.975134
30min_MT.	Q14511	-0.13873	0.282227	0.286756	NEDD9	Enhancer o	CASL_HUM	1	0.908316
30min_MT.	Q96BP2	0.460394	0.282247	0.286756	CHCHD1	Small ribos	CHCH1_HU	1	1.375917
15min_MT.	O95373	0.016912	0.282467	0.286942	IPO7	Importin-7	IPO7_HUM	23	1.011791
15min_MT.	Q5VSY0	-0.50983	0.282582	0.287022	GKAP1	G kinase-an	GKAP1_HU	1	0.702307
15min_MT.	Q5JVS0	0.160611	0.28301	0.287345	HABP4	Intracellula	HABP4_HU	2	1.11776
15min_MT.	Q8N5A5	-0.06658	0.283057	0.287355	ZGPAT	Zinc finger	ZGPAT_HU	3	0.954899
30min_MT.	Q9HCJ6	0.083178	0.283141	0.287397	VAT1L	Synaptic ve	VAT1L_HU	12	1.059349
15min_MT.	P46736	0.059543	0.283209	0.287398	BRCC3	Lys-63-spec	BRCC3_HU	8	1.042136
15min_MT.	Q56P03	-0.15951	0.28338	0.287534	EAPP	E2F-associa	EAPP_HUM	1	0.895326
15min_MT.	O75439	0.158548	0.283462	0.287547	PMPCB	Mitochond	MPPB_HUM	12	1.116163
15min_MT.	P08579	0.110184	0.283476	0.287547	SNRPB2	U2 small nu	RU2B_HUM	8	1.079366
15min_MT.	Q15025	0.038137	0.28354	0.287547	TNIP1	TNFAIP3-in	TNIP1_HUM	2	1.026787
15min_MT.	Q9BUH6	-0.14257	0.283533	0.287547	PAXX	Protein PA	PAXX_HUM	8	0.905906
15min_MT.	Q16890	-0.16593	0.283581	0.287551	TPD52L1	Tumor prot	TPD53_HU	1	0.891353
15min_MT.	P00491	-0.01308	0.283743	0.287641	PNP	Purine nucl	PNPH_HUM	16	0.990977
30min_MT.	Q15007	-0.05125	0.283737	0.287641	WTAP	Pre-mRNA-	FL2D_HUM	6	0.965101
15min_MT.	Q8NC69	0.111434	0.283871	0.287733	KCTD6	BTB/POZ do	KCTD6_HU	3	1.080301
30min_MT.	Q15545	-0.45073	0.284078	0.287906	TAF7	Transcripti	TAF7_HUM	1	0.731671
15min_MT.	P40306	0.141783	0.284176	0.287968	PSMB10	Proteasom	PSB10_HU	3	1.103268
30min_MT.	O43741	0.321311	0.284357	0.288017	PRKAB2	5'-AMP-acti	AAKB2_HU	1	1.249465
15min_MT.	O43818	0.037882	0.284482	0.288017	RRP9	U3 small nu	U3IP2_HUM	10	1.026605
30min_MT.	Q8IYI6	-0.17685	0.284467	0.288017	EXOC8	Exocyst cor	EXOC8_HU	13	0.884633

30min_MT.	Q8TBF2	0.593972	0.284344	0.288017	PRXL2B	Prostamide	PXL2B_HUI	1	1.509397
30min_MT.	Q9BY49	-0.28493	0.284397	0.288017	PECR	Peroxisoma	PECR_HUM	4	0.820779
15min_MT.	O95881	-0.17843	0.284548	0.288046	TXNDC12	Thioredoxi	TXD12_HUI	7	0.883665
30min_MT.	P05455	0.075574	0.285191	0.288429	SSB	Lupus La pr	LA_HUMAN	29	1.05378
15min_MT.	P53985	-0.41323	0.285215	0.288429	SLC16A1	Monocarbc	MOT1_HUN	2	0.750941
30min_MT.	Q13620	-0.02205	0.285295	0.288429	CUL4B	Cullin-4B	CUL4B_HUI	37	0.98483
30min_MT.	Q9BWF3	-0.18631	0.285202	0.288429	RBM4	RNA-bindir	RBM4_HUN	3	0.878853
30min_MT.	O14974	0.126527	0.285441	0.288462	PPP1R12A	Protein phc	MYPT1_HU	23	1.091663
30min_MT.	Q13542	-0.06428	0.285548	0.288462	EIF4EBP2	Eukaryotic	4EBP2_HUI	2	0.956419
30min_MT.	Q9BRX2	-0.06845	0.285412	0.288462	PELO	Protein pel	PELO_HUM	7	0.953661
15min_MT.	Q9H9B1	0.004352	0.285538	0.288462	EHMT1	Histone-lys	EHMT1_HU	3	1.003021
15min_MT.	P35580	-0.10615	0.285717	0.288595	MYH10	Myosin-10	MYH10_HU	55	0.929063
30min_MT.	Q14653	-0.09038	0.28583	0.288671	IRF3	Interferon r	IRF3_HUM/	8	0.939273
15min_MT.	Q5T013	0.427108	0.285949	0.288755	HYI	Putative hy	HYI_HUMAI	3	1.344535
15min_MT.	P15144	-0.01027	0.286246	0.288815	ANPEP	Aminopept	AMPN_HUI	17	0.992905
30min_MT.	P40306	-0.36504	0.286379	0.288815	PSMB10	Proteasom	PSB10_HUI	3	0.776446
30min_MT.	P55199	-0.17708	0.286326	0.288815	ELL	RNA polym	ELL_HUMAI	4	0.884491
30min_MT.	P84101	0.128283	0.286202	0.288815	SERF2	Small EDRK	SERF2_HUN	3	1.092992
30min_MT.	Q13123	0.077014	0.286274	0.288815	IK	Protein Rec	RED_HUMA	6	1.054833
15min_MT.	Q6ZVF9	-0.04813	0.28619	0.288815	GPRIN3	G protein-r	GRIN3_HUI	4	0.96719
15min_MT.	Q96JB3	0.316949	0.286238	0.288815	HIC2	Hypermeth	HIC2_HUM	1	1.245693
30min_MT.	Q13507;Q9	-0.59305	0.286502	0.288851	TRPC3;TRP	Short trans	TRPC3_HUI	1	0.662941
30min_MT.	Q06124	0.049315	0.286747	0.288888	PTPN11	Tyrosine-pr	PTN11_HUI	17	1.034774
15min_MT.	Q14126	-0.12326	0.286852	0.288888	DSG2	Desmoglein	DSG2_HUM	18	0.918109
30min_MT.	Q8N584	0.070601	0.286608	0.288888	TTC39C	Tetratricop	TT39C_HUN	18	1.050154
15min_MT.	Q96I25	0.056432	0.286731	0.288888	RBM17	Splicing fac	SPF45_HUN	11	1.03989
30min_MT.	Q9GZT9	-0.09541	0.286868	0.288888	EGLN1	Egl nine ho	EGLN1_HUI	5	0.936005
30min_MT.	O95433	0.019318	0.286931	0.288888	AHSA1	Activator o	AHSA1_HUI	15	1.01348
15min_MT.	Q9UJX3	0.246102	0.287011	0.288932	ANAPC7	Anaphase-p	APC7_HUM	6	1.185998
30min_MT.	P98160	0.0962	0.28719	0.289029	HSPG2	Basement n	PGBM_HUN	19	1.068954
15min_MT.	Q9Y3I0	0.067124	0.287219	0.289029	RTCB	RNA-splicir	RTCB_HUM	16	1.047626
15min_MT.	Q16626	-0.21706	0.287421	0.289196	MEA1	Male-enhar	MEA1_HUN	1	0.860315
30min_MT.	Q8N3X6	0.380298	0.287638	0.28934	LCORL	Ligand-dep	LCORL_HUI	1	1.301611
30min_MT.	P06400	-0.04061	0.287705	0.28937	RB1	Retinoblast	RB_HUMAN	3	0.972244
15min_MT.	O95758	0.117473	0.287844	0.289398	PTBP3	Polypyrimi	PTBP3_HUI	3	1.084833
15min_MT.	Q15436	-0.10353	0.288141	0.289517	SEC23A	Protein trai	SC23A_HUI	21	0.930755
15min_MT.	Q15652	0.744407	0.288147	0.289517	JMJD1C	Probable Jr	JHD2C_HUI	1	1.675285
15min_MT.	A6NDG6	0.138767	0.288401	0.289698	PGP	Glycerol-3-	PGP_HUMA	14	1.100964
15min_MT.	P29083	-0.34991	0.288573	0.289833	GTF2E1	General tra	T2EA_HUM	6	0.784636
30min_MT.	P49356	0.083837	0.288675	0.289861	FNTB	Protein farr	FNTB_HUM	6	1.059833
30min_MT.	Q07283	0.490948	0.289169	0.290208	TCHH	Trichohyali	TRHY_HUM	2	1.405368
15min_MT.	Q15286	-0.16678	0.289348	0.29035	RAB35	Ras-related	RAB35_HUI	4	0.890827
15min_MT.	Q8IWS0	-0.25838	0.289523	0.290488	PHF6	PHD finger	PHF6_HUM	4	0.836028
30min_MT.	O95336	0.062483	0.2896	0.290529	PGLS	6-phospho	6PGL_HUM	13	1.044261
30min_MT.	Q8N0U4	0.274074	0.289804	0.290659	FAM185A	Protein FAI	F185A_HUI	6	1.209217
15min_MT.	P31948	0.122623	0.290043	0.290861	STIP1	Stress-indu	STIP1_HUM	39	1.088712
30min_MT.	Q14997	-0.29906	0.290133	0.290914	PSME4	Proteasom	PSME4_HU	8	0.812781
15min_MT.	P05388	0.040644	0.290215	0.290959	RPLP0	Large ribos	RLA0_HUM	11	1.028573
30min_MT.	Q93008	-0.03849	0.290265	0.290972	USP9X	Ubiquitin c	USP9X_HUI	47	0.973673
30min_MT.	Q9Y6W5	0.040669	0.290513	0.291146	WASF2	Actin-bindi	WASF2_HU	7	1.028591
30min_MT.	P30837	0.058335	0.290631	0.291189	ALDH1B1	Aldehyde d	AL1B1_HUI	14	1.041263
15min_MT.	Q9H444	0.165895	0.290625	0.291189	CHMP4B	Charged mu	CHM4B_HU	10	1.121862

15min_MT.	Q9NRN9	-0.05807	0.290699	0.29122	METTL5	rRNA N6-ad	METL5_HUI		8	0.960551
30min_MT.	Q6P5S2	-0.16857	0.29087	0.291354	LEG1	Protein LEC	LEG1H_HUI		1	0.889723
15min_MT.	Q96G61	0.10525	0.290908	0.291355	NUDT11	Diphospho	NUD11_HU		1	1.075681
30min_MT.	P51570	-0.1083	0.291084	0.291382	GALK1	Galactokin:	GALK1_HUI		12	0.927678
15min_MT.	P51687	0.022967	0.291036	0.291382	SUOX	Sulfite oxid	SUOX_HUM		14	1.016047
30min_MT.	Q9Y315	0.188414	0.291051	0.291382	DERA	Deoxyribos	DEOC_HUM		11	1.13951
15min_MT.	P61018	-0.20516	0.291245	0.291414	RAB4B	Ras-related	RAB4B_HUI		2	0.867443
15min_MT.	Q3MIT2	0.345106	0.291314	0.291414	PUS10	tRNA pseuc	PUS10_HUI		3	1.270245
30min_MT.	Q8N1B4	-0.3748	0.29134	0.291414	VPS52	Vacuolar pı	VPS52_HUI		4	0.771214
15min_MT.	Q6GYQ0	-0.01536	0.291507	0.291507	RALGAPA1	Ral GTPase-	RGPA1_HU		12	0.989408
30min_MT.	Q9NWU5	0.135996	0.291482	0.291507	MRPL22	Large ribos	RM22_HUM		1	1.098851
30min_MT.	O60333	-0.09456	0.29182	0.291617	KIF1B	Kinesin-like	KIF1B_HUM		4	0.936558
30min_MT.	P37108	-0.03753	0.291694	0.291617	SRP14	Signal reco	SRP14_HUI		7	0.974324
15min_MT.	Q8IWJ2	0.138659	0.291665	0.291617	GCC2	GRIP and cc	GCC2_HUM		40	1.100881
15min_MT.	Q9UKU7	0.107634	0.291752	0.291617	ACAD8	Isobutyryl-	ACAD8_HU		13	1.07746
30min_MT.	Q9H9B1	-0.35867	0.291943	0.291676	EHMT1	Histone-lys	EHMT1_HU		3	0.779881
30min_MT.	E9PAV3;Q1	0.056803	0.29231	0.291937	NACA	Nascent po	NACAM_HU		7	1.040158
15min_MT.	Q9Y305	0.05228	0.292558	0.292147	ACOT9	Acyl-coenzy	ACOT9_HUI		11	1.036903
15min_MT.	E9PAV3;Q1	0.103526	0.29299	0.292429	NACA	Nascent po	NACAM_HU		7	1.074396
15min_MT.	P60903	0.091578	0.292985	0.292429	S100A10	Protein S1(	S10AA_HUI		4	1.065535
15min_MT.	Q9NZQ3	0.15497	0.292963	0.292429	NCKIPSD	NCK-intera	SPN90_HUI		2	1.113399
30min_MT.	Q96CP2	-0.12297	0.293062	0.292439	FLYWCH2	FLYWCH far	FWCH2_HU		7	0.918293
15min_MT.	P00390	-0.06252	0.293219	0.292439	GSR	Glutathion	GSHR_HUM		19	0.957587
15min_MT.	P55735	-0.03875	0.293253	0.292439	SEC13	Protein SEC	SEC13_HUI		11	0.973499
30min_MT.	Q14739	-0.06804	0.293129	0.292439	LBR	Delta(14)-s	LBR_HUMA		3	0.953932
15min_MT.	O14980	-0.12304	0.293637	0.292644	XPO1	Exportin-1	XPO1_HUM		24	0.918251
15min_MT.	Q9BUK0	-0.04598	0.293648	0.292644	CHCHD7	Coiled-coil	CHCH7_HU		1	0.968629
30min_MT.	Q9Y2H0	0.124836	0.293654	0.292644	DLGAP4	Disks large-	DLGP4_HU		9	1.090384
30min_MT.	O43768	0.195759	0.293766	0.292718	ENSA	Alpha-endc	ENSA_HUM		7	1.145327
30min_MT.	P55789	0.001778	0.29402	0.292934	GFER	FAD-linked	ALR_HUMA		4	1.001233
30min_MT.	Q9NX40	0.175073	0.29418	0.293056	OCIAD1	OCIA doma	OCAD1_HU		1	1.129021
15min_MT.	O95999	0.211601	0.29422	0.293058	BCL10	B-cell lymp	BCL10_HUI		6	1.157973
30min_MT.	P41250	0.086821	0.294451	0.293165	GARS1	Glycine--tR	GARS_HUM		36	1.062028
15min_MT.	Q6UB35	0.087622	0.294477	0.293165	MTHFD1L	Monofunct	C1TM_HUM		14	1.062617
15min_MT.	Q96C86	-0.1233	0.294417	0.293165	DCPS	m7GpppX (	DCPS_HUM		11	0.918086
15min_MT.	Q0VAQ4	-0.04959	0.294622	0.293272	SMAGP	Small cell a	SMAGP_HU		1	0.966211
30min_MT.	Q92734	-0.12345	0.294772	0.293384	TFG	Protein TFC	TFG_HUMA		10	0.917988
15min_MT.	Q96JM3	-0.01951	0.294881	0.293456	CHAMP1	Chromosor	CHAP1_HU		9	0.986565
30min_MT.	P19022	0.052781	0.294946	0.293468	CDH2	Cadherin-2	CADH2_HU		4	1.037262
15min_MT.	Q9P270	0.000323	0.295162	0.293586	SLAIN2	SLAIN moti	SLAI2_HUM		4	1.000224
30min_MT.	Q8IZP0	-0.05619	0.295267	0.293615	ABI1	Abl interac	ABI1_HUM		6	0.9618
15min_MT.	Q09013	0.237055	0.295886	0.294156	DMPK	Myotonin-p	DMPK_HUM		1	1.178585
30min_MT.	Q04206	-0.0734	0.29669	0.294731	RELA	Transcripti	TF65_HUM		11	0.950394
30min_MT.	Q8IVS2	-0.22998	0.296829	0.294757	MCAT	Malonyl-Co	FABD_HUM		8	0.852647
30min_MT.	Q93034	-0.019	0.296786	0.294757	CUL5	Cullin-5	CUL5_HUM		26	0.986916
15min_MT.	P26639	-0.03415	0.29729	0.295176	TARS1	Threonine--	SYTC_HUM		29	0.976607
30min_MT.	P62906	-0.37926	0.297539	0.295237	RPL10A	Large ribos	RL10A_HUI		6	0.768829
30min_MT.	Q8N684	0.089155	0.297484	0.295237	CPSF7	Cleavage ar	CPSF7_HUI		13	1.063747
15min_MT.	Q9Y5K6	0.105389	0.297439	0.295237	CD2AP	CD2-associ	CD2AP_HU		21	1.075785
30min_MT.	Q14683	-0.03405	0.298052	0.295596	SMC1A	Structural	SMC1A_HU		46	0.976675
15min_MT.	P14324	-0.03785	0.298143	0.295623	FDPS	Farnesyl py	FPPS_HUM		8	0.974109
30min_MT.	O75844	0.049922	0.298456	0.295759	ZMPSTE24	CAAX preny	FACE1_HUI		4	1.035209

15min_MT.	P33176	-0.02275	0.298484	0.295759	KIF5B	Kinesin-1 h	KINH_HUM	43	0.984352
15min_MT.	Q9BVA1	0.231481	0.298528	0.295759	TUBB2B	Tubulin bet	TBB2B_HUI	1	1.17404
15min_MT.	Q9NP55	0.341163	0.298546	0.295759	BPIFA1	BPI fold-co	BPIA1_HUN	2	1.266777
30min_MT.	P06396	0.106583	0.298809	0.295934	GSN	Gelsolin	GELS_HUM	22	1.076675
30min_MT.	Q9Y450	-0.2102	0.298786	0.295934	HBS1L	HBS1-like p	HBS1L_HUI	15	0.86442
15min_MT.	Q6ULP2	0.139311	0.298931	0.296017	AFTPH	Aftiphilin	AFTIN_HUN	2	1.101379
30min_MT.	P59665;P5	0.757194	0.298984	0.296032	DEFA1;DEF.	Neutrophil	DEF1_HUM	1	1.6902
15min_MT.	P49406	0.255676	0.299235	0.296168	MRPL19	Large ribos	RM19_HUN	2	1.193895
15min_MT.	P62312	0.105319	0.299538	0.296318	LSM6	U6 snRNA-a	LSM6_HUN	5	1.075732
15min_MT.	Q96EC8	-0.11303	0.299878	0.296579	YIPF6	Protein YIP	YIPF6_HUN	2	0.924645
15min_MT.	Q9NV06	-0.25024	0.300072	0.296733	DCAF13	DDB1- and	DCA13_HU	1	0.840758
15min_MT.	P41240	0.37516	0.300337	0.296921	CSK	Tyrosine-pr	CSK_HUMA	9	1.296983
15min_MT.	P04040	0.081321	0.300431	0.296976	CAT	Catalase	CATA_HUM	23	1.057986
15min_MT.	P60880	0.473196	0.300648	0.297084	SNAP25	Synaptosor	SNP25_HUI	1	1.388181
30min_MT.	Q96P16	-0.24688	0.300654	0.297084	RPRD1A	Regulation	RPR1A_HUI	5	0.842718
30min_MT.	Q9BT78	0.056435	0.300611	0.297084	COPS4	COP9 signa	CSN4_HUM	20	1.039893
15min_MT.	P25311	0.303676	0.300943	0.297231	AZGP1	Zinc-alpha-	ZA2G_HUM	11	1.234285
30min_MT.	Q9BS26	0.029635	0.300891	0.297231	ERP44	Endoplasm	ERP44_HUI	13	1.020754
30min_MT.	O75600	-0.12546	0.301326	0.297299	GCAT	2-amino-3-	KBL_HUMA	1	0.916713
30min_MT.	P00367	0.070499	0.301166	0.297299	GLUD1	Glutamate	DHE3_HUM	27	1.05008
30min_MT.	P28370	-0.23195	0.301373	0.297299	SMARCA1	Probable gl	SMCA1_HU	4	0.851485
15min_MT.	Q6KC79	0.256559	0.301134	0.297299	NIPBL	Nipped-B-li	NIPBL_HUN	4	1.194626
15min_MT.	Q86X27	0.084956	0.301442	0.297299	RALGPS2	Ras-specific	RGPS2_HUI	1	1.060656
30min_MT.	Q8NBF6	-0.09898	0.301357	0.297299	AVL9	Late secret	AVL9_HUM	10	0.933695
15min_MT.	Q9H1A4	0.281183	0.301169	0.297299	ANAPC1	Anaphase-p	APC1_HUM	5	1.215191
15min_MT.	O00423	-0.04534	0.301503	0.297321	EML1	Echinoderm	EMAL1_HU	13	0.96906
15min_MT.	P21817	-0.25718	0.301679	0.297334	RYR1	Ryanodine	RYR1_HUM	1	0.836721
30min_MT.	Q12929	-0.07422	0.301662	0.297334	EPS8	Epidermal g	EPS8_HUM	8	0.949857
30min_MT.	Q9UJ83	0.131557	0.301705	0.297334	HACL1	2-hydroxya	HACL1_HUI	7	1.095476
15min_MT.	O00154	0.094727	0.301819	0.297408	ACOT7	Cytosolic a	BACH_HUM	13	1.067863
30min_MT.	P57086	0.279229	0.302086	0.29756	SCAND1	SCAN doma	SCND1_HU	1	1.213546
15min_MT.	Q9NYK5	-0.67707	0.302063	0.29756	MRPL39	Large ribos	RM39_HUN	2	0.625436
30min_MT.	Q6NYC8	-0.00718	0.302412	0.29767	PPP1R18	Phostensin	PPR18_HUI	7	0.995034
30min_MT.	Q14C86	-0.08307	0.3027	0.297826	GAPVD1	GTPase-acti	GAPD1_HU	32	0.944046
30min_MT.	Q9C005	0.140271	0.302691	0.297826	DPY30	Protein dpy	DPY30_HUI	1	1.102112
30min_MT.	Q9Y5L4	-0.15722	0.302792	0.297879	TIMM13	Mitochond	TIM13_HUI	7	0.896754
30min_MT.	Q9H8Y5	0.452954	0.302863	0.297912	ANKZF1	tRNA endor	ANKZ1_HUI	2	1.36884
30min_MT.	Q08378	-0.06891	0.302952	0.297925	GOLGA3	Golgin subf	GOGA3_HU	49	0.953355
30min_MT.	P25398	0.120094	0.303149	0.298011	RPS12	Small ribos	RS12_HUM	8	1.086805
30min_MT.	Q7Z6Z6	0.258332	0.30308	0.298011	PNPLA5	Patatin-like	PLPL5_HUN	1	1.196095
30min_MT.	Q7Z7F0	-0.04006	0.303192	0.298011	KHDC4	KH homolo	KHDC4_HU	2	0.972613
15min_MT.	P62942	0.162436	0.303281	0.298024	FKBP1A	Peptidyl-pr	FKB1A_HUI	7	1.119175
30min_MT.	P19440	0.11245	0.303552	0.298252	GGT1	Glutathion	GGT1_HUM	6	1.081062
15min_MT.	P11940	0.048468	0.303857	0.298364	PABPC1	Polyadenyl	PABP1_HUI	23	1.034166
15min_MT.	Q00688	0.106575	0.303743	0.298364	FKBP3	Peptidyl-pr	FKBP3_HUI	8	1.076669
30min_MT.	Q9H5Q4	-0.51856	0.303828	0.298364	TFB2M	Dimethylac	TFB2M_HU	2	0.698069
15min_MT.	Q9HCJ6	0.044624	0.30385	0.298364	VAT1L	Synaptic ve	VAT1L_HUN	12	1.031414
15min_MT.	Q8WXX5	0.188583	0.303971	0.298438	DNAJC9	DnaJ homo	DNJC9_HUI	5	1.139644
15min_MT.	Q9BQ67	0.190592	0.304271	0.298658	GRWD1	Glutamate-	GRWD1_HU	3	1.141232
15min_MT.	P07108	-0.20943	0.304577	0.298846	DBI	Acyl-CoA-bi	ACBP_HUM	6	0.864878
15min_MT.	Q9Y3E0	-0.18307	0.304738	0.298966	GOLT1B	Vesicle tran	GOT1B_HU	1	0.880826
15min_MT.	Q9Y5J9	0.141178	0.304786	0.298976	TIMM8B	Mitochond	TIM8B_HUI	5	1.102805

30min_MT.	O95453	-0.07529	0.304932	0.299081	PARN	Poly(A)-spe	PARN_HUM	20	0.949149
30min_MT.	Q12979	-0.13253	0.305277	0.299271	ABR	Active brea	ABR_HUMA	5	0.91223
30min_MT.	Q6ZUT6	-0.67072	0.305265	0.299271	CCDC9B	Coiled-coil	CCD9B_HU	1	0.628193
30min_MT.	Q8TEH3	-0.31023	0.305265	0.299271	DENND1A	DENN doma	DEN1A_HU	3	0.806515
30min_MT.	Q5JS54	0.00502	0.305359	0.299313	PSMG4	Proteasom	PSMG4_HU	5	1.003486
15min_MT.	P31150	-0.04837	0.305415	0.299331	GDI1	Rab GDP di	GDIA_HUM	14	0.967031
15min_MT.	Q13561	0.158251	0.305827	0.299659	DCTN2	Dynactin su	DCTN2_HU	18	1.115933
30min_MT.	P51553	-0.05292	0.305904	0.299697	IDH3G	Isocitrate d	IDH3G_HU	3	0.963986
30min_MT.	P16220	0.103701	0.30616	0.29991	CREB1	Cyclic AMP	CREB1_HU	3	1.074527
30min_MT.	P30566	0.044893	0.306414	0.300084	ADSL	Adenylosuc	PUR8_HUM	17	1.031607
30min_MT.	P82909	-0.18816	0.306379	0.300084	KGD4	Alpha-keto	KGD4_HUM	2	0.877724
15min_MT.	Q2VPK5	0.286999	0.306574	0.300115	CTU2	Cytoplasmi	CTU2_HUM	3	1.2201
15min_MT.	Q9C0C9	-0.01838	0.30667	0.300115	UBE2O	(E3-indeper	UBE2O_HU	36	0.987344
30min_MT.	Q9H2P0	-0.05287	0.306687	0.300115	ADNP	Activity-de	ADNP_HUM	6	0.964019
15min_MT.	Q9NQR4	-0.01272	0.306749	0.300115	NIT2	Omega-ami	NIT2_HUM	14	0.991223
30min_MT.	Q9UHB4	-0.41093	0.306849	0.300172	NDOR1	NADPH-dep	NDOR1_HU	5	0.75214
30min_MT.	Q12906	0.063089	0.30705	0.300331	ILF3	Interleukin	ILF3_HUMA	32	1.0447
30min_MT.	Q92945	-0.03632	0.307139	0.300381	KHSRP	Far upstrea	FUBP2_HU	28	0.975137
30min_MT.	Q03154	0.093008	0.307188	0.300391	ACY1	Aminoacyla	ACY1_HUM	19	1.066592
30min_MT.	Q96MH2	0.500528	0.307488	0.300609	HEXIM2	Protein HE	HEXI2_HUM	1	1.414731
30min_MT.	Q9Y2S7	0.043975	0.307561	0.300642	POLDIP2	Polymerase	PDIP2_HUM	14	1.03095
15min_MT.	Q96EY8	0.161358	0.307718	0.300759	MMAB	Corrinoid a	MMAB_HU	6	1.118339
30min_MT.	O95372	0.278135	0.30788	0.300808	LYPLA2	Acyl-protei	LYPA2_HUM	6	1.212627
30min_MT.	P35754	0.161318	0.307851	0.300808	GLRX	Glutaredox	GLRX1_HU	4	1.118308
15min_MT.	P22629	0.70117	0.30798	0.300864	NaN	Streptavidi	SAV_STRAV	2	1.625822
15min_MT.	P11182	0.079273	0.308078	0.300923	DBT	Lipoamide	ODB2_HUM	15	1.056486
15min_MT.	Q8N7H5	-0.11902	0.308242	0.301045	PAF1	RNA polym	PAF1_HUM	11	0.920816
15min_MT.	Q5JTD0	0.034635	0.308405	0.301166	TJAP1	Tight juncti	TJAP1_HUM	11	1.024297
30min_MT.	P31040	0.056473	0.309096	0.301472	SDHA	Succinate d	SDHA_HUM	22	1.03992
30min_MT.	Q0VDG4	0.044768	0.309136	0.301472	SCRN3	Secernin-3	SCRN3_HU	7	1.031517
15min_MT.	Q9Y3F4	-0.0917	0.309007	0.301472	STRAP	Serine-thre	STRAP_HUM	20	0.938419
30min_MT.	Q86XA9	-0.3252	0.309313	0.301564	HEATR5A	HEAT repea	HTR5A_HU	2	0.798186
30min_MT.	Q9Y3C8	0.11779	0.309304	0.301564	UFC1	Ubiquitin-f	UFC1_HUM	7	1.085072
15min_MT.	O60235;Q8	0.26035	0.309462	0.30161	TMPRSS11I	Transmemb	TM11D_HU	1	1.197769
15min_MT.	P49006	-0.22475	0.309476	0.30161	MARCKSL1	MARCKS-re	MRP_HUMA	5	0.855743
30min_MT.	Q9P206	-0.07305	0.30963	0.301647	NHSL3	NHS-like pr	NHSL3_HU	5	0.950627
15min_MT.	Q9UMR5	0.041004	0.309586	0.301647	PPT2	Lysosomal	PPT2_HUM	2	1.028829
15min_MT.	Q96A72	0.202577	0.309967	0.301825	MAGOHB	Protein ma	MGN2_HU	7	1.150752
30min_MT.	Q9H0A9	0.061057	0.309941	0.301825	SPATC1L	Speriolin-li	SPC1L_HUM	1	1.04323
30min_MT.	Q3SY69	-0.15471	0.310235	0.301846	ALDH1L2	Mitochond	AL1L2_HUM	15	0.898312
30min_MT.	Q5QJE6	0.001943	0.310176	0.301846	DNTTIP2	Deoxynucle	TDIF2_HUM	3	1.001348
30min_MT.	Q86TP1	-0.01417	0.310212	0.301846	PRUNE1	Exopolyph	PRUN1_HU	9	0.990229
30min_MT.	Q9NRR5	0.03058	0.310117	0.301846	UBQLN4	Ubiquilin-4	UBQL4_HU	5	1.021423
30min_MT.	Q9UHB6	-0.09657	0.310062	0.301846	LIMA1	LIM domai	LIMA1_HU	18	0.935255
30min_MT.	Q9Y6Q2	0.005476	0.310258	0.301846	STON1	Stonin-1	STON1_HU	1	1.003803
15min_MT.	P61960	-0.18895	0.310323	0.301871	UFM1	Ubiquitin-f	UFM1_HUM	4	0.877245
15min_MT.	Q7L266	-0.0069	0.310521	0.301989	ASRGL1	Isoaspartyl	ASGL1_HU	7	0.995225
30min_MT.	Q99735	0.552745	0.310653	0.30208	MGST2	Microsoma	MGST2_HU	1	1.466874
30min_MT.	O15305	0.058734	0.310706	0.302093	PMM2	Phosphoma	PMM2_HU	15	1.041551
30min_MT.	Q96AJ9	-0.30337	0.310905	0.30225	VTI1A	Vesicle tran	VTI1A_HUM	1	0.810358
30min_MT.	Q92686	-0.21198	0.310962	0.302267	NRGN	Neurograni	NEUG_HUM	1	0.863353
30min_MT.	O00443	-1.79741	0.311064	0.302291	PIK3C2A	Phosphatic	P3C2A_HU	1	0.28769

15min_MT.	Q92979	0.122034	0.31118	0.302328	EMG1	Ribosomal	NEP1_HUM	4	1.088268
15min_MT.	P37198	0.274102	0.311229	0.302339	NUP62	Nuclear po	NUP62_HU	4	1.209241
30min_MT.	Q9H9A5	-0.4179	0.31128	0.302351	CNOT10	CCR4-NOT t	CNO10_HU	4	0.748512
30min_MT.	P61018	-0.03793	0.311612	0.302416	RAB4B	Ras-related	RAB4B_HU	2	0.974055
15min_MT.	P05114	-0.07815	0.311709	0.302429	HMGN1	Non-histon	HMGN1_HU	6	0.94727
30min_MT.	P07947	-0.03342	0.312358	0.302909	YES1	Tyrosine-pr	YES_HUMA	10	0.977103
15min_MT.	Q13485	-0.07219	0.312348	0.302909	SMAD4	Mothers ag	SMAD4_HU	10	0.951195
15min_MT.	Q8TF01	0.051184	0.312413	0.302925	PNISR	Arginine/se	PNISR_HUM	8	1.036115
15min_MT.	Q9NSD9	0.105526	0.312551	0.303021	FARSB	Phenylalan	SYFB_HUMA	18	1.075887
30min_MT.	O75414	0.081199	0.312708	0.303135	NME6	Nucleoside	NDK6_HUM	6	1.057897
15min_MT.	Q9H2K8	0.039204	0.31287	0.303255	TAOK3	Serine/thre	TAOK3_HU	6	1.027547
30min_MT.	Q08209	0.055811	0.313099	0.303402	PPP3CA	Protein pho	PP2BA_HU	7	1.039443
30min_MT.	Q00577	0.040721	0.31318	0.303443	PURA	Transcripti	PURA_HUM	6	1.028628
30min_MT.	Q9Y2Q9	-0.0183	0.313373	0.303555	MRPS28	Small ribos	RT28_HUM	4	0.987394
15min_MT.	Q96K17	0.113057	0.313796	0.303897	BTF3L4	Transcripti	BT3L4_HUM	3	1.081517
15min_MT.	O15379	0.045712	0.313892	0.303906	HDAC3	Histone dea	HDAC3_HU	2	1.032193
30min_MT.	Q6DN90	0.112177	0.313991	0.303928	IQSEC1	IQ motif an	IQEC1_HUM	2	1.080858
30min_MT.	P32969	-0.0971	0.314079	0.303975	RPL9	Large ribos	RL9_HUMA	3	0.934909
30min_MT.	P02679	-0.06517	0.31414	0.303996	FGG	Fibrinogen	FIBG_HUMA	14	0.955834
15min_MT.	Q71RC2	0.288978	0.314195	0.304012	LARP4	La-related p	LARP4_HU	5	1.221775
15min_MT.	O15145	-0.15418	0.314503	0.304235	ARPC3	Actin-relate	ARPC3_HU	10	0.898642
15min_MT.	Q96ST3	0.103929	0.314732	0.304418	SIN3A	Paired amp	SIN3A_HUM	12	1.074696
30min_MT.	P11387	0.091325	0.314811	0.304427	TOP1	DNA topois	TOP1_HUM	9	1.065348
30min_MT.	Q8NEU8	-0.04015	0.315145	0.304667	APPL2	DCC-intera	DP13B_HU	2	0.972556
30min_MT.	P07902	-0.14139	0.315537	0.305009	GALT	Galactose-1	GALT_HUM	8	0.906643
15min_MT.	Q14197	-0.4185	0.315779	0.305129	MRPL58	Large ribos	ICT1_HUMA	2	0.748202
30min_MT.	P07741	-0.04153	0.315904	0.305212	APRT	Adenine ph	APT_HUMA	5	0.971625
15min_MT.	Q7Z7F0	-0.03857	0.316207	0.305399	KHDC4	KH homolo	KHDC4_HU	2	0.973619
15min_MT.	P28074	-0.04097	0.316307	0.305451	PSMB5	Proteasom	PSB5_HUM	12	0.972001
15min_MT.	P15311	0.070051	0.316362	0.305467	EZR	Ezrin	EZRI_HUMA	21	1.049754
15min_MT.	C9JRZ8	-0.22992	0.316505	0.305491	AKR1B15	Aldo-keto r	AK1BF_HU	1	0.852684
15min_MT.	P56945	0.385124	0.316474	0.305491	BCAR1	Breast canc	BCAR1_HU	4	1.305972
15min_MT.	Q7Z406	0.25257	0.316487	0.305491	MYH14	Myosin-14	MYH14_HU	10	1.191327
15min_MT.	O95182	0.261158	0.316717	0.305546	NDUFA7	NADH dehy	NDUA7_HU	1	1.19844
15min_MT.	O95865	0.026276	0.316703	0.305546	DDAH2	Putative hy	DDAH2_HU	10	1.01838
15min_MT.	P25205	-0.02031	0.316674	0.305546	MCM3	DNA replica	MCM3_HU	27	0.986018
30min_MT.	Q6VN20	0.130186	0.316671	0.305546	RANBP10	Ran-bindin	RBP10_HU	5	1.094435
15min_MT.	P51159	0.50956	0.317145	0.305901	RAB27A	Ras-related	RB27A_HU	2	1.423616
30min_MT.	Q99797	-0.00186	0.317519	0.306206	MIPEP	Mitochond	MIPEP_HU	2	0.99871
30min_MT.	Q8N573	-0.16776	0.31791	0.306469	OXR1	Oxidation r	OXR1_HUM	16	0.890225
15min_MT.	Q96K76	0.04437	0.317902	0.306469	USP47	Ubiquitin c	UBP47_HU	32	1.031233
15min_MT.	Q9H1P3	0.028189	0.317956	0.306474	OSBPL2	Oxysterol-b	OSBL2_HU	2	1.019731
15min_MT.	P53041	0.045078	0.318578	0.306946	PPP5C	Serine/thre	PPP5_HUM	18	1.031739
30min_MT.	P62760	-0.35132	0.318612	0.306946	VSNL1	Visinin-like	VISL1_HUM	4	0.783866
15min_MT.	P05062	0.127417	0.318885	0.307083	ALDOB	Fructose-bi	ALDOB_HU	1	1.092336
30min_MT.	Q9NYU2	-0.04459	0.3189	0.307083	UGGT1	UDP-glucos	UGGG1_HU	46	0.969569
15min_MT.	O43679;Q8	0.205116	0.31908	0.307181	LDB2;LDB1	LIM domair	LDB2_HUM	1	1.152779
15min_MT.	Q969U7	-0.07264	0.319376	0.307428	PSMG2	Proteasom	PSMG2_HU	10	0.950897
15min_MT.	Q8NBS9	0.12475	0.319445	0.307456	TXNDC5	Thioredoxi	TXND5_HU	19	1.090319
30min_MT.	P25325	0.059123	0.319679	0.307616	MPST	3-mercapto	THTM_HUM	13	1.041832
30min_MT.	Q6GMV3	0.143794	0.319689	0.307616	PTRHD1	Putative pe	PTRD1_HU	5	1.104806
15min_MT.	P54886	-0.01355	0.319963	0.307831	ALDH18A1	Delta-1-pyr	P5CS_HUM	33	0.990649

30min_MT.	Q92665	-0.72083	0.319992	0.307831	MRPS31	Small ribos	RT31_HUM	2	0.606747
15min_MT.	Q96PU5	0.202769	0.320071	0.307862	NEDD4L	E3 ubiquiti	NED4L_HUI	4	1.150906
15min_MT.	Q8WWI1	0.045486	0.320461	0.307971	LMO7	LIM domair	LMO7_HUN	20	1.032031
30min_MT.	Q96HC4	-0.05294	0.320431	0.307971	PDLIM5	PDZ and LIN	PDLI5_HUN	23	0.96397
30min_MT.	Q9HCD5	-0.00802	0.320464	0.307971	NCOA5	Nuclear rec	NCOA5_HU	6	0.994456
30min_MT.	Q9NUP9	0.234509	0.320465	0.307971	LIN7C	Protein lin-	LIN7C_HUN	4	1.176507
15min_MT.	Q9NXV2	0.17611	0.320492	0.307971	KCTD5	BTB/POZ dc	KCTD5_HUI	5	1.129834
15min_MT.	Q9H0E9	0.438918	0.320739	0.308095	BRD8	Bromodom	BRD8_HUN	1	1.355587
30min_MT.	O75792	-0.35021	0.320907	0.308181	RNASEH2A	Ribonuclea	RNH2A_HU	2	0.784471
30min_MT.	Q99536	0.038766	0.321077	0.308244	VAT1	Synaptic ve	VAT1_HUM	16	1.027235
15min_MT.	O94985	-0.14511	0.32168	0.308507	CLSTN1	Calsynteni	CSTN1_HUI	2	0.904308
30min_MT.	P15144	0.017796	0.321657	0.308507	ANPEP	Aminopept	AMPN_HUN	17	1.012411
30min_MT.	Q9BQE3	0.339633	0.321571	0.308507	TUBA1C	Tubulin alp	TBA1C_HUI	2	1.265435
15min_MT.	Q9NX24	-0.06704	0.321612	0.308507	NHP2	H/ACA ribo	NHP2_HUN	4	0.954592
30min_MT.	Q9NUJ1	0.028622	0.322116	0.308812	ABHD10	Palmitoyl-p	ABHDA_HU	14	1.020037
30min_MT.	P35244	-0.04466	0.322214	0.308868	RPA3	Replicatior	RFA3_HUM	6	0.969519
30min_MT.	Q6NZY4	-0.16307	0.322361	0.308877	ZCCHC8	Zinc finger	ZCHC8_HUI	8	0.893121
15min_MT.	Q96AH0;Q	0.355991	0.322311	0.308877	NABP1;NAE	SOSS comp	SOSB2_HUI	1	1.279864
15min_MT.	Q9Y365	-0.02302	0.322382	0.308877	STARD10	START dom	STA10_HUN	6	0.984168
30min_MT.	Q00536	0.420085	0.322624	0.309033	CDK16	Cyclin-depe	CDK16_HU	1	1.338006
30min_MT.	Q9H8Y8	-0.08544	0.322715	0.309083	GORASP2	Golgi reass	GORS2_HU	9	0.942495
30min_MT.	Q9NUB1	-0.02598	0.322867	0.309191	ACSS1	Acetyl-coer	ACS2L_HUN	5	0.982152
15min_MT.	P35268	0.104221	0.323063	0.30934	RPL22	Large ribos	RL22_HUM	2	1.074914
15min_MT.	P10909	0.137266	0.323184	0.309343	CLU	Clusterin	CLUS_HUM	5	1.099819
15min_MT.	Q9Y2I8	-0.24684	0.323157	0.309343	WDR37	WD repeat-	WDR37_HU	1	0.842739
30min_MT.	P51114	-0.37489	0.323409	0.30946	FXR1	RNA-bindir	FXR1_HUM	6	0.771166
15min_MT.	P04062	-0.08756	0.323583	0.309535	GBA1	Lysosomal	GBA1_HUN	7	0.94111
15min_MT.	Q86YH6	-0.16776	0.323784	0.30968	PDSS2	All trans-pc	DLP1_HUN	11	0.890224
15min_MT.	P20290	0.17958	0.323855	0.309681	BTF3	Transcripti	BTF3_HUM	6	1.132554
15min_MT.	Q9BY42	-0.08953	0.323976	0.309759	RTF2	Replicatior	RTF2_HUM	4	0.939827
15min_MT.	O95059	0.180014	0.324166	0.309859	RPP14	Ribonuclea	RPP14_HUI	2	1.132895
15min_MT.	P62424	-0.05546	0.3242	0.309859	RPL7A	Large ribos	RL7A_HUM	8	0.962285
15min_MT.	Q99805	0.019706	0.324153	0.309859	TM9SF2	Transmemb	TM9S2_HU	2	1.013753
15min_MT.	Q8WWM7	0.094927	0.324257	0.309876	ATXN2L	Ataxin-2-lik	ATX2L_HUN	30	1.068011
15min_MT.	O43290	0.127819	0.32437	0.309909	SART1	U4/U6.U5 t	SNUT1_HUI	13	1.092641
30min_MT.	O00273	0.120233	0.324473	0.309969	DFFA	DNA fragme	DFFA_HUM	11	1.086911
30min_MT.	A1X283	-0.04982	0.324747	0.310155	SH3PXD2B	SH3 and PX	SPD2B_HUI	10	0.966059
15min_MT.	P09234	0.110829	0.324956	0.310279	SNRPC	U1 small nu	RU1C_HUM	2	1.079848
30min_MT.	Q86UA1	0.475964	0.324952	0.310279	PRPF39	Pre-mRNA-p	PRP39_HUI	2	1.390847
30min_MT.	Q06587	-0.23555	0.325066	0.310308	RING1	E3 ubiquiti	RING1_HUI	4	0.84936
30min_MT.	Q9NQT4	-0.08923	0.325056	0.310308	EXOSC5	Exosome cc	EXOS5_HUI	4	0.940023
15min_MT.	P09211	0.144993	0.325777	0.310887	GSTP1	Glutathion	GSTP1_HUI	14	1.105725
15min_MT.	O15047	-0.39248	0.325952	0.310965	SETD1A	Histone-lys	SET1A_HUN	1	0.761817
15min_MT.	Q16204	0.081832	0.325927	0.310965	CCDC6	Coiled-coil	CCDC6_HU	14	1.058361
15min_MT.	Q66K74	-0.04863	0.326109	0.311076	MAP1S	Microtubul	MAP1S_HU	3	0.966853
30min_MT.	Q14241	-0.03382	0.326158	0.311085	ELOA	Elongin-A	ELOA1_HUI	1	0.976829
30min_MT.	Q9Y547	-0.28834	0.326306	0.311189	IFT25	Intraflagell	IFT25_HUN	2	0.818844
30min_MT.	Q13347	-0.04484	0.326647	0.311429	EIF3I	Eukaryotic	EIF3I_HUM	10	0.9694
30min_MT.	Q69YN4	0.329253	0.326797	0.311429	VIRMA	Protein viri	VIR_HUMAI	10	1.256363
15min_MT.	Q6UX04	0.220232	0.326784	0.311429	CWC27	Spliceosom	CWC27_HU	3	1.164921
30min_MT.	Q6UXD5	0.138234	0.326874	0.311464	SEZ6L2	Seizure 6-lil	SE6L2_HUN	5	1.100557
30min_MT.	Q49B96	0.178807	0.32747	0.311842	COX19	Cytochrom	COX19_HU	2	1.131947

15min_MT.	P01008	0.105682	0.327775	0.312095	SERPINC1	Antithroml	ANT3_HUM	6	1.076003
30min_MT.	P78417	0.096359	0.327816	0.312095	GSTO1	Glutathion	GSTO1_HUI	17	1.069072
30min_MT.	O43809	-0.05241	0.327941	0.312177	NUDT21	Cleavage ar	CPSF5_HUN	11	0.964325
15min_MT.	P83916	0.180129	0.32806	0.3122	CBX1	Chromobo:	CBX1_HUM	1	1.132986
30min_MT.	Q9UBL3	0.019844	0.328109	0.3122	ASH2L	Set1/Ash2 l	ASH2L_HUI	3	1.01385
15min_MT.	Q9UDR5	-0.10338	0.328126	0.3122	AASS	Alpha-amir	AASS_HUM	10	0.930849
15min_MT.	Q16342	-0.05004	0.328179	0.312213	PDCD2	Programme	PDCD2_HU	3	0.965909
30min_MT.	O43715	0.180326	0.328497	0.312364	TRIAP1	TP53-regul	TRIA1_HUN	5	1.13314
30min_MT.	O60522	0.246004	0.328481	0.312364	TDRD6	Tudor dom	TDRD6_HU	1	1.185918
15min_MT.	Q9H1I8	0.007525	0.328388	0.312364	ASCC2	Activating	ASCC2_HUI	1	1.005229
30min_MT.	O94913	0.307225	0.328822	0.312635	PCF11	Pre-mRNA	PCF11_HUI	2	1.237326
15min_MT.	Q8IUD2	0.090761	0.328941	0.31271	ERC1	ELKS/Rab6-	RB6I2_HUN	32	1.064932
15min_MT.	Q8TD30	0.123889	0.329196	0.312838	GPT2	Alanine am	ALAT2_HUN	7	1.089668
30min_MT.	P17676	0.227148	0.329537	0.313056	CEBPB	CCAAT/enh	CEBPB_HUI	1	1.170519
30min_MT.	P36957	0.018046	0.329614	0.313081	DLST	Dihydrolip	ODO2_HUN	16	1.012587
30min_MT.	Q15067	0.150452	0.329984	0.313359	ACOX1	Peroxisom	ACOX1_HU	5	1.109917
15min_MT.	P41091	-0.03556	0.330179	0.313506	EIF2S3	Eukaryotic	IF2G_HUM	23	0.975651
30min_MT.	P30044	0.053798	0.330295	0.313577	PRDX5	Peroxiredo	PRDX5_HU	11	1.037994
15min_MT.	P10746	-0.11558	0.330538	0.31373	UROS	Uroporphy	HEM4_HUN	5	0.923014
30min_MT.	P49585	0.037513	0.330576	0.31373	PCYT1A	Choline-ph	PCY1A_HUI	12	1.026343
30min_MT.	Q96HR9	-0.00346	0.330758	0.313827	REEP6	Receptor e	REEP6_HUN	5	0.997601
15min_MT.	Q86WP2	-0.38921	0.331608	0.314366	GPBP1	Vasculin	GPBP1_HU	1	0.76355
30min_MT.	P08670	-0.07009	0.331774	0.314471	VIM	Vimentin	VIME_HUM	36	0.952579
15min_MT.	Q5BKU9	-0.05482	0.331948	0.314574	OXLD1	Oxidoreduc	OXLD1_HU	1	0.962712
30min_MT.	O75380	0.126547	0.33212	0.314661	NDUFS6	NADH dehy	NDUS6_HU	3	1.091678
15min_MT.	Q5MNZ6	-0.12535	0.332376	0.314866	WDR45B	WD repeat	WIPI3_HUN	5	0.916779
30min_MT.	Q7L5Y9	-0.08081	0.332462	0.314909	MAEA	E3 ubiquiti	MAEA_HUN	2	0.945528
15min_MT.	P11021	0.083177	0.332641	0.315033	HSPA5	Endoplasm	BIP_HUMA	36	1.059348
15min_MT.	Q8N371	0.097232	0.332714	0.315033	KDM8	Bifunctiona	KDM8_HUN	6	1.069719
15min_MT.	Q96DC9	-0.01816	0.332735	0.315033	OTUB2	Ubiquitin t	OTUB2_HU	1	0.98749
15min_MT.	Q66K14	-0.02675	0.332907	0.315106	TBC1D9B	TBC1 doma	TBC9B_HUI	14	0.98163
30min_MT.	Q9BZQ6	-0.08438	0.332911	0.315106	EDEM3	ER degrada	EDEM3_HU	5	0.94319
15min_MT.	Q13740	0.106849	0.333628	0.315708	ALCAM	CD166 anti	CD166_HU	7	1.076874
15min_MT.	Q96FV9	0.114782	0.333735	0.315736	THOC1	THO compl	THOC1_HU	20	1.082812
30min_MT.	P45973	-0.02504	0.333855	0.315769	CBX5	Chromobo:	CBX5_HUM	6	0.982794
15min_MT.	Q16740	0.109161	0.334126	0.315912	CLPP	ATP-depen	CLPP_HUM	10	1.078601
15min_MT.	O75494	-0.17825	0.334314	0.316051	SRSF10	Serine/argi	SRS10_HUN	4	0.883773
30min_MT.	Q96ST3	-0.06491	0.334577	0.316261	SIN3A	Paired amp	SIN3A_HUN	12	0.956003
15min_MT.	Q9H6E4	-0.3322	0.334681	0.316283	CCDC134	Coiled-coil	CC134_HUI	7	0.794322
15min_MT.	Q9Y3C0	0.48319	0.334651	0.316283	WASHC3	WASH com	WASC3_HU	2	1.397831
30min_MT.	P05204	0.189356	0.334823	0.316379	HMGN2	Non-histon	HMGN2_HU	2	1.140255
15min_MT.	O43707	-0.03616	0.334903	0.316417	ACTN4	Alpha-actir	ACTN4_HUI	46	0.975246
30min_MT.	O75170	-0.10386	0.33501	0.316479	PPP6R2	Serine/thre	PP6R2_HUI	24	0.930539
30min_MT.	P35606	-0.04717	0.335509	0.316637	COPB2	Coatomer s	COPB2_HU	39	0.967832
15min_MT.	P46087	-0.0133	0.335459	0.316637	NOP2	28S rRNA (c	NOP2_HUN	12	0.990824
15min_MT.	Q06830	0.105949	0.335605	0.316637	PRDX1	Peroxiredo	PRDX1_HU	16	1.076202
30min_MT.	Q5HYJ3	-0.13622	0.335309	0.316637	FAM76B	Protein FA	FA76B_HUI	1	0.909898
15min_MT.	Q86TB9	-0.41227	0.335354	0.316637	PATL1	Protein PA	PATL1_HUN	1	0.751441
15min_MT.	Q9Y2S2	0.077628	0.335351	0.316637	CRYL1	Lambda-cry	CRYL1_HUN	10	1.055282
15min_MT.	Q15118	0.15222	0.335739	0.316671	PDK1	[Pyruvate d	PDK1_HUN	1	1.111278
15min_MT.	Q5TAQ9	0.049936	0.336057	0.316785	DCAF8	DDB1- and	DCAF8_HUI	10	1.035219
30min_MT.	Q8WX92	-0.1365	0.335984	0.316785	NELFB	Negative el	NELFB_HUN	10	0.909721

15min_MT.	Q96DI7	-0.08655	0.336103	0.316785	SNRNP40	U5 small nu	SNR40_HUI	11	0.941774
30min_MT.	Q99575	0.035734	0.336089	0.316785	POP1	Ribonuclea	POP1_HUM	12	1.025078
30min_MT.	Q9BW71	0.310116	0.336056	0.316785	HIRIP3	HIRA-intera	HIRP3_HUI	5	1.239808
30min_MT.	P08579	0.095228	0.336308	0.31694	SNRPB2	U2 small nu	RU2B_HUM	8	1.068234
15min_MT.	Q6ZS30	0.387639	0.336403	0.316991	NBEAL1	Neurobeacl	NBEL1_HUI	4	1.30825
15min_MT.	Q8NFZ8	0.185302	0.336767	0.31722	CADM4	Cell adhesio	CADM4_HU	4	1.137055
15min_MT.	Q13190	0.015918	0.336972	0.317374	STX5	Syntaxin-5	STX5_HUM	5	1.011094
15min_MT.	P07099	-0.22946	0.337683	0.317471	EPHX1	Epoxide hy	HYEP_HUM	8	0.852952
30min_MT.	P24534	0.17578	0.337451	0.317471	EEF1B2	Elongation	EF1B_HUM	9	1.129575
15min_MT.	P46926	0.104916	0.337565	0.317471	GNPDA1	Glucosamir	GNPI1_HUI	14	1.075432
15min_MT.	P84085	-0.08002	0.337345	0.317471	ARF5	ADP-ribosy	ARF5_HUM	4	0.946043
30min_MT.	Q15785	0.215987	0.337635	0.317471	TOMM34	Mitochond	TOM34_HU	2	1.161498
30min_MT.	Q16643	0.063182	0.337608	0.317471	DBN1	Drebrin	DREB_HUM	22	1.044767
30min_MT.	Q32P44	-0.08823	0.337417	0.317471	EML3	Echinoderm	EMAL3_HU	12	0.940675
30min_MT.	Q58FF6	0.389523	0.337564	0.317471	HSP90AB4I	Putative he	H90B4_HU	1	1.30996
30min_MT.	Q86W34	-0.01867	0.337665	0.317471	AMZ2	Archaemet:	AMZ2_HUM	1	0.987145
15min_MT.	Q9NVZ3	-0.05999	0.337615	0.317471	NECAP2	Adaptin ear	NECP2_HUI	5	0.959268
15min_MT.	Q9Y484	-0.03783	0.337521	0.317471	WDR45	WD repeat	WIPI4_HUM	6	0.974121
15min_MT.	Q9Y580	0.030077	0.33756	0.317471	RBM7	RNA-bindir	RBM7_HUM	1	1.021067
30min_MT.	Q9Y265	0.039884	0.337753	0.317499	RUVBL1	RuvB-like 1	RUVB1_HU	19	1.028031
30min_MT.	Q96BP3	-0.14687	0.337915	0.317613	PPWD1	Peptidylpro	PPWD1_HU	11	0.90321
30min_MT.	P09429	-0.13214	0.338057	0.317696	HMGB1	High mobil	HMGB1_HU	12	0.912475
30min_MT.	Q8WTV0	-0.01618	0.338186	0.317696	SCARB1	Scavenger r	SCRB1_HUI	5	0.988847
15min_MT.	Q9Y3A5	0.031851	0.338135	0.317696	SBDS	Ribosome r	SBDS_HUM	17	1.022323
15min_MT.	Q12974	-0.00336	0.338295	0.317697	PTP4A2	Protein tyr	TP4A2_HUI	3	0.997677
15min_MT.	Q7L8W6	0.256953	0.33837	0.317697	DPH6	Diphthine--	DPH6_HUM	1	1.194952
30min_MT.	Q96K21	-0.02818	0.338306	0.317697	ZFYVE19	Abscission/	ANCHR_HU	6	0.980658
15min_MT.	Q6PJT7	0.071442	0.338507	0.317749	ZC3H14	Zinc finger (	ZC3HE_HUI	5	1.050767
15min_MT.	P13797	0.043559	0.338625	0.317822	PLS3	Plastin-3	PLST_HUMA	36	1.030653
30min_MT.	Q9H2G2	0.050451	0.338703	0.317856	SLK	STE20-like s	SLK_HUMA	31	1.035588
15min_MT.	Q8NFD5	0.132407	0.338829	0.317899	ARID1B	AT-rich inte	ARI1B_HUM	11	1.096121
15min_MT.	P40222	0.076904	0.338923	0.317949	TXLNA	Alpha-taxili	TXLNA_HUI	18	1.054752
15min_MT.	Q68E01	-0.10012	0.339149	0.318123	INTS3	Integrator (	INT3_HUMA	13	0.932957
15min_MT.	Q8WZA0	0.165769	0.33919	0.318123	LZIC	Protein LZI(	LZIC_HUMA	6	1.121764
15min_MT.	P12270	0.092284	0.339378	0.318261	TPR	Nucleoprot	TPR_HUMA	83	1.066057
30min_MT.	Q99549	-0.23751	0.33942	0.318262	MPHOSPH8	M-phase ph	MPP8_HUM	1	0.848209
30min_MT.	Q15942	-0.0524	0.339488	0.318288	ZYX	Zyxin	ZYX_HUMA	10	0.964328
30min_MT.	O00471	-0.08395	0.339722	0.318419	EXOC5	Exocyst cor	EXOC5_HUI	13	0.943473
30min_MT.	P00883	0.440784	0.339722	0.318419	ALDOA	Fructose-bi	ALDOA_RAI	1	1.357341
30min_MT.	Q9BSJ8	-0.04539	0.339791	0.318419	ESYT1	Extended sy	ESYT1_HUM	16	0.969025
15min_MT.	O75674	0.108074	0.339836	0.318423	TOM1L1	TOM1-like p	TM1L1_HU	9	1.077788
15min_MT.	O00571	0.197933	0.340004	0.318437	DDX3X	ATP-depend	DDX3X_HU	13	1.147054
30min_MT.	P51398	-0.01702	0.340184	0.318444	DAP3	Small ribos	RT29_HUM	8	0.988269
15min_MT.	Q92878	-0.03103	0.340064	0.318444	RAD50	DNA repair	RAD50_HU	39	0.978721
30min_MT.	Q99638	0.218281	0.340179	0.318444	RAD9A	Cell cycle cl	RAD9A_HU	4	1.163347
15min_MT.	Q9Y3C8	0.013315	0.340129	0.318444	UFC1	Ubiquitin-f	UFC1_HUM	7	1.009272
30min_MT.	Q9BWW4	-0.76365	0.340337	0.318517	SSBP3	Single-strar	SSBP3_HUI	1	0.589003
15min_MT.	Q07960	0.076004	0.340527	0.318568	ARHGAP1	Rho GTPase	RHG01_HU	14	1.054094
30min_MT.	Q7Z406	0.210282	0.3405	0.318568	MYH14	Myosin-14	MYH14_HU	10	1.156914
15min_MT.	Q9UGI8	0.077216	0.340588	0.318568	TES	Testin	TES_HUMAI	21	1.05498
15min_MT.	Q9BX68	-0.03403	0.34072	0.318641	HINT2	Adenosine !	HINT2_HUI	5	0.976687
30min_MT.	Q9UL16	-0.15464	0.340913	0.318783	CFAP45	Cilia- and fl	CFA45_HUI	1	0.898359

30min_MT.	Q96BR5	-0.16658	0.341	0.318788	COA7	Cytochrom	COA7_HUM	8	0.890954
30min_MT.	P55196	-0.04752	0.341052	0.318799	AFDN	Afadin	AFAD_HUM	59	0.967595
15min_MT.	O14737	0.123859	0.34127	0.318965	PDCD5	Programme	PDCD5_HU	10	1.089645
30min_MT.	P16401	0.14953	0.341328	0.318975	H1-5	Histone H1	H15_HUMA	6	1.109208
15min_MT.	Q13614	0.400038	0.341434	0.319004	MTMR2	Myotubula	MTMR2_HU	4	1.319542
30min_MT.	Q9Y4W6	-0.24842	0.341561	0.319079	AFG3L2	Mitochond	AFG32_HU	2	0.841819
30min_MT.	Q8NAV1	-0.04661	0.341738	0.319173	PRPF38A	Pre-mRNA-s	PR38A_HU	2	0.968208
15min_MT.	Q9BXY0	0.038893	0.342099	0.319396	MAK16	Protein MA	MAK16_HU	5	1.027325
30min_MT.	P42166	-0.04028	0.342167	0.319414	TMPO	Lamina-ass	LAP2A_HU	17	0.972466
30min_MT.	Q9H063	0.241437	0.342201	0.319414	MAF1	Repressor c	MAF1_HUM	2	1.182169
30min_MT.	A0A0B4J2D	0.021759	0.342414	0.319442	GATD3B;GA	Putative glu	GAL3B_HU	12	1.015196
15min_MT.	Q02413	0.126115	0.34235	0.319442	DSG1	Desmoglein	DSG1_HUM	20	1.091351
30min_MT.	Q10471	-0.33173	0.34239	0.319442	GALNT2	Polypeptid	GALT2_HU	8	0.794585
30min_MT.	Q99747	0.04503	0.342316	0.319442	NAPG	Gamma-sol	SNAG_HUM	11	1.031705
15min_MT.	Q9UBI1	0.586143	0.342475	0.319442	COMMD3	COMM dom	COMD3_HU	3	1.501228
15min_MT.	Q15477	-0.48645	0.342756	0.319649	SKIC2	Superkiller	SKI2_HUMA	3	0.71378
30min_MT.	P21399	-0.08517	0.342998	0.319777	ACO1	Cytoplasmi	ACOHC_HU	30	0.942672
15min_MT.	P02812	1.042348	0.343348	0.319951	PRB2	Basic saliva	PRB2_HUM	1	2.059577
15min_MT.	Q6PL24	0.181729	0.34334	0.319951	TMED8	Protein TM	TMED8_HU	3	1.134243
30min_MT.	Q9NQW7	0.091232	0.34348	0.32001	XPNPEP1	Xaa-Pro am	XPP1_HUM	19	1.065279
15min_MT.	Q9NYJ1	0.093886	0.343517	0.32001	COA4	Cytochrom	COA4_HUM	6	1.067241
15min_MT.	P78560	0.160893	0.343597	0.32003	CRADD	Death dom	CRADD_HU	3	1.117979
15min_MT.	O15027	0.017274	0.343859	0.32016	SEC16A	Protein tra	SC16A_HU	16	1.012046
15min_MT.	Q9UMZ2	0.136436	0.34384	0.32016	SYNRG	Synergin ga	SYNRG_HU	10	1.099187
15min_MT.	P49903	0.090255	0.344015	0.320193	SEPHS1	Selenide, w	SPS1_HUM	7	1.064558
15min_MT.	Q9NP81	-0.04504	0.344017	0.320193	SARS2	Serine--tRN	SYSM_HUM	7	0.969263
15min_MT.	Q9Y6D9	-0.2527	0.343957	0.320193	MAD1L1	Mitotic spi	MD1L1_HU	5	0.839323
15min_MT.	Q8N9V7	-0.05535	0.344118	0.320249	TOPAZ1	Protein TO	TOPZ1_HU	1	0.962361
30min_MT.	Q14257	0.181541	0.344347	0.320347	RCN2	Reticulocal	RCN2_HUM	5	1.134094
15min_MT.	P06493	0.054588	0.344648	0.320552	CDK1	Cyclin-depe	CDK1_HUM	3	1.038562
15min_MT.	O14964	0.089495	0.344908	0.320679	HGS	Hepatocyte	HGS_HUMA	13	1.063998
30min_MT.	P42766	0.082893	0.34495	0.320679	RPL35	Large ribos	RL35_HUM	2	1.05914
30min_MT.	Q92900	-0.08124	0.344909	0.320679	UPF1	Regulator c	RENT1_HU	29	0.945243
30min_MT.	Q9UPN9	0.040589	0.344834	0.320679	TRIM33	E3 ubiquiti	TRI33_HUM	6	1.028534
30min_MT.	O60841	-0.06418	0.345002	0.32069	EIF5B	Eukaryotic	IF2P_HUMA	45	0.956487
30min_MT.	Q9Y2W1	0.054188	0.34508	0.320724	THRAP3	Thyroid ho	TR150_HU	23	1.038275
15min_MT.	Q9NVA1	-0.03869	0.345218	0.320814	UQCC1	Ubiquinol-	UQCC1_HU	5	0.973539
15min_MT.	Q07812	-0.0646	0.345351	0.3209	BAX	Apoptosis r	BAX_HUMA	7	0.95621
15min_MT.	Q96BM9	0.050304	0.345408	0.320905	ARL8A	ADP-ribosy	ARL8A_HU	3	1.035483
30min_MT.	Q00013	-0.11061	0.345527	0.320947	MPP1	55 kDa eryt	EM55_HUM	5	0.926199
30min_MT.	P22681	-0.10915	0.345735	0.321025	CBL	E3 ubiquiti	CBL_HUMA	22	0.927131
15min_MT.	Q5HYJ3	-0.46599	0.345773	0.321025	FAM76B	Protein FA	FA76B_HU	1	0.723976
30min_MT.	Q9NR30	0.155875	0.34572	0.321025	DDX21	Nucleolar R	DDX21_HU	19	1.114097
30min_MT.	Q08AM6	-0.04706	0.346246	0.321312	VAC14	Protein VA	VAC14_HU	14	0.967905
15min_MT.	Q96PU8	0.134084	0.346342	0.321362	QKI	KH domain	QKI_HUMA	6	1.097396
30min_MT.	Q29RF7	-0.01251	0.346472	0.321445	PDS5A	Sister chro	PDS5A_HU	4	0.991369
15min_MT.	A6NIH7	0.417239	0.346561	0.321489	UNC119B	Protein un	U119B_HU	1	1.33537
15min_MT.	Q9HCE1	-0.01543	0.346692	0.321524	MOV10	Helicase M	MOV10_HU	20	0.98936
30min_MT.	O00567	-0.0247	0.346988	0.321623	NOP56	Nucleolar p	NOP56_HU	7	0.983026
30min_MT.	P14550	0.162501	0.346892	0.321623	AKR1A1	Aldo-keto r	AK1A1_HU	13	1.119226
30min_MT.	P49821	0.027524	0.346993	0.321623	NDUFV1	NADH dehy	NDUV1_HU	6	1.019261
15min_MT.	P56277	0.040995	0.346955	0.321623	CMC4	Cx9C motif	CMC4_HUM	3	1.028823

30min_MT.	Q5T8P6	-0.14057	0.347224	0.3218	RBM26	RNA-bindir	RBM26_HU	12	0.907162
30min_MT.	O75554	0.110957	0.347707	0.32204	WBP4	WW domai	WBP4_HUI	4	1.079944
30min_MT.	P33908	-0.60105	0.347694	0.32204	MAN1A1	Mannosyl-c	MA1A1_HU	2	0.659274
30min_MT.	Q96EQ0	0.037325	0.347694	0.32204	SGTB	Small gluta	SGTB_HUM	5	1.02621
15min_MT.	P25774	0.067118	0.347828	0.322092	CTSS	Cathepsin S	CATS_HUM	7	1.047622
15min_MT.	P05141;P1	-0.49018	0.347956	0.322134	SLC25A5;SI	ADP/ATP tr	ADT2_HUM	2	0.711939
30min_MT.	Q6V1X1	0.08778	0.348154	0.322279	DPP8	Dipeptidyl	DPP8_HUM	3	1.062734
15min_MT.	Q8WVY7	0.1629	0.348472	0.322497	UBLCP1	Ubiquitin-l	UBCP1_HU	8	1.119535
15min_MT.	P00742	-0.30267	0.348708	0.322605	F10	Coagulatio	FA10_HUM	5	0.810753
30min_MT.	P17096	0.227042	0.348781	0.322605	HMGA1	High mobil	HMGA1_HU	4	1.170433
30min_MT.	Q6JQN1	-0.01936	0.348722	0.322605	ACAD10	Acyl-CoA de	ACD10_HU	18	0.986669
30min_MT.	Q6P2P2	0.285167	0.349001	0.322605	PRMT9	Protein arg	ANM9_HUM	6	1.218551
15min_MT.	Q8WXF1	-0.14169	0.348878	0.322605	PSPC1	Paraspeckle	PSPC1_HUI	13	0.906454
15min_MT.	Q9H098	-0.03769	0.348973	0.322605	FAM107B	Protein FAI	F107B_HUI	7	0.974215
30min_MT.	Q9HD15	0.117759	0.348929	0.322605	SRA1	Steroid rec	SRA1_HUM	10	1.085048
30min_MT.	P28161	-0.06039	0.349448	0.322849	GSTM2	Glutathion	GSTM2_HU	9	0.959003
30min_MT.	Q9H3H3	0.146544	0.349455	0.322849	C11orf68	UPF0696 p	CK068_HUI	9	1.106915
15min_MT.	P51965	-0.11411	0.350085	0.323216	UBE2E1	Ubiquitin-c	UB2E1_HUI	1	0.923954
30min_MT.	O00423	0.099367	0.350635	0.32339	EML1	Echinoderm	EMAL1_HU	13	1.071303
15min_MT.	O75153	-0.00847	0.350586	0.32339	CLUH	Clustered n	CLU_HUMA	29	0.994145
15min_MT.	Q8WVJ2	0.076092	0.350619	0.32339	NUDCD2	NudC doma	NUDC2_HU	5	1.054159
30min_MT.	Q9NSD9	-0.09203	0.350618	0.32339	FARSB	Phenylalan	SYFB_HUMA	18	0.938201
30min_MT.	Q9BTY7	0.0826	0.350749	0.323392	HGH1	Protein HG	HGH1_HUM	3	1.058925
15min_MT.	Q9Y530	0.183008	0.350761	0.323392	OARD1	ADP-ribose	OARD1_HU	3	1.135249
15min_MT.	Q12959	0.055172	0.351326	0.323611	DLG1	Disks large	DLG1_HUM	14	1.038983
15min_MT.	Q8IV48	0.00351	0.35133	0.323611	ERI1	3'-5' exorib	ERI1_HUMA	4	1.002436
15min_MT.	Q8NEB9	0.229351	0.351443	0.323639	PIK3C3	Phosphatic	PK3C3_HUI	4	1.172308
15min_MT.	Q96SI9	0.085959	0.351414	0.323639	STRBP	Spermatid	STRBP_HUI	8	1.061393
30min_MT.	P52565	0.030605	0.351986	0.323865	ARHGDIA	Rho GDP-di	GDIR1_HUI	9	1.02144
30min_MT.	Q2TAL8	-0.26687	0.352056	0.323865	QRICH1	Transcripti	QRIC1_HUI	1	0.831121
30min_MT.	Q58WW2	0.081228	0.352056	0.323865	DCAF6	DDB1- and	DCAF6_HU	14	1.057918
30min_MT.	Q99570	-0.19974	0.351953	0.323865	PIK3R4	Phosphoin	PI3R4_HUI	1	0.870706
15min_MT.	Q9P2M7	0.081916	0.351955	0.323865	CGN	Cingulin	CING_HUM	35	1.058423
30min_MT.	P50395	0.020498	0.352248	0.323923	GDI2	Rab GDP di	GDIB_HUM	31	1.01431
15min_MT.	Q8NBK3	0.063455	0.352182	0.323923	SUMF1	Formylglyc	SUMF1_HU	6	1.044965
30min_MT.	Q9BUL9	0.091663	0.352301	0.323933	RPP25	Ribonuclea	RPP25_HUI	4	1.065597
15min_MT.	Q8N3P4	-0.13308	0.352397	0.323941	VPS8	Vacuolar pi	VPS8_HUM	4	0.911881
15min_MT.	P04004	0.150228	0.352549	0.324008	VTN	Vitronectir	VTNC_HUM	11	1.109745
30min_MT.	O95573	-0.39402	0.352772	0.324039	ACSL3	Fatty acid C	ACSL3_HUI	2	0.761006
30min_MT.	P13010	0.027258	0.352833	0.324039	XRCC5	X-ray repair	XRCC5_HUI	26	1.019073
30min_MT.	P21580	0.006763	0.352905	0.324039	TNFAIP3	Tumor necr	TNAP3_HUI	4	1.004699
30min_MT.	Q15021	0.027831	0.352739	0.324039	NCAPD2	Condensin	CND1_HUM	10	1.019478
15min_MT.	Q8NHH9	-0.14236	0.352879	0.324039	ATL2	Atlastin-2	ATLA2_HUI	7	0.906038
30min_MT.	Q9Y520	0.054991	0.352672	0.324039	PRRC2C	Protein PRI	PRC2C_HUI	32	1.038853
30min_MT.	O95208	0.047563	0.353117	0.32415	EPN2	Epsin-2	EPN2_HUM	1	1.033517
30min_MT.	Q9P289	0.096573	0.353101	0.32415	STK26	Serine/thre	STK26_HUI	11	1.069231
30min_MT.	Q15334	-0.11289	0.353314	0.324293	LLGL1	Lethal(2) gi	L2GL1_HUI	11	0.924732
15min_MT.	P25705	0.044561	0.353449	0.32434	ATP5F1A	ATP synthas	ATPA_HUM	17	1.031369
15min_MT.	O00628	0.169951	0.354084	0.324648	PEX7	Peroxisoma	PEX7_HUM	1	1.12502
30min_MT.	P13073	0.181082	0.354158	0.324648	COX4I1	Cytochrom	COX41_HU	5	1.133734
30min_MT.	P14384	0.254979	0.353896	0.324648	CPM	Carboxypep	CBPM_HUM	1	1.193318
15min_MT.	Q13636;Q9	0.202392	0.354066	0.324648	RAB31;RAB	Ras-related	RAB31_HUI	2	1.150605

15min_MT.	Q15084	0.082128	0.354094	0.324648	PDIA6	Protein dis	PDIA6_HUI	13	1.058579
15min_MT.	Q8IWZ8	0.291514	0.353929	0.324648	SUGP1	SURP and G	SUGP1_HU	8	1.223924
30min_MT.	P00167	0.127823	0.354282	0.324686	CYB5A	Cytochrom	CYB5_HUM	4	1.092643
15min_MT.	P52789	0.136442	0.354251	0.324686	HK2	Hexokinase	HXK2_HUM	20	1.099191
30min_MT.	P53396	0.072596	0.354376	0.324716	ACLY	ATP-citrate	ACLY_HUM	30	1.051607
15min_MT.	Q14696	0.118789	0.354398	0.324716	MESD	LRP chaper	MESD_HUM	11	1.085823
15min_MT.	Q5SSJ5	0.176657	0.354636	0.324858	HP1BP3	Heterochro	HP1B3_HU	3	1.130262
30min_MT.	P20962	-0.01364	0.355048	0.325197	PTMS	Parathymo	PTMS_HUM	5	0.990589
30min_MT.	Q96FW1	0.035754	0.35522	0.32528	OTUB1	Ubiquitin t	OTUB1_HU	12	1.025092
15min_MT.	Q9BTE7	0.075136	0.355221	0.32528	DCUN1D5	DCN1-like p	DCNL5_HU	4	1.053461
15min_MT.	Q8NB15	0.314518	0.355386	0.325355	ZNF511	Zinc finger	ZN511_HUI	2	1.243596
30min_MT.	P62273	-0.51501	0.355496	0.325417	RPS29	Small ribos	RS29_HUM	1	0.699788
30min_MT.	Q5U5X0	0.252932	0.3556	0.325444	LYRM7	Complex III	LYRM7_HU	3	1.191626
15min_MT.	Q96QZ7	0.007799	0.355608	0.325444	MAGI1	Membrane-	MAGI1_HU	7	1.00542
15min_MT.	P51970	0.082898	0.355717	0.325505	NDUFA8	NADH dehy	NDUA8_HU	4	1.059144
15min_MT.	Q9H0U6	0.112305	0.355765	0.325511	MRPL18	Large ribos	RM18_HUM	2	1.080954
15min_MT.	Q9ULX6	-0.09108	0.355946	0.325524	AKAP8L	A-kinase an	AKP8L_HUI	3	0.938823
30min_MT.	Q9UGV2	0.072857	0.356108	0.325634	NDRG3	Protein ND	NDRG3_HU	12	1.051798
15min_MT.	P49792	-0.01846	0.356392	0.325813	RANBP2	E3 SUMO-p	RBP2_HUM	68	0.987289
15min_MT.	Q9H993	0.169575	0.356581	0.325813	ARMT1	Damage-co	ARMT1_HU	7	1.124727
15min_MT.	Q9Y490	-0.02363	0.356545	0.325813	TLN1	Talin-1	TLN1_HUM	115	0.983757
15min_MT.	O00459	0.173013	0.356869	0.325949	PIK3R2	Phosphatid	P85B_HUM	1	1.127411
30min_MT.	Q9P2M7	-0.05283	0.356869	0.325949	CGN	Cingulin	CING_HUM	35	0.964043
30min_MT.	O60931	0.0081	0.357309	0.326032	CTNS	Cystinosin	CTNS_HUM	1	1.00563
30min_MT.	Q9H3G5	0.049986	0.357254	0.326032	CPVL	Probable se	CPVL_HUM	8	1.035255
15min_MT.	Q9UHK6	0.189552	0.357124	0.326032	AMACR	Alpha-meth	AMACR_HU	4	1.14041
30min_MT.	P28676	0.014791	0.357746	0.326127	GCA	Grancalcin	GRAN_HUM	4	1.010305
30min_MT.	Q9NZ63	-0.16411	0.357797	0.326127	C9orf78	Splicing fac	TLS1_HUM	2	0.89248
30min_MT.	O00459	-0.81514	0.35802	0.326138	PIK3R2	Phosphatid	P85B_HUM	1	0.568354
15min_MT.	P51114	-0.46258	0.357979	0.326138	FXR1	RNA-bindir	FXR1_HUM	6	0.725687
15min_MT.	Q6DD88	-0.09547	0.358362	0.326303	ATL3	Atlastin-3	ATLA3_HUI	13	0.93597
30min_MT.	Q6PII5	0.381827	0.358412	0.326303	HAGHL	Hydroxyacy	HAGHL_HU	2	1.302991
30min_MT.	Q7Z6K5	0.044884	0.358401	0.326303	ARPIN	Arpin	ARPIN_HUI	4	1.0316
30min_MT.	Q96DT6	0.197339	0.358424	0.326303	ATG4C	Cysteine pr	ATG4C_HUI	1	1.146581
15min_MT.	Q15642	0.199676	0.359282	0.326969	TRIP10	Cdc42-inte	CIP4_HUM	9	1.14844
15min_MT.	P62993	-0.07689	0.359621	0.327164	GRB2	Growth fac	GRB2_HUM	10	0.948098
30min_MT.	Q8N1G2	0.044838	0.359581	0.327164	CMTR1	Cap-specifi	CMTR1_HU	2	1.031567
30min_MT.	P09493	0.116026	0.35986	0.32719	TPM1	Tropomyos	TPM1_HUM	7	1.083745
15min_MT.	P29558	-0.30647	0.359901	0.32719	RBMS1	RNA-bindir	RBMS1_HU	2	0.808616
15min_MT.	Q7Z417	0.118769	0.359735	0.32719	NUFIP2	FMR1-inter	NUFP2_HU	11	1.085808
15min_MT.	Q96P70	-0.09561	0.35982	0.32719	IPO9	Importin-9	IPO9_HUM	31	0.935878
15min_MT.	Q9BTE1	0.05901	0.359824	0.32719	DCTN5	Dynactin su	DCTN5_HU	3	1.041751
30min_MT.	O15355	-0.14817	0.360043	0.327281	PPM1G	Protein pho	PPM1G_HU	18	0.902396
30min_MT.	O75340	0.143874	0.360124	0.327317	PDCD6	Programme	PDCD6_HU	5	1.104868
30min_MT.	Q9NVS9	0.024996	0.360196	0.327337	PNPO	Pyridoxine-	PNPO_HUM	8	1.017477
30min_MT.	Q9HD42	0.07719	0.360504	0.327545	CHMP1A	Charged mu	CHM1A_HU	6	1.054962
15min_MT.	O14530	0.065384	0.360659	0.327587	TXNDC9	Thioredoxi	TXND9_HU	4	1.046363
15min_MT.	P62249	0.194156	0.360672	0.327587	RPS16	Small ribos	RS16_HUM	7	1.144055
15min_MT.	Q8TCT9	-0.26789	0.361004	0.32785	HM13	Minor histo	HM13_HUM	3	0.830534
30min_MT.	Q9BTK6	-0.65515	0.361243	0.327991	PAGR1	PAXIP1-ass	PAGR1_HU	1	0.63501
15min_MT.	A6NKH3;P6	0.098644	0.361469	0.328158	RPL37AP8;	Putative rib	RL37L_HUM	1	1.070767
30min_MT.	Q9Y217	-0.65635	0.361658	0.328216	MTMR6	Myotubula	MTMR6_HU	2	0.634483

15min_MT.	Q9NNW5	0.004399	0.361728	0.328241	WDR6	tRNA (34-2'	WDR6_HUI		6	1.003054
15min_MT.	P18085	0.020466	0.362086	0.32832	ARF4	ADP-ribosy	ARF4_HUM		5	1.014287
15min_MT.	P36578	-0.11798	0.362004	0.32832	RPL4	Large ribos	RL4_HUMA		13	0.921478
15min_MT.	P61106	-0.05295	0.361903	0.32832	RAB14	Ras-related	RAB14_HUI		13	0.963966
15min_MT.	Q13439	0.10935	0.362151	0.32832	GOLGA4	Golgin subf	GOGA4_HU		59	1.078742
30min_MT.	Q9HA47	-0.17433	0.362002	0.32832	UCK1	Uridine-cyt	UCK1_HUM		1	0.886177
30min_MT.	Q9Y570	0.001252	0.361967	0.32832	PPME1	Protein pho	PPME1_HU		13	1.000868
15min_MT.	Q9BTK6	-0.47817	0.362311	0.328427	PAGR1	PAXIP1-ass	PAGR1_HU		1	0.717889
15min_MT.	P09496	0.083597	0.362389	0.32846	CLTA	Clathrin lig	CLCA_HUM		7	1.059657
15min_MT.	Q96PK6	0.062477	0.362637	0.328512	RBM14	RNA-bindir	RBM14_HU		15	1.044257
15min_MT.	Q8WXB1	-0.3138	0.363273	0.328902	METTL21A	Protein N-l	MT21A_HU		4	0.804522
15min_MT.	P0C0L4	0.143961	0.363348	0.328909	C4A	Compleme	CO4A_HUM		62	1.104934
30min_MT.	O75190	-0.05043	0.363792	0.329083	DNAJB6	DnaJ homo	DNJB6_HUI		3	0.965647
15min_MT.	Q2NKX8	-0.03796	0.363778	0.329083	ERCC6L	DNA excisic	ERC6L_HUI		9	0.97403
30min_MT.	Q969Q5	-0.27881	0.363702	0.329083	RAB24	Ras-related	RAB24_HUI		1	0.824273
30min_MT.	Q6PKG0	-0.07406	0.363858	0.329104	LARP1	La-related p	LARP1_HUI		9	0.949958
30min_MT.	Q96EY1	-0.28147	0.363982	0.329179	DNAJA3	DnaJ homo	DNJA3_HUI		5	0.82275
30min_MT.	Q9NVD7	-0.01081	0.364086	0.329235	PARVA	Alpha-parvi	PARVA_HUI		4	0.992532
15min_MT.	Q8ND56	0.311374	0.364621	0.329509	LSM14A	Protein LSM	LS14A_HUI		4	1.240889
30min_MT.	Q9Y2V7	-0.12888	0.364642	0.329509	COG6	Conserved	COG6_HUM		10	0.914543
15min_MT.	P33240	0.123737	0.365163	0.329789	CSTF2	Cleavage sti	CSTF2_HUI		13	1.089554
30min_MT.	Q15814	0.107708	0.366097	0.330534	TBCC	Tubulin-spe	TBCC_HUM		10	1.077515
30min_MT.	Q96AT1	0.250717	0.366156	0.330534	KIAA1143	Uncharacte	K1143_HUI		3	1.189799
30min_MT.	Q9HAN9	0.165078	0.366154	0.330534	NMNAT1	Nicotinami	NMNA1_HU		2	1.121227
30min_MT.	Q5VSY0	-0.46071	0.366223	0.33055	GKAP1	G kinase-an	GKAP1_HU		1	0.726626
30min_MT.	Q96GS4	0.123883	0.366259	0.33055	BORCS6	BLOC-1-rel	BORC6_HU		12	1.089664
15min_MT.	Q6SZW1	0.000764	0.366413	0.330575	SARM1	NAD(+) hyd	SARM1_HU		4	1.00053
30min_MT.	P82094	0.00634	0.366571	0.330641	TMF1	TATA eleme	TMF1_HUM		24	1.004404
15min_MT.	O43795	-0.06203	0.366774	0.330786	MYO1B	Unconvent	MYO1B_HU		19	0.957915
30min_MT.	Q15084	0.032798	0.366817	0.330787	PDIA6	Protein dis	PDIA6_HUI		13	1.022994
30min_MT.	O15379	-0.38662	0.366972	0.330837	HDAC3	Histone dea	HDAC3_HU		2	0.764922
30min_MT.	P00742	-0.37095	0.366999	0.330837	F10	Coagulatio	FA10_HUM		5	0.773275
30min_MT.	Q15149	0.0565	0.367424	0.331143	PLEC	Plectin	PLEC_HUM		144	1.03994
15min_MT.	Q9P0L0	0.056352	0.367395	0.331143	VAPA	Vesicle-ass	VAPA_HUM		11	1.039833
30min_MT.	P49458	-0.0564	0.36766	0.331318	SRP9	Signal reco	SRP09_HUI		6	0.961659
30min_MT.	P09211	0.089725	0.368024	0.331451	GSTP1	Glutathion	GSTP1_HUI		14	1.064167
15min_MT.	P62263	0.003408	0.368147	0.331451	RPS14	Small ribos	RS14_HUM		2	1.002365
15min_MT.	Q14684	0.073102	0.368126	0.331451	RRP1B	Ribosomal	RRP1B_HUI		2	1.051976
15min_MT.	Q9UNX4	0.529769	0.368064	0.331451	WDR3	WD repeat-	WDR3_HUI		1	1.443698
30min_MT.	P02656	0.376443	0.368222	0.331481	APOC3	Apolipopro	APOC3_HU		1	1.298137
15min_MT.	P61978	-0.05771	0.368352	0.331547	HNRNPK	Heterogene	HNRPK_HU		23	0.960789
15min_MT.	Q96AY3	0.088382	0.368381	0.331547	FKBP10	Peptidyl-pr	FKB10_HUI		18	1.063177
30min_MT.	P43487	0.137174	0.368704	0.33174	RANBP1	Ran-specifi	RANG_HUM		6	1.099749
15min_MT.	P35573	-0.04198	0.368894	0.33179	AGL	Glycogen d	GDE_HUMA		29	0.971319
15min_MT.	P78347	0.071135	0.368919	0.33179	GTF2I	General tra	GTF2I_HUM		24	1.050543
30min_MT.	Q9H0U6	0.038078	0.369295	0.331924	MRPL18	Large ribos	RM18_HUM		2	1.026745
15min_MT.	Q9HAU0	0.115078	0.369231	0.331924	PLEKHA5	Pleckstrin	PKHA5_HU		7	1.083033
30min_MT.	P15735	0.225574	0.369406	0.331973	PHKG2	Phosphoryl	PHKG2_HU		3	1.169242
30min_MT.	P35659	0.086363	0.369648	0.332076	DEK	Protein DEI	DEK_HUMA		15	1.06169
15min_MT.	Q9UKZ1	0.019684	0.369717	0.3321	CNOT11	CCR4-NOT t	CNO11_HU		2	1.013737
15min_MT.	P13674	0.0682	0.369903	0.332229	P4HA1	Prolyl 4-hy	P4HA1_HU		15	1.048408
30min_MT.	Q9BXF6	-0.23187	0.369995	0.332236	RAB11FIP5	Rab11 fami	RFIP5_HUM		2	0.851532

15min_MT.	Q9H8H3	0.011136	0.370157	0.332304	TMT1A	Thiol S-met	TMT1A_HU		3	1.007749
15min_MT.	Q9BRZ2	-0.94755	0.370374	0.332461	TRIM56	E3 ubiquiti	TRI56_HUN		2	0.518512
15min_MT.	P62937	0.096988	0.370535	0.332568	PPIA	Peptidyl-pr	PPIA_HUMA		13	1.069538
15min_MT.	P29144	-0.07122	0.370757	0.332728	TPP2	Tripeptidyl	TPP2_HUM		34	0.951834
15min_MT.	O95336	-0.0584	0.370812	0.332732	PGLS	6-phospho	6PGL_HUM		13	0.960328
30min_MT.	Q96HQ2	-0.2399	0.370846	0.332732	CDKN2AIPN	CDKN2AIP I	C2AIL_HUN		2	0.846806
15min_MT.	P46783	0.002759	0.371236	0.333044	RPS10	Small ribos	RS10_HUM		4	1.001914
15min_MT.	P62760	0.194774	0.371327	0.333087	VSNL1	Visinin-like	VISL1_HUN		4	1.144544
30min_MT.	O43896	0.456475	0.371422	0.333116	KIF1C	Kinesin-like	KIF1C_HUN		2	1.372185
15min_MT.	O60341	-0.07586	0.371629	0.333205	KDM1A	Lysine-spec	KDM1A_HU		7	0.948778
15min_MT.	Q6YN16	0.060392	0.371608	0.333205	HSDL2	Hydroxyste	HSDL2_HUI		9	1.042749
30min_MT.	P05787	-0.0653	0.372136	0.333552	KRT8	Keratin, typ	K2C8_HUM		29	0.955745
30min_MT.	Q06787	-0.12688	0.372143	0.333552	FMR1	Fragile X m	FMR1_HUN		6	0.915808
30min_MT.	P62879	-0.151	0.372312	0.333665	GNB2	Guanine nu	GBB2_HUM		9	0.900629
30min_MT.	Q12986	-0.2609	0.372452	0.3337	NFX1	Transcripti	NFX1_HUM		4	0.834566
30min_MT.	Q2TAA2	0.015054	0.372721	0.3337	IAH1	Isoamyl ace	IAH1_HUM		10	1.010489
30min_MT.	Q562E7	0.114427	0.372734	0.3337	WDR81	WD repeat-	WDR81_HU		4	1.082545
15min_MT.	Q6IQ49	0.019107	0.372585	0.3337	SDE2	Splicing reg	SDE2_HUM		4	1.013332
15min_MT.	Q7Z3K3	-0.18937	0.372655	0.3337	POGZ	Pogo trans	POGZ_HUN		1	0.876988
30min_MT.	Q8TCD5	0.035837	0.372735	0.3337	NT5C	5'(3')-deox	NT5C_HUM		8	1.025151
15min_MT.	Q92805	0.369516	0.372566	0.3337	GOLGA1	Golgin subf	GOGA1_HU		9	1.291919
30min_MT.	Q13085	-0.07564	0.372945	0.333776	ACACA	Acetyl-CoA	ACACA_HUI		14	0.948924
30min_MT.	Q8WWM7	-0.03684	0.372948	0.333776	ATXN2L	Ataxin-2-lik	ATX2L_HUN		30	0.974787
15min_MT.	Q96A65	0.172555	0.372925	0.333776	EXOC4	Exocyst cor	EXOC4_HUI		7	1.127053
30min_MT.	Q9Y587	0.108504	0.373149	0.333917	AP4S1	AP-4 compl	AP4S1_HUI		3	1.07811
15min_MT.	Q9NYL9	0.127772	0.373351	0.334022	TMOD3	Tropomodu	TMOD3_HU		17	1.092605
15min_MT.	Q10567	-0.03637	0.374027	0.334469	AP1B1	AP-1 compl	AP1B1_HUI		20	0.975103
15min_MT.	Q14738	0.044786	0.374065	0.334469	PPP2R5D	Serine/thre	2A5D_HUM		7	1.03153
15min_MT.	Q96G74	0.454468	0.374051	0.334469	OTUD5	OTU domai	OTUD5_HU		1	1.370277
30min_MT.	Q5JXB2	-0.86977	0.374292	0.33452	UBE2NL	Putative ub	UE2NL_HUI		1	0.547234
30min_MT.	Q9NR31	0.010967	0.374229	0.33452	SAR1A	Small COPII	SAR1A_HUI		3	1.00763
30min_MT.	P05937;P2	-0.42822	0.374531	0.334618	CALB1;CALI	Calbindin;C	CALB1_HUI		1	0.743181
30min_MT.	P42858	-0.0853	0.374587	0.334618	HTT	Huntingtin	HD_HUMAI		6	0.942591
30min_MT.	P98082	-0.138	0.374616	0.334618	DAB2	Disabled hc	DAB2_HUM		1	0.908779
30min_MT.	O60826	-0.08829	0.374794	0.334739	CCDC22	Coiled-coil	CCD22_HU		2	0.940636
15min_MT.	P15374	-0.10362	0.375066	0.33481	UCHL3	Ubiquitin c	UCHL3_HU		10	0.930697
30min_MT.	Q08170	-0.06816	0.375089	0.33481	SRSF4	Serine/argi	SRSF4_HUN		12	0.953856
30min_MT.	Q9BRJ2	0.632993	0.37493	0.33481	MRPL45	Large ribos	RM45_HUN		1	1.550779
15min_MT.	Q9H1K0	0.044372	0.375097	0.33481	RBSN	Rabenosyn-	RBNS5_HUI		2	1.031234
15min_MT.	P06748	-0.12664	0.375319	0.334901	NPM1	Nucleopho	NPM_HUM		10	0.915964
15min_MT.	Q8N9V3	0.094119	0.375434	0.334966	WDSUB1	WD repeat,	WSDU1_HU		1	1.067413
15min_MT.	P02533	0.225708	0.375702	0.335142	KRT14	Keratin, typ	K1C14_HUI		33	1.169351
15min_MT.	Q5JRA6	-0.2417	0.375717	0.335142	MIA3	Transport a	TGO1_HUN		10	0.845746
30min_MT.	Q96F63	0.131552	0.375779	0.335159	CCDC97	Coiled-coil	CCD97_HU		2	1.095471
30min_MT.	Q9NYV4	0.47764	0.375861	0.335194	CDK12	Cyclin-depe	CDK12_HU		3	1.392464
30min_MT.	Q3KRA6	-0.40052	0.376182	0.335407	C2orf76	UPF0538 p	CB076_HUI		2	0.757585
15min_MT.	Q9NZD8	0.131057	0.376302	0.335472	SPG21	Maspardin	SPG21_HUI		1	1.095096
30min_MT.	P34932	0.03209	0.376432	0.335474	HSPA4	Heat shock	HSP74_HUI		49	1.022492
15min_MT.	Q9H4M9	0.022792	0.376416	0.335474	EHD1	EH domain-	EHD1_HUM		11	1.015924
15min_MT.	O75718	0.234155	0.376583	0.335526	CRTAP	Cartilage-as	CRTAP_HUI		8	1.176218
30min_MT.	Q13257	-0.06226	0.37662	0.335526	MAD2L1	Mitotic spi	MD2L1_HU		5	0.957762
15min_MT.	Q9BVM2	0.03106	0.376578	0.335526	DPCD	Protein DP	DPCD_HUN		7	1.021763

15min_MT.	Q9Y2I1	-0.45139	0.376795	0.335606	NISCH	Nischarin	NISCH_HUM	2	0.731338
30min_MT.	P06748	0.055774	0.377053	0.335677	NPM1	Nucleopho	NPM_HUM	10	1.039417
30min_MT.	P26583	-0.139	0.377132	0.335677	HMGB2	High mobil	HMGB2_HU	5	0.90815
15min_MT.	P78537	-0.05701	0.377007	0.335677	BLOC1S1	Biogenesis	BL1S1_HUM	3	0.961255
30min_MT.	Q9UKM9	0.171016	0.376948	0.335677	RALY	RNA-bindir	RALY_HUM	5	1.125851
30min_MT.	Q9UKN8	-0.08066	0.377107	0.335677	GTF3C4	General tra	TF3C4_HUM	16	0.945626
30min_MT.	P60510	0.104013	0.377202	0.3357	PPP4C	Serine/thre	PP4C_HUM	10	1.074759
30min_MT.	O76014	0.412789	0.377362	0.335767	KRT37	Keratin, typ	KRT37_HUI	2	1.331257
15min_MT.	Q03113	-0.65719	0.377467	0.335822	GNA12	Guanine nu	GNA12_HU	1	0.634113
30min_MT.	P02792	0.073673	0.377739	0.335937	FTL	Ferritin ligh	FRIL_HUMA	4	1.052393
15min_MT.	P31939	0.077617	0.377735	0.335937	ATIC	Bifunctiona	PUR9_HUM	29	1.055274
30min_MT.	Q13554	0.396779	0.377775	0.335937	CAMK2B	Calcium/ca	KCC2B_HUI	3	1.316565
15min_MT.	Q9P2K8	0.154795	0.377854	0.335937	EIF2AK4	eIF-2-alpha	E2AK4_HUI	12	1.113264
15min_MT.	O14908	-0.22814	0.378029	0.335993	GIPC1	PDZ domaii	GIPC1_HUM	5	0.853737
15min_MT.	Q9H1Y0	0.15561	0.378087	0.335993	ATG5	Autophagy	ATG5_HUM	10	1.113893
30min_MT.	Q70CQ2	0.146472	0.378192	0.336046	USP34	Ubiquitin c	UBP34_HU	7	1.10686
15min_MT.	Q8WUX2	0.023131	0.378391	0.336185	CHAC2	Glutathion	CHAC2_HU	1	1.016163
15min_MT.	Q86WR0	-0.07212	0.378673	0.336321	CCDC25	Coiled-coil	CCD25_HU	6	0.95124
30min_MT.	Q9P1Y5	-0.11971	0.379558	0.336941	CAMSAP3	Calmodulir	CAMP3_HU	8	0.920373
15min_MT.	O94760	0.074403	0.379955	0.337057	DDAH1	N(G),N(G)-c	DDAH1_HU	15	1.052925
15min_MT.	Q15036	0.036913	0.379957	0.337057	SNX17	Sorting nex	SNX17_HUI	4	1.025916
15min_MT.	Q15393	-0.01697	0.379861	0.337057	SF3B3	Splicing fac	SF3B3_HUM	41	0.988307
15min_MT.	Q562R1	-0.36793	0.379885	0.337057	ACTBL2	Beta-actin-l	ACTBL_HUM	3	0.774893
15min_MT.	Q96SY0	0.004536	0.38025	0.337263	INTS14	Integrator	INT14_HUM	9	1.003149
15min_MT.	P22307	0.057738	0.380443	0.337396	SCP2	Sterol carri	SCP2_HUM	14	1.040832
15min_MT.	P55786	-0.04054	0.380489	0.337398	NPEPPS	Puromycin	PSA_HUMA	38	0.972294
15min_MT.	Q9BR11	-0.14185	0.3811	0.337863	ZSWIM1	Zinc finger	ZSWM1_HU	1	0.906354
30min_MT.	Q99538	0.175231	0.381261	0.337929	LGMN	Legumain	LGMN_HUM	6	1.129145
15min_MT.	P51956	0.128769	0.381613	0.3382	NEK3	Serine/thre	NEK3_HUM	3	1.09336
15min_MT.	Q9H4Z3	0.253172	0.381653	0.3382	PCIF1	mRNA (2'-O	CAPAM_HU	5	1.191825
30min_MT.	P62249	0.167149	0.381793	0.338285	RPS16	Small ribos	RS16_HUM	7	1.122837
15min_MT.	Q99653	-0.04721	0.381843	0.338291	CHP1	Calcineurin	CHP1_HUM	1	0.967806
30min_MT.	Q04760	0.031469	0.38223	0.338557	GLO1	Lactoylglut	LGUL_HUM	10	1.022052
15min_MT.	O95340	-0.05561	0.38255	0.33865	PAPSS2	Bifunctiona	PAPS2_HUI	4	0.962187
15min_MT.	Q9UBT7	-0.13816	0.382549	0.33865	CTNNAL1	Alpha-catul	CTNL1_HUI	1	0.908677
15min_MT.	P38159	0.124082	0.382716	0.338758	RBMX	RNA-bindir	RBMX_HUM	13	1.089814
30min_MT.	O94927	0.268886	0.382819	0.338778	HAUS5	HAUS augm	HAUS5_HU	1	1.204877
15min_MT.	Q9HCD5	0.138022	0.382825	0.338778	NCOA5	Nuclear rec	NCOA5_HU	6	1.100395
30min_MT.	P30086	-0.10205	0.383091	0.338955	PEBP1	Phosphatic	PEBP1_HUI	13	0.931707
15min_MT.	P54725	0.133601	0.383112	0.338955	RAD23A	UV excisior	RD23A_HU	6	1.097029
30min_MT.	P62495	0.046163	0.383337	0.339116	ETF1	Eukaryotic	ERF1_HUM	17	1.032515
30min_MT.	Q5VYK3	-0.08566	0.383472	0.339197	ECPAS	Proteasom	ECM29_HU	17	0.942351
15min_MT.	Q92785	0.041975	0.384076	0.339578	DPF2	Zinc finger	REQU_HUM	7	1.029523
30min_MT.	P09496	0.136759	0.384169	0.33958	CLTA	Clathrin lig	CLCA_HUM	7	1.099432
15min_MT.	Q10471	-0.25493	0.384202	0.33958	GALNT2	Polypeptid	GALT2_HUI	8	0.838028
30min_MT.	Q9Y2R5	-0.3071	0.384384	0.339696	MRPS17	Small ribos	RT17_HUM	1	0.808267
15min_MT.	O00443	-1.22106	0.3845	0.339722	PIK3C2A	Phosphatic	P3C2A_HUI	1	0.428969
30min_MT.	P98170	0.011031	0.385215	0.340205	XIAP	E3 ubiquiti	XIAP_HUM	12	1.007675
30min_MT.	Q13098	0.032732	0.385338	0.340205	GPS1	COP9 signa	CSN1_HUM	13	1.022948
15min_MT.	Q17R98	0.100399	0.385172	0.340205	ZNF827	Zinc finger	ZN827_HUI	2	1.07207
15min_MT.	Q96P47	0.290233	0.385237	0.340205	AGAP3	Arf-GAP wit	AGAP3_HU	1	1.222838
15min_MT.	Q9P0P8	0.009086	0.385296	0.340205	MTRES1	Mitochond	MRES1_HU	1	1.006318

30min_MT.	Q99650	-0.06399	0.385451	0.340245	OSMR	Oncostatin	OSMR_HUM	2	0.956615
30min_MT.	Q9Y3P9	-0.046	0.385484	0.340245	RABGAP1	Rab GTPase	RBGP1_HU	28	0.968621
30min_MT.	Q9P265	-0.0113	0.385565	0.340279	DIP2B	Disco-inter	DIP2B_HUM	33	0.992195
30min_MT.	Q99961	-0.09723	0.385814	0.340421	SH3GL1	Endophilin	SH3G1_HU	11	0.934829
30min_MT.	Q8WUM0	-0.17868	0.38592	0.340477	NUP133	Nuclear po	NU133_HU	9	0.883508
15min_MT.	Q15185	0.085922	0.386155	0.340646	PTGES3	Prostagland	TEBP_HUM	7	1.061366
15min_MT.	Q13112	-0.29885	0.386403	0.34077	CHAF1B	Chromatin	CAF1B_HUI	3	0.812899
30min_MT.	Q9BXS5	-0.08033	0.386342	0.34077	AP1M1	AP-1 compl	AP1M1_HU	18	0.94584
15min_MT.	Q9BYB4	-0.27243	0.386426	0.34077	GNB1L	Guanine nu	GNB1L_HU	10	0.827925
30min_MT.	Q9NNW5	0.114259	0.38667	0.340908	WDR6	tRNA (34-2'	WDR6_HUI	6	1.082419
15min_MT.	O60701	0.085379	0.386726	0.340919	UGDH	UDP-glucos	UGDH_HUM	17	1.060966
15min_MT.	Q6Y7W6	0.134652	0.386924	0.341054	GIGYF2	GRB10-inte	GGYF2_HUI	14	1.097828
15min_MT.	Q13868	0.178935	0.387138	0.341205	EXOSC2	Exosome cc	EXOS2_HUI	6	1.132048
30min_MT.	Q8NDI1	-0.03148	0.387267	0.34128	EHBP1	EH domain	EHBP1_HUI	13	0.978418
15min_MT.	O14776	-0.05299	0.38738	0.341307	TCERG1	Transcripti	TCRG1_HUI	14	0.963937
30min_MT.	P49005	-0.04623	0.387385	0.341307	POLD2	DNA polym	DPOD2_HU	11	0.968464
30min_MT.	P14314	0.106329	0.387639	0.341493	PRKCSH	Glucosidas	GLU2B_HU	23	1.076486
30min_MT.	P27487	0.081209	0.387912	0.341656	DPP4	Dipeptidyl	DPP4_HUM	8	1.057904
15min_MT.	P10321	-0.23466	0.388096	0.34178	HLA-C	HLA class I	HLAC_HUM	5	0.849883
15min_MT.	P36954	0.165023	0.388304	0.341847	POLR2I	DNA-direct	RPB9_HUM	4	1.121184
30min_MT.	Q9UKZ1	-0.34694	0.38825	0.341847	CNOT11	CCR4-NOT t	CNO11_HU	2	0.786248
30min_MT.	Q9BWH6	0.064095	0.388374	0.34187	RPAP1	RNA polym	RPAP1_HUI	5	1.045429
30min_MT.	P55327	0.07929	0.388494	0.341938	TPD52	Tumor prot	TPD52_HUI	7	1.056498
30min_MT.	Q9P035	0.056355	0.388594	0.341987	HACD3	Very-long-c	HACD3_HU	4	1.039835
30min_MT.	Q8TBC4	0.014463	0.388823	0.342073	UBA3	NEDD8-acti	UBA3_HUM	14	1.010076
15min_MT.	Q9NVX7	-0.00061	0.389023	0.342211	KBTBD4	Kelch repea	KBTB4_HUI	3	0.999575
15min_MT.	P35998	0.049609	0.389138	0.342273	PSMC2	26S protea	PRS7_HUM	24	1.034985
15min_MT.	Q7Z3Y8	-0.93855	0.389192	0.342282	KRT27	Keratin, typ	K1C27_HUI	1	0.521759
30min_MT.	Q96GK7	0.088005	0.389482	0.34244	FAHD2A	Oxaloaceta	FAH2A_HUI	8	1.062899
15min_MT.	P17987	-0.05186	0.389862	0.342717	TCP1	T-complex	TCPA_HUM	29	0.964695
15min_MT.	Q12905	-0.00756	0.389996	0.342797	ILF2	Interleukin	ILF2_HUMA	14	0.994775
30min_MT.	Q9Y295	-0.12452	0.390065	0.342819	DRG1	Developme	DRG1_HUM	14	0.917307
15min_MT.	P35613	0.000561	0.390285	0.34287	BSG	Basigin	BASI_HUMA	7	1.000389
15min_MT.	P54687	-0.01095	0.390298	0.34287	BCAT1	Branched-c	BCAT1_HUI	10	0.992442
30min_MT.	Q08830	0.149541	0.390265	0.34287	FGL1	Fibrinogen	FGL1_HUM	2	1.109217
30min_MT.	Q9Y597	-0.00428	0.390278	0.34287	KCTD3	BTB/POZ dc	KCTD3_HUI	5	0.99704
30min_MT.	O95777	0.089067	0.390362	0.342887	LSM8	U6 snRNA-a	LSM8_HUM	4	1.063682
15min_MT.	P52701	0.125623	0.390407	0.342889	MSH6	DNA misma	MSH6_HUM	5	1.090979
30min_MT.	O00303	0.094131	0.390556	0.34298	EIF3F	Eukaryotic	EIF3F_HUM	12	1.067422
30min_MT.	Q8N954	0.259394	0.390641	0.343017	GPATCH11	G patch do	GPT11_HUI	2	1.196976
30min_MT.	Q9Y5H5	-1.12871	0.390732	0.34302	PCDHA9	Protocadhe	PCDA9_HU	1	0.457324
30min_MT.	Q9H0B6	-0.11321	0.390905	0.343056	KLC2	Kinesin ligh	KLC2_HUM	5	0.924529
30min_MT.	Q969J3	-0.14125	0.390969	0.343073	BORCS5	BLOC-1-rela	BORC5_HU	4	0.906733
15min_MT.	P49023	-0.04084	0.391073	0.343099	PXN	Paxillin	PAXI_HUMA	9	0.972091
30min_MT.	P62753	-0.01875	0.391085	0.343099	RPS6	Small ribos	RS6_HUMA	5	0.987088
15min_MT.	Q9H9P8	0.259463	0.391319	0.343265	L2HGDH	L-2-hydroxy	L2HDH_HU	5	1.197033
30min_MT.	Q14240	0.107494	0.391486	0.343373	EIF4A2	Eukaryotic	IF4A2_HUM	11	1.077355
15min_MT.	P51649	0.280082	0.391712	0.343533	ALDH5A1	Succinate-s	SSDH_HUM	4	1.214264
30min_MT.	P05062	0.142237	0.391812	0.343544	ALDOB	Fructose-bi	ALDOB_HU	1	1.103615
30min_MT.	Q7Z4G1	-0.12368	0.391892	0.343572	COMMD6	COMM don	COMD6_HU	2	0.917846
15min_MT.	P12109	-0.0442	0.391982	0.343577	COL6A1	Collagen al	CO6A1_HU	14	0.96983
30min_MT.	P51809	-0.12128	0.392491	0.343831	VAMP7	Vesicle-assc	VAMP7_HU	3	0.919374

15min_MT.	Q9NP97	-0.02552	0.392418	0.343831	DYNLRB1	Dynein ligh	DLRB1_HU	7	0.982468
30min_MT.	Q9Y580	-0.31825	0.392477	0.343831	RBM7	RNA-bindir	RBM7_HUN	1	0.802043
15min_MT.	O14745	0.076992	0.392684	0.343923	NHERF1	Na(+)/H(+) e	NHRF1_HU	23	1.054817
15min_MT.	Q7Z3E2	0.417217	0.392672	0.343923	CCDC186	Coiled-coil	CC186_HU	8	1.335349
15min_MT.	O95081	0.067278	0.393015	0.344089	AGFG2	Arf-GAP do	AGFG2_HU	5	1.047738
30min_MT.	Q5VU43	-0.05044	0.39305	0.344089	PDE4DIP	Myomegali	MYOME_HU	11	0.965641
15min_MT.	Q68CZ2	-0.11593	0.393038	0.344089	TNS3	Tensin-3	TENS3_HUI	11	0.922787
15min_MT.	Q5VWJ9	0.249013	0.393314	0.344282	SNX30	Sorting nex	SNX30_HU	1	1.188394
15min_MT.	Q9BQ04	0.039411	0.393813	0.344641	RBM4B	RNA-bindir	RBM4B_HU	13	1.027694
30min_MT.	Q9NVZ3	-0.09988	0.39395	0.344723	NECAP2	Adaptin ea	NECP2_HU	5	0.933111
15min_MT.	Q9H6T3	-0.14774	0.394289	0.344904	RPAP3	RNA polym	RPAP3_HU	12	0.902663
15min_MT.	P62277	0.061468	0.394547	0.345091	RPS13	Small ribos	RS13_HUM	5	1.043527
15min_MT.	A0MZ66	0.084462	0.394764	0.345183	SHTN1	Shootin-1	SHOT1_HU	34	1.060292
15min_MT.	P51608	0.005784	0.394829	0.345183	MECP2	Methyl-CpG	MECP2_HU	4	1.004018
30min_MT.	Q99996	-0.13016	0.39506	0.345308	AKAP9	A-kinase an	AKAP9_HU	35	0.913728
15min_MT.	P31943	0.068086	0.39538	0.345472	HNRNPH1	Heterogene	HNRH1_HU	15	1.048325
15min_MT.	P04275	-0.2284	0.395546	0.345578	VWF	von Willeb	VWF_HUM	2	0.853583
15min_MT.	P02792	0.00972	0.395764	0.345614	FTL	Ferritin ligh	FRIL_HUM	4	1.00676
15min_MT.	Q16637	-0.03742	0.395745	0.345614	SMN1	Survival mc	SMN_HUM	1	0.974397
30min_MT.	P22670	-0.1338	0.395857	0.345617	RFX1	MHC class I	RFX1_HUM	11	0.911427
30min_MT.	Q9UJU6	0.119028	0.396056	0.345617	DBNL	Drebrin-lik	DBNL_HUM	17	1.086003
15min_MT.	Q04760	0.018965	0.396297	0.345631	GLO1	Lactoylglut	LGUL_HUM	10	1.013232
30min_MT.	Q9UHB9	0.274747	0.396312	0.345631	SRP68	Signal reco	SRP68_HU	6	1.209782
15min_MT.	Q9NU23	-0.03786	0.396666	0.345896	LYRM2	LYR motif-c	LYRM2_HU	2	0.974099
15min_MT.	O95376	-0.03697	0.39689	0.345941	ARIH2	E3 ubiquiti	ARI2_HUM	11	0.9747
30min_MT.	Q8WV24	-0.23024	0.396958	0.345961	PHLDA1	Pleckstrin h	PHLA1_HU	1	0.852495
30min_MT.	Q6UX53	-0.05285	0.397328	0.346168	TMT1B	Thiol S-met	TMT1B_HU	2	0.964032
15min_MT.	Q92733	-0.03428	0.397327	0.346168	PRCC	Proline-ricl	PRCC_HUM	3	0.976518
15min_MT.	Q6UXH1	0.06553	0.397578	0.346309	CRELD2	Protein dis	CREL2_HU	7	1.046469
30min_MT.	O15498	-0.11304	0.397722	0.346396	YKT6	Synaptobre	YKT6_HUM	11	0.924639
15min_MT.	Q13825	0.090435	0.397884	0.346468	AUH	Methylglut	AUHM_HU	7	1.064691
30min_MT.	P27540	0.35431	0.398009	0.346484	ARNT	Aryl hydroc	ARNT_HUM	3	1.278374
15min_MT.	Q6WKZ4	0.135519	0.398044	0.346484	RAB11FIP1	Rab11 fami	RFIP1_HUN	4	1.098488
15min_MT.	Q9H6T0	0.241656	0.398234	0.346571	ESRP2	Epithelial s	ESRP2_HUI	2	1.182349
30min_MT.	P48147	0.07462	0.398325	0.346612	PREP	Prolyl endc	PPCE_HUM	28	1.053084
30min_MT.	Q5SRH9	-0.09808	0.39854	0.346761	TTC39A	Tetratricop	TT39A_HUI	1	0.934277
30min_MT.	Q12959	-0.05918	0.398611	0.346784	DLG1	Disks large	DLG1_HUM	14	0.959809
15min_MT.	Q70UQ0	0.30859	0.398668	0.346795	IKBIP	Inhibitor o	IKIP_HUM	1	1.238496
15min_MT.	P60660	-0.09526	0.398871	0.346895	MYL6	Myosin ligh	MYL6_HUM	7	0.936101
15min_MT.	Q9Y5V0	-0.14521	0.399032	0.346996	ZNF706	Zinc finger	ZN706_HU	2	0.904246
30min_MT.	P07099	-0.10771	0.399181	0.347049	EPHX1	Epoxide hy	HYEP_HUM	8	0.928063
30min_MT.	O00244	0.109972	0.399824	0.347492	ATOX1	Copper tra	ATOX1_HU	5	1.079207
30min_MT.	P84103	-0.11975	0.399808	0.347492	SRSF3	Serine/argi	SRSF3_HUN	7	0.920348
30min_MT.	Q15311	-0.08411	0.399914	0.347493	RALBP1	RalA-bindir	RBP1_HUM	8	0.943363
15min_MT.	Q13619	-0.02784	0.400088	0.347605	CUL4A	Cullin-4A	CUL4A_HU	10	0.980888
30min_MT.	Q8N0X7	-0.07755	0.400141	0.347612	SPART	Spartin	SPART_HUI	10	0.947664
15min_MT.	Q9NY27	0.047817	0.400345	0.347751	PPP4R2	Serine/thre	PP4R2_HU	13	1.0337
30min_MT.	P02787	0.036074	0.400607	0.347824	TF	Serotransfe	TRFE_HUM	41	1.02532
15min_MT.	Q9UPN7	0.011512	0.400606	0.347824	PPP6R1	Serine/thre	PP6R1_HU	14	1.008012
15min_MT.	O94967	0.035573	0.40087	0.347878	WDR47	WD repeat-	WDR47_HU	6	1.024964
30min_MT.	P07360	0.207426	0.400784	0.347878	C8G	Compleme	CO8G_HUM	2	1.154626
30min_MT.	P17900	0.239042	0.40104	0.347878	GM2A	Ganglioside	SAP3_HUM	4	1.180209

15min_MT.	Q6I9Y2	0.172218	0.400879	0.347878	THOC7	THO compl	THOC7_HU	3	1.126789
30min_MT.	Q7Z6V5	-0.18829	0.401029	0.347878	ADAT2	tRNA-speci	ADAT2_HU	3	0.877648
15min_MT.	Q96SW2	-0.04631	0.401114	0.347878	CRBN	Protein cer	CRBN_HUM	4	0.968409
15min_MT.	Q9H1E1	-0.05354	0.40108	0.347878	RNASE7	Ribonuclea	RNAS7_HU	1	0.963569
30min_MT.	Q9UNQ2	0.431004	0.400969	0.347878	DIMT1	Dimethylac	DIM1_HUM	4	1.348172
30min_MT.	Q9UPT6	-0.22578	0.401038	0.347878	MAPK8IP3	C-Jun-amin	JIP3_HUMA	7	0.85513
30min_MT.	O43903	0.019478	0.401294	0.347971	GAS2	Growth arr	GAS2_HUM	8	1.013592
30min_MT.	Q8NC96	0.242118	0.401309	0.347971	NECAP1	Adaptin ear	NECP1_HU	4	1.182727
15min_MT.	Q9NZJ9	0.11286	0.401377	0.347981	NUDT4	Diphospho	NUDT4_HU	5	1.08137
30min_MT.	P50914	-0.28518	0.401905	0.348255	RPL14	Large ribos	RL14_HUM	3	0.820641
15min_MT.	P40616	-0.12848	0.402384	0.348416	ARL1	ADP-ribosy	ARL1_HUM	4	0.914795
30min_MT.	P55060	0.021111	0.402198	0.348416	CSE1L	Exportin-2	XPO2_HUM	34	1.014741
30min_MT.	Q92520	-0.38949	0.402352	0.348416	FAM3C	Protein FAM	FAM3C_HU	1	0.763401
30min_MT.	Q96K76	-0.03154	0.402338	0.348416	USP47	Ubiquitin c	UBP47_HU	32	0.978373
30min_MT.	Q5JSH3	-0.00404	0.402488	0.348451	WDR44	WD repeat-	WDR44_HU	32	0.997202
15min_MT.	Q13557	-0.00158	0.402936	0.348667	CAMK2D	Calcium/ca	KCC2D_HU	4	0.998903
30min_MT.	Q9H4A3	-0.0705	0.402959	0.348667	WNK1	Serine/thre	WNK1_HU	31	0.952311
30min_MT.	Q9P2K8	0.159436	0.403073	0.348688	EIF2AK4	eIF-2-alpha	E2AK4_HU	12	1.116851
30min_MT.	Q9H2C0	-0.13793	0.403146	0.348712	GAN	Gigaxonin	GAN_HUMA	15	0.908824
30min_MT.	Q5BKU9	0.363022	0.403217	0.348735	OXLD1	Oxidoreduc	OXLD1_HU	1	1.286117
30min_MT.	O95757	0.01171	0.403422	0.348874	HSPA4L	Heat shock	HS74L_HU	25	1.00815
30min_MT.	P02666	0.031736	0.403903	0.349179	CSN2	Beta-casein	CASB_BOVI	1	1.022241
30min_MT.	P20930	0.314339	0.404223	0.349374	FLG	Filaggrin	FILA_HUMA	16	1.243442
30min_MT.	Q6IAA8	0.091843	0.404217	0.349374	LAMTOR1	Ragulator c	LTOR1_HU	3	1.065731
30min_MT.	O94830	-0.00284	0.404288	0.349392	DDHD2	Phospholip	DDHD2_HU	9	0.99803
15min_MT.	Q9BWH6	-0.02385	0.404352	0.349408	RPAP1	RNA polym	RPAP1_HU	5	0.983602
15min_MT.	Q8NFH3	-0.01668	0.404596	0.349542	NUP43	Nucleopori	NUP43_HU	5	0.988508
15min_MT.	Q7Z2T5	-0.0341	0.404781	0.349586	TRMT1L	TRMT1-like	TRM1L_HU	5	0.976642
30min_MT.	Q9Y230	0.063118	0.404765	0.349586	RUVBL2	RuvB-like 2	RUVB2_HU	23	1.044721
30min_MT.	O00267	0.051595	0.405023	0.349708	SUPT5H	Transcripti	SPT5H_HU	25	1.03641
15min_MT.	Q13123	0.055161	0.405021	0.349708	IK	Protein Rec	RED_HUMA	6	1.038975
30min_MT.	Q14847	0.112008	0.405293	0.349786	LASP1	LIM and SH	LASP1_HU	16	1.080732
30min_MT.	Q9NRY5	-0.09716	0.4053	0.349786	FAM114A2	Protein FAM	F1142_HU	21	0.934874
30min_MT.	Q04724	0.004536	0.405514	0.349909	TLE1	Transducin	TLE1_HUMA	11	1.003149
15min_MT.	Q9BWF3	-0.10741	0.405693	0.350025	RBM4	RNA-bindin	RBM4_HUM	3	0.928253
15min_MT.	P17096	-0.03671	0.405764	0.350048	HMGA1	High mobil	HMGA1_HU	4	0.974873
15min_MT.	O75821	-0.02817	0.405889	0.350094	EIF3G	Eukaryotic	EIF3G_HUM	17	0.980662
15min_MT.	P55084	0.027257	0.406102	0.350193	HADHB	Trifunction	ECHB_HUM	17	1.019073
30min_MT.	Q96IF1	-0.29456	0.406111	0.350193	AJUBA	LIM domain	AJUBA_HU	8	0.815321
15min_MT.	O14841	-0.18738	0.406528	0.350475	OPLAH	5-oxoprolin	OPLA_HUM	3	0.878197
15min_MT.	O75113	-0.1893	0.406715	0.350541	N4BP1	NEDD4-bin	N4BP1_HU	5	0.877031
30min_MT.	P24864	0.375907	0.406715	0.350541	CCNE1	G1/S-specif	CCNE1_HU	1	1.297655
30min_MT.	P54920	0.071779	0.406794	0.350541	NAPA	Alpha-solul	SNAA_HUM	15	1.051012
15min_MT.	Q12907	0.118627	0.406828	0.350541	LMAN2	Vesicular in	LMAN2_HU	8	1.085701
30min_MT.	P14649	0.102562	0.407185	0.350809	MYL6B	Myosin ligh	MYL6B_HU	6	1.073678
30min_MT.	Q9NT62	0.112547	0.407373	0.350933	ATG3	Ubiquitin-l	ATG3_HUM	6	1.081135
15min_MT.	Q9UNI6	0.013158	0.407815	0.351197	DUSP12	Dual specifi	DUS12_HU	8	1.009162
15min_MT.	Q9BT30	-0.75987	0.408107	0.35141	ALKBH7	Alpha-keto	ALKB7_HU	1	0.590548
30min_MT.	Q4G0F5	-0.02917	0.40835	0.351504	VPS26B	Vacuolar pr	VP26B_HU	10	0.979986
30min_MT.	O43390	0.087666	0.4087	0.351766	HNRNPR	Heterogene	HNRPR_HU	22	1.06265
15min_MT.	P78509	-0.05377	0.408748	0.351769	RELN	Reelin	RELN_HUM	23	0.963413
30min_MT.	O75674	-0.09114	0.409173	0.352018	TOM1L1	TOM1-like	TM1L1_HU	9	0.938783

15min_MT.	P43155	-0.0207	0.409159	0.352018	CRAT	Carnitine O	CACP_HUM	17	0.985754
15min_MT.	P08758	-0.00363	0.409393	0.35213	ANXA5	Annexin A5	ANXA5_HU	23	0.997487
15min_MT.	Q96FS4	-0.04892	0.409651	0.35227	SIPA1	Signal-indu	SIPA1_HUM	3	0.966658
30min_MT.	Q9H467	0.283388	0.409913	0.352423	CUEDC2	CUE domai	CUED2_HU	2	1.217049
15min_MT.	Q96JB2	0.051121	0.409972	0.352435	COG3	Conserved	COG3_HUM	19	1.036069
30min_MT.	P01034	0.296881	0.410046	0.352459	CST3	Cystatin-C	CYTC_HUM	2	1.228486
15min_MT.	Q00536	-0.05962	0.410132	0.352495	CDK16	Cyclin-depe	CDK16_HU	1	0.959517
15min_MT.	Q9UM54	0.060823	0.410219	0.352498	MYO6	Unconvent	MYO6_HUM	14	1.04306
15min_MT.	P60174	0.086284	0.410302	0.352524	TPI1	Triosephos	TPIS_HUMA	21	1.061632
30min_MT.	Q8IVT2	-0.12198	0.410393	0.352564	MISP	Mitotic int	MISP_HUM	3	0.918926
30min_MT.	Q15637	0.082694	0.41094	0.352995	SF1	Splicing fac	SF01_HUM	9	1.058993
30min_MT.	P57764	-0.04484	0.411182	0.353125	GSDMD	Gasdermin	GSDMD_HU	4	0.969396
15min_MT.	P11413	-0.01647	0.41166	0.353381	G6PD	Glucose-6-p	G6PD_HUM	19	0.988651
30min_MT.	Q9H1E3	0.163382	0.411769	0.353435	NUCKS1	Nuclear ubi	NUCKS_HU	5	1.11991
15min_MT.	P55268	0.034876	0.411843	0.35346	LAMB2	Laminin sul	LAMB2_HU	21	1.024469
15min_MT.	O15084	-0.25319	0.411908	0.353477	ANKRD28	Serine/thre	ANR28_HU	5	0.83904
15min_MT.	P49916	0.742468	0.412149	0.353645	LIG3	DNA ligase	DNLI3_HUM	1	1.673036
30min_MT.	O60271	0.039865	0.412387	0.353655	SPAG9	C-Jun-amin	JIP4_HUMA	52	1.028017
15min_MT.	Q13490	0.216493	0.412324	0.353655	BIRC2	Baculoviral	BIRC2_HUM	9	1.161906
30min_MT.	Q8IY67	-0.12682	0.412286	0.353655	RAVER1	Ribonucleo	RAVR1_HU	1	0.915848
30min_MT.	Q9UNE7	0.064765	0.41235	0.353655	STUB1	E3 ubiquiti	CHIP_HUM	9	1.045915
30min_MT.	Q8WXE9	0.14135	0.412643	0.353758	STON2	Stonin-2	STON2_HU	1	1.102937
30min_MT.	O14579	-0.04202	0.41324	0.353965	COPE	Coatomer s	COPE_HUM	11	0.971297
30min_MT.	O14907	-0.14884	0.413164	0.353965	TAX1BP3	Tax1-bindir	TX1B3_HU	2	0.901977
30min_MT.	P23246	-0.05106	0.413044	0.353965	SFPQ	Splicing fac	SFPQ_HUM	28	0.965226
15min_MT.	P46976	-0.01983	0.413246	0.353965	GYG1	Glycogenin	GLYG_HUM	10	0.98635
15min_MT.	Q9NP79	0.090739	0.413108	0.353965	VTA1	Vacuolar pr	VTA1_HUM	11	1.064915
30min_MT.	Q8NEF9	-0.03142	0.413482	0.35409	SRFBP1	Serum resp	SRFB1_HUM	3	0.978454
30min_MT.	P34096	0.120044	0.413816	0.354238	RNASE4	Ribonuclea	RNAS4_HU	5	1.086768
15min_MT.	Q9H0N5	-0.30805	0.413836	0.354238	PCBD2	Pterin-4-alp	PHS2_HUM	3	0.807733
30min_MT.	P23258	-0.35828	0.414058	0.35435	TUBG1	Tubulin gar	TBG1_HUM	4	0.780092
15min_MT.	P05165	0.055379	0.414296	0.354399	PCCA	Propionyl-(	PCCA_HUM	17	1.039132
30min_MT.	Q9H3P7	0.036559	0.414392	0.354442	ACBD3	Golgi reside	GCP60_HU	13	1.025664
15min_MT.	Q12986	-0.02234	0.414699	0.354666	NFX1	Transcripti	NFX1_HUM	4	0.984633
15min_MT.	Q01968	-0.03215	0.414896	0.354796	OCRL	Inositol pol	OCRL_HUM	13	0.977961
15min_MT.	Q9UMY4	0.030937	0.415187	0.355006	SNX12	Sorting nex	SNX12_HUI	6	1.021676
15min_MT.	Q5F1R6	-0.26647	0.415249	0.35502	DNAJC21	DnaJ homo	DJC21_HUI	4	0.831354
15min_MT.	Q9UK76	-0.05256	0.415965	0.355574	JPT1	Jupiter mic	JUPI1_HUM	8	0.964226
30min_MT.	Q9Y2S2	0.067511	0.416529	0.35592	CRYL1	Lambda-cry	CRYL1_HUM	10	1.047908
30min_MT.	O14641;Q9	0.005223	0.416768	0.356046	DVL2;DVL3	Segment pc	DVL2_HUM	1	1.003627
15min_MT.	P67775	0.005834	0.41694	0.356154	PPP2CA	Serine/thre	PP2AA_HUI	2	1.004052
30min_MT.	Q96FQ6	-0.00213	0.417278	0.356288	S100A16	Protein S1(	S10AG_HUI	6	0.998522
30min_MT.	Q15075	0.071632	0.417511	0.356409	EEA1	Early endos	EEA1_HUM	65	1.050905
15min_MT.	O60716	0.030244	0.417898	0.356661	CTNND1	Catenin del	CTND1_HU	20	1.021185
15min_MT.	O75347	0.083849	0.418091	0.356727	TBCA	Tubulin-spe	TBCA_HUM	9	1.059842
30min_MT.	O95747	-0.01409	0.418112	0.356727	OXSR1	Serine/thre	OXSR1_HUI	12	0.990281
30min_MT.	Q70E73	-0.17136	0.418079	0.356727	RAPH1	Ras-associa	RAPH1_HU	4	0.888002
15min_MT.	Q16610	0.283774	0.418215	0.356769	ECM1	Extracellula	ECM1_HUM	1	1.217375
30min_MT.	Q96P70	-0.01587	0.418253	0.356769	IPO9	Importin-9	IPO9_HUM	31	0.989062
30min_MT.	Q13464	-0.00846	0.418584	0.356935	ROCK1	Rho-associa	ROCK1_HU	46	0.994154
30min_MT.	P62304	0.191937	0.418838	0.357074	SNRPE	Small nucle	RUXE_HUM	2	1.142296
15min_MT.	O75367	0.152209	0.419188	0.357216	MACROH2A	Core histor	H2AY_HUM	2	1.11127

15min_MT.	Q15582	0.140911	0.41932	0.357253	TGFBI	Transformi	BGH3_HUM	11	1.102601
15min_MT.	Q6JQN1	0.161736	0.419457	0.357289	ACAD10	Acyl-CoA de	ACD10_HU	18	1.118632
30min_MT.	P35249	-0.13144	0.41965	0.357396	RFC4	Replication	RFC4_HUM	6	0.91292
30min_MT.	Q6P1M3	0.027354	0.419673	0.357396	LLGL2	LLGL scribb	L2GL2_HUI	6	1.019141
30min_MT.	O60447	-0.00639	0.419981	0.357478	EVI5	Ecotropic v	EVI5_HUM/	11	0.995579
15min_MT.	O75781	-0.2105	0.419978	0.357478	PALM	Paralemmi	PALM_HUM	4	0.864237
15min_MT.	Q8IXB1	0.070744	0.420181	0.357478	DNAJC10	DnaJ homo	DJC10_HUI	9	1.050258
15min_MT.	Q96QA5	0.174464	0.420024	0.357478	GSDMA	Gasdermin	GSDMA_HU	5	1.128545
15min_MT.	P14618	-0.03346	0.420473	0.357649	PKM	Pyruvate ki	KPYM_HUM	35	0.977073
15min_MT.	Q70CQ2	0.330485	0.420465	0.357649	USP34	Ubiquitin c	UBP34_HU	7	1.257436
30min_MT.	Q99590	0.010232	0.420648	0.357759	SCAF11	Protein SC/	SCAFB_HUI	11	1.007118
15min_MT.	P54252	0.350426	0.420696	0.357761	ATXN3	Ataxin-3	ATX3_HUM	5	1.274937
15min_MT.	O95202	-0.08549	0.420752	0.357769	LETM1	Mitochond	LETM1_HUI	7	0.942465
30min_MT.	Q16864	-0.12024	0.420819	0.357787	ATP6V1F	V-type prot	VATF_HUM	4	0.920037
30min_MT.	Q9UBC2	-0.05768	0.420874	0.357796	EPS15L1	Epidermal g	EP15R_HUI	28	0.960806
15min_MT.	Q09161	-0.00557	0.421408	0.358094	NCBP1	Nuclear cap	NCBP1_HU	9	0.996148
30min_MT.	Q9UPM8	-0.15128	0.421378	0.358094	AP4E1	AP-4 compl	AP4E1_HUI	3	0.90045
30min_MT.	Q8WUD4	-0.03779	0.421607	0.358224	CCDC12	Coiled-coil	CCD12_HU	6	0.974143
15min_MT.	Q14203	0.013653	0.421698	0.358225	DCTN1	Dynactin su	DCTN1_HU	45	1.009509
30min_MT.	Q9NVR2	-0.73005	0.421832	0.358298	INTS10	Integrator c	INT10_HUM	1	0.602884
30min_MT.	P78537	-0.02984	0.421917	0.358331	BLOC1S1	Biogenesis	BL1S1_HUI	3	0.97953
15min_MT.	O95479	-0.03193	0.422149	0.358346	H6PD	GDH/6PGL	G6PE_HUM	26	0.978112
30min_MT.	Q01581	0.017634	0.422042	0.358346	HMGCS1	Hydroxyme	HMCS1_HU	13	1.012298
15min_MT.	Q7L1V2	-0.09598	0.422163	0.358346	MON1B	Vacuolar fu	MON1B_HU	3	0.935637
30min_MT.	Q7L266	0.021289	0.422138	0.358346	ASRGL1	Isoaspartyl	ASGL1_HUI	7	1.014866
15min_MT.	Q9C075	0.181487	0.422126	0.358346	KRT23	Keratin, typ	K1C23_HUI	8	1.134052
30min_MT.	Q7L099	0.144803	0.422503	0.358518	RUFY3	Protein RUI	RUFY3_HUI	7	1.10558
30min_MT.	Q9H4B0	-0.57335	0.422482	0.358518	OSGEPL1	tRNA N6-ad	OSGL1_HUI	2	0.672055
15min_MT.	Q5VWQ8;C	0.031249	0.422765	0.358651	DAB2IP;RA:	Disabled hc	DAB2P_HU	1	1.021897
30min_MT.	Q9BU89	0.354443	0.422797	0.358651	DOHH	Deoxyhypu	DOHH_HUI	4	1.278492
30min_MT.	Q9UFN0	-0.19826	0.422715	0.358651	NIPSNAP3A	Protein Nip	NPS3A_HUI	10	0.871599
15min_MT.	Q86WQ0	-0.14641	0.423015	0.358797	NR2C2AP	Nuclear rec	NR2CA_HU	4	0.903493
30min_MT.	O94906	-0.11935	0.423155	0.358798	PRPF6	Pre-mRNA-|	PRP6_HUM	8	0.920602
30min_MT.	P19404	-0.10378	0.423113	0.358798	NDUFV2	NADH dehy	NDUV2_HU	7	0.930589
15min_MT.	Q9H9G7	-0.22774	0.423152	0.358798	ago-03	Protein arg	AGO3_HUM	3	0.853971
30min_MT.	P00568	-0.00567	0.42331	0.358869	AK1	Adenylate k	KAD1_HUM	9	0.996077
15min_MT.	Q2TAY7	-0.05757	0.423473	0.358869	SMU1	WD40 repe	SMU1_HUM	14	0.960882
15min_MT.	Q9BU76	-0.38457	0.423514	0.358869	MMTAG2	Multiple m	MMTA2_HU	3	0.766007
15min_MT.	O60671	-0.04623	0.423876	0.359073	RAD1	Cell cycle c	RAD1_HUM	5	0.968466
15min_MT.	Q9NZU5	0.101892	0.423937	0.359073	LMCD1	LIM and cys	LMCD1_HU	14	1.07318
30min_MT.	Q13232	0.022863	0.424197	0.359137	NME3	Nucleoside	NDK3_HUM	8	1.015974
30min_MT.	Q7Z6E9	0.131682	0.424114	0.359137	RBBP6	E3 ubiquiti	RBBP6_HUI	8	1.095571
30min_MT.	Q8N371	0.094569	0.424174	0.359137	KDM8	Bifunctiona	KDM8_HUM	6	1.067746
30min_MT.	Q9NW64	-0.11567	0.424259	0.359151	RBM22	Pre-mRNA-s	RBM22_HU	8	0.922956
30min_MT.	A6NHL2	-0.34494	0.424602	0.35929	TUBAL3	Tubulin alp	TBAL3_HUM	1	0.787342
30min_MT.	O14976	-0.01089	0.424628	0.35929	GAK	Cyclin-G-as	GAK_HUM/	6	0.992478
15min_MT.	Q8NFW8	0.139746	0.424528	0.35929	CMAS	N-acylneur	NEUA_HUM	5	1.101711
15min_MT.	Q96CS3	0.000883	0.424699	0.35929	FAF2	FAS-associa	FAF2_HUM	4	1.000612
30min_MT.	Q9H2M9	-0.09105	0.424684	0.35929	RAB3GAP2	Rab3 GTPas	RBGPR_HU	17	0.938839
15min_MT.	P20810	0.063358	0.424903	0.359423	CAST	Calpastatin	ICAL_HUM/	27	1.044895
15min_MT.	O76041	0.21431	0.424993	0.359461	NEBL	Nebulette	NEBL_HUM	2	1.160149
15min_MT.	Q92820	0.045309	0.425256	0.35963	GGH	Gamma-glu	GGH_HUM/	10	1.031904

15min_MT.	O95070	-0.20964	0.425596	0.359815	YIF1A	Protein YIF:	YIF1A_HUM	1	0.864754
15min_MT.	Q6NYC1	0.215085	0.425662	0.359832	JMJD6	Bifunctiona	JMJD6_HUI	6	1.160772
30min_MT.	Q13188	-0.11418	0.425853	0.359955	STK3	Serine/thre	STK3_HUM	4	0.923905
15min_MT.	Q15645	0.00868	0.425936	0.359981	TRIP13	Pachytene (	PCH2_HUM	9	1.006035
30min_MT.	Q16352	-0.15519	0.425976	0.359981	INA	Alpha-inter	AINX_HUM	2	0.898013
15min_MT.	Q16527	0.083348	0.426072	0.360021	CSRP2	Cysteine an	CSRP2_HUI	7	1.059474
30min_MT.	Q8N4F0	1.374275	0.426206	0.360059	BPIFB2	BPI fold-co	BPIB2_HUM	1	2.592376
15min_MT.	Q9BXB5	-0.01579	0.426492	0.360184	OSBPL10	Oxysterol-b	OSB10_HUI	4	0.989111
15min_MT.	Q9UGJ0	0.165761	0.426449	0.360184	PRKAG2	5'-AMP-acti	AAKG2_HU	2	1.121757
30min_MT.	P04075	-0.09897	0.42677	0.36038	ALDOA	Fructose-bi	ALDOA_HU	35	0.933697
30min_MT.	O95671	-0.05821	0.427152	0.360534	ASMTL	Probable bi	ASML_HUM	22	0.960458
15min_MT.	Q9GZY8	0.169132	0.427183	0.360534	MFF	Mitochond	MFF_HUMA	1	1.124382
30min_MT.	Q9ULC6	0.463415	0.427184	0.360534	PADI1	Protein-arg	PADI1_HUM	1	1.378802
30min_MT.	O95544	-0.03082	0.427511	0.360771	NADK	NAD kinase	NADK_HUM	8	0.978865
30min_MT.	P15121	0.120729	0.427893	0.360938	AKR1B1	Aldo-keto r	ALDR_HUM	15	1.087284
30min_MT.	P15516	0.038335	0.428166	0.361052	HTN3	Histatin-3	HIS3_HUMA	1	1.026928
30min_MT.	Q01085	-0.03804	0.428149	0.361052	TIAL1	Nucleolysir	TIAR_HUMA	7	0.973978
30min_MT.	P11166	0.462657	0.428961	0.361464	SLC2A1	Solute carri	GTR1_HUM	1	1.378078
15min_MT.	P49959	0.024105	0.428944	0.361464	MRE11	Double-stra	MRE11_HU	22	1.016849
30min_MT.	Q7RTP6	0.30757	0.428851	0.361464	MICAL3	[F-actin]-m	MICA3_HUI	2	1.237621
30min_MT.	Q9UKV8	0.029644	0.428935	0.361464	ago-02	Protein arg	AGO2_HUM	11	1.02076
30min_MT.	Q04323	0.068043	0.429087	0.361517	UBXN1	UBX domai	UBXN1_HU	12	1.048294
30min_MT.	P26885	0.048073	0.429184	0.361559	FKBP2	Peptidyl-pr	FKBP2_HUI	6	1.033883
30min_MT.	Q96L92	-0.03565	0.429269	0.361592	SNX27	Sorting nex	SNX27_HUI	8	0.975594
30min_MT.	P49006	0.046353	0.429495	0.361729	MARCKSL1	MARCKS-re	MRP_HUMA	5	1.032651
30min_MT.	Q8NFC6	0.054489	0.42957	0.361729	BOD1L1	Biorientati	BD1L1_HUI	29	1.038491
15min_MT.	Q9P2I0	-0.03799	0.429566	0.361729	CPSF2	Cleavage ar	CPSF2_HUM	11	0.974012
15min_MT.	P47985	0.16742	0.429744	0.361836	UQCRFS1	Cytochrom	UCRI_HUM	5	1.123048
15min_MT.	P26640	-0.03607	0.429925	0.361937	VARS1	Valine--tRN	SYVC_HUM	35	0.975308
15min_MT.	P63279	0.023132	0.429957	0.361937	UBE2I	SUMO-conj	UBC9_HUM	9	1.016163
15min_MT.	Q14202	0.033245	0.430391	0.362186	ZMYM3	Zinc finger	ZMYM3_HU	9	1.023311
15min_MT.	Q9H223	-0.01912	0.430383	0.362186	EHD4	EH domain-	EHD4_HUM	6	0.986835
15min_MT.	Q9NQG6	-0.33977	0.430333	0.362186	MIEF1	Mitochond	MID51_HU	2	0.790168
15min_MT.	P04632	0.040357	0.431026	0.362308	CAPNS1	Calpain sma	CPNS1_HUI	5	1.028368
30min_MT.	P28715	-0.25914	0.430905	0.362308	ERCC5	DNA excisic	ERCC5_HUI	6	0.835584
30min_MT.	Q15628	0.207342	0.431006	0.362308	TRADD	Tumor necr	TRADD_HU	3	1.154559
30min_MT.	Q86YT6	-0.10257	0.430932	0.362308	MIB1	E3 ubiquiti	MIB1_HUM	1	0.931374
30min_MT.	Q96JY6	0.206353	0.430794	0.362308	PDLIM2	PDZ and LIN	PDLI2_HUM	4	1.153768
15min_MT.	Q9NR30	0.002841	0.431004	0.362308	DDX21	Nucleolar R	DDX21_HU	19	1.001972
30min_MT.	Q9NZZ3	-0.07588	0.430674	0.362308	CHMP5	Charged mu	CHMP5_HU	4	0.948762
15min_MT.	P11766	0.073906	0.431322	0.362446	ADH5	Alcohol deh	ADHX_HUM	16	1.052563
15min_MT.	Q02818	0.242379	0.431377	0.362446	NUCB1	Nucleobinc	NUCB1_HU	8	1.182942
30min_MT.	Q07065	0.085158	0.431283	0.362446	CKAP4	Cytoskeleto	CKAP4_HUI	4	1.060804
30min_MT.	Q8IZ13	-0.21675	0.431395	0.362446	FAM200C	Protein FAN	F200C_HUI	1	0.860501
30min_MT.	P83369	-0.12215	0.431542	0.362495	LSM11	U7 snRNA-a	LSM11_HU	1	0.918818
15min_MT.	Q02218	-0.043	0.431546	0.362495	OGDH	2-oxoglutar	ODO1_HUM	9	0.970635
15min_MT.	Q8WYN0	-0.01939	0.43163	0.362526	ATG4A	Cysteine pr	ATG4A_HUI	4	0.986653
30min_MT.	Q8TE77	0.02192	0.431727	0.362541	SSH3	Protein pho	SSH3_HUM	8	1.01531
30min_MT.	Q9NRW7	-0.12171	0.43174	0.362541	VPS45	Vacuolar pr	VPS45_HUI	4	0.919097
30min_MT.	Q8WU79	-0.11587	0.431809	0.36256	SMAP2	Stromal me	SMAP2_HU	12	0.922824
30min_MT.	Q96EE3	-0.13246	0.431864	0.362567	SEH1L	Nucleopori	SEH1_HUM	8	0.912277
15min_MT.	Q8N8A2	0.129002	0.431953	0.362603	ANKRD44	Serine/thre	ANR44_HU	3	1.093537

15min_MT.	P16949	0.05268	0.432328	0.362866	STMN1	Stathmin	STMN1_HU	8	1.03719
15min_MT.	P61313	0.027388	0.432405	0.362866	RPL15	Large ribos	RL15_HUM	7	1.019165
15min_MT.	Q96JJ3	-0.18749	0.432641	0.362931	ELMO2	Engulfment	ELMO2_HU	2	0.878135
30min_MT.	Q9BW27	-0.09709	0.432629	0.362931	NUP85	Nuclear po	NUP85_HU	6	0.934914
15min_MT.	Q9NZ32	-0.04551	0.432663	0.362931	ACTR10	Actin-relate	ARP10_HU	11	0.968944
30min_MT.	O60885	0.036531	0.432966	0.363064	BRD4	Bromodom	BRD4_HUM	22	1.025645
30min_MT.	O95340	-0.44084	0.433118	0.363125	PAPSS2	Bifunctiona	PAPS2_HU	4	0.736703
30min_MT.	Q9Y6A4	0.093948	0.433132	0.363125	CFAP20	Cilia- and fl	CFA20_HU	5	1.067287
30min_MT.	P61964	0.03667	0.433299	0.363188	WDR5	WD repeat-	WDR5_HU	11	1.025743
15min_MT.	Q00013	0.12918	0.433283	0.363188	MPP1	55 kDa eryt	EM55_HUM	5	1.093672
15min_MT.	P35754	0.330525	0.433487	0.363306	GLRX	Glutaredox	GLRX1_HU	4	1.25747
30min_MT.	Q04864	0.012725	0.433883	0.363482	REL	Proto-onco	REL_HUMA	5	1.008859
15min_MT.	Q9H0D6	-0.02478	0.433839	0.363482	XRN2	5'-3' exorib	XRN2_HUM	33	0.98297
15min_MT.	P63092;Q5	-0.11591	0.433992	0.363496	GNAS	Guanine nu	GNAS2_HU	7	0.9228
30min_MT.	Q9HAV7	0.097801	0.433955	0.363496	GRPEL1	GrpE protei	GRPE1_HU	9	1.070141
30min_MT.	Q6PID6	0.125505	0.434426	0.363742	TTC33	Tetratricop	TTC33_HU	3	1.09089
30min_MT.	O14828	-0.32943	0.434495	0.363761	SCAMP3	Secretory c	SCAM3_HU	4	0.795849
15min_MT.	P16278	0.045372	0.435304	0.364283	GLB1	Beta-galact	BGAL_HUM	11	1.031949
15min_MT.	Q16181	0.036008	0.435277	0.364283	SEPTIN7	Septin-7	SEPT7_HU	12	1.025273
15min_MT.	P52306	0.090098	0.435659	0.364501	RAP1GDS1	Rap1 GTPas	GDS1_HUM	21	1.064443
30min_MT.	Q9BUK0	0.25147	0.435634	0.364501	CHCHD7	Coiled-coil-	CHCH7_HU	1	1.190419
30min_MT.	Q8IYL3	-0.08706	0.435866	0.364636	C1orf174	UPF0688 p	CA174_HU	3	0.941439
15min_MT.	O95831	-0.01692	0.43647	0.365024	AIFM1	Apoptosis-i	AIFM1_HU	22	0.988339
30min_MT.	Q9H3U1	0.062837	0.436445	0.365024	UNC45A	Protein unc	UN45A_HU	29	1.044518
15min_MT.	O95777	0.087213	0.436692	0.365171	LSM8	U6 snRNA-a	LSM8_HUM	4	1.062316
15min_MT.	O00468	-0.06304	0.437016	0.365187	AGRN	Agrin	AGRIN_HU	36	0.957245
30min_MT.	P45984	0.062674	0.436915	0.365187	MAPK9	Mitogen-ac	MK09_HUM	9	1.0444
15min_MT.	P46379	-0.07159	0.436946	0.365187	BAG6	Large prolir	BAG6_HUM	30	0.951588
15min_MT.	Q8N4Q1	0.121001	0.436915	0.365187	CHCHD4	Mitochond	MIA40_HU	2	1.087489
15min_MT.	Q96C90	-0.07505	0.437038	0.365187	PPP1R14B	Protein pho	PP14B_HU	5	0.949312
15min_MT.	P54920	0.042695	0.437244	0.365319	NAPA	Alpha-solul	SNAA_HUM	15	1.030036
15min_MT.	O15230	0.031904	0.437599	0.365538	LAMA5	Laminin sul	LAMA5_HU	41	1.02236
30min_MT.	O75822	0.117437	0.437723	0.365603	EIF3J	Eukaryotic	EIF3J_HUM	12	1.084806
30min_MT.	O95865	-0.05767	0.437978	0.365738	DDAH2	Putative hy	DDAH2_HU	10	0.960817
15min_MT.	Q14694	-0.16562	0.438145	0.365746	USP10	Ubiquitin c	UBP10_HU	14	0.891546
30min_MT.	Q9BW92	-0.02386	0.43816	0.365746	TARS2	Threonine--	SYTM_HUM	9	0.983601
15min_MT.	Q9NP72	0.008345	0.438175	0.365746	RAB18	Ras-related	RAB18_HU	8	1.005801
15min_MT.	Q9NS86	0.049816	0.438174	0.365746	LANCL2	LanC-like pr	LANC2_HU	13	1.035133
15min_MT.	Q96DR5	0.496892	0.438723	0.366008	BPIFA2	BPI fold-co	BPIA2_HUM	3	1.411171
15min_MT.	Q9UHJ6	-0.25968	0.438679	0.366008	SHPK	Sedoheptul	SHPK_HUM	6	0.835272
30min_MT.	Q8TEW0	0.171291	0.439767	0.366683	PARD3	Partitionin	PARD3_HU	3	1.126066
30min_MT.	Q92990	-0.03883	0.439726	0.366683	GLMN	Glomulin	GLMN_HUN	10	0.973442
30min_MT.	Q9UHN1	-0.08266	0.439887	0.366745	POLG2	DNA polym	DPOG2_HU	6	0.944316
30min_MT.	Q13523	-0.15979	0.440122	0.366901	PRP4K	Serine/thre	PRP4K_HU	7	0.895157
15min_MT.	P08236	-0.07984	0.440898	0.367157	GUSB	Beta-glucur	BGLR_HUM	13	0.946163
15min_MT.	P13667	-0.05125	0.440652	0.367157	PDIA4	Protein dis	PDIA4_HUN	41	0.965099
15min_MT.	P15735	0.385027	0.440734	0.367157	PHKG2	Phosphoryl	PHKG2_HU	3	1.305884
15min_MT.	Q14789	0.206989	0.440779	0.367157	GOLGB1	Golgin subf	GOGB1_HU	13	1.154277
15min_MT.	Q92771	-0.30838	0.440863	0.367157	DDX12P	Putative AT	DDX12_HU	1	0.807548
15min_MT.	Q99538	0.007242	0.44083	0.367157	LGMN	Legumain	LGMN_HUN	6	1.005032
30min_MT.	O43657	-0.02491	0.440977	0.367184	TSPAN6	Tetraspanir	TSN6_HUM	1	0.982885
30min_MT.	Q7Z6K3	0.065816	0.441097	0.367205	PTAR1	Protein pre	PTAR1_HU	9	1.046677

15min_MT.	Q9UI08	-0.06562	0.441242	0.367248	EVL	Ena/VASP-li	EVL_HUMA	6	0.955535
15min_MT.	O75351	0.27007	0.441359	0.36728	VPS4B	Vacuolar pr	VPS4B_HUI	5	1.205866
15min_MT.	P02768	0.080422	0.441431	0.36728	ALB	Albumin	ALBU_HUM	28	1.057327
30min_MT.	Q8WYP5	-0.54222	0.441468	0.36728	AHCTF1	Protein ELY	ELYS_HUM/	1	0.686712
30min_MT.	O14933	-0.04277	0.441796	0.367333	UBE2L6	Ubiquitin/I	UB2L6_HUI	1	0.970789
30min_MT.	P35613	-0.08295	0.441653	0.367333	BSG	Basigin	BASI_HUM/	7	0.944126
30min_MT.	P48681	-0.10382	0.441656	0.367333	NES	Nestin	NEST_HUM	1	0.930566
15min_MT.	Q969S3	0.083686	0.441734	0.367333	ZNF622	Cytoplasmi	ZN622_HUI	5	1.059722
30min_MT.	Q9NUJ3	-0.23877	0.441674	0.367333	TCP11L1	T-complex	T11L1_HUN	3	0.847469
30min_MT.	Q6IN84	0.082702	0.442523	0.367805	MRM1	rRNA meth	MRM1_HUI	7	1.058999
30min_MT.	O43679;Q8	0.125786	0.442593	0.367824	LDB2;LDB1	LIM domair	LDB2_HUM	1	1.091102
15min_MT.	O75312	0.076267	0.443004	0.367972	ZPR1	Zinc finger	ZPR1_HUM	16	1.054286
30min_MT.	P12111	0.117314	0.442825	0.367972	COL6A3	Collagen al	CO6A3_HU	1	1.084714
15min_MT.	P42166	0.096805	0.442891	0.367972	TMPO	Lamina-ass	LAP2A_HUI	17	1.069403
30min_MT.	P78347	0.02858	0.443005	0.367972	GTF2I	General tra	GTF2I_HUN	24	1.020008
30min_MT.	Q96K17	0.033249	0.442937	0.367972	BTF3L4	Transcripti	BT3L4_HUN	3	1.023314
30min_MT.	Q9UIV1	-0.09718	0.443056	0.367975	CNOT7	CCR4-NOT t	CNOT7_HU	3	0.934857
30min_MT.	O15344	-0.09038	0.443317	0.368102	MID1	E3 ubiquiti	TRI18_HUN	17	0.939277
30min_MT.	P08697	0.130755	0.443417	0.368102	SERPINF2	Alpha-2-an	A2AP_HUM	15	1.094867
15min_MT.	P11233	0.15773	0.443435	0.368102	RALA	Ras-related	RALA_HUM	3	1.11553
15min_MT.	P22570	-0.02268	0.443459	0.368102	FDXR	NADPH:adr	ADRO_HUN	7	0.984403
30min_MT.	Q15046	-0.06328	0.44359	0.368105	KARS1	Lysine--tRN	SYK_HUMA	20	0.957086
15min_MT.	Q5BJH7	0.221681	0.443543	0.368105	YIF1B	Protein YIF	YIF1B_HUN	1	1.166092
15min_MT.	Q5R372	-0.03616	0.443978	0.368349	RABGAP1L	Rab GTPase	RBG1L_HUI	13	0.975249
30min_MT.	P49589	-0.09153	0.444222	0.368512	CARS1	Cysteine--tl	SYCC_HUM	25	0.938526
15min_MT.	P22891	0.209669	0.444651	0.368517	PROZ	Vitamin K-c	PROZ_HUM	1	1.156423
30min_MT.	Q14554	0.056729	0.444365	0.368517	PDIA5	Protein dis	PDIA5_HUN	21	1.040105
15min_MT.	Q567U6	0.178135	0.444404	0.368517	CCDC93	Coiled-coil	CCD93_HU	2	1.13142
30min_MT.	Q5JRA6	-0.11301	0.44454	0.368517	MIA3	Transport a	TGO1_HUN	10	0.924659
30min_MT.	Q96TA1	-0.08975	0.444564	0.368517	NIBAN2	Protein Nib	NIBA2_HUN	8	0.939685
15min_MT.	Q9HAU5	0.831123	0.444555	0.368517	UPF2	Regulator c	RENT2_HUI	2	1.77907
30min_MT.	O00330	-0.04516	0.445048	0.368768	PDHX	Pyruvate de	ODPX_HUN	16	0.969184
30min_MT.	Q7L9L4	-0.16142	0.44539	0.368779	MOB1B	MOB kinase	MOB1B_HU	7	0.894142
30min_MT.	Q8N5M1	-0.0565	0.44519	0.368779	ATPAF2	ATP syntha:	ATPF2_HUN	5	0.961597
30min_MT.	Q9NRX4	0.031497	0.44528	0.368779	PHPT1	14 kDa pho	PHP14_HU	6	1.022072
15min_MT.	O43805	0.711762	0.445679	0.368809	SSNA1	Microtubul	SSNA1_HUI	2	1.637803
15min_MT.	P63220	0.109093	0.445692	0.368809	RPS21	Small ribos	RS21_HUM	4	1.07855
30min_MT.	Q5T5C0	0.020384	0.445811	0.368809	STXBP5	Syntaxin-bi	STXB5_HUN	4	1.014229
30min_MT.	Q9H1D9	0.190447	0.445764	0.368809	POLR3F	DNA-direct	RPC6_HUM	6	1.141117
30min_MT.	Q9Y303	0.069352	0.445715	0.368809	AMDHD2	N-acetylglu	NAGA_HUN	4	1.049246
30min_MT.	P51948	0.043354	0.446031	0.368879	MNAT1	CDK-activa	MAT1_HUN	3	1.030507
30min_MT.	Q5GLZ8	-0.14983	0.446356	0.368991	HERC4	Probable E3	HERC4_HUI	14	0.901358
15min_MT.	Q99541	0.130183	0.4463	0.368991	PLIN2	Perilipin-2	PLIN2_HUN	7	1.094432
30min_MT.	Q658Y4	-0.13137	0.446583	0.369101	FAM91A1	Protein FAN	F91A1_HUI	17	0.912962
15min_MT.	P49585	0.04261	0.446814	0.369253	PCYT1A	Choline-ph	PCY1A_HUI	12	1.029976
30min_MT.	Q01970	0.046742	0.446942	0.369281	PLCB3	1-phosphat	PLCB3_HUI	18	1.032929
15min_MT.	Q8WUH6	-0.69988	0.447152	0.369377	TMEM263	Transmemb	TM263_HU	2	0.615622
30min_MT.	Q16513	-0.04229	0.447303	0.369462	PKN2	Serine/thre	PKN2_HUN	20	0.971112
15min_MT.	Q15907	0.021213	0.447672	0.369728	RAB11B	Ras-related	RB11B_HUI	13	1.014812
30min_MT.	P00387	-0.15165	0.448186	0.369845	CYB5R3	NADH-cyto	NB5R3_HU	6	0.900223
15min_MT.	P49441	-0.33295	0.448253	0.369845	INPP1	Inositol pol	INPP_HUM	3	0.793914
15min_MT.	P62714	-0.11104	0.447975	0.369845	PPP2CB	Serine/thre	PP2AB_HUI	13	0.92592

15min_MT.	Q5JTV8	0.112322	0.448072	0.369845	TOR1AIP1	Torsin-1A-i	TOIP1_HUM	4	1.080967
30min_MT.	Q86UE4	0.107973	0.44808	0.369845	MTDH	Protein LYF	LYRIC_HUM	10	1.077713
30min_MT.	Q92466	-0.14688	0.447922	0.369845	DDB2	DNA damag	DDB2_HUM	9	0.903203
30min_MT.	Q9NSK7	0.260708	0.448217	0.369845	C19orf12	Protein C19	CS012_HUI	3	1.198067
30min_MT.	Q92530	-0.04037	0.448657	0.370111	PSMF1	Proteasom	PSMF1_HU	7	0.972405
15min_MT.	Q9ULR3	0.064179	0.44878	0.370173	PPM1H	Protein pho	PPM1H_HU	1	1.04549
30min_MT.	A6NHQ2	-0.07473	0.448956	0.37028	FBLL1	rRNA/tRNA	FBLL1_HUM	1	0.949517
15min_MT.	Q13347	0.106516	0.449359	0.370573	EIF3I	Eukaryotic	EIF3I_HUM	10	1.076625
30min_MT.	O95155	-0.13965	0.449422	0.370586	UBE4B	Ubiquitin c	UBE4B_HUI	20	0.907737
15min_MT.	Q14847	0.074905	0.449616	0.370707	LASP1	LIM and SH	LASP1_HUM	16	1.053291
15min_MT.	A6NKN8	0.436567	0.450063	0.370997	PCP4L1	Purkinje ce	PC4L1_HUI	1	1.35338
15min_MT.	Q96SZ5	0.188113	0.450132	0.371015	ADO	2-aminoeth	AEDO_HUM	5	1.139272
15min_MT.	Q09472;Q9	-0.01502	0.45036	0.371125	EP300;CREI	Histone ace	EP300_HUI	1	0.989642
15min_MT.	Q92783	0.006088	0.450335	0.371125	STAM	Signal trans	STAM1_HU	8	1.004229
15min_MT.	Q96PE2	0.707952	0.450485	0.371157	ARHGEF17	Rho guanin	ARHGH_HU	1	1.633484
30min_MT.	A3KMH1	-0.12652	0.450772	0.371209	VWA8	von Willebi	VWA8_HUM	2	0.916038
15min_MT.	P98179	0.164291	0.450795	0.371209	RBM3	RNA-bindin	RBM3_HUM	3	1.120615
15min_MT.	Q9HD15	0.105276	0.450636	0.371209	SRA1	Steroid rec	SRA1_HUM	10	1.0757
15min_MT.	Q9Y295	-0.01192	0.450777	0.371209	DRG1	Developme	DRG1_HUM	14	0.991773
15min_MT.	P00441	-0.0953	0.450875	0.371236	SOD1	Superoxide	SODC_HUM	6	0.93608
30min_MT.	Q9BQP7	-0.11631	0.451132	0.37133	MGME1	Mitochond	MGME1_HU	12	0.922545
30min_MT.	Q9UK59	0.092193	0.451125	0.37133	DBR1	Lariat debr	DBR1_HUM	10	1.065989
15min_MT.	Q15643	0.109599	0.451289	0.3714	TRIP11	Thyroid rec	TRIPB_HUM	17	1.078929
30min_MT.	O43815	-0.14186	0.451478	0.37142	STRN	Striatin	STRN_HUM	9	0.906353
30min_MT.	O75886	-0.05319	0.451439	0.37142	STAM2	Signal trans	STAM2_HU	10	0.963805
30min_MT.	Q5VV43	0.083099	0.451385	0.37142	KIAA0319	Dyslexia-as	K0319_HUI	2	1.059291
15min_MT.	Q6VN20	0.221309	0.451526	0.37142	RANBP10	Ran-bindin	RBP10_HUI	5	1.165791
30min_MT.	P48454	0.169123	0.451686	0.371474	PPP3CC	Serine/thre	PP2BC_HUI	1	1.124375
15min_MT.	O43924	-0.20091	0.451928	0.371478	PDE6D	Retinal rod	PDE6D_HU	4	0.869999
30min_MT.	P23434	-0.17109	0.451887	0.371478	GCSH	Glycine cle	GCSH_HUM	4	0.888173
15min_MT.	Q96BP2	-0.06747	0.451839	0.371478	CHCHD1	Small ribos	CHCH1_HU	1	0.954313
30min_MT.	Q9BQI0	-0.07904	0.452314	0.371756	AIF1L	Allograft in	AIF1L_HUM	4	0.946687
15min_MT.	P35558	0.034425	0.452461	0.371802	PCK1	Phosphoen	PCKGC_HU	22	1.024148
30min_MT.	Q15011	-0.55573	0.452465	0.371802	HERPUD1	Homocyste	HERP1_HUI	1	0.680313
30min_MT.	Q8IWJ2	0.058987	0.452732	0.371943	GCC2	GRIP and co	GCC2_HUM	40	1.041734
15min_MT.	Q0VDF9	0.003763	0.453163	0.372219	HSPA14	Heat shock	HSP7E_HUI	15	1.002612
30min_MT.	Q9C0E8	-0.14504	0.453139	0.372219	LNPK	Endoplasm	LNP_HUMA	6	0.904355
15min_MT.	Q9HAT2	-0.01718	0.453384	0.372322	SIAE	Sialate O-ac	SIAE_HUMA	12	0.988166
15min_MT.	Q9BWW4	-0.36826	0.453523	0.372397	SSBP3	Single-strar	SSBP3_HUM	1	0.774716
15min_MT.	O75052	-0.01059	0.453901	0.372666	NOS1AP	Carboxyl-te	CAPON_HU	3	0.992688
30min_MT.	P54259	0.376682	0.453994	0.372666	ATN1	Atrophin-1	ATN1_HUM	1	1.298352
30min_MT.	Q9Y6B6	-0.41846	0.453966	0.372666	SAR1B	Small COPII	SAR1B_HUI	2	0.748221
30min_MT.	Q15047	-0.4601	0.454361	0.372791	SETDB1	Histone-lys	SETB1_HUM	4	0.726934
30min_MT.	Q9NP81	-0.13928	0.454309	0.372791	SARS2	Serine--tRN	SYSM_HUM	7	0.907974
15min_MT.	Q9UIM3	0.601144	0.454371	0.372791	FKBPL	FK506-binc	FKBPL_HUM	2	1.516919
30min_MT.	Q96AC1	-0.05219	0.454537	0.372831	FERMT2	Fermitin fa	FERM2_HU	22	0.964474
30min_MT.	Q9HCK4	0.074964	0.4545	0.372831	ROBO2	Roundabou	ROBO2_HU	4	1.053335
30min_MT.	Q9Y4P8	-0.07093	0.454576	0.372831	WIPI2	WD repeat	WIPI2_HUM	4	0.952024
30min_MT.	O00541	-0.0904	0.45483	0.372883	PES1	Pescadillo I	PESC_HUM	4	0.93926
15min_MT.	P78346	0.053704	0.454789	0.372883	RPP30	Ribonuclea	RPP30_HUI	7	1.037927
15min_MT.	P78386	0.083369	0.454783	0.372883	KRT85	Keratin, typ	KRT85_HUI	1	1.059489
30min_MT.	Q5R372	-0.09287	0.455072	0.372964	RABGAP1L	Rab GTPase	RBG1L_HUI	13	0.937653

15min_MT.	A6NHL2	-0.42256	0.455209	0.372999	TUBAL3	Tubulin alp	TBAL3_HUI	1	0.746099
30min_MT.	Q9BVK6	-0.2399	0.455325	0.373054	TMED9	Transmemb	TMED9_HU	1	0.846805
15min_MT.	O75208	0.054238	0.455689	0.373102	COQ9	Ubiquinon	COQ9_HUN	4	1.038311
15min_MT.	Q8N5M1	0.033831	0.455717	0.373102	ATPAF2	ATP syntha	ATPF2_HUI	5	1.023727
30min_MT.	Q9Y4E5	0.136195	0.455483	0.373102	ZNF451	E3 SUMO-p	ZN451_HUI	3	1.099002
30min_MT.	P35250	-0.27283	0.455779	0.373114	RFC2	Replication	RFC2_HUM	7	0.827697
15min_MT.	Q9NWV4	0.021231	0.455975	0.373235	CZIB	CXXC motif	CZIB_HUM	9	1.014825
30min_MT.	Q96DC9	0.282469	0.456192	0.373374	OTUB2	Ubiquitin t	OTUB2_HU	1	1.216275
30min_MT.	Q8WVV9	-0.44507	0.456486	0.373576	HNRNPLL	Heterogene	HNRLL_HUI	2	0.734547
30min_MT.	Q92804	0.084385	0.456594	0.373624	TAF15	TATA-bindi	RBP56_HUI	10	1.060236
15min_MT.	P57081	-0.16804	0.456738	0.373704	WDR4	tRNA (guan	WDR4_HUI	8	0.890053
15min_MT.	P62847	0.029545	0.456835	0.373743	RPS24	Small ribos	RS24_HUM	4	1.02069
30min_MT.	Q13813	0.071651	0.457028	0.373793	SPTAN1	Spectrin al	SPTN1_HUI	125	1.050919
15min_MT.	P30622	0.089548	0.457183	0.373794	CLIP1	CAP-Gly do	CLIP1_HUN	65	1.064037
15min_MT.	Q9BVP2	-0.0944	0.457151	0.373794	GNL3	Guanine nu	GNL3_HUM	3	0.936663
15min_MT.	Q9P0J7	-0.3303	0.457316	0.373863	KCMF1	E3 ubiquiti	KCMF1_HU	1	0.795369
15min_MT.	Q9P206	0.095075	0.457392	0.373886	NHSL3	NHS-like pr	NHSL3_HUI	5	1.068121
15min_MT.	O75874	0.117518	0.457715	0.374073	IDH1	Isocitrate d	IDHC_HUM	20	1.084867
15min_MT.	Q9UJX4	0.172822	0.458317	0.374408	ANAPC5	Anaphase-p	APC5_HUM	2	1.127261
30min_MT.	Q7Z6B7	-0.10215	0.458545	0.374555	SRGAP1	SLIT-ROBO	SRGP1_HUI	10	0.931641
15min_MT.	A3KMH1	-0.4093	0.458704	0.374606	VWA8	von Willebi	VWA8_HUN	2	0.752987
15min_MT.	Q7L8L6	-0.17066	0.458694	0.374606	FASTKD5	FAST kinase	FAKD5_HUI	6	0.888433
15min_MT.	O95678	0.085199	0.458783	0.374632	KRT75	Keratin, typ	K2C75_HUI	1	1.060834
15min_MT.	O43809	0.071298	0.45899	0.374762	NUDT21	Cleavage ar	CPSF5_HUI	11	1.050662
15min_MT.	P23396	0.073681	0.459091	0.374805	RPS3	Small ribos	RS3_HUMA	15	1.052398
30min_MT.	Q5VT52	-0.28301	0.459624	0.375202	RPRD2	Regulation	RPRD2_HU	8	0.821877
30min_MT.	Q13217	-0.12235	0.459829	0.37529	DNAJC3	DnaJ homo	DNJC3_HUI	14	0.918691
15min_MT.	Q9HA47	-0.20974	0.460763	0.375935	UCK1	Uridine-cyt	UCK1_HUM	1	0.864695
30min_MT.	P12645	-0.22785	0.460886	0.375957	BMP3	Bone morp	BMP3_HUN	1	0.853908
30min_MT.	Q9H9T3	0.0952	0.460946	0.375967	ELP3	Elongator c	ELP3_HUM	9	1.068214
15min_MT.	P28066	0.039184	0.461076	0.376022	PSMA5	Proteasom	PSA5_HUM	12	1.027532
30min_MT.	Q9UKL6	0.04166	0.46111	0.376022	PCTP	Phosphatic	PPCT_HUM	8	1.029297
30min_MT.	Q96T37	-0.09759	0.461455	0.376264	RBM15	RNA-bindir	RBM15_HU	8	0.934591
30min_MT.	P24928	-0.0814	0.461599	0.376342	POLR2A	DNA-direct	RPB1_HUM	16	0.945143
15min_MT.	Q3KQV9	0.133357	0.461867	0.376403	UAP1L1	UDP-N-acet	UAP1L_HUI	1	1.096843
30min_MT.	Q9UMZ2	-0.20838	0.461782	0.376403	SYNRG	Synergin ga	SYNRG_HUI	10	0.86551
30min_MT.	O95684	0.011571	0.462177	0.376549	CEP43	Centrosom	CEP43_HUI	6	1.008053
30min_MT.	Q00059	0.130673	0.46216	0.376549	TFAM	Transcripti	TFAM_HUM	7	1.094804
15min_MT.	Q07817	0.224781	0.46219	0.376549	BCL2L1	Bcl-2-like p	B2CL1_HUI	1	1.1686
30min_MT.	P10636	0.152725	0.462563	0.376775	MAPT	Microtubul	TAU_HUMA	5	1.111667
15min_MT.	P55199	0.096706	0.462993	0.37698	ELL	RNA polym	ELL_HUMAI	4	1.069329
15min_MT.	Q00325	-0.02798	0.462954	0.37698	SLC25A3	Solute carri	S25A3_HUI	3	0.98079
15min_MT.	Q16775	-0.01685	0.463007	0.37698	HAGH	Hydroxyacy	GLO2_HUM	10	0.988389
15min_MT.	Q9BXB4	-0.37351	0.462931	0.37698	OSBPL11	Oxysterol-b	OSB11_HUI	2	0.771901
30min_MT.	Q9BTE6	0.025679	0.463119	0.377032	AARSD1	Alanyl-tRN	AASD1_HUI	6	1.017959
30min_MT.	Q9H649	0.100644	0.463193	0.377052	NSUN3	tRNA (cytos	NSUN3_HU	1	1.072252
30min_MT.	P31948	0.081984	0.463593	0.377338	STIP1	Stress-indu	STIP1_HUM	39	1.058473
15min_MT.	P10809	0.039179	0.463712	0.377396	HSPD1	60 kDa hea	CH60_HUM	40	1.027529
15min_MT.	P04150	0.140205	0.463901	0.377511	NR3C1	Glucocorti	GCR_HUMA	5	1.102061
30min_MT.	P49441	-0.26562	0.464227	0.377711	INPP1	Inositol pol	INPP_HUM	3	0.831843
15min_MT.	Q5T447	0.10337	0.464376	0.37774	HECTD3	E3 ubiquiti	HECD3_HU	5	1.07428
30min_MT.	P13727	-1.1294	0.464526	0.377818	PRG2	Bone marro	PRG2_HUM	2	0.457107

30min_MT.	Q9UMR5	0.073212	0.464569	0.377818	PPT2	Lysosomal	PPT2_HUM	2	1.052057
15min_MT.	Q9BQE5	0.217842	0.464759	0.377895	APOL2	Apolipoprc	APOL2_HUI	7	1.162993
30min_MT.	Q9NRA2	-0.01104	0.464868	0.377944	SLC17A5	Sialin	S17A5_HUI	1	0.99238
30min_MT.	Q96RU3	0.049134	0.464979	0.377994	FNBP1	Formin-bin	FNBP1_HUI	5	1.034644
30min_MT.	P0DPD7;P(	0.145422	0.465086	0.378011	EEF1AKMT4	EEF1A lysin	EFMT4_HUI	4	1.106054
30min_MT.	P08708	-0.01002	0.465351	0.378179	RPS17	Small ribos	RS17_HUM	5	0.993076
30min_MT.	O43242	-0.04564	0.465518	0.378183	PSMD3	26S protea:	PSMD3_HU	27	0.968862
30min_MT.	P31749	0.233825	0.465493	0.378183	AKT1	RAC-alpha s	AKT1_HUM	5	1.175948
15min_MT.	Q15417	0.058311	0.465526	0.378183	CNN3	Calponin-3	CNN3_HUN	12	1.041246
30min_MT.	Q7L5Y1	0.042393	0.465549	0.378183	ENOSF1	Mitochond	ENOF1_HUI	10	1.029821
30min_MT.	P21953	0.089165	0.465795	0.378226	BCKDHB	2-oxoisoval	ODBB_HUN	5	1.063754
30min_MT.	P30084	0.025466	0.46574	0.378226	ECHS1	Enoyl-CoA l	ECHM_HUN	16	1.017808
15min_MT.	P39880	0.092878	0.465658	0.378226	CUX1	Homeobox	CUX1_HUN	1	1.066495
30min_MT.	P49770	0.074199	0.465768	0.378226	EIF2B2	Translation	EI2BB_HUN	10	1.052777
15min_MT.	O75635	-0.1891	0.465978	0.378335	SERPINB7	Serpin B7	SPB7_HUM	3	0.877153
15min_MT.	Q12965	-0.09331	0.466077	0.378376	MYO1E	Unconvent	MYO1E_HU	12	0.937373
15min_MT.	Q96JP5	0.009631	0.466193	0.378431	ZFP91	E3 ubiquiti	ZFP91_HUN	2	1.006698
30min_MT.	O75208	0.139653	0.466356	0.378524	COQ9	Ubiquinon	COQ9_HUN	4	1.10164
30min_MT.	Q63HM1	-0.04309	0.466595	0.378639	AFMID	Kynurenine	KFA_HUMA	3	0.970571
15min_MT.	Q9UJY5	0.142293	0.466668	0.378659	GGA1	ADP-ribosy	GGA1_HUN	8	1.103658
30min_MT.	P49841	0.322705	0.466913	0.37876	GSK3B	Glycogen sy	GSK3B_HUI	6	1.250674
30min_MT.	P63000	-0.03978	0.466986	0.37876	RAC1	Ras-related	RAC1_HUM	7	0.972802
30min_MT.	O95857	0.003722	0.46717	0.37887	TSPAN13	Tetraspanir	TSN13_HUI	2	1.002583
15min_MT.	P52209	-0.05966	0.467442	0.378973	PGD	6-phospho	6PGD_HUN	23	0.95949
15min_MT.	Q7Z739	-0.05306	0.467846	0.379183	YTHDF3	YTH domair	YTHD3_HUI	12	0.963893
15min_MT.	O14936	-0.07269	0.468096	0.379197	CASK	Peripheral	CSKP_HUM	23	0.950864
15min_MT.	P32321	-0.05	0.468083	0.379197	DCTD	Deoxycytid	DCTD_HUN	4	0.965935
30min_MT.	Q8N183	0.216451	0.468106	0.379197	NDUFAF2	NADH dehy	NDUF2_HU	1	1.161872
30min_MT.	Q99470	-0.36439	0.468079	0.379197	SDF2	Stromal cel	SDF2_HUM	4	0.776798
15min_MT.	O00139	0.139584	0.468357	0.379283	KIF2A	Kinesin-like	KIF2A_HUN	10	1.101588
30min_MT.	Q7Z422	0.117332	0.46835	0.379283	SZRD1	SUZ RNA-bi	SZRD1_HUI	6	1.084727
15min_MT.	P51665	0.099215	0.468593	0.379356	PSMD7	26S protea:	PSMD7_HU	7	1.071191
15min_MT.	O43759	0.216314	0.468859	0.379514	SYNGR1	Synaptogyr	SNG1_HUN	1	1.161761
30min_MT.	P21817	-0.04647	0.469147	0.379608	RYR1	Ryanodine	RYR1_HUM	1	0.968304
15min_MT.	Q93052	0.054037	0.469132	0.379608	LPP	Lipoma-pre	LPP_HUMA	13	1.038166
15min_MT.	Q969X5	-0.13077	0.469135	0.379608	ERGIC1	Endoplasm	ERGI1_HUN	5	0.913345
30min_MT.	B7ZAP0	0.016256	0.469505	0.37974	RABGAP1L	Rab GTPase	RBG10_HU	5	1.011331
15min_MT.	Q9BYD6	0.060065	0.469431	0.37974	MRPL1	Large ribos	RM01_HUN	2	1.042512
15min_MT.	P63244	-0.09025	0.46961	0.379766	RACK1	Small ribos	RACK1_HUI	18	0.93936
15min_MT.	Q15555	0.041409	0.469844	0.379782	MAPRE2	Microtubul	MARE2_HU	3	1.029118
15min_MT.	Q7Z4G1	0.063451	0.469946	0.379782	COMMD6	COMM don	COMD6_HU	2	1.044962
30min_MT.	Q96MG8	-0.50288	0.469905	0.379782	PCMTD1	Protein-L-is	PCMD1_HU	1	0.705698
30min_MT.	Q9BPY3	0.039085	0.469793	0.379782	FAM118B	Protein FAI	F118B_HUI	5	1.027462
30min_MT.	P42785	0.039055	0.470177	0.37987	PRCP	Lysosomal	PCP_HUMA	12	1.027441
30min_MT.	Q7Z2K8	-0.16529	0.470249	0.37987	GPRIN1	G protein-r	GRIN1_HUI	2	0.891752
15min_MT.	Q9P0J1	0.273516	0.470221	0.37987	PDP1	[Pyruvate d	PDP1_HUN	3	1.20875
30min_MT.	P21980	-0.03639	0.470622	0.380093	TGM2	Protein-glu	TGM2_HUN	24	0.975094
30min_MT.	P37235	-0.21343	0.47058	0.380093	HPCAL1	Hippocalci	HPCL1_HUI	4	0.862486
15min_MT.	O00233	0.069922	0.47091	0.380169	PSMD9	26S protea:	PSMD9_HU	9	1.04966
30min_MT.	P07203	-0.06909	0.471023	0.380183	GPX1	Glutathion	GPX1_HUN	3	0.953239
15min_MT.	Q9BX67	-0.18994	0.471025	0.380183	JAM3	Junctional	JAM3_HUM	1	0.87664
30min_MT.	Q8WW33	0.158044	0.47139	0.38036	GTSF1	Gametocyt	GTSF1_HUI	6	1.115773

15min_MT.	P02766	0.106682	0.4718	0.380534	TTR	Transthyret	TTHY_HUM	10	1.076749
30min_MT.	Q13151	0.160869	0.471796	0.380534	HNRNPA0	Heterogene	ROA0_HUM	5	1.11796
15min_MT.	P09132	0.20984	0.471907	0.380581	SRP19	Signal reco	SRP19_HUI	2	1.15656
30min_MT.	P20248	0.688114	0.472286	0.380769	CCNA2	Cyclin-A2	CCNA2_HU	2	1.611176
30min_MT.	Q9P021	0.044539	0.47224	0.380769	CRIPT	Cysteine-ric	CRIPT_HUM	1	1.031354
15min_MT.	Q15274	0.013445	0.472373	0.3808	QPRT	Nicotinate-	NADC_HUM	10	1.009363
30min_MT.	O00161	0.00299	0.472752	0.381027	SNAP23	Synaptosor	SNP23_HUI	8	1.002075
15min_MT.	Q8IWD4	-0.05342	0.472752	0.381027	CCDC117	Coiled-coil	CC117_HUI	2	0.963646
15min_MT.	O15405;O9	0.099652	0.472851	0.381055	TOX3;TOX4	TOX high m	TOX3_HUM	1	1.071515
15min_MT.	Q08378	0.093416	0.472884	0.381055	GOLGA3	Golgin subf	GOGA3_HU	49	1.066894
30min_MT.	Q8NHG7	0.038464	0.472942	0.381062	SVIP	Small VCP/	SVIP_HUM/	1	1.02702
30min_MT.	Q9C0I1	-0.10098	0.473435	0.381351	MTMR12	Myotubula	MTMRC_HU	9	0.932397
30min_MT.	P61088	0.00289	0.473518	0.381369	UBE2N	Ubiquitin-c	UBE2N_HU	9	1.002005
15min_MT.	Q00577	-0.08079	0.473582	0.381382	PURA	Transcripti	PURA_HUM	6	0.945537
30min_MT.	O43175	-0.05036	0.473893	0.381435	PHGDH	D-3-phosph	SERA_HUM	17	0.965692
30min_MT.	P16333	0.049705	0.473862	0.381435	NCK1	SH2/SH3 ac	NCK1_HUM	6	1.035053
15min_MT.	P57737	0.054352	0.473753	0.381435	CORO7	Coronin-7	CORO7_HU	12	1.038393
30min_MT.	Q06330	-0.05106	0.474056	0.381488	RBPJ	Recombinii	SUH_HUMA	3	0.96523
15min_MT.	Q96HR9	-0.13528	0.474129	0.381508	REEP6	Receptor ex	REEP6_HUI	5	0.910492
15min_MT.	O14639	0.115119	0.474455	0.381692	ABLIM1	Actin-bindi	ABLM1_HU	12	1.083064
30min_MT.	Q93052	-0.06622	0.474539	0.38172	LPP	Lipoma-pre	LPP_HUMA	13	0.955139
15min_MT.	Q9H2P0	0.042787	0.474743	0.381805	ADNP	Activity-de	ADNP_HUM	6	1.030102
30min_MT.	C9JLW8	0.128762	0.475279	0.382119	MCRIP1	Mapk-regul	MCRI1_HUI	5	1.093355
15min_MT.	Q9NVS9	-0.14256	0.475249	0.382119	PNPO	Pyridoxine-	PNPO_HUM	8	0.90591
30min_MT.	Q9UDY4	-0.19644	0.475269	0.382119	DNAJB4	DnaJ homo	DNJB4_HUI	7	0.8727
15min_MT.	Q8NDC0	-0.03533	0.475477	0.382199	MAPK1IP1I	MAPK-inter	MISSL_HUM	1	0.975805
30min_MT.	Q15118	0.058069	0.475533	0.382205	PDK1	[Pyruvate d	PDK1_HUM	1	1.041071
15min_MT.	Q9BRK5	-0.23819	0.475705	0.382264	SDF4	45 kDa calc	CAB45_HUI	5	0.847809
30min_MT.	A8MWD9;I	-0.14315	0.47586	0.38235	SNRPGP15;	Putative sm	RUXGL_HU	3	0.905541
15min_MT.	P54652	0.042938	0.475925	0.38235	HSPA2	Heat shock	HSP72_HUI	24	1.03021
15min_MT.	Q9Y2S7	0.008861	0.476901	0.382951	POLDIP2	Polymerase	PDIP2_HUM	14	1.006161
15min_MT.	Q9UN86	-0.04332	0.477552	0.383434	G3BP2	Ras GTPase-	G3BP2_HU	6	0.970421
30min_MT.	Q9GZQ3	0.163582	0.477715	0.383447	COMMD5	COMM don	COMD5_HU	6	1.120065
15min_MT.	Q9H8S9	0.166691	0.477914	0.383567	MOB1A	MOB kinase	MOB1A_HU	1	1.122481
15min_MT.	O14950;P1	0.039066	0.478148	0.383676	MYL12B;MY	Myosin regi	ML12B_HU	6	1.027448
30min_MT.	A0JNW5	-0.24875	0.478499	0.383918	BLTP3B	Bridge-like	BLT3B_HUM	13	0.841626
15min_MT.	Q9UBR2	-0.06026	0.478595	0.383946	CTSZ	Cathepsin Z	CATZ_HUM	7	0.959088
30min_MT.	Q9UKJ3	-0.0416	0.478632	0.383946	GPATCH8	G patch doi	GPTC8_HUI	17	0.971574
30min_MT.	P16403	-0.01179	0.478991	0.384195	H1-2	Histone H1	H12_HUMA	1	0.99186
15min_MT.	P20226	0.050631	0.479121	0.38423	TBP	TATA-box-b	TBP_HUMA	1	1.035718
30min_MT.	Q13428	0.049947	0.479133	0.38423	TCOF1	Treacle pro	TCOF_HUM	50	1.035227
15min_MT.	Q14574	0.132085	0.479425	0.384354	DSC3	Desmocolli	DSC3_HUM	5	1.095877
15min_MT.	Q86UE4	0.089194	0.479411	0.384354	MTDH	Protein LYF	LYRIC_HUM	10	1.063776
30min_MT.	Q9H8K7	-0.19839	0.479435	0.384354	PAAT	ATPase PAA	PAAT_HUM	5	0.871522
30min_MT.	P58546	0.079298	0.479659	0.384416	MTPN	Myotrophi	MTPN_HUM	4	1.056504
15min_MT.	Q9NTX5	0.044059	0.47966	0.384416	ECHDC1	Ethylmalor	ECHD1_HU	13	1.03101
15min_MT.	P62280	0.016476	0.480093	0.384513	RPS11	Small ribos	RS11_HUM	5	1.011486
15min_MT.	Q07157	0.007464	0.480043	0.384513	TJP1	Tight juncti	ZO1_HUMA	32	1.005187
30min_MT.	Q15262	-0.01313	0.480064	0.384513	PTPRK	Receptor-ty	PTPRK_HUI	10	0.99094
15min_MT.	Q6P996	0.04445	0.479918	0.384513	PDXDC1	Pyridoxal-d	PDXD1_HU	17	1.03129
30min_MT.	Q8WXA9	-0.08315	0.480126	0.384513	SREK1	Splicing reg	SREK1_HUI	5	0.943994
15min_MT.	Q9BXP5	0.070226	0.47993	0.384513	SRRT	Serrate RN/	SRRT_HUM	17	1.049881

30min_MT.	P06899	0.117254	0.48027	0.38459	H2BC11	Histone H2	H2B1J_HUI		2	1.084669
30min_MT.	Q8WZA0	0.099563	0.480459	0.384701	LZIC	Protein LZI(	LZIC_HUMA		6	1.071449
30min_MT.	Q13636;Q9	0.262142	0.480693	0.38481	RAB31;RAB	Ras-related	RAB31_HUI		2	1.199258
30min_MT.	O15294	-0.00458	0.480859	0.384855	OGT	UDP-N-acet	OGT1_HUM		1	0.996833
15min_MT.	P51398	0.256509	0.480813	0.384855	DAP3	Small ribos	RT29_HUM		8	1.194584
30min_MT.	Q9BZL4	-0.05679	0.480988	0.384855	PPP1R12C	Protein pho	PP12C_HUI		7	0.961399
15min_MT.	Q9NSB2	0.209719	0.480994	0.384855	KRT84	Keratin, typ	KRT84_HUI		1	1.156463
15min_MT.	Q9UBK8	0.006259	0.480914	0.384855	MTRR	Methionine	MTRR_HUM		10	1.004347
30min_MT.	P67936	0.070875	0.481082	0.384885	TPM4	Tropomyos	TPM4_HUM		19	1.050353
15min_MT.	Q6V1X1	-0.21875	0.481607	0.385266	DPP8	Dipeptidyl	DPP8_HUM		3	0.859309
30min_MT.	Q92733	-0.09498	0.481997	0.385485	PRCC	Proline-rich	PRCC_HUM		3	0.936282
15min_MT.	P12110	0.030576	0.482137	0.385532	COL6A2	Collagen al	CO6A2_HU		3	1.02142
30min_MT.	Q9HAU0	0.139517	0.482346	0.38566	PLEKHA5	Pleckstrin h	PKHA5_HU		7	1.101536
30min_MT.	Q14126	-0.10956	0.482516	0.385717	DSG2	Desmoglein	DSG2_HUM		18	0.926871
15min_MT.	A2RUC4	-0.09264	0.482754	0.385818	TYW5	tRNA wybu	TYW5_HUM		2	0.937802
30min_MT.	P29323	0.182547	0.48322	0.386004	EPHB2	Ephrin type	EPHB2_HUI		3	1.134886
15min_MT.	Q15181	0.10007	0.483453	0.386114	PPA1	Inorganic p	IPYR_HUMA		19	1.071825
15min_MT.	P26196	0.067759	0.483717	0.386251	DDX6	Probable AT	DDX6_HUM		12	1.048087
15min_MT.	P55209	-0.02897	0.483808	0.386251	NAP1L1	Nucleosom	NP1L1_HUI		7	0.980121
15min_MT.	Q8N6T3	0.045513	0.483864	0.386251	ARFGAP1	ADP-ribosy	ARFG1_HUI		9	1.03205
30min_MT.	Q8NBK3	0.056226	0.483875	0.386251	SUMF1	Formylglyc	SUMF1_HU		6	1.039742
15min_MT.	P60842	-0.04228	0.484013	0.386322	EIF4A1	Eukaryotic	IF4A1_HUM		25	0.971117
15min_MT.	P10253	0.123971	0.484513	0.386619	GAA	Lysosomal a	LYAG_HUM		16	1.08973
30min_MT.	Q6ICG6	-0.03483	0.484581	0.386619	KIAA0930	Uncharacte	K0930_HUI		7	0.976144
15min_MT.	Q9H0W9	0.023466	0.485032	0.386898	C11orf54	Ester hydro	CK054_HUI		10	1.016399
15min_MT.	P18827	-0.16318	0.485296	0.386981	SDC1	Syndecan-1	SDC1_HUM		1	0.893057
15min_MT.	P21580	-0.21876	0.485334	0.386981	TNFAIP3	Tumor necr	TNAP3_HUI		4	0.859302
15min_MT.	Q8N573	-0.12287	0.485317	0.386981	OXR1	Oxidation r	OXR1_HUM		16	0.91836
15min_MT.	Q9BW83	0.117475	0.48533	0.386981	IFT27	Intraflagell	IFT27_HUM		5	1.084835
30min_MT.	Q5JTJ3	0.0877	0.485396	0.386991	COA6	Cytochrom	COA6_HUM		5	1.062675
30min_MT.	P05154	0.325859	0.485581	0.38706	SERPINA5	Plasma seri	IPSP_HUMA		2	1.253411
30min_MT.	Q9P0M2	-0.12638	0.485561	0.38706	AKAP7	A-kinase an	AKA7G_HU		3	0.916128
15min_MT.	P48735	0.104432	0.485694	0.38711	IDH2	Isocitrate d	IDHP_HUM		23	1.075071
15min_MT.	Q15637	0.031502	0.485744	0.387111	SF1	Splicing fac	SF01_HUM		9	1.022076
30min_MT.	P20839	0.123663	0.486142	0.387309	IMPDH1	Inosine-5'-r	IMDH1_HU		6	1.089498
30min_MT.	Q96DC7	-0.05987	0.486116	0.387309	TMCO6	Transmemb	TMCO6_HU		1	0.959353
30min_MT.	Q13561	0.010374	0.48648	0.387539	DCTN2	Dynactin su	DCTN2_HU		18	1.007217
30min_MT.	Q96CB8	-0.22827	0.486566	0.387568	INTS12	Integrator (	INT12_HUM		3	0.853658
30min_MT.	Q15303	-0.74549	0.48726	0.388042	ERBB4	Receptor ty	ERBB4_HUI		1	0.596467
15min_MT.	P50542	0.05067	0.48737	0.38805	PEX5	Peroxisoma	PEX5_HUM		3	1.035746
30min_MT.	Q96F86	-0.02716	0.487353	0.38805	EDC3	Enhancer o	EDC3_HUM		9	0.981348
30min_MT.	O60573	-0.07056	0.487475	0.388055	EIF4E2	Eukaryotic	IF4E2_HUM		2	0.952267
15min_MT.	P99999	-0.07957	0.48762	0.388092	CYCS	Cytochrom	CYC_HUMA		6	0.94634
30min_MT.	Q9Y5Q8	-0.14955	0.487588	0.388092	GTF3C5	General tra	TF3C5_HUI		5	0.901532
30min_MT.	P10114	0.030102	0.488009	0.388243	RAP2A	Ras-related	RAP2A_HUI		1	1.021085
30min_MT.	Q53S33	0.09428	0.487865	0.388243	BOLA3	BolA-like pr	BOLA3_HUI		4	1.067533
15min_MT.	Q5W0V3	-0.00484	0.487977	0.388243	FHIP2A	FHF comple	FHI2A_HUM		14	0.996652
15min_MT.	P48454	0.091201	0.488306	0.3884	PPP3CC	Serine/thre	PP2BC_HUI		1	1.065257
15min_MT.	Q9C0B7	-0.42499	0.488287	0.3884	TANGO6	Transport a	TNG6_HUM		2	0.744845
30min_MT.	Q96T23	0.053923	0.4884	0.388436	RSF1	Remodeling	RSF1_HUM		6	1.038084
15min_MT.	Q9NYV4	0.57889	0.488693	0.38859	CDK12	Cyclin-depe	CDK12_HU		3	1.4937
15min_MT.	P11142	-0.02966	0.489116	0.388865	HSPA8	Heat shock	HSP7C_HUI		24	0.979652

15min_MT.	Q3LXA3	0.113358	0.489139	0.388865	TKFC	Triokinase/	TKFC_HUM	11	1.081743
30min_MT.	P08962	-0.07039	0.489305	0.388958	CD63	CD63 antig	CD63_HUM	2	0.952382
30min_MT.	Q16762	-0.10461	0.489831	0.389228	TST	Thiosulfate	THTR_HUM	12	0.930054
15min_MT.	Q7Z4G4	-0.14375	0.489843	0.389228	TRMT11	tRNA (guan	TRM11_HU	3	0.905162
30min_MT.	P61160	0.008774	0.490248	0.389312	ACTR2	Actin-relate	ARP2_HUM	13	1.0061
15min_MT.	Q13404	0.110845	0.490059	0.389312	UBE2V1	Ubiquitin-c	UB2V1_HU	9	1.07986
30min_MT.	Q9NWZ5	-0.14265	0.490203	0.389312	UCKL1	Uridine-cyt	UCKL1_HU	4	0.905854
30min_MT.	Q8WWI1	0.046266	0.49033	0.389337	LMO7	LIM domair	LMO7_HUM	20	1.032589
15min_MT.	P35080	0.006473	0.490775	0.389572	PFN2	Profilin-2	PROF2_HU	5	1.004497
30min_MT.	Q8IVM0	0.091449	0.490704	0.389572	CCDC50	Coiled-coil	CCD50_HU	10	1.06544
15min_MT.	Q96T60	0.156464	0.490757	0.389572	PNKP	Bifunctiona	PNKP_HUM	1	1.114552
30min_MT.	Q9BQ13	-0.06509	0.490833	0.389579	KCTD14	BTB/POZ dc	KCD14_HU	7	0.955887
15min_MT.	O75323	0.156137	0.490971	0.389649	NIPSNAP2	Protein Nip	NIPS2_HUM	4	1.1143
30min_MT.	Q9Y6M1	0.045008	0.491134	0.389699	IGF2BP2	Insulin-like	IF2B2_HUM	11	1.031689
30min_MT.	O75489	0.016991	0.491454	0.389865	NDUFS3	NADH dehy	NDUS3_HU	5	1.011847
15min_MT.	P23258	-0.07388	0.491455	0.389865	TUBG1	Tubulin gar	TBG1_HUM	4	0.950078
15min_MT.	Q9NUQ3	0.098392	0.491744	0.389946	TXLNG	Gamma-tax	TXLNG_HU	10	1.070579
15min_MT.	P31350	0.051965	0.491913	0.389961	RRM2	Ribonuclec	RIR2_HUM	7	1.036676
15min_MT.	Q14764	0.066604	0.492049	0.390029	MVP	Major vault	MVP_HUM	33	1.047249
15min_MT.	P22695	-0.32708	0.492255	0.390124	UQCRC2	Cytochrom	QCR2_HUM	2	0.797148
30min_MT.	Q9H871	0.062829	0.492269	0.390124	RMND5A	E3 ubiquiti	RMD5A_HU	4	1.044512
15min_MT.	P08243	0.03577	0.492477	0.39025	ASNS	Asparagine	ASNS_HUM	11	1.025104
15min_MT.	P63241	-0.06646	0.492735	0.390335	EIF5A	Eukaryotic	IF5A1_HUM	13	0.954979
30min_MT.	O00159	0.11445	0.493283	0.390453	MYO1C	Unconvent	MYO1C_HU	15	1.082562
15min_MT.	P35606	-0.00241	0.492946	0.390453	COPB2	Coatomer s	COPB2_HU	39	0.99833
30min_MT.	Q13642	-0.12943	0.493233	0.390453	FHL1	Four and a l	FHL1_HUM	3	0.914192
30min_MT.	Q9BTM9	-0.03125	0.493244	0.390453	URM1	Ubiquitin-r	URM1_HUM	2	0.97857
15min_MT.	Q9Y2H2	-0.16804	0.493198	0.390453	INPP5F	Phosphatic	SAC2_HUM	1	0.890052
30min_MT.	P68363	-0.09472	0.49348	0.39057	TUBA1B	Tubulin alp	TBA1B_HU	20	0.936451
30min_MT.	P46781	0.145122	0.493936	0.390793	RPS9	Small ribos	RS9_HUMA	10	1.105824
15min_MT.	Q5SW79	0.115924	0.494184	0.390793	CEP170	Centrosom	CE170_HU	7	1.083669
30min_MT.	Q8IYD1	-0.09406	0.494143	0.390793	GSPT2	Eukaryotic	ERF3B_HUI	6	0.936882
30min_MT.	Q92572	-0.09215	0.494303	0.390793	AP3S1	AP-3 compl	AP3S1_HU	5	0.938121
30min_MT.	Q93096	-0.17275	0.494267	0.390793	PTP4A1	Protein tyr	TP4A1_HU	7	0.88715
15min_MT.	O00161	-0.0866	0.494529	0.390794	SNAP23	Synaptosor	SNP23_HU	8	0.941742
30min_MT.	O00505	0.118845	0.494452	0.390794	KPNA3	Importin su	IMA4_HUM	10	1.085865
30min_MT.	P49848	-0.27834	0.494544	0.390794	TAF6	Transcripti	TAF6_HUM	1	0.824538
30min_MT.	Q6PJT7	-0.01734	0.494563	0.390794	ZC3H14	Zinc finger	ZC3HE_HU	5	0.98805
15min_MT.	P23381	-0.01766	0.494993	0.391033	WARS1	Tryptophar	SYWC_HUM	27	0.987832
15min_MT.	Q6Q0C0	-0.28395	0.495015	0.391033	TRAF7	E3 ubiquiti	TRAF7_HUI	3	0.821342
30min_MT.	Q8NDX5	-0.23153	0.494921	0.391033	PHC3	Polyhomec	PHC3_HUM	1	0.851729
15min_MT.	Q9UJU6	0.123214	0.495405	0.391258	DBNL	Drebrin-like	DBNL_HUM	17	1.089158
15min_MT.	O75882	0.064492	0.496359	0.391897	ATRN	Attractin	ATRN_HUM	11	1.045717
15min_MT.	P10636	0.159471	0.496967	0.392337	MAPT	Microtubul	TAU_HUMA	5	1.116878
15min_MT.	O43396	-0.05545	0.497613	0.392689	TXNL1	Thioredoxi	TXNL1_HU	13	0.962291
30min_MT.	P68371	-0.02981	0.497704	0.392721	TUBB4B	Tubulin bet	TBB4B_HU	18	0.979552
30min_MT.	Q9Y2G5	-0.0969	0.497844	0.392791	POFUT2	GDP-fucose	OFUT2_HU	9	0.935037
15min_MT.	Q9C0J8	0.000682	0.498354	0.392996	WDR33	pre-mRNA 3	WDR33_HU	7	1.000473
30min_MT.	P45985	-0.11913	0.498424	0.393011	MAP2K4	Dual specifi	MP2K4_HU	4	0.92074
30min_MT.	Q9C0C2	-0.01356	0.498692	0.393183	TNKS1BP1	182 kDa tar	TB182_HU	37	0.990648
15min_MT.	P62854;Q5	0.017579	0.499121	0.393433	RPS26;RPS:	Small ribos	RS26_HUM	1	1.01226
15min_MT.	Q32P28	-0.00991	0.499159	0.393433	P3H1	Prolyl 3-hy	P3H1_HUM	18	0.993157

30min_MT.	Q86X27	0.389515	0.499129	0.393433	RALGPS2	Ras-specific	RGPS2_HUI	1	1.309953
30min_MT.	Q15041	0.037355	0.499501	0.393622	ARL6IP1	ADP-ribosy	AR6P1_HUI	1	1.026231
15min_MT.	Q9BZF9	-0.36163	0.499906	0.393863	UACA	Uveal autoa	UACA_HUM	3	0.778284
15min_MT.	Q9H081	0.284295	0.500072	0.393953	MIS12	Protein MIS	MIS12_HUI	4	1.217815
30min_MT.	O15160	-0.08813	0.500537	0.394111	POLR1C	DNA-direct	RPAC1_HUI	6	0.94074
30min_MT.	Q5T6V5	-0.01689	0.500693	0.394111	QNG1	Queuosine	QNG1_HUM	12	0.988358
15min_MT.	Q9UHP3	0.010239	0.501051	0.394248	USP25	Ubiquitin c	UBP25_HU	28	1.007122
30min_MT.	Q7Z434	0.160779	0.501308	0.394401	MAVS	Mitochond	MAVS_HUM	8	1.117891
30min_MT.	Q02878	0.078366	0.501655	0.394455	RPL6	Large ribos	RL6_HUMA	9	1.055821
30min_MT.	Q12972	-0.08827	0.501667	0.394455	PPP1R8	Nuclear inh	PP1R8_HUI	7	0.940649
15min_MT.	Q5JSH3	-0.01174	0.501527	0.394455	WDR44	WD repeat-	WDR44_HU	32	0.991897
15min_MT.	Q8WTV0	-0.14524	0.501563	0.394455	SCARB1	Scavenger r	SCRB1_HUI	5	0.90423
15min_MT.	Q92620	0.028024	0.501999	0.394637	DHX38	Pre-mRNA-	PRP16_HUI	5	1.019615
30min_MT.	Q8NBL1	-0.08272	0.502089	0.394668	POGLUT1	Protein O-g	PGLT1_HUI	7	0.944274
15min_MT.	P05387	0.073153	0.50226	0.394698	RPLP2	Large ribos	RLA2_HUM	6	1.052014
15min_MT.	Q14999;Q8	-0.51092	0.502299	0.394698	CUL7;CUL9	Cullin-7;Cu	CUL7_HUM	2	0.701773
30min_MT.	O14530	0.116423	0.502531	0.394751	TXNDC9	Thioredoxi	TXND9_HU	4	1.084044
15min_MT.	Q8N1N4	0.116092	0.502464	0.394751	KRT78	Keratin, typ	K2C78_HUI	12	1.083795
30min_MT.	P50995	-0.01074	0.502654	0.394755	ANXA11	Annexin A1	ANX11_HU	15	0.99258
30min_MT.	Q15427	0.109857	0.502648	0.394755	SF3B4	Splicing fac	SF3B4_HUM	3	1.079121
30min_MT.	P07307	-1.31835	0.503066	0.39496	ASGR2	Asialoglyco	ASGR2_HUI	2	0.400993
15min_MT.	P51116	-0.31281	0.503001	0.39496	FXR2	RNA-bindir	FXR2_HUM	4	0.805071
30min_MT.	A0PJZ3	-0.16208	0.503431	0.395061	GXYLT2	Glucoside x	GXLT2_HUI	1	0.893733
15min_MT.	A5YKK6	-0.07116	0.503566	0.395061	CNOT1	CCR4-NOT t	CNOT1_HU	20	0.951876
15min_MT.	O60506	0.049665	0.503677	0.395061	SYNCRIP	Heterogene	HNRPQ_HU	15	1.035025
15min_MT.	O75177	0.596672	0.503407	0.395061	SS18L1	Calcium-res	CREST_HUM	1	1.512224
15min_MT.	Q7Z6M1	-0.03318	0.503574	0.395061	RABEPK	Rab9 effect	RABEK_HUI	15	0.977264
15min_MT.	Q8WWY3	-0.05505	0.503655	0.395061	PRPF31	U4/U6 sma	PRP31_HUI	6	0.962559
30min_MT.	Q8WY54	0.195076	0.503762	0.395061	PPM1E	Protein pho	PPM1E_HU	3	1.144784
30min_MT.	Q92623	0.191932	0.503632	0.395061	TTC9	Tetratricop	TTC9A_HUM	2	1.142293
30min_MT.	Q9ULV4	-0.06225	0.503289	0.395061	CORO1C	Coronin-1C	COR1C_HU	20	0.957772
30min_MT.	P27707	0.191497	0.504541	0.395482	DCK	Deoxycytid	DCK_HUMA	5	1.141948
15min_MT.	Q96L92	0.027173	0.504518	0.395482	SNX27	Sorting nex	SNX27_HUI	8	1.019013
30min_MT.	Q92871	0.517429	0.50467	0.395504	PMM1	Phosphoma	PMM1_HUI	2	1.431402
15min_MT.	Q15067	0.111262	0.504828	0.395549	ACOX1	Peroxisoma	ACOX1_HU	5	1.080173
15min_MT.	P13929	0.340829	0.505092	0.395716	ENO3	Beta-enolas	ENOB_HUM	2	1.266484
15min_MT.	Q9NRF8	0.094211	0.50515	0.395722	CTPS2	CTP syntha:	PYRG2_HUI	17	1.067481
15min_MT.	O60684	0.100919	0.505382	0.395836	KPNA6	Importin su	IMA7_HUM	8	1.072457
30min_MT.	O95999	0.103925	0.505647	0.395953	BCL10	B-cell lymp	BCL10_HUI	6	1.074693
30min_MT.	Q9NZI7	0.015024	0.505843	0.396067	UBP1	Upstream-b	UBIP1_HUM	1	1.010468
30min_MT.	Q9NPJ1	-0.16725	0.50598	0.396081	MKKS	Molecular c	MKKS_HUM	2	0.89054
30min_MT.	Q9P0J1	0.038425	0.506065	0.396081	PDP1	[Pyruvate d	PDP1_HUM	3	1.026992
30min_MT.	O95273	0.004119	0.506369	0.39628	CCNDBP1	Cyclin-D1-b	CCDB1_HU	5	1.002859
15min_MT.	Q8NBJ5	0.1102	0.506466	0.396316	COLGALT1	Procollager	GT251_HUI	13	1.079378
30min_MT.	P49916	-0.04866	0.506557	0.396348	LIG3	DNA ligase 3	DNLI3_HUM	1	0.966836
15min_MT.	Q14232	-0.02683	0.506903	0.396539	EIF2B1	Translation	EI2BA_HUM	17	0.981577
15min_MT.	Q69YN4	0.268381	0.506888	0.396539	VIRMA	Protein viri	VIR_HUMAI	10	1.204456
30min_MT.	O60264	0.065661	0.507116	0.396638	SMARCA5	SWI/SNF-re	SMCA5_HU	7	1.046564
15min_MT.	Q9UII2	0.100817	0.507264	0.396638	ATP5IF1	ATPase inhi	ATIF1_HUM	4	1.072381
15min_MT.	P49321	-0.01842	0.507388	0.396679	NASP	Nuclear aut	NASP_HUM	32	0.987313
15min_MT.	Q29RF7	-0.13224	0.507436	0.396679	PDS5A	Sister chror	PDS5A_HUI	4	0.912412
30min_MT.	Q07955	0.07344	0.50756	0.396736	SRSF1	Serine/argi	SRSF1_HUM	14	1.052223

15min_MT.	Q9NWU5	0.254795	0.508085	0.397042	MRPL22	Large ribos	RM22_HUN	1	1.193166
30min_MT.	Q9P270	-0.1777	0.508466	0.397285	SLAIN2	SLAIN moti	SLAI2_HUN	4	0.884114
30min_MT.	Q9Y2H5	-0.35847	0.508549	0.39731	PLEKHA6	Pleckstrin h	PKHA6_HU	2	0.779991
30min_MT.	P20340	0.006868	0.508659	0.397354	RAB6A	Ras-related	RAB6A_HUI	9	1.004772
30min_MT.	P23378	-0.03839	0.508998	0.397463	GLDC	Glycine del	GCSP_HUM	15	0.973743
30min_MT.	Q86TX2	0.032181	0.509543	0.397729	ACOT1	Acyl-coenzy	ACOT1_HUI	18	1.022557
15min_MT.	Q9H7N4	-0.32449	0.509766	0.397784	SCAF1	Splicing fac	SFR19_HUN	1	0.798579
15min_MT.	O95822	-0.01426	0.510382	0.398206	MLYCD	Malonyl-Co	DCMC_HUN	11	0.990163
15min_MT.	Q9BXF6	-0.5696	0.511265	0.398755	RAB11FIP5	Rab11 fami	RFIP5_HUN	2	0.673803
15min_MT.	Q16774	0.026905	0.511456	0.398864	GUK1	Guanylate l	KGUA_HUN	6	1.018824
15min_MT.	P52272	0.063052	0.512153	0.399354	HNRNPM	Heterogene	HNRPM_HU	20	1.044674
30min_MT.	Q9BYB4	-0.2394	0.512186	0.399354	GNB1L	Guanine nu	GNB1L_HU	10	0.847099
30min_MT.	Q14118	0.183488	0.512808	0.399662	DAG1	Dystroglyca	DAG1_HUN	5	1.135626
30min_MT.	Q9UNM6	-0.02926	0.512888	0.399662	PSMD13	26S protea	PSD13_HUI	21	0.979925
15min_MT.	P40692	-0.46864	0.51321	0.399748	MLH1	DNA misma	MLH1_HUN	5	0.722647
15min_MT.	Q15154	0.102634	0.513075	0.399748	PCM1	Pericentrio	PCM1_HUN	36	1.073732
30min_MT.	Q9UQ35	-0.03233	0.513119	0.399748	SRRM2	Serine/argi	SRRM2_HU	68	0.977837
30min_MT.	Q15154	-0.02852	0.513412	0.399832	PCM1	Pericentrio	PCM1_HUN	36	0.980423
30min_MT.	Q9BZL1	0.084677	0.513583	0.399925	UBL5	Ubiquitin-l	UBL5_HUM	3	1.06045
30min_MT.	Q13492	0.008045	0.513692	0.399931	PICALM	Phosphatic	PICAL_HUN	14	1.005592
30min_MT.	Q86VQ6	0.101811	0.513693	0.399931	TXNRD3	Thioredoxi	TRXR3_HUI	8	1.073119
30min_MT.	Q6ULP2	-0.38454	0.51392	0.400028	AFTPH	Aftiphilin	AFTIN_HUN	2	0.766022
15min_MT.	P51153	-0.05708	0.514198	0.400165	RAB13	Ras-related	RAB13_HUI	1	0.961205
30min_MT.	Q9UK61	-0.24369	0.514354	0.400247	TASOR	Protein TAS	TASOR_HUI	1	0.84458
15min_MT.	Q9Y3Z3	-0.04395	0.514436	0.400271	SAMHD1	Deoxynucle	SAMH1_HU	3	0.969997
15min_MT.	Q7L2H7	-0.11331	0.514651	0.400358	EIF3M	Eukaryotic	EIF3M_HUN	13	0.924462
15min_MT.	P28332	-0.00498	0.514884	0.400485	ADH6	Alcohol del	ADH6_HUN	12	0.996553
15min_MT.	Q13303	-0.14305	0.514916	0.400485	KCNAB2	Voltage-gat	KCAB2_HUI	3	0.905602
30min_MT.	O60888	0.108846	0.515791	0.401036	CUTA	Protein Cut	CUTA_HUM	4	1.078365
15min_MT.	Q99567	0.004252	0.51583	0.401036	NUP88	Nuclear por	NUP88_HU	16	1.002952
15min_MT.	Q99798	0.000352	0.515993	0.401123	ACO2	Aconitate h	ACON_HUN	30	1.000244
30min_MT.	Q03113	0.441084	0.516063	0.401137	GNA12	Guanine nu	GNA12_HU	1	1.357624
30min_MT.	P62072	-0.02166	0.516273	0.401224	TIMM10	Mitochond	TIM10_HUI	3	0.985102
30min_MT.	Q96ER3	-0.08804	0.516278	0.401224	SAAL1	Protein SA/	SAAL1_HUN	12	0.940799
30min_MT.	Q2M389	0.119479	0.516482	0.401343	WASHC4	WASH com	WASC4_HU	6	1.086343
30min_MT.	Q2TAZ0	-0.0758	0.516746	0.401469	ATG2A	Autophagy-	ATG2A_HUI	14	0.948814
15min_MT.	Q15057	0.055912	0.517115	0.401659	ACAP2	Arf-GAP wit	ACAP2_HUI	10	1.039516
30min_MT.	Q16773	0.062134	0.51714	0.401659	KYAT1	Kynurenine	KAT1_HUM	10	1.044009
30min_MT.	Q16851	0.035769	0.517284	0.401727	UGP2	UTP--glucos	UGPA_HUN	24	1.025103
30min_MT.	Q96C19	0.072208	0.517572	0.401911	EFHD2	EF-hand do	EFHD2_HUI	13	1.051324
15min_MT.	P09382	0.106094	0.518058	0.402129	LGALS1	Galectin-1	LEG1_HUM	11	1.07631
30min_MT.	O76041	0.090784	0.518232	0.402144	NEBL	Nebulette	NEBL_HUM	2	1.064949
30min_MT.	Q7L5N1	0.054553	0.518226	0.402144	COPS6	COP9 signa	CSN6_HUM	10	1.038537
15min_MT.	Q9H3P7	0.004833	0.518515	0.402324	ACBD3	Golgi reside	GCP60_HU	13	1.003356
30min_MT.	Q9NQZ5	-0.1856	0.518689	0.402419	STARD7	StAR-relate	STAR7_HUN	4	0.879286
15min_MT.	O00746	0.027564	0.518891	0.402495	NME4	Nucleoside	NDKM_HUI	3	1.01929
30min_MT.	Q8IXB1	-0.05207	0.518976	0.402522	DNAJC10	DnaJ homo	DJC10_HUI	9	0.964551
30min_MT.	Q96H20	-0.00906	0.519164	0.402627	SNF8	Vacuolar-sc	SNF8_HUM	6	0.993737
15min_MT.	Q13153	0.026685	0.519388	0.402762	PAK1	Serine/thre	PAK1_HUM	13	1.018669
30min_MT.	Q9BSH4	-0.00186	0.519513	0.40279	TACO1	Translation	TACO1_HUI	7	0.998711
15min_MT.	Q9NVR2	-0.76049	0.51958	0.40279	INTS10	Integrator (	INT10_HUN	1	0.590294
30min_MT.	O00178	0.453011	0.519703	0.402807	GTPBP1	GTP-bindin	GTPB1_HUI	2	1.368894

15min_MT.	P11117	0.050465	0.519705	0.402807	ACP2	Lysosomal a	PPAL_HUM	11	1.035599
30min_MT.	Q8WWH5	-0.24802	0.51976	0.40281	TRUB1	Pseudouric	TRUB1_HUI	5	0.842052
30min_MT.	O15173	0.051893	0.520095	0.402952	PGRMC2	Membrane-	PGRC2_HU	4	1.036624
30min_MT.	Q8WUM4	-0.06053	0.520098	0.402952	PDCD6IP	Programme	PDC6I_HUI	38	0.958914
30min_MT.	Q13405	0.250518	0.520251	0.403031	MRPL49	Large ribos	RM49_HUM	2	1.189634
30min_MT.	P55210	0.088612	0.52033	0.403052	CASP7	Caspase-7	CASP7_HUI	6	1.063347
15min_MT.	Q96CN9	0.133064	0.52078	0.403321	GCC1	GRIP and cc	GCC1_HUM	5	1.096621
15min_MT.	O14981	0.276054	0.520967	0.403353	BTAF1	TATA-bindi	BTAF1_HUI	6	1.210878
15min_MT.	P08651	-0.07767	0.520894	0.403353	NFIC	Nuclear fac	NFIC_HUM.	3	0.947587
15min_MT.	Q9Y2B9	-0.11158	0.520977	0.403353	PKIG	cAMP-depe	IPKG_HUM	1	0.925573
30min_MT.	Q9BV57	0.028906	0.52158	0.40378	ADI1	Acireducto	MTND_HUI	8	1.020238
30min_MT.	P00966	-0.06642	0.521762	0.403882	ASS1	Argininosu	ASSY_HUM.	18	0.955005
30min_MT.	Q9Y314	0.072813	0.521964	0.403998	NOSIP	Nitric oxide	NOSIP_HUI	3	1.051765
30min_MT.	O95070	-0.19374	0.522288	0.404085	YIF1A	Protein YIF	YIF1A_HUM	1	0.874334
15min_MT.	P48426	0.061694	0.522438	0.404085	PIP4K2A	Phosphatid	PI42A_HUI	8	1.043691
15min_MT.	Q8N0U4	0.134899	0.522389	0.404085	FAM185A	Protein FAI	F185A_HUI	6	1.098016
30min_MT.	Q9Y237	0.124617	0.522335	0.404085	PIN4	Peptidyl-pr	PIN4_HUM	7	1.090219
15min_MT.	Q9GZU8	-0.12332	0.523596	0.4049	PSME3IP1	PSME3-inte	PIP30_HUI	6	0.918074
15min_MT.	Q9BQG2	-0.03413	0.523661	0.404904	NUDT12	NAD-cappe	NUD12_HU	3	0.976623
30min_MT.	Q9NX74	0.037586	0.523704	0.404904	DUS2	tRNA-dihyc	DUS2L_HUI	2	1.026395
30min_MT.	Q9Y5M8	-0.03199	0.523871	0.404993	SRPRB	Signal reco	SRPRB_HUI	5	0.978069
30min_MT.	A6NKH3;P6	-0.09178	0.523992	0.405047	RPL37AP8;	Putative rib	RL37L_HUI	1	0.938363
15min_MT.	P34949	0.072681	0.524111	0.405058	MPI	Mannose-6	MPI_HUMA	8	1.05167
30min_MT.	Q9C0C4	0.394654	0.524209	0.405094	SEMA4C	Semaphori	SEM4C_HU	2	1.314627
15min_MT.	Q9UPT8	-0.10593	0.524371	0.405139	ZC3H4	Zinc finger	ZC3H4_HUI	13	0.929205
15min_MT.	Q9Y371	-0.12558	0.524335	0.405139	SH3GLB1	Endophilin	SHLB1_HUI	4	0.916636
30min_MT.	Q9NTZ6	0.017997	0.524488	0.40519	RBM12	RNA-bindir	RBM12_HU	18	1.012552
15min_MT.	Q9UL15	0.048494	0.524686	0.405303	BAG5	BAG family	BAG5_HUM	8	1.034185
15min_MT.	Q8TBC5	0.640097	0.524842	0.405383	ZSCAN18	Zinc finger	ZSC18_HUI	1	1.558434
30min_MT.	Q8N9N2	-0.01381	0.525232	0.405571	ASCC1	Activating s	ASCC1_HUI	3	0.990476
15min_MT.	Q9NX02	-0.23883	0.525241	0.405571	NLRP2	NACHT, LRF	NALP2_HUI	6	0.847432
30min_MT.	P56211	0.094446	0.525997	0.405949	ARPP19	cAMP-regul	ARP19_HUI	4	1.067655
15min_MT.	Q86UY8	0.072341	0.526016	0.405949	NT5DC3	5'-nucleoti	NT5D3_HU	9	1.051421
30min_MT.	Q9NVM4	-0.23446	0.526047	0.405949	PRMT7	Protein arg	ANM7_HUI	2	0.850001
30min_MT.	P49591	0.053601	0.526165	0.405964	SARS1	Serine--tRN	SYSC_HUM.	22	1.037852
15min_MT.	Q14527	-0.00968	0.526823	0.406271	HLTF	Helicase-lik	HLTF_HUM	1	0.993311
30min_MT.	P63313	0.132605	0.526924	0.406309	TMSB10	Thymosin b	TYB10_HUI	2	1.096271
15min_MT.	P17174	-0.04641	0.527082	0.406351	GOT1	Aspartate a	AATC_HUM	24	0.968342
15min_MT.	P48634	-0.19062	0.527227	0.406385	PRRC2A	Protein PRI	PRC2A_HUI	20	0.876228
30min_MT.	Q96EB1	-0.00973	0.527571	0.406607	ELP4	Elongator c	ELP4_HUM	8	0.993279
30min_MT.	Q9UKY7	0.090334	0.527655	0.406632	CDV3	Protein CD'	CDV3_HUM	15	1.064617
30min_MT.	A6NK58	-0.16034	0.527967	0.406752	LIPT2	Octanoyl-[a	LIPT2_HUM	4	0.894815
15min_MT.	O43837	0.177779	0.52794	0.406752	IDH3B	Isocitrate d	IDH3B_HUI	7	1.131141
15min_MT.	O43464	0.041557	0.528282	0.406955	HTRA2	Serine prot	HTRA2_HUI	6	1.029224
15min_MT.	Q9Y281	-0.1221	0.528578	0.407143	CFL2	Cofilin-2	COF2_HUM	11	0.918847
30min_MT.	P35520	-0.01205	0.528714	0.407208	CBS	Cystathioni	CBS_HUMA	22	0.99168
15min_MT.	P10644	0.144461	0.528903	0.40722	PRKAR1A	cAMP-depe	KAP0_HUM	7	1.105317
15min_MT.	P29966	-0.03949	0.528925	0.40722	MARCKS	Myristoylat	MARCS_HU	15	0.972999
30min_MT.	Q6ZUJ8	-0.04976	0.528938	0.40722	PIK3AP1	Phosphoin	BCAP_HUM	6	0.966097
15min_MT.	P61221	-0.04435	0.52904	0.407258	ABCE1	ATP-bindin	ABCE1_HUI	18	0.969727
30min_MT.	O75937	0.061546	0.529291	0.407387	DNAJC8	DnaJ homo	DNJC8_HUI	7	1.043583
30min_MT.	Q9H1H9	-0.10205	0.529449	0.407453	KIF13A	Kinesin-like	KI13A_HUM	9	0.931709

15min_MT.	P58546	0.059165	0.529766	0.407656	MTPN	Myotrophi	MTPN_HUM	4	1.041863
15min_MT.	Q9GZT8	-0.01379	0.529923	0.407737	NIF3L1	NIF3-like pr	NIF3L_HUM	15	0.990488
30min_MT.	Q8N3X1	-0.0891	0.530242	0.407903	FNBP4	Formin-bin	FNBP4_HUI	11	0.940107
15min_MT.	O43684	0.087822	0.530962	0.408257	BUB3	Mitotic che	BUB3_HUM	10	1.062765
15min_MT.	O95793	0.178139	0.530879	0.408257	STAU1	Double-stra	STAU1_HUI	7	1.131424
30min_MT.	P55081	-0.09651	0.531124	0.408271	MFAP1	Microfibrill	MFAP1_HU	3	0.935294
30min_MT.	Q4J6C6	0.170907	0.531191	0.408271	PREPL	Prolyl endc	PPCEL_HUI	4	1.125766
15min_MT.	Q86U44	0.162191	0.53119	0.408271	METTL3	N6-adenosi	MTA70_HU	10	1.118985
15min_MT.	P40855	-0.2621	0.531673	0.408403	PEX19	Peroxisoma	PEX19_HUI	7	0.833871
15min_MT.	Q0VDG4	0.027932	0.53178	0.408403	SCRN3	Secernin-3	SCRN3_HUI	7	1.01955
15min_MT.	Q9P2B2	-0.01966	0.531755	0.408403	PTGFRN	Prostaglanc	FPRP_HUM	2	0.986467
15min_MT.	Q9Y3A6	0.039561	0.531681	0.408403	TMED5	Transmemb	TMED5_HU	3	1.027801
30min_MT.	Q13769	-0.29194	0.531879	0.408439	THOC5	THO compl	THOC5_HU	7	0.816801
30min_MT.	Q9BWJ5	-0.16073	0.531934	0.408441	SF3B5	Splicing fac	SF3B5_HUI	2	0.894571
30min_MT.	Q02218	-0.26963	0.532038	0.408481	OGDH	2-oxoglutai	ODO1_HUM	9	0.82953
15min_MT.	Q92616	0.0085	0.532459	0.408724	GCN1	Stalled ribc	GCN1_HUM	40	1.005909
30min_MT.	P11169	-0.10382	0.532752	0.408868	SLC2A3	Solute carri	GTR3_HUM	6	0.930568
15min_MT.	O75886	0.014126	0.532875	0.408883	STAM2	Signal trans	STAM2_HU	10	1.009839
15min_MT.	Q96IU4	0.019685	0.532832	0.408883	ABHD14B	Putative pr	ABHEB_HUI	9	1.013738
15min_MT.	P16152	0.026537	0.533077	0.408998	CBR1	Carbonyl re	CBR1_HUM	16	1.018564
30min_MT.	O43665	0.178582	0.533176	0.409033	RGS10	Regulator c	RGS10_HUI	3	1.131771
15min_MT.	Q99575	-0.03427	0.533261	0.409058	POP1	Ribonuclea	POP1_HUM	12	0.976526
15min_MT.	Q12979	0.11231	0.533314	0.409059	ABR	Active brea	ABR_HUMA	5	1.080958
15min_MT.	Q92871	0.20242	0.533881	0.409333	PMM1	Phosphoma	PMM1_HUI	2	1.150627
30min_MT.	P21281	-0.00457	0.534133	0.40935	ATP6V1B2	V-type prot	VATB2_HUI	14	0.996838
30min_MT.	P36915	0.014691	0.534145	0.40935	GNL1	Guanine nu	GNL1_HUM	11	1.010235
30min_MT.	Q96A49	-0.14017	0.534165	0.40935	SYAP1	Synapse-ass	SYAP1_HUI	8	0.907412
15min_MT.	Q96G46	0.096759	0.534029	0.40935	DUS3L	tRNA-dihyd	DUS3L_HUI	11	1.069369
30min_MT.	Q9BQS8	-0.20762	0.534518	0.409541	FYCO1	FYVE and cc	FYCO1_HUI	21	0.865965
30min_MT.	Q9UMS0	0.075015	0.53459	0.409555	NFU1	NFU1 iron-s	NFU1_HUM	6	1.053372
15min_MT.	P07205	0.102459	0.534778	0.409659	PGK2	Phosphogly	PGK2_HUM	5	1.073602
15min_MT.	P11387	-0.06165	0.534982	0.409776	TOP1	DNA topois	TOP1_HUM	9	0.958167
30min_MT.	P20936	-0.73648	0.535585	0.409796	RASA1	Ras GTPase-	RASA1_HUI	2	0.600202
15min_MT.	P61201	-0.04546	0.535158	0.409796	COPS2	COP9 signa	CSN2_HUM	18	0.96898
30min_MT.	Q13243	0.075201	0.535447	0.409796	SRSF5	Serine/argi	SRSF5_HUM	6	1.053508
15min_MT.	Q8N6M0	-0.21509	0.535264	0.409796	OTUD6B	Deubiquitii	OTU6B_HU	6	0.861491
30min_MT.	Q8TEA8	0.057495	0.535468	0.409796	DTD1	D-aminoac	DTD1_HUM	6	1.040657
15min_MT.	Q92845	0.010738	0.535265	0.409796	KIFAP3	Kinesin-ass	KIFA3_HUM	4	1.007471
15min_MT.	Q9BUJ2	0.01333	0.535536	0.409796	HNRNPUL1	Heterogene	HNRL1_HU	14	1.009283
30min_MT.	Q9NWZ3	0.26691	0.535514	0.409796	IRAK4	Interleukin	IRAK4_HUM	6	1.203228
15min_MT.	Q9UDY2	-0.03861	0.535808	0.409927	TJP2	Tight juncti	ZO2_HUMA	23	0.973595
30min_MT.	O43805	0.583221	0.53601	0.410041	SSNA1	Microtubul	SSNA1_HUI	2	1.49819
15min_MT.	Q14353	0.099243	0.53621	0.410154	GAMT	Guanidinoa	GAMT_HUM	6	1.071211
30min_MT.	O43169	0.047721	0.536557	0.410339	CYB5B	Cytochrom	CYB5B_HUI	3	1.033631
30min_MT.	P48506	0.045972	0.536698	0.410407	GCLC	Glutamate-	GSH1_HUM	19	1.032378
30min_MT.	Q5JTD0	-0.05815	0.53698	0.410462	TJAP1	Tight juncti	TJAP1_HUM	11	0.960493
15min_MT.	Q99471	0.036253	0.536979	0.410462	PFDN5	Prefoldin su	PFD5_HUM	7	1.025447
15min_MT.	Q9HDC9	0.068564	0.53732	0.410617	APMAP	Adipocyte p	APMAP_HU	1	1.048672
30min_MT.	Q86WW8	0.247175	0.537475	0.41068	COA5	Cytochrom	COA5_HUM	1	1.186881
30min_MT.	Q9UBP0	-0.04086	0.537748	0.410739	SPAST	Spastin	SPAST_HUM	1	0.972075
15min_MT.	O00399	-0.15526	0.53791	0.410812	DCTN6	Dynactin su	DCTN6_HU	3	0.897967
30min_MT.	O95721	-0.03838	0.538187	0.410863	SNAP29	Synaptosor	SNP29_HUI	6	0.973746

30min_MT.	P50402	0.082359	0.538112	0.410863	EMD	Emerin	EMD_HUMA	3	1.058747
30min_MT.	Q9BT23	-0.06887	0.538151	0.410863	LIMD2	LIM domair	LIMD2_HUI	3	0.953383
15min_MT.	Q9NQZ5	0.005142	0.53811	0.410863	STARD7	StAR-relate	STAR7_HUN	4	1.003571
30min_MT.	Q8N9N5	0.245895	0.538598	0.411096	BANP	Protein BAI	BANP_HUM	1	1.185828
15min_MT.	Q3KRA6	-0.45349	0.538709	0.411141	C2orf76	UPF0538 p	CB076_HUI	2	0.730273
30min_MT.	Q04656	0.000605	0.539258	0.411479	ATP7A	Copper-trai	ATP7A_HUI	11	1.000419
30min_MT.	P04626	0.045682	0.539645	0.411614	ERBB2	Receptor ty	ERBB2_HUI	1	1.032171
30min_MT.	Q6P4F2	-0.55154	0.53963	0.411614	FDX2	Ferredoxin-	FDX2_HUM	1	0.682291
15min_MT.	Q9UL46	0.09446	0.539816	0.411651	PSME2	Proteasome	PSME2_HU	15	1.067666
30min_MT.	P18754	-0.11152	0.540848	0.41233	RCC1	Regulator c	RCC1_HUM	7	0.925615
30min_MT.	O43290	-0.01217	0.541118	0.412496	SART1	U4/U6.U5 t	SNUT1_HUI	13	0.991597
15min_MT.	Q6ICG6	0.089921	0.541234	0.412545	KIAA0930	Uncharacte	K0930_HUI	7	1.064312
30min_MT.	Q9UMX0	-0.10661	0.541401	0.412632	UBQLN1	Ubiquilin-1	UBQL1_HU	11	0.928771
15min_MT.	O60306	-0.00273	0.541654	0.412785	AQR	RNA helicas	AQR_HUMA	2	0.998111
15min_MT.	Q15018	-0.00511	0.541842	0.412807	ABRAXAS2	BRISC comp	ABRX2_HUI	17	0.996467
30min_MT.	P05556	0.168311	0.541983	0.412874	ITGB1	Integrin be	ITB1_HUMA	11	1.123742
15min_MT.	P14868	-0.02981	0.542194	0.412954	DARS1	Aspartate--t	SYDC_HUM	28	0.979551
15min_MT.	P09417	-0.10405	0.542691	0.413209	QDPR	Dihydropte	DHPR_HUM	7	0.930418
15min_MT.	P17612	-0.34152	0.543033	0.413209	PRKACA	cAMP-depe	KAPCA_HUI	3	0.789207
15min_MT.	P83369	0.066833	0.542815	0.413209	LSM11	U7 snRNA-a	LSM11_HU	1	1.047415
30min_MT.	Q13501	-0.0644	0.542929	0.413209	SQSTM1	Sequestoso	SQSTM_HU	3	0.956345
30min_MT.	Q52LW3	-0.05701	0.543056	0.413209	ARHGAP29	Rho GTPase	RHG29_HU	29	0.961257
30min_MT.	Q8WUF8	-0.26813	0.543055	0.413209	ARB2A	Cotranscrip	ARB2A_HUI	1	0.830395
30min_MT.	Q96B45	0.086325	0.542726	0.413209	BORCS7	BLOC-1-rela	BORC7_HU	3	1.061662
30min_MT.	Q9H4G0	0.070629	0.542909	0.413209	EPB41L1	Band 4.1-lil	E41L1_HUN	4	1.050175
30min_MT.	Q96SW2	-0.06117	0.54332	0.413369	CRBN	Protein cer	CRBN_HUM	4	0.958484
30min_MT.	A6NIH7	0.18015	0.543554	0.413431	UNC119B	Protein unc	U119B_HU	1	1.133002
15min_MT.	O95352	0.120737	0.543612	0.413431	ATG7	Ubiquitin-l	ATG7_HUM	10	1.08729
30min_MT.	Q15532	0.132646	0.543476	0.413431	SS18	Protein SSX	SSXT_HUMA	1	1.096302
30min_MT.	Q8IUQ4	0.012755	0.54356	0.413431	SIAH1	E3 ubiquiti	SIAH1_HUN	1	1.00888
30min_MT.	Q9BU02	0.039622	0.543754	0.413498	THTPA	Thiamine-ti	THTPA_HUI	4	1.027844
15min_MT.	P30740	-0.03622	0.543809	0.413499	SERPINB1	Leukocyte e	ILEU_HUMA	17	0.975206
30min_MT.	Q9H1B7	0.239738	0.543877	0.413499	IRF2BPL	Probable E3	I2BPL_HUN	1	1.180778
15min_MT.	O75170	-0.03912	0.544534	0.413729	PPP6R2	Serine/thre	PP6R2_HUI	24	0.97325
30min_MT.	Q13619	-0.06819	0.544524	0.413729	CUL4A	Cullin-4A	CUL4A_HUI	10	0.953836
15min_MT.	P49366	0.053051	0.544994	0.413979	DHPS	Deoxyhypu	DHYS_HUM	10	1.037456
30min_MT.	Q96PE2	-0.07709	0.544967	0.413979	ARHGEF17	Rho guanin	ARHGH_HU	1	0.947971
30min_MT.	Q9H2U1	0.030862	0.545026	0.413979	DHX36	ATP-depenc	DHX36_HU	10	1.021623
15min_MT.	P17936	0.165902	0.545275	0.413985	IGFBP3	Insulin-like	IBP3_HUMA	6	1.121868
15min_MT.	Q9H4A3	0.025448	0.545274	0.413985	WNK1	Serine/thre	WNK1_HUI	31	1.017796
30min_MT.	Q15121	0.112831	0.545484	0.414069	PEA15	Astrocytic p	PEA15_HUI	2	1.081348
15min_MT.	Q9NP61	0.071918	0.54551	0.414069	ARFGAP3	ADP-ribosy	ARFG3_HUI	15	1.051113
30min_MT.	O94992	0.108225	0.54605	0.414398	HEXIM1	Protein HEX	HEXI1_HUN	6	1.077901
30min_MT.	Q6PL24	-0.08438	0.546204	0.414444	TMED8	Protein TM	TMED8_HU	3	0.943187
30min_MT.	Q9BV38	-0.06099	0.546269	0.414444	WDR18	WD repeat-	WDR18_HU	6	0.958608
15min_MT.	Q7Z7C8	-0.34471	0.546475	0.41456	TAF8	Transcripti	TAF8_HUM	1	0.787469
15min_MT.	Q99797	-0.16042	0.5466	0.414592	MIPEP	Mitochond	MIPEP_HUI	2	0.894766
15min_MT.	Q9BRS2	-0.4422	0.546624	0.414592	RIOK1	Serine/thre	RIOK1_HUN	1	0.736013
15min_MT.	P06744	-0.06798	0.547337	0.415093	GPI	Glucose-6-p	G6PI_HUM	22	0.953972
30min_MT.	Q86V21	0.022378	0.547749	0.415158	AACS	Acetoacety	AACS_HUM	4	1.015632
30min_MT.	Q8TEX9	0.046986	0.547795	0.415158	IPO4	Importin-4	IPO4_HUM	23	1.033105
15min_MT.	Q92989	0.013593	0.547716	0.415158	CLP1	Polyribonu	CLP1_HUM	4	1.009466

30min_MT.	Q9NPF4	-0.00357	0.547711	0.415158	OSGEP	tRNA N6-ad	OSGEP_HUI	8	0.997525
30min_MT.	Q9NWT6	-0.08399	0.54763	0.415158	HIF1AN	Hypoxia-in	HIF1N_HUI	5	0.943442
15min_MT.	Q9NWX6	0.176063	0.547594	0.415158	THG1L	Probable tF	THG1_HUM	1	1.129797
15min_MT.	P12004	0.055898	0.548186	0.415289	PCNA	Proliferatin	PCNA_HUM	13	1.039506
30min_MT.	Q96HP4	-0.30346	0.548256	0.415289	OXNAD1	Oxidoreduc	OXND1_HU	1	0.810305
30min_MT.	Q9HD26	0.086133	0.548286	0.415289	GOPC	Golgi-assoc	GOPC_HUM	11	1.061521
30min_MT.	Q86TI2	-0.08186	0.548647	0.415482	DPP9	Dipeptidyl	DPP9_HUM	10	0.944838
30min_MT.	O43504	0.189885	0.548923	0.415651	LAMTOR5	Ragulator c	LTOR5_HUI	2	1.140673
30min_MT.	Q9HC21	0.302163	0.549083	0.415692	SLC25A19	Mitochond	TPC_HUMA	1	1.232992
15min_MT.	Q16254	-0.07502	0.549179	0.415724	E2F4	Transcripti	E2F4_HUM	3	0.94933
30min_MT.	P17844	-0.03307	0.549298	0.415774	DDX5	Probable A	DDX5_HUM	15	0.977342
15min_MT.	Q9H063	0.085889	0.549397	0.415809	MAF1	Repressor c	MAF1_HUM	2	1.061342
15min_MT.	Q9UJY4	0.085223	0.549628	0.415943	GGA2	ADP-ribosy	GGA2_HUM	2	1.060852
30min_MT.	P15289	0.03073	0.549974	0.416125	ARSA	Arylsulfatas	ARSA_HUM	10	1.021529
30min_MT.	Q9NVM6	-0.13433	0.550192	0.416209	DNAJC17	DnaJ homo	DJC17_HUI	3	0.911094
15min_MT.	Q14241	0.055284	0.550475	0.416302	ELOA	Elongin-A	ELOA1_HUI	1	1.039064
30min_MT.	Q8IZA0	0.009333	0.550816	0.416516	KIAA0319L	Dyslexia-as	K319L_HUI	2	1.00649
30min_MT.	Q6IQ22	0.04183	0.551003	0.416546	RAB12	Ras-related	RAB12_HUI	1	1.029419
30min_MT.	Q9Y2Y0	-0.04013	0.55101	0.416546	ARL2BP	ADP-ribosy	AR2BP_HUI	3	0.97257
30min_MT.	O00628	0.431765	0.551483	0.416823	PEX7	Peroxisoma	PEX7_HUM	1	1.348883
30min_MT.	O75165	-0.07093	0.551876	0.416831	DNAJC13	DnaJ homo	DJC13_HUI	13	0.952027
15min_MT.	P37235	-0.15577	0.551721	0.416831	HPCAL1	Hippocalci	HPCL1_HUI	4	0.897653
15min_MT.	P61769	0.106412	0.551798	0.416831	B2M	Beta-2-mici	B2MG_HUM	2	1.076547
15min_MT.	Q58WW2	0.084153	0.552066	0.416831	DCAF6	DDB1- and	DCAF6_HUI	14	1.060065
15min_MT.	Q5T6V5	-0.02726	0.552065	0.416831	QNG1	Queuosine	QNG1_HUM	12	0.981284
30min_MT.	Q68E01	-0.03772	0.55208	0.416831	INTS3	Integrator	INT3_HUMA	13	0.974197
15min_MT.	Q9BZL4	-0.02905	0.551699	0.416831	PPP1R12C	Protein pho	PP12C_HUI	7	0.980068
15min_MT.	Q9P2B4	0.448675	0.551954	0.416831	CTTNBP2NI	CTTNBP2 N	CT2NL_HUI	4	1.364787
15min_MT.	P27694	-0.03279	0.552413	0.416841	RPA1	Replicatior	RFA1_HUM	26	0.977525
15min_MT.	P52294	-0.07844	0.552362	0.416841	KPNA1	Importin su	IMA5_HUM	4	0.947084
30min_MT.	P62314	-0.2033	0.552374	0.416841	SNRPD1	Small nucle	SMD1_HUM	4	0.868562
15min_MT.	Q15942	0.01064	0.552407	0.416841	ZYX	Zyxin	ZYX_HUMA	10	1.007402
15min_MT.	O94979	-0.00968	0.552566	0.416852	SEC31A	Protein trai	SC31A_HUI	34	0.993309
30min_MT.	Q9UHQ1	-0.16522	0.552587	0.416852	NARF	Nuclear pre	NARF_HUM	3	0.891792
15min_MT.	P43487	0.061357	0.552856	0.416871	RANBP1	Ran-specifi	RANG_HUM	6	1.043447
15min_MT.	Q7Z3U7	-0.01263	0.552697	0.416871	MON2	Protein MC	MON2_HUI	30	0.991282
15min_MT.	Q9Y3D2	0.033143	0.552743	0.416871	MSRB2	Methionine	MSRB2_HU	4	1.023239
30min_MT.	Q6BDS2	-0.17359	0.552993	0.416917	BLTP3A	Bridge-like	BLT3A_HUI	3	0.886631
15min_MT.	P27695	0.049849	0.553226	0.417052	APEX1	DNA repair	APEX1_HUI	9	1.035156
30min_MT.	Q14061	-0.06899	0.553387	0.417133	COX17	Cytochrom	COX17_HU	3	0.953308
15min_MT.	P08195	0.046203	0.553609	0.417254	SLC3A2	Amino acid	4F2_HUMA	17	1.032544
30min_MT.	Q53GS9	-0.05281	0.554034	0.417358	USP39	Ubiquitin c	UBP39_HU	10	0.964059
15min_MT.	Q86VP1	-0.44337	0.553862	0.417358	TAX1BP1	Tax1-bindir	TAXB1_HUI	6	0.735413
15min_MT.	Q8IWQ3	0.200517	0.554165	0.417358	BRSK2	Serine/thre	BRSK2_HUI	1	1.14911
15min_MT.	Q92636	0.048929	0.553906	0.417358	NSMAF	Protein FAI	FAN_HUMA	1	1.034497
15min_MT.	Q9NR09	0.052217	0.553991	0.417358	BIRC6	Baculoviral	BIRC6_HUM	47	1.036857
30min_MT.	Q9NVU0	-0.15471	0.554109	0.417358	POLR3E	DNA-direct	RPC5_HUM	1	0.898313
15min_MT.	P30043	0.039613	0.554571	0.417503	BLVRB	Flavin redu	BLVRB_HUI	7	1.027838
15min_MT.	Q86TI2	0.044586	0.554559	0.417503	DPP9	Dipeptidyl	DPP9_HUM	10	1.031387
15min_MT.	Q9P253	-0.2794	0.554494	0.417503	VPS18	Vacuolar pi	VPS18_HUI	4	0.823933
15min_MT.	Q9P2E9	-0.01449	0.554567	0.417503	RRBP1	Ribosome-l	RRBP1_HUI	57	0.990007
15min_MT.	Q01813	0.091676	0.554676	0.417542	PFKP	ATP-depend	PFKAP_HUI	13	1.065607

30min_MT.	Q86YH6	-0.24299	0.555104	0.417824	PDSS2	All trans-pc	DLP1_HUM	11	0.844993
30min_MT.	O76071	-0.00061	0.555205	0.417859	CIAO1	Probable cy	CIAO1_HUI	7	0.999577
30min_MT.	Q5VUM1	-0.20786	0.555541	0.417991	SDHAF4	Succinate d	SDHF4_HUI	3	0.865818
15min_MT.	Q9Y3D0	0.056299	0.555523	0.417991	CIAO2B	Cytosolic ir	CIA2B_HUN	3	1.039795
30min_MT.	Q9UL40	-0.13396	0.555658	0.418	ZNF346	Zinc finger	ZN346_HUI	1	0.911327
30min_MT.	P53680	-0.23183	0.555728	0.418008	AP2S1	AP-2 compl	AP2S1_HUI	3	0.851552
15min_MT.	P84101	-0.02005	0.55593	0.418061	SERF2	Small EDRK	SERF2_HUN	3	0.986199
30min_MT.	Q9C010	0.018058	0.555953	0.418061	PKIB	cAMP-depe	IPKB_HUMA	3	1.012595
15min_MT.	Q86YS3	0.093923	0.55638	0.418261	RAB11FIP4	Rab11 fami	RFIP4_HUN	6	1.067269
15min_MT.	Q9BQ61	0.001895	0.556618	0.418359	TRIR	Telomerase	TRIR_HUMA	5	1.001314
15min_MT.	Q05682	-0.03007	0.556682	0.418367	CALD1	Caldesmon	CALD1_HUI	28	0.979372
30min_MT.	O75306	-0.06133	0.556774	0.418396	NDUFS2	NADH dehy	NDUS2_HU	7	0.958381
30min_MT.	Q9BT30	0.094946	0.55754	0.41869	ALKBH7	Alpha-keto	ALKB7_HUI	1	1.068026
15min_MT.	Q14677	-0.06437	0.557993	0.41899	CLINT1	Clathrin int	EPN4_HUM	15	0.956363
30min_MT.	Q8N0X4	-0.03217	0.558102	0.419032	CLYBL	Citramalyl-	CLYBL_HUN	14	0.977947
30min_MT.	Q96BW1	0.005902	0.558429	0.419197	UPRT	Uracil phos	UPP_HUMA	6	1.004099
30min_MT.	Q9C0C9	0.06781	0.558602	0.419246	UBE2O	(E3-indeper	UBE2O_HU	36	1.048125
30min_MT.	Q9NY61	-0.48969	0.558893	0.419384	AATF	Protein AA	AATF_HUM	2	0.71218
30min_MT.	A4D1P6	0.18487	0.559031	0.419438	WDR91	WD repeat-	WDR91_HU	10	1.136715
15min_MT.	Q2KHT3	-0.02445	0.559125	0.419438	CLEC16A	Protein CLE	CL16A_HUI	10	0.983195
30min_MT.	Q96BY7	-0.31924	0.559116	0.419438	ATG2B	Autophagy-	ATG2B_HUI	14	0.801494
15min_MT.	P13073	0.126303	0.559719	0.419793	COX4I1	Cytochrom	COX41_HU	5	1.091493
15min_MT.	P31944	-0.10049	0.559771	0.419793	CASP14	Caspase-14	CASPE_HUN	9	0.932718
30min_MT.	Q2NKX8	-0.08027	0.55966	0.419793	ERCC6L	DNA excisic	ERC6L_HUN	9	0.945879
30min_MT.	Q14166	-0.14082	0.560033	0.419917	TTLL12	Tubulin--ty	TTL12_HUN	7	0.907005
15min_MT.	Q12768	-0.22224	0.560322	0.420094	WASHC5	WASH com	WASC5_HU	12	0.857232
30min_MT.	P08651	-0.22659	0.560766	0.420117	NFIC	Nuclear fac	NFIC_HUMA	3	0.854653
15min_MT.	P49591	0.057586	0.560836	0.420117	SARS1	Serine--tRN	SYSC_HUMA	22	1.040723
15min_MT.	P49773	0.039883	0.560781	0.420117	HINT1	Adenosine	HINT1_HUN	4	1.02803
30min_MT.	Q4VC05;Q8	0.156107	0.560787	0.420117	BCL7A;BCL	B-cell CLL/l	BCL7A_HUI	1	1.114276
15min_MT.	Q7Z422	-0.17333	0.56049	0.420117	SZRD1	SUZ RNA-bi	SZRD1_HUI	6	0.886793
30min_MT.	Q9UNX4	0.22882	0.560725	0.420117	WDR3	WD repeat-	WDR3_HUI	1	1.171876
15min_MT.	P53384	-0.07276	0.561279	0.420408	NUBP1	Cytosolic F	NUBP1_HU	5	0.950816
30min_MT.	Q07666	-0.04377	0.561794	0.420582	KHDRBS1	KH domain	KHDR1_HU	6	0.970114
15min_MT.	Q8NI27	-0.10365	0.561771	0.420582	THOC2	THO compl	THOC2_HU	14	0.930673
30min_MT.	Q9UGJ0	-0.04673	0.561833	0.420582	PRKAG2	5'-AMP-acti	AAKG2_HU	2	0.968125
15min_MT.	Q9UHY1	-0.05363	0.561687	0.420582	NRBP1	Nuclear rec	NRBP_HUN	8	0.963507
30min_MT.	O95049	-0.54517	0.562029	0.420614	TJP3	Tight juncti	ZO3_HUMA	2	0.685311
15min_MT.	O95857	0.211423	0.562236	0.420642	TSPAN13	Tetraspanir	TSN13_HUI	2	1.15783
15min_MT.	Q9ULT8	-0.00657	0.562501	0.420719	HECTD1	E3 ubiquiti	HECD1_HU	44	0.995458
30min_MT.	Q9BTE3	-0.06044	0.56264	0.420777	MCMBP	Mini-chron	MCMBP_HU	5	0.958969
30min_MT.	P83916	0.201759	0.562796	0.420779	CBX1	Chromobo:	CBX1_HUN	1	1.150099
30min_MT.	Q9P2D7	0.131195	0.562784	0.420779	DNAH1	Dynein axo	DYH1_HUN	1	1.0952
30min_MT.	P78562	0.466934	0.562976	0.420874	PHEX	Phosphate-	PHEX_HUN	1	1.382169
30min_MT.	Q9BU61	0.200874	0.563499	0.421144	NDUFAF3	NADH dehy	NDUF3_HU	4	1.149394
30min_MT.	P22234	0.045383	0.563674	0.421194	PAICS	Bifunctiona	PUR6_HUN	20	1.031957
15min_MT.	O00244	-0.10403	0.563916	0.421329	ATOX1	Copper trar	ATOX1_HUI	5	0.930433
30min_MT.	O14545	-0.05967	0.564107	0.421329	TRAFD1	TRAF-type z	TRAD1_HUI	6	0.959482
15min_MT.	O43399	0.028635	0.564132	0.421329	TPD52L2	Tumor prot	TPD54_HUI	12	1.020047
30min_MT.	P00403	0.331232	0.564319	0.421329	MT-CO2	Cytochrom	COX2_HUN	1	1.258087
30min_MT.	Q9BVS5	0.000177	0.564302	0.421329	TRMT61B	tRNA (aden	TR61B_HUI	5	1.000123
15min_MT.	Q9NZB8	0.203428	0.564216	0.421329	MOCS1	Molybdenu	MOCS1_HU	3	1.151431

30min_MT.	Q9NSB2	-0.15015	0.564454	0.421374	KRT84	Keratin, typ	KRT84_HUI	1	0.901155
15min_MT.	P22626	0.046398	0.564819	0.421485	HNRNPA2B	Heterogene	ROA2_HUM	19	1.032683
30min_MT.	Q9P0L0	-0.04386	0.564711	0.421485	VAPA	Vesicle-asso	VAPA_HUM	11	0.970053
15min_MT.	Q9UBN7	0.291793	0.564993	0.421576	HDAC6	Histone dea	HDAC6_HU	2	1.224161
15min_MT.	O43324	0.183018	0.565275	0.421746	EEF1E1	Eukaryotic	MCA3_HUM	6	1.135256
30min_MT.	P56277	0.202701	0.565348	0.421756	CMC4	Cx9C motif	CMC4_HUM	3	1.150851
30min_MT.	P63241	0.038806	0.565588	0.421818	EIF5A	Eukaryotic	IF5A1_HUM	13	1.027263
15min_MT.	Q9UL16	-0.21291	0.565897	0.421928	CFAP45	Cilia- and fl	CFA45_HUI	1	0.862795
15min_MT.	Q8IZA0	-0.2153	0.566191	0.422026	KIAA0319L	Dyslexia-as	K319L_HUI	2	0.86137
15min_MT.	Q16270	0.34782	0.566387	0.422132	IGFBP7	Insulin-like	IBP7_HUMA	4	1.272636
30min_MT.	P60006	0.678374	0.566478	0.42216	ANAPC15	Anaphase-p	APC15_HUI	1	1.600335
30min_MT.	P19971	-0.08461	0.566684	0.422234	TYMP	Thymidine	TYPH_HUM	16	0.94304
30min_MT.	P22087	-0.10044	0.566793	0.422234	FBL	rRNA 2'-O-n	FBRL_HUM	2	0.93275
30min_MT.	P13929	0.099415	0.56704	0.422377	ENO3	Beta-enolas	ENOB_HUM	2	1.071339
15min_MT.	Q04864	0.001641	0.56725	0.422453	REL	Proto-oncc	REL_HUMA	5	1.001138
30min_MT.	O60493	-0.05693	0.567434	0.42247	SNX3	Sorting nex	SNX3_HUM	8	0.961306
15min_MT.	Q5SYE7	0.014804	0.567431	0.42247	NHSL1	NHS-like pr	NHSL1_HUI	7	1.010314
30min_MT.	Q9BZE1	0.224019	0.56735	0.42247	MRPL37	Large ribos	RM37_HUM	4	1.167983
30min_MT.	O60934	0.030658	0.567752	0.42255	NBN	Nibrin	NBN_HUMA	4	1.021478
30min_MT.	O95059	0.102372	0.568119	0.422698	RPP14	Ribonuclea	RPP14_HUI	2	1.073537
15min_MT.	Q8NAV1	0.163565	0.568793	0.42301	PRPF38A	Pre-mRNA-:	PR38A_HUI	2	1.120052
15min_MT.	Q96GW9	0.06041	0.568758	0.42301	MARS2	Methionine	SYMM_HUM	14	1.042762
15min_MT.	P17900	0.070263	0.568864	0.423011	GM2A	Ganglioside	SAP3_HUM	4	1.049908
30min_MT.	P56381;Q5	-0.20799	0.569129	0.423168	ATP5F1E;A1	ATP synthas	ATP5E_HUM	2	0.865741
15min_MT.	P14735	0.004494	0.569598	0.423396	IDE	Insulin-deg	IDE_HUMAI	37	1.00312
30min_MT.	Q9Y3C6	0.021595	0.570018	0.423628	PPIL1	Peptidyl-pr	PPIL1_HUM	5	1.015081
15min_MT.	Q14739	0.21069	0.570362	0.423843	LBR	Delta(14)-st	LBR_HUMA	3	1.157241
15min_MT.	P63010	-0.01303	0.570638	0.424008	AP2B1	AP-2 compl	AP2B1_HUI	31	0.991008
30min_MT.	P02769	0.605915	0.570713	0.424023	ALB	Serum albu	ALBU_BOVI	39	1.521944
15min_MT.	P05546	-0.15232	0.570806	0.424052	SERPIND1	Heparin co	HEP2_HUM	3	0.899803
30min_MT.	P31146	-0.17615	0.571397	0.424297	CORO1A	Coronin-1A	COR1A_HU	3	0.885062
30min_MT.	Q9NR12	0.039179	0.571401	0.424297	PDLIM7	PDZ and LIN	PDLI7_HUM	8	1.027529
30min_MT.	Q9Y606	0.008248	0.571406	0.424297	PUS1	Pseudourid	PUS1_HUM	10	1.005733
30min_MT.	O60508	-0.02679	0.571491	0.424319	CDC40	Pre-mRNA-|	PRP17_HUI	7	0.981601
15min_MT.	Q5T1M5	-0.02989	0.57174	0.424424	FKBP15	FK506-binc	FKB15_HUI	22	0.979492
15min_MT.	Q16773	-0.00508	0.571859	0.424472	KYAT1	Kynurenine	KAT1_HUM	10	0.996482
30min_MT.	Q86XP3	-0.20593	0.57196	0.424506	DDX42	ATP-depenc	DDX42_HU	6	0.866981
15min_MT.	P21912	0.034548	0.572109	0.424576	SDHB	Succinate d	SDHB_HUM	4	1.024236
30min_MT.	O43716	-0.16082	0.57301	0.425044	GATC	Glutamyl-tl	GATC_HUM	6	0.894515
15min_MT.	Q9UI42	0.003616	0.573317	0.425191	CPA4	Carboxypep	CBPA4_HUI	3	1.00251
30min_MT.	P53677	-0.1128	0.573448	0.425206	AP3M2	AP-3 compl	AP3M2_HU	3	0.924792
30min_MT.	Q9GZX9	-0.34392	0.573445	0.425206	TWSG1	Twisted gas	TWSG1_HU	1	0.787897
15min_MT.	O75391	0.096184	0.573583	0.425227	SPAG7	Sperm-asso	SPAG7_HUI	5	1.068942
15min_MT.	O95433	0.003753	0.574017	0.425508	AHSA1	Activator o	AHSA1_HUI	15	1.002605
15min_MT.	P62191	-0.05393	0.574723	0.425829	PSMC1	26S protea	PRS4_HUM	18	0.963309
15min_MT.	Q6P2Q9	0.056638	0.57471	0.425829	PRPF8	Pre-mRNA-|	PRP8_HUM	46	1.040039
15min_MT.	Q9Y3B7	-0.3283	0.574573	0.425829	MRPL11	Large ribos	RM11_HUM	5	0.796476
15min_MT.	Q8WTS6	-0.05107	0.574866	0.425896	SETD7	Histone-lys	SETD7_HUM	5	0.965219
15min_MT.	O75879	0.150506	0.574944	0.425913	GATB	Glutamyl-tl	GATB_HUM	4	1.109959
30min_MT.	Q15165	-0.06466	0.575017	0.425926	PON2	Serum para	PON2_HUM	3	0.956171
15min_MT.	Q6IN85	-0.03496	0.575624	0.426231	PPP4R3A	Serine/thre	P4R3A_HUI	15	0.976057
30min_MT.	Q9NZM3	0.204517	0.575646	0.426231	ITSN2	Intersectin-	ITSN2_HUM	8	1.152301

30min_MT.	Q96C90	-0.0578	0.575755	0.426271	PPP1R14B	Protein pho	PP14B_HUI	5	0.960731
15min_MT.	P82094	0.130195	0.576066	0.426381	TMF1	TATA eleme	TMF1_HUM	24	1.094442
30min_MT.	Q96EN8	-0.03775	0.575963	0.426381	MOCOS	Molybdenu	MOCOS_HU	21	0.974173
15min_MT.	Q9BZL1	0.013744	0.576047	0.426381	UBL5	Ubiquitin-l	UBL5_HUM	3	1.009572
30min_MT.	Q16777;Q6	0.356056	0.576417	0.42656	H2AC20;H2	Histone H2	H2A2C_HU	1	1.279922
30min_MT.	Q07157	0.021234	0.57677	0.426624	TJP1	Tight juncti	ZO1_HUMA	32	1.014827
30min_MT.	Q7KZF4	0.00879	0.576628	0.426624	SND1	Staphyloco	SND1_HUM	37	1.006111
30min_MT.	Q92888	-0.12707	0.576683	0.426624	ARHGEF1	Rho guanin	ARHG1_HU	4	0.915688
15min_MT.	Q9BU89	0.196053	0.57677	0.426624	DOHH	Deoxyhypu	DOHH_HUM	4	1.14556
15min_MT.	P42765	0.04194	0.577172	0.426876	ACAA2	3-ketoacyl-	THIM_HUM	16	1.029497
15min_MT.	P05937;P2	-0.05573	0.577445	0.426982	CALB1;CALI	Calbindin;C	CALB1_HUI	1	0.96211
15min_MT.	P35579	-0.03595	0.577954	0.427252	MYH9	Myosin-9	MYH9_HUM	74	0.975392
15min_MT.	Q7Z6K5	0.173356	0.578438	0.427283	ARPIN	Arpin	ARPIN_HUM	4	1.127678
30min_MT.	Q8NCN5	-0.00926	0.578272	0.427283	PDPR	Pyruvate de	PDPR_HUM	12	0.993605
15min_MT.	Q8TB03	0.005587	0.578417	0.427283	CXorf38	Uncharacte	CX038_HUI	5	1.00388
30min_MT.	Q92905	-0.03716	0.578346	0.427283	COPS5	COP9 signa	CSN5_HUM	12	0.97457
15min_MT.	Q96AB6	-0.39237	0.578487	0.427283	NTAN1	Protein N-t	NTAN1_HU	1	0.761875
30min_MT.	Q9ULC5	-0.039	0.578401	0.427283	ACSL5	Long-chain	ACSL5_HUI	4	0.973331
30min_MT.	Q9Y3U8	-0.06485	0.57821	0.427283	RPL36	Large ribos	RL36_HUM	3	0.956045
30min_MT.	P05388	-0.07439	0.578826	0.427412	RPLP0	Large ribos	RLA0_HUM	11	0.949741
15min_MT.	O76061	0.17778	0.578944	0.427418	STC2	Stanniocalc	STC2_HUM	1	1.131142
30min_MT.	P62269	0.037043	0.578906	0.427418	RPS18	Small ribos	RS18_HUM	8	1.026009
15min_MT.	Q9UEY8	0.007848	0.579275	0.427582	ADD3	Gamma-ad	ADDG_HUM	16	1.005455
15min_MT.	Q9BZQ6	-0.25054	0.579414	0.42764	EDEM3	ER degrada	EDEM3_HU	5	0.840581
15min_MT.	Q99576	-0.26536	0.579533	0.427652	TSC22D3	TSC22 dom	T22D3_HUI	1	0.831991
15min_MT.	P47813	0.042115	0.579672	0.427674	EIF1AX	Eukaryotic	IF1AX_HUM	5	1.029622
30min_MT.	O75764	0.096855	0.580141	0.427843	TCEA3	Transcripti	TCEA3_HUM	1	1.06944
30min_MT.	Q5T6F2	-0.05233	0.580077	0.427843	UBAP2	Ubiquitin-a	UBAP2_HU	11	0.964379
15min_MT.	Q6UN15	0.21302	0.580229	0.427843	FIP1L1	Pre-mRNA 3	FIP1_HUMA	3	1.159112
30min_MT.	Q9NTX7	0.341949	0.580015	0.427843	RNF146	E3 ubiquiti	RN146_HU	2	1.267468
30min_MT.	P52434	-0.05575	0.580353	0.427858	POLR2H	DNA-direct	RPAB3_HUI	5	0.962095
30min_MT.	Q14677	-0.03247	0.58036	0.427858	CLINT1	Clathrin int	EPN4_HUM	15	0.977742
30min_MT.	Q8NC56	-0.20447	0.580523	0.427904	LEMD2	LEM domai	LEMD2_HU	1	0.86786
15min_MT.	Q9NPH2	-0.02159	0.580531	0.427904	ISYNA1	Inositol-3-p	INO1_HUM	15	0.985146
30min_MT.	P12814	0.000247	0.580785	0.42797	ACTN1	Alpha-actin	ACTN1_HUI	57	1.000171
15min_MT.	Q8WVN8	0.006321	0.580721	0.42797	UBE2Q2	Ubiquitin-c	UB2Q2_HU	2	1.004391
30min_MT.	Q8IUR7	-0.01638	0.581169	0.428172	ARMC8	Armadillo r	ARMC8_HU	12	0.988713
30min_MT.	O75410	0.207139	0.581351	0.428225	TACC1	Transformi	TACC1_HUI	7	1.154396
30min_MT.	O00401	-0.07856	0.581763	0.428368	WASL	Actin nucle	WASL_HUM	8	0.947003
15min_MT.	P51570	0.136407	0.581651	0.428368	GALK1	Galactokina	GALK1_HUI	12	1.099165
30min_MT.	Q8WZ42	-0.0241	0.581696	0.428368	TTN	Titin	TITIN_HUM	8	0.983435
15min_MT.	Q9H267	-0.11686	0.581839	0.428383	VPS33B	Vacuolar pr	VP33B_HUI	6	0.922195
30min_MT.	P52756	0.083122	0.581942	0.428419	RBM5	RNA-bindin	RBM5_HUM	1	1.059308
15min_MT.	Q9BQP7	-0.1186	0.582302	0.428503	MGME1	Mitochond	MGME1_HU	12	0.92108
30min_MT.	Q9H3K6	-0.08074	0.582587	0.428571	BOLA2	BolA-like pr	BOLA2_HUI	6	0.945572
15min_MT.	Q9UI10	-0.09847	0.582571	0.428571	EIF2B4	Translation	EI2BD_HUM	4	0.934024
30min_MT.	Q9NSI2	-0.04273	0.58284	0.428636	SLX9	Ribosome b	SLX9_HUM	6	0.970814
15min_MT.	Q96CU9	-0.37908	0.582977	0.428697	FOXRED1	FAD-depen	FXRD1_HUI	1	0.76893
15min_MT.	A2RTX5	-0.15711	0.583363	0.428798	TARS3	Threonine-	SYTC2_HUM	4	0.896819
15min_MT.	Q13615	-0.1192	0.583444	0.428798	MTMR3	Myotubula	MTMR3_HU	6	0.920696
30min_MT.	P15336	-0.217	0.583719	0.42888	ATF2	Cyclic AMP	ATF2_HUM	4	0.860354
15min_MT.	Q6P1R3	0.118132	0.583665	0.42888	MSANTD2	Myb/SANT-	MSD2_HUM	4	1.085328

30min_MT.	Q96BX8	-0.1235	0.584138	0.429066	MOB3A	MOB kinase	MOB3A_HU	5	0.91796
15min_MT.	P16455	0.006242	0.58428	0.42913	MGMT	Methylated	MGMT_HU	5	1.004336
15min_MT.	Q8NBJ7	-0.02721	0.584359	0.429148	SUMF2	Inactive C-a	SUMF2_HU	7	0.981316
15min_MT.	Q6NZY4	0.018987	0.584538	0.429239	ZCCHC8	Zinc finger	ZCHC8_HU	8	1.013248
15min_MT.	P42167	-0.14971	0.584792	0.429284	TMPO	Lamina-ass	LAP2B_HU	3	0.901429
15min_MT.	Q8NEF9	0.000823	0.584828	0.429284	SRFBP1	Serum resp	SRFB1_HUI	3	1.000571
15min_MT.	P29353	-0.0818	0.584932	0.429287	SHC1	SHC-transfc	SHC1_HUM	11	0.944877
15min_MT.	Q9UKX7	0.037298	0.585117	0.429382	NUP50	Nuclear po	NUP50_HU	10	1.02619
15min_MT.	Q7Z478	0.17926	0.585236	0.429429	DHX29	ATP-depen	DHX29_HU	7	1.132303
15min_MT.	P29323	0.155909	0.58539	0.429475	EPHB2	Ephrin type	EPHB2_HU	3	1.114123
30min_MT.	Q9BU76	-0.32941	0.585408	0.429475	MMTAG2	Multiple m	MMTA2_HU	3	0.795864
30min_MT.	Q641Q2	-0.02609	0.585534	0.429527	WASHC2A	WASH com	WAC2A_HU	14	0.982078
15min_MT.	O76071	-0.03623	0.585705	0.429572	CIAO1	Probable cy	CIAO1_HUI	7	0.975201
15min_MT.	P02786	0.022522	0.586047	0.429626	TFRC	Transferrin	TFR1_HUM	26	1.015733
15min_MT.	Q8WWV3	0.016862	0.58601	0.429626	RTN4IP1	Reticulon-4	RT4I1_HUM	6	1.011757
15min_MT.	Q9UKB1	-0.23983	0.586529	0.429935	FBXW11	F-box/WD r	FBW1B_HU	2	0.846843
30min_MT.	O60216	-0.1007	0.586637	0.429959	RAD21	Double-stra	RAD21_HU	13	0.932579
15min_MT.	Q92576	-0.00352	0.586782	0.429959	PHF3	PHD finger	PHF3_HUM	25	0.997563
30min_MT.	Q9H777	0.434792	0.586675	0.429959	ELAC1	Zinc phospl	RNZ1_HUM	3	1.351716
15min_MT.	Q9HB21	0.013442	0.586771	0.429959	PLEKHA1	Pleckstrin h	PKHA1_HU	5	1.009361
30min_MT.	A0MZ66	0.046774	0.587113	0.43004	SHTN1	Shootin-1	SHOT1_HUI	34	1.032952
15min_MT.	Q9Y224	0.028481	0.587051	0.43004	RTRAF	RNA transci	RTRAF_HUI	11	1.019938
15min_MT.	Q12904	0.008941	0.587338	0.430084	AIMP1	Aminoacyl	AIMP1_HU	10	1.006217
30min_MT.	O75146	-0.16312	0.587531	0.430145	HIP1R	Huntingtin	HIP1R_HUI	13	0.893091
30min_MT.	Q9ULU4	0.097619	0.587687	0.430219	ZMYND8	MYND-type	ZMYD8_HU	3	1.070006
30min_MT.	Q96CN9	0.118062	0.58797	0.430386	GCC1	GRIP and cc	GCC1_HUM	5	1.085276
15min_MT.	Q6DN90	-0.35651	0.588347	0.430581	IQSEC1	IQ motif an	IQEC1_HUI	2	0.781051
15min_MT.	Q9HCE6	-0.00636	0.588645	0.430718	ARHGEF10I	Rho guanin	ARGAL_HU	12	0.995603
15min_MT.	Q6FIF0	-0.22203	0.588708	0.430724	ZFAND6	AN1-type zi	ZFAN6_HU	3	0.857357
15min_MT.	P00966	0.057745	0.588836	0.430778	ASS1	Argininosu	ASSY_HUM	18	1.040838
15min_MT.	P30566	0.038232	0.589666	0.431161	ADSL	Adenylosuc	PUR8_HUM	17	1.026855
15min_MT.	P54253	0.004114	0.589744	0.431161	ATXN1	Ataxin-1	ATX1_HUM	1	1.002855
15min_MT.	Q06136	-0.20886	0.589745	0.431161	KDSR	3-ketodihy	KDSR_HUM	2	0.865223
30min_MT.	Q15424	-0.08109	0.589553	0.431161	SAFB	Scaffold att	SAFB1_HUI	15	0.945345
30min_MT.	Q9UPN3	0.037071	0.589861	0.431165	MACF1	Microtubul	MACF1_HU	142	1.026028
15min_MT.	Q99523	-0.00091	0.590096	0.431238	SORT1	Sortilin	SORT_HUM	12	0.99937
30min_MT.	Q9NZJ6	-0.06761	0.590035	0.431238	COQ3	Ubiquinon	COQ3_HUM	3	0.95422
30min_MT.	Q8IXH7	0.251931	0.590327	0.431344	NELFCD	Negative el	NELFD_HUI	8	1.1908
15min_MT.	Q8ND24	0.010528	0.590388	0.431348	RNF214	RING finger	RN214_HU	7	1.007324
30min_MT.	O14787	-0.08888	0.591053	0.431586	TNPO2	Transportir	TNPO2_HU	5	0.940252
15min_MT.	O75937	0.075428	0.591243	0.431586	DNAJC8	DnaJ homo	DNJC8_HUI	7	1.053674
30min_MT.	Q16718	-0.03914	0.591268	0.431586	NDUFA5	NADH dehy	NDUA5_HU	3	0.973232
15min_MT.	Q9BQ13	0.002594	0.591011	0.431586	KCTD14	BTB/POZ dc	KCD14_HU	7	1.0018
30min_MT.	Q9P1Y6	-0.01056	0.591304	0.431586	PHRF1	PHD and RI	PHRF1_HUI	6	0.992707
15min_MT.	Q9Y3I1	0.142108	0.591164	0.431586	FBXO7	F-box only	FBX7_HUM	5	1.103516
15min_MT.	Q9Y6E0	0.038284	0.591049	0.431586	STK24	Serine/thre	STK24_HUI	6	1.026891
30min_MT.	Q9UDR5	-0.15516	0.591614	0.43176	AASS	Alpha-amir	AASS_HUM	10	0.898031
15min_MT.	Q9NTJ3	-0.04089	0.591811	0.431864	SMC4	Structural r	SMC4_HUM	20	0.972058
15min_MT.	Q7L5D6	0.007621	0.591886	0.431878	GET4	Golgi to ER	GET4_HUM	7	1.005296
15min_MT.	Q9HCG8	0.017556	0.592031	0.431943	CWC22	Pre-mRNA-s	CWC22_HU	8	1.012243
30min_MT.	Q96Q05	0.076333	0.592518	0.432148	TRAPPC9	Trafficking	TPPC9_HUI	6	1.054335
30min_MT.	Q9BYD3	-0.15416	0.592588	0.432148	MRPL4	Large ribos	RM04_HUM	1	0.898655

15min_MT.	P53597	0.009188	0.592729	0.432211	SUCLG1	Succinate--	SUCA_HUM	5	1.006389
15min_MT.	Q86X67	-0.01872	0.592948	0.43229	NUDT13	NAD(P)H py	NUD13_HU	5	0.987108
30min_MT.	Q15019	0.026571	0.593257	0.432435	SEPTIN2	Septin-2	SEPT2_HUM	14	1.018588
15min_MT.	P09668	0.09919	0.593412	0.432476	CTSH	Pro-cathep	CATH_HUM	5	1.071172
30min_MT.	Q5VZK9	0.023653	0.593424	0.432476	CARMIL1	F-actin-unc	CARL1_HUI	27	1.01653
30min_MT.	Q92879	0.001903	0.593789	0.432637	CELF1	CUGBP Elav	CELF1_HUM	4	1.00132
15min_MT.	Q5JTJ3	-0.10072	0.593884	0.43265	COA6	Cytochrom	COA6_HUM	5	0.932565
30min_MT.	P46019	0.161784	0.59397	0.432672	PHKA2	Phosphoryl	KPB2_HUM	4	1.11867
30min_MT.	Q8N7H5	0.008146	0.594333	0.432889	PAF1	RNA polym	PAF1_HUM	11	1.005662
15min_MT.	Q08830	-0.26166	0.594503	0.432939	FGL1	Fibrinogen-	FGL1_HUM	2	0.834128
30min_MT.	O95490	0.073501	0.594834	0.433059	ADGRL2	Adhesion G	AGRL2_HUI	9	1.052267
30min_MT.	P45974	0.026369	0.594768	0.433059	USP5	Ubiquitin c	UBP5_HUM	27	1.018446
15min_MT.	Q8TBF2	0.308358	0.594904	0.43307	PRXL2B	Prostamide	PXL2B_HUI	1	1.238298
15min_MT.	P51858	-0.08295	0.595499	0.433383	HDGF	Hepatoma-	HDGF_HUM	19	0.944122
15min_MT.	Q8TEH3	0.01322	0.595496	0.433383	DENND1A	DENN dom	DEN1A_HU	3	1.009206
15min_MT.	Q96QK1	-0.01996	0.595741	0.433518	VPS35	Vacuolar pr	VPS35_HUI	27	0.986261
30min_MT.	P53778	0.072649	0.595806	0.433525	MAPK12	Mitogen-ac	MK12_HUM	6	1.051646
30min_MT.	O75116	-0.02457	0.596086	0.433576	ROCK2	Rho-associ	ROCK2_HU	40	0.983115
15min_MT.	Q9H4G0	0.200492	0.596098	0.433576	EPB41L1	Band 4.1-lil	E41L1_HUM	4	1.14909
15min_MT.	O15160	0.10068	0.59627	0.43362	POLR1C	DNA-direct	RPAC1_HUI	6	1.072279
15min_MT.	O43719	-0.08148	0.596238	0.43362	HTATSF1	17S U2 SnR	HTSF1_HUM	25	0.945085
15min_MT.	Q8WW12	0.097216	0.59635	0.433639	PCNP	PEST protec	PCNP_HUM	8	1.069707
15min_MT.	Q14166	-0.02706	0.597162	0.43418	TTLL12	Tubulin--ty	TTL12_HUM	7	0.981416
15min_MT.	P00568	0.042206	0.597835	0.434369	AK1	Adenylate k	KAD1_HUM	9	1.029687
30min_MT.	Q13268	0.265066	0.597691	0.434369	DHRS2	Dehydroge	DHRS2_HU	1	1.201691
15min_MT.	Q9NVM9	-0.0219	0.597855	0.434369	INTS13	Integrator	INT13_HUM	9	0.984935
30min_MT.	Q15102	0.077543	0.597927	0.434382	PAFAH1B3	Platelet-act	PA1B3_HUI	7	1.055219
15min_MT.	P05783	-0.01936	0.598045	0.434427	KRT18	Keratin, typ	K1C18_HUI	26	0.986668
15min_MT.	Q96QC0	0.08131	0.598209	0.434506	PPP1R10	Serine/thre	PP1RA_HUI	3	1.057978
30min_MT.	P37275	-0.63483	0.598694	0.434616	ZEB1	Zinc finger	ZEB1_HUM	1	0.644017
15min_MT.	P52948	0.090171	0.598613	0.434616	NUP98	Nuclear por	NUP98_HU	18	1.064496
15min_MT.	Q96EV8	0.047138	0.598429	0.434616	DTNBP1	Dysbindin	DTBP1_HUI	1	1.033214
15min_MT.	Q9NWM8	-0.65673	0.598658	0.434616	FKBP14	Peptidyl-pr	FKB14_HUI	2	0.634315
15min_MT.	Q9H0B6	-0.02055	0.598973	0.434778	KLC2	Kinesin ligh	KLC2_HUM	5	0.985854
15min_MT.	P68363	0.031637	0.599236	0.434929	TUBA1B	Tubulin alp	TBA1B_HUI	20	1.022171
15min_MT.	Q6ZVK8	0.113754	0.599698	0.435224	NUDT18	8-oxo-dGD	NUD18_HU	3	1.08204
15min_MT.	Q06330	0.109193	0.600063	0.435376	RBPJ	Recombinii	SUH_HUMA	3	1.078625
30min_MT.	Q71UM5	-0.08564	0.599981	0.435376	RPS27L	Ribosomal	RS27L_HUM	3	0.942365
30min_MT.	Q14141	0.029846	0.600179	0.435407	SEPTIN6	Septin-6	SEPT6_HUM	5	1.020903
15min_MT.	Q562E7	0.117685	0.600395	0.435487	WDR81	WD repeat-	WDR81_HU	4	1.084993
30min_MT.	Q9UPT8	-0.0183	0.600835	0.435747	ZC3H4	Zinc finger	ZC3H4_HUI	13	0.987394
15min_MT.	Q86VQ6	0.02045	0.601163	0.43592	TXNRD3	Thioredoxi	TRXR3_HUI	8	1.014276
30min_MT.	Q14746	0.07495	0.601385	0.435962	COG2	Conserved	COG2_HUM	9	1.053325
30min_MT.	Q9UJX3	-0.17967	0.601371	0.435962	ANAPC7	Anaphase-p	APC7_HUM	6	0.882904
15min_MT.	P25440	0.09332	0.601612	0.436086	BRD2	Bromodom	BRD2_HUM	6	1.066822
30min_MT.	Q49A26	0.468082	0.601723	0.436086	GLYR1	Cytokine-lil	GLYR1_HUI	1	1.383269
15min_MT.	Q9NUG6	-0.12182	0.601827	0.436121	PDRG1	p53 and DN	PDRG1_HU	1	0.919028
15min_MT.	E9PRG8	0.164846	0.601923	0.43615	C11orf98	Characte	CK098_HUI	2	1.121047
30min_MT.	P51572	-0.05696	0.602366	0.436323	BCAP31	B-cell recep	BAP31_HUI	5	0.961288
30min_MT.	Q12904	0.05271	0.602347	0.436323	AIMP1	Aminoacyl	AIMP1_HUI	10	1.037212
30min_MT.	Q9UJX6	0.171916	0.602385	0.436323	ANAPC2	Anaphase-p	ANC2_HUM	9	1.126554
30min_MT.	Q9H0D6	0.036356	0.60265	0.436435	XRN2	5'-3' exorib	XRN2_HUM	33	1.02552

15min_MT.	Q9ULP9	-0.13274	0.602619	0.436435	TBC1D24	TBC1 doma	TBC24_HUI	6	0.912096
30min_MT.	Q05D60	0.700253	0.602841	0.436532	DEUP1	Deuterosor	DEUP1_HU	1	1.624789
30min_MT.	P30154	0.009102	0.603057	0.436568	PPP2R1B	Serine/thre	2AAB_HUM	13	1.006329
15min_MT.	P37275	0.248332	0.603001	0.436568	ZEB1	Zinc finger	ZEB1_HUM	1	1.187833
15min_MT.	Q8NDI1	-0.00658	0.603005	0.436568	EHBP1	EH domain-	EHBP1_HUI	13	0.995447
15min_MT.	Q13188	-0.4096	0.603195	0.436601	STK3	Serine/thre	STK3_HUM	4	0.75283
30min_MT.	O75935	0.020517	0.603344	0.436654	DCTN3	Dynactin su	DCTN3_HU	8	1.014323
15min_MT.	O75306	0.084676	0.603623	0.436775	NDUFS2	NADH dehy	NDUS2_HU	7	1.06045
15min_MT.	Q96I59	-0.12306	0.60361	0.436775	NARS2	Asparaginyl	SYNM_HUM	6	0.918238
30min_MT.	P27694	-0.02309	0.603779	0.436807	RPA1	Replication	RFA1_HUM	26	0.984121
30min_MT.	P13797	0.031856	0.60409	0.43683	PLS3	Plastin-3	PLST_HUMA	36	1.022326
15min_MT.	P31689	0.043165	0.603993	0.43683	DNAJA1	DnaJ homo	DNJA1_HUI	11	1.030372
15min_MT.	Q9NRR5	0.066507	0.60406	0.43683	UBQLN4	Ubiquilin-4	UBQL4_HU	5	1.047178
15min_MT.	O43505	-0.30859	0.604519	0.436949	B4GAT1	Beta-1,4-gl	B4GA1_HU	2	0.807432
30min_MT.	Q6P158	0.017949	0.604534	0.436949	DHX57	Putative AT	DHX57_HU	4	1.012519
15min_MT.	Q9Y4K3	-0.07697	0.604423	0.436949	TRAF6	TNF recepto	TRAF6_HUI	3	0.948045
30min_MT.	Q9NVA2	0.045063	0.604724	0.436984	SEPTIN11	Septin-11	SEP11_HUI	14	1.031728
15min_MT.	Q86X10	0.0843	0.604937	0.436998	RALGAPB	Ral GTPase-	RLGPB_HUI	10	1.060173
15min_MT.	Q8NCE2	-0.03662	0.604929	0.436998	MTMR14	Myotubula	MTMRE_HU	7	0.974936
15min_MT.	Q9NQT4	0.040695	0.604929	0.436998	EXOSC5	Exosome cc	EXOS5_HUI	4	1.028609
15min_MT.	Q9BY89	0.216682	0.60564	0.437385	KIAA1671	Uncharacte	K1671_HUI	2	1.162058
30min_MT.	Q9H0N5	-0.08029	0.605569	0.437385	PCBD2	Pterin-4-alp	PHS2_HUM	3	0.945869
30min_MT.	P61006	0.090554	0.605714	0.437398	RAB8A	Ras-related	RAB8A_HUI	6	1.064779
15min_MT.	A6NDB9	0.099878	0.60581	0.437427	PALM3	Paralemmi	PALM3_HU	4	1.071683
30min_MT.	Q5TA45	-0.2482	0.605934	0.437436	INTS11	Integrator c	INT11_HUM	1	0.841945
15min_MT.	Q86SQ0	0.190316	0.605886	0.437436	PHLDB2	Pleckstrin h	PHLB2_HUI	2	1.141014
15min_MT.	Q9Y3C6	0.045772	0.606115	0.437526	PPIL1	Peptidyl-pr	PPIL1_HUM	5	1.032235
30min_MT.	Q9Y692	-0.0781	0.606278	0.437564	GMEB1	Glucocortic	GMEB1_HU	4	0.947307
30min_MT.	P62310	0.076787	0.606403	0.437613	LSM3	U6 snRNA-a	LSM3_HUM	3	1.054666
30min_MT.	Q13144	0.024611	0.606527	0.437662	EIF2B5	Translation	EI2BE_HUM	11	1.017206
15min_MT.	Q16401	0.071745	0.606619	0.437688	PSMD5	26S protea	PSMD5_HU	22	1.050987
30min_MT.	Q99442	0.410421	0.607291	0.438052	SEC62	Translocati	SEC62_HUI	2	1.329073
30min_MT.	P50579	-0.03793	0.607514	0.43815	METAP2	Methionine	MAP2_HUM	7	0.974051
30min_MT.	Q15029	0.021744	0.607539	0.43815	EFTUD2	116 kDa U5	U5S1_HUM	30	1.015186
15min_MT.	Q8TC07	0.014546	0.607622	0.438169	TBC1D15	TBC1 doma	TBC15_HUI	19	1.010134
30min_MT.	Q9H993	-0.09395	0.608029	0.438422	ARMT1	Damage-co	ARMT1_HU	7	0.936951
15min_MT.	O15144	-0.01486	0.608174	0.438486	ARPC2	Actin-relate	ARPC2_HUI	15	0.989755
30min_MT.	P52735	-0.03507	0.608247	0.438498	VAV2	Guanine nu	VAV2_HUM	5	0.975982
30min_MT.	P41240	0.14164	0.608382	0.438515	CSK	Tyrosine-pr	CSK_HUMA	9	1.103158
15min_MT.	P08134	0.024361	0.608523	0.438531	RHOC	Rho-relatec	RHOC_HUM	5	1.017029
30min_MT.	Q9Y294	-0.05203	0.608793	0.43865	ASF1A	Histone cha	ASF1A_HUI	1	0.964578
30min_MT.	Q7Z4H8	0.021597	0.60897	0.438736	POGLUT3	Protein O-g	PLGT3_HUI	9	1.015082
30min_MT.	Q9Y3I1	0.214164	0.609031	0.43874	FBXO7	F-box only	FBX7_HUM	5	1.160032
30min_MT.	Q8N6N6	0.093801	0.609117	0.438757	NATD1	Protein NA	NATD1_HU	2	1.067178
30min_MT.	Q7L775	-0.31115	0.609745	0.439093	EPM2AIP1	EPM2A-inte	EPMIP_HUI	2	0.806
30min_MT.	A0A024R1I	0.008643	0.609886	0.439154	TMA7B;TM.	Translation	TMA7B_HU	1	1.006009
30min_MT.	P50053	0.022653	0.610118	0.43928	KHK	Ketohexoki	KHK_HUMA	10	1.015826
30min_MT.	P49588	-0.00033	0.610447	0.439407	AARS1	Alanine--tR	SYAC_HUM	43	0.99977
30min_MT.	Q9NTJ3	-0.18686	0.610462	0.439407	SMC4	Structural	SMC4_HUM	20	0.878517
15min_MT.	O15347	-0.07525	0.610647	0.439459	HMGB3	High mobil	HMGB3_HU	5	0.94918
15min_MT.	Q15262	0.113494	0.610626	0.439459	PTPRK	Receptor-ty	PTPRK_HUI	10	1.081845
15min_MT.	Q5T1C6	0.128751	0.610899	0.4396	THEM4	Acyl-coenzy	THEM4_HU	5	1.093347

30min_MT.	P40616	0.082803	0.611024	0.439649	ARL1	ADP-ribosy	ARL1_HUM	4	1.059074
15min_MT.	P46939	0.04878	0.611298	0.439806	UTRN	Utrophin	UTRN_HUM	89	1.03439
15min_MT.	Q8N1G4	-0.05227	0.611461	0.439843	LRRC47	Leucine-ric	LRC47_HUI	21	0.96442
15min_MT.	Q8N5N7	-0.07496	0.61144	0.439843	MRPL50	Large ribos	RM50_HUM	2	0.949371
15min_MT.	P0DTE7;P0	0.770844	0.612501	0.440441	AMY1B;AM	Alpha-amyl	AMY1B_HU	15	1.706268
15min_MT.	P43363	0.097608	0.612533	0.440441	MAGEA10	Melanoma-	MAGAA_HU	3	1.069998
15min_MT.	Q9Y6R4	0.186454	0.612575	0.440441	MAP3K4	Mitogen-ac	M3K4_HUM	1	1.137964
15min_MT.	P39687	0.05036	0.612795	0.440519	ANP32A	Acidic leuc	AN32A_HU	10	1.035523
15min_MT.	P62310	-0.09368	0.612762	0.440519	LSM3	U6 snRNA-a	LSM3_HUM	3	0.937127
30min_MT.	O95848	0.112704	0.613296	0.440643	NUDT14	Uridine dip	NUD14_HU	2	1.081253
15min_MT.	P62826	-0.02258	0.613136	0.440643	RAN	GTP-bindin	RAN_HUMA	9	0.984467
30min_MT.	Q99832	0.020417	0.613443	0.440643	CCT7	T-complex	TCPH_HUM	29	1.014253
15min_MT.	Q9NQP4	0.028451	0.613415	0.440643	PFDN4	Prefoldin s	PFD4_HUM	3	1.019916
15min_MT.	Q9NYM9	0.395313	0.613237	0.440643	BET1L	BET1-like p	BET1L_HUM	2	1.315228
15min_MT.	Q15029	0.020236	0.613582	0.440679	EFTUD2	116 kDa U5	U5S1_HUM	30	1.014125
30min_MT.	O15127	-0.26987	0.614147	0.440912	SCAMP2	Secretory c	SCAM2_HU	1	0.829394
30min_MT.	P53365	0.047414	0.61415	0.440912	ARFIP2	Arfaptin-2	ARFP2_HUI	10	1.033411
30min_MT.	Q5VWJ9	0.355066	0.614268	0.440929	SNX30	Sorting nex	SNX30_HUI	1	1.279044
30min_MT.	P12270	0.034523	0.614339	0.440939	TPR	Nucleoprot	TPR_HUMA	83	1.024218
30min_MT.	O95182	-0.3228	0.61491	0.441228	NDUFA7	NADH dehy	NDUA7_HU	1	0.799517
15min_MT.	P53677	0.082244	0.615057	0.441252	AP3M2	AP-3 compl	AP3M2_HU	3	1.058664
15min_MT.	Q96AX1	-0.13314	0.615054	0.441252	VPS33A	Vacuolar p	VP33A_HUI	1	0.911845
30min_MT.	Q6PIJ6	-0.18635	0.615593	0.441593	FBXO38	F-box only	FBX38_HUI	1	0.878827
30min_MT.	Q00535	-0.12863	0.615809	0.44167	CDK5	Cyclin-dep	CDK5_HUM	5	0.914702
30min_MT.	Q9UI26	-0.00238	0.616191	0.441863	IPO11	Importin-1	IPO11_HUN	14	0.998349
30min_MT.	O75955	0.114255	0.616496	0.442001	FLOT1	Flotillin-1	FLOT1_HUN	4	1.082416
15min_MT.	Q96AT1	0.208672	0.616778	0.442082	KIAA1143	Uncharacte	K1143_HUI	3	1.155624
15min_MT.	O14641;Q9	0.14204	0.616909	0.442094	DVL2;DVL3	Segment pc	DVL2_HUM	1	1.103465
15min_MT.	Q7Z6Z6	-0.07785	0.61686	0.442094	PNPLA5	Patatin-like	PLPL5_HUN	1	0.947471
30min_MT.	P68402	-0.01641	0.617045	0.442144	PAFAH1B2	Platelet-act	PA1B2_HUI	6	0.988688
30min_MT.	P78560	0.038508	0.617204	0.442144	CRADD	Death dom	CRADD_HU	3	1.027051
30min_MT.	P46108	0.009795	0.617692	0.442302	CRK	Adapter mc	CRK_HUMA	17	1.006812
15min_MT.	Q9UK55	-0.25278	0.617708	0.442302	SERPINA10	Protein Z-d	ZPI_HUMAI	2	0.839278
30min_MT.	P00441	-0.09301	0.617986	0.442407	SOD1	Superoxide	SODC_HUM	6	0.937564
30min_MT.	Q13796	-0.31445	0.618033	0.442407	SHROOM2	Protein Shr	SHRM2_HU	4	0.804156
30min_MT.	Q8IWQ3	0.387369	0.618072	0.442407	BRSK2	Serine/thre	BRSK2_HUI	1	1.308006
15min_MT.	Q9Y606	0.102961	0.61808	0.442407	PUS1	Pseudourid	PUS1_HUM	10	1.073975
15min_MT.	P36543	-0.03247	0.618204	0.442413	ATP6V1E1	V-type prot	VATE1_HUN	9	0.977747
30min_MT.	Q5XKL5	0.287928	0.618258	0.442413	BTBD8	BTB/POZ dc	BTBD8_HUI	2	1.220886
15min_MT.	Q05D60	0.194905	0.618334	0.442426	DEUP1	Deuterosor	DEUP1_HU	1	1.144649
30min_MT.	Q9UH77	-0.02124	0.618441	0.442463	KLHL3	Kelch-like p	KLHL3_HUI	1	0.985383
30min_MT.	O14929	-0.06047	0.618696	0.442604	HAT1	Histone ace	HAT1_HUM	7	0.958954
15min_MT.	Q9UJV9	0.185015	0.6188	0.442638	DDX41	Probable A	DDX41_HU	1	1.136829
15min_MT.	Q8N4F0	0.553099	0.618917	0.442682	BPIFB2	BPI fold-co	BPIB2_HUM	1	1.467234
30min_MT.	O75391	-0.03329	0.619178	0.442828	SPAG7	Sperm-asso	SPAG7_HUI	5	0.97719
15min_MT.	Q13796	-0.27142	0.61928	0.442861	SHROOM2	Protein Shr	SHRM2_HU	4	0.828502
15min_MT.	P49327	-0.01891	0.619409	0.442912	FASN	Fatty acid s	FAS_HUMA	73	0.986976
15min_MT.	O43548	0.217644	0.62007	0.443128	TGM5	Protein-glu	TGM5_HUM	1	1.162833
30min_MT.	P53999	0.030138	0.620071	0.443128	SUB1	Activated R	TCP4_HUM	10	1.02111
30min_MT.	Q02809	-0.01306	0.620035	0.443128	PLOD1	Procollage	PLOD1_HU	15	0.990989
15min_MT.	Q9C0B5	0.092203	0.620107	0.443128	ZDHHC5	Palmitoyltr	ZDHC5_HU	1	1.065997
30min_MT.	Q6P3W7	-0.02775	0.620292	0.44322	SCYL2	SCY1-like p	SCYL2_HUM	12	0.980947

15min_MT.	L0R8F8	-0.31881	0.620605	0.443362	MIEF1	Mitochond	MIDUO_HU	1	0.801734
30min_MT.	O95758	-0.04674	0.620836	0.443446	PTBP3	Polypyrimi	PTBP3_HU	3	0.968123
15min_MT.	Q02447	-0.25428	0.6213	0.443615	SP3	Transcripti	SP3_HUMA	2	0.838406
15min_MT.	Q9BRP1	0.101888	0.621242	0.443615	PDCD2L	Programme	PDD2L_HU	5	1.073177
15min_MT.	Q15382	0.110512	0.621507	0.443684	RHEB	GTP-bindin	RHEB_HUM	4	1.079611
30min_MT.	Q8NB46	-0.30589	0.62152	0.443684	ANKRD52	Serine/thre	ANR52_HU	1	0.808946
15min_MT.	Q9NYY3	0.561155	0.621566	0.443684	PLK2	Serine/thre	PLK2_HUM	1	1.47545
15min_MT.	P18206	0.033713	0.621787	0.44372	VCL	Vinculin	VINC_HUM	74	1.023643
15min_MT.	Q2VIR3	-0.07356	0.621774	0.44372	EIF2S3B	Eukaryotic	IF2GL_HUM	1	0.95029
15min_MT.	Q9BUE6	0.100651	0.621678	0.44372	ISCA1	Iron-sulfur	ISCA1_HUM	4	1.072257
15min_MT.	P02654	-0.66418	0.622087	0.443732	APOC1	Apolipopro	APOC1_HU	1	0.631048
30min_MT.	P04156	-0.00545	0.622225	0.443732	PRNP	Major prior	PRIO_HUM	3	0.996232
15min_MT.	P32322	0.010528	0.622203	0.443732	PYCR1	Pyrroline-5	P5CR1_HU	10	1.007324
30min_MT.	Q5VWQ0	-0.01183	0.621965	0.443732	RSBN1	Lysine-spec	RSBN1_HU	9	0.99183
30min_MT.	Q9NUS5	0.175075	0.622283	0.443732	AP5S1	AP-5 compl	AP5S1_HU	1	1.129023
15min_MT.	Q9NWZ5	0.17232	0.621906	0.443732	UCKL1	Uridine-cyt	UCKL1_HU	4	1.126869
15min_MT.	O60885	-0.01727	0.622402	0.443755	BRD4	Bromodom	BRD4_HUM	22	0.9881
30min_MT.	P33992	-0.2329	0.622534	0.443768	MCM5	DNA replica	MCM5_HU	12	0.850924
30min_MT.	P50990	0.00464	0.622803	0.443919	CCT8	T-complex	TCPQ_HUM	36	1.003222
15min_MT.	Q96QD9	-0.45394	0.622884	0.443936	FYTTD1	UAP56-inte	UIF_HUMA	1	0.730046
15min_MT.	P82664	-0.16993	0.623302	0.444153	MRPS10	Small ribos	RT10_HUM	3	0.888888
15min_MT.	P12645	-0.03098	0.623427	0.444202	BMP3	Bone morp	BMP3_HUM	1	0.978756
15min_MT.	Q15293	0.095647	0.623491	0.444207	RCN1	Reticulocal	RCN1_HUM	10	1.068545
30min_MT.	O60524	0.067222	0.623717	0.444327	NEMF	Ribosome c	NEMF_HUM	6	1.047697
15min_MT.	A8K2U0	0.402522	0.623922	0.444372	A2ML1	Alpha-2-ma	A2ML1_HU	4	1.321817
15min_MT.	Q92665	-0.42903	0.62395	0.444372	MRPS31	Small ribos	RT31_HUM	2	0.742759
30min_MT.	Q9P1U1	0.074983	0.623867	0.444372	ACTR3B	Actin-relate	ARP3B_HU	7	1.053349
30min_MT.	Q9HB71	0.02808	0.624777	0.44488	CACYBP	Calcyclin-b	CYBP_HUM	20	1.019654
15min_MT.	P11177	0.078116	0.624985	0.444962	PDHB	Pyruvate de	ODPB_HUM	11	1.055639
30min_MT.	P26374	0.489371	0.62525	0.445095	CHML	Rab proteir	RAE2_HUM	2	1.403832
15min_MT.	Q7L592	0.157149	0.625488	0.445224	NDUFAF7	Protein arg	NDUF7_HU	6	1.115081
30min_MT.	Q9NW82	0.036542	0.625559	0.445234	WDR70	WD repeat-	WDR70_HU	11	1.025653
15min_MT.	P04080	-0.11336	0.625867	0.445372	CSTB	Cystatin-B	CYTB_HUM	9	0.924433
15min_MT.	Q92609	0.066775	0.625847	0.445372	TBC1D5	TBC1 doma	TBCD5_HU	15	1.047373
15min_MT.	O15294	0.078819	0.626072	0.445477	OGT	UDP-N-acet	OGT1_HUM	1	1.056153
15min_MT.	Q9UBC2	0.071255	0.62616	0.4455	EPS15L1	Epidermal g	EP15R_HU	28	1.050631
15min_MT.	P78527	0.12348	0.626658	0.445732	PRKDC	DNA-depen	PRKDC_HU	77	1.08936
30min_MT.	Q6WKZ4	-0.03889	0.627253	0.446075	RAB11FIP1	Rab11 fami	RFIP1_HUM	4	0.973403
15min_MT.	Q9UBK9	-0.05232	0.627501	0.44617	UXT	Protein UX	UXT_HUMA	2	0.964382
30min_MT.	Q15555	0.02266	0.627706	0.446193	MAPRE2	Microtubul	MARE2_HU	3	1.015831
15min_MT.	Q96FV2	0.091465	0.627763	0.446193	SCRN2	Secernin-2	SCRN2_HU	7	1.065452
15min_MT.	P18031	-0.02509	0.627925	0.446268	PTPN1	Tyrosine-pr	PTN1_HUM	8	0.982757
30min_MT.	Q96M96	-0.15851	0.628102	0.446326	FGD4	FYVE, RhoG	FGD4_HUM	10	0.895948
15min_MT.	O14773	-0.15206	0.628665	0.446655	TPP1	Tripeptidyl	TPP1_HUM	5	0.899962
15min_MT.	Q13247	-0.05236	0.628698	0.446655	SRSF6	Serine/argi	SRSF6_HUM	15	0.964355
15min_MT.	Q04837	-0.02538	0.629154	0.446817	SSBP1	Single-strar	SSBP_HUM	6	0.982559
30min_MT.	O14841	-0.03138	0.629444	0.446942	OPLAH	5-oxoprolir	OPLA_HUM	3	0.978485
15min_MT.	P05023	-0.07728	0.629797	0.447009	ATP1A1	Sodium/po	AT1A1_HU	23	0.947844
30min_MT.	P52701	0.134526	0.629926	0.447009	MSH6	DNA misma	MSH6_HUM	5	1.097732
30min_MT.	Q99816	-0.14423	0.629881	0.447009	TSG101	Tumor susc	TS101_HUM	7	0.904865
30min_MT.	Q9Y5J9	0.125256	0.629939	0.447009	TIMM8B	Mitochond	TIM8B_HU	5	1.090701
15min_MT.	P61077	0.067527	0.630175	0.447136	UBE2D3	Ubiquitin-c	UB2D3_HU	1	1.047919

15min_MT.	Q14966	-0.04887	0.630486	0.447235	ZNF638	Zinc finger	ZN638_HUI	13	0.966691
15min_MT.	Q14814	0.117191	0.630576	0.447255	MEF2D	Myocyte-sp	MEF2D_HU	6	1.084621
15min_MT.	Q9Y314	-0.14203	0.630685	0.447255	NOSIP	Nitric oxide	NOSIP_HUI	3	0.906246
30min_MT.	P48444	-0.0337	0.630884	0.447315	ARCN1	Coatomer s	COPD_HUM	25	0.97691
15min_MT.	Q8IZL8	-0.10932	0.631045	0.447347	PELP1	Proline-, gl	PELP1_HUI	9	0.927027
30min_MT.	A6NH11	-0.03344	0.631244	0.447434	GLTPD2	Glycolipid	GLTD2_HUI	3	0.97709
30min_MT.	P62136	-0.04051	0.631338	0.447434	PPP1CA	Serine/thre	PP1A_HUM	15	0.972309
15min_MT.	Q9BT23	0.061511	0.631314	0.447434	LIMD2	LIM domair	LIMD2_HUI	3	1.043558
30min_MT.	Q6IPR3	0.128615	0.631725	0.447667	TYW3	tRNA wybu	TYW3_HUM	1	1.093244
30min_MT.	O15164	-0.04419	0.632245	0.447676	TRIM24	Transcripti	TIF1A_HUM	4	0.969831
30min_MT.	P20339	-0.08218	0.632253	0.447676	RAB5A	Ras-related	RAB5A_HUI	5	0.944628
30min_MT.	Q8TD19	-0.06982	0.63204	0.447676	NEK9	Serine/thre	NEK9_HUM	18	0.952759
15min_MT.	Q9NR50	-0.05378	0.632212	0.447676	EIF2B3	Translation	EI2BG_HUM	16	0.963411
15min_MT.	P50402	0.203457	0.63236	0.447712	EMD	Emerin	EMD_HUMA	3	1.151454
15min_MT.	P50895	0.137014	0.633708	0.448585	BCAM	Basal cell a	BCAM_HUM	12	1.099627
15min_MT.	P31146	0.098548	0.633784	0.448598	CORO1A	Coronin-1A	COR1A_HU	3	1.070695
30min_MT.	Q9NXV2	0.0873	0.634015	0.448721	KCTD5	BTB/POZ dc	KCTD5_HUI	5	1.06238
30min_MT.	O14786	0.32981	0.634428	0.448932	NRP1	Neuropilin-	NRP1_HUM	1	1.256848
30min_MT.	Q8IWS0	-0.07598	0.634603	0.449016	PHF6	PHD finger	PHF6_HUM	4	0.948697
30min_MT.	Q96JB5	-0.05775	0.635064	0.449151	CDK5RAP3	CDK5 regul	CK5P3_HUI	13	0.960762
30min_MT.	Q9P0J7	-0.64991	0.635081	0.449151	KCMF1	E3 ubiquiti	KCMF1_HU	1	0.63732
30min_MT.	O00399	0.077617	0.635327	0.449179	DCTN6	Dynactin su	DCTN6_HU	3	1.055274
30min_MT.	P16949	0.044904	0.635296	0.449179	STMN1	Stathmin	STMN1_HU	8	1.031615
15min_MT.	P21953	0.185665	0.635422	0.449179	BCKDHB	2-oxoisoval	ODBB_HUM	5	1.137341
15min_MT.	Q5T440	0.130786	0.63526	0.449179	IBA57	Putative tra	CAF17_HUI	4	1.09489
15min_MT.	Q8IYB5	0.042901	0.635465	0.449179	SMAP1	Stromal me	SMAP1_HU	4	1.030183
30min_MT.	Q8NDV3	-0.17898	0.635388	0.449179	SMC1B	Structural	SMC1B_HU	2	0.883328
30min_MT.	Q9UK22	-0.02139	0.63559	0.449226	FBXO2	F-box only	FBX2_HUM	8	0.985282
15min_MT.	Q15438	-0.11827	0.635702	0.449264	CYTH1	Cytohesin-1	CYH1_HUM	9	0.921294
30min_MT.	O43615	-0.01126	0.635939	0.449338	TIMM44	Mitochond	TIM44_HUI	16	0.992226
30min_MT.	Q9Y4F1	-0.04212	0.635872	0.449338	FARP1	FERM, ARH	FARP1_HUI	7	0.971226
30min_MT.	P31150	0.046106	0.63647	0.449526	GDI1	Rab GDP di	GDIA_HUM	14	1.032475
15min_MT.	P61218	0.011005	0.636454	0.449526	POLR2F	DNA-direct	RPAB2_HUI	2	1.007657
15min_MT.	Q5VTQ0	0.236062	0.636473	0.449526	TTC39B	Tetratricop	TT39B_HUI	1	1.177773
15min_MT.	O75570	-0.09776	0.636589	0.449526	MTRF1	Peptide cha	RF1M_HUM	1	0.934483
15min_MT.	P02679	0.054787	0.636564	0.449526	FGG	Fibrinogen	FIBG_HUMA	14	1.038705
30min_MT.	A2RTX5	0.011119	0.636794	0.44963	TARS3	Threonine--	SYTC2_HUM	4	1.007737
30min_MT.	P28340	0.013122	0.637043	0.449739	POLD1	DNA polym	DPOD1_HU	31	1.009137
15min_MT.	Q13642	-0.01207	0.637063	0.449739	FHL1	Four and a	FHL1_HUM	3	0.99167
15min_MT.	O96033	0.122779	0.637464	0.449981	MOCS2	Molybdopt	MOC2A_HU	4	1.08883
30min_MT.	P50897	0.049616	0.637759	0.450149	PPT1	Palmitoyl-p	PPT1_HUM	5	1.034989
30min_MT.	P40692	0.00264	0.638126	0.450196	MLH1	DNA misma	MLH1_HUM	5	1.001831
30min_MT.	P62280	0.026607	0.63801	0.450196	RPS11	Small ribos	RS11_HUM	5	1.018613
30min_MT.	Q99459	-0.08336	0.638206	0.450196	CDC5L	Cell divisio	CDC5L_HUI	6	0.943859
15min_MT.	Q9BPX7	0.19529	0.638268	0.450196	C7orf25	UPF0415 p	CG025_HU	3	1.144955
30min_MT.	P35269	0.018699	0.638708	0.450412	GTF2F1	General tra	T2FA_HUM	15	1.013045
30min_MT.	Q6DKK2	0.079441	0.63884	0.450424	TTC19	Tetratricop	TTC19_HUI	5	1.056608
15min_MT.	Q9H8M7	-0.07073	0.63884	0.450424	MINDY3	Ubiquitin c	MINY3_HUI	5	0.952153
15min_MT.	Q9UKD2	0.008299	0.638928	0.450446	MRTO4	mRNA turn	MRT4_HUM	9	1.005769
15min_MT.	P12931	0.124618	0.639187	0.450507	SRC	Proto-onco	SRC_HUMA	10	1.090219
30min_MT.	Q07960	0.003886	0.639108	0.450507	ARHGAP1	Rho GTPase	RHG01_HU	14	1.002697
15min_MT.	Q9Y5P6	0.065918	0.639154	0.450507	GMPPB	Mannose-1	GMPPB_HU	9	1.046751

30min_MT.	P43897	-0.09285	0.639821	0.450913	TSFM	Elongation	EFTS_HUM/	8	0.937666
15min_MT.	Q92615	0.104371	0.639937	0.450954	LARP4B	La-related p	LAR4B_HUI	8	1.075025
30min_MT.	Q9UBQ5	0.057339	0.640026	0.450976	EIF3K	Eukaryotic	EIF3K_HUM	7	1.040545
30min_MT.	P40855	-0.25781	0.640366	0.450978	PEX19	Peroxisoma	PEX19_HUI	7	0.836358
30min_MT.	Q9BVA1	-0.08757	0.640326	0.450978	TUBB2B	Tubulin bet	TBB2B_HUI	1	0.941105
15min_MT.	Q9P0K7	-0.27214	0.640408	0.450978	RAI14	Ankycorbin	RAI14_HUM	4	0.82809
30min_MT.	Q9UPY8	0.001592	0.64042	0.450978	MAPRE3	Microtubul	MARE3_HU	4	1.001104
30min_MT.	Q9Y5K8	0.108921	0.640433	0.450978	ATP6V1D	V-type prot	VATD_HUM	6	1.078422
30min_MT.	P51991	0.061577	0.640558	0.451025	HNRNPA3	Heterogene	ROA3_HUM	14	1.043606
30min_MT.	P49638	0.105157	0.641239	0.451384	TTPA	Alpha-toco	TTPA_HUM	2	1.075612
30min_MT.	O60828	-0.03371	0.641558	0.451527	PQBP1	Polyglutam	PQBP1_HU	6	0.976907
15min_MT.	Q96DG6	-0.02582	0.641633	0.451539	CMBL	Carboxyme	CMBL_HUM	12	0.98226
15min_MT.	Q8NC51	-0.01237	0.641854	0.451613	SERBP1	SERPINE1 m	SERB1_HUM	18	0.991463
15min_MT.	Q15836	-0.00581	0.642106	0.45175	VAMP3	Vesicle-asso	VAMP3_HU	4	0.99598
15min_MT.	O00592	0.233446	0.642288	0.451837	PODXL	Podocalyxi	PODXL_HU	1	1.17564
30min_MT.	Q9UM13	-0.07968	0.642363	0.451849	ANAPC10	Anaphase-p	APC10_HUI	3	0.94627
30min_MT.	O95487	-0.2147	0.642595	0.451855	SEC24B	Protein tra	SC24B_HUI	3	0.861728
30min_MT.	Q05048	-0.01881	0.642742	0.451913	CSTF1	Cleavage sti	CSTF1_HUM	13	0.987045
30min_MT.	P30039	-0.01329	0.64296	0.452025	PBLD	Phenazine l	PBLD_HUM	1	0.990831
15min_MT.	Q9UKG1	-0.01035	0.643108	0.452089	APPL1	DCC-intera	DP13A_HU	20	0.992851
15min_MT.	Q9UGC7	-0.0804	0.64318	0.452099	MTRF1L	Peptide cha	RF1ML_HU	3	0.945796
30min_MT.	Q96A65	-0.37555	0.64356	0.452139	EXOC4	Exocyst cor	EXOC4_HUI	7	0.770812
30min_MT.	Q96TA2	-0.27731	0.643397	0.452139	YME1L1	ATP-depend	YMEL1_HUI	5	0.825126
30min_MT.	O60502	0.028309	0.644493	0.452574	OGA	Protein O-G	OGA_HUM/	30	1.019816
30min_MT.	P26368	-0.07649	0.644641	0.452637	U2AF2	Splicing fac	U2AF2_HUI	12	0.94836
15min_MT.	A5PLN9	0.020457	0.645258	0.452853	TRAPPC13	Trafficking	TPC13_HUI	2	1.014281
30min_MT.	Q13485	-0.02609	0.645013	0.452853	SMAD4	Mothers ag	SMAD4_HU	10	0.982078
30min_MT.	Q14558	-0.00141	0.645493	0.452951	PRPSAP1	Phosphorib	KPRA_HUM	7	0.999021
30min_MT.	Q96EL2	0.040641	0.646012	0.453234	MRPS24	Small ribos	RT24_HUM	1	1.02857
15min_MT.	Q9BYW2	0.228478	0.64622	0.453335	SETD2	Histone-lys	SETD2_HUI	3	1.171598
30min_MT.	Q9P2B2	0.120284	0.646374	0.453335	PTGFRN	Prostagland	FPRP_HUM	2	1.086949
15min_MT.	P98175	0.130012	0.646603	0.453368	RBM10	RNA-bindir	RBM10_HU	11	1.094303
30min_MT.	Q05682	0.067963	0.646609	0.453368	CALD1	Caldesmon	CALD1_HUI	28	1.048236
15min_MT.	O43708	0.086924	0.647155	0.453583	GSTZ1	Maleylacet	MAAI_HUM	4	1.062103
15min_MT.	Q6ZSZ5	-0.01105	0.647178	0.453583	ARHGEF18	Rho guanin	ARHGI_HUI	1	0.992372
15min_MT.	Q8IYI6	0.033733	0.6474	0.453638	EXOC8	Exocyst cor	EXOC8_HUI	13	1.023657
15min_MT.	A0PJZ3	-0.23377	0.647653	0.453693	GXYLT2	Glucoside x	GXLT2_HUI	1	0.85041
30min_MT.	Q9Y2I8	-0.06667	0.64781	0.453763	WDR37	WD repeat-	WDR37_HU	1	0.954837
15min_MT.	Q99996	0.080819	0.648035	0.45388	AKAP9	A-kinase an	AKAP9_HUI	35	1.057618
30min_MT.	P0DP23;PC	0.090381	0.648159	0.453885	CALM1;CAL	Calmodulir	CALM1_HU	10	1.064651
30min_MT.	Q9Y3Z3	-0.16663	0.648116	0.453885	SAMHD1	Deoxynucle	SAMH1_HU	3	0.89092
15min_MT.	O95249	0.076918	0.648225	0.453891	GOSR1	Golgi SNAP	GOSR1_HU	2	1.054762
15min_MT.	Q03154	0.040229	0.648701	0.454184	ACY1	Aminoacyla	ACY1_HUM	19	1.028277
15min_MT.	P55795	0.058324	0.649025	0.454283	HNRNPH2	Heterogene	HNRH2_HU	5	1.041256
30min_MT.	P68871	-0.21241	0.649042	0.454283	HBB	Hemoglobi	HBB_HUM/	3	0.863094
30min_MT.	Q99798	0.032894	0.649075	0.454283	ACO2	Aconitate h	ACON_HUM	30	1.023062
15min_MT.	Q8IY67	0.137116	0.649426	0.454488	RAVER1	Ribonuclec	RAVR1_HUI	1	1.099705
30min_MT.	Q5BKY9	0.168746	0.649615	0.454579	FAM133B	Protein FAI	F133B_HUI	2	1.124081
15min_MT.	P16870	0.046555	0.649732	0.454621	CPE	Carboxype	CBPE_HUM	8	1.032796
30min_MT.	Q92817	-0.07871	0.649799	0.454627	EVPL	Envoplakin	EVPL_HUM	1	0.946902
15min_MT.	O00499	-0.03781	0.650115	0.454645	BIN1	Myc box-de	BIN1_HUM	5	0.974131
30min_MT.	Q9BUL5	-0.09254	0.650068	0.454645	PHF23	PHD finger	PHF23_HUI	3	0.93787

15min_MT.	Q9H2D6	0.134701	0.649896	0.454645	TRIOBP	TRIO and F-	TARA_HUM	12	1.097865
15min_MT.	Q9Y4X5	-0.03929	0.650111	0.454645	ARIH1	E3 ubiquiti	ARI1_HUM.	10	0.973133
15min_MT.	O75554	0.02563	0.650251	0.454699	WBP4	WW domai	WBP4_HUI	4	1.017924
30min_MT.	P56962	0.304821	0.650803	0.455004	STX17	Syntaxin-1	STX17_HUI	1	1.235266
15min_MT.	Q8TBZ3	-0.22695	0.650929	0.455051	WDR20	WD repeat-	WDR20_HU	4	0.85444
15min_MT.	Q92844	0.01385	0.651007	0.455065	TANK	TRAF family	TANK_HUM	2	1.009646
30min_MT.	Q9NZW5	0.024678	0.651449	0.455292	PALS2	Protein PAI	PALS2_HUI	9	1.017253
30min_MT.	Q9ULD2	-0.07058	0.651786	0.455488	MTUS1	Microtubul	MTUS1_HU	4	0.952252
30min_MT.	Q7L592	0.023118	0.652014	0.455524	NDUFAF7	Protein arg	NDUF7_HU	6	1.016153
30min_MT.	Q9UN86	0.005064	0.652071	0.455524	G3BP2	Ras GTPase-	G3BP2_HU	6	1.003516
15min_MT.	P67809	-0.05014	0.652175	0.455542	YBX1	Y-box-bindi	YBOX1_HUI	16	0.965843
15min_MT.	Q7Z434	0.074528	0.652223	0.455542	MAVS	Mitochond	MAVS_HUM	8	1.053016
30min_MT.	Q96RU2	-0.01269	0.65228	0.455542	USP28	Ubiquitin c	UBP28_HU	14	0.991241
30min_MT.	Q9NV35	0.589654	0.652329	0.455542	NUDT15	Nucleotide	NUD15_HU	1	1.504886
30min_MT.	P61224	0.02307	0.652669	0.455698	RAP1B	Ras-related	RAP1B_HUI	7	1.01612
15min_MT.	P62072	0.063476	0.652668	0.455698	TIMM10	Mitochond	TIM10_HUI	3	1.04498
15min_MT.	P57740	-0.07695	0.652765	0.455724	NUP107	Nuclear po	NU107_HU	8	0.94806
15min_MT.	O14929	-0.03109	0.652957	0.455727	HAT1	Histone ace	HAT1_HUM	7	0.978681
30min_MT.	P04181	-0.02738	0.652865	0.455727	OAT	Ornithine a	OAT_HUMA	12	0.981204
15min_MT.	Q14CN4	0.061305	0.653003	0.455727	KRT72	Keratin, typ	K2C72_HUI	2	1.043409
15min_MT.	Q13576	-0.05069	0.653127	0.455756	IQGAP2	Ras GTPase-	IQGA2_HUI	48	0.965472
30min_MT.	Q9NWT1	0.112849	0.653161	0.455756	PAK1IP1	p21-activat	PK1IP_HUM	6	1.081361
30min_MT.	O76094	0.019482	0.653431	0.455822	SRP72	Signal reco	SRP72_HUI	7	1.013596
15min_MT.	Q96Q05	-0.05829	0.653488	0.455822	TRAPPC9	Trafficking	TPPC9_HUI	6	0.960403
30min_MT.	Q9UKB1	0.058566	0.653479	0.455822	FBXW11	F-box/WD r	FBW1B_HU	2	1.04143
30min_MT.	Q8NFQ8	0.058176	0.65357	0.455838	TOR1AIP2	Torsin-1A-i	TOIP2_HUM	5	1.041148
15min_MT.	Q8NEZ2	0.219371	0.653658	0.455859	VPS37A	Vacuolar pr	VP37A_HUI	7	1.164226
30min_MT.	Q9UI14	-0.24456	0.654708	0.456509	RABAC1	Prenylated	PRAF1_HUI	4	0.844076
30min_MT.	O00231	0.032855	0.655075	0.456684	PSMD11	26S protea:	PSD11_HUI	22	1.023034
30min_MT.	Q12857	0.084941	0.65531	0.456726	NFIA	Nuclear fac	NFIA_HUM.	1	1.060644
30min_MT.	Q13315	0.310366	0.655293	0.456726	ATM	Serine-prot	ATM_HUM.	2	1.240022
30min_MT.	Q9UBS4	0.006587	0.655241	0.456726	DNAJB11	DnaJ homo	DJB11_HUI	11	1.004576
30min_MT.	P16435	-0.04747	0.655676	0.456818	POR	NADPH--cy	NCPR_HUM	14	0.967635
15min_MT.	P40818	-0.02957	0.655904	0.456936	USP8	Ubiquitin c	UBP8_HUM	8	0.979715
15min_MT.	P18846	0.69441	0.65613	0.457012	ATF1	Cyclic AMP	ATF1_HUM	1	1.618222
15min_MT.	O95834	0.063377	0.656623	0.457242	EML2	Echinoderm	EMAL2_HU	14	1.044909
15min_MT.	Q7Z5L9	-0.05567	0.656635	0.457242	IRF2BP2	Interferon r	I2BP2_HUM	14	0.962148
30min_MT.	Q96AT9	-0.04602	0.656817	0.457328	RPE	Ribulose-pl	RPE_HUMA	4	0.968605
15min_MT.	Q9NRP2	-0.06606	0.656901	0.457345	CMC2	COX assemb	COXM2_HU	2	0.955246
30min_MT.	Q6UB28	-0.09109	0.657218	0.457444	METAP1D	Methionine	MAP12_HU	4	0.938814
30min_MT.	Q96HN2	-0.02913	0.657669	0.457718	AHCYL2	Adenosylhc	SAHH3_HU	9	0.98001
15min_MT.	P04264	0.079159	0.658451	0.458017	KRT1	Keratin, typ	K2C1_HUM	42	1.056402
15min_MT.	P60228	0.07221	0.658348	0.458017	EIF3E	Eukaryotic	EIF3E_HUM	19	1.051326
15min_MT.	Q9P2D7	0.047271	0.658433	0.458017	DNAH1	Dynein axo	DYH1_HUM	1	1.033309
30min_MT.	Q14145	0.071195	0.659237	0.458338	KEAP1	Kelch-like E	KEAP1_HUI	7	1.050586
15min_MT.	Q8NFI3	-0.23286	0.659188	0.458338	ENGASE	Cytosolic e	ENASE_HUI	2	0.850948
30min_MT.	A4D126	0.100258	0.659448	0.458385	CRPPA	D-ribitol-5-	ISPD_HUM.	6	1.071965
30min_MT.	O75534	0.00883	0.659547	0.458413	CSDE1	Cold shock	CSDE1_HUI	31	1.006139
30min_MT.	O94985	-0.10441	0.659932	0.458517	CLSTN1	Calsynteni	CSTN1_HUI	2	0.930184
15min_MT.	O43678	-0.58255	0.660238	0.458648	NDUFA2	NADH dehy	NDUA2_HU	3	0.667784
15min_MT.	O43768	-0.1072	0.660829	0.458648	ENSA	Alpha-endc	ENSA_HUM	7	0.92839
15min_MT.	O75131	-0.04352	0.660331	0.458648	CPNE3	Copine-3	CPNE3_HUI	18	0.970288

15min_MT.	P00734	0.020898	0.661048	0.458648	F2	Prothromb	THRB_HUM	22	1.014591
30min_MT.	P01042	0.041413	0.661028	0.458648	KNG1	Kininogen-:	KNG1_HUM	7	1.029121
15min_MT.	P28827	-0.02694	0.660554	0.458648	PTPRM	Receptor-ty	PTPRM_HU	11	0.981501
15min_MT.	P33993	0.125121	0.660971	0.458648	MCM7	DNA replica	MCM7_HUI	6	1.090599
30min_MT.	Q3MHD2	-0.05511	0.660186	0.458648	LSM12	Protein LSM	LSM12_HU	4	0.962521
30min_MT.	Q99471	0.049329	0.66063	0.458648	PFDN5	Prefoldin su	PFD5_HUM	7	1.034784
15min_MT.	Q9BSH5	0.029041	0.660752	0.458648	HDHD3	Haloacid de	HDHD3_HU	9	1.020334
30min_MT.	Q9GZN8	0.062024	0.661058	0.458648	ADISSP	Adipose-sec	ADSSP_HUI	3	1.04393
15min_MT.	Q9UPN9	0.232598	0.661141	0.458665	TRIM33	E3 ubiquiti	TRI33_HUM	6	1.174949
30min_MT.	Q9UBF2	-0.05267	0.661459	0.458813	COPG2	Coatomer s	COPG2_HU	8	0.96415
30min_MT.	Q7Z4V5	0.040828	0.662133	0.45919	HDGFL2	Hepatoma-	HDGR2_HU	19	1.028704
15min_MT.	Q15031	-0.10749	0.662431	0.459284	LARS2	Leucine--tR	SYLM_HUM	4	0.928201
15min_MT.	Q9H0X4	0.440625	0.662445	0.459284	FAM234A	Protein FAM	F234A_HUI	1	1.357192
30min_MT.	O95232	-0.04164	0.662772	0.45947	LUC7L3	Luc7-like pr	LC7L3_HUM	13	0.971551
30min_MT.	Q92620	0.058586	0.662898	0.459517	DHX38	Pre-mRNA-s	PRP16_HUI	5	1.041445
30min_MT.	P37198	0.127772	0.663124	0.459581	NUP62	Nuclear por	NUP62_HU	4	1.092605
30min_MT.	Q07812	0.043239	0.663076	0.459581	BAX	Apoptosis r	BAX_HUMA	7	1.030425
15min_MT.	Q92900	0.063389	0.663217	0.459581	UPF1	Regulator c	RENT1_HUI	29	1.044918
15min_MT.	Q9Y5K8	-0.08553	0.663226	0.459581	ATP6V1D	V-type prot	VATD_HUM	6	0.942436
30min_MT.	O60347	0.086525	0.663397	0.459641	TBC1D12	TBC1 doma	TBC12_HUI	2	1.06181
15min_MT.	Q9NRD1	-0.03578	0.663637	0.459689	FBXO6	F-box only p	FBX6_HUM	6	0.975502
15min_MT.	P54136	0.01888	0.663927	0.459782	RARS1	Arginine--tI	SYRC_HUM	31	1.013172
15min_MT.	O94855	0.003726	0.664054	0.459782	SEC24D	Protein trai	SC24D_HUI	25	1.002586
15min_MT.	Q8IYB7	0.029799	0.66428	0.459782	DIS3L2	DIS3-like ex	DI3L2_HUM	23	1.02087
30min_MT.	Q8N8R5	-0.00225	0.664149	0.459782	C2orf69	Mitochond	CB069_HUI	8	0.998444
30min_MT.	Q9Y4G6	-0.00392	0.664267	0.459782	TLN2	Talin-2	TLN2_HUM	68	0.99729
15min_MT.	O60563	0.160791	0.664427	0.459802	CCNT1	Cyclin-T1	CCNT1_HUI	1	1.1179
30min_MT.	Q15527	0.283456	0.664417	0.459802	SURF2	Surfeit locu	SURF2_HUI	2	1.217107
30min_MT.	P09234	-0.04432	0.664588	0.459842	SNRPC	U1 small nu	RU1C_HUM	2	0.969748
15min_MT.	Q5SXM8	0.260379	0.664859	0.459979	DNLZ	DNL-type zi	DNLZ_HUM	2	1.197794
15min_MT.	Q14980	0.079526	0.665239	0.46012	NUMA1	Nuclear mit	NUMA1_HU	32	1.056671
30min_MT.	Q7Z3K3	-0.43149	0.665572	0.460229	POGZ	Pogo transp	POGZ_HUM	1	0.741498
15min_MT.	P09493	-0.06388	0.665812	0.460273	TPM1	Tropomyos	TPM1_HUM	7	0.956688
15min_MT.	P51151	-0.18428	0.665761	0.460273	RAB9A	Ras-related	RAB9A_HUI	2	0.880086
15min_MT.	Q7Z569	0.23681	0.666211	0.460386	BRAP	BRCA1-assc	BRAP_HUM	11	1.178384
15min_MT.	Q9UBB6	-0.12991	0.666155	0.460386	NCDN	Neurochon	NCDN_HUM	4	0.913888
30min_MT.	O75317	0.164704	0.666452	0.460471	USP12	Ubiquitin c	UBP12_HU	2	1.120936
15min_MT.	P61011	0.056839	0.666419	0.460471	SRP54	Signal reco	SRP54_HUI	8	1.040184
30min_MT.	Q9BTE7	-0.12493	0.66702	0.460741	DCUN1D5	DCN1-like p	DCNL5_HU	4	0.91705
15min_MT.	O75175	0.106313	0.667387	0.460763	CNOT3	CCR4-NOT t	CNOT3_HU	8	1.076474
30min_MT.	P02647	0.068884	0.667179	0.460763	APOA1	Apolipoprc	APOA1_HU	13	1.048905
15min_MT.	P50897	-0.07916	0.667319	0.460763	PPT1	Palmitoyl-p	PPT1_HUM	5	0.946607
30min_MT.	Q8IYB1	-0.00335	0.667343	0.460763	MB21D2	Nucleotidy	M21D2_HU	10	0.997679
30min_MT.	Q12874	0.072222	0.667887	0.461014	SF3A3	Splicing fac	SF3A3_HUM	13	1.051335
15min_MT.	O94763	0.110849	0.668065	0.461015	URI1	Unconvent	RMP_HUMA	5	1.079864
15min_MT.	Q5T749	0.014769	0.667959	0.461015	KPRP	Keratinocy	KPRP_HUM	9	1.010289
15min_MT.	Q9NRX4	-0.0815	0.668059	0.461015	PHPT1	14 kDa pho	PHP14_HU	6	0.945074
15min_MT.	O60508	-0.09167	0.668176	0.461051	CDC40	Pre-mRNA-p	PRP17_HUI	7	0.938433
30min_MT.	P32929	-0.0375	0.668406	0.461073	CTH	Cystathioni	CGL_HUMA	16	0.974342
15min_MT.	Q8IXL7	0.275977	0.668434	0.461073	MSRB3	Methionine	MSRB3_HU	2	1.210814
30min_MT.	P28070	-0.00913	0.668614	0.4611	PSMB4	Proteasome	PSB4_HUM	7	0.99369
15min_MT.	Q13618	-0.00938	0.66866	0.4611	CUL3	Cullin-3	CUL3_HUM	27	0.99352

15min_MT.	P62269	0.037236	0.669062	0.461296	RPS18	Small ribos	RS18_HUM	8	1.026146
30min_MT.	O60506	0.043305	0.669279	0.461354	SYNCRIP	Heterogene	HNRPQ_HU	15	1.030472
15min_MT.	P46459	0.010059	0.669416	0.461354	NSF	Vesicle-fusi	NSF_HUMA	26	1.006997
30min_MT.	Q9UQB8	-0.13164	0.669359	0.461354	BAIAP2	BAR/IMD d	BAIP2_HUM	2	0.912795
30min_MT.	P04350	0.116705	0.669647	0.461446	TUBB4A	Tubulin bet	TBB4A_HUI	2	1.084256
30min_MT.	Q15061	0.074976	0.669943	0.461578	WDR43	WD repeat-	WDR43_HU	8	1.053343
15min_MT.	Q16851	0.005456	0.670151	0.46168	UGP2	UTP--gluco:	UGPA_HUM	24	1.003789
30min_MT.	O14657	0.105901	0.670716	0.461709	TOR1B	Torsin-1B	TOR1B_HUI	3	1.076166
15min_MT.	P24390;P3	-0.28238	0.670635	0.461709	KDELR1;KD	ER lumen p	ERD21_HUI	1	0.822235
15min_MT.	P28161	-0.01615	0.670725	0.461709	GSTM2	Glutathion	GSTM2_HU	9	0.988871
30min_MT.	P38606	-0.03393	0.670374	0.461709	ATP6V1A	V-type prot	VATA_HUM	23	0.976754
30min_MT.	Q9BRP1	-0.06145	0.670593	0.461709	PDCD2L	Programme	PDD2L_HU	5	0.9583
15min_MT.	Q9UNH7	0.051235	0.670674	0.461709	SNX6	Sorting nex	SNX6_HUM	7	1.036152
30min_MT.	O14893	0.046085	0.671769	0.46211	GEMIN2	Gem-associ	GEMI2_HUI	2	1.03246
15min_MT.	P61981	0.046728	0.671779	0.46211	YWHAG	14-3-3 prot	1433G_HU	11	1.03292
15min_MT.	Q9NWV8	-0.10203	0.671701	0.46211	BABAM1	BRISC and E	BABA1_HUI	7	0.931722
15min_MT.	O60313	0.12549	0.672205	0.46218	OPA1	Dynamin-li	OPA1_HUM	6	1.090878
30min_MT.	P06132	-0.07697	0.672273	0.46218	UROD	Uroporphy	DCUP_HUM	13	0.948047
15min_MT.	Q16658	-0.01756	0.672294	0.46218	FSCN1	Fascin	FSCN1_HUI	23	0.987902
15min_MT.	Q8IWL3	0.131109	0.672224	0.46218	HSCB	Iron-sulfur	HSC20_HUI	9	1.095135
15min_MT.	Q96EY9	0.183028	0.672153	0.46218	ADAT3	Probable ir	ADAT3_HUI	3	1.135264
30min_MT.	Q9H814	-0.10741	0.672041	0.46218	PHAX	Phosphoryl	PHAX_HUM	8	0.928253
15min_MT.	Q96T37	-0.1994	0.672695	0.462374	RBM15	RNA-bindir	RBM15_HU	8	0.870915
15min_MT.	Q96GC5	-0.19305	0.672879	0.462419	MRPL48	Large ribos	RM48_HUM	4	0.874754
30min_MT.	Q5VW32	0.081816	0.673023	0.462478	BROX	BRO1 doma	BROX_HUM	5	1.058349
30min_MT.	P04908;P0	-0.01368	0.673543	0.462795	H2AC4;H2A	Histone H2	H2A1B_HU	4	0.990565
15min_MT.	Q9UPR3	0.051063	0.673703	0.462823	SMG5	Nonsense-n	SMG5_HUM	1	1.036028
30min_MT.	P39060	0.19377	0.67384	0.462877	COL18A1	Collagen al	COIA1_HUM	7	1.143748
30min_MT.	Q9NW68	0.23735	0.674684	0.463375	BSDC1	BSD domai	BSDC1_HUI	4	1.178825
15min_MT.	O60825	-0.10507	0.674963	0.463434	PFKFB2	6-phospho	F262_HUM	5	0.929761
15min_MT.	O60828	0.040787	0.674921	0.463434	PQBP1	Polyglutam	PQBP1_HU	6	1.028675
15min_MT.	P36405	0.049064	0.675007	0.463434	ARL3	ADP-ribosy	ARL3_HUM	8	1.034594
15min_MT.	Q969G3	0.085728	0.674854	0.463434	SMARCE1	SWI/SNF-re	SMCE1_HU	7	1.061223
30min_MT.	Q4G0J3	0.13494	0.675115	0.463461	LARP7	La-related p	LARP7_HUI	10	1.098047
15min_MT.	P28330	-0.31614	0.675333	0.463473	ACADL	Long-chain	ACADL_HUI	5	0.803213
30min_MT.	P46976	0.000766	0.675466	0.463473	GYG1	Glycogenin	GLYG_HUM	10	1.000531
15min_MT.	Q6P1M3	0.043025	0.67545	0.463473	LLGL2	LLGL scribb	L2GL2_HUI	6	1.030272
30min_MT.	Q9UJC5	-0.08878	0.675486	0.463473	SH3BGRL2	SH3 domai	SH3L2_HUI	5	0.940316
30min_MT.	P49903	0.042584	0.675866	0.463576	SEPHS1	Selenide, w	SPS1_HUM	7	1.029957
30min_MT.	Q6UWE0	0.038989	0.67608	0.463622	LRSAM1	E3 ubiquiti	LRSM1_HU	14	1.027393
15min_MT.	Q8IWW8	0.305174	0.676254	0.463639	ADHFE1	Hydroxyaci	HOT_HUMA	7	1.235568
30min_MT.	Q13283	0.068304	0.676452	0.463734	G3BP1	Ras GTPase-	G3BP1_HU	10	1.048484
15min_MT.	Q9H910	-0.0583	0.676883	0.463867	JPT2	Jupiter mic	JUPI2_HUM	10	0.960396
15min_MT.	O95372	0.099995	0.677185	0.463993	LYPLA2	Acyl-protei	LYPA2_HUM	6	1.07177
30min_MT.	O14874	0.068815	0.677466	0.464084	BCKDK	Branched-c	BCKD_HUM	8	1.048855
15min_MT.	Q8TCD5	-0.08176	0.6774	0.464084	NT5C	5'(3')-deoxy	NT5C_HUM	8	0.944904
30min_MT.	Q96F24	-0.02384	0.677577	0.464099	NRBF2	Nuclear rec	NRBF2_HUI	4	0.983614
30min_MT.	Q99615	-0.02986	0.678658	0.464758	DNAJC7	DnaJ homo	DNJC7_HUI	5	0.979517
15min_MT.	O43169	0.094351	0.679188	0.464999	CYB5B	Cytochrom	CYB5B_HUI	3	1.067585
15min_MT.	Q9Y4P1	-0.0809	0.679339	0.465061	ATG4B	Cysteine pr	ATG4B_HUI	3	0.945469
15min_MT.	O00159	-0.15578	0.679541	0.465159	MYO1C	Unconvent	MYO1C_HU	15	0.897645
15min_MT.	Q9BQI0	-0.10947	0.679825	0.465312	AIF1L	Allograft in	AIF1L_HUM	4	0.926927

15min_MT.	Q9NRF9	0.131914	0.680096	0.465452	POLE3	DNA polym	DPOE3_HU	2	1.095746
30min_MT.	O75570	0.234001	0.680274	0.465498	MTRF1	Peptide cha	RF1M_HUN	1	1.176092
15min_MT.	P62495	-0.013	0.681024	0.465889	ETF1	Eukaryotic	ERF1_HUM	17	0.991032
15min_MT.	Q9H3S4	0.058494	0.680905	0.465889	TPK1	Thiamine p	TPK1_HUM	3	1.041378
30min_MT.	Q9BTT0	-0.00871	0.68115	0.465934	ANP32E	Acidic leuci	AN32E_HUI	2	0.993984
15min_MT.	P51003	0.041931	0.681304	0.465999	PAPOLA	Poly(A) poly	PAPOA_HU	18	1.029491
15min_MT.	Q9NUP7	-0.06946	0.681523	0.466104	TRMT13	tRNA:m(4)X	TRM13_HU	3	0.952994
30min_MT.	Q9Y6H1	-0.00325	0.681578	0.466104	CHCHD2	Coiled-coil-	CHCH2_HU	3	0.997748
15min_MT.	Q6PKG0	-0.08158	0.681894	0.46628	LARP1	La-related p	LARP1_HUI	9	0.945022
30min_MT.	O14730	0.206383	0.682058	0.466311	RIOK3	Serine/thre	RIOK3_HUN	2	1.153792
30min_MT.	O14562	0.018946	0.682466	0.46634	UBFD1	Ubiquitin d	UBFD1_HU	10	1.013219
30min_MT.	O15085	0.007698	0.682389	0.46634	ARHGEF11	Rho guanin	ARHGB_HU	4	1.00535
30min_MT.	Q5JTH9	-0.16447	0.682497	0.46634	RRP12	RRP12-like	RRP12_HUI	5	0.892254
30min_MT.	Q9NTJ4	-0.16817	0.682511	0.46634	MAN2C1	Alpha-mani	MA2C1_HU	3	0.889969
30min_MT.	Q9Y224	-0.07532	0.682578	0.46634	RTRAF	RNA transcı	RTRAF_HUN	11	0.949133
15min_MT.	Q86V21	-0.16295	0.682793	0.466405	AACS	Acetoacety	AACS_HUM	4	0.893196
15min_MT.	P06132	0.064859	0.682987	0.466484	UROD	Uroporphy	DCUP_HUN	13	1.045982
15min_MT.	O94901	0.028957	0.683242	0.466523	SUN1	SUN domai	SUN1_HUN	3	1.020274
15min_MT.	Q6ZUT1	-0.14563	0.683273	0.466523	NKAPD1	Uncharacte	NKAP1_HU	1	0.903983
30min_MT.	Q9BX93	0.222537	0.683323	0.466523	PLA2G12B	Group XIIB	PG12B_HU	3	1.166783
30min_MT.	Q9NRD1	-0.0065	0.68408	0.466999	FBXO6	F-box only p	FBX6_HUM	6	0.995506
30min_MT.	O15347	-0.08955	0.6843	0.467065	HMGB3	High mobil	HMGB3_HU	5	0.939818
30min_MT.	Q9ULP9	-0.00522	0.684356	0.467065	TBC1D24	TBC1 doma	TBC24_HUI	6	0.996388
15min_MT.	O14732	0.041987	0.684583	0.467098	IMPA2	Inositol mc	IMPA2_HUI	3	1.029531
30min_MT.	Q9UHP3	-0.06208	0.684576	0.467098	USP25	Ubiquitin c	UBP25_HU	28	0.957885
30min_MT.	O14964	0.013937	0.685267	0.467483	HGS	Hepatocyte	HGS_HUMA	13	1.009707
30min_MT.	Q9Y508	-0.03799	0.68524	0.467483	RNF114	E3 ubiquiti	RN114_HU	8	0.974009
15min_MT.	P35900	-0.70258	0.685658	0.467668	KRT20	Keratin, typ	K1C20_HUI	1	0.614471
15min_MT.	A8MWD9;F	0.205981	0.685797	0.467681	SNRPGP15;	Putative sm	RUXGL_HU	3	1.15347
30min_MT.	O95359	-0.01733	0.685747	0.467681	TACC2	Transformi	TACC2_HUI	61	0.988057
15min_MT.	Q96PE3	0.118258	0.686596	0.468104	INPP4A	Inositol pol	INP4A_HUN	8	1.085423
30min_MT.	Q01105	0.056195	0.686854	0.468143	SET	Protein SET	SET_HUMA	9	1.03972
30min_MT.	Q15758	-0.08458	0.686842	0.468143	SLC1A5	Neutral am	AAAT_HUM	6	0.943058
30min_MT.	Q96EL3	0.173905	0.686798	0.468143	MRPL53	Large ribos	RM53_HUN	2	1.128108
15min_MT.	Q9Y5R8	-0.38962	0.687518	0.468406	TRAPPC1	Trafficking p	TPPC1_HUI	4	0.763331
30min_MT.	Q9NYB0	0.120411	0.687906	0.468629	TERF2IP	Telomeric r	TE2IP_HUN	6	1.087044
30min_MT.	P19387	0.021147	0.688036	0.468677	POLR2C	DNA-direct	RPB3_HUN	3	1.014766
15min_MT.	P52888	0.005277	0.688366	0.46882	THOP1	Thimet olig	THOP1_HU	32	1.003665
15min_MT.	P60891	0.012289	0.688607	0.46887	PRPS1	Ribose-pho	PRPS1_HUI	16	1.008554
30min_MT.	P31483	-0.04671	0.689161	0.469117	TIA1	Cytotoxic g	TIA1_HUMA	6	0.968139
30min_MT.	Q9UIM3	0.339758	0.689312	0.469178	FKBPL	FK506-binc	FKBPL_HUN	2	1.265544
15min_MT.	Q9BPX3	-0.14175	0.689377	0.469182	NCAPG	Condensin	CND3_HUN	7	0.906418
30min_MT.	Q86W42	0.029615	0.689799	0.469428	THOC6	THO compl	THOC6_HU	4	1.02074
15min_MT.	P51149	0.05547	0.690039	0.46948	RAB7A	Ras-related	RAB7A_HUI	11	1.039197
15min_MT.	Q07666	-0.04477	0.690115	0.46948	KHDRBS1	KH domain	KHDR1_HU	6	0.969443
15min_MT.	Q9BQE3	0.10486	0.689967	0.46948	TUBA1C	Tubulin alp	TBA1C_HUI	2	1.07539
15min_MT.	P07951	0.053743	0.690594	0.469765	TPM2	Tropomyos	TPM2_HUN	6	1.037954
15min_MT.	P22830	0.056813	0.691364	0.46988	FECH	Ferrochelat	HEMH_HUN	8	1.040165
30min_MT.	Q92896	-0.00994	0.691287	0.46988	GLG1	Golgi appar	GSLG1_HUI	20	0.993137
15min_MT.	Q8N6V4	0.337329	0.691578	0.469984	C10orf53	UPF0728 p	CJ053_HUN	1	1.263415
15min_MT.	Q7Z6E9	-0.02427	0.692197	0.470323	RBBP6	E3 ubiquiti	RBBP6_HUI	8	0.983316
30min_MT.	Q86W50	0.040072	0.69228	0.470339	METTL16	RNA N6-ade	MET16_HU	8	1.028165

15min_MT.	P23786	0.016032	0.692538	0.470414	CPT2	Carnitine O	CPT2_HUM	5	1.011175
15min_MT.	P46109	0.018185	0.692523	0.470414	CRKL	Crk-like pro	CRKL_HUM	13	1.012685
15min_MT.	Q13085	-0.00071	0.692571	0.470414	ACACA	Acetyl-CoA	ACACA_HU	14	0.999508
30min_MT.	P53634	0.106576	0.692994	0.470474	CTSC	Dipeptidyl	CATC_HUM	11	1.07667
30min_MT.	P68431;P8	0.210042	0.69302	0.470474	H3C1;H3-3	Histone H3	H31_HUMA	4	1.156722
15min_MT.	Q8TBC3	0.039809	0.692999	0.470474	SHKBP1	SH3KBP1-b	SHKB1_HU	8	1.027977
30min_MT.	P61201	-0.03572	0.693102	0.470489	COPS2	COP9 signa	CSN2_HUM	18	0.975542
15min_MT.	Q8TD16	0.072703	0.693261	0.470515	BICD2	Protein bic	BICD2_HUI	15	1.051685
30min_MT.	O75879	-0.097	0.693674	0.470673	GATB	Glutamyl-tl	GATB_HUM	4	0.934975
15min_MT.	O14867	0.02675	0.69398	0.470693	BACH1	Transcripti	BACH1_HU	6	1.018715
15min_MT.	P16035	0.058476	0.694035	0.470693	TIMP2	Metallopro	TIMP2_HUI	2	1.041365
15min_MT.	P30533	-0.02748	0.694301	0.470693	LRPAP1	Alpha-2-ma	AMRP_HUM	15	0.98113
15min_MT.	P63313	-0.05345	0.694271	0.470693	TMSB10	Thymosin b	TYB10_HUI	2	0.963627
30min_MT.	P82930	0.059272	0.69413	0.470693	MRPS34	Small ribos	RT34_HUM	3	1.04194
15min_MT.	Q15047	-0.17726	0.694235	0.470693	SETDB1	Histone-lys	SETB1_HUI	4	0.884382
30min_MT.	Q9BY44	-0.03334	0.694328	0.470693	EIF2A	Eukaryotic	EIF2A_HUM	21	0.977159
15min_MT.	Q9UPU5	-0.03982	0.694201	0.470693	USP24	Ubiquitin c	UBP24_HU	28	0.972777
15min_MT.	Q9UQN3	-0.02533	0.694761	0.470879	CHMP2B	Charged mu	CHM2B_HU	3	0.982596
30min_MT.	P27144	-0.0108	0.695302	0.471022	AK4	Adenylate k	KAD4_HUM	8	0.992539
15min_MT.	P46781	0.096405	0.695332	0.471022	RPS9	Small ribos	RS9_HUMA	10	1.069106
30min_MT.	Q96PE7	-0.08038	0.695492	0.471089	MCEE	Methylmal	MCEE_HUM	3	0.945809
30min_MT.	Q8TB61	0.527807	0.695683	0.471137	SLC35B2	Adenosine	S35B2_HUI	2	1.441736
30min_MT.	Q14152	-0.06208	0.695861	0.471176	EIF3A	Eukaryotic	EIF3A_HUM	39	0.957882
15min_MT.	Q9NUJ3	-0.03097	0.695957	0.4712	TCP11L1	T-complex	T11L1_HUI	3	0.978759
15min_MT.	A3KN83	0.001596	0.696219	0.471337	SBNO1	Protein stra	SBNO1_HU	3	1.001107
30min_MT.	P33240	-0.02552	0.696544	0.471475	CSTF2	Cleavage sti	CSTF2_HUM	13	0.982464
30min_MT.	P17706	-0.01528	0.69678	0.471594	PTPN2	Tyrosine-pr	PTN2_HUM	1	0.989465
15min_MT.	O14657	0.125305	0.696867	0.471612	TOR1B	Torsin-1B	TOR1B_HUI	3	1.090738
30min_MT.	O95817	-0.08048	0.697124	0.471678	BAG3	BAG family	BAG3_HUM	15	0.945745
15min_MT.	P50579	-0.05827	0.697145	0.471678	METAP2	Methionine	MAP2_HUM	7	0.960414
30min_MT.	Q8N5I2	-0.23637	0.697046	0.471678	ARRDC1	Arrestin do	ARRD1_HU	1	0.848878
15min_MT.	O14874	0.090933	0.69764	0.471905	BCKDK	Branched-c	BCKD_HUM	8	1.065059
30min_MT.	Q13185	-0.07436	0.697722	0.471905	CBX3	Chromobo	CBX3_HUM	7	0.949763
30min_MT.	Q8NC51	0.07557	0.697817	0.471928	SERBP1	SERPINE1 n	SERB1_HUI	18	1.053778
30min_MT.	P53985	0.153982	0.698044	0.472041	SLC16A1	Monocarbo	MOT1_HUM	2	1.112636
30min_MT.	P63092;Q5	-0.11244	0.698526	0.472295	GNAS	Guanine nu	GNAS2_HU	7	0.925022
30min_MT.	Q14157	-0.06225	0.69854	0.472295	UBAP2L	Ubiquitin-a	UBP2L_HUI	16	0.95777
15min_MT.	O94826	0.265588	0.698614	0.472304	TOMM70	Mitochond	TOM70_HU	1	1.202126
15min_MT.	O15067	0.031992	0.698962	0.472458	PFAS	Phosphorib	PUR4_HUM	13	1.022423
30min_MT.	Q9Y2Z0	0.059526	0.699305	0.472608	SUGT1	Protein SG	SGT1_HUM	23	1.042123
30min_MT.	P62875	-0.32896	0.699525	0.472663	POLR2L	DNA-direct	RPAB5_HUI	1	0.796111
30min_MT.	P16383	0.000906	0.699754	0.472748	GCFC2	Intron Larg	GCFC2_HUI	6	1.000628
30min_MT.	O75312	-0.00985	0.700034	0.472774	ZPR1	Zinc finger	ZPR1_HUM	16	0.993193
30min_MT.	O95396	-0.1946	0.700045	0.472774	MOCS3	Adenylyltra	MOCS3_HU	2	0.873818
30min_MT.	P60903	-0.03365	0.699903	0.472774	S100A10	Protein S1(	S10AA_HUI	4	0.976948
15min_MT.	Q15208	0.610922	0.700064	0.472774	STK38	Serine/thre	STK38_HUI	2	1.527235
30min_MT.	Q14376	0.015454	0.700208	0.472809	GALE	UDP-glucos	GALE_HUM	3	1.010769
30min_MT.	P04632	-0.07069	0.700363	0.472823	CAPNS1	Calpain sm	CPNS1_HUI	5	0.952183
15min_MT.	P55145	-0.0796	0.700564	0.472823	MANF	Mesenceph	MANF_HUM	8	0.946321
30min_MT.	Q13439	-0.03403	0.700384	0.472823	GOLGA4	Golgin subf	GOGA4_HU	59	0.976686
15min_MT.	Q6ZRP7	-0.06141	0.700652	0.472823	QSOX2	Sulfhydryl	QSOX2_HU	3	0.958327
30min_MT.	P49189	0.014181	0.70093	0.47297	ALDH9A1	4-trimethyl	AL9A1_HUI	20	1.009878

15min_MT.	O14545	0.033123	0.701514	0.473149	TRAFD1	TRAF-type z	TRAD1_HUI	6	1.023225
30min_MT.	P53990	-0.01402	0.701613	0.473149	IST1	IST1 homol	IST1_HUMA	8	0.990326
15min_MT.	Q2TAL8	0.132196	0.701452	0.473149	QRICH1	Transcripti	QRIC1_HUI	1	1.09596
30min_MT.	Q5VZE5	-0.28254	0.701403	0.473149	NAA35	N-alpha-ace	NAA35_HU	2	0.822142
30min_MT.	Q6P3X3	0.220432	0.701424	0.473149	TTC27	Tetratricop	TTC27_HUI	5	1.165082
30min_MT.	Q9UKE5	-0.17755	0.701484	0.473149	TNIK	TRAF2 and I	TNIK_HUMA	9	0.884203
30min_MT.	P27361	0.000349	0.702055	0.473187	MAPK3	Mitogen-ac	MK03_HUI	8	1.000242
15min_MT.	Q14554	0.005457	0.702097	0.473187	PDIA5	Protein dis	PDIA5_HUI	21	1.003789
15min_MT.	Q96CF2	0.104563	0.702017	0.473187	CHMP4C	Charged mu	CHM4C_HU	3	1.075169
30min_MT.	Q9NZT2	-0.04458	0.701908	0.473187	OGFR	Opioid grov	OGFR_HUM	15	0.969572
30min_MT.	Q9BVP2	0.116529	0.702342	0.47327	GNL3	Guanine nu	GNL3_HUM	3	1.084124
30min_MT.	Q9UII2	0.087243	0.702606	0.473397	ATP5IF1	ATPase inhi	ATIF1_HUM	4	1.062338
15min_MT.	P10599	0.047689	0.702842	0.473485	TXN	Thioredoxi	THIO_HUM	7	1.033608
15min_MT.	P40227	0.051558	0.703003	0.473552	CCT6A	T-complex	TCPZ_HUM	21	1.036383
15min_MT.	P39019	-0.05776	0.703498	0.473723	RPS19	Small ribos	RS19_HUM	4	0.960752
15min_MT.	Q92614	-0.12131	0.703407	0.473723	MYO18A	Unconvent	MY18A_HU	11	0.91935
30min_MT.	Q99417	-0.05146	0.703492	0.473723	MYCBP	c-Myc-bind	MYCBP_HU	4	0.964957
15min_MT.	P59780	0.414034	0.703835	0.473909	AP3S2	AP-3 compl	AP3S2_HUI	1	1.332406
30min_MT.	O60613	0.074403	0.704063	0.473998	SELENOF	Selenoprot	SEP15_HUI	1	1.052925
30min_MT.	Q16890	-0.11567	0.704259	0.473998	TPD52L1	Tumor prot	TPD53_HUI	1	0.922953
15min_MT.	Q9BYM8	0.192851	0.70427	0.473998	RBCK1	RanBP-type	HOIL1_HUI	7	1.14302
30min_MT.	P61163	-0.06691	0.704443	0.474073	ACTR1A	Alpha-centi	ACTZ_HUM	4	0.954684
30min_MT.	P30038	-0.20374	0.704544	0.474101	ALDH4A1	Delta-1-pyr	AL4A1_HUI	4	0.868296
30min_MT.	P62330	-0.01481	0.704639	0.474124	ARF6	ADP-ribosy	ARF6_HUM	2	0.989787
15min_MT.	P07360	0.064494	0.705561	0.474336	C8G	Complemei	CO8G_HUM	2	1.045718
15min_MT.	P61019	0.002166	0.70553	0.474336	RAB2A	Ras-related	RAB2A_HUI	11	1.001503
30min_MT.	P61247	0.012763	0.705451	0.474336	RPS3A	Small ribos	RS3A_HUM	11	1.008886
15min_MT.	Q92973	-0.01467	0.705364	0.474336	TNPO1	Transportir	TNPO1_HU	25	0.989885
15min_MT.	Q9NR12	0.124128	0.705273	0.474336	PDLIM7	PDZ and LII	PDLI7_HUI	8	1.089849
15min_MT.	Q3SY69	-0.0346	0.705725	0.474405	ALDH1L2	Mitochond	AL1L2_HUI	15	0.976301
15min_MT.	O14907	-0.08762	0.705899	0.474427	TAX1BP3	Tax1-bindir	TX1B3_HUI	2	0.941074
30min_MT.	Q96TC7	-0.11705	0.705917	0.474427	RMDN3	Regulator c	RMD3_HUI	3	0.922068
30min_MT.	P47755	-0.03467	0.70615	0.474517	CAPZA2	F-actin-cap	CAZA2_HUI	13	0.976255
15min_MT.	Q8IY47	-0.02881	0.706195	0.474517	KBTBD2	Kelch repea	KBTB2_HUI	3	0.980231
30min_MT.	P0C870	0.008157	0.707112	0.47497	JMJD7	Bifunctiona	JMJD7_HUI	3	1.00567
15min_MT.	P17066	0.050438	0.707081	0.47497	HSPA6	Heat shock	HSP76_HUI	3	1.035579
30min_MT.	Q6P2E9	-0.05924	0.707314	0.475066	EDC4	Enhancer o	EDC4_HUM	31	0.959767
30min_MT.	O15260	-0.53042	0.707412	0.475091	SURF4	Surfeit locu	SURF4_HUI	1	0.692355
30min_MT.	P35813	0.006673	0.708272	0.475586	PPM1A	Protein pho	PPM1A_HU	11	1.004636
15min_MT.	Q5T4S7	0.052388	0.708743	0.475821	UBR4	E3 ubiquiti	UBR4_HUM	66	1.03698
15min_MT.	O43157	0.158229	0.708869	0.475845	PLXNB1	Plexin-B1	PLXB1_HUI	3	1.115916
15min_MT.	O43310	0.276215	0.709416	0.475845	CTIF	CBP80/20-c	CTIF_HUMA	3	1.211014
15min_MT.	P18124	-0.01011	0.70951	0.475845	RPL7	Large ribos	RL7_HUMA	10	0.99302
15min_MT.	P23528	0.036587	0.709507	0.475845	CFL1	Cofilin-1	COF1_HUM	20	1.025684
30min_MT.	Q6I9Y2	-0.2285	0.709491	0.475845	THOC7	THO compl	THOC7_HU	3	0.853523
30min_MT.	Q6L8Q7	-0.09449	0.709141	0.475845	PDE12	2',5'-phosp	PDE12_HUI	11	0.936603
30min_MT.	Q8N2M8	-0.23396	0.709031	0.475845	CLASRP	CLK4-assoc	CLASR_HUI	3	0.8503
15min_MT.	Q9UKN8	0.003154	0.709707	0.475937	GTF3C4	General tra	TF3C4_HUI	16	1.002188
30min_MT.	P13796	-0.02398	0.710007	0.476097	LCP1	Plastin-2	PLSL_HUMA	10	0.983516
15min_MT.	Q9NTJ4	-0.09004	0.71008	0.476106	MAN2C1	Alpha-mani	MA2C1_HU	3	0.939497
15min_MT.	P05556	0.00724	0.710228	0.476164	ITGB1	Integrin be	ITB1_HUMA	11	1.005031
30min_MT.	O00487	-0.09502	0.710305	0.476175	PSMD14	26S protea	PSDE_HUM	7	0.93626

30min_MT.	P31350	-0.19039	0.710944	0.476562	RRM2	Ribonuclec	RIR2_HUMA	7	0.876371
30min_MT.	P07437	0.02913	0.711065	0.476577	TUBB	Tubulin bet	TBB5_HUM	4	1.020397
30min_MT.	Q96JG6	-0.14479	0.711125	0.476577	VPS50	Syndetin	VPS50_HUI	3	0.904509
15min_MT.	O14974	0.073171	0.71162	0.476578	PPP1R12A	Protein pho	MYPT1_HU	23	1.052026
15min_MT.	O43447	-0.0095	0.711709	0.476578	PPIH	Peptidyl-pr	PPIH_HUM	6	0.993434
15min_MT.	O96006	-0.5134	0.711942	0.476578	ZBED1	E3 SUMO-p	ZBED1_HUI	1	0.700567
15min_MT.	P19338	0.007253	0.71152	0.476578	NCL	Nucleolin	NUCL_HUM	30	1.00504
30min_MT.	P36578	0.091988	0.711877	0.476578	RPL4	Large ribos	RL4_HUMA	13	1.065838
15min_MT.	Q15334	0.130085	0.711645	0.476578	LLGL1	Lethal(2) gi	L2GL1_HUI	11	1.094358
30min_MT.	Q5W111	0.431638	0.711405	0.476578	SPRYD7	SPRY doma	SPRY7_HUI	1	1.348764
15min_MT.	Q96LA8	0.248309	0.71122	0.476578	PRMT6	Protein arg	ANM6_HUI	3	1.187814
15min_MT.	Q9BX66	0.03323	0.712273	0.476758	SORBS1	Sorbin and	SRBS1_HUI	30	1.023301
30min_MT.	Q92552	-0.02331	0.712734	0.477026	MRPS27	Small ribos	RT27_HUM	5	0.983971
30min_MT.	Q86Y46	0.548021	0.713111	0.477197	KRT73	Keratin, typ	K2C73_HUI	2	1.462079
30min_MT.	Q15599	0.136385	0.713342	0.477311	NHERF2	Na(+)/H(+) e	NHRF2_HU	7	1.099147
15min_MT.	O43149	-0.17857	0.713452	0.477323	ZZEF1	Zinc finger	ZZEF1_HUI	6	0.883575
15min_MT.	P61457	0.042386	0.713483	0.477323	PCBD1	Pterin-4-alp	PHS_HUMA	5	1.029815
15min_MT.	Q15363	-0.05694	0.713793	0.477353	TMED2	Transmemb	TMED2_HU	1	0.961304
15min_MT.	Q8IVS2	-0.11125	0.713816	0.477353	MCAT	Malonyl-Co	FABD_HUM	8	0.925789
30min_MT.	Q8WWX9	-0.154	0.713832	0.477353	SELENOM	Selenoprot	SELM_HUM	1	0.898753
30min_MT.	Q13825	0.183997	0.713978	0.477409	AUH	Methylglut	AUHM_HUI	7	1.136027
30min_MT.	O95782	-0.0331	0.71414	0.477443	AP2A1	AP-2 compl	AP2A1_HUI	32	0.977318
15min_MT.	Q92841	0.113364	0.714151	0.477443	DDX17	Probable A	DDX17_HU	15	1.081747
15min_MT.	P02753	-0.05268	0.714362	0.4775	RBP4	Retinol-bin	RET4_HUM	8	0.964141
15min_MT.	Q13564	0.063611	0.714419	0.4775	NAE1	NEDD8-acti	ULA1_HUM	16	1.045078
30min_MT.	P53611	-0.0065	0.714903	0.477701	RABGGTB	Geranylger	PGTB2_HUI	8	0.995508
15min_MT.	Q8WX92	0.05576	0.714851	0.477701	NELFB	Negative el	NELFB_HUI	10	1.039407
30min_MT.	P07737	0.004796	0.715735	0.477807	PFN1	Profilin-1	PROF1_HUI	10	1.00333
30min_MT.	Q15653	-0.06628	0.715275	0.477807	NFKBIB	NF-kappa-B	IKBB_HUMA	3	0.9551
30min_MT.	Q4V328	0.088057	0.71552	0.477807	GRIPAP1	GRIP1-asso	GRAP1_HU	22	1.062938
15min_MT.	Q96BJ3	-0.06594	0.715479	0.477807	AIDA	Axin intera	AIDA_HUM	7	0.955322
30min_MT.	Q9BVA0	0.095778	0.715474	0.477807	KATNB1	Katanin p8	KTNB1_HUI	3	1.068642
15min_MT.	Q9Y5T5	-0.20219	0.715716	0.477807	USP16	Ubiquitin c	UBP16_HU	1	0.869227
15min_MT.	O75363	-0.04713	0.716499	0.478117	BCAS1	Breast carci	BCAS1_HUI	3	0.96786
15min_MT.	P31930	0.258538	0.716345	0.478117	UQCRC1	Cytochrom	QCR1_HUM	4	1.196266
30min_MT.	Q13557	-0.00314	0.716388	0.478117	CAMK2D	Calcium/ca	KCC2D_HU	4	0.997824
30min_MT.	Q8N3D4	-0.01347	0.716564	0.478117	EHBP1L1	EH domain-	EH1L1_HUI	9	0.99071
30min_MT.	Q9UBQ7	-0.03876	0.717089	0.478345	GRHPR	Glyoxylate	GRHPR_HU	12	0.97349
30min_MT.	P62854;Q5	0.063123	0.717246	0.478375	RPS26;RPS	Small ribos	RS26_HUM	1	1.044725
15min_MT.	O43252	-0.08347	0.717581	0.478498	PAPSS1	Bifunctiona	PAPS1_HUI	5	0.943782
30min_MT.	Q6ZSZ5	0.436091	0.71754	0.478498	ARHGEF18	Rho guanin	ARHGI_HUI	1	1.352934
30min_MT.	Q9BPX3	-0.03206	0.717624	0.478498	NCAPG	Condensin	CND3_HUM	7	0.978021
15min_MT.	P42330	-0.0227	0.717763	0.47855	AKR1C3	Aldo-keto r	AK1C3_HUI	13	0.984387
15min_MT.	Q9H773	0.086376	0.718135	0.478716	DCTPP1	dCTP pyrop	DCTP1_HUI	10	1.0617
15min_MT.	O43716	-0.05392	0.718645	0.478829	GATC	Glutamyl-t	GATC_HUM	6	0.963317
15min_MT.	O95295	0.005726	0.718726	0.478829	SNAPIN	SNARE-asso	SNAPN_HU	4	1.003977
30min_MT.	P08648	0.134276	0.718614	0.478829	ITGA5	Integrin alp	ITA5_HUMA	9	1.097542
30min_MT.	P09958	0.521961	0.718494	0.478829	FURIN	Furin	FURIN_HUI	4	1.435906
30min_MT.	Q96FV9	-0.00917	0.718525	0.478829	THOC1	THO compl	THOC1_HU	20	0.993667
30min_MT.	Q99805	0.43809	0.718793	0.478829	TM9SF2	Transmemb	TM9S2_HU	2	1.35481
30min_MT.	P21127	0.11414	0.718945	0.478848	CDK11B	Cyclin-depe	CD11B_HU	11	1.082329
30min_MT.	Q2VIR3	-0.25163	0.719651	0.479207	EIF2S3B	Eukaryotic	IF2GL_HUI	1	0.839949

15min_MT.	Q96DX4	0.211999	0.719559	0.479207	RSPRY1	RING finger	RSPRY_HUM	3	1.158292
15min_MT.	P01031	0.059818	0.720313	0.479351	C5	Complemen	CO5_HUMA	16	1.042334
15min_MT.	P19440	0.138273	0.72031	0.479351	GGT1	Glutathion	GGT1_HUM	6	1.100587
30min_MT.	Q96J02	-0.1018	0.720189	0.479351	ITCH	E3 ubiquiti	ITCH_HUMA	4	0.931868
30min_MT.	Q9Y2X7	0.101083	0.720474	0.479417	GIT1	ARF GTPase	GIT1_HUMA	1	1.072578
30min_MT.	P62256	0.348841	0.72093	0.47968	UBE2H	Ubiquitin-c	UBE2H_HU	1	1.273537
15min_MT.	Q9NVN3	0.06761	0.721263	0.47982	RIC8B	Synembryn	RIC8B_HUM	2	1.047979
30min_MT.	Q9NWS0	-0.20952	0.721394	0.479866	PIH1D1	PIH1 doma	PIHD1_HUI	1	0.864826
30min_MT.	Q92771	-0.08085	0.721464	0.479872	DDX12P	Putative AT	DDX12_HU	1	0.9455
15min_MT.	Q4LE39	-0.01158	0.72175	0.480021	ARID4B	AT-rich inte	ARI4B_HUM	6	0.992007
30min_MT.	O15405;O9	-0.10477	0.722582	0.480441	TOX3;TOX4	TOX high m	TOX3_HUM	1	0.929956
30min_MT.	P12259	-0.10602	0.722466	0.480441	F5	Coagulation	FA5_HUMA	3	0.929145
15min_MT.	P42785	-0.02818	0.722626	0.480441	PRCP	Lysosomal	PCP_HUMA	12	0.980657
30min_MT.	Q16143	0.097189	0.722518	0.480441	SNCB	Beta-synucl	SYUB_HUM	1	1.069687
30min_MT.	Q96I25	-0.12357	0.722912	0.480585	RBM17	Splicing fac	SPF45_HUM	11	0.917913
30min_MT.	O14639	-0.06037	0.723165	0.480635	ABLIM1	Actin-bindi	ABLM1_HU	12	0.959015
15min_MT.	O75449	0.212522	0.723156	0.480635	KATNA1	Katanin p60	KTNA1_HUI	2	1.158712
30min_MT.	Q9Y5K6	0.024049	0.723373	0.480733	CD2AP	CD2-associa	CD2AP_HU	21	1.016809
15min_MT.	Q9BSY4	-0.0623	0.723806	0.480898	CHCHD5	Coiled-coil-	CHCH5_HU	5	0.957736
15min_MT.	Q96HC4	-0.00088	0.72403	0.48093	PDLIM5	PDZ and LIM	PDLI5_HUM	23	0.999391
30min_MT.	Q9BQ61	0.006975	0.724088	0.48093	TRIR	Telomerase	TRIR_HUMA	5	1.004846
30min_MT.	Q7Z3Y8	-0.35245	0.724438	0.481024	KRT27	Keratin, typ	K1C27_HUI	1	0.783251
30min_MT.	Q99439	0.045587	0.724447	0.481024	CNN2	Calponin-2	CNN2_HUM	9	1.032103
30min_MT.	Q49AR2	-0.01027	0.724827	0.481208	C5orf22	UPF0489 p	CE022_HUI	6	0.99291
15min_MT.	Q92888	-0.05643	0.7249	0.481216	ARHGEF1	Rho guanin	ARHG1_HU	4	0.96164
30min_MT.	Q14108	-0.27986	0.725163	0.481286	SCARB2	Lysosome m	SCRB2_HUI	5	0.82367
30min_MT.	Q8TCA0	-0.20166	0.725229	0.481286	LRRC20	Leucine-ric	LRC20_HUI	7	0.869547
15min_MT.	Q92696	0.058604	0.72509	0.481286	RABGGTA	Geranylgera	PGTA_HUM	20	1.041457
30min_MT.	O14773	-0.035	0.725425	0.481291	TPP1	Tripeptidyl	TPP1_HUM	5	0.976031
15min_MT.	P31431	0.125341	0.725443	0.481291	SDC4	Syndecan-4	SDC4_HUM	2	1.090765
15min_MT.	Q9BSE5	0.018811	0.7256	0.481313	AGMAT	Guanidino	GDAH_HUM	14	1.013124
15min_MT.	Q6ZVX7	0.281391	0.725763	0.48134	NCCRP1	F-box only	FBX50_HUI	8	1.215366
15min_MT.	O00151	-0.02692	0.725922	0.481343	PDLIM1	PDZ and LIM	PDLI1_HUM	18	0.981511
30min_MT.	P46777	0.066244	0.725952	0.481343	RPL5	Large ribos	RL5_HUMA	13	1.046987
15min_MT.	P35609	-0.18214	0.726132	0.48138	ACTN2	Alpha-actin	ACTN2_HUI	2	0.881395
30min_MT.	Q6PKC3	0.348482	0.72609	0.48138	TXNDC11	Thioredoxi	TXD11_HUI	3	1.27322
15min_MT.	P17480	0.156302	0.726249	0.481417	UBTF	Nucleolar t	UBF1_HUM	1	1.114427
15min_MT.	Q8WV24	-0.32542	0.726642	0.481577	PHLDA1	Pleckstrin h	PHLA1_HUI	1	0.798065
30min_MT.	Q9GZP4	0.049917	0.727024	0.481726	PITHD1	PITH domai	PITH1_HUM	11	1.035205
15min_MT.	P38606	-0.04341	0.727281	0.481775	ATP6V1A	V-type prot	VATA_HUM	23	0.97036
15min_MT.	P49915	0.024109	0.727204	0.481775	GMPS	GMP synth	GUAA_HUM	18	1.016852
15min_MT.	P13798	-0.00137	0.727372	0.481794	APEH	Acylamino-	ACPH_HUM	20	0.999049
30min_MT.	O75368	0.096132	0.727512	0.481824	SH3BGRL	Adapter SH	SH3L1_HUI	7	1.068904
15min_MT.	P38919	0.041062	0.727957	0.481955	EIF4A3	Eukaryotic	IF4A3_HUM	12	1.028871
15min_MT.	Q5VIR6	-0.23022	0.727917	0.481955	VPS53	Vacuolar pr	VPS53_HUI	8	0.852503
15min_MT.	Q7Z7E8	-0.02021	0.728407	0.482141	UBE2Q1	Ubiquitin-c	UB2Q1_HU	9	0.986088
30min_MT.	Q9Y296	0.040569	0.728451	0.482141	TRAPPC4	Trafficking	TPPC4_HUI	7	1.028519
15min_MT.	P48507	-0.01117	0.728547	0.482164	GCLM	Glutamate-	GSH0_HUM	11	0.992286
15min_MT.	Q13907	-0.0415	0.72872	0.482237	IDI1	Isopenteny	IDI1_HUMA	8	0.971642
15min_MT.	O95801	-0.58678	0.729497	0.482607	TTC4	Tetratricop	TTC4_HUM	1	0.665828
15min_MT.	P0C870	0.114016	0.729525	0.482607	JMJD7	Bifunctiona	JMJD7_HUI	3	1.082237
15min_MT.	Q9BPW8	-0.00978	0.72976	0.482689	NIPSNAP1	Protein Nip	NIPS1_HUM	6	0.993244

30min_MT.	P25440	0.00946	0.730278	0.482983	BRD2	Bromodom	BRD2_HUM	6	1.006579
15min_MT.	Q9NW68	0.08721	0.730402	0.483024	BSDC1	BSD domai	BSDC1_HUI	4	1.062314
15min_MT.	O00584	0.052704	0.730743	0.483127	RNASET2	Ribonuclea	RNT2_HUM	9	1.037207
15min_MT.	O15085	-0.40862	0.730684	0.483127	ARHGEF11	Rho guanin	ARHGB_HU	4	0.753346
15min_MT.	O43670	-0.29113	0.731348	0.483188	ZNF207	BUB3-inter	ZN207_HUI	3	0.81726
15min_MT.	O75414	0.061009	0.731454	0.483188	NME6	Nucleoside	NDK6_HUM	6	1.043195
15min_MT.	P45877	-0.81313	0.731118	0.483188	PPIC	Peptidyl-pr	PPIC_HUM.	2	0.569147
30min_MT.	P60880	-0.10699	0.731449	0.483188	SNAP25	Synaptosor	SNP25_HUI	1	0.928522
30min_MT.	Q8NBJ7	0.061301	0.73128	0.483188	SUMF2	Inactive C-a	SUMF2_HU	7	1.043407
30min_MT.	Q9H9Q4	-0.02647	0.73102	0.483188	NHEJ1	Non-homol	NHEJ1_HUI	1	0.981817
30min_MT.	Q9Y624	-0.0402	0.731233	0.483188	F11R	Junctional	JAM1_HUM	8	0.97252
30min_MT.	Q6FIF0	-0.11519	0.731616	0.483189	ZFAND6	AN1-type zi	ZFAN6_HUI	3	0.923259
15min_MT.	Q96DX5	-0.00868	0.731523	0.483189	ASB9	Ankyrin rep	ASB9_HUM	2	0.994004
30min_MT.	Q99576	-0.02146	0.73164	0.483189	TSC22D3	TSC22 dom	T22D3_HUI	1	0.985238
30min_MT.	P36543	0.087981	0.731975	0.483342	ATP6V1E1	V-type prot	VATE1_HUI	9	1.062881
30min_MT.	P62873	-0.00921	0.731995	0.483342	GNB1	Guanine nu	GBB1_HUM	3	0.99364
15min_MT.	O75794	0.079231	0.732317	0.483379	CDC123	Translation	CD123_HU	2	1.056455
15min_MT.	P33908	-0.10249	0.732199	0.483379	MAN1A1	Mannosyl-c	MA1A1_HU	2	0.931424
15min_MT.	Q13151	0.191052	0.732237	0.483379	HNRNPA0	Heterogene	ROA0_HUM	5	1.141596
30min_MT.	Q13951	-0.07447	0.73236	0.483379	CBFB	Core-bindir	PEBB_HUM	5	0.949693
15min_MT.	Q99459	-0.00183	0.732257	0.483379	CDC5L	Cell divisior	CDC5L_HUI	6	0.998731
15min_MT.	Q14957	0.160313	0.732824	0.483563	GRIN2C	Glutamate	NMDE3_HU	1	1.11753
30min_MT.	Q15645	-0.10859	0.733392	0.483897	TRIP13	Pachytene c	PCH2_HUM	9	0.927497
30min_MT.	O60645	-0.19227	0.733703	0.483938	EXOC3	Exocyst cor	EXOC3_HUI	2	0.875228
15min_MT.	P21127	0.127707	0.733699	0.483938	CDK11B	Cyclin-depe	CD11B_HU	11	1.092556
30min_MT.	Q9HB90;Q!	-0.03439	0.733552	0.483938	RRAGC;RRA	Ras-related	RRAGC_HU	5	0.976441
15min_MT.	Q8NEU8	-0.35232	0.734088	0.48407	APPL2	DCC-intera	DP13B_HU	2	0.783325
15min_MT.	P30084	0.047149	0.734422	0.484168	ECHS1	Enoyl-CoA I	ECHM_HUM	16	1.033221
30min_MT.	P46060	0.040291	0.734762	0.48431	RANGAP1	Ran GTPase	RAGP1_HU	21	1.028321
15min_MT.	Q07020	0.02052	0.734984	0.484415	RPL18	Large ribos	RL18_HUM	4	1.014325
30min_MT.	P78318	0.056893	0.735142	0.484438	IGBP1	Immunoglc	IGBP1_HUI	11	1.040223
15min_MT.	Q14974	-0.08596	0.735322	0.484495	KPNB1	Importin su	IMB1_HUM	30	0.942158
30min_MT.	Q8IVH8	0.155066	0.735352	0.484495	MAP4K3	Mitogen-ac	M4K3_HUM	1	1.113472
30min_MT.	O75054	-0.08189	0.735487	0.484525	IGSF3	Immunoglc	IGSF3_HUM	4	0.94482
15min_MT.	Q16625	-0.01293	0.735521	0.484525	OCLN	Occludin	OCLN_HUM	5	0.991076
15min_MT.	Q9NX55	0.164406	0.735731	0.484622	HYPK	Huntingtin	HYPK_HUM	2	1.120704
30min_MT.	Q92974	-0.037	0.735808	0.484632	ARHGEF2	Rho guanin	ARHG2_HU	4	0.974677
15min_MT.	Q9NRX5	0.337381	0.736164	0.484785	SERINC1	Serine inco	SERC1_HUI	1	1.26346
15min_MT.	P49448	-0.01659	0.73629	0.484792	GLUD2	Glutamate	DHE4_HUM	4	0.988565
30min_MT.	Q86X55	0.023386	0.736391	0.484792	CARM1	Histone-arg	CARM1_HU	11	1.016342
15min_MT.	Q8IY31	0.368017	0.736384	0.484792	IFT20	Intraflagell	IFT20_HUM	1	1.290578
30min_MT.	Q8N6N3	0.133079	0.736539	0.484828	C1orf52	UPF0690 p	CA052_HUI	5	1.096631
30min_MT.	P42285	-0.02537	0.737152	0.485065	MTREX	Exosome RI	MTREX_HU	20	0.982568
15min_MT.	P82650	-0.06691	0.737396	0.485065	MRPS22	Small ribos	RT22_HUM	5	0.954679
30min_MT.	Q12933	0.019819	0.737366	0.485065	TRAF2	TNF recepto	TRAF2_HUI	18	1.013832
30min_MT.	Q4G0N4	-0.02567	0.737338	0.485065	NADK2	NAD kinase	NAKD2_HU	16	0.982366
30min_MT.	Q8N5V2	0.019945	0.737152	0.485065	NGEF	Ephexin-1	NGEF_HUM	6	1.013921
15min_MT.	Q8N9V6	-0.10841	0.737276	0.485065	ANKRD53	Ankyrin rep	ANR53_HU	1	0.927611
15min_MT.	Q9H1Z4	-0.01508	0.737513	0.485102	WDR13	WD repeat-	WDR13_HU	10	0.989601
15min_MT.	P25490	-0.30191	0.737997	0.485297	YY1	Transcripti	TYY1_HUM.	2	0.81118
15min_MT.	Q8IZ69	0.12365	0.73797	0.485297	TRMT2A	tRNA (uraci	TRM2A_HU	3	1.089488
30min_MT.	P78324	-0.15491	0.738077	0.485309	SIRPA	Tyrosine-pr	SHPS1_HUI	2	0.898187

30min_MT.	P53597	0.009998	0.738207	0.485331	SUCLG1	Succinate--	SUCA_HUM	5	1.006954
30min_MT.	P61353	-0.11023	0.738395	0.485358	RPL27	Large ribos	RL27_HUM	5	0.926443
30min_MT.	Q96SB4	0.022155	0.738399	0.485358	SRPK1	SRSF protei	SRPK1_HU	3	1.015475
15min_MT.	Q08J23	0.003022	0.739014	0.485598	NSUN2	RNA cytosi	NSUN2_HU	37	1.002097
30min_MT.	O95479	0.00609	0.739107	0.485619	H6PD	GDH/6PGL	G6PE_HUM	26	1.00423
15min_MT.	Q9Y3D9	0.044399	0.739257	0.485677	MRPS23	Small ribos	RT23_HUM	5	1.031253
15min_MT.	Q8IX03	-0.27614	0.739491	0.485726	WWC1	Protein KIB	KIBRA_HUM	4	0.8258
30min_MT.	A3KN83	-0.23398	0.74013	0.485788	SBNO1	Protein stra	SBNO1_HU	3	0.850285
30min_MT.	A6NKN8	-0.01933	0.74026	0.485788	PCP4L1	Purkinje ce	PC4L1_HU	1	0.986694
15min_MT.	P33991	0.004515	0.740098	0.485788	MCM4	DNA replica	MCM4_HU	6	1.003135
15min_MT.	P42229;P5	0.030082	0.739943	0.485788	STAT5A;STA	Signal trans	STA5A_HUM	1	1.02107
30min_MT.	P48643	0.013111	0.73999	0.485788	CCT5	T-complex	TCPE_HUM	31	1.009129
15min_MT.	P49755	-0.06077	0.740254	0.485788	TMED10	Transmemb	TMEDA_HU	3	0.95875
30min_MT.	Q8NBS9	0.027721	0.740296	0.485788	TXNDC5	Thioredoxi	TXND5_HU	19	1.019401
15min_MT.	Q13428	0.000291	0.740654	0.48586	TCOF1	Treacle pro	TCOF_HUM	50	1.000202
15min_MT.	Q8N9N8	-0.34089	0.740654	0.48586	EIF1AD	Probable R	EIF1A_HUM	5	0.789552
15min_MT.	Q96AC1	-0.03322	0.741189	0.485966	FERMT2	Fermitin fa	FERM2_HU	22	0.977237
15min_MT.	Q9UL63	-0.04597	0.741279	0.485985	MKLN1	Muskelin	MKLN1_HU	7	0.968636
30min_MT.	Q8IWT0	0.112773	0.741434	0.486046	ZBTB8OS	Protein arc	ARCH_HUM	6	1.081304
15min_MT.	P06730	0.084574	0.741625	0.48613	EIF4E	Eukaryotic	IF4E_HUM	8	1.060374
30min_MT.	P02654	-0.22577	0.74204	0.48628	APOC1	Apolipopro	APOC1_HU	1	0.85514
30min_MT.	P85037	-0.0202	0.742118	0.48629	FOXK1	Forkhead b	FOXK1_HU	7	0.986097
30min_MT.	Q15382	-0.21584	0.742328	0.486364	RHEB	GTP-bindin	RHEB_HUM	4	0.861048
15min_MT.	Q9ULA0	-0.02937	0.742356	0.486364	DNPEP	Aspartyl am	DNPEP_HU	13	0.979847
30min_MT.	Q96SI9	0.083092	0.74242	0.486366	STRBP	Spermatid	STRBP_HUM	8	1.059286
30min_MT.	Q9NP79	0.055392	0.742703	0.48647	VTA1	Vacuolar pr	VTA1_HUM	11	1.039141
15min_MT.	Q99436	-0.02125	0.742932	0.486539	PSMB7	Proteasom	PSB7_HUM	9	0.985379
30min_MT.	Q6ZUT1	0.397363	0.743286	0.486729	NKAPD1	Uncharacte	NKAP1_HU	1	1.317099
15min_MT.	Q04726	0.103394	0.743368	0.486735	TLE3	Transducin	TLE3_HUM	10	1.074298
15min_MT.	Q05639	0.05291	0.743419	0.486735	EEF1A2	Elongation	EF1A2_HUM	5	1.037355
15min_MT.	P41567	0.034022	0.743548	0.486752	EIF1	Eukaryotic	EIF1_HUM	4	1.023862
15min_MT.	Q8TBZ6	-0.17016	0.743921	0.486941	TRMT10A	tRNA meth	TM10A_HU	1	0.888746
15min_MT.	Q9UBS4	0.005292	0.744166	0.487061	DNAJB11	DnaJ homo	DJB11_HU	11	1.003675
15min_MT.	Q9NTK5	-0.13102	0.744346	0.487138	OLA1	Obg-like AT	OLA1_HUM	14	0.913185
30min_MT.	Q96EM0	0.067027	0.744569	0.487243	L3HYPDH	Trans-3-hy	T3HPD_HU	3	1.047556
30min_MT.	Q15054	-0.00012	0.744716	0.487258	POLD3	DNA polym	DPOD3_HU	1	0.999914
30min_MT.	Q0VGL1	-0.13682	0.744888	0.487289	LAMTOR4	Ragulator c	LTOR4_HU	1	0.909523
30min_MT.	Q53F19	-0.16904	0.745385	0.48743	NCBP3	Nuclear ca	NCBP3_HU	6	0.889435
15min_MT.	Q7L523	0.072576	0.745348	0.48743	RRAGA	Ras-related	RRAGA_HU	5	1.051593
15min_MT.	Q86VN1	-0.03601	0.745415	0.48743	VPS36	Vacuolar pr	VPS36_HU	14	0.975347
30min_MT.	P42330	-0.02013	0.745537	0.487469	AKR1C3	Aldo-keto r	AK1C3_HU	13	0.986143
15min_MT.	Q14558	-0.14353	0.745616	0.48748	PRPSAP1	Phosphorib	KPRA_HUM	7	0.905299
15min_MT.	P17858	0.050333	0.746244	0.487686	PFKL	ATP-depen	PFKAL_HUM	14	1.035504
30min_MT.	P23919	-0.01932	0.746062	0.487686	DTYMK	Thymidylat	KTHY_HUM	12	0.9867
30min_MT.	Q7Z4G4	0.277668	0.746124	0.487686	TRMT11	tRNA (guan	TRM11_HU	3	1.212234
30min_MT.	Q9NYL9	0.091271	0.746375	0.487732	TMOD3	Tropomod	TMOD3_HU	17	1.065308
30min_MT.	Q6ZRP7	-0.10607	0.746503	0.487775	QSOX2	Sulfhydryl	QSOX2_HU	3	0.929115
30min_MT.	P46779	0.052932	0.747034	0.48804	RPL28	Large ribos	RL28_HUM	3	1.037371
30min_MT.	Q3B726	0.129967	0.747215	0.488118	POLR1F	DNA-direct	RPA43_HU	2	1.094269
30min_MT.	O15067	-0.06219	0.747449	0.488188	PFAS	Phosphorib	PUR4_HUM	13	0.957806
15min_MT.	O14672	-0.44435	0.747917	0.488452	ADAM10	Disintegrin	ADA10_HU	2	0.734914
30min_MT.	O15145	-0.04035	0.74821	0.488481	ARPC3	Actin-relate	ARPC3_HU	10	0.972418

15min_MT.	Q9UMS0	0.092481	0.748166	0.488481	NFU1	NFU1 iron-s	NFU1_HUM	6	1.066202
30min_MT.	Q14651	-0.03984	0.74834	0.488485	PLS1	Plastin-1	PLSI_HUMA	19	0.972763
30min_MT.	Q9NYL2	0.085315	0.748309	0.488485	MAP3K20	Mitogen-ac	M3K20_HU	1	1.060919
30min_MT.	Q6ZVM7	-0.05635	0.748412	0.488491	TOM1L2	TOM1-like p	TM1L2_HU	3	0.961695
15min_MT.	Q9Y6H1	0.034729	0.748483	0.488497	CHCHD2	Coiled-coil-	CHCH2_HU	3	1.024364
30min_MT.	Q7Z3U7	-0.06367	0.74858	0.48852	MON2	Protein MC	MON2_HUI	30	0.956826
30min_MT.	P52732	0.099453	0.748906	0.488599	KIF11	Kinesin-like	KIF11_HUM	6	1.071367
30min_MT.	Q9H098	0.127252	0.749011	0.488599	FAM107B	Protein FAM	F107B_HUI	7	1.092212
30min_MT.	Q9H8T0	-0.02016	0.748989	0.488599	AKTIP	AKT-interac	AKTIP_HUM	2	0.986126
30min_MT.	Q9NYM9	0.069302	0.748833	0.488599	BET1L	BET1-like pr	BET1L_HUM	2	1.049209
15min_MT.	P60006	0.416047	0.749325	0.48872	ANAPC15	Anaphase-p	APC15_HUI	1	1.334266
30min_MT.	Q9NQT8	-0.08298	0.749479	0.488739	KIF13B	Kinesin-like	KI13B_HUM	7	0.944105
30min_MT.	O75356	-0.02824	0.750021	0.488929	ENTPD5	Nucleoside	ENTP5_HUI	5	0.980613
30min_MT.	P07225	-0.0335	0.749978	0.488929	PROS1	Vitamin K-c	PROS_HUM	20	0.977044
15min_MT.	O14730	0.240692	0.750364	0.488949	RIOK3	Serine/thre	RIOK3_HUI	2	1.181559
15min_MT.	Q9NR31	0.08263	0.750308	0.488949	SAR1A	Small COPII	SAR1A_HUI	3	1.058947
30min_MT.	Q9NSE4	-0.01826	0.750338	0.488949	IARS2	Isoleucine--	SYIM_HUM	28	0.987423
15min_MT.	Q96RP9	-0.01348	0.750583	0.489051	GFM1	Elongation	EFGM_HUM	19	0.990699
15min_MT.	P56381;Q5	-0.04547	0.750888	0.489209	ATP5F1E;AT	ATP syntha:	ATP5E_HUM	2	0.968976
15min_MT.	Q9BVA0	0.068446	0.751688	0.489608	KATNB1	Katanin p8(	KTNB1_HUI	3	1.048587
30min_MT.	P82970	-0.12937	0.752039	0.489714	HMGN5	High mobil	HMGN5_HU	10	0.914229
30min_MT.	Q8NFI3	0.059323	0.751931	0.489714	ENGASE	Cytosolic er	ENASE_HUM	2	1.041977
15min_MT.	Q9BT09	0.026206	0.752037	0.489714	CNPY3	Protein can	CNPY3_HUI	5	1.01833
30min_MT.	Q56P03	0.240357	0.752354	0.489838	EAPP	E2F-associa	EAPP_HUM	1	1.181285
30min_MT.	Q15293	0.055975	0.75293	0.490172	RCN1	Reticulocal	RCN1_HUM	10	1.039561
30min_MT.	Q9UJY4	-0.0291	0.753145	0.49019	GGA2	ADP-ribosy	GGA2_HUM	2	0.980034
30min_MT.	P20810	0.068818	0.753775	0.490206	CAST	Calpastatin	ICAL_HUMA	27	1.048857
30min_MT.	P41091	-0.01937	0.754156	0.490206	EIF2S3	Eukaryotic	IF2G_HUMA	23	0.986663
30min_MT.	Q5VSL9;Q9	-0.01687	0.753918	0.490206	STRIP1;STR	Striatin-int	STRP1_HUM	1	0.988374
30min_MT.	Q7Z569	0.05446	0.753538	0.490206	BRAP	BRCA1-asso	BRAP_HUM	11	1.038471
15min_MT.	Q9BXI6	0.116401	0.754088	0.490206	TBC1D10A	TBC1 doma	TB10A_HUI	7	1.084027
30min_MT.	Q9UDY2	0.008991	0.753421	0.490206	TJP2	Tight juncti	ZO2_HUMA	23	1.006252
15min_MT.	Q9Y2Q9	-0.00113	0.754173	0.490206	MRPS28	Small ribos	RT28_HUM	4	0.99922
15min_MT.	Q9H3U1	0.012411	0.754293	0.490244	UNC45A	Protein unc	UN45A_HU	29	1.00864
15min_MT.	Q9NV35	0.672075	0.754499	0.490337	NUDT15	Nucleotide	NUD15_HU	1	1.593363
15min_MT.	P80723	-0.00535	0.754893	0.490552	BASP1	Brain acid s	BASP1_HUI	14	0.996301
30min_MT.	O43760	0.073155	0.75522	0.490707	SYNGR2	Synaptogyr	SNG2_HUM	3	1.052015
15min_MT.	Q7Z4S6	-0.0607	0.755499	0.490782	KIF21A	Kinesin-like	KI21A_HUM	16	0.958799
15min_MT.	Q15121	-0.06832	0.75575	0.490905	PEA15	Astrocytic p	PEA15_HUI	2	0.953748
30min_MT.	P19388	-0.09393	0.755992	0.490995	POLR2E	DNA-direct	RPAB1_HUI	5	0.936964
15min_MT.	P62244	-0.05234	0.756022	0.490995	RPS15A	Small ribos	RS15A_HUI	4	0.964368
15min_MT.	P42285	0.028182	0.756281	0.491086	MTREX	Exosome RI	MTREX_HU	20	1.019726
30min_MT.	Q8NFV4	0.060716	0.756418	0.491135	ABHD11	sn-1-specifi	ABHDB_HU	6	1.042983
30min_MT.	O75817	0.005212	0.756594	0.491208	POP7	Ribonuclea	POP7_HUM	1	1.003619
15min_MT.	P11441	0.048645	0.756783	0.491288	UBL4A	Ubiquitin-l	UBL4A_HUI	8	1.034293
30min_MT.	Q969S9	-0.0656	0.756842	0.491288	GFM2	Ribosome-r	RRF2M_HU	13	0.955548
30min_MT.	P46937	-0.04453	0.756923	0.4913	YAP1	Transcripti	YAP1_HUM	7	0.969605
30min_MT.	A1L170	-0.09492	0.757578	0.491602	C1orf226	Uncharacte	CA226_HUI	2	0.936327
30min_MT.	Q8N6M0	-0.07716	0.758094	0.491815	OTUD6B	Deubiquitii	OTU6B_HU	6	0.947924
15min_MT.	Q13045	-0.01467	0.758459	0.491922	FLII	Protein flig	FLII_HUMA	14	0.989884
15min_MT.	Q2TAZ0	0.013252	0.758402	0.491922	ATG2A	Autophagy-	ATG2A_HUI	14	1.009228
15min_MT.	O43665	0.222107	0.758638	0.491923	RGS10	Regulator c	RGS10_HUI	3	1.166436

30min_MT.	Q96CB9	-0.02968	0.758602	0.491923	NSUN4	5-methylcy	NSUN4_HU	3	0.97964
15min_MT.	Q8TBA6	-0.03672	0.759049	0.492108	GOLGA5	Golgin subf	GOGA5_HU	6	0.974872
15min_MT.	P04114	-0.00158	0.759379	0.49224	APOB	Apolipopro	APOB_HUM	97	0.998905
15min_MT.	P84090	-0.12642	0.759376	0.49224	ERH	Enhancer o	ERH_HUMA	4	0.916103
30min_MT.	P56377	0.051159	0.759596	0.49234	AP1S2	AP-1 compl	AP1S2_HUI	4	1.036097
30min_MT.	Q6KC79	0.00016	0.760091	0.49262	NIPBL	Nipped-B-li	NIPBL_HUM	4	1.000111
30min_MT.	O43255	-0.01074	0.76035	0.492706	SIAH2	E3 ubiquiti	SIAH2_HUM	2	0.992581
30min_MT.	Q06136	0.170052	0.760634	0.492818	KDSR	3-ketodihy	KDSR_HUM	2	1.125099
15min_MT.	Q15843	-0.01084	0.760669	0.492818	NEDD8	NEDD8	NEDD8_HU	4	0.992518
30min_MT.	Q9UI10	-0.2365	0.760711	0.492818	EIF2B4	Translation	EI2BD_HUM	4	0.848804
30min_MT.	Q6ZSJ8	0.115434	0.760956	0.492895	C1orf122	Uncharacte	CA122_HUI	2	1.083301
15min_MT.	Q99832	0.014833	0.760924	0.492895	CCT7	T-complex	TCPH_HUM	29	1.010335
15min_MT.	O43766	-0.07765	0.761355	0.49303	LIAS	Lipoyl synt	LIAS_HUMA	4	0.947602
15min_MT.	P27816	-0.02583	0.761298	0.49303	MAP4	Microtubul	MAP4_HUM	55	0.982257
30min_MT.	Q8WZB0	-1.03481	0.761416	0.49303	ERCC6L2-AS	Putative ur	CI130_HUM	1	0.488079
30min_MT.	Q9BSY4	-0.03988	0.761417	0.49303	CHCHD5	Coiled-coil	CHCH5_HU	5	0.972733
30min_MT.	Q9NX62	0.493571	0.761593	0.493103	BPNT2	Golgi-resid	IMPA3_HUI	2	1.407925
30min_MT.	Q86Y82	0.196437	0.761879	0.493207	STX12	Syntaxin-12	STX12_HUM	4	1.145865
30min_MT.	Q13395	-0.12431	0.761942	0.493207	TARBP1	Probable m	TARB1_HUI	11	0.917441
15min_MT.	Q6ZUT6	0.555414	0.762088	0.493229	CCDC9B	Coiled-coil	CCD9B_HU	1	1.46959
15min_MT.	Q14746	0.330626	0.762222	0.493265	COG2	Conserved	COG2_HUM	9	1.257559
15min_MT.	P33992	-0.01783	0.762417	0.493351	MCM5	DNA replica	MCM5_HUI	12	0.987719
30min_MT.	Q96GW9	0.027896	0.763043	0.493633	MARS2	Methionine	SYMM_HUM	14	1.019524
15min_MT.	Q96HJ9	0.199755	0.762999	0.493633	FMC1	Protein FM	FMC1_HUM	1	1.148503
30min_MT.	Q9H9G7	0.146457	0.762982	0.493633	ago-03	Protein arg	AGO3_HUM	3	1.106848
30min_MT.	P80723	0.063731	0.763252	0.49371	BASP1	Brain acid s	BASP1_HUI	14	1.045165
30min_MT.	Q8TD16	0.123549	0.763414	0.49371	BICD2	Protein bic	BICD2_HUM	15	1.089412
30min_MT.	Q9Y6E0	0.059415	0.763383	0.49371	STK24	Serine/thre	STK24_HUM	6	1.042043
30min_MT.	Q9GZV4	0.098477	0.763608	0.493754	EIF5A2	Eukaryotic	IF5A2_HUM	3	1.070642
15min_MT.	P57772	-0.21832	0.763891	0.493854	EEFSEC	Selenocyste	SELB_HUMA	6	0.859568
15min_MT.	O14786	0.260761	0.764064	0.493885	NRP1	Neuropilin-	NRP1_HUM	1	1.19811
15min_MT.	Q6VMQ6	0.154162	0.76461	0.494034	ATF7IP	Activating t	MCAF1_HU	1	1.112775
15min_MT.	Q9C0C2	0.082702	0.764546	0.494034	TNKS1BP1	182 kDa tar	TB182_HUI	37	1.059
15min_MT.	Q9NX08	-0.38033	0.764572	0.494034	COMMD8	COMM dom	COMD8_HU	2	0.768262
30min_MT.	Q9UM22	0.063287	0.764576	0.494034	EPDR1	Mammaliar	EPDR1_HUI	4	1.044844
30min_MT.	Q9HAS0	-0.06448	0.764948	0.4941	C17orf75	Protein Njn	NJMU_HUM	13	0.956289
30min_MT.	Q9Y3B7	-0.28955	0.764961	0.4941	MRPL11	Large ribos	RM11_HUM	5	0.818158
30min_MT.	Q96ME1	-0.00747	0.765221	0.494225	FBXL18	F-box/LRR-	FXL18_HUM	10	0.994833
15min_MT.	Q15042	-0.03103	0.765342	0.494263	RAB3GAP1	Rab3 GTPas	RB3GP_HU	18	0.978725
15min_MT.	P10515	0.033436	0.765535	0.494346	DLAT	Dihydrolip	ODP2_HUM	17	1.023446
15min_MT.	O60888	-0.03092	0.766081	0.494576	CUTA	Protein Cut	CUTA_HUM	4	0.978797
30min_MT.	Q8N3P4	0.00142	0.76606	0.494576	VPS8	Vacuolar pr	VPS8_HUM	4	1.000985
15min_MT.	Q9HB90;Q9	0.13842	0.765974	0.494576	RRAGC;RRA	Ras-related	RRAGC_HU	5	1.100699
15min_MT.	O75843	-0.02481	0.766257	0.494649	AP1G2	AP-1 compl	AP1G2_HU	1	0.982953
30min_MT.	A6NED2	-0.18977	0.7665	0.494765	RCCD1	RCC1 doma	RCCD1_HU	4	0.876744
30min_MT.	Q86X95	-0.53925	0.76674	0.494838	CIR1	Corepresso	CIR1_HUMA	1	0.688128
30min_MT.	O43395	-0.06001	0.767324	0.494967	PRPF3	U4/U6 sma	PRPF3_HUI	6	0.959256
15min_MT.	O95155	-0.05949	0.767506	0.494967	UBE4B	Ubiquitin c	UBE4B_HUI	20	0.959605
15min_MT.	O96007	-0.11379	0.767197	0.494967	MOCS2	Molybdopt	MOC2B_HU	4	0.92416
15min_MT.	P00480	-0.07655	0.76734	0.494967	OTC	Ornithine t	OTC_HUMA	2	0.94832
30min_MT.	P62333	0.05635	0.767459	0.494967	PSMC6	26S protea	PRS10_HUI	15	1.039831
30min_MT.	Q14353	0.078469	0.767508	0.494967	GAMT	Guanidino	GAMT_HUM	6	1.055897

30min_MT.	P35609	0.140516	0.767856	0.495071	ACTN2	Alpha-actir	ACTN2_HUI		2	1.102299
30min_MT.	Q14165	0.044942	0.767778	0.495071	MLEC	Malectin	MLEC_HUM		2	1.031642
15min_MT.	Q8TCX1	-0.0139	0.76786	0.495071	DYNC2LI1	Cytoplasmi	DC2L1_HUI		2	0.99041
15min_MT.	O94916	-0.0674	0.767985	0.495111	NFAT5	Nuclear fac	NFAT5_HUI		6	0.954354
30min_MT.	O60568	0.054228	0.768236	0.495232	PLOD3	Multifuncti	PLOD3_HU		22	1.038303
30min_MT.	P61011	-0.07359	0.768468	0.4953	SRP54	Signal reco	SRP54_HUI		8	0.950268
30min_MT.	P28328	-0.1528	0.769044	0.495592	PEX2	Peroxisome	PEX2_HUM		1	0.899501
30min_MT.	Q15738	-0.08789	0.769108	0.495592	NSDHL	Sterol-4-alp	NSDHL_HU		1	0.940899
15min_MT.	Q96KP4	0.04207	0.769112	0.495592	CNDP2	Cytosolic n	CNDP2_HU		18	1.02959
30min_MT.	Q9UBF6	-0.05932	0.769325	0.495689	RNF7	RING-box p	RBX2_HUM		3	0.959717
15min_MT.	Q05086	0.027961	0.769872	0.495959	UBE3A	Ubiquitin-p	UBE3A_HUI		21	1.01957
30min_MT.	O00748	-0.03455	0.769996	0.495983	CES2	Cocaine est	EST2_HUM.		7	0.976334
15min_MT.	P60953	0.029489	0.770036	0.495983	CDC42	Cell divisio	CDC42_HU		6	1.02065
15min_MT.	Q8TE02	0.129555	0.770267	0.496091	ELP5	Elongator c	ELP5_HUM		3	1.093956
15min_MT.	Q8IZ83	0.046111	0.770438	0.496161	ALDH16A1	Aldehyde d	A16A1_HUI		15	1.032478
30min_MT.	O00151	0.057799	0.770706	0.496292	PDLIM1	PDZ and LII	PDLI1_HUN		18	1.040877
15min_MT.	O00462	-0.00102	0.771153	0.496539	MANBA	Beta-mann	MANBA_HU		16	0.999291
30min_MT.	O43491	-0.00853	0.771392	0.496595	EPB41L2	Band 4.1-lil	E41L2_HUN		51	0.994106
15min_MT.	Q8NB46	-1.19228	0.77143	0.496595	ANKRD52	Serine/thre	ANR52_HU		1	0.437612
15min_MT.	Q9UP83	0.024766	0.771375	0.496595	COG5	Conserved	COG5_HUN		8	1.017315
30min_MT.	Q9UBI1	0.119184	0.771866	0.496835	COMMD3	COMM don	COMD3_HU		3	1.08612
15min_MT.	P62834	-0.23136	0.772104	0.49692	RAP1A	Ras-related	RAP1A_HUI		2	0.851829
30min_MT.	Q8WUA2	-0.0003	0.772222	0.496941	PPIL4	Peptidyl-pr	PPIL4_HUN		13	0.999791
15min_MT.	P61158	-0.02123	0.772333	0.496972	ACTR3	Actin-relate	ARP3_HUM		15	0.98539
30min_MT.	P02790	0.407531	0.773005	0.49724	HPX	Hemopexir	HEMO_HUN		3	1.326414
15min_MT.	P55210	-0.05194	0.773592	0.497495	CASP7	Caspase-7	CASP7_HUI		6	0.964641
30min_MT.	P51946	-0.11656	0.773896	0.497609	CCNH	Cyclin-H	CCNH_HUN		3	0.922384
30min_MT.	P30533	0.020205	0.774364	0.497828	LRPAP1	Alpha-2-ma	AMRP_HUN		15	1.014104
15min_MT.	Q9NUW8	-0.05799	0.774978	0.498182	TDP1	Tyrosyl-DN.	TYDP1_HUI		1	0.9606
30min_MT.	Q5JTZ9	-0.22448	0.77538	0.498399	AARS2	Alanine--tR	SYAM_HUN		6	0.855901
30min_MT.	Q96QC0	-0.03302	0.775847	0.498577	PPP1R10	Serine/thre	PP1RA_HUI		3	0.97737
30min_MT.	P11234	0.160453	0.775999	0.498634	RALB	Ras-related	RALB_HUM		6	1.117638
15min_MT.	P61160	-0.01706	0.776302	0.498664	ACTR2	Actin-relate	ARP2_HUM		13	0.988241
15min_MT.	Q14139	-0.02264	0.776148	0.498664	UBE4A	Ubiquitin c	UBE4A_HUI		13	0.984432
15min_MT.	Q9NVD7	0.029273	0.776222	0.498664	PARVA	Alpha-parvi	PARVA_HUI		4	1.020498
30min_MT.	P08709	-0.23803	0.776412	0.498694	F7	Coagulatio	FA7_HUMA		3	0.847901
30min_MT.	Q9NRF8	0.062254	0.77655	0.498742	CTPS2	CTP syntha	PYRG2_HUI		17	1.044096
30min_MT.	P46109	0.093277	0.777351	0.498928	CRKL	Crk-like prc	CRKL_HUM		13	1.066791
15min_MT.	P49356	-0.16948	0.777198	0.498928	FNTB	Protein fari	FNTB_HUM		6	0.889164
30min_MT.	Q69YN2	0.033309	0.777289	0.498928	CWF19L1	CWF19-like	C19L1_HUI		10	1.023357
30min_MT.	Q92764	0.024772	0.777277	0.498928	KRT35	Keratin, typ	KRT35_HUI		8	1.017319
30min_MT.	Q9BSL1	-0.09809	0.777131	0.498928	UBAC1	Ubiquitin-a	UBAC1_HU		3	0.934269
30min_MT.	Q9Y5V0	-0.01442	0.77729	0.498928	ZNF706	Zinc finger	ZN706_HUI		2	0.990054
30min_MT.	P42898	0.06909	0.777545	0.498971	MTHFR	Methylenet	MTHR_HUN		7	1.049055
30min_MT.	Q3YEC7	-0.04119	0.778169	0.499207	RABL6	Rab-like prc	RABL6_HUI		13	0.971856
15min_MT.	Q9NRS6	0.097532	0.778521	0.499392	SNX15	Sorting nex	SNX15_HUI		2	1.069941
30min_MT.	Q12907	0.023299	0.779104	0.499543	LMAN2	Vesicular ir	LMAN2_HU		8	1.01628
15min_MT.	Q9H2J4	0.436309	0.779422	0.499643	PDCL3	Phosducin-	PDCL3_HUI		8	1.353138
15min_MT.	Q9H8Y5	0.326354	0.779562	0.499692	ANKZF1	tRNA endor	ANKZ1_HUI		2	1.253841
15min_MT.	Q96MW5	0.01804	0.779961	0.499865	COG8	Conserved	COG8_HUN		3	1.012583
30min_MT.	Q9Y221	0.327299	0.780061	0.499888	NIP7	60S ribosor	NIP7_HUM		1	1.254662
15min_MT.	Q9ULC5	-0.15224	0.780204	0.499939	ACSL5	Long-chain	ACSL5_HUN		4	0.899851

30min_MT.	O76075	0.193336	0.780522	0.50003	DFFB	DNA fragme	DFFB_HUM	2	1.143404
30min_MT.	P05026	0.174496	0.780537	0.50003	ATP1B1	Sodium/po	AT1B1_HUI	4	1.12857
30min_MT.	Q15691	0.018469	0.780455	0.50003	MAPRE1	Microtubul	MARE1_HU	15	1.012884
30min_MT.	Q8N8N7	-0.16227	0.781177	0.500316	PTGR2	Prostaglan	PTGR2_HUI	7	0.893618
15min_MT.	P78368;Q9	-0.13314	0.782238	0.500914	CSNK1G2;C	Casein kina	KC1G2_HU	2	0.911842
30min_MT.	Q9H2K8	0.19468	0.7822	0.500914	TAOK3	Serine/thre	TAOK3_HUI	6	1.144471
15min_MT.	Q6UWE0	0.002166	0.782502	0.500936	LRSAM1	E3 ubiquiti	LRSM1_HU	14	1.001503
30min_MT.	Q96CM8	-0.02033	0.782461	0.500936	ACSF2	Medium-ch	ACSF2_HUI	15	0.986006
30min_MT.	P25686	-0.0436	0.78301	0.501091	DNAJB2	DnaJ homo	DNJB2_HUI	6	0.97023
30min_MT.	P28330	-0.01486	0.783072	0.501091	ACADL	Long-chain	ACADL_HUI	5	0.989755
30min_MT.	Q9BY32	-0.01679	0.783219	0.501091	ITPA	Inosine trip	ITPA_HUM	7	0.988428
15min_MT.	Q9NPJ3	0.010269	0.783196	0.501091	ACOT13	Acyl-coenzy	ACO13_HU	5	1.007143
30min_MT.	Q9NZB8	0.179194	0.783328	0.50112	MOCS1	Molybdenu	MOCS1_HU	3	1.132251
30min_MT.	O43181	0.069642	0.783553	0.501141	NDUFS4	NADH dehy	NDUS4_HU	2	1.049456
15min_MT.	P09622	-0.06152	0.783535	0.501141	DLD	Dihydrolip	DLDH_HUM	17	0.958251
30min_MT.	Q96EY4	-0.02258	0.783429	0.501141	TMA16	Translation	TMA16_HU	3	0.984468
30min_MT.	Q53H96	-0.14093	0.783632	0.50115	PYCR3	Pyrroline-5	P5CR3_HUI	3	0.906935
30min_MT.	P50542	-0.1099	0.78517	0.501929	PEX5	Peroxisom	PEX5_HUM	3	0.926655
30min_MT.	P51159	0.498186	0.785088	0.501929	RAB27A	Ras-related	RB27A_HUI	2	1.412436
15min_MT.	Q9NZI7	0.141727	0.785153	0.501929	UBP1	Upstream-b	UBIP1_HUI	1	1.103225
30min_MT.	P24752	-0.00326	0.785411	0.501947	ACAT1	Acetyl-CoA	THIL_HUM	19	0.99774
15min_MT.	P51148	0.026285	0.785456	0.501947	RAB5C	Ras-related	RAB5C_HUI	4	1.018386
15min_MT.	Q03393	0.108957	0.785596	0.501996	PTS	6-pyruvoyl	PTPS_HUM	4	1.078448
30min_MT.	P62253	0.165527	0.785767	0.502	UBE2G1	Ubiquitin-c	UB2G1_HU	4	1.121576
15min_MT.	Q8NBF6	-0.00153	0.785795	0.502	AVL9	Late secret	AVL9_HUM	10	0.998942
15min_MT.	Q9HCU8	0.053291	0.786262	0.502176	POLD4	DNA polym	DPOD4_HU	2	1.037629
15min_MT.	O95544	0.099439	0.786473	0.502269	NADK	NAD kinase	NADK_HUN	8	1.071357
15min_MT.	O15371	-0.04683	0.786798	0.502435	EIF3D	Eukaryotic	EIF3D_HUN	8	0.968058
15min_MT.	Q6QNY1	-0.07025	0.7869	0.50246	BLOC1S2	Biogenesis	BL1S2_HUN	4	0.952473
30min_MT.	P55010	-0.01181	0.787183	0.502558	EIF5	Eukaryotic	IF5_HUMAI	19	0.991847
15min_MT.	Q32MZ4	0.016764	0.787146	0.502558	LRRFIP1	Leucine-ric	LRRF1_HUN	19	1.011687
15min_MT.	P07148	-0.10229	0.787539	0.502717	FABP1	Fatty acid-b	FABPL_HUN	6	0.931555
30min_MT.	Q14CX7	-0.00495	0.787617	0.502717	NAA25	N-alpha-ac	NAA25_HU	12	0.996574
30min_MT.	Q13867	-0.06145	0.787923	0.502866	BLMH	Bleomycin	BLMH_HUN	18	0.958302
30min_MT.	O43505	-0.00995	0.788129	0.502943	B4GAT1	Beta-1,4-gl	B4GA1_HU	2	0.993124
15min_MT.	Q96T76	-0.01375	0.788171	0.502943	MMS19	MMS19 nu	MMS19_HU	8	0.990512
30min_MT.	Q86UA6	-0.11761	0.788326	0.503001	RPAIN	RPA-intera	RIP_HUMAI	1	0.921711
30min_MT.	P50613	-0.03801	0.78874	0.503183	CDK7	Cyclin-dep	CDK7_HUN	10	0.973996
30min_MT.	Q09472;Q9	-0.15529	0.788904	0.503205	EP300;CREI	Histone ace	EP300_HUI	1	0.897954
30min_MT.	O60664	0.009068	0.789055	0.50326	PLIN3	Perilipin-3	PLIN3_HUN	25	1.006305
15min_MT.	Q8N108	-0.04793	0.789418	0.503451	MIER1	Mesoderm	MIER1_HUI	5	0.96732
30min_MT.	Q9BRG1	0.092232	0.789584	0.503516	VPS25	Vacuolar pr	VPS25_HUI	8	1.066018
30min_MT.	Q13057	0.030738	0.789829	0.50359	COASY	Bifunctiona	COASY_HUI	7	1.021534
30min_MT.	Q9NVH6	0.074135	0.789829	0.50359	TMLHE	Trimethylly	TMLH_HUN	11	1.05273
30min_MT.	P45877	-0.77374	0.790139	0.503705	PPIC	Peptidyl-pr	PPIC_HUM	2	0.584901
15min_MT.	Q9NW64	-0.02612	0.790202	0.503705	RBM22	Pre-mRNA-	RBM22_HU	8	0.982061
30min_MT.	O43852	-0.02522	0.790344	0.503754	CALU	Calumenin	CALU_HUM	14	0.982669
30min_MT.	O94763	0.049498	0.790917	0.503904	URI1	Unconvent	RMP_HUM	5	1.034905
30min_MT.	P21283	0.002239	0.790934	0.503904	ATP6V1C1	V-type prot	VATC1_HUI	21	1.001553
30min_MT.	Q02318	0.109665	0.791089	0.503904	CYP27A1	Sterol 26-h	CP27A_HUI	7	1.078977
15min_MT.	Q14157	0.006389	0.791094	0.503904	UBAP2L	Ubiquitin-a	UBP2L_HUI	16	1.004438
30min_MT.	Q15438	-0.07305	0.790829	0.503904	CYTH1	Cytohesin-1	CYH1_HUM	9	0.950623

30min_MT.	Q16222	0.103477	0.790713	0.503904	UAP1	UDP-N-acet	UAP1_HUM	8	1.07436
30min_MT.	Q86SX6	-0.06572	0.791083	0.503904	GLRX5	Glutaredox	GLRX5_HUI	3	0.955468
30min_MT.	Q5T447	0.136715	0.791176	0.503915	HECTD3	E3 ubiquiti	HECD3_HU	5	1.099399
30min_MT.	Q8IUC4	0.067902	0.791463	0.503975	RHPN2	Rhophilin-2	RHPN2_HU	3	1.048191
30min_MT.	Q14197	-0.20212	0.791901	0.50413	MRPL58	Large ribos	ICT1_HUMA	2	0.869275
15min_MT.	Q9BV57	0.061828	0.791895	0.50413	ADI1	Acireducto	MTND_HUN	8	1.043788
15min_MT.	Q9NQ92	0.104421	0.792096	0.504157	COPRS	Coordinatc	COPRS_HUI	1	1.075063
15min_MT.	Q9NQT8	0.106922	0.792137	0.504157	KIF13B	Kinesin-like	KI13B_HUN	7	1.076928
30min_MT.	Q9UJ41	0.009247	0.792356	0.504256	RABGEF1	Rab5 GDP/(	RABX5_HUI	11	1.00643
30min_MT.	O14802	-0.24365	0.792421	0.504256	POLR3A	DNA-direct	RPC1_HUM	5	0.844608
15min_MT.	Q9UPM8	-0.189	0.792717	0.504404	AP4E1	AP-4 compl	AP4E1_HUI	3	0.877214
15min_MT.	Q8IUC4	-0.20861	0.793039	0.504567	RHPN2	Rhophilin-2	RHPN2_HU	3	0.865369
15min_MT.	Q1KMD3	0.045621	0.793301	0.504663	HNRNPUL2	Heterogene	HNRL2_HU	15	1.032127
30min_MT.	Q9HCC0	-0.03177	0.793841	0.504872	MCCC2	Methylcrot	MCCB_HUN	21	0.978219
15min_MT.	O60870	-0.25066	0.794061	0.50488	KIN	DNA/RNA-b	KIN17_HUN	3	0.840512
15min_MT.	P20340	-0.00518	0.794111	0.50488	RAB6A	Ras-related	RAB6A_HUI	9	0.996418
30min_MT.	P78346	0.083888	0.794488	0.504996	RPP30	Ribonuclea	RPP30_HUI	7	1.05987
30min_MT.	Q6ZVK8	0.172832	0.794438	0.504996	NUDT18	8-oxo-dGDI	NUD18_HU	3	1.127269
30min_MT.	O43172	-0.09682	0.794778	0.505	PRPF4	U4/U6 sma	PRP4_HUM	9	0.935094
15min_MT.	Q8TD19	0.079313	0.794817	0.505	NEK9	Serine/thre	NEK9_HUM	18	1.056515
30min_MT.	Q9Y3A6	-0.58632	0.795584	0.505446	TMED5	Transmemb	TMED5_HU	3	0.666042
15min_MT.	O43301	0.02181	0.79593	0.505502	HSPA12A	Heat shock	HS12A_HUI	8	1.015233
15min_MT.	Q86Y46	0.541025	0.79581	0.505502	KRT73	Keratin, typ	K2C73_HUI	2	1.455006
30min_MT.	L0R8F8	0.270021	0.796222	0.505605	MIEF1	Mitochond	MIDUO_HU	1	1.205826
30min_MT.	Q6NYC1	0.254716	0.796212	0.505605	JMJD6	Bifunctiona	JMJD6_HUI	6	1.193101
30min_MT.	Q16854	-0.13088	0.796412	0.505643	DGUOK	Deoxyguan	DGUOK_HU	4	0.913277
15min_MT.	Q4G0N4	0.095728	0.796475	0.505643	NADK2	NAD kinase	NAKD2_HU	16	1.068604
15min_MT.	Q86Y82	0.109337	0.796414	0.505643	STX12	Syntaxin-12	STX12_HUN	4	1.078733
30min_MT.	O60825	-0.25312	0.796613	0.505689	PFKFB2	6-phospho	F262_HUM	5	0.839082
30min_MT.	P15151	0.280634	0.796805	0.505771	PVR	Poliovirus r	PVR_HUMA	2	1.214729
15min_MT.	Q9UJX5	-0.03805	0.797002	0.505854	ANAPC4	Anaphase-p	APC4_HUM	14	0.973969
30min_MT.	P17987	-0.01599	0.797238	0.505922	TCP1	T-complex	TCPA_HUM	29	0.98898
15min_MT.	Q12864	0.12361	0.797805	0.506101	CDH17	Cadherin-1	CAD17_HU	4	1.089457
30min_MT.	Q5W0V3	-0.02044	0.797712	0.506101	FHIP2A	FHF comple	FHI2A_HUN	14	0.985935
15min_MT.	Q9H977	0.039056	0.798064	0.506159	WDR54	WD repeat-	WDR54_HU	3	1.027441
30min_MT.	Q9NV56	0.262496	0.798145	0.506169	MRGBP	MRG/MORI	MRGBP_HU	1	1.199552
15min_MT.	Q8TF74	-0.04161	0.79869	0.506474	WIPF2	WAS/WASL	WIPF2_HUI	6	0.971572
30min_MT.	P62487	0.023927	0.799089	0.506686	POLR2G	DNA-direct	RPB7_HUM	5	1.016723
15min_MT.	Q9Y587	-0.14383	0.799629	0.506987	AP4S1	AP-4 compl	AP4S1_HUI	3	0.905111
30min_MT.	Q96EY9	0.045015	0.799715	0.507001	ADAT3	Probable ir	ADAT3_HUI	3	1.031694
15min_MT.	O95394	0.004506	0.799854	0.507007	PGM3	Phosphoac	AGM1_HUN	21	1.003128
30min_MT.	Q96CW1	-0.07711	0.800205	0.507147	AP2M1	AP-2 compl	AP2M1_HU	16	0.947953
30min_MT.	O14617	-0.04348	0.801441	0.507149	AP3D1	AP-3 compl	AP3D1_HU	18	0.970309
30min_MT.	O75915	-0.14331	0.801496	0.507149	ARL6IP5	PRA1 famil	PRAF3_HUI	1	0.905438
15min_MT.	O95163	0.033836	0.800945	0.507149	ELP1	Elongator c	ELP1_HUM	18	1.023731
15min_MT.	P08670	0.036689	0.800588	0.507149	VIM	Vimentin	VIME_HUM	36	1.025757
30min_MT.	P54725	0.071479	0.80093	0.507149	RAD23A	UV excisior	RD23A_HU	6	1.050793
30min_MT.	P83111	0.197635	0.801462	0.507149	LACTB	Serine beta	LACTB_HUN	2	1.146817
30min_MT.	Q14202	-0.0504	0.801238	0.507149	ZMYM3	Zinc finger	ZMYM3_HU	9	0.965666
30min_MT.	Q8N0W3	0.2216	0.800413	0.507149	FCSK	L-fucose kir	FCSK_HUM	1	1.166026
30min_MT.	Q96AE4	0.023399	0.800319	0.507149	FUBP1	Far upstrea	FUBP1_HUI	29	1.016351
15min_MT.	Q9H1D9	0.221114	0.800914	0.507149	POLR3F	DNA-direct	RPC6_HUM	6	1.165634

15min_MT.	Q9UHB9	0.315959	0.801226	0.507149	SRP68	Signal reco	SRP68_HUI	6	1.244839
15min_MT.	Q9UID3	-0.13777	0.800825	0.507149	VPS51	Vacuolar pr	VPS51_HUI	9	0.90892
15min_MT.	Q9ULU4	-0.0484	0.800517	0.507149	ZMYND8	MYND-type	ZMYD8_HU	3	0.967011
30min_MT.	P09012	0.010805	0.802356	0.507399	SNRPA	U1 small nu	SNRPA_HUI	10	1.007517
30min_MT.	P34949	0.114657	0.802126	0.507399	MPI	Mannose-6	MPI_HUMA	8	1.082717
30min_MT.	P51149	0.015813	0.802397	0.507399	RAB7A	Ras-related	RAB7A_HUI	11	1.011021
30min_MT.	P84077	-0.01702	0.802126	0.507399	ARF1	ADP-ribosy	ARF1_HUM	8	0.988272
15min_MT.	Q9P265	-0.03328	0.802419	0.507399	DIP2B	Disco-inter	DIP2B_HUI	33	0.977196
15min_MT.	P49411	0.030961	0.802896	0.507536	TUFM	Elongation	EFTU_HUM	23	1.021693
15min_MT.	Q14320	0.026584	0.80284	0.507536	FAM50A	Protein FAI	FA50A_HUI	11	1.018598
30min_MT.	Q13155	-0.14932	0.80363	0.507855	AIMP2	Aminoacyl	AIMP2_HUI	4	0.901674
30min_MT.	Q16539	0.122005	0.80366	0.507855	MAPK14	Mitogen-ac	MK14_HUM	7	1.088246
15min_MT.	Q7Z5Q5	-0.19173	0.80381	0.507909	POLN	DNA polym	DPOLN_HU	1	0.875556
30min_MT.	Q9GZU8	-0.14457	0.804121	0.508064	PSME3IP1	PSME3-inte	PIP30_HUI	6	0.904649
30min_MT.	Q9H9A6	-0.13992	0.80422	0.508085	LRRC40	Leucine-ric	LRC40_HUI	14	0.907571
15min_MT.	Q08188	0.081639	0.80451	0.508176	TGM3	Protein-glu	TGM3_HUI	16	1.058219
15min_MT.	Q6NYC8	-0.08042	0.804457	0.508176	PPP1R18	Phostensin	PPR18_HUI	7	0.945783
15min_MT.	P36871	-0.01477	0.805034	0.508395	PGM1	Phosphoglu	PGM1_HUI	25	0.989813
30min_MT.	O95562	-0.1097	0.805534	0.508601	SFT2D2	Vesicle trar	SFT2B_HUI	1	0.92678
30min_MT.	O94919	0.120797	0.805998	0.508798	ENDOD1	Endonuclea	ENDD1_HU	3	1.087335
15min_MT.	Q86UN3	-0.1876	0.806081	0.50881	RTN4RL2	Reticulon-4	R4RL2_HUI	1	0.878063
15min_MT.	O95347	-0.01877	0.806223	0.508858	SMC2	Structural r	SMC2_HUI	21	0.987076
15min_MT.	Q6VY07	0.040191	0.8065	0.508992	PACS1	Phosphofur	PACS1_HUI	19	1.02825
15min_MT.	P61599	-0.07978	0.807065	0.509184	NAA20	N-alpha-ace	NAA20_HU	5	0.946201
15min_MT.	Q96IF1	0.17953	0.807398	0.509353	AJUBA	LIM domair	AJUBA_HUI	8	1.132515
15min_MT.	Q9NPJ1	0.195542	0.807632	0.509459	MKKS	Molecular c	MKKS_HUI	2	1.145155
30min_MT.	Q00613	-0.29787	0.80771	0.509468	HSF1	Heat shock	HSF1_HUM	1	0.813455
30min_MT.	P15428	0.135567	0.808197	0.509631	HPGD	15-hydroxy	PGDH_HUI	5	1.098524
30min_MT.	Q6VMQ6	0.314344	0.808229	0.509631	ATF7IP	Activating t	MCAF1_HU	1	1.243446
15min_MT.	O75717	-0.18427	0.809027	0.509846	WDHD1	WD repeat	WDHD1_HI	11	0.880096
30min_MT.	P15880	0.097406	0.808931	0.509846	RPS2	Small ribos	RS2_HUMA	10	1.069848
15min_MT.	P49790	-0.25796	0.808704	0.509846	NUP153	Nuclear po	NU153_HU	7	0.836268
15min_MT.	P55265	0.077482	0.808987	0.509846	ADAR	Double-stra	DSRAD_HU	14	1.055175
15min_MT.	Q9H0N0	0.099955	0.808963	0.509846	RAB6C	Ras-related	RAB6C_HUI	1	1.07174
15min_MT.	P15291	0.063665	0.809407	0.510044	B4GALT1	Beta-1,4-ga	B4GT1_HUI	1	1.045117
15min_MT.	Q9NQT5	0.083567	0.809641	0.51011	EXOSC3	Exosome cc	EXOS3_HUI	2	1.059635
15min_MT.	O43164	0.098388	0.810053	0.510238	PJA2	E3 ubiquiti	PJA2_HUM	4	1.070577
15min_MT.	P51809	-0.02932	0.810082	0.510238	VAMP7	Vesicle-assc	VAMP7_HU	3	0.979884
30min_MT.	Q96I59	-0.0457	0.81017	0.510238	NARS2	Asparaginyl	SYNM_HUI	6	0.968817
15min_MT.	P18615	-0.09716	0.810441	0.510367	NELFE	Negative el	NELFE_HUI	8	0.934869
15min_MT.	P62256	-0.15809	0.810929	0.510592	UBE2H	Ubiquitin-c	UBE2H_HU	1	0.896209
30min_MT.	Q15036	-0.15353	0.8109	0.510592	SNX17	Sorting nex	SNX17_HUI	4	0.89905
30min_MT.	Q96A35	0.420762	0.811113	0.510626	MRPL24	Large ribos	RM24_HUI	1	1.338635
30min_MT.	Q5H9R7	0.033935	0.811471	0.51081	PPP6R3	Serine/thre	PP6R3_HUI	30	1.023801
15min_MT.	P50440	0.029212	0.811673	0.510855	GATM	Glycine am	GATM_HUI	19	1.020454
30min_MT.	Q8WUQ7	0.151044	0.811761	0.510869	CACTIN	Splicing fac	CATIN_HUI	2	1.110373
15min_MT.	Q12931	0.01115	0.811994	0.510904	TRAP1	Heat shock	TRAP1_HUI	29	1.007759
15min_MT.	Q86X95	-0.30806	0.81193	0.510904	CIR1	Corepresso	CIR1_HUM.	1	0.807729
30min_MT.	Q9UDY8	-0.00537	0.812669	0.511112	MALT1	Mucosa-ass	MALT1_HU	12	0.996288
30min_MT.	O75390	-0.04498	0.813075	0.511159	CS	Citrate synt	CISY_HUMA	13	0.969306
15min_MT.	P46779	-0.01838	0.813299	0.511159	RPL28	Large ribos	RL28_HUM	3	0.98734
30min_MT.	P57076	-0.13371	0.813091	0.511159	CFAP298	Cilia- and fl	CF298_HUI	1	0.911487

15min_MT.	Q14116	-0.03482	0.813593	0.511159	IL18	Interleukin	IL18_HUMA	10	0.976156
15min_MT.	Q8N4T8	-0.02778	0.812928	0.511159	CBR4	3-oxoacyl-[	CBR4_HUM	10	0.980932
15min_MT.	Q8NFC6	-0.06098	0.813541	0.511159	BOD1L1	Biorientati	BD1L1_HU	29	0.958613
15min_MT.	Q9BUF5	0.002577	0.813367	0.511159	TUBB6	Tubulin bet	TBB6_HUM	8	1.001788
30min_MT.	Q9P2R7	-0.03207	0.813583	0.511159	SUCLA2	Succinate--	SUCB1_HU	12	0.978019
15min_MT.	Q9NR45	0.026117	0.813865	0.511288	NANS	Sialic acid s	SIAS_HUMA	10	1.018268
15min_MT.	P05026	0.085337	0.814074	0.511367	ATP1B1	Sodium/po	AT1B1_HU	4	1.060936
15min_MT.	P07902	-0.03673	0.814241	0.511367	GALT	Galactose-1	GALT_HUM	8	0.974863
15min_MT.	Q10713	0.011474	0.814252	0.511367	PMPCA	Mitochond	MPPA_HUM	13	1.007985
15min_MT.	Q9H649	0.146111	0.814159	0.511367	NSUN3	tRNA (cytos	NSUN3_HU	1	1.106583
15min_MT.	Q14019	0.015459	0.814973	0.511738	COTL1	Coactosin-l	COTL1_HU	11	1.010773
15min_MT.	Q9BUR4	-0.00734	0.815532	0.512007	WRAP53	Telomerase	TCAB1_HU	3	0.994925
30min_MT.	Q9BUR4	0.148784	0.815916	0.512174	WRAP53	Telomerase	TCAB1_HU	3	1.108635
30min_MT.	Q9NUQ8	-0.20335	0.81606	0.512174	ABCF3	ATP-bindin	ABCF3_HU	5	0.868533
30min_MT.	Q9H6V9	0.13594	0.816671	0.512393	LDAH	Lipid dropl	LDAH_HUM	1	1.098809
30min_MT.	Q8N9V3	0.043969	0.817127	0.512521	WDSUB1	WD repeat,	WSDU1_HU	1	1.030946
15min_MT.	Q96AT9	0.060112	0.817203	0.512521	RPE	Ribulose-pl	RPE_HUMA	4	1.042546
15min_MT.	P49207	-0.10666	0.817814	0.51278	RPL34	Large ribos	RL34_HUM	3	0.928735
30min_MT.	Q6P1X6	0.023186	0.817859	0.51278	C8orf82	UPF0598 p	CH082_HU	4	1.016201
30min_MT.	Q969M3	-0.16596	0.817694	0.51278	YIPF5	Protein YIP	YIPF5_HUM	1	0.891338
15min_MT.	Q9UDT6	0.029824	0.817878	0.51278	CLIP2	CAP-Gly do	CLIP2_HUM	26	1.020888
30min_MT.	Q96I51	-0.02866	0.818046	0.512844	RCC1L	RCC1-like G	RCC1L_HU	9	0.980331
15min_MT.	Q9UPT6	-0.23153	0.818169	0.51288	MAPK8IP3	C-Jun-amin	JIP3_HUMA	7	0.851733
30min_MT.	Q86VX9	-0.03878	0.818599	0.513108	MON1A	Vacuolar fu	MON1A_HU	1	0.97348
30min_MT.	Q15428	0.007368	0.818789	0.513187	SF3A2	Splicing fac	SF3A2_HUM	5	1.00512
15min_MT.	Q13315	0.290665	0.818881	0.513203	ATM	Serine-prot	ATM_HUMA	2	1.223204
15min_MT.	O43414	0.083135	0.819053	0.513209	ERI3	ERI1 exorib	ERI3_HUMA	1	1.059317
30min_MT.	Q9BYJ9	-0.01126	0.819284	0.513209	YTHDF1	YTH domair	YTHD1_HU	1	0.992229
30min_MT.	Q9H9E3	0.069837	0.819272	0.513209	COG4	Conserved	COG4_HUM	12	1.049598
30min_MT.	Q9Y3B4	-0.09032	0.819119	0.513209	SF3B6	Splicing fac	SF3B6_HUM	1	0.939316
15min_MT.	Q15599	0.058736	0.81945	0.513231	NHERF2	Na(+)/H(+) e	NHRF2_HU	7	1.041553
30min_MT.	P18859	-0.27145	0.819744	0.51325	ATP5PF	ATP syntha	ATP5J_HUM	2	0.828489
30min_MT.	P40818	-0.20747	0.819663	0.51325	USP8	Ubiquitin c	UBP8_HUM	8	0.866057
15min_MT.	Q9BUL5	0.086583	0.819883	0.513255	PHF23	PHD finger	PHF23_HU	3	1.061852
30min_MT.	Q9H1A4	0.120242	0.819863	0.513255	ANAPC1	Anaphase-p	APC1_HUM	5	1.086917
30min_MT.	Q9UBB4	-0.00479	0.819986	0.513279	ATXN10	Ataxin-10	ATX10_HU	18	0.996684
30min_MT.	O43548	-0.07371	0.820124	0.513283	TGM5	Protein-glu	TGM5_HUM	1	0.950195
15min_MT.	Q9Y4G6	0.019399	0.820123	0.513283	TLN2	Talin-2	TLN2_HUM	68	1.013538
15min_MT.	Q5T160	0.055852	0.820399	0.513391	RARS2	Probable ar	SYRM_HUM	6	1.039473
15min_MT.	Q96S38	-0.05527	0.820428	0.513391	RPS6KC1	Ribosomal	KS6C1_HU	3	0.962412
15min_MT.	P26440	-0.00606	0.820593	0.513401	IVD	Isovaleryl-C	IVD_HUMA	11	0.995808
15min_MT.	P53582	0.073367	0.820682	0.513401	METAP1	Methionine	MAP11_HU	5	1.052169
15min_MT.	Q04724	0.134945	0.820521	0.513401	TLE1	Transducin	TLE1_HUMA	11	1.098051
15min_MT.	Q8WYP5	-0.43928	0.82123	0.513605	AHCTF1	Protein ELY	ELYS_HUMA	1	0.737503
15min_MT.	Q12756	0.133991	0.821348	0.513638	KIF1A	Kinesin-like	KIF1A_HUM	1	1.097325
30min_MT.	P17480	0.571449	0.821602	0.513715	UBTF	Nucleolar t	UBF1_HUM	1	1.486015
15min_MT.	O43390	0.060403	0.822867	0.513724	HNRNPR	Heterogene	HNRPR_HU	22	1.042757
30min_MT.	P02751	0.002683	0.821853	0.513724	FN1	Fibronectir	FINC_HUMA	84	1.001861
30min_MT.	P08195	0.030316	0.822149	0.513724	SLC3A2	Amino acid	4F2_HUMA	17	1.021236
30min_MT.	P11802	0.027848	0.822865	0.513724	CDK4	Cyclin-depe	CDK4_HUM	2	1.01949
15min_MT.	P18887	0.097136	0.822802	0.513724	XRCC1	DNA repair	XRCC1_HU	5	1.069648
30min_MT.	P45954	0.035645	0.823057	0.513724	ACADSB	Short/bran	ACDSB_HU	13	1.025015

30min_MT.	P49748	-0.00071	0.822473	0.513724	ACADVL	Very long-c	ACADV_HU	30	0.999507
30min_MT.	P60660	-0.00191	0.823193	0.513724	MYL6	Myosin ligh	MYL6_HUM	7	0.998675
30min_MT.	P78540	0.021723	0.82179	0.513724	ARG2	Arginase-2,	ARGI2_HUI	13	1.015171
15min_MT.	P85037	-0.18937	0.822793	0.513724	FOXK1	Forkhead b	FOXK1_HUI	7	0.876991
30min_MT.	Q15417	-0.02925	0.821872	0.513724	CNN3	Calponin-3	CNN3_HUM	12	0.979928
30min_MT.	Q15642	-0.03592	0.822207	0.513724	TRIP10	Cdc42-inte	CIP4_HUMA	9	0.975408
30min_MT.	Q99614	-0.07216	0.822968	0.513724	TTC1	Tetratricop	TTC1_HUM	10	0.951215
30min_MT.	Q9BRV8	0.064135	0.822435	0.513724	SIKE1	Suppressor	SIKE1_HUM	3	1.045458
30min_MT.	Q9Y680	-0.18208	0.822811	0.513724	FKBP7	Peptidyl-pr	FKBP7_HUI	5	0.881432
30min_MT.	Q07021	-0.02491	0.823433	0.513833	C1QBP	Compleme	C1QBP_HU	5	0.982882
15min_MT.	Q96PC3	-0.14787	0.824037	0.514087	AP1S3	AP-1 compl	AP1S3_HUI	2	0.902582
15min_MT.	Q7Z4Q2	-0.04268	0.824768	0.514388	HEATR3	HEAT repea	HEAT3_HUI	12	0.970848
15min_MT.	Q9H5V9	-0.33756	0.824784	0.514388	STEEP1	STING ER ex	STEEP_HUM	1	0.791381
30min_MT.	P30419	0.015471	0.825392	0.514563	NMT1	Glycylpepti	NMT1_HUM	11	1.010782
15min_MT.	P47897	-0.008	0.825284	0.514563	QARS1	Glutamine-	SYQ_HUMA	24	0.994471
30min_MT.	Q15057	0.003449	0.825306	0.514563	ACAP2	Arf-GAP wit	ACAP2_HUI	10	1.002393
30min_MT.	Q9HA65	-0.04154	0.825182	0.514563	TBC1D17	TBC1 doma	TBC17_HUI	6	0.971615
30min_MT.	P14678	-0.02348	0.825859	0.514731	SNRPB	Small nucle	RSMB_HUM	9	0.983855
15min_MT.	P50747	-0.15334	0.826308	0.514846	HLCS	Biotin--pro	BPL1_HUM	8	0.899168
30min_MT.	P55735	-0.01933	0.826298	0.514846	SEC13	Protein SEC	SEC13_HUM	11	0.986688
30min_MT.	Q9Y3B9	0.175023	0.826779	0.515058	RRP15	RRP15-like	RRP15_HUI	3	1.128983
15min_MT.	Q01081	-0.0065	0.82693	0.515111	U2AF1	Splicing fac	U2AF1_HUI	5	0.995504
30min_MT.	P61019	-0.0257	0.827125	0.51515	RAB2A	Ras-related	RAB2A_HUI	11	0.982341
15min_MT.	Q5T2W1	0.004925	0.827117	0.51515	PDZK1	Na(+)/H(+) e	NHRF3_HU	24	1.00342
30min_MT.	P05141;P1	-0.02182	0.827265	0.515172	SLC25A5;SI	ADP/ATP tr	ADT2_HUM	2	0.984986
15min_MT.	Q9NZ09	0.095361	0.827442	0.515225	UBAP1	Ubiquitin-a	UBAP1_HU	1	1.068332
30min_MT.	O75439	0.135748	0.827938	0.515328	PMPCB	Mitochond	MPPB_HUM	12	1.098662
30min_MT.	P37840	0.019199	0.827845	0.515328	SNCA	Alpha-synu	SYUA_HUM	5	1.013397
15min_MT.	Q14653	-0.01781	0.827801	0.515328	IRF3	Interferon r	IRF3_HUMA	8	0.987729
30min_MT.	Q15126	-0.10575	0.827758	0.515328	PMVK	Phosphomo	PMVK_HUM	9	0.929323
15min_MT.	Q8IUR0	1.650392	0.827931	0.515328	TRAPPC5	Trafficking	TPPC5_HUI	1	3.139189
30min_MT.	Q9H267	-0.11618	0.828248	0.515398	VPS33B	Vacuolar pr	VP33B_HUI	6	0.922628
30min_MT.	P35670	-0.11625	0.828571	0.51541	ATP7B	Copper-trai	ATP7B_HUI	3	0.922584
30min_MT.	P36954	0.132128	0.82854	0.51541	POLR2I	DNA-direct	RPB9_HUM	4	1.095909
15min_MT.	Q15596	-0.06702	0.828584	0.51541	NCOA2	Nuclear rec	NCOA2_HU	5	0.954611
30min_MT.	Q8TCX1	-0.15541	0.828596	0.51541	DYNC2LI1	Cytoplasmi	DC2L1_HUI	2	0.897874
15min_MT.	Q9NZ52	0.112289	0.828381	0.51541	GGA3	ADP-ribosy	GGA3_HUM	10	1.080942
15min_MT.	Q99598	-0.04661	0.828669	0.515414	TSNAX	Translin-as	TSNAX_HUI	14	0.968207
15min_MT.	Q9Y262	-0.00486	0.828774	0.515438	EIF3L	Eukaryotic	EIF3L_HUM	24	0.996636
30min_MT.	Q9BRS2	0.064413	0.828857	0.515449	RIOK1	Serine/thre	RIOK1_HUI	1	1.045659
30min_MT.	Q6UXN9	0.099046	0.829674	0.515702	WDR82	WD repeat-	WDR82_HU	11	1.071065
15min_MT.	O75396	0.055913	0.830011	0.515709	SEC22B	Vesicle-traf	SC22B_HUI	10	1.039516
30min_MT.	Q0VAQ4	0.088367	0.830067	0.515709	SMAGP	Small cell a	SMAGP_HU	1	1.063166
30min_MT.	Q7Z4S6	-0.04392	0.830265	0.515781	KIF21A	Kinesin-like	KI21A_HUM	16	0.970015
15min_MT.	O60216	-0.0185	0.830845	0.515788	RAD21	Double-stra	RAD21_HU	13	0.987257
15min_MT.	P01042	-0.02867	0.830798	0.515788	KNG1	Kininogen-:	KNG1_HUM	7	0.980322
15min_MT.	P49748	0.019388	0.830755	0.515788	ACADVL	Very long-c	ACADV_HU	30	1.01353
15min_MT.	P84022	-0.09675	0.830733	0.515788	SMAD3	Mothers ag	SMAD3_HU	4	0.935139
30min_MT.	Q9BZG8	0.018004	0.830853	0.515788	DPH1	2-(3-amino	DPH1_HUM	11	1.012558
15min_MT.	Q9NRV9	0.052829	0.830468	0.515788	HEBP1	Heme-bind	HEBP1_HUI	9	1.037297
30min_MT.	O60499	0.308075	0.830991	0.515833	STX10	Syntaxin-1(	STX10_HUI	1	1.238055
30min_MT.	P20290	0.022748	0.831437	0.515987	BTF3	Transcripti	BTF3_HUM	6	1.015892

15min_MT.	P78540	-0.06	0.831377	0.515987	ARG2	Arginase-2,	ARGI2_HUM	13	0.959263
15min_MT.	Q9HD26	0.085064	0.831964	0.516232	GOPC	Golgi-assoc	GOPC_HUM	11	1.060735
30min_MT.	Q96T51	0.03863	0.832197	0.516336	RUFY1	RUN and FY	RUFY1_HUI	22	1.027138
30min_MT.	Q9H2J4	0.385568	0.832529	0.516481	PDCL3	Phosducin-	PDCL3_HUI	8	1.306374
15min_MT.	O60499	0.030773	0.832715	0.516493	STX10	Syntaxin-1(	STX10_HUM	1	1.021559
30min_MT.	Q5SXM8	0.325094	0.832673	0.516493	DNLZ	DNL-type zi	DNLZ_HUM	2	1.252746
30min_MT.	P11310	0.056742	0.832788	0.516498	ACADM	Medium-ch	ACADM_HU	15	1.040114
15min_MT.	Q6P1X6	-0.02765	0.833033	0.516608	C8orf82	UPF0598 p	CH082_HU	4	0.981017
15min_MT.	Q58FG1	0.071253	0.833277	0.51671	HSP90AA4I	Putative he	HS904_HUI	2	1.050629
30min_MT.	Q8TBF4	0.002868	0.833329	0.51671	ZCRB1	Zinc finger (	ZCRB1_HUI	1	1.00199
15min_MT.	Q15311	0.090303	0.833727	0.516793	RALBP1	RalA-bindir	RBP1_HUM	8	1.064594
30min_MT.	Q8IXW5	-0.08386	0.833714	0.516793	RPAP2	Putative RN	RPAP2_HUI	2	0.94353
15min_MT.	Q9Y237	-0.00113	0.834049	0.516952	PIN4	Peptidyl-pr	PIN4_HUM	7	0.999218
15min_MT.	Q9P2R3	-0.00891	0.834119	0.516954	ANKFY1	Rabankyrin	ANFY1_HUI	7	0.993841
15min_MT.	Q13813	0.047694	0.834515	0.517118	SPTAN1	Spectrin alp	SPTN1_HUI	125	1.033611
30min_MT.	Q6QNY0	0.356513	0.834486	0.517118	BLOC1S3	Biogenesis (	BL1S3_HUN	1	1.280328
30min_MT.	Q9UJX4	0.052471	0.835048	0.517325	ANAPC5	Anaphase-p	APC5_HUM	2	1.03704
30min_MT.	Q14738	-0.16725	0.835128	0.517334	PPP2R5D	Serine/thre	2A5D_HUM	7	0.890537
30min_MT.	Q9Y3D2	-0.02095	0.835401	0.517461	MSRB2	Methionine	MSRB2_HU	4	0.985581
15min_MT.	Q9NWU2	0.152741	0.836039	0.517775	GID8	Glucose-ind	GID8_HUM	3	1.111679
30min_MT.	Q16881	0.029577	0.836177	0.517819	TXNRD1	Thioredoxi	TRXR1_HUI	22	1.020713
15min_MT.	Q93034	-0.01742	0.836642	0.518066	CUL5	Cullin-5	CUL5_HUM	26	0.987999
30min_MT.	P18827	-0.04849	0.837299	0.518303	SDC1	Syndecan-1	SDC1_HUM	1	0.966947
15min_MT.	Q01469	0.031892	0.837227	0.518303	FABP5	Fatty acid-b	FABP5_HUI	11	1.022352
30min_MT.	Q6DD88	-0.07965	0.837356	0.518303	ATL3	Atlastin-3	ATLA3_HUN	13	0.946287
30min_MT.	P49590	0.012975	0.837439	0.518314	HARS2	Histidine--t	SYHM_HUM	9	1.009034
30min_MT.	P61962	-0.07721	0.837506	0.518314	DCAF7	DDB1- and	DCAF7_HUI	6	0.947886
15min_MT.	C9JLW8	0.034847	0.837912	0.518401	MCRIP1	Mapk-regul	MCRI1_HUI	5	1.024448
15min_MT.	O75380	-0.15893	0.837873	0.518401	NDUFS6	NADH dehy	NDUS6_HU	3	0.89569
15min_MT.	Q9Y296	0.090748	0.837844	0.518401	TRAPPC4	Trafficking	TPPC4_HUI	7	1.064922
30min_MT.	P01871	-0.68333	0.838797	0.518443	IGHM	Immunoglc	IGHM_HUM	3	0.622726
30min_MT.	Q13425	-0.13505	0.83847	0.518443	SNTB2	Beta-2-synt	SNTB2_HUI	5	0.91064
30min_MT.	Q6GYQ0	-0.05419	0.83869	0.518443	RALGAPA1	Ral GTPase-	RGPA1_HU	12	0.963137
15min_MT.	Q7LBC6	0.040577	0.838908	0.518443	KDM3B	Lysine-spec	KDM3B_HU	7	1.028525
30min_MT.	Q7Z3B4	0.039784	0.838814	0.518443	NUP54	Nucleopori	NUP54_HU	3	1.02796
30min_MT.	Q8IWD4	-0.28323	0.838173	0.518443	CCDC117	Coiled-coil	CC117_HUI	2	0.821749
15min_MT.	Q8NB37	0.142214	0.838325	0.518443	GATD1	Glutamine	GALD1_HU	3	1.103598
30min_MT.	Q92599	-0.00111	0.838795	0.518443	SEPTIN8	Septin-8	SEPT8_HUN	5	0.99923
30min_MT.	Q9H832	-0.08128	0.838866	0.518443	UBE2Z	Ubiquitin-c	UBE2Z_HUI	8	0.945219
30min_MT.	Q9HCN8	-0.02154	0.838398	0.518443	SDF2L1	Stromal cel	SDF2L_HUN	6	0.985183
15min_MT.	Q9ULC6	0.034794	0.838482	0.518443	PADI1	Protein-arg	PADI1_HUN	1	1.024411
30min_MT.	B5ME19;Q	0.019845	0.839029	0.518467	EIF3CL;EIF3	Eukaryotic	EIFCL_HUM	23	1.01385
15min_MT.	Q15046	0.056298	0.839266	0.518483	KARS1	Lysine--tRN	SYK_HUMA	20	1.039794
30min_MT.	Q13618	0.022888	0.840061	0.518828	CUL3	Cullin-3	CUL3_HUM	27	1.015991
30min_MT.	O75608	0.022271	0.841024	0.51889	LYPLA1	Acyl-protei	LYPA1_HUN	5	1.015557
15min_MT.	P48506	0.024853	0.840633	0.51889	GCLC	Glutamate-	GSH1_HUM	19	1.017376
15min_MT.	Q04206	0.015396	0.841005	0.51889	RELA	Transcripti	TF65_HUM	11	1.010729
30min_MT.	Q13490	-0.04164	0.84054	0.51889	BIRC2	Baculoviral	BIRC2_HUN	9	0.971552
15min_MT.	Q15758	-0.14968	0.840814	0.51889	SLC1A5	Neutral am	AAAT_HUM	6	0.901448
30min_MT.	Q96G46	0.046121	0.840567	0.51889	DUS3L	tRNA-dihyd	DUS3L_HUI	11	1.032485
15min_MT.	Q96QG7	-0.24073	0.840955	0.51889	MTMR9	Myotubula	MTMR9_HU	3	0.84632
30min_MT.	O14980	-0.03108	0.841292	0.518991	XPO1	Exportin-1	XPO1_HUM	24	0.97869

15min_MT.	Q6IPR3	0.151132	0.841321	0.518991	TYW3	tRNA wybu	TYW3_HUM	1	1.11044
15min_MT.	P22087	0.054616	0.841644	0.519073	FBL	rRNA 2'-O-n	FBRL_HUM	2	1.038582
15min_MT.	P63000	0.093527	0.841652	0.519073	RAC1	Ras-related	RAC1_HUM	7	1.066976
15min_MT.	Q8TAQ2	0.102117	0.841878	0.519171	SMARCC2	SWI/SNF cc	SMRC2_HU	7	1.073347
30min_MT.	Q00534	0.068711	0.842083	0.519216	CDK6	Cyclin-depe	CDK6_HUM	9	1.048779
15min_MT.	Q96EQ0	0.009961	0.842204	0.51925	SGTB	Small gluta	SGTB_HUM	5	1.006928
15min_MT.	Q13042	0.053239	0.842402	0.519283	CDC16	Cell divisio	CDC16_HU	11	1.037592
15min_MT.	Q99584	0.075836	0.842533	0.519283	S100A13	Protein S1(	S10AD_HU	7	1.053971
15min_MT.	P40939	0.008152	0.842952	0.519352	HADHA	Trifunction	ECHA_HUM	20	1.005666
30min_MT.	Q6IA69	-0.00269	0.842967	0.519352	NADSYN1	Glutamine-	NADE_HUM	10	0.998134
30min_MT.	P50502	0.036223	0.843072	0.519375	ST13	Hsc70-inte	F10A1_HU	10	1.025426
30min_MT.	Q96LR5	-0.16111	0.843309	0.51944	UBE2E2	Ubiquitin-c	UB2E2_HU	3	0.894336
30min_MT.	O95376	0.040538	0.843427	0.519471	ARIH2	E3 ubiquiti	ARI2_HUM.	11	1.028497
30min_MT.	P13693	0.030446	0.843681	0.519546	TPT1	Translation	TCTP_HUM	9	1.021328
30min_MT.	Q96DB5	-0.11875	0.843683	0.519546	RMDN1	Regulator c	RMD1_HUM	11	0.920983
30min_MT.	Q8IZ07	0.033615	0.844434	0.519574	ANKRD13A	Ankyrin rep	AN13A_HU	4	1.023574
30min_MT.	Q969Q0	-0.03422	0.844129	0.519574	RPL36AL	Ribosomal	RL36L_HUM	1	0.97656
15min_MT.	Q96BP3	-0.06502	0.844519	0.519574	PPWD1	Peptidylpr(	PPWD1_HU	11	0.955934
15min_MT.	Q9BUT9	-0.40168	0.844287	0.519574	MCRIP2	MAPK regul	MCRI2_HU	1	0.756977
30min_MT.	Q9Y3C0	0.556376	0.844267	0.519574	WASHC3	WASH com	WASC3_HU	2	1.470571
30min_MT.	Q13451	0.049282	0.845127	0.519823	FKBP5	Peptidyl-pr	FKBP5_HU	16	1.03475
30min_MT.	Q9GZY8	-0.16777	0.845127	0.519823	MFF	Mitochond	MFF_HUM/	1	0.89022
15min_MT.	Q9Y2G5	-0.02282	0.845007	0.519823	POFUT2	GDP-fucose	OFUT2_HU	9	0.984305
30min_MT.	O94888	0.013203	0.845733	0.52008	UBXN7	UBX domai	UBXN7_HU	12	1.009194
30min_MT.	O00468	0.007859	0.846254	0.52027	AGRN	Agrin	AGRIN_HU	36	1.005463
15min_MT.	Q14204	0.006864	0.846211	0.52027	DYNC1H1	Cytoplasmi	DYHC1_HU	101	1.004769
30min_MT.	Q99733	0.038955	0.846708	0.520413	NAP1L4	Nucleosom	NP1L4_HU	8	1.02737
30min_MT.	O43670	-0.17151	0.84728	0.520492	ZNF207	BUB3-inter	ZN207_HU	3	0.887915
15min_MT.	Q12982	-0.10359	0.847225	0.520492	BNIP2	BCL2/aden	BNIP2_HUM	3	0.930711
15min_MT.	Q9BSH4	-0.08463	0.847253	0.520492	TACO1	Translation	TACO1_HU	7	0.943026
30min_MT.	Q9UBP6	0.178215	0.847133	0.520492	METTL1	tRNA (guan	TRMB_HUM	3	1.131483
30min_MT.	Q9Y6X3	0.005883	0.847502	0.520562	MAU2	MAU2 chro	SCC4_HUM	3	1.004086
30min_MT.	Q13637	0.090032	0.848255	0.52073	RAB32	Ras-related	RAB32_HU	6	1.064394
15min_MT.	Q9BTY2	0.038277	0.848161	0.52073	FUCA2	Plasma alpl	FUCO2_HU	15	1.026887
30min_MT.	O95347	-0.06363	0.848389	0.520764	SMC2	Structural r	SMC2_HUM	21	0.956853
30min_MT.	P17405	-0.20412	0.849018	0.521031	SMPD1	Sphingomy	ASM_HUM/	4	0.86807
30min_MT.	Q15366	0.013282	0.849023	0.521031	PCBP2	Poly(rC)-bir	PCBP2_HU	15	1.009249
30min_MT.	Q6YP21	-0.02273	0.849007	0.521031	KYAT3	Kynurenine	KAT3_HUM	19	0.984365
15min_MT.	Q9H1B7	0.247424	0.849433	0.52116	IRF2BPL	Probable E3	I2BPL_HUM	1	1.187086
30min_MT.	P04792	-0.01543	0.849601	0.521222	HSPB1	Heat shock	HSPB1_HU	12	0.989361
15min_MT.	Q06787	0.011259	0.849852	0.521335	FMR1	Fragile X m	FMR1_HUM	6	1.007834
30min_MT.	Q8WVJ2	0.019973	0.850068	0.521426	NUDCD2	NudC doma	NUDC2_HU	5	1.01394
30min_MT.	P34741	-0.1287	0.850576	0.521657	SDC2	Syndecan-2	SDC2_HUM	3	0.914658
15min_MT.	P67936	-0.03768	0.850794	0.521709	TPM4	Tropomyos	TPM4_HUM	19	0.974224
15min_MT.	P22392	0.014376	0.850908	0.521737	NME2	Nucleoside	NDKB_HUM	14	1.010015
30min_MT.	Q15172	-0.21238	0.850978	0.521739	PPP2R5A	Serine/thre	2A5A_HUM	4	0.863113
30min_MT.	Q5F1R6	0.083373	0.851243	0.521861	DNAJC21	DnaJ homo	DJC21_HU	4	1.059492
30min_MT.	P12109	0.059319	0.851332	0.521874	COL6A1	Collagen al	CO6A1_HU	14	1.041974
15min_MT.	P12955	-0.0005	0.851896	0.521982	PEPD	Xaa-Pro dip	PEPD_HUM	19	0.999656
15min_MT.	P18754	-0.00671	0.851974	0.521982	RCC1	Regulator c	RCC1_HUM	7	0.995363
30min_MT.	P30519	0.184781	0.851935	0.521982	HMOX2	Heme oxyg	HMOX2_HU	5	1.136645
15min_MT.	Q13888;Q(	-0.04437	0.851643	0.521982	GTF2H2;GT	General tra	TF2H2_HU	4	0.969713

15min_MT.	Q9UHD8	-0.01663	0.851946	0.521982	SEPTIN9	Septin-9	SEPT9_HUM	23	0.988537
15min_MT.	Q96AJ9	-0.47596	0.852162	0.522056	VTI1A	Vesicle tran	VTI1A_HUM	1	0.71899
30min_MT.	O15514	0.120425	0.852453	0.522193	POLR2D	DNA-direct	RPB4_HUM	4	1.087055
30min_MT.	P78344	-0.04506	0.852736	0.522285	EIF4G2	Eukaryotic	IF4G2_HUM	26	0.969249
15min_MT.	P46934	0.130368	0.852822	0.522296	NEDD4	E3 ubiquiti	NEDD4_HU	3	1.094573
15min_MT.	P78332	0.020603	0.853233	0.522426	RBM6	RNA-bindir	RBM6_HUM	5	1.014383
15min_MT.	Q9Y696	-0.01909	0.853218	0.522426	CLIC4	Chloride in	CLIC4_HUM	15	0.986854
30min_MT.	O60341	-0.11519	0.853414	0.522426	KDM1A	Lysine-spec	KDM1A_HU	7	0.923263
30min_MT.	P98179	0.13197	0.853434	0.522426	RBM3	RNA-bindir	RBM3_HUM	3	1.095789
30min_MT.	O60437	0.135172	0.853709	0.522513	PPL	Periplakin	PEPL_HUM	23	1.098223
15min_MT.	Q96DB5	-0.11089	0.853861	0.522565	RMDN1	Regulator c	RMD1_HUM	11	0.926014
15min_MT.	P62081	-0.00481	0.85413	0.522624	RPS7	Small ribos	RS7_HUMA	8	0.99667
15min_MT.	Q6UXD5	0.018815	0.854225	0.522624	SEZ6L2	Seizure 6-lil	SE6L2_HUM	5	1.013127
30min_MT.	O15031	0.035596	0.854326	0.522645	PLXNB2	Plexin-B2	PLXB2_HUI	21	1.02498
30min_MT.	P18124	0.126058	0.854838	0.522917	RPL7	Large ribos	RL7_HUMA	10	1.091308
15min_MT.	Q8N0X7	0.027653	0.855191	0.523051	SPART	Spartin	SPART_HUM	10	1.019352
30min_MT.	Q2KHT3	-0.00999	0.855342	0.523103	CLEC16A	Protein CLE	CL16A_HUI	10	0.993101
30min_MT.	Q9UPN7	0.052124	0.855533	0.523179	PPP6R1	Serine/thre	PP6R1_HUI	14	1.03679
15min_MT.	O15514	0.07501	0.855696	0.523197	POLR2D	DNA-direct	RPB4_HUM	4	1.053368
15min_MT.	O00429	-0.03657	0.856128	0.52342	DNM1L	Dynamin-1	DNM1L_HU	28	0.974967
15min_MT.	P55011	0.146571	0.856401	0.523478	SLC12A2	Solute carri	S12A2_HUI	6	1.106935
30min_MT.	Q8N5F7	-0.08758	0.856326	0.523478	NKAP	NF-kappa-B	NKAP_HUM	4	0.941099
15min_MT.	Q9BV86	0.222545	0.856424	0.523478	NTMT1	N-terminal	NTM1A_HU	2	1.16679
30min_MT.	P51151	-0.04202	0.856705	0.523609	RAB9A	Ras-related	RAB9A_HUI	2	0.971295
15min_MT.	Q14444	0.031265	0.8571	0.523664	CAPRIN1	Caprin-1	CAPR1_HUI	11	1.021908
15min_MT.	Q5T8P6	0.004808	0.857093	0.523664	RBM26	RNA-bindir	RBM26_HU	12	1.003338
30min_MT.	Q86XE5	-0.36826	0.857129	0.523664	HOGA1	4-hydroxy-2	HOGA1_HU	1	0.774718
15min_MT.	Q9BRJ7	-0.04082	0.857065	0.523664	NUDT16L1	Tudor-inter	TIRR_HUM/	2	0.972103
15min_MT.	Q9NQW7	0.02336	0.856921	0.523664	XPNPEP1	Xaa-Pro am	XPP1_HUM	19	1.016324
30min_MT.	P05198	-0.02615	0.857979	0.524019	EIF2S1	Eukaryotic	IF2A_HUM/	22	0.982041
15min_MT.	P50991	0.009332	0.858155	0.524052	CCT4	T-complex	TCPD_HUM	32	1.00649
15min_MT.	Q13442	-0.03442	0.858234	0.524052	PDAP1	28 kDa heat	HAP28_HU	7	0.976426
30min_MT.	Q9BUF5	-0.14448	0.858207	0.524052	TUBB6	Tubulin bet	TBB6_HUM	8	0.904706
30min_MT.	O94901	0.010538	0.858806	0.524157	SUN1	SUN domai	SUN1_HUM	3	1.007331
15min_MT.	Q5ST30	0.155835	0.858774	0.524157	VARS2	Valine--tRN	SYVM_HUM	5	1.114067
15min_MT.	Q7Z6V5	-0.38094	0.858809	0.524157	ADAT2	tRNA-speci	ADAT2_HUI	3	0.767938
30min_MT.	Q8N4Q1	0.174708	0.85864	0.524157	CHCHD4	Mitochond	MIA40_HUI	2	1.128736
30min_MT.	Q7Z3E2	0.226614	0.858896	0.52417	CCDC186	Coiled-coil	CC186_HUI	8	1.170086
30min_MT.	Q13310	0.039351	0.85899	0.524186	PABPC4	Polyadenyl	PABP4_HUI	8	1.027651
15min_MT.	O15382	0.035056	0.859203	0.524276	BCAT2	Branched-c	BCAT2_HUI	18	1.024597
30min_MT.	Q9Y5T5	-0.15219	0.859581	0.524465	USP16	Ubiquitin c	UBP16_HU	1	0.899885
15min_MT.	Q13643	-0.10161	0.859948	0.524567	FHL3	Four and a	FHL3_HUM	4	0.931994
30min_MT.	Q66K74	0.12517	0.860083	0.524567	MAP1S	Microtubul	MAP1S_HU	3	1.090636
30min_MT.	Q9BSR8	0.37377	0.860024	0.524567	YIPF4	Protein YIP	YIPF4_HUM	2	1.295735
15min_MT.	Q7L9L4	-0.10911	0.860247	0.524626	MOB1B	MOB kinase	MOB1B_HU	7	0.927157
15min_MT.	Q16643	0.035349	0.860756	0.524799	DBN1	Drebrin	DREB_HUM	22	1.024805
15min_MT.	Q99497	0.012414	0.860782	0.524799	PARK7	Parkinson c	PARK7_HUI	14	1.008642
15min_MT.	P55081	0.064333	0.860895	0.524817	MFAP1	Microfibrill	MFAP1_HU	3	1.045602
15min_MT.	Q9UNQ2	0.025964	0.861093	0.524896	DIMT1	Dimethylac	DIM1_HUM	4	1.01816
15min_MT.	Q01844	-0.01921	0.86152	0.52501	EWSR1	RNA-bindir	EWS_HUM/	6	0.986771
15min_MT.	Q7KZ85	-0.01355	0.861548	0.52501	SUPT6H	Transcripti	SPT6H_HUI	17	0.990655
15min_MT.	Q9Y2V2	-0.07813	0.861518	0.52501	CARHSP1	Calcium-re	CHSP1_HUI	6	0.947288

30min_MT.	P39023	0.078041	0.862381	0.525313	RPL3	Large ribos	RL3_HUMA	13	1.055583
15min_MT.	Q13287	-0.0304	0.862735	0.525455	NMI	N-myc-inte	NMI_HUMA	10	0.979147
15min_MT.	O00232	0.01432	0.862829	0.525463	PSMD12	26S protea	PSD12_HU	20	1.009975
15min_MT.	P21266	-0.02825	0.863071	0.525492	GSTM3	Glutathion	GSTM3_HU	16	0.980612
15min_MT.	Q16822	0.02142	0.863077	0.525492	PCK2	Phosphoen	PCKGM_HU	30	1.014958
15min_MT.	Q5BKZ1	-0.22744	0.863014	0.525492	ZNF326	DBIRD com	ZN326_HU	5	0.85415
15min_MT.	Q9NVM6	-0.15251	0.863339	0.525569	DNAJC17	DnaJ homo	DJC17_HU	3	0.899682
30min_MT.	Q9Y223	0.182478	0.863572	0.525629	GNE	Bifunctiona	GLCNE_HU	10	1.134831
15min_MT.	P49821	0.118577	0.86387	0.525729	NDUFV1	NADH dehy	NDUV1_HU	6	1.085664
30min_MT.	P51970	-0.04419	0.863966	0.525732	NDUFA8	NADH dehy	NDUA8_HU	4	0.969837
15min_MT.	O75822	0.084781	0.864091	0.52574	EIF3J	Eukaryotic	EIF3J_HUM	12	1.060527
15min_MT.	O00505	0.072119	0.86419	0.525755	KPNA3	Importin su	IMA4_HUM	10	1.05126
30min_MT.	P51956	0.17297	0.864414	0.525755	NEK3	Serine/thre	NEK3_HUM	3	1.127377
30min_MT.	P62244	0.013635	0.864299	0.525755	RPS15A	Small ribos	RS15A_HU	4	1.009496
30min_MT.	Q6ZWJ1	-0.26343	0.864536	0.525755	STXBP4	Syntaxin-bi	STXB4_HUM	5	0.833104
15min_MT.	Q9Y4B6	-0.18111	0.864586	0.525755	DCAF1	DDB1- and	DCAF1_HU	2	0.882026
30min_MT.	Q6PGP7	-0.18296	0.864785	0.525816	SKIC3	Superkiller	SKI3_HUMA	8	0.880893
30min_MT.	Q9NUG6	0.322614	0.86482	0.525816	PDRG1	p53 and DN	PDRG1_HU	1	1.250594
30min_MT.	Q9Y5Y2	0.224529	0.864937	0.525846	NUBP2	Cytosolic F	NUBP2_HU	2	1.168396
30min_MT.	P33993	-0.15724	0.865158	0.52594	MCM7	DNA replica	MCM7_HU	6	0.896741
30min_MT.	P61960	-0.03124	0.865252	0.525956	UFM1	Ubiquitin-f	UFM1_HUM	4	0.97858
30min_MT.	P54252	0.367933	0.865421	0.526018	ATXN3	Ataxin-3	ATX3_HUM	5	1.290503
15min_MT.	P26641	-0.00942	0.86573	0.526124	EEF1G	Elongation	EF1G_HUM	23	0.99349
30min_MT.	Q8IUR0	1.278261	0.865697	0.526124	TRAPPC5	Trafficking	TPPC5_HU	1	2.425464
15min_MT.	O75534	0.029559	0.865866	0.526166	CSDE1	Cold shock	CSDE1_HU	31	1.0207
30min_MT.	O95197	0.005429	0.866142	0.52617	RTN3	Reticulon-3	RTN3_HUM	4	1.00377
30min_MT.	O00139	-0.03817	0.866234	0.526185	KIF2A	Kinesin-like	KIF2A_HUM	10	0.973893
30min_MT.	P42025	0.046455	0.866552	0.526312	ACTR1B	Beta-centra	ACTY_HUM	6	1.032724
15min_MT.	Q13405	0.297796	0.866951	0.526444	MRPL49	Large ribos	RM49_HUM	2	1.229265
15min_MT.	Q86V81	0.016526	0.866996	0.526444	ALYREF	THO compl	THOC4_HU	6	1.011521
30min_MT.	Q9H4Z3	0.063442	0.866921	0.526444	PCIF1	mRNA (2'-O	CAPAM_HU	5	1.044956
30min_MT.	O14497	-0.02531	0.867331	0.526544	ARID1A	AT-rich inte	ARI1A_HUM	17	0.982611
15min_MT.	P35658	0.016571	0.867431	0.526544	NUP214	Nuclear po	NU214_HU	36	1.011552
30min_MT.	P55145	-0.04397	0.867394	0.526544	MANF	Mesenceph	MANF_HUM	8	0.969985
15min_MT.	Q96R27	-0.31804	0.86783	0.526745	OR2M4	Olfactory re	OR2M4_HU	1	0.802157
30min_MT.	P11498	0.011788	0.86832	0.526952	PC	Pyruvate ca	PYC_HUMA	24	1.008204
15min_MT.	P27708	0.087266	0.868339	0.526952	CAD	Multifuncti	PYR1_HUM	23	1.062355
30min_MT.	Q96SY0	-0.02765	0.868372	0.526952	INTS14	Integrator c	INT14_HUM	9	0.981014
30min_MT.	P01036	-0.06183	0.868745	0.526978	CST4	Cystatin-S	CYTS_HUMA	3	0.958049
30min_MT.	P56945	0.247204	0.868743	0.526978	BCAR1	Breast canc	BCAR1_HU	4	1.186905
15min_MT.	Q5JTZ9	0.08631	0.868618	0.526978	AARS2	Alanine--tR	SYAM_HUM	6	1.061651
30min_MT.	P0C7T5	-0.03593	0.86934	0.527293	ATXN1L	Ataxin-1-lik	ATX1L_HUM	3	0.975401
15min_MT.	Q9HCE5	-0.2346	0.869463	0.527327	METTL14	N6-adenosi	MET14_HU	2	0.849921
30min_MT.	P57088	-0.20848	0.869939	0.527508	TMEM33	Transmemb	TMM33_HU	4	0.865452
15min_MT.	Q7LG56	0.035069	0.869964	0.527508	RRM2B	Ribonucleo	RIR2B_HUM	5	1.024606
15min_MT.	Q96T23	-0.08659	0.870927	0.527888	RSF1	Remodeling	RSF1_HUM	6	0.941748
30min_MT.	Q9BQG2	-0.00629	0.870894	0.527888	NUDT12	NAD-cappe	NUD12_HU	3	0.995651
15min_MT.	Q8TDY4	-0.37143	0.871431	0.528063	ASAP3	Arf-GAP wit	ASAP3_HU	2	0.773017
30min_MT.	Q9H1K0	0.020218	0.871409	0.528063	RBSN	Rabenosyn-	RBNS5_HU	2	1.014113
30min_MT.	Q9ULT8	0.003816	0.871486	0.528063	HECTD1	E3 ubiquiti	HECD1_HU	44	1.002648
15min_MT.	Q14141	0.026066	0.871586	0.528082	SEPTIN6	Septin-6	SEPT6_HUM	5	1.018231
30min_MT.	P62891;Q5	-0.03899	0.871941	0.528257	RPL39;RPL	Large ribos	RL39_HUM	1	0.973339

15min_MT.	Q13451	0.023734	0.872202	0.528333	FKBP5	Peptidyl-pr	FKBP5_HU	16	1.016587
15min_MT.	Q9H3S7	0.086779	0.872522	0.528376	PTPN23	Tyrosine-pr	PTN23_HU	16	1.061996
30min_MT.	Q9H977	0.020375	0.872552	0.528376	WDR54	WD repeat-	WDR54_HU	3	1.014223
30min_MT.	Q9BZE9	-0.07138	0.872681	0.528378	ASPSCR1	Tether cont	ASPC1_HU	7	0.95173
15min_MT.	Q7Z6Z7	-0.01144	0.87292	0.528482	HUWE1	E3 ubiquiti	HUWE1_HU	109	0.9921
30min_MT.	Q96HJ9	0.066108	0.873278	0.528555	FMC1	Protein FM	FMC1_HUM	1	1.046888
30min_MT.	Q9H501	0.051961	0.873244	0.528555	ESF1	ESF1 homo	ESF1_HUM	12	1.036673
30min_MT.	Q9Y3B8	-0.01065	0.873145	0.528555	REXO2	Oligoribon	ORN_HUM	11	0.992643
15min_MT.	Q04941	0.1063	0.873579	0.528643	PLP2	Proteolipid	PLP2_HUM	1	1.076464
30min_MT.	Q13162	-0.01897	0.873593	0.528643	PRDX4	Peroxiredo	PRDX4_HU	11	0.986936
15min_MT.	Q9NPI6	0.021641	0.873798	0.528726	DCP1A	mRNA-deca	DCP1A_HU	4	1.015113
15min_MT.	Q9UDY4	0.080496	0.873993	0.528762	DNAJB4	DnaJ homo	DNJB4_HU	7	1.057381
30min_MT.	Q9H9P8	0.118807	0.874697	0.528984	L2HGDH	L-2-hydrox	L2HDH_HU	5	1.085837
15min_MT.	Q9UMX0	0.010459	0.874581	0.528984	UBQLN1	Ubiquilin-1	UBQL1_HU	11	1.007276
15min_MT.	Q96I99	0.023504	0.875087	0.528985	SUCLG2	Succinate--	SUCB2_HU	20	1.016425
30min_MT.	Q9HCG8	0.08199	0.875171	0.528985	CWC22	Pre-mRNA-	CWC22_HU	8	1.058477
30min_MT.	P46821	-0.01175	0.875657	0.529073	MAP1B	Microtubul	MAP1B_HU	3	0.991889
30min_MT.	Q96D71	0.000566	0.875606	0.529073	REPS1	RalBP1-ass	REPS1_HU	15	1.000393
15min_MT.	Q9BRP4	0.053212	0.875905	0.529142	PAAF1	Proteasom	PAAF1_HU	12	1.037572
15min_MT.	Q15011	-0.10576	0.876184	0.529228	HERPUD1	Homocyste	HERP1_HU	1	0.929318
30min_MT.	Q9BSU1	0.137446	0.876147	0.529228	PHAF1	Phagosome	PHAF1_HU	3	1.099956
15min_MT.	P30154	0.020287	0.876469	0.529281	PPP2R1B	Serine/thre	2AAB_HUM	13	1.014161
15min_MT.	Q6P6C2	0.271028	0.876798	0.529285	ALKBH5	RNA demet	ALKB5_HU	4	1.206667
15min_MT.	Q8NEZ5	0.044437	0.876818	0.529285	FBXO22	F-box only	FBX22_HU	10	1.03128
30min_MT.	Q9HCE7	0.015975	0.876557	0.529285	SMURF1	E3 ubiquiti	SMUF1_HU	2	1.011135
15min_MT.	Q9Y285	-0.04453	0.876688	0.529285	FARSA	Phenylalan	SYFA_HUM	10	0.969604
30min_MT.	Q9NWY4	-0.05651	0.876967	0.529334	HPF1	Histone PA	HPF1_HUM	2	0.961585
15min_MT.	Q13425	-0.11081	0.877087	0.529365	SNTB2	Beta-2-synt	SNTB2_HU	5	0.92607
30min_MT.	Q92783	-0.12263	0.877412	0.52948	STAM	Signal trans	STAM1_HU	8	0.918512
30min_MT.	Q8N7Z5	-0.12027	0.87749	0.529486	ANKRD31	Ankyrin rep	ANR31_HU	1	0.920018
15min_MT.	O00471	0.122158	0.877702	0.529491	EXOC5	Exocyst cor	EXOC5_HU	13	1.088361
15min_MT.	Q8TEU7;Q9	-0.208	0.877677	0.529491	RAPGEF6;R	Rap guanin	RPGF6_HU	1	0.865737
15min_MT.	Q8IUQ4	0.048235	0.877895	0.529567	SIAH1	E3 ubiquiti	SIAH1_HUM	1	1.033999
15min_MT.	Q7Z6B0	0.015818	0.878186	0.529702	CCDC91	Coiled-coil	CCD91_HU	2	1.011024
15min_MT.	Q14C86	-0.00361	0.878385	0.52974	GAPVD1	GTPase-acti	GAPD1_HU	32	0.997498
30min_MT.	Q9H081	0.223726	0.878336	0.52974	MIS12	Protein MIS	MIS12_HU	4	1.167746
15min_MT.	Q9NZZ3	-0.04772	0.8786	0.529763	CHMP5	Charged mu	CHMP5_HU	4	0.967465
15min_MT.	Q9Y4L1	0.012651	0.878575	0.529763	HYOU1	Hypoxia up	HYOU1_HU	44	1.008808
15min_MT.	A0JNW5	-0.00614	0.878898	0.529886	BLTP3B	Bridge-like	BLT3B_HUM	13	0.995756
30min_MT.	Q9H910	0.033339	0.879811	0.530314	JPT2	Jupiter mic	JUPI2_HUM	10	1.023378
30min_MT.	P63151	0.04625	0.880052	0.530399	PPP2R2A	Serine/thre	2ABA_HUM	11	1.032577
15min_MT.	Q00534	0.104724	0.880089	0.530399	CDK6	Cyclin-depe	CDK6_HUM	9	1.075289
15min_MT.	Q5T0F9	0.230403	0.880317	0.530457	CC2D1B	Coiled-coil	C2D1B_HU	1	1.173163
15min_MT.	O94925	0.03791	0.880673	0.530507	GLS	Glutamina	GLSK_HUM	4	1.026625
30min_MT.	P41208;Q1	-0.00689	0.880915	0.530507	CETN2;CETI	Centrin-2;C	CETN2_HU	1	0.995239
15min_MT.	P62807	0.016017	0.881013	0.530507	H2BC4	Histone H2	H2B1C_HU	8	1.011164
30min_MT.	Q71RC2	-0.12091	0.880884	0.530507	LARP4	La-related	LARP4_HU	5	0.919609
30min_MT.	Q8NBQ5	0.156599	0.880752	0.530507	HSD17B11	Estradiol 1	DHB11_HU	4	1.114657
15min_MT.	Q6GQQ9	-0.05294	0.881293	0.530594	OTUD7B	OTU domai	OTU7B_HU	2	0.963971
30min_MT.	Q8IWL3	0.044861	0.8815	0.530678	HSCB	Iron-sulfur	HSC20_HU	9	1.031584
15min_MT.	P62070	0.260843	0.881881	0.530784	RRAS2	Ras-related	RRAS2_HU	6	1.198179
15min_MT.	O00268	0.12749	0.882308	0.530796	TAF4	Transcripti	TAF4_HUM	1	1.092392

30min_MT.	P63244	0.010533	0.882041	0.530796	RACK1	Small ribos	RACK1_HU	18	1.007328
15min_MT.	Q9UEE9	0.005164	0.882185	0.530796	CFDP1	Craniofacia	CFDP1_HU	5	1.003586
15min_MT.	Q9Y2W2	0.036019	0.88227	0.530796	WBP11	WW domai	WBP11_HU	15	1.025281
30min_MT.	Q5T653	0.376559	0.882495	0.530868	MRPL2	Large ribos	RM02_HUN	1	1.298242
30min_MT.	Q96AB6	-0.09011	0.882643	0.530916	NTAN1	Protein N-t	NTAN1_HU	1	0.939452
15min_MT.	O94929	0.003711	0.883297	0.531216	ABLIM3	Actin-bindi	ABLM3_HU	5	1.002576
30min_MT.	Q9NNW7	-0.00208	0.883548	0.531297	TXNRD2	Thioredoxi	TRXR2_HU	15	0.998557
15min_MT.	Q9NY93	0.297744	0.883763	0.531304	DDX56	Probable A	DDX56_HU	1	1.229221
30min_MT.	Q9HBI1	0.150598	0.884033	0.531425	PARVB	Beta-parvir	PARVB_HU	5	1.11003
15min_MT.	P57721	-0.03946	0.884455	0.531516	PCBP3	Poly(rC)-bir	PCBP3_HU	2	0.973021
30min_MT.	P22314	-0.02687	0.884621	0.531575	UBA1	Ubiquitin-l	UBA1_HUN	35	0.981544
15min_MT.	Q7Z5K2	-0.02263	0.885148	0.53185	WAPL	Wings apar	WAPL_HUN	16	0.984439
30min_MT.	P62834	-0.12898	0.886099	0.53221	RAP1A	Ras-related	RAP1A_HU	2	0.914478
15min_MT.	Q96LR5	-0.1165	0.886185	0.53221	UBE2E2	Ubiquitin-c	UB2E2_HU	3	0.922426
15min_MT.	Q9UNP9	-0.02908	0.886223	0.53221	PPIE	Peptidyl-pr	PPIE_HUM	10	0.980046
15min_MT.	A6NHQ2	0.055943	0.886294	0.532212	FBLL1	rRNA/tRNA	FBLL1_HUN	1	1.039538
15min_MT.	P63104	0.031089	0.886572	0.532338	YWHAZ	14-3-3 prot	1433Z_HU	14	1.021783
30min_MT.	Q9Y5B6	0.02685	0.88672	0.532345	PAXBP1	PAX3- and I	PAXB1_HU	1	1.018785
30min_MT.	Q08257	0.02463	0.886867	0.532392	CRYZ	Quinone ox	QOR_HUM	18	1.017219
15min_MT.	O75792	0.193985	0.887158	0.532397	RNASEH2A	Ribonuclea	RNH2A_HU	2	1.143919
15min_MT.	Q13263	0.016306	0.887216	0.532397	TRIM28	Transcripti	TIF1B_HUN	27	1.011366
30min_MT.	Q99523	0.021642	0.887022	0.532397	SORT1	Sortilin	SORT_HUM	12	1.015114
30min_MT.	P05023	0.059834	0.887289	0.5324	ATP1A1	Sodium/po	AT1A1_HU	23	1.042346
15min_MT.	Q53SF7	-0.13322	0.887378	0.532413	COBLL1	Cordon-ble	COBL1_HU	1	0.911797
15min_MT.	P50990	0.005751	0.887485	0.532429	CCT8	T-complex	TCPQ_HUM	36	1.003994
15min_MT.	Q7Z6B7	0.08002	0.887829	0.532521	SRGAP1	SLIT-ROBO	SRGP1_HU	10	1.057033
30min_MT.	O75635	0.005526	0.887907	0.532527	SERPINB7	Serpin B7	SPB7_HUM	3	1.003838
30min_MT.	P22059	0.000391	0.888247	0.532584	OSBP	Oxysterol-b	OSBP1_HU	28	1.000271
15min_MT.	Q75N03	0.261996	0.888276	0.532584	CBLL1	E3 ubiquiti	HAKAI_HUN	2	1.199137
15min_MT.	O95817	-0.03734	0.888503	0.532639	BAG3	BAG family	BAG3_HUM	15	0.974451
30min_MT.	Q9NUQ3	0.045424	0.888732	0.532664	TXLNG	Gamma-tax	TXLNG_HU	10	1.031986
30min_MT.	Q9Y530	0.154393	0.888624	0.532664	OARD1	ADP-ribose	OARD1_HU	3	1.112954
30min_MT.	Q96FN9	-0.40439	0.888878	0.5327	DTD2	D-aminoac	DTD2_HUM	3	0.755554
30min_MT.	Q8NCA5	0.196654	0.889082	0.532753	FAM98A	Protein FAI	FA98A_HU	2	1.146037
15min_MT.	O95429	0.283743	0.88921	0.532777	BAG4	BAG family	BAG4_HUM	1	1.217349
15min_MT.	O14802	-0.14881	0.889347	0.532818	POLR3A	DNA-direct	RPC1_HUM	5	0.901993
15min_MT.	O95071	0.048076	0.88989	0.532952	UBR5	E3 ubiquiti	UBR5_HUM	28	1.033886
15min_MT.	O95782	0.003026	0.889843	0.532952	AP2A1	AP-2 compl	AP2A1_HU	32	1.0021
30min_MT.	Q01658	0.055506	0.889799	0.532952	DR1	Protein Dr1	NC2B_HUM	4	1.039223
15min_MT.	Q12888	-0.00236	0.890211	0.532952	TP53BP1	TP53-bindi	TP53B_HU	55	0.998368
30min_MT.	Q6IA86	0.010508	0.890251	0.532952	ELP2	Elongator c	ELP2_HUM	14	1.00731
15min_MT.	Q96IJ6	0.035505	0.890134	0.532952	GMPPA	Mannose-1	GMPPA_HU	9	1.024916
30min_MT.	Q6NSW5;Q	-0.17259	0.890769	0.53318	DENND10P	Putative DI	DE10B_HU	2	0.887246
30min_MT.	O95163	0.061646	0.890926	0.533233	ELP1	Elongator c	ELP1_HUM	18	1.043656
30min_MT.	Q86YS7	-0.05116	0.891279	0.533363	C2CD5	C2 domain	C2CD5_HU	15	0.965158
30min_MT.	Q8IV38	-0.01005	0.891241	0.533363	ANKMY2	Ankyrin rep	ANKY2_HU	4	0.99306
30min_MT.	O95159	-0.09023	0.891493	0.533394	ZFPL1	Zinc finger	ZFPL1_HUN	3	0.939376
15min_MT.	P11908	0.183409	0.891536	0.533394	PRPS2	Ribose-pho	PRPS2_HU	4	1.135564
30min_MT.	Q9NQ84	0.053277	0.891894	0.533567	GPRC5C	G-protein c	GPC5C_HU	4	1.037619
15min_MT.	Q08211	0.021364	0.892409	0.533671	DHX9	ATP-depen	DHX9_HUN	36	1.014919
15min_MT.	Q8N3X6	0.116349	0.892227	0.533671	LCORL	Ligand-dep	LCORL_HU	1	1.083988
30min_MT.	Q9Y4X5	-0.03624	0.892376	0.533671	ARIH1	E3 ubiquiti	ARI1_HUM	10	0.975193

30min_MT.	Q9P0K7	-0.10083	0.892644	0.533771	RAI14	Ankycorbir	RAI14_HUM	4	0.932496
15min_MT.	P26373	0.015685	0.892964	0.533876	RPL13	Large ribos	RL13_HUM	8	1.010931
15min_MT.	Q13277	-0.00204	0.893023	0.533876	STX3	Syntaxin-3	STX3_HUM	3	0.998586
15min_MT.	O95861	0.040553	0.893344	0.533911	BPNT1	3'(2'),5'-bis	BPNT1_HU	14	1.028508
30min_MT.	P04899	-0.03245	0.893277	0.533911	GNAI2	Guanine nu	GNAI2_HU	3	0.977758
30min_MT.	Q14789	-0.01326	0.893344	0.533911	GOLGB1	Golgin subf	GOGB1_HU	13	0.990851
30min_MT.	Q96P20	-0.03427	0.893356	0.533911	NLRP3	NACHT, LRF	NLRP3_HU	1	0.976523
30min_MT.	Q12800	0.104541	0.893493	0.533952	TFCP2	Alpha-globi	TFCP2_HUN	2	1.075153
15min_MT.	P46108	0.065193	0.893732	0.534021	CRK	Adapter mo	CRK_HUMA	17	1.046225
15min_MT.	Q14151	-0.07621	0.893942	0.534069	SAFB2	Scaffold att	SAFB2_HUN	15	0.948546
15min_MT.	P10768	-0.03321	0.894282	0.534162	ESD	S-formylglu	ESTD_HUM	10	0.97724
15min_MT.	Q3MHD2	-0.01272	0.894335	0.534162	LSM12	Protein LSN	LSM12_HU	4	0.991223
15min_MT.	Q92922	0.049887	0.894194	0.534162	SMARCC1	SWI/SNF cc	SMRC1_HU	12	1.035184
30min_MT.	P49368	0.011216	0.894577	0.534177	CCT3	T-complex	TCPG_HUM	36	1.007804
15min_MT.	O95620	-0.07303	0.894855	0.534277	DUS4L	tRNA-dihyd	DUS4L_HU	1	0.950641
30min_MT.	Q9BZM5	0.126879	0.895332	0.53452	ULBP2	UL16-bindi	ULBP2_HU	5	1.091929
15min_MT.	O15031	0.077048	0.895677	0.534565	PLXNB2	Plexin-B2	PLXB2_HU	21	1.054857
30min_MT.	O43447	0.008431	0.895667	0.534565	PPIH	Peptidyl-pr	PPIH_HUM	6	1.005861
15min_MT.	Q99496	-0.13541	0.895681	0.534565	RNF2	E3 ubiquiti	RING2_HU	2	0.91041
15min_MT.	Q49AR2	-0.0506	0.895863	0.534593	C5orf22	UPF0489 p	CE022_HU	6	0.965531
30min_MT.	Q9NQ94	0.00103	0.895993	0.534629	A1CF	APOBEC1 c	A1CF_HUM	15	1.000714
30min_MT.	Q13153	-0.0148	0.896175	0.534667	PAK1	Serine/thre	PAK1_HUM	13	0.989791
15min_MT.	Q5XPI4	0.0442	0.896193	0.534667	RNF123	E3 ubiquiti	RN123_HU	2	1.031112
30min_MT.	O75781	0.176918	0.896393	0.534744	PALM	Paralemmi	PALM_HUN	4	1.130467
30min_MT.	O43324	-0.03665	0.896931	0.534904	EEF1E1	Eukaryotic	MCA3_HUN	6	0.974914
30min_MT.	P78371	0.007451	0.897554	0.535193	CCT2	T-complex	TCPB_HUM	27	1.005178
30min_MT.	O43684	0.047282	0.898235	0.535446	BUB3	Mitotic che	BUB3_HUN	10	1.033316
15min_MT.	Q86W50	0.002742	0.898319	0.535446	METTL16	RNA N6-ad	MET16_HU	8	1.001902
15min_MT.	Q9UHQ1	-0.33155	0.898309	0.535446	NARF	Nuclear pre	NARF_HUN	3	0.794681
30min_MT.	Q05086	0.021525	0.898896	0.535708	UBE3A	Ubiquitin-p	UBE3A_HU	21	1.015032
30min_MT.	Q8NB15	-0.07349	0.899099	0.535788	ZNF511	Zinc finger	ZN511_HU	2	0.950339
30min_MT.	Q5T4S7	-0.00495	0.89925	0.535796	UBR4	E3 ubiquiti	UBR4_HUN	66	0.996575
30min_MT.	Q8WXX5	-0.03451	0.899408	0.535824	DNAJC9	DnaJ homo	DNJC9_HU	5	0.976363
15min_MT.	Q8NHU6	-0.33527	0.89974	0.535925	TDRD7	Tudor dom	TDRD7_HU	1	0.792636
15min_MT.	Q86YB8	-0.18952	0.899876	0.535965	ERO1B	ERO1-like p	ERO1B_HU	6	0.8769
30min_MT.	Q86YS3	-0.00549	0.900878	0.536439	RAB11FIP4	Rab11 fami	RFIP4_HUN	6	0.996201
30min_MT.	Q8IWV7	0.045055	0.900778	0.536439	UBR1	E3 ubiquiti	UBR1_HUN	7	1.031723
15min_MT.	Q9BUP3	0.384927	0.900819	0.536439	HTATIP2	Oxidoredu	HTAI2_HUN	3	1.305794
15min_MT.	Q9NRA2	-0.37098	0.900996	0.536469	SLC17A5	Sialin	S17A5_HU	1	0.773255
15min_MT.	Q9NZN5	-0.05567	0.901101	0.536491	ARHGEF12	Rho guanin	ARHGC_HU	33	0.962151
15min_MT.	Q8WUH1	-0.09655	0.901179	0.536496	CHURC1	Protein Chu	CHUR_HUN	2	0.935267
30min_MT.	P27695	0.02514	0.901313	0.536535	APEX1	DNA repair	APEX1_HU	9	1.017578
30min_MT.	O14981	0.164396	0.901501	0.536606	BTAF1	TATA-bindi	BTAF1_HUN	6	1.120697
30min_MT.	P21741	0.062922	0.902279	0.536935	MDK	Midkine	MK_HUMA	2	1.044579
15min_MT.	O60934	0.034868	0.902726	0.537131	NBN	Nibrin	NBN_HUMA	4	1.024463
30min_MT.	P17936	0.098591	0.902947	0.537183	IGFBP3	Insulin-like	IBP3_HUMA	6	1.070728
15min_MT.	P18859	-0.3826	0.903083	0.537183	ATP5PF	ATP syntha	ATP5J_HUN	2	0.767052
15min_MT.	Q15369	0.003369	0.903129	0.537183	ELOC	Elongin-C	ELOC_HUM	7	1.002338
15min_MT.	Q7KZF4	0.001454	0.903208	0.537183	SND1	Staphyloco	SND1_HUN	37	1.001008
15min_MT.	O14818	-0.01933	0.903456	0.537198	PSMA7	Proteasom	PSA7_HUM	12	0.986688
15min_MT.	Q8IZ13	0.117758	0.903801	0.537358	FAM200C	Protein FAN	F200C_HU	1	1.085047
15min_MT.	Q00535	-0.20171	0.904446	0.537534	CDK5	Cyclin-depe	CDK5_HUN	5	0.86952

30min_MT.	Q0VDF9	-0.01181	0.904502	0.537534	HSPA14	Heat shock	HSP7E_HUI	15	0.991847
30min_MT.	P30043	0.021551	0.904799	0.537546	BLVRB	Flavin redu	BLVRB_HUI	7	1.01505
30min_MT.	Q13564	0.03002	0.90469	0.537546	NAE1	NEDD8-acti	ULA1_HUM	16	1.021027
15min_MT.	Q9P2J5	0.044041	0.904671	0.537546	LARS1	Leucine--tR	SYLC_HUM.	21	1.030997
15min_MT.	Q16222	0.007514	0.905624	0.537914	UAP1	UDP-N-acet	UAP1_HUM	8	1.005222
30min_MT.	Q9NTG7	-0.02903	0.905731	0.537937	SIRT3	NAD-depen	SIR3_HUM/	2	0.980079
15min_MT.	P12694	-0.03888	0.905921	0.537968	BCKDHA	2-oxoisoval	ODBA_HUM	7	0.973408
15min_MT.	P62701	-0.03048	0.906029	0.537975	RPS4X	Small ribos	RS4X_HUM	10	0.979094
15min_MT.	Q9HAD4	-0.07077	0.90607	0.537975	WDR41	WD repeat-	WDR41_HU	3	0.952131
30min_MT.	P12268	-0.02712	0.906612	0.538174	IMPDH2	Inosine-5'-r	IMDH2_HU	26	0.981378
30min_MT.	O75044	0.059339	0.907051	0.538302	SRGAP2	SLIT-ROBO I	SRGP2_HUI	1	1.041988
30min_MT.	P49959	-0.02156	0.906965	0.538302	MRE11	Double-stra	MRE11_HU	22	0.985166
30min_MT.	Q86VN1	-0.03123	0.907103	0.538302	VPS36	Vacuolar pı	VPS36_HUI	14	0.978588
30min_MT.	P15291	0.108094	0.907452	0.538436	B4GALT1	Beta-1,4-ga	B4GT1_HUI	1	1.077804
30min_MT.	P63010	-0.04144	0.907487	0.538436	AP2B1	AP-2 compl	AP2B1_HUI	31	0.971686
15min_MT.	Q9GZX9	0.234632	0.907535	0.538436	TWSG1	Twisted gas	TWSG1_HU	1	1.176607
15min_MT.	Q92599	0.051612	0.907888	0.538552	SEPTIN8	Septin-8	SEPT8_HUM	5	1.036422
15min_MT.	Q15370	-0.03276	0.908184	0.538616	ELOB	Elongin-B	ELOB_HUM	8	0.977546
15min_MT.	Q6ZWJ1	-0.11703	0.908751	0.538799	STXBP4	Syntaxin-bi	STXB4_HUI	5	0.922084
30min_MT.	Q96S97	0.312363	0.908767	0.538799	MYADM	Myeloid-as:	MYADM_HU	1	1.24174
30min_MT.	P05114	0.052593	0.908926	0.538853	HMGN1	Non-histon	HMGN1_HI	6	1.037127
15min_MT.	P49590	0.008447	0.909338	0.538943	HARS2	Histidine--t	SYHM_HUM	9	1.005872
30min_MT.	Q03111	0.11176	0.909354	0.538943	MLLT1	Protein ENI	ENL_HUMA	3	1.080545
15min_MT.	Q14108	-0.25114	0.909153	0.538943	SCARB2	Lysosome n	SCRB2_HUI	5	0.840231
15min_MT.	P00883	-0.19621	0.909474	0.538952	ALDOA	Fructose-bi	ALDOA_RAI	1	0.872839
30min_MT.	P68036	-0.00853	0.909717	0.539035	UBE2L3	Ubiquitin-c	UB2L3_HUI	7	0.994105
30min_MT.	O95716	0.54945	0.910237	0.539118	RAB3D	Ras-related	RAB3D_HU	3	1.463527
30min_MT.	P18085	-0.07198	0.910235	0.539118	ARF4	ADP-ribosy	ARF4_HUM	5	0.951328
30min_MT.	P26641	-0.00122	0.910126	0.539118	EEF1G	Elongation	EF1G_HUM	23	0.999153
15min_MT.	P36404	-0.049	0.910545	0.539118	ARL2	ADP-ribosy	ARL2_HUM	4	0.966605
15min_MT.	Q53S33	-0.09352	0.910074	0.539118	BOLA3	BolA-like pı	BOLA3_HUI	4	0.937233
15min_MT.	Q86WA6	-0.07057	0.910459	0.539118	BPHL	Valacyclovi	BPHL_HUM	8	0.952259
30min_MT.	Q9NRP2	0.101764	0.910498	0.539118	CMC2	COX assemI	COXM2_HU	2	1.073085
15min_MT.	Q9Y3X0	0.057852	0.910775	0.539172	CCDC9	Coiled-coil	CCDC9_HU	5	1.040915
15min_MT.	Q96S97	0.21812	0.911122	0.539337	MYADM	Myeloid-as:	MYADM_HI	1	1.163217
30min_MT.	Q9Y490	0.008122	0.911871	0.539657	TLN1	Talin-1	TLN1_HUM	115	1.005645
30min_MT.	Q9Y5J7	0.032175	0.91194	0.539658	TIMM9	Mitochond	TIM9_HUM	6	1.022553
15min_MT.	Q9P2T1	-0.00294	0.91318	0.54035	GMPR2	GMP reduc	GMPR2_HU	17	0.997963
30min_MT.	O75052	0.129499	0.91363	0.54051	NOS1AP	Carboxyl-te	CAPON_HU	3	1.093914
15min_MT.	Q68EM7	0.075336	0.913657	0.54051	ARHGAP17	Rho GTPase	RHG17_HU	7	1.053607
15min_MT.	O15355	-0.08345	0.913819	0.540565	PPM1G	Protein pho	PPM1G_HU	18	0.9438
15min_MT.	Q8WU79	-0.05254	0.913946	0.540599	SMAP2	Stromal me	SMAP2_HU	12	0.96424
30min_MT.	Q9BZ67	0.213743	0.91406	0.540626	FRMD8	FERM doma	FRMD8_HU	6	1.159693
30min_MT.	P13807	-0.10521	0.914433	0.540674	GYS1	Glycogen [s	GYS1_HUM	7	0.92967
15min_MT.	P55039	-0.21036	0.914276	0.540674	DRG2	Developme	DRG2_HUM	4	0.864322
15min_MT.	Q9Y2Z9	0.030149	0.914662	0.540696	COQ6	Ubiquinon	COQ6_HUM	1	1.021117
30min_MT.	Q9Y4P1	0.060847	0.914902	0.540797	ATG4B	Cysteine pr	ATG4B_HUI	3	1.043078
15min_MT.	O94992	0.070826	0.915545	0.541058	HEXIM1	Protein HE)	HEXI1_HUM	6	1.050318
30min_MT.	Q6ZS30	-0.25826	0.91569	0.541058	NBEAL1	Neurobeacl	NBEL1_HUI	4	0.836097
30min_MT.	Q92845	0.037962	0.915645	0.541058	KIFAP3	Kinesin-ass	KIFA3_HUM	4	1.026663
30min_MT.	Q99598	-0.03023	0.916255	0.54127	TSNAX	Translin-as:	TSNAX_HUI	14	0.979264
30min_MT.	Q8TBA6	-0.10999	0.916558	0.541407	GOLGA5	Golgin subf	GOGA5_HU	6	0.926596

15min_MT.	P32119	-0.007	0.916651	0.541422	PRDX2	Peroxiredo	PRDX2_HU	12	0.99516
15min_MT.	O75165	0.163137	0.916792	0.541464	DNAJC13	DnaJ homo	DJC13_HUI	13	1.119719
15min_MT.	P07237	-0.00552	0.917616	0.541503	P4HB	Protein dis	PDIA1_HUN	40	0.996181
30min_MT.	P31939	-0.00741	0.917286	0.541503	ATIC	Bifunctiona	PUR9_HUM	29	0.99488
30min_MT.	P46199	0.041786	0.91744	0.541503	MTIF2	Translation	IF2M_HUM	10	1.029388
30min_MT.	P48735	0.03116	0.917554	0.541503	IDH2	Isocitrate d	IDHP_HUM	23	1.021834
30min_MT.	P53367	-0.06187	0.917147	0.541503	ARFIP1	Arfaptin-1	ARFP1_HUI	9	0.958024
30min_MT.	Q13190	-0.20562	0.916964	0.541503	STX5	Syntaxin-5	STX5_HUM	5	0.867163
15min_MT.	Q9H814	-0.11983	0.917609	0.541503	PHAX	Phosphoryl	PHAX_HUM	8	0.920296
30min_MT.	Q9NQG5	-0.0522	0.917197	0.541503	RPRD1B	Regulation	RPR1B_HUI	10	0.964466
15min_MT.	P49189	0.029238	0.917904	0.541535	ALDH9A1	4-trimethyl	AL9A1_HUI	20	1.020473
30min_MT.	Q15208	0.33234	0.917963	0.541535	STK38	Serine/thre	STK38_HUN	2	1.259053
30min_MT.	Q9UGC7	0.052579	0.917927	0.541535	MTRF1L	Peptide cha	RF1ML_HU	3	1.037117
15min_MT.	O75475	-0.22504	0.918187	0.54158	PSIP1	PC4 and SFI	PSIP1_HUN	7	0.85557
30min_MT.	P29692	-0.02151	0.918235	0.54158	EEF1D	Elongation	EF1D_HUM	11	0.985201
30min_MT.	O00142	0.10961	0.918396	0.541634	TK2	Thymidine	KITM_HUM	4	1.078937
15min_MT.	P43490	0.05782	0.919576	0.542085	NAMPT	Nicotinami	NAMPT_HU	25	1.040891
30min_MT.	Q9GZT3	0.041606	0.919488	0.542085	SLIRP	SRA stem-lo	SLIRP_HUN	6	1.029259
30min_MT.	Q96JJ3	-0.10113	0.919904	0.542196	ELMO2	Engulfment	ELMO2_HU	2	0.932301
15min_MT.	Q9Y3B9	0.360034	0.919875	0.542196	RRP15	RRP15-like	RRP15_HUI	3	1.283456
30min_MT.	Q8NHZ8	0.003152	0.920122	0.542246	CDC26	Anaphase-p	CDC26_HU	1	1.002187
30min_MT.	P23193	0.006787	0.920835	0.542271	TCEA1	Transcripti	TCEA1_HUN	16	1.004715
30min_MT.	P41235	-0.06003	0.921235	0.542271	HNF4A	Hepatocyte	HNF4A_HU	6	0.959247
30min_MT.	P62316	0.004493	0.921035	0.542271	SNRPD2	Small nucle	SMD2_HUN	9	1.003119
30min_MT.	Q06830	0.007964	0.92065	0.542271	PRDX1	Peroxiredo	PRDX1_HU	16	1.005536
30min_MT.	Q14151	-0.07187	0.920644	0.542271	SAFB2	Scaffold att	SAFB2_HUN	15	0.951405
30min_MT.	Q6Q0C0	0.103545	0.921679	0.542271	TRAF7	E3 ubiquiti	TRAF7_HUN	3	1.07441
30min_MT.	Q6UW63	-0.02002	0.921773	0.542271	POGLUT2	Protein O-g	PLGT2_HUI	7	0.986222
30min_MT.	Q8IX04	0.030834	0.92153	0.542271	UEVLD	Ubiquitin-c	UEVLD_HUI	11	1.021603
30min_MT.	Q8TDZ2	-0.14702	0.92175	0.542271	MICAL1	[F-actin]-m	MICA1_HUI	11	0.903116
15min_MT.	Q969J3	-0.04325	0.920969	0.542271	BORCS5	BLOC-1-rela	BORC5_HU	4	0.970469
30min_MT.	Q9NRL3	0.037348	0.921835	0.542271	STRN4	Striatin-4	STRN4_HUI	10	1.026226
30min_MT.	O43399	-0.02199	0.922212	0.542327	TPD52L2	Tumor prot	TPD54_HUI	12	0.984873
30min_MT.	P32456	0.012612	0.922275	0.542327	GBP2	Guanylate-l	GBP2_HUM	17	1.00878
15min_MT.	Q8IVM0	0.064288	0.922079	0.542327	CCDC50	Coiled-coil	CCD50_HU	10	1.045569
15min_MT.	Q9UL25	0.05307	0.922141	0.542327	RAB21	Ras-related	RAB21_HUI	7	1.037471
15min_MT.	Q9UMX5	0.031426	0.922448	0.542388	NENF	Neudesin	NENF_HUM	7	1.022021
30min_MT.	Q96T60	-0.39123	0.922607	0.542441	PNKP	Bifunctiona	PNKP_HUM	1	0.762477
30min_MT.	P36404	-0.10962	0.923107	0.542612	ARL2	ADP-ribosy	ARL2_HUM	4	0.926834
15min_MT.	O60443	-0.02736	0.923363	0.542722	GSDME	Gasdermin-	GSDME_HU	4	0.981216
30min_MT.	Q16204	-0.04633	0.923487	0.542731	CCDC6	Coiled-coil	CCDC6_HU	14	0.968395
15min_MT.	Q8IUR7	0.088299	0.923517	0.542731	ARMC8	Armadillo r	ARMC8_HU	12	1.063116
15min_MT.	Q9BZG8	-0.00956	0.923888	0.542883	DPH1	2-(3-amino	DPH1_HUM	11	0.993394
15min_MT.	Q9UL12	0.031067	0.923964	0.542883	SARDH	Sarcosine d	SARDH_HU	9	1.021768
30min_MT.	O95749	-0.01848	0.924502	0.543024	GGPS1	Geranylgera	GGPPS_HU	7	0.987272
15min_MT.	Q9UKE5	0.057754	0.924494	0.543024	TNIK	TRAF2 and I	TNIK_HUM.	9	1.040844
15min_MT.	P12277	-0.00748	0.924682	0.543025	CKB	Creatine kir	KCRB_HUM	18	0.994831
30min_MT.	Q15437	-0.00458	0.924789	0.54303	SEC23B	Protein trai	SC23B_HUI	29	0.99683
30min_MT.	P62699	-0.08187	0.925231	0.543236	YPEL5	Protein yip	YPEL5_HUN	4	0.944832
30min_MT.	Q9Y5P4	-0.02482	0.92528	0.543236	CERT1	Ceramide tı	CERT_HUM	14	0.982946
30min_MT.	Q9UK41	-0.06875	0.925603	0.543385	VPS28	Vacuolar pı	VPS28_HUI	5	0.953463
30min_MT.	Q9NR45	0.059175	0.926213	0.543703	NANS	Sialic acid s	SIAS_HUMA	10	1.04187

30min_MT.	P02810	0.239492	0.927626	0.543941	PRH1	Salivary aci	PRPC_HUM	3	1.180577
15min_MT.	P60900	0.018007	0.927306	0.543941	PSMA6	Proteasome	PSA6_HUM	13	1.01256
15min_MT.	Q15165	0.172534	0.927544	0.543941	PON2	Serum para	PON2_HUM	3	1.127037
30min_MT.	Q32MZ4	0.033068	0.92769	0.543941	LRRFIP1	Leucine-ric	LRRF1_HUM	19	1.023186
30min_MT.	Q5TEC6	-0.10594	0.927732	0.543941	H3-7	Histone H3	H37_HUMA	1	0.929197
30min_MT.	Q9NUW8	-0.01992	0.927492	0.543941	TDP1	Tyrosyl-DN.	TYDP1_HUI	1	0.986291
30min_MT.	Q9Y2V2	0.017908	0.927403	0.543941	CARHSP1	Calcium-re	CHSP1_HUI	6	1.01249
15min_MT.	Q93062	-0.04917	0.92848	0.544306	RBPMS	RNA-bindir	RBPMS_HU	2	0.966492
30min_MT.	Q92805	-0.10788	0.928831	0.544463	GOLGA1	Golgin subf	GOGA1_HU	9	0.927948
15min_MT.	Q03001	-0.14007	0.929303	0.544699	DST	Dystonin	DYST_HUM	33	0.907475
15min_MT.	Q86SQ4	0.194493	0.929447	0.544731	ADGRG6	Adhesion G	AGRG6_HU	1	1.144322
30min_MT.	Q9NWV8	-0.10155	0.92983	0.544845	BABAM1	BRISC and E	BABA1_HUI	7	0.93203
30min_MT.	Q8TEM1	0.040793	0.929941	0.544869	NUP210	Nuclear poi	PO210_HU	4	1.028679
15min_MT.	P08709	-0.11132	0.930034	0.544882	F7	Coagulatio	FA7_HUMA	3	0.92574
15min_MT.	Q9Y3E7	-0.13333	0.930117	0.544891	CHMP3	Charged mu	CHMP3_HU	3	0.911725
30min_MT.	P24385	0.038354	0.93043	0.544951	CCND1	G1/S-specif	CCND1_HU	3	1.026942
15min_MT.	Q8N183	0.099033	0.930372	0.544951	NDUFAF2	NADH dehy	NDUF2_HU	1	1.071055
15min_MT.	Q99570	-0.15977	0.93042	0.544951	PIK3R4	Phosphoin	PI3R4_HUM	1	0.89517
15min_MT.	O15083	0.023406	0.930813	0.545135	ERC2	ERC proteir	ERC2_HUM	1	1.016356
15min_MT.	Q12788	-0.11998	0.931337	0.54536	TBL3	Transducin	TBL3_HUM	1	0.920201
30min_MT.	P10515	-0.02416	0.931608	0.545478	DLAT	Dihydrolip	ODP2_HUM	17	0.983391
15min_MT.	Q9UPT5	0.148943	0.931829	0.545526	EXOC7	Exocyst cor	EXOC7_HUI	8	1.108757
30min_MT.	Q8WXD5	-0.13707	0.93267	0.545878	GEMIN6	Gem-associ	GEMI6_HUI	3	0.909367
15min_MT.	Q6PGP7	-0.16215	0.932832	0.545879	SKIC3	Superkiller	SKI3_HUMA	8	0.893695
30min_MT.	P83731	0.016004	0.93308	0.545896	RPL24	Large ribos	RL24_HUM	4	1.011155
15min_MT.	Q8IYD1	-0.03611	0.93309	0.545896	GSPT2	Eukaryotic	ERF3B_HUM	6	0.975281
15min_MT.	Q8N0W3	-0.04736	0.932993	0.545896	FCSK	L-fucose kir	FCSK_HUM	1	0.967708
30min_MT.	O43490	0.205895	0.933241	0.545903	PROM1	Prominin-1	PROM1_HU	2	1.153402
30min_MT.	Q92890	0.018252	0.933181	0.545903	UFD1	Ubiquitin r	UFD1_HUM	10	1.012732
15min_MT.	O95359	0.017035	0.933445	0.545918	TACC2	Transformi	TACC2_HUI	61	1.011878
15min_MT.	P31937	-0.0029	0.933419	0.545918	HIBADH	3-hydroxyi	3HIDH_HUI	10	0.997989
30min_MT.	P09327	-0.001	0.933564	0.545928	VIL1	Villin-1	VILI_HUMA	21	0.999306
15min_MT.	P11234	0.050939	0.933729	0.545984	RALB	Ras-related	RALB_HUM	6	1.035939
30min_MT.	Q7Z5K2	-0.17182	0.933882	0.545992	WAPL	Wings apar	WAPL_HUM	16	0.887723
30min_MT.	O15143	-0.00427	0.934166	0.546047	ARPC1B	Actin-relate	ARC1B_HUI	19	0.997045
15min_MT.	O75143	0.072594	0.934378	0.546047	ATG13	Autophagy-	ATG13_HUI	6	1.051605
30min_MT.	Q14657	-0.15324	0.934832	0.546262	LAGE3	EKC/KEOPS	LAGE3_HUI	2	0.89923
30min_MT.	Q8IWB7	0.008694	0.934972	0.546302	WDFY1	WD repeat	WDFY1_HU	11	1.006045
15min_MT.	P51659	0.033233	0.935134	0.546356	HSD17B4	Peroxisoma	DHB4_HUM	12	1.023303
30min_MT.	O95881	-0.00365	0.935296	0.546395	TXNDC12	Thioredoxi	TXD12_HUI	7	0.997476
30min_MT.	P54802	0.142519	0.935339	0.546395	NAGLU	Alpha-N-ac	ANAG_HUM	15	1.103831
30min_MT.	O96007	-0.07153	0.93541	0.546395	MOCS2	Molybdopt	MOC2B_HU	4	0.951631
15min_MT.	Q9BTT0	-0.18073	0.935567	0.546446	ANP32E	Acidic leuci	AN32E_HUI	2	0.882254
15min_MT.	P49789	0.011427	0.936044	0.546562	FHIT	Bis(5'-aden	FHIT_HUMA	5	1.007952
15min_MT.	Q6QNY0	0.42138	0.935857	0.546562	BLOC1S3	Biogenesis	BL1S3_HUM	1	1.339208
15min_MT.	Q9Y6K9	0.119609	0.936041	0.546562	IKBKG	NF-kappa-B	NEMO_HUM	6	1.086441
30min_MT.	P62861	-0.11615	0.936127	0.546569	FAU	Ubiquitin-l	RS30_HUM	1	0.922648
15min_MT.	O94913	0.130137	0.936521	0.546638	PCF11	Pre-mRNA c	PCF11_HUI	2	1.094397
30min_MT.	P60953	-0.01163	0.936525	0.546638	CDC42	Cell divisio	CDC42_HU	6	0.991969
30min_MT.	Q13303	0.183707	0.936447	0.546638	KCNAB2	Voltage-gat	KCAB2_HUI	3	1.135798
15min_MT.	Q92520	0.194192	0.937117	0.546902	FAM3C	Protein FAI	FAM3C_HU	1	1.144083
15min_MT.	P12830	0.013888	0.937531	0.547103	CDH1	Cadherin-1	CADH1_HU	12	1.009673

15min_MT.	Q9Y4P8	0.013279	0.937804	0.547187	WIPI2	WD repeat	WIPI2_HUI		4	1.009247
15min_MT.	Q9HA77	0.014991	0.93819	0.547242	CARS2	Probable cy	SYCM_HUM		16	1.010445
30min_MT.	Q12905	-0.03181	0.938335	0.547287	ILF2	Interleukin	ILF2_HUMA		14	0.978193
15min_MT.	O60256	0.019609	0.938517	0.547351	PRPSAP2	Phosphoribo	KPRB_HUM		13	1.013685
30min_MT.	Q9NRX5	0.32333	0.939502	0.547844	SERINC1	Serine inco	SERC1_HUI		1	1.251215
30min_MT.	Q8WVM7	-0.00146	0.9396	0.547861	STAG1	Cohesin sul	STAG1_HUI		6	0.998986
30min_MT.	Q9H6T0	0.057308	0.939994	0.54805	ESRP2	Epithelial s	ESRP2_HUI		2	1.040522
30min_MT.	A5YM69;Q:	0.152287	0.940234	0.548149	ARHGEF35;	Rho guanin	ARG35_HU		3	1.11133
15min_MT.	A4D2B0	-0.1516	0.941073	0.54839	MBLAC1	Metallo-bet	MBLC1_HU		1	0.900249
30min_MT.	P32320	0.283849	0.941094	0.54839	CDA	Cytidine de	CDD_HUMA		2	1.217438
15min_MT.	P46782	-0.02415	0.941139	0.54839	RPS5	Small ribos	RS5_HUMA		6	0.983402
15min_MT.	Q6PI48	0.016909	0.94108	0.54839	DARS2	Aspartate--t	SYDM_HUM		15	1.011789
15min_MT.	Q86YT6	-0.13839	0.940866	0.54839	MIB1	E3 ubiquiti	MIB1_HUM		1	0.908535
15min_MT.	P23246	-0.01464	0.941386	0.548402	SFPQ	Splicing fac	SFPQ_HUM		28	0.989903
15min_MT.	P36551	-0.00292	0.941546	0.548402	CPOX	Oxygen-dep	HEM6_HUM		18	0.997979
30min_MT.	Q96AG4	0.206331	0.941446	0.548402	LRRC59	Leucine-ric	LRC59_HUI		6	1.153751
30min_MT.	Q8NFH3	-0.11638	0.942003	0.548526	NUP43	Nucleopori	NUP43_HU		5	0.922502
15min_MT.	P06756	0.077041	0.942765	0.548643	ITGAV	Integrin alp	ITAV_HUMA		2	1.054852
30min_MT.	P12830	-0.00246	0.942461	0.548643	CDH1	Cadherin-1	CADH1_HU		12	0.998299
15min_MT.	P60983	0.058341	0.942696	0.548643	GMFB	Glia matura	GMFB_HUM		5	1.041268
30min_MT.	Q92989	-0.22016	0.942547	0.548643	CLP1	Polyribonu	CLP1_HUM		4	0.858471
15min_MT.	Q96GG9	0.006219	0.942606	0.548643	DCUN1D1	DCN1-like p	DCNL1_HU		8	1.00432
15min_MT.	Q86TJ2	0.211232	0.943028	0.548755	TADA2B	Transcripti	TAD2B_HUI		1	1.157676
15min_MT.	O95684	0.095542	0.943331	0.54885	CEP43	Centrosom	CEP43_HUI		6	1.068466
30min_MT.	P50552	-0.06731	0.94345	0.548878	VASP	Vasodilator	VASP_HUM		12	0.954419
30min_MT.	Q7Z4Q2	-0.12173	0.943712	0.548949	HEATR3	HEAT repea	HEAT3_HUI		12	0.919086
30min_MT.	P51858	-0.00536	0.94409	0.549006	HDGF	Hepatoma-	HDGF_HUM		19	0.996292
15min_MT.	Q07866	0.064719	0.944056	0.549006	KLC1	Kinesin ligh	KLC1_HUM		11	1.045881
30min_MT.	Q99436	-0.00781	0.944052	0.549006	PSMB7	Proteasome	PSB7_HUM		9	0.994599
30min_MT.	Q9NVX7	0.022981	0.944008	0.549006	KBTBD4	Kelch repea	KBTB4_HUI		3	1.016057
30min_MT.	P62993	0.043348	0.944352	0.549117	GRB2	Growth fac	GRB2_HUM		10	1.030502
30min_MT.	Q04941	0.138313	0.944721	0.54925	PLP2	Proteolipid	PLP2_HUM		1	1.100617
15min_MT.	P08582	-0.13731	0.944901	0.549276	MELTF	Melanotrar	TRFM_HUM		7	0.909215
15min_MT.	Q9UKV3	-0.01208	0.944907	0.549276	ACIN1	Apoptotic c	ACINU_HUI		31	0.991665
30min_MT.	Q8IYB5	0.000882	0.945237	0.549386	SMAP1	Stromal me	SMAP1_HU		4	1.000612
15min_MT.	Q9UBF2	0.032758	0.945454	0.549472	COPG2	Coatomer s	COPG2_HU		8	1.022966
30min_MT.	P07108	-0.04669	0.945883	0.549572	DBI	Acyl-CoA-bi	ACBP_HUM		6	0.968155
15min_MT.	P20618	0.012642	0.945807	0.549572	PSMB1	Proteasome	PSB1_HUM		9	1.008801
15min_MT.	P54802	0.066513	0.945907	0.549572	NAGLU	Alpha-N-ac	ANAG_HUM		15	1.047182
15min_MT.	O43251;Q9	0.147382	0.946332	0.54971	RBFOX2;RB	RNA bindin	RFOX2_HUI		2	1.107558
15min_MT.	Q9BRF8	0.000423	0.946356	0.54971	CPPED1	Serine/thre	CPPED_HUI		14	1.000293
15min_MT.	O95721	0.01966	0.946763	0.549783	SNAP29	Synaptosor	SNP29_HUI		6	1.01372
15min_MT.	P02763	0.143337	0.947266	0.549953	ORM1	Alpha-1-aci	A1AG1_HU		3	1.104457
30min_MT.	Q13496	-0.10981	0.947217	0.549953	MTM1	Myotubula	MTM1_HUI		11	0.926709
30min_MT.	Q5SYC1	0.011431	0.947901	0.550281	CLVS2	Clavesin-2	CLVS2_HUM		9	1.007955
30min_MT.	Q9H7Z7	0.065229	0.948126	0.55033	PTGES2	Prostagland	PGES2_HUI		1	1.046251
30min_MT.	Q9BZE2	-0.19653	0.948626	0.550534	PUS3	tRNA pseud	PUS3_HUM		2	0.872647
15min_MT.	Q2TAA2	-0.00869	0.948872	0.550559	IAH1	Isoamyl ace	IAH1_HUM		10	0.993992
15min_MT.	Q969S9	0.06233	0.949652	0.550847	GFM2	Ribosome-r	RRF2M_HU		13	1.04415
15min_MT.	Q00613	0.10376	0.950177	0.551111	HSF1	Heat shock	HSF1_HUM		1	1.07457
30min_MT.	Q96JB2	-0.01374	0.950508	0.551222	COG3	Conserved c	COG3_HUM		19	0.990522
30min_MT.	Q96B36	-0.03907	0.950966	0.551446	AKT1S1	Proline-rich	AKTS1_HUI		2	0.973282

15min_MT.	Q8TDY2	-0.1109	0.951215	0.551489	RB1CC1	RB1-induci	RBCC1_HUI	17	0.92601
30min_MT.	Q9BSH5	0.082388	0.951536	0.551531	HDHD3	Haloacid de	HDHD3_HU	9	1.058769
15min_MT.	Q5TA45	-0.23796	0.952003	0.55164	INTS11	Integrator c	INT11_HUM	1	0.847946
15min_MT.	Q9Y5L4	0.029386	0.951986	0.55164	TIMM13	Mitochond	TIM13_HUI	7	1.020577
15min_MT.	Q8TEQ6	-0.02054	0.952455	0.551818	GEMIN5	Gem-associ	GEMI5_HUI	22	0.985863
15min_MT.	Q969M3	-0.321	0.952535	0.551824	YIPF5	Protein YIP	YIPF5_HUM	1	0.800513
30min_MT.	O94880	-0.11849	0.952916	0.551963	PHF14	PHD finger	PHF14_HUI	1	0.92115
15min_MT.	O14828	-0.36837	0.9532	0.552087	SCAMP3	Secretory c	SCAM3_HU	4	0.77466
15min_MT.	Q9Y5U2	0.143593	0.953316	0.552113	TSSC4	U5 small nu	TSSC4_HUM	4	1.104653
15min_MT.	Q86X76	0.019547	0.95374	0.552276	NIT1	Deaminate	NIT1_HUMA	13	1.013641
30min_MT.	Q9Y6B7	0.140337	0.95397	0.552369	AP4B1	AP-4 compl	AP4B1_HUI	2	1.102162
30min_MT.	Q9BXP5	0.001169	0.954252	0.552451	SRRT	Serrate RNA	SRRT_HUM	17	1.00081
15min_MT.	Q07283	-0.34819	0.954463	0.552453	TCHH	Trichohyali	TRHY_HUM	2	0.78557
15min_MT.	Q8IWZ3	0.135688	0.954343	0.552453	ANKHD1	Ankyrin rep	ANKH1_HU	18	1.098617
30min_MT.	Q9H6Q4	0.003425	0.954468	0.552453	CIAO3	Cytosolic ir	CIAO3_HUI	5	1.002377
30min_MT.	Q12981	-0.29856	0.95458	0.552477	BNIP1	Vesicle trar	SEC20_HUI	1	0.813066
15min_MT.	Q9HAV7	-0.03569	0.954834	0.552583	GRPEL1	GrpE protei	GRPE1_HUI	9	0.975563
30min_MT.	O96019	-0.05907	0.954984	0.552629	ACTL6A	Actin-like p	ACL6A_HUI	10	0.959885
30min_MT.	Q96GM5	0.010573	0.955358	0.552805	SMARCD1	SWI/SNF-re	SMRD1_HU	2	1.007356
15min_MT.	O15173	0.129715	0.95554	0.552828	PGRMC2	Membrane	PGRC2_HU	4	1.094077
15min_MT.	P68104	0.013408	0.956378	0.55304	EEF1A1	Elongation	EF1A1_HUI	25	1.009337
15min_MT.	P26927	0.062945	0.956496	0.553054	MST1	Hepatocyte	HGFL_HUM	8	1.044596
30min_MT.	Q96EV8	-0.22702	0.956609	0.553078	DTNBP1	Dysbindin	DTBP1_HUI	1	0.854396
15min_MT.	P30405	-0.02036	0.957094	0.553309	PPIF	Peptidyl-pr	PPIF_HUMA	7	0.985986
30min_MT.	Q8NI27	-0.10397	0.95746	0.553407	THOC2	THO compl	THOC2_HU	14	0.930469
30min_MT.	O75396	-0.00709	0.957665	0.553484	SEC22B	Vesicle-traf	SC22B_HUI	10	0.995097
30min_MT.	O75821	0.001216	0.958452	0.553575	EIF3G	Eukaryotic	EIF3G_HUM	17	1.000843
15min_MT.	P02771	0.133733	0.95817	0.553575	AFP	Alpha-fetop	FETA_HUMA	2	1.097129
30min_MT.	Q1KMD3	0.069418	0.958516	0.553575	HNRNPUL2	Heterogene	HNRL2_HU	15	1.049293
15min_MT.	Q99543	0.057567	0.958288	0.553575	DNAJC2	DnaJ homo	DNJC2_HUI	7	1.040709
15min_MT.	Q9BUL9	0.050503	0.958541	0.553575	RPP25	Ribonuclea	RPP25_HUI	4	1.035626
30min_MT.	P47985	0.148637	0.958702	0.553592	UQCRFS1	Cytochrom	UCRI_HUM	5	1.108522
30min_MT.	Q9BQ52	-0.02421	0.958858	0.553594	ELAC2	Zinc phospl	RNZ2_HUM	25	0.98336
15min_MT.	Q9H477	-0.00964	0.958874	0.553594	RBKS	Ribokinase	RBSK_HUM	10	0.993337
30min_MT.	Q9BT09	0.108096	0.959414	0.553799	CNPY3	Protein car	CNPY3_HUI	5	1.077805
15min_MT.	P62258	0.009067	0.959529	0.553825	YWHAE	14-3-3 prot	1433E_HUI	22	1.006305
15min_MT.	Q15545	-0.12638	0.9596	0.553825	TAF7	Transcripti	TAF7_HUM	1	0.916129
30min_MT.	O43318	0.161353	0.960261	0.554029	MAP3K7	Mitogen-ac	M3K7_HUM	2	1.118335
15min_MT.	P16383	-0.03294	0.960307	0.554029	GCFC2	Intron Larg	GCFC2_HUI	6	0.977428
30min_MT.	Q7Z7A4	-0.22636	0.960145	0.554029	PXK	PX domain	PXK_HUMA	3	0.85479
15min_MT.	P51946	0.157588	0.960384	0.554032	CCNH	Cyclin-H	CCNH_HUM	3	1.115421
30min_MT.	P33991	0.05488	0.960652	0.554091	MCM4	DNA replica	MCM4_HUI	6	1.038773
30min_MT.	Q5VTL8	-0.11806	0.960698	0.554091	PRPF38B	Pre-mRNA-	PR38B_HUI	14	0.921426
15min_MT.	O00178	0.13755	0.960931	0.554143	GTPBP1	GTP-bindin	GTPB1_HUI	2	1.100035
15min_MT.	Q5VSL9;Q9	0.506915	0.960893	0.554143	STRIP1;STR	Striatin-int	STRP1_HUM	1	1.421008
30min_MT.	O00232	0.005652	0.961342	0.554299	PSMD12	26S protea	PSD12_HUI	20	1.003925
30min_MT.	Q709C8	-0.05778	0.961498	0.554348	VPS13C	Intermemb	VP13C_HUI	34	0.960738
30min_MT.	Q01650	-0.00746	0.961676	0.554369	SLC7A5	Large neutr	LAT1_HUM	4	0.994841
15min_MT.	Q15691	0.002831	0.961635	0.554369	MAPRE1	Microtubul	MARE1_HU	15	1.001964
30min_MT.	Q96QD9	-0.54198	0.961816	0.554409	FYTTD1	UAP56-inte	UIF_HUMA	1	0.686826
15min_MT.	O43776	0.009841	0.961918	0.554426	NARS1	Asparagine	SYNC_HUM	15	1.006845
15min_MT.	Q9H2U2	-0.03453	0.962098	0.55449	PPA2	Inorganic p	IPYR2_HUM	9	0.976353

30min_MT.	Q9UK76	0.073418	0.962528	0.554656	JPT1	Jupiter mic	JUPI1_HUM	8	1.052207
30min_MT.	O43747	-0.02527	0.963018	0.554692	AP1G1	AP-1 compl	AP1G1_HU	22	0.982633
30min_MT.	P48449	0.005175	0.962906	0.554692	LSS	Lanosterol	LSS_HUMA	1	1.003593
30min_MT.	Q8N1G4	-0.05251	0.962985	0.554692	LRRC47	Leucine-ric	LRC47_HUI	21	0.964256
30min_MT.	Q8N4P3	0.209767	0.963088	0.554692	HDDC3	Guanosine-	MESH1_HU	5	1.156502
15min_MT.	Q8NFH5	-0.59254	0.962768	0.554692	NUP35	Nucleopori	NUP35_HU	1	0.663173
15min_MT.	P61970	0.002012	0.963369	0.554772	NUTF2	Nuclear tra	NTF2_HUM	5	1.001396
30min_MT.	O14967	-0.33632	0.964051	0.554985	CLGN	Calmegin	CLGN_HUM	1	0.792058
15min_MT.	P29372	0.005637	0.96406	0.554985	MPG	DNA-3-met	3MG_HUM	2	1.003915
15min_MT.	Q8IVT2	0.099717	0.964033	0.554985	MISP	Mitotic int	MISP_HUM	3	1.071563
30min_MT.	Q9UI30	0.107289	0.964001	0.554985	TRMT112	Multifuncti	TR112_HUI	2	1.077202
30min_MT.	Q13643	-0.03309	0.964277	0.55505	FHL3	Four and a l	FHL3_HUM	4	0.977324
15min_MT.	Q9NZJ6	-0.01172	0.96444	0.555103	COQ3	Ubiquinon	COQ3_HUM	3	0.991907
30min_MT.	P00480	-0.21236	0.964675	0.55511	OTC	Ornithine t	OTC_HUMA	2	0.863126
30min_MT.	P55011	-0.05601	0.964867	0.55511	SLC12A2	Solute carri	S12A2_HUI	6	0.961919
15min_MT.	Q49A26	0.678634	0.964873	0.55511	GLYR1	Cytokine-lil	GLYR1_HUI	1	1.600623
15min_MT.	Q5TGL8	0.031801	0.964584	0.55511	PXDC1	PX domain-	PXDC1_HU	1	1.022287
15min_MT.	Q99757	0.044271	0.964614	0.55511	TXN2	Thioredoxi	THIOM_HU	2	1.031162
15min_MT.	Q14061	-0.01782	0.965667	0.555441	COX17	Cytochrom	COX17_HU	3	0.987721
30min_MT.	Q00325	0.015514	0.966205	0.555476	SLC25A3	Solute carri	S25A3_HUI	3	1.010812
15min_MT.	Q5JXB2	-0.3149	0.965983	0.555476	UBE2NL	Putative ub	UE2NL_HUI	1	0.803907
30min_MT.	Q96I99	0.021108	0.966224	0.555476	SUCLG2	Succinate--	SUCB2_HUI	20	1.014738
30min_MT.	Q9ULJ6	-0.12186	0.966063	0.555476	ZMIZ1	Zinc finger	ZMIZ1_HUI	6	0.919001
30min_MT.	Q9UMS4	-0.00464	0.966161	0.555476	PRPF19	Pre-mRNA-|	PRP19_HUI	15	0.99679
15min_MT.	P04350	0.219545	0.96665	0.555552	TUBB4A	Tubulin bet	TBB4A_HUI	2	1.164367
30min_MT.	P49321	0.062562	0.966731	0.555552	NASP	Nuclear aut	NASP_HUM	32	1.044318
30min_MT.	P78310	-0.1208	0.96684	0.555552	CXADR	Coxsackievi	CXAR_HUM	7	0.919675
15min_MT.	Q69YN2	0.063522	0.967067	0.555552	CWF19L1	CWF19-like	C19L1_HUI	10	1.045014
15min_MT.	Q8NCN5	0.0267	0.966858	0.555552	PDPR	Pyruvate de	PDPR_HUM	12	1.018679
30min_MT.	O95801	-0.1373	0.967355	0.555595	TTC4	Tetratricop	TTC4_HUM	1	0.90922
15min_MT.	P52756	0.320119	0.967906	0.555796	RBM5	RNA-bindir	RBM5_HUM	1	1.248434
30min_MT.	P21589	-0.08489	0.967997	0.555801	NT5E	5'-nucleoti	5NTD_HUM	2	0.942857
30min_MT.	Q9UKL0	0.163908	0.96847	0.555991	RCOR1	REST corep	RCOR1_HU	4	1.120318
15min_MT.	Q9GZN8	0.042664	0.968686	0.556074	ADISSP	Adipose-sec	ADSSP_HUI	3	1.030014
30min_MT.	O00391	0.015022	0.968776	0.556084	QSOX1	Sulfhydryl c	QSOX1_HU	2	1.010467
15min_MT.	Q6NSW5;Q	0.134277	0.969133	0.556249	DENND10P	Putative DE	DE10B_HUI	2	1.097543
30min_MT.	Q3T906	0.038407	0.969244	0.556272	GNPTAB	N-acetylglu	GNPTA_HU	1	1.026979
15min_MT.	P53609	0.028526	0.969436	0.556331	PGGT1B	Geranylger	PGTB1_HUI	9	1.01997
30min_MT.	Q9P2I0	-0.08167	0.969561	0.556331	CPSF2	Cleavage ar	CPSF2_HUI	11	0.944966
15min_MT.	P00918	0.008876	0.969711	0.556376	CA2	Carbonic ar	CAH2_HUM	12	1.006172
15min_MT.	Q8IVH8	-0.03847	0.970036	0.556522	MAP4K3	Mitogen-ac	M4K3_HUM	1	0.973689
30min_MT.	Q9UJX5	-0.00691	0.971308	0.557089	ANAPC4	Anaphase-p	APC4_HUM	14	0.995223
30min_MT.	Q7LG56	0.029425	0.971444	0.557126	RRM2B	Ribonuclec	RIR2B_HUM	5	1.020605
30min_MT.	Q06203	0.00084	0.97167	0.557214	PPAT	Amidophos	PUR1_HUM	18	1.000583
30min_MT.	Q86WA6	0.085646	0.971933	0.557324	BPHL	Valacyclovi	BPHL_HUM	8	1.061163
15min_MT.	Q9Y3B8	-0.03333	0.972079	0.557367	REXO2	Oligoribon	ORN_HUMA	11	0.977159
15min_MT.	P48444	-0.01988	0.972589	0.557547	ARCN1	Coatomer s	COPD_HUM	25	0.986312
15min_MT.	Q9Y697	0.018151	0.972607	0.557547	NFS1	Cysteine de	NFS1_HUM	6	1.01266
15min_MT.	Q96C36	-0.00588	0.973052	0.557721	PYCR2	Pyrroline-5	P5CR2_HUI	9	0.995932
15min_MT.	O75884	0.050611	0.973201	0.557765	RBBP9	Serine hydr	RBBP9_HUI	7	1.035704
15min_MT.	O43847	-0.00819	0.973586	0.557875	NRDC	Nardilysin	NRDC_HUM	26	0.99434
30min_MT.	O95620	-0.4627	0.973934	0.55794	DUS4L	tRNA-dihyd	DUS4L_HUI	1	0.725629

30min_MT.	Q14444	0.012383	0.973827	0.55794	CAPRIN1	Caprin-1	CAPR1_HUI	11	1.00862
15min_MT.	P14314	0.000318	0.974247	0.558038	PRKCSH	Glucosidas	GLU2B_HU	23	1.000221
15min_MT.	Q14CX7	-0.03228	0.97436	0.558061	NAA25	N-alpha-ac	NAA25_HU	12	0.977874
30min_MT.	Q5VTQ0	0.512061	0.974517	0.558111	TTC39B	Tetratricop	TT39B_HUI	1	1.426086
15min_MT.	Q2M2I8	0.252763	0.974698	0.558173	AAK1	AP2-associa	AAK1_HUM	13	1.191487
15min_MT.	P15289	-0.02888	0.974992	0.558219	ARSA	Arylsulfata	ARSA_HUM	10	0.980182
30min_MT.	P61077	0.102168	0.974966	0.558219	UBE2D3	Ubiquitin-c	UB2D3_HU	1	1.073385
15min_MT.	Q9Y5A9	-0.21039	0.975071	0.558223	YTHDF2	YTH domair	YTHD2_HUI	7	0.864306
15min_MT.	Q12769	-0.03963	0.97602	0.558725	NUP160	Nuclear poi	NU160_HU	6	0.972903
30min_MT.	P40937	-0.20123	0.976749	0.559102	RFC5	Replicatior	RFC5_HUM	6	0.869806
30min_MT.	Q9BPX1	0.213613	0.976872	0.559132	HSD17B14	17-beta-hy	DHB14_HU	2	1.159588
15min_MT.	P31151	0.218199	0.977122	0.559234	S100A7	Protein S1(	S10A7_HUI	2	1.163281
15min_MT.	Q01105	0.014722	0.977651	0.559414	SET	Protein SET	SET_HUMA	9	1.010257
15min_MT.	O43760	0.286793	0.978011	0.559497	SYNGR2	Synaptogyr	SNG2_HUM	3	1.219925
30min_MT.	Q16563	0.133438	0.977959	0.559497	SYPL1	Synaptophy	SYPL1_HUI	2	1.096905
15min_MT.	O96019	0.094545	0.978127	0.559523	ACTL6A	Actin-like p	ACL6A_HUI	10	1.067729
15min_MT.	Q13884	0.043056	0.978354	0.559612	SNTB1	Beta-1-synt	SNTB1_HUI	10	1.030294
30min_MT.	Q8IWZ8	0.039335	0.978747	0.559754	SUGP1	SURP and G	SUGP1_HU	8	1.02764
15min_MT.	O75390	-0.00138	0.979034	0.559878	CS	Citrate synt	CISY_HUMA	13	0.999045
30min_MT.	Q96KP1	-0.0762	0.979411	0.560052	EXOC2	Exocyst cor	EXOC2_HUI	4	0.948552
30min_MT.	P49757	0.141821	0.979988	0.560136	NUMB	Protein nur	NUMB_HUI	7	1.103297
15min_MT.	Q96KP1	0.220704	0.979938	0.560136	EXOC2	Exocyst cor	EXOC2_HUI	4	1.165302
30min_MT.	Q9Y484	-0.01754	0.979905	0.560136	WDR45	WD repeat	WIPI4_HUI	6	0.987916
15min_MT.	Q9C0E2	0.038969	0.980212	0.560223	XPO4	Exportin-4	XPO4_HUM	17	1.027379
30min_MT.	Q8N5K1	-0.03033	0.980429	0.560306	CISD2	CDGSH iror	CISD2_HUI	2	0.979193
15min_MT.	Q9NVU0	-0.16831	0.980499	0.560306	POLR3E	DNA-direct	RPC5_HUM	1	0.889887
30min_MT.	P04080	-0.07154	0.980595	0.560319	CSTB	Cystatin-B	CYTB_HUM	9	0.951619
15min_MT.	Q9NSA3	0.288909	0.980666	0.560319	CTNNBIP1	Beta-cateni	CNBP1_HU	1	1.221716
15min_MT.	Q08AI8	-0.23215	0.980781	0.560344	MAB21L4	Protein ma	MB214_HU	3	0.851366
15min_MT.	Q5JRX3	-0.00042	0.980995	0.560425	PITRM1	Presequenc	PREP_HUM	30	0.999708
15min_MT.	O14893	0.164696	0.981419	0.560463	GEMIN2	Gem-associ	GEMI2_HUI	2	1.12093
30min_MT.	P05387	0.029446	0.981336	0.560463	RPLP2	Large ribos	RLA2_HUM	6	1.02062
30min_MT.	P51812	0.001855	0.981337	0.560463	RPS6KA3	Ribosomal	KS6A3_HUI	19	1.001287
15min_MT.	P68402	-0.04916	0.981347	0.560463	PAFAH1B2	Platelet-act	PA1B2_HUI	6	0.966497
15min_MT.	Q96EY7	-0.04731	0.981764	0.560619	PTCD3	Small ribos	PTCD3_HUI	2	0.967738
30min_MT.	O43759	0.068226	0.982067	0.560632	SYNGR1	Synaptogyr	SNG1_HUM	1	1.048427
30min_MT.	Q9BRJ7	0.034392	0.981979	0.560632	NUDT16L1	Tudor-inter	TIRR_HUMA	2	1.024125
30min_MT.	Q9BUQ8	-0.00653	0.981986	0.560632	DDX23	Probable A	DDX23_HU	16	0.995482
30min_MT.	O75874	0.041427	0.982766	0.560863	IDH1	Isocitrate d	IDHC_HUM	20	1.029131
30min_MT.	P05783	0.003023	0.983006	0.560944	KRT18	Keratin, typ	K1C18_HUI	26	1.002098
15min_MT.	P83731	-0.03305	0.983486	0.560968	RPL24	Large ribos	RL24_HUM	4	0.97735
30min_MT.	Q13885	-0.02939	0.983385	0.560968	TUBB2A	Tubulin bet	TBB2A_HUI	5	0.979832
15min_MT.	Q9H7Z7	0.090113	0.983397	0.560968	PTGES2	Prostagland	PGES2_HUI	1	1.064453
15min_MT.	P54753	0.067903	0.983966	0.561029	EPHB3	Ephrin type	EPHB3_HUI	2	1.048192
30min_MT.	Q9Y3A5	-0.02569	0.983958	0.561029	SBDS	Ribosome r	SBDS_HUM	17	0.982354
30min_MT.	Q96LD4	0.179512	0.984234	0.561066	TRIM47	E3 ubiquiti	TRI47_HUM	1	1.132501
30min_MT.	Q8TBZ3	-0.14563	0.984355	0.561075	WDR20	WD repeat-	WDR20_HU	4	0.903987
15min_MT.	Q6PCE3	0.024695	0.984442	0.561083	PGM2L1	Glucose 1,6	PGM2L_HU	17	1.017264
15min_MT.	O15040	0.227215	0.984753	0.561138	TECPR2	Tectonin be	TCPR2_HUI	5	1.170573
30min_MT.	Q9BY42	0.019501	0.984657	0.561138	RTF2	Replicatior	RTF2_HUM	4	1.013609
30min_MT.	Q9NZD8	-0.28291	0.984703	0.561138	SPG21	Maspardin	SPG21_HUI	1	0.821929
15min_MT.	P61964	0.048779	0.985215	0.56127	WDR5	WD repeat-	WDR5_HUI	11	1.034389

30min_MT.	Q02952	-0.00943	0.985196	0.56127	AKAP12	A-kinase an	AKA12_HUI	70	0.993482
15min_MT.	Q9NQX3	0.054908	0.985158	0.56127	GPHN	Gephyrin	GEPH_HUM	23	1.038793
15min_MT.	A1X283	-0.02274	0.985729	0.561408	SH3PXD2B	SH3 and PX	SPD2B_HUI	10	0.984363
30min_MT.	Q86VW0	0.028241	0.985613	0.561408	SESTD1	SEC14 dom	SESD1_HUI	9	1.019768
30min_MT.	Q9UBT2	-0.01549	0.985725	0.561408	UBA2	SUMO-activ	SAE2_HUM	24	0.98932
30min_MT.	Q9C0J8	-0.00432	0.985938	0.561445	WDR33	pre-mRNA 3	WDR33_HU	7	0.997007
15min_MT.	Q8N954	0.089607	0.986395	0.561623	GPATCH11	G patch do	GPT11_HUI	2	1.06408
30min_MT.	Q96CV9	0.048779	0.986668	0.561738	OPTN	Optineurin	OPTN_HUM	16	1.034389
15min_MT.	Q15796	0.194642	0.986801	0.561773	SMAD2	Mothers ag	SMAD2_HU	1	1.14444
30min_MT.	P49750	-0.00188	0.987142	0.561844	YLPM1	YLP motif-c	YLPM1_HU	17	0.998695
30min_MT.	Q6P179	0.169731	0.987081	0.561844	ERAP2	Endoplasm	ERAP2_HUI	5	1.124848
15min_MT.	P22314	0.005237	0.98745	0.561938	UBA1	Ubiquitin-l	UBA1_HUM	35	1.003637
30min_MT.	P35270	0.097216	0.987396	0.561938	SPR	Sepiapterin	SPRE_HUM	10	1.069707
30min_MT.	A4FU69	0.53252	0.988271	0.561948	EFCAB5	EF-hand cal	EFCB5_HUI	1	1.446454
15min_MT.	O14579	0.01753	0.987841	0.561948	COPE	Coatomer s	COPE_HUM	11	1.012225
15min_MT.	O43741	0.177401	0.988539	0.561948	PRKAB2	5'-AMP-acti	AAKB2_HUI	1	1.130845
30min_MT.	O94776	-0.22905	0.988347	0.561948	MTA2	Metastasis-	MTA2_HUM	1	0.853194
15min_MT.	O94906	0.095515	0.988248	0.561948	PRPF6	Pre-mRNA-p	PRP6_HUM	8	1.068447
30min_MT.	O95260	-0.03796	0.98818	0.561948	ATE1	Arginyl-tRN	ATE1_HUM	8	0.974032
15min_MT.	P35637	0.02288	0.987908	0.561948	FUS	RNA-bindin	FUS_HUMA	10	1.015986
15min_MT.	P45984	0.017496	0.98829	0.561948	MAPK9	Mitogen-ac	MK09_HUM	9	1.012201
30min_MT.	P46087	0.09312	0.988481	0.561948	NOP2	28S rRNA (c	NOP2_HUM	12	1.066675
15min_MT.	P61006	-0.10964	0.988081	0.561948	RAB8A	Ras-related	RAB8A_HUI	6	0.926816
30min_MT.	Q16626	-0.0012	0.987605	0.561948	MEA1	Male-enhar	MEA1_HUM	1	0.999172
15min_MT.	Q7Z6J8	-0.22129	0.988476	0.561948	UBE3D	E3 ubiquiti	UBE3D_HU	2	0.857798
30min_MT.	O43414	0.088312	0.989016	0.562093	ERI3	ERI1 exorib	ERI3_HUMA	1	1.063126
30min_MT.	P08754	0.064479	0.989008	0.562093	GNAI3	Guanine nu	GNAI3_HUI	8	1.045707
30min_MT.	Q9HAD4	-0.20423	0.988938	0.562093	WDR41	WD repeat-	WDR41_HU	3	0.867999
15min_MT.	P57764	0.221333	0.989369	0.562252	GSDMD	Gasdermin-	GSDMD_HU	4	1.165811
30min_MT.	Q9UNS2	-0.02391	0.989868	0.56244	COPS3	COP9 signa	CSN3_HUM	14	0.983561
30min_MT.	Q9Y3X0	0.217083	0.989915	0.56244	CCDC9	Coiled-coil	CCDC9_HU	5	1.162381
15min_MT.	Q92882	-0.24502	0.990018	0.562458	OSTF1	Osteoclast-	OSTF1_HUI	4	0.843804
30min_MT.	P29966	-0.0156	0.99044	0.56254	MARCKS	Myristoylat	MARCS_HU	15	0.989247
15min_MT.	Q5VU43	0.121609	0.990379	0.56254	PDE4DIP	Myomegali	MYOME_HU	11	1.087948
30min_MT.	Q08379	-0.11346	0.990777	0.562644	GOLGA2	Golgin subf	GOGA2_HU	12	0.924368
15min_MT.	P07814	0.010382	0.991519	0.562729	EPRS1	Bifunctiona	SYEP_HUMA	59	1.007222
15min_MT.	Q6UW63	0.036446	0.991702	0.562729	POGLUT2	Protein O-g	PLGT2_HUI	7	1.025584
15min_MT.	Q8N1A0	-0.44008	0.991729	0.562729	KRT222	Keratin-like	KT222_HUI	1	0.737093
30min_MT.	Q96PC3	-0.2204	0.991089	0.562729	AP1S3	AP-1 compl	AP1S3_HUI	2	0.858327
30min_MT.	Q96S55	-0.16724	0.991553	0.562729	WRNIP1	ATPase WRI	WRIP1_HU	5	0.890544
30min_MT.	Q99653	0.184753	0.99179	0.562729	CHP1	Calcineurin	CHP1_HUM	1	1.136622
15min_MT.	Q9NPQ8	-0.03306	0.991679	0.562729	RIC8A	Synembryn	RIC8A_HUM	13	0.977345
15min_MT.	Q9Y4F1	-0.04295	0.991508	0.562729	FARP1	FERM, ARH	FARP1_HUI	7	0.970669
15min_MT.	Q9BTE6	0.032938	0.991908	0.562755	AARSD1	Alanyl-tRN	AASD1_HUI	6	1.023093
30min_MT.	Q9H2U2	0.013477	0.992125	0.562768	PPA2	Inorganic p	IPYR2_HUM	9	1.009385
30min_MT.	P14324	0.031009	0.992927	0.562829	FDPS	Farnesyl py	FPPS_HUM	8	1.021726
15min_MT.	P42224	0.002365	0.992798	0.562829	STAT1	Signal trans	STAT1_HUM	24	1.001641
30min_MT.	Q01130	0.002348	0.992779	0.562829	SRSF2	Serine/argi	SRSF2_HUM	10	1.001629
30min_MT.	Q8IYS1	-0.09622	0.992974	0.562829	PM20D2	Xaa-Arg dip	P20D2_HU	4	0.935478
15min_MT.	Q9GZT3	0.019881	0.992849	0.562829	SLIRP	SRA stem-lo	SLIRP_HUM	6	1.013876
15min_MT.	Q9NYL2	0.285054	0.99321	0.562881	MAP3K20	Mitogen-ac	M3K20_HU	1	1.218456
30min_MT.	Q9UL46	0.018115	0.99316	0.562881	PSME2	Proteasom	PSME2_HU	15	1.012635

30min_MT.	P61599	0.116941	0.993735	0.562971	NAA20	N-alpha-ace	NAA20_HU	5	1.084433
15min_MT.	Q01970	0.051819	0.993725	0.562971	PLCB3	1-phosphat	PLCB3_HUI	18	1.036571
15min_MT.	Q8N5I2	-0.34752	0.994257	0.563149	ARRDC1	Arrestin do	ARRD1_HU	1	0.785936
15min_MT.	Q9BUI4	0.087676	0.994624	0.563275	POLR3C	DNA-direct	RPC3_HUM	7	1.062657
15min_MT.	O75643	-0.0179	0.994949	0.563312	SNRNP200	U5 small nu	U520_HUM	40	0.98767
15min_MT.	Q14008	0.045037	0.995122	0.563312	CKAP5	Cytoskeleto	CKAP5_HUI	33	1.03171
15min_MT.	Q6L8Q7	0.024554	0.994893	0.563312	PDE12	2',5'-phosp	PDE12_HUI	11	1.017165
30min_MT.	Q8NHU6	-0.16824	0.995116	0.563312	TDRD7	Tudor dom	TDRD7_HU	1	0.88993
15min_MT.	O43615	0.001432	0.995447	0.563455	TIMM44	Mitochond	TIM44_HUI	16	1.000993
30min_MT.	Q14244	0.175122	0.995523	0.563458	MAP7	Ensconsin	MAP7_HUM	1	1.12906
30min_MT.	O94979	-0.00154	0.995927	0.563605	SEC31A	Protein trai	SC31A_HUI	34	0.998932
15min_MT.	Q9NY12	0.265344	0.996045	0.563631	GAR1	H/ACA ribo	GAR1_HUM	2	1.201922
30min_MT.	P05161	-0.04045	0.996164	0.563657	ISG15	Ubiquitin-l	ISG15_HUM	8	0.972351
15min_MT.	Q9NVM4	0.056278	0.996936	0.564012	PRMT7	Protein arg	ANM7_HUM	2	1.03978
15min_MT.	Q8IYX8	-0.15277	0.997382	0.564224	CEP57L1	Centrosom	CE57L_HUM	1	0.899524
15min_MT.	P52732	0.1386	0.997892	0.56439	KIF11	Kinesin-like	KIF11_HUM	6	1.100837
15min_MT.	Q96BR5	-0.16603	0.998348	0.564566	COA7	Cytochrom	COA7_HUM	8	0.891292
30min_MT.	Q9BWD1	0.022709	0.998524	0.564625	ACAT2	Acetyl-CoA	THIC_HUMA	13	1.015865
15min_MT.	P49458	-0.02375	0.998658	0.56466	SRP9	Signal reco	SRP09_HUI	6	0.983671
15min_MT.	O60613	-0.00234	0.998805	0.564661	SELENOF	Selenoprot	SEP15_HUM	1	0.998381
30min_MT.	O60784	-0.01354	0.998908	0.564678	TOM1	Target of M	TOM1_HUM	8	0.990661
15min_MT.	Q9BZE2	-0.09995	0.999042	0.564713	PUS3	tRNA pseud	PUS3_HUM	2	0.933063
15min_MT.	Q641Q2	-0.01991	0.999444	0.5649	WASHC2A	WASH com	WAC2A_HU	14	0.986295
