# Supplementary material for: Decoding the Cellular Response to Methylthioadenosine Uncovers Novel Targets for Chronic Liver Disease
Source: Cells. 2026 Sep 22;15(19):1720. doi: 10.3390/cells15191720 (PMC13664110; doi:10.3390/cells15191720)
Supplement: Supplementary file 1 [file cells-15-01720-s001.zip › Table S4_PISA_Extracts_LiverWT.pdf]

Compariso	ProteinGro	AVG Log2 F	Pvalue	Qvalue	Genes	ProteinDes	ProteinNar	# Unique To	FC
15_MTA / C	P50247	1.912071	9.04E-10	3.25E-06	Ahcy	Adenosylhc	SAHH_MOU	29	3.763489
30_MTA / C	Q9CQ65	1.300215	2.91E-09	5.23E-06	Mtap	S-methyl-5'	MTAP_MOU	16	2.462656
15_MTA / C	P50446	0.863602	7.32E-09	8.78E-06	Krt6a	Keratin, typ	K2C6A_MO	19	1.819576
15_MTA / C	Q9NSB2	0.781641	2.24E-08	2.01E-05	KRT84	Keratin, typ	KRT84_HUI	7	1.719085
15_MTA / C	P16331	1.333608	3.67E-08	2.25E-05	Pah	Phenylalan	PH4H_MOU	28	2.520321
15_MTA / C	Q9CQ65	1.237339	3.74E-08	2.25E-05	Mtap	S-methyl-5'	MTAP_MOU	16	2.357633
15_MTA / C	Q61781	0.770901	5.01E-08	2.58E-05	Krt14	Keratin, typ	K1C14_MO	19	1.706335
30_MTA / C	P50247	2.129339	2.43E-07	0.0001	Ahcy	Adenosylhc	SAHH_MOU	29	4.375169
30_MTA / C	Q9ER35	2.413096	2.51E-07	0.0001	Fn3k	Fructosami	FN3K_MOU	1	5.32616
30_MTA / C	P16331	1.340393	4.68E-07	0.000169	Pah	Phenylalan	PH4H_MOU	28	2.532203
15_MTA / C	Q64339	-0.50816	1.01E-06	0.000289	Isg15	Ubiquitin-l	ISG15_MOU	3	0.703117
30_MTA / C	Q8VC85	0.108309	1.81E-06	0.000434	Lsm1	U6 snRNA-a	LSM1_MOU	1	1.077964
15_MTA / C	Q9ER35	2.036274	1.78E-06	0.000434	Fn3k	Fructosami	FN3K_MOU	1	4.101849
15_MTA / C	P04264	0.819881	3.55E-06	0.0008	KRT1	Keratin, typ	K2C1_HUM	34	1.76526
15_MTA / C	Q64674	-1.50977	5.81E-06	0.001163	Srm	Spermidine	SPEE_MOUS	1	0.351166
15_MTA / C	Q15323	-0.17914	7.17E-06	0.00129	KRT31	Keratin, typ	K1H1_HUM	10	0.883228
15_MTA / C	Q922U2	1.208554	7.00E-06	0.00129	Krt5	Keratin, typ	K2C5_MOU	9	2.31106
15_MTA / C	P13645	0.875081	9.80E-06	0.00168	KRT10	Keratin, typ	K1C10_HUI	30	1.834111
15_MTA / C	P21300	-1.40986	2.28E-05	0.003039	Akr1b7	Aldo-keto r	ALD1_MOU	1	0.376347
15_MTA / C	P06683	-0.72328	2.55E-05	0.003166	C9	Complemer	CO9_MOUS	2	0.60572
15_MTA / C	Q76LS9	-0.56931	2.54E-05	0.003166	Mindy1	Ubiquitin c	MINY1_MO	2	0.67394
15_MTA / C	P31938	-0.7851	2.82E-05	0.003215	Map2k1	Dual specifi	MP2K1_MO	2	0.580314
15_MTA / C	Q6IFX2	0.891014	2.75E-05	0.003215	Krt42	Keratin, typ	K1C42_MO	5	1.85448
15_MTA / C	Q91VR8	-1.288	3.10E-05	0.003296	Brk1	Protein BRI	BRK1_MOU	1	0.40952
15_MTA / C	Q9D7M1	-1.62717	3.18E-05	0.003296	Gid8	Glucose-inc	GID8_MOU	1	0.323722
15_MTA / C	O35737	-1.1136	4.16E-05	0.004051	Hnrnph1	Heterogene	HNRH1_MO	3	0.462139
15_MTA / C	Q8CGK3	-0.18764	4.34E-05	0.004069	Lonp1	Lon proteas	LONM_MOU	10	0.878038
15_MTA / C	Q921M7	-1.46608	4.61E-05	0.004148	Cyrib	CYFIP-relat	CYRIB_MOU	2	0.361965
15_MTA / C	Q9CQZ1	-1.52379	6.97E-05	0.005703	Hsbp1	Heat shock	HSBP1_MO	1	0.347771
15_MTA / C	P35908	0.607776	8.41E-05	0.006474	KRT2	Keratin, typ	K22E_HUM	38	1.523908
15_MTA / C	Q7TQG1	-0.8084	8.45E-05	0.006474	Plekha6	Pleckstrin h	PKHA6_MO	1	0.571013
15_MTA / C	P17225	-0.66443	0.000119	0.008556	Ptbp1	Polypyrimi	PTBP1_MO	5	0.630937
15_MTA / C	Q5I2A0	-0.79401	0.000119	0.008556	Serpina3g	Serine prot	SPA3G_MO	1	0.57674
15_MTA / C	P70444	-1.07135	0.000145	0.009829	Bid	BH3-intera	BID_MOUSI	4	0.475874
15_MTA / C	O55060	0.603142	0.000186	0.012199	Tpmt	Thiopurine	TPMT_MOU	16	1.519021
15_MTA / C	P60867	-0.40503	0.00022	0.013188	Rps20	Small ribos	RS20_MOU	2	0.755221
30_MTA / C	O55060	0.516921	0.000224	0.0132	Tpmt	Thiopurine	TPMT_MOU	16	1.430898
15_MTA / C	Q9QZ23	-0.70906	0.000234	0.013366	Nfu1	NFU1 iron-s	NFU1_MOU	5	0.61172
30_MTA / C	P35908	0.404238	0.000244	0.013533	KRT2	Keratin, typ	K22E_HUM	38	1.32339
15_MTA / C	Q3V1L4	-0.45741	0.000243	0.013533	Nt5c2	Cytosolic p	5NTC_MOU	10	0.72829
15_MTA / C	Q62318	-1.01947	0.000272	0.014435	Trim28	Transcripti	TIF1B_MOU	7	0.493297
15_MTA / C	Q9CQE8	-1.3174	0.000281	0.014435	RTRAF	RNA transc	RTRAF_MO	5	0.401258
30_MTA / C	Q9ERH7	0.055565	0.000289	0.014636	Hipk3	Homeodon	HIPK3_MOI	1	1.039266
15_MTA / C	P54822	-1.10002	0.000315	0.014846	Adsl	Adenylosuc	PUR8_MOU	1	0.46651
15_MTA / C	P56812	-0.78687	0.00031	0.014846	Pdcd5	Programme	PDCD5_MC	4	0.5796
15_MTA / C	P62627	-2.7961	0.000318	0.014846	Dynlrb1	Dynein ligh	DLRB1_MO	1	0.143976
15_MTA / C	Q6ZQK5	-1.36073	0.000297	0.014846	Acap2	Arf-GAP wit	ACAP2_MO	1	0.389384
30_MTA / C	Q9Z0K8	-1.01665	0.000309	0.014846	Vnn1	Pantethein	VNN1_MOU	1	0.494262
15_MTA / C	Q61655	-1.59767	0.000335	0.015275	Ddx19a	ATP-depenc	DD19A_MC	2	0.330411
30_MTA / C	P01872	0.256648	0.000369	0.016397	Ighm	Immunoglc	IGHM_MOU	10	1.1947
15_MTA / C	Q8K1Z0	-0.51244	0.000377	0.016539	Coq9	Ubiquinon	COQ9_MOU	5	0.701037

15_MTA / C	Q78J03	-0.63983	0.000399	0.016628	Msrb2	Methionine	MSRB2_MO	1	0.641788
15_MTA / C	Q9ERE7	-0.45536	0.000395	0.016628	Mesd	LRP chaper	MESD_MOU	7	0.729328
30_MTA / C	Q91WS4	-0.13136	0.000433	0.017331	Bhmt2	S-methylm	BHMT2_MO	19	0.912968
15_MTA / C	Q921M4	-1.65861	0.00045	0.017623	Golga2	Golgin subf	GOGA2_MO	2	0.316744
15_MTA / C	P26350	0.33803	0.000472	0.01787	Ptma	Prothymos	PTMA_MOU	3	1.264029
30_MTA / C	Q923D2	-0.14994	0.000471	0.01787	Blvrb	Flavin redu	BLVRB_MO	11	0.901286
15_MTA / C	Q9QXS1	-1.16652	0.00047	0.01787	Plec	Plectin	PLEC_MOU	2	0.445496
15_MTA / C	Q06770	-1.15465	0.000568	0.020876	Serpina6	Corticoster	CBG_MOUS	3	0.449175
15_MTA / C	Q8BKZ9	-0.47237	0.00062	0.022115	Pdhx	Pyruvate de	ODPX_MOU	7	0.720778
15_MTA / C	P62141	-0.23735	0.000643	0.022628	Ppp1cb	Serine/thre	PP1B_MOU	2	0.848301
15_MTA / C	O08529	-1.24943	0.000677	0.022976	Capn2	Calpain-2 c	CAN2_MOU	4	0.420615
15_MTA / C	Q99KN2	-2.18011	0.000669	0.022976	Ciao1	Probable cy	CIAO1_MO	2	0.220658
15_MTA / C	Q9CR51	-1.10609	0.000671	0.022976	Atp6v1g1	V-type prot	VATG1_MO	2	0.46455
30_MTA / C	Q8BT07	-0.73353	0.000711	0.023485	Cep55	Centrosom	CEP55_MO	1	0.601429
15_MTA / C	Q9DAW9	-0.18822	0.000705	0.023485	Cnn3	Calponin-3	CNN3_MOU	2	0.87769
30_MTA / C	Q9ERB0	0.788373	0.000707	0.023485	Snap29	Synaptosor	SNP29_MO	2	1.727125
15_MTA / C	Q8CIM7	-1.5614	0.000746	0.024431	Cyp2d26	Cytochrom	CP2DQ_MO	5	0.338822
15_MTA / C	P99028	-1.34442	0.000769	0.024955	Uqcrh	Cytochrom	QCR6_MOU	1	0.393812
15_MTA / C	O35593	-1.48007	0.000873	0.027183	Psmd14	26S protea	PSDE_MOU	4	0.358472
15_MTA / C	P35527	0.757976	0.000876	0.027183	KRT9	Keratin, typ	K1C9_HUM	30	1.691117
15_MTA / C	P62827	-1.63703	0.000911	0.028044	Ran	GTP-bindin	RAN_MOUS	5	0.321518
15_MTA / C	P40336	-0.1303	0.00098	0.028111	Vps26a	Vacuolar pr	VP26A_MO	12	0.913644
15_MTA / C	P58137	0.079349	0.000976	0.028111	Acot8	Acyl-coenzy	ACOT8_MO	3	1.056541
30_MTA / C	P61089	-0.16932	0.000999	0.028111	Ube2n	Ubiquitin-c	UBE2N_MO	8	0.889262
15_MTA / C	Q8BT07	-0.43215	0.000931	0.028111	Cep55	Centrosom	CEP55_MO	1	0.741155
30_MTA / C	Q7TNG8	0.572755	0.001038	0.028568	Ldhd	Probable D	LDHD_MOU	18	1.487361
15_MTA / C	Q9QWL7	0.356964	0.001052	0.028687	Krt17	Keratin, typ	K1C17_MO	14	1.280728
30_MTA / C	O77727	2.683295	0.001106	0.028939	KRT15	Keratin, typ	K1C15_SHE	3	6.423211
15_MTA / C	Q60710	-0.54574	0.001104	0.028939	Samhd1	Deoxynucle	SAMH1_MO	3	0.685041
15_MTA / C	Q9WVE8	-0.62432	0.001109	0.028939	Pacsin2	Protein kin	PACN2_MO	5	0.648725
15_MTA / C	P35979	-1.384	0.001121	0.029035	Rpl12	Large ribos	RL12_MOU	5	0.383156
15_MTA / C	P47753	0.017386	0.001222	0.029232	Capza1	F-actin-cap	CAZA1_MO	7	1.012124
15_MTA / C	Q02257	0.524772	0.001195	0.029232	Jup	Junction pl	PLAK_MOU	16	1.438706
15_MTA / C	Q99JY0	-0.64845	0.001209	0.029232	Hadhb	Trifunction	ECHB_MOU	7	0.637964
15_MTA / C	Q9DBZ5	-1.46612	0.001145	0.029232	Eif3k	Eukaryotic	EIF3K_MOU	2	0.361955
30_MTA / C	Q6GQT1	-1.37635	0.001243	0.029256	A2m	Alpha-2-ma	A2MG_MOU	1	0.385191
15_MTA / C	Q9CXW4	-0.70679	0.001294	0.029717	Rpl11	Large ribos	RL11_MOU	2	0.61268
30_MTA / C	Q6P8I4	0.93885	0.001378	0.030946	Pcnp	PEST protec	PCNP_MOU	2	1.916999
15_MTA / C	P97470	-1.02581	0.001476	0.032199	Ppp4c	Serine/thre	PP4C_MOU	3	0.491135
15_MTA / C	P56395	-1.09918	0.001506	0.032423	Cyb5a	Cytochrom	CYB5_MOU	4	0.466783
30_MTA / C	Q6P9R2	-1.41064	0.001513	0.032423	Oxsr1	Serine/thre	OXSR1_MO	2	0.376145
15_MTA / C	P01887	-0.68891	0.001524	0.032471	B2m	Beta-2-micr	B2MG_MOU	2	0.620323
15_MTA / C	Q9D142	-0.3488	0.001554	0.03272	Nudt14	Uridine dip	NUD14_MO	5	0.785237
15_MTA / C	O77727	2.670844	0.001688	0.034531	KRT15	Keratin, typ	K1C15_SHE	3	6.368014
15_MTA / C	Q9Z204	-0.60181	0.00176	0.035794	Hnrnpc	Heterogene	HNRPC_MO	5	0.658926
15_MTA / C	Q9D8B3	-0.94326	0.001797	0.035939	Chmp4b	Charged mu	CHM4B_MO	4	0.520058
15_MTA / C	P40936	0.417092	0.001862	0.036029	Inmt	Indolethyla	INMT_MOU	12	1.335233
15_MTA / C	Q99MI1	-1.43874	0.001831	0.036029	Erc1	ELKS/Rab6-	RB6I2_MOU	9	0.368891
15_MTA / C	Q9WUR2	-0.02189	0.001918	0.036339	Eci2	Enoyl-CoA d	ECI2_MOUS	4	0.984945
15_MTA / C	Q62523	-0.45865	0.001957	0.036673	Zyx	Zyxin	ZYX_MOUSE	4	0.727666
15_MTA / C	Q9CZN7	-0.83151	0.001957	0.036673	Shmt2	Serine hydr	GLYM_MOU	6	0.56194
15_MTA / C	P22315	-0.74663	0.002152	0.036767	Fech	Ferrochelat	HEMH_MOU	4	0.595993

15_MTA / C	P52503	-1.11944	0.002132	0.036767	Ndufs6	NADH dehy	NDUS6_MC	3	0.460273
30_MTA / C	P58137	-0.64658	0.002033	0.036767	Acot8	Acyl-coenzy	ACOT8_MC	3	0.638793
15_MTA / C	P97447	-0.53589	0.002022	0.036767	Fhl1	Four and a l	FHL1_MOU	5	0.689732
30_MTA / C	P97770	-0.84324	0.002045	0.036767	Thumpd3	tRNA (guan	THUM3_MC	1	0.55739
15_MTA / C	Q8BP47	-1.25055	0.002136	0.036767	Nars1	Asparagine-	SYNC_MOU	1	0.420288
15_MTA / C	Q9JI46	-0.25337	0.002038	0.036767	Nudt3	Diphospho	NUDT3_MC	2	0.838936
15_MTA / C	Q9JLZ3	-1.47127	0.002029	0.036767	Auh	Methylglut	AUHM_MO	5	0.360664
15_MTA / C	Q9Z0P5	-1.59805	0.002078	0.036767	Twf2	Twinfilin-2	TWF2_MOU	4	0.330323
15_MTA / C	Q8BGS2	-0.8274	0.002199	0.03734	Bola2	BolA-like pr	BOLA2_MO	3	0.563545
30_MTA / C	P30115	-0.20015	0.002285	0.037532	Gsta3	Glutathion	GSTA3_MO	11	0.870459
15_MTA / C	P60710	-0.16483	0.002283	0.037532	Actb	Actin, cyto	ACTB_MOU	21	0.892032
15_MTA / C	Q923G2	-0.78812	0.002243	0.037532	Polr2h	DNA-direct	RPAB3_MO	3	0.579099
15_MTA / C	Q9CVB6	-0.3379	0.002304	0.037532	Arpc2	Actin-relate	ARPC2_MO	14	0.791195
30_MTA / C	Q9JLT4	-0.25331	0.002363	0.037869	Txnrd2	Thioredoxi	TRXR2_MO	17	0.838967
15_MTA / C	Q8K2Q0	-0.71704	0.00246	0.038336	Commd9	COMM don	COMD9_M	3	0.608343
15_MTA / C	Q99N87	-0.82465	0.002483	0.038532	Mrps5	Small ribos	RT05_MOU	3	0.564621
15_MTA / C	O70362	-0.54678	0.002511	0.038792	Gpld1	Phosphatic	PHLD_MOU	6	0.684544
15_MTA / C	Q91WS4	-0.0997	0.00254	0.038965	Bhmt2	S-methylm	BHMT2_MC	19	0.933225
15_MTA / C	Q91XL9	-0.49458	0.002566	0.039139	Osbpl1a	Oxysterol-b	OSBL1_MO	3	0.709767
15_MTA / C	P83741	-0.26209	0.002608	0.039618	Wnk1	Serine/thre	WNK1_MO	2	0.833879
15_MTA / C	Q8VC97	-0.36426	0.002648	0.039745	Upb1	Beta-ureidc	BUP1_MOU	6	0.776865
15_MTA / C	P02668	-2.31597	0.002711	0.04006	CSN3	Kappa-case	CASK_BOVI	2	0.200828
15_MTA / C	Q9D8Y0	-0.84145	0.002694	0.04006	Efhd2	EF-hand do	EFHD2_MO	5	0.55808
15_MTA / C	P28474	0.147636	0.002826	0.040914	Adh5	Alcohol deh	ADHX_MOU	21	1.107752
15_MTA / C	P61965	-0.82845	0.002837	0.040914	Wdr5	WD repeat-	WDR5_MO	4	0.563135
15_MTA / C	Q03249	-0.87383	0.00281	0.040914	Galt	Galactose-1	GALT_MOU	7	0.545695
15_MTA / C	Q8VED9	-2.20777	0.0028	0.040914	Lgalsl	Galectin-re	LEGL_MOU	1	0.216469
30_MTA / C	P10518	-0.16764	0.002894	0.041316	Alad	Delta-amin	HEM2_MOU	23	0.890297
30_MTA / C	P48024	0.411814	0.002904	0.041316	Eif1	Eukaryotic	EIF1_MOUS	5	1.330358
30_MTA / C	O54962	0.302149	0.003025	0.042703	Banf1	Barrier-to-a	BAF_MOUS	1	1.23298
30_MTA / C	Q03958	1.653911	0.003071	0.042855	Pfdn6	Prefoldin su	PFD6_MOU	4	3.146856
15_MTA / C	Q921M3	0.007811	0.003065	0.042855	Sf3b3	Splicing fac	SF3B3_MO	18	1.005429
15_MTA / C	O70318	0.03202	0.003096	0.043038	Epb41l2	Band 4.1-lil	E41L2_MO	3	1.022443
30_MTA / C	Q99JF8	0.452204	0.003175	0.043708	Psip1	PC4 and SFI	PSIP1_MOU	2	1.368128
15_MTA / C	P68037	-0.12119	0.003225	0.043868	Ube2l3	Ubiquitin-c	UB2L3_MO	9	0.91943
15_MTA / C	P97865	-1.06921	0.003254	0.043868	Pex7	Peroxisoma	PEX7_MOU	4	0.476579
15_MTA / C	Q571F8	-0.48271	0.003253	0.043868	Gls2	Glutaminas	GLSL_MOU	1	0.715632
15_MTA / C	Q3THS6	-0.61877	0.003496	0.045637	Mat2a	S-adenosylr	METK2_MC	6	0.651227
15_MTA / C	Q8VCG4	-0.58257	0.003486	0.045637	C8g	Complemer	CO8G_MOU	7	0.667775
15_MTA / C	P49962	-0.05574	0.003607	0.046585	Srp9	Signal reco	SRP09_MO	3	0.962099
15_MTA / C	Q3ULW8	-1.10817	0.003603	0.046585	Parp3	Protein mo	PARP3_MO	2	0.463883
15_MTA / C	Q99LL5	-1.14267	0.003635	0.046585	Pwp1	Periodic try	PWP1_MOU	3	0.45292
15_MTA / C	Q9JJU8	-0.39163	0.003625	0.046585	Sh3bgrl	Adapter Sh3	SH3L1_MO	6	0.762266
30_MTA / C	O88653	1.072887	0.003715	0.046942	Lamtor3	Ragulator c	LTOR3_MO	1	2.103639
15_MTA / C	Q3UJB0	-0.40886	0.00371	0.046942	Sf3b2	Splicing fac	SF3B2_MOU	3	0.753219
15_MTA / C	Q8BKC5	-1.03842	0.003719	0.046942	Ipo5	Importin-5	IPO5_MOU	4	0.48686
30_MTA / C	Q9R0M5	1.800167	0.003729	0.046942	Tpk1	Thiamine p	TPK1_MOU	2	3.482605
15_MTA / C	Q00PI9	-0.95891	0.003821	0.047765	Hnrnpul2	Heterogene	HNRL2_MC	5	0.514445
30_MTA / C	P17225	-0.18493	0.00389	0.048452	Ptbp1	Polypyrimi	PTBP1_MO	5	0.879689
15_MTA / C	O08808	-0.90839	0.003937	0.048541	Diaph1	Protein dia	DIAP1_MOU	2	0.532781
15_MTA / C	Q05421	-1.59911	0.00391	0.048541	Cyp2e1	Cytochrom	CP2E1_MO	1	0.33008
30_MTA / C	O54983	0.500524	0.003985	0.048844	Crym	Ketimine re	CRYM_MOU	1	1.414728

15_MTA / C	Q8BVQ5	-1.58644	0.003989	0.048844	Ppme1	Protein pho	PPME1_MO	2	0.332993
30_MTA / C	Q9DD18	0.999862	0.00408	0.049617	Dtd1	D-aminoac	DTD1_MOU	1	1.999808
15_MTA / C	Q3ULD5	-0.15833	0.004131	0.050073	Mccc2	Methylcrot	MCCB_MOU	21	0.896062
15_MTA / C	P58771	0.184043	0.004218	0.050617	Tpm1	Tropomyos	TPM1_MOU	4	1.136063
15_MTA / C	Q99KK2	-0.0825	0.004208	0.050617	Cmas	N-acylneur	NEUA_MOU	4	0.944423
15_MTA / C	P14152	0.141216	0.004392	0.051841	Mdh1	Malate deh	MDHC_MO	15	1.102834
15_MTA / C	Q64471	-0.47222	0.004405	0.051841	Gstt1	Glutathion	GSTT1_MO	8	0.720854
30_MTA / C	P04264	0.844569	0.004451	0.051983	KRT1	Keratin, typ	K2C1_HUM	34	1.795729
15_MTA / C	P48024	-0.1054	0.004441	0.051983	Eif1	Eukaryotic	EIF1_MOUS	5	0.929546
15_MTA / C	Q6PE15	-0.49365	0.004583	0.052541	Abhd10	Palmitoyl-p	ABHDA_MO	1	0.710228
15_MTA / C	Q9DCB8	-0.72248	0.004614	0.05273	Isca2	Iron-sulfur	ISCA2_MOU	3	0.606055
15_MTA / C	Q569Z6	-0.97312	0.00468	0.053294	Thrap3	Thyroid hor	TR150_MO	4	0.509403
15_MTA / C	P43274	0.363614	0.004826	0.054042	H1-4	Histone H1	H14_MOUS	2	1.286645
15_MTA / C	P62334	-0.19227	0.004811	0.054042	Psmc6	26S protea	PRS10_MO	5	0.87523
15_MTA / C	P97823	-2.60287	0.004809	0.054042	Lypla1	Acyl-protei	LYPA1_MO	4	0.16461
15_MTA / C	P09411	-0.13757	0.004863	0.054204	Pgk1	Phosphogly	PGK1_MOU	23	0.909049
30_MTA / C	Q61656	1.748586	0.004922	0.054228	Ddx5	Probable A	DDX5_MOU	2	3.36029
15_MTA / C	Q8BND5	-1.05629	0.004918	0.054228	Qsox1	Sulfhydryl o	QSOX1_MO	5	0.480868
15_MTA / C	Q8R121	-0.35362	0.004926	0.054228	Serpina10	Protein Z-d	ZPI_MOUSE	6	0.782618
30_MTA / C	Q9D115	-0.48365	0.004925	0.054228	Znf706	Zinc finger	ZN706_MO	1	0.715165
30_MTA / C	E9Q5C9	0.086734	0.005184	0.05554	Nolc1	Nucleolar a	NOLC1_MO	2	1.061963
30_MTA / C	P57746	0.588836	0.005156	0.05554	Atp6v1d	V-type prot	VATD_MOU	3	1.504033
30_MTA / C	Q9D8B3	1.003943	0.005131	0.05554	Chmp4b	Charged mu	CHM4B_MO	4	2.005474
15_MTA / C	P07356	0.300934	0.005287	0.055981	Anxa2	Annexin A2	ANXA2_MO	10	1.231942
15_MTA / C	Q7TQI3	-0.294	0.005279	0.055981	Otub1	Ubiquitin t	OTUB1_MO	6	0.81564
15_MTA / C	Q9D7J9	-1.22171	0.005342	0.0564	Echdc3	Enoyl-CoA h	ECHD3_MO	2	0.428774
15_MTA / C	Q8QZY2	-3.3569	0.00548	0.057516	Glyctk	Glycerate k	GLCTK_MO	2	0.097605
15_MTA / C	Q9D1G1	-1.83521	0.00553	0.057873	Rab1b	Ras-related	RAB1B_MO	1	0.280251
15_MTA / C	Q80YC5	-1.02146	0.005576	0.058182	F12	Coagulation	FA12_MOU	4	0.492618
30_MTA / C	P58771	0.209628	0.005619	0.058291	Tpm1	Tropomyos	TPM1_MOU	4	1.15639
15_MTA / C	Q9CQW1	-1.16831	0.005611	0.058291	Ykt6	Synaptobre	YKT6_MOU	5	0.444943
15_MTA / C	Q8BFQ8	-0.72661	0.005743	0.059237	Gatd1	Glutamine	GALD1_MO	4	0.604322
15_MTA / C	P14069	0.104785	0.005806	0.059722	S100a6	Protein S10	S10A6_MO	1	1.075334
15_MTA / C	Q8BHG1	-0.45512	0.00587	0.060031	Nrdc	Nardilysin	NRDC_MOU	10	0.729451
30_MTA / C	Q9DCH4	1.16442	0.005902	0.060192	Eif3f	Eukaryotic	EIF3F_MOU	2	2.241431
15_MTA / C	Q921W4	1.558291	0.00598	0.060611	Cryzl1	Quinone ox	QORL1_MO	2	2.945048
15_MTA / C	Q99K30	-1.1913	0.005975	0.060611	Eps8l2	Epidermal g	ES8L2_MOU	2	0.437908
15_MTA / C	P28271	-0.45044	0.006056	0.061069	Aco1	Cytoplasmi	ACOHC_MO	29	0.731818
15_MTA / C	Q8R3P0	-1.57573	0.006128	0.061524	Aspa	Aspartoacy	ACY2_MOU	2	0.335474
15_MTA / C	Q7TNG8	0.538328	0.00625	0.0625	Ldhd	Probable D	LDHD_MOU	18	1.452289
15_MTA / C	P16294	0.868385	0.006336	0.063122	F9	Coagulation	FA9_MOUS	3	1.825619
15_MTA / C	Q9QUI0	-0.46413	0.006347	0.063122	Rhoa	Transformi	RHOA_MOU	3	0.724907
15_MTA / C	P01630	0.316937	0.006479	0.063181	NaN	Ig kappa ch	KV2A6_MO	2	1.245683
15_MTA / C	Q8CG76	-1.06283	0.006596	0.063491	Akr7a2	Aflatoxin B	ARK72_MO	6	0.478693
30_MTA / C	P17563	-0.11831	0.006659	0.063756	Selenbp1	Methaneth	SBP1_MOU	36	0.921269
15_MTA / C	Q61656	1.179884	0.006784	0.064172	Ddx5	Probable A	DDX5_MOU	2	2.265585
15_MTA / C	Q6PIP5	-0.97461	0.006772	0.064172	Nudcd1	NudC doma	NUDC1_MO	9	0.508879
15_MTA / C	Q62448	-0.34648	0.006945	0.065114	Eif4g2	Eukaryotic	IF4G2_MOU	8	0.7865
15_MTA / C	Q8R1Q9	-0.52586	0.006938	0.065114	Rbks	Ribokinase	RBSK_MOU	6	0.694546
15_MTA / C	Q9WUL7	-0.50168	0.006972	0.065194	Arl3	ADP-ribosy	ARL3_MOU	1	0.706286
15_MTA / C	Q8BWR2	-1.06254	0.007014	0.06542	Pithd1	PITH domai	PITH1_MOU	6	0.478788
15_MTA / C	P0DP26;PC	0.170929	0.007061	0.065683	Calm1;Caln	Calmodulin	CALM1_MO	9	1.125783

15_MTA / C	Q9CQM5	-0.44033	0.007148	0.066323	Txndc17	Thioredoxi	TXD17_MO	5	0.736965
15_MTA / C	Q8BG73	-1.37021	0.007218	0.0668	Sh3bgrl2	SH3 domai	SH3L2_MO	5	0.386836
30_MTA / C	Q06138	0.10245	0.007306	0.067254	Cab39	Calcium-bi	CAB39_MO	8	1.073595
15_MTA / C	Q8BMA6	-0.33327	0.007356	0.067254	Srp68	Signal reco	SRP68_MO	5	0.793733
30_MTA / C	Q9CWW6	-1.29133	0.007463	0.067672	Pin4	Peptidyl-pr	PIN4_MOU	1	0.408573
30_MTA / C	Q9JI75	0.527195	0.007448	0.067672	Nqo2	Ribosyldihy	NQO2_MOU	4	1.441124
15_MTA / C	C0HKG5;C(	-0.64331	0.007512	0.067676	Rnaset2a;R	Ribonuclea	RNT2A_MO	1	0.640242
15_MTA / C	Q80SY6	-0.99496	0.007555	0.067676	Adal	Adenosine	ADAL_MOU	2	0.50175
15_MTA / C	O88451	-0.2234	0.007609	0.0678	Rdh7	Retinol deh	RDH7_MOU	9	0.856547
15_MTA / C	P52840	-1.40445	0.007753	0.068747	Sult1a1	Sulfotransf	ST1A1_MO	7	0.377763
15_MTA / C	Q9D8T7	-1.81388	0.007796	0.068956	Slirp	SRA stem-lo	SLIRP_MOU	1	0.284424
15_MTA / C	P46061	-0.54919	0.00782	0.068997	Rangap1	Ran GTPase	RAGP1_MO	12	0.683406
15_MTA / C	Q91V12	-0.76818	0.007868	0.069251	Acot7	Cytosolic a	BACH_MOU	6	0.587157
15_MTA / C	Q62452	-2.27751	0.008126	0.06989	Ugt1a9	UDP-glucur	UD19_MOU	4	0.206254
30_MTA / C	Q64471	-0.40806	0.008112	0.06989	Gstt1	Glutathion	GSTT1_MO	8	0.753637
15_MTA / C	Q6ZWU9;Q	-0.80253	0.008112	0.06989	Rps27;Rps2	Small ribos	RS27_MOU	2	0.573342
15_MTA / C	Q8BPB0;Q9	-1.78618	0.008034	0.06989	Mob1b;Mo	MOB kinase	MOB1B_M(	1	0.289939
15_MTA / C	Q99J99	-0.81055	0.008096	0.06989	Mpst	3-mercapto	THTM_MOU	3	0.570164
15_MTA / C	P26645	-0.49108	0.008207	0.070182	Marcks	Myristoylat	MARCS_MC	4	0.711493
15_MTA / C	P50429	-2.53758	0.008239	0.070284	Arsb	Arylsulfatas	ARSB_MOU	2	0.172232
15_MTA / C	Q61035	-1.6052	0.008332	0.070743	Hars1	Histidine--t	HARS1_MO	3	0.328691
15_MTA / C	Q9ERB0	1.170016	0.008428	0.071104	Snap29	Synaptosor	SNP29_MO	2	2.250142
30_MTA / C	Q9JHU2	2.341976	0.008434	0.071104	Palmd	Palmdelphi	PALMD_MC	2	5.069967
15_MTA / C	Q9QZS3	-0.67059	0.008413	0.071104	Numb	Protein nur	NUMB_MO	3	0.62825
15_MTA / C	A0JNU3	-0.40142	0.008458	0.071142	Aspg	60 kDa lyso	LPP60_MO	4	0.75711
15_MTA / C	P0C7N9	-1.60155	0.008558	0.071472	Psmg4	Proteasom	PSMG4_MC	1	0.329524
30_MTA / C	Q8CAY6	-0.14073	0.008577	0.071472	Acat2	Acetyl-CoA	THIC_MOUS	17	0.907062
30_MTA / C	P34928	-0.64657	0.008617	0.071643	Apoc1	Apolipopro	APOC1_MC	1	0.638797
15_MTA / C	P16858	-0.43021	0.008737	0.072359	Gapdh	Glyceraldel	G3P_MOUS	16	0.742155
15_MTA / C	Q9D8S9	-0.6426	0.008743	0.072359	Bola1	BolA-like pr	BOLA1_MO	5	0.640556
30_MTA / C	P56395	-0.68434	0.008784	0.072531	Cyb5a	Cytochrom	CYB5_MOU	4	0.622289
15_MTA / C	Q58A65	-0.19474	0.008824	0.072696	Spag9	C-Jun-amin	JIP4_MOUS	15	0.873733
15_MTA / C	O88342	-0.18647	0.008943	0.073248	Wdr1	WD repeat-	WDR1_MO	19	0.878753
15_MTA / C	P56380	-0.67644	0.008958	0.073248	Nudt2	Bis(5'-nucle	AP4A_MOU	2	0.625705
30_MTA / C	Q62264	0.960491	0.008973	0.073248	Thrsp	Thyroid ho	THRSP_MO	3	1.945972
15_MTA / C	Q91YP2	-0.34181	0.008944	0.073248	Nln	Neurolysin,	NEUL_MOU	10	0.789053
30_MTA / C	Q9D1F4	0.699341	0.009035	0.073588	Akt1s1	Proline-ricl	AKTS1_MO	2	1.623762
30_MTA / C	P61079	0.165192	0.009058	0.073607	Ube2d3	Ubiquitin-c	UB2D3_MC	1	1.121316
15_MTA / C	Q8BTI8	0.767876	0.009182	0.073703	Srrm2	Serine/argi	SRRM2_MC	5	1.702762
15_MTA / C	Q9DD20	-0.81765	0.009192	0.073703	Tmt1b	Thiol S-met	TMT1B_MC	4	0.567365
15_MTA / C	Q9D0I9	-1.46783	0.009291	0.07416	Rars1	Arginine--tl	SYRC_MOU	1	0.361525
15_MTA / C	Q8BFY9	-0.76349	0.009382	0.074648	Tnpo1	Transportir	TNPO1_MC	4	0.589068
15_MTA / C	Q3UNZ8	-0.21116	0.009424	0.074725	Cryzl2	Quinone ox	QORL2_MC	7	0.863841
15_MTA / C	Q99LT0	-0.32958	0.00953	0.075237	Dpy30	Protein dpy	DPY30_MO	2	0.795771
30_MTA / C	Q78JE5	1.672293	0.009668	0.075562	Fbxo22	F-box only	FBX22_MO	2	3.187207
15_MTA / C	O88685	-0.08042	0.009806	0.076244	Psmc3	26S protea	PRS6A_MO	8	0.945782
15_MTA / C	Q9QZ88	-0.65669	0.009829	0.076262	Vps29	Vacuolar pr	VPS29_MO	5	0.634332
30_MTA / C	P24549	-0.35541	0.010088	0.076944	Aldh1a1	Aldehyde d	AL1A1_MO	21	0.781649
30_MTA / C	P47968	-0.72346	0.010072	0.076944	Rpia	Ribose-5-pl	RPIA_MOUS	1	0.605645
15_MTA / C	Q922D8	-1.75592	0.010068	0.076944	Mthfd1	C-1-tetrahy	C1TC_MOU	11	0.296083
15_MTA / C	O35488	1.909524	0.010187	0.077045	Slc27a2	Long-chain	S27A2_MO	1	3.756851
30_MTA / C	Q8VBW6	0.468621	0.010171	0.077045	Nae1	NEDD8-acti	ULA1_MOU	5	1.383787

15_MTA / C	Q9CYA0	-0.77428	0.010141	0.077045	Creld2	Protein dis	CREL2_MOU	4	0.584681
30_MTA / C	O88668	0.209611	0.01035	0.077946	Creg1	Protein CRE	CREG1_MO	4	1.156376
15_MTA / C	Q8K3H0	-1.76074	0.010456	0.078139	Appl1	DCC-intera	DP13A_MO	2	0.295096
15_MTA / C	Q9DCL8	-0.81587	0.010516	0.078217	Ppp1r2	Protein pho	IPP2_MOUS	1	0.568066
15_MTA / C	Q02780	-0.93377	0.010611	0.078598	Nfia	Nuclear fac	NFIA_MOUS	3	0.52349
30_MTA / C	P47941	0.217434	0.010704	0.078702	Crkl	Crk-like pro	CRKL_MOU	7	1.162664
15_MTA / C	Q9CQI3	-0.58733	0.010715	0.078702	Gmfb	Glia matura	GMFB_MOU	4	0.665573
15_MTA / C	Q9CZY3;Q9	-0.53619	0.010734	0.078702	Ube2v1;Ub	Ubiquitin-c	UB2V1_MO	3	0.689589
15_MTA / C	P62320	-0.51377	0.01083	0.078807	Snrpd3	Small nucle	SMD3_MOU	5	0.700391
15_MTA / C	P99025	-0.36278	0.010812	0.078807	Gchfr	GTP cycloh	GFRP_MOU	6	0.777667
15_MTA / C	Q61189	-0.39507	0.010831	0.078807	Clns1a	Methylosor	ICLN_MOUS	1	0.760452
15_MTA / C	Q78ZA7	-0.98111	0.010858	0.078807	Nap1l4	Nucleosom	NP1L4_MO	3	0.506589
15_MTA / C	Q5FW60	-0.01615	0.0109	0.078956	Mup20	Major urina	MUP20_MO	3	0.988865
15_MTA / C	P45952	-0.15058	0.010974	0.079171	Acadm	Medium-ch	ACADM_MO	11	0.900887
30_MTA / C	P05202	-0.22498	0.01101	0.079275	Got2	Aspartate a	AATM_MOU	32	0.855609
15_MTA / C	Q8BVY0	-0.81666	0.011047	0.079379	Rsl1d1	Ribosomal	RL1D1_MO	2	0.567754
30_MTA / C	Q6IRU5	0.298707	0.011188	0.079916	Cltb	Clathrin lig	CLCB_MOU	5	1.230042
15_MTA / C	Q923D2	-0.07448	0.011176	0.079916	Blvrb	Flavin redu	BLVRB_MO	11	0.949686
30_MTA / C	Q80WJ7	0.693925	0.011334	0.080481	Mtdh	Protein LYF	LYRIC_MOU	5	1.617678
15_MTA / C	Q8R1B4	-0.56547	0.011312	0.080481	Eif3c	Eukaryotic	EIF3C_MOU	6	0.675734
15_MTA / C	Q9CQX8	-0.75495	0.011334	0.080481	Kgd4	Alpha-keto	KGD4_MOU	4	0.592565
30_MTA / C	P56394	0.325018	0.011461	0.080945	Cox17	Cytochrom	COX17_MO	1	1.25268
15_MTA / C	P70694	-0.36649	0.011477	0.080945	Akr1c6	Estradiol 17	DHB5_MOU	16	0.775665
15_MTA / C	Q2TPA8	-1.12675	0.011463	0.080945	Hsdl2	Hydroxyste	HSDL2_MO	2	0.457946
15_MTA / C	Q62188	-0.7914	0.011513	0.080945	Dpysl3	Dihydropyr	DPYL3_MO	2	0.577783
15_MTA / C	Q99KP3	0.199742	0.011535	0.080945	Cryl1	Lambda-cry	CRYL1_MO	9	1.148493
15_MTA / C	Q6P8I4	0.544112	0.011633	0.081401	Pcnp	PEST protec	PCNP_MOU	2	1.458122
30_MTA / C	Q99020	0.256965	0.011941	0.08267	Hnrnpab	Heterogene	ROAA_MOU	5	1.194962
15_MTA / C	E9Q5C9	0.269355	0.012073	0.083105	Nolc1	Nucleolar a	NOLC1_MO	2	1.205269
30_MTA / C	O35215	-0.41072	0.012175	0.083105	Ddt	D-dopachro	DOPD_MOU	13	0.752246
30_MTA / C	P27546	0.391133	0.012115	0.083105	Map4	Microtubul	MAP4_MOU	19	1.311423
15_MTA / C	P56387	-0.82459	0.012169	0.083105	Dynlt3	Dynein ligh	DYLT3_MO	3	0.564642
15_MTA / C	Q64458	-0.72597	0.012194	0.083105	Cyp2c29	Cytochrom	CP2CT_MO	2	0.604589
15_MTA / C	Q60994	-0.09346	0.012417	0.083864	Adipoq	Adiponecti	ADIPO_MO	1	0.937274
15_MTA / C	Q91XE4	-0.23192	0.012394	0.083864	Acy3	N-acyl-aron	ACY3_MOU	6	0.851499
30_MTA / C	P46938	0.471219	0.012544	0.084143	Yap1	Transcripti	YAP1_MOU	2	1.38628
15_MTA / C	P99024	-0.9035	0.012662	0.084143	Tubb5	Tubulin bet	TBB5_MOU	2	0.534588
30_MTA / C	Q7TPC1	1.235958	0.012716	0.084143	Cdsn	Corneodesr	CDSN_MOU	1	2.355378
15_MTA / C	Q8BGD9	0.256767	0.012647	0.084143	Eif4b	Eukaryotic	IF4B_MOUS	17	1.194798
15_MTA / C	Q91X44	-1.23359	0.012731	0.084143	Gckr	Glucokinas	GCKR_MOU	7	0.425257
15_MTA / C	Q6IFZ6	1.267674	0.012841	0.084511	Krt77	Keratin, typ	K2C1B_MO	3	2.407731
15_MTA / C	Q8VDD5	-0.7137	0.012833	0.084511	Myh9	Myosin-9	MYH9_MOU	20	0.609756
30_MTA / C	P70122	0.889501	0.013021	0.085539	Sbds	Ribosome r	SBDS_MOU	5	1.852536
15_MTA / C	P58710	-2.52739	0.013251	0.086372	Gulo	L-gulonolac	GGLO_MOU	3	0.173452
15_MTA / C	Q501J6	-1.02444	0.013222	0.086372	Ddx17	Probable AT	DDX17_MO	3	0.491603
30_MTA / C	Q91V64	0.18185	0.013343	0.086393	Isoc1	Isochorism	ISOC1_MOU	9	1.134337
30_MTA / C	Q9JKX6	0.920396	0.013342	0.086393	Nudt5	ADP-sugar p	NUDT5_MO	2	1.892635
15_MTA / C	P51881	-1.90199	0.013514	0.087345	Slc25a5	ADP/ATP tr	ADT2_MOU	5	0.267574
15_MTA / C	P41317	-0.72407	0.013544	0.087382	Mbl2	Mannose-b	MBL2_MOU	4	0.605386
30_MTA / C	Q9DCN1	0.11488	0.013673	0.087744	Nudt12	NAD-cappe	NUD12_MO	7	1.082885
15_MTA / C	P62313	-1.07156	0.013728	0.087908	Lsm6	U6 snRNA-a	LSM6_MOU	3	0.475806
15_MTA / C	Q07417	-0.28298	0.013772	0.087908	Acads	Short-chair	ACADS_MO	11	0.821894

30_MTA / C	Q8BTI8	1.331976	0.013762	0.087908	Srrm2	Serine/argi	SRRM2_MO	5	2.517472
30_MTA / C	P56380	-1.81928	0.013868	0.088051	Nudt2	Bis(5'-nucle	AP4A_MOU	2	0.283362
15_MTA / C	P58774	0.284237	0.013858	0.088051	Tpm2	Tropomyos	TPM2_MOU	8	1.217766
30_MTA / C	Q3TLH4	0.411331	0.013898	0.088088	Prrc2c	Protein PRI	PRC2C_MO	5	1.329912
15_MTA / C	Q9JIL4	-0.11359	0.014076	0.089056	Pdzk1	Na(+)/H(+) e	NHRF3_MO	26	0.924287
15_MTA / C	Q3B7Z2	-0.09986	0.014473	0.090204	Osbp	Oxysterol-b	OSBP1_MO	6	0.933122
30_MTA / C	Q8QZR5	0.147798	0.014445	0.090204	Gpt	Alanine am	ALAT1_MOU	25	1.107877
30_MTA / C	Q9D172	-0.28126	0.014483	0.090204	Gatd3	Glutamine	GAL3A_MO	12	0.822873
15_MTA / C	O35841	-0.03837	0.014917	0.090865	Api5	Apoptosis i	API5_MOUS	7	0.973754
30_MTA / C	P13645	1.088319	0.014797	0.090865	KRT10	Keratin, typ	K1C10_HUI	30	2.126262
15_MTA / C	P30115	-0.15505	0.014756	0.090865	Gsta3	Glutathion	GSTA3_MO	11	0.898102
15_MTA / C	P62311	-0.35861	0.014887	0.090865	Lsm3	U6 snRNA-a	LSM3_MOU	2	0.779917
30_MTA / C	Q14CH1	0.282103	0.014899	0.090865	Mocos	Molybdenu	MOCOS_MO	20	1.215966
15_MTA / C	Q6PB66	-0.33844	0.01477	0.090865	Lrpprc	Leucine-ric	LPPRC_MO	4	0.790895
30_MTA / C	Q8JZR0	-1.5836	0.014887	0.090865	Acsl5	Long-chain	ACSL5_MOI	1	0.333648
30_MTA / C	Q921W4	1.797733	0.014854	0.090865	Cryzl1	Quinone ox	QORL1_MO	2	3.476735
15_MTA / C	Q9WUM5	-0.52969	0.014676	0.090865	Suclg1	Succinate--	SUCA_MOU	6	0.692701
30_MTA / C	Q64674	2.091891	0.014959	0.09097	Srm	Spermidine	SPEE_MOUS	1	4.263064
15_MTA / C	O35405	-2.9002	0.015031	0.091249	Pld3	5'-3' exonu	PLD3_MOU	2	0.133953
15_MTA / C	P19157	-0.51112	0.015107	0.091489	Gstp1	Glutathion	GSTP1_MO	11	0.701676
15_MTA / C	P70265;P7	-1.92735	0.015212	0.091885	Pfkfb2;Pfkfl	6-phosphof	F262_MOU	1	0.262911
30_MTA / C	P36552	0.220838	0.015381	0.092207	Cpox	Oxygen-dep	HEM6_MOU	21	1.16541
30_MTA / C	Q6IFZ6	1.096821	0.015355	0.092207	Krt77	Keratin, typ	K2C1B_MO	3	2.138829
15_MTA / C	Q9JM14	-1.50806	0.015393	0.092207	Nt5c	5'(3')-deoxy	NT5C_MOU	6	0.351585
15_MTA / C	Q9R112	-1.12468	0.015365	0.092207	Sqor	Sulfide:qui	SQOR_MOU	5	0.458604
15_MTA / C	P53612	-1.98206	0.015881	0.094341	Rabggtb	Geranylger	PGTB2_MO	2	0.253129
30_MTA / C	Q9QXD6	-0.08344	0.015839	0.094341	Fbp1	Fructose-1,	F16P1_MO	28	0.943802
30_MTA / C	P21300	0.298966	0.016131	0.094664	Akr1b7	Aldo-keto r	ALD1_MOU	1	1.230263
15_MTA / C	P35235	-0.88195	0.016145	0.094664	Ptpn11	Tyrosine-pr	PTN11_MO	4	0.542632
15_MTA / C	Q61937	0.118754	0.016112	0.094664	Npm1	Nucleopho	NPM_MOU	9	1.085797
15_MTA / C	Q91YE6	-0.35384	0.016087	0.094664	Ipo9	Importin-9	IPO9_MOU	6	0.782501
15_MTA / C	Q9D0M5	-0.95777	0.015972	0.094664	Dynll2	Dynein ligh	DYL2_MOU	5	0.51485
15_MTA / C	Q9WV55	-0.25344	0.016094	0.094664	Vapa	Vesicle-asso	VAPA_MOU	3	0.838893
15_MTA / C	Q9Z0S1	-0.63148	0.016111	0.094664	Bpnt1	3'(2'),5'-bis	BPNT1_MO	8	0.645514
30_MTA / C	P06151	-0.19147	0.016308	0.095309	Ldha	L-lactate de	LDHA_MOU	23	0.875715
15_MTA / C	P02662	-2.85592	0.016572	0.09614	CSN1S1	Alpha-S1-ca	CASA1_BOV	3	0.138128
15_MTA / C	Q64514	-1.12363	0.016545	0.09614	Tpp2	Tripeptidyl	TPP2_MOU	6	0.458937
15_MTA / C	P51125	-0.63297	0.016872	0.096158	Cast	Calpastatin	ICAL_MOUS	6	0.644846
15_MTA / C	P55302	-0.40724	0.016615	0.096158	Lrpap1	Alpha-2-ma	AMRP_MOU	5	0.754063
30_MTA / C	P62858	0.453877	0.016918	0.096158	Rps28	Small ribos	RS28_MOU	3	1.369716
30_MTA / C	Q91V92	0.259451	0.016711	0.096158	Acly	ATP-citrate	ACLY_MOU	30	1.197023
15_MTA / C	Q9CWW6	-1.64165	0.016787	0.096158	Pin4	Peptidyl-pr	PIN4_MOU	1	0.32049
30_MTA / C	Q9R1Z7	0.392086	0.016934	0.096158	Pts	6-pyruvoyl	PTPS_MOU	5	1.31229
15_MTA / C	P19639	-0.4132	0.017039	0.096449	Gstm3	Glutathion	GSTM3_MO	12	0.750956
15_MTA / C	P23780	-0.22368	0.017108	0.096684	Glb1	Beta-galact	BGAL_MOU	3	0.85638
15_MTA / C	Q8BH35	-0.61852	0.017166	0.096862	C8b	Compleme	CO8B_MOU	5	0.65134
15_MTA / C	P16406	-0.14157	0.017375	0.097472	Enpep	Glutamyl a	AMPE_MOU	11	0.906535
15_MTA / C	P84089	-1.13665	0.017417	0.097472	Erh	Enhancer o	ERH_MOUS	2	0.454816
15_MTA / C	Q78PY7	-0.35398	0.01745	0.097472	Snd1	Staphyloco	SND1_MOU	13	0.782422
15_MTA / C	Q922R8	-0.17354	0.017403	0.097472	Pdia6	Protein dis	PDIA6_MO	12	0.886665
30_MTA / C	Q9D1P4	-0.44368	0.017464	0.097472	Chordc1	Cysteine an	CHRD1_MO	8	0.735258
15_MTA / C	Q99JX3	-0.52759	0.017522	0.097645	Gorasp2	Golgi reass	GORS2_MO	5	0.693712

30_MTA / C	P99025	-0.38631	0.017583	0.097692	Gchfr	GTP cycloh	GFRP_MOU	6	0.765082
30_MTA / C	Q9CRD0	-1.25535	0.01772	0.097692	Ociad1	OCIA doma	OCAD1_MC	2	0.418893
15_MTA / C	Q9JM62	-0.50986	0.017654	0.097692	Reep6	Receptor e:	REEP6_MOI	2	0.70229
15_MTA / C	Q9Z1R2	-0.44116	0.017696	0.097692	Bag6	Large prolii	BAG6_MOU	7	0.73654
30_MTA / C	Q9CQE1	-0.10307	0.017959	0.098581	Nipsnap3b	Protein Nip	NPS3B_MO	12	0.93105
15_MTA / C	Q9CQ89	-0.42945	0.018214	0.099502	Cuta	Protein Cut	CUTA_MOU	4	0.742547
15_MTA / C	P14206	-0.85156	0.018432	0.100385	Rpsa	Small ribos	RSSA_MOU	2	0.554185
15_MTA / C	P84091	-0.41901	0.018797	0.101912	Ap2m1	AP-2 compl	AP2M1_MC	12	0.747939
15_MTA / C	Q01730	-4.27191	0.018837	0.101977	Rsu1	Ras suppres	RSU1_MOU	1	0.051764
30_MTA / C	P45952	-0.31756	0.019033	0.102824	Acadm	Medium-ch	ACADM_MC	11	0.802427
30_MTA / C	Q4VAA2	0.372053	0.019054	0.102824	Cdv3	Protein CD'	CDV3_MOU	8	1.294193
15_MTA / C	P57746	0.360435	0.019162	0.102934	Atp6v1d	V-type prot	VATD_MOU	3	1.283813
30_MTA / C	Q9R062	1.045199	0.019186	0.102934	Gyg1	Glycogenin	GLYG_MOU	2	2.06365
15_MTA / C	Q64105	-0.87609	0.019404	0.103643	Spr	Sepiapterir	SPRE_MOU	7	0.544842
15_MTA / C	P17426	-0.15854	0.019501	0.103854	Ap2a1	AP-2 compl	AP2A1_MO	17	0.895933
15_MTA / C	Q9CZS1	-0.92507	0.01954	0.103904	Aldh1b1	Aldehyde d	AL1B1_MO	8	0.526653
15_MTA / C	Q9D404	-0.2359	0.019605	0.104097	Oxsm	3-oxoacyl-[	OXSM_MOI	14	0.849155
30_MTA / C	P16406	-0.03426	0.019754	0.104274	Enpep	Glutamyl ai	AMPE_MOU	11	0.976534
15_MTA / C	Q8BWF0	-0.05408	0.019737	0.104274	Aldh5a1	Succinate-s	SSDH_MOU	16	0.96321
15_MTA / C	Q8K2Q9	-0.40457	0.019752	0.104274	Shtn1	Shootin-1	SHOT1_MO	5	0.755462
15_MTA / C	Q9QX60	-1.15664	0.019699	0.104274	Dguok	Deoxyguan	DGUOK_MC	2	0.448556
30_MTA / C	Q8BWG8	0.132659	0.01989	0.104683	Arrb1	Beta-arresti	ARRB1_MO	2	1.096312
30_MTA / C	Q9NSB2	3.719668	0.019948	0.104683	KRT84	Keratin, typ	KRT84_HUI	7	13.17443
15_MTA / C	O35660	-0.21512	0.020325	0.106353	Gstm6	Glutathion	GSTM6_MC	6	0.861473
30_MTA / C	Q9CYI0	-0.1068	0.020413	0.106657	NaN	Protein Njn	NJMU_MOI	2	0.928644
15_MTA / C	Q9DCS2	-0.51393	0.020504	0.106976	Mettl26	Methyltran	MTL26_MC	5	0.700314
15_MTA / C	Q8VEM8	-2.22863	0.020831	0.108126	Slc25a3	Solute carri	S25A3_MO	1	0.213361
15_MTA / C	Q9D6Y7	-0.27359	0.021032	0.108945	Msra	Mitochond	MSRA_MOI	9	0.827258
15_MTA / C	Q9Z1N5	-0.05931	0.021078	0.109026	Ddx39b	Spliceosom	DX39B_MC	14	0.959721
15_MTA / C	Q9D7P6	0.280977	0.021199	0.109494	Iscu	Iron-sulfur	ISCU_MOU!	4	1.215017
15_MTA / C	Q8BZW8	-0.06004	0.02169	0.111548	Nhlrc2	NHL repeat	NHLC2_MC	6	0.959241
15_MTA / C	O08547	-3.00651	0.021799	0.111816	Sec22b	Vesicle-traf	SC22B_MO	2	0.124437
15_MTA / C	P70274	-0.46628	0.02193	0.111822	Selenop	Selenoprot	SEPP1_MOI	4	0.723827
30_MTA / C	Q8BP40	0.162202	0.021882	0.111822	Acp6	Lysophospl	PPA6_MOU	14	1.118993
15_MTA / C	P57759	-0.26331	0.022055	0.111873	Erp29	Endoplasm	ERP29_MO	5	0.833175
15_MTA / C	Q9WV32	0.632838	0.022051	0.111873	Arpc1b	Actin-relate	ARC1B_MO	8	1.550612
15_MTA / C	Q99JF8	-0.11741	0.022232	0.11241	Psip1	PC4 and SFI	PSIP1_MOU	2	0.921839
15_MTA / C	Q9DBA8	-0.82384	0.022207	0.11241	Amdhd1	Probable in	HUTI_MOU	11	0.564935
15_MTA / C	P42932	0.040068	0.022276	0.112471	Cct8	T-complex		TCPQ_MOU	30	1.028162
15_MTA / C	Q9CQ62	-0.42586	0.022477	0.113328	Decr1	2,4-dienoyl	DECR_MOU	8	0.744394
30_MTA / C	O35343	0.511612	0.022515	0.113363	Kpna4	Importin su	IMA3_MOU	9	1.425642
15_MTA / C	Q9D0E1	-0.98668	0.022548	0.113372	Hnrnpm	Heterogene	HNRPM_M(	4	0.504637
15_MTA / C	Q99L27	-0.24794	0.022662	0.113627	Gmpr2	GMP reduc	GMPR2_M(	7	0.8421
15_MTA / C	P01644;P0	-1.02185	0.022748	0.113655	NaN	Ig kappa ch	KV5AB_MO	3	0.492484
30_MTA / C	P35505	-0.14666	0.022763	0.113655	Fah	Fumarylace	FAAA_MOU	28	0.90334
15_MTA / C	Q00724	-0.20021	0.022727	0.113655	Rbp4	Retinol-bin	RET4_MOU	8	0.870426
15_MTA / C	Q9CR41	-1.923	0.023176	0.11556	Hypk	Huntingtin	HYPK_MOU	1	0.263706
30_MTA / C	P70671	0.626128	0.023244	0.115608	Irf3	Interferon r	IRF3_MOUS	2	1.543417
15_MTA / C	Q9R0M5	1.916564	0.02325	0.115608	Tpk1	Thiamine p	TPK1_MOU	2	3.775229
15_MTA / C	P52480	-0.01827	0.023351	0.115886	Pkm	Pyruvate ki	KPYM_MOL	14	0.987416
15_MTA / C	Q9CZL5	-0.19465	0.02337	0.115886	Pcbd2	Pterin-4-alı	PHS2_MOU	5	0.873787
30_MTA / C	Q01730	-1.01271	0.023485	0.115974	Rsu1	Ras suppres	RSU1_MOU	1	0.495614

15_MTA / C	Q9CQR6	-0.6848	0.023579	0.11614	Ppp6c	Serine/thre	PPP6_MOU	7	0.622092
30_MTA / C	Q9D7M1	-0.51739	0.023583	0.11614	Gid8	Glucose-in(	GID8_MOU	1	0.698636
15_MTA / C	O70475	-1.1513	0.023738	0.116443	Ugdh	UDP-glucos	UGDH_MO	7	0.450219
15_MTA / C	Q78JE5	-0.27423	0.023895	0.116443	Fbxo22	F-box only	FBX22_MO	2	0.826892
15_MTA / C	Q922Y1	0.080741	0.023769	0.116443	Ubxn1	UBX domai	UBXN1_MC	9	1.057561
15_MTA / C	Q99KI0	-0.48152	0.023856	0.116443	Aco2	Aconitate h	ACON_MOU	14	0.716222
15_MTA / C	Q9JJH1	-0.92061	0.024189	0.117361	Rnase4	Ribonuclea	RNAS4_MO	4	0.528285
15_MTA / C	Q61823	-0.47654	0.02437	0.11776	Pdcd4	Programme	PDCD4_MC	4	0.718698
15_MTA / C	P53810	-0.13586	0.024432	0.117903	Pitpna	Phosphatic	PIPNA_MO	5	0.910127
30_MTA / C	Q9JHR7	0.423799	0.024643	0.118604	Ide	Insulin-deg	IDE_MOUSI	22	1.341455
15_MTA / C	Q9JKV1	-0.8145	0.024626	0.118604	Adrm1	Proteasom	ADRM1_M(	1	0.568604
15_MTA / C	Q8R1K4	-1.07264	0.024975	0.11972	Phykpl	5-phosphol	AT2L2_MO	3	0.475449
30_MTA / C	Q9CPY7	-0.0808	0.024971	0.11972	Lap3	Cytosol am	AMPL_MOU	30	0.945534
15_MTA / C	Q8CAY6	-0.10836	0.02514	0.120274	Acat2	Acetyl-CoA	THIC_MOUS	17	0.927642
15_MTA / C	Q9D6F9	-3.43942	0.025157	0.120274	Tubb4a	Tubulin bet	TBB4A_MO	1	0.092179
15_MTA / C	Q8CAK1	0.392832	0.025472	0.121456	Iba57	Putative tra	CAF17_MO	3	1.312968
15_MTA / C	Q9EPL9	-0.53132	0.025615	0.121978	Acox3	Peroxisoma	ACOX3_MC	3	0.691919
30_MTA / C	Q61206	0.57751	0.025872	0.12239	Pafah1b2	Platelet-act	PA1B2_MO	4	1.492271
15_MTA / C	Q9D7P9	0.73833	0.025772	0.12239	Serpinb12	Serpin B12	SPB12_MO	1	1.668244
15_MTA / C	Q9JHU2	1.506777	0.025841	0.12239	Palmd	Palmdelphi	PALMD_MC	2	2.841746
15_MTA / C	O35295	-0.14438	0.025942	0.122435	Purb	Transcripti	PURB_MOU	8	0.904767
15_MTA / C	Q920E5	-0.84455	0.026035	0.12268	Fdps	Farnesyl py	FPPS_MOU	11	0.556886
30_MTA / C	P11725	-0.18105	0.026429	0.122825	Otc	Ornithine t	OTC_MOUS	25	0.882064
15_MTA / C	P53808	0.998208	0.026501	0.122825	Pctp	Phosphatic	PPCT_MOU	1	1.997517
15_MTA / C	P61358	-0.25823	0.026578	0.122825	Rpl27	Large ribos	RL27_MOU	6	0.836111
15_MTA / C	P68372	-0.18755	0.026308	0.122825	Tubb4b	Tubulin bet	TBB4B_MO	18	0.878094
30_MTA / C	P97372	-0.19642	0.026412	0.122825	Psme2	Proteasom	PSME2_MC	15	0.872713
30_MTA / C	Q91YR1	0.068481	0.026408	0.122825	Twf1	Twinfilin-1	TWF1_MOU	7	1.048612
15_MTA / C	Q921I2	-2.04729	0.026109	0.122825	Klhdc4	Kelch doma	KLDC4_MO	1	0.241938
30_MTA / C	Q922U2	1.975052	0.026547	0.122825	Krt5	Keratin, typ	K2C5_MOU	9	3.931425
30_MTA / C	Q93092	-0.02643	0.026466	0.122825	Taldo1	Transaldola	TALDO_MO	16	0.981847
30_MTA / C	Q9D0B5	-0.73658	0.026378	0.122825	Tstd3	Thiosulfate	TSTD3_MO	2	0.600162
15_MTA / C	Q9JMH6	-0.07314	0.026274	0.122825	Txnrd1	Thioredoxi	TRXR1_MO	21	0.95057
30_MTA / C	Q9QXF8	-0.23683	0.026551	0.122825	Gnmt	Glycine N-n	GNMT_MO	20	0.848607
15_MTA / C	Q9CY97	-1.58945	0.026771	0.123423	Ssu72	RNA polym	SSU72_MO	1	0.332298
30_MTA / C	Q9DBF1	-0.05577	0.026831	0.123521	Aldh7a1	Alpha-amir	AL7A1_MO	27	0.962083
15_MTA / C	P70697	-0.24509	0.026925	0.123521	Urod	Uroporphy	DCUP_MOU	3	0.843764
30_MTA / C	Q8R164	-0.09269	0.026934	0.123521	Bphl	Valacyclovi	BPHL_MOU	19	0.937771
30_MTA / C	O35864	0.499185	0.027168	0.124116	Cops5	COP9 signa	CSN5_MOU	4	1.413415
15_MTA / C	Q9CYR0	-0.33541	0.027254	0.124351	Ssbp1	Single-strar	SSBP_MOU	5	0.79256
30_MTA / C	Q80VP1	0.392435	0.027676	0.125957	Epn1	Epsin-1	EPN1_MOU	2	1.312607
30_MTA / C	P19157	-0.51169	0.028113	0.127223	Gstp1	Glutathion	GSTP1_MO	11	0.701399
30_MTA / C	Q9QXT0	0.302114	0.028101	0.127223	Cnpy2	Protein car	CNPY2_MO	6	1.23295
30_MTA / C	P60521	-0.4295	0.028422	0.127896	Gabarapl2	Gamma-am	GBRL2_MO	2	0.742519
30_MTA / C	Q99KP3	0.290118	0.028457	0.127896	Cryl1	Lambda-cry	CRYL1_MO	9	1.22274
30_MTA / C	Q9Z1Q5	-1.05369	0.028386	0.127896	Clic1	Chloride in	CLIC1_MOU	3	0.481734
15_MTA / C	P11438	-0.19805	0.02864	0.128559	Lamp1	Lysosome-a	LAMP1_MC	2	0.871728
30_MTA / C	Q922Y1	0.47982	0.028911	0.129615	Ubxn1	UBX domai	UBXN1_MC	9	1.39457
15_MTA / C	Q8VBV7	-1.51917	0.029028	0.129815	Cops8	COP9 signa	CSN8_MOU	2	0.348887
30_MTA / C	Q9EQS3	1.285208	0.029075	0.129866	Mycbp	c-Myc-bind	MYCBP_MC	2	2.437171
30_MTA / C	P54726	0.358067	0.029151	0.130001	Rad23a	UV excisior	RD23A_MC	6	1.281708
15_MTA / C	Q9ET22	-1.58101	0.029178	0.130001	Dpp7	Dipeptidyl	DPP2_MOU	2	0.334248

15_MTA / C	P28063	-0.08669	0.029277	0.13012	Psmb8	Proteasom	PSB8_MOU	12	0.941679
30_MTA / C	P48428	0.438931	0.029437	0.130573	Tbca	Tubulin-spe	TBCA_MOU	6	1.3556
30_MTA / C	Q9JLQ0	0.58655	0.029451	0.130573	Cd2ap	CD2-associ	CD2AP_MC	5	1.501652
15_MTA / C	P62983	0.320797	0.029533	0.130776	Rps27a	Ubiquitin-r	RS27A_MO	6	1.249021
15_MTA / C	O88696	-0.64364	0.029571	0.130782	Clpp	ATP-depend	CLPP_MOU	4	0.640098
15_MTA / C	O88545	-0.08905	0.029752	0.130861	Cops6	COP9 signa	CSN6_MOU	6	0.940144
30_MTA / C	P51855	0.238465	0.029673	0.130861	Gss	Glutathion	GSHB_MOU	13	1.179737
30_MTA / C	Q3UGC7;Q	0.284865	0.02964	0.130861	Eif3j1;Eif3j	Eukaryotic	EI3JA_MOU	8	1.218296
30_MTA / C	O08807	-0.18326	0.030029	0.131035	Prdx4	Peroxiredo	PRDX4_MC	11	0.880714
15_MTA / C	P05063	-0.75406	0.029886	0.131035	Aldoc	Fructose-bi	ALDOC_MC	5	0.592931
30_MTA / C	P34884	-0.59046	0.029971	0.131035	Mif	Macrophag	MIF_MOUS	5	0.664133
15_MTA / C	Q8VCX1	-0.2439	0.029976	0.131035	Akr1d1	Aldo-keto r	AK1D1_MC	18	0.844461
30_MTA / C	Q9CPV4	0.207031	0.030004	0.131035	Glod4	Glyoxalase	GLOD4_MC	16	1.154311
30_MTA / C	P70694	-0.32588	0.030148	0.131236	Akr1c6	Estradiol 1	DHB5_MOU	16	0.797814
15_MTA / C	P62257	-0.68715	0.030287	0.131525	Ube2h	Ubiquitin-c	UBE2H_MC	1	0.621081
15_MTA / C	Q9EST5	-0.28597	0.030387	0.131642	Anp32b	Acidic leuci	AN32B_MC	4	0.820191
30_MTA / C	Q9WVE8	-1.10215	0.030588	0.132139	Pacsin2	Protein kin	PACN2_MC	5	0.465822
15_MTA / C	O08539	-0.45986	0.031347	0.133078	Bin1	Myc box-de	BIN1_MOU	3	0.727054
30_MTA / C	P02769	-0.49464	0.031049	0.133078	ALB	Serum albu	ALBU_BOVI	77	0.70974
15_MTA / C	P51150	-1.57253	0.031076	0.133078	Rab7a	Ras-related	RAB7A_MO	3	0.336218
15_MTA / C	Q3UPH1	-0.15756	0.031237	0.133078	Prrc1	Protein PRI	PRRC1_MO	8	0.89654
15_MTA / C	Q8BHG2	-0.0608	0.031373	0.133078	Czib	CXXC motif	CZIB_MOUS	6	0.958732
15_MTA / C	Q8C0J2	-0.6344	0.031384	0.133078	Atg16l1	Autophagy-	A16L1_MO	3	0.644209
15_MTA / C	Q8R238	-0.10837	0.031323	0.133078	Sdsl	Serine dehy	SDSL_MOU	9	0.927633
15_MTA / C	Q9CPY7	-0.03338	0.03129	0.133078	Lap3	Cytosol am	AMPL_MOU	30	0.977131
30_MTA / C	Q9D8U8	0.775115	0.031162	0.133078	Snx5	Sorting nex	SNX5_MOU	2	1.711327
15_MTA / C	Q9WTM5	-0.24422	0.03113	0.133078	Ruvbl2	RuvB-like 2	RUVB2_MC	7	0.844274
30_MTA / C	P47791	-0.07626	0.031668	0.133968	Gsr	Glutathion	GSHR_MOU	18	0.948512
15_MTA / C	P24547	-0.1842	0.03181	0.133975	Impdh2	Inosine-5'-r	IMDH2_MC	19	0.880136
30_MTA / C	P62305	0.49827	0.031856	0.133975	Snrpe	Small nucle	RUXE_MOU	1	1.412518
30_MTA / C	P63005	0.129533	0.031757	0.133975	Pafah1b1	Platelet-act	LIS1_MOUS	17	1.09394
15_MTA / C	P26231	-0.87161	0.032136	0.134368	Ctnna1	Catenin alp	CTNA1_MO	6	0.546535
15_MTA / C	P62317	-0.15997	0.032116	0.134368	Snrpd2	Small nucle	SMD2_MOU	7	0.895046
30_MTA / C	Q64737	0.595233	0.032124	0.134368	Gart	Trifunction	PUR2_MOU	4	1.510716
15_MTA / C	P50428	0.050436	0.032282	0.134818	Arsa	Arylsulfatas	ARSA_MOU	2	1.035578
15_MTA / C	Q9DBD0	-0.33693	0.032442	0.135331	Inhca	Inhibitor of	ICA_MOUSI	27	0.791724
15_MTA / C	O35386	-0.78494	0.032677	0.135683	Phyh	Phytanoyl-	PAHX_MOU	6	0.580378
30_MTA / C	Q61792	0.289978	0.032751	0.135833	Lasp1	LIM and SH	LASP1_MO	13	1.222622
15_MTA / C	Q91X34	0.102377	0.033104	0.136982	Baat	Bile acid-Co	BAAT_MOU	5	1.073541
15_MTA / C	O08807	-0.15721	0.033483	0.137126	Prdx4	Peroxiredo	PRDX4_MC	11	0.896759
15_MTA / C	O55013	0.561559	0.033528	0.137126	Trappc3	Trafficking	TPPC3_MO	8	1.475864
15_MTA / C	P24456	-0.40063	0.033488	0.137126	Cyp2d10	Cytochrom	CP2DA_MC	2	0.757528
15_MTA / C	P28656	-0.49985	0.033597	0.137126	Nap1l1	Nucleosom	NP1L1_MO	5	0.707181
15_MTA / C	Q60631	-0.42322	0.033443	0.137126	Grb2	Growth fac	GRB2_MOU	4	0.745758
15_MTA / C	Q61703	-0.20363	0.033291	0.137126	Itih2	Inter-alpha	ITIH2_MOU	17	0.868365
30_MTA / C	Q9D273	1.075996	0.033603	0.137126	Mmab	Corrinoid a	MMAB_MO	5	2.108177
30_MTA / C	P24472	-0.14191	0.033726	0.137153	Gsta4	Glutathion	GSTA4_MO	9	0.906319
15_MTA / C	Q64737	0.387396	0.033835	0.137153	Gart	Trifunction	PUR2_MOU	4	1.308031
30_MTA / C	Q91WK2	0.136419	0.033945	0.137153	Eif3h	Eukaryotic	EIF3H_MOU	15	1.099173
15_MTA / C	P21107	0.238461	0.034039	0.137376	Tpm3	Tropomyos	TPM3_MOU	4	1.179733
15_MTA / C	Q9CQ92	-0.0712	0.034309	0.138108	Fis1	Mitochond	FIS1_MOUS	3	0.951843
15_MTA / C	Q8BWG8	0.063213	0.034658	0.139249	Arrb1	Beta-arresti	ARRB1_MO	2	1.04479

30_MTA / C	Q9DCH6	-0.91429	0.034822	0.139755	Zfand6	AN1-type zi	ZFAN6_MO	1	0.530604
30_MTA / C	P97315	-0.37608	0.035001	0.14016	Csrp1	Cysteine an	CSRP1_MO	6	0.770526
15_MTA / C	P26883	-0.47195	0.035385	0.140294	Fkbp1a	Peptidyl-pr	FKB1A_MO	5	0.72099
30_MTA / C	P26883	-0.33661	0.035187	0.140294	Fkbp1a	Peptidyl-pr	FKB1A_MO	5	0.791903
15_MTA / C	Q03958	1.158983	0.035154	0.140294	Pfdn6	Prefoldin su	PFD6_MOU	4	2.232999
30_MTA / C	Q07797	0.104796	0.035347	0.140294	Lgals3bp	Galectin-3-	LG3BP_MO	8	1.075343
30_MTA / C	Q5EBG8	0.308654	0.03534	0.140294	NaN	Uncharacte	CA050_MO	5	1.238552
30_MTA / C	Q8CGK3	0.340626	0.035204	0.140294	Lonp1	Lon protea	LONM_MOU	10	1.266306
15_MTA / C	Q8CIE0	-0.18455	0.035313	0.140294	Serpina11	Serpin A11	SPA11_MO	1	0.879924
30_MTA / C	Q8R238	-0.08497	0.035309	0.140294	Sdsl	Serine dehy	SDSL_MOU	9	0.942807
15_MTA / C	Q8VDK1	-0.07433	0.035496	0.140578	Nit1	Deaminate	NIT1_MOUS	19	0.949786
30_MTA / C	P62983	0.24229	0.035579	0.140753	Rps27a	Ubiquitin-r	RS27A_MO	6	1.182869
15_MTA / C	P61089	-0.02137	0.035915	0.141614	Ube2n	Ubiquitin-c	UBE2N_MC	8	0.985296
15_MTA / C	Q61553	-0.59369	0.035909	0.141614	Fscn1	Fascin	FSCN1_MO	2	0.662645
30_MTA / C	Q8R0V5	-0.69906	0.036205	0.141673	Ido2	Indoleamir	I23O2_MO	1	0.615973
30_MTA / C	Q06890	0.078189	0.03626	0.141735	Clu	Clusterin	CLUS_MOU	6	1.055692
30_MTA / C	Q6IFX2	2.756213	0.036303	0.141748	Krt42	Keratin, typ	K1C42_MO	5	6.756205
15_MTA / C	P33267	-1.93324	0.036662	0.14284	Cyp2f2	Cytochrom	CP2F2_MO	6	0.26184
30_MTA / C	P35527	0.443308	0.03698	0.143303	KRT9	Keratin, typ	K1C9_HUM	30	1.359718
30_MTA / C	Q9JLF6	3.197908	0.036899	0.143303	Tgm1	Protein-glu	TGM1_MOU	5	9.176273
30_MTA / C	O35295	-0.15276	0.037046	0.143348	Purb	Transcripti	PURB_MOU	8	0.899526
15_MTA / C	O55125	-0.04996	0.037699	0.143578	Nipsnap1	Protein Nip	NIPS1_MOU	11	0.965961
15_MTA / C	P32020	-0.10668	0.037519	0.143578	Scp2	Sterol carri	SCP2_MOU	25	0.928723
15_MTA / C	P60335	-0.1149	0.037414	0.143578	Pcbp1	Poly(rC)-bir	PCBP1_MO	10	0.923446
15_MTA / C	P62075	-0.1535	0.037791	0.143578	Timm13	Mitochond	TIM13_MO	5	0.899064
30_MTA / C	P84084	-0.3735	0.037374	0.143578	Arf5	ADP-ribosy	ARF5_MOU	1	0.771905
15_MTA / C	Q8BI84	-0.39141	0.037591	0.143578	Mia3	Transport a	TGO1_MOU	2	0.762386
15_MTA / C	Q9CRB3	-0.2468	0.037314	0.143578	Urah	5-hydroxyi	HIUH_MOU	10	0.842761
15_MTA / C	Q9D114	-1.16564	0.037821	0.143578	Hddc3	Guanosine-	MESH1_MC	1	0.445765
15_MTA / C	Q9JKX6	0.584983	0.037739	0.143578	Nudt5	ADP-sugar	NUDT5_MC	2	1.500021
30_MTA / C	Q9JMH6	-0.14839	0.037849	0.143578	Txnrd1	Thioredoxi	TRXR1_MO	21	0.902254
15_MTA / C	P23116	-0.85548	0.038203	0.144158	Eif3a	Eukaryotic	EIF3A_MOU	4	0.552681
15_MTA / C	Q9JLB0	-2.19778	0.038506	0.14476	Pals2	Protein PAl	PALS2_MO	3	0.217973
30_MTA / C	Q9EQU5	0.211777	0.038706	0.145151	Set	Protein SET	SET_MOUS	5	1.158114
15_MTA / C	P70460	-0.21688	0.038836	0.145331	Vasp	Vasodilator	VASP_MOU	1	0.860426
30_MTA / C	Q01405	0.098791	0.039083	0.145955	Sec23a	Protein tra	SC23A_MO	12	1.070876
30_MTA / C	Q62189	-0.57913	0.039175	0.146144	Snrpa	U1 small nu	SNRPA_MO	1	0.66937
30_MTA / C	B2RXS4	0.365242	0.039223	0.146174	Plxnb2	Plexin-B2	PLXB2_MO	2	1.288097
15_MTA / C	P97429	-0.25238	0.039495	0.146968	Anxa4	Annexin A4	ANXA4_MC	4	0.839508
15_MTA / C	Q7TPC1	0.823164	0.039518	0.146968	Cdsn	Corneodesı	CDSN_MOU	1	1.769282
15_MTA / C	Q8QZR1	-0.59955	0.039686	0.147438	Tat	Tyrosine an	ATTY_MOUS	12	0.659959
30_MTA / C	Q9CRB3	-0.30411	0.039961	0.148004	Urah	5-hydroxyi	HIUH_MOU	10	0.809941
15_MTA / C	P61087	-0.1725	0.04008	0.14814	Ube2k	Ubiquitin-c	UBE2K_MO	7	0.887302
15_MTA / C	Q5SWU9	-0.89432	0.040334	0.148622	Acaca	Acetyl-CoA	ACACA_MO	5	0.538002
15_MTA / C	Q99KJ8	-0.23473	0.040331	0.148622	Dctn2	Dynactin su	DCTN2_MC	7	0.849841
15_MTA / C	Q3UUI3	-0.21299	0.040436	0.148844	Them4	Acyl-coenzy	THEM4_MC	6	0.862748
15_MTA / C	Q922V4	-0.67368	0.040599	0.149192	Plrg1	Pleiotropic	PLRG1_MO	5	0.626908
15_MTA / C	Q9JII6	-0.89512	0.040655	0.149192	Akr1a1	Aldo-keto r	AK1A1_MO	11	0.537702
15_MTA / C	Q7TSV4	-0.42708	0.04102	0.149768	Pgm2	Phosphope	PGM2_MOU	6	0.743764
30_MTA / C	Q9D404	-0.13605	0.041002	0.149768	Oxsm	3-oxoacyl-[	OXSM_MOU	14	0.910007
15_MTA / C	Q9JLI8	-0.05346	0.040939	0.149768	Sart3	Squamous	SART3_MO	3	0.963619
30_MTA / C	P53808	0.931615	0.041169	0.149839	Pctp	Phosphatid	PPCT_MOU	1	1.90741

15_MTA / C	Q06890	-0.39431	0.041247	0.149839	Clu	Clusterin	CLUS_MOU	6	0.760852
15_MTA / C	Q3ULJ0	-0.29985	0.041136	0.149839	Gpd1l	Glycerol-3-	GPD1L_MC	9	0.812339
15_MTA / C	Q6P6M7	-0.27808	0.041215	0.149839	Sepsecs	O-phospho	SPCS_MOU	11	0.824686
15_MTA / C	O54754	-0.08919	0.041512	0.150192	Aox1	Aldehyde o	AOXA_MOU	50	0.940053
30_MTA / C	P09411	-0.07256	0.041642	0.150515	Pgk1	Phosphogly	PGK1_MOU	23	0.950948
15_MTA / C	Q9D9H8	-0.35652	0.042086	0.151483	NaN	Mitochond	CB069_MC	5	0.781045
30_MTA / C	O55125	-0.24973	0.042416	0.15195	Nipsnap1	Protein Nip	NIPS1_MOU	11	0.841055
15_MTA / C	Q9JK38	-0.06895	0.042957	0.153419	Gnpnat1	Glucosamir	GNA1_MOU	9	0.953332
15_MTA / C	Q9WU84	-0.18911	0.043234	0.154254	Ccs	Copper cha	CCS_MOUS	5	0.877146
15_MTA / C	Q9CQ60	-0.3696	0.043618	0.155315	Pgls	6-phospho	6PGL_MOU	11	0.773997
30_MTA / C	P33267	-1.89784	0.044084	0.155855	Cyp2f2	Cytochrom	CP2F2_MO	6	0.268345
30_MTA / C	P58774	0.326618	0.044159	0.155855	Tpm2	Tropomyos	TPM2_MOU	8	1.25407
15_MTA / C	P63276	-1.30769	0.044134	0.155855	Rps17	Small ribos	RS17_MOU	2	0.403966
30_MTA / C	P70670;Q6	0.201078	0.043919	0.155855	Naca	Nascent po	NACAM_MC	5	1.149557
30_MTA / C	P70677	0.146713	0.044002	0.155855	Casp3	Caspase-3	CASP3_MO	11	1.107044
30_MTA / C	P01630	0.446755	0.044339	0.156336	NaN	Ig kappa ch	KV2A6_MO	2	1.362971
15_MTA / C	Q61074	0.239773	0.044569	0.15684	Ppm1g	Protein pho	PPM1G_MC	1	1.180806
30_MTA / C	Q04447	0.203386	0.044985	0.157383	Ckb	Creatine kir	KCRB_MOU	2	1.151398
15_MTA / C	Q9DCY0	-0.52341	0.044805	0.157383	Keg1	Glycine N-a	KEG1_MOU	7	0.695726
15_MTA / C	Q9JLV5	-0.76878	0.04494	0.157383	Cul3	Cullin-3	CUL3_MOU	8	0.586915
30_MTA / C	Q62348	0.08347	0.045181	0.157608	Tsn	Translin	TSN_MOUS	10	1.059563
15_MTA / C	Q8K4Z3	-0.20216	0.04517	0.157608	Naxe	NAD(P)H-hy	NNRE_MOU	9	0.869246
15_MTA / C	Q9DAR7	-0.08018	0.045177	0.157608	Dcps	m7GpppX c	DCPS_MOU	13	0.945942
15_MTA / C	P53996	0.17088	0.045275	0.157631	Cnbp	CCHC-type	CNBP_MOU	4	1.125745
30_MTA / C	O70591	0.518663	0.045731	0.158757	Pfdn2	Prefoldin su	PFD2_MOU	3	1.432627
15_MTA / C	Q99LB7	-0.48694	0.045798	0.158773	Sardh	Sarcosine d	SARDH_MC	17	0.713536
15_MTA / C	O55234	-0.09754	0.046065	0.159135	Psmb5	Proteasom	PSB5_MOU	15	0.934626
15_MTA / C	O88958	0.091982	0.046308	0.159135	Gnpda1	Glucosamir	GNPI1_MO	14	1.065833
30_MTA / C	P21107	0.45169	0.04637	0.159135	Tpm3	Tropomyos	TPM3_MOU	4	1.367641
15_MTA / C	Q9QXL2	-0.78003	0.04621	0.159135	Kif21a	Kinesin-like	KI21A_MOU	4	0.582354
15_MTA / C	P05214	0.129132	0.046594	0.159751	Tuba3a	Tubulin alp	TBA3_MOU	2	1.093635
15_MTA / C	Q8CDM8	-0.99026	0.046783	0.160093	Fhip2a	FHF comple	FHI2A_MOU	2	0.503389
30_MTA / C	Q9D6S7	0.869676	0.047165	0.16079	Mrrf	Ribosome-r	RRFM_MOU	3	1.827253
30_MTA / C	Q9WU28	0.082928	0.047571	0.162022	Pfdn5	Prefoldin su	PFD5_MOU	3	1.059166
30_MTA / C	Q8VDM4	0.180208	0.047713	0.162351	Psmd2	26S proteas	PSMD2_MC	30	1.133047
30_MTA / C	P23953	-0.14044	0.047987	0.16313	Ces1c	Carboxyles	EST1C_MOU	9	0.907245
15_MTA / C	Q62422	0.097154	0.048158	0.163556	Ostf1	Osteoclast-	OSTF1_MO	7	1.069661
15_MTA / C	P24549	-0.21898	0.048337	0.163853	Aldh1a1	Aldehyde d	AL1A1_MO	21	0.85917
15_MTA / C	Q8BTZ7	-0.47499	0.048454	0.164095	Gmppb	Mannose-1	GMPPB_MC	3	0.719473
15_MTA / C	Q99KK7	-0.62415	0.048939	0.164962	Dpp3	Dipeptidyl	DPP3_MOU	5	0.648802
15_MTA / C	Q9QUR6	-0.56886	0.048913	0.164962	Prep	Prolyl endo	PPCE_MOU	7	0.674147
30_MTA / C	P58389	0.419667	0.049227	0.165774	Ptpa	Serine/thre	PTPA_MOU	6	1.337619
15_MTA / C	Q8R317	-0.20021	0.049272	0.165774	Ubqln1	Ubiquilin-1	UBQL1_MC	7	0.870423
30_MTA / C	O08529	-0.31941	0.049509	0.166263	Capn2	Calpain-2 c	CAN2_MOU	4	0.801395
15_MTA / C	Q8R3Q6	-0.09306	0.049632	0.166389	Mix23	Protein MI)	MIX23_MO	6	0.937533
15_MTA / C	P38060	-0.27814	0.049805	0.166632	Hmgcl	Hydroxyme	HMGCL_MC	8	0.824652
15_MTA / C	Q8QZR5	0.127235	0.049788	0.166632	Gpt	Alanine am	ALAT1_MOU	25	1.092199
15_MTA / C	Q9CPX6	-0.45449	0.049972	0.167038	Atg3	Ubiquitin-l	ATG3_MOU	5	0.729767
30_MTA / C	P01674	-0.0548	0.050642	0.16912	NaN	Ig kappa ch	KV3AM_MC	1	0.962725
30_MTA / C	Q02257	2.018498	0.050753	0.1693	Jup	Junction pl	PLAK_MOU	16	4.051618
30_MTA / C	Q8BIJ6	-0.06278	0.051104	0.170189	Iars2	Isoleucine-	SYIM_MOU	8	0.957416
15_MTA / C	P97872	-2.49548	0.051225	0.170435	Fmo5	Flavin-cont	FMO5_MOU	6	0.177331

15_MTA / C	Q8BFQ4	-0.51417	0.051313	0.17057	Wdr82	WD repeat-	WDR82_M(	5	0.700193
15_MTA / C	O35381	0.156449	0.051428	0.170767	Anp32a	Acidic leuci	AN32A_MC	8	1.11454
15_MTA / C	P10649	-0.07002	0.051562	0.170767	Gstm1	Glutathion	GSTM1_MC	13	0.952627
15_MTA / C	Q8K274	-0.53054	0.051822	0.171469	Fn3krp	Ketosamine	KT3K_MOU	1	0.692294
30_MTA / C	Q9D0M1	2.016881	0.051938	0.17154	Prpsap1	Phosphorik	KPRA_MOU	2	4.04708
15_MTA / C	O89106	-0.09358	0.052265	0.172302	Fhit	Bis(5'-aden	FHIT_MOUS	8	0.937193
15_MTA / C	Q9CZ13	-1.53707	0.05241	0.172463	Uqcrc1	Cytochrom	QCR1_MOU	2	0.344584
15_MTA / C	Q91XE0	-0.04515	0.05254	0.172735	Glyat	Glycine N-a	GLYAT_MOU	18	0.969191
30_MTA / C	P97872	-2.29214	0.052784	0.173378	Fmo5	Flavin-cont	FMO5_MOU	6	0.204173
30_MTA / C	Q9WTL4	2.129958	0.052835	0.173387	Insrr	Insulin rece	INSRR_MOU	2	4.377048
15_MTA / C	Q61792	0.216923	0.052992	0.173745	Lasp1	LIM and SH	LASP1_MOU	13	1.162252
15_MTA / C	P28651	-0.16731	0.053196	0.173951	Ca8	Carbonic ar	CAH8_MOU	8	0.890499
15_MTA / C	P70362	-0.03824	0.0532	0.173951	Ufd1	Ubiquitin r	UFD1_MOU	7	0.973843
30_MTA / C	Q61081	0.472266	0.053349	0.17413	Cdc37	Hsp90 co-c	CDC37_MC	6	1.387287
15_MTA / C	Q3V4B5	-0.05237	0.053495	0.174323	Commd6	COMM don	COMD6_M(	1	0.964354
15_MTA / C	Q8K0E8	-0.81853	0.053541	0.174323	Fgb	Fibrinogen	FIBB_MOUS	5	0.56702
15_MTA / C	Q9CYZ2	-0.1506	0.053709	0.174348	Tpd52l2	Tumor prot	TPD54_MO	5	0.900874
15_MTA / C	Q8BHA3	-0.7272	0.054084	0.175408	Dtd2	D-aminoac'	DTD2_MOU	6	0.604075
30_MTA / C	P50446	2.129147	0.054354	0.175525	Krt6a	Keratin, typ	K2C6A_MO	19	4.374587
15_MTA / C	P63325	-0.82705	0.054267	0.175525	Rps10	Small ribos	RS10_MOU	3	0.563681
15_MTA / C	Q9QZ08	-0.33554	0.054291	0.175525	Nagk	N-acetyl-D-	NAGK_MOU	4	0.792485
15_MTA / C	P48193	-0.239	0.054876	0.17702	Epb41	Protein 4.1	EPB41_MO	4	0.84733
15_MTA / C	O35945	-0.13443	0.055122	0.177365	Aldh1a7	Aldehyde d	AL1A7_MO	31	0.911032
15_MTA / C	Q9CQV6	-0.60835	0.05514	0.177365	Map1lc3b	Microtubul	MLP3B_MC	2	0.655945
15_MTA / C	Q9Z2M7	-0.35984	0.055179	0.177365	Pmm2	Phosphoma	PMM2_MO	7	0.779253
15_MTA / C	P42208	0.010262	0.055716	0.178076	Septin2	Septin-2	SEPT2_MOU	10	1.007138
30_MTA / C	Q9JMA1	-0.0203	0.055934	0.178513	Usp14	Ubiquitin c	UBP14_MC	15	0.98603
15_MTA / C	P61082	-0.16249	0.05623	0.178825	Ube2m	NEDD8-cor	UBC12_MC	4	0.89348
15_MTA / C	Q9D8C4	-1.2888	0.056222	0.178825	Ifi35	Interferon-i	IN35_MOU	3	0.409291
15_MTA / C	Q9D115	-0.09675	0.056495	0.179342	Znf706	Zinc finger	ZN706_MO	1	0.935139
15_MTA / C	Q9DD18	-0.14083	0.056542	0.179342	Dtd1	D-aminoac'	DTD1_MOU	1	0.906996
30_MTA / C	Q8BGD8	0.405309	0.056638	0.179488	Coa6	Cytochrom	COA6_MOU	1	1.324373
30_MTA / C	P11983	0.207149	0.056871	0.179643	Tcp1	T-complex	TCPA_MOU	19	1.154404
30_MTA / C	P28076	0.207377	0.056944	0.179643	Psmb9	Proteasome	PSB9_MOU	7	1.154587
15_MTA / C	Q05793	-0.37017	0.0569	0.179643	Hspg2	Basement n	PGBM_MOU	27	0.773693
15_MTA / C	Q9CQA3	-0.84583	0.056946	0.179643	Sdhb	Succinate d	SDHB_MOU	7	0.55639
15_MTA / C	Q9JI90	-1.89826	0.056979	0.179643	Rnf14	E3 ubiquiti	RNF14_MO	1	0.268267
15_MTA / C	Q8CIF4	-0.33514	0.057178	0.180088	Btd	Biotinidase	BTD_MOUS	6	0.792705
30_MTA / C	O35955	0.433427	0.057361	0.18035	Psmb10	Proteasome	PSB10_MO	5	1.350438
15_MTA / C	Q9JL35	0.117422	0.057496	0.180615	Hmgn5	High mobil	HMGN5_M	8	1.084795
15_MTA / C	P54869	-0.22701	0.057563	0.180667	Hmgcs2	Hydroxyme	HMCS2_MC	16	0.854406
30_MTA / C	P10639	-0.11804	0.057796	0.180994	Txn	Thioredoxi	THIO_MOU	6	0.921438
15_MTA / C	P68254	0.002237	0.057803	0.180994	Ywhaq	14-3-3 prot	1433T_MO	11	1.001552
30_MTA / C	P97379	0.331392	0.057818	0.180994	G3bp2	Ras GTPase-	G3BP2_MC	7	1.258226
15_MTA / C	Q99MD9	-2.05556	0.057982	0.181052	Nasp	Nuclear aut	NASP_MOU	2	0.240556
15_MTA / C	Q9DBR1	-1.0339	0.058031	0.181052	Xrn2	5'-3' exorib	XRN2_MOU	4	0.488387
30_MTA / C	Q9DCL8	0.357988	0.058037	0.181052	Ppp1r2	Protein pho	IPP2_MOUS	1	1.281638
30_MTA / C	O08573	-0.08992	0.05814	0.181183	Lgals9	Galectin-9	LEG9_MOU	9	0.939575
30_MTA / C	P99028	1.487403	0.05818	0.181183	Uqcrh	Cytochrom	QCR6_MOU	1	2.803838
15_MTA / C	Q9D7G0	-0.30045	0.058252	0.181252	Prps1	Ribose-pho	PRPS1_MO	7	0.811999
15_MTA / C	P52623	0.451446	0.058953	0.182997	Uck1	Uridine-cyt	UCK1_MOU	2	1.36741
15_MTA / C	P62301	-0.48442	0.058966	0.182997	Rps13	Small ribos	RS13_MOU	5	0.714784

30_MTA / C	Q6P069	-0.38521	0.059162	0.183449	Sri	Sorcin	SORCN_MC	1	0.765666
15_MTA / C	P08607	0.018911	0.059268	0.183459	C4bpa	C4b-bindin	C4BPA_MO	8	1.013194
30_MTA / C	P26350	0.167002	0.059225	0.183459	Ptma	Prothymos	PTMA_MOU	3	1.122723
15_MTA / C	P68373	-0.30437	0.059323	0.183472	Tuba1c	Tubulin alp	TBA1C_MO	1	0.809795
15_MTA / C	Q9Z223	-0.24861	0.059691	0.184296	Mocs2	Molybdopt	MOC2B_M(	3	0.841709
15_MTA / C	P48428	0.353586	0.059804	0.184393	Tbca	Tubulin-spe	TBCA_MOU	6	1.277732
15_MTA / C	Q8BK64	-0.15192	0.059825	0.184393	Ahsa1	Activator o	AHSA1_MO	18	0.900049
15_MTA / C	Q9CR09	-0.77182	0.059993	0.184473	Ufc1	Ubiquitin-f	UFC1_MOU	4	0.58568
30_MTA / C	Q9DCT8	0.205103	0.060524	0.185467	Crip2	Cysteine-ric	CRIP2_MOU	9	1.152769
30_MTA / C	Q91VH2	0.164211	0.060644	0.185644	Snx9	Sorting nex	SNX9_MOU	1	1.120553
30_MTA / C	Q61599	0.521187	0.060789	0.185819	Arhgdib	Rho GDP-di	GDIR2_MO	3	1.435135
15_MTA / C	Q6ZQ58	-0.20866	0.060804	0.185819	Larp1	La-related p	LARP1_MO	4	0.865338
30_MTA / C	Q91YP3	0.192682	0.061149	0.185925	Dera	Deoxyribos	DEOC_MOU	14	1.142886
15_MTA / C	Q91ZA3	-0.56109	0.061129	0.185925	Pcca	Propionyl-(	PCCA_MOU	15	0.677789
30_MTA / C	Q99JY0	-0.18551	0.061068	0.185925	Hadhb	Trifunction	ECHB_MOU	7	0.879338
30_MTA / C	Q9QXS1	1.3409	0.060989	0.185925	Plec	Plectin	PLEC_MOU	2	2.533092
15_MTA / C	Q8BGT5	-0.61955	0.061289	0.185974	Gpt2	Alanine am	ALAT2_MO	6	0.650873
30_MTA / C	Q8CBW3	0.559326	0.06132	0.185974	Abi1	Abl interact	ABI1_MOUS	2	1.473581
15_MTA / C	Q61545	-0.78462	0.06154	0.186329	Ewsr1	RNA-bindir	EWS_MOUS	3	0.580504
15_MTA / C	Q99LU0;Q9	-0.06056	0.061502	0.186329	Chmp1b1;(	Charged mu	CH1B1_MC	2	0.958893
30_MTA / C	O35490	-0.07786	0.061661	0.186537	Bhmt	Betaine--hc	BHMT1_MC	29	0.947462
15_MTA / C	P26262	-0.12751	0.061852	0.186644	Klkb1	Plasma kall	KLKB1_MO	6	0.915409
30_MTA / C	P14069	0.195417	0.061952	0.18673	S100a6	Protein S1(	S10A6_MO	1	1.145055
15_MTA / C	P61202	-1.03509	0.062039	0.186741	Cops2	COP9 signa	CSN2_MOU	9	0.487985
30_MTA / C	P61759	0.439053	0.0622	0.187068	Vbp1	Prefoldin su	PFD3_MOU	3	1.355714
15_MTA / C	Q9DBT5	-0.06642	0.062252	0.187068	Ampd2	AMP deami	AMPD2_MC	6	0.955005
15_MTA / C	Q91WP6	-0.54833	0.06241	0.187231	Serpina3n	Serine prot	SPA3N_MO	2	0.683811
15_MTA / C	Q62093	0.056505	0.062675	0.187869	Srsf2	Serine/argi	SRSF2_MOU	3	1.039943
30_MTA / C	Q91XC8	-0.35272	0.062777	0.188019	Dap	Death-asso	DAP1_MOU	2	0.783109
30_MTA / C	P63087	0.617895	0.06306	0.188238	Ppp1cc	Serine/thre	PP1G_MOU	2	1.534634
30_MTA / C	Q99LT0	0.8032	0.063038	0.188238	Dpy30	Protein dpy	DPY30_MO	2	1.744967
30_MTA / C	P97350	3.757284	0.06336	0.188977	Pkp1	Plakophilin	PKP1_MOU	4	13.52245
15_MTA / C	Q3TNA1	-0.59384	0.063542	0.189364	Xylb	Xylulose kir	XYLB_MOU	4	0.662579
30_MTA / C	P62748	-1.56082	0.063634	0.189481	Hpcal1	Hippocalcir	HPCL1_MO	1	0.338958
15_MTA / C	Q9EST4	0.050564	0.063722	0.189586	Psmg2	Proteasome	PSMG2_MC	2	1.03567
30_MTA / C	P46061	-0.53364	0.063817	0.189712	Rangap1	Ran GTPase	RAGP1_MC	12	0.690809
15_MTA / C	Q9JLT4	-0.2358	0.064195	0.19052	Txnrd2	Thioredoxir	TRXR2_MO	17	0.849211
15_MTA / C	Q8BGF3	-0.31396	0.064436	0.190608	Dnaaf10	Dynein axo	DAA10_MC	5	0.80443
30_MTA / C	Q9R0H0	-0.06801	0.064344	0.190608	Acox1	Peroxisoma	ACOX1_MC	37	0.953953
30_MTA / C	Q9D7P6	0.269174	0.064615	0.19098	Iscu	Iron-sulfur	ISCU_MOUS	4	1.205118
15_MTA / C	Q8R164	-0.07719	0.06473	0.191162	Bphl	Valacyclovi	BPHL_MOU	19	0.947904
30_MTA / C	Q9JLV1	0.326229	0.064844	0.191343	Bag3	BAG family	BAG3_MOU	20	1.253732
30_MTA / C	O70456	2.812317	0.065031	0.191579	Sfn	14-3-3 prot	1433S_MO	5	7.024115
30_MTA / C	P32020	-0.12138	0.065021	0.191579	Scp2	Sterol carri	SCP2_MOU	25	0.919309
30_MTA / C	Q61768	-0.05066	0.065105	0.191641	Kif5b	Kinesin-1 h	KINH_MOU	13	0.965498
15_MTA / C	E9Q912	-0.75993	0.065446	0.191932	Rap1gds1	Rap1 GTPas	GDS1_MOU	11	0.590523
30_MTA / C	Q61781	2.047573	0.06562	0.191932	Krt14	Keratin, typ	K1C14_MO	19	4.1341
15_MTA / C	Q91VS7	-1.2671	0.065413	0.191932	Mgst1	Microsoma	MGST1_MC	2	0.415495
15_MTA / C	Q9CXZ1	-0.34345	0.065523	0.191932	Ndufs4	NADH dehy	NDUS4_MC	2	0.788156
15_MTA / C	Q9QZE7	-0.89153	0.065593	0.191932	Tsnax	Translin-ass	TSNAX_MO	7	0.539041
30_MTA / C	P50396	0.105557	0.065697	0.191973	Gdi1	Rab GDP di	GDIA_MOU	11	1.075909
15_MTA / C	P62774	-0.06148	0.06585	0.192106	Mtpn	Myotrophir	MTPN_MOU	6	0.958283

30_MTA / C	Q80X90	0.105406	0.06609	0.192496	Flnb	Filamin-B	FLNB_MOU	74	1.075797
15_MTA / C	P47941	-0.1149	0.066823	0.194004	Crkl	Crk-like prc	CRKL_MOU	7	0.923446
15_MTA / C	P70194	-1.43556	0.066963	0.194095	Clec4f	C-type lecti	CLC4F_MOU	3	0.369703
15_MTA / C	P24270	-0.48245	0.06713	0.194159	Cat	Catalase	CATA_MOU	19	0.715763
15_MTA / C	P62838	-0.00306	0.067129	0.194159	Ube2d2	Ubiquitin-c	UB2D2_MC	3	0.997882
30_MTA / C	Q91X34	0.412374	0.067342	0.194567	Baat	Bile acid-Cc	BAAT_MOU	5	1.330874
15_MTA / C	Q8CIM3	-0.61883	0.067407	0.194599	D2hgdh	D-2-hydrox	D2HDH_MC	5	0.651198
30_MTA / C	Q6ZWZ2	0.182529	0.06775	0.19512	Ube2r2	Ubiquitin-c	UB2R2_MC	2	1.134871
15_MTA / C	P56389	-1.41455	0.067814	0.195123	Cda	Cytidine de	CDD_MOUS	1	0.375126
15_MTA / C	P11031	0.067935	0.069018	0.197193	Sub1	Activated R	TCP4_MOU	6	1.048215
15_MTA / C	P61971	-0.34613	0.069102	0.197236	Nutf2	Nuclear tra	NTF2_MOU	5	0.78669
15_MTA / C	Q9ERG0	-0.55979	0.069142	0.197236	Lima1	LIM domair	LIMA1_MO	5	0.678403
30_MTA / C	Q8K3W0	-0.18144	0.069302	0.197536	Babam2	BRISC and E	BABA2_MO	1	0.88182
30_MTA / C	A2ATU0	-0.24431	0.070393	0.198649	Dhtkd1	2-oxoadipa	DHTK1_MC	10	0.844219
30_MTA / C	O43790	2.192389	0.070611	0.198649	KRT86	Keratin, typ	KRT86_HUI	21	4.570617
15_MTA / C	P47754	0.148609	0.070296	0.198649	Capza2	F-actin-cap	CAZA2_MO	9	1.1085
15_MTA / C	Q8CHT0	-0.40977	0.070378	0.198649	Aldh4a1	Delta-1-pyr	AL4A1_MO	8	0.752741
15_MTA / C	Q91W50	-0.5948	0.069817	0.198649	Csde1	Cold shock	CSDE1_MO	6	0.662136
30_MTA / C	Q9QWL7	2.946543	0.070666	0.198649	Krt17	Keratin, typ	K1C17_MO	14	7.708994
15_MTA / C	Q9WVA2	-0.13463	0.069903	0.198649	Timm8a1	Mitochond	TIM8A_MO	5	0.910901
30_MTA / C	Q9Z0S1	-0.59806	0.070686	0.198649	Bpnt1	3'(2'),5'-bis	BPNT1_MO	8	0.660641
15_MTA / C	Q9Z224	-0.49958	0.070648	0.198649	Mocs2	Molybdopt	MOC2A_MC	4	0.707315
30_MTA / C	Q921T2	0.242964	0.071253	0.199932	Tor1aip1	Torsin-1A-ii	TOIP1_MOU	1	1.183422
15_MTA / C	P10630	-0.06591	0.071652	0.20027	Eif4a2	Eukaryotic	IF4A2_MOU	10	0.955343
15_MTA / C	Q9QXT0	0.218159	0.071486	0.20027	Cnpy2	Protein car	CNPY2_MO	6	1.163249
15_MTA / C	P50462	-0.21951	0.071785	0.200329	Csrp3	Cysteine an	CSRP3_MO	5	0.858857
15_MTA / C	Q9CZD3	-0.28074	0.071844	0.200339	Gars1	Glycine--tR	GARS_MOU	3	0.823171
15_MTA / C	P01872	0.151392	0.072366	0.201017	Ighm	Immunoglc	IGHM_MOU	10	1.11064
30_MTA / C	P62313	-0.25944	0.072422	0.201017	Lsm6	U6 snRNA-a	LSM6_MOU	3	0.835411
30_MTA / C	Q64176	0.192252	0.072168	0.201017	Ces1e	Carboxyles	EST1E_MOU	13	1.142546
30_MTA / C	Q8R086	-0.17381	0.072281	0.201017	Suox	Sulfite oxid	SUOX_MOU	23	0.886498
30_MTA / C	Q9EST4	1.018266	0.072252	0.201017	Psmg2	Proteasom	PSMG2_MC	2	2.025483
15_MTA / C	P26638	0.181092	0.072722	0.201283	Sars1	Serine--tRN	SYSC_MOUS	2	1.133742
30_MTA / C	P11930	0.174548	0.072978	0.201306	Nudt19	Acyl-coenz	NUD19_MC	5	1.128611
15_MTA / C	Q99N42	-0.36992	0.073252	0.201358	Tymp	Thymidine	TYPH_MOU	6	0.773824
15_MTA / C	Q9CR16	-0.02114	0.073211	0.201358	Ppid	Peptidyl-pr	PPID_MOU	11	0.985454
15_MTA / C	Q9D9V3	0.11877	0.073213	0.201358	Echdc1	Ethylmalor	ECHD1_MC	17	1.085808
15_MTA / C	Q9JJI8	-0.60868	0.073322	0.201358	Rpl38	Large ribos	RL38_MOU	3	0.655795
30_MTA / C	P19639	-0.34662	0.073618	0.202001	Gstm3	Glutathion	GSTM3_MC	12	0.786426
15_MTA / C	P42227	-1.36303	0.073896	0.202609	Stat3	Signal trans	STAT3_MOU	5	0.388766
30_MTA / C	P60867	0.931717	0.074024	0.202805	Rps20	Small ribos	RS20_MOU	2	1.907545
15_MTA / C	Q9D8U8	0.573169	0.074379	0.203622	Snx5	Sorting nex	SNX5_MOU	2	1.487788
15_MTA / C	P70670;Q6	0.191041	0.074574	0.203906	Naca	Nascent po	NACAM_MC	5	1.141587
15_MTA / C	Q62264	-1.80878	0.074739	0.203906	Thrsp	Thyroid hor	THRSP_MO	3	0.285432
15_MTA / C	Q80X90	-0.04087	0.074687	0.203906	Flnb	Filamin-B	FLNB_MOU	74	0.972071
30_MTA / C	Q9CQW1	-0.27824	0.074635	0.203906	Ykt6	Synaptobre	YKT6_MOU	5	0.824596
30_MTA / C	Q9WVQ5	0.012988	0.074765	0.203906	Apip	Methylthio	MTNB_MOU	9	1.009044
30_MTA / C	Q9WTM5	0.336624	0.075017	0.204283	Ruvbl2	RuvB-like 2	RUVB2_MC	7	1.262798
15_MTA / C	P34884	-0.42476	0.075265	0.204803	Mif	Macrophag	MIF_MOUS	5	0.74496
30_MTA / C	Q9EPU0	-0.112	0.075714	0.205714	Upf1	Regulator c	RENT1_MO	16	0.925306
30_MTA / C	O55013	0.466333	0.076823	0.20606	Trappc3	Trafficking	TPPC3_MO	8	1.381593
30_MTA / C	O70274;Q6	-0.78736	0.076848	0.20606	Ptp4a2;Ptp	Protein tyr	TP4A2_MO	1	0.579404

15_MTA / C	P09813	-0.24935	0.076701	0.20606	Apoa2	Apolipoprc	APOA2_MC	2	0.841277
30_MTA / C	P48678	1.407445	0.076237	0.20606	Lmna	Prelamin-A	LMNA_MOU	15	2.652669
30_MTA / C	P55264	0.226926	0.076354	0.20606	Adk	Adenosine l	ADK_MOUS	18	1.170339
15_MTA / C	P62858	0.425418	0.076749	0.20606	Rps28	Small ribos	RS28_MOU	3	1.342962
30_MTA / C	P68368	0.291259	0.076567	0.20606	Tuba4a	Tubulin alp	TBA4A_MO	3	1.223708
15_MTA / C	Q11136	-0.08215	0.076549	0.20606	Pepd	Xaa-Pro dip	PEPD_MOU	17	0.944649
30_MTA / C	Q60994	1.532778	0.07668	0.20606	Adipoq	Adiponecti	ADIPO_MO	1	2.893425
15_MTA / C	Q8R0N6	-0.09518	0.076311	0.20606	Adhfe1	Hydroxyaci	HOT_MOUS	16	0.936156
30_MTA / C	Q9DBM2	0.142173	0.076041	0.20606	Ehhadh	Peroxisoma	ECHP_MOU	24	1.103566
15_MTA / C	Q9QXD1	-0.19387	0.07634	0.20606	Acox2	Peroxisoma	ACOX2_MC	19	0.874259
15_MTA / C	Q9QXG4	-0.01018	0.076466	0.20606	Acss2	Acetyl-coer	ACSA_MOU	11	0.992966
15_MTA / C	P07724	-0.0876	0.077104	0.206374	Alb	Albumin	ALBU_MOU	49	0.941085
15_MTA / C	Q8VI75	0.079561	0.077351	0.206575	Ipo4	Importin-4	IPO4_MOU	7	1.056697
15_MTA / C	P62309	0.182646	0.077543	0.206629	Snrpg	Small nucle	RUXG_MOU	2	1.134964
30_MTA / C	Q8BMS1	-0.19806	0.077617	0.206672	Hadha	Trifunction	ECHA_MOU	10	0.871724
15_MTA / C	O70274;Q6	-0.78866	0.07779	0.20698	Ptp4a2;Ptp	Protein tyr	TP4A2_MO	1	0.57888
30_MTA / C	Q9CY58	0.143167	0.07787	0.207039	Serbp1	SERPINE1 m	SERB1_MOU	10	1.104326
15_MTA / C	Q80Y55	0.118747	0.078286	0.207685	Bsdc1	BSD domai	BSDC1_MO	2	1.085791
15_MTA / C	Q6P069	-0.21408	0.078526	0.208089	Sri	Sorcin	SORCN_MC	1	0.862097
15_MTA / C	O08553	-0.10244	0.078701	0.208174	Dpysl2	Dihydropyr	DPYL2_MO	16	0.931459
15_MTA / C	Q6ZWZ2	-0.41783	0.078685	0.208174	Ube2r2	Ubiquitin-c	UB2R2_MC	2	0.748547
30_MTA / C	O35129	1.371716	0.078801	0.208211	Phb2	Prohibitin-	PHB2_MOU	1	2.587782
15_MTA / C	P68040	-1.90843	0.078831	0.208211	Rack1	Small ribos	RACK1_MO	6	0.266382
30_MTA / C	P70296	-0.13167	0.07924	0.208985	Pebp1	Phosphatic	PEBP1_MO	14	0.912777
30_MTA / C	Q9JHS4	0.515837	0.079352	0.209127	Clpx	ATP-depenc	CLPX_MOU	3	1.429824
15_MTA / C	Q8BMJ3	0.224753	0.079428	0.209174	Eif1ax	Eukaryotic	IF1AX_MOU	2	1.168577
15_MTA / C	P70122	0.485475	0.080576	0.211732	Sbds	Ribosome r	SBDS_MOU	5	1.400047
15_MTA / C	Q9Z2K1	0.405181	0.080661	0.211801	Krt16	Keratin, typ	K1C16_MO	5	1.324255
30_MTA / C	E9PV24	0.278871	0.080818	0.212057	Fga	Fibrinogen	FIBA_MOUS	7	1.213245
30_MTA / C	P46412	0.317838	0.081095	0.212397	Gpx3	Glutathion	GPX3_MOU	2	1.246462
15_MTA / C	P62137	0.153609	0.081124	0.212397	Ppp1ca	Serine/thre	PP1A_MOU	12	1.112348
15_MTA / C	O54962	-0.11235	0.081517	0.212878	Banf1	Barrier-to-a	BAF_MOUS	1	0.92508
30_MTA / C	P53996	0.266856	0.081544	0.212878	Cnbp	CCHC-type	CNBP_MOU	4	1.203183
15_MTA / C	Q62261	-0.27901	0.081433	0.212878	Sptbn1	Spectrin be	SPTB2_MOU	14	0.824154
30_MTA / C	P01660	0.995754	0.081727	0.213201	NaN	Ig kappa ch	KV3A8_MO	1	1.994123
30_MTA / C	P27048;P6	0.077999	0.082256	0.213479	Snrpb;Snrp	Small nucle	RSMB_MOU	3	1.055553
30_MTA / C	P49182	0.41659	0.082303	0.213479	Serpind1	Heparin cof	HEP2_MOU	9	1.334769
30_MTA / C	Q60631	-0.31433	0.082556	0.213479	Grb2	Growth fac	GRB2_MOU	4	0.804225
30_MTA / C	Q61699	-0.01171	0.082493	0.213479	Hsph1	Heat shock	HS105_MO	12	0.991918
30_MTA / C	Q8BGZ7	2.647643	0.082032	0.213479	Krt75	Keratin, typ	K2C75_MO	2	6.266428
15_MTA / C	Q8BYK6	-0.77543	0.08219	0.213479	Ythdf3	YTH domair	YTHD3_MO	2	0.584214
15_MTA / C	Q8CBW3	0.282593	0.082605	0.213479	Abi1	Abl interact	ABI1_MOUS	2	1.216379
30_MTA / C	Q8VE97	0.351893	0.082445	0.213479	Srsf4	Serine/argi	SRSF4_MOU	3	1.276234
15_MTA / C	Q91WK2	0.130524	0.082522	0.213479	Eif3h	Eukaryotic	EIF3H_MOU	15	1.094691
15_MTA / C	Q9Z2Y8	-0.23216	0.082323	0.213479	Plpbp	Pyridoxal p	PLPHP_MO	10	0.851361
30_MTA / C	P32261	-0.27471	0.083095	0.214286	Serpinc1	Antithroml	ANT3_MOU	16	0.826614
30_MTA / C	Q9CQ60	-0.33312	0.083061	0.214286	Pgls	6-phospho	6PGL_MOU	11	0.793819
30_MTA / C	P97328	-0.09947	0.083229	0.214478	Khk	Ketohexoki	KHK_MOUS	22	0.933379
15_MTA / C	O54983	-0.57254	0.083559	0.214867	Crym	Ketimine re	CRYM_MOU	1	0.672432
30_MTA / C	P70349	-0.34699	0.083505	0.214867	Hint1	Adenosine !	HINT1_MO	9	0.786222
15_MTA / C	Q8BGZ7	0.294745	0.084056	0.215836	Krt75	Keratin, typ	K2C75_MO	2	1.226668
30_MTA / C	Q9Z2K1	2.656031	0.084654	0.217061	Krt16	Keratin, typ	K1C16_MO	5	6.302965

15_MTA / C	Q8BMS1	-0.301	0.084746	0.217142	Hadha	Trifunction	ECHA_MOU	10	0.811692
15_MTA / C	O35551	-0.90728	0.084914	0.217265	Rabep1	Rab GTPase	RABE1_MO	3	0.533191
15_MTA / C	Q9WVJ3	-0.04427	0.084858	0.217265	Cpq	Carboxypep	CBPQ_MOU	9	0.969782
30_MTA / C	Q9D8X1	0.371476	0.085217	0.217576	Cutc	Copper hor	CUTC_MOU	2	1.293675
15_MTA / C	P18524	0.435029	0.085471	0.217924	NaN	Ig heavy ch	HVM53_MO	1	1.351938
15_MTA / C	P26369	-0.38375	0.086142	0.218387	U2af2	Splicing fac	U2AF2_MO	3	0.766445
30_MTA / C	Q3U4I7	0.170754	0.086026	0.218387	Pyroxd2	Pyridine nu	PYRD2_MO	6	1.125647
30_MTA / C	Q61703	-0.18611	0.086113	0.218387	Itih2	Inter-alpha	ITIH2_MOU	17	0.878971
30_MTA / C	Q9D358	-0.52422	0.085859	0.218387	Acp1	Low molecu	PPAC_MOU	3	0.695337
30_MTA / C	Q9DBT5	0.602099	0.086109	0.218387	Ampd2	AMP deami	AMPD2_MO	6	1.517924
15_MTA / C	Q80X50	-0.63482	0.086578	0.218722	Ubap2l	Ubiquitin-a	UBP2L_MO	3	0.644023
15_MTA / C	Q8BMD5	-1.03415	0.086567	0.218722	Cdadc1	Cytidine an	CDAC1_MO	4	0.488304
30_MTA / C	P61458	-0.19237	0.086764	0.218972	Pcbd1	Pterin-4-alp	PHS_MOUS	7	0.875169
15_MTA / C	Q80XU3	-0.34913	0.086826	0.218972	Nucks1	Nuclear ubi	NUCKS_MO	2	0.78506
15_MTA / C	Q8BH43	-0.87624	0.087088	0.219277	Wasf2	Actin-bindi	WASF2_MO	3	0.544786
15_MTA / C	Q8JZR0	-1.27657	0.08749	0.219759	Acsl5	Long-chain	ACSL5_MOU	1	0.412776
30_MTA / C	Q8K370	0.140796	0.087457	0.219759	Acad10	Acyl-CoA de	ACD10_MO	10	1.102513
15_MTA / C	Q9CZ42	-0.14054	0.087366	0.219759	Naxd	ATP-depend	NNRD_MOU	12	0.907179
15_MTA / C	P07361	-0.80902	0.087723	0.219765	Orm2	Alpha-1-aci	A1AG2_MO	3	0.57077
15_MTA / C	Q8VCH8	-0.11381	0.087719	0.219765	Ubxn4	UBX domai	UBXN4_MO	2	0.924146
15_MTA / C	P02301;P6	-0.34488	0.087911	0.219929	H3-5;H3c1;	Histone H3	H3C_MOUS	3	0.787372
15_MTA / C	Q8CHP5	0.779767	0.087885	0.219929	Pym1	Partner of Y	PYM1_MOU	2	1.716853
15_MTA / C	P47964	-1.01705	0.088091	0.220228	Rpl36	Large ribos	RL36_MOU	5	0.494125
30_MTA / C	P47757	0.058244	0.088222	0.220249	Capzb	F-actin-cap	CAPZB_MO	11	1.041198
30_MTA / C	Q9DBA8	-0.4897	0.088301	0.220294	Amdhd1	Probable in	HUTI_MOU	11	0.712173
15_MTA / C	P10126	-0.02839	0.088822	0.221126	Eef1a1	Elongation	EF1A1_MO	26	0.980511
15_MTA / C	P31786	0.136708	0.088828	0.221126	Dbi	Acyl-CoA-bi	ACBP_MOU	6	1.099394
30_MTA / C	Q61990	0.028424	0.088754	0.221126	Pcbp2	Poly(rC)-bir	PCBP2_MO	8	1.019897
30_MTA / C	P04117	0.034718	0.090011	0.222088	Fabp4	Fatty acid-b	FABP4_MO	8	1.024357
30_MTA / C	P17742	-0.16865	0.090059	0.222088	Ppia	Peptidyl-pr	PPIA_MOUS	11	0.889676
30_MTA / C	P49962	0.68691	0.089819	0.222088	Srp9	Signal reco	SRP09_MO	3	1.609831
15_MTA / C	Q8BH86	-0.43635	0.090069	0.222088	Dglucy	D-glutamat	GLUCM_MO	6	0.739003
30_MTA / C	Q99KR7	0.268505	0.090061	0.222088	Ppif	Peptidyl-pr	PPIF_MOUS	7	1.204559
15_MTA / C	Q9D1I5	-0.32759	0.089815	0.222088	Mcee	Methylmal	MCEE_MOU	3	0.796867
30_MTA / C	Q9QUH0	-0.43358	0.089744	0.222088	Glrx	Glutaredox	GLRX1_MO	5	0.74042
15_MTA / C	Q9QXE0	-0.06276	0.089834	0.222088	Hacl1	2-hydroxya	HACL1_MO	30	0.95743
30_MTA / C	Q9Z223	0.178231	0.089674	0.222088	Mocs2	Molybdopt	MOC2B_MO	3	1.131495
30_MTA / C	P49722	-0.16459	0.090279	0.222453	Psma2	Proteasom	PSA2_MOU	14	0.892183
15_MTA / C	P50431	-0.09371	0.090798	0.22312	Shmt1	Serine hydr	GLYC_MOU	15	0.937113
15_MTA / C	O89110	-0.33912	0.091059	0.223238	Casp8	Caspase-8	CASP8_MO	5	0.790524
15_MTA / C	P12265	-0.27175	0.09103	0.223238	Gusb	Beta-glucur	BGLR_MOU	17	0.828314
15_MTA / C	P19001	1.057481	0.091218	0.223238	Krt19	Keratin, typ	K1C19_MO	1	2.081294
15_MTA / C	P61255	-0.02992	0.091112	0.223238	Rpl26	Large ribos	RL26_MOU	7	0.979473
15_MTA / C	Q9D379	-1.38653	0.091083	0.223238	Ephx1	Epoxide hy	HYEP_MOU	5	0.382485
15_MTA / C	Q9WTP7	-0.20939	0.092104	0.22434	Ak3	GTP:AMP p	KAD3_MOU	10	0.864901
15_MTA / C	Q9WTX5	-0.201	0.092057	0.22434	Skp1	S-phase kin	SKP1_MOU	5	0.869946
30_MTA / C	P54869	-0.23985	0.092475	0.224748	Hmgcs2	Hydroxyme	HMCS2_MO	16	0.846834
30_MTA / C	P62254	-1.08445	0.092584	0.224748	Ube2g1	Ubiquitin-c	UB2G1_MO	2	0.471573
15_MTA / C	Q8BH69	-0.39944	0.09254	0.224748	Sephs1	Selenide, w	SPS1_MOU	5	0.75815
15_MTA / C	Q91W90	-0.0731	0.092529	0.224748	Txndc5	Thioredoxi	TXND5_MO	13	0.950595
15_MTA / C	Q9R0N0	-0.79849	0.092392	0.224748	Galk1	Galactokin	GALK1_MO	3	0.574949
15_MTA / C	Q99J77	-0.69449	0.0928	0.224882	Nans	Sialic acid s	SIAS_MOUS	1	0.617928

30_MTA / C	Q99LB7	-0.60908	0.092792	0.224882	Sardh	Sarcosine d	SARDH_MC	17	0.655614
30_MTA / C	Q8VCR7	-0.14782	0.092933	0.224988	Abhd14b	Putative pr	ABHEB_MC	11	0.902615
15_MTA / C	P60122	-0.4755	0.093014	0.225034	Ruvbl1	RuvB-like 1	RUVB1_MC	6	0.719218
15_MTA / C	Q923B0	-0.84464	0.093357	0.225713	Ggact	Gamma-glu	GGACT_MC	3	0.556849
30_MTA / C	P40936	0.253222	0.094152	0.227023	Inmt	Indolethyla	INMT_MOU	12	1.191866
15_MTA / C	P62843	-0.60033	0.094131	0.227023	Rps15	Small ribos	RS15_MOU	2	0.659604
30_MTA / C	P97494	0.096869	0.094134	0.227023	Gclc	Glutamate-	GSH1_MOU	23	1.06945
30_MTA / C	P62075	-0.18564	0.094533	0.227033	Timm13	Mitochond	TIM13_MO	5	0.879259
30_MTA / C	Q3UMF0	0.21366	0.094506	0.227033	Cobll1	Cordon-ble	COBL1_MC	19	1.159626
15_MTA / C	Q9CQ10	-0.27979	0.094493	0.227033	Chmp3	Charged mu	CHMP3_MC	4	0.823712
30_MTA / C	Q9ERE7	-0.25606	0.094255	0.227033	Mesd	LRP chaper	MESD_MOU	7	0.837372
15_MTA / C	Q9ESX5	-0.78957	0.094534	0.227033	Dkc1	H/ACA ribo	DKC1_MOU	7	0.578516
15_MTA / C	P62849	-0.13481	0.094647	0.227152	Rps24	Small ribos	RS24_MOU	4	0.91079
30_MTA / C	P07356	1.949566	0.094895	0.227294	Anxa2	Annexin A2	ANXA2_MC	10	3.862583
15_MTA / C	Q9Z2W0	0.158375	0.095322	0.228014	Dnpep	Aspartyl am	DNPEP_MC	16	1.116029
30_MTA / C	P42208	0.051445	0.095695	0.228298	Septin2	Septin-2	SEPT2_MOU	10	1.036302
15_MTA / C	Q99JT9	-0.33092	0.095579	0.228298	Adi1	Acireducto	MTND_MOU	7	0.795029
30_MTA / C	Q60605	0.347924	0.095869	0.228563	Myl6	Myosin ligh	MYL6_MOU	7	1.272728
15_MTA / C	O54774	-0.40428	0.096524	0.229233	Ap3d1	AP-3 compl	AP3D1_MC	3	0.755615
30_MTA / C	P97822	0.235121	0.096466	0.229233	Anp32e	Acidic leuci	AN32E_MC	2	1.177005
15_MTA / C	Q8BIJ6	-0.3353	0.096532	0.229233	Iars2	Isoleucine--	SYIM_MOU	8	0.792621
15_MTA / C	O09061	-0.0956	0.09797	0.231798	Psmb1	Proteasome	PSB1_MOU	14	0.935882
15_MTA / C	Q68FD5	-0.69149	0.097994	0.231798	Cltc	Clathrin he	CLH1_MOU	18	0.619213
30_MTA / C	Q9JJU8	-0.30281	0.098063	0.231798	Sh3bgrl	Adapter Sh:	SH3L1_MO	6	0.810669
30_MTA / C	P62869	-0.21711	0.098328	0.232207	Elob	Elongin-B	ELOB_MOU	8	0.860284
30_MTA / C	O35660	0.107943	0.098588	0.232451	Gstm6	Glutathion	GSTM6_MC	6	1.077691
30_MTA / C	P05214	-0.00756	0.098598	0.232451	Tuba3a	Tubulin alp	TBA3_MOU	2	0.994773
15_MTA / C	Q9Z2L6	0.412071	0.098782	0.232669	Minpp1	Multiple in	MINP1_MC	1	1.330594
15_MTA / C	A6X935	-0.10427	0.099777	0.234149	Itih4	Inter alpha-	ITIH4_MOU	35	0.930274
15_MTA / C	P46656	0.204473	0.099652	0.234149	Fdx1	Adrenodox	ADX_MOUS	5	1.152265
30_MTA / C	Q05D44	0.23683	0.099838	0.234149	Eif5b	Eukaryotic	IF2P_MOUS	14	1.178401
30_MTA / C	Q8BWP5	-0.11744	0.099715	0.234149	Ttpa	Alpha-toco	TTPA_MOU	15	0.921823
30_MTA / C	Q91VF2	-0.27666	0.099593	0.234149	Hnmt	Histamine I	HNMT_MOU	8	0.825498
30_MTA / C	Q62426	-0.14798	0.100038	0.23416	Cstb	Cystatin-B	CYTB_MOU	7	0.902515
15_MTA / C	O89079	-0.01162	0.100817	0.235327	Cope	Coatomer s	COPE_MOU	8	0.991975
30_MTA / C	P24270	-0.41406	0.100685	0.235327	Cat	Catalase	CATA_MOU	19	0.750507
15_MTA / C	Q8C5H8	-0.43597	0.100618	0.235327	Nadk2	NAD kinase	NAKD2_MC	12	0.739195
30_MTA / C	Q8CEI1	0.490154	0.100864	0.235327	Bola3	BolA-like pr	BOLA3_MO	3	1.404595
15_MTA / C	Q9D6Y9	-1.94405	0.100826	0.235327	Gbe1	1,4-alpha-g	GLGB_MOU	6	0.259886
15_MTA / C	P23953	-0.18291	0.101454	0.235338	Ces1c	Carboxyles	EST1C_MOU	9	0.880926
15_MTA / C	P26443	-0.55702	0.101348	0.235338	Glud1	Glutamate	DHE3_MOU	24	0.679705
15_MTA / C	P48036	-0.23662	0.101421	0.235338	Anxa5	Annexin A5	ANXA5_MC	13	0.848732
30_MTA / C	Q60668	0.322681	0.101457	0.235338	Hnrnpd	Heterogene	HNRPD_MC	7	1.250652
30_MTA / C	Q9D1A2	0.071153	0.101368	0.235338	Cndp2	Cytosolic n	CNDP2_MC	23	1.050556
15_MTA / C	Q9D1Q6	-0.17072	0.101438	0.235338	Erp44	Endoplasm	ERP44_MO	13	0.8884
15_MTA / C	Q9QZE5	-0.09724	0.101134	0.235338	Copg1	Coatomer s	COPG1_MC	33	0.934817
15_MTA / C	Q9D051	-0.85517	0.101547	0.235395	Pdhb	Pyruvate de	ODPB_MOU	2	0.552799
15_MTA / C	Q9CPT4	-0.02752	0.10284	0.23778	Mydgf	Myeloid-de	MYDGF_MC	6	0.981107
30_MTA / C	Q9WUP7	-0.03551	0.10359	0.239361	Uchl5	Ubiquitin c	UCHL5_MC	6	0.975684
30_MTA / C	Q9CR21	0.712215	0.103895	0.239775	Ndufab1	Acyl carrier	ACPM_MOU	3	1.638318
30_MTA / C	Q99J77	-0.6258	0.103976	0.23979	Nans	Sialic acid s	SIAS_MOUS	1	0.648062
30_MTA / C	Q3THE2	0.196025	0.104194	0.240007	Myl12b	Myosin regu	ML12B_MC	5	1.145538

15_MTA / C	P62869	-0.14462	0.104346	0.240073	Elob	Elongin-B	ELOB_MOU	8	0.904618
15_MTA / C	Q60866	0.019435	0.104365	0.240073	Pter	Phosphotri	PTER_MOU	19	1.013563
15_MTA / C	Q8R5I6	-0.09674	0.10448	0.240184	Gstm4	Glutathion	GSTM4_MC	4	0.935142
15_MTA / C	Q6PDY2	-0.59118	0.104724	0.240437	Ado	2-aminoetl	AEDO_MOU	5	0.663798
15_MTA / C	Q9DCJ9	-0.42752	0.104963	0.240679	Npl	N-acetylneu	NPL_MOUS	11	0.74354
30_MTA / C	Q9CY62	-1.84892	0.105355	0.240812	Rnf181	E3 ubiquiti	RN181_MC	2	0.2776
15_MTA / C	Q8CH18	-1.02455	0.106204	0.24229	Ccar1	Cell divisio	CCAR1_MC	3	0.491562
15_MTA / C	O88456	-0.41379	0.106583	0.242846	Capns1	Calpain sma	CPNS1_MC	4	0.750647
15_MTA / C	Q3THE2	-0.29834	0.107207	0.243352	Myl12b	Myosin regu	ML12B_MC	5	0.813189
15_MTA / C	Q6ZWM4	-0.11313	0.107056	0.243352	Lsm8	U6 snRNA-a	LSM8_MOU	3	0.924579
15_MTA / C	Q8VE09	-0.05176	0.107337	0.243487	Ttc39c	Tetratricop	TT39C_MOU	13	0.964758
30_MTA / C	P52623	1.278215	0.10744	0.243524	Uck1	Uridine-cyt	UCK1_MOU	2	2.425386
15_MTA / C	O88844	-0.37862	0.10756	0.243531	Idh1	Isocitrate d	IDHC_MOU	18	0.76917
30_MTA / C	Q9D8T7	-0.84765	0.108109	0.244314	Slirp	SRA stem-lo	SLIRP_MOU	1	0.555689
15_MTA / C	Q920A5	-0.17994	0.108392	0.244494	Scpep1	Retinoid-in	RISC_MOUS	4	0.88274
30_MTA / C	P62309	0.547688	0.109166	0.24593	Snrpg	Small nucle	RUXG_MOU	2	1.461742
30_MTA / C	Q61655	0.497674	0.10937	0.246208	Ddx19a	ATP-depend	DD19A_MC	2	1.411935
15_MTA / C	Q9JHH9	-1.09986	0.109426	0.246208	Copz2	Coatomer s	COPZ2_MC	3	0.466561
15_MTA / C	Q8BJW6	0.193741	0.109818	0.246628	Eif2a	Eukaryotic	EIF2A_MOU	13	1.143726
15_MTA / C	Q9R0H0	-0.0614	0.109781	0.246628	Acox1	Peroxisoma	ACOX1_MC	37	0.958333
30_MTA / C	Q9WV68	0.195463	0.110083	0.246914	Decr2	Peroxisoma	DECR2_MC	5	1.145092
15_MTA / C	O09172	-0.11963	0.110511	0.247463	Gclm	Glutamate-	GSH0_MOU	10	0.920423
15_MTA / C	Q9D172	-0.19466	0.110506	0.247463	Gatd3	Glutamine	GAL3A_MC	12	0.873778
15_MTA / C	Q6NVF9	-0.26318	0.110949	0.24809	Cpsf6	Cleavage an	CPSF6_MO	3	0.833252
15_MTA / C	Q9Z1K5	-0.31158	0.111045	0.24809	Arih1	E3 ubiquiti	ARI1_MOUS	3	0.805761
15_MTA / C	Q9CQI6	0.114633	0.111237	0.248266	Cotl1	Coactosin-l	COTL1_MO	7	1.0827
15_MTA / C	Q14CH1	0.071035	0.111322	0.248268	Mocos	Molybdenu	MOCOS_MC	20	1.05047
15_MTA / C	Q99MN1	-0.57758	0.111376	0.248268	Kars1	Lysine--tRN	SYK_MOUSI	8	0.670089
15_MTA / C	P04186	-0.15518	0.111573	0.248554	Cfb	Compleme	CFAB_MOU	15	0.89802
15_MTA / C	P07901	0.114696	0.112068	0.248893	Hsp90aa1	Heat shock	HS90A_MO	23	1.082747
15_MTA / C	P08207	-0.28815	0.112047	0.248893	S100a10	Protein S1(	S10AA_MO	1	0.818951
30_MTA / C	Q3TLP5	0.032478	0.112543	0.249633	Echdc2	Enoyl-CoA I	ECHD2_MC	7	1.022768
30_MTA / C	P01942	-0.25639	0.112671	0.249763	Hba	Hemoglobi	HBA_MOUS	9	0.837183
15_MTA / C	Q9QXF8	-0.10807	0.113024	0.250237	Gnmt	Glycine N-n	GNMT_MO	20	0.927828
30_MTA / C	P62897	-0.30757	0.113408	0.250779	Cycs	Cytochrom	CYC_MOUS	8	0.808003
15_MTA / C	Q571E4	-0.38417	0.113594	0.251036	Galns	N-acetylgal	GALNS_MO	7	0.766218
15_MTA / C	P18242	-0.15512	0.114096	0.251374	Ctsd	Cathepsin D	CATD_MOU	8	0.898061
15_MTA / C	Q9JII5	-0.51555	0.114051	0.251374	Dazap1	DAZ-associa	DAZP1_MO	8	0.699528
15_MTA / C	P18528	-0.59673	0.114262	0.251432	NaN	Ig heavy cha	HVM57_MC	3	0.66125
15_MTA / C	Q8BTM8	-0.38204	0.114211	0.251432	Flna	Filamin-A	FLNA_MOU	16	0.767353
30_MTA / C	Q9JL35	0.185048	0.114361	0.251496	Hmgn5	High mobil	HMGN5_M	8	1.136855
30_MTA / C	O35381	0.286684	0.114655	0.251989	Anp32a	Acidic leuci	AN32A_MC	8	1.219834
15_MTA / C	P51880	0.104295	0.115109	0.252832	Fabp7	Fatty acid-b	FABP7_MO	4	1.074969
30_MTA / C	Q5SW19	0.07465	0.115219	0.252919	Cluh	Clustered m	CLU_MOUS	6	1.053106
15_MTA / C	O35459	-0.26858	0.115354	0.252938	Ech1	Delta(3,5)-I	ECH1_MOU	12	0.830134
30_MTA / C	P97493	-0.27114	0.115368	0.252938	Txn2	Thioredoxi	THIOM_MC	3	0.828663
15_MTA / C	P09103	-0.06152	0.115505	0.253084	P4hb	Protein dis	PDIA1_MO	35	0.958255
30_MTA / C	P16254	0.338406	0.116034	0.253934	Srp14	Signal reco	SRP14_MO	5	1.264359
15_MTA / C	P27612	0.014119	0.116216	0.254062	Plaa	Phospholip	PLAP_MOU	22	1.009835
30_MTA / C	Q99K51	-0.06277	0.116347	0.254155	Pls3	Plastin-3	PLST_MOUS	35	0.957423
30_MTA / C	P42932	-0.03712	0.116719	0.254386	Cct8	T-complex	TCPQ_MOU	30	0.974598
30_MTA / C	Q921G7	0.175621	0.11665	0.254386	Etfdh	Electron tra	ETFD_MOU	7	1.129451

15_MTA / C	P05064	-0.1256	0.117183	0.254438	Aldoa	Fructose-bi	ALDOA_MC	22	0.916625
15_MTA / C	Q8K4F5	-0.22582	0.117176	0.254438	Abhd11	sn-1-specifi	ABHDB_MC	7	0.85511
15_MTA / C	Q91X91	-0.12834	0.117135	0.254438	Qprt	Nicotinate-	NADC_MOU	13	0.914886
15_MTA / C	Q91Z53	-0.21065	0.116891	0.254438	Grhpr	Glyoxylate	GRHPR_MC	14	0.864146
15_MTA / C	Q9D1P4	-0.10014	0.117013	0.254438	Chordc1	Cysteine an	CHRD1_MC	8	0.932944
15_MTA / C	P10639	0.210199	0.117464	0.254552	Txn	Thioredoxi	THIO_MOU	6	1.156848
30_MTA / C	Q5I2A0	-0.25684	0.117518	0.254552	Serpina3g	Serine prot	SPA3G_MC	1	0.836917
30_MTA / C	Q99JX4	0.006598	0.117465	0.254552	Eif3m	Eukaryotic	EIF3M_MOU	5	1.004584
30_MTA / C	Q9CQ48	0.174588	0.117599	0.254573	Nudcd2	NudC doma	NUDC2_MC	1	1.128642
30_MTA / C	Q8CJG0	0.154379	0.117715	0.254673	ago-02	Protein arg	AGO2_MOU	8	1.112942
30_MTA / C	O35226	0.194993	0.118011	0.255005	Psmd4	26S protea	PSMD4_MC	6	1.144719
30_MTA / C	Q91ZA3	-0.09781	0.118009	0.255005	Pcca	Propionyl-(	PCCA_MOU	15	0.934451
30_MTA / C	Q9DAW9	0.346051	0.118242	0.255351	Cnn3	Calponin-3	CNN3_MOU	2	1.271076
15_MTA / C	Q9D6S7	-0.16412	0.118482	0.255717	Mrrf	Ribosome-r	RRFM_MOU	3	0.892474
30_MTA / C	O88545	0.330261	0.118826	0.255999	Cops6	COP9 signa	CSN6_MOU	6	1.257241
30_MTA / C	Q78ZA7	0.449375	0.118806	0.255999	Nap1l4	Nucleosom	NP1L4_MC	3	1.365449
15_MTA / C	Q9DCL9	-0.05084	0.118815	0.255999	Paics	Bifunctiona	PUR6_MOU	18	0.965374
15_MTA / C	Q8CIH9	0.024604	0.118928	0.256065	Ppat	Amidophos	PUR1_MOU	11	1.017201
30_MTA / C	P61971	-0.24406	0.119336	0.25652	Nutf2	Nuclear tra	NTF2_MOU	5	0.844366
15_MTA / C	Q4VAA2	0.260487	0.119353	0.25652	Cdv3	Protein CD'	CDV3_MOU	8	1.197883
15_MTA / C	Q61247	-0.18286	0.120523	0.258418	Serpinf2	Alpha-2-ant	A2AP_MOU	12	0.880954
15_MTA / C	Q7TMK9	-0.17406	0.120456	0.258418	Syncrip	Heterogene	HNRPQ_MC	14	0.886342
15_MTA / C	Q8JZQ9	-0.16771	0.120402	0.258418	Eif3b	Eukaryotic	EIF3B_MOU	19	0.890255
30_MTA / C	P43276	1.247059	0.120677	0.258593	H1-5	Histone H1	H15_MOUS	3	2.37357
30_MTA / C	Q9D711	0.259715	0.121261	0.259075	Pir	Pirin	PIR_MOUSI	2	1.197243
30_MTA / C	P62743	0.196302	0.121426	0.259273	Ap2s1	AP-2 compl	AP2S1_MO	5	1.145758
15_MTA / C	Q9DBC0	-0.06528	0.121539	0.25936	Selenoo	Protein ade	SELO_MOU	1	0.955761
15_MTA / C	P04247	-1.17795	0.122114	0.259632	Mb	Myoglobin	MYG_MOUS	1	0.441979
15_MTA / C	P06151	-0.04376	0.122143	0.259632	Ldha	L-lactate de	LDHA_MOU	23	0.970126
15_MTA / C	P34927	-0.6252	0.122061	0.259632	Asgr1	Asialoglyco	ASGR1_MO	5	0.648328
15_MTA / C	Q8C0D5	-0.6811	0.122171	0.259632	Efl1	Elongation	EFL1_MOUS	2	0.623692
15_MTA / C	Q922H9	-0.12653	0.121961	0.259632	Znf330	Zinc finger	ZN330_MO	1	0.916035
15_MTA / C	Q9QYI5	-0.78902	0.122131	0.259632	Dnajb2	DnaJ homo	DNJB2_MO	2	0.578737
30_MTA / C	Q91X83	-0.07485	0.122833	0.260576	Mat1a	S-adenosylr	METK1_MC	20	0.949438
15_MTA / C	Q7TMM9	0.001807	0.123046	0.260875	Tubb2a	Tubulin bet	TBB2A_MO	4	1.001253
15_MTA / C	Q9EQG9	0.906119	0.123207	0.26091	Cert1	Ceramide tı	CERT_MOU	1	1.873997
30_MTA / C	P0DP26;PC	0.172113	0.123432	0.261232	Calm1;Caln	Calmodulir	CALM1_MC	9	1.126708
30_MTA / C	P63001	-0.73921	0.123774	0.261801	Rac1	Ras-related	RAC1_MOU	5	0.599067
30_MTA / C	Q9DBJ1	-0.17546	0.124038	0.262097	Pgam1	Phosphogly	PGAM1_MC	22	0.885485
30_MTA / C	Q9WU84	-0.16387	0.124059	0.262097	Ccs	Copper cha	CCS_MOUS	5	0.892626
30_MTA / C	P62307	0.017682	0.124447	0.262763	Snrpf	Small nucle	RUXF_MOU	3	1.012331
15_MTA / C	Q61768	0.097821	0.124788	0.263046	Kif5b	Kinesin-1 h	KINH_MOU	13	1.070156
15_MTA / C	Q8BL66	-0.01733	0.12508	0.263135	Eea1	Early endos	EEA1_MOU	35	0.988062
30_MTA / C	Q8BL97	0.894038	0.124928	0.263135	Srsf7	Serine/argi	SRSF7_MOU	3	1.85837
15_MTA / C	Q9D7I5	-0.70369	0.125022	0.263135	Lhpp	Phospholys	LHPP_MOU	2	0.613999
15_MTA / C	P42125	-0.11781	0.125329	0.263389	Eci1	Enoyl-CoA (	ECI1_MOUS	16	0.921589
15_MTA / C	P47962	-0.16048	0.125551	0.263394	Rpl5	Large ribos	RL5_MOUS	11	0.894729
30_MTA / C	Q99LP6	0.268652	0.125522	0.263394	Grpel1	GrpE protei	GRPE1_MO	11	1.204682
15_MTA / C	Q9D8N0	-0.17016	0.125548	0.263394	Eef1g	Elongation	EF1G_MOU	11	0.888745
30_MTA / C	O70493	-0.15785	0.125964	0.263952	Snx12	Sorting nex	SNX12_MO	4	0.896359
15_MTA / C	P63028	-0.41091	0.126591	0.264651	Tpt1	Translation	TCTP_MOU	8	0.752148
15_MTA / C	Q8CIB5	-0.12427	0.126549	0.264651	Fermt2	Fermitin fa	FERM2_MC	6	0.917468

15_MTA / C	Q8VBV3	-0.11636	0.126591	0.264651	Exosc2	Exosome cc	EXOS2_MO	4	0.922514
15_MTA / C	Q9Z1K6	0.261976	0.126772	0.264875	Arih2	E3 ubiquiti	ARI2_MOUS	1	1.19912
30_MTA / C	Q8C0M9	0.048359	0.127077	0.265357	Asrgl1	Isoaspartyl	ASGL1_MO	8	1.034088
30_MTA / C	O88451	-0.07676	0.127489	0.265733	Rdh7	Retinol deh	RDH7_MOU	9	0.948185
30_MTA / C	Q9CQZ1	-0.1358	0.127552	0.265733	Hsbp1	Heat shock	HSBP1_MO	1	0.910166
15_MTA / C	Q00623	-0.22756	0.127816	0.265975	Apoa1	Apolipoprc	APOA1_MC	17	0.854077
30_MTA / C	P52840	-0.79727	0.128388	0.266905	Sult1a1	Sulfotransf	ST1A1_MO	7	0.575439
15_MTA / C	Q99L20	-0.11856	0.128411	0.266905	Gstt3	Glutathion	GSTT3_MO	11	0.921104
15_MTA / C	Q64010	-0.23559	0.128777	0.267203	Crk	Adapter mc	CRK_MOUS	6	0.849335
15_MTA / C	Q9CQU0	-0.01448	0.128737	0.267203	Txndc12	Thioredoxi	TXD12_MO	4	0.990014
15_MTA / C	Q9DBL1	-1.43342	0.128739	0.267203	Acadsb	Short/bran	ACDSB_MO	3	0.370251
30_MTA / C	Q8BHG1	-0.09875	0.129307	0.267686	Nrdc	Nardilysin	NRDC_MOU	10	0.933843
15_MTA / C	Q9JLJ2	-0.31886	0.129293	0.267686	Aldh9a1	4-trimethyl	AL9A1_MO	15	0.801703
30_MTA / C	Q9JMD0	0.258992	0.129399	0.267722	Znf207	BUB3-inter	ZN207_MO	2	1.196643
15_MTA / C	Q9CY62	-1.38267	0.12962	0.268025	Rnf181	E3 ubiquiti	RN181_MC	2	0.383507
15_MTA / C	P18760	-0.13155	0.129714	0.268066	Cfl1	Cofilin-1	COF1_MOU	14	0.912852
30_MTA / C	Q99JW4	0.373214	0.130249	0.268744	Lims1	LIM and ser	LIMS1_MO	4	1.295235
15_MTA / C	Q9CY58	0.107526	0.130266	0.268744	Serbp1	SERPINE1 n	SERB1_MO	10	1.077379
15_MTA / C	Q9DBM2	-0.0773	0.13018	0.268744	Ehhadh	Peroxisom	ECHP_MOU	24	0.94783
15_MTA / C	Q64726	-0.37146	0.130462	0.268995	Azgp1	Zinc-alpha-	ZA2G_MOU	3	0.773002
15_MTA / C	P11862	-0.18128	0.130858	0.269501	Gas2	Growth arr	GAS2_MOU	4	0.881917
15_MTA / C	O35900	0.03769	0.131079	0.269802	Lsm2	U6 snRNA-a	LSM2_MOU	4	1.026469
15_MTA / C	Q9CX34	-0.06534	0.131277	0.26999	Sugt1	Protein SG	SGT1_MOU	8	0.955721
15_MTA / C	Q9DCH4	0.528016	0.131371	0.26999	Eif3f	Eukaryotic	EIF3F_MOU	2	1.441945
15_MTA / C	O09131	-0.15617	0.131684	0.270342	Gsto1	Glutathion	GSTO1_MO	15	0.897406
15_MTA / C	Q9D892	-0.17326	0.131849	0.270459	Itpa	Inosine trip	ITPA_MOUS	7	0.886839
30_MTA / C	P46737	0.454701	0.131963	0.270461	Brcc3	Lys-63-spec	BRCC3_MO	1	1.370499
15_MTA / C	P62962	-0.24279	0.132075	0.270461	Pfn1	Profilin-1	PROF1_MO	8	0.84511
30_MTA / C	Q9D0M5	-0.44387	0.13203	0.270461	Dynll2	Dynein ligh	DYL2_MOU	5	0.735159
15_MTA / C	Q8BX09	-0.23196	0.13236	0.270729	Rbbp5	Retinoblast	RBBP5_MO	3	0.851475
30_MTA / C	Q9CT10	-0.00602	0.132431	0.270729	Ranbp3	Ran-bindin	RANB3_MC	9	0.995837
30_MTA / C	P19001	4.352364	0.132564	0.270845	Krt19	Keratin, typ	K1C19_MO	1	20.42641
30_MTA / C	P53612	-1.04635	0.133715	0.272643	Rabggtb	Geranylger	PGTB2_MO	2	0.484192
30_MTA / C	Q8CHP5	0.630039	0.133746	0.272643	Pym1	Partner of Y	PYM1_MOU	2	1.547607
30_MTA / C	Q9DAR7	0.06225	0.134466	0.2738	Dcps	m7GpppX c	DCPS_MOU	13	1.044093
15_MTA / C	P22599	-0.18351	0.134955	0.274419	Serpina1b	Alpha-1-ant	A1AT2_MO	7	0.880559
30_MTA / C	Q64213	0.356057	0.134999	0.274419	Sf1	Splicing fac	SF01_MOU	8	1.279923
30_MTA / C	Q9CR00	0.041069	0.134921	0.274419	Psmd9	26S protea	PSMD9_MC	9	1.028876
15_MTA / C	Q61207	0.228774	0.135239	0.274598	Psap	Prosaposin	SAP_MOUS	9	1.171839
30_MTA / C	Q62452	-0.87541	0.135217	0.274598	Ugt1a9	UDP-glucur	UD19_MOU	4	0.5451
15_MTA / C	P85094	-0.06883	0.135517	0.275007	Isoc2a	Isochorism	ISC2A_MOU	9	0.953408
15_MTA / C	P61079	0.465655	0.135878	0.275584	Ube2d3	Ubiquitin-c	UB2D3_MC	1	1.380944
15_MTA / C	Q8R574	-0.04056	0.136128	0.275896	Prpsap2	Phosphorib	KPRB_MOU	8	0.97228
30_MTA / C	Q9EPL9	0.188113	0.136262	0.275896	Acox3	Peroxisom	ACOX3_MC	3	1.139273
30_MTA / C	Q99L27	-0.06721	0.136375	0.275969	Gmpr2	GMP reduc	GMPR2_MC	7	0.954485
30_MTA / C	P35492	-0.10874	0.136931	0.276473	Hal	Histidine ar	HUTH_MOU	37	0.927397
30_MTA / C	Q9QX60	0.230537	0.136856	0.276473	Dguok	Deoxyguan	DGUOK_MC	2	1.173272
15_MTA / C	P16045	-0.43999	0.137072	0.276602	Lgals1	Galectin-1	LEG1_MOU	5	0.737139
30_MTA / C	Q3UEG6	-0.09054	0.137621	0.27724	Agxt2	Alanine--gly	AGT2_MOU	28	0.939169
30_MTA / C	Q8R016	-0.04943	0.137795	0.27724	Blmh	Bleomycin	BLMH_MOU	20	0.96632
15_MTA / C	P43276	0.909608	0.138198	0.277459	H1-5	Histone H1	H15_MOUS	3	1.878535
15_MTA / C	Q60737	-1.5102	0.138165	0.277459	Csnk2a1	Casein kina	CSK21_MO	3	0.351062

30_MTA / C	Q8BUV3	0.139884	0.138284	0.277459	Gphn	Gephyrin	GEPH_MOU	27	1.101816
15_MTA / C	P11276	-0.0547	0.138573	0.277609	Fn1	Fibronectir	FINC_MOUS	30	0.962797
15_MTA / C	P43277	0.212642	0.138503	0.277609	H1-3	Histone H1	H13_MOUS	5	1.158809
30_MTA / C	P10605	-0.08902	0.138937	0.277719	Ctsb	Cathepsin E	CATB_MOU	10	0.940161
15_MTA / C	P50172	-0.25031	0.138757	0.277719	Hsd11b1	11-beta-hy	DHI1_MOU	4	0.840717
30_MTA / C	P59325	0.096122	0.138908	0.277719	Eif5	Eukaryotic	IF5_MOUSE	13	1.068896
15_MTA / C	Q9QUH0	-0.29493	0.138925	0.277719	Glrx	Glutaredox	GLRX1_MO	5	0.815113
15_MTA / C	P17563	-0.05719	0.139073	0.277838	Selenbp1	Methaneth	SBP1_MOU	36	0.961133
15_MTA / C	Q8CIN4	-0.26503	0.139297	0.277975	Pak2	Serine/thre	PAK2_MOU	2	0.83218
15_MTA / C	Q91VA6	-1.71079	0.139716	0.278503	Poldip2	Polymerase	PDIP2_MO	4	0.305493
30_MTA / C	Q99PT1	-0.08478	0.139646	0.278503	Arhgdia	Rho GDP-di	GDIR1_MO	12	0.942929
15_MTA / C	Q8K010	-0.01873	0.140102	0.278708	Oplah	5-oxoprolir	OPLA_MOU	26	0.987103
30_MTA / C	Q9Z204	1.101215	0.140128	0.278708	Hnrnpc	Heterogene	HNRPC_MC	5	2.145354
15_MTA / C	Q61160	-0.30932	0.140569	0.27943	Fadd	FAS-associa	FADD_MOU	1	0.807021
15_MTA / C	Q91VI7	-0.13954	0.140673	0.279483	Rnh1	Ribonuclea	RINI_MOUS	25	0.907808
15_MTA / C	P29595	-0.0305	0.14142	0.280194	Nedd8	NEDD8	NEDD8_MC	3	0.979082
30_MTA / C	P63085	0.743491	0.141382	0.280194	Mapk1	Mitogen-ac	MK01_MOU	7	1.674222
15_MTA / C	Q9CXF0	-0.16496	0.141377	0.280194	Kynu	Kynurenina	KYNU_MOU	12	0.891955
15_MTA / C	Q9JIX0	-1.07699	0.141171	0.280194	Eny2	Transcripti	ENY2_MOU	2	0.474017
30_MTA / C	Q3TC72	0.081616	0.141711	0.280279	Fahd2a	Oxaloaceta	FAH2A_MO	1	1.058203
30_MTA / C	Q8K4M5	-0.63473	0.141774	0.280279	Commd1	COMM don	COMD1_M	2	0.644059
30_MTA / C	Q921I1	-0.10341	0.141658	0.280279	Tf	Serotransfe	TRFE_MOUS	51	0.93083
30_MTA / C	P40142	-0.10091	0.14192	0.280414	Tkt	Transketola	TKT_MOUSI	39	0.932444
30_MTA / C	P16460	-0.11716	0.142046	0.280507	Ass1	Argininosu	ASSY_MOUS	27	0.922004
15_MTA / C	O88544	-0.12646	0.142175	0.280546	Cops4	COP9 signa	CSN4_MOU	14	0.916073
30_MTA / C	Q5FW60	0.031364	0.142221	0.280546	Mup20	Major urina	MUP20_MC	3	1.021978
30_MTA / C	P02089	-0.24171	0.142822	0.280802	Hbb-b2	Hemoglobi	HBB2_MOU	6	0.84574
30_MTA / C	Q5RKZ7	-0.10161	0.142701	0.280802	Mocs1	Molybdenu	MOCS1_MC	6	0.931995
30_MTA / C	Q80W21	-0.41665	0.142498	0.280802	Gstm7	Glutathion	GSTM7_MC	5	0.74916
30_MTA / C	Q9CQH7	0.16169	0.142836	0.280802	Btf3l4	Transcripti	BT3L4_MO	4	1.118597
15_MTA / C	Q9D358	-0.04593	0.142759	0.280802	Acp1	Low molecu	PPAC_MOU	3	0.968666
15_MTA / C	Q921I1	-0.07581	0.143134	0.281114	Tf	Serotransfe	TRFE_MOUS	51	0.94881
15_MTA / C	Q9WTP6	-0.09422	0.143953	0.282261	Ak2	Adenylate k	KAD2_MOU	17	0.936778
15_MTA / C	Q9Z2V4	-0.56975	0.14394	0.282261	Pck1	Phosphoen	PCKGC_MC	5	0.673735
15_MTA / C	Q91ZX7	-0.25349	0.144139	0.282362	Lrp1	Prolow-der	LRP1_MOU	30	0.838865
15_MTA / C	Q8BTE0	0.261989	0.144367	0.282449	Sdhaf4	Succinate c	SDHF4_MO	1	1.199131
15_MTA / C	Q8R0P4	0.711095	0.144381	0.282449	Aamdc	Mth938 do	AAMDC_M	1	1.637046
30_MTA / C	Q8K4L4	1.537487	0.144547	0.282502	Pof1b	Protein PO	POF1B_MO	5	2.902885
15_MTA / C	P58058	-0.12561	0.14472	0.28252	Nadk	NAD kinase	NADK_MOU	8	0.916618
30_MTA / C	Q60865	0.158648	0.145873	0.284168	Caprin1	Caprin-1	CAPR1_MO	7	1.116241
15_MTA / C	Q99J36	-1.07656	0.145802	0.284168	Thumpd1	THUMP do	THUM1_M	3	0.474158
30_MTA / C	P56593	-1.39961	0.146125	0.284351	Cyp2a12	Cytochrom	CP2AC_MO	1	0.379032
30_MTA / C	P19096	-0.31402	0.146239	0.284419	Fasn	Fatty acid s	FAS_MOUS	78	0.804396
30_MTA / C	P10649	-0.1414	0.146536	0.284421	Gstm1	Glutathion	GSTM1_MC	13	0.906641
15_MTA / C	P24472	-0.07441	0.146415	0.284421	Gsta4	Glutathion	GSTA4_MO	9	0.949732
15_MTA / C	P62245	-0.47731	0.146495	0.284421	Rps15a	Small ribos	RS15A_MO	7	0.718313
15_MTA / C	Q9DBG3	-0.11583	0.147289	0.285537	Ap2b1	AP-2 compl	AP2B1_MO	9	0.922851
15_MTA / C	B5X0G2	-0.39929	0.147558	0.285659	Mup17	Major urina	MUP17_M	2	0.758229
15_MTA / C	P15327	-0.31132	0.147633	0.285659	Bpgm	Bisphosphc	PMGE_MOU	6	0.805903
15_MTA / C	P39039	-0.47089	0.147793	0.285659	Mbl1	Mannose-b	MBL1_MOU	2	0.721518
30_MTA / C	Q61035	-0.61034	0.147828	0.285659	Hars1	Histidine--t	HARS1_MO	3	0.655041
30_MTA / C	Q8BGD9	0.24762	0.147829	0.285659	Eif4b	Eukaryotic	IF4B_MOUS	17	1.187247

30_MTA / C	Q8JZL3	0.235142	0.14775	0.285659	Thtpa	Thiamine-ti	THTPA_MO	4	1.177023
30_MTA / C	Q921M3	0.171134	0.148072	0.285823	Sf3b3	Splicing fac	SF3B3_MOU	18	1.125943
15_MTA / C	P56399	0.120277	0.148532	0.285948	Usp5	Ubiquitin c	UBP5_MOU	29	1.086944
30_MTA / C	P70362	0.545863	0.148347	0.285948	Ufd1	Ubiquitin r	UFD1_MOU	7	1.459894
30_MTA / C	Q3UIA2	-0.79376	0.14847	0.285948	Arhgap17	Rho GTPase	RHG17_MO	1	0.576839
15_MTA / C	Q9CQC9	-1.24934	0.148534	0.285948	Sar1b	Small COPII	SAR1B_MO	2	0.420641
30_MTA / C	Q9DCY0	-0.43772	0.148477	0.285948	Keg1	Glycine N-a	KEG1_MOU	7	0.738302
30_MTA / C	Q9R112	-0.58675	0.148794	0.286143	Sqor	Sulfide:qui	SQOR_MOU	5	0.665841
15_MTA / C	Q8VEB4	0.08681	0.149107	0.286439	Pla2g15	Lysosomal	PAG15_MO	6	1.062019
15_MTA / C	Q91VT4	-0.27733	0.149331	0.286715	Cbr4	3-oxoacyl-[	CBR4_MOU	7	0.825115
15_MTA / C	Q921F2	0.407198	0.149475	0.28684	Tardbp	TAR DNA-bi	TADBP_MO	2	1.326108
30_MTA / C	Q3UM45	0.202276	0.149583	0.286893	Ppp1r7	Protein pho	PP1R7_MO	11	1.150512
30_MTA / C	Q9D6F9	-1.75417	0.149963	0.287468	Tubb4a	Tubulin bet	TBB4A_MO	1	0.296443
15_MTA / C	Q8R1X6	-0.62002	0.150275	0.287915	Spart	Spartin	SPART_MOU	3	0.650664
15_MTA / C	Q62465	-0.05222	0.150545	0.288228	Vat1	Synaptic ve	VAT1_MOU	7	0.964452
15_MTA / C	Q91YH6	1.543859	0.150599	0.288228	Atp6v1b1	V-type prot	VATB1_MO	1	2.915733
15_MTA / C	O35129	-0.38599	0.151665	0.289186	Phb2	Prohibitin-	PHB2_MOU	1	0.765255
15_MTA / C	O35490	-0.0138	0.151656	0.289186	Bhmt	Betaine--hc	BHMT1_MO	29	0.990479
30_MTA / C	O35658	0.184023	0.151742	0.289186	C1qbp	Complemen	C1QBP_MO	6	1.136047
15_MTA / C	O88543	-0.11557	0.15168	0.289186	Cops3	COP9 signa	CSN3_MOU	11	0.92302
15_MTA / C	Q8QZT1	-0.03961	0.151602	0.289186	Acat1	Acetyl-CoA	THIL_MOUS	20	0.972921
15_MTA / C	Q9EQ20	-0.17566	0.151502	0.289186	Aldh6a1	Methylmalo	MMSA_MO	21	0.885361
15_MTA / C	Q9WV54	-1.23422	0.152099	0.289406	Asah1	Acid ceram	ASAH1_MO	4	0.425073
15_MTA / C	P34928	-0.19211	0.152411	0.289657	Apoc1	Apolipoprc	APOC1_MO	1	0.875324
15_MTA / C	Q05915	-0.32952	0.152757	0.289738	Gch1	GTP cycloh	GCH1_MOU	9	0.7958
30_MTA / C	Q8BMA6	0.593408	0.153177	0.290382	Srp68	Signal reco	SRP68_MO	5	1.508807
15_MTA / C	Q9D8X1	-0.73182	0.153376	0.290457	Cutc	Copper hor	CUTC_MOU	2	0.602144
30_MTA / C	Q9DCM2	-0.27698	0.153377	0.290457	Gstk1	Glutathion	GSTK1_MO	11	0.825314
30_MTA / C	Q8K4F5	-0.05632	0.153782	0.291071	Abhd11	sn-1-specifi	ABHDB_MO	7	0.961717
15_MTA / C	P48758	-0.02855	0.154287	0.291446	Cbr1	Carbonyl re	CBR1_MOU	20	0.980408
30_MTA / C	P56812	0.575433	0.154151	0.291446	Pdcd5	Programme	PDCD5_MO	4	1.490125
30_MTA / C	Q99KK2	0.303978	0.154304	0.291446	Cmas	N-acylneur	NEUA_MOU	4	1.234543
15_MTA / C	Q9EQ32	-0.07847	0.154528	0.291716	Pik3ap1	Phosphoin	BCAP_MOU	3	0.947064
30_MTA / C	P63028	-0.40033	0.154995	0.292137	Tpt1	Translation	TCTP_MOU	8	0.757686
15_MTA / C	Q3UIA2	-1.11978	0.154954	0.292137	Arhgap17	Rho GTPase	RHG17_MO	1	0.460164
15_MTA / C	P26039	-0.20952	0.155358	0.292363	Tln1	Talin-1	TLN1_MOU	55	0.864823
30_MTA / C	P80318	0.146181	0.155335	0.292363	Cct3	T-complex	TCPG_MOU	19	1.106636
15_MTA / C	Q80W21	-0.54643	0.155263	0.292363	Gstm7	Glutathion	GSTM7_MO	5	0.684715
15_MTA / C	Q8K183	-0.07879	0.155849	0.292979	Pdxk	Pyridoxal k	PDXK_MOU	11	0.94685
15_MTA / C	P17182	-0.12244	0.156047	0.293199	Eno1	Alpha-enol	ENOA_MOU	21	0.918631
15_MTA / C	Q99LD8	-0.49639	0.156266	0.293304	Ddah2	Putative hy	DDAH2_MO	2	0.708877
15_MTA / C	Q9D662	-0.02186	0.156232	0.293304	Sec23b	Protein tra	SC23B_MO	12	0.984963
15_MTA / C	P06728	-0.16579	0.156447	0.293366	Apoa4	Apolipoprc	APOA4_MO	23	0.891438
15_MTA / C	Q9CPW4	-0.63502	0.156543	0.293366	Arpc5	Actin-relate	ARPC5_MO	3	0.64393
30_MTA / C	Q91XE4	0.0306	0.156667	0.293445	Acy3	N-acyl-aron	ACY3_MOU	6	1.021437
30_MTA / C	P52760	-0.17204	0.156997	0.293888	Rida	2-iminobut	RIDA_MOU	10	0.887584
15_MTA / C	Q8K3C3	0.041232	0.157394	0.294347	Lzic	Protein LZI	LZIC_MOUS	3	1.028992
30_MTA / C	Q61937	0.164207	0.157478	0.294351	Npm1	Nucleopho	NPM_MOU	9	1.12055
30_MTA / C	O35945	-0.11658	0.157828	0.294382	Aldh1a7	Aldehyde d	AL1A7_MO	31	0.922374
30_MTA / C	P47955	0.244539	0.158041	0.294382	Rplp1	Large ribos	RLA1_MOU	3	1.184715
30_MTA / C	P61082	-0.07129	0.158009	0.294382	Ube2m	NEDD8-cor	UBC12_MO	4	0.951786
15_MTA / C	Q8VC85	0.435134	0.158067	0.294382	Lsm1	U6 snRNA-a	LSM1_MOU	1	1.352036

30_MTA / C	Q3UZZ6	-0.35008	0.15817	0.294422	Sult1d1	Sulfotransf	ST1D1_MO	4	0.784543
30_MTA / C	Q00623	-0.18675	0.158509	0.294709	Apoa1	Apolipoprc	APOA1_MC	17	0.878582
15_MTA / C	P12246	-0.9031	0.158954	0.294897	Apcs	Serum amy	SAMP_MOU	4	0.534736
15_MTA / C	P15532	-0.09975	0.158773	0.294897	Nme1	Nucleoside	NDKA_MOU	7	0.933197
30_MTA / C	Q9QUR6	0.443064	0.159081	0.294897	Prep	Prolyl endc	PPCE_MOU	7	1.359489
15_MTA / C	Q3UEG6	-0.05192	0.159182	0.294934	Agxt2	Alanine--gly	AGT2_MOU	28	0.96465
30_MTA / C	O08738	0.152232	0.159521	0.295258	Casp6	Caspase-6	CASP6_MO	4	1.111287
30_MTA / C	Q15323	1.390071	0.159755	0.295538	KRT31	Keratin, typ	K1H1_HUM	10	2.620915
30_MTA / C	Q8R4N0	0.079112	0.159878	0.295614	Clybl	Citramalyl-	CLYBL_MOU	10	1.056368
30_MTA / C	P62962	-0.1744	0.160003	0.295694	Pfn1	Profilin-1	PROF1_MO	8	0.886138
15_MTA / C	Q7TPR4	-0.02737	0.160167	0.295844	Actn1	Alpha-actin	ACTN1_MO	45	0.981206
30_MTA / C	P56212	0.193724	0.160585	0.296465	Arpp19	cAMP-regul	ARP19_MO	1	1.143713
15_MTA / C	O43790	0.128783	0.161236	0.296753	KRT86	Keratin, typ	KRT86_HUI	21	1.093371
15_MTA / C	P27773	-0.02372	0.161214	0.296753	Pdia3	Protein dis	PDIA3_MOU	28	0.983691
30_MTA / C	P54728	0.196493	0.161129	0.296753	Rad23b	UV excision	RD23B_MC	10	1.145909
30_MTA / C	Q9CZ04	0.377544	0.161084	0.296753	Cops7a	COP9 signa	CSN7A_MO	5	1.299129
30_MTA / C	P62838	-0.08103	0.161507	0.297101	Ube2d2	Ubiquitin-c	UB2D2_MC	3	0.945383
15_MTA / C	Q91WG8	-0.74276	0.162257	0.29827	Gne	Bifunctiona	GLCNE_MO	5	0.597596
30_MTA / C	Q9CQR4	0.954379	0.163025	0.299282	Acot13	Acyl-coenzy	ACO13_MC	2	1.937745
30_MTA / C	Q9Z2L6	0.362837	0.163128	0.299317	Minpp1	Multiple in	MINP1_MC	1	1.285952
30_MTA / C	Q9CQQ8	-0.26988	0.164157	0.301053	Lsm7	U6 snRNA-a	LSM7_MOU	2	0.82939
30_MTA / C	Q9CQI3	-0.38168	0.16425	0.30107	Gmfb	Glia matura	GMFB_MOU	4	0.767545
15_MTA / C	P47738	-0.13165	0.164573	0.301508	Aldh2	Aldehyde d	ALDH2_MC	20	0.912786
30_MTA / C	Q8VI36	0.304161	0.164943	0.302032	Pxn	Paxillin	PAXI_MOUS	1	1.2347
30_MTA / C	Q3UHX2	0.078055	0.16532	0.302415	Pdap1	28 kDa heat	HAP28_MC	4	1.055594
30_MTA / C	Q8BMF4	0.22885	0.165608	0.302788	Dlat	Dihydrolipo	ODP2_MOU	18	1.1719
15_MTA / C	Q60854	0.060884	0.165765	0.302921	Serpinb6	Serpin B6	SPB6_MOU	10	1.043105
30_MTA / C	P50462	0.452637	0.166386	0.303563	Csrp3	Cysteine an	CSRP3_MO	5	1.368539
30_MTA / C	P28650	0.302839	0.167998	0.305241	Adss1	Adenylosuc	PURA1_MC	1	1.233569
15_MTA / C	P39061	-0.37165	0.167945	0.305241	Col18a1	Collagen alp	COIA1_MOU	3	0.772898
15_MTA / C	P63001	-0.71397	0.167964	0.305241	Rac1	Ras-related	RAC1_MOU	5	0.60964
15_MTA / C	Q8VIJ6	-0.20536	0.168033	0.305241	Sfpq	Splicing fac	SFPQ_MOU	10	0.86732
30_MTA / C	Q9CPU0	-0.1402	0.168137	0.305241	Glo1	Lactoylglut	LGUL_MOU	13	0.907392
30_MTA / C	Q9D0A3	-0.13372	0.167832	0.305241	Arpin	Arpin	ARPIN_MOU	3	0.91148
30_MTA / C	Q9QYB1	-0.07208	0.168108	0.305241	Clic4	Chloride in	CLIC4_MOU	14	0.951266
30_MTA / C	O35857	0.577236	0.169314	0.305565	Timm44	Mitochond	TIM44_MO	4	1.491988
30_MTA / C	P05201	-0.06955	0.169097	0.305565	Got1	Aspartate a	AATC_MOU	27	0.952932
30_MTA / C	P16125	0.187588	0.169189	0.305565	Ldhb	L-lactate de	LDHB_MOU	8	1.138858
15_MTA / C	Q8N9S3	1.096	0.169262	0.305565	Ahsa2	Activator o	AHSA2_MO	1	2.137612
30_MTA / C	Q8R574	-0.14701	0.169078	0.305565	Prpsap2	Phosphorib	KPRB_MOU	8	0.903122
30_MTA / C	Q8VBV3	-0.164	0.168433	0.305565	Exosc2	Exosome cc	EXOS2_MO	4	0.892546
30_MTA / C	Q9QUI0	-0.02363	0.169281	0.305565	Rhoa	Transformi	RHOA_MOU	3	0.983756
15_MTA / C	Q9WV85	-0.06567	0.16891	0.305565	Nme3	Nucleoside	NDK3_MOU	5	0.955504
30_MTA / C	P60670	-0.90079	0.169665	0.305856	Nploc4	Nuclear prc	NPL4_MOU	3	0.535594
15_MTA / C	Q8CEI1	-0.94208	0.169658	0.305856	Bola3	BolA-like pr	BOLA3_MO	3	0.520482
15_MTA / C	A2ADY9	-0.07097	0.169882	0.305903	Ddi2	Protein DD	DDI2_MOU	16	0.951997
30_MTA / C	P34022	0.121961	0.169946	0.305903	Ranbp1	Ran-specific	RANG_MOU	5	1.088213
30_MTA / C	O09167	0.238779	0.170147	0.306009	Rpl21	Large ribos	RL21_MOU	4	1.179993
15_MTA / C	Q9DCM2	-0.23594	0.170175	0.306009	Gstk1	Glutathion	GSTK1_MO	11	0.849134
30_MTA / C	P26443	-0.44934	0.170629	0.306061	Glud1	Glutamate	DHE3_MOU	24	0.732377
15_MTA / C	P49722	-0.11939	0.17056	0.306061	Psma2	Proteasom	PSA2_MOU	14	0.920576
30_MTA / C	Q9R060	-1.22774	0.170604	0.306061	Nubp1	Cytosolic F	NUBP1_MC	1	0.426987

15_MTA / C	P26041	-0.03446	0.170736	0.3061	Msn	Moesin	MOES_MOU		10	0.976399
15_MTA / C	Q5SW19	-0.39137	0.17168	0.307074	Cluh	Clustered n	CLU_MOUS		6	0.762406
15_MTA / C	Q9D1E6	-0.13318	0.171706	0.307074	Tbcb	Tubulin-fol	TBCB_MOU		6	0.911817
15_MTA / C	Q9DCV4	-0.61688	0.171925	0.307314	Rmdn1	Regulator c	RMD1_MOI		3	0.652078
30_MTA / C	P29391	-0.28289	0.172856	0.308003	Ftl1	Ferritin ligh	FRIL1_MOU		14	0.82194
30_MTA / C	P62320	-0.32938	0.172649	0.308003	Snrpd3	Small nucle	SMD3_MOI		5	0.79588
15_MTA / C	P68368	0.484642	0.172681	0.308003	Tuba4a	Tubulin alp	TBA4A_MO		3	1.399239
15_MTA / C	Q8K2I4	-0.4397	0.172622	0.308003	Manba	Beta-manni	MANBA_M(		9	0.737287
30_MTA / C	Q91VU6	0.274944	0.17298	0.308003	Dcaf11	DDB1- and	DCA11_MC		2	1.209947
30_MTA / C	Q9JHI7	0.650665	0.173103	0.308043	Exosc9	Exosome cc	EXOS9_MO		3	1.569892
30_MTA / C	Q60648	0.338163	0.173772	0.30849	Gm2a	Ganglioside	SAP3_MOU		3	1.264146
30_MTA / C	Q8BWW4	0.477644	0.17359	0.30849	Larp4	La-related p	LARP4_MO		3	1.392467
30_MTA / C	Q922Q8	0.065935	0.173959	0.308499	Lrrc59	Leucine-ric	LRC59_MO		8	1.046763
15_MTA / C	Q80VJ3	-0.51283	0.174052	0.308512	Dnph1	5-hydroxyn	DNPH1_M(		3	0.700848
15_MTA / C	Q8R016	-0.0071	0.174179	0.308584	Blmh	Bleomycin	BLMH_MOI		20	0.995088
15_MTA / C	P27005	-0.40876	0.174589	0.308713	S100a8	Protein S1(	S10A8_MO		2	0.753271
30_MTA / C	Q921I2	-1.58601	0.174516	0.308713	Klhdc4	Kelch doma	KLDC4_MO		1	0.333091
30_MTA / C	Q9WUU7	-0.06545	0.175829	0.31059	Ctsz	Cathepsin Z	CATZ_MOU		7	0.95565
15_MTA / C	Q5XG73	0.296661	0.176009	0.310756	Acbd5	Acyl-CoA-bi	ACBD5_MC		3	1.228298
15_MTA / C	Q9CQE1	-0.13991	0.176375	0.31125	Nipsnap3b	Protein Nip	NPS3B_MO		12	0.907574
30_MTA / C	P28843	0.154533	0.176635	0.311557	Dpp4	Dipeptidyl	DPP4_MOU		5	1.113061
15_MTA / C	Q811D0	-0.15423	0.176926	0.311764	Dlg1	Disks large	DLG1_MOU		2	0.898614
30_MTA / C	Q9WVA4	0.120158	0.176866	0.311764	Tagln2	Transgelin-:	TAGL2_MO		8	1.086854
15_MTA / C	Q8QZR3	0.121049	0.177496	0.312615	Ces2a	Pyrethroid	EST2A_MOI		16	1.087525
15_MTA / C	P35700	-0.13478	0.178721	0.314004	Prdx1	Peroxiredo	PRDX1_MC		21	0.910811
30_MTA / C	Q99MD9	-0.49575	0.178546	0.314004	Nasp	Nuclear aut	NASP_MOU		2	0.709194
15_MTA / C	Q9DCC4	-0.91514	0.17866	0.314004	Pycr3	Pyrroline-5	P5CR3_MO		4	0.530292
30_MTA / C	O88587	-0.02782	0.17885	0.314078	Comt	Catechol O-	COMT_MOI		4	0.980898
15_MTA / C	Q9CXW3	-0.2039	0.179012	0.31421	Cacybp	Calcyclin-b	CYBP_MOU		14	0.868197
15_MTA / C	P19096	-0.27193	0.179557	0.314468	Fasn	Fatty acid s	FAS_MOUSI		78	0.828209
15_MTA / C	P97350	0.307906	0.179712	0.314468	Pkp1	Plakophilin	PKP1_MOU		4	1.23791
15_MTA / C	Q60605	0.183482	0.179716	0.314468	Myl6	Myosin ligh	MYL6_MOU		7	1.135621
30_MTA / C	Q61171	0.005249	0.179663	0.314468	Prdx2	Peroxiredo	PRDX2_MC		15	1.003645
30_MTA / C	Q99LI8	-0.60387	0.179379	0.314468	Hgs	Hepatocyte	HGS_MOUS		4	0.657986
30_MTA / C	Q9EQG9	0.528279	0.179708	0.314468	Cert1	Ceramide tı	CERT_MOU		1	1.442207
30_MTA / C	P35278;P6	0.639291	0.180233	0.315124	Rab5c;Rab!	Ras-related	RAB5C_MO		2	1.557563
15_MTA / C	Q8CJG0	-0.19231	0.180371	0.315199	ago-02	Protein arg	AGO2_MOU		8	0.875204
30_MTA / C	Q8JZQ9	-0.01582	0.180469	0.315199	Eif3b	Eukaryotic	EIF3B_MOI		19	0.989096
30_MTA / C	Q922D8	-0.81747	0.180539	0.315199	Mthfd1	C-1-tetrahy	C1TC_MOU		11	0.567435
30_MTA / C	P62192	0.339518	0.180648	0.315236	Psmc1	26S protea:	PRS4_MOU		9	1.265334
30_MTA / C	O88844	-0.36269	0.181428	0.316355	Idh1	Isocitrate d	IDHC_MOU		18	0.777715
30_MTA / C	Q9CR86	0.243715	0.181465	0.316355	Carhsp1	Calcium-re	CHSP1_MO		3	1.184038
30_MTA / C	P08071	-0.25165	0.181912	0.316521	Ltf	Lactotransf	TRFL_MOU:		16	0.839937
30_MTA / C	Q9QY76	-0.42492	0.181757	0.316521	Vapb	Vesicle-assc	VAPB_MOU		1	0.744879
15_MTA / C	O70370	-0.33784	0.1823	0.317044	Ctss	Cathepsin S	CATS_MOU		6	0.791228
30_MTA / C	O55229	1.323078	0.182822	0.317644	Chkb	Choline/eth	CHKB_MOU		2	2.501994
15_MTA / C	P30416	-0.03388	0.183304	0.318329	Fkbp4	Peptidyl-pr	FKBP4_MO		19	0.976788
30_MTA / C	P41105	0.293198	0.183689	0.318843	Rpl28	Large ribos	RL28_MOU		6	1.225353
15_MTA / C	Q8BIQ5	-0.67153	0.184415	0.319795	Cstf2	Cleavage sti	CSTF2_MOI		1	0.62784
30_MTA / C	Q6ZWM4	0.054993	0.184798	0.320226	Lsm8	U6 snRNA-a	LSM8_MOU		3	1.038854
30_MTA / C	Q9D9H8	0.138997	0.184841	0.320226	NaN	Mitochond	CB069_MO		5	1.10114
30_MTA / C	P07758	-0.10633	0.185012	0.320367	Serpina1a	Alpha-1-ant	A1AT1_MO		17	0.928949

30_MTA / C	Q6NSR8	-0.01871	0.185242	0.320458	Npepl1	Probable ar	PEPL1_MOU	12	0.987113
30_MTA / C	Q9Z0J0	0.146492	0.18519	0.320458	Npc2	NPC intrace	NPC2_MOU	5	1.106874
30_MTA / C	Q9DCJ9	-0.30702	0.1854	0.320577	Npl	N-acetylneu	NPL_MOUS	11	0.80831
30_MTA / C	Q6P5E4	0.051731	0.187026	0.322613	Uggt1	UDP-glucos	UGGG1_MO	20	1.036508
30_MTA / C	Q6A028	0.215941	0.187773	0.323128	Swap70	Switch-asso	SWP70_MO	1	1.161461
30_MTA / C	Q91V76	-0.20693	0.187865	0.323132	NaN	Ester hydro	CK054_MO	16	0.866379
15_MTA / C	Q60598	0.072937	0.188323	0.323765	Cttn	Src substrat	SRC8_MOU	11	1.051856
15_MTA / C	P57780	-0.11434	0.188822	0.323961	Actn4	Alpha-actin	ACTN4_MO	38	0.923806
15_MTA / C	Q8C1A5	-0.02005	0.188887	0.323961	Thop1	Thimet olig	THOP1_MO	10	0.986198
30_MTA / C	Q8CHT0	-0.41695	0.188874	0.323961	Aldh4a1	Delta-1-pyr	AL4A1_MO	8	0.749008
15_MTA / C	Q91V76	-0.19437	0.188754	0.323961	NaN	Ester hydro	CK054_MO	16	0.873955
15_MTA / C	Q9DAK9	-0.1905	0.189073	0.324125	Phpt1	14 kDa pho	PHP14_MO	4	0.876302
15_MTA / C	P00761	0.456224	0.190354	0.324812	NaN	Trypsin (Lal	TRYP_PIG	3	1.371946
15_MTA / C	P63005	0.014235	0.190257	0.324812	Pafah1b1	Platelet-act	LIS1_MOUS	17	1.009916
15_MTA / C	Q3UZZ6	0.074991	0.190172	0.324812	Sult1d1	Sulfotransf	ST1D1_MO	4	1.053354
30_MTA / C	Q61702	-0.08342	0.190466	0.324812	Itih1	Inter-alpha	ITIH1_MOU	14	0.943819
30_MTA / C	Q69ZR2	0.404618	0.189655	0.324812	Hectd1	E3 ubiquiti	HECD1_MO	2	1.323739
15_MTA / C	Q9CQH7	0.02802	0.190123	0.324812	Btf3l4	Transcripti	BT3L4_MOU	4	1.019611
30_MTA / C	P16015	-0.13494	0.191001	0.325415	Ca3	Carbonic ar	CAH3_MOU	17	0.910705
30_MTA / C	Q9Z2X1	0.429298	0.191194	0.325448	Hnrnpf	Heterogene	HNRPF_MO	5	1.346578
15_MTA / C	P70168	-0.03794	0.191903	0.32618	Kpnb1	Importin su	IMB1_MOU	25	0.974042
30_MTA / C	Q923G2	-0.34281	0.19212	0.326395	Polr2h	DNA-direct	RPAB3_MO	3	0.788504
15_MTA / C	O70493	-0.28373	0.192699	0.326701	Snx12	Sorting nex	SNX12_MO	4	0.821465
15_MTA / C	P61961	-0.09431	0.192754	0.326701	Ufm1	Ubiquitin-f	UFM1_MOU	4	0.936721
30_MTA / C	Q9CYR0	-0.16707	0.192521	0.326701	Ssbp1	Single-strar	SSBP_MOU	5	0.890651
15_MTA / C	Q9Z1Z0	-0.10605	0.19324	0.327371	Uso1	General ves	USO1_MOU	31	0.929126
15_MTA / C	P56391	-0.3712	0.193336	0.327379	Cox6b1	Cytochrom	CX6B1_MO	4	0.773141
15_MTA / C	O08583	-0.51483	0.193705	0.327697	Alyref	THO compl	THOC4_MO	3	0.699876
30_MTA / C	P63101	0.139819	0.194143	0.328129	Ywhaz	14-3-3 prot	1433Z_MO	12	1.101767
30_MTA / C	Q61207	0.218578	0.195749	0.329452	Psap	Prosaposin	SAP_MOUS	9	1.163586
15_MTA / C	Q76MZ3	0.037925	0.195697	0.329452	Ppp2r1a	Serine/thre	2AAA_MOU	24	1.026636
30_MTA / C	Q9CZU6	-1.25907	0.19562	0.329452	Cs	Citrate synt	CISY_MOUS	2	0.417814
15_MTA / C	Q9WV68	-0.1099	0.195672	0.329452	Decr2	Peroxisoma	DECR2_MO	5	0.926652
15_MTA / C	P50396	0.035093	0.195868	0.329498	Gdi1	Rab GDP di	GDIA_MOU	11	1.024623
15_MTA / C	Q8VC12	-0.04197	0.195993	0.329555	Uroc1	Urocanate l	HUTU_MOU	30	0.971325
30_MTA / C	Q8BWF0	0.063959	0.196395	0.329922	Aldh5a1	Succinate-s	SSDH_MOU	16	1.045331
30_MTA / C	Q8VCN5	-0.04818	0.196656	0.330206	Cth	Cystathioni	CGL_MOUS	22	0.967157
30_MTA / C	Q9DAK9	-0.16509	0.196852	0.33038	Phpt1	14 kDa pho	PHP14_MO	4	0.891873
30_MTA / C	Q05793	-0.3265	0.197784	0.331636	Hspg2	Basement n	PGBM_MOU	27	0.797468
15_MTA / C	Q4KML4	-0.02907	0.198484	0.332655	Abracl	Costars fam	ABRAL_MO	3	0.980054
30_MTA / C	Q91Y97	-0.11261	0.19877	0.332825	Aldob	Fructose-bi	ALDOB_MO	29	0.924915
15_MTA / C	O08759	-0.74418	0.199296	0.333278	Ube3a	Ubiquitin-p	UBE3A_MO	1	0.597006
30_MTA / C	P51410	0.342785	0.199904	0.33397	Rpl9	Large ribos	RL9_MOUS	6	1.268202
15_MTA / C	P60766	-0.64567	0.199918	0.33397	Cdc42	Cell divisio	CDC42_MO	3	0.639197
15_MTA / C	P16254	0.087278	0.200395	0.334456	Srp14	Signal reco	SRP14_MO	5	1.062364
30_MTA / C	Q8R1F5	-0.14079	0.200984	0.335128	Hyi	Putative hy	HYI_MOUSI	13	0.907023
30_MTA / C	P28474	0.047452	0.201245	0.335399	Adh5	Alcohol deh	ADHX_MOU	21	1.033438
15_MTA / C	Q61029	-0.26766	0.201333	0.335399	Tmpo	Lamina-ass	LAP2B_MO	3	0.830665
15_MTA / C	P12710	0.229434	0.201499	0.335522	Fabp1	Fatty acid-l	FABPL_MO	11	1.172375
15_MTA / C	A2AS89	-0.08546	0.20166	0.335635	Agmat	Guanidino	GDAH_MOU	8	0.942484
30_MTA / C	P26516	0.096359	0.202151	0.336296	Psmd7	26S proteas	PSMD7_MO	5	1.069072
30_MTA / C	P18760	-0.12074	0.202292	0.336375	Cfl1	Cofilin-1	COF1_MOU	14	0.919719

15_MTA / C	Q60676	-0.00162	0.202476	0.336525	Ppp5c	Serine/thre	PPP5_MOU	13	0.998877
15_MTA / C	Q8K023	-0.23617	0.202769	0.336856	Akr1c18	Aldo-keto r	AKC1H_MC	5	0.848999
30_MTA / C	Q9CPW4	0.526396	0.202972	0.337038	Arpc5	Actin-relate	ARPC5_MO	3	1.440327
15_MTA / C	Q80UG5	-0.43844	0.20321	0.337278	Septin9	Septin-9	SEPT9_MOU	5	0.73793
15_MTA / C	P70195	-0.06943	0.20356	0.337447	Psmb7	Proteasome	PSB7_MOU	10	0.953011
15_MTA / C	Q9QXC1	-0.0593	0.203593	0.337447	Fetub	Fetuin-B	FETUB_MOU	8	0.959729
30_MTA / C	A2AKK5	0.225499	0.203787	0.337613	Acnat1	Acyl-coenzy	ACNT1_MO	5	1.169182
30_MTA / C	O54879	0.460563	0.204281	0.33821	Hmgb3	High mobil	HMGB3_M	1	1.376079
15_MTA / C	Q9JLQ0	-0.6905	0.204429	0.33821	Cd2ap	CD2-associ	CD2AP_MC	5	0.619639
15_MTA / C	Q9CQM9	-0.00847	0.204666	0.338447	Glrx3	Glutaredox	GLRX3_MO	10	0.994147
30_MTA / C	P52430	-0.70586	0.20514	0.339074	Pon1	Serum para	PON1_MOU	2	0.613078
30_MTA / C	Q3THS6	0.308212	0.205669	0.339637	Mat2a	S-adenosylr	METK2_MC	6	1.238172
30_MTA / C	Q9JI90	0.424338	0.205962	0.339965	Rnf14	E3 ubiquiti	RNF14_MO	1	1.341957
15_MTA / C	Q9QXA5	0.001847	0.206288	0.340348	Lsm4	U6 snRNA-a	LSM4_MOU	2	1.001281
15_MTA / C	Q63880	0.102399	0.206715	0.340896	Ces3a	Carboxyles	EST3A_MOU	10	1.073557
30_MTA / C	Q61510	0.906396	0.20685	0.340962	Trim25	E3 ubiquiti	TRI25_MOU	2	1.874357
30_MTA / C	P13634	0.316572	0.207111	0.341031	Ca1	Carbonic ar	CAH1_MOU	9	1.245368
30_MTA / C	P47754	0.068905	0.207176	0.341031	Capza2	F-actin-cap	CAZA2_MO	9	1.04892
30_MTA / C	P61290	0.166868	0.207626	0.341335	Psme3	Proteasome	PSME3_MC	6	1.122619
30_MTA / C	P97447	-0.25604	0.207681	0.341335	Fhl1	Four and a l	FHL1_MOU	5	0.837384
15_MTA / C	Q3TLH4	-0.01395	0.207813	0.341335	Prrc2c	Protein PRI	PRC2C_MO	5	0.990374
30_MTA / C	Q9WU19	-0.13344	0.207835	0.341335	Hao1	2-Hydroxya	HAOX1_MC	20	0.911655
30_MTA / C	Q921M7	-0.15481	0.208001	0.341452	Cyrib	CYFIP-relat	CYRIB_MOU	2	0.898251
30_MTA / C	Q61598	0.098416	0.208347	0.341848	Gdi2	Rab GDP di	GDIB_MOU	18	1.070598
15_MTA / C	O70400	-0.13568	0.208592	0.341953	Pdlim1	PDZ and LI	PDLI1_MOU	8	0.910241
30_MTA / C	O09131	-0.1254	0.208737	0.341989	Gsto1	Glutathion	GSTO1_MO	15	0.916752
15_MTA / C	Q99JW2	-0.21357	0.208873	0.341989	Acy1	Aminoacyla	ACY1_MOU	20	0.862403
30_MTA / C	Q9DBX2	0.232975	0.208898	0.341989	Pdcl	Phosducin-	PHLP_MOU	1	1.175256
30_MTA / C	P39447	-0.14111	0.209386	0.342166	Tjp1	Tight juncti	ZO1_MOUS	7	0.906819
15_MTA / C	Q61699	-0.22921	0.2092	0.342166	Hsph1	Heat shock	HS105_MO	12	0.853101
30_MTA / C	Q76LS9	-0.1328	0.209312	0.342166	Mindy1	Ubiquitin c	MINY1_MO	2	0.91206
30_MTA / C	Q99J99	-0.27505	0.209506	0.342206	Mpst	3-mercapto	THTM_MOU	3	0.826424
30_MTA / C	P09528	-0.19341	0.2098	0.34253	Fth1	Ferritin hea	FRIH_MOUS	14	0.874537
15_MTA / C	P07758	-0.17503	0.210063	0.342729	Serpina1a	Alpha-1-ant	A1AT1_MO	17	0.885752
30_MTA / C	Q8BTZ7	-0.17571	0.210473	0.343143	Gmppb	Mannose-1	GMPPB_M	3	0.885329
15_MTA / C	Q8JZS0	-0.38484	0.210556	0.343143	Lin7a	Protein lin-	LIN7A_MOU	4	0.765862
30_MTA / C	Q80XI3	-0.00579	0.21078	0.343352	Eif4g3	Eukaryotic	IF4G3_MOU	3	0.995995
15_MTA / C	Q8R1F5	-0.12152	0.211847	0.344777	Hyi	Putative hy	HYI_MOUSI	13	0.919217
15_MTA / C	Q9CXI5	0.06197	0.211951	0.344792	Manf	Mesenceph	MANF_MOU	9	1.04389
30_MTA / C	P62264	0.351063	0.212593	0.345368	Rps14	Small ribos	RS14_MOU	3	1.2755
30_MTA / C	O88456	-0.2241	0.212854	0.345371	Capns1	Calpain sma	CPNS1_MO	4	0.85613
15_MTA / C	Q01405	-0.01959	0.212918	0.345371	Sec23a	Protein tra	SC23A_MO	12	0.98651
30_MTA / C	Q99KI0	-0.23909	0.212979	0.345371	Aco2	Aconitate h	ACON_MOU	14	0.847277
15_MTA / C	Q99KN9	-0.04334	0.212967	0.345371	Clint1	Clathrin int	EPN4_MOU	5	0.970405
15_MTA / C	O88587	-0.04348	0.213534	0.345848	Comt	Catechol O	COMT_MOU	4	0.970313
30_MTA / C	Q02819	0.273588	0.214068	0.345848	Nucb1	Nucleobinc	NUCB1_MC	12	1.20881
30_MTA / C	Q4KML4	0.023601	0.214137	0.345848	Abracl	Costars fam	ABRAL_MO	3	1.016494
15_MTA / C	Q8BH00	-0.43352	0.214083	0.345848	Aldh8a1	2-aminomu	AL8A1_MO	15	0.740452
30_MTA / C	Q05421	-0.78783	0.214792	0.346427	Cyp2e1	Cytochrom	CP2E1_MO	1	0.579213
30_MTA / C	Q9CQJ6	0.263904	0.214639	0.346427	Denr	Density-reg	DENR_MOU	6	1.200724
15_MTA / C	Q9CYR6	-0.3075	0.214733	0.346427	Pgm3	Phosphoac	AGM1_MOU	4	0.80804
15_MTA / C	P97315	-0.52778	0.21503	0.346512	Csrp1	Cysteine an	CSRP1_MO	6	0.693619

30_MTA / C	P14152	-0.02904	0.215133	0.346523	Mdh1	Malate deh	MDHC_MO		15	0.980072
15_MTA / C	O08601	-0.03924	0.215451	0.346726	Mttp	Microsoma	MTP_MOUS		28	0.973169
30_MTA / C	Q91VS7	-0.98788	0.215447	0.346726	Mgst1	Microsoma	MGST1_MO		2	0.504217
30_MTA / C	P70335	1.01195	0.216484	0.348076	Rock1	Rho-associa	ROCK1_MO		3	2.016635
30_MTA / C	Q5XJY5	0.068808	0.216795	0.34842	Arcn1	Coatomer s	COPD_MOU		26	1.04885
15_MTA / C	P26043	-0.03873	0.21726	0.348746	Rdx	Radixin	RADI_MOU		38	0.973511
15_MTA / C	P35505	-0.05912	0.217728	0.348746	Fah	Fumarylace	FAAA_MOU		28	0.959848
15_MTA / C	Q3TEA8	-0.29433	0.217317	0.348746	Hp1bp3	Heterochro	HP1B3_MO		4	0.815451
30_MTA / C	Q3UUI3	0.075375	0.217611	0.348746	Them4	Acyl-coenzy	THEM4_MO		6	1.053635
15_MTA / C	Q60936	-0.36419	0.217799	0.348746	Coq8a	Atypical kir	COQ8A_MO		3	0.776906
15_MTA / C	Q8K003	0.1627	0.217966	0.348746	Tma7	Translation	TMA7_MOU		1	1.11938
30_MTA / C	Q9CQT1	-0.09596	0.218278	0.349008	Mri1	Methylthio	MTNA_MOU		15	0.935651
30_MTA / C	P56376	0.471743	0.218558	0.349072	Acyp1	Acylphosph	ACYP1_MO		3	1.386784
30_MTA / C	F6ZDS4	-0.19303	0.219237	0.349691	Tpr	Nucleoprot	TPR_MOUS		8	0.874765
15_MTA / C	Q61419	-0.06295	0.219192	0.349691	Cmah	Cytidine m	CMAH_MO		11	0.957303
30_MTA / C	Q8K4H1	0.105029	0.219107	0.349691	Afmid	Kynurenine	KFA_MOUS		14	1.075516
30_MTA / C	Q6P542	0.11687	0.219361	0.349735	Abcf1	ATP-bindin	ABCF1_MO		3	1.08438
30_MTA / C	Q8CH18	0.064382	0.21957	0.349757	Ccar1	Cell divisio	CCAR1_MO		3	1.045637
30_MTA / C	P11859	0.027337	0.219743	0.349829	Agt	Angiotensir	ANGT_MOU		4	1.019129
30_MTA / C	P29699	0.187008	0.219809	0.349829	Ahsg	Alpha-2-HS	FETUA_MO		8	1.1384
30_MTA / C	P68040	-1.16492	0.220176	0.350104	Rack1	Small ribos	RACK1_MO		6	0.445988
30_MTA / C	Q8QZR3	0.068519	0.220134	0.350104	Ces2a	Pyrethroid	EST2A_MOU		16	1.04864
15_MTA / C	Q921H8	-0.06122	0.220406	0.35016	Acaa1a	3-ketoacyl-	THIKA_MOU		8	0.958452
15_MTA / C	Q9EQH3	-0.12899	0.220556	0.350244	Vps35	Vacuolar pr	VPS35_MO		23	0.914472
30_MTA / C	Q8VEM8	-0.90849	0.221244	0.350871	Slc25a3	Solute carri	S25A3_MO		1	0.532744
30_MTA / C	Q60676	-0.0915	0.221565	0.351225	Ppp5c	Serine/thre	PPP5_MOU		13	0.938544
30_MTA / C	Q9D1Q6	-0.07435	0.221804	0.351451	Erp44	Endoplasm	ERP44_MO		13	0.94977
30_MTA / C	Q8K2I4	0.233196	0.222119	0.35164	Manba	Beta-mann	MANBA_MO		9	1.175436
30_MTA / C	Q9CXW3	-0.0584	0.222041	0.35164	Cacybp	Calcyclin-b	CYBP_MOU		14	0.960332
30_MTA / C	Q61074	0.120171	0.222485	0.351909	Ppm1g	Protein pho	PPM1G_MO		1	1.086864
30_MTA / C	Q9D114	-1.14072	0.222403	0.351909	Hddc3	Guanosine-	MESH1_MO		1	0.453534
30_MTA / C	P80313	0.112922	0.222965	0.352359	Cct7	T-complex p	TCPH_MOU		16	1.081417
30_MTA / C	Q9D9V3	0.019974	0.223335	0.352789	Echdc1	Ethylmalon	ECHD1_MO		17	1.013941
15_MTA / C	P46638	0.000924	0.223738	0.352961	Rab11b	Ras-related	RB11B_MO		8	1.000641
15_MTA / C	Q80WQ2	-0.45512	0.223556	0.352961	Vac14	Protein VAC	VAC14_MO		4	0.729451
30_MTA / C	Q8VCT3	0.131203	0.223674	0.352961	Rnpep	Aminopept	AMPB_MOU		10	1.095207
15_MTA / C	P70445	-1.03255	0.224273	0.35365	Eif4ebp2	Eukaryotic	4EBP2_MO		2	0.488847
30_MTA / C	P43883	-0.14535	0.224587	0.353991	Plin2	Perilipin-2	PLIN2_MOU		9	0.904158
15_MTA / C	Q9JLV1	0.158298	0.224887	0.354307	Bag3	BAG family	BAG3_MOU		20	1.11597
30_MTA / C	P35700	-0.13179	0.225163	0.354588	Prdx1	Peroxiredo	PRDX1_MO		21	0.912696
30_MTA / C	O35887	0.292082	0.225998	0.355353	Calu	Calumenin	CALU_MOU		7	1.224406
30_MTA / C	O88947	0.349984	0.226044	0.355353	F10	Coagulatio	FA10_MOU		2	1.274546
15_MTA / C	Q8VCH0	-0.07333	0.225978	0.355353	Acaa1b	3-ketoacyl-	THIKB_MOU		23	0.950444
30_MTA / C	Q8C0E2	-0.03493	0.226162	0.355384	Vps26b	Vacuolar pr	VP26B_MO		6	0.976079
30_MTA / C	P51885	-0.04024	0.226283	0.355418	Lum	Lumican	LUM_MOUS		2	0.972492
15_MTA / C	Q64727	-0.01348	0.226774	0.356015	Vcl	Vinculin	VINC_MOU		65	0.990701
15_MTA / C	Q9D7X8	-0.61402	0.227158	0.356015	Ggct	Gamma-glu	GGCT_MOU		4	0.653375
15_MTA / C	Q9ER72	-0.84011	0.227038	0.356015	Cars1	Cysteine--tl	SYCC_MOU		2	0.558601
30_MTA / C	O35943	0.118054	0.227363	0.356028	Fxn	Frataxin, m	FRDA_MOU		2	1.08527
30_MTA / C	P62257	0.728985	0.227321	0.356028	Ube2h	Ubiquitin-c	UBE2H_MO		1	1.657473
30_MTA / C	Q921F2	0.666405	0.22835	0.357262	Tardbp	TAR DNA-bi	TADBP_MO		2	1.587113
30_MTA / C	P62245	-0.19328	0.228804	0.357763	Rps15a	Small ribos	RS15A_MO		7	0.874616

15_MTA / C	P56394	-0.06856	0.229045	0.357882	Cox17	Cytochrom	COX17_MC	1	0.953588
30_MTA / C	Q8VI75	0.630286	0.229378	0.358248	Ipo4	Importin-4	IPO4_MOU	7	1.547872
30_MTA / C	P07724	-0.11655	0.229526	0.358323	Alb	Albumin	ALBU_MOU	49	0.922388
30_MTA / C	Q8BIQ5	-0.06628	0.229655	0.358369	Cstf2	Cleavage sti	CSTF2_MOU	1	0.955095
30_MTA / C	P99027	0.085957	0.230189	0.359047	Rplp2	Large ribos	RLA2_MOU	6	1.061392
15_MTA / C	Q9R257	-0.08557	0.231013	0.359902	Hebp1	Heme-bind	HEBP1_MO	10	0.942409
15_MTA / C	P55264	0.190306	0.23221	0.361573	Adk	Adenosine	ADK_MOUS	18	1.141006
15_MTA / C	Q64516	-0.01684	0.23271	0.362194	Gk	Glycerol kir	GLPK_MOU	29	0.988393
30_MTA / C	P14115	0.226982	0.23282	0.362209	Rpl27a	Large ribos	RL27A_MO	4	1.170384
15_MTA / C	P17427	-0.13022	0.233538	0.362543	Ap2a2	AP-2 compl	AP2A2_MO	22	0.913693
15_MTA / C	Q9CX56	-0.45783	0.233358	0.362543	Psmd8	26S protea	PSMD8_MC	3	0.728082
30_MTA / C	Q8VCM7	-0.2114	0.234143	0.363325	Fgg	Fibrinogen	FIBG_MOUS	6	0.863702
30_MTA / C	Q5U5V2	0.287669	0.234819	0.363806	Hykk	Hydroxylys	HYKK_MOU	3	1.220667
30_MTA / C	P62830	0.217353	0.235302	0.364025	Rpl23	Large ribos	RL23_MOU	3	1.162598
15_MTA / C	Q9D967	-1.88104	0.235229	0.364025	Mdp1	Magnesium	MGDP1_MC	2	0.271487
30_MTA / C	Q9JHI5	-0.01957	0.235702	0.364057	Ivd	Isovaleryl-C	IVD_MOUSI	15	0.98653
15_MTA / C	Q9WVM8	-0.11985	0.235429	0.364057	Aadat	Kynurenine	AADAT_MO	13	0.920281
30_MTA / C	P53395	0.067998	0.235879	0.364136	Dbt	Lipoamide	ODB2_MOU	22	1.048261
15_MTA / C	P61982	-0.00881	0.236911	0.365415	Ywhag	14-3-3 prot	1433G_MC	16	0.993913
30_MTA / C	E9Q414	0.062507	0.237303	0.365766	Apob	Apolipoprc	APOB_MOU	44	1.044279
15_MTA / C	Q9WUM4	-0.74239	0.237342	0.365766	Coro1c	Coronin-1C	COR1C_MC	5	0.59775
15_MTA / C	P27048;P6	0.072269	0.238399	0.366764	Snrpb;Snrp	Small nucle	RSMB_MOU	3	1.051369
30_MTA / C	P49429	-0.01287	0.238563	0.366764	Hpd	4-hydroxyp	HPPD_MOU	25	0.991117
15_MTA / C	Q99KV1	-0.14384	0.238243	0.366764	Dnajb11	DnaJ homo	DJB11_MO	4	0.905108
30_MTA / C	Q9CR16	-0.12462	0.239386	0.367501	Ppid	Peptidyl-pr	PPID_MOU	11	0.917248
15_MTA / C	Q9ET26	-0.48243	0.240355	0.368517	Rnf114	E3 ubiquiti	RN114_MC	7	0.715771
30_MTA / C	Q9Z1Z2	-0.04642	0.240321	0.368517	Strap	Serine-thre	STRAP_MO	13	0.968334
15_MTA / C	P05202	-0.0969	0.240603	0.36874	Got2	Aspartate a	AATM_MOU	32	0.935042
15_MTA / C	P19253	0.368957	0.241135	0.369255	Rpl13a	Large ribos	RL13A_MO	8	1.291419
15_MTA / C	Q3UZ39	-1.07497	0.242187	0.369595	Lrrfip1	Leucine-ric	LRRF1_MO	2	0.47468
30_MTA / C	Q61419	0.21199	0.242039	0.369595	Cmah	Cytidine m	CMAH_MO	11	1.158285
30_MTA / C	Q8BJY1	0.106809	0.241872	0.369595	Psmd5	26S protea	PSMD5_MC	6	1.076844
15_MTA / C	Q91Y97	-0.09855	0.2415	0.369595	Aldob	Fructose-bi	ALDOB_MC	29	0.933971
30_MTA / C	Q99KR3	0.00844	0.241988	0.369595	Lactb2	Endoribonu	LACB2_MO	16	1.005867
30_MTA / C	Q9JK38	0.164596	0.242036	0.369595	Gnpnat1	Glucosamir	GNA1_MOU	9	1.120852
30_MTA / C	G3X982	-0.12713	0.242978	0.370488	Aox3	Aldehyde o	AOXC_MOU	65	0.915653
30_MTA / C	P02088	-0.16659	0.243397	0.370969	Hbb-b1	Hemoglobi	HBB1_MOU	12	0.890947
30_MTA / C	Q80UG5	0.186902	0.243616	0.371145	Septin9	Septin-9	SEPT9_MOU	5	1.138317
30_MTA / C	P23116	-0.49722	0.243808	0.371196	Eif3a	Eukaryotic	EIF3A_MOU	4	0.708472
30_MTA / C	P09405	0.140145	0.244375	0.371829	Ncl	Nucleolin	NUCL_MOU	24	1.102016
15_MTA / C	P39447	0.340808	0.24506	0.372147	Tjp1	Tight juncti	ZO1_MOUS	7	1.266466
15_MTA / C	Q91YR1	-0.01909	0.24483	0.372147	Twf1	Twinfilin-1	TWF1_MOU	7	0.986852
15_MTA / C	Q9CR86	0.205728	0.2451	0.372147	Carhsp1	Calcium-re	CHSP1_MC	3	1.153268
30_MTA / C	Q8WTY4	-0.22651	0.245556	0.372525	Ciapin1	Anamorsin	CPIN1_MO	7	0.8547
30_MTA / C	Q9DD20	-0.47668	0.24546	0.372525	Tmt1b	Thiol S-met	TMT1B_MC	4	0.71863
30_MTA / C	O70310	0.53577	0.245941	0.372736	Nmt1	Glycylpepti	NMT1_MOU	4	1.449716
15_MTA / C	Q9R1P1	0.135275	0.24611	0.372736	Psmb3	Proteasom	PSB3_MOU	12	1.098302
30_MTA / C	P43274	0.196357	0.246419	0.372955	H1-4	Histone H1	H14_MOUS	2	1.145801
15_MTA / C	Q61730	0.071415	0.246512	0.372955	Il1rap	Interleukin	IL1AP_MOU	4	1.050747
15_MTA / C	P14685	-0.18185	0.247128	0.37365	Psmd3	26S protea	PSMD3_MC	12	0.881571
15_MTA / C	P17918	-0.73587	0.247247	0.373673	Pcna	Proliferatir	PCNA_MOU	7	0.600455
30_MTA / C	O89020	0.299234	0.247594	0.373727	Afm	Afamin	AFAM_MOU	12	1.230491

15_MTA / C	Q8VE97	0.390849	0.24752	0.373727	Srsf4	Serine/argi	SRSF4_MOU	3	1.311165
30_MTA / C	Q9CZN7	-0.41839	0.247391	0.373727	Shmt2	Serine hydr	GLYM_MOU	6	0.74826
30_MTA / C	Q8R121	-0.14465	0.248704	0.375246	Serpina10	Protein Z-d	ZPI_MOUSE	6	0.9046
30_MTA / C	O35841	0.276377	0.248828	0.375274	Api5	Apoptosis i	API5_MOU	7	1.211149
15_MTA / C	O88533	-0.06971	0.248987	0.375358	Ddc	Aromatic-L	DDC_MOUS	15	0.952833
30_MTA / C	Q6P1F6	0.439058	0.249407	0.375833	Ppp2r2a	Serine/thre	2ABA_MOU	2	1.355719
30_MTA / C	P61961	-0.10033	0.250394	0.376218	Ufm1	Ubiquitin-f	UFM1_MOU	4	0.932818
30_MTA / C	Q8VCI0	0.070095	0.249814	0.376218	Plbd1	Phospholip	PLBL1_MO	10	1.049786
15_MTA / C	Q91XD4	-0.13445	0.250257	0.376218	Ftcd	Formimido	FTCD_MOU	23	0.911016
15_MTA / C	Q9CPW2	-0.64197	0.250252	0.376218	Fdx2	Ferredoxin-	FDX2_MOU	1	0.640838
30_MTA / C	Q91YE6	-0.09788	0.25069	0.376506	Ipo9	Importin-9	IPO9_MOU	6	0.934403
15_MTA / C	P01631	-0.79509	0.251013	0.376528	NaN	Ig kappa ch	KV2A7_MO	3	0.576308
30_MTA / C	P62702	0.25057	0.251019	0.376528	Rps4x	Small ribos	RS4X_MOU	13	1.189677
30_MTA / C	P09103	-0.08843	0.251348	0.376727	P4hb	Protein disu	PDIA1_MO	35	0.940543
15_MTA / C	P47968	-0.61458	0.252527	0.377532	Rpia	Ribose-5-pl	RPIA_MOUS	1	0.653119
15_MTA / C	P56565	0.157827	0.252664	0.37758	S100a1	Protein S10	S10A1_MO	1	1.115606
30_MTA / C	Q3UPL0	-0.15673	0.252843	0.377691	Sec31a	Protein tra	SC31A_MO	32	0.897055
30_MTA / C	Q99PL5	0.103019	0.253548	0.37843	Rrbp1	Ribosome-b	RRBP1_MO	58	1.074018
15_MTA / C	Q9DCN1	-0.37698	0.254348	0.379309	Nudt12	NAD-cappe	NUD12_MO	7	0.770046
30_MTA / C	Q91W90	0.153418	0.254606	0.379352	Txndc5	Thioredoxi	TXND5_MO	13	1.112201
30_MTA / C	Q99N87	0.339164	0.254903	0.379352	Mrps5	Small ribos	RT05_MOU	3	1.265023
15_MTA / C	Q9CQQ8	-0.2606	0.254502	0.379352	Lsm7	U6 snRNA-a	LSM7_MOU	2	0.834739
30_MTA / C	Q501J7	0.367322	0.255434	0.379909	Phactr4	Phosphatas	PHAR4_MO	2	1.289956
30_MTA / C	Q9DCL2	-0.12422	0.255541	0.379909	Ciao2a	Cytosolic ir	CIA2A_MOU	1	0.917502
15_MTA / C	O54950	0.180984	0.256556	0.381015	Prkag1	5'-AMP-acti	AAKG1_MO	7	1.133657
30_MTA / C	Q9DBP5	-0.10489	0.256695	0.381015	Cmpk1	UMP-CMP k	KCY_MOUS	14	0.929875
15_MTA / C	P01942	-0.15205	0.257873	0.382349	Hba	Hemoglobi	HBA_MOUS	9	0.89997
30_MTA / C	O88986	-0.06619	0.258398	0.382497	Gcat	2-amino-3-	KBL_MOUS	16	0.955158
15_MTA / C	P13020	-0.09031	0.258364	0.382497	Gsn	Gelsolin	GELS_MOU	3	0.939324
30_MTA / C	Q3UGR5	-0.09169	0.258168	0.382497	Hdhd2	Haloacid de	HDHD2_MO	11	0.938425
15_MTA / C	P01633	-0.41251	0.259216	0.383468	Igkv6-17	Immunoglo	KV5A1_MO	3	0.751314
15_MTA / C	Q62426	0.018395	0.259826	0.384137	Cstb	Cystatin-B	CYTB_MOU	7	1.012832
15_MTA / C	Q9R062	-0.62144	0.259966	0.384186	Gyg1	Glycogenin	GLYG_MOU	2	0.650021
15_MTA / C	Q80XI3	-0.15716	0.26024	0.384433	Eif4g3	Eukaryotic	IF4G3_MOU	3	0.896787
15_MTA / C	Q6NSR8	-0.01585	0.260555	0.384741	Npepl1	Probable ar	PEPL1_MO	12	0.989075
30_MTA / C	Q9WTP7	0.018487	0.261281	0.385184	Ak3	GTP:AMP p	KAD3_MOU	10	1.012897
15_MTA / C	Q9D1L0	-0.38833	0.261637	0.385547	Chchd2	Coiled-coil-	CHCH2_MO	3	0.764011
30_MTA / C	Q8C5H8	-0.23894	0.261746	0.385551	Nadk2	NAD kinase	NAKD2_MO	12	0.847368
30_MTA / C	P10853;P1	0.119664	0.261867	0.385572	H2bc7;H2b	Histone H2	H2B1F_MO	5	1.086482
15_MTA / C	P29341	-0.13841	0.262636	0.385782	Pabpc1	Polyadenyl	PABP1_MO	16	0.908518
15_MTA / C	P62830	-0.00246	0.262622	0.385782	Rpl23	Large ribos	RL23_MOU	3	0.998293
30_MTA / C	P97352	0.367828	0.262517	0.385782	S100a13	Protein S10	S10AD_MO	3	1.290409
15_MTA / C	Q9WV98	1.072541	0.262566	0.385782	Timm9	Mitochond	TIM9_MOU	1	2.103134
30_MTA / C	P01671	0.239765	0.263012	0.385994	NaN	Ig kappa ch	KV3AJ_MO	3	1.1808
15_MTA / C	O54984	-0.12659	0.263324	0.386136	Get3	ATPase GET	GET3_MOU	5	0.915992
30_MTA / C	Q9JII6	-0.57922	0.264027	0.38701	Akr1a1	Aldo-keto r	AK1A1_MO	11	0.669326
30_MTA / C	Q8BL66	0.181056	0.264867	0.388084	Eea1	Early endos	EEA1_MOU	35	1.133713
15_MTA / C	Q8C0E2	-0.10024	0.265046	0.388161	Vps26b	Vacuolar pr	VP26B_MO	6	0.932878
30_MTA / C	Q8K1Z0	0.019437	0.265136	0.388161	Coq9	Ubiquinone	COQ9_MOU	5	1.013564
30_MTA / C	A3KMP2	0.156597	0.266771	0.389012	Ttc38	Tetratricop	TTC38_MO	16	1.114655
15_MTA / C	P50544	-0.02249	0.266762	0.389012	Acadvl	Very long-c	ACADV_MO	32	0.984531
30_MTA / C	P51125	0.245582	0.266905	0.389012	Cast	Calpastatin	ICAL_MOUS	6	1.185571

15_MTA / C	Q61704	-0.23081	0.266858	0.389012	Itih3	Inter-alpha	ITIH3_MOU	10	0.852157
30_MTA / C	Q9DCU9	-0.09621	0.266492	0.389012	Hoga1	4-hydroxy-:	HOGA1_MC	16	0.93549
15_MTA / C	Q9JHI5	-0.03308	0.266524	0.389012	Ivd	Isovaleryl-C	IVD_MOUSI	15	0.977332
15_MTA / C	Q9JLI6	-0.13116	0.267187	0.389108	Scly	Selenocyste	SCLY_MOUS	16	0.913099
30_MTA / C	P12367	-0.18125	0.267775	0.389287	Prkar2a	cAMP-depe	KAP2_MOU	9	0.881939
30_MTA / C	P16858	-0.2292	0.267598	0.389287	Gapdh	Glyceraldel	G3P_MOUS	16	0.85311
30_MTA / C	P22315	0.206452	0.267881	0.389287	Fech	Ferrochelat	HEMH_MOI	4	1.153847
30_MTA / C	P97855	-0.11005	0.268006	0.389287	G3bp1	Ras GTPase-	G3BP1_MC	6	0.926553
30_MTA / C	Q922H9	-0.00435	0.268199	0.389287	Znf330	Zinc finger	ZN330_MO	1	0.996988
15_MTA / C	Q99LX0	-0.04963	0.26773	0.389287	Park7	Parkinson c	PARK7_MO	14	0.966186
15_MTA / C	Q9CR98	0.086651	0.268116	0.389287	Fam136a	Protein FAI	F136A_MO	8	1.061902
30_MTA / C	Q91WG8	-0.52801	0.268954	0.390103	Gne	Bifunctiona	GLCNE_MO	5	0.69351
15_MTA / C	Q91VF2	-0.11065	0.2697	0.390713	Hnmt	Histamine I	HNMT_MOI	8	0.926168
15_MTA / C	Q9D753	0.207288	0.270192	0.391268	Exosc8	Exosome cc	EXOS8_MO	1	1.154516
15_MTA / C	A2ATU0	-0.17813	0.270411	0.39127	Dhtkd1	2-oxoadipa	DHTK1_MC	10	0.883848
15_MTA / C	P50516	-0.10449	0.271187	0.391518	Atp6v1a	V-type prot	VATA_MOU	18	0.930136
15_MTA / C	P53657	0.129438	0.271143	0.391518	Pklr	Pyruvate ki	KPYR_MOU	37	1.093868
15_MTA / C	Q8VCR7	-0.06311	0.271344	0.391518	Abhd14b	Putative pr	ABHEB_MO	11	0.957199
30_MTA / C	Q91W43	-0.22053	0.27107	0.391518	Gldc	Glycine del	GCSP_MOU	10	0.858248
30_MTA / C	Q91X91	-0.08751	0.271586	0.39171	Qprt	Nicotinate-	NADC_MOU	13	0.941144
30_MTA / C	P14685	0.066472	0.272101	0.392033	Psmd3	26S proteas	PSMD3_MC	12	1.047152
30_MTA / C	P61922	-0.08197	0.272245	0.392033	Abat	4-aminobu	GABT_MOU	27	0.944769
30_MTA / C	Q62093	0.035905	0.272543	0.392148	Srsf2	Serine/argi	SRSF2_MOU	3	1.0252
15_MTA / C	O35309	-0.10356	0.27302	0.392483	Nmi	N-myc-inte	NMI_MOUS	7	0.930732
30_MTA / C	P10630	-0.05256	0.27343	0.392483	Eif4a2	Eukaryotic	IF4A2_MOU	10	0.964223
30_MTA / C	P46638	0.15066	0.273213	0.392483	Rab11b	Ras-related	RB11B_MO	8	1.110078
15_MTA / C	P62743	0.215275	0.272949	0.392483	Ap2s1	AP-2 compl	AP2S1_MO	5	1.160925
15_MTA / C	Q3UHX2	0.118677	0.273108	0.392483	Pdap1	28 kDa heat	HAP28_MC	4	1.085739
15_MTA / C	Q8BGD8	0.329549	0.273388	0.392483	Coa6	Cytochrom	COA6_MOU	1	1.256621
15_MTA / C	Q6NZJ6	0.061336	0.273828	0.392898	Eif4g1	Eukaryotic	IF4G1_MOI	21	1.043431
30_MTA / C	Q91WG0	-0.13478	0.274173	0.393237	Ces2c	Acylcarniti	EST2C_MOU	9	0.910809
30_MTA / C	Q60936	-0.20612	0.274307	0.393263	Coq8a	Atypical kir	COQ8A_MC	3	0.866865
30_MTA / C	Q9D1E6	-0.07548	0.27441	0.393263	Tbcb	Tubulin-fol	TBCB_MOU	6	0.949023
30_MTA / C	E9Q557	1.593003	0.274735	0.393302	Dsp	Desmoplak	DESP_MOU	32	3.016767
15_MTA / C	P47757	0.030643	0.274765	0.393302	Capzb	F-actin-cap	CAPZB_MO	11	1.021467
15_MTA / C	Q91VR2	-0.71353	0.274673	0.393302	Atp5f1c	ATP synthas	ATPG_MOU	4	0.609826
15_MTA / C	P52792	-0.13798	0.275279	0.393881	Gck	Hexokinase	HXK4_MOU	17	0.908792
15_MTA / C	P97494	0.091787	0.275535	0.39409	Gclc	Glutamate-	GSH1_MOU	23	1.065689
30_MTA / C	O55234	-0.11495	0.275937	0.394509	Psmb5	Proteasom	PSB5_MOU	15	0.923414
30_MTA / C	O88428	0.067634	0.276439	0.394623	Papss2	Bifunctiona	PAPS2_MO	12	1.047996
30_MTA / C	P99024	-0.98402	0.276674	0.394623	Tubb5	Tubulin bet	TBB5_MOU	2	0.505567
30_MTA / C	Q60854	0.028635	0.276784	0.394623	Serpinb6	Serpin B6	SPB6_MOU	10	1.020046
15_MTA / C	Q8CDN6	0.015414	0.276251	0.394623	Txnl1	Thioredoxi	TXNL1_MO	14	1.010741
15_MTA / C	Q9CWS0	-0.02196	0.276465	0.394623	Ddah1	N(G),N(G)-c	DDAH1_MC	16	0.984893
15_MTA / C	Q9DBL7	-0.90771	0.276667	0.394623	Coasy	Bifunctiona	COASY_MO	4	0.533029
30_MTA / C	Q9JM14	-0.51559	0.276768	0.394623	Nt5c	5'(3')-deoxy	NT5C_MOU	6	0.699506
30_MTA / C	P18528	-0.72365	0.277136	0.394748	NaN	Ig heavy ch	HVM57_MC	3	0.605563
30_MTA / C	Q9D7G0	-0.13363	0.276997	0.394748	Prps1	Ribose-pho	PRPS1_MO	7	0.911533
30_MTA / C	P20108	-0.11514	0.277381	0.394769	Prdx3	Thioredoxi	PRDX3_MC	10	0.92329
30_MTA / C	P62908	0.192433	0.277435	0.394769	Rps3	Small ribos	RS3_MOUS	15	1.142689
15_MTA / C	Q63836	-0.16551	0.277568	0.394803	Selenbp2	Selenium-b	SBP2_MOU	3	0.891614
15_MTA / C	P54071	-1.26161	0.277899	0.395118	Idh2	Isocitrate d	IDHP_MOU	7	0.417077

30_MTA / C	P10922	0.229981	0.278024	0.395138	H1-0	Histone H1	H10_MOUS	6	1.172819
15_MTA / C	Q6ZWV7	0.209202	0.278288	0.395202	Rpl35	Large ribos	RL35_MOU	2	1.156049
30_MTA / C	Q80Y14	0.592516	0.278186	0.395202	Glrx5	Glutaredox	GLRX5_MO	1	1.507874
15_MTA / C	C0HKE1;C0	-0.35007	0.278536	0.395242	H2ac4;H2a	Histone H2	H2A1B_MC	3	0.784546
15_MTA / C	Q6IRU5	0.152624	0.279182	0.396002	Cltb	Clathrin lig	CLCB_MOU	5	1.111589
30_MTA / C	Q9EPL8	0.149023	0.280078	0.396805	Ipo7	Importin-7	IPO7_MOU	8	1.108818
30_MTA / C	Q8BK48	0.01417	0.280879	0.39747	Ces2e	Pyrethroid	EST2E_MOU	18	1.00987
15_MTA / C	Q93092	0.021503	0.280831	0.39747	Taldo1	Transaldola	TALDO_MO	16	1.015016
30_MTA / C	Q99KN9	0.086431	0.280658	0.39747	Clint1	Clathrin int	EPN4_MOU	5	1.06174
30_MTA / C	Q9ET26	0.297931	0.281103	0.397631	Rnf114	E3 ubiquiti	RN114_MC	7	1.22938
15_MTA / C	P62307	0.005776	0.281864	0.398551	Snrpf	Small nucle	RUXF_MOU	3	1.004011
30_MTA / C	P28063	-0.12824	0.282159	0.39869	Psmb8	Proteasom	PSB8_MOU	12	0.914945
30_MTA / C	Q9R1P3	-0.10389	0.282184	0.39869	Psmb2	Proteasom	PSB2_MOU	11	0.930521
30_MTA / C	Q8CIN4	0.300629	0.282515	0.399001	Pak2	Serine/thre	PAK2_MOU	2	1.231681
30_MTA / C	Q6PDM2	0.322981	0.282653	0.39904	Srsf1	Serine/argi	SRSF1_MOU	5	1.250913
15_MTA / C	P10853;P1	0.113969	0.283521	0.399475	H2bc7;H2b	Histone H2	H2B1F_MO	5	1.082201
30_MTA / C	P61982	-0.07185	0.283415	0.399475	Ywhag	14-3-3 prot	1433G_MC	16	0.951415
15_MTA / C	Q8QZS1	-0.10647	0.283627	0.399475	Hibch	3-hydroxyi	HIBCH_MO	4	0.92886
30_MTA / C	Q91VR8	-0.07178	0.283336	0.399475	Brk1	Protein BRI	BRK1_MOU	1	0.951467
30_MTA / C	P14131	0.244775	0.283829	0.399603	Rps16	Small ribos	RS16_MOU	7	1.184908
15_MTA / C	Q3UM45	0.025857	0.284056	0.399766	Ppp1r7	Protein pho	PP1R7_MO	11	1.018084
15_MTA / C	P54726	0.130205	0.284426	0.40013	Rad23a	UV excision	RD23A_MC	6	1.094449
30_MTA / C	Q80VJ3	0.297275	0.284662	0.400149	Dnph1	5-hydroxyn	DNPH1_MC	3	1.228821
15_MTA / C	Q8BP40	0.065506	0.285194	0.40074	Acp6	Lysophospl	PPA6_MOU	14	1.046452
30_MTA / C	P62814	0.219162	0.285505	0.401021	Atp6v1b2	V-type prot	VATB2_MO	14	1.164057
15_MTA / C	P01878	-0.07132	0.286088	0.401196	NaN	Ig alpha cha	IGHA_MOU	7	0.951769
15_MTA / C	Q3TCN2	-0.74947	0.286223	0.401196	Plbd2	Putative ph	PLBL2_MO	6	0.594822
15_MTA / C	Q80VJ2	-0.07218	0.286331	0.401196	Sra1	Steroid rec	SRA1_MOU	2	0.951203
15_MTA / C	Q8BGA8	0.065145	0.287032	0.401603	Acsm5	Acyl-coenzy	ACSM5_MC	22	1.04619
30_MTA / C	Q9CYW4	-0.09504	0.287146	0.401603	Hdhd3	Haloacid de	HDHD3_MC	11	0.936247
15_MTA / C	Q9DBJ1	-0.09095	0.287485	0.401614	Pgam1	Phosphogly	PGAM1_MC	22	0.938904
15_MTA / C	P17751	-0.07483	0.288326	0.402316	Tpi1	Triosephos	TPIS_MOUS	16	0.949455
30_MTA / C	Q8BK64	-0.04864	0.288201	0.402316	Ahsa1	Activator o	AHSA1_MO	18	0.966851
15_MTA / C	A2AJL3	-0.00825	0.289698	0.402462	Fggy	FGGY carbc	FGGY_MOU	22	0.9943
15_MTA / C	P01819;P0	-1.23891	0.288822	0.402462	NaN	Ig heavy cha	HVM43_MC	1	0.423693
30_MTA / C	P08207	1.354555	0.289354	0.402462	S100a10	Protein S10	S10AA_MO	1	2.557182
15_MTA / C	P15626	-0.1862	0.289469	0.402462	Gstm2	Glutathion	GSTM2_MC	17	0.878918
30_MTA / C	Q7TQG1	0.905503	0.288963	0.402462	Plekha6	Pleckstrin h	PKHA6_MC	1	1.873197
30_MTA / C	Q8K3H0	-0.43785	0.289104	0.402462	Appl1	DCC-intera	DP13A_MC	2	0.738235
15_MTA / C	Q8VDG5	-0.02405	0.289772	0.402462	Ppcs	Phosphopa	PPCS_MOU	15	0.983466
30_MTA / C	Q9EQ20	-0.13717	0.288862	0.402462	Aldh6a1	Methylmal	MMSA_MO	21	0.909299
15_MTA / C	Q9JM76	-0.40268	0.288707	0.402462	Arpc3	Actin-relate	ARPC3_MO	2	0.756454
15_MTA / C	Q9Z1Z2	-0.02641	0.289896	0.402478	Strap	Serine-thre	STRAP_MO	13	0.981859
15_MTA / C	O55201	0.106193	0.290566	0.402942	Supt5h	Transcripti	SPT5H_MO	3	1.076384
15_MTA / C	Q61233	-0.04781	0.290477	0.402942	Lcp1	Plastin-2	PLSL_MOUS	31	0.967405
15_MTA / C	Q8BHN3	-0.52673	0.290388	0.402942	Ganab	Neutral alp	GANAB_MC	10	0.694125
30_MTA / C	P80317	0.146049	0.290993	0.403069	Cct6a	T-complex	TCPZ_MOU	17	1.106535
15_MTA / C	Q61166	-0.64948	0.290961	0.403069	Mapre1	Microtubul	MARE1_MC	4	0.637509
15_MTA / C	P20108	-0.1318	0.292696	0.404808	Prdx3	Thioredoxi	PRDX3_MC	10	0.912694
15_MTA / C	P84104	-0.09421	0.292517	0.404808	Srsf3	Serine/argi	SRSF3_MOU	4	0.936783
30_MTA / C	Q05915	-0.14911	0.292699	0.404808	Gch1	GTP cycloh	GCH1_MOU	9	0.901805
15_MTA / C	Q3TLS3	0.339135	0.292935	0.404979	Gdpgp1	GDP-D-gluc	GDPP1_MC	2	1.264998

30_MTA / C	Q62167	0.335044	0.293308	0.405163	Ddx3x	ATP-depend	DDX3X_MO	9	1.261416
30_MTA / C	Q8BH43	-0.4187	0.29366	0.405163	Wasf2	Actin-bindi	WASF2_MO	3	0.748098
30_MTA / C	Q8QZS1	-0.84369	0.293729	0.405163	Hibch	3-hydroxyi	HIBCH_MO	4	0.557217
15_MTA / C	Q91YY4	-0.10257	0.294881	0.406265	Atpaf2	ATP synthas	ATPF2_MO	1	0.931374
15_MTA / C	Q922E4	0.090526	0.295767	0.407174	Pcyt2	Ethanolami	PCY2_MOU	20	1.064758
30_MTA / C	Q9CZ13	-1.0592	0.29588	0.407174	Uqcrc1	Cytochrom	QCR1_MOU	2	0.479897
30_MTA / C	Q9Z1K5	0.098734	0.295824	0.407174	Arih1	E3 ubiquiti	ARI1_MOUS	3	1.070833
30_MTA / C	P83741	0.322044	0.296947	0.40833	Wnk1	Serine/thre	WNK1_MO	2	1.250101
15_MTA / C	Q01339	-0.32222	0.29723	0.408563	Apoh	Beta-2-glyc	APOH_MOU	11	0.799837
15_MTA / C	P11983	0.098879	0.298512	0.409857	Tcp1	T-complex p	TCPA_MOU	19	1.070941
15_MTA / C	P29391	-0.17181	0.298857	0.410174	Ftl1	Ferritin ligh	FRIL1_MOU	14	0.887728
30_MTA / C	Q80XU3	-0.36123	0.299348	0.410691	Nucks1	Nuclear ubi	NUCKS_MO	2	0.778498
30_MTA / C	O35423	-0.08263	0.300121	0.411285	Agxt	Alanine--gly	AGT1_MOU	20	0.944331
30_MTA / C	Q9CQC6	-0.18732	0.300124	0.411285	Bzw1	eIF5-mimic	5MP2_MOU	10	0.878234
15_MTA / C	P38647	-0.01583	0.300488	0.411615	Hspa9	Stress-70 pr	GRP75_MO	38	0.989085
30_MTA / C	Q07417	-0.0828	0.300764	0.411615	Acads	Short-chain	ACADS_MO	11	0.944226
15_MTA / C	Q9D5J6	-0.04316	0.300843	0.411615	Shpk	Sedoheptul	SHPK_MOU	16	0.970528
30_MTA / C	Q9JMD3	-0.13332	0.30064	0.411615	Stard10	START dom	STA10_MO	13	0.911732
30_MTA / C	P15864	0.74375	0.301942	0.412587	H1-2	Histone H1	H12_MOUS	1	1.674523
15_MTA / C	Q9D1A2	0.007511	0.302002	0.412587	Cndp2	Cytosolic n	CNDP2_MO	23	1.00522
15_MTA / C	Q9R1P4	-0.09891	0.302947	0.412797	Psma1	Proteasome	PSA1_MOU	19	0.93374
30_MTA / C	O55201	-0.05944	0.303316	0.413141	Supt5h	Transcripti	SPT5H_MO	3	0.959635
15_MTA / C	P40124	-0.36988	0.303429	0.413141	Cap1	Adenylyl cy	CAP1_MOU	12	0.773848
30_MTA / C	Q811D0	-0.07352	0.303607	0.413227	Dlg1	Disks large	DLG1_MOU	2	0.950314
30_MTA / C	P11862	-0.35161	0.303882	0.413289	Gas2	Growth arr	GAS2_MOU	4	0.783708
15_MTA / C	P67984	-0.19482	0.304509	0.413985	Rpl22	Large ribos	RL22_MOU	3	0.873684
15_MTA / C	O88851	-0.48723	0.304633	0.413998	Rbbp9	Putative hy	RBBP9_MO	7	0.713392
15_MTA / C	P61957;Q9	0.076139	0.305095	0.414218	Sumo2;Sum	Small ubiq	SUMO2_MO	1	1.054193
15_MTA / C	P62918	-0.09865	0.30537	0.414218	Rpl8	Large ribos	RL8_MOUS	13	0.933905
15_MTA / C	P80315	0.032271	0.305352	0.414218	Cct4	T-complex p	TCPD_MOU	22	1.022621
15_MTA / C	P54728	0.112582	0.30628	0.414827	Rad23b	UV excision	RD23B_MO	10	1.081162
30_MTA / C	Q8BHG2	0.221299	0.306146	0.414827	Czib	CXXC motif	CZIB_MOUS	6	1.165783
30_MTA / C	P60843	-0.02715	0.306731	0.415206	Eif4a1	Eukaryotic	IF4A1_MOU	25	0.981354
30_MTA / C	Q8VC12	-0.08019	0.306791	0.415206	Uroc1	Urocanate l	HUTU_MOU	30	0.945933
15_MTA / C	P63158	-0.12931	0.306998	0.415331	Hmgb1	High mobil	HMGB1_MO	11	0.914267
15_MTA / C	Q61129	-0.05419	0.307501	0.415698	Cfi	Complemen	CFAI_MOUS	14	0.963137
15_MTA / C	Q9D0J8	0.225955	0.307804	0.415951	Ptms	Parathymo	PTMS_MOU	4	1.169551
30_MTA / C	Q3UMU9	0.256579	0.308098	0.416036	Hdgfl2	Hepatoma-	HDGR2_MO	4	1.194642
15_MTA / C	Q9D0B5	-0.26037	0.30832	0.416179	Tstd3	Thiosulfate	TSTD3_MO	2	0.834875
30_MTA / C	Q9D379	-0.57837	0.308461	0.416214	Ephx1	Epoxide hy	HYEP_MOU	5	0.66972
30_MTA / C	Q9Z2M7	-0.03231	0.308831	0.416557	Pmm2	Phosphoma	PMM2_MO	7	0.977851
30_MTA / C	Q99JI4	0.128266	0.309051	0.416698	Psmd6	26S protea	PSMD6_MO	13	1.092979
15_MTA / C	Q922D4	-0.10668	0.309247	0.416806	Ppp6r3	Serine/thre	PP6R3_MO	20	0.928721
15_MTA / C	P62267	-0.2697	0.30987	0.417333	Rps23	Small ribos	RS23_MOU	6	0.829494
30_MTA / C	P84104	0.225552	0.31017	0.417581	Srsf3	Serine/argi	SRSF3_MOU	4	1.169225
15_MTA / C	Q9DCG6	-0.13231	0.310794	0.418265	Pbld1	Phenazine b	PBLD1_MO	9	0.912372
15_MTA / C	Q99PT1	-0.0236	0.311019	0.418411	Arhgdia	Rho GDP-di	GDIR1_MO	12	0.983773
15_MTA / C	Q6PDM2	0.091098	0.311897	0.419332	Srsf1	Serine/argi	SRSF1_MOU	5	1.065181
15_MTA / C	Q9R1Z7	-0.02882	0.311937	0.419332	Pts	6-pyruvoyl	PTPS_MOU	5	0.980221
15_MTA / C	P13439	-0.43143	0.312354	0.419736	Umps	Uridine 5'-r	UMPS_MOU	4	0.741527
30_MTA / C	P48036	-0.23551	0.312979	0.420118	Anxa5	Annexin A5	ANXA5_MO	13	0.849382
30_MTA / C	O70318	-0.23474	0.313601	0.420327	Epb41l2	Band 4.1-lil	E41L2_MO	3	0.849836

15_MTA / C	P20918	-0.04539	0.313819	0.420327	Plg	Plasminoge	PLMN_MOU	19	0.969027
15_MTA / C	P56375	-0.04702	0.31337	0.420327	Acyp2	Acylphosph	ACYP2_MO	1	0.967932
30_MTA / C	P58871	-0.30436	0.313727	0.420327	Tnks1bp1	182 kDa tar	TB182_MO	3	0.809802
30_MTA / C	Q9DB15	0.070461	0.313844	0.420327	Mrpl12	Large ribos	RM12_MOU	5	1.050052
15_MTA / C	O08663	-1.36569	0.314444	0.420784	Metap2	Methionine	MAP2_MOU	5	0.388048
30_MTA / C	P11588	-0.15294	0.314382	0.420784	Mup1	Major urina	MUP1_MOU	1	0.899414
30_MTA / C	P18524	-0.61995	0.314612	0.420784	NaN	Ig heavy cha	HVM53_MO	1	0.650691
30_MTA / C	P26262	0.026237	0.314653	0.420784	Klkb1	Plasma kall	KLKB1_MO	6	1.018353
15_MTA / C	Q9CXN7	-0.01581	0.314821	0.420853	Pbld2	Phenazine b	PBLD2_MO	14	0.989099
15_MTA / C	Q9R1T2	-0.24481	0.315012	0.420951	Sae1	SUMO-activ	SAE1_MOU	7	0.84393
30_MTA / C	P62627	-0.26215	0.315346	0.421086	Dynlrb1	Dynein ligh	DLRB1_MO	1	0.833847
30_MTA / C	Q9JLI8	-0.12552	0.315255	0.421086	Sart3	Squamous c	SART3_MOU	3	0.916671
30_MTA / C	O55023	0.122426	0.317948	0.424245	Impa1	Inositol mo	IMPA1_MO	8	1.088564
15_MTA / C	P58389	-0.35135	0.318513	0.42437	Ptpa	Serine/thre	PTPA_MOU	6	0.78385
15_MTA / C	Q99KQ4	0.075119	0.318479	0.42437	Nampt	Nicotinami	NAMPT_MO	21	1.053448
30_MTA / C	Q9R257	-0.13713	0.318483	0.42437	Hebp1	Heme-bind	HEBP1_MO	10	0.909325
30_MTA / C	Q8BMD5	0.773433	0.318749	0.424398	Cdadc1	Cytidine an	CDAC1_MO	4	1.709332
15_MTA / C	Q8K2B3	-0.22732	0.318993	0.424538	Sdha	Succinate d	SDHA_MOU	23	0.85422
30_MTA / C	Q8CG76	-0.64818	0.32012	0.425409	Akr7a2	Aflatoxin B	ARK72_MO	6	0.638085
15_MTA / C	Q3UQ44	0.063614	0.321255	0.425973	Iqgap2	Ras GTPase-	IQGA2_MO	35	1.04508
30_MTA / C	P58252	-0.0191	0.321395	0.426002	Eef2	Elongation	EF2_MOUSE	57	0.986845
30_MTA / C	Q8K2Q9	0.112226	0.321922	0.426229	Shtn1	Shootin-1	SHOT1_MO	5	1.080894
15_MTA / C	Q8VI36	0.10181	0.321919	0.426229	Pxn	Paxillin	PAXI_MOUS	1	1.073119
30_MTA / C	O08709	-0.06782	0.322815	0.426893	Prdx6	Peroxiredo	PRDX6_MO	18	0.95408
30_MTA / C	P08226	-0.10247	0.32291	0.426893	Apoe	Apolipopro	APOE_MOU	14	0.931435
15_MTA / C	Q60865	0.02112	0.323121	0.426893	Caprin1	Caprin-1	CAPR1_MO	7	1.014747
15_MTA / C	Q8VCI0	-0.15649	0.322886	0.426893	Plbd1	Phospholip	PLBL1_MOU	10	0.897204
15_MTA / C	Q91ZJ5	0.122773	0.323135	0.426893	Ugp2	UTP--gluco	UGPA_MOU	28	1.088826
30_MTA / C	P27612	0.045644	0.325371	0.427934	Plaa	Phospholip	PLAP_MOU	22	1.032144
15_MTA / C	P35492	-0.02273	0.325407	0.427934	Hal	Histidine ar	HUTH_MOU	37	0.984369
30_MTA / C	P58044	-0.15673	0.325891	0.427934	Idi1	Isopenteny	IDI1_MOUS	4	0.897057
15_MTA / C	Q5EBG8	-0.1548	0.325137	0.427934	NaN	Uncharacte	CA050_MO	5	0.898259
15_MTA / C	Q62446	-0.27775	0.325596	0.427934	Fkbp3	Peptidyl-pr	FKBP3_MO	7	0.824877
15_MTA / C	Q64374	0.113316	0.324317	0.427934	Rgn	Regucalcin	RGN_MOUS	23	1.081711
30_MTA / C	Q6PE15	0.179541	0.325655	0.427934	Abhd10	Palmitoyl-p	ABHDA_MO	1	1.132524
15_MTA / C	Q80WJ7	0.299611	0.324912	0.427934	Mtdh	Protein LYR	LYRIC_MOU	5	1.230813
30_MTA / C	Q8K385	-0.13609	0.325947	0.427934	FRRS1	Ferric-chela	FRRS1_MO	3	0.909983
30_MTA / C	Q8R3Q6	-0.01397	0.324652	0.427934	Mix23	Protein MIX	MIX23_MO	6	0.990362
15_MTA / C	Q91VH2	0.003113	0.324689	0.427934	Snx9	Sorting nex	SNX9_MOU	1	1.00216
30_MTA / C	Q922V4	0.13078	0.325871	0.427934	Plrg1	Pleiotropic	PLRG1_MO	5	1.094885
15_MTA / C	Q9DB27	-0.34824	0.325429	0.427934	Mcts1	Malignant T	MCTS1_MO	3	0.785544
30_MTA / C	Q9WV32	0.085454	0.325801	0.427934	Arpc1b	Actin-relate	ARC1B_MO	8	1.061021
15_MTA / C	Q9Z1X4	-0.57334	0.325694	0.427934	Ilf3	Interleukin	ILF3_MOUS	2	0.67206
15_MTA / C	Q91V09	0.152874	0.326407	0.428075	Wdr13	WD repeat-	WDR13_MO	2	1.111782
30_MTA / C	Q9DCB8	0.498086	0.326405	0.428075	Isca2	Iron-sulfur	ISCA2_MOU	3	1.412338
30_MTA / C	P16294	1.059776	0.326666	0.428259	F9	Coagulation	FA9_MOUSE	3	2.084608
30_MTA / C	P31938	-0.45743	0.327899	0.429562	Map2k1	Dual specifi	MP2K1_MO	2	0.728283
30_MTA / C	Q8R050	0.049819	0.328624	0.430199	Gspt1	Eukaryotic	ERF3A_MOU	18	1.035135
30_MTA / C	O08553	-0.03873	0.329033	0.430464	Dpysl2	Dihydropyr	DPYL2_MO	16	0.973514
15_MTA / C	P02088	-0.11391	0.329474	0.430841	Hbb-b1	Hemoglobi	HBB1_MOU	12	0.924079
15_MTA / C	Q8BZB2	-0.54695	0.329883	0.43122	Ppcdc	Phosphopa	COAC_MOU	3	0.684466
30_MTA / C	P70195	-0.07018	0.330434	0.431263	Psmb7	Proteasom	PSB7_MOU	10	0.95252

30_MTA / C	Q8BX09	0.669791	0.330515	0.431263	Rbbp5	Retinoblast	RBBP5_MO	3	1.590843
30_MTA / C	Q91XL9	0.471823	0.330241	0.431263	Osbpl1a	Oxysterol-b	OSBL1_MO	3	1.386861
15_MTA / C	Q9D8S4	0.074602	0.330403	0.431263	Rexo2	Oligoribon	ORN_MOUS	3	1.053071
15_MTA / C	Q5FW57	-0.48568	0.330673	0.431313	Gm4952	Glycine N-a	GLYAL_MO	1	0.71416
30_MTA / C	P47911	0.192889	0.331389	0.43151	Rpl6	Large ribos	RL6_MOUS	14	1.143051
30_MTA / C	P62806	0.343611	0.33141	0.43151	H4c1	Histone H4	H4_MOUSE	6	1.268928
15_MTA / C	Q9CZ04	0.062054	0.331528	0.43151	Cops7a	COP9 signa	CSN7A_MO	5	1.043951
15_MTA / C	Q9Z0J0	0.112874	0.331062	0.43151	Npc2	NPC intrace	NPC2_MOU	5	1.081381
30_MTA / C	P15626	-0.15968	0.332056	0.432021	Gstm2	Glutathion	GSTM2_MO	17	0.895225
15_MTA / C	P97493	-0.29066	0.332478	0.432055	Txn2	Thioredoxi	THIOM_MO	3	0.817527
30_MTA / C	Q9CPR4	0.121924	0.332563	0.432055	Rpl17	Large ribos	RL17_MOU	5	1.088185
15_MTA / C	Q8N7N5	-0.18789	0.33335	0.432766	Dcaf8	DDB1- and	DCAF8_MO	4	0.877886
15_MTA / C	P62192	-0.3931	0.333763	0.433146	Psmc1	26S protea	PRS4_MOU	9	0.761492
15_MTA / C	Q5RKZ7	-0.09132	0.334233	0.433443	Mocs1	Molybdenu	MOCS1_MO	6	0.938666
15_MTA / C	P31725	-0.42106	0.334899	0.4335	S100a9	Protein S1(	S10A9_MO	6	0.746878
30_MTA / C	Q61166	-0.44397	0.33512	0.4335	Mapre1	Microtubul	MARE1_MO	4	0.735109
30_MTA / C	Q61316	0.065308	0.334829	0.4335	Hspa4	Heat shock	HSP74_MO	23	1.046308
30_MTA / C	O08997	0.035108	0.335404	0.433712	Atox1	Copper trar	ATOX1_MO	6	1.024634
15_MTA / C	P06909	-0.12261	0.337026	0.43534	Cfh	Compleme	CFAH_MOU	34	0.918524
15_MTA / C	O88428	-0.07171	0.337855	0.435496	Papss2	Bifunctiona	PAPS2_MO	12	0.951506
15_MTA / C	P12787	-1.26191	0.337615	0.435496	Cox5a	Cytochrom	COX5A_MO	2	0.416992
30_MTA / C	P56213	-0.04919	0.338099	0.435496	Gfer	FAD-linked	ALR_MOUS	4	0.966481
15_MTA / C	Q9QY76	-0.04755	0.338057	0.435496	Vapb	Vesicle-ass	VAPB_MOU	1	0.967581
15_MTA / C	Q8R0F9	-0.06735	0.338654	0.435879	Sec14l4	SEC14-like	S14L4_MO	15	0.954392
15_MTA / C	P80318	0.154597	0.339096	0.435981	Cct3	T-complex	TCPG_MOU	19	1.113111
15_MTA / C	Q9CQC6	-0.21223	0.338856	0.435981	Bzw1	eIF5-mimic	5MP2_MOU	10	0.863199
30_MTA / C	Q9ET22	-0.54152	0.339096	0.435981	Dpp7	Dipeptidyl	DPP2_MOU	2	0.687047
15_MTA / C	Q91WQ3	-0.02153	0.339436	0.436261	Yars1	Tyrosine--t	SYYC_MOUS	15	0.985188
30_MTA / C	Q9DBD0	-0.14656	0.339959	0.436778	Inhca	Inhibitor o	ICA_MOUSE	27	0.903403
30_MTA / C	Q99N42	-0.26203	0.340224	0.436962	Tymp	Thymidine	TYPH_MOU	6	0.833916
30_MTA / C	Q9D8T2	-0.06399	0.340604	0.437139	Gsdmd	Gasdermin	GSDMD_M	12	0.956613
15_MTA / C	O09167	-0.25112	0.341342	0.437774	Rpl21	Large ribos	RL21_MOU	4	0.840246
15_MTA / C	P10518	-0.03321	0.34165	0.438013	Alad	Delta-amin	HEM2_MOU	23	0.977243
15_MTA / C	P41105	0.189	0.341856	0.438102	Rpl28	Large ribos	RL28_MOU	6	1.139973
30_MTA / C	Q9DBZ5	-0.49	0.341963	0.438102	Eif3k	Eukaryotic	EIF3K_MOU	2	0.712025
30_MTA / C	P70444	-0.26397	0.342632	0.438513	Bid	BH3-intera	BID_MOUSE	4	0.832792
30_MTA / C	Q03734	-0.08653	0.342649	0.438513	Serpina3m	Serine prot	SPA3M_MO	9	0.941782
30_MTA / C	Q8BIW1	0.110005	0.342922	0.4387	Prune1	Exopolyph	PRUN1_MO	1	1.079232
15_MTA / C	Q8R050	-0.10662	0.343039	0.4387	Gspt1	Eukaryotic	ERF3A_MO	18	0.928764
30_MTA / C	Q80WQ2	-0.90838	0.343423	0.439026	Vac14	Protein VA(	VAC14_MO	4	0.532784
30_MTA / C	Q9JLV5	-0.14453	0.343538	0.439026	Cul3	Cullin-3	CUL3_MOU	8	0.904676
15_MTA / C	P23589	-0.03066	0.344102	0.439054	Ca5a	Carbonic ar	CAH5A_MO	10	0.978971
30_MTA / C	Q7M6Y3	-0.11774	0.344135	0.439054	Picalm	Phosphatic	PICAL_MOU	15	0.921632
30_MTA / C	Q8VBV7	-0.52357	0.344117	0.439054	Cops8	COP9 signa	CSN8_MOU	2	0.695648
15_MTA / C	Q9DCQ2	-0.03288	0.343792	0.439054	Aspdh	Aspartate d	ASPDH_MO	18	0.977468
30_MTA / C	Q9QZ23	-0.00132	0.34417	0.439054	Nfu1	NFU1 iron-	NFU1_MOU	5	0.999085
30_MTA / C	Q8VEB4	-0.08191	0.344393	0.439183	Pla2g15	Lysosomal	PAG15_MO	6	0.944805
30_MTA / C	O35488	-0.4197	0.34465	0.4392	Slc27a2	Long-chain	S27A2_MO	1	0.747579
15_MTA / C	Q9Z2U1	-0.01045	0.344588	0.4392	Psma5	Proteasom	PSA5_MOU	14	0.99278
30_MTA / C	O09159	0.125355	0.344991	0.439324	Man2b1	Lysosomal	MA2B1_MO	12	1.090776
30_MTA / C	P14211	-0.02703	0.345544	0.439353	Calr	Calreticulir	CALR_MOU	20	0.981436
15_MTA / C	Q02819	0.15194	0.345442	0.439353	Nucb1	Nucleobinc	NUCB1_MO	12	1.111062

15_MTA / C	Q8BJY1	0.093554	0.346113	0.439353	Psmd5	26S protea:	PSMD5_MO	6	1.066995
30_MTA / C	Q8BK30	0.34927	0.34584	0.439353	Ndufv3	NADH dehy	NDUV3_MO	1	1.273916
30_MTA / C	Q91ZX7	-0.22082	0.345162	0.439353	Lrp1	Prolow-der	LRP1_MOU	30	0.858077
30_MTA / C	Q9CR57	0.171953	0.34547	0.439353	Rpl14	Large ribos	RL14_MOU	6	1.126583
30_MTA / C	Q9CR98	0.295269	0.346005	0.439353	Fam136a	Protein FAI	F136A_MO	8	1.227114
15_MTA / C	Q9DB15	-0.19393	0.345912	0.439353	Mrpl12	Large ribos	RM12_MOU	5	0.874224
30_MTA / C	Q9Z1D1	0.09796	0.345972	0.439353	Eif3g	Eukaryotic	EIF3G_MOU	11	1.070259
15_MTA / C	P61759	-0.37214	0.346831	0.4398	Vbp1	Prefoldin su	PFD3_MOU	3	0.772635
15_MTA / C	Q8CGC7	-0.11161	0.347287	0.440223	Eprs1	Bifunctiona	SYEP_MOUS	19	0.925552
30_MTA / C	P00761	0.276269	0.347553	0.440364	NaN	Trypsin (Lal	TRYP_PIG	3	1.211059
30_MTA / C	Q62465	-0.17336	0.347643	0.440364	Vat1	Synaptic ve	VAT1_MOU	7	0.886773
15_MTA / C	P01637;P0	-0.18641	0.348724	0.441058	NaN	Ig kappa ch	KV5A5_MO	2	0.878788
30_MTA / C	P62849	-0.03124	0.348803	0.441058	Rps24	Small ribos	RS24_MOU	4	0.978577
30_MTA / C	Q8BTE0	0.399315	0.348615	0.441058	Sdhaf4	Succinate d	SDHF4_MO	1	1.318882
15_MTA / C	Q8VCM7	-0.51959	0.348798	0.441058	Fgg	Fibrinogen	FIBG_MOUS	6	0.69757
30_MTA / C	Q9JLJ2	-0.23306	0.349178	0.441377	Aldh9a1	4-trimethyl	AL9A1_MO	15	0.850831
30_MTA / C	Q8VC97	-0.14962	0.349391	0.44149	Upb1	Beta-ureidc	BUP1_MOU	6	0.901487
30_MTA / C	Q3ULD5	-0.04826	0.350225	0.442234	Mccc2	Methylcrot	MCCB_MOU	21	0.967103
30_MTA / C	P23589	0.038589	0.351097	0.442816	Ca5a	Carbonic ar	CAH5A_MO	10	1.027109
30_MTA / C	P24456	-0.45181	0.351011	0.442816	Cyp2d10	Cytochrom	CP2DA_MO	2	0.731124
30_MTA / C	Q6ZQK5	0.318389	0.350994	0.442816	Acap2	Arf-GAP wit	ACAP2_MO	1	1.246937
15_MTA / C	Q9JHW2	0.009519	0.351301	0.442816	Nit2	Omega-ami	NIT2_MOUS	20	1.00662
30_MTA / C	O55135	0.015339	0.351789	0.443098	Eif6	Eukaryotic	IF6_MOUSE	7	1.010689
30_MTA / C	P01657	0.0149	0.351894	0.443098	NaN	Ig kappa ch	KV3A5_MO	1	1.010381
30_MTA / C	Q9D7X3	-0.24954	0.351648	0.443098	Dusp3	Dual specifi	DUS3_MOU	1	0.841162
30_MTA / C	Q9CQV8	-0.01623	0.35211	0.443216	Ywhab	14-3-3 prot	1433B_MO	7	0.988811
30_MTA / C	P01635	0.356159	0.352765	0.443697	Igkv12-41	Immunoglo	KV5A3_MO	2	1.280013
30_MTA / C	P70194	-0.64247	0.352701	0.443697	Clec4f	C-type lecti	CLC4F_MOU	3	0.640613
15_MTA / C	Q9QYY9	0.143234	0.35325	0.444029	Adh4	All-trans-re	ADH4_MOU	18	1.104378
30_MTA / C	Q60872	0.034282	0.354424	0.445039	Eif1a	Eukaryotic	IF1A_MOUS	6	1.024047
30_MTA / C	O09172	0.003244	0.354909	0.445102	Gclm	Glutamate-	GSH0_MOU	10	1.002251
15_MTA / C	P08071	-0.37251	0.354926	0.445102	Ltf	Lactotransf	TRFL_MOUS	16	0.772436
30_MTA / C	P28271	-0.1362	0.354957	0.445102	Aco1	Cytoplasmi	ACOHC_MO	29	0.909912
30_MTA / C	Q9CQX8	0.511906	0.355125	0.445143	Kgd4	Alpha-keto	KGD4_MOU	4	1.425933
15_MTA / C	P02802	-0.59797	0.355346	0.445265	Mt1	Metallothi	MT1_MOUS	2	0.660685
30_MTA / C	P68037	-0.0106	0.356003	0.445623	Ube2l3	Ubiquitin-c	UB2L3_MO	9	0.992683
30_MTA / C	Q64520	0.011242	0.355934	0.445623	Guk1	Guanylate l	KGUA_MOU	1	1.007823
15_MTA / C	P09528	-0.11912	0.35725	0.446407	Fth1	Ferritin hea	FRIH_MOUS	14	0.920747
15_MTA / C	Q01279	-0.22787	0.357058	0.446407	Egfr	Epidermal g	EGFR_MOU	9	0.853893
30_MTA / C	Q9CQR2	-0.12719	0.357911	0.446923	Rps21	Small ribos	RS21_MOU	4	0.915613
30_MTA / C	Q64374	-0.08611	0.358098	0.447002	Rgn	Regucalcin	RGN_MOUS	23	0.942062
15_MTA / C	Q05D44	0.218803	0.358357	0.447107	Eif5b	Eukaryotic	IF2P_MOUS	14	1.163768
15_MTA / C	Q8BL97	0.529727	0.358829	0.447449	Srsf7	Serine/argi	SRSF7_MOU	3	1.443656
15_MTA / C	P10922	0.115533	0.359091	0.447621	H1-0	Histone H1	H10_MOUS	6	1.083375
15_MTA / C	P11352	0.142934	0.359592	0.448089	Gpx1	Glutathion	GPX1_MOU	16	1.104148
15_MTA / C	Q924Y0	-0.23275	0.35985	0.448256	Bbox1	Gamma-bu	BODG_MOU	6	0.851013
15_MTA / C	P80317	0.017708	0.360196	0.448531	Cct6a	T-complex	TCPZ_MOU	17	1.01235
15_MTA / C	O88986	-0.09277	0.361434	0.449153	Gcat	2-amino-3-	KBL_MOUS	16	0.93772
15_MTA / C	P99029	-0.00041	0.36109	0.449153	Prdx5	Peroxiredo	PRDX5_MO	13	0.999717
30_MTA / C	Q8C0J2	-0.60546	0.360828	0.449153	Atg16l1	Autophagy-	A16L1_MO	3	0.657264
15_MTA / C	Q9CRD0	-0.59701	0.361316	0.449153	Ociad1	OCIA doma	OCAD1_MO	2	0.661124
15_MTA / C	Q9JHI7	0.208729	0.361443	0.449153	Exosc9	Exosome cc	EXOS9_MO	3	1.15567

30_MTA / C	Q80VJ2	-0.24091	0.362104	0.449664	Sra1	Steroid rec	SRA1_MOU	2	0.84621
30_MTA / C	P62281	0.10472	0.362738	0.450295	Rps11	Small ribos	RS11_MOU	12	1.075285
30_MTA / C	O08808	0.600521	0.363432	0.450361	Diaph1	Protein dia	DIAP1_MO	2	1.516264
15_MTA / C	P48678	-0.1259	0.363759	0.450361	Lmna	Prelamin-A	LMNA_MOU	15	0.91643
15_MTA / C	P62196	-0.21346	0.363331	0.450361	Psmc5	26S protea	PRS8_MOU	9	0.862467
15_MTA / C	Q60590	-0.16297	0.363148	0.450361	Orm1	Alpha-1-aci	A1AG1_MC	1	0.893183
30_MTA / C	Q8R1Q8	-0.29069	0.364123	0.450361	Dync1li1	Cytoplasmi	DC1L1_MO	4	0.817509
15_MTA / C	Q9ESP1	0.072732	0.364057	0.450361	Sdf2l1	Stromal cel	SDF2L_MO	2	1.051706
15_MTA / C	Q9WU19	-0.05513	0.364077	0.450361	Hao1	2-Hydroxya	HAOX1_MC	20	0.962507
30_MTA / C	P00493	-0.03671	0.364723	0.450739	Hprt1	Hypoxanth	HPRT_MOU	12	0.974874
15_MTA / C	Q04447	-0.61852	0.364653	0.450739	Ckb	Creatine kir	KCRB_MOU	2	0.651338
30_MTA / C	A2BIM8;P0	-0.05765	0.36495	0.450865	Mup18;Mu	Major urina	MUP18_MC	16	0.960827
15_MTA / C	P16332	-0.04241	0.366055	0.451919	Mmut	Methylmal	MUTA_MOU	12	0.97103
30_MTA / C	Q6PGH2	0.177343	0.367082	0.452743	Jpt2	Jupiter mic	JUPI2_MOU	2	1.130799
15_MTA / C	Q8BJU0	-0.50588	0.367292	0.452743	Sgta	Small gluta	SGTA_MOU	5	0.704232
30_MTA / C	Q8R5I6	0.235422	0.36735	0.452743	Gstm4	Glutathion	GSTM4_MC	4	1.177251
30_MTA / C	Q07456	0.195065	0.36815	0.453418	Ambp	Protein AM	AMBP_MOU	11	1.144776
30_MTA / C	Q8VE70	-0.17666	0.368656	0.45373	Pdcd10	Programme	PDC10_MC	4	0.884747
15_MTA / C	Q9CQR2	-0.12989	0.368554	0.45373	Rps21	Small ribos	RS21_MOU	4	0.913904
30_MTA / C	P23492	-0.04078	0.369154	0.454188	Pnp	Purine nucl	PNPH_MOU	21	0.972126
30_MTA / C	Q91X52	0.342341	0.369549	0.454518	Dcxr	L-xylulose r	DCXR_MOU	4	1.267812
30_MTA / C	Q01768	-0.0744	0.370474	0.455501	Nme2	Nucleoside	NDKB_MOU	13	0.94974
15_MTA / C	P17742	-0.07388	0.371023	0.455864	Ppia	Peptidyl-pr	PPIA_MOUS	11	0.950077
30_MTA / C	Q64442	-0.00264	0.370919	0.455864	Sord	Sorbitol de	DHSO_MOU	18	0.99817
30_MTA / C	P32067	0.067746	0.37143	0.456209	Ssb	Lupus La pr	LA_MOUSE	10	1.048078
15_MTA / C	Q9D711	-0.29698	0.371654	0.456328	Pir	Pirin	PIR_MOUSI	2	0.813955
30_MTA / C	Q9QZ08	-0.01651	0.372503	0.456903	Nagk	N-acetyl-D-	NAGK_MOU	4	0.988619
30_MTA / C	Q9WVM8	-0.05222	0.372774	0.45708	Aadat	Kynurenine	AADAT_MO	13	0.964448
30_MTA / C	P40124	-0.02511	0.37336	0.457643	Cap1	Adenylyl cy	CAP1_MOU	12	0.982743
30_MTA / C	Q3B7Z2	-0.07461	0.373524	0.457688	Osbp	Oxysterol-b	OSBP1_MO	6	0.9496
30_MTA / C	Q8VED9	-0.12717	0.373808	0.45788	Lgalsl	Galectin-re	LEGL_MOU	1	0.915624
30_MTA / C	Q8CIM7	-0.49467	0.374256	0.458273	Cyp2d26	Cytochrom	CP2DQ_MC	5	0.709726
30_MTA / C	P97470	0.025754	0.374807	0.458635	Ppp4c	Serine/thre	PP4C_MOU	3	1.018011
30_MTA / C	P10711	0.181439	0.375936	0.459843	Tcea1	Transcripti	TCEA1_MO	9	1.134015
30_MTA / C	Q8VC28	-0.11698	0.376257	0.459882	Akr1c13	Aldo-keto r	AK1CD_MC	11	0.922115
30_MTA / C	P04939	-0.09331	0.376665	0.459971	Mup3	Major urina	MUP3_MOU	12	0.937367
15_MTA / C	P62702	0.251666	0.376625	0.459971	Rps4x	Small ribos	RS4X_MOU	13	1.190581
15_MTA / C	Q8BK30	-1.03796	0.377306	0.460442	Ndufv3	NADH dehy	NDUV3_MC	1	0.487015
30_MTA / C	Q9CZ42	0.089939	0.377438	0.460446	Naxd	ATP-depend	NNRD_MOU	12	1.064325
15_MTA / C	P01806;P0	-0.43913	0.37875	0.460641	NaN	Ig heavy ch	HVM36_MC	3	0.737579
15_MTA / C	P14869	-0.12716	0.37825	0.460641	Rplp0	Large ribos	RLA0_MOU	9	0.915635
30_MTA / C	Q9D6Y9	-0.42336	0.378352	0.460641	Gbe1	1,4-alpha-g	GLGB_MOU	6	0.745687
30_MTA / C	Q9DBL7	-0.5891	0.377881	0.460641	Coasy	Bifunctiona	COASY_MO	4	0.664756
15_MTA / C	Q9DCH6	-0.33735	0.37871	0.460641	Zfand6	AN1-type zi	ZFAN6_MO	1	0.791495
15_MTA / C	P58044	-0.12479	0.379567	0.461079	Idi1	Isopenteny	IDI1_MOUS	4	0.917139
15_MTA / C	Q8VC30	0.113914	0.37975	0.461079	Tkfc	Triokinase/	TKFC_MOU	27	1.08216
15_MTA / C	Q8VCF0	-0.0355	0.379539	0.461079	Mavs	Mitochond	MAVS_MOU	7	0.975695
15_MTA / C	Q91V92	0.196371	0.380222	0.461306	Acly	ATP-citrate	ACLY_MOU	30	1.145812
30_MTA / C	Q921M4	-0.05656	0.380449	0.461306	Golga2	Golgin subf	GOGA2_MC	2	0.961552
15_MTA / C	Q9JIX8	0.052543	0.380345	0.461306	Acin1	Apoptotic c	ACINU_MO	3	1.037092
15_MTA / C	Q9JIY5	-0.23001	0.38041	0.461306	Htra2	Serine prot	HTRA2_MO	4	0.852627
30_MTA / C	Q9Z2U0	-0.07468	0.380938	0.461743	Psma7	Proteasom	PSA7_MOU	15	0.949556

30_MTA / C	P51910	0.755557	0.381331	0.461909	Apod	Apolipopro	APOD_MOU	1	1.688283
15_MTA / C	P56959	0.085059	0.381227	0.461909	Fus	RNA-bindir	FUS_MOUS	5	1.060731
15_MTA / C	P97352	-0.16477	0.382126	0.46256	S100a13	Protein S1(	S10AD_MO	3	0.892069
15_MTA / C	P28665	-0.06328	0.382607	0.462656	Mug1	Murinoglol	MUG1_MO	59	0.957087
30_MTA / C	Q9Z2Y8	-0.04038	0.38272	0.462656	Plpbp	Pyridoxal p	PLPHP_MO	10	0.972402
15_MTA / C	P52430	-0.83936	0.383762	0.463606	Pon1	Serum para	PON1_MOU	2	0.558892
30_MTA / C	Q9D967	-1.23926	0.383756	0.463606	Mdp1	Magnesium	MGDP1_M	2	0.42359
30_MTA / C	P63242	-0.04894	0.385166	0.464989	Eif5a	Eukaryotic	IF5A1_MOU	11	0.966647
15_MTA / C	P15105	0.055443	0.386132	0.465576	Glul	Glutamine	GLNA_MOU	10	1.039178
15_MTA / C	Q99KB8	-0.0483	0.386265	0.465576	Hagh	Hydroxyac	GLO2_MOU	12	0.967078
15_MTA / C	P62270	-0.34146	0.386855	0.466091	Rps18	Small ribos	RS18_MOU	6	0.789244
15_MTA / C	Q8VCT3	-0.02365	0.387128	0.466263	Rnpep	Aminopept	AMPB_MOU	10	0.983737
15_MTA / C	Q921G7	-0.62941	0.387974	0.46697	Etfdh	Electron tra	ETFD_MOU	7	0.646441
30_MTA / C	P23198	0.100887	0.388147	0.467022	Cbx3	Chromobo:	CBX3_MOU	3	1.072433
30_MTA / C	Q9Z0N2	-0.05644	0.388872	0.467737	Eif2s3y	Eukaryotic	IF2H_MOU	14	0.961634
30_MTA / C	Q8VEK3	0.048306	0.389646	0.4682	Hnrnpu	Heterogene	HNRPU_M(	17	1.03405
15_MTA / C	Q91W43	-0.51051	0.389536	0.4682	Gldc	Glycine del	GCSP_MOU	10	0.701976
15_MTA / C	Q61316	0.077732	0.389843	0.46828	Hspa4	Heat shock	HSP74_MO	23	1.055358
15_MTA / C	P97372	-0.06286	0.390643	0.468901	Psme2	Proteasom	PSME2_MC	15	0.957367
15_MTA / C	Q8C0M9	-0.2144	0.390751	0.468901	Asrgl1	Isoaspartyl	ASGL1_MO	8	0.861903
30_MTA / C	O54950	0.511031	0.391037	0.468931	Prkag1	5'-AMP-acti	AAKG1_MC	7	1.425068
30_MTA / C	Q8CAK1	0.252762	0.391872	0.46962	Iba57	Putative tra	CAF17_MO	3	1.191486
30_MTA / C	Q9CYA0	-0.22084	0.392351	0.470037	Creld2	Protein dis	CREL2_MO	4	0.858068
15_MTA / C	P21981	-0.05543	0.392614	0.470196	Tgm2	Protein-glu	TGM2_MOU	27	0.96231
30_MTA / C	O88851	-0.46957	0.392828	0.470268	Rbbp9	Putative hy	RBBP9_MO	7	0.722182
30_MTA / C	Q8BGC4	-0.08265	0.392935	0.470268	Ptgr3	Prostaglan	PTGR3_MO	6	0.944324
15_MTA / C	P60670	-1.57321	0.394159	0.471577	Nploc4	Nuclear pr	NPL4_MOU	3	0.33606
30_MTA / C	P03987	0.554399	0.394396	0.471669	NaN	Ig gamma-3	IGHG3_MO	3	1.468556
30_MTA / C	Q8BFQ4	0.220295	0.394499	0.471669	Wdr82	WD repeat-	WDR82_M	5	1.164971
15_MTA / C	O70251	0.052876	0.395062	0.471742	Eef1b	Elongation	EF1B_MOU	7	1.037331
30_MTA / C	P62754	0.115919	0.395138	0.471742	Rps6	Small ribos	RS6_MOUS	7	1.083665
15_MTA / C	Q6PGH2	0.016118	0.395127	0.471742	Jpt2	Jupiter mic	JUPI2_MOU	2	1.011235
30_MTA / C	P29788	0.500695	0.395614	0.471905	Vtn	Vitronectir	VTNC_MOU	3	1.414895
15_MTA / C	Q02053	-0.11472	0.396266	0.472057	Uba1	Ubiquitin-l	UBA1_MOU	15	0.923559
30_MTA / C	Q9WVL0	0.032385	0.396168	0.472057	Gstz1	Maleylacet	MAAI_MOU	17	1.022702
30_MTA / C	P50428	-0.23289	0.396734	0.472459	Arsa	Arylsulfata	ARSA_MOU	2	0.850927
15_MTA / C	Q8K4H1	0.045891	0.398071	0.473738	Afmid	Kynurenine	KFA_MOUS	14	1.03232
30_MTA / C	P39039	0.06962	0.398686	0.473999	Mbl1	Mannose-b	MBL1_MOU	2	1.04944
30_MTA / C	P61202	-0.60641	0.398442	0.473999	Cops2	COP9 signa	CSN2_MOU	9	0.656831
30_MTA / C	O08759	-0.68636	0.39902	0.474083	Ube3a	Ubiquitin-p	UBE3A_MO	1	0.621422
15_MTA / C	P01660	0.153782	0.398988	0.474083	NaN	Ig kappa ch	KV3A8_MO	1	1.112482
15_MTA / C	Q9JHS4	-0.68532	0.399483	0.474476	Clpx	ATP-depenc	CLPX_MOU	3	0.621869
15_MTA / C	O55137;Q9	0.104873	0.401279	0.475982	Acot1;Acot	Acyl-coenzy	ACOT1_MO	3	1.0754
30_MTA / C	Q9Z2U1	-0.02804	0.401216	0.475982	Psma5	Proteasom	PSA5_MOU	14	0.98075
15_MTA / C	P61458	-0.16791	0.402633	0.477239	Pcbd1	Pterin-4-alp	PHS_MOUS	7	0.890129
15_MTA / C	P70335	0.417498	0.402737	0.477239	Rock1	Rho-associa	ROCK1_MC	3	1.33561
30_MTA / C	Q9CQ62	-0.0962	0.403445	0.477607	Decr1	2,4-dienoyl	DECR_MOU	8	0.935494
15_MTA / C	Q9DCT8	0.068391	0.403594	0.477626	Crip2	Cysteine-ric	CRIP2_MO	9	1.048547
30_MTA / C	Q9CZ44	0.058179	0.403841	0.477762	Nsfl1c	NSFL1 cofa	NSF1C_MO	15	1.041151
15_MTA / C	O88947	-0.25097	0.404783	0.478562	F10	Coagulatio	FA10_MOU	2	0.840332
30_MTA / C	Q91WT9	-0.00471	0.405349	0.478805	Cbs	Cystathion	CBS_MOUS	26	0.996741
30_MTA / C	Q9ESB3	0.09549	0.405521	0.478805	Hrg	Histidine-ri	HRG_MOUS	10	1.068428

15_MTA / C	Q9JMD3	-0.10676	0.405379	0.478805	Stard10	START dom	STA10_MOI	13	0.928669
30_MTA / C	Q99JT9	0.045333	0.405914	0.478955	Adi1	Acireducto	MTND_MOI	7	1.031922
15_MTA / C	P13634	0.111631	0.406364	0.479328	Ca1	Carbonic ar	CAH1_MOU	9	1.080449
30_MTA / C	Q9ER72	-1.39321	0.406642	0.4795	Cars1	Cysteine--tI	SYCC_MOU	2	0.380716
30_MTA / C	Q8CIH9	0.122229	0.407128	0.479915	Ppat	Amidophos	PUR1_MOU	11	1.088415
15_MTA / C	E9QAT4	0.398113	0.409275	0.480095	Sec16a	Protein trai	SC16A_MO	6	1.317783
15_MTA / C	O55131	-0.02383	0.407495	0.480095	Septin7	Septin-7	SEPT7_MOI	3	0.983619
30_MTA / C	O70475	-0.47692	0.409129	0.480095	Ugdh	UDP-glucos	UGDH_MOI	7	0.718509
30_MTA / C	P00329	-0.02167	0.408617	0.480095	Adh1	Alcohol del	ADH1_MOU	24	0.985094
30_MTA / C	Q3UZ39	0.388589	0.407761	0.480095	Lrrfip1	Leucine-ric	LRRF1_MOI	2	1.309113
15_MTA / C	Q60759	-0.11151	0.409281	0.480095	Gcdh	Glutaryl-Cc	GCDH_MOU	10	0.925619
15_MTA / C	Q8CI51	-0.18469	0.409222	0.480095	Pdlim5	PDZ and LIM	PDLI5_MOU	15	0.879836
15_MTA / C	Q9CR00	-0.03108	0.408863	0.480095	Psmd9	26S protea:	PSMD9_MC	9	0.97869
15_MTA / C	Q9CZU6	-1.16934	0.408566	0.480095	Cs	Citrate synt	CISY_MOUS	2	0.444624
30_MTA / C	Q9D0J8	0.176258	0.408087	0.480095	Ptms	Parathymo	PTMS_MOU	4	1.129949
30_MTA / C	Q9D8S4	-0.03797	0.408499	0.480095	Rexo2	Oligoribon	ORN_MOUS	3	0.974027
30_MTA / C	A0JNU3	0.02565	0.411254	0.482108	Aspg	60 kDa lyso	LPP60_MO	4	1.017938
15_MTA / C	Q91YP3	0.121976	0.411338	0.482108	Dera	Deoxyribos	DEOC_MOU	14	1.088224
15_MTA / C	P40142	-0.02694	0.412229	0.482767	Tkt	Transketola	TKT_MOUSI	39	0.981502
30_MTA / C	P56399	0.024214	0.412214	0.482767	Usp5	Ubiquitin c	UBP5_MOU	29	1.016925
30_MTA / C	Q8C1A5	-0.02348	0.412763	0.483077	Thop1	Thimet olig	THOP1_MC	10	0.983857
15_MTA / C	P10648	-0.15019	0.413123	0.483342	Gsta2	Glutathion	GSTA2_MO	7	0.901133
15_MTA / C	P68510	0.096123	0.414401	0.483423	Ywhah	14-3-3 prot	1433F_MO	8	1.068897
15_MTA / C	Q61133	-0.0455	0.414328	0.483423	Gstt2	Glutathion	GSTT2_MOI	13	0.968954
15_MTA / C	Q64459	-1.96991	0.414011	0.483423	Cyp3a11	Cytochrom	CP3AB_MO	2	0.255268
15_MTA / C	Q8BK48	0.024847	0.413575	0.483423	Ces2e	Pyrethroid	EST2E_MOU	18	1.017372
15_MTA / C	Q8K182	0.240543	0.414282	0.483423	C8a	Complemer	CO8A_MOU	4	1.181437
15_MTA / C	Q99LI8	-0.27272	0.413783	0.483423	Hgs	Hepatocyte	HGS_MOUS	4	0.827757
30_MTA / C	Q9WTX6	0.094647	0.414751	0.483518	Cul1	Cullin-1	CUL1_MOU	16	1.067804
30_MTA / C	Q6PB66	-0.09932	0.414887	0.483519	Lrpprc	Leucine-ric	LPPRC_MO	4	0.93347
30_MTA / C	P61965	-0.38204	0.415113	0.483627	Wdr5	WD repeat-	WDR5_MO	4	0.767351
30_MTA / C	Q8CIB5	0.335303	0.415791	0.48426	Fermt2	Fermitin fai	FERM2_MC	6	1.261643
15_MTA / C	Q60864	0.031687	0.415952	0.484266	Stip1	Stress-indu	STIP1_MOU	30	1.022207
15_MTA / C	O55229	0.839328	0.416779	0.48494	Chkb	Choline/etl	CHKB_MOU	2	1.789217
30_MTA / C	Q9JJH1	-0.43388	0.417146	0.485054	Rnase4	Ribonuclea	RNAS4_MO	4	0.740271
15_MTA / C	E9PV24	-0.07252	0.418209	0.485201	Fga	Fibrinogen	FIBA_MOUS	7	0.950973
15_MTA / C	O08738	-0.18279	0.418546	0.485201	Casp6	Caspase-6	CASP6_MO	4	0.880998
30_MTA / C	P42125	-0.08165	0.418404	0.485201	Eci1	Enoyl-CoA c	ECI1_MOUS	16	0.944975
30_MTA / C	P46471	-0.22208	0.418611	0.485201	Psmc2	26S protea:	PRS7_MOU	17	0.857329
30_MTA / C	P97371	0.092236	0.41802	0.485201	Psme1	Proteasom	PSME1_MC	15	1.066021
30_MTA / C	Q60710	0.198139	0.418619	0.485201	Samhd1	Deoxynucl	SAMH1_MC	3	1.147218
30_MTA / C	Q9D826	-0.05774	0.417545	0.485201	Pipox	Peroxisom	SOX_MOUS	22	0.96077
30_MTA / C	O08749	-0.05292	0.419387	0.485324	Dld	Dihydrolip	DLDH_MOU	21	0.963987
30_MTA / C	P17426	-0.05939	0.419512	0.485324	Ap2a1	AP-2 compl	AP2A1_MO	17	0.959668
30_MTA / C	P60335	-0.03676	0.420075	0.485324	Pcbp1	Poly(rC)-bir	PCBP1_MO	10	0.974843
30_MTA / C	Q06770	-0.30448	0.419985	0.485324	Serpina6	Corticoster	CBG_MOUS	3	0.809732
15_MTA / C	Q3TXS7	-0.08839	0.419377	0.485324	Psmd1	26S protea:	PSMD1_MC	34	0.940572
30_MTA / C	Q8R0F8	0.15464	0.419071	0.485324	Fahd1	Oxaloaceta	FAHD1_MC	11	1.113143
15_MTA / C	Q8R1Q8	-0.25895	0.419891	0.485324	Dync1li1	Cytoplasmi	DC1L1_MO	4	0.835696
15_MTA / C	Q9DBG5	-0.37499	0.419846	0.485324	Plin3	Perilipin-3	PLIN3_MOI	5	0.771109
30_MTA / C	P53810	-0.0153	0.420402	0.485546	Pitpna	Phosphatid	PIPNA_MOI	5	0.989453
30_MTA / C	P61027	0.642855	0.422316	0.487444	Rab10	Ras-related	RAB10_MO	1	1.561416

30_MTA / C	Q8VCH0	-0.01628	0.422759	0.487486	Acaa1b	3-ketoacyl-	THIKB_MOU	23	0.988777
30_MTA / C	O70194	0.505063	0.423178	0.487679	Eif3d	Eukaryotic	EIF3D_MOU	5	1.419185
15_MTA / C	P14148	0.150628	0.423266	0.487679	Rpl7	Large ribos	RL7_MOUS	17	1.110052
30_MTA / C	Q60598	0.126397	0.423332	0.487679	Cttn	Src substrat	SRC8_MOU	11	1.091564
15_MTA / C	Q99L45	0.154211	0.423841	0.487953	Eif2s2	Eukaryotic	IF2B_MOUS	7	1.112813
30_MTA / C	P24547	-0.02504	0.42419	0.488198	Impdh2	Inosine-5'-r	IMDH2_MO	19	0.982794
15_MTA / C	O35643	0.000577	0.425298	0.488636	Ap1b1	AP-1 compl	AP1B1_MO	35	1.0004
15_MTA / C	O70435	0.09429	0.425385	0.488636	Psma3	Proteasom	PSA3_MOU	13	1.06754
15_MTA / C	P28798	-0.39511	0.425233	0.488636	Grn	Progranulir	GRN_MOUS	5	0.760429
30_MTA / C	Q61425	-0.05802	0.42506	0.488636	Hadh	Hydroxyacy	HCDH_MOU	9	0.96058
30_MTA / C	Q9DCV4	0.394509	0.424722	0.488636	Rmdn1	Regulator c	RMD1_MOU	3	1.314495
15_MTA / C	Q9WVQ5	-0.1004	0.425112	0.488636	Apip	Methylthio	MTNB_MOU	9	0.932772
30_MTA / C	P47964	-0.2066	0.425642	0.488776	Rpl36	Large ribos	RL36_MOU	5	0.866576
15_MTA / C	P29699	0.05893	0.426321	0.488931	Ahsg	Alpha-2-HS	FETUA_MOU	8	1.041693
30_MTA / C	Q8BKC5	-0.34559	0.42596	0.488931	Ipo5	Importin-5	IPO5_MOU	4	0.786986
15_MTA / C	Q91YI0	-0.04674	0.426134	0.488931	Asl	Argininosu	ARLY_MOU	21	0.968122
15_MTA / C	O88668	-0.10622	0.427534	0.49001	Creg1	Protein CRI	CREG1_MO	4	0.929018
15_MTA / C	Q99KP6	0.148266	0.427432	0.49001	Prpf19	Pre-mRNA-p	PRP19_MO	11	1.108236
15_MTA / C	Q99KR7	0.193089	0.427746	0.490097	Ppif	Peptidyl-pr	PPIF_MOUS	7	1.143209
15_MTA / C	P62754	0.137392	0.428869	0.490381	Rps6	Small ribos	RS6_MOUS	7	1.099915
30_MTA / C	Q9D7A8	0.080064	0.428596	0.490381	Armc1	Armadillo r	ARMC1_MO	1	1.057065
30_MTA / C	Q9DCL9	0.09053	0.428483	0.490381	Paics	Bifunctiona	PUR6_MOU	18	1.064761
30_MTA / C	Q9WUM4	-0.10418	0.428947	0.490381	Coro1c	Coronin-1C	COR1C_MO	5	0.930334
30_MTA / C	P43277	0.121331	0.429552	0.490909	H1-3	Histone H1	H13_MOUS	5	1.087738
15_MTA / C	P56212	0.228774	0.429682	0.490909	Arpp19	cAMP-regul	ARP19_MO	1	1.171838
30_MTA / C	Q00519	-0.02035	0.430428	0.491605	Xdh	Xanthine de	XDH_MOUS	51	0.985991
15_MTA / C	P47911	0.143835	0.431182	0.491998	Rpl6	Large ribos	RL6_MOUS	14	1.104838
15_MTA / C	P62751	-0.06259	0.432103	0.492425	Rpl23a	Large ribos	RL23A_MO	6	0.957545
15_MTA / C	P12367	-0.50493	0.432344	0.492544	Prkar2a	cAMP-depe	KAP2_MOU	9	0.704696
30_MTA / C	P63323	0.016095	0.43255	0.492568	Rps12	Small ribos	RS12_MOU	8	1.011219
15_MTA / C	Q9D6J6	-0.4341	0.432775	0.492568	Ndufv2	NADH dehy	NDUV2_MO	2	0.740157
30_MTA / C	Q03249	-0.17781	0.433295	0.492848	Galt	Galactose-1	GALT_MOU	7	0.884041
30_MTA / C	P51174	-0.08563	0.433829	0.492886	Acadl	Long-chain	ACADL_MO	22	0.942372
30_MTA / C	Q3ULJ0	-0.21988	0.433724	0.492886	Gpd1l	Glycerol-3-	GPD1L_MO	9	0.858637
30_MTA / C	Q9DBC0	-0.12114	0.433554	0.492886	Selenoo	Protein ade	SELO_MOU	1	0.919462
30_MTA / C	Q78JW9	-0.16749	0.434075	0.492956	Ubfd1	Ubiquitin d	UBFD1_MO	3	0.890393
30_MTA / C	Q61176	-0.07112	0.434517	0.493302	Arg1	Arginase-1	ARGI1_MO	23	0.951898
15_MTA / C	P08228	-0.02518	0.434857	0.493402	Sod1	Superoxide	SODC_MOU	12	0.9827
30_MTA / C	Q8BFR5	0.138024	0.435709	0.493722	Tufm	Elongation	EFTU_MOU	22	1.100397
15_MTA / C	Q8BT60	-0.05501	0.435615	0.493722	Cpne3	Copine-3	CPNE3_MO	8	0.962587
30_MTA / C	Q9QZE7	-0.14852	0.435514	0.493722	Tsnax	Translin-ass	TSNAX_MO	7	0.902175
30_MTA / C	Q8BZB2	0.069579	0.436456	0.494125	Ppcdc	Phosphopa	COAC_MOU	3	1.049411
15_MTA / C	Q9D0R2	0.030374	0.43719	0.494621	Tars1	Threonine--	SYTC_MOUS	25	1.021277
30_MTA / C	Q3V1L4	-0.01208	0.437617	0.494949	Nt5c2	Cytosolic p	5NTC_MOU	10	0.991659
15_MTA / C	Q9WUZ9	0.186774	0.437853	0.49506	Entpd5	Ectonucleo	ENTP5_MO	9	1.138215
30_MTA / C	P62196	-0.02574	0.438327	0.495284	Psmc5	26S protea	PRS8_MOU	9	0.982315
15_MTA / C	Q9JI75	0.127779	0.438282	0.495284	Nqo2	Ribosyldihy	NQO2_MOU	4	1.092611
30_MTA / C	Q9WUZ9	-0.08045	0.43848	0.495302	Entpd5	Ectonucleo	ENTP5_MO	9	0.945761
30_MTA / C	P70699	-0.00677	0.438987	0.495409	Gaa	Lysosomal a	LYAG_MOU	3	0.995321
30_MTA / C	Q8R3P0	0.295797	0.438814	0.495409	Aspa	Aspartoacy	ACY2_MOU	2	1.227563
15_MTA / C	P62821	-0.07947	0.439388	0.495706	Rab1a	Ras-related	RAB1A_MO	7	0.946405
15_MTA / C	P50580	-0.05098	0.439828	0.495891	Pa2g4	Proliferatic	PA2G4_MO	16	0.96528

30_MTA / C	P80316	-0.02931	0.439769	0.495891	Cct5	T-complex	TCPE_MOU	20	0.979891
30_MTA / C	Q922D4	0.060544	0.440155	0.496105	Ppp6r3	Serine/thre	PP6R3_MO	20	1.042859
15_MTA / C	P11859	-0.2943	0.441027	0.496136	Agt	Angiotensi	ANGT_MOU	4	0.815465
30_MTA / C	P61161	0.00788	0.441088	0.496136	Actr2	Actin-relate	ARP2_MOU	13	1.005477
15_MTA / C	P62281	0.039434	0.440575	0.496136	Rps11	Small ribos	RS11_MOU	12	1.027711
15_MTA / C	Q61702	-0.15434	0.440632	0.496136	Itih1	Inter-alpha	ITIH1_MOU	14	0.898546
30_MTA / C	Q8VCG4	-0.07481	0.440337	0.496136	C8g	Compleme	CO8G_MOU	7	0.949465
30_MTA / C	Q9EST5	-0.17935	0.441148	0.496136	Anp32b	Acidic leuci	AN32B_MO	4	0.883103
30_MTA / C	O55142	0.17547	0.441466	0.496225	Rpl35a	Large ribos	RL35A_MO	7	1.129332
15_MTA / C	P53395	0.103069	0.44229	0.496955	Dbt	Lipoamide	ODB2_MOU	22	1.074056
30_MTA / C	P22599	0.119981	0.442978	0.497399	Serpina1b	Alpha-1-ant	A1AT2_MO	7	1.086721
15_MTA / C	P49182	-0.08476	0.443011	0.497399	Serpind1	Heparin co	HEP2_MOU	9	0.94294
15_MTA / C	O35343	0.003843	0.443973	0.497913	Kpna4	Importin su	IMA3_MOU	9	1.002667
30_MTA / C	P01636	0.027209	0.445273	0.498034	NaN	Ig kappa ch	KV5A4_MO	1	1.019039
15_MTA / C	P62737;P6	-0.09687	0.444786	0.498034	Acta2;Actg	Actin, aorti	ACTA_MOU	2	0.935062
15_MTA / C	Q00519	0.012522	0.445325	0.498034	Xdh	Xanthine d	XDH_MOUS	51	1.008717
30_MTA / C	Q6ZWU9;Q	0.004894	0.444721	0.498034	Rps27;Rps2	Small ribos	RS27_MOU	2	1.003398
30_MTA / C	Q8BVQ5	0.00469	0.444531	0.498034	Ppme1	Protein pho	PPME1_MO	2	1.003256
15_MTA / C	Q924M7	-0.36404	0.445316	0.498034	Mpi	Mannose-6	MPI_MOUS	6	0.776983
15_MTA / C	Q9CR21	0.123994	0.444953	0.498034	Ndufab1	Acyl carrier	ACPM_MOU	3	1.089748
15_MTA / C	Q9DBT9	0.098577	0.44431	0.498034	Dmgdh	Dimethylgl	M2GD_MO	36	1.070717
30_MTA / C	Q9WV54	0.1078	0.445043	0.498034	Asah1	Acid ceram	ASAH1_MO	4	1.077584
15_MTA / C	Q8WTY4	0.047705	0.445805	0.498415	Ciapin1	Anamorsin	CPIN1_MOU	7	1.033619
15_MTA / C	P63085	0.329571	0.447038	0.49902	Mapk1	Mitogen-ac	MK01_MOU	7	1.25664
30_MTA / C	P70445	0.010971	0.446746	0.49902	Eif4ebp2	Eukaryotic	4EBP2_MO	2	1.007634
15_MTA / C	Q05816	-0.06507	0.446765	0.49902	Fabp5	Fatty acid-b	FABP5_MO	9	0.955896
15_MTA / C	Q3TC72	-0.07616	0.446924	0.49902	Fahd2a	Oxaloaceta	FAH2A_MO	1	0.948578
30_MTA / C	Q8BND5	0.491738	0.447281	0.499135	Qsox1	Sulfhydryl	QSOX1_MO	5	1.406137
30_MTA / C	P28651	0.331931	0.448873	0.500137	Ca8	Carbonic ar	CAH8_MOU	8	1.258697
15_MTA / C	P70699	0.026634	0.448668	0.500137	Gaa	Lysosomal	LYAG_MOU	3	1.018633
30_MTA / C	Q8BWR2	-0.06424	0.448821	0.500137	Pithd1	PITH domai	PITH1_MOU	6	0.956447
15_MTA / C	Q8BUV3	-0.0009	0.449323	0.500329	Gphn	Gephyrin	GEPH_MOU	27	0.999377
15_MTA / C	Q8R086	-0.08638	0.449229	0.500329	Suox	Sulfite oxid	SUOX_MOU	23	0.941886
15_MTA / C	P01754;P0	-0.10794	0.450734	0.500353	Ighv1-62-3	Ig heavy ch	HVM10_MO	1	0.927915
15_MTA / C	Q64152	-0.46354	0.450443	0.500353	Btf3	Transcripti	BTF3_MOU	4	0.725204
15_MTA / C	Q8K157	0.090537	0.449931	0.500353	Galm	Galactose n	GALM_MOU	10	1.064767
30_MTA / C	Q8R146	0.081708	0.450456	0.500353	Apeh	Acylamino-	ACPH_MOU	20	1.05827
30_MTA / C	Q9JIX8	0.115372	0.450631	0.500353	Acin1	Apoptotic	ACINU_MO	3	1.083254
15_MTA / C	O08756	0.033358	0.451469	0.501014	Hsd17b10	3-hydroxya	HCD2_MOU	10	1.023392
15_MTA / C	P11725	-0.05358	0.452209	0.501102	Otc	Ornithine t	OTC_MOUS	25	0.963543
30_MTA / C	P84089	-0.48315	0.4521	0.501102	Erh	Enhancer o	ERH_MOUS	2	0.715412
15_MTA / C	Q501J7	0.123514	0.451694	0.501102	Phactr4	Phosphata	PHAR4_MO	2	1.089385
30_MTA / C	Q9D3D9	0.448358	0.452858	0.501165	Atp5f1d	ATP syntha	ATPD_MOU	4	1.364486
30_MTA / C	Q91X44	-0.60535	0.453181	0.501368	Gckr	Glucokinas	GCKR_MOU	7	0.657311
30_MTA / C	P26369	0.108639	0.454685	0.502569	U2af2	Splicing fac	U2AF2_MO	3	1.078211
30_MTA / C	Q60866	-0.02983	0.454534	0.502569	Pter	Phosphotri	PTER_MOU	19	0.979533
15_MTA / C	P97765	-0.0255	0.455005	0.502768	Wbp2	WW domai	WBP2_MOU	1	0.982482
30_MTA / C	Q9D7I5	-0.29957	0.455188	0.502816	Lhpp	Phospholys	LHPP_MOU	2	0.812495
30_MTA / C	Q9EQ32	0.324454	0.456131	0.503549	Pik3ap1	Phosphoin	BCAP_MOU	3	1.25219
15_MTA / C	P01674	-1.01008	0.456428	0.503582	NaN	Ig kappa ch	KV3AM_MO	1	0.496518
30_MTA / C	P84099	0.209891	0.456441	0.503582	Rpl19	Large ribos	RL19_MOU	4	1.156601
30_MTA / C	O09174	0.002052	0.458312	0.503831	Amacr	Alpha-meth	AMACR_MO	13	1.001423

15_MTA / C	P29758	-0.15658	0.458011	0.503831	Oat	Ornithine a	OAT_MOUS	8	0.897148
30_MTA / C	P54071	-0.70297	0.457443	0.503831	Idh2	Isocitrate d	IDHP_MOU	7	0.614305
15_MTA / C	P55050	0.085731	0.457813	0.503831	Fabp2	Fatty acid-b	FABPI_MOU	5	1.061226
15_MTA / C	P61205;P8	-0.06333	0.457469	0.503831	Arf3;Arf1	ADP-ribosy	ARF3_MOU	8	0.957052
15_MTA / C	Q711T7	0.007504	0.45741	0.503831	Nadsyn1	Glutamine-	NADE_MOU	14	1.005215
30_MTA / C	Q8BP47	0.340449	0.456974	0.503831	Nars1	Asparagine-	SYNC_MOU	1	1.26615
15_MTA / C	Q91XC8	-0.3157	0.457968	0.503831	Dap	Death-asso	DAP1_MOU	2	0.80346
30_MTA / C	Q99LC5	-0.01593	0.458552	0.503831	Etfa	Electron tra	ETFA_MOUS	17	0.989021
30_MTA / C	Q9DBB8	-0.06189	0.4579	0.503831	Dhdh	Trans-1,2-d	DHDH_MOU	20	0.95801
15_MTA / C	P28650	0.213587	0.461077	0.505905	Adss1	Adenylosuc	PURA1_MC	1	1.159568
30_MTA / C	O70362	-0.01379	0.461605	0.506331	Gpld1	Phosphatid	PHLD_MOU	6	0.990487
15_MTA / C	P62911	0.174499	0.46312	0.507374	Rpl32	Large ribos	RL32_MOU	8	1.128572
30_MTA / C	Q9CZS1	0.165709	0.462926	0.507374	Aldh1b1	Aldehyde d	AL1B1_MO	8	1.121718
30_MTA / C	Q9R111	-0.0791	0.463082	0.507374	Gda	Guanine de	GUAD_MOU	27	0.94665
15_MTA / C	Q9DCX2	-0.28476	0.464727	0.50898	Atp5pd	ATP synthas	ATP5H_MO	3	0.820879
30_MTA / C	P62073	0.021277	0.46591	0.50981	Timm10	Mitochond	TIM10_MO	1	1.014857
30_MTA / C	Q8R0P4	0.082609	0.466015	0.50981	Aamdc	Mth938 do	AAMDC_MC	1	1.058931
30_MTA / C	Q923B0	-0.6773	0.466051	0.50981	Ggact	Gamma-glu	GGACT_MC	3	0.625336
30_MTA / C	Q64514	-0.33514	0.466364	0.509997	Tpp2	Tripeptidyl	TPP2_MOU	6	0.792705
15_MTA / C	P14131	0.157086	0.466585	0.510052	Rps16	Small ribos	RS16_MOU	7	1.115032
30_MTA / C	P19253	0.180372	0.467131	0.510052	Rpl13a	Large ribos	RL13A_MO	8	1.133176
15_MTA / C	P49429	-0.00953	0.467323	0.510052	Hpd	4-hydroxyp	HPPD_MOU	25	0.993414
15_MTA / C	Q6ZQ38	0.021702	0.467406	0.510052	Cand1	Cullin-asso	CAND1_MC	37	1.015156
15_MTA / C	Q8R0V5	-0.11524	0.467086	0.510052	Ido2	Indoleamin	I23O2_MO	1	0.923226
15_MTA / C	Q8R0Y6	-0.04035	0.466954	0.510052	Aldh1l1	Cytosolic 1	AL1L1_MO	53	0.972422
15_MTA / C	P08226	-0.00886	0.46782	0.510276	Apoe	Apolipopro	APOE_MOU	14	0.993874
30_MTA / C	Q9WUM5	-0.14781	0.468337	0.510449	Suclg1	Succinate--	SUCA_MOU	6	0.902618
30_MTA / C	Q8K3C3	-0.31871	0.468511	0.510485	Lzic	Protein LZI	LZIC_MOUS	3	0.801789
15_MTA / C	Q64520	-0.0308	0.468654	0.510485	Guk1	Guanylate I	KGUA_MOU	1	0.978875
30_MTA / C	Q9DCX2	0.035875	0.469272	0.510777	Atp5pd	ATP synthas	ATP5H_MO	3	1.025178
30_MTA / C	Q9Z1J3	0.84381	0.469243	0.510777	Nfs1	Cysteine de	NFS1_MOU	4	1.794784
15_MTA / C	P35980	0.180081	0.469972	0.510912	Rpl18	Large ribos	RL18_MOU	7	1.132947
30_MTA / C	Q8QZY2	-0.31582	0.469938	0.510912	Glyctk	Glycerate k	GLCTK_MO	2	0.803395
30_MTA / C	Q91VA6	-0.50036	0.470229	0.510912	Poldip2	Polymerase	PDIP2_MO	4	0.70693
30_MTA / C	O08547	-0.74912	0.470831	0.511155	Sec22b	Vesicle-traf	SC22B_MO	2	0.594965
15_MTA / C	Q9Z2U0	-0.07736	0.471318	0.511493	Psma7	Proteasom	PSA7_MOU	15	0.947788
30_MTA / C	P62774	0.008264	0.472076	0.511634	Mtpn	Myotrophi	MTPN_MOU	6	1.005744
30_MTA / C	P70441	0.101776	0.47207	0.511634	Nherf1	Na(+)/H(+) e	NHRF1_MC	19	1.073094
30_MTA / C	Q61189	0.056384	0.472235	0.511634	Clns1a	Methylosor	ICLN_MOUS	1	1.039856
30_MTA / C	P00920	0.191778	0.472988	0.512014	Ca2	Carbonic ar	CAH2_MOU	9	1.142171
15_MTA / C	P09405	0.138614	0.473044	0.512014	Ncl	Nucleolin	NUCL_MOU	24	1.100847
30_MTA / C	Q8BWT1	-0.00306	0.472856	0.512014	Acaa2	3-ketoacyl-	THIM_MOU	27	0.997883
30_MTA / C	P97429	0.128785	0.474338	0.51319	Anxa4	Annexin A4	ANXA4_MC	4	1.093373
15_MTA / C	E9Q557	0.751054	0.475614	0.513435	Dsp	Desmoplak	DESP_MOU	32	1.683022
30_MTA / C	O35405	-0.38829	0.475957	0.513435	Pld3	5'-3' exonu	PLD3_MOU	2	0.764036
15_MTA / C	P83940	0.084014	0.475791	0.513435	Eloc	Elongin-C	ELOC_MOU	5	1.059963
30_MTA / C	Q3UNZ8	-0.10352	0.476273	0.513435	Cryzl2	Quinone ox	QORL2_MC	7	0.930757
15_MTA / C	Q8BWT1	-0.01375	0.476618	0.513435	Acaa2	3-ketoacyl-	THIM_MOU	27	0.990516
30_MTA / C	Q8C0D5	-0.20395	0.476238	0.513435	Efl1	Elongation	EFL1_MOUS	2	0.868173
15_MTA / C	Q8K4M5	-0.29105	0.475877	0.513435	Commd1	COMM dom	COMD1_MC	2	0.817306
15_MTA / C	Q9D1F4	-0.50919	0.476531	0.513435	Akt1s1	Proline-rich	AKTS1_MO	2	0.702617
15_MTA / C	Q9JHS9	-0.93518	0.476639	0.513435	Cwc15	Spliceosom	CWC15_MC	1	0.522976

30_MTA / C	O89110	-0.09103	0.477199	0.513731	Casp8	Caspase-8	CASP8_MOU	5	0.938852
15_MTA / C	Q8K385	0.083905	0.477673	0.51374	FRRS1	Ferric-chela	FRRS1_MOU	3	1.059883
30_MTA / C	Q9D8E6	0.128011	0.47772	0.51374	Rpl4	Large ribos	RL4_MOUS	19	1.092786
15_MTA / C	A2BIM8;P0	-0.05802	0.478635	0.513861	Mup18;Mu	Major urina	MUP18_MO	16	0.960583
30_MTA / C	Q91V12	0.055949	0.478518	0.513861	Acot7	Cytosolic a	BACH_MOU	6	1.039542
15_MTA / C	Q922Q8	0.220905	0.478709	0.513861	Lrrc59	Leucine-ric	LRC59_MO	8	1.165464
30_MTA / C	Q9CR95	0.042635	0.478614	0.513861	Necap1	Adaptin ear	NECP1_MO	6	1.029994
30_MTA / C	Q9JJI8	-0.17335	0.478419	0.513861	Rpl38	Large ribos	RL38_MOU	3	0.88678
15_MTA / C	Q61425	0.134652	0.481384	0.515769	Hadh	Hydroxyacy	HCDH_MOU	9	1.097828
15_MTA / C	Q8BWP5	-0.00117	0.481588	0.515834	Ttpa	Alpha-toco	TTPA_MOU	15	0.999187
15_MTA / C	P21614	-0.03059	0.482164	0.516297	Gc	Vitamin D-b	VTDB_MOU	25	0.979018
15_MTA / C	P02089	-0.08644	0.48333	0.517238	Hbb-b2	Hemoglobi	HBB2_MOU	6	0.941846
15_MTA / C	Q8BFS6	-0.00225	0.483293	0.517238	Cpped1	Serine/thre	CPPED_MO	9	0.998439
15_MTA / C	Q06185	-0.28711	0.483908	0.517399	Atp5me	ATP syntha	ATP5I_MOU	1	0.819541
30_MTA / C	Q99MI1	-0.21544	0.483955	0.517399	Erc1	ELKS/Rab6-	RB6I2_MOU	9	0.861285
15_MTA / C	Q9Z1J3	0.59362	0.484009	0.517399	Nfs1	Cysteine de	NFS1_MOU	4	1.509028
15_MTA / C	Q00915	-0.1901	0.48442	0.517414	Rbp1	Retinol-bin	RET1_MOU	7	0.876543
15_MTA / C	Q8BWY3	-0.12004	0.484761	0.517414	Etf1	Eukaryotic	ERF1_MOU	10	0.920164
30_MTA / C	Q9QXE0	-0.03103	0.484788	0.517414	Hacl1	2-hydroxya	HACL1_MO	30	0.978722
15_MTA / C	Q8JZL3	-0.24438	0.485206	0.517675	Thtpa	Thiamine-tr	THTPA_MO	4	0.844179
30_MTA / C	P62137	0.307812	0.486067	0.518318	Ppp1ca	Serine/thre	PP1A_MOU	12	1.237829
30_MTA / C	Q61704	-0.45699	0.486706	0.518692	Itih3	Inter-alpha	ITIH3_MOU	10	0.728506
30_MTA / C	Q9D0R8	0.01826	0.486682	0.518692	Lsm12	Protein LSM	LSM12_MO	3	1.012737
15_MTA / C	Q91V64	0.009027	0.487012	0.518864	Isoc1	Isochorism	ISOC1_MOU	9	1.006276
30_MTA / C	P62082	0.266152	0.487861	0.519308	Rps7	Small ribos	RS7_MOUS	7	1.202596
30_MTA / C	Q8VCT4	-0.05327	0.487647	0.519308	Ces1d	Carboxyles	EST1D_MO	33	0.963752
15_MTA / C	O35857	0.258677	0.488547	0.519432	Timm44	Mitochond	TIM44_MO	4	1.196381
15_MTA / C	Q03734	-0.06595	0.488309	0.519432	Serpina3m	Serine prot	SPA3M_MO	9	0.955313
30_MTA / C	P12970	0.134151	0.489505	0.519846	Rpl7a	Large ribos	RL7A_MOU	13	1.097447
30_MTA / C	Q9CX34	-0.02072	0.489522	0.519846	Sugt1	Protein SGT	SGT1_MOU	8	0.985741
30_MTA / C	P61205;P8	-0.06057	0.490055	0.520142	Arf3;Arf1	ADP-ribosy	ARF3_MOU	8	0.958886
30_MTA / C	Q9JKB1	0.060358	0.490559	0.520487	Uchl3	Ubiquitin c	UCHL3_MO	8	1.042724
30_MTA / C	Q8CIM3	-0.26663	0.490791	0.520576	D2hgdh	D-2-hydrox	D2HDH_MO	5	0.831261
30_MTA / C	P09671	-0.03297	0.49178	0.521014	Sod2	Superoxide	SODM_MOU	12	0.977404
15_MTA / C	P46737	0.004984	0.491567	0.521014	Brcc3	Lys-63-spec	BRCC3_MO	1	1.00346
30_MTA / C	Q03265	-0.12571	0.491663	0.521014	Atp5f1a	ATP syntha	ATPA_MOU	20	0.916551
30_MTA / C	Q8VC30	0.11826	0.492157	0.52126	Tkfc	Triokinase/	TKFC_MOU	27	1.085425
30_MTA / C	Q9DBG5	-0.38686	0.492844	0.521835	Plin3	Perilipin-3	PLIN3_MOU	5	0.764792
15_MTA / C	O08997	-0.04146	0.493598	0.521937	Atox1	Copper tra	ATOX1_MO	6	0.971672
30_MTA / C	O55131	-0.01972	0.493377	0.521937	Septin7	Septin-7	SEPT7_MOU	3	0.986423
30_MTA / C	Q64726	0.010807	0.493666	0.521937	Azgp1	Zinc-alpha-	ZA2G_MOU	3	1.007519
30_MTA / C	Q9D7J9	-0.00232	0.493477	0.521937	Echdc3	Enoyl-CoA h	ECHD3_MO	2	0.998392
15_MTA / C	P11588	-0.1217	0.493843	0.521972	Mup1	Major urina	MUP1_MOU	1	0.919106
30_MTA / C	Q99K30	-0.10691	0.494031	0.522017	Eps8l2	Epidermal g	ES8L2_MOU	2	0.928572
30_MTA / C	P20065;Q6	-0.24634	0.494513	0.522373	Tmsb4x;Tm	Thymosin b	TYB4_MOU	1	0.843034
15_MTA / C	Q9CQR4	0.647224	0.49487	0.522596	Acot13	Acyl-coenzy	ACO13_MO	2	1.566151
30_MTA / C	P62242	0.064237	0.495666	0.523131	Rps8	Small ribos	RS8_MOUS	10	1.045532
30_MTA / C	P14148	0.152075	0.498124	0.52347	Rpl7	Large ribos	RL7_MOUS	17	1.111167
30_MTA / C	P17918	0.186416	0.496875	0.52347	Pcna	Proliferatin	PCNA_MOU	7	1.137933
30_MTA / C	P27005	-0.25111	0.498169	0.52347	S100a8	Protein S10	S10A8_MO	2	0.840249
15_MTA / C	P32067	-0.0343	0.498166	0.52347	Ssb	Lupus La pr	LA_MOUSE	10	0.976509
30_MTA / C	Q60590	-0.14898	0.498049	0.52347	Orm1	Alpha-1-aci	A1AG1_MO	1	0.901886

30_MTA / C	Q80SY6	0.040867	0.49685	0.52347	Adal	Adenosine (	ADAL_MOU	2	1.028732
15_MTA / C	Q8BWW4	-0.04633	0.496187	0.52347	Larp4	La-related p	LARP4_MO	3	0.968399
15_MTA / C	Q8JZK9	-0.23908	0.497483	0.52347	Hmgcs1	Hydroxyme	HMCS1_MC	7	0.847285
30_MTA / C	Q8K003	-0.3235	0.497764	0.52347	Tma7	Translation	TMA7_MOU	1	0.799128
30_MTA / C	Q9CXZ1	0.217399	0.496463	0.52347	Ndufs4	NADH dehy	NDUS4_MC	2	1.162635
30_MTA / C	Q8JZS0	-0.13821	0.498558	0.523573	Lin7a	Protein lin-	LIN7A_MOU	4	0.908644
30_MTA / C	Q9CXA2	-0.25709	0.498457	0.523573	L3hypdh	Trans-L-3-h	T3HPD_MC	1	0.836777
15_MTA / C	Q64176	0.08555	0.498709	0.523579	Ces1e	Carboxyles	EST1E_MOU	13	1.061092
30_MTA / C	O88543	-0.0994	0.499066	0.523801	Cops3	COP9 signa	CSN3_MOU	11	0.933422
30_MTA / C	Q8BVI4	0.127298	0.499775	0.52424	Qdpr	Dihydropte	DHPR_MOU	8	1.092246
15_MTA / C	P24369	0.029055	0.500355	0.52439	Ppib	Peptidyl-pr	PPIB_MOUS	11	1.020343
30_MTA / C	Q9CQN1	0.118649	0.50035	0.52439	Trap1	Heat shock	TRAP1_MO	21	1.085718
15_MTA / C	P01868;P0	0.208883	0.500904	0.524812	Ighg1	Ig gamma-1	IGHG1_MO	11	1.155793
15_MTA / C	Q8CGA0	0.108132	0.501668	0.525061	Ppm1f	Protein pho	PPM1F_MC	2	1.077832
15_MTA / C	Q9Z0K8	-0.31257	0.501659	0.525061	Vnn1	Pantethein	VNN1_MOU	1	0.805208
30_MTA / C	P35980	0.184378	0.502637	0.5251	Rpl18	Large ribos	RL18_MOU	7	1.136327
15_MTA / C	Q9WUK2	0.114621	0.502473	0.5251	Eif4h	Eukaryotic	IF4H_MOUS	7	1.08269
30_MTA / C	O08663	-0.3158	0.50434	0.52644	Metap2	Methionine	MAP2_MOU	5	0.803404
30_MTA / C	P97450	0.231024	0.504071	0.52644	Atp5pf	ATP syntha	ATP5J_MOU	4	1.173668
15_MTA / C	Q8JZN5	-0.10479	0.504359	0.52644	Acad9	Complex I a	ACAD9_MC	16	0.929941
15_MTA / C	Q9Z1Q5	-1.09643	0.504761	0.526555	Clic1	Chloride in	CLIC1_MOU	3	0.467672
15_MTA / C	P62897	-0.10214	0.505502	0.526655	Cycs	Cytochrom	CYC_MOUS	8	0.93165
15_MTA / C	Q61838	-0.06364	0.505539	0.526655	Pzp	Pregnancy	PZP_MOUS	66	0.956846
30_MTA / C	Q8R0Y6	-0.10266	0.505455	0.526655	Aldh1l1	Cytosolic 1	AL1L1_MO	53	0.931315
30_MTA / C	Q01279	-0.46296	0.505978	0.526755	Egfr	Epidermal g	EGFR_MOU	9	0.725497
30_MTA / C	Q8BZW8	0.145841	0.506205	0.526839	Nhlrc2	NHL repeat	NHLC2_MC	6	1.106376
30_MTA / C	Q61838	-0.06796	0.506542	0.527038	Pzp	Pregnancy	PZP_MOUS	66	0.953989
15_MTA / C	P03987	-0.28715	0.507134	0.52723	NaN	Ig gamma-3	IGHG3_MO	3	0.819517
15_MTA / C	P14115	0.267603	0.507299	0.52723	Rpl27a	Large ribos	RL27A_MO	4	1.203806
30_MTA / C	Q8VE09	-0.04729	0.507312	0.52723	Ttc39c	Tetratricop	TT39C_MOU	13	0.967754
15_MTA / C	Q9R060	0.452134	0.507245	0.52723	Nubp1	Cytosolic F	NUBP1_MC	1	1.368062
15_MTA / C	P10605	0.064066	0.507787	0.527266	Ctsb	Cathepsin E	CATB_MOU	10	1.045408
30_MTA / C	Q9EQH2	0.004433	0.507702	0.527266	Erap1	Endoplasm	ERAP1_MO	32	1.003078
30_MTA / C	Q9R1P4	-0.03244	0.507724	0.527266	Psma1	Proteasom	PSA1_MOU	19	0.977768
30_MTA / C	P57759	0.011444	0.508235	0.527427	Erp29	Endoplasm	ERP29_MO	5	1.007964
15_MTA / C	Q8BFZ3	-0.02751	0.508179	0.527427	Actbl2	Beta-actin-l	ACTBL_MOU	1	0.98111
15_MTA / C	O88653	0.061648	0.508718	0.527496	Lamtor3	Ragulator c	LTOR3_MO	1	1.043657
15_MTA / C	Q9D0S9	0.094492	0.508741	0.527496	Hint2	Adenosine 5	HINT2_MOU	5	1.067689
30_MTA / C	Q9CQV6	0.397733	0.509529	0.528161	Map1lc3b	Microtubul	MLP3B_MC	2	1.317436
30_MTA / C	O88533	0.049805	0.509908	0.528386	Ddc	Aromatic-L	DDC_MOUS	15	1.035125
30_MTA / C	P11247	-0.92662	0.51004	0.528386	Mpo	Myelopero	PERM_MOU	3	0.526091
15_MTA / C	P04117	-0.03372	0.511066	0.529	Fabp4	Fatty acid-b	FABP4_MO	8	0.976899
30_MTA / C	P52480	0.342607	0.511111	0.529	Pkm	Pyruvate ki	KPYM_MOU	14	1.268046
30_MTA / C	Q7TMM9	0.079934	0.5118	0.529	Tubb2a	Tubulin bet	TBB2A_MO	4	1.056969
30_MTA / C	Q80Y55	0.432198	0.511374	0.529	Bsdc1	BSD domai	BSDC1_MO	2	1.349288
15_MTA / C	Q8VCU1	0.118387	0.511808	0.529	Ces3b	Carboxyles	EST3B_MOU	16	1.08552
15_MTA / C	Q9CR57	0.144236	0.51161	0.529	Rpl14	Large ribos	RL14_MOU	6	1.105146
30_MTA / C	Q9CZD3	0.001615	0.511154	0.529	Gars1	Glycine--tR	GARS_MOU	3	1.00112
15_MTA / C	Q9WU28	-0.13463	0.511531	0.529	Pfdn5	Prefoldin su	PFD5_MOU	3	0.9109
30_MTA / C	Q8BYK6	-0.69517	0.512339	0.529397	Ythdf3	YTH domai	YTHD3_MO	2	0.617639
15_MTA / C	O88487	-0.10262	0.513621	0.530196	Dync1i2	Cytoplasmi	DC1I2_MOU	9	0.931342
30_MTA / C	O88696	-0.34045	0.513995	0.530196	Clpp	ATP-depend	CLPP_MOU	4	0.789794

15_MTA / C	Q9CZM2	-0.05212	0.51395	0.530196	Rpl15	Large ribos	RL15_MOU	10	0.964519
30_MTA / C	Q8BFQ8	-0.09062	0.514934	0.531012	Gatd1	Glutamine	GALD1_MC	4	0.939117
15_MTA / C	P62717	-0.03007	0.515247	0.531182	Rpl18a	Large ribos	RL18A_MO	9	0.979373
15_MTA / C	Q91VU6	-0.38212	0.515612	0.531254	Dcaf11	DDB1- and	DCA11_MC	2	0.767307
30_MTA / C	Q9ESP1	-0.01505	0.51552	0.531254	Sdf2l1	Stromal cel	SDF2L_MO	2	0.989622
15_MTA / C	Q9CYI0	0.199363	0.515823	0.53132	NaN	Protein Njn	NJMU_MOU	2	1.148191
15_MTA / C	Q99PL5	0.05124	0.516388	0.531664	Rrbp1	Ribosome-l	RRBP1_MO	58	1.036155
30_MTA / C	P31786	-0.03537	0.516889	0.53192	Dbi	Acyl-CoA-bi	ACBP_MOU	6	0.975779
30_MTA / C	Q9QXL2	-0.06439	0.516997	0.53192	Kif21a	Kinesin-like	KI21A_MO	4	0.95635
30_MTA / C	Q9D8C4	0.204506	0.518132	0.53246	Ifi35	Interferon-i	IN35_MOU	3	1.152292
30_MTA / C	Q9Z1X4	0.119848	0.518229	0.53246	Ilf3	Interleukin	ILF3_MOUS	2	1.086621
30_MTA / C	P97364	-0.05027	0.518713	0.532772	Sephs2	Selenide, w	SPS2_MOU	18	0.965757
30_MTA / C	P70265;P7	-0.01947	0.520728	0.53408	Pfkfb2;Pfkfl	6-phospho	F262_MOU	1	0.986594
30_MTA / C	Q8BGA8	0.063504	0.520701	0.53408	Acsm5	Acyl-coenzy	ACSM5_MC	22	1.045001
15_MTA / C	Q99K51	-0.01408	0.520599	0.53408	Pls3	Plastin-3	PLST_MOUS	35	0.990286
30_MTA / C	Q9D8X2	-0.29534	0.520188	0.53408	Ccdc124	Coiled-coil	CC124_MO	3	0.814882
15_MTA / C	P62082	0.178504	0.521292	0.534347	Rps7	Small ribos	RS7_MOUS	7	1.13171
15_MTA / C	P62814	0.139377	0.521363	0.534347	Atp6v1b2	V-type prot	VATB2_MO	14	1.101429
15_MTA / C	P63037	-0.11026	0.522732	0.535069	Dnaja1	DnaJ homo	DNJA1_MO	8	0.926421
30_MTA / C	P80314	0.069017	0.522693	0.535069	Cct2	T-complex	TCPB_MOU	22	1.049001
30_MTA / C	Q3UQ44	0.111768	0.523577	0.53519	Iqgap2	Ras GTPase-	IQGA2_MO	35	1.080551
30_MTA / C	Q9Z2V4	-0.47977	0.523571	0.53519	Pck1	Phosphoen	PCKGC_MC	5	0.717092
30_MTA / C	Q8R5H1	-0.12301	0.524122	0.535577	Usp15	Ubiquitin c	UBP15_MC	9	0.918271
30_MTA / C	Q921X9	-0.02615	0.524661	0.535976	Pdia5	Protein dis	PDIA5_MO	13	0.982034
30_MTA / C	Q8BH35	-0.07426	0.525066	0.536085	C8b	Complemen	CO8B_MOU	5	0.949827
30_MTA / C	Q9QXK3	-0.05979	0.524939	0.536085	Copg2	Coatomer s	COPG2_MC	5	0.959406
30_MTA / C	P63325	-0.45633	0.525636	0.536515	Rps10	Small ribos	RS10_MOU	3	0.728838
30_MTA / C	Q9QYG0	0.016642	0.52631	0.536899	Ndrg2	Protein ND	NDRG2_MC	16	1.011602
15_MTA / C	P01898	-0.00613	0.526862	0.53731	H2-Q10	H-2 class I h	HA10_MOU	10	0.99576
30_MTA / C	P01741	-0.10408	0.527252	0.537518	NaN	Ig heavy ch	HVM00_M	1	0.930395
15_MTA / C	Q8CIE6	-0.03342	0.527365	0.537518	Copa	Coatomer s	COPA_MOU	33	0.977104
15_MTA / C	Q9EQ80	-0.01773	0.52794	0.537703	Nif3l1	NIF3-like p	NIF3L_MOU	7	0.987787
30_MTA / C	Q00915	0.157039	0.528706	0.537972	Rbp1	Retinol-bin	RET1_MOU	7	1.114996
30_MTA / C	Q9DCD0	0.080243	0.528559	0.537972	Pgd	6-phospho	6PGD_MOU	20	1.057196
30_MTA / C	P22907	-0.00789	0.529595	0.538669	Hmbs	Porphobili	HEM3_MOU	14	0.994546
30_MTA / C	Q91X72	-0.08493	0.529691	0.538669	Hpx	Hemopexin	HEMO_MOU	24	0.942831
30_MTA / C	P12710	0.176189	0.53006	0.538892	Fabp1	Fatty acid-b	FABPL_MO	11	1.129895
30_MTA / C	O88685	0.141661	0.530537	0.539072	Psmc3	26S protea	PRS6A_MO	8	1.103174
30_MTA / C	P52503	0.47651	0.531224	0.539162	Ndufs6	NADH dehy	NDUS6_MC	3	1.391374
15_MTA / C	Q8BFW7	-0.04763	0.531046	0.539162	Lpp	Lipoma-pre	LPP_MOUS	13	0.967528
30_MTA / C	Q9CR09	-0.02041	0.53243	0.540081	Ufc1	Ubiquitin-f	UFC1_MOU	4	0.985954
30_MTA / C	Q9Z0N1	0.021683	0.532301	0.540081	Eif2s3x	Eukaryotic	IF2G_MOUS	4	1.015143
30_MTA / C	O70370	-0.16777	0.533371	0.540731	Ctss	Cathepsin S	CATS_MOU	6	0.890219
15_MTA / C	P35585	-0.09883	0.533731	0.540791	Ap1m1	AP-1 compl	AP1M1_MC	11	0.933792
15_MTA / C	P62900	-0.01198	0.534055	0.540968	Rpl31	Large ribos	RL31_MOU	3	0.991732
15_MTA / C	O08573	-0.06977	0.534549	0.541116	Lgals9	Galectin-9	LEG9_MOU	9	0.952789
30_MTA / C	O35386	-0.01207	0.535261	0.541116	Phyh	Phytanoyl-	PAHX_MOU	6	0.991666
30_MTA / C	O70172	-0.05598	0.535666	0.541116	Pip4k2a	Phosphatic	PI42A_MOU	1	0.961939
15_MTA / C	P22907	-0.0484	0.535922	0.541116	Hmbs	Porphobili	HEM3_MOU	14	0.967007
30_MTA / C	P31725	0.329596	0.535883	0.541116	S100a9	Protein S1C	S10A9_MO	6	1.256662
15_MTA / C	Q80VP1	0.098302	0.536156	0.541116	Epn1	Epsin-1	EPN1_MOU	2	1.070513
15_MTA / C	Q8VCW8	-0.25506	0.535333	0.541116	Acsf2	Medium-ch	ACSF2_MO	13	0.837952

30_MTA / C	Q91ZJ5	-0.03108	0.535871	0.541116	Ugp2	UTP--glucos	UGPA_MOU	28	0.978689
30_MTA / C	Q9CQI6	0.022554	0.534429	0.541116	Cotl1	Coactosin-l	COTL1_MO	7	1.015756
15_MTA / C	Q9D0F9	0.054807	0.535993	0.541116	Pgm1	Phosphoglu	PGM1_MOU	36	1.03872
30_MTA / C	Q9EQ80	0.187599	0.536113	0.541116	Nif3l1	NIF3-like pr	NIF3L_MOU	7	1.138867
15_MTA / C	P11679	-0.04159	0.53687	0.541645	Krt8	Keratin, typ	K2C8_MOU	18	0.971585
15_MTA / C	Q6PDN3	-0.13637	0.537804	0.542171	Mylk	Myosin ligh	MYLK_MOU	8	0.909807
15_MTA / C	P56376	-0.4773	0.540919	0.544854	Acyp1	Acylphosph	ACYP1_MO	3	0.718321
30_MTA / C	P07361	0.010513	0.543134	0.546341	Orm2	Alpha-1-aci	A1AG2_MC	3	1.007313
15_MTA / C	P43883	-0.05736	0.543609	0.546341	Plin2	Perilipin-2	PLIN2_MOU	9	0.961022
30_MTA / C	Q11136	0.073426	0.543303	0.546341	Pepd	Xaa-Pro dip	PEPD_MOU	17	1.052212
30_MTA / C	Q8K023	-0.09539	0.543176	0.546341	Akr1c18	Aldo-keto r	AKC1H_MC	5	0.936021
30_MTA / C	Q8K182	0.072652	0.54334	0.546341	C8a	Compleme	CO8A_MOU	4	1.051648
30_MTA / C	Q62523	0.006077	0.545081	0.547515	Zyx	Zyxin	ZYX_MOUS	4	1.004221
30_MTA / C	Q9R0N0	-0.75222	0.546039	0.548324	Galk1	Galactokina	GALK1_MO	3	0.59369
30_MTA / C	P50431	-0.07476	0.546628	0.548513	Shmt1	Serine hydr	GLYC_MOU	15	0.949497
30_MTA / C	Q00897	0.112249	0.546685	0.548513	Serpina1d	Alpha-1-ant	A1AT4_MO	3	1.080912
30_MTA / C	Q9WVJ2	-0.09919	0.546513	0.548513	Psmd13	26S proteas	PSD13_MO	14	0.933555
30_MTA / C	Q99KJ8	-0.08227	0.547144	0.548798	Dctn2	Dynactin su	DCTN2_MC	7	0.94457
30_MTA / C	E9QAT4	0.786759	0.547752	0.548976	Sec16a	Protein tra	SC16A_MO	6	1.725194
30_MTA / C	O35459	-0.17236	0.548366	0.548976	Ech1	Delta(3,5)-l	ECH1_MOU	12	0.88739
15_MTA / C	O70591	0.009039	0.548025	0.548976	Pfdn2	Prefoldin su	PFD2_MOU	3	1.006285
15_MTA / C	P01642	-0.13188	0.54788	0.548976	Igkv5-48	Immunoglo	KV5A9_MO	2	0.91264
15_MTA / C	Q9R0P5	0.029127	0.548233	0.548976	Dstn	Destrin	DEST_MOU	11	1.020394
15_MTA / C	P51660	-0.06278	0.548714	0.549019	Hsd17b4	Peroxisoma	DHB4_MOU	21	0.957415
30_MTA / C	P06909	-0.04629	0.548973	0.549126	Cfh	Compleme	CFAH_MOU	34	0.96842
30_MTA / C	A2ADY9	0.012141	0.549661	0.549356	Ddi2	Protein DD	DDI2_MOU	16	1.008451
15_MTA / C	P49586	-0.74615	0.549554	0.549356	Pcyt1a	Choline-ph	PCY1A_MO	5	0.596192
30_MTA / C	Q9D8Y0	0.115912	0.550209	0.549602	Efhd2	EF-hand do	EFHD2_MC	5	1.08366
15_MTA / C	Q99L47	0.054063	0.551168	0.550404	St13	Hsc70-inter	F10A1_MO	9	1.038185
30_MTA / C	Q91WU0	-0.06845	0.552509	0.551267	Ces1f	Carboxyles	EST1F_MOU	29	0.953663
30_MTA / C	Q99J36	0.159951	0.552741	0.551267	Thumpd1	THUMP dor	THUM1_MC	3	1.117249
30_MTA / C	P01887	0.119125	0.55316	0.551475	B2m	Beta-2-micr	B2MG_MOU	2	1.086076
30_MTA / C	P11352	0.089765	0.553834	0.551536	Gpx1	Glutathion	GPX1_MOU	16	1.064197
30_MTA / C	Q3UJB0	-0.47371	0.553814	0.551536	Sf3b2	Splicing fac	SF3B2_MO	3	0.72011
30_MTA / C	Q91VR2	-0.48003	0.554338	0.55158	Atp5f1c	ATP synthas	ATPG_MOU	4	0.716964
30_MTA / C	Q9DBE0	-0.02766	0.554128	0.55158	Csad	Cysteine su	CSAD_MOU	23	0.981008
30_MTA / C	Q05920	0.114392	0.554807	0.551892	Pc	Pyruvate ca	PYC_MOUS	30	1.082519
15_MTA / C	Q8BWN8	-0.1783	0.555292	0.551892	Acot4	Peroxisoma	ACOT4_MO	3	0.883745
30_MTA / C	Q8CGA0	0.114957	0.555122	0.551892	Ppm1f	Protein pho	PPM1F_MC	2	1.082943
30_MTA / C	Q91VA0	0.028164	0.555418	0.551892	Acsm1	Acyl-coenzy	ACSM1_MC	25	1.019714
15_MTA / C	Q91WU0	-0.0466	0.55497	0.551892	Ces1f	Carboxyles	EST1F_MOU	29	0.968215
30_MTA / C	G5E8V9	-0.15364	0.555999	0.552164	Arfip1	Arfaptin-1	ARFP1_MO	1	0.898976
15_MTA / C	Q3U4I7	-0.01088	0.556187	0.552199	Pyroxd2	Pyridine nu	PYRD2_MO	6	0.992486
30_MTA / C	P12787	-0.13733	0.557142	0.552995	Cox5a	Cytochrom	COX5A_MC	2	0.9092
15_MTA / C	Q8VCC2	-0.05814	0.557922	0.553008	Ces1	Liver carbo	EST1_MOUS	14	0.960503
15_MTA / C	Q9EQK5	-0.08678	0.557584	0.553008	Mvp	Major vault	MVP_MOUS	34	0.94162
15_MTA / C	Q9QXK3	0.346168	0.557924	0.553008	Copg2	Coatomer s	COPG2_MC	5	1.27118
15_MTA / C	P58252	0.012783	0.558152	0.553082	Eef2	Elongation	EF2_MOUSI	57	1.0089
15_MTA / C	O08692	0.155555	0.558674	0.553142	Ngp	Neutrophil	NGP_MOUS	3	1.11385
30_MTA / C	P06728	-0.12759	0.558453	0.553142	Apoa4	Apolipopro	APOA4_MC	23	0.915356
30_MTA / C	Q80X50	-0.36809	0.558668	0.553142	Ubap2l	Ubiquitin-a	UBP2L_MO	3	0.774806
30_MTA / C	Q8BGT5	0.073233	0.559484	0.553792	Gpt2	Alanine am	ALAT2_MOI	6	1.052072

15_MTA / C	O08709	0.095132	0.560183	0.554129	Prdx6	Peroxiredo	PRDX6_MC	18	1.068163
30_MTA / C	P63037	-0.01563	0.56044	0.554129	Dnaja1	DnaJ homo	DNJA1_MC	8	0.989225
15_MTA / C	Q9DC50	0.237465	0.560287	0.554129	Crot	Peroxisoma	OCTC_MOU	2	1.17892
15_MTA / C	Q61599	0.063192	0.561618	0.554894	Arhgdib	Rho GDP-di	GDIR2_MC	3	1.044775
15_MTA / C	Q9CQ86	-0.16919	0.561676	0.554894	Mien1	Migration a	MIEN1_MC	3	0.889343
30_MTA / C	Q64727	-0.03689	0.561905	0.554968	Vcl	Vinculin	VINC_MOU	65	0.974752
30_MTA / C	Q2TPA8	-0.08599	0.562812	0.555559	Hsdl2	Hydroxyste	HSDL2_MC	2	0.942138
30_MTA / C	Q9D7X8	-0.05939	0.562727	0.555559	Ggct	Gamma-glu	GGCT_MOU	4	0.959672
30_MTA / C	P62737;P6	-0.00961	0.563029	0.555621	Acta2;Actg	Actin, aorti	ACTA_MOU	2	0.993363
30_MTA / C	Q7TQI3	-0.0743	0.563256	0.555693	Otub1	Ubiquitin t	OTUB1_MC	6	0.949805
15_MTA / C	O35943	-0.185	0.563496	0.555777	Fxn	Frataxin, m	FRDA_MOU	2	0.87965
30_MTA / C	Q9Z2W0	0.066425	0.564278	0.556396	Dnpep	Aspartyl am	DNPEP_MC	16	1.047118
30_MTA / C	P15532	-0.00963	0.564899	0.556521	Nme1	Nucleoside	NDKA_MOU	7	0.993347
15_MTA / C	P28658	-0.77051	0.564676	0.556521	Atxn10	Ataxin-10	ATX10_MO	5	0.586212
15_MTA / C	Q61171	0.085488	0.565229	0.556521	Prdx2	Peroxiredo	PRDX2_MC	15	1.061047
30_MTA / C	Q9D753	0.197325	0.565333	0.556521	Exosc8	Exosome cc	EXOS8_MO	1	1.14657
30_MTA / C	P17427	-0.01275	0.566268	0.55729	Ap2a2	AP-2 compl	AP2A2_MC	22	0.9912
30_MTA / C	Q61545	-0.26006	0.567192	0.55788	Ewsr1	RNA-bindir	EWS_MOUS	3	0.835051
30_MTA / C	Q9JIF7	0.049015	0.567333	0.55788	Copb1	Coatomer s	COPB_MOU	32	1.034558
15_MTA / C	P63017	0.098867	0.568506	0.558582	Hspa8	Heat shock	HSP7C_MC	37	1.070932
30_MTA / C	Q61029	0.454965	0.568295	0.558582	Tmpo	Lamina-ass	LAP2B_MO	3	1.370749
30_MTA / C	Q99MN9	0.070797	0.568823	0.558584	Pccb	Propionyl-(	PCCB_MOU	25	1.050297
30_MTA / C	P48758	0.067442	0.569355	0.5588	Cbr1	Carbonyl re	CBR1_MOU	20	1.047857
15_MTA / C	Q9WUP7	-0.37039	0.569341	0.5588	Uchl5	Ubiquitin c	UCHL5_MC	6	0.773576
15_MTA / C	P06745	0.011708	0.570039	0.559017	Gpi	Glucose-6-p	G6PI_MOU	23	1.008148
30_MTA / C	Q6NZJ6	0.101975	0.570042	0.559017	Eif4g1	Eukaryotic	IF4G1_MOU	21	1.073242
15_MTA / C	Q61176	-0.05262	0.570269	0.559088	Arg1	Arginase-1	ARGI1_MOI	23	0.964186
30_MTA / C	Q6IRU2	0.269896	0.570806	0.559157	Tpm4	Tropomyos	TPM4_MOU	6	1.205721
15_MTA / C	P14211	-0.00549	0.572098	0.559661	Calr	Calreticulir	CALR_MOU	20	0.996205
15_MTA / C	Q3UGC7;Q	0.038536	0.572078	0.559661	Eif3j1;Eif3j	Eukaryotic	EI3JA_MOU	8	1.027071
30_MTA / C	Q8VCX1	-0.11245	0.572095	0.559661	Akr1d1	Aldo-keto r	AK1D1_MC	18	0.925014
15_MTA / C	Q91WT9	0.070849	0.571631	0.559661	Cbs	Cystathioni	CBS_MOUS	26	1.050335
30_MTA / C	Q99LD8	-0.40456	0.572275	0.559682	Ddah2	Putative hy	DDAH2_MC	2	0.755469
30_MTA / C	P02798	0.427735	0.573083	0.560231	Mt2	Metallothi	MT2_MOUS	2	1.34512
15_MTA / C	Q8VBT2	-0.0439	0.5738	0.560564	Sds	L-serine del	SDHL_MOU	12	0.970029
15_MTA / C	P60521	0.146764	0.57544	0.561557	Gabarapl2	Gamma-am	GBRL2_MO	2	1.107083
15_MTA / C	Q8R4N0	0.048907	0.575395	0.561557	Clybl	Citramalyl-	CLYBL_MOI	10	1.034481
15_MTA / C	Q9DCU9	-0.02224	0.575917	0.561871	Hoga1	4-hydroxy-	HOGA1_MC	16	0.984705
30_MTA / C	P58710	-0.55154	0.576195	0.561989	Gulo	L-gulonolac	GGLO_MOU	3	0.682291
15_MTA / C	O55023	-0.11624	0.576701	0.562122	Impa1	Inositol mc	IMPA1_MO	8	0.922587
15_MTA / C	Q99020	0.14189	0.5772	0.56223	Hnrnpab	Heterogene	ROAA_MOU	5	1.103349
30_MTA / C	Q9D6J6	0.717282	0.577222	0.56223	Ndufv2	NADH dehy	NDUV2_MC	2	1.644082
30_MTA / C	A6X935	-0.07969	0.577808	0.562236	Itih4	Inter alpha	ITIH4_MOU	35	0.946261
30_MTA / C	Q64459	-1.01129	0.577743	0.562236	Cyp3a11	Cytochrom	CP3AB_MO	2	0.496103
15_MTA / C	Q6WVG3	0.041443	0.577853	0.562236	Kctd12	BTB/POZ dc	KCD12_MC	11	1.029142
30_MTA / C	Q91WU5	0.140794	0.577758	0.562236	As3mt	Arsenite me	AS3MT_MC	13	1.102512
30_MTA / C	Q8BH95	-0.0454	0.578308	0.562526	Echs1	Enoyl-CoA l	ECHM_MOU	16	0.96902
30_MTA / C	P83882	0.095246	0.578734	0.562636	Rpl36a	Large ribos	RL36A_MO	4	1.068248
15_MTA / C	Q921T2	-0.7919	0.578621	0.562636	Tor1aip1	Torsin-1A-i	TOIP1_MOU	1	0.577583
30_MTA / C	Q9D1I5	0.127544	0.579034	0.56269	Mcee	Methylmal	MCEE_MOU	3	1.092432
30_MTA / C	P53026	-0.07803	0.581426	0.564474	Rpl10a	Large ribos	RL10A_MO	9	0.947353
30_MTA / C	O35737	-0.32241	0.581944	0.564689	Hnrnph1	Heterogene	HNRH1_MC	3	0.799735

30_MTA / C	Q9JLI6	-0.03098	0.582191	0.564777	Scly	Selenocyste	SCLY_MOUS	16	0.978755
30_MTA / C	Q9D6Y7	0.08388	0.582427	0.564854	Msra	Mitochond	MSRA_MOU	9	1.059864
15_MTA / C	P97855	-0.06882	0.582723	0.564989	G3bp1	Ras GTPase-	G3BP1_MC	6	0.953418
15_MTA / C	Q61081	0.2112	0.583059	0.565162	Cdc37	Hsp90 co-c	CDC37_MC	6	1.15765
30_MTA / C	Q9CYZ2	0.121108	0.583386	0.565327	Tpd52l2	Tumor prot	TPD54_MO	5	1.08757
15_MTA / C	P99026	0.082534	0.583696	0.565475	Psmb4	Proteasom	PSB4_MOU	8	1.058877
15_MTA / C	Q99LC5	-0.0521	0.584458	0.566061	Etfa	Electron tra	ETFA_MOUS	17	0.964534
15_MTA / C	P01629	0.38509	0.58488	0.566105	NaN	Ig kappa ch	KV2A4_MO	1	1.305941
15_MTA / C	P16460	-0.00251	0.584759	0.566105	Ass1	Argininosu	ASSY_MOUS	27	0.998259
15_MTA / C	P97822	-0.03564	0.585245	0.566105	Anp32e	Acidic leuci	AN32E_MO	2	0.975602
15_MTA / C	Q6P1F6	-0.55158	0.585145	0.566105	Ppp2r2a	Serine/thre	2ABA_MOU	2	0.682274
30_MTA / C	Q8BH69	-0.24891	0.586282	0.56676	Sephs1	Selenide, w	SPS1_MOU	5	0.84153
15_MTA / C	O55142	0.17726	0.586523	0.566841	Rpl35a	Large ribos	RL35A_MO	7	1.130734
30_MTA / C	Q922E4	-0.02548	0.587058	0.566972	Pcyt2	Ethanolami	PCY2_MOU	20	0.982494
30_MTA / C	Q9JLB0	-0.73075	0.587131	0.566972	Pals2	Protein PAI	PALS2_MO	3	0.602591
30_MTA / C	Q9CPT4	-0.06081	0.587478	0.567155	Mydgf	Myeloid-de	MYDGF_MC	6	0.958725
30_MTA / C	O54754	-0.01749	0.588463	0.567801	Aox1	Aldehyde o	AOXA_MOU	50	0.987952
30_MTA / C	O88569	-0.05132	0.590012	0.568488	Hnrnpa2b1	Heterogene	ROA2_MOU	16	0.965056
30_MTA / C	P70274	-0.29076	0.589578	0.568488	Selenop	Selenoprot	SEPP1_MO	4	0.817471
30_MTA / C	Q9D8S9	-0.30029	0.58971	0.568488	Bola1	BolA-like pr	BOLA1_MO	5	0.812089
15_MTA / C	Q9ERH7	-0.25904	0.589862	0.568488	Hipk3	Homeodom	HIPK3_MO	1	0.835644
30_MTA / C	P34927	0.056244	0.590718	0.56854	Asgr1	Asialoglyco	ASGR1_MO	5	1.039755
30_MTA / C	P61979	0.022198	0.591811	0.56854	Hnrnpk	Heterogene	HNRPK_MC	20	1.015506
15_MTA / C	P97351	0.003809	0.591614	0.56854	Rps3a	Small ribos	RS3A_MOU	15	1.002644
30_MTA / C	Q8CDM8	-0.21091	0.59053	0.56854	Fhip2a	FHF comple	FHI2A_MOU	2	0.863991
30_MTA / C	Q99KB8	-0.0102	0.59187	0.56854	Hagh	Hydroxyacy	GLO2_MOU	12	0.992956
30_MTA / C	Q9D0R2	0.074668	0.591913	0.56854	Tars1	Threonine--	SYTC_MOUS	25	1.053118
30_MTA / C	P57780	-0.00473	0.592542	0.56878	Actn4	Alpha-actin	ACTN4_MO	38	0.996724
30_MTA / C	Q11011	0.077515	0.592542	0.56878	Npepps	Puromycin	PSA_MOUS	21	1.055199
30_MTA / C	Q9JI46	0.410801	0.592638	0.56878	Nudt3	Diphospho	NUDT3_MC	2	1.329424
30_MTA / C	P01878	0.054417	0.593913	0.569398	NaN	Ig alpha cha	IGHA_MOU	7	1.03844
15_MTA / C	P61161	-0.01476	0.593796	0.569398	Actr2	Actin-relate	ARP2_MOU	13	0.989822
15_MTA / C	Q9CR95	-0.05765	0.593704	0.569398	Necap1	Adaptin ear	NECP1_MO	6	0.960826
30_MTA / C	P24369	-0.0984	0.59476	0.56954	Ppib	Peptidyl-pr	PPIB_MOUS	11	0.934066
15_MTA / C	Q11011	-0.21507	0.594853	0.56954	Npepps	Puromycin	PSA_MOUS	21	0.861504
15_MTA / C	Q99MN9	0.099969	0.594817	0.56954	Pccb	Propionyl-(	PCCB_MOU	25	1.071751
30_MTA / C	Q9D7B6	-0.14412	0.594461	0.56954	Acad8	Isobutyryl-(	ACAD8_MC	5	0.904929
15_MTA / C	Q9WUU7	-0.00949	0.595095	0.56962	Ctsz	Cathepsin Z	CATZ_MOU	7	0.993442
30_MTA / C	Q3V4B5	0.191857	0.596267	0.570509	Commd6	COMM dom	COMD6_M	1	1.142233
30_MTA / C	Q8R1X6	-0.37072	0.596341	0.570509	Spart	Spartin	SPART_MO	3	0.773395
30_MTA / C	P05064	0.020373	0.597186	0.570818	Aldoa	Fructose-bi	ALDOA_MC	22	1.014222
30_MTA / C	Q9WUL7	0.114363	0.597298	0.570818	Arl3	ADP-ribosy	ARL3_MOU	1	1.082497
15_MTA / C	O09174	0.029019	0.598059	0.571394	Amacr	Alpha-meth	AMACR_MC	13	1.020318
15_MTA / C	P51855	0.044367	0.59842	0.571435	Gss	Glutathion	GSHB_MOU	13	1.031231
30_MTA / C	P49443	0.01167	0.598716	0.571516	Ppm1a	Protein phc	PPM1A_MC	10	1.008122
15_MTA / C	P00493	0.081167	0.599481	0.571539	Hprt1	Hypoxanth	HPRT_MOU	12	1.057873
15_MTA / C	P62264	0.331025	0.599418	0.571539	Rps14	Small ribos	RS14_MOU	3	1.257906
15_MTA / C	P97864	0.123116	0.599205	0.571539	Casp7	Caspase-7	CASP7_MO	3	1.089084
30_MTA / C	Q7TPR4	0.005401	0.599475	0.571539	Actn1	Alpha-actin	ACTN1_MO	45	1.003751
15_MTA / C	P12382	-0.32984	0.599881	0.571697	Pfkl	ATP-depenc	PFKAL_MO	4	0.795627
30_MTA / C	Q62048	0.56533	0.599964	0.571697	Pea15	Astrocytic p	PEA15_MO	3	1.479726
30_MTA / C	Q9CZL5	0.181823	0.600669	0.572217	Pcbd2	Pterin-4-alp	PHS2_MOU	5	1.134317

30_MTA / C	Q3TMH2	-0.12749	0.601064	0.572255	Scrn3	Secernin-3	SCRN3_MO	16	0.915421
15_MTA / C	Q91YR9	0.11358	0.601186	0.572255	Ptgr1	Prostaglanc	PTGR1_MO	2	1.08191
30_MTA / C	Q8BTY1	-0.05627	0.601613	0.57251	Kyat1	Kynurenine	KAT1_MOU	17	0.961745
30_MTA / C	Q7TSV4	0.141835	0.60293	0.573157	Pgm2	Phosphope	PGM2_MO	6	1.103308
15_MTA / C	Q91X52	0.464267	0.60266	0.573157	Dcxr	L-xylulose r	DCXR_MOU	4	1.379617
15_MTA / C	Q9Z0M5	-0.18831	0.603337	0.573393	Lipa	Lysosomal a	LICH_MOUS	7	0.877631
30_MTA / C	Q9WVJ3	0.037952	0.603624	0.573515	Cpq	Carboxypep	CBPQ_MOU	9	1.026656
30_MTA / C	O55137;Q9	0.426219	0.604808	0.574195	Acot1;Acot	Acyl-coenzy	ACOT1_MO	3	1.343707
15_MTA / C	P01636	-1.19517	0.604818	0.574195	NaN	Ig kappa ch	KV5A4_MO	1	0.436735
30_MTA / C	Q9CVB6	-0.0144	0.604699	0.574195	Arpc2	Actin-relate	ARPC2_MO	14	0.990067
15_MTA / C	Q9QXD6	0.06239	0.605186	0.57432	Fbp1	Fructose-1,	F16P1_MO	28	1.044194
30_MTA / C	B5X0G2	-0.28234	0.605477	0.574366	Mup17	Major urina	MUP17_MO	2	0.822256
30_MTA / C	Q9CQR6	-0.23102	0.605903	0.574495	Ppp6c	Serine/thre	PPP6_MOU	7	0.85203
15_MTA / C	P47955	0.012205	0.606798	0.575164	Rplp1	Large ribos	RLA1_MOU	3	1.008495
30_MTA / C	P47738	-0.05167	0.607618	0.575336	Aldh2	Aldehyde d	ALDH2_MO	20	0.964822
30_MTA / C	Q9D051	-0.10943	0.607568	0.575336	Pdhb	Pyruvate de	ODPB_MOU	2	0.926952
15_MTA / C	Q9D394	-0.34329	0.607971	0.575518	Rufy3	Protein RUI	RUFY3_MO	4	0.788241
15_MTA / C	P62242	0.16456	0.610094	0.576466	Rps8	Small ribos	RS8_MOUS	10	1.120824
30_MTA / C	P08607	0.045456	0.610464	0.576665	C4bpa	C4b-bindin	C4BPA_MO	8	1.032009
15_MTA / C	P01867	0.172505	0.610702	0.576738	Ighg2b	Immunoglo	IGG2B_MO	6	1.127013
30_MTA / C	Q8CC86	0.069924	0.611296	0.576996	Naprt	Nicotinate	PNCB_MOU	25	1.049662
15_MTA / C	Q62348	-0.06934	0.611476	0.577015	Tsn	Translin	TSN_MOUS	10	0.953077
15_MTA / C	P06801	0.133759	0.611924	0.577287	Me1	NADP-depe	MAOX_MO	34	1.097148
30_MTA / C	P47753	-0.02992	0.614113	0.579009	Capza1	F-actin-cap	CAZA1_MO	7	0.979473
15_MTA / C	Q03265	0.074897	0.613973	0.579009	Atp5f1a	ATP synthas	ATPA_MOU	20	1.053286
30_MTA / C	Q99L45	0.187189	0.614232	0.579009	Eif2s2	Eukaryotic	IF2B_MOUS	7	1.138544
30_MTA / C	Q99JX3	-0.0714	0.614921	0.579507	Gorasp2	Golgi reass	GORS2_MO	5	0.951714
30_MTA / C	Q91YR9	-0.00764	0.615464	0.579866	Ptgr1	Prostaglanc	PTGR1_MO	2	0.994721
30_MTA / C	P50171	0.171938	0.6161	0.580028	Hsd17b8	(3R)-3-hydr	DHB8_MOU	9	1.12657
15_MTA / C	Q9D3D9	0.184996	0.616118	0.580028	Atp5f1d	ATP synthas	ATPD_MOU	4	1.136814
30_MTA / C	Q9Z0M5	-0.2292	0.615827	0.580028	Lipa	Lysosomal a	LICH_MOUS	7	0.853107
15_MTA / C	Q9QYG0	0.043761	0.617445	0.580698	Ndrg2	Protein ND	NDRG2_MO	16	1.030798
30_MTA / C	P49312	-0.01639	0.617693	0.580751	Hnrnpa1	Heterogene	ROA1_MOU	8	0.988701
30_MTA / C	O54984	0.094339	0.619621	0.5809	Get3	ATPase GET	GET3_MOU	5	1.067576
15_MTA / C	P05213	0.032397	0.61923	0.5809	Tuba1b	Tubulin alp	TBA1B_MO	19	1.02271
30_MTA / C	P83940	0.09343	0.618728	0.5809	Eloc	Elongin-C	ELOC_MOU	5	1.066904
15_MTA / C	Q62418	-0.02536	0.618927	0.5809	Dbnl	Drebrin-like	DBNL_MOU	9	0.982576
30_MTA / C	Q78J03	0.713048	0.619626	0.5809	Msrb2	Methionine	MSRB2_MO	1	1.639264
30_MTA / C	Q8QZT1	0.000671	0.61826	0.5809	Acat1	Acetyl-CoA	THIL_MOUS	20	1.000465
15_MTA / C	Q91VM9	0.066509	0.619582	0.5809	Ppa2	Inorganic p	IPYR2_MOU	10	1.04718
15_MTA / C	Q9CPR4	0.056096	0.618457	0.5809	Rpl17	Large ribos	RL17_MOU	5	1.039648
15_MTA / C	Q9CZX8	0.296023	0.619378	0.5809	Rps19	Small ribos	RS19_MOU	3	1.227755
15_MTA / C	P51859	0.126598	0.620247	0.581179	Hdgf	Hepatoma-	HDGF_MOU	15	1.091717
30_MTA / C	P56959	0.099149	0.620188	0.581179	Fus	RNA-bindin	FUS_MOUS	5	1.071142
30_MTA / C	Q60972	-0.22548	0.621106	0.581832	Rbbp4	Histone-bir	RBBP4_MO	10	0.855309
15_MTA / C	Q8BFR5	0.010203	0.622602	0.582636	Tufm	Elongation	EFTU_MOU	22	1.007097
30_MTA / C	Q78JT3	-0.05743	0.623458	0.583022	Haao	3-hydroxya	3HAO_MOU	22	0.960973
30_MTA / C	Q8K2K6	0.219779	0.623672	0.583022	Agfg1	Arf-GAP do	AGFG1_MO	1	1.164555
15_MTA / C	Q9D7B6	-0.33161	0.623649	0.583022	Acad8	Isobutyryl-	ACAD8_MO	5	0.794651
30_MTA / C	P15105	0.096183	0.624106	0.583079	Glul	Glutamine	GLNA_MOU	10	1.068941
30_MTA / C	P27659	0.144053	0.624219	0.583079	Rpl3	Large ribos	RL3_MOUS	17	1.105005
15_MTA / C	Q9JIF7	-0.0144	0.623967	0.583079	Copb1	Coatomer s	COPB_MOU	32	0.990067

15_MTA / C	Q9EQU5	0.109284	0.625241	0.58358	Set	Protein SET	SET_MOUSE	5	1.078693
15_MTA / C	G3X982	-0.02996	0.625934	0.584075	Aox3	Aldehyde o	AOXC_MOU	65	0.979445
30_MTA / C	P63073	0.331372	0.626375	0.584184	Eif4e	Eukaryotic	IF4E_MOUS	1	1.258209
15_MTA / C	P62077	-0.14781	0.627302	0.58438	Timm8b	Mitochond	TIM8B_MO	3	0.902618
15_MTA / C	Q3UPL0	-0.09177	0.627302	0.58438	Sec31a	Protein tra	SC31A_MO	32	0.938374
15_MTA / C	Q9CQT1	0.066357	0.627197	0.58438	Mri1	Methylthio	MTNA_MOU	15	1.047069
30_MTA / C	Q7TMK9	0.090265	0.628014	0.584653	Syncrip	Heterogene	HNRPQ_MO	14	1.064566
15_MTA / C	P24527	0.020026	0.628436	0.584894	Lta4h	Leukotrien	LKHA4_MO	30	1.013978
30_MTA / C	Q9DCQ2	0.010129	0.628737	0.585022	Aspdh	Aspartate d	ASPDH_MO	18	1.007046
30_MTA / C	P60710	-0.0166	0.629297	0.585392	Actb	Actin, cyto	ACTB_MOU	21	0.988558
30_MTA / C	O70251	0.038326	0.63083	0.586473	Eef1b	Elongation	EF1B_MOU	7	1.026922
30_MTA / C	Q9CQ92	-0.01075	0.630947	0.586473	Fis1	Mitochond	FIS1_MOUS	3	0.992578
30_MTA / C	P26039	0.010069	0.631438	0.586762	Tln1	Talin-1	TLN1_MOU	55	1.007004
15_MTA / C	Q922B2	-0.73663	0.631584	0.586762	Dars1	Aspartate--	SYDC_MOU	7	0.60014
30_MTA / C	P38060	-0.16006	0.632706	0.587198	Hmgcl	Hydroxyme	HMGCL_MO	8	0.894986
30_MTA / C	Q9JIL4	-0.07671	0.632609	0.587198	Pdzk1	Na(+)/H(+) e	NHRF3_MO	26	0.948215
30_MTA / C	P50544	0.054194	0.633587	0.587561	Acadvl	Very long-c	ACADV_MO	32	1.038279
30_MTA / C	Q8K4Z3	0.052144	0.633547	0.587561	Naxe	NAD(P)H-hy	NNRE_MOU	9	1.036805
30_MTA / C	P62141	0.027403	0.633815	0.587621	Ppp1cb	Serine/thre	PP1B_MOU	2	1.019176
30_MTA / C	Q8CDN6	0.001084	0.63453	0.587982	Txnl1	Thioredoxi	TXNL1_MO	14	1.000752
30_MTA / C	Q9CZM2	0.118783	0.634842	0.588097	Rpl15	Large ribos	RL15_MOU	10	1.085819
30_MTA / C	P07309	-0.05005	0.635244	0.588189	Ttr	Transthyret	TTHY_MOU	6	0.9659
30_MTA / C	P08003	0.082819	0.635938	0.588227	Pdia4	Protein dis	PDIA4_MO	28	1.059085
15_MTA / C	P12970	0.118045	0.635493	0.588227	Rpl7a	Large ribos	RL7A_MOU	13	1.085263
30_MTA / C	P20029	0.087843	0.635733	0.588227	Hspa5	Endoplasm	BIP_MOUSE	32	1.06278
15_MTA / C	P97364	-0.02643	0.635806	0.588227	Sephs2	Selenide, w	SPS2_MOU	18	0.981845
30_MTA / C	O70435	-0.01029	0.636574	0.588401	Psma3	Proteasom	PSA3_MOU	13	0.992891
15_MTA / C	P23492	0.032249	0.636885	0.588401	Pnp	Purine nucl	PNPH_MOU	21	1.022605
30_MTA / C	Q99LL5	-0.18246	0.636728	0.588401	Pwp1	Periodic try	PWP1_MO	3	0.881202
30_MTA / C	Q99LX0	-0.02721	0.636944	0.588401	Park7	Parkinson c	PARK7_MO	14	0.981316
30_MTA / C	Q8BMJ3	-0.13909	0.637483	0.588748	Eif1ax	Eukaryotic	IF1AX_MOU	2	0.908094
15_MTA / C	P05201	-0.01797	0.637776	0.588854	Got1	Aspartate a	AATC_MOU	27	0.98762
30_MTA / C	Q9DCM0	-0.04888	0.637925	0.588854	Ethe1	Persulfide c	ETHE1_MO	11	0.966684
15_MTA / C	Q99J08	-0.01886	0.63842	0.589008	Sec14l2	SEC14-like	S14L2_MO	24	0.98701
30_MTA / C	P01899	-0.12353	0.638993	0.589294	H2-D1	H-2 class I h	HA11_MOU	2	0.917942
30_MTA / C	P21614	-0.00867	0.639384	0.589294	Gc	Vitamin D-l	VTDB_MOU	25	0.994009
30_MTA / C	P07759	-0.00978	0.639683	0.589418	Serpina3k	Serine prot	SPA3K_MO	14	0.993244
15_MTA / C	Q9DCW4	0.055885	0.639936	0.589501	Etfb	Electron tra	ETFB_MOUS	16	1.039496
15_MTA / C	Q9WVJ2	-0.00135	0.640685	0.589889	Psmd13	26S protea	PSD13_MO	14	0.999067
30_MTA / C	P25444	0.119746	0.641034	0.59006	Rps2	Small ribos	RS2_MOUS	17	1.086544
30_MTA / C	Q63836	0.021659	0.641405	0.59025	Selenbp2	Selenium-b	SBP2_MOU	3	1.015126
15_MTA / C	P16015	-0.04302	0.642192	0.590267	Ca3	Carbonic ar	CAH3_MOU	17	0.970619
15_MTA / C	Q60692	-0.01193	0.642244	0.590267	Psmb6	Proteasom	PSB6_MOU	7	0.991765
15_MTA / C	Q9D1M0	0.000294	0.642098	0.590267	Sec13	Protein SEC	SEC13_MO	7	1.000204
15_MTA / C	Q9JMD0	-0.03819	0.641755	0.590267	Znf207	BUB3-inter	ZN207_MO	2	0.973879
15_MTA / C	Q7M6Y3	-0.12175	0.643123	0.590701	Picalm	Phosphatic	PICAL_MOU	15	0.919075
30_MTA / C	O35309	-0.27696	0.644111	0.590881	Nmi	N-myc-inte	NMI_MOUS	7	0.825326
15_MTA / C	Q60972	-0.14743	0.644221	0.590881	Rbbp4	Histone-bir	RBBP4_MO	10	0.902857
30_MTA / C	Q80W22	0.004675	0.644224	0.590881	Thnsl2	Threonine s	THNS2_MO	23	1.003246
30_MTA / C	Q8VDK1	-0.02137	0.643781	0.590881	Nit1	Deaminate	NIT1_MOUS	19	0.985297
15_MTA / C	Q9Z0N1	-0.25851	0.643808	0.590881	Eif2s3x	Eukaryotic	IF2G_MOUS	4	0.835948
15_MTA / C	P01657	-0.20415	0.644887	0.591338	NaN	Ig kappa ch	KV3A5_MO	1	0.868047

30_MTA / C	Q9D819	-0.02306	0.645057	0.591343	Ppa1	Inorganic p	IPYR_MOUS	16	0.984142
15_MTA / C	Q99MZ7	0.015898	0.645717	0.591798	Pecr	Peroxisoma	PECR_MOU	8	1.01108
30_MTA / C	Q8VE62	0.341319	0.64626	0.591903	Paip1	Polyadenyl	PAIP1_MOU	5	1.266914
30_MTA / C	P25688	-0.07091	0.64791	0.5926	Uox	Uricase	URIC_MOU	22	0.952039
15_MTA / C	P51410	0.218917	0.64767	0.5926	Rpl9	Large ribos	RL9_MOUS	6	1.16386
15_MTA / C	Q64433	0.022066	0.648616	0.592945	Hspe1	10 kDa hea	CH10_MOU	9	1.015413
15_MTA / C	Q9D273	0.728184	0.648456	0.592945	Mmab	Corrinoid a	MMAB_MO	5	1.656553
30_MTA / C	P01801	0.691645	0.649058	0.593048	NaN	Ig heavy ch	HVM32_M	3	1.615124
30_MTA / C	P12382	0.471494	0.648953	0.593048	Pfkl	ATP-depend	PFKAL_MO	4	1.386545
30_MTA / C	P16045	-0.11544	0.64967	0.593456	Lgals1	Galectin-1	LEG1_MOU	5	0.923099
15_MTA / C	P62908	-0.03533	0.651856	0.593744	Rps3	Small ribos	RS3_MOUS	15	0.97581
15_MTA / C	P63087	-0.56729	0.651071	0.593744	Ppp1cc	Serine/thre	PP1G_MOU	2	0.674885
15_MTA / C	Q8K2K6	-0.35046	0.651793	0.593744	Agfg1	Arf-GAP do	AGFG1_MC	1	0.784331
30_MTA / C	Q9WTX5	-0.07063	0.651335	0.593744	Skp1	S-phase kin	SKP1_MOU	5	0.952221
30_MTA / C	P15327	-0.16685	0.652612	0.594034	Bpgm	Bisphosphc	PMGE_MOU	6	0.890784
30_MTA / C	Q8BVY0	-0.20141	0.653539	0.594727	Rsl1d1	Ribosomal	RL1D1_MO	2	0.869701
15_MTA / C	O35955	0.188664	0.653916	0.59492	Psmb10	Proteasom	PSB10_MO	5	1.139707
15_MTA / C	O35226	-0.12482	0.654128	0.594931	Psmd4	26S protea	PSMD4_MC	6	0.917119
30_MTA / C	P62751	-0.03416	0.654424	0.594931	Rpl23a	Large ribos	RL23A_MO	6	0.976597
30_MTA / C	Q8BFW7	0.05942	0.654408	0.594931	Lpp	Lipoma-pre	LPP_MOUS	13	1.042047
15_MTA / C	O08677	0.057303	0.655171	0.595006	Kng1	Kininogen-	KNG1_MOU	21	1.040519
30_MTA / C	P53811	-0.05571	0.655151	0.595006	Pitpnb	Phosphatid	PIPNB_MO	9	0.962121
15_MTA / C	Q3TMH2	-0.0723	0.655333	0.595006	Scrn3	Secernin-3	SCRN3_MO	16	0.95112
15_MTA / C	Q9DBB9	-0.18634	0.656413	0.595686	Cpn2	Carboxype	CPN2_MOU	7	0.878833
30_MTA / C	P18242	-0.00869	0.657033	0.596098	Ctsd	Cathepsin D	CATD_MOU	8	0.993994
15_MTA / C	P62254	0.905222	0.657467	0.596342	Ube2g1	Ubiquitin-c	UB2G1_MC	2	1.872833
30_MTA / C	P10648	-0.07076	0.657992	0.596367	Gsta2	Glutathion	GSTA2_MO	7	0.952135
15_MTA / C	P46471	0.00774	0.657821	0.596367	Psmc2	26S protea	PRS7_MOU	17	1.00538
30_MTA / C	Q9EQK5	0.122057	0.657845	0.596367	Mvp	Major vault	MVP_MOUS	34	1.088285
30_MTA / C	C0HKE1;C0	-0.21197	0.658642	0.596757	H2ac4;H2a	Histone H2	H2A1B_MC	3	0.863356
30_MTA / C	Q6A0A9	0.077969	0.659112	0.596757	FAM120A	Constitutiv	F120A_MO	5	1.055531
30_MTA / C	Q71RI9	-0.06903	0.66008	0.596757	Kyat3	Kynurenine	KAT3_MOU	20	0.953278
30_MTA / C	Q9JII5	0.067472	0.659392	0.596757	Dazap1	DAZ-associa	DAZP1_MO	8	1.047879
15_MTA / C	Q9QZ85	0.019317	0.659797	0.596757	Iigp1	Interferon-i	IIGP1_MOU	16	1.01348
15_MTA / C	Q9WVA4	-0.01356	0.659779	0.596757	Tagln2	Transgelin-	TAGL2_MO	8	0.990644
30_MTA / C	O55029	0.058542	0.660784	0.597094	Copb2	Coatomer s	COPB2_MC	40	1.041413
15_MTA / C	O89020	0.103567	0.661794	0.597557	Afm	Afamin	AFAM_MOU	12	1.074426
30_MTA / C	Q9DBT9	0.013866	0.661742	0.597557	Dmgdh	Dimethylgl	M2GD_MO	36	1.009657
30_MTA / C	Q8BFZ3	-0.07591	0.662336	0.597896	Actbl2	Beta-actin-l	ACTBL_MOU	1	0.948746
30_MTA / C	P62843	0.120247	0.662862	0.598221	Rps15	Small ribos	RS15_MOU	2	1.086921
30_MTA / C	P56480	0.102943	0.664109	0.59863	Atp5f1b	ATP synthas	ATPB_MOU	21	1.073962
30_MTA / C	Q3UPH1	0.236684	0.664168	0.59863	Prrc1	Protein PRI	PRRC1_MO	8	1.178281
30_MTA / C	Q62446	-0.14081	0.664154	0.59863	Fkbp3	Peptidyl-pr	FKBP3_MO	7	0.907013
30_MTA / C	Q8VCC2	0.081689	0.664761	0.59863	Ces1	Liver carbo	EST1_MOUS	14	1.058256
30_MTA / C	Q99KK7	0.240903	0.664521	0.59863	Dpp3	Dipeptidyl	DPP3_MOU	5	1.181732
30_MTA / C	Q9CYR6	-0.05592	0.664812	0.59863	Pgm3	Phosphoac	AGM1_MOU	4	0.961982
30_MTA / C	Q9QZE5	0.012575	0.663866	0.59863	Copg1	Coatomer s	COPG1_MC	33	1.008755
15_MTA / C	Q8BGL3	0.021468	0.665024	0.598671	Sult2a8	Sulfotransf	ST2A8_MO	14	1.014992
15_MTA / C	Q9R0P3	0.011918	0.665414	0.598873	Esd	S-formylglu	ESTD_MOU	13	1.008295
30_MTA / C	Q8VCA8	0.091365	0.666043	0.599181	Scrn2	Secernin-2	SCRN2_MO	14	1.065377
30_MTA / C	Q9WV55	0.152584	0.66609	0.599181	Vapa	Vesicle-assc	VAPA_MOU	3	1.111558
15_MTA / C	Q3U1J4	0.033698	0.666591	0.599183	Ddb1	DNA damag	DDB1_MOU	55	1.023633

30_MTA / C	Q9CPW2	-0.29172	0.66645	0.599183	Fdx2	Ferredoxin-	FDX2_MOU	1	0.816926
30_MTA / C	O08583	-0.04646	0.666913	0.599322	Alyref	THO compl	THOC4_MC	3	0.968309
15_MTA / C	Q00897	0.046985	0.667209	0.599439	Serpina1d	Alpha-1-ant	A1AT4_MO	3	1.033103
30_MTA / C	P07901	0.042739	0.668166	0.599517	Hsp90aa1	Heat shock	HS90A_MO	23	1.030068
15_MTA / C	P11589	-0.06052	0.668371	0.599517	Mup2	Major urina	MUP2_MOU	2	0.958915
30_MTA / C	P26041	0.050358	0.668345	0.599517	Msn	Moesin	MOES_MOU	10	1.035522
30_MTA / C	P61358	0.009036	0.668372	0.599517	Rpl27	Large ribos	RL27_MOU	6	1.006283
15_MTA / C	Q99K28	0.142097	0.668461	0.599517	Arfgap2	ADP-ribosy	ARFG2_MO	12	1.103508
15_MTA / C	Q61206	-0.16465	0.668632	0.59952	Pafah1b2	Platelet-act	PA1B2_MO	4	0.892143
15_MTA / C	O70456	0.328065	0.669595	0.600085	Sfn	14-3-3 prot	1433S_MO	5	1.255328
30_MTA / C	P54775	0.134253	0.66992	0.600227	Psmc4	26S protea	PRS6B_MO	8	1.097525
30_MTA / C	P17182	-0.02951	0.670362	0.600473	Eno1	Alpha-enol	ENOA_MOU	21	0.979754
15_MTA / C	G5E8V9	-0.15263	0.672575	0.601629	Arfip1	Arfaptin-1	ARFP1_MO	1	0.899608
15_MTA / C	P99027	0.05586	0.672978	0.601629	Rplp2	Large ribos	RLA2_MOU	6	1.039478
30_MTA / C	Q3TNA1	-0.29294	0.672989	0.601629	Xylb	Xylulose kir	XYLB_MOU	4	0.816236
30_MTA / C	P62911	0.028028	0.673645	0.601767	Rpl32	Large ribos	RL32_MOU	8	1.019618
30_MTA / C	Q3U1J4	0.03788	0.673863	0.601812	Ddb1	DNA damag	DDB1_MOU	55	1.026604
15_MTA / C	P11499	0.070513	0.674534	0.602234	Hsp90ab1	Heat shock	HS90B_MO	38	1.05009
15_MTA / C	Q06138	-0.0587	0.67467	0.602234	Cab39	Calcium-bi	CAB39_MO	8	0.960131
30_MTA / C	P38647	0.089354	0.676743	0.602779	Hspa9	Stress-70 p	GRP75_MC	38	1.063894
15_MTA / C	Q61598	-0.01297	0.676807	0.602779	Gdi2	Rab GDP di	GDIB_MOU	18	0.99105
15_MTA / C	Q8VBW6	-0.46625	0.675945	0.602779	Nae1	NEDD8-acti	ULA1_MOU	5	0.723845
15_MTA / C	Q91WU5	-0.24324	0.676662	0.602779	As3mt	Arsenite me	AS3MT_MC	13	0.844847
30_MTA / C	Q9WUM3	-0.12795	0.677051	0.602779	Coro1b	Coronin-1E	COR1B_MC	6	0.915131
30_MTA / C	P05063	0.221946	0.677751	0.603041	Aldoc	Fructose-bi	ALDOC_MC	5	1.166306
30_MTA / C	P01837	-0.1164	0.678174	0.603268	Igkc	Immunoglo	IGKC_MOU	6	0.922485
30_MTA / C	P11276	-0.01219	0.678757	0.60339	Fn1	Fibronectir	FINC_MOUS	30	0.991588
15_MTA / C	Q5XJY5	0.062307	0.678671	0.60339	Arcn1	Coatomer s	COPD_MOU	26	1.044134
30_MTA / C	P13020	0.153138	0.680282	0.604094	Gsn	Gelsolin	GELS_MOU	3	1.111985
30_MTA / C	Q62422	0.286561	0.680612	0.604094	Ostf1	Osteoclast-	OSTF1_MO	7	1.219729
15_MTA / C	P46412	-0.08264	0.682482	0.605157	Gpx3	Glutathion	GPX3_MOU	2	0.944331
15_MTA / C	Q8VE70	-0.55274	0.683353	0.605488	Pdcd10	Programme	PDC10_MC	4	0.681726
15_MTA / C	P61290	-0.09128	0.684291	0.605963	Psme3	Proteasom	PSME3_MC	6	0.938691
30_MTA / C	Q91YH6	0.906961	0.684446	0.605963	Atp6v1b1	V-type prot	VATB1_MO	1	1.875091
30_MTA / C	Q9Z1N5	-0.07724	0.684486	0.605963	Ddx39b	Spliceosom	DX39B_MC	14	0.947873
15_MTA / C	Q9CWK8	-0.22987	0.685009	0.606203	Snx2	Sorting nex	SNX2_MOU	12	0.852714
30_MTA / C	P13707	-0.02316	0.685525	0.606212	Gpd1	Glycerol-3-	GPDA_MOU	18	0.984074
30_MTA / C	P62878	-0.03633	0.685428	0.606212	Rbx1	E3 ubiquiti	RBX1_MOU	2	0.975134
30_MTA / C	Q9WUK2	0.304337	0.685492	0.606212	Eif4h	Eukaryotic	IF4H_MOUS	7	1.234851
15_MTA / C	P51174	0.00145	0.68634	0.606635	Acadl	Long-chain	ACADL_MO	22	1.001005
30_MTA / C	P11499	-0.00135	0.686793	0.606886	Hsp90ab1	Heat shock	HS90B_MO	38	0.999063
30_MTA / C	P04247	-0.40154	0.687559	0.606915	Mb	Myoglobin	MYG_MOUS	1	0.75705
30_MTA / C	P41317	0.265695	0.687278	0.606915	Mbl2	Mannose-b	MBL2_MOU	4	1.202215
15_MTA / C	P56213	-0.18238	0.687837	0.606915	Gfer	FAD-linked	ALR_MOUS	4	0.881248
15_MTA / C	P63073	0.169515	0.687758	0.606915	Eif4e	Eukaryotic	IF4E_MOUS	1	1.124681
15_MTA / C	Q921X9	0.049415	0.687646	0.606915	Pdia5	Protein dis	PDIA5_MO	13	1.034845
15_MTA / C	F6ZDS4	-0.54867	0.688147	0.60704	Tpr	Nucleoprot	TPR_MOUS	8	0.68365
30_MTA / C	Q5SWU9	-0.33993	0.6889	0.607406	Acaca	Acetyl-CoA	ACACA_MO	5	0.790077
15_MTA / C	P97450	-0.00782	0.690158	0.607772	Atp5pf	ATP synthas	ATP5J_MOU	4	0.994596
15_MTA / C	Q62048	0.348894	0.689862	0.607772	Pea15	Astrocytic p	PEA15_MO	3	1.273584
30_MTA / C	Q571E4	0.295635	0.690402	0.607837	Galns	N-acetylgal	GALNS_MO	7	1.227425
30_MTA / C	A2AJL3	-0.00442	0.690659	0.607915	Fggy	FGGY carbc	FGGY_MOU	22	0.996944

30_MTA / C	Q91XD4	-0.04837	0.690933	0.608007	Ftcd	Formimido	FTCD_MOU	23	0.96703
15_MTA / C	Q9Z1D1	0.142259	0.691876	0.608689	Eif3g	Eukaryotic	EIF3G_MOU	11	1.103632
15_MTA / C	A3KMP2	0.006698	0.692579	0.608702	Ttc38	Tetratricop	TTC38_MO	16	1.004653
30_MTA / C	Q8VDD5	-0.01738	0.692935	0.608728	Myh9	Myosin-9	MYH9_MOU	20	0.988028
30_MTA / C	P56375	0.199651	0.693625	0.609037	Acyp2	Acylphosph	ACYP2_MO	1	1.148421
30_MTA / C	Q9CQM9	-0.04633	0.693815	0.609055	Glrx3	Glutaredox	GLRX3_MO	10	0.968399
15_MTA / C	P47791	0.004087	0.695115	0.609898	Gsr	Glutathion	GSHR_MOU	18	1.002837
15_MTA / C	O35344	0.187848	0.698815	0.61038	Kpna3	Importin su	IMA4_MOU	2	1.139064
15_MTA / C	O70172	-0.18269	0.69836	0.61038	Pip4k2a	Phosphatic	PI42A_MOU	1	0.88106
30_MTA / C	O89079	0.082371	0.700416	0.61038	Cope	Coatomer s	COPE_MOU	8	1.058757
15_MTA / C	P01027	0.011854	0.697818	0.61038	C3	Complemer	CO3_MOUS	100	1.008251
30_MTA / C	P16332	0.107538	0.697635	0.61038	Mmut	Methylmal	MUTA_MOU	12	1.077388
15_MTA / C	P46664	0.080875	0.699706	0.61038	Adss2	Adenylosuc	PURA2_MO	18	1.057659
15_MTA / C	P62305	0.005752	0.701089	0.61038	Snrpe	Small nucle	RUXE_MOU	1	1.003995
15_MTA / C	Q71RI9	0.039695	0.700947	0.61038	Kyat3	Kynurenine	KAT3_MOU	20	1.027897
30_MTA / C	Q78PY7	-0.01782	0.700299	0.61038	Snd1	Staphyloco	SND1_MOU	13	0.987726
15_MTA / C	Q7TQH0	-0.04843	0.700474	0.61038	Atxn2l	Ataxin-2-lik	ATX2L_MOU	5	0.966989
30_MTA / C	Q8BG32	0.247266	0.699021	0.61038	Psmd11	26S proteas	PSD11_MO	13	1.186956
30_MTA / C	Q8BT60	0.312007	0.698293	0.61038	Cpne3	Copine-3	CPNE3_MO	8	1.241433
15_MTA / C	Q8CFX1	0.37402	0.700954	0.61038	H6pd	GDH/6PGL	G6PE_MOU	11	1.295959
15_MTA / C	Q8R146	0.037876	0.698666	0.61038	Apeh	Acylamino-	ACPH_MOU	20	1.026601
30_MTA / C	Q8R1B4	0.131794	0.697277	0.61038	Eif3c	Eukaryotic	EIF3C_MOU	6	1.095656
30_MTA / C	Q91Z53	-0.05442	0.699479	0.61038	Grhpr	Glyoxylate	GRHPR_MO	14	0.962982
30_MTA / C	Q99KV1	0.285511	0.700449	0.61038	Dnajb11	DnaJ homo	DJB11_MO	4	1.218842
30_MTA / C	Q9CQE8	-0.60492	0.69735	0.61038	RTRAF	RNA transc	RTRAF_MOU	5	0.657509
30_MTA / C	Q9D7P9	-0.02242	0.699271	0.61038	Serpinb12	Serpin B12	SPB12_MO	1	0.984583
30_MTA / C	Q9JHU9	-0.0401	0.699682	0.61038	Isyna1	Inositol-3-p	INO1_MOU	11	0.97259
30_MTA / C	Q9QXG4	0.09108	0.698671	0.61038	Acss2	Acetyl-coer	ACSA_MOU	11	1.065167
30_MTA / C	Q9WTP6	-0.06397	0.69831	0.61038	Ak2	Adenylate k	KAD2_MOU	17	0.956625
30_MTA / C	Q9WV98	-0.75814	0.697262	0.61038	Timm9	Mitochond	TIM9_MOU	1	0.591257
30_MTA / C	P01868;P0	0.208446	0.701944	0.610976	Ighg1	Ig gamma-1	IGHG1_MO	11	1.155443
30_MTA / C	Q91VM9	-0.09603	0.702419	0.611094	Ppa2	Inorganic p	IPYR2_MOU	10	0.935606
30_MTA / C	Q99KQ4	0.140918	0.702398	0.611094	Nampt	Nicotinami	NAMPT_MO	21	1.102606
30_MTA / C	P01629	0.324974	0.702919	0.611234	NaN	Ig kappa ch	KV2A4_MO	1	1.252642
30_MTA / C	P19221	-0.03798	0.704276	0.61197	F2	Prothromb	THRB_MOU	14	0.974019
30_MTA / C	P17751	-0.01547	0.705468	0.612415	Tpi1	Triosephos	TPIS_MOUS	16	0.989333
30_MTA / C	Q02780	-0.31038	0.705373	0.612415	Nfia	Nuclear fac	NFIA_MOUS	3	0.806428
15_MTA / C	O54879	0.476931	0.708148	0.61243	Hmgb3	High mobil	HMGB3_MO	1	1.39178
30_MTA / C	O88544	-0.06106	0.70669	0.61243	Cops4	COP9 signa	CSN4_MOU	14	0.95856
15_MTA / C	P32261	-0.07344	0.707928	0.61243	Serpinc1	Antithromb	ANT3_MOU	16	0.950366
30_MTA / C	Q58A65	-0.15807	0.708101	0.61243	Spag9	C-Jun-amin	JIP4_MOUS	15	0.896226
30_MTA / C	Q60759	-0.20154	0.707703	0.61243	Gcdh	Glutaryl-Cc	GCDH_MOU	10	0.869622
30_MTA / C	Q62448	0.159802	0.707915	0.61243	Eif4g2	Eukaryotic	IF4G2_MOU	8	1.117134
30_MTA / C	Q8BGQ7	-0.09296	0.705918	0.61243	Aars1	Alanine--tR	SYAC_MOU	27	0.937595
30_MTA / C	Q8CI51	0.04991	0.705944	0.61243	Pdlim5	PDZ and LIN	PDLI5_MOU	15	1.0352
30_MTA / C	Q8K157	-0.01344	0.707252	0.61243	Galm	Galactose n	GALM_MOU	10	0.990729
30_MTA / C	Q922J3	0.251424	0.707005	0.61243	Clip1	CAP-Gly do	CLIP1_MOU	4	1.190382
15_MTA / C	Q9EPU0	-0.63965	0.708367	0.61243	Upf1	Regulator c	RENT1_MO	16	0.641868
30_MTA / C	Q9R0Q7	-0.0737	0.708378	0.61243	Ptges3	Prostaglan	TEBP_MOU	7	0.9502
15_MTA / C	P20029	0.025119	0.709094	0.612608	Hspa5	Endoplasm	BIP_MOUSI	32	1.017563
15_MTA / C	P62259	0.027126	0.708944	0.612608	Ywhae	14-3-3 prot	1433E_MO	20	1.01898
15_MTA / C	O09159	-0.12481	0.70935	0.612682	Man2b1	Lysosomal	MA2B1_MO	12	0.917125

15_MTA / C	Q01853	0.040005	0.71011	0.613191	Vcp	Transitiona	TERA_MOU	56	1.028118
15_MTA / C	O70570	-0.19296	0.710954	0.613303	Pigr	Polymeric i	PIGR_MOU	8	0.874811
30_MTA / C	P47199	0.036558	0.71091	0.613303	Cryz	Quinone ox	QOR_MOUS	16	1.025664
15_MTA / C	Q07797	-0.35563	0.711091	0.613303	Lgals3bp	Galectin-3-	LG3BP_MO	8	0.781528
15_MTA / C	Q07813	-0.10024	0.710805	0.613303	Bax	Apoptosis r	BAX_MOUS	5	0.932876
30_MTA / C	Q8JZK9	0.020098	0.710601	0.613303	Hmgcs1	Hydroxyme	HMCS1_MO	7	1.014028
30_MTA / C	Q99L47	0.002851	0.711456	0.613324	St13	Hsc70-inte	F10A1_MO	9	1.001978
30_MTA / C	P01867	0.110136	0.712624	0.613605	Ighg2b	Immunoglo	IGG2B_MO	6	1.07933
15_MTA / C	P02769	0.132951	0.712634	0.613605	ALB	Serum albu	ALBU_BOVI	77	1.096534
15_MTA / C	P16125	0.27918	0.71251	0.613605	Ldhb	L-lactate de	LDHB_MOU	8	1.213505
30_MTA / C	Q99L20	0.001743	0.712555	0.613605	Gstt3	Glutathion	GSTT3_MO	11	1.001209
30_MTA / C	Q9Z224	-0.23241	0.712425	0.613605	Mocs2	Molybdopt	MOC2A_MO	4	0.851212
15_MTA / C	P45376	-0.01013	0.712835	0.613632	Akr1b1	Aldo-keto r	ALDR_MOU	20	0.993004
30_MTA / C	Q6PDN3	-0.01683	0.713302	0.613887	Mylk	Myosin ligh	MYLK_MOU	8	0.988399
15_MTA / C	Q9D0M1	1.451308	0.713882	0.614125	Prpsap1	Phosphorib	KPRA_MOU	2	2.734558
30_MTA / C	Q9WUR2	-0.57917	0.71427	0.614279	Eci2	Enoyl-CoA d	ECI2_MOUS	4	0.669351
30_MTA / C	Q9R0P5	-0.03219	0.71459	0.614407	Dstn	Destrin	DEST_MOU	11	0.977931
30_MTA / C	P62821	-0.00438	0.714905	0.614496	Rab1a	Ras-related	RAB1A_MO	7	0.996968
30_MTA / C	Q64152	0.10656	0.715034	0.614496	Btf3	Transcripti	BTF3_MOU	4	1.076658
30_MTA / C	O08601	0.001458	0.715804	0.614591	Mttp	Microsoma	MTP_MOUS	28	1.001011
30_MTA / C	Q9DCG6	-0.04041	0.715827	0.614591	Pbld1	Phenazine b	PBLD1_MO	9	0.97238
15_MTA / C	Q9ESB3	0.046638	0.715544	0.614591	Hrg	Histidine-ri	HRG_MOUS	10	1.032855
30_MTA / C	Q9JLZ3	-0.42371	0.715378	0.614591	Auh	Methylglut	AUHM_MO	5	0.745505
15_MTA / C	P01741	0.069634	0.716827	0.615009	NaN	Ig heavy ch	HVM00_MO	1	1.04945
30_MTA / C	P50429	-0.59461	0.716732	0.615009	Arsb	Arylsulfatas	ARSB_MOU	2	0.662223
30_MTA / C	Q8BP67	-0.0537	0.716643	0.615009	Rpl24	Large ribos	RL24_MOU	6	0.963463
30_MTA / C	Q8BH00	-0.22777	0.717011	0.61502	Aldh8a1	2-aminomu	AL8A1_MO	15	0.853954
15_MTA / C	P80313	0.091764	0.71771	0.615326	Cct7	T-complex p	TCPH_MOU	16	1.065672
30_MTA / C	Q99KC8	0.061076	0.718502	0.615712	Vwa5a	von Willebr	VMA5A_MO	29	1.043243
30_MTA / C	Q8BFY9	-0.1816	0.719135	0.615961	Tnpo1	Transportir	TNPO1_MO	4	0.881724
30_MTA / C	P24527	0.027299	0.72011	0.616503	Lta4h	Leukotrien	LKHA4_MO	30	1.019102
30_MTA / C	P01631	0.273599	0.72067	0.616836	NaN	Ig kappa ch	KV2A7_MO	3	1.20882
30_MTA / C	P61087	-0.16962	0.721622	0.617211	Ube2k	Ubiquitin-c	UBE2K_MO	7	0.889074
15_MTA / C	P61924	0.192279	0.721984	0.617227	Copz1	Coatomer s	COPZ1_MO	7	1.142567
30_MTA / C	Q99JW2	-0.10169	0.721956	0.617227	Acy1	Aminoacyla	ACY1_MOU	20	0.931939
30_MTA / C	P58058	-0.16615	0.722173	0.617242	Nadk	NAD kinase	NADK_MOU	8	0.891218
15_MTA / C	P01671	-0.13328	0.723351	0.617955	NaN	Ig kappa ch	KV3AJ_MO	3	0.911758
30_MTA / C	Q3TXS7	0.004869	0.724518	0.618659	Psmd1	26S protea	PSMD1_MO	34	1.003381
15_MTA / C	P63242	-0.01226	0.724877	0.618819	Eif5a	Eukaryotic	IF5A1_MOU	11	0.991541
15_MTA / C	Q60648	0.189241	0.725435	0.619002	Gm2a	Ganglioside	SAP3_MOU	3	1.140164
15_MTA / C	Q9D1J3	0.087805	0.725616	0.619009	Sarnp	SAP domain	SARNP_MO	4	1.062752
30_MTA / C	Q91V09	0.173118	0.725925	0.619126	Wdr13	WD repeat-	WDR13_MO	2	1.127492
30_MTA / C	P80315	0.046063	0.727228	0.619551	Cct4	T-complex p	TCPD_MOU	22	1.032444
30_MTA / C	Q07813	0.184415	0.727284	0.619551	Bax	Apoptosis r	BAX_MOUS	5	1.136356
30_MTA / C	Q68FD5	0.049555	0.727248	0.619551	Cltc	Clathrin he	CLH1_MOU	18	1.034946
30_MTA / C	Q6WVG3	-0.01791	0.726957	0.619551	Kctd12	BTB/POZ do	KCD12_MO	11	0.987665
15_MTA / C	Q9CQJ6	0.009442	0.728032	0.619885	Denr	Density-reg	DENR_MOU	6	1.006566
15_MTA / C	P13707	0.074703	0.730213	0.620332	Gpd1	Glycerol-3-	GPDA_MOU	18	1.053144
30_MTA / C	Q8BI84	0.176878	0.730003	0.620332	Mia3	Transport a	TGO1_MOU	2	1.130435
30_MTA / C	Q8VCF0	-0.09003	0.729636	0.620332	Mavs	Mitochond	MAVS_MOU	7	0.939502
30_MTA / C	Q99J08	-0.0648	0.730036	0.620332	Sec14l2	SEC14-like p	S14L2_MO	24	0.956077
15_MTA / C	Q9CQN1	0.120655	0.730268	0.620332	Trap1	Heat shock	TRAP1_MO	21	1.087228

30_MTA / C	Q9D1G1	0.045456	0.730166	0.620332	Rab1b	Ras-related	RAB1B_MO	1	1.03201
15_MTA / C	Q6A0A9	-0.44394	0.731626	0.621046	FAM120A	Constitutiv	F120A_MO	5	0.735123
15_MTA / C	P00920	0.081009	0.731865	0.621102	Ca2	Carbonic ar	CAH2_MOU	9	1.057757
30_MTA / C	Q9CXF0	-0.03099	0.732802	0.621457	Kynu	Kynurenina	KYNU_MOU	12	0.978746
15_MTA / C	Q9D7X3	-0.767	0.733037	0.62151	Dusp3	Dual specifi	DUS3_MOU	1	0.587638
30_MTA / C	Q64433	0.016939	0.733255	0.621549	Hspe1	10 kDa heat	CH10_MOU	9	1.01181
30_MTA / C	P47963	0.140918	0.734009	0.621603	Rpl13	Large ribos	RL13_MOU	8	1.102606
30_MTA / C	Q9CR51	0.238513	0.733874	0.621603	Atp6v1g1	V-type prot	VATG1_MO	2	1.179776
15_MTA / C	Q62167	-0.21478	0.734587	0.621946	Ddx3x	ATP-depend	DDX3X_MC	9	0.861677
30_MTA / C	Q61133	-0.10423	0.735687	0.622584	Gstt2	Glutathion	GSTT2_MO	13	0.930298
30_MTA / C	P68372	0.008623	0.736354	0.62281	Tubb4b	Tubulin bet	TBB4B_MO	18	1.005995
30_MTA / C	P68373	-0.10617	0.736645	0.62281	Tuba1c	Tubulin alp	TBA1C_MO	1	0.929054
30_MTA / C	Q3U0V1	0.173253	0.736502	0.62281	Khsrp	Far upstrea	FUBP2_MO	14	1.127598
30_MTA / C	P62317	-0.11247	0.736886	0.622867	Snrpd2	Small nucle	SMD2_MOU	7	0.925
30_MTA / C	P28665	-0.03176	0.738046	0.623582	Mug1	Murinoglol	MUG1_MO	59	0.978226
30_MTA / C	P28798	0.028862	0.739244	0.624141	Grn	Progranulir	GRN_MOUS	5	1.020207
15_MTA / C	P63323	0.011443	0.739405	0.624141	Rps12	Small ribos	RS12_MOU	8	1.007963
30_MTA / C	Q9JM62	-0.01395	0.740016	0.624193	Reep6	Receptor ex	REEP6_MO	2	0.990378
30_MTA / C	Q02053	-0.11487	0.740368	0.624344	Uba1	Ubiquitin-l	UBA1_MOU	15	0.923467
30_MTA / C	P46656	0.107986	0.74247	0.624421	Fdx1	Adrenodox	ADX_MOUS	5	1.077722
15_MTA / C	P59325	0.088439	0.741849	0.624421	Eif5	Eukaryotic	IF5_MOUSE	13	1.063219
15_MTA / C	Q05920	0.120443	0.742613	0.624421	Pc	Pyruvate ca	PYC_MOUS	30	1.087069
30_MTA / C	Q8K183	0.073985	0.741087	0.624421	Pdxk	Pyridoxal k	PDXK_MOU	11	1.05262
30_MTA / C	Q920E5	-0.44081	0.742714	0.624421	Fdps	Farnesyl py	FPPS_MOU	11	0.736723
15_MTA / C	Q99JW4	0.355735	0.741974	0.624421	Lims1	LIM and ser	LIMS1_MO	4	1.279637
15_MTA / C	P35441	-0.25829	0.744347	0.625268	Thbs1	Thrombosp	TSP1_MOU	4	0.836078
15_MTA / C	Q6P5E4	0.1117	0.744309	0.625268	Uggt1	UDP-glucos	UGGG1_MC	20	1.080501
15_MTA / C	Q9CPU0	-0.02068	0.744813	0.625455	Glo1	Lactoylglut	LGUL_MOU	13	0.985766
30_MTA / C	O88342	-0.02079	0.746941	0.626219	Wdr1	WD repeat-	WDR1_MO	19	0.985691
15_MTA / C	P25444	0.065653	0.746263	0.626219	Rps2	Small ribos	RS2_MOUS	17	1.046559
30_MTA / C	P51859	0.189377	0.746188	0.626219	Hdgf	Hepatoma-	HDGF_MOU	15	1.140271
30_MTA / C	P23591	0.139182	0.747379	0.626295	Gfus	GDP-L-fuco	FCL_MOUSI	10	1.10128
30_MTA / C	P01675;P0	0.015423	0.748528	0.626443	NaN	Ig kappa ch	KV6A1_MO	1	1.010748
15_MTA / C	P19536	-0.0316	0.748579	0.626443	Cox5b	Cytochrom	COX5B_MC	2	0.978336
15_MTA / C	P70441	0.073516	0.74805	0.626443	Nherf1	Na(+)/H(+) e	NHRF1_MC	19	1.052278
15_MTA / C	Q80W22	0.084629	0.748728	0.626443	Thnsl2	Threonine s	THNS2_MO	23	1.060415
30_MTA / C	Q8CIE6	0.077064	0.748453	0.626443	Copa	Coatomer s	COPA_MOU	33	1.054869
15_MTA / C	Q9DBB8	0.012767	0.748774	0.626443	Dhdh	Trans-1,2-d	DHDH_MO	20	1.008889
15_MTA / C	P10711	-0.11367	0.749113	0.626492	Tcea1	Transcripti	TCEA1_MO	9	0.924232
30_MTA / C	P16546	-0.34072	0.750146	0.626559	Sptan1	Spectrin alp	SPTN1_MO	32	0.789645
30_MTA / C	P35585	0.017708	0.751001	0.626559	Ap1m1	AP-1 compl	AP1M1_MC	11	1.01235
15_MTA / C	P51910	-0.42535	0.750233	0.626559	Apod	Apolipoprc	APOD_MOU	1	0.74466
30_MTA / C	Q76MZ3	0.029432	0.750742	0.626559	Ppp2r1a	Serine/thre	2AAA_MOU	24	1.02061
30_MTA / C	Q8K2Q0	-0.24395	0.750563	0.626559	Commd9	COMM don	COMD9_M	3	0.844432
30_MTA / C	Q9CPX6	0.016531	0.749818	0.626559	Atg3	Ubiquitin-l	ATG3_MOU	5	1.011524
15_MTA / C	Q9DBX2	-0.90096	0.74955	0.626559	Pdcl	Phosducin-	PHLP_MOU	1	0.535529
30_MTA / C	Q63880	0.023389	0.751349	0.626705	Ces3a	Carboxyles	EST3A_MOU	10	1.016344
30_MTA / C	P62717	0.144342	0.753774	0.627804	Rpl18a	Large ribos	RL18A_MO	9	1.105226
30_MTA / C	Q6ZWV3	-0.00612	0.753658	0.627804	Rpl10	Large ribos	RL10_MOU	9	0.99577
30_MTA / C	Q91YP2	-0.19713	0.753936	0.627804	Nln	Neurolysin,	NEUL_MOU	10	0.872286
15_MTA / C	Q9ET54	-0.53126	0.75396	0.627804	Palld	Palladin	PALLD_MO	5	0.691949
30_MTA / C	Q60737	-0.23285	0.754263	0.627826	Csnk2a1	Casein kina	CSK21_MO	3	0.85095

15_MTA / C	P62748	0.624948	0.754695	0.62789	Hpcal1	Hippocalci	HPCL1_MO	1	1.542156
30_MTA / C	Q8BPB0;Q9	0.504521	0.754935	0.62789	Mob1b;Mo	MOB kinase	MOB1B_M	1	1.418653
15_MTA / C	Q8C196	0.065042	0.755226	0.627902	Cps1	Carbamoyl-	CPSM_MOU	85	1.046116
15_MTA / C	P20065;Q6	-0.01665	0.755641	0.627956	Tmsb4x;Tm	Thymosin b	TYB4_MOU	1	0.988523
30_MTA / C	Q8VBT2	-0.03762	0.75556	0.627956	Sds	L-serine deh	SDHL_MOU	12	0.974263
15_MTA / C	O09173	0.00243	0.756361	0.628198	Hgd	Homogenti	HGD_MOUS	27	1.001686
30_MTA / C	P20918	-0.05574	0.756455	0.628198	Plg	Plasminoge	PLMN_MOU	19	0.962099
30_MTA / C	Q9DBB9	-0.1723	0.757051	0.628548	Cpn2	Carboxypep	CPN2_MOU	7	0.887424
15_MTA / C	E9Q414	0.053122	0.759425	0.628726	Apob	Apolipopro	APOB_MOU	44	1.037508
30_MTA / C	P02802	-0.35782	0.757905	0.628726	Mt1	Metallothio	MT1_MOUS	2	0.780341
15_MTA / C	P05784	0.06017	0.75814	0.628726	Krt18	Keratin, typ	K1C18_MO	18	1.042588
30_MTA / C	P21981	-0.03504	0.759317	0.628726	Tgm2	Protein-glu	TGM2_MOU	27	0.976002
30_MTA / C	P46664	-0.03572	0.758294	0.628726	Adss2	Adenylosuc	PURA2_MO	18	0.975548
30_MTA / C	P62259	0.056182	0.759299	0.628726	Ywhae	14-3-3 prot	1433E_MO	20	1.039711
15_MTA / C	P62960	0.05077	0.758136	0.628726	Ybx1	Y-box-bindi	YBOX1_MO	10	1.035818
30_MTA / C	Q8R1Q9	0.062145	0.759145	0.628726	Rbks	Ribokinase	RBSK_MOU	6	1.044017
30_MTA / C	Q99JZ7	0.908854	0.759885	0.628726	Errfi1	ERBB recep	ERRFI_MOU	2	1.877553
15_MTA / C	Q9JLF6	0.454388	0.759391	0.628726	Tgm1	Protein-glu	TGM1_MOU	5	1.370201
30_MTA / C	Q9QZS3	-0.08256	0.75852	0.628726	Numb	Protein nur	NUMB_MO	3	0.944381
15_MTA / C	Q9R1P3	-0.05546	0.758953	0.628726	Psmb2	Proteasom	PSB2_MOU	11	0.962291
15_MTA / C	Q9Z2I9	0.038746	0.757833	0.628726	Sucla2	Succinate--	SUCB1_MO	17	1.027221
30_MTA / C	P19536	-0.19592	0.760996	0.62918	Cox5b	Cytochrom	COX5B_MO	2	0.873018
30_MTA / C	Q5XG73	0.521284	0.762239	0.62918	Acbd5	Acyl-CoA-bi	ACBD5_MO	3	1.435232
30_MTA / C	Q60864	0.076405	0.762505	0.62918	Stip1	Stress-indu	STIP1_MOU	30	1.054387
30_MTA / C	Q61730	-0.11816	0.761771	0.62918	Il1rap	Interleukin	IL1AP_MOU	4	0.921365
15_MTA / C	Q7TMY8	-0.43086	0.761635	0.62918	Huwe1	E3 ubiquiti	HUWE1_M	7	0.74182
15_MTA / C	Q8VDM6	-0.50318	0.761445	0.62918	Hnrnpul1	Heterogene	HNRL1_MO	2	0.705548
15_MTA / C	Q99KR3	0.064056	0.762668	0.62918	Lactb2	Endoribonu	LACB2_MO	16	1.0454
15_MTA / C	Q9DCN2	-0.55098	0.762706	0.62918	Cyb5r3	NADH-cyto	NB5R3_MO	6	0.682555
30_MTA / C	A2AS89	-0.05229	0.763131	0.629189	Agmat	Guanidino	GDAH_MOU	8	0.964406
15_MTA / C	Q3UMU9	-0.02028	0.763015	0.629189	Hdgfl2	Hepatoma-	HDGR2_MO	4	0.986042
30_MTA / C	Q99MZ7	0.022565	0.763241	0.629189	Pecr	Peroxisoma	PECR_MOU	8	1.015764
30_MTA / C	Q91YY4	-0.5304	0.763918	0.629459	Atpaf2	ATP syntha	ATPF2_MOU	1	0.692363
30_MTA / C	P51150	0.141419	0.764515	0.629662	Rab7a	Ras-related	RAB7A_MO	3	1.102989
30_MTA / C	Q62418	-0.04892	0.765283	0.630006	Dbnl	Drebrin-like	DBNL_MOU	9	0.966661
30_MTA / C	P23249	0.073685	0.766036	0.630482	Mov10	Putative he	MOV10_M	15	1.052401
15_MTA / C	Q8K370	0.039818	0.767022	0.63115	Acad10	Acyl-CoA de	ACD10_MO	10	1.027984
15_MTA / C	P27659	0.093626	0.767965	0.631492	Rpl3	Large ribos	RL3_MOUS	17	1.067049
15_MTA / C	Q99JX4	-0.47497	0.767886	0.631492	Eif3m	Eukaryotic	EIF3M_MOU	5	0.719483
15_MTA / C	Q61147	-0.03129	0.769255	0.632264	Cp	Ceruloplasr	CERU_MOU	39	0.978547
15_MTA / C	O09012	-1.2581	0.770438	0.632306	Pex5	Peroxisoma	PEX5_MOU	1	0.418094
30_MTA / C	P42227	-0.01291	0.769666	0.632306	Stat3	Signal trans	STAT3_MOU	5	0.991093
30_MTA / C	P62301	-0.23703	0.769917	0.632306	Rps13	Small ribos	RS13_MOU	5	0.848489
30_MTA / C	P45376	-0.00147	0.771122	0.632542	Akr1b1	Aldo-keto r	ALDR_MOU	20	0.998982
30_MTA / C	P51880	-0.0109	0.771526	0.632542	Fabp7	Fatty acid-b	FABP7_MO	4	0.992476
15_MTA / C	P52760	-0.01214	0.771267	0.632542	Rida	2-iminobut	RIDA_MOU	10	0.991623
15_MTA / C	P62073	0.008079	0.771446	0.632542	Timm10	Mitochond	TIM10_MO	1	1.005615
30_MTA / C	Q920A5	0.196155	0.772353	0.633076	Scpep1	Retinoid-in	RISC_MOUS	4	1.145641
15_MTA / C	P63038	0.030123	0.773185	0.63347	Hspd1	60 kDa heat	CH60_MOU	45	1.021099
30_MTA / C	Q9JIX0	-0.43965	0.773708	0.633754	Eny2	Transcripti	ENY2_MOU	2	0.737313
30_MTA / C	Q8BWY3	0.180878	0.774116	0.6338	Etf1	Eukaryotic	ERF1_MOU	10	1.133574
15_MTA / C	Q8BKX1	0.250157	0.774358	0.633818	Baiap2	BAR/IMD do	BAIP2_MOU	3	1.189336

30_MTA / C	Q9D2G2	0.072604	0.77449	0.633818	Dlst	Dihydrolip	ODO2_MOU	15	1.051613
15_MTA / C	P54775	0.04929	0.77528	0.634032	Psmc4	26S protea	PRS6B_MO	8	1.034755
30_MTA / C	P29341	-0.04745	0.776034	0.634126	Pabpc1	Polyadenyl	PABP1_MO	16	0.967644
15_MTA / C	P53026	0.126752	0.775982	0.634126	Rpl10a	Large ribos	RL10A_MO	9	1.091833
30_MTA / C	Q8K2B3	-0.08148	0.776049	0.634126	Sdha	Succinate d	SDHA_MOU	23	0.945088
15_MTA / C	Q91VA0	0.061252	0.7761	0.634126	Acsm1	Acyl-coenzy	ACSM1_MO	25	1.043371
30_MTA / C	P36993	0.02458	0.776759	0.634235	Ppm1b	Protein pho	PPM1B_MO	9	1.017184
15_MTA / C	Q8VCA8	-0.14154	0.776447	0.634235	Scrn2	Secernin-2	SCRN2_MO	14	0.906552
15_MTA / C	Q9DCM0	-0.06622	0.776938	0.634235	Ethe1	Persulfide d	ETHE1_MOU	11	0.955135
30_MTA / C	P03976	-0.20451	0.777949	0.634267	NaN	Ig kappa ch	KV2A5_MO	1	0.867834
15_MTA / C	P59999	0.088041	0.778163	0.634267	Arpc4	Actin-relate	ARPC4_MO	8	1.062926
15_MTA / C	P61967	0.242194	0.778562	0.634267	Ap1s1	AP-1 compl	AP1S1_MO	1	1.18279
15_MTA / C	P62315	0.016527	0.777808	0.634267	Snrpd1	Small nucle	SMD1_MOU	5	1.011521
30_MTA / C	Q8BGS2	0.12833	0.778449	0.634267	Bola2	BolA-like pr	BOLA2_MO	3	1.093028
15_MTA / C	Q8R1G2	-0.06366	0.778317	0.634267	Cmbl	Carboxyme	CMBL_MOU	12	0.956836
30_MTA / C	Q91VI7	0.001045	0.777605	0.634267	Rnh1	Ribonuclea	RINI_MOUS	25	1.000725
15_MTA / C	Q9QUM9	0.088005	0.778485	0.634267	Psma6	Proteasom	PSA6_MOU	16	1.062899
30_MTA / C	P29758	-0.09263	0.780381	0.634743	Oat	Ornithine a	OAT_MOUS	8	0.937811
15_MTA / C	P53811	0.017167	0.780145	0.634743	Pitpnb	Phosphatid	PIPNB_MO	9	1.01197
15_MTA / C	P62889	0.064581	0.779719	0.634743	Rpl30	Large ribos	RL30_MOU	6	1.045781
15_MTA / C	Q8K4L4	-0.06353	0.780207	0.634743	Pof1b	Protein POI	POF1B_MO	5	0.956918
30_MTA / C	Q91XE0	0.074974	0.780219	0.634743	Glyat	Glycine N-a	GLYAT_MO	18	1.053342
15_MTA / C	Q9D0A3	-1.06119	0.779905	0.634743	Arpin	Arpin	ARPIN_MO	3	0.479238
30_MTA / C	Q9D1M0	0.025204	0.780801	0.634852	Sec13	Protein SEC	SEC13_MO	7	1.017623
30_MTA / C	Q9QXA5	-0.07069	0.781045	0.634852	Lsm4	U6 snRNA-a	LSM4_MOU	2	0.952183
15_MTA / C	P42669	0.003946	0.781963	0.635312	Pura	Transcripti	PURA_MOU	9	1.002739
15_MTA / C	O08795	-0.00805	0.786824	0.635356	Prkcsh	Glucosidas	GLU2B_MO	13	0.994432
30_MTA / C	O09061	-0.02246	0.785861	0.635356	Psmb1	Proteasom	PSB1_MOU	14	0.984554
15_MTA / C	O35658	-0.03668	0.784245	0.635356	C1qbp	Complemen	C1QBP_MO	6	0.974897
15_MTA / C	O35685	-0.01111	0.784703	0.635356	Nudc	Nuclear mi	NUDC_MOU	13	0.992329
15_MTA / C	P29788	0.445562	0.782222	0.635356	Vtn	Vitronectin	VTNC_MOU	3	1.361845
15_MTA / C	P47199	-0.04075	0.784136	0.635356	Cryz	Quinone ox	QOR_MOUS	16	0.972151
30_MTA / C	P51660	-0.0101	0.785303	0.635356	Hsd17b4	Peroxisom	DHB4_MOU	21	0.993025
30_MTA / C	P59999	0.082595	0.785847	0.635356	Arpc4	Actin-relate	ARPC4_MO	8	1.058921
15_MTA / C	P61922	-0.00173	0.783763	0.635356	Abat	4-aminobu	GABT_MOU	27	0.998803
15_MTA / C	P97379	-0.06447	0.78458	0.635356	G3bp2	Ras GTPase	G3BP2_MO	7	0.956298
30_MTA / C	P97461	0.238066	0.782789	0.635356	Rps5	Small ribos	RS5_MOUS	5	1.17941
30_MTA / C	P99029	0.042116	0.786167	0.635356	Prdx5	Peroxiredo	PRDX5_MO	13	1.029623
30_MTA / C	Q3TLS3	0.555224	0.785892	0.635356	Gdpgp1	GDP-D-gluc	GDPP1_MO	2	1.469397
30_MTA / C	Q8R317	0.032753	0.78696	0.635356	Ubqln1	Ubiquilin-1	UBQL1_MO	7	1.022963
30_MTA / C	Q91YI0	-0.05637	0.786412	0.635356	Asl	Argininosu	ARLY_MOU	21	0.961682
15_MTA / C	Q99KC8	0.035505	0.786677	0.635356	Vwa5a	von Willebr	VMA5A_MO	29	1.024916
30_MTA / C	Q9CY97	-0.23818	0.786311	0.635356	Ssu72	RNA polym	SSU72_MO	1	0.847813
15_MTA / C	Q9D2G2	0.058979	0.783124	0.635356	Dlst	Dihydrolip	ODO2_MOU	15	1.041728
30_MTA / C	Q9D8W5	0.039718	0.786125	0.635356	Psmd12	26S protea	PSD12_MO	9	1.027913
30_MTA / C	Q9WV85	-0.025	0.784334	0.635356	Nme3	Nucleoside	NDK3_MOU	5	0.982824
30_MTA / C	O54774	0.28948	0.787379	0.635552	Ap3d1	AP-3 compl	AP3D1_MO	3	1.2222
30_MTA / C	P85094	0.024762	0.787793	0.635578	Isoc2a	Isochorism	ISC2A_MOU	9	1.017312
30_MTA / C	Q9CWK8	-0.11074	0.787805	0.635578	Snx2	Sorting nex	SNX2_MOU	12	0.926112
30_MTA / C	P61957;Q9	0.08185	0.788925	0.635948	Sumo2;Sun	Small ubiqu	SUMO2_MO	1	1.058374
30_MTA / C	Q8BGF3	0.023374	0.789283	0.635948	Dnaaf10	Dynein axo	DAA10_MO	5	1.016333
30_MTA / C	Q3ULW8	-0.21505	0.789939	0.636334	Parp3	Protein mo	PARP3_MO	2	0.861517

30_MTA / C	D3Z4S3	-0.19227	0.791558	0.636456	Ptrhd1	Putative pe	PTRD1_MO	1	0.875228
30_MTA / C	P09813	-0.06147	0.790406	0.636456	Apoa2	Apolipoprc	APOA2_MC	2	0.958285
30_MTA / C	P28658	-0.2318	0.791545	0.636456	Atxn10	Ataxin-10	ATX10_MO	5	0.851571
30_MTA / C	P50580	-0.02798	0.79245	0.636456	Pa2g4	Proliferatic	PA2G4_MC	16	0.980793
15_MTA / C	Q60872	0.115714	0.792446	0.636456	Eif1a	Eukaryotic	IF1A_MOUS	6	1.083511
30_MTA / C	Q61129	0.128015	0.790974	0.636456	Cfi	Complemei	CFAI_MOUS	14	1.092789
30_MTA / C	Q64516	-0.04998	0.791601	0.636456	Gk	Glycerol kir	GLPK_MOU	29	0.965947
15_MTA / C	Q8BH95	0.027625	0.792422	0.636456	Echs1	Enoyl-CoA I	ECHM_MOU	16	1.019333
30_MTA / C	Q8BJW6	0.198811	0.792002	0.636456	Eif2a	Eukaryotic	EIF2A_MOU	13	1.147752
30_MTA / C	Q99KP6	0.093232	0.792068	0.636456	Prpf19	Pre-mRNA-|	PRP19_MO	11	1.066758
30_MTA / C	O08795	0.044984	0.794225	0.636458	Prkcsh	Glucosidas	GLU2B_MC	13	1.031672
15_MTA / C	O70310	-0.24156	0.793904	0.636458	Nmt1	Glycylpepti	NMT1_MOU	4	0.845831
15_MTA / C	P01029	0.039328	0.793763	0.636458	C4b	Complemei	CO4B_MOU	44	1.027635
30_MTA / C	Q62318	-0.38484	0.794511	0.636458	Trim28	Transcripti	TIF1B_MOU	7	0.765863
30_MTA / C	Q8CFX1	0.266313	0.79413	0.636458	H6pd	GDH/6PGL	G6PE_MOU	11	1.20273
30_MTA / C	Q91XF0	0.059752	0.792991	0.636458	Pnpo	Pyridoxine	PNPO_MOU	10	1.042286
30_MTA / C	Q9R1T2	-0.01943	0.793477	0.636458	Sae1	SUMO-activ	SAE1_MOU	7	0.986624
30_MTA / C	Q9D5J6	-0.02399	0.794707	0.636473	Shpk	Sedoheptul	SHPK_MOU	16	0.983512
30_MTA / C	P97865	0.001403	0.795106	0.636528	Pex7	Peroxisoma	PEX7_MOU	4	1.000973
30_MTA / C	Q6NVF9	-0.06589	0.795307	0.636528	Cpsf6	Cleavage ar	CPSF6_MO	3	0.955359
30_MTA / C	Q8CGC7	-0.05536	0.796269	0.636759	Eprs1	Bifunctiona	SYEP_MOUS	19	0.962356
15_MTA / C	Q9DCL2	-0.77334	0.796302	0.636759	Ciao2a	Cytosolic ir	CIA2A_MOU	1	0.585063
30_MTA / C	Q8K010	-0.07089	0.796541	0.636808	Oplah	5-oxoprolir	OPLA_MOU	26	0.952054
30_MTA / C	O70400	0.075584	0.797361	0.636862	Pdlim1	PDZ and LIN	PDLI1_MOU	8	1.053788
30_MTA / C	P53657	0.08766	0.798178	0.636862	Pklr	Pyruvate ki	KPYR_MOU	37	1.062645
15_MTA / C	Q62189	-0.20968	0.798554	0.636862	Snrpa	U1 small nu	SNRPA_MO	1	0.864726
30_MTA / C	Q8VCU1	0.063104	0.798505	0.636862	Ces3b	Carboxyles	EST3B_MOU	16	1.044711
30_MTA / C	Q9CQ10	0.124492	0.797566	0.636862	Chmp3	Charged mu	CHMP3_MC	4	1.090124
30_MTA / C	Q9DCW4	0.053705	0.798517	0.636862	Etfb	Electron tra	ETFB_MOUS	16	1.037927
30_MTA / C	Q6PDY2	-0.41332	0.798816	0.636875	Ado	2-aminoeth	AEDO_MOU	5	0.750896
30_MTA / C	P68510	0.0898	0.799282	0.637019	Ywhah	14-3-3 prot	1433F_MO	8	1.064223
30_MTA / C	Q61823	0.380855	0.800249	0.637415	Pdcd4	Programme	PDCD4_MC	4	1.302114
15_MTA / C	Q9R0Q7	-0.13654	0.801196	0.637839	Ptges3	Prostaglanc	TEBP_MOU	7	0.909696
15_MTA / C	P70671	-0.57535	0.802382	0.638218	Irf3	Interferon r	IRF3_MOUS	2	0.671122
30_MTA / C	Q6ZQ58	0.087388	0.802354	0.638218	Larp1	La-related p	LARP1_MO	4	1.062445
15_MTA / C	P17047	0.042632	0.802784	0.638397	Lamp2	Lysosome-a	LAMP2_MC	4	1.029991
15_MTA / C	Q60668	0.075589	0.803636	0.638756	Hnrnpd	Heterogene	HNRPD_MC	7	1.053792
30_MTA / C	Q9CXN7	-0.00131	0.804082	0.638756	Pbld2	Phenazine I	PBLD2_MO	14	0.999091
15_MTA / C	Q9D8E6	0.083868	0.804686	0.639063	Rpl4	Large ribos	RL4_MOUS	19	1.059856
30_MTA / C	P70168	0.080116	0.805198	0.63912	Kpnb1	Importin su	IMB1_MOU	25	1.057103
15_MTA / C	P97461	-0.34476	0.805563	0.63912	Rps5	Small ribos	RS5_MOUS	5	0.787437
30_MTA / C	Q99LD4	-0.02197	0.805647	0.63912	Gps1	COP9 signa	CSN1_MOU	10	0.984887
30_MTA / C	Q9CR41	-0.56051	0.805159	0.63912	Hypk	Huntingtin	HYPK_MOU	1	0.67806
30_MTA / C	Q9R0P3	-0.02699	0.806045	0.639253	Esd	S-formylglu	ESTD_MOU	13	0.981467
30_MTA / C	Q9Z0X1	0.073916	0.806169	0.639253	Aifm1	Apoptosis-i	AIFM1_MO	17	1.05257
30_MTA / C	P01642	-0.00566	0.807668	0.639431	Igkv5-48	Immunoglc	KV5A9_MO	2	0.996082
30_MTA / C	P01806;P0	0.089525	0.80817	0.639431	NaN	Ig heavy ch	HVM36_MC	3	1.06402
30_MTA / C	P01898	-0.01273	0.807624	0.639431	H2-Q10	H-2 class I h	HA10_MOU	10	0.991213
30_MTA / C	P55302	0.362994	0.807049	0.639431	Lrpap1	Alpha-2-ma	AMRP_MOU	5	1.286093
30_MTA / C	Q6PIP5	-0.30962	0.806969	0.639431	Nudcd1	NudC doma	NUDC1_MC	9	0.806852
30_MTA / C	Q9DCN2	-0.17019	0.807602	0.639431	Cyb5r3	NADH-cyto	NB5R3_MC	6	0.888728
15_MTA / C	Q9ET01	0.03153	0.80793	0.639431	Pygl	Glycogen p	PYGL_MOU	41	1.022096

30_MTA / C	Q8BKZ9	0.021099	0.809771	0.640557	Pdhx	Pyruvate de	ODPX_MOU	7	1.014732
30_MTA / C	P26231	-0.13154	0.810115	0.64065	Ctnna1	Catenin alp	CTNA1_MO	6	0.912857
30_MTA / C	P35979	-0.46002	0.810618	0.640724	Rpl12	Large ribos	RL12_MOU	5	0.726977
15_MTA / C	Q9R111	-0.0163	0.811341	0.641095	Gda	Guanine de	GUAD_MOU	27	0.988767
15_MTA / C	P01863;P0	0.092395	0.812615	0.641701	Ighg;Ighg2a	Ig gamma-2	GCAA_MOU	6	1.066139
30_MTA / C	Q9DC50	-0.17602	0.812643	0.641701	Crot	Peroxisoma	OCTC_MOU	2	0.885142
30_MTA / C	P62315	-0.04383	0.81304	0.641733	Snrpd1	Small nucle	SMD1_MOU	5	0.970075
15_MTA / C	Q9Z2I8	0.041789	0.813945	0.642025	Suclg2	Succinate--	SUCB2_MO	20	1.02939
30_MTA / C	O35344	-0.29423	0.814326	0.642044	Kpna3	Importin su	IMA4_MOU	2	0.815506
30_MTA / C	P01819;P0	-0.05431	0.81424	0.642044	NaN	Ig heavy ch	HVM43_M	1	0.963056
30_MTA / C	Q99LB2	0.016817	0.814655	0.642163	Dhrs4	Dehydroge	DHRS4_MO	11	1.011725
15_MTA / C	Q3UGR5	0.101799	0.815016	0.642307	Hdhd2	Haloacid de	HDHD2_M	11	1.07311
15_MTA / C	Q9WTX6	-0.05408	0.81585	0.642542	Cul1	Cullin-1	CUL1_MOU	16	0.96321
15_MTA / C	O08749	0.015873	0.816115	0.642596	Dld	Dihydrolip	DLDH_MOU	21	1.011063
30_MTA / C	Q9CQK7	0.078257	0.816275	0.642596	Rwdd1	RWD doma	RWDD1_M	3	1.055742
30_MTA / C	Q9CZX8	0.154038	0.817029	0.642956	Rps19	Small ribos	RS19_MOU	3	1.112679
30_MTA / C	P56389	0.231144	0.817682	0.643145	Cda	Cytidine de	CDD_MOUS	1	1.173766
30_MTA / C	Q8BFS6	-0.02658	0.817687	0.643145	Cpped1	Serine/thre	CPPED_MO	9	0.981742
15_MTA / C	Q69ZR2	0.383104	0.818182	0.643364	Hectd1	E3 ubiquiti	HECD1_MO	2	1.304145
30_MTA / C	Q9DCS2	-0.22352	0.818527	0.643384	Mettl26	Methyltran	MTL26_MO	5	0.856472
30_MTA / C	Q9ET54	-0.12733	0.819093	0.643585	Palld	Palladin	PALLD_MO	5	0.915521
15_MTA / C	P50171	-0.00835	0.81952	0.643743	Hsd17b8	(3R)-3-hydr	DHB8_MOU	9	0.99423
30_MTA / C	O08692	0.32741	0.820626	0.643993	Ngp	Neutrophil	NGP_MOUS	3	1.254759
15_MTA / C	P49443	0.049525	0.820473	0.643993	Ppm1a	Protein pho	PPM1A_M	10	1.034924
30_MTA / C	Q9CWS0	-0.02211	0.820166	0.643993	Ddah1	N(G),N(G)-d	DDAH1_M	16	0.984789
15_MTA / C	Q9DCD0	0.091693	0.820733	0.643993	Pgd	6-phospho	6PGD_MOU	20	1.06562
30_MTA / C	Q924M7	-0.00842	0.820995	0.644058	Mpi	Mannose-6	MPI_MOUS	6	0.99418
30_MTA / C	Q62188	-0.367	0.82149	0.64427	Dpysl3	Dihydropy	DPYL3_MO	2	0.775391
15_MTA / C	Q9DBP5	-0.05594	0.821624	0.64427	Cmpk1	UMP-CMP k	KCY_MOUS	14	0.961966
30_MTA / C	P97290	-0.04623	0.82207	0.64434	Serping1	Plasma pro	IC1_MOUS	10	0.968467
30_MTA / C	Q9Z2I8	0.082808	0.823367	0.645076	Suclg2	Succinate--	SUCB2_MO	20	1.059077
15_MTA / C	D3Z4S3	0.206666	0.824309	0.645532	Ptrhd1	Putative pe	PTRD1_MO	1	1.154018
30_MTA / C	C0HKG5;C0	-0.255	0.824489	0.645533	Rnaset2a;R	Ribonuclea	RNT2A_MO	1	0.837986
15_MTA / C	Q99LP6	-0.02955	0.824845	0.645619	Grpel1	GrpE protei	GRPE1_MO	11	0.979729
15_MTA / C	P23198	-0.00222	0.825465	0.645735	Cbx3	Chromobo:	CBX3_MOU	3	0.998462
30_MTA / C	P11589	-0.07925	0.826213	0.645899	Mup2	Major urina	MUP2_MOU	2	0.946552
30_MTA / C	Q8VDM6	0.025895	0.82616	0.645899	Hnrnpul1	Heterogene	HNRL1_MO	2	1.018111
30_MTA / C	Q9D394	0.118991	0.826141	0.645899	Rufy3	Protein RU	RUFY3_MO	4	1.085975
15_MTA / C	Q5U5V2	-0.14354	0.826403	0.645908	Hykk	Hydroxylys	HYKK_MOU	3	0.905298
30_MTA / C	Q571I9	-0.01751	0.827567	0.646541	Aldh16a1	Aldehyde d	A16A1_MO	18	0.987936
15_MTA / C	P01837	-0.02938	0.828943	0.646663	Igkc	Immunoglo	IGKC_MOU	6	0.97984
30_MTA / C	Q3UEB3	-0.73256	0.828326	0.646663	Puf60	Poly(U)-bin	PUF60_MO	2	0.601836
15_MTA / C	Q6GQT1	0.007945	0.828937	0.646663	A2m	Alpha-2-ma	A2MG_MO	1	1.005523
15_MTA / C	Q8BVI4	0.158505	0.828763	0.646663	Qdpr	Dihydropte	DHPR_MOU	8	1.11613
15_MTA / C	Q9EQH2	-0.02561	0.828986	0.646663	Erap1	Endoplasm	ERAP1_MO	32	0.982403
30_MTA / C	Q9DBR1	0.07391	0.830035	0.647201	Xrn2	5'-3' exorib	XRN2_MOU	4	1.052565
15_MTA / C	Q99P30	0.039635	0.831188	0.647508	Nudt7	Peroxisoma	NUDT7_MO	16	1.027854
30_MTA / C	Q9CQU0	-0.00812	0.831378	0.647508	Txndc12	Thioredoxi	TXD12_MO	4	0.994385
15_MTA / C	P23249	0.394813	0.831908	0.647539	Mov10	Putative he	MOV10_M	15	1.314772
30_MTA / C	P40336	0.001205	0.832382	0.647628	Vps26a	Vacuolar pr	VP26A_MO	12	1.000836
30_MTA / C	Q00PI9	0.417638	0.832921	0.647849	Hnrnpul2	Heterogene	HNRL2_MO	5	1.335739
15_MTA / C	Q9Z0N2	-0.03973	0.833025	0.647849	Eif2s3y	Eukaryotic	IF2H_MOUS	14	0.97284

30_MTA / C	P61255	0.131577	0.834821	0.648685	Rpl26	Large ribos	RL26_MOU	7	1.095491
15_MTA / C	P46938	0.105561	0.83546	0.648776	Yap1	Transcripti	YAP1_MOU	2	1.075913
30_MTA / C	Q8R0N6	-0.00511	0.835479	0.648776	Adhfe1	Hydroxyaci	HOT_MOUS	16	0.996462
30_MTA / C	Q9DCC4	-0.40954	0.83529	0.648776	Pycr3	Pyrroline-5	P5CR3_MO	4	0.752865
15_MTA / C	Q8BTY1	0.046665	0.835836	0.648837	Kyat1	Kynurenine	KAT1_MOU	17	1.032874
30_MTA / C	Q9D1L0	-0.01013	0.835918	0.648837	Chchd2	Coiled-coil	CHCH2_MO	3	0.993002
30_MTA / C	Q05816	0.004594	0.836245	0.64895	Fabp5	Fatty acid-b	FABP5_MO	9	1.003189
15_MTA / C	P56480	0.08061	0.83703	0.649241	Atp5f1b	ATP synthas	ATPB_MOU	21	1.057465
30_MTA / C	Q8BHN3	-0.07028	0.83734	0.649241	Ganab	Neutral alp	GANAB_MO	10	0.952452
15_MTA / C	Q9R0Q6	0.012309	0.838308	0.649766	Arpc1a	Actin-relate	ARC1A_MO	6	1.008568
30_MTA / C	Q9Z1Z0	0.00895	0.838378	0.649766	Uso1	General ves	USO1_MOU	31	1.006223
30_MTA / C	P01027	-0.02419	0.838898	0.649946	C3	Complemen	CO3_MOUS	100	0.983371
15_MTA / C	Q64442	-0.01586	0.838973	0.649946	Sord	Sorbitol de	DHSO_MOU	18	0.989065
30_MTA / C	Q9D0E1	-0.13464	0.839849	0.650346	Hnrnpm	Heterogene	HNRPM_MO	4	0.910898
30_MTA / C	Q62261	0.1433	0.840571	0.650625	Sptbn1	Spectrin be	SPTB2_MO	14	1.104428
30_MTA / C	Q8JZN5	-0.18344	0.840978	0.6508	Acad9	Complex I a	ACAD9_MO	16	0.880603
15_MTA / C	Q6ZWV3	-0.0675	0.841559	0.651109	Rpl10	Large ribos	RL10_MOU	9	0.954293
15_MTA / C	P34022	0.118748	0.843239	0.651161	Ranbp1	Ran-specific	RANG_MOU	5	1.085792
30_MTA / C	P56387	-0.05813	0.842907	0.651161	Dynlt3	Dynein ligh	DYLT3_MO	3	0.960506
15_MTA / C	P80316	0.01974	0.842381	0.651161	Cct5	T-complex p	TCPE_MOU	20	1.013777
30_MTA / C	P99026	0.037469	0.842862	0.651161	Psmb4	Proteasome	PSB4_MOU	8	1.026312
30_MTA / C	Q8VBW8	-0.00923	0.842838	0.651161	Ttc36	Tetratricop	TTC36_MO	8	0.993624
30_MTA / C	Q99LU0;Q9	0.084439	0.842697	0.651161	Chmp1b1;C	Charged mu	CH1B1_MO	2	1.060275
15_MTA / C	Q9D826	-0.02712	0.843066	0.651161	Pipox	Peroxisoma	SOX_MOUS	22	0.981375
30_MTA / C	Q9D892	-0.00523	0.843442	0.651161	Itpa	Inosine trip	ITPA_MOUS	7	0.996379
30_MTA / C	Q9JM76	-0.09587	0.843635	0.651161	Arpc3	Actin-relate	ARPC3_MO	2	0.935705
30_MTA / C	Q9QYI5	-0.64969	0.843391	0.651161	Dnajb2	DnaJ homo	DNJB2_MO	2	0.637418
15_MTA / C	P06330	-0.04952	0.844771	0.651216	NaN	Ig heavy ch	HVM51_MO	5	0.966259
30_MTA / C	Q8BJU0	-0.18988	0.844301	0.651216	Sgta	Small gluta	SGTA_MOU	5	0.876677
30_MTA / C	Q91WQ3	0.036771	0.844138	0.651216	Yars1	Tyrosine--tI	SYYC_MOUS	15	1.025816
15_MTA / C	Q91XF0	0.0032	0.844681	0.651216	Pnpo	Pyridoxine-	PNPO_MOU	10	1.00222
30_MTA / C	Q924Y0	-0.05884	0.844665	0.651216	Bbox1	Gamma-bu	BODG_MOU	6	0.960034
30_MTA / C	P63276	-0.1047	0.845156	0.651373	Rps17	Small ribos	RS17_MOU	2	0.929999
15_MTA / C	Q3UEB3	-0.80118	0.84547	0.651475	Puf60	Poly(U)-bin	PUF60_MO	2	0.573878
30_MTA / C	P08113	-0.00482	0.847391	0.652397	Hsp90b1	Endoplasm	ENPL_MOU	40	0.996668
30_MTA / C	Q9CX56	-0.03941	0.849263	0.653419	Psmd8	26S proteas	PSMD8_MO	3	0.973049
30_MTA / C	P62077	0.09003	0.849525	0.65348	Timm8b	Mitochond	TIM8B_MO	3	1.064393
15_MTA / C	O35864	0.157295	0.851514	0.653811	Cops5	COP9 signa	CSN5_MOU	4	1.115194
30_MTA / C	P14869	-0.07166	0.850739	0.653811	Rplp0	Large ribos	RLA0_MOU	9	0.951545
30_MTA / C	P63038	0.003324	0.85165	0.653811	Hspd1	60 kDa heat	CH60_MOU	45	1.002307
15_MTA / C	Q9DBE0	0.0295	0.85177	0.653811	Csad	Cysteine su	CSAD_MOU	23	1.020658
30_MTA / C	Q9ET01	0.08665	0.850436	0.653811	Pygl	Glycogen p	PYGL_MOU	41	1.061902
15_MTA / C	Q9QYJ0	0.137774	0.851374	0.653811	Dnaja2	DnaJ homo	DNJA2_MO	8	1.100206
30_MTA / C	Q9QXC1	0.006664	0.852245	0.653896	Fetub	Fetuin-B	FETUB_MO	8	1.004629
15_MTA / C	Q78JT3	0.000317	0.852506	0.653958	Haao	3-hydroxya	3HAO_MOU	22	1.000219
30_MTA / C	P05213	0.003117	0.856347	0.655526	Tuba1b	Tubulin alp	TBA1B_MO	19	1.002163
15_MTA / C	P84084	-0.58448	0.856543	0.655526	Arf5	ADP-ribosy	ARF5_MOU	1	0.66689
30_MTA / C	Q99P30	-0.01277	0.85594	0.655526	Nudt7	Peroxisoma	NUDT7_MO	16	0.991189
30_MTA / C	Q9CQC9	-0.34924	0.858504	0.656367	Sar1b	Small COPII	SAR1B_MO	2	0.784997
15_MTA / C	Q9EQS3	-1.31777	0.858564	0.656367	Mycbp	c-Myc-bind	MYCBP_MO	2	0.401156
30_MTA / C	O09173	0.028858	0.858769	0.656384	Hgd	Homogenti	HGD_MOUS	27	1.020204
15_MTA / C	Q6IRU2	0.054014	0.859175	0.656555	Tpm4	Tropomyos	TPM4_MOU	6	1.03815

15_MTA / C	P62852	0.059842	0.859528	0.656685	Rps25	Small ribos	RS25_MOU	4	1.042351
15_MTA / C	Q8VCN5	0.050842	0.859799	0.656753	Cth	Cystathioni	CGL_MOUS	22	1.035869
15_MTA / C	Q3TLP5	0.033336	0.860316	0.656869	Echdc2	Enoyl-CoA I	ECHD2_MC	7	1.023376
30_MTA / C	Q8CIF4	-0.0619	0.860276	0.656869	Btd	Biotinidase	BTD_MOUS	6	0.958003
30_MTA / C	P01754;P0	0.085826	0.86111	0.656944	Ighv1-62-3;	Ig heavy ch	HVM10_MC	1	1.061295
15_MTA / C	P28843	-0.11394	0.860991	0.656944	Dpp4	Dipeptidyl	DPP4_MOU	5	0.924061
15_MTA / C	P50136	-0.16252	0.86122	0.656944	Bckdha	2-oxoisoval	ODBA_MOU	10	0.893463
30_MTA / C	Q8BG05	0.002035	0.861004	0.656944	Hnrnpa3	Heterogene	ROA3_MOU	11	1.001411
15_MTA / C	Q99JI4	-0.06094	0.861509	0.656944	Psmd6	26S protea	PSMD6_MC	13	0.958637
15_MTA / C	Q91X72	0.095564	0.861791	0.65702	Hpx	Hemopexin	HEMO_MOU	24	1.068483
15_MTA / C	Q6P9R2	-0.17923	0.8622	0.657192	Oxsr1	Serine/thre	OXSR1_MO	2	0.883176
15_MTA / C	B2RXS4	-0.66996	0.864578	0.658169	Plxnb2	Plexin-B2	PLXB2_MO	2	0.628525
30_MTA / C	P62267	-0.1903	0.864303	0.658169	Rps23	Small ribos	RS23_MOU	6	0.876421
15_MTA / C	Q8K3W0	0.333111	0.864398	0.658169	Babam2	BRISC and E	BABA2_MO	1	1.259727
15_MTA / C	Q8VDJ3	-0.05429	0.864394	0.658169	Hdlbp	Vigilin	VIGLN_MOI	33	0.96307
30_MTA / C	Q9Z1K6	0.023646	0.864276	0.658169	Arih2	E3 ubiquiti	ARI2_MOUS	1	1.016525
30_MTA / C	Q52KI8	0.065542	0.864815	0.658183	Srrm1	Serine/argi	SRRM1_MC	2	1.046478
15_MTA / C	Q99JY9	0.083967	0.864962	0.658183	Actr3	Actin-relate	ARP3_MOU	22	1.059929
15_MTA / C	P09671	0.033314	0.865185	0.658213	Sod2	Superoxide	SODM_MOI	12	1.02336
30_MTA / C	P06683	-0.09232	0.865567	0.658365	C9	Compleme	CO9_MOUS	2	0.938013
15_MTA / C	O35887	0.126538	0.867103	0.659394	Calu	Calumenin	CALU_MOU	7	1.091671
15_MTA / C	Q3UMF0	-0.08634	0.867378	0.659464	Cobll1	Cordon-ble	COBL1_MO	19	0.94191
30_MTA / C	Q9JKV1	-0.13555	0.868821	0.660282	Adrm1	Proteasom	ADRM1_MC	1	0.910322
30_MTA / C	P01863;P0	0.146928	0.869861	0.660671	Ighg;Ighg2a	Ig gamma-2	GCAA_MOU	6	1.107209
15_MTA / C	Q8C1B7	-0.03119	0.86953	0.660671	Septin11	Septin-11	SEP11_MOI	14	0.978616
30_MTA / C	P55050	0.027889	0.870991	0.661175	Fabp2	Fatty acid-k	FABPI_MOU	5	1.019519
30_MTA / C	Q61160	-0.10457	0.870987	0.661175	Fadd	FAS-associa	FADD_MOU	1	0.93008
30_MTA / C	O70570	0.187238	0.872198	0.661195	Pigr	Polymeric i	PIGR_MOU	8	1.138582
15_MTA / C	P08003	0.048203	0.872857	0.661195	Pdia4	Protein dis	PDIA4_MO	28	1.033976
30_MTA / C	Q01339	-0.01961	0.873742	0.661195	Apoh	Beta-2-glyc	APOH_MOU	11	0.986499
30_MTA / C	Q64010	-0.07207	0.872616	0.661195	Crk	Adapter mc	CRK_MOUS	6	0.951275
30_MTA / C	Q8R0F9	-0.01034	0.873538	0.661195	Sec14l4	SEC14-like	S14L4_MO	15	0.99286
30_MTA / C	Q8R1G2	0.023426	0.872122	0.661195	Cmbl	Carboxyme	CMBL_MOU	12	1.01637
15_MTA / C	Q8VEK3	-0.00567	0.872403	0.661195	Hnrnpu	Heterogene	HNRPU_MC	17	0.996075
30_MTA / C	Q91WP6	0.257843	0.873129	0.661195	Serpina3n	Serine prot	SPA3N_MO	2	1.19569
15_MTA / C	Q9CXA2	-0.79132	0.87388	0.661195	L3hypdh	Trans-L-3-h	T3HPD_MC	1	0.577817
30_MTA / C	Q9DBG3	-0.08867	0.873527	0.661195	Ap2b1	AP-2 compl	AP2B1_MO	9	0.940391
30_MTA / C	Q9EQF5	0.025576	0.87138	0.661195	Dpys	Dihydropyr	DPYS_MOU	26	1.017886
15_MTA / C	Q9DBF1	0.04436	0.874307	0.661379	Aldh7a1	Alpha-amin	AL7A1_MO	27	1.031226
30_MTA / C	P26645	0.068607	0.874626	0.661482	Marcks	Myristoylat	MARCS_MC	4	1.048703
30_MTA / C	O35593	-0.32276	0.875798	0.661812	Psmd14	26S protea	PSDE_MOU	4	0.799538
30_MTA / C	P57776	0.013221	0.875613	0.661812	Eef1d	Elongation	EF1D_MOU	13	1.009206
30_MTA / C	P62311	-0.40939	0.875657	0.661812	Lsm3	U6 snRNA-a	LSM3_MOU	2	0.752942
30_MTA / C	Q99K28	0.341758	0.875738	0.661812	Arfgap2	ADP-ribosy	ARFG2_MO	12	1.2673
15_MTA / C	O55029	-0.03502	0.877754	0.662279	Copb2	Coatomer s	COPB2_MC	40	0.976017
30_MTA / C	O89106	-0.00341	0.877163	0.662279	Fhit	Bis(5'-aden	FHIT_MOUS	8	0.99764
15_MTA / C	P01635	-0.21354	0.876896	0.662279	Igkv12-41	Immunoglo	KV5A3_MO	2	0.862419
30_MTA / C	P01637;P0	0.285433	0.878808	0.662279	NaN	Ig kappa ch	KV5A5_MO	2	1.218776
30_MTA / C	P06745	0.030049	0.880095	0.662279	Gpi	Glucose-6-p	G6PI_MOU	23	1.021047
15_MTA / C	P19221	0.023137	0.880019	0.662279	F2	Prothromb	THRB_MOU	14	1.016167
30_MTA / C	P34914	-0.05466	0.877906	0.662279	Ephx2	Bifunctiona	HYES_MOU	26	0.96282
15_MTA / C	P60843	0.016086	0.878419	0.662279	Eif4a1	Eukaryotic	IF4A1_MOU	25	1.011213

30_MTA / C	P61924	0.023253	0.879051	0.662279	Copz1	Coatomer s	COPZ1_MO	7	1.016248
30_MTA / C	P97765	0.041163	0.878777	0.662279	Wbp2	WW domai	WBP2_MO	1	1.028943
15_MTA / C	Q8CHR6	0.118169	0.87927	0.662279	Dpyd	Dihydropyı	DPYD_MOU	37	1.085356
30_MTA / C	Q99JY9	0.044602	0.877716	0.662279	Actr3	Actin-relate	ARP3_MOU	22	1.031398
30_MTA / C	Q9ERG0	-0.11356	0.87996	0.662279	Lima1	LIM domair	LIMA1_MO	5	0.924306
30_MTA / C	P70460	0.210781	0.880311	0.662303	Vasp	Vasodilator	VASP_MOU	1	1.157314
30_MTA / C	P50516	0.142839	0.883388	0.663517	Atp6v1a	V-type prot	VATA_MOU	18	1.104076
30_MTA / C	Q3TCN2	-0.13371	0.883196	0.663517	Plbd2	Putative ph	PLBL2_MO	6	0.911486
30_MTA / C	Q64339	0.190238	0.883261	0.663517	Isg15	Ubiquitin-l	ISG15_MOU	3	1.140952
30_MTA / C	Q8VCH8	0.248601	0.882805	0.663517	Ubxn4	UBX domai	UBXN4_MC	2	1.188054
15_MTA / C	P00329	0.046578	0.884148	0.663558	Adh1	Alcohol del	ADH1_MOU	24	1.032812
15_MTA / C	Q8BGQ7	0.050386	0.884304	0.663558	Aars1	Alanine--tR	SYAC_MOU	27	1.035542
30_MTA / C	Q9DBL1	-0.41816	0.884215	0.663558	Acadsb	Short/bran	ACDSB_MO	3	0.748378
30_MTA / C	Q9Z0P5	-0.32398	0.884364	0.663558	Twf2	Twinfilin-2	TWF2_MOU	4	0.798865
30_MTA / C	Q9CQM5	-0.0087	0.884616	0.663601	Txndc17	Thioredoxi	TXD17_MO	5	0.993986
30_MTA / C	Q922B2	-0.3011	0.885928	0.664308	Dars1	Aspartate--	SYDC_MOU	7	0.811634
30_MTA / C	Q64105	-0.36041	0.886591	0.664528	Spr	Sepiapterin	SPRE_MOU	7	0.778945
15_MTA / C	Q9JKB1	-0.11516	0.886424	0.664528	Uchl3	Ubiquitin c	UCHL3_MO	8	0.923282
30_MTA / C	P01644;P0	0.149207	0.887275	0.664773	NaN	Ig kappa ch	KV5AB_MO	3	1.10896
30_MTA / C	P45591	-0.03354	0.887349	0.664773	Cfl2	Cofilin-2	COF2_MOU	11	0.977018
30_MTA / C	P62852	0.054577	0.887841	0.664773	Rps25	Small ribos	RS25_MOU	4	1.038555
30_MTA / C	P84091	0.063279	0.887733	0.664773	Ap2m1	AP-2 compl	AP2M1_MC	12	1.044838
30_MTA / C	Q7TMY8	-0.52292	0.887654	0.664773	Huwe1	E3 ubiquiti	HUWE1_M	7	0.69596
15_MTA / C	Q9Z0X1	0.080772	0.888916	0.665166	Aifm1	Apoptosis-i	AIFM1_MO	17	1.057583
15_MTA / C	O70194	-0.15413	0.889446	0.665283	Eif3d	Eukaryotic	EIF3D_MOU	5	0.898675
30_MTA / C	Q5FW57	0.847673	0.889273	0.665283	Gm4952	Glycine N-a	GLYAL_MO	1	1.799597
30_MTA / C	Q8K0E8	-0.05692	0.889772	0.665346	Fgb	Fibrinogen	FIBB_MOUS	5	0.961316
30_MTA / C	Q9JHH9	0.381557	0.890269	0.665346	Copz2	Coatomer s	COPZ2_MO	3	1.302747
30_MTA / C	Q9QUM9	0.045553	0.890194	0.665346	Psma6	Proteasom	PSA6_MOU	16	1.032079
30_MTA / C	P04186	-0.20304	0.89046	0.66535	Cfb	Complemer	CFAB_MOU	15	0.868715
15_MTA / C	P02798	-0.19692	0.890761	0.665437	Mt2	Metallothi	MT2_MOUS	2	0.872413
15_MTA / C	P61027	-0.06169	0.89204	0.66555	Rab10	Ras-related	RAB10_MO	1	0.958138
30_MTA / C	P62334	0.214994	0.891639	0.66555	Psmc6	26S protea	PRS10_MO	5	1.160699
30_MTA / C	P97864	0.409837	0.892334	0.66555	Casp7	Caspase-7	CASP7_MO	3	1.328536
30_MTA / C	Q283N4	-0.10127	0.891593	0.66555	Urad	2-oxo-4-hy	URAD_MOU	8	0.932213
15_MTA / C	Q80Y14	0.738541	0.891922	0.66555	Glrx5	Glutaredox	GLRX5_MO	1	1.668488
30_MTA / C	Q8VDJ3	0.00214	0.891373	0.66555	Hdlbp	Vigilin	VIGLN_MOU	33	1.001485
30_MTA / C	P12246	0.086627	0.893776	0.665893	Apcs	Serum amy	SAMP_MOU	4	1.061884
15_MTA / C	Q52KI8	-0.05239	0.893628	0.665893	Srrm1	Serine/argi	SRRM1_MC	2	0.964336
15_MTA / C	Q64213	-0.03315	0.893333	0.665893	Sf1	Splicing fac	SF01_MOU	8	0.977282
15_MTA / C	Q8BMF4	0.209155	0.893683	0.665893	Dlat	Dihydrolip	ODP2_MOU	18	1.156011
30_MTA / C	Q9CXI5	-0.03598	0.894224	0.666089	Manf	Mesenceph	MANF_MOU	9	0.975369
15_MTA / C	P01801	0.263669	0.894507	0.666162	NaN	Ig heavy ch	HVM32_M	3	1.200528
15_MTA / C	P51885	-0.21423	0.895088	0.666456	Lum	Lumican	LUM_MOUS	2	0.862008
30_MTA / C	P06330	-0.1562	0.895404	0.666554	NaN	Ig heavy ch	HVM51_M	5	0.897386
15_MTA / C	Q8VCT4	0.027145	0.895638	0.66659	Ces1d	Carboxyles	EST1D_MO	33	1.018994
30_MTA / C	O88487	0.056333	0.898594	0.66686	Dync1i2	Cytoplasmi	DC1I2_MO	9	1.039819
30_MTA / C	P35235	-0.46521	0.897577	0.66686	Ptpn11	Tyrosine-pr	PTN11_MO	4	0.724366
30_MTA / C	P50136	-0.01279	0.897753	0.66686	Bckdha	2-oxoisoval	ODBA_MOU	10	0.991175
15_MTA / C	P70296	0.077181	0.896605	0.66686	Pebp1	Phosphatid	PEBP1_MO	14	1.054955
15_MTA / C	P97807	-0.21495	0.898312	0.66686	Fh	Fumarate h	FUMH_MOU	10	0.861576
30_MTA / C	Q3TEA8	0.314761	0.898057	0.66686	Hp1bp3	Heterochro	HP1B3_MC	4	1.243805

15_MTA / C	Q6ZWX6	0.166515	0.897528	0.66686	Eif2s1	Eukaryotic	IF2A_MOUS	16	1.122344
30_MTA / C	Q8VIJ6	0.034387	0.897734	0.66686	Sfpq	Splicing fac	SFPQ_MOU	10	1.024121
15_MTA / C	Q9CZ44	-0.0053	0.897704	0.66686	Nsfl1c	NSFL1 cofa	NSF1C_MO	15	0.996332
15_MTA / C	Q9D819	0.043963	0.898514	0.66686	Ppa1	Inorganic p	IPYR_MOUS	16	1.030942
30_MTA / C	O08756	0.013952	0.899058	0.666929	Hsd17b10	3-hydroxya	HCD2_MOU	10	1.009717
30_MTA / C	Q80YC5	-0.54186	0.898959	0.666929	F12	Coagulatio	FA12_MOU	4	0.686886
30_MTA / C	P14824	0.094122	0.900563	0.667253	Anxa6	Annexin A6	ANXA6_MC	36	1.067416
30_MTA / C	P97823	-0.11094	0.900496	0.667253	Lypla1	Acyl-protei	LYPA1_MO	4	0.925985
30_MTA / C	Q9JHW2	0.032289	0.900269	0.667253	Nit2	Omega-ami	NIT2_MOUS	20	1.022633
30_MTA / C	Q61247	-0.04533	0.901011	0.667415	Serpinf2	Alpha-2-an	A2AP_MOU	12	0.969071
30_MTA / C	P30416	-0.00687	0.901563	0.667607	Fkbp4	Peptidyl-pr	FKBP4_MO	19	0.995251
15_MTA / C	Q8CC86	-0.01559	0.901641	0.667607	Naprt	Nicotinate	PNCB_MOU	25	0.989255
30_MTA / C	Q61646	-0.04555	0.902419	0.667934	Hp	Haptoglobi	HPT_MOUS	17	0.968921
30_MTA / C	Q8CHP8	0.048895	0.903916	0.668605	Pgp	Glycerol-3-	PGP_MOUS	11	1.034472
30_MTA / C	Q501J6	0.124538	0.905128	0.669053	Ddx17	Probable A	DDX17_MC	3	1.090158
15_MTA / C	Q6ZWN5	0.101797	0.905803	0.669053	Rps9	Small ribos	RS9_MOUS	15	1.07311
15_MTA / C	Q78JW9	-0.32959	0.904811	0.669053	Ubfd1	Ubiquitin d	UBFD1_MC	3	0.795763
30_MTA / C	Q8C1B7	-0.02255	0.905559	0.669053	Septin11	Septin-11	SEP11_MO	14	0.98449
30_MTA / C	Q9D1R9	0.084387	0.905634	0.669053	Rpl34	Large ribos	RL34_MOU	6	1.060237
30_MTA / C	Q9D8N0	0.108424	0.905824	0.669053	Eef1g	Elongation	EF1G_MOU	11	1.07805
15_MTA / C	P04939	0.081735	0.907459	0.669106	Mup3	Major urina	MUP3_MO	12	1.05829
15_MTA / C	P22892	-0.16233	0.906965	0.669106	Ap1g1	AP-1 compl	AP1G1_MC	8	0.89358
30_MTA / C	P62918	-0.02772	0.907113	0.669106	Rpl8	Large ribos	RL8_MOUS	13	0.980972
30_MTA / C	P67984	-0.0418	0.907754	0.669106	Rpl22	Large ribos	RL22_MOU	3	0.971444
15_MTA / C	Q571I9	-0.00734	0.906311	0.669106	Aldh16a1	Aldehyde d	A16A1_MO	18	0.994923
15_MTA / C	Q8BG05	-0.00761	0.907469	0.669106	Hnrnpa3	Heterogene	ROA3_MOU	11	0.994737
30_MTA / C	Q9CQA3	-0.1956	0.906655	0.669106	Sdhb	Succinate d	SDHB_MOU	7	0.873207
30_MTA / C	Q9QYY9	0.007786	0.907687	0.669106	Adh4	All-trans-re	ADH4_MOU	18	1.005411
30_MTA / C	P17047	-0.01015	0.908048	0.669186	Lamp2	Lysosome-a	LAMP2_MC	4	0.992992
15_MTA / C	P58871	-0.25069	0.908365	0.669283	Tnks1bp1	182 kDa tar	TB182_MO	3	0.840496
15_MTA / C	P15864	0.054763	0.909384	0.669622	H1-2	Histone H1	H12_MOUS	1	1.038688
30_MTA / C	P56391	-0.00934	0.909236	0.669622	Cox6b1	Cytochrom	CX6B1_MO	4	0.993549
15_MTA / C	P70677	0.025028	0.9102	0.669949	Casp3	Caspase-3	CASP3_MO	11	1.0175
30_MTA / C	P05784	0.118036	0.9111	0.67033	Krt18	Keratin, typ	K1C18_MO	18	1.085256
30_MTA / C	P13439	0.24382	0.911734	0.67033	Umps	Uridine 5'-r	UMPS_MOU	4	1.184124
15_MTA / C	P61979	-0.00568	0.911726	0.67033	Hnrnpk	Heterogene	HNRPK_MC	20	0.996069
30_MTA / C	Q8QZR1	0.237711	0.911834	0.67033	Tat	Tyrosine an	ATTY_MOUS	12	1.179121
30_MTA / C	P11438	0.358157	0.912408	0.670336	Lamp1	Lysosome-a	LAMP1_MC	2	1.281788
15_MTA / C	P23591	0.0666	0.912659	0.670336	Gfus	GDP-L-fuco	FCL_MOUS	10	1.047246
15_MTA / C	Q3U0V1	0.130118	0.913072	0.670336	Khsrp	Far upstrea	FUBP2_MO	14	1.094383
15_MTA / C	Q922J3	0.23576	0.913003	0.670336	Clip1	CAP-Gly do	CLIP1_MOU	4	1.177527
15_MTA / C	Q9CQK7	-0.559	0.913505	0.670336	Rwdd1	RWD doma	RWDD1_M	3	0.678772
30_MTA / C	Q9CZY3;Q9	0.096544	0.912375	0.670336	Ube2v1;Ub	Ubiquitin-c	UB2V1_MC	3	1.069209
15_MTA / C	Q9D8X2	-0.05469	0.91352	0.670336	Ccdc124	Coiled-coil	CC124_MO	3	0.962803
15_MTA / C	Q9EQF5	0.019319	0.912466	0.670336	Dpys	Dihydropyr	DPYS_MOU	26	1.013481
30_MTA / C	Q9DB29	-0.01197	0.91397	0.670394	Iah1	Isoamyl ace	IAH1_MOU	14	0.991736
30_MTA / C	Q9JHS9	0.183374	0.913847	0.670394	Cwc15	Spliceosom	CWC15_M	1	1.135537
30_MTA / C	Q8BGL3	0.027037	0.914531	0.670556	Sult2a8	Sulfotransf	ST2A8_MO	14	1.018918
30_MTA / C	P35441	-0.05425	0.915134	0.670565	Thbs1	Thrombosp	TSP1_MOU	4	0.963096
15_MTA / C	Q9WTL4	-0.06958	0.915424	0.67064	Insrr	Insulin rece	INSRR_MO	2	0.952914
30_MTA / C	P54822	-0.57346	0.91622	0.670814	Adsl	Adenylosuc	PUR8_MOU	1	0.672003
30_MTA / C	Q64458	-0.0556	0.916046	0.670814	Cyp2c29	Cytochrom	CP2CT_MO	2	0.962193

30_MTA / C	P26043	0.009967	0.917159	0.671269	Rdx	Radixin	RADI_MOU		38	1.006932
15_MTA / C	P26516	-0.10939	0.917921	0.671403	Psmd7	26S protea:	PSMD7_MC		5	0.926981
15_MTA / C	Q61646	0.106161	0.918128	0.671403	Hp	Haptoglobi	HPT_MOUS		17	1.07636
15_MTA / C	P97371	0.038103	0.91869	0.671666	Psme1	Proteasom	PSME1_MC		15	1.026763
15_MTA / C	Q8CHP8	-0.02569	0.919584	0.672047	Pgp	Glycerol-3-	PGP_MOUS		11	0.982352
30_MTA / C	P23780	0.403934	0.920334	0.672194	Glb1	Beta-galact	BGAL_MOU		3	1.323111
15_MTA / C	Q6A028	-0.13657	0.920238	0.672194	Swap70	Switch-assc	SWP70_MC		1	0.90968
15_MTA / C	Q91X83	0.08197	0.920346	0.672194	Mat1a	S-adenosyl	METK1_MC		20	1.058462
15_MTA / C	Q9D8T2	-0.06319	0.921477	0.672208	Gsdmd	Gasdermin	GSDMD_M		12	0.957147
30_MTA / C	P26638	0.03154	0.922433	0.672339	Sars1	Serine--tRN	SYSC_MOU		2	1.022103
30_MTA / C	P97351	0.013349	0.922014	0.672339	Rps3a	Small ribos	RS3A_MOU		15	1.009295
15_MTA / C	Q8VC28	0.020256	0.922598	0.672339	Akr1c13	Aldo-keto r	AK1CD_MC		11	1.014139
15_MTA / C	Q9Z2X1	-0.1583	0.922307	0.672339	Hnrnpf	Heterogene	HNRPF_MC		5	0.896081
15_MTA / C	Q9R1P0	0.050279	0.923596	0.672791	Psma4	Proteasom	PSA4_MOU		13	1.035465
30_MTA / C	Q9QZ85	-0.02825	0.924611	0.672988	Iigp1	Interferon-i	IIGP1_MOU		16	0.98061
30_MTA / C	O35551	-0.24781	0.926015	0.673066	Rabep1	Rab GTPase	RABE1_MO		3	0.842174
30_MTA / C	P28656	-0.25649	0.925543	0.673066	Nap1l1	Nucleosom	NP1L1_MO		5	0.837124
30_MTA / C	P42669	0.034261	0.926754	0.673066	Pura	Transcripti	PURA_MOU		9	1.024032
30_MTA / C	P62855	0.064463	0.926615	0.673066	Rps26	Small ribos	RS26_MOU		2	1.045696
30_MTA / C	Q01853	-0.02889	0.926376	0.673066	Vcp	Transitiona	TERA_MOU		56	0.980177
15_MTA / C	Q07456	0.213315	0.925995	0.673066	Ambp	Protein AM	AMBP_MOU		11	1.159349
30_MTA / C	Q61233	-0.00282	0.925146	0.673066	Lcp1	Plastin-2	PLSL_MOUS		31	0.998046
30_MTA / C	Q8R1K4	-0.0595	0.926451	0.673066	Phykpl	5-phosphol	AT2L2_MO		3	0.959599
30_MTA / C	Q9DB27	0.270644	0.926962	0.673066	Mcts1	Malignant	MCTS1_MC		3	1.206346
30_MTA / C	Q9ESX5	0.170771	0.926868	0.673066	Dkc1	H/ACA ribo	DKC1_MOU		7	1.12566
30_MTA / C	Q61147	-0.05112	0.92716	0.673075	Cp	Ceruloplas	CERU_MOU		39	0.96519
30_MTA / C	P63017	0.041709	0.9274	0.673113	Hspa8	Heat shock	HSP7C_MO		37	1.029333
15_MTA / C	P01899	0.061745	0.928763	0.673567	H2-D1	H-2 class I h	HA11_MOU		2	1.043727
15_MTA / C	P08249	0.048985	0.928897	0.673567	Mdh2	Malate deh	MDHM_MC		19	1.034537
30_MTA / C	P39061	0.078833	0.929838	0.673931	Col18a1	Collagen al	COIA1_MO		3	1.056163
30_MTA / C	Q60692	-0.02129	0.930358	0.673989	Psmb6	Proteasom	PSB6_MOU		7	0.985349
30_MTA / C	P97821	0.383408	0.931127	0.674178	Ctsc	Dipeptidyl	CATC_MOU		3	1.30442
30_MTA / C	P27773	0.005993	0.932129	0.674641	Pdia3	Protein dis	PDIA3_MO		28	1.004163
15_MTA / C	P47963	0.117664	0.932854	0.67503	Rpl13	Large ribos	RL13_MOU		8	1.084977
15_MTA / C	P97290	-0.15764	0.93325	0.675034	Serping1	Plasma pro	IC1_MOUS		10	0.896492
15_MTA / C	Q8R0F8	0.093382	0.933292	0.675034	Fahd1	Oxaloaceta	FAHD1_MC		11	1.066868
15_MTA / C	Q99JZ7	0.128794	0.933422	0.675034	Errfi1	ERBB recep	ERRFI_MOU		2	1.093379
30_MTA / C	P52196	0.036368	0.934443	0.67543	Tst	Thiosulfate	THTR_MOU		11	1.025529
30_MTA / C	Q8N7N5	0.055838	0.934401	0.67543	Dcaf8	DDB1- and	DCAF8_MO		4	1.039463
30_MTA / C	Q9Z1R2	0.212748	0.934595	0.67543	Bag6	Large prolir	BAG6_MOU		7	1.158893
15_MTA / C	O88569	-0.02316	0.935487	0.675713	Hnrnpa2b1	Heterogene	ROA2_MOU		16	0.984074
15_MTA / C	P07759	0.045018	0.936366	0.675941	Serpina3k	Serine prot	SPA3K_MO		14	1.031696
30_MTA / C	P50172	-0.09789	0.93623	0.675941	Hsd11b1	11-beta-hy	DHI1_MOU		4	0.934397
30_MTA / C	O08539	-0.23857	0.937367	0.676007	Bin1	Myc box-de	BIN1_MOU		3	0.847582
30_MTA / C	P02301;P6	0.120384	0.937088	0.676007	H3-5;H3c1;	Histone H3	H3C_MOUS		3	1.087024
30_MTA / C	Q8BTM8	0.324091	0.937397	0.676007	Flna	Filamin-A	FLNA_MOU		16	1.251876
30_MTA / C	Q99L13	-0.01746	0.93727	0.676007	Hibadh	3-hydroxyi:	3HIDH_MO		13	0.987967
15_MTA / C	P80314	0.08601	0.938045	0.676204	Cct2	T-complex	TCPB_MOU		22	1.061431
30_MTA / C	P02662	-0.05304	0.938922	0.676254	CSN1S1	Alpha-S1-ca	CASA1_BOV		3	0.963902
15_MTA / C	Q9CQ48	-0.51406	0.938981	0.676254	Nudcd2	NudC doma	NUDC2_MC		1	0.700251
30_MTA / C	Q9EQH3	0.024298	0.938708	0.676254	Vps35	Vacuolar pr	VPS35_MO		23	1.016984
15_MTA / C	Q9WVL0	0.017973	0.939081	0.676254	Gstz1	Maleylacet	MAAI_MOU		17	1.012536

15_MTA / C	P01680	0.050226	0.9399	0.676458	NaN	Ig kappa ch	KV4A1_MO	1	1.035427
30_MTA / C	P62960	0.02651	0.939803	0.676458	Ybx1	Y-box-bindi	YBOX1_MO	10	1.018545
30_MTA / C	O08677	0.031568	0.940198	0.676523	Kng1	Kininogen-1	KNG1_MOU	21	1.022123
30_MTA / C	Q8C196	0.035273	0.940743	0.676523	Cps1	Carbamoyl-	CPSM_MOU	85	1.02475
15_MTA / C	Q9CPV4	0.042497	0.940742	0.676523	Glod4	Glyoxalase	GLOD4_MO	16	1.029895
15_MTA / C	P07309	-0.01069	0.94548	0.676613	Ttr	Transthyret	TTHY_MOU	6	0.992616
15_MTA / C	P08113	0.024433	0.943358	0.676613	Hsp90b1	Endoplasm	ENPL_MOU	40	1.01708
30_MTA / C	P11031	0.146715	0.943382	0.676613	Sub1	Activated R	TCP4_MOU	6	1.107046
15_MTA / C	P11247	-0.83755	0.943214	0.676613	Mpo	Myeloperox	PERM_MOU	3	0.559593
15_MTA / C	P28076	0.114407	0.945952	0.676613	Psmb9	Proteasome	PSB9_MOU	7	1.08253
30_MTA / C	P47962	-0.00968	0.943695	0.676613	Rpl5	Large ribos	RL5_MOUS	11	0.993311
30_MTA / C	P52792	-0.06119	0.946111	0.676613	Gck	Hexokinase	HXK4_MOU	17	0.958471
15_MTA / C	P97770	-0.74762	0.94235	0.676613	Thumpd3	tRNA (guan	THUM3_MO	1	0.595585
30_MTA / C	Q61553	0.060997	0.946058	0.676613	Fscn1	Fascin	FSCN1_MO	2	1.043187
30_MTA / C	Q8BHA3	-0.04509	0.946194	0.676613	Dtd2	D-aminoacy	DTD2_MOU	6	0.96923
30_MTA / C	Q8BKX1	-0.42237	0.945452	0.676613	Baiap2	BAR/IMD do	BAIP2_MOU	3	0.746197
30_MTA / C	Q91W50	0.189691	0.941892	0.676613	Csde1	Cold shock	CSDE1_MO	6	1.14052
30_MTA / C	Q921H8	0.027345	0.94497	0.676613	Acaa1a	3-ketoacyl-	THIKA_MOU	8	1.019135
30_MTA / C	Q99KN2	-0.19614	0.94222	0.676613	Ciao1	Probable cy	CIAO1_MOU	2	0.872884
15_MTA / C	Q9CT10	-0.06347	0.943844	0.676613	Ranbp3	Ran-bindin	RANB3_MO	9	0.956958
30_MTA / C	Q9D142	0.069372	0.944	0.676613	Nudt14	Uridine dip	NUD14_MO	5	1.04926
15_MTA / C	Q9D1R9	-0.0188	0.943845	0.676613	Rpl34	Large ribos	RL34_MOU	6	0.987056
15_MTA / C	Q9QYB1	-0.01136	0.946318	0.676613	Clic4	Chloride in	CLIC4_MOU	14	0.992156
30_MTA / C	Q9QYJ0	0.024434	0.941515	0.676613	Dnaja2	DnaJ homo	DNJA2_MO	8	1.017081
30_MTA / C	Q9R1P1	0.023699	0.94214	0.676613	Psmb3	Proteasome	PSB3_MOU	12	1.016563
15_MTA / C	Q9WUM3	-0.34284	0.94329	0.676613	Coro1b	Coronin-1B	COR1B_MO	6	0.788489
15_MTA / C	O35215	0.010185	0.947074	0.676635	Ddt	D-dopachro	DOPD_MOU	13	1.007085
30_MTA / C	P14206	0.099644	0.94696	0.676635	Rpsa	Small ribos	RSSA_MOU	2	1.071509
15_MTA / C	P97328	0.054763	0.946768	0.676635	Khk	Ketohexoki	KHK_MOUS	22	1.038688
30_MTA / C	Q8VDG5	-0.10657	0.947101	0.676635	Ppcs	Phosphopa	PPCS_MOU	15	0.928792
30_MTA / C	P01029	0.041978	0.948018	0.676899	C4b	Complemen	CO4B_MOU	44	1.029524
15_MTA / C	P62878	0.000875	0.947856	0.676899	Rbx1	E3 ubiquiti	RBX1_MOU	2	1.000607
30_MTA / C	Q6ZWN5	0.051103	0.948515	0.676899	Rps9	Small ribos	RS9_MOUS	15	1.036057
15_MTA / C	Q8BIW1	0.128138	0.948359	0.676899	Prune1	Exopolypho	PRUN1_MO	1	1.092882
30_MTA / C	Q8BH86	0.025685	0.949611	0.677218	Dglucy	D-glutamat	GLUCM_MO	6	1.017963
30_MTA / C	Q6ZWV7	0.025781	0.950186	0.677264	Rpl35	Large ribos	RL35_MOU	2	1.018031
30_MTA / C	Q9QXD1	-0.03454	0.950239	0.677264	Acox2	Peroxisoma	ACOX2_MO	19	0.97634
30_MTA / C	E9Q912	-0.05569	0.952412	0.677346	Rap1gds1	Rap1 GTPas	GDS1_MOU	11	0.962137
15_MTA / C	O35423	0.015545	0.95214	0.677346	Agxt	Alanine--gly	AGT1_MOU	20	1.010833
30_MTA / C	P01633	0.127733	0.953578	0.677346	Igkv6-17	Immunoglo	KV5A1_MO	3	1.092575
15_MTA / C	P01675;P0	0.070744	0.950785	0.677346	NaN	Ig kappa ch	KV6A1_MO	1	1.050258
15_MTA / C	P27546	0.120843	0.953948	0.677346	Map4	Microtubul	MAP4_MOU	19	1.08737
15_MTA / C	P36993	0.042344	0.952568	0.677346	Ppm1b	Protein pho	PPM1B_MO	9	1.029786
30_MTA / C	P45878	-0.26691	0.954117	0.677346	Fkbp2	Peptidyl-pr	FKBP2_MO	5	0.831096
15_MTA / C	P52196	0.107077	0.953554	0.677346	Tst	Thiosulfate	THTR_MOU	11	1.077044
30_MTA / C	P63158	-0.02956	0.951437	0.677346	Hmgb1	High mobil	HMGB1_MO	11	0.979716
30_MTA / C	Q8CHR6	0.016474	0.950877	0.677346	Dpyd	Dihydropyr	DPYD_MOU	37	1.011484
30_MTA / C	Q8CIE0	0.166861	0.952539	0.677346	Serpina11	Serpin A11	SPA11_MO	1	1.122613
15_MTA / C	Q8VBW8	-0.00805	0.951657	0.677346	Ttc36	Tetratricop	TTC36_MO	8	0.994438
15_MTA / C	Q99LB2	0.026128	0.952999	0.677346	Dhrs4	Dehydroge	DHRS4_MO	11	1.018275
30_MTA / C	Q9D0F9	0.040733	0.952759	0.677346	Pgm1	Phosphoglu	PGM1_MOU	36	1.028636
15_MTA / C	Q9D0R8	0.0522	0.953432	0.677346	Lsm12	Protein LSM	LSM12_MO	3	1.036845

30_MTA / C	Q9R1P0	0.026372	0.951436	0.677346	Psma4	Proteasom	PSA4_MOU	13	1.018448
15_MTA / C	Q9JHU9	0.178918	0.954668	0.677604	Isyna1	Inositol-3-p	INO1_MOU	11	1.132035
30_MTA / C	O35643	0.028101	0.95536	0.677961	Ap1b1	AP-1 compl	AP1B1_MO	35	1.019669
30_MTA / C	P62827	-0.04713	0.955776	0.67804	Ran	GTP-bindin	RAN_MOUS	5	0.967861
30_MTA / C	P22892	0.026293	0.956924	0.678087	Ap1g1	AP-1 compl	AP1G1_MO	8	1.018392
15_MTA / C	Q01768	-0.01227	0.956248	0.678087	Nme2	Nucleoside	NDKB_MOU	13	0.991529
30_MTA / C	P10126	0.000357	0.958695	0.678275	Eef1a1	Elongation	EF1A1_MO	26	1.000248
30_MTA / C	P29595	-0.03967	0.958472	0.678275	Nedd8	NEDD8	NEDD8_MO	3	0.972877
30_MTA / C	Q711T7	-0.08363	0.958817	0.678275	Nadsyn1	Glutamine-	NADE_MOU	14	0.943679
30_MTA / C	Q8K274	0.246163	0.957851	0.678275	Fn3krp	Ketosamine	KT3K_MOU	1	1.186049
15_MTA / C	Q8VE62	0.100982	0.958775	0.678275	Paip1	Polyadenyl	PAIP1_MOU	5	1.072503
30_MTA / C	P49586	-0.22403	0.960159	0.678894	Pcyt1a	Choline-ph	PCY1A_MO	5	0.856168
30_MTA / C	Q8N9S3	0.299983	0.960046	0.678894	Ahsa2	Activator o	AHSA2_MO	1	1.23113
30_MTA / C	P0C7N9	-0.1189	0.960703	0.678942	Psmg4	Proteasom	PSMG4_MO	1	0.920892
30_MTA / C	Q6P3A8	0.082261	0.960665	0.678942	Bckdhb	2-oxoisoval	ODBB_MOU	10	1.058676
30_MTA / C	P01680	0.171988	0.966476	0.67956	NaN	Ig kappa ch	KV4A1_MO	1	1.12661
30_MTA / C	P12265	-0.00972	0.96529	0.67956	Gusb	Beta-glucur	BGLR_MOU	17	0.993288
15_MTA / C	P25688	-0.00467	0.963258	0.67956	Uox	Uricase	URIC_MOU	22	0.996768
15_MTA / C	P34914	0.012349	0.962153	0.67956	Ephx2	Bifunctiona	HYES_MOU	26	1.008597
15_MTA / C	P35278;P6	-0.08811	0.963981	0.67956	Rab5c;Rab5	Ras-related	RAB5C_MO	2	0.940754
15_MTA / C	P62806	0.138013	0.964838	0.67956	H4c1	Histone H4	H4_MOUSE	6	1.100388
15_MTA / C	Q61990	-0.08287	0.963062	0.67956	Pcbp2	Poly(rC)-bir	PCBP2_MO	8	0.944175
30_MTA / C	Q7TQH0	0.347702	0.966459	0.67956	Atxn2l	Ataxin-2-lik	ATX2L_MO	5	1.272532
15_MTA / C	Q8BP67	-0.10941	0.966196	0.67956	Rpl24	Large ribos	RL24_MOU	6	0.926968
30_MTA / C	Q9CQ89	0.088428	0.965693	0.67956	Cuta	Protein Cut	CUTA_MOU	4	1.063211
15_MTA / C	Q9CQV8	0.073068	0.963007	0.67956	Ywhab	14-3-3 prot	1433B_MO	7	1.051952
30_MTA / C	Q9D1J3	-0.03521	0.965383	0.67956	Sarnp	SAP domair	SARNP_MO	4	0.975889
15_MTA / C	Q9D7A8	0.846647	0.965212	0.67956	Armc1	Armadillo r	ARMC1_MO	1	1.798317
15_MTA / C	Q9D8W5	-0.08546	0.963914	0.67956	Psmd12	26S protea	PSD12_MO	9	0.942482
15_MTA / C	Q9DB29	-0.03764	0.962883	0.67956	Iah1	Isoamyl ace	IAH1_MOU	14	0.974247
30_MTA / C	Q9QZ88	0.039586	0.962627	0.67956	Vps29	Vacuolar pr	VPS29_MO	5	1.027819
15_MTA / C	A2AKK5	-0.24798	0.969365	0.680059	Acnat1	Acyl-coenzy	ACNT1_MO	5	0.842072
30_MTA / C	O09012	0.077964	0.968945	0.680059	Pex5	Peroxisoma	PEX5_MOU	1	1.055527
30_MTA / C	P11679	-0.07801	0.968866	0.680059	Krt8	Keratin, typ	K2C8_MOU	18	0.947364
15_MTA / C	P11930	-0.45184	0.970217	0.680059	Nudt19	Acyl-coenzy	NUD19_MO	5	0.73111
15_MTA / C	P14824	0.035386	0.969601	0.680059	Anxa6	Annexin A6	ANXA6_MO	36	1.024831
30_MTA / C	P48193	0.241742	0.97015	0.680059	Epb41	Protein 4.1	EPB41_MO	4	1.18242
30_MTA / C	P61967	1.059078	0.969254	0.680059	Ap1s1	AP-1 compl	AP1S1_MO	1	2.083599
15_MTA / C	P62855	-0.73297	0.967916	0.680059	Rps26	Small ribos	RS26_MOU	2	0.601665
15_MTA / C	Q99LD4	-0.14492	0.968893	0.680059	Gps1	COP9 signa	CSN1_MOU	10	0.904429
15_MTA / C	Q9EPL8	-0.18529	0.968765	0.680059	Ipo7	Importin-7	IPO7_MOU	8	0.87947
30_MTA / C	Q9JIY5	-0.00186	0.969423	0.680059	Htra2	Serine prot	HTRA2_MO	4	0.99871
30_MTA / C	Q8VCW8	-0.22369	0.970754	0.680303	Acsf2	Medium-ch	ACSF2_MO	13	0.856374
30_MTA / C	Q8BG73	-0.20456	0.971481	0.680547	Sh3bgrl2	SH3 domair	SH3L2_MO	5	0.867805
30_MTA / C	P08249	0.04391	0.972468	0.680576	Mdh2	Malate deh	MDHM_MO	19	1.030904
15_MTA / C	P84099	0.276927	0.97233	0.680576	Rpl19	Large ribos	RL19_MOU	4	1.211611
15_MTA / C	Q8BG32	0.317287	0.971849	0.680576	Psmd11	26S protea	PSD11_MO	13	1.245985
30_MTA / C	P60122	0.168712	0.974186	0.681514	Ruvbl1	RuvB-like 1	RUVB1_MO	6	1.124054
15_MTA / C	Q283N4	-0.06658	0.97415	0.681514	Urad	2-oxo-4-hy	URAD_MOU	8	0.954901
30_MTA / C	Q00724	-0.02199	0.974507	0.681606	Rbp4	Retinol-bin	RET4_MOU	8	0.984873
15_MTA / C	P03976	-1.2794	0.979079	0.682578	NaN	Ig kappa ch	KV2A5_MO	1	0.411966
30_MTA / C	P56565	-0.10216	0.977955	0.682578	S100a1	Protein S1(	S10A1_MO	1	0.931639

15_MTA / C	P70349	0.034044	0.978268	0.682578	Hint1	Adenosine !	HINT1_MOU	9	1.023878
15_MTA / C	Q6P3A8	-0.12138	0.976812	0.682578	Bckdhb	2-oxoisoval	ODBB_MOU	10	0.919311
30_MTA / C	Q6P6M7	-0.10735	0.977937	0.682578	Sepsecs	O-phospho	SPCS_MOU	11	0.928292
30_MTA / C	Q922R8	-0.04911	0.979065	0.682578	Pdia6	Protein dis	PDIA6_MO	12	0.966531
30_MTA / C	Q9WVA2	0.056429	0.979051	0.682578	Timm8a1	Mitochond	TIM8A_MO	5	1.039889
30_MTA / C	Q9Z2I9	0.010532	0.977127	0.682578	Sucla2	Succinate--	SUCB1_MO	17	1.007327
15_MTA / C	O89017	0.225809	0.980271	0.683016	Lgmn	Legumain	LGMN_MO	3	1.169433
15_MTA / C	Q61510	0.464337	0.980225	0.683016	Trim25	E3 ubiquiti	TRI25_MOU	2	1.379683
30_MTA / C	Q9CQ86	-0.22145	0.980507	0.683016	Mien1	Migration a	MIEN1_MO	3	0.857705
30_MTA / C	Q9D0S9	0.071564	0.980383	0.683016	Hint2	Adenosine !	HINT2_MO	5	1.050855
30_MTA / C	Q06185	0.29153	0.980826	0.683084	Atp5me	ATP syntha	ATP5I_MOU	1	1.223937
30_MTA / C	P62270	-0.15641	0.981205	0.683105	Rps18	Small ribos	RS18_MOU	6	0.897255
30_MTA / C	O35685	0.045786	0.982052	0.683167	Nudc	Nuclear mi	NUDC_MOU	13	1.032245
30_MTA / C	P70697	-0.18202	0.981986	0.683167	Urod	Uroporphy	DCUP_MOU	3	0.881467
30_MTA / C	P97807	-0.23257	0.981906	0.683167	Fh	Fumarate h	FUMH_MO	10	0.851119
30_MTA / C	Q91VT4	-0.0249	0.981985	0.683167	Cbr4	3-oxoacyl-[	CBR4_MOU	7	0.982886
30_MTA / C	P51881	0.09326	0.982939	0.683651	Slc25a5	ADP/ATP tr	ADT2_MOU	5	1.066778
15_MTA / C	P83882	-0.04334	0.983783	0.683974	Rpl36a	Large ribos	RL36A_MO	4	0.970405
30_MTA / C	P08228	0.053093	0.984455	0.684047	Sod1	Superoxide	SODC_MOU	12	1.037487
15_MTA / C	P16546	-0.45526	0.98864	0.684047	Sptan1	Spectrin al	SPTN1_MO	32	0.729378
15_MTA / C	P36552	0.091749	0.987731	0.684047	Cpox	Oxygen-de	HEM6_MOU	21	1.065661
15_MTA / C	P45591	0.026461	0.986122	0.684047	Cfl2	Cofilin-2	COF2_MOU	11	1.018511
15_MTA / C	P45878	0.02962	0.986662	0.684047	Fkbp2	Peptidyl-pr	FKBP2_MO	5	1.020743
15_MTA / C	P56593	-0.67887	0.987283	0.684047	Cyp2a12	Cytochrom	CP2AC_MO	1	0.624652
15_MTA / C	P57776	-0.04904	0.987903	0.684047	Eef1d	Elongation	EF1D_MOU	13	0.96658
30_MTA / C	P62889	0.011348	0.986822	0.684047	Rpl30	Large ribos	RL30_MOU	6	1.007897
30_MTA / C	P62900	0.120568	0.988366	0.684047	Rpl31	Large ribos	RL31_MOU	3	1.087163
30_MTA / C	Q569Z6	0.032898	0.985673	0.684047	Thrap3	Thyroid hor	TR150_MO	4	1.023065
15_MTA / C	Q8BGC4	-0.09718	0.987325	0.684047	Ptgr3	Prostagland	PTGR3_MO	6	0.934859
15_MTA / C	Q91WG0	0.052471	0.986266	0.684047	Ces2c	Acylcarniti	EST2C_MOU	9	1.03704
30_MTA / C	Q99MN1	-0.43902	0.986015	0.684047	Kars1	Lysine--tRN	SYK_MOUSE	8	0.737637
30_MTA / C	Q9CXW4	0.033145	0.987992	0.684047	Rpl11	Large ribos	RL11_MOU	2	1.02324
30_MTA / C	Q9D662	0.227977	0.986782	0.684047	Sec23b	Protein tra	SC23B_MO	12	1.171192
15_MTA / C	Q9JHR7	0.067043	0.988801	0.684047	Ide	Insulin-deg	IDE_MOUSE	22	1.047568
15_MTA / C	O55135	0.084199	0.989867	0.684317	Eif6	Eukaryotic	IF6_MOUSE	7	1.060099
30_MTA / C	P60766	0.082309	0.989941	0.684317	Cdc42	Cell divisio	CDC42_MC	3	1.058711
15_MTA / C	Q9CYW4	0.096617	0.990905	0.684703	Hdhd3	Haloacid de	HDHD3_MO	11	1.069263
30_MTA / C	O88958	0.064584	0.991371	0.684754	Gnpda1	Glucosamin	GNPI1_MO	14	1.045783
15_MTA / C	Q9JMA1	-0.07854	0.991282	0.684754	Usp14	Ubiquitin c	UBP14_MC	15	0.947014
30_MTA / C	Q9R0Q6	0.372638	0.992311	0.685271	Arpc1a	Actin-relate	ARC1A_MO	6	1.294718
30_MTA / C	O35900	0.133974	0.993208	0.685759	Lsm2	U6 snRNA-a	LSM2_MOU	4	1.097312
30_MTA / C	P02668	0.076562	0.994829	0.686029	CSN3	Kappa-case	CASK_BOVI	2	1.054502
30_MTA / C	P68254	0.027833	0.994275	0.686029	Ywhaq	14-3-3 prot	1433T_MO	11	1.01948
15_MTA / C	Q6P542	-0.3646	0.994932	0.686029	Abcf1	ATP-bindin	ABCF1_MO	3	0.776684
30_MTA / C	Q8BWN8	-0.14589	0.994876	0.686029	Acot4	Peroxisoma	ACOT4_MO	3	0.903819
15_MTA / C	Q8VDM4	0.043876	0.993934	0.686029	Psmd2	26S protea	PSMD2_MC	30	1.03088
30_MTA / C	Q9D0I9	0.658832	0.994571	0.686029	Rars1	Arginine--tl	SYRC_MOU	1	1.578804
15_MTA / C	P63101	0.034957	0.995879	0.68655	Ywhaz	14-3-3 prot	1433Z_MO	12	1.024527
30_MTA / C	Q6ZWX6	0.12991	0.996109	0.686577	Eif2s1	Eukaryotic	IF2A_MOUS	16	1.094226
30_MTA / C	O89017	0.166233	0.997281	0.687073	Lgmn	Legumain	LGMN_MO	3	1.122125
15_MTA / C	Q8R5H1	-0.37428	0.997418	0.687073	Usp15	Ubiquitin c	UBP15_MC	9	0.771488
15_MTA / C	Q99L13	-0.05775	0.997591	0.687073	Hibadh	3-hydroxyi:	3HIDH_MO	13	0.960764

15_MTA / C	P49312	0.057832	0.998004	0.687226	Hnrnpa1	Heterogene	ROA1_MOU	8	1.0409
15_MTA / C	P97821	0.068374	0.99847	0.687415	Ctsc	Dipeptidyl	CATC_MOU	3	1.048535
30_MTA / C	P06801	0.012684	0.998751	0.687477	Me1	NADP-depe	MAOX_MOU	34	1.008831
30_MTA / C	Q6ZQ38	0.031545	0.99895	0.687482	Cand1	Cullin-asso	CAND1_MC	37	1.022106
30_MTA / C	Q571F8	-0.01608	0.999766	0.687912	Gls2	Glutaminas	GLSL_MOU	1	0.988919
