# Supplementary material for: Decoding the Cellular Response to Methylthioadenosine Uncovers Novel Targets for Chronic Liver Disease
Source: Cells. 2026 Sep 22;15(19):1720. doi: 10.3390/cells15191720 (PMC13664110; doi:10.3390/cells15191720)
Supplement: Supplementary file 1 [file cells-15-01720-s001.zip › Table S5_PISA_Extracts_LiverKO.xlsx.pdf]

Compariso	ProteinGro	AVG Log2 F	Pvalue	Qvalue	Genes	ProteinDes	ProteinNar	# Unique To	FC
15min / C	P50247	3.250934	9.36E-11	4.47E-07	Ahcy	Adenosylhc	SAHH_MOU	35	9.519819
30min / C	P50247	3.374686	3.28E-10	7.85E-07	Ahcy	Adenosylhc	SAHH_MOU	35	10.37246
15min / C	O55060	0.806844	2.22E-07	0.000353	Tpmt	Thiopurine	TPMT_MOU	16	1.74938
30min / C	O55060	0.757798	3.35E-07	0.000365	Tpmt	Thiopurine	TPMT_MOU	16	1.690908
15min / C	Q62419	2.307278	3.82E-07	0.000365	Sh3gl1	Endophilin	SH3G1_MC	9	4.949483
30min / C	Q9CQ65	0.518712	2.91E-06	0.001986	Mtap	S-methyl-5'	MTAP_MOU	18	1.432675
30min / C	Q99LB7	-1.00515	5.31E-06	0.003005	Sardh	Sarcosine d	SARDH_MC	45	0.498218
30min / C	P68040	-0.5791	6.67E-06	0.003187	Rack1	Small ribos	RACK1_MO	19	0.66938
15min / C	P16331	0.797528	2.63E-05	0.01145	Pah	Phenylalan	PH4H_MOU	34	1.73812
30min / C	Q8CHP5	-0.73885	3.60E-05	0.014327	Pym1	Partner of Y	PYM1_MOU	7	0.599218
15min / C	Q6GQT9	2.275207	3.91E-05	0.014383	Nomo1	BOS compl	NOMO1_M	8	4.84067
30min / C	Q64737	0.949893	4.41E-05	0.015073	Gart	Trifunction	PUR2_MOU	30	1.931729
15min / C	Q3UMC0	1.410709	4.81E-05	0.015342	Afg2a	ATPase fam	AFG2A_MO	3	2.658679
15min / C	Q9CQ65	0.554847	6.23E-05	0.018601	Mtap	S-methyl-5'	MTAP_MOU	18	1.469012
15min / C	P02663	-0.79071	8.52E-05	0.021704	CSN1S2	Alpha-S2-ca	CASA2_BOV	4	0.578059
15min / C	Q64737	0.952495	9.08E-05	0.021704	Gart	Trifunction	PUR2_MOU	30	1.935216
15min / C	P02668	0.883217	0.000104	0.022293	CSN3	Kappa-case	CASK_BOVI	2	1.844484
30min / C	P16331	0.882903	0.000107	0.022293	Pah	Phenylalan	PH4H_MOU	34	1.844082
30min / C	Q9DBF1	-0.26203	0.0001	0.022293	Aldh7a1	Alpha-amin	AL7A1_MO	28	0.833913
15min / C	P60335	-0.44763	0.000124	0.024285	Pcbp1	Poly(rC)-bir	PCBP1_MO	11	0.733247
30min / C	Q3UMC0	1.3747	0.000127	0.024285	Afg2a	ATPase fam	AFG2A_MO	3	2.593139
15min / C	Q4KWH5	-3.08686	0.000149	0.026435	Plch1	1-phosphat	PLCH1_MO	3	0.117696
30min / C	Q6ZWQ0	-4.68276	0.000166	0.028417	Syne2	Nesprin-2	SYNE2_MO	4	0.038936
30min / C	Q6GQT9	2.232842	0.000245	0.035034	Nomo1	BOS compl	NOMO1_M	8	4.700589
30min / C	Q91V41	-0.57709	0.000227	0.035034	Rab14	Ras-related	RAB14_MO	13	0.670315
15min / C	P08207	-1.06407	0.000288	0.036201	S100a10	Protein S1(	S10AA_MO	3	0.47828
30min / C	P17742	-0.27449	0.000273	0.036201	Ppia	Peptidyl-pr	PPIA_MOUS	12	0.82674
30min / C	P43276	-0.34663	0.000282	0.036201	H1-5	Histone H1	H15_MOUS	4	0.786417
15min / C	Q61990	-0.32826	0.000338	0.041382	Pcbp2	Poly(rC)-bir	PCBP2_MO	16	0.796495
30min / C	P26350	-0.5985	0.000362	0.0423	Ptma	Prothymos	PTMA_MOU	7	0.66044
30min / C	P62259	-0.13362	0.000363	0.0423	Ywhae	14-3-3 prot	1433E_MO	18	0.911543
30min / C	Q6PDY2	2.45289	0.000381	0.043408	Ado	2-aminoeth	AEDO_MOU	5	5.475119
30min / C	Q7TNC4	-1.90866	0.000428	0.045885	Luc7l2	Putative RN	LC7L2_MO	7	0.266341
15min / C	Q8K1R3	-2.36036	0.000418	0.045885	Pnpt1	Polyribonu	PNPT1_MO	16	0.194742
30min / C	P52760	-0.28498	0.000465	0.04634	Rida	2-iminobut	RIDA_MOU	9	0.820751
15min / C	Q6ZPJ3	0.761468	0.000458	0.04634	Ube2o	(E3-indeper	UBE2O_MC	13	1.695215
30min / C	Q99K67	-1.25947	0.000452	0.04634	Aass	Alpha-amin	AASS_MOU	37	0.417698
15min / C	Q8R4N0	0.642941	0.000477	0.04654	Clybl	Citramalyl-	CLYBL_MO	19	1.561509
30min / C	P17563	-0.37659	0.000568	0.048191	Selenbp1	Methaneth	SBP1_MOU	9	0.770254
30min / C	P29595	-1.0141	0.000524	0.048191	Nedd8	NEDD8	NEDD8_MC	4	0.495136
30min / C	P82198	1.045827	0.00055	0.048191	Tgfbi	Transformi	BGH3_MOU	12	2.06455
30min / C	Q3UTQ7	-1.20569	0.000604	0.048191	Prdm10	PR domain	PRD10_MC	1	0.433561
30min / C	Q9EQ80	0.352122	0.000586	0.048191	Nif3l1	NIF3-like pr	NIF3L_MOU	10	1.276436
15min / C	Q9R257	-0.44557	0.000562	0.048191	Hebp1	Heme-bind	HEBP1_MO	11	0.734296
30min / C	Q9D8T2	-0.30071	0.000629	0.04848	Gsdmd	Gasdermin-	GSDMD_M	17	0.811855
15min / C	P15864	-0.274	0.000644	0.048586	H1-2	Histone H1	H12_MOUS	4	0.827021
30min / C	P08226	-0.30575	0.000673	0.049472	Apoe	Apolipopr	APOE_MOU	17	0.809019
30min / C	P97765	-0.93739	0.000695	0.050332	Wbp2	WW domai	WBP2_MO	5	0.522175
30min / C	Q99M28	1.740083	0.000791	0.056402	Rnps1	RNA-bindir	RNPS1_MO	4	3.340543
15min / C	Q9D8T2	-0.21914	0.000803	0.056472	Gsdmd	Gasdermin-	GSDMD_M	17	0.859078
30min / C	Q8K4Z3	-0.26135	0.000831	0.056737	Naxe	NAD(P)H-hy	NNRE_MOU	10	0.834305

15min / C	Q06890	-0.31098	0.000844	0.056802	Clu	Clusterin	CLUS_MOU	13	0.806095
15min / C	P09405	0.235806	0.000909	0.058488	Ncl	Nucleolin	NUCL_MOU	26	1.177565
15min / C	Q9JII5	-0.41719	0.000918	0.058488	Dazap1	DAZ-associa	DAZP1_MO	7	0.748881
15min / C	Q8CF02	-0.57677	0.000951	0.059041	Fam25a	Protein FAI	FM25C_MC	2	0.670463
30min / C	P01887	-0.46154	0.000992	0.060025	B2m	Beta-2-mici	B2MG_MOU	2	0.726212
15min / C	Q8JZV7	0.933769	0.001008	0.060227	Amdhd2	N-acetylglu	NAGA_MOU	11	1.91026
30min / C	P55264	0.363142	0.001108	0.063795	Adk	Adenosine I	ADK_MOUS	23	1.286224
30min / C	P62301	-0.84042	0.001173	0.065957	Rps13	Small ribos	RS13_MOU	8	0.55848
15min / C	Q8K274	1.231825	0.001172	0.065957	Fn3krp	Ketosamine	KT3K_MOU	7	2.34864
30min / C	P61089	-0.18229	0.001224	0.066072	Ube2n	Ubiquitin-c	UBE2N_MC	9	0.881302
30min / C	P83882	-1.05545	0.001199	0.066072	Rpl36a	Large ribos	RL36A_MO	4	0.481148
30min / C	Q06138	-0.2027	0.001232	0.066072	Cab39	Calcium-bi	CAB39_MO	13	0.868921
15min / C	Q8K2Q0	-2.01641	0.001244	0.066072	Commd9	COMM don	COMD9_M	9	0.247172
30min / C	Q9EST4	0.6971	0.001223	0.066072	Psmg2	Proteasom	PSMG2_MC	7	1.621243
15min / C	P01837	-0.16798	0.00129	0.067275	Igkc	Immunoglc	IGKC_MOU	6	0.890089
30min / C	P11725	-0.22355	0.001361	0.068095	Otc	Ornithine t	OTC_MOUS	27	0.856453
30min / C	Q3TC93	-1.11716	0.001327	0.068095	Hs1bp3	HCLS1-binc	H1BP3_MC	10	0.460999
15min / C	Q8VCR7	-0.19174	0.001363	0.068095	Abhd14b	Putative pr	ABHEB_MO	12	0.875546
15min / C	P62743	-1.28914	0.001396	0.068099	Ap2s1	AP-2 compl	AP2S1_MO	5	0.409195
15min / C	Q91VT4	-0.37362	0.001412	0.068186	Cbr4	3-oxoacyl-[	CBR4_MOU	9	0.771841
30min / C	P62960	-0.59717	0.001472	0.069679	Ybx1	Y-box-bindi	YBOX1_MO	17	0.661051
30min / C	D3Z4S3	-0.58695	0.001539	0.071156	Ptrhd1	Putative pe	PTRD1_MO	2	0.665748
15min / C	P43274	-0.31522	0.001548	0.071156	H1-4	Histone H1	H14_MOUS	3	0.803728
15min / C	O70591	0.348022	0.001864	0.072021	Pfdn2	Prefoldin su	PFD2_MOU	4	1.272814
30min / C	P05202	-0.2164	0.001719	0.072021	Got2	Aspartate a	AATM_MOU	35	0.860712
30min / C	P12710	-0.42008	0.001659	0.072021	Fabp1	Fatty acid-k	FABPL_MO	10	0.747383
30min / C	P15864	-0.25046	0.001692	0.072021	H1-2	Histone H1	H12_MOUS	4	0.840629
15min / C	P28798	-0.49278	0.001843	0.072021	Grn	Progranulir	GRN_MOUS	5	0.710657
30min / C	P49718	-0.98005	0.001838	0.072021	Mcm5	DNA replica	MCM5_MO	9	0.506961
30min / C	P53996	-1.37915	0.001686	0.072021	Cnbp	CCHC-type	CNBP_MOU	6	0.384445
15min / C	P70297	-0.97713	0.00165	0.072021	Stam	Signal trans	STAM1_MC	7	0.507988
15min / C	P84104	-0.36373	0.001616	0.072021	Srsf3	Serine/argi	SRSF3_MOI	4	0.777152
15min / C	Q3U0V1	-0.24424	0.001789	0.072021	Khsrp	Far upstrea	FUBP2_MO	21	0.844258
30min / C	Q6ZWZ2	-0.22986	0.001695	0.072021	Ube2r2	Ubiquitin-c	UB2R2_MC	4	0.852718
15min / C	Q8CIN4	1.496819	0.001807	0.072021	Pak2	Serine/thre	PAK2_MOU	15	2.822197
15min / C	Q8K4Z3	-0.24592	0.001839	0.072021	Naxe	NAD(P)H-hy	NNRE_MOU	10	0.843277
15min / C	Q8VCI5	-0.54304	0.00177	0.072021	Pex19	Peroxisoma	PEX19_MO	10	0.686324
30min / C	Q9D6Y9	-0.89731	0.001786	0.072021	Gbe1	1,4-alpha-g	GLGB_MOU	32	0.536888
30min / C	Q9Z0S1	0.23038	0.001798	0.072021	Bpnt1	3'(2'),5'-bis	BPNT1_MO	16	1.173144
15min / C	B1AY13	-1.93086	0.001915	0.073244	Usp24	Ubiquitin c	UBP24_MC	27	0.262273
30min / C	Q9DBL7	-1.16681	0.001996	0.073954	Coasy	Bifunctiona	COASY_MO	18	0.445406
15min / C	P54728	-0.15685	0.002043	0.075017	Rad23b	UV excisior	RD23B_MC	13	0.896979
15min / C	Q8CHP5	-0.42627	0.002056	0.075017	Pym1	Partner of Y	PYM1_MOU	7	0.744183
30min / C	P62313	-0.38778	0.002108	0.07506	Lsm6	U6 snRNA-a	LSM6_MOU	2	0.764306
30min / C	P97807	-0.48742	0.002099	0.07506	Fh	Fumarate h	FUMH_MOI	21	0.713299
30min / C	Q9DCH6	-0.40771	0.00212	0.07506	Zfand6	AN1-type zi	ZFAN6_MO	4	0.753818
30min / C	Q9D819	0.20294	0.002193	0.077081	Ppa1	Inorganic p	IPYR_MOUS	19	1.151041
30min / C	Q9WVL0	-0.29467	0.002243	0.077912	Gstz1	Maleylacet	MAAI_MOU	18	0.81526
15min / C	Q9Z1D1	-0.1217	0.002249	0.077912	Eif3g	Eukaryotic	EIF3G_MOI	12	0.919106
30min / C	Q8C5H8	-0.27324	0.002308	0.079373	Nadk2	NAD kinase	NAKD2_MC	21	0.827459
30min / C	P17182	0.353916	0.002407	0.08161	Eno1	Alpha-enol	ENOA_MOU	24	1.278025
30min / C	Q7TMC8	-0.32576	0.002477	0.083112	Fcsk	L-fucose kir	FCSK_MOU	11	0.79788

30min / C	Q921W4	0.285758	0.002521	0.083112	Cryzl1	Quinone ox	QORL1_MC	5	1.21905
30min / C	Q9R0H5	2.025799	0.002515	0.083112	Krt71	Keratin, typ	K2C71_MO	3	4.072173
30min / C	P14206	-1.34944	0.002562	0.083678	Rpsa	Small ribos	RSSA_MOU	14	0.392443
15min / C	Q99JX4	-0.83419	0.002573	0.083678	Eif3m	Eukaryotic	EIF3M_MO	11	0.560897
30min / C	P49443	-0.48051	0.002646	0.08389	Ppm1a	Protein phc	PPM1A_MC	8	0.716723
30min / C	Q62426	-0.16456	0.00263	0.08389	Cstb	Cystatin-B	CYTB_MOU	8	0.892199
15min / C	Q61166	-2.71328	0.002739	0.08473	Mapre1	Microtubul	MARE1_MC	12	0.152483
15min / C	Q9DBF1	-0.1676	0.002729	0.08473	Aldh7a1	Alpha-amir	AL7A1_MO	28	0.890321
15min / C	P82198	1.222071	0.002802	0.085427	Tgfbi	Transformi	BGH3_MOU	12	2.332813
30min / C	Q9DBT9	-0.09484	0.002821	0.085427	Dmgdh	Dimethylgl	M2GD_MO	49	0.936375
30min / C	Q3U0V1	-0.27141	0.00289	0.086558	Khsrp	Far upstrea	FUBP2_MO	21	0.82851
30min / C	Q9D7A8	2.173065	0.002897	0.086558	Armc1	Armadillo r	ARMC1_MC	4	4.509806
30min / C	P61979	-0.12817	0.003002	0.088709	Hnrnpk	Heterogene	HNRPK_MC	24	0.914993
15min / C	Q9JIX8	0.62896	0.003013	0.088709	Acin1	Apoptotic c	ACINU_MO	14	1.54645
30min / C	Q61990	-0.3086	0.003093	0.089589	Pcbp2	Poly(rC)-bir	PCBP2_MO	16	0.807427
30min / C	P11352	-0.27133	0.003148	0.090011	Gpx1	Glutathion	GPX1_MOU	16	0.828554
30min / C	Q99J77	1.590653	0.003164	0.090011	Nans	Sialic acid s	SIAS_MOUS	10	3.011857
15min / C	P53996	-1.20264	0.00321	0.090796	Cnbp	CCHC-type	CNBP_MOU	6	0.434479
30min / C	Q9WVJ2	-0.17146	0.003261	0.091148	Psmd13	26S protea:	PSD13_MO	23	0.887941
30min / C	P01639	-0.93008	0.003479	0.094494	Igkv9-120	Immunoglc	KV5A7_MO	1	0.52483
30min / C	P46414	-1.76852	0.003425	0.094494	Cdkn1b	Cyclin-depe	CDN1B_MC	4	0.29351
30min / C	Q3UMU9	-0.3215	0.003416	0.094494	Hdgfl2	Hepatoma-	HDGR2_MC	10	0.80024
30min / C	Q9Z172	-0.25828	0.003466	0.094494	Sumo3	Small ubiqu	SUMO3_MC	2	0.836082
15min / C	Q99JW4	-0.61391	0.00352	0.095063	Lims1	LIM and ser	LIMS1_MO	7	0.653424
30min / C	P61290	-0.36796	0.003561	0.095236	Psme3	Proteasom	PSME3_MC	11	0.774878
15min / C	O08638	-1.38503	0.003702	0.096287	Myh11	Myosin-11	MYH11_MC	18	0.382882
30min / C	P11930	-0.75474	0.00364	0.096287	Nudt19	Acyl-coenzy	NUD19_MC	11	0.592654
30min / C	P62267	-0.86301	0.003737	0.096287	Rps23	Small ribos	RS23_MOU	6	0.549805
30min / C	P63330	0.250151	0.003665	0.096287	Ppp2ca	Serine/thre	PP2AA_MO	14	1.189331
30min / C	Q9JII5	-0.06077	0.003747	0.096287	Dazap1	DAZ-associa	DAZP1_MO	7	0.95875
15min / C	Q9WU84	0.103403	0.003673	0.096287	Ccs	Copper cha	CCS_MOUS	10	1.074304
30min / C	P84089	-0.45205	0.003795	0.097016	Erh	Enhancer o	ERH_MOUS	3	0.731004
30min / C	Q60676	0.417262	0.003826	0.097217	Ppp5c	Serine/thre	PPP5_MOU	15	1.33539
30min / C	Q9QY93	-0.37964	0.003891	0.097375	Dctpp1	dCTP pyrop	DCTP1_MO	3	0.76863
30min / C	Q9WV54	-0.37562	0.003883	0.097375	Asah1	Acid ceram	ASAH1_MO	8	0.770773
30min / C	P24270	-0.64841	0.003966	0.097956	Cat	Catalase	CATA_MOU	35	0.637982
30min / C	Q8CH18	-0.31579	0.003955	0.097956	Ccar1	Cell divisio	CCAR1_MO	14	0.803412
15min / C	Q8VDK1	-0.12044	0.003976	0.097956	Nit1	Deaminate	NIT1_MOU:	18	0.919909
30min / C	P97470	-0.90689	0.004012	0.098357	Ppp4c	Serine/thre	PP4C_MOU	4	0.533332
15min / C	P36993	0.063815	0.004069	0.099243	Ppm1b	Protein phc	PPM1B_MC	13	1.045226
15min / C	P15508	-1.36844	0.004236	0.100399	Sptb	Spectrin be	SPTB1_MO	14	0.387309
15min / C	P17427	-0.37622	0.00427	0.100399	Ap2a2	AP-2 compl	AP2A2_MO	32	0.770452
30min / C	Q62465	-0.18955	0.004285	0.100399	Vat1	Synaptic ve	VAT1_MOU	12	0.876882
30min / C	Q6PEB6	-1.75402	0.004225	0.100399	Mob4	MOB-like pı	PHOCN_MC	5	0.296474
30min / C	Q8BWT1	-0.22364	0.004272	0.100399	Acaa2	3-ketoacyl-	THIM_MOU	27	0.856403
30min / C	Q9JIX8	0.724766	0.004172	0.100399	Acin1	Apoptotic c	ACINU_MO	14	1.652632
30min / C	Q9R0P5	0.255985	0.00414	0.100399	Dstn	Destrin	DEST_MOU	14	1.194151
30min / C	P09055	1.209098	0.004337	0.101136	Itgb1	Integrin be	ITB1_MOUS	10	2.31193
15min / C	Q60865	-0.30766	0.00438	0.101139	Caprin1	Caprin-1	CAPR1_MO	9	0.807948
15min / C	P97364	-0.25909	0.004401	0.101142	Sephs2	Selenide, w	SPS2_MOU	17	0.835617
30min / C	Q8N7N5	0.762374	0.004489	0.102259	Dcaf8	DDB1- and	DCAF8_MO	9	1.69628
30min / C	P43274	-0.40927	0.004583	0.103342	H1-4	Histone H1	H14_MOUS	3	0.753002

30min / C	Q99K48	-1.03553	0.004689	0.104739	Nono	Non-POU d	NONO_MOU		21	0.487838
30min / C	Q9QUI0	0.600193	0.00467	0.104739	Rhoa	Transformi	RHOA_MOU		3	1.515919
15min / C	Q8N7N5	0.75793	0.004724	0.105025	Dcaf8	DDB1- and	DCAF8_MO		9	1.691063
15min / C	Q99M28	2.456934	0.00491	0.108164	Rnps1	RNA-bindir	RNPS1_MO		4	5.490487
30min / C	Q99020	-0.22753	0.004958	0.108707	Hnrnpab	Heterogene	ROAA_MOU		8	0.854097
15min / C	Q4LDG0	1.076289	0.005085	0.109245	Slc27a5	Long-chain	S27A5_MO		18	2.108605
15min / C	Q8BPB0;Q9	-0.79643	0.005124	0.109334	Mob1b;Mo	MOB kinase	MOB1B_MO		4	0.575773
30min / C	Q8BH43	-0.15137	0.005209	0.109687	Wasf2	Actin-bindi	WASF2_MO		9	0.900393
15min / C	Q8BWN8	1.076289	0.005189	0.109687	Acot4	Peroxisoma	ACOT4_MO		7	2.108605
15min / C	O08573	0.347663	0.005236	0.10978	Lgals9	Galectin-9	LEG9_MOU		9	1.272498
30min / C	P20918	0.310682	0.005344	0.111462	Plg	Plasminoge	PLMN_MOU		32	1.240294
15min / C	Q9CQI3	0.223583	0.005363	0.111462	Gmfb	Glia matura	GMFB_MOU		5	1.16763
30min / C	E9PV24	-0.7034	0.005402	0.111776	Fga	Fibrinogen	FIBA_MOUS		25	0.614122
15min / C	Q9CQ48	-0.47428	0.005462	0.112535	Nudcd2	NudC doma	NUDC2_MO		5	0.719828
30min / C	Q7TMK9	0.406205	0.0055	0.112837	Syncrip	Heterogene	HNRPQ_MO		20	1.325196
15min / C	O54804	1.22077	0.005562	0.113614	Chka	Choline kin	CHKA_MOU		3	2.330711
30min / C	Q9CQ60	-0.25679	0.005699	0.11495	Pgls	6-phospho	6PGL_MOU		13	0.836951
30min / C	Q3UTJ2	-0.62923	0.005774	0.115964	Sorbs2	Sorbin and	SRBS2_MO		16	0.64652
15min / C	Q91YR9	0.219602	0.005801	0.11602	Ptgr1	Prostagland	PTGR1_MO		11	1.164412
30min / C	P61961	-0.17214	0.005863	0.116286	Ufm1	Ubiquitin-f	UFM1_MOU		4	0.887528
15min / C	Q6PDY2	3.028994	0.005866	0.116286	Ado	2-aminoeth	AEDO_MOU		5	8.162403
15min / C	Q91V41	-0.53611	0.005925	0.116286	Rab14	Ras-related	RAB14_MO		13	0.68963
15min / C	Q9QUI0	0.870244	0.005936	0.116286	Rhoa	Transformi	RHOA_MOU		3	1.827972
15min / C	P28843	0.061549	0.00605	0.11782	Dpp4	Dipeptidyl	DPP4_MOU		16	1.043586
30min / C	P53994	-0.72341	0.006284	0.119275	Rab2a	Ras-related	RAB2A_MO		11	0.605663
30min / C	Q61207	0.632665	0.006281	0.119275	Psap	Prosaposin	SAP_MOUS		13	1.550427
15min / C	Q64010	0.169901	0.006245	0.119275	Crk	Adapter mo	CRK_MOUS		13	1.124981
30min / C	Q99L27	0.150308	0.006211	0.119275	Gmpr2	GMP reduc	GMPR2_MO		8	1.109806
30min / C	Q9CQI3	-0.45469	0.006288	0.119275	Gmfb	Glia matura	GMFB_MOU		5	0.729667
15min / C	P12367	-0.46509	0.006339	0.119394	Prkar2a	cAMP-depe	KAP2_MOU		11	0.724426
30min / C	P62315	-0.3656	0.006428	0.119785	Snrpd1	Small nucle	SMD1_MOU		5	0.776148
30min / C	Q9CZ44	-0.09484	0.006432	0.119785	Nsfl1c	NSFL1 cofa	NSF1C_MO		18	0.936373
15min / C	Q9Z255	-2.7651	0.00644	0.119785	Ube2a	Ubiquitin-c	UBE2A_MO		2	0.147103
15min / C	P07091	-1.09138	0.006484	0.120138	S100a4	Protein S10	S10A4_MO		2	0.469314
30min / C	Q64291	1.905432	0.006577	0.12091	Krt12	Keratin, typ	K1C12_MO		1	3.746212
30min / C	Q7TNE3	1.405975	0.006686	0.121409	Spag7	Sperm-asso	SPAG7_MO		2	2.649968
15min / C	Q9Z184	-1.1274	0.006705	0.121409	Padi3	Protein-arg	PADI3_MOU		1	0.457739
15min / C	P39061	1.37557	0.006828	0.12224	Col18a1	Collagen al	COIA1_MO		9	2.594704
15min / C	P57776	-0.13769	0.006809	0.12224	Eef1d	Elongation	EF1D_MOU		13	0.908976
15min / C	Q9WUM4	0.870575	0.006927	0.123026	Coro1c	Coronin-1C	COR1C_MO		14	1.828392
15min / C	O35295	-0.03848	0.007021	0.123832	Purb	Transcripti	PURB_MOU		7	0.973677
30min / C	P70335	-0.48359	0.007155	0.124247	Rock1	Rho-associa	ROCK1_MO		29	0.715195
15min / C	Q5I2A0	-0.2745	0.007174	0.124247	Serpina3g	Serine prot	SPA3G_MO		2	0.826739
30min / C	Q9JK38	0.358124	0.007139	0.124247	Gnpnat1	Glucosamir	GNA1_MOU		12	1.281758
30min / C	Q8VCM7	0.674567	0.007327	0.125725	Fgg	Fibrinogen	FIBG_MOUS		21	1.596117
30min / C	O88587	-0.13868	0.007581	0.125966	Comt	Catechol O-	COMT_MOU		14	0.908347
15min / C	P01887	-0.29059	0.007467	0.125966	B2m	Beta-2-mici	B2MG_MOU		2	0.81757
30min / C	P60843	-0.10767	0.0076	0.125966	Eif4a1	Eukaryotic	IF4A1_MOU		25	0.928085
30min / C	P97315	-0.38803	0.0076	0.125966	Csrp1	Cysteine an	CSRP1_MO		8	0.764171
30min / C	Q64727	-0.21464	0.007616	0.125966	Vcl	Vinculin	VINC_MOU		69	0.861759
15min / C	Q8CI32	-1.2199	0.007466	0.125966	Bag5	BAG family	BAG5_MOU		9	0.429313
15min / C	Q9ER00	-1.45203	0.007571	0.125966	Stx12	Syntaxin-12	STX12_MO		4	0.365506

30min / C	Q9WUM4	0.665951	0.007539	0.125966	Coro1c	Coronin-1C	COR1C_MO	14	1.586614
15min / C	P63085	0.338855	0.007826	0.127312	Mapk1	Mitogen-ac	MK01_MOU	12	1.264753
30min / C	Q8BGC0	-1.19008	0.00783	0.127312	Htatsf1	17S U2 SnR	HTSF1_MO	6	0.438279
15min / C	Q8R0N6	-0.17628	0.007785	0.127312	Adhfe1	Hydroxyaci	HOT_MOUS	18	0.884982
15min / C	Q9CXA2	0.219855	0.00795	0.128816	L3hypdh	Trans-L-3-h	T3HPD_MO	11	1.164616
30min / C	P11588	-0.33116	0.008012	0.128948	Mup1	Major urina	MUP1_MOU	1	0.794898
30min / C	Q8BWN8	0.348678	0.007992	0.128948	Acot4	Peroxisoma	ACOT4_MO	7	1.273394
15min / C	Q9D1A2	-0.21768	0.008116	0.129934	Cndp2	Cytosolic n	CNDP2_MO	24	0.859949
15min / C	Q9EQ80	0.210223	0.008267	0.13128	Nif3l1	NIF3-like pr	NIF3L_MOU	10	1.156867
30min / C	Q9DBB8	-0.1521	0.008405	0.133039	Dhdh	Trans-1,2-d	DHDH_MOU	21	0.899941
30min / C	Q60974	-1.64321	0.008444	0.133213	Ncor1	Nuclear rec	NCOR1_MO	6	0.320144
15min / C	Q9EST4	0.837937	0.008534	0.133315	Psmg2	Proteasome	PSMG2_MO	7	1.787492
15min / C	Q9QZQ1	-0.41176	0.008517	0.133315	Afdn	Afadin	AFAD_MOU	33	0.751708
30min / C	P17427	-0.72755	0.008655	0.13391	Ap2a2	AP-2 compl	AP2A2_MO	32	0.60393
30min / C	Q8VBT2	-0.18342	0.008657	0.13391	Sds	L-serine del	SDHL_MOU	16	0.880614
30min / C	P00329	-0.188	0.009121	0.134666	Adh1	Alcohol del	ADH1_MOU	24	0.877819
15min / C	P09055	1.603891	0.009128	0.134666	Itgb1	Integrin be	ITB1_MOUS	10	3.03962
30min / C	P35505	-0.20481	0.008945	0.134666	Fah	Fumarylace	FAAA_MOU	23	0.867652
15min / C	P58871	-0.08901	0.009108	0.134666	Tnks1bp1	182 kDa tar	TB182_MO	33	0.940169
30min / C	P62320	-0.09166	0.009012	0.134666	Snrpd3	Small nucle	SMD3_MOU	5	0.938445
30min / C	P99025	-0.12689	0.008846	0.134666	Gchfr	GTP cycloh	GFRP_MOU	6	0.915801
15min / C	Q8BMQ8	-0.43765	0.009098	0.134666	Mon1b	Vacuolar fu	MON1B_M	1	0.738337
30min / C	Q8R059	-0.83973	0.008826	0.134666	Gale	UDP-glucos	GALE_MOU	12	0.558749
30min / C	Q9CPT4	-0.31145	0.008856	0.134666	Mydgf	Myeloid-de	MYDGF_MO	6	0.805834
30min / C	Q9CPY7	-0.2741	0.009127	0.134666	Lap3	Cytosol am	AMPL_MOU	28	0.826965
30min / C	Q9CZ42	-0.19189	0.009112	0.134666	Naxd	ATP-depend	NNRD_MOU	14	0.87546
30min / C	Q9CZU3	-1.11382	0.009009	0.134666	Mtrex	Exosome RI	MTREX_MO	10	0.462068
15min / C	O35639	0.873501	0.009445	0.134866	Anxa3	Annexin A3	ANXA3_MO	13	1.832104
30min / C	P30115	-0.52508	0.009357	0.134866	Gsta3	Glutathion	GSTA3_MO	10	0.694922
30min / C	P32020	-0.18662	0.009363	0.134866	Scp2	Sterol carri	SCP2_MOU	27	0.878663
15min / C	P47955	-0.2991	0.009374	0.134866	Rplp1	Large ribos	RLA1_MOU	3	0.812757
30min / C	Q62093	-0.26707	0.00944	0.134866	Srsf2	Serine/argi	SRSF2_MOU	7	0.831007
15min / C	Q8CHW4	-0.18939	0.009264	0.134866	Eif2b5	Translation	EI2BE_MOU	8	0.876975
30min / C	Q9D1A2	-0.21823	0.009355	0.134866	Cndp2	Cytosolic n	CNDP2_MO	24	0.859617
30min / C	P07091	-1.45255	0.009589	0.135712	S100a4	Protein S10	S10A4_MO	2	0.365375
15min / C	P00761	-0.28092	0.00972	0.135955	NaN	Trypsin (Lal	TRYP_PIG	2	0.823069
15min / C	P20918	0.234082	0.009734	0.135955	Plg	Plasminoge	PLMN_MOU	32	1.176158
30min / C	Q8R016	0.016196	0.009653	0.135955	Blmh	Bleomycin	BLMH_MOU	19	1.01129
15min / C	Q9D855	1.596996	0.00969	0.135955	Uqcrb	Cytochrom	QCR7_MOU	5	3.025127
30min / C	P06151	-0.17045	0.009871	0.135974	Ldha	L-lactate de	LDHA_MOU	26	0.888565
30min / C	Q8QZT1	-0.16613	0.009854	0.135974	Acat1	Acetyl-CoA	THIL_MOUS	25	0.891228
30min / C	Q9CR70	-1.55607	0.009856	0.135974	Lage3	EKC/KEOPS	LAGE3_MO	1	0.340077
30min / C	O08810	0.200274	0.010008	0.13668	Eftud2	116 kDa U5	U5S1_MOU	23	1.148917
15min / C	Q99KJ8	-0.19218	0.009988	0.13668	Dctn2	Dynactin su	DCTN2_MO	16	0.875285
15min / C	Q9JKX6	0.5445	0.010006	0.13668	Nudt5	ADP-sugar p	NUDT5_MO	10	1.458515
30min / C	O35295	-0.21961	0.010191	0.137179	Purb	Transcripti	PURB_MOU	7	0.8588
30min / C	P62331	1.844555	0.010094	0.137179	Arf6	ADP-ribosy	ARF6_MOU	6	3.591421
30min / C	Q8BFW7	0.160682	0.010219	0.137179	Lpp	Lipoma-pre	LPP_MOUS	17	1.117815
15min / C	Q8BH61	-0.38666	0.010137	0.137179	F13a1	Coagulatio	F13A_MOU	4	0.764896
15min / C	Q8BRG8	-1.10569	0.010311	0.137179	Tmem209	Transmemb	TM209_MO	1	0.464681
30min / C	Q8BTJ4	-1.23646	0.010407	0.137179	Enpp4	Bis(5'-aden	ENPP4_MO	2	0.424413
30min / C	Q8CGA0	-1.16889	0.010417	0.137179	Ppm1f	Protein pho	PPM1F_MO	6	0.444762

30min / C	Q9JMG1	0.362897	0.010418	0.137179	Edf1	Endothelial	EDF1_MOU	6	1.286006
15min / C	Q9R0H5	1.176799	0.010409	0.137179	Krt71	Keratin, typ	K2C71_MO	3	2.260746
15min / C	Q9D1I5	-0.70893	0.010481	0.137262	Mcee	Methylmal	MCEE_MOU	5	0.611774
30min / C	Q4U2R1	1.32816	0.010566	0.137399	Herc2	E3 ubiquiti	HERC2_MO	2	2.510822
30min / C	P11499	-0.18993	0.010792	0.138685	Hsp90ab1	Heat shock	HS90B_MO	39	0.876646
30min / C	Q3TIU4	-0.28715	0.010822	0.138685	Pde12	2',5'-phosp	PDE12_MO	8	0.819518
30min / C	P12367	0.442003	0.011033	0.139906	Prkar2a	cAMP-depe	KAP2_MOU	11	1.358489
30min / C	P56375	-0.57142	0.010954	0.139906	Acyp2	Acylphosph	ACYP2_MO	1	0.672955
30min / C	Q9D0E1	0.880828	0.010989	0.139906	Hnrnpm	Heterogene	HNRPM_M	28	1.841432
30min / C	P68373	1.290527	0.011329	0.142809	Tuba1c	Tubulin alp	TBA1C_MO	2	2.446174
15min / C	Q6ZWZ2	0.022424	0.011353	0.142809	Ube2r2	Ubiquitin-c	UB2R2_MC	4	1.015665
30min / C	Q62419	1.625354	0.011385	0.14284	Sh3gl1	Endophilin	SH3G1_MC	9	3.085178
15min / C	P11352	-0.2037	0.011523	0.143062	Gpx1	Glutathion	GPX1_MOU	16	0.868321
30min / C	Q8CDM8	-1.25049	0.011716	0.144449	Fhip2a	FHF comple	FHI2A_MOU	6	0.420306
15min / C	Q61702	0.172471	0.011926	0.145793	Itih1	Inter-alpha	ITIH1_MOU	20	1.126987
30min / C	Q9R0X4	-0.97503	0.011969	0.145946	Acot9	Acyl-coenzy	ACOT9_MO	12	0.50873
30min / C	Q3TXS7	-0.20957	0.012093	0.146439	Psmd1	26S protea	PSMD1_M	35	0.864798
30min / C	Q61598	0.165477	0.01226	0.146439	Gdi2	Rab GDP di	GDIB_MOU	31	1.121537
15min / C	Q6P8I4	-0.33298	0.012264	0.146439	Pcnp	PEST prote	PCNP_MOU	7	0.793896
30min / C	Q99MD9	-0.7687	0.012171	0.146439	Nasp	Nuclear aut	NASP_MOU	10	0.586947
30min / C	P19096	-0.70645	0.012563	0.14665	Fasn	Fatty acid s	FAS_MOUS	130	0.612828
30min / C	P99027	-0.17388	0.01264	0.14665	Rplp2	Large ribos	RLA2_MOU	6	0.886454
30min / C	Q08189	1.290517	0.012541	0.14665	Tgm3	Protein-glu	TGM3_MOU	1	2.446156
15min / C	Q8VCC1	-0.6961	0.012497	0.14665	Hpgd	15-hydroxy	PGDH_MOU	9	0.617237
30min / C	Q8VI75	-0.61673	0.012355	0.14665	Ipo4	Importin-4	IPO4_MOU	14	0.652148
30min / C	Q91VT4	-0.5158	0.012623	0.14665	Cbr4	3-oxoacyl-[	CBR4_MOU	9	0.699407
30min / C	Q91Z53	0.244946	0.012384	0.14665	Grhpr	Glyoxylate	GRHPR_MC	18	1.185049
30min / C	Q9QXG4	-0.72876	0.012607	0.14665	Acss2	Acetyl-coer	ACSA_MOU	24	0.603423
15min / C	Q9Z1R2	-0.6255	0.012608	0.14665	Bag6	Large prolir	BAG6_MOU	18	0.648195
30min / C	P52196	0.193726	0.012675	0.146699	Tst	Thiosulfate	THTR_MOU	16	1.143714
30min / C	Q8BG94	1.041485	0.012808	0.147528	Commd7	COMM dom	COMD7_M	5	2.058345
15min / C	P16330	-0.96456	0.013039	0.147993	Cnp	2',3'-cyclic-	CN37_MOU	7	0.512436
30min / C	P49722	-0.32043	0.012899	0.147993	Psma2	Proteasom	PSA2_MOU	15	0.800833
15min / C	P56394	-0.11549	0.012928	0.147993	Cox17	Cytochrom	COX17_MC	1	0.923066
30min / C	Q9D0J8	-0.33662	0.013066	0.147993	Ptms	Parathymo	PTMS_MOU	4	0.791895
15min / C	Q7TPZ8	-0.63576	0.013142	0.148505	Cpa1	Carboxype	CBPA1_MO	3	0.643604
15min / C	Q62465	-0.16638	0.013196	0.14877	Vat1	Synaptic ve	VAT1_MOU	12	0.891074
15min / C	O08583	-0.1931	0.013393	0.149374	Alyref	THO compl	THOC4_MC	6	0.874726
30min / C	P62245	0.843703	0.013409	0.149374	Rps15a	Small ribos	RS15A_MO	7	1.794651
30min / C	Q06890	-0.38721	0.013403	0.149374	Clu	Clusterin	CLUS_MOU	13	0.764609
30min / C	Q8R570	-1.29591	0.013437	0.149374	Snap47	Synaptosor	SNP47_MO	9	0.407279
15min / C	Q9CWQ0	1.989712	0.013377	0.149374	Dph5	Diphthine r	DPH5_MOU	2	3.971578
30min / C	P10922	-0.33365	0.013501	0.149694	H1-0	Histone H1	H10_MOUS	7	0.793527
30min / C	Q6PB66	0.123794	0.0136	0.149694	Lrpprc	Leucine-ric	LPPRC_MO	58	1.089597
30min / C	Q9CQ92	-0.48875	0.013623	0.149694	Fis1	Mitochond	FIS1_MOUS	4	0.712644
15min / C	Q9QWK4	-0.49857	0.013574	0.149694	Cd5l	CD5 antige	CD5L_MOU	3	0.707809
30min / C	Q8JZV7	1.652862	0.013832	0.150428	Amdhd2	N-acetylglu	NAGA_MOU	11	3.144569
15min / C	Q9CZ42	-0.20253	0.013907	0.150428	Naxd	ATP-depen	NNRD_MOU	14	0.869025
30min / C	Q8BRG8	-0.20184	0.014009	0.150821	Tmem209	Transmemb	TM209_MC	1	0.869443
30min / C	O35381	0.218327	0.014169	0.151484	Anp32a	Acidic leuci	AN32A_MC	9	1.163384
15min / C	Q8BHG1	1.036943	0.014123	0.151484	Nrdc	Nardilysin	NRDC_MOU	25	2.051875
15min / C	Q8BQ47	-0.45171	0.014247	0.151484	Cnpy4	Protein can	CNPY4_MO	3	0.731177

15min / C	Q9DBL1	0.907422	0.014164	0.151484	Acadsb	Short/bran	ACDSB_MO	14	1.875691
30min / C	P34884	-0.14503	0.014475	0.152466	Mif	Macrophag	MIF_MOUS	6	0.904363
15min / C	Q61598	0.184345	0.01452	0.152466	Gdi2	Rab GDP di	GDIB_MOU	31	1.136301
30min / C	Q8BFY9	-0.66518	0.014609	0.152466	Tnpo1	Transportir	TNPO1_MC	15	0.630612
30min / C	Q9CPZ8	-0.33988	0.014558	0.152466	Cmc1	COX assemb	COXM1_MO	2	0.790105
15min / C	Q9QXY6	1.9482	0.014603	0.152466	Ehd3	EH domain-	EHD3_MOU	26	3.858928
30min / C	Q9DCN1	0.5428	0.014673	0.152802	Nudt12	NAD-cappe	NUD12_MC	17	1.456797
30min / C	P00493	-0.18471	0.014882	0.153308	Hprt1	Hypoxanth	HPRT_MOU	13	0.879828
30min / C	P06537	-1.24578	0.014917	0.153308	Nr3c1	Glucocorti	GCR_MOUS	9	0.421679
15min / C	P62878	-0.16503	0.014975	0.153308	Rbx1	E3 ubiquiti	RBX1_MOU	2	0.89191
30min / C	Q9CQU5	2.239325	0.014978	0.153308	Zwint	ZW10 inter	ZWINT_MO	1	4.72176
30min / C	Q8CI08	-1.45575	0.015018	0.153391	Slain2	SLAIN moti	SLAI2_MOU	5	0.364565
30min / C	A2ADY9	-0.07999	0.015296	0.153659	Ddi2	Protein DD	DDI2_MOU	16	0.946066
30min / C	O54984	-0.59925	0.015302	0.153659	Get3	ATPase GET	GET3_MOU	9	0.660096
15min / C	P30416	0.066877	0.015156	0.153659	Fkbp4	Peptidyl-pr	FKBP4_MO	28	1.047447
30min / C	Q5M8N0	-1.4191	0.015126	0.153659	Cnrip1	CB1 cannab	CNRP1_MC	2	0.373945
30min / C	Q8K0V4	1.208699	0.015285	0.153659	Cnot3	CCR4-NOT t	CNOT3_MC	5	2.311291
30min / C	Q9JMD0	-0.58789	0.01529	0.153659	Znf207	BUB3-inter	ZN207_MO	3	0.665313
30min / C	P33267	-0.97359	0.015371	0.154037	Cyp2f2	Cytochrom	CP2F2_MO	25	0.509236
30min / C	P47791	-0.08243	0.015484	0.154837	Gsr	Glutathion	GSHR_MOU	19	0.944465
30min / C	Q5SGK3	0.463192	0.015549	0.15487	Aox2	Aldehyde o	AOXB_MOU	2	1.378589
15min / C	Q62318	0.282937	0.015552	0.15487	Trim28	Transcripti	TIF1B_MOU	23	1.216669
30min / C	P50518	-0.67704	0.015712	0.155529	Atp6v1e1	V-type prot	VATE1_MO	9	0.625446
30min / C	Q3TMH2	0.073554	0.015733	0.155529	Scrn3	Secernin-3	SCRN3_MO	14	1.052306
15min / C	Q9CR95	-0.09998	0.015748	0.155529	Necap1	Adaptin ear	NECP1_MO	9	0.933049
15min / C	Q80WQ2	1.069537	0.015817	0.155884	Vac14	Protein VA(	VAC14_MO	9	2.09876
15min / C	P10630	-0.30255	0.01601	0.156523	Eif4a2	Eukaryotic	IF4A2_MOU	12	0.810818
15min / C	P97379	-0.71861	0.016103	0.156523	G3bp2	Ras GTPase-	G3BP2_MC	12	0.607682
15min / C	Q8BUV3	-0.0539	0.016161	0.156523	Gphn	Gephyrin	GEPH_MOU	34	0.963332
30min / C	Q8BX09	-0.46593	0.016137	0.156523	Rbbp5	Retinoblast	RBBP5_MO	2	0.724004
30min / C	Q8R164	-0.15846	0.016022	0.156523	Bphl	Valacyclovi	BPHL_MOU	17	0.895982
30min / C	Q91VX2	-0.84148	0.016242	0.156523	Ubap2	Ubiquitin-a	UBAP2_MC	6	0.558069
30min / C	Q9CWQ0	2.101903	0.016234	0.156523	Dph5	Diphthine r	DPH5_MOU	2	4.292753
30min / C	Q9DBH5	-0.68809	0.016373	0.156523	Lman2	Vesicular ir	LMAN2_MC	11	0.620673
30min / C	Q9JM62	-0.21445	0.016159	0.156523	Reep6	Receptor e	REEP6_MO	5	0.861876
15min / C	Q7TNE3	1.275503	0.016494	0.157369	Spag7	Sperm-asso	SPAG7_MO	2	2.420832
15min / C	Q9WTL7	-0.75577	0.016528	0.157382	Lypla2	Acyl-protei	LYPA2_MO	7	0.592231
15min / C	P61290	-0.28701	0.016583	0.157585	Psme3	Proteasom	PSME3_MC	11	0.819596
15min / C	Q9CZ44	-0.10219	0.016981	0.160418	Nsfl1c	NSFL1 cofa	NSF1C_MO	18	0.931621
30min / C	P06330	-0.17758	0.017256	0.161169	NaN	Ig heavy ch	HVM51_MC	5	0.884183
30min / C	P08607	0.315372	0.017322	0.161169	C4bpa	C4b-bindin	C4BPA_MO	12	1.244332
30min / C	P54728	-0.13456	0.017331	0.161169	Rad23b	UV excisior	RD23B_MC	13	0.910949
15min / C	Q8QZT1	-0.12422	0.017329	0.161169	Acat1	Acetyl-CoA	THIL_MOUS	25	0.917502
15min / C	Q64291	1.089956	0.017373	0.161253	Krt12	Keratin, typ	K1C12_MO	1	2.128676
15min / C	O88271	-0.46878	0.017489	0.162007	Cfdp1	Craniofacia	CFDP1_MO	2	0.722576
15min / C	Q61581	0.264261	0.017553	0.162292	Igfbp7	Insulin-like	IBP7_MOUS	3	1.201021
30min / C	Q8VCI5	-0.43302	0.017871	0.164275	Pex19	Peroxisoma	PEX19_MO	10	0.740709
30min / C	Q9JJU8	-0.28062	0.017854	0.164275	Sh3bgrl	Adapter Sh	SH3L1_MO	10	0.823236
15min / C	P04186	-0.14615	0.017944	0.164287	Cfb	Compleme	CFAB_MOU	29	0.903656
15min / C	P55264	0.535455	0.01801	0.164287	Adk	Adenosine	ADK_MOUS	23	1.449399
30min / C	P62862	-0.44109	0.018004	0.164287	Fau	Ubiquitin-l	RS30_MOU	2	0.736576
30min / C	Q6A028	-0.07331	0.018044	0.164287	Swap70	Switch-asso	SWP70_MC	6	0.950457

15min / C	P00329	-0.13576	0.018103	0.164506	Adh1	Alcohol del	ADH1_MOU	24	0.910189
15min / C	P47964	-0.43189	0.018242	0.165053	Rpl36	Large ribos	RL36_MOU	4	0.741288
15min / C	Q4U2R1	-0.31787	0.018266	0.165053	Herc2	E3 ubiquiti	HERC2_MO	2	0.802256
30min / C	Q60865	-0.1976	0.018218	0.165053	Caprin1	Caprin-1	CAPR1_MO	9	0.871999
30min / C	P97855	-1.31517	0.018389	0.165269	G3bp1	Ras GTPase	G3BP1_MC	12	0.401879
15min / C	Q60676	0.314494	0.018382	0.165269	Ppp5c	Serine/thre	PPP5_MOU	15	1.243575
30min / C	Q9CR98	-0.20962	0.018429	0.165269	Fam136a	Protein FAI	F136A_MO	8	0.864767
15min / C	P02662	0.062084	0.018739	0.165772	CSN1S1	Alpha-S1-ca	CASA1_BOV	3	1.043972
15min / C	P68373	0.590179	0.018652	0.165772	Tuba1c	Tubulin alp	TBA1C_MO	2	1.505434
30min / C	Q6P1F6	0.190591	0.018565	0.165772	Ppp2r2a	Serine/thre	2ABA_MOU	10	1.141231
15min / C	Q8K1L5	-0.61815	0.018524	0.165772	Ppp1r11	E3 ubiquiti	PP1RB_MO	3	0.651506
30min / C	Q9Z0N1	-0.37485	0.018762	0.165772	Eif2s3x	Eukaryotic	IF2G_MOUS	5	0.771183
15min / C	Q9CQU5	1.248219	0.018837	0.166123	Zwint	ZW10 inter	ZWINT_MO	1	2.37548
30min / C	Q8CF02	-0.29053	0.019008	0.167326	Fam25a	Protein FAI	FM25C_MC	2	0.817603
15min / C	Q9Z1N5	-0.16313	0.019218	0.168243	Ddx39b	Spliceosom	DX39B_MC	18	0.893083
15min / C	Q925B0	-0.14914	0.019444	0.169605	Pawr	PRKC apop	PAWR_MOU	9	0.901788
30min / C	P58058	0.287645	0.019628	0.170584	Nadk	NAD kinase	NADK_MOU	9	1.220646
15min / C	Q99J08	-0.14237	0.019787	0.171354	Sec14l2	SEC14-like	S14L2_MO	26	0.906027
30min / C	Q64010	0.140365	0.020382	0.173018	Crk	Adapter mc	CRK_MOUS	13	1.102184
30min / C	Q6PEE2	-0.50544	0.020451	0.173018	Ctif	CBP80/20-	CTIF_MOUS	8	0.704446
30min / C	Q8VE09	-0.26582	0.020443	0.173018	Ttc39c	Tetratricop	TT39C_MO	20	0.831725
30min / C	Q9CQM5	-0.38575	0.020214	0.173018	Txndc17	Thioredoxi	TXD17_MO	7	0.765381
30min / C	Q9DAW9	-0.07383	0.020291	0.173018	Cnn3	Calponin-3	CNN3_MOU	10	0.950111
30min / C	Q9EQF5	-0.22854	0.020383	0.173018	Dpys	Dihydropyı	DPYS_MOU	22	0.853496
30min / C	Q9JI75	0.175343	0.020322	0.173018	Nqo2	Ribosyldihy	NQO2_MOU	4	1.129232
30min / C	Q9Z2W0	-0.23754	0.02009	0.173018	Dnpep	Aspartyl am	DNPEP_MC	18	0.848187
30min / C	P60824	-0.84225	0.020573	0.173557	Cirbp	Cold-induc	CIRBP_MOU	2	0.557771
15min / C	Q8VE09	-0.24231	0.020587	0.173557	Ttc39c	Tetratricop	TT39C_MO	20	0.845389
30min / C	Q61702	0.210311	0.020674	0.173981	Itih1	Inter-alpha	ITIH1_MOU	20	1.156938
15min / C	Q8C1D8	-0.80967	0.020713	0.174004	Iws1	Protein IW	IWS1_MOU	2	0.570512
30min / C	P52430	-0.54278	0.020795	0.174082	Pon1	Serum para	PON1_MOU	6	0.686447
30min / C	Q61545	-0.46366	0.020771	0.174082	Ewsr1	RNA-bindir	EWS_MOUS	5	0.725145
30min / C	P70441	-0.14641	0.021126	0.17557	Nherf1	Na(+)/H(+)	NHRF1_MC	23	0.903497
15min / C	Q80Y81	-0.36544	0.02123	0.17557	Elac2	Zinc phospl	RNZ2_MOU	12	0.776232
30min / C	Q9D8C4	-0.41345	0.021055	0.17557	Ifi35	Interferon-	IN35_MOU	8	0.750824
15min / C	Q9QZE7	0.26414	0.021138	0.17557	Tsnax	Translin-as	TSNAX_MO	10	1.20092
30min / C	Q8VE95	-0.60791	0.021294	0.175798	NaN	UPF0598 p	CH082_MC	4	0.656145
15min / C	Q641P0	-0.28337	0.021442	0.176102	Actr3b	Actin-relate	ARP3B_MO	2	0.821669
15min / C	P84084	-0.51586	0.021481	0.176124	Arf5	ADP-ribosy	ARF5_MOU	9	0.699376
30min / C	P61458	-0.26395	0.02165	0.17662	Pcbd1	Pterin-4-alı	PHS_MOUS	7	0.832804
30min / C	P62858	-0.30012	0.021646	0.17662	Rps28	Small ribos	RS28_MOU	3	0.812184
30min / C	Q07813	-0.68197	0.021653	0.17662	Bax	Apoptosis r	BAX_MOUS	5	0.623313
15min / C	P17182	0.472549	0.02179	0.177133	Eno1	Alpha-enol	ENOA_MOU	24	1.387559
15min / C	P10126	-0.31864	0.021896	0.177697	Eef1a1	Elongation	EF1A1_MO	32	0.801825
15min / C	P49962	0.143035	0.021961	0.177922	Srp9	Signal reco	SRP09_MO	4	1.104226
30min / C	Q8VEM8	-1.04203	0.022517	0.180893	Slc25a3	Solute carri	S25A3_MO	11	0.485642
15min / C	P61089	-0.14577	0.022881	0.182585	Ube2n	Ubiquitin-c	UBE2N_MC	9	0.903897
30min / C	P84084	-0.66492	0.022955	0.182585	Arf5	ADP-ribosy	ARF5_MOU	9	0.630724
30min / C	Q8R4N0	0.592409	0.022881	0.182585	Clybl	Citramalyl-	CLYBL_MOU	19	1.507763
15min / C	Q9Z0S1	0.477876	0.022957	0.182585	Bpnt1	3'(2'),5'-bis	BPNT1_MO	16	1.392692
15min / C	P10711	-0.12266	0.023168	0.183023	Tcea1	Transcripti	TCEA1_MO	13	0.918495
30min / C	P31725	-0.6583	0.023082	0.183023	S100a9	Protein S1(	S10A9_MO	3	0.633625

15min / C	P61965	-0.4636	0.023203	0.183023	Wdr5	WD repeat-	WDR5_MO	7	0.725176
30min / C	Q6ZPJ3	0.529887	0.023183	0.183023	Ube2o	(E3-indeper	UBE2O_MC	13	1.443816
30min / C	Q9WUM5	-0.2872	0.023168	0.183023	Suclg1	Succinate--	SUCA_MOU	12	0.819493
30min / C	P62488	-1.18228	0.023291	0.183409	Polr2g	DNA-direct	RPB7_MOU	2	0.440656
15min / C	P61924	-0.2346	0.023555	0.184881	Copz1	Coatomer s	COPZ1_MO	6	0.849923
15min / C	Q78J03	-0.43313	0.02366	0.185401	Msrb2	Methionine	MSRB2_MC	3	0.740652
15min / C	P16110	-0.28177	0.024036	0.186094	Lgals3	Galectin-3	LEG3_MOU	3	0.822582
15min / C	Q3UQ84	-0.80705	0.024009	0.186094	Tars2	Threonine--	SYTM_MOU	3	0.57155
15min / C	Q6PDL0	0.64371	0.023951	0.186094	Dync1li2	Cytoplasmi	DC1L2_MO	10	1.562341
15min / C	Q8BVE3	-0.48818	0.02406	0.186094	Atp6v1h	V-type prot	VATH_MOU	10	0.712922
15min / C	O88543	-0.34425	0.024498	0.187329	Cops3	COP9 signa	CSN3_MOU	13	0.787715
15min / C	P63323	-0.16512	0.024422	0.187329	Rps12	Small ribos	RS12_MOU	8	0.891855
30min / C	Q9D1J3	-0.10703	0.024415	0.187329	Sarnp	SAP domair	SARNP_MO	6	0.928498
15min / C	Q9DCS2	1.161103	0.024565	0.187329	Mettl26	Methyltran	MTL26_MC	13	2.236283
30min / C	Q9QUH0	-0.15171	0.024481	0.187329	Glrx	Glutaredox	GLRX1_MO	6	0.90018
15min / C	Q9DCS3	-0.99309	0.024651	0.187633	Mecr	Enoyl-[acyl-	MECR_MOU	14	0.5024
30min / C	P10711	-0.20791	0.024798	0.188149	Tcea1	Transcripti	TCEA1_MO	13	0.865792
30min / C	Q9JHU4	-0.47279	0.024863	0.188341	Dync1h1	Cytoplasmi	DYHC1_MC	145	0.720569
30min / C	Q5EBG8	0.394948	0.025034	0.189337	NaN	Uncharacte	CA050_MO	6	1.314895
30min / C	P56394	-0.40517	0.025167	0.189711	Cox17	Cytochrom	COX17_MC	1	0.755145
15min / C	Q99MI1	-1.23822	0.025202	0.189711	Erc1	ELKS/Rab6-	RB6I2_MOU	25	0.423895
15min / C	Q8CHP8	0.405558	0.025359	0.190588	Pgp	Glycerol-3-	PGP_MOUS	14	1.324601
15min / C	Q60972	0.293798	0.025426	0.190798	Rbbp4	Histone-bir	RBBP4_MO	11	1.225863
15min / C	Q9R059	-0.86057	0.025494	0.191006	Fhl3	Four and a l	FHL3_MOU	2	0.550734
30min / C	P17751	0.100097	0.02554	0.191051	Tpi1	Triosephos	TPIS_MOUS	18	1.071845
30min / C	Q8CAK1	0.366944	0.025644	0.191444	Iba57	Putative tra	CAF17_MO	6	1.289619
30min / C	Q9D892	-0.23111	0.025673	0.191444	Itpa	Inosine trip	ITPA_MOUS	9	0.851982
15min / C	P17225	-0.23178	0.025727	0.191549	Ptbp1	Polypyrimi	PTBP1_MO	18	0.851584
15min / C	P49443	-0.4296	0.026189	0.193582	Ppm1a	Protein pho	PPM1A_MO	8	0.742465
15min / C	Q9QX60	0.896586	0.026202	0.193582	Dguok	Deoxyguan	DGUOK_MO	3	1.861655
15min / C	Q9D1L0	-0.17484	0.026482	0.195347	Chchd2	Coiled-coil-	CHCH2_MC	4	0.885869
30min / C	P35441	0.540374	0.026797	0.196846	Thbs1	Thrombosp	TSP1_MOU	18	1.454349
30min / C	P10649	-0.25008	0.027142	0.198376	Gstm1	Glutathion	GSTM1_MC	15	0.840852
15min / C	P45878	0.12492	0.027224	0.198676	Fkbp2	Peptidyl-pr	FKBP2_MO	6	1.090447
15min / C	Q8BZW8	-0.30245	0.027376	0.199171	Nhlrc2	NHL repeat	NHLC2_MC	12	0.810874
15min / C	Q3UZ39	-0.03335	0.027631	0.200416	Lrrfip1	Leucine-ric	LRRF1_MO	16	0.97715
15min / C	Q9DCM0	-0.13009	0.027706	0.200656	Ethe1	Persulfide c	ETHE1_MO	13	0.913773
30min / C	P08032	-0.84038	0.027994	0.201317	Spta1	Spectrin al	SPTA1_MO	16	0.558495
30min / C	Q8BWU8	0.503078	0.028189	0.201466	Etnppl	Ethanolami	AT2L1_MO	19	1.417234
30min / C	Q9CQR6	-0.59401	0.028197	0.201466	Ppp6c	Serine/thre	PPP6_MOU	8	0.662498
30min / C	Q91VH2	-0.10746	0.02842	0.202759	Snx9	Sorting nex	SNX9_MOU	9	0.92822
30min / C	Q64339	-0.58382	0.028562	0.203146	Isg15	Ubiquitin-l	ISG15_MOU	6	0.667193
30min / C	P62996	-0.25084	0.02886	0.203765	Tra2b	Transforme	TRA2B_MO	4	0.840407
30min / C	Q3UGR5	0.265457	0.028821	0.203765	Hdhd2	Haloacid de	HDHD2_MO	10	1.202017
30min / C	Q9WVQ5	0.302776	0.028785	0.203765	Apip	Methylthio	MTNB_MOU	11	1.233515
30min / C	P30416	0.181078	0.02902	0.203767	Fkbp4	Peptidyl-pr	FKBP4_MO	28	1.133731
30min / C	Q8BKC5	-0.16885	0.02903	0.203767	Ipo5	Importin-5	IPO5_MOU	25	0.889553
15min / C	Q8CDM8	-1.07825	0.028969	0.203767	Fhip2a	FHF comple	FHI2A_MOU	6	0.473602
30min / C	O08709	-0.0819	0.029307	0.204434	Prdx6	Peroxiredo	PRDX6_MC	19	0.944815
15min / C	P17426	-0.57109	0.029201	0.204434	Ap2a1	AP-2 compl	AP2A1_MO	34	0.673107
30min / C	P47915	-0.34198	0.029347	0.204434	Rpl29	Large ribos	RL29_MOU	5	0.78896
30min / C	Q6IFZ6	1.983009	0.029425	0.204434	Krt77	Keratin, typ	K2C1B_MO	4	3.953168

15min / C	Q8K0C8	-0.77495	0.029297	0.204434	Cox19	Cytochrom	COX19_MC	2	0.58441
15min / C	Q8K0Y7	-0.57424	0.029418	0.204434	Paxx	Protein PA)	PAXX_MOU	3	0.671641
30min / C	Q9R112	-0.72926	0.029365	0.204434	Sqor	Sulfide:qui	SQOR_MOU	28	0.603213
30min / C	P54869	-0.42668	0.029652	0.204583	Hmgcs2	Hydroxyme	HMCS2_MC	24	0.743971
30min / C	Q3TCN2	-0.30375	0.02966	0.204583	Plbd2	Putative ph	PLBL2_MOU	10	0.810145
30min / C	Q3UPL0	-0.25295	0.029506	0.204583	Sec31a	Protein tra	SC31A_MO	43	0.839179
30min / C	Q9CQR2	-0.21423	0.029647	0.204583	Rps21	Small ribos	RS21_MOU	4	0.862007
15min / C	P11031	0.271173	0.029727	0.204689	Sub1	Activated R	TCP4_MOU	7	1.206789
30min / C	P62317	-0.02978	0.029761	0.204689	Snrpd2	Small nucle	SMD2_MOU	9	0.979566
30min / C	Q9DCS3	-0.80955	0.029922	0.205499	Mecr	Enoyl-[acyl	MECR_MOU	14	0.570561
30min / C	P62827	-0.7239	0.029991	0.205678	Ran	GTP-bindin	RAN_MOUS	9	0.60546
15min / C	P52479	0.216764	0.030242	0.2071	Usp10	Ubiquitin c	UBP10_MC	16	1.162124
30min / C	P62737	-0.45965	0.030563	0.208405	Acta2	Actin, aorti	ACTA_MOU	6	0.727163
30min / C	Q3UJB0	0.594156	0.03078	0.208989	Sf3b2	Splicing fac	SF3B2_MOU	18	1.509589
15min / C	O35737	-0.90453	0.030909	0.209051	Hnrnph1	Heterogene	HNRH1_MC	13	0.534206
30min / C	P16045	0.217352	0.030906	0.209051	Lgals1	Galectin-1	LEG1_MOU	10	1.162598
30min / C	Q9CZL5	0.025707	0.030933	0.209051	Pcbd2	Pterin-4-alp	PHS2_MOU	5	1.017979
15min / C	Q9JJH1	0.54939	0.031175	0.209885	Rnase4	Ribonuclea	RNAS4_MO	4	1.463467
30min / C	Q99L45	-0.4839	0.031235	0.209992	Eif2s2	Eukaryotic	IF2B_MOUS	17	0.71504
15min / C	Q9R060	1.203682	0.031859	0.21299	Nubp1	Cytosolic F	NUBP1_MC	5	2.303267
15min / C	Q61545	-0.41154	0.031981	0.213206	Ewsr1	RNA-bindir	EWS_MOUS	5	0.751819
15min / C	P01754;P0	-0.91544	0.03222	0.213907	Ighv1-62-3;	Ig heavy ch	HVM10_MC	1	0.53018
30min / C	P23589	0.267193	0.03219	0.213907	Ca5a	Carbonic ar	CAH5A_MC	12	1.203464
15min / C	Q99KB8	-0.24208	0.032132	0.213907	Hagh	Hydroxyacy	GLO2_MOU	11	0.845525
15min / C	P52760	-0.1512	0.032365	0.214007	Rida	2-iminobut	RIDA_MOU	9	0.900504
30min / C	Q62048	-0.50825	0.03237	0.214007	Pea15	Astrocytic	PEA15_MO	6	0.703073
30min / C	Q99MI1	-1.29031	0.032368	0.214007	Erc1	ELKS/Rab6-	RB6I2_MOU	25	0.408864
30min / C	Q8BG32	-0.55801	0.032429	0.214101	Psmd11	26S protea	PSD11_MO	23	0.679238
15min / C	Q8VDM6	0.618755	0.032568	0.214727	Hnrnpul1	Heterogene	HNRL1_MC	11	1.53555
30min / C	P62806	-0.46239	0.032625	0.214803	H4c1	Histone H4	H4_MOUSE	6	0.725782
30min / C	P04247	1.078663	0.032775	0.215199	Mb	Myoglobin	MYG_MOUS	3	2.112077
15min / C	P52196	0.485475	0.032945	0.21566	Tst	Thiosulfate	THTR_MOU	16	1.400046
30min / C	Q9CQZ1	1.129538	0.032981	0.21566	Hsbp1	Heat shock	HSBP1_MO	2	2.187887
15min / C	P45377	0.53144	0.033174	0.21604	Akr1b8	Aldose redu	ALD2_MOU	15	1.445371
15min / C	P50518	-0.87414	0.033169	0.21604	Atp6v1e1	V-type prot	VATE1_MO	9	0.545578
30min / C	P63276	-0.36914	0.033171	0.21604	Rps17	Small ribos	RS17_MOU	7	0.774246
30min / C	Q8CFX1	-0.24431	0.033454	0.217562	H6pd	GDH/6PGL	G6PE_MOU	22	0.844218
30min / C	O88492	-0.03211	0.033638	0.217672	Plin4	Perilipin-4	PLIN4_MOU	9	0.977991
30min / C	Q570Y9	-0.66967	0.033647	0.217672	Deptor	DEP domai	DPTOR_MC	11	0.628653
15min / C	Q9JIF7	-0.27405	0.033562	0.217672	Copb1	Coatomer s	COPB_MOU	41	0.826992
15min / C	Q9JK92	-1.51158	0.033653	0.217672	Hspb8	Heat shock	HSPB8_MO	4	0.350728
15min / C	P63001	1.29393	0.033721	0.217728	Rac1	Ras-related	RAC1_MOU	8	2.45195
30min / C	O88543	-0.36476	0.034214	0.218348	Cops3	COP9 signa	CSN3_MOU	13	0.776598
30min / C	P26883	-0.13684	0.034147	0.218348	Fkbp1a	Peptidyl-pr	FKB1A_MO	6	0.909511
15min / C	Q6PGH2	-0.43829	0.03402	0.218348	Jpt2	Jupiter mic	JUPI2_MOU	8	0.738007
15min / C	Q8K3D3	1.259858	0.033934	0.218348	Swi5	DNA repair	SWI5_MOU	1	2.394722
15min / C	Q91YP2	0.769694	0.034171	0.218348	Nln	Neurolysin,	NEUL_MOU	23	1.704908
30min / C	Q9JII6	-0.97715	0.034052	0.218348	Akr1a1	Aldo-keto r	AK1A1_MO	21	0.507984
30min / C	Q9CX34	-0.10837	0.034432	0.219446	Sugt1	Protein SG	SGT1_MOU	14	0.927638
15min / C	Q8R016	0.050729	0.034614	0.220312	Blmh	Bleomycin	BLMH_MOU	19	1.035788
15min / C	P61979	-0.10273	0.03479	0.220844	Hnrnpk	Heterogene	HNRPK_MC	24	0.931271
30min / C	Q80WQ2	1.647594	0.035184	0.222753	Vac14	Protein VA	VAC14_MO	9	3.133107

30min / C	Q8BJU0	-0.2756	0.035171	0.222753	Sgta	Small gluta	SGTA_MOU	10	0.826109
30min / C	O70456	-0.12377	0.035272	0.222886	Sfn	14-3-3 prot	1433S_MO	1	0.917788
15min / C	Q922Q8	0.386205	0.035298	0.222886	Lrrc59	Leucine-ric	LRC59_MO	10	1.306951
15min / C	P70665	0.186197	0.03536	0.222984	Siae	Sialate O-ac	SIAE_MOUS	7	1.137761
30min / C	P01680	-0.22311	0.035472	0.223264	NaN	Ig kappa ch	KV4A1_MO	1	0.856715
15min / C	P31230	0.850818	0.035573	0.22336	Aimp1	Aminoacyl	AIMP1_MO	19	1.803523
30min / C	P09405	0.04433	0.036095	0.223614	Ncl	Nucleolin	NUCL_MOU	26	1.031204
30min / C	P35235	-0.75585	0.036115	0.223614	Ptpn11	Tyrosine-pr	PTN11_MO	14	0.592196
30min / C	P61514	-1.05335	0.035888	0.223614	Rpl37a	Large ribos	RL37A_MO	4	0.481849
30min / C	P62754	-0.51423	0.035856	0.223614	Rps6	Small ribos	RS6_MOUS	8	0.700164
15min / C	Q93092	0.184217	0.035859	0.223614	Taldo1	Transaldola	TALDO_MO	17	1.1362
15min / C	Q9CR09	-0.25776	0.035699	0.223614	Ufc1	Ubiquitin-f	UFC1_MOU	4	0.836383
30min / C	Q9DB29	0.32309	0.036045	0.223614	Iah1	Isoamyl ace	IAH1_MOU	14	1.251007
15min / C	P46737	-0.65513	0.036479	0.223839	Brcc3	Lys-63-spec	BRCC3_MO	6	0.635018
15min / C	Q569Z6	0.293357	0.036369	0.223839	Thrap3	Thyroid ho	TR150_MO	19	1.225489
30min / C	Q6ZWY3	1.129742	0.036445	0.223839	Rps27l	Small ribos	RS27L_MO	3	2.188196
15min / C	P30681	0.359753	0.036592	0.224074	Hmgb2	High mobil	HMGB2_M	3	1.283206
30min / C	P50429	-0.76047	0.036611	0.224074	Arsb	Arylsulfata	ARSB_MOU	5	0.590304
30min / C	O08692	0.881851	0.036692	0.224279	Ngp	Neutrophil	NGP_MOUS	6	1.842738
15min / C	Q9D1K2	1.313234	0.036777	0.224511	Atp6v1f	V-type prot	VATF_MOU	3	2.48498
30min / C	P21614	0.195107	0.036838	0.2246	Gc	Vitamin D-l	VTDB_MOU	23	1.144809
15min / C	O08759	0.351384	0.036922	0.224803	Ube3a	Ubiquitin-p	UBE3A_MO	15	1.275784
15min / C	P35441	0.425823	0.037229	0.225847	Thbs1	Thrombosp	TSP1_MOU	18	1.343339
15min / C	O35344	-0.22007	0.037335	0.226184	Kpna3	Importin su	IMA4_MOU	11	0.858524
15min / C	P62196	0.936666	0.037451	0.226184	Psmc5	26S protea	PRS8_MOU	21	1.9141
30min / C	Q9CQC6	0.426923	0.037571	0.226184	Bzw1	eIF5-mimic	5MP2_MOU	16	1.344363
30min / C	Q9CQI6	0.067267	0.037514	0.226184	Cotl1	Coactosin-l	COTL1_MO	8	1.04773
30min / C	Q9CWW6	-0.45999	0.037539	0.226184	Pin4	Peptidyl-pr	PIN4_MOU	3	0.72699
15min / C	Q9D8S9	-0.16371	0.037539	0.226184	Bola1	BolA-like pr	BOLA1_MO	6	0.892727
15min / C	Q7M6Y3	-0.21712	0.037726	0.226548	Picalm	Phosphatid	PICAL_MOU	17	0.860278
30min / C	P36993	0.129427	0.037866	0.226814	Ppm1b	Protein ph	PPM1B_M	13	1.093859
30min / C	Q9ET26	0.455044	0.037821	0.226814	Rnf114	E3 ubiquiti	RN114_MC	8	1.370824
30min / C	P02089	-0.32695	0.03849	0.228265	Hbb-b2	Hemoglobi	HBB2_MOU	7	0.79722
30min / C	P84099	-0.47102	0.038354	0.228265	Rpl19	Large ribos	RL19_MOU	3	0.721452
30min / C	Q8CIN4	0.936722	0.038318	0.228265	Pak2	Serine/thre	PAK2_MOU	15	1.914174
15min / C	Q9D8X1	-0.43242	0.038443	0.228265	Cutc	Copper hor	CUTC_MOU	8	0.741019
30min / C	Q9JKB3	-0.78313	0.038488	0.228265	Ybx3	Y-box-bindi	YBOX3_MO	5	0.581106
15min / C	O08795	-0.24889	0.038692	0.228927	Prkcsh	Glucosidas	GLU2B_MC	18	0.841543
15min / C	Q8VE47	0.319014	0.038697	0.228927	Uba5	Ubiquitin-l	UBA5_MOU	11	1.247478
15min / C	O08585	-0.08878	0.038852	0.229556	Clta	Clathrin lig	CLCA_MOU	5	0.940316
30min / C	O35226	0.040433	0.039255	0.230515	Psmd4	26S protea	PSMD4_M	11	1.028422
15min / C	P51125	-0.17207	0.039921	0.233281	Cast	Calpastatin	ICAL_MOUS	28	0.887568
30min / C	P17225	-0.697	0.04004	0.233407	Ptbp1	Polypyrimi	PTBP1_MO	18	0.616853
15min / C	P28650	0.301142	0.040004	0.233407	Adss1	Adenylosuc	PURA1_MC	17	1.232119
30min / C	Q6P069	-0.72758	0.04012	0.233585	Sri	Sorcin	SORCN_MC	11	0.603917
30min / C	P39061	1.383616	0.040335	0.234464	Col18a1	Collagen al	COIA1_MO	9	2.609216
30min / C	P97364	-0.16588	0.040562	0.235013	Sephs2	Selenide, w	SPS2_MOU	17	0.891385
15min / C	P16254	0.468314	0.040721	0.23511	Srp14	Signal reco	SRP14_MO	4	1.383491
30min / C	P63038	-0.239	0.040747	0.23511	Hspd1	60 kDa hea	CH60_MOU	38	0.847335
30min / C	P97822	0.596884	0.040696	0.23511	Anp32e	Acidic leuci	AN32E_MO	3	1.512447
30min / C	Q8BUV3	-0.05313	0.040829	0.235135	Gphn	Gephyrin	GEPH_MOU	34	0.963844
30min / C	Q9ERL9	-0.74612	0.041118	0.235948	Gucy1a1	Guanylate	GCYA1_MO	8	0.596205

15min / C	Q8BP40	-0.10151	0.041184	0.236045	Acp6	Lysophospl	PPA6_MOU	12	0.932054
15min / C	P12710	-0.23831	0.041424	0.237048	Fabp1	Fatty acid-b	FABPL_MOU	10	0.847738
30min / C	Q99LI8	-0.23777	0.041508	0.237048	Hgs	Hepatocyte	HGS_MOUS	12	0.848058
30min / C	P60335	-0.19531	0.041659	0.237248	Pcbp1	Poly(rC)-bir	PCBP1_MO	11	0.873386
15min / C	P35700	-0.11487	0.042085	0.237738	Prdx1	Peroxiredo	PRDX1_MO	19	0.923464
30min / C	P40936	0.376876	0.042182	0.237738	Inmt	Indolethyla	INMT_MOU	13	1.298527
30min / C	P59017	-0.89536	0.042025	0.237738	Bcl2l13	Bcl-2-like p	B2L13_MO	7	0.537613
15min / C	Q8CAY6	-0.32766	0.041899	0.237738	Acat2	Acetyl-CoA	THIC_MOUS	18	0.79683
15min / C	Q99PV0	-2.03868	0.042265	0.237738	Prpf8	Pre-mRNA-|	PRP8_MOU	33	0.243386
15min / C	Q9CXW3	-0.00047	0.041834	0.237738	Cacybp	Calcyclin-b	CYBP_MOU	13	0.999673
15min / C	Q9D6Z1	0.819167	0.042288	0.237738	Nop56	Nucleolar p	NOP56_MO	9	1.764387
30min / C	Q6PE15	0.623424	0.042653	0.239016	Abhd10	Palmitoyl-p	ABHDA_MO	2	1.540527
15min / C	Q922V4	-0.75247	0.042637	0.239016	Plrg1	Pleiotropic	PLRG1_MO	3	0.593585
15min / C	Q9EQG9	0.604549	0.042769	0.239105	Cert1	Ceramide tı	CERT_MOU	9	1.520503
30min / C	Q7TMY8	-0.50131	0.042922	0.239684	Huwe1	E3 ubiquiti	HUWE1_M	58	0.706463
30min / C	P61922	-0.07698	0.043116	0.240201	Abat	4-aminobu	GABT_MOU	29	0.94804
15min / C	P62331	0.981494	0.043076	0.240201	Arf6	ADP-ribosy	ARF6_MOU	6	1.974509
30min / C	P00761	-0.21836	0.043514	0.241307	NaN	Trypsin (Lal	TRYP_PIG	2	0.859542
30min / C	Q8BHG1	0.901261	0.043617	0.241307	Nrdc	Nardilysin	NRDC_MOU	25	1.867698
15min / C	Q8VDD5	-0.39084	0.043577	0.241307	Myh9	Myosin-9	MYH9_MOU	90	0.762685
15min / C	O70133	-0.55759	0.044123	0.242365	Dhx9	ATP-depenc	DHX9_MOU	25	0.679436
15min / C	O70172	0.058153	0.044081	0.242365	Pip4k2a	Phosphatid	PI42A_MOU	5	1.041132
30min / C	P62702	1.26787	0.043962	0.242365	Rps4x	Small ribos	RS4X_MOU	15	2.408058
30min / C	P70274	0.09718	0.044093	0.242365	Selenop	Selenoprot	SEPP1_MO	6	1.069681
15min / C	Q9CX00	-0.80765	0.044214	0.242365	Ist1	IST1 homol	IST1_MOUS	6	0.57131
30min / C	Q9D394	-0.72803	0.044168	0.242365	Rufy3	Protein RUI	RUFY3_MO	7	0.603727
15min / C	P10107	1.317564	0.044374	0.242685	Anxa1	Annexin A1	ANXA1_MO	15	2.492449
15min / C	Q3UGR5	0.087529	0.044327	0.242685	Hdhd2	Haloacid de	HDHD2_M	10	1.062549
30min / C	Q6ZWY8	-0.15133	0.044592	0.242792	Tmsb10	Thymosin b	TYB10_MO	2	0.90042
15min / C	Q9DAW9	-0.11168	0.044726	0.24322	Cnn3	Calponin-3	CNN3_MOU	10	0.925507
30min / C	Q9CR95	-0.15352	0.044851	0.243344	Necap1	Adaptin ear	NECP1_MO	9	0.899052
30min / C	P27546	0.013547	0.044912	0.243402	Map4	Microtubul	MAP4_MOU	48	1.009434
30min / C	Q9D9V3	-0.14967	0.045032	0.243773	Echdc1	Ethylmalor	ECHD1_MO	16	0.901457
15min / C	P56375	-0.29011	0.045334	0.24419	Acyp2	Acylphosph	ACYP2_MO	1	0.81784
15min / C	Q9EQF5	-0.17929	0.04522	0.24419	Dpys	Dihydropyr	DPYS_MOU	22	0.883135
15min / C	Q9D819	0.146635	0.045416	0.244196	Ppa1	Inorganic p	IPYR_MOUS	19	1.106985
30min / C	P42125	-0.11727	0.046002	0.245719	Eci1	Enoyl-CoA c	ECI1_MOUS	14	0.921928
30min / C	Q6P4T2	0.069487	0.045983	0.245719	Snrnp200	U5 small nu	U520_MOU	48	1.049344
30min / C	Q8BGQ7	-0.06636	0.046059	0.245719	Aars1	Alanine--tR	SYAC_MOU	43	0.955045
15min / C	Q8BWT1	-0.1731	0.045768	0.245719	Acaa2	3-ketoacyl-	THIM_MOU	27	0.886933
30min / C	Q9CYR6	-0.60114	0.046023	0.245719	Pgm3	Phosphoac	AGM1_MOU	20	0.659232
30min / C	Q9DBC0	-0.61922	0.045882	0.245719	Selenoo	Protein ade	SELO_MOU	16	0.651025
30min / C	P30681	0.243513	0.046237	0.245862	Hmgb2	High mobil	HMGB2_M	3	1.183872
30min / C	Q01853	-0.16899	0.046195	0.245862	Vcp	Transitiona	TERA_MOU	57	0.889463
30min / C	Q91WP6	-0.20426	0.046256	0.245862	Serpina3n	Serine prot	SPA3N_MO	11	0.867985
30min / C	P57780	-0.01976	0.046492	0.246105	Actn4	Alpha-actir	ACTN4_MO	40	0.986396
30min / C	Q61191	-0.01932	0.046616	0.246213	Hcfc1	Host cell fa	HCFC1_MO	27	0.9867
30min / C	Q9WTP7	-0.12654	0.046787	0.246573	Ak3	GTP:AMP p	KAD3_MOU	16	0.916025
30min / C	P06683	-0.08274	0.046971	0.246617	C9	Complemer	CO9_MOUS	15	0.944265
30min / C	P11714	-0.16528	0.047102	0.246617	Cyp2d9	Cytochrom	CP2D9_MO	16	0.891758
30min / C	Q62418	-0.20906	0.046928	0.246617	Dbnl	Drebrin-like	DBNL_MOU	12	0.865099
15min / C	Q64727	-0.12729	0.047105	0.246617	Vcl	Vinculin	VINC_MOU	69	0.91555

30min / C	Q9Z1J3	-0.80146	0.047029	0.246617	Nfs1	Cysteine de	NFS1_MOU		15	0.573767
15min / C	P13020	0.573147	0.047281	0.247269	Gsn	Gelsolin	GELS_MOU		16	1.487765
30min / C	Q9QXL2	0.853465	0.047511	0.248201	Kif21a	Kinesin-like	KI21A_MOU		12	1.806835
15min / C	P31786	-0.21927	0.047881	0.249366	Dbi	Acyl-CoA-bi	ACBP_MOU		7	0.858998
30min / C	Q9D7I5	1.023463	0.048296	0.250688	Lhpp	Phospholys	LHPP_MOU		6	2.032792
15min / C	Q9DBA8	0.10301	0.048268	0.250688	Amdhd1	Probable in	HUTI_MOU		23	1.074012
30min / C	O88271	0.412503	0.048489	0.250983	Cfdp1	Craniofacia	CFDP1_MO		2	1.330993
30min / C	P15508	-1.39964	0.048539	0.250983	Sptb	Spectrin be	SPTB1_MOU		14	0.379022
15min / C	Q9CQS8	-0.89672	0.048437	0.250983	Sec61b	Protein tra	SC61B_MO		3	0.537107
30min / C	Q9Z1G3	-0.49772	0.048569	0.250983	Atp6v1c1	V-type prot	VATC1_MO		9	0.708227
15min / C	P00493	-0.11966	0.049083	0.252013	Hprt1	Hypoxanth	HPRT_MOU		13	0.920402
15min / C	Q7TSV4	0.116205	0.049019	0.252013	Pgm2	Phosphope	PGM2_MOU		19	1.08388
15min / C	Q9QYI5	-1.11018	0.049208	0.252376	Dnajb2	DnaJ homo	DNJB2_MO		3	0.463237
30min / C	P52479	-0.20115	0.049424	0.252672	Usp10	Ubiquitin c	UBP10_MC		16	0.869857
30min / C	Q3TGF2	0.641223	0.049694	0.25378	Fam107b	Protein FAI	F107B_MO		3	1.559651
30min / C	P55849	1.015792	0.050119	0.254911	Dsc1	Desmocolli	DSC1_MOU		1	2.022013
30min / C	P56959	-0.18256	0.050297	0.254911	Fus	RNA-bindir	FUS_MOUS		7	0.881136
30min / C	Q501J7	-0.90557	0.050244	0.254911	Phactr4	Phosphatas	PHAR4_MO		5	0.533822
15min / C	Q8BP56	0.383816	0.050056	0.254911	Pgghg	Protein-glu	PGGHG_M(		7	1.304788
30min / C	Q8C0J2	0.276646	0.050196	0.254911	Atg16l1	Autophagy-	A16L1_MO		6	1.211375
15min / C	Q8QZY2	-0.54937	0.050342	0.254911	Glyctk	Glycerate k	GLCTK_MO		13	0.683318
30min / C	Q99PT1	-0.20546	0.050325	0.254911	Arhgdia	Rho GDP-di	GDIR1_MO		14	0.867259
30min / C	Q91XL9	0.0443	0.050545	0.255668	Osbpl1a	Oxysterol-b	OSBL1_MO		11	1.031182
15min / C	Q99M51	-0.60143	0.050643	0.255893	Nck1	SH2/SH3 ac	NCK1_MOU		8	0.6591
30min / C	O70591	-0.14401	0.051232	0.256938	Pfdn2	Prefoldin su	PFD2_MOU		4	0.905003
30min / C	P40142	-0.06131	0.050992	0.256938	Tkt	Transketola	TKT_MOUS		41	0.958396
30min / C	Q60590	-0.55421	0.051203	0.256938	Orm1	Alpha-1-aci	A1AG1_MC		2	0.681032
30min / C	Q8R0F3	0.787579	0.051222	0.256938	Sumf1	Formylglyc	SUMF1_MC		1	1.726175
30min / C	Q8VDM6	0.329276	0.051252	0.256938	Hnrnpul1	Heterogene	HNRL1_MC		11	1.256383
30min / C	Q9QYJ0	-0.57301	0.051103	0.256938	Dnaja2	DnaJ homo	DNJA2_MO		17	0.672213
30min / C	Q62167	0.868192	0.051578	0.257619	Ddx3x	ATP-depenc	DDX3X_MC		30	1.825374
30min / C	Q9Z1Z0	-0.32808	0.051885	0.25805	Uso1	General ves	USO1_MOU		34	0.796597
30min / C	Q9ERF3	-1.21843	0.052009	0.258147	Skic8	Superkiller	SKI8_MOUS		7	0.429751
30min / C	Q8K274	0.579633	0.052328	0.258758	Fn3krp	Ketosamine	KT3K_MOU		7	1.494469
30min / C	P20108	0.096314	0.052422	0.258859	Prdx3	Thioredoxi	PRDX3_MC		10	1.069039
15min / C	P10853;P1	-0.31145	0.052502	0.258986	H2bc7;H2b	Histone H2	H2B1F_MO		7	0.805834
15min / C	Q920Q4	-1.19124	0.052643	0.259414	Vps16	Vacuolar pı	VPS16_MO		6	0.437927
15min / C	Q7TMY8	-0.65625	0.052751	0.259683	Huwe1	E3 ubiquiti	HUWE1_M(		58	0.634523
15min / C	A2A974	0.536705	0.052841	0.259854	Cyp4a12b	Cytochrom	CP4CB_MO		20	1.450656
15min / C	Q811J3	-0.75196	0.053052	0.260184	Ireb2	Iron-respor	IREB2_MOU		2	0.593795
15min / C	Q9Z1T1	-1.10189	0.053071	0.260184	Ap3b1	AP-3 compl	AP3B1_MO		21	0.465906
30min / C	Q3TRJ4	-1.96551	0.053182	0.26046	Krt26	Keratin, typ	K1C26_MO		1	0.256048
30min / C	Q9D0M5	-0.24307	0.053624	0.262356	Dynll2	Dynein ligh	DYL2_MOU		4	0.844946
15min / C	Q9CQI6	0.094591	0.053681	0.262368	Cotl1	Coactosin-l	COTL1_MO		8	1.067763
30min / C	Q62417	-0.96425	0.053759	0.262389	Sorbs1	Sorbin and	SRBS1_MOU		12	0.512544
15min / C	Q8BK75	-0.5502	0.053795	0.262389	Elp6	Elongator c	ELP6_MOU		2	0.682927
30min / C	P01671;P0	0.268826	0.054005	0.262617	NaN	Ig kappa ch	KV3AJ_MOU		1	1.204827
15min / C	Q9JMD0	-0.4786	0.053965	0.262617	Znf207	BUB3-inter	ZN207_MO		3	0.717675
15min / C	Q9WUL7	-1.04799	0.054007	0.262617	Arl3	ADP-ribosy	ARL3_MOU		4	0.483643
30min / C	O88685	-0.40224	0.054159	0.26298	Psmc3	26S protea	PRS6A_MO		22	0.756682
15min / C	Q3TXS7	-0.23416	0.054194	0.26298	Psmd1	26S protea	PSMD1_M(		35	0.850179
15min / C	Q8BTI8	0.236165	0.054314	0.26298	Srrm2	Serine/argi	SRRM2_M(		30	1.177858

30min / C	Q8CHW4	-0.0979	0.054453	0.263181	Eif2b5	Translation	EI2BE_MOU	8	0.934393
15min / C	P62960	-0.23335	0.055089	0.265182	Ybx1	Y-box-bindi	YBOX1_MO	17	0.850656
15min / C	Q5XJE5	-0.52563	0.055445	0.26556	Leo1	RNA polym	LEO1_MOU	1	0.694655
30min / C	Q6NZJ6	-0.16743	0.055433	0.26556	Eif4g1	Eukaryotic	IF4G1_MOU	47	0.890426
15min / C	Q9DBT9	-0.07909	0.055359	0.26556	Dmgdh	Dimethylgl	M2GD_MO	49	0.946652
15min / C	Q9D1C8	-1.1241	0.055562	0.265584	Vps28	Vacuolar pr	VPS28_MO	4	0.458788
30min / C	D3Z6Q9	-0.76633	0.056133	0.266626	Bin2	Bridging int	BIN2_MOU	4	0.587912
15min / C	P62259	-0.08267	0.056226	0.266626	Ywhae	14-3-3 prot	1433E_MO	18	0.944305
30min / C	Q9D0R8	-0.71315	0.055989	0.266626	Lsm12	Protein LSM	LSM12_MC	4	0.609985
15min / C	Q60994	-0.79943	0.056507	0.26743	Adipoq	Adiponecti	ADIPO_MO	1	0.574577
15min / C	Q8BJU0	-0.50648	0.056739	0.267775	Sgta	Small gluta	SGTA_MOU	10	0.703936
30min / C	Q91YP3	-0.17116	0.056748	0.267775	Dera	Deoxyribos	DEOC_MOU	13	0.888128
30min / C	Q8K0Y7	-0.89103	0.056809	0.267799	Paxx	Protein PA)	PAXX_MOU	3	0.539229
30min / C	P19157	-1.00335	0.057011	0.267903	Gstp1	Glutathion	GSTP1_MO	18	0.49884
15min / C	P26369	0.280915	0.056888	0.267903	U2af2	Splicing fac	U2AF2_MO	9	1.214965
15min / C	Q9QUR6	0.651975	0.057055	0.267903	Prep	Prolyl endo	PPCE_MOU	32	1.571318
30min / C	O70435	-0.21097	0.057268	0.26811	Psma3	Proteasom	PSA3_MOU	14	0.863955
30min / C	Q9CYW4	0.379322	0.057246	0.26811	Hdhd3	Haloacid de	HDHD3_M(	11	1.300731
30min / C	Q9D8S9	-0.46693	0.057217	0.26811	Bola1	BolA-like pr	BOLA1_MO	6	0.723504
30min / C	P48776	-0.43646	0.057362	0.268287	Tdo2	Tryptophar	T23O_MOU	16	0.738945
15min / C	Q9D892	-0.16047	0.057431	0.268349	Itpa	Inosine trip	ITPA_MOUS	9	0.894734
30min / C	Q924T3	-1.89031	0.058015	0.270021	Xrcc4	DNA repair	XRCC4_MO	1	0.26975
30min / C	P02668	3.524899	0.058109	0.270072	CSN3	Kappa-case	CASK_BOVI	2	11.51066
15min / C	Q8C5H8	-0.05661	0.058139	0.270072	Nadk2	NAD kinase	NAKD2_MC	21	0.961524
30min / C	P54726	-0.02608	0.058694	0.271858	Rad23a	UV excisior	RD23A_MC	10	0.982085
30min / C	P28667	-0.43892	0.059052	0.272172	Marcksl1	MARCKS-re	MRP_MOUS	2	0.737689
15min / C	P35505	-0.16533	0.058922	0.272172	Fah	Fumarylace	FAAA_MOU	23	0.891725
15min / C	P70699	0.668937	0.058955	0.272172	Gaa	Lysosomal a	LYAG_MOU	12	1.589901
30min / C	P60710	-0.19802	0.059293	0.272518	Actb	Actin, cyto	ACTB_MOU	24	0.871748
30min / C	P97872	-1.72415	0.059284	0.272518	Fmo5	Flavin-cont	FMO5_MOU	30	0.302678
15min / C	P58058	0.181573	0.059604	0.273283	Nadk	NAD kinase	NADK_MOU	9	1.134119
30min / C	Q9D051	0.964142	0.059722	0.27342	Pdhb	Pyruvate de	ODPB_MOU	17	1.950903
15min / C	P14869	-0.19913	0.060036	0.273612	Rplp0	Large ribos	RLA0_MOU	11	0.871075
30min / C	Q8CHT0	-2.27488	0.060046	0.273612	Aldh4a1	Delta-1-pyr	AL4A1_MO	26	0.20663
30min / C	Q91W50	-1.09726	0.060003	0.273612	Csde1	Cold shock	CSDE1_MO	30	0.467403
30min / C	Q99KQ4	-0.13718	0.060029	0.273612	Nampt	Nicotinami	NAMPT_M(	31	0.909294
30min / C	P28661	-0.39232	0.060665	0.274938	Septin4	Septin-4	SEPT4_MOU	4	0.761902
30min / C	P80316	0.010128	0.060619	0.274938	Cct5	T-complex	TCPE_MOU	30	1.007045
15min / C	Q5M8N0	-1.05067	0.060671	0.274938	Cnrip1	CB1 cannat	CNRP1_MC	2	0.482745
30min / C	Q8BK64	-0.17106	0.060682	0.274938	Ahsa1	Activator o	AHSA1_MO	19	0.888188
15min / C	Q8VHK9	1.713351	0.060614	0.274938	Dhx36	ATP-depen	DHX36_MC	6	3.279216
30min / C	Q6ZPJ0	1.085403	0.06086	0.275079	Tex2	Testis-expr	TEX2_MOU	5	2.121968
15min / C	Q80UG2	-0.25818	0.060985	0.275079	Plxna4	Plexin-A4	PLXA4_MO	1	0.836141
15min / C	Q99K28	-0.25499	0.060921	0.275079	Arfgap2	ADP-ribosy	ARFG2_MO	21	0.837995
30min / C	O70172	0.48708	0.061137	0.275435	Pip4k2a	Phosphatic	PI42A_MOU	5	1.401605
15min / C	Q9D8X2	-0.50186	0.061226	0.275573	Ccdc124	Coiled-coil	CC124_MO	6	0.706195
15min / C	P01680	-0.17422	0.061495	0.276101	NaN	Ig kappa ch	KV4A1_MO	1	0.886244
15min / C	P47968	-0.81251	0.061845	0.276101	Rpia	Ribose-5-pl	RPIA_MOUS	3	0.56939
30min / C	P52792	-0.18038	0.061833	0.276101	Gck	Hexokinase	HXK4_MOU	20	0.88247
30min / C	P61924	-0.15485	0.061516	0.276101	Copz1	Coatomer s	COPZ1_MO	6	0.898225
15min / C	P97765	-0.32419	0.061577	0.276101	Wbp2	WW domai	WBP2_MOU	5	0.798745
15min / C	Q66JS6	-0.06709	0.061863	0.276101	Eif3j2	Eukaryotic	EI3JB_MOU	12	0.954559

15min / C	Q9Z1Z0	-0.27886	0.061683	0.276101	Uso1	General ves	USO1_MOU	34	0.82424
30min / C	O55201	0.124308	0.062044	0.276652	Supt5h	Transcripti	SPT5H_MO	17	1.089985
30min / C	Q9QXY6	1.145888	0.062174	0.27697	Ehd3	EH domain-	EHD3_MOU	26	2.212822
15min / C	P63242	-0.29373	0.063041	0.278413	Eif5a	Eukaryotic	IF5A1_MOU	11	0.815788
15min / C	P97470	-0.49143	0.063012	0.278413	Ppp4c	Serine/thre	PP4C_MOU	4	0.711319
15min / C	Q9D8C4	0.316639	0.062804	0.278413	Ifi35	Interferon-i	IN35_MOU	8	1.245426
30min / C	Q6ZWM4	0.313203	0.063392	0.278562	Lsm8	U6 snRNA-a	LSM8_MOU	5	1.242463
15min / C	Q91WQ3	-0.03291	0.063405	0.278562	Yars1	Tyrosine--tI	SYYC_MOUS	32	0.977446
15min / C	Q99J39	-0.14422	0.063297	0.278562	Mlycd	Malonyl-Co	DCMC_MOU	17	0.904868
30min / C	Q7TMM9	-0.2972	0.064	0.280134	Tubb2a	Tubulin bet	TBB2A_MO	7	0.813833
30min / C	Q8CAY6	-0.34055	0.064344	0.280195	Acat2	Acetyl-CoA	THIC_MOUS	18	0.78974
30min / C	Q8CIE6	-0.26745	0.064318	0.280195	Copa	Coatomer s	COPA_MOU	56	0.830785
15min / C	Q99J77	0.965592	0.064363	0.280195	Nans	Sialic acid s	SIAS_MOUS	10	1.952865
15min / C	Q9D9V3	-0.15292	0.064276	0.280195	Echdc1	Ethylmalon	ECHD1_MO	16	0.899426
30min / C	P02662	3.747131	0.064665	0.281001	CSN1S1	Alpha-S1-ca	CASA1_BOV	3	13.42762
15min / C	P45591	0.147308	0.064871	0.281611	Cfl2	Cofilin-2	COF2_MOU	11	1.107501
30min / C	Q9JKX6	0.5693	0.064924	0.281611	Nudt5	ADP-sugar p	NUDT5_MO	10	1.483804
15min / C	Q11136	0.016635	0.065444	0.282389	Pepd	Xaa-Pro dip	PEPD_MOU	20	1.011598
30min / C	Q8BH00	-0.66989	0.065285	0.282389	Aldh8a1	2-aminomu	AL8A1_MO	26	0.628554
30min / C	P56812	-0.59787	0.065523	0.282419	Pdcd5	Programme	PDCD5_MO	6	0.660727
30min / C	P10853;P1	-0.37426	0.065618	0.282571	H2bc7;H2b	Histone H2	H2B1F_MO	7	0.771502
30min / C	Q811J3	0.000888	0.065698	0.282661	Ireb2	Iron-respor	IREB2_MOU	2	1.000615
15min / C	Q6A028	0.222604	0.066235	0.283441	Swap70	Switch-assc	SWP70_MO	6	1.166838
15min / C	Q7TQG1	-1.74531	0.066164	0.283441	Plekha6	Pleckstrin h	PKHA6_MO	2	0.29827
30min / C	Q9JHW2	-0.17132	0.066207	0.283441	Nit2	Omega-ami	NIT2_MOUS	19	0.888032
30min / C	Q9DCN2	-0.94056	0.066437	0.283796	Cyb5r3	NADH-cyto	NB5R3_MO	16	0.52103
30min / C	P51859	-0.17002	0.066684	0.283836	Hdgf	Hepatoma-	HDGF_MOU	13	0.888828
15min / C	Q6P5F9	-0.4253	0.066669	0.283836	Xpo1	Exportin-1	XPO1_MOU	26	0.744685
15min / C	Q9DBB8	-0.11194	0.066684	0.283836	Dhdh	Trans-1,2-d	DHDH_MOU	21	0.92534
15min / C	Q9JHH9	0.504853	0.06661	0.283836	Copz2	Coatomer s	COPZ2_MO	3	1.418979
30min / C	O88456	-0.33297	0.067077	0.284044	Capns1	Calpain sma	CPNS1_MO	8	0.793902
30min / C	P20852	-0.34217	0.067216	0.284044	Cyp2a5	Cytochrom	CP2A5_MO	18	0.788853
30min / C	P26638	0.481878	0.067267	0.284044	Sars1	Serine--tRN	SYSC_MOUS	22	1.396561
15min / C	Q3UTJ2	0.312628	0.06687	0.284044	Sorbs2	Sorbin and	SRBS2_MOU	16	1.241968
30min / C	Q80YC5	-0.29753	0.066996	0.284044	F12	Coagulatio	FA12_MOU	8	0.813645
30min / C	Q9CQJ6	-0.05135	0.06719	0.284044	Denr	Density-reg	DENR_MOU	7	0.965033
30min / C	Q9CQW1	-0.32834	0.067225	0.284044	Ykt6	Synaptobre	YKT6_MOU	9	0.796454
15min / C	P02089	-0.33972	0.067411	0.28406	Hbb-b2	Hemoglobi	HBB2_MOU	7	0.790195
15min / C	P40936	0.33403	0.067449	0.28406	Inmt	Indolethyla	INMT_MOU	13	1.26053
15min / C	Q9D394	-0.6547	0.067358	0.28406	Rufy3	Protein RUI	RUFY3_MO	7	0.635208
30min / C	E9Q5C9	0.008279	0.067756	0.284181	Nolc1	Nucleolar a	NOLC1_MO	12	1.005755
15min / C	P01819;P0	0.610652	0.067666	0.284181	NaN	Ig heavy cha	HVM43_MO	1	1.526949
30min / C	P01837	-0.08287	0.067807	0.284181	Igkc	Immunoglo	IGKC_MOU	6	0.944177
30min / C	Q91ZJ5	-0.10562	0.067587	0.284181	Ugp2	UTP--glucos	UGPA_MOU	31	0.929402
30min / C	Q2TPA8	-0.60441	0.06804	0.284293	Hsdl2	Hydroxyste	HSDL2_MO	23	0.657739
30min / C	Q7M6Y3	-0.21983	0.06804	0.284293	Picalm	Phosphatic	PICAL_MOU	17	0.858668
30min / C	Q60597	0.373706	0.068226	0.284736	Ogdh	2-oxoglutar	ODO1_MOU	42	1.295677
30min / C	Q9JMH6	-0.07849	0.068265	0.284736	Txnrd1	Thioredoxi	TRXR1_MO	22	0.947048
15min / C	Q62167	0.738215	0.068537	0.285123	Ddx3x	ATP-depend	DDX3X_MO	30	1.668111
30min / C	Q91WS4	-0.08014	0.068505	0.285123	Bhmt2	S-methylme	BHMT2_MO	18	0.945968
15min / C	Q9D7A8	1.260079	0.068755	0.285123	Armc1	Armadillo r	ARMC1_MO	4	2.395089
15min / C	Q9JHR7	0.119108	0.068621	0.285123	Ide	Insulin-deg	IDE_MOUSI	31	1.086063

30min / C	P33622	-0.68468	0.069135	0.285186	Apoc3	Apolipopro	APOC3_MO	3	0.622144
30min / C	P47964	-0.34175	0.068992	0.285186	Rpl36	Large ribos	RL36_MOU	4	0.789081
15min / C	O35343	-0.58302	0.069458	0.285621	Kpna4	Importin su	IMA3_MOU	5	0.667565
30min / C	O54804	0.978216	0.069349	0.285621	Chka	Choline kin	CHKA_MOU	3	1.970028
30min / C	Q8VDG5	-0.10869	0.069547	0.285621	Ppcs	Phosphopa	PPCS_MOU	12	0.927431
30min / C	Q9JIF7	-0.30563	0.069553	0.285621	Copb1	Coatomer s	COPB_MOU	41	0.809091
15min / C	P70303	1.230199	0.069739	0.285894	Ctps2	CTP syntha	PYRG2_MO	8	2.345993
30min / C	Q6P6M7	0.000346	0.069689	0.285894	Sepsecs	O-phospho	SPCS_MOU	15	1.00024
30min / C	G3X982	-0.17728	0.070022	0.28665	Aox3	Aldehyde o	AOXC_MOU	68	0.884367
30min / C	Q8CI51	-0.22331	0.070043	0.28665	Pdlim5	PDZ and LIN	PDLI5_MOU	20	0.856598
30min / C	P09671	-0.20066	0.070197	0.286923	Sod2	Superoxide	SODM_MOU	9	0.870155
15min / C	Q9WUP7	-0.42389	0.07023	0.286923	Uchl5	Ubiquitin c	UCHL5_MO	9	0.74541
15min / C	Q8VBX6	-0.11921	0.070749	0.2876	Mpdz	Multiple PD	MPDZ_MOU	12	0.92069
15min / C	Q91YE6	-0.23538	0.07077	0.2876	Ipo9	Importin-9	IPO9_MOU	12	0.849461
30min / C	Q99LS3	0.337032	0.070817	0.2876	Psph	Phosphoser	SERB_MOU	3	1.263155
30min / C	Q9CRD0	-0.39846	0.070803	0.2876	Ociad1	OCIA doma	OCAD1_MO	4	0.758669
15min / C	Q9Z2H5	-0.86018	0.070791	0.2876	Epb41l1	Band 4.1-lik	E41L1_MOU	18	0.550883
15min / C	P47857	0.282014	0.071091	0.288376	Pfkm	ATP-depend	PFKAM_MO	11	1.215891
15min / C	Q9DBX2	-0.38853	0.071503	0.289649	Pdcl	Phosducin-	PHLP_MOU	3	0.763908
30min / C	O70200	0.844909	0.072033	0.291469	Aif1	Allograft in	AIF1_MOUS	2	1.796152
15min / C	P31725	-0.43374	0.072126	0.291469	S100a9	Protein S10	S10A9_MO	3	0.740338
30min / C	Q7TPC1	0.805561	0.072185	0.291469	Cdsn	Corneodesn	CDSN_MOU	1	1.747825
15min / C	Q921M4	-1.15293	0.072302	0.291469	Golga2	Golgin subf	GOGA2_MO	13	0.44971
15min / C	Q8BTM8	-0.13108	0.072466	0.291819	Flna	Filamin-A	FLNA_MOU	82	0.913146
30min / C	P61202	-0.56394	0.072734	0.292405	Cops2	COP9 signa	CSN2_MOU	15	0.676454
15min / C	Q8BFY9	-0.57901	0.073012	0.292536	Tnpo1	Transportin	TNPO1_MO	15	0.669422
30min / C	Q9D727	-0.14217	0.072997	0.292536	NaN	Uncharacte	CF226_MO	2	0.906156
30min / C	Q9Z1R2	-0.43142	0.072926	0.292536	Bag6	Large prolin	BAG6_MOU	18	0.741532
15min / C	Q8BVU5	-0.97101	0.073384	0.293127	Nudt9	ADP-ribose	NUDT9_MO	2	0.510148
30min / C	P63280	0.719691	0.073489	0.293166	Ube2i	SUMO-conj	UBC9_MOU	6	1.646829
30min / C	Q9CZS1	-0.33591	0.073537	0.293166	Aldh1b1	Aldehyde d	AL1B1_MO	28	0.792284
30min / C	O54909	-0.55264	0.073643	0.293236	Rdh16	Retinol deh	RDH16_MO	7	0.681773
15min / C	P56565	0.273904	0.073677	0.293236	S100a1	Protein S10	S10A1_MO	1	1.209075
30min / C	P09411	-0.1644	0.073915	0.293306	Pgk1	Phosphogly	PGK1_MOU	30	0.892301
30min / C	P43277	-0.18534	0.073826	0.293306	H1-3	Histone H1	H13_MOUS	10	0.879439
15min / C	P62245	1.123654	0.073763	0.293306	Rps15a	Small ribos	RS15A_MO	7	2.178981
30min / C	Q9DCG6	-0.20204	0.07394	0.293306	Pbld1	Phenazine b	PBLD1_MO	12	0.86932
30min / C	P62849	-0.30954	0.074082	0.293462	Rps24	Small ribos	RS24_MOU	5	0.806898
30min / C	Q61704	0.327149	0.074102	0.293462	Itih3	Inter-alpha	ITIH3_MOU	12	1.254532
15min / C	Q9DCC4	-0.17461	0.074458	0.294628	Pycr3	Pyrroline-5	P5CR3_MO	9	0.886007
30min / C	P62751	-0.14342	0.074611	0.294987	Rpl23a	Large ribos	RL23A_MO	9	0.905372
15min / C	P55258	-0.08799	0.074768	0.295119	Rab8a	Ras-related	RAB8A_MO	6	0.940832
15min / C	Q3UPH1	-0.18048	0.074734	0.295119	Prrc1	Protein PRR	PRRC1_MO	8	0.882411
15min / C	A2ADY9	-0.12984	0.075047	0.295293	Ddi2	Protein DD	DDI2_MOU	16	0.913934
30min / C	Q3UD01	-0.32119	0.07514	0.295293	Atxn7l3b	Ataxin-7-lik	A7L3B_MO	2	0.800408
30min / C	Q3UZ39	-0.04252	0.075062	0.295293	Lrrfip1	Leucine-ric	LRRF1_MO	16	0.970955
30min / C	Q9CR09	-0.18292	0.075169	0.295293	Ufc1	Ubiquitin-f	UFC1_MOU	4	0.880921
30min / C	Q9CYR0	-0.17446	0.075182	0.295293	Ssbp1	Single-stran	SSBP_MOU	5	0.886097
15min / C	Q9D2V7	0.540395	0.074997	0.295293	Coro7	Coronin-7	CORO7_MO	19	1.454371
30min / C	Q8BTM8	-0.09795	0.075244	0.295294	Flna	Filamin-A	FLNA_MOU	82	0.934361
30min / C	Q0P678	-0.71473	0.07562	0.295595	Zc3h18	Zinc finger	ZCH18_MO	6	0.609319
30min / C	Q8VCR7	-0.07506	0.07563	0.295595	Abhd14b	Putative pr	ABHEB_MO	12	0.949303

15min / C	Q9D0E1	0.538736	0.075455	0.295595	Hnrnpm	Heterogene	HNRPM_M(	28	1.4527
15min / C	Q9WUZ9	-0.24571	0.075437	0.295595	Entpd5	Ectonucleo	ENTP5_MO	14	0.843403
30min / C	Q8R0Y6	-0.25832	0.075896	0.29639	Aldh1l1	Cytosolic 1	AL1L1_MOU	70	0.83606
15min / C	Q8K2K6	-0.284	0.075972	0.296445	Agfg1	Arf-GAP do	AGFG1_MC	6	0.82131
30min / C	Q8CIM3	-0.28792	0.076057	0.296537	D2hgdh	D-2-hydrox	D2HDH_M(	15	0.81908
15min / C	O09131	-0.11814	0.076266	0.296663	Gsto1	Glutathion	GSTO1_MO	15	0.921372
30min / C	Q61768	-0.55834	0.076199	0.296663	Kif5b	Kinesin-1 h	KINH_MOU	39	0.679085
15min / C	Q8CGA0	-1.43449	0.076276	0.296663	Ppm1f	Protein pho	PPM1F_MC	6	0.369978
30min / C	O35639	0.390567	0.076525	0.297167	Anxa3	Annexin A3	ANXA3_MC	13	1.310908
15min / C	P26039	-0.26342	0.07653	0.297167	Tln1	Talin-1	TLN1_MOU	99	0.833111
30min / C	Q8VBV3	-0.0996	0.076626	0.2973	Exosc2	Exosome cc	EXOS2_MO	5	0.933291
15min / C	O54865	0.405854	0.076822	0.297632	Gucy1b1	Guanylate (	GCYB1_MO	5	1.324873
30min / C	P52840	-0.65374	0.077283	0.299119	Sult1a1	Sulfotransf	ST1A1_MO	15	0.63563
30min / C	Q9WUL7	-0.8848	0.07747	0.299586	Arl3	ADP-ribosy	ARL3_MOU	4	0.541563
15min / C	G5E8K5	0.942634	0.078042	0.299606	Ank3	Ankyrin-3	ANK3_MOU	14	1.922034
30min / C	O08638	-0.85278	0.077937	0.299606	Myh11	Myosin-11	MYH11_M(	18	0.553717
30min / C	O54786	-0.93226	0.078058	0.299606	Dffa	DNA fragme	DFFA_MOU	2	0.524035
15min / C	P52792	-0.1272	0.078589	0.299606	Gck	Hexokinase	HXK4_MOU	20	0.915609
15min / C	P57746	0.413834	0.078662	0.299606	Atp6v1d	V-type prot	VATD_MOU	6	1.332222
15min / C	Q6PAM1	0.53328	0.07793	0.299606	Txlna	Alpha-taxili	TXLNA_MO	8	1.447216
30min / C	Q8BRK8	0.712704	0.078639	0.299606	Prkaa2	5'-AMP-acti	AAPK2_MO	13	1.638873
30min / C	Q91VA0	0.163418	0.078243	0.299606	Acsm1	Acyl-coenzy	ACSM1_MC	26	1.119937
30min / C	Q921H9	-0.04986	0.078284	0.299606	Coa7	Cytochrom	COA7_MOU	7	0.966027
30min / C	Q922D8	-1.60635	0.07851	0.299606	Mthfd1	C-1-tetrahy	C1TC_MOU	52	0.328429
30min / C	Q9DBG9	-0.84819	0.078266	0.299606	Tax1bp3	Tax1-bindir	TX1B3_MO	3	0.555483
15min / C	Q9JLF6	-0.89322	0.078043	0.299606	Tgm1	Protein-glu	TGM1_MOU	5	0.53841
30min / C	Q9WTX5	-0.5188	0.077967	0.299606	Skp1	S-phase kin	SKP1_MOU	9	0.697954
15min / C	P07309	-0.18225	0.07914	0.30103	Ttr	Transthyret	TTHY_MOU	6	0.881328
15min / C	P60843	-0.02043	0.079162	0.30103	Eif4a1	Eukaryotic	IF4A1_MOU	25	0.98594
15min / C	O55042	-0.97499	0.079708	0.301699	Snca	Alpha-synu	SYUA_MOU	2	0.508743
30min / C	Q9Z1N5	-0.18071	0.079743	0.301699	Ddx39b	Spliceosom	DX39B_MC	18	0.882266
30min / C	P16110	-0.20329	0.080131	0.302347	Lgals3	Galectin-3	LEG3_MOU	3	0.868567
15min / C	Q91YY4	-0.26527	0.080141	0.302347	Atpaf2	ATP syntha:	ATPF2_MO	4	0.832045
15min / C	P63276	-0.18333	0.080205	0.30235	Rps17	Small ribos	RS17_MOU	7	0.880667
15min / C	O08808	0.783398	0.081181	0.303393	Diaph1	Protein dia	DIAP1_MO	32	1.721179
15min / C	O70493	-0.79441	0.081118	0.303393	Snx12	Sorting nex	SNX12_MO	4	0.576578
30min / C	P28798	0.62849	0.081233	0.303393	Grn	Progranulir	GRN_MOUS	5	1.545946
15min / C	P70194	0.578177	0.081309	0.303393	Clec4f	C-type lecti	CLC4F_MO	10	1.492961
15min / C	Q8BH69	0.201992	0.081227	0.303393	Sephs1	Selenide, w	SPS1_MOU	9	1.150286
30min / C	Q8CDN6	-0.08439	0.080583	0.303393	Txnl1	Thioredoxi	TXNL1_MO	15	0.943182
15min / C	Q91X44	-0.08373	0.08118	0.303393	Gckr	Glucokinas	GCKR_MOU	26	0.943617
30min / C	Q9CQU0	0.274153	0.081362	0.303393	Txndc12	Thioredoxi	TXD12_MO	5	1.209284
15min / C	Q9WUK2	0.004583	0.081211	0.303393	Eif4h	Eukaryotic	IF4H_MOUS	10	1.003182
30min / C	Q6P9R2	-0.43095	0.081884	0.304597	Oxsr1	Serine/thre	OXSR1_MO	11	0.741775
30min / C	Q9CZU6	-0.11717	0.082018	0.304858	Cs	Citrate synt	CISY_MOUS	17	0.921994
30min / C	P70303	1.138468	0.082539	0.306132	Ctps2	CTP syntha:	PYRG2_MO	8	2.201471
15min / C	Q9JJT9	0.438115	0.082553	0.306132	Phax	Phosphoryl	PHAX_MOU	3	1.354833
30min / C	Q9QZE5	-0.20399	0.082511	0.306132	Copg1	Coatomer s	COPG1_MC	33	0.868147
30min / C	Q61035	-0.80603	0.082678	0.306357	Hars1	Histidine--t	HARS1_MO	18	0.571954
30min / C	Q99N42	0.184406	0.083152	0.307875	Tymp	Thymidine	TYPH_MOU	20	1.136349
30min / C	P35492	-0.1223	0.083501	0.307962	Hal	Histidine ai	HUTH_MOU	35	0.918722
30min / C	P61982	-0.11922	0.08408	0.307962	Ywhag	14-3-3 prot	1433G_MC	15	0.920688

15min / C	Q3UPL0	-0.16933	0.08407	0.307962	Sec31a	Protein tra	SC31A_MO	43	0.889254
30min / C	Q6PAM1	0.682144	0.083969	0.307962	Txlna	Alpha-taxili	TXLNA_MO	8	1.604523
30min / C	Q99P72	0.415043	0.083994	0.307962	Rtn4	Reticulon-4	RTN4_MOU	10	1.333339
30min / C	Q9CR00	0.064637	0.083302	0.307962	Psmd9	26S protea	PSMD9_MO	9	1.045821
15min / C	Q9CR39	1.15858	0.083337	0.307962	Wdr45b	WD repeat	WIPI3_MO	4	2.232375
30min / C	Q9CW03	-3.00983	0.083718	0.307962	Smc3	Structural	SMC3_MOU	19	0.124151
15min / C	Q9D358	-0.04401	0.084142	0.307962	Acp1	Low molecu	PPAC_MOU	4	0.969958
30min / C	P63328	0.681331	0.084237	0.307974	Ppp3ca	Protein pho	PP2BA_MO	7	1.603619
30min / C	Q61696	-0.07967	0.084274	0.307974	Hspa1a	Heat shock	HS71A_MO	21	0.946275
30min / C	O35900	-0.23132	0.085786	0.31189	Lsm2	U6 snRNA-a	LSM2_MOU	4	0.851853
30min / C	O88947	0.269939	0.085802	0.31189	F10	Coagulatio	FA10_MOU	6	1.205757
30min / C	Q61425	-0.39296	0.08587	0.311898	Hadh	Hydroxyacy	HCDH_MOU	17	0.761567
30min / C	Q9D0M1	-0.48547	0.086372	0.313008	Prpsap1	Phosphorib	KPRA_MOU	13	0.714267
30min / C	Q91X44	-0.32499	0.086514	0.313049	Gckr	Glucokinas	GCKR_MOU	26	0.798302
30min / C	Q9WVA2	0.314862	0.08645	0.313049	Timm8a1	Mitochond	TIM8A_MO	5	1.243893
15min / C	Q8CF31	0.778155	0.086636	0.313254	Frey1	Protein Fre	FREY_MOUS	1	1.714937
30min / C	O35215	-0.13867	0.087598	0.314084	Ddt	D-dopachro	DOPD_MOU	13	0.908357
30min / C	O35685	0.0328	0.087117	0.314084	Nudc	Nuclear mi	NUDC_MOU	15	1.022995
15min / C	P80316	-0.11954	0.087231	0.314084	Cct5	T-complex	TCPE_MOU	30	0.920483
15min / C	Q61656	-3.0339	0.087943	0.314084	Ddx5	Probable A	DDX5_MOU	16	0.122097
15min / C	Q7TSI3	-0.40682	0.087521	0.314084	Ppp6r1	Serine/thre	PP6R1_MO	12	0.754286
30min / C	Q8K1B8	-0.93516	0.087338	0.314084	Fermt3	Fermitin fa	URP2_MOU	7	0.522985
15min / C	Q922Y1	-0.20908	0.087845	0.314084	Ubxn1	UBX domai	UBXN1_MO	14	0.865087
30min / C	Q99J99	-0.45233	0.087144	0.314084	Mpst	3-mercapto	THTM_MOU	14	0.730864
15min / C	Q99KQ4	-0.08656	0.087896	0.314084	Nampt	Nicotinami	NAMPT_MO	31	0.941764
30min / C	Q99MR8	0.490829	0.087352	0.314084	Mccc1	Methylcrot	MCCA_MOU	24	1.405252
15min / C	Q9CQV8	-0.11243	0.086966	0.314084	Ywhab	14-3-3 prot	1433B_MO	7	0.92503
30min / C	Q9QXD6	-0.08132	0.087605	0.314084	Fbp1	Fructose-1,	F16P1_MO	27	0.945192
15min / C	Q64213	-0.42118	0.088053	0.3141	Sf1	Splicing fac	SF01_MOU	12	0.746816
30min / C	O88958	-0.04372	0.089088	0.315585	Gnpda1	Glucosamir	GNPI1_MO	13	0.970148
30min / C	P24369	-0.20546	0.088713	0.315585	Ppib	Peptidyl-pr	PPIB_MOUS	10	0.86726
15min / C	P61458	-0.13547	0.088875	0.315585	Pcbd1	Pterin-4-alp	PHS_MOUS	7	0.910371
30min / C	P99026	-0.31466	0.088807	0.315585	Psmb4	Proteasom	PSB4_MOU	8	0.804043
30min / C	Q3B7Z2	-0.2475	0.089215	0.315585	Osbp	Oxysterol-b	OSBP1_MO	21	0.842357
15min / C	Q6P1F6	0.042215	0.089291	0.315585	Ppp2r2a	Serine/thre	2ABA_MOU	10	1.029694
15min / C	P26443	0.489929	0.089707	0.316024	Glud1	Glutamate	DHE3_MOU	35	1.404376
30min / C	P63254	0.740462	0.089549	0.316024	Crip1	Cysteine-ric	CRIP1_MOU	3	1.670711
30min / C	Q3UNZ8	-0.54365	0.089716	0.316024	Cryzl2	Quinone ox	QORL2_MO	12	0.686031
15min / C	O35593	-0.28842	0.089895	0.316194	Psmd14	26S protea	PSDE_MOU	12	0.818797
30min / C	Q9CVB6	-0.13487	0.089897	0.316194	Arpc2	Actin-relate	ARPC2_MO	22	0.910752
15min / C	Q61247	-0.23492	0.089971	0.316221	Serpinf2	Alpha-2-ant	A2AP_MOU	12	0.84973
30min / C	P22907	-0.09217	0.090856	0.316533	Hmbs	Porphobilir	HEM3_MOU	14	0.938113
15min / C	P46414	-0.81508	0.09088	0.316533	Cdkn1b	Cyclin-depe	CDN1B_MO	4	0.568377
30min / C	P56395	-0.54463	0.090511	0.316533	Cyb5a	Cytochrom	CYB5_MOU	5	0.685567
15min / C	P62313	-0.0916	0.090942	0.316533	Lsm6	U6 snRNA-a	LSM6_MOU	2	0.938484
30min / C	Q8VCW8	-0.66413	0.090532	0.316533	Acsf2	Medium-ch	ACSF2_MO	24	0.631068
30min / C	Q9CR86	0.505256	0.090447	0.316533	Carhsp1	Calcium-re	CHSP1_MO	4	1.419375
30min / C	Q9D967	-0.18831	0.090987	0.316533	Mdp1	Magnesium	MGDP1_M	3	0.877631
30min / C	Q9DBJ1	-0.21345	0.090265	0.316533	Pgam1	Phosphogly	PGAM1_M	18	0.862474
30min / C	Q9DBL1	0.412404	0.090425	0.316533	Acadsb	Short/bran	ACDSB_MO	14	1.330902
15min / C	Q9CQ75	-0.34064	0.091453	0.317231	Ndufa2	NADH dehy	NDUA2_MO	6	0.789693
15min / C	P11862	-0.05014	0.091667	0.317744	Gas2	Growth arr	GAS2_MOU	15	0.965844

15min / C	O35685	0.131834	0.0921	0.317885	Nudc	Nuclear mi	NUDC_MOU		15	1.095686
30min / C	P0DP26;PC	-0.1251	0.092052	0.317885	Calm1;Caln	Calmodulir	CALM1_MC		11	0.916943
15min / C	Q80X19	-0.1479	0.092078	0.317885	Col14a1	Collagen al	COEA1_MO		37	0.902563
30min / C	Q91YI0	-0.14926	0.091935	0.317885	Asl	Argininosu	ARLY_MOU		27	0.901714
30min / C	Q62318	0.273446	0.092734	0.319358	Trim28	Transcripti	TIF1B_MOU		23	1.208691
15min / C	Q9Z224	0.407194	0.09273	0.319358	Mocs2	Molybdopt	MOC2A_M		3	1.326104
30min / C	O08573	0.088067	0.093277	0.319522	Lgals9	Galectin-9	LEG9_MOU		9	1.062945
30min / C	O55013	-0.21118	0.093193	0.319522	Trappc3	Trafficking	TPPC3_MO		9	0.863829
30min / C	P68372	-0.26438	0.093346	0.319522	Tubb4b	Tubulin bet	TBB4B_MO		23	0.832556
30min / C	Q922R8	-0.22557	0.093279	0.319522	Pdia6	Protein dis	PDIA6_MO		12	0.855256
15min / C	Q9WTU6	0.46258	0.093383	0.319522	Mapk9	Mitogen-ac	MK09_MOU		9	1.378004
15min / C	O08756	0.167447	0.094178	0.319833	Hsd17b10	3-hydroxya	HCD2_MOU		12	1.12307
30min / C	O35423	-0.10203	0.094612	0.319833	Agxt	Alanine--gl	AGT1_MOU		18	0.931721
30min / C	P26039	-0.26202	0.093986	0.319833	Tln1	Talin-1	TLN1_MOU		99	0.833918
30min / C	P28651	0.361929	0.09406	0.319833	Ca8	Carbonic ai	CAH8_MOU		8	1.285143
15min / C	P58389	0.975604	0.094533	0.319833	Ptpa	Serine/thre	PTPA_MOU		10	1.966464
30min / C	P67984	-0.02025	0.094026	0.319833	Rpl22	Large ribos	RL22_MOU		3	0.986062
15min / C	Q61937	-0.08043	0.094583	0.319833	Npm1	Nucleopho	NPM_MOU		9	0.945778
30min / C	Q8BKZ9	-0.22442	0.094396	0.319833	Pdhx	Pyruvate de	ODPX_MOU		13	0.855939
15min / C	Q8BTV2	0.48483	0.094362	0.319833	Cpsf7	Cleavage ar	CPSF7_MO		8	1.399421
30min / C	Q8BWG8	0.665261	0.094565	0.319833	Arrb1	Beta-arresti	ARRB1_MO		6	1.585855
30min / C	Q91VU6	-0.42346	0.093739	0.319833	Dcaf11	DDB1- and	DCA11_MC		13	0.745633
30min / C	Q9JHR7	-0.02685	0.093752	0.319833	Ide	Insulin-deg	IDE_MOUSI		31	0.981563
30min / C	O08784	0.249199	0.095124	0.320659	Tcof1	Treacle pro	TCOF_MOU		10	1.188547
15min / C	Q3TRJ4	-0.7472	0.094988	0.320659	Krt26	Keratin, typ	K1C26_MO		1	0.595757
15min / C	Q9CR86	-0.23386	0.095103	0.320659	Carhsp1	Calcium-re	CHSP1_MO		4	0.850357
15min / C	Q3ULW8	0.491522	0.095313	0.320903	Parp3	Protein mo	PARP3_MO		16	1.405928
30min / C	Q9D0A3	-0.60613	0.095331	0.320903	Arpin	Arpin	ARPIN_MO		5	0.656955
30min / C	P63101	-0.04861	0.095728	0.321787	Ywhaz	14-3-3 prot	1433Z_MO		11	0.966867
30min / C	Q4LDG0	0.443485	0.095833	0.321911	Slc27a5	Long-chain	S27A5_MO		18	1.359885
15min / C	Q9D8B3	-0.31631	0.095912	0.321952	Chmp4b	Charged mu	CHM4B_M		6	0.803122
15min / C	Q5SWU9	0.682937	0.096152	0.322304	Acaca	Acetyl-CoA	ACACA_MO		101	1.605405
30min / C	P13020	0.402332	0.096595	0.322434	Gsn	Gelsolin	GELS_MOU		16	1.321642
15min / C	P26350	-0.31792	0.096582	0.322434	Ptma	Prothymos	PTMA_MOU		7	0.802227
15min / C	P28271	0.076761	0.096401	0.322434	Aco1	Cytoplasmi	ACOHC_MC		49	1.054647
15min / C	Q8BHL8	-0.38849	0.09632	0.322434	Psmf1	Proteasom	PSMF1_MC		4	0.763928
15min / C	Q9D7P6	-0.3366	0.096339	0.322434	Iscu	Iron-sulfur	ISCU_MOUS		4	0.791907
15min / C	Q9D0A3	-0.50988	0.097005	0.323124	Arpin	Arpin	ARPIN_MO		5	0.702282
15min / C	Q9QZ23	-0.60029	0.09711	0.323249	Nfu1	NFU1 iron-	NFU1_MOU		7	0.65962
30min / C	Q60866	-0.17407	0.097208	0.323277	Pter	Phosphotri	PTER_MOU		19	0.886336
15min / C	A2A7S8	0.24058	0.098291	0.324019	Nhsl3	NHS-like pr	NHSL3_MO		6	1.181467
30min / C	E9Q912	-0.18423	0.098609	0.324019	Rap1gds1	Rap1 GTPas	GDS1_MOU		13	0.880122
30min / C	P10107	1.341666	0.098909	0.324019	Anxa1	Annexin A1	ANXA1_MC		15	2.534438
30min / C	P16858	-0.4103	0.099025	0.324019	Gapdh	Glyceraldel	G3P_MOUS		22	0.752467
15min / C	P17563	-0.14701	0.097848	0.324019	Selenbp1	Methaneth	SBP1_MOU		9	0.90312
15min / C	Q61316	0.118164	0.098742	0.324019	Hspa4	Heat shock	HSP74_MO		46	1.085352
15min / C	Q8BWG8	0.204868	0.099017	0.324019	Arrb1	Beta-arresti	ARRB1_MO		6	1.152581
30min / C	Q8C7E7	0.69814	0.098515	0.324019	Stbd1	Starch-binc	STBD1_MO		11	1.622411
15min / C	Q8K0Z7	0.920258	0.097903	0.324019	Taco1	Translation	TACO1_MO		7	1.892454
15min / C	Q923D2	0.157129	0.098359	0.324019	Blvrb	Flavin redu	BLVRB_MO		10	1.115066
30min / C	Q923D2	-0.00052	0.098702	0.324019	Blvrb	Flavin redu	BLVRB_MO		10	0.99964
15min / C	Q924T3	-0.39709	0.099036	0.324019	Xrcc4	DNA repair	XRCC4_MO		1	0.759387

15min / C	Q99JY3	-0.18231	0.098943	0.324019	Gimap4	GTPase IMA	GIMA4_MC	10	0.881291
15min / C	Q99LD8	-0.66901	0.098037	0.324019	Ddah2	Putative hy	DDAH2_MC	6	0.628937
15min / C	Q9D924	-2.35557	0.097725	0.324019	Isca1	Iron-sulfur	ISCA1_MOU	1	0.19539
30min / C	Q9EQG9	-0.03309	0.098939	0.324019	Cert1	Ceramide tı	CERT_MOU	9	0.977323
15min / C	O35326	0.225981	0.099387	0.32428	Srsf5	Serine/argi	SRSF5_MOU	4	1.169573
15min / C	P68040	1.826263	0.099322	0.32428	Rack1	Small ribos	RACK1_MO	19	3.546172
15min / C	Q61207	0.216198	0.099368	0.32428	Psap	Prosaposin	SAP_MOUS	13	1.161668
15min / C	P46664	0.042843	0.099692	0.324832	Adss2	Adenylosuc	PURA2_MC	17	1.030142
15min / C	Q9JHS4	-0.32544	0.099625	0.324832	Clpx	ATP-depenc	CLPX_MOU	17	0.798055
15min / C	Q8BH86	0.758836	0.100007	0.325415	Dglucy	D-glutamat	GLUCM_MC	16	1.692125
15min / C	P46935	0.582664	0.100334	0.325959	Nedd4	E3 ubiquiti	NEDD4_MC	18	1.497612
30min / C	P70362	-0.30394	0.10038	0.325959	Ufd1	Ubiquitin r	UFD1_MOU	9	0.810037
30min / C	Q921M7	-0.07835	0.100525	0.325959	Cyrib	CYFIP-relat	CYRIB_MOU	7	0.947137
15min / C	Q9JLJ2	0.135188	0.100534	0.325959	Aldh9a1	4-trimethyl	AL9A1_MO	27	1.098236
30min / C	Q3V4B5	-0.09432	0.100683	0.326061	Commd6	COMM don	COMD6_MC	2	0.936717
30min / C	Q68FD5	-0.48815	0.100984	0.326815	Cltc	Clathrin he	CLH1_MOU	81	0.712938
15min / C	Q9CWX4	-0.43962	0.101061	0.326841	Rpusd4	Pseudourid	RUSD4_MC	1	0.737326
30min / C	Q3UPH1	-0.24667	0.101343	0.327533	Prrc1	Protein PRI	PRRC1_MC	8	0.842839
15min / C	Q9D6J6	-0.49144	0.101584	0.327676	Ndufv2	NADH dehy	NDUV2_MC	8	0.711317
30min / C	Q9QWL7	0.351865	0.101496	0.327676	Krt17	Keratin, typ	K1C17_MO	17	1.276209
30min / C	Q9Z2I9	-0.04228	0.101593	0.327676	Sucla2	Succinate--	SUCB1_MO	25	0.971116
30min / C	O89112	0.425731	0.101795	0.327884	Lancl1	Glutathion	LANC1_MO	8	1.343253
30min / C	Q6P8I4	-0.12628	0.10175	0.327884	Pcnp	PEST protec	PCNP_MOU	7	0.916191
15min / C	P68254	0.052585	0.102077	0.328573	Ywhaq	14-3-3 prot	1433T_MO	11	1.037121
15min / C	O08692	1.935085	0.102326	0.328774	Ngp	Neutrophil	NGP_MOUS	6	3.824005
30min / C	O70362	-0.22395	0.102553	0.328774	Gpld1	Phosphatid	PHLD_MOU	12	0.856218
30min / C	P50136	0.049915	0.102521	0.328774	Bckdha	2-oxoisoval	ODBA_MOU	20	1.035204
15min / C	Q61074	0.359396	0.102538	0.328774	Ppm1g	Protein pho	PPM1G_MC	9	1.282889
30min / C	Q9D1K2	0.699276	0.102247	0.328774	Atp6v1f	V-type prot	VATF_MOU	3	1.62369
15min / C	P27612	-0.26837	0.103015	0.32966	Plaa	Phospholip	PLAP_MOU	29	0.830255
30min / C	A2AKK5	-0.37814	0.103933	0.331197	Acnat1	Acyl-coenzy	ACNT1_MO	12	0.769429
30min / C	P02666	3.218136	0.104001	0.331197	CSN2	Beta-casein	CASB_BOVI	3	9.305836
15min / C	Q91Z53	0.218017	0.104781	0.332573	Grhpr	Glyoxylate	GRHPR_MC	18	1.163134
15min / C	Q922H4	0.112455	0.105055	0.333219	Gmppa	Mannose-1	GMPPA_MC	10	1.081067
30min / C	Q8BTY1	-0.16419	0.105148	0.333295	Kyat1	Kynurenine	KAT1_MOU	18	0.892431
30min / C	Q9R060	0.514512	0.105283	0.3335	Nubp1	Cytosolic F	NUBP1_MC	5	1.42851
15min / C	Q9QZE5	-0.17341	0.105457	0.333831	Copg1	Coatomer s	COPG1_MC	33	0.886743
30min / C	G5E8K5	0.476001	0.106028	0.334258	Ank3	Ankyrin-3	ANK3_MOU	14	1.390883
30min / C	O35864	-0.43473	0.105782	0.334258	Cops5	COP9 signa	CSN5_MOU	11	0.739832
15min / C	P20108	0.012057	0.105725	0.334258	Prdx3	Thioredoxi	PRDX3_MC	10	1.008393
15min / C	P32020	-0.10977	0.106081	0.334258	Scp2	Sterol carri	SCP2_MOU	27	0.926739
30min / C	Q9ESZ8	1.360819	0.106076	0.334258	Gtf2i	General tra	GTF2I_MOU	9	2.568309
15min / C	P32233	0.632784	0.106888	0.335144	Drg1	Developme	DRG1_MOU	8	1.550554
30min / C	P39447	0.160032	0.107684	0.335144	Tjp1	Tight juncti	ZO1_MOUS	28	1.117312
15min / C	P55302	-1.78416	0.107714	0.335144	Lrpap1	Alpha-2-ma	AMRP_MOU	5	0.290345
15min / C	Q3TIU4	0.897631	0.107733	0.335144	Pde12	2',5'-phosp	PDE12_MC	8	1.863004
30min / C	Q60648	-0.52312	0.10701	0.335144	Gm2a	Ganglioside	SAP3_MOU	5	0.695864
15min / C	Q6WVG3	-0.1062	0.107371	0.335144	Kctd12	BTB/POZ dc	KCD12_MC	10	0.929032
15min / C	Q7TNV0	0.565033	0.10736	0.335144	Dek	Protein DEI	DEK_MOUS	6	1.479421
15min / C	Q8BGD9	-0.18016	0.107855	0.335144	Eif4b	Eukaryotic	IF4B_MOUS	19	0.882608
15min / C	Q8BTE0	0.481742	0.107808	0.335144	Sdhaf4	Succinate c	SDHF4_MC	2	1.396429
30min / C	Q8C1A5	0.197714	0.107815	0.335144	Thop1	Thimet olig	THOP1_MC	18	1.14688

15min / C	Q8K2Q9	-0.20959	0.106805	0.335144	Shtn1	Shootin-1	SHOT1_MO	23	0.864782
15min / C	Q9CQ45	0.097487	0.106907	0.335144	Nenf	Neudesin	NENF_MOU	5	1.069908
30min / C	Q9D6Z1	0.904358	0.107212	0.335144	Nop56	Nucleolar p	NOP56_MC	9	1.871712
15min / C	Q9JKF1	-0.29407	0.107886	0.335144	Iqgap1	Ras GTPase-	IQGA1_MO	37	0.815599
30min / C	P10126	-0.18109	0.108733	0.336407	Eef1a1	Elongation	EF1A1_MO	32	0.882038
15min / C	Q62452	1.057197	0.108707	0.336407	Ugt1a9	UDP-glucur	UD19_MOU	17	2.080885
15min / C	Q9CXF0	0.151072	0.108734	0.336407	Kynu	Kynurenina	KYNU_MOU	20	1.110394
30min / C	Q9CQ89	-0.31859	0.10882	0.336456	Cuta	Protein Cut	CUTA_MOU	3	0.801856
15min / C	P08032	-0.47462	0.109015	0.336673	Spta1	Spectrin alp	SPTA1_MO	16	0.719654
30min / C	P61255	0.68337	0.10934	0.336825	Rpl26	Large ribos	RL26_MOU	9	1.605886
15min / C	P62821	-0.25346	0.109503	0.336825	Rab1a	Ras-related	RAB1A_MO	12	0.838884
15min / C	P97822	-0.23204	0.109478	0.336825	Anp32e	Acidic leuci	AN32E_MO	3	0.85143
15min / C	Q8C196	0.399367	0.109414	0.336825	Cps1	Carbamoyl-	CPSM_MOU	98	1.318929
15min / C	Q91Z58	-0.56637	0.109473	0.336825	NaN	Uncharacte	CF132_MO	14	0.675313
30min / C	Q9DD20	-0.63413	0.109388	0.336825	Tmt1b	Thiol S-met	TMT1B_MC	11	0.644328
30min / C	Q5SWU9	0.410336	0.109622	0.336973	Acaca	Acetyl-CoA	ACACA_MO	101	1.328996
30min / C	Q61581	0.109074	0.10988	0.337333	Igfbp7	Insulin-like	IBP7_MOUS	3	1.078536
15min / C	Q8BJY1	0.054895	0.10988	0.337333	Psmd5	26S protea	PSMD5_MC	19	1.038783
30min / C	Q91VR8	-0.49088	0.110108	0.3376	Brk1	Protein BRI	BRK1_MOU	3	0.71159
15min / C	P02666	-0.02194	0.110655	0.33864	CSN2	Beta-casein	CASB_BOVI	3	0.984908
15min / C	P08226	-0.12906	0.110542	0.33864	Apoe	Apolipoprc	APOE_MOU	17	0.91443
15min / C	Q8R317	-0.3221	0.11066	0.33864	Ubqln1	Ubiquilin-1	UBQL1_MC	13	0.799902
30min / C	Q99PV0	-1.72704	0.110746	0.338685	Prpf8	Pre-mRNA-p	PRP8_MOU	33	0.302071
30min / C	O54931	-0.76239	0.1109	0.338725	Pakap	PALM2-AKA	AKAP2_MO	7	0.58952
15min / C	Q8BWR2	0.205727	0.111427	0.340115	Pithd1	PITH domai	PITH1_MOU	3	1.153267
15min / C	Q9D7X3	-0.68537	0.111687	0.340691	Dusp3	Dual specifi	DUS3_MOU	5	0.621844
15min / C	Q9QXL2	0.80273	0.112103	0.341742	Kif21a	Kinesin-like	KI21A_MOU	12	1.744399
30min / C	Q52KI8	-0.17417	0.112337	0.342237	Srrm1	Serine/argi	SRRM1_MC	5	0.886275
30min / C	P35700	-0.11023	0.112553	0.342459	Prdx1	Peroxiredo	PRDX1_MC	19	0.926442
15min / C	Q58A65	-0.14954	0.112498	0.342459	Spag9	C-Jun-amin	JIP4_MOUS	31	0.901539
15min / C	Q9DCY0	0.243953	0.112642	0.342511	Keg1	Glycine N-a	KEG1_MOU	15	1.184233
30min / C	Q8BMJ3	0.114165	0.112776	0.342612	Eif1ax	Eukaryotic	IF1AX_MOU	2	1.082349
30min / C	Q9CXN7	-0.12126	0.112818	0.342612	Pbld2	Phenazine I	PBLD2_MO	19	0.919385
30min / C	A0JNU3	-0.10811	0.113147	0.3427	Aspg	60 kDa lyso	LPP60_MO	12	0.927805
30min / C	O35344	-0.29703	0.113081	0.3427	Kpna3	Importin su	IMA4_MOU	11	0.813925
15min / C	Q6NVE8	0.892979	0.113141	0.3427	Wdr44	WD repeat-	WDR44_M	6	1.857007
30min / C	Q8BTI8	0.043667	0.113114	0.3427	Srrm2	Serine/argi	SRRM2_MC	30	1.03073
30min / C	Q99KB8	-0.07929	0.113206	0.3427	Hagh	Hydroxyacy	GLO2_MOU	11	0.946521
15min / C	O70589	-0.92802	0.113807	0.344085	Cask	Peripheral	CSKP_MOU	5	0.525581
15min / C	P47738	0.218855	0.114092	0.344345	Aldh2	Aldehyde d	ALDH2_MC	29	1.16381
15min / C	Q9JKB3	-0.51617	0.11404	0.344345	Ybx3	Y-box-bindi	YBOX3_MO	5	0.699224
15min / C	P62320	-0.04278	0.114326	0.344347	Snrpd3	Small nucle	SMD3_MOU	5	0.97078
30min / C	Q9QZB7	-0.19902	0.114326	0.344347	Actr10	Actin-relate	ARP10_MO	5	0.87114
30min / C	P02663	2.285391	0.114523	0.344505	CSN1S2	Alpha-S2-ca	CASA2_BOV	4	4.874961
30min / C	P28656	-0.24042	0.114891	0.344961	Nap1l1	Nucleosom	NP1L1_MO	5	0.846498
15min / C	Q9EPL8	-0.57178	0.114886	0.344961	Ipo7	Importin-7	IPO7_MOU	18	0.672784
15min / C	P56391	-0.4509	0.114985	0.345026	Cox6b1	Cytochrom	CX6B1_MO	6	0.731585
30min / C	O35841	-0.10546	0.115491	0.345462	Api5	Apoptosis i	API5_MOUS	14	0.929509
30min / C	Q9CR39	0.326708	0.115289	0.345462	Wdr45b	WD repeat	WIPI3_MO	4	1.254148
15min / C	Q8K182	0.174307	0.116124	0.346487	C8a	Compleme	CO8A_MOU	13	1.128422
30min / C	P32921	0.599532	0.116311	0.346828	Wars1	Tryptophar	SYWC_MOU	18	1.515225
15min / C	Q8K2Q7	0.497377	0.116519	0.347017	Brox	BRO1 doma	BROX_MOU	5	1.411645

15min / C	Q8BIW1	-0.40879	0.116799	0.347633	Prune1	Exopolyphc	PRUN1_MC	11	0.753255
30min / C	P48758	0.081919	0.117059	0.348192	Cbr1	Carbonyl re	CBR1_MOU	16	1.058425
15min / C	Q3TGF2	0.742035	0.117455	0.3485	Fam107b	Protein FAI	F107B_MO	3	1.672534
15min / C	Q3TNA1	0.636127	0.117379	0.3485	Xylb	Xylulose kir	XYLB_MOU	21	1.554151
15min / C	Q8K4F5	-0.39681	0.117434	0.3485	Abhd11	sn-1-specifi	ABHDB_MC	6	0.759534
15min / C	P54071	1.138695	0.117631	0.348806	Idh2	Isocitrate d	IDHP_MOU	26	2.201818
30min / C	Q9D1G1	-0.15749	0.117704	0.348808	Rab1b	Ras-related	RAB1B_MC	5	0.896585
30min / C	O08759	0.723892	0.117983	0.348896	Ube3a	Ubiquitin-p	UBE3A_MC	15	1.651631
15min / C	P62702	0.906274	0.118164	0.348896	Rps4x	Small ribos	RS4X_MOU	15	1.874199
30min / C	Q00915	0.245305	0.118245	0.348896	Rbp1	Retinol-bin	RET1_MOU	10	1.185343
30min / C	Q8VBX6	0.023653	0.118127	0.348896	Mpdz	Multiple PI	MPDZ_MOI	12	1.01653
30min / C	Q8K354	-0.32592	0.118382	0.349084	Cbr3	Carbonyl re	CBR3_MOU	7	0.797791
15min / C	P53395	-0.09491	0.118761	0.349286	Dbt	Lipoamide	ODB2_MOU	24	0.936331
15min / C	Q62264	0.557593	0.118889	0.349286	Thrsp	Thyroid hor	THRSP_MO	5	1.471812
15min / C	Q8R0F8	0.194871	0.118857	0.349286	Fahd1	Oxaloaceta	FAHD1_MC	11	1.144621
30min / C	Q9CZD3	0.27695	0.118687	0.349286	Gars1	Glycine--tR	GARS_MOU	17	1.211631
15min / C	Q5XG73	-1.99087	0.119099	0.349475	Acbd5	Acyl-CoA-bi	ACBD5_MC	13	0.251587
30min / C	Q8BHL8	-0.05171	0.119033	0.349475	Psmf1	Proteasom	PSMF1_MC	4	0.964793
30min / C	P62843	-1.42348	0.119586	0.350688	Rps15	Small ribos	RS15_MOU	7	0.372813
15min / C	Q60737	0.373502	0.119693	0.350786	Csnk2a1	Casein kina	CSK21_MO	8	1.295494
15min / C	O08709	-0.09756	0.119894	0.350787	Prdx6	Peroxiredo	PRDX6_MC	19	0.93461
30min / C	P01027	-0.05241	0.119913	0.350787	C3	Compleme	CO3_MOUS	101	0.964327
15min / C	P35293	0.760753	0.119901	0.350787	Rab18	Ras-related	RAB18_MC	10	1.694374
30min / C	P10630	-0.16739	0.120039	0.350941	Eif4a2	Eukaryotic	IF4A2_MOU	12	0.890449
30min / C	P06909	0.199441	0.120687	0.351497	Cfh	Compleme	CFAH_MOU	43	1.148253
30min / C	P61082	-0.24291	0.121112	0.351497	Ube2m	NEDD8-cor	UBC12_MC	7	0.845039
15min / C	P70158	0.392795	0.120944	0.351497	Smpdl3a	Cyclic GMP	ASM3A_MC	3	1.312934
30min / C	Q3V1L4	0.475848	0.120574	0.351497	Nt5c2	Cytosolic p	5NTC_MOU	16	1.390736
15min / C	Q8BWZ3	-0.26865	0.120973	0.351497	Naa25	N-alpha-ace	NAA25_MC	5	0.830097
30min / C	Q8K2K6	-0.44519	0.120952	0.351497	Agfg1	Arf-GAP dor	AGFG1_MC	6	0.73449
15min / C	Q8VCM7	1.308314	0.12059	0.351497	Fgg	Fibrinogen	FIBG_MOUS	21	2.47652
15min / C	Q9CZY3	-0.12359	0.120391	0.351497	Ube2v1	Ubiquitin-c	UB2V1_MC	7	0.917898
15min / C	Q61129	-0.27669	0.121247	0.351675	Cfi	Compleme	CFAI_MOUS	18	0.825482
15min / C	Q91ZI0	-0.97781	0.121533	0.352077	Celsr3	Cadherin E(	CELR3_MO	1	0.50775
30min / C	Q8VC30	-0.10549	0.121881	0.352872	Tkfc	Triokinase/	TKFC_MOU	31	0.929492
30min / C	Q8K182	0.157896	0.121988	0.352921	C8a	Compleme	CO8A_MOU	13	1.115659
15min / C	Q9ES00	0.427441	0.122046	0.352921	Ube4b	Ubiquitin c	UBE4B_MC	11	1.344846
30min / C	Q6PDM2	0.085001	0.122283	0.352967	Srsf1	Serine/argi	SRSF1_MOI	14	1.060688
15min / C	E9Q5C9	0.034655	0.122438	0.353029	Nolc1	Nucleolar a	NOLC1_MC	12	1.024312
30min / C	Q283N4	-0.13913	0.122452	0.353029	Urad	2-oxo-4-hy	URAD_MOU	8	0.908066
15min / C	Q9JK38	0.177965	0.122704	0.353464	Gnpnat1	Glucosamir	GNA1_MOU	12	1.131287
15min / C	P26883	-0.09196	0.122911	0.353633	Fkbp1a	Peptidyl-pr	FKB1A_MO	6	0.938249
30min / C	P97792	-1.11285	0.123106	0.353633	Cxadr	Coxsackievi	CXAR_MOU	3	0.462379
15min / C	Q99KK2	0.177822	0.123094	0.353633	Cmas	N-acylneur	NEUA_MOU	12	1.131175
30min / C	P10518	-0.18591	0.123677	0.353787	Alad	Delta-amin	HEM2_MOU	23	0.879095
30min / C	Q3UI43	-0.80083	0.123453	0.353787	Babam1	BRISC and E	BABA1_MC	5	0.574018
30min / C	Q60692	-0.13796	0.123538	0.353787	Psmb6	Proteasom	PSB6_MOU	8	0.908801
30min / C	Q8K1Z0	-0.48082	0.123628	0.353787	Coq9	Ubiquinon	COQ9_MOU	7	0.716568
30min / C	Q99KR3	-0.24355	0.123504	0.353787	Lactb2	Endoribonu	LACB2_MO	15	0.844665
15min / C	Q99MN1	-0.06541	0.123414	0.353787	Kars1	Lysine--tRN	SYK_MOUS	23	0.955672
15min / C	P11725	-0.09161	0.12397	0.354413	Otc	Ornithine t	OTC_MOUS	27	0.938475
30min / C	O35943	0.030748	0.124092	0.354549	Fxn	Frataxin, m	FRDA_MOU	3	1.021542

15min / C	P23506	0.684729	0.12466	0.355747	Pcmt1	Protein-L-is	PIMT_MOU	8	1.6074
15min / C	P28656	-0.75751	0.125202	0.355837	Nap1l1	Nucleosom	NP1L1_MO	5	0.591516
15min / C	P49722	-0.19815	0.125213	0.355837	Psma2	Proteasom	PSA2_MOU	15	0.871667
30min / C	P51125	-0.19482	0.125189	0.355837	Cast	Calpastatin	ICAL_MOUS	28	0.873683
15min / C	Q8BH43	-0.08546	0.12488	0.355837	Wasf2	Actin-bindi	WASF2_MC	9	0.942481
30min / C	Q9JHS4	0.813533	0.125031	0.355837	Clpx	ATP-depend	CLPX_MOU	17	1.75751
30min / C	Q9EPL8	-0.46003	0.125366	0.35585	Ipo7	Importin-7	IPO7_MOU	18	0.726969
15min / C	Q9DB29	0.256959	0.12617	0.357283	Iah1	Isoamyl ace	IAH1_MOU	14	1.194958
30min / C	E9Q414	-0.16945	0.126407	0.357529	Apob	Apolipoprc	APOB_MOU	98	0.88918
15min / C	Q62189	0.222389	0.126396	0.357529	Snrpa	U1 small nu	SNRPA_MO	9	1.166664
30min / C	O88844	-0.58165	0.126644	0.357717	Idh1	Isocitrate d	IDHC_MOU	32	0.668198
15min / C	P43276	-0.19598	0.126639	0.357717	H1-5	Histone H1	H15_MOUS	4	0.872978
30min / C	Q9JLQ0	-0.53419	0.126698	0.357717	Cd2ap	CD2-associ	CD2AP_MC	14	0.690547
15min / C	Q8VCG4	-0.06316	0.127208	0.358945	C8g	Complemer	CO8G_MOU	7	0.957164
30min / C	Q8BP40	-0.00806	0.127485	0.359515	Acp6	Lysophospl	PPA6_MOU	12	0.99443
15min / C	A2AJL3	0.00085	0.127818	0.359607	Fggy	FGGY carbc	FGGY_MOU	22	1.000589
15min / C	Q6PB66	0.639084	0.127693	0.359607	Lrpprc	Leucine-ric	LPPRC_MO	58	1.55734
30min / C	P54227	0.179323	0.12805	0.360015	Stmn1	Stathmin	STMN1_MC	4	1.132353
15min / C	Q91W50	-0.68144	0.128114	0.360015	Csde1	Cold shock	CSDE1_MO	30	0.623543
15min / C	Q8VBW6	0.083488	0.128282	0.360274	Nae1	NEDD8-acti	ULA1_MOU	11	1.059577
30min / C	Q8BX80	0.523452	0.128624	0.360958	Engase	Cytosolic er	ENASE_MO	6	1.437391
30min / C	Q9D0F9	-0.09917	0.128676	0.360958	Pgm1	Phosphoglu	PGM1_MOU	35	0.933573
30min / C	P01631	-0.12119	0.129219	0.361421	NaN	Ig kappa ch	KV2A7_MO	3	0.919432
15min / C	Q2TPA8	-0.40733	0.129158	0.361421	Hsdl2	Hydroxyste	HSDL2_MO	23	0.754016
15min / C	Q8BRK8	0.489909	0.129129	0.361421	Prkaa2	5'-AMP-acti	AAPK2_MO	13	1.404357
30min / C	Q9CYA0	-0.19316	0.129143	0.361421	Creld2	Protein disu	CREL2_MO	8	0.874687
30min / C	Q9CPV4	0.123163	0.130581	0.363952	Glod4	Glyoxalase	GLOD4_MC	18	1.08912
30min / C	Q8VE97	-0.12838	0.130792	0.364326	Srsf4	Serine/argi	SRSF4_MOU	4	0.914861
15min / C	Q6AW69	-0.48662	0.130945	0.36433	Cgnl1	Cingulin-lik	CGNL1_MC	7	0.713696
15min / C	Q9CW03	-2.69788	0.13087	0.36433	Smc3	Structural r	SMC3_MOU	19	0.15412
15min / C	Q00898	-0.19464	0.131387	0.364922	Serpina1e	Alpha-1-ant	A1AT5_MO	1	0.873793
15min / C	Q78ZA7	-0.47142	0.131385	0.364922	Nap1l4	Nucleosom	NP1L4_MO	8	0.721255
30min / C	Q9Z1K5	0.342651	0.131266	0.364922	Arih1	E3 ubiquiti	ARI1_MOUS	4	1.268085
30min / C	Q91VH6	0.360113	0.131507	0.365042	Memo1	Protein ME	MEMO1_M	9	1.283526
15min / C	O88696	0.446409	0.131814	0.365063	Clpp	ATP-depend	CLPP_MOU	8	1.362644
15min / C	P70677	-0.07578	0.131879	0.365063	Casp3	Caspase-3	CASP3_MO	10	0.948827
15min / C	Q9D8S3	0.561324	0.131865	0.365063	Arfgap3	ADP-ribosy	ARFG3_MO	7	1.475623
30min / C	P51881	-0.83647	0.132053	0.365284	Slc25a5	ADP/ATP tr	ADT2_MOU	18	0.560011
15min / C	P70195	0.052017	0.132854	0.366654	Psmb7	Proteasom	PSB7_MOU	9	1.036714
30min / C	Q9JJT9	0.385884	0.132771	0.366654	Phax	Phosphoryl	PHAX_MOU	3	1.306661
30min / C	P49962	0.529645	0.133287	0.367342	Srp9	Signal reco	SRP09_MO	4	1.443574
15min / C	Q9JJU8	-0.24085	0.133334	0.367342	Sh3bgrl	Adapter Sh3	SH3L1_MO	10	0.846246
15min / C	P99029	-0.05616	0.133622	0.367922	Prdx5	Peroxiredo	PRDX5_MC	12	0.961823
15min / C	O35658	-0.20898	0.134135	0.368329	C1qbp	Complemer	C1QBP_MC	5	0.865151
15min / C	Q6A0A9	-0.16524	0.134145	0.368329	FAM120A	Constitutiv	F120A_MO	32	0.891783
30min / C	Q9CX00	-0.52571	0.134076	0.368329	Ist1	IST1 homol	IST1_MOUS	6	0.694616
30min / C	Q61656	-2.2591	0.134327	0.36859	Ddx5	Probable AT	DDX5_MOU	16	0.208902
15min / C	P26262	0.055781	0.136018	0.370019	Klkb1	Plasma kall	KLKB1_MO	11	1.039422
30min / C	P27612	-0.19693	0.1351	0.370019	Plaa	Phospholip	PLAP_MOU	29	0.872404
15min / C	P40142	-0.09374	0.136396	0.370019	Tkt	Transketola	TKT_MOUSI	41	0.937089
30min / C	P47968	-0.79268	0.135606	0.370019	Rpia	Ribose-5-pl	RPIA_MOUS	3	0.577271
30min / C	P62082	-0.65788	0.135181	0.370019	Rps7	Small ribos	RS7_MOUS	11	0.633808

15min / C	P63005	0.061486	0.136282	0.370019	Pafah1b1	Platelet-act	LIS1_MOUS	19	1.04354
30min / C	P70699	0.320972	0.135137	0.370019	Gaa	Lysosomal a	LYAG_MOU	12	1.249172
30min / C	Q06770	0.220618	0.13629	0.370019	Serpina6	Corticoster	CBG_MOUS	6	1.165233
15min / C	Q8BYK6	-0.01548	0.13636	0.370019	Ythdf3	YTH domair	YTHD3_MO	14	0.989325
30min / C	Q8C878	0.29881	0.135848	0.370019	Uba3	NEDD8-acti	UBA3_MOU	10	1.230129
30min / C	Q8CG76	-0.86209	0.136252	0.370019	Akr7a2	Aflatoxin B	ARK72_MO	14	0.550153
30min / C	Q8VCI0	-0.11857	0.135438	0.370019	Plbd1	Phospholip	PLBL1_MO	10	0.921103
30min / C	Q9QXF8	-0.2204	0.135483	0.370019	Gnmt	Glycine N-n	GNMT_MO	21	0.85833
30min / C	O54754	-0.1715	0.136807	0.370922	Aox1	Aldehyde o	AOXA_MOU	50	0.887922
15min / C	A2ATU0	0.401951	0.137147	0.371633	Dhtkd1	2-oxoadipa	DHTK1_MC	36	1.321293
30min / C	Q8R1X6	0.16557	0.137243	0.371682	Spart	Spartin	SPART_MO	6	1.121609
30min / C	P45377	0.566433	0.137459	0.372058	Akr1b8	Aldose redu	ALD2_MOU	15	1.480858
15min / C	P08249	-0.08738	0.138518	0.372766	Mdh2	Malate deh	MDHM_MC	19	0.94123
15min / C	P97371	-0.12989	0.138247	0.372766	Psme1	Proteasom	PSME1_MC	18	0.913903
15min / C	Q283N4	-0.16904	0.138597	0.372766	Urad	2-oxo-4-hy	URAD_MOU	8	0.889433
30min / C	Q5FW60	0.216657	0.1386	0.372766	Mup20	Major urina	MUP20_M	9	1.162038
15min / C	Q8BL97	0.177993	0.138743	0.372766	Srsf7	Serine/argi	SRSF7_MO	5	1.131309
15min / C	Q8VC28	0.469969	0.13881	0.372766	Akr1c13	Aldo-keto r	AK1CD_MC	18	1.38508
15min / C	Q99JY0	0.220953	0.138098	0.372766	Hadhb	Trifunction	ECHB_MOU	23	1.165504
30min / C	Q9Z1Q5	-0.74637	0.138119	0.372766	Clic1	Chloride in	CLIC1_MO	9	0.596103
30min / C	Q9WTL7	-0.62936	0.139067	0.37324	Lypla2	Acyl-protei	LYPA2_MO	7	0.646465
30min / C	Q64459	-0.57856	0.139406	0.373939	Cyp3a11	Cytochrom	CP3AB_MO	21	0.669631
30min / C	O70370	0.568003	0.139604	0.374083	Ctss	Cathepsin S	CATS_MOU	5	1.48247
30min / C	Q08093	-0.42813	0.139616	0.374083	Cnn2	Calponin-2	CNN2_MOU	5	0.743225
15min / C	P19157	-0.62672	0.139838	0.374418	Gstp1	Glutathion	GSTP1_MO	18	0.647647
30min / C	P45878	0.108863	0.140136	0.374637	Fkbp2	Peptidyl-pr	FKBP2_MO	6	1.078378
30min / C	Q8BK30	0.600431	0.140405	0.374935	Ndufv3	NADH dehy	NDUV3_M	2	1.516169
30min / C	O35326	0.531537	0.141072	0.375465	Srsf5	Serine/argi	SRSF5_MO	4	1.445469
30min / C	P13645	0.49611	0.140727	0.375465	KRT10	Keratin, typ	K1C10_HU	27	1.410406
30min / C	P49222	-0.64565	0.141231	0.375465	Epb42	Protein 4.2	EPB42_MO	2	0.639204
30min / C	Q9D6J6	-0.53858	0.141011	0.375465	Ndufv2	NADH dehy	NDUV2_M	8	0.68845
15min / C	E9QAT4	0.438414	0.141318	0.375487	Sec16a	Protein tra	SC16A_MO	31	1.355114
15min / C	O08788	0.161544	0.142119	0.376766	Dctn1	Dynactin su	DCTN1_MC	30	1.118484
15min / C	Q99MR8	-0.52382	0.142194	0.376766	Mccc1	Methylcrot	MCCA_MOU	24	0.695526
15min / C	Q6ZWQ0	-0.86052	0.142547	0.376948	Syne2	Nesprin-2	SYNE2_MO	4	0.550752
15min / C	O55201	0.556091	0.143068	0.377351	Supt5h	Transcripti	SPT5H_MO	17	1.47028
30min / C	Q64213	-0.19055	0.143196	0.377351	Sf1	Splicing fac	SF01_MOU	12	0.87627
30min / C	Q99LD9	-1.55632	0.14291	0.377351	Eif2b2	Translation	EI2BB_MO	7	0.340018
30min / C	Q99P31	0.22061	0.143204	0.377351	Hspbp1	Hsp70-binc	HPBP1_MC	7	1.165226
30min / C	P20060	0.859446	0.143465	0.377623	Hexb	Beta-hexos	HEXB_MOU	11	1.814341
30min / C	P68254	0.064509	0.143457	0.377623	Ywhaq	14-3-3 prot	1433T_MO	11	1.045729
15min / C	Q9QXA5	0.164345	0.143812	0.378281	Lsm4	U6 snRNA-a	LSM4_MOU	5	1.120658
30min / C	O08915	0.797536	0.14443	0.379326	Aip	AH recepto	AIP_MOUSI	8	1.738129
30min / C	Q9WVA3	0.00499	0.144604	0.379367	Bub3	Mitotic che	BUB3_MOU	9	1.003465
30min / C	O55135	-0.104	0.144812	0.379552	Eif6	Eukaryotic	IF6_MOUSE	11	0.930451
15min / C	Q8VED9	-0.05874	0.144833	0.379552	Lgalsl	Galectin-re	LEGL_MOU	4	0.960101
15min / C	P27546	0.032076	0.145145	0.379566	Map4	Microtubul	MAP4_MOU	48	1.022483
30min / C	Q60759	-0.11947	0.145156	0.379566	Gcdh	Glutaryl-Cc	GCDH_MOU	16	0.920528
15min / C	Q920A5	0.324254	0.145269	0.379654	Scpep1	Retinoid-in	RISC_MOUS	7	1.252017
30min / C	Q8BZW8	-0.23483	0.145877	0.38082	Nhlrc2	NHL repeat	NHLC2_MC	12	0.849788
30min / C	Q8JZV9	0.112145	0.145955	0.38082	Bdh2	Dehydroger	DHRS6_MC	6	1.080834
15min / C	Q60876	-0.74866	0.146178	0.380878	Eif4ebp1	Eukaryotic	4EBP1_MO	2	0.595156

15min / C	Q9DB34	-0.94694	0.146358	0.381041	Chmp2a	Charged mu	CHM2A_MO	4	0.518731
15min / C	Q8BML9	0.335997	0.146654	0.381225	Qars1	Glutamine-	SYQ_MOUS	34	1.262249
30min / C	Q8BMS1	0.014559	0.146668	0.381225	Hadha	Trifunction	ECHA_MOU	36	1.010142
30min / C	Q9DCB8	-0.23202	0.147065	0.381924	Isca2	Iron-sulfur	ISCA2_MOU	4	0.851443
30min / C	Q9JMH9	-0.70475	0.147097	0.381924	Myo18a	Unconvent	MY18A_MO	42	0.61355
15min / C	P02769	-0.18607	0.147753	0.382389	ALB	Serum albu	ALBU_BOVI	65	0.878998
15min / C	P09671	-0.10577	0.147654	0.382389	Sod2	Superoxide	SODM_MO	9	0.929306
30min / C	P80315	0.153771	0.147756	0.382389	Cct4	T-complex	TCPD_MOU	26	1.112473
30min / C	Q9JL35	-0.16172	0.147486	0.382389	Hmgn5	High mobil	HMGN5_M	21	0.893959
30min / C	O35309	0.477494	0.147952	0.382484	Nmi	N-myc-inte	NMI_MOUS	13	1.392323
15min / C	Q9CQM9	0.01934	0.14835	0.383244	Glrx3	Glutaredox	GLRX3_MO	11	1.013495
15min / C	D3Z4S3	-0.18574	0.149367	0.383652	Ptrhd1	Putative pe	PTRD1_MO	2	0.879197
30min / C	O88696	0.347804	0.148919	0.383652	Clpp	ATP-depend	CLPP_MOU	8	1.272622
15min / C	P08228	-0.10871	0.149007	0.383652	Sod1	Superoxide	SODC_MOU	10	0.92742
30min / C	P16125	-0.29057	0.149343	0.383652	Ldhb	L-lactate de	LDHB_MOU	12	0.817578
15min / C	P22315	-0.31856	0.148959	0.383652	Fech	Ferrochelat	HEMH_MO	16	0.80187
30min / C	Q9D115	-1.31029	0.149361	0.383652	Znf706	Zinc finger	ZN706_MO	2	0.403239
15min / C	Q8BWF0	0.035557	0.149731	0.384083	Aldh5a1	Succinate-s	SSDH_MOU	19	1.024952
30min / C	Q9D5V6	-0.78801	0.149664	0.384083	Syap1	Synapse-ass	SYAP1_MO	8	0.579143
15min / C	Q9D281	0.652222	0.149998	0.384445	Fam114a1	Protein No	NXP20_MO	13	1.571586
15min / C	Q9DCE9	-0.40128	0.150474	0.385253	Igtp	Immunity-r	IRGM3_MO	6	0.757186
15min / C	E9Q912	-0.30185	0.150986	0.385943	Rap1gds1	Rap1 GTPas	GDS1_MOU	13	0.81121
30min / C	P07724	0.236298	0.150851	0.385943	Alb	Albumin	ALBU_MOU	48	1.177966
30min / C	Q91V76	0.156622	0.151394	0.386669	NaN	Ester hydro	CK054_MO	16	1.114674
30min / C	P41317	-0.12611	0.151966	0.387302	Mbl2	Mannose-b	MBL2_MOU	6	0.916302
30min / C	Q99M51	0.954461	0.152151	0.387302	Nck1	SH2/SH3 ac	NCK1_MOU	8	1.937856
15min / C	Q9D0R8	-0.52785	0.151877	0.387302	Lsm12	Protein LSM	LSM12_MO	4	0.693589
30min / C	Q8BJW6	-0.25862	0.152653	0.387429	Eif2a	Eukaryotic	EIF2A_MOU	22	0.835889
15min / C	Q9D7M1	-0.24739	0.152553	0.387429	Gid8	Glucose-ind	GID8_MOU	3	0.842422
15min / C	Q9D7X8	0.100206	0.152817	0.387429	Ggct	Gamma-glu	GGCT_MOU	6	1.071926
15min / C	Q9D8U8	0.6703	0.152705	0.387429	Snx5	Sorting nex	SNX5_MOU	17	1.591404
30min / C	Q8VCM4	-0.83674	0.15301	0.387594	Lipt1	Lipoyl amic	LIPT_MOUS	4	0.559908
30min / C	Q9D1L0	-0.27495	0.153166	0.387783	Chchd2	Coiled-coil-	CHCH2_MO	4	0.826481
30min / C	O89020	-0.30909	0.153433	0.387975	Afm	Afamin	AFAM_MOU	12	0.807148
30min / C	Q9CQ48	-0.26835	0.153772	0.388494	Nudcd2	NudC doma	NUDC2_MO	5	0.830271
30min / C	Q9D1M0	0.275783	0.153934	0.388698	Sec13	Protein SEC	SEC13_MO	10	1.210651
30min / C	A1L317	1.129807	0.154288	0.389181	Krt24	Keratin, typ	K1C24_MO	1	2.188295
30min / C	P26369	0.44231	0.154598	0.389552	U2af2	Splicing fac	U2AF2_MO	9	1.358778
15min / C	Q9DBG9	-0.69903	0.155127	0.390472	Tax1bp3	Tax1-bindir	TX1B3_MO	3	0.615985
30min / C	P34928	-0.0072	0.155474	0.390933	Apoc1	Apolipopro	APOC1_MO	2	0.995024
30min / C	Q9CQF4	0.436514	0.155793	0.391325	Mtres1	Mitochond	MRES1_MO	2	1.35333
30min / C	Q58A65	-0.06499	0.156378	0.39185	Spag9	C-Jun-amin	JIP4_MOUS	31	0.955953
30min / C	Q80X19	-0.11214	0.156412	0.39185	Col14a1	Collagen al	COEA1_MO	37	0.925214
15min / C	P70460	-0.13082	0.156532	0.391944	Vasp	Vasodilator	VASP_MOU	10	0.913315
15min / C	O54833	-0.6499	0.157102	0.392026	Csnk2a2	Casein kina	CSK22_MO	5	0.637325
30min / C	P62983	-0.1413	0.156969	0.392026	Rps27a	Ubiquitin-r	RS27A_MO	7	0.906701
15min / C	Q07076	0.392219	0.156694	0.392026	Anxa7	Annexin A7	ANXA7_MO	16	1.31241
30min / C	Q8VCN5	-0.07241	0.157221	0.392026	Cth	Cystathioni	CGL_MOUS	21	0.95105
15min / C	Q8VEM8	-0.40793	0.156868	0.392026	Slc25a3	Solute carri	S25A3_MO	11	0.753705
15min / C	P63087	-0.70693	0.15749	0.392288	Ppp1cc	Serine/thre	PP1G_MOU	1	0.612624
15min / C	Q9CZL5	-0.14814	0.157458	0.392288	Pcbd2	Pterin-4-alp	PHS2_MOU	5	0.902413
30min / C	P70318	-0.10966	0.157743	0.39251	Tial1	Nucleolysir	TIAR_MOUS	7	0.926805

15min / C	Q9Z2N8	-0.83078	0.157998	0.392735	Actl6a	Actin-like p	ACL6A_MO	8	0.562225
15min / C	Q99LS3	0.43193	0.158363	0.393121	Psph	Phosphose	SERB_MOU	3	1.349037
15min / C	Q3UFF7	0.606974	0.158639	0.393306	Lyplal1	Lysophospl	LYPL1_MOU	8	1.523062
15min / C	Q0VBK2	0.75045	0.159018	0.393586	Krt80	Keratin, typ	K2C80_MO	1	1.682317
30min / C	Q99LD8	-0.71623	0.15892	0.393586	Ddah2	Putative hy	DDAH2_MO	6	0.608686
30min / C	P70670;Q6	0.074205	0.159322	0.393978	Naca	Nascent po	NACAM_MO	5	1.052781
30min / C	Q9D8X1	-0.33249	0.160019	0.395241	Cutc	Copper hor	CUTC_MOU	8	0.794163
15min / C	Q5SW19	0.321757	0.160262	0.395483	Cluh	Clustered m	CLU_MOUS	48	1.249852
15min / C	Q9JIX0	-0.48357	0.160692	0.395728	Eny2	Transcripti	ENY2_MOU	2	0.715207
15min / C	P99028	0.475394	0.160988	0.396252	Uqcrh	Cytochrom	QCR6_MOU	1	1.390298
15min / C	Q3TMH2	-0.0061	0.16139	0.396964	Scrn3	Secernin-3	SCRN3_MO	14	0.995781
15min / C	Q6P9R2	-0.49864	0.161616	0.39698	Oxsr1	Serine/thre	OXSR1_MO	11	0.707773
30min / C	Q7TSI3	-0.41537	0.161921	0.397322	Ppp6r1	Serine/thre	PP6R1_MO	12	0.749829
15min / C	Q9JM14	-0.12054	0.161847	0.397322	Nt5c	5'(3')-deoxy	NT5C_MOU	7	0.919846
15min / C	P14069	-0.24789	0.162102	0.397357	S100a6	Protein S1(	S10A6_MO	4	0.842126
30min / C	Q9QUM9	-0.06092	0.162091	0.397357	Psma6	Proteasom	PSA6_MOU	17	0.958653
30min / C	Q99LB6	0.195156	0.162199	0.397392	Mat2b	Methionine	MAT2B_MO	10	1.144848
15min / C	P50429	-0.21638	0.162718	0.397571	Arsb	Arylsulfatas	ARSB_MOU	5	0.860724
15min / C	Q62193	0.600145	0.162719	0.397571	Rpa2	Replication	RFA2_MOU	4	1.515869
30min / C	Q8VC49	-0.33137	0.162534	0.397571	Ifi27l2b	Interferon a	IF27B_MOU	4	0.794781
15min / C	Q9JI46	-0.23976	0.162771	0.397571	Nudt3	Diphospho	NUDT3_MO	3	0.846888
15min / C	P35396	1.545884	0.16302	0.397976	Ppard	Peroxisome	PPARD_MO	1	2.919829
30min / C	P70460	-0.3685	0.163665	0.398127	Vasp	Vasodilator	VASP_MOU	10	0.774589
30min / C	Q91XE0	0.250926	0.163602	0.398127	Glyat	Glycine N-a	GLYAT_MO	22	1.189971
15min / C	P01671;P0	0.071012	0.163891	0.398473	NaN	Ig kappa ch	KV3AJ_MO	1	1.050454
15min / C	Q91VF2	0.08453	0.164379	0.398849	Hnmt	Histamine N	HNMT_MO	10	1.060342
30min / C	Q9Z224	0.323971	0.164927	0.399772	Mocs2	Molybdopt	MOC2A_MO	3	1.251771
15min / C	O89112	0.431597	0.165688	0.400398	Lancl1	Glutathion	LANC1_MO	8	1.348726
15min / C	P26638	0.444312	0.165545	0.400398	Sars1	Serine--tRN	SYSC_MOU	22	1.360665
30min / C	Q6IRU2	-0.17454	0.165533	0.400398	Tpm4	Tropomyos	TPM4_MOU	12	0.886047
30min / C	Q9CWZ3	0.769505	0.165402	0.400398	Rbm8a	RNA-bindir	RBM8A_MO	2	1.704685
15min / C	Q9CZM2	0.472782	0.165679	0.400398	Rpl15	Large ribos	RL15_MOU	9	1.387783
30min / C	O77727	0.426943	0.16639	0.400676	KRT15	Keratin, typ	K1C15_SHE	10	1.344382
15min / C	P35979	-0.16483	0.166513	0.400676	Rpl12	Large ribos	RL12_MOU	8	0.892035
15min / C	Q60597	0.289357	0.166308	0.400676	Ogdh	2-oxoglutar	ODO1_MOU	42	1.222095
30min / C	Q9D7P6	-0.41114	0.166185	0.400676	Iscu	Iron-sulfur	ISCU_MOUS	4	0.752027
30min / C	Q9DBJ3	0.473437	0.16658	0.400676	Baiap2l1	BAR/IMD do	BI2L1_MOU	11	1.388413
15min / C	Q9EPK6	-0.51703	0.165996	0.400676	Sil1	Nucleotide	SIL1_MOUS	3	0.698807
30min / C	P61079	-0.11675	0.16722	0.40109	Ube2d3	Ubiquitin-c	UB2D3_MO	1	0.92226
30min / C	Q3UIA2	0.553039	0.167158	0.40109	Arhgap17	Rho GTPase	RHG17_MO	9	1.467173
15min / C	Q61749	-0.03306	0.166962	0.40109	Eif2b4	Translation	EI2BD_MOU	11	0.977347
15min / C	Q64261	-0.15644	0.167319	0.40109	Cdk6	Cyclin-depe	CDK6_MOU	11	0.897234
30min / C	Q8BH86	0.952466	0.167247	0.40109	Dglucy	D-glutamat	GLUCM_MO	16	1.935178
30min / C	Q9CQR4	0.351855	0.167017	0.40109	Acot13	Acyl-coenzy	ACO13_MO	6	1.276201
15min / C	O35900	-0.16999	0.168009	0.401117	Lsm2	U6 snRNA-a	LSM2_MOU	4	0.888849
30min / C	P02301;P8	-0.4492	0.168424	0.401117	H3-5;H3-3a	Histone H3	H3C_MOUS	4	0.732451
15min / C	P57759	0.40017	0.167806	0.401117	Erp29	Endoplasm	ERP29_MO	9	1.319663
15min / C	Q3U4I7	-0.03842	0.168467	0.401117	Pyroxd2	Pyridine nu	PYRD2_MO	11	0.973724
15min / C	Q60668	0.118251	0.1677	0.401117	Hnrnpd	Heterogene	HNRPD_MO	10	1.085418
30min / C	Q8BHA3	-0.31574	0.168351	0.401117	Dtd2	D-aminoac	DTD2_MOU	4	0.803437
30min / C	Q8BIW1	0.253944	0.16832	0.401117	Prune1	Exopolyph	PRUN1_MO	11	1.192463
15min / C	Q8CI51	-0.19002	0.168457	0.401117	Pdlim5	PDZ and LIM	PDLI5_MOU	20	0.876596

30min / C	Q9WUK2	-0.07241	0.168498	0.401117	Eif4h	Eukaryotic	IF4H_MOUS	10	0.951048
15min / C	P99026	-0.2254	0.168662	0.401295	Psmb4	Proteasom	PSB4_MOU	8	0.855358
30min / C	Q91XF0	0.093162	0.17	0.403175	Pnpo	Pyridoxine	PNPO_MOU	8	1.066706
30min / C	Q9JJH1	0.134926	0.170211	0.403175	Rnase4	Ribonuclea	RNAS4_MO	4	1.098036
15min / C	Q9QZM0	-0.45882	0.170062	0.403175	Ubqln2	Ubiquilin-2	UBQL2_MO	3	0.727583
15min / C	P56376	-0.27965	0.170337	0.403275	Acyp1	Acylphosph	ACYP1_MO	3	0.823789
30min / C	O89106	-0.15881	0.170783	0.403929	Fhit	Bis(5'-aden	FHIT_MOUS	8	0.895762
30min / C	Q5XJY5	-0.10152	0.170754	0.403929	Arcn1	Coatomer s	COPD_MOU	25	0.932049
15min / C	Q8K0V4	0.706932	0.170936	0.404092	Cnot3	CCR4-NOT t	CNOT3_MO	5	1.63233
15min / C	O35841	-0.18361	0.17153	0.40453	Api5	Apoptosis i	API5_MOUS	14	0.880499
15min / C	Q9WV98	-0.15912	0.171506	0.40453	Timm9	Mitochond	TIM9_MOU	3	0.895572
15min / C	Q8R1X6	0.01045	0.172313	0.405741	Spart	Spartin	SPART_MO	6	1.007269
30min / C	Q8BGD9	-0.1482	0.17255	0.4061	Eif4b	Eukaryotic	IF4B_MOUS	19	0.902377
30min / C	Q62159	0.376814	0.173047	0.406112	Rhoc	Rho-relatec	RHOC_MOU	8	1.298471
15min / C	Q6PEE2	-0.13028	0.172909	0.406112	Ctif	CBP80/20-	CTIF_MOUS	8	0.913653
30min / C	Q8CIF4	-0.35294	0.172781	0.406112	Btd	Biotinidase	BTD_MOUS	8	0.782989
30min / C	Q8VE47	0.186178	0.173175	0.406112	Uba5	Ubiquitin-l	UBA5_MOU	11	1.137745
15min / C	Q9CR21	-0.23242	0.173302	0.406112	Ndufab1	Acyl carrier	ACPM_MOU	3	0.851206
30min / C	P01633	-0.30539	0.173847	0.406173	Igkv6-17	Immunoglo	KV5A1_MO	2	0.809224
30min / C	P46737	0.81135	0.173855	0.406173	Brcc3	Lys-63-spec	BRCC3_MO	6	1.754853
30min / C	P70665	0.615731	0.173789	0.406173	Siae	Sialate O-ac	SIAE_MOUS	7	1.532335
30min / C	Q8CI32	-0.20309	0.173718	0.406173	Bag5	BAG family	BAG5_MOU	9	0.868685
30min / C	Q91VM9	-0.07082	0.173763	0.406173	Ppa2	Inorganic p	IPYR2_MOU	13	0.952097
30min / C	Q99J08	-0.11565	0.174138	0.406436	Sec14l2	SEC14-like	S14L2_MO	26	0.922968
15min / C	Q99L27	0.184903	0.174117	0.406436	Gmpr2	GMP reduc	GMPR2_MO	8	1.136741
15min / C	P84089	-0.40003	0.174395	0.406524	Erh	Enhancer o	ERH_MOUS	3	0.757841
15min / C	Q9CPV4	0.139039	0.174431	0.406524	Glod4	Glyoxalase	GLOD4_MO	18	1.101171
15min / C	Q9Z1P6	0.278016	0.174647	0.406829	Ndufa7	NADH dehy	NDUA7_MO	5	1.212526
15min / C	P63328	0.509018	0.17478	0.406941	Ppp3ca	Protein pho	PP2BA_MO	7	1.423081
15min / C	Q64674	0.088337	0.175046	0.40736	Srm	Spermidine	SPEE_MOUS	14	1.063144
15min / C	P28661	-0.57468	0.17541	0.407786	Septin4	Septin-4	SEPT4_MOU	4	0.671433
15min / C	P59017	-0.57159	0.175588	0.407786	Bcl2l13	Bcl-2-like p	B2L13_MO	7	0.672875
30min / C	Q8R317	-0.14201	0.17535	0.407786	Ubqln1	Ubiquilin-1	UBQL1_MO	13	0.906255
30min / C	Q99JX3	-0.41646	0.175581	0.407786	Gorasp2	Golgi reass	GORS2_MO	9	0.74926
15min / C	E9Q4Z2	0.923573	0.176537	0.409238	Acacb	Acetyl-CoA	ACACB_MO	32	1.896807
30min / C	Q9QXG2	-0.65556	0.17647	0.409238	Chm	Rab protein	RAE1_MOU	7	0.634829
15min / C	Q8BK64	-0.12066	0.176853	0.409572	Ahsa1	Activator o	AHSA1_MO	19	0.919764
15min / C	Q8R086	-0.21558	0.176833	0.409572	Suox	Sulfite oxid	SUOX_MOU	21	0.861199
30min / C	P99028	2.205073	0.17751	0.410101	Uqcrh	Cytochrom	QCR6_MOU	1	4.610979
30min / C	Q8BWZ3	-0.36429	0.177349	0.410101	Naa25	N-alpha-ace	NAA25_MO	5	0.776851
30min / C	Q9CWM4	0.909959	0.177442	0.410101	Pfdn1	Prefoldin su	PFD1_MOU	5	1.878992
15min / C	Q9CQU0	-0.10479	0.177627	0.410137	Txndc12	Thioredoxi	TXD12_MO	5	0.92994
15min / C	Q8C4B4	-1.75322	0.177804	0.410185	Unc119b	Protein unc	U119B_MO	5	0.296639
15min / C	Q8BWU5	0.424433	0.178061	0.410381	Osgep	tRNA N6-ad	OSGEP_MO	12	1.342045
15min / C	P99027	-0.0648	0.178171	0.410438	Rplp2	Large ribos	RLA2_MOU	6	0.956081
30min / C	P13745	-0.3635	0.178362	0.410482	Gsta1	Glutathion	GSTA1_MO	13	0.777276
30min / C	P28658	-0.59622	0.178312	0.410482	Atxn10	Ataxin-10	ATX10_MO	16	0.661484
30min / C	P63087	-0.69406	0.17902	0.411102	Ppp1cc	Serine/thre	PP1G_MOU	1	0.618114
15min / C	Q8VDG5	-0.14534	0.178937	0.411102	Ppcs	Phosphopa	PPCS_MOU	12	0.904164
15min / C	Q9Z1Q9	0.813015	0.178909	0.411102	Vars1	Valine--tRN	SYVC_MOU	44	1.756879
15min / C	P48774	0.222648	0.179592	0.412122	Gstm5	Glutathion	GSTM5_MO	16	1.166873
30min / C	Q8K1R3	0.256794	0.179828	0.412268	Pnpt1	Polyribonu	PNPT1_MO	16	1.19482

15min / C	Q8VCN5	-0.07007	0.179938	0.412322	Cth	Cystathioni	CGL_MOUS	21	0.952589
15min / C	P51881	-0.57583	0.18035	0.413067	Slc25a5	ADP/ATP tr	ADT2_MOU	18	0.670901
15min / C	Q8R0F3	1.071882	0.180517	0.413253	Sumf1	Formylglyc	SUMF1_MC	1	2.102174
30min / C	P62743	0.723387	0.181708	0.415581	Ap2s1	AP-2 compl	AP2S1_MO	5	1.651053
30min / C	P31230	-1.06417	0.182231	0.416379	Aimp1	Aminoacyl	AIMP1_MO	19	0.478247
15min / C	Q07813	-0.43508	0.182186	0.416379	Bax	Apoptosis r	BAX_MOUS	5	0.739652
30min / C	Q9Z1Z2	0.11723	0.182329	0.416404	Strap	Serine-thre	STRAP_MO	16	1.084651
15min / C	Q6NVF9	-0.20644	0.182462	0.416508	Cpsf6	Cleavage ar	CPSF6_MO	7	0.866671
15min / C	O89017	0.310017	0.183177	0.416746	Lgmn	Legumain	LGMN_MO	4	1.239722
15min / C	P23116	0.46354	0.182762	0.416746	Eif3a	Eukaryotic	EIF3A_MOU	50	1.378921
15min / C	P62983	-0.13384	0.18314	0.416746	Rps27a	Ubiquitin-r	RS27A_MO	7	0.911403
30min / C	Q9CQS8	-0.54817	0.182909	0.416746	Sec61b	Protein trai	SC61B_MO	3	0.683886
30min / C	Q9EQ20	-0.13036	0.183502	0.417091	Aldh6a1	Methylmal	MMSA_MO	28	0.913606
15min / C	Q62523	-0.28454	0.183654	0.417236	Zyx	Zyxin	ZYX_MOUS	14	0.821003
15min / C	P39447	0.217246	0.184494	0.418747	Tjp1	Tight juncti	ZO1_MOUS	28	1.162512
30min / C	C0HKE1;C0	-0.85287	0.184865	0.418848	H2ac4;H2a	Histone H2	H2A1B_MC	5	0.553683
30min / C	P62889	0.537253	0.184781	0.418848	Rpl30	Large ribos	RL30_MOU	7	1.451207
15min / C	Q8BX80	-0.1753	0.184802	0.418848	Engase	Cytosolic e	ENASE_MO	6	0.885583
30min / C	P35396	0.449243	0.185201	0.418959	Ppard	Peroxisome	PPARD_MC	1	1.365323
15min / C	Q6P6M7	0.066281	0.185106	0.418959	Sepsecs	O-phospho	SPCS_MOU	15	1.047014
15min / C	Q9CR00	0.150647	0.185174	0.418959	Psmd9	26S protea	PSMD9_MC	9	1.110067
30min / C	Q9DC61	0.498034	0.186085	0.420163	Pmpca	Mitochond	MPPA_MOU	19	1.412287
30min / C	Q9WVE8	0.005826	0.18625	0.420185	Pacsin2	Protein kin	PACN2_MC	13	1.004047
15min / C	Q7TMM9	-0.17116	0.186556	0.420368	Tubb2a	Tubulin bet	TBB2A_MO	7	0.888126
15min / C	Q9CQW1	-0.00731	0.186615	0.420368	Ykt6	Synaptobre	YKT6_MOU	9	0.994947
30min / C	P47857	0.398143	0.186856	0.420513	Pfkm	ATP-depen	PFKAM_MC	11	1.317811
30min / C	Q8BG73	-0.94018	0.186965	0.420561	Sh3bgrl2	SH3 domai	SH3L2_MO	5	0.521168
30min / C	O70475	-0.25653	0.187335	0.420992	Ugdh	UDP-glucos	UGDH_MO	30	0.837098
30min / C	Q8VC28	-0.23407	0.18742	0.420992	Akr1c13	Aldo-keto r	AK1CD_MC	18	0.850233
15min / C	E9Q4P1	0.5787	0.188399	0.421726	Wdfy1	WD repeat	WDFY1_MC	5	1.493502
15min / C	P16294	0.409375	0.188449	0.421726	F9	Coagulatio	FA9_MOUS	7	1.32811
30min / C	P38060	-0.30055	0.188014	0.421726	Hmgcl	Hydroxyme	HMGCL_MC	15	0.81194
30min / C	Q91ZX7	0.111457	0.188217	0.421726	Lrp1	Prolow-der	LRP1_MOU	63	1.080319
30min / C	Q9CQD1	0.007521	0.187922	0.421726	Rab5a	Ras-related	RAB5A_MO	5	1.005227
30min / C	Q9DCD0	-0.06951	0.1883	0.421726	Pgd	6-phospho	6PGD_MOU	20	0.952962
30min / C	Q9D753	-0.12309	0.1887	0.42208	Exosc8	Exosome co	EXOS8_MO	5	0.918217
30min / C	P29699	0.111681	0.188856	0.422102	Ahsg	Alpha-2-HS	FETUA_MO	10	1.080487
15min / C	P62838	-0.14591	0.189126	0.422102	Ube2d2	Ubiquitin-c	UB2D2_MC	4	0.903806
30min / C	Q91YR9	0.109896	0.189191	0.422102	Ptgr1	Prostagland	PTGR1_MO	11	1.079151
15min / C	Q9CZU6	0.80479	0.18924	0.422102	Cs	Citrate synt	CISY_MOUS	17	1.746891
30min / C	Q9EST5	0.091429	0.18913	0.422102	Anp32b	Acidic leuci	AN32B_MC	5	1.065425
30min / C	Q9CZY3	-0.22835	0.189555	0.422412	Ube2v1	Ubiquitin-c	UB2V1_MC	7	0.85361
30min / C	Q7TNG8	0.220228	0.189858	0.422824	Ldhd	Probable D	LDHD_MOU	18	1.164918
15min / C	Q91WT9	-0.1042	0.189917	0.422824	Cbs	Cystathioni	CBS_MOUS	24	0.930318
15min / C	P04247	0.809264	0.190383	0.423468	Mb	Myoglobin	MYG_MOUS	3	1.752317
15min / C	P24547	0.082374	0.191006	0.424458	Impdh2	Inosine-5'-r	IMDH2_MC	19	1.058759
15min / C	Q80X50	0.615641	0.191199	0.424689	Ubap2l	Ubiquitin-a	UBP2L_MO	14	1.532239
30min / C	Q99K28	-0.19813	0.191484	0.425125	Arfgap2	ADP-ribosy	ARFG2_MO	21	0.871677
15min / C	Q8QZR5	-0.13672	0.192061	0.426207	Gpt	Alanine am	ALAT1_MO	22	0.909582
15min / C	P20852	0.93035	0.192619	0.427051	Cyp2a5	Cytochrom	CP2A5_MO	18	1.905738
15min / C	Q61599	-0.07724	0.192904	0.427211	Arhgdib	Rho GDP-di	GDIR2_MO	7	0.947869
15min / C	Q921W4	-0.21513	0.192864	0.427211	Cryzl1	Quinone ox	QORL1_MC	5	0.861471

15min / C	Q99J36	-0.06323	0.19296	0.427211	Thumpd1	THUMP dor	THUM1_MO	8	0.957121
15min / C	Q6NSR8	0.061571	0.193325	0.42782	Npepl1	Probable ar	PEPL1_MOU	16	1.043601
30min / C	Q61879	-0.10681	0.193441	0.42788	Myh10	Myosin-10	MYH10_MO	44	0.92864
15min / C	P34928	-0.23455	0.19354	0.4279	Apoc1	Apolipoprc	APOC1_MO	2	0.849953
15min / C	Q9QXF8	-0.16344	0.193861	0.428412	Gnmt	Glycine N-n	GNMT_MOU	21	0.892895
15min / C	Q3THS6	-0.05275	0.194038	0.428555	Mat2a	S-adenosyl	METK2_MO	11	0.964098
15min / C	Q9JIL4	0.173584	0.194195	0.428555	Pdzk1	Na(+)/H(+) e	NHRF3_MO	22	1.127857
30min / C	Q8C0E2	-0.00367	0.194768	0.429226	Vps26b	Vacuolar pr	VP26B_MO	10	0.997458
30min / C	P70194	0.703645	0.19488	0.429274	Clec4f	C-type lecti	CLC4F_MOU	10	1.628615
30min / C	Q91WD5	0.69829	0.195343	0.429898	Ndufs2	NADH dehy	NDUS2_MO	12	1.62258
15min / C	Q3TWW8	0.185187	0.195446	0.429927	Srsf6	Serine/argi	SRSF6_MOU	6	1.136964
15min / C	O88544	-0.26879	0.196708	0.430987	Cops4	COP9 signa	CSN4_MOU	17	0.830018
30min / C	P23492	0.065372	0.196334	0.430987	Pnp	Purine nucl	PNPH_MOU	19	1.046355
30min / C	Q8QZR5	-0.16402	0.196143	0.430987	Gpt	Alanine am	ALAT1_MOU	22	0.892537
15min / C	Q9D967	-0.16109	0.196533	0.430987	Mdp1	Magnesium	MGDP1_MO	3	0.894347
15min / C	Q9WVJ2	0.011037	0.196159	0.430987	Psmd13	26S protea	PSD13_MO	23	1.007679
15min / C	Q9Z2U0	-0.04965	0.196678	0.430987	Psma7	Proteasom	PSA7_MOU	15	0.966169
15min / C	Q5F226	0.601374	0.197351	0.431021	Fat2	Protocadhe	FAT2_MOU	2	1.51716
15min / C	Q8K370	-0.13271	0.197476	0.431021	Acad10	Acyl-CoA de	ACD10_MO	31	0.912116
30min / C	Q9D7V9	-1.23739	0.197413	0.431021	Naaa	N-acylethar	NAAA_MOU	2	0.424139
30min / C	P47199	0.145103	0.197846	0.431291	Cryz	Quinone ox	QOR_MOUS	18	1.105809
30min / C	Q6WVG3	-0.21716	0.19787	0.431291	Kctd12	BTB/POZ dc	KCD12_MO	10	0.860258
30min / C	Q8VHK9	0.443317	0.197844	0.431291	Dhx36	ATP-depenc	DHX36_MO	6	1.359727
15min / C	P41317	-0.07242	0.198403	0.432255	Mbl2	Mannose-b	MBL2_MOU	6	0.951041
30min / C	Q7TQG1	-0.33831	0.198549	0.432376	Plekha6	Pleckstrin h	PKHA6_MO	2	0.790966
30min / C	Q8BVE3	0.157633	0.199005	0.43258	Atp6v1h	V-type prot	VATH_MOU	10	1.115456
30min / C	Q9Z223	0.055854	0.199177	0.432758	Mocs2	Molybdopt	MOC2B_MO	4	1.039474
30min / C	P01796;P0	-0.32829	0.199673	0.432852	NaN	Ig heavy ch	HVM27_MO	1	0.796483
15min / C	Q00724	0.099513	0.199667	0.432852	Rbp4	Retinol-bin	RET4_MOU	8	1.071412
15min / C	Q9JHW2	-0.12557	0.199924	0.433002	Nit2	Omega-ami	NIT2_MOUS	19	0.916639
15min / C	Q05920	0.911463	0.200301	0.433035	Pc	Pyruvate ca	PYC_MOUS	58	1.880952
30min / C	Q80TY0	0.464402	0.20017	0.433035	Fnbp1	Formin-bin	FNBP1_MO	4	1.379746
30min / C	Q8CHP8	0.212996	0.200285	0.433035	Pgp	Glycerol-3-	PGP_MOUS	14	1.159093
30min / C	Q924Y0	0.031679	0.200238	0.433035	Bbox1	Gamma-bu	BODG_MOU	14	1.022201
30min / C	P97328	-0.07289	0.200418	0.433091	Khk	Ketohexoki	KHK_MOUS	24	0.950733
15min / C	Q9CR16	0.050339	0.200964	0.433878	Ppid	Peptidyl-pr	PPID_MOU	20	1.035508
30min / C	Q9Z2U0	-0.1335	0.201635	0.434738	Psma7	Proteasom	PSA7_MOU	15	0.911615
15min / C	P17047	0.026709	0.202	0.435318	Lamp2	Lysosome-a	LAMP2_MO	5	1.018686
30min / C	Q921M4	-0.74384	0.202086	0.435318	Golga2	Golgin subf	GOGA2_MO	13	0.597149
30min / C	P07356	0.093128	0.202375	0.435548	Anxa2	Annexin A2	ANXA2_MO	22	1.06668
30min / C	P06745	-0.06868	0.202531	0.435558	Gpi	Glucose-6-p	G6PI_MOU	28	0.953512
30min / C	Q93092	0.106493	0.202562	0.435558	Taldo1	Transaldola	TALDO_MO	17	1.076608
15min / C	Q9CVB6	-0.08435	0.202679	0.435614	Arpc2	Actin-relate	ARPC2_MO	22	0.943212
30min / C	Q9D7X8	0.196657	0.203141	0.436019	Ggct	Gamma-glu	GGCT_MOU	6	1.146039
30min / C	Q9R0Q6	-0.03122	0.203072	0.436019	Arpc1a	Actin-relate	ARC1A_MO	13	0.97859
15min / C	Q9CQR4	0.299398	0.20389	0.437119	Acot13	Acyl-coenzy	ACO13_MO	6	1.230631
30min / C	P48024	-0.23942	0.204202	0.43751	Eif1	Eukaryotic	EIF1_MOUS	5	0.847088
30min / C	O70325	0.358753	0.204338	0.437525	Gpx4	Phospholip	GPX4_MOU	8	1.282317
15min / C	P63260	1.272831	0.204392	0.437525	Actg1	Actin, cyto	ACTG_MOU	1	2.416353
30min / C	Q02780	-0.67598	0.205323	0.43898	Nfia	Nuclear fac	NFIA_MOUS	5	0.625907
30min / C	Q9R1P3	-0.15728	0.205397	0.43898	Psmb2	Proteasom	PSB2_MOU	10	0.896714
15min / C	P97328	-0.08377	0.206605	0.44088	Khk	Ketohexoki	KHK_MOUS	24	0.943591

15min / C	P29595	-0.50443	0.206889	0.441227	Nedd8	NEDD8	NEDD8_MC	4	0.70494
15min / C	Q9Z2G9	-1.86417	0.206952	0.441227	Htatip2	Oxidoreduc	HTAI2_MOU	11	0.274682
30min / C	Q61233	-0.14727	0.207199	0.441557	Lcp1	Plastin-2	PLSL_MOUS	33	0.90296
30min / C	P01898	0.029571	0.207865	0.441598	H2-Q10	H-2 class I h	HA10_MOU	8	1.020709
15min / C	P29699	-0.11311	0.207744	0.441598	Ahsg	Alpha-2-HS	FETUA_MOU	10	0.924594
15min / C	Q3UQ44	-0.03083	0.207613	0.441598	Iqgap2	Ras GTPase-	IQGA2_MO	63	0.978859
30min / C	Q80WT5	-0.5565	0.207789	0.441598	Aftph	Aftiphilin	AFTIN_MOU	5	0.679949
30min / C	Q9JJL8	-0.28116	0.207503	0.441598	Sars2	Serine--tRN	SYSM_MOU	7	0.822926
30min / C	Q64261	-0.10187	0.208263	0.441937	Cdk6	Cyclin-depe	CDK6_MOU	11	0.931821
15min / C	Q9CQJ6	0.042698	0.208427	0.441937	Denr	Density-reg	DENR_MOU	7	1.030038
30min / C	Q9CXZ1	-0.01075	0.208487	0.441937	Ndufs4	NADH dehy	NDUS4_MC	5	0.992573
30min / C	Q9R1P4	-0.19057	0.208347	0.441937	Psma1	Proteasom	PSA1_MOU	16	0.87626
15min / C	P05064	-0.04668	0.208844	0.442301	Aldoa	Fructose-bi	ALDOA_MC	25	0.968159
15min / C	O35215	0.15035	0.208949	0.442327	Ddt	D-dopachro	DOPD_MOU	13	1.109838
30min / C	P14115	-0.23147	0.209551	0.442872	Rpl27a	Large ribos	RL27A_MO	6	0.851765
30min / C	P50171	-0.26001	0.209409	0.442872	Hsd17b8	(3R)-3-hydr	DHB8_MOU	11	0.835084
30min / C	Q9ET22	-0.04111	0.209636	0.442872	Dpp7	Dipeptidyl	DPP2_MOU	12	0.971905
30min / C	P46061	-0.45623	0.21023	0.443468	Rangap1	Ran GTPase	RAGP1_MC	18	0.728888
30min / C	P61164	-0.38848	0.210201	0.443468	Actr1a	Alpha-centr	ACTZ_MOU	13	0.763935
30min / C	P35279	-0.10134	0.211381	0.444027	Rab6a	Ras-related	RAB6A_MO	8	0.932169
15min / C	P62267	-0.30808	0.211112	0.444027	Rps23	Small ribos	RS23_MOU	6	0.807714
15min / C	Q61206	-0.16837	0.211237	0.444027	Pafah1b2	Platelet-act	PA1B2_MO	5	0.88985
30min / C	Q924M7	0.664271	0.211099	0.444027	Mpi	Mannose-6	MPI_MOUS	12	1.584767
30min / C	Q9DCQ2	-0.09119	0.211175	0.444027	Aspdh	Aspartate d	ASPDH_MC	16	0.938749
30min / C	Q9QX60	0.14343	0.211524	0.444027	Dguok	Deoxyguan	DGUOK_M(	3	1.104528
15min / C	P11714	0.086275	0.212355	0.445032	Cyp2d9	Cytochrom	CP2D9_MC	16	1.061625
30min / C	P16294	0.022199	0.212368	0.445032	F9	Coagulatior	FA9_MOUS	7	1.015506
15min / C	P14847	-0.50294	0.212744	0.445039	Crp	C-reactive p	CRP_MOUS	4	0.705667
30min / C	Q9WV98	-0.23871	0.212661	0.445039	Timm9	Mitochond	TIM9_MOU	3	0.847506
15min / C	P63038	-0.18771	0.213219	0.445645	Hspd1	60 kDa heat	CH60_MOU	38	0.877999
30min / C	Q64674	0.354615	0.213415	0.445663	Srm	Spermidine	SPEE_MOUS	14	1.278645
30min / C	O54950	0.5216	0.214233	0.44658	Prkag1	5'-AMP-acti	AAKG1_MC	12	1.435546
15min / C	P23492	0.115764	0.214102	0.44658	Pnp	Purine nucl	PNPH_MOU	19	1.083549
15min / C	Q62426	-0.1139	0.214321	0.44658	Cstb	Cystatin-B	CYTB_MOU	8	0.924086
15min / C	P97823	-0.50536	0.214798	0.44704	Lypla1	Acyl-protei	LYPA1_MOI	8	0.704483
15min / C	Q8C0J2	0.131408	0.214989	0.447063	Atg16l1	Autophagy-	A16L1_MO	6	1.095362
15min / C	Q9QZB7	-0.4065	0.215021	0.447063	Actr10	Actin-relate	ARP10_MO	5	0.754451
15min / C	O89079	0.334541	0.215122	0.44708	Cope	Coatomer s	COPE_MOU	11	1.260976
15min / C	Q99JI4	0.047276	0.215356	0.447371	Psmd6	26S protea	PSMD6_M(	24	1.033312
15min / C	Q91XE4	0.048888	0.21556	0.447601	Acy3	N-acyl-aron	ACY3_MOU	12	1.034468
15min / C	P70670;Q6	0.039691	0.215939	0.448192	Naca	Nascent po	NACAM_M(	5	1.027894
15min / C	Q91VR2	0.266198	0.216252	0.448648	Atp5f1c	ATP synthas	ATPG_MOU	11	1.202634
15min / C	O88685	0.163604	0.216485	0.448937	Psmc3	26S protea	PRS6A_MO	22	1.120082
15min / C	P62305	-0.89119	0.216798	0.44939	Snrpe	Small nucle	RUXE_MOU	3	0.539169
30min / C	Q9ERG0	-0.05017	0.217433	0.450317	Lima1	LIM domair	LIMA1_MO	28	0.965823
15min / C	P01868;P0	-0.24544	0.217531	0.450325	Ighg1	Ig gamma-1	IGHG1_MO	7	0.843561
30min / C	P26262	0.028069	0.217702	0.450484	Klkb1	Plasma kall	KLKB1_MO	11	1.019646
30min / C	Q8JZN5	-0.14586	0.217988	0.450489	Acad9	Complex I a	ACAD9_MC	24	0.90384
30min / C	Q9D1I5	-0.5611	0.217921	0.450489	Mcee	Methylmal	MCEE_MOU	5	0.677786
30min / C	Q91WQ3	-0.00784	0.218359	0.451061	Yars1	Tyrosine--tI	SYYC_MOUS	32	0.994578
30min / C	Q99KJ8	-0.13504	0.218701	0.451378	Dctn2	Dynactin su	DCTN2_MC	16	0.910647
30min / C	Q8BL97	0.036124	0.219726	0.452571	Srsf7	Serine/argi	SRSF7_MOU	5	1.025356

30min / C	Q99LP6	0.02403	0.219649	0.452571	Grpel1	GrpE protei	GRPE1_MO	11	1.016796
30min / C	P61358	0.315366	0.220011	0.452796	Rpl27	Large ribos	RL27_MOU	8	1.244327
15min / C	Q60598	-0.20374	0.220092	0.452796	Cttn	Src substra	SRC8_MOU	17	0.868296
15min / C	O35423	-0.0345	0.220501	0.452938	Agxt	Alanine--gl	AGT1_MOU	18	0.976369
30min / C	P57746	0.416043	0.220577	0.452938	Atp6v1d	V-type prot	VATD_MOU	6	1.334263
30min / C	Q3U1J4	-0.04671	0.220594	0.452938	Ddb1	DNA damag	DDB1_MOU	55	0.968142
30min / C	P56376	-0.19995	0.220783	0.45313	Acyp1	Acylphosph	ACYP1_MO	3	0.870579
15min / C	P60824	-0.69401	0.221386	0.453269	Cirbp	Cold-induc	CIRBP_MOU	2	0.618133
15min / C	P80313	-0.01793	0.221567	0.453269	Cct7	T-complex	TCPH_MOU	27	0.98765
15min / C	Q8BWU8	-0.03898	0.221014	0.453269	Etnppl	Ethanolami	AT2L1_MOU	19	0.973346
30min / C	Q91VM5	0.890556	0.221609	0.453269	Rbmxl1	RNA bindin	RMXL1_MC	10	1.85389
15min / C	Q9DB15	-0.40571	0.221321	0.453269	Mrpl12	Large ribos	RM12_MOU	9	0.754865
15min / C	P56399	0.040515	0.222558	0.453923	Usp5	Ubiquitin c	UBP5_MOU	31	1.028481
30min / C	Q9DBD0	0.074977	0.222376	0.453923	Inhca	Inhibitor of	ICA_MOUSI	30	1.053344
30min / C	Q9R111	-0.0876	0.222614	0.453923	Gda	Guanine de	GUAD_MOU	20	0.941085
30min / C	P12265	-0.21392	0.222999	0.454178	Gusb	Beta-glucur	BGLR_MOU	21	0.862188
30min / C	Q922D4	-0.24389	0.223003	0.454178	Ppp6r3	Serine/thre	PP6R3_MO	22	0.844467
15min / C	Q61699	0.020068	0.223314	0.454545	Hsph1	Heat shock	HS105_MO	29	1.014007
15min / C	Q9WVM8	0.103241	0.22365	0.454914	Aadat	Kynurenine	AADAT_MO	19	1.074184
15min / C	O55125	0.337087	0.224526	0.455533	Nipsnap1	Protein Nip	NIPS1_MOU	13	1.263203
15min / C	P16332	0.170019	0.224292	0.455533	Mmut	Methylmal	MUTA_MOU	25	1.125073
15min / C	P50580	0.247954	0.224244	0.455533	Pa2g4	Proliferatic	PA2G4_MC	18	1.187522
30min / C	P53395	-0.04147	0.224495	0.455533	Dbt	Lipoamide	ODB2_MOU	24	0.971662
30min / C	Q9JLF6	0.376361	0.22443	0.455533	Tgm1	Protein-glu	TGM1_MOU	5	1.298063
30min / C	Q9JHI5	-0.14836	0.225015	0.455945	Ivd	Isovaleryl-C	IVD_MOUSI	18	0.902278
30min / C	Q9CQE1	-0.10959	0.225586	0.456714	Nipsnap3b	Protein Nip	NPS3B_MO	13	0.926853
15min / C	D3Z6Q9	-0.26339	0.226337	0.456879	Bin2	Bridging in	BIN2_MOU	4	0.833129
15min / C	O09061	-0.11046	0.22613	0.456879	Psmb1	Proteasom	PSB1_MOU	11	0.926295
30min / C	P05213	-0.18244	0.226161	0.456879	Tuba1b	Tubulin alp	TBA1B_MO	21	0.881209
30min / C	P10605	-0.09716	0.226315	0.456879	Ctsb	Cathepsin E	CATB_MOU	12	0.934874
15min / C	P19001	0.572743	0.225963	0.456879	Krt19	Keratin, typ	K1C19_MO	1	1.487349
30min / C	Q8BH69	0.043117	0.226087	0.456879	Sephs1	Selenide, w	SPS1_MOU	9	1.030338
30min / C	P54731	-0.67054	0.227088	0.457675	Faf1	FAS-associa	FAF1_MOU	7	0.62827
15min / C	P61971	-0.17478	0.227046	0.457675	Nutf2	Nuclear tra	NTF2_MOU	6	0.885905
30min / C	Q00898	-0.13205	0.227401	0.457675	Serpina1e	Alpha-1-an	A1AT5_MO	1	0.912535
15min / C	O88947	-0.04508	0.227629	0.457941	F10	Coagulatio	FA10_MOU	6	0.969237
15min / C	Q9CQT1	-0.07275	0.227888	0.458077	Mri1	Methylthio	MTNA_MOU	19	0.950823
15min / C	Q3TCJ1	-0.50781	0.228273	0.458499	Abraxas2	BRISC comp	ABRX2_MO	8	0.703289
30min / C	Q62452	-0.20334	0.22829	0.458499	Ugt1a9	UDP-glucur	UD19_MOU	17	0.868536
30min / C	Q8BPB0;Q9	-0.16704	0.228813	0.459164	Mob1b;Mo	MOB kinase	MOB1B_MC	4	0.890671
15min / C	Q91XC8	0.567063	0.229214	0.459389	Dap	Death-asso	DAP1_MOU	3	1.481504
15min / C	Q9CPW2	-0.77726	0.229158	0.459389	Fdx2	Ferredoxin	FDX2_MOU	3	0.583473
15min / C	Q9CXZ1	-0.28437	0.229548	0.459865	Ndufs4	NADH dehy	NDUS4_MC	5	0.821102
15min / C	P46061	-0.30787	0.229982	0.460542	Rangap1	Ran GTPase	RAGP1_MC	18	0.807834
30min / C	P19639	-0.18667	0.230183	0.460665	Gstm3	Glutathion	GSTM3_MC	17	0.878632
30min / C	Q61703	-0.12562	0.230333	0.460665	Itih2	Inter-alpha	ITIH2_MOU	17	0.91661
15min / C	Q6ZWV3	0.489621	0.230539	0.460865	Rpl10	Large ribos	RL10_MOU	7	1.404076
15min / C	P62827	0.604131	0.231031	0.46129	Ran	GTP-bindin	RAN_MOUS	9	1.520063
30min / C	Q91ZI0	-0.94718	0.231343	0.461584	Celsr3	Cadherin E	CELR3_MOI	1	0.518644
15min / C	O54950	-0.12247	0.232221	0.462893	Prkag1	5'-AMP-acti	AAKG1_MC	12	0.918615
30min / C	P16546	-0.1378	0.232582	0.463418	Sptan1	Spectrin al	SPTN1_MO	106	0.908906
30min / C	P02769	-0.12125	0.232747	0.463554	ALB	Serum albu	ALBU_BOVI	65	0.919392

30min / C	Q9D9H8	-0.14287	0.232868	0.463603	NaN	Mitochond	CB069_MO	2	0.905713
15min / C	Q3T9E4;Q6	0.384214	0.233532	0.463958	Tgtp2;Tgtp	T-cell-speci	TGTP2_MO	5	1.305149
30min / C	Q8R574	0.337851	0.233531	0.463958	Prpsap2	Phosphorib	KPRB_MOU	7	1.263872
30min / C	Q8VCG4	-0.08244	0.233742	0.463989	C8g	Compleme	CO8G_MOU	7	0.944462
15min / C	P01027	-0.02461	0.234079	0.464093	C3	Compleme	CO3_MOUS	101	0.983089
30min / C	Q80Y14	-0.4136	0.234558	0.464837	Glrx5	Glutaredox	GLRX5_MO	3	0.750746
30min / C	P06728	-0.16043	0.234802	0.464868	Apoa4	Apolipopro	APOA4_MO	27	0.894758
30min / C	Q9DB16	0.6998	0.234764	0.464868	Cab39l	Calcium-bi	CB39L_MO	5	1.62428
30min / C	Q8R519	-0.28738	0.235089	0.465118	Acmsd	2-amino-3-	ACMSD_MO	11	0.81939
30min / C	Q9CQQ8	0.360268	0.235741	0.466023	Lsm7	U6 snRNA-a	LSM7_MOU	2	1.283664
15min / C	P61087	-0.0167	0.236121	0.466548	Ube2k	Ubiquitin-c	UBE2K_MO	10	0.988492
30min / C	Q9D1E6	0.177488	0.23648	0.466548	Tbcb	Tubulin-fol	TBCB_MOU	10	1.130913
15min / C	O70200	0.151043	0.237333	0.46743	Aif1	Allograft in	AIF1_MOUS	2	1.110372
30min / C	Q8CEI1	-0.3273	0.237768	0.467708	Bola3	BolA-like pr	BOLA3_MO	4	0.797026
30min / C	Q8VCU1	-0.01854	0.237639	0.467708	Ces3b	Carboxylest	EST3B_MOU	8	0.987233
15min / C	P05208	-0.2367	0.238388	0.467715	Cela2a	Chymotryp	CEL2A_MOU	2	0.848683
30min / C	P17047	0.133968	0.239127	0.467715	Lamp2	Lysosome-a	LAMP2_MO	5	1.097308
15min / C	P34022	-0.00275	0.23904	0.467715	Ranbp1	Ran-specific	RANG_MOU	4	0.998098
15min / C	Q570Y9	0.136785	0.237935	0.467715	Deptor	DEP domain	DPTOR_MO	11	1.099453
30min / C	Q5U5M8	-0.36886	0.238426	0.467715	Bloc1s3	Biogenesis	BL1S3_MOU	4	0.774393
30min / C	Q8CC86	-0.19713	0.238311	0.467715	Naprt	Nicotinate	PNCB_MOU	22	0.872282
15min / C	Q8WTY4	-0.09826	0.237994	0.467715	Ciapin1	Anamorsin	CPIN1_MOU	12	0.934157
15min / C	Q9CQD1	1.410634	0.238607	0.467715	Rab5a	Ras-related	RAB5A_MO	5	2.65854
30min / C	Q9D8T7	-1.06129	0.238125	0.467715	Slirp	SRA stem-lo	SLIRP_MOU	3	0.479203
15min / C	Q9DC63	-1.1018	0.239099	0.467715	Fbxo3	F-box only p	FBX3_MOU	9	0.465936
15min / C	Q9ET54	0.538268	0.238618	0.467715	Palld	Palladin	PALLD_MO	17	1.452228
15min / C	Q5FWK3	-0.26353	0.240127	0.46788	Arhgap1	Rho GTPase	RHG01_MO	8	0.833048
15min / C	Q8C7G5	-0.63064	0.240059	0.46788	Apoa5	Apolipopro	APOA5_MO	4	0.645889
15min / C	Q8VBV3	-0.45003	0.239581	0.46788	Exosc2	Exosome co	EXOS2_MO	5	0.732028
15min / C	Q9CYW4	0.221621	0.239496	0.46788	Hdhd3	Haloacid de	HDHD3_MO	11	1.166043
30min / C	Q9EQU5	-0.24932	0.239947	0.46788	Set	Protein SET	SET_MOUSE	7	0.841292
15min / C	Q9JJF9	1.018867	0.239696	0.46788	Sppl2a	Signal pept	SPP2A_MO	1	2.026327
30min / C	Q9R1Z7	-0.0561	0.239929	0.46788	Pts	6-pyruvoyl	PTPS_MOU	5	0.961863
30min / C	P35278	-0.79675	0.240757	0.467966	Rab5c	Ras-related	RAB5C_MO	9	0.575645
30min / C	Q0VBK2	1.0832	0.240819	0.467966	Krt80	Keratin, typ	K2C80_MO	1	2.118731
30min / C	Q80UG2	0.951662	0.240836	0.467966	Plxna4	Plexin-A4	PLXA4_MO	1	1.934099
15min / C	Q922Q1	0.133273	0.240438	0.467966	Mtarc2	Mitochond	MARC2_MO	8	1.096779
30min / C	Q9R0H0	-0.10026	0.240816	0.467966	Acox1	Peroxisoma	ACOX1_MO	39	0.932868
30min / C	P14869	-0.15371	0.241071	0.468232	Rplp0	Large ribos	RLA0_MOU	11	0.898936
30min / C	P26443	0.169993	0.24143	0.468549	Glud1	Glutamate	DHE3_MOU	35	1.125053
15min / C	Q9D6Y9	1.152763	0.241424	0.468549	Gbe1	1,4-alpha-g	GLGB_MOU	32	2.223392
15min / C	O88746	-0.49673	0.24155	0.46859	Tom1	Target of M	TOM1_MOU	12	0.70871
15min / C	P14115	-0.27173	0.241772	0.468811	Rpl27a	Large ribos	RL27A_MO	6	0.828326
15min / C	P61358	0.435303	0.241859	0.468811	Rpl27	Large ribos	RL27_MOU	8	1.352194
30min / C	P01878	0.303372	0.242343	0.469155	NaN	Ig alpha cha	IGHA_MOU	7	1.234025
15min / C	Q03249	0.136614	0.242238	0.469155	Galt	Galactose-1	GALT_MOU	10	1.099322
15min / C	Q9JHU9	-0.08072	0.242171	0.469155	Isyna1	Inositol-3-p	INO1_MOU	10	0.945583
30min / C	Q07076	-0.33474	0.242968	0.469264	Anxa7	Annexin A7	ANXA7_MO	16	0.792929
30min / C	Q9D8Y0	0.197961	0.242809	0.469264	Efhd2	EF-hand do	EFHD2_MO	9	1.147076
30min / C	Q9DCL9	-0.15401	0.243008	0.469264	Paics	Bifunctiona	PUR6_MOU	19	0.898747
15min / C	Q9JHJ0	0.137296	0.242685	0.469264	Tmod3	Tropomodu	TMOD3_MO	11	1.099842
15min / C	A1L317	0.001526	0.244122	0.469768	Krt24	Keratin, typ	K1C24_MO	1	1.001059

15min / C	P10493	0.460964	0.24356	0.469768	Nid1	Nidogen-1	NID1_MOU	2	1.376461
15min / C	P61961	0.011657	0.243781	0.469768	Ufm1	Ubiquitin-f	UFM1_MOU	4	1.008113
15min / C	P62141	0.389293	0.244062	0.469768	Ppp1cb	Serine/thre	PP1B_MOU	5	1.309752
30min / C	Q920E5	-0.396	0.24388	0.469768	Fdps	Farnesyl py	FPPS_MOU	19	0.759963
30min / C	Q9Z0P5	0.386062	0.243754	0.469768	Twf2	Twinfilin-2	TWF2_MOU	5	1.306821
15min / C	O35943	0.111078	0.245023	0.471123	Fxn	Frataxin, m	FRDA_MOU	3	1.080035
30min / C	P35282	0.261076	0.245219	0.471147	Rab21	Ras-related	RAB21_MO	6	1.198372
15min / C	Q64471	-0.26238	0.245233	0.471147	Gstt1	Glutathion	GSTT1_MO	16	0.833714
30min / C	P00405	-1.0224	0.245812	0.471476	Mtco2	Cytochrom	COX2_MOU	6	0.492298
30min / C	Q8R0J8	0.866982	0.245553	0.471476	Idnk	Probable gl	GNTK_MOU	5	1.823843
30min / C	Q91XE4	-0.00354	0.245716	0.471476	Acy3	N-acyl-aron	ACY3_MOU	12	0.997548
15min / C	P20152	0.05004	0.247631	0.471772	Vim	Vimentin	VIME_MOU	21	1.035294
30min / C	Q01405	-0.0505	0.247043	0.471772	Sec23a	Protein tra	SC23A_MO	28	0.9656
30min / C	Q61074	0.236575	0.246361	0.471772	Ppm1g	Protein pho	PPM1G_M	9	1.178192
30min / C	Q64471	-0.29977	0.246627	0.471772	Gstt1	Glutathion	GSTT1_MO	16	0.81238
30min / C	Q66JS6	0.01175	0.247499	0.471772	Eif3j2	Eukaryotic	EI3JB_MOU	12	1.008178
15min / C	Q80XI3	0.116624	0.246926	0.471772	Eif4g3	Eukaryotic	IF4G3_MOU	30	1.084195
30min / C	Q8VEK3	0.019064	0.247164	0.471772	Hnrnpu	Heterogene	HNRPU_M	24	1.013302
15min / C	Q99L47	-0.10626	0.246324	0.471772	St13	Hsc70-inte	F10A1_MO	10	0.928993
15min / C	Q99LD4	-0.1864	0.246494	0.471772	Gps1	COP9 signa	CSN1_MOU	11	0.878793
30min / C	Q9D358	-0.15049	0.247434	0.471772	Acp1	Low molec	PPAC_MOU	4	0.900945
15min / C	Q9DBG5	-0.18566	0.246719	0.471772	Plin3	Perilipin-3	PLIN3_MOU	14	0.879244
15min / C	Q9ET26	0.141963	0.247183	0.471772	Rnf114	E3 ubiquiti	RN114_MC	8	1.103406
15min / C	Q9JK81	-0.49632	0.247626	0.471772	Myg1	MYG1 exon	MYG1_MOU	13	0.708914
30min / C	P00920	-0.107	0.247936	0.471978	Ca2	Carbonic a	CAH2_MOU	11	0.928515
30min / C	P17717	0.874134	0.247935	0.471978	Ugt2b17	UDP-glucu	UDB17_MC	16	1.832908
30min / C	Q64516	-0.05485	0.248143	0.472111	Gk	Glycerol ki	GLPK_MOU	28	0.962696
15min / C	P60867	-0.26413	0.249306	0.472531	Rps20	Small ribos	RS20_MOU	5	0.832701
30min / C	Q00519	-0.13517	0.249469	0.472531	Xdh	Xanthine d	XDH_MOUS	52	0.910562
15min / C	Q6IME9	-0.53104	0.248599	0.472531	Krt72	Keratin, typ	K2C72_MO	1	0.692055
15min / C	Q6P8M1	0.408336	0.248933	0.472531	Tatdn1	Deoxyribor	TATD1_MO	7	1.327154
15min / C	Q78PY7	1.039567	0.249517	0.472531	Snd1	Staphyloco	SND1_MOU	51	2.055611
15min / C	Q8BFW7	0.043402	0.249414	0.472531	Lpp	Lipoma-pre	LPP_MOUS	17	1.030541
15min / C	Q8BVI4	0.330634	0.249037	0.472531	Qdpr	Dihydropte	DHPR_MOU	12	1.257566
30min / C	Q99LD4	-0.28514	0.24909	0.472531	Gps1	COP9 signa	CSN1_MOU	11	0.820662
15min / C	P35908	-0.38594	0.250855	0.47264	KRT2	Keratin, typ	K22E_HUM	39	0.765281
30min / C	P42567	0.444361	0.250272	0.47264	Eps15	Epidermal g	EPS15_MO	23	1.360712
15min / C	P60122	-0.23769	0.250266	0.47264	Ruvbl1	RuvB-like 1	RUVB1_MC	18	0.848101
30min / C	Q11011	-0.13795	0.250818	0.47264	Npepps	Puromycin	PSA_MOUS	30	0.908809
30min / C	Q3UEB3	0.12612	0.250683	0.47264	Puf60	Poly(U)-bin	PUF60_MO	13	1.091355
15min / C	Q6ZWY3	0.594802	0.250491	0.47264	Rps27l	Small ribos	RS27L_MO	3	1.510266
15min / C	Q7TMK9	-0.09226	0.250409	0.47264	Syncrip	Heterogene	HNRPQ_M	20	0.938053
30min / C	Q8BVU5	-0.37564	0.250732	0.47264	Nudt9	ADP-ribose	NUDT9_MC	2	0.770763
30min / C	Q91WK2	-0.07805	0.249777	0.47264	Eif3h	Eukaryotic	EIF3H_MOU	14	0.947339
15min / C	Q9CR98	-0.06672	0.250766	0.47264	Fam136a	Protein FA	F136A_MO	8	0.954807
15min / C	Q9JMG1	-0.24872	0.251018	0.472761	Edf1	Endothelial	EDF1_MOU	6	0.841641
30min / C	Q6ZWN5	0.255125	0.251435	0.473172	Rps9	Small ribos	RS9_MOUS	12	1.193439
30min / C	Q9Z2V4	0.288937	0.251379	0.473172	Pck1	Phosphoen	PCKGC_MC	34	1.221739
15min / C	P47915	0.300193	0.251865	0.473184	Rpl29	Large ribos	RL29_MOU	5	1.231309
15min / C	Q00623	-0.12323	0.251865	0.473184	Apoa1	Apolipopro	APOA1_MC	18	0.918132
30min / C	Q922H4	-0.14393	0.252315	0.473184	Gmppa	Mannose-1	GMPPA_M	10	0.905053
15min / C	Q9R1A8	-0.3964	0.251637	0.473184	Cop1	E3 ubiquiti	COP1_MOU	1	0.759751

15min / C	Q9Z2V4	0.545637	0.252086	0.473184	Pck1	Phosphoen	PCKGC_MO	34	1.459664
30min / C	P97314	0.22611	0.252626	0.473339	Csrp2	Cysteine an	CSRP2_MO	5	1.169677
30min / C	Q9D379	-0.56841	0.252515	0.473339	Ephx1	Epoxide hy	HYEP_MOU	22	0.674359
15min / C	Q05816	-0.27993	0.253139	0.473605	Fabp5	Fatty acid-b	FABP5_MO	9	0.823632
30min / C	Q9JLZ3	-0.16023	0.253136	0.473605	Auh	Methylglut	AUHM_MO	7	0.894885
30min / C	Q8K3D3	0.12496	0.253538	0.473958	Swi5	DNA repair	SWI5_MOU	1	1.090478
30min / C	Q9DB34	-0.63431	0.253465	0.473958	Chmp2a	Charged mu	CHM2A_MO	4	0.644251
15min / C	Q8BG05	-0.12071	0.253673	0.474025	Hnrnpa3	Heterogene	ROA3_MOU	14	0.919733
30min / C	Q9DBG5	-0.26711	0.254229	0.474509	Plin3	Perilipin-3	PLIN3_MOU	14	0.830981
15min / C	Q91VA6	0.646461	0.254809	0.475215	Poldip2	Polymerase	PDIP2_MOU	10	1.565324
30min / C	Q9WUZ9	-0.24786	0.254906	0.475215	Entpd5	Ectonucleo	ENTP5_MO	14	0.842146
15min / C	Q924Y0	0.620009	0.255369	0.475707	Bbox1	Gamma-bu	BODG_MOU	14	1.536885
30min / C	Q9JIF0	-0.50453	0.255664	0.475885	Prmt1	Protein arg	ANM1_MOU	11	0.704889
15min / C	P63017	-0.02214	0.255803	0.475959	Hspa8	Heat shock	HSP7C_MO	28	0.984773
15min / C	Q80Y14	0.315541	0.256142	0.476245	Glrx5	Glutaredox	GLRX5_MO	3	1.244479
30min / C	Q8K2I4	-0.15359	0.256156	0.476245	Manba	Beta-mann	MANBA_MO	8	0.899013
15min / C	P97872	-0.72365	0.256302	0.476332	Fmo5	Flavin-cont	FMO5_MOU	30	0.605563
15min / C	O35226	0.087982	0.257691	0.477867	Psmd4	26S protea	PSMD4_MO	11	1.062882
15min / C	P01629	0.209969	0.257707	0.477867	NaN	Ig kappa ch	KV2A4_MO	1	1.156663
15min / C	P62852	0.216585	0.258428	0.477867	Rps25	Small ribos	RS25_MOU	6	1.16198
30min / C	Q711T7	0.030998	0.2577	0.477867	Nadsyn1	Glutamine-	NADE_MOU	12	1.021719
30min / C	Q7TNV0	0.244219	0.258032	0.477867	Dek	Protein DE	DEK_MOUS	6	1.184451
30min / C	Q8BVI4	0.128592	0.258164	0.477867	Qdpr	Dihydropte	DHPR_MOU	12	1.093227
15min / C	Q8JZR0	0.439453	0.25787	0.477867	Acsl5	Long-chain	ACSL5_MO	27	1.35609
30min / C	Q9CQN1	-0.23627	0.258317	0.477867	Trap1	Heat shock	TRAP1_MO	31	0.84894
15min / C	Q9WTX5	0.115677	0.258847	0.47826	Skp1	S-phase kin	SKP1_MOU	9	1.083483
15min / C	O35660	0.043763	0.260767	0.478315	Gstm6	Glutathion	GSTM6_MO	11	1.030799
15min / C	P01631	-0.08498	0.259243	0.478315	NaN	Ig kappa ch	KV2A7_MO	3	0.942798
15min / C	P11859	-0.82112	0.260021	0.478315	Agt	Angiotensi	ANGT_MOU	6	0.566002
30min / C	P23506	-0.07099	0.260199	0.478315	Pcmt1	Protein-L-is	PIMT_MOU	8	0.951985
30min / C	P70677	0.031071	0.260505	0.478315	Casp3	Caspase-3	CASP3_MO	10	1.021771
15min / C	Q08879	0.134093	0.259915	0.478315	Fbln1	Fibulin-1	FBLN1_MO	9	1.097403
30min / C	Q71RI9	-0.08017	0.260222	0.478315	Kyat3	Kynurenine	KAT3_MOU	19	0.945949
30min / C	Q7TPZ8	0.080913	0.260321	0.478315	Cpa1	Carboxype	CBPA1_MO	3	1.057687
30min / C	Q80W22	-0.1558	0.260555	0.478315	Thnsl2	Threonine s	THNS2_MO	25	0.897637
30min / C	Q8BML9	0.574535	0.260215	0.478315	Qars1	Glutamine-	SYQ_MOUS	34	1.489197
30min / C	Q9DCE9	-0.2707	0.259261	0.478315	Igtp	Immunity-r	IRGM3_MO	6	0.828918
15min / C	Q9EQH3	0.151684	0.260187	0.478315	Vps35	Vacuolar p	VPS35_MO	26	1.110866
15min / C	Q9JKR6	-0.94815	0.260755	0.478315	Hyou1	Hypoxia up	HYOU1_MO	40	0.518295
15min / C	P19536	0.204438	0.261975	0.479785	Cox5b	Cytochrom	COX5B_MO	3	1.152238
30min / C	Q05915	-0.16719	0.26194	0.479785	Gch1	GTP cycloh	GCH1_MOU	11	0.890573
15min / C	C0HKE1;C0	-0.79439	0.262722	0.480601	H2ac4;H2a	Histone H2	H2A1B_MO	5	0.576588
15min / C	P01796;P0	-0.29401	0.263489	0.481665	NaN	Ig heavy ch	HVM27_MO	1	0.815634
30min / C	Q3ULW8	0.128477	0.263505	0.481665	Parp3	Protein mo	PARP3_MO	16	1.093139
30min / C	P62774	-0.05991	0.263679	0.481665	Mtpn	Myotrophi	MTPN_MOU	6	0.959321
30min / C	P70349	-0.11014	0.263826	0.481665	Hint1	Adenosine	HINT1_MO	7	0.926501
30min / C	Q569Z6	0.418698	0.263908	0.481665	Thrap3	Thyroid ho	TR150_MO	19	1.33672
15min / C	P61082	-0.03937	0.264275	0.482151	Ube2m	NEDD8-con	UBC12_MO	7	0.973082
15min / C	Q61176	0.049432	0.26464	0.482294	Arg1	Arginase-1	ARGI1_MO	21	1.034857
30min / C	Q91VF2	0.101714	0.264656	0.482294	Hnmt	Histamine	HNMT_MOU	10	1.073047
15min / C	P97298	-1.02761	0.265301	0.483101	Serpinf1	Pigment ep	PEDF_MOU	10	0.490522
30min / C	P15626	-0.01913	0.265758	0.483131	Gstm2	Glutathion	GSTM2_MO	21	0.98683

30min / C	P24527	-0.13523	0.267864	0.483131	Lta4h	Leukotrien	LKHA4_MO	26	0.910526
15min / C	P60710	-0.13031	0.267153	0.483131	Actb	Actin, cyto	ACTB_MOU	24	0.913637
15min / C	P97429	0.473783	0.267309	0.483131	Anxa4	Annexin A4	ANXA4_MC	18	1.388746
15min / C	Q3B7Z2	-0.19998	0.267946	0.483131	Osbp	Oxysterol-b	OSBP1_MO	21	0.870562
15min / C	Q8BIQ5	-0.68798	0.267737	0.483131	Cstf2	Cleavage sti	CSTF2_MOI	9	0.62072
30min / C	Q8BJY1	-0.03156	0.266775	0.483131	Psmd5	26S protea	PSMD5_MC	19	0.978359
15min / C	Q8VE97	-0.00558	0.267939	0.483131	Srsf4	Serine/argi	SRSF4_MOI	4	0.99614
15min / C	Q922R8	-0.23126	0.267445	0.483131	Pdia6	Protein dis	PDIA6_MOI	12	0.851893
30min / C	Q9CZH3	0.456103	0.26629	0.483131	Psmg3	Proteasom	PSMG3_MC	2	1.371831
15min / C	Q9D8W5	-0.38248	0.266967	0.483131	Psmd12	26S protea	PSD12_MO	23	0.767116
30min / C	Q9ER35	-0.74105	0.266263	0.483131	Fn3k	Fructosami	FN3K_MOU	4	0.598306
15min / C	Q9R092	0.353715	0.265785	0.483131	Hsd17b6	17-beta-hy	H17B6_MC	9	1.277847
15min / C	Q9WVA2	0.036195	0.267275	0.483131	Timm8a1	Mitochond	TIM8A_MO	5	1.025406
30min / C	Q9Z0M5	-0.2324	0.266338	0.483131	Lipa	Lysosomal	LICH_MOUS	9	0.851219
15min / C	Q91V12	0.024945	0.268065	0.483164	Acot7	Cytosolic a	BACH_MOU	10	1.017441
15min / C	P58137	0.03202	0.268484	0.483191	Acot8	Acyl-coenz	ACOT8_MO	7	1.022443
15min / C	Q8CAK1	-0.23738	0.268251	0.483191	Iba57	Putative tra	CAF17_MO	6	0.848286
15min / C	Q9CY64	0.738764	0.268302	0.483191	Blvra	Biliverdin r	BIEA_MOUS	13	1.668745
15min / C	Q9Z1Q5	0.149899	0.268695	0.48324	Clic1	Chloride in	CLIC1_MOU	9	1.109492
15min / C	Q8R570	-0.80647	0.269107	0.483766	Snap47	Synaptosor	SNP47_MO	9	0.571779
30min / C	Q922J3	-0.25573	0.269235	0.483813	Clip1	CAP-Gly do	CLIP1_MOU	23	0.83756
30min / C	Q8R0F8	0.061685	0.269736	0.48435	Fahd1	Oxaloaceta	FAHD1_MC	11	1.043684
15min / C	Q9D727	0.218944	0.269729	0.48435	NaN	Uncharacte	CF226_MO	2	1.163881
30min / C	O54833	-0.67985	0.270661	0.484373	Csnk2a2	Casein kina	CSK22_MO	5	0.62423
30min / C	Q3TCJ1	0.133288	0.270339	0.484373	Abraxas2	BRISC com	ABRX2_MO	8	1.096791
15min / C	Q8VD04	-0.04971	0.27054	0.484373	Gripap1	GRIP1-asso	GRAP1_MC	14	0.96613
15min / C	P06728	-0.11374	0.271015	0.484414	Apoa4	Apolipoprc	APOA4_MC	27	0.924189
30min / C	P08249	-0.10202	0.271034	0.484414	Mdh2	Malate deh	MDHM_MC	19	0.93173
15min / C	Q91X72	-0.07227	0.270814	0.484414	Hpx	Hemopexir	HEMO_MO	23	0.95114
15min / C	Q99P72	-0.10989	0.27109	0.484414	Rtn4	Reticulon-4	RTN4_MOU	10	0.92666
30min / C	P47941	0.062396	0.27137	0.484734	Crkl	Crk-like prc	CRKL_MOU	8	1.044199
30min / C	O08997	-0.04979	0.271632	0.485021	Atox1	Copper trai	ATOX1_MO	5	0.966075
30min / C	P04186	0.020819	0.271786	0.485047	Cfb	Compleme	CFAB_MOU	29	1.014535
30min / C	P14069	-0.16597	0.272053	0.485047	S100a6	Protein S1C	S10A6_MO	4	0.891332
15min / C	Q62446	0.044496	0.27198	0.485047	Fkbp3	Peptidyl-pr	FKBP3_MO	8	1.031323
30min / C	Q9DB05	-0.19802	0.271989	0.485047	Napa	Alpha-solul	SNAA_MOU	12	0.871747
30min / C	Q9D172	-0.00048	0.272278	0.485269	Gatd3	Glutamine	GAL3A_MO	12	0.999671
30min / C	Q8BFR5	-0.01956	0.272602	0.485664	Tufm	Elongation	EFTU_MOU	25	0.986531
30min / C	Q8BWP5	-0.21821	0.272862	0.485946	Ttpa	Alpha-toco	TTPA_MOU	17	0.859629
30min / C	P49182	-0.07971	0.273466	0.486129	Serpind1	Heparin co	HEP2_MOU	15	0.946246
15min / C	P62774	-0.02607	0.273151	0.486129	Mtpn	Myotrophi	MTPN_MOU	6	0.982093
30min / C	Q8VE62	0.127881	0.27318	0.486129	Paip1	Polyadenyl	PAIP1_MOU	8	1.092688
15min / C	Q9DCL9	-0.1504	0.273575	0.486129	Paics	Bifunctiona	PUR6_MOU	19	0.900999
15min / C	P22907	-0.11295	0.273903	0.486158	Hmbs	Porphobili	HEM3_MOU	14	0.924693
30min / C	Q61133	0.00275	0.273874	0.486158	Gstt2	Glutathion	GSTT2_MOI	13	1.001908
30min / C	Q9JHI7	0.299324	0.273998	0.486158	Exosc9	Exosome cc	EXOS9_MO	6	1.230568
30min / C	Q99J39	0.144529	0.274189	0.486316	Mlycd	Malonyl-Cc	DCMC_MOI	17	1.10537
30min / C	Q8VCH8	-0.7349	0.274557	0.486789	Ubxn4	UBX domai	UBXN4_MC	7	0.60086
30min / C	Q9JHJ0	-0.231	0.274785	0.487013	Tmod3	Tropomodu	TMOD3_MC	11	0.852043
15min / C	Q9Z1G3	-0.32339	0.27499	0.487015	Atp6v1c1	V-type prot	VATC1_MO	9	0.799187
30min / C	Q91X52	-0.24573	0.275149	0.487116	Dcxr	L-xylulose r	DCXR_MOU	14	0.84339
30min / C	Q99MN9	-0.05775	0.275899	0.488263	Pccb	Propionyl-(	PCCB_MOU	25	0.960761

30min / C	Q01339	0.17002	0.276061	0.488369	Apoh	Beta-2-glyc	APOH_MOU	13	1.125074
30min / C	P49586	-0.22706	0.278157	0.48926	Pcyt1a	Choline-ph	PCY1A_MO	10	0.854373
15min / C	P50543	-0.0814	0.27798	0.48926	S100a11	Protein S10	S10AB_MO	4	0.945138
30min / C	Q64520	-0.3577	0.277185	0.48926	Guk1	Guanylate k	KGUA_MOU	4	0.780408
15min / C	Q80X95	-0.07793	0.277493	0.48926	Rraga	Ras-related	RRAGA_MC	6	0.947413
30min / C	Q8BMQ8	2.936859	0.278099	0.48926	Mon1b	Vacuolar fu	MON1B_M	1	7.657422
30min / C	Q8BWU5	-0.21632	0.2775	0.48926	Osgep	tRNA N6-ad	OSGEP_MO	12	0.86076
15min / C	Q8VE62	0.036525	0.277879	0.48926	Paip1	Polyadenyl	PAIP1_MOU	8	1.025641
15min / C	Q91ZA3	0.507238	0.277209	0.48926	Pcca	Propionyl-C	PCCA_MOU	34	1.421326
30min / C	Q9DBG3	0.099355	0.277072	0.48926	Ap2b1	AP-2 compl	AP2B1_MO	21	1.071294
30min / C	Q9ERB0	-0.08505	0.278065	0.48926	Snap29	Synaptosor	SNP29_MO	7	0.942754
15min / C	P42567	-0.30806	0.278477	0.489562	Eps15	Epidermal g	EPS15_MOU	23	0.807726
30min / C	P14824	0.037002	0.278832	0.489603	Anxa6	Annexin A6	ANXA6_MC	37	1.02598
30min / C	Q9D281	-0.24168	0.278858	0.489603	Fam114a1	Protein No:	NXP20_MC	13	0.845761
30min / C	Q9JLV1	-0.17925	0.278845	0.489603	Bag3	BAG family	BAG3_MOU	28	0.883162
15min / C	P17742	-0.14321	0.279889	0.48981	Ppia	Peptidyl-pr	PPIA_MOUS	12	0.905504
30min / C	P19536	0.088619	0.27995	0.48981	Cox5b	Cytochrom	COX5B_MC	3	1.063352
15min / C	P45952	0.103528	0.279751	0.48981	Acadm	Medium-ch	ACADM_M	22	1.074397
15min / C	Q501J7	-0.10996	0.279518	0.48981	Phactr4	Phosphatas	PHAR4_MC	5	0.926613
15min / C	Q9WTP7	0.138593	0.2794	0.48981	Ak3	GTP:AMP p	KAD3_MOU	16	1.100831
30min / C	P07759	-0.14159	0.280155	0.489811	Serpina3k	Serine prot	SPA3K_MO	19	0.906517
15min / C	Q9DBM2	0.742294	0.280082	0.489811	Ehhadh	Peroxisoma	ECHP_MOU	46	1.672834
30min / C	P63085	0.228063	0.280366	0.490001	Mapk1	Mitogen-ac	MK01_MOU	12	1.171261
15min / C	Q61187	0.292032	0.280679	0.490367	Tsg101	Tumor susc	TS101_MO	9	1.224363
15min / C	P47757	-0.13028	0.281199	0.490613	Capzb	F-actin-cap	CAPZB_MO	14	0.913651
30min / C	Q9CXW3	-0.02389	0.28123	0.490613	Cacybp	Calcyclin-b	CYBP_MOU	13	0.983576
15min / C	Q9WTM5	-0.10478	0.281208	0.490613	Ruvbl2	RuvB-like 2	RUVB2_MC	17	0.929946
30min / C	Q3UM45	0.228741	0.282472	0.491855	Ppp1r7	Protein pho	PP1R7_MO	15	1.171812
15min / C	Q8CGC7	0.168483	0.282559	0.491855	Eprs1	Bifunctiona	SYEP_MOUS	61	1.123876
30min / C	Q9D8S3	0.360991	0.282806	0.491961	Arfgap3	ADP-ribosy	ARFG3_MO	7	1.284308
15min / C	O35459	0.079613	0.282962	0.492018	Ech1	Delta(3,5)-I	ECH1_MOU	15	1.056735
15min / C	Q99KR3	-0.22927	0.283306	0.492258	Lactb2	Endoribonu	LACB2_MO	15	0.853066
30min / C	Q9QZ23	-0.65911	0.283247	0.492258	Nfu1	NFU1 iron-s	NFU1_MOU	7	0.633269
15min / C	P24456	0.001543	0.283813	0.492899	Cyp2d10	Cytochrom	CP2DA_MC	25	1.00107
30min / C	Q91X91	-0.06626	0.283984	0.492899	Qprt	Nicotinate-	NADC_MOU	14	0.955108
30min / C	P03976	0.270821	0.284577	0.493213	NaN	Ig kappa ch	KV2A5_MO	1	1.206494
15min / C	Q8CIG8	0.46287	0.285303	0.494234	Prmt5	Protein arg	ANM5_MO	11	1.378281
15min / C	P50544	-0.15339	0.285599	0.494445	Acadvl	Very long-c	ACADV_MC	35	0.899136
30min / C	Q61495;Q7	0.6933	0.285859	0.494717	Dsg1a;Dsg1	Desmoglein	DSG1A_MC	1	1.616978
15min / C	P42208	0.136889	0.286724	0.495032	Septin2	Septin-2	SEPT2_MOU	17	1.099532
30min / C	P99024	-0.51238	0.286273	0.495032	Tubb5	Tubulin bet	TBB5_MOU	4	0.701063
30min / C	Q8BG05	-0.16178	0.286714	0.495032	Hnrnpa3	Heterogene	ROA3_MOU	14	0.893921
30min / C	Q923D5	0.469223	0.286513	0.495032	Wbp11	WW domai	WBP11_M	4	1.384364
15min / C	Q99K48	-0.36785	0.286333	0.495032	Nono	Non-POU d	NONO_MO	21	0.774935
30min / C	Q9JHS9	-0.81981	0.286552	0.495032	Cwc15	Spliceosom	CWC15_M	2	0.566515
30min / C	Q9QYG0	0.088924	0.28687	0.495032	Ndrg2	Protein ND	NDRG2_M	17	1.063577
30min / C	P07901	0.00295	0.287055	0.49509	Hsp90aa1	Heat shock	HS90A_MO	25	1.002047
30min / C	Q6IME9	0.517955	0.287629	0.49509	Krt72	Keratin, typ	K2C72_MO	1	1.431924
30min / C	Q9CR21	0.029791	0.287261	0.49509	Ndufab1	Acyl carrier	ACPM_MOU	3	1.020865
30min / C	Q9R008	-0.14121	0.287244	0.49509	Mvk	Mevalonate	KIME_MOU	13	0.906759
30min / C	O70589	-0.91839	0.287948	0.495461	Cask	Peripheral	CSKP_MOU	5	0.529101
30min / C	Q8K411	0.005036	0.288193	0.495526	Pitrm1	Presequenc	PREP_MOU	31	1.003497

15min / C	Q9R0Q6	-0.01612	0.288134	0.495526	Arpc1a	Actin-relate	ARC1A_MO	13	0.988889
30min / C	P05201	-0.11645	0.289568	0.496001	Got1	Aspartate a	AATC_MOU	26	0.922452
15min / C	P62996	-0.34788	0.289191	0.496001	Tra2b	Transforme	TRA2B_MO	4	0.785738
30min / C	P80313	-0.21743	0.289388	0.496001	Cct7	T-complex	TCPH_MOU	27	0.860098
15min / C	Q63880	0.077266	0.289103	0.496001	Ces3a	Carboxyles	EST3A_MOU	20	1.055016
15min / C	Q7TQI3	-0.18667	0.288741	0.496001	Otub1	Ubiquitin t	OTUB1_MO	12	0.878628
30min / C	Q8R1H0	0.225519	0.28961	0.496001	Hopx	Homeodon	HOP_MOUS	3	1.169198
30min / C	Q91YY4	0.585675	0.28868	0.496001	Atpaf2	ATP syntha	ATPF2_MO	4	1.500741
30min / C	Q9CXI5	-0.20555	0.289192	0.496001	Manf	Mesenceph	MANF_MOU	11	0.867206
30min / C	Q9D8U8	-0.05004	0.289355	0.496001	Snx5	Sorting nex	SNX5_MOU	17	0.965909
30min / C	Q3THS6	0.052494	0.289759	0.496076	Mat2a	S-adenosyl	METK2_MO	11	1.037056
15min / C	Q9DB05	-0.27537	0.289867	0.496076	Napa	Alpha-solul	SNAA_MOU	12	0.82624
15min / C	Q9Z223	0.154423	0.289966	0.496076	Mocs2	Molybdopt	MOC2B_MO	4	1.112977
30min / C	Q9QZQ1	-0.18039	0.290611	0.496824	Afdn	Afadin	AFAD_MOU	33	0.882466
30min / C	Q9ERE7	0.419259	0.290826	0.497013	Mesd	LRP chaper	MESD_MOU	7	1.337241
30min / C	Q9DCT8	-0.34064	0.291069	0.497158	Crip2	Cysteine-ric	CRIP2_MOU	12	0.78969
15min / C	Q9DCN1	0.183181	0.291442	0.497533	Nudt12	NAD-cappe	NUD12_MO	17	1.135384
15min / C	Q9R0Y5	-0.12922	0.291837	0.49803	Ak1	Adenylate k	KAD1_MOU	4	0.914327
30min / C	Q5U5V2	-0.33925	0.292105	0.498132	Hykk	Hydroxylys	HYKK_MOU	15	0.790453
15min / C	P62082	-0.32914	0.292251	0.498202	Rps7	Small ribos	RS7_MOUS	11	0.79601
15min / C	Q3U1J4	-0.04069	0.292494	0.498439	Ddb1	DNA damag	DDB1_MOU	55	0.972193
15min / C	Q99K30	-0.30317	0.292757	0.498532	Eps8l2	Epidermal g	ES8L2_MOU	23	0.810467
15min / C	Q99N42	0.26246	0.292888	0.498576	Tymp	Thymidine	TYPH_MOU	20	1.199522
30min / C	Q3UMF0	-0.37551	0.293004	0.498597	Cobll1	Cordon-ble	COBL1_MO	47	0.770831
30min / C	Q8K0E8	-0.18603	0.293167	0.498606	Fgb	Fibrinogen	FIBB_MOUS	30	0.879018
30min / C	Q8R1G2	0.054995	0.293218	0.498606	Cmbl	Carboxyme	CMBL_MOU	15	1.038855
30min / C	A2AS89	-0.19847	0.29399	0.498879	Agmat	Guanidino	GDAH_MOU	7	0.871476
30min / C	P46471	0.186298	0.294245	0.498879	Psmc2	26S protea	PRS7_MOU	25	1.13784
15min / C	P54227	0.309595	0.294166	0.498879	Stmn1	Stathmin	STMN1_MO	4	1.23936
15min / C	Q8CIE0	-0.30676	0.293678	0.498879	Serpina11	Serpin A11	SPA11_MO	1	0.808457
15min / C	Q9CYA0	-0.54716	0.293992	0.498879	Creld2	Protein dis	CREL2_MO	8	0.684368
15min / C	Q9CXY6	-0.17029	0.294781	0.499133	Ilf2	Interleukin	ILF2_MOUS	10	0.888663
30min / C	P47738	0.03062	0.295286	0.499458	Aldh2	Aldehyde d	ALDH2_MO	29	1.021451
15min / C	Q91VM9	-0.03503	0.295876	0.499924	Ppa2	Inorganic p	IPYR2_MOU	13	0.976011
15min / C	Q80UM3	0.146431	0.296015	0.499984	Naa15	N-alpha-ac	NAA15_MO	18	1.106828
15min / C	Q91YH6	-0.18565	0.296706	0.500797	Atp6v1b1	V-type prot	VATB1_MO	1	0.879253
30min / C	O09159	-0.09114	0.297127	0.500976	Man2b1	Lysosomal	MA2B1_MO	18	0.938778
15min / C	Q8BX09	-0.20738	0.297102	0.500976	Rbbp5	Retinoblast	RBBP5_MO	2	0.86611
15min / C	Q8VI36	-0.08905	0.29765	0.501504	Pxn	Paxillin	PAXI_MOUS	15	0.940142
15min / C	O55142	-0.66103	0.298991	0.501728	Rpl35a	Large ribos	RL35A_MO	8	0.632425
30min / C	O70194	0.184692	0.299206	0.501728	Eif3d	Eukaryotic	EIF3D_MOU	16	1.136574
15min / C	P26041	-0.04877	0.298686	0.501728	Msn	Moesin	MOES_MOU	19	0.966758
30min / C	P45952	-0.43379	0.298379	0.501728	Acadm	Medium-ch	ACADM_MO	22	0.740313
30min / C	P84104	-0.18537	0.298187	0.501728	Srsf3	Serine/argi	SRSF3_MOU	4	0.879426
15min / C	Q3UEB3	-0.20855	0.298677	0.501728	Puf60	Poly(U)-bin	PUF60_MO	13	0.865407
30min / C	Q5FWK3	-0.24588	0.299234	0.501728	Arhgap1	Rho GTPase	RHG01_MO	8	0.843301
30min / C	Q6ZQ38	-0.15809	0.298761	0.501728	Cand1	Cullin-asso	CAND1_MO	45	0.896213
15min / C	Q6ZWN5	0.620683	0.298241	0.501728	Rps9	Small ribos	RS9_MOUS	12	1.537603
15min / C	Q8R1G2	0.026254	0.299085	0.501728	Cmbl	Carboxyme	CMBL_MOU	15	1.018364
15min / C	Q922D8	-0.6942	0.297935	0.501728	Mthfd1	C-1-tetrahy	C1TC_MOU	52	0.618053
15min / C	Q9CQZ1	0.68919	0.2988	0.501728	Hsbp1	Heat shock	HSBP1_MO	2	1.612378
15min / C	Q9QXD6	-0.07978	0.299252	0.501728	Fbp1	Fructose-1,	F16P1_MO	27	0.946201

30min / C	Q9WVS8	0.291302	0.29804	0.501728	Mapk7	Mitogen-ac	MK07_MOU	1	1.223744
30min / C	Q60994	0.013885	0.299592	0.501743	Adipoq	Adiponecti	ADIPO_MO	1	1.00967
30min / C	Q8BL66	-0.09253	0.299496	0.501743	Eea1	Early endos	EEA1_MOU	53	0.937873
30min / C	Q8CI94	-0.01304	0.300065	0.501743	Pygb	Glycogen p	PYGB_MOU	19	0.991001
15min / C	P05784	-0.09941	0.303978	0.50177	Krt18	Keratin, typ	K1C18_MO	29	0.933418
30min / C	P31786	-0.02541	0.300958	0.50177	Dbi	Acyl-CoA-bi	ACBP_MOU	7	0.982539
30min / C	P40124	-0.00747	0.302163	0.50177	Cap1	Adenylyl cy	CAP1_MOU	21	0.994834
15min / C	P48776	0.665139	0.30425	0.50177	Tdo2	Tryptophar	T23O_MOU	16	1.585721
30min / C	P58389	0.169215	0.303902	0.50177	Ptpa	Serine/thre	PTPA_MOU	10	1.124446
15min / C	P61922	-0.04395	0.301784	0.50177	Abat	4-aminobu	GABT_MOU	29	0.969992
30min / C	P68510	-0.07015	0.302645	0.50177	Ywhah	14-3-3 prot	1433F_MO	9	0.952536
15min / C	P97449	-0.21148	0.303574	0.50177	Anpep	Aminopept	AMPN_MOU	16	0.863649
15min / C	Q3V4B5	-0.20997	0.304192	0.50177	Commd6	COMM don	COMD6_M	2	0.864553
15min / C	Q80TY0	0.462251	0.303917	0.50177	Fnbp1	Formin-bin	FNBP1_MO	4	1.377689
30min / C	Q811D0	-0.16733	0.303148	0.50177	Dlg1	Disks large	DLG1_MOU	14	0.89049
15min / C	Q8R059	0.524414	0.301861	0.50177	Gale	UDP-glucos	GALE_MOU	12	1.438349
15min / C	Q9D7I5	0.520738	0.301403	0.50177	Lhpp	Phospholys	LHPP_MOU	6	1.434689
15min / C	Q9DAR7	-0.16186	0.303065	0.50177	Dcps	m7GpppX c	DCPS_MOU	11	0.893872
15min / C	Q9DB27	0.30544	0.302784	0.50177	Mcts1	Malignant T	MCTS1_MC	7	1.235796
15min / C	Q9DCG6	-0.11619	0.303149	0.50177	Pbld1	Phenazine b	PBLD1_MO	12	0.922619
15min / C	Q9DCL8	-0.34982	0.304143	0.50177	Ppp1r2	Protein pho	IPP2_MOUS	6	0.784684
15min / C	Q9DCM2	0.519793	0.304511	0.50177	Gstk1	Glutathion	GSTK1_MO	17	1.43375
15min / C	Q9EPC1	-0.155	0.303519	0.50177	Parva	Alpha-parvi	PARVA_MO	10	0.89813
30min / C	Q9JLM9	0.077051	0.303493	0.50177	Grb14	Growth fac	GRB14_MC	12	1.05486
30min / C	Q9QYB1	0.159176	0.30479	0.50177	Clic4	Chloride in	CLIC4_MOU	15	1.116649
15min / C	Q9R1P4	-0.10183	0.303323	0.50177	Psma1	Proteasome	PSA1_MOU	16	0.931852
15min / C	Q7TMC8	0.093626	0.305106	0.501899	Fcsk	L-fucose kir	FCSK_MOU	11	1.067048
30min / C	Q9WUB3	-0.23404	0.305129	0.501899	Pygm	Glycogen p	PYGM_MOU	12	0.850251
30min / C	Q9CY58	-0.06452	0.305266	0.501951	Serbp1	SERPINE1 m	SERB1_MO	20	0.956266
30min / C	O55029	-0.1352	0.306452	0.502416	Copb2	Coatomer s	COPB2_MC	40	0.910541
15min / C	P43883	0.117567	0.3066	0.502416	Plin2	Perilipin-2	PLIN2_MOU	11	1.084904
15min / C	Q5SUF2	0.615	0.306284	0.502416	Luc7l3	Luc7-like pr	LC7L3_MO	8	1.531558
15min / C	Q91V92	0.772344	0.305844	0.502416	Acly	ATP-citrate	ACLY_MOU	56	1.708042
15min / C	Q91V09	0.447428	0.307102	0.502932	Wdr13	WD repeat-	WDR13_M	7	1.363607
15min / C	Q9CWZ3	0.14963	0.307125	0.502932	Rbm8a	RNA-bindir	RBM8A_MC	2	1.109285
15min / C	Q91WP6	-0.11145	0.307292	0.503033	Serpina3n	Serine prot	SPA3N_MO	11	0.925655
15min / C	Q9CQ92	-0.344	0.307681	0.503497	Fis1	Mitochond	FIS1_MOUS	4	0.787856
30min / C	P28063	-0.20229	0.307877	0.503646	Psmb8	Proteasome	PSB8_MOU	12	0.86917
15min / C	P21614	-0.07094	0.30831	0.503661	Gc	Vitamin D-b	VTDB_MOU	23	0.952017
30min / C	P56593	-0.05268	0.308325	0.503661	Cyp2a12	Cytochrom	CP2AC_MO	24	0.964142
30min / C	Q8R1S4	-0.25267	0.308217	0.503661	Mtss1	Protein MT	MTSS1_MO	10	0.83934
30min / C	P42208	0.071848	0.309076	0.504226	Septin2	Septin-2	SEPT2_MOU	17	1.051062
15min / C	Q9Z2W0	-0.11525	0.309242	0.504325	Dnpep	Aspartyl am	DNPEP_MC	18	0.923222
30min / C	E9QAT4	0.201226	0.311029	0.504591	Sec16a	Protein tra	SC16A_MO	31	1.149675
15min / C	G3X982	-0.09332	0.309652	0.504591	Aox3	Aldehyde o	AOXC_MOU	68	0.937365
15min / C	O70274	-0.81451	0.310613	0.504591	Ptp4a2	Protein tyr	TP4A2_MO	2	0.568602
30min / C	P16332	-0.0839	0.310392	0.504591	Mmut	Methylmal	MUTA_MOU	25	0.943502
15min / C	P56395	-0.27558	0.311305	0.504591	Cyb5a	Cytochrom	CYB5_MOU	5	0.82612
15min / C	P62315	-0.12079	0.31059	0.504591	Snrpd1	Small nucle	SMD1_MOU	5	0.919683
30min / C	P62838	-0.17773	0.309566	0.504591	Ube2d2	Ubiquitin-c	UB2D2_MC	4	0.884091
15min / C	P63254	0.043495	0.310718	0.504591	Crip1	Cysteine-ric	CRIP1_MOU	3	1.030608
30min / C	Q3TEA8	0.454833	0.310933	0.504591	Hp1bp3	Heterochro	HP1B3_MC	13	1.370624

30min / C	Q3TLP5	-0.13901	0.310532	0.504591	Echdc2	Enoyl-CoA I	ECHD2_MO	11	0.908145
15min / C	Q61704	0.198641	0.309993	0.504591	Itih3	Inter-alpha	ITIH3_MOU	12	1.147617
15min / C	Q91VA0	0.011742	0.310582	0.504591	Acsm1	Acyl-coenzy	ACSM1_MO	26	1.008172
30min / C	O54983	-0.12644	0.312189	0.504654	Crym	Ketimine re	CRYM_MOU	8	0.916092
15min / C	P70441	-0.09718	0.312066	0.504654	Nherf1	Na(+)/H(+) e	NHRF1_MO	23	0.934857
30min / C	Q60737	-0.06617	0.311666	0.504654	Csnk2a1	Casein kina	CSK21_MO	8	0.955169
15min / C	Q9CQC6	0.198059	0.31165	0.504654	Bzw1	eIF5-mimic	5MP2_MOU	16	1.147154
30min / C	Q9Z1X4	0.324213	0.311718	0.504654	Ilf3	Interleukin	ILF3_MOUS	16	1.251982
30min / C	P15532	-0.03604	0.312439	0.504888	Nme1	Nucleoside	NDKA_MOU	7	0.975326
30min / C	Q91YP2	0.570871	0.313662	0.50635	Nln	Neurolysin,	NEUL_MOU	23	1.48542
30min / C	O09061	-0.13892	0.314318	0.506554	Psmb1	Proteasome	PSB1_MOU	11	0.9082
30min / C	Q8C196	0.251045	0.314124	0.506554	Cps1	Carbamoyl-	CPSM_MOU	98	1.190069
30min / C	A2ATU0	0.030012	0.314956	0.506718	Dhtkd1	2-oxoadipa	DHTK1_MO	36	1.021021
30min / C	P62322	-0.26489	0.315087	0.506718	Lsm5	U6 snRNA-a	LSM5_MOU	1	0.832263
15min / C	Q8JZQ9	0.035817	0.314961	0.506718	Eif3b	Eukaryotic	EIF3B_MOU	31	1.025138
15min / C	Q8VCM4	-0.76147	0.315666	0.506718	Lipt1	Lipoyl amic	LIPT_MOUS	4	0.589897
15min / C	Q9ERB0	-0.24719	0.315325	0.506718	Snap29	Synaptosor	SNP29_MO	7	0.842534
30min / C	Q9Z2G9	-0.96782	0.315692	0.506718	Htatip2	Oxidoreduc	HTAI2_MOU	11	0.511279
30min / C	P10493	0.353838	0.31645	0.506736	Nid1	Nidogen-1	NID1_MOU	2	1.277956
30min / C	P28650	0.146015	0.316116	0.506736	Adss1	Adenylosuc	PURA1_MO	17	1.106509
15min / C	P54775	-0.68258	0.316832	0.506736	Psmc4	26S proteas	PRS6B_MO	19	0.623049
30min / C	P56399	0.006512	0.316562	0.506736	Usp5	Ubiquitin c	UBP5_MOU	31	1.004524
15min / C	Q7TPC1	0.016529	0.316772	0.506736	Cdsn	Corneodesn	CDSN_MOU	1	1.011523
15min / C	Q8BZQ7	-0.13454	0.316815	0.506736	Anapc2	Anaphase-p	ANC2_MOU	4	0.910961
30min / C	Q9DCC4	-0.0299	0.316794	0.506736	Pycr3	Pyrroline-5	P5CR3_MO	9	0.979489
15min / C	Q76LS9	-0.38617	0.31761	0.507322	Mindy1	Ubiquitin c	MINY1_MO	10	0.765156
15min / C	P85094	-0.11794	0.317887	0.507516	Isoc2a	Isochorism	ISC2A_MOU	11	0.921499
30min / C	P63325	0.03983	0.318207	0.507741	Rps10	Small ribos	RS10_MOU	11	1.027993
15min / C	Q8K2I4	0.141321	0.318241	0.507741	Manba	Beta-mann	MANBA_MO	8	1.102915
30min / C	P62141	0.37706	0.318789	0.50792	Ppp1cb	Serine/thre	PP1B_MOU	5	1.298692
15min / C	Q8BVQ5	-0.58955	0.318867	0.50792	Ppme1	Protein pho	PPME1_MO	9	0.664552
15min / C	Q9CQH7	-0.06586	0.31898	0.50792	Btf3l4	Transcripti	BT3L4_MOU	6	0.955376
15min / C	Q9CRD0	-0.42557	0.318806	0.50792	Ociad1	OCIA doma	OCAD1_MO	4	0.744546
15min / C	Q9R0H0	-0.04107	0.318487	0.50792	Acox1	Peroxisoma	ACOX1_MO	39	0.971934
15min / C	O89086	0.59509	0.320014	0.508871	Rbm3	RNA-bindir	RBM3_MOU	4	1.510567
15min / C	P35235	-0.22204	0.320528	0.50935	Ptpn11	Tyrosine-pr	PTN11_MO	14	0.85735
15min / C	P47934	0.110397	0.320704	0.509374	Crat	Carnitine O	CACP_MOU	12	1.079525
30min / C	O55234	-0.096	0.321303	0.509872	Psmb5	Proteasome	PSB5_MOU	14	0.935622
15min / C	Q07797	0.090377	0.322044	0.510232	Lgals3bp	Galectin-3-	LG3BP_MO	12	1.064649
15min / C	Q9D273	0.056051	0.321838	0.510232	Mmab	Corrinoid a	MMAB_MO	7	1.039616
15min / C	Q9WU28	-0.25729	0.321825	0.510232	Pfdn5	Prefoldin su	PFD5_MOU	7	0.83666
15min / C	O35945	0.070165	0.322219	0.510341	Aldh1a7	Aldehyde d	AL1A7_MO	30	1.049837
15min / C	Q9D0J8	0.175582	0.323582	0.511821	Ptms	Parathymo	PTMS_MOU	4	1.12942
30min / C	P26041	-0.05101	0.323972	0.512267	Msn	Moesin	MOES_MOU	19	0.965259
15min / C	O70325	0.658635	0.325598	0.513989	Gpx4	Phospholip	GPX4_MOU	8	1.578588
15min / C	Q9DCV4	0.440163	0.325918	0.514155	Rmdn1	Regulator c	RMD1_MOU	8	1.356757
15min / C	P0DP26;PC	0.087343	0.326472	0.514585	Calm1;Caln	Calmodulir	CALM1_MO	11	1.062412
15min / C	P27773	0.160163	0.326671	0.514585	Pdia3	Protein disu	PDIA3_MOU	28	1.117413
30min / C	Q91XD4	-0.09073	0.326729	0.514585	Ftcd	Formimido	FTCD_MOU	31	0.939048
30min / C	Q9CXW4	-0.04384	0.326575	0.514585	Rpl11	Large ribos	RL11_MOU	7	0.970066
15min / C	P12246	0.67209	0.327047	0.514747	Apcs	Serum amy	SAMP_MOU	3	1.59338
15min / C	Q9CPY7	-0.13173	0.327327	0.515018	Lap3	Cytosol am	AMPL_MOU	28	0.912735

15min / C	P48024	-0.37841	0.327507	0.515132	Eif1	Eukaryotic	EIF1_MOUS	5	0.769285
15min / C	P16045	-0.17921	0.327735	0.515321	Lgals1	Galectin-1	LEG1_MOU	10	0.883184
15min / C	P47754	-0.11985	0.328508	0.51635	Capza2	F-actin-cap	CAZA2_MO	15	0.920284
15min / C	Q06138	0.038961	0.328606	0.51635	Cab39	Calcium-bi	CAB39_MO	13	1.027374
30min / C	Q6NVF9	-0.22427	0.32927	0.517223	Cpsf6	Cleavage ar	CPSF6_MO	7	0.856027
30min / C	Q9CQ45	0.200851	0.32941	0.517273	Nenf	Neudesin	NENF_MOU	5	1.149376
15min / C	Q3UTQ7	0.173793	0.329588	0.517382	Prdm10	PR domain	PRD10_MC	1	1.128021
15min / C	P35278	-0.2758	0.32985	0.517624	Rab5c	Ras-related	RAB5C_MO	9	0.825992
15min / C	Q8BFR4	-0.02206	0.329982	0.517661	Gns	N-acetylglu	GNS_MOUS	6	0.984825
30min / C	Q9DBZ5	-0.44238	0.330607	0.518131	Eif3k	Eukaryotic	EIF3K_MOU	9	0.735919
15min / C	Q9DCL2	0.317753	0.330561	0.518131	Ciao2a	Cytosolic ir	CIA2A_MOU	2	1.246387
15min / C	Q923G2	0.010138	0.33111	0.518411	Polr2h	DNA-direct	RPAB3_MO	3	1.007052
15min / C	P08752	0.049814	0.331261	0.518477	Gnai2	Guanine nu	GNAI2_MO	10	1.035132
15min / C	P48036	0.202448	0.331821	0.519074	Anxa5	Annexin A5	ANXA5_MC	22	1.150649
15min / C	Q99LD9	-0.9448	0.331991	0.519109	Eif2b2	Translation	EI2BB_MOU	7	0.519502
15min / C	Q9CRB3	-0.07061	0.332127	0.519152	Urah	5-hydroxyi	HIUH_MOU	9	0.952237
30min / C	P02088	-0.16165	0.332651	0.519801	Hbb-b1	Hemoglobi	HBB1_MOU	13	0.894001
30min / C	A2A974	0.289242	0.33402	0.520409	Cyp4a12b	Cytochrom	CP4CB_MO	20	1.221998
15min / C	P21460	0.214428	0.333572	0.520409	Cst3	Cystatin-C	CYTC_MOU	3	1.160243
30min / C	Q00623	-0.11282	0.333964	0.520409	Apoa1	Apolipopro	APOA1_MC	18	0.924778
30min / C	Q60631	0.23562	0.333677	0.520409	Grb2	Growth fac	GRB2_MOU	5	1.177413
30min / C	Q60854	-0.16033	0.333972	0.520409	Serpinb6	Serpin B6	SPB6_MOU	19	0.894818
30min / C	Q62393	-0.01992	0.333997	0.520409	Tpd52	Tumor prot	TPD52_MO	5	0.986284
30min / C	Q9D2V7	0.320634	0.334133	0.520416	Coro7	Coronin-7	CORO7_MC	19	1.248879
30min / C	P01819;P0	0.166965	0.334658	0.520574	NaN	Ig heavy ch	HVM43_MC	1	1.122694
15min / C	P62900	0.268079	0.334753	0.520574	Rpl31	Large ribos	RL31_MOU	7	1.204203
30min / C	Q5SUF2	0.684945	0.334478	0.520574	Luc7l3	Luc7-like p	LC7L3_MO	8	1.60764
15min / C	Q9DBH5	0.974021	0.334491	0.520574	Lman2	Vesicular ir	LMAN2_MC	11	1.964308
15min / C	P46471	0.432488	0.335419	0.520671	Psmc2	26S protea	PRS7_MOU	25	1.349559
30min / C	P62878	-0.07448	0.335411	0.520671	Rbx1	E3 ubiquiti	RBX1_MOU	2	0.949681
30min / C	Q8VHZ7	0.114611	0.335142	0.520671	Imp4	U3 small nu	IMP4_MOU	1	1.082683
30min / C	Q91Y97	-0.04171	0.335327	0.520671	Aldob	Fructose-bi	ALDOB_MC	29	0.9715
30min / C	Q922V4	-0.23129	0.335222	0.520671	Plrg1	Pleiotropic	PLRG1_MO	3	0.85187
30min / C	Q9CZN7	-0.02027	0.336212	0.521445	Shmt2	Serine hydr	GLYM_MOU	30	0.986051
15min / C	P14685	0.198608	0.336901	0.522174	Psmd3	26S protea	PSMD3_MC	28	1.14759
15min / C	O55029	-0.06938	0.337218	0.522328	Copb2	Coatomer s	COPB2_MC	40	0.953049
30min / C	Q9D7J9	0.374581	0.337431	0.522489	Echdc3	Enoyl-CoA l	ECHD3_MC	3	1.296463
30min / C	P55302	-1.20819	0.33766	0.522619	Lrpap1	Alpha-2-ma	AMRP_MOU	5	0.432813
30min / C	Q9Z0K8	-0.12307	0.337735	0.522619	Vnn1	Pantethein	VNN1_MOU	1	0.918233
15min / C	Q9D711	0.19072	0.337965	0.522637	Pir	Pirin	PIR_MOUSI	11	1.141333
30min / C	Q5I2A0	-0.08666	0.338212	0.522682	Serpina3g	Serine prot	SPA3G_MO	2	0.941702
30min / C	P60122	-0.19596	0.33837	0.522757	Ruvbl1	RuvB-like 1	RUVB1_MC	18	0.872991
15min / C	Q99LB7	1.179034	0.338782	0.522903	Sardh	Sarcosine d	SARDH_MC	45	2.264251
30min / C	Q07417	-0.17945	0.33937	0.523455	Acads	Short-chair	ACADS_MO	16	0.883039
30min / C	Q9JKR6	-1.34358	0.340185	0.524374	Hyou1	Hypoxia up	HYOU1_MC	40	0.394041
15min / C	Q9ER35	-0.5442	0.340323	0.524418	Fn3k	Fructosami	FN3K_MOU	4	0.685773
30min / C	O35350	-0.52229	0.340917	0.524788	Capn1	Calpain-1 c	CAN1_MOU	23	0.696264
30min / C	O35643	-0.09956	0.341441	0.524788	Ap1b1	AP-1 compl	AP1B1_MO	42	0.933319
30min / C	P01637;P0	-0.14924	0.340678	0.524788	NaN	Ig kappa ch	KV5A5_MO	2	0.901723
30min / C	P51174	-0.1117	0.341151	0.524788	Acadl	Long-chain	ACADL_MO	21	0.925498
15min / C	P61164	-0.20123	0.341514	0.524788	Actr1a	Alpha-centr	ACTZ_MOU	13	0.869811
30min / C	P97379	-0.26278	0.341661	0.524788	G3bp2	Ras GTPase-	G3BP2_MC	12	0.833481

30min / C	Q3ULJ0	0.014245	0.341268	0.524788	Gpd1l	Glycerol-3-	GPD1L_MC	17	1.009923
15min / C	Q99LX0	-0.09537	0.34082	0.524788	Park7	Parkinson c	PARK7_MO	15	0.936032
30min / C	Q9CR51	0.026146	0.341643	0.524788	Atp6v1g1	V-type prot	VATG1_MO	3	1.018288
15min / C	P97792	0.140929	0.341966	0.524919	Cxadr	Coxsackievi	CXAR_MOU	3	1.102615
15min / C	Q91Y97	-0.03895	0.341894	0.524919	Aldob	Fructose-bi	ALDOB_MC	29	0.973364
15min / C	Q8CHT0	-0.75935	0.342098	0.524953	Aldh4a1	Delta-1-pyr	AL4A1_MO	26	0.590761
15min / C	Q01768	-0.11274	0.342418	0.525276	Nme2	Nucleoside	NDKB_MOU	12	0.924829
30min / C	P42232	-0.32063	0.342666	0.525487	Stat5b	Signal trans	STA5B_MO	18	0.800718
15min / C	Q9CWW6	0.287363	0.342982	0.525802	Pin4	Peptidyl-pr	PIN4_MOU	3	1.220407
30min / C	P24472	-0.08985	0.343101	0.525817	Gsta4	Glutathion	GSTA4_MO	10	0.939621
15min / C	P34927	-0.24875	0.343662	0.52593	Asgr1	Asialoglyco	ASGR1_MO	8	0.841628
15min / C	Q1HFZ0	-0.12675	0.343632	0.52593	Nsun2	RNA cytosir	NSUN2_MC	20	0.915895
15min / C	Q9JLM9	1.057778	0.343725	0.52593	Grb14	Growth fac	GRB14_MC	12	2.081724
30min / C	O55137	-0.02602	0.343993	0.526172	Acot1	Acyl-coenzy	ACOT1_MO	11	0.982124
15min / C	Q61768	-0.26277	0.344189	0.526303	Kif5b	Kinesin-1 h	KINH_MOU	39	0.833487
15min / C	P47941	0.085448	0.345387	0.527488	Crkl	Crk-like prc	CRKL_MOU	8	1.061017
30min / C	Q8CIG8	0.263887	0.34526	0.527488	Prmt5	Protein arg	ANM5_MO	11	1.20071
15min / C	Q9D6Y7	0.409423	0.345288	0.527488	Msra	Mitochond	MSRA_MOU	14	1.328155
15min / C	P50428	-0.19876	0.345779	0.527889	Arsa	Arylsulfatas	ARSA_MOU	3	0.871299
30min / C	Q8BZB2	0.122057	0.346393	0.528332	Ppcdc	Phosphopa	COAC_MOU	3	1.088285
15min / C	P26645	-0.0784	0.347039	0.528773	Marcks	Myristoylat	MARCS_MC	13	0.947108
30min / C	Q9DC50	-0.09984	0.347062	0.528773	Crot	Peroxisoma	OCTC_MOU	26	0.933138
15min / C	P13439	-0.28015	0.347345	0.528807	Umps	Uridine 5'-r	UMPS_MOU	20	0.823503
30min / C	G5E8V9	-0.10822	0.348491	0.530167	Arfip1	Arfaptin-1	ARFP1_MO	8	0.927734
30min / C	P20152	0.068866	0.348896	0.530567	Vim	Vimentin	VIME_MOU	21	1.048892
30min / C	O70274	-0.68656	0.350578	0.530886	Ptp4a2	Protein tyr	TP4A2_MO	2	0.621332
15min / C	P11588	0.098915	0.34956	0.530886	Mup1	Major urina	MUP1_MO	1	1.070967
30min / C	P13439	-0.31322	0.34977	0.530886	Umps	Uridine 5'-r	UMPS_MOU	20	0.804841
30min / C	P46935	-0.17719	0.350728	0.530886	Nedd4	E3 ubiquiti	NEDD4_MC	18	0.884427
30min / C	P58044	0.120652	0.350715	0.530886	Idi1	Isopenteny	IDI1_MOUS	10	1.087226
30min / C	P80317	-0.04041	0.35045	0.530886	Cct6a	T-complex	TCPZ_MOU	23	0.972378
30min / C	Q11136	0.046287	0.350211	0.530886	Pepd	Xaa-Pro dip	PEPD_MOU	20	1.032604
30min / C	Q60739	-0.69083	0.349534	0.530886	Bag1	BAG family	BAG1_MOU	4	0.619499
15min / C	Q8BG94	0.410111	0.349526	0.530886	Commd7	COMM don	COMD7_M	5	1.328788
30min / C	Q99PG2	-0.03885	0.350068	0.530886	Ogfr	Opioid grov	OGFR_MOU	11	0.97343
30min / C	Q9WTU6	-0.05561	0.350261	0.530886	Mapk9	Mitogen-ac	MK09_MOU	9	0.962189
30min / C	P22315	-0.40606	0.351722	0.531699	Fech	Ferrochelat	HEMH_MO	16	0.754679
15min / C	Q6P4T2	0.036126	0.351667	0.531699	Snrnp200	U5 small nu	U520_MOU	48	1.025357
15min / C	Q6PEB6	-0.28826	0.35238	0.532002	Mob4	MOB-like pr	PHOCN_MC	5	0.818892
30min / C	Q99P30	0.088271	0.352083	0.532002	Nudt7	Peroxisoma	NUDT7_MC	17	1.063096
30min / C	Q9CWK8	-0.02233	0.352264	0.532002	Snx2	Sorting nex	SNX2_MOU	17	0.984643
30min / C	Q9R1P0	-0.12336	0.352479	0.532002	Psma4	Proteasom	PSA4_MOU	14	0.918046
30min / C	Q61553	0.099652	0.352614	0.532037	Fscn1	Fascin	FSCN1_MO	8	1.071515
15min / C	A0JNU3	0.414517	0.352831	0.532197	Aspg	60 kDa lyso	LPP60_MO	12	1.332852
30min / C	P51660	-0.15526	0.353943	0.533202	Hsd17b4	Peroxisoma	DHB4_MOU	37	0.89797
15min / C	Q9QYR9	0.300475	0.354094	0.53326	Acot2	Acyl-coenzy	ACOT2_MO	5	1.23155
15min / C	Q99K70	0.591708	0.354247	0.533322	Rragc	Ras-related	RRAGC_MC	4	1.50703
15min / C	P26516	-0.40569	0.354581	0.533657	Psmd7	26S protea	PSMD7_MC	13	0.754876
15min / C	Q99PG0	-0.00535	0.354796	0.533814	Aadac	Arylacetam	AAAD_MOU	11	0.996296
15min / C	P41105	0.728779	0.355536	0.534736	Rpl28	Large ribos	RL28_MOU	7	1.657235
30min / C	Q8BQ47	0.155135	0.355745	0.534736	Cnpy4	Protein car	CNPY4_MO	3	1.113526
15min / C	Q3UHX2	-0.18029	0.356361	0.53535	Pdap1	28 kDa hea	HAP28_MC	6	0.882525

15min / C	Q5RKZ7	-0.14974	0.356489	0.53535	Mocs1	Molybdenu	MOCS1_MO	14	0.901413
15min / C	P13645	-0.29417	0.35718	0.536008	KRT10	Keratin, typ	K1C10_HU	27	0.81554
15min / C	Q00915	0.137602	0.357678	0.536008	Rbp1	Retinol-bin	RET1_MOU	10	1.100075
15min / C	Q60854	0.062083	0.357274	0.536008	Serpinb6	Serpin B6	SPB6_MOU	19	1.043972
15min / C	Q9ESZ8	1.075465	0.357712	0.536008	Gtf2i	General tra	GTF2I_MOU	9	2.107401
15min / C	P62889	0.385312	0.358936	0.537051	Rpl30	Large ribos	RL30_MOU	7	1.306142
30min / C	Q8BH61	0.01447	0.35897	0.537051	F13a1	Coagulatio	F13A_MOU	4	1.01008
30min / C	P17918	0.061273	0.359497	0.537055	Pcna	Proliferatin	PCNA_MOU	10	1.043386
30min / C	P50580	0.039539	0.359594	0.537055	Pa2g4	Proliferatic	PA2G4_MO	18	1.027785
15min / C	P61967	0.150777	0.359872	0.537055	Ap1s1	AP-1 compl	AP1S1_MO	2	1.110167
15min / C	P63024	-0.32662	0.359472	0.537055	Vamp3	Vesicle-asso	VAMP3_MO	3	0.797402
30min / C	Q60598	-0.16876	0.359849	0.537055	Cttn	Src substra	SRC8_MOU	17	0.889607
15min / C	Q811D0	-0.15024	0.359214	0.537055	Dlg1	Disks large	DLG1_MOU	14	0.901099
15min / C	P54726	-0.04971	0.360615	0.537158	Rad23a	UV excision	RD23A_MO	10	0.966127
15min / C	Q9CPW4	-0.00842	0.360123	0.537158	Arpc5	Actin-relate	ARPC5_MO	5	0.994181
30min / C	Q9CQH7	0.063011	0.360536	0.537158	Btf3l4	Transcripti	BT3L4_MO	6	1.044644
30min / C	Q8BSL7	-0.06262	0.361234	0.537577	Arf2	ADP-ribosy	ARF2_MOU	5	0.957523
15min / C	P38647	-0.12659	0.362446	0.537856	Hspa9	Stress-70 p	GRP75_MO	40	0.915992
30min / C	Q62264	0.854275	0.362556	0.537856	Thrsp	Thyroid ho	THRSP_MO	5	1.80785
15min / C	Q8C0E2	0.109024	0.362296	0.537856	Vps26b	Vacuolar p	VP26B_MO	10	1.078498
30min / C	Q8R086	-0.105	0.36178	0.537856	Suox	Sulfite oxid	SUOX_MOU	21	0.929806
30min / C	Q91WK1	0.580987	0.362443	0.537856	Spryd4	SPRY doma	SPRY4_MO	12	1.495873
15min / C	Q9D1P4	-0.14728	0.361556	0.537856	Chordc1	Cysteine an	CHRD1_MO	13	0.902954
15min / C	Q9DCQ2	-0.0793	0.363109	0.537856	Aspdh	Aspartate d	ASPDH_MO	16	0.946515
30min / C	Q9QZE7	0.019159	0.362717	0.537856	Tsnax	Translin-ass	TSNAX_MO	10	1.013369
30min / C	Q9R069	-0.39589	0.362419	0.537856	Bcam	Basal cell a	BCAM_MOU	4	0.760021
15min / C	Q9Z1J3	-0.09318	0.362914	0.537856	Nfs1	Cysteine de	NFS1_MOU	15	0.937453
15min / C	P01639	-0.11538	0.36326	0.537902	Igkv9-120	Immunoglo	KV5A7_MO	1	0.923142
30min / C	P18528	-0.037	0.364265	0.537902	NaN	Ig heavy ch	HVM57_MO	2	0.974679
30min / C	P62814	0.110744	0.363743	0.537902	Atp6v1b2	V-type prot	VATB2_MO	18	1.079785
15min / C	Q8BH57	-0.24117	0.363603	0.537902	Wdr48	WD repeat-	WDR48_MO	3	0.84606
30min / C	Q8R1Q9	-0.02594	0.36345	0.537902	Rbks	Ribokinase	RBSK_MOU	4	0.982178
30min / C	Q99LT0	-0.26451	0.363851	0.537902	Dpy30	Protein dpy	DPY30_MO	3	0.832484
15min / C	Q9EST5	-0.25554	0.364052	0.537902	Anp32b	Acidic leuci	AN32B_MO	5	0.837671
30min / C	O88533	-0.05493	0.364802	0.538362	Ddc	Aromatic-L	DDC_MOUS	15	0.962641
15min / C	Q78JT3	-0.02446	0.365329	0.538931	Haao	3-hydroxya	3HAO_MOU	21	0.983186
15min / C	Q91VW3	-0.15397	0.365413	0.538931	Sh3bgrl3	SH3 domai	SH3L3_MO	3	0.898776
30min / C	P28843	-0.19865	0.365672	0.539146	Dpp4	Dipeptidyl	DPP4_MOU	16	0.871366
30min / C	Q8K019	-0.04086	0.365805	0.539176	Bclaf1	Bcl-2-assoc	BCLF1_MO	7	0.972075
15min / C	P06745	0.039261	0.366056	0.53938	Gpi	Glucose-6-p	G6PI_MOU	28	1.027587
30min / C	Q91X83	0.060652	0.366452	0.539686	Mat1a	S-adenosyl	METK1_MO	17	1.042937
30min / C	Q8BH95	0.056112	0.366928	0.53986	Echs1	Enoyl-CoA	ECHM_MOU	18	1.03966
15min / C	P97493	-0.16638	0.367834	0.540501	Txn2	Thioredoxi	THIOM_MO	4	0.891079
15min / C	Q9CQR2	-0.12549	0.36766	0.540501	Rps21	Small ribos	RS21_MOU	4	0.916694
30min / C	P05064	0.031686	0.368457	0.540866	Aldoa	Fructose-bi	ALDOA_MO	25	1.022206
15min / C	Q8K003	-0.47564	0.369049	0.541122	Tma7	Translation	TMA7_MOU	1	0.719148
30min / C	Q9D404	0.168624	0.36938	0.54144	Oxsm	3-oxoacyl-[	OXSM_MOU	14	1.123986
30min / C	Q9Z2K1	-0.26673	0.370601	0.542632	Krt16	Keratin, typ	K1C16_MO	4	0.831203
30min / C	Q80VJ3	-0.16329	0.370818	0.542715	Dnph1	5-hydroxym	DNPH1_MO	5	0.892988
30min / C	P60867	-0.18929	0.371661	0.543616	Rps20	Small ribos	RS20_MOU	5	0.877037
15min / C	P61202	0.333473	0.372162	0.544016	Cops2	COP9 signa	CSN2_MOU	15	1.260043
15min / C	Q8K183	0.228831	0.372114	0.544016	Pdxk	Pyridoxal k	PDXK_MOU	12	1.171885

15min / C	O08529	-0.68431	0.372593	0.544314	Capn2	Calpain-2 c	CAN2_MOU	22	0.622303
30min / C	Q9CXY6	-0.13392	0.37295	0.544669	Ilf2	Interleukin	ILF2_MOUS	10	0.911352
15min / C	Q9ERF3	-0.1767	0.373164	0.544816	Skic8	Superkiller	SKI8_MOUS	7	0.884726
15min / C	P62192	-0.13943	0.373313	0.544842	Psmc1	26S protea	PRS4_MOU	23	0.907877
30min / C	Q04447	-0.03486	0.373514	0.544842	Ckb	Creatine ki	KCRB_MOU	13	0.976127
30min / C	Q4KWH5	0.31057	0.373638	0.544842	Plch1	1-phosphat	PLCH1_MO	3	1.240198
30min / C	Q4VAA2	0.014928	0.374361	0.545462	Cdv3	Protein CD	CDV3_MOU	14	1.010401
15min / C	E9Q414	-0.03949	0.37781	0.546279	Apob	Apolipopro	APOB_MOU	98	0.973
15min / C	O70400	-0.05007	0.377117	0.546279	Pdlim1	PDZ and LIM	PDLI1_MOU	20	0.965888
15min / C	P09103	-0.09382	0.378992	0.546279	P4hb	Protein dis	PDIA1_MO	37	0.937036
15min / C	P35282	-0.11304	0.376537	0.546279	Rab21	Ras-related	RAB21_MO	6	0.924636
30min / C	P47962	0.289393	0.377852	0.546279	Rpl5	Large ribos	RL5_MOUS	14	1.222126
30min / C	P54071	0.168037	0.379086	0.546279	Idh2	Isocitrate d	IDHP_MOU	26	1.123529
15min / C	P59325	-0.09203	0.376248	0.546279	Eif5	Eukaryotic	IF5_MOUSE	17	0.938202
30min / C	P62137	0.117068	0.376274	0.546279	Ppp1ca	Serine/thre	PP1A_MOU	15	1.084528
30min / C	P62869	-0.07149	0.377623	0.546279	Elob	Elongin-B	ELOB_MOU	10	0.951657
30min / C	P63242	-0.196	0.377386	0.546279	Eif5a	Eukaryotic	IF5A1_MOU	11	0.872968
15min / C	P68368	-0.06569	0.377237	0.546279	Tuba4a	Tubulin alp	TBA4A_MO	4	0.955489
15min / C	Q08093	-0.4092	0.3786	0.546279	Cnn2	Calponin-2	CNN2_MOU	5	0.753043
15min / C	Q5SGK3	0.317239	0.375886	0.546279	Aox2	Aldehyde o	AOXB_MOU	2	1.245944
30min / C	Q6GQT1	0.039045	0.378558	0.546279	A2m	Alpha-2-ma	A2MG_MO	1	1.027434
15min / C	Q8CFE2	-0.12846	0.376352	0.546279	Hpf1	Histone PA	HPF1_MOU	2	0.914809
15min / C	Q8K0E8	0.629697	0.37713	0.546279	Fgb	Fibrinogen	FIBB_MOUS	30	1.54724
15min / C	Q8K1Z0	-0.36284	0.376649	0.546279	Coq9	Ubiquinon	COQ9_MOU	7	0.777631
30min / C	Q8K3H0	-0.20972	0.378742	0.546279	Appl1	DCC-intera	DP13A_MC	14	0.864705
30min / C	Q8QZS1	0.071707	0.378142	0.546279	Hibch	3-hydroxyi	HIBCH_MO	21	1.050959
30min / C	Q8VD04	0.090156	0.378342	0.546279	Gripap1	GRIP1-asso	GRAP1_MC	14	1.064485
15min / C	Q8VDJ3	-0.31755	0.378149	0.546279	Hdlbp	Vigilin	VIGLN_MO	65	0.802432
15min / C	Q9CWJ9	0.524562	0.376313	0.546279	Atic	Bifunctiona	PUR9_MOU	28	1.438497
15min / C	Q9D0R2	0.286651	0.379285	0.546279	Tars1	Threonine-	SYTC_MOUS	32	1.219806
15min / C	Q9D662	0.121094	0.379193	0.546279	Sec23b	Protein tra	SC23B_MO	18	1.087559
15min / C	Q9DB16	-0.04405	0.37913	0.546279	Cab39l	Calcium-bi	CB39L_MO	5	0.969929
15min / C	Q9DCN2	-0.27689	0.376626	0.546279	Cyb5r3	NADH-cyto	NB5R3_MC	16	0.825367
15min / C	Q9JKV1	-0.42619	0.37625	0.546279	Adrm1	Proteasom	ADRM1_M	7	0.744223
30min / C	Q9QZM0	-0.27941	0.378865	0.546279	Ubqln2	Ubiquilin-2	UBQL2_MC	3	0.823928
30min / C	Q9R0P3	-0.05333	0.376152	0.546279	Esd	S-formylglu	ESTD_MOU	14	0.963708
30min / C	Q9R257	-0.14377	0.379589	0.546352	Hebp1	Heme-bind	HEBP1_MO	11	0.905151
15min / C	Q8BKC5	0.062554	0.379914	0.546656	Ipo5	Importin-5	IPO5_MOU	25	1.044313
30min / C	P23249	-0.49471	0.381073	0.54709	Mov10	Putative he	MOV10_M	23	0.709706
30min / C	P27005	1.478348	0.380877	0.54709	S100a8	Protein S10	S10A8_MO	2	2.786295
30min / C	Q02053	-0.14841	0.381301	0.54709	Uba1	Ubiquitin-l	UBA1_MOU	43	0.902241
30min / C	Q60872	0.081401	0.381369	0.54709	Eif1a	Eukaryotic	IF1A_MOUS	4	1.058045
30min / C	Q8K0C8	-0.07547	0.381132	0.54709	Cox19	Cytochrom	COX19_MC	2	0.949035
15min / C	Q99KN9	-0.08738	0.38123	0.54709	Clint1	Clathrin int	EPN4_MOU	12	0.941234
15min / C	Q99LU0	0.065032	0.381455	0.54709	Chmp1b1	Charged mu	CH1B1_MC	2	1.046108
15min / C	Q9CZD3	0.1518	0.38058	0.54709	Gars1	Glycine--tR	GARS_MOU	17	1.110955
30min / C	Q9WTM5	-0.07043	0.38159	0.54709	Ruvbl2	RuvB-like 2	RUVB2_MC	17	0.952357
15min / C	Q9CWM4	0.005977	0.382076	0.547597	Pfdn1	Prefoldin su	PFD1_MOU	5	1.004151
30min / C	Q571I9	-0.15984	0.382607	0.547731	Aldh16a1	Aldehyde d	A16A1_MO	21	0.895124
30min / C	Q8JZR0	-0.12011	0.382724	0.547731	Acsl5	Long-chain	ACSL5_MO	27	0.920118
15min / C	Q9CQQ8	0.265732	0.382688	0.547731	Lsm7	U6 snRNA-a	LSM7_MOU	2	1.202246
30min / C	P14152	-0.06578	0.383551	0.548585	Mdh1	Malate deh	MDHC_MO	18	0.955428

30min / C	Q9D898	0.271222	0.38355	0.548585	Arpc5l	Actin-relate	ARP5L_MO	4	1.206829
30min / C	P70122	0.079692	0.383801	0.548663	Sbds	Ribosome r	SBDS_MOU	13	1.056792
15min / C	Q8CI94	0.369397	0.383834	0.548663	Pygb	Glycogen p	PYGB_MOU	19	1.291812
15min / C	G3X9C2	-0.19729	0.384172	0.548817	Nccrp1	F-box only	FBX50_MO	1	0.872186
30min / C	O88668	-0.34171	0.384293	0.548827	Creg1	Protein CRI	CREG1_MO	4	0.789106
15min / C	Q9DCD0	-0.0119	0.384561	0.549045	Pgd	6-phospho	6PGD_MOU	20	0.991786
15min / C	O88958	0.098143	0.385611	0.549262	Gnpda1	Glucosamir	GNPI1_MO	13	1.070395
30min / C	P17426	0.192256	0.38613	0.549262	Ap2a1	AP-2 compl	AP2A1_MO	34	1.142549
15min / C	P62322	0.443748	0.386241	0.549262	Lsm5	U6 snRNA-a	LSM5_MOU	1	1.360134
30min / C	Q5XG73	-0.81427	0.386322	0.549262	Acbd5	Acyl-CoA-bi	ACBD5_MC	13	0.568697
30min / C	Q61187	0.26303	0.385508	0.549262	Tsg101	Tumor susc	TS101_MO	9	1.199996
30min / C	Q9D924	-1.79128	0.385113	0.549262	Isca1	Iron-sulfur	ISCA1_MOU	1	0.288916
15min / C	Q9ERL9	0.403715	0.386292	0.549262	Gucy1a1	Guanylate	GCYA1_MO	8	1.32291
30min / C	Q9R0Y5	-0.06496	0.3856	0.549262	Ak1	Adenylate k	KAD1_MOU	4	0.955973
15min / C	Q9WVS8	0.316564	0.386171	0.549262	Mapk7	Mitogen-ac	MK07_MOU	1	1.245361
15min / C	Q64459	-0.25353	0.386582	0.549304	Cyp3a11	Cytochrom	CP3AB_MO	21	0.838843
15min / C	Q9WU78	0.025086	0.386523	0.549304	Pdcd6ip	Programme	PDC6I_MOU	38	1.01754
30min / C	Q00897	0.11232	0.387203	0.549861	Serpina1d	Alpha-1-an	A1AT4_MO	2	1.080965
15min / C	P62862	-0.14958	0.387895	0.549865	Fau	Ubiquitin-l	RS30_MOU	2	0.90151
30min / C	Q01730	-0.16124	0.387766	0.549865	Rsu1	Ras suppres	RSU1_MOU	10	0.894255
30min / C	Q99KR7	0.13219	0.387399	0.549865	Ppif	Peptidyl-pr	PPIF_MOUS	10	1.095956
15min / C	Q9QYB1	-0.07545	0.387965	0.549865	Clic4	Chloride in	CLIC4_MOU	15	0.949045
30min / C	Q6AW69	-0.027	0.388963	0.550202	Cgnl1	Cingulin-lik	CGNL1_MC	7	0.981459
30min / C	Q921M3	-0.20025	0.388392	0.550202	Sf3b3	Splicing fac	SF3B3_MO	28	0.870398
30min / C	Q9QXK3	-0.25829	0.388675	0.550202	Copg2	Coatomer s	COPG2_MC	24	0.836079
15min / C	Q91XF0	0.066561	0.389626	0.550847	Pnpo	Pyridoxine-	PNPO_MOU	8	1.047218
15min / C	Q9Z2X1	0.37011	0.390034	0.551261	Hnrnpf	Heterogene	HNRPF_MC	11	1.292451
30min / C	P29758	0.469637	0.390494	0.551747	Oat	Ornithine a	OAT_MOUS	19	1.384761
15min / C	Q9QYG0	0.045961	0.390849	0.551922	Ndrg2	Protein ND	NDRG2_MC	17	1.032371
30min / C	O88428	0.316314	0.391198	0.552049	Papss2	Bifunctiona	PAPS2_MO	23	1.245145
15min / C	P07901	0.01865	0.391457	0.552049	Hsp90aa1	Heat shock	HS90A_MO	25	1.013011
30min / C	Q07797	-0.00509	0.391631	0.552049	Lgals3bp	Galectin-3-	LG3BP_MO	12	0.996477
15min / C	Q8VDM4	-0.13188	0.391504	0.552049	Psmd2	26S protea	PSMD2_MC	40	0.91264
15min / C	Q9DBR7	-0.38883	0.391571	0.552049	Ppp1r12a	Protein phc	MYPT1_MC	16	0.763749
30min / C	Q9QUR6	0.432876	0.391428	0.552049	Prep	Prolyl endc	PPCE_MOU	32	1.349922
30min / C	P50446	0.400155	0.392349	0.552897	Krt6a	Keratin, typ	K2C6A_MO	17	1.319649
30min / C	Q9R1T2	0.055549	0.39275	0.5533	Sae1	SUMO-activ	SAE1_MOU	17	1.039255
15min / C	O88653	0.010167	0.393741	0.553623	Lamtor3	Ragulator c	LTOR3_MO	3	1.007072
15min / C	P07141	-0.82596	0.39338	0.553623	Csf1	Macrophag	CSF1_MOU	1	0.564106
30min / C	P11859	-1.00762	0.394022	0.553623	Agt	Angiotensir	ANGT_MOU	6	0.497365
15min / C	P20060	0.540245	0.393975	0.553623	Hexb	Beta-hexos	HEXB_MOU	11	1.45422
30min / C	P61967	0.1216	0.393323	0.553623	Ap1s1	AP-1 compl	AP1S1_MO	2	1.087941
30min / C	Q02257	0.566049	0.393678	0.553623	Jup	Junction pl	PLAK_MOU	18	1.480463
15min / C	Q9CZH3	0.396816	0.393815	0.553623	Psmg3	Proteasom	PSMG3_MC	2	1.316599
15min / C	O09159	0.070522	0.394216	0.553628	Man2b1	Lysosomal	MA2B1_MC	18	1.050097
30min / C	P01642	-0.71639	0.394257	0.553628	Igkv5-48	Immunoglc	KV5A9_MO	2	0.608617
15min / C	Q91XE0	0.167689	0.394425	0.553701	Glyat	Glycine N-a	GLYAT_MO	22	1.123258
15min / C	P61255	0.463542	0.394649	0.553837	Rpl26	Large ribos	RL26_MOU	9	1.378923
15min / C	Q8BGC0	0.660716	0.394969	0.553837	Htatsf1	17S U2 SnR	HTSF1_MO	6	1.580867
30min / C	Q8VDD5	-0.7073	0.395689	0.554336	Myh9	Myosin-9	MYH9_MOU	90	0.612463
30min / C	Q3TRM8	1.45508	0.39629	0.554586	Hk3	Hexokinase	HXK3_MOU	8	2.741717
30min / C	Q7TPR4	-0.02177	0.396431	0.554586	Actn1	Alpha-actir	ACTN1_MO	48	0.985025

15min / C	Q91X91	-0.03601	0.397405	0.555437	Qprt	Nicotinate-	NADC_MOU	14	0.97535
30min / C	P97371	-0.06548	0.397616	0.555569	Psme1	Proteasom	PSME1_MC	18	0.955629
30min / C	P83940	0.065871	0.397769	0.555622	Eloc	Elongin-C	ELOC_MOU	6	1.046717
15min / C	P26043	0.069771	0.398019	0.555808	Rdx	Radixin	RADI_MOU	42	1.04955
15min / C	O54984	0.016071	0.400203	0.55642	Get3	ATPase GET	GET3_MOU	9	1.011202
30min / C	P26516	0.387893	0.399622	0.55642	Psmd7	26S protea	PSMD7_MC	13	1.308481
15min / C	P58771	0.028164	0.400117	0.55642	Tpm1	Tropomyos	TPM1_MOU	5	1.019714
15min / C	P60521	0.170146	0.398574	0.55642	Gabarapl2	Gamma-am	GBRL2_MO	2	1.125173
15min / C	P62137	0.26324	0.399811	0.55642	Ppp1ca	Serine/thre	PP1A_MOU	15	1.200171
15min / C	Q01853	-0.07665	0.399881	0.55642	Vcp	Transitiona	TERA_MOU	57	0.948256
30min / C	Q64105	-0.39729	0.399888	0.55642	Spr	Sepiapterin	SPRE_MOU	14	0.759285
30min / C	Q8VE70	0.087732	0.399654	0.55642	Pdcd10	Programme	PDC10_MC	6	1.062698
15min / C	Q921M3	-0.13669	0.399316	0.55642	Sf3b3	Splicing fac	SF3B3_MO	28	0.909607
15min / C	Q99KV1	-0.18891	0.400044	0.55642	Dnajb11	DnaJ homo	DJB11_MO	10	0.877271
30min / C	Q9JK81	0.120745	0.399873	0.55642	Myg1	MYG1 exon	MYG1_MOU	13	1.087296
15min / C	O88456	-0.31315	0.4005	0.55667	Capns1	Calpain sma	CPNS1_MO	8	0.80488
15min / C	Q80X90	0.039446	0.401839	0.558218	Flnb	Filamin-B	FLNB_MOU	110	1.027719
30min / C	Q60972	-0.03013	0.403004	0.558365	Rbbp4	Histone-bir	RBBP4_MO	11	0.979329
30min / C	Q76MZ3	-0.1025	0.402933	0.558365	Ppp2r1a	Serine/thre	2AAA_MOU	26	0.931415
15min / C	Q8BT60	0.190935	0.402848	0.558365	Cpne3	Copine-3	CPNE3_MO	16	1.141503
15min / C	Q922B2	0.06345	0.402908	0.558365	Dars1	Aspartate--	SYDC_MOU	27	1.044962
30min / C	Q9CT10	-0.0647	0.402912	0.558365	Ranbp3	Ran-bindin	RANB3_MC	13	0.956147
15min / C	Q7TNG5	0.262503	0.403365	0.558414	Eml2	Echinoderm	EMAL2_MC	16	1.199558
15min / C	P68372	-0.11366	0.404521	0.559817	Tubb4b	Tubulin bet	TBB4B_MO	23	0.924238
30min / C	Q9DCJ9	-0.15167	0.404659	0.559846	Npl	N-acetylneu	NPL_MOUS	11	0.90021
15min / C	P27005	1.660972	0.405194	0.560425	S100a8	Protein S1C	S10A8_MO	2	3.162295
15min / C	P00920	-0.11409	0.405625	0.560469	Ca2	Carbonic ar	CAH2_MOU	11	0.923963
30min / C	P11031	0.167175	0.405611	0.560469	Sub1	Activated R	TCP4_MOU	7	1.122858
30min / C	P35980	-0.35133	0.405695	0.560469	Rpl18	Large ribos	RL18_MOU	7	0.783859
15min / C	Q9WV32	-0.14649	0.405495	0.560469	Arpc1b	Actin-relate	ARC1B_MO	17	0.903445
15min / C	P24270	0.824176	0.406487	0.560915	Cat	Catalase	CATA_MOU	35	1.770523
15min / C	P33267	-0.0572	0.40639	0.560915	Cyp2f2	Cytochrom	CP2F2_MO	25	0.961127
15min / C	Q6PDM2	0.030061	0.407306	0.56172	Srsf1	Serine/argi	SRSF1_MOU	14	1.021055
30min / C	O09131	-0.03776	0.407697	0.561935	Gsto1	Glutathion	GSTO1_MO	15	0.974163
15min / C	Q9R0P3	0.446692	0.407978	0.56216	Esd	S-formylglu	ESTD_MOU	14	1.362912
30min / C	Q3THK3	0.604628	0.408934	0.563316	Gtf2f1	General tra	T2FA_MOU	4	1.520587
15min / C	P17918	0.095021	0.409304	0.56365	Pcna	Proliferatin	PCNA_MOU	10	1.068081
30min / C	P03987	0.341251	0.409629	0.563785	NaN	Ig gamma-3	IGHG3_MO	8	1.266855
30min / C	P27773	0.064131	0.410199	0.563854	Pdia3	Protein disu	PDIA3_MOU	28	1.045455
30min / C	Q61699	0.076728	0.410639	0.563854	Hsph1	Heat shock	HS105_MO	29	1.054623
15min / C	Q8BL66	-0.1452	0.410624	0.563854	Eea1	Early endos	EEA1_MOU	53	0.904254
15min / C	Q8VHZ7	-0.23136	0.410626	0.563854	Imp4	U3 small nu	IMP4_MOU	1	0.85183
30min / C	Q9QXA5	0.037536	0.410741	0.563854	Lsm4	U6 snRNA-a	LSM4_MOU	5	1.02636
15min / C	Q14CH1	-0.26495	0.41125	0.564229	Mocos	Molybdenu	MOCOS_MO	23	0.832228
30min / C	Q99P91	-0.21521	0.411217	0.564229	Gpnmb	Transmemb	GPNMB_MO	1	0.861421
30min / C	O88653	-0.28709	0.411386	0.564254	Lamtor3	Ragulator c	LTOR3_MO	3	0.819553
15min / C	P05063	-0.01928	0.411686	0.564264	Aldoc	Fructose-bi	ALDOC_MC	14	0.986726
15min / C	Q9WVL0	-0.1023	0.411748	0.564264	Gstz1	Maleylacet	MAAI_MOU	18	0.931548
15min / C	O08784	-0.01121	0.412311	0.564551	Tcof1	Treacle pro	TCOF_MOU	10	0.992263
30min / C	Q9EPC1	0.015133	0.412308	0.564551	Parva	Alpha-parvi	PARVA_MO	10	1.010544
30min / C	Q8BFZ3	-0.30939	0.412779	0.56503	Actbl2	Beta-actin-l	ACTBL_MOU	3	0.80698
15min / C	P11679	-0.06417	0.412988	0.565031	Krt8	Keratin, typ	K2C8_MOU	27	0.956495

15min / C	Q8VC49	-0.12651	0.413135	0.565031	Ifi27l2b	Interferon a	IF27B_MOU	4	0.916042
30min / C	P56382	-0.2329	0.413502	0.565313	Atp5f1e	ATP synthas	ATP5E_MOU	2	0.850924
30min / C	P62821	-0.38903	0.413696	0.565313	Rab1a	Ras-related	RAB1A_MO	12	0.763642
15min / C	Q9DBC0	-0.13553	0.413963	0.565517	Selenoo	Protein ade	SELO_MOU	16	0.910335
30min / C	P58871	-0.2176	0.414303	0.565582	Tnks1bp1	182 kDa tar	TB182_MO	33	0.859992
30min / C	Q9DAR7	-0.09553	0.414532	0.565647	Dcps	m7GpppX c	DCPS_MOU	11	0.93593
15min / C	P55849	-0.84125	0.414837	0.565836	Dsc1	Desmocolli	DSC1_MOU	1	0.558159
30min / C	P57776	-0.01169	0.415083	0.565836	Eef1d	Elongation	EF1D_MOU	13	0.99193
30min / C	P97865	0.582884	0.415183	0.565836	Pex7	Peroxisoma	PEX7_MOU	4	1.49784
15min / C	Q60872	0.068887	0.415869	0.565836	Eif1a	Eukaryotic	IF1A_MOUS	4	1.048907
15min / C	Q8CHR6	0.091335	0.415838	0.565836	Dpyd	Dihydropyr	DPYD_MOU	48	1.065356
30min / C	Q8CIE0	-0.11996	0.415855	0.565836	Serpina11	Serpin A11	SPA11_MO	1	0.920215
15min / C	Q91YR1	-0.02945	0.415759	0.565836	Twf1	Twinfilin-1	TWF1_MOU	12	0.979795
30min / C	P50516	0.063031	0.416386	0.566122	Atp6v1a	V-type prot	VATA_MOU	27	1.044658
15min / C	Q9CQF4	0.231328	0.416766	0.566271	Mtres1	Mitochond	MRES1_MO	2	1.173915
15min / C	Q8R3Q6	-0.04038	0.417204	0.566543	Mix23	Protein MI)	MIX23_MO	5	0.972397
30min / C	Q921I1	-0.08484	0.417172	0.566543	Tf	Serotransfe	TRFE_MOUS	51	0.942892
30min / C	Q99JX4	-0.32995	0.417665	0.566903	Eif3m	Eukaryotic	EIF3M_MOU	11	0.795566
30min / C	Q9Z0X1	-0.10109	0.417706	0.566903	Aifm1	Apoptosis-i	AIFM1_MO	22	0.932327
30min / C	Q9Z0U1	-0.58821	0.418363	0.567635	Tjp2	Tight juncti	ZO2_MOUS	23	0.665167
30min / C	P38647	-0.09878	0.41854	0.567713	Hspa9	Stress-70 pr	GRP75_MO	40	0.933825
30min / C	A2A7S8	0.537538	0.418659	0.567714	Nhsl3	NHS-like pr	NHSL3_MO	6	1.451493
15min / C	P83940	-0.36403	0.418783	0.567721	Eloc	Elongin-C	ELOC_MOU	6	0.776992
30min / C	P37804	0.248598	0.419042	0.56791	Tagln	Transgelin	TAGL_MOU	11	1.188052
15min / C	P47199	0.144467	0.419407	0.56822	Cryz	Quinone ox	QOR_MOUS	18	1.105323
15min / C	Q61879	0.100755	0.419508	0.56822	Myh10	Myosin-10	MYH10_MO	44	1.072334
15min / C	O88451	-0.15771	0.420618	0.568741	Rdh7	Retinol deh	RDH7_MOU	18	0.896449
15min / C	P35279	0.059495	0.42229	0.568741	Rab6a	Ras-related	RAB6A_MO	8	1.042101
30min / C	P56565	-0.06655	0.420932	0.568741	S100a1	Protein S10	S10A1_MO	1	0.954919
15min / C	P63158	-0.05585	0.420461	0.568741	Hmgb1	High mobil	HMGB1_MO	10	0.962026
15min / C	Q02780	-0.02055	0.421067	0.568741	Nfia	Nuclear fac	NFIA_MOUS	5	0.985857
30min / C	Q3T9E4;Q6	-0.37129	0.42138	0.568741	Tgtp2;Tgtp	T-cell-speci	TGTP2_MO	5	0.773089
15min / C	Q8BJW6	-0.23487	0.421992	0.568741	Eif2a	Eukaryotic	EIF2A_MOU	22	0.849763
15min / C	Q8CIF4	-0.15012	0.421964	0.568741	Btd	Biotinidase	BTD_MOUS	8	0.901178
30min / C	Q8K4M5	0.088127	0.421607	0.568741	Commd1	COMM don	COMD1_MO	3	1.062989
30min / C	Q921H8	-0.13119	0.422269	0.568741	Acaa1a	3-ketoacyl-	THIKA_MOU	9	0.91308
15min / C	Q9CQN1	-0.07844	0.421133	0.568741	Trap1	Heat shock	TRAP1_MO	31	0.947084
15min / C	Q9Z2Y8	0.04414	0.420141	0.568741	Plpbp	Pyridoxal p	PLPHP_MO	12	1.031068
15min / C	Q99JW2	0.008049	0.422788	0.568794	Acy1	Aminoacyla	ACY1_MOU	18	1.005595
30min / C	Q61655	0.277404	0.423338	0.568894	Ddx19a	ATP-depenc	DD19A_MO	14	1.212012
15min / C	P19096	0.731051	0.423854	0.569267	Fasn	Fatty acid s	FAS_MOUS	130	1.659848
30min / C	P62918	0.087918	0.423804	0.569267	Rpl8	Large ribos	RL8_MOUS	11	1.062836
15min / C	Q9WUM5	1.331381	0.424109	0.56929	Suclg1	Succinate--	SUCA_MOU	12	2.516434
15min / C	Q8VCA8	0.074355	0.424277	0.569356	Scrn2	Secernin-2	SCRN2_MO	16	1.05289
30min / C	Q6PIP5	0.392876	0.424532	0.569538	Nudcd1	NudC doma	NUDC1_MO	14	1.313009
15min / C	P06151	-0.0592	0.425512	0.570474	Ldha	L-lactate de	LDHA_MOU	26	0.959798
30min / C	P08752	-0.04012	0.426181	0.570474	Gnai2	Guanine nu	GNAI2_MO	10	0.972573
30min / C	P39039	-0.328	0.426304	0.570474	Mbl1	Mannose-b	MBL1_MOU	3	0.796642
30min / C	P60766	0.30618	0.425969	0.570474	Cdc42	Cell divisio	CDC42_MO	8	1.236429
30min / C	P62242	-0.20487	0.425954	0.570474	Rps8	Small ribos	RS8_MOUS	8	0.86762
30min / C	Q62165	-0.34415	0.426199	0.570474	Dag1	Dystroglyca	DAG1_MOU	8	0.787773
15min / C	Q8R0P4	-0.00995	0.425828	0.570474	Aamdc	Mth938 do	AAMDC_MO	2	0.993127

30min / C	Q9CQ10	0.037988	0.425706	0.570474	Chmp3	Charged mu	CHMP3_MO	5	1.026681
15min / C	P97807	0.748483	0.426803	0.570982	Fh	Fumarate h	FUMH_MOU	21	1.680025
30min / C	P97821	-0.15515	0.427303	0.571491	Ctsc	Dipeptidyl	CATC_MOU	8	0.898038
30min / C	P12382	-0.14148	0.428485	0.572091	Pfkl	ATP-depend	PFKAL_MO	25	0.90659
15min / C	P47962	0.410734	0.4284	0.572091	Rpl5	Large ribos	RL5_MOUS	14	1.329362
15min / C	Q8BFZ3	0.404847	0.428371	0.572091	Actbl2	Beta-actin-l	ACTBL_MO	3	1.323948
15min / C	Q9DBE0	0.141544	0.429159	0.572421	Csad	Cysteine su	CSAD_MOU	28	1.103085
15min / C	Q8BWP5	-0.16192	0.429575	0.572607	Ttpa	Alpha-toco	TTPA_MOU	17	0.893836
15min / C	Q8CG76	0.166427	0.429466	0.572607	Akr7a2	Aflatoxin B	ARK72_MO	14	1.122276
30min / C	A2A432	0.19203	0.43082	0.573808	Cul4b	Cullin-4B	CUL4B_MO	19	1.14237
30min / C	P01029	-0.11379	0.430903	0.573808	C4b	Complemen	CO4B_MOU	47	0.924156
15min / C	Q9EQH2	0.156099	0.430956	0.573808	Erap1	Endoplasm	ERAP1_MO	33	1.11427
30min / C	O88851	0.299768	0.432457	0.574048	Rbbp9	Putative hy	RBBP9_MO	8	1.230946
30min / C	P01629	-0.1993	0.432438	0.574048	NaN	Ig kappa ch	KV2A4_MO	1	0.870974
30min / C	P04939	0.101167	0.4321	0.574048	Mup3	Major urina	MUP3_MO	10	1.072641
15min / C	Q61553	0.072839	0.432329	0.574048	Fscn1	Fascin	FSCN1_MO	8	1.051784
15min / C	Q91WK1	0.247747	0.431389	0.574048	Spryd4	SPRY doma	SPRY4_MO	12	1.187351
30min / C	Q9D2G2	-0.07572	0.431762	0.574048	Dlst	Dihydrolipo	ODO2_MOU	16	0.948868
15min / C	Q9Z0K8	-0.05942	0.432205	0.574048	Vnn1	Pantethein	VNN1_MOU	1	0.959651
15min / C	Q64516	-0.03575	0.432681	0.574185	Gk	Glycerol kin	GLPK_MOU	28	0.975525
30min / C	O88451	-0.26224	0.432871	0.574278	Rdh7	Retinol deh	RDH7_MOU	18	0.833791
30min / C	D3YXK2	0.173279	0.433133	0.574403	Safb	Scaffold att	SAFB1_MO	10	1.127618
15min / C	O70305	0.24271	0.433566	0.574403	Atxn2	Ataxin-2	ATX2_MOU	16	1.183213
15min / C	Q3UMF0	-0.01199	0.433312	0.574403	Cobll1	Cordon-ble	COBL1_MO	47	0.991721
30min / C	Q91VS7	-0.41685	0.43347	0.574403	Mgst1	Microsoma	MGST1_MO	4	0.749061
30min / C	P56212	-0.31533	0.434183	0.574902	Arpp19	cAMP-regul	ARP19_MO	4	0.803668
15min / C	P53657	0.141664	0.434858	0.575212	Pklr	Pyruvate ki	KPYR_MOU	35	1.103176
30min / C	Q9D8W5	-0.36074	0.434899	0.575212	Psmd12	26S protea	PSD12_MO	23	0.778764
30min / C	P04264	0.29032	0.43535	0.575547	KRT1	Keratin, typ	K2C1_HUM	38	1.222911
15min / C	Q02248	-0.12053	0.435514	0.575547	Ctnnb1	Catenin bet	CTNB1_MO	18	0.919853
15min / C	Q3UMU9	0.071467	0.435453	0.575547	Hdgfl2	Hepatoma-	HDGR2_MO	10	1.050785
15min / C	O35887	-0.09372	0.436817	0.576453	Calu	Calumenin	CALU_MOU	10	0.937101
30min / C	P09813	-0.16689	0.436922	0.576453	Apoa2	Apolipopro	APOA2_MO	2	0.890762
15min / C	Q9DD20	0.086235	0.43674	0.576453	Tmt1b	Thiol S-met	TMT1B_MO	11	1.061596
30min / C	P58774	-0.14552	0.438097	0.576774	Tpm2	Tropomyos	TPM2_MOU	11	0.904052
15min / C	Q9WV68	-0.09166	0.437782	0.576774	Decr2	Peroxisoma	DECR2_MO	13	0.93844
15min / C	P08607	0.222486	0.438266	0.576792	C4bpa	C4b-bindin	C4BPA_MO	12	1.166742
15min / C	Q9D0M1	0.137523	0.439253	0.577548	Prpsap1	Phosphorib	KPRA_MOU	13	1.100015
15min / C	Q9JJL8	-0.35216	0.439323	0.577548	Sars2	Serine--tRN	SYSM_MOU	7	0.783408
30min / C	O35490	-0.08012	0.439675	0.577852	Bhmt	Betaine--ho	BHMT1_MO	28	0.945979
30min / C	Q8BFQ4	0.599757	0.439808	0.577868	Wdr82	WD repeat-	WDR82_MO	4	1.515461
30min / C	Q5XJE5	-0.36792	0.440244	0.578281	Leo1	RNA polym	LEO1_MOU	1	0.774897
15min / C	Q6PIP5	0.065855	0.4409	0.578984	Nudcd1	NudC doma	NUDC1_MO	14	1.046705
15min / C	Q9JII6	0.531009	0.441177	0.579097	Akr1a1	Aldo-keto r	AK1A1_MO	21	1.444939
30min / C	P62281	0.519743	0.441796	0.57927	Rps11	Small ribos	RS11_MOU	12	1.433699
15min / C	Q8C0M9	0.002236	0.441793	0.57927	Asrgl1	Isoaspartyl	ASGL1_MO	13	1.001551
15min / C	Q9JJI8	0.375357	0.441824	0.57927	Rpl38	Large ribos	RL38_MOU	3	1.29716
30min / C	P29341	-0.06059	0.443179	0.5807	Pabpc1	Polyadenyl	PABP1_MO	27	0.958871
15min / C	Q9CR70	-0.40623	0.443383	0.580809	Lage3	EKC/KEOPS	LAGE3_MO	1	0.754593
30min / C	Q8K2Q9	-0.13687	0.44502	0.582275	Shtn1	Shootin-1	SHOT1_MO	23	0.909491
30min / C	Q9Z255	-0.41517	0.445321	0.58239	Ube2a	Ubiquitin-c	UBE2A_MO	2	0.749931
30min / C	Q922Q8	0.011968	0.445601	0.582596	Lrrc59	Leucine-ric	LRC59_MO	10	1.00833

30min / C	Q61646	-0.07991	0.446261	0.5833	Hp	Haptoglobi	HPT_MOUS	17	0.946118
15min / C	Q9JMA2	-0.05815	0.44675	0.583779	Qtrt1	Queuine tR	TGT_MOUS	1	0.960497
30min / C	P01635	-0.14987	0.447239	0.583909	Igkv12-41	Immunoglo	KV5A3_MO	2	0.901329
30min / C	P70445	-0.26754	0.44726	0.583909	Eif4ebp2	Eukaryotic	4EBP2_MO	4	0.830734
30min / C	Q61129	-0.18302	0.447297	0.583909	Cfi	Complemen	CFAI_MOUS	18	0.880858
15min / C	Q63918	-0.04067	0.447338	0.583909	Cavin2	Caveolae-as	CAVN2_MO	4	0.972201
15min / C	Q99P91	0.210678	0.447528	0.583997	Gpnmb	Transmemb	GPNMB_MO	1	1.157232
15min / C	Q9QUH0	-0.07241	0.447929	0.584362	Glrx	Glutaredox	GLRX1_MO	6	0.951051
30min / C	P01868;P0	-0.25141	0.44816	0.584504	Ighg1	Ig gamma-1	IGHG1_MO	7	0.840072
15min / C	Q80UG5	0.016128	0.449019	0.585463	Septin9	Septin-9	SEPT9_MOU	26	1.011242
30min / C	B2RQA1	0.042039	0.449273	0.585571	Kcna10	Potassium v	KCA10_MO	1	1.029568
30min / C	Q8BK48	-0.06327	0.449346	0.585571	Ces2e	Pyrethroid	EST2E_MOU	21	0.957092
15min / C	Q9DBL7	0.508916	0.450619	0.58691	Coasy	Bifunctiona	COASY_MO	18	1.422981
30min / C	Q05920	-0.26733	0.451941	0.587569	Pc	Pyruvate ca	PYC_MOUS	58	0.830856
15min / C	P01806;P0	0.260841	0.452767	0.588105	NaN	Ig heavy ch	HVM36_MO	2	1.198177
15min / C	P17717	0.831911	0.452682	0.588105	Ugt2b17	UDP-glucur	UDB17_MO	16	1.780042
30min / C	P62311	-0.24063	0.452946	0.588177	Lsm3	U6 snRNA-a	LSM3_MOU	3	0.846378
30min / C	O08529	-0.22793	0.453275	0.588414	Capn2	Calpain-2 c	CAN2_MOU	22	0.853861
15min / C	O88487	0.03573	0.453864	0.588591	Dync1i2	Cytoplasmi	DC1I2_MO	14	1.025075
15min / C	O88545	0.300103	0.454495	0.588591	Cops6	COP9 signa	CSN6_MOU	8	1.231233
30min / C	P00375	-0.5306	0.454283	0.588591	Dhfr	Dihydrofola	DYR_MOUS	10	0.692264
30min / C	P48678	-0.15885	0.454216	0.588591	Lmna	Prelamin-A	LMNA_MOU	30	0.895737
15min / C	Q8BFS6	0.12771	0.454448	0.588591	Cpped1	Serine/thre	CPPED_MO	8	1.092558
30min / C	Q9WU28	0.076321	0.454064	0.588591	Pfdn5	Prefoldin su	PFD5_MOU	7	1.054326
30min / C	Q05816	-0.16503	0.454679	0.588606	Fabp5	Fatty acid-b	FABP5_MO	9	0.891909
15min / C	Q91VS7	0.41327	0.454753	0.588606	Mgst1	Microsoma	MGST1_MO	4	1.331701
15min / C	P10605	-0.03363	0.454895	0.58863	Ctsb	Cathepsin B	CATB_MOU	12	0.97696
30min / C	O08553	0.137653	0.455731	0.589232	Dpysl2	Dihydropyr	DPYL2_MO	22	1.100114
30min / C	P35979	-0.20765	0.456528	0.589625	Rpl12	Large ribos	RL12_MOU	8	0.865948
30min / C	Q01768	-0.0664	0.456404	0.589625	Nme2	Nucleoside	NDKB_MOU	12	0.955017
15min / C	Q5EBG8	-0.07387	0.456907	0.589796	NaN	Uncharacte	CA050_MO	6	0.950088
30min / C	P63028	0.113911	0.457565	0.590195	Tpt1	Translation	TCTP_MOU	8	1.082158
15min / C	Q4KML4	-0.14462	0.457696	0.590195	Abracl	Costars fam	ABRAL_MO	3	0.904615
15min / C	Q9JLQ0	-0.15949	0.457994	0.590195	Cd2ap	CD2-associ	CD2AP_MO	14	0.895341
15min / C	Q9Z1X4	0.10103	0.457341	0.590195	Ilf3	Interleukin	ILF3_MOUS	16	1.072539
15min / C	Q8R1B4	-0.27336	0.458401	0.59029	Eif3c	Eukaryotic	EIF3C_MOU	23	0.827392
30min / C	Q99K70	-0.39223	0.458356	0.59029	Rragc	Ras-related	RRAGC_MO	4	0.761949
15min / C	Q8K411	0.321476	0.458767	0.590603	Pitrm1	Presequenc	PREP_MOU	31	1.249609
30min / C	P27659	-0.22435	0.459468	0.590827	Rpl3	Large ribos	RL3_MOUS	20	0.85598
30min / C	P58771	0.068801	0.45956	0.590827	Tpm1	Tropomyos	TPM1_MOU	5	1.048845
30min / C	Q8VDM4	-0.20484	0.459464	0.590827	Psmd2	26S protea	PSMD2_MO	40	0.867635
30min / C	Q9DCL8	0.327565	0.459462	0.590827	Ppp1r2	Protein pho	IPP2_MOUS	6	1.254893
15min / C	P97315	-0.46786	0.460813	0.59212	Csrp1	Cysteine an	CSRP1_MO	8	0.723037
30min / C	P18760	0.033796	0.461244	0.592202	Cfl1	Cofilin-1	COF1_MOU	17	1.023702
15min / C	Q9D142	0.12658	0.461805	0.592667	Nudt14	Uridine dip	NUD14_MO	7	1.091702
30min / C	P61087	-0.1082	0.462284	0.592894	Ube2k	Ubiquitin-c	UBE2K_MO	10	0.927744
15min / C	Q80SY6	-0.48539	0.462854	0.593467	Adal	Adenosine d	ADAL_MOU	4	0.714302
15min / C	Q8R1F5	-0.01583	0.463446	0.594066	Hyi	Putative hy	HYI_MOUSE	10	0.98909
30min / C	Q9JLT4	0.038256	0.463626	0.594137	Txnrd2	Thioredoxi	TRXR2_MO	14	1.026871
15min / C	P17156	0.001508	0.463862	0.59428	Hspa2	Heat shock	HSP72_MO	21	1.001046
30min / C	P27048;P6	-0.03174	0.464047	0.594358	Snrpb;Snrp	Small nucle	RSMB_MOU	5	0.978239
15min / C	P49182	-0.02723	0.465008	0.59511	Serpind1	Heparin co	HEP2_MOU	15	0.9813

30min / C	E9Q4Z2	0.192048	0.465314	0.59533	Acacb	Acetyl-CoA	ACACB_MO	32	1.142384
15min / C	P52503	-0.69706	0.465553	0.59533	Ndufs6	NADH dehy	NDUS6_MC	4	0.616829
30min / C	Q9CR35	-0.2869	0.465547	0.59533	Ctrb1	Chymotryp	CTRB1_MO	6	0.819659
30min / C	P58252	-0.01319	0.46611	0.595405	Eef2	Elongation	EF2_MOUSI	58	0.990898
15min / C	Q8BTY1	-0.11069	0.466081	0.595405	Kyat1	Kynurenine	KAT1_MOU	18	0.926144
30min / C	Q8VBW6	-0.08454	0.466479	0.595559	Nae1	NEDD8-acti	ULA1_MOU	11	0.943086
30min / C	P82343	-0.14993	0.466737	0.595691	Renbp	N-acylgluco	RENBP_MO	2	0.901296
15min / C	P97864	0.060632	0.466832	0.595691	Casp7	Caspase-7	CASP7_MO	8	1.042923
30min / C	Q06185	-0.2936	0.467615	0.596053	Atp5me	ATP synthas	ATP5I_MOU	4	0.815863
15min / C	Q920E5	0.574067	0.467562	0.596053	Fdps	Farnesyl py	FPPS_MOU	19	1.488714
15min / C	Q9CQ10	0.109512	0.467401	0.596053	Chmp3	Charged mu	CHMP3_MC	5	1.078864
15min / C	Q9CWS0	-0.03856	0.467867	0.596215	Ddah1	N(G),N(G)-d	DDAH1_MC	19	0.973624
30min / C	Q6P5E4	-0.21236	0.468657	0.596668	Uggt1	UDP-glucos	UGGG1_MC	38	0.863122
30min / C	Q8VCA8	0.110217	0.468722	0.596668	Scrn2	Secernin-2	SCRN2_MO	16	1.07939
15min / C	P29341	0.110138	0.469452	0.596962	Pabpc1	Polyadenyl	PABP1_MO	27	1.079331
15min / C	P70362	-0.25902	0.469223	0.596962	Ufd1	Ubiquitin r	UFD1_MOU	9	0.835656
30min / C	Q9DCX2	0.259391	0.469639	0.597041	Atp5pd	ATP synthas	ATP5H_MO	11	1.196973
15min / C	P70296	0.007624	0.469859	0.597162	Pebp1	Phosphatid	PEBP1_MO	12	1.005299
30min / C	Q6PDL0	-0.31479	0.470452	0.597594	Dync1li2	Cytoplasmi	DC1L2_MO	10	0.803967
30min / C	Q91YH6	1.183129	0.471359	0.597955	Atp6v1b1	V-type prot	VATB1_MO	1	2.270688
15min / C	Q922U2	-0.24036	0.471625	0.598018	Krt5	Keratin, typ	K2C5_MOU	9	0.846532
15min / C	O88428	-0.31529	0.472537	0.598956	Papss2	Bifunctiona	PAPS2_MO	23	0.803687
15min / C	Q60866	-0.11121	0.472899	0.598956	Pter	Phosphotri	PTER_MOU	19	0.925811
30min / C	Q63918	-0.08387	0.472726	0.598956	Cavin2	Caveolae-as	CAVN2_MC	4	0.943523
30min / C	E9Q4P1	0.293114	0.473166	0.599135	Wdfy1	WD repeat	WDFY1_MC	5	1.225282
30min / C	O70133	0.322108	0.474098	0.599199	Dhx9	ATP-depend	DHX9_MOU	25	1.250156
30min / C	P23591	0.076372	0.474161	0.599199	Gfus	GDP-L-fuco	FCL_MOUSI	13	1.054363
15min / C	P29788	-0.23808	0.473822	0.599199	Vtn	Vitronectin	VTNC_MOU	9	0.847875
15min / C	Q6IRU2	-0.02917	0.474219	0.599199	Tpm4	Tropomyos	TPM4_MOU	12	0.979985
30min / C	Q8BK75	-0.27214	0.473764	0.599199	Elp6	Elongator c	ELP6_MOU	2	0.828088
15min / C	Q6IFZ6	0.565081	0.474948	0.59985	Krt77	Keratin, typ	K2C1B_MO	4	1.479471
30min / C	Q99JY9	0.080412	0.475096	0.59985	Actr3	Actin-relate	ARP3_MOU	22	1.05732
30min / C	Q9WVM8	-0.05715	0.475111	0.59985	Aadat	Kynurenine	AADAT_MO	19	0.961164
30min / C	P85094	-0.07433	0.475959	0.600762	Isoc2a	Isochorism	ISC2A_MOU	11	0.949785
15min / C	P28667	-0.18434	0.476265	0.600831	Marcksl1	MARCKS-re	MRP_MOUS	2	0.880049
30min / C	O55023	-0.09609	0.476649	0.600839	Impa1	Inositol mc	IMPA1_MO	13	0.935566
30min / C	Q8BGZ7	0.676626	0.476557	0.600839	Krt75	Keratin, typ	K2C75_MO	2	1.598397
15min / C	P13745	-0.22817	0.476987	0.601106	Gsta1	Glutathion	GSTA1_MO	13	0.853717
15min / C	Q06770	-0.07638	0.477368	0.60127	Serpina6	Corticoster	CBG_MOUS	6	0.948434
15min / C	O55101	-0.79243	0.477999	0.601431	Syngr2	Synaptogyr	SNG2_MOU	1	0.577369
30min / C	Q3ULD5	-0.09306	0.478087	0.601431	Mccc2	Methylcrot	MCCB_MOU	25	0.93753
15min / C	P58774	0.104346	0.478355	0.601501	Tpm2	Tropomyos	TPM2_MOU	11	1.075007
15min / C	Q9D7S9	-0.0851	0.478432	0.601501	Chmp5	Charged mu	CHMP5_MC	3	0.942717
15min / C	Q11011	-0.00503	0.479177	0.601507	Npepps	Puromycin	PSA_MOUS	30	0.996517
30min / C	Q6NXH9	0.569721	0.479	0.601507	Krt73	Keratin, typ	K2C73_MO	1	1.484237
30min / C	Q922U2	0.353916	0.47917	0.601507	Krt5	Keratin, typ	K2C5_MOU	9	1.278025
30min / C	Q9CWS0	-0.00436	0.479192	0.601507	Ddah1	N(G),N(G)-d	DDAH1_MC	19	0.996984
30min / C	P10637	0.026503	0.479743	0.601711	Mapt	Microtubul	TAU_MOUS	15	1.018541
15min / C	P62281	0.65051	0.479917	0.601711	Rps11	Small ribos	RS11_MOU	12	1.569723
30min / C	P97447	0.031945	0.479702	0.601711	Fhl1	Four and a l	FHL1_MOU	5	1.02239
15min / C	P97494	0.088424	0.480173	0.601711	Gclc	Glutamate-	GSH1_MOU	26	1.063208
15min / C	Q3TC93	-0.10144	0.480362	0.601711	Hs1bp3	HCLS1-binc	H1BP3_MC	10	0.932102

30min / C	Q9JJF9	-0.09704	0.480048	0.601711	Sppl2a	Signal pept	SPP2A_MO	1	0.934951
15min / C	Q9WTX6	0.04329	0.480315	0.601711	Cul1	Cullin-1	CUL1_MOU	23	1.030461
30min / C	Q8BH57	-0.42512	0.480568	0.601811	Wdr48	WD repeat-	WDR48_M	3	0.744777
30min / C	O88545	0.045188	0.481307	0.602106	Cops6	COP9 signa	CSN6_MOU	8	1.031818
30min / C	Q60876	-0.59179	0.481181	0.602106	Eif4ebp1	Eukaryotic	4EBP1_MO	2	0.663521
30min / C	Q8R238	0.127974	0.481048	0.602106	Sdsl	Serine dehy	SDSL_MOU	11	1.092758
15min / C	P63280	0.611982	0.482162	0.602545	Ube2i	SUMO-conj	UBC9_MOU	6	1.528358
15min / C	P04919	-0.48837	0.483344	0.602666	Slc4a1	Band 3 anic	B3AT_MOU	12	0.712829
30min / C	P23198	0.346612	0.482468	0.602666	Cbx3	Chromobo:	CBX3_MOU	6	1.271571
15min / C	P32067	-0.00049	0.483369	0.602666	Ssb	Lupus La pr	LA_MOUSE	16	0.999661
30min / C	Q05D44	0.208484	0.483256	0.602666	Eif5b	Eukaryotic	IF2P_MOUS	27	1.155473
30min / C	Q9CWJ9	-0.17049	0.483181	0.602666	Atic	Bifunctiona	PUR9_MOU	28	0.88854
30min / C	Q9ESB3	-0.04965	0.482724	0.602666	Hrg	Histidine-ri	HRG_MOUS	12	0.966174
15min / C	Q9Z0M5	-0.05289	0.482846	0.602666	Lipa	Lysosomal	LICH_MOUS	9	0.964003
15min / C	Q5XJY5	-0.05467	0.484259	0.602801	Arcn1	Coatomer s	COPD_MOU	25	0.962815
30min / C	Q62193	0.491178	0.484231	0.602801	Rpa2	Replicatior	RFA2_MOU	4	1.405592
15min / C	Q8R519	0.098817	0.484204	0.602801	Acmsd	2-amino-3-	ACMSD_M	11	1.070895
15min / C	Q60902	0.756732	0.484459	0.602893	Eps15l1	Epidermal	EP15R_MO	18	1.689659
30min / C	Q8BZF8	-0.20721	0.484813	0.60302	Pgm5	Phosphoglu	PGM5_MO	1	0.866213
15min / C	Q99L20	0.087524	0.484805	0.60302	Gstt3	Glutathion	GSTT3_MO	10	1.062545
30min / C	O09172	0.069398	0.485167	0.603231	Gclm	Glutamate-	GSH0_MOU	11	1.049279
15min / C	Q61425	-0.155	0.485235	0.603231	Hadh	Hydroxyacy	HCDH_MOU	17	0.89813
30min / C	P62852	0.519464	0.485476	0.603374	Rps25	Small ribos	RS25_MOU	6	1.433422
30min / C	Q9WTP6	-0.12016	0.485865	0.603512	Ak2	Adenylate k	KAD2_MOU	17	0.920087
30min / C	Q9D3D9	0.220171	0.486566	0.603787	Atp5f1d	ATP synthas	ATPD_MOU	5	1.164872
30min / C	Q91X72	-0.02966	0.4872	0.603809	Hpx	Hemopexir	HEMO_MO	23	0.979652
15min / C	Q9CYK1	0.252408	0.486873	0.603809	Wars2	Tryptophar	SYWM_MO	5	1.191193
30min / C	Q9JMD3	0.092952	0.487216	0.603809	Stard10	START dom	STA10_MO	15	1.06655
30min / C	Q8BYK6	-0.12241	0.487368	0.603841	Ythdf3	YTH domair	YTHD3_MO	14	0.918651
15min / C	P50171	-0.06893	0.487502	0.603851	Hsd17b8	(3R)-3-hydr	DHB8_MOU	11	0.953345
15min / C	Q571I9	-0.01745	0.488395	0.604174	Aldh16a1	Aldehyde d	A16A1_MO	21	0.987978
30min / C	Q8R3F9	0.698144	0.488255	0.604174	Tut1	Speckle tar	STPAP_MO	1	1.622416
30min / C	Q02248	-0.45631	0.488564	0.604227	Ctnnb1	Catenin bet	CTNB1_MO	18	0.728849
15min / C	Q62159	0.05605	0.48907	0.60454	Rhoc	Rho-relatec	RHOC_MOU	8	1.039615
15min / C	Q9CQA3	-0.24619	0.489032	0.60454	Sdhb	Succinate c	SDHB_MOU	10	0.843119
15min / C	P35492	-0.04336	0.48921	0.604556	Hal	Histidine ar	HUTH_MOU	35	0.970392
15min / C	O88851	0.023931	0.490097	0.605339	Rbbp9	Putative hy	RBBP9_MO	8	1.016726
15min / C	Q64514	0.170289	0.490024	0.605339	Tpp2	Tripeptidyl	TPP2_MOU	54	1.125284
15min / C	O08663	-0.20943	0.491003	0.605455	Metap2	Methionine	MAP2_MOU	10	0.864877
30min / C	P54823	0.211835	0.490853	0.605455	Ddx6	Probable A	DDX6_MOU	14	1.15816
15min / C	P56212	0.1322	0.490941	0.605455	Arpp19	cAMP-regul	ARP19_MO	4	1.095964
15min / C	Q3THK3	-0.00089	0.490403	0.605455	Gtf2f1	General tra	T2FA_MOU	4	0.999383
30min / C	Q8CCS6	0.810199	0.490573	0.605455	Pabpn1	Polyadenyl	PABP2_MO	4	1.753454
15min / C	Q64152	0.119423	0.491492	0.605746	Btf3	Transcripti	BTF3_MOU	5	1.0863
15min / C	Q9ESB3	-0.02672	0.491933	0.605854	Hrg	Histidine-ri	HRG_MOUS	12	0.981647
15min / C	Q9WUM3	-0.07747	0.492034	0.605854	Coro1b	Coronin-1E	COR1B_MC	10	0.947717
30min / C	O70570	0.0772	0.492442	0.605929	Pigr	Polymeric i	PIGR_MOU	22	1.054969
30min / C	P08207	-0.27195	0.492978	0.605929	S100a10	Protein S1C	S10AA_MO	3	0.828198
15min / C	Q6NXN1	-0.05914	0.492525	0.605929	Szrd1	SUZ RNA-bi	SZRD1_MO	4	0.959839
15min / C	Q8BIJ6	-0.03251	0.492983	0.605929	Iars2	Isoleucine-	SYIM_MOU	31	0.977721
30min / C	Q8BTE0	0.429068	0.49256	0.605929	Sdhaf4	Succinate c	SDHF4_MO	2	1.346363
30min / C	Q9DBG1	-0.09659	0.4928	0.605929	Cyp27a1	Sterol 26-h	CP27A_MO	14	0.935239

30min / C	O55131	0.07931	0.493304	0.606169	Septin7	Septin-7	SEPT7_MOU	16	1.056513
30min / C	P19221	0.207734	0.493837	0.606625	F2	Prothromb	THRB_MOU	22	1.154873
15min / C	P99024	-0.15691	0.49393	0.606625	Tubb5	Tubulin bet	TBB5_MOU	4	0.896946
30min / C	Q8BTZ7	0.019763	0.494158	0.60675	Gmppb	Mannose-1	GMPPB_MO	10	1.013793
30min / C	Q9JHE3	-0.57534	0.494551	0.607076	Asah2	Neutral cer	ASAH2_MO	1	0.67113
15min / C	P19221	0.052472	0.494719	0.607126	F2	Prothromb	THRB_MOU	22	1.037041
15min / C	Q571E4	0.083652	0.494911	0.607206	Galns	N-acetylgal	GALNS_MO	11	1.059697
15min / C	Q9ET01	0.034931	0.495127	0.607315	Pygl	Glycogen p	PYGL_MOU	46	1.024508
15min / C	Q9D5J6	-0.0508	0.495351	0.607435	Shpk	Sedoheptul	SHPK_MOU	17	0.965402
30min / C	P01806;P0	0.079775	0.496191	0.607529	NaN	Ig heavy cha	HVM36_MO	2	1.056854
30min / C	P14211	0.111927	0.496184	0.607529	Calr	Calreticulin	CALR_MOU	25	1.080671
15min / C	P84099	-0.02395	0.496016	0.607529	Rpl19	Large ribos	RL19_MOU	3	0.983533
15min / C	Q62393	-0.27932	0.495903	0.607529	Tpd52	Tumor prot	TPD52_MO	5	0.823978
30min / C	P48428	0.024263	0.496491	0.607585	Tbca	Tubulin-spe	TBCA_MOU	7	1.01696
30min / C	P54822	-0.06101	0.496377	0.607585	Adsl	Adenylosuc	PUR8_MOU	19	0.958591
30min / C	Q91WT9	-0.10959	0.49687	0.607893	Cbs	Cystathioni	CBS_MOUS	24	0.926854
30min / C	P01872	0.215073	0.497428	0.608109	Ighm	Immunoglo	IGHM_MOU	14	1.160763
30min / C	Q9CQ86	-0.01439	0.497401	0.608109	Mien1	Migration a	MIEN1_MO	3	0.990076
30min / C	Q99LX0	-0.07174	0.497802	0.608411	Park7	Parkinson c	PARK7_MO	15	0.95149
15min / C	P53810	0.157071	0.498196	0.608428	Pitpna	Phosphatic	PIPNA_MO	9	1.115021
30min / C	Q9JHU9	-0.01327	0.498326	0.608428	Isyna1	Inositol-3-p	INO1_MOU	10	0.990841
15min / C	Q7TNG8	0.121585	0.49864	0.608657	Ldhd	Probable D	LDHD_MOU	18	1.087929
15min / C	Q921I1	-0.06481	0.499485	0.609066	Tf	Serotransfe	TRFE_MOUS	51	0.956068
15min / C	P49312	-0.0706	0.499942	0.609158	Hnrnpa1	Heterogene	ROA1_MOU	10	0.952239
15min / C	P97821	0.024485	0.499886	0.609158	Ctsc	Dipeptidyl	CATC_MOU	8	1.017116
30min / C	Q9DBA8	0.000472	0.50044	0.609369	Amdhd1	Probable in	HUTI_MOU	23	1.000327
30min / C	Q07456	0.072377	0.50132	0.609746	Ambp	Protein AM	AMBP_MOU	12	1.051448
15min / C	Q60759	0.128066	0.501446	0.609746	Gcdh	Glutaryl-Co	GCDH_MOU	16	1.092828
30min / C	Q63836	-0.09207	0.501427	0.609746	Selenbp2	Selenium-b	SBP2_MOU	36	0.938176
30min / C	Q8CGC7	0.205082	0.501278	0.609746	Eprs1	Bifunctiona	SYEP_MOUS	61	1.152752
15min / C	F6ZDS4	-0.10424	0.502304	0.6098	Tpr	Nucleoprot	TPR_MOUS	37	0.930298
15min / C	P47791	-0.00959	0.503016	0.6098	Gsr	Glutathion	GSHR_MOU	19	0.993374
15min / C	Q07968	0.193664	0.502762	0.6098	F13b	Coagulatio	F13B_MOU	1	1.143665
15min / C	Q64339	0.232626	0.502981	0.6098	Isg15	Ubiquitin-l	ISG15_MOU	6	1.174972
30min / C	Q6PGH2	-0.09408	0.502962	0.6098	Jpt2	Jupiter mic	JUPI2_MOU	8	0.936868
15min / C	Q924M7	0.723552	0.503021	0.6098	Mpi	Mannose-6	MPI_MOUS	12	1.651243
30min / C	Q9CWX4	-0.00977	0.50276	0.6098	Rpusd4	Pseudourid	RUSD4_MO	1	0.993253
15min / C	Q8CC86	-0.07714	0.50348	0.610201	Naprt	Nicotinate	PNCB_MOU	22	0.947934
30min / C	P01867	-0.0862	0.503832	0.61041	Ighg2b	Immunoglo	IGG2B_MO	5	0.942001
15min / C	Q9QZN4	-0.34027	0.503908	0.61041	Fbxo6	F-box only	FBX6_MOU	8	0.789895
15min / C	P11983	-0.07974	0.504037	0.610412	Tcp1	T-complex	TCPA_MOU	29	0.946231
15min / C	Q8BWW4	-0.30376	0.505027	0.611146	Larp4	La-related p	LARP4_MO	10	0.810138
15min / C	Q8BGA8	-0.02896	0.505683	0.611435	Acsm5	Acyl-coenzy	ACSM5_MO	22	0.980124
30min / C	Q8BLY2	0.403275	0.505712	0.611435	Tars3	Threonine--	SYTC2_MOU	13	1.322507
15min / C	Q8K2B3	-0.011	0.505777	0.611435	Sdha	Succinate d	SDHA_MOU	27	0.992407
30min / C	O35887	0.07177	0.506118	0.611485	Calu	Calumenin	CALU_MOU	10	1.051005
15min / C	P14152	0.034776	0.506237	0.611485	Mdh1	Malate deh	MDHC_MO	18	1.024398
15min / C	Q9QXG4	0.830017	0.506005	0.611485	Acss2	Acetyl-coer	ACSA_MOU	24	1.777706
15min / C	P28658	-0.50128	0.5067	0.611623	Atxn10	Ataxin-10	ATX10_MO	16	0.70648
15min / C	O70194	-0.33392	0.508907	0.612215	Eif3d	Eukaryotic	EIF3D_MOU	16	0.793378
30min / C	P03958	0.152989	0.507627	0.612215	Ada	Adenosine	ADA_MOUS	9	1.111871
30min / C	P14847	0.207631	0.50864	0.612215	Crp	C-reactive p	CRP_MOUS	4	1.15479

15min / C	P62962	0.141736	0.508949	0.612215	Pfn1	Profilin-1	PROF1_MOU	9	1.103232
15min / C	P70349	0.033124	0.508103	0.612215	Hint1	Adenosine 5	HINT1_MOU	7	1.023225
30min / C	Q3UUI3	-0.07803	0.508017	0.612215	Them4	Acyl-coenzy	THEM4_MO	5	0.947351
15min / C	Q62093	0.016167	0.507766	0.612215	Srsf2	Serine/argi	SRSF2_MOU	7	1.011269
15min / C	Q64726	-0.11304	0.508081	0.612215	Azgp1	Zinc-alpha-	ZA2G_MOU	12	0.924637
30min / C	Q80SY6	-0.54066	0.508984	0.612215	Adal	Adenosine c	ADAL_MOU	4	0.687456
15min / C	Q80VP1	0.106899	0.508623	0.612215	Epn1	Epsin-1	EPN1_MOU	10	1.076911
30min / C	Q9DCY0	0.254109	0.508303	0.612215	Keg1	Glycine N-a	KEG1_MOU	15	1.192599
15min / C	P05202	-0.05296	0.50975	0.612869	Got2	Aspartate a	AATM_MOU	35	0.963959
15min / C	O54983	0.279185	0.510185	0.612886	Crym	Ketimine re	CRYM_MOU	8	1.213509
15min / C	Q05793	0.084205	0.510252	0.612886	Hspg2	Basement m	PGBM_MOU	37	1.060103
15min / C	Q8K1B8	0.148554	0.510544	0.612886	Fermt3	Fermitin fa	URP2_MOU	7	1.108458
30min / C	Q9QXE0	0.118083	0.51098	0.613073	Hacl1	2-hydroxya	HACL1_MO	30	1.085292
15min / C	P10922	0.147696	0.512036	0.613703	H1-0	Histone H1	H10_MOUS	7	1.107799
30min / C	P14685	-0.18765	0.512091	0.613703	Psmd3	26S proteas	PSMD3_MO	28	0.878036
30min / C	Q9QZ08	0.112884	0.512147	0.613703	Nagk	N-acetyl-D-	NAGK_MOU	9	1.081388
30min / C	Q9WVJ3	-0.0015	0.511775	0.613703	Cpq	Carboxypep	CBPQ_MOU	11	0.998958
15min / C	O89020	-0.21635	0.513166	0.614612	Afm	Afamin	AFAM_MOU	12	0.860739
30min / C	P01897	-0.3954	0.51334	0.614612	H2-L	H-2 class I h	HA1L_MOU	9	0.760277
15min / C	P62830	0.394074	0.513435	0.614612	Rpl23	Large ribos	RL23_MOU	7	1.314099
15min / C	Q8K3K8	-0.22311	0.513548	0.614612	Optn	Optineurin	OPTN_MOU	6	0.856716
15min / C	D3YXK2	1.123555	0.514193	0.614737	Safb	Scaffold att	SAFB1_MO	10	2.178832
15min / C	P56382	-0.21328	0.513953	0.614737	Atp5f1e	ATP synthas	ATP5E_MO	2	0.862572
30min / C	P63158	0.063706	0.514055	0.614737	Hmgb1	High mobil	HMGB1_M	10	1.045147
30min / C	Q8K2Q7	0.150273	0.514295	0.614737	Brox	BRO1 doma	BROX_MOU	5	1.10978
15min / C	P97350	0.144917	0.514897	0.614918	Pkp1	Plakophilin	PKP1_MOU	4	1.105667
15min / C	Q52KI8	-0.13151	0.514697	0.614918	Srrm1	Serine/argi	SRRM1_MO	5	0.912877
30min / C	Q91V09	0.221969	0.514962	0.614918	Wdr13	WD repeat-	WDR13_M	7	1.166325
15min / C	Q8BH97	0.015063	0.515632	0.615412	Rcn3	Reticulocal	RCN3_MOU	3	1.010496
15min / C	P61514	-0.02998	0.516107	0.615609	Rpl37a	Large ribos	RL37A_MO	4	0.979432
30min / C	P97290	0.021143	0.516043	0.615609	Serping1	Plasma pro	IC1_MOUSE	12	1.014763
15min / C	Q9JMD3	0.116263	0.516433	0.615609	Stard10	START dom	STA10_MO	15	1.083924
15min / C	P16858	-0.0631	0.516877	0.615667	Gapdh	Glyceraldeh	G3P_MOUS	22	0.957207
30min / C	Q9D8S4	-0.13289	0.516786	0.615667	Rexo2	Oligoribonu	ORN_MOUS	6	0.912004
15min / C	P97865	0.428774	0.517154	0.615844	Pex7	Peroxisoma	PEX7_MOU	4	1.346089
30min / C	P35908	0.258601	0.517813	0.616475	KRT2	Keratin, typ	K22E_HUM	39	1.196318
30min / C	Q99MZ7	-0.25417	0.51838	0.616704	Pecr	Peroxisoma	PECR_MOU	13	0.838472
15min / C	Q9D5V6	-0.4369	0.518392	0.616704	Syap1	Synapse-ass	SYAP1_MO	8	0.738722
30min / C	P16460	-0.07762	0.519032	0.617004	Ass1	Argininosuc	ASSY_MOUS	26	0.947621
15min / C	P67984	0.115879	0.518925	0.617004	Rpl22	Large ribos	RL22_MOU	3	1.083635
15min / C	Q9Z0J0	-0.00874	0.518905	0.617004	Npc2	NPC intrace	NPC2_MOU	6	0.993963
30min / C	B1AY13	0.537108	0.519617	0.617199	Usp24	Ubiquitin c	UBP24_MO	27	1.45106
15min / C	P68510	-0.10057	0.519377	0.617199	Ywhah	14-3-3 prot	1433F_MO	9	0.932664
30min / C	Q6ZWX6	0.025726	0.520274	0.617199	Eif2s1	Eukaryotic	IF2A_MOUS	21	1.017992
15min / C	Q80YC5	-0.13012	0.520276	0.617199	F12	Coagulation	FA12_MOU	8	0.913757
30min / C	Q8BIJ6	-0.02952	0.519714	0.617199	Iars2	Isoleucine--	SYIM_MOU	31	0.979743
15min / C	Q8BND5	0.112212	0.520345	0.617199	Qsox1	Sulfhydryl c	QSOX1_MO	15	1.080884
15min / C	O55131	-0.01443	0.52144	0.61777	Septin7	Septin-7	SEPT7_MOU	16	0.990047
15min / C	Q9D8N0	0.098901	0.521333	0.61777	Eef1g	Elongation	EF1G_MOU	25	1.070957
15min / C	Q64433	-0.08077	0.521836	0.618032	Hspe1	10 kDa heat	CH10_MOU	9	0.945556
15min / C	P82343	0.176757	0.521972	0.61804	Renbp	N-acylgluco	RENBP_MO	2	1.13034
30min / C	Q9JHH9	0.289056	0.522549	0.618263	Copz2	Coatomer s	COPZ2_MO	3	1.22184

30min / C	Q9JKF1	-0.27473	0.52232	0.618263	Iqgap1	Ras GTPase-	IQGA1_MO	37	0.826602
30min / C	E9Q0S6	-0.73661	0.522945	0.618468	Tns1	Tensin-1	TENS1_MO	21	0.600149
30min / C	O08795	-0.16903	0.522981	0.618468	Prkcsh	Glucosidase	GLU2B_MO	18	0.889438
15min / C	Q00897	0.087777	0.52436	0.618644	Serpina1d	Alpha-1-ant	A1AT4_MO	2	1.062731
15min / C	Q6NXH9	1.698321	0.524189	0.618644	Krt73	Keratin, typ	K2C73_MO	1	3.24523
30min / C	Q8CIH9	-0.01047	0.523928	0.618644	Ppat	Amidophos	PUR1_MOU	11	0.992767
15min / C	Q9JLV1	-0.24407	0.523434	0.618644	Bag3	BAG family	BAG3_MOU	28	0.844357
30min / C	P25688	-0.02756	0.525003	0.619022	Uox	Uricase	URIC_MOU	22	0.981079
30min / C	Q9R1P1	-0.12832	0.525408	0.619347	Psmb3	Proteasom	PSB3_MOU	10	0.914894
15min / C	O35350	-0.36133	0.527423	0.619426	Capn1	Calpain-1 c	CAN1_MOU	23	0.778447
15min / C	O70370	0.478502	0.527479	0.619426	Ctss	Cathepsin S	CATS_MOU	5	1.393296
15min / C	P28063	-0.11805	0.526321	0.619426	Psmb8	Proteasom	PSB8_MOU	12	0.921435
30min / C	P32261	0.214825	0.526984	0.619426	Serpinc1	Antithromb	ANT3_MOU	26	1.160563
15min / C	P56959	-0.07445	0.52742	0.619426	Fus	RNA-bindin	FUS_MOUS	7	0.949703
30min / C	P62270	-0.14188	0.527678	0.619426	Rps18	Small ribos	RS18_MOU	11	0.906337
15min / C	P62751	0.248886	0.526983	0.619426	Rpl23a	Large ribos	RL23A_MO	9	1.188289
30min / C	P62908	-0.19125	0.527583	0.619426	Rps3	Small ribos	RS3_MOUS	19	0.875844
15min / C	Q7TQH0	-0.20724	0.526432	0.619426	Atxn2l	Ataxin-2-lik	ATX2L_MO	21	0.866195
15min / C	Q9CZS1	0.112548	0.527267	0.619426	Aldh1b1	Aldehyde d	AL1B1_MO	28	1.081136
30min / C	Q9D1Q6	0.223006	0.52739	0.619426	Erp44	Endoplasm	ERP44_MO	15	1.167163
30min / C	Q9QXD1	-0.17563	0.526955	0.619426	Acox2	Peroxisoma	ACOX2_MO	31	0.88538
15min / C	Q9Z2K1	0.440325	0.526167	0.619426	Krt16	Keratin, typ	K1C16_MO	4	1.35691
30min / C	Q99L13	-0.02741	0.528574	0.620248	Hibadh	3-hydroxyis	3HIDH_MO	13	0.981178
15min / C	P51174	-0.01411	0.529742	0.621023	Acadl	Long-chain	ACADL_MO	21	0.990267
30min / C	Q9D7G0	0.086901	0.529688	0.621023	Prps1	Ribose-pho	PRPS1_MO	13	1.062086
30min / C	Q9CQW3	-0.09214	0.52995	0.621025	Proz	Vitamin K-c	PROZ_MOU	2	0.938127
15min / C	Q91VI7	0.032543	0.530231	0.621202	Rnh1	Ribonuclea	RINI_MOUS	26	1.022813
30min / C	D3YZP9	0.113371	0.530507	0.621374	Ccdc6	Coiled-coil	CCDC6_MO	7	1.081753
30min / C	Q9D5J6	0.153663	0.531386	0.622098	Shpk	Sedoheptul	SHPK_MOU	17	1.11239
15min / C	Q6P5E4	-0.06148	0.531566	0.622156	Uggt1	UDP-glucos	UGGG1_MO	38	0.958279
15min / C	Q91VH6	-0.00753	0.531701	0.622162	Memo1	Protein ME	MEMO1_M	9	0.994797
30min / C	Q9DAK9	0.025004	0.531903	0.622246	Phpt1	14 kDa pho	PHP14_MO	6	1.017482
30min / C	Q8VBW8	-0.07849	0.533161	0.623412	Ttc36	Tetratricop	TTC36_MO	7	0.947047
30min / C	P34927	-0.73079	0.534659	0.624095	Asgr1	Asialoglyco	ASGR1_MO	8	0.602575
30min / C	Q61937	-0.00944	0.535212	0.624283	Npm1	Nucleopho	NPM_MOU	9	0.993477
30min / C	P23116	0.104617	0.535907	0.624561	Eif3a	Eukaryotic	EIF3A_MOU	50	1.075209
30min / C	P49312	-0.02297	0.535973	0.624561	Hnrnpa1	Heterogene	ROA1_MOU	10	0.984202
15min / C	Q9DBD0	0.049509	0.535597	0.624561	Inhca	Inhibitor of	ICA_MOUSI	30	1.034913
30min / C	P42932	-0.06651	0.53612	0.624581	Cct8	T-complex p	TCPQ_MOU	32	0.954947
30min / C	Q91WK5	0.060743	0.536484	0.624852	Gcsh	Glycine clea	GCSH_MOU	1	1.043003
15min / C	P56380	0.055298	0.53677	0.625001	Nudt2	Bis(5'-nucle	AP4A_MOU	7	1.039074
30min / C	Q9Z0J0	0.014217	0.536873	0.625001	Npc2	NPC intrace	NPC2_MOU	6	1.009903
15min / C	P70274	-0.00158	0.537313	0.625056	Selenop	Selenoprot	SEPP1_MO	6	0.998909
15min / C	Q61081	-0.0699	0.537211	0.625056	Cdc37	Hsp90 co-c	CDC37_MO	10	0.952707
30min / C	Q9WUM3	0.460927	0.537054	0.625056	Coro1b	Coronin-1B	COR1B_MO	10	1.376426
30min / C	Q8K2C6	-0.35663	0.537536	0.625163	Sirt5	NAD-depen	SIR5_MOUS	11	0.780985
30min / C	P58137	0.020158	0.538144	0.625395	Acot8	Acyl-coenzy	ACOT8_MO	7	1.014071
15min / C	Q9D1Q6	-0.03667	0.538316	0.625395	Erp44	Endoplasm	ERP44_MO	15	0.974903
30min / C	Q9JK92	0.238329	0.53825	0.625395	Hspb8	Heat shock	HSPB8_MO	4	1.179625
15min / C	P46656	-0.11284	0.538677	0.625425	Fdx1	Adrenodox	ADX_MOUS	4	0.924763
30min / C	P60229	-0.02417	0.539169	0.625693	Eif3e	Eukaryotic	EIF3E_MOU	19	0.983384
15min / C	Q9QZ85	-0.10771	0.539064	0.625693	Iigp1	Interferon-i	IIGP1_MOU	19	0.928063

30min / C	Q62189	-0.11304	0.540273	0.626764	Snrpa	U1 small nu	SNRPA_MO	9	0.924636
15min / C	Q9JHS9	0.615477	0.540355	0.626764	Cwc15	Spliceosom	CWC15_MO	2	1.532065
15min / C	P63037	-0.00389	0.541798	0.627981	Dnaja1	DnaJ homo	DNJA1_MO	13	0.997308
15min / C	Q62048	0.084435	0.542123	0.628053	Pea15	Astrocytic p	PEA15_MO	6	1.060273
15min / C	O70456	1.059547	0.542932	0.628557	Sfn	14-3-3 prot	1433S_MO	1	2.084277
30min / C	Q99MN1	-0.17749	0.543084	0.628557	Kars1	Lysine--tRN	SYK_MOUSI	23	0.884241
15min / C	O88533	-0.05819	0.543642	0.629051	Ddc	Aromatic-L	DDC_MOUS	15	0.960471
30min / C	Q99JI4	-0.17958	0.544482	0.629718	Psmd6	26S proteas	PSMD6_MO	24	0.882961
15min / C	P10518	-0.08044	0.545309	0.62976	Alad	Delta-amin	HEM2_MOU	23	0.945772
15min / C	P14131	0.044092	0.544831	0.62976	Rps16	Small ribos	RS16_MOU	9	1.031034
15min / C	P70335	0.15175	0.545166	0.62976	Rock1	Rho-associa	ROCK1_MO	29	1.110916
30min / C	P97493	-0.05502	0.545224	0.62976	Txn2	Thioredoxi	THIOM_MO	4	0.962583
15min / C	P02088	-0.10304	0.545539	0.629874	Hbb-b1	Hemoglobi	HBB1_MOU	13	0.931072
30min / C	Q62523	-0.07133	0.546088	0.630203	Zyx	Zyxin	ZYX_MOUSI	14	0.951763
15min / C	P70697	0.288525	0.547431	0.631144	Urod	Uroporphy	DCUP_MOU	13	1.221391
15min / C	O55229	-0.20838	0.547824	0.631445	Chkb	Choline/eth	CHKB_MOU	11	0.865511
15min / C	P02301;P8	-0.28866	0.548483	0.631601	H3-5;H3-3a	Histone H3	H3C_MOUS	4	0.818663
30min / C	Q3UFF7	0.270127	0.548361	0.631601	Lyplal1	Lysophospl	LYPL1_MOU	8	1.205914
30min / C	Q8R5I6	-0.11889	0.548145	0.631601	Gstm4	Glutathion	GSTM4_MO	9	0.920894
30min / C	Q9D855	0.211549	0.548488	0.631601	Uqcrb	Cytochrom	QCR7_MOU	5	1.157931
15min / C	Q6P3A8	0.133223	0.548852	0.631867	Bckdhb	2-oxoisoval	ODBB_MOU	13	1.096741
30min / C	Q60967	0.088246	0.548999	0.631884	Papss1	Bifunctiona	PAPS1_MO	10	1.063077
15min / C	O77727	-0.19773	0.550591	0.631948	KRT15	Keratin, typ	K1C15_SHE	10	0.871922
30min / C	P26231	-0.24493	0.551204	0.631948	Ctnna1	Catenin alp	CTNA1_MO	26	0.843858
30min / C	P28076	-0.15062	0.549474	0.631948	Psmb9	Proteasom	PSB9_MOU	7	0.900865
30min / C	P50396	0.019744	0.549933	0.631948	Gdi1	Rab GDP di	GDIA_MOU	17	1.01378
15min / C	Q78JE5	0.031661	0.55048	0.631948	Fbxo22	F-box only p	FBX22_MO	9	1.022188
15min / C	Q8CIH9	-0.14696	0.550534	0.631948	Ppat	Amidophos	PUR1_MOU	11	0.903153
30min / C	Q8VC97	0.205345	0.550472	0.631948	Upb1	Beta-ureido	BUP1_MOU	16	1.152962
15min / C	Q99KC8	-0.10192	0.550312	0.631948	Vwa5a	von Willebr	VMA5A_MO	25	0.931789
15min / C	Q9CZ04	0.237073	0.551302	0.631948	Cops7a	COP9 signa	CSN7A_MO	11	1.178599
30min / C	Q9Z2N8	-0.85509	0.549966	0.631948	Actl6a	Actin-like p	ACL6A_MO	8	0.552833
30min / C	Q99JW2	-0.09888	0.551674	0.632223	Acy1	Aminoacyla	ACY1_MOU	18	0.933755
15min / C	O35490	-0.07702	0.552485	0.632282	Bhmt	Betaine--ho	BHMT1_MO	28	0.948011
30min / C	O89079	0.304741	0.553064	0.632282	Cope	Coatomer s	COPE_MOU	11	1.235197
30min / C	P47963	-0.50311	0.552713	0.632282	Rpl13	Large ribos	RL13_MOU	10	0.705583
30min / C	P50172	-0.09007	0.552898	0.632282	Hsd11b1	11-beta-hy	DHI1_MOU	12	0.939475
15min / C	P62814	0.026054	0.553098	0.632282	Atp6v1b2	V-type prot	VATB2_MO	18	1.018223
15min / C	P70122	0.06742	0.553116	0.632282	Sbds	Ribosome r	SBDS_MOU	13	1.047841
30min / C	P70444	0.237878	0.552419	0.632282	Bid	BH3-intera	BID_MOUSI	5	1.179257
15min / C	Q63836	-0.08605	0.552259	0.632282	Selenbp2	Selenium-b	SBP2_MOU	36	0.942102
15min / C	Q8CCS6	0.839842	0.553181	0.632282	Pabpn1	Polyadenyl	PABP2_MO	4	1.789854
30min / C	Q80XI4	-0.51347	0.553966	0.632405	Pip4k2b	Phosphatid	PI42B_MOU	1	0.700533
30min / C	Q8K4H1	0.003908	0.554082	0.632405	Afmid	Kynurenine	KFA_MOUS	14	1.002713
15min / C	Q8QZY1	-0.18932	0.553844	0.632405	Eif3l	Eukaryotic	EIF3L_MOU	22	0.87702
30min / C	P24549	0.004503	0.554977	0.632822	Aldh1a1	Aldehyde d	AL1A1_MO	21	1.003126
15min / C	P84091	0.159969	0.555592	0.632961	Ap2m1	AP-2 compl	AP2M1_MO	15	1.117263
15min / C	Q61035	-0.51911	0.555502	0.632961	Hars1	Histidine--t	HARS1_MO	18	0.697803
15min / C	Q99LB2	0.308966	0.555442	0.632961	Dhrs4	Dehydroger	DHRS4_MO	15	1.23882
15min / C	Q99P31	0.040253	0.555761	0.632961	Hspbp1	Hsp70-binc	HPBP1_MO	7	1.028294
15min / C	Q9CQ89	-0.36039	0.555758	0.632961	Cuta	Protein Cut	CUTA_MOU	3	0.778953
15min / C	P62806	-0.03588	0.556304	0.633277	H4c1	Histone H4	H4_MOUSE	6	0.975435

15min / C	O08915	-0.15782	0.556658	0.633529	Aip	AH recepto	AIP_MOUSI	8	0.896375
15min / C	Q8BHN3	0.307259	0.557265	0.633918	Ganab	Neutral alp	GANAB_MC	39	1.237355
15min / C	P48678	0.218843	0.558071	0.634124	Lmna	Prelamin-A	LMNA_MOU	30	1.1638
15min / C	Q9D1F4	0.205646	0.557861	0.634124	Akt1s1	Proline-ricl	AKTS1_MOI	4	1.153203
30min / C	Q9QZS3	0.261336	0.558064	0.634124	Numb	Protein nur	NUMB_MO	8	1.198588
30min / C	Q9WU78	0.356067	0.557866	0.634124	Pdcd6ip	Programme	PDC6I_MO	38	1.279932
15min / C	P00375	0.528096	0.559079	0.634623	Dhfr	Dihydrofol	DYR_MOUS	10	1.442025
15min / C	Q9Z1Z2	0.068024	0.558863	0.634623	Strap	Serine-thre	STRAP_MO	16	1.04828
15min / C	P59242	-0.02509	0.559956	0.635166	Cgn	Cingulin	CING_MOU	32	0.982759
30min / C	Q64152	0.12429	0.560643	0.635644	Btf3	Transcripti	BTF3_MOU	5	1.089971
15min / C	P48193	-0.33883	0.560942	0.635832	Epb41	Protein 4.1	EPB41_MO	11	0.790684
15min / C	E9Q557	-0.24464	0.561246	0.635875	Dsp	Desmoplak	DESP_MOU	33	0.844025
15min / C	Q80VJ3	-0.20085	0.561173	0.635875	Dnph1	5-hydroxyn	DNPH1_MC	5	0.870039
30min / C	Q62261	-0.02043	0.562017	0.636597	Sptbn1	Spectrin be	SPTB2_MO	78	0.985936
15min / C	P01897	0.028536	0.562494	0.636986	H2-L	H-2 class I h	HA1L_MOU	9	1.019976
30min / C	P63024	0.074844	0.562848	0.637236	Vamp3	Vesicle-assc	VAMP3_MC	3	1.053247
15min / C	P50462	0.734299	0.563242	0.637381	Csrp3	Cysteine an	CSRP3_MO	5	1.663589
15min / C	O35405	-0.12396	0.563989	0.637493	Pld3	5'-3' exonu	PLD3_MOU	4	0.917666
15min / C	P06683	0.337562	0.563654	0.637493	C9	Compleme	CO9_MOUS	15	1.26362
30min / C	Q8K2Q0	-0.45973	0.563514	0.637493	Commd9	COMM don	COMD9_M	9	0.727123
30min / C	P99029	0.051456	0.565549	0.638565	Prdx5	Peroxiredo	PRDX5_MC	12	1.03631
30min / C	Q64442	0.052338	0.566025	0.638565	Sord	Sorbitol de	DHSO_MOU	23	1.036944
15min / C	Q8BZB2	0.06946	0.566025	0.638565	Ppcdc	Phosphopa	COAC_MOU	3	1.049324
15min / C	Q8K023	0.070909	0.565695	0.638565	Akr1c18	Aldo-keto r	AKC1H_MC	6	1.050378
30min / C	P04919	-0.58612	0.568671	0.639162	Slc4a1	Band 3 anic	B3AT_MOU	12	0.666131
15min / C	P11438	0.137802	0.569036	0.639162	Lamp1	Lysosome-a	LAMP1_MC	5	1.100228
15min / C	P15532	0.022428	0.569498	0.639162	Nme1	Nucleoside	NDKA_MOU	7	1.015667
30min / C	P43883	0.23315	0.567432	0.639162	Plin2	Perilipin-2	PLIN2_MOU	11	1.175399
15min / C	P50516	0.083066	0.56963	0.639162	Atp6v1a	V-type prot	VATA_MOU	27	1.059267
15min / C	P70318	-0.33959	0.569357	0.639162	Tial1	Nucleolysir	TIAR_MOUS	7	0.790265
15min / C	Q6IFX2	-0.17246	0.568535	0.639162	Krt42	Keratin, typ	K1C42_MO	4	0.887327
15min / C	Q8BGT5	0.448458	0.567821	0.639162	Gpt2	Alanine am	ALAT2_MO	21	1.364581
15min / C	Q8C0L6	0.119842	0.569607	0.639162	Paox	Peroxisoma	PAOX_MOU	6	1.086616
30min / C	Q8K3C3	-0.17418	0.568028	0.639162	Lzic	Protein LZI	LZIC_MOUS	5	0.886273
15min / C	Q99PG2	0.527896	0.568259	0.639162	Ogfr	Opioid grov	OGFR_MOU	11	1.441825
30min / C	Q9CPU0	-0.08051	0.568123	0.639162	Glo1	Lactoylglut	LGUL_MOU	13	0.945724
15min / C	Q9D051	0.546748	0.568109	0.639162	Pdhb	Pyruvate de	ODPB_MOU	17	1.460789
15min / C	Q9QYJ0	-0.07619	0.569547	0.639162	Dnaja2	DnaJ homo	DNJA2_MO	17	0.94856
15min / C	Q9Z2I9	0.166192	0.566936	0.639162	Sucla2	Succinate--	SUCB1_MO	25	1.122093
15min / C	P20029	-0.12158	0.569922	0.63934	Hspa5	Endoplasm	BIP_MOUSI	38	0.919178
30min / C	Q8K003	0.014315	0.570066	0.639351	Tma7	Translation	TMA7_MOU	1	1.009972
30min / C	P62073	0.126822	0.570404	0.63943	Timm10	Mitochond	TIM10_MO	2	1.091886
15min / C	Q01405	-0.02724	0.570401	0.63943	Sec23a	Protein trai	SC23A_MO	28	0.981294
15min / C	P09411	0.16134	0.570936	0.639727	Pgk1	Phosphogly	PGK1_MOU	30	1.118326
15min / C	P39039	0.544185	0.570923	0.639727	Mbl1	Mannose-b	MBL1_MOU	3	1.458196
30min / C	P05208	0.287347	0.571116	0.639779	Cela2a	Chymotryp	CEL2A_MO	2	1.220394
30min / C	Q9CR16	0.198828	0.571422	0.639971	Ppid	Peptidyl-pr	PPID_MOU	20	1.147766
30min / C	Q6A0A9	-0.38371	0.571903	0.640343	FAM120A	Constitutiv	F120A_MO	32	0.766464
15min / C	Q9WUU7	0.132817	0.572022	0.640343	Ctsz	Cathepsin Z	CATZ_MOU	8	1.096432
30min / C	P70694	-0.08783	0.572338	0.640538	Akr1c6	Estradiol 17	DHB5_MOU	20	0.940936
15min / C	Q8R0Y6	0.200933	0.572687	0.64063	Aldh1l1	Cytosolic 1	AL1L1_MO	70	1.149441
30min / C	Q8BGT5	-0.03122	0.573159	0.640715	Gpt2	Alanine am	ALAT2_MO	21	0.978595

15min / C	Q8BK30	0.368315	0.57439	0.641942	Ndufv3	NADH dehy	NDUV3_MC	2	1.290845
30min / C	Q99K30	-0.33298	0.574632	0.642062	Eps8l2	Epidermal g	ES8L2_MOU	23	0.793897
15min / C	P12382	-0.01106	0.57488	0.642189	Pfkl	ATP-depend	PFKAL_MOU	25	0.992362
30min / C	Q61508	0.194078	0.575789	0.642271	Ecm1	Extracellula	ECM1_MOU	4	1.143993
30min / C	Q8BHN3	0.162956	0.57595	0.642271	Ganab	Neutral alp	GANAB_MC	39	1.119578
15min / C	Q8CEI1	-0.43775	0.575616	0.642271	Bola3	BolA-like pr	BOLA3_MO	4	0.738287
15min / C	Q99MP8	0.114525	0.575547	0.642271	Brap	BRCA1-asso	BRAP_MOU	4	1.082618
15min / C	Q9WV85	0.116548	0.575847	0.642271	Nme3	Nucleoside	NDK3_MOU	5	1.084138
15min / C	Q91XL9	-0.00607	0.57637	0.642503	Osbpl1a	Oxysterol-b	OSBL1_MO	11	0.995801
30min / C	Q9D0B5	-0.31886	0.576742	0.642583	Tstd3	Thiosulfate	TSTD3_MO	3	0.801705
15min / C	Q99K51	-0.00743	0.577661	0.642891	Pls3	Plastin-3	PLST_MOUS	39	0.994861
30min / C	Q9QZ88	0.018353	0.578354	0.643363	Vps29	Vacuolar pr	VPS29_MO	8	1.012802
30min / C	O08738	0.379589	0.579329	0.643998	Casp6	Caspase-6	CASP6_MO	8	1.300971
15min / C	Q99KP6	-0.16867	0.57921	0.643998	Prpf19	Pre-mRNA-p	PRP19_MO	14	0.889663
15min / C	P01635	-0.09306	0.580054	0.644355	Igkv12-41	Immunoglo	KV5A3_MO	2	0.937534
30min / C	Q9WUU7	0.053493	0.579948	0.644355	Ctsz	Cathepsin Z	CATZ_MOU	8	1.037775
30min / C	P53811	-0.01909	0.581132	0.645102	Pitpnb	Phosphatid	PIPNB_MO	14	0.986858
15min / C	P62311	-0.20537	0.581437	0.645142	Lsm3	U6 snRNA-a	LSM3_MOU	3	0.867318
30min / C	Q03265	-0.12841	0.581591	0.645162	Atp5f1a	ATP syntha	ATPA_MOU	32	0.914839
30min / C	O08601	-0.00866	0.582105	0.645433	Mttp	Microsoma	MTP_MOUS	42	0.994019
30min / C	P09103	-0.07758	0.582921	0.645629	P4hb	Protein dis	PDIA1_MO	37	0.947647
30min / C	P63001	0.236404	0.582825	0.645629	Rac1	Ras-related	RAC1_MOU	8	1.178053
15min / C	Q3ULD5	0.010352	0.583113	0.645629	Mccc2	Methylcrot	MCCB_MOU	25	1.007201
15min / C	Q91VR8	-0.14326	0.583227	0.645629	Brk1	Protein BRI	BRK1_MOU	3	0.905473
15min / C	Q9D753	0.071221	0.582826	0.645629	Exosc8	Exosome co	EXOS8_MO	5	1.050605
30min / C	Q99JY3	-0.68817	0.583363	0.64563	Gimap4	GTPase IMA	GIMA4_MC	10	0.62064
15min / C	P54823	-0.07798	0.584112	0.645645	Ddx6	Probable AT	DDX6_MOU	14	0.947381
15min / C	Q61189	0.201172	0.584041	0.645645	Clns1a	Methylosor	ICLN_MOUS	1	1.149632
30min / C	Q61206	0.082863	0.584323	0.645645	Pafah1b2	Platelet-act	PA1B2_MO	5	1.059118
30min / C	Q9CQA3	-0.21804	0.584284	0.645645	Sdhb	Succinate d	SDHB_MOU	10	0.859734
15min / C	Q9R008	-0.23632	0.583894	0.645645	Mvk	Mevalonate	KIME_MOU	13	0.84891
30min / C	P11983	-0.02745	0.585678	0.645914	Tcp1	T-complex p	TCPA_MOU	29	0.981156
30min / C	Q80X90	0.029549	0.585345	0.645914	Flnb	Filamin-B	FLNB_MOU	110	1.020693
30min / C	Q8K010	0.117268	0.585034	0.645914	Oplah	5-oxoprolir	OPLA_MOU	42	1.084679
15min / C	Q91ZE0	0.266566	0.585297	0.645914	Tmlhe	Trimethylly	TMLH_MOU	7	1.202941
15min / C	Q9CT10	0.055071	0.585074	0.645914	Ranbp3	Ran-bindin	RANB3_MC	13	1.03891
30min / C	Q9DBR7	-0.18607	0.58537	0.645914	Ppp1r12a	Protein pho	MYPT1_MC	16	0.878995
30min / C	Q9JMA1	-0.05411	0.585917	0.645914	Usp14	Ubiquitin c	UBP14_MC	19	0.96319
30min / C	Q99L20	-0.07755	0.586061	0.645924	Gstt3	Glutathion	GSTT3_MO	10	0.947664
15min / C	P05213	-0.0566	0.586481	0.646089	Tuba1b	Tubulin alp	TBA1B_MO	21	0.961527
15min / C	Q9JMH6	0.038975	0.586768	0.646256	Txnrd1	Thioredoxi	TRXR1_MO	22	1.027383
15min / C	P51880	-0.31449	0.586981	0.646341	Fabp7	Fatty acid-b	FABP7_MO	4	0.804134
30min / C	P13634	-0.11322	0.58748	0.646723	Ca1	Carbonic ar	CAH1_MOU	14	0.924523
30min / C	P24547	0.007036	0.587598	0.646723	Impdh2	Inosine-5'-r	IMDH2_MC	19	1.004889
15min / C	Q8QZR3	0.123824	0.588052	0.646925	Ces2a	Pyrethroid	EST2A_MOU	15	1.08962
30min / C	Q7TQH0	-0.31216	0.588471	0.647088	Atxn2l	Ataxin-2-lik	ATX2L_MO	21	0.805437
15min / C	O70362	-0.00627	0.58938	0.647416	Gpld1	Phosphatid	PHLD_MOU	12	0.995666
15min / C	P61759	0.221758	0.589124	0.647416	Vbp1	Prefoldin su	PFD3_MOU	7	1.166154
15min / C	Q78JW9	0.058583	0.589447	0.647416	Ubfd1	Ubiquitin d	UBFD1_MC	5	1.041442
15min / C	Q8VC97	-0.17138	0.589442	0.647416	Upb1	Beta-ureido	BUP1_MOU	16	0.887992
15min / C	Q91YD9	0.254025	0.589418	0.647416	Wasl	Actin nucle	WASL_MOU	10	1.19253
15min / C	P24369	-0.00144	0.590752	0.648161	Ppib	Peptidyl-pr	PPIB_MOUS	10	0.998999

30min / C	Q9QYC0	-0.09752	0.590803	0.648161	Add1	Alpha-addu	ADDA_MOU	11	0.934638
30min / C	P59999	-0.05123	0.59107	0.648305	Arpc4	Actin-relate	ARPC4_MO	11	0.965111
15min / C	Q9QUM9	-0.03222	0.591487	0.648465	Psma6	Proteasom	PSA6_MOU	17	0.977916
30min / C	P47757	-0.07367	0.591781	0.648639	Capzb	F-actin-cap	CAPZB_MO	14	0.95022
30min / C	P01657	0.015395	0.592444	0.648902	NaN	Ig kappa ch	KV3A5_MO	2	1.010728
30min / C	Q8K3J1	-0.12326	0.592314	0.648902	Ndufs8	NADH dehy	NDUS8_MC	4	0.91811
30min / C	Q9DD18	-0.46022	0.592564	0.648902	Dtd1	D-aminoac	DTD1_MOU	3	0.726878
15min / C	P14211	0.063448	0.59303	0.649263	Calr	Calreticulir	CALR_MOU	25	1.04496
30min / C	Q9R0M5	0.505526	0.593228	0.649332	Tpk1	Thiamine p	TPK1_MOU	7	1.419641
30min / C	Q922E4	-0.19868	0.593802	0.649811	Pcyt2	Ethanolami	PCY2_MOU	23	0.871344
15min / C	Q8R1S4	-0.10775	0.594857	0.650424	Mtss1	Protein MT	MTSS1_MO	10	0.928036
15min / C	Q99L45	0.221621	0.594753	0.650424	Eif2s2	Eukaryotic	IF2B_MOUS	17	1.166043
30min / C	Q8VIJ6	0.008211	0.595343	0.650752	Sfpq	Splicing fac	SFPQ_MOU	23	1.005708
30min / C	Q91YR1	-0.05995	0.595654	0.650795	Twf1	Twinfilin-1	TWF1_MOU	12	0.959298
30min / C	P46638	-0.16909	0.59607	0.650951	Rab11b	Ras-related	RB11B_MO	12	0.889402
15min / C	P52430	-0.61591	0.596231	0.650951	Pon1	Serum para	PON1_MOU	6	0.652519
15min / C	Q8BH95	0.014086	0.596342	0.650951	Echs1	Enoyl-CoA I	ECHM_MOU	18	1.009811
15min / C	Q99PT1	-0.03462	0.596263	0.650951	Arhgdia	Rho GDP-di	GDIR1_MO	14	0.976286
30min / C	Q99JB8	0.206645	0.597453	0.651687	Pacsin3	Protein kin	PACN3_MC	10	1.154001
30min / C	Q99LU0	0.022743	0.597562	0.651687	Chmp1b1	Charged mu	CH1B1_MC	2	1.015889
15min / C	Q9R0M5	0.30879	0.597305	0.651687	Tpk1	Thiamine p	TPK1_MOU	7	1.238668
30min / C	P62307	-0.12722	0.598604	0.652228	Snrpf	Small nucle	RUXF_MOU	4	0.915592
15min / C	Q3THE2	0.092511	0.598584	0.652228	Myl12b	Myosin regi	ML12B_MC	7	1.066224
15min / C	Q99LP6	0.034368	0.598207	0.652228	Grpel1	GrpE protei	GRPE1_MO	11	1.024108
15min / C	Q9DBP5	0.029978	0.598535	0.652228	Cmpk1	UMP-CMP I	KCY_MOUS	12	1.020996
30min / C	Q8K1L5	0.21532	0.598784	0.652276	Ppp1r11	E3 ubiquiti	PP1RB_MO	3	1.160962
30min / C	P70697	-0.13835	0.599142	0.652369	Urod	Uroporphy	DCUP_MOU	13	0.908558
30min / C	Q9ES00	0.181801	0.599047	0.652369	Ube4b	Ubiquitin c	UBE4B_MO	11	1.134299
30min / C	P17156	-0.00574	0.599499	0.652609	Hspa2	Heat shock	HSP72_MO	21	0.99603
15min / C	P13707	0.000772	0.600617	0.652934	Gpd1	Glycerol-3-	GPDA_MOU	24	1.000535
30min / C	Q99K51	0.024153	0.599979	0.652934	Pls3	Plastin-3	PLST_MOUS	39	1.016882
30min / C	Q9D8N0	-0.24509	0.600359	0.652934	Eef1g	Elongation	EF1G_MOU	25	0.843762
15min / C	Q9DCU9	0.025621	0.600265	0.652934	Hoga1	4-hydroxy-	HOGA1_MC	16	1.017918
15min / C	P62754	-0.10524	0.600978	0.653046	Rps6	Small ribos	RS6_MOUS	8	0.929651
30min / C	Q02819	-0.0072	0.601581	0.653387	Nucb1	Nucleobinc	NUCB1_MC	22	0.995019
30min / C	Q8R1B4	-0.2228	0.601933	0.653621	Eif3c	Eukaryotic	EIF3C_MOU	23	0.856903
15min / C	O70318	-0.27899	0.603835	0.654517	Epb41l2	Band 4.1-lil	E41L2_MOU	19	0.82417
15min / C	P43277	-0.03753	0.603199	0.654517	H1-3	Histone H1	H13_MOUS	10	0.97432
15min / C	P47963	-0.01386	0.603603	0.654517	Rpl13	Large ribos	RL13_MOU	10	0.990438
15min / C	Q8VCF0	-0.17394	0.603662	0.654517	Mavs	Mitochond	MAVS_MOU	13	0.886416
15min / C	P33622	-0.27128	0.604056	0.654573	Apoc3	Apolipopro	APOC3_MC	3	0.828586
15min / C	Q64105	-0.08741	0.604179	0.654573	Spr	Sepiapterin	SPRE_MOU	14	0.941212
30min / C	O35405	-0.04559	0.604713	0.654706	Pld3	5'-3' exonu	PLD3_MOU	4	0.968892
15min / C	Q76MZ3	0.006336	0.604632	0.654706	Ppp2r1a	Serine/thre	2AAA_MOU	26	1.004401
30min / C	P53810	0.157279	0.604861	0.654718	Pitpna	Phosphatic	PIPNA_MO	9	1.115182
30min / C	Q3V0K9	-0.1932	0.605389	0.65483	Pls1	Plastin-1	PLSI_MOUS	22	0.874662
15min / C	Q60631	0.127575	0.6063	0.65483	Grb2	Growth fac	GRB2_MOU	5	1.092456
15min / C	Q91WG0	0.166557	0.606036	0.65483	Ces2c	Acylcarniti	EST2C_MOU	11	1.122377
30min / C	Q61781	0.213699	0.606951	0.655017	Krt14	Keratin, typ	K1C14_MO	6	1.159658
15min / C	Q8R0F9	0.073724	0.606832	0.655017	Sec14l4	SEC14-like	S14L4_MOU	23	1.052429
30min / C	Q9Z1D1	-0.05457	0.607056	0.655017	Eif3g	Eukaryotic	EIF3G_MOU	12	0.962884
15min / C	Q9QZS3	0.129384	0.607339	0.655175	Numb	Protein nur	NUMB_MO	8	1.093826

15min / C	A2AKK5	0.216117	0.608764	0.656116	Acnat1	Acyl-coenzy	ACNT1_MO	12	1.161603
15min / C	O35309	0.316242	0.608725	0.656116	Nmi	N-myc-inte	NMI_MOUS	13	1.245083
30min / C	O88746	-0.59658	0.610471	0.656116	Tom1	Target of M	TOM1_MOU	12	0.661321
15min / C	P07724	-0.05365	0.609965	0.656116	Alb	Albumin	ALBU_MOU	48	0.963498
15min / C	P08071	0.025524	0.608513	0.656116	Ltf	Lactotransf	TRFL_MOUS	8	1.01785
15min / C	P62849	0.494808	0.610099	0.656116	Rps24	Small ribos	RS24_MOU	5	1.409133
15min / C	P97314	-0.29979	0.610482	0.656116	Csrp2	Cysteine an	CSRP2_MO	5	0.812369
15min / C	Q61133	-0.06674	0.610261	0.656116	Gstt2	Glutathion	GSTT2_MO	13	0.954795
30min / C	Q61247	-0.07999	0.609981	0.656116	Serpinf2	Alpha-2-ant	A2AP_MOU	12	0.946066
30min / C	Q99KK2	0.188036	0.609983	0.656116	Cmas	N-acylneura	NEUA_MOU	12	1.139212
15min / C	Q9ERG0	0.033754	0.610659	0.656116	Lima1	LIM domair	LIMA1_MO	28	1.023672
30min / C	Q9QYI5	-0.31883	0.610333	0.656116	Dnajb2	DnaJ homo	DNJB2_MO	3	0.801721
30min / C	Q8CBW3	0.260493	0.610891	0.656193	Abi1	Abl interact	ABI1_MOUS	10	1.197888
15min / C	P18760	-0.07124	0.611105	0.656274	Cfl1	Cofilin-1	COF1_MOU	17	0.951818
30min / C	Q3TW96	0.147481	0.611241	0.656274	Uap1l1	UDP-N-acet	UAP1L_MO	20	1.107634
15min / C	Q6ZPJ0	-0.01949	0.611801	0.656728	Tex2	Testis-expre	TEX2_MOU	5	0.986578
15min / C	P62869	0.010215	0.612282	0.657097	Elob	Elongin-B	ELOB_MOU	10	1.007106
15min / C	O54754	-0.07669	0.613085	0.657363	Aox1	Aldehyde o	AOXA_MOU	50	0.948228
15min / C	P01786;P0	-0.28057	0.613027	0.657363	NaN	Ig heavy cha	HVM17_M	1	0.823265
15min / C	P21981	0.034795	0.61322	0.657363	Tgm2	Protein-glu	TGM2_MOU	34	1.024411
30min / C	P46412	-0.2329	0.613493	0.657363	Gpx3	Glutathion	GPX3_MOU	4	0.850923
30min / C	Q8R0N6	-0.18806	0.613165	0.657363	Adhfe1	Hydroxyaci	HOT_MOUS	18	0.877788
15min / C	P97447	0.214174	0.613978	0.657462	Fhl1	Four and a l	FHL1_MOU	5	1.160039
15min / C	P14206	-0.14664	0.615373	0.65805	Rpsa	Small ribos	RSSA_MOU	14	0.903351
30min / C	Q8BWW4	-0.14863	0.615126	0.65805	Larp4	La-related p	LARP4_MO	10	0.902109
30min / C	Q8C1B7	-0.07081	0.615334	0.65805	Septin11	Septin-11	SEP11_MO	15	0.952104
30min / C	Q9EPL9	0.568089	0.614975	0.65805	Acox3	Peroxisoma	ACOX3_MC	11	1.482559
30min / C	O55142	-0.05724	0.616074	0.658178	Rpl35a	Large ribos	RL35A_MO	8	0.9611
30min / C	P01786;P0	-0.21092	0.615936	0.658178	NaN	Ig heavy cha	HVM17_M	1	0.863988
15min / C	P21107	-0.03317	0.615834	0.658178	Tpm3	Tropomyos	TPM3_MOU	6	0.977272
15min / C	Q61655	-0.49829	0.616181	0.658178	Ddx19a	ATP-depend	DD19A_MC	14	0.707945
15min / C	Q8JZV9	-0.58052	0.615854	0.658178	Bdh2	Dehydroge	DHRS6_MC	6	0.668725
30min / C	O70493	0.083033	0.617509	0.659302	Snx12	Sorting nex	SNX12_MO	4	1.059243
30min / C	Q3TWW8	0.166406	0.617767	0.659429	Srsf6	Serine/argi	SRSF6_MOU	6	1.122259
30min / C	P63260	0.738869	0.618077	0.659466	Actg1	Actin, cyto	ACTG_MOU	1	1.668867
15min / C	Q99P27	-0.51073	0.617942	0.659466	Pla2g12b	Group XIIB	PG12B_MC	6	0.701865
15min / C	Q9WVA3	0.163106	0.618528	0.6598	Bub3	Mitotic che	BUB3_MOU	9	1.119695
15min / C	P16125	-0.1001	0.61924	0.660264	Ldhb	L-lactate de	LDHB_MOU	12	0.932968
30min / C	Q8C6I2	-0.0897	0.619434	0.660325	Sdhaf2	Succinate d	SDHF2_MO	1	0.939719
15min / C	P97450	-0.03547	0.619647	0.660404	Atp5pf	ATP syntha:	ATP5J_MOU	5	0.975712
30min / C	Q9WVA4	-0.00626	0.620296	0.660801	Tagln2	Transgelin-	TAGL2_MO	13	0.995673
15min / C	Q99JX3	-0.49398	0.620511	0.660883	Gorasp2	Golgi reass	GORS2_MC	9	0.710062
30min / C	O54879	0.303534	0.620943	0.661039	Hmgb3	High mobil	HMGB3_M	4	1.234164
15min / C	O88844	0.286545	0.622232	0.661039	Idh1	Isocitrate d	IDHC_MOU	32	1.219716
30min / C	P35585	-0.00402	0.622907	0.661039	Ap1m1	AP-1 compl	AP1M1_MC	17	0.997218
30min / C	P61161	-0.11478	0.62327	0.661039	Actr2	Actin-relate	ARP2_MOU	15	0.923526
30min / C	P70158	-0.02168	0.62161	0.661039	Smpdl3a	Cyclic GMP	ASM3A_MC	3	0.985088
15min / C	P80315	-0.10119	0.622614	0.661039	Cct4	T-complex	TCPD_MOU	26	0.932261
15min / C	Q60864	-0.01357	0.621682	0.661039	Stip1	Stress-indu	STIP1_MOU	29	0.990638
15min / C	Q6NZR5	0.542225	0.62099	0.661039	Skic2	Superkiller	SKI2_MOUS	11	1.456216
15min / C	Q8CIE6	-0.20077	0.621446	0.661039	Copa	Coatomer s	COPA_MOU	56	0.870084
15min / C	Q8R146	0.049265	0.623045	0.661039	Apeh	Acylamino-	ACPH_MOU	26	1.034737

15min / C	Q91WS4	-0.00993	0.621545	0.661039	Bhmt2	S-methylm	BHMT2_MO		18	0.993143
30min / C	Q9WTX6	-0.01755	0.623424	0.661039	Cul1	Cullin-1	CUL1_MOU		23	0.987907
15min / C	P11589	0.077566	0.623764	0.661253	Mup2	Major urina	MUP2_MO		2	1.055236
30min / C	P83741	0.219958	0.624292	0.661475	Wnk1	Serine/thre	WNK1_MO		7	1.1647
30min / C	Q920A5	0.156586	0.624875	0.661698	Scpep1	Retinoid-in	RISC_MOUS		7	1.114646
30min / C	P07309	-0.09914	0.625068	0.661755	Ttr	Transthyret	TTHY_MOU		6	0.933587
30min / C	Q921G7	-0.48504	0.626463	0.662206	Etfdh	Electron tra	ETFD_MOU		24	0.714479
15min / C	Q9CQK7	0.171722	0.626226	0.662206	Rwdd1	RWD doma	RWDD1_M		3	1.126402
15min / C	Q91VX2	-0.25562	0.627053	0.662583	Ubap2	Ubiquitin-a	UBAP2_MO		6	0.837628
15min / C	Q9CX34	-0.03174	0.627097	0.662583	Sugt1	Protein SG	SGT1_MOU		14	0.978242
30min / C	Q8BP56	0.286517	0.627333	0.662685	Pgghg	Protein-glu	PGGHG_MO		7	1.219692
15min / C	Q60967	0.287871	0.627849	0.662938	Papss1	Bifunctiona	PAPS1_MO		10	1.220838
30min / C	Q9Z1Q9	0.152342	0.628202	0.663164	Vars1	Valine--tRN	SYVC_MOU		44	1.111373
15min / C	Q9CPT4	-0.01242	0.62835	0.663174	Mydgf	Myeloid-de	MYDGF_MO		6	0.991426
15min / C	Q9D8T7	0.124419	0.628645	0.663338	Slirp	SRA stem-lo	SLIRP_MOU		3	1.090068
15min / C	P18242	-0.08502	0.629091	0.663356	Ctsd	Cathepsin D	CATD_MOU		13	0.942772
30min / C	P56380	0.280458	0.629217	0.663356	Nudt2	Bis(5'-nucle	AP4A_MOU		7	1.21458
15min / C	O35643	-0.0438	0.629421	0.663425	Ap1b1	AP-1 compl	AP1B1_MO		42	0.970094
30min / C	P14131	-0.00877	0.629732	0.663606	Rps16	Small ribos	RS16_MOU		9	0.993942
15min / C	Q8R164	-0.01432	0.630121	0.66387	Bphl	Valacyclovi	BPHL_MOU		17	0.990127
30min / C	O09173	0.001883	0.630619	0.663956	Hgd	Homogenti	HGD_MOUS		27	1.001306
15min / C	P51859	-0.06095	0.630582	0.663956	Hdgf	Hepatoma-	HDGF_MOU		13	0.958636
30min / C	Q8BWF0	0.157646	0.630548	0.663956	Aldh5a1	Succinate-s	SSDH_MOU		19	1.115466
30min / C	Q78JE5	0.140899	0.630777	0.663976	Fbxo22	F-box only	FBX22_MO		9	1.102592
15min / C	Q3V1L4	0.293142	0.631412	0.664206	Nt5c2	Cytosolic p	5NTC_MOU		16	1.225306
30min / C	Q8C4B4	-0.60338	0.631388	0.664206	Unc119b	Protein unc	U119B_MO		5	0.658209
15min / C	P42125	-0.03726	0.631607	0.664264	Eci1	Enoyl-CoA d	ECI1_MOUS		14	0.974503
30min / C	P62627	-0.30036	0.632436	0.664325	Dynlrb1	Dynein ligh	DLRB1_MO		5	0.812049
30min / C	Q8VDK1	-0.0025	0.632411	0.664325	Nit1	Deaminated	NIT1_MOUS		18	0.998271
30min / C	Q922B2	-0.43063	0.632328	0.664325	Dars1	Aspartate--t	SYDC_MOU		27	0.741935
30min / C	Q9JK23	-0.25923	0.632498	0.664325	Psmg1	Proteasome	PSMG1_MO		4	0.835536
15min / C	P40336	-0.21889	0.633368	0.664386	Vps26a	Vacuolar pr	VP26A_MO		16	0.859225
15min / C	P62918	0.098499	0.635327	0.664386	Rpl8	Large ribos	RL8_MOUS		11	1.070659
15min / C	Q03265	-0.04649	0.634153	0.664386	Atp5f1a	ATP syntha	ATPA_MOU		32	0.96829
30min / C	Q60864	0.060885	0.634062	0.664386	Stip1	Stress-indu	STIP1_MOU		29	1.043106
15min / C	Q61029	-0.18928	0.634746	0.664386	Tmpo	Lamina-ass	LAP2B_MO		11	0.877041
30min / C	Q62446	-0.08125	0.634828	0.664386	Fkbp3	Peptidyl-pr	FKBP3_MO		8	0.945238
30min / C	Q6ZWV7	0.002785	0.634259	0.664386	Rpl35	Large ribos	RL35_MOU		4	1.001932
15min / C	Q8VI75	0.268369	0.635065	0.664386	Ipo4	Importin-4	IPO4_MOU		14	1.204446
30min / C	Q91W89	-0.0664	0.634774	0.664386	Man2c1	Alpha-man	MA2C1_MO		13	0.955018
15min / C	Q91YI0	-0.05481	0.634557	0.664386	Asl	Argininosu	ARLY_MOU		27	0.962722
30min / C	Q920Q4	-0.27885	0.634736	0.664386	Vps16	Vacuolar pr	VPS16_MO		6	0.824251
30min / C	Q9D826	-0.04226	0.635099	0.664386	Pipox	Peroxisoma	SOX_MOUS		20	0.971131
30min / C	Q9QXT0	0.077344	0.632864	0.664386	Cnpy2	Protein can	CNPY2_MO		8	1.055074
15min / C	Q62165	-0.29503	0.635717	0.664638	Dag1	Dystroglyca	DAG1_MOU		8	0.815056
15min / C	P36552	0.035112	0.636028	0.664673	Cpox	Oxygen-dep	HEM6_MOU		20	1.024636
15min / C	Q62422	-0.15958	0.635933	0.664673	Ostf1	Osteoclast-	OSTF1_MO		8	0.895283
15min / C	P12265	-0.10577	0.636943	0.664824	Gusb	Beta-glucur	BGLR_MOU		21	0.929306
30min / C	Q8BMF4	0.051059	0.63697	0.664824	Dlat	Dihydrolipo	ODP2_MOU		19	1.036025
30min / C	Q8VCH0	-0.12476	0.637365	0.664824	Acaa1b	3-ketoacyl-	THIKB_MOU		22	0.917158
15min / C	Q921H8	-0.21549	0.636716	0.664824	Acaa1a	3-ketoacyl-	THIKA_MOU		9	0.861254
15min / C	Q9CZN7	-0.03051	0.636346	0.664824	Shmt2	Serine hydr	GLYM_MOU		30	0.979076

30min / C	Q9D8X2	-0.08273	0.637425	0.664824	Ccdc124	Coiled-coil	CC124_MO	6	0.944268
30min / C	Q9QY76	-0.23365	0.636936	0.664824	Vapb	Vesicle-ass	VAPB_MOU	6	0.850481
15min / C	Q9R1P3	-0.03996	0.637225	0.664824	Psmb2	Proteasom	PSB2_MOU	10	0.972681
30min / C	Q9WUP7	-0.1275	0.637198	0.664824	Uchl5	Ubiquitin c	UCHL5_MC	9	0.915416
15min / C	Q9DBR1	-0.02773	0.638733	0.665753	Xrn2	5'-3' exorib	XRN2_MOU	8	0.980965
15min / C	Q62188	0.136982	0.639318	0.666072	Dpysl3	Dihydropyr	DPYL3_MO	17	1.099602
30min / C	P08030	-0.0526	0.639501	0.666118	Aprt	Adenine ph	APT_MOUS	8	0.964199
30min / C	Q7TNG5	0.068755	0.640125	0.666416	Eml2	Echinoderm	EMAL2_MC	16	1.048811
30min / C	Q8R0F9	0.010854	0.639956	0.666416	Sec14l4	SEC14-like	S14L4_MO	23	1.007552
30min / C	P21460	0.014191	0.640703	0.666643	Cst3	Cystatin-C	CYTC_MOU	3	1.009885
15min / C	Q6ZWX6	0.028419	0.641392	0.667211	Eif2s1	Eukaryotic	IF2A_MOUS	21	1.019894
15min / C	Q9CR57	0.181467	0.641528	0.667211	Rpl14	Large ribos	RL14_MOU	5	1.134037
30min / C	P29391	-0.07218	0.642116	0.667303	Ftl1	Ferritin ligh	FRIL1_MOU	12	0.951199
15min / C	O88668	-0.11712	0.64294	0.667456	Creg1	Protein CRI	CREG1_MO	4	0.922029
30min / C	P60521	0.148827	0.64302	0.667456	Gabarapl2	Gamma-am	GBRL2_MO	2	1.108668
15min / C	Q61781	-0.18905	0.642939	0.667456	Krt14	Keratin, typ	K1C14_MO	6	0.877182
15min / C	P63330	0.073625	0.643307	0.667609	Ppp2ca	Serine/thre	PP2AA_MO	14	1.052357
30min / C	P51410	0.056623	0.643532	0.667651	Rpl9	Large ribos	RL9_MOUS	10	1.040028
15min / C	O08997	-0.08304	0.644013	0.667738	Atox1	Copper tran	ATOX1_MO	5	0.944065
15min / C	Q61249	-0.19508	0.64427	0.667738	Igbp1	Immunoglo	IGBP1_MO	10	0.873526
15min / C	Q8BMJ3	0.035088	0.644204	0.667738	Eif1ax	Eukaryotic	IF1AX_MOU	2	1.024619
15min / C	Q9D379	-0.28136	0.645066	0.668129	Ephx1	Epoxide hy	HYEP_MOU	22	0.822815
15min / C	Q8BGL3	-0.05645	0.645455	0.668387	Sult2a8	Sulfotransf	ST2A8_MO	13	0.961628
15min / C	Q8BJF9	0.185627	0.646394	0.668848	Chmp2b	Charged mu	CHM2B_M	2	1.137311
30min / C	Q9EQS3	0.307321	0.646281	0.668848	Mycbp	c-Myc-bind	MYCBP_MC	6	1.237408
15min / C	Q9JLT4	0.038817	0.646271	0.668848	Txnrd2	Thioredoxi	TRXR2_MO	14	1.027271
15min / C	Q9CQ86	0.070736	0.647255	0.668978	Mien1	Migration a	MIEN1_MO	3	1.050252
30min / C	Q9JM14	0.367699	0.64716	0.668978	Nt5c	5'(3')-deoxy	NT5C_MOU	7	1.290293
15min / C	Q9JMH9	0.177705	0.64709	0.668978	Myo18a	Unconvent	MY18A_MC	42	1.131083
30min / C	P21107	0.084994	0.647739	0.669302	Tpm3	Tropomyos	TPM3_MOU	6	1.060683
30min / C	Q01279	-0.03928	0.648284	0.669574	Egfr	Epidermal g	EGFR_MOU	23	0.973142
30min / C	Q8R050	0.120131	0.648422	0.669574	Gspt1	Eukaryotic	ERF3A_MO	21	1.086834
30min / C	P61971	0.089971	0.648603	0.669616	Nutf2	Nuclear tra	NTF2_MOU	6	1.064349
30min / C	Q9D6Y7	0.166331	0.649	0.669881	Msra	Mitochond	MSRA_MOU	14	1.122201
15min / C	O54931	-0.09239	0.649392	0.670141	Pakap	PALM2-AKA	AKAP2_MO	7	0.937965
15min / C	Q91X83	-0.10124	0.65015	0.670779	Mat1a	S-adenosyl	METK1_MC	17	0.932229
15min / C	P45376	-0.04936	0.650456	0.670804	Akr1b1	Aldo-keto r	ALDR_MOU	24	0.966367
30min / C	Q8BH97	0.293504	0.650348	0.670804	Rcn3	Reticulocal	RCN3_MOU	3	1.225613
15min / C	O70435	-0.01366	0.652225	0.67117	Psma3	Proteasom	PSA3_MOU	14	0.990578
15min / C	Q3UI43	-0.48888	0.6522	0.67117	Babam1	BRISC and E	BABA1_MO	5	0.712577
15min / C	Q61508	0.121453	0.652025	0.67117	Ecm1	Extracellula	ECM1_MOU	4	1.08783
15min / C	Q7TMB8	0.359985	0.651974	0.67117	Cyfip1	Cytoplasmi	CYFP1_MO	32	1.283413
15min / C	Q921X9	0.113487	0.651858	0.67117	Pdia5	Protein dis	PDIA5_MO	14	1.08184
30min / C	Q9CXA2	0.064293	0.651614	0.67117	L3hypdh	Trans-L-3-h	T3HPD_MC	11	1.045573
15min / C	Q9EPV8	-0.23387	0.651449	0.67117	Ubl5	Ubiquitin-l	UBL5_MOU	3	0.850353
15min / C	Q9Z1F9	-0.15533	0.652369	0.67117	Uba2	SUMO-activ	SAE2_MOU	15	0.897926
15min / C	Q99KI0	0.051061	0.652875	0.671272	Aco2	Aconitate h	ACON_MOU	39	1.036027
15min / C	Q9D2G2	-0.02597	0.652743	0.671272	Dlst	Dihydrolip	ODO2_MOU	16	0.98216
15min / C	O08601	0.21255	0.653244	0.671493	Mttp	Microsoma	MTP_MOUS	42	1.158735
30min / C	Q9Z1T1	0.117121	0.653539	0.671521	Ap3b1	AP-3 compl	AP3B1_MO	21	1.084568
30min / C	Q60902	-0.04065	0.653916	0.67162	Eps15l1	Epidermal g	EP15R_MO	18	0.972214
30min / C	O89017	0.084045	0.654542	0.671918	Lgmn	Legumain	LGMN_MO	4	1.059986

15min / C	Q3TRM8	0.986282	0.654628	0.671918	Hk3	Hexokinase	HXK3_MOU	8	1.981073
15min / C	P62075	-0.01067	0.655094	0.672158	Timm13	Mitochond	TIM13_MO	6	0.992634
30min / C	P11276	0.02329	0.656673	0.672165	Fn1	Fibronectir	FINC_MOUS	60	1.016275
15min / C	Q5U5V2	-0.12986	0.655351	0.672165	Hykk	Hydroxylys	HYKK_MOU	15	0.913919
15min / C	Q9CX80	0.003976	0.656339	0.672165	Cygb	Cytoglobin	CYGB_MOU	12	1.00276
15min / C	Q9JHI7	-0.36143	0.655999	0.672165	Exosc9	Exosome cc	EXOS9_MO	6	0.778392
15min / C	Q9WVQ5	0.089433	0.655514	0.672165	Apip	Methylthio	MTNB_MOU	11	1.063952
30min / C	O88544	-0.16099	0.657196	0.672403	Cops4	COP9 signa	CSN4_MOU	17	0.89441
15min / C	Q61160	-0.30666	0.658743	0.67325	Fadd	FAS-associa	FADD_MOU	3	0.808512
15min / C	O88986	-0.02621	0.6599	0.674145	Gcat	2-amino-3-	KBL_MOUS	13	0.981993
15min / C	Q9CPX6	0.22072	0.660075	0.67418	Atg3	Ubiquitin-l	ATG3_MOU	8	1.165315
15min / C	P50172	0.315115	0.660387	0.674187	Hsd11b1	11-beta-hy	DHI1_MOU	12	1.244111
15min / C	Q9CXI5	0.066003	0.660506	0.674187	Manf	Mesenceph	MANF_MOU	11	1.046812
15min / C	P48428	0.024435	0.661527	0.674593	Tbca	Tubulin-spe	TBCA_MOU	7	1.017081
15min / C	Q9WV54	-0.11604	0.66175	0.674593	Asah1	Acid ceram	ASAH1_MO	8	0.922714
15min / C	P11276	-0.02842	0.664905	0.674638	Fn1	Fibronectir	FINC_MOUS	60	0.980493
30min / C	P13707	0.014896	0.663159	0.674638	Gpd1	Glycerol-3-	GPDA_MOU	24	1.010379
15min / C	P34884	0.00369	0.665313	0.674638	Mif	Macrophag	MIF_MOUS	6	1.002561
15min / C	P49429	0.069527	0.664679	0.674638	Hpd	4-hydroxyp	HPPD_MOU	23	1.049372
30min / C	P49429	0.058051	0.665024	0.674638	Hpd	4-hydroxyp	HPPD_MOU	23	1.041058
15min / C	P56387	-0.22979	0.66283	0.674638	Dynlt3	Dynein ligh	DYLT3_MO	2	0.852756
15min / C	P70445	-0.22554	0.665323	0.674638	Eif4ebp2	Eukaryotic	4EBP2_MO	4	0.855275
15min / C	Q8VBT2	0.068987	0.663015	0.674638	Sds	L-serine del	SDHL_MOU	16	1.04898
30min / C	Q91YD9	-0.51403	0.664455	0.674638	Wasl	Actin nucle	WASL_MOU	10	0.700264
30min / C	Q99PG0	-0.18149	0.66465	0.674638	Aadac	Arylacetam	AAAD_MOU	11	0.881793
30min / C	Q9CPW2	-0.01201	0.664897	0.674638	Fdx2	Ferredoxin-	FDX2_MOU	3	0.991713
15min / C	Q9DBT5	-0.07715	0.662243	0.674638	Ampd2	AMP deami	AMPD2_MO	11	0.947931
15min / C	Q9R0X4	-0.1885	0.66207	0.674638	Acot9	Acyl-coenzy	ACOT9_MO	12	0.877518
30min / C	Q6NVE8	-0.01104	0.665716	0.674674	Wdr44	WD repeat-	WDR44_M	6	0.992376
15min / C	Q91W89	0.35213	0.666476	0.674674	Man2c1	Alpha-mani	MA2C1_MO	13	1.276444
30min / C	Q9Z1P6	-0.24922	0.665982	0.674674	Ndufa7	NADH dehy	NDUA7_MO	5	0.841352
30min / C	P56391	0.399667	0.666998	0.674974	Cox6b1	Cytochrom	CX6B1_MO	6	1.319203
30min / C	P63017	0.045039	0.667567	0.675112	Hspa8	Heat shock	HSP7C_MO	28	1.031711
30min / C	Q64374	-0.0316	0.667369	0.675112	Rgn	Regucalcin	RGN_MOUS	24	0.978336
30min / C	Q9DCL2	-0.20287	0.667626	0.675112	Ciao2a	Cytosolic ir	CIA2A_MOU	2	0.868822
30min / C	Q8VCC1	0.143002	0.668461	0.675527	Hpgd	15-hydroxy	PGDH_MOU	9	1.104201
15min / C	Q922E4	-0.13146	0.668241	0.675527	Pcyt2	Ethanolami	PCY2_MOU	23	0.912905
30min / C	Q9EPK6	0.000332	0.668407	0.675527	Sil1	Nucleotide	SIL1_MOUS	3	1.00023
15min / C	Q8C1B7	-0.06729	0.668648	0.675573	Septin11	Septin-11	SEP11_MOU	15	0.95443
30min / C	Q99J36	-0.11232	0.668919	0.675704	Thumpd1	THUMP dor	THUM1_MO	8	0.925097
30min / C	P05784	-0.0272	0.669705	0.675837	Krt18	Keratin, typ	K1C18_MO	29	0.981323
30min / C	Q61171	0.082647	0.66947	0.675837	Prdx2	Peroxiredo	PRDX2_MO	14	1.058959
30min / C	Q7TQI3	-0.01287	0.669593	0.675837	Otub1	Ubiquitin t	OTUB1_MO	12	0.991122
15min / C	Q9D115	0.082274	0.669722	0.675837	Znf706	Zinc finger	ZN706_MO	2	1.058686
15min / C	O55135	0.060298	0.670386	0.676168	Eif6	Eukaryotic	IF6_MOUSE	11	1.042681
15min / C	Q9CQR6	0.098628	0.67024	0.676168	Ppp6c	Serine/thre	PPP6_MOU	8	1.070755
30min / C	Q9QZN4	-0.26223	0.67051	0.676168	Fbxo6	F-box only	FBX6_MOU	8	0.833797
30min / C	O35459	-0.02953	0.671114	0.676207	Ech1	Delta(3,5)-I	ECH1_MOU	15	0.979743
15min / C	P40124	-0.00858	0.670737	0.676207	Cap1	Adenylyl cy	CAP1_MOU	21	0.99407
30min / C	Q571E4	0.114366	0.670996	0.676207	Galns	N-acetylgal	GALNS_MO	11	1.0825
30min / C	Q8VCX1	0.124831	0.672167	0.676982	Akr1d1	Aldo-keto r	AK1D1_MO	21	1.09038
15min / C	Q9EQU5	-0.09021	0.672541	0.677074	Set	Protein SET	SET_MOUSI	7	0.939387

30min / C	Q9DB15	-0.22522	0.673217	0.677469	Mrpl12	Large ribos	RM12_MOU	9	0.855466
30min / C	O88986	0.003102	0.673443	0.677554	Gcat	2-amino-3-	KBL_MOUS	13	1.002153
30min / C	P51150	-0.3106	0.674336	0.67779	Rab7a	Ras-related	RAB7A_MO	13	0.806304
15min / C	Q8CBW3	0.021025	0.67465	0.677912	Abi1	Abl interact	ABI1_MOUS	10	1.01468
30min / C	P63323	-0.02822	0.675329	0.678001	Rps12	Small ribos	RS12_MOU	8	0.98063
30min / C	Q8CIB5	-0.2041	0.675726	0.678001	Fermt2	Fermitin fa	FERM2_MO	25	0.868081
15min / C	Q8VE70	-0.02728	0.675731	0.678001	Pdcd10	Programme	PDC10_MO	6	0.981269
30min / C	Q99LB2	0.228462	0.675677	0.678001	Dhrs4	Dehydroge	DHRS4_MO	15	1.171586
15min / C	Q9CQW3	-0.22909	0.675643	0.678001	Proz	Vitamin K-c	PROZ_MOU	2	0.853174
15min / C	Q9DC61	0.031807	0.675388	0.678001	Pmpca	Mitochond	MPPA_MOU	19	1.022292
30min / C	P04117	-0.13586	0.676639	0.678627	Fabp4	Fatty acid-b	FABP4_MO	9	0.910128
30min / C	O55229	-0.14059	0.676873	0.678718	Chkb	Choline/eth	CHKB_MOU	11	0.90715
15min / C	Q9CXN7	-0.03641	0.677045	0.678749	Pbld2	Phenazine b	PBLD2_MO	19	0.975081
30min / C	Q80XI3	-0.03923	0.677792	0.679355	Eif4g3	Eukaryotic	IF4G3_MOU	30	0.973173
15min / C	Q8VCH0	-0.10656	0.678179	0.679446	Acaa1b	3-ketoacyl-	THIKB_MOU	22	0.928802
30min / C	Q9R092	-0.1817	0.678309	0.679446	Hsd17b6	17-beta-hy	H17B6_MO	9	0.881666
30min / C	O35593	-0.12366	0.679219	0.679548	Psmd14	26S protea	PSDE_MOU	12	0.917855
15min / C	P32921	0.147608	0.679127	0.679548	Wars1	Tryptophar	SYWC_MOU	18	1.107732
15min / C	P60766	0.091432	0.678927	0.679548	Cdc42	Cell divisio	CDC42_MO	8	1.065427
30min / C	Q03958	-0.1967	0.679023	0.679548	Pfdn6	Prefoldin su	PFD6_MOU	5	0.872541
30min / C	Q8C7G5	0.135711	0.679264	0.679548	Apoa5	Apolipoprc	APOA5_MO	4	1.098634
15min / C	Q9D7B6	0.354147	0.679491	0.679633	Acad8	Isobutyryl-	ACAD8_MO	20	1.27823
30min / C	Q9D142	0.046803	0.679664	0.679664	Nudt14	Uridine dip	NUD14_MO	7	1.032973
30min / C	Q9JLI6	-0.0965	0.679811	0.679668	Scly	Selenocyste	SCLY_MOUS	20	0.935299
15min / C	Q91WU5	-0.10728	0.680269	0.679842	As3mt	Arsenite me	AS3MT_MO	17	0.928337
30min / C	Q03249	0.088595	0.680835	0.680124	Galt	Galactose-1	GALT_MOU	10	1.063334
30min / C	Q7TSV4	-0.0031	0.680702	0.680124	Pgm2	Phosphope	PGM2_MOU	19	0.997855
15min / C	O08749	0.00762	0.681066	0.680152	Dld	Dihydrolipo	DLDH_MOU	22	1.005296
30min / C	P57784	-0.17896	0.681338	0.6802	Snrpa1	U2 small nu	RU2A_MOU	6	0.883341
15min / C	Q8K3C3	-0.10821	0.681709	0.680428	Lzic	Protein LZIC	LZIC_MOUS	5	0.927736
30min / C	Q64176	-0.04986	0.682099	0.680675	Ces1e	Carboxyles	EST1E_MOU	19	0.966029
15min / C	Q80W21	0.264824	0.682419	0.680853	Gstm7	Glutathion	GSTM7_MO	8	1.201489
30min / C	Q80Y81	-0.03983	0.682575	0.680866	Elac2	Zinc phosph	RNZ2_MOU	12	0.972767
15min / C	Q9DBG1	0.21582	0.682747	0.680895	Cyp27a1	Sterol 26-h	CP27A_MO	14	1.161364
15min / C	Q9R1P0	0.043734	0.682944	0.68095	Psma4	Proteasom	PSA4_MOU	14	1.030778
30min / C	P42669	-0.03496	0.683703	0.681422	Pura	Transcripti	PURA_MOU	8	0.976061
30min / C	Q9DCM2	0.039304	0.683893	0.681469	Gstk1	Glutathion	GSTK1_MO	17	1.027618
30min / C	P50462	0.230042	0.684298	0.681731	Csrp3	Cysteine an	CSRP3_MO	5	1.172869
30min / C	O55125	0.34947	0.685101	0.681923	Nipsnap1	Protein Nip	NIPS1_MOU	13	1.274093
30min / C	P62900	-0.05348	0.684887	0.681923	Rpl31	Large ribos	RL31_MOU	7	0.96361
15min / C	Q8BH35	0.242224	0.685205	0.681923	C8b	Compleme	CO8B_MOU	12	1.182815
30min / C	Q8K370	0.047957	0.685029	0.681923	Acad10	Acyl-CoA de	ACD10_MO	31	1.0338
30min / C	A2AQ19	-0.32757	0.687235	0.682207	Rtf1	RNA polym	RTF1_MOU	4	0.796876
30min / C	B2RXS4	0.12607	0.685973	0.682207	Plxnb2	Plexin-B2	PLXB2_MO	6	1.091317
15min / C	O35386	0.036907	0.687109	0.682207	Phyh	Phytanoyl-	PAHX_MOU	17	1.025912
30min / C	P16015	-0.02315	0.686704	0.682207	Ca3	Carbonic ar	CAH3_MOU	20	0.98408
15min / C	P29758	0.276346	0.687345	0.682207	Oat	Ornithine a	OAT_MOUS	19	1.211124
15min / C	P48758	0.019697	0.686525	0.682207	Cbr1	Carbonyl re	CBR1_MOU	16	1.013746
15min / C	Q8VEB4	0.141161	0.685967	0.682207	Pla2g15	Lysosomal	PAG15_MO	6	1.102792
15min / C	Q9DC50	0.041727	0.687276	0.682207	Crot	Peroxisoma	OCTC_MOU	26	1.029346
15min / C	Q9R111	-0.00011	0.686079	0.682207	Gda	Guanine de	GUAD_MOU	20	0.999924
15min / C	Q9WVA4	0.023735	0.68727	0.682207	Tagln2	Transgelin-	TAGL2_MO	13	1.016588

15min / C	O88587	0.260608	0.687752	0.682327	Comt	Catechol O-	COMT_MOU		14	1.197984
15min / C	Q61696	-0.02595	0.688217	0.682506	Hspa1a	Heat shock	HS71A_MO		21	0.982175
30min / C	P12970	-0.12281	0.690194	0.682509	Rpl7a	Large ribos	RL7A_MOU		13	0.918399
15min / C	P23249	-0.175	0.692177	0.682509	Mov10	Putative he	MOV10_MO		23	0.885765
30min / C	P24456	-0.20388	0.691346	0.682509	Cyp2d10	Cytochrom	CP2DA_MO		25	0.868214
15min / C	P50136	-0.00436	0.690345	0.682509	Bckdha	2-oxoisoval	ODBA_MOU		20	0.996985
30min / C	P50431	-0.01335	0.692058	0.682509	Shmt1	Serine hydr	GLYC_MOU		24	0.99079
15min / C	P53811	0.085273	0.693361	0.682509	Pitpnb	Phosphatid	PIPNB_MO		14	1.060889
15min / C	Q00519	-0.019	0.692752	0.682509	Xdh	Xanthine d	XDH_MOUS		52	0.986916
15min / C	Q60936	0.215663	0.691417	0.682509	Coq8a	Atypical kir	COQ8A_MO		20	1.161237
15min / C	Q61191	0.232083	0.689642	0.682509	Hcfc1	Host cell fa	HCFC1_MO		27	1.174529
15min / C	Q62348	-0.01976	0.69322	0.682509	Tsn	Translin	TSN_MOUS		13	0.986399
15min / C	Q64520	-0.37186	0.689452	0.682509	Guk1	Guanylate k	KGUA_MOU		4	0.772787
15min / C	Q6PE15	0.872401	0.688737	0.682509	Abhd10	Palmitoyl-p	ABHDA_MO		2	1.830707
15min / C	Q80W22	-0.05252	0.692843	0.682509	Thnsl2	Threonine s	THNS2_MO		25	0.964248
30min / C	Q8BND5	-0.00061	0.691274	0.682509	Qsox1	Sulfhydryl c	QSOX1_MO		15	0.999578
30min / C	Q91VA6	0.26402	0.691369	0.682509	Poldip2	Polymerase	PDIP2_MO		10	1.20082
15min / C	Q9JIF0	0.865073	0.691833	0.682509	Prmt1	Protein arg	ANM1_MO		11	1.821432
15min / C	Q9QY76	-0.23679	0.692755	0.682509	Vapb	Vesicle-asso	VAPB_MOU		6	0.848634
15min / C	Q9R1Z8	-0.03417	0.689865	0.682509	Sorbs3	Vinexin	VINEX_MOU		5	0.976595
15min / C	Q9WVE8	0.081158	0.691065	0.682509	Pacsin2	Protein kin	PACN2_MO		13	1.057867
30min / C	O70318	-0.16838	0.693815	0.682816	Epb41l2	Band 4.1-lil	E41L2_MO		19	0.889844
30min / C	Q9Z2M7	0.059114	0.694103	0.682846	Pmm2	Phosphoma	PMM2_MO		12	1.041826
30min / C	Q62266	-0.07295	0.694369	0.682939	Sprr1a	Cornifin-A	SPR1A_MO		9	0.950691
30min / C	Q78JT3	-0.01946	0.694528	0.682955	Haao	3-hydroxya	3HAO_MOU		21	0.986605
15min / C	P24527	0.014705	0.695537	0.683196	Lta4h	Leukotrien	LKHA4_MO		26	1.010245
30min / C	P70195	-0.1254	0.695631	0.683196	Psmb7	Proteasom	PSB7_MOU		9	0.91675
30min / C	P01646	0.470783	0.697159	0.683852	NaN	Ig kappa ch	KV5AD_MO		1	1.385861
15min / C	P58044	0.086479	0.697541	0.683852	Idi1	Isopenteny	IDI1_MOUS		10	1.061776
30min / C	Q61730	0.016918	0.69759	0.683852	Il1rap	Interleukin	IL1AP_MOU		5	1.011796
15min / C	Q62384	-0.23719	0.698497	0.683852	Zpr1	Zinc finger p	ZPR1_MOU		8	0.848394
30min / C	Q8CF31	0.106527	0.698555	0.683852	Frey1	Protein Fre	FREY_MOUS		1	1.076633
15min / C	Q8CIM3	0.185525	0.698537	0.683852	D2hgdh	D-2-hydrox	D2HDH_MO		15	1.137231
15min / C	Q9D172	-0.0994	0.696691	0.683852	Gatd3	Glutamine	GAL3A_MO		12	0.933419
30min / C	Q9JKV1	-0.48664	0.697928	0.683852	Adrm1	Proteasom	ADRM1_MO		7	0.713687
30min / C	Q9Z2Y8	-0.03608	0.697448	0.683852	Plpbp	Pyridoxal p	PLPHP_MO		12	0.975304
30min / C	Q8VEB4	0.131642	0.698943	0.68406	Pla2g15	Lysosomal p	PAG15_MO		6	1.09554
15min / C	Q9CPU0	-0.02363	0.699172	0.684143	Glo1	Lactoylglut	LGUL_MOU		13	0.983753
15min / C	Q9CQM5	-0.10835	0.700139	0.684886	Txndc17	Thioredoxi	TXD17_MO		7	0.92765
30min / C	A3KMP2	-0.01082	0.701152	0.685107	Ttc38	Tetratricop	TTC38_MO		24	0.992532
15min / C	E9PV24	-0.43035	0.701999	0.685107	Fga	Fibrinogen	FIBA_MOUS		25	0.742081
30min / C	O08808	0.282119	0.702455	0.685107	Diaph1	Protein dia	DIAP1_MO		32	1.21598
30min / C	O70404	0.185298	0.70205	0.685107	Vamp8	Vesicle-asso	VAMP8_MO		4	1.137051
30min / C	O88342	0.020216	0.701493	0.685107	Wdr1	WD repeat-	WDR1_MO		27	1.014111
30min / C	P32233	-0.32227	0.701335	0.685107	Drg1	Developme	DRG1_MOU		8	0.79981
30min / C	Q91ZE0	-0.16436	0.702153	0.685107	Tmlhe	Trimethylly	TMLH_MOU		7	0.892323
15min / C	Q91ZJ5	-0.03374	0.700744	0.685107	Ugp2	UTP--gluco	UGPA_MOU		31	0.976884
15min / C	Q9D1R9	0.377446	0.701939	0.685107	Rpl34	Large ribos	RL34_MOU		5	1.299041
15min / C	Q9EQK5	0.115491	0.70258	0.685107	Mvp	Major vault	MVP_MOUS		43	1.083344
30min / C	Q9JMA2	0.217843	0.703531	0.685624	Qtrt1	Queuine tR	TGT_MOUS		1	1.162994
30min / C	Q8CJG0	0.178746	0.704199	0.685834	ago-02	Protein arg	AGO2_MOU		16	1.1319
30min / C	O55101	0.428768	0.70518	0.68633	Syngr2	Synaptogyr	SNG2_MOU		1	1.346084

15min / C	P97930	0.069229	0.7055	0.686357	Dtymk	Thymidylat	KTHY_MOU	5	1.049156
15min / C	Q91WK5	0.181281	0.705816	0.686357	Gcsh	Glycine cle	GCSH_MOU	1	1.133891
30min / C	Q9CR68	0.239264	0.705885	0.686357	Uqcrfs1	Cytochrom	UCRI_MOU	11	1.180391
30min / C	Q8VI36	0.031644	0.706925	0.686949	Pxn	Paxillin	PAXI_MOUS	15	1.022176
15min / C	Q80WT5	-0.00216	0.707078	0.686958	Aftph	Aftiphilin	AFTIN_MOU	5	0.998501
15min / C	Q9Z2M7	0.064066	0.707396	0.686988	Pmm2	Phosphoma	PMM2_MO	12	1.045408
30min / C	P84091	0.059199	0.707824	0.687264	Ap2m1	AP-2 compl	AP2M1_MO	15	1.041887
30min / C	P47955	-0.00018	0.708034	0.687328	Rplp1	Large ribos	RLA1_MOU	3	0.999877
15min / C	O09173	0.001926	0.709569	0.687592	Hgd	Homogenti	HGD_MOUS	27	1.001336
30min / C	O35658	-0.06656	0.708798	0.687592	C1qbp	Complemen	C1QBP_MO	5	0.954913
30min / C	O35737	-0.01711	0.709503	0.687592	Hnrnph1	Heterogene	HNRH1_MO	13	0.98821
15min / C	O88952	0.032534	0.710032	0.687592	Lin7c	Protein lin-	LIN7C_MOU	2	1.022807
30min / C	Q07968	0.320354	0.709571	0.687592	F13b	Coagulatio	F13B_MOU	1	1.248637
15min / C	Q3V0K9	-0.2846	0.709859	0.687592	Pls1	Plastin-1	PLSI_MOUS	22	0.820972
30min / C	Q8VED9	-0.13524	0.709283	0.687592	Lgalsl	Galectin-re	LEGL_MOU	4	0.910515
15min / C	Q9ESP1	-0.38669	0.708936	0.687592	Sdf2l1	Stromal cel	SDF2L_MOU	4	0.764885
15min / C	P01648;P0	0.432485	0.710344	0.687754	NaN	Ig kappa ch	KV5AF_MO	1	1.349556
15min / C	O08553	0.065993	0.710852	0.687838	Dpysl2	Dihydropyr	DPYL2_MO	22	1.046805
15min / C	P28076	-0.096	0.71179	0.688458	Psmb9	Proteasom	PSB9_MOU	7	0.935621
15min / C	Q05915	-0.11547	0.712019	0.688539	Gch1	GTP cycloh	GCH1_MOU	11	0.923084
15min / C	Q9DCX2	0.099228	0.712535	0.68876	Atp5pd	ATP synthas	ATP5H_MO	11	1.0712
30min / C	Q9R020	0.231942	0.712932	0.689004	Zranb2	Zinc finger	ZRAB2_MO	7	1.174415
30min / C	G3X9C2	0.407299	0.714819	0.689449	Nccrp1	F-box only	FBX50_MO	1	1.3262
15min / C	P07759	-0.03231	0.716315	0.689449	Serpina3k	Serine prot	SPA3K_MO	19	0.97785
30min / C	P28271	-0.03068	0.715406	0.689449	Aco1	Cytoplasmi	ACOHC_MO	49	0.97896
30min / C	P35527	0.115685	0.716583	0.689449	KRT9	Keratin, typ	K1C9_HUM	34	1.083489
30min / C	Q00724	-0.02886	0.715833	0.689449	Rbp4	Retinol-bin	RET4_MOU	8	0.980195
15min / C	Q5FW60	-0.06063	0.716567	0.689449	Mup20	Major urina	MUP20_MO	9	0.958844
30min / C	Q6IFX2	0.271694	0.714534	0.689449	Krt42	Keratin, typ	K1C42_MO	4	1.207224
30min / C	Q80UM3	-0.02861	0.715091	0.689449	Naa15	N-alpha-ace	NAA15_MO	18	0.980361
30min / C	Q8VC12	0.007597	0.714775	0.689449	Uroc1	Urocanate	HUTU_MOU	34	1.00528
30min / C	Q91V92	0.211883	0.71605	0.689449	Acly	ATP-citrate	ACLY_MOU	56	1.158199
15min / C	Q9CPZ8	-0.04203	0.714088	0.689449	Cmc1	COX assem	COXM1_MO	2	0.971288
30min / C	Q9ET54	-0.15026	0.716471	0.689449	Palld	Palladin	PALLD_MO	17	0.901089
15min / C	P25444	0.141549	0.71747	0.689764	Rps2	Small ribos	RS2_MOUS	15	1.103089
15min / C	P42669	0.024223	0.717466	0.689764	Pura	Transcripti	PURA_MOU	8	1.016932
30min / C	Q8K385	0.049508	0.718082	0.690074	FRRS1	Ferric-chela	FRRS1_MO	3	1.034912
30min / C	O35386	-0.14754	0.718495	0.690333	Phyh	Phytanoyl-	PAHX_MOU	17	0.902788
30min / C	P08228	-0.01624	0.718845	0.690392	Sod1	Superoxide	SODC_MOU	10	0.988807
15min / C	Q8K4M5	0.443166	0.719794	0.690887	Commd1	COMM dom	COMD1_M	3	1.359584
30min / C	O08583	0.106241	0.720412	0.69105	Alyref	THO compl	THOC4_MO	6	1.07642
30min / C	O88487	0.039863	0.720687	0.69105	Dync1i2	Cytoplasmi	DC1I2_MO	14	1.028016
15min / C	Q60605	-0.04754	0.720146	0.69105	Myl6	Myosin ligh	MYL6_MOU	10	0.967582
15min / C	Q8K385	0.026015	0.720275	0.69105	FRRS1	Ferric-chela	FRRS1_MO	3	1.018196
30min / C	Q9Z2U1	-0.08097	0.720834	0.691053	Psma5	Proteasom	PSA5_MOU	13	0.94542
30min / C	P97494	0.057508	0.72176	0.691399	Gclc	Glutamate-	GSH1_MOU	26	1.040667
30min / C	Q63880	-0.03382	0.721593	0.691399	Ces3a	Carboxyles	EST3A_MOU	20	0.976833
30min / C	Q9QZ85	-0.01547	0.722283	0.691609	Iigp1	Interferon-i	IIGP1_MOU	19	0.989335
30min / C	P70168	-0.15772	0.722799	0.691965	Kpnb1	Importin su	IMB1_MOU	34	0.896438
30min / C	Q9CZ04	-0.0184	0.723297	0.692164	Cops7a	COP9 signa	CSN7A_MO	11	0.987328
30min / C	Q9CZX8	-0.03173	0.723486	0.692207	Rps19	Small ribos	RS19_MOU	8	0.978246
30min / C	Q91W43	0.047537	0.723713	0.692285	Gldc	Glycine deh	GCSP_MOU	31	1.033499

15min / C	O70404	0.045466	0.724446	0.692694	Vamp8	Vesicle-asso	VAMP8_MO	4	1.032016
15min / C	P04264	-0.09727	0.724575	0.692694	KRT1	Keratin, typ	K2C1_HUM	38	0.934798
30min / C	Q99KP3	0.217053	0.724311	0.692694	Cryl1	Lambda-cry	CRYL1_MOU	14	1.162357
15min / C	P60229	-0.2815	0.725089	0.692769	Eif3e	Eukaryotic	EIF3E_MOU	19	0.822736
30min / C	Q91WU5	-0.05018	0.724941	0.692769	As3mt	Arsenite me	AS3MT_MO	17	0.965815
15min / C	Q61495;Q7	-0.3715	0.725364	0.692894	Dsg1a;Dsg1	Desmoglein	DSG1A_MO	1	0.772978
30min / C	P01648;P0	0.183427	0.725882	0.693071	NaN	Ig kappa ch	KV5AF_MO	1	1.135578
15min / C	Q99MZ7	-0.14247	0.72583	0.693071	Pecr	Peroxisoma	PECR_MOU	13	0.905964
30min / C	Q62422	0.070764	0.727042	0.693793	Ostf1	Osteoclast-	OSTF1_MOU	8	1.050273
15min / C	Q9D1J3	-0.03631	0.727267	0.693793	Sarnp	SAP domain	SARNP_MO	6	0.975146
15min / C	Q9Z2U1	-0.06204	0.72748	0.693806	Psma5	Proteasome	PSA5_MOU	13	0.95791
30min / C	P22892	0.063103	0.728125	0.694006	Ap1g1	AP-1 compl	AP1G1_MO	19	1.044711
30min / C	Q05793	-0.05331	0.727913	0.694006	Hspg2	Basement m	PGBM_MOU	37	0.96372
15min / C	A3KMP2	0.04821	0.72841	0.694096	Ttc38	Tetratricop	TTC38_MOU	24	1.033981
15min / C	P09813	-0.05483	0.72851	0.694096	Apoa2	Apolipopro	APOA2_MO	2	0.962708
30min / C	Q3UQ84	-0.14207	0.728666	0.694106	Tars2	Threonine--	SYTM_MOU	3	0.906216
15min / C	Q9CY58	-0.05128	0.729316	0.694448	Serbp1	SERPINE1 m	SERB1_MOU	20	0.965078
15min / C	Q8BSL7	0.068313	0.729935	0.694622	Arf2	ADP-ribosy	ARF2_MOU	5	1.04849
15min / C	D3YZP9	-0.20339	0.730973	0.69475	Ccdc6	Coiled-coil	CCDC6_MO	7	0.868505
15min / C	O70475	0.249468	0.730744	0.69475	Ugdh	UDP-glucos	UGDH_MOU	30	1.188769
30min / C	P62717	0.296698	0.731486	0.69475	Rpl18a	Large ribos	RL18A_MO	9	1.228329
15min / C	Q8K010	0.178312	0.730835	0.69475	Oplah	5-oxoprolir	OPLA_MOU	42	1.131559
30min / C	Q8VCF0	-0.08348	0.730428	0.69475	Mavs	Mitochond	MAVS_MOU	13	0.943779
15min / C	Q9D898	0.1748	0.731522	0.69475	Arpc5l	Actin-relate	ARP5L_MO	4	1.128808
30min / C	Q60936	0.109491	0.73173	0.694809	Coq8a	Atypical kir	COQ8A_MO	20	1.078847
15min / C	Q3TW96	0.120522	0.731932	0.694863	Uap1l1	UDP-N-acet	UAP1L_MO	20	1.087128
15min / C	O35381	0.095022	0.733723	0.695199	Anp32a	Acidic leuci	AN32A_MO	9	1.068082
30min / C	P34914	-0.00775	0.733831	0.695199	Ephx2	Bifunctiona	HYES_MOU	38	0.994644
30min / C	P97449	-0.09042	0.733738	0.695199	Anpep	Aminopept	AMPN_MOU	16	0.939249
30min / C	Q61029	-0.07961	0.734153	0.695199	Tmpo	Lamina-asso	LAP2B_MO	11	0.946311
15min / C	Q80WJ7	0.029363	0.734416	0.695199	Mtdh	Protein LYR	LYRIC_MOU	15	1.020562
15min / C	Q8BHG2	0.297523	0.734611	0.695199	Czib	CXXC motif	CZIB_MOUS	7	1.229033
15min / C	Q8BKZ9	0.187131	0.734904	0.695199	Pdhx	Pyruvate de	ODPX_MOU	13	1.138498
30min / C	Q8C1D8	-0.21583	0.7343	0.695199	Iws1	Protein IWS	IWS1_MOU	2	0.861048
30min / C	Q9CQ62	-0.12453	0.732593	0.695199	Decr1	2,4-dienoyl	DECR_MOU	18	0.917303
15min / C	Q8VCX1	0.088735	0.735464	0.695453	Akr1d1	Aldo-keto r	AK1D1_MO	21	1.063437
15min / C	Q9WVJ3	0.02506	0.735861	0.695561	Cpq	Carboxypep	CBPQ_MOU	11	1.017522
15min / C	Q9Z0N2	0.062577	0.736014	0.695561	Eif2s3y	Eukaryotic	IF2H_MOUS	17	1.04433
30min / C	Q61599	-0.06181	0.736406	0.69576	Arhgdib	Rho GDP-di	GDIR2_MO	7	0.958062
15min / C	A2AS89	0.075646	0.737764	0.696514	Agmat	Guanidino	GDAH_MOU	7	1.053833
15min / C	Q91W90	-0.12071	0.738026	0.696514	Txndc5	Thioredoxin	TXND5_MO	14	0.919734
15min / C	O88342	0.045708	0.73931	0.697022	Wdr1	WD repeat-	WDR1_MO	27	1.03219
30min / C	Q80VP1	0.321197	0.739198	0.697022	Epn1	Epsin-1	EPN1_MOU	10	1.249366
15min / C	Q8BFQ8	-0.22116	0.739096	0.697022	Gatd1	Glutamine	GALD1_MO	4	0.857876
30min / C	Q8BTV2	0.119746	0.738887	0.697022	Cpsf7	Cleavage an	CPSF7_MOU	8	1.086544
15min / C	A2BIM8;P0	0.061686	0.739508	0.697071	Mup18;Mu	Major urina	MUP18_MO	14	1.043685
30min / C	Q99KP6	-0.02961	0.740652	0.697737	Prpf19	Pre-mRNA-p	PRP19_MO	14	0.979687
15min / C	Q9JI75	0.184266	0.740634	0.697737	Nqo2	Ribosyldihy	NQO2_MOU	4	1.136239
15min / C	P51885	0.101367	0.741117	0.697764	Lum	Lumican	LUM_MOUS	11	1.072789
30min / C	Q80X95	-0.31781	0.741143	0.697764	Rraga	Ras-related	RRAGA_MO	6	0.802287
30min / C	Q99JT9	-0.05953	0.741156	0.697764	Adi1	Acireducto	MTND_MOU	10	0.959576
15min / C	Q9ERE7	0.150121	0.741265	0.697764	Mesd	LRP chaper	MESD_MOU	7	1.109662

30min / C	Q5SW19	0.141051	0.741679	0.697988	Cluh	Clustered m	CLU_MOUS	48	1.102708
30min / C	Q91WJ8	-0.1486	0.742155	0.698053	Fubp1	Far upstrea	FUBP1_MO	23	0.902125
30min / C	Q8BGF3	-0.06261	0.742319	0.698069	Dnaaf10	Dynein axo	DAA10_MC	4	0.957533
15min / C	Q60739	-0.58512	0.742798	0.698127	Bag1	BAG family	BAG1_MOU	4	0.666595
30min / C	Q8WTY4	0.078775	0.742748	0.698127	Ciapin1	Anamorsin	CPIN1_MO	12	1.056121
15min / C	P62843	-0.20396	0.743081	0.698213	Rps15	Small ribos	RS15_MOU	7	0.868165
15min / C	Q5U5M8	0.154612	0.744143	0.698822	Bloc1s3	Biogenesis	BL1S3_MO	4	1.113122
30min / C	P28665	0.091615	0.744296	0.698828	Mug1	Murinoglol	MUG1_MO	63	1.065562
15min / C	O70570	0.097567	0.746311	0.699028	Pigr	Polymeric i	PIGR_MOU	22	1.069967
30min / C	P97864	-0.01805	0.745154	0.699028	Casp7	Caspase-7	CASP7_MO	8	0.987569
15min / C	Q04447	-0.04726	0.746936	0.699028	Ckb	Creatine kir	KCRB_MOU	13	0.967772
15min / C	Q3ULJ0	-0.06285	0.746545	0.699028	Gpd1l	Glycerol-3-	GPD1L_MC	17	0.957373
15min / C	Q61838	-0.03778	0.745702	0.699028	Pzp	Pregnancy :	PZP_MOUS	65	0.974156
15min / C	Q6P2K6	-0.3909	0.746647	0.699028	Ppp4r3a	Serine/thre	P4R3A_MO	11	0.762653
30min / C	Q78JW9	-0.03815	0.746393	0.699028	Ubfd1	Ubiquitin d	UBFD1_MC	5	0.973905
15min / C	Q8BGF3	-0.19438	0.745619	0.699028	Dnaaf10	Dynein axo	DAA10_MC	4	0.873946
30min / C	Q91W90	-0.12015	0.745431	0.699028	Txndc5	Thioredoxi	TXND5_MC	14	0.920095
15min / C	Q9CR51	0.255384	0.746488	0.699028	Atp6v1g1	V-type prot	VATG1_MO	3	1.193653
15min / C	Q9D3D9	0.100511	0.746896	0.699028	Atp5f1d	ATP synthas	ATPD_MOU	5	1.072153
30min / C	Q3UEG6	-0.03334	0.747705	0.699282	Agxt2	Alanine--gly	AGT2_MOU	29	0.977159
30min / C	Q9D711	-0.19286	0.747608	0.699282	Pir	Pirin	PIR_MOUSI	11	0.874869
15min / C	A2A432	0.602542	0.748502	0.6993	Cul4b	Cullin-4B	CUL4B_MO	19	1.518389
30min / C	O54865	-0.66669	0.748897	0.6993	Gucy1b1	Guanylate c	GCYB1_MO	5	0.629951
15min / C	P11499	-0.00674	0.749166	0.6993	Hsp90ab1	Heat shock	HS90B_MO	39	0.995342
15min / C	P52480	0.090626	0.749004	0.6993	Pkm	Pyruvate ki	KPYM_MOU	33	1.064832
15min / C	Q8VCI0	0.164443	0.747926	0.6993	Plbd1	Phospholip	PLBL1_MO	10	1.120733
15min / C	Q9CQW2	-1.1193	0.748466	0.6993	Arl8b	ADP-ribosy	ARL8B_MO	3	0.460317
30min / C	Q9DCW4	-0.00819	0.749453	0.6993	Etfb	Electron tra	ETFB_MOUS	16	0.994342
15min / C	Q9QXE0	0.057538	0.748262	0.6993	Hacl1	2-hydroxya	HACL1_MO	30	1.040688
15min / C	P14824	0.015852	0.75021	0.699504	Anxa6	Annexin A6	ANXA6_MC	37	1.011048
30min / C	P52503	-0.32394	0.75116	0.699504	Ndufs6	NADH dehy	NDUS6_MC	4	0.798884
15min / C	P62858	-0.1438	0.75111	0.699504	Rps28	Small ribos	RS28_MOU	3	0.90513
15min / C	Q02053	0.351842	0.750523	0.699504	Uba1	Ubiquitin-l	UBA1_MOU	43	1.276189
30min / C	Q3UHX2	0.028769	0.750048	0.699504	Pdap1	28 kDa heat	HAP28_MC	6	1.020141
15min / C	Q3UUI3	0.104229	0.750601	0.699504	Them4	Acyl-coenzy	THEM4_MC	5	1.07492
15min / C	Q8BWY3	0.031578	0.7512	0.699504	Etf1	Eukaryotic	ERF1_MOU	19	1.022129
15min / C	Q91VH2	-0.24892	0.751309	0.699504	Snx9	Sorting nex	SNX9_MOU	9	0.841528
30min / C	Q9D8B3	-0.29991	0.750169	0.699504	Chmp4b	Charged mu	CHM4B_MC	6	0.812303
30min / C	Q9Z2I8	-0.01516	0.751493	0.69954	Suclg2	Succinate--	SUCB2_MO	21	0.989545
15min / C	P56213	0.194447	0.751693	0.699589	Gfer	FAD-linked	ALR_MOUS	8	1.144285
30min / C	P11862	0.095016	0.752216	0.699754	Gas2	Growth arr	GAS2_MOU	15	1.068077
30min / C	Q9D7X3	-0.25609	0.752308	0.699754	Dusp3	Dual specifi	DUS3_MOU	5	0.837355
30min / C	P11438	-0.17075	0.752603	0.699807	Lamp1	Lysosome-a	LAMP1_MC	5	0.88838
30min / C	Q8JZK9	0.115014	0.753017	0.699807	Hmgcs1	Hydroxyme	HMCS1_MC	18	1.082986
15min / C	Q91WU0	0.036941	0.752756	0.699807	Ces1f	Carboxylest	EST1F_MOU	29	1.025936
15min / C	Q99LC5	0.076242	0.753026	0.699807	Etfa	Electron tra	ETFA_MOUS	19	1.054269
15min / C	Q8CIB5	-0.09086	0.754025	0.70026	Fermt2	Fermitin fai	FERM2_MC	25	0.93896
15min / C	O55137	0.004037	0.754304	0.700353	Acot1	Acyl-coenzy	ACOT1_MO	11	1.002802
15min / C	P05201	-0.0552	0.754878	0.700353	Got1	Aspartate a	AATC_MOU	26	0.962464
15min / C	Q05117	0.228885	0.755075	0.700353	Acp5	Tartrate-res	PPA5_MOU	6	1.171929
30min / C	Q3THE2	0.069516	0.754841	0.700353	Myl12b	Myosin regi	ML12B_MC	7	1.049364
15min / C	Q9CYR6	0.14437	0.75515	0.700353	Pgm3	Phosphoac	AGM1_MO	20	1.105248

15min / C	Q61239	0.105578	0.755853	0.70054	Fnta	Protein farı	FNTA_MOU	8	1.075925
30min / C	Q8K3K8	-0.16961	0.755938	0.70054	Optn	Optineurin	OPTN_MOU	6	0.889083
15min / C	Q61171	0.049646	0.756436	0.700665	Prdx2	Peroxiredo	PRDX2_MC	14	1.035011
30min / C	Q99LC5	-0.04746	0.756513	0.700665	Etfa	Electron tra	ETFA_MOU!	19	0.967635
30min / C	Q9WV85	0.031289	0.756755	0.700754	Nme3	Nucleoside	NDK3_MOU	5	1.021924
15min / C	P01942	-0.09804	0.759329	0.7011	Hba	Hemoglobi	HBA_MOUS	10	0.934304
15min / C	P06537	0.362155	0.759007	0.7011	Nr3c1	Glucocortic	GCR_MOUS	9	1.285344
30min / C	P48193	-0.39638	0.757897	0.7011	Epb41	Protein 4.1	EPB41_MO	11	0.759762
15min / C	P49710	0.131511	0.759136	0.7011	Hcls1	Hematopoi	HCLS1_MO	5	1.095441
30min / C	Q62188	0.066163	0.759108	0.7011	Dpysl3	Dihydropyı	DPYL3_MO	17	1.046928
15min / C	Q8BFR5	-0.09867	0.758558	0.7011	Tufm	Elongation	EFTU_MOU	25	0.933895
30min / C	Q8BHG2	0.193379	0.757893	0.7011	Czib	CXXC motif	CZIB_MOUS	7	1.143438
15min / C	Q91ZX7	0.009386	0.757461	0.7011	Lrp1	Prolow-der	LRP1_MOU	63	1.006527
30min / C	Q9R062	0.133132	0.759093	0.7011	Gyg1	Glycogenin	GLYG_MOU	5	1.096672
30min / C	Q60605	0.016355	0.759716	0.701322	Myl6	Myosin ligh	MYL6_MOU	10	1.011401
15min / C	Q02819	-0.02484	0.760818	0.701526	Nucb1	Nucleobinc	NUCB1_MC	22	0.982929
30min / C	Q7TMB8	-0.01996	0.760321	0.701526	Cyfip1	Cytoplasmi	CYFP1_MOU	32	0.986257
30min / C	Q8BGS2	0.095266	0.760743	0.701526	Bola2	BolA-like pı	BOLA2_MO	4	1.068263
15min / C	Q8K3H0	-0.12346	0.760656	0.701526	Appl1	DCC-intera	DP13A_MC	14	0.917983
15min / C	Q91YN5	0.177241	0.760458	0.701526	Uap1	UDP-N-acet	UAP1_MOU	12	1.130719
30min / C	P46664	0.035988	0.761548	0.701826	Adss2	Adenylosuc	PURA2_MC	17	1.025258
15min / C	P49222	-0.53327	0.761584	0.701826	Epb42	Protein 4.2	EPB42_MO	2	0.690987
15min / C	Q9JIY5	-0.08251	0.761432	0.701826	Htra2	Serine prot	HTRA2_MO	7	0.944414
15min / C	P61079	-0.03466	0.762467	0.702082	Ube2d3	Ubiquitin-c	UB2D3_MC	1	0.976259
30min / C	Q8R0P4	-0.01713	0.76289	0.702082	Aamdc	Mth938 do	AAMDC_M	2	0.988196
30min / C	Q9D1C8	-0.01877	0.762767	0.702082	Vps28	Vacuolar pı	VPS28_MO	4	0.987071
30min / C	Q9D6S7	0.021869	0.762811	0.702082	Mrrf	Ribosome-ı	RRFM_MOU	5	1.015274
30min / C	Q99JW4	-0.06228	0.763165	0.7022	Lims1	LIM and ser	LIMS1_MO	7	0.957748
15min / C	O54909	-0.23255	0.763793	0.702642	Rdh16	Retinol deh	RDH16_MC	7	0.85113
30min / C	P62192	-0.34832	0.764242	0.70265	Psmc1	26S protea	PRS4_MOU	23	0.7855
30min / C	Q64514	-0.0408	0.764197	0.70265	Tpp2	Tripeptidyl	TPP2_MOU	54	0.972118
15min / C	Q99JY9	-0.02472	0.764525	0.702775	Actr3	Actin-relate	ARP3_MOU	22	0.983009
30min / C	P61027	0.233987	0.764817	0.702908	Rab10	Ras-related	RAB10_MO	5	1.176081
15min / C	Q921G7	0.012837	0.765178	0.703104	Etfdh	Electron tra	ETFD_MOU	24	1.008937
15min / C	Q8C1A5	0.235923	0.765445	0.703149	Thop1	Thimet olig	THOP1_MC	18	1.17766
15min / C	Q8CJG0	0.062953	0.76552	0.703149	ago-02	Protein arg	AGO2_MOU	16	1.044602
15min / C	Q9QYY9	0.003193	0.765967	0.703424	Adh4	All-trans-re	ADH4_MOU	19	1.002216
15min / C	P51150	0.123734	0.7665	0.703431	Rab7a	Ras-related	RAB7A_MO	13	1.089551
15min / C	Q9DBB9	0.011447	0.766144	0.703431	Cpn2	Carboxypep	CPN2_MOU	11	1.007966
30min / C	Q9D8E6	0.063115	0.766874	0.703489	Rpl4	Large ribos	RL4_MOUS	22	1.044719
30min / C	Q9DBE0	0.056922	0.767067	0.703489	Csad	Cysteine su	CSAD_MOU	28	1.040244
30min / C	Q5F226	0.643276	0.768587	0.704341	Fat2	Protocadhe	FAT2_MOU	2	1.561871
30min / C	O08788	-0.38582	0.768868	0.704464	Dctn1	Dynactin su	DCTN1_MC	30	0.765341
30min / C	Q8BP67	0.485015	0.769433	0.704711	Rpl24	Large ribos	RL24_MOU	9	1.3996
30min / C	Q9DB27	0.288347	0.769409	0.704711	Mcts1	Malignant	MCTS1_MC	7	1.22124
15min / C	P24549	-0.02571	0.770914	0.705662	Aldh1a1	Aldehyde d	AL1A1_MO	21	0.982336
30min / C	Q9QYY9	0.007352	0.771265	0.705849	Adh4	All-trans-re	ADH4_MOU	19	1.005109
30min / C	Q9CQK7	0.341939	0.771898	0.706022	Rwdd1	RWD doma	RWDD1_M	3	1.267459
30min / C	P01644;P0	-0.27781	0.772367	0.706172	NaN	Ig kappa ch	KV5AB_MO	2	0.824841
15min / C	Q62418	-0.0222	0.772273	0.706172	Dbnl	Drebrin-lik	DBNL_MOU	12	0.98473
15min / C	Q9CQX8	-0.15843	0.772505	0.706172	Kgd4	Alpha-keto	KGD4_MOU	3	0.895997
30min / C	O08756	-0.01405	0.772984	0.70634	Hsd17b10	3-hydroxya	HCD2_MOU	12	0.990309

30min / C	P32067	-0.05426	0.772944	0.70634	Ssb	Lupus La pr	LA_MOUSE	16	0.963088
15min / C	Q8VC30	0.00029	0.773466	0.706511	Tkfc	Triokinase/	TKFC_MOU	31	1.000201
15min / C	Q9DD18	0.12703	0.77339	0.706511	Dtd1	D-aminoac	DTD1_MOU	3	1.092044
30min / C	P97350	0.513949	0.773698	0.706587	Pkp1	Plakophilir	PKP1_MOU	4	1.427954
15min / C	Q9D8Y0	0.177703	0.773926	0.70659	Efhd2	EF-hand do	EFHD2_MO	9	1.131082
30min / C	P01942	-0.05684	0.774471	0.706835	Hba	Hemoglobi	HBA_MOUS	10	0.96137
15min / C	Q06185	0.095531	0.774709	0.706835	Atp5me	ATP synthas	ATP5I_MOU	4	1.068458
15min / C	P52840	0.055751	0.775059	0.70702	Sult1a1	Sulfotransf	ST1A1_MO	15	1.0394
30min / C	P16254	0.493525	0.776511	0.707276	Srp14	Signal reco	SRP14_MO	4	1.40788
15min / C	P47911	-0.02439	0.776278	0.707276	Rpl6	Large ribos	RL6_MOUS	13	0.983236
15min / C	Q711T7	0.104076	0.776874	0.707276	Nadsyn1	Glutamine-	NADE_MOU	12	1.074806
15min / C	Q8C7E7	0.90017	0.775751	0.707276	Stbd1	Starch-binc	STBD1_MO	11	1.866286
30min / C	Q8R3Q6	0.15757	0.775805	0.707276	Mix23	Protein MI	MIX23_MO	5	1.115407
30min / C	Q9D0R2	-0.0137	0.776125	0.707276	Tars1	Threonine-	SYTC_MOU	32	0.990546
15min / C	Q9DCJ9	0.158143	0.775662	0.707276	Npl	N-acetylne	NPL_MOUS	11	1.11585
15min / C	Q9JLI6	0.077301	0.776532	0.707276	Scly	Selenocyste	SCLY_MOU	20	1.055042
30min / C	Q6P3A8	0.153614	0.777688	0.707366	Bckdhb	2-oxoisoval	ODBB_MOU	13	1.112352
30min / C	Q8BWS5	0.072764	0.777702	0.707366	Gprin3	G protein-r	GRIN3_MO	11	1.05173
15min / C	Q8CDN6	0.036181	0.777228	0.707366	Txnl1	Thioredoxi	TXNL1_MO	15	1.025396
15min / C	Q9DAK9	0.025076	0.777807	0.707366	Phpt1	14 kDa pho	PHP14_MC	6	1.017533
30min / C	Q9WU84	0.059209	0.777794	0.707366	Ccs	Copper cha	CCS_MOUS	10	1.041895
30min / C	Q60710	-0.07012	0.777978	0.707388	Samhd1	Deoxynucle	SAMH1_MC	23	0.95256
15min / C	Q6ZWM4	0.064856	0.778516	0.707535	Lsm8	U6 snRNA-a	LSM8_MOU	5	1.045981
30min / C	Q8QZR3	0.046688	0.778597	0.707535	Ces2a	Pyrethroid	EST2A_MOU	15	1.032891
30min / C	Q91VR2	-0.07149	0.77835	0.707535	Atp5f1c	ATP synthas	ATPG_MOU	11	0.951656
30min / C	Q9JHU2	0.15127	0.779505	0.707968	Palmd	Palmdelphi	PALMD_MC	12	1.110546
15min / C	Q9R1P1	0.056671	0.779424	0.707968	Psmb3	Proteasom	PSB3_MOU	10	1.040063
15min / C	P57784	0.049456	0.780111	0.708384	Snrpa1	U2 small nu	RU2A_MOU	6	1.034875
15min / C	Q9JK23	-0.34883	0.780357	0.708472	Psmg1	Proteasom	PSMG1_MC	4	0.785222
15min / C	Q8C6I2	0.18046	0.780729	0.708541	Sdhaf2	Succinate d	SDHF2_MO	1	1.133245
30min / C	Q80UG5	0.071208	0.781067	0.708713	Septin9	Septin-9	SEPT9_MOU	26	1.050596
15min / C	Q91XD4	0.069265	0.781317	0.708806	Ftcd	Formimido	FTCD_MOU	31	1.049182
15min / C	Q91VU6	-0.09165	0.781651	0.708974	Dcaf11	DDB1- and	DCA11_MC	13	0.93845
15min / C	P04939	0.089242	0.782251	0.709114	Mup3	Major urina	MUP3_MOU	10	1.063811
15min / C	P16675	0.236319	0.782016	0.709114	Ctsa	Lysosomal	PPGB_MOU	9	1.177983
15min / C	Q9CYZ2	-0.02077	0.782158	0.709114	Tpd52l2	Tumor prot	TPD54_MO	9	0.985703
15min / C	P51855	0.087609	0.782855	0.70924	Gss	Glutathion	GSHB_MOU	24	1.062607
15min / C	Q64374	0.084903	0.782854	0.70924	Rgn	Regucalcin	RGN_MOUS	24	1.060617
15min / C	Q8VBV7	0.061594	0.783005	0.70924	Cops8	COP9 signa	CSN8_MOU	5	1.043618
30min / C	Q9CQE8	-0.71274	0.783132	0.70924	RTRAF	RNA transc	RTRAF_MO	14	0.61016
15min / C	Q9QYC0	0.049946	0.782553	0.70924	Add1	Alpha-addu	ADDA_MOU	11	1.035226
30min / C	Q5RKZ7	0.011708	0.784807	0.709414	Mocs1	Molybdenu	MOCS1_MC	14	1.008148
30min / C	Q6ZWV3	0.059109	0.784258	0.709414	Rpl10	Large ribos	RL10_MOU	7	1.041822
15min / C	Q99JB8	0.041938	0.784387	0.709414	Pacsin3	Protein kin	PACN3_MC	10	1.029495
30min / C	Q99L47	-0.09015	0.784063	0.709414	St13	Hsc70-inte	F10A1_MO	10	0.939423
15min / C	Q99LB6	0.087538	0.78465	0.709414	Mat2b	Methionine	MAT2B_MC	10	1.062555
15min / C	Q9R1T2	0.103939	0.78433	0.709414	Sae1	SUMO-activ	SAE1_MOU	17	1.074704
15min / C	P55050	0.014964	0.785107	0.70955	Fabp2	Fatty acid-k	FABPI_MOU	6	1.010426
30min / C	Q76LS9	-0.02515	0.786435	0.710347	Mindy1	Ubiquitin c	MINY1_MO	10	0.982722
15min / C	Q9Z0N1	-0.13347	0.786291	0.710347	Eif2s3x	Eukaryotic	IF2G_MOU	5	0.911635
15min / C	Q61730	0.190339	0.786746	0.710494	Il1rap	Interleukin	IL1AP_MOU	5	1.141032
30min / C	Q9WV32	0.059607	0.787371	0.71079	Arpc1b	Actin-relate	ARC1B_MO	17	1.042182

15min / C	P50446	-0.05319	0.788124	0.710948	Krt6a	Keratin, typ	K2C6A_MO	17	0.963803
30min / C	Q8R1F5	-0.02226	0.787858	0.710948	Hyi	Putative hy	HYI_MOUSI	10	0.984691
15min / C	Q8R238	0.060919	0.787821	0.710948	Sdsl	Serine dehy	SDSL_MOU	11	1.04313
30min / C	Q9JKB1	0.171304	0.788141	0.710948	Uchl3	Ubiquitin c	UCHL3_MC	8	1.126076
15min / C	O89110	0.024482	0.788524	0.711075	Casp8	Caspase-8	CASP8_MO	13	1.017114
15min / C	Q08189	-0.17887	0.78858	0.711075	Tgm3	Protein-glu	TGM3_MOU	1	0.883397
15min / C	Q9JHE3	0.068127	0.79029	0.711678	Asah2	Neutral cer	ASAH2_MO	1	1.048355
15min / C	O55023	-0.01001	0.791116	0.712028	Impa1	Inositol mc	IMPA1_MO	13	0.993088
15min / C	Q8VBW8	-0.0589	0.791424	0.712028	Ttc36	Tetratricop	TTC36_MO	7	0.959999
15min / C	Q91WJ8	0.071549	0.791287	0.712028	Fubp1	Far upstrea	FUBP1_MO	23	1.050844
30min / C	Q99KC8	0.012245	0.791207	0.712028	Vwa5a	von Willebr	VMA5A_M(	25	1.008524
15min / C	Q60692	0.014819	0.791931	0.712248	Psmb6	Proteasom	PSB6_MOU	8	1.010324
15min / C	O35864	-0.19681	0.792526	0.712256	Cops5	COP9 signa	CSN5_MOU	11	0.872475
15min / C	P62073	0.174564	0.792283	0.712256	Timm10	Mitochond	TIM10_MO	2	1.128623
15min / C	Q60590	-0.07132	0.792571	0.712256	Orm1	Alpha-1-aci	A1AG1_MC	2	0.951769
30min / C	Q6IRU5	0.04436	0.792474	0.712256	Cltb	Clathrin lig	CLCB_MOU	9	1.031226
30min / C	O35955	0.051878	0.79289	0.712275	Psmb10	Proteasom	PSB10_MO	9	1.036613
30min / C	P19001	1.114325	0.792832	0.712275	Krt19	Keratin, typ	K1C19_MO	1	2.164936
30min / C	P21981	0.003879	0.79325	0.71228	Tgm2	Protein-glu	TGM2_MOU	34	1.002692
30min / C	P22599	0.03021	0.793852	0.712374	Serpina1b	Alpha-1-an	A1AT2_MO	7	1.021161
30min / C	Q9R059	0.032777	0.793895	0.712374	Fhl3	Four and a	FHL3_MOU	2	1.02298
15min / C	P19253	0.228379	0.794437	0.712593	Rpl13a	Large ribos	RL13A_MO	8	1.171518
30min / C	Q9QWK4	-0.57321	0.794318	0.712593	Cd5l	CD5 antigen	CD5L_MOU	3	0.67212
15min / C	Q8BZF8	0.212233	0.794871	0.712755	Pgm5	Phosphoglu	PGM5_MO	1	1.15848
30min / C	P12246	0.421661	0.795293	0.712959	Apcs	Serum amy	SAMP_MOU	3	1.339469
15min / C	O54879	-0.01418	0.795542	0.713007	Hmgb3	High mobil	HMGB3_M	4	0.990222
15min / C	Q7TNC4	-0.45259	0.795656	0.713007	Luc7l2	Putative RN	LC7L2_MO	7	0.730731
15min / C	Q8R3F9	0.502979	0.796389	0.713007	Tut1	Speckle tar	STPAP_MO	1	1.417137
15min / C	Q9D6S7	0.008756	0.796375	0.713007	Mrrf	Ribosome-r	RRFM_MOU	5	1.006088
15min / C	Q9D7G0	-0.05283	0.796179	0.713007	Prps1	Ribose-pho	PRPS1_MO	13	0.964042
15min / C	Q9D8E6	0.122294	0.796128	0.713007	Rpl4	Large ribos	RL4_MOUS	22	1.088464
15min / C	Q9R069	-0.55779	0.796821	0.713097	Bcam	Basal cell ac	BCAM_MOU	4	0.67934
15min / C	P50431	0.054578	0.799276	0.713475	Shmt1	Serine hydr	GLYC_MOU	24	1.038556
15min / C	P56480	-0.06965	0.798932	0.713475	Atp5f1b	ATP syntha	ATPB_MOU	26	0.952867
15min / C	Q810U5	-0.29901	0.799131	0.713475	Ccdc50	Coiled-coil	CCD50_MC	2	0.812809
30min / C	Q8BFS6	0.003359	0.798958	0.713475	Cpped1	Serine/thre	CPPED_MO	8	1.002331
15min / C	Q8BHA3	-0.04887	0.798902	0.713475	Dtd2	D-aminoacy	DTD2_MOU	4	0.966693
30min / C	Q9CX80	-0.06451	0.799076	0.713475	Cygb	Cytoglobin	CYGB_MOU	12	0.956271
15min / C	Q9CXW4	0.411223	0.799451	0.713475	Rpl11	Large ribos	RL11_MOU	7	1.329813
30min / C	Q9EQH2	0.051132	0.799239	0.713475	Erap1	Endoplasm	ERAP1_MO	33	1.036077
15min / C	Q9JL35	-0.08273	0.799335	0.713475	Hmgn5	High mobil	HMGN5_M	21	0.944268
30min / C	Q9Z2X1	0.308459	0.799067	0.713475	Hnrnpf	Heterogene	HNRPF_MC	11	1.238384
15min / C	P34914	0.001581	0.80043	0.713683	Ephx2	Bifunctiona	HYES_MOU	38	1.001096
30min / C	P59242	0.005909	0.800422	0.713683	Cgn	Cingulin	CING_MOU	32	1.004104
30min / C	Q62384	0.310582	0.800146	0.713683	Zpr1	Zinc finger	ZPR1_MOU	8	1.240208
15min / C	Q923D5	-0.08448	0.800113	0.713683	Wbp11	WW domai	WBP11_M(	4	0.943123
15min / C	P01867	0.008564	0.800754	0.713706	Ighg2b	Immunoglo	IGG2B_MO	5	1.005954
15min / C	P18527	0.031607	0.801176	0.713949	NaN	Ig heavy ch	HVM56_M(	1	1.02215
15min / C	P16015	-0.00662	0.801575	0.713987	Ca3	Carbonic ar	CAH3_MOU	20	0.995421
15min / C	P28474	0.02603	0.801667	0.713987	Adh5	Alcohol del	ADHX_MOU	20	1.018206
15min / C	Q9CR68	-0.17797	0.801444	0.713987	Uqcrfs1	Cytochrom	UCRI_MOU	11	0.883947
15min / C	P10639	-0.00427	0.802209	0.714089	Txn	Thioredoxi	THIO_MOU	7	0.997045

30min / C	Q9DCM0	0.038343	0.802379	0.714089	Ethe1	Persulfide c	ETHE1_MOU	13	1.026934
15min / C	P28651	0.006353	0.803591	0.714635	Ca8	Carbonic ar	CAH8_MOU	8	1.004413
30min / C	Q9D0S9	0.05602	0.803826	0.714712	Hint2	Adenosine 5	HINT2_MOU	5	1.039594
30min / C	P48774	0.083235	0.804126	0.71482	Gstm5	Glutathion	GSTM5_MO	16	1.059391
15min / C	O70251	-0.04048	0.806943	0.714823	Eef1b	Elongation	EF1B_MOU	10	0.972332
30min / C	P18242	-0.10109	0.805475	0.714823	Ctsd	Cathepsin D	CATD_MOU	13	0.932328
30min / C	P34022	-0.06604	0.80661	0.714823	Ranbp1	Ran-specific	RANG_MOU	4	0.955258
30min / C	P55258	-0.0134	0.806731	0.714823	Rab8a	Ras-related	RAB8A_MO	6	0.990753
15min / C	Q01339	-0.04598	0.805866	0.714823	Apoh	Beta-2-glyc	APOH_MOU	13	0.968632
30min / C	Q61189	-0.27754	0.804444	0.714823	Clns1a	Methylosor	ICLN_MOUS	1	0.824998
30min / C	Q99P27	-0.40285	0.805869	0.714823	Pla2g12b	Group XIIB	PG12B_MO	6	0.756363
15min / C	Q9R0Q7	-0.15879	0.806419	0.714823	Ptges3	Prostagland	TEBP_MOU	7	0.895779
15min / C	O54786	0.175319	0.809741	0.714917	Dffa	DNA fragme	DFFA_MOU	2	1.129214
30min / C	P11679	-0.08836	0.808839	0.714917	Krt8	Keratin, typ	K2C8_MOU	27	0.94059
15min / C	P19639	0.008957	0.807968	0.714917	Gstm3	Glutathion	GSTM3_MO	17	1.006228
30min / C	P63037	-0.08994	0.808925	0.714917	Dnaja1	DnaJ homo	DNJA1_MO	13	0.939564
30min / C	Q60668	0.035041	0.809188	0.714917	Hnrnpd	Heterogene	HNRPD_MO	10	1.024586
15min / C	Q8BFQ4	0.00301	0.809624	0.714917	Wdr82	WD repeat-	WDR82_MO	4	1.002088
30min / C	Q8BWR2	0.047933	0.807489	0.714917	Pithd1	PITH domai	PITH1_MOU	3	1.033783
30min / C	Q8K2B3	-0.01625	0.809489	0.714917	Sdha	Succinate d	SDHA_MOU	27	0.9888
15min / C	Q9DCH6	-0.28716	0.809416	0.714917	Zfand6	AN1-type zi	ZFAN6_MO	4	0.819514
30min / C	Q9EQH3	-0.13703	0.807499	0.714917	Vps35	Vacuolar pr	VPS35_MO	26	0.909392
30min / C	Q9Z184	0.288689	0.809242	0.714917	Padi3	Protein-arg	PADI3_MOU	1	1.22153
15min / C	Q9D0S9	0.005037	0.810082	0.715087	Hint2	Adenosine 5	HINT2_MOU	5	1.003498
15min / C	Q9CQE8	-0.42716	0.811007	0.715771	RTRAF	RNA transcr	RTRAF_MOU	14	0.743727
15min / C	Q64176	-0.05013	0.811994	0.716192	Ces1e	Carboxylest	EST1E_MOU	19	0.965847
15min / C	Q71RI9	-0.03901	0.812084	0.716192	Kyat3	Kynurenine	KAT3_MOU	19	0.973326
15min / C	Q91WK2	-0.09992	0.811802	0.716192	Eif3h	Eukaryotic	EIF3H_MOU	14	0.933088
15min / C	Q9WU19	-0.07834	0.811727	0.716192	Hao1	2-Hydroxya	HAOX1_MO	17	0.947147
30min / C	O09174	0.063794	0.815474	0.716557	Amacr	Alpha-meth	AMACR_MO	20	1.045211
15min / C	P08030	0.20002	0.81478	0.716557	Aprt	Adenine ph	APT_MOUS	8	1.148714
30min / C	P08071	-0.01871	0.81535	0.716557	Ltf	Lactotransf	TRFL_MOUS	8	0.987114
15min / C	P23780	0.221487	0.81324	0.716557	Glb1	Beta-galact	BGAL_MOU	9	1.165935
30min / C	P40336	-0.14068	0.814294	0.716557	Vps26a	Vacuolar pr	VP26A_MO	16	0.907089
30min / C	P50544	-0.04418	0.812994	0.716557	Acadvl	Very long-c	ACADV_MO	35	0.969839
15min / C	P53994	-0.28536	0.813862	0.716557	Rab2a	Ras-related	RAB2A_MO	11	0.820534
15min / C	P62309	-0.25164	0.815628	0.716557	Snrpg	Small nucle	RUXG_MOU	2	0.839943
15min / C	P62717	0.25025	0.815188	0.716557	Rpl18a	Large ribos	RL18A_MO	9	1.189414
30min / C	P97372	0.020576	0.813133	0.716557	Psme2	Proteasome	PSME2_MO	18	1.014364
30min / C	Q1HFZ0	0.123855	0.813278	0.716557	Nsun2	RNA cytosir	NSUN2_MO	20	1.089642
30min / C	Q61316	0.024169	0.815623	0.716557	Hspa4	Heat shock	HSP74_MO	46	1.016894
15min / C	Q8VIJ6	0.127637	0.813726	0.716557	Sfpq	Splicing fac	SFPQ_MOU	23	1.092503
15min / C	Q9CYR0	0.064178	0.815882	0.716557	Ssbp1	Single-strar	SSBP_MOU	5	1.045489
15min / C	Q9D8S4	0.040311	0.814645	0.716557	Rexo2	Oligoribonu	ORN_MOUS	6	1.028336
15min / C	Q9Z0X1	-0.05795	0.816125	0.716583	Aifm1	Apoptosis-i	AIFM1_MO	22	0.960627
15min / C	A6X935	0.015115	0.816738	0.716833	Itih4	Inter alpha-	ITIH4_MOU	31	1.010532
15min / C	P01863;P0	-0.0531	0.819112	0.716833	Ighg;Ighg2a	Ig gamma-2	GCAA_MOU	5	0.963864
15min / C	P16546	-0.03497	0.819011	0.716833	Sptan1	Spectrin alp	SPTN1_MO	106	0.976055
30min / C	P19253	0.000326	0.819931	0.716833	Rpl13a	Large ribos	RL13A_MO	8	1.000226
15min / C	P30115	-0.09778	0.818154	0.716833	Gsta3	Glutathion	GSTA3_MO	10	0.934472
30min / C	P45376	0.028569	0.818011	0.716833	Akr1b1	Aldo-keto r	ALDR_MOU	24	1.02
15min / C	P62737	-0.08156	0.817579	0.716833	Acta2	Actin, aorti	ACTA_MOU	6	0.945036

30min / C	P68037	-0.04713	0.819841	0.716833	Ube2l3	Ubiquitin-c	UB2L3_MO	9	0.967863
15min / C	Q62417	-0.25678	0.817656	0.716833	Sorbs1	Sorbin and	SRBS1_MO	12	0.836954
30min / C	Q8BFR4	0.205013	0.819367	0.716833	Gns	N-acetylglu	GNS_MOUS	6	1.152697
15min / C	Q8BG73	0.106412	0.818796	0.716833	Sh3bgrl2	SH3 domai	SH3L2_MO	5	1.076548
30min / C	Q8BT60	0.106766	0.816957	0.716833	Cpne3	Copine-3	CPNE3_MO	16	1.076812
15min / C	Q8K019	-0.19835	0.818352	0.716833	Bclaf1	Bcl-2-assoc	BCLF1_MO	7	0.871549
15min / C	Q8K157	0.017078	0.820586	0.716833	Galm	Galactose n	GALM_MOU	12	1.011908
30min / C	Q8K157	0.065311	0.818224	0.716833	Galm	Galactose n	GALM_MOU	12	1.04631
30min / C	Q91VI7	0.026384	0.819282	0.716833	Rnh1	Ribonuclea	RINI_MOUS	26	1.018456
30min / C	Q922Y1	0.052168	0.819086	0.716833	Ubxn1	UBX domai	UBXN1_MO	14	1.036822
30min / C	Q9DBR1	-0.21895	0.820171	0.716833	Xrn2	5'-3' exorib	XRN2_MOU	8	0.859188
15min / C	Q6ZWY8	0.086744	0.821104	0.717004	Tmsb10	Thymosin b	TYB10_MO	2	1.061971
30min / C	Q9QYR9	0.229526	0.822305	0.717922	Acot2	Acyl-coenzy	ACOT2_MO	5	1.172449
15min / C	Q8K3J1	0.51543	0.822648	0.718003	Ndufs8	NADH dehy	NDUS8_MO	4	1.429421
30min / C	Q8VCT4	0.014939	0.823741	0.718388	Ces1d	Carboxyles	EST1D_MO	30	1.010409
15min / C	Q9DBJ3	0.074226	0.823402	0.718388	Baiap2l1	BAR/IMD d	BI2L1_MOU	11	1.052796
15min / C	Q8R5I6	0.085153	0.824232	0.718685	Gstm4	Glutathion	GSTM4_MO	9	1.0608
15min / C	Q8BWS5	-0.12854	0.825001	0.718882	Gprin3	G protein-r	GRIN3_MO	11	0.914756
30min / C	Q923G2	-0.19366	0.825661	0.718882	Polr2h	DNA-direct	RPAB3_MO	3	0.874384
30min / C	O35660	0.070513	0.828622	0.719021	Gstm6	Glutathion	GSTM6_MO	11	1.05009
30min / C	O88952	-0.1678	0.826493	0.719021	Lin7c	Protein lin-	LIN7C_MOU	2	0.890197
30min / C	P23780	0.071589	0.828785	0.719021	Glb1	Beta-galact	BGAL_MOU	9	1.050873
15min / C	P25688	0.016274	0.826929	0.719021	Uox	Uricase	URIC_MOU	22	1.011344
30min / C	P47911	0.0947	0.82883	0.719021	Rpl6	Large ribos	RL6_MOUS	13	1.067843
30min / C	P62196	0.060209	0.828827	0.719021	Psmc5	26S protea	PRS8_MOU	21	1.042617
15min / C	Q8R574	0.320961	0.827066	0.719021	Prpsap2	Phosphorib	KPRB_MOU	7	1.249163
15min / C	Q91V64	-0.09886	0.828558	0.719021	Isoc1	Isochorism	ISOC1_MOU	15	0.933769
15min / C	Q99PL5	-0.02751	0.82716	0.719021	Rrbp1	Ribosome-b	RRBP1_MO	82	0.981109
15min / C	P38060	-0.10163	0.829324	0.71917	Hmgcl	Hydroxyme	HMGCL_MO	15	0.931979
15min / C	Q921M7	0.094395	0.829453	0.71917	Cyrib	CYFIP-relat	CYRIB_MOU	7	1.067618
30min / C	Q8C0M9	-0.03335	0.82986	0.719262	Asrgl1	Isoaspartyl	ASGL1_MO	13	0.977147
30min / C	Q9CQX8	0.064381	0.829755	0.719262	Kgd4	Alpha-keto	KGD4_MOU	3	1.045636
15min / C	Q8JZN5	-0.19311	0.830158	0.71939	Acad9	Complex I a	ACAD9_MO	24	0.874717
30min / C	P46656	-0.1073	0.830579	0.719556	Fdx1	Adrenodox	ADX_MOUS	4	0.928326
15min / C	Q9D154	0.437305	0.83117	0.719745	Serpinb1a	Leukocyte e	ILEUA_MOU	12	1.354072
30min / C	P50428	-0.05781	0.832163	0.720214	Arsa	Arylsulfatas	ARSA_MOU	3	0.960722
15min / C	P62270	-0.03475	0.831944	0.720214	Rps18	Small ribos	RS18_MOU	11	0.976198
15min / C	Q6PDN3	-0.03925	0.832064	0.720214	Mylk	Myosin ligh	MYLK_MOU	39	0.973159
15min / C	P26231	-0.23118	0.832467	0.720216	Ctnna1	Catenin alp	CTNA1_MO	26	0.851936
15min / C	Q9QXK3	-0.06661	0.83243	0.720216	Copg2	Coatomer s	COPG2_MO	24	0.954877
30min / C	Q61081	0.070493	0.833199	0.720463	Cdc37	Hsp90 co-c	CDC37_MO	10	1.050075
30min / C	Q91VW3	0.001225	0.833067	0.720463	Sh3bgrl3	SH3 domai	SH3L3_MO	3	1.00085
30min / C	Q9CPW4	-0.17962	0.833355	0.720463	Arpc5	Actin-relate	ARPC5_MO	5	0.882938
30min / C	O35551	0.100427	0.833804	0.72072	Rabep1	Rab GTPase	RABE1_MO	15	1.072091
30min / C	P08113	-0.03898	0.834172	0.720744	Hsp90b1	Endoplasm	ENPL_MOU	43	0.973341
15min / C	Q99LT0	0.032318	0.834288	0.720744	Dpy30	Protein dp	DPY30_MO	3	1.022654
30min / C	Q9R0N0	-0.17972	0.834434	0.720744	Galk1	Galactokin	GALK1_MO	19	0.882874
30min / C	F6ZDS4	-0.3666	0.834693	0.720769	Tpr	Nucleoprot	TPR_MOUS	37	0.77561
30min / C	O70305	-0.25458	0.834916	0.720769	Atxn2	Ataxin-2	ATX2_MOU	16	0.838231
30min / C	O08539	0.144421	0.835493	0.721082	Bin1	Myc box-de	BIN1_MOU	8	1.105287
30min / C	A6X935	-0.01965	0.835788	0.721131	Itih4	Inter alpha	ITIH4_MOU	31	0.986471
15min / C	Q6ZQ38	-0.01391	0.836328	0.721467	Cand1	Cullin-asso	CAND1_MO	45	0.990407

15min / C	P97855	0.149608	0.837482	0.721741	G3bp1	Ras GTPase-	G3BP1_MO	12	1.109268
15min / C	Q9DBG3	-0.30266	0.837687	0.721741	Ap2b1	AP-2 compl	AP2B1_MO	21	0.810758
15min / C	Q61646	-0.01893	0.838486	0.722026	Hp	Haptoglobi	HPT_MOUS	17	0.986967
15min / C	Q9JLZ3	0.225712	0.83842	0.722026	Auh	Methylglut	AUHM_MO	7	1.169354
30min / C	Q9EQK5	0.09245	0.838828	0.72219	Mvp	Major vault	MVP_MOUS	43	1.066179
30min / C	P35293	0.102267	0.839169	0.722353	Rab18	Ras-related	RAB18_MO	10	1.073459
30min / C	Q62348	-0.05929	0.839346	0.722375	Tsn	Translin	TSN_MOUS	13	0.959735
30min / C	Q9Z1F9	0.022345	0.839758	0.7226	Uba2	SUMO-activ	SAE2_MOU	15	1.015609
15min / C	E9Q0S6	-0.11664	0.840204	0.722656	Tns1	Tensin-1	TENS1_MO	21	0.922336
15min / C	Q3UEG6	0.017036	0.840428	0.722656	Agxt2	Alanine--gly	AGT2_MOU	29	1.011878
15min / C	Q99K67	0.558481	0.840311	0.722656	Aass	Alpha-amir	AASS_MOU	37	1.472717
15min / C	Q9Z1K5	-0.05053	0.840069	0.722656	Arih1	E3 ubiquiti	ARI1_MOUS	4	0.965583
15min / C	Q9JMA1	0.002363	0.840632	0.722702	Usp14	Ubiquitin c	UBP14_MO	19	1.001639
30min / C	O35343	0.100167	0.84082	0.722733	Kpna4	Importin su	IMA3_MOU	5	1.071898
15min / C	Q3UD01	-0.03219	0.841947	0.723572	Atxn7l3b	Ataxin-7-lik	A7L3B_MO	2	0.977933
15min / C	P01633	0.105075	0.845369	0.723625	Igkv6-17	Immunoglo	KV5A1_MO	2	1.07555
15min / C	P07758	-0.02904	0.845246	0.723625	Serpina1a	Alpha-1-ant	A1AT1_MO	16	0.980074
15min / C	P23198	0.070591	0.845294	0.723625	Cbx3	Chromobo:	CBX3_MOU	6	1.050147
30min / C	P48036	0.031658	0.844103	0.723625	Anxa5	Annexin A5	ANXA5_MO	22	1.022186
30min / C	P62309	-0.26936	0.842562	0.723625	Snrpg	Small nucle	RUXG_MOU	2	0.829685
15min / C	P62317	-0.00124	0.843886	0.723625	Snrpd2	Small nucle	SMD2_MOU	9	0.999141
15min / C	P70168	-0.01822	0.844303	0.723625	Kpnb1	Importin su	IMB1_MOU	34	0.987451
30min / C	P97298	-0.37189	0.843825	0.723625	Serpinf1	Pigment ep	PEDF_MOU	10	0.772767
15min / C	P97351	0.221542	0.843417	0.723625	Rps3a	Small ribos	RS3A_MOU	16	1.165979
15min / C	Q62266	0.047038	0.845342	0.723625	Sprr1a	Cornifin-A	SPR1A_MO	9	1.033142
15min / C	Q6GQT1	0.020189	0.844389	0.723625	A2m	Alpha-2-ma	A2MG_MO	1	1.014092
15min / C	Q8BTJ4	-0.10156	0.845369	0.723625	Enpp4	Bis(5'-aden	ENPP4_MO	2	0.932023
30min / C	Q8CCH2	0.4633	0.84549	0.723625	Nhlrc3	NHL repeat	NHLC3_MO	1	1.378691
15min / C	Q9D1E6	0.190558	0.843433	0.723625	Tbcb	Tubulin-fol	TBCB_MOU	10	1.141205
15min / C	Q9QXG2	-0.51134	0.845404	0.723625	Chm	Rab proteir	RAE1_MOU	7	0.701569
30min / C	O70400	-0.0965	0.846778	0.724308	Pdlim1	PDZ and LIM	PDLI1_MOU	20	0.935297
30min / C	O89110	-0.23081	0.846898	0.724308	Casp8	Caspase-8	CASP8_MO	13	0.852158
15min / C	Q4VAA2	-0.02479	0.847247	0.72435	Cdv3	Protein CD'	CDV3_MOU	14	0.982964
30min / C	P54775	-0.61961	0.848245	0.724782	Psmc4	26S protea:	PRS6B_MO	19	0.650845
30min / C	Q08879	0.338832	0.848358	0.724782	Fbln1	Fibulin-1	FBLN1_MO	9	1.264732
30min / C	Q61166	0.013234	0.84831	0.724782	Mapre1	Microtubul	MARE1_MO	12	1.009215
15min / C	P46638	0.023506	0.849974	0.724883	Rab11b	Ras-related	RB11B_MO	12	1.016427
15min / C	Q3TCN2	-0.00632	0.849764	0.724883	Plbd2	Putative ph	PLBL2_MOU	10	0.995631
15min / C	Q60710	0.108851	0.849777	0.724883	Samhd1	Deoxynucle	SAMH1_MO	23	1.078369
30min / C	Q64726	-0.05383	0.849099	0.724883	Azgp1	Zinc-alpha-	ZA2G_MOU	12	0.963374
30min / C	Q9JIX0	-0.12528	0.849811	0.724883	Eny2	Transcripti	ENY2_MOU	2	0.916824
15min / C	Q9QXD1	-0.0177	0.849741	0.724883	Acox2	Peroxisoma	ACOX2_MO	31	0.987805
15min / C	Q9QZ88	-0.03037	0.849993	0.724883	Vps29	Vacuolar pr	VPS29_MO	8	0.979167
30min / C	P97352	-0.07971	0.850206	0.724935	S100a13	Protein S10	S10AD_MO	3	0.946246
15min / C	P62264	0.039895	0.851491	0.725378	Rps14	Small ribos	RS14_MOU	5	1.028039
30min / C	Q8VCC2	0.073744	0.852387	0.725378	Ces1	Liver carbo	EST1_MOUS	19	1.052444
15min / C	Q8VE95	0.058004	0.851224	0.725378	NaN	UPF0598 p	CH082_MO	4	1.041025
15min / C	Q91X34	0.103203	0.852395	0.725378	Baat	Bile acid-Co	BAAT_MOU	15	1.074156
30min / C	Q91YS8	-0.54433	0.85183	0.725378	Camk1	Calcium/ca	KCC1A_MO	5	0.685708
30min / C	O08807	-0.00607	0.852908	0.725546	Prdx4	Peroxiredo	PRDX4_MO	11	0.995805
15min / C	P62488	0.145834	0.853048	0.725546	Polr2g	DNA-direct	RPB7_MOU	2	1.10637
15min / C	P06909	0.058663	0.853226	0.725567	Cfh	Complemer	CFAH_MOU	43	1.0415

15min / C	Q8CCH2	0.615827	0.853506	0.725567	Nhlrc3	NHL repeat	NHLC3_MO	1	1.532436
30min / C	P07141	-0.15363	0.853716	0.725598	Csf1	Macrophag	CSF1_MOU	1	0.898985
30min / C	A2AJL3	-0.02757	0.855385	0.726267	Fggy	FGGY carbo	FGGY_MOU	22	0.981071
15min / C	P70444	0.010584	0.8555	0.726267	Bid	BH3-intera	BID_MOUS	5	1.007363
15min / C	Q91W43	-0.02672	0.855201	0.726267	Gldc	Glycine del	GCSP_MOU	31	0.981649
30min / C	Q91YN5	0.079652	0.855706	0.726267	Uap1	UDP-N-acet	UAP1_MOU	12	1.056763
15min / C	Q91YP3	0.007585	0.855719	0.726267	Dera	Deoxyribos	DEOC_MOU	13	1.005272
30min / C	Q99KI0	0.056313	0.855366	0.726267	Aco2	Aconitate h	ACON_MOU	39	1.039805
15min / C	B2RXS4	0.388206	0.856077	0.726386	Plxnb2	Plexin-B2	PLXB2_MO	6	1.308765
30min / C	P07758	0.059692	0.856894	0.726409	Serpina1a	Alpha-1-ant	A1AT1_MO	16	1.042243
15min / C	Q01730	-0.02529	0.85695	0.726409	Rsu1	Ras suppres	RSU1_MOU	10	0.982623
15min / C	Q8BG32	-0.14443	0.856619	0.726409	Psmd11	26S protea	PSD11_MO	23	0.904736
15min / C	P01878	0.027913	0.85728	0.72656	NaN	Ig alpha cha	IGHA_MOU	7	1.019536
15min / C	Q07456	0.04565	0.857751	0.72683	Ambp	Protein AM	AMBP_MOU	12	1.032148
15min / C	Q99020	0.068822	0.858209	0.72709	Hnrnpab	Heterogene	ROAA_MOU	8	1.04886
30min / C	Q3U4I7	0.176289	0.858729	0.727144	Pyroxd2	Pyridine nu	PYRD2_MO	11	1.129974
15min / C	Q8K354	-0.02831	0.858648	0.727144	Cbr3	Carbonyl re	CBR3_MOU	7	0.980565
30min / C	Q8BGA8	-0.00575	0.859166	0.727163	Acsm5	Acyl-coenzy	ACSM5_MC	22	0.996023
30min / C	Q8QZY2	-0.06967	0.859208	0.727163	Glyctk	Glycerate k	GLCTK_MO	13	0.952858
30min / C	Q9DCU9	0.017919	0.85936	0.727163	Hoga1	4-hydroxy-2	HOGA1_MC	16	1.012498
15min / C	P37804	0.152685	0.860656	0.727408	Tagln	Transgelin	TAGL_MOU	11	1.111637
30min / C	P50543	-0.0758	0.859816	0.727408	S100a11	Protein S10	S10AB_MO	4	0.948817
15min / C	P54869	-0.10736	0.860715	0.727408	Hmgcs2	Hydroxyme	HMCS2_MC	24	0.928282
30min / C	Q80W21	-0.01353	0.860341	0.727408	Gstm7	Glutathion	GSTM7_MC	8	0.990668
30min / C	Q8CFE2	0.258392	0.860222	0.727408	Hpf1	Histone PA	HPF1_MOU	2	1.196145
15min / C	Q9CQ62	0.021708	0.860324	0.727408	Decr1	2,4-dienoyl	DECR_MOU	18	1.015161
15min / C	P03976	-0.19843	0.863075	0.728032	NaN	Ig kappa ch	KV2A5_MO	1	0.871501
30min / C	P10639	0.011646	0.862712	0.728032	Txn	Thioredoxi	THIO_MOU	7	1.008105
15min / C	Q62261	-0.05299	0.86292	0.728032	Sptbn1	Spectrin be	SPTB2_MO	78	0.963938
30min / C	Q6NZR5	0.440932	0.863253	0.728032	Skic2	Superkiller	SKI2_MOUS	11	1.357481
30min / C	Q80VJ2	0.065316	0.863434	0.728032	Sra1	Steroid rec	SRA1_MOU	4	1.046314
15min / C	Q8BMS1	0.354324	0.86343	0.728032	Hadha	Trifunction	ECHA_MOU	36	1.278386
30min / C	Q9CQM9	-0.00932	0.861935	0.728032	Glrx3	Glutaredox	GLRX3_MO	11	0.99356
30min / C	Q8K0Z7	-0.04868	0.864167	0.728393	Taco1	Translation	TACO1_MO	7	0.96682
15min / C	P97352	-0.10316	0.864816	0.728438	S100a13	Protein S10	S10AD_MO	3	0.930992
30min / C	P97450	-0.01827	0.865199	0.728438	Atp5pf	ATP synthas	ATP5J_MOU	5	0.987417
30min / C	Q61239	-0.45171	0.865116	0.728438	Fnta	Protein farr	FNTA_MOU	8	0.731176
30min / C	Q61838	0.029722	0.865287	0.728438	Pzp	Pregnancy z	PZP_MOUS	65	1.020815
30min / C	Q8QZY1	-0.25229	0.865258	0.728438	Eif3l	Eukaryotic	EIF3L_MOU	22	0.839565
15min / C	Q6ZWV7	-0.10241	0.865518	0.728504	Rpl35	Large ribos	RL35_MOU	4	0.931474
15min / C	Q8CH18	0.181128	0.865806	0.728618	Ccar1	Cell divisio	CCAR1_MO	14	1.13377
30min / C	Q3TLH4	-0.25485	0.866551	0.728619	Prrc2c	Protein PRI	PRC2C_MO	26	0.838071
30min / C	Q91WU0	0.020716	0.866583	0.728619	Ces1f	Carboxyles	EST1F_MOU	29	1.014463
15min / C	Q9CQ60	-0.03683	0.866097	0.728619	Pgls	6-phospho	6PGL_MOU	13	0.974792
30min / C	Q9CQV8	-0.04104	0.866744	0.728619	Ywhab	14-3-3 prot	1433B_MO	7	0.971953
15min / C	P35527	0.07383	0.867847	0.728773	KRT9	Keratin, typ	K1C9_HUM	34	1.052507
30min / C	Q8BGC4	-0.08795	0.867689	0.728773	Ptgr3	Prostagland	PTGR3_MO	12	0.940859
30min / C	Q8BVQ5	-0.27286	0.867806	0.728773	Ppme1	Protein pho	PPME1_MC	9	0.827676
15min / C	Q8VCW8	-0.26201	0.86725	0.728773	Acsf2	Medium-ch	ACSF2_MO	24	0.833923
15min / C	Q9DBJ1	0.031639	0.867971	0.728773	Pgam1	Phosphogly	PGAM1_MC	18	1.022173
30min / C	P68368	-0.21759	0.868126	0.728775	Tuba4a	Tubulin alp	TBA4A_MO	4	0.859999
15min / C	P23591	0.112599	0.869049	0.729064	Gfus	GDP-L-fuco	FCL_MOUS	13	1.081174

30min / C	Q9DCV4	0.091322	0.869233	0.729064	Rmdn1	Regulator c	RMD1_MOU	8	1.065346
30min / C	P51885	-0.11193	0.870312	0.729237	Lum	Lumican	LUM_MOUS	11	0.925351
30min / C	P62897	0.012371	0.870217	0.729237	Cycs	Cytochrom	CYC_MOUS	8	1.008612
15min / C	Q6NZJ6	-0.02414	0.870003	0.729237	Eif4g1	Eukaryotic	IF4G1_MOU	47	0.98341
15min / C	Q9CWK8	0.095335	0.870355	0.729237	Snx2	Sorting nex	SNX2_MOU	17	1.068314
30min / C	Q9D1R9	0.279429	0.869715	0.729237	Rpl34	Large ribos	RL34_MOU	5	1.213715
15min / C	Q02257	0.184816	0.870716	0.729411	Jup	Junction pl	PLAK_MOU	18	1.136672
30min / C	Q8BGL3	-0.05814	0.871284	0.729681	Sult2a8	Sulfotransf	ST2A8_MOU	13	0.9605
30min / C	Q8BZQ7	-0.05096	0.871343	0.729681	Anapc2	Anaphase-p	ANC2_MOU	4	0.965295
15min / C	Q3TLP5	0.015657	0.871615	0.729781	Echdc2	Enoyl-CoA	ECHD2_MC	11	1.010912
30min / C	P51855	0.004846	0.871888	0.729882	Gss	Glutathion	GSHB_MOU	24	1.003365
15min / C	O08738	0.114477	0.872527	0.730086	Casp6	Caspase-6	CASP6_MO	8	1.082583
15min / C	Q9R0N0	0.297743	0.87259	0.730086	Galk1	Galactokin	GALK1_MO	19	1.22922
15min / C	P01657	-0.08923	0.873145	0.730323	NaN	Ig kappa ch	KV3A5_MO	2	0.940022
30min / C	Q9R0Q7	-0.08561	0.873178	0.730323	Ptges3	Prostaglan	TEBP_MOU	7	0.942384
15min / C	Q8JZK9	0.104681	0.87353	0.730488	Hmgcs1	Hydroxyme	HMCS1_MC	18	1.075257
15min / C	Q8JZS0	0.153357	0.873793	0.730581	Lin7a	Protein lin-	LIN7A_MOU	7	1.112155
15min / C	Q9D0B5	-0.18382	0.874034	0.730654	Tstd3	Thiosulfate	TSTD3_MO	3	0.880368
15min / C	P15327	0.062128	0.874756	0.730981	Bpgm	Bisphosph	PMGE_MOU	7	1.044004
15min / C	P61027	1.005021	0.874976	0.730981	Rab10	Ras-related	RAB10_MO	5	2.006973
30min / C	P61759	0.15794	0.875801	0.730981	Vbp1	Prefoldin s	PFD3_MOU	7	1.115693
15min / C	Q61233	-0.04892	0.875678	0.730981	Lcp1	Plastin-2	PLSL_MOUS	33	0.966658
15min / C	Q9CR35	0.198857	0.875484	0.730981	Ctrb1	Chymotryp	CTRB1_MO	6	1.147788
30min / C	E9Q557	0.038609	0.877475	0.731379	Dsp	Desmoplak	DESP_MOU	33	1.027123
30min / C	P15327	-0.08268	0.877349	0.731379	Bpgm	Bisphosph	PMGE_MOU	7	0.944304
15min / C	P22599	0.103285	0.878909	0.731379	Serpina1b	Alpha-1-an	A1AT2_MO	7	1.074217
15min / C	Q8R1H0	0.118948	0.878061	0.731379	Hopx	Homeodon	HOP_MOUS	3	1.085942
30min / C	Q99JY0	-0.12183	0.878564	0.731379	Hadhb	Trifunction	ECHB_MOU	23	0.919018
15min / C	Q99KK7	0.071457	0.878859	0.731379	Dpp3	Dipeptidyl	DPP3_MOU	26	1.050778
30min / C	Q9CYZ2	-0.00726	0.876481	0.731379	Tpd52l2	Tumor prot	TPD54_MO	9	0.994982
30min / C	Q9D273	-0.00749	0.877379	0.731379	Mmab	Corrinoid a	MMAB_MO	7	0.994823
15min / C	Q9JM76	0.059814	0.877788	0.731379	Arpc3	Actin-relate	ARPC3_MO	6	1.042332
30min / C	Q99MP8	-0.18281	0.879705	0.731492	Brap	BRCA1-assc	BRAP_MOU	4	0.880984
30min / C	Q9D662	-0.29817	0.879691	0.731492	Sec23b	Protein tra	SC23B_MO	18	0.813281
15min / C	Q8VCC2	0.061173	0.879953	0.731509	Ces1	Liver carbo	EST1_MOUS	19	1.043314
15min / C	Q8BK48	0.131024	0.880133	0.731531	Ces2e	Pyrethroid	EST2E_MOU	21	1.095071
15min / C	P23589	0.189142	0.881185	0.731896	Ca5a	Carbonic ar	CAH5A_MC	12	1.140085
15min / C	P62897	-0.0491	0.88105	0.731896	Cycs	Cytochrom	CYC_MOUS	8	0.96654
15min / C	Q91VM5	0.073725	0.881471	0.732007	Rbmxl1	RNA bindin	RMXL1_MC	10	1.05243
30min / C	P01592	-0.06239	0.882524	0.732552	Jchain	Immunoglc	IGJ_MOUSE	4	0.957677
15min / C	Q8QZS1	0.040692	0.882468	0.732552	Hibch	3-hydroxyi	HIBCH_MO	21	1.028607
30min / C	Q8VBV7	0.274295	0.882587	0.732552	Cops8	COP9 signa	CSN8_MOU	5	1.209403
30min / C	P62500	-0.27405	0.883343	0.732693	Tsc22d1	TSC22 dom	T22D1_MO	1	0.826994
15min / C	P80317	-0.06706	0.88429	0.732693	Cct6a	T-complex	TCPZ_MOU	23	0.954582
15min / C	Q01279	-0.13262	0.883409	0.732693	Egfr	Epidermal	EGFR_MOU	23	0.91217
30min / C	Q9DBP5	-0.01701	0.884263	0.732693	Cmpk1	UMP-CMP	KCY_MOUS	12	0.988279
30min / C	Q8C0L6	-0.042	0.885266	0.732704	Paox	Peroxisom	PAOX_MOU	6	0.971308
15min / C	O88492	0.417294	0.885709	0.732725	Plin4	Perilipin-4	PLIN4_MOU	9	1.335421
30min / C	P06801	-0.00344	0.886607	0.733341	Me1	NADP-depe	MAOX_MO	33	0.99762
15min / C	P97372	-0.02574	0.887133	0.733523	Psme2	Proteasom	PSME2_MC	18	0.98232
15min / C	Q9QXT0	-0.01385	0.887089	0.733523	Cnpy2	Protein car	CNPY2_MO	8	0.990446
30min / C	O55042	0.562315	0.887753	0.733839	Snca	Alpha-synu	SYUA_MOU	2	1.476637

30min / C	P63005	-0.06139	0.888466	0.733839	Pafah1b1	Platelet-act	LIS1_MOUS	19	0.95834
15min / C	Q8BGC4	-0.03142	0.888646	0.733839	Ptgr3	Prostaglanc	PTGR3_MO	12	0.978459
15min / C	Q9D404	0.133614	0.888744	0.733839	Oxsm	3-oxoacyl-[	OXSM_MOU	14	1.097038
15min / C	Q9ET22	0.049157	0.888023	0.733839	Dpp7	Dipeptidyl	DPP2_MOU	12	1.03466
15min / C	P03987	-0.02164	0.889703	0.733905	NaN	Ig gamma-3	IGHG3_MO	8	0.985112
15min / C	P08003	0.011437	0.891768	0.733905	Pdia4	Protein disu	PDIA4_MO	31	1.007959
30min / C	P08003	0.04506	0.892153	0.733905	Pdia4	Protein disu	PDIA4_MO	31	1.031726
15min / C	P53026	0.236598	0.891135	0.733905	Rpl10a	Large ribos	RL10A_MO	10	1.178211
30min / C	P56213	0.15502	0.892134	0.733905	Gfer	FAD-linked	ALR_MOUS	8	1.113437
15min / C	P97290	-0.02586	0.891102	0.733905	Serping1	Plasma pro	IC1_MOUSI	12	0.982232
30min / C	Q80X50	-0.07733	0.892201	0.733905	Ubap2l	Ubiquitin-a	UBP2L_MO	14	0.947807
15min / C	Q91YS8	-0.2758	0.891766	0.733905	Camk1	Calcium/ca	KCC1A_MO	5	0.825993
15min / C	Q922D4	-0.00903	0.890525	0.733905	Ppp6r3	Serine/thre	PP6R3_MO	22	0.99376
15min / C	Q9D1M0	0.093296	0.891184	0.733905	Sec13	Protein SEC	SEC13_MO	10	1.066805
15min / C	Q9Z0P5	0.067725	0.889475	0.733905	Twf2	Twinfilin-2	TWF2_MOU	5	1.048063
15min / C	Q9Z0U1	-0.2295	0.889498	0.733905	Tjp2	Tight juncti	ZO2_MOUS	23	0.852933
15min / C	O89106	-0.07931	0.892567	0.734047	Fhit	Bis(5'-aden	FHIT_MOUS	8	0.94651
30min / C	Q9ESP1	-0.46133	0.89268	0.734047	Sdf2l1	Stromal cel	SDF2L_MO	4	0.726317
30min / C	P09528	0.021365	0.893592	0.734417	Fth1	Ferritin hea	FRIH_MOUS	13	1.014919
30min / C	P62075	-0.03353	0.893508	0.734417	Timm13	Mitochond	TIM13_MO	6	0.977028
15min / C	Q9DBZ5	-0.38932	0.895007	0.735074	Eif3k	Eukaryotic	EIF3K_MOU	9	0.76349
30min / C	Q9JIY5	0.036227	0.894937	0.735074	Htra2	Serine prot	HTRA2_MO	7	1.025429
15min / C	Q9JKB1	0.042386	0.894995	0.735074	Uchl3	Ubiquitin c	UCHL3_MC	8	1.029815
30min / C	O08749	0.024648	0.895987	0.735382	Dld	Dihydrolip	DLDH_MOU	22	1.017231
30min / C	P16406	0.024456	0.895997	0.735382	Enpep	Glutamyl a	AMPE_MOU	12	1.017096
15min / C	O08677	0.049296	0.896485	0.735441	Kng1	Kininogen-	KNG1_MOU	21	1.03476
30min / C	P16675	0.503002	0.89653	0.735441	Ctsa	Lysosomal	PPGB_MOU	9	1.417159
30min / C	P25444	-0.10718	0.896294	0.735441	Rps2	Small ribos	RS2_MOUS	15	0.928401
15min / C	Q3UJB0	0.466546	0.896975	0.735679	Sf3b2	Splicing fac	SF3B2_MO	18	1.381797
30min / C	Q9JJI8	0.033054	0.897507	0.735989	Rpl38	Large ribos	RL38_MOU	3	1.023175
30min / C	O08663	-0.11085	0.901288	0.736313	Metap2	Methionine	MAP2_MOU	10	0.926043
30min / C	P05063	-0.13941	0.901104	0.736313	Aldoc	Fructose-bi	ALDOC_MC	14	0.907892
30min / C	P20029	-0.05065	0.89978	0.736313	Hspa5	Endoplasm	BIP_MOUSI	38	0.965503
30min / C	P47753	-0.22561	0.9013	0.736313	Capza1	F-actin-cap	CAZA1_MO	9	0.855234
15min / C	P61161	0.017538	0.898811	0.736313	Actr2	Actin-relate	ARP2_MOU	15	1.012231
15min / C	Q61792	0.048518	0.902329	0.736313	Lasp1	LIM and SH	LASP1_MO	14	1.034202
30min / C	Q810U5	-0.20011	0.9024	0.736313	Ccdc50	Coiled-coil	CCD50_MC	2	0.870485
15min / C	Q8BLY2	0.752058	0.899731	0.736313	Tars3	Threonine-	SYTC2_MOU	13	1.684194
15min / C	Q8VCT3	0.095276	0.89895	0.736313	Rnpep	Aminopept	AMPB_MOU	19	1.06827
30min / C	Q925B0	0.062255	0.901356	0.736313	Pawr	PRKC apop	PAWR_MO	9	1.044097
15min / C	Q99L13	-0.00672	0.902444	0.736313	Hibadh	3-hydroxyi	3HIDH_MO	13	0.995351
15min / C	Q99MD9	-0.1035	0.900784	0.736313	Nasp	Nuclear aut	NASP_MOU	10	0.93077
30min / C	Q9CQ75	0.138227	0.898628	0.736313	Ndufa2	NADH dehy	NDUA2_MC	6	1.100552
30min / C	Q9DBM2	-0.10723	0.90238	0.736313	Ehhadh	Peroxisoma	ECHP_MOU	46	0.928371
30min / C	Q9QXC1	0.19606	0.901789	0.736313	Fetub	Fetuin-B	FETUB_MO	9	1.145566
15min / C	Q9R1Z7	-0.07843	0.900579	0.736313	Pts	6-pyruvoyl	PTPS_MOU	5	0.947087
15min / C	P01872	0.045499	0.903465	0.736411	Ighm	Immunoglo	IGHM_MOU	14	1.03204
30min / C	P29788	-0.25781	0.902894	0.736411	Vtn	Vitronectin	VTNC_MOU	9	0.836359
15min / C	Q07417	0.084725	0.903567	0.736411	Acads	Short-chain	ACADS_MO	16	1.060486
30min / C	Q99PL5	-0.00119	0.903868	0.736412	Rrbp1	Ribosome-b	RRBP1_MO	82	0.999178
15min / C	P59999	0.049111	0.9045	0.736715	Arpc4	Actin-relate	ARPC4_MO	11	1.034627
30min / C	Q8VDJ3	-0.46301	0.904556	0.736715	Hdlbp	Vigilin	VIGLN_MO	65	0.725472

15min / C	O55234	-0.02736	0.904795	0.736784	Psmb5	Proteasom	PSB5_MOU	14	0.981212
30min / C	P52480	0.037455	0.905515	0.736803	Pkm	Pyruvate ki	KPYM_MOU	33	1.026302
15min / C	P68037	-0.11125	0.905466	0.736803	Ube2l3	Ubiquitin-c	UB2L3_MO	9	0.925783
15min / C	P27659	0.500363	0.9066	0.737024	Rpl3	Large ribos	RL3_MOUS	20	1.41457
15min / C	P63101	0.010714	0.906256	0.737024	Ywhaz	14-3-3 prot	1433Z_MO	11	1.007454
30min / C	Q8BFQ8	0.186352	0.906535	0.737024	Gatd1	Glutamine	GALD1_MC	4	1.137883
15min / C	Q91WD5	-0.27095	0.9066	0.737024	Ndufs2	NADH dehy	NDUS2_MC	12	0.828775
30min / C	Q9CXF0	-0.20968	0.906632	0.737024	Kynu	Kynurenina	KYNU_MOU	20	0.864731
15min / C	A2AQ19	-0.43213	0.909417	0.73708	Rtf1	RNA polym	RTF1_MOU	4	0.741167
15min / C	P01898	0.023417	0.907327	0.73708	H2-Q10	H-2 class I h	HA10_MOU	8	1.016364
15min / C	P17751	0.007126	0.90869	0.73708	Tpi1	Triosephos	TPIS_MOUS	18	1.004952
15min / C	P46938	0.107369	0.909516	0.73708	Yap1	Transcripti	YAP1_MOU	6	1.077262
30min / C	P56480	-0.09157	0.909712	0.73708	Atp5f1b	ATP syntha	ATPB_MOU	26	0.938502
15min / C	P56593	0.065103	0.907511	0.73708	Cyp2a12	Cytochrom	CP2AC_MO	24	1.04616
15min / C	P56812	-0.01663	0.909193	0.73708	Pdcd5	Programme	PDCD5_MC	6	0.988537
15min / C	Q3TEA8	-0.7302	0.908032	0.73708	Hp1bp3	Heterochrc	HP1B3_MC	13	0.60282
15min / C	Q64442	-0.02406	0.90944	0.73708	Sord	Sorbitol de	DHSO_MOU	23	0.983461
30min / C	Q78ZA7	0.173725	0.910226	0.73708	Nap1l4	Nucleosom	NP1L4_MO	8	1.127967
30min / C	Q91WG0	0.207731	0.910289	0.73708	Ces2c	Acylcarniti	EST2C_MOU	11	1.154871
15min / C	Q9CPR4	-0.1133	0.909367	0.73708	Rpl17	Large ribos	RL17_MOU	6	0.924473
15min / C	Q9D1G1	-0.34872	0.908467	0.73708	Rab1b	Ras-related	RAB1B_MO	5	0.78528
30min / C	Q9DBB9	0.027247	0.910384	0.73708	Cpn2	Carboxype	CPN2_MOU	11	1.019065
30min / C	Q9EPU0	-0.18042	0.909444	0.73708	Upf1	Regulator c	RENT1_MO	34	0.882443
15min / C	Q9R062	0.173255	0.906885	0.73708	Gyg1	Glycogenin	GLYG_MOU	5	1.1276
15min / C	Q7TPR4	0.003381	0.91108	0.737261	Actn1	Alpha-actir	ACTN1_MO	48	1.002346
30min / C	P11589	0.011099	0.911303	0.737272	Mup2	Major urina	MUP2_MO	2	1.007723
15min / C	P54731	-0.30001	0.911411	0.737272	Faf1	FAS-associa	FAF1_MOU	7	0.81225
15min / C	P12787	0.289058	0.911975	0.737448	Cox5a	Cytochrom	COX5A_MC	4	1.221843
15min / C	Q99LI8	0.138357	0.911828	0.737448	Hgs	Hepatocyte	HGS_MOUS	12	1.100651
30min / C	P41105	0.130935	0.913601	0.73751	Rpl28	Large ribos	RL28_MOU	7	1.095003
15min / C	P62627	0.009472	0.913597	0.73751	Dynlrb1	Dynein ligh	DLRB1_MO	5	1.006587
30min / C	P80318	0.171704	0.912968	0.73751	Cct3	T-complex	TCPG_MOU	34	1.126388
15min / C	Q8R1Q9	-0.18831	0.913089	0.73751	Rbks	Ribokinase	RBSK_MOU	4	0.877633
15min / C	Q8VCH8	-0.20763	0.913503	0.73751	Ubxn4	UBX domai	UBXN4_MC	7	0.865959
15min / C	Q99P30	0.016455	0.912808	0.73751	Nudt7	Peroxisoma	NUDT7_MC	17	1.011471
15min / C	Q9CZX8	-0.05396	0.91371	0.73751	Rps19	Small ribos	RS19_MOU	8	0.963286
15min / C	Q0P678	0.216457	0.914236	0.7376	Zc3h18	Zinc finger	ZCH18_MO	6	1.161877
30min / C	Q78J03	-0.18605	0.914284	0.7376	Msrb2	Methionine	MSRB2_MC	3	0.879008
30min / C	Q80WJ7	0.171545	0.914245	0.7376	Mtdh	Protein LYF	LYRIC_MOU	15	1.126264
30min / C	Q9JIL4	0.021298	0.914483	0.737635	Pdzk1	Na(+)/H(+) e	NHRF3_MC	22	1.014872
30min / C	Q6NSR8	-0.22242	0.9158	0.73854	Npepl1	Probable ar	PEPL1_MO	16	0.857124
15min / C	Q9EQ20	0.026853	0.915913	0.73854	Aldh6a1	Methylmalc	MMSA_MO	28	1.018788
15min / C	Q9JHU2	0.01274	0.916751	0.738738	Palmd	Palmdelphi	PALMD_MC	12	1.00887
15min / C	Q68FD5	0.386876	0.917775	0.739168	Cltc	Clathrin he	CLH1_MOU	81	1.307559
30min / C	P57759	-0.04271	0.919043	0.73994	Erp29	Endoplasm	ERP29_MO	9	0.97083
30min / C	Q9WU19	-0.077	0.919699	0.740253	Hao1	2-Hydroxya	HAOX1_MC	17	0.948029
30min / C	Q91ZA3	0.149972	0.920154	0.740461	Pcca	Propionyl-(	PCCA_MOU	34	1.109548
15min / C	P42232	0.052625	0.920636	0.740599	Stat5b	Signal trans	STA5B_MO	18	1.037151
30min / C	Q8BJF9	-0.06318	0.920539	0.740599	Chmp2b	Charged mu	CHM2B_MC	2	0.957153
15min / C	P16406	0.219841	0.921313	0.740856	Enpep	Glutamyl ai	AMPE_MOU	12	1.164605
30min / C	Q8BH35	0.039442	0.92142	0.740856	C8b	Compleme	CO8B_MOU	12	1.027716
15min / C	Q8K4H1	0.093455	0.921935	0.740995	Afmid	Kynurenine	KFA_MOUS	14	1.066922

30min / C	Q9CQW2	-0.71731	0.921981	0.740995	Arl8b	ADP-ribosy	ARL8B_MO	3	0.608232
15min / C	P50396	0.029401	0.922459	0.741068	Gdi1	Rab GDP di	GDIA_MOU	17	1.020589
15min / C	P07356	0.012896	0.922939	0.741245	Anxa2	Annexin A2	ANXA2_MC	22	1.008979
30min / C	Q3UQ44	-0.22006	0.923156	0.741245	Iqgap2	Ras GTPase·	IQGA2_MO	63	0.858529
15min / C	P51410	0.302113	0.924308	0.74125	Rpl9	Large ribos	RL9_MOUS	10	1.232949
15min / C	P51660	0.046887	0.92413	0.74125	Hsd17b4	Peroxisoma	DHB4_MOU	37	1.033034
15min / C	P58252	0.019009	0.923981	0.74125	Eef2	Elongation	EF2_MOUSI	58	1.013263
30min / C	P97429	0.092966	0.924245	0.74125	Anxa4	Annexin A4	ANXA4_MC	18	1.066561
15min / C	Q61703	-0.04024	0.924118	0.74125	Itih2	Inter-alpha	ITIH2_MOU	17	0.97249
15min / C	Q80XI4	0.044881	0.924391	0.74125	Pip4k2b	Phosphatic	PI42B_MOI	1	1.031598
30min / C	P97930	-0.01334	0.924722	0.74139	Dtymk	Thymidylat	KTHY_MOU	5	0.990797
30min / C	P01863;P0	0.099201	0.925415	0.741822	Ighg;Ighg2a	Ig gamma-2	GCAA_MOU	5	1.07118
15min / C	Q91X52	-0.0129	0.925631	0.74187	Dcxr	L-xylulose r	DCXR_MOU	14	0.991102
15min / C	Q8BGZ7	-0.04343	0.925987	0.741907	Krt75	Keratin, typ	K2C75_MO	2	0.970346
30min / C	P14148	0.022716	0.92648	0.742178	Rpl7	Large ribos	RL7_MOUS	16	1.01587
30min / C	P62264	-0.13411	0.926919	0.742289	Rps14	Small ribos	RS14_MOU	5	0.911229
30min / C	Q9D7S9	0.229645	0.927085	0.742289	Chmp5	Charged mu	CHMP5_MO	3	1.172546
15min / C	P08113	0.002533	0.928225	0.742953	Hsp90b1	Endoplasm	ENPL_MOU	43	1.001757
15min / C	O09174	0.059138	0.9284	0.742968	Amacr	Alpha-meth	AMACR_MO	20	1.041843
30min / C	Q8CHR6	0.015287	0.928823	0.743059	Dpyd	Dihydropyr	DPYD_MOU	48	1.010652
15min / C	Q9DCT8	-0.05017	0.928799	0.743059	Crip2	Cysteine-ric	CRIP2_MOI	12	0.965822
15min / C	P61982	-0.03413	0.92934	0.743348	Ywhag	14-3-3 prot	1433G_MC	15	0.976619
15min / C	P62077	-0.16007	0.930746	0.743601	Timm8b	Mitochond	TIM8B_MO	4	0.894981
15min / C	P83882	0.168972	0.930724	0.743601	Rpl36a	Large ribos	RL36A_MO	4	1.124257
30min / C	Q641P0	-0.1301	0.930602	0.743601	Actr3b	Actin-relate	ARP3B_MO	2	0.913771
15min / C	P06330	-0.02403	0.931343	0.743705	NaN	Ig heavy ch	HVM51_MO	5	0.983482
30min / C	Q91XC8	0.726359	0.931273	0.743705	Dap	Death-asso	DAP1_MOU	3	1.654459
15min / C	O08807	0.015784	0.931855	0.74377	Prdx4	Peroxiredo	PRDX4_MC	11	1.011
15min / C	O08810	0.049389	0.932522	0.74377	Eftud2	116 kDa U5	U5S1_MOU	23	1.034826
15min / C	O35955	0.119335	0.93303	0.74377	Psmb10	Proteasom	PSB10_MO	9	1.086234
30min / C	P26645	0.257402	0.932882	0.74377	Marcks	Myristoylat	MARCS_MC	13	1.195325
30min / C	P49710	-0.05886	0.932408	0.74377	Hcls1	Hematopoi	HCLS1_MO	5	0.960022
15min / C	Q99MN9	0.019021	0.93201	0.74377	Pccb	Propionyl-C	PCCB_MOU	25	1.013272
30min / C	Q9Z0N2	0.193091	0.93308	0.74377	Eif2s3y	Eukaryotic	IF2H_MOUS	17	1.14321
15min / C	Q922J3	-0.14479	0.933675	0.744064	Clip1	CAP-Gly do	CLIP1_MOU	23	0.904511
30min / C	Q64433	-0.04318	0.933983	0.744073	Hspe1	10 kDa heat	CH10_MOU	9	0.970516
30min / C	Q9DBT5	-0.1848	0.934653	0.744335	Ampd2	AMP deami	AMPD2_MO	11	0.87977
30min / C	Q9DCS2	0.091549	0.93481	0.744335	Mettl26	Methyltran	MTL26_MC	13	1.065514
30min / C	Q9R1Z8	-0.18857	0.934935	0.744335	Sorbs3	Vinexin	VINEX_MOU	5	0.877474
30min / C	Q8BIQ5	-0.21833	0.935376	0.744562	Cstf2	Cleavage sti	CSTF2_MOU	9	0.859559
15min / C	O08539	0.171647	0.936786	0.744752	Bin1	Myc box-de	BIN1_MOU	8	1.126344
15min / C	P01644;P0	0.260856	0.936847	0.744752	NaN	Ig kappa ch	KV5AB_MO	2	1.198189
15min / C	P35980	0.02185	0.936325	0.744752	Rpl18	Large ribos	RL18_MOU	7	1.015261
30min / C	P36552	-0.05673	0.93704	0.744752	Cpox	Oxygen-dep	HEM6_MOU	20	0.961443
15min / C	P70694	0.128092	0.936588	0.744752	Akr1c6	Estradiol 17	DHB5_MOU	20	1.092847
15min / C	Q03958	-0.31454	0.937167	0.744752	Pfdn6	Prefoldin su	PFD6_MOU	5	0.804106
30min / C	Q61792	0.053631	0.93613	0.744752	Lasp1	LIM and SH	LASP1_MOU	14	1.037874
30min / C	Q8R146	0.037566	0.937173	0.744752	Apeh	Acylamino-	ACPH_MOU	26	1.026381
30min / C	Q91Z58	-0.12723	0.935918	0.744752	NaN	Uncharacte	CF132_MO	14	0.915591
30min / C	Q9Z2H5	0.262193	0.936549	0.744752	Epb41l1	Band 4.1-lil	E41L1_MOU	18	1.199301
30min / C	O70251	-0.15579	0.938647	0.745208	Eef1b	Elongation	EF1B_MOU	10	0.897641
15min / C	P15105	0.086425	0.940108	0.745208	Glul	Glutamine	GLNA_MOU	19	1.061736

30min / C	P23953	-0.03114	0.940293	0.745208	Ces1c	Carboxyles	EST1C_MOU	10	0.978646
15min / C	P29391	-0.00636	0.938644	0.745208	Ftl1	Ferritin ligh	FRIL1_MOU	12	0.995604
15min / C	P32261	-0.00408	0.938339	0.745208	Serpinc1	Antithromb	ANT3_MOU	26	0.997173
15min / C	P54822	-0.0659	0.939308	0.745208	Adsl	Adenylosuc	PUR8_MOU	19	0.95535
15min / C	P62307	-0.21516	0.940443	0.745208	Snrpf	Small nucle	RUXF_MOU	4	0.861448
15min / C	P62908	-0.2206	0.938632	0.745208	Rps3	Small ribos	RS3_MOUS	19	0.858211
15min / C	P63028	-0.05179	0.940448	0.745208	Tpt1	Translation	TCTP_MOU	8	0.964741
30min / C	P70297	0.068815	0.93977	0.745208	Stam	Signal trans	STAM1_MC	7	1.048855
15min / C	Q8BMF4	-0.05896	0.940553	0.745208	Dlat	Dihydrolip	ODP2_MOU	19	0.959958
15min / C	Q8VCT4	0.041897	0.940249	0.745208	Ces1d	Carboxyles	EST1D_MO	30	1.029466
15min / C	Q9DCB8	-0.1062	0.940528	0.745208	Isca2	Iron-sulfur	ISCA2_MOU	4	0.929032
15min / C	Q9EPL9	0.067668	0.938792	0.745208	Acox3	Peroxisoma	ACOX3_MC	11	1.048021
15min / C	Q9QY93	0.400317	0.939543	0.745208	Dctpp1	dCTP pyrop	DCTP1_MO	3	1.319798
30min / C	P97351	0.161801	0.941118	0.74542	Rps3a	Small ribos	RS3A_MOU	16	1.118683
15min / C	Q8R050	-0.03143	0.941313	0.74542	Gspt1	Eukaryotic	ERF3A_MO	21	0.978447
15min / C	Q91V76	0.036197	0.941405	0.74542	NaN	Ester hydro	CK054_MO	16	1.025407
30min / C	Q9ET01	-0.02442	0.941444	0.74542	Pygl	Glycogen p	PYGL_MOU	46	0.983216
15min / C	Q3UNZ8	-0.04977	0.941901	0.745659	Cryzl2	Quinone ox	QORL2_MC	12	0.966093
15min / C	P03958	0.035095	0.942166	0.745745	Ada	Adenosine	ADA_MOUS	9	1.024624
30min / C	Q91V12	-0.07018	0.942594	0.745837	Acot7	Cytosolic a	BACH_MOU	10	0.95252
30min / C	A2BIM8;P0	0.048787	0.943035	0.745884	Mup18;Mu	Major urina	MUP18_MC	14	1.034395
30min / C	P01754;P0	0.036133	0.943331	0.745884	Ighv1-62-3;	Ig heavy cha	HVM10_MC	1	1.025361
15min / C	P11930	0.057669	0.944004	0.745884	Nudt19	Acyl-coenzy	NUD19_MC	11	1.040783
15min / C	P57780	0.047738	0.943471	0.745884	Actn4	Alpha-actin	ACTN4_MO	40	1.033643
15min / C	Q9CZU3	-0.11801	0.943868	0.745884	Mtrex	Exosome RI	MTREX_MC	10	0.92146
15min / C	Q9Z172	-0.03429	0.944214	0.745884	Sumo3	Small ubiqu	SUMO3_MC	2	0.976512
15min / C	P06801	0.030434	0.944776	0.745996	Me1	NADP-depe	MAOX_MO	33	1.02132
30min / C	P16330	-0.49812	0.944981	0.745996	Cnp	2',3'-cyclic-	CN37_MOU	7	0.70803
30min / C	P45591	-0.00718	0.9449	0.745996	Cfl2	Cofilin-2	COF2_MOU	11	0.995034
30min / C	P62962	0.015687	0.945389	0.746071	Pfn1	Profilin-1	PROF1_MO	9	1.010933
30min / C	Q9CZM2	-0.19824	0.94566	0.746071	Rpl15	Large ribos	RL15_MOU	9	0.871616
15min / C	Q9JHI5	0.02658	0.945699	0.746071	Ivd	Isovaleryl-C	IVD_MOUS	18	1.018595
15min / C	P10649	0.008943	0.946337	0.746451	Gstm1	Glutathion	GSTM1_MC	15	1.006218
15min / C	Q9EQS3	0.476711	0.946544	0.746491	Mycbp	c-Myc-bind	MYCBP_MC	6	1.391568
30min / C	Q61176	-0.00729	0.947793	0.746823	Arg1	Arginase-1	ARGI1_MO	21	0.994957
30min / C	Q6P8M1	-0.12451	0.947362	0.746823	Tatdn1	Deoxyribor	TATD1_MO	7	0.917316
30min / C	P28474	0.017357	0.948682	0.747202	Adh5	Alcohol del	ADHX_MOU	20	1.012104
30min / C	Q61160	-0.22045	0.948841	0.747202	Fadd	FAS-associa	FADD_MOU	3	0.858299
15min / C	Q9D826	0.009072	0.948852	0.747202	Pipox	Peroxisoma	SOX_MOUS	20	1.006308
15min / C	P24472	0.101566	0.951008	0.747274	Gsta4	Glutathion	GSTA4_MO	10	1.072938
15min / C	P99025	-0.04038	0.94975	0.747274	Gchfr	GTP cycloh	GFRP_MOU	6	0.972401
30min / C	Q61147	0.029414	0.951015	0.747274	Cp	Ceruloplasr	CERU_MOU	47	1.020597
15min / C	Q8BP67	-0.01416	0.950508	0.747274	Rpl24	Large ribos	RL24_MOU	9	0.990236
30min / C	Q8BWY3	0.065627	0.950102	0.747274	Etf1	Eukaryotic	ERF1_MOU	19	1.04654
15min / C	Q8VC12	0.03828	0.950761	0.747274	Uroc1	Urocanate	HUTU_MOU	34	1.026889
15min / C	Q8VCU1	0.036227	0.950281	0.747274	Ces3b	Carboxyles	EST3B_MOU	8	1.025429
30min / C	Q9CPR4	-0.12713	0.95044	0.747274	Rpl17	Large ribos	RL17_MOU	6	0.915651
15min / C	Q9CQE1	-0.00483	0.949795	0.747274	Nipsnap3b	Protein Nip	NPS3B_MO	13	0.996658
15min / C	Q9JHU4	0.729901	0.950564	0.747274	Dync1h1	Cytoplasmi	DYHC1_MC	145	1.658525
30min / C	Q9JLJ2	-0.03606	0.950548	0.747274	Aldh9a1	4-trimethyl	AL9A1_MO	27	0.975314
15min / C	P16460	0.03551	0.951563	0.747489	Ass1	Argininosu	ASSY_MOUS	26	1.024919
30min / C	Q99KK7	0.115609	0.952003	0.747589	Dpp3	Dipeptidyl	DPP3_MOU	26	1.083432

15min / C	Q9R020	0.155782	0.951952	0.747589	Zranb2	Zinc finger	ZRAB2_MO	7	1.114026
30min / C	Q8JZQ9	0.008901	0.952475	0.747714	Eif3b	Eukaryotic	EIF3B_MOU	31	1.006189
30min / C	Q921X9	0.127619	0.952459	0.747714	Pdia5	Protein dis	PDIA5_MO	14	1.092489
30min / C	O08585	-0.06171	0.953153	0.747825	Clta	Clathrin lig	CLCA_MOU	5	0.958129
15min / C	P01029	-0.00233	0.953555	0.747825	C4b	Compleme	CO4B_MOU	47	0.998384
15min / C	Q3TLH4	-0.10512	0.953269	0.747825	Prrc2c	Protein PR	PRC2C_MO	26	0.929726
15min / C	Q9QWL7	-0.01613	0.953436	0.747825	Krt17	Keratin, typ	K1C17_MO	17	0.988883
15min / C	O88569	-0.04909	0.95486	0.748608	Hnrnpa2b1	Heterogene	ROA2_MOU	19	0.966547
30min / C	Q6P5F9	-0.20058	0.954959	0.748608	Xpo1	Exportin-1	XPO1_MOU	26	0.870202
30min / C	Q8JZS0	-0.03231	0.95518	0.748608	Lin7a	Protein lin-	LIN7A_MO	7	0.977852
30min / C	Q9D1F4	0.440662	0.955356	0.748624	Akt1s1	Proline-ricl	AKTS1_MO	4	1.357227
30min / C	Q9CPX6	-0.2715	0.956002	0.748761	Atg3	Ubiquitin-l	ATG3_MOU	8	0.828458
30min / C	Q9D7B6	0.303684	0.9558	0.748761	Acad8	Isobutyryl-	ACAD8_MC	20	1.234293
30min / C	Q99KV1	-0.23367	0.956337	0.748901	Dnajb11	DnaJ homo	DJB11_MO	10	0.850469
15min / C	Q60648	-0.32031	0.956627	0.748972	Gm2a	Ganglioside	SAP3_MOU	5	0.800898
30min / C	Q8K183	0.214535	0.956873	0.748972	Pdxk	Pyridoxal k	PDXK_MOU	12	1.16033
15min / C	P62242	0.228835	0.957295	0.749064	Rps8	Small ribos	RS8_MOUS	8	1.171889
15min / C	P04117	-0.03346	0.95803	0.749245	Fabp4	Fatty acid-l	FABP4_MO	9	0.977076
15min / C	O35551	0.071703	0.959094	0.749587	Rabep1	Rab GTPase	RABE1_MO	15	1.050957
15min / C	P01646	0.543038	0.958801	0.749587	NaN	Ig kappa ch	KV5AD_MC	1	1.457037
30min / C	P15105	0.080851	0.958851	0.749587	Glul	Glutamine	GLNA_MOU	19	1.057642
15min / C	P42932	-0.00387	0.959817	0.749642	Cct8	T-complex	TCPQ_MOU	32	0.99732
30min / C	Q9CR57	-0.11204	0.95995	0.749642	Rpl14	Large ribos	RL14_MOU	5	0.925276
30min / C	P53657	0.054375	0.960668	0.749696	Pklr	Pyruvate ki	KPYR_MOU	35	1.038409
15min / C	P80314	0.009189	0.960735	0.749696	Cct2	T-complex	TCPB_MOU	28	1.006389
30min / C	Q4KML4	-0.05235	0.96174	0.75006	Abracl	Costars fam	ABRAL_MO	3	0.964367
15min / C	O55013	0.010817	0.962933	0.750096	Trappc3	Trafficking	TPPC3_MO	9	1.007526
30min / C	P12787	0.497538	0.962266	0.750096	Cox5a	Cytochrom	COX5A_MC	4	1.411802
15min / C	P35585	0.13356	0.962049	0.750096	Ap1m1	AP-1 compl	AP1M1_MC	17	1.096998
15min / C	P63325	-0.3242	0.963389	0.750096	Rps10	Small ribos	RS10_MOU	11	0.798739
30min / C	Q6P2K6	-0.26748	0.963251	0.750096	Ppp4r3a	Serine/thre	P4R3A_MO	11	0.83077
15min / C	Q8R0J8	-0.00868	0.962848	0.750096	Idnk	Probable gl	GNTK_MOU	5	0.993999
15min / C	Q9D9H8	0.12589	0.963263	0.750096	NaN	Mitochond	CB069_MO	2	1.091181
15min / C	Q9QZ08	0.032435	0.963133	0.750096	Nagk	N-acetyl-D-	NAGK_MOU	9	1.022737
15min / C	Q3UIA2	0.027194	0.964412	0.750674	Arhgap17	Rho GTPase	RHG17_MC	9	1.019028
15min / C	Q3UM45	-0.11767	0.96486	0.750873	Ppp1r7	Protein pho	PP1R7_MO	15	0.921676
15min / C	Q9EPU0	-0.15682	0.965219	0.750936	Upf1	Regulator c	RENT1_MO	34	0.896997
30min / C	O35945	-0.01903	0.96608	0.750952	Aldh1a7	Aldehyde d	AL1A7_MO	30	0.986895
15min / C	P18528	-0.08899	0.965877	0.750952	NaN	Ig heavy ch	HVM57_MC	2	0.940184
15min / C	Q8BTZ7	0.009613	0.966039	0.750952	Gmppb	Mannose-1	GMPPB_MC	10	1.006686
15min / C	Q99JT9	-0.0815	0.9661	0.750952	Adi1	Acireducto	MTND_MO	10	0.945074
30min / C	Q9D1P4	-0.10654	0.96634	0.750952	Chordc1	Cysteine an	CHRD1_MC	13	0.928815
15min / C	Q6P069	-0.17997	0.966716	0.751	Sri	Sorcin	SORCN_MC	11	0.882719
30min / C	Q99KN9	-0.23711	0.966704	0.751	Clint1	Clathrin int	EPN4_MOU	12	0.848443
15min / C	Q99J99	0.151189	0.967223	0.751028	Mpst	3-mercapto	THTM_MOU	14	1.110484
15min / C	Q921W0	-0.15961	0.967539	0.751151	Chmp1a	Charged mu	CHM1A_MC	1	0.895266
15min / C	P00405	-0.50945	0.969278	0.751588	Mtco2	Cytochrom	COX2_MOU	6	0.702492
15min / C	P12970	-0.12249	0.969121	0.751588	Rpl7a	Large ribos	RL7A_MOU	13	0.918598
30min / C	Q3TNA1	-0.06996	0.968936	0.751588	Xylb	Xylulose kir	XYLB_MOU	21	0.952664
30min / C	Q61249	0.027246	0.969359	0.751588	Igbp1	Immunoglo	IGBP1_MO	10	1.019065
15min / C	Q8CI08	-0.06058	0.96833	0.751588	Slain2	SLAIN moti	SLAI2_MOU	5	0.958876
15min / C	Q8R121	-0.04555	0.96919	0.751588	Serpina10	Protein Z-d	ZPI_MOUSE	6	0.968919

15min / C	Q9QXC1	-0.03419	0.968886	0.751588	Fetub	Fetuin-B	FETUB_MO	9	0.976578
15min / C	G5E8V9	0.016707	0.970649	0.751856	Arfip1	Arfaptin-1	ARFP1_MO	8	1.011648
15min / C	P49718	0.042058	0.970181	0.751856	Mcm5	DNA replica	MCM5_MO	9	1.029581
30min / C	Q9D154	-0.04192	0.97034	0.751856	Serpinb1a	Leukocyte e	ILEUA_MOU	12	0.971362
15min / C	P47753	-0.32347	0.971763	0.752109	Capza1	F-actin-cap	CAZA1_MO	9	0.799144
30min / C	P80314	-0.05511	0.971528	0.752109	Cct2	T-complex p	TCPB_MOU	28	0.962518
15min / C	B2RQA1	-0.02286	0.974762	0.752285	Kcna10	Potassium v	KCA10_MO	1	0.984282
15min / C	P15626	0.148931	0.973721	0.752285	Gstm2	Glutathion	GSTM2_MC	21	1.108747
30min / C	P26043	0.029593	0.973922	0.752285	Rdx	Radixin	RADI_MOU	42	1.020724
30min / C	P46938	-0.02448	0.973958	0.752285	Yap1	Transcripti	YAP1_MOU	6	0.983178
30min / C	P53026	0.190529	0.974779	0.752285	Rpl10a	Large ribos	RL10A_MO	10	1.141182
15min / C	P60334	0.503702	0.973772	0.752285	Cdo1	Cysteine di	CDO1_MOU	7	1.417847
30min / C	P60334	0.437621	0.974351	0.752285	Cdo1	Cysteine di	CDO1_MOU	7	1.354369
15min / C	Q8CFX1	0.392681	0.973675	0.752285	H6pd	GDH/6PGL	G6PE_MOU	22	1.312831
30min / C	Q8VCT3	0.123136	0.975137	0.752285	Rnpep	Aminopept	AMPB_MOU	19	1.089099
15min / C	Q9D964	0.026398	0.974905	0.752285	Gatm	Glycine am	GATM_MOU	10	1.018466
30min / C	Q9D964	0.475449	0.972616	0.752285	Gatm	Glycine am	GATM_MOU	10	1.390351
30min / C	Q9EPV8	-0.12276	0.975011	0.752285	Ubl5	Ubiquitin-l	UBL5_MOU	3	0.918428
15min / C	Q9WUB3	-0.05512	0.974497	0.752285	Pygm	Glycogen p	PYGM_MOU	12	0.962514
30min / C	Q9JM76	-0.03449	0.975525	0.752462	Arpc3	Actin-relate	ARPC3_MO	6	0.976374
15min / C	P13634	-0.00906	0.975799	0.752524	Ca1	Carbonic ar	CAH1_MOU	14	0.993741
30min / C	P62305	-0.17312	0.97592	0.752524	Snrpe	Small nucle	RUXE_MOU	3	0.88692
15min / C	Q9DCW4	0.007887	0.976337	0.752724	Etfb	Electron tra	ETFB_MOUS	16	1.005482
15min / C	P46412	-0.23366	0.977551	0.752834	Gpx3	Glutathion	GPX3_MOU	4	0.850474
30min / C	P47934	-0.02175	0.977081	0.752834	Crat	Carnitine O	CACP_MOU	12	0.98504
15min / C	Q61147	0.022471	0.977392	0.752834	Cp	Ceruloplasr	CERU_MOU	47	1.015698
30min / C	Q8K023	-0.05598	0.977725	0.752834	Akr1c18	Aldo-keto r	AKC1H_MC	6	0.96194
30min / C	Q921W0	-0.04672	0.976904	0.752834	Chmp1a	Charged mu	CHM1A_M	1	0.968133
30min / C	Q9ER00	0.006457	0.97774	0.752834	Stx12	Syntaxin-12	STX12_MO	4	1.004485
15min / C	O09172	0.142295	0.97884	0.752911	Gclm	Glutamate-	GSH0_MOU	11	1.103659
30min / C	P47754	-0.06958	0.978342	0.752911	Capza2	F-actin-cap	CAZA2_MO	15	0.952913
30min / C	P55050	0.087763	0.978145	0.752911	Fabp2	Fatty acid-b	FABPI_MOU	6	1.062721
30min / C	P62077	0.195383	0.979257	0.752911	Timm8b	Mitochond	TIM8B_MO	4	1.145028
15min / C	Q9D7J9	0.601163	0.979249	0.752911	Echdc3	Enoyl-CoA h	ECHD3_MC	3	1.516939
30min / C	Q9DBX2	0.710692	0.978965	0.752911	Pdcl	Phosducin-	PHLP_MOU	3	1.636589
30min / C	Q9R1A8	0.199506	0.978659	0.752911	Cop1	E3 ubiquiti	COP1_MOU	1	1.148305
30min / C	Q9WV68	-0.10425	0.979098	0.752911	Decr2	Peroxisoma	DECR2_MO	13	0.93029
15min / C	Q6IRU5	0.076424	0.979618	0.752947	Cltb	Clathrin lig	CLCB_MOU	9	1.054401
15min / C	Q8BGQ7	0.047499	0.979495	0.752947	Aars1	Alanine--tR	SYAC_MOU	43	1.033472
30min / C	Q9DC63	0.029696	0.980471	0.753481	Fbxo3	F-box only p	FBX3_MOU	9	1.020797
15min / C	Q8BGS2	0.169751	0.981025	0.75369	Bola2	BolA-like pr	BOLA2_MO	4	1.124864
30min / C	Q91X34	-0.13818	0.981373	0.75369	Baat	Bile acid-Co	BAAT_MOU	15	0.908662
30min / C	Q91YE6	0.057163	0.981268	0.75369	Ipo9	Importin-9	IPO9_MOU	12	1.040418
30min / C	Q9CQT1	-0.06721	0.981308	0.75369	Mri1	Methylthio	MTNA_MOU	19	0.954482
15min / C	Q9D0F9	0.006797	0.981789	0.753767	Pgm1	Phosphoglu	PGM1_MOU	35	1.004722
15min / C	P28665	0.024705	0.982006	0.753812	Mug1	Murinoglol	MUG1_MO	63	1.017272
15min / C	P10637	-0.10391	0.983021	0.754132	Mapt	Microtubul	TAU_MOUS	15	0.930511
30min / C	Q9D7M1	-0.12607	0.982771	0.754132	Gid8	Glucose-inc	GID8_MOU	3	0.916322
15min / C	Q9Z2I8	-0.00915	0.983004	0.754132	Suclg2	Succinate--	SUCB2_MO	21	0.99368
30min / C	Q9CY64	-0.11767	0.983939	0.754569	Blvra	Biliverdin r	BIEA_MOUS	13	0.921676
15min / C	P14148	0.052725	0.98625	0.754724	Rpl7	Large ribos	RL7_MOUS	16	1.037222
15min / C	P62301	-0.23376	0.986184	0.754724	Rps13	Small ribos	RS13_MOU	8	0.850417

30min / C	P97823	-0.04275	0.985378	0.754724	Lypla1	Acyl-protei	LYPA1_MOU	8	0.970803
30min / C	Q05117	0.106864	0.985621	0.754724	Acp5	Tartrate-res	PPA5_MOU	6	1.076885
15min / C	Q05D44	0.194606	0.984674	0.754724	Eif5b	Eukaryotic	IF2P_MOUS	27	1.144412
15min / C	Q60974	-0.10775	0.985218	0.754724	Ncor1	Nuclear rec	NCOR1_MO	6	0.928034
15min / C	Q99KR7	-0.01662	0.984594	0.754724	Ppif	Peptidyl-pr	PPIF_MOUS	10	0.988548
30min / C	Q9CYK1	-0.02871	0.985071	0.754724	Wars2	Tryptophar	SYWM_MO	5	0.980299
15min / C	Q9D7V9	-0.36888	0.986285	0.754724	Naaa	N-acylethar	NAAA_MOU	2	0.774384
15min / C	Q9R112	-0.01834	0.986303	0.754724	Sqor	Sulfide:qui	SQOR_MOU	28	0.987369
30min / C	O08677	0.010496	0.986677	0.754852	Kng1	Kininogen-	KNG1_MOU	21	1.007302
15min / C	P01642	0.112156	0.987426	0.754942	Igkv5-48	Immunoglo	KV5A9_MO	2	1.080842
15min / C	P62500	-0.61271	0.987307	0.754942	Tsc22d1	TSC22 dom	T22D1_MO	1	0.653969
30min / C	P51880	0.036548	0.987846	0.755043	Fabp7	Fatty acid-b	FABP7_MO	4	1.025657
30min / C	Q9CRB3	0.011684	0.987914	0.755043	Urah	5-hydroxyi	HIUH_MOU	9	1.008132
30min / C	O88569	-0.02967	0.991367	0.755084	Hnrnpa2b1	Heterogene	ROA2_MOU	19	0.979647
15min / C	P01637;P0	0.076682	0.991212	0.755084	NaN	Ig kappa ch	KV5A5_MO	2	1.05459
15min / C	P09528	0.001139	0.988977	0.755084	Fth1	Ferritin hea	FRIH_MOUS	13	1.00079
30min / C	P18527	0.001069	0.991304	0.755084	NaN	Ig heavy cha	HVM56_MO	1	1.000741
15min / C	P49586	-0.02745	0.989191	0.755084	Pcyt1a	Choline-ph	PCY1A_MO	10	0.981154
30min / C	P59325	-0.03432	0.990868	0.755084	Eif5	Eukaryotic	IF5_MOUSE	17	0.976489
15min / C	P80318	0.157878	0.989455	0.755084	Cct3	T-complex	TCPG_MOU	34	1.115645
30min / C	Q6PDN3	0.01835	0.990238	0.755084	Mylk	Myosin ligh	MYLK_MOU	39	1.012801
30min / C	Q78PY7	0.171391	0.990752	0.755084	Snd1	Staphyloco	SND1_MOU	51	1.126144
15min / C	Q8BH00	0.065545	0.989244	0.755084	Aldh8a1	2-aminomu	AL8A1_MO	26	1.046481
15min / C	Q8K2C6	-0.20425	0.990556	0.755084	Sirt5	NAD-depen	SIR5_MOUS	11	0.867991
15min / C	Q921H9	0.103998	0.991365	0.755084	Coa7	Cytochrom	COA7_MOU	7	1.074748
30min / C	Q922Q1	-0.01391	0.991785	0.755084	Mtarc2	Mitochond	MARC2_MO	8	0.990408
15min / C	Q99KP3	0.020376	0.988744	0.755084	Cryl1	Lambda-cry	CRYL1_MO	14	1.014224
15min / C	Q9JM62	0.041665	0.990484	0.755084	Reep6	Receptor ex	REEP6_MO	5	1.029301
15min / C	Q9R0P5	0.05735	0.991876	0.755084	Dstn	Destrin	DEST_MOU	14	1.040553
30min / C	O89086	0.189962	0.993028	0.755102	Rbm3	RNA-bindir	RBM3_MOU	4	1.140734
15min / C	P23953	0.000384	0.993322	0.755102	Ces1c	Carboxyles	EST1C_MOU	10	1.000266
15min / C	P27048;P6	0.083949	0.992995	0.755102	Snrpb;Snrp	Small nucle	RSMB_MOU	5	1.059915
30min / C	P70296	0.069676	0.99212	0.755102	Pebp1	Phosphatid	PEBP1_MO	12	1.049481
15min / C	P83741	0.171071	0.992666	0.755102	Wnk1	Serine/thre	WNK1_MO	7	1.125894
15min / C	Q80VJ2	-0.03115	0.992648	0.755102	Sra1	Steroid rec	SRA1_MOU	4	0.978639
30min / C	Q8K4F5	-0.08549	0.992488	0.755102	Abhd11	sn-1-specifi	ABHDB_MO	6	0.942466
15min / C	Q8VEK3	-0.03105	0.992813	0.755102	Hnrnpu	Heterogene	HNRPU_MO	24	0.978711
30min / C	Q61749	0.25789	0.994098	0.755442	Eif2b4	Translation	EI2BD_MOU	11	1.195729
30min / C	Q14CH1	-0.07677	0.997088	0.756444	Mocos	Molybdenu	MOCOS_MO	23	0.948176
30min / C	Q6NXN1	0.073181	0.996389	0.756444	Szrd1	SUZ RNA-bi	SZRD1_MO	4	1.052034
30min / C	Q8R121	-0.28664	0.996726	0.756444	Serpina10	Protein Z-d	ZPI_MOUSE	6	0.819808
30min / C	Q91V64	-0.08426	0.996618	0.756444	Isoc1	Isochorism	ISOC1_MOU	15	0.94327
15min / C	Q9D0M5	0.030076	0.996038	0.756444	Dynll2	Dynein ligh	DYL2_MOU	4	1.021066
15min / C	Q9WTP6	-0.0608	0.995824	0.756444	Ak2	Adenylate k	KAD2_MOU	17	0.958735
15min / C	P01592	-0.00122	0.998375	0.75683	Jchain	Immunoglo	IGJ_MOUSE	4	0.999157
15min / C	P22892	0.044734	0.999358	0.75683	Ap1g1	AP-1 compl	AP1G1_MO	19	1.031493
30min / C	P56387	-0.72417	0.998304	0.75683	Dynlt3	Dynein ligh	DYLT3_MOU	2	0.605345
30min / C	P61965	-0.17034	0.998927	0.75683	Wdr5	WD repeat-	WDR5_MO	7	0.888634
30min / C	P62830	0.083357	0.999209	0.75683	Rpl23	Large ribos	RL23_MOU	7	1.05948
